# A comprehensive comparative genomic analysis of the endogenous Florendovirus-1 in *Prunus*

**DOI:** 10.3389/fpls.2026.1886618

**Published:** 2026-09-29

**Authors:** Carlos M. Vicient

**Affiliations:** 1 Centre for Research in Agricultural Genomics (CRAG), CSIC-IRTA-UAB-UB, Edifici CRAG, Barcelona, Spain; 2 Consejo Superior de Investigaciones Científicas (CSIC), Barcelona, Spain

**Keywords:** almond, endogenous caulimovirid elements (ECVs), endogenous viral elements (EVEs), florendovirus, *Prunus*

## Abstract

Endogenous caulimovirid elements (ECVs) are widespread components of plant genomes and provide valuable information on the long-term evolution of plant viruses and their hosts. However, the mechanisms underlying their integration and subsequent genomic reorganization remain poorly understood. Here, we investigated the diversity, distribution, and structural organization of ECVs in 30 chromosome-level genome assemblies representing 15 species of the genus *Prunus*. A genome-wide search based on conserved reverse transcriptase (RT) domains identified 781 caulimovirid sequences, which were classified into 18 clusters belonging to the operational taxonomic units Florendovirus and Yendovirus. Florendovirus-1 (Flo-1) was the predominant lineage, accounting for 532 sequences and 68% of all RT sequences detected. To characterize this lineage in detail, we reconstructed a 7,193-bp Flo-1 consensus sequence from seven complete elements identified in a high-quality almond genome assembly. The reconstructed Flo-1 genome contained two open reading frames and the canonical caulimovirid movement protein, coat protein, aspartic protease, reverse transcriptase, and RNase H domains. A detailed nucleotide-level search identified 757 Flo-1-derived fragments in *P. dulcis* genome spanning 0.91 Mb, corresponding to 0.37% of the almond genome. These fragments were organized into 176 loci, of which 36% contained multiple insertions comprising complete or fragmented elements in different orientations. The inferred endogenization breakpoints of 77% of complete elements were located near the Met-tRNA binding site. Consistently, few fragments spanned this region, whereas fragment termini showed a pronounced enrichment around it, suggesting preferential viral genome linearization at or near this position. Phylogenetic analysis of RT-coding sequences within multiple-insertion loci revealed both closely related and divergent elements. These findings indicate that the present-day organization of Flo-1 loci cannot be explained by a single process and probably reflects the combined effects of concatemer integration, recurrent insertion, local duplication, recombination, fragmentation, and post-integration rearrangement. Overall, this study reveals the extensive contribution and complex evolutionary history of endogenous florendoviruses in *Prunus* genomes.

## 1 Introduction

Endogenous viral elements (EVEs) are virus-derived DNA sequences integrated into the genomes of eukaryotic organisms (Chu et al., 2014). Many types of viruses can produce EVEs (Feschotte and Gilbert, 2012). Among these, the most studied EVEs are those that originated from retroviruses because integration is a mandatory stage in their life cycle through a retrovirus-encoded integrase (Katzourakis and Gifford, 2010). However, although retrovirus-like sequences have been found in plant genomes (Vicient et al., 2001; Vicient, 2025), *sensu-stricto* retroviruses are restricted to vertebrates (Zheng et al., 2022). EVEs have also been observed for many other virus types for which integration in the host genome is not part of their usual life cycle, such as Hepadnavirus, SARS-CoV-2, or giant viruses (Kojima et al., 2021; Zhang et al., 2021; Erazo-Garcia et al., 2025). In plants, EVEs have been found derived from viruses with either DNA or RNA genomes (Takahashi et al., 2019). The first reported plant EVE was a Geminivirus-related sequence (Bejarano et al., 1996), but those derived from members of the family *Caulimoviridae*, called Endogenous Caulimovirid Elements (ECVs), are the most abundant (de Tomás and Vicient, 2022).

In plants, viruses belonging to the family *Caulimoviridae* (order *Ortervirales*), also known as pararetroviruses, are reverse-transcribing non-covalently closed circular dsDNA viruses with genomes ranging from 6 to 10 kb. Virions of caulimovirids are non-enveloped and either isometric or bacilliform in shape. They comprise 11 recognized genera (Teycheney et al., 2020). Depending on the genus, caulimovirid genomes may comprise one to nine open reading frames (ORFs). All members of the family *Caulimoviridae* encode a common set of proteins: a movement protein (MP), a coat protein (CP), a pepsin-like aspartic protease (AP), a reverse transcriptase (RT), and an RNase H (RH) (Teycheney et al., 2020). Some members of the family *Caulimoviridae* encode additional proteins.

Although integration into the host genome is not part of the caulimovirid life cycle (they do not encode an integrase), advances in genomic sequencing have contributed to the detection of numerous ECVs in plant genomes (Mushegian and Elena, 2015; Diop et al., 2018; Serfraz et al., 2021; de Tomás and Vicient, 2022). ECVs include some of the 11 *Caulimoviridae* genera and diverse ECV genera for which no episomal counterparts have yet been described and which have been assigned tentative names such as Florendovirus, Zendovirus, Yendovirus, Xendovirus, Wendovirus and others (Geering et al., 2014; Chen and Kishima, 2016; Diop et al., 2018; de Tomás and Vicient, 2022). Florendoviruses are the most abundant and widely distributed ECVs (de Tomás and Vicient, 2022).

Insertion mechanisms of the ECVs remain largely uncharacterized, and different mechanisms have been proposed. The most accepted theory is that endogenization results from non-homologous recombination occurring during the repair of DNA double-strand breaks (Richert-Pöggeler et al., 2003; Hohn et al., 2008; Geering et al., 2014), but it has also been proposed that illegitimate recombination events or transposon-mediated integration are possible (Harper et al., 2002; Staginnus and Richert-Pöggeler, 2006; Liu et al., 2012; Chu et al., 2014; Richert-Pöggeler et al., 2021).

Most characterized ECV insertions do not appear to have an evident effect on host genes, and they are degraded by the accumulation of disruptive mutations, insertions, or deletions. Due to the random nature of these mutations, it is possible to reconstruct the sequences of the original viruses based on the inserted sequences, particularly for high-copy-number ECVs (Aiewsakun and Katzourakis, 2015).

The genus *Prunus* belongs to *Rosaceae* and comprises 250–400 species mainly found in the temperate regions of the northern hemisphere and in subtropical and tropical regions (Shi et al., 2013). *Prunus* species are classified into five or six subgenera (Lee and Wen, 2001). Most cultivated species are diploid (2n = 2x = 16) except *P. pseudocerasus* (2n = 4x = 32) and *P. cerasus* (2n = 4x = 32) (Wang et al., 2022). There are substantial genomic resources for the genus *Prunus*, with good-quality genome assemblies (complete or chromosome-level assemblies according to NCBI criteria; https://www.ncbi.nlm.nih.gov/datasets/docs/v2/glossary/#assembly-level) available for 15 species (Hodel et al., 2021).

Here, advanced genomics was used to investigate the presence, distribution, and conservation of endogenous caulimovirid genome fragments in the genus *Prunus*. Florendovirus- and Yendovirus-related sequences were identified, with florendovirus being the most abundant. Florendovirus can be inserted as isolated elements (complete or fragmented) or as multiple insertions. The possible mechanisms of insertion are discussed.

## 2 Methods

### 2.1 Detection and analysis of inserted endogenous *Caulimoviridae* RT-coding sequences

A tBLASTn search was performed (e-value ≤ 1e−30 and at least 250 aa) to identify RT sequences of the different *Caulimoviridae* genera present in 30 high-quality genome assemblies (chromosome quality level according to NCBI standards) corresponding to 15 species of the genus *Prunus* (**Supplementary Material 1**). We used the amino acid sequences corresponding to the conserved reverse transcriptase (RT) domain of 12 representatives of the major *Caulimoviridae* genera and Florendovirus as queries (**Supplementary Material 2**). Only elements present in chromosome assemblies were selected (not in contigs or in scaffolds). The selected twelve RT sequences were sufficient to detect the diversity of genera, including potential endogenous operational taxonomic units (OTUs). The search also identified RTs from LTR-retrotransposons. The results of the search were aligned with the representative RT sequences of *Caulimoviridae* and of the main groups of Ty3-LTR retrotransposons (*Metaviridae*) (**Supplementary Material 3**). Sequences showing higher similarity with *Metaviridae* were removed. Finally, we obtained 781 RT sequences (**Supplementary Materials 4**, **5**). The RT sequences were grouped into clusters with CD-HIT using a sequence identity cutoff of 80%. One sequence was then selected to be representative of each cluster (sequences in red in **Supplementary Material 4**), yielding a total of 18 clusters.

### 2.2 Detection of Florendovirus-1 sequences in almond

Genomic regions extending 5,000 bp upstream and downstream of each RT-coding sequence were extracted. The choice of a 5 kb extension was based on the fact that florendovirus genomes are known to range between 7.2 and 8 kb in length (Geering et al., 2014). Therefore, extending the sequences by 5 kb on both sides of the region encoding the RT domain, which is approximately 0.9 kb long, was considered sufficient to encompass a potentially complete genome. Admittedly, some complete elements may not have been recovered, such as full-length copies disrupted by the insertion of a transposable element, or copies that became linearized and integrated near the RT domain. Nevertheless, the identification of seven sequences was sufficient for the construction of a reliable consensus sequence, and the subsequent analyses (**Figure 4**; **Supplementary Material 10**) demonstrated that the choice of a 5 kb extension was appropriate.

Potential ORFs were predicted using ORF Finder (https://www.ncbi.nlm.nih.gov/orffinder/) and the presence of Pfam domains in their encoded polypeptides was confirmed using MOTIF Search (https://www.genome.jp/tools/motif/).

### 2.3 Phylogenetic analyses

Sequence alignments were performed using MAFFT. Phylogenetic analyses were conducted using MEGA-X (Kumar et al., 2018) under the maximum likelihood (ML) method and 1,000 bootstrap replicates.

## 3 Results

### 3.1 Endogenous caulimovirids in *Prunus*

We screened 30 genome assemblies corresponding to 15 species of the genus *Prunus*, all assembled at chromosome-level quality (**Supplementary Material 1**), for the presence of endogenous caulimovirid RT protein-coding sequences using a tBLASTn approach and a collection of 12 RT domain amino acid sequences representative of the *Caulimoviridae* family (**Supplementary Material 2**). The genome dataset included representatives of three subgenera: *Prunus*, *Amygdalus*, and *Cerasus*. This screening identified 781 RT amino acid sequences across all analyzed species, with numbers ranging from 3 (Prunus_pseudocerasus_1) to 58 (Prunus_persica_5). Using a minimum amino acid identity threshold of 80%, these sequences were grouped into 18 clusters (**Supplementary Materials 1**–**5**).

To determine the phylogenetic placement of the 18 clusters, we performed a phylogenetic analysis using one representative RT sequence from each cluster, together with 45 RT sequences representing the genera of the family *Caulimoviridae*, including endogenous elements, and 60 RT sequences from Ty3 LTR retrotransposons used as outgroups (**Supplementary Material 3**). The maximum likelihood (ML) phylogenetic tree indicated that the RT sequences identified in *Prunus* genomes were divided into two major groups (**Figure 1**). Seven clusters were assigned to the operational taxonomic unit (OTU) Florendovirus, whereas the remaining eleven clusters belonged to Yendovirus.

**Figure 1:**
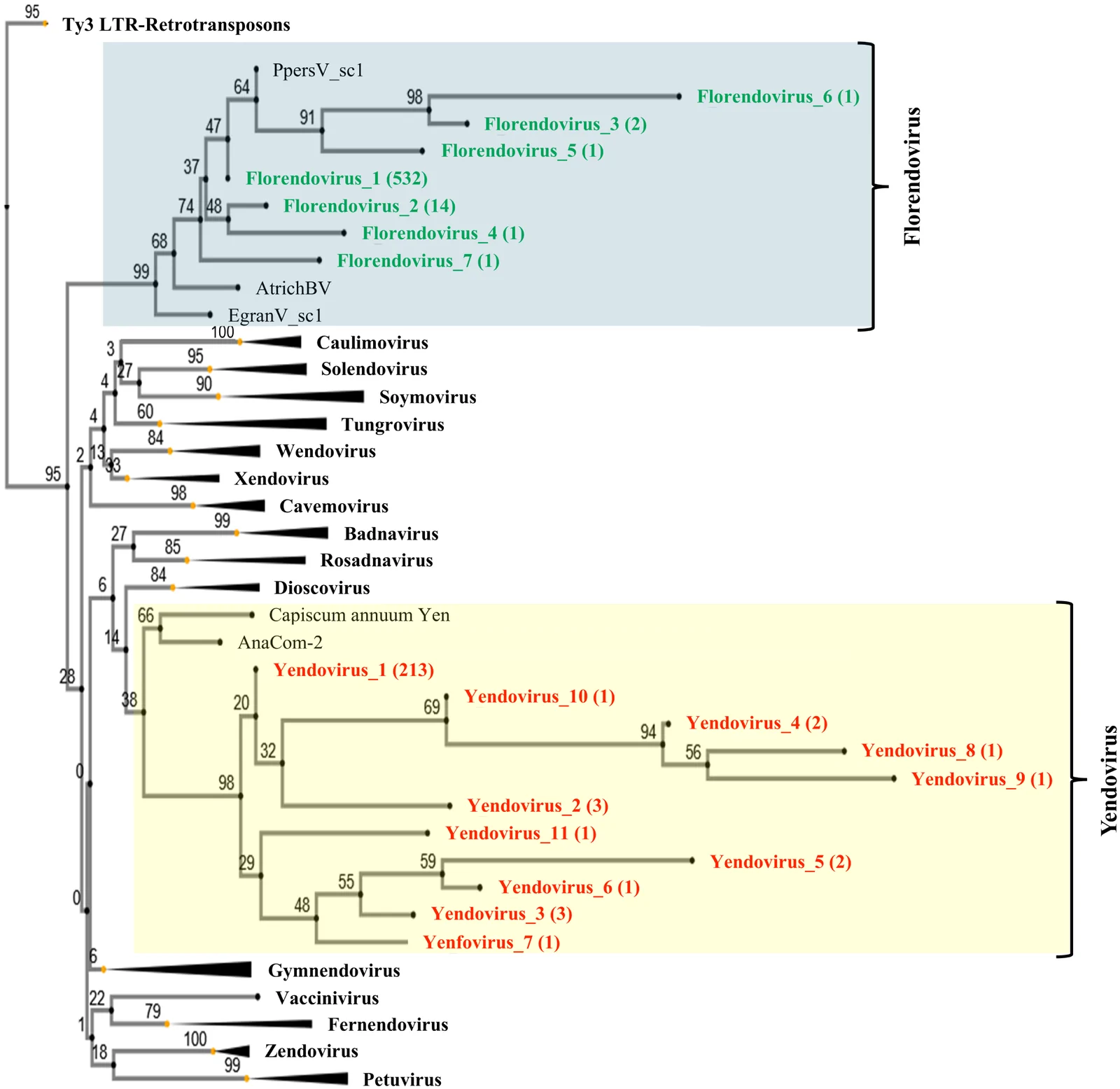
Phylogenetic analysis of RT sequences identified in 30 *Prunus* genome assemblies. The maximum likelihood (ML) phylogenetic tree was constructed using amino acid sequences of the conserved reverse transcriptase (RT) domain of representatives of the *Prunus* element clusters and representatives of the previously discovered sequences from the family *Caulimoviridae* (including endogenous elements) and *Metaviridae* (Ty3 LTR-retrotransposons). The Florendovirus lineage is highlighted in blue (green labels), whereas the Yendovirus lineage is highlighted in yellow (red labels). The bootstrap consensus tree inferred from 1,000 replicates is shown. Numbers in parentheses indicate the number of sequences assigned to each cluster. For clarity, branches containing a single operational taxonomic unit (OTU) were collapsed.

To assess the distribution of RT sequences, we calculated the number of sequences detected in each *Prunus* genome assembly normalized by genome size, distinguishing between sequences assigned to Florendovirus and Yendovirus (**Figure 2**).

**Figure 2:**
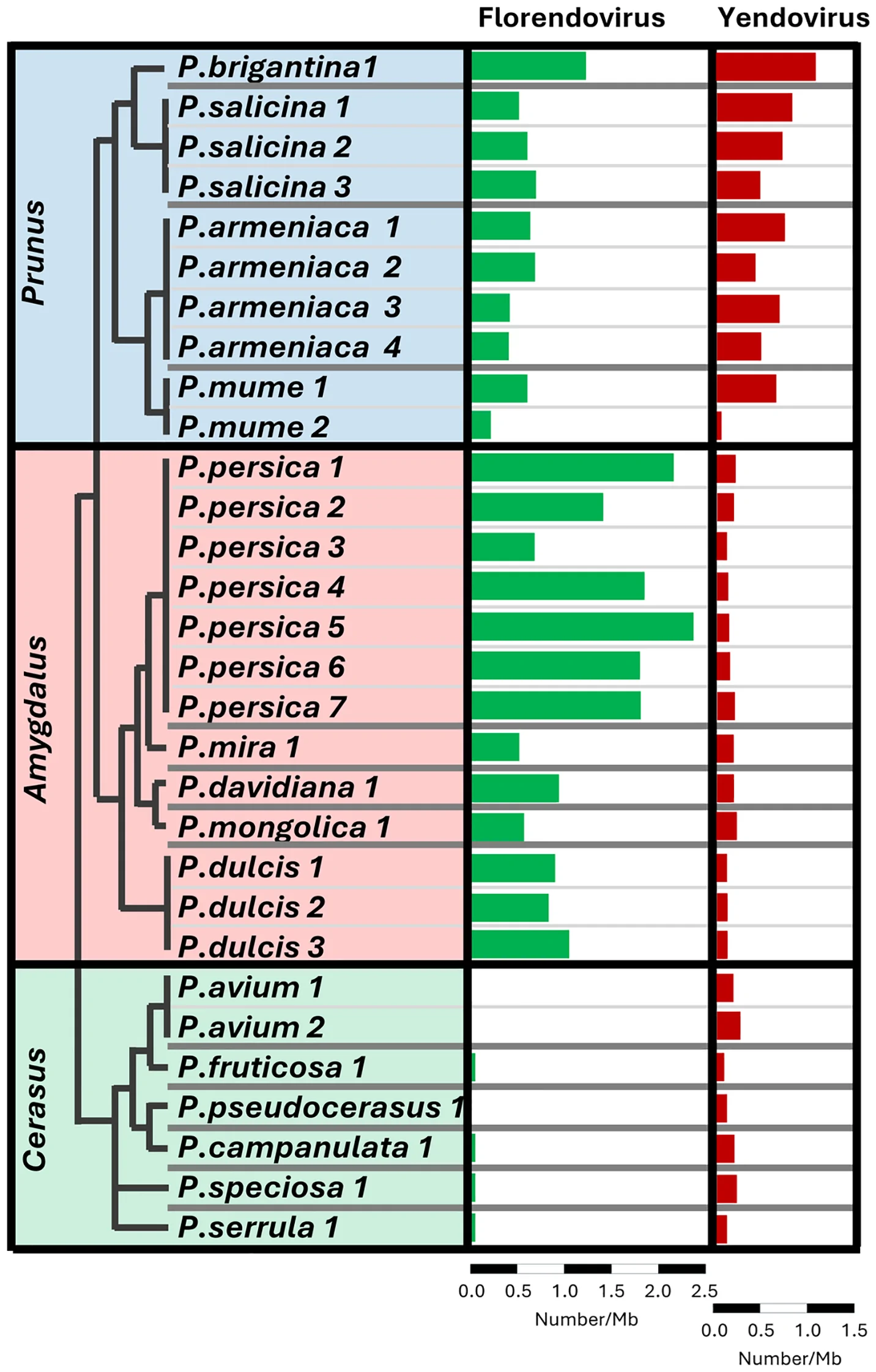
Distribution of Florendovirus and Yendovirus RT sequences across *Prunus* genome assemblies. Values are expressed as copies per megabase. Labels on the left indicate the three *Prunus* subgenera. The phylogenetic relationships within the genus *Prunus* are based on Hodel et al. (2021); Shi et al. (2013), and the references cited therein. The genome assemblies analyzed in this study are listed in **Supplementary Material 1**.

Regarding florendoviruses, similar abundances were observed among species belonging to the *Prunus* and *Amygdalus* subgenera, whereas they were much less frequent, or even absent, in species of the *Cerasus* subgenus. In two species, *Prunus brigantina* and *Prunus persica*, a markedly higher abundance was detected compared with the other assemblies within their respective subgenera. Comparisons among genome assemblies of the same species generally produced consistent results. However, two exceptions were identified, namely Prunus_mume_2 and Prunus_persica_3, which exhibited substantially lower values. This reduced abundance of florendoviruses, as well as yendoviruses, may be explained by the lower assembly quality metrics of these genomes (**Supplementary Material 1**).

With respect to yendoviruses, their frequency was comparable to that of florendoviruses in the *Prunus* subgenus but markedly lower in the *Amygdalus* subgenus. Frequencies observed in genomes belonging to the *Cerasus* subgenus were similar to those found in *Amygdalus* and, consequently, were higher than those observed for florendoviruses.

Among the 552 sequences assigned to Florendovirus, 532 belonged to the Florendovirus-1 cluster, whereas 213 of the 229 sequences assigned to Yendovirus belonged to the Yendovirus-1 cluster (**Figure 1**). Thus, 68% of all RT sequences identified were concentrated in a single cluster, Florendovirus-1 (Flo-1). It is well established that florendoviruses are the most abundant and taxonomically widespread endogenous caulimovirids described to date (de Tomás and Vicient, 2022). Therefore, Flo-1 was selected for further analysis, and the remainder of this study focuses on this cluster.

### 3.2 Genomic organization of Florendovirus-1 in almond

We reconstructed a representative Flo-1 genome in almond by generating a full-length consensus sequence. All sequences encoding Flo-1 RTs in the Prunus_dulcis_3 genome assembly were extended by 5 kb upstream and downstream and examined for the presence of uninterrupted coding regions corresponding to the movement protein (MP), coat protein (CP), aspartic protease (AP), and RNase H (RH) domains. Nine sequences encoded all these domains; however, following manual curation and pairwise sequence comparisons, two highly divergent sequences were excluded (they were less than 82% similar to the consensus whereas the other seven were more than 87%). A consensus Flo-1 sequence was subsequently generated from the remaining seven sequences (**Supplementary Material 6**).

The reconstructed Flo-1 genome was 7,193 bp in length and contained a putative Met-tRNA binding site at position 1 (**Figure 3**). A predicted hairpin-like structure was identified at position 257. Flo-1 contains two open reading frames (ORFs) located in different translational frames. ORF1 encodes a 215-kDa polyprotein comprising the canonical caulimovirid domains MP, CP, AP, RT, and RH. ORF2 encodes a 35-kDa protein of unknown function showing similarity to the DUF8797 motif (PF31063; domain of unknown function). This genomic organization is consistent with that previously described for florendoviruses (Geering et al., 2014).

**Figure 3:**
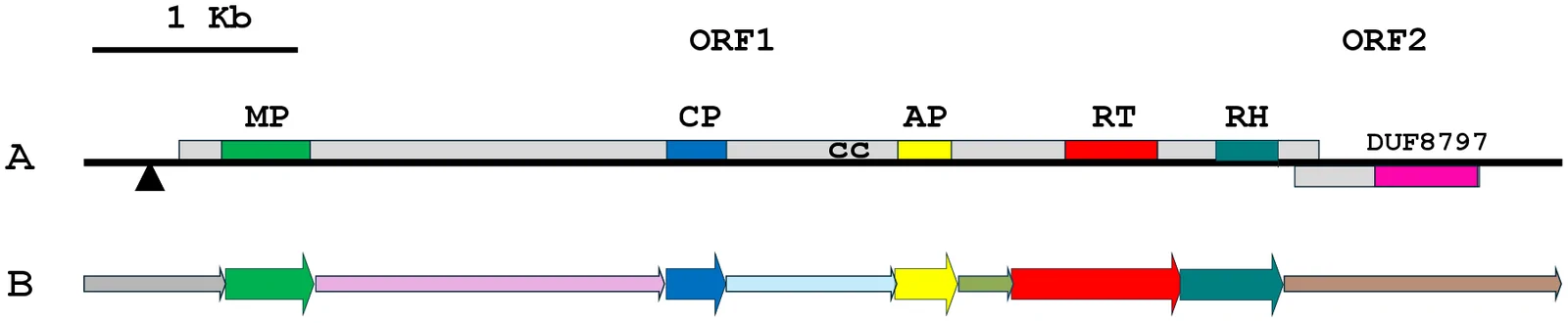
Schematic representation of Florendovirus-1 in almond. **(A)** Scaled linear representation of the Florendovirus-1 genome. Light gray boxes indicate open reading frames (ORFs), and colored regions within the ORFs correspond to conserved protein domains: green, movement protein (MP; PF01107); blue, coat protein (CP; PF22909); yellow, aspartyl protease (AP; PF00077); red, reverse transcriptase (RT; PF00078); blue-green, RNase H (RH; PF17917); dark pink, DUF8797. The label “cc” denotes a predicted coiled-coil domain. A black triangle marks a predicted hairpin-like secondary structure. Additional details are provided in **Supplementary Material 6**. **(B)** Schematic representation of the linearized Florendovirus-1 genome. The same color scheme is used throughout the following figures.

To perform a more comprehensive search for Flo-1-derived sequences in the Prunus_dulcis_3 genome, the consensus Flo-1 sequence was used as a BLASTn query. This analysis was designed to identify homologous regions regardless of whether they corresponded to complete endogenous viral elements. A total of 757 genomic fragments of at least 100 bp showing significant similarity to the Flo-1 consensus sequence (e-value ≤ 1 × 10⁻^10^) were identified. Together, these sequences span 0.91 Mb, corresponding to 0.37% of the almond genome.

Close inspection of the genomic positions of these fragments revealed that many were located in close proximity to one another, suggesting the presence of tandem or nested structures. Indeed, it has been described that ECVs are frequently organized into tandem arrays and complex multicopy rearrangements (Vassilieff et al., 2023). Accordingly, two fragments were assigned to the same Flo-1 locus when the distance between them did not exceed 1,000 bp, except when the intervening sequence corresponded to a recognizable transposable element (TE). The 1-kb threshold was considered an operational clustering criterion rather than a biologically defined boundary. In most cases, distances between consecutive fragments greatly exceeded 1,000 bp, and only a small number of loci could potentially be affected by this cutoff (**Supplementary Materials 7**, **8**).

Using these criteria, 176 Flo-1 loci were identified (**Supplementary Materials 7**, **8**), of which 36% contained multiple insertions. In total, 53 complete or near-complete elements were detected, including 27 occurring as isolated insertions. A near-complete element was defined as one exhibiting sequence similarity across all regions of the Flo-1 consensus genome (**Figure 3**), although it does not necessarily retain intact ORFs or represent a potentially functional element. Multiple-insertion loci may contain complete sequences and fragments arranged in either the same or opposite orientations. The largest locus, Chr6-05, spans 59,352 bp and contains four complete elements together with nine additional fragments arranged in both sense and antisense orientations (**Supplementary Material 7**). Furthermore, 37% of Flo-1 loci were flanked by TA dinucleotide repeats, a value consistent with previous reports for other plant species, including *Vitis vinifera* (14%), *Glycine max* (46%), and *Oryza sativa* (51%) (Geering et al., 2014).

Members of the family *Caulimoviridae* possess circular double-stranded DNA genomes, whereas ECVs occur as linear sequences integrated into plant chromosomes. Endogenization of a complete viral genome therefore requires linearization of the circular viral DNA at a specific position. We operationally define this position as the endogenization breakpoint. This term refers to the inferred point at which continuity of the circular viral genome was disrupted during integration and does not necessarily correspond to the precise molecular breakpoint or underlying integration mechanism.

The positions of the endogenization breakpoints were determined for all complete Flo-1 elements relative to the consensus Flo-1 sequence. The observed distribution appears to be non-random, as the breakpoint was located adjacent to the Met-tRNA binding site in 77% of cases. This pattern was evident both for complete elements occurring as isolated insertions (**Figure 4A**; 81%) and for complete elements embedded within multiple-insertion loci (**Figure 4B**; 73%).

**Figure 4:**
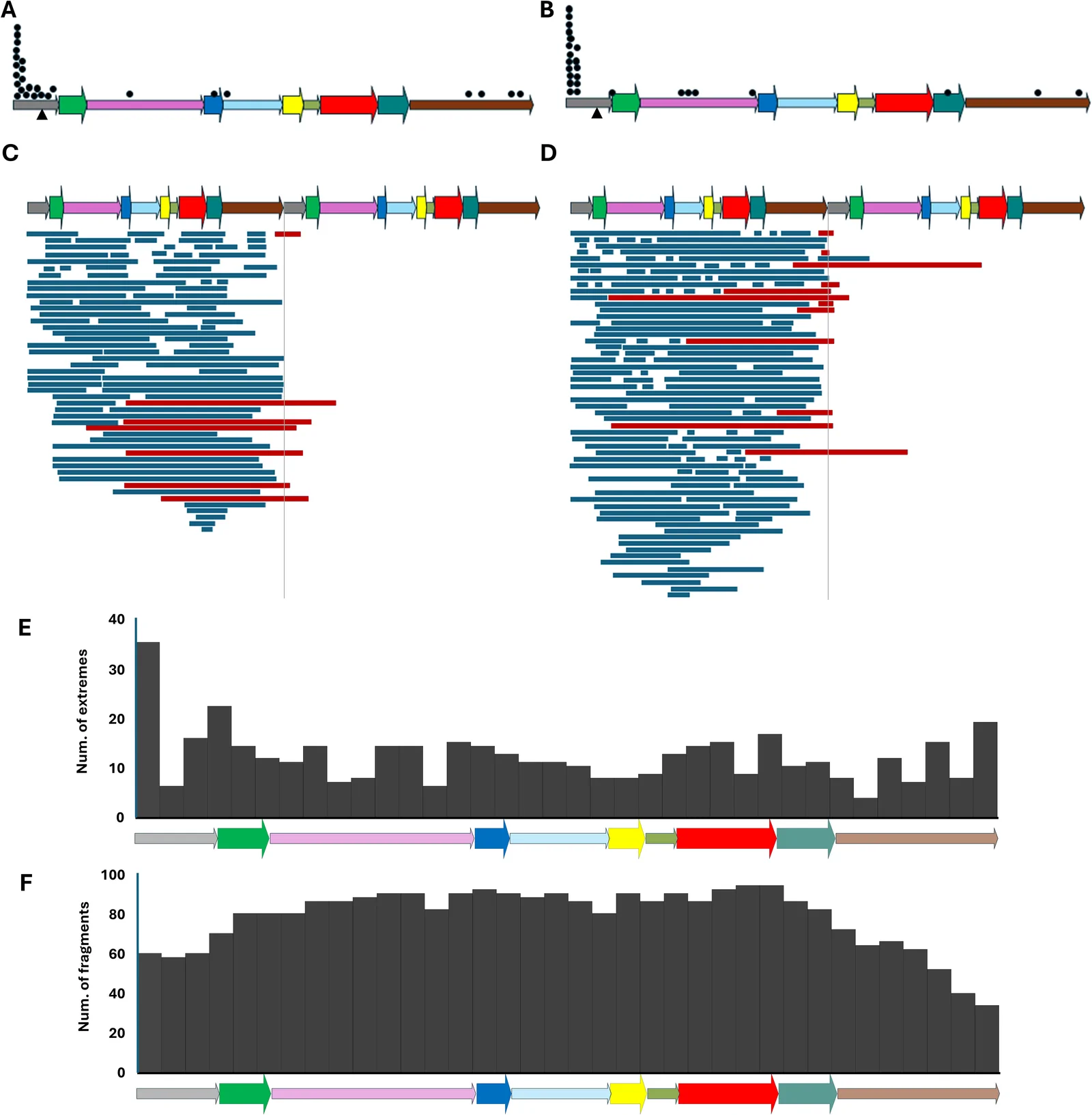
Structural organization of complete and fragmentary Florendovirus-1 elements in the almond genome. **(A, B)** Distribution of endogenization breakpoints (black dots) in complete Flo-1 elements. **(A)** Isolated complete elements. **(B)** Complete elements located within multiple-insertion loci. **(C, D)** Distribution of Flo-1 fragments (lines) relative to the Flo-1 consensus sequence. Red lines indicate fragments containing the Met-tRNA binding site. **(C)** Isolated fragments. **(D)** Fragments located within multiple-insertion loci. **(E)** Distribution of fragment termini along the Flo-1 consensus sequence. **(F)** Number of genomic fragments showing similarity to each region of the Flo-1 consensus sequence. Colors and symbols are as described in **Figure 3**.

We next analyzed the distribution of Flo-1 fragments relative to the consensus sequence. Fragment lengths varied considerably, and fragments were distributed throughout the consensus genome. However, only a small proportion overlapped with the Met-tRNA binding site (8.2%). This pattern was observed both for isolated fragments (**Figure 4C**; 7.5%) and for fragments located within multiple-insertion loci (**Figure 4D**; 8.7%).

We also examined the distribution of fragment termini along the consensus sequence. To this end, the consensus genome was divided into 36 segments, and the number of fragment ends within each segment was determined (**Figure 4E**). A pronounced peak was observed in the region encompassing the Met-tRNA binding site, whereas the remainder of the sequence displayed a relatively uniform distribution. Finally, we evaluated the representation of different regions of the consensus genome among the integrated fragments (**Figure 4F**). Central regions of the Flo-1 genome were represented more frequently than terminal regions.

Detailed examination of multiple-insertion loci revealed several structures closely resembling LTR retrotransposons. These structures contain protein domains similar to those encoded by retrotransposons and are flanked by long direct repeats (**Supplementary Material 10**). Nevertheless, closer inspection readily distinguishes them from bona fide LTR retrotransposons. In particular, ECVs encode an MP domain but lack an integrase domain. Moreover, phylogenetic analysis of reverse transcriptase (RT) sequences can reliably discriminate between members of *Caulimoviridae* and LTR retrotransposon lineages. Consequently, such misclassification is unlikely when modern annotation pipelines are employed. However, errors may persist in genomes annotated using earlier methodologies, and some ECVs may therefore have been classified as LTR retrotransposons of unknown lineage because of the absence of an identifiable integrase domain.

### 3.3 Phylogenetic analysis of Flo-1 elements

All sequences encoding the conserved Flo-1 RT domain present in the almond reference genome were extracted, aligned, and used to construct a phylogenetic tree. The resulting tree revealed the presence of two clearly differentiated groups, designated A and B, together with several subordinate clades, with group A being considerably more abundant than group B (**Figure 5**).

**Figure 5:**
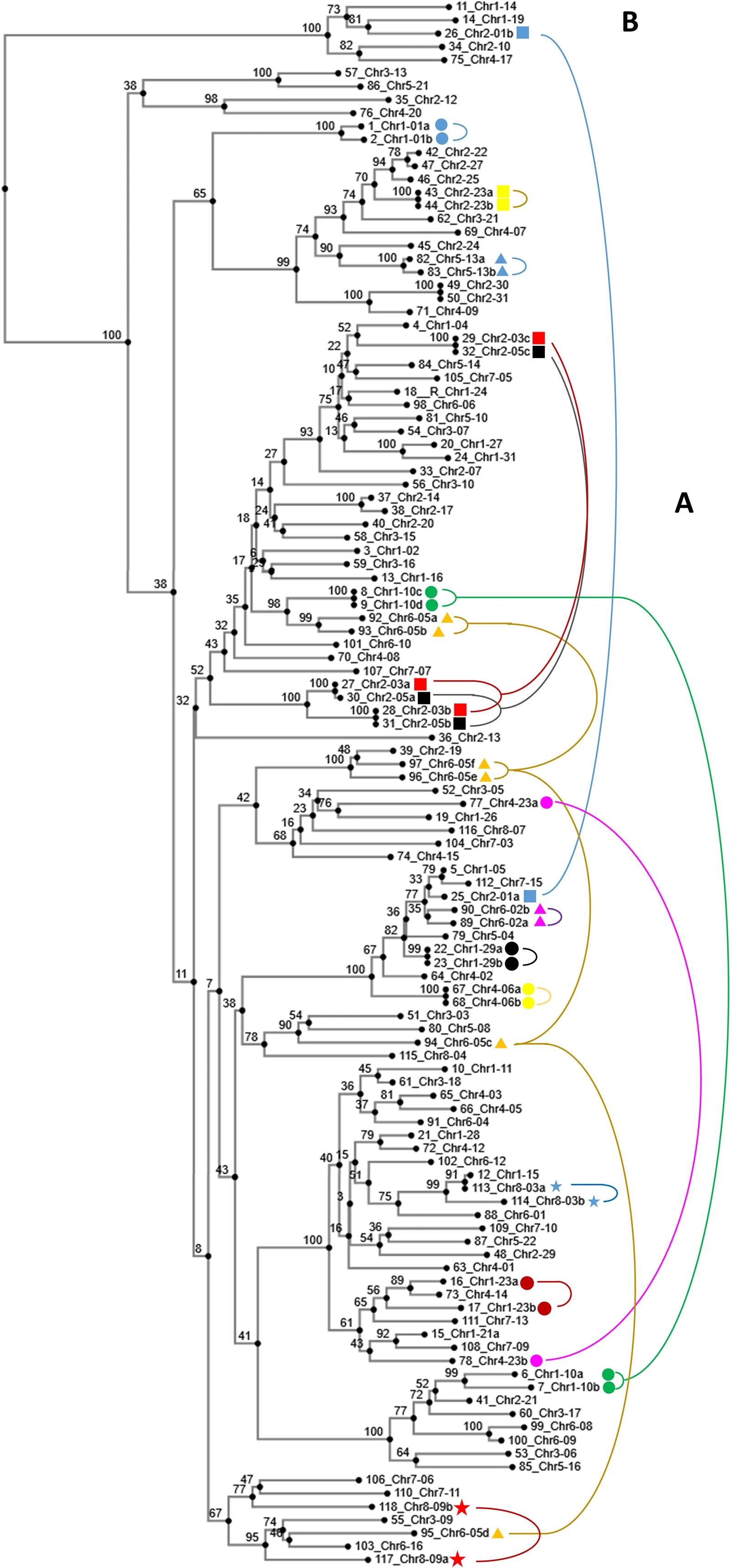
Phylogenetic analysis of Florendovirus-1 RT-coding sequences in the almond genome. The maximum likelihood (ML) phylogenetic tree was constructed using the nucleotide sequences encoding the reverse transcriptase (RT) domain of Florendovirus-1 elements identified in the almond genome. Sequences connected by lines and marked with the same symbol correspond to elements located within the same genomic locus. The bootstrap consensus tree inferred from 1,000 replicates is shown. The two major phylogenetic groups identified in the analysis are designated **(A, B)**.

Particular attention was given to the RT sequences associated with the 15 multiple-insertion loci identified in the genome (indicated by colored symbols in **Figure 5**). In nine loci, all RT sequences clustered within the same phylogenetic branch. In two loci, the RT sequences were distributed between two distinct branches of the tree. Finally, in four loci, some RT sequences clustered within closely related branches, whereas other RT sequences from the same locus were assigned to distantly related branches.

## 4 Discussion

Endogenous caulimovirid elements (ECVs) are a widespread component of plant genomes (Diop et al., 2018) that can play significant roles in evolution, serving as long-term genetic repositories, driving diversification through recombination, and, in some cases, giving rise to transcriptionally active and infectious episomal forms. In addition, ECVs act as molecular fossils, and the analysis of ECVs provides insights into the deep evolutionary history, long-term evolution, and diversification of plant viruses (Vassilieff et al., 2023).

The systematic screening of 30 chromosome-level genome assemblies representing 15 species revealed that endogenous caulimovirid sequences in *Prunus* are associated with two operational taxonomic units, Florendovirus and Yendovirus (**Figure 2**). The distribution of sequences among clusters shows that florendoviruses are more abundant and, in particular, Florendovirus-1 (Flo-1) alone accounted for 532 of the 781 RT sequences identified, whereas Yendovirus-1 contained 213 of the 229 sequences. The predominance of Flo-1 is consistent with previous evidence that florendoviruses are the most abundant genus of ECVs in plants (Geering et al., 2014; de Tomás and Vicient, 2022). Their abundance may reflect differences in historical exposure, integration propensity, post-integration amplification, or long-term retention. However, the relative contribution of these processes remains unknown.

The relative abundance of Florendovirus and Yendovirus sequences differed among the three examined subgenera. Florendoviruses occurred at broadly similar frequencies in the subgenera *Prunus* and *Amygdalus* but were less frequent or absent in *Cerasus*. By contrast, yendoviruses occurred at frequencies comparable to those of florendoviruses in the subgenus *Prunus*, were less abundant in *Amygdalus*, and showed frequencies in *Cerasus* similar to those observed in *Amygdalus*. These contrasting distributions may reflect recent lineage-specific amplification events of endogenous viral sequences.

Given the predominance of Flo-1, we focused on this element and performed an in-depth characterization using the Prunus_dulcis_3 genome assembly. This assembly was selected because of its high quality and contiguity, particularly in repetitive parts of the genome (Castanera et al., 2024). Reconstruction of a 7,193-bp Flo-1 consensus sequence from seven almond elements provided a reference with which to investigate the genomic organization of this lineage in greater detail. The method used here is similar to those applied to the succesfull reconstitution of the transposable element Sleeping Beauty (Ivics et al., 1997) and the retrovirus HERV-K (Lee and Bieniasz, 2007), both present only in defective forms today. The reconstructed genome contains a putative Met-tRNA binding site, a predicted hairpin-like structure, and two open reading frames. ORF1 encodes a 215 kDa polyprotein with the canonical caulimovirid MP, CP, AP, RT, and RH domains, whereas ORF2 encodes a 35 kDa protein containing the domain of unknown function DUF8797. This architecture resembles that previously reported for florendoviruses (Geering et al., 2014), supporting the classification of Flo-1 within this group.

Flo-1-derived sequences represent 0.37% of the almond reference genome, including complete (or near-complete) elements and fragments. The distribution of Flo-1 sequences relative to the consensus sequence does not appear to be entirely random (**Figure 1**). Most complete elements are linearized near the Met-tRNA binding site, whereas very few fragments span this region. This bias may reflect features of the integration process. Caulimovirid genomes enter the host cell nucleus as circular double-stranded DNA molecules containing discontinuities, or gaps in one of the two DNA strands (Hohn and Rothnie, 2013). The number and positions of these discontinuities vary among viral species. For example, members of the genera *Caulimovirus* and *Soymovirus* contain three discontinuities, whereas those of *Badnavirus* and *Tungrovirus* contain only one (Teycheney et al., 2020). Although these discontinuities are repaired in the nucleus during viral minichromosome formation, they are transiently present following nuclear import. They could therefore constitute regions of increased susceptibility to breakage and subsequent linearization of the viral genome. Under this hypothesis, if episomal florendovirus genomes contain a single discontinuity near the Met-tRNA binding site, the preferential linearization of viral DNA at this position could account for the non-random distribution of Flo-1 sequences integrated into the almond genome. Complete copies may have been preferentially linearized at or near this site, whereas partial elements may have arisen predominantly through subsequent fragmentation of these linearized viral genomes. Nevertheless, the structure of the original episomal Flo-1 genome remains unknown, and this model should therefore be regarded as a working hypothesis requiring further experimental support.

Insertion mechanisms of the ECVs remain largely uncharacterized, and different mechanisms have been proposed. The most accepted theory is that endogenization results from non-homologous recombination occurring during the repair of DNA double-strand breaks (Richert-Pöggeler et al., 2003; Hohn et al., 2008; Geering et al., 2014), but it has also been proposed that illegitimate recombination events or transposon-mediated integration are possible (Harper et al., 2002; Staginnus and Richert-Pöggeler, 2006; Liu et al., 2012; Chu et al., 2014; Richert-Pöggeler et al., 2021). Transposon-mediated insertion appears unlikely in the case of Flo-1, because this mechanism typically generates target-site duplications, which were not detected at the Flo-1 insertion boundaries. The presence of AT-rich sequences in 37% of the flanking regions is consistent with the possible involvement of a DNA repair-associated mechanism in Flo-1 integration, given the susceptibility of AT-rich regions to DNA breakage. The presence of TA dinucleotide repeats in ECV flanking regions is consistent with previous reports for other plant species, including *Glycine max* (46%), and *Oryza sativa* (51%) (Geering et al., 2014).

Notably, 36% of Flo-1 loci contain multiple insertions, comprising complete or fragmented elements arranged in either the same or opposite orientations. Similar multicopy architectures have previously been documented for endogenous caulimovirids (ECVs) (Lockhart et al., 2000; Richert-Pöggeler et al., 2003; Liu et al., 2012; Yu et al., 2019; Schmidt et al., 2021). Several mechanisms could account for these complex structures, including the integration of preassembled viral DNA concatemers; the simultaneous or successive insertion of multiple viral copies into the same chromosomal region, potentially reflecting a genomic fragile site; and post-integration remodeling through local duplication or recombination between pre-existing ECVs, or between an ECV and an episomal viral genome.

The architecture of different Flo-1 loci resembles tandem arrays. Consistent with this interpretation, phylogenetic analysis of the RT-coding sequences showed that domains located within the same locus were often closely related. Together, these observations support two plausible and non-mutually exclusive scenarios: the simultaneous integration of multiple, closely related viral copies and the local amplification of a pre-existing insertion. In contrast, adjacent Flo-1 sequences arranged in opposite orientations are less readily explained by straightforward tandem duplication. Such configurations more strongly support recurrent integration into the same genomic region, although inversion, recombination, and other post-integration rearrangements remain possible alternative explanations.

Additional evidence for recurrent integration comes from the identification of RT-coding sequences from the same locus that cluster in distantly related branches of the phylogenetic tree. Their marked evolutionary divergence is difficult to reconcile with recent local duplication alone and instead suggests that some loci accumulated Flo-1 sequences through independent integration events involving genetically distinct viral variants or lineages. Nevertheless, recombination between divergent endogenous copies, or between an endogenous element and an episomal Flo-1 genome, could generate a comparable phylogenetic pattern and therefore cannot be ruled out.

Taken together, our findings indicate that Florendovirus-1, the most abundant endogenous caulimovirus in the almond genome, could be inserted in the genome forming multicopy loci that cannot be explained by a single universal mechanism. Rather, their present-day architecture most likely records the cumulative action of several processes, including concatemer integration, recurrent insertion, local amplification, recombination, inversion, and broader post-integration genomic rearrangement. The relative contribution of each mechanism probably varies among loci, highlighting the dynamic and potentially prolonged evolution of Flo-1-containing genomic regions.

## 5 Conclusions

Endogenous caulimovirids are widespread in *Prunus*, with Flo-1 representing the predominant lineage. Its particularly detailed characterization in almond has revealed a complex genomic landscape comprising isolated, fragmented, and multicopy insertions. The non-random distribution of endogenization breakpoints near the Met-tRNA binding site suggests preferential linearization at this region. Phylogenetic and structural evidence indicates that multiple-insertion loci arose through a combination of concatemer integration, recurrent insertion, duplication, recombination, fragmentation, and post-integration rearrangement rather than through a single mechanism.

## Data Availability

The original contributions presented in the study are included in the article/**Supplementary Material**. Further inquiries can be directed to the corresponding author.
