# Supplementary material for: A comprehensive comparative genomic analysis of the endogenous Florendovirus-1 in Prunus
Source: Front Plant Sci. 2026 Sep 29;17:1886618. doi: 10.3389/fpls.2026.1886618 (PMC13658181; doi:10.3389/fpls.2026.1886618)
Supplement: Supplementary file 1 [file SupplementaryFile1.zip › Supplementary Material 1 - Genome assemblies.docx]

Supplemental File 1 – Genome assemblies.

**Species	Variety	Assembly	GenBank	Level	Scaffold N50 (Mb)	Contig N50 (Mb)	Coverage (x)**

Prunus armeniaca 1	Meihua	ASM1852499v1	GCA_018524995.1	Chromosome	30.1	1.3	100

Prunus armeniaca 2	GSYX	GSYX_assembly	GCA_020226305.1	Chromosome	30.9	2.1	470

Prunus armeniaca 3	Yinxiangbai Xing	ASM2042406v1	GCA_020424065.1	Chromosome	31.0	3.2	294

Prunus armeniaca 4	Rojo Pasion	CUR.v1	GCA_964259005.1	Chromosome	26.9	2.6	20

Prunus avium 1	Big Star	Prunus_avium_v1.0	GCA_013416215.1	Chromosome	186.0	21.0	105

Prunus avium 2	Tieton	NE-ME-NP-PH	GCA_014155035.1	Chromosome	42.6	3.2	1

Prunus brigantina 1	-	drPruBrig1.1	GCA_964267205.1	Chromosome	29.3	12.7	273

Prunus campanulata 1	-	ASM3419096v1	GCA_034190965.1	Chromosome	30.7	12.0	97

Prunus davidiana 1	ST	ST_assembly	GCA_020226225.1	Chromosome	28.1	1.6	390

Prunus dulcis 1	Nonpareil	OSU_Pdul_2.5	GCA_021292205.2	Chromosome	30.6	1.7	571

Prunus dulcis 2	Wanfeng	ASM4058150v1	GCA_040581505.1	Chromosome	31.4	29.4	126

Prunus dulcis 3	Texas	Texas_F0	GCA_963978805.1	Chromosome	30.5	1.2	260

Prunus fruticosa 1	JH20200508	JKI_Pf_1.0	GCA_018703695.1	Chromosome	43.8	530.9	97

Prunus mira 1	GHT-1	ASM3363506v1	GCA_033635065.1	Chromosome	27.1	2.4	216

Prunus mongolica 1	pm01	ASM3034539v1	GCA_030345395.1	Chromosome	26.5	24.3	42

Prunus mume 1	Tortuosa	ASM2933915v1	GCA_029339155.1	Chromosome	29.4	2.7	50

Prunus mume 2	4	P.mume_V1.0	GCA_000346735.1	Chromosome	636.9	32.3	180

Prunus persica 1	Rui You Pan 1	ASM1573044v1	GCA_015730445.1	Chromosome	28.9	11.5	590

Prunus persica 2	Long Hua Shui Mi	ASM1834083v1	GCA_018340835.1	Chromosome	29.0	2.4	120

Prunus persica 3	Sovetskiy	PSovetskiy_1.0	GCA_022343065.3	Chromosome	24.0	0.6	100

Prunus persica 4	Tianjin Shui Mi	ASM2433755v1	GCA_024337555.1	Chromosome	28.6	23.7	103

Prunus persica 5	Feng Hua Yu Lu	ASM4563546v1	GCA_045635465.1	Chromosome	28.8	4.1	106

Prunus persica 6	HJML	ASM4563551v1	GCA_045635515.1	Chromosome	27.1	7.0	533

Prunus persica 7	Lovell	Prunus_persica_NCBIv2	GCA_000346465.2	Chromosome	27.4	0.3	8

Prunus pseudocerasus 1	Duiying	ASM4122247v1	GCA_041222475.1	Chromosome	29.0	2.4	35

Prunus salicina 1	YHH-2019	SCAU_Psal_1.0	GCA_014863905.1	Chromosome	1.8	1.8	255

Prunus salicina 2	Sanyueli	ASM1927791v1	GCA_019277915.1	Chromosome	34.5	1.0	91

Prunus salicina 3	ZGL	ZGL_assembly	GCA_020226455.1	Chromosome	30.7	1.5	480

Prunus serrula 1	Tibet	ASM4646409v1	GCA_046464095.1	Chromosome	32.4	9.5	120

Prunus speciosa 1	IZO01	CerSpe_IZO01_v1.0_hap1	GCA_046000225.1	Chromosome	30.8	30.8	132
