# Supplementary material for: A comprehensive comparative genomic analysis of the endogenous Florendovirus-1 in Prunus
Source: Front Plant Sci. 2026 Sep 29;17:1886618. doi: 10.3389/fpls.2026.1886618 (PMC13658181; doi:10.3389/fpls.2026.1886618)
Supplement: Supplementary file 1 [file SupplementaryFile1.zip › Supplementary Material 3 - Representative RT sequences.docx]

Supplemental File 3 - Representative RT sequences.

>Badnavirus-1; Blackberry virus F; NC_029303.1;

LERLKEQGYVGEEPMRHWSKNQVKCKLEIINPDITIQDKPMKHVTPAMKDQFQKHTQALLKLGVIRPSKSRHRTMAMIVYSGTSVDEKTGKEVKGKERMVFNYKTLNDNTFKDQYSLPGINTILQRIGKSKIYSKFDLKSGFHQVAMDEESIPWTAFITPEGLYEWLVMPFGLKNAPAIFQRKMDNCFRGTEEFIAVYIDDILVFSETEEQHAKHLQIMLEICEKNGLVLSANKMKIAVKEVEFLGAVICDRKIKLQPHIIKKITSVPEDSLKEKKGLRSWLGILNYARTYIPKLGTLLGPLYEKTSPHGDKRM

>Badnavirus-2; Cacao mild mosaic virus KX276640;

LLQLSDMGYIGEEPLKHWKKNQVECKLEIINPQLKIEDKPLKHITPQMQESFNKQVESLLKLGVIRPSKSQHRTTAFLVKSGTTVDPKTGKETKGKERMVFNYQRLNDNTEKDQYPLPGINTILQRIGKTKIYSKFDLKSGFHQVAMAEESIPWTAFAIPGKGLFEWMVMPFGLKNAPSVFQRKMDLCFTGLEKFVAVYIDDVLIFSNSEQEHLEHLDRFFERVQQHGLVLSPTKMKIGVRKVDFLGTVISNGLLHMQEHILKKIAAFGPEQYKSKKDLRSWLGLINYARNHIPNVGRMLGPIYAKTSPQGEPKM

>Badnavirus-3; Banana streak UL virus HQ593109;

INEMKELGYIGDEPLRYWANNKVKCHIRIKNPELTIQDKPHKFVTPQMKEQMQKHMDELLKRKVIRPSSSRHRTNAFIVNSGTTVDPLTKKEIKGKPRLVFNYKRLNDNTEKDQYSLPGINGLLKSVGNAKIYSKFDLKSGFHQVAMEEESIEWTAFWAFTGLYEWLVMPFGLKNAPAIFQRKMDNCFRGTEKFITVYIDDILVFSETEEEHAIHLTKMLEICKRNGLILSPTKMKIGVKVIDFLGSTIGQNQLDLQPHIVQKIVDFSEEKLKTKEGLKSWLAILNYARGHIKNLGRTLGPLYPKTSEKGERSL

>Caulimovirus-1; Cauliflower mosaic virus M90543;

IEELLEKVCSENPLDPNKTKQWMKASIKLSDPNKAIKVKPMKYSPMDREEFDKQIKELLDLKVIKPSKSPHMAPAFLVNNEAEKRRGKKRMVVNYKAMNKATVGDAYNLPNKDELLTLIRGKKIFSSFDCKSGFWQVLLDQESRPLTAFTCPQGHYEWNVVPFGLKQAPSIFQRHMDEAFRVFRKFCCVYVDDILVFSNNEEDHLLHVAMILQKCNQHGIILSKKKAQLFKKKINFLGLEIDEGTHKPQGHILEHINKFPDTLEDKKQLQRFLGILTYASDYIPKLAQIRKPLQAKLKENVPWKW

>Caulimovirus-2; Atractylodes mild mottle virus KR080327;

IEKLLDEVCSENPIDPHKSKKWMTASIKLKDPNTLVQVKPMQYCPEDRKEFAVQIKELLDLKIIIPSKSPHRSPAFLVENEAERRRGKKRMVVNYKKLNEVTLGDSHNLPNKDELLTLIRGKTIYSSFDCKSGFWQVLLDQESQLLTAFTCPTGHYQWKVVPFGLKQAPSIFQRHMNNAFREFEEFCCVYVDDILVFSKNETEHRKHVIAILEQCKNLGIILSKKKAHLFKTKINFLGLEIDQGSHKPQNHILEHIHKFPDRLEDKKQLQRFLGILTYASDYIPKLAQIRKPFQAKLKKDVTWSW

>Caulimovirus-3; Carnation etched ring virus AJ853858;

IEEMLERVSSENPIDPEKSKQWMTATIELIDPKTIVEVKPMSYSPSDREEFDKQIKEPLDLKVIKPSKSPHMSPAFLVENEAERRRGKKRMVVNYKAMNKATKGDAHNLPNKDELLALVRGKKIYSSFDCKSGFWQVLLDKESQLLTAFTCPQGHYQWNVVPFGLKQAPSIFQRHMQTAFNQHSKYCCVYVDDILVFSNTEEEHYIHVLNILRRCEKLGIILSKKKAQLFKEKINFLGLEIDQGTHCPQNHILEHIHKFPDRIEDKKQLQRSLGILTYASDYIPKLASIRKPLQSKLKEDSTWTW

>Cavemovirus-1; Sweet potato collusive virus HQ694978;

LNNILGEDIYGDNPLKHWEKHKTYAKIELKNPDDHIYKPPINYQESDYKEFKMHIDEMVKEGFIEECKNLENKKYSSPAFIVNKHSEIKRGKSRMVIDYKDLNKKAKVIKHPIPNKDILINRGIKANYFSKFDCKSGFYHIKLEEDSKKYTAFTVPQGYYVWIVLPFGYHNSPSIYQQFMDGIFRPYYDFILVYIDDILIFSKTYEEHKIHLEIFRNIIIKHGIVLSKKKAEIGKQKIEFLGVKIEQGGIELQPHIIDKILEKHIKIKSKKELQSILGLVNQIRNFLPNLSKILLPIQKKLKIKNEEVW

>Cavemovirus-2; Chicory mosaic cavemovirus MW811396;

LLQILGEEIFNENPLQGWEKHKTYAKIELKNPNDEIYKPPMIYKEEDYKEFELHITELIKGDYIEQKDKYENKKYSSPAFIVNNHSEQKRGKTRMVIDYKDLNKKAKIIKHPIPNKDILLHRGIKAKIFSKFDCKSGFYHIKLEEESKKYTAFTVPQGYYQWKVLPFGYHNAPSIFQKFMDIIFRRYYEFIIVYIDDILIFSNNINEHKYHLKIFKEIVKENGISLSKKKAELGKEKIEFLGMQIEQGGLKIQQHIIDKILTKHIKIKNKKELQSVLGLLNQIRNFIPRLAEILQPLQKKLKIKKEEYW

>Cavemovirus-3; Epiphyllum virus 4 NC_055588;

INKVLGSDVFGEHPLQGWEKHKTFAKIELKNDDEEIYKPPLHYQPSDLPEFKMHIDEMLRDDYIEEKTNFENKKYSSPAFIVNKHSEQKRGKTRLVIDYKDLNKKAKIIKHPIPNKDILIIRGIKANYFSKFDCKSGFYHIKLDKESKKYTAFTVPQGYYQWKVLPFGYHNSPSIFQQFMDKIFRPYYEFILVYIDDILIFSHTEEDHLKHIKIFKECILQNGITLSNKKSELGKTTIHFLGVIITHGGIELQPHIIDKILEKHIKIKNKTELQSILGLLNQIRNFVPNLAKLLKPLQKRLKIKPEEQW

>Dioscovirus-1; Cajanus cajan CajCaj-05 de Tomas and Vicient, 2022;

IDRLNKSSVIGEDPTRYWEKDPVICKLDIINHDFIIKTKDIPYGNFEQEECRIHIKALLDLKVIEPSTSPHRSPTFIVNKHSEQKRGKTRMVYNYKHLNDNTHIDGYTIPSKDVLINRIQKAKWFSKFDIKSGFHQVKMHPDSIKWTAFSCSEGLFEWKVMPFGLKNAPQIFQRKMDNIFGDYKEFTCTYIDDVLVFSKTKEEHFLHLKQVLHLFEKYGLIVSKSKMEICKTHISFLGTEIGNGKIKLQPHVSQKILAFPEKMEDVKTLRAFLGLLNYARTFVKDLGKFTAPLYNKTSLTGQRKF

>Dioscovirus-2; Cynara cardunculus CynCar-16 de Tomas and Vicient, 2022;

VDHLQKIKIIGEIPTKFWERNKIFCKLEIINPDLVIKTGMPKYDNWTIEEFKMHISQLLKLGVITRSTSSHRTAAFIVQKESKRKRGQSRMVFNYKRLNDNTHIDGYCIPTKEILLNKIQGAKWFSKFDLKSGFWQVKMHPESIPWTAFTCSEGHFEWKVMSFGLKNAPQIFQRKMDNCFQHLSHFTCVYIDDVLVFSKDKNEHYAHLYIVFKIFEKEGIVISLKKMQLLREHIEFLGVEIEKGRIKLQPHISRKISEFPDKIENLKQLQSFLGFLNYARPFIKDLSKYTAPLYNKTSVKGTRKF

>Fernendovirus-1; ENQF-2084799-Lycopodium annotinum; Issa Diop et al., 2018;

FTELFKTQSTMNPLEFWDKEQYMVTLPEVPLVNPTKATYMYMSPEDRILCEAEIAELLKMKLIEPSTSNWACQAFYVNKHSEQRRGKKRMVINYKPLNKYLVSVKYPLPLKDNLIDRIQGATVFSKFDLKSGFWQFKIHPKDRHKTAFTVPQGMYQWIVMPFGLKNAPSEFQKRIDLILKPVAKFTIGYIDDILVFSKSFALHDDHLTQVHNLFWNHGTALSEAKTVLFQPEIQFLGVTICQGKIKLQPHVLAKIAAMPATFSTLQEVQKFLGCLNWIHWNIPYLAELTRPLCKLLRKQQQPHQ

>Fernendovirus-2; 1319 Dipteris conjugata; Issa Diop et al., 2018;

EQQLITNCSSTSPQAFKHIFHHQIGLPIKEGSKPYKESPYGFTHEDRLLCTAEITDLLQQGLIRESKSAWAAAAFYVNKYSEQKRGKKRLVINYKPLNKCLINIAYPLPNKEYLMQKISGATVFSKLDLKAGFWQIGLFPEDQHKTGFVVPQGFYEWTVLPFGLKTAPVEFQKFMDSIFSSIHEFCLVYIDDILIYSKDQHQHLQHLQRVHDLLQQHGLVLSPTKVQVGKASIDFLGVIIQHGAIQLQDHVLKALAAFPTEIHDKQQLQRFLGCLNYISPFYQDLAKDRYLLQLCLRKDGPKWG

>Fernendovirus-3; NOKI-2097008-Lindsaea linearis; Issa Diop et al., 2018;

VSTKMLTYTSDHPLQFWNHTQHLITLPMVPDANPTKAGHIGMIPEDLHLCQQEIRELLTKALIRPSNSPWASPAFYVNKHSEKRGCKRLVINYKPLTKYLICPKFPLPLKDLLLPQKIAGKTIFSKFDLKSGFWQFKIHHNHCYKTSFIISFGQYEWLVLPFGLNLAPSLCQEKVSQIFITLDQCCIVYMDDILIYSKTIAAHLLHLEAFYVLCKSGLVLSITKEICKSQIQFLGVILCNNTIKCEHVPKKIMEFPPQLTDVSQLCSLFGCCNSIWQYPFTILELSPFHAYLKKVSSLQW

>Florendovirus-1; PpersV_sc1; Geering et al. 2014;

EQRLKEEICSELPTAFWYRKQHVVRLPYIKTFSEKNIPTKARPIQMSQEMMEFCKKEIEELLQKKIIRKSKSPWSCPAFYVQKNAELERGVPRLVINYKPLNAVLEWIRYPIPNKRDLINRLEKAVVYSKFDMKSGFWQIQIDESDRYKTAFVTPFGHYEWNVMPFGLKNAPSEFQNIMNDIFNPYSQFSIVYIDDVLIFSESIEQHWKHLNKFLQVVKQNGLVVSAKKIKLFQTNIRFLGFNICQSQISPIDRVIQFADKFPDQILDKSQLQRFLGSLNYISDFYQNLRKQCKPLFDRLQSNPPPWS

>Florendovirus-2; AtrichBV; Geering et al. 2014;

KNFIISEICNELPNAFWHRKQHIVGLPYEPNFNETKIPTKARPIQMNTELLQTCHHEIQDLLQKKLIRPSSSPWSCSAFYVNKNAEQERGVPRLVINYKPLNKVLQWIRYPIPNKKDLLDRLLHAVIFSKFDLKSGFWQIQIKEEDKYKTAFNVPFGQYEWNVMPFGLKNAPSEFQKIMNDIFNPFYKFMIVYIDDVLVFSESLEQHFKHLHTFIHTVKNAGLVVSAKKLNLFQTKIRFLGHNINQGTIIPIDRSILFADKFPDKILDKTELQRFLGCLNYISDFYQNLALDAKPLYDRLKKNPPPWT

>Florendovirus-3; EgranV_sc1; Geering et al. 2014;

QKHIEKTICSTLPNAFWERKKHIVDLPYEHDFSENKIPTKARPTQMNQEVLKFCQAEIQDLLNKKLIRKSQSPWSCSAFYVNKNAELERGVPRLVINYKPLNTALRWIRYPIPNKKDLLNRLCRSKIFSKFDMKSGFWQIQISEKDKYKTAFTVPFGQYEWNVMPFGLKNAPSEFQRIMNEIFNPYSEYIIVYIDDVLVFSSNIEQHFKHLSTFIKIIIQNGLVVSPTKIALFKTKIRFLGHYIHLSTITPIERSISFTDKFPDEIKDKTQLQRFLGSLNYILDFFPNINILCKPLHQRLRKDPPPWT

>Gymnendovirus-1; Picea glauca; Issa Diop et al., 2018;

ILEKLLKYCSSDPQAFWDVASPSMEIETLHDNGVTGKLIPYTPADEQEIKNQIEELMAMKLIEPSNSHYCCSAFIVRNHAEVVRGKARLVVNYKPLNAITKDFHYPLPRQEVLMQKLQNSKMFSKFDMKSGYYQVQIRPEDRHKTTFTCPAGLFQWKVVPFGLKNAPSYFQRRMDSIFNKYDFVIVYIDDILVHSQNEEDHVQHIAVFTSECEKHGIVLSDKKIELFRTTMEFLGICIIDGKIQMQPHIVQKIVGFPDQLENKNQIQNFWGILNYVHKYIPRLAEKTVAIRQHLSGGWSPTA

>Gymnendovirus-2; Gingko biloba; Issa Diop et al., 2018;

ITKQLEECCSMNPLAFWDKDQPEAEIELTDQNAKVYEKPIPCPEKYRKEMESQLEELLKLDLIEESKSPFCCPAFMVYKHSEIKRGKGRMVINYKRLNSVTKSFNYPLPHKETLFERIAGCQYFSKFDCKSGFYQIMVKAEDRWKTAFSALNGQFQWKVMPFGLKNAPAYFQRRMDKIFRHLDWIFVYIDDMLIFSRTLEDHVQHLKEFYEICKNHGIVLSSTKMVICKKEMEFLGFNIQGGKIKLQGHVLEKIDQFPDKMEDKKLITKIFRMFKLYWEFLFFGCQRPARITEFVKKGCSILD

>Gymnendovirus-3; Pinus nigra virus 1 MH551472;

IYQRLLKSCSDNPQQFWETESPMQEIVTLHDNGVKGKMIPCTPADEQEMRNQIQELLKMQLIEPSESHYACSAFLVRNHSEIVRGKPRMVINYKPLNAITQNFNYPLPRPEVIMQKIQHSKVFSKFDMKSGYYQIQIQPEDRHKTAFICPAGFYQWKVVPFGLKNAPAFFQRRMDYIFAKYDFIVTYIDDILIHSPDVQNHLKHLEIFLEEVKKHGIVLSERKMSLFQDNIDFLGINVANGSIQMQPHVLTKLTQFPDKLKDKKEIQRFIGVLNYLHKYIPNLSEKTAPIRRHNNGGWSDEA

>Petuvirus-1; Petunia vein clearing virus; NC_001839.2;

CADSHVDFLSKCSHPLWLNQDFFIQLPFKKNENINPTKASHSGMNPEHLQLAIKECDELQQFDLIEPSDSQWACEAFYVNKRSEQVRGKLRLVINYQPLNHFLQDDKFPIPNKLTLFSHLSKAKLFSKFDLKSGFWQLGIHPNERPKTGFCIPDRHFQWKVMPFGLKTAPSLFQKAMIKIFQPILFSALVYIDDILLFSETLEDHIKLLNQFISLVKKFGVMLSAKKMILAQNKIQFLGMDFADGTFSPAGHISLELQKFPDTNLSVKQIQQFLGIVNYIRDFIPEVTEHISPLSDMLKKKPPAWG

>Petuvirus-2; Citrus blight-associated pararetrovirus MN814438;

CPESHSEFTHPNPLWKNKSFFIKLPFKLNEDINPTKAALPRMSPSDLLLAQKECSQLLVQGLIEPTSSQWACQAFYVEKHYEIVREKKRLVIDYQPLNMFLQDDKFPFPRRQSMFTFLKNAQIFSKFDLKSGFWQLGIEPSECYKTTFCIPNAHFQWTVLPFGLKTSSSIFQKSMVQIFQPILHHALIYIDDILLFSGSYNEHRQLLTQFYDIIQSHGIMLSAKKSTIATDNIEFLGMIIKDRHYQLGKHIAQELLHFPDQHLSKKQVQQLLGIINYIRDFIPHVDHYTHHLSALLKKKPPEWN

>Petuvirus-3; Durio zibethinus DurZiv-1; de Tomas and Vicient, 2022;

CAETHSDFLQKCDHPLWQNPEFFIFLPFKKDENANPTKASHSGMNPDHFKQAIQECSLLQAQGLIEPTSSPWACEAFYVNKRSEQVRGKLRLVINYKPLNIFLADDKFPLPDRKTLFVNLANAKVFSKFDLKAGFWQLGIHPQDRYKTAFCIPQHHFQWTVMPFGLKVAPSLFQKAMTKIFAPILDNALIYIDDILLFSPDDTSHAQLLDKFQRIVQQYGIMLSEKKMLIGATEIDFLGMHLKDGQYIAQPHIAQSLADFFDANLSKKQIQQFLGIINYMNEFLPDIAQLTYPLRQMLKKEPPTWS

>Rosadnavirus-1; Viola yellow mottle virus; MZ911846.1;

VKEILHESFGVHPQQFYKEGQPKCVLFLKNTRMEPIRVKPIPLTPVDSKEMERQINELLNSGLIRVTSSPWSSPAFLVRNHSEIKRGKARMVINYAPLNAHLVKDAYRLPNMFGLIQKLRYSKWYTKLDCTSGYWQVRMDEASIPLTAFSTPVGSYEWLVMPFGLLTAPAIFQRKMDAIFNEHSEYCSAYIDDIIIFSNSRAEHLVHLEKIALTIKEAGLVLSEKKAEICKSKIQFLGCEIEGGKISLQPHVLVKLANYPEVLKSKKELSSFLGIVNFAAKDSVQNIAAIKRPLQEKLKKNVVFTW

>Rosadnavirus-2; Rose yellow vein virus strain RYVV-MN1; NC_020999.1;

VEEILLDTFSDNPLALYNPRHPKCQIELLPQDQLDRLKLKCPVRCKPIGANPIDMEEFHNQINELLRLKLIRKTNSPWSFQAFMVRNHAEIVRGKARMVINYKPLNLRIRKNAYRIPNKDSLFLAIRESQFYSKFDCKSGFYQVPMEQDSIQLTAFSTPIGSYEWLVMPFGLATAPSIFQAKMDNVLEDHHDYCLVYIDDIIVFSRTLEEHKIHVITIAKTLKKNGIVISKKKMELGLTKINFLGCEIENGRIILQNHVLENLSKFPSEIKDKKELQSFLGIINYAASHYSIEVTKLRVPLQKKLKKNYIWSW

>Solendovirus-1; Tobacco vein-clearing virus AF190123;

IQKKLEKLYNDNPLQGWEKHKTKVKIELIEENSIITQKPLKYNFNDLTEFKIHIKDLLDNKYIQESNSKHTSPAFIVNKHSEQKRGKSRMVIDYRNLNAKTKTYNYPIPNKILKIRQIQGYNYFSKFDCKSGFYHLKLEDESKKLTAFTVPQGFYEWNVLPFGYKNAPGRYQHFMDNYFNQLENCIVYIDDILLYSRTQDEHIKLLEKFAHIIENSGISLSKTKAEIMKNQIEFLGIQIDKNGIKMQTHIVQKIINLDENIDTKKKLQSFLGIVNQVREYIPKLAENLKPLQKKLKKDVEYSF

>Solendovirus-2; Bacopa monnieri virus 3 BK014481;

LAHRLDDFYTDNPLTNWKKHKTICNIELINKDSIIQQKPFFNTEKDINEFKMHIDELIKMEFIQNSNSKHSSSAFIVNKHSEIKRGKSRMVIDYRILNAKTKTYNYPIPNKILKIKQIQGYKWFSKFDCKSGFHHIKLSEDSKELTAFSVPNGFYEWNVLPFGYKNAPGIFQSFMDKYFNQLENCVVYIDDILLFTKTEDEHIQLLKNFIEIVDKSGIVLSKKKTELFKNQIEFLGHDIDIAGIKMQSHIAIKLLEFTENIETRKQLQSFLGLINQLREFIPKLSKHLVPLQKKLKKDIQWSW

>Solendovirus-3; Sweet potato vein clearing virus HQ694979;

VRNQIKTLFSENPLEFWDKHRTEVKIELINPDSIVYQKPLRWNFEDIEEFKLHIDELLKGGFIRPSNSKHSSPAFIVNKHSEQKRGKSRMVIDYRNLNAKTKTYNYPLPNKILRVRQVQGYNWFSKFDCKSGFYHLKLTEESKHLSAFNVPQGFYEFNVLMFGYKNAPGRYQCYMDSYFSKLENCIVYIDDILLYSKTKDEHETLLKKFYHIVKEAGVSLSEKKAIIGVNQIEFLGIEIDKSGVKMQNHIVTKIVQCEEVLDTKKKLQSFLGLINQVREYVPNIAKELLFLQKKLKKDVEYHF

>Soymovirus-1; Soybean chlorotic mottle virus NC_001739;

IEEQLEEVCSEHPLDETKNKNGLLIEIRLKDPLQEINVTNRIPYTIRDVQEFKEECEDLLKKGLIRESQSPHSAPAFYVENHNEIKRGKRRMVINYKKMNEATIGDSYKLPRKDFILEKIKGSLWFSSLDAKSGYYQLRLHENTKPLTAFSCPPQKHYEWNVLSFGLKQAPSIYQRFMDQSLKGLEHICLAYIDDILIFTKGSKEQHVNDVRIVLQRIKEKGIIISKKKSKLIQQEIEYLGLKIQGNGEIDLSPHTQEKILQFPDELEDRKQIQRFLGCINYIANEGFFKNLALERKHLQKKISVKNPWKW

>Soymovirus-2; Blueberry red ringspot virus; AF404509.2;

VNKILQERFSLDLLGEKNKNKELIEIKLKDPNAEIFVPNNIPYTQRDIEEFKEDMEDLINKGLIRPSKSPHSAPAFYVENHSEIKRAKRRIVINYKAMNEATIGTPKTLPRADYIMNRLKGKIWFSTLDVKSAYWQLRLTEESKPLTAFSYPPPKHYEWNVLPMGLKQAPGIFQEFMNRSLHNLEHICLVYVDDIIIFSEKDKNDHLSKVLQVLKRCEDEGIILSQPKAKIAHKEIDFFGLHISEGEIILQPHILEKLVLFPDEIENRKQLQRFLGNLNYISEKGFFKDFAKYRKELQKKVSEKVPWKW

>Soymovirus-3; Water chestnut soymovirus 1 KU365408;

VWNQIDEMSSDNPLDPVKNTNSELVEIKLKDPKQEVNVVNRIPYTEQDVLEFVEETSMMLEKGIIEIANSPHSAPAFYVNNHAEQKRGKRRMVINYKAMNNATVGEAYKLPRQDYLFERIKDADWFSSLDAKSGYWQLRLAENTKPLTAFSCPPQKHFQFKVMPFGLKQAPSIYQRFMDKTLKGLESNCLAYIDDVIIFTRGNKIEHLKVVSGILKQIKLAGLILSKKKCQIGKEEVQFLGMKILKKGKIKPQEHLLEKLQDFSDQLEDRKQIQKFLGCLNYLCDKGFIKNIVEKKKILQDLLSTKKLWKW

>Tungrovirus-1; Rice tungro bacilliform virus; X57924.1;

VKELEALGFIGDDITKNRTTWVCDFKIINPDINITCATIPYTPADKEVFEKQIKELLDNKLIKKADPTCRHRTAAFIVRNHSEEVAQKPRIVYNYKRLNDNMHTDPFNIPHKISMINLIQKANIFSKFDLKAGFHHMKLKDDFKDWTTFTCSEGLYTWNVCPFGIANAPCAFQRFMQESFGDLKFALLYIDDILIASNNEKEHIEHLKIFFNRVKEVGCVLSKKKSKMFLKEVEYLGVEIKEGKISLQPHIVDKIKKFDKNKLNTLKGLQAYLGLLNYARGYIKDLSKLVGPLYKKTGKNGQRIF

>Tungrovirus-2; Agapanthus tungrovirus_MT501687;

IKDLETLGFLGDDITKCQSNWVCKFKVLNPDIKITCKGIDETPADKEEFRKQIAELLNQGLIYQAKPDCRHRSAAFIVRNHSEEKRGKARIVYNYKRLNDNMETDPFNLPHKNSLMNLIQGAKVFSKFDLKSGFHQMKLEEWFQPWTTFTCSEGLYTWKVCPFGIANAPCAFQRFMQDVFGHLKFCLVYIDDILVMSKSRDQHKKHLLEFFDTVKNNGIVLSKKKTELFKEEIDYLGLKINKGKIELQPHISQKILTFDKKTLESKKGLQSYLGLLNYARPFFKDLSKLIKPLYSKTGKNGQSYF

>Tungrovirus-3; Piper DNA virus 1 JX406741.1;

MRELEKLGLIGEDLAKNNCQHEFSLNIKNPDLIIRTANLEYTPEDIEEFKKQIKELLDLKLIRRSTSPHRSAAFIVRNHAEQKRGKARIVYNYKRLNDNTHDDAYNLPHKDSILNLIQNKKIFSKFDLKSGYNQIKMKEEDRPWTAFTCPEGLFEWNVLSFGLKNAPAIFQRFMDSLFKKYEFCIVYIDDILVASDTVQEHIKHLELVFKTIKEAGIVISKKKTEIAKTYINFLGLKIGKGQIELQPHIVTKALEYPDKIENKNKLQSFLGLLNYARKFIPNLSKLVGPLYSKLRKNGQIYF

>Vaccinivirus-1; Blueberry fruit drop associated virus; NC_028462.1;

LTKQLEPNISDNPIHKWDVTQTYADIQLKDPYALIRVKPMPYSAEDEAEFERQLEEQLKLELIQPSKSPHSSPAFCVRNHAEIKRQKARIVINYKELNRVTKDDGYFLPNLETLVYQVAEARVFSKFDCKSGYWQIKLTPESIPLTAFSTPKGQYEWKVLPFGLKNAPNIFQRRMDKIFKDCYQFCGVYVDDILVFSKDKEEHLLHLDIIINKIVQNGIIIGRTKYELVRESIDFLGVTICKGKIKLQPHILTHLMEFPDKLKDRLQVQQFLGCVNYAGKFIQRLAELCSVLHTKLKKDHKWKF

>Wendovirus-1; Citrus màxima; Vicient and de Tomas, 2022;

IKSQLEKVYKENPLALWKADHPRAKIELIENRIIRLKPMMYTEEDREEFKIQIKELLNLNLIRNSNSPHNSPAFMVRKRSEQVRGKARMVINYKELNKYTKFDGYFLPNKEVLINLVKNKTYYSKFDCKSGFWQIKMEEDSIPYTTFSTPQGHYEWLVMPFGLKNAPQIFQNRMDKVFKDYRYIIVYVDDILIASETLEDHRRHLKEFVNICIKEGIVLSERKAVIEHKKIEFLGMILDKQGIRLQSHIGKKISEFPDKLENKQQIQKFLGCLNYAEGFIKDLAKKRNTLQKLLRKNNTRGW

>Wendovirus-2; Portulaca amilis PorAmi-2; Vicient and de Tomas, 2022;

LLTKLKQIYSENPLQFWKPNHPRAKIELTQEVYIKEKPMLYTPEDIKELSVQIKELLEKGLIRPSKGSYLSPAFMVMNEAEKRRGKARMIINYKKLNQFTKTDNYFLPNKQVLINLVKNKNYFSKFDCKSGFWQIKMEEDSIKYTGFSTPQEFYEWIVMPFGLKNAPRIFQRRMDDAFRHLNSFLIVYVDDILISSRTLEEHREHLKIFIETAIKEGISLSEKKAIIEKDEIEFLGFKLGKNGISLQPHITRKILEYPDELRTKKQIQGFLGLLNYAGDYIEKLAEEKKDLQKLLRKNNTKQW

>Wendovirus-3; Atalantia buxifolia; Vicient and de Tomas, 2022;

IKSQLEPIYKENPLALWKPEHPRAKIELTIERIIRLKPMMYTEEDREEFKIQIKELLNLKLIRHSNSPHNSPAFMVRKRSEQLRGKARMVINYKELNKYTKFDGYYLPNKEVLINLVKNKTYYSKFDCKSGFWQIKMEEKSIPYTAFSTPQRHYKWLVMPFGLKNAPQIFQSRMDKIFKEYKYIIVNVDDILIASETIEEHRKHLKEFVNVCIKEGIVLSERKVVIEHKKIEFLGMILDKNGIRLQNHIGKKIIEFPDKLDNKQQIQKFLGCLNYTEGFIRDLAKKRNLLQKLLRKNNTRGW

>Xendovirus-1; Olea europaea OleEur-33; de Tomas and Vicient, 2022;

LKKEISETYSENPLEKWNIDKVKAKIELTNPDTIVRIKPMKYSVDDQKEFRNQIKELLEAKLIRPSKSPHSSPAFMVRKHSEIKRGKVRMVINYKKLNEVTKFDGYYLPNKDTLIKQVQNKKIFSKFDCKSGFWQIMLTEESKPLTAFSTPNPLGQYEWNVMPFGLKNAPQIFQRRMDEVIKPITHFCIAYVDDLLIHSNNIDEHIEHLEKFCYIMKKHGITLSQKKSEIAKTEIEFLGLFIKEGELQLQSHIGEKLQEFPEEIKDKKQLQSFLGILNYAEGFIENCAKLKNYFLPKLRKNIMWSW

>Xendovirus-2; Ipomoea nil IpoNil-01; de Tomas and Vicient, 2022;

YLELLKINFGENPITLWDKEKIYADIKLINPNDIIRSKPIRYSPTDKKEFEIQIKELLELGIIKESISPHSSPAFLVRKHSEQKRGKTRMVIDYRELNKKIIFDGYFLPYKRNLINRIASKKWFSKFDCKSGFWQIKLTKEAQPLTAFSAPQRQYEWKVLPFGLKNAPQIYQRRMDQVFRNLNEFCIVYVDDILIYSSTLKDHVEHLNEFIKTVRKHGILLSEKKSEIFKNKIEYLGYIISEEGLQLQEHIVTKIKDFKEKLDNKKEVQQFLGIVNYASDYIKDLPTLRKPLQSLIKKSSIFDW

>Yendovirus-1; Capiscum annuum; Issa Diop et al., 2018;

IQILDKLVIIREKPLQHWDSNQITCKLEIINPECTIKTTSIEAKNEDLKDFEIQIKELLEGIIRRATSRHRSTTFIVKNHSELVREKARMVINYKRLNDNTVMDGCKLPDKIELINKIQGRKVFSKFDCKSGYQIKMHEDSIETAFTCPEGHFEWLVMPFGLKTACPIFQRKMNSIFRDYKKFVLVYVDDILVFSNNMRKNLGHLQVFKLFVDNGIIISRKKMELFKNSINFLGVVIGDSRIKLQPHIAKKVLEMPDKFEKTKDLQNFLGLLNYARAFIKDLGKVAGPLYSKTGSTFQKSF

>Yendovirus-2; Ananas comosus AnaCom-2; de Tomas and Vicient, 2022;

LHKLDKLEIMGDNPIKYWENDKIYCKLDIINPEYIVRTASIDFTNEEREQFKLHINELLKLGVIRKSISKHRSTAFIVMKHSEIVRGKSRMVINYKRLNDNTKFDGYTIPDKSQLINSIQDKKVFSKFDCKSGFWQVKMDKDSIEWTAFTCPEGHFEWLVMPFGLKNAPSIFQRKMDTIFNEHRDFVLVYVDDILVFSKNMQEHLGHLQIVFHEFIKHGIIISKNKVELCKKYINFLGVTIGEGKIKLQPHIAKKILDMPDKLETLKELQSFLGLVNYARTFIKDLGKIAGPLYAKTGKSGQRHF

>Zendovirus-1; Rosa rugosa RosRug-160; de Tomas and Vicient, 2022;

IKKLLEQNIDVDPQKFWEKDPVVCELRLHDMNAICHVKAIPQYKEEDQKEFRKDIEDLLNKRLIQHSSSPHHAPAFYVRNHAETLRGKARMVIDYRDVNKKTVKDGYQIAQVRVLINQLRGAKVFSKFGAKSGFWQVKMHPESVPLTAFGTPQGHYEWLVMPFGLKQAPSIFQRKMDNIFKHVAEFCVVYIDDILVFSKNREEHMKHLHEVVKLIVQHGIILGEKKIFFILDEVDFLGINIKNGVIKLQPHILEKIWKFPDKIPDAKSLERFLGVINYGRDFIPKISGLTAMLSPKTSSKRKWNF

>Zendovirus-2; Potentilla micrantha PotMic-1; de Tomas and Vicient, 2022;

IENLLKPIISKDPQLHWERDPVYCQLKMBDANAICHVKVIPHYRDEDRKEIENQIQEFLEKRLIRPSDSPHHAPAFLVRNHAEQLRGKARMVIDYRYVNKKTVKDGYQIAQVRVLINRLKGAKIFSKFDAKSGFWQVKMHPDSIALTAFGTPQGHYEWLVMLFGLKQAPSIFQRKMDNIFKPFSDFCIVYIDDIFVFSRTMKEHLKHLEQICKIIVQKGIILGQKKIHLIKGEIDFLGIHIKDGEIRLQEYIVEKISQFSDNIPDAKSLQRFLGVVNFARDFVPQVSGLTALLSLKTSSKRKWSF

>Zendovirus-3; Potentilla micrantha PotMic-1; de Tomas and Vicient, 2022;

IENLLKPIISKDPQLHWERDPVYCQLKMBDANAICHVKVIPHYRDEDRKEIENQIQEFLEKRLIRPSDSPHHAPAFLVRNHAEQLRGKARMVIDYRYVNKKTVKDGYQIAQVRVLINRLKGAKIFSKFDAKSGFWQVKMHPDSIALTAFGTPQGHYEWLVMLFGLKQAPSIFQRKMDNIFKPFSDFCIVYIDDIFVFSRTMKEHLKHLEQICKIIVQKGIILGQKKIHLIKGEIDFLGIHIKDGEIRLQEYIVEKISQFSDNIPDAKSLQRFLGVVNFARDFVPQVSGLTALLSLKTSSKRKWSF

>Ty3_Chromov-Tekay-1; Peabody;

VKKKEGTMRLCVDYRQLNKVTIKNRYPLPRIDDLMDQLVGASVFSKIDLRSGYHQIRVKTEDIQKTAFRTRYGHYEYSVMPSGVTNAPGVFMEYMNRIFHPYLDKFVVVFIDDILVYSKSEEEHVEHLRVVLGVLREKKLFAKLSKCEFWLEEVSFLGH

>Ty3_Chromov-Tekay-2; Tekay;

VSKKDGSRRMCVDYRSLNEVTIKNKYPLPRIEDLFDRMKGAKIVSKIDLRLGYHQLKIRAEDVPKIAFTTRYGLYEFLVMSFGLTNAPAYFMNLMNKAFMEYLDQFVVVFIDDILIYSLNEEAHEDHLRLVLQKLRDNQLYAKFSKCDFWLKEVAFLRT

>Ty3_Chromov-Tekay-3; Xilon-Diguus;

VEKKDGTKRMCIDYRALNEVTIKNKYPLPRIEDLFDQLRGASVFSKIDLRSGYHQLRIRPSDIPKTAFITKYGLYEFTVMSFGLTNAPAFFMNLMNSVFMDYLDKFVVVFIDDILVYSQSEEEHADHLRMVLQRLREHQLYAKLSKCEFWISEVLFLGH

>Ty3_Chromov-Tekay-4; Gyma;

VKKKDQTLRMCVDYRPLNEVTIKNKYPLPRIDILFDQLTGARVFSKIDLRSGYHQIRIRPEDIPKTAFTTRYGLFEYLVMSFGLTNAPAHFTYLMNSVFMPELDKFVVVFIDDILIYSKNEEEHAQHLRIVLTRLREHQLYAKFSKCAFWLEEIQFLGH

>Ty3_Chromov-Chlamvir-1; Vcarteri199-102;

VGKKDGSLRMCIDYRGLNSMTTKNRYPLPRVDDLLDKLRGSAYFSSIDLQQGYNQIRIAESDIPKTAFRTPFGHFEYTVLSFGLVNAPATFQAVIDRMFRPYIDKFVVCYLDDILVYSKTKEEHLQHLRLVLDVLRREQLYAKQSKCHWAKSEVEYLGH

>Ty3_Chromov-Chlamvir-2; Vcarteri199-4__Chromov-Chlamvir;

EKANGQGLRMVLDYRALNKLTIKRRYPMPNITELFDQLQGANLFSSLDLQQGYNQIRISKEDIEKTAFIALGLGQYQFKVLCFGLTNAPATFQAVMNRIFAAYIGKFVLVYLDDILVFSKTPAEHEQHIRTVLSLLREHQFKAKMSKCQFNQTELHFLGV

>Ty3_Chromov-Chlamvir-3; REM1__Chromov-Chlamvir;

VEKADGSLRMVLDYRALNKITRKRRYPMPNITELFDQLAGAKVFSSLDLQQGYNQIRIHPDDVPKTGFIAPGMGQFEYKVLCFGLTNAPATFQSVMNNMFGPHIGKFVLVYLDDILVFSKNAEEHKEHLRTVLEILRKNQFKAKRSKCDFNRPELHFLGH

>Ty3_Chromov-Chlamvir-4; Ty3-RT__REXdb_ID9791;

VGKKDGSLRMCIDYRGLNSMTTKNQYPLPRVDDLLDKLRGSAYFSSIDLQQGYNQIRIAESDIPKTAFRTPFGHFEYTVLSFGLVNAPATFQAVIDRMFRPYIDKFVVCYLDDILVYSKTKEEHLQHLRLVLDVLRREQLYAKQSKCHWAKSEVEYLGH

>Ty3_Chromov-Reina-1; Magellan;

VKKKDQTYRFCVDYRHLNALTIKTKFPVPIIDEFLDELCGAAWFSTLDLRAGFHQIRMSPKDQHKTAFQTHHGHFEFRVMPFGLSGAPATFQGAMNTTLAPLLRKCVLVFFDDILVYSHTWEDHLSHLQQVLTLLAKDQWYIKMSKCAFAKKQIAYLGH

>Ty3_Chromov-Reina-2; Boshoe;

VKKPDGSWRFCVDYRALNALTIKDAFPIPVVEELLDELHGAKFFTKLDLRSGYHQVRMRPEDVHKTAFRTHDGLYEFLVMAFGLCNAPATFQALMNDVLRPFLRRFVLVFFDDILIYSSTWADHLRHLRAVLDELQRHQLFIKRSKCSFGAPSVAYLGH

>Ty3_Chromov-Reina-3; Apil;

VKKSDGTWRFCVDYRELNGKTVKDRYPIPVVDELLDELHGSIFFTKLDLRSGYHQVRMHPEDVDKTAFQTHRGHFEFLVMPFGLTNAPSTFQSLMNDILNPFIRKFVLVFFDDILIYSKTWAAHLQQVKQVFQTLRAHKLALKRSKCFFGVTNVSYLGH

>Ty3_Chromov-Reina-4; Gloin;

VKKKDGSWRFCVDYRALNRVTVLDKFPIPMIDELLDELHGTTIFSKLDLCLGYHQIRMREDDIEKTAFRTHDGHFEFLVMPFGLTNAPASFQSLMNELFGPFLGKFVLVFFDDILIYSNNLTNHVKHLTLVMEVLAKHQLFANRKKCLLRQSQIDYLGH

>Ty3_Chromov-TCN1-1; Ppatens251-111;

VKKKDGSLRMCVDYRGLNKITIKNRYPLPLISGLLDQLNQAKIYTKIDLRGAYNLVRIKEGDEWKTAFRTRYGHFEYNVMPFGLTNAPAAFQHLMNDVFREFLDNFVVCYLDDILIYSKNVEEHEIHVRQVLQKLRDAGLYAKMEKCVFHTTQVDFLGY

>Ty3_Chromov-TCN1-2; Ty3-RT__REXdb_ID9841;

VKKKDGSLRMCVDYRGLNKITIKNRYPLPLISGLLDQLNQAKIYTKIDLRGAYNLVRIKEGDEWKTAFRTRYGHFEYNVMPFGLTNAPTAFQHLMNDVFREFLDNFVVCYLDDILIYSKNVKEHEIHVRQVLQKLRDAGLYAKMEKCVFHTTQVDFLGY

>Ty3_Chromov-TCN1-3; Ty3-RT__REXdb_ID10309;

VKKKDGSLRMCVDYRGLNKITIKNRYPLPLISGLLDQLNQAKIYTKIDLRGAYNLVRIKEGDEWKTAFRTRYGHFEYNVMPFGLTNAPAAFQHLMNDVFREFLDNFVVCYLDDILIYSKNVEEHEIHVRQVLQKLRDAGLYAKMEKCVFHTTQVEFLGY

>Ty3_Chromov-TCN1-4; Ty3-RT__REXdb_ID10885

VKKKDGSXQMCVDYRGLNKITIKNRYPLSLISGLLDHLNQAKIYTKIDLRRAYNLVCIKEGDEWKTAFRTKYGHFEYNVMPFGLTNAPVAFQHLMNDVFREFLDDFVVCYLDDILIYSKKPKEHELHVRHVLQKLRDARLYAKIEKFVFHTTQVNFLGY

>Ty3_Chromov-Galadriel-1; Galadriel__Chromov-Galadriel;

QKKQDGTMRMCVDYRALNKATIKNKYSVPLVQDLMDRLSKACWFTKLDLRAGYWQVRIAEGDEPKTTCVTRYGSYEFLVMPFGLTNAPATFCNLMNNVLFDYLDDFVVVYLDDIVIYSRTLEEHVNHLSLVLSQLRKYTLYVKMEKCEFAQQEIKFLGH

>Ty3_Chromov-Galadriel-2; Monkey__Chromov-Galadriel;

QKKQDGSLRLCVDYRALNKVTVKNKYPIPLIADLFDQLGKAKYFSKLDLRSGYWQVCIAEGDEAKITCVTRYGAFEFLVMPFGLTNAPATFCTLMNQLFKEYLDKFVVVYLDDIVVYSQTLEEHVKHLWMIFKVLRENTLFVKREKCYFAQTEILFLGH

>Ty3_Chromov-Galadriel-3; PtGaladriel;

QKKADGSLRMCVDYRALNKVTIKNKYPVPLVQDLMDRLSGASVFTKLDLRSGYWQVRIAEGDEHKTTCVTRYGSYEFLVMPFGLTNAPATFCNLMNDVLYEFLDDFVVVYLDDIVVFSRSMNEHVVHLSRVLSRLREHKLFVKREKCDFASAEIMFLGH

>Ty3_Chromov-Galadriel-4; Ty3-RT__REXdb_ID12313

QKKADGSLRMCVDYRALNKVTIKNKYPVPLIQDLMDRLCGASIFTKLDLRSGYWQVRVADGDEHKTTCVTRYGSYEFLVMSFGLTNAPATFCNLMNDVLYDFLDNFVVVYLDDIVIYSRGIEEHVTHLSKVLGRLREHELYVKKEKCEFAKSEIMFLGH

>Ty3_Chromov-CRM-1; CRM;

VPKKDGTWRMCVDCRAINNITIRYRHPIPRLDDMLDELSGAIVFSKVDLRSGYHQIRMKLGDEWKTAFKTKFGLYEWLVMPFGLTNAPSTFMRLMNEVLRAFIGKFVVVYFDDILIYSKSMDEHVDHMRAVFNALRDARLFGNLEKCTFCTDRVSFLGY

>Ty3_Chromov-CRM-2; PetI1__Chromov-CRM;

VPKKDGSWRMCVDCRAVNKITVKYRHPIPRLDDMLDELSGSCVFSKIDLRSGYHRIRMKPGDEWKTAFKTKFGLYEWLVMPFGLSNAPSTFMRLMNHVLKPFINKFVVVYFYDILIYSKTIDEHIGHLSQVLEALRAEKLYANLKKCAFCVEKVVLLGF

>Ty3_Chromov-CRM-3; CRA1__Chromov-CRM;

VPKKDGSWRMCVDCRAINNITVKYRHPIPRLDDMLDELHGSCVFSKIDLKSGYHQIRMKEGDEWKTAFKTKHGLYEWLVMPFGLTNAPSTFMRLMNHVLRAFIGVFVVVYFDDILIYSRSLDDHVEHLKTVLDVLRREQLFANFKKCTFCTDNLVFLGF

>Ty3_Chromov-CRM-4; VitV6__Chromov-CRM;

TPKKYGSxCMCVDSRAINKITVKYHFPISRLNDMLDRLEGAVVFTKLDLRSGYRQIRIRQGDEWKTDFKTKDGLYEWLVMPFVLSNAPSTFMRLMNYILHSFIGKFVVVCFDDILIYSKDEEEHLSHLREVLLALQANKLYINLKKCSFMTSHLLFLGF

>Ty3_Non-chromo_TatI-1; Smoellend91-784;

ARKANGKLRLCVDFRDLNKATAKDHFPTPFIDAIVDEMAGHWYYSFMDGFSGYNQIFIHPDHRDLMAFRTPWGVFAFNVLPFGLCNAGATFQRFMTHAFHDLIGGALRVYMDDLCTTSDSFEDHLMHLDQIFTRCRHHRFSLNPAKCKFGVTEGRLLGF

>Ty3_Non-chromo_TatI-2; Ty3-RT__REXdb_ID6782;

ARKANGKLRLCIDFRDLNKATVKDHFPTPFIDAIVDEMAGHRYYSFMDGFSGYNQIFIHPDHRDLTAFRTPXGVFAFNVLPFGLCNAGATFQRFMTHAFHDLIGGALRVYMDDLCTTSDSFEDHLMHLEQIFTRCRHHRFSLNPAKCKFGVTEGRLLGF

>Ty3_Non-chromo_TatI-3; Ty3-RT__REXdb_ID6783;

ARKANGKLHLCIDFRDLNKATAKDHFPTPFIDAIVDEMAGHRYYSFMDGFSGYNQIFIHPDLTAFQTPWGIFAFNVLPFGLCNAGATFQRFMTHTFHDLIGGALRVYMDDLCTTSDSFEDHLMHLEQIFTRCRHHRFSLNPAKCKFGVTEGRLLGF

>Ty3_Non-chromo_TatI-4; Ty3-RT__REXdb_ID6784;

ARKANGKLRLCVDFRDLNKATAKDHFPTPFIDAIVDEMAGHWYYSFMDGFSGYNQIFIHPDHRDLMAFRTPWGVFAFNVLPFGLCNAGATFQRFMTHAFHDLIGGALRVYMDDLCTTSDSFEDHLMHLDQIFTRCRHHRFSLNPAKCKFGVTEGRLLGF

>Ty3_Non-chromo_TatII-1; Ty3-RT__REXdb_ID6786;

VRKKSGEIRLCVDFRNLNRSSKKDNYPLPKMEHILQRVIGASRISMIDGFSGYNQISIMPEDREKTTFTTPWGTFMYAKMPFGLMNAGATFQRAMDITFIGEKDQFVVIYLDDITVFSRTNKEHRCHMKRVFLKCRRFGLSLNPKKSLFAMREGKLLGH

>Ty3_Non-chromo_TatII-2; Ty3-RT__REXdb_ID6794;

VRKKNRKIQLCVDFRNLNKASDKDNYPVPSMEQILQQVSGSERLSLLDGFSGYNQVLMSPPDQLKTTFRTPWGTYAYCKMPFGLINADATFQRAMEISFRGLINHSVAIYLDDVTIYSKNKDDHLVHLRAVLLRCRKYGISLNPKKSIFAVEXGKLLGF

>Ty3_Non-chromo_TatII-3; Ty3-RT__REXdb_ID6800;

VWKKSGEIRFCIDFXNLNRVSNKDNYPVPPMEQILQQVSGSKRLSLLDGFSGYNQVLMSPPNQLKTTFRTPWGTYAYRKMPFGLINAGATFQRVMDITFXGLINQSIIVYLYDVTVFSKNKNDHLAHLRVVLQXCRKYDISLNPKKSIFAVEXGKILGF

>Ty3_Non-chromo_TatII-4; Ty3-RT__REXdb_ID6806;

ARKKNGEIRLCVDFRNLNKCSLKDNYPLPKMDHVLEKVVGANRMSMIDGFSGYNQITMNExDREKTAFTTPWGTFMYDKMPFGLMNAGATFQRAMDIDFVGERDKFVVIYLDDLTVFSKNDDEHLIHLKHTFEKCRRYGLSLNPKKSHFAMQEGKLLGH

>Ty3_Non-chromo_TatIII-1; Pabies2;

IDKKGGSIRVCVDYRDINKACPKDNFPTPFVDQIVHDCTRSEIFSLMDGFSGYNQINIAPEDQHKMAFICPWGTFAYRKLPFGLKNAGATFQRAMSYAFHDIKHIVQPYLDDLPAHSLHRVDHPNHLRAIFVRCXFYRIHLNPHKCVFCVESARLLGF

>Ty3_Non-chromo_TatIII-2; Ty3-RT__REXdb_ID6814;

IDKKQGMIRVCVDYRDINKACPKDNFLTPFVDQIVDDCARSEIFSLMDGFFGYNQINIVPEDQHKTAFICPWGTFPYRKLPFGLKNVGTTFQRAMSYAFHDIKHIVQPYLDDLPAHSMHRVDHPTHLRAIFICYRFYRIRLNPHKCVFYVESDRLLGF

>Ty3_Non-chromo_TatIII-3; Ty3-RT__REXdb_ID6824;

IDKKGDTIHLCIDYRDINKACPKDNFPTPFIDQIVDDCVGSEIFSLMNGFSDYNQINIAPKDQHKTAFICPWGTFAYRKLPFGLKNACAMFQRAMSYAFHDIKHIIQPYLDDLPTHSMHRVDHPTHLXAIFVCCRFYRIRLNPHKCIFCVESGRLLGF

>Ty3_Non-chromo_TatIII-4; Ty3-RT__REXdb_ID6835;

IDKKGGSIRVCVDYRDINKSCPKDNFPTPFVNQVVDDCADSEIFSLMDGFSGYNQINIAPEDQHKMDFICPWGTFAYKKLPFSLKNAGATFQRAMSYAFHNIKHIVQPYLDNLSAHSLRRVDHPNHLQAIFIRCWFYRIHLNPHKCVFCVEFGRLLGF

>Ty3_Non-chromo_Ogre-1; Fvesca226-108;

VRKKNGKMRICVDYRDLNNATPKDIYPMPVADLLIDAAAGHEVLSFMDGTAGYHQILVAEEDRHKTAFRCPGFAGAFEYVVMPFGLKNAGATYQRAMNLIFHDILGKLIEVYIDDVVVKTKTRATHVADLRQVFTRMRRHNLKMNPAKCVFFAEAGDFLGF

>Ty3_Non-chromo_Ogre-2; Mdomestica1;

VLKKNGALRICTDFRNLNLATPKDEYTMPISDLLIDAAANHAILSFMDGHAGYNQIFIAEADVHKTAFRCPGALGTYEWVVMPFGLKNAGATYQRAMNTIFHDLIGTIVEVYIDDVVVKSKRRQTHLDDLRQAFLRMRQHNLKMNPAKCAFGVSAGNFLGF

>Ty3_Non-chromo_Ogre-3; Atlantys2;

VIKKNGKLRVCINFRDLNKAIPKDEYPMPVADQLVDAASGHKIISFMDGNAGYNQIFMAEEDIHKTAFRCPGAIGLYEWVVMTFGLRSAGATYQRAMNYIFHDLIGMLVEIYIddvvvkskevdeHIADLRQVLDRARKYGLKMNPTKCAFGVSAGQFLGF

>Ty3_Non-chromo_Ogre-4; Ty3-RT__REXdb_ID6996;

VYKKNGKLRVCVDFRNLNQATPMDGYPMPTADVLIDAAAGHKIISFMDGNAGYNQILMAEEDIPKTAFRCPGHLGLFEWVVMTFGLKNAGATYQRAMNYIFHKLIGLLVEIYIDDVVVKSKSHEEHLADLRRVLECTKKHGLKMNPNKCAFGVSAGQFLGF

>Ty3_Non-chromo_Phygy-1; >Ty3-RT__REXdb_ID5434;

VPKKNGSLRFIQDMQPVNAVTVRNAGIGPIVDEFAEAFAGRAIYSMGDLYSGYDQFQLAEGSRDMTTMRTPLGLVRMCTLPQGATNSVAHMMNGMNKVLRDFIPEKTMPFLDDVPIKGCREGDKDETLDTRGCRRYVVDHIQDCEKILTRLGEVHLTLSGMKSVFGVREVVIVGH

>Ty3_Non-chromo_Phygy-2; >Ty3-RT__REXdb_ID5470;

VPKKNGSLRFIQDMQPVNALTVRNAGIGPIVDEFAEAFAGRAIYSMGDLYSGYDQFQLAEGSRDMTTMRTPLGLVRMCTLPQGATNSVAHMMNGMNKVLRDFISEKTMPFLDDVPIKGCREGDKDETLDTRGCRRYVVDHIQDCEKILTRLGEVHLTLSGMKSVFGVREVVIVGH

>Ty3_Non-chromo_Phygy-3; >Ty3-RT__REXdb_ID5542;

IPKKNGSLRFIQDMQPVNALTVRNAGIGPIVDEFAEAFAGRVIYSMDDLYSGYDQFQLAEGSRDMTTMTTPLRLVRMCTLPQGATNSVAHMMNGMNKVLRDFIPEKTMPFLDDVPIKGCKEGDKDETLDTRGCRRYVVDHIQDCEKILTRLGEVHLTLSGMKSVFGVREVVIVGH

>Ty3_Non-chromo_Phygy-4; >Ty3-RT__REXdb_ID5579;

VPKKNGSLRFIQDMQPVNAVTVKNVGIGPIVDEFAEVFARRAIYSMGDLYSGYDQFQLVEGSRDMTTMRTPLGLVRMCTLPQGATNSVAYMMNGMNKVLRDFIPKKTMPFLDDVPIKGCREGDKDETLDTRGCRRYVVDHIQDCEKILTRLGEVHLTLSGMKSVFGVREVIIVGN

>Ty3_Non-chromo_Selgy-1; Smoellend91-110;

VTKPDGSIRFCIDYRPLNEVTIPDVYPLPHTTDLLDRLGRSRWFTKLDLASGYWQVPMAEEDIPKTAFRTPFGLYEFLVMPFGLNNAPATFQRMMDEVLCDMPEVTADFIDDVLIHSEGTWEGHVRDVCKVLDRLRDVGLKLKRSKCVFGSKDTTYLGY

>Ty3_Non-chromo_Selgy-2; Smoellend91-571;

VTKPDGSIRFCIDYRPLNEVTIPDVYPLPHTADLLDRLGRSRWFTKLDLASGYWQVPMAEDDIPKTAFRTPFGLYEFLVMPFGLNNAPATFQRMMDEVLCDMPEVTADFIDDVLIHSEGTWEEHVRDVCKVLDRLRDVGLKLKRSKCVFGSKDTTYLGY

>Ty3_Non-chromo_Selgy-3; Gmr1;

VPKPDGTHRFCNDFRRLNEVSDFDSYPMPRVDELIERLGPARYLSTLDLTKGYWQVPLAPSSREKTAFATPGGLFQYTVLPFGVHGAPATFQRMMDQVLRPHSSYAAAYIDDIIIHSASWDEHVKHVRAVLNGLRAAGLTANPAKCRLGREETAYLGY

>Ty3_Non-chromo_Selgy-4; rGmr1;

VPKPDGTLRFCNDFRKLNEASSFDGYPMPRVDELLDRLGSARFISTLDLTKGYWQVPLAPGAKEKTAFTTPSGHWHYRVLPFGLHGAPATFQRMMDILLRPHQSYAAAYLDDVVVHSMCWEEHLTRLRRVLLELRRAGLTANPKKCHLGLAEAKYLGY

>Ty3_Non-chromo_Athila-1; Athila4-1;

PTRTITGHRMCIDYRKLNAASRKDHFPLPFIDQMLERLANHPYYCFLDGYSGFFQIPIHPNDQEKTTFTCPYGTFAYKRMPFGLCNAPATFQRCMTSIFSDLIEEMVEVFMDDFSVYGPSFSSCLLNLGRVLTRCEETNLVLNWEKCHFMVKEGIVLDH

>Ty3_Non-chromo_Athila-2; Diaspora;

PTRVQNNWRVCIHYRRLNQVTKKDHFPLPFIDQILECLAGKSHYCFLDGFSGYMQITIALEDQEKTTFTCLFGTFAYRRMSFGLCNAPGTFQRCMISIFSDFLENCIEEFMDDFTVYGSSFDGCLDSLEKVLNRRIETNLVLNFEKCHFMVEQGIVLGH

>Ty3_Non-chromo_Athila-3; Ty3-RT__REXdb_ID5751;

PTRTITGHRMCIDYRKLNSASGKDHFPLPFIDQMLERLANHPYYCFLDGYSGFFQIPIHPNDQEKTTFTCPYGTFAYKRMPFGLCNAPATFQRCMTSIFSDLIEEMVEVFMDDFSVYGSSFSSCLSNLCRVLFRCEETNLVLNWEKCHFMVKEGIVLGH

>Ty3_Non-chromo_Athila-4; Ty3-RT__REXdb_ID5932

PTRTVTGHRMCIDYRKLNAATRKDHFPLPFIDQMLERLASHPYYCFLDGYSGFFQIPIHPDDQEKTTFTCPYGTFAYRRMSFGLCNAPATFQRCMMSIFTDMIEDFMEIFMDDFSVYGSSFKDCLDNLCKVLARCEEKHLVLNWEKCHFMVRDGIVLGH

>Ty3_Non-chromo_Retand-1; Tat4-1;

VKKKNDKWRVCIDFTDLNKACPKDSFPLPHIDRMVEATTGNELLSFMDAFSGYNQIPMHKDDQEKTSFIIDRGTYCYKVMPFGLKNVGARYQRLVNQMFAPQLGKTMEVYIDDMLVKSTRSADHIDHLKACFETLNKYNMKLNPAKCLFGVTSGEFLGY

>Ty3_Non-chromo_Retand-2; Gret1;

VPKKEGKWRVCVDYTNLNNACPKDSFPLPRIDQIVDSTSGQGMLSFLDAFSGYHQIPMSPDDEEKIAFITPHDLYCYKVMPFGLKNAGATYQRLMTKIFKPLIGHSVEVYIDDIVVKSKTREQHILHLQEVFYLLRRYGMKLNPSKCAFGVSARKFLGF

>Ty3_Non-chromo_Retand-3; Cinful-1;

VKKANGKWRMCIDFTDLNKACPKDEFPLPRIDSLVDATASSELMSLLDCYSGYHQIWMKREDEPKTSFITPSGTYCYLRMPEGLKNAGGSFSRMTAKVLQSQIGRNVLTYVDDIIVKSTKQENHIADLQETFASFRQAGLKLNPEKCVFGVKKGKFLGC

>Ty3_Non-chromo_Retand-4; Grande1-4;

VRKKTGQWRMCVDYTDLNKSCPKKDPFGLPRIDQVVDSTAGCELLSFLDCYSGYHQIRLKESDCLKTSFITPFGAYCYVTMPFGLKNAGATYQRMIQRCFSTQIGRNVEAYVDDVVVKTKQKDDLISDLEETFASIRAFRMKLNPEKCTFGVPSGKLLGV
