# Supplementary material for: A comprehensive comparative genomic analysis of the endogenous Florendovirus-1 in Prunus
Source: Front Plant Sci. 2026 Sep 29;17:1886618. doi: 10.3389/fpls.2026.1886618 (PMC13658181; doi:10.3389/fpls.2026.1886618)
Supplement: Supplementary file 1 [file SupplementaryFile1.zip › Supplementary Material 5 - RT-ECVs sequences found in the Prunus genome assemblies (FASTA).docx]

>Parm1-Chr1-01; Yendovirus; Prunus armeniaca 1;

IIGEDPQLHWNKNKTLAKLDIINPDLTIKTADMPCNLLDKQEFEQQIKELLNLKVIRPSKSRHRSTAFVVRKHSELKRGKARIVYNYRRLNDNTYEDSYKLPNKDELMNKIQESKYFSKFDCKSGFWQIRLAEESIHWTAFTCPEGHFEWLVMPFGLKNAPSIFQRKMDQIFKKYDKFCSVYVDDILVHSKNKNEHRKHLEIILKEFINNGIIISRNKVELEKQDIEFLGMYIKSGTIQLQEHIAKKVLEFPDKLEDKKQLQAFXDLLNYARSFIPD

>Parm1-Chr1-02; Yendovirus; Prunus armeniaca 1;

LEELLQLAEQTRIIGEDPQLHWNKNRILAKLDIINPDLTIKTADMPCNLLDKQEFEQQIKELLNLKVIRPSKSRHRSAAFVVRKHSELKRGKARIVYNYRRLNDNTYEDSYKLPNKDELMNKIQESKYFSKFDCKSGFWQIRLAEESIHWIAFTCPEGHFVWLVMPFGLKNAPSIFQRKMDQIFKKYDNFCSVYVDDILVHSKNKNEHRRHLEIILKEFIDNGIIISRNKVELEKQDIEFLGMYIKSGTIQLQEHIAKKVLEFPDKLEDKKQLQAFLGLLNYARSFIPD

>Parm1-Chr1-03; Yendovirus; Prunus armeniaca 1;

WNKNKILAKLDIINLDLTIKTTYMLCNLLDEXEFEQKIKELLNLKVIRPSKSRHRSSTPFVVRKHSKLKIGKARIVYNYRRLNDNTYEDSYKLPNKDELLNKIQENKYFSKFDCKSGFWQIKLTEESIQWTTFTCVEXYFKWLVMPFALKNAPLIFQRKMDHISKKYDKFFSVDDVLVMIIFVLYMQTIYLFIEMNLENTXKXFXKNLLIMYSNKXEXSRAXKTRHTIFRYVYXARNYTITRTHSXKSVRISRXIKDKKXLQVFLGLLNYAR

>Parm1-Chr1-04; Yendovirus; Prunus armeniaca 1;

LEELLQLAEQTRIIGEDPQLHWNKNKILAKLDIINPDLTIKTADMSCNLLDKQEFEQQIKELLNLKVIRPSKSRHRSAAFVVRKHSELKRGKARIVYNYRRLNDNTYEDSYKLPNKDELMNKIQESKYFSKFDCKSGFWQIRLTEESIHWTTFTCPEGHFEWLVMPFGLKNAPSIFQRKMDQIFKKYDNFCSVYVVDILVHSKNKNEHRKHLEIILKEFINNGIIISRNKVELEKQDIEFLGMYIKSGTIQLQEHIAKKVLEFPDKLEDKKQLQAFLGLLNYARNFIPD

>Parm1-Chr1-05; Florendovirus; Prunus armeniaca 1;

KLKNEICSELPNAFWYRKQHVVCLPYIKTFNEKNIPTKARPIQMSQEIMEFCKKEIHELLQKKIIRKSKSPWSCPAFYVQKNAELERGVPRLVINYKPLNSVLEWIRYPVPNKRDLINRLEKAVVYSKFDMKSGFWQIQIDKSDRYKTAFVTPFGHYEWNVMPFGLKNAPSKFQNIMNEIFNPYSHFSIVYIDDVLIFSESIEQHWKHLHKFLQIVKRNGLVVSAKKIKLFQTNIRFLGFNICQSQISPIDRVIQFANKFPDQILDKSQLQRFLGSLNYVSDF

>Parm1-Chr1-06; Florendovirus; Prunus armeniaca 1;

KLIADVCSDLPTAFWHRKNHMVSLPYIKHFSEKNIPTKARPIQMNPEMLEFCKTEINDLLQKRIIRRSKSPWSCPAFYVQKNAELERGSPRLVINYKPLNTVLEWIRYPIPNKRDLINRLSKAVVFSKFDLKSGFWQIQITESDRYKTAFVTPFGHYEWNVMPFGLKNAPSEFQNIMNEIFNPYSQFSIVYIDDVLIFSESIEQHWKHLHKFLHVIKTNGLVVSAKKIKLFQMHVRFLGFNINQSQIQPIDRSIQFADKFPDQITDKNQLQRFLGSLNY

>Parm1-Chr1-07; Florendovirus; Prunus armeniaca 1;

KLIADVCSDLPTAFWHRKNHMVSLPYIKHFSEKNIPTKARPIQMNPEMLEFCKTEINDLLQKRIIRRSKSPWSCPAFYVQKNAELERGSPRLVINYKPLNTVLEWIRYPIPNKRDLINRLSKAVVFSKFDLKSGFWQIQITESDRYKTAFVTPFGHYEWNVMPFGLKNAPSEFQNIMNEIFNPYSQFSIVYIDDVLIFSESIEQHWKHLHKFLHVIKTNGLVVSAKKIKLFQMHVRFLGFNINQSQIQPIDRSIQFADKFPDQITDKNQLQRFLGSLNY

>Parm1-Chr2-01; Florendovirus; Prunus armeniaca 1;

KLKNEICSELPNVFWYRKQHVVCLPYIKTFNEKNIPTKAMPIQMSQEIMEFCKKEIHELLQKKIIRKSKSPWSCPAFYVQKNVELERGVPRLVINYKPLNSVLEWIRYPIPNKRDLINRLEKAVVYSKFDMKSGFWQIQIDESDIYKTAFVTPFGHYEWNVMPFGLKNSPSEFQNIMNEIFNPYSHFSIVYIDDVLIFSESIEQHWKHLHKFLQIVKQNGLVVSAKKIKLFQTNIRFLGFNICQSQISPIDRVIQFADKFPDQILDKSQLQRFLGSLNYVSDF

>Parm1-Chr2-02; Florendovirus; Prunus armeniaca 1;

KLKNEICSELPNAFWYRKQHVACLPYIKTFNEKNIPTKARPIQMSQEIMEFCKKEIHELLQKKIIRKSKSPWSCPAFYVQKNAELERGVPRLLINYKPLNSVLEWIRFPIPNKRDLINRLEKAVVYSKFDMKSGFWQIQIDESDRYKTAFVTPFGHYEWNVMPFGLKNAPSEFQNIMNEIFHPYSHFSIVYIDDVLIFSESIEQHWKHLHKFLQIVKQNGLDVSAKKIKLFQTNIRFLGFNICQSQISLIDRVIQFADKFPDQILDKSQLQRFLGSLNYV

>Parm1-Chr2-03; Yendovirus; Prunus armeniaca 1;

HWNKNKILAKLDIINPYSTIKTADMPCNLLDKQKFXQQIKELLNIVIXVSKSRHRSTAFVVRKHSELKRGKARIMYNYKRXNDNTYEDSYKLPNKDELIMNKIEESKYSSKLDCKSRFCEIRLPEXSIQXTTFTCTXAYFEWFVRPFGLKNAPSIFXRKMNXIVKKYDNFGSIYVDDMLVCSKNKNEHRKHXEIILKEFIDNGIVISMNKVELEKQGIXFLGMYIKXETIQLQEHIGKKVLXFPDKXEDKKXLQAFLGLLNYARSFI

>Parm1-Chr2-04; Yendovirus; Prunus armeniaca 1;

HWNKNKILAKLDIINPYSTIKIADMPCNLLDKQKFXQQIKELLNIVIXPSKSRHRSTTFVVRKHSELKRGKARIVYNYKRXNDNTYEDSYKLPNKDELIMNKIEESKYSSKLDCKSRFCEIRLPEXSIQXTTFTCTXAYFEWLVRPFGLKNAPSIFXRKMNXIVKKYDNFGSIYVDDILVCSKNKNEHRKHXEIILKEFIDNGIVISMNKVELEKQGIXFLGMYIKSETIQLQEHIGKKVLXFPDKXEDKKXLQAFLGLLNYARSFI

>Parm1-Chr2-05; Florendovirus; Prunus armeniaca 1;

DKLIADVCSDLPTAFWHRKNHIVSLPYIKHFSEKNIPTKARPIQMNPETLEFCKSEINDLLQKCIIRRSKSPWSCPAFYVQKNAELERGSPRLVINYKPLNTVLEWIRYPIPNKRDLINRLSKAIIFSKFDLKSEFWQIQITESDRYKTAFVTPFGHYEWNVMPFGLKNAPSEFQNIMNEIFNPYSQFAIVYIDDVLIFSESIEQHWKHLYKFLHVIKVNGLVVSAKKIKLFQTHVRFLGFNISQSQIQPIDRSIQFADKFPDQIIDKNQLQRFLGSLNY

>Parm1-Chr2-06; Florendovirus; Prunus armeniaca 1;

KLKNEIFSELPNAFWYRKQHVVCLPYIKTFSEKNIPTKARPIQMSQEIMEFCKKEIHELLEKKIICKSKSPWSCPAFYVQKNAELERGVPRLVINYKPLNSVLEWIRYPIPNKRDLINRLERAVVYSKFDMKSGFWQIQIHESNRYKTAFVTPFGHYEWNVMPFGLKNAPSEFQNIMNEIFNPYSHFSIVYIDDVLIFSESIEQHRKHLHKFLQIVKQNGLVVSARKIKLFQTNIRFLGFNICQSQISPIDRVIQFADKFPNQILDRSQLXRFLGSLNYVSDF

>Parm1-Chr2-07; Florendovirus; Prunus armeniaca 1;

KLIAEVCSDLPTAFWHRKNHIVSLPYIQNFSEKNIPTKARPIQMNPEMLEFCKNEINDLLQKNIIRSSKSPWSCPAFYVQKNAELERGSPRLVINYKPLNTVLEWIRYPIPNKRDLINRLSKAIIFSKFDLKSGFWQIQITESDRYKTAFVTPFGHYEWNVMPFGLKNAPSEFQNIMNEIFNPYSQFAIVYIDDVLIFSESIEQHWKHLYKFLHVIKANGLVVSAKKIKLFQTHVRFLGFNISQSQIRPIDRAIQFADKFPDQITKKNQLQRFLGSLNY

>Parm1-Chr2-08; Florendovirus; Prunus armeniaca 1;

KLIAEVCSDLPTAFWHRKNHIVSLPYIQNFSEKNIPTKARPIQMNPEMLEFCKNEINDLLQKNIIRSSKSPWSCPAFYVQKNAELERGSPRLVINYKPLNTVLEWIRYPIPNKRDLINRLSKAIIFSKFDLKSGFWQIQITESDRYKTAFVTPFGHYEWNVMPFGLKNAPSEFQNIMNEIFNPYSQFAIVYIDDVLIFSESIEQHWKHLYKFLHVIKANGLVVSAKKIKLFQTHVRFLGFNISQSQIRPIDRAIQFADKFPDQITEKKSITEIFRI

>Parm1-Chr2-09; Yendovirus; Prunus armeniaca 1;

LEELLQLAEQTRIMGEDPQLHWNKNKILAKLDIINPDLTIKTADMPCNLLDKQEFEQQIKELLNLKVIRPSKSRHRSAAFVVRKHSELKRGKARIVYNYRRLNDNTYEDSYKLPNKDELMNKIQESKYFSKFDCKSGFWQIILAEESIHWTTFTCPEGHFEWLVMPFGLKNAPSIFQRKMDQIFKKYDKFCSVYVDDILVHSKNKNEHKKHLEIILKEFIDNGIVISRNKVELEKXDIEFLGMYIKSGTIQLQEHIAKKVLEFPDKLEDKKQLQAFLGLLNYARSFIPD

>Parm1-Chr3-01; Florendovirus; Prunus armeniaca 1;

KLKNEICSELPNAFWYRKQHVVCLPYIKTFSEKNIPTKARPIQMSQEIMEFCKKEIHELLEKKIIRKSKSPWSCPAFYVQKNAELKRGVPRLVINYKPLNSALEWIRYPILNKRDLINRLEKAVVYSKFDMKSGFWQIQIDXSDRYKTAFVTPFGHYEWNVMPFGLKNAPSEFQNIMNEIFNPYSHFSIVYIDDVLIFSESIEQHWKHLHKFLQIVKQNGLVVSAKKIKLFQTNIRFLGFNICQSQI

>Parm1-Chr3-02; Florendovirus; Prunus armeniaca 1;

DKLKNEICSELPNAFWYRKQHVVCLPYIKTFSEKNIPTKARPIQMSQEIMEFCKKEIHEXLEKKIIRKSKSPWSCPAFYVQKNAELERGVPRLVINYKPLNSVLEWIRYPIPNKRDLINRLEKAVVYSKFDMKSGFWQIQIDESDRYKTAFVTPFGHYEWNVMPFGMKNAPSEFQNIMNEIFNPYSHFSIVYIDDVLIFSESIEQHWKHLHKFLQIVKQNGLVVSAKKIKLFQTNIRFLGFNICQSQISPIDRVIQFADKFPDQILDKSQLQRFLGSLNYV

>Parm1-Chr3-03; Florendovirus; Prunus armeniaca 1;

EKLKQEVCSDLPNAFWHRKQHVVSLPYVKTFNEKHIPTKARPIQMSQEVMEFCKKEITELLQKGIIRKSKSPRSCPAFYVQKNAELERGAPRLVINYKSLNTALEWIRYPIPNKRDLINRLDKAIIFSKFDMKNGFWQIQIQKSDKYKTAFVTSFGHYEXNVMPFGLKNAPSEFQNIINEIFNPFSDFCIVYIDDVLIFSXSIEQHWKHLYKFLQIVKRNGLVISTKKIKLFQTNIRFLGFNICQSLISPIDQVIQFADKFPDQILAKSQLQRFLGSLNYVSDF

>Parm1-Chr4-01; Yendovirus; Prunus armeniaca 1;

LEELLQLAEQTRIIGEDPQLHWNKNKILAKLDIINPYLTIKTADMPCNLLDKQEFEQEIKELLNLKVIRPSKSRQRSAAFVVRKHSELKRGKARIVYNYRRLNDNTYEDSYKLPNKDELMNKIQESKYFSKFDCKSGFWQIRLAEESIHWTTFTCPKGHFEWLVMPFELKNAPSIFQRKMDQIFKKYDKFCSVYVDDILVHSKNKNEHRKHLEIILKEFIDMVXLXVEIKXSLKNKIXNSXVCILSQELYNYRSTXLKKVLEFPDKLEDKKQLK

>Parm1-Chr4-02; Yendovirus; Prunus armeniaca 1;

LEELLQLAEQTRIIGEDPQLHWNKNKILAKLDIINPDLTIKTADMPCNLLDKQEFEQQIKELLNLKVIRPSKSRHRSAAFVVRKHSELKRGKARIVYNYRRLNDNTYEDSYKLPNKDELMNKIQESKYFSKFDCKSGFWQIRLAEESIHWTTFTCLEGHFEWLVMPFGLKNAPSIFQRKMDQIFKKYDKFCSVYVDDILVHSKNKNEHRKHLEIILKEFINNGIIISRNKVELEKQDIEFLGMYIKSGTIQLQEHIAKKVLEFPDKLEDKRQLQAFLGLLNYARSFIPD

>Parm1-Chr4-03; Yendovirus; Prunus armeniaca 1;

HWNKNKIVANLHIIYPDLFIKPADMPCNLLDKQEFXTKIKKLLNLKVIRPSKSRHRSTSFVVRKHSXLKIGKARVLDNYRRLNDNTYKDSYKLPNKDEFINKVXESKYFSKFDCKLGFWKIRLAEESIEWTSFTCLDGQFEWLVMPFGLKNAPSIFQRKMDQIFKKYDNFCSVYVDDILLHSKNNNDHRKHLEIILHKFIKNNIVISKNKIELEKQDIEFFRMYIKSGTIELQEHIAKKVLEFPNKLEDKKK

>Parm1-Chr4-04; Florendovirus; Prunus armeniaca 1;

KLIADVCSDLPTAFWHRKNHIVSLPYIKNFSEKNIPTKARPIQMNPEMLEFCKNEINDLLQKSIIRRSKSPWSCPAFYVQKNAELERGSPRLVINYKPLNTVLEWIRYPIPNKRDLINRLSKAIVFSKFDLKSGFWQIQITESDRYKTAFVTPFGHYEWNVMPFALKNAPSEFQNIMNEIFNPYSQFSIVYIDDVLIFSESIEQHWKHLHKFLHVIKTNGLVVSAKKIKLFQMHVRFLGFNISQSQIQPIDRSLQFADKFPDQIIDKNQLQRFLGSLNY

>Parm1-Chr4-05; Florendovirus; Prunus armeniaca 1;

KLIADVCSDLPTAFWHRKNHIVSLPYIKNFSEKNIPTKARPIQMNPEMLEFCKNEINDLLQKSIIRRSKSPWSCPAFYVQKNAELERGSPRLVINYKPLNTVLEWIRYPIPNKRDLINRLSKAIVFSKFDLKSGFWQIQITESDRYKTAFVTPFGHYEWNVMPFALKNAPSEFQNIMNEIFNPYSQFSIVYIDDVLIFSESIEQHWKHLHKFLHVIKTNGLVVSAKKIKLFQMHVRFLGFNISQSQIQPIDRSLQFADKFPDQIIDKNQLQRFLGSLNY

>Parm1-Chr4-06; Yendovirus; Prunus armeniaca 1;

IFGEDPQLHWNKNXILKKLDIINTDLTIKMTDMTCNLLDKQEFEQQIKELLNLKVIRPSKSRHRLAAFVVRKXLELERGKARIVYNYRRLNNNTYEDSYKLPIKMNLXIKLKRVNILVNLIVSQDFDKLDXQKNQLNGXHLLVHKDILNCLLCHLDLKKCTIDLSKKDISKFXKYDNFCSVYVDDILVHSKNKNEHRKHLEIILQEFIKNGIVISRNKVELEKQDIEYLGMYIKSGTVQLQEYIAKKVLEFPDKLEDKKQLQAFLG

>Parm1-Chr4-07; Yendovirus; Prunus armeniaca 1;

HWNKNKILAKLDIINQDLTIKIANMPCNLLDKQEFEQXIKELLNLKVIRPSKSRHRSTAFVVRKHSELKRGKARIVYNYRRLNDNTYEDRYKLPNKDELMNKIQESKYFSKFDCKTEFWQIRLAEESIQWTTFTCPKGHFEXLVMPFGLKNAPLIFQRKMDQIFKKYDNFCSVYVDDILVHSKNKNEHKKHLEIILIEFIDNGIVISRNKGELEKQDIEFLGMYIKSGTIQLQEHVSKKVLEFLDKLEDKKQLQTFLGLLNYSRCFIPD

>Parm1-Chr4-08; Florendovirus; Prunus armeniaca 1;

KLKNEICSELPNAFWYRKHVVCLPYIKTFNEKNIPTKARPIQMSQEIMEFCKKEIHELLQKKIIRKSKSPWSCPAFYVQKNAELERGVPRLVINYKPLNSVLEWIRYPIPNKRDLINRLEKAVVYSKFDMKSGFWQIQIDESDRYKTAFVTAFGHYEWNVMPFGLKNAPSEFQNIMNEIFNPYSHFSIVYIDDVLIFSESIEQHWKHLHKFLQIVKQNGLVVSAKKIKLFQTNIRFLGFNICQSQISPIDRVIXFADKFPDQILDKSQLQRFLGSLNYVSDF

>Parm1-Chr5-01; Yendovirus; Prunus armeniaca 1;

LEELLQLAEQTRIIGEDPQLHWNKNKILAKLDIINPDLTIKTADMPCNLLDKQEFEQQIKELLNLKVIRPSKSRHRSAAFVVRKHSELKRGKARIVYNYRRLNDNTYEDSYKLPNKDELMNKIQESKYFSKFDCKSGFWQIRLAEESIHWTAFTCPEGHFEWLVMPFGLKNAPSIFQRKMDQIFKKYDKFCSVYVDDILVHSKNKNEHRKHLEIILKEFINNGIIISRNKVELEKQDIEFLGMYIKXGTIQLQEHIAKKVLEFPDKLEDKKQLQAFLGLLNYARSFIPD

>Parm1-Chr5-02; Yendovirus; Prunus armeniaca 1;

IIGEDPQLHWNKNKILAKLDIINPDLTIKTTDMPCNLLDKQEFEQQIKELLNLKVIRPSKSRHRSAAFIVRKHSELKRGKARIMYNYRRLNDNTYEDSYKLPNKDELMNKIQESKYFSKFDCKSGFWQIRLAEESIHWTAFTCPEGHFEWLVMPFGLKNAPSIFQRKMDQIFKKYDKFCSVYVDDILVHSKNKNEHRKHLEIILKEFIDNGIVISRNKVELEKQDIEFLGMYIKSGTIQLQEHIAKKVLEFPDKLEDKKQLQTFLGLLNYVRSFI

>Parm1-Chr6-01; Florendovirus; Prunus armeniaca 1;

KLKNEICSELPNAFWYRKQHVVCLPYIKTFNEKNIPTKARPIQMSQEMMEFCKKEIHELLQKKIIRKSKSPWSCPAFYVQKNAELERGVPRLVINYKPLNSVLEWIRYPIPNKRDLINRLEKAVVYSKFDMKSGFWQIQIDESDRYKTAFVTPFGHYEWNVMPFGLKNAPSEFQNIMNEIFNPYSHFSIVYIDDVLIFSESIEQHWKHLHKFLQIIKQNGLVVSAKKIKLFQTNIRFLGFNICQSQISPIDRVIQFADKFLDQILDKSQLQRFLGSLNYV

>Parm1-Chr6-02; Yendovirus; Prunus armeniaca 1;

IIGEDPQLHWNKNKILPKLNIINQDLTIKTTDMPCNLLDKXEFXQQIKELLNLKVIRPSKSRHRSAAFVVRKHLELKRGKARIVYNYRRLNDNTYEDSYKLLNKDELMNKIQESKYFSKFDCKLGFWQIRLAEESIQWTTFTCPXGHFEWLVMPFGLKNAPLIFQRKMDQIFKKYDNFCSVYVDDILVHSKNKNEHRKHIEIILKEFIDNGIDISRNKVELEKQDIEFLGMYIKSGTIQLQEHIAKKVLEFLDKLEDKKQLQAFLGLLNYARSFIP

>Parm1-Chr6-03; Yendovirus; Prunus armeniaca 1;

LEELLQLVEQTRIIGEDPQLHWNKNKILPKLNIINQDLTIKTTDMPCNLLDKXEFXQQIKELLNLKVIRPSKSRHRSAAFVVRKHLELKRGKARIVYNYRRLNDNTYEDSYKLLNKDELMNKIQESKYFSKFDCKLGFWQIRLAEESIQWTTFTCPXGHFEWLVMPFGLKNAPLIFQRKMDQIFKKYDNFCSVYVDDILVHSKNKNEHRKHIEIILKEFIDNGIDISRNKVELEKQDIEFLGMYIKSGTIQLQEHIAKKVLEFLDKLEDKKQLQAFLGLLNYARSFIP

>Parm1-Chr6-04; Yendovirus; Prunus armeniaca 1;

LEELLQLAEQTRIIGEDPQLHWNKNKILAKLDIINPDLTIKTADMPCNLLDKQEFEQQIKELLNLKVIRPSKSRHRSAAFVVRKHSELKRGKARIVYNYRRLNDNTYEDSYKLPNKDELMNKIQESKYFSKFDCKSGFWQIRLAEESIHWTAFTCPEGHFEWLVMPFGLKNAPSIFQRKMDQIFKKYDNFCSVYVDDILVHSKNKNEHRRHLEIILKEFIDNGIVISRNKVELEKQDIEFLGMYIKSGTIQLQEHIAKKVLEFPDKLEDKKQLQAFLGLLNYARSFIP

>Parm2-Chr1-01; Yendovirus; Prunus armeniaca 2;

IIGEDPQLHWNKNKILAKLDIINPDLTIKTADMPCNLLDKQEFEQQIKELLNLKVIRPSKSRHRSAAFVVRKHSELKRGKARIVYNYRRLNDNTYEDSYKLPNKDELMNKIQESKYFSKFDCKSGFWQIRLAEESIHWTTFTCPEGHFEWLVMPFGLKNAPSIFQRKMDQIFKKYDKFCSVYVDDILVHSKNKNEHRKHLEIILKEFINNGIIISRNKVELEKQDIEFLGMYIKSTIQLQEHIAKKVLEFPDKLEDKKQLQAFLGLLNYARSFIP

>Parm2-Chr1-02; Yendovirus; Prunus armeniaca 2;

LEELLQLAEQTRIIGEDPQLHWNKNKILAKLDIINPDLTIKTADMPCNLLDKQEFEQQIKELLNLKVIRPSKSRHRSAAFVVRKHSELKRGKARIVYNYRRLNDNTYEDSYKLPNKDELMNKIQESKYFSKFDCKSGFWQIRLAEESIHWTAFTCPEGHFEWLVMPFGLKNAPSIFQRKMDQIFKKYDKFCSVYVDDILVHSKNKNEHRKHLEIILKEFINNGIIISRNKVELEKQDIEFLGMYIKSGTIQLQEHIAKKVLEFPDKLEDKKQLQAFLGLLNYARSFIP

>Parm2-Chr1-03; Florendovirus; Prunus armeniaca 2;

KKLIDEIFSDLPTAFWHRKHHVVKLPYIKNFSEKNIPTKARPIQMSQEIVEYCKTEINELLQKGIIMKSKSPWSCPAFYVQKNAEIERGSPRLVINYKPLNTVLEWIRYPIPNKRDLINHLSNAVIFSKFDLKSGFWQIQIDESDRYKTAFVTPFGHYEWNVMPFGLKNAPSEFQNIMNEIFNPYSHFSIVYIDDVLIFSKSIDQHWKHLYKFLQVVKTNGLVVSAKKIKLFQTHIRFLGYSISQSQIRPIDRAIQFADKFPDQITDKNQLQRFLGSLNYVSDF

>Parm2-Chr1-04; Yendovirus; Prunus armeniaca 2;

LEELLQLAEQTRIIGEDPQLHWNKNKILAKLDIINPDLTIKTADMPCNLLDKQEFEQQIKELLNLKVIRLSKSRHRSATFVVRKHSELKRGKARIVYNYRRLNDNTYEDSYKLPNKDELMNKIQESKYFSKFDCKSGFWQIRLTEESIHWTAFTCPEGHFEWLVMPFGLKNAPSIFQRKMDQIFKKYDNFCSVYVVDILVHSKNKNEHRKHFEIILKEFINNGIIISRNKVELEKQDIEFLGMYIKSGTIQLQEHIAKKVLEFPDKLEDKKQLQAFLGLLNYARNFIPD

>Parm2-Chr1-05; Florendovirus; Prunus armeniaca 2;

KLKNEICSELPNAFWYRKQHVVCLPYIKTFNEKNIPTKARPIQMSQEIMEFCKKEIHELLQKKIIRKSKSPWSCPAFYVXKNAELERGVPRLVINYKPLNSVLEWIRYPIPNKRDLINRLEKAVVYSKFDMKSGFWQIQIDESDRYKTAFVTPFGHYEWNVMPFGLKNAPSEFQNIMNEIFNPYSHFSIVYIDDVLIFSESIEQHWKHLHKFLQIVKRNGLVVSAKKIKLFQTNIRFLGFNICQSQISPIDRVIQFADKFPDQILDKSQLQRFLGSLNYV

>Parm2-Chr2-01; Florendovirus; Prunus armeniaca 2;

KLKNEICSELPNAFWYRKQHVACLPYIKTFNEKNIPTKARPIQMSQEIMEFCKKEIHELLQKKIIRKSKSPWSCPAFYVQKNAELERGVPRLVINYNPLNLVLEWIRYPIPNKRDLINRLEKAVVYSKFDMKSGFWQIQIDESDRYKTAFVTPFGHYEWNVMPFGLKNAPSEFQNIMNEIFHPYSHFSIVYIDDVLIFSESIEQHWKHLHKFLQIVKQNGLVVSAKKIKLFQTNIRFLGFNICQSQISPIDRVIQFADKFPDQILDKSQLXRFLGSLNYV

>Parm2-Chr2-02; Yendovirus; Prunus armeniaca 2;

LEELLQLAEQTRIIGEDPQLHWNKNKILAKLDIINPDLTIKTADMPCNLLDKQEFEQQIKELLNLKVIRPSKSRHRSAAFVVRKHSELKRGKARIVYNYRRLNDNTYEDSYKLPNKDELMNKIQESKYFSKFDCKSGFWQIRLAEESIHWTTFTCPEGHFEWLVMPFGLKNAPSIFQRKMDQIFKKYDNFCSVYVDDILVHSKNKNEHRKHLEIILKEFINNGIIISRNKVELEKQDIEFLGMYIKSGTIQLQEHIAKKVLEFPDKLEDKKQLQAFLSLLNYARSFIPD

>Parm2-Chr2-03; Yendovirus; Prunus armeniaca 2;

LEELLQLAEQTRIIGEDPQLHWNKNKILAKLDIINPDLTIKTADMPCNLLDKQEFEQQIKELLNLKVIRPSKSRHRSAAFVVRKHSELKRGKARIVYNYRRLNDNTYEDSYKLPNKDELMNKIQESKYFSKFDCKSGFWQIRLAEESIHWTAFTCPEGHFEWLVMPFGLKNAPSIFQRKMDQIFKKYDKFCSVYVDDILVHSKNKNEHRKHLEIILKEFINNGIIISRNKVELEKQDIEFLGMYIKSGTIQLQEHIAKKVLEFPDKLEDKKQLQAFLGLLNYARSFIPD

>Parm2-Chr2-04; Florendovirus; Prunus armeniaca 2;

KLIAEVCSDLPTAFWHRKNHIVSLPYIQNFSEKNIPTKARPIQMNPEMLEFCKNEINDLLQKNIIRSSKSPWSCPAFYVQKNAELERGSPRLVINYKPLNTVLEWIRYPIPNKRDLINRLSKAIIFSKFDLKSGFWQIQITESDRYKTAFVTPFGHYEWNVMPFGLKNAPSEFQNIMNEIFNPYSQFAIVYIDDVLIFSESIEQHWKHLYKFLHVIKANGLVVSAKKIKLFQTHVRFLGFNISQSQIRPIDRAIQFADKFPDQITEKNQLQRFLGSLNY

>Parm2-Chr2-05; Florendovirus; Prunus armeniaca 2;

KLIAEVCSDLPTAFWHRKNHIVSLPYIQNFSEKNIPTKARPIQMNPEMLEFCKNEINDLLQKNIIRSSKSPWSCPAFYVQKNAELERGSPRLVINYKPLNTVLEWIRYPIPNKRDLINRLSKAIIFSKFDLKSGFWQIQITESDRYKTAFVTPFGHYEWNVMPFGLKNAPSEFQNIMNEIFNPYSQFAIVYIDDVLIFSESIEQHWKHLYKFLHVIKANGLVVSAKKIKLFQTHVRFLGFNISQSQIRPIDRAIQFADKFPDQITEKNQLQRFLGSLNY

>Parm2-Chr3-01; Florendovirus; Prunus armeniaca 2;

KLKNEICSELPNAFWYRKQHVVCLPYIKTFSEKNIPTKARPIQMSQEIMEFCKKEIHELLEKKIIRKSKSPWSCPAFYVHKNAELERGVPRLVINYKPLNFVLEWIRYPIPNKRDLINRLEKAVVYSKFDMKSGFWQIQIHESDRYKTAFVTPFGHYEWNVMPFGLKNAPSEFQNIMNEIFNPYSHFSIVYIDDVLIFSESIEQHWKHLHKFLQIVKQNGLVVSAKKIKLFQTNIRFLGFNICRSQISPIDRVIQFADKFLDQILDKSQLHRFLGSL

>Parm2-Chr3-02; Florendovirus; Prunus armeniaca 2;

EKLKQEVCSDLPNAFWHRKQHVVSLPYVKTFNEKHIPTKARPIQMSQEVMEFCKKEITELLQKGIIRKSKSPRSCPAFYVQKNAELERGAPRLVINXKSLNMALEWIRYLIPNKRDLINRLDKAIIFSKFDMKNGFWQIQIQKSDKYKTAFVTSFGHYEXNVMPFGLKNAPSEFQNIINEIFNPFSDFCIVYIDDVLIFSXSIEQHWKHLYKFLQIVKRNGLVISAKKIKLFQTNIRFLGFNICQSLISPIDQVIQFADKFPDQILAKSQLQRFLGSLNYVSDF

>Parm2-Chr4-01; Yendovirus; Prunus armeniaca 2;

IIGEDPQLHWNKNKILPKLNIINQDLTIKTTDMPCNLLDKXEFXQQIKELLNLKVIRPSKSRHRSAAFVVRKHLELKRGKARIVYNYRRLNDNTYEDSYKLLNKDELMNKIQESKYFSKFDCKLGFWQIRLAEESIQWTTFTCPXGHFEWLVMPFGLKNAPLIFQRKMDQIFKKYDNFCSVYVDDILVHSKNKNEHRKHLEIILKEFIDNGIVISRNKVELEKQDIEFLGMYIKSRTIQLQEHIAKKVLEFPDKLEDKKQLQAFLGLLNYARSFIP

>Parm2-Chr4-02; Florendovirus; Prunus armeniaca 2;

KLKNEICSELPNAFWYRKQHVVCLPYIKTFNEKNIPTKARPIQMSQEMMEFCKKEIHELLQKKIIRKSKSPWSCPAFYVQKNAELERGVPRLVINYKPLNSVLEWIRYPIPNKRDLINRLEKAVVYSKFDMKSGFWQIQIDESDRYKTAFVTPFGHYEWNVMPFGLKNAPSEFQNIMNEIFNPYSHFSIVYIDDVLIFSESIEQHWKHLHKFLQIIKQNGLVVSAKKIKLFQTNIRFLGFNICQSQISPIDRVIQFADKFLDQILDKSQLQRFLGSLNYV

>Parm2-Chr5-01; Florendovirus; Prunus armeniaca 2;

KLKNEICSELPNAFWYRKQHVVCLPYIKTFNEKNIPTKARPIQMSQEIMEFCKKEIQELLQKKIIRKSKSPWSCPAFYVQKNAKLERGVPRLVINYKPLNSVLEWIRYPIPNKRDLINRLEKAVVYSKFDMKSGFWQIQINESDRYKTAFVTPFGHYEWNIMPFGLKNAPSKFQNIMNEIFNPYSHFSIVNIDDVLIFSESIEQHWKHLHKFLQIVKQNGLVVSAKKIKLFQTNIRFLGFNICQSQISPIDRVIQFADKFLDQILDKSQLQRFLGSLNYVSDF

>Parm2-Chr5-02; Florendovirus; Prunus armeniaca 2;

KLKNEICSELPNAFWYRKQHVVCLPYIKTFSEKNIPTKARPIQMSQEIMEFCKKEIHELLEKKIIRKSKSPWSCPAFYVQKNAELERGVSRLVINYKPLNSVLEWIRYPIPNKRDLINRLERAVVYSKFDMKSGFWQIQIHESDRYKTAFVTPFGHYEWNVMSFGLKNAPSEFQNIMNEIFNPYSHFSIVYIDDVLIFSESIEQHWKHLHKFLQIVKQNGLVVSAKKIKLFQTNIRFLGFNICQSQISPIDRVIQFADKFPDQILDKSQLQRFLGSLNYV

>Parm2-Chr6-01; Florendovirus; Prunus armeniaca 2;

KLIADVCSDLPTAFWHRKNHIVSLPYIKNFSEKNIPTKARPIQMNPEMLEFCKNEINDLLQKSIIRRSKSPWSCPAFYVQKNAELERGSPRLVINYKPLNTVLEWIRYPIPNKRDLINRLSKAIVFSKFDLKSGFWQIQITESDRYKTAFVTPFGHYEWNVMPFGLKNAPSEFQNIMNEIFNPYSQFSIVYIDDVLIFSESIEQHWKHLHKFLHVIKTNGLVVSAKKIKLFQMHVRFLGFNISQSQIQPIDRSQQIADKFPDQIIDKNQLQRFLGSLNY

>Parm2-Chr6-02; Yendovirus; Prunus armeniaca 2;

HWNKNKIVANLHIIYPDLFIKPADMPCNLLDKQEFXTKIKKLLNLKVIRPSKSRHRSTSFIVRKHSXLKRGKARVLDNYRRLNDNTYKDSYKLPNKDEFINKVXESKYFSKFDCKLGFWKIRLAEESIEWTSFTCLDGQFEWLVMPFGLKNAPSIFLRKMDQIFKKYDNFCSVYVDDILLHSKNNNDHRKHLEIILHKFIKNNIVISKNKIELEKQDIEFFRMYIKSGTIELQEHIAKKVLEFPDKLEDKKK

>Parm2-Chr6-03; Florendovirus; Prunus armeniaca 2;

KLRNEISSELPNAFWYRKQHVVCLPYIKTFSEKNIPTKARPIQMSQEMMEFCKKEIHELLEKKIIRKSKSPWSCPAFYVQKNAELERGVPRLVINYKPLNSVLEWIRYPIPNKRDLINRLEKAVVYSKFDMKSGFWQIQIHESDRYKTAFVTPFGHYEWNVMPFGLKNAPSEFQNIMNEIFNPYSHFSIVYIDDVLIFSEFIEQHWKHLHKFLQIVKQNGLVVSAKKIKLFQTNIRFLGFNICQSQISPIDRVIQFADKFPDQILDKSQLQRFLGSLNYV

>Parm2-Chr6-04; Yendovirus; Prunus armeniaca 2;

LEELLQLVEQTRIIGEDPQLHWNKNKILAKLDIINPDLTIKTADMPCNLLDKQEFEQQIKELLNLKVIRPSKSRHRSAAFVVRKHSELKRGKARIVYNYRRLNDNTYEDSYKLPNKDELMNKIQESKYFSKFDCKLGFWQIRLAEESIHWTAFTCPEGHFEWLVMPFGLKNAPSIFQRKMDQIFKKYDKFCSVYVDDILVHSKNKNEHRKHLEIILKEFINNGIIISRNKVELEKQDIEFLGMYIKSGTIQLQEHIAKKVLEFPDKLEDKKQLQAFLGLLNYARSFIPD

>Parm2-Chr6-05; Florendovirus; Prunus armeniaca 2;

KLITDVCSDLPTAFWHRKNHIVSLPYIQNFSEKNIPTKARPIQMSPEMLEFCKNEINDLLQKNIIRRSKSPWSCSAFYVQKNAELERGSPRLVINYKPLNTVLKWIRYPIPNKRDLINRLSKAIIFSKFDLKSGFWQIQITESDRYKTAFVTPFGHYEWNVMPFGLKNAPSEFXNIMNEIFNSYSQFAIVYIDDVLIFSESIEQHWKHLYKFLHVIKANGLVVSAKKIKLFQMHIRFLGFNISQSQIQPINRSIQFADKFPDQIIDKNQLQRFLGSLNY

>Parm2-Chr7-01; Florendovirus; Prunus armeniaca 2;

KLINEICSELPNAFWYRKQHVVCLPYIKTFNEKNIPTKARPIQMSQEIMEFCKKEIQELLQKKIIRKSKSPWSCPAFYVXKNVELERGVPRLVINYKPLNSVLEWIRYPIPNKRDLINRLEKAVVYSKFDMKSGFWQIQIDESDRYKTAFVTPFGHYEWNVMPFGLKNAPSEFQNIMNEIFNPYSHFSIVYIDDVLIFSESIEQHWKHLHKFLQIVKQNGLVVSAKKIKLFQTNIRFLGFNICQSQISPIDRVIQFADKFPNQILDKSQLHRFLGSLNYVSDF

>Parm2-Chr7-02; Florendovirus; Prunus armeniaca 2;

KLINEICSELPNAFWYRKQHVVCLPYIKTFNEKNIPTKARPIQMSQEIMEFCKKEIQELLQKKIIRKSKSPWSCPAFYVXKNVELERGVPRLVINYKPLNSVLEWIRYPIPNKRDLINRLEKAVVYSKFDMKSGFWQIQIDESDRYKTAFVTPFGHYEWNVMPFGLKNAPSEFQNIMNEIFNPYSHFSIVYIDDVLIFSESIEQHWKHLHKFLQIVKQNGLVVSAKKIKLFQTNIRFLGFNICQSQISPIDRVIQFADKFPNQILDKSQLHRFLGSLNYV

>Parm2-Chr7-03; Florendovirus; Prunus armeniaca 2;

KLINEICSELPNAFWYRKQHVVCLPYIKTFNEKNIPTKARPIQMSQEIMEFCKKEIQELLQKKIIRKSKSPWSCPAFYVXKNVELERGVPRLVINYKPLNSVLEWIRYPIPNKRDLINRLEKAVVYSKFDMKSGFWQIQIDESDRYKTAFVTPFGHYEWNVMPFGLKNAPSEFQNIMNEIFNPYSHFSIVYIDDVLIFSESIEQHWKHLHKFLQIVKQNGLVVSAKKIKLFQTNIRFLGFNICQSQISPIDRVIQFADKFPNQILDKSQLHRFLGSLNYV

>Parm2-Chr7-04; Florendovirus; Prunus armeniaca 2;

KLINEICSELPNAFWYRKQHVVCLPYIKTFNEKNIPTKARPIQMSQEIMEFCKKEIQELLQKKIIRKSKSPWSCPAFYVXKNVELERGVPRLVINYKPLNSVLEWIRYPIPNKTDLINRLEKAVVYSKFDMKSGFWQIQIDESDRYKTAFVTPFGHYEWNVMPFGLKNAPSEFQNIMNEIFNPYSHFSIVYIDDVLIFSESIEQHWKHLHKFLQIVKQNGLVVSAKKIKLFQTNIRFLGFNICQSQISPIDRVIQFADKFPDQILDKSQLHRFLGSLNYVSDF

>Parm2-Chr8-01; Yendovirus; Prunus armeniaca 2;

IIGEDPQLHWNKNKILAKLDIINPDLTIKTIDMPCSLLDKQEFEEQIKELLNLKVIRPLKSRHRSAVFVVIKHSELKRCKARIVYNYRRLNDNTYEDNYKLPNKDELMNKIQESKYFSKFDCKSGFRQIRLAEESIQWTAFTCPKGHFEWLVMPFGLKNAPSIFQRKMDQIFKKYDNFCSVYVDDILVHSKNKNEHRKHLEIILKEFMNNGIVISRNKVELGKQDIEFLCMYIKSGTIQLQEHIAKKVLEFPDKLEDKKQLQAFLGLLNYSRSFIPD

>Parm2-Chr8-02; Yendovirus; Prunus armeniaca 2;

IIGEDPQLHWNKNKILEKLDIINPDLTIKTAYMPCSLLDKQEFEEQIKELLNLKVIRPSKSRHRSAAFVVIKHSELKRGKARIVYNYRRLNDNTYEDSYKLPNKDELMNKIQESKYFSKFDCKSGFWQIRLAEESIQWTTFTCPKDILNGLXCHLDXKMHHRSFKERWTKSLRSMIIFVVYMXMIYXFIVKIKMNIENTXKLFXKNLLTMVXLXVEIKXSLKNKIXFLCMYIKSGTIQLQEHIAKKMLEFPDKLEDKKQLQAFLGLLNYSRSFIP

>Parm3-Chr1-01; Yendovirus; Prunus armeniaca 3;

NKNKILAKLDIINPDLTIKTTDMPCNLLDKQEFXQQIKELLNLKVIRPSKSRHRSAAFIVRKHLKLKRGKAKIVYNXMIILMKIVINYQTKMNLXIKFKKVNILVNLIVNQDFGKLDXQKNQFKWTTFTYPEGHFEWLIMPFGLKNAPSIFQKKMDQIFKKYDNFCSVYVDDILVHSKNKNEHRKHLEIILKNFIDNVIVINRNKVELEKQDIEFLGMYIKSGTIQLXEDMAKKVLEFPGKLEDKKQLQAFLRLLNCARSFILD

>Parm3-Chr1-02; Florendovirus; Prunus armeniaca 3;

KLIADVCSDLPTAFWHRKNHIVSLPYIKNFSEKNIPTKARPIQMNPEMLEFCKNEINDLLQKNIIRRSKSPWSCPAFYVQKNAELERGSPRLVINYKPLNTVLEWIRYPIPNKRDLINCLSKAIIFSKFDLKSGFWQVQITESDRYKTAFVTPFGHYEWNVMPFGLKNAPSEFQNIMNEIFNSYSQFAIVYIDDVLIFSESIEQHWKHLYKFLHVIKTNGLVVSAKKIKLFQTHIRFLGFNISQSQIQSIDRSIQFADKFPDQITDKNQLQRFLGSLNY

>Parm3-Chr2-01; Yendovirus; Prunus armeniaca 3;

LEELLQLAEQTRIIGEDPQLHWNKNKILAKLDIINPDLTIKTADMPCNLLDKQEFEQQIKELLNLKVIRPSKSRHRSAAFVVRKHSELKRGKARIVYNYRRLNDNTYEDSYKLPNKDELMNKIQESKYFSKFDCKSGFWQIRLAEESIHWTAFTCPEGHFEWLVMPFGLKNAPSIFQRKMDQIFKKYDKFCSVYVDDILVHSNNKNEHRKHLEIILKEFINNGIIISRNKVELEKQDIEFLGMYIKSGTIQLQEHIAKKVLEFPDKLEDKKQLQAFLGLLNYARSFIPD

>Parm3-Chr2-02; Yendovirus; Prunus armeniaca 3;

LEELLQLAEQTRIIGEDPQLHWNKNKILAKLDIINPDLTIKTADMPCNLLDKQEFEQQIKELLNLKVIRPSKSRHRSAAFVVRKHSELKRGKARIVYNYRRLNDNTYEDSYKLPNKDELMNKIQESKYFSKFDCKFGFWKIRLTEESIHWTSFTCPEGHFEWLVMPFGLKNAPSIFQRKMDQIFKKYDNFCSVYVVDILVHSKNKNEHRKHLEIILKEFINNGIIISRNKVELEKQYIEFLGMYIKSGTIQLQEHIAKNVLEFPDKLEDKKQLQAFLGLLNYARNFIPD

>Parm3-Chr3-01; Florendovirus; Prunus armeniaca 3;

KLIADVCSDLPTAFWHRKNHIVSLPYIKNFSEKNIPTKARPIQMNPEMLEFCKNEINDLLQKSIIRRSKSPWSCPAFYVQKNAELERGSPRLVINYKPLNTVLEWIRYPIPNKRDLINRLSKAIVFSKFDLKSGFWQIQITESDRYKTAFVTPFGHYEWNVMPFALKNAPSEFQNIMNEIFNPYSQFSIVYIDDVLIFSESIEQHWKHLHKFLHVIKTNGLVVSAKKIKLFQMHVRFLGFNISQSQIQPIDRSLQFADKFPDQIIDKNQLQRFLGSLNY

>Parm3-Chr3-02; Florendovirus; Prunus armeniaca 3;

KLIADVCSDLPTAFWHRKNHIVSLPYIKNFSEKNIPTKARPIQMNPEMLEFCKNEINDLLQKSIIRRSKSPWSCPAFYVQKNAELERGSPRLVINYKPLNTVLEWIRYPIPNKRDLINRLSKAIVFSKFDLKSGFWQIQITESDRYKTAFVTPFGHYEWNVMPFALKNAPSEFQNIMNEIFNPYSQFSIVYIDDVLIFSESIEQHWKHLHKFLHVIKTNGLVVSAKKIKLFQMHVRFLGFNISQSQIQPIDRSLQFADKFPDQIIDKNQLQRFLGSLNY

>Parm3-Chr3-03; Yendovirus; Prunus armeniaca 3;

LEELLQLAEQTRIIGEDPQLHWNKNKILAKLDIINPDLTIKTADMPCNLLDKQEFEQQIKELLNLKVIRPSKSRHRSAAFVVIKHSELKRGKARIVYNYRRLNDNTYEDSYKLPNKDELMNKIQESKYFSKFDCKSGFWQIRLAEESIHWTAFTCPEGHFEWLVMPFGLKNAPSIFQRKMDQIFKKYDKFCSVYVDDILVHSKNKNEHRKHLEIILKEFIDNGIIISRNKVELEKQDIEFLGMYIKSGTIQLQEHIAKKVLEFPDKLEDKKQLQAFLGLLNYARSFIPD

>Parm3-Chr3-04; Florendovirus; Prunus armeniaca 3;

KLKNEICSELPNAFWYRKQHVVCLPYIKTFSEKNIPTKARPIQMSQEIMEFCKKEIHELLQKKIIRKSKSPWYCPAFYVQKNAELERGVPRLVINYKPLNSVLEWIRYPIPNKRDLINRLGKAVVYSKFDMKSGFWQIQIHESDRYKTAFVTPFGHYEWNVMPFGLKNAPSEFQNIMNEIFNPYSHFSIVYIDDVLIFSESIEQHWKHLHKFLQIVKQNGLVVSAKKIKLFQTNIRFLGFNIHQSQISPIDRVVQFADKFPDQILDKSQLQRFLGSLNYV

>Parm3-Chr3-05; Yendovirus; Prunus armeniaca 3;

HWNKNKILAKLDIINQDLTIKIADMPCNLLDKQEFEQXIKELLNLKVIRPSKSRYRSTAFVVRKHSELKRGKARIVYNYRRLNDNTYEDRYKLPNKDELMNKIQESKYFSKFDCKTEFWQIRLAEESIRWTTFTCPKGHFEXLVMPFGLKNAPLIFQRKMDQIFKKYDNFCSVYVDDILVHSKNKNEHKKHLEIILIEFIDNGIVISRNKGELEKQDIEFLGMYIKSGTIQLQEHVSKKVLEFLDKLEDKKQLQTFLGLLNYSRCFIPD

>Parm3-Chr3-06; Yendovirus; Prunus armeniaca 3;

HWNKNKILAKLDIINQDLTIKIADMPCNLLDKQEFEQXIKELLNLKVIRPSKSRHRSTAFVVRKHLELKRGKARIVYNYRRLNDNAYEDRYKLTNKDELMNKIQESKYFSKFDCKTEFWKIRLAEESIQWTTFTCPKGHFEWLVMPFGLKNAPLIFQRKMNQIFKKYDNFCSVYVDDILVHSKNKNEHKKHLEIILIEFIDNGIVISRNKGELEKQDIEFLGMYIKSGTIQLQEHVSKKVLEFLDKLEDKKQLQTFLGLLNYSRSFIPD

>Parm3-Chr4-01; Florendovirus; Prunus armeniaca 3;

EKLKQEVCSDLPNAFWHRKQHVVSLPYVKTFNEKHIPTKARPIQMSQEVMEFCKKEITELLQKGIIRKSKSPRSCPAFYVQKNAELERGAPRLVINYKSLNTALEWIRYPIPNKRDLINRLDKAIIFSKFDMKSGFWQIQIQKSDKYKTAFVTSFGHYEXNVMPFGLKNAPSEFQNIINEIFNPFSDFCIVYIDDVLIFSXSIEQHWKHLYKFLQIVKRNGLVISAKKIKLFQTNIRFLGFNICQSLISPIDQVIQFADKFPDQILAKSQLQRFLGSLNYVSDF

>Parm3-Chr4-02; Florendovirus; Prunus armeniaca 3;

DKLKNEICSELPNAFWYRKQHVVCLPYIKTFSEKNIPTKARPIQMSQEIMEFCKKEIHELLEKKIIRKSKSPWSCPAFYVQKNAELERGVPRLVINYKPLNSVLEWIRYPIPNKRDLINRLEKAVVYSKFDMKSGFWQIQIDESDRYKTAFITPFGHYEWNVMPFGMKNAPSEFQNIMNEIFNPYSHFSIVYIDDVLIFSESIEQHWKHLHKFLQIVKQNGLVVSAKKIKLFQTNIRFLGFNICQSQISPIDRVIQFADKFPDQILDKSQLQRFLGSLN

>Parm3-Chr4-03; Florendovirus; Prunus armeniaca 3;

KLKNEICSELPNAFWYRKQHVVCLPYIKTFSEKNIPTKARPIQMSQEIMEFCKKEIHELLEKKIIRKSKSPWSCPAFYVQKNAELERGVPRLVINYKPLNSALEWIRYPIPNKRDLINRLEKAVVYSKFDMKSGFWQIQIDESDRYKTAFVTPFGHYEWNVMPFGLKNAPSEFQNIMNEIFNPYSHFSIVYIDDVLIFSESIEQHWKHLHKFLQIVKQNGLVVSAKKIKLFQTNIRFLGFNICQSQI

>Parm3-Chr4-04; Florendovirus; Prunus armeniaca 3;

KLKNEICSELPNAFWYRKQHVVCLPYIKTFSEKNIPTKARPIQMSQEIMEFCKKEIHELLEKKIIRKSKSPWSCPAFYVQKNAELERGVPRLVINYKPLNSVLEWIRYPIPNKRDLINRLEKAVVYSKFDMKSGFWQIQIHESDRYKTAFVTPFGHYEWNVMPFGLKNAPSEFQNIMNEIFNPYSHFSIVYIDDVLIFSESIEQHWKHLHKFLQIVKQNGLVVSAKKIKLFQTNIRFLGFNICQSQISPIDRVIQFADKFLDQILDKSQLQRFLGSL

>Parm3-Chr5-01; Yendovirus; Prunus armeniaca 3;

LEELLQLAEQTRIIGEDPQLHWNKNKILAKLDIINLDLTIKTAYMPCNLLDKQEFEQQIKELLNLKVIRPSKSRHRSAAFVVRKHSEVKRGKARIVYNYRRLNDNTYEDSYKLPNKDELMNKIQESKYFSKFDCKSGFWQIILAEESIHWTTFTCPEGHFEWLLMPFGLKNAPSIFQRKMDQIFKKYDKFCSVYVDDILVHSKNKNEHRKHLEIILKEFIDNGIVISRNKVELEKQDIEFLGMYIKSGTIQLQEHIAKKVLEFPDKLEDKKQLQAFLGLLNYARSFIPD

>Parm3-Chr5-02; Yendovirus; Prunus armeniaca 3;

LEELLQLAEQTRIIGEDPQLHWNKNKILAKLDIINPDLTIKTADMPCNLLDKQEFEQQIKELLNLKVIRPSKSRHRSAAFVVRKHSELKRGKARIVYNYRRLNDNTYEDSYKLPNKDELMNKIQESKYFSKFDCKSGFWQIRLAEESIIWTAFTCPEGHFEWLVMPFGLKNAPSIFQRKMDQIFKKYDKFCSVYVDDILVHSKNKNEHRKHLEIILKEFINNGIIISRNKVELEKQDIXFLGMYIKSGTIQLQEHIAKKVLEFPDKLEDKKQLQAFLGLLNYARSFIPD

>Parm3-Chr5-03; Yendovirus; Prunus armeniaca 3;

HWNKNKMLAKLDIVNPYSTIKTADMPCNLLDKQKFXQQIKELLNIVIXPSKSRHKSTAFVVRKHSELKRGKARIVYNYKRXNDNTYEDSYKLPNKDELIMNKIEESKYSSKLDCKSRFCEIRLPEXSIQXTTFTCTXAYFEWLVRPFGLKNAPSIFQRKMNXIVKKYDNFGSIYVDDILVCSKNKNEHRKHXEIILKEFIDNGIVISMNKVELEKQGIXFLGMYIKSETIQLQEHIGKKVLXFLDKXEDKKXLQAFLGLLNYARSFI

>Parm3-Chr5-04; Yendovirus; Prunus armeniaca 3;

LEELLQLAEQTRIIGEDPQLHWNKNKILAKLDIINPDLTIKTADMPCNLLDKQEFEQQIKELLNLKVIRPSKSRHRSAAFVVRKHSELKRGKARIVYNYRRLNDNTYEDSYKLPNKDELMNKIQESKYFSKFDCKSGFWQIRLAEESIHWTAFTCPEGHFEWLVMPFGLKNAPSIFQRKMDQIFKKYDKFCSVYVDDILVHSKNKNEHRKHLEIILKEFINNGIIISRNKVELEKQDIEFLGMYIKSGTIQLQEHIAKKVLEFPDKLEDKKQLQAFLGLLNYARSFIPD

>Parm3-Chr5-05; Florendovirus; Prunus armeniaca 3;

KLKNEICSELPNAFWYRKQHVVCLPYIKTFNEKNIPTKAMPIQMSQEIMEFCKKEIHELLQKKIIRKSKSPWSCPAFYVQKNVELERGVPRLVINYKPLNSVLEWIRYPIPNKRDLINRLEKAVVYSKFDMKSGFWQIQIDESDRYKTAFVTPFGHYEWNVMPFGLKNSPSEFQNIMNEIFNPYSHFSIVYIDDVLIFSESIEQHWKHLHKFLQIVKQNGLVVSAKKIKLFQTNIRFLGFNICQSQISPIDRVIQFADKFPDQILDKSQLQRFLGSLNYVSDF

>Parm3-Chr5-06; Yendovirus; Prunus armeniaca 3;

LEELLQLAEQTRIIREDPQLHWNKNKILAKLDIINPDLTTKTTDMPCNLLDKQEFEQQIKELLNLKVIRPPKSRHRSAAFVVRKHSELKRGKARIIYNYRRLNDNTYEDSYKLPNKDELMNKIQESKYFSKFDCKSGFWQIRLAEESIQWTAFTCPEGHFEWLVMPFGLKNASLIFERKMDQIFKKYDNFCSVYVDDILVHSKNKNEHRKHLEIVLKEFIDNGIVISRNKVELEKQDIEFLGMYIKSGSIQLQEHIAKKVLEFPDKLEDKKQLQAFLGLLNYARSFIPD

>Parm3-Chr5-07; Yendovirus; Prunus armeniaca 3;

LEELLQLAEQTRIIGEDPQLHWNKNKILAKLDIINPDLTIKTADMPCNLLDKQEFEQQIKELLNLKAIRPSKSRHRSAAFVVRKHSELKRGKARIVYNYRRLNDNTYEDSYKLPNKDELMNKIQESKYFSKFDCKSGFWQIRLAEESIHWTSFTCPEGHFEWLVMPFGLKNAPSIFQRKMDQIFKKYDNFCSVYVDDILVHSKNKNEHRKHLEIILKEFINNGIIISRNKVELEKQDIEFLGMYIKSGTIQLQEHIAKKVLEFPDKLEDKKQLQAFLSLLNYARSFIPD

>Parm3-Chr5-08; Yendovirus; Prunus armeniaca 3;

LEELLQLAEQTRIIGEDPQLHWNKNKILAKLDIINPDLTIKTADMPCNLLDKQEFEQQIKELLNLKAIRPSKSRHRSAAFVVRKHSELKRGKARIVYNYRRLNDNTYEDSYKLPNKDELMNKIQESKYFSKFDCKSGFWQIRLAEESIHWTSFTCPEGHFEWLVMPFGLKNAPSIFQRKMDQIFKKYDNFCSVYVDDILVHSKNKNEHRKHLEIILKEFINNGIIISRNKVELEKQDIEFLGMYIKSGTIQLQEHIAKKVLEFPDKLEDKKQLQAFLSLLNYARSFIPD

>Parm3-Chr6-01; Yendovirus; Prunus armeniaca 3;

LEELLQLAEQTRIIGEDPQLHWNKNKILAKLDIINPDLTIKTADMPCNLLDKQEFEQQIKELLNLKVIRPSKSRHRSAAFVVRKHSELKRGKARIVYNYRRLNDNTYEDSYKLPNKDELMNKIQESKYFSKFDCKSGFWQIRLAEESIHWTAFTCPEGHFEWLVMPFGLKNAPSIFQRKMDQIFKKYDKFCSVYVDDILVHSKNKNEHRKHLEIILKEFINNGIIISRNKVELEKQDIEFLGMYIKSGTIQLQEHIAKKVLEFLDKLEDKKQLQAFLGLLNYARSFIPD

>Parm3-Chr6-02; Florendovirus; Prunus armeniaca 3;

KLKNEICSKLPNAFWYRKQHVVCLPYIKTFNEKNIPTKARPIQMSQEIMEFCKKEIHELLQKKIIRKSKSPWLCPAFYVQKNAELERGVPRLVINYKPLNSVLEWIRYPIPNKRDLINRLEKAIVYSKFDMKSGFWQIQIDESDRYKTAFVTPFGHYEWNVMPFGLKNSPSEFQNIMNEIFNPYNHFSIVYIDDVLIFSESIEQHWKHLHKFLQIVKQNGLVVSAKKIKLFQTNIRFLGFNICQSQISPIDRVIQFADKFPDQIIDKSQLQRFLGSLNYV

>Parm3-Chr7-01; Yendovirus; Prunus armeniaca 3;

IIGEDPQLHWNKNKILAKLDIINPDLTIKTIDMPCSLLDKQEFEEQIKELLNLKVIRPLKSRHRSAVFVVIKHSELKRCKARIVYNYRRLNDNTYEDSYKLPNKDELMNKIQESKYFSKFDCKSGFRQIRLAEESIQWTAFTCPKGHFEWLVMPFGLKNAPSIFQRKMDQIFKKYDNFCSVYVDDILVHSKNKNEHRKHLEIILKEFMNNGIVISRNKVELGKQDIEFLCMYIKSGTIQLQEHIAKKVLEFPDKLEDKKQLQAFLGLLNYSRSFIPD

>Parm3-Chr7-02; Florendovirus; Prunus armeniaca 3;

KLIADVCSDLPTAFWHRKNHIVSLPYIKNFSEKNIPTKARPIQMNPEILEFCKNEINDLLQKQIIRRSKSPWSCPAFYVQKNAELERGSPRLVINYKPLNTVLKWIRYPIPNKRDLINRLSKAVVFSKFDLKSGFWQIQITESDRYKTAFVTPFGHYEWNVMPFGLKNAPSEFQNIMNEIFNPYSQFSIVYIDDVLIFSESIEQHWKHLHKFLHVIKANGLVVSAKKIKLFQMHVRFLGFNISQSQIQPIDRSIQFADKFPDQITDKNQLQRFLGSLNY

>Parm3-Chr8-01; Yendovirus; Prunus armeniaca 3;

LEELLQLAEQTRIIGEDPQLHWNKNKILAKLDIINPDLTIKTADMPCNLLDKQEFEQQIKELLNLKVIRPSKSRHRSTAFVVRKHSELKRGKARIVYNYRRLNDNTYEDSYKLPNKDELMNKIQESKYFSKFDCKSXFWQIRLAEESIHWTSFTCPEGHFEWLVMPFGLKNAPSIFQRKMDQIFKKYDKFCSVYVDDILVHSKNKNEHRKHLEIILKEFIDNGIVISRNKVELEKQDIEFLGMYIKSGTIQLQEHIAKKVLEFPDKLEDKKQLQAFLGLLNYARSFI

>Parm4-Chr1-01; Florendovirus; Prunus armeniaca 4;

KLIADVCSDLPTAFWHRKNHMVSLPYIKHFSEKNIPTKARPIQMNPEMLEFCKTEINDLLQKRIIRRSKSPWSCPAFYVQKNAELERGSPRLVINYKPLNTVLEWIRYPIPNKRDLINRLSKAVVFSKFDLKSGFWQIQITESDRYKTAFVTPFGHYEWNVMPFGLKNAPSEFQNIMNEIFNPYSQFSIVYIDDVLIFSESIEQHWKHLHKFLHVIKTNGLVVSAKKIKLFQMHVRFLGFNINQSQIQPIDRSIQFADKFPDQITDKNQLQRFLGSLNY

>Parm4-Chr1-02; Florendovirus; Prunus armeniaca 4;

KLIADVCSDLPTAFWHRKNHMVSLPYIKHFSEKNIPTKARPIQMNPEMLEFCKTEINDLLQKRIIRRSKSPWSCPAFYVQKNAELERGSPRLVINYKPLNTVLEWIRYPIPNKRDLINRLSKAVVFSKFDLKSGFWQIQITESDRYKTAFVTPFGHYEWNVMPFGLKNAPSEFQNIMNEIFNPYSQFSIVYIDDVLIFSESIEQHWKHLHKFLHVIKTNGLVVSAKKIKLFQMHVRFLGFNINQSQIQPIDRSIQFADKFPDQITDKNQLQRFLGSLNY

>Parm4-Chr1-03; Florendovirus; Prunus armeniaca 4;

KLKNEICSELPNAFWYRKQHVVCLPYIKTFNEKNIPTKARPIQMSQEIMEFCKKEIHELLQKKIIRKSKSPWSCPAFYVQKNAELERGVPRLVINYKPLNSVLEWIRYPIPNKRDLINRLEKAVVYSKFDMKSGFWQIQIDKSDRYKTAFVTPFGHYEWNVMPFGLKNAHSKFQNIMNEIFNPYSHFSIVYIDDVLIFSESIEQHWKHLHKFLQIVKRNGLVVSAKKIKLFQTNIRFLGFNICQSQISPIDRVIQFANKFPDQILDKSQLQRFLGSLNYVSDF

>Parm4-Chr1-04; Florendovirus; Prunus armeniaca 4;

KLKNEICSELPNVFWYRKQHVVCLPYIKTFSEKNIPTKARPIQMSREIMEFCKKEIHELLEKKIIRKSKSPWSCPAFYVQKNAELKRGVPRLVINYKPLNSVLEWIRYPIPNKRDLFNRLERAVVYSKFDMKSCFWQIQIDESDRYKTAFVTPFGHYEWNVMPFGLKNAPSEFQNIMNEIFNPYSHFSIVYIDDVLIFSESIEQHWKHLHKFLQIIKQNGLVVSAKKIKLFQTNIRFLGFNICQSQISPIDRVIQFADKFPDQILDKSQLQRFLGSLNYVSDF

>Parm4-Chr1-05; Yendovirus; Prunus armeniaca 4;

LEELLQLAEQTRIIGEDPQLHWNKNKILAKLDIINPDLTIKTADMPCNLLDKQEFEQQIKELLNLKVIRPSKSRHRSAAFVVRKHSELKRGKARIVYNYRRLNDNTYEDSYKLPNKDELMNKIQESKYFSKFDCKSGFWQIRLAEESIHWTAFTCPEGHFEWLVMPFGLKNAPSIFQRKMDQIFKKYDKFCSVYVDDILVHSKNKNEHRKHLEIILKEFINNGIIISRNKVELEKQDIEFLGMYIKSGTIQLQEHIAKKVLEFPDKLEDKKQLQAFLGLLNYARSFIP

>Parm4-Chr2-01; Yendovirus; Prunus armeniaca 4;

LEELLQLAEQTRIIGEDPQLHWNKNKILAKLDIINPDLTIKTADMPCNLLDKQEFEQQIKELLNLKVIRPSKSRHRSAAFVVRKHSELKRGKARIVYNYRRLNDNTYEDSYKLPNKDELMNKIQESKYFSKFDCKSGFWQIRLAEESIHWTAFTCPEGHFEWLVMPFGLKNAPSIFQRKMDQIFKKYDKFCSVYVDDILVHSKNKNEHRKHLEIILKEFINNGIIISRNKVELEKQDIEFLGMYIKSGTIQLQEHIAKKVLEFPDKLEDKKQLQAFLGLLNYARSFIPD

>Parm4-Chr2-02; Florendovirus; Prunus armeniaca 4;

KLKNEICSELPNVFWYRKQHVVCLPYIKTFNEKNIPTKAMPIQMSQEIMEFCKKEIHELLQKKIIRKSKSPWSCPAFYVQKNVELERGVPRLVINYKPLNSVLEWIRYPIPNKRDLINRLEKAVVYSKFDMKSGFWQIQIDESDIYKTAFVTPFGHYEWNVMPFGLKNSPSEFQNIMNEIFNPYSHFSIVYIDDVLIFSESIEQHWKHLHKFLQIVKQNGLVVSAKKIKLFQTNIRFLGFNICQSQISPIDRVIQFADKFPDQILDKSQLQRFLGSLNYVSDF

>Parm4-Chr2-03; Yendovirus; Prunus armeniaca 4;

IIGEDPQLHWNKNKILAKLDIINPDLTIKTADMPCNLLDKQEFEQQIKELLNLKVIRPSKSRHRSAAFVVRKYSELKRGKARIVYNYRRLNDNTYEDSYKLPNKDELMNKIQESKYFSKFDCKSGFWQIRLAEESIHWTAFTCPEGHFEWLVMPFGLKNAPSIFQRKMDQIFKKYDKFCSVYVDDILVHSKNKNEHRKHLEIILKEFINNGIIISRNKVELEKQDIEFLGMYIKSGTIQLQEHIAKKVLEFPDKLEDKKQLQAFLGLLNYARSFIP

>Parm4-Chr2-04; Yendovirus; Prunus armeniaca 4;

HWNKNKILAKLDIINPYSTIKTADMPCNLLDKQKFXQQIKELLNIVIXPSKSRHRSTAFVVRKHSELKRGKARIVYNYKRXNDNTYEDSYKLPNKDELIMNKIEESKYSSKLDCKSRFCEIRLPEXSIQXTTFTCTXAYFEWLVRPFGLKNAPSIFXRKMNXIVKKYDNFGSIYVDDILVCSKNKNEHRKHXEIILKEFIDNGIVISMNKVELEKQGIXFLGMYIKSETIQLQEHIGKKVLXFPDKXEDKKXLQAFLGLLNYARSFI

>Parm4-Chr3-01; Yendovirus; Prunus armeniaca 4;

LEELLQLAEQTRIIGEDPQLHWNKNKILAKLDIINPDLTIKTADMPCNLLDKQEFEQQIKELLNLKVIRPSKSKHRSAAFVVRKHSELKRGKARIVYNYRRLNDNTYEDSYKLPNKDELMNKIQESKYFSKFDCKSGFWQIRLAEESIHWTAFTCPEGHFEWLVMPFGLKNALSIFQRKMDQIFKKYDKFCSVYVDDKLVHSKNKNEHRKHLEIILKEFIDNGIIISRNKVELEKQDIEFLGMYIKLGTIQLQEHIAKKVFEFPDKLEDKKQLQAFLGLLNYARSFIP

>Parm4-Chr3-02; Florendovirus; Prunus armeniaca 4;

KLKNEICSELPNAFWYRKQHVVCLPYIKTFSEKNIPTKARPIQMSQEIMEFCKKEIHELLEKKIIRKSKSPWSCPAFYVHKNAELERGVPRLVINYKPLNFVLEWIRYPIPNKRDLINRLEKAVVYSKFDMKSGFWQIQIHESDRYKTAFVTPFGHYEWNVMPFGLKNAPSEFQNIMNEIFNPYSHFSIVYIDDVLIFSESIEQHWKHLHKFLQIVKQNGLVVSAKKIKLFQTNIRFLGFNICQSQISPIDRVIQFADKFLDQILDKSQLQRFLGSL

>Parm4-Chr4-01; Yendovirus; Prunus armeniaca 4;

HWNKNKILAKLDIINQDLTIKIADMPCNLLDKQEFEQXIKELLNLKVIRPSKSRYRSTAFVVRKHSELKRGKARIVYNYRRLNDNTYEDRYKLPNKDELMNKIQESKYFSKFDCKTEFWQIRLAEESIRWTTFTCPKGHFEXLVMPFGLKNAPLIFQRKMDQIFKKYDNFCSVYVDDILVHSKNKNEHKKHLEIILIEFIDNGIVISRNKGELEKQDIEFLGMYIKXGTIQLQEHVSKKVLEFLDKLEDKKQLQTFLGLLNYSRCFIPD

>Parm4-Chr4-02; Florendovirus; Prunus armeniaca 4;

vKLKNEICSELPNAFWYRKQHVVCLPYIKTFSEKNIPTKARPIQMSQEIMEFCKKEIHELLQKKIIRKSKSPWYCPAFYVQKNAELERGVPRLVINYKPLNSVLEWIRYPIPNKRDLINRLGKAVVYSKFDMKSGFWQIQIHESDRYKTAFVTPFGHYEWNVMPFGLKNAPSEFQNIMNEIFNPYSHFSIVYIDDVLIFSESIEQHWKHLHKFLQIVKQNGLVVSAKKIKLFQTNIRFLGFNIHQSQISPIDRVVQFADKFPDQILDKSQLQRFLGSLNYV

>Parm4-Chr5-01; Yendovirus; Prunus armeniaca 4;

LEELLQLAEQTRIIGEDPQLHWNKNKILAKLNIINPDLTIKTEDMPCNLLDKQEFEQQIKELLNLKVIRPSKSRHRSAAFIVRKHSELKRGKARIMYNYRRLNDNTYEDSYKLPNKDELMNKIQESKYFSKFDCKSGFWQIRLAKESIHWTAFTCPEGHFEWLVMPFGLKNAPSIFQRKMDQIFKKYDKFCSVYVDDILVHGKNKNEHRKHLEIILKEFIDNGIVISRNKVELEKQDIEFLGMYIKSGTIQLQEHMAKKVLEFLDKLEDKKQLQAFLGLLNYARSFI

>Parm4-Chr5-02; Yendovirus; Prunus armeniaca 4;

LEELLQLAEPTRIIGEDPQLHWNTNKILAKLDIINPDLTIKTANMPCSLLDKQEFEEQIKELLNLKVIRPSKSRHRSAAFVVIKHSXLKRGKARIVYNYRRLNDNTYEDSYKLPNKDELMNKIQESKYFSEFDCKXGFWQIRLAEESIQWTTFTCPKGHFEWLVMPFGLKNAPSIFQRKMDQIFNKYDNFCSVYVDDILVHSKNKNEHRKHLEIILKEFINNGIVISRNKLELEKQDIEFLCMYIKSGTIQLQEHIAKKVLEFPDKLEDKKQLQAFLGLLNYSRSFIPD

>Parm4-Chr5-03; Yendovirus; Prunus armeniaca 4;

LEELLQLAEPTRIIGEDPQLHWNKNKILAKLDIINPYLTIKTADMPCSLLDKQEFEEQIKELLNLKVIRPLKSXHRSAAFVVIKHSELKRGKAKIVYNYRRLNDNTYEDSYKLPNKDELMNKIQESKYFSKFDCKSGFWQIRLAEESIQWTAFTCPKGHFEWLVMPFGLKNAPSIFQRKMDXIFKNYDNFCSVYVDDILVHSKNKNEHRKHIEIILKEFINNGIVISRNKVELEKQDIEFLCMYIKSGTIQLQEHIAKKVLEFPDKLEDKKQLQAFLGLLNYSRSFIPD

>Parm4-Chr6-01; Florendovirus; Prunus armeniaca 4;

KLIADVCSDFPTAFWHRKNHIVSLPYIQNFSEKNIPTKARPIQMSPEMLEFCKNEINDLLQKNIIRRSKSPWSCSAFYVQKNAELERGSPRLVINYKPLNNVLEWIRYPIPNKRDLINRLSKAIIFSKFDLKSGFWQIQITESDRYKTAFVTPFGHYEWNVMPFGLKNAPSEFQNIMNEIFNSYSQFAIVYIDDVLIFSESIEQHWKHLYKFLHVIKANGLVVSAKKIKLFQMHIRFLGFNISQSQIQPIDRSIQFADKFPDQIIDKNQLQRFLGSLNY

>Parm4-Chr6-02; Yendovirus; Prunus armeniaca 4;

LEELLQLVEQTRIIGEDPQLHWNKNKILPKLNIINQDLTIKTTDMPCNLLDKXEFXQQIKELLNLKVIRPSKSRHRSAAFVVRKHLELKRGKARIVYNYRRLNDNTYEDSYKLLNKDELMNKIQESKYFSKFDCKLGFWQIRLAEESIQWTTFTCPXGHFEWLVMPFGLKNAPLIFQRKMDQIFKKYDNFCSVYVDDILVHSKNKNEHRKHIEIILKEFIDNGIDISRNKVELEKQDIEFLGMYIKSGTIQLQEHIAKKVLEFLDKLEDKKQLQAFLGLLNYARSFIP

>Parm4-Chr6-03; Florendovirus; Prunus armeniaca 4;

KLKNEICSELPNAFWYRKQHVVCLPYIKTFNEKNIPTKARPIQMSQEMMEFCKKEIHELLQKKIIRKSKSPWSCPAFYVQKNAELERGVPRLVINYKPLNSVLEWIRYPIPNKRDLINRLEKAVVYSKFDMKSGFWQIQIDESDRYKTAFVTPFGHYEWNVMPFGLKNAPSEFQNIMNEIFNPYSHFSIVYIDDVLIFSESIEQHWKHLHKFLQIIKQNGLVVSAKKIKLFQTNIRFLGFNICQSQISPIDRVIQFADKFLDQILDKSQLQRFLGSLNYV

>Parm4-Chr7-01; Yendovirus; Prunus armeniaca 4;

LEELLQLAEQTRIIGEDPQLHWNKNKILAKLDIINPDLTIKTADMPCNLLDKQEFEQQIKELLNLKVIRPSKSRHRSTAFVVRKHSELKRGKARIVYNYRRLNDNTYEDSYKLPNKDELMNKIQESKYFSKFDCKSXFWQIRLAEESIHWTSFTCPEGHFEWLVMPFGLKNAPSIFQRKMDQIFKKYDKFCSVYVDDILVHSKNKNEHRKHLEIILKEFIDNGIVISRNKVELEKQDIEFLGMYIKSGTIQLQEHIAKKVLEFPDKLEDKKQLQAFLGLLNYARSFI

>Parm4-Chr7-02; Florendovirus; Prunus armeniaca 4;

KLVTDVCSDLPTAFWHRKNHIVSLPYIQNFSEKNIPTKARPIQMSPEMLEFCKNEINDLLQKNIIRRSKSPWSCSAFYVQKNAELERGSPRLVINYKPLNTVLEWIRYPIPNKRDLINRLSKAIIFSKFDLKSGFWQIQIAESDRYKTAFVTPFGHYEWNVMPFGLKNAPSEFQNIMNEIFNSYSQFAIVYIDDVLIFSESIEQHWKHLYKFLHVIKANGLVVSAKKIKLFQMHIRFLGFNISQSQIQPIDRSIQFADKFPDQIIDKNQLQRFLGSLNY

>Pavi1-Chr2-01; Yendovirus; Prunus avium 1;

LEELLQLVEQTRIIGEDPQLHWNKNKILAKLDIINPDLTIKTANMPCNLLDKQEFEQQIKELLNLKVIRPSKSRHRSPLXLENIQNLREVKLEXCTIIGDXMIILMKIVINYQIKMNLXIKFKKVNILVNLIVNQDFGKLDQQKSQSNGQHLLVQKDILNGLXCHLDXKNATSIFQXKMDQIFKKYDTFFSVYVDDMLVHSKNKNEHRKHLEIILKEFIDNGIVISRNKVELEKQDIEFLSMYIKSGTIQLQEHIAKKVLEFLDKLEDKKQLQAFLGLLNYARSFIPD

>Pavi1-Chr4-01; Yendovirus; Prunus avium 1;

NKNNILAKLDIINPDLTIKTVDMSCNLLYKQEFEQQIKELLNLNAIRSSKSRHRSATFVFRKHSKLKRGKARIVYNYRRLNDNTCEDSYKLPNKAELMNKIQESKYFSKFDCNSGFWQIRLAEESIQWTTFTCPEGHFKWLVMPFGLKNAPLIFQRKLDQIFKKYDNFCSEYVDDILVHSKNKNEISKHLEIILKEFIDNGIVINRNKVELEKXDMEFLGMYIKSGTIQLQEHIAQKVLKFPDKLEDKKQLQAFLGLLNYARSFIPD

>Pavi1-Chr5-01; Yendovirus; Prunus avium 1;

HWNKILAKLDIINPDLTIKTTYMPYNLLDKQEFEXXIKELLNLKVIRPSKFKHRSVAFVVRKNLEFKRGKARIVYNHRRLNDATYEDSXKLPNKDELINIIQESKYFSKFDCKSGFWQIRLAEESIQWTSFTCPKGHFEWLVMPFGLKNAKSIFQRKMDQIFKKYDNFCSVYVDDILVHSKNKNEHRKHLXIILKEFIDNGIVISRNKVKLEKQDIKFLGMYIKSGTIQLQEHIAKKALXFPDKLEDKKQLXAFLGLLNYARSFIPD

>Pavi1-Chr5-02; Yendovirus; Prunus avium 1;

AKLDIINPYLTIKTTDMPCNLLDKQEFEQHIKELLNLKVIRPSKSRHISTAFVVKKHSELKRGKARIVYDYRRLNDNTYEGSYKPPNKDELMNKIQESKYFSKFDCKSGFWQIRLAEESIQWTTFACLEGHFEWLVMSFGLKNAPSIFQRKMDQIFKKCDNFCSVYVDDILVPSKNKNEHRKHLEIILKEFINNGIIISRNKVELEKQDIEFLGMYIKPXTIQLQEHIAKKVLEFPDKLEDKKQLQAFLGLLNYARSFIP

>Pavi2-Chr1-01; Yendovirus; Prunus avium 2;

LEKLLQLAEQTRIIGEDPQLHXSKNKILAKLDIINPDLTIKTADMPCNLLDKQEFEQQIKELLILKVIRPSKSRHRSAAFVVRKHSKLKRGKARIVYNYRRLNDNTYEDSYKLPNKDELMNKIQESKYSSKFDCKSGFWQIRLAEESIQWTGFTCPEGHFEWLVMPFGLKNAPSIFQRKMDQIFKKYDNFCSVYVDDILVHSENKNEHRKHLEIILEEFIDNGIVISRNKVELEKQDIEFLGMYIKSGTIQLQEHITKKVLEFPDKLEDKKQLQAFLGLLNYARSFIPD

>Pavi2-Chr2-01; Yendovirus; Prunus avium 2;

LEELLQLVEQTRIIGEDPQLHWNKNKILAKLDIINPDLTIKTANMPCNLLDKQEFEQQIKELLNLKVIRPSKSRHRSPLXLENIQNLREVKLEXCTIIGDXMIILMKIVINYQIKMNLXIKFKKVNILVNLIVNQDFGKLDQQKSQSNGQHLLVQKDILNGLXCHLDXKNATSIFQXKMDQIFKKYDTFFSVYVDDMLVHSKNKNEHRKHLEIILKEFIDNGIVISRNKVELEKQDIEFLSMYIKSGTIQLQEHIAKKVLEFLDKLEDKKQLQAFLGLLNYARSFIPD

>Pavi2-Chr4-01; Yendovirus; Prunus avium 2;

NKNNILAKLDIINPDLTIKTVDMSCNLLYKQEFEQQIKELLNLNAIRSSKSRHRSATFVFRKHSKLKRGKARIVYNYRRLNDNTCEDSYKLPNKAELMNKIQESKYFSKFDCNSGFWQIRLAEESIQWTTFTCPEGHFKWLVMPFGLKNAPLIFQRKLDQIFKKYDNFCSEYVDDILVHSKNKNEISKHLEIILKEFIDNGIVINRNKVELEKXDMEFLGMYIKSGTIQLQEHIAQKVLKFPDKLEDKKQLQAFLGLLNYARSFIPD

>Pavi2-Chr4-02; Yendovirus; Prunus avium 2;

LEELLQLAEQTRIIGEDPQLHWNKNKILAKLDIINPDLTIKTADMPCNLLGKQEFEQQIKELLNLKVIRPSKSRHRSAAFVVRKYSELKRGKARIVYNYRRLNNNTYEDSYKLPNKDELMNKIQESKYFSKFDCKSGFWQIRLAEESIQWTAFTCPEGHFEWLVMPFGLKNAPSIFQRKMDXIFKKYDNFCSVYVDDILVHSKNKDEHIKHLEIILKEFIDNGIVISRNKVELEKQDIEFLGMYIKSGTIQLQXHIAKKVLEFPDKLEDKKQLQAFLGLLNYARSFISD

>Pavi2-Chr5-01; Yendovirus; Prunus avium 2;

HWNKILAKLDIINPDLTIKTTYMHYNLLDKQEFEXXIKELLNLKVIRPSKFKHRSVAFVVRKNLEFKRGKARIVYNHRRLNDATYEDSXKLPNKDELINIIQESKYFSKFDCKSGFWQIRLAEESIQWTSFTCPKGHFEWLVMPFGLKNAKSIFQRXMDQIFKKYDNFCSVYVDDILVHSKNKNEHRKHLXIILKEFIDNGIVISRNKVKLEKQDIKFLGMYIKSGTIQLQEHIAKKALXFPDKLEDKKQLXAFLGLLNYARSFIPD

>Pavi2-Chr6-01; Yendovirus; Prunus avium 2;

LEELLQLAEQTRIIGEDPQLHWNKNKILAKLDIINPDLTIKTADMPCNLLDKQEFEQQIKELLNLKVIRPSKSRHRSAAFVVRKHSELKRGKARIVYNYRRLNDNTYEDSYKLPNKDELMNKIQESKYFSKFDCKLGFWQIRLAEESIQWTAFTCPEGHFEWLVMPFGLKNAPSIFQRKMDQIFKKYDNFCSVYVDDILVHGKNKNEHRKHLEIILKEFIDNGIVISRNKVELEKQDIEFLGMYIKSGTIQLQEHITKKVLEFPDKLEDKKQLQAFLGLLNYARSFIPD

>Pavi2-Chr6-02; Yendovirus; Prunus avium 2;

LEELLQLTEQTRIIGEDPQLHGNKNKILAKLDIINPYLTIKTAYMPCNLLDKQEFEQQIKELLNLKVIRPSKSRHRLATFVVRKHSELKRGKAKIVYNYRRLNDNTYVDSYKLPNKVQESEYSSKFDCKSGFWQIKLAEESIQWTSFTCPEGHFEWLVMPFELKNAPLIFQRSFKERLIKSLRNIIIFVVYMXMIYLFMVKIKMNIENTXKLFXKNLSIMVXSXVGLKXSLKNKTYNFXVCILSQELYNYKNIXLKSVRISRXIRRLKKKLQAFLELLNYAGSFIPD

>Pavi2-Chr7-01; Yendovirus; Prunus avium 2;

HWNKNKILAKLDIINPDLTIKTADMPCNLLDKQEFEQQIKELLNLKVIRPSKSRHRSATFVVRKHSKLKRGKARIVYNYRRLNDNTYEDSYKLPNKDELMNKIQESKYFSKFDCKSGFWQIRLAKESIQWTAFTCPEGHFEWLVMPFGLKNAPSIFQRKMDQIFKKYDNFCSVYVDDILVDSKDKIEHRKHLXIILKEFIDNGIVISRNKVELEKQDIEFLGMNIKSGTIQLQEHIAKKVLEFPDKLEDKKQLQAFLGLLNYARSFIPD

>Pavi2-Chr7-02; Yendovirus; Prunus avium 2;

AKLDIINPYLTIKTTDMPCNLLDKQEFEQHIKELLNLKVIRPSKSRHISTAFVVKKHSELKRGKARIVYDYRRLNDNTYEGSYKPPNKDELMNKIQESKYFSKFDCKSGFWQIRLAEESIQWTTFACLEGHFEWLVMSFGLKNAPSIFQRKMDQIFKKCDNFCSVYVDDILVPSKNKNEHRKHLEIILKEFINNGIIISRNKVELEKQDIEFLGMYIKPXTIQLQEHIAKKVLEFPDKLEDKKQLQAFLGLLNYARSFIP

>Pbri1-Chr1-01; Yendovirus; Prunus brigantina 1;

LEELLQLAXQTRIIGEDPQLHWNKNKILAKLDIINPDLTIKTADMPCNLLDKQEFEQQIKELLNLKVIRPSKSRHRSAAFVVRKHSELKRGKARIVYNYRRLNDNTYEDSYKLPNKDELMNKIQESKYFSKFDCKSGFWQIRLAEESIHWTTFTCPGGHFEWLVMPFGLKNAPSIFQRKMDQIFKKYDKFCSVYVDDILVHSKNKNEHRKHLEIILKEFIDNGIVISRNKVELEKQDIEFLGMYIKSGTIQLQEHITKKVLEFPDKLEDKKQLQAFLGLLNYARNFIPD

>Pbri1-Chr1-02; Yendovirus; Prunus brigantina 1;

LEELLQLAEQTRIIGEDPQLHWNKNKILAKLDIINPDLTIKTADMPCNLLDKQEFEQQIKELLNLKVIRPSKSRHRSAAFVVRKHSELKRGKARIVYNYRRLNDNTYEDSYKLPNKDELMNKIQESKYFSKFDCKSGFWQIRLAEESIHWTAFTCPEGHFEWLVMPFGLKNAPSIFQRKMDQIFKKYDNFCSVYVDDILVHSKNKNEHRKHLEIILKEFIDNGIIISRNKVELEKQDIEFLGMYIKSGTIQLQEHITKKVLEFPDKLEDKKQLQAFLGLLNYARNFIPD

>Pbri1-Chr1-03; Yendovirus; Prunus brigantina 1;

LEELLQLAEQTRIIGEDPQLHWNKNKILAKLDIINPDLTIKIADMPCNLLDKQEFEQQIKELLNLKVIRPSKSRHRSAAFVVRKHSELKRGKTRIVYNYRRLNDNTYEDSYKLPNKDELMNKIQESKYFSKFDCKSGFWQIRLAEESIHWTAFTCPEGHFEWLVMPFGLKNAPSIFQRKMDQIFKKYDNFCSVYVDDILVHSKNKNEHRKHLEIILKEFIDNGIIISRNKVELEKQDIEFLGMYIKSGTIQLQEHITKKVLEFPDKLEDKKQLQAFLGLLNYARNFIPD

>Pbri1-Chr1-04; Florendovirus; Prunus brigantina 1;

EKLKNEICSDLPNAFWYRKQHVVCLPYIKTFSEKNIPTKARPIQMSQEIMEFCKNEINELLQKKIIRRSKSPWSCPAFYVQKNGELERGVPRLVINYKPLNSVLEWIRYPIPNKRDLINRLGRAVIFSKFDLKSGFWQIQIDESDRYKTAFVTPFGHYEWNVMPFGLKNAPSEFQNIMNEIFNPYSHFSIVYIDDVLIFSESIEQHWKHLHKFLQVVKQNGLVVSAKKIKLFQTKVRFLGFNICQSQITPIDRVIQFADKFPDQILDKSQLQRFLGSLNYV

>Pbri1-Chr1-05; Florendovirus; Prunus brigantina 1;

EKLKIEICSELPNAFWYRKQHVVCLPYIKTFSEKNIPTKARPIQMSQEIMEFCKNEINELLQKKIIRRSKSPWSCPAFYVQKNAELERGVPRLVINYKPLNSVLEWIRYPIPNKRDLINRLGKAVIFSKFDMKSGFWQIQIDESDRYKTAFVTPFGHYEWNVMPFGLKNAPSEFQNIMNEIFNPYSHFSIVYIDDVLIFSESIEQHWKHLHKFFQVIKQNGLVVSAKKIKLFQTKVRFLGFNICQSQITPIDRVIQFADKFPDQILDKSQLHRFLGSLNYV

>Pbri1-Chr1-06; Florendovirus; Prunus brigantina 1;

DKLKNEICSDLPNAFWYRKQHVVCLPYIKTFSEKNIPTKARPIQMSQEIMEFCKNEINELLQKKIIRRSKSPWSCPAFYVQKNAELERGVPRLVINYKPLNSVLEWIRYPIPNKRDLINRLGRAVIFSKFDMKSGFWQIQIDESDRYKTAFVTPFGHYEWNVMPFGLKNAPSEFQNIMNEIFNPYSHFSIVYIDDVLIFSESIEQHWKHLHKFLQVVKQNGLVVSAKKIKLFQTKVRFLGFNICQSQITPIDRVIQFADKFPDQILDKSQLQRFLGSLNYV

>Pbri1-Chr1-07; Yendovirus; Prunus brigantina 1;

LEELLQLAEQTRIIGEDPQLHWNKNKILAKLDIINPDLTIKTADMPCNLLDKQEFEQQIKELLNLKVIRPSKSRHRSAASVVRKHSELKRGKARIVYNYRRLNDNTYEDSYKLPNKDELMNKIQESKYFSKFDCKSGFWQIXLAEESIHWTAFTCPERHFEWLVMPFGLKNAPSIFQRKMDQIFKKYDNFCSVYVDDILVHSKNKNEHRKHLEIILKEFIDNGIIISRNKVELEKQDIEFLGMYIKSGTIQLQEHITKKVLEFPDKLEDKKQLQAFLGLLNYARNFIPD

>Pbri1-Chr1-08; Florendovirus; Prunus brigantina 1;

DKLKNEICSDLPNAFWYRKQHVVCLPYIKTFSEKNIPTKARPIQMSQEIMEFCKNEIHELLQKKIIRKSKSPWSCPAFYVQKNAELERGVPRLVINYKPLNSVLEWIRYPIPNKRDLINRLGKAVVFSKFDMKSGFWQIQIEESDRYKTAFVTPFGHYEWNVMPFGLKNAPSEFQNIMNEIFNPYSHFSIVYIDDVLIFSESIEQHWKHLHKFLQIVKQNGLVVSAKKIKLFQTKIRFLGFHICQSQIAPIDRVIQFADKFPDQILDKSQLQRFLGSLNYV

>Pbri1-Chr1-09; Florendovirus; Prunus brigantina 1;

QRLISEVCSDLPTAFWHRKNHVVCLPYIKNFSEKHIPTKARPIQMNHEIMEFCKTEIDDLLQKNIIRKSKSPWSCPAFYVQKNAELERGSPRLVINYKPLNSVLEWIRYPIPNKRDLIKRLDKALVFSKFDMKSGFWQIQINESDRYKTAFVTPFGHYEWNVMPFGLKNAPSEFQNIMNEIFNSYSHFSIVYIDDVLIFSESIEQHWKHLHKFLHIIKSNGLVVSAKKIKLFQMHIRFLGYNISQSQIHPIDRVIHFADKFPDQITDKNQLQRFLGSLNYVSDF

>Pbri1-Chr1-10; Florendovirus; Prunus brigantina 1;

QRLISEVCSDLPTAFWHRKNHVVCLPYIKNFSEKHIPTKARPIQMNHEIMEFCKTEIDDLLQKNIIRKSKSPWSCPAFYVQKNAELERGSPRLVINYKPLNSVLEWIRYPIPNKRDLIKRLDKALVFSKFDMKSGFWQIQINESDRYKTAFVTPFGHYEWNVMSFGLKNAPSEFQNIMNEIFNSYSHFSIVYIDDVLIFSESIEQHWKHLHKFLHIIKSNGLVVSAKKIKLFQMHIRFLGYNISQSQIHPIDRVIHFADKFPDQITDKNQLQRFLGSLNYVSDF

>Pbri1-Chr1-11; Yendovirus; Prunus brigantina 1;

LEELLQLAEQTRIIGEDPQLHWNKNKILAKLDIFNPDLTIKTADMPCNLLDKQEFEQQIKELLNLKVIRPSKSRHRSAAFVVRKHSELKRGKARIVYNYRRLNDNTYEDSYKLPNKDELMNKIQESKYFSKFDCKSGFWQIRLAEESIHWTAFTCPEGHFEWLVMPFGLKNAPSIFQRKMDQIFKKYDKFCSVYVDDILVHSKNKNEHRKHLEIILKEFIDNGIVISRNKVELEKQDIEFLGMYIKSGTIQLQEHITKKVLEFPDKLEDKKQLQAFLGLLNYARSFIPD

>Pbri1-Chr2-01; Florendovirus; Prunus brigantina 1;

DKLKNEICSDLPNAFWYRKQHVVCLPYIKTFSEKNIPTKARPIQMSQEIMEFCKKEINELLQKKIIRKSKSPWSCPAFYVQKNAELERGVPRLVINYKPLNSVLEWIRYPIPNKRDLINRLGKAVIFSKFDMKSGFWQIQIDESDRYKTAFVTPFGHYEWNVMPFGLKNAPSEFQNIMNEIFNPYSHFSIVYIDDVLIFSESIGQHWKHLHKFLQVVKQNGLVVSAKKIKLFQTKVRFLGFNICQSQIAPIDRVIQFADKFADQILDKSQLQRFLGSLNYVSDF

>Pbri1-Chr2-02; Florendovirus; Prunus brigantina 1;

DKLKNEICSELPNAFWYRKQHVVCLPYIKTFSEKNIPTKARPIQMSQEVMEFCKNEIHELLQKKIIRKSKSSWSCPAFYVQKNAELERGVPRLVINYKPLNSVLEWIRYPIPNKRDLINRLGKAVVFSKFDMKSGFWQIQIDESDKYKTAFVTPFGHYEWNVMPFGLKNAPSEFQNIMNEIFNPYSHFSIVYIDDVLIFSKSIEQHWKHLHKFLQVVKQNGLVVSAKKIKLFQTKVRFLGFNICQSQFTPIDRVIQFADKFPDQILDKSQLQRFLGSLNYVSDF

>Pbri1-Chr2-03; Yendovirus; Prunus brigantina 1;

HWNKNKILAKLDIINPYSTIKTADMSCNLLDKQNFXQQIKELLNIVIXPSKSRHRSTAFVVRKHSELKRGKARIVYNYKRXNDNTYEDSYKLPNKDELMNKIEESKYSSKLDCKSRFCEIRLAEXSIQXTTFTCTEAYFEWLVRPFGLKNAPSIFQRKMNXIFKKYDNFGSIYVDDILVCSKNKNEHRKHXEIILKEFIDNGIVISMNKVELEKQGIXFLGMYIKLETIQLQEHIGKKVLXFPDKXKDKKQLQAFLGLLNYARSFYP

>Pbri1-Chr2-04; Florendovirus; Prunus brigantina 1;

QKLISEICSDLPTAFWHRKNHVVCLPYIKNFSEKNIPTKARPIQMNHEMMEFCKTEINDLLQKNIIRKSKSPWSCPAFYVQKNAELERGSPRLVINYKPLNSVLEWIRYPIPNKRDLIKRLDKAIIFSKFDMKSGFWQIQITESDRYKTAFVTPFGHYEWNVMPFGIKNAPSEFQNIMNEIFNAYSHFSIVYIDDVLIFSESIEQHWKHLHKFLHIIKSNGLVVSAKKIKLFQTNIRFLGYNISQSQIHPIDRVIHFADKFPDQITDKNQLQRFLGSLNYVSDF

>Pbri1-Chr2-05; Florendovirus; Prunus brigantina 1;

EKLKNEICSDLPNAFWYRKQHVVCLPYIKTFSEKNIPTKARPIQMSQEIMEFCKNEINELLQKKIIRRSKSPWSCPAFYVQKNAELERGVPRLVINYKPLNSVLEWIRYPIPNKRDLINRLGKAVIFSKFDMKSGFWQIQIDESDRYKTAFVTPFGHYEWNVMPFGLKNAPSEFQNIMNEIFNPYSHFSIVYIDDVLIFSESIEQHWKHLHKFFQVVKQNGLVVSAKKIKLFQTKVKFLGFNICQSQITPIDRVIQFADKFPDQILDKSQLQRFLGSLNYV

>Pbri1-Chr3-01; Florendovirus; Prunus brigantina 1;

DKLKNEICSDLPNAFWYRKQHVVCLPYIKTFSEKNIPTKARPIQMSQEIMEFCKNEINELLQKKIIRRSKSPWSCPAFYVQKNAELERGVPRLVINYKPLNSVLEWIRYPIPNKRDLINRLGRAVIFSKFDMKSGFWQIQIDESDRYKTAFVTPFGHYEWNVMPFVLKNAPSEFQNIMNEIFNPYSHFSIVYIDDVLIFSESIEQHWKHLHKFLQVVKQNGLVVSAKKIKLFQTKVRFLGFNICQSQITPIDXVIQFADKFPDQILDKSQLQRFLGSLNYV

>Pbri1-Chr3-02; Yendovirus; Prunus brigantina 1;

LEELLQLAEQTRIIGEDPQLHWNKNKILAKLDIINPDLTIKTADMPCNLLDKQEFEQQIKELLNLKVIRPSKSRHRSAAFVVRKHSELKRGKARIVYNYRRLNDNTYEDSYKLPNKDELMNKIQESKYFSKFDCKSGFWQIRLAEESIHWTAFTCPEGHFEWLVMPFGLKNAPSIFQRKMDQIFKKYDNFCSVYVDDILVHSKNKNEHRKHLEIILKEFIDNGIVISRNKVELEKQDIEFLGMYIKSGTIQLQEHITKKVLEFPDKLEDKKQLQAFLGLLNYARSFIPD

>Pbri1-Chr3-03; Florendovirus; Prunus brigantina 1;

QRLISEVCSDLPTAFWHRKNHVVCLPYIKNFSEKHIPTKARPIQMNHEIMEFCKTEIDDLLQKNIIRKSKSPWSCPAFYVQKNAELERGSPRLVINYKPLNAVLEWIRYPIPNKRDLIKRLDKAPVFSKFDMKSGFWQIQINESDRYKTAFVTPFGHYEWNVMPFGLKNAPSEFQNIMNEIFNSYSHFSIVYIDDVLIFSESIEQHWKHLHKFLHIIKSNGLVVSAKKIKLFQTNIRFLGYNISQSQIHPIDRVIHFADKFPDQITDKNQLQRFLGSLNYVSDF

>Pbri1-Chr3-04; Florendovirus; Prunus brigantina 1;

KRLISEVCSDLPTAFWHRKNHVVSLPYIKHFSKKNIPTKARPIQMNHEMMEFCKFEINDLLQKKIIRKSKSPWSCPAFYVQKNAELERGSPRLVINYKPLNTVLEWIRYPIPNKRDLIKRLDRAIIFSTFDMKSGFWQIQIHESDRYKTAFVTPFVHYEWNVMPFGLKNAPSEFQNIMNEIFNSYSHFSIVYIDDVLIFSESIEQHWKHLHKFLQIIKSNGLVVSAKKIKLFQTNIRFLGYNISQSQIHPIDRVIHFADIFPDQITDKNQLQRFLGSLNYVSDF

>Pbri1-Chr3-05; Yendovirus; Prunus brigantina 1;

LEELLQLAEQTRIIGEDPQLHWNKNKILAKLDIINPDLTIKTADMPCNLLDKQEFEQQIKELLNLKVIRPSKSRHRSAAFVVRKHSELKRGKARVVYNYRRLNDNTYEDSYKLPNKDELMNKIQESKYFSKFDCKSGFWQIRLAEESIHWTAFTCPEGHFEWLVMPFGLKNAPSIFQRKMDQIFKKYDKFCSVYVDDILVHSKNKNEHRKHLEIILKEFIDNGIVISRNKVELEKQDIEFLGMYIKSGTIQLQEHITKKVLEFPDKLEDKKQLQAFLGLLNYARSFIPD

>Pbri1-Chr3-06; Yendovirus; Prunus brigantina 1;

LEELLQLAEQTRIIGEDPQLHWNKNKILAKLDIINPDLTIKTANMPCNLLDKQEFEQQIKELLNLKVIRPSKSTHRSAAFVVRKHSELKRGKARIVYNYRRLNDNTYEDSYKLPNKDELMNKIQERKYFSKFDCKSGFWQIRLAEESIHWTAFTCPEGHFEWLVMPFGLKNAPSIFQRKMDQIFKKYDNFCSVYVDDILVHSKNKNEHRKHLEIILKEFIDNGIVISRNKVELEKQDIEFLGIYIKSGTIQLQEHITKKVLEFPDKLEDKKQLQAFLGLLNYARNFIPD

>Pbri1-Chr3-07; Yendovirus; Prunus brigantina 1;

LEELLQLAEQTRIIGEDPQLHWNKNKILAKLDIINPDLTIKTADMPCNLLDXQEFEQQIKELLNLKVIRPSKSRHRSAAFVVRKHSELKRGKARIVYNYRRLNDNTYEDSYKLPNKDELMNKIQESKHFSKFDCKSGFWQIRLAEESIHWTAFTCPEGHFEWLVMPFGLKNAPSIFQRKMDQIFKKYDKFCSVYVDDILVHSKNKNEHRKHLEIILKEFIDNGIVISRNKVELEKQDIEFLGMYIKSGTIQLQGHITKKVLEFPDKLEDKKQLQAFLGLLNYARSFIPD

>Pbri1-Chr3-08; Yendovirus; Prunus brigantina 1;

IIGEDPQLHWNKNKILAKLDIINPDLTIKTADMPCNLLDKQEFEQQIKELLNLKVIRPSKSRHRSAAFVVRKHSELKRGKARIVYNYRRLNDNTYEDSYKLPNKDELMNKIQESKHFSKFDCKSGFWQIRLAEESIHWTAFTCPEGHFEWLVMPFGLKNAPSIFQRKMDQIFKKYDKFCSAYVDDILVHSKNKNEHRKHLEIILKEFIDNGIVISRNKVELEKQDIEFLGMYIKSGTIQLQGHITKKSIRI

>Pbri1-Chr3-09; Yendovirus; Prunus brigantina 1;

LEELLQLAEQTRIIGEDPQLHWNKNKILAKLDIINPDLTIKTADMPCNLLDKQEFEQQIKELLNLKVIRPSKSRHRSAAFVVRKHSELKRGKARIVYNYRRLNDNTYEDSYKLPNKDELMNKIQESKYFSKFDCKSGFXQIRLAEESIHWTAFTCPEGHFEWLVMPFGLKNAPSIFQRKMDQIFKKYDKFCSVYVDDILVHSKNKNEHRKHLEIILKEFIDNGIVISRNKVELEKQDIEFLGMYIKSGTIQLQEHITKKVLEFPDKLEDKKQLQAFLGLLNYARNFIPD

>Pbri1-Chr3-10; Florendovirus; Prunus brigantina 1;

QRLISEVCSDLPTAFWHRKNHVVCLPYIKNFSEKHISTKARPIQMNHEIMEFCKTEINDLLQKNIIRKSKSPWSCPAFYVQKNAELERGSPRLVINYKPLNSVLEWIRYPIPNKRDLIKRLDKALVFSKFDMKSGFWQIQINESDRYKTAFVTPFGHYEWNVMPFGLKNAPSEFQNIMNEIFNSYSHFSIVYIDDVLIFSESVEQHWKHLHTFLHIIKSNGLVVSAKKIKLFQMNIRFLGYNISQSQIHPIDRVINFADKFPDQITDKNQLQRFLGSLNYVSDF

>Pbri1-Chr3-11; Florendovirus; Prunus brigantina 1;

DKLKNEICSDLPNAFWYRKQHVVCLPYIKTFSEKNIPTKARPIQMSQEIMEFCKNEINELLQKKIIRRSKSPWSCPAFYVQKNAELERGVPRLVINYKPLNSVLEWIRYPIPNKRDLINRLGRAVIFSKFDMKSGFWQIQIDESDRYKTAFVTPFGHYEWNVMPFGLKNAPSEFQNIMNEIFNPYSHFSIVYIDDVLIFSESIEQHWKHLHKFFQVVKQNGLVVSAKKIKLFQTKVRFLGFNICQSQITPIDRVIQFADKFPDQILDKNQLQRFLGSLNYV

>Pbri1-Chr3-12; Florendovirus; Prunus brigantina 1;

EKLKIEICSDLPNAFWYRKQHVVCLPYIKTFSEKNIPTKARPIQMSQEIMEFCKNEINELLQKKIIRRSKSPWSCPAFYVQKNAELERGVPRLVINYKPLNSVLEWIRYPIPNKRDLINRLGKAVIFSKFDMKSGFWQIQIDESDRYKTAFVTPFGHYEWNVMPFGLKNAPSEFQNIMNEIFNPYSHFSIVYIDDVLIFSESIEQHWKHLHKFFQVVKQNGLVVSAKKIKLFQTKVRFLGFNICQSQITPIDRVIQFADKFPDQILDKSQLQRFLGSLNYV

>Pbri1-Chr3-13; Florendovirus; Prunus brigantina 1;

DKLKNEICSDLPNAFWYRKQHVVCLPYIKTFSEKNIPTKARPIQMSQEIMEFCKNEINELLQKKIIRRSKSPWSCPAFYVQKNAELERGVPILVINYKPLNSVLEWIRYPIPNKRDLINRLGKAVIFSKFDMKSGFWQIQIDESDRYKTAFVTPFGHYEWNVMPFGLKNAPSEFQNIMNEIFNPYSHFSIVYIDDVLIFSESIEQHWKHLHKFFQVVKQNGLVVSAKKIKLFQTNVRFLGFNICQSQITPIDRVIQFADKFADQILDKSQLQRFLGSLNYV

>Pbri1-Chr3-14; Florendovirus; Prunus brigantina 1;

QKIISEVCSDLPTAFWYRKNHVVSLPYIKNFSEKNIPTKARPIQMNHETMEFCKSEINDLLQKNIIRKSKSPWSCPAFYVQKNAELERGSPRLVINYKPLNTVLEWIRYPIPNKRDLIKRLDKAIIFSKFDMKSGFWQIQIAESDKYKTAFVTPFGHYEWNVMPFGLKNAPSEFQNIMNEIFNSYSHFSIVYIDDVLIFSDSIEQHWKHLHKFLHIIKSNGLVVSAKKIKLFQTHIRFLGYNISQSQIHPIDRVILFADKFPDQITDKTQLQRFLGSLNYVSDF

>Pbri1-Chr4-01; Florendovirus; Prunus brigantina 1;

QRLISEVCSDLPTAFWHRKNHVVCLPYIKNFSEKHIPTKARPIQMNHEVMEFCKTEIDDLLQKNIIRKSKSPWSCPAFYVQKNAELERGSPRLVINYKPLNSVLEWIRYPIPNKRDLIKRLDKALVFSKFDMKSGFWQIQINESDRYKTAFVTPFGHYEWNVMPFGLKNAPSEFQNIMNEIFNSYSHFSIVYIDDVLIFSESIEQHWKHLHMFLHIIKSNGLVVSAKKIKLFQMHIRFLGYNISQSQIHPIDRAIHFADKFPDQITDKNQLQRFLGSLNY

>Pbri1-Chr4-02; Florendovirus; Prunus brigantina 1;

QRLISEVCSDLPTAFWHRKNHVVCLPYIKNFSEKHIPTKARPIQMNHEIMEFCKTEINDLLQKNIIRKSKSPWSCPAFYVQKNAELERGSPRLVINYKPLNSVLEWIRYPIPNKRDLIKRLDKALVFSKFDMKSGFWQIQIKESDRYKTAFVTPFGHYEWNVMPFGLKNAPSEFQNIMNEIFNSYSHFSIVYIDDVLIFSESIEQHWKHLHKFLHIIKSNGLVVSAKKIKLFQTNIRFLGYNISQSQIHPIDRVIHFADKFPDQITDK

>Pbri1-Chr4-03; Yendovirus; Prunus brigantina 1;

LEELLQLVEQTRIIGEDPQLYWNKNKILAKLDIINPDLTIKTADMPCNLLDKQEFEQQIKELLNLKVIRPSKSRHRSAAFVVRKHSELKRGKARIVYNYRRLNDNTYEDSYKLPNEDELMNKIQESKYFSKFDCKSGFWQIRLAEESIHWTAFTYPEGHFEWLVMPFGLKNAPSIFQRKMDQIFKKYDKFCSVYVDDILVHSKNKNEHRKHLEIILKEFIENGIVISRNKVELEKQNIEFLGMYIKSGTIQLQEHITKKVLEFPDKLEDKKQLQAFLGLLNYARNFIPD

>Pbri1-Chr4-04; Yendovirus; Prunus brigantina 1;

IIGEDPQLHWNKNKILAKLDIINQDLTIKTAYMPCNLLDKQEFEQXIKELLNLKVIRPSKSKHRSTAFVVRKHSELKRGKARIVYNYRRLNDNTYEDSYKLPNKDELMNKIQXSKYFSKFDCKSGFWQIRLAEESIQWTTFTCPEGHFEWLVMPFGLKNAPLIFQRKIDQIFKKYDNFCSVYVDDILVHSKNKNEHRKHLEIILIEFIDNGIVISRNKGELEKQDIEFLGMYIKSXTIQLQERIAKKVLEFLDKLEDKKXLPAFLGLLNYARSFIPD

>Pbri1-Chr4-05; Yendovirus; Prunus brigantina 1;

LEELLQLAEQTRIIGEDPQLHWNKNKILAKLDIINSDLTIKTADMPRNLLDKQEFEQQIKELLNLKVIRPSKSRHRSVAFVVRKHSELKRGKARIVYNYRRLNDNTYEDSYKLPNKDELMNKIQESKYFSKFDCKSGFWQIRLAEESIHWTTFTCLEGHFEWLVMPFGLKNAPSIFQRKMDQIFKKYDKFCSVYVDDILVHSKNKNEHRKHLEIILKEFIDNGIVISRNKVELEKQDIKFLGMYIKSGTIQLQEHITKKVLEFPDKLEDKKQLQAFLGLLNYARSFIPE

>Pbri1-Chr4-06; Yendovirus; Prunus brigantina 1;

LEELLQLAEQTRIIGEDPQLHWNKNKILAKXDIINPDLTIKTADMPCNLLDKQEFEQXIKELLNLKVIRPSKSRHRSAAFVVRKHSELKRGKARIVYNYRRLNDNTYEDSYKLPNKDELMNKIQESKYFSKFDCKSGFWQIRLAEESIHWTAFTCPEGHFEWLVMPFGLKNAPSIFQRKMDQIFKKYDNFCNVYVDDILVHSKNKNEHRKHLEIILKEFIDNGIVISRNKVELEKQDIEFLGMYIKSGTIQLQEHITKKVLEFPDKLEDKKQLQAFLGLLNYARSFIPD

>Pbri1-Chr5-01; Florendovirus; Prunus brigantina 1;

DKLKNEICSELPNAFWYRKQHVVCLPYIKTFSEKNIPTKARPIQMSQEIMEFCKNEINELLQKKIIRRSKSPWSCPAFYVQKNAELERGVPRLVINYKPLNSVLEWIRYPIPNKRDLINRLGRAVIFSKFDMKSGFWQIQIDESDRYKTAFVTPFGHYEWNVMPFGLKNAPSEFQNIMNEIFNPYSHFSIVYIDDVLIFSESIEQHWKHLHKFLQIVKQNGLVVSAKKIKLFQTKVRFLGFNICQSQITPIDRVIQFADKFPDQILDKSQLQRFLGSLNYVSDF

>Pbri1-Chr5-02; Florendovirus; Prunus brigantina 1;

KRLISEVCSDLPTAFWHRKNHVVSLPYIKNFSEKNIPTKARPIQMNHEMMEFCKSEINDLLQKNIIRKSKSPWSCPAFYVQKNAELERGSPRLVINYKPLNTVLEWIRYPIPNKRDLIKRLDKAIIFSKFDMKSGFWQIQIAEHDRYKTAFVTPFGHYEWNVMPFGLKNAPSEFQNIMNEIFNSYSHFSIVYIDDVLIFSESIDQHWKHLHKFLHIIKSNGLVVSAKKIKLFQTNIRFLGYNISQSQIHPIDRVIHFADKFPDQITDKNQLQRFLGSLNYVSDF

>Pbri1-Chr5-03; Florendovirus; Prunus brigantina 1;

KRLISEVCSDLPTAFWHRKNHVVSLPYIKNFSEKNIQTKARPIQMNHEMMEFCKSEINDLLQKNIIRKSKSPWSCPAFYVQKNAELERGSPRLVINYKPLNTVLEWIRYPIPNKRDLIKRLDKAIIFSKFDMKSGFWQIQIAEHDRYKTAFVTPFGHYEWNVMPFGLKNAPSEFQNIMNEIFNSSSYFSIVYIDDVLIFSESIDQHWKHLHKFLHIIKSNGLVVSAKKIKLFQTNIRFLGYNISQSQIHPIDRVIHFADKFPDQITDKNQLQRFLGSLNYVSDF

>Pbri1-Chr5-04; Yendovirus; Prunus brigantina 1;

ILEELLQLAEQTRIIGEDPQLHWNKNKILAKLDIINPDLTIKTADMPCNLLDKQEFEQQIKELLNLKVIRPSKSRHRSAAFVVRKHSELKRGKARIVYNYRRLNDNTYEDSYKLPNKDELMNKIQESKYFSKFDCKSGFWQIRLAEESIHWTAFTCPEGHFEWLVMPFGLKNAPSIFQRKMDQIFKKYDNFCSVYVDDILVHSKNKNEHRKHLEIILKEFIDNGIIISRNKVELEKQDIEFLGMYIKSGTIQLQEHITKKVLEFPDKLEDKKQLQAFLGLLNYARNFIPD

>Pbri1-Chr6-01; Yendovirus; Prunus brigantina 1;

HWNKNKILAKLDIINPNLTIKTADMPCNLLDKQEFEQQIKELLNLKVIRPSKSMHRSAAFVVRKHSELKRGKARIVYNYRRLNDNTYEDSYKLPNKDELMNKIQESKYFSKFDCKSGFWQIRLAEESIQWTSFTCPQGHFEWLVMPFGLKNAPSIFQRKMDQICKKYDNFCSVYVDDILVHSKNKNEHRKHLEIILKEFIDNCIVISRNKVELEKQDIEFLGMYIKSGTIQLQEHIAKKVLEFPYKLEDKKQLQAFLGLLNYARSFIPD

>Pbri1-Chr6-02; Yendovirus; Prunus brigantina 1;

LEELLQLAEQTRIIGEDPQLHWNKNKILAKLDIINPDLTIKTPDMPCNLLDKQEFEQQIKELLNLKVIRPSKSRHRSAAFVVRKHSELKRGKARIVYNYRRLNDNTYEDSYKLPNKDELINKIQESKYFSKFDCKSGFWQIRLAEESIHWIAFTCLEGHFEWLVMPFGLKNASSIFQRKMDQIFKKYDKFCSVYVDDILVHSKNKNEHRKHLEIILKEFIDNGIVISRNKVELEKQDIEFLGMYIKSGTIQLQERITKKVLEFPDKLEDKKQLQAFLGLLNYARSFI

>Pbri1-Chr6-03; Florendovirus; Prunus brigantina 1;

DKLKNEICSDLPNAFWYRKQHVVCLPYIKTFSEKNIPTKARPIQMSQEIMEFCKNEIHELLQKKIIRKSKSPWSCPAFYVQKNAELERGVPRLVINYKPLNSVLEWIRYPIPNKRDLINRLGKAVIFSKFDMKSGFWQIQIEESDRYKTAFVTPFGHYEWNVMPFGLKNAPSEFQNIMNEIFNPYSHFSIVYIDDVLIFSESIEQHWKHLHKFLQIVKQNGLVVSAKKIKLFQTKIRILGFHICQSQIAPIDRVIQFADKFPDQILDKSQLQRFLGSLNYV

>Pbri1-Chr6-04; Yendovirus; Prunus brigantina 1;

LEELLQLVEQTRIIGEDPQLHWNKNKILAKLDIINPDLTIKTEDMPCNLLDKQEFEQQIKELLNLKVIRPSKSRHRSAAFVVRKHSELKRGKARIVYNYRRLNDNTYEDSYKLPNKDELMNKIQESKYFSKFDCKSRFWQIRLVEESIHWTAFTYPEGHFEWLVMPFGLKNAPSIFQRKMDQIFKKYDKFCGVYVDDILVHSKNKNEHRKHLEIILKEFIDNGIVISRNKVELEKQDIEFLGMYIKSGTIQLQEHIAKKVLEFPDKLEDKKQLQAFLGLLNYARSFIPD

>Pbri1-Chr6-05; Florendovirus; Prunus brigantina 1;

EKLKIEICSELPNAFWYRKQHVVCLPYIKTFSEKNIPTKARPIQMSQEIMEFCKNEINELLQKKIIRRSKSPWSCPAFYVQKNAELERGVPRLVINYKPLNSVLEWIRYPIPNKRDLINRLGKAVIFSKFDMKSGFWQIQIDESDRYKTAFVTPFGHYEWNVMPFGLKNAPSEFQNIMNEIFNPYSHFSIVYIDDVLIFSESIEQHWKHLHKFFQVVKQNGLVVSAKKIKLFQTKVRFLGFNICQSQITPIDRVIQFADKFPDQILDKSQLQRFLGSLNYV

>Pbri1-Chr6-06; Florendovirus; Prunus brigantina 1;

EKLKNEICSDLPNAFWYRKQHVVCLPYIKTFSEKNIPTKARPIQMSQEIMEFCKNEIHELLQKKIIRKSKSPWSCPAFYVQKNAELERGVPRLVINYKPLNSVLEWIRYPIPNKRDLINRLGKAVIFSKFDMKSGFWKIQIEESDRYKTAFVTPFGHYEXNVMPFGLKNAPSEFQNIMNEIFNPYSHFSIVYIDDVLIFSESIEQHWKHLHKFLQIVRQNGLVVSAKKIKLFQTKVRFLGFHICQSQISPIDRVIQFADKFPDQILDKSQLHRFLGSLNYVSDF

>Pbri1-Chr6-07; Florendovirus; Prunus brigantina 1;

EKLKNEICSDLPNAFWYRKQHVVCLPYIKTFSEKNIPTKARPIQMSQEIMEFCKNEIHELLQKKIIRKSKSPWSCPAFYVQKNAELERGVPRLVINYKPLNSVLEWIRYLIPNKRDLINRLGKAVIFSKFDMKSGFWKIQIEESDRYKTAFVTPFGHYEWNVMPFGLKNAPSEFQNIMNEIFNPYSHFSIVYIDDVLIFSESIEQHWKHLHKFLQIVKQNGLVVSAKKIKLFQTKVRFLGFLICQSQISPIDRVIQFADKFPDQILDKSQLQRFLGSLNYVSDF

>Pbri1-Chr6-08; Florendovirus; Prunus brigantina 1;

QKIISEVCSDLPTAFWHRKNHVVCLPYIKNFSEKNIPTKARPIQMNHEMMEFCKTEINDLLQKNIIRKSKSPWSCPAFYVQKNAELERGSPRLVINYKPLNSVLEWIRYPIPNKRDLIKRLDKATVFSKFDMKSGFWQIQIHESDRYKTAFVTPFGHYEWNVMSFGLKNAPSEFQNIMNEIFNSYSHFSIVYIDDVLIFSESIEQHWKHLHKFLHIIKSNGLVVSAKKIKLFQMHIRFLGYNISQSQIHPIDRVIHFADKFPDQITDKNQLQRFLGSLNYVSDF

>Pbri1-Chr7-01; Yendovirus; Prunus brigantina 1;

LEELLQLAEQTRIIGEDPQLHWNKNKILAKLDIINPDLTIKIADMPCNLLDKQEFEQQIKELLNLKVIRPSKSRHRSATFVVRKHSELKRGKARIMYNYRRLNDNTYEDSYKLPNKDELMNKIQESKYFSKFDCKSGFWQIRLAEESIHWTTFTCPEGHFEWLVMPFGLKNAPSIFQRKMDQIFKKYDNFCSVYVDDILIHSKNKNEHRKHLEIILKEFIDNGIIISRNKVELEKQDIEFLGMYIKSGTIQLQEHITKKVLEFPDKLEDKKQLQAFLGLLNYAINFIPD

>Pbri1-Chr7-02; Florendovirus; Prunus brigantina 1;

QKLISEVCSDLPTTFWHRKNHVVCLPYIKNFSEKHIPTKSRPIQMNHEIMEFCKTEIDDLLQKNIIRKSKSPWSCPAFYVQKNAELERGSPRLVINYKPLNSVLEWIRYPIPNKRDLIKRLDKALVFSKFDMKSGFWQIQINESDRYKTAFVTPFGHYEWNVMPFGLKNAPSEFQNIMNEIFNSYSHFSIVYIDDVLIFSESIEQHWKHLHKFLHIIKSNGLVVSAKKIRLFQTNIRFLGYNISQSQIHPIDRVFHFADKFPDQITDKNQLQRFLGSLNYVS

>Pbri1-Chr7-03; Yendovirus; Prunus brigantina 1;

IIGEDPQLHWNKNKILAKLDIINPDLTIKTTDMPCNLLDKQEFEQQIKELLNLKVIRPSKSRHRSTTFVVRKHSKLKRGKTRIVYNYRRLNDNTYEDSYKLPNKDELMNKIQESKYFSKFDCKSGFWQIRLAEESIQWTTLTCPEGLFEWLVMPFGLKNAPSIFQRKMDQIFKKYDNFCSVYVDDILVNSKNKNEHRKLLXIILKEFIDNGIVISRNKVELEKQDIEILGMYIKSGIIQLQEHIAKKVLEFPDKLEDKKQLQAFLGLLNYA

>Pbri1-Chr8-01; Yendovirus; Prunus brigantina 1;

LEELLQLAEQTRIIGEDPQLHWNKNKILAKLDIINPDLTIKTADMPCKLLDKQEFEQQIKELLNLKVIRPSKSRHRSAAFVVRKHSELKRGKARIVYNYRRLNDNTYEDSYKLPNKDELMNKIQESKYFSKFDCKSGFWQIRLAEESIHWTAFTCPEGHFEWLVMPFGLKNAPSIFQRKMDQIFKKYDKFCSVYVDDILVHSKNKNEHRKHLEIILKEFIDNGIVISRNKVELEKQDIEFLGMYIKSGTIQLQEHITKKVLEFPDKLEDRKQLQAFLGLLNHARSFIPD

>Pbri1-Chr8-02; Florendovirus; Prunus brigantina 1;

QRLISEVCSDLPTAFWHRKNHVVCLPYIKNFSEKHIPTKARPIQMNHEIMKFCKTEIDDLLQKNIIRKSKSPWSCPAFYVQKNAELERGSPRLVINYKPLNSVLEWIRYPIPNKRDLIKRLDKALVFSKFDMKSGFWQIQINESDRYKTAFVTPFGHYEWNVMPFGLKNAPSEFQNIMNEIFNSYSHFSIVYIDDVLIFSESIEQHWKHLHKFLHIIKSNGLVVSAKKIKLFQTNIRFLGYNISQSQIHPIDRVIHFADKFPDQITDKNQLQRFLGSLNY

>Pbri1-Chr8-03; Yendovirus; Prunus brigantina 1;

LEELLQLAEQTRIIGEDPQLHWNKNKILAKLDIINPDLTIKTADMPCNLLDKQEFEQQIKELLNLKVIRPSKSRHRSATFVVRKHSELKRGKARIMYNYRRLNDNTYEDSYKLPNKDELMNKIFQESKYFSKFDCKSRFWQIRLAEESIHWTAFTCPEGHFEWLVMPFGLKNAPSIFQRKMDQIFKKYDKFCSVYVDDILVHSKNKNEHRKHLEIILKEFIDNGIVISRNKVELEKQDIEFLGMYIKSGTIQLQEHIAKKVLEFPDKLEDKKQLQAFLGLLNYA

>Pbri1-Chr8-04; Yendovirus; Prunus brigantina 1;

LEELLQLAEQTRIIGEDPQLHWNKNKILAKLDIINPDLTIKTADMSCNLLDKQEFEQQIKELLNLKVIRPSKSRHRSAGFVVRKHSELKRGKARIVYNYRRLNDNTYEDSYKLPNKDELMNKIQESKYFSKFDCKSGFWQIRLAEESIDWTAFTYPEGHFEWLVMPFGLKNAPSIFQRMMDQIFKKYDKFCNVYVDDILVHSKNKNEHRKHLEIILKKFIDNGIVISRNKVELEKQDIEFLGMYIKSGTIQLQEHIAKKVLEFPDKLEDNKQLQAFLGLLNYARSFIPD

>Pbri1-Chr8-05; Yendovirus; Prunus brigantina 1;

LEELLQLAEQTRIIGEDPQLHWNKNKILAKLDIINPDLTIKTADMPCNLLDKQEFEQEIKELLNLKVIRPSKSRHRSAAFVVRKHSELKRGKARIVYNYRRLNDNTYEDSYKLPNKDELMNKIQESKYFSKFDCKSGFWQIRLAEESIHWTAFTCPEGHFEWLVMPFGLKNAQSIFQRKMDQIFKKYDNLCSVYVDDILVHSKNKNEHRKHLEIILKEFIDNGIIISRNKVELEKQDIEFLGMYIKSGTIQLQEHITKKVLEFPDKLEDKKQLQAFLGLLNYARNFIPD

>Pbri1-Chr8-06; Florendovirus; Prunus brigantina 1;

EKLKIEICSDLPNAFWYRKQHVVCLPYIKTFSEKNIPTKARPIQMSQEIMEFCKNEINELLQKKIIRKSKSPWSCPAFYVQKNAELERGVPRLVINYKPLNSVLEWIRYPIPNKRDLINRLEKAVIFSKFDMKSGFWQIQIDESDRYKTAFVTPFGHYEWNVMPFGLKNAPSEFQNIMNEIFNPYSHFSIVYIDDVLIFSESIEQHWKHLHKFFQVVKQNGLVVSAKKIKLFQTKVRFLGFNICQSQITPIDRVIQFADKFPDQILDKSQLQRFLGSLNYV

>Pcam1-Chr2-01; Yendovirus; Prunus campanulata 1;

LEELLQLAEQTRIIGEDPQLHWNKNKILAKLDIINPDLTIKTADMPCNLLDKQEFEQQIKELLNLKVIRPSKSRHRSAAFVVRKHSELKRGKARIVYNYRRLNDNTYEDSYKLPNKDELMNKIQESKYFSKFDCKSGFWQIRLTEESIQWTTFTCPEGHFEWLVMPFGLKNAPSIFQRKMDQIFKKYDNFCSVYVDDILVHSKNKNEHRKHLEIILKEFIDNGIVISRNKVELEKQDIEFLGMYIKSGTIQLQEHITKKVLEFRDKLEDKKQLQAFLGLLNYARSFIPD

>Pcam1-Chr6-01; Yendovirus; Prunus campanulata 1;

IIGEDPQLHWNKNKILAKLDIINPDLTIKTADMPCNLLDKQEFEQQIKELLNLKVIRPSKSRHRSAAFVVRKHSXLKRGKARIVYNYRRLNDNTYEDSYKLPNKDELMNKIQESKYFSKFDCKSGFWQIRLAEESIQWTTFTCPEGHFEWLVMPFGLKNAPSIFRRKMDQIFKKYDNFCSVYVDDILVHSKNKNEHRKHLEIILKEFIDNGIVISRNKVELEKQDIEFLGMYIKSGTIQLQEHITKKVLEFPNKLEDKKQLQAFLGLLNYARSFIPD

>Pcam1-Chr7-01; Yendovirus; Prunus campanulata 1;

HWNKNKILAKLDIINPDLTIKTTDMPCNLLDKQEFEQYIKELLNLKVIRPSKSRHRSAAFVVRKHSELKRGKARIVYDYRRLNDNSYEDSYKLPNKDELMNKIQESKYFSKFDCKSEFWQIRLVEESIQWTTFVCPEGHFEXLTMPFGLKNAPSIFQRKMDQIFKKYDNFFSVYVDDILIHSKNKNEHRKHLEIILKEFINNGIIISRNKVELEKQDIEFLGMYIKSRTIQLQEHIAKKVLEFPDKLKDKKQLQAFLGLLNYARSFIP

>Pcam1-Chr7-02; Yendovirus; Prunus campanulata 1;

HWNKNKILAKLDIINPDLTIKTVDMPCNLLDKQKFEQHIKELLNLKVIRPSKSRHRSAAFVVRKHSELKRGKARIVYNYRRLNDNTYEDSYKLPNKDELMNKIQEIKYFSKFDCKSGFWQIRLAEESIQWIAFTCPEGHFEWLVMPFGLKNAPSIFQRKMDQIFKKCDNFCSVYVDDILVHSKNKNEHRKHLEIILKEFIDNGIVISRNKVELQKQDIEFLGMYIKSGIIQLQEHITKKVLEFPDKLEDKKQLQAFLGLLNYARSFIPD

>Pcam1-Chr8-01; Florendovirus; Prunus campanulata 1;

EKLKNEVCSDMPNAFWHRKQHVVSLPYVKTFSEKNIPTKARLIQMSQELMELCKAEVTDLVNKGIVRKSKSHWSCSAFYVQKNAELERGAPRLVINYKPLNSVLEWIRYPIPNKRDLVNRLEKAIIFSKFDMKSGFWQIQVKESDRYKTAFVTPFGHYEWNVMPFGLKNAPSEFQNIMNEIFNDYSHFSIVYIDDVLIFSESIEQHWKHLHKFLQIVKTNGLVISAKKIKLFQVNVRFLGFNISQSQISPINRVIQFADKFPDQILEKSQLQRFLGSLNYVSDF

>Pcam1-Chr8-02; Yendovirus; Prunus campanulata 1;

LEELLQLAEQTRIIGEDPQLHWNKNKILAKLDIINPDLTIKTADMPCNLLDKQEFEQQQKELLNLKVIRPSKSRHRSAAFVVRKHSKLKRGKARIVYNYRRLNDNTYEDSYKLPNKDELMNKIQESKYFSKFDCKSGFWQIGLVEESIQWTTFTCPEGHFEWLVMPFGLKNAPSIFQRKMDQIFKKYDNFCSVYVDDILVHSKNKNEHRKHLEIILKEFIDTGIVISRNKVELEKQDIEFLGMYIKSGTIQLQEHIAKKVLEFPDKLEDKKQLQAFLGLLNYARSFIPD

>Pdav1-Chr1-01; Florendovirus; Prunus davidiana 1;

QKLKDAVCSELPNAFWYRKQHVVCLPYIKTFSEKNIPTKARPIQMNQEMLEFCRKEINELLQKKIIRKSKSPWSCPAFYVQKNAELERGVPRLVINYKPLNAVLEWIRYPIPNKRDLINRLGKAVVYSKFDMKSGFWQIQIDESDRYKTAFVTPFGHYEWNVMPFGLKNAPSEFQNIMNDIFNPYSLFSIVYIDDVLIFSESVEQHWKHLHQFLQIVKHNGLVVSAKKIKLFQTNIRFLGFNICQSQISPIDRVIQFADKFPDQILDKSQLQRFLGSLNYVSDF

>Pdav1-Chr1-02; Florendovirus; Prunus davidiana 1;

QKLKDAVCSELPNAFWYRKQHVVCLPYIKTFSEKNIPTKARPIQMNQEMLEFCRKEINELLQKKIIRKSKSPWSCPAFYVQKNAELERGVPRLVINYKPLNAVLEWIRYPIPNKRDLINRLGKAVVYSKFDMKSGFWQIQIDESDRYKTAFVTPFGHYEWNVMPFGLKNAPSEFQNIMNDIFNPYSLFSIVYIDDVLIFSESVEQHWKHLHQFLQIVKHNGLVVSAKKIKLFQTNIRFLGFNICQSQISPIDRVIQFADKFPDQILDKSQLQRFLGSLNYVSDF

>Pdav1-Chr1-03; Florendovirus; Prunus davidiana 1;

QKIKEEICSELPTAFWYRKQHVVRLPYIKTFSEKNIPTKARPIQMSQEIMEFCKKEIHELLQKKIIRKSKSPRSCPAFYVQKNAELERGVPRLVINYKPLNAVLEWIRYPIPNKRDLINRLGKAVVYSKFDLKSGFWQIQIDESDRYKTAFVTPFGHYEWNVMPFGLKNAPSEFQNIMNDIFNPYSQFSIVYIDDVLIFSESIEQHWKHLYKFLQVIKQNGLVVSAKKIKLFQTNIRFLGFNICQSQISPIDRVIQFADKFPDQILDKSQLQRFLGSLNY

>Pdav1-Chr1-04; Florendovirus; Prunus davidiana 1;

EKLKQEVCSELPNAFWHRKQHVVNIPYVKSFSEKNIPTKARPIQMSQELMEFCKTEITELLEKGIIRKSKSPWSCPAFYVQKNAELERGVPRLAINYKPLNTVLEWIRYPIPNKRDLINRLEKSVIFSKFDMKSGFWQIQICESDRYKIAFVTPFGHYEWNVMPFGLKNAPSEFQNRMNEIFIPFNNFSIVYIDDVLIFSESIEQHWKNLHKFLQIVKQNGLVVSAKKIKLFQTNVRFLGFNICQSQISPIDRVIQFVDKFPNQILDKSQLQRFLGSLNYV

>Pdav1-Chr2-01; Florendovirus; Prunus davidiana 1;

QKLKDEICSELPNAFWYRKQHVVCLPYIKTFSEKNIPTKARPIQMSQDMMEFCKKEIDELLNKKIIRKSKSPWSCPAFYVQKNAELERGVPRLVINYKPLNAVLEWIRYPIPNKRDLTNRLGKAVVYSKFDMKSGFWQIQIDESDRYKTAFVTPFGHYEWNVMPFGLKNAPSEFQNIMNDIFNPYSQFSIVYIDDVLIFSESVEQHWKHLHKFLQIVKNNGLVVSAKKIKLFQTNVRFLGFNICQSQISPIDRVIQFADKFPDQILDKSQLQRFLGSLNY

>Pdav1-Chr2-02; Florendovirus; Prunus davidiana 1;

EKLKQEVCSELPNAFWHRKQHVVSLPYVKSFSEKNIPTKARPIQMSQELMEFCKIEINDVLKKGIIRKSKSPWSCPAFYVQKNAELERGVPRLVINYKPLNTVLEWIRYPIPNKRDLINRLENSVIFSKFDMKSGFWKIQISESDRYKTAFVTPFGHYEWNVMLFGLKNAPSEFXNIMNEIFNPFSHFFIVYIDDVLIFSESIEQHWKHLHKFLQIVKQNGLVVSAKKIKLFQINVRFLRFNICXSQIIPIDRVIQFADKFPDQILDKSQLQRFLGSLNYV

>Pdav1-Chr3-01; Florendovirus; Prunus davidiana 1;

EKLKQEVCSELPKAFWHRKQHVVSLPYVKTFSEKNIPTKARPIQMSQELMEFCKTEITELLEKGIIRKSKSPWSCPAFYVQKNAELERGVPILVINYKPINTVLEWIRYLIPNKRDLINRSEKSVIFSKFNMKSGLWQIQIRESDRYKTAFVTPFGHYEWNVMPFRLKNAPSEFQNIMNEIFNPYGHFSIVYIDDVLIFSESIEQHXKHLHKFLQIVKQNGLVVSAKKIKLFQTNVRFLGFNICQSQISPINRIIXFADKFLDQITDKSQLHRFLESLNYVSDF

>Pdav1-Chr3-02; Florendovirus; Prunus davidiana 1;

KLKNEICSELPNAFWYRKQHVVCLPYIKTFSEKNIPTKAQPIQMNQEMLEFCKKEINELLDKKIIRKSKSPWSCPAFYVQKNAELERGVPRLVINYKPLNTVLEWIRYPIPNKRDLINRLEKAVVYSKFDLKSGFWQIQIDESDRYKTAFVTPFGHYEWNVMPFGLKNAPSEFQNIMNDIFNPYSQFSIVYIDDVLIFSESIEQHWKHLHKFLQVVKQNGLAVSAKKIKLFQTNIRFLGFNICQSQISPIDRVIQFAEKFPDQILDKNQLHRFLGSLNY

>Pdav1-Chr4-01; Yendovirus; Prunus davidiana 1;

NKNKILAKLDIINPDLTIKTADMPCNLLDKQEFEQKIKELLNLKVIRPSKSRHRSVAFVVRKHSELKRVKARIVYNYRRLNDNTYEDSYKLPNKDELMNKIQESKYFSKFDCKSGFXQIRLAEESIQXITFTCPEGHFEXLVMSFGLKNAPSIFQRKMDQILKKYNNFYSVYVDDILVHSKNKNEHRKHLEIILKEFIDNGIVISRNKVKLEKQDIEFLGMYIKSGTIQLQXHIAKKVLEFPDKLEDKKQLXAFLGLLNYARSFI

>Pdav1-Chr4-02; Florendovirus; Prunus davidiana 1;

QKIKNEICSELPNAFWYRKQHVVCLPYIKTFSEKNIPTKARPIQMSQEMMELCKKEINELLQQKIIRKSKSPWSCPAFYVQKNAELERGVPRLVINYKPLNAVLEWIRYPIPNKRDLINRLGNAVVYSKFDLKSGFWQIQIDESDRYKTAFVTPFGHYEWNVMPFGLKNAPSEFQNIMNDIFNPYSQFSIVYIDDVLIFSESVEQHWKHLHKFFQIVKQNGLVVSAKKIKLFQTNVRFLGFNICQSQISPIDRVIQFADKFPDQILDKSQLQRFLGSLNY

>Pdav1-Chr4-03; Yendovirus; Prunus davidiana 1;

LEELLQLAEQTRIVGEDPQLHWNKNKILAKLDIINPDLTIKTADMPCNLLDKQEFEQQIKELLNLKVIRPSKSRHRSVAFVVRKHSELKRGKARIVYNYRRLNDNTYEDSYKLPNKDELMNKIQESKYFSKFDCKSGFWQTRLAEESIQWTTFTCTEGHFEWLVMPFGLKNAPSIFQRKMDQSFKKYDNFCSVYVDDILVHSKNKNEHSKHLEIILKEFIDNGVVISRNKIKFEKQDIEFLGMYIKSGTIQLQEHIAKKVLEFSDRLEDKKQLQAFLGLLNYVSSFIPD

>Pdav1-Chr4-04; Florendovirus; Prunus davidiana 1;

EKLKQEVCSDLPNVFWHRKQHVVSLPYVKNFSEKNILTKARPIQMSQEIMEFYKTEITELLQKGIIRKSKSPWSCPAFYVXKNAELERGAPRLVINYKPLNTTLEWIRYPIPNKRDLINRLDKSVIFSKFDMKIGFWQIQILESDRYKTAFVTPFGHYEWNVMPFGLKNAPSEFQNIMNEIFNPFSHFSIVYIDDVFIFSQSIEQHWKHLHKFLKIVKQNGLVVLAKKIKLFQTNIRFLGFNICQSQISLIDRVIQFADKFSYQILDKSQLHRFLGSLNY

>Pdav1-Chr4-05; Florendovirus; Prunus davidiana 1;

EKLKQEVCSELPNAFWHRKQHVVSLPYIKSFSEKNIPTKARPIQMSQELMEFCKIEITELLEKGIIRKSKSPWSCPAFYVQKNAELERGVPRLVINYKPLNTVLEXIRYSIPNKRDLINRLEKSVIFSKFDMKSGFWQIQIHESDRYKTAFVTPFGHYEWNVMPFGLKNAPSEFQNIMNEIFNHFSHFSIVYIDDVLIFSQSIEQHWKHLNQFLQIVKQNGLVVSAKKIKLFQTNVRFLGFNICQSQISPIDRVIQFADKFPDLDKIQLQRFLGSLNYVSDF

>Pdav1-Chr4-06; Florendovirus; Prunus davidiana 1;

QKLKDEICSELPNAFWYRKQHVVCLPYIKTFSEKNIPTKARPIQMSQEMMEFCKKEINELLQKKIIRKSKSPWSCPAFYVQKNAELERGVPRLVINYKPLNAVLEWIRYPIPNKRDLINRLERAVVYSKFDLKSGFWQIQIDESDRYKTAFVTPFGHYEWNVMPFGLKNAPSEFQNIMNDIFNPYSQFSIVYIDDVLIFSESIEQHWKHLHKFFQVVKQNGLVVSAKKIKLFQTNIRFLGFNICQSQISPIDRVIQFADKFPDQILDKSQLQRFLGSLNY

>Pdav1-Chr4-07; Florendovirus; Prunus davidiana 1;

QKLKDEICSELPNAFWYRKQHVVCLPYIKTFSEKNIPTKARPIQMSQEMMEFCKKEINELLQKKIIRKSKSPWSCPAFYVQKNAELERGVPRLVINYKPLNAVLEWIRYPIPNKRDLINRLERAVVYSKFDLKSGFWQIQIDESDRYKTAFVTPFGHYEWNVMPFGLKNAPSEFQNIMNDIFNPYSQFSIVYIDDVLIFSESIEQHWKHLHKFFQVVKQNGLVVSAKKIKLFQTNIRFLGFNICQSQISPIDRVIQFADKFPDQILDKSQLQRFLGSLNY

>Pdav1-Chr5-01; Florendovirus; Prunus davidiana 1;

QKIKNEICSELPNAFWYRKQHVVCLPYIKTFSEKNIPTKARPIQMSQEIMEFCKTEISELLQKKIIRKSKSPWSCPAFYVQKNAELERGVPRLVINYKSLNSVLEWIRYPIPNKRDLINRLGKAVVYSKFDMKSGFWQIQIEESDRYKTAFVTPFGHYEWNVMPFGLKNAPSEFQNIMNEIFNPYSHFSIVYIDDVLIFSESIEQHWKHLHKFFQIVKQNGLVVSAKKTKLFQTNIRFLGFNICQSQISPIDRVIQFANKFPDQILDKSQLKRVLGSLNY

>Pdav1-Chr5-02; Florendovirus; Prunus davidiana 1;

QKIKDEICSELPNAFWYRKQHVVCLPYIKTFSEKNIPTKARPIQMSQEMMEFCKKEINELLQKKIIRKSKSPWSCPAFYVQKNAELERGVPRLVINYKPLNAVLEWIRYPIPNKRDLINRLEKAVVYSKFDMKSGFWQIQIEESDRYNTAFVTPFGHYEWNVMPFGLKNAPSEFQNIMNDIFNLYSQFSIVYIDDVLIFSESIEQHWKHLHKFLQIVKHNGLVVSAKKIKLFQTNIRFLCFNICQSQLSPIDRVIQFADKFPDQILDKSQLQRFLGSLNYV

>Pdav1-Chr5-03; Florendovirus; Prunus davidiana 1;

KLKQEVCSELPNAFWSRKQHVVCLPYIKTFNEKNIPTKARPIXMSQEIMEFCKKEINDLLQKKIIRKSKSPWSCPAFYVQKNAELERGVPRLVINYKPLNSILEWIRYPIPNKRDLINRLRKAVVFSKFDMKSGFLQIQIDESDKYKTAFVTPFGHYEWNVMPFGLKNAPSEFQNIMNDIFNPYNHFSIVYIDDVLIFSESIEQHWKHLHKFLQVVKQNGLVVSAKKIKLFQTNIRFLDFNIYQSQISPIDRVIQFADKFPDEIIDKSQLQRFLGSLNY

>Pdav1-Chr5-04; Florendovirus; Prunus davidiana 1;

KKIKDEICSELPNAFWYRKQHVVCLPYIKTFSEKNIPTKARPIQMSQEIMEFCKKEIEELLQKKIIRKSKSPWSCPAFYVQKNAELERGVPRLVINYKPLNAVLEWIRYPIPNKRDLINRLEKAVVYSKFDMKSGFWQIQIEESDRYKTAFVTPFGHYEWNVMPFGLKNAPSEFQNIMNDIFNPYSQFSIVYIDDVLIFSESIEQHWKHLHKFLQVVKQNGLVVSAKKIKLFQTNVHFLGFNICQSXISPIDRVIXFADKFPDQILDKNQLQRLLGSLNY

>Pdav1-Chr6-01; Florendovirus; Prunus davidiana 1;

QKIVAEICSESPNAFWYRKQYVVCLPYIKTFSEKNIPTKARPIQMSQEMMEFCKKEINELLQKKIIRKSKSPWSCPAFYVQKNAELERGVPRLVINYKPLNAVLEWIRYPIPNKRDLINRLGNAVVYSKFDLKSGFWQIQIDESDRYKTAFVTPFGHYEWNVMPFGLKNAPSEFQNIMNEIFNPYSQFSIVYIDDVLIFSELIEQHWKHLHKFLQIVKQNGLVVSAKKIKLFQTNIRFLGFNISQSQIAPIDRVIQFADKFPDQILDKSQLQRFLGSLNY

>Pdav1-Chr6-02; Florendovirus; Prunus davidiana 1;

EKLKHEVCSDLPNAFWHQKQRVVTLPYIKTFSEKNIRTKARPIQMSQELMELCKQEVTELVQKGIVRKSKSPWSCPAVYVQKNAELKRGAPHLVINYKPFNAVLXWIRYPIPNKRDLVNRLEKAVVFSKFDMKSRFWQIQIKESDRYKTAFVTPFGYYECNVMPFGLKNAPSEFQNIMNEIFNQYSQFFVVYIDDVLIFSESIEQHWKHLNKFLQAKWSSYLNQENQIISSQCXIFRIQHWAIPNKPNQSSYPVCRXISDQILDKSQLQRFLGSLNYVSDF

>Pdav1-Chr6-03; Yendovirus; Prunus davidiana 1;

LEELLQLAEQTKIIGEDPQLHWSKNKILTKLDIINQDLTIKTADMPCNLLDKQEFEQXIKELLNLKVIRPSKSRHRSAAFVVRKHSELKRARIVYNYRRLNDNTYEDSYKLPNKDELMNKIQESKYFSKFDCKSGFWQIRLAEESIQWTTFTCLEGHFEWLVMPFGLKNAPLIFQRKMDQIFKKYDNLCSVYVDDILVHSKNKNEHRKHLEIILKEFTDNGIVISRNKVELQKQDIEFLGMYIKSGTIQLQEHVAKKVLEFPDKLEDKKQLQAFLDLLNYARNFFPD

>Pdav1-Chr7-01; Florendovirus; Prunus davidiana 1;

QKIKDEICSELPNAFWYRKQHVVCLPYIKTFSEKNIPTKARPIQMNQEMLEFCKKEIDELLQKKIIRKSKSPWSCPAFYVQKNAELERGVPRLVINYKPLNAVLEWIRYPIPNKRDLINRLGKAVIYSKFDMKSGFWQIQIDESDRYKTAFVTPFGHYEWNVMPFGLKNAPSEFQNIMNDIFNPYSQFSIVYIDDVLIFSESVEQHWKHLHKFLQIVKQNGLVVSAKKIKLFQTNVRFLGFNICQSQISPIDRVIQFADKFPDQILDKSQLQRFLGSLNY

>Pdav1-Chr7-02; Florendovirus; Prunus davidiana 1;

EKLKQEICSDLPNAFWHRKQHVVKLPYVKNFSEKNIPTKAKPIQMSQEIMEFLKTEITKLLQKGIIRKSKSPWSCPAFYVQKNAELERGAPRLVINYINLLIQSXNGLRIQFLIKEIXLIDXTKQSSFLSLIXTVVSGKSKSGNLIGIKSLLXPHSVTMSGIMPFGLKNAHSEFQNIMNEIFNHFSHFSIVYIDDVLIFSQSVEQHWKHLHKFLQIVKQNGLVVSTKKIKLFQTNIRFIGFNICQSQISPIDRVIQFSDKFSDQILDKGQLHRFLGSLNYV

>Pdav1-Chr8-01; Yendovirus; Prunus davidiana 1;

LEELLQLAKQTRIIGEDPQLHWNKNKILAKLDIINLDLTIKTADMPCNLLDKQEFYQQIKQILNLKVIRPSKSRHRSTAFVVRKHLELKRGKVRIVYNYRRLNDNTYEDSYKLPNKDELMNKIQESKYFSKFDCKSGFXQIRLAEESIXLTTFTCPEGNFEWLVMPFGLKNAPSIFQRKMDQIFKKYDNFCSVYVEDILVHSKNKNKHRKYLEIILKEFIDNGIVISRNKVELEKQDIEFLGIYIKSGTIQLQEHIAKKVLEFPDKLENKKQLQEFLGLLNYARSFIPD

>Pdav1-Chr8-02; Florendovirus; Prunus davidiana 1;

QKIVAEICSELPNAFWYRKQHVVCLPYIKTFSEKNIPTKARPIQMNQEMLEFCRKEINELLQKKIIRKSKSPWSCPAFYVQKNAELERGVPRLVINYKPLNTVLEWIRYPIPNKRDLINRLEKAVVYSKFDMKSGFWQIQIDESDRYKTAFVTPFGHYEWNVMPFGLKNAPSEFQNIMNEIFNPYSQFSIVYIDDVLIFSESVEQHWKHLHKFLQVVKQNGLVVSAKKIKLFQTNIRFLGFNICQSQISPIDRVIQFADKFPDQILDKSQLQRFLGSLNY

>Pdul1-Chr1-01; Florendovirus; Prunus dulcis 1;

KLKEEICSELPTAFWYRKQHVVCLPYIKTFSEKNIPTKARPIQMNQELMEFCKKEIHELLQKNIIHKSKSPWSCPAFYVQKNAELERGVPRLVINYKPLNSVLEWIRYPIPNKRDLINRLGKAVIFSKFDMKSGFWQIQIDESDRYKTAFVTPFGHYEWNVMPFGLKNAPSEFQNIMNDIFNPYSHFSIVYIDDVLIFSESVEQHWKHLHKFLSIVKQNGLVVSAKKIKLFQTNIRFLGFNISQSSISPINRVIQFADKFPDQILDRSQLQRFLGSLNYISDFYP

>Pdul1-Chr1-02; Florendovirus; Prunus dulcis 1;

EKLKQEVCSELPNAFWHRKQLVVSLPYVKSFSEKNIPTKARPIQMSQELMEFCKTEITELLEKGIIRKSKSRWSYPTFYVQKNAELERGVSRLVINYKPLNTVIEWIRYPIPNKRDLINRLEKSVIFSKFDMKSGFWQIQICESDRXKTAFVTPFGHYEWNVMPFGLKNAPGEFQNIMNEIFNPFSHFSIVYIDDVLIFSQSIEQHWKHLHKFLRIVKQNGLVVSAKKIKLFQTNVRFLRFNICQSQISPIDRVIQFADKFPDQILDKSQLHRFLGSLNYVSDF

>Pdul1-Chr1-03; Florendovirus; Prunus dulcis 1;

EKLKQEVCSELPNAFWHRKQHVVSLPYVKSFGVKNIPTKARPIQMSQELMEFCKLEIANLLXKGIIRKSKSPWSCPAFYVQKNAELERGVPRLVINYKPLNTVLKWIRYPIPNKRDLINRLEKSVILSKFDMKSGFWQIQISESDRYKTAFVTPFGHYEWNVMPFGLKNAPSEFQNIMNEIFNPFSHLSIVYIDDVLIFSESIEQHWKHLYKFLQIIKQNGLVVSSKKIKLFQTNVRFLGFNICQSQISPIDRVIQFADKFPDQILDKSQLXRFLGSLNYVSDF

>Pdul1-Chr1-04; Florendovirus; Prunus dulcis 1;

LKNEICSDLPTAFWYRKQHVVCLPYIKTFSEKNIPTKARPIQMNQELMEFCKKEIQELLQKNIIRKSKSPWSCFAFYVQKNAELERGVPRFVINYKPLNSVLEWIRYHIPNKRDLINRLGNAVVYSKFDMKSGFWQIQIDESDRYKTAFVTPFGHCEWNVMPFGLKNAPSEFQNIMNDIFNPYSHFSIVYIDDFLIFSESVQQHWKHLHKFLHIVKRNGLVVSAKKIKLFQTNIRFLGFNICQSQISPIDRVIQFADKFPDQILDKSQLQRFLGSLNYVS

>Pdul1-Chr2-01; Yendovirus; Prunus dulcis 1;

SKNKILTKPDIINPYLTIKTVDMPCSLLDKQEFEHQIKELQNLKVIRPSKSRHRSAAFIVIKHSELKRGKARILYNYRRLKDNTYEDSYKLLNKDELINKIQDSKYFSKFDCKFGFWQIRLAKESIEWTIFTCPEXHFEWLVMPFGLKNAPSIFQRKMDQTFKKYDSFCRCXXYTYTXXNKNDHKKTLRNNFAGIYXKXSLVKIKLEKQDKNFXEYMSGTIKLQEHI

>Pdul1-Chr2-02; Florendovirus; Prunus dulcis 1;

QKLVSEICSDLPTAFWHRKNHVVKLPYIKEFSEKNISTKSRPIQMNHEMMEFCKNEINDLLQKNIIRKSKSPWSCPAFYVQKNAELERGSPRLVINYKPLNSVLEWIRYPIPNKRDLIKRLDKAIVFSKFDMKSGFWQIQIHESDRYKTAFVTPFGHYEWNVMPFGLKNAPSEFQNIMNEIFNPYSHFSIVYIDDVLIFSESIEQHWKHLHSFLHIIKTNGLVVSAKKIKLFQTAIRFLGYNISQSQIHPIDRVIQFADKFPDQITDKNQLQRFLGSLNYVSDF

>Pdul1-Chr2-03; Florendovirus; Prunus dulcis 1;

QKLVSEICSDLPTAFWHRKNHVVKLPYIKEFSEKNISTKSRPIQMNHEMMEFCKNEINDLLQKNIIRKSKSPWSCPAFYVQKNAELERGSPRLVINYKPLNSVLEWIRYPIPNKRDLIKRLDKAIVFSKFDMKSGFWQIQIHESDRYKTAFVTPFGHYEWNVMPFGLKNAPSEFQNIMNEIFNPYSHFSIVYIDDVLIFSESIEQHWKHLHSFLHIIKTNGLVVSAKKIKLFQTAIRFLGYNISQSQIHPIDRVIQFADKFPDQITDKNQLQRFLGSLNYVSDF

>Pdul1-Chr2-04; Florendovirus; Prunus dulcis 1;

DKIKAEICSELPTAFWYRKQHVVCLPYIKTFSEKNIPTKARPIQMSQELMEVCKEEIQELLQKNIIRKSKSPWSCPAFYVQKNAELERGVPRLVINYKPLNSVLEWIRYPIPNKRDLINRLEKAVVFSKFDMKSGFWQIQIDEPNRYKTAFVTPFGHYEWNVMPFGLKNAPSEFQNIMNDIFNPYSHFSIVYIDDVLIFSESVEQHWKHLHKFLHIVKHNGLVVSVKKIKLFQTTVRFLGFNINQSQISPIDRVIQFADKFPDQILDKSQLQRFLGSLNFVSDF

>Pdul1-Chr2-05; Florendovirus; Prunus dulcis 1;

KKLISEVCSDFPTAFWHRKNHVVHLPYIKDFSEKNIPTKARPIQMNHEMMEFCKTEINELLQKNIIRKSKSPWSCPAFYVQKNAELERGSPRLVINYKPLNSVLAWIRYPIPNKRDLIKRLDKAVIFSKFDMKSGFWQIQIHESNRYKTAFVTPFGHYEWNVMPFGLKNAPSEFQNIMNEIFNPYSHFSIVYIDDVLIFSESLEQHXKHLHTFLHIIKTNGLVVSAKKIKLFQTHIRFLGYNIHHLQIHPIDRAIHFADKFPDQITDKNQLQRFLGSLNY

>Pdul1-Chr2-06; Florendovirus; Prunus dulcis 1;

KKLISEVCSDFPTAFWHRKNHVVHLPYIKDFSEKNIPTKARPIQMNHEMMEFCKTEINELLQKNIIRKSKSPWSCPAFYVQKNAELERGSPRLVINYKPLNSVLAWIRYPIPNKRDLIKRLDKAVIFSKFDMKSGFWQIQIHESNRYKTAFVTPFGHYEWNVMPFGLKNAPSEFQNIMNEIFNPYSHFSIVYIDDVLIFSESLEQHXKHLHTFLHIIKTNGLVVSAKKIKLFQTHIRFLGYNIHHLQIHPIDRAIHFADKFPDQITDKNQLQRFLGSLNY

>Pdul1-Chr3-01; Florendovirus; Prunus dulcis 1;

EKLKQEVCSEFPNAFWHRKQHVICLPYVKTFSEKNIPTKARPIQMSQELMEFCKIEITDLLKKGIIRKSKSPWSCPAFYVQKNAELERGVPRLVINYKPLNTVLEWIRYPIPNKRDLINRLEKSVIFFKFDMKSGFWQIQISEADRYKTAFVTPFGHYEWNVMHFGLKNAPSEFQNIMNEIFNPFSHFSIVYIDDVLIFSESIEQHWKHLYKFLQIVKQNGLVISAKKIKLFQTNVRFLGFNICQSQISPIDRVIQFADKFPDQIVDKSQLQRFLGSLNYV

>Pdul1-Chr3-02; Florendovirus; Prunus dulcis 1;

EKIKNEICSELRNAFWYRKQHVVCLPYIKTFSEKNILTRARPIQMNQELMEFCKKEIHELLQKNIIRKSKSPWSCPAFYVQKNAELERGVPRLVINYKPLNSVLEWIRYPIPNKRDLINRLGKAVIVFSKFDMKSGFWQIQIDESDRYKTAFVTHFGHYEWNVMPFGLKNAPSEFQNIMNDIFNHYSHFSIVYIDDVLIFSESVEQHWKHLHQFFHIVKHNGLVVSARKIKLFQTNVRFLGFNISQSQISPTDRVIQFADKFPDQILDKSQLQRFLGSLNYVSDF

>Pdul1-Chr3-03; Florendovirus; Prunus dulcis 1;

DKLKEEICSDLPTAFWYRKQHVVCLPYIKTFSEKNIPTKARPIQMNQELMEFCKKEIHELLQKNIIRKSKSPWSCPAFYVQKNAELERGVPRLVINYKPLNSVLEWIRYPIPNKRDLINRLGKAVVFSKFDMKSGFWQIQIDESDMYKTAFVTPFGHYEWNVMPFGLKNAPSEFQNIMNDIFNPYSHFSIVYIDDVLIFSESVEQHWKHLHKFLHIVKQNGLVVSAKKIKLFQINIRFLGFNISQSQITPIDRVIQFTDKFPDQILDKNQLQRFLGSLNYISDFYP

>Pdul1-Chr4-01; Florendovirus; Prunus dulcis 1;

RLQVEICSNLPTAFWYRKQHVVCLPYIKTFSEKNIPTKARPIQMNQELMEICKKEIQELLHKNIIRKSKSPWSCPAFYVQKNAELERGVPRLVINYKPLNSVLEWIRYPIPNKRDLINRLEKAVVFSKFDMKSGFWQIQIDESDRYKTAFVTPFGHYEWNVMPFGLKNAPSEFXNIMNDIFNPYSHFSIVYIDDVLIFSESVKQHWKHLHKFLHIVKQNGLVVSAKKIKLLQTNIRFLGFNISQSQIAPIDRVIQFADKFPDQILDKSQLQRFLGSLNYV

>Pdul1-Chr4-02; Florendovirus; Prunus dulcis 1;

KLKDEICSDLPTAFWYRKQHVVCLPYIKTFSEKNIPTKARPIQMNQELMEFCKKEIQELLQKNIIRKSKSPWSCPAFYVQKNAELERGVPRLVINYKPLNSVLEWIRYPIPNKSDLINGLEKAVVFSKFDMKSGFWQIQIDESDRYKTAFVTPFGHYEWNVMPFGLKNAPSEFQNIMNDIFNPYSHFSIVYIDDVLIFSESVEQHWKHLHKFLYIVKQNGLVVSAKKIKLFQTNIRFLGFNICQSQISPIDRVIQFADKFPDQILDKSQLQRFLGSLNYISDFYP

>Pdul1-Chr4-03; Florendovirus; Prunus dulcis 1;

KLKDEICSELPTAFRYRKQHVVCLPYIKTFSEKNIPTKARPIQMNQELMEVCKKEIHELLQKNIIRKSKSPWSCPAFYVQKNAEVERGVPRLVINYKPLNSVLEWIRYPIPNKRDLINRLGKAVVFSKFDMKSGFWQIQIDQSDRYKTAFVTPFGHYEWNVMPFGLKNAPSEFQNIMNDIFNPYSHFSIVYIDDVLIFSESVEQHWKHLHKFLHIVKQNGLVVSAKKIKLFQTNIRFLGFNICQSQISPIDRVIQFADKFPDQILDKSQLQRFLGSLNYISDFYP

>Pdul1-Chr4-04; Yendovirus; Prunus dulcis 1;

LEELLQLAEQTKIIGEDPQLHWTKNKILAKLDIINQDLTIKTADMPCNLLDKQKFKQXIKELLNLKVIRPSKSRHRSAAFVVRKHPELKRGKARIVYNYRRLNDNTYEDSYKLPNKDELMNKIQESKYFSKFDCKSGFWQIRLAEESIQWTTFTCPKGHFEWPVMPFGLKNAPLIFQRKMDQIFKKYDNLCSVYVDDILVHSKNKNEHRKHLEIILKEIIDNGRVTSRNKVELEKQDIEFLGMYIKXGTIQLQEHIAKKVLEFPDKLEDKKQLQVFLGLLNYARNFFPD

>Pdul1-Chr5-01; Florendovirus; Prunus dulcis 1;

KLINDICSDLPNAFWSRKQHIVSLPYAKTFSEKNIPIKARPIQMNQETMEFCKKEITELLLKGIIRKSKSPWSCPAFYVQKNAELERGVPRLVINYKPLNAVLEWIRYPIPNKRDLINRLEKAVVFSKFDLKSGFWQIQIEESDRYKTAFVTPFGHYEWNVMPFGLKNAPSEFQNIMNEIFNAYSHFSIVYIDDVLIYSQSIEQHWKHLSIFLQVVKQNGLVVSAKKIKLFQTNVRFLGFNICQSQISPIDRVIQFADKFPDEIVDKSQLQRFLGSLNYVSDF

>Pdul1-Chr5-02; Florendovirus; Prunus dulcis 1;

KLKNEICSELPTTFWYRKQHVVCLPYIKTFSEKNIPTKSRPIQMNQELMEVCKKEILELLQKNIIRRSKSPWSCPAFYVQKNAELERGVPRLVINYKPLNSVLEWIRYPIPNKRDLINQLEKAVVYSKFDMKSGFWQIQIDESDRYKTAFVTPFGHYEWNIMPFGLKNAPSEFQNIMNDIFNPYSHFSIVYIDDVLIFSESVEQHWKHLYKFLHIAKQNGLVVSAKKIKLFQTNIRFLGFNISQSQISPIDRVIQFADKFPDQILDKSQLQRFLGSLNYVSDF

>Pdul1-Chr6-01; Florendovirus; Prunus dulcis 1;

KLKEEICSDLPTAFWYRKQHVVCLPYIKTFSEKNIPTKARPIQMNQELMEFCKEEIHELLQKNIIRKSKSPWSCPAFYVQKNAELERGVPRLVINYKPLNSVLEWIRYPIPNKRDLINRLGKAVVFSKFDMKSGFWQIQIDESDRYKTAFVTPFGHYEWNVMPFGLKNAPSEFQNIMNDIFNPYSHFSIVYIDDVLIFSESVEQHWKHLHKFLHIVKQNGLVVSAKKIKLFQTNIRFLGFNICQSQISPIDRVIQFADKFPDQILDKSQLQRFLGSLNYISDFYP

>Pdul1-Chr6-02; Florendovirus; Prunus dulcis 1;

EKLKQEVCSELPNAFWHRKQHVVSLPYVKSFSKKNIPTKARPIQMSQELMEFCKIEITDLLKKGIIRKSKSPXSCPAFYVQKNAELERGVPRLVINYKPLNTVLEWIRYPIPNKRDLINRLENSVIFSKFDMKSGFWQIQISESDRYKTAFVTPFGHYEWKVMPFSLKNAPSEFQNIMNEIFNPFSHFSIVYIDDVLIFSESIEQHWKHLYKFLQIVKQNGLVVSTKNIKLFQTNVRFLRFNICQSQISLIDRVIQFADKFPDQILDKSQLQRFLGSLNYVSDF

>Pdul1-Chr6-03; Yendovirus; Prunus dulcis 1;

IIGEDPQLHWNKNKILAKLDIINPDLTIKIADMPCTLLDKQEFEQQIKELLNLKVIRPSKSRHRSAAFVVRKHSELKRGKARIMYNYRRLNDNTYENSYKLPNKDELMNKIQXSKYFSKFDCKSGFWQIRLAEESIXXTAFTCPEGHFEWLVMSFGLKKMHHRSFKERWTKYLKSMIVFVVYMXVIYXFIVKIKMNKKHLEIILKEFIDNGIVISRNKVELKKQDIEFLGMYIKSGTIXLQEHIAKKVLEFPDKLDDKKQLQAFLGLLNYAXSFIPD

>Pdul1-Chr6-04; Florendovirus; Prunus dulcis 1;

KLKEEICSDLPTAFWYRKQHVVCLPYIKTFSEKNIPTKARPIQMSQELMEVCKKEIHELLQKNIIRKSKSPWSCPAFYVQKNAELERGVPRLVINYKPLNSVLEWIRYPIPNKRDLINRLGKAVVFSKFDLKSGFWQIQINESDRYKTAFVTPFGHYEWNVMPFGLKNAPSEFQNIMNDIFNPYSHFSIVYIDDVLIFSESVEQHWKHLHKFLHIVKQNGLVVSAKKIKLFQTNIRFLGFNICQSQVAPINRVIQFADKFPNQILDKSQLXRFLGSLNYVSDF

>Pdul1-Chr7-01; Florendovirus; Prunus dulcis 1;

EKLKQEVCSDLPNAFWHRKQHVVKLPYVKNFSEKNIPTKARPIQMSQEIMEFLKTEITELLQKGIIRKSKSPWSCPAFYVXKNAELGRGAPRLVTNYKPLNTVLEWIRYPIPNKRDLINRLDKAVIFSKFDMNSGFWQIQIQESDRYKIAFLTPFGHYEWNVMPFGLKNAPSEFQNIMNEIFNPFSHFSIVYIDDVLIFSQSIEQHWKHLHKFLQIVKQNGLVVSAKKMKLFQTNIRFLGFNIYXSQISPIDRVIQFANKFPDQILDKGQLQRFLGSLNYV

>Pdul1-Chr7-02; Florendovirus; Prunus dulcis 1;

KLQDEICSDLPTAFWYRKQHVVCLPYIKTFSEKNIPTKARPIQMNQEFMEVCKNEIHELLQKNIIRKSKSPWSCPAFYVQKNAELERGVPRLVINYKPLNSALEWLRYPIPNKRDLINRLEKAIVFSKFDMKSGFWQIQIDESDRYKTAFVTPFGHYEWNVMPFGLKNAPSEFQNIMNDIFNPYSHFSIVYIDDVLIYSESVEXHWKHLHKFLHIVKQNGLVVSAKKIKLFQTNIRFLGFNISQSQISPIDRVIQFADKFPDQILDKSQLQRFLGSLNYVSDF

>Pdul1-Chr7-03; Florendovirus; Prunus dulcis 1;

KLKNEICSELPTAFWYRKQHVVCLPYIKTFSEKNIPTKARPIQMNQELMEFCKKEIQELLQKNIIRKSKSPWSCPAFYVQKNAELERGVPRLVINYKPLNSVLEWIRYPIPNKRDLINRSGNAVVYSKFDMKSGFWQIQIDESDRYKTAFVTPFGHYEWNVMPFGLKNAPSEFQNIMNDIFNPYSHFSIVYIDDVLIFSESVEQHWKHLHKFLHIVKQNGLVVSAKKIKLFQTNIRFLGFQICQSQISPIDRVIQFADKFPDQILDKSQLQRFLGSLNYVSDF

>Pdul2-Chr1-01; Florendovirus; Prunus dulcis 2;

KLKEEICSDLPTAFWYRKQHVVCLPYIKTFSEKNIPNKARPIQMNQELMEFCKKEIHELLQKNIIRKSKSPWSCPAFYVQKNAELERGVPRLVINYKPLNSVLEWIRYPIPNKRDLINRLEKAVVFSKFDMKSGFWQIQIDESDRCKTAFVTPFGHYEWTVMPFGLKNAPSEFQNIMNDIFNPYSHFSIVYIDDVLIFSESVEQHWKHLHKFLQIVKQNGLVVSAKKIKLFQTNIRFLGFNISQSHISPIDRVIQFADKFPDQILDKSQLQRFLGSLNYV

>Pdul2-Chr1-02; Florendovirus; Prunus dulcis 2;

KLKEEIFFDLPTAFWYRKQHVVCLPYIKTFSEKNIPTKARPIQMNQELMEFCKKEIHELLQKNIIRKSKSPXSCPAFYVQKNAELERGVPRLVINYKPLNSVLEWIRYPIPNKRNLINRLEKAVVFSKFDMKSGFWQIQIDESDRYKTAFVTPFGHYEWNVMPFGLKNAPSEFQNIMNDIFNPYSHFSIVYIDDVLIFSESVEQHWKHLHKFLQIVKQNGLVVFAKKIKLFQTNIRFLGFNISQSHISPIDRVIQFADKFPDQILDKSQLQRFLGSLNYV

>Pdul2-Chr1-03; Yendovirus; Prunus dulcis 2;

NKNKILAKLDIISPDLTIKTTDMPYNLLDKQEFEQQIKELLNLKVIRPSKSRHRSAAFVVRKYSKLKRGKAKXCIIIGDXMIKLMKIVINYKIKMNLRIKFKKVNILVNLIVNQDFAKLDXQNNQFNGRHSVIQKDILNGLSCHFRXRMHHRSFKERWIKILRSMTIFVVYMXMIYLFIVKKKKKEHRKHLEIILKXFIDNGIVISRNKVELEKQDIEFLGTYIKSGTIQLQXHIAKKVLKFPNKLEDKKQLQVFLGXLNYARSFIPK

>Pdul2-Chr1-04; Florendovirus; Prunus dulcis 2;

EKLKQEVCSELPNAFWHRKQLVVSLPYVKSFSEKNIPTKARPIQMSQELMEFCKTEITELLEKGIIRKSKSPWSYPTFYVQKNAELERGVSRLVINYKPLNTVLEWIRYLIPNKRDLINRLEKSVIFSKFDMKSGFWXIQICESDRYKTAFVTPFGHYEWNVMPFSLKNAPGEFQNIMNEIFNPFSHFSIVYIDDVLIFSQSIEQHWKHLHKFLQIVKHNGLVVSAKKIKLFQTNVRFLGFNICQSQISPIDRVIQFTDKF

>Pdul2-Chr1-05; Florendovirus; Prunus dulcis 2;

EKLKQEVCSELPNAFWHRKQHVVSLPYVKSFSVKNIPTKARPIQMSQELMEFCKLEIADLLXKGIIRKSKSPWSCPAFYVQKNAELERGVPRLVINYKPLNTVLKWIRYPIPNKRDLINRLEKSVILSKFDMKSGFWQIQISESDRYKTAFVTPFGHYEWNVMPFGLKNAPSEFQNIMNEIFNPFSHLSIVYIDDVLIFSESIEQHWKHLYKFLQIIKQNGLVVSAKKIKLFQTNVRFLGFNICQSQISPIDRVIQFADKFPDQILDKSQLXRFLGSLNYV

>Pdul2-Chr1-06; Florendovirus; Prunus dulcis 2;

LKNEICSDLPTAFWYRKQHVVCLPYIKTFSEKNIPTKARPIQMNQELMEFCKKEIQELLQKNIIRKSKSPWSCLAFYVQKNAELERGVPRFVINYKPLNSVLEWIRYHIPNKRDLINRLGNAVVYSKFDMKSGFWQIQIDESDRYKTAFVTPFGHYEWNVMPFGLKNAPSEFQNIMNDIFNPYSHFSIVYIDDFLIFSESVQQHWKHLHKFLHIVKRNGLVVSAKKIKLFQTNIRFLGFNICQSQISPIDRVIQFADKFPDQILDKSQLQRFLGSLNYVSDF

>Pdul2-Chr1-07; Florendovirus; Prunus dulcis 2;

EKLKQEVCSELPTAFWHRKQHVVSLSYVKSFSEKNIPTKAKPIQMSQELMEFCKLEIANLLXKRIIRKSKSPXSCPAFYVQKNAELERGLPRLFINYKPLNTVLEWIRYPIPNKRDLINILEKSVIFSKFDMKSGFWQIQISEPDRYKTAFVTPFGHYEWNVMPFGLKNAPSEFQNIMNEIFNPFSHFSIVYIDDVLIFSESIEQHWKHLYKFLQIVKQNGLVVSAKKIKLFQTNVRFLRFSICXSQISPIDRVIQFADKFPDQILDKSQLQRFLGSLNYVSDF

>Pdul2-Chr2-01; Florendovirus; Prunus dulcis 2;

DKIKAEICSELPTAFWYRKQHVVCLPYVKTFSEKNIPTKARPIQMSQELMEVCKEEIQELLQKNIIRKSKSPWSCPAFYVQKNAELERGVPRLVINYKPLNSVLEWIRYPIPNKRDLINRLEKAVVFSKFDMKSGFWQIQIDEPNRYKTAFVTPFGHYEWNVMPFGLKNAPSEFQNIMNDIFNPYSHFSIVYIDDVLIFSESVEQHWKHLHKFLHIVKHNGLVVSVKKIKLFQTTVRFLGFNINQSQISPIDRVIQFADKFPDQILDKSQLQRFLGSLNFVSDF

>Pdul2-Chr2-02; Florendovirus; Prunus dulcis 2;

KKLISEVCSDFPTAFWHRKNHVVHLPYIKDFSEKNIPTKARPIQMNHEMMEFCKTEINELLQKNIIRKSKSPWSCPAFYVQKNAELERGSPRLVINYKPLNSVLAWIRYPIPNKRDLIKRLDKAVIFSKFDMKSGFWQIQIHESNRYKTAFVTPFGHYEWNVMPFGLKNAPSEFQNIMNEIFNPYSHFSIVYIDDVLIFSESLEQHXKHLHTFLHIIKTNGLVVSAKKIKLFQTHIRFLGYNIHHLQIHPIDRAIHFADKFPDQITDKNQLQRFLGSLNY

>Pdul2-Chr3-01; Florendovirus; Prunus dulcis 2;

EKLKQEVCTEFPNAFWHRKQHVICLPYVKTFSEKNIPTKARPIQMSQELMEFCKIEITDLLKKGIIRKSKSPWSCPAFYVQKNAELERGVPRLVINYKPLNTVLEWIRYPIPNKRDLINRLEKSVIFFKFDMKSGFWXIQISEADRYKTAFVTPFGHYEWNVMPFGLKNAPSEFQNIMNEIFNPFSHFSIVYIDDVLIFSESIEQHWKHLYKFLQIVKQNGLVISAKKIKLFQTNVRFLGFNICQSQISPIDRVIQFADKFPDQIVDKSQLQRFLGSLNYV

>Pdul2-Chr3-02; Florendovirus; Prunus dulcis 2;

EKLKQEVCSELPNAFWHRKQHVVSLPYVKSFSEKNIPTKARSIQMSQELMEFCKTEITELIEKRIIRKSNSPWSCPAFYVQKNAELERGVPRLVINYKPLNIVLEWIRYPIPNKRDLINRLEKSVIFSKFDMNSGFWQIQIHESDRYKTAFVTPFGHYEWNVMSFGLKNAPNEFQNIMNEIFNPYSHFSIVYIDDMLIFSQSIEQHWKHLHKFLQIVKQNGLVVSAKKIKLFQTNVRFLGFNICHTQISPIDRVIXFVVKFPNQILDKNQLQRYLGSLNYVSDF

>Pdul2-Chr3-03; Florendovirus; Prunus dulcis 2;

EKIKNEICSELRNAFWYRKQHVVCLPYIKTFSEKNIPTRARPIQMNQELMEFCKKEIHELLQKNIIRKSKSPWSCPAFYVQKNAELERGVPRLVINYKPLNSVLEWIRYPIPNKRDLINRLGKAVIVFSKFDMKSGFWQIQIDESDRYKTAFVTHFGHYEWNVMPFGLKNAPSEFQNIMNDIFNHYSHFSIVYIDDVLIFSESVEQHWKHLHQFFHIVKHNGLVVSARKIKLFQTNVRFLGFNISQSQISPTDRVIQFADKFPDQILDKSQLQRFLGSLNYVSDF

>Pdul2-Chr3-04; Florendovirus; Prunus dulcis 2;

DKLKEEICSDLPTAFWYRKQHVVCLPYIKTFSEKNIPTKARPIQMNQELMEFCKKEIHELLQKNIIRKSKSPWSCPAFYVQKNAELERGVPRLVINYKPLNSVLEWIRYPIPNKRDLINRLGKAVVFSKFDMKSGFWQIQIDESDRYKTAFVTPFGHYEWNVMPFGLKNAPSEFQNIMNDIFNPYSHFSIVYIDDVLIFSESVEQHWKHLHKFLHIVKQNGLVVSAKKIKLFQINIRFLGFNISQSQITPIDRVIQFADKFPDQILDKNQLQRFLGSLNYISDFYP

>Pdul2-Chr4-01; Florendovirus; Prunus dulcis 2;

RLQVEICSNLPTAFWYRKQHVVCLPYIKTFSEKNIPTKARPIQMNQELMEICKKEIQELLHKNIIRKSKSPWSCPAFYVQKNAELERGVPRLVINYKPLNSVLEWIRYPIPNKRDLINRLEKAVVFSKFDMKSGFWQIQIDESDRYKTAFVTPFGHYEWNVMPFGLKNAPSEFXNIMNDIFNPYSHFSIVYIDDVLIFSESVKQHWKHLHKFLHIVKQNGLVVSAKKIKLLQTNIRFLGFNISQSQIAPIDRVIQFADKFPDQILDKSQLQRFLGSLNYV

>Pdul2-Chr4-02; Florendovirus; Prunus dulcis 2;

KLKDEICSDLPTAFWYRKQHVVCLPYIKTFSEKNIPTKARPIQMNQELMEFCKKEIQELLQKNIIRKSKSPWSCPAFYVQKNAELERGVPRLVINYKPLNSVLEWIRYPIPNKRDLINGLEKAVVFSKFDMKSGFWQIQIDESDRYKTAFVTPFGHYEWNVMPFGLKNAPSEFQNIMNDIFNPYSHFSIVYIDDVLIFSESVEQHWKHLHKFLYIVKQNGLVVSAKKIKLFQTNIRFLGFNICQSQISPIDRVVQFADKFPDQILDKSQLQRFLGSLNYISDFYP

>Pdul2-Chr4-03; Florendovirus; Prunus dulcis 2;

KIKEEICSDLPTAFWYRKQHVVCLPYIKTFSEKNIPTKARPIQMNQELMEVCKKEIHELLQKNIIRKSKSPWSCPAFYVQKNAELERGVPRLVINYKPLNSVLEWIRYPIPNKRDLINRLGKSVVFSKFDMKSGFWQIQIDESDRYKTAFVTPFGHYEWNVMPFGLKNAPSEFXNIMNDIFNPYSHFLIVYIDDVLIFSESVEQHWKHLHTFLHIVKHNGLVVSAKKIKLFQTNVRFLGFNISQSQISPIDSVIQFADKFPDQILDKSQLQRFLGSLNYVSDF

>Pdul2-Chr4-04; Yendovirus; Prunus dulcis 2;

LEELLQLAEQTKIIGEDPQLHWTKNKILAKLDIINQDLTIKTADMPCNLLDKQKFKQXIKELLNLKVIRPSKSRHRSAAFVVRKHPELKRGKARIVYNYRRLNDNTYEDSYKLPNKDELMNKIQESKYFSKFDCKSGFWQIRLAEESIQWTTFTCPKGHFEWPVMPFGLKNAPLIFQRKMDQIFKKYDNLCSVYVDDILVHSKNKNEHRKHLEIILKEIIDNGRVTSRNKVELEKQDIEFLGMYIKXGTIQLQEHIAKKVLEFPDKLEDKKQLQVFLGLLNYARNFFPD

>Pdul2-Chr4-05; Florendovirus; Prunus dulcis 2;

EKIKQEIYSDLSNAFWHRKNHSVTLPYIKEFSEKNIPTKVRPIQISQEVMEFCKSEITELLQKKIIRKSKSPWSCPAFNVQKNAKLKQGGTRLVINYKPINSILEWIRYPIPNKRDLIKRLNNSVVFSKFYTKGGFXQIQIQECDRYKIAFVTPFGHSKWNVMPFGLKNAPSEFQNIMNEIFDPFSEFSIVYIDDFLIFSQSIEQHWKHLHKFLQIVKSNGLVVFAKKIKLFQSNVRFLGYNIHQSQISPVDRVIQFGNNFPYEILEKSQLQRFLGSLSYVSDF

>Pdul2-Chr4-06; Florendovirus; Prunus dulcis 2;

ELKSEICSELPTAFWYRKQHVVCLPYIKTFSEKNIPIKARPIQMNQELMEFCKKEIHELLQKNIIRKSKSPWSCPGFYVQKNAELERGVPRLVINYKPLNSVLEWIRYPIPNKRDLINRLEKAVVYSKFDMKSGFWQIQIDESDRYKTAFVTPFGHYEWNVMSFSLKNAPSEFQNIMNDIFNPYSHFSIVYIDDVLIFSESIEQHWKHLQKFLYIVKQNGLVVSAKKIKLFQTNIRFLGFNISQSQIAPIDRVIQFADKFPDPILDK

>Pdul2-Chr5-01; Florendovirus; Prunus dulcis 2;

QKLISEICSDLPTAFWYRKNHVVNLPYIKDFSEKNILTKSRPIQMNHEMMEFCKNEICDLLQKNIIRKSKSPWSCPTFYVQKNAELERGSPRLVINYKPLNSVLEWIRYPIPNKRDLIKRLDKAVVFSKFDMKSGFWQIQIHESGRYKTAFVTPFGHYEWNVMPFGLKNAPSEFQNIMNEIFNTHSHFSIVYIDDVLIFSESIEQHWKHLHTFLHIIKTNGLVVSAKKIKLFQTAIRFLGYNISQSQIHPIDRVIHFADKFPDQITDKNQLQRFLGSLNYVSDF

>Pdul2-Chr5-02; Yendovirus; Prunus dulcis 2;

LEELLQLAKQTRIIGEDPQLHWNKNKILAKLDIINLDLTIKTADMPCNLLDKQEFYQQIKQILNSKVIRPSKSRHRSTAFVVRKHLELKRGKVRIVYNYRRLNDNTYEDSYKLPNKDELMNKIQESKYFSKFDCKSRFXQIRLAEESIXLTTFTCPEGNFEWLVMPFGLKNAPSIFQRKMDQIFKKYDNFCSVYVEDILVHSKNKNKHRKYLEIILKEFIDNGIVISRNKVELEKQDIEFLGIYIKSGTIQLQEHIAKKVLEFPDKLENKKHYKNF

>Pdul2-Chr6-01; Florendovirus; Prunus dulcis 2;

LKNEICSELPTAFWYRKQHVVCLPYIKTFSEKNIPTKARPIQMNQELMLYCKKEIQELLQKNIIRKSKSPWSCPAFYVQKNAELERGVPTLVINYKPLNSVLEWIRYPIPNKRDLINRLEKAVVYSKFDMKSAFSRIHIDESDRYKTAFVTPFGHYEWNVMPFGLKNAPSEFQNIMNDIFNPYSHFSIVYIDDVLIFFESVAQRWKHLHKFLHIVKQNGLVVFAKKIKLFQTNTRFLGFNICQSQISPIDRVIQFADKFPNQILDKSQLQRFLGSLNYVSDF

>Pdul2-Chr6-02; Florendovirus; Prunus dulcis 2;

KLKDEICSELPTAFWYRKQHVVCLPYIKTFSEKNIPTKAQPIQMNQELMEVCKKEIHELLQKNIIRKSKSPWSCPAFYVQKNAELERGVPRLVINYKSLNSVLEWIRYPIPNKRDLINRLEKAVVFSKFDMKSGFWQIQIDESDRYKTAFVTPFGHYEWNVMPFGLKNAPSEFQNIMNDIFNPYSHFSIVYIDHVLIFSESVEQHWKHLHKFLHIVKQNGLVVSAKKIKLFQTNIRFLGFNICQSQISPIDRVIQFADKFPDQILDKSQLQRFLGSLNY

>Pdul2-Chr6-03; Florendovirus; Prunus dulcis 2;

KLIHDICSDLPNAFWSKKQHIVSLPYTKTFSETNIPTKARPIQMNQETMEFCKKEITELLLKGIIRKSKSPWSCPAFYVQKNAELERGVPRLVINYKPLNAVLEWIRYPIPNKRDLINSLEKAVVFSKFDLKSGFWQIQIKETDRYKTAFVTPFGHYEWNVMPFGLKNAPSEFQNIMNEIFNAHSHFSIVYIDDVLIYSQSIDQHWKHLSIFLQIIKQNGLVVSAKKIKLFQTNIRFLGFNICQSQISPIDRVIQFADKFPDEIIDKSQLQRFLGSLNYVSDF

>Pdul2-Chr6-04; Florendovirus; Prunus dulcis 2;

KLKEEICSDLPTAFWYRKQHVVCLPYIKSFSEKNIPTKARPIQMSQELMEICKNEIHELLQKNIIRKSKSPWSCPAFYVQKNAELERGVPRLVINYKPLNSVLEWIRYPIPNKRDLINRLGKAVVFSKFDMKSGFWQIQIDESDKYKTAFVTPFGHFEWNVMPFGLKNAPSEFQNIMNDIFNPYSHFSIVYIDDVLIFSESVEHHWKHLYQFLSIIKLNGLVVSAKKIKLFQVNIRFLGFHISQSQISPIDRVIQFADKFPDQILDKNQLXRFLGSLNYISDFYP

>Pdul3-Chr1-01; Florendovirus; Prunus dulcis 3a;

EKLKQEVCFELPNAFWHRKQLVVSLPYVKSFSEKNIPTKARPIQMSQELMEFCKTEITELLEKGIIRKSKSPWSYPTFYVQKNAELERGVLRLVINYKPLNTVLEWIRYLIPNKRDLINRLEKSIIFSKFDMKSGFWXIQICESDRYKTAFVTPFGHYEWNVMPFSLKNAPGEFQNIMNEIFNPFSHFSIVYIDDVLIFSQSIEQHWKHLHKFLQIVKHNGLVVSAKKIKLFQTNVRFLGFNICQSQISPIDRVIQFTNKFPDQILDKSQLHRFLGSLNYVSDF

>Pdul3-Chr1-02; Florendovirus; Prunus dulcis 3a;

KLKEEICSELPTAFWYRKQHVVCLPYIKTFSEKNIPTKARPIQMNQELMEFCKKEIHELLQKNIISKSKSPWSCPAFYVQKIAELERGVPRLVINYKPLNSVLEWIRYPIPNKRDLINRLGKAVVFSKFDMKSGFWQIQIDESDRYKTAFVTPFGHYEWNVMPFGLKNAPSEFQNIMNDTFNPYSHFSIVYIDDVLIFSESVEQHWKHLHKFLYIVKQNGLVVSAKKIKLFQTNIRFLGFNISQSHISPIDXVIQFADKFPDQILDKSQLQRFLGSLNYISDFYP

>Pdul3-Chr1-04; Florendovirus; Prunus dulcis 3a;

EKLKQEVCSELPNAFWHRKQHVVSLPYVKSFGVKNIPTKARPIQMSQELMEFCKLEIANLLXKGIIRKSKSPWSCPAFYVQKNAELERGVPRLVINYKPLNTVLKWIRYPIPNKRDLINRLEKSVILSKFDMKSGFWQIQISESDRYKTAFVTPFGHYEWNVMPFGLKNAPSEFQNIMNEIFNPFSHLSIVYIDDVLIFSESIEQHWKHLYKFLQIIKQNGLVVSSKKIKLFQTNVRFLGFNICQSQISPIDRVIQFADKFPDQILDKSQLXRFLGSLNYVSDF

>Pdul3-Chr1-05; Florendovirus; Prunus dulcis 3a;

LKNEICSDLPTAFWYRKQHVVCLPYIKTFSEKNIPTKARPIQMNQELMEFCKKEIQELLQKNIIRKSKSPWSCLAFYVQKNAELERGVPRLVINYKPLNSVLEWIRYPIPNKRDLINRLGNAVVYSKFDMKSGFWQIQIDESNRYKTAFVTPFGHYEWNVMPFGLKNAPSEFQNIMNDIFNPHSHFSIVYIDAFLIFSESVEQHLKHLHKFLHIVKRNGLVVSAKKIKLFQTNIRFLGFNICXSQISPIDRVIQFADKFPDHILD

>Pdul3-Chr1-06; Florendovirus; Prunus dulcis 3a;

KLKEEICSDLPTAFWYRKQHVVCLPYIKSFSEKNIPTKARPIQMSQELMEICKKEIHELLQKNIIRKSKSPWSCPAFYVQKNAELERGVPRLVINYNPLNSVLEWIRYPIPNKRDLINRLGKAVVFSKFDMKSGFWQIQINESDRYKTAFVTPFGHYEWNVMPFGLKNAPSEFQNIMNDIFNPHSHFSIVYIDDVLIFSESVEQHWKHLHKFLHIVKQNGLVVSAKKIKLFQTNIRFLGFNISQSQITPIDRVIQFADKFPDQILDKNQLQRFLGSLNYISDFYP

>Pdul3-Chr1-07; Florendovirus; Prunus dulcis 3a;

EKLKQEVCSELPTAFWHRKQHVVSLSYVKSFSEKNIPTKAKPIQMSQELMEFCKLEIANLLXKRIIRKSKSPXSCPAFYVQKNAELERGLPRLFINYKPLNTVLEWIRYPIPNKRDLINILEKSVIFSKFDMKSGFWQIQISEPDRYKTAFVTPFGHYEWNVMPFGLKNAPSEFQNIMNEIFNPFSHFSIVYIDDVLIFSESIEQHWKHLYKFLQIVKQNGLVVSAKKIKLFQTNVRFLWFSICXSQISPIDRVIQFADKFPDQILDKSQLQRFLGSLNYVSDF

>Pdul3-Chr2-01; Florendovirus; Prunus dulcis 3a;

EKLKQEVCSELPNAFWHRKQHVVNLPCVKTFSEKNIPTKARPIQMSQELMEFCKIDITDLLKKGIIRKSKSPWSCPAFYVQKNTKLECGVPRLVINYKPLNTVLEWIRYPIPNKRDLINILEKSVIFSKFDMKSGFWQIQIYESDRYKTAFVTPFGHYEWNVMPFGLKNAPREFQNIMNEIFNPFSHFSIVYIDEVLIFSESVEQHWKHLYKFLQIVKQNGLVVSAKKIKLFQTNVRFLGFNICQSQISPIYQVIQFADKFPDQILDKSQLQRFLGPLNYVSDF

>Pdul3-Chr2-03; Florendovirus; Prunus dulcis 3a;

QKLVSEICSDLPTAFWHRKNHVVKLPYIKEFSEKNISTKSRPIQMNHEMMEFCKNEINDLLQKNIIRKSKSPWSCPAFYVQKNAELERGSPRLVINYKPLNSVLEWIRYPIPNKRDLIKRLDKAIVFSKFDMKSGFWQIQIHESDRYKTAFVTPFGHYEWNVMPFGLKNAPSEFQNIMNEIFNPYSHFSIVYIDDVLIFSESIEQHWKHLHSFLHIIKTNGLVVSAKKIKLFQTAIRFLGYNISQSQIHPIDRVIQFADKFPDQITDKNQLQRFLGSLNYVSDF

>Pdul3-Chr2-04; Florendovirus; Prunus dulcis 3a;

QKLVSEICSDLPTAFWHRKNHVVKLPYIKEFSEKNISTKSRPIQMNHEMMEFCKNEINDLLQKNIIRKSKSPWSCPAFYVQKNAELERGSPRLVINYKPLNSVLEWIRYPIPNKRDLIKRLDKAIVFSKFDMKSGFWQIQIHESDRYKTAFVTPFGHYEWNVMPFGLKNAPSEFQNIMNEIFNPYSHFSIVYIDDVLIFSESIEQHWKHLHSFLHIIKTNGLVVSAKKIKLFQTAIRFLGYNISQSQIHPIDRVIQFADKFPDQITDKNQLQRFLGSLNYVSDF

>Pdul3-Chr2-05; Florendovirus; Prunus dulcis 3a;

QKLVSEVCSDLPTAFWHRKNHVVNLPYIKDFSEKNIPTKSRPIQMNHEMMEFCKNEINDLLQKNIIRKSKSPWSCPAFYVQKNAELERGSPRLVINYKPLNSVLQWIRYPIPNKRDLIKRLDKAVVFSKFDMKSGFWQIQIHESDRYKTAFVTPFGHYEWNVMPFGLKNAPSEFQNIMNEIFNTYSHFSIVYIDDVLIFSESIDQHWKHLHTFLHIIKTNGLVVSAKKIKLFQTDIRFLGYNISQSQIHPIDRVIHFADKFPDQITDKNQLQRFLGSLNYV

>Pdul3-Chr2-06; Florendovirus; Prunus dulcis 3a;

DKIKAEICSELPTAFWYRKQHVVCLPYIKTFSEKNIPTKARPIQMSQELMEVCKEEIQELLQKNIIRKSKSPWSCPAFYVQKNAELERGVPRLVINYKPLNSVLEWIRYPIPNKRDLINRLEKAVVFSKFDMKSGFWQIQIDEPNRYKTAFVTPFGHYEWNVMPFGLKNAPSEFQNIMNDIFNPYSHFSIVYIDDVLIFSESVEQHWKHLHKFLHIVKHNGLVVSVKKIKLFQTTVRFLGFNINQSQISPIDRVIQFANKFPDQILDKSQLQRFLGSLNFVSDF

>Pdul3-Chr2-07; Florendovirus; Prunus dulcis 3a;

LISEVCSDFPTAFWHRKNHVVHLPYIKDFSEKNIPTKARPIQMNHEMMEFCKTEINELLQKNIIRKSKSPWSCPAFYVQKNAELERGSPRLVINYKPLNSVLAWIRYPIPNKRDLIKRLDKAVIFSKFDMKSGFWQIQIHESNRYKTAFVTPFGHYEWNVMPFGLKNAPSEFQNIMNEIFNPYSHFSIVYIDDVLIFSESLEQHXKHLHTFLHIIKTNGLVVSAKKIKLFQTHIRFLGYNIHHLQIHPIDRAIHFADKFPDQITDKNQLQRFLGSLNY

>Pdul3-Chr3-03; Florendovirus; Prunus dulcis 3a;

EKLKQEVCSELPNAFWHRKQHFVSLPYVKSFSEKNIPTKVRPIQMSQELMEFCKTEITDLLKKGIIRKSKSPWSCPAFHVQKNAELECGVPRLVINCKPLNIVLEXIRYQIPNKRDLINRLGKSVIFSKFDMKSGFWQIQISESDKYKTAFVTPFGHYEWNVMPFGLKNAPSEFQNIMNEIFNPFSHFSIVXIDDVLIFSESIEQHWKHLHKFLEMVKQSGLVVSAKKIKLFQTNVRFLGFNICXSQISPIDRVVQFADKFADQILDKSQLQRFLGSLNYVSDF

>Pdul3-Chr3-06; Florendovirus; Prunus dulcis 3a;

EKLKQEVCSELPNAFWHRKQHVVSLPYVKSFSEKNIPTKARSIQMSQELMEFCKTEITELIEKRIIRKSNSPWSCPAFYVQKNAELERGVPRLVINYKPLNIVLEWIRYPIPNKRDLINRLEKSVIFSKFDMNSGFWQIQIHESDRYKTAFVTPFGHYEWNVMSFGLKNAPNEFQNIMNEIFNPYSHFSIVYIDDMLIFSQLIEQHWKHLHKFLQIVKQNGLVVSAKKIKLFQTNVRFLGFNICHTQISPIDRVIXFVVKFPNQILDKNQLQRYLGSLNYVSDF

>Pdul3-Chr3-07; Florendovirus; Prunus dulcis 3a;

DKLKEEICSDLPTAFWYRKQHVVCLPYIKTFSEKNIPTKARPIQMNQELMEFCKKEIHELLQKNIIRKSKSPWSCPAFYVQKNAELERGVPRLVINYKPLNSVLEWIRYPIPNKRDLINRLGKAVVFSKFDMKSGFWQIQIDESDRYKTAFVTPFGHYEWNVMPFGLKNAPSEFQNIMNDIFNPYSHFSIVYIDDVLIFSESVEQHWKHLHKFLHIVKQNGLVVSAKKIKLFQINIRFLGFNISQSQITPIDRVIQFADKFPDQILDKNQLQRFLGSLNYIFDFYP

>Pdul3-Chr4-01; Florendovirus; Prunus dulcis 3a;

RLQVEICSNLPTAFWYRKQHVVCLPYIKTFSEKNIPTKARPIQMNQELMEICKKEIQELLHKNIIRKSKSPWSCPAFYVQKNAELERGVPRLVINYKPLNSVLEWIRYPIPNKRDLINRLEKAVVFSKFDMKSGFWQIQIDESDRYKTAFVTPFGHYEWNVMPFGLKNAPSEFXNIMNDIFNPYSHFSIVYIDDVLIFSESVKQHWKHLHKFLHIVKQNGLVVSAKKIKLLQTNIRFLGFNISQSQIAPIDRVIQFADKFPDQILDKSQLQRFLGSLNYV

>Pdul3-Chr4-02; Florendovirus; Prunus dulcis 3a;

KLKDEICSDLPTAFWYRKQHVVCLPYIKTFSEKNIPTKARPIQMNQELMEFCKKEIQELLQKNIIRKSKSPWSCPAFYVQKNAELERGVPRLVINYKPLNSVLEWIRYPIPNKRDLINRLEKAVVFSKFDMKSGFWQIQIDESDRYKTAFVTPFGHYEWNVMPFGLKNAPSEFQNIMNDIFNPYSHFSNVYIDDVLIFSESVEQHWKHLHKFLYIVKQNGLVVSAKKIKLFQTNIRFLGFNICQSQISPIDRVIQFADKFPDQILDKSQLQRFLGSLNYISDFYP

>Pdul3-Chr4-05; Florendovirus; Prunus dulcis 3a;

KLKEEICSDLPTAFWYRKQHVVCLPYIKSFSEKNIPTKARPIQMSQELMEICKKEIHELLQKNIIRKSKSPWSCPAFYVQKNAELERGVPRLVINYKPLNSVLEWIRYPIPNKRDLINRLGKAIIFSKFDMKSGFWQIQIDESDRYKTAFVTPFGHYEWNVMPFGFKNAPSEFQNIMNDIFNPYSHFSIVYIDDVLIFSESVEQHWKHLHKFLHIVKQNGLVVSAKKIKLFQTNIRFLGFNICQSQITPIDRVIQFADKFPDQILDKNQLQRFLGSLNYISDFYP

>Pdul3-Chr4-07; Yendovirus; Prunus dulcis 3a;

KIIGEDPQLHWSKNKILAKLDIINQDLTIKTVDMPCNLLDKQKFEQXIKELLNLKVIRPSKSRHRSAAFVVRKHSELKRGKARIVYNYRRLNDNTYEDSYKLPNKDELMNKIQESKYFSKFDCKSGFWQIRLAEESIQWTTFTCPEGHFEWPVMPFGLKNAPLIFQRKMDQIFKKYDNLCSVYVDDILVHSKNKNEHRKHLEIILKEIIDNGRVISRNKVELEKQDIEFLGMYIKXGTIQLQEHIAKKVLEFPDKLEDKKQLQVFLGLLNYARNFFPD

>Pdul3-Chr4-08; Florendovirus; Prunus dulcis 3a;

KLKNEICSELPTAFWYRKQHVVCLPYIKTFSEKNIPIKARPIQMNQELMEFCKKEIHELLQKNIIRKSKSPWSCPDFYVQKNAELERGVPRLVINYKPLNSVLEWIRYPIPNKRDLINRLEKAVVYSKFDMKSGFWQIQIDESDRYKTAFVTPFGHYEWNVMPFGLKNAPSEFQNIMNDIFNPYSHFSIVYIDDVLIFSESIEQHWKHLQKFLHIVKQNGLVVSAKKIKLFQTNIRFLGFNISQSQIAPIDRVIQFADKFPDXILDK

>Pdul3-Chr5-04; Yendovirus; Prunus dulcis 3a;

LEELLQLAKQTRIIGEDPQLHWNKNKILAKLDIINLDLTIKTADMPCNLLDKQEFYQQIKQILNSKVIRPSKSRHRSTAFVVRKHLELKRGKVRIVYNYRRLNDNTYEDSYKLPNKDELMNKIQESKYFSKFDCKSRFXQIRLAEESIXLTTFTCPEGNFEWLVMPFGLKNAPSIFQRKMDQIFKKYDNFCSVYVEDILVHSKNKNKHRKYLEIILKEFIDNGIVISRNKVELEKQDIEFLGIYIKSGTIQLQEHIAKKVLEFPDKLENKKHYKNF

>Pdul3-Chr5-05; Florendovirus; Prunus dulcis 3a;

QKLISEVCSDLPTAFWHRKNHVVNLPYIKDFSEKNIPTKARPIQMNHEMMEFCKNEINDLLQKNIIRKSKSHWSCPAFYVQKNAELERGSPRLVINYKPLNTVLEWIRYPIPNKRDLIKRLDKAVIFSKFDMKSGFWQIQIHEFDKYKIAFVTPFGHYEWNVMPFGLKNAPSEFQNIMNEIFNSYSHFSIVYIDDVLIFSESLEQHWKHLHKFLHIIKTNGLVVSAKKIKLFQTDIRFLGYNIRQSQIHPVDRVINFADKFPDQIIDKNQLQRFLGSLNYVSDF

>Pdul3-Chr6-02; Florendovirus; Prunus dulcis 3a;

KLKEEICSDLPTAFWYRKQHVVCLPYIKTFSEKNIPTKARPIQMNQELMEICKNEIHELLQKNIIRKSKSPWSCPAFYVQKNAELERGVPRLVINYKPLNSVLEWIRYPIPNKRDLINRLGKAVVFSKFDMKSGFWQIQIDESDRYKTAFVTPFGHYEWNVMPFGLKNAPSEFQNIMNDIFNPYSHFSIVYIDDVLIFSESVEHHWKHLHQFLSIIKKNGLVVSAKKIKLFQTNIRFLGFHISQSQISPIDRVIQFADKFPDQILDKNSSRGF

>Pdul3-Chr6-03; Florendovirus; Prunus dulcis 3a;

KLKEEICSDLPTAFWYRKQHVVCLPYIKTFSEKNIPTKARPIQMNQELMEFCKKEIHELLQKNIIRKSKSPWSCPAFYVQKNAELERGVPRLVINYKPLNSVLEWIRYPIPNKRDLINRLGKAVVFSKFDMKSGFWQIQIDESDRYKTAFVTPFGHYEWNVIPFGLKNAPSEFQNIMNDIFNPYSHFSIVYIDDVLIFSESVEQHWKHLHKFLHIVKQNGLVVSAKKIKLFQTNIRFLGFNICQSQISPIDRVIQFADKFPDQILDKSQLQRFLGSLNHISDFYP

>Pdul3-Chr6-06; Florendovirus; Prunus dulcis 3a;

KLKEEICSDLPTAFWYRKQHVVCLPYIKTFSEKNIPTKARPIQMSQELMEVCKKEIHELLQKNIIRKSKSPWSCPAFYVQKNAELERGVPRLVINYKPLNSVLEWIRYPIPNKRDLINRLGKAVVFSKFDLKSGFWQIQINESDRYKTAFVTPFGHYEWNVMPFGLKNAPSEFQNIMNDIFNPYSHFSIVYIDDVLIFSESVEQHWKHLHKFLHIVKQNGLVVSAKKIKLFQTNIRFLGFNICQSQVAPINRVIQFADKFPNQILDKSQLXRFLGSLNYVSDF

>Pdul3-Chr7-01; Yendovirus; Prunus dulcis 3a;

IIGEDPQLHWSKNKILAKLDIINQDLTIKTVDMPCNLLDKQKFEQXIKELLNLKVIRPSKSRHRSAAFVVRKHSELKRGKARIVYNYRRLNDNTYEDSYKLPNKDELMNKIQESKYFSKFDCKSGFWQIRLAEESIQWTTFTCPEGHFEWPVMPFGLKNAPLIFQRKMDQIFKKYDNLCSVYVDDILVHSKNKNEHRKHLEIILKEIIDNGRVISRNKVELEKQDIEFLGMYIKXGTIQLQEHIAKKVLEFPDKLEDKKQLQVFLGLLNYARNFFPD

>Pdul3-Chr7-04; Florendovirus; Prunus dulcis 3a;

KLQDEICSDLPTAFWYRKQHVVCLPYIKTFSEKNIPTKARPIQMNQEFMEVCKNEIHELLQKNIIRKSKSPWSCPAFYVQKNAELERGVPRLVINYKPLNSALEWLRYPIPNKRDLINRLEKAIVFSKFDMKSGFWQIQIDESDRYKTAFVTPFGHYEWNVMPFGLKNAPSEFQNIMNDIFNPYSHFSIVYIDDVLIYSESVEXHWKHLHKFLHIVKQNGLVVSAKKIKLFQTNIRFLGFNISQSQISPIDRVIQFADKFPDQILDKSQLQRFLGSLNYVSDF

>Pdul3-Chr7-05; Florendovirus; Prunus dulcis 3a;

KLKNEICSELPTAFWYRKQHVVCLPYIKTFSEKNIPTKARPIQMNQELMEFCKKEIQELLQKNIIRKSKSPWSCPAFYVQKNAELERGVPRLFINYKPLNSVLEWIRYPIPNKRDLINRLGNAVVYSKFDMKSGFWQIQIDESDRYKTAFVTPFGHYEWNVMPFGLKNAHGEFQNIMNDIFNPYSHFSIVYIDDVLIFSESVEQHWKHLHKFLHIVKQNGLVVSAKKIKLFQTNIRFLGFQICQSQISPIDRVIQFADKSPDQILDKSQLQKFLGSLNYVSDF

>Pdul3-Chr8-02; Florendovirus; Prunus dulcis 3a;

KLKEEICSDLPTAFWYRKQHLVCLPYIKTFSEKNIPTKARPIQMSQELMEVCKKEIHELLQKNIIRKSKSPWSCPAFYVQKNAELERGVPRLVINYKPLNSVLEWIRYPIPNKRDLINRLGKAVVFSKFDMKSGFWQIQIEESDRYKTAFVTPFGHYEWNVMPFGLKNAPSEFQNIMNDIFNPYSHFSIVYIDDVLVFSESVEQHWKHLHQFLSIIKKNGLAISAKKIKLFQVNIRFLGFHICQSQISPIDRVIQFADKFPDQILDKNQLQRFLGSLNYISDFYP

>Pfru1-Chr1-01; Yendovirus; Prunus fruticosa 1;

LEELLQLAEQTRIIGEDPQLHWNKNKILAKLDIINPDLTIKTADIPCNLLDKQEFEQQIKELSNLRVIRPSKSRHRSAAFVVRKHSELKRGKARIVYNYRRLNYNTYEDSYKLPNKDELMNKIQESKYFSKFDCKSGFWQIRLAEESIQWTVFTCPEGHFEWLVMPFGLKKCTIDLSKKDGPNFKKYDNFCSVYVDDILIHSKNKNEHRKHLEIILKEFIDNGVVISRNKVELEKDIEFLGMYIKSGTIQLQEHITKKVLEFPDKLEDKKQLQAFLGLLNYARSFIPD

>Pfru1-Chr3-01; Florendovirus; Prunus fruticosa 1;

EKLKNEVCSDMPYTFWHRKQHVVSLPYVRXFSEKNIPTKARPIQMSQELMELCKAEVTDLVNKGIVRKSKSPWSCPAFYVQKNAELERGAPRLVINYKPLNSVLEWIRYPIPNKRDLVNRLEKTVIFSKFDMKSGFLQIQIKESDRYKIAFVTHFGHYEWNVMPFGLKNAPSEFQNIMNEIFNSYSHFSVVYIDDVLIFXVNXAAWKHLSKFLQIVKSNVLVLSTKKIKLFQVNIRFLGFNISQSQISPINRVIQFADKFPDQILEKSQLQRFLGSLNYVSDF

>Pfru1-Chr4-01; Yendovirus; Prunus fruticosa 1;

HWNKNKILAKLDIIKPDLAIKTIDMPCNLLYKQEFEQQIKELLNLKVIISSKSRHRSATFVFRKHSELKRGKARIMYNYRRLNDNTYEDSYKLPNKAELMNKIQESKYFSKFDCKSGFWQIRLAEESIQWTTFTCPEGHFKWLVMPFGLKNAPLSFQRKMDQMFKKYDNFCSVYVDDILVHSKNKNELRKHLEIILKEFIDNGIVINRNKVELEKQDMEFLGMYIKSGTIQLQEHIAQKVLKFLDKLEDKKQLQVFLGLLNYARSFIPD

>Pfru1-Chr5-01; Yendovirus; Prunus fruticosa 1;

LEELLQLAXQTRIIGEDPQLHWNKNKILAKLDIINPELTIKTTDMPCNLLDKQEFEQQIKELLNLKVIRPSKSRHRSAAFVVRKHSELKRGKARIVYNYKRLNDNTYEDSYKLPNKDELMNKIQESKYFSKFDCKSGFWQIRLAEESIXXTSFTCPEGHFEWLVMPFGLKNAPSIFQRKMDQIFKKYDNFCSVYVDDILVYSKNKNEHRKHLEIILKEFIDNGIVISRNKVELEKQDIEFLGMYIKSGTIQLQEHITKKVLEFPDKLEDKKQLQAFLGLLNYARSFIPD

>Pmir1-Chr1-01; Florendovirus; Prunus mira 1;

KIVKEICSELPNAFWYRKQHVVCLPYIKTFSEKNIPTKARPIQMNQEMLEFCKKEISELLQKKIIRKSKSPWSCPAFYVQKNAELERGVPRLVINYKPLNAVLEWIRYPIPNKRDLINRLGKAVVYSKFDLKSGFWQIQIDESDRYKTAFVTPFGHYEWNVMPFGLKNAPSEFQNIMNDIFNPYSQFSIVYIDDVLIFSESIEQHWKHLHKFFQVVKQNGLVVSAKKIKLFQTNVRFLGFNICQSQISPIDRVIQFADKFPDQILDKSQLQRFLGSLNY

>Pmir1-Chr2-01; Florendovirus; Prunus mira 1;

KLKIEICSELPNAFWYRKPHVVCLPYIKTFNEKHIPIKARPIQMSQDIMEFCKKEIHELLQKKIIRKSKSPWSCPAFYVQKNAELERGVPRLVINYKPLNSVLEWIRYPIPNKRDLINRLEKAVVYSKFDMKSGFWQIQIEELDRYKTAFVTPFGHYEWNVMPFGLKNAPSEFQNIMNEIFNPYSHFSIVYIDDVLIFSESIEQHWKHLHKFFQIVKQNGLVVSAKKIKLFQTNVRFLGFNISQSQISPIDRVIQFADKFPDQILDKSQLQRFLGSLNY

>Pmir1-Chr2-01; Florendovirus; Prunus mira 1;

EKLRREVCSELPNAFWHRKQHVVRLPYIKSFNEKNILTKARPIQMSQELMELCKTEITELLEKRIIRKSKSPWSCPAFYVHKNVELERGVPRLVINYKPLNTVLEWIRYPIPNKRDLINRLEKSVIFSKFDMKSGFWQIQIHESDRYKTAFVTPFGHYEWNVMPFGLKNAPSEFQNIMNEIFNPYSHFSIVYIDDVLIFSQSIEQHWKHLHKFLQIVKQNGLVVSAKKIKLFQTNVRFLGFNIYQSQISPIDRVIQFADKFADQILDKSQLQRFLGFLNYVSDF

>Pmir1-Chr2-01; Florendovirus; Prunus mira 1;

EKLQREVYSELPNAFWHRKQHVVRLPYIKSFNEKNIPTKARPIQMSQELMELCKTEITELLEKRIIRKSKSPWSCPAFYVQKNVELERGVPRLVINYKPLNTVLEWIRYPIPNKRDLINRLEKSVIFSKFDMKSGFWQIQIHESDRYKTAFVTPFGHYEWNVMPFGLKNAPSEFQNKMNEIFNPYSHFSIVYIDDVLIFSQSIEQHWKHLHKFLQIVKQNGLVVSAKKIKLFQTNVRFLGFNICQSHISPIDRVISLLTSF

>Pmir1-Chr2-01; Florendovirus; Prunus mira 1;

KIVNEICSELPNAFWYRKQHAVCLSYIKTFSEKNIPTKARPIQMNQEMLEFCKKEISELLQKNIIRKSKSPWSCPAFYVQKNAELERGVPRLVINYKPLNAVLEWIRYPIPNKRDLINRLGKAVVYSKFDLKSGFWQIQIDESDRYKTAFVTPFGHYEWNVMPFGLKNAPSEFQNIMNDIFNPYSQFSIVYIDDVLIFSESIEQHWKHLHKFFQVVKQNGLVVSAKKIKLFQTNVRFLGFNICQSQISPIDRVIQFADKFPDQILDKSQLQRFLGSLNY

>Pmir1-Chr2-01; Florendovirus; Prunus mira 1;

QKLKNEICSELPIAFWYRKQHVVRLPYIKSFSEKNIPTKARPIQMSQEMMEFCKIEIHELLQKKIIRKSKSPWSCPAFYVQKNAELERGVPRLVINYKPLNAVLEWIRYPIPNKRDLINRLEKAVVYSKFDMKSGFWQIQIDESDRYKTAFVTPFGHYEWNVMPFGLKNAPSEFQNIMNDIFNPQSQFSIVYIDDVLIFSESIEQHWKHLHRFLQVVKQNGLVVSAKKIKLFQTNIRFLGFNICQSQISPIDRVIQFADKFPDQILEKSQLQRFLGSLNY

>Pmir1-Chr3-01; Florendovirus; Prunus mira 1;

KIKNEICSELPNAFWYRKQHVVCLPYIKTFSEKNIPTKARPIQMNQEMLEFCKKEIHELLQKKIIRTSKSPWSCPAFYVQKNAELERGVPRLVINYKPLNAVLEWIRYPIPNKRDLINRLGKAVVYSKFDMKSGFWHIQIDESDRYKTAFVTPFGHYEWNVMPFGLKNAPSEFQNIMNDIFNPYSQFSIVYIDDILIFSESIEQHWKHLHKFFQVVKQNGLVVSAKKIKLFQTNIRFLGFNICQSQISPIDRVIQFADKFPYQILDKSQLQRFLGSLNY

>Pmir1-Chr3-01; Yendovirus; Prunus mira 1;

LQLHWNKNKILAILDIINPDLTIKTADMPCNLLDKQEFEQQIKELLNLKVIIPSKSRHRSTSFVVRKHSELKRGKARIVYNYKRLNYNTYEDSYKLPNKDELMNKIQESKYFSKFDCKSGFWQIRLAEESIQTTFTCPEGHFEWLIMPFGLKNAPSIFQRKMDQIFKKYDNFCSVYVDDIPVHSKNKNEHRKHLEIILKKFIDNGIVISRKKVELEKDIEFLGMYIKSGAIQLQDIAKKVFEFSDKLEDKKLQAFFGLLNY

>Pmir1-Chr4-01; Florendovirus; Prunus mira 1;

EKLKQEVCSDLPNAFWHRKQHVVSLPYVKNFSEKNILTKARPIQMSQEIMEFYKTEITELLQKGIIRKSKSPWSCPAFYVKNTELERGAPRLVINYKPLNTTLEWIRYPIPNKRDLINRLDKSVIFSKFDMKIGFWQIQILESDRYKTAFVTPFGHYEWNVMPFGLKNAPSEFQNIMNEIFNHFSHFSIVYIDDVLIFSQSIEHWKHLHKFLQIVKQNGLVVLAKKIKLFQTNIRFLGFNICQSQISLIDRVIQFADKFSYQILDKSQLH

>Pmir1-Chr4-01; Yendovirus; Prunus mira 1;

NKNKILAKLDIINPDLTIKTADMPCNLLDKQEFEQKIKELLNLKVIRPSKSRHRSVAFVVSKHSELKRGKARIVYNYRRLNDNTYEDSYKLPNKDELMNKIQESKYFSKFDCKSGFQIRLAEESIQWIAFTCPEGHFQLVMSFGLKNAPSIFQRKMDQILKKYNNFYSVYVDDILVHSKNKNEHREHLEIILKEFIDNGIVISRNKVKLEKQDIEFLGMYIKSGTIQLQHIAKKVLEFPDKLEDKKQLAFLGLLNYARSFIPD

>Pmir1-Chr4-01; Florendovirus; Prunus mira 1;

EKLKQEVCSELPNAFWHRKQHVVSLPYVKSFSEKNIPTKARSIQMSQELMKFCKTEITELLEKRIIRKSKSPWSCPAFYVQKNAELERGVSRLVINYKPLNIVLEWIRYPIPNKRDLINRLEKSVIFSKFDMKSGFWQIQIHESDRYKTAFVTPFGHYEWNVMPFGLKNAPSEFQNIMNEIFNPYSHFSIVYIDDVLIFSQSIEQHWKHLHKFLQIVKQNGLVVPAKKIKLFQTNVRFLGFNICHSQISPIDRVIQFADKFPNQILDKSQLQRFLGSLNYVSDF

>Pmir1-Chr5-01; Yendovirus; Prunus mira 1;

LEELLQLAEQTKIIGEDPQLHWSKNKILTKLDIINQDLTIKTADMPCNLLDKQEFEQIKELLNLKVIRPSKSRHRSAAFVVRKHSELKRARIVYNYRRLNDNTYEVSYKLPNKDELMNKIQESKYFSKFDCKLGFWQIRLAEESIQWTTFTCPEGHFEWLVMPFGLKNAPLIFQRKMDQIFKKYDNLCSVYVDDILVHSKNKNEHRKHLEIILKEFTDNGIVISRNKVELQKQDIEFLGMYIKSGTIQLQEHVAKKVLEFPDKLEDKKQLQAFLGLLNYARNFFPD

>Pmir1-Chr6-01; Florendovirus; Prunus mira 1;

KIVNEICSELPNAFWYRKQHVVCLPYIKTFSEKNIPTKARPIQMNQEMLEFCKKEISELLQKKIIRKSKSPWSCPAFYVQKNAELERGVPRLVINYKPLNAVLEWIRYPIPNKRDLINRLGKAVVYSKFDLKSGFWQIQIDESDRDKTAFVTPFGHYEWNVMPFGLKNAPSEFQNIMNDIFNPYSQFSIVYIDDVLIFSESIEQHWKHLHKFFQVVKQNGLVVSAKKIKLFQTNVRFLGFNICQSQISPIDRVIQFADKFPDQILDKSQLQRFLGSLNY

>Pmir1-Chr6-01; Florendovirus; Prunus mira 1;

EKLKQEVCSELPNAFWHRKQYVVSLPYVKTFSEKNIPTKARPIQMNQELMEFCKIEITDLLKKGIIRKSKSPSYPAFHVQKNAELERGVPRLVINYKPLNTVLEWIRYPIPNKRDLINRLEKSVIFSKFDMKSGFWQIQISESDRYKTAFVTPFGHYEWNVMPFGLKNAPSEFQNIMNEIFNHFSHFSIIYIDDVLIFSESIEQHWKHLYKFLQIIKQNGLVISAKKIKLFQTNVRFLEFNICQSQISPIDRVIFADKFPDQILDKSQLRFLGSLNYVSDF

>Pmir1-Chr7-01; Florendovirus; Prunus mira 1;

QKIKNEICSDMPNAFWYRKQHVVCLPYIKTCSEKNIPTKARPIQMSQEIMEFCKTEISELLQKKIIRKSKSPWSCPSFYVQKNAELERGVPRLVINYKSLNSVLELIRYPIPNKRDLINRLGKAVVYSKFDMKNGFWQIQIEESDRYKTAFVTPFGHYEWNVMPFGLKNAPSEFQNIMNEIFNPYRHFSIVYIDDVLIFSESIEQHWKHLHKFFQIVKQNGLVVSAKKIKLFQTNIRFLGFNICQSQISPIDRVIQFADKFPDQILDKSQLQRFLGSLNY

>Pmir1-Chr8-01; Yendovirus; Prunus mira 1;

LEELLQLAKQTRIIGEDPQLHWNKNKILAKLDIINLDLTIKTADMPCNLLDKQEFYQQIKQILNLKVIRPSKSRHRSTAFVVRKHLELKRGKVRIVYNYRRLNDNTYEDSYKLPNKDELMNKIQESKYFSKFDCKSGFQIRLAEESILTTFTCPEGNFEWLVMPFGLKNAPSIFQRKMDQIFKKYDNFCSVYVEDILVHSKNKNKHRKYLEIILKEFIDNGIVISRNKVELEKQDIEFLGIYIKSGTIQLQEHIAKKVLEFPDKLENKKQLQEFLGLLNYARSFIPD

>Pmon1-Chr2-01; Florendovirus; Prunus mongolica 1;

EKLKQEVCSELPKAFWHRKQHVVSLPYVKTFREKNIPTKARPIQMSQELMEFCKTEITELLEKGIIRKSKSPWSCPAFYVQKNAELERGVPILVINYKPINTVLEWIRYLIPNKRDLINRSEKSVIFSKFNMKSGLWQIQIRESDRYKTAFVTPFGHYEWNVMPFRLKNAPSEFQNIMNEIFNPYGHFSIVYIDDVLIFSESIEQHXKHLHKFLQIVKQNGLVVSAKKIKLFQTNVRFLGFNICQSQISPINRIIXFADKFLDQITDKSQLHRFLESLNYVSDF

>Pmon1-Chr2-02; Florendovirus; Prunus mongolica 1;

EKLQREVYSELPNAFWHRKQHVVRLPYIKSFNEKNIPTKARPIQMSQELMELCKTEITEMLEKRIIRKSKSPWSCPAFYVQKNVELERAVPRLVINYKPLNTVLEWIRYPIPNKRDLINRLEKSVIFSKFDMKSGFWQIQIHESDRYKTAFVTPFGHYEWNVMPFGLKNAPSEFQNIMNEIFNPYSHFSIVYIDDVLIFSQSIEQHWKHLHKFLQIVKQNGLVVSAKKIKLFQTNVRFLGFNICQSHISPIDRVIQFADKFPDQILEKSQLQRFLGSLNYVSDF

>Pmon1-Chr2-03; Florendovirus; Prunus mongolica 1;

KLKTEIYSELPNAFWYRKQHVVCLPYIRTFSEKNIPTKARPIQMSQELMEFCKKEISELLEKKIIRKSKSPWSCPAFYVQKNAELERGVPRLVINYKPLNAILEWIRYPIPNKRDLINRLEKAVVYSKFDMKSGFWQIQIEESDRYKTAFVTPFGHYEWNVMPFDLKNAPSEFQNIMNDIFNPYSHFSIVYIDDVLIYSESVEQHWKHLHKFLQIVKQNGLVVSAKKIKLFQTSIRFLGFNICQSQISPIDRVIQFADKFPDQILDKSQLQRFLGSLNY

>Pmon1-Chr2-04; Yendovirus; Prunus mongolica 1;

HWNKNKILAKLDIINPYSTIKIAYMPYNLLDKQNFXQQIKELLNIVLRPSKSRHRSTAFVIRKHSKVKRGKARIVYNYKRXNDNTYEDSYKLPNKDEFMNKIEESKYSSKLYCKSRFWKIRLAEXSIQXTTFTCTESHFEWLVMPFGFKNAPSIFQRKMNQIFKKYDNFGSIYVDDKLVCSKNKMNIENTEKLFXKNLLIMVXLXVGIKXSLKNKAYNFKVCILSQKLYNYKNTXVKKCYNFQINEKIKKKLQAFLGLLNYARSFILD

>Pmon1-Chr2-05; Yendovirus; Prunus mongolica 1;

SKNKILTKLDIINPYLTIKTVDMPCSLLDKQEFEHQIKELQNLKVIRPSKSRHRSAAFIVIKHSELKRGKARILYNYRRLNDNTYEDSYKLLNKDELINKIQDSKYFSKFDCKFGFWQIRLAKESIEWTIFTCPEXHFEWLVMPFGLKNAPSIFQRKMDQTFKKYDSFCRYXXDTYTXXNKNDHRKEIILQGFIKNGIVISKNKIELEKQEIEFLGIYIQSGTIKLQEHIAKKVLEFPNKLVDKKQLQAFLGLLNYARHFIPD

>Pmon1-Chr2-06; Florendovirus; Prunus mongolica 1;

QKIKDEICSELPNAFWYRKQHVVCLPYIKNFSEKNIPTKARPIQMNQEIMEFCKKEIDELLQKKIIRKSKSPWSCPAFYVQKNAEIERGVPXLVINYKPLNAVLEWIRYPIPNKRDLINRLGKAVVYSKFDLKSGFWQIQIDESDRYKTAFVTPFGHYEWNVMPFGLKNAPSEFQNIMNDIFNPYSQFSIVYINDVLIFSESIEQHWKHLHKFFQVVKQNGLVVSAKKIKLFQTNVRFLGFNICQSQISPIDRVIQFANKFPDQILDKNQLQRFLGSLNY

>Pmon1-Chr3-01; Florendovirus; Prunus mongolica 1;

EKLKQEVCSELPNAFWHRKQHVVNLPYIKSFSEKNIPTKARPIQMSQELMEFCKIEITELLEKGIIRKSKSPWSCPTIYVQKNAELERGVPRLVINYKPLNTVLEWIRYSIPNKRDLINRLEKSVIFSKFDMKSGFWQIQIHESDRYKTAFVTPFGHYEWNVMPFGLKKAPSEFQNIMNEIFNPFSHFSIVYIDDVLIFSQSIEQHWKHLNQFLQIVKQNGLVVSAKKIKLFQTNVRFLGFNICQSQISLIDRVIQFADKFPDLDKIQLQRFLGSLNYVSDF

>Pmon1-Chr3-02; Florendovirus; Prunus mongolica 1;

QKIKDEICSELPNAFWYRKQHVVCLPYIKTFSEKNIPTKARPIQMSQEMMEFCKKEINELLQKKIIRKSKSPWSCPAFYVXKNAELERGVPRLVINYKPLNAVLEWIRYPIPNKRDLINRLEKSVVFSKFDMKSGFWQIQIEESDRYKTAFVTPFGHYEWNVMPFGLKNAPSEFQNIMNDIFNPYSQFSIVYIDDVLIFSESVEQHWKHLHKFFQIVKQNGLVVSAKKIKLFQTNVRFLGFNICQSQISPIDRVIQFADKFPDQILDKSQLQRFLGSLNY

>Pmon1-Chr3-03; Yendovirus; Prunus mongolica 1;

NKNKILAKLDIINPDLTIKTADMPCNLLDKQEFEQKIKELLNLKVIRPSKSRHRSVAFVVRKHSELKRVKARIVYNYRRLNDNTYEDSYKLPNKDELMNKIQESKYYSKFDCKSRFXQIRLAEESIQWIAFTCPEGHFEXLVMSFGLKNAPSIFQRKMDQILKKYNNFYSVYVDDILVHSKNKNEHRKHLEIILKEFIDNGIVISRNKVKLEKQDIEFLGMYIKSGTIQLQXHIAKKVLEFPDKLEDKKQLXAFLGLLNYARSFIPD

>Pmon1-Chr3-04; Florendovirus; Prunus mongolica 1;

QKIKNEICSELPNAFWYRKQHVVCLPYIKTFSEKNIPTKARPIQMSQEIMEFCKTEISELLQKKIIRKSKSPWSCPAFYVQKNAELERGVPRLVINYKPLNSVLEWIRYPIPNKRDLINRLEKAVVYSKFDMKSGFWQIQIEESDIYKTAFVTPFGHYEWNVMSFGLKNAPSEFQNIMNEIFNPYSHFSIVYIDNVLIFYESIEQHWKHLHKFFQIVKQSGLVVSAKKIKLFQTNIRFLGFNICKSQISPIGRVIQFVDKFPDQILDKSQLXRFLGSLNY

>Pmon1-Chr4-01; Florendovirus; Prunus mongolica 1;

EKLKHEICSELPNAFWHRKQHVVSLPYVKSFSENNIPTKAKPIQMSQELMEFCKTEITELLEKGIIRKSKSTWSCPAFYVQKNAELERGVLRLVINYKPLNTVLEWIRYPIPNKRDLINRLEKSLIFSKFDMKSGFWQIQICESDRYKTAFVTLFGHYEWNVMPFGLKNAHSEFQNIMNEIFNPYNHFSIIYIDDVLIFSQSIEQHWKHLYKFLQIVKQNGLVVSAKKIKLFQTNVRFLGFNICQSEISPIDRVIQFADKFPDQILDKSQLQRFLGSLNYV

>Pmon1-Chr5-01; Yendovirus; Prunus mongolica 1;

LEELLQLAKQTRIIGEDPQLHWNKNKILAKLDIINLDLTIKTADMPCNLLDKQEFYQQIKXILNLKVIRPSKSRHRSTAFVVRKHLELKRGKVRIVYNYRRLNDNTYEDSYKLPNKDELMNKIQESKYFSKFDCKSGFXQIRLAEESIXLTTFTCPEGNFEWLVMPFGLKNAPSIFQRKMDQIFKKYDNFCSVYVEDILVHSKNKNKHRKYLEIILKEFIDNGIVISRNKVELEKQDIEFLGIYIKSGTIQLQEHIAKKVLEFPDKLENKKQLQEFLGLLNYARSFIPD

>Pmon1-Chr6-01; Florendovirus; Prunus mongolica 1;

QKIKAEICSELPNAFWYRKQHVVCLPYIKTFSEKNIPTKARPIQMSQEIMEFCKKEISELLQKKIIRKSKSPWSCPAFYVQKNAELERGVPRLVINYKPLNAVLEWIRYPFPNKRDLINRLEKTVVYFKFDMKSGFWQIQIEESDRYKTAFVTPFGHYEWNVMPFGLKNAPSEFQNIMNEIFNPYSHFSIVYIDDVLIFSESIEQHWKHLHKFYQIVKQNGLVVSAKKIKLFQTNVRFLSFNICQSQICPIDRVIQFADKFPDQNLDKSQLXRFLGSLNY

>Pmon1-Chr6-02; Florendovirus; Prunus mongolica 1;

LKNEICSELPTTFWSRKQHVVCLPYVKTFNEKNIPTKARPIQMSQELMEVCKKEIDELLQKKIIRKSKSPWSCPAFHVQKNAELERGVPRLVINYKPLNSVLEWIRYPIPNKRDLINRLRNAVVYSKFDMKSGFWQIQIDESDRYKTAFVTPFGHYEWNVMPFGLKNAPSEFQNIMNDIFNPYSQFSIVYIDDVLVFSESLEQHWKHLHKFLQIVKQNGLVVSAKKIKLFQTNIRFLGFNICQSQISPIDRVIQFADKFPDQILDKSQLXRFLGSLNY

>Pmon1-Chr7-01; Yendovirus; Prunus mongolica 1;

LEELLQLAEQTRIIGEDPQLHWNKNKILAKLDIINPDLTIKTADMPCNLLDKQEFEQQIKELLNLKVIRPSKSRHRSAAFVVRQHSELKRGKARIMYNYRRLNDNTYEDSYKLPNKDELMNKIQESKYFSKFDCKSGFWQIRLAEESIQWTAFTCLEGHFEWLVMPFGLKNASSIFQRKMDQIFKKYDSFCNIYVDDILVHSKNKNEHRKHLEIILKEFIDNGIIISRNKVXLEKQYIEFLGMYIKSGTIELQEHIAKKVLEFPDKLEDKKQLQAFLGLLNYARSFIPD

>Pmon1-Chr7-02; Florendovirus; Prunus mongolica 1;

EKLKQEVCSELPNAFWHRKQHVVSLPYVKTFSEKNIPTKARPFQMNQELMEFCKIEITDLLKKGIIRKSKSLXSYPAFYVXKNAELERGVPRLVINYKPLNTILEWIRYPIPNKRDLINRLEKSVIFSKFDMKSRFWQIQISESDRYKTAFVTPFGHYEWNFMPFDLKNAPSEFQNIMNEIFNPFSHFSIIYIDDVLIFSESIEQHWKHLYKFLQIIKQNGLVISAKKIKLFQTNVRFLGFNICKSQISPIDRVILFADKFPDQILDKSQLXRFLGSLNYV

>Pmon1-Chr7-03; Florendovirus; Prunus mongolica 1;

QKIKDEICSELPNAFWYRKQHVVCLPYIKTFSEKNIPTKARPIQMSQDMMEFCKKEIEDLLQKKIIRKSKSPWSCPAFYVQKNAELERGVPRLVINYKPLNAVLEWIRYPIPNKRDLINRLEKSVVFSKFDMKSGFWQIQIDESDRYKTAFVTPFGHYEWNVMPFGLKNAPSEFQNIMNDIFNPYSQFSIVYIDDVLIFSESVEQHWKHLHKFFQIVKQNGLVVSAKKIKLFQTNIRFLGFNICQSQISPIDRVIQFADKFPDQILDKSQLQRFLGSLNY

>Pmon1-Chr8-01; Florendovirus; Prunus mongolica 1;

QKIKNEICSELPNAFWYRKQHVVCLPYIKTFSEKNIPTKARPIQMNQEMMEFCKKEISELLQKKIIRTSKSPWSCPAFYVQKNAELGRGVPRLVINYKPLNAVLEWIRYPIPNKRDLIKRLEKAVVYSKFDMKSGFWQIQIEESDRYKTAFVTPFGHYEWNVMPFGLKNAPSEFQNIMNDIFNPYSQFSIVYIDDVLIFFESIEQHWEHLHKFLQIVKQNGLVVSAKKIKLFQTNVRFLGFNICQSHISPIDRVIQFADKFPDQILDRSQLLRFLGSLNY

>Pmum1-Chr1-01; Florendovirus; Prunus mume 1;

KKLIADVCSDLPTAFWHRKNHIVSLPYIQNFSEKNIPTKARPIQMSPEMLEFCKNEINDLLQKNIIRRSKSPWSCSAFYVQKNAELERGSPRLVINYKPLNTVLEWIRYPIPNKRDLINRLSKAIIFSKFDLKSGFWQIQITESDRYKTAFVTPFGHYEWNVMPFGLKNAPSEFQNIMNEIFNSYSQFAIVYIDDVLIFSESIEQHWKHLYKFLHVIKANGLVVSAKKIKLFQLHVRFLGFNISQSQIQPIDRSIQFADKFPDQIIDKNQLQRFLGSLNY

>Pmum1-Chr1-02; Florendovirus; Prunus mume 1;

KKLIADVCSDLPTAFWHRKNHIVSLPYIQNFSEKNIPTKARPIQMSPEMLEFCKNEINDLLQKNIIRRSKSPWSCSAFYVQKNAELERGSPRLVINYKPLNTVLEWIRYPIPNKRDLINRLSKAIIFSKFDLKSGFWQIQITESDRYKTAFVTPFGHYEWNVMPFGLKNAPSEFQNIMNEIFNSYSQFAIVYIDDVLIFSESIEQHWKHLYKFLHVIKANGLVVSAKKIKLFQLHVRFLGFNISQSQIQPIDRSIQFADKFPDQIIDKNQLQRF

>Pmum1-Chr1-03; Yendovirus; Prunus mume 1;

LEELLQLAEQTRIIGEDPQLHWNKNKILAKLDIINPDLTIKTADMPCNLLDKXEFEQQIKELLNLKVIRPSKSRHRSAAFVVRKHSELKRGKARIVYNYRRLNDNTYEDSYKLPNKDELMNTIQESKYFSKFDCKSRFWQIRLAEESIHWTAFTYPEGHFEWLVMPFGLKNAPSIFQRKMDQIFKKYDKFCSVYVDDILVHSKNKNEHRKHLEIILKEFIDNGIIISRNKVELEKQDIEFLGMYIKSGTIQLQKHIAKKVLEFPDKLEDKKQLQAFLGLLNYARSFIP

>Pmum1-Chr1-04; Florendovirus; Prunus mume 1;

KLIADVCSDLPTAFWHRKNHIVSLPYIQKFSEKNIPTKARPIQMSPEMLEFCKNEINDLLQKNIIRRSKSPWSCSAFYVQKNAELERGSPRLVINYKPLNTVLEWIRYPIPNKRDLINRLSKAIIFSKFDLKSGFWQIQITESDRYKTAFVTPFGHYEWNVMPFGLKNAPSEFQNIMNEIFNSYSQFAIVYIDDVLIFSESIEQHWKHLYKFLHVIKANGLVVSAKKIKLFQMHIRFLGFNISQSQIQPIDRSIQFADKFPDQIIDRNQLQRFLGSLNY

>Pmum1-Chr1-05; Yendovirus; Prunus mume 1;

WNKNNILAKLDIINLDLTIKTADMPCNLLDKQEFEQQIKELLNLKVIRPSKSRHRLAAFVVRKHSELKRGKARIVYNYRRLNDNTYEDSYKLPNKDELMNKIQESKYFSKFDCKXGFWQIRLAEESIQWTTFTCPEGHFEWLVMPFGLKNAPSIFQRKMDQIFKKYDNFCSVYVDDILVPSKNKNEHRKHLEIILKEFIDNGIVTSRNKVELEKQDIEFLGMYIKSGTIQLQEQIAKKVLEFPNKLEDKKQLQAFLGLLNYS

>Pmum1-Chr1-06; Florendovirus; Prunus mume 1;

KLINDVCSDLPTAFWHRKNHIVSLPYIQNFSEKNIPTKARPIQMSPEMLEFCKNEINDLLQKNIIRRSKSPWSCSAFYVQKNAELERGSPRLVINYKPLNTVLEWIRYPIPNKRDLINRLSKAIIFSKFDLKSGFWQIQITESDRYKTAFVTPFGHYEWNVMPFGLKNAPSEFQNIMNEIFNSYSQFAIVYIDDVLIFSESIEQHWKHLYKFLHVIKANRLVVSAKKIKLFQMHIRFLGFNISQSQIQPIDRSIQFADKFPDQIIDKNQLQRFLGSLNY

>Pmum1-Chr2-01; Yendovirus; Prunus mume 1;

LEELLQLVEQTRIIGEDPQLHWNKNKILAKLDIINPDLTIKTADMPCNLLDKQEFEQQIKELLNLKVIRPSKSRHRSAAFVVRKHSELKRGKARIVYNYRRLNDNTYEDSYKLPNKDELMNKIQESKYFSKFDCKSGFWQIRLAEESIHWTAFTCPEGHFEWLVMPFGLKNAPSIFQRKMDQIFKKYDKFCSVYVDDILVHSKNKNEHRKHLEIILKEFIDNGIIISRNKVELEKQDIEFLGMYIKSGTIQLQEHIAKKVLEFPDKLEDKKQLQAFLSLLNYARSFVPD

>Pmum1-Chr2-02; Yendovirus; Prunus mume 1;

HWNKNKILAKLDIINPDLTIKTADMPCNLLDKQEFEQQIKELLNLKVIRPSKSRHRSAAFVVRKHSELKKGKARIVYNYRRLNDNTYEDSYKLPNKDELMNKIQESKYFSKFDCKSGFWQIRLAEESIHXTTFTCPEGHSEWLVMPFGLKNAPSIFQRKMDQIFKKYDKFCSVYVDDILVHSKNKNEHRKHLEIILKEFIDDGIVISRNKVELEKQDIEFLGMYIKPGTIQLQEHIAKKVLEFSDKLEDKKQLQAFLGLLNYARSFIPD

>Pmum1-Chr2-03; Yendovirus; Prunus mume 1;

IIGEDPQLHWNKNKILAKLDIINPDLTIKTADIPCNLLDKQEFEQQIKELLNLKVIRPSKSRHRSAAFVVRKHSELKRGKARIVYNYRRLNDNTYEDSYKLPNKDELMNKIQESKYFSKFDCKSGFWQIRLAEESIHWTAFTCPEGHFEWLVMPFGLKNAPSIFQRKMDQIFKKYDKFCSVYVDDILVHSKNKNEHRKHLEIILKEFINNGIIISRNKVELEKQDIEFLGMYIKSGTIQLQEHIAKKVLEFLDKLEDKKQLQAFLGLLNYARSFI

>Pmum1-Chr2-04; Yendovirus; Prunus mume 1;

ILGEDPQLHWNKNKILAKLDIINPDLTIKTTDMPCNLLDKQEFEQQIKELLNLKVIRPSKSRHRSAAFVVRKHSELKRGKARIVYNYRRLNDNTYEDSYKLPNKDELINKIQESKYFSKFDCKSGFWQIRLAEESIHWTAFTCPEGHFEWLVMPFGLKNAPSIFQRKMDQIFKKYDKFCSVYVDDILVHSKNKNEHRKHLEIILKEFIDNGIIISRNKVELEKQDIEFLGMYIKSGTIQLQEHIAKKVLEFPDKLEDKKQLQAFLGLLNYARSFIPD

>Pmum1-Chr2-05; Florendovirus; Prunus mume 1;

QKLIEEICSDLPTTFWHRKHHVVKLPYIKNFSEKNIPTKARPIQMNQEMVSYCKTEINELLQKNIIRKSKSPWSCPAFYVQKNAELERGSPRLVINYKPLNTVLEWIRYPIPNKRDLINRLSNAVIFSKFDLKSGFWQIQIDESDRYKTAFVTPFGHYEWNVMSFGLKNAPSEFQNIMNEIFNPYSHFSIVYIDDVLIFSKSIEQHWKHLHKFLQVVKTNGLVVSAKKIKLFQTSIRFLGFNISHYQIHPIDRAIQFADKFPDQITDKNQLQRFLGSLNYVSDF

>Pmum1-Chr2-06; Florendovirus; Prunus mume 1;

KKLIADVCSDLPTAFWHRKNHIVSLPYIQNFSEKNIPTKARPIQMSPEMLEFCKNEINDLLQKNIIRRSKSPWSCSAFYVQKNAELERGSPRLVINYKPLNTVLEWIRYPIPNKRDLINRLSRAIIFSKFDLKSGFWQIQITESDRYKTAFVTPFGHYEWNVMPFGLKNAPSEFQNIMNEIFNSYSQFAIVYIDDVLIFSESIEQHWKHLYKFLHVIKANGLVVSAKKIKLFQMHIRFLGFNISQSQIQPIDRSIQFADKFPDQIIDKNQLQRFLGSLNY

>Pmum1-Chr2-07; Yendovirus; Prunus mume 1;

LEELLQLAEQTRIIGEDPQLHWNKNKILAKLDIINLDLTIKIADMPCNLLDKQEFEQQIKELLNLKVIRPSKSRHRSAAFVVRKHSELKRGKTRIVYNYRRLNDNTYEDSYKLPNKDELMNKIQESKYFSKFDCKSGFWQIRLAEESIHWTTFTCPEGHFEWLVMPFGLKNAPSIFQRKMDQIFKKYDNFCSVYVDDILVHSKNKNEHRKHLEIILKEFIDNGIVISRNKVELEKQDIEFLGMYIKSGTIQLXEHIAKKVLEFPDKLEDKKQLQAFLGLLNYARSFI

>Pmum1-Chr2-08; Florendovirus; Prunus mume 1;

KLKNEIYSDLPNAFWYRKQHVVCLPYIKTFSEKNIPTKARPIQMSQEIMEFCKKEIHKLLQKKIIRKSKSPWSCPAFYVQKNAELERGVPRLVINYKPLNLVLEWIRYPIPNKRDLINRLEKAVVFSKFDMKSGFWQIQIDEXDRYKTAFVTPFSHYEWNVMSFGLKNAHSEFQNIMNEIFNLHSHFSIVYIDDVLIFSESIEQHWKHLHKFLQIVKHNGLVVYANKIKLFQTNIRFLGFNICXSQISPIDRVIQFAGKFPDQILDKSQLQRFLGSLNYVSDF

>Pmum1-Chr2-09; Florendovirus; Prunus mume 1;

KLITDVCSDLPTAFWHRKNHIVSLPYIQNFSEKNIPTKARPIQMSPEMLEFCKNEINDLLQKNIIRRSKSPWSCSAFYVQKNAELERGSPRLVINYKPLNTVLEWIRYPIPNKRDLINRLSKAIIFSKFDLKSGFWQIQITESDXYKTAFVTPFGHYEWNVMPFGLKNAPSEFQNIMNEIFNPYSQFAIVYIDDVLIFSESIEQHWKHLYKFLHVIKANGLVVSAKKIKLFQMHVRFLGFNISQSQIQPIDRSIQFADKFPDQIIDKNQLQRFLGSLNY

>Pmum1-Chr2-10; Florendovirus; Prunus mume 1;

KLINDVCSDLPTAFWHRKNHIVSLPYIQNFSEKNIPTKARPIQMSPEMLEFCKNEINDLLQKNIIRRSKSPWSCSAFYVQKNAELERGSPRLVINYKPLNTVLEWIRYPIPDKRDLINRLSKAIIFSKFDLKSGFWQIQITESDRYKTAFVTPFGHYEWNVMPFGLKNTPSEFQNIMNEIFNSYSQFAIVYIDDVLIFFETIEQHWKHLYKFLHVIKANGLVVSAKKIKLFQMHIRFLGFNISQSQIQPIDRSIQFADKFPDQIIDKNQLQRFLGSLNY

>Pmum1-Chr3-01; Yendovirus; Prunus mume 1;

LEELLQLAEQTRIIGEDPQLHWNKNKILAKLDIINPDLTIKTADMPCNLLDKQEFEQQIKELLNLKVIRPSKSRHRSAAFVVRKHSELKRGKARIVYNYRRLNDNTYEDSYKLPNEDELMNKIQESKYFSKFDCKSGFWQIRLAEESIHWTTFTCLEGHFEWLVMPFGLKNAPSIFQRKMDQIFKKYDQFCSVYVDDILVHSKNKNEHRKHLEIILKEFIDNGIIISRNKVELEKQDIEFLGMYIKSRTIQLQEHIAKKVLEFPDKLEDKKQLQAFLGLLNYARSFIPD

>Pmum1-Chr3-02; Florendovirus; Prunus mume 1;

KLINDVCSDLPTAFWHRKNHIVSLPYIQNFSEKNIPTKARPIQMSPEMLEFCKNEINDLLQKNIIRRSKSPWSCSAFYVQKNAELERGSPRLVINYKPLNTVLEWIRYPIPNKRDLINRLSKAIIFSKFDLKSGFWQIQITESDRYKTAFVTPFGHYEWNVMPFGLKNAPSEFQNIMNEIFNSYSQFAIVYIDDVLIFSESIEQHWKHLYKFLHVIKANGLVVSAKKIKLFQMHIRFLGFNISQSQIQPIDRSIQFADKFPDQIIDKNQLQRFLGSLNY

>Pmum1-Chr3-03; Florendovirus; Prunus mume 1;

KKLIDEICSDLPTAFWHRKQHVVKLPYIKNFSEKNIPTKARPIQMNQEMVSYCKTEINELLQKNIIRKSKSPWSCPAFYVQKNAELERGSPRLVINYKPLNTVLEWIQYPIPNKRDLINRLSNAVIFSKFDLKSGFWQIQIDESDRYKTAFVTPFGHYEWNVMPFGLKNAPSEFQNIMNEIFNPYSHFSIVYIDDVLIFSKSIEQHWKHLHKFLQIVKTNGLVVSEKKIKLFQTSIRFLGFNISHSQIHPIDRAIQFADKFPDQITDKSQLQRFLGSLNYVSDF

>Pmum1-Chr3-04; Florendovirus; Prunus mume 1;

KKLITDVCSDLPTAFWHRKNHIVSLPYIQNFSEKNIPTKARPIQMSPEMLEFCKNEINDLLQKNIIRRSKSPWSCSAFYVQKNAELERGSPRLVINYKPLNTVLEWIRYPIPNKRDLINRLSKAIIFSKFDLKSGFWQIQITESDRYKTAFVTPFGHYEWNVMPFGLKNAPSEFQNIMNEIFNSYSQFAIVYIDDVLIFSESIEQHWKHLYKFLHVIKANGLVVSAKKIKLFQMHIRFLGFNISQSQIQPIDRSIQFADKFPDQIIDKNQLQRFLGSLNY

>Pmum1-Chr5-01; Yendovirus; Prunus mume 1;

IIGEDPQLHWNKNKILAKLDIINPDLTIKTAYMPCNLLDKQEFEQQIKELLNLKVIXPSKSRHRSAAFVVRKHSELKRGKARIVYNYRRLNDNTYEDSYKLPNKDELMNKIQESKYFSKFDCKSGFWQIRLAKNQSIGQIYLYEGHFEWLVMPFGLKNAPSIFQRKMDQIFKKYDKFCSVYVDDILVHSKNKNEHRKHLEIILKEFIDNGIIISRNKVELEKQDIEFLGMYIKSGTIQLQEHIAKKVLEFPDKLEDKKQLQAFLGLLNYARSFIP

>Pmum1-Chr5-02; Yendovirus; Prunus mume 1;

HWNKNKILAKLDIINPYSTIKTADMSCNLLDKQKFXQQIKELLNIVIXPSKSRHRSTAFVVRKHSELKRGKARIVYNYKRXNDNTYEDSYKLPNKDELIMNKIEESKYSSKLDCKSRFCEIRLPEXSIQXTTFTCTXAYFEWLVRPFGLKNAPSIFXRKMNXIVKKYDNFGSIHVDDILVCSKNKNEHRKHXEIILKEFIDNGIVISMNKVELEKQGIQFLGMYIKSETIQLQEHIGKKVLXFPDKXEDKKXLQEFLGLLNYARSFI

>Pmum1-Chr6-01; Yendovirus; Prunus mume 1;

LEELLQLAEQTRIIGEDPQLHWNKNKILAKLDIINLDLTIKTIDMPCNLLNKQEFEQQIKELLNLKVIRPSKSRHRSAAFVVRKHSELKRGKARIVYNYRRLNDNTYEDSYKLPNKDELMNKIQESKYFNKFDCKSGFWQIRLAEESIHWTAFTCLEGHFEWLVMPFGLKNAPSIFQRKMDQIFKKYDNFCSVYVDDILVHSKNKNEHRKHLEIFXKNLLKMVXLXVEIKXSLKNKIXNSXVCILSQVLYNYKNTXLKKCXNFQISXRKKKQLQAFLGLLNYARSFIPD

>Pmum1-Chr6-02; Florendovirus; Prunus mume 1;

KLITDVCSDLPTAFWHRKNHIVSLPYIQNFSEKNIPTKARPIQMSPEMLEFCKNEINDLLQKNIIRRSKSPWSCSAFYVQKNAELERGSPRLVINYKPLNTVLEWIRYPIPNKRDLINRLNKAIIFSKFDLKSGFWQIQITESDRYKTAFVTPFGHYEWNVMPFGLKNAPSEFQNIMNEIFNSYSQFAIVYIDDVLIFSESIEQHWKHLYKFLHVIKANGLVVSAKKIKLFQMHIRFLGFNISQSQIQPIDRSIQFADKFPDQIIDKNQLQRFLGSLNY

>Pmum1-Chr6-03; Yendovirus; Prunus mume 1;

LEELLQLAEQTRIIGEDPQLHWNKNKILAKLDIINPDLTIKTTDMPCNLLDKQEFGQQIKELLNLKVIRPSKSRHRSAAFVVRKHSELKRGKARIVYNYRRLNDNTYEDSYKLPNKDELMNKIQESKYFSKFDCKSGFWQIRLAEESIHWTTFTCPEGHFEWLVMPFGLKNAPSIFQRKMDQIFKKYDKFCSVYVDDILVHSKNKNEHRKHLEIILKEFIDNGIVISRNKVELEKQDIGFLGMYIKSGTIQLQEHIAKKVLEFPDKLEDKKQLQAFLGLLNYARSFIP

>Pmum1-Chr6-04; Florendovirus; Prunus mume 1;

KLKNEICSELPNAFWYRKQHVVCLPYIKTFSEKNIPTKARPIQMSQEIMEFCKKEIHELLEKKIIRKSKSPWSCPAFYVQKNAELERGVPRLVINYKALNSVLEWIRYPIPNKRDLINRLERAIVYSKFDMKSGFWQIQIHESDRYKTAFVTPFGHYEWNVMSFGLKNAPSEFQNIMNEIFNPYSHFSIVYIDDVLIFSESIEQHWKHLHKFLQIVKQNGLVVSAKKIKLFQTNIRFLGFNICQSQISPIDRVIQFADKFPDQILDKSQLQRFLESLNYVSDF

>Pmum1-Chr7-01; Yendovirus; Prunus mume 1;

LEELLQLAEQTRIIGEDPQLHWNKNKILAKLDIINPDLTIKTADMPCNLLDKQEFEQQIKELLNLKVIRPSKSRHRSAAFVVRKHSELKRGKARIVYNYRRLNDNTYEDSYKLPNKDELMNKIQESKYFSKFDCKSGFWQIRLAEESIHWTTFTCPEGHFEWLVMPFGLKNAPSIFQRKMDQIFKKYDKFCSVYVDDILVHSKNKNEHRKHLEIILKEFIDNGIIISRNKVELEKQDIEFLGMYIKSGTIQLQEHIAKKVLEFPDKLEDKKQLQAFLGLLNYARSFIPD

>Pmum1-Chr7-02; Florendovirus; Prunus mume 1;

KLMADVCSDLPTAFWHRKNHIVSLPYIQNFSEKNIPTKARPIQMSPEMLEFCKIEINDLLQKNIIRRSKSPWSCSAFYVQKNAELERGSPRLVINYKPLNTVLEWIRYPIPNKRDLINRLSKAIIFSKFDLKSGFWQIQITESDRYKTAFVTPFGHYEWNVMPFGLKNAPSEFQNIMNEIFNPYSQFAIVYIDDVLIFSESIEQHWKHLYKFXHVIKANGLVVSAKKIKLFQMHVRFLGFNISQSQIQPIDRSIQFADKFPDQIIDKNQLQRFLGSLNY

>Pmum1-Chr7-03; Yendovirus; Prunus mume 1;

LEELLQLGEQTRIIGEDPQLHWNKNKILAKLDIINPDLTIKTAYMTCNLLDKQEFEEQMXELLNLKVIRPSKSRHRSVTFVVIKHSELKRGKARIMYNYRRLNDNTYEDSYKLPNKDELMNKIQESKYFSKFDCKSGFWQIRLAEESIQWTTFTCPEGHFEWLVMPFSLKNAPLIFQRKMDQIFKKYDNFCSVYVDDILVHSKNKNEHRKHLEMILKEFINNGIVISRNKVELEKQDIEFLCMYIKSGTIQLQEHIAKKVLEFPDKLEDKKQLQAFLGLLNYSRSFIPD

>Pmum1-Chr8-01; Yendovirus; Prunus mume 1;

LEELLQLAEQTRIIGEDPQLHWNKNKTLAKLDIINPDLTIKTADMPCNLLDKQEFEQQIKELLNLKVIRPSKSRHRSAAFVVRKHSELKRGKARIVYNYRRLNDNTYEDSYKLPNKDKLVNKIQESKYFSKFDCKSGFWQIRLAKESIHWTTFTYPEGHFEWLVMPFGLKNAPSIFQRKMDQIFKKYDNFCSVYVDDILVHSKNKNEHRKHLEIILKEFIDNGIVISRNKVELEKQDIEFLGMYIKSGTIQLQEHIAKKVLEFPDKLEDKRQLQAFLGLLNYARSFIPD

>Pmum2-Chr2-01; Florendovirus; Prunus mume 2;

KLKNEICYELPNAFWFRKQHVVCLPYIKTFNEKNIPTKAKPIQMSQEIMEFCKKEIHELLQKKIIRKSKSPWSCPAFYVQKNAELERRVPRLVINYKPLNSVLEWIRYPIPNKRDLINRLEKAVVYSKFDMKSGFWQIQIDESDRYKTAFVTLFGHYEWNVMSFGLKNAPSEFQNIMNEIFNPYSHFSIVYIDDVLIFSESIEQHWKHLHKFLQVVKQNGLVVSAKKIKLFQTNIRFLGFNICQSQISPIDRVIQFADKFPDQIFDKSQLQRFLXSHNYV

>Pmum2-Chr3-01; Florendovirus; Prunus mume 2;

KLKNEICSDLPNAFWYRKQHVVCLPYIKTFSEKNIATKAKPIQMSQEIMEFCKKEINELLXKKIIRKSKSSWSCPAFYVXKNAELERGVPRLVINYKPLNSVLEWIRYPIPNKRDLINRLEKAVVYSEFDMKSGFWQIQIDESDRYKTAFFTPFGHYEWNVIPFGLKNAPSEFQNIINEIFNPYSHFSIVYTDDVLIFSESIEQHWKHLHKFLQIVKQNGLLVSAKKIKLFQTNIRFLGFTIYQSQISPIDRVIXFADKFSNQILDKSQLQRFLGSLNYVSDF

>Pmum2-Chr5-01; Yendovirus; Prunus mume 2;

HWNKNKILAKLDIINPYSTIKTADMPCNLLDKQNFXQQIKELLNIVIXPSKSRHRSTAFVVRKHSELKRGKARIVYNYKRXNDNTYEDSYKLPNKDELIMNKIEESKYSCKLDCKSRFCEIRLPEXSIQXTTFTCTXAYFEWLVRPFGLKNAPSIFXRKMNKKYDNFGSIYVDDILVCSKNKNEHRKHXEIILKKFIDNGIVISMNKVELEKQGIXFLGMYIKSXTTQLQEHIGKKVLXFPDKXEDKKXLQAFLGLLNYARSFI

>Pmum2-Chr6-01; Florendovirus; Prunus mume 2;

KLKNEICSELPNAFWYRKQHVVCLPYIKTFSEKNIPTKARPIQMSQEIMEFCKKEIHELLEKKIIRKSKSPWSCPAFYVQKNAELERGVPRLVINYKPLNSVLEWIRYLIPNKRDLINRLERAIVYSKFDMKSGFWQIQIHESDRYKTAFVTPFGHYEWNVMSFGLKNAPSEFQNIMNEIFNPYSHFSIVYIDDVLIFSESIEQHWKHLHKFLQIVKQNGLVVSAKKIKLFQTNIRFLGFNICQSQISPIDRVIQFADKFPNQILDKSQLQRFLGSLNYV

>Pmum2-Chr6-02; Florendovirus; Prunus mume 2;

KKLIDEICSDLPTAFWHRKQHVVKLPYIKNFSKKNIPTKARPIEMNQEMVSYCKTEVNELLQKNIIRKSKSPWSCPAFYVQKNAELERGSPRLVINYKPLNTVLEWIRYPIPNKRDLINRLSNAVIFSKFDLKSGFWKIQIDESDRYKTAFVTPFGHYEWNVMPFGLKNAPSEFQNIMNDIFNPYSHFSIVYIDDVLIFSKSIEQHWKHLHKFLQIVKTNGLVVSEKKIKLFQTSIRFLGFNISHSQIHPIDRAIQFADKFPDQITDKSQLQRFLGSLNYVSDF

>Pmum2-Chr7-01; Florendovirus; Prunus mume 2;

KLKNEICSELPNAFWYRKQHVVCLPYIKTFSEKNIPTKAKPIQMSQEIMEFCKKEINELLQKKIIRKNKSPWXCPTFYVQKNAELERGVPRLVINYKPLNSVLEWIRYPIPNKRDLINRLEKAVVYSKFDKKSGFWQIQIDESDRYKTAFVTPFGHYEWNLMPFGLKNAPSEFQNIMNEIFNPYSHFSIVYIDDVLIFSESIEQHWKHLHKFLQIVKQNGLAVSAKKIKLFQTNIRFLGFNICQSQISPIDRVIQFADKFPDQILDKSQLHRFLGSLNYVSDF

>Pper1-Chr1-01; Florendovirus; Prunus persica 1;

EKLKQEVCSELPNAFWHRKQHVVSLPYVKTSSEKNIPTKARPIQISQELMKFCKIEITDLLKKGIIRKSRSPWSCPAFYVQKNAELERGVPRLVINYKPLNTVLEWIRYPIPNKRDIINRFEKSVIFSKFDMKSGFWQIQISETDRYKTAFVTPFGHYEWNVMPFDLKNAPSEFQNIINVIFNPFSHFSIVYIDDVLIFSASIEQHWKHLYKFLQIVKQNGLVVSAKKIKLFQTNVRFLGFNIXQSQISPIDRVIQFADKFPDQILEKGQLQRFLGSLNYVSDF

>Pper1-Chr1-02; Florendovirus; Prunus persica 1;

QRLKEEICSELPTAFWYRKQHVVRLPYIKTFSEKNIPTKARPIQMSQEMMEFCKKEIEELLQKKIIRKSKSPWSCPAFYVQKNAELERGVPRLVINYKPLNAVLEWIRYPIPNKRDLINRLEKAVVYSKFDMKSGFWQIQIDESDRYKTAFVTPFGHYEWNVMPFGLKNAPSEFQNIMNDIFNPYSQFSIVYIDDVLIFSESIEQHWKHLNKFLQVVKQNGLVVSAKKIKLFQTNIRFLGFNICQSQISPIDRVIQLADKFPDQILDKSQLQRFLGSLNY

>Pper1-Chr1-03; Florendovirus; Prunus persica 1;

EKLKQEVCSELPNAFWHRKQHVVSLPYVKSFSEKNIPTKARPIQMSQELMKFCKTEITELLEKGIIRKSKSLWSCPAFYVQKNVELEHGVPRLVINYKPLNTVLEWIRYPIPNKRDLINRLEKSVIFSKFDMKSGFWQIQIRESFSYKTAFVTPFGHYEWNVMPFGLKNAPSEFENIMNEIFNPFSHFSIVYIDDVLIFSESIEQHWKHLHKFLQIVKRNGLVVSAKKIKLFQTNVRFFGFNICQSQISPIDRVIQFADKFPDQILDKSQLHRFLGSLNYVSDF

>Pper1-Chr1-04; Florendovirus; Prunus persica 1;

QKIKNEICSELPNAFWYRKQHVVCLPYIKTFSEKNIPTKARPIQMSQDMMEFYKKEIDELLQKKIIRKSKSLWSCPAFYVQKNAELERGVPRLVINYKPLNAVLEWIRYPIPNKRDLINRLEKAVVYSKFDMKSGFWQIQIEESDRYKTAFVTPFSHYEWNVMPFGLKNAPSEFQNIMNDIFNPYSQFSIVYVDDVLIFSESIEQHWKHLHKFFHVIKQNGLVVSAKKIKLFQTNVRFLGFNICQSQISPIDRVIQFADKFPDQILDKSQLQRFLGSFNY

>Pper1-Chr1-05; Florendovirus; Prunus persica 1;

QRLKEEICSEVPTAFWYRKQHVVRLPYIKTFSEKNIPTKARPIQMNQEMMEFCKKEIEELLQKKIIRKSKSPWSCPAFYVQKNAELERGVPRLVINYKPLNAVLEWIRYPIPNKRDLINRLEKAVVYSKFDMKSGFWQIQIDESDRYKTAFVTPFGHYEWNVMPFGLKNAPSEFQNIMNDIFNPYSQFSIVYIDDVLIFSESIEQHWKHLNKFLQVVKQNGLVVSAKKIKLFQTNIRFLGFNICQSQISPIDRVIQFADKFLDQILDKSQLQRFLGSLNY

>Pper1-Chr2-01; Florendovirus; Prunus persica 1;

QRLKEEICSELPTAFWYRKQHVVRLPYIKTFSEKNIPTKARPIQMSQEMMEFCKKEIEELLQKKIIRKSKSPWSCPAFYVQKNAELERGVPRLVINYKPLNAVLEWIRYPIPNKRDLINRLEKAVVYSKFDLKSGFWQIQIDESDRYKTAFVTPFGHYEWNVMPFGLKNAPSEFQNIMNDIFNPYSQFSIVYIDDVLIFSESIEQHWKHLNKFLQVVKQNGLVVSAKKIKLFQTNIRFLGFNICQSQISPIDRVIQFADKFPDQILDKSQLQRFLGSLNY

>Pper1-Chr2-02; Florendovirus; Prunus persica 1;

QRLKEEICSELPTAFWYRKQHVVRLPYIKTFSEKNIPTKARPIQMSQEMMEFCKKEIEELLQKKIIRKSKSPWSCPAFYVQKNAELERGVPRLVINYKPLNAVLEWIRYPIPNKRDLINRLEKAVVYSKFDLKSGFWQIQIDESDRYKTAFVTPFGHYEWNVMPFGLKNAPSEFQNIMNDIFNPYSQFSIVYIDDVLIFSESIEQHWKHLNKFLQVVKQNGLVVSAKKIKLFQTNIRFLGFNICQSQISPIDRVIQFADKFPDQILDKSQLQRFLGSLNY

>Pper1-Chr2-03; Yendovirus; Prunus persica 1;

HWNKNKILAKLDIINPYSTIKIAYMPYNLLDKQNFXQQIKELLNIVLRPSKSRHRSTAFVIRKHSKVKRGKARIVYNYKRXNDNTYEDSYKLPNKDEFMNKIEESKYSSKLYCKSRFWKIRLAEXSIQXTTFTCTESHFEWLVMPFGFKNAPSIFQRKMNQIFKKYDNFGSIYVDDKLVCSKNKMNIENTEKLFXKNLLIMVXLXVGIKXSLKNKAYNFKVCILSQKLYNYKNTXVKKCYNFQINEKIKKKLQAFLGLLNYARSFI

>Pper1-Chr2-04; Yendovirus; Prunus persica 1;

HWNKNKILAKLDIINPYSTIKIAYMPCNLLDKQNFXQQIKELLNIVLRPSKSRHRSTAFVIRKHSKVKRGKARIVYNYKRXNDNTYEDSYKLPNKDELMNKIEESKYSSKLYCKSRFWQIRLAEXSIQXTTFTRTKAHFEWLVMPFGLMNAPSIFQRKMNQIFEKYDNFGSIYVDDKLVCSKNKNEHRKHXENILKEFIDNGIVISRNKVELEKXGIXFXGMYIKSETIXLQEHIGKKVLXFPNKXEDKKQLQAFLGLLNYARSFI

>Pper1-Chr2-05; Florendovirus; Prunus persica 1;

EKLKQEVCSELPNAFWHRKQHVVSLLYVKSFSEKNIPTKARPIQMSQELMEFCKTEITDLLKKGIIRKSKSPWSCPTFYVQKNTELERGVSRLVINYKPLNTVLEWIRYPIPNKRDLINRLEKSVIFSKFDMKSGFWQIQISESDRYKTAFVTPFGHYEWNVMPFGLKNAPSKFQNIMNEIFNPYSHFSIVYIDDVLIFSESIEQHWKHLYKFLQIVKQNGLVVSAKKIKLFQTNVRFLGFNIYQSQVSPIDQVSPMNQLQRFLGSLNYV

>Pper1-Chr2-06; Florendovirus; Prunus persica 1;

QRLKEEICSELPTAFWYRKQHVVRLPYIKTFSEKNIPTKARPIQMSQEMMEFCKKEIEELLQKKIIRKSKSPWSCPAFYVQKNAELERGVPRLVINYKPLNAILEWIRYPIPNKRDLINRLEKAVVYSKFDMKSGFWQIQIDESDRYKTAFVTPFGHYEWNVMPFGLKNAPSEFQNIMNDIFNPYSQFSIVYIDDVLIFSESIEQHWKHLNKFLQVVKRNGLVVSAKKIKLFQTNIRFLGFNICQSQISPIDRVIQFADKFPDQILDKSQLQRFLGSLNY

>Pper1-Chr3-01; Florendovirus; Prunus persica 1;

QRLKEEICSELPTAFWYRKQHVVRLPYIKTFSEKNIPTKARPIQMSQEMMEFCMKEIKELLQKKIIRKSKSPWSCPAFYVQKNAELERGVPRLVINYKPLNAVLEWIRYPIPNKRDLINRLEKAVVYSKFDLKSGFWQIQIDESDRYKTAFVTPFGHYEWNVMPFGLKNAPSEFQNIMNDIFNPYSQFSIVYIDDVLIFSESIEQHWKHLNKFLQVVKQNGLVVSAKKIKLFQINIRFLGFNICQSQISPIDRVIQFADKFPDQILDKSQLQRFLGSLNY

>Pper1-Chr3-02; Florendovirus; Prunus persica 1;

EKLKQEVCSELPNAFWHRKQHVICLPYVKTFSEKNIPTKARPIQMSQELMEFCKIEITDLLKKGIIRKSKSPWSCPAFYVQKNAELERGVPRLVINYKPLNTVLEWIRYPIPNKRDLINRLEKSVIFSKFDMKSGFWQIQISEADRYKTAFVTHFGHYEWNVMPFGLKNAPSEFQNIMNETFNHFSHFSIVYIDDVLIFSESIEQHWKHLYKFLQIVKQNGLVISAKKIKLLQTNVRFLGFNICQSXISPIDRVIQFVDKFLDQILD

>Pper1-Chr3-03; Florendovirus; Prunus persica 1;

EKLKQEVCSDLPNAFWHRKQHVVSLPYVKNFSEKNILTKARPIQMSQEIMEFYKTEITELLQKGIIRKSKSPWSCPAFYVXKNAELERGAPRLVINYKPLNTTLEWIRYPIPNKRDLINRLDKSVIFSKFDMKIGFWXIQILESDRYKTAFVTPFGHYEWNVMPFGLKNAHSEFQNIMNEIFNPFSHFSIVYIDDALIFSQSIEQHWKHLHKFLKIVKQNGLVVLAKKIKLFQTNICFLGFNICQSQISLIDRVIQFADKFSYQILDKSQLQRFLGSLNY

>Pper1-Chr3-04; Florendovirus; Prunus persica 1;

EKLKQEICSDLPTAFWHRKQHVVSLPYVKTFNEKHIPTKSRPIQMSREVMEFCKKEIAELLYKGIIRKSKSPWSCPAFYVQKNAELERGAPRLVINFKPLNTVLEWIRYPIPNKRDLINRLSSAVIFSKFHMKSGFWHIQIQERDRYKTAFVTPFGHYEWNVMHFGLKNAPSEFQNIMNEIFNPFSEFSIVYIDDILIFSSSIEQHWKHLHKFLQIVKKNGLVLSAKKIKLFQTNIKFLGFNIHQS

>Pper1-Chr3-05; Florendovirus; Prunus persica 1;

EKLKQEVCSELPNAFWHRKQHVVNLPYVKSFSDKNFSTKARPIQISQELMEFCKTEITELLEKGIIRKSKSPXSCPAFYVQENAELERGDPRLVIDYKPLNIILEWIRYPIHNKRDLINRLEKSVIFSKFDMKSGFWQIQIHESDRYKTAFITSFGHYEWNVMPFGLXNASSEFQNIMNEILNHFSHFFIVYIDDVLIFSQSIEQHWKHLHXFLQIVKQNGLVVSAXKIKLFQTNVRFLGFNICQSQISPIDQVIQFADKFPDQILDKSQLXRFLGSLNYV

>Pper1-Chr3-06; Yendovirus; Prunus persica 1;

LEELLQLAEQTRIVGEDPQLHWNKNKILAKLDIINPDLTIKTTDMPCNLLDKQEFEHQIKELLNLKVIRPSKSRHRSVAFVVRKHSELKRGKARIVYNYRRLNDNTYEDSYKLPNKDELVNKIQESKYFSKFDCKSGFWQTRLAEESIQWTTFTCTEGHFEWLVMPFGLKNAPSIFQRKMDQSFKKYDNFCSVYVDDILVHSKNKNEHSKHLEIILKEFIDNGVVISRNKIEFEKQDIEFLGMYIKSGTIQLQEHIAKKVLKFLDRLEDKKQLQAFLGLLNYVSSFIPD

>Pper1-Chr4-01; Florendovirus; Prunus persica 1;

QRLKEEICSELPTAFWYRKQHVVRLPYIKTFSEKNIPTKARPIQMSQEMMEFCKKEIEELLHKKIIRKSKSPWSCPAFYVQKNAELERGVPRLVINYKPLNAVLEWIRYPIPNKRDLINRLEKAVVYSKFDLKSGFWQIQIDESDRYKTAFVTPFGHYEWNVMPFGLKNAPSEFQNIMNDIFNPYSQFSIVYIDDVLIFSESLEQHWKHLNKFLQVVKQNGLVVSAKKIKLFQTNIRFLGFNICQSQISPIDRVIQFADKFPDQILDKSQLQRFLGSLNY

>Pper1-Chr5-01; Florendovirus; Prunus persica 1;

QRLKEEICSELPTAFWYRKQHVVRLPYIKTFSEKNIPTKARPIQMSQEMMEFCKKEIEELLQKKIIRKSKSPWSCPAFYVQKNAELERGVPRLVINYKPLNAVLEWIRYPIPNKRDLINRLEKAVVYSKFDMKSGFWQIQIDKSDRYKTAFVTPFGHYEWNVMPFGLKNAPSEFQNIMNDIFNPYSQFSIVYIDDVLIFSESIEQHWKHLNKFLQVVKQNGLVVSAKKIKLFQTNIRFLGFNICQSQISPIDRVIQFADKFPDQILDKSQLQRFLGSLNY

>Pper1-Chr5-02; Yendovirus; Prunus persica 1;

LEELLQLAKQTRIIGEDPQLHWNKNKILAKLDIINLDLTIKTADMPCNLLDKQEFYQQIKQILNLKVIRPSKSRHRSTAFVVRKHLELKRGKVRIVYNYRRLNDNTYEDSYKLPNKDELMNKIQESKYFSKFDCKSGFXQIRLAEESIXLTTFTCPEGNFEWLVMPFGLKNAPSIFQRKMDQIFKKYDNFCSVYVEDILVHSKNKNKHRKYLEIILKEFIDNGIVISRNKVELEKQDIEFLGIYIKSGTIQLQEHIAKKVLEFPDKLENKKQLQEFLGLLNYARSFIPD

>Pper1-Chr5-03; Florendovirus; Prunus persica 1;

QKLKEEICSELPTAFWYRKQHVVCLPYIKTFSEKNIPTKARPIQMSQEMMEFCKKEIEELLQKKIIRKSKSPWLCPAFYVQKNAELERGVPRLVINYKPLNAVLEWIRYPIPNKRDLINRLEKAVVYSKFDMKSGFWQIQIDESDRYKTVFVTPFGHYEWNVMPFGLKNAPSEFQNIMNDIFNPYSQFSIVYIDDVLIFSESIEQHWKHLYKFLQIVKQNGLVVSAKKIKLFQTNIRFLGFNICQSQISPIDRVIQFADKFPDQILDKSQLQRFLGSLNY

>Pper1-Chr5-04; Florendovirus; Prunus persica 1;

QKIKSEICSELPNAFWYRKQHVVCLPYMKTFSEKNIPTKARPIQMSQEMMEFCKQEIKELLQKKIIRQSKSPWSCPAFYVQKNAELERGVPRLVINYKPLNAVLEWIRYPIPNKRDLINRLEKAVVYSKFDMKSGFWQIQIEESDRYKTAFVTPFGHYEWNVMPFGLKNAPSEFQNIMNDIFNPYSQFSIVYIDDVLIFSESIEQHWKHLYKFLQVVKQNGLVVSAKKIKLFQTNVRFLGFNICQSQISPIDRVIQFADKFPDQILDKSQLQRFLGSLNY

>Pper1-Chr6-01; Florendovirus; Prunus persica 1;

QRLKEEICSELPTAFWYRKQHVVRLPYIKTFSENNIPTKARPIQMSQEMMEFCKKEIEELLQKKIIRKSKSPWSCPAFYVQKNAELERGVPRLVINFKPLNAVLEWIRYPIPNKRDLINRLEKAVVYSKFDLKSGFWQIQIDESDRYKTAFVTPFGHYEWNVMPFGLKNAPSEFQNIMNDIFNPYSQFSIVYIDDVLIFSESIEQHWKHLNKFLQVVKQNGLVVSAKKIKLFQTNIRFLGFNICQSQISPIDRVIQFADKFPDQILDKSQLQRFLGSLNY

>Pper1-Chr6-02; Florendovirus; Prunus persica 1;

QRLKEEICSELPTAFWYRKQHVVRLPYIKTFSENNIPTKARPIQMSQEMMEFCKKEIEELLQKKIIRKSKSPWSCPAFYVQKNAELERGVPRLVINYKPLNAVLEWIRYPIPNKRDLINRLEKAVVYSKFDLKSGFWQIQIDESDRYKTAFVTPFGHYEWNVMPFGLKNAPSEFQNIMNDIFNPYSQFSIVYIDDVLIFSESIEQHWKHLNKFLQVVKQNGLVVSAKKIKLFQTNIRFLGFNICQSQISPIDRVIQFADKFPDQILDKSQLQRFLGSLNY

>Pper1-Chr6-03; Florendovirus; Prunus persica 1;

EKLKQEVCSELPNAFWHRKQHVVSLPCVKSFSEKNIPTKARPIQMSQELMEFCKIEITDLLKKGIIRKSKSPWSCPDFYVQKNAELECGVPRLVINYKPLNTVLEWIRYPIPNKRDLINRLENSVIFSKFDMKSGFWQIQISESDRYKTAFVTPFGHYEWNVMPFGLKNAPSEFQNIMNEIFNPFSHFSIVYIDDVLIFSESIEQHXKHLHKFLQIVKQNGLVVSAKKIKLFQTNVRFLRFNIYQSQISPIDRVIXIADKFPDQILDKSQLQRFLGSLN

>Pper1-Chr6-04; Florendovirus; Prunus persica 1;

QRLKEEICSELPTAFWYRKQHVVRLPYIKTFSEKNIPTKARPIQMSQEMMEFCKKEIEELLQKKIIRKSKSPWSCPAFYVQKNAELERGVPRLVINYKPLNAVLEWIRYPIPNKRDLINRLEKAVVYSKFDMKSGFWQIQIDESDRYKTAFVTPFGHYEWNVMPFGLKNAPSEFQNIMNDIFNPYSQFSIVYIDDVLIFSESLEQHWKHLNKFLQVVKQNGLVVSAKKIKLFQTNIRFLGFNICQSQISPIDRVIQFADKFPDQILDKSQLQRFLGSLNY

>Pper1-Chr6-05; Florendovirus; Prunus persica 1;

QRLKEEICSELPTAFWYRKQHVVRLPYIKTFSEKNIPTKARPIQMSQEMMEFCKKEIEELLQKKIIRKSKSPWSCPAFYVQKNAELERGVPRLVINYKPLNAVLEWIRYPIPNKRDLINRLEKAVVYSKFDMKSGFWQIQIDESDRYKTAFVTPFGHYEWNVMPFGLKNAPSEFQNIMNDIFNPYSQFSIVYIDDVLIFSESLEQHWKHLNKFLQVVKQNGLVVSAKKIKLFQTNIRFLGFNICQSQISPIDRVIQFADKFPDQILDKSQLQRFLGSLNY

>Pper1-Chr6-06; Florendovirus; Prunus persica 1;

QRLKEEICSELPTAFWYRKQHVVCLPYIKSFSEKNIPTKARPIQMSQEMMEFCKKEIEELLQKKIIRKSKSPWSCPAFYVQKNAELERGVPRLVINYKPLNAVLEWIRYPIPNKRDLINRLEKAVVYSKFDMKSGFWQIQIDESDRYKTAFVTPFGHYEWNVMPFGLKNAPSEFQNIMNDIFNPYSQFLIVYIDDVLIFSESIEQHWKHLNKFLQVVKQNGLVVSAKKIKLFQTNIRFLGFNICQSQISPIDRVIQFVDKFPDQILDKSQLQRFLGSLNY

>Pper1-Chr7-01; Florendovirus; Prunus persica 1;

QRLKEEICSELPTAFWYRKQHVVRLPYIKTFSEKNIPTKARPIQMSQEMMEFCKKEIEELLQKKIIRKSKSPWSCPAFYVQKNAELERGVPRLVINYKPLNAVLEWIRYPIPNKRDLINRLEKAVVYSKFDMKSGFWQIQIDESDRYKTAFVTPFGHYEWNVMPFGLKNAPSEFQNIMNDIFNPYSQFSIVYIDDVLIFSESIEQHWKHLNKFLQVVKQNGLVVSAKKIKLFQTNIRFLGFNICQSQISPIDRVIQFADKFPDQILDKSQLQRFLGSLNY

>Pper1-Chr7-02; Florendovirus; Prunus persica 1;

QRLKEEICSELPTAFWYRKQHVVRLPYIKTFSEKNIPTKARPIQMSQEMMEFCKKEIEELLQKKIIRKSKSPWSCPAFYVQKNAELERGVPRLVINYKPLNAVLEWIRYPIPNKRDLINRLEKAVVYSKFDMKSGFWQIQIDESDRYKTAFVTPFGHYEWNVMPFGLKNAPSEFQNIMNDIFNPYSQFSIVYIDDVLIFSESIEQHWKHLNKFLQVVKQNGLVVSAKKIKLFQTNIRFLGFNICQSQISPIDRVIQFADKFPDQILDKSQLQRFLGSLNY

>Pper1-Chr7-03; Florendovirus; Prunus persica 1;

QRLKEEICSELPTAFWYRKQHVVRLPYIKTFSEKNIPTKARPIQMSQEMMEFCKKEIEELLQKKIIRKSKSPWSCPAFYVQKNAELERGVPRLVINYKPLNAVLEWIRYPIPNKRDLINRLEKAVVYSKFDMKSGFWQIQIDESDRYKTAFVTPFGHYEWNVMPFGLKNAPSEFQNIMNDIFNPYSQFLIVYIDDVLIFSESIEQHWKHLNKFLQVVKQNGLVVSAKKIKLFQTNIRFLGFNICQSQISPIDRVIQFADKFPDQILDKSQLQRFLGSLNY

>Pper1-Chr7-04; Florendovirus; Prunus persica 1;

QKLKEEVCSDLPTAFWYRKQHVVCLPYIKNFSEKNIPIKARPIQMSQEMMEFCKTEIHEQLQKKIIRKSRSPWSCPAFYVQKNAELERGVPRLVINYKPLNAVLEWIRYPIPNKRDLINRLEKAVVYSKFDMKSDFWQIQIDESDRYKTAFVTPFGHYEWNVMPFGLKNAPSEFQNIMNDIFNPYNQFTIVYIDDVLIFSESIEQHWKHLNKFLQVVIKNGLVVSAKKIKLFQTNIRFLGFNICQSQISPIDRVIQFADKFPDQILDKSQLQRFLGSLNY

>Pper1-Chr7-05; Florendovirus; Prunus persica 1;

QKLKEEICSELPTAFWYRKQHVVRLPYIKSFSEKNIPTKARPIQMSQEMMEFCKKEIEELLQKKIIRKSKSPWSCPAFYVQKNAELERGVPRLVINYKPLNAVLEWIRYPIPNKRDLINRLEKAVVYSKFDLKSGFWQIQIDESDRYKTAFVTPFGHYEWNVMPFGLKNAPSEFQNIMNDIFNPYSQFSIVYIDDVLIFSESIKQHWKHLHKFLQVFKQNGLVVSAKKIKLFQTNIRFLGFNICQSQISPIDRVIQFADKFLDQILDKSQLQRFLGSLNY

>Pper1-Chr7-06; Florendovirus; Prunus persica 1;

QKLKEEICSELPTTFWYRKQHVVCLPYIKTFSEKNIPTKARPIQMSQEMMEFCKKEIEELLQKKIIRKSKSPWSCPAFYVQKNAELERGVPRLVINYKPLNAVLEWIRYPIPNKRDLINRLEKAFVYSKFDLKSGFWQIQIDESDRYKIAFVTPFGHYEWNVMPFGLKNAPSEFQNIMNDIFNPYSQFSIVYIDDVLIFSESIEQHWKHLNKFLQVVKQNGLVVSAKKIKLFQTNIRFLGFNICQSQISPIDRVIQFADKFPDQILDKSQLQRFLRSLNY

>Pper1-Chr8-01; Florendovirus; Prunus persica 1;

QRLKEEICSELPTAFWYRKQHVVRLPYIKTFSEKNIPTKARPIQMSQEMMEFCKKEIEELLQKKIIRKSKSPWSCPAFYVQKNAELERGVPRLVINYKPLNAVLEWIRYPIPNKRDLINRLEKAVVYSKFDMKSGFWQIQIDESDRYKTAFVTPFGHYEWNVMPFGLKNAPSEFQNIMNDIFNPYSQFSIVYIDDVLIFSESIEQHWKHLYKFLQVVKQNGLVVSAKKIKLFQTNIRFLGFNICQSQISPIDRVIQFADKFPDQILDKSQLQRFLGSLNY

>Pper1-Chr8-02; Florendovirus; Prunus persica 1;

QRLKEEICSELPTAFWYRKQHVVRLPYIKTFSEKNIPTKARPIQMSQEMMEFCKKEIEELLQKKIIRKSKSPWSCPAFYVQKNAELERGVPRLVINYKPLNAVLEWIRYPIPNKRDLINRLEKAVVYSKFDMKSGFWQIQIDESDRYKTAFVTPFGHYEWNVMPFGLKNAPSEFQNIMNDIFNPYSQFSIVYIDDVLIFSESIEQHWKHLNKFLQVVKRNGLVVSAKKIKLFQTNIRFLGFNICQSQISPIDRVIQFADKFPDQILDKSQLQRFLGSLNY

>Pper1-Chr8-03; Florendovirus; Prunus persica 1;

QRLKEEICSELPTAFWYRKQHVVRLPYIKTFSEKNIPTKARPIQMSQEMMEFCKKEIEELLQKKIIRKSKSPWSCPAFYVQKNAELERGVPRLVINYKPLNAVLEWIRYPIPNKRDLINRLEKAVVYSKFDMKSGFWQIQIDESDRYKTAFVTPFGHYEWNVMPFGLKNAPSEFQNIMNDIFNPYSQFSIVYIDDVLIFSESIEQHLKHLNKFLQVVKRNGLVVSAKKIKLFQTNIRFLGFNICQSQISPIDRVIQFADKFPDQILDKSQLQRFLGSLNY

>Pper1-Chr8-04; Florendovirus; Prunus persica 1;

QRLKEEICSELPTAFWYRKQHVVRLPYIKTFSEKNIPTKARPIQMSQEMMEFCKKEIEELLQKKIIRKSKSPWSCPAFYVQKNAELERGVPRLVINYKPLNAVLEWIRYPIPNKRDLINRLEKAVVYSKFDMKSGFWQIQIDESDRYKTAFVTPFGHYEWNVMPFGLKNAPSEFQNIMNDIFNPYSQFSIVYIDDVLIFSESIEQHWKHLNKFLQVVKRNGLVVSAKKIKLFQTNIRFLGFNICQSQISPIDRVIQFADKFPDQILDKSQLQRFLGSLNY

>Pper1-Chr8-05; Florendovirus; Prunus persica 1;

QRLKEEICSELPTAFWYRKQHVVRLPYIKTFSEKNIPTKARPIQMSQEMMEFCKKEIEELLQKKIIRKSKSPWSCPAFYVQKNAELERGVPRLVINYKPLNAVLEWIRYPIPNKRDLINRLEKAVVYSKFDMKSGFWQIQIDESDRYKTAFVTPFGHYEWNVMPFGLKNAPSEFQNIMNDIFNPYSQFSIVYIDDVLIFSESIEQHWKHLNKFLQVVKRNGLVVSAKKIKLFQTNIRFLGFNICQSQISPIDRVIQFADKFPDQILDKSQLQRFLGSLNY

>Pper1-Chr8-06; Florendovirus; Prunus persica 1;

QRLKEEICSELPTAFWYRKQHVVRLPYIKTFSEKNIPTKARPIQMSQEMMEFCKKEIEELLQKKIIRKSKSPWSCPAFYVQKNAELERGVPRLVINYKPLNAVLEWIRYPIPNKRDLINRLEKAVVYSKFDMKSGFWQIQIDESDRYKTAFVTPFGHYEWNVMPFGLKNAPSEFQNIMNDIFNPYSQFSIVYIDDVLIFSESIEQHWKHLNKFLQVVKRNGLVVSAKKIKLFQTNIRFLGFNICQSQISPIDRVIQFADKFPDQILDKSQLQRFLGSLNY

>Pper1-Chr8-07; Florendovirus; Prunus persica 1;

QRLKEEICSELPTAFWYRKQHVVRLPYIKTFSEKNIPTKARPIQMSQEMMEFCKKEIEELLQKKIIRKSKSPWSCPAFYVQKNAELERGVPRLVINYKPLNAVLEWIRYPIPNKRDLINRLEKAVVYSKFDMKSGFWQIQIDESDRYKTAFVTPFGHYEWNVMPFGLKNAPSEFQNIMNDIFNPYSQFSIVYIDDVLIFSESIEQHWKHLNKFLQVVKRNGLVVSAKKIKLFQTNIRFLGFNICQSQISPIDRVIQFADKFPDQILDKSQLQRFLGSLNY

>Pper1-Chr8-08; Florendovirus; Prunus persica 1;

QRLKEEICSELPTAFWYRKQHVVRLPYIKTFSEKNIPTKARPIQMSQEMMEFCKKEIEELLQKKIIRKSKSPWSCPAFYVQKNAELERGVPRLVINYKPLNAVLEWIRYPIPNKRDLINRLEKAVVYSKFDMKSGFWQIQIDESDRYKTAFVTPFGHYEWNVMPFGLKNAPSEFQNIMNDIFNPYSQFSIVYIDDVLIFSESIEQHWKHLNKFLQVVKRNGLVVSAKKIKLFQTNIRFLGFNICQSQISPIDRVIQFADKFPDQILDKSQLQRFLGSLNY

>Pper1-Chr8-09; Florendovirus; Prunus persica 1;

QRLKEEICSELPTAFWYRKQHVVRLPYIKTFSEKNIPTKARPIQMSQEMMEFCKKEIEELLQKKIIRKSKSPWSCPAFYVQKNAELERGVPRLVINYKPLNAVLEWIRYPIPNKRDLINRLEKAVVYSKFDMKSGFWQIQIDESDRYKTAFVTPFGHYEWNVMPFGLKNAPSEFQNIMNDIFNPYSQFSIVYIDDVLIFSESIEQHWKHLNKFLQVVKRNGLVVSAKKIKLFQTNIRFLGFNICQSQISPIDRVIQFADKFPDQILDKSQLQRFLGSLNY

>Pper1-Chr8-10; Florendovirus; Prunus persica 1;

QRLKEEICSELPTAFWYRKQHVVRLPYIKTFSEKNIPTKARPIQMSQEMMEFCKKEIEELLQKKIIRKSKSPWSCPAFYVQKNAELERGVPRLVINYKPLNAVLEWIRYPIPNKRDLINRLEKAVVYSKFDMKSGFWQIQIDESDRYKTAFVTPFGHYEWNVMPFGLKNAPSEFQNIMNDIFNPYSQFSIVYIDDVLIFSESIEQHWKHLNKFLQVVKRNGLVVSAKKIKLFQTNIRFLGFNICQSQISPIDRVIQFADKFPDQILDKSQLQRFLGSLNY

>Pper1-Chr8-11; Florendovirus; Prunus persica 1;

QRLKEEICSELPTAFWYRKQHVVRLPYIKTFSEKNIPTKARPIQMSQEMMEFCKKEIEELLQKKIIRKSKSPWSCPAFYVQKNAELERGVPRLVINYKPLNAVLEWIRYPIPNKRDLINRLEKAVVYSKFDMKSGFWQIQIDESDRYKTAFVTPFGHYEWNVMPFGLKNAPSEFQNIMNDIFNPYSQFSIVYIDDVLIFSESIEQHWKHLNKFLQVVKRNGLVVSAKKIKLFQTNIRFLGFNICQSQISPIDRVIQFADKFPDQILDKSQLQRFLGSLNY

>Pper1-Chr8-12; Florendovirus; Prunus persica 1;

QKIKNEICSELPNAFWYRKQHVVCLPYIKTFSEKNIPTKARPIQMSQEIMEFCKTEISELLQKKIIRKSKSSWSCPAFYVQKNAELERGVPRLVINYKSLNSVLEWIWYPIPNKRDLINRLGKAVVYSKFDMKSGFWQIQIEESDRYKTAFNTPFGHYEWNVMPFGLKNAPSEFQNIMNEIFNPYSHFSIVYIDDVLIFSESIEQYWKHLHKFFQIVKQNGLVVSAKKIKLFQTNIRFLGFNICQSQISPIDRVIQFADKFPDQILDKSQLQRFLGSLNY

>Pper1-Chr8-13; Florendovirus; Prunus persica 1;

QKIKNEICSELPNAFWYRKQHVVCLPYIKTFSEKNIPTKARPIQMSQEIMEFCKTEISELLQKKIIRKSKSPWSCPAFYVQKNAELERGVPRLVINYKSLNSVLEWIWYPIPNKRDLINRLGKAVVYSKFDMKSEFWQIQIEESDRYKTAFVTPFGHYEWNVMPFGLKNAPSEFQNIMNEIFNPYSHFSIVYIDDVLIFSESIEQHWKHLHKFFQIVKQNGLVVSAKKTKLFQTNIKFLGFNICQSQISPIDRVIQFANKFPDQILDKSQLMRVLGSLNY

>Pper2-Chr1-01; Florendovirus; Prunus persica 2;

EKLKQEVCSELPNAFWHRKQHVVSLPYVKTSSEKNIPTKARPIQISQELMKFCKIEITDLLKKGIIRKSRSPWSCPAFYVQKNAELERGVPRLVINYKPLNTVLEWIRYPIPNKRDIINRLEKSVIFSKFDMKSGFWQIQISETDRYKTAFVTPFGHYEWNVMPFDLKNAPSEFQNIINVIFNPFSHFSIVYIDDVLIFSASIEQHWKHLYKFLQIVKQNGLVVSAKKIKLFQTNVRFLGFNIXQSQISPIDRVIQFADKFPDQILEKGQLQRFLGSLNYVSDF

>Pper2-Chr1-02; Florendovirus; Prunus persica 2;

QKLKEEVCSELPTAFWYRKQHVVRLPYIKTFSEKNIPTKARPIQMSQEMMEFCKKEIEELLQKRIIRKSKSPWSCPAFYVQKNVELERGVPRLVINYKPLNAVLEWIRYPIPNKRDLINRLEKAVVYSKFDMKSGFWQIQIDESDRYKTAFVTPFGHYEWNVMPFGLKNAPSEFQNIMNDIFNPYSQFSIVYIDDVLIFSESIEQHWKHLYKFLQVVKQNGLVVSAKKIKLFQTNIRFFGFNICQSQISPIDRVIQFADKFPDQILDKSQLQRFLGSLNY

>Pper2-Chr1-03; Florendovirus; Prunus persica 2;

EKLKQEVCSELPNAFWHRKQHVVSLPYVKSFSEKNIPTKARPIQMSQELMKFCKTEITELLEKGIIRKSKSLWSCPAFYVQKNVELEHGVPRLVINYKPLNTVLEWIRYPIPNKRDLINRLEKSVIFSKFDMKSGFWQIQIRESFSYKTAFVTPFGHYEWNVMPFGLKNAPSEFENIMNEIFNHFSHFSIVYIDDVLIFSESIEQHWKHLHKFLQIVKRNGLVVSAKKIKLFQTNVRFLGFNICQSQISPIDRVIQFADKFPDQILDKSQLHRFLGSLNYVSDF

>Pper2-Chr2-01; Yendovirus; Prunus persica 2;

HWNKNKILAKLDIINPYSTIKIAYMPYNLLDKQNFXQQIKELLNIVLRPSKSRHRSTAFVIRKHSKVKRGKARIVYNYKRXNDNTYEDSYKLPNKDEFMNKIEESKYSSKLYCKSRFWKIRLAEXSIQXTTFTCTESHFEWLVMPFGFKNAPSIFQRKMNQIFKKYDNFGSIYVDDKLVCSKNKMNIENTEKLFXKNLLIMVXLXVGIKXSLKNKAYNFKVCILSQKLYNYKNTXVKKCYNFQINEKIKKKLQAFLGLLNYARSFI

>Pper2-Chr2-02; Florendovirus; Prunus persica 2;

KLKTEIYSELPNAFWYRKQHVVCLPYIRTFSEKNIPTKARPIQMSQELMEFCKKEISELLEKKIIRKSKSPWSCPAFYVQKNAELERGVPRLVINYKPLNAILEWIRYPIPNKRDLINRLEKAVVYSKFDMKSGFWQIQIEESDRYKTAFVTPFGHYEWNVMPFDLKNAPSEFQNIMNDIFNPYSHFSIVYIDDVLIYSESVEQHWKHLHKFLQIVKQNGLVVSAKKIKLFQTSIRFLGFNICQSQISPIDRVIQFADKFPDQILDKSQLQRFLGSLNY

>Pper2-Chr2-03; Yendovirus; Prunus persica 2;

HWNKNKILAKLDIINPYSTIKIAYMPCNLLDKQNFXQQIKELLNIVLRPSKSRHRSTAFVIRKHSKVKRGKARIVYNYKRXNDNTYEDSYKLPNKDELMNKIEESKYSSKLYCKSRFWQIRLAEXSIQXTTFTRTKAHFEWLVMPFGLMNAPSIFQRKMNQIFEKYDNFGSIYVDDKLVCSKNKNEHRKHXENILKEFIDNGIVISRNKVELEKXGIXFXGMYIKSETIXLQEHIGKKVLXFPNKXEDKKQLQAFLGLLNYARSFI

>Pper2-Chr2-04; Florendovirus; Prunus persica 2;

EKLKQEVCSELPNAFWHRKQHVVSLLYVKSFSEKNIPTKARPIQMSQELMEFCKTEITDLLKKGIIRKSKSPWSCPTFYVQKNTELERGVSRLVINYKPLNTVLEWIRYPIPNKRDLINRLEKSVIFSKFDMKSGFWQIQISESDRYKTAFVTPFGHYEWNVMPFGLKNAPSKFQNIMNEIFNPYSHFSIVYIDDVLIFSESIEQHWKHLYKFLQIVKQNGLVVSAKKIKLFQTNVRFLGFNIYQSQVSPIDQVSPMNQLQRFLGSLNYV

>Pper2-Chr2-05; Florendovirus; Prunus persica 2;

QKLKEEICSELPTAFWYRKQHVVRLPYIKSFSEKNIPTKARPIQMSQEMMEFCKKEIHELLQKKIIRKSKSPWSCPAFYVQKNAELERGVPRLVINYKPLNAVLEWIRYPIPNKRDLINRLEKAVVYSKFDLKSGFWQIQIDESDRYKTAFVTPFGHYEWNVMPFGLKNAPSEFQNIMNDIFNHFSQFSIVYIDDVLIFSESIEQHWKHLNKFLQVVKQNGLVVSAKKIKLFQTNIRFLGFNICQSQISPIDRVIQFADKFPDQILDKSQLQRFLGSLNY

>Pper2-Chr2-06; Florendovirus; Prunus persica 2;

QRLKEEICSELPTAFWYRKQHVVRLPYIKTFSEKNIPTKARPIQMSQEMMEFCKKEIEELLQKKIIRKSKSPWSCPAFYVQKNAELERGVPRLVINYKPLNAILEWIRYPIPNKRDLINRLEKAVVYSKFDMKSGFWQIQIDESDRYKTAFVTPFGHYEWNVMPFGLKNAPSEFQNIMNDIFNPYSQFSIVYIDDVLIFSESIEQHWKHLNKFLQVVKRNGLVVSAKKIKLFQTNIRFLGFNICQSQISPIDRVIQFADKFPDQILDKSQLQRFLGSLNY

>Pper2-Chr2-07; Florendovirus; Prunus persica 2;

QRLKEEICSELPTAFWYRKQHVVRLPYIKTFSEKNIPTKARPIQMSQEMMEFCKKEIEELLQKKIIRKSKSPWSCPAFYVQKNAELERGVPRLVINYKPLNAVLEWIRYPIPNKRDLINRLEKAVVYSKFDMKSGFWQIQIDESDRYKTAFVTPFGHYEWNVMPFGLKNAPSEFQNIMNDIFNPYSQFSIVYIDDVLIFSESIEQHWKHLNKFLQVVKQNGLVVSAKKIKLFQTNIRFLGFNICQSQISPIDRVIQFADKFPDQILDKSQLQRFLGSLNY

>Pper2-Chr3-01; Florendovirus; Prunus persica 2;

EKLKQEVCSELPNAFWHRKQHVICLPYVKTFSEKNIPTKARPIQMSQELMEFCKIEITDLLKKGIIRKSKSPWSCPAFYVQKNAELERGVPRLVINYKPLNTVLEWIRYPIPNKRDLINRLEKSVIFSKFDMKSGFWQIQISEADRYKTAFVTHFGHYEWNVMPFGLKNAPSEFQNIMNETFNHFSHFSIVYIDDVLIFSESIEQHWKHLYKFLQIVKQNGLVISAKKIKLLQTNVRFLGFNICQSXISPIDRVIQFVDKFLDQILD

>Pper2-Chr3-02; Florendovirus; Prunus persica 2;

QRLKEEICSELPTAFWYRKQHVVRLPYIKTFSEKNIPTKARPIQMSQEMMEFCMKEIKELLQKKIIRKSKSPWSCPAFYVQKNAELERGVPRLVINYKPLNAVLEWIRYPIPNKRDLINRLEKAVVYSKFDLKSGFWQIQIDESDRYKTAFVTPFGHYEWNVMPFGLKNAPSEFQNIMNDIFNPYSQFSIVYIDDVLIFSESIEQHWKHLNKFLQVVKQNGLVVSAKKIKLFQINIRFLGFNICQSQISPIDRVIQFADKFPDQILDKSQLQRFLGSLNY

>Pper2-Chr3-03; Florendovirus; Prunus persica 2;

EKLKQEVCSDLPNAFWHRKQHVVSLPYVKNFSEKNILTKARPIQMSQEIMEFYKTEITELLQKGIIRKSKSPWSCPAFYVXKNAELERGAPRLVINYKPLNTTLEWIRYPIPNKRDLINRLDKSVIFSKFDMKIGFWXIQILESDRYKTAFVTPFGHYEWNVMPFGLKNAHSEFQNIMNEIFNPFSHFSIVYIDDALIFSQSIEQHWKHLHKFLKIVKQNGLVVLAKKIKLFQTNICFLGFNICQSQISLIDRVIQFADKFSYQILDKSQLQRFLGSLNY

>Pper2-Chr3-04; Florendovirus; Prunus persica 2;

EKLKQEICSDLPTAFWHRKQHVVSLPYVKTFNEKHIPTKSRPIQMSREVMEFCKKEIAELLYKGIIRKSKSPWSCPAFYVQKNAELERGAPRLVINFKPLNTVLEWIRYPIPNKRDLINRLSSAVIFSKFHMKSGFWHIQIQERDRYKTAFVTPFGHYEWNVMHFGLKNAPSEFQNIMNEIFNPFSEFSIVYIDDILIFSSSIEQHWKHLHKFLQIVKKNGLVLSAKKIKLFQTNIKFLGFNIHQS

>Pper2-Chr3-05; Yendovirus; Prunus persica 2;

LEELLQLAEQTRIVGEDPQLHWNKNKILAKLDIINPDLTIKTTDMPCNLLDKQEFEHQIKELLNLKVIRPSKSRHRSVAFVVRKHSELKRGKARIVYNYRRLNDNTYEDSYKLPNKDELVNKIQESKYFSKFDCKSGFWQTRLAEESIQWTTFTCTEGHFEWLVMPFGLKNAPSIFQRKMDQSFKKYDNFCSVYVDDILVHSKNKNEHSKHLEIILKEFIDNGVVISRNKIEFEKQDIEFLGMYIKSGTIQLQEHIAKKVLKFLDRLEDKKQLQAFLGLLNYVSSFIPD

>Pper2-Chr3-06; Florendovirus; Prunus persica 2;

EKLKQEVCSELPNAFWHRKQHVVNLPYVKSFSDKNFSTKARPIQISQELMEFCKTEITELLEKGIIRKSKSPXSCPAFYVQENAELERGDPRLVIDYKPLNIILEWIRYPIHNKRDLINRLEKSVIFSKFDMKSGFWQIQIHESDRYKTAFITSFGHYEWNVMPFGLXNASSEFQNIMNEILNHFSHFFIVYIDDVLIFSQSIEQHWKHLHXFLQIVKQNGLVVSAXKIKLFQTNVRFLGFNICQSQISPIDQVIQFADKFPDQILDKSQLXRFLGSLNYV

>Pper2-Chr4-01; Florendovirus; Prunus persica 2;

QRLKEEICSELPTAFWYRKQHVVRLPYIKTFSEKNIPTKARPIQMSQEMMEFCKKEIEELLHKKIIRKSKSPWSCPAFYVQKNAELERGVPRLVINYKPLNAVLEWIRYPIPNKRDLINRLEKAVVYSKFDLKSGFWQIQIDESDRYKTAFVTPFGHYEWNVMPFGLKNAPSEFQNIMNDIFNPYSQFSIVYIDDVLIFSESLEQHWKHLNKFLQVVKQNGLVVSAKKIKLFQTNIRFLGFNICQSQISPIDRVIQFADKFPDQILDKSQLQRFLGSLNY

>Pper2-Chr4-02; Florendovirus; Prunus persica 2;

QRLKEEICSELPTAFWYRKQHVVRLPYIKSFSEKNIPTKARPIQMSQEMMEFCKKEIEELLQKKIIRKSKSPWSCPAFYVQKNAELERGVPRLVINYKPLNAVLEWIRYPIPNKRDLINRLEKAVVYSKFDMKSGFWQIQIDESDRYKTAFVTPFGHYEWNVMPFGLKNAPSEFQNIMNDIFNPYSQFSIVYIDDVLIFSESIEQHWKHLNKFLQVVKRNGLVVSAKKIKLFQTNIRFLGFNICQSQISPIDRVIQFADKFPDQILDKSQLQRFLGSLNY

>Pper2-Chr4-03; Florendovirus; Prunus persica 2;

QRLKEEICSELPTAFWYRKQHVVRLPYIKSFSEKNIPTKARPIQMSQEMMEFCKKEIEELLQKKIIRKSKSPWSCPAFYVQKNAELERGVPRLVINYKPLNAVLEWIRYPIPNKRDLINRLEKAVVYSKFDMKSGFWQIQIDESDRYKTAFVTPFGHYEWNVMPFGLKNAPSEFQNIMNDIFNPYSQFSIVYIDDVLIFSESIEQHWKHLNKFLQVVKRNGLVVSAKKIKLFQTNIRFLGFNICQSQISPIDRVIQFADKFPDQILDKSQLQRFLGSLNY

>Pper2-Chr5-01; Florendovirus; Prunus persica 2;

QRLKEEICSELPTAFWYRKQHVVRLPYIKTFSEKNIPTKARPIQMSQEMMEFCKKEIEELLQKKIIRKSKSPWSCPAFYVQKNAELERGVPRLVINYKPLNAVLEWIRYPIPNKRDLINRLEKAVVYSKFDMKSGFWQIQIDKSDRYKTAFVTPFGHYEWNVMPFGLKNAPSEFQNIMNDIFNPYSQFSIVYIDDVLIFSESIEQHWKHLNKFLQVVKQNGLVVSAKKIKLFQTNIRFLGFNICQSQISPIDRVIQFADKFPDQILDKSQLQRFLGSLNY

>Pper2-Chr5-02; Yendovirus; Prunus persica 2;

LEELLQLAKQTRIIGEDPQLHWNKNKILAKLDIINLDLTIKTADMPCNLLDKQEFYQQIKQILNLKVIRPSKSRHRSTAFVVRKHLELKRGKVRIVYNYRRLNDNTYEDSYKLPNKDELMNKIQESKYFSKFDCKSGFXQIRLAEESIXLTTFTCPEGNFEWLVMPFGLKNAPSIFQRKMDQIFKKYDNFCSVYVEDILVHSKNKNKHRKYLEIILKEFIDNGIVISRNKVELEKQDIEFLGIYIKSGTIQLQEHIAKKVLEFPDKLENKKQLQEFLGLLNYARSFIPD

>Pper2-Chr5-03; Florendovirus; Prunus persica 2;

QKLKEEICSELPTAFWYRKQHVVCLPYIKTFSEKNIPTKARPIQMSQEMMEFCKKEIEELLQKKIIRKSKSPWLCPAFYVQKNAELERGVPRLVINYKPLNAVLEWIRYPIPNKRDLINRLEKAVVYSKFDMKSGFWQIQIDESDRYKTVFVTPFGHYEWNVMPFGLKNAPSEFQNIMNDIFNPYSQFSIVYIDDVLIFSESIEQHWKHLYKFLQIVKQNGLVVSAKKIKLFQTNIRFLGFNICQSQISPIDRVIQFADKFPDQILDKSQLQRFLGSLNY

>Pper2-Chr5-04; Florendovirus; Prunus persica 2;

QKIKEEICSELPTAFWYRKQHVVRLPYIKTFSEKNIPTKARPIQMSQEMMEFCKTEINELLQKKIIRKSKSPWSCPAFYVQKNAELERGVPRLVINYKPLNAVLEWIRYLIPNKRDLINRLEKAIVYSKFDMKSGFWQIQIDESDRYKTAFVTPFGHYEWNVMPFGLKNAPSEFQNIMNDIFNPYSQFSIVYIDDVLIFSESIEQHWKHLYKFLQVVKQNGLVVSAKKIKLFQTNVRFLGFNICQSQISPIDRVIQFADRFPDQILDKSQLQRFLGSLNY

>Pper2-Chr5-05; Florendovirus; Prunus persica 2;

QKIKSEICSELPNAFWYRKQHVVCLPYMKTFSEKNIPTKARPIQMSQEMMEFCKQEIKELLQKKIIRQSKSPWSCPAFYVQKNAELERGVPRLVINYKPLNAVLEWIRYPIPNKRDLINRLEKAVVYSKFDMKSGFWQIQIEESDRYKTAFVTPFGHYEWNVMPFGLKNAPSEFQNIMNDIFNPYSQFSIVYIDDVLIFSESIEQHWKHLYKFLQVVKQNGLVVSAKKIKLFQTNVRFLGFNICQSQISPIDRVIQFADKFPDQILDKSQLQRFLGSLNY

>Pper2-Chr6-01; Florendovirus; Prunus persica 2;

QKLKEEVCSELPTAFWYRKQHVVRLPYIKTFSEKNIPTKARPIQMSQEMMEFCKKEIEELLQKRIIRKSKSPXSCPAFYVQKNAELERGVPRLVINYKPLNAVLEWIRYPIPNKRDLINRLEKAVVYSKFDMKSGFWQIQIDESDRYKTAFVTPFGHYEWNVMPFGLKNAPSEFQNIMNDIFNPYSQFSIVYIDDVLIFSESIEQHWKHLYKFLQVVKQNGLVVSAKKIKLFQTNIRFLGFNICQSQISPIDRVIQFADKFPDQILDKSQLQRFLGSLNY

>Pper2-Chr6-02; Florendovirus; Prunus persica 2;

QRLKEEICSELPTAFWYRKQHVVRLPYIKTFSENNIPTKARPIQMSQEMMEFCKKEIEELLQKKIIRKSKSPWSCPAFYVQKNAELERGVPRLVINFKPLNAVLEWIRYPIPNKRDLINRLEKAVVYSKFDLKSGFWQIQIDESDRYKTAFVTPFGHYEWNVMPFGLKNAPSEFQNIMNDIFNPYSQFSIVYIDDVLIFSESIEQHWKHLNKFLQVVKQNGLVVSAKKIKLFQTNIRFLGFNICQSQISPIDRVIQFADKFPDQILDKSQLQRFLGSLNY

>Pper2-Chr6-03; Florendovirus; Prunus persica 2;

QRLKEEICSELPTAFWYRKQHVVRLPYIKTFSENNIPTKARPIQMSQEMMEFCKKEIEELLQKKIIRKSKSPWSCPAFYVQKNAELERGVPRLVINYKPLNAVLEWIRYPIPNKRDLINRLEKAVVYSKFDLKSGFWQIQIDESDRYKTAFVTPFGHYEWNVMPFGLKNAPSEFQNIMNDIFNPYSQFSIVYIDDVLIFSESIEQHWKHLNKFLQVVKQNGLVVSAKKIKLFQTNIRFLGFNICQSQISPIDRVIQFADKFPDQILDKSQLQRFLGSLNY

>Pper2-Chr6-04; Florendovirus; Prunus persica 2;

EKLKQEVCSELPNAFWHRKQHVVSLPCVKSFSEKNIPTKARPIQMSQELMEFCKIEITDLLKKGIIRKSKSPWSCPDFYVQKNAELECGVPRLVINYKPLNTVLEWIRYPIPNKRDLINRLENSVIFSKFDMKSGFWQIQISESDRYKTAFVTPFGHYEWNVMPFGLKNAPSEFQNIMNEIFNPFSHFSIVYIDDVLIFSESIEQHXKHLHKFLQIVKQNGLVVSAKKIKLFQTNVRFLRFNIYQSQISPIDRVIXIADKFPDQILDKSQLQRFLGSLN

>Pper2-Chr6-05; Florendovirus; Prunus persica 2;

QRLKEEICSELPTAFWYRKQHVVRLPYIKTFSEKNIPTKARPIQMSQEMMEFCKKEIEELLQKKIIRKSKSPWSCPAFYVQKNAELERGVPRLVINYKPLNAVLEWIRYPIPNKRDLINRLEKAVVYSKFDMKSGFWQIQIDESDRYKTAFVTPFGHYEWNVMPFGLKNAPSEFQNIMNDIFNPYSQFSIVYIDDVLIFSESIEQHWKHLNKFLQVVKQNGLVVSAKKIKLFQTDIRFLGFNICQSQISPIDRVIQFADKFPDQILDKSQLQRFLGSLNY

>Pper2-Chr7-01; Florendovirus; Prunus persica 2;

QRLKEEICSELPTAFWYRKQHVVRLPYIKTFSEKNIPTKARPIQMSQEMMEFCKKEIEELLQKKIIRKSKSPWSCPAFYVQKNAELERGVPRLVINYKPLNAVLEWIRYPIPNKRDLINRLEKAVVYSKFDMKSGFWQIQIDESDRYKTAFVTPFGHYEWNVMPFGLKNAPSEFQNIMNDIFNPYSQFLIVYIDDVLIFSESIEQHWKHLNKFLQVVKQNGLVVSAKKIKLFQTNIRFLGFNICQSQISPIDRVIQFADKFPDQILDKSQLQRFLGSLNY

>Pper2-Chr7-02; Florendovirus; Prunus persica 2;

EKLKNEVCSDLPNAFWHRKQHVVTLPYVNSFNEKNIPTKARLIQMSQELMDLCKQEVTELVQKGIVRKSKSPWSCPAFYVQKNAXLERGDPRLVINYKALNAVLEWIRYPIPNKRDLVNRLEQVVFFSKFDMKSGFWQIQIKESNRYKTAFVTPFGHYEWNVMPFGLKNAPSEFQNVMNEIFNQCSHFSIVYIDDVLIFSESIEQHWKHLNKFLQIVKQNGLVISATKIKLFQANVRFXGFNIGQSQ

>Pper2-Chr7-03; Florendovirus; Prunus persica 2;

QRLKEEICSELPTAFWYRKQHVVRLPYIKTFSEKNIPTKARPIQMSQEMMEFCKKEIEELLQKKIIRKSKSPWSCPAFYVQKNAELERGVPRLVINYKPLNAVLEWIRYPIPNKRDLINRLEKAVVYSKFDMKSGFWQIQIDESDRYKTAFVTPFGHYEWNVMPFGLKNAPSEFQNIMNDIFNPYSQFSIVYIDDVLIFSESIEQHWKHLNKFLQVVKQNGLVVSAKKIKLFQTNIRFLGFNICQSQISPIDRVIQFADKFPDQILDKSQLQRFLGSLNY

>Pper2-Chr7-04; Florendovirus; Prunus persica 2;

QRLKEEICSELPTAFWYRKQHVVRLPYIKTFSEKNIPTKARPIQMSQEMMEFCKKEIEELLQKKIIRKSKSPWSCPAFYVQKNAELERGVPRLVINYKPLNAVLEWIRYPIPNKRDLINRLEKAVVYSKFDMKSGFWQIQIDESDRYKTAFVTPFGHYEWNVMPFGLKNAPSEFQNIMNDIFNPYSQFSIVYIDDVLIFSESIEQHWKHLNKFLQVVKQNGLVVSAKKIKLFQTNIRFLGFNICQSQISPIDRVIQFADKFPDQILDKSQLQRFLGSLNY

>Pper2-Chr7-05; Florendovirus; Prunus persica 2;

QKLKEEICSELPTAFWYRKQHVVRLPYIKSFSEKNIPTKARPIQMSQEMMEFCKKEIEELLQKKIIRKSKSPWSCPAFYVQKNAELERGVPRLVINYKPLNAVLEWIRYPIPNKRDLINRLEKAVVYSKFDLKSGFWQIQIDESDRYKTAFVTPFGHYEWNVMPFGLKNAPSEFQNIMNDIFNPYSQFSIVYIDDVLIFSESIKQHWKHLHKFLQVFKQNGLVVSAKKIKLFQTNIRFLGFNICQSQISPIDRVIQFADKFLDQILDKSQLQRFLGSLNY

>Pper2-Chr7-06; Florendovirus; Prunus persica 2;

QKLKEEICSELPTAFWYRKQHVVCLPYIKTFSEKNIPTKARPIQMSQEMMEFCKKEIEELLQKKIIRKSKSPWSCPAFYVQKNAELERGVPRLVINYKPLNAVLEWIRYPIPNKRDLINRLEKAFVYSKFDLKSGFWQIQIDESDRYKIAFVTPFGHYEWNVMPFGLKNSPSEFQNIMNDIFNPYSQFSIVYIDDVLIFSESIEQHWKHLNKFLQVVKQNGLVVSAKKIKLFQTNIRFLGFNICQSQISPIDRVIQFADKFPDQILDKSQLQRFLRSLNY

>Pper2-Chr8-01; Florendovirus; Prunus persica 2;

QRLKEEICSELPTAFWYRKQHVVRLPYIKTFSEKNIPTKARPIQMSQEMMEFCKKEIEELLQKKIIRKSKSPWSCPAFYVQKNAELERGVPRLVINYKPLNAVLEWIRYPIPNKRDLINRLEKAVVYSKFDMKSGFWQIQIDESDRYKTAFVTPFGHYEWNVMPFGLKNAPSEFQNIMNDIFNPYSQFSIVYIDDVLIFSESIEQHWKHLNKFLQVVKRNGLVVSAKKIKLFQTNIRFLGFNICQSQISPIDRVIQFADKFP

>Pper2-Chr8-02; Florendovirus; Prunus persica 2;

QRLKEEICSELPTAFWYRKQHVVRLPYIKTFSEKNIPTKARPIQMSQEMMEFCKKEIEELLQKKIIRKSKSPWSCPAFYVQKNAELERGVPRLVINYKPLNAVLEWIRYPIPNKRDLINRLEKAVVYSKFDLKSGFWQIQIDESDRYKTAFVTPFGHYEWNVMPFGLKNAPSEFQNIMNDIFNPYSQFSIVYIDDVLIFSESIEQHWKHLHKFLQVVKQNGLVVSAKKIKLFQTNIRFLGFNICQSQI

>Pper2-Chr8-03; Florendovirus; Prunus persica 2;

QKIKNEICSELPNAFWYRKQHVVCLPYIKTFSEKNIPTKARPIQMSQEIMEFCKTEISELLQKKIIRKSKSSWSCPAFYVQKNAELERGVPRLVINYKSLNSVLEWIWYPIPNKRDLINRLGKAVVYSKFDMKSGFWQIQIEESDRYKTAFNTPFGHYEWNVMPFGLKNAPSEFQNIMNEIFNPYSHFSIVYIDDVLIFSESIEQYWKHLHKFFQIVKQNGLVVSAKKIKLFQTNIRFLGFNICQSQISPIDRVIQFADKFPDQILDKSQLQRFLGSLNY

>Pper2-Chr8-04; Florendovirus; Prunus persica 2;

QKIKNEICSELPNAFWYRKQHVVCLPYIKTFSEKNIPTKARPIQMSQEIMEFCKTEISELLQKKIIRKSKSPWSCPAFYVQKNAELERGVPRLVINYKSLNSVLEWIWYPIPNKRDLINRLGKAVVYSKFDMKSEFWQIQIEESDRYKTAFVTPFGHYEWNVMPFGLKNAPSEFQNIMNEIFNPYSHFSIVYIDDVLIFSESIEQHWKHLHKFFQIVKQNGLVVSAKKTKLFQTNIKFLGFNICQSQISPIDRVIQFANKFPDQILDKSQLMRVLGSLNY

>Pper3-Chr1-01; Florendovirus; Prunus persica 3;

EKLKQEVCSELPNAFWHRKQHVVSLPYVKSFSEKNIPTKARPIQMSQELMEFCKTEITELLEKGIIRKSKSPWSCPAFYVQKNAELEHRVPRLAINYKLLNTVLEWIRYPIPNKRDLINRLEKSVIFSKFDMKSGFWQIQICESDRYKTAFVTPFGYYEWNVMSFGLKNAPSEFQNRMNEIFIPFNHFSIVYIDDVLIFSESIEQHWKNLHKFLQIVKQNGLVVSAKKIKLFQTNVR FLGFNICQSQISPIDRVIQFVDKFPNQILDKSQLQRFLGSLNYV

>Pper3-Chr1-02; Florendovirus; Prunus persica 3;

EKLKQEVCSELPNAFWHRKQHVVSLPYVKTSSEKNIPTKARPIQISQELMKFCKIEITDLLKKGIIRKSRSPWSCPAFYVQKNAELERGVPRLVINYKPLNTVLEWIRYPIPNKRDIINRLEKSVIFSKFDMKSGFWQIQISETDRYKTTFVTPFGHYEWNVMPFDLKNAPSEFQNIINVIFNPFSHFSIVYIDDVLIFSASIEQHWKYLYKFLQIVKQNGLVVSAKKIKLFQTNVRFLGFNIXQSQISPIDRVIQFADKFPDQILEKGQLQRFLGSLNYVSDF

>Pper3-Chr1-03; Florendovirus; Prunus persica 3;

QRLKEEICSELPTAFWYRKQHVVRLPYIKTFSEKNIPTKARPIQMSQEMMEFCKKEIEELLQKKIIRKSKSPWSCPAFYVQKNAELERGVPRLVINYKPLNAVLEWIRYPIPNKRDLINRLEKAVVYSKFDMKSGFGKSKXMSRQRDIRLHLSLPWSLRMECNALWFEKCSXXIPNIMNDIFNPYNGFDCLYXXYFDLFSESIEQHWKHLNKFLQVVKQNGLVVYAKKIKLFQTNIRFLGFNICQSQISPIDRVIRLGDKFP

>Pper3-Chr1-04; Florendovirus; Prunus persica 3;

EKLKQEVCSELPNAFWHRKQHVVSLPYVKSFSEKNIPTKARPIQMSQELMKFCKTEITELLEKGIIRKSKSLWSCPAFYVQKNVELEHGVPRLVINYKPLNTVLEWIRYPIPNKRDLINRLEKSVIFSKFDMKSGFWQIQIRESFSYKTAFVTPFGHYEWNVMPFGLKNAPSEFENIMNEIFNHFSHFSIVYIDDVLIFSESIEQHWKHLHKFLQIVKRNGLVVSAKKIKLFQTNVRFLGFNICQSQISPIDRVIQFADKFPDQILDKSQLHRFLGSLNYVSDF

>Pper3-Chr2-01; Florendovirus; Prunus persica 3;

EKLKNEVCSDLPNASWYRKQHVVTLPYVKSFNEKNIPTKARPIQMSQELMELCKQEVTELVQKGIVRKGKSPWSCPAFYVQKNAELERXAPRLVINYKPLNAVLEWIRYPIPNKRELLNRLEKAVIFSKFDMKSGFWQIQIKDSDRHKTAFVTPFGHYEWNVMPFGLKNAPSEFLNIMNEIFNQYSQFFIVYIDDVLIFSVSIEQHWKHLNKFLQIVKQNGLVISAKKIKLFQVNVRFLGFNIGQSQISPINRVIQFADKFPDQILDKSQLQRFFGSLNYVSDF

>Pper3-Chr2-02; Florendovirus; Prunus persica 3;

QKLKEEICSELPTAFWYRKQHVVRLPYIKSFSEKNIPTKARPIQMSQEMMEFCKKEIHELLQKKIIRKSKSPWSCPAFYVQKNAELERGVPRLVINYKPLNAVLEWIRYPIPNKRDLINRLEKAVVYSKFDLKSGFWQIQIDESDRYKTAFVTPFGHYEWNVMPFGLKNAPSEFQNIMNDIFNHFSQFSIVYIDDVLIFSESIEQHWKHLNKFLQVVKQNGLVVSAKKIKLFQTNIRFLGFNICQSQISPIDRVIQFADKFPDQILDKSQLQRFLGSLNY

>Pper3-Chr3-01; Florendovirus; Prunus persica 3;

EKLKQEVCSELPNAFWHRKQHVICLPYVKTFSEKNIPTKARPIQMSQELMEFCKIEITDLLKKGIIRKSKSPWSCPAFYVQKNAELERGVPRLVINYKPLNTVLEWIRYPIPNKRDLINRLEKSVIFSKFDMKSGFWQIQISEADRYKTAFVTHFGHYEWNVMPFGLKNAPSEFQNIMNETFNHFSHFSIVYIDDVLIFSESIEQHWKHLYKFLQIVKQNGLVISAKKIKLLQTNVRFLGFNICQSXISPIDRVIQFVDKFLDQILD

>Pper3-Chr3-02; Florendovirus; Prunus persica 3;

EKLKQEVCSDLPNAFWHRKQHVVSLPYVKNFSEKNILTKARPIQMSQEIMEFYKTEITELLQKGIIRKSKSPWSCPAFYVXKNAELERGAPRLVINYKPLNTTLEWIRYPIPNKRDLINRLDKSVIFSKFDMKIGFWXIQILESDRYKTAFVTPFGHYEWNVMPFGLKNAHSEFQNIMNEIFNPFSHFSIVYIDDALIFSQSIEQHWKHLHKFLKIVKQNGLVVLAKKIKLFQTNICFLGFNICQSQISLIDRVIQFADKFSYQILDKSQLQRFLGSLNY

>Pper3-Chr3-03; Florendovirus; Prunus persica 3;

EKLKQEICSDLPTAFWHRKQHVVSLPYVKTFNEKHIPTKSRPIQMSREVMEFCKKEIAELLYKGIIRKSKSPWSCPAFYVQKNAELERGAPRLVINFKPLNTVLEWIRYPIPNKRDLINRLSSAVIFSKFHMKSGFWHIQIQERDRYKTAFVTPFGHYEWNVMHFGLKNAPSEFQNIMNEIFNPFSEFSIVYIDDILIFSSSIEQHWKHLHKFLQIVKKNGLVLSAKKIKLFQTNIKFLGFNIHQS

>Pper3-Chr3-04; Florendovirus; Prunus persica 3;

KLKNEICSELPNAFWYRKQHVVCLPYIKTFNEKNIPTKARPIQMSQEIMEFCKKEISELLQKKIIRKSKSPWSCPAFYVQKNAELEGGVPRLVINYKPLNAVLEWIRYLIPNKRDLINRLEKAVVFSKFDMKSGFWQIQIEESNRYKTAFVTPFGPYEWNVMPFGLKNAPSEFQNIMNDIFMPCSHFSIVYIDDVLIFSESIEHHWKHLHKFVQIIKQNGLVVSAKKIKLFQTNIRFLGFNICQSQISPIDRVIQFADKFPDQILDKSQLQRFLGSLNY

>Pper3-Chr4-01; Yendovirus; Prunus persica 3;

LEELLQLAEQTKIIGEDPQLHWSKNKILTKLDIINQDLTIKTADMPCNLLDKQEFEQXIKELLNLKVIRPSKSRHRSAAFVVRKHSELKRARIVYNYRRLNDNTYEDSYKLPNKDELMNKIQESKYFSKFDCKSGFWQIRLAEESIQWTTFTCLEGHFEWLVMPFGLKNAPLIFQRKMDQIFKKYDNLCSVYVDDILVHSKNKNEHRKHLEIILKEFTDNGIVISRNKVELQKQDIEFLGMYIKSGTIQLQEHVAKKVLEFPDKLEDKKQLQAFLDLLNYARNFFPD

>Pper3-Chr5-01; Yendovirus; Prunus persica 3;

LEELLQLAKQTRIIGEDPQLHWNKNKILAKLDIINLDLTIKTADMPCNLLDKQEFYQQIKQILNLKVIRPSKSRHRSTAFVVRKHLELKRGKVRIVYNYRRLNDNTYEDSYKLPNKDELMNKIQESKYFSKFDCKSGFXQIRLAEESIXLTTFTCPEGNFEWLVMPFGLKNAPSIFQRKMDQIFKKYDNFCSVYVEDILVHSKNKNKHRKYLEIILKEFIDNGIVISRNKVELEKQDIEFLGIYIKSGTIQLQEHIAKKVLEFPDKLENKKQLQEFLGLLNYARSFIPD

>Pper3-Chr5-02; Florendovirus; Prunus persica 3;

QKIKSEICSELPNAFWYRKQHVVCLPYMKTFSEKNIPTKARPIQMSQEMMEFCKQEIKELLQKKIIRQSKSPWSCPAFYVQKNAELERGVPRLVINYKPLNAVLEWIRYPIPNKRDLINRLEKAVVYSKFDMKSGFWQIQIEESDRYKTAFVTPFGHYEWNVMPFGLKNAPSEFQNIMNDIFNPYSQFSIVYIDDVLIFSESIEQHWKHLYKFLQVVKQNGLVVSAKKIKLFQTNVRFLGFNICQSQISPIDRVIQFADKFPDQILDKSQLQRFLGSLNY

>Pper3-Chr7-01; Florendovirus; Prunus persica 3;

QKLKEEVCSDLPTAFWYRKQHVVCLPYIKNFSEKNIPIKARPIQMSQEMMEFCKTEIHEQLQKKIIRKSRSPWSCPAFYVQKNAELERGVPRLVINYKPLNAVLEWIRYPIPNKRDLINRLEKAVVYSKFDMKSDFWQIQIDESDRYKTAFVTPFGHYEWNVMPFGLKNAPSEFQNIMNDIFNPYNQFTIVYIDDVLIFSESIEQHWKHLNKFLQVVIKNGLVVSAKKIKLFQTNIRFLGFNICQSQISPIDRVIQFADKFPDQILDKSQLQRFLGSLNY

>Pper3-Chr7-02; Florendovirus; Prunus persica 3;

QRLKEEICSELPTAFWYRKQHVVRLPYIKTFSEKNIPTKARPIQMSQEMMEFCKKEIEELLQKKIIRKSKSPWSCPAFYVQKNAELERGVPRLVINYKPLNAVLEWIRYPIPNKRDLINRLEKAVVYSKFDLKSGFWQIQIDESDRYKTAFVTPFGHYEWNVMPFGLKNAPSEFQNIMNDIFNPYSQFSIVYIDDVLIFSESIEQHWKHLNKFLQVVKQNGLVVSAKKIKLFQTNIRFLGFNICQSQISPIDRVIQFADKFPDQILDKSQLQRFLRSLNY

>Pper3-Chr7-03; Florendovirus; Prunus persica 3;

QKLKEEICSELPTAFWYRKQHVVCLPYIKTFSEKNIPTKARPIQMSQEMMEFCKKEIEELLQKKIIRKSKSPWSCPAFYVQKNAELERGVPRLVINYKPLNAVLEWIRYPIPNKRDLINRLEKAFVYSKFDLKSGFWQIQIDESDRYKIAFVTPFGHYEWNVMPFGLKNAPSEFQNIMNDIFNPYSQFSIVYIDDVLIFSESIEQHWKHLNKFLQVVKQNGLVVSAKKIKLFQTNIRFLGFNICQSQISPIDRVIQFADKFPDQILDKSQLQRFLRSLNY

>Pper4-Chr1-01; Florendovirus; Prunus persica 4;

EKLKQEVCSELPNAFWHRKQHVVSLPYVKTSSEKNIPTKARPIQISQELMKFCKIEITDLLKKGIIRKSRSPWSCPAFYVQKNAELERGVPRLVINYKPLNTVLEWIRYPIPNKRDIINRLEKSVIFSKFDMKSGFWQIQISETDRYKTAFVTPFGHYEWNVMPFDLKNAPSEFQNIINVIFNPFSHFSIVYIDDVLIFSASIEQHWKHLYKFLQIVKQNGLVVSAKKIKLFQTNVRFLGFNIXQSQISPIDRVIQFADKFPDQILEKGQLQRFLGSLNYVSDF

>Pper4-Chr1-02; Florendovirus; Prunus persica 4;

EKLKQEVCSELPNAFWHRKQHVVSLPYVKSFSEKNIPTKARPIQMSQELMKFCKTEITELLEKGIIRKSKSLWSCPAFYVQKNVELEHGVPRLVINYKPLNTVLEWIRYPIPNKRDLINRLEKSVIFSKFDMKSGFWQIQIRESFSYKTAFVTPFGHYEWNVMPFGLKNAPSEFENIMNEIFNHFSHFSIVYIDDVLIFSESIEQHWKHLHKFLQIVKRNGLVVSAKKIKLFQTNVRFLGFNICQSQISPIDRVIQFADKFPDQILDKSQLHRFLGSLNYVSDF

>Pper4-Chr1-03; Florendovirus; Prunus persica 4;

QRLKEEICSELPTAFWYRKQHVVRLPYIKTFSEKNIPTKARPIQMSQEMMEFCKKEIEELLQKKIIRKSKSPWSCPAFYVQKNAELERGVPRLVINYKPLNAVLEWIRYPIPNKRDLINRLEKAVVYSKFDLKSGFWQIQIDESDRYKTAFVTPFGHYEWNVMPFGLKNAPSEFQNIMNDIFNPYSQFSIVYIDDVLIFSESIEQHWKHLNKFLQVVKQNGLVVSAKKIKLFQTNIRFLGFNICQSQISPIDRVIQFADKFPDQILDKSQLQRFLGSLNY

>Pper4-Chr1-04; Florendovirus; Prunus persica 4;

QRLKEEICSELPTAFWYRKQHVVRLPYIKTFSEKNIPTKARPIQMSQEMMEFCMKEIEELLQKKIIRKSKSPWSCPAFYVQKNAELERGVPRLVINYKPLNAVLEWIRYPIPNKRDLINRLEKAVVYSKFDLKSGFWQIQIDESDRYKTAFVTPFGHYEWNVMPFGLKNAPSEFQNIMNDIFNPYSQFSIVYIDDVLIFSESIEQHWKHLNKFLQVVKQNGLVVSAKKIKLFQINIRFLGFNICQSQISPIDRVIQFADKFPDQILDKSQLQRFLGSLNY

>Pper4-Chr1-05; Florendovirus; Prunus persica 4;

QRLKEEICSELPTAFWYRKQHVVRLPYIKTFSEKNIPTKARPIQMSQEMMEFCKKEIEELLQKKIIRKSKSPWSCPAFYVQKNAELERGVPRLVINYKPLNAVLEWIRYPIPNKRDLINRLEKAVVYSKFDLKSGFWQIQIDESDRYKTAFVTPFGHYEWNVMPFGLKNAPSEFQNIMNDIFNPYSQFSIVYIDDVLIFSESIEQHWKHLNKFLQVVKQNGLVVSAKKIKLFQTNIRFLGFNICQSQISPIDRVIQFADKFPDQILDKSQLQRFLGSLNY

>Pper4-Chr1-06; Florendovirus; Prunus persica 4;

QRLKEEICSELPTAFWYRKQHVVRLPYIKTFSEKNIPTKARPIQMSQEMMEFCKKEIEELLQKKIIRKSKSPWSCPAFYVQKNAELERGVPRLVINYKPLNAVLEWIRYPIPNKRDLINRLEKAVVYSKFDLKSGFWQIQIDESDRYKTAFVTPFGHYEWNVMPFGLKNAPSEFQNIMNDIFNPYSQFSIVYIDDVLIFSESIEQHWKHLNKFLQVVKQNGLVVSAKKIKLFQTNIRFLGFNICQSQISPIDRVIQFADKFPDQILDKSQLQRFLGSLNY

>Pper4-Chr1-07; Florendovirus; Prunus persica 4;

QRLKEEICSELPTAFWYRKQHVVRLPYIKTFSEKNIPTKARPIQMSQEMMEFCKKEIEELLQKKIIRKSKSPWSCPAFYVQKNAELERGVPRLVINYKPLNAVLEWIRYPIPNKRDLINRLEKAVVYSKFDLKSGFWQIQIDESDRYKTAFVTPFGHYEWNVMPFGLKNAPSEFQNIMNDIFNPYSQFSIVYIDDVLIFSESIEQHWKHLIRFLQVVKQNGLVVSAKKIKLFQTNIRFLGFNICQSQISPIDRVIQFADKFPDQILDKSQLQRFLGSLNY

>Pper4-Chr1-08; Florendovirus; Prunus persica 4;

QRLKEEICSELPTAFWYRKQHVVRLPYIKTFSEKNIPTKARPIQMSQEMMEFCKKEIEELLQKKIIRKSKSPWSCPAFYVQKNAELERGVPRLVINYKPLNAVLEWIRYPIPNKRDLINRLEKAVVYSKFDLKSGFWQIQIDESDRYKTAFVTPFGHYEWNVMPFGLKNAPSEFQNIMNDIFNPYSQFSIVYIDDVLIFSESLEQHWKHLNKFLQVVKQNGLVVSAKKIKLFQTNIRFLGFNICQSQISPIDRVIQFADKFPDQILDKSQLQRFLGSLNY

>Pper4-Chr2-01; Florendovirus; Prunus persica 4;

QRLKEEICSELPTAFWYRKQHVVRLPYIKTFSEKNIPTKARPIQMSQEMMEFCKKEIEELLQKKIIRKSKSPWSCPAFYVQKNAELERGVPRLVINYKPLNAVLEWIRYPIPNKRDLINRLEKAVVYSKFDLKSGFWQIQIDESDRYKTAFVTPFGHYEWNVMPFGLKNAPSEFQNIMNDIFNPYSQFSIVYIDDVLIFSESIEQHWKHLNKFLQVVKQNGLVVSAKKIKLFQTNIRFLGFNICQSQISPIDRVIQFADKFPDQILDKSQLQRFLGSLNY

>Pper4-Chr2-02; Florendovirus; Prunus persica 4;

QRLKEEICSELPTAFWYRKQHVVRLPYIKTFSEKNIPTKARPIQMSQEMMEFCKKEIEELLQKKIIRKSKSPWSCPAFYVQKNAELERGVPRLVINYKPLNAVLEWIRYPIPNKRDLINRLEKAVVYSKFDLKSGFWQIQIDESDRYKTAFVTPFGHYEWNVMPFGLKNAPSEFQNIMNDIFNPYSQFSIVYIDDVLIFSESIEQHWKHLNKFLQVVKQNGLVVSAKKIKLFQTNIRFLGFNICQSQISPIDRVIQFADKFPDQILDKSQLQRFLGSLNY

>Pper4-Chr2-03; Yendovirus; Prunus persica 4;

HWNKNKILAKLDIINPYSTIKIAYMPCNLLDKQNFXQQIKELLNIVLRPSKSRHRSTAFVIRKHSKVKRGKARIVYNYKRXNDNTYEDSYKLPNKDELMNKIEESKYSSKLYCKSRFWQIRLAEXSIQXTTFTRTKAHFEWLVMPFGLMNAPSIFQRKMNQIFEKYDNFGSIYVDDKLVCSKNKNEHRKHXENILKEFIDNGIVISRNKVELEKXGIXFXGMYIKSETIXLQEHIGKKVLXFPNKXEDKKQLQAFLGLLNYARSFI

>Pper4-Chr2-04; Florendovirus; Prunus persica 4;

QKLKEEICSELPTAFWYRKQHVVRLPYIKSFSEKNIPTKARPIQMSQEMMEFCKKEIHELLQKKIIRKSKSPWSCPAFYVQKNAELERGVPRLVINYKPLNAVLEWIRYPIPNKRDLINRLEKAVVYSKFDLKSGFWQIQIDESDRYKTAFVTPFGHYEWNVMPFGLKNAPSEFQNIMNDIFNHFSQFSIVYIDDVLIFSESIEQHWKHLNKFLQVVKQNGLVVSAKKIKLFQTNIRFLGFNICQSQISPIDRVIQFADKFPDQILDKSQLQRFLGSLNY

>Pper4-Chr2-05; Florendovirus; Prunus persica 4;

QRLKEEICSELPTAFWYRKQHVVRLPYIKTFSEKNIPTKARPIQMSQEMMEFCMKEIEELLQKKIIRKSKSPWSCPAFYVQKNAELERGVPRLVINYKPLNAVLEWIRYPIPNKRDLINRLEKAVVYSKFDLKSGFWQIQIDESDRYKTAFVTPFGHYEWNVMPFGLKNAPSEFQNIMNDIFNPYSQFSIVYIDDVLIFSESIEQHWKHLNKFLQVVKQNGLVVSAKKIKLFQINIRFLGFNICQSQISPIDRVIQFADKFPDQILDKSQLQRFLGSLNY

>Pper4-Chr2-06; Florendovirus; Prunus persica 4;

QRLKEEICSELPTAFWYRKQHVVRLPYIKTFSEKNIPTKARPIQMSQEMMEFCMKEIEELLQKKIIRKSKSPWSCPAFYVQKNAELERGVPRLVINYKPLNAVLEWIRYPIPNKRDLINRLEKAVVYSKFDLKSGFWQIQIDESDRYKTAFVTPFGHYEWNVMPFGLKNAPSEFQNIMNDIFNPYSQFSIVYIDDVLIFSESIEQHWKHLNKFLQVVKQNGLVVSAKKIKLFQINIRFLGFNICQSQISPIDRVIQFADKFPDQILDKSQLQRFLGSLNY

>Pper4-Chr2-07; Florendovirus; Prunus persica 4;

QRLKEEICSELPTAFWYRKQHVVRLPYIKTFSEKNIPTKARPIQMSQEMMEFCMKEIEELLQKKIIRKSKSPWSCPAFYVQKNAELERGVPRLVINYKPLNAVLEWIRYPIPNKRDLINRLEKAVVYSKFDLKSGFWQIQIDESDRYKTAFVTPFGHYEWNVMPFGLKNAPSEFQNIMNDIFNPYSQFSIVYIDDVLIFSESIEQHWKHLNKFLQVVKQNGLVVSAKKIKLFQINIRFLGFNICQSQISPIDRVIQFADKFPDQILDKSQLQRFLGSLNY

>Pper4-Chr2-08; Florendovirus; Prunus persica 4;

QRLKEEICSELPTAFWYRKQHVVRLPYIKTFSEKNIPTKARPIQMSQEMMEFCMKEIEELLQKKIIRKSKSPWSCPAFYVQKNAELERGVPRLVINYKPLNAVLEWIRYPIPNKRDLINRLEKAVVYSKFDLKSGFWQIQIDESDRYKTAFVTPFGHYEWNVMPFGLKNAPSEFQNIMNDIFNPYSQFSIVYIDDVLIFSESIEQHWKHLNKFLQVVKQNGLVVSAKKIKLFQINIRFLGFNICQSQISPIDRVIQFADKFPDQILDKSQLQRFLGSLNY

>Pper4-Chr3-01; Florendovirus; Prunus persica 4;

EKLKQEVCSELPNAFWHRKQHVICLPYVKTFSEKNIPTKARPIQMSQELMEFCKIEITDLLKKGIIRKSKSPWSCPAFYVQKNAELERGVPRLVINYKPLNTVLEWIRYPIPNKRDLINRLEKSVIFSKFDMKSGFWQIQISEADRYKTAFVTHFGHYEWNVMPFGLKNAPSEFQNIMNEIFNHFSHFSIVYIDDVLIFSESIEQHWKHLYKFLQIVKQNGLVISAKKIKLLQTNVRFLGFNICQSXISPIDRVIQFVDKFLDQILD

>Pper4-Chr3-02; Florendovirus; Prunus persica 4;

EKLKQEVCSDLPNAFWHRKQHVVSLPYVKNFSEKNILTKARPIQMSQEIMEFYKTEITELLQKGIIRKSKSPWSCPAFYVXKNAELERGAPRLVINYKPLNTTLEWIRYPIPNKRDLINRLDKSVIFSKFDMKIGFWXIQILESDRYKTAFVTPFGHYEWNVMPFGLKNAHSEFQNIMNEIFNPFSHFSIVYIDDALIFSQSIEQHWKHLHKFLKIVKQNGLVVLAKKIKLFQTNICFLGFNICQSQISLIDRVIQFADKFSYQILDKSQLQRFLGSLNY

>Pper4-Chr3-03; Florendovirus; Prunus persica 4;

EKLKQEICSDLPTAFWHRKQHVVSLPYVKTFNEKHIPTKSRPIQMSREVMEFCKKEIAELLYKGIIRKSKSPWSCPAFYVQKNAELERGAPRLVINFKPLNTVLEWIRYPIPNKRDLINRLSSAVIFSKFHMKSGFWHIQIQERDRYKTAFVTPFGHYEWNVMHFGLKNAPSEFQNIMNEIFNPFSEFSIVYIDDILIFSSSIEQHWKHLHKFLQIVKKNGLVLSAKKIKLFQTNIKFLGFNIHQS

>Pper4-Chr3-04; Yendovirus; Prunus persica 4;

LEELLQLAEQTRIVGEDPQLHWNKNKILAKLDIINPDLTIKTTDMPCNLLDKQEFEHQIKELLNLKVIRPSKSRHRSVAFVVRKHSELKRGKARIVYNYRRLNDNTYEDSYKLPNKDELVNKIQESKYFSKFDCKSGFWQTRLAEESIQWTTFTCTEGHFEWLVMPFGLKNAPSIFQRKMDQSFKKYDNFCSVYVDDILVHSKNKNEHSKHLEIILKEFIDNGVVISRNKIEFEKQDIEFLGMYIKSGTIQLQEHIAKKVLKFLDRLEDKKQLQAFLGLLNYVSSFIPD

>Pper4-Chr4-01; Florendovirus; Prunus persica 4;

QRLKEEICSELPTAFWYRKQHVVRLPYIKTFSEKNIPTKARPIQMSQEMMEFCKKEIEELLHKKIIRKSKSPWSCPAFYVQKNAELERGVPRLVINYKPLNAVLEWIRYPIPNKRDLINRLEKAVVYSKFNLKSGFWQIQIDESDRYKTAFVTPFGHYEWNVMPFGLKNAPSEFQNIMNDIFNPYSQFSIVYIDDVLIFSESLEQHWKHLNKFLQVVKQNGLVVSAKKIKLFQTNIRFLGFNICQSQISPIDRVIQFADKFPDQILDKSQLQRFLGSLNY

>Pper4-Chr4-02; Florendovirus; Prunus persica 4;

QRLKEEICSELPTAFWYRKQHVVRLPYIKTFSEKNIPTKARPIQMSQEMMEFCKKEIEELLQKKIIRKSKSPWSCPAFYVQKNAELERGVPRLVINYKPLNAVLEWIRYPIPNKRDLINRLEKAVVYSKFDMKSGFWQIQIDESDRYKTAFVTPFGHYEWNVMPFGLKNAPSEFQNIMNDIFNPYSQFSIVYIDDVLIFSESIEQHWKHLNKFLQVVKQNGLVVSAKKIKLFQTNIRFLGFNICQSQISPIDRVIQFADKFPDQILDKSQLQRFLGSLNY

>Pper4-Chr5-01; Florendovirus; Prunus persica 4;

QRLKEEICSELPTAFWYRKQHVVRLPYIKTFSEKNIPTKARPIQMSQEMMEFCKKEIEELLQKKIIRKSKSPWSCPAFYVQKNAELERGVPRLVINYKPLNAVLEWIRYPIPNKRDLINRLEKAVVYSKFDMKSGFWQIQIDKSDRYKTAFVTPFGHYEWNVMPFGLKNAPSEFQNIMNDIFNPYSQFSIVYIDDVLIFSESIEQHWKHLNKFLQVVKQNGLVVSAKKIKLFQTNIRFLGFNICQSQISPIDRVIQFADKFPDQILDKSQLQRFLGSLNY

>Pper4-Chr5-02; Yendovirus; Prunus persica 4;

LEELLQLAKQTRIIGEDPQLHWNKNKILAKLDIINLDLTIKTADMPCNLLDKQEFYQQIKQILNLKVIRPSKSRHRSTAFVVRKHLELKRGKVRIVYNYRRLNDNTYEDSYKLPNKDELMNKIQESKYFSKFDCKSGFXQIRLAEESIXLTTFTCPEGNFEWLVMPFGLKNAPSIFQRKMDQIFKKYDNFCSVYVEDILVHSKNKNKHRKYLEIILKEFIDNGIVISRNKVELEKQDIEFLGIYIKSGTIQLQEHIAKKVLEFPDKLENKKQLQEFLGLLNYARSFIPD

>Pper4-Chr5-03; Florendovirus; Prunus persica 4;

QKIKEEICSELPTAFWYRKQHVVRLPYIKTFSEKNIPTKARPIQMSQEMMEFCKTEINELLQKKIIRKSKSPWSCPAFYVQKNAELERGVPRLVINYKPLNAVLEWIRYLIPNKRDLINRLEKAIVYSKFDMKSGFWQIQIDESDRYKTAFVTPFGHYEWNVMPFGLKNAPSEFQNIMNDIFNPYSQFSIVYIDDVLIFSESIEQHWKHLYKFLQVVKQNGLVVSAKKIKLFQTNVRFLGFNICQSQISPIDRVIQFADRFPDQILDKSQLQRFLGSLNY

>Pper4-Chr5-04; Florendovirus; Prunus persica 4;

QKIKSEICSELPNAFWYRKQHVVCLPYMKTFSEKNIPTKARPIQMSQEMMEFCKQEIKELLQKKIIRQSKSPWSCPAFYVQKNAELERGVPRLVINYKPLNAVLEWIRYPIPNKRDLINRLEKAVVYSKFDMKSGFWQIQIEESDRYKTAFVTPFGHYEWNVMPFGLKNAPSEFQNIMNDIFNPYSQFSIVYIDDVLIFSESIEQHWKHLYKFLQVVKQNGLVVSAKKIKLFQTNVRFLGFNICQSQISPIDRVIQFADKFPDQILDKSQLQRFLGSLNY

>Pper4-Chr6-01; Florendovirus; Prunus persica 4;

QRLKEEICSELPTAFWYRKQHVVRLPYIKTFSENNIPTKARPIQMSQEMMEFCKKEIEELLQKKIIRKSKSPWSCPAFYVQKNAELERGVPRLVINFKPLNAVLEWIRYPIPNKRDLINRLEKAVVYSKFDLKSGFWQIQIDESDRYKTAFVTPFGHYEWNVMPFGLKNAPSEFQNIMNDIFNPYSQFSIVYIDDVLIFSESIEQHWKHLNKFLQVVKQNGLVVSAKKIKLFQTNIRFLGFNICQSQISPIDRVIQFADKFPDQILDKSQLQRFLGSLNY

>Pper4-Chr6-02; Florendovirus; Prunus persica 4;

QRLKEEICSELPTAFWYRKQHVVRLPYIKTFSENNIPTKARPIQMSQEMMEFCKKEIEELLQKKIIRKSKSPWSCPAFYVQKNAELERGVPRLVINYKPLNAVLEWIRYPIPNKRDLINRLEKAVVYSKFDLKSGFWQIQIDESDRYKTAFVTPFGHYEWNVMPFGLKNAPSEFQNIMNDIFNPYSQFSIVYIDDVLIFSESIEQHWKHLNKFLQVVKQNGLVVSAKKIKLFQTNIRFLGFNICQSQISPIDRVIQFADKFPDQILDKSQLQRFLGSLNY

>Pper4-Chr6-03; Florendovirus; Prunus persica 4;

EKLKQEVCSELPNAFWHRKQHVVSLPYVKSFSEKNIPTKARPIQMSQELMEFCKIEITDLLKKGIIRKSKSPWSCPDFYVQKNAELECGVPRLVINYKPLNTVLEWIRYPIPNKRDLINRLENSVIFSKFDMKSGFWQIQISESDRYKTAFVTPFGHYEWNVMPFGLKNAPSEFQNIMNEIFNPFSHFSIVYIDDVLIFSESIEQHXKHLHKFLQIVKQNGLVVSAKKIKLFQTNVRFLRFNIYQSQISPIDRVIXIADKFPDQILDKSQLQRFLGSLN

>Pper4-Chr7-01; Florendovirus; Prunus persica 4;

QRLKEEICSELPTAFWYRKQHVVRLPYIKTFSEKNIPTKARPIQMSQEMMEFCKKEIEELLQKKIIRKSKSPWSCPAFYVQKNAELERGVPRLVINYKPLNAVLEWIRYPIPNKRDLINRLEKAVVYSKFDMKSGFWQIQIDESDRYKTAFVTPFGHYEWNVMPFGLKNAPSEFQNIMNDIFNPYSQFSIVYIDDVLIFSESIEQHWKHLNKFLQVVKQNGLVVSAKKIKLFQTNIRFLGFNICQSQISPIDRVIQFADKFPDQILDKSQLQRFLGSLNY

>Pper4-Chr7-02; Florendovirus; Prunus persica 4;

QRLKEEICSELPTAFWYRKQHVVRLPYIKTFSEKNIPTKARPIQMSQEMMEFCKKEIEELLQKKIIRKSKSPWSCPAFYVQKNAELERGVPRLVINYKPLNAVLEWIRYPIPNKRDLINRLEKAVVYSKFDMKSGFWQIQIDESDRYKTAFVTPFGHYEWNVMPFGLKNAPSEFQNIMNDIFNPYSQFLIVYIDDVLIFSESIEQHWKHLNKFLQVVKQNGLVVSAKKIKLFQTNIRFLGFNICQSQISPIDRVIQFADKFPDQILDKSQLQRFLGSLNY

>Pper4-Chr7-03; Florendovirus; Prunus persica 4;

QKLKEEICSELPTAFWYRKQHVVCLPYIKTFSEKNIPTKARPIQMSQEMMEFCKKEIEELLQKKIIRKSKSPWSCPAFYVQKNAELERGVPRLVINYKPLNAVLEWIRYPIPNKRDLINRLEKAFVYSKFDLKSGFWQIQIDESDRYKIAFVTPFGHYEWNVMPFGLKNSPSEFQNIMNDIFNPYSQFSIVYIDDVLIFSESIEQHWKHLNKFLQVVKQNGLVVSAKKIKLFQTNIRFLGFNICQSQISPIDRVIQFADKFPDQILDKSQLQRFLRSLNY

>Pper4-Chr8-01; Florendovirus; Prunus persica 4;

QRLKEEICSELPTAFWYRKQHVVRLPYIKTFSEKNIPTKARPIQMSQEMMEFCKKEIEELLQKKIIRKSKSPWSCPAFYVQKNAELERGVPRLVINYKPLNAVLEWIRYPIPNKRDLINRLEKAVVYSKFDMKSGFWQIQIDESDRYKTAFVTPFGHYEWNVMPFGLKNAPSEFQNIMNDIFNPYSQFSIVYIDDVLIFSESIEQHWKHLYKFLQVVKQNGLVVSAKKIKLFQTNIRFLGFNICQSQISPIDRVIQFADKFPDQILDKSQLQRFLGSLNY

>Pper4-Chr8-02; Florendovirus; Prunus persica 4;

QRLKEEICSELPTAFWYRKQHVVRLPYIKTFSEKNIPTKARPIQMSQEMMEFCKKEIEELLQKKIIRKSKSPWSCPAFYVQKNAELERGVPRLVINYKPLNAVLEWIRYPIPNKRDLINRLEKAVVYSKFDMKSGFWQIQIDESDRYKTAFVTPFGHYEWNVMPFGLKNAPSEFQNIMNDIFNPYSQFSIVYIDDVLIFSESIEQHWKHLNKFLQVVKRNGLVVSAKKIKLFRTNIRFLGFNICQSQISPIDRVIQFADKFPDQILDKSQLQRFLGSLNY

>Pper4-Chr8-03; Florendovirus; Prunus persica 4;

QRLKEEICSELPTAFWYRKQHVVRLPYIKTFSEKNIPTKARPIQMSQEMMEFCKKEIEELLQKKIIRKSKSPWSCPAFYVQKNAELERGVPRLVINYKPLNAVLEWIRYPIPNKRDLINRLEKAVVYSKFDMKSGFWQIQIDESDRYKTAFVTPFGHYEWNVMPFGLKNAPSEFQNIMNDIFNPYSQFSIVYIDDVLIFSESIEQHWKHLNKFLQVVKRNGLVVSAKKIKLFQTNIRFLGFNICQSQISPIDRVIQFADKFPDQILDKSQLQRFLGSLNY

>Pper4-Chr8-04; Florendovirus; Prunus persica 4;

QRLKEEICSELPTAFWYRKQHVVRLPYIKTFSEKNIPTKARPIQMSQEMMEFCKKEIEELLQKKIIRKSKSPWSCPAFYVQKNAELERGVPRLVINYKPLNAVLEWIRYPIPNKRDLINRLEKAVVYSKFDMKSGFWQIQIDESDRYKTAFVTPFGHYEWNVMPFGLKNAPSEFQNIMNDIFNPYSQFSIVYIDDVLIFSESIEQHWKHLNKFLQVVKRNGLVVSAKKIKLFQTNIRFLGFNICQSQISPIDRVIQFADKFPDQILDKSQLQRFLGSLNY

>Pper4-Chr8-05; Florendovirus; Prunus persica 4;

QRLKEEICSELPTAFWYRKQHVVRLPYIKTFSEKNIPTKARPIQMSQEMMEFCKKEIEELLQKKIIRKSKSPWSCPAFYVQKNAELERGVPRLVINYKPLNAVLEWIRYPIPNKRDLINRLEKAVVYSKFDMKSGFWQIQIDESDRYKTAFVTPFGHYEWNVMPFGLKNAPSEFQNIMNDIFNPYSQFSIVYIDDVLIFSESIEQHWKHLNKFLQVVKRNGLVVSAKKIKLFQTNIRFLGFNICQSQISPIDRVIQFADKFPDQILDKSQLQRFLGSLNY

>Pper4-Chr8-06; Florendovirus; Prunus persica 4;

QRLKEEICSELPTAFWYRKQHVVRLPYIKTFSEKNIPTKARPIQMSQEMMEFCKKEIEELLQKKIIRKSKSPWSCPAFYVQKNAELERGVPRLVINYKPLNAVLEWIRYPIPNKRDLINRLEKAVVYSKFDMKSGFWQIQIDESDRYKTAFVTPFGHYEWNVMPFGLKNAPSEFQNIMNDIFNPYSQFSIVYIDDVLIFSESIEQHWKHLNKFLQVVKRNGLVVSAKKIKLFQTNIRFLGFNICQSQISPIDRVIQFADKFPDQILDKSQLQRFLGSLNY

>Pper4-Chr8-07; Florendovirus; Prunus persica 4;

QRLKEEICSELPTAFWYRKQHVVRLPYIKTFSEKNIPTKARPIQMSQEMMEFCKKEIEELLQKKIIRKSKSPWSCPAFYVQKNAELERGVPRLVINYKPLNAVLEWIRYPIPNKRDLINRLEKAVVYSKFDMKSGFWQIQIDESDRYKTAFVTPFGHYEWNVMPFGLKNAPSEFQNIMNDIFNPYSQFSIVYIDDVLIFSESIEQHWKHLNKFLQVVKRNGLVVSAKKIKLFQTNIRFLGFNICQSQISPIDRVIQFADKFPDQILDKSQLQRFLGSLNY

>Pper4-Chr8-08; Florendovirus; Prunus persica 4;

QRLKEEICSELPTAFWYRKQHVVRLPYIKTFSEKNIPTKARPIQMSQEMMEFCKKEIEELLQKKIIRKSKSPWSCPAFYVQKNAELERGVPRLVINYKPLNAVLEWIRYPIPNKRDLINRLEKAVVYSKFDMKSGFWQIQIDESDRYKTAFVTPFGHYEWNVMPFGLKNAPSEFQNIMNDIFNPYSQFSIVYIDDVLIFSESIEQHWKHLNKFLQVVKRNGLVVSAKKIKLFQTNIRFLGFNICQSQISPIDRVIQFADKFPDQILDKSQLQRFLGSLNY

>Pper4-Chr8-09; Florendovirus; Prunus persica 4;

QRLKEEICSELPTAFWYRKQHVVRLPYIKTFSEKNIPTKARPIQMSQEMMKFCKKEIEELLQKKIIRKSKSPWSCPAFYVQKNAELERGVPRLVINYKPLNAVLEWIRYPIPNKRDLINRLEKAVVYSKFDMKSGFWQIQIDESDRYKTAFVTPFGHYEWNVMPFGLKNAPSEFQNIMNDIFNPYSQFSIVYIDDVLIFSESIEQHWKHLNKFLQVVKRNGLVVSAKKIKLFQTNIRFLGFNICQSQISPIDRVIQFADKFPDQILDKSQLQRFLGSLNY

>Pper4-Chr8-10; Florendovirus; Prunus persica 4;

QRLKEEICSELPTAFWYRKQHVVRLPYIKTFSEKNIPTKARPIQMSQEMMEFCKKEIEELLQKKIIRKSKSPWSCPAFYVQKNAELERGVPRLVINYKPLNAVLEWIRYPIPNKRDLINRLEKAVVYSKFDLKSGFWQIQIDESDRYKTAFVTPFGHYEWNVMPFGLKNAPSEFQNIMNDIFNPYSQFSIVYIDDVLIFSESIEQHWKHLHKFLQVVKQNGLVVSAKKIKLFQTNIRFLGFNICQSQISPIDRVIQFADKFPDQILDKSQLQRFLGSLNY

>Pper4-Chr8-11; Florendovirus; Prunus persica 4;

QRLKEEICSELPTAFWYRKQHVVRLPYIKTFSEKNIPTKARPIQMSQEMMEFCKKEIEELLQKKIIRKSKSPWSCPAFYVQKNAELERGVPRLVINYKPLNAVLEWIRYPIPNKRDLINRLEKAVVYSKFDMKSGFWQIQIDESDRYKTAFVTPFGHYEWNVMPFGLKNAPSEFQNIMNDIFNPYSQFSIVYIDDVLIFSESIEQHWKHLHKFLQVVKQNGLVVSAKKIKLFQTNIRFLGFNICQSQISPIDRVIQFADKFPDQILDKSQLQRFLGSLNY

>Pper4-Chr8-12; Florendovirus; Prunus persica 4;

QRLKEEICSELPTAFWYRKQHVVRLPYIKTFSEKNIPTKARPIQMSQEMMEFCKKEIEELLQKKIIRKSKSPWSCPAFYVQKNAELERGVPRLVINYKPLNAVLEWIRYPIPNKRDLINRLEKAVVYSKFDLKSGFWQIQIDESDRYKTAFVTPFGHYEWNVMPFGLKNAPSEFQNIMNDTFNPYSQFSIVYIDDVLIFSESIEQHWKHLHKFLQVVKQNGLVVSAKKIKLFQTNIRFLGFNICQSQISPIDRVIQFADKFPDQILDKSQLQRFLGSLNY

>Pper4-Chr8-13; Florendovirus; Prunus persica 4;

QRLKEEICSELPTAFWYRKQHVVRLPYIKTFSEKNIPTKARPIQMSQEMMEFCKKEIEELLQKKIIRKSKSPWSCPAFYVQKNAELERGVPRLVINYKPLNAVLEWIRYPIPNKRDLINRLEKAVVYSKFDLKSGFWQIQIDESDRYKTAFVTPFGHYEWNVMPFGLKNAPSEFQNIMNDIFNPYSQFSIVYIDDVLIFSESIEQHWKHLHKFLQVVKQNGLVVSAKKIKLFQTNIRFLGFNICQSQISPIDRVIQFADKFPDQILDKSQLQRFLGSLNY

>Pper4-Chr8-14; Florendovirus; Prunus persica 4;

QKIKNEICSELPNAFWYRKQHVVCLPYIKTFSEKNIPTKARPIQMSQEIMEFCKTEISELLQKKIIRKSKSSWSCPAFYVQKNAELERGVPRLVINYKSLNSVLEWIWYPIPNKRDLINRLGKAVVYSKFDMKSGFWQIQIEESDRYKTAFNTPFGHYEWNVMPFGLKNAPSEFQNIMNEIFNPYSHFSIVYIDDVLIFSESIEQYWKHLHKFFQIVKQNGLVVSAKKIKLFQTNIRFLGFNICQSQISPIDRVIQFADKFPDQILDKSQLQRFLGSLNY

>Pper4-Chr8-15; Florendovirus; Prunus persica 4;

QKIKNEICSELPNAFWYRKQHVVCLPYIKTFSEKNIPTKARPIQMSQEIMEFCKTEISELLQKKIIRKSKSPWSCPAFYVQKNAELERGVPRLVINYKSLNSVLEWIWYPIPNKRDLINRLGKAVVYSKFDMKSEFWQIQIEESDRYKTAFVTPFGHYEWNVMPFGLKNAPSEFQNIMNEIFNPYSHFSIVYIDDVLIFSESIEQHWKHLHKFFQIVKQNGLVVSAKKTKLFQTNIKFLGFNICQSQISPIDRVIQFANKFPDQILDKSQLMRVLGSLNY

>Pper5-Chr1-01; Florendovirus; Prunus persica 5;

EKLKQEVCSELPNAFWHRKQHVVSLPYVKTSSEKNIPTKARPIQISQELMKFCKIEITDLLKKGIIRKSRSPWSCPAFYVQKNAELERGVPRLVINYKPLNTVLEWIRYPIPNKRDIINRLEKSVIFSKFDMKSGFWQIQISETDRYKTAFVTPFGHYEWNVMPFDLKNAPSEFQNIINVIFNPFSHFSIVYIDDVLIFSASIEQHWKHLYKFLQIVKQNGLVVSAKKIKLFQTNVRFLGFNIXQSQISPIDRVIQFADKFPDQILEKGQLQRFLGSLNYVSDF

>Pper5-Chr1-02; Florendovirus; Prunus persica 5;

EKLKQEVCSELPNAFWHRKQHVVSLPYVKSFSEKNIPTKARPIQMSQELMKFCKTEITELLEKGIIRKSKSLWSCPAFYVQKNVELEHGVPRLVINYKPLNTVLEWIRYPIPNKRDLINRLEKSVIFSKFDMKSGFWQIQIRESFSYKTAFVTPFGHYEWNVMPFGLKNAPSEFENIMNEIFNPFSHFSIVYIDDVLIFSESIEQHWKHLHKFLQIVKRNGLVVSAKKIKLFQTNVRFFGFNICQSQISPIDRVIQFADKFPDQILDKSQLHRFLGSLNYVSDF

>Pper5-Chr1-03; Florendovirus; Prunus persica 5;

QKLKEEVCSELPTAFWYRKQHVVRLPYIKTFSEKNIPTKARPIQMSQEMMEFCKKEIEELLQKRIIRKSKSPWSCPAFYVQKNVELERGVPRLVINYKPLNAVLEWIRYPIPNKRDLINRLEKAVVYSKFDMKSGFWQIQIDESDRYKTAFVTPFGHYEWNVMPFGLKNAPSEFQNIMNDIFNPYSQFSIVYIDDVLIFSESIEQHWKHLYKFLQVVKQNGLVVSAKKIKLFQTNIRFFGFNICQSQISPIDRVIQFADKFPDQILDKSQLQRFLGSLNY

>Pper5-Chr1-04; Florendovirus; Prunus persica 5;

QKLKEEVCSELPTAFWYRKQHVVRLPYIKTFSEKNIPTKARPIQMSQEMMEFCKKEIEELLQKRIIRKSKSPWSCPAFYVQKNVELERGVPRLVINYKPLNAVLEWIRYPIPNKRDLINRLEKAVVYSKFDMKSGFWQIQIDESDRYKTAFVTPFGHYEWNVMPFGLKNAPSEFQNIMNDIFNPYSQFSIVYIDDVLIFSESIEQHWKHLYKFLQVVKQNGLVVSAKKIKLFQTNIRFFGFNICQSQISPIDRVIQFADKFPDQILDKSQLQRFLGSLNY

>Pper5-Chr1-05; Florendovirus; Prunus persica 5;

QKIKNEICSELPNAFWYRKQHVVCLPYIKTFSEKNIPTKARPIQMSQDMMEFYKKEIDELLQKKIIRKSKSLWSCPAFYVQKNAELERGVPRLVINYKPLNAVLEWIRYPIPNKRDLINRLEKAVVYSKFDMKSGFWQIQIEESDRYKTAFVTPFSHYEWNVMPFGLKNAPSEFQNIMNDIFNPYSQFSIVYVDDVLIFSESIEQHWKHLHKFFHVIKQNGLVVSAKKIKLFQTNVRFLGFNICQSQISPIDRVIQFADKFPDQILDKSQLQRFLGSFNY

>Pper5-Chr1-06; Florendovirus; Prunus persica 5;

QRLKEEICSELPTAFWYRKQHVVRLPYIKTFSEKNIPTKARPIQMSQEMMEFCKKEIEELLQKKIIRKSKSPWSCPAFYVQKNAELERGVPRLVINYKPLNAVLEWIRYPIPNKRDLINRLEKAVVYSKFDLKSGFWQIQIDESDRYKTAFVTPFGHYEWNVMPFGLKNAPSEFQNIMNDIFNPYSQFSIVYIDDVLIFSESIEQHWKHLIRFLQVVKQNGLVVSAKKIKLFQTNIRFLGFNICQSQISPIDRVIQFADKFPDQILDKSQLQRFLGSLNY

>Pper5-Chr2-01; Florendovirus; Prunus persica 5;

QKLKEEICSELPTAFWYRKQHVVRLPYIKSFSEKNIPTKARPIQMSQEMMEFCKLEIHELLQKKIIRKSKSPWSCPAFYVQKNAELERGVPRLVINYKPLNAVLEWIRYPIPNKRDLINRLEKAVVYSKFDMKSGFWQIQIDESDRYKTAFVTPFGHYEWNVMPFGLKNAPSEFQNIMNDIFNPYSQFSIVYINDVLIFSESIEQHWKHLHKFLHVVKQNGLVVSAKKIKLFQTNIRFLGFNICQSQISPIDRVIQFADKFPDQILEKSQLQRFLGSLNY

>Pper5-Chr2-02; Florendovirus; Prunus persica 5;

QKLKEEICSELPTAFWYRKQHVVRLPYIKSFSEKNIPTKARPIQMSQEMMEFCKLEIHELLQKKIIRKSKSPWSCPAFYVQKNAELERGVPRLVINYKPLNAVLEWIRYPIPNKRDLINRLEKAVVYSKFDMKSGFWQIQIDESDRYKTAFVTPFGHYEWNVMPFGLKNAPSEFQNIMNDIFNPYSQFSIVYIDDVLIFSESIEQHWKHLHKFLHVVKQNGLVVSAKKIKLFQTNIRFLGFNICQSQISPIDRVIQFADKFPDQILEKSQLQRFLGSLNY

>Pper5-Chr2-03; Florendovirus; Prunus persica 5;

QKLKEEICSELPTAFWYRKQHVVRLPYIKSFSEKNIPTKARPIQMSQEMMEFCKLEIHELLQKKIIRKSKSPWSCPAFYVQKNAELERGVPRLVINYKPLNAVLEWIRYPIPNKRDLINRLEKAVVYSKFDMKSGFXQIQIDESDRYKTAFVTPFGHYEWNVMPFGLKNAPSEFQNIMNDIFNPYSQFSIVYIDDVLIFSESIEQHWKHLHKFLHVVKQNGLVVSAKKIKLFQTNIRFLGFNICQSQISPIDRVIQFADKFPDQILEKSQLQRFLGSLNY

>Pper5-Chr2-04; Florendovirus; Prunus persica 5;

QKLKEEICSELPTAFWYRKQHVVRLPYIKSFSEKNIPTKARPIQMSQEMMEFCKLEIHELLQKKIIRKSKSPWSCPAFYVQKNAELERGVPRLVINYKPLNAVLEWIRYPIPNKRDLINRLEKAVVYSKFDMKSGFWQIQIDESDRYKTAFVTPFGHYEWNVMPFGLKNAPSEFQNIMNDIFNPYSQFSIVYIDDVLIFSESIEQHWKHLHKFLHVVKQNGLVVSAKKIKLFQTNIRFLGFNICQSQISPIDRVIQFADKFPDQILEKSQLQRFLGSLNY

>Pper5-Chr2-05; Florendovirus; Prunus persica 5;

QKLKEEICSELPTAFWYRKQHVVRLPYIKSFSEKNIPTKARPIQMSQEMMEFCKLEIHELLQKKIIRKSKSPWSCPAFYVQKNAELERGVPRLVINYKPLNAVLEWIRYPIPNKRDLINRLEKAVVYSKFDMKSGFWQIQLDESERYKTAFVTPFGHYEWNVMPFGLKNAPSEFQNIMIDIFNPYSQFSIVYIDDVLIFSESIEQHWKHLHKFLHVVKQNGLVVSAKKIKLFQTNIRFLGFNICQSQISPIDRVIQFADKFPDQILEKSQLQRFLGSLNY

>Pper5-Chr2-06; Florendovirus; Prunus persica 5;

QKLKEEICSELPTAFWYRKQHVVRLPYIKSFSEKNIPTKARPIQMSQEMMEFCKLEIHELLQKKIIRKSKSPWSCPAFYVQKNAELERGVPRLVINYKPLNAVLEWIRYPIPNKRDLINRLEKAVVYSKFDMKSGFWQIQLDESERYKTAFVTPFGHYEWNVMPFGLKNAPSEFQNIMNDIFNPYSQFSIVYIDDVLIFSESIEQHWKHLHKFLHVVKQNGLVVSAKKIKLFQTNIRFLGFNICQSQISPIDRVIQFADKFPDQILEKSQLQRFLGSLNY

>Pper5-Chr2-07; Florendovirus; Prunus persica 5;

QKLKEEICSELPTAFWYRKQHVVRLPYIKSFSEKNIPTKARPIQMSQEMMEFCKLEIHELLQKKIIRKSKSPWSCPAFYVQKNAELERGVPRLVINYKPLNAVLEWIRYPIPNKRDLINRLEKAVVYSKFDMKSGFWQIQIDESDRYKTAFVTPFGHYEWNVMPFGLKNAPSEFQNIMNDIFNPYSQFSIVYIDDVLIFSESIEQHWKHLHKFLHVVKQNGLVVSAKKIKLFQTNIRFLGFNICQSQISPIDRVIQFADKFPDQILEKSQLQRFLGSLNY

>Pper5-Chr2-08; Florendovirus; Prunus persica 5;

EKLKNEVCSDLPNAFWHRKQHVVTLPYVNSFNEKNIPTKARLIQMSQELMDLCKQEVTELVQKGIVRKSKSPWSCPAFYVQKNAXLERGDPRLVINYKALNAVLEWIRYPIPNKRDLVNRLEQVVFFSKFDMKSGFWQIQIKESNRYKTAFVTPFGHYEWNVMPFGLKNAPSEFQNVMNEIFNQCSHFSIVYIDDVLIFSESIEQHWKHLNKFLQIVKQNGLVISATKIKLFQANVRFXGFNIGQSQ

>Pper5-Chr2-09; Florendovirus; Prunus persica 5;

EKLKNEVCSDLPNASWYRKQHVVTLPYVKSFNEKNIPTKARPIQMSQELMELCKQELTELVQKGIVRKSKSPWSCPAFYVQKNVELERGAPRLVINYQPLNAVLEWIRYPIHNKRDLVNRLEKAVIFSKFDMKSDFWQIQIKDSDRYKTAFVTPFCHYEWNVMPFGLKNAPSEFQNIMNEIFNQYRQFSIVYIDDVLIFSESIEQHWKHLNKFLQIVKQNGLVISAKKIKLFQVNVRFLGFNIGQSQISPINPVIQFADKFPDQILDKSQLQRFLGSLNYVSDF

>Pper5-Chr2-10; Yendovirus; Prunus persica 5;

HWNKNKILAKLDIINPYSTIKIAYMPCNLLDKQNFXQQIKELLNIVLRPSKSRHRSTAFVIRKHSKVKRGKARIVYNYKRXNDNTYEDSYKLPNKDEFMNKIEESKYSSKLYCKSRFWQIRLAEXSIQXTTFTRTESHFEWLVMPFGFKNAPSIFQRKMNQIFKKYDNFGSIYVDDKLVCSKNKNEHRKHXENILKEFIDNGIVISRNKVELEKXGIXFXGMYIKSETIXLQEHIGKKVLXFPNKXEDKKQLQAFLGLLNYARSFI

>Pper5-Chr2-11; Florendovirus; Prunus persica 5;

EKLKQEVCSELPNAFWHRKQHVVSLLYVKSFSEKNIPTKARPIQMSQELMEFCKTEITDLLKKGIIRKSKSPWSCPTFYVQKNTELERGVSRLVINYKPLNTVLEWIRYPIPNKRDLINRLEKSVIFSKFDMKSGFWQIQISESDRYKTAFVTPFGHYEWNVMPFGLKNAPSKFQNIMNEIFNPYSHFSIVYIDDVLIFSESIEQHWKHLYKFLQIVKQNGLVVSAKKIKLFQTNVRFLGFNIYQSQVSPIDQVSPMNQLQRFLGSLNYV

>Pper5-Chr2-12; Florendovirus; Prunus persica 5;

QKLKEEICSELPTAFWYRKQHVVRLPYIKSFSEKNIPTKARPIQMSQEMMEFCKKEIHELLQKKIIRKSKSPWSCPAFYVQKNAELERGVPRLVINYKPLNAVLEWIRYPIPNKRDLINRLEKAVVYSKFDLKSGFWQIQIDESDRYKTAFVTPFGHYEWNVMPFGLKNAPSEFQNIMNDIFNHFSQFSIVYIDDVLIFSESIEQHWKHLNKFLQVVKQNGLVVSAKKIKLFQTNIRFLGFNICQSQISPIDRVIQFADKFPDQILDKSQLQRFLGSLNY

>Pper5-Chr2-13; Florendovirus; Prunus persica 5;

QRLKEEICSELPTAFWYRKQHVVRLPYIKTFSEKNIPTKARPIQMSQEMMEFCKKEIEELLQKKIIRKSKSPWSCPAFYVQKNAELERGVPRLVINYKPLNAILEWIRYPIPNKRDLINRLEKAVVYSKFDMKSGFWQIQIDESDRYKTAFVTPFGHYEWNVMPFGLKNAPSEFQNIMNDIFNPYSQFSIVYIDDVLIFSESIEQHWKHLNKFLQVVKRNGLVVSAKKIKLFQTNIRFLGFNICQSQISPIDRVIQFADKFPDQILDKSQLQRFLGSLNY

>Pper5-Chr2-14; Florendovirus; Prunus persica 5;

QRLKEEICSELPTAFWYRKQHVVRLPYIKTFSEKNIPTKARPIQMSQEMMEFCKKEIEELLQKKIIRKSKSPWSCPAFYVQKNAELERGVPRLVINYKPLNAVLEWIRYPIPNKRDLINRLEKAVVYSKFDMKSGFWQIQIDESDRYKTAFVTPFGHYEWNVMPFGLKNAPSEFQNIMNDIFNPYSQFSIVYIDDVLIFSESIEQHWKHLNKFLQVVKQNGLVVSAKKIKLFQTNIRFLGFNICQSQISPIDRVIQFADKFPDQILDKSQLQRFLGSLNY

>Pper5-Chr3-01; Florendovirus; Prunus persica 5;

EKLKQEVCSELPNAFWHRKQHVICLPYVKTFSEKNIPTKARPIQMSQELMEFCKIEITDLLKKGIIRKSKSPWSCPAFYVQKNAELERGVPRLVINYKPLNTVLEWIRYPIPNKRDLINRLEKSVIFSKFDMKSGFWQIQISEADRYKTAFVTHFGHYEWNVMPFGLKNAPSEFQNIMNETFNHFSHFSIVYIDDVLIFSESIEQHWKHLYKFLQIVKQNGLVISAKKIKLLQTNVRFLGFNICQSXISPIDRVIQFVDKFLDQILD

>Pper5-Chr3-02; Florendovirus; Prunus persica 5;

QRLKEEICSELPTAFWYRKQHVVRLPYIKTFSEKNIPTKARPIQMSQEMMEFCMKEIKELLQKKIIRKSKSPWSCPAFYVQKNAELERGVPRLVINYKPLNAVLEWIRYPIPNKRDLINRLEKAVVYSKFDLKSGFWQIQIDESDRYKTAFVTPFGHYEWNVMPFGLKNAPSEFQNIMNDIFNPYSQFSIVYIDDVLIFSESIEQHWKHLNKFLQVVKQNGLVVSAKKIKLFQINIRFLGFNICQSQISPIDRVIQFADKFPDQILDKSQLQRFLGSLNY

>Pper5-Chr3-03; Florendovirus; Prunus persica 5;

EKLKQEVCSDLPNAFWHRKQHVVSLPYVKNFSEKNILTKARPIQMSQEIMEFYKTEITELLQKGIIRKSKSPWSCPAFYVXKNAELERGAPRLVINYKPLNTTLEWIRYPIPNKRDLINRLDKSVIFSKFDMKIGFWXIQILESDRYKTAFVTPFGHYEWNVMPFGLKNAHSEFQNIMNEIFNPFSHFSIVYIDDALIFSQSIEQHWKHLHKFLKIVKQNGLVVLAKKIKLFQTNICFLGFNICQSQISLIDRVIQFADKFSYQILDKSQLQRFLGSLNY

>Pper5-Chr3-04; Florendovirus; Prunus persica 5;

EKLKQEICSDLPTAFWHRKQHVVSLPYVKTFNEKHIPTKSRPIQMSREVMEFCKKEIAELLYKGIIRKSKSPWSCPAFYVQKNAELERGAPRLVINFKPLNTVLEWIRYPIPNKRDLINRLSSAVIFSKFHMKSGFWHIQIQERDRYKTAFVTPFGHYEWNVMHFGLKNAPSEFQNIMNEIFNPFSEFSIVYIDDILIFSSSIEQHWKHLHKFLQIVKKNGLVLSAKKIKLFQTNIKFLGFNIHQS

>Pper5-Chr3-05; Yendovirus; Prunus persica 5;

LEELLQLAEQTRIVGEDPQLHWNKNKILAKLDIINPDLTIKTTDMPCNLLDKQEFEHQIKELLNLKVIRPSKSRHRSVAFVVRKHSELKRGKARIVYNYRRLNDNTYEDSYKLPNKDELVNKIQESKYFSKFDCKSGFWQTRLAEESIQWTTFTCTEGHFEWLVMPFGLKNAPSIFQRKMDQSFKKYDNFCSVYVDDILVHSKNKNEHSKHLEIILKEFIDNGVVISRNKIEFEKQDIEFLGMYIKSGTIQLQEHIAKKVLKFLDRLEDKKQLQAFLGLLNYVSSFIPD

>Pper5-Chr3-06; Florendovirus; Prunus persica 5;

EKLKQEVCSELPNAFWHRKQHVVNLPYVKSFSDKNFSTKARPIQISQELMEFCKTEITELLEKGIIRKSKSPXSCPAFYVQENAELERGDPRLVIDYKPLNIILEWIRYPIHNKRDLINRLEKSVIFSKFDMKSGFWQIQIHESDRYKTAFITSFGHYEWNVMPFGLXNASSEFQNIMNEILNHFSHFFIVYIDDVLIFSQSIEQHWKHLHXFLQIVKQNGLVVSAXKIKLFQTNVRFLGFNICQSQISPIDQVIQFADKFPDQILDKSQLXRFLGSLNYV

>Pper5-Chr4-01; Florendovirus; Prunus persica 5;

QRLKEEICSELPTAFWYRKQHVVRLPYIKTFSEKNIPTKARPIQMSQEMMEFCKKEIEELLHKKIIRKSKSPWSCPAFYVQKNAELERGVPRLVINYKPLNAVLEWIRYPIPNKRDLINRLEKAVVYSKFDLKSGFWQIQIDESDRYKTAFVTPFGHYEWNVMPFGLKNAPSEFQNIMNDIFNPYSQFSIVYIDDVLIFSESLEQHWKHLNKFLQVVKQNGLVVSAKKIKLFQTNIRFLGFNICQSQISPIDRVIQFADKFPDQILDKSQLQRFLGSLNY

>Pper5-Chr4-02; Florendovirus; Prunus persica 5;

QRLKEEICSELPTAFWYRKQHVVRLPYIKSFSEKNIPTKARPIQMSQEMMEFCKKEIEELLQKKIIRKSKSPWSCPAFYVQKNAELERGVPRLVINYKPLNAVLEWIRYPIPNKRDLINRLEKAVVYSKFDMKSGFWQIQIDESDRYKTAFVTPFGHYEWNVMPFGLKNAPSEFQNIMNDIFNPYSQFSIVYIDDVLIFSESIEQHWKHLNKFLQVVKRNGLVVSAKKIKLFQTNIRFLGFNICQSQISPIDRVIQFADKFPDQILDKSQLQRFLGSLNY

>Pper5-Chr4-03; Florendovirus; Prunus persica 5;

QRLKEEICSELPTAFWYRKQHVVRLPYIKSFSEKNIPTKARPIQMSQEMMEFCKKEIEELLQKKIIRKSKSPWSCPAFYVQKNAELERGVPRLVINYKPLNAVLEWIRYPIPNKRDLINRLEKAVVYSKFDMKSGFWQIQIDESDRYKTAFVTPFGHYEWNVMPFGLKNAPSEFQNIMNDIFNPYSQFSIVYIDDVLIFSESIEQHWKHLNKFLQVVKRNGLVVSAKKIKLFQTNIRFLGFNICQSQISPIDRVIQFADKFPDQILDKSQLQRFLGSLNY

>Pper5-Chr5-01; Florendovirus; Prunus persica 5;

QRLKEEICSELPTAFWYRKQHVVRLPYIKTFSEKNIPTKARPIQMSQEMMEFCKKEIEELLQKKIIRKSKSPWSCPAFYVQKNAELERGVPRLVINYKPLNAVLEWIRYPIPNKRDLINRLEKAVVYSKFDMKSGFWQIQIDKSDRYKTAFVTPFGHYEWNVMPFGLKNAPSEFQNIMNDIFNPYSQFSIVYIDDVLIFSESIEQHWKHLNKFLQVVKQNGLVVSAKKIKLFQTNIRFLGFNICQSQISPIDRVIQFADKFPDQILDKSQLQRFLGSLNY

>Pper5-Chr5-02; Yendovirus; Prunus persica 5;

LEELLQLAKQTRIIGEDPQLHWNKNKILAKLDIINLDLTIKTADMPCNLLDKQEFYQQIKQILNLKVIRPSKSRHRSTAFVVRKHLELKRGKVRIVYNYRRLNDNTYEDSYKLPNKDELMNKIQESKYFSKFDCKSGFXQIRLAEESIXLTTFTCPEGNFEWLVMPFGLKNAPSIFQRKMDQIFKKYDNFCSVYVEDILVHSKNKNKHRKYLEIILKEFIDNGIVISRNKVELEKQDIEFLGIYIKSGTIQLQEHIAKKVLEFPDKLENKKQLQEFLGLLNYARSFIPD

>Pper5-Chr5-03; Florendovirus; Prunus persica 5;

QKLKEEICSELPTAFWYRKQHVVCLPYIKTFSEKNIPTKARPIQMSQEMMEFCKKEIEELLQKKIIRKSKSPWLCPAFYVQKNAELERGVPRLVINYKPLNAVLEWIRYPIPNKRDLINRLEKAVVYSKFDMKSGFWQIQIDESDRYKTVFVTPFGHYEWNVMPFGLKNAPSEFQNIMNDIFNPYSQFSIVYIDDVLIFSESIEQHWKHLYKFLQIVKQNGLVVSAKKIKLFQTNIRFLGFNICQSQISPIDRVIQFADKFPDQILDKSQLQRFLGSLNY

>Pper5-Chr6-01; Florendovirus; Prunus persica 5;

QRLKEEICSELPTAFWYRKQHVVRLPYIKTFSENNIPTKARPIQMSQEMMEFCKKEIEELLQKKIIRKSKSPWSCPAFYVQKNAELERGVPRLVINFKPLNAVLEWIRYPIPNKRDLINRLEKAVVYSKFDLKSGFWQIQIDESDRYKTAFVTPFGHYEWNVMPFGLKNAPSEFQNIMNDIFNPYSQFSIVYIDDVLIFSESIEQHWKHLNKFLQVVKQNGLVVSAKKIKLFQTNIRFLGFNICQSQISPIDRVIQFADKFPDQILDKSQLQRFLGSLNY

>Pper5-Chr6-02; Florendovirus; Prunus persica 5;

QRLKEEICSELPTAFWYRKQHVVRLPYIKTFSENNIPTKARPIQMSQEMMEFCKKEIEELLQKKIIRKSKSPWSCPAFYVQKNAELERGVPRLVINYKPLNAVLEWIRYPIPNKRDLINRLEKAVVYSKFDLKSGFWQIQIDESDRYKTAFVTPFGHYEWNVMPFGLKNAPSEFQNIMNDIFNPYSQFSIVYIDDVLIFSESIEQHWKHLNKFLQVVKQNGLVVSAKKIKLFQTNIRFLGFNICQSQISPIDRVIQFADKFPDQILDKSQLQRFLGSLNY

>Pper5-Chr6-03; Florendovirus; Prunus persica 5;

EKLKQEVCSELPNAFWHRKQHVVSLPYVKSFSEKNIPTKARPIQMSQELMEFCKIEITDLLKKGIIRKSKSPWSCPDFYVQKNAELECGVPRLVINYKPLNTVLEWIRYPIPNKRDLINRLENSVIFSKFDMKSGFWQIQISESDRYKTAFVTPFGHYEWNVMPFGLKNAPSEFQNIMNEIFNPFSHFSIVYIDDVLIFSESIEQHXKHLHKFLQIVKQNGLVVSAKKIKLFQTNVRFLRFNIYQSQISPIDRVIXIADKFPDQILDKSQLQRFLGSLN

>Pper5-Chr6-04; Florendovirus; Prunus persica 5;

QRLKEEICSELPTAFWYRKQHVVRLPYIKTFSEKNIPTKARPIQMSQEMMEFCKKEIEELLQKKIIRKSKSPWSCPAFYVQKNAELERGVPRLVINYKPLNAVLEWIRYPIPNKRDLINRLEKAVVYSKFDMKSGFWQIQIDESDRYKTAFVTPFGHYEWNVMPFGLKNAPSEFQNIMNDIFNPYSQFSIVYIDDVLIFSESIEQHWKHLNKFLQVVKQNGLVVSAKKIKLFQTDIRFLGFNICQSQISPIDRVIQFADKFPDQILDKSQLQRFLGSLNY

>Pper5-Chr6-05; Florendovirus; Prunus persica 5;

QRLKEEICSELPTAFWYRKQHVVRLPYIKTFSEKNIPTKARPIQMSQEMMEFCKKEIEELLQKKIIRKSKSPWSCPAFYVQKNAELERGVPRLVINYKPLNAVLEWIRYPIPNKRDLINRLEKAVVYSKFDMKSGFWQIQIDESDRYKTAFVTPFGHYEWNVMPFGLKNAPSEFQNIMNDIFNPYSQFSIVYIDDVLIFSESIEQHWKHLNKFLQVVKQNGLVVSAKKIKLFQTDIRFLGFNICQSQISPIDRVIQFADKFPDQILDKSQLQRFLGSLNY

>Pper5-Chr7-01; Florendovirus; Prunus persica 5;

QRLKEEICSELPTAFWYRKQHVVRLPYIKTFSEKNIPTKARPIQMSQEMMEFCKKEIEELLQKKIIRKSKSPWSCPAFYVQKNAELERGVPRLVINYKPLNAVLEWIRYPIPNKRDLINRLEKAVVYSKFDMKSGFWQIQIDESDRYKTAFVTPFGHYEWNVMPFGLKNAPSEFQNIMNDIFNPYSQFSIVYIDDVLIFSESIEQHWKHLNKFLQVVKQNGLVVSAKKIKLFQTNIRFLGFNICQSQISPIDRVIQFADKFPDQILDKSQLQRFLGSLNY

>Pper5-Chr7-02; Florendovirus; Prunus persica 5;

QRLKEEICSELPTAFWYRKQHVVRLPYIKTFSEKNIPTKARPIQMSQEMMEFCKKEIEELLQKKIIRKSKSPWSCPAFYVQKNAELERGVPRLVINYKPLNAVLEWIRYPIPNKRDLINRLEKAVVYSKFDMKSGFWQIQIDESDRYKTAFVTPFGHYEWNVMPFGLKNAPSEFQNIMNDIFNPYSQFLIVYIDDVLIFSESIEQHWKHLNKFLQVVKQNGLVVSAKKIKLFQTNIRFLGFNICQSQISPIDRVIQFADKFPDQILDKSQLQRFLGSLNY

>Pper5-Chr7-03; Florendovirus; Prunus persica 5;

QRLKEEICSELPTAFWYRKQHVVRLPYIKTFSEKNIPTKARPIQMSQEMMEFCKKEIEELLQKKIIRKSKSPWSCPAFYVQKNAELERGVPRLVINYKPLNAVLEWIRYPIPNKRDLINRLEKAVVYSKFDMKSGFWQIQIDESDRYKTAFVTPFGHYEWNVMPFGLKNAPSEFQNIMNDIFNPYSQFSIVYIDDVLIFSESIEQHWKHLNKFLQVVKQNGLVVSAKKIKLFQTNIRFLGFNICQSQISPIDRVIQFADKFPDQILDKSQLQRFLGSLNY

>Pper5-Chr7-04; Florendovirus; Prunus persica 5;

QRLKEEICSELPTAFWYRKQHVVRLPYIKTFSEKNIPTKARPIQMSQEMMEFCKKEIEELLQKKIIRKSKSPWSCPAFYVQKNAELERGVPRLVINYKPLNAVLEWIRYPIPNKRDLINRLEKAVVYSKFDMKSGFWQIQIDESDRYKTAFVTPFGHYEWNVMPFGLKNAPSEFQNIMNDIFNPYSQFSIVYIDDVLIFSESIEQHWKHLNKFLQVVKQNGLVVSAKKIKLFQTNIRFLGFNICQSQISPIDRVIQFADKFPDQILDKSQLQRFLGSLNY

>Pper5-Chr7-05; Florendovirus; Prunus persica 5;

QRLKEEICSELPTAFWYRKQHVVRLPYIKTFSEKNIPTKARPIQMSQEMMEFCKKEIEELLQKKIIRKSKSPWSCPAFYVQKNAELERGVPRLVINYKPLNAVLEWIRYPIPNKRDLINRLEKAVVYSKFDMKSGFWQIQIDESDRYKTAFVTPFGHYEWNVMPFGLKNAPSEFQNIMNDIFNPYSQFSIVYIDDVLIFSESIEQHWKHLNKFLQVVKQNGLVVSAKKIKLFQTNIRFLGFNICQSQISPIDRVIQFADKFPDQILDKSQLQRFLGSLNY

>Pper5-Chr7-06; Florendovirus; Prunus persica 5;

QKLKEEICSELPTAFWYRKQHVVRLPYIKSFSEKNIPTKARPIQMSQEMMEFCKKEIEELLQKKIIRKSKSPWSCPAFYVQKNAELERGVPRLVINYKPLNAVLEWIRYPIPNKRDLINRLEKAVVYSKFDLKSGFWQIQIDESDRYKTAFVTPFGHYEWNVMPFGLKNAPSEFQNIMNDIFNPYSQFSIVYIDDVLIFSESIKQHWKHLHKFLQVFKQNGLVVSAKKIKLFQTNIRFLGFNICQSQISPIDRVIQFADKFLDQILDKSQLQRFLGSLNY

>Pper5-Chr7-07; Florendovirus; Prunus persica 5;

QKLKEEICSELPTAFWYRKQHVVCLPYIKTFSEKNIPTKARPIQMSQEMMEFCKKEIEELLQKKIIRKSKSPWSCPAFYVQKNAELERGVPRLVINYKPLNAVLEWIRYPIPNKRDLINRLEKAFVYSKFDLKSGFWQIQIDESDRYKIAFVTPFGHYEWNVMPFGLKNSPSEFQNIMNDIFNPYSQFSIVYIDDVLIFSESIEQHWKHLNKFLQVVKQNGLVVSAKKIKLFQTNIRFLGFNICQSQISPIDRVIQFADKFPDQILDKSQLQRFLRSLNY

>Pper5-Chr8-01; Florendovirus; Prunus persica 5;

QRLKEEICSELPTAFWYRKQHVVRLPYIKTFSEKNIPTKARPIQMSQEMMEFCKKEIEELLQKKIIRKSKSPWSCPAFYVQKNAELERGVPRLVINYKPLNAVLEWIRYPIPNKRDLINRLEKAVVYSKFDMKSGFWQIQIDESDRYKTAFVTPFGHYEWNVMPFGLKNAPSEFQNIMNDIFNPYSQFSIVYIDDVLIFSESIEQHWKHLNKFLQVVKRNGLVVSAKKIKLFQTNIRFLGFNICQSQISPIDRVIQFADKFPDQILDKSQLQRFLGSLNY

>Pper5-Chr8-02; Florendovirus; Prunus persica 5;

QRLKEEICSELPTAFWYRKQHVVRLPYIKTFSEKNIPTKARPIQMSQEMMEFCKKEIEELLQKKIIRKSKSPWSCPAFYVQKNAELERGVPRLVINYKPLNAVLEWIRYPIPNKRDLINRLEKAVVYSKFDMKSGFWQIQIDESDRYKTAFVTPFGHYEWNVMPFGLKNAPSEFQNIMNDIFNPYSQFSIVYIDDVLIFSESIEQHLKHLNKFLQVVKRNGLVVSAKKIKLFQTNIRFLGFNICQSQISPIDRVIQFADKFPDQILDKSQLQRFLGSLNY

>Pper5-Chr8-03; Florendovirus; Prunus persica 5;

QRLKEEICSELPTAFWYRKQHVVRLPYIKTFSEKNIPTKARPIQMSQEMMEFCKKEIEELLQKKIIRKSKSPWSCPAFYVQKNAELERGVPRLVINYKPLNAVLEWIRYPIPNKRDLINRLEKAVVYSKFDMKSGFWQIQIDESDRYKTAFVTPFGHYEWNVMPFGLKNAPSEFQNIMNDIFNPYSQFSIVYIDDVLIFSESIEQHWKHLNKFLQVVKRNGLVVSAKKIKLFQTNIRFLGFNICQSQISPIDRVIQFADKFPDQILDKSQLQRFLGSLNY

>Pper5-Chr8-04; Florendovirus; Prunus persica 5;

QRLKEEICSELPTAFWYRKQHVVRLPYIKTFSEKNIPTKARPIQMSQEMMEFCKKEIEELLQKKIIRKSKSPWSCPAFYVQKNAELERGVPRLVINYKPLNAVLEWIRYPIPNKRDLINRLEKAVVYSKFDMKSGFWQIQIDESDRYKTAFVTPFGHYEWNVMPFGLKNAPSEFQNIMNDIFNPYSQFSIVYIDDVLIFSESIEQHWKHLNKFLQVVKRNGLVVSAKKIKLFQTNIRFLGFNICQSQISPIDRVIQFADKFPDQILDKSQLQRFLGSLNY

>Pper5-Chr8-05; Florendovirus; Prunus persica 5;

QRLKEEICSELPTAFWYRKQHVVRLPYIKTFSEKNIPTKARPIQMSQEMMEFCKKEIEELLQKKIIRKSKSPWSCPAFYVQKNAELERGVPRLVINYKPLNAVLEWIRYPIPNKRDLINRLEKAVVYSKFDMKSGFWQIQIDESDRYKTAFVTPFGHYEWNVMPFGLKNAPSEFQNIMNDIFNPYSQFSIVYIDDVLIFSESIEQHWKHLNKFLQVVKRNGLVVSAKKIKLFQTNIRFLGFNICQSQISPIDRVIQFADKFPDQILDKSQLQRFLGSLNY

>Pper5-Chr8-06; Florendovirus; Prunus persica 5;

QRLKEEICSELPTAFWYRKQHVVRLPYIKTFSEKNIPTKARPIQMSQEMMEFCKKEIEELLQKKIIRKSKSPWSCPAFYVQKNAELERGVPRLVINYKPLNAVLEWIRYPIPNKRDLINRLEKAVVYSKFDMKSGFWQIQIDESDRYKTAFVTPFGHYEWNVMPFGLKNAPSEFQNIMNDIFNPYSQFSIVYIDDVLIFSESIEQHWKHLNKFLQVVKRNGLVVSAKKIKLFQTNIRFLGFNICQSQISPIDRVIQFADKFPDQILDKSQLQRFLGSLNY

>Pper5-Chr8-07; Florendovirus; Prunus persica 5;

QRLKEEICSELPTAFWYRKQHVVRLPYIKTFSEKNIPTKARPIQMSQEMMEFCKKEIEELLQKKIIRKSKSPWSCPAFYVQKNAELERGVPRLVINYKPLNAVLEWIRYPIPNKRDLINRLEKAVVYSKFDMKSGFWQIQIDESDRYKTAFVTPFGHYEWNVMPFGLKNAPSEFQNIMNDIFNPYSQFSIVYIDDVLIFSESIEQHWKHLNKFLQVVKRNGLVVSAKKIKLFQTNIRFLGFNICQSQISPIDRVIQFADKFPDQILDKSQLQRFLGSLNY

>Pper5-Chr8-08; Florendovirus; Prunus persica 5;

QRLKEEICSELPTAFWYRKQHVVRLPYIKTFSEKNIPTKARPIQMSQEMMEFCKKEIEELLQKKIIRKSKSPWSCPAFYVQKNAELERGVPRLVINYKPLNAVLEWIRYPIPNKRDLINRLEKAVVYSKFDMKSGFWQIQIDESDRYKTAFVTPFGHYEWNVMPFGLKNAPSEFQNIMNDIFNPYSQFSIVYIDDVLIFSESIEQHWKHLNKFLQVVKRNGLVVSAKKIKLFQTNIRFLGFNICQSQISPIDRVIQFADKFPDQILDKSQLQRFLGSLNY

>Pper5-Chr8-09; Florendovirus; Prunus persica 5;

QRLKEEICSELPTAFWYRKQHVVRLPYIKTFSEKNIPTKARPIQMSQEMMEFCKKEIEELLQKKIIRKSKSPWSCPAFYVQKNAELERGVPRLVINYKPLNAVLEWIRYPIPNKRDLINRLEKAVVYSKFDLKSGFWQIQIDESDRYKTAFVTPFGHYEWNVMPFGLKNAPSEFQNIMNDTFNPYSQFSIVYIDDVLIFSESIEQHWKHLHKFLQVVKQNGLVVSAKKIKLFQTNIRFLGFNICQSQISPIDRVIQFADKFPDQILDKSQLQRFLGSLNY

>Pper5-Chr8-10; Florendovirus; Prunus persica 5;

QRLKEEICSELPTAFWYRKQHVVRLPYIKTFSEKNIPTKARPIQMSQEMMEFCKKEIEELLQKKIIRKSKSPWSCPAFYVQKNAELERGVPRLVINYKPLNAVLEWIRYPIPNKRDLINRLEKAVVYSKFDLKSGFWQIQIDESDRYKTAFVTPFGHYEWNVMPFGLKNAPSEFQNIMNDIFNPYSQFSIVYIDDVLIFSESIEQHWKHLHKFLQVVKQNGLVVSAKKIKLFQTNIRFLGFNICQSQISPIDRVIQFADKFPDQILDKSQLQRFLGSLNY

>Pper5-Chr8-11; Florendovirus; Prunus persica 5;

QRLKEEICSELPTAFWYRKQHVVRLPYIKTFSEKNIPTKARPIQMSQEMMEFCKKEIEELLQKKIIRKSKSPWSCPAFYVQKNAELERGVPRLVINYKPLNAVLEWIRYPIPNKRDLINRLEKAVVYSKFDLKSGFWQIQIDESDRYKTAFVTPFGHYEWNVMPFGLKNAPSEFQNIMNDIFNPYSQFSIVYIDDVLIFSESIEQHWKHLHKFLQVVKQNGLVVSAKKIKLFQTNIRFLGFNICQSQISPIDRVIQFADKFPDQILDKSQLQRFLGSLNY

>Pper5-Chr8-12; Florendovirus; Prunus persica 5;

QRLKEEICSELPTAFWYRKQHVVRLPYIKTFSEKNIPTKARPIQMSQEMMEFCKKEIEELLQKKIIRKSKSPWSCPAFYVQKNAELERGVPRLVINYKPLNAVLEWIRYPIPNKRDLINRLEKAVVYSKFDMKSGFWQIQIDESDRYKTAFVTPFGHYEWNVMPFGLKNAPSEFQNIMNDIFNPYSQFSIVYIDDVLIFSESIEQHWKHLHKFLQVVKQNGLVVSAKKIKLFQTNIRFLGFNICQSQISPIDRVIQFADKFPDQILDKSQLQRFLGSLNY

>Pper5-Chr8-13; Florendovirus; Prunus persica 5;

QKIKNEICSELPNAFWYRKQHVVCLPYIKTFSEKNIPTKARPIQMSQEIMEFCKTEISELLQKKIIRKSKSSWSCPAFYVQKNAELERGVPRLVINYKSLNSVLEWIWYPIPNKRDLINRLGKAVVYSKFDMKSGFWQIQIEESDRYKTAFNTPFGHYEWNVMPFGLKNAPSEFQNIMNEIFNPYSHFSIVYIDDVLIFSESIEQYWKHLHKFFQIVKQNGLVVSAKKIKLFQTNIRFLGFNICQSQISPIDRVIQFADKFPDQILDKSQLQRFLGSLNY

>Pper5-Chr8-14; Florendovirus; Prunus persica 5;

QKIKNEICSELPNAFWYRKQHVVCLPYIKTFSEKNIPTKARPIQMSQEIMEFCKTEISELLQKKIIRKSKSPWSCPAFYVQKNAELERGVPRLVINYKSLNSVLEWIWYPIPNKRDLINRLGKAVVYSKFDMKSEFWQIQIEESDRYKTAFVTPFGHYEWNVMPFGLKNAPSEFQNIMNEIFNPYSHFSIVYIDDVLIFSESIEQHWKHLHKFFQIVKQNGLVVSAKKTKLFQTNIKFLGFNICQSQISPIDRVIQFANKFPDQILDKSQLMRVLGSLNY

>Pper6-Chr1-01; Florendovirus; Prunus persica 6;

EKLKQEVCSELPNAFWHRKQHVVSLPYVKTSSEKNIPTKARPIQISQELMKFCKIEITDLLKKGIIRKSRSPWSCPAFYVQKNAELERGVPRLVINYKPLNTVLEWIRYPIPNKRDIINRLEKSVIFSKFDMKSGFWQIQISETDRYKTAFVTPFGHYEWNVMPFDLKNAPSEFQNIINVIFNPFSHFSIVYIDDVLIFSASIEQHWKHLYKFLQIVKQNGLVVSAKKIKLFQTNVRFLGFNIXQSQISPIDRVIQFADKFPDQILEKGQLQRFLGSLNYVSDF

>Pper6-Chr1-02; Florendovirus; Prunus persica 6;

EKLKQEVCSELPNAFWHRKQHVVSLPYVKSFSEKNIPTKARPIQMSQELMKFCKTEITELLEKGIIRKSKSLWSCPAFYVQKNVELEHGVPRLVINYKPLNTVLEWIRYPIPNKRDLINRLEKSVIFSKFDMKSGFWQIQIRESFSYKTAFVTPFGHYEWNVMPFGLKNAPSEFENIMNEIFNHFSHFSIVYIDDVLIFSESIEQHWKHLHKFLQIVKRNGLVVSAKKIKLFQTNVRFLGFNICQSQISPIDRVIQFADKFPDQILDKSQLHRFLGSLNYVSDF

>Pper6-Chr1-03; Florendovirus; Prunus persica 6;

QRLKEEICSELPTAFWYRKQHVVRLPYIKTFSEKNIPTKARPIQMSQEMMEFCKKEIEELLQKKIIRKSKSPWSCPAFYVQKNAELERGVPRLVINYKPLNAVLEWIRYPIPNKRDLINRLEKAVVYSKFDLKSGFWQIQIDESDRYKTAFVTPFGHYEWNVMPFGLKNAPSEFQNIMNDIFNPYSQFSIVYIDDVLIFSESIEQHWKHLNKFLQVVKQNGLVVSAKKIKLFQTNIRFLGFNICQSQISPIDRVIQFADKFPDQILDKSQLQRFLGSLNY

>Pper6-Chr1-04; Florendovirus; Prunus persica 6;

QRLKEEICSELPTAFWYRKQHVVRLPYIKTFSEKNIPTKARPIQMSQEMMEFCMKEIEELLQKKIIRKSKSPWSCPAFYVQKNAELERGVPRLVINYKPLNAVLEWIRYPIPNKRDLINRLEKAVVYSKFDLKSGFWQIQIDESDRYKTAFVTPFGHYEWNVMPFGLKNAPSEFQNIMNDIFNPYSQFSIVYIDDVLIFSESIEQHWKHLNKFLQVVKQNGLVVSAKKIKLFQINIRFLGFNICQSQISPIDRVIQFADKFPDQILDKSQLQRFLGSLNY

>Pper6-Chr1-05; Florendovirus; Prunus persica 6;

QRLKEEICSELPTAFWYRKQHVVRLPYIKTFSEKNIPTKARPIQMSQEMMEFCKKEIEELLQKKIIRKSKSPWSCPAFYVQKNAELERGVPRLVINYKPLNAVLEWIRYPIPNKRDLINRLEKAVVYSKFDLKSGFWQIQIDESDRYKTAFVTPFGHYEWNVMPFGLKNAPSEFQNIMNDIFNPYSQFSIVYIDDVLIFSESIEQHWKHLNKFLQVVKQNGLVVSAKKIKLFQTNIRFLGFNICQSQISPIDRVIQFADKFPDQILDKSQLQRFLGSLNY

>Pper6-Chr1-06; Florendovirus; Prunus persica 6;

QRLKEEICSELPTAFWYRKQHVVRLPYIKTFSEKNIPTKARPIQMSQEMMEFCKKEIEELLQKKIIRKSKSPWSCPAFYVQKNAELERGVPRLVINYKPLNAVLEWIRYPIPNKRDLINRLEKAVVYSKFDLKSGFWQIQIDESDRYKTAFVTPFGHYEWNVMPFGLKNAPSEFQNIMNDIFNPYSQFSIVYIDDVLIFSESIEQHWKHLNKFLQVVKQNGLVVSAKKIKLFQTNIRFLGFNICQSQISPIDRVIQFADKFPDQILDKSQLQRFLGSLNY

>Pper6-Chr1-07; Florendovirus; Prunus persica 6;

QRLKEEICSEVPTAFWYRKQHVVRLPYIKTFSEKNIPTKARPIQMNQEMMEFCKKEIEELLQKKIIRKSKSPWSCPAFYVQKNAELERGVPRLVINYKPLNAVLEWIRYPIPNKRDLINRLEKAVVYSKFDMKSGFWQIQIDESDRYKTAFVTPFGHYEWNVMPFGLKNAPSEFQNIMNDIFNPYSQFSIVYIDDVLIFSESIEQHWKHLNKFLQVVKQNGLVVSAKKIKLFQTNIRFLGFNICQSQISPIDRVIQFADKFLDQILDKSQLQRFLGSLNY

>Pper6-Chr2-01; Florendovirus; Prunus persica 6;

QRLKEEICSELPTAFWYRKQHVVRLPYIKTFSEKNIPTKARPIQMSQEMMEFCKKEIEELLQKKIIRKSKSPWSCPAFYVQKNAELERGVPRLVINYKPLNAVLEWIRYPIPNKRDLINRLEKAVVYSKFDLKSGFWQIQIDESDRYKTAFVTPFGHYEWNVMPFGLKNAPSEFQNIMNDIFNPYSQFSIVYIDDVLIFSESIEQHWKHLNKFLQVVKQNGLVVSAKKIKLFQTNIRFLGFNICQSQISPIDRVIQFADKFPDQILDKSQLQRFLGSLNY

>Pper6-Chr2-02; Florendovirus; Prunus persica 6;

QRLKEEICSELPTAFWYRKQHVVRLPYIKTFSEKNIPTKARPIQMSQEMMEFCKKEIEELLQKKIIRKSKSPWSCPAFYVQKNAELERGVPRLVINYKPLNAVLEWIRYPIPNKRDLINRLEKAVVYSKFDLKSGFWQIQIDESDRYKTAFVTPFGHYEWNVMPFGLKNAPSEFQNIMNDIFNPYSQFSIVYIDDVLIFSESIEQHWKHLNKFLQVVKQNGLVVSAKKIKLFQTNIRFLGFNICQSQISPIDRVIQFADKFPDQILDKSQLQRFLGSLNY

>Pper6-Chr2-03; Florendovirus; Prunus persica 6;

EKLQREVCSELPNAFWHRKQHVVRLPYIKSFNEKNIPTKARPIQMSQELMELCKTKITELLEKRIIRKSKSPWSCPAFYVQKNAELERGVPRLVINYKPLNTVLEWIRYPILNKRDLINRLEKSVIFSKFDMKSGFWQIQIYETDRHKIAFVTPFGHYEWNVMPFGLKNAPSEFQNIMNEIFNPYSHFSIVYIDDVLIFSQSIEQHWKHLHKFLQIVKQNGLVVSAKKIKLFQTNVRFLGFNICQSQISPIDXVIQFADKFPDQILDKSQLQRFLGSLNYVSDF

>Pper6-Chr2-04; Yendovirus; Prunus persica 6;

HWNKNKILAKLDIINPYSTIKIAYMPCNLLDKQNFXQQIKELLNIVLRPSKSRHRSTAFVIRKHSKVKRGKARIVYNYKRXNDNTYEDSYKLPNKDELMNKIEESKYSSKLYCKSRFWQIRLAEXSIQXTTFTRTKAHFEWLVMPFGLMNAPSIFQRKMNQIFEKYDNFGSIYVDDKLVCSKNKNEHRKHXENILKEFIDNGIVISRNKVELEKXGIXFXGMYIKSETIXLQEHIGKKVLXFPNKXEDKKQLQAFLGLLNYARSFI

>Pper6-Chr2-05; Florendovirus; Prunus persica 6;

QKLKEEICSELPTAFWYRKQHVVRLPYIKSFSEKNIPTKARPIQMSQEMMEFCKKEIHELLQKKIIRKSKSPWSCPAFYVQKNAELERGVPRLVINYKPLNAVLEWIRYPIPNKRDLINRLEKAVVYSKFDLKSGFWQIQIDESDRYKTAFVTPFGHYEWNVMPFGLKNAPSEFQNIMNDIFNHFSQFSIVYIDDVLIFSESIEQHWKHLNKFLQVVKQNGLVVSAKKIKLFQTNIRFLGFNICQSQISPIDRVIQFADKFPDQILDKSQLQRFLGSLNY

>Pper6-Chr2-06; Florendovirus; Prunus persica 6;

QRLKEEICSELPTAFWYRKQHVVRLPYIKTFSEKNIPTKARPIQMSQEMMEFCKKEIEELLQKKIIRKSKSPWSCPAFYVQKNAELERGVPRLVINYKPLNAILEWIRYPIPNKRDLINRLEKAVVYSKFDMKSGFWQIQIDESDRYKTAFVTPFGHYEWNVMPFGLKNAPSEFQNIMNDIFNPYSQFSIVYIDDVLIFSESIEQHWKHLNKFLQVVKRNGLVVSAKKIKLFQTNIRFLGFNICQSQISPIDRVIQFADKFPDQILDKSQLQRFLGSLNY

>Pper6-Chr3-01; Florendovirus; Prunus persica 6;

EKLKQEVCSELPNAFWHRKQHVICLPYVKTFSEKNIPTKARPIQMSQELMEFCKIEITDLLKKGIIRKSKSPWSCPAFYVQKNAELERGVPRLVINYKPLNTVLEWIRYPIPNKRDLINRLEKSVIFSKFDMKSGFWQIQISEADRYKTAFVTHFGHYEWNVMPFGLKNAPSEFQNIMNETFNHFSHFSIVYIDDVLIFSESIEQHWKHLYKFLQIVKQNGLVISAKKIKLLQTNVRFLGFNICQSXISPIDRVIQFVDKFLDQILD

>Pper6-Chr3-02; Florendovirus; Prunus persica 6;

EKLKQEVCSDLPNAFWHRKQHVVSLPYVKNFSEKNILTKARPIQMSQEIMEFYKTEITELLQKGIIRKSKSPWSCPAFYVXKNAELERGAPRLVINYKPLNTTLEWIRYPIPNKRDLINRLDKSVIFSKFDMKIGFWXIQILESDRYKTAFVTPFGHYEWNVMPFGLKNAHSEFQNIMNEIFNPFSHFSIVYIDDALIFSQSIEQHWKHLHKFLKIVKQNGLVVLAKKIKLFQTNICFLGFNICQSQISLIDRVIQFADKFSYQILDKSQLQRFLGSLNY

>Pper6-Chr3-03; Florendovirus; Prunus persica 6;

EKLKQEICSDLPTAFWHRKQHVVSLPYVKTFNEKHIPTKSRPIQMSREVMEFCKKEIAELLYKGIIRKSKSPWSCPAFYVQKNAELERGAPRLVINFKPLNTVLEWIRYPIPNKRDLINRLSSAVIFSKFHMKSGFWHIQIQERDRYKTAFVTPFGHYEWNVMHFGLKNAPSEFQNIMNEIFNPFSEFSIVYIDDILIFSSSIEQHWKHLHKFLQIVKKNGLVLSAKKIKLFQTNIKFLGFNIHQS

>Pper6-Chr3-04; Yendovirus; Prunus persica 6;

LEELLQLAEQTRIVGEDPQLHWNKNKILAKLDIINPDLTIKTTDMPCNLLDKQEFEHRIKELLNLKVIRPSKSRHRSVAFVVRKHSELKRGKARIVYNYRRLNDNTYEDSYKLPNKDELMNKIQESKYFSKFDCKSGFWQTRLAEESIQWTTFTCTEGHFEWLVMPFGLKNAPSIFQRKMDQSFKKYDNFCSVYVDDILVHSKNKNEHSKHLEIILKEFIDNGVVISRNKIEFEKQDIEFLGMYIKSGTIQLQEHIAKKVLKFLDRLEDKKQLQAFLGLLNYVSSFIPD

>Pper6-Chr3-05; Florendovirus; Prunus persica 6;

KLKNEICSELPNAFWYRKQHVVCLPYIKTFNEKNIPTKARPIQMSQEIMEFCKKXISELLQKKIIRKSKSPWSCPTFYVQKNAELERGVPRLVINYKPLNAVLEWIRYPIPNKRDLINRLEKAVVFFPNFTXRVVSGKSKXKSQIDIRLHLLLLLVITNGMLCHLDXKNAPSEFQNIMNDIFMPCSHFSIVYIDDVLIFFESIEHHWKHLHKFVQIIKQNGLVVSAKKIKLFQTNIRFIGFNICXSKISPIDRVIQFADKFPDQILDKSQLQRFLGSLNY

>Pper6-Chr4-01; Florendovirus; Prunus persica 6;

QRLKEEICSELPTAFWYRKQHVVRLPYIKTFSEKNIPTKARPIQMSQEMMEFCKKEIEELLHKKIIRKSKSPWSCPAFYVQKNAELERGVPRLVINYKPLNAVLEWIRYPIPNKRDLINRLEKAVVYSKFDLKSGFWQIQIDESDRYKTAFVTPFGHYEWNVMPFGLKNAPSEFQNIMNDIFNPYSQFSIVYIDDVLIFSESLEQHWKHLNKFLQVVKQNGLVVSAKKIKLFQTNIRFLGFNICQSQISPIDRVIQFADKFPDQILDKSQLQRFLGSLNY

>Pper6-Chr5-01; Florendovirus; Prunus persica 6;

QRLKEEICSELPTAFWYRKQHVVRLPYIKTFSEKNIPTKARPIQMSQEMMEFCKKEIEELLQKKIIRKSKSPWSCPAFYVQKNAELERGVPRLVINYKPLNAVLEWIRYPIPNKRDLINRLEKAVVYSKFDMKSGFWQIQIDKSDRYKTAFVTPFGHYEWNVMPFGLKNAPSEFQNIMNDIFNPYSQFSIVYIDDVLIFSESIEQHWKHLNKFLQVVKQNGLVVSAKKIKLFQTNIRFLGFNICQSQISPIDRVIQFADKFPDQILDKSQLQRFLGSLNY

>Pper6-Chr5-02; Yendovirus; Prunus persica 6;

LEELLQLAKQTRIIGEDPQLHWNKNKILAKLDIINLDLTIKTADMPCNLLDKQEFYQQIKQILNLKVIRPSKSRHRSTAFVVRKHLELKRGKVRIVYNYRRLNDNTYEDSYKLPNKDELMNKIQESKYFSKFDCKSGFXQIRLAEESIXLTTFTCPEGNFEWLVMPFGLKNAPSIFQRKMDQIFKKYDNFCSVYVEDILVHSKNKNKHRKYLEIILKEFIDNGIVISRNKVELEKQDIEFLGIYIKSGTIQLQEHIAKKVLEFPDKLENKKQLQEFLGLLNYARSFIPD

>Pper6-Chr5-03; Florendovirus; Prunus persica 6;

QKLKEEICSELPTAFWYRKQHVVCLPYIKTFSEKNIPTKARPIQMSQEMMEFCKKEIEELLQKKIIRKSKSPWLCPAFYVQKNAELERGVPRLVINYKPLNAVLEWIRYPIPNKRDLINRLEKAVVYSKFDMKSGFWQIQIDESDRYKTVFVTPFGHYEWNVMPFGLKNAPSEFQNIMNDIFNPYSQFSIVYIDDVLIFSESIEQHWKHLYKFLQIVKQNGLVVSAKKIKLFQTNIRFLGFNICQSQISPIDRVIQFADKFPDQILDKSQLQRFLGSLNY

>Pper6-Chr5-04; Florendovirus; Prunus persica 6;

QKIKEEICSELPTAFWYRKQHVVRLPYIKTFSEKNIPTKARPIQMSQEMMEFCKTEINELLQKKIIRKSKSPWSCPAFYVQKNAELERGVPRLVINYKPLNAVLEWIRYLIPNKRDLINRLEKAIVYSKFDMKSGFWQIQIDESDRYKTAFVTPFGHYEWNVMPFGLKNAPSEFQNIMNDIFNPYSQFSIVYIDDVLIFSESIEQHWKHLYKFLQVVKQNGLVVSAKKIKLFQTNVRFLGFNICQSQISPIDRVIQFADRFPDQILDKSQLQRFLGSLNY

>Pper6-Chr5-05; Florendovirus; Prunus persica 6;

QKIKSEICSELPNAFWYRKQHVVCLPYMKTFSEKNIPTKARPIQMSQEMMEFCKQEIKELLQKKIIRQSKSPWSCPAFYVQKNAELERGVPRLVINYKPLNAVLEWIRYPIPNKRDLINRLEKAVVYSKFDMKSGFWQIQIEESDRYKTAFVTPFGHYEWNVMPFGLKNAPSEFQNIMNDIFNPYSQFSIVYIDDVLIFSESIEQHWKHLYKFLQVVKQNGLVVSAKKIKLFQTNVRFLGFNICQSQISPIDRVIQFADKFPDQILDKSQLQRFLGSLNY

>Pper6-Chr6-01; Florendovirus; Prunus persica 6;

QRLKEEICSELPTAFWYRKQHVVRLPYIKTFSENNIPTKARPIQMSQEMMEFCKKEIEELLQKKIIRKSKSPWSCPAFYVQKNAELERGVPRLVINFKPLNAVLEWIRYPIPNKRDLINRLEKAVVYSKFDLKSGFWQIQIDESDRYKTAFVTPFGHYEWNVMPFGLKNAPSEFQNIMNDIFNPYSQFSIVYIDDVLIFSESIEQHWKHLNKFLQVVKQNGLVVSAKKIKLFQTNIRFLGFNICQSQISPIDRVIQFADKFPDQILDKSQLQRFLGSLNY

>Pper6-Chr6-02; Florendovirus; Prunus persica 6;

QRLKEEICSELPTAFWYRKQHVVRLPYIKTFSENNIPTKARPIQMSQEMMEFCKKEIEELLQKKIIRKSKSPWSCPAFYVQKNAELERGVPRLVINYKPLNAVLEWIRYPIPNKRDLINRLEKAVVYSKFDLKSGFWQIQIDESDRYKTAFVTPFGHYEWNVMPFGLKNAPSEFQNIMNDIFNPYSQFSIVYIDDVLIFSESIEQHWKHLNKFLQVVKQNGLVVSAKKIKLFQTNIRFLGFNICQSQISPIDRVIQFADKFPDQILDKSQLQRFLGSLNY

>Pper6-Chr6-03; Florendovirus; Prunus persica 6;

QRLKEEICSELPTAFWYRKQHVVRLPYIKSFSEKNIPTKARPIQMSQEMMEFCKKEIEELLQKKIIRKSKSPWSCPAFYVQKNAELERGVPRLVINYKPLNAVLEWIRYPIPNKRDLINRLEKAVVYSKFDMKSGFWQIQIDESDRYKTAFVTPFGHYEWNVMPFGLKNAPSEFQNIMNDIFNPYSQFSIVYIDDVLIFSESIEQHWKHLYKFLQVVKQNGLVVSAKKIKLFQTNIRFLGFNICQSQISPIDRVIQFADKFPDQILDKSQLQRFLGSLNY

>Pper6-Chr6-04; Florendovirus; Prunus persica 6;

EKLKQEVCSELPNAFWHRKQHVVSLPCVKSFSEKNIPTKARPIQMSQELMEFCKIEITDLLKKGIIRKSKSPWSCPDFYVQKNAELECGVPRLVINYKPLNTVLEWIRYPIPNKRDLINRLENSVIFSKFDMKSGFWQIQISESDRYKTAFVTPFGHYEWNVMPFGLKNAPSEFQNIMNEIFNPFSHFSIVYIDDVLIFSESIEQHXKHLHKFLQIVKQNGLVVSAKKIKLFQTNVRFLRFNIYQSQISPIDRVIXIADKFPDQILDKSQLQRFLGSLN

>Pper6-Chr6-05; Florendovirus; Prunus persica 6;

QRLKEEICSELPTAFWYRKQHVVRLPYIKTFSEKNIPTKARPIQMSQEMMEFCKKEIEELLQKKIIRKSKSPWSCPAFYVQKNAELERGVPRLVINYKPLNAVLEWIRYPIPNKRDLINRLEKAVVYSKFDMKSGFWQIQIDESDRYKTAFVTPFGHYEWNVMPFGLKNAPSEFQNIMNDIFNPYSQFSIVYIDDVLIFSESLEQHWKHLNKFLQVVKQNGLVVSAKKIKLFQTNIRFLGFNICQSQISPIDRVIQFADKFPDQILDKSQLQRFLGSLNY

>Pper6-Chr6-06; Florendovirus; Prunus persica 6;

QRLKEEICSELPTAFWYRKQHVVRLPYIKTFSEKNIPTKARPIQMSQEMMEFCKKEIEELLQKKIIRKSKSPWSCPAFYVQKNAELERGVPRLVINYKPLNAVLEWIRYPIPNKRDLINRLEKAVVYSKFDMKSGFWQIQIDESDRYKTAFVTPFGHYEWNVMPFGLKNAPSEFQNIMNDIFNPYSQFSIVYIDDVLIFSESLEQHWKHLNKFLQVVKQNGLVVSAKKIKLFQTNIRFLGFNICQSQISPIDRVIQFADKFPDQILDKSQLQRFLGSLNY

>Pper6-Chr7-01; Florendovirus; Prunus persica 6;

QKLKEEICSELPTAFWYRKQHVVCLPYIKTFSEKNIPTKARPIQMSQEMMEFCKKEIEELLQKKIIRKSKSPWSCPAFYVQKNAELERGVPRLVINYKPLNAVLEWIRYPIPNKRDLINRLEKAFVYSKFDLKSGFWQIQIDESDRYKIAFVTPFGHYEWNVMPFGLKNSPSEFQNIMNDIFNPYSQFSIVYIDDVLIFSESIEQHWKHLNKFLQVVKQNGLVVSAKKIKLFQTNIRFLGFNICQSQISPIDRVIQFADKFPDQILDKSQLQRFLRSLNY

>Pper6-Chr8-01; Florendovirus; Prunus persica 6;

QRLKEEICSELPTAFWYRKQHVVRLPYIKTFSEKNIPTKARPIQMSQEMMEFCKKEIEELLQKKIIRKSKSPWSCPAFYVQKNAELERGVPRLVINYKPLNAVLEWIRYPIPNKRDLINRLEKAVVYSKFDMKSGFWQIQIDESDRYKTAFVTPFGHYEWNVMPFGLKNAPSEFQNIMNDIFNPYSQFSIVYIDDVLIFSESIEQHWKHLNKFLQVVKRNGLVVSAKKIKLFQTNIRFLGFNICQSQISPIDRVIQFADKFPDQILDKSQLQRFLGSLNY

>Pper6-Chr8-02; Florendovirus; Prunus persica 6;

QRLKEEICSELPTAFWYRKQHVVRLPYIKTFSEKNIPTKARPIQMSQEMMEFCKKEIEELLQKKIIRKSKSPXSCPTFYVQKNAELERGVPRLVINYKPLNAVLEWIRYPIPNKRDLINRLEKAVVYSKFDMKSGFWQIQIDESDRYKTAFVTPFGHYEWNVMPFGLKNAPSEFQNIMNDIFNPYSQFSIVYIDDVLIFSESIEQHWKHLNKFLQVVKRNGLVVSAKKIKLFQTNIRFLGFNICQSQISPIDRVIQFADKFPDQILDKSQLQRFLGSLNY

>Pper6-Chr8-03; Florendovirus; Prunus persica 6;

QRLKEEICSELPTAFWYRKQHVVRLPYIKTFSEKNIPTKARPIQMSQEMMEFCKKEIEELLQKKIIRKSKSPWSCPAFYVQKNAELERGVPRLVINYKPLNAVLEWIRYPIPNKRDLINRLEKAVVYSKFDMKSGFWQIQIDESDRYKTAFVTPFGHYEWNVMPFGLKNAPSEFQNIMNDIFNPYSQFSIVYIDDVLIFSESIEQHWKHLNKFLQVVKRNGLVVSAKKIKLFQTNIRFLGFNICQSQISPIDRVIQFADKFPDQILDKSQLQRFLGSLNY

>Pper6-Chr8-04; Florendovirus; Prunus persica 6;

QRLKEEICSELPTAFWYRKQHVVRLPYIKTFSEKNIPTKARPIQMSQEMMEFCKKEIEELLQKKIIRKSKSPWSCPAFYVQKNAELERGVPRLVINYKPLNAVLEWIRYPIPNKRDLINRLEKAVVYSKFDMKSGFWQIQIDESDRYKTAFVTPFGHYEWNVMPFGLKNAPSEFQNIMNDIFNPYSQFSIVYIDDVLIFSESIEQHWKHLNKFLQVVKRNGLVVSAKKIKLFQTNIRFLGFNICQSQISPIDRVIQFADKFPDQILDKSQLQRFLGSLNY

>Pper6-Chr8-05; Florendovirus; Prunus persica 6;

QRLKEEICSELPTAFWYRKQHVVRLPYIKTFSEKNIPTKARPIQMSQEMMEFCKKEIEELLQKKIIRKSKSPWSCPAFYVQKNAELERGVPRLVINYKPLNAVLEWIRYPIPNKRDLINRLEKAVVYSKFDMKSGFWQIQIDESDRYKTAFVTPFGHYEWNVMPFGLKNAPSEFQNIMNDIFNPYSQFSIVYIDDVLIFSESIEQHWKHLNKFLQVVKRNGLVVSAKKIKLFQTNIRFLGFNICQSQISPIDRVIQFADKFPDQILDKSQLQRFLGSLNY

>Pper6-Chr8-06; Florendovirus; Prunus persica 6;

QRLKEEICSELPTAFWYRKQHVVRLPYIKTFSEKNIPTKARPIQMSQEMMEFCKKEIEELLQKKIIRKSKSPWSCPAFYVQKNAELERGVPRLVINYKPLNAVLEWIRYPIPNKRDLINRLEKAVVYSKFDMKSGFWQIQIDESDRYKTAFVTPFGHYEWNVMPFGLKNAPSEFQNIMNDIFNPYSQFSIVYIDDVLIFSESIEQHWKHLNKFLQVVKRNGLVVSAKKIKLFQTNIRFLGFNICQSQISPIDRVIQFADKFPDQILDKSQLQRFLGSLNY

>Pper6-Chr8-07; Florendovirus; Prunus persica 6;

QRLKEEICSELPTAFWYRKQHVVRLPYIKTFSEKNIPTKARPIQMSQEMMEFCKKEIEELLQKKIIRKSKSPWSCPAFYVQKNAELERGVPRLVINYKPLNAVLEWIRYPIPNKRDLINRLEKAVVYSKFDMKSGFWQIQIDESDRYKTAFVTPFGHYEWNVMPFGLKNAPSEFQNIMNDIFNPYSQFSIVYIDDVLIFSESIEQHWKHLNKFLQVVKRNGLVVSAKKIKLFQTNIRFLGFNICQSQISPIDRVIQFADKFPDQILDKSQLQRFLGSLNY

>Pper6-Chr8-08; Florendovirus; Prunus persica 6;

QRLKEEICSELPTAFWYRKQHVVRLPYIKTFSEKNIPTKARPIQMSQEMMEFCKKEIEELLQKKIIRKSKSPWSCPAFYVQKNAELERGVPRLVINYKPLNAVLEWIRYPIPNKRDLINRLEKAVVYSKFDMKSGFWQIQIDESDRYKTAFVTPFGHYEWNVMPFGLKNAPSEFQNIMNDIFNPYSQFSIVYIDDVLIFSESIEQHWKHLNKFLQVVKRNGLVVSAKKIKLFQTNIRFLGFNICQSQISPIDRVIQFADKFPDQILDKSQLQRFLGSLNY

>Pper6-Chr8-09; Florendovirus; Prunus persica 6;

QRLKEEICSELPTAFWYRKQHVVRLPYIKTFSEKNIPTKARPIQMSQEMMEFCKKEIEELLQKKIIRKSKSPWSCPAFYVQKNAELERGVPRLVINYKPLNAVLEWIRYPIPNKRDLINRLEKAVVYSKFDLKSGFWQIQIDESDRYKTAFVTPFGHYEWNVMPFGLKNAPSEFQNIMNDIFNPYSQFSIVYIDDVLIFSESIEQHWKHLHKFLQVVKQNGLVVSAKKIKLFQTNIRFLGFNICQSQISPIDRVIQFADKFPDQILDKSQLQRFLGSLNY

>Pper6-Chr8-10; Florendovirus; Prunus persica 6;

QRLKEEICSELPTAFWYRKQHVVRLPYIKTFSEKNIPTKARPIQMSQEMMEFCKKEIEELLQKKIIRKSKSPWSCPAFYVQKNAELERGVPRLVINYKPLNAVLEWIRYPIPNKRDLINRLEKAVVYSKFDMKSGFWQIQIDESDRYKTAFVTPFGHYEWNVMPFGLKNAPSEFQNIMNDIFNPYSQFSIVYIDDVLIFSESIEQHWKHLHKFLQVVKQNGLVVSAKKIKLFQTNIRFLGFNICQSQISPIDRVIQFADKFPDQILDKSQLQRFLGSLNY

>Pper6-Chr8-11; Florendovirus; Prunus persica 6;

QKIKNEICSELPNAFWYRKQHVVCLPYIKTFSEKNIPTKARPIQMSQEIMEFCKTEISELLQKKIIRKSKSSWSCPAFYVQKNAELERGVPRLVINYKSLNSVLEWIWYPIPNKRDLINRLGKAVVYSKFDMKSGFWQIQIEESDRYKTAFNTPFGHYEWNVMPFGLKNAPSEFQNIMNEIFNPYSHFSIVYIDDVLIFSESIEQYWKHLHKFFQIVKQNGLVVSAKKIKLFQTNIRFLGFNICQSQISPIDRVIQFADKFPDQILDKSQLQRFLGSLNY

>Pper6-Chr8-12; Florendovirus; Prunus persica 6;

QKIKNEICSELPNAFWYRKQHVVCLPYIKTFSEKNIPTKARPIQMSQEIMEFCKTEISELLQKKIIRKSKSPWSCPAFYVQKNAELERGVPRLVINYKSLNSVLEWIWYPIPNKRDLINRLGKAVVYSKFDMKSEFWQIQIEESDRYKTAFVTPFGHYEWNVMPFGLKNAPSEFQNIMNEIFNPYSHFSIVYIDDVLIFSESIEQHWKHLHKFFQIVKQNGLVVSAKKTKLFQTNIKFLGFNICQSQISPIDRVIQFANKFPDQILDKSQLMRVLGSLNY

>Pper7-Chr1-01; Florendovirus; Prunus persica 7;

EKLKQEVCSELPNAFWHRKQHVVSLPYVKTSSEKNIPTKARPIQISQELMKFCKIEITDLLKKGIIRKSRSPWSCPAFYVQKNAELERGVPRLVINYKPLNTVLEWIRYPIPNKRDIINRLEKSVIFSKFDMKSGFWQIQISETDRYKTTFVTPFGHYEWNVMPFDLKNAPSEFQNIINVIFNPFSHFSIVYIDDVLIFSASIEQHWKYLYKFLQIVKQNGLVVSAKKIKLFQTNVRFLGFNIXQSQISPIDRVIQFADKFPDQILEKGQLQRFLGSLNYVSDF

>Pper7-Chr1-02; Florendovirus; Prunus persica 7;

QRLKEEICSELPTAFWYRKQHVVRLPYIKTFSEKNIPTKARPIQMSQEMMEFCKKEIEELLQKKIIRKSKSPWSCPAFYVQKNAELERGVPRLVINYKPLNAVLEWIRYPIPNKRDLINRLEKAVVYSKFDMKSGFWQIQIDESDRYKTAFVTPFGHYEWNVMPFGLKNAPSEFQNIMNDIFNPYSQFSIVYIDDVLIFSESIEQHWKHLNKFLQVVKQNGLVVSAKKIKLFQTNIRFLGFNICQSQISPIDRVIQLADKFPDQILDKSQLQRFLGSLNY

>Pper7-Chr1-03; Florendovirus; Prunus persica 7;

EKLKQEVCSELPNAFWHRKQHVVSLPYVKSFSEKNIPTKARPIQMSQELMKFCKTEITELLEKGIIRKSKSLWSCPAFYVQKNVELEHGVPRLVINYKPLNTVLEWIRYPIPNKRDLINRLEKSVIFSKFDMKSGFWQIQIRESFSYKTAFVTPFGHYEWNVMPFGLKNAPSEFENIMNEIFNHFSHFSIVYIDDVLIFSESIEQHWKHLHKFLQIVKRNGLVVSAKKIKLFQTNVRFLGFNICQSQISPIDRVIQFADKFPDQILDKSQLHRFLGSLNYVSDF

>Pper7-Chr2-01; Florendovirus; Prunus persica 7;

QRLKEEICSELPTAFWYRKQHVVRLPYIKTFSEKNIPTKARPIQMSQEMMEFCKKEIEELLQKKIIRKSKSPWSCPAFYVQKNAELERGVPRLVINYKPLNAVLEWIRYPIPNKRDLINRLEKAVVYSKFDLKSGFWQIQIDESDRYKTAFVTPFGHYEWNVMPFGLKNAPSEFQNIMNDIFNPYSQFSIVYIDDVLIFSESIEQHWKHLNKFLQVVKQNGLVVSAKKIKLFQTNIRFLGFNICQSQISPIDRVIQFADKFPDQILDKSQLQRFLGSLNY

>Pper7-Chr2-02; Florendovirus; Prunus persica 7;

QRLKEEICSELPTAFWYRKQHVVRLPYIKTFSEKNIPTKARPIQMSQEMMEFCKKEIEELLQKKIIRKSKSPWSCPAFYVQKNAELERGVPRLVINYKPLNAVLEWIRYPIPNKRDLINRLEKAVVYSKFDLKSGFWQIQIDESDRYKTAFVTPFGHYEWNVMPFGLKNAPSEFQNIMNDIFNPYSQFSIVYIDDVLIFSESIEQHWKHLNKFLQVVKQNGLVVSAKKIKLFQTNIRFLGFNICQSQISPIDRVIQFADKFPDQILDKSQLQRFLGSLNY

>Pper7-Chr2-03; Florendovirus; Prunus persica 7;

EKLQREVCSELPNAFWHRKQHVVRLPYIKSFNEKNIPTKTRPIQMSQELMELCKTEITKLLEKRIIRKSKSPWSCPAFYVQKNVELERGVPRLVINYKPLNTVLEWIRYPIPNKRDLINRLEKSVIFSKFDMKSGFWQIQIHESDRYKTAFLNPFGHYEWNVMPFGLKNAPSEFQNIMNEIFNPYSHFSIVYIDDVLIFSQSIEQHWKHLHKFLQIVKQNGLVVSAKKIKLFQTNVRFLGFNICQSQISPIDRVIQFADKFSDQILDKSQLQRFLGFLNYV

>Pper7-Chr2-04; Florendovirus; Prunus persica 7;

EKLQREVCSELPNAFWHRKQHVVRLPYIKSFNEKNILTKARPIQMSQELMKLCKTEITELLEKRIICKSKSPWSCPAFYVQKNVELERGVPRLVINYKPLNTVLEWIRYPIPNKRDLINRLEKSVIFSKFDMKSGFWQIQIHESDRYKTAFVTPFGHYEWNVMPFGLKNAPSEFQNIMNEIFNPYSHFSIVYIDDVLIFSQSIEQHWKHLHKFLQIVKQNGLVVSAKKIKLFQTNVRFLGFNICQSXISPIDRVIQFADKFPDQILDKSQLQRFLGFLNYVSDF

>Pper7-Chr2-05; Yendovirus; Prunus persica 7;

HWNKNKILAKLDIINPYSTIKIAYMPCNLLDKQNFXQQIKELLNIVLRPSKSRHRSTAFVIRKHSKVKRGKARIVYNYKRXNDNTYEDSYKLPNKDELMNKIEESKYSSKLYCKSRFWQIRLAEXSIQXTTFTRTKAHFEWLVMPFGLMNAPSIFQRKMNQIFEKYDNFGSIYVDDKLVCSKNKNEHRKHXENILKEFIDNGIVISRNKVELEKXGIXFXGMYIKSETIXLQEHIGKKVLXFPNKXEDKKQLQAFLGLLNYARSFI

>Pper7-Chr2-06; Florendovirus; Prunus persica 7;

EKLKQEVCSELPNAFWHRKQHVVSLLYVKSFSEKNIPTKARPIQMSQELMEFCKTEITDLLKKGIIRKSKSPWSCPTFYVQKNTELERGVSRLVINYKPLNTVLEWIRYPIPNKRDLINRLEKSVIFSKFDMKSGFWQIQISESDRYKTAFVTPFGHYEWNVMPFGLKNAPSKFQNIMNEIFNPYSHFSIVYIDDVLIFSESIEQHWKHLYKFLQIVKQNGLVVSAKKIKLFQTNVRFLGFNIYQSQVSPIDQVSPMNQLQRFLGSLNYV

>Pper7-Chr2-07; Florendovirus; Prunus persica 7;

QRLKEEICSELPTAFWYRKQHVVRLPYIKTFSEKNIPTKARPIQMSQEMMEFCKKEIEELLQKKIIRKSKSPXSCPAFYVQKNAELERGVPRLVINYKPLNAILEWIRYPIPNKRDLINRLEKAVVYSKFDMKSGFWQIQIDESDRYKTAFVTPFGHYEWNVMPFGLKNAPSEFQNIMNDIFNPYSQFSIVYIDDVLIFSESIEQHWKHLNKFLQVVKRNGLVVSAKKIKLFQTNIRFLGFNICQSQISPIDRVIQFADKFPDQILDKSQLQRFLGSLNY

>Pper7-Chr3-01; Florendovirus; Prunus persica 7;

EKLKQEVCSELPNAFWHRKQHVICLPYVKTFSEKNIPTKARPIQMSQELMEFCKIEITDLLKKGIIRKSKSPWSCPAFYVQKNAELERGVPRLVINYKPLNTVLEWIRYPIPNKRDLINRLEKSVIFSKFDMKSGFWQIQISEADRYKTAFVTHFGHYEWNVMPFGLKNAPSEFQNIMNETFNHFSHFSIVYIDDVLIFSESIEQHWKHLYKFLQIVKQNGLVISAKKIKLLQTNVRFLGFNICQSXISPIDRVIQFVDKFLDQILD

>Pper7-Chr3-02; Florendovirus; Prunus persica 7;

EKLKQEVCSDLPNAFWHRKQHVVSLPYVKNFSEKNILTKARPIQMSQEIMEFYKTEITELLQKGIIRKSKSPWSCPAFYVXKNAELERGAPRLVINYKPLNTTLEWIRYPIPNKRDLINRLDKSVIFSKFDMKIGFWXIQILESDRYKTAFVTPFGHYEWNVMPFGLKNAHSEFQNIMNEIFNPFSHFSIVYIDDALIFSQSIEQHWKHLHKFLKIVKQNGLVVLAKKIKLFQTNICFLGFNICQSQISLIDRVIQFADKFSYQILDKSQLQRFLGSLNY

>Pper7-Chr3-03; Florendovirus; Prunus persica 7;

EKLKQEICSDLPTAFWHRKQHVVSLPYVKTFNEKHIPTKSRPIQMSREVMEFCKKEIAELLYKGIIRKSKSPWSCPAFYVQKNAELERGAPRLVINFKPLNTVLEWIRYPIPNKRDLINRLSSAVIFSKFHMKSGFWHIQIQEHDRYKTAFVTPFGHYEWNVMHFGLKNAPSEFQNIMNEIFNPFSEFSIVYIDDILIFSSSIEQHWKHLHKFLQIVKKNGLVLSAKKIKLFQTNIKFLGFNIHQS

>Pper7-Chr3-04; Florendovirus; Prunus persica 7;

QRLKEEICSELPTAFWYRKQHVVRLPYIKTFSEKNIPTKARSIQMSQEMMEFCKKEIDELLQKKIIRKSKSPWSCPAFYVQKNAELERGVPRLVINYKPLNAVLEWIRYPIPNKRDLINRLEKAVVYSKFDMKSGFGQIQIDESDRYKTAFVTPFGHYEWNVMPFGLKNAPSEFQNIMNDIFNPYSQFSIVYIDDVLIFSESIEQHWKHLNKFLQVVKQNGLVVSVKKIKLFQTNIRFLGFNICQSQISPIDRVIQFADKFPDQILDKSQLQRFLGSLNY

>Pper7-Chr3-05; Florendovirus; Prunus persica 7;

KLKNEICSELPNAFWYRKQHVVCLPYIKTFNEKNIPTKARPIQMSQEIMEFCKKXISELLQKKIIRKSKSPWSCPTFYVQKNAELERGVPRLVINYKPLNAVLEWIRYPIPNKRDLINRLEKAVVFFPNFTXRVVSGKSKXKSQIDIRLHLLLLLVITNGMLCHLDXKNAPSEFQNIMNDIFMPCSHFSIVYIDDVLIFFESIEHHWKHLHKFVQIIKQNGLVVPPRKSNYSQTNIRFIGFNICXSKISPIDRVIQFADKFPDQILDKSQLQRFLGSLNY

>Pper7-Chr3-06; Yendovirus; Prunus persica 7;

LEELLQLAEQTRIVGEDPQLHWNKNKILAKLDIINPDLTIKTTDMPCNLLDKQEFEHRIKELLNLKVIRPSKSRHRSVAFVVRKHSELKRGKARIVYNYRRLNDNTYEDSYKLPNKDELMNKIQESKYFSKFDCKSGFWQTRLAEESIQWTTFTCTEGHFEWLVMPFGLKNAPSIFQRKMDQSFKKYDNFCSVYVDDILVHSKNKNEHSKHLEIILKEFIDNGVVISRNKIEFEKQDIEFLGMYIKSGTIQLQEHIAKKVLKFLDRLEDKKQLQAFLGLLNYVSSFIPD

>Pper7-Chr4-01; Florendovirus; Prunus persica 7;

QRLKEEICSELPTAFWYRKQHVVRLPYIKTFSEKNIPTKARPIQMSQEMMEFCKKEIEELLQKKIIRKSKSPWSCPAFYVQKNAELERGVPRLVINYKPLNAVLEWIRYPIPNKRDLINRLEKAVVYSKFDMKSGFWQIQIDESDRYKTAFVTPFGHYEWNVMPFGLKNAPSEFQNIMNDIFNPYSQFSIVYIDDVLIFSESIEQHWKHLNKFLQVVKQNGLVVSAKKIKLFQTNIRFLGFNICQSQISPIDRVIQFADKFPDQILDKSQLQRFLGSLNY

>Pper7-Chr4-02; Yendovirus; Prunus persica 7;

LEELLQLAEQTKIIGEDPQLHWSKNKILTKLDIINQDLTIKTADMPCNLLDKQEFEQXIKELLNLKVIRPSKSRHRSAAFVVRKHSELKRARIVYNYRRLNDNTYEDSYKLPNKDELMNKIQESKYFSKFDCKSGFWQIRLAEESIQWTTFTCLEGHFEWLVMPFGLKNAPLIFQRKMDQIFKKYDNLCSVYVDDILVHSKNKNEHRKHLEIILKEFTDNGIVISRNKVELQKQDIEFLGMYIKSGTIQLQEHVAKKVLEFPDKLEDKKQLQAFLDLLNYARNFFPD

>Pper7-Chr5-01; Florendovirus; Prunus persica 7;

QRLKEEICSELPTAFWYRKQHVVRLPYIKTFSEKNIPTKARPIQMSQEMMEFCKKEIEELLQKKIIRKSKSPWSCPAFYVQKNAELERGVPRLVINYKPLNAVLEWIRYPIPNKRDLINRLEKAVVYSKFDMKSGFWQIQIDKSDRYKTAFVTPFGHYEWNVMPFGLKNAPSEFQNIMNDIFNPYSQFSIVYIDDVLIFSESIEQHWKHLNKFLQVVKQNGLVVSAKKIKLFQTNIRFLGFNICQSQISPIDRVIQFADKFPDQILDKSQLQRFLGSLNY

>Pper7-Chr5-01; Yendovirus; Prunus persica 7;

LEELLQLAKQTRIIGEDPQLHWNKNKILAKLDIINLDLTIKTADMPCNLLDKQEFYQQIKQILNLKVIRPSKSRHRSTAFVVRKHLELKRGKVRIVYNYRRLNDNTYEDSYKLPNKDELMNKIQESKYFSKFDCKSGFXQIRLAEESIXLTTFTCPEGNFEWLVMPFGLKNAPSIFQRKMDQIFKKYDNFCSVYVEDILVHSKNKNKHRKYLEIILKEFIDNGIVISRNKVELEKQDIEFLGIYIKSGTIQLQEHIAKKVLEFPDKLENKKQLQEFLGLLNYARSFIPD

>Pper7-Chr5-02; Florendovirus; Prunus persica 7;

QKLKEEICSELPTAFWYRKQHVVCLPYIKTFSEKNIPTKARPIQMSQEMMEFCKKEIEELLQKKIIRKSKSPWSCPAFYVQKNAELERGVPRLVINYKPLNAVLEWIRYPIPNKRDLINRLEKAVVYSKFDMKSGFWQIQIDESDRYKTVFVTPFGHYEWNVMPFGLKNAPSEFQNIMNDIFNPYSQFSIVYIDDVLIFSESIEQHWKHLYKFLQIVKQNGVVVSAKKIKLFQTNIRFLGFNICQSQISPIDRVIQFADKFPDQILDKSQLQRFLGSLNY

>Pper7-Chr5-03; Florendovirus; Prunus persica 7;

QKIKEEICSELPTAFWYRKQHVVRLPYIKTFSEKNIPTKARPIQMSQEMMEFCKTEINELLQKKIIRKSKSPWSCPAFYVQKNAELERGVPRLVINYKPLNAVLEWIRYLIPNKRDLINRLEKAIVYSKFDMKSGFWQIQIDESDRYKTAFVTPFGHYEWNVMPFGLKNAPSEFQNIMNDIFNPYSQFSIVYIDDVLIFSESIEQHWKHLYKFLQVVKQNGLVVSAKKIKLFQTNVRFLGFNICQSQISPIDRVIQFADRFPDQILDKSQLQRFLGSLNY

>Pper7-Chr5-04; Florendovirus; Prunus persica 7;

QKIKSEICSELPNAFWYRKQHVVCLPYMKTFSEKNIPTKARPIQMSQEMMEFCKQEIKELLQKKIIRQSKSPWSCPAFYVQKNAELERGVPRLVINYKPLNAVLEWIRYPIPNKRDLINRLEKAVVYSKFDMKSGFWQIQIEESDRYKTAFVTPFGHYEWNVMPFGLKNAPSEFQNIMNDIFNPYSQFSIVYIDDVLIFSESIEQHWKHLYKFLQVVKQNGLVVSAKKIKLFQTNVRFLGFNICQSQISPIDRVIQFADKFPDQILDKSQLQRFLGSLNY

>Pper7-Chr6-01; Florendovirus; Prunus persica 7;

QRLKEEICSELPTAFWYRKQHVVRLPYIKTFSENNIPTKARPIQMSQEMMEFCKKEIEELLQKKIIRKSKSPWSCPAFYVQKNAELERGVPRLVINFKPLNAVLEWIRYPIPNKRDLINRLEKAVVYSKFDLKSGFWQIQIDESDRYKTAFVTPFGHYEWNVMPFGLKNAPSEFQNIMNDIFNPYSQFSIVYIDDVLIFSESIEQHWKHLNKFLQVVKQNGLVVSAKKIKLFQTNIRFLGFNICQSQISPIDRVIQFADKFPDQILDKSQLQRFLGSLNY

>Pper7-Chr6-02; Florendovirus; Prunus persica 7;

QRLKEEICSELPTAFWYRKQHVVRLPYIKTFSENNIPTKARPIQMSQEMMEFCKKEIEELLQKKIIRKSKSPWSCPAFYVQKNAELERGVPRLVINYKPLNAVLEWIRYPIPNKRDLINRLEKAVVYSKFDLKSGFWQIQIDESDRYKTAFVTPFGHYEWNVMPFGLKNAPSEFQNIMNDIFNPYSQFSIVYIDDVLIFSESIEQHWKHLNKFLQVVKQNGLVVSAKKIKLFQTNIRFLGFNICQSQISPIDRVIQFADKFPDQILDKSQLQRFLGSLNY

>Pper7-Chr6-03; Florendovirus; Prunus persica 7;

EKLKQEVCSELPNAFWHRKQHVVSLPCVKSFSEKNIPTKARPIQMSQELMEFCKIEITDLLKKGIIRKSKSPWSCPDFYVQKNAELECGVPRLVINYKPLNTVLEWIRYPIPNKRDLINRLENSVIFSKFDMKSGFWQIQISESDRYKTAFVTPFGHYEWNVMPFGLKNAPSEFQNIMNEIFNPFSHFSIVYIDDVLIFSESIEQHXKHLHKFLQIVKQNGLVVSAKKIKLFQTNVRFLRFNIYQSQISPIDRVIXIADKFPDQILDKSQLQRFLGSLN

>Pper7-Chr6-04; Florendovirus; Prunus persica 7;

QRLKEEICSELPTAFWYRKQHVVRLPYIKTFSEKNIPTKARPIQMSQEMMEFCKKEIEELLQKKIIRKSKSPWSCPAFYVQKNAELERGVPRLVINYKPLNAVLEWIRYPIPNKRDLINRLEKAVVYSKFDMKSGFWQIQIDESDRYKTAFVTPFGHYEWNVMPFGLKNAPSEFQNIMNDIFNPYSQFSIVYIDDVLIFSESLEQHWKHLNKFLQVVKQNGLVVSAKKIKLFQTNIRFLGFNICQSQISPIDRVIQFADKFPDQILDKSQLQRFLGSLNY

>Pper7-Chr6-05; Florendovirus; Prunus persica 7;

QRLKEEICSELPTAFWYRKQHVVCLPYIKSFSEKNIPTKARPIQMSQEMMEFCKKEIEELLQKKIIRKSKSPWSCPAFYVQKNAELERGVPRLVINYKPLNAVLEWIRYPIPNKRDLINRLEKAVVYSKFDMKSGFWQIQIDESDRYKTAFVTPFGHYEWNVMPFGLKNAPSEFQNIMNDIFNPYSQFLIVYIDDVLIFSESIEQHWKHLNKFLQVVKQNGLVVSAKKIKLFQTNIRFLGFNICQSQISPIDRVIQFVDKFPDQILDKSQLQRFLGSLNY

>Pper7-Chr6-06; Florendovirus; Prunus persica 7;

QRLKEEICSELPTAFWYRKQHVVRLPYIKTFSEKNIPTKARPIQMSQEMMEFCKKEIEELLQKKIIRKSKSPWSCPAFYVQKNAELERGVPRLVINYKPLNAVLEWIRYPIPNKRDLINRLEKAVVYSKFDMKSGFWQIQIDESDRYKTAFVTPFGHYEWNVMPFGLKNAPSEFQNIMNDIFNPYSQFSIVYIDDVLIFSESIEQHWKHLNKFLQVVKQNGLVVSAKKIKLFQTDIRFLGFNICQSQISPIDRVIQFADKFPDQILDKSQLQRFLGSLNY

>Pper7-Chr7-01; Florendovirus; Prunus persica 7;

QKLKEEVCSDLPTAFWYRKQHVVCLPYIKNFSEKNIPIKARPIQMSQEMMEFCKTEIHEQLQKKIIRKSRSPWSCPAFYVQKNAELERGVPRLVINYKPLNAVLEWIRYPIPNKRDLINRLEKAVVYSKFDMKSDFWQIQIDESDRYKTAFVTPFGHYEWNVMPFGLKNAPSEFQNIMNDIFNPYNQFTIVYIDDVLIFSESIEQHWKHLNKFLQVVIKNGLVVSAKKIKLFQTNIRFLGFNICQSQISPIDRVIQFADKFPDQILDKSQLQRFLGSLNY

>Pper7-Chr7-02; Florendovirus; Prunus persica 7;

QRLKEEICSELPTAFWYRKQHVVRLPYIKTFSEKNIPTKARPIQMSQEMMEFCKKEIEELLQKKIIRKSKSPWSCPAFYVQKNAELERGVPRLVINYKPLNAVLEWIRYPIPNKRDLINRLEKAVVYSKFDMKSGFWQIQIDESDRYKTAFVTPFGHYEWNVMPFGLKNAPSEFQNIMNDIFNPYSQFSIVYIDDVLIFSESIEQHWKHLNKFLQVVKQNGLVVSAKKVKLFQTNIRFLGFNICQSQISPIDRVIQFADKFPDQILDKSQLQRFLGSLNY

>Pper7-Chr7-03; Florendovirus; Prunus persica 7;

QRLKEEICSELPTAFWYRKQHVVRLPYIKTFSEKNIPTKARPIQMSQEMMEFCKKEIEELLQKKIIRKSKSPWSCPAFYVQKNAELERGVPRLVINYKPLNAVLEWIRYPIPNKRDLINRLEKAVVYSKFDMKSGFWQIQIDESDRYKTAFVTPFGHYEWNVMPFGLKNAPSEFQNIMNDIFNPYSQFSIVYIDDVLIFSESIEQHWKHLNKFLQVVKQNGLVVSAKKVKLFQTNIRFLGFNICQSQI

>Pper7-Chr7-04; Florendovirus; Prunus persica 7;

QKLKEEICSELPTAFWYRKQHVVRLPYIKSFSEKNIPTKARPIQMSQEMMEFCKKEIEELLQKKIIRKSKSPWSCPAFYVQKNAELERGVPRLVINYKPLNAVLEWIRYPIPNKRDLINRLEKAVVYSKFDLKSGFWQIQIDESDRYKTAFVTPFGHYEWNVMPFGLKNAPSEFQNIMNDIFNPYSQFSIVYIDDVLIFSESIKQHWKHLHKFLQVFKQNGLVVSAKKIKLFQTNIRFLGFNICQSQISPIDRVIQFADKFLDQILDKSQLQRFLGSLNY

>Pper7-Chr7-05; Florendovirus; Prunus persica 7;

QKLKEEICSELPTAFWYRKQHVVCLPYIKTFSEKNIPTKARPIQMSQEMMEFCKKEIEELLQKKIIRKSKSPWSCPAFYVQKNAELERGVPRLVINYKPLNAVLEWIRYPIPNKRDLINRLEKAFVYSKFDLKSGFWQIQIDESDRYKIAFVTPFGHYEWNVMPFGLKNSPSEFQNIMNDIFNPYSQFSIVYIDDVLIFSESIEQHWKHLNKFLQVVKQNGLVVSAKKIKLFQTNIRFLGFNICQSQISPIDRVIQFADKFPDQILDKSQLQRFLRSLNY

>Pper7-Chr8-01; Florendovirus; Prunus persica 7;

QRLKEEICSELPTAFWYRKQHVVRLPYIKTFSEKNIPTKARPIQMSQEMMEFCKKEIEELLQKKIIRKSKSPWSCPAFYVQKNAELERGVPRLVINYKPLNAVLEWIRYPIPNKRDLINRLEKAVVYSKFDMKSGFWQIQIDESDRYKTAFVTPFGHYEWNVMPFGLKNAPSEFQNIMNDIFNPYSQFSIVYIDDVLIFSESIEQHWKHLYKFLQVVKQNGLVVSAKKIKLFQTNIRFLGFNICQSQISPIDRVIQFADKFPDQILDKSQLQRFLGSLNY

>Pper7-Chr8-02; Florendovirus; Prunus persica 7;

QRLKEEICSELPTAFWYRKQHVVRLPYIKTFSEKNIPTKARPIQMSQEMMEFCKKEIEELLQKKIIRKSKSPWSCPAFYVQKNAELERGVPRLVINYKPLNAVLEWIRYPIPNKRDLINRLEKAVVYSKFDMKSGFWQIQIDESDRYKTAFVTPFGHYEWNVMPFGLKNAPSEFQNIMNDIFNPYSQFSIVYIDDVLIFSESIEQHWKHLNKFLQVVKRNGLVVSAKKIKLFQTNIRFLGFNICQSQISPIDRVIQFADKFPDQILDKSQLQRFLGSLNY

>Pper7-Chr8-03; Florendovirus; Prunus persica 7;

QRLKEEICSELPTAFWYRKQHVVRLPYIKTFSEKNIPTKARPIQMSQEMMEFCKKEIEELLQKKIIRKSKSPXSCPTFYVQKNAELERGVPRLVINYKPLNAVLEWIRYPIPNKRDLINRLEKAVVYSKFDMKSGFWQIQIDESDRYKTAFVTPFGHYEWNVMPFGLKNAPSEFQNIMNDIFNPYSQFSIVYIDDVLIFSESIEQHWKHLNKFLQVVKRNGLVVSAKKIKLFQTNIRFLGFNICQSQISPIDRVIQFADKFPDQILDKSQLQRFLGSLNY

>Pper7-Chr8-04; Florendovirus; Prunus persica 7;

QRLKEEICSELPTAFWYRKQHVVRLPYIKTFSEKNIPTKARPIQMSQEMMEFCKKEIEELLQKKIIRKSKSPWSCPAFYVQKNAELERGVPRLVINYKPLNAVLEWIRYPIPNKRDLINRLEKAVVYSKFDMKSGFWQIQIDESDRYKTAFVTPFGHYEWNVMPFGLKNAPSEFQNIMNDIFNPYSQFSIVYIDDVLIFSESIEQHWKHLNKFLQVVKRNGLVVSAKKIKLFQTNIRFLGFNICQSQISPIDRVIQFADKFPDQILDKSQLQRFLGSLNY

>Pper7-Chr8-05; Florendovirus; Prunus persica 7;

QRLKEEICSELPTAFWYRKQHVVRLPYIKTFSEKNIPTKARPIQMSQEMMEFCKKEIEELLQKKIIRKSKSPWSCPAFYVQKNAELERGVPRLVINYKPLNAVLEWIRYPIPNKRDLINRLEKAVVYSKFDMKSGFWQIQIDESDRYKTAFVTPFGHYEWNVMPFGLKNAPSEFQNIMNDIFNPYSQFSIVYIDDVLIFSESIEQHWKHLNKFLQVVKRNGLVVSAKKIKLFQTNIRFLGFNICQSQISPIDRVIQFADKFPDQILDKSQLQRFLGSLNY

>Pper7-Chr8-06; Florendovirus; Prunus persica 7;

QRLKEEICSELPTAFWYRKQHVVRLPYIKTFSEKNIPTKARPIQMSQEMMEFCKKEIEELLQKKIIRKSKSPWSCPAFYVQKNAELERGVPRLVINYKPLNAVLEWIRYPIPNKRDLINRLEKAVVYSKFDMKSGFWQIQIDESDRYKTAFVTPFGHYEWNVMPFGLKNAPSEFQNIMNDIFNPYSQFSIVYIDDVLIFSESIEQHLKHLNKFLQVVKRNGLVVSAKKIKLFQTNIRFLGFNICQSQISPIDRVIQFADKFPDQILDKSQLQRFLGSLNY

>Pper7-Chr8-07; Florendovirus; Prunus persica 7;

QRLKEEICSELPTAFWYRKQHVVRLPYIKTFSEKNIPTKARPIQMSQEMMEFCKKEIEELLQKKIIRKSKSPWSCPAFYVQKNAELERGVPRLVINYKPLNAVLEWIRYPIPNKRDLINRLEKAVVYSKFDMKSGFWQIQIDESDRYKTAFVTPFGHYEWNVMPFGLKNAPSEFQNIMNDIFNPYSQFSIVYIDDVLIFSESIEQHWKHLNKFLQVVKRNGLVVSAKKIKLFQTNIRFLGFNICQSQISPIDRVIQFADKFPDQILDKSQLQRFLGSLNY

>Pper7-Chr8-08; Florendovirus; Prunus persica 7;

QRLKEEICSELPTAFWYRKQHVVRLPYIKTFSEKNIPTKARPIQMSQEMMEFCKKEIEELLQKKIIRKSKSPWSCPAFYVQKNAELERGVPRLVINYKPLNAVLEWIRYPIPNKRDLINRLEKAVVYSKFDLKSGFWQIQIDESDRYKTAFVTPFGHYEWNVMPFGLKNAPSEFQNIMNDIFNPYSQFSIVYIDDVLIFSESIEQHWKHLHKFLQVVKQNGLVVSAKKIKLFQTNIRFLGFNICQSQISPIDRVIQFADKFPDQILDKSQLQRFLGSLNY

>Pper7-Chr8-09; Florendovirus; Prunus persica 7;

QRLKEEICSELPTAFWYRKQHVVRLPYIKTFSEKNIPTKARPIQMSQEMMEFCKKEIEELLQKKIIRKSKSPWSCPAFYVQKNAELERGVPRLVINYKPLNAVLEWIRYPIPNKRDLINRLEKAVVYSKFDLKSGFWQIQIDESDRYKTAFVTPFGHYEWNVMPFGLKNAPSEFQNIMNDIFNPYSQFSIVYIDDVLIFSESIEQHWKHLHKFLQVVKQNGLVVSAKKIKLFQTNIRFLGFNICQSQISPIDRVIQFADKFPDQILDKSQLQRFLGSLNY

>Pper7-Chr8-10; Florendovirus; Prunus persica 7;

QKIKNEICSELPNAFWYRKQHVVCLPYIKTFSEKNIPTKARPIQMSQEIMEFCKTEISELLQKKIIRKSKSSWSCPAFYVQKNAELERGVPRLVINYKSLNSVLEWIWYPIPNKRDLINRLGKAVVYSKFDMKSGFWQIQIEESDRYKTAFNTPFGHYEWNVMPFGLKNAPSEFQNIMNEIFNPYSHFSIVYIDDVLIFSESIEQYWKHLHKFFQIVKQNGLVVSAKKIKLFQTNIRFLGFNICQSQISPIDRVIQFADKFPDQILDKSQLQRFLGSLNY

>Pper7-Chr8-11; Florendovirus; Prunus persica 7;

QKIKNEICSELPNAFWYRKQHVVCLPYIKTFSEKNIPTKARPIQMSQEIMEFCKTEISELLQKKIIRKSKSPWSCPAFYVQKNAELERGVPRLVINYKSLNSVLEWIWYPIPNKRDLINRLGKAVVYSKFDMKSEFWQIQIEESDRYKTAFVTPFGHYEWNVMPFGLKNAPSEFQNIMNEIFNPYSHFSIVYIDDVLIFSESIEQHWKHLHKFFQIVKQNGLVVSAKKTKLFQTNIKFLGFNICQSQISPIDRVIQFANKFPDQILDKSQLMRVLGSLNY

>Ppse1-Chr4-01; Yendovirus; Prunus pseudocerasus 1;

LEELLQLAEQTRIIGEDPQLHWNKNKILAKLDIINPDLTIKTADMPCNLLDKQEFEQQIKELLNLKVIRPSKSRHRSATFVVRKHSELKRGKARIVYNYRRLNDNTYEDSYKLPNKDELMNKIQESKYFSKFDCKSGFWQIRLAEESIQWTAFTCPEGHFEWLVMPFGLKNAPXIFQRKMDQIFKKYDNFCSVYVDDILVHSKNKNEHRKHLEIILKEFIDNGIVISRNKVELEKQDIEFLGMYIKSGTIQLQEHITKKVLEFPDKLEDKKQLQAFLGLLNYARSFIPD

>Ppse1-Chr6-01; Yendovirus; Prunus pseudocerasus 1;

IIGEDPQLHWNKNKILANLDIINPDLTIKTTYMPYNLLDKQEFEQQIQELLNLKVIRPSKSRHISATFVVRKHLGLKKGKARIVYNYRRLNDNTXENSYKLPNKDELMNKIQESKYFSKFDCKSGFWQIRLAEESIQWTTFTCPEGHFEXLVTPFGLKNAPSIFKRKMDQIFKKYDNFCSVYVDDILVQSKKKKNIENTXKLFXKNLLIMVYSXVAIKXSLKNKTXFLVMYIKSXAIQLQEHIVKKVLEFSDKLEDKKQLKAFLGLLNYARSFIPD

>Ppse1-Chr8-01; Yendovirus; Prunus pseudocerasus 1;

LEELLQFAEQTRIIGEDPQLHWNKNNILAKLYIINPDLTIKTADMPWNLLDKQEFEQQIKELLNLKVIGPSKSRHRSTTFVVRKHSELKRGKARIVYNYRRLNDNTYEDRYKLPNKDELMNKIQESKYFSKFNCKSGFWKIGLVEEXIKWTTFTCLEGHFEXLVKPFGLKNAPSIFQRKMDQIFKKYDNFCSVYVDDILVHSKNKNEHRKHLEIILKEFIDIGIVISRNKVELEKQDIEFLGMYIKSGTIQLQEHIAKKVLEFPDKLEDKKXLQAFLGLLNYARSFIPD

>Psal1-Chr1-01; Yendovirus; Prunus salicina 1;

LEELLQLAEQTRIIGEDPQLHWNKNKILAKLDIINPDLTIKTADMPCNLLDKQEFEQQIKELLNLKVIRPSKSRHRSAAFVVRKHSELKRGKARIVYNYRRLNDNTYEDSYKLPNKDELMNKIQESKYFSKFDCKSGFWQIRLAEESIHWTAFTCPEGHFEWLVMPFGLKNAPSIFQRKMDQIFKKYDTFCSVYVDDILVHSKNKNEHRKHLEIILKEFIDNGIVISRNKVELEKQDIEFLGMYIKSGTIQLQEHITKKVLEFPDKLEDKKQLQAFLGLLNYARNFIPD

>Psal1-Chr1-02; Florendovirus; Prunus salicina 1;

KKLISEVCSDLPTAFWHRKNHVVCLPYIKNFSEKNIPTKARPIQMNHEMMEFCKSEIDDLLQKNIIRKSKSPWSCPAFYVQKNAELERGSPRLVINYKPLNAVLEWIRYPIPNKRDLIKRLDKAVIFSKFDMKSGFWQIQIDESDRYKTAFVTPFGHYEWNVMPFGLKNAPSEFQNIMNEIFNAYSHFSIVYIDDVLIFSESLEQHWKHLHKFLHIIKSNGLVVSAKKIKLFQPNIRFLGYIISQSQIQPIDRVIHFADKFPDQITDKNQLQRFLGSLNYVSDF

>Psal1-Chr1-03; Yendovirus; Prunus salicina 1;

LEELLQLAEQTRIIGEDPQLHWNKNKILAKLDIINPDLTIKTADMPCNLLDKQEFEQQIKELLNLKVIRPSKSRHRSAAFVVRKHSELKRGKARIVYNYRRLNDNTYEDSYKLPNKDELMNKIQESKYFSKFDCKSGFWQIRLAEESIHWTAFTCPEGHFEWLVMPFGLKNAPSIFQRKMDQIFKKYDNFYSVYVDDILVHSKNKNEHRKHLEIILKEFIDNGIVISRNKVELEKQDIEFLGMYIKSGTIQLQEHITKKVLEFPDKLEDKKQLQAFLGLLNYARNFIPD

>Psal1-Chr1-04; Florendovirus; Prunus salicina 1;

KKLISEVCSDLPTAFWHRKNHVVCLPYIKNFSEKNIPTKARPIQMSHEMMEFCKTEINDLLQKNIIRKSKSPWSCPAFYVQKNAELERGSPRLVINYKPLNVVLEWIRYPIPNKRDLIKRLDKAVIFSKFDMKSGFWQIQIDESDRYKTAFVTPFGHYEWNVMPFGLKNAPSEFQNIMNEIFNAYSHFSIVYIDDVLIFSESLDQHWKHLHTFLHIIKSNGLVVSAKKIKLFQTNNRFLGYNISQSQIQPIDRVIHFADKFPDQITDKNQLQRFLGSLNYVSDF

>Psal1-Chr1-05; Yendovirus; Prunus salicina 1;

LEELLQLDEQTRIIGEDPQLHWNKNKILAKLDIINPDLTIKTADMPCNLLDKQEFEQQIKELLNLKVIRPSKSRHRSAAFVVRKHSELKRGKARIVYNYRRLNDNTYEDSYKLPNKDELMNKIQESKYFSKFDCKSGFWQIRLAEESIQWTAFTCPEGHFEWLVMPFGLKNAPSIFQRKMDQIFKKYDNFCSVYVDDILVHSKNKNEHRKHLEIILKEFIDNGIVISRNKVELEKQDIEFLGMYIKSGTIQLQEHITKKVLEFPDKLEDKKQLQAFLGLLNYARNFIPD

>Psal1-Chr1-06; Yendovirus; Prunus salicina 1;

IIGEDPQLHWNKNKILAKLDIINQNLTTKTADMSRNLLDKQEFEQQIKELLNLKVIRPSKSRHRSAAFVVRKHXELKRGKARIVYNYRRLNDNTYEDSYKLPNKDELMNKIQESKYFSKFDCKSGFWQIRLAEESIQWTTFTCLEGHFEWLVMPFGLKNAPSIFQRKMDQIFKKYDNFCSVXVDDILVHSKNKNEHRKHLEIILKEFIDNGIVISRNKVELEKQDIEFLGMYIKLGNIQLQEHMAKKVLEFPDKLEDKKQLQAFLGLLNYARNFIP

>Psal1-Chr1-07; Yendovirus; Prunus salicina 1;

IIGEDPQLHWNKNKILAKLDIINQNLTTKTADMSRNLLDKQEFEQQIKELLNLKVIRPSKSRHRSAAFVVRKHXELKRGKARIVYNYRRLNDNTYEDSYKLPNKDELMNKIQESKYFSKFDCKSGFWQIRLAEESIQWTTFTCLEGHFEWLVMPFGLKNAPSIFQRKMDQIFKKYDNFCSVXVDDILVHSKNKNEHRKHLEIILKEFIDNGIVISRNKVELEKQDIEFLGMYIKLGNIQLQEHMAKKVLEFPDKLEDKKQLQAFLGLLNYARNFIP

>Psal1-Chr1-08; Yendovirus; Prunus salicina 1;

LEELLQLAEQTRIIGEDPQLHWNKNKILAKLDIINPDLTIKTADMPCNLLDKQEFEQQIKELLNLKVIRPSKSRHRSAAFVVRKHSELKRGKARIVYNYRRLNDNTYEDSYKLPNKDELMNKIQESKYFSKFDCKSGFWQIRLAEESIHWTAFTCPEGHFEWLVMPFGLKNAPSIFQRKMDQIFKKYDNFCSVYVDDILVHSKNKNEHRKHLEIILKEFIDNGIVISRNKVELEKQDIEFLGMYIKSGTIQLQEHITKKVLEFPDKLEDKKQLQAFLGLLNYARSFIPD

>Psal1-Chr1-10; Yendovirus; Prunus salicina 1;

IIGEDPRLHWNKNKILETLDIINPDLTIKTTYMLCNLLDKXEFEQQIKELLNLKVIRPSKSRHRSSTPFVVRKHSELKIGKARIVYNYRRLNDNTYEDSYKLPNKDELLNKIQESKYFSKFDCKSGFWQIKLAEELIQWTTFTCLEXYFKWLVMSFALKICSIDLSKKDGSDFXEVXXILXCICRXCTCYDHFCSVYANDILVHRNEHRKHLEIILQEFIDNGIVISRNKVELEKKDIQFLSMYIKSGTIQLQEHIAKKVLEFPDKLKIKNNYKYF

>Psal1-Chr2-01; Yendovirus; Prunus salicina 1;

LEELLQLAEQTRIIGEDPQLHWNKNKILAKLDIINPDLTIKTADMPCNLLDKEEFEQQIKELLNLXVIRPSKSRHRSATFVVRKHSELKRGKARIVYNYRRLNDNTYEDSYRLPNKDELMNKIQESKYFRKFDCKSGFWQIRLAEESIHWTTFTCPEGHFEWLVMPFGLKNAPSIFQRKMDQIFKKYDNFCSVYVDDILVHSKNKNEHRKHLEIILKEFIDNGIVISRNKVELEKQDIEFLGMYIKSGTIQLQEHITKKVLEFPDKLEDKKQLQAFLGLLNYARNFIPD

>Psal1-Chr2-02; Yendovirus; Prunus salicina 1;

IIGEDPQLHWNKNKILAKLDIINPDLTIKTADMPCNLLDKQEFEQQIKELLNLKVIRPSKSRHRSAAFVVRKHSELKRGKARIVYNYRRLNDNTYEDSYKLPNKDELMNKIQESKYFSKFDCKSGFWQIRLAEESIHWTTFTCPEGHFEWLVMPFGLKNAPSIFQRKMDQIFKKYDKFCSVYVDDILVHSKNKNEHRKHLEIILKEFIDNGIVISRNKVELEKQDIEFLGMYIKSGTIQLQEHITKKVLEFPDKLEDKKTI

>Psal1-Chr2-03; Yendovirus; Prunus salicina 1;

LEELLQLAEQTRIIGEDPQLHWNKNKILAKLDIINPDLTIKTADMPCNLLDKQEFEQQIKELLNLKVIRPSKSRHRTAAFVVRKHSELKRGKARIVYNYRRLNDNTYEDSYKLPNKDELMNKIQESKYFSKFDCKSGFWQIRLAEESIHXTAFTCPEGHFEWLVMPFGLKNAPSIFQRKMDQIFKKYDNFCSVYVDDILVHSKNKNEHRKHLEIILKEFIDNGIVISRNKVELEKQDIEFLGMYIKSGTIQLQEHITKKVLEFPDKLEDKKQLQAFLGLLNYARSFIPD

>Psal1-Chr2-04; Florendovirus; Prunus salicina 1;

DKLKNEICSDLPNAFWYRKQHVVCLPYIKTFSEKNIPTKARPIQMSQEIMEFCKNEINELLQKKIIRKSKSPWSCPAFYVQKNAELERGVPRLVINYKPLNSVLEWIRYPIPNKRDLINRLGKAVIFSKFDMKSGLWQIQIEESDRYKTAFVTPFGHYEWNVMPFGLKNAPSEFQNIMNEIFNPYSHFSIVYIDDVLIFSESVEQHWKHLHKFLQIVKQNGLVVSAKKIKLFQTKVRFLGFNICQSQITPIDRVIQFADKFPDQILDKNQLQRFLGSLNYVSDF

>Psal1-Chr2-05; Yendovirus; Prunus salicina 1;

LEELLQLAEQTRIIGEDPQLHWNKNKILAKLDIINPDLTIKTADMPCNLLDKQEFEQQIKELLNLKVIRPSKSRHRSAAFVVRKHSELKRGKARIVYNYRRLNDNTYEDSYKLPNKDELMNKIQESKYFSKFDCKSGFWQIRLAEESIHWIAFTCPEGHFEWLVMPFGLKNAPSIFQRKMDQIFKKYDNFCSVYVDDILVHSKNKNEHRKHLEIILKEFIDNGIVISRNKVELEKQDIEFLGMYIKSGTIQLQEHITKKVLEFPDKLEDKKQLQAFLGLLNYARNFIPD

>Psal1-Chr2-06; Yendovirus; Prunus salicina 1;

LEELLQLAEQTRIIGEDPQLHWNKNKILAKLDIINPDLTIKTADMPCNLLDKQEFEQQIKELLNLKVIIPSKSRHRSAAFVVRKHSELKRGKARIVYNYRRLNDNTYEDSYKLPNKDELMNKIQESKYFSKFDCKSGFWQIRLAEESIHWTAFTCPEGHFEWLVMPFGLKNAPSIFQRKMDQIFKKYDNFCSVYVDDILVHNKNKNEHRKHLEIILKEFIDNGIVISRNKVELEKQDIEFLVMYIKSGTIQLQEHITKKVLEFPDKLEDKKQLQAFLGLLNYARSFIPD

>Psal1-Chr2-07; Yendovirus; Prunus salicina 1;

LEELLQLAEQTRIIGEDPQLHWNKNKILAKLDIINPDLTIKTADMPCYFFDKQEFEQQIKELLNLKVIRPSKSRHRSTAFVVRKHSELKRGKARIVYNYRRLNDNTYEDSYKLPNKDELMNKIQESKYFSKFDCKXGFWQIRLEEESIHWTAFTCPEGHFEWLVMPFGLKNAPSIFQRKMDQIFKKYDNFCSVYVDDILVHSKNKNEYRKHLEIILKEFIDNGIVISRNKVELEKQDIEFLGMYIKSGTIQLQEHIAKKVLEFPDKLEDKQR

>Psal1-Chr2-08; Yendovirus; Prunus salicina 1;

QKIIGEDPQLHWNKNKILAKLDIINPDLTIKTADMPCNLLDKQEFEQQIKELLNLKVIRPSKSRHRSAAFVVRKHSELKRGKARIVYNYRRLNDNTYEDSYKLPNKDELMNKIQESKYFSKFGCKXGFWQIRLEEESIHWIAFIYPEGHFEWLVMPFGLQNAPSIFQRKMDQIFKKYDNFCSVYVDDILVHSKNKNEHRKHLEIILKEFIDKGIVISGNKVELEKQDIEFLGMYIKSGTIQLQEHIVKKVLEFPDKLEDKKQLQAFLGLLNYARSFIPD

>Psal1-Chr3-01; Florendovirus; Prunus salicina 1;

EKLKQEVFSDPPNAFWHRKQHVVSLPHVKTFNEKHIPTKARPIQMSQEVMEFCKKEITELLQKWIICKSKSPWSYPAFYVQKNADLERGALRLVINYKSLNTALEWIRYPIPNKRDLINRLDKAIIFSKFDMKSGFWQIQIQKSDKYXTAFVTPFGHYEXNVMPFGLKNAPSEFQNIMNEIFNPFSDFSIVYIDDVLIFSQSIEQNLKHLYKFLQIVKRNGLVISAKKIKLFQTNIRFLGFNICQSQISPIDQVIQFADKFSDQILAKSQLQRFLGSLNYVSDF

>Psal1-Chr4-01; Yendovirus; Prunus salicina 1;

LEELLQLAEQTRIIGEDPQLHWNKNKILAKLDIINPDLTIKTADMPCNLLDKQEFEQQIKELLNLKVIRPSKSRHRSAAFVVRKHSELKRGKARIVYNYRRLNDNTYEDSYKLPNKDELMNKIQESKYFSKFDCKSGFWQIRLAEESIHWTAFTCPEGHFEWLVMPFGLKNAPSIFQRKMDQIFKKYDTFCSVYVDDILVHSKNKNEHRKHLEIILKEFIDNGIVISRNKVELEKQDIEFLGMYIKSGTIQLQEHITKKVLEFPDKLEDKKQLQAFLGLLNYARNFIPD

>Psal1-Chr4-02; Florendovirus; Prunus salicina 1;

KKLISEVCSDLPTAFWHRKNHVVCLPYIKHFSEKNIPTKARPIQMSHEMMEFCKAEINDLLHKNIIRKSKSPWSCPAFYVQKNAELERGSPRLVINYKPLNAVLEWIRYPIPNKRDLIKRLDKAAVFSKFDMKSGFWQIQIDESDRYKTAFVTPFGHYEWNVMPFGLKNAPSEFQNIMNEIFNAYSHFSIVYIDDVLIFSESLEQHWKHLHKFLHIIKSNGLVVSAKKIKLFQTNIRFLGYNISQSQIQPIDRVIHFADKFPDQITDKNQLQRFLGSLNYVSDF

>Psal1-Chr4-03; Florendovirus; Prunus salicina 1;

KKLISEVCSDFPTAFWHRKNHVVCLPYIKDFSEKNIPTKARPIQMSHEMMEFCKTEINDLLHKNIIRKSKSPWSCPAFYVQKNAELERGSPRLVINYKPLNAVLEWIRYPIPNKRDLIKRLDKAVIFSKFDMKSGFWQIQINESDRYKTAFVTPFGHYEWNVMPFGLKNAPSEFQNIMNEILNAYSHFSIVYIDDVLIFSESIEQHWKHLHKFLHTIKSNGLVISAKKIKLFQTNIRFLGYNISQSQIQPIDRVIHFADKFPDQITDKNQLQRFLGSLNYVSDF

>Psal1-Chr4-04; Florendovirus; Prunus salicina 1;

KKLISEVCSDLPTAFWHRKNHVVCLPYIKNFSEKNIPTKARPIQMNHEMMEFCKSEINDLLQKNIIRKSKSPWSCPAFYVQKNAELERGSPRLVINYKPLNAVLEWIRYPIPNKRDLIKRLDKAVIFSKFDMKSGFWQIQIDESDRYKTAFVTPFGHYEWNVMPFGLKNAPSEFQNIMNEIFNAYSQFSIVYIDDVLIFSESLEQHWKHLHKFLHIIKSNGLVVSAKKIKLFQTNIRFLGYNISQSQIQPIDRVIHFADKFPDQITDKNQLQRFLGSLNYVSDF

>Psal1-Chr4-05; Florendovirus; Prunus salicina 1;

KKLISEVCSDLPTAFWHRKNHVVCLPYIKNFSEKNIPTKARPIQMNHEMMEFCKSEINDLLQKNIIRKSKSPWSCPAFYVQKNAELERGSPRLVINYKPLNAVLEWIRYPIPNKRDLIKRLDKAVIFSKFDMKSGFWQIQIDESDRYKTAFVTPFGHYEWNVMPFGLKNAPSEFQNIMNEIFNAYSQFSIVYIDDVLIFSESLEQHWKHLHKFLHIIKSNGLVVSAKKIKLFQTNIRFLGYNISQSQIQPIDRVIHFADKFPDQITDKNQLQRFLGSLNYVSDF

>Psal1-Chr4-06; Yendovirus; Prunus salicina 1;

LEELLQLAEQTRIIGEDPQLHWNKNKILAKLDIINPDLTIKTSYMPCNLLDKQEFEQQIKELLNLKVIRPSKSRHRSAAFVVRKHSELKRGKARIVYNYRRLNDNTYEDSYKLPNKNELMNXIQESKYFSKFDCKSGFWQIRLAEESIQWTSFTCPEGHFEWLVMPFGLKNAPSIFQRKMDQIFKKYDKFCSVYVDDILVHSKNKNEHRKHLEIILKEFIDNGIVISRNKVELEKQEIEFLGMYIKSGTIQLQEHIAKKVLEFPDKLEDKKELQAFLGLLNYARSFIPD

>Psal1-Chr4-07; Yendovirus; Prunus salicina 1;

LEELLQLAEQTRIIGEDPQLHWNKNKILAKLDIINPDLTIKTADMPCNLLDKQEFEQQIKELLNLKVIRPSKSRHRSAAFVVRKHSELKRGKARIVYNYRRLNDNTYEDSYKLPNKDELMNKIQESKYFSKFDCKSGFWQIRLAEESIHWTAFTCPEGHFEWLVMPFGLKNAPSIFQRKMDQIFKKYDTFCSVYVDDILVHSKNKNEHRKHLEIILKEFIDNGIVISRNKVELEKQDIEFLGMYIKSGTIQLQEHITKKVLEFPDKLEDKKQLQAFLGLLNYARNFIPD

>Psal1-Chr4-08; Florendovirus; Prunus salicina 1;

DKLKNEICSELPNVFWYRKQHVVCLPYIKTFSEKNIPTKARPIQMSQEIMEFCKNEINELLQRKIIRRSKSPWSCPAFYVQKNAELERGVPRLVINYKPLNSVLEWIRYPIPNKRDLINRLGKAVIFSKFDMKSGFWQIQIDESDRYKTAFVTPFGHYEWNVMPFGLKNAPSEFQNIMNEIFNPYSHFSIVYIDDVLIFSESIEQHWKHLHKFFQVVKQNGLVVSAKKIKLFXTKVRFLGFNIYQSQIAPIDRVIQFADKFPDQILDKSQLQRFLGSLKYYVSDF

>Psal1-Chr4-09; Yendovirus; Prunus salicina 1;

LEELLQLAEQTRIIGEDPQLHWNKNKILAKLDIINPDLTIKTADMPCNLLDKQEFEQQIKELLNLKVIRPSKSRHRSAAFVVRKHSELKRGKARIVYNYRRLNDNTYEDSYKLPNKDELMNKIQESKYFSKFDCKSGFWQIRLAEESIHWTAFTCPEGHFEWLVMPFGLKNAPSIFQRKMDQIFKKYDTFCSVYVDDILVHSKNKNEHRKHLEIILKEFIDNGIVISRNKVELEKQDIEFLGMYIKSGTIQLQEHITKKVLEFPDKLEDKKQLQAFLGLLNYARSFIPD

>Psal1-Chr4-10; Florendovirus; Prunus salicina 1;

KKLISEVCSDLPTAFWHRKNHVVCLPYIKHFSEKNIPTKARPIQMSHEMMEFCKSEIDDLLHKNIIRRSKSPWSCPAFYVQKNAELERGSPRLVINYKSLNAVLEWIRYPIPNKRDLIKRLDKAVVFSKFDMKSGLWQIQIDESDRYKTAFVTPFGHYEWNVMPFGLKNAPSEFQNIMNEIFNAYSHFSIVYIDDVLIFSESLEQHWKHLHKFLHIIKSNGLVVSAKKIKLFQTNIRFLGYNISQSQIQPIDRVIHFADKFPDQITDKNQLQRFLGSLNYVSDF

>Psal1-Chr4-11; Florendovirus; Prunus salicina 1;

KKLISEVCSDLPTAFWHRKNHVVCLPYIKHFSEKNIPTKARPIQMSHEMMEFCKYEIDDLLHKNIIRRSKSPWSCPAFYVQKNAELERGSPRLVINYKSLNAVLEWIRYPIPNKRDLIKRLDKAVVFSKFDMKSGLWQIQIDESDRYKTAFVTPFGHYEWNVMPFGLKNAPSEFQNIMNEIFNAYSHFSIVYIDDVLIFSESLEQHWKHLHKFLHIIKSNGLVVYAKKIKLFQTNIRFLGYNISQSQIQPIDRVIHFADKFPDQITDKNQLQRFLGSLNYVSDF

>Psal1-Chr4-12; Yendovirus; Prunus salicina 1;

LEELLQLVEQTRIIGEDPXLHWNKKKLLTKLDIINPDLTIKIADMPCNLLNKQEFEQQIKELLNLKVIRPSKSRHRSTAFAVRKHSELKRGKARIVYNYRRLNDNTYEDSYKLPNKDELMNKIQESKYFSKFDCKSGFWKIRLTXESIQXTAFICPXGHFEWLVMPFGLKNAPSIFQRKMDQIFKKYDNFCSVYVDDILVHSKNKNEHRKHLEIILKEFIDNGIVISRNKVELEKQDIEFLGMYIKSGTIQLQEHIAKKVLEFPYKLEDKKQLQAFLGLLNYARSFIP

>Psal1-Chr4-13; Florendovirus; Prunus salicina 1;

KKLISEVCSDFPTPFWHRKNHVVCLPYIKNFSKKNIPTKARPIQMSHEMMEFCKSEINDLLQKNIIRKSKSPWSCPAFYVQKNAELERGSPRLVINYKPLNAILEWIRYPIPNKRDLIKRLNKAVIFSKFYMKSGFWQIQIDESDRYKTAFVTPFGHYEWNVMPFGLKNAPSEFQNIMNEIFNSYSHFSIVYIDDVLIFSESIEQHWKHLHKFLQIIKSNGLVVSAKKIKLFQTNIRFLGFNISQSXIHPIDRVIQFADKFPDQIVDKNQLQRFLGSLNYVSDF

>Psal1-Chr5-01; Yendovirus; Prunus salicina 1;

LEELLQLAEQSRIIGEDPQLHWNKNKILAKLDIINPDLTIKTANMPCNLLDKQEFEXQIKELLNLKVIRPPKSRHRLAAFVVRKHSELKRGKARIVYNYRRLNDNTYEDSYKLPNKDELMNKIQESKYFSKFECKSGFCQIRLAEESIQWTTFTCPEEHFEWLVMPFGLKNAPSIFQRKMDQIFKKYDNFCSVYVDDILVHSKNKHLEIILKEFINNGIVISRNKVELEKQDIEFLGMYIKSETIRTHGXKSVRISKXTRRXNAITSIFSLLNYARSFIPD

>Psal1-Chr6-01; Yendovirus; Prunus salicina 1;

LEELLQLAEQTRIIGEDPQLHWNKNKILAKLDIINPDLTIKTADMPCNLLDKQEFEQQIKELLNLKVIRPSKSRHRSAAFVVRKHSELKRGKARIVYNYRRLNDNTYEDSYKLPNKDELMNKIQESKYFSKFDCKSGFWQIRLAEESIHWTAFTCPEGHFEWLVMPFGLKNAPSIFQRKMDQIFKKYDNFCSVYVDDILVHSKNKNEHRKHLEIILKEFIDNGIVISRNKVELEKQDIEFLGMYIKSGTIQLQEHITKKVLEFPDKLEDKKQLQAFLGLLNYARSFIPD

>Psal1-Chr6-02; Florendovirus; Prunus salicina 1;

KKLISEVCSDLPTAFWHRKNHVVCLPYIKHFSEKNIPTKARPIQMSHEMMEFCKSEINDLLHKNIIRKSKSPWSCPAFYVQKNAELERGSPRLVINYKPLNAVLEWIRYPIPNKRDLIKHLDKAVVFSKFDMKSGFWQIQIDESDRYKTAFVTPFGHYEWNVMPFGLKNAPSEFQNIMNEIFNAYSHFSIVYIDDVLIFSESLEQHWKHLHKFLHIIKSNGLVVSAKKIKLFQTNIRFLGYNISQSQIQPIDRVIHFADKFPDQITDKNQLQRFLGSLNYV

>Psal1-Chr6-03; Florendovirus; Prunus salicina 1;

KKLISEVCSDLPTAFWHRKNHVVCLPYIKHFSEKNIPTKARPIQMSHEMMEFCKYEINDLLHKNIIRKSKSPWSCPAFYVQKNAELERGSPRLVINYKPLNAVLEWIRYPIPNKRDLIKRLDKAVVFSKFDMKSGFWQIQIDESDRYKTAFVTPFGHYEWNVMPFGLKNAPSEFQNIMNEIFNAYSHFSIVYIDDVLIFSESLEQHWKHLHKFLHIIKSNGLVVSAKKIKLFQTNIRFLGYNISQSQIQPIDRVIHFADKFPDQITDKNQLQRFLGSLNYV

>Psal1-Chr7-01; Florendovirus; Prunus salicina 1;

KKLISEVCSDLPIAFWHRKNHVVCLTYIKNFSEKNIPTKARPIQMSHEMMEFCKSEINDLLQKNIIRKSKSPWSCLAFYVQKNAELERGSPRLVINYKPLNAVLEXIRYPIPNKRDLIKRLDKAVVFSKFDMKSGFWQIQIDESDRYKTAFVTPFGHYEWNVMPFGLKNAPSEFQNIMNEIFNTYSHFSIVYIDDVLIFSESIEQHWKHLHKFLHIIKSNGLVVSAKKIKLFQTKIRFLGYNISQSHIQPIDRVIHFADKFPDQITDKNQLQRFLGSLNYVSDF

>Psal1-Chr7-02; Yendovirus; Prunus salicina 1;

IIGEDPQLHWNKNKILAKLDIINPDLTIKTADMPCNLLDKQEFEQQIKELLNLKVIRPSKSRHRSAAFVVRKHSELKRGKARIVYNYRRLNDNTYEDSYKLPNKDELMNKIQESKYFSKFDCKSGFWQIRLAEESIHWTAFTCPEGHFEWLVMPFGLKNAPSIFQRKMDQIFKKYDKFCSVYVDDILVHSKNKNEHRKHLEIILKEFIDNGIVISRNKVELEKQDIEFLGMYIKSGTIQLQEHITKKVLEFPDKLEDKKQLQAFLGLLNYARSFIPD

>Psal2-Chr1-01; Yendovirus; Prunus salicina 2;

LEELLQLAEQTRIIGEDPQLHWNKNKILAKLDIINPDLTIKTADMPCNLLDKQEFEQQIKELLNLKVIRPSKSRHRSAAFVVRKHSELKRGKARIVYNYRRLNDNTYEDSYKLPNKDELMNKIQESKYFSKFDCKSGFWQIRLAEESIHWTAFTCPEGHFEWLVMPFGLKNAPSIFQRKMDQIFKKYDTFCSVYVDDILVHSKNKNEHRKHLEIILKEFIDNGIVISRNKVELEKQDIEFLGMYIKSGTIQLQEHITKKVLEFPDKLEDKKQLQAFLGLLNYARNFIPD

>Psal2-Chr1-02; Florendovirus; Prunus salicina 2;

KKLISEVCSDLPTAFWHRKNHVVCLPYIKNFSEKNIPTKARPIQMNHEMMEFCKSEIDDLLQKNIIRKSKSPWSCPAFYVQKNAELERGSPRLVINYKPLNAVLEWIRYPIPNKRDLIKRLDKAVIFSKFDMKSGFWQIQIDESDRYKTAFVTPFGHYEWNVMPFGLKNAPSEFQNIMNEIFNAYSHFSIVYIDDVLIFSESLEQHWKHLHKFLHIIKSNGLVVSAKKIKLFQPNIRFLGYIISQSQIQPIDRVIHFADKFPDQITDKNQLQRFLGSLNYVSDF

>Psal2-Chr1-03; Florendovirus; Prunus salicina 2;

KKLISEVCSDLPTAFWHRKNHVVCLPYIKNFSEKNIPTKARPIQMSHEMMEFCKTEINDLLQKNIIRKSKSPWSCPAFYVQKNAELERGSPRLVINYKPLNVVLEWIRYPIPNKRDLIKRLDKAVIFSKFDMKSGFWQIQIDESDRYKTAFVTPFGHYEWNVMPFGLKNAPSEFQNIMNEIFNAYSHFSIVYIDDVLIFSESLDQHWKHLHTFLHIIKSNGLVVSAKKIKLFQTNNRFLGYNISQSQIQPIDRVIHFADKFPDQITDKNQLQRFLGSLNYVSDF

>Psal2-Chr1-04; Yendovirus; Prunus salicina 2;

LEELLQLDEQTRIIGEDPQLHWNKNKILAKLDIINPDLTIKTADMPCNLLDKQEFEQQIKELLNLKVIRPSKSRHRSAAFVVRKHSELKRGKARIVYNYRRLNDNTYEDSYKLPNKDELMNKIQESKYFSKFDCKSGFWQIRLAEESIQWTAFTCPEGHFEWLVMPFGLKNAPSIFQRKMDQIFKKYDNFCSVYVDDILVHSKNKNEHRKHLEIILKEFIDNGIVISRNKVELEKQDIEFLGMYIKSGTIQLQEHITKKVLEFPDKLEDKKQLQAFLGLLNYARNFIPD

>Psal2-Chr1-05; Yendovirus; Prunus salicina 2;

IIGEDPQLHWNKNKILAKLDIINQNLTTKTADMSRNLLDKQEFEQQIKELLNLKVIRPSKSRHRSAAFVVRKHXELKRGKARIVYNYRRLNDNTYEDSYKLPNKDELMNKIQESKYFSKFDCKSGFWQIRLAEESIQWTTFTCLEGHFEWLVMPFGLKNAPSIFQRKMDQIFKKYDNFCSVXVDDILVHSKNKNEHRKHLEIILKEFIDNGIVISRNKVELEKQDIEFLGMYIKLGNIQLQEHMAKKVLEFPDKLEDKKQLQAFLGLLNYARNFIP

>Psal2-Chr1-06; Yendovirus; Prunus salicina 2;

LEELLQLAEQTRIIGEDPQLHWNKNKILAKLDIINPDLTIKTADMPCNLLDKQEFEQQIKELLNLKVIRPSKSRHRSAAFVVRKHSELKRGKARIVYNYRRLNDNTYEDSYKLPNKDELMNKIQESKYFSKFDCKSGFWQIRLAEESIHWTAFTCPEGHFEWLVMPFGLKNAPSIFQRKMDQIFKKYDNFCSVYVDDILVHSKNKNEHRKHLEIILKEFIDNGIVISRNKVELEKQDIEFLGMYIKSGTIQLQEHITKKVLEFPDKLEDKKQLQAFLGLLNYARSFIPD

>Psal2-Chr1-07; Yendovirus; Prunus salicina 2;

HWNKNKILEKLDIINPDLTIKTTYMLCNLLDKXEFEQQIKELLNLKVIRPSKSRHRSSTPFVVRKHSELKIGKARIVYNYRRLNDNTYEDSYKLPNKDELLNKIQESKYFSKFACKSGFWQIKLAEELIQWTTFTCLEXYFKWLVMSFALKICSIDLSKKDGSDFXEVXXILXCICRXCTCYDHFCSVYANDILVHRNEHRKHLEIILQEFIDNGLVISRNKVELEKKDIXFLGMYIKSGTIQLQEHIAKKVLEFPDKLKIKNNYKYF

>Psal2-Chr1-08; Florendovirus; Prunus salicina 2;

KKLISEVCSDLPIAFWHRKNHVVCLPYIKNFSEKNIPTKARPIQMSHEMMEFCKTEINDLLHKNIIRKSKSPWSCLAFYVQKNAELERGSPRLVINYKPLNAVLQWIRYPIPNKRDLIKRLDKAVVFSKFDMKSGLWQIQIDESDRYKTEFVTPFGHYEWNVMPFGLKNAPSEFQNIMNEIFNTYSHFSIVYIDDVLIFSESLEQHWKHLHKFLHIIKSNGLVVSAKKIKLFQTHIRFLGYNISQSQIQPIDRVIHFADKFPDQIIDKNQLQRFLGSLNYVSDF

>Psal2-Chr2-01; Yendovirus; Prunus salicina 2;

IIGEDPQLHWNKNKILAKLDIINPDLTIKTADMPCNLLDKQEFEQQIKELLNLKVIRPSKSRHRSAAFVVRKHSELKRGKARIVYNYRRLNDNTYEDSYKLPNKDELMNKIQESKYFSKFDCKSGFWQIRLAEESIHWTTFTCPEGHFEWLVMPFGLKNAPSIFQRKMDQIFKKYDKFCSVYVDDILVHSKNKNEHRKHLEIILKEFIDNGIVISRNKVELEKQDIEFLGMYIKSGTIQLQEHITKKVLEFPDKLEDKKTI

>Psal2-Chr2-02; Yendovirus; Prunus salicina 2;

LEELLQLAEQTRIIGEDPQLHWNKNKILAKLDIINPDLTIKTADMPCNLLDKQEFEQQIKELLNLKVIRPSKSRHRTAAFVVRKHSELKRGKARIVYNYRRLNDNTYEDSYKLPNKDELMNKIQESKYFSKFDCKSGFWQIRLAEESIHXTAFTCPEGHFEWLVMPFGLKNAPSIFQRKMDQIFKKYDNFCSVYVDDILVHSKNKNEHRKHLEIILKEFIDNGIVISRNKVELEKQDIEFLGMYIKSGTIQLQEHITKKVLEFPDKLEDKKQLQAFLGLLNYARSFIPD

>Psal2-Chr2-03; Yendovirus; Prunus salicina 2;

IIGEDPQLHWNKNKILAKLDIINPDLTIKTADMPCYFFDKQEFEQQIKELLNLKVIRPSKSRHRSTAFVVRKHSELKRGKARIVYNYRRLNDNTYEDSYKLPNKDELMNKIQESKYFSKFGCKXGFWQIRLEEESIHWIAFIYPEGHFEWLVMPFGLQNAPSIFQRKMDQIFKKYDNFCSVYVDDILVHSKNKNEHRKHLEIILKEFIDKGIVISRNKVELEKQDIEFLGMYIKSGTIQLQEHIAKKVLEFPDKLEDKQR

>Psal2-Chr3-01; Florendovirus; Prunus salicina 2;

EKLKQEVFSDPPNAFWHRKQHVVSLPHVKTFNEKHIPTKARPIQMSQEVMEFCKKEITELLQKWIICKSKSPWSYPAFYVQKNADLERGALRLVINYKSLNTALEWIRYPIPNKRDLINRLDKAIIFSKFDMKSGFWQIQIQKSDKYKTAFVTPLGHYEXNVMPFGLKNAPSEFQNIMNEIFNPFSDFSIVYIDDVLIFSQSIEQNLKHLYKFLQIVKRNGLVISAKKIKLFQTNIRFLGFNICQSQISPIDQVIQFADKFSDQILAKSQLQRFLGSLNYVSDF

>Psal2-Chr4-01; Florendovirus; Prunus salicina 2;

KKLISEVCSDLPTAFWHRKNHVVCLPYIKHFSEKNIPTKARPIQMSHEMMEFCKSEIDDLLHKNIIRRSKSPWSCPAFYVQKNAELERGSPRLVINYKSLNAVLEWIRYPIPNKRDLIKRLDKAVVFSKFDMKSGLWQIQIDESDRYKTAFVTPFGHYEWNVMPFGLKNAPSEFQNIMNEIFNAYSHFSIVYIDDVLIFSESLEQHWKHLHKFLHIIKSNGLVVSAKKIKLFQTNIRFLGYNISQSQIQPIDRVIHFADKFPDQITDKNQLQRFLGSLNYVSDF

>Psal2-Chr4-02; Florendovirus; Prunus salicina 2;

KKLISEVCSDFPTPFWHRKNHVVCLPYIKNFSKKNIPTKARPIQMSHEMMEFCKSEINDLLQKNIIRKSKSPWSCPAFYVQKNAELERGSPRLVINYKPLNAILEWIRYPIPNKRDLIKRLNKAVIFSKFYMKSGFWQIQIDESDRYKTAFVTPFGHYEWNVMPFGLKNAPSEFQNIMNEIFNSYSHFSIVYIDDVLIFSESIEQHWKHLHKFLQIIKSNGLVVSAKKIKLFQTNIRFLGFNISQSXIHPIDRVIQFADKFPDQIVDKNQLQRFLGSLNYVSDF

>Psal2-Chr4-03; Florendovirus; Prunus salicina 2;

KKLISEVCSDLPTAFWHRKNHVVCLPYIKHFSEKNIPTKARPIQMSHEMMEFCKYEIDDLLHKNIIRRSKSPWSCPAFYVQKNAELERGSPRLVINYKSLNAVLEWIRYPIPNKRDLIKRLDKAVVFSKFDMKSGLWQIQIDESDRYKTAFVTPFGHYEWNVMPFGLKNAPSEFQNIMNEIFNAYSHFSIVYIDDVLIFSESLEQHWKHLHKFLHIIKSNGLVVYAKKIKLFQTNIRFLGYNISQSQIQPIDRVIHFADKFPDQITDKNQLQRFLGSLNYVSDF

>Psal2-Chr4-04; Yendovirus; Prunus salicina 2;

LEELLQLVEQTRIIGEDPXLHWNKKKLLTKLDIINPDLTIKIADMPCNLLNKQEFEQQIKELLNLKVIRPSKSRHRSTAFAVRKHSELKRGKARIVYNYRRLNDNTYEDSYKLPNKDELMNKIQESKYFSKFDCKSGFWKIRLTXESIQXTAFICPXGHFEWLVMPFGLKNAPSIFQRKMDQIFKKYDNFCSVYVDDILVHSKNKNEHRKHLEIILKEFIDNGIVISRNKVELEKQDIEFLGMYIKSGTIQLQEHIAKKVLEFPYKLEDKKQLQAFLGLLNYARSFIP

>Psal2-Chr4-05; Florendovirus; Prunus salicina 2;

DKLKNEICSELPNVFWYRKQHVVCLPYIKTFSEKNIPTKARPIQMSQEIMEFCKNEINELLQRKIIRRSKSPWSCPAFYVQKNAELERGVSRLVINYKPLNSVLEWIRYPIPNKRDLINRLGKAVIFSKFDMKSGFWQIQIDESDRYKTAFVTHFGHYEWNVMPFGLKNAPSEFQNIMNEIFNPYSHFSIVYIDDVLIFSESIEQHWKHLHKFFQVFKQNGLVVSAKKIKLFQTKVRFLGFNIYQSQIAPIDRVIQFADKFPDQILNKSQLQRFLGSLKYYVSDF

>Psal2-Chr4-06; Yendovirus; Prunus salicina 2;

LEELLQLAEQTRIIGEDPQLHWNKNKILAKLDIINPDLTIKTSYMPCNLLDKQEFEQQIKELLNLKVIRPSKSRHRSAAFVVRKHSELKRGKARIVYNYRRLNDNTYEDSYKLPNKNELMNXIQESKYFSKFDCKSGFWQIRLAEESIQWTSFTCPEGHFEWLVMPFGLKNAPSIFQRKMDQIFKKYDKFCSVYVDDILVHSKNKNEHRKHLEIILKEFIDNGIVISRNKVELEKQEIEFLGMYIKSGTIQLQEHIAKKVLEFPDKLEDKKELQAFLGLLNYARSFIPD

>Psal2-Chr4-07; Yendovirus; Prunus salicina 2;

ILANLDIINPDLTIKTADIPCNLLDKQEFEQQIKELLNLKVIRPSKSRHRSAAFVVRKHSELKRGKARIVYNYRRLNDNTYEDSYKLPNKDELMNXIQESKYFSKFDCKSGFWKIRLAEESIQWTTFTGLEGHFEWLVMPFGLKKMHHRSFKERWTKSLRSMIILVVYMXMIYXFIVKIKMNIENTXKLFXKNLLIMVXLXVRIKXSLKNKIXNSXVCILSQETIQLQEHIAKKVIEFPDKLEDKKQLQAFLGLLNYVRSFI

>Psal2-Chr4-08; Florendovirus; Prunus salicina 2;

KKLISEVCSDLPTAFWHRKNHVVCLPYIKHFSEKNIPTKARPIQMSHEMMEFCKAEINDLLHKNIIRKSKSPWSCPAFYVQKNAELERGSPRLVINYKPLNAVLEWIRYPIPNKRDLIKRLDKAAVFSKFDMKSGFWQIQIDESDRYKTAFVTPFGHYEWNVMPFGLKNAPSEFQNIMNEIFNAYSHFSIVYIDDVLIFSESLEQHWKHLHKFLHIIKSNGLVVSAKKIKLFQTNIRFLGYNISQSQIQPIDRVIHFADKFPDQITDKNQLQRFLGSLNYVSDF

>Psal2-Chr4-09; Yendovirus; Prunus salicina 2;

LEELLQLAEQTRIIGEDPQLHWNKNKILAKLDIINPDLTIKTADMPCNLLDKQEFEQQIKELLNLKVIRPSKSRHRSAAFVVRKHSELKRGKARIVYNYRRLNDNTYEDSYKLPNKDELMNKIQESKYFSKFDCKSGFWQIRLAEESIHWTAFTCPEGHFEWLVMPFGLKNAPSIFQRKMDQIFKKYDTFCSVYVDDILVHSKNKNEHRKHLEIILKEFIDNGIVISRNKVELEKQDIEFLGMYIKSGTIQLQEHITKKVLEFPDKLEDKKQLQAFLGLLNYARNFIPD

>Psal2-Chr5-01; Florendovirus; Prunus salicina 2;

KKLISEVCSDLPTAFWHRKNHVVCLPYIKNFSEKNIPTKARPIQMNHEMMEFCKSEINDLLQKNIIRKSKSPWSCPAFYVQKNAELERGSPRLVINYKPLNAVLEWIRYPIPNKRDLIKRLDKAVIFSKFDMKSGFWQIQIDESDRYKTAFVTPFGHYEWNVMPFGLKNAPSEFQNIMNEIFNAYSQFSIVYIDDVLIFSESLEQHWKHLHKFLHIIKSNGLVVSAKKIKLFQTNIRFLGYNISQSQIQPIDRVIHFADKFPDQITDKNQLQRFLGSLNYVSDF

>Psal2-Chr5-02; Florendovirus; Prunus salicina 2;

KKLIYEVCSDLPTAFWHRKNHVVCLPYIKNFSEKNIPTKARPIQMNHEMMEFCKSEINDLLQKNIIRKSKSPWSCPAFYVQKNAELERGSPRLVINYKPLNAVLEWIRYPIPNKRDLIKRLDKAVIFSKFDMKSGFWQIQIDESDRYKTAFVTPFGHYEWNVMPFGLKNAPSEFQNIMNEIFNAYSQFSIVYIDDVLIFSESLEQHWKHLHKFLHIIKSNGLVVSAKKIKLFQTNIRFLGYNISQSQIQPIDRVIHFADKFPDQITDKNQLQRFLGSLNYVSDF

>Psal2-Chr5-03; Yendovirus; Prunus salicina 2;

LEELLQLAEQTRIIGEDPQLHWNKNKILAKLDIINPDLTIKTADMPCNLLDKQEFEQQIKELLNLKVIRPSKSRHRSAAFVVRKHSELKRGKARIVYNYRRLNDNTYEDSYKLPNKDELMNKIQESKYFSKFDCKSGFWQIRLAEESIHWTAFTCPEGHFEWLVMPFGLKNAPSIFQRKMDQIFKKYDNFCSVYVDDILVHSKNKNEHRKHLEIILKEFIDNGIVISRNKVELEKQDIEFLGMYIKSGTIQLQEHITKKVLEFPDKLEDKKQLQAFLGLLNYARSFIPD

>Psal2-Chr5-04; Yendovirus; Prunus salicina 2;

LEELLQLAEQSRIIGEDPQLHWNKNKILAKLDIINPDLTIKTANMPCNLLDKQEFEXQIKELLNLKVIRPPKSRHRLAAFVVRKHSELKRGKARIVYNYRRLNDNTYEDSYKLPNKDELMNKIQESKYFSKFECKSGFCQIRLAEESIQWTTFTCPEEHFEWLVMPFGLKNAPSIFQRKMDQIFKKYDNFCSVYVDDILVHSKNKHLEIILKEFINNGIVISRNKVELEKQDIEFLGMYIKSETIRTHGXKSVRISKXTRRXNAITSIFSLLNYARSFIPD

>Psal2-Chr6-01; Yendovirus; Prunus salicina 2;

LEELLQLAEQTRIIGEDPQLHWNKNKILAKLDIINPDLTIKTADMPCNLLDKQEFEQQIKELLNLKVIRPSKSRHRSAAFVVRKHSELKRGKARIVYNYRRLNDNTYEDSYKLPNKDELMNKIQESKYFSKFDCKSGFWQIRLAEESIHWTAFTCPEGHFEWLVMPFGLKNAPSIFQRKMDQIFKKYDNFCSVYVDDILVHSKNKNEHRKHLEIILKEFIDNGIVISRNKVELEKQDIEFLGMYIKSGTIQLQEHITKKVLEFPDKLEDKKQLQAFLGLLNYARSFIPD

>Psal2-Chr6-02; Florendovirus; Prunus salicina 2;

KKLISEVCSDLPTAFWHRKNHVVCLPYIKHFSEKNIPTKARPIQMSHEMMEFCKSEINDLLHKNIIRKSKSPWSCPAFYVQKNAELERGSPRLVINYKPLNAVLEWIRYPIPNKRDLIKHLDKAVVFSKFDMKSGFWQIQIDESDRYKTAFVTPFGHYEWNVMPFGLKNAPSEFQNIMNEIFNAYSHFSIVYIDDVLIFSESLEQHWKHLHKFLHIIKSNGLVVSAKKIKLFQTNIRFLGYNISQSQIQPIDRVIHFADKFPDQITDKNQLQRFLGSLNYV

>Psal2-Chr6-03; Florendovirus; Prunus salicina 2;

KKLISEVCSDLPTAFWHRKNHVVCLPYIKHFSEKNIPTKARPIQMSHEMMEFCKYEINDLLHKNIIRKSKSPWSCPAFYVQKNAELERGSPRLVINYKPLNAVLEWIRYPIPNKRDLIKRLDKAVVFSKFDMKSGFWQIQIDESDRYKTAFVTPFGHYEWNVMPFGLKNAPSEFQNIMNEIFNAYSHFSIVYIDDVLIFSESLEQHWKHLHKFLHIIKSNGLVVSAKKIKLFQTNIRFLGYNISQSQIQPIDRVIHFADKFPDQITDKNQLQRFLGSLNYV

>Psal2-Chr6-04; Florendovirus; Prunus salicina 2;

KKLISEVCSDLPTAFWHRKNHVVCLPYIKHFSEKNIPTKARPIQMSHEMMEFCKSEINDLLHKNIIRKSKSPWSCPAFYVQKNAELERGSPRLVINYKPLNAVLEWIRYPIPNKRDLIKHLDKAVVFSKFDMKSGFWQIQIDESDRYKTAFVTPFGHYEWNVMPFGLKNAPSEFQNIMNEIFNAYSHFSIVYIDDVLIFSESLEQHWKHLHKFLHIIKSNGLVVSAKKIKLFQTNIRFLGYNISQSQIQPIDRVIHFADKFPDQITDKNQLQRFLGSLNYV

>Psal2-Chr6-05; Florendovirus; Prunus salicina 2;

KKLISEVCSDLPTAFWHRKNHVVCLPYIKHFSEKNIPTKARPIQMSHEMMEFCKYEINDLLHKNIIRKSKSPWSCPAFYVQKNAELERGSPRLVINYKPLNAVLEWIRYPIPNKRDLIKRLDKAVVFSKFDMKSGFWQIQIDESDRYKTAFVTPFGHYEWNVMPFGLKNAPSEFQNIMNEIFNAYSHFSIVYIDDVLIFSESLEQHWKHLHKFLHIIKSNGLVVSAKKIKLFQTNIRFLGYNISQSQIQPIDRVIHFADKFPDQITDKNQLQRFLGSLNYV

>Psal2-Chr7-01; Yendovirus; Prunus salicina 2;

LEELLQLAEQTRIIGEDPQLHWNKNKILAKLDIINPDLTIKTADMPCNLLVKQEFEQQIKELLNLKVIRPSKSRHRSAAFVVRKHSELKRGKARIVYNYRRLNDNTYEDSYKLPNKDELMNKIQESKYFSKFDCKSGFWQIRLAEESIHWTAFTCPEGHFEWLVMPFGLKNAPSIFQRKMDQIFKKYDNFCSVYVDDILVHSKNKNEHRKHLEIILKEFIDNGIVISRNKVELEKQDIEFLGMYIKSGTIQLQEHITKKVLEFPDKLEDKKQLQAFLGLLNYARSFIPD

>Psal2-Chr7-02; Florendovirus; Prunus salicina 2;

KKLISEVCSDLPIAFWHRKNHVVCLTYIKNFSEKNIPTKARPIQMSHEMMEFCKSEINDLLQKNIIRKSKSPWSCLAFYVQKNAELERGSPRLVINYKPLNAVLEXIRYPIPNKRDLIKRLDKAVVFSKFDMKSGFWQIQIDESDRYKTAFVTPFGHYEWNVMPFGLKNAPSEFQNIMNEIFNTYSHFSIVYIDDVLIFSESIEQHWKHLHKFLHIIKSNGLVVSAKKIKLFQTKIRFLGYNISQSHIQPIDRVIHFADKFPDQITDKNQLQRFLGSLNYVSDF

>Psal2-Chr7-03; Florendovirus; Prunus salicina 2;

KKLISEVCSDFPTAFWHRKNHVVCLPYIKDFSEKNIPTKARPIQMSHEMMEFCKTEINDLLHKNIIRKSKSPWSCPAFYVQKNAELERGSPRLVINYKPLNAVLEWIRYPIPNKRDLIKRLDKAIIFSKFDMKSGFWQIQIDESDRYKTAFVTPFGHYEWNVMPFGLKNAPSEFQNIMNEIFNAYSHFSIVYIDDVLIFSESLEQHWKHLHTFLHIIKSNGLVVSAKKIKLFQTNIRFLGYNISQSQIQPIDRVIHFADKFPDQITDKNQLQRFLGSLNYVSDF

>Psal2-Chr7-04; Yendovirus; Prunus salicina 2;

LEELLQLAEQTRIIGEDPQLHWNKNKILAKLDIINPDLTIKTADMPCNLLDKQEFEQQIKELLNLKVIRPSKSRHRSAAFVVRKHSELKRGKARIVYNYRRLNDNTYEDSYKLPNKDELMNKIQESKYFSKFDCKSGFWQIRLAEESIHWTAFTCPEGHFEWLVMPFGLKNAPSIFQRKMDQIFKKYDNFYSVYVDDILVHSKNKNEHRKHLEIILKEFIDNGIVISRNKVELEKQDIEFLGMYIKSGTIQLQEHITKKVLEFPDKLEDKKQLQAFLGLLNYARNFIPD

>Psal2-Chr7-05; Yendovirus; Prunus salicina 2;

LEELLQLAEQTRIIGEDPQLHWNKNKILAKLDIINPDLTIKTADMPCNLLDKQEFEQQIKELLNLKVIRPSKSRHRSAAFVVRKHSELKRGKARIVYNYRRLNDNTYEDSYKLPNKDELMNKIQESKYFSKFDCKSGFWQIRLAEESIHWIAFTCPEGHFEWLVMPFGLKNAPSIFQRKMDQIFKKYDNFCSVYVDDILVHSKNKNEHRKHLEIILKEFIDNGIVISRNKVELEKQDIEFLGMYIKSGTIQLQEHITKKVLEFPDKLEDKKQLQAFLGLLNYARNFIPD

>Psal2-Chr7-06; Yendovirus; Prunus salicina 2;

IIGEDPQLHWNKNKILAKLDIINPDLTIKTTDMPCNLLDKQEFEQQIKELLNLKVIRPSKSRHRSTTFVVRKHSKLKRGKTRIVYNYRRLNDNTYEDSYKLPNKDELMNKIQESKYFSKFDCKLGFWQIRLAEESIQWTTFTCPEGLFEWLVMPFGLKNAPSIFQRKMDQIFKKYDNFCSVYVDDILVNSKNKNEHRKLLXIILKEFIDNGIVISRNKVELEKQDIEILGMYIKSGTIQLQEHIAKKVLEFPDKLEDKKQLQAFLGLLNYA

>Psal2-Chr8-01; Florendovirus; Prunus salicina 2;

KKLISEVCSDLPTAFWHRKNHVVCLPYIKNFSEKNIPTKARPIQMSHEMMEFCKTEINDLLHKNIIRKSKSSWSCPAFYVQKNAELERGSPRLVINYKPLNTVLEWIRYPIPNKRDLIKRLDKAVIFSKFDMKSGFWQIQIDESDRYKTAFVTPFGHYEWNVMPFGLKNAPSEFQNIMNEIFNAYSHFSIVYIDDVLIFSESLEQHWKHLHKFLHIIKSNGLVVSAKKIKLFQTNIRFLGYNISQSQIQPIDRVIHFADKFPDQITDKNQLQRFLGSLNYVSDF

>Psal2-Chr8-02; Yendovirus; Prunus salicina 2;

LEELLQLAEQTRIIGEDPQLHWNKNKILAKLDIINPDLTIKTADMPCNLLDKQEFEQQIKELLNLKVIRPSKSRHRSAAFVVRKHSELKRGKARIVYNYRRLNDNTYEDSYKLPNKDELMNKIQESKYFSKFDCKSGFWQIRLAEESIHWAAFTCPEGHFEWLVMPFGLKNAPSIFQRKMDQIFKKCDKFCSVYVDDILVHSKNKNEHRKHLEIILKEFIDNGIVISRNKVELEKQDIEFLGMYIKSGTIQLQEHITKKVLEFPDKLEDKKQLQAFLGLLNYARSFIPD

>Psal3-Chr1-01; Florendovirus; Prunus salicina 3;

KKLISEVCSDLPTAFWHRKHHVVCLPYFKNFSEKNIPTKARPIQMNHEMMEFCKSEIDDLLQKNIIRKSKSPWSCPAFYVQKNAELERGSPRLVINYKPLNTVLEWIRYPIPNKRDLIKRLDKAVIFSKFDMKSGFWQIQIDESDRYKTAFVTPFGHYEWNVMPFGLKNAPSEFQNIMNEIFNTYSHFSIVYIDDVLIFSESLEQHWKHLHKFLHIIKSNGLVVSAKKIKLFQTNIRF LGYNISQSHIQPIDRVIHFADKFPDQITDKNQLQRFLGSLNYVSDF

>Psal3-Chr1-02; Florendovirus; Prunus salicina 3;

KKLVSEVCSDLPTAFWHRKNHVVCLPYIKKFSEKNIPTKARPIQMNHEMMEFCKSEINDLLHKNIIRKSKSPWSCPAFYVQKNAELERGSPRLVINYKPLNTVLEWIRYPIPNKRDLIKRLDKAVIFSKFDMKSGFWQIQINEADRYKTAFVTPFGHYEWNVMPFGLKNAPSEFQNIMNEIFNAYSYFSIVYIDDVLIFSESLEQHWKHLHKFLHIIKSNGLVVSAKKIKLFQTNIRFLGYNISQSQIQPIDRVIHFADKFPDQITDKNQLQRFLGSLNYVSDF

>Psal3-Chr1-03; Yendovirus; Prunus salicina 3;

IIGEDPRLHWNKNKILEKLDIINPDLTIKTTYMLCNLLDKXEFEQQIKELLNLKVIRPSKSRHRSSTPFVVRKHSELKIGKARIVYNYRRLNDNTYEDSYKLPNKDELLNKIQESKYFSKFDCKSGFWQIKLAEELIQWTTFTCLEXYFKWLVMSFALKICSIDLSKKDGXDFXEVXXILXCICRXCTCYDHFCSVYANDILVHRNEHRKHLKIILQEFIDNGIVISRNKVELEKKDIQFLSMYIKSGTIQLQEHIAKKVLEFPDKLKIKNNYKYF

>Psal3-Chr1-04; Yendovirus; Prunus salicina 3;

LEELLQLAEQTRIIGEDPQLHWNKNKILAKLDIINPDLTIKTADMPCNLLDKQEFEQQIKELLNLKVIRPSKSRHRSAAFVVRKHSELKRDKARIVYNYRRLNDNTYEDSYKLPNKDELMNKIQESKYFSKFDCKSGFWQIRLAEESIHWTAFTCPEGHFEWLVMPFGLKNAPSIFQRKMDQIFKKYDNFCSVYVDDILVHSKNKNEHRKHLEIILKEFIDNGIVISRNKVELEKQDIEFLGMYIKLGTIQLQEHITKKILEFPDKLEDKKQLQAFLGLLNYARNFIPD

>Psal3-Chr1-05; Florendovirus; Prunus salicina 3;

DKLRNEICSDLPNAFWYRKQHVVCLPYIKTFSEKNIPTKARPIQMSQEIMEFCKNEINELLQKKIIRKSKSPWSCPAFYVQKNAELERGVPRLVINYKPLNSVLEWIRYPIPNKRDLINRLGKAVIFSKFDMKSGFWQIQIEESDRYKTAFVTPFGHYEWNVMPFGLKNAPSEFQNIMNEIFNPYSHFSIVYIDDVLIFSESIEQHWKHLHKFLQIVKQNGLVVSAKKIKLFQTKVRFLGFNICQSQITPIHRVIQFADKFPDQILDKNQLQRFLGSLNYVSDF

>Psal3-Chr2-01; Florendovirus; Prunus salicina 3;

KKLISEVCSDLPTALWHRKNHVVCLPYIKHFSEKNIPTKARPIQMSHEMMEFCKYEINDLLHKNIIRKSKSPWSCPAFYVQKNAELERGSPRLVINYKPLNAVLEWIRYPIPNKRDLIKRLDKAVVFSKFDMKSGFWQIXIDESDRYKTAFVTPFGHYEWNVMPFGLKNAPSEFQNIMNEIFNAYSHFSIVYIDDVLIFSESLEQHWKHLHKFLHIIKSNGLVVSAKKIKLFQTNIRFLGYNISQSQIQPIDRVIHFADKFPDQITDKNQLQRFLGSLNYV

>Psal3-Chr2-02; Florendovirus; Prunus salicina 3;

KKLISEVCSDLPTALWHRKNHVVCLPYIKHFSEKNIPTKARPIQMSHEMMEFCKYEINDLLHKNIIRKSKSPWSCPAFYVQKNAELERGSPRLVINYKPLNAVLEWIRYPIPNKRDLIKRLDKAVVFSKFDMKSGFWQIXIDESDRYKTAFVTPFGHYEWNVMPFGLKNAPSEFQNIMNEIFNAYSHFSIVYIDDVLIFSESLEQHWKHLHKFLHIIKSNGLVVSAKKIKLFQTNIRFLGYNISQSQIQPIDRVIHFADKFPDQITDKNQLQRFLGSLNYV

>Psal3-Chr2-03; Florendovirus; Prunus salicina 3;

KKLISEVCSDLPTAFWHRKNHVVCLPYIKNFSEKNIPTKARPIQMSHEMMEFCKTEINNLLHKNIIRKSKSPWSCPAFYVQKNAELERGSPRLVINYKPLNAVLEWIRYPIPNKRDLIKRLDKAVIFSKFDMKSGFWQIQIDESDRYKTAFVTPFGHYEWNVMPFGLKNAPSEFQNIMNEIFNAYSHFSIVYIDDVLIFSESLEQHWKHLHKFLHIIKSNGLVVSAKKIKLFQTNIRFLGYNISQSQIQPIDRVIHFADKFPDQITDKNQLQRFLGSLNYVSDF

>Psal3-Chr2-04; Yendovirus; Prunus salicina 3;

LEELLQLAEKTRIIGEDPQLHWNKNKILAKLDIINPDLTIKTADMPCNLLDKQEFEQQIKELLNLKVIRPSKSRHRSAAFVVRKHSKLKRGKARIVYNYRRLNDNTYEDSYKLPNKDKLMNKIQESKYFSKFDCKSGLWQIRLAEESIHWTAFTYPEGHFEWLVMPFGLKNAPSIFQRKMDQIFKKYDKFCSVYVDDILVHSKNKNEHRKHLEIILKEFIDNGIVISRNKVELEKQDLEFLGMYIKSGTIQLQEHIAKKVLEFPDKLEDKKQLQAFLGLLNYARNFIPD

>Psal3-Chr2-05; Florendovirus; Prunus salicina 3;

DKLKNEICSELPNAFWYRKQHVVCLPYIKTFSEKNIPTKARPIQMSQEIMEFCKNEINELLQKKIIRKSKSPWSCPAFYVQKNVELERGVPRLVINYKPLNSVLEWIRYPIPNKRDLINRLGKAVIFSKFDMKSGFWQIQIDESDRYKTAFVTPFGHYEWNVMPFGLKNAPSEFQNIMNEIFNPYSHFSIVYIDDVLIFSESIEQHWKHLHKFLQIVXQNGLVVSAKKIKLFQTKVRFLGFNICQSQIAPIDRVIQFADKFRDQILDKSQLXRFLGSLNYV

>Psal3-Chr2-06; Florendovirus; Prunus salicina 3;

KKLISEVCSDLPTAFWHRKNHVVCLPYIKNFSEKNIPTKARPIQMNHEMMEFCKTEINDLLQKNIIRKSKSPWSCPAFYVQKNAELERGSPRLVINYKPLNAVLEWIRYPIPNKRDLIKRLDKAVVFSKFDMKSGFWQIQIDESDRYKTAFVTPFGHYEWNVMPFGLKNAPSEFQNIMNEIFNAYSHFSIVYIDDVLVFSESVEQHWKHLHKFLHIIKSNGLVVSAKKIKLFQTNVRFLGYNISQSQIQPIDRVIHFADKFPDQIIDKNQLQRFLGSLNYVSDF

>Psal3-Chr3-01; Florendovirus; Prunus salicina 3;

KKLISEICSDSPTAFWHRKNHVVRLPYIKNFSEKNIPTKARPIQMNHEMMEFCKSEINDLLQKNIIRKSKSPWSCPAFYVQKNAELERGSPRLVINYKPLNAVLEWIRYPIPNKRDLIKRLDKAVVFSKFDMKSGFWQIQIDESDRYKTAFVTPFGHYEWNVMPFGLKNAPSEFQNIMNEIFNAYSHFSIVYIDDVLIFSESLEQHWKHLHKFLHIIKSNGLVVSAKKIKLFQTNIRFLGYNISQSQIQPIDRVIYFADKFPDQITDKNQLQRFLGSLNYVSDF

>Psal3-Chr3-02; Yendovirus; Prunus salicina 3;

IIGEDPQLHWNKNKILAKLDIINPDLTIKTAYMPCNLLDKQEFEXQIKELLNLKVIRPSKSRHRSAAFVVRKHSELKRGKARIVYNYRRLNDNTYEDSYKLPNKDELMNKIQESKYFSKFGCKXGFWQIRLEEESIHWIAFTCPEGHFEWLVMPFGLQNAPLIFQRKMDQIFKKYDNFCSVYVDDILVHSKNKNEHRKHLEIILKEFIDNGIVISRNKVELEKQDIEFLGMYIKLGTIQLQEHIAKKVLEFPDKLEDKKQLQAFLGLLNYARSFIPD

>Psal3-Chr3-03; Florendovirus; Prunus salicina 3;

KKLISEVCSDLPTAFWHRKNHVICLPYIKNFSEKNIPTKARPIQMSHEMMEFCKTEINDLLHKNIIRKSESPWSCPAFYVQKNAELERGSPRLVINYKPLNAVLKWIRYPIPNKRDLIKRLDKAVVFSKFDMKSGFWQIQIDESDRYKTAFVTPFGHYEWNVMPFGLKNAPSEFQNIMNEIFNAYSHFSIVYIDDVLIFSESLEQHWKHLHKFLHIIKSNGLVVSAKKIKLFQTNIRFLGYNISQSQIQPIDRVIHFADKFPDQITDKNQLQRFLGSLNYVSDF

>Psal3-Chr3-04; Yendovirus; Prunus salicina 3;

LEELLQLAEQTRIIGEDPQLHWNKNKILAKLDIINPDLTIKTADMPCNLLDKQEFEQQIKELLNLKVIRPSKSRHRSAAFVVRKHSELKRGKARIVYNYRRLNDNTYEDSYKLPNKDELMNKIQESKYFSKFDCKSGFWQIRLAEESIHWTAFTCPEGHFEWLVMPFGLKNAPSIFQRKMDQIFKKYDNFCSVYVDDILVHSKNKNEHRKHLEIILKEFIDNGIVISRNKVELEKQDIEFLGMYIKSGTIQLQEHITKKVLEFPDKLEDKKQLQAFLGLLNYARNFIPD

>Psal3-Chr3-05; Florendovirus; Prunus salicina 3;

DKLKNEICSDLPNAFWYRKQHVVCLPYIKTFSEKNIPTKARPIQMSQEIMEFCKNEINELLQKKIIRKSKSPWSCPAFYVQKNAELERGVPRLVINYKPLNSVLEWIRYPIPNKRDLINRLGKAVIFSKFDMKSGFWQIQIEESDRYKTAFVTPFGHYEWNVMPFGLKNAPSEFQNIMNEIFNPYSHFSIVYIDDVLIFSESVEQHWKHLHKFLQIVKQNGLVVSAKKIKLFQTKVRFLGFNICQSQITPIDRVIQFADKFPDQILDKNQLQRFLGSLNYV

>Psal3-Chr4-01; Florendovirus; Prunus salicina 3;

EKLKQEVFSDPPNAFWHRKQHVVSLPHVKTFNEKHIPTKARPIQMSQEVMEFCKKEITELLQKWIICKSKSPWSYPAFYVQKNADLERGALRLVINYKSLNTALEWIRYPIPNKRDLINRLDKAIIFSKFDMKSGFWQIQIQKSDKYKTAFVTPFGHYEXNVMPFGLKNAPSEFQNIMNEIFNPFSDFSIIYIDDVLIFSQSIEQHLKHLYKFLQIVKRNGLVISAKKIKLFQTNIRFLGFNICQSQISPIDQVIQFADKFSGQILAKSQLQRFLGSLNYVSDF

>Psal3-Chr4-02; Florendovirus; Prunus salicina 3;

EKLKQEVFSDPPNAFWHRKQHVVSLPHVKTFNEKHIPTKARPIQMSQEVMEFCKKEITELLQKWIICKSKSPWSYPAFYVQKNADLERGALRLVINYKSLNTALEWIRYPIPNKRDLINRLDKAIIFSKFDMKSGFWQIQIQKSDKYXTAFVTPFGHYEXNVMPFGLKHAPSEFQNIMNEIFNPFSDFSIVYIDDVLIFSQSIEQNLKHLYKFLQIVKRNGLVISAKKIKLFQTNIRFLGFNICQSQISPIDQVIQFADKFSDQILAKSQLQRFLGSLNYVSDF

>Psal3-Chr4-03; Florendovirus; Prunus salicina 3;

EKLKQEVFSDPPNAFWHRKQHVVSLPHVKTFNEKHIPTKARPIQMSQEVMEFCKKEITELLQKWIICKSKSPWSYPAFYVQKNADLERGALRLVINYKSLNTALEWIRYPIPNKRDLINRLDKAIIFSKFDMKSGFWQIQIQKSDKYXTAFVTPFGHYEXNVMPFGLKHAPSEFQNIMNEIFNPFSDFSIVYIDDVLIFSQSIEQNLKHLYKFLQIVKRNGLVISAKKIKLFQTNIRFLGFNICQSQISPIDQVIQFADKFSDQILAKSQLQRFLGSLNYVSDF

>Psal3-Chr4-04; Yendovirus; Prunus salicina 3;

LEELLQLAEQTRIIGEDPQLHWNKNKILAKLDIINPDLTIKTADMPCNLLDKQEFEQQIKELLNLKVIRPSKSKHRSAAFVVRKHSELKRGKARIVYNYRRLNDNTYEDSYKLPNKDELMNKIQESKYFSKFDCKSGFWQIRLAEESIHWTAFTCPEGHFEWLVMPFGLKNAPSIFQRKMDQIFKKYDNFCSVYVDDILVHSKNKNEHRKHLEIILKEFIDNGIVISRNKVELEKQDIEFLGMYIKLGTIQLQEHITKKVLEFPDKLEDKKQLQAFLGLLNYARSFIPD

>Psal3-Chr5-01; Florendovirus; Prunus salicina 3;

KKLISEVCSDLPTAFWHRKNHIVYLPYIKNFSEKNIPIKARPIQMNHEMMEFCKSEINDLLHKNIIRKSKSPWSCPAFYVHKNAELERGSPRLVINYKPLNAVLEWIRYPIPNKRDLIKRLDKAVVFSKFDMKSGFWQIQIDESDRYKTAFVTPFGHYEWNVMPFGLKNAPSEFQNIMNEIFNAYSHFSIVYIDDVLIFSESLEQHWKHLHKFLHIIKSNGLVVSAKKIKLFQTNIRFLGYNISQSQIQPIDRVIHFADKFPDQITDKNQLQRFLGSLNYVSDF

>Psal3-Chr5-02; Florendovirus; Prunus salicina 3;

KKLISEVCSDFPTAFWHRKNHVVCLPYIKNFSEKNIPTKARPIQMSHEMMEFCKYEINDLLQKNIIRKSKSPWSCPAFYVQKNAELERGSPRLVINYKPLNAVLEWIRYPIPNKRDLIKRLNKAVIFSKFDMKSGFWQIQIDESDRYKTAFVTPFGHYEWNVMPFGLKNAPSEFQNIMNEIFNSYSHFSIVYIDDVLIFSESIEQHWKHLHKFLQIIKSNGLVVSAKKIKLFQTNIRFLGFNISQSXIHPIDRVIQFADKFPDQIVDKNQLQRFLGSLNYVSDF

>Psal3-Chr5-03; Yendovirus; Prunus salicina 3;

IIGEDPQLHWNKNKILAKLDIINPDLTIKTADMPCNLLDKQEFEQQIKELLNLKVIRPSKSRHRSAAFVVRKHSELKRGKARIVYNYRRLNDNTYEDSYKLPNKDELMNKIQESKYFSKFDCKSGFWQIRLAEESIHWTAFTCPEGHFEWLVMPFGLKNAPSIFQRKMDQIFKKYDQFCSVYVDDILVHSKNKNEHRKHLEIILKEFIDNGIVISRNKVELEKQDIEFLGMYIKSGTIQLQEHITKKVLEFP

>Psal3-Chr5-04; Florendovirus; Prunus salicina 3;

KKLISEVCSDLPTAFWHRKNHVVCLPYIKNLCEKNIPTKARPIQMSHEMMEFCKSEINDLLQKNIIRKSKSPWSCPAFYVQKNAELERGSPRLVINYKPLNAVLEWIRYPIPNKRDLIKRLDKAVIFSKFDMKSGFWQIQIDELDRYKTAFVTPFGHYEWNVMPFGLKNAPSEFQNMMNEIFNVYGHFSIVYIDDVPIFSESLEQHWKHLHKFLHIIKSNGLVVSAKKIKLFQTNIRFLGYNISQSQIQPIDRVIHFGDKFPDQITDKNQLQRFLGSLNYVSDF

>Psal3-Chr5-05; Florendovirus; Prunus salicina 3;

KKLISEVCSNLPTAFWHRKNHVVCLPYIKNFSEKNIPTKARPIQMSHEMMEFCKSEINDLLQKNIIRKSKSPWSCPAFYVQKNAELERGSPRLVINYKPLNAVLEWIRYPIPNKRDLIKRLDKAVIFSKFDMKSGFWQIQIDELDRYKTAFVTPFGHYEWNVMPFGLKNAPSEFQNIMNEIFNVYSHFSIVYIDDVLIFSESLEQHWKHLHKFLHIIKSNGLVVSAKKIKLFQTNIRFLGYNISQSQIQPIDRVLHFGDKFPDQITDKNQLQRFLGSLNYVSDF

>Psal3-Chr5-06; Florendovirus; Prunus salicina 3;

KLVSEVCSDLPTAFWHRKNHVVCLPYIKNFSEKNIPTKARPIQMNHEMMEFCKSEINDLLHKNIIRKSKSPWSCPAFYVQKNAELERGSPRLVINYKPLNAVLEWIRYPIPNKRDLIKRLDKAVIFSKFDMKSGFWQIQIDESDRYKTAFVTPFGHYEWNVMPFGLKNAPSEFQNIMNEIFNAYSHFSIVYIDDVLIFSESLEQHWKHLHKFLHIIKSNGLVVSAKKIKLFQTNIRFLGYNISQSQIQPIDRVIHFADKFPDQITDKNQLQRFLGSLNYVSDF

>Psal3-Chr5-07; Yendovirus; Prunus salicina 3;

LEELLQLAEQTRIIGEDPQLHWNKNKILAKLDIINPDLTIKTADMPCNLLDKQEFEQQIKELLNLKVIRPSKSRHRSAAFVVRKHSELKRGKARIVYNYRRLNDNTYEDSYKLPNKDELMNKIQESKYFSKFDCKSGFWQIRLAEESIQWTSFTCPEGHFEWLVMTFGLKNAPSIFQRKMDQIFKKYDKFCSVFVDDILVHSKNKNEHRKHLEIILKEFIDNGIVISRNKVELEKQEIEFLGMYIKSGTIQLQEHIAKKVLEFPDKLEDKKELQAFLGLLNYARSFIPD

>Psal3-Chr5-08; Yendovirus; Prunus salicina 3;

ILANLDIINPDLTIKTADIPCNLLDKQEFKQQIKELLNLKVIRPSKSRHRSAAFVVRKHSELKRGKARIVYNYRRLNDNTYEDSYKLPNKDELMNXIQESKYFSKFDCKSGFWKIRLAEESIQWTTFTGLEGHFEWLLMPFGLKKNAPSIFQRKMDQIFKKYDNFCSVYVDDILVHSKNKNEHRKHLEIILKEFINNGIVISKNKVELEKQDIEFLGMYIKSGTIQLQEHIAKKVIEFPDKLEDKKQLQAFLGLLNYVRSFIPD

>Psal3-Chr6-01; Florendovirus; Prunus salicina 3;

KKLIYEVCSDLPTAFWHRKNHVVCLPYIKNFSEKNIPTKARPIQMSHEMMEFCKSEINDLLQKNIIRKSKSPWSCPAFYVQKNAELERGSPRLVINYKPLNAVLEWIRYPIPNKRDLIKRLDKAVVFSKFDMKSGFWQIQIDESDRYKTAFVTPFGHYEWNVIPFGLKNAPSEFQNIMNEIFNAYSYFSIVYIDDVFIFSESLEQHWKHLHKFLHIIRSNGLVVSAKKIKLFQTNIRFLGYNISQSQIQPIDRVIHFADKFPDQITDKNQLQRFLGSLNYVSDF

>Psal3-Chr6-02; Yendovirus; Prunus salicina 3;

IIGEDPQLHWNKNKILAKLDIINPDLTIKTADMPCNLLDKQEFEQQIKELLNLKVIRPSKSRHRSAAFVVRKHSELKRGKARIVYNYRRLNDNTYEDSYKLPNKDELMNKIQESKYFSKFDCKSGFWQIRLAEESIHWTAFTCPEGHFEWLVMPFGLKNAPSIFQRKMDQIFKKYDKFCSVYVDDILVHSKNKNEHRKHLEIILKEFIDNGIVISRNKVELEKQDIEFLGMYIKSGTIQLQEHITKKVLEFPDKLEDKKQLQAFLGLLNYARSFIPD

>Psal3-Chr6-03; Yendovirus; Prunus salicina 3;

IIGEDPQLHWNKNKILAKLDIINPDLTIKTADMPCNLLDKQEFEQQIKELLNLKVIRPSKSRHRSAAFVVRKHSELKRGKARIVYNYRRLNDNTYEDSYKLPNKDELMNKIQESKYFSKFDCKSGFWQIRLAEESIHWTAFTCPEGHFEWLVMPFGLKNAPSIFQRKMDQIFKKYDKFCSVYVDDILVHSKNKNEHRKHLEIILKEFIDNGIVISRNKVELEKQDIEFLGMYIKSGTIQLQEHITKKVLEFPDKLEIKNNYKHF

>Psal3-Chr6-04; Yendovirus; Prunus salicina 3;

LEELLQLAEQTRIIGEDPQLHWNKNKILAKLDIINPDLTIKTADMPCNLLDKQEFEQQIKELLNLKVIRPSKSRHRSAAFVVRKHSELKRGKARIVYNYRRLNDNTYEDSYKLPNKDELMNKIQESKYFSKFDCKSGFWQIRLAEESIHWTAFTCPEGHFEWLVMPFGLKNAPSIFQRKMDQIFKKYDNFCSVYVDDILVHSKNKNEHRKHLEIILKEFIDNGIVISRNKVELEKQDIEFLGMYIKSGTIQLQEHITKKVLEFPDKLEDKKQLQAFLGLLNYARSFIPD

>Psal3-Chr8-01; Yendovirus; Prunus salicina 3;

LEELLQLAEQTRIIGEDPQLHWNKNKILAKLDIINPDLTIKTADMPCNLLDKQEFEQQIKELLNLKVIRPSKSRHRSAAFVVRKHSELKRGKARIVYNYRRLNDNTYEDSYKLPNKDELMNKIQESKYFSKFDCKSGFWQIRLAEESIHWTAFTCPERHFEWLVMPFRLKNAPSIFQRKMDQIFKKYDNFCSVYVDDILVHSKNKNEHRKHLEIILKEFIDNGIVISRNKVELEKQDIEFLGMYIKSGTIQLQEHITKKVLEFPDKLEHKKQLQAFLGLLNYARNFIPD

>Pser1-Chr2-01; Yendovirus; Prunus serrula 1;

LEELLQLAEQTRIIGEDPQLHWNKNKILAKLDIINPDLTIKTTDMPCNLLDKQEFEQQIKELLNLKVIRPSKSRHRSAAFVVRKHSELKRGKARIVYNYRRLNDNTYEDSYKLPNKDELMNKIQESKYFSKFDCKSGFWQIRLAEESIQWTTFTCPEGHFEWLVMPFGLKNAPSIFQRKMDQIFKKYDNFCSVYVDDILVHSKNKNEHRKHLEIILKEFIDNGIVISRNKVELEKQDIEFLRMYIKSGTIQLQEHIAKKVLEFPDKLEDKKQLQAFLGLLNYARSFIPD

>Pser1-Chr6-01; Florendovirus; Prunus serrula 1;

DKLKNEVCSDLPNAFWHRKQHVFTLHYVKSFNKKNIPTKARPIQMNQELMELCKQEVTDLVKRGIVRKSKSPWSCPTFFVQKNAKLERGAPRLVINYKPLNSVLEWIRYPIPNKRDLVNKLEKAVIFSKFDMKSDFLQIHIKDSDRYKTTFVTPFGHYEWNVMPFGLKNAPSEFQNIMNEIFNAYSEFSIVYIDDVLIYSQSIEQHWKHLHKFFQIVKANGLVLSAKKIKLFQTKVRFLGFNISQSQISPIDRVIXFADKFPNQILEKSQLQRFLGSLNYVSDF

>Pser1-Chr7-01; Yendovirus; Prunus serrula 1;

LEELLQLAEQTRIIGEDPQLHWNKNKILAKLDIINPDLTIKTANMPCNLLDKQEFEQQIKELLNLKVIRPSKSRHRSAAFVVRKHSELKRGKARIVYNYRRLNDNTYEDSYKLPNKDELTNKIQESKYFSKFDYKSGFWQIRLAEKSIQWTTFTCLEGHFEWLVMPFGLKNAPSIFQRKMDQIFKKYDNFCSVYVDDILVHSKNKNEHRKHLEIILKEFIDNGIVISRNKVELEKQDIEFLGMYIKSGTIQLQEHITKKVLEFPDKLEDKKKLQAFLGLLNYARSFIPD

>Pser1-Chr7-02; Yendovirus; Prunus serrula 1;

LEELLQLAEQTRIIGEDPQLHWNKNKILAKLDIINPDLTIKTADMPCNLLDKQEFEQQIKELLNLKVIRPSKSRHRSAAFVVRKHSELKRGKARIVYNYRRLNDNTYEDSYKLPNKDELMNKIQESKYFSKFDCKSGFWQIRLAEESIQWTTFTCPKGHFEWLVMSFGLKNAPSIFQRKMDQIFKKYDNFCSVYVDDILVHSKNKNEHRKHLEIILKEFIDNGIVISRNKVELEKQDIEFLGMYIKSGTIQLQEHITKKVLESPDKLEDKKQLQAFLGLLNYARSFIPD

>Pspe1-Chr1-01; Yendovirus; Prunus speciosa 1a;

LEELLQLAEQTRIIGEDPQLHWNKNKILAKLDIINSDLTIKTADMPCNLLDKQEFEQQIKELLNLKVIRPSKSRHRSAAFVVRKHSELKRGKATIVYNYRRLNDNTXEDSYKLPNKDELMNKIQESKYFSKFDCKSGFWQIILAEESIQWTTFTCPEGHFEWLVMPFGLKNAQSIFQRKMHQIFKKYDNFCSVYADDILVHSKNKNEHRKCLENILKXFIDNGIVISRDKVELEKQDIEFLGMYIKSGTIXLQEHIAKKVLEFPDKLEDKKQLQAFLGLLNYARSFIPD

>Pspe1-Chr2-01; Yendovirus; Prunus speciosa 1a;

IIGEDPQSHWNKNKIVSKLGIINPDLTIKIAYMPYNLLDKQXFEQQKKELLNLKVIRPSNLGIYQQPLXLENTRNLNGNTYEDSYKLPNKDVLINKIQESKYFTKFDCKLGFWQIRLAEESIQWTTFTCPEGHFEWLVMPFGLKNAPSIFQRKMDQIFKKYDNFCSVHVDDILVHSKNKNEHRKHLEIILKEFIDNGIVISRNKVELEKXDIQFLGMYIKSGTIQLQEHIPKKVLEFPDKLEDKKELQAFLGLLNYARSLIPD

>Pspe1-Chr2-02; Yendovirus; Prunus speciosa 1a;

LEELLQLAEQTRIIGEDPQLHWNKNKILAKLDIINPDLTIKTADMPCNLLDKQEFEQQIKELLNLKVIRPSKSRHRSAAFVVRKHSELKRGKARIVYNYRRLNDNTYEDKDELMNKIQESKYFSKFDCKSGFWQIKLAEESIQWTAFTCPEGHFEWLVMPFGLKNAPSIFQRKMDQIFKKYDNFCSVYVDDILVHSKNKNEHRKHLEIILKEFIDNGIVISRNKVELEKQDIEFLGMYIKSGTIQLQEHITKKVLEFPDKLEDKKQLQAFLGLLNYARSFI

>Pspe1-Chr4-01; Yendovirus; Prunus speciosa 1a;

HWNKNKILAKLGIINPDLTIKTVDMSCNLLYKQEFEQQIXELLNLKVIRSSKSRHRSATFVFRKHSELKRCKARIVYNYRRLNDNTYEDSYKLPNKAELMNKIQESKYFSKFDCKSGFWQIRLAEESIQWTTFTCLEGHFKWLVMPFGLKNAPLSFQRKMDQIFKKYDNFCSVYVDDILVHNKNKNELRKHLEIILKEFIDNGIVKNRNKVELEKQDMEFLGMYIKSGTIQLQEHIAQKVLKFPDKLEDKKQLQAFLGLLNYARSFIPD

>Pspe1-Chr5-01; Florendovirus; Prunus speciosa 1a;

EKLKNEVCSDMPNAFWHRKQHVVSLPYVKSFSVKNIPTKAHPIQMSQELMELCKTEVTDLVNKGIVRKSKSPWSCPAFYVQKNAELERGAPRLVINYKPLNSVLEWIRYPIPNKRDLVNRLEKAVIFSKFDMKSGFWQIQIKEXDRYKTAFVTPFGHYEWNVMPFGLKNAFSEFQNIMNEIFNDYSHFSIVYIDDVLIFSKSIEQHWKHLHKFLQIVKTKGLVISAKKIKLFQVNVRFLGFNISQNQISPISRVIQFADKFPDQIFEKSQLQRFLGSLNYVSDF

>Pspe1-Chr6-01; Yendovirus; Prunus speciosa 1a;

LEELLQLAEQTRIIGEDPQLHWNKNKILAKLDIINPNLTIKTADMPCNLLDKQEFEQQIKELLNLKVIRPSKSRHRSAAFVVRKHSELKRGKARIVYNYRRLNDNTYEDSYKLPNKDELMKKIQESKYFSKFDCKSGFWQIRLAEESIQWTTFTCPEGHFEWLVIPFGLKNAPSIFQRNMDQIFKKYDNFCSVYVDDILVHSKNKNEHRKHLEIILKEFFDNGIVISRNKVELEKQDIEFLGMYIKSGTIQLQEHIAQKVLEFPDKLEDKKQLQAFLGLLNYARSFIPD

>Pspe1-Chr7-01; Yendovirus; Prunus speciosa 1a;

LEELLQLAEQTRIIGEDPQLHWNKNKILAKLDIINPDLTIKTADMPCNLLDKQEFEQQIKELLNLKVIRPSKSRHRSAAFVVRKHSELKRGKARIVYNYRRLNDNTYEDSYKLPNKDELMNKIQESKYFSKFDCKSGFWQIRLAEESIQWTTFTCPEGHFEWLVMPFGLKNAPSIFQRKMDQIFKKYDNFCSVYVDDILVHSKNKNEHRKHLEIILKEFIDNGIVISRNKVELEKQDIEFLGMYIKSGTTQLQEHITKKVLEFSDKLEDKKQLQAFLGLLNYARSFIPD
