# Supplementary material for: A comprehensive comparative genomic analysis of the endogenous Florendovirus-1 in Prunus
Source: Front Plant Sci. 2026 Sep 29;17:1886618. doi: 10.3389/fpls.2026.1886618 (PMC13658181; doi:10.3389/fpls.2026.1886618)
Supplement: Supplementary file 1 [file SupplementaryFile1.zip › Supplementary Material 6 - Florendovirus-1 consensus sequence.docx]

Supplemental File 6 – Florendovirus-1 consensus sequence.

>Consensus

TGAAAGCATTATTTTAATGCATTATCATTATTTTCTGCATTAGTCTGAAAGTTGCTTAGGTTGCATCCTTTTCTAATTTCTTTCTGATCTTCATTCTGTATTATATTCAGTTGCTTGTTTGTTCTATCTCTTTGTCCCTCTTTCTTGGTATCAGAGCCGTAAGTGTTGCGGTCTTTGTCTAAGTCTGCTACTCATCCCCGCCCAAGTGGCTGGTTTAAGCTTCTGAGTAGGAGGTTTTCATTTTTTCCTTTTTCTTCCCTTTTATTCTTTGAGTCATCTGTTGGTTTTCTCTAAGGTCTGACCCGTGAGTCCTTTTTTCTAGATCAGTAAGTCCCCAGGGAGGGCGTGTGTGCTGGGCAGGAGACAGAAAATTGAGAAAATTAATAGGTTGCTTAGAGTCTAAACGGTCGGTTTAGGATCTACTGTTCATCTTCTTCCTCAGTCTGCCAAACCCAGTTTCTTGAGTAGTCATGAGTCGTTTATTTAGAACAAAGTCTTGTAATTCTACCAGTTCTAGTGCCAGTATAAGTCGACTCCCAGAGATAGTTAATGAAGAACAATTTGAATATAATTCCAGTAGTAAAGAACAATTAGATTTTAGAGATTGGAATATACCTAAAGTCCCAAGTCAGAAAATCTATAAGAAACATTGGTTGCCTTCTAGTTTTAATAGCACAACACATGTCAAAACAGTTGAACAAGTTTACGCCTTAAGTAAAGAACATGAAACTTGTCAGTTGCTTAATCTTGAGTCAATTTTAAAACATAAGAAAGATGGTAATAATTTCCTGCATATTGGTTTAGTTCAAGTTGCTGTTAAGCCCCTGACACGGTTAGGATTGAAAGCTTCAATCCTGCTATGTCTGCGTGATGCTAGATTCACTGAATTTAGCGATAGTACCCTTGGAATCATTGAGTCCAGTCTGTGTAATGGCCCTGTCCATTTTGATTGCTATCCTGATTTCACTGTAAGCCTTAGTGATCCTCATATCCTGAAAACTTTGACTCTGAATATTAAGACTGAAGGTTATAATGTTCTGCCAGGAACCCAACCTTTAGCCTTAGTTTACAGAATCTATTATAAGGTCACAGGAACTAATATGAATTTCCAAGCCCTTAATAAAAGCCCAAAGGATCAAACCGTCTTGATCCAGAGTCATACCTCAGATGCCCATATACAAGTTCCTCATACTATTAAGTGGTCAGAAGTTGCCCTTCCAAAAGATTGGACCTTAGTCACTGAGAGCCAACCAGCTCCTATCCAGCGTAGTCTAAACAATTTAGACTACATCCAACAATACTTAGATGGCACAGTTAAAATTAAGTTTGATAGTCATCCTTTACGAAAATCTAATGTCCAGTTACAACAGTTGCCAACTCCTGGTCAATCCCAGAGGCATGTCCCTGCTCGGCATTCCTTTGCTGCCTCTTCCTCCACTCTAGAACGAGACCTAGAGTTAGAAAAGGCTATGATTGATTTCAAACTTGAGTCCCTGAGAAAAACCTCTCAGGTTACCCAACCTTGCTACGGCAAGGTTCCAGTTCAAGAAGATAAGTCTGAATCACCTGAGTCACCTACCCAGTCTGACTTCGTAGTTGAAAATCAGCTTAGAACCCTTAATAAAAAATTTGAAATTAATTGGGTAAGATTAAATCGTCATCTAAAAGCCCCTGAAAACCAGGCAAGAAGAGATAATTATCATCAAGCCTACCCTAATAGCAAACACCGAGAAATGATCTTTAAACAATGGAAAGATTATATGCGGTCAGCCAAAGTTGAAATTTATTACCTTGATTTTGTAGAAAGCCGTTATATGTCTAATGATGAATTAAAAACTTTAACTAAAGAAAAATGGAAAATAATAGATAAATCTGTAGTTGAGTCTAGTCATCCTCCAGTAGAAACTATAATAATAGAACACCAGAAAGCACCAGTTCCTGCCACTCCTTTCAAAACCTTTGAAAGCAATGACCCTAATAGAAAACTAATTGAACAAAATAATTACACCAACCAGAGTCTAATCACCATAGGAAAACAATTAGATACAATTGAAACCAAAGTTGATATGTTGAGTCCGCCTGTTGCAGACTCCAGACCCAAACCTAAAGTTGAAAAACCAATAGTTCATTTCCAAGAACTAAAGTCTAGTCCAGCTTTTAAAACTAATTCTACAATGAAAAAGATAGAAGAAATGTTAGAACAATTGACCCCTGTCAAACCCGAGAAAGGTGAAAAATCTGGTATTAAAACACTAGATGCTTTTGCTGCTTCTCAATCTGAGTCTGAATCAGTAGCAGAAACTAGTAATATTTCTAAAATTGAAAATGCTTTCAAAAATCTAGAAGTCCAAACTGAGCCCAAAGTCAAAAGAATAGAAAAACATATAAGCCCTACTAGCCTAACAAAAAATTGGTATCCCAGACCAACACCTCCAGATATCCAATTTGAAGAAAGAAACTTTCAAACCCAGTTTTCTGTTTCTTCTGATAAATTGTATGAATGGAATATTGATGGTCTTTCTGAGCAAGAAATCCTAAATAAACTGCAACATATGTCTATGGTCGCCAATAGTTACATCACCAATCACAGTTTCAGACAGTCTGAAATTGTTCCTCTGTTAGTTACTGGTTTCACAGGTACTCTCAGATCTTGGTGGGATAAACACCTAACCCCTGAGTCTAAAAATCGAATAATTTATGCTGTAAAACTTAATGAAGACGGTCTCCCTATTTTTGATGAACAAATAGGTCAAGGTATAGAAGACGGTGTCAACACTCTGTTTTACACAATAATTGAACATTTCATTGGAACCCCTAGTAATACCACTGCTAGGATACATGATCAGTTGAGTAATCTTAGATGCCCAAAGTTGTCTGATTTCAGATGGTATAAAGATGTTTTTATTTCTAGAGTCATGCTTAGAGATGATAGTAATCAACCATTTTGGAAAGAAAAGTTTATTAATGGTCTGCCTAATTTATTTGCTCATAAAATACGCACAACCCTAAGTAATGAACAAGGTCAAATTGATTGGGATAATTTAACTTATGGAAATATTATAAGCACAATTAATCAAGTTGGTATGAAAATGTGTATTGACTTTAAAATTAATAAACAAATCCAGTCTGATAGAAAGTCAGCCAAGTATGAGTTAGGTAATTTCTGTGAACAATATGGTCTCATTACTATTCCTCCTTCTAGAAAAAATAAGCCTAGTCATCTTAGAAAACGCCATTATCCGAGTAGAAAGAAACATTTTCTTGAGAAAGATGAATTCTATAAGAAAAGAAAGTTCTCCCCTAAGAAAAATTGGTCAAAACGTCCCAAACAAAATCATTATGCTTCTGAATGTAAAGTAAAAGATGCTATTAAACAATTACAAATTACAGAAGAAGATAAGAACAAATTAATCCAAGTTTTGGAGTTGCGAGACTCTGAAATAAGTGATGTTGAATCCATGGTTACTAGTTCTGAGTCTGAGTATAGCCAGTCTTCAGATTCACAACCCAGTTCACCTAGAATCCAATTTGGTTGCACAGATAAATGTTGTAATAGGTTAAAGTCTATTTTTGTTCTCACTAAACAAGAAGAACAAGAAGAATTATTAATAGATTTAATAGGTAAAGTTGAAAACCCTGAGTTAAAATCTGAATATTTAAAAAAATTAAAAAAGATCATAAGTCAGGAAGGCAGTTCAAATTATCCTACTCCCCAGCCAATTAGCATCAACACTACTTTAGAAAAATTCAATCGTAAGAAAGATGTTACCTTACATGATCTACAATTAGAAGTCAAACAAGTTAAAAAAGAAATAGTTGAGTTAAAACAAATAAGTCAACAATTGCAAACTGAGAATTATGAGATCAAACAAGATTTATTTGACTTAGAAAAAGAAACTTCTAAGTCTAATAACCATTCAGAAAGTCCGCATCATTCTGATGATGAACCTTCTGAAGAACAGCCAGTTGTCAATTTAATTAAACAAGTTCATCTTAGAAAATGGTATTCTAAAGTCACGATATTTGTTCAAGATTTTGAATTTACCACAGTTGCCTTATTTGATTCTGGTGCAGATCTTAACTGTATTCAAGAAGGTCTAATCCCTACTAAGTATTACCAGAAGTCTAGAGAATCCCTTAGCACTGCCTCAGGGAAATCTCTCCAGTTAAATTTTGAAATACCAAAAGCCCATATTTGTCAAAACAAAGTCTGTTTTAAAACTTCATTCGTCTTAGTTAAAAATATTACTGATGAAGTCATCTTAGGATTACCTTTCCTTGCCCTGTTGTATCCTTTCCAGGTAGAATACGATGGTGTCATTTCTACCTATTTAGGAGAAAAGGTCAAGTTTGAATTCTTGTCTAAACCTGAGTTGCATAATTTAAAAGCTTTACAAAAACAGTCAGTCTCTAAATCTGTCCAAATTATAAGAAAAACTAATCAATTGAATTTTCTTAAAGAAGAAGTTAAATTTAAAAGAATTGAACAACAACTTAGTGATAAAGCTTTACAGTTAAAAATTCAGTCTTTTGAAAACAAATTAAAAGAAGAAATATGTTCTGATTTGCCTACTGCTTTTTGGTATAGAAAACAGCATGTTGTCTGTCTTCCTTATATCAAAACCTTTAGTGAGAAAAATATCCCCACTAAAGCCAGGCCAATTCAGATGAATCAAGAATTAATGGAATTTTGTAAAAAAGAAATTCATGAGTTGCTTCAAAAAAATATAATTCGCAAAAGTAAGTCCCCCTGGTCTTGTCCTGCTTTTTATGTCCAGAAAAATGCTGAATTAGAAAGAGGAGTCCCCAGATTAGTTATCAATTATAAGCCTCTTAATTCGGTCTTAGAATGGATTAGGTATCCCATACCTAATAAAAGAGATCTTATTAATCGATTAGAAAAAGCAGTTGTCTTCTCTAAATTTGATATGAAAAGTGGTTTCTGGCAAATCCAAATAGATGAGTCAGATAGGTATAAAACTGCATTTGTTACTCCTTTCGGTCATTATGAATGGAATGTTATGCCGTTTGGTTTTAAAAATGCCCCTAGTGAATTCCAGAATATTATGAATGATATCTTCAATCCTTACAGTCATTTCTCGATTGTTTATATTGATGATGTTCTTATATTTTCTGAGTCAGTAGAACAACATTGGAAACATTTGCATAAATTCCTTCACATAGTCAAACATTCTGCTAAGAAAATTAAGTTATTTCAAACTAATATTAGATTTCTTGGTTTTAATATCTGCCAGTCACAAATTACTCCAATAGATCGAGTCATCCAATTTGCTGATAAGTTCCCTGATCAGATTCTTGATAAAAGTCAACTCCAGAGGTTTTTAGGATCTCTAAATTATGTTTCTGATTTTTATCAAAATCTGAGAAAACAATGTAAGCCTCTATTTGATAGATTGCAGAATAATCCTCCTCCTTGGTCAACTATTCATACTGAAATTGTCAAACAGATCAAAGCCCATGTCAAGACACTTCCCTGTCTTGGTATTCCTTCAGTTGATTCTTTCAAAATAGTTGAAACTGATGCCTCTGACATTGGTTATGGTGGTATTCTCAAACAGAAAGTCAGTCCAGATTCTCCAGAGCAAATTGTTCGTTTCCATTCTGGAACCTGGACTCAGTCGCAATATAATTATAGTACTATTAAAAAAGAAATATTAGCTATTGTATTATGCATTAGTAAATTTCAAGATGATTTATTAAATCAAAAGTTTTTAATCCGAGTTGATTGCAAATCTGCAAAACATGTTTTAGAAAAAGATGTTCAAAACATTGCATCTAAACAGATTTTTGCACGATGGCAAAGTATATTAAGTATATTTGATTTTGATATTGAATATTTGAGAGGTAGTGAGAATTCAGTCCCTGATTTTCTTACTCGAGAATTTTTGCAGGAACCACAATGGCTCCCAAAAAAGACAAAAACAAAGAAGTTGCCAGTCAACCCAAGTCAACCCAGTCGCAACCCAAGTCATCCCAGTTGCAACCATCTCAGTCGCCATCCATGAGTCCAGCCAAGCCAGAAATGCCCAAACAGATAGTTCCCTATCCTGGTTGCACCCCTCTTACAGTTGCCACCCCATTATCAGTTGCCAACAGATTCTCTTCCTTAGGATCTACAGTTGGCCAAATCTGCCCCAGTTACCAATCCACATTAGTTGCCAGTTATGACCCTTTTTCAGTAGATGTCCCAGCAGGACCGTCAATTGCTTTGAAAAAATCTTCTCCTTATTTGCCAAAGAGTAATTCCCATCTATTTGTCCTTGAGCCTTTGTATGATATTCATACTAACCCAGTTGAAATTGCCAAACATTATTTCCCTCCTGGATTCCATTATATGCCTTCCAGTCCTTACAAGTCCCTCAAGTATTATAGAGATATATTGCTTGAAACTAAGTCAGTTGAAATTAAGCTGATAAAGGATCGAGATCACCCTGAGATCATTCTTTATCATTCAATGTACATCCACCATATCATAAGCCAAGAAGCCTTCAGTAGTCGCCCTTATGAGTTAAAAACCCTTCAATCCAAATTACAGTATAATTATGCTGATTATATTGATGCCTGGTACACTATTTTCCTTCACCAATCTGAGGATTTTAGTCATTCCTGGTTCATCAATTTCGATAATAAGTTCAAATGTTCTTTCCCCTATTGGTTCCTCCATTGGTGGGAAAAACATGGCCCAGTTGATGAAATTCTGCCTGTATCAGTTCTTGAATTAATCCGCCATTATACGGAGACTGCCAAGTTCTCCACCGTTGATTTATTCTTCCCCAAACAATTGCTTTTCCTTGCCAGATAAAAAGTTCCTTGGATCCTCAAATGGTCCTACAGAGTCACTAAGGATGTTCGTATCTTTGCCCGCCAATTTTCAGTCAAATGGTGGGACAAATTTGAAGTTCAAAGAATAGCCAAGTATGTTTACAATGATCTCCCTGCTGAGCCTCAGTCAACTTCTCCAGTTGGTTCTGTTCGATCCTCCCTCTCAGTCGAAGGAAAGTCGAAAGCAGAACTCCAAGAAATTGCTCGCCAGTTGATGATGCAGGCCTCTCAGATGGATGATGATGAGGACAACGACTCTCCTTCGTCGTCCAGTTGTCAACCCATTCCTGATCCTGTTAAGCCTAAGTCCTGGTATGATGACAGCCAGGATCCCTATGATGCCTATGATC

Lettres in red: hairpin-like structure.

HAIRPIN

32/50(64%)

Query 2179 CAGGAGACAGAAAATTGAGAAAATTAATAGGTTGCTTAGAGTCTAAACGG 2228

|| | | |||| | ||||||| || || | || | || |||| ||

Sbjct 2128 CACGGGTCAGACCTTAGAGAAAACCAACAGATGACTCAAAGAATAAAAGG 2079

>ORF1 (DNA)

ATGAGTCGTTTATTTAGAACAAAGTCTTGTAATTCTACCAGTTCTAGTGCCAGTATAAGTCGACTCCCAGAGATAGTTAATGAAGAACAATTTGAATATAATTCCAGTAGTAAAGAACAATTAGATTTTAGAGATTGGAATATACCTAAAGTCCCAAGTCAGAAAATCTATAAGAAACATTGGTTGCCTTCTAGTTTTAATAGCACAACACATGTCAAAACAGTTGAACAAGTTTACGCCTTAAGTAAAGAACATGAAACTTGTCAGTTGCTTAATCTTGAGTCAATTTTAAAACATAAGAAAGATGGTAATAATTTCCTGCATATTGGTTTAGTTCAAGTTGCTGTTAAGCCCCTGACACGGTTAGGATTGAAAGCTTCAATCCTGCTATGTCTGCGTGATGCTAGATTCACTGAATTTAGCGATAGTACCCTTGGAATCATTGAGTCCAGTCTGTGTAATGGCCCTGTCCATTTTGATTGCTATCCTGATTTCACTGTAAGCCTTAGTGATCCTCATATCCTGAAAACTTTGACTCTGAATATTAAGACTGAAGGTTATAATGTTCTGCCAGGAACCCAACCTTTAGCCTTAGTTTACAGAATCTATTATAAGGTCACAGGAACTAATATGAATTTCCAAGCCCTTAATAAAAGCCCAAAGGATCAAACCGTCTTGATCCAGAGTCATACCTCAGATGCCCATATACAAGTTCCTCATACTATTAAGTGGTCAGAAGTTGCCCTTCCAAAAGATTGGACCTTAGTCACTGAGAGCCAACCAGCTCCTATCCAGCGTAGTCTAAACAATTTAGACTACATCCAACAATACTTAGATGGCACAGTTAAAATTAAGTTTGATAGTCATCCTTTACGAAAATCTAATGTCCAGTTACAACAGTTGCCAACTCCTGGTCAATCCCAGAGGCATGTCCCTGCTCGGCATTCCTTTGCTGCCTCTTCCTCCACTCTAGAACGAGACCTAGAGTTAGAAAAGGCTATGATTGATTTCAAACTTGAGTCCCTGAGAAAAACCTCTCAGGTTACCCAACCTTGCTACGGCAAGGTTCCAGTTCAAGAAGATAAGTCTGAATCACCTGAGTCACCTACCCAGTCTGACTTCGTAGTTGAAAATCAGCTTAGAACCCTTAATAAAAAATTTGAAATTAATTGGGTAAGATTAAATCGTCATCTAAAAGCCCCTGAAAACCAGGCAAGAAGAGATAATTATCATCAAGCCTACCCTAATAGCAAACACCGAGAAATGATCTTTAAACAATGGAAAGATTATATGCGGTCAGCCAAAGTTGAAATTTATTACCTTGATTTTGTAGAAAGCCGTTATATGTCTAATGATGAATTAAAAACTTTAACTAAAGAAAAATGGAAAATAATAGATAAATCTGTAGTTGAGTCTAGTCATCCTCCAGTAGAAACTATAATAATAGAACACCAGAAAGCACCAGTTCCTGCCACTCCTTTCAAAACCTTTGAAAGCAATGACCCTAATAGAAAACTAATTGAACAAAATAATTACACCAACCAGAGTCTAATCACCATAGGAAAACAATTAGATACAATTGAAACCAAAGTTGATATGTTGAGTCCGCCTGTTGCAGACTCCAGACCCAAACCTAAAGTTGAAAAACCAATAGTTCATTTCCAAGAACTAAAGTCTAGTCCAGCTTTTAAAACTAATTCTACAATGAAAAAGATAGAAGAAATGTTAGAACAATTGACCCCTGTCAAACCCGAGAAAGGTGAAAAATCTGGTATTAAAACACTAGATGCTTTTGCTGCTTCTCAATCTGAGTCTGAATCAGTAGCAGAAACTAGTAATATTTCTAAAATTGAAAATGCTTTCAAAAATCTAGAAGTCCAAACTGAGCCCAAAGTCAAAAGAATAGAAAAACATATAAGCCCTACTAGCCTAACAAAAAATTGGTATCCCAGACCAACACCTCCAGATATCCAATTTGAAGAAAGAAACTTTCAAACCCAGTTTTCTGTTTCTTCTGATAAATTGTATGAATGGAATATTGATGGTCTTTCTGAGCAAGAAATCCTAAATAAACTGCAACATATGTCTATGGTCGCCAATAGTTACATCACCAATCACAGTTTCAGACAGTCTGAAATTGTTCCTCTGTTAGTTACTGGTTTCACAGGTACTCTCAGATCTTGGTGGGATAAACACCTAACCCCTGAGTCTAAAAATCGAATAATTTATGCTGTAAAACTTAATGAAGACGGTCTCCCTATTTTTGATGAACAAATAGGTCAAGGTATAGAAGACGGTGTCAACACTCTGTTTTACACAATAATTGAACATTTCATTGGAACCCCTAGTAATACCACTGCTAGGATACATGATCAGTTGAGTAATCTTAGATGCCCAAAGTTGTCTGATTTCAGATGGTATAAAGATGTTTTTATTTCTAGAGTCATGCTTAGAGATGATAGTAATCAACCATTTTGGAAAGAAAAGTTTATTAATGGTCTGCCTAATTTATTTGCTCATAAAATACGCACAACCCTAAGTAATGAACAAGGTCAAATTGATTGGGATAATTTAACTTATGGAAATATTATAAGCACAATTAATCAAGTTGGTATGAAAATGTGTATTGACTTTAAAATTAATAAACAAATCCAGTCTGATAGAAAGTCAGCCAAGTATGAGTTAGGTAATTTCTGTGAACAATATGGTCTCATTACTATTCCTCCTTCTAGAAAAAATAAGCCTAGTCATCTTAGAAAACGCCATTATCCGAGTAGAAAGAAACATTTTCTTGAGAAAGATGAATTCTATAAGAAAAGAAAGTTCTCCCCTAAGAAAAATTGGTCAAAACGTCCCAAACAAAATCATTATGCTTCTGAATGTAAAGTAAAAGATGCTATTAAACAATTACAAATTACAGAAGAAGATAAGAACAAATTAATCCAAGTTTTGGAGTTGCGAGACTCTGAAATAAGTGATGTTGAATCCATGGTTACTAGTTCTGAGTCTGAGTATAGCCAGTCTTCAGATTCACAACCCAGTTCACCTAGAATCCAATTTGGTTGCACAGATAAATGTTGTAATAGGTTAAAGTCTATTTTTGTTCTCACTAAACAAGAAGAACAAGAAGAATTATTAATAGATTTAATAGGTAAAGTTGAAAACCCTGAGTTAAAATCTGAATATTTAAAAAAATTAAAAAAGATCATAAGTCAGGAAGGCAGTTCAAATTATCCTACTCCCCAGCCAATTAGCATCAACACTACTTTAGAAAAATTCAATCGTAAGAAAGATGTTACCTTACATGATCTACAATTAGAAGTCAAACAAGTTAAAAAAGAAATAGTTGAGTTAAAACAAATAAGTCAACAATTGCAAACTGAGAATTATGAGATCAAACAAGATTTATTTGACTTAGAAAAAGAAACTTCTAAGTCTAATAACCATTCAGAAAGTCCGCATCATTCTGATGATGAACCTTCTGAAGAACAGCCAGTTGTCAATTTAATTAAACAAGTTCATCTTAGAAAATGGTATTCTAAAGTCACGATATTTGTTCAAGATTTTGAATTTACCACAGTTGCCTTATTTGATTCTGGTGCAGATCTTAACTGTATTCAAGAAGGTCTAATCCCTACTAAGTATTACCAGAAGTCTAGAGAATCCCTTAGCACTGCCTCAGGGAAATCTCTCCAGTTAAATTTTGAAATACCAAAAGCCCATATTTGTCAAAACAAAGTCTGTTTTAAAACTTCATTCGTCTTAGTTAAAAATATTACTGATGAAGTCATCTTAGGATTACCTTTCCTTGCCCTGTTGTATCCTTTCCAGGTAGAATACGATGGTGTCATTTCTACCTATTTAGGAGAAAAGGTCAAGTTTGAATTCTTGTCTAAACCTGAGTTGCATAATTTAAAAGCTTTACAAAAACAGTCAGTCTCTAAATCTGTCCAAATTATAAGAAAAACTAATCAATTGAATTTTCTTAAAGAAGAAGTTAAATTTAAAAGAATTGAACAACAACTTAGTGATAAAGCTTTACAGTTAAAAATTCAGTCTTTTGAAAACAAATTAAAAGAAGAAATATGTTCTGATTTGCCTACTGCTTTTTGGTATAGAAAACAGCATGTTGTCTGTCTTCCTTATATCAAAACCTTTAGTGAGAAAAATATCCCCACTAAAGCCAGGCCAATTCAGATGAATCAAGAATTAATGGAATTTTGTAAAAAAGAAATTCATGAGTTGCTTCAAAAAAATATAATTCGCAAAAGTAAGTCCCCCTGGTCTTGTCCTGCTTTTTATGTCCAGAAAAATGCTGAATTAGAAAGAGGAGTCCCCAGATTAGTTATCAATTATAAGCCTCTTAATTCGGTCTTAGAATGGATTAGGTATCCCATACCTAATAAAAGAGATCTTATTAATCGATTAGAAAAAGCAGTTGTCTTCTCTAAATTTGATATGAAAAGTGGTTTCTGGCAAATCCAAATAGATGAGTCAGATAGGTATAAAACTGCATTTGTTACTCCTTTCGGTCATTATGAATGGAATGTTATGCCGTTTGGTTTTAAAAATGCCCCTAGTGAATTCCAGAATATTATGAATGATATCTTCAATCCTTACAGTCATTTCTCGATTGTTTATATTGATGATGTTCTTATATTTTCTGAGTCAGTAGAACAACATTGGAAACATTTGCATAAATTCCTTCACATAGTCAAACATTCTGCTAAGAAAATTAAGTTATTTCAAACTAATATTAGATTTCTTGGTTTTAATATCTGCCAGTCACAAATTACTCCAATAGATCGAGTCATCCAATTTGCTGATAAGTTCCCTGATCAGATTCTTGATAAAAGTCAACTCCAGAGGTTTTTAGGATCTCTAAATTATGTTTCTGATTTTTATCAAAATCTGAGAAAACAATGTAAGCCTCTATTTGATAGATTGCAGAATAATCCTCCTCCTTGGTCAACTATTCATACTGAAATTGTCAAACAGATCAAAGCCCATGTCAAGACACTTCCCTGTCTTGGTATTCCTTCAGTTGATTCTTTCAAAATAGTTGAAACTGATGCCTCTGACATTGGTTATGGTGGTATTCTCAAACAGAAAGTCAGTCCAGATTCTCCAGAGCAAATTGTTCGTTTCCATTCTGGAACCTGGACTCAGTCGCAATATAATTATAGTACTATTAAAAAAGAAATATTAGCTATTGTATTATGCATTAGTAAATTTCAAGATGATTTATTAAATCAAAAGTTTTTAATCCGAGTTGATTGCAAATCTGCAAAACATGTTTTAGAAAAAGATGTTCAAAACATTGCATCTAAACAGATTTTTGCACGATGGCAAAGTATATTAAGTATATTTGATTTTGATATTGAATATTTGAGAGGTAGTGAGAATTCAGTCCCTGATTTTCTTACTCGAGAATTTTTGCAGGAACCACAATGGCTCCCAAAAAAGACAAAAACAAAGAAGTTGCCAGTCAACCCAAGTCAACCCAGTCGCAACCCAAGTCATCCCAGTTGCAACCATCTCAGTCGCCATCCATGA

>ORF2 (DNA)

ATGGCTCCCAAAAAAGACAAAAACAAAGAAGTTGCCAGTCAACCCAAGTCAACCCAGTCGCAACCCAAGTCATCCCAGTTGCAACCATCTCAGTCGCCATCCATGAGTCCAGCCAAGCCAGAAATGCCCAAACAGATAGTTCCCTATCCTGGTTGCACCCCTCTTACAGTTGCCACCCCATTATCAGTTGCCAACAGATTCTCTTCCTTAGGATCTACAGTTGGCCAAATCTGCCCCAGTTACCAATCCACATTAGTTGCCAGTTATGACCCTTTTTCAGTAGATGTCCCAGCAGGACCGTCAATTGCTTTGAAAAAATCTTCTCCTTATTTGCCAAAGAGTAATTCCCATCTATTTGTCCTTGAGCCTTTGTATGATATTCATACTAACCCAGTTGAAATTGCCAAACATTATTTCCCTCCTGGATTCCATTATATGCCTTCCAGTCCTTACAAGTCCCTCAAGTATTATAGAGATATATTGCTTGAAACTAAGTCAGTTGAAATTAAGCTGATAAAGGATCGAGATCACCCTGAGATCATTCTTTATCATTCAATGTACATCCACCATATCATAAGCCAAGAAGCCTTCAGTAGTCGCCCTTATGAGTTAAAAACCCTTCAATCCAAATTACAGTATAATTATGCTGATTATATTGATGCCTGGTACACTATTTTCCTTCACCAATCTGAGGATTTTAGTCATTCCTGGTTCATCAATTTCGATAATAAGTTCAAATGTTCTTTCCCCTATTGGTTCCTCCATTGGTGGGAAAAACATGGCCCAGTTGATGAAATTCTGCCTGTATCAGTTCTTGAATTAATCCGCCATTATACGGAGACTGCCAAGTTCTCCACCGTTGATTTATTCTTCCCCAAACAATTGCTTTTCCTTGCCAGATAA

>ORF1 (aa)

MSRLFRTKSCNSTSSSASISRLPEIVNEEQFEYNSSSKEQLDFRDWNIPKVPSQKIYKKHWLPSSFNSTTHVKTVEQVYALSKEHETCQLLNLESILKHKKDGNNFLHIGLVQVAVKPLTRLGLKASILLCLRDARFTEFSDSTLGIIESSLCNGPVHFDCYPDFTVSLSDPHILKTLTLNIKTEGYNVLPGTQPLALVYRIYYKVTGTNMNFQALNKSPKDQTVLIQSHTSDAHIQVPHTIKWSEVALPKDWTLVTESQPAPIQRSLNNLDYIQQYLDGTVKIKFDSHPLRKSNVQLQQLPTPGQSQRHVPARHSFAASSSTLERDLELEKAMIDFKLESLRKTSQVTQPCYGKVPVQEDKSESPESPTQSDFVVENQLRTLNKKFEINWVRLNRHLKAPENQARRDNYHQAYPNSKHREMIFKQWKDYMRSAKVEIYYLDFVESRYMSNDELKTLTKEKWKIIDKSVVESSHPPVETIIIEHQKAPVPATPFKTFESNDPNRKLIEQNNYTNQSLITIGKQLDTIETKVDMLSPPVADSRPKPKVEKPIVHFQELKSSPAFKTNSTMKKIEEMLEQLTPVKPEKGEKSGIKTLDAFAASQSESESVAETSNISKIENAFKNLEVQTEPKVKRIEKHISPTSLTKNWYPRPTPPDIQFEERNFQTQFSVSSDKLYEWNIDGLSEQEILNKLQHMSMVANSYITNHSFRQSEIVPLLVTGFTGTLRSWWDKHLTPESKNRIIYAVKLNEDGLPIFDEQIGQGIEDGVNTLFYTIIEHFIGTPSNTTARIHDQLSNLRCPKLSDFRWYKDVFISRVMLRDDSNQPFWKEKFINGLPNLFAHKIRTTLSNEQGQIDWDNLTYGNIISTINQVGMKMCIDFKINKQIQSDRKSAKYELGNFCEQYGLITIPPSRKNKPSHLRKRHYPSRKKHFLEKDEFYKKRKFSPKKNWSKRPKQNHYASECKVKDAIKQLQITEEDKNKLIQVLELRDSEISDVESMVTSSESEYSQSSDSQPSSPRIQFGCTDKCCNRLKSIFVLTKQEEQEELLIDLIGKVENPELKSEYLKKLKKIISQEGSSNYPTPQPISINTTLEKFNRKKDVTLHDLQLEVKQVKKEIVELKQISQQLQTENYEIKQDLFDLEKETSKSNNHSESPHHSDDEPSEEQPVVNLIKQVHLRKWYSKVTIFVQDFEFTTVALFDSGADLNCIQEGLIPTKYYQKSRESLSTASGKSLQLNFEIPKAHICQNKVCFKTSFVLVKNITDEVILGLPFLALLYPFQVEYDGVISTYLGEKVKFEFLSKPELHNLKALQKQSVSKSVQIIRKTNQLNFLKEEVKFKRIEQQLSDKALQLKIQSFENKLKEEICSDLPTAFWYRKQHVVCLPYIKTFSEKNIPTKARPIQMNQELMEFCKKEIHELLQKNIIRKSKSPWSCPAFYVQKNAELERGVPRLVINYKPLNSVLEWIRYPIPNKRDLINRLEKAVVFSKFDMKSGFWQIQIDESDRYKTAFVTPFGHYEWNVMPFGFKNAPSEFQNIMNDIFNPYSHFSIVYIDDVLIFSESVEQHWKHLHKFLHIVKHSAKKIKLFQTNIRFLGFNICQSQITPIDRVIQFADKFPDQILDKSQLQRFLGSLNYVSDFYQNLRKQCKPLFDRLQNNPPPWSTIHTEIVKQIKAHVKTLPCLGIPSVDSFKIVETDASDIGYGGILKQKVSPDSPEQIVRFHSGTWTQSQYNYSTIKKEILAIVLCISKFQDDLLNQKFLIRVDCKSAKHVLEKDVQNIASKQIFARWQSILSIFDFDIEYLRGSENSVPDFLTREFLQEPQWLPKKTKTKKLPVNPSQPSRNPSHPSCNHLSRHP

>ORF2 (aa)

MAPKKDKNKEVASQPKSTQSQPKSSQLQPSQSPSMSPAKPEMPKQIVPYPGCTPLTVATPLSVANRFSSLGSTVGQICPSYQSTLVASYDPFSVDVPAGPSIALKKSSPYLPKSNSHLFVLEPLYDIHTNPVEIAKHYFPPGFHYMPSSPYKSLKYYRDILLETKSVEIKLIKDRDHPEIILYHSMYIHHIISQEAFSSRPYELKTLQSKLQYNYADYIDAWYTIFLHQSEDFSHSWFINFDNKFKCSFPYWFLHWWEKHGPVDEILPVSVLELIRHYTETAKFSTVDLFFPKQLLFLAR

MP	PF01107, Viral movement protein (MP)	69..211

CP	PF22909, Caulimovirus coat protein, conserved domain	791..887

AP	PF00077, Retroviral aspartyl protease	1181..1267

RVT_1	PF00078, Reverse transcriptase (RNA-dependent DNA polymerase)	1443..1593

RT_RnaseH	PF17917, RNase H-like domain found in reverse transcriptase	1656..1756

DUF8797	PF31063, Domain of unknown function

In red letters, coiled-coil prediction according to Waggawagga (https://waggawagga.motorprotein.de/)

The different regions in which the consensesus sequence has been divided according to the encoded protein domains.

>5’-MP

TGAAAGCATTATTTTAATGCATTATCATTATTTTCTGCATTAGTCTGAAAGTTGCTTAGGTTGCATCCTTTTCTAATTTCTTTCTGATCTTCATTCTGTATTATATTCAGTTGCTTGTTTGTTCTATCTCTTTGTCCCTCTTTCTTGGTATCAGAGCCGTAAGTGTTGCGGTCTTTGTCTAAGTCTGCTACTCATCCCCGCCCAAGTGGCTGGTTTAAGCTTCTGAGTAGGAGGTTTTCATTTTTTCCTTTTTCTTCCCTTTTATTCTTTGAGTCATCTGTTGGTTTTCTCTAAGGTCTGACCCGTGAGTCCTTTTTTCTAGATCAGTAAGTCCCCAGGGAGGGCGTGTGTGCTGGGCAGGAGACAGAAAATTGAGAAAATTAATAGGTTGCTTAGAGTCTAAACGGTCGGTTTAGGATCTACTGTTCATCTTCTTCCTCAGTCTGCCAAACCCAGTTTCTTGAGTAGTCATGAGTCGTTTATTTAGAACAAAGTCTTGTAATTCTACCAGTTCTAGTGCCAGTATAAGTCGACTCCCAGAGATAGTTAATGAAGAACAATTTGAATATAATTCCAGTAGTAAAGAACAATTAGATTTTAGAGATTGGAATATACCTAAAGTCCCAAGTCAGAAAATCTATAAGAAACATTGGTTGCCTTCTAGTTTTAATAGC

>MP

ACAACACATGTCAAAACAGTTGAACAAGTTTACGCCTTAAGTAAAGAACATGAAACTTGTCAGTTGCTTAATCTTGAGTCAATTTTAAAACATAAGAAAGATGGTAATAATTTCCTGCATATTGGTTTAGTTCAAGTTGCTGTTAAGCCCCTGACACGGTTAGGATTGAAAGCTTCAATCCTGCTATGTCTGCGTGATGCTAGATTCACTGAATTTAGCGATAGTACCCTTGGAATCATTGAGTCCAGTCTGTGTAATGGCCCTGTCCATTTTGATTGCTATCCTGATTTCACTGTAAGCCTTAGTGATCCTCATATCCTGAAAACTTTGACTCTGAATATTAAGACTGAAGGTTATAATGTTCTGCCAGGAACCCAACCTTTAGCCTTAGTTTACAGAATCTATTATAAGGTCACAGGAACTAATATG

>MP-CP

AATTTCCAAGCCCTTAATAAAAGCCCAAAGGATCAAACCGTCTTGATCCAGAGTCATACCTCAGATGCCCATATACAAGTTCCTCATACTATTAAGTGGTCAGAAGTTGCCCTTCCAAAAGATTGGACCTTAGTCACTGAGAGCCAACCAGCTCCTATCCAGCGTAGTCTAAACAATTTAGACTACATCCAACAATACTTAGATGGCACAGTTAAAATTAAGTTTGATAGTCATCCTTTACGAAAATCTAATGTCCAGTTACAACAGTTGCCAACTCCTGGTCAATCCCAGAGGCATGTCCCTGCTCGGCATTCCTTTGCTGCCTCTTCCTCCACTCTAGAACGAGACCTAGAGTTAGAAAAGGCTATGATTGATTTCAAACTTGAGTCCCTGAGAAAAACCTCTCAGGTTACCCAACCTTGCTACGGCAAGGTTCCAGTTCAAGAAGATAAGTCTGAATCACCTGAGTCACCTACCCAGTCTGACTTCGTAGTTGAAAATCAGCTTAGAACCCTTAATAAAAAATTTGAAATTAATTGGGTAAGATTAAATCGTCATCTAAAAGCCCCTGAAAACCAGGCAAGAAGAGATAATTATCATCAAGCCTACCCTAATAGCAAACACCGAGAAATGATCTTTAAACAATGGAAAGATTATATGCGGTCAGCCAAAGTTGAAATTTATTACCTTGATTTTGTAGAAAGCCGTTATATGTCTAATGATGAATTAAAAACTTTAACTAAAGAAAAATGGAAAATAATAGATAAATCTGTAGTTGAGTCTAGTCATCCTCCAGTAGAAACTATAATAATAGAACACCAGAAAGCACCAGTTCCTGCCACTCCTTTCAAAACCTTTGAAAGCAATGACCCTAATAGAAAACTAATTGAACAAAATAATTACACCAACCAGAGTCTAATCACCATAGGAAAACAATTAGATACAATTGAAACCAAAGTTGATATGTTGAGTCCGCCTGTTGCAGACTCCAGACCCAAACCTAAAGTTGAAAAACCAATAGTTCATTTCCAAGAACTAAAGTCTAGTCCAGCTTTTAAAACTAATTCTACAATGAAAAAGATAGAAGAAATGTTAGAACAATTGACCCCTGTCAAACCCGAGAAAGGTGAAAAATCTGGTATTAAAACACTAGATGCTTTTGCTGCTTCTCAATCTGAGTCTGAATCAGTAGCAGAAACTAGTAATATTTCTAAAATTGAAAATGCTTTCAAAAATCTAGAAGTCCAAACTGAGCCCAAAGTCAAAAGAATAGAAAAACATATAAGCCCTACTAGCCTAACAAAAAATTGGTATCCCAGACCAACACCTCCAGATATCCAATTTGAAGAAAGAAACTTTCAAACCCAGTTTTCTGTTTCTTCTGATAAATTGTATGAATGGAATATTGATGGTCTTTCTGAGCAAGAAATCCTAAATAAACTGCAACATATGTCTATGGTCGCCAATAGTTACATCACCAATCACAGTTTCAGACAGTCTGAAATTGTTCCTCTGTTAGTTACTGGTTTCACAGGTACTCTCAGATCTTGGTGGGATAAACACCTAACCCCTGAGTCTAAAAATCGAATAATTTATGCTGTAAAACTTAATGAAGACGGTCTCCCTATTTTTGATGAACAAATAGGTCAAGGTATAGAAGACGGTGTCAACACTCTGTTTTACACAATAATTGAACATTTCATTGGAACCCCTAGTAATACCACTGCTAGGATACAT

>CP

GATCAGTTGAGTAATCTTAGATGCCCAAAGTTGTCTGATTTCAGATGGTATAAAGATGTTTTTATTTCTAGAGTCATGCTTAGAGATGATAGTAATCAACCATTTTGGAAAGAAAAGTTTATTAATGGTCTGCCTAATTTATTTGCTCATAAAATACGCACAACCCTAAGTAATGAACAAGGTCAAATTGATTGGGATAATTTAACTTATGGAAATATTATAAGCACAATTAATCAAGTTGGTATGAAAATGTGTATTGACTTTAAAATTAATAAACAAATCCAGTCTGAT

>CP-AP

AGAAAGTCAGCCAAGTATGAGTTAGGTAATTTCTGTGAACAATATGGTCTCATTACTATTCCTCCTTCTAGAAAAAATAAGCCTAGTCATCTTAGAAAACGCCATTATCCGAGTAGAAAGAAACATTTTCTTGAGAAAGATGAATTCTATAAGAAAAGAAAGTTCTCCCCTAAGAAAAATTGGTCAAAACGTCCCAAACAAAATCATTATGCTTCTGAATGTAAAGTAAAAGATGCTATTAAACAATTACAAATTACAGAAGAAGATAAGAACAAATTAATCCAAGTTTTGGAGTTGCGAGACTCTGAAATAAGTGATGTTGAATCCATGGTTACTAGTTCTGAGTCTGAGTATAGCCAGTCTTCAGATTCACAACCCAGTTCACCTAGAATCCAATTTGGTTGCACAGATAAATGTTGTAATAGGTTAAAGTCTATTTTTGTTCTCACTAAACAAGAAGAACAAGAAGAATTATTAATAGATTTAATAGGTAAAGTTGAAAACCCTGAGTTAAAATCTGAATATTTAAAAAAATTAAAAAAGATCATAAGTCAGGAAGGCAGTTCAAATTATCCTACTCCCCAGCCAATTAGCATCAACACTACTTTAGAAAAATTCAATCGTAAGAAAGATGTTACCTTACATGATCTACAATTAGAAGTCAAACAAGTTAAAAAAGAAATAGTTGAGTTAAAACAAATAAGTCAACAATTGCAAACTGAGAATTATGAGATCAAACAAGATTTATTTGACTTAGAAAAAGAAACTTCTAAGTCTAATAACCATTCAGAAAGTCCGCATCATTCTGATGATGAACCTTCTGAAGAA

>AP

CAGCCAGTTGTCAATTTAATTAAACAAGTTCATCTTAGAAAATGGTATTCTAAAGTCACGATATTTGTTCAAGATTTTGAATTTACCACAGTTGCCTTATTTGATTCTGGTGCAGATCTTAACTGTATTCAAGAAGGTCTAATCCCTACTAAGTATTACCAGAAGTCTAGAGAATCCCTTAGCACTGCCTCAGGGAAATCTCTCCAGTTAAATTTTGAAATACCAAAAGCCCATATTTGTCAAAACAAAGTCTGTTTTAAAACTTCATTCGTCTTAGTTAAAAATATTACTGATGAAGTCATCTTAGGA

>AP-RT

TTACCTTTCCTTGCCCTGTTGTATCCTTTCCAGGTAGAATACGATGGTGTCATTTCTACCTATTTAGGAGAAAAGGTCAAGTTTGAATTCTTGTCTAAACCTGAGTTGCATAATTTAAAAGCTTTACAAAAACAGTCAGTCTCTAAATCTGTCCAAATTATAAGAAAAACTAATCAATTGAATTTTCTTAAAGAAGAAGTTAAATTTAAAAGAATTGAACAACAACTTAGTGATAAAGCTTTACAGTTAAAAATTCAGTCTTTTGAA

>RT

AACAAATTAAAAGAAGAAATATGTTCTGATTTGCCTACTGCTTTTTGGTATAGAAAACAGCATGTTGTCTGTCTTCCTTATATCAAAACCTTTAGTGAGAAAAATATCCCCACTAAAGCCAGGCCAATTCAGATGAATCAAGAATTAATGGAATTTTGTAAAAAAGAAATTCATGAGTTGCTTCAAAAAAATATAATTCGCAAAAGTAAGTCCCCCTGGTCTTGTCCTGCTTTTTATGTCCAGAAAAATGCTGAATTAGAAAGAGGAGTCCCCAGATTAGTTATCAATTATAAGCCTCTTAATTCGGTCTTAGAATGGATTAGGTATCCCATACCTAATAAAAGAGATCTTATTAATCGATTAGAAAAAGCAGTTGTCTTCTCTAAATTTGATATGAAAAGTGGTTTCTGGCAAATCCAAATAGATGAGTCAGATAGGTATAAAACTGCATTTGTTACTCCTTTCGGTCATTATGAATGGAATGTTATGCCGTTTGGTTTTAAAAATGCCCCTAGTGAATTCCAGAATATTATGAATGATATCTTCAATCCTTACAGTCATTTCTCGATTGTTTATATTGATGATGTTCTTATATTTTCTGAGTCAGTAGAACAACATTGGAAACATTTGCATAAATTCCTTCACATAGTCAAACATTCTGCTAAGAAAATTAAGTTATTTCAAACTAATATTAGATTTCTTGGTTTTAATATCTGCCAGTCACAAATTACTCCAATAGATCGAGTCATCCAATTTGCTGATAAGTTCCCTGATCAGATTCTTGATAAAAGTCAACTCCAGAGGTTTTTAGGATCTCTAAATTATGTTTCTGATTTT

>RNASEH

TATCAAAATCTGAGAAAACAATGTAAGCCTCTATTTGATAGATTGCAGAATAATCCTCCTCCTTGGTCAACTATTCATACTGAAATTGTCAAACAGATCAAAGCCCATGTCAAGACACTTCCCTGTCTTGGTATTCCTTCAGTTGATTCTTTCAAAATAGTTGAAACTGATGCCTCTGACATTGGTTATGGTGGTATTCTCAAACAGAAAGTCAGTCCAGATTCTCCAGAGCAAATTGTTCGTTTCCATTCTGGAACCTGGACTCAGTCGCAATATAATTATAGTACTATTAAAAAAGAAATATTAGCTATTGTATTATGCATTAGTAAATTTCAAGATGATTTATTAAATCAAAAGTTTTTAATCCGAGTTGATTGCAAATCTGCAAAACATGTTTTAGAAAAAGATGTTCAAAACATTGCATCTAAACAGATTTTTGCACGATGGCAAAGTATATTAAGTATAT

>RH-3’

TTGATTTTGATATTGAATATTTGAGAGGTAGTGAGAATTCAGTCCCTGATTTTCTTACTCGAGAATTTTTGCAGGAACCACAATGGCTCCCAAAAAAGACAAAAACAAAGAAGTTGCCAGTCAACCCAAGTCAACCCAGTCGCAACCCAAGTCATCCCAGTTGCAACCATCTCAGTCGCCATCCATGAGTCCAGCCAAGCCAGAAATGCCCAAACAGATAGTTCCCTATCCTGGTTGCACCCCTCTTACAGTTGCCACCCCATTATCAGTTGCCAACAGATTCTCTTCCTTAGGATCTACAGTTGGCCAAATCTGCCCCAGTTACCAATCCACATTAGTTGCCAGTTATGACCCTTTTTCAGTAGATGTCCCAGCAGGACCGTCAATTGCTTTGAAAAAATCTTCTCCTTATTTGCCAAAGAGTAATTCCCATCTATTTGTCCTTGAGCCTTTGTATGATATTCATACTAACCCAGTTGAAATTGCCAAACATTATTTCCCTCCTGGATTCCATTATATGCCTTCCAGTCCTTACAAGTCCCTCAAGTATTATAGAGATATATTGCTTGAAACTAAGTCAGTTGAAATTAAGCTGATAAAGGATCGAGATCACCCTGAGATCATTCTTTATCATTCAATGTACATCCACCATATCATAAGCCAAGAAGCCTTCAGTAGTCGCCCTTATGAGTTAAAAACCCTTCAATCCAAATTACAGTATAATTATGCTGATTATATTGATGCCTGGTACACTATTTTCCTTCACCAATCTGAGGATTTTAGTCATTCCTGGTTCATCAATTTCGATAATAAGTTCAAATGTTCTTTCCCCTATTGGTTCCTCCATTGGTGGGAAAAACATGGCCCAGTTGATGAAATTCTGCCTGTATCAGTTCTTGAATTAATCCGCCATTATACGGAGACTGCCAAGTTCTCCACCGTTGATTTATTCTTCCCCAAACAATTGCTTTTCCTTGCCAGATAAAAAGTTCCTTGGATCCTCAAATGGTCCTACAGAGTCACTAAGGATGTTCGTATCTTTGCCCGCCAATTTTCAGTCAAATGGTGGGACAAATTTGAAGTTCAAAGAATAGCCAAGTATGTTTACAATGATCTCCCTGCTGAGCCTCAGTCAACTTCTCCAGTTGGTTCTGTTCGATCCTCCCTCTCAGTCGAAGGAAAGTCGAAAGCAGAACTCCAAGAAATTGCTCGCCAGTTGATGATGCAGGCCTCTCAGATGGATGATGATGAGGACAACGACTCTCCTTCGTCGTCCAGTTGTCAACCCATTCCTGATCCTGTTAAGCCTAAGTCCTGGTATGATGACAGCCAGGATCCCTATGATGCCTATGATC

Individual sequences used for the consensus (Colours are as in previous page; non-highlighted letters correspond to TE insertions).

>1; Chr1; OZ022206.1; 19418907-19426420; dir;

TACAGAAGAAGATAAGAACAAATTAATCCAAGTTTTAGAGTTGCGAGACTCTGAAATGAGTGAAGTTGAGACCTTTGCACCCAATTCTGAGTCTGAGTCTGCCCAGTCTTCAGATTCACAGCCCAGTTCTCCTAATATTCAAGTTGGTTGTACTGATAAATGTTGTAATAGGTTAAAATCTATTTTAGTTCTTACTAAACAAGAGGAACAAGAAGAGTTACTTGTAGATTTAATAGGAAAGGTTCAAAACCCTGAGTTAAAATCTGAATATTTGAAAAAATTAAAAAAGATCTTAAGTCAGGAAGGTCAGAGTTCACCATCTCCCATTCCTCAGCCAATCAGCATCAATACAACATTAGAAAAATTCAATCGTAGAAAAGATGTCACCCTTCATGACCTCCAAACGGAAGTCAAGCAGATTAAAAAAGAATTAGTTGAATTAAAACAGACAAGCCAACAATTGCAAGTCGAAAATAATATCATTAAGCAAGACTTATATGACCTAAAATAAGAAACCTGCAAGCCGAGTAACCTTTCTGAAAGTCCTCATCAATCTGATGATGAACCTTCTGAAGAACAACCAGTTGTCAATTTAATTAAACAAGTTCATCTTAGAAAATGGTATTCAAAAGTCACGATATTCGTTCATGATTTTGAATTTCCCACAGTGGCTTTATTCGATTCTGGTGCAGATCTTAACTGTATTCAGGAAGGTCTCATCCCTACTAAGTATTACCAGAAGTCAAGAGAATCCCTTAGCACTGCCTCCGGGAAGTCTCTCCAGTTAAATTTTGAAATACCAAAAGCCCATATTTGCCAAAACAAAATCTGTTTTAAAACTTCATTCGTTTTAGTTAAAAATATCATTGATGATGTCATTTTAGGACTACTCTTCCTAGCTTTGCTGTATCCTTTCCAGGTAGAATACGATAGCCTCACCTCTACCCATTTAGGAGAAACAGTCAAATTTAAATTCTTGTCAAAACCTGAGTTGCATAATTTGAAAGCTTTACAAAAACAGTCAGTAGCTAAGTCTATCCAAGTTATCCGAAAAACCAACCAATTGAATTTTCTTAAAGAAGAAATTAAGTTTAAAAGGGTTGAACAACAACTTACTGATAAGGCTCTACAGTTAAAAATCCAGTCGTTTGAAAACAAATTAAAAGAAGAAATATGTTCCGAATTACCTACTGCTTTTTGGTATAGAAAACAGCATGTTGTCTGTCTTCCTTATATCAAAACCTTTAGTGAGAAAAATATCCCCACAAAAGCCAGGCCAATACAAATGAATCAAGAGTTGATGGAATTTTGCAAGAAAGAAATTCATGAGTTGCTTCAGAAAAATATAATTAGCAAAAGTAAGTCCCCCTGGTCTTGTCCTGCTTTTTATGTCCAGAAAATTGCTGAACTAGAAAGAGGAGTCCCCAGGTTAGTCATCAATTATAAGCCCCTTAATTCGGTCCTAGAATGGATTAGGTATCCCATACCTAATAAAAGAGACCTTATTAATCGGTTAGGGAAAGCAGTTGTCTTCTCCAAATTTGATATGAAAAGTGGTTTCTGGCAAATCCAAATAGATGAGTCAGATAGGTATAAAACTGCATTTGTTACTCCTTTCGGTCATTATGAATGGAATGTCATGCCGTTTGGATTAAAAAATGCCCCCAGTGAGTTTCAGAATATTATGAATGATACCTTCAATCCTTACAGTCATTTCTCGATTGTCTATATTGATGATGTTCTGATTTTTTCTGAGTCAGTTGAACAGCATTGGAAACATTTGCATAAATTCCTTTACATAGTCAAACAAAATGGTCTAGTTGTCTCTGCTAAGAAAATTAAGTTGTTTCAAACAAATATAAGATTTCTTGGTTTCAATATTAGTCAATCTCATATCTCTCCAATTGATTGAGTCATTCAGTTTGCTGATAAATTCCCTGATCAGATTCTTGATAAAAGTCAACTCCAGAGGTTTTTAGGATCTCTAAATTATATCTCTGACTTCTATCCAAATCTGAGAAAACAATGTAAGCCCCTATTTGATAGGTTGAAAAATAATCCTCCTCCTTGGTCAAATATTCATACTGAAGTTGTCAAACAGATCAAAGCCCATGTCAAGACACTTCCCTGTCTTGGTATTCCTTCAGTCGATTCTTTCAAAATAATTGAAACTGATGCCTCTGACATTGGTTATGGTGGCATCCTCAAACAGAAAGTCAGTCCAGAATCTCCAGAACAAATTGTTCGTTTCCATTCTGGAACCTGGACTCAGTCGCAATATAATTATAGTACTATTAAAAAAGAAATATTAGCTATTGTATTATGCATTAGTAAATTTCAAGATGATTTACTTAATCAAAAGTTTTTAATACGAGTTGACTGCAAATCTGCAAAACATGTTTTAGAAAAAGATGTTCATAACATTGCATCTAAACAGATTTTTGCACGATGGCAAAGTATATTAAGTATATATAAATTTTGATATTGAATATTTGAGAGGCAGTGAGAATTCAGTTCCTGATTTTCTTACTCGAGAATTCCTGCAGGAACCACAATGGCTCCCAAAAAAGACAAAAACAAAGAAGTTGTCAGTCAGCCCAAGTCAAGCCAGTCGCAACCCAAATCATCCCAGTTGCAACCATCTCAGTCGCCATCCATGAGTCCAGCCAAGCCAGAAATGTCCAAACAGATAGTTCCCTATCCTGGTTGTACCCCCATCAAAGTTGCCACCCCTCTATCAGTTGCCAATAGATTTTCCTCTCTAGGATCCACAGTTGGCCAAATTCGCCCCAGTTACCAGTCTACTCTAGTTGCCAGTTATGATCCCTTTGCAGTTGATATCCCTGCTGAACCCTCGGTACCTGCAGTTATATATAAAAAATCATCCCCCTATTTGCCCAAAAGTAATTCCCATCTGTTTGTCATTGAGCCCATTTACGATATCCATACCAACCCAATTGAAATTGCCCGTCATTATTTCCCTCCTGGATTTCATTATATGCCTTCCAGTCCCTACAAATTTCTCAAGTATTATAGAGATATATTGCTTGAGACCAAATCAGTTGAAATCAAACCCATAAAGGACCGTGACAACCCTGGCACTATCCTTTATCATTCTTTGTACATCCATCAAATTATAAGCCAAGAAGCCTTCAGCAGTCGCCCTTATGAACTCAAGTTACTCCAGTCCCAATTGCAATACAATTATGCTGATTATATTGATGCCTGGTATACTATTTGTCTTCATCAATCTGAAGATTTTAGTCATTCCTGGTTCATTAATTTTGATAACAAGTTCAAATGTTCTTTCCCCTATTGGTTTCTCCATTGGTGGGAAAAACATGGTCCAGTTGAAGAAATTTTACCAGCATCAGTTCTTGCACTCATCCACCATTACACCAAGAAGGCCAAGTTCTCTGCAGTTGACTTATTTTTCCCCAAGCAATTGTTATTTATTGCCAGATACAAAGTGCCCTGGATATTGAAATGGTCCTACAGGGTCACTAAAGATGTTCGTTTTTTTTGCCCGCCAATTCTCTGTTAAATGGTGGGACAAATTTGAAGTTGACAGAATTCAACAGAGAAGAACACAGAGAAGATAAGGAAGACAACTAAATACAATACAGAGAGAAAGAAATTAGAAATTAGAAATGAATGCAACCTAAGCAACTTTCAGACTAATGCAGAAAATAATGATAATGCATCAAATTAGTGCTTTCAAGAAATAGATAATTTGAAAATAACTTTTATTTATACTCACAAAGATTGCAACTGCAATTCTTCAAAGCACAATTGCACGCTATTTACAAGAGTGTTTACAAAGATGAATTTCTCCGAGGGATGTATCAGAGAGATTCTTTAAAACTGAAGAAAGGAAAATTACATTCTCAACAGAGAAGAACACAGAGAAGATAAGGAAGACAACTGAATATAATACAGAGAGAAAGAAATTAGAAATTAGAAATGAATGCAACCTAAGCAACTTTCAGACTAATGCAGAAAATAATGATAATGCATCAAATTAGTGCTTTCAAGAAATAGATAATTTTAAAAGTTATTTTCTTAAAATTATCTATTTCTTGAAAGCACTAATTTGATGCATTATCATTATTTTCTGCATTAGTCTGAAAGTTGCTTAGGTTGCATTCATTTCTAATTTCTAATTTCTTTCTCTCTGTATTATATTCAGTTGTCTTCCTTATCTTCTCTGTGTTCTTCTCTGTTGGTATCAGAGCCGTAAGTGTTACGGTCTTTGTCTAAGTTTGCTACTCATCCCCCCCAAGTGGCTGGTTTAAGCATCTGAGTAGAAGGTTTTCATTCTTTCCTTTTCTTCCCTTTTAGCCCTCGAGTCCTCTGTTGGTTTTCTCTAAGGTCTGACCCGTGAGTCCTTTTTACTAGATCAGTAAGTCCTCAGGGAGGGCGTGTGTGCTAGGCAGGAGACAGAAAATTGAGAAAATTAATAGGTTACTTAGAGTCTAAACGGTCGGTTTAGGATATACTGTTCATCTTCTTCCTCAGTCTGCTGAATCCAGTTTCTTGAGTAGTCATGAGTCACTTATTTAGAACAAAGTCTTGTAATTCTGCCAGTTCTAGTGCCAGTATAAGTCGACTCCCAGAGATAGTTAATGAAGAACAATTTGAATATAATTCCAGCAGTAAAGAACAATTAGATTTCAGAGATTGGAACATACCTAAAGTCCCCAGCCAGAAAATCTATAAGAAACATTGGTTGCCTTCTAGTTTTAATAGCACAACACATGTCAAAACAGTTGAACAAGTATACGCCTTAAGTAAAGAACATGAAACTTGTCAGTTGCTTAATCTTGAGTCTATTTTAAAACATAAGAAAGATGGTAATAATTTCCTGCATATTGGCTTAGTTCAAGTTGCTGTTAAACCCCTGACACGATTAGGGTTGAAAGCCTCAATCCTACTTTGTCTACGTGATGCTAGATTCACCGAATTTAGCGATAGTACCCTCGGAGTCATTGAGTCCAGTCTGTGTAATGGCCCTGTCCATTTTGACTGCTATCCCGATTTCACTGTAAGCCTTAGTGATCCCCATATCCTGAAAACTTTGACTCTGAACATTAAGACTGAAGGTTATAATGTCCTTCCAGGAACCCAACCTTTAGCCTTAGTTTACAGAATCTATTATAAGGTCACAGGAACTAATATGAATTTCCAAGCCCTTAATAAAAGCCCAAAGGATCAAACCGTCTTGATCCAGAGTCACACCTCAGATGCCCATATACAAGTCCCCCATACTATCAAGTGGTCTGAAGTCGCCCTCCCAAAAGATTGGACCCTAGTTACTGAGAGCCACCCAGCTCCAATCCAGTGTAGTCTGAACAATTTAGACTACATCCAACAATACTTAGATGGTACAGTTAAAATCAAATTTGACAGTCTTCCCCTACGCAAGTCTAATGTCCAGTTACAACAGTTGCCAACTCCTGGTCAGTCCCAAAGGCATGTCCCTGCTCGACATTCCTTTGCTGCCTCTTCCTCCACTCTAGAACGAGACCTAGAGTTAGAAAAGGCCATGATCGATTTCAAACTTGAGTCCCTGAGAAAAACCTCTCAGGTTACCCAACCTTGCTACGGCATGATTCCAGTTCAAGAAGACAAGTCAGAATCACCAGAGTCACCTACCCAGTCTGACTTTGTAGTTGAAAATCAGCTTAGAACCCTTAATAAAGAATTTAAAATTGATTGGATTTACCTAAATCGTCATCTAAAAGCCCCCGAAAACAAGTCAAGGCGAGATCTTTATCACCAAGCCTATCCTAATTGCAAACACCGAGAAATGATCTTTAAACAATGGAAAGATTATATGCGGTCAGCCAAAGTTGAAATTTTCTACCTTGATTTTATTGAAAGCCGTTATATGTCTAGTGATGAATTAAAAACCCTGACTAAAGAAAAATGGAAATTAGTTGACAAATCTGTAGTTGAATCTAGTCACCCTCCAGTAGAAACTATAATTATAGAACATCAGAAAGCCCCAGTTCCTGCCACTCCTTTTCAAAACCTTTGAAAGCAATGATCCTCATAGGAAACTAATTGAACAAAATAATTATACGAACCAGAGTTTAATCACCATAGGTAAACAATTAGATACAATTGAAACCAAGGTTGACCTGCTGAGTCCGCCTGTTGCAGACTCCAGACCCAGACCCAAAGTTGAAAAACCAATAGTTCAGTTTCAAGAACTAAAGACAAGTCCAGTTTTAAAAGTTAATTCCACAATGAAAAAGATAGAAGACATGTTAGCACAATTGACCCCTGTCAAGCCCGAGAAAGGTGAACAATCTGGTATTAAAACGCTAGATGCTTTTGCCACTTCTCAATCTGAGTCAGAATCCGTCAGCTCCGAAACTAGTGACATTTCTAAAATTGAAAATGCCTTCAAAAATTTAGAAATCCAAACTGAGCCCAAAGTCAAAAGAATTGAAAAACATATAAGCCCTACTAGTCTTACAAAAAATTGGTACCCCAGGCCAACACCTCCAGATATCCAATTTGAAGAAAGAAACTTCCAAACTCAGTTTTCTGTTTCTTCTGATAAATTGTATGAATGGAATATTGATGGTCTCTCTGAGCAAGAAATTCTGAATAAATTGCAACATATGTCTATGGTCGCAAATAGTTACATCACCAATCACAGTTTCAGACAATCTGAAATTGTTCCTCTGTTAGTTACTGGATTTACTGGTACTCTCAGATATTGGTGGGATAAGCACCTCACCCCTGAGTCTAAAAATCGTGTCATCTATGCTGTAAAACTTAATGAAGACGGTCTCCCTATATTTGACGAACAAATAGGTCAAGGTATAGAAGACGGTGTCAACACTTTGTTTTACACAATAATTGAACATTTCATCGGAACCCCTAGTAACACCACCGCCAGGATCCATGATCAGTTAAGCAATCTTAGATGCCCAAAGTTGTCTGATTTCAGATGGTATAAAGACGTTTTCATTTCTAGAGTCATGCTTAGAGATGATAGTAATCAACCATTCTGGAAAGAAAAGTTCATTAATGGTCTACCTAATTTGTTTGCTCACAAAATACGCACGACACTAAGTAATGAACAAGGCCAAATTGATTGGGATAATTTAACGTATGGAAACATTATAAGCACAATTAATCAAGTCGGCATGAAAATGTGTATTGACTTTAAAATTAATCGTCAAATACAGTCTGATAGGAAGTCGGCCAAGTATGAGTTAGGAAACTTTTGTGAACAATATGGTCTCATTAGTACTCCTCCCTCCAGGAAACATAAGCCCAGTCACCTTAGAAAACGCCATCATCCGAAGAAGCAATTTCTCCGGAAAGATGAATTCTATAAGAAAAGAAAGTTCTCCCCTAAGAAGAATTGGTCAAAACACCCAAAGCAGAGTCGCCCTAAGAAAGATAAAAGCAAAGTCAAATGTTTTAAATGCCAAAAATTTGGCCATTATGCCTCTGAATGCAAAATAAAAGATGTTATTAAACAATTACAGATC

>2; Chr1; OZ022206.1; 30340244-30332593; rev;

TGAAAGCATTATTTTGATGTATTATCATTATTTTCTGCATTAGTCTGAAAGTTGTTTAGGCTGCATCCATCTCTACTCTACTGTTCTTTCTTTTCCTAGTTCTTCTTTTGCCCTTCCTTTCTATTTCACTGGTTCTTCGTCTTGTTGGTATCAGAGCCGTAAGAGTTACGGTCTTTGTCTAAGTTTGCTACTCATTCCCGCCCAAGTGGCTGGTTTAAGCCTCTGAGTAGAAGGTTTTCATTTCTTCCTTTTCTTCCCTTTTATTCTTTGAGTCATCTGTTACTTTTCTCTAAGTTCTGACCCGTGAGTCCTTTTTTCTATATCAGTAAGTCCCCAGGGAGGGCATGTGTGGTGGGCAGGAGACAGAAAATTGAGAGAAGTTAATAGGTTCCTTAGAGTCTAGACGGTCGGTTTAGGATCTACTGTTTATCTTCTTCCTCAGACAGCCAAACCCAGTTATTTGAGTAGTTATGAGTCACTTATTTAGAACAAAGTCTTGTAGTTCAAACAGTTCTAATATCAGTCGACTCCCAAAAATAGTTAATGAAGAACAATTTGAATATAATTCCAGTAGCAAAGAACAATTAGATTTTAGAGATTGGAATATACCTAAAGTCCCCAGTCAGAAAATTTATAAGAAACATTGGTTGCCTTCCAGTTTTAATAGCACAACACATGTCAAAACAGTTGAACAAGTTTACGCCTTAAGTAAAGAACATGAAACTTGTCAGTTGCTTAATCTTGAGTCTATTTTAAAACATAAGAAAGATGGTAATAATTTCCTGCATATTGGTTTAGTTCAAGTGTCCGTTAAGCCCTTGACACGGTTAGGATTGAAAGCCTCAATCCTGCTCTGTCTGCGTGATGCTAGATTCACTGAATTTAGCGACAGTACACTTGGAGTCATTGAGTCCAGTCTGTGTAATGGCCCTGTCCATTTTGACTGCTATCCTGACTTCACTGTAAGCCTTAGTGATCCTCATATCCTGAGAACTTTGACTCTGAATATTAAGACTGAAGGTTATAATGTCCTTCCCGGGACACAACCGTTAGCCTTAGTTTACAGAATCTACTATAAGGTCATAGGAACAAATATGAACTTCCAAGCCCTCAATAAAAGCCCAAAGGATCAAACCGTCCTGATCCAGAGTCATACTTCAGACGCCCATATACAAGTTCCCCATACCATTAAGTGGTCTGAAGTTGCCCTTCCAAAAGATTGGACCCTAGTTACAGAGAGCCAACCAACTCCAATCCAGCATAGTCTAAACAATTTAGACTACATCCAGCAATACCTAGACAGCACAGTAAAAATTAAATTTGACAGTCATCCCTTACGAAAATCAAATGTCCAGTTACAACAGTTACCAACTCCTGGTCAGTCACAAAGGCATGTTCCTGCTCGGCATTCCTTTGCTGCTTCGTCCTCCACTCTAGAACGAGACCTAGAGTTAGAAAAAGCTATGATAGATTTCAAACTTGAGTCCCTGAGAAAAACCTCTCAGGTTACCCAACCTTGCTACGGCCAGGTTCCAGTTCCAGAAGATAAGTCTGACTTGCCTGAGTCTCCAACCCAGTCTGACTTCCTAGTAGAAAATCAACTTAGAACCCTAAATAAGAAATTTGAAATTAATTGGGTACGTCTAAATCGCCATTTAAAAGCCCCGGAAAACCAATCTAGAAGAGATAGTTACCACCAAGCCTATCCTGATAGTAAGCACCGTGAACAAATCTTTAGTAAATGGAAAGATTACATGCGGTCAGCCAGAGTTGAAATATTCTACCTAGATTTTGTTGAAAGCCGTTACATGTCTAATGATGAGTTAAAAACCCTAACTAAAGAAAAGTGGAATTTGATAGATAGGTCGGTAGTTAAAGCCAGTCATCCTCCAGTAGAAACTATAATAATAGATCATAAGAAAGCACCAATACCTGCCACCCCTTTCAAAACCTTTGAAAGTAGTGATCCAAATAGAAAACTAATCGAACAAAGTAATTACACCAACCAGAGTCTAATTGTCATAGGAAAACAGTTGGATACAATTGAAACTAAGATTGACAAGTTGCATCCCCAAGAGACAGATCTCAAGCCCAAATCTAGGGTTGAAAAACCAATAGTTCAGTTTCAAGAGCTTAAGTCCAGTCCTGCACTTAAAGTCAATTCTACAATGAAAAAGATAGAAGAAATGCTAGAACAGTTGACCCCAACTAAAACTGAGAAAGCCGAAAAATCTGGTCTTAAAACACTAGATTCTTTTGCTGTTGCTTCATCTTCTGAGTCTGAGTCAATAAGTTCTGAAACTAGTAACATCTCAAAAATTGAAAATGCTTTCAAAAATTTAGAAGTCCAAACTGAACCCAGAATCAAAAGATTAGACAAACATGTAAGTCCCACTAGCTTAACAAAAAATTGGTATCCCAGACCAACACCTCCAGATATGCAGTTTGAAGAAAGAAGCTTCCAAACCCAGTTTTCAGTTTCTTCTGATAAATTGTATGAATGGAATATTGATGGTCTTTCTGAGCAAGAAATCTTGAATAAGTTACAACATATGTCCATGGTTGCCAATAGTTACATCACCAATCACAGTTTCAGACAGTCTGAAATTGTTCCTCTGCTAGTTACTGGTTTTACCGGTACTCCCAGATATTGGTGGGATAAGCACCTCACCCCTGAGTCTAAGAACCAGATCACTTATGCCGTAAAACTTAATGAAGACGGTCTCCCTATATTTGACGAACAAATAGGTCAAGGTATAGAAGACGGTGTCAACACTCTGTTTTACACAATCATTGAGCATTTCATTGGAACCCCTAGTAATACCACTGCTAGGATCCATGATCAGTTGAGTAACCTTAGATGCCCAAAGTTGTCTGATTTCAGATGGTACAAAGATGTTTTCATTTCTAGAGTCATGCTTAGAGATGATAGTAATTAGCCATTCTGGAAAGAAAAATTTATTAATGGTTTGCCAAACTTATTTGCCCACAAAATACGTACAACCCTGAGTAATGAACAAGGTCAAATTGATTGGGATAATTTAACATATGGAAATATTATAAGCACAATTAATCAAGTCGGTATGAACATGTGTATTGACTTTAAAATTAATAGGCAAATCCAGTCTGATAGAAAGTCAGCCAAATATGAGTTAGGAAATTTCTGTGAACAATACGGTCTTACCAGCATCCCTCCTTCTAGGAAAAATAAGCCCAGTCATCTTAGAAAAAATCGTTATTCTTTTCCTAAGAAACAATTTTCCCGAAAAAATGAGTATTATAAGAAAAGAAAGTTCTCCCCCAAAAAGAATTGGTCGAAACACCCAGATAGTAAGAAAAGTCGCCCTAAGAAAGATAAAAGCAAAGTTAAATGTTTTAAATGTCAGAAGATCGGTCATTTTGCCTCTGAATGCAAAGTAAAAGATGTGATTAGACAGTTGCAAATTACTGAAGAGGAGAAGAACAAGCTGATACAAGTTTTAGAGTTGCGAGACTCAGAAGCAAGTTCTGCTGAAACCTTCGTTCCCAGTTCTGAGTCCGATTCTGACCAATCTTCATAATCACAACCCAGTTCTCCTAGAGTCCAAATTGGTTGCACAGATAAATGTTGTAATAGATTAAAATCTATATTTGTTCTTACTAAACAGGAAGAACAAGAAGAGCTGCTAATAGATTTAATAGGTAAAGTTGAAAATCCTGAGTTGAAATCTGAATATTTGAAGAAGTTAAAAAAGGTCCTTAGTCAAGACAGTTCAAACCACTCTAACCCCCAGCCTATAAGTATCAATACTACTTTAGAAAAATTCAATAGCAGAAAAGAAGTAACCCTCCATGACCTCCAGTTAGAAGTCAAGAAAGTTAAAAAAGAATTAGTTGAGTTAAAACAAATAAGTCAACAATTGCAGACTGAAAATTATACGATCAAACAAGATATAACTGCCCTTTTAAGACAGGAAGCCTTTAAGCTTAAAAGCCATTCAGAGAGCCCTCACCATTCTGATGATGAACCTCCTGAAGACCAACCAGTTATCAATTTAATTAAACAAGTTCACCTTAGGAAATGGTATTCGAAAGTAACGATATTTGTTCAAAATTTTGAACTCACCACAGTCGCCTTGTTTGACTCAGGAGCAGATCTTAACTGTATCCAAGAAGGTCTAATCCCCACTAAGTATTGTCAAAAGTCTAGAGAATCCCTTAGTACTGCCTTCGGAAAATCTCTTCAGTTGAATTTTGAGTTATCAAAAGCCCATATTTACCAAAACAAAGTCTGTTTTAAAACCTCGTTTGTCCTAGTCAGAAACATCACTGACGAAGTCATCTTAGGTTTGCCATTCCTTGCTTTATTGTATCCTTTTCAGGTAGAATACGATGGTATCATTTCTACCTATTTAGGTGAACAGGTTAAGTTTGAGTTCTTAACGAAACCCGAGTTGCACAATCTAAAAGCTTTACAAAAACAGTCAGTCTCTAAATCTGTTCAAGTTATCCAAAAATCTAATCAGTTGAAATTCCTTAAAGAAGAAATTAAATTTAAACGAGTCGAACAACAGCTTAATAATAAAGCCTTCCAATTGCAAATACAGTCCTTTGAAAACCATTTGAAAAATGAAATTTGTTCTGATTTGCCTACTGCTTTTTGGTATAGAAAACAGCACGTCGTCTGTCTTCCCTATATCAAAACCTTTAGTGAAAAGAATATCCCCACTAAAGCCCGGCCAATTCAGATGAATCAAGAACTAATGGAATTTTGTAAGAAAGAAATTCAAGAGTTGCTTCAAAAGAACATAATACGCAAAAGTAAGTCTCCCTGGTCGTGTCTTGCTTTTTATGTCCAGAAAAATGCTGAACTAGAAAGAGGAGTCCCAAGATTAGTTATCAATTATAAGCCTCTTAATTCGGTTTTGGAATGGATAAGGTATCCTATTCCTAACAAGAGAGATCTCATCAACCGATTAGGAAATGCAGTTGTCTATTCCAAATTTGATATGAAAAGTGGCTTCTGGCAAATCCAAATAGATGAGTCAAATAGGTATAAGACTGCATTTGTTACTCCTTTCGGTCATTATGAATGGAATGTCATGCCGTTTGGTTTGAAAAATGCCCCTAGTGAGTTTCAGAATATTATGAATGATATCTTCAATCCTCACAGTCATTTCTCGATTGTTTATATTGATGCTTTTCTCATCTTTTCTGAGTCAGTTGAACAGCATTTGAAGCATTTGCATAAATTCCTTCACATAGTCAAACGAAATGGTTTAGTTGTCTCTGCCAAGAAAATTAAGTTGTTCCAAACCAATATTCGATTTCTTGGTTTCAATATTTGCTAGTCTCAAATTTCCCCAATTGATCGAGTTATCCAGTTTGCTGATAAATTTCCTGATCATATCCTTGATATAAGCCCCTATTCGAAAGGTTGCAGAATAATCCTCCTCCTTGGTCAGATATTCATACTGAAGTTGTCAAACAAATCAAAGCCCATGTCAAGACCCTTCCCTGTCTTGGTATACCTTCAGTTGATTCTTTCAAAATAGTTGAACCAATGCCTCTGACATAGGTTATGGTGGCATTCTCAAACAGAAAGTCAGTCCAGATTCTCCAGAACAAATTGTTCGTTTTCATTCTGGAACCTGGACTCAGTCACAATATAATTATAGTACTATTAAAAAAGAAATATTAGCTATTGTATTATGCATTAGTAAATTTCAAGATGATTTACTTAATCAAAAGTTTTTAATACGAGTTGATTGCAAATCTGCAAAACATGTTTTAGAAAAAGATGTTCATAACATTGCATCTAAACAGATTTTTGCACGATGGCAAAGTATATTAAGTATATTTGATTTTGACATTGAATATTTGAGAGGTAGTGAGAATTCAGTCCCTGATTTTCTTACTCGAGAATTTTTGCAGGAACCACAATGGCTCCCAAAAAAGACAAACACAAAGAGTCAGCCAGTCAACCTAAGCCAACCCAGTCGCAACCATCTCAATCTCCATCCATGAGTCAAAATAAGCCAGAATTGCCCAAACAGATAGTTCCTTATCCCGGTCATCCCCCTCTTACAGTTGCTACCCCATTATCAGTTGCAAACATGTTCTCTTCCTTAGGTTCCACAATTGGCCAAATTTGCCCCAGTTATCAATCCACATTAGTTGCCAGTTATGACCCTTTTTCAGTTGATGTACCAGCAGGACCTTCAGTAGCCGTTAAAAAATCATCCCCTATTTGCCCAAAAGTAATTCCCATTTATTTGTCATTGAGCCAGTTTATGATGTTCATGCCAACCCAGTTGAAATCGCCAAACACTATTTCCCTCCTGGATTCCATTATATGCCTTCCAGTCCTTATAAGTCTCTCAAGTATTATAGAGATATATTACTTGAAACCAAGTCAGTTGAAATCAAACCAATAAAGTATCGTGATCACCCCGAGATCATCCTTTATCATTCTCTCTATATTCACCAAATTATAAGCCAAGAAGCATTCAGGTCGCCCTTATGAACTCAAGTTGCTCCAGTCAAAATTACAGTATAATTATTCTGATTATATTGATGCTTGGTATACTATATTTCTTCACCAATCTGAAGATTTTAGTCATTCCTGGTTCATCAATTTTGATAATAAGTTCAAAAGTTCTTTCCCCTACTGGTTTCTCCATTGGTGGGAAAAGCATGGTCCAGTTGATGAAATATTGCCTGTTTCAGTCCTTGAGTTAATCCGCCACTACACCAAGAAGGCTAAGTTCTCCAAAGTTGATTTATTTTTCCCAAAGCAGTTGTTATTTGTTGCCAGATATAAGGTTCCATGGATCCTCAAATGGTCCTACAGAATCACCAAAGACGCCCGAATCTTTGCCCGCCAATTCTCTGTCAAGTGGTGGGACAAATTTGAAGTTGATCGAATTGCTAGATATGTCTACAATGATCTCCCAGTTGAACCAATGCCGCGCCCAGTTTCCCCTTCTGGTTCTATCCGATCTTCCCTCTCAGTCGAAGGAAAATCCAAAGCAGAACTCCAGGAAATTGCTCGCCAATTGATAATGCAGGCCTCCCAGATGGATGATGATGAGGACAACGACTCTCCTTCGTCGTCCAGTTGTCAGCCCATTCAGAATCCTGCTAAGCCCAAGTCTTGGTACGAAGACAGTCAAGACCCCTATGATGCCTATGATCTGCACTCTGACTAGGCTTCATTATGGTTGCCGACACCTTCACATGCCAAAAGTTGCTGACACCTTCACATGCCAAAGTCGCCCAACATCATAATTGCCAAGAAGAAGCATCTGCTTTTACTGTTCGCTTTTCCACCGAAAAGCTGCCTCAGTAAAAGAATCTTCCGAATAAAGCCAAATTCAAAGTTGAATCAGGTTGCTTTTCCGTGACCCAACAGTTGCAGCAAACCTCAGTTACTTTACTATAGTTAATCTAAATCATAGTTGCTTCTCTCTTTATAAGGAGGCTTCGGCCTCAGTTGTAATCAGAATGTAATTTTTCATTTTCTAAGTTTTCTTCAGTTTTAAAGATTTCCTCTGAAACATCCCTCGGAGAAATCATCTTTGTAAATACGGAGTGCAGTTGTTTGAAGTTGCAGTAGCAATCTTTGTAAGTATAAATTAAAGTTGCTATTTTCAAAATTATTTATTTTCTGA

>3; Chr1; OZ022206.1; 44710876-44718651; dir;

TGAAAGCATTATTTTAAATGCATTATCATTATTTTGCTGCATTAGTCTGAAAGTTGCTTAGGTTGCATCCATTCCTAATTTCCTTCTGATCTTCGTCTCTGTATTATATTCAGTTGTCTTTCATATTCTCTTTCTGTTCTTTGTTCTTCCTCTGTTGGTATCAAAGCCGTAAGTGTTACGGTCTTTGTCTAAGTCTGCTACTCATCCCCGCCCAAGTGGCTGGTTTAAGCATCTGAGTAGAAGGTTTTCAATTCTTCCTTTTCTTCCCTTTTATTCTTTGAGTCCTCTGTTGGTTTTCTCTAAGGTCTGACTCGTGAGTCCTTTTTGCTAGATCAGTAAGTCCCCAGGGAGGGCGTGTGTGCTGGGCAGGAGACAGAAAATTGAGAAAATTAATAGGTTGCTTAGAGTCTAAACGGTCGGTTTAGGATCTACTGTTCATCTTCTTTCTCAGTCTGTTTAACCTAGTTTCTTGAGTAGTCATGAATAGTCTATTTAGAACAAAGTCTTGTAATTCTGCCAGTTCTAGTACCAGTATAGGTCGTCTTCCAGAGATAGTTAATGAAGAACAATTTGAATATAATTCCAGCAGTAGAGAACAATTAGATTTTAGAGATTGGAATATACCTAAAGTCCCCAGTCAAAAAATCTATAAGAAACATTGGTTGCCTTCCAGTTTTAATAGTACAACACATGTCAAAACAGTTGAACAAGTGTACGCTTTAAGTAAAGAACATGAAACCTGTCAGTTGCTTAATCTTGAGTCTATTTTAAAACATAAGAAAGATGGTAATAATTTCCTACATATTGGTTTAGTTCAAGTTGCTGTCAAACCCCTGACACGGTTAGGATTGAAAGCTTCGATCCTGCTATGTCTACGCGATGCTAGATTCACTGAATTTAGCGATAGTACCCTTGGAATCATTGAGTCCAGTCTGTGTAATGGCCCTGTCCATTTTGACTGCTATCCTGATTTCACTGTAAGTCTTAGTGATCCTCATATCCTGAGAACTTTGACTCTGAATATTAAGACTGAAGGTTATAATGTCCTCCCAGGAACCCAACCTTTAGCCTTAGTTTACAGAATCTATTATAAGGTCACAGGAACCAATATGAATTTCCAAGCCCTTAACAAGAGCCCAAATGATCAAACCGTCTTGATCCAGAGTCATACCTCAGACGCCCATATACAGGTCCCCCATACTATCAAGTGGTCCGAAGTTGCCCTTCCAAAAGATTGGACCTTAGTTACCGAGAGCCAACCAGCTCCCATCCAGCGTAGTCTAAACAATTTAAACTACATCCAACAATACTTAGATGGCACAGTTAAGATTCAGTTCGACAGTCATCCTTTACGAAAGTCTAATGTCCAGTTACAGCAGTTGCCAACCCCTGGTCAGTCCCAGAGGCATGCCCCTGCTCGGCATTCCTTTGCTGCCTCTTCCTCCACTCTAGAACGAGACCTAGAGTTAGAAAAGGCCATGATCGATTTCAAACTTGAGTCTCTGAGAAAAACCTCTCAGGTCACCCAACCTTGCTACAGCACGGTTCCAGTTCAAGAAGACAAGTCAGAGTCACCTGAGTCACCTACCCAGTCTGACTTTGTAGTTGAAAATCAGCTTAGAACCCTCAGTAAAGAATTTGAAATTAATTGGGTAAGTCTAAATCGTCATCTAAAAGCCCCTGAATACAAGCCAAGGCGAGATCTTTATCATCAAGCCTATCCTAATAGCAAACACCGAGATATGATCTTTAAACAATGGAAAGATTATATGCGGTCAGCCAAAGTTGAAATTTTCTACCTTGATTTTATTGAAAGCCGTTACATGTCTAATGATGAATTGAAAACTCTAAATAAAGAAAAATGGAAACTAGTTGACAAGTCTGTAGTAGAATCGAGTCACCCTCCAGAAGAAACAATTATAATAGAACACCAGAAAGCGCCAGTTGCCGCCACTCCTTTCAAAACCTTTGAAAGCAGTGATCCTCATAGAAAACTAATTGAACAAAATAATTACACGAACCAAAGTCTAATCACCATAGGAAAACAGTTAGACACAATCGAAACCAAGGTTGATCTGCTGAGTCCGCCTGTTGCAGACTCCAAACCCAAACCCAAAGTAGAAAAGCCCATAGTACAATTCCAGGAACTTAAGTCCAGTCCAGTCCTCAAAGTTAATTCCACAATGAAAAAGATAGAAGACATGTTAGCACAATTGACCCCCGTCAAATCCGAGAAAGGCGAACAATCTGGTATTAAAACACTAGATTCTTTTGCCGCTTCTCATTCTGAGTCCGAGTCAGAATCCGTAAGTTCAGAAACTAGTAACATTTCTAAAATTGAAAATGCTTTTCAGAATTTAGAAATCCAACCTGAGCCCAGAATTAAAAAGATAGAAAAACATATAAGCCCTGCTAGCCTAACAAAGAATTGGTACCCCAGACCAACACCTCCAGATATCCAATTTGAAGAAAGAAACTCTCAAACCCAGTTTTCGGTTTCTTCTGATAAATTGTATGAATGGAATATTGATGGTCTTTCTGAGTAAGAAATCTTAAATAAACTGCAACATATGTCTATGGTCGCAAATAGTTATATCACCAATCACAGTTTCAGACAGTCTGAAATTGTCCCTTTGTTAGTTACCGGTTTCACTGGTACTCTCAGATGTTGGTGGGATAAGCACCTCACTCCTAAGGCTAAAAATCGAATCATTTATGCTGTAAAACTTAATGAAGACGGTCTCCCTATATTTGACGAACAAATAGGTCAAGGTATAGAAGACGGTGTCAACACTCTGTTTTACACAATAATTGAACATTTCGTCGGAACCCCTAGTAACACCACTGCTAGGATCCATGACCAATTAAGCAATCTTAGATGCCCAAAGTTGTCTGACTTCAGATGGTATAAAGATGTTTTCATTTCTAGAGTCATGCTTAGAGATGATAGTAATCAACCATTTTGGAAAGAAAAGTTCATTAATGGTCTGCCTAATCTATTTGCTCACAAAATACGCACGACACTAAGTAATGAACAAGGTCAAATTGATTGGGATAGTTTAACTTATGGAAACATTATAAGCACAATTAATCAAGTTGGTATGAAAATGTGTATCGACTTTAAAATCAATCGCCAAATCCAGTCTGATAGAAAGTCAGCCAAATACGAGTTAGGGAATTTCTGTGAACAATATGGTCTCACCAGTATCCCTCCTTCTCGGAAACACAAGCCTAGTCATCTTAGAAAGCGTCATCTCCCGAAGAAACAATTTCTTCAGAAAGATGAATTCTACAAGAAAAGAAAGTTCTCTCCTAAGAAAAATTGGTCTAAACGTCCAAAACAAAGTCATTCAAAACAAAGTCGTTCTAAGAAAGATAAAAGCAAAGTTAAATGTTTTAAATGTCAAAAATTTGGTCATTATGCTTCTGAATGTAAAGTAAAAGATGTTATTAAACAATTACAGATTACAGAAGATGATAAGAACAAGTTAATTCAAGTTTTGGAGTTGCGAGACTCTGAAATGAGTGATGTTGAGTCTCTAGTATCCAGTTCTGAGTCTGAGTCTAGCCGGTCTTCAGATTCACAACCCGGTTCCCCTAAAGTTCAATTTGGTTGTACTGACAAATGCTGTAATAGATTAAAATCTGTCTTAGTCCTCACTAAGCAAGAAGAACTCTTAGTAGACATAATAGGTAGAGTCCAAAACCCTGAGTTAAAAGCTGAATATTTGAGAAAATGAAAAAAATCTTAAGCCAGGATAGCCAGAGTTCTCCCTCGCCTATTCCTCAGCCAATTAGCATCAACACTACGTTAGAAAAATTCAATCGTAGGAAAGATGTCACCATTCATGATCTCCAGTCAGAAGTCAAACAAGTTAAAAAAGAATTAGTTGAACTTAAACAGATAAGCCAACAATTGCAAACTGATAATTATAAGATCAAACAAGACCTGTTCGAATTAAAACAAGATTCCTGTAAACCTAGTAACCCTTCAGAAAGTCCGCATCATTCTGATGATGAACCTTATGAAGAACAGCCAGTTGTCAACTTAATCAAACAAGTTCACCTTAGGAAATGGTATTCCAAAGTCACGATTTATGTTCATGATTTTGAATTTACCACAGTTGCATTATTTGATTCCGGTGCAGATCTTAACTGTATTCAGGAAGGTCTCATCCCTACTAGGTACTTCCAGAAGTCTAGAGAATCCCTTAGCACCGCTTCAGGGAAATCTCTCCAGTTGAATTTTGAGTTACCAAAAGCCCATATTTGCCAAAACAAAGTTTGTTTTAAAACTTCATTCGTCCTAGTTAAAAATATTACTGATGAAGTCATTCTAGGATTACCCTTCCTTGCCCTACTGTATCCAATCCAAGTAGAATACGATGGTATCATTTCTACCCACCTTGGAGAGTCGGTCAAGTTTAGCTTTCTGTCTAAACTAGAGTTGCATAATCTAAAAGCTTTGCAGAAACAGTCAATTTCTAAATCTGTCCAAGTTTTATGAAAGACCAATCAGTTAAATTTTCTTAAAGAAGAAGTTAAGTTTAAACGAATTGAACAACAACTTAGTGATAAAACTTTACAGTTGAAAATCCAGACTTTTGAAAGCAAACTAAAAGAAGAAATATGCTCCGATTTACCTACTGCCTTTTGGTATAGAAAACAGCACGTTGTCTGTCTTCCTTATATCAAATCTTTTAGTGAGAAAAATATCCCCACTAAAGCAAGGCCGATACAGATGAGTCAAGAATTAATGGAAATTTGTAAAAAAGAAATTCATGAGTTGCTTCAGAAAAATATTATTCGCAAAAGTAAATCCCCCTGGTCGTGTCCTGCTTTTTATGTCCAGAAAAATGCTGAACTAGAAAGAGGAGTCCCCAGATTAGTTATCAATTATAATCCTCTTAATTCGGTCTTAGAATGGATTAGGTACCCCATACCTAATAAAAGAGACCTTATTAATCGCTTAGGAAAAGCAGTTGTCTTCTCCAAATTTGATATGAAAAGTGGTTTCTGGCAAATCCAAATTAATGAGTCAGATAGGTATAAGACTGCATTTGTTACCCCTTTCGGTCATTATGAATGGAATGTCATGCCGTTTGGTTTAAAAAATGCCCCTAGTGAATTCCAGAATATTATGAATGATATCTTCAATCCTCACAGTCATTTTTCGATTGTTTATATTGATGATGTTCTAATATTCTCTGAGTCAGTAGAGCAACATTGGAAGCATTTGCATAAGTTCCTTCATATAGTCAAACAAAATGGTCTAGTTGTCTCTGCTAAGAAAATTAAATTGTTTCAAACCAATATTAGATTTCTTGGTTTTAATATCAGTCAGTCTCAAATTACTCCAATTGATCGAGTCATCCAATTTGCTGATAAGTTCCCTGATCAGATTCTTGATAAAAATCAGCTCCAGAGGTTTTTAGGATCTCTAAATTATATTTCTGATTTTTATCCAAATTTACGAAAACAATGTAAGCCCCTATTTGACAGACTAAAGGATAATCCTCCTCCTTGGTCTAGTATCCATACTGAAGTTGTCAAACAAATCAAAACCCATGTCAAGACACTTCCCTGTCTTGGTATTCCTTCAGTCGACTCTTTCAAAATAGTTGAAACAGATGCCTCTGACATTGGTTATGGTGGCATTCTCAAACAGAAAGTCAGTCCAGAATCCCCAGAGCAAATTGTTCGTTTCCACTCTGGAACCTGGACCCAGTCGCAATATAATTATAGTACTATTAAAAAAGAAATATTAGCTATTGTATTATGCATTAGTAAATTTCAAGATGATTTATTAAATCAAAAGTTTTTAATACGAGTTGATTGCAAATCTGCAAAACATGTTTTAGAAAAGGATGTTCATAACATTGCATCTAAACAGATTTTTGCACGATGGCAAAGTATATTAAGTATATTTGATTTTGATATTGAGTATCTGAGAGGTAGTGAGAATTCAGTCCCTGATTTTCTTACTCGAGAATTTCTGCAGGAACCACAATGGCTCCCAAAAAAGACAAAAACAAAGAAGTTGCCAATCAATCCAAGTCATCCCAGTCGCAACCCAAGTCATCCCAGTTGCAACCATCTCAGTCGCCATCCATGAGTCCAGCCAAACCAGAAATGCCCAAACAGATAGTTCCCTATCCTAGTTGCACCCCCATCAAAGTTGCCACTCCTCTATCAGTTGCCAATAGATTTTCCTCCTTAGGATCTACAGTCGGCCAAATCCGCCCCAGTTATCAGTCTACTCTAGTTGCCAGTTATGATCCCTTTGCAGTAGACATCCCTGCCGGCACTGCTGGCCCCTCAGTTGCATTCAAAAAATCATCTCCTTATTTGCCTAAAAGTAATTCTCATTTATTTGTAATTGAGCCCTTGTATGACATCAATACCAGTCCAGTTGAAATTGCCAGACATTATTTCCCTCCTGGATTCCATTATATGCCTTCCAATCCTTACAAGTCCCTCAAGTATTATAGAGATATACTACTTGAAACCAAATCAGTTGAAATTAAGCCGATCAAGGATCGTGACAATCCTGCCACTATCCTTTATCATTCTTTATATATTCACCAAATTATAAGCCAAGAAGCCTTCAGCAGTCGCCCTTATGAACTCAAGTTACTTCAGTCCCAGTTGCAATACAATTATGCTGACTATATTGATGCATGGTATACTATTTTTCTTCATCAATCTGAAGATTTTAGTCATTCCTGGTTCATCAATTTTGATAACAAGTTCAAAAGCCATTTCCCCTACTGGTTCCTCCATTGGTGGGAAAAACATGGTCCAGTTGAAGAAATTTTACCGGTATCAGTCCTTGAGCTCATCCGCCATTACACCCAGAAGGCCAAGTTCTCTGCAGTTGATTTATTTTTTCCCAAACAGTTGTTATTTATTGCCAGATACAAGGTACCCTGGATACTAAAATGGTCTTACAGGGTCACTAAAGATGTTCGCATTCTTGCCCGCCAATTCTCTGTCAAGTGGTGGGACAAATTTGAAGTTGATAGAATTGCCAGATACGTCTACAACGACCTCCCAGTTGCACCAGTACCACTGCCGGTCTCTCCTTCTGCATCTGTTCGATCTTCCCTCTCAGTCGAAGGAAAATCAAAAGCAGAACTTCGAGAAATCGCCACCCAGTTGCTCAGACAAGCCTCCCAGATGGATGATGATGAAGACAACGGGTCCCCCTCGTCTTCCAGTTGTCAGCCCATCCCTGTCAAGCCAAAGACCTGGTATGAAGACAGTCAAGACCCCTATGACGCCTACGATTTGAACTCTGACTAGGCCTCATGATGTTCCTGATATCTTCACATGCCAAAGTAGCCGAAAGTAGCCGGTCCTACAAAAGTTGTCATTAGTCGCCGACATGATCATTGTCTTCTGTGAAGAATCTGCTTTTACTATCTACTTTCCACCGAAAGCTCCATCAGTAAAAGAATTCCCCACAAACAGACAAAATTGAAAGTTGAATTAGGTTGCTTTCTTGTGACCTAGCAGTAGCCTCCGTTCCAGTAACTCTACCATAGTTGAACCTTAGTTAATTTTCTCTTTATAAGCAGGCTTCGGCCTCAGTTGTAAAAGGAATGCAATTTTTCCTTTCTTCAGTTTCAAAGAAATCTCTCTGATACATCCTTCGGAGAATTTCACTTTTGTAAACACTCTTGTAAATAGCGTGCAGTTGTGCTTTGAAGAATTGCAGTTGCAATCTTTGTAAGTATAAATAAAAAGTTATTTTCAAAATTATCTATGTTGAAA

>4; Chr2; OZ022207.1; 25198039-25205707; dir;

TGAAAGCATTATTTTAAAAGCATTTTCATTATTTTGCTGCATTAGTCTGAAAGTTGTTTAGGTTGCATCCAATCTATTTATCAGTCACTCTCTCTCTTGTTTATATTCAGTTGTCTTGTTCTCTATTTCTGTTGGTCCTTCTTTTGTTGGTATCAGAGCCGTAAGTGTTGCGGTCTTTGTCTAAGTTTGCTACTCATCCCCGCCCAAGTGGCTGGTTTAAGCATCTGAGTAGAAGGTTTCATTTCTTCCTTTTCTTCCCTTTTATCCTTTAAGTCATCTGTTGGTTTTCTCTAAGGTTTGACCCGTGAGTCCTTTTTCCTAGATCAGTAAGTCCCCAGGGAGGGCGTGTGTGGTGGGCAGGAGACAGAAAACTGAGAAAATTAATAGGTTACTTAGAGTCTAAACGGTTGGTTTAGGATCCACTGTTTATCTTCTTCCTCAGATAGCCAAACCCAGTTATTTGAGTAGTCATGAGTCGTCTGTTTCGAGCAAAGTCTTGTAATTCTCCCAGTTCTAGTACCAGTATTAGTCGACTCCCAGAAATAGTTAATGAAGAACAATTTGAATATAATTCTAGTAGTAAAGAGCAATTAGATTTCAGAGATTGGAACATACCTAGAGTCCCAAGTCAGAAAATCTATAAGAAACATTGGTTGCCTTTTAGTTTTAATAGCACAACACATGTCAAAACAGTTGAACAAGTGTACGCCTTAAGCAAAGAACACGAAACATGCCAGTTGCTTAATCTTGAGTCAATTGCAAAACATAAGAAAGATGGTAATAATTTCCTTCACATTGGATTAGTTCAAGTTGCAGTCAAGCCACTGACACGGCTAGGATTGAAAGCTTCAATTCTACTTTGTCTTCGTGATGCTAGATTCACTGAATTTAGCGATAGCACCTTAGGAGTCATTGAGTCTAGCCTGTGTAATGGCCCTGTCCATTTGGATTGTTATCCTGACTTCACTGTAAGCCTTAGTGATCCTCATATCCTGAGAACCTTGACTCTGAATATTAAGACTGAAGGTTATAATGTTCTCCCGGGAACCCAACCTTTAGCCTTAGTCTACAGAATCTATTATAAGGTTACAGGAACAAATATGAACTTCCAAGCCCTTAACAAAAGCCCAAAGGATCAAACATTCCTGAGCCAGAGTCACACCTCAGATGCCCATATACAAGTTCCCCATACAATCAAGTGGTCCGAAGTTGCCCTTCCAAAAGATTGGACCTTAGTTACCGAGAGCCAACCAGCTCCAATCCAACGTAGTCTAAACAATCTAGACTACATCCAACAATATCTTGATGGCACGGTTAAAATAAAATTTGATAATCATCCTTTACGCAAATCTAATGTCCAGCTACAACAGTTGCCAACCCCTGATAGGCATTCCTTTGCTGCTTCCTCCTCCACTCTAGAACGAGACCTAGAGCTAGAAAAAGCCATGATTGATTTCAAACTTGAGTCCTTGAGAAAAACTTCTCAAGTGACACAACCGTGCTACGGTGCGGTTCCAACCCAAGATGACAAGTCTGACTCGCCCAAGTCTCCAACTCAGTCCGATTTCCTAGTTGAAAATCAATTAAGAACCCTTCATAAGAAATTTGAAATTAATTGGGTACGTTTAAATCACCACCTAAAGGCCCCAGAAAACCAAGCTAGAAGAGACAGTTATCACCAAGCTTATCCGAACAACAAACACCGAGAATTAATCTTTAAACAATGGAAAGATTATATGCGGTCAGCTAAAGTTGAAGTTTTCTACCTTGATTTTGTTGAAAGCCGTTATATGGCCACGGATGAATTAAAAACTATAAATAAAGAAAAATGGAAATTAGTAGACAAGTCGGTTGTTGAGTCCAGCCATCCTCCAGTAGAAACCATTATAATTGATCACCAGAAAGCCCCAGTTCCTGCTACTCCCTTCAAAACCTTTGAAAGCAGTGACCCCCATAGGAAACTAATTGAACAGAATAATTATACGAATCAGAGTCTAATCACAATAGGAACACAATTAGATGCAATTGAATCTAAGGTCGATTTGTTAAGTCCACCAGTTGCGGATTCCAGACCTAGATCTAAAATTGAAAAGCCGATAGTTCAGTTTCAAGAACTTAAGTCTAGTCCTGTCCTTAAAGTTAATTCCACAATGAAAAAGATAGAAGAAATGTTAGAGCAGTTAACCCCGGCCAAGTTAGAAAAAGGTGAAAAATCTGGTATTAAAACATTAGATTCTTTTGCTACTTCTCTATCTGAGTCTGAATTAGTAAATTCTGAAATCAGCGATATTTCAAAAATTGAAAGTGCCTTCAAAAATTTAGAAGTCCAAACTAAGCCCAGAATCCAAAAAGTTGACAGACATATAAGTCCTACTAGTTTAACAAAAAATTGGTATCCCAGACCAACACCACCAGACATCCAATTTGAAGAAAGAAACTTTCAAACCCAGTTTTCAGTTTCTTCTGATAAATTGTATAAATGGAACATTGATGGTCTTTCTGAACAAGAAATCTTAAATAAGCTGCAACATATGTCTATGGTCGCCAATAGTTATATCACCAATCACAGTTTCAGGCATTCTGAAATTCCTCTGTTAGTCACCGGTTTCACTGGTACTCTCAGATCTTGGTGGGATAAACATCGTACCCCTGAGTCTAAAAGTCACATCATTTATGCTGTAAAACTCAATGAAGACGGTCTTCCTATCTTTGATGAACAATTAGGTCAAGGCATAGAGGACGGTGTCAACACTCTGTTTTACACAATAATTGAACATTTCGTTGGAACCCCTAGTAACACTACTGCTAGGATACATGATCAGTTAAGTAATCTTAGATGCCCAAAGTTGTCTGATTTCAGATGGTACAAAGATGTCTTTATTTCCAGAGTCATGCTTAGGGAAGATAGTAATCAACCCTTCTGGAAAGAAAAGTTCATTAATGGTCTCCCAAACTTAGTCGCCCATAAAATACGTACAACCCTGAGTAATGAACAAGGTCAAATAGATTGGGATAATTTAACCTATGGAAACATTATAAGCACAATTAATCAAGTCGGAATGAAAATGTGTATTGACTTTAAAATCAATAAACAAATTCAGTCTGACAGAAAATCAGCCAAGTACGAACTAGGTAATTTCTGTGAACAATATGGCCTTACTAGTGTTCCTCCTTCTAGAAGAAGTAAGCCCAGTCATCTTAGAAAACGCCATCATCCGAAGAAACAATTTTTTCGGAAAAATGAATTCTATAAGAAAAGAAAGTTATCCCCTAAGAAGAATTGGTCGAAACACCCCAAACAGAGTCGCACTAAAAAAGATAAAAGCAAAGTTAAGTGTTTTAAATGTCAAAAATTTGGTCATTATGCTTCTGAGTGTAAAGTAAAAGATGTTATTAAGCAATTACAGATTACAGAAGAAGATAAGAACAAATTAATCCAAGTTTTAGAGTTGCAAGACTCTGAAATAAGTGATGCTGAATCCTTATTGTCCAGTTCTGAGTCTGAATTTATCCAGTCTTCAGATTCTCAACCCAGTTCACCTAGAATCTAATTTGGTTGTACAGATAAATATTGTAATAGACTGAAATCTGTCTCGGTTCTCACTAAACAAGAAGAACAAGAAGAACTTTTAATAGACCTAATAGGACGAGTCCAAAACCCCGAACTAAAAGCTGAATATTTGCAAAAGTTGAAAAAAGTCTTGAGTCATGAAAGCCCAAGTTTAGTTGCTCCTCCTCCCCAGGCCATTAGTATTAATACTACATTAGAAAAATTTTAAAAAAGAAATCACCCTTCATGATCTCCAATTGGAAGTCAAGAAAGTCAAACAAGAATTGGTTGAGTTAAAACAAGTCAGCCAACAACTGCAAAATGAGAATTACACGATCAAACAAGATTTATCTAGTCTTCCACATACAGGCCCTAGTTCGTTCAAAGCCCATTCTGAAAGTCCGCATCATTCTAATGACGAACCATTAGAGGAACAACCAGTCGTCAACTTAATTAAACAAGTTCATCTTAGAAAATGGTATTCAAAAGTAACGATATTTGTTAATGATTTTGAATTTTCCACAGTTGCCCTGTTTGATTCAGGTGCCGACCTTAATTGTATTCAAGAAAGCCTAACCCCACCAAATATTACCACAAGTCTAGAGAATCTTTGCGTACGGCCTCAGGGAAATCTCTCCAGTTGAATTATGAATTACCAAAGGTCCATGTTTGCCAAAACAAAGTCTGCTTCAAAACTTCATTTGTCTTAGTTAAAAATATAACTGACAGTGTCATCTTAGGATTACCCTTTCTACCTTTATTATATCCTTTCCAGGTGGAATACGATGGCCTCGTCTCTACCTATTTAGATGAAAAAATCAAGTTTCAATTTTTGTCTAAACCAGAGTTGCATAACGTGAAAGCTTTACAAGAACAGTCAGTCTCCAAGTCTATCCAAATAATTAGAAAAACTAATCAGTTGAATTTCCTTAAAGAAGAAATTAAATTTAAAAGAATTGAACGCCAACTTACTGATAAAGATTTACAGATGCAAATACAATCCTTTGAGGACAAAATAAAAGCAGAAATTTGTTCTGAGTTGCCTACTGCTTTTTGGTATAGAAAACAGCATGTTGTTTGTCTTCCTTATATCAAAACCTTTAGTGAAAAAAATATCCCTACAAAAGCCCGACCAATTCAGATGAGTCAAGAATTGATGGAAGTCTGTAAAGAAGAAATTCAAGAATTGCTACAAAAGAATATTATTCGCAAAAGTAAATCCCCGTGGTCATGTCCTGCTTTTTATGTTCAGAAAAATGCTGAATTAGAAAGAGGAGTCCCCAGATTAGTAATCAACTACAAACCCCTTAATTCGGTCTTAGAATGGATTAGGTATCCCATCCCTAATAAAAGAGACCTTATTAATCGATTAGAGAAAGCAGTTGTCTTCTCTAAATTTGATATGAAGAGTGGTTTCTGGCAAATCCAAATAGATGAGCCAAACAGGTATAAGACTGCATTTGTCACCCCTTTCGGACATTATGAATGGAATGTCATGCCGTTTGGTTTGAAAAATGCTCCTAGTGAATTCCAGAATATTATGAATGATATCTTCAATCCTTATAGTCATTTCTCCATTGTTTACATTGATGATGTCCTTATATTTTCTGAGTCAGTCGAACAGCATTGGAAACATTTGCACAAATTCCTTCACATAGTTAAACACAATGGTTTAGTTGTCTCTGTCAAGAAAATTAAGTTGTTTCAAACTACAGTTAGGTTTCTTGGTTTTAATATCAATCAATCCCAAATTTCCCCAATAGATAGAGTCATTCAATTTGCTAATAAATTTCCTGATCAGATTCTTGATAAAAGTCAACTCCAGAGGTTTTTAGGATCTCTAAATTTTGTTTCTGACTTCTACCAAAATCTGCGAAAGCAATGTAAGCCTCTGTTTGATAGATTGCAAAATAATCCGCCCCCTTGGACAAATATCCATACTGAGGTTGTCAAACAAATCAAAACCCATGTCAAGACACTTCCCTGTCTTGGTATTCCTTCAGTTAATTCCTTCAAAATAGTAGAAACAGATGCCTCTGACATTGGTTATGGTGGCATTCTCAAACAGAAAGTTAATCCAGATTCCCCAGAGCAAATTGTTCGCTTCCACTCTGGAACCTGGACTCAGTCGCAATACAATTATAGTACTATTAAGAAAGAAATATTAGCTATTGTATTATGCATTAGTAAATTTCAAGATGATTTACTCAATCAAAAGTTTTTAATACGAGTTGATTGCAAATATGCAAAACATGTTTTAGAAAAAGATGTTCATAACATTGCATCTAAACAGATTTTTGCACGATGGAAAAGTATATTAAGTATATTTGATTTTGAAATTGAATATTTGAGAGGTAGTGAGAACTCAGTTCCTGATTTTCTTACTCGAGAATTTTTTCAGGATACACAATGGCCCCTAAAAAAGACAAACACAAAGCAACCAGTCAACCTGAGCCATCCCAGTCGCAACCCAAGCCTTCTCAATTGCCACAACTCCAGTCCTCATCCATAAGTCCCATTAAGCCAGAATTGCCCAAACAGATAGTTCTGTATCCTGGTTGCACCCCCCTTTCAGTCGTCACCCCATTATCAGTTGCCAATCGATACTCCTCTCTAGGATCCACAGTTGGCCAAATTCGACCCAGTTATCAGTCTACCTTAGTGGCTAGTTATGATCCCTTTTCCATAGAAGTCCCTGCTAGACCATCAGTTGCATACAAAAAATCATCTCCCTATTTGCCCAAAAGTAACTCCTATTTATTTGTCATTGAGCCTGTATACGATGTTCATGCTAATCTAGTTGAAATTGCTCGACACTACTTCCCTCCTAGGTTTCATTATATGCCTTCCAGTCCCTACAAGTCTCTCAAGTATTATAGAGATATATTACTTGAAACAAAATCAGTTGAAATTAAGCCAATAAAGGATTGGGATCATCCCGAAATCATTCTTTATCATTCTTTGTATATCCACCAGATCATAAGCCAAGAATCTTTCAGCAGTCGCCCTTATGAATTAAAGTTGCTCCAGTCAAAACTGCAATATAACTATTCTGATTATATTGATGCCTGGTACACTATTTTCCTTCACCAATCTGAAGATTTTAGTCATTCCTGGTTCATCAATTTTGATAATAAGTTCAAGTGTTCTTTCCCTTACTGGTTCCTCCATTGGTGGGAAAAGCACGGCCCAGTTGATGAAATATTACCAGTATCAATTCATGAGTTAATTCGCCATTTTACCAAGAAAGCCAAGTTCTCTAAAGTTGATTCATTCTTCCCCAAACAGTTGCTGTTCGTCGCTAGGTACAAAGTGCCATGGATCCTGAAATGGTCCTATAAGATCACTAAATTGCCAAGTATGTCTACAATGACCTTCTAGTTGAGCCAGCTCATCCAATTTCTCCTACTGGTTCTGTCAGATCTTCCCTCTCAGTCGAAGGAAAGTCCAAATCAGAACTACAGGAAATTGCACGCCAGTTGATCATGCAAGCTTCCCAGATGGATGATGATGAAGACAATGGGTCTCCTTCGTCGTCTAGCTGCCAGCCCATACAAGCCCAAGTCAAGCCCAAATCATGGTATGAGGATAGTCAAGATCCCTACGACGCCTATGATTTGCATTCTGACTAGAAGCCAAAATTTGCCGTAATTATCACCAGACAAAGCATTTGCTTTCCACCGAAAGAAGCCACCAGTCGTCCCAGATGCTAGCTGCTCCAGCCATTGCATCTGCCTTTTGTCAAAAGTTGCCAACCGACAGACTTAAAAGATGAATCAGCATCTGCTCCTTACTTTTCGCCGAAAGTTGCCAGAAGATGCCAACCGACAAAATTGAAAGTTGAATTAGGTTGCATGTGACTCCACTGTTGCAACCCTACCATAGTTAGCCAACTATAGTTGATTAAACCCAAGTTGATTTTCTCTTTATAAGTAGGCTTCGGCCTCAGTTGAAAACAGAATGTAATTTTCCCTTTCTTTAGTTTTAAAGATTTCCTCTGATACATCCTTCGGAGAAATCACCTTTGTAAACACCTTGTGAATAGCGTGCTGTTCGAAGAATTGCAGTTGCAATCTTTTGTGAGTATAAATAAAAGTTATTTTCAAATTATCTATTTTC

>5; Chr4, OZ022209.1; 1386818-1379779; rev;

AGTGTTGCGGTCTTTGTCTAAGTTTGCTACTCATTCCCGCCCAATTGGCTGGTTTAAGCATCTGAGTAGGAGGTTTTCATTCTTTCCTTTTCTTATCTTTTATTCTTTGAGTCCTCTGTTGGTTTTCTCTAAGGTCTGACCCGTGAGTCCTTTTTGCTAGATCAGTAAGTCCCCAGGGAGGGCGTGTGTGCTGGGTAGGAGACAGAAAATTGAGAAAATTAATAGGTTGCTTAGAGTCTAAACGGTTGGTTTAGGATCTACTGTTTATCTTCTTCCTCAGACAGCCAAACCCAGTTATTTGAGTAGTCATGAGTCGTCTATTTAGAACAAAGTCATGTAATTCTGCCAGTTCTAGTACCAATATAAGCCGACTCCCAGAGATAGTTAATGAAGAACAATTTGAATATAACTCCAGTAGTAAAGAACAATTAGATTTTAGATATTGGAATATACCTAAAGTCCCCAGTCAGAAAATTTATAAGAAACATTGGTTGCCTTCTAGTTTTAATAGCACAACACATGTCAAAACAGTTGAACAAGTTTATGCATTAAGCAAAGAACATGAAACTTGCCAGTTGCTTAATCTTGAGTCTATTTTAAGACATAAGAAAGATGGTAATAATTTTCTGCATATTGGATTAGTTCAAGTTGCCGTAAAACCTCTGACACGGTTAGGATTGAAAGCTTCAATTCTGCTATGTCTCCGCGATGCTAGATTCACTGAATTTAGCGATAGTACCTTAGGAGTCATTGAGTCCAGTCTGTGTAATGGTCCTGTCCATTTTGACTGCTATCCTGATTTCACTGTAAGCCTAAGTGATCCTCATATCCTGAGAACTTTGACTCTGAACATTAAGACTGAAGGTTATAATGTTCTTCCAGGACTCAACCTCTAGCCTTAGTATACAGAATCTATTATAAGGTCACAGGAACAAATATGAATTTCCAAGCCCTCAACAAAAGCCCAAAGGATCAAACCGTCCTGATTCAGAGTCATACCTCAGACGCCCATATACAAGTTCCTCATACGATTAAGTGGTCTGAAGTTGCCCTTCCAAAAGATTGGACCCTAGTCACCGAGAGCTAACCAGCTCCAATCCAGCGTAGTCTAAACAATTTAGACTATATCCAGCAATACTTAGATGGCACAGTTAAGATTCAGTTTGATAGTCATCCTTTACGAAAGTCTAATGTCCAGTTACAACAATTGCCAACCCTGGTCAATCATAAAGGCATGTCCCTGCCAGACAATCCTTTGCGGCCTCTTCCTCCACTCTAGAACGAGACCTAGAGTTAGAAAAGGCTATGATCGACTTCAAACTTGAGTCCCTGAGAAAAATCTCTCAGGTCACCCAACCTTGCTACGGCCAGGTTCCAGTTCCGGACGATAAGTCAGAATCACCTGAGTCACCTACCCAGTCTGACTTCGTAGTTGAAAATCAACTTAGAACCTTGAATAAGAAATTTGAAATTAATTGGGTCAGCCTAAATCGTCATCTAAAAGCCCCCGAAAACAAGCCAAGGCGAGATCTTTATCATCAAGCCTATCCTAATAGCAAGCACCGTGAAATGATCTTTAAACAATGGAAAGATATGCGGTCAGCCAAAGTTGAAATTTACTACCTTGATTTTATTGAAAGCCGTTATATGACTAATGATGAGTTAAAAACATTGACTAAAGAAAAATGGAAATTAGCCGATAAGTCTGTTGTAGAGTCTAGTCACCCTCCAGTAGAAACCATCATAATAGAACACCAGAAAGCCCCAGTTCCTACCACTCCTTTCAAAACCTTTGAAAGCAGTGACCCTCATAGAAAACTAATTGAACAAAATAATTATACGAACTAAAGCCTAGTCACCCTAGGAAAACAATTATACACAATTGAAACCAAAGTTGATCTGCTGAGTCCGCCAGTTGCAGACTCCAGTCCCAAACCTAAAATTGAAAGACCAATAGTTCAGTTCCAAGAACTTAAGTCTAGTCCAGCTTTAAAAGTCAATTCTACAATGAAAAAGATAGAAGATATGTTAGCACAGTTGACCCCTGTTAAACCCGAGAAAGATGATCAGTCTGGTATTAAAACACTGGATGCTTTTGCCGTTTCTCAATCTGAGTCGGAATCTTTAAGTTCAGAAACTAGTGATATCACTAAAATCGAAAATGCTTTCAAATATCTAGAATTCCAACCTGAGCCCAGAGTCAAAAAGATAGAAAAACATATAAGTCCTACTAGTCTAACAAAAAATTGGTATCCCAGACCAACACCTCCAGATATCCAATTTGAAGAAAGAAACTTCCAAACCCAGTTTTCTGTTTCTTCTGATAAATTGTATGAATGGAATATTGATGGTCTTTCTGAGCAAGAAGTCTTGAACAAATTACAACATATGTCTATGGTTGCCAATAGTTACATCACCAATCACAGTTTCAGGCAGTCTGAAATTGTCCCTCTGTTAGTTACCGGGTTTACCGGTACTCTCAGATATTGGTGGGATAAGCACCTTACCTCTGAGTCTAAAAGTCAAATTATTTATGCCGTAAAACTTAATGAAGATGGTCTCCCTATATTTGACGAACAAATAGGTCAAGGTATAGAAGACGGTGTCAACACTCTGTTTTACACAATAATTGAACATTTTATTGGAACCCCTAGCAACACTACGGCTAGAATCCATGACCAGTTGAGTAATCTTAGATGTCCAAAGTTGTCTGATTTCAGATGGTATAAAGATGTTTTCATTTCTAGAGTCATGCTTTGAGATGATAGTAATCAACCATTCTGGAAAGAAAAGTTTATTAATGGTCTGCCTAATCTGTTTTCCCACAAAATACGCACAACGTTAAGCAATGAACAAGGTCAAATTGAATGGGATAATTTAACTTATGGTAATATTATAAGCACAATTAATCAAGTTGGTATGAAAATGTGTATTGACTTTAAAATTAATCGACAAATTCAATCTGATAGAAAGTCAGCCAAGTATGAGTTAGGAAACTTTTGTGAACAATATGGTCTCACTAGTATTCCTCCCTCCAGGAAACATAAGCCTAGTCATCTTAGAAAACGTCATTACCCGAAGAAGCAGTTTCTCCGGCAAGACGAATTCTATAAGAAAAGAAAGTTCTCCCCTAAGAAAAATTGGTCGAAACGCCCCAAGCAGAGTCGCCATAAGAAAGATAAAAGCAAAGTTAAATGCTTTAAATGTCAAAAATTTGGTCATTACGCTTCTGATTGTAAGGTAAAAGATGTAATTAAACAGTTGCAGATTACAGAAGAAGATAAGAACAAATTGATCCAAGTTTTGGAGTTGCGAAACTCTGAAATGAGTGATGTTGAGTCCCTAGTATCCAGTTCTGCGTCTGATTCTGACCAGTCTTCAGAATCACAATCCAGTTCCCCTAATGTCCAATTTGGTTGTACTGATAAATGTTGTAATAGGTTAAATTTTGTTTCAGTTCTTACTAAGCAGGAAGAACAAGAAGAGTTGCTTGTAGACATAATTGGTAAAGTCCAAAACCCTGAGTTAAAATCTGAATATTTGAAAAAGCTAAAAAAGATCCTAAGTCGAGATAGCCAGAGTTCACCCTCTCCTACTCCCCAGCCAATTAGCATCAACACTACGTTAGAAAAATTCAACCGTAGAAAAGATGTCACCCTTCATGACCTCCATTCGGAAGTCAAGCAGGTTAAAAAAGAAATAGTTGAGCTTAAACAATTAAGTCAACAATTGCAAGCCGAGAATTATGTTATCAAACAAGATTTATTTGACTTAAGACAAGAAACCTATAAGCCTAGCAACCACTCGGAAAGTCCGCATCACTCTGATGAGGAACCTTCTGAAGAACAGCCAGTTGTCAATTTAATTAAACAAGTTCATCTTAGAAAATGGTATTCACAAGTCACGATATTTGTTCATGATTTTGAATTTACCACAGTTGCTTTATTTGATTCTGGTGCCGACCTTAACTGTATTCAGGAAGGTCTCATCCCTACTAAGTACTACCAGAAGTCTAGAGAATCCCTTAGCACTGCCTCTGGGAAATCTCTCCAGTTGAATTTTGAAATACCAAAAGCCCATATTTGCCAAAATAAAGTCTGCTTTAAAACTTCATTCGTCTTAGTTAAAAATATTACTGATGAAGTTATTCTAGGATTACCTTTCCTAGCCTTATTATATCCTTTCCAGGTAGAATACGATGGCCTCATTTTTACCCATTTAGGGGAACAAGTTAAATTCAAATTCCTGTCAAAACCTGAGTTGCATAATTTAAAAATTTTACAAAAACAATCAGTTTCTAAATCTGTTTAAATTATTCGAAAAACCAACCAATTGAATTTCCTTAAAGAAGAAATTAGATTTAAACGAATTGAACAACAACTTTCTGATAAAGCCTTGCAATTGAAAATTCAGTCTTTTGAGAATAGATTACAAGTTGAAATATGTTCTAATTTGCCTACTGCTTTTTGGTATCGAAAACAGCATGTTGTTTGTCTTCCTTATATCAAGACCTTTAGTGAGAAAAATATCCCCACTAAAGCCAGGCCAATCCAGATGAATCAAGAATTGATGGAAATCTGTAAGAAAGAAATCCAAGAGTTGCTTCACAAAAATATCATTCGCAAAAGTAAATCCCCCTGGTCATGTCCTGCTTTTTATGTCCAGAAAAATGCTGAACTAGAAAGAGGAGTTCCCAGATTAGTTATCAACTATAAGCCTCTTAATTCGGTCTTAGAATGGATTAGGTACCCTATACCTAATAAAAGAGATCTTATTAATCGGTTAGAAAAAGCAGTTGTCTTCTCTAAGTTTGATATGAAAAGTGGTTTCTGGCAAATCCAGATAGATGAGTCAGATAGGTATAAGACTGCATTTGTCACTCCTTTTGGTCATTATGAATGGAATGTTATGCCATTCGGTTTAAAAAATGCCCCTAGTGAATTCTAGAATATTATGAATGATATCTTCAATCCTTACAGTCATTTCTCGATTGTTTATATTGATGATGTTCTAATATTCTCTGAGTCAGTTAAACAACATTGGAAGCATTTGCATAAATTCCTTCACATAGTCAAACAAAATGGTTTAGTTGTCTCTGCTAAGAAAATCAAGTTATTACAAACCAATATTAGATTTCTTGGTTTTAATATTTCCCAGTCCCAAATTGCTCCAATTGATCGAGTCATTCAGTTTGCTGACAAATTCCCTGACCAGATTCTTGATAAAAGTCAACTCCAGAGGTTTTTAGGATCTCTAAATTATGTTTTTGACTTCTACCAAAATCTGCGAAAACAATGTAAGCCGCTATTCGATAGATTACAGAATAATCCTCCTCCTTGGTCGAATATCCATACTGATGTGGTTAAACAAATTAAGTCCCATGTCAAGACACTTCCCTGTCTTGGTATTCCTTCAGTTGATTCTTTCAAAATAGTTGAAACTGATGCCTCTGACATTGGTTATGGTGGCATTCTTAAACAGAGAGTCAGCCCAGAATCTCCAGAACAAATTGTTCGTTTCCATTCTGGAACCTGGACCCAGTCGCAATATAATTATAGTATGATTAAAAAAGAAATATTAGCTATTGTATTATGCATTAGTAAATTTCAGGATGATTTATTAAATCAAAAGTTTTTAATCAGAGTTGACTGCAAATCTGCAAAGCATGTTTTAGAAAAAGATGTTCATAACATTGCATCTAAACAGATTTTTGCACGATGGCAAAGTATATTAAGTATATTTGATTTTGATATTGAATACTTGAGAGGTAGTGAGAATTCAATCCCTGATTTTCTTACTCGTGAGTTTTTACAGGAACCACAATGGCTCCCAAAAAAGACAAGAATAAACAAGTCGCCAGTCAGCCAATTGCCAGCCAGCCAAAACATTCCCAGTCGCCACAGTCACAACAGGCCCAATCCATGAGTCCATCTAAGCCAGAAATTCCTAAGCAAATAGTTCCCCATCCTAGTTGCACCCCTCTTTCCATTGGCTCCCCCTTATCAGTCGCAAACAGATTTTCCTCCTTACGATCCACAGTTGGCCAAATTCGTCCTAGTTATCAGTCAACCCTAGTTGCCAGTTACGACCCCTTTTCAGTTGATGTCCCTGCCGGACCCTCAGTCACGTATAGAAAGTCATCTCCTTACTTGCCCAAAAGTGAGTCGCATCTATTTGTCATCGAACCCGTATATGACATTATTGCCAATCCAGTTGAAATTGCCAGACATTATTTCCCTCCTGGATTTCACTATATGCCTCCAAGTCCTTACAAATCCCTCAAGTATTATAGAGATATATTACTTGAAACCAAATTAGTTGAAATTAAGCCCATAAAGGATCGTGACAACCCTGGAACTATCCTTTATCATTCTCTATATATCCATCAGATTATAAGCTAAGCAGCCTTTGCAAGTCGTCCTTTTGAGTTAAAGTTGCTCCAATCAAAATTGCAATATAATTATTCTGATTATATTGAAGCCTGGTACACTATCTTTCTTCACCAATCTGAAGATTTTAGTCATTCCTGGTTCATCAATTTTGATAATAAATTTAAATGCCCTTTCCCCTACTGGTTCTTCCATTGGTGGGAAAAGCATGGTCCAGTTGATGATATCCTGCTAGTATCAGTTCATGAGTTGATTATCCATTATACTAAGAAGGCCAAGTTCTCCGCAGTTGACTTATTTTTTTCCAAAGCAAATGTTATTTATTGCCAGGTACAAAGTTCCCTGGATTCTGAAGTGGTCTTATAGAGTCACCAAAGATGCTCGCATTTTTGCCCGCCAATTTTCAGTCAAATGGTGGGATAAGTTTGAAGTTGACAGAATTGCCAAATATGTCTACAATGATCTCCCAAATGAACCAATACCGCATCCTATTTCTCGTACTGGATCTGTTCGATCTTCCCTCTCAGTCGAAGGAAAATCCAAATCAGAGCTTCAAGAAATTGCCTGCCAGTTGATCCTACAAGCCTCTCAAATGGATGATGATGAAGACAATGGGTCTCCTTCGTCCTCTAGTTGCCAGCCCATATCTGA

>6; Chr6; OZ022211.1; 22443999-22451536; dir;

TGAAAGCATTATTTTAATGCATTATCATTATTTTCTGCATTAGTCTGAAAGTTGCTTAGGTTGCATCCTTTTCTAATTTCTTTCTGATCTTCATTCTGTATTATATTCAGTTGCTTGTTTGTTCTATCTCTTTGTCCCTCTTTCTTGGTATCAGAGCCGTAAGTGTTACGGTCTTTGTCTAAGTCTGCTACTCATTCCCGCCCAAGTGGCTGGTTTAAGCTTCTGAGTAGAAGGTTTTCATTTCTTCCTTTTCTTCCCCTTTATTCTTTGAGTCCTCTGTTAGTCTTCTCTAAGGTCTGACCCGTGAGTCCTTTTTGCTAGATCACGTAAATCCCAGAGAGGTAGTGTGTGTTGGGCAGGAGACAGAAAATTGAGAAAATTAATAGGTTGCTTAGAGTCTAAACGGTCAGTTTAGGATCTACTGTTCATCTTCTTCCTCAGACAGTTCAACCCAGTTTCTTGAGTAGTCATGAGTCGTTTCCTTAGGTCAAGGTTTAGTAATTCTGCCAGTTCTAGTGCCAGTATAAGTCGACTCCCAGAGATAGTAAATGAAGAACAATTTGAATATAATTCCAGTAGCAAAGAACAGTTAGATTTTAGAGATTGGAACATACCTAAAATCCCTAGTCAGAAAATCTATAAGAAACATTGGTTGCCTTCTAGTTTCAATAGCACCACACATGTCAAAACAGTTGAACAAGTATACGCCTTAAGCAAAGAACACGAAACTTGCCAGTTGCTTAATCTTGAGTCTATTTTAAAACATAAGAAAGATGGTAATAATTTCCTGCATATTGGTCTAGTTCAAGTTGCAGTAAAGCCTCTGACACGGTTAGGGTTGAAAGCCTCAATCCTGCTATGTCTTCGTGATGCTAGATTCACTGAATTTTGCGATAATACCCTTGGAATCATTGAGTCCAGTCTGTGTAATGGCCCTGTCCATTTTGACTGCTATCCTGATTTCACTGTAAGCCTTAGTGATCCCCATATTCTGAGAACTTTGACTCTAAATATTAAGACTGAAGGTTATAATGTTCTCCCGGGAACCCAACCTCTAGCCTTGGTATATAGAATCTACTATAAGGTCACAGGAACTAATATGAACTTCCAAGCCCTTAATAAAAGCCCAAAGGATCAGACAGTCCTGATCCAGAGTCATACCTCAGATGCCCATATCCAAGTTCCCCATACTATTAAGTGGTCAGAAGTCACCCTTCCAAAAGATTGGACCTTAGTCACTGAGAGCCAACCAGCTCCCATTCAGCGTAGTCTAAACAATTTAGACTATATCCAACAATATTTAGACGGCACAGTTAAGATTCAGTTTGATAGTCATCCCTTACGCAAGTCCAATGTCCAATTACAACAGTTGCCAACCCCTGGTCAGTCCCAAAGGCATGCCCCTGCTCGACATTCCTTTGCTGCCTCTTCCTCCACTCTAGAACGAGACCTAGAGTTAGAAAAGGCTATGATTGATTTTAAACTTGAGTCCCTGAGAAAAACCTCTCAGGTTACCCAACCTTGCTACGGCCAGGTTCCAGTTCCTGATGATAAGTCAGAATCACCTGAGTCACCTACCCAGTCTGACTTCGTAGTTGAAAATCAGCTTAGAACCCTCAATAAAGAATTTAAAATTGATTGGGTTTACTTAAATCGTCACCTAAAAGACCCTGAGAACAAGCCCAGGCGAGATCTTTATCATCAAGCCTACCCTAATAGCAAACACCGAGATATGATCTTTCAACAATGGAAAGATTATATGCGGTCAGCCAAAGTTGAAATATTTTACCTTGATTTCATAGAAAGCCGTTATTTGACTAATGATGAGTTAAAAACTTTGACTAAAGAAAAGTGGAAATTAGCTGATAAATCTGTTGTAGAGTCTAGTCACCCTCCAGTAGAAACTATAGTCATAGAACACCAGAACACCAGAAAACCTTTGAAAGCAGTGACCCTCATAGGAAATTAATCGAACAAAATAATTACACCAACCAGAGTCTAATCACCATAGGAAAACAGTTAGACACAATTGAAACCAAAGTTGATCTGCTGAGTCCGCCTGTTGCAGACTCCAGACCCAAACCTAAAGTTGAAAAGCCGATAGCTCACTTCCAAGAACTTAAGTCTGGTCCTGCACTCAAGACCAATTCCACGATAGAAAAAATAGAAGAAATGCTAAAACAATTGACTCCTGTCAAGTCTGAGAAAGGTAATCAGTCTGGTATTAAAACACTAGACGCCTTTGCCGCTTCTCAATCTGAGTCTGAATCTGTAAGTACTGAAATTAGTGATATTTCTAAAATTGAAAAAGCTTTTCAAAATATAGAAATCCAACCTGAGCCTAGAGTCAAGAAAATTGAGAAACATGTAAGTCCTACTAGCCTTACCAAGAATTGGTACCCCAGACCAACACCTCCAGATATCCAATTTGAAGAAAGAAACTTTCAAACCCAGTTTTCTGTTTCTTCCGACAAATTGTATGAATGGAATATTGATGGTCTTTCTGAGCAAGAAATATTAAATAAATTGCAACACATGTCAATGGTCGCCAATAGTTATATCACCAATCACAGTTTTAGACAGTCTGAAATTGTCCCTCTGTTAGTTACTGGTTTCACCGGTACTCTCAGATATTGGTGGGATAAACACCTTACCCTTGAAAATAAAAATCAAATTATTTATGCTGTAAAACTTAATGAAGACGGTCTCCCTATTTTTGACGAACAAATAGGACAAGGTATAGAAGACGGTGTTAACACTTTGTTTTACACGATAATTGAACATTTCATTGGAACCCCTAGTAACACCACTGCTAGGATCCATGATCAGCTGAGTAACCTTAGATGTCCCAAGTTGTCTGATTTCAGATGGTATAAAGACGTTTTCATCTCTAGAGTCATGCTTAGAGATGATAGTAATCAACCATTCTGGAAAGAAAAGTTTATTAATGGACTGCCAAACTTATTTGCTCATAAAATCCGCACAACTCTAAGTAATGAACAAGGTCAAATTGATTGGGATTCATTAACTTATGGTAATATTATAAGCACAATTAATCAAGTTGGTATGAAAATGTGTATCGATTTTAAAATTAATCGTCAAATCCAGTCTGATAGAAAGTCAGCCAAATATGAGTTAGGAAACTTCTGTGAACAATATGGTCTCACTAGTGTTCCTCCCTCTAGAAGACAAAAGCCTAGCCATCTGAGAAAGCGTCATTTCTCGAAGAAGCAATTTCTTTGAAAAGACGAATTCTACAAGAAGAGAAAGTTCTCTCCTAAGAAGAGCTGGTCTAAACGCCCTAAGCAGAGTCGCTCCAAGAAAGATAAAAGCAAAGTTAAATGTTTTAAATGTCAAAAATTTGGTCATTATGCTTCTGAATGTAAAGTTAAAGATACTATTAAACAGTTGCAGATTTCAGAAGAAGATAAGAACAAATTGATCCAAGTTTTGGAGTTGCGAGACTCTGAAATGAGCGATGTTGAGTCTCTAGTACCCAGTTCTGAATCTGAGTCAAGCCAGTCTTCAGATTCACAACCCAGTTCACCTAGAATTCAATTTGGTTGCACTGATAAATGCTGTAATAGATTAAAGTCTGTTCTTGTTCTTACTAAACAGGAAGAACAAGAAGAGTTGCTTGTAGATCTAATAGGAAGAGTTCAGAACCCTGAATTAAAAGCTGAATATTTAAAGAAGTTGAAAAAGATCCTAAGTCAGGATAGTCAGAGTTCATCCTCTCCTAATCCTCAGCCAATTAACATCTCCACTACGTTAGAAAAATTCAATCGTAGGAAAGATGTTACCCTGCATGATCTCCAGCTGGAAGTCAAGCAGGTTAAGAAAGAAATAGTTGAACTCAAACAGATAAATCAACAATTGCAAGCCGAGAATTATACAATCAAACAAGACTTAGTTGATTTACAACAAGCCACTCGCCAGCCTAGTACCCATTCGGAAAGCCCACATCATTCTGATGAGGAACCTTCTAAAGAACAACCAGTTGTCAATCTGATCAAACAAGTACACCTTAGAAAATGGTATTCCAAAGTCACGATTTTTGTTCATGATTTTGAATTTACCACAGTCGCTTTATTTGATTCTGGTGCAGATCTTAATTGTATTCAGGAAGGTCTCATCCCTACTAAGTACTACCAAAAGTCCAGAGAAGCCCTTAGCACCGCTTCAGGGAAATCTCTCCAATTGAACTTTGAGCTACCAAAAGCCCATATTTGTCAAAACAAAGTTTGTTTTAAAACCTCATTCGTCTTAGTTAAAAATATTACTGATGAAGTCATATTAGGATTGCCTTTCCTTGCTCTGTTATATCCTTTCCAGGTAGATTACGATGGCCTCATTTCTACCCATTTAGGAGAAACGGTCAAGTTTAGTTTCCTGTCTAAACCAGAGTTGCATAATTTAAAAACCTTACAAAAACAATCAGTCTTTAAATCTGTCCAAGTTGTTTGGAAAACCAACCAGTTGAATTTTCTTAAAGAAGAAGTTAAATTTAAACGAGTTGAACAACAACTTAGTGATAAAACTTTACAGTTGAAAATTCAGTCTTTTGAGAACAAATTAAAAGAAGAAATATGTTCTGATTTGCCTACTGCCTTTTGGTATAGAAAACAGCATGTCGTCTGTCTTCCCTATATCAAAACCTTTAGTGAGAAAAATATCCCTACTAAAGCCAGGCCAATCCAGATGAGTCAAGAGTTAATGGAAGTCTGTAAGAAAGAAATTCATGAGTTGTTACAGAAAAATATAATTCGCAAAAGTAAGTCCCCCTGGTCATGTCCTGCTTTTTACGTCCAGAAAAATGCTGAACTAGAAAGAGGAGTCCCTAGATTAGTAATCAACTATAAGCCTCTTAATTCGGTCCTAGAATGGATTAGGTATCCCATACCTAATAAAAGAGACCTTATTAACCGATTAGGTAAAGCAGTTGTCTTCTCAAAATTTGATTTGAAAAGTGGTTTCTGGCAAATCCAAATAAATGAGTCAGATAGGTATAAAACTGCATTTGTTACTCCTTTCGGTCATTATGAATGGAATGTCATGCCGTTTGGTTTAAAAAATGCCCCTAGTGAATTCCAGAATATTATGAATGATATCTTCAATCCTTACAGCCATTTTTCGATTGTTTATATTGATGATGTTCTAATATTCTCTGAGTCAGTTGAACAACATTGGAAACATTTGCATAAGTTCCTTCATATAGTCAAACAGAACGGTCTAGTTGTTTCTGCCAAAAAGATCAAGTTGTTTCAAACCAATATTCGATTTCTTGGTTTTAACATTTGCCAGTCTCAAGTTGCTCCAATCAATCGAGTCATTCAGTTTGCTGATAAGTTCCCTAATCAGATTCTTGATAAAAGTCAACTCTAGAGGTTTTTAGGATCTCTAAATTATGTTTCTGACTTCTACCAAAATCTGCGAAAGCAATGTAAGCCTTTATTTGACAGATTGCAAAGTAATCCTCCTCCGTGGACAGAGCTCCACACTGAAATTGTCAAACAGATTAAGTCCCATGTCAAGACACTTCCCTGTCTTGGTATTCCCTCAGTTGATTCCTTCAAGATAGTTGAAACAGATGCCTCTGACATTGGTTATGGTGGCATTCTCAAACAGAGAGTCAGTCCAGATTCTCCAGAACAAATCGTTCGTTTCCATTCTGGAACCTGGACCCAGTCGCAATATAATTATAGTACTATTAAAAAAGAGATATTAGCTATTATATTATGCATCAGTAAATTTCAAGATGATTTACTAAATCAAAAGTTTTTAATCAGAGTTGATTGTTTATCTGCAAAACATGTTTTAGAAAAAGATGTTCAAAACATTGCATCTAAACAGATTTTTGCACGATGGCAAAGTATATTAAGCATATTTGATTTTGATATTGAATATTTGAGAGGTAGTGAGAATTCAGTTCCTGATTTTCTTACTCGAGAATTCCTGCAGGAACCACAATGGCTCCCAAAAAAGACAAAAACAAAGAAGTTGCTAAGTCAAGCCAGTTGCCCAAACCATCCTAGTCGCAACAATCTCAGTCGCCATCCATGAGTCCAGCCAAGCCTGAATTGCCCAAACAGATAGTTCCCTATCCTGGTTGCCCCCCTATCAGAGTTGCTACCCCACTATCAGTTGCCAACAGATTTTCCTCTTTAGGATCCACAGTTGGCCAAATCCGTCCTAGTTATCAGTCCACTCTAGTTGCCAGTTATGACCCTTTTGCAGTTGACATCCCTACTGGATCCTCTGCACCTACAGTTGTCCATAAAAAATCGTCTCCCTATTTGCCTAAGAGTAACTCCCATTTATTTGTCATTGAGCCCATTTATGATATTAATACCAACCCAGTCGAAATCGCCAGGCATTATTTTCCTCCTGGTTTTCATTATATGCCCTCTAATCCCTATAAGTCCCTCAAGTATTATAGAGATATTTTGCTTGAAACCAAATCAGTTGAAATCAAGCCCATAAAAGATCGTGATAATCCCAGCACTATCCTTTATCATTCATTGTACATTCACCAGATTGTAAGCCAAGAAGCCTTCAGCAGTCGCCCTTATGAACTTAAGTTGCTTCAATCTCAGTTGCAATACAATTATGCAGATTATATTGATGCATGGTATACTATTTTCCTTCATCAGTCTGAGGATTTTAGTCATTCCTGGTTCATCAATTTTGATAATAAGTTCAAAAGTCCTTTCCCATATTGGTTTCTCCATTGGTGGGAAAAACATGGTCCAGTTGAAGAAATTCTACCAGCTTCGGTTCTCCAGCTCATTCACCATTACACTCAGAAGGCCAAGTTCTCCGCAGTTGATTTATTTTTCCCCAAACAGTTATTATTTATTGCCCGATACAAGGTGCCCTGGATACTAAAATGGTCTTACAGGGTTACTAAAGATGCTCGCATTTTTGCCCGCCAATTTTCAGTCAAATGGTGGGATAAATTTGAAGTTGACAGAATCGCCAAATATGTTTACAATGATCTTCCAGTTGCACAAGTCCCTCTGCCGGTCTCTCCCTCTGCATCTGTCCGATCTTCCCTCTCAGTCGAAGGAAAATCCAAGGCAGAACTCCGAGAAATCGCCACTCAGTTGCTCCGACAAGCCTCCCAGATGGATGATGATGAAGACAACGGGTCCCCCTCGTCTTCCAGCTGTCAGCCCATCTCTGTCAAGCCCAAAACCTGGTTTGAAGACAGTCAAGATCCTTATGATGCCTATGAGACAAAATTGAAAGTTGAATTAGGTTGCTTTCAGGTGACTCCACTGTGACAAGCAAACTGCAGTTGCTTCACCATAGTTAGCAAACCCTAGTTGCTTATCTATTTAAGCTGGCTTCGGCCTCAGTTGCAGGCAGAATGTAATTTTCCTTTCTTCAGTTTTTTTACAGAATCTCTCTGATATATCCTTCGGAGAAATTCACTTTTGTAAACACTCTTGTAAATAGCGTGCAGTTGTGTTTGAAGAATTGCAGTTGCAATCTTTGTGAGTATAAATAAAAGTTATTTTCAATTATCTATTTTC

>7; Chr7; OZ022212.1; 8574482-8582769; dir;

TGAAAAGCATTATTTTGATGCTTTGTTGGTATCAGAGCCGTAAGCATTACGGTCTTTGTCTAAGTTTGCTACTCATTCCCGCCCAAGTGGCTGGTTTAAGCATCTGAGTAGAAGGTTTTCATTCCTTCCTTTTCTTCCCTTTTATTCTTTGAGTCCTCTGTTGGTCTTCTCTAAGGTCTGACCCGTGAGTCCTTTTTCCTAGATCAGTAAGTCCCCAGGGAGGGCGTGTGTGGTGGGCAGGAGACAGAAAATTGAGAAAATTAATAGGTTGCTTAGAGTTTAAGCGGTCGGTTTGGGATCTACTGTTTATCTTCTTCCTCAGATAGCCAAACCGAGTTATTTGAGTAGTTATGAGTCGTCTGTTTAGAACAAAGTCTTGTAATTCTGCCAGTTCTAGTACCAGTATAAGCCGACTCCCAGAGATAGTTAATGAAGAACAATTTGAATATAATTCCAGTAGTAAAGAACAATTGGATTTTAGAGATTGGAATATACCTAAAATCCCGAGTCAAAAAATATATAAGAAACATTGGTTGCTGTGGGAGCAAATTTCCCCACGATAATATTCGCCCAAGATTCCTTCGTCTTCTCCGCCTCTCTTTCTCCCTGCAAAACAGATCGGAGTAAAAGGACCACACCCGGGGGGTGTTGGCCAAAGGCCCTCCGATGCCTAAGTTAGATAGGGTAATTCAAGGAAAACCGGGTGGCCGGAGCCGTGTGTGGTGGCCGGAGCCTTGTGTAAGAAAGAGAAAGAGAGGAGGTGGCTAGGGTTTTTGAGAAATAACTTATGCAGAGTTTGAGTAGGAATTCTGACGTACCTCATCCTTGTGCGTAACCAAGTATTTATAGTGGCCTTGAAGAGGTTGGTGTAGTTGGTGAGGTTGGTGTGGTTGGTGTAGTTGGTGAGGTTGGTGTGGTTGGTGAGGTTGGTGTATTTAGGGATTATGGATTTAACCGAATCCCTAATTAAGGCGACGATCAAGTAACCCCCAATTTGATTAGGTAATTCCCAATTAGGTTAGGTAACTCCCAATTAGGTTAGGTAACTCATAATTAGGTCAAATAATTCCCAAATTGATTGGCCTAATTATTTAACCTAGGGTTTTAATTAGGTTCCCACAGTTGCCTTCCAGTTTTAATAGCACAACACATGTCAAAACAGTTGAACAAGTATACGCCCTAAGTAAAGAACATGAAACTTGTCAGTTGCTTAATCTTGAGTCTATTTTAAAATATAAGAAAGATGGTAATAATTTCCTGCATATTGGTTTAGTTCAAGTAGCTGTCAAACCCTTGATACGGTTAGGGTTGAAAGCCTCAATCCTACTCTGTCTGCGTGATGCTAGATTCACCGAATTTAGCGATAGTACCCTTGGAGTCATTGAGTCCAGTATGTGTAATGGCCCTGTCCATTTTGACTGCTATCCTGATTTCACTGTAAGCCTTAGTGATCCTCATATCCTGAGAACTTTGACTCTGAATATTAAGACTGAAGGTTATAATGTCCTTCCGGGAACCCAACCTTTAGCCTTAGTTTACAGAATCTACTATAAGGTCACAGGAACTAATATGAATTTCCAAGCCCTTAATAAAAGCCCGAAGGATCAAACCGTCCTGATCCAGAGTCACACCTCAGACGCCCATATACAAGTTCCCCATACCATCAAATGGTCTGAAGTTGCCCTTCCAAAAGATTGGACCTTAGTTACTGAGAGCCAACCGGCTCCGATCCAGCGTAGTCTAAAAAATTTAGACTACATCCAACAATACTTAGATGGCACAGTTAAAATTAAGTTCGACAGTCATCCCCTCCGCAAGTCTAATGTCTAGTTACAACAATTGCCAACTCCTGGTCAATCTCAGAGGCATGCCCCTGCTCGGCATTCCTTTGCTGCCTCTTCCTCCACTCTAGAACGAGACCTAGAGTTAGAAAAGGCCATGATCGACTTCAAACTTGAGTCCCTGAGAAAAACCTCTCAAGTTACCCAACCTTGCTGCGGCACGATTCCAGTCCAAGACGATAAATCAGAATCCCCTGAGTCTCCGACCCAGTCTGATTTCCTAGTTGACAACCAACTCAGAACCCTAAATAAGAAATTTGAAATTAATTGGGTAAGTCTAAATCGCCATTTAAAAGCCCCTGAAAACCAGCACAGAAGAGATAATTATCACCAAGCCTACCCTAATAGCAAACACCGCGAAATGATCTTTAGTAAATGGAAAGATTACATGCGGTCTGCCAAAGTTGAAATTTTCTACCCTGATTTCGTTGAAAGCCATTACATGTCTAGTGATGAATTGAAAACTCTGACTAAAGAAAAATTGAAACTAATTGATAAATCTGTAGTTGAGTCGAGTCATCCTCTAGTAGAAACTATCATAATAGAACACCAGAAAGCACCAGTAGCTGCCACCCCTTTCAAAACCTTTGAAAGCAGTGACCCGAATAGAAAAATGATTGAACAAAACAATTACACCAATCAGAGTTTAATCACCATAGGAAAACAATTAGATACAATCGAAACCAAGGTCGACATGCTGAGTCCGCCTGTTGCAGACTCCAGACCCATACCTAAAATTGAAAAGCCGATAGTTCAATTCCAAGAGCTTAAGTCCAGTCCGGTCTTAAAAGTCAATTCTACAATGAAAAAGATAGAAGAAATGCTAGAACAGTTGACCTCTGCCAAGCCCGAGAAAAGTGAAAAATCTGGTATTAAAACACTAGATTCTTTTGCTGCTTCTCAATCTGAGTCCGAATCAGTAAGTTCAAAAACCAGTGACATTTCCAAAATTGAAAACGCCTTCAAAAATTTAGAAGTCCAAACTAAGCCCAGAGTCAAAAGAATAGAAAAACATATAAGTCCCACTAGCCTAACAAAGAATTGGTATCCCGGACCAACACCTCCAGATATCCAATTTGAAGAACGAAACTTTCAAACCCAGTTTTCAGTTTCTTCTAATAAATTGTATGAATGGAATATTGATGGTCTTTCTGAGCAAGAAATTCTAAATAAGTTGCAACATATGTCTATGGTCGCTAATAGTTATATCACCAATCATAGTTTCAGACAGTCTGAAATTGTTCCTCTGTTAGTTACCGGTTTCACTGGTACTCTCAGATATTGGTGGGATAAGCATCTTACCCCTGAGTCTAAAAATTGAATAATCTATGCTGTAAAACTTAATGAAGACAGTCTCCCTATATTTGACGAACAAATAGGTCAAGGTATAGAAGACGGTGTCAACACTTTGTTTTACACTATTATTGAACATTTCATAGGAACCCCTAGTAATACCACTGCTAGGATACATGATCAGTTGAGTAATCTTAGATGCCCAAAGTTGTCTGATTTCAGATGGTACAAAGATGTCTTTATTTCCAGAGTCATGCTTAGGGACGATAGTAATCAACCCTTCTGGAAAGAAAAGTTCATTAATGGTTTACCAAATCTATTCGCTCATAAAATACGCACAACCCTGAGTAATGAACAAGGTCAAATTGATTGGGATAATTTAACTTATGGTAACATTATAAGCACGATTAATCAAGTTGGTATGAAAATATGTATCGACTTTAAAATCAATCACCAAATCCAGTTTGATAGAAAGTCAGCTAAGTATGAATTAGGAAATTTCTGTGAACAATATGGTCTTACTACTACCCCTCCTTCTAGGAAACATAAGCCTAGTCAACTTAGAAAGCGCCATTTCCCGAAGAAGCAATTTCTCCGGAAAGATGAATTCTATAAGAAAAGAAAGTTCTCCCCTAAAAAGAATTGGTCAAAACGTCCCGAGCAAAAACAAAGTCGCCCTACGAAAGATAAAAGCAAAGTCAAATGTTTTAAATGCCAAAAATTTGGTCATTATGCTTCTGAATGCAAGGTAAAAGATGTTATTAAACAATTGCAGATTACAGAAGAGGATAAGAACAAATTAATCCAAGTTTTAGAGTTGCGAGACTCTGAAATAAGTAATGTTGAGTCTCTGCTTTCCAGTTCTGAGTCTGATTCTGACCAGTCTTCAGATTCACAACCCAGTTCTCCTAGAGTCCAATTTGGTTGTACTGATAAATGTTGTAATAGGTTAAAATCTGTCTTAGTCCTCACTAAACAAGAAGAACAAGAAGAACTTTTAATAGATTTAATAAGAAGAGTTCAAAACCCTGAGTTAAAATCTGAATACTTGAAAAAATTGAAAAAGGACCTAAGTCAGGATAGCCAGAGTTCACTCTCTCCTACTCCCCAGCCAATTAGCATCAACACTACTTTAGAAAAATTCAATCGTAGAAAAGATGTCACCCTCCATGATCTCCAGATAGAAGTTAAATAGGTCAAAAAAGAAATAGTTGAGCTAAAACAGATAAGTCAACAATTGCAAACCGAGAATTATACTATCAAACAAGAATTATTTGATTTAAAACAAGAAACCTGTACGCCTAACAACCACTCAGAAAGTCTGCATCACTCTGATGACGAACCTTCTGAAGAACAGCCAGTTGTCAATTTAATTAAATAAGTTCACCTTAGAAAATGGTATTCAAAAGTCACAATATTTGTCCATGATTTTGAATTCACCACAGTCACCTTATTTGATTCAGGAGCAGACCTTAACTGTATCCAGGAAGGTCTCATCCCCACTAAGTATTACCAGAAGTCCAGAGAATCCCTTAGCACTGCCTCAGGGAAGTCCCTCCAGTTAAATTTTGAAATACCAAAAGCCCATATTTGCCAAAACAAAGTCTGTTTTAAAACTTCATTCGTCTTAGTTAAAAACACTACTGATGAAGTCATCTTAGGTTTGCCTTTCCTCGCCCTGCTTTATCCTTTCCAGGTAGAATACGATGGTATCATTTCTACCCATCTTGGAGAAAAGGTCAAATTTGGCTTCCTGTCGAAACCTGAGTTGCATAACCTGAAAACTTTACAAAAACAGTCAATCTCTAAATTTGTCCAAGTTATCAGAAAAACTAATCAGTTGAATTTTCTTAAAGAAGAAATTAAGTTTAAAAGAATTGAACAACAGCTTACTGATAAAGCCTTACAGTTGAAAATCCAGTCCTTTGAAACCAAACTACAAGATGAAATATGTTCTGATTTACCTACTGCTTTTTGGTATAGAAAACAACATGTTGTCTGTCTTCCCTATATCAAAACCTTTAGTGAGAAAAATATCCCCACTAAAGCTAGGCCAATCCAGATGAATCAAGAGTTTATGGAAGTCTGTAAAAACGAAATTCATGAGTTGCTCCAAAAGAATATTATTCGAAAAAGTAAATCCCCTTGGTCGTGCCCTGCTTTTTATGTTCAGAAAAATGCTGAATTAGAAAGAGGAGTCCCCAGATTAGTTATCAATTATAAACCCCTTAATTCGGCCTTAGAATGGCTTAGGTACCCTATACCTAATAAAAGAGATCTCATTAATCGGTTAGAAAAAGCAATTGTCTTCTCCAAATTTGATATGAAAAGTGGTTTCTGGCAAATCCAAATAGATGAGTCAGATAGGTATAAGACTGCATTTGTTACTCCTTTCGGTCATTATGAATGGAATGTCATGCCGTTTGGTTTAAAAAATGCTCCTAGTGAATTCCAGAATATTATGAATGACATCTTCAATCCTTACAGTCATTTTTCGATTGTTTATATTGATGATGTTCTTATTTATTCTGAGTCAGTTGAATAGCACTGGAAGCATCTGCATAAATTCCTTCATATTGTCAAACAGAATGGTCTAGTTGTTTCTGCCAAGAAAATTAAGTTGTTTCAAACTAATATAAGGTTTCTAGGTTTCAATATTAGTCAATCTCAAATTTCCCCAATTGACAGAGTCATTCAATTTGCCGATAAATTCCCTGATCAGATTCTTGATAAAAGTCAACTCCAGAGGTTTCTTGGATCTCTAAATTATGTTTCTGACTTCTACCAAAATCTGCGAAAGCAATGTAAGCCTCTGTTTGATAGATTACAAAATAATCCCCCTCCATGGTCGAATGTCCATACTGAAGTTGTCAAACAGATTAAGACCCATGTCAAGACACTTCCCTGTCTTGGTATTCCTTCAGTTAACTCTTTCAAAATAGTTGAAACTGATGCCTCTGACATAGGTTATGGTGGCATTCTCAAACAGAAATTCAGTCCAGAATCTCCAGAACAAATTGTTCGTTTCCATTCTGGAACCTGGACTCAGTCGCAATATAATTATAGTACTATTAAAAAAGAAATATTAGCTATTGTATTATGCATTAGTAAATTTCAAGATGATTTATTAAATCAAAAGTTTTTAATACGAGTTGATTGCAAATCTGCAAAACATGTTTTAGAAAAAGATGTTCATAACATTGCATCTAAACAAATTTTGCACGATGGCAAAGTATATTAAGTATTTTTGATTTTGATATTGAATATTTGAAAGGTAGTGAGAATTCGGTCCCTGATTTTCTTACTCGAGAATTTTTGCAGGAACCTCAATGGCTCCCAAAAAAGACAAACACAAAGAAGTTGCCAGTCAACTCAAGTCACCCCAGTTGCAACCATCCCAGTCACCCCAGTTGCAACCATCCCAGTCACCATCCATGAGTCCAGCCAAACCAGAAATGCCCAAACAGATAGTTCCTTATCCTGGTCATCCCCCTCTTCCAATTGCCACCCATTATCAGTTGCAAACAGATTCTCTTCCTTAGGATCTACAGTTGGCCAAATCTGCCCCAGTTACCAATCCACATTAGTTGCCAGTTATGACCCTTTTTCAGTAGATGTCCCAGCAGGACCGTCAATTGCTTTGAAAAAATCTTCTCCTTATTTGCCAAAGAGTAATTCCCATCTATTTGTCCTTGAGCCTTTGTATGATATTCATACTAACCCAGTTGAAATTGCCAAACATTATTTCCCTCCTGGATTCCATTATATGCCTTCCAGTCCTTACAAGTCCCTCAAGTATTATAGAGATATATTGCTTGAAACTAAGTCAGTTGAAATTAAGCTGATAAAGGATCGAGATCACCCTGAGATCATTCTTTATCATTCAATGTACATCCACCATATCATAAGCCAAGAAGCCTTCAGTAGTCGCCCTTATGAGTTAAAAACCCTTCAATCCAAATTACAGTATAATTATGCTGATTATATTGATGCCTGGTACACTATTTTCCTTCACCAATCTGAGGATTTTAGTCATTCCTGGTTCATCAATTTCGATAATAAGTTCAAATGTTCTTTCCCCTATTGGTTCCTCCATTGGTGGGAAAAACATGGCCCAGTTGATGAAATTCTGCCTGTATCAGTTCTTGAATTAATCCGCCATTATACGGAGACTGCCAAGTTCTCCACCGTTGATTTATTCTTCCCCAAACAATTGCTTTTCCTTGCCAGATAAAAAGTTCCTTGGATCCTCAAATGGTCCTACAGAGTCACTAAGGATGTTCGTATCTTTGCCCGCCAATTTTCAGTCAAATGGTGGGACAAATTTGAAGTTCAAAGAATAGCCAAGTATGTTTACAATGATCTCCCTGCTGAGCCTCAGTCAACTTCTCCAGTTGGTTCTGTTCGATCCTCCCTCTCAGTCGAAGGAAAGTCGAAAGCAGAACTCCAAGAAATTGCTCGCCAGTTGATGATGCAGGCCTCTCAGATGGATGATGATGAGGACAACGACTCTCCTTCGTCGTCCAGTTGTCAACCCATTCCTGATCCTGTTAAGCCTAAGTCCTGGTATGATGACAGCCAGGATCCCTATGATGCCTATGATCTGCATTCTGACTAGGCCTCATAATTTCTGCTGACATCTCCACATGCAAAAGTTGCCCGACACCATAATTGCCACAGGAAGAAGCATCTGCTTTTTCTGTTTACTTTTCCACCGAAAGAAGCCATCAGTTCCAGACAAAGTCGCTCAGACCGCCAGCTGTCATCTGCCTTTCCACCGAAAGGAGCCAGCCGACAAAATTGAAAGTTGAATCAGGTTGCATGTGACTCCACTGTTGCAACCAACCATAGTTTAGCCAACCATAGTTGAAAGAACTCTAGTTGATTTTTCTCTTTATAAGCCGGCTTCGGCCTCAGTTGAAAACAGAATGTAAATTTTCCCTTCCTTCAGTTTCAAAGATTCTCTCTGATACATCCTTCGGAGAAAATCATCTTTGTAAATAGCGTGTGAGTTTGAAGTGTTGCAGTTGCAATCCTTTGTGAGTATAATAAAAGTTATTTTCAATTATCTATTTTC
