# Supplementary material for: A comprehensive comparative genomic analysis of the endogenous Florendovirus-1 in Prunus
Source: Front Plant Sci. 2026 Sep 29;17:1886618. doi: 10.3389/fpls.2026.1886618 (PMC13658181; doi:10.3389/fpls.2026.1886618)
Supplement: Supplementary file 1 [file SupplementaryFile1.zip › Supplementary Material 7 - Sequences of Florendovirus-1 in Prunus dulcis 3.docx]

Supplementary File 7 - Sequences of Florendovirus-1 in Prunus dulcis 3.

Non-highlighted letters are the flanking sequences (1000 bp).

Blue letters indicate that the Florendovirus 1 is in the reverse sense.

Grey letters are transposable elements or other insertions.

The NCBI Accession number, the positions and the sense respect the sequence are indicated.

A – Isolated complete element.

B – Isolated fragment.

C – Multiple loci with at least one complet element.

D - Multiple loci with no complet elements.

>Consensus

TGAAAGCATTATTTTAATGCATTATCATTATTTTCTGCATTAGTCTGAAAGTTGCTTAGGTTGCATCCTTTTCTAATTTCTTTCTGATCTTCATTCTGTATTATATTCAGTTGCTTGTTTGTTCTATCTCTTTGTCCCTCTTTCTTGGTATCAGAGCCGTAAGTGTTGCGGTCTTTGTCTAAGTCTGCTACTCATCCCCGCCCAAGTGGCTGGTTTAAGCTTCTGAGTAGGAGGTTTTCATTTTTTCCTTTTTCTTCCCTTTTATTCTTTGAGTCATCTGTTGGTTTTCTCTAAGGTCTGACCCGTGAGTCCTTTTTTCTAGATCAGTAAGTCCCCAGGGAGGGCGTGTGTGCTGGGCAGGAGACAGAAAATTGAGAAAATTAATAGGTTGCTTAGAGTCTAAACGGTCGGTTTAGGATCTACTGTTCATCTTCTTCCTCAGTCTGCCAAACCCAGTTTCTTGAGTAGTCATGAGTCGTTTATTTAGAACAAAGTCTTGTAATTCTACCAGTTCTAGTGCCAGTATAAGTCGACTCCCAGAGATAGTTAATGAAGAACAATTTGAATATAATTCCAGTAGTAAAGAACAATTAGATTTTAGAGATTGGAATATACCTAAAGTCCCAAGTCAGAAAATCTATAAGAAACATTGGTTGCCTTCTAGTTTTAATAGCACAACACATGTCAAAACAGTTGAACAAGTTTACGCCTTAAGTAAAGAACATGAAACTTGTCAGTTGCTTAATCTTGAGTCAATTTTAAAACATAAGAAAGATGGTAATAATTTCCTGCATATTGGTTTAGTTCAAGTTGCTGTTAAGCCCCTGACACGGTTAGGATTGAAAGCTTCAATCCTGCTATGTCTGCGTGATGCTAGATTCACTGAATTTAGCGATAGTACCCTTGGAATCATTGAGTCCAGTCTGTGTAATGGCCCTGTCCATTTTGATTGCTATCCTGATTTCACTGTAAGCCTTAGTGATCCTCATATCCTGAAAACTTTGACTCTGAATATTAAGACTGAAGGTTATAATGTTCTGCCAGGAACCCAACCTTTAGCCTTAGTTTACAGAATCTATTATAAGGTCACAGGAACTAATATGAATTTCCAAGCCCTTAATAAAAGCCCAAAGGATCAAACCGTCTTGATCCAGAGTCATACCTCAGATGCCCATATACAAGTTCCTCATACTATTAAGTGGTCAGAAGTTGCCCTTCCAAAAGATTGGACCTTAGTCACTGAGAGCCAACCAGCTCCTATCCAGCGTAGTCTAAACAATTTAGACTACATCCAACAATACTTAGATGGCACAGTTAAAATTAAGTTTGATAGTCATCCTTTACGAAAATCTAATGTCCAGTTACAACAGTTGCCAACTCCTGGTCAATCCCAGAGGCATGTCCCTGCTCGGCATTCCTTTGCTGCCTCTTCCTCCACTCTAGAACGAGACCTAGAGTTAGAAAAGGCTATGATTGATTTCAAACTTGAGTCCCTGAGAAAAACCTCTCAGGTTACCCAACCTTGCTACGGCAAGGTTCCAGTTCAAGAAGATAAGTCTGAATCACCTGAGTCACCTACCCAGTCTGATTCATAGTTGAAAATCAGCTTAGAACCCTTAATAAAAAATTTGAAATTAATTGGGTAAGATTAAATCGTCATCTAAAAGCCCCTGAAAACCAGGCAAGAAGAGATAATTATCATCAAGCCTACCCTAATAGCAAACACCGAGAAATGATCTTTAAACAATGGAAAGATTATATGCGGTCAGCCAAAGTTGAAATTTATTACCTTGATTTTGTAGAAAGCCGTTATATGTCTAATGATGAATTAAAAACTTTAACTAAAGAAAAATGGAAAATAATAGATAAaTCTGTAGTTGAGTCTAGTCATCCTCCAGTAGAAACTATAATAATAGAACACCAGAAAGCACCAGTTCCTGCCACTCCTTTCAAAACCTTTGAAAGCAATGACCCTAATAGAAAACTAATTGAACAAAATAATTACACCAACCAGAGTCTAATCACCATAGGAAAACAATTAGATACAATTGAAACCAAAGTTGATATGtTGAGTCCGCCTGTTGCAGACTCCAGACCCAAACCTAAAGTTGAAAAACCAATAGTTCATTTCCAAGAACTAAAGTCTAGTCCAGCTTTTAAAACTAATTCTACAATGAAAAAGATAGAAGAAATGTTAGAACAATTGACCCCTGTCAAACCCGAGAAAGGTGAAAAATCTGGTATTAAAACACTAGATGCTTTTGCTGCTTCTCAATCTGAGTCTGAATCAGTAGAGATACAGAAACTAGTAATATTTCTAAAATTGAAAATGCTTTCAAAAATCTAGAAGTCCAAACTGAGCCCAAAGTCAAAAGAATAGAAAAACATATAAGCCCTACTAGCCTAACAAAAAATTGGTATCCCAGACCAACACCTCCAGATATCCAATTTGAAGAAAGAAACTTTCAAACCCAGTTTTCTGTTTCTTCTGATAAATTGTATGAATGGAATATTGATGGTCTTTCTGAGCAAGAAATCCTAAATAAACTGCAACATATGTCTATGGTCGCCAATAGTTACATCACCAATCACAGTTTCAGACAGTCTGAAATTGTTCCTCTGTTAGTTACTGGTTTCACAGGTACTCTCAGATCTTGGTGGGATAAACACCTAACCCCTGAGTCTAAAAATCGAATAATTTATGCTGTAAAACTTAATGAAGACGGTCTCCCTATTTTTGATGAACAAATAGGTCAAGGTATAGAAGACGGTGTCAACACTCTGTTTTACACAATAATTGAACATTTCATTGGAACCCCTAGTAATACCACTGCTAGGATACATGATCAGTTGAGTAATCTTAGATGCCCAAAGTTGTCTGATTTCAGATGGTATAAAGATGTTTTTATTTCTAGAGTCATGCTTAGAGATGATAGTAATCAACCATTTTGGAAAGAAAAGTTTATTAATGGTCTGCCTAATTTATTTGCTCATAAAATACGCACAACCCTAAGTAATGAACAAGGTCAAATTGATTGGGATAATTTAACTTATGGAAATATTATAAGCACAATTAATCAAGTTGGTATGAAAATGTGTATTGACTTTAAAATTAATAAACAAATCCAGTCTGATAGAAAGTCAGCCAAGTATGAGTTAGGTAATTTCTGTGAACAATATGGTCTCATTACTATTCCTCCTTCTAGAAAAAATAAGCCTAGTCATCTTAGAAAACGCCATTATCCGAGTAGAAAGAAACATTTTCTTGAGAAAGATGAATTCTATAAGAAAAGAAAGTTCTCCCCTAAGAAAAATTGGTCAAAACGTCCCAAACAAAATGCTCTAAGAAAAGTAAAGATAAAAGCAAAGTTAAATGTTTTAAATGTCAAAAATTTGGTCATTATGCTTCTGAATGTAAAGTAAAAGATGCTATTAAACAATTACAAATTACAGAAGAAGATAAGAACAAATTAATCCAAGTTTTGGAGTTGCGAGACTCTGAAATAAGTGATGTTGAATCCATGGTTACTAGTTCTGAGTCTGAGTATAGCCAGTCTTCAGATTCACAACCCAGTTCACCTAGAATCCAATTTGGTTGCACAGATAAATGTTGTAATAGGTTAAAGTCTATTTTTGTTCTCACTAAACAAGAAGAACAAGAAGAATTATTAATAGATTTAATAGGTAAAGTTGAAAACCCTGAGTTAAAATCTGAATATTTAAAAAAATTAAAAAAGATCATAAGTCAGGAAGGCAGTTCAAATTATCCTACTCCCCAGCCAATTAGCATCAACACTACTTTAGAAAAATTCAATCGTAAGAAAGATGTTACCTTACATGATCTACAATTAGAAGTCAAACAAGTTAAAAAAGAAATAGTTGAGTTAAAACAAATAAGTCAACAATTGCAAACTGAGAATTATGAGATCAAACAAGATTTATTTGACTTAGAAAAAGAAACTTCTAAGTCTAATAACCATTCAGAAAGTCCGCATCATTCTGATGATGAACCTTCTGAAGAACAGCCAGTTGTCAATTTAATTAAACAAGTTCATCTTAGAAAATGGTATTCTAAAGTCACGATATTTGTTCAAGATTTTGAATTTACCACAGTTGCCTTATTTGATTCTGGTGCAGATCTTAACTGTATTCAAGAAGGTCTAATCCCTACTAAGTATTACCAGAAGTCTAGAGAATCCCTTAGCACTGCCTCAGGGAAATCTCTCCAGTTAAATTTTGAAATACCAAAAGCCCATATTTGTCAAAACAAAGTCTGTTTTAAAACTTCATTCGTCTTAGTTAAAAATATTACTGATGAAGTCATCTTAGGATTACCTTTCCTTGCCCTGTTGTATCCTTTCCAGGTAGAATACGATGGTGTCATTTCTACCTATTTAGGAGAAAAGGTCAAGTTTGAATTCTTGTCTAAACCTGAGTTGCATAATTTAAAAGCTTTACAAAAACAGTCAGTCTCTAAATCTGTCCAAATTATAAGAAAAACTAATCAATTGAATTTTCTTAAAGAAGAAGTTAAATTTAAAAGAATTGAACAACAACTTAGTGATAAAGCTTTACAGTTAAAAATTCAGTCTTTTGAAAACAAATTAAAAGAAGAAATATGTTCTGATTTGCCTACTGCTTTTTGGTATAGAAAACAGCATGTTGTCTGTCTTCCTTATATCAAAACCTTTAGTGAGAAAAATATCCCCACTAAAGCCAGGCCAATTCAGATGAATCAAGAATTAATGGAATTTTGTAAAAAAGAAATTCATGAGTTGCTTCAAAAAAATATAATTCGCAAAAGTAAGTCCCCCTGGTCTTGTCCTGCTTTTTATGTCCAGAAAAATGCTGAATTAGAAAGAGGAGTCCCCAGATTAGTTATCAATTATAAGCCTCTTAATTCGGTCTTAGAATGGATTAGGTATCCCATACCTAATAAAAGAGATCTTATTAATCGATTAGAAAAAGCAGTTGTCTTCTCTAAATTTGATATGAAAAGTGGTTTCTGGCAAATCCAAATAGATGAGTCAGATAGGTATAAAACTGCATTTGTTACTCCTTTCGGTCATTATGAATGGAATGTTATGCCGTTTGGTTTTAAAAATGCCCCTAGTGAATTCCAGAATATTATGAATGATATCTTCAATCCTTACAGTCATTTCTCGATTGTTTATATTGATGATGTTCTTATATTTTCTGAGTCAGTAGAACAACATTGGAAACATTTGCATAAATTCCTTCACATAGTCAAACAtTCTGCTAAGAAAATTAAGTTATTTCAAACTAATATTAGATTTCTTGGTTTTAATATCTGCCAGTCACAAATTACTCCAATAGATCGAGTCATCCAATTTGCTGATAAGTTCCCTGATCAGATTCTTGATAAAAGTCAACTCCAGAGGTTTTTAGGATCTCTAAATTATGTTTCTGATTTTTATCAAAATCTGAGAAAACAATGTAAGCCTCTATTTGATAGATTGCAGAATAATCCTCCTCCTTGGTCAACTATTCATACTGAAATTGTCAAACAGATCAAAGCCCATGTCAAGACACTTCCCTGTCTTGGTATTCCTTCAGTTGATTCTTTCAAAATAGTTGAAACTGATGCCTCTGACATTGGTTATGGTGGTATTCTCAAACAGAAAGTCAGTCCAGATTCTCCAGAGCAAATTGTTCGTTTCCATTCTGGAACCTGGACTCAGTCGCAATATAATTATAGTACTATTAAAAAAGAAATATTAGCTATTGTATTATGCATTAGTAAATTTCAAGATGATTTATTAAATCAAAAGTTTTTAATCCGAGTTGATTGCAAATCTGCAAAACATGTTTTAGAAAAAGATGTTCAAAACATTGCATCTAAACAGATTTTTGCACGATGGCAAAGTATATTAAGTATATTTTTGATTTTGATATTGAATATTTGAAAGGTAGTGAGAATTCGGTCCCTGATTTTCTTACTCGAGAATTTTTGCAGGAACCTCAATGGCTCCCAAAAAAGACAAACACAAAGAAGTTGCCAGTCAACTCAAGTCACCCCAGTTGCAACCATCCCAGTCACCCCAGTTGCAACCATCCCAGTCACCATCCATGAGTCCAGCCAAACCAGAAATGCCCAAACAGATAGTTCCTTATCCTGGTCATCCCCCTCTTCCAATTGCCACCCATTATCAGTTGCAAACAGATTCTCTTCCTTAGGATCTACAGTTGGCCAAATCTGCCCCAGTTACCAATCCACATTAGTTGCCAGTTATGACCCTTTTTCAGTAGATGTCCCAGCAGGACCGTCAATTGCTTTGAAAAAATCTTCTCCTTATTTGCCAAAGAGTAATTCCCATCTATTTGTCCTTGAGCCTTTGTATGATATTCATACTAACCCAGTTGAAATTGCCAAACATTATTTCCCTCCTGGATTCCATTATATGCCTTCCAGTCCTTACAAGTCCCTCAAGTATTATAGAGATATATTGCTTGAAACTAAGTCAGTTGAAATTAAGCTGATAAAGGATCGAGATCACCCTGAGATCATTCTTTATCATTCAATGTACATCCACCATATCATAAGCCAAGAAGCCTTCAGTAGTCGCCCTTATGAGTTAAAAACCCTTCAATCCAAATTACAGTATAATTATGCTGATTATATTGATGCCTGGTACACTATTTTCCTTCACCAATCTGAGGATTTTAGTCATTCCTGGTTCATCAATTTCGATAATAAGTTCAAATGTTCTTTCCCCTATTGGTTCCTCCATTGGTGGGAAAAACATGGCCCAGTTGATGAAATTCTGCCTGTATCAGTTCTTGAATTAATCCGCCATTATACGGAGACTGCCAAGTTCTCCACCGTTGATTTATTCTTCCCCAAACAATTGCTTTTCCTTGCCAGATAAAAAGTTCCTTGGATCCTCAAATGGTCCTACAGAGTCACTAAGGATGTTCGTATCTTTGCCCGCCAATTTTCAGTCAAATGGTGGGACAAATTTGAAGTTCAAAGAATAGCCAAGTATGTTTACAATGATCTCCCTGCTGAGCCTCAGTCAACTTCTCCAGTTGGTTCTGTTCGATCCTCCCTCTCAGTCGAAGGAAAGTCGAAAGCAGAACTCCAAGAAATTGCTCGCCAGTTGATGATGCAGGCCTCTCAGATGGATGATGATGAGGACAACGACTCTCCTTCGTCGTCCAGTTGTCAACCCATTCCTGATCCTGTTAAGCCTAAGTCCTGGTATGATGACAGCCAGGATCCCTATGATGCCTATGATCTGCATTCTGACTAGGCCTCATAATTTCTGCTGACATCTCCACATGCAAAAGTTGCCCGACACCATAATTGCCACAGGAAGAAGCATCTGCTTTTTCTGTTTACTTTTCCACCGAAAGAAGCCATCAGTTCCAGACAAAGTCGCTCAGACCGCCAGCTGTCATCTGCCTTTCCACCGAAAGGAGCCAGCCGACAAAATTGAAAGTTGAATCAGGTTGCATGTGACTCCACTGTTGCAACCAACCATAGTTTAGCCAACCATAGTTGAAAGAACTCTAGTTGATTTTTCTCTTTATAAGCCGGCTTCGGCCTCAGTTGAAAACAGAATGTAAATTTTCCCTTCCTTCAGTTTCAAAGATTCTCTCTGATACATCCTTCGGAGAAAATCATCTTTGTAAATAGCGTGTGAGTTTGAAGTGTTGCAGTTGCAATCCTTTGTGAGTATAATAAAAGTTATTTTCAATTATCTATTTTCTGA

>5’-MP

TGAAAGCATTATTTTAATGCATTATCATTATTTTCTGCATTAGTCTGAAAGTTGCTTAGGTTGCATCCTTTTCTAATTTCTTTCTGATCTTCATTCTGTATTATATTCAGTTGCTTGTTTGTTCTATCTCTTTGTCCCTCTTTCTTGGTATCAGAGCCGTAAGTGTTGCGGTCTTTGTCTAAGTCTGCTACTCATCCCCGCCCAAGTGGCTGGTTTAAGCTTCTGAGTAGGAGGTTTTCATTTTTTCCTTTTTCTTCCCTTTTATTCTTTGAGTCATCTGTTGGTTTTCTCTAAGGTCTGACCCGTGAGTCCTTTTTTCTAGATCAGTAAGTCCCCAGGGAGGGCGTGTGTGCTGGGCAGGAGACAGAAAATTGAGAAAATTAATAGGTTGCTTAGAGTCTAAACGGTCGGTTTAGGATCTACTGTTCATCTTCTTCCTCAGTCTGCCAAACCCAGTTTCTTGAGTAGTCATGAGTCGTTTATTTAGAACAAAGTCTTGTAATTCTACCAGTTCTAGTGCCAGTATAAGTCGACTCCCAGAGATAGTTAATGAAGAACAATTTGAATATAATTCCAGTAGTAAAGAACAATTAGATTTTAGAGATTGGAATATACCTAAAGTCCCAAGTCAGAAAATCTATAAGAAACATTGGTTGCCTTCTAGTTTTAATAGC

>MP

ACAACACATGTCAAAACAGTTGAACAAGTTTACGCCTTAAGTAAAGAACATGAAACTTGTCAGTTGCTTAATCTTGAGTCAATTTTAAAACATAAGAAAGATGGTAATAATTTCCTGCATATTGGTTTAGTTCAAGTTGCTGTTAAGCCCCTGACACGGTTAGGATTGAAAGCTTCAATCCTGCTATGTCTGCGTGATGCTAGATTCACTGAATTTAGCGATAGTACCCTTGGAATCATTGAGTCCAGTCTGTGTAATGGCCCTGTCCATTTTGATTGCTATCCTGATTTCACTGTAAGCCTTAGTGATCCTCATATCCTGAAAACTTTGACTCTGAATATTAAGACTGAAGGTTATAATGTTCTGCCAGGAACCCAACCTTTAGCCTTAGTTTACAGAATCTATTATAAGGTCACAGGAACTAATATG

>MP-CP

AATTTCCAAGCCCTTAATAAAAGCCCAAAGGATCAAACCGTCTTGATCCAGAGTCATACCTCAGATGCCCATATACAAGTTCCTCATACTATTAAGTGGTCAGAAGTTGCCCTTCCAAAAGATTGGACCTTAGTCACTGAGAGCCAACCAGCTCCTATCCAGCGTAGTCTAAACAATTTAGACTACATCCAACAATACTTAGATGGCACAGTTAAAATTAAGTTTGATAGTCATCCTTTACGAAAATCTAATGTCCAGTTACAACAGTTGCCAACTCCTGGTCAATCCCAGAGGCATGTCCCTGCTCGGCATTCCTTTGCTGCCTCTTCCTCCACTCTAGAACGAGACCTAGAGTTAGAAAAGGCTATGATTGATTTCAAACTTGAGTCCCTGAGAAAAACCTCTCAGGTTACCCAACCTTGCTACGGCAAGGTTCCAGTTCAAGAAGATAAGTCTGAATCACCTGAGTCACCTACCCAGTCTGATTCATAGTTGAAAATCAGCTTAGAACCCTTAATAAAAAATTTGAAATTAATTGGGTAAGATTAAATCGTCATCTAAAAGCCCCTGAAAACCAGGCAAGAAGAGATAATTATCATCAAGCCTACCCTAATAGCAAACACCGAGAAATGATCTTTAAACAATGGAAAGATTATATGCGGTCAGCCAAAGTTGAAATTTATTACCTTGATTTTGTAGAAAGCCGTTATATGTCTAATGATGAATTAAAAACTTTAACTAAAGAAAAATGGAAAATAATAGATAAaTCTGTAGTTGAGTCTAGTCATCCTCCAGTAGAAACTATAATAATAGAACACCAGAAAGCACCAGTTCCTGCCACTCCTTTCAAAACCTTTGAAAGCAATGACCCTAATAGAAAACTAATTGAACAAAATAATTACACCAACCAGAGTCTAATCACCATAGGAAAACAATTAGATACAATTGAAACCAAAGTTGATATGtTGAGTCCGCCTGTTGCAGACTCCAGACCCAAACCTAAAGTTGAAAAACCAATAGTTCATTTCCAAGAACTAAAGTCTAGTCCAGCTTTTAAAACTAATTCTACAATGAAAAAGATAGAAGAAATGTTAGAACAATTGACCCCTGTCAAACCCGAGAAAGGTGAAAAATCTGGTATTAAAACACTAGATGCTTTTGCTGCTTCTCAATCTGAGTCTGAATCAGTAGAGATACAGAAACTAGTAATATTTCTAAAATTGAAAATGCTTTCAAAAATCTAGAAGTCCAAACTGAGCCCAAAGTCAAAAGAATAGAAAAACATATAAGCCCTACTAGCCTAACAAAAAATTGGTATCCCAGACCAACACCTCCAGATATCCAATTTGAAGAAAGAAACTTTCAAACCCAGTTTTCTGTTTCTTCTGATAAATTGTATGAATGGAATATTGATGGTCTTTCTGAGCAAGAAATCCTAAATAAACTGCAACATATGTCTATGGTCGCCAATAGTTACATCACCAATCACAGTTTCAGACAGTCTGAAATTGTTCCTCTGTTAGTTACTGGTTTCACAGGTACTCTCAGATCTTGGTGGGATAAACACCTAACCCCTGAGTCTAAAAATCGAATAATTTATGCTGTAAAACTTAATGAAGACGGTCTCCCTATTTTTGATGAACAAATAGGTCAAGGTATAGAAGACGGTGTCAACACTCTGTTTTACACAATAATTGAACATTTCATTGGAACCCCTAGTAATACCACTGCTAGGATACAT

>CP

GATCAGTTGAGTAATCTTAGATGCCCAAAGTTGTCTGATTTCAGATGGTATAAAGATGTTTTTATTTCTAGAGTCATGCTTAGAGATGATAGTAATCAACCATTTTGGAAAGAAAAGTTTATTAATGGTCTGCCTAATTTATTTGCTCATAAAATACGCACAACCCTAAGTAATGAACAAGGTCAAATTGATTGGGATAATTTAACTTATGGAAATATTATAAGCACAATTAATCAAGTTGGTATGAAAATGTGTATTGACTTTAAAATTAATAAACAAATCCAGTCTGAT

>CP-AP

AGAAAGTCAGCCAAGTATGAGTTAGGTAATTTCTGTGAACAATATGGTCTCATTACTATTCCTCCTTCTAGAAAAAATAAGCCTAGTCATCTTAGAAAACGCCATTATCCGAGTAGAAAGAAACATTTTCTTGAGAAAGATGAATTCTATAAGAAAAGAAAGTTCTCCCCTAAGAAAAATTGGTCAAAACGTCCCAAACAAAATGCTCTAAGAAAAGTAAAGATAAAAGCAAAGTTAAATGTTTTAAATGTCAAAAATTTGGTCATTATGCTTCTGAATGTAAAGTAAAAGATGCTATTAAACAATTACAAATTACAGAAGAAGATAAGAACAAATTAATCCAAGTTTTGGAGTTGCGAGACTCTGAAATAAGTGATGTTGAATCCATGGTTACTAGTTCTGAGTCTGAGTATAGCCAGTCTTCAGATTCACAACCCAGTTCACCTAGAATCCAATTTGGTTGCACAGATAAATGTTGTAATAGGTTAAAGTCTATTTTTGTTCTCACTAAACAAGAAGAACAAGAAGAATTATTAATAGATTTAATAGGTAAAGTTGAAAACCCTGAGTTAAAATCTGAATATTTAAAAAAATTAAAAAAGATCATAAGTCAGGAAGGCAGTTCAAATTATCCTACTCCCCAGCCAATTAGCATCAACACTACTTTAGAAAAATTCAATCGTAAGAAAGATGTTACCTTACATGATCTACAATTAGAAGTCAAACAAGTTAAAAAAGAAATAGTTGAGTTAAAACAAATAAGTCAACAATTGCAAACTGAGAATTATGAGATCAAACAAGATTTATTTGACTTAGAAAAAGAAACTTCTAAGTCTAATAACCATTCAGAAAGTCCGCATCATTCTGATGATGAACCTTCTGAAGAA

>AP

CAGCCAGTTGTCAATTTAATTAAACAAGTTCATCTTAGAAAATGGTATTCTAAAGTCACGATATTTGTTCAAGATTTTGAATTTACCACAGTTGCCTTATTTGATTCTGGTGCAGATCTTAACTGTATTCAAGAAGGTCTAATCCCTACTAAGTATTACCAGAAGTCTAGAGAATCCCTTAGCACTGCCTCAGGGAAATCTCTCCAGTTAAATTTTGAAATACCAAAAGCCCATATTTGTCAAAACAAAGTCTGTTTTAAAACTTCATTCGTCTTAGTTAAAAATATTACTGATGAAGTCATCTTAGGA

>AP-RT

TTACCTTTCCTTGCCCTGTTGTATCCTTTCCAGGTAGAATACGATGGTGTCATTTCTACCTATTTAGGAGAAAAGGTCAAGTTTGAATTCTTGTCTAAACCTGAGTTGCATAATTTAAAAGCTTTACAAAAACAGTCAGTCTCTAAATCTGTCCAAATTATAAGAAAAACTAATCAATTGAATTTTCTTAAAGAAGAAGTTAAATTTAAAAGAATTGAACAACAACTTAGTGATAAAGCTTTACAGTTAAAAATTCAGTCTTTTGAA

>RT

AACAAATTAAAAGAAGAAATATGTTCTGATTTGCCTACTGCTTTTTGGTATAGAAAACAGCATGTTGTCTGTCTTCCTTATATCAAAACCTTTAGTGAGAAAAATATCCCCACTAAAGCCAGGCCAATTCAGATGAATCAAGAATTAATGGAATTTTGTAAAAAAGAAATTCATGAGTTGCTTCAAAAAAATATAATTCGCAAAAGTAAGTCCCCCTGGTCTTGTCCTGCTTTTTATGTCCAGAAAAATGCTGAATTAGAAAGAGGAGTCCCCAGATTAGTTATCAATTATAAGCCTCTTAATTCGGTCTTAGAATGGATTAGGTATCCCATACCTAATAAAAGAGATCTTATTAATCGATTAGAAAAAGCAGTTGTCTTCTCTAAATTTGATATGAAAAGTGGTTTCTGGCAAATCCAAATAGATGAGTCAGATAGGTATAAAACTGCATTTGTTACTCCTTTCGGTCATTATGAATGGAATGTTATGCCGTTTGGTTTTAAAAATGCCCCTAGTGAATTCCAGAATATTATGAATGATATCTTCAATCCTTACAGTCATTTCTCGATTGTTTATATTGATGATGTTCTTATATTTTCTGAGTCAGTAGAACAACATTGGAAACATTTGCATAAATTCCTTCACATAGTCAAACAtTCTGCTAAGAAAATTAAGTTATTTCAAACTAATATTAGATTTCTTGGTTTTAATATCTGCCAGTCACAAATTACTCCAATAGATCGAGTCATCCAATTTGCTGATAAGTTCCCTGATCAGATTCTTGATAAAAGTCAACTCCAGAGGTTTTTAGGATCTCTAAATTATGTTTCTGATTTT

>RNASEH

TATCAAAATCTGAGAAAACAATGTAAGCCTCTATTTGATAGATTGCAGAATAATCCTCCTCCTTGGTCAACTATTCATACTGAAATTGTCAAACAGATCAAAGCCCATGTCAAGACACTTCCCTGTCTTGGTATTCCTTCAGTTGATTCTTTCAAAATAGTTGAAACTGATGCCTCTGACATTGGTTATGGTGGTATTCTCAAACAGAAAGTCAGTCCAGATTCTCCAGAGCAAATTGTTCGTTTCCATTCTGGAACCTGGACTCAGTCGCAATATAATTATAGTACTATTAAAAAAGAAATATTAGCTATTGTATTATGCATTAGTAAATTTCAAGATGATTTATTAAATCAAAAGTTTTTAATCCGAGTTGATTGCAAATCTGCAAAACATGTTTTAGAAAAAGATGTTCAAAACATTGCATCTAAACAGATTTTTGCACGATGGCAAAGTATATTAAGTATA

>RH-3’

TTTTGATTTTGATATTGAATATTTGAAAGGTAGTGAGAATTCGGTCCCTGATTTTCTTACTCGAGAATTTTTGCAGGAACCTCAATGGCTCCCAAAAAAGACAAACACAAAGAAGTTGCCAGTCAACTCAAGTCACCCCAGTTGCAACCATCCCAGTCACCCCAGTTGCAACCATCCCAGTCACCATCCATGAGTCCAGCCAAACCAGAAATGCCCAAACAGATAGTTCCTTATCCTGGTCATCCCCCTCTTCCAATTGCCACCCATTATCAGTTGCAAACAGATTCTCTTCCTTAGGATCTACAGTTGGCCAAATCTGCCCCAGTTACCAATCCACATTAGTTGCCAGTTATGACCCTTTTTCAGTAGATGTCCCAGCAGGACCGTCAATTGCTTTGAAAAAATCTTCTCCTTATTTGCCAAAGAGTAATTCCCATCTATTTGTCCTTGAGCCTTTGTATGATATTCATACTAACCCAGTTGAAATTGCCAAACATTATTTCCCTCCTGGATTCCATTATATGCCTTCCAGTCCTTACAAGTCCCTCAAGTATTATAGAGATATATTGCTTGAAACTAAGTCAGTTGAAATTAAGCTGATAAAGGATCGAGATCACCCTGAGATCATTCTTTATCATTCAATGTACATCCACCATATCATAAGCCAAGAAGCCTTCAGTAGTCGCCCTTATGAGTTAAAAACCCTTCAATCCAAATTACAGTATAATTATGCTGATTATATTGATGCCTGGTACACTATTTTCCTTCACCAATCTGAGGATTTTAGTCATTCCTGGTTCATCAATTTCGATAATAAGTTCAAATGTTCTTTCCCCTATTGGTTCCTCCATTGGTGGGAAAAACATGGCCCAGTTGATGAAATTCTGCCTGTATCAGTTCTTGAATTAATCCGCCATTATACGGAGACTGCCAAGTTCTCCACCGTTGATTTATTCTTCCCCAAACAATTGCTTTTCCTTGCCAGATAAAAAGTTCCTTGGATCCTCAAATGGTCCTACAGAGTCACTAAGGATGTTCGTATCTTTGCCCGCCAATTTTCAGTCAAATGGTGGGACAAATTTGAAGTTCAAAGAATAGCCAAGTATGTTTACAATGATCTCCCTGCTGAGCCTCAGTCAACTTCTCCAGTTGGTTCTGTTCGATCCTCCCTCTCAGTCGAAGGAAAGTCGAAAGCAGAACTCCAAGAAATTGCTCGCCAGTTGATGATGCAGGCCTCTCAGATGGATGATGATGAGGACAACGACTCTCCTTCGTCGTCCAGTTGTCAACCCATTCCTGATCCTGTTAAGCCTAAGTCCTGGTATGATGACAGCCAGGATCCCTATGATGCCTATGATCTGCATTCTGACTAGGCCTCATAATTTCTGCTGACATCTCCACATGCAAAAGTTGCCCGACACCATAATTGCCACAGGAAGAAGCATCTGCTTTTTCTGTTTACTTTTCCACCGAAAGAAGCCATCAGTTCCAGACAAAGTCGCTCAGACCGCCAGCTGTCATCTGCCTTTCCACCGAAAGGAGCCAGCCGACAAAATTGAAAGTTGAATCAGGTTGCATGTGACTCCACTGTTGCAACCAACCATAGTTTAGCCAACCATAGTTGAAAGAACTCTAGTTGATTTTTCTCTTTATAAGCCGGCTTCGGCCTCAGTTGAAAACAGAATGTAAATTTTCCCTTCCTTCAGTTTCAAAGATTCTCTCTGATACATCCTTCGGAGAAAATCATCTTTGTAAATAGCGTGTGAGTTTGAAGTGTTGCAGTTGCAATCCTTTGTGAGTATAATAAAAGTTATTTTCAATTATCTATTTTCTGA

>Chr1-01; OZ022206.1; 3065967-3074484; rev; D;

TTTCTCGGCAGCTCCCTGCTGTAGTACTCAGACTTCACCTCCCTTGGCAGCTCGTGGAACCCACGCGCCGCCGCCAACATCTCATCCAACACTCTCTTGGGTATCCCATGGTTCACCACCTGGAAGAACCCAACAGTCTCCGCGGCACGCCGAACTCCGGCAACCACGTCAGCACGCTGACCGACTGTGTCGGCGAGGTCCACAACAGGAACACTGAACTGGGTTCGAGTTCGATGGCCGGAATTTGGTTTGTCGCCGCCAGAAAATTCCGGCGGCCGAACGAAAATTGGTGGGACTTTTGTGATGCCTTTGTCGACGATCCCTTTGACACCAGATTTGGACTCGTCAAAGGCTTTTAGCTGTTGCAGACGGTCTCCGTTTGCTGTGTACCCGGAGATCATTTTCATGCACCTGTTTTTCCTGTAAAATCTTGCAAACTTTTTTAGCACTTGGTTGAAAATGCGGAGACTTTTCTTGAAGGCAAGGACACGTGTTTCTATTTTGTGCCACATATGAGCTTTGCAATTTACGTTAGTCTTTGATTGGATTTGATCATGTCATTTCCTCTGTCATTTAGCGGGAAAGGGGCAGACAGGTTTAAGGATTGAGTTTTGCTCTCATTGTGCCCTTTCTAAAAAAATTGGAATGTTGCTATCAAGATAAAGTCACAATGATGTTAAATGAAGCAATTGGAATTAGTTGGTGAAATGGTGAAATGGTGGACCAGGAATATTTACAGGGATGCGGGCTATTTTTTTTTGTCGGTTGGAATCAATTATAAGATCAATTATAAGTCTTATTTTCATATATATATATATATATACATAATGATATTTGCTCAAATTACTCAAAATATATTGATAACTTTTAAAATAAGCATAGGCTTATGAGAATGGCTTACAAAAGATAGCTCCTAGTTGCGTTCTTCATATCATTGTTTCTGAGAGAACATTTTAAACTGAGAAACTGAAATTTTTCGATTGCTGGGATGTCAGACTTA

GGACCATGGGTATCCCACCAATGGAGGAACCAGTAGGGAAGCTCACTTCGAAACTTACTGTCAAAATTGATGAACCAGGAATGGGAAAACTTAGGTGTCTGGTGCAGGAAGATATAATACCAGGACTCAACATAATCAGCATAATTGTATTGGAATTTTGAAGGAAGGGTTCTTAGCTCATGAGGACGGGATCCCCAACTTTTTTGGCTTAAAACTTGGTGGATGTAAAGGGAGTGATAAATAATTTTTGTAGGGTCTTTTTTATCACGAATGGGCTTAATATCAACAGATTTTGTTTCATGAAGAATTTCTCTGTAAAATTTAAGGGATTTATATGGACTGTGAGGAAGGTAATGGAAACCATTAGGGAAATGGTGATCTGCAATCATGGCAGAGCTCATTGTCTTAGAAATATGAGGTTCAAGAATTAAAAGATTAGTTGTATGCCTAAGGACATAGGGGGATGACTTAGGATAACACTGGGATGAGGAAATAGGTGTAGCAGTTGGTGGCAACTGGAAAGGATCATAACTAGAGACTAAAGCTGACTGGTAATTAGGTCTTATTTGGGCAATTGTAGATCCTAAGGGGCTGAAACGATTAAGTACTGGAATAGGAGAGTGACCAGGGTAGGGGATAACAGAAGAAGGCTCTGGTTGAACTATTTTTTGACTCAATGAGGCAGGAAGGATTCTGGGTGACAGTGGCTTTTTGACTTGACTCATTTTCTGCTTATCTTTACTTCCGGCCATTGCTTATCCTGCAAAAACTCTCTAGTAAGAAAATCAGGAACAGAATTCTCACTGCCTCTCAGATATTCAATTTCAAAATCAAATATACTTAAAATAGCTTGCCAACGTGCAAAAATCTGTTTTGATGCAATATTTTGAACATCTTTTCTAATATGTGCTTAGCAGACTTGCAATCAACTCGTATTAAAAATTTCTGGTTTAATAAATCATCTTGAAATTTAGTAATGCATAGTACAATAGCTAATATTTCTTTTTTAATAGTACTATAATTAGATTGCGACTTAGTCCAAATATCAGAATGGAAACGAACAATTTGCTCAGAAGGAATGGAATTAGATTTTTGCTTAAGAATACCACCATAACCTAGCTCAGAAGCATCTGTTTCGACGATTTTAAAAGCATTTGGAGCAAGAATGCCAAGGCAAGGAAGTGTCCTGACATGACTCTTGATTTGTTTAACAATCTCAGTATGAACAGATGACCAAGGAGGAGGATTACTCAGCAATCTGTCAAACAAAGGCTTACACTGTTTTCTTAGATTTTGATAAAAATCAGAAACATAGTTAAGGGATCCTAAGAATCTTTGCAATTGATTTTTATCAATTATTTGATCAGGAAATTTATCGGCAAATTGGATGACTTGGTCTATTGGAAGAATATGGGCTTTGCAAATGTTAAAACCAAGAAATCTAATATTATTCTGAAATAACTTAATCTTTGTAGCAGAGACTACTAAACCATTTGACTTAATTATGTGCAAGAATTTATGCAGATGTTTCCAGTGCTGTTCTATAGACTCTGAAAAAATAAGAACATCATCGATATAGACTATGAAAAAGTGACTGTAAGAATTAAAAATCTCACTCATTATATTTTGAAATTCACTCGGTGCATTCTTAAGACCAAAAGGCATGACGTTCCATTCATAATGACCAAAATGAGTAACAAAAGCAGTTTTATATTTATCAGACTCATCAATCTGTATTTGCCAAAATCCACTCTTCATATCAAACTTGGAGAAAACAACAGCTCTACCTAAACGATTAATCAAATCTCTTTTATTAGGAATAGGGTAACGAATCCATTCTAACACAGTATTAAGTGGCTTATAATTAATAACTAATCGTGGTGATCCTCTTTCTAACTCAGCATTTTTCTGGACGTAAAAGCCAGGCCAAGACCAAGGAGACTTACTCTTCCTAATTATATTTTTCTGTAATAAATCATTAATCTCAGTTTTGCAAAATTCCATCATTTCGTGGCTCATTTGAATTGGTCTAGCTTTAGTAGGTATATTTTTTTCAGAAAAGTTCTTGATGTAAGGTAATCTCACAACATGATTCTTTCTGTGCCAAAAAGCAGTAGGTAAATCAGAACATATTTCAGATATTAGTTTTTTCTCAAATCTTTCAATTTCTTGTCGTAAAGTAGGACTACTTAATTGGGACTCAAATCTTTTAGTTTTAACTTCTTCTTGAAGGTGACTTAAATGATTAGTCTTATCATTAATTAAAGTAATTGTCTTAGAAAATTCATCTTTTTGTAAGTGCTTTAGTTTATGTAACTCAGATTGAGTTAAAAATTCAAACTTAACTCTTTCTCCTAAATGATGTGAAACAACACCATCAAATTCAACTTGAAGAGGATAAAGTAGGCTTATGAAAGGTAAACCTAGGATGACTTCATCAGTTATGTTTTTGATTAAAACAAAAGGGGTTTTAAAACAAATCTTATTTTGACATACATGGGCTTTTGGAATCTCATAATTTAATTGAAGTAATTTTCCTGAAGCAGTGCTGAAAGATTCCTTAGATTTCTTATAATACTTAGTGGGAATTAGTCATTCTTTAATACAATTAAGATCAGCTCCTGAATCAAATAAAGCAACTGTGTTAAACTCAAAGTTTTTAACAACTATGGTAACTTTGGAATACCATTTCCTAAAACTTACTTGTTTAATCAAATTAATAGTTTGTTCTTCGTGGAAGGACCTATCAGAATGTACTGGGCTACCAGGTTGACTGTTTGACTCAAAGGATTCTTTTTCTAACAAGGAAGTTAAACTCTGCTTAATTTCATAATTTTCAGTTTGTAACCTGCGACTTAATTGTTTTAATTCAATTATTTCTTTTTTAATTATTTTTACCTCAGACTGCAAGTCTTGTAAAGACAACTCTTTTTTCTTATTAAACCGTTCTAAAGTAGTATTGAGACTTATTGAGGGACTCAGAAGATGACTCGTACTTGTTTCCTGGGTAATAATTTTTTTTAGCTTTTTTAGGTATTCGGATTTTAACTCGGGGTTTTCTATTTACTAGTTAAATCGATTAATAACTCCATTATGTTTATATTCCATATATTTATATTTTCATTAATAAGATTCATGCATAATTTGTGACTTTTTTATTATCTCTGTTTTCATACTTACATTAATATGTTTCCCTGGTTCTAGACCCCTTCTGGTATCAATGCCATAAGAGTTGTTAGAGTTTTTTCTAGGTCGCAACTCATCCAACCCAAGTGGCTGGTTTAGGTTTCTGAGTTGAAGTTCTTTTGGTTTCTTCTTCTTCTTCTTTTTATTCTTCAATTTATTTCTTGGTTTCTTCTCTTAAGGTCTGACCCTGAATCCTTTTTCCAAACGATAAGTCCCGATGATAGGGCCCGAGCGAGTAGGAGACAGAAAACTAGAGAGAAATCTAGTAAGTTTCTTGTTGAGTAATTAGTAAGTTAGGAGTCACTATTTATCTTCTTCTTCAGTCATCATTTCCCAAACACTTAAGTAAAAATGAGTCGCCTTTTTAGAACAAATTCTTGTAGTTCGAGCAGCTCTAGAACCCCTATAAGTCGTTTACCTGAGATTGTAAATGAGGAACAATTTGAATATAATTCTAGTAATGATGAACAATTAGATTTTCGAGACTGGAATATACCTAGAGTTCCAAGCCAAAATATATATAAGAAAAAATGGTCACTTTCTAGTTTTAATTCTACTACACACGTTAAGACAGTAGAACAAGTTTATGCCCTAAGTAAAGAACATGAGTCGTGTCAATTACTTAATCTTGAGTCAATTAAGAAACATAAACAAGATATGGTAATAATTTCTTGCACATAGGTCTTGTCCAAGTTGCTGTTAAGCCTCTAACTAGATTAGGATTGCAAGCTTCTATTCTGTTATATTTACGTGATGCTAGATTTACTGAGTTTAATGATAGTGTTCTTGGAATGATTGAGTCAAGTCTGTGTAATGGCCCTGTCCATTTTGATTGCTACCCTGATTTCACAGTAAGCCTAAATGATCCACATATTCTTAAGACTTTAACTTTGAATATTAAGACTTCTGGTTATAATGTTCTTCCAGGTACACAGCCTTTAGCCTTAGTTTATCGAATTTATTATAAAGTCACATGCACTAATATGAATTTCCAAGCTTTAAATAAGAGTCCTAAAGATCAAACACTTTTAATACAAAGTTGCACACCTGATGCTAATATTAGGATCTCACATACAGTTAAGTGGTCTGAGATTAGTTTACCTATTGATTGGACTTTAGTTACTGAGAATCAACCTGCACCAATTCAACGAAGTATAAATAATTTAGATTTTATTCAACAATATCCAGATGGTACAGTTAAGATTAATTTTGGAAATCAACGCGAACCTTTAGTTCAACCTAAACCTAATATCCAGTTGCAACAGTTACCTACACCAAGCCAATCTAAAAGACATGTTCCTGCTAGACATTCTTTCACTGCTTCTTCTTCTACTGATATAAGGGATCTAGAAGTAGAAAAGAATTTAATTGATTTAAAATTGGAATCCCTAATAAAAGCAGCCCAAGTGAGTCACCCCTTTACAGAGCTCCTCCAGCTCTTCCAGTTCAAAATGAGCTTAAAACTGAGTCCCCTCCTACTTCACCCACTCAGTCAGATTTTCTTGCTAGTGAAATTACTGCCCATGTGTTAGATCAACAATTAAGAGTTCTTAAGAAATCCAAGCCTTTCAAGATTAATTGGAAAAAATTAGATGATCACTTTCATCATCCTGAGCATAAGTCTAGAAAAGATAACTACCATAGGATAATTCCTGATAAAAATGAAAGACAAAAAATCTTTAATAATAGGAAAGATTATATGTGATCTGCTAGAGTTGAGATTTGTTACCTTGATTTTGTAGAAAGCCGTTATATTTCAAATGAGTTAAAAACTTTAACTAAGGAAAAATGGGTAAAAGAAGACCAATCTGAAATTATTTCTAGTCACCCACCAGTTGAAAAAATAGTCATTCCTTATGGTACGTCCAAGGTCATTGCAGCCCCCTTCAAAATAGCTGGTCAAGACAATGATAAAAAATTAATTGAGCAAAATAATTACACCAACCAAAGCTTAGTTGTCATAGGAAGACAGTTAGACAAGATAGAAACCAAAGTTGATAAGTTTCTTCCTTCTGAGCCAAAACTTAAGATTAAAGAAAAACCTTTAGTTCATTTCCAAGATTTGAAACCTGCACATTCACTTAGGACCTCTACCACTATGAAAAAAATTGAAGGAATGCTTGAACAATTAACTCCTGTTAAACCTGAGAAGTCTGGTCTTAAAATCCTAGAATCTTCTACAGCCTTAGTCATTCATTCGGACAGTGAGTCTAATGAGAGCATGGAAACCATTGAGTCTAGTAATATTTCCAAAATTGAAAATGCATTCAAAAATTTAGAACTAGAGTCTGAGTTAAAAGTCAAAAGACTAACTAATAGAGTCAATCCTACTAGTTTAACAAAAAACTAGTATCCTAAGCCTACACCTCCTGATATCCAATTCGAAGAAAGAAATAATTTGAGTCAATTTTCTGTTTCTTCTGATAAATTATATGAATGGAATATAGATGGTTTATCTGAGCAAGAGATAATAAATAAATTAACTCATATGTCCATGGTTGCTAATAGTTATATAACTAATCACAGTTTGCGTCAATCTGAGATAGTTCCATTATTAGAAACTGGTTTCACCGGAACCTTACGTTCTTGGTGGGATAAGCATTTAACCAATGAATCGCGAAATAATTTAATTCATGCAGTTAAGTCTGATGAAGAAGGTCTTCCTTTATTTGACCAAAATATAGGCCGCGGTATAAAAGACAGAGTCAACTGCCTTTTGTACACCATAGTTGAACATTTTATAGAACACCTAGTCATACACAGGCCAGAATACATGATCAACTAAGTAATTTAAGATTCCCTCAATTAAGCGATTTTAGATGGTATAAAGATGTCTTTATTTCCAGAGTAATGTTAAGAGAAGATAGTAATCAAGCCTTTTGGAAAGAAAATTTATTAATGGTTTACCACATTTATTTGCCCATAAAATTAGAACAGTCTTGAGTAATGAGTCCGGACACATTGATTATGATAATTTAACTTATGGTAATATAATTAGTACAATCAACCAAGAAGGTATGAAAATATGTATAGATATGAAAATTAATAAACAAATTCAATCTGAACGAAAATCTGCTAAATATGAGCTAGGTAATTTCTGTGAACAATATGGTCTAATTGCCACCCCTCCTTCCAGAAAAAATAAGTATAGTTCTCCCCGTCATATCCTTCAAAAACGCCATTATGAAAGAAAAAGAAAATCTTATCGACACAATAATGAGTCTAATGATTTTTACACGAAAAGAAAATATTATCCTAAGAGAAAGAGGTCCAATGTACCAAAAGATAAGAAAGGTAGCCACTTTAAGAAAAGAACCGACAAGAGTAAAGTAAAATGTTTTAAATGTCAAAAATTTGGTCATTATGCTAATAAGTGTAAAGTAAAAGATGATATCCGACAATTACAAATTACAGAAGAGGAAAGAGCTAATCTAATTAAAGTCCTTGAATTATGTAACTTTGCGTCAAGGGAAAAAGAAATCATGGTTAACAATACTAAGTCAGACAACTATGAGTCCTCGACATCTCAATTAAGTTCTCCCCGAGTCCAATTAGGATGTAGAGATAAATGCTGTAACACTCTAAACTCACTCTCTATCTTAACTAAGCAAGAAGAACAAGAAGAGTTATTAATCGATTTAATTAGTAAAATAGAAAACCCCGAGTTAAAATCCGAATACCTAAAAAAGCTAAAAAAAATTATTACCCAGGAAACAAGTACGAGTCATCTTCTGAGTCCCTCAATAAGTCTCAATACTACTTTAGAACGGATTAATAAGAAAAAAGAGTTGTCTTTACAAGACTTGCAGTCTGAGGTAAAAATAATTAAAAAAGAAATAATTGAATTAAAACAATTAAGTTGCAGGTTACAAACTGAAAATTATGAAATTAAGCAGAGTTTAACTTCCTTGTTAGAAAAAGAATCCTCTGAGTCAAACAGTCAACCTGGTAGCCCAATACATTCTGACAGGTCCTTCCACGAAGAACAAACTATTAATTTGATTAAACAAGTAAGTTTTAGGAAATGGTATTCCAAAGTTACCATAGTTGTTAAAAACTTTGAGTTTAACACAGTTGCTTTATTTGATTCAGGAGCTGATCTTAATTGTATTAAAGAAGGACTAATTCCCACTAAGTATTATAAGAAATCTAAGGAATCTTTAAGCACTGCTTCAGGAAAATTACTTCAATTAAATTATGAGATTCCAAAAGCCCATGTATGTCAAAATAAGATTTGTTTTAAAACCCCTTTTGTTTTAATCAAAAACATAACTGATGAAGTCATCCTAGGTTTACCTTTCATAAGATTACTTTATCCTCTTCAAGTTGAATTTGATGGTGTTGTTTCACATCATTTAGGAGAAAGAGTTAAGTTTGAATTTTTAACTCAATCTGAGTTACATAAACTAAAGCACTTACAAAAAGATGAATTTTCTAAGACAATTACTTTAATTAATGATAAGACTAATCATTTAAGTCACCTTCAAGAAGAAGTTAAAACTAAAAGATTTGAGTCCCAATTAAGTAGTCCTACTTTACGACAAGAAATTGAAAGATTTGAGAAAAAACTAATATCTGAAATATGTTCTAATTTACCTACTGCTTTTTGGCACAGAAAGAATCATGTTGTGAGATTACCTTACATCAAGAACTTTTCTGAAAAAAATAGACCTACTAAAGCTAGACCAATTCAAATGAGCCACGAAATGATAGAATTTTGCAAAACTGAGATTAATGATTTATTACAGAAAAATATAATTAGGAAGAGTAAGTCTCCTTGGTCCTGTCCTGGCTTTTACGTCCAGAAAAATGCTGAGTTAGAAAGAGGATCACCACGATTAGTTATTAATTATAAGCCACTTAATACTGTGTTAGAATGGATTCGTTACCCTATTCCTAATAAAAGAGATTTGATTAATCGTTTAGGTAGAGCTGTTGTTTTCTCCAAGTTTGATGTGAAGAGTGGATTTTGGCAAATACAGATTGATGAGTCTGATAAATATAAAACTGATTTTGTTACTCCTTTTGGCCATTATGAATGGAACGTCATGCCTTTTGGTCTTAAGAATGCACCGAGTGAATTTCAAAATATAATGAATGAGATTTTTAATTCTTACAGTCACTTTTCCATAGTCTATATTGATGATGTTCTTATTTTTTCAGAGTCTATAGAACAGCACTGGAAACATCTGCATAAATTCTTGCACATAATTAAGTCAAATGGTTTAGTAGTCTCTGCTACAAAGATTAAGTTATTTCAGAATAATATTAGATTTCTTGGTTTTAACATTTGCAAAGCCCAGATTCTTCCAATAGACCGAGTCATCCAATT

TATATATATATATATATACACACACATACAGTGAGCTTCGATTGAGGGATTCTTTGAATAAGTTTATTTGAAAGACATACTTGTAGGGCCCACCATATATTGTATTTCATTAATCCAAATCGTCTATTTTTGTAGATATTCATTCAAAGATAATCTCCACAAAAAATCACTTGAATCCGATATCATTTAACCACTTAATTAAATTATATGTGTATATATATATATATATAAGATTTTTCTCTTATCCGAACATCCGGATTGTTTAGTGTGCGGATTCCAATATTTGCAGTTGTGTTTCATTGTAAAAAACGATTTGAAGATGGATACTTCTACAGCACCGACTGGATATTTTTATATAACAGTGTAGTGGCTTGTGATTTGGCAGCATATATAAGAGGAGAAAACTGAAGAAACATTGAGGTACGCATGCTAAACAATCTGGACATCCGGATAAGAGAAAGACTCTATATATATATATATAGTCCTGATATCTTAGACTACAGGGGTCCAAGAGATTTGTGTTCACTCACCGTTGGTTCACTCACTCTTGCACTCCTTTTAAGAAATTTTTTTGAACCATTGGATTAATATCCAACGGTGGGTGACCACAATCTCTTGGACTCCTGTGGTCCAAGAGATCGGGACTGTCTATATATATACTGTGAGCTTCGGTTGAGGGATTCTTCGAATAAGTTTATTTGAGGGACACCCTTGTATGGCCCATCATATATTGTATTTCATTAATCAAAACCGTCTATTTTTGTAGATATTCATTCAAAGATAATCTCTACAAAAATCACTTGAATCCAATATAATTTGATCACTTAATTAAATTATTGAAATTTTAGTACTTTTTTGAAGCACCGTGTTCATTGATTTTGTAGGACACCATTAGATGTCTAAATGGTTTCTGATTTGTCTAATTTTTTTCAAGGATGATCTATAAAAAAAAGACTTAAAAAATAGATGGTTTCAATTGTTGGAAAAAAACATTTGTAGG

>Chr1-02; OZ022206.1; 3163222-3164502; dir; B;

ATTGACCCTTAAAATAGGGTCTAGTCCAGTGGAAAAGAACATTGACTTGCTGGTTAGTTGTCTCATGTTTGAATATAGATGGCACCTTAGTAGTGTGGATGTGAGAAACCCTTTTGTAGGACTTAGACTATCGCTTATATTAAAATAATACTAATAAAAACTGAAAAACTTTTCATTCATAATACTGCACAAACCACACATAGGAAAACGAGAAAATGGCTTTTCCTAATTCTCACTTCCCAGCTTTCCCAATCATGGGAATGAACGTCCGTCATTCCCATCTCGTAGGATTAGACATGGAAATTTCATTTTCCATGAAATCTGCAAACCAAACGGGCCAGGGAGTTGGAACAATGCAAAGATTTTGTTTATCTTTTGGTTGGGCCAATAAGTTTCTGGGCCGTTTGATTTCGGCCTCCCTGTTTTGTTTGCAACCCAGCGGAGGTTGGAGCCCTTTTCTTGTTCTTTGGGCTTAGTTATATTGCCTTGTTCTTTGGGCTATTTCTAAATTATTTGGGTTGTGCCTCTTTCTGCGTTTGACGTCGTTTAATGTTTAATTTGTTTGATTTTTGAGTTGGAGCTGGTTGGGTCCTTCTCATTTGAAGGTAGGGTCAGTCTCAAATGGAAAATATTATTGCTCGATGTGAAAAATAAAGAAATTCGGTTTTGTGGGCTTATGGCCTAAATTAATTGAGGAATAATGCCATTCAAAAGACTGAATTAACATGACCCACATTTTTCCATCCTAGAAAATTTGTTCCTTAAGCAGATCATGGCCACTGATGAAGTAAAGAAGAAAGGGATGCTTAAAAAGAGATGTACAAATATATATATATATATATATATATCAAATGAGCCAAGAATTAATGGAATTTTGTAAAATTGAGATTACTGAATTACTTGAAAAAGGAATAATCCGTAAGAGTAAGTCATCCTGGTCTTGTCCTGCTTTTTATGTTCAGAAAAATGCTGAATTAGAATGCGGAGTCCCAAGATTAGT

CAAACCAAGTGGCTGGTTTAGGTTTACTGAGTCAAAAGAGTCTTTGAGCACATCTTCAGGAAAGTCACTCCAGTTGAATTATGAGATTCCTAAAGCCCATGTCTGTCAAAATAAAATCTGTTTTAAGACCTCTTTTGTCTTAGTTAAGAACATTACTGATGAAGTCATCTTAGGTTTACCCTTTATTGCTCTTTTATATCCTTTCCAAGTAGAGCACGATGGAGTCATTTCCACCTGTTTAGGAGAAAAGGTTAAGTTTGAATTCCTGAGTAAACCAGAGTTACATAATTTAAAAGAATTACAAAAGAATGCAGTCACTAAAACAGTCAGAGTCATTCAAAATGAAGATAAGCAACTAAAATTCCTCCAGGACGAAGTTAAATAAAAACGAATTGAGCAACAGCTTAGTTATGAGTCTTTACAGTCACAAATTTGAGAGTTTGAAGAAAAATTAAAATAAGAAGTTTGTTCTGAGTTGCCCAATACCTTTTGGCATAGGAAACAACATGTAGTCAGCCTTTCTTATGTCAAATCCTTTAGTGAAAAAAATATTCCCACTAAAGCCAGACCAATTCAAATGAGCCAAGAATTAATGGAATTTTGTAAAACTGAGATTACTGAATTACTTGAAAAAGGAATAATCCGTAAAAGTAAGTCACCTTGGTCTTGTCCTGCTTTCTATGTCCAGAAAAATGCTGAATTAGAACGCGAAGTCCCAAGATTAGTCATTAATTACAAACCGCTTAATACTATTTTAGAATGGATTAGATATCCAATCCCCAATAAAAGAGATTTAATTAATCGTTTAGAGAAGTCAGTCATCTTTTCTAAATTTGATATGAAGAGTGGATTTTGGCAAATCCAAATTTGTGAGTCTGATAGATATAAAACTGCTTTTGTTACCCCATTTGGTCATTATGAGTGGAATGTTATGCCATTTGGTCTTAAAAATGCATCCAGTGAGTTTCAAAATATAATGAATGAGATTTTTATTCCATTGAACAGCACTAGAAAAATTTGCATAAGTTCTTACAAATAGTCAAACAAAATGGATTAGTCATATCTGCTAAGAAAATTAAGTTATTCCAAACTAATGTGAGATTTCTTGGGTTTAATATCTGTCAGTCACAGATTAGCCCCATAGATCAAGTTATCCAATTTGCTGATAAATTTCCTGATCAAATCCTAGACAAAAGTCAACTCCAGAGATTTCTAGGATCTCTAAATTATGTTTCTGATTTCTATAAGAATTTGAGAAAACAATGTAAGCCTTTATTTGGT

CATGTATGGAGACGTCTTATGATAAGAGGCAACTGGAAGATTAGACGGAGCAATATCTTGGAAGGGATCATGACTGGATATTAGGGCAGACTAAAAACTGGGGCGAACTTGGCCGACTGTGGCTCCTAGGTTGGCAAATTTGTTACTAATTTGAATGGGAGAACATCCCGGAAAGGGGATGATCTGAGCAGGTGACTCTTGTTTGGCTTGACTCAATGATGGAGGGAGGACTCGGAGTGACTAGGGTTGACTAGACTGACCAACTTGACTCTTTTGCTTGTCTTTTTCTTTTCTTCTTTCTGCCATTGTTTTCCATGCAGGAATTCTCTAGTAAGAAAATCAGTGACAGAATATATATATGGAGTTTGAGTTGAGGGATCTCTTAAATAAGCTTATTTGAGAGACACCTTGTAGGGTACACTCTTTGTTGTATTTCACTAATCCTAACCGTCTATTTTTTAGATATTCATTCAAAGATCATCTCTACAAAAAATCACTTGAATCCGATATCATTTGACAATTCAATTGAAATATTGAAATTTTAGTACTTTCTTGAAGCACTGTGTTAATTAATTTTGTATGACACAATTGAATATTGAAATAGTTTCCGATTTGTCCAATTTTTTTTTTCAAGGATGATCTATAATTGTAGGCTTAAGAAATAGATGGTTTGGATCGTTGAAAAACAATTCACAGGGGACCCTATATATATAAAGAGAGCTTCCATTGAGGGATCCCTCAAATAATCTTATTTAAGAGACACCTTTGTAGGGCTCACTCCACATTGTATTTCACTAATCTAAACCGTTTATTTTTTAGATAATCATTCAAAGATCACCTCTACAAAAAATCACTTGAATTCGATATCACTTGACCATTCAATTGAGTTATTGAAATTTTTGTACTTTCTTAAACTACCATGTTGATTGATTTTGAAGAACATAATTGGATTTCGGTTTCTATTTGTCAAAATATATATATATATATATATATATATTGA

>Chr1-03; OZ022206.1; 7750757-7755782; rev; B;

TCTCAAAAAACACTTGAAAAAGCTCAAGACAGCAATCTGCCAATATTTCCAGATAACCAGTTGACAACTCCTGCGTTTATCTCTCGTTGCTCTGCCATCTTCATGGTATGATTTTCTTTTAAAATAATCCACTTTAATTCATGTAATCACCTAATCGGATAGAGAAATTTCCAGCCCTTTTTCCTACTCTATAATTTGGATTTAAAGATTGATTAAATGGATTAGTTAAAAAAGGCTCTGCTCTTTACTTTCATTCACGATTAGACCGATTGAAAATTGTAGAAACTGTCTTAAACTATTTCATTTAATCACTTAAAAGCGAATCACGGATCACTCTAATACATAAATTTTAATTCTCACCCTCGTCCTTTTAGAAAAATCCAAAGGACCATATCGTTCGGGCTCGAACAGGAACCTGGGCCTGAATAGAATAGTGATAGTAGAAGGGATAGGAGGTTGAATCAGGCCCAAACCATATCATTGGGCTCGAATTAACTGTTAATCCAACCCATGGATGATATACATATTGAATCCGGTTGTTAAAACCCAGACAGGGTCTTCCAAACCACGATCTTGGTATCTTCCCCTAGCCTGCTCAGGTATCGCTCATGCTTGTCAGGCTGACGTACGCGAACGAAAAAATATTGAAGTCCTCTTAATTGAGGCATAGAAAAGAATCAATCCAAGTCAGTATCACACTTGGAGCACAATGGCTTGAGAAGAAGTCCAGAAAAAGACGCGTGAACCTCACCAAAGTTCATTACAACATCCCTCTATCACAAGACCAGCCCTGAATTCCTCGGAACCCGAGACGAATCCTATGGAAATTTTGACATGAGACCAATGCCGGGTCCACTTCAAATACTATATCCTCTTTTCCAAAATTAAGGAAAAAGGACACAAGACTTTCTGCTGAATTTTCAGCAGTCTCCACTGAAAAATGGACATTTCCTAAAATTTCTCAACCTGTTAAAACACATTTAATATCACCAAGCAGCAA

TGAGTTCCAAAATATTATGAATGAGATATTTAACCCCTACAGTCACTTTTCCATAATTTATATCGATGATATTCTCATCTTTTCTGAGTCTTTAGGGCAACATTGGAAACATTTACATAAGTTCTTACAGGTAGTCAAACAAAATGGTCTAGTCGTTTCTGCTAAGAAAATCAAGTTGTTTCAAACTAATATTTGTTTCCTTGGTTTAATATATGCCAATCCCATATTAGTCCAATAGATCGAGTTATCCAATTTGCTGACAAATTTCCTGATCAAATCCTTGATAAAAGTCAGTTGCAAAGATTTTTAGGTTCTTTAAATTATATTTTTTATTTTTACCAAAATTTGAGAAAGCAATGTAAGCCTTTATTTGACAGATTACAGTGTAATCCTCCCCCTTGGTCTTCTATTCATACTGAGATAGTCAAACAAATTAAAATTCATGTCAAGACACTTCCTTGTCTTGGTATCCCTTCAGGTGATTCATTCAAAATAGTTGAAACATATGCTTCTAATATTGGTTATGGAGGCATCCTCAAACAAAAAATCAATTCAAGTCATGCTGAGCAAATTGTCCGTTTTCATTTTAGTCTATGGACCCAGTCACAGAGCAATTATAGTACTATCAAAAAAGAAATATTATCTATTGCATTATGCATTAGTAAATTTCAAGATGATTTATTAAATCAAAAATTTTTATTACGAGTTGATTGTAAGTCTGCTAAACATGTATTAGAAAAATATGTTCAAAATATTGCATCCATACAAATATTTGCAAGATGGCATGACAGGACCCGACCCAATTTCCACTTTGGAATTCGGGCCAAGTCCTGTGCGCGTCCGATACCTGGCTGATGTCGGGCACAGATGACCTTTGTACCCTTTTTATCTCAATTTCTTTTCAAATTTCTCTTCGACTTCTGCCGAAATTTCGGCAGAGTCTCCCCTGTATTTTGACCAATCCCAAAATTTTCTACCTGTTAAACAAACAATAAATTCATCCCAACAGCCAGAATGTCTCAAGTGACAGATCTCAACTTCGTTTTTCCACTAATACTAGATATCAGAGCATATTCTAAAGTTTTCGAGGTTTTAAGGATTTTCTACTAAACTCTACCTTACTTGATGGCGCGGAAGCTATGAATGGCCCGGTGGTGGATCTCGCGTACTTCTACGGCCCGGGGTCGAAAAACAAGTTTTAAACTGTGAGTGGACAAAATAAAATTCTTAAAAACAAGCCAAAATATAATACCCCCGTTTTGAAATAACTAAGGATATATATCTATATATCGATAATTTAACTATATTTCAATATAAGAAATTCTATAAGCATGAATCAGTATGCTGATGAATAAACTTACTTAATTGATCAAACATATCACTGCCTGGAGAAATCAAAGAACTGGTATAACCGAACAAATATACAAAGATATAAATGTGCAAATACTAAGAAACCAATATAAAATAACTGAAAGTCATCCTTGAATAAAAGAAAACTCAAAATCCTTTGGAAATACCACCTATTTGTACCCCTGTCATATCCGTCAATTCCCTGGCAGGTCTCGGGGGCCACGCAGTCTACCCGAGCCGCAAACTGGCGAATCAGGGGACTATGATCAGCCTGTCCCGCCGGCAGATTCCTCGATGACACCAAGTCAGCTCGAGTCGCTCTGGCATGATGCAGGGGACCGTAGTCAACCTGATCCGCAATCCTGGCAGGTCTCGGGGACACAGAGTCAGCCGAGCCGCAATCCTGGCAGGTCTCGGGACACCAAGTCTGCCGAGCCGCAAATCCTGGCACTCACGGTCCGAGCGTCCCCGTAACTCGTGAGGCAAAGTCAAGTGCACTGACGTAACTGAAATCAGACTGGATGTCAATCAGACTGGATGTCCGTAGACATCGGTCCGACTCTGGATAATCACCATAAATAAACGGGTACATGGTGGTTTAATTAGAAAATCCGATAACTTCTGAAATCTAATAAATTAATTGTAGTCTCAATGCTGTTGCTATTCTGTTATATAAAACTCAAGGTATGCTTCTAGATATAACCTTCATCATATATGCATCTCCAAATATTTAACAGTATAAAACATGCTCAAAGGCTTACAAAATACTTATTCAAGATCAAATAATAAAATTCGCTTATAATAACTTATTTATATAAAATTATGATAAAATCCTTTGTGTAAATTTATCTGTATAACTCATTTATATAACTTATTTATATCAAACATTTATGGAAGAAAGTCCACTCACAGATGGTCCGAACTAATTTGACCCTTTGAAGGTTCCTCCTGCTGGTCGGCTGGGCCTCTGATGCTTGATTAGCCATGAATAATAAATCAATAAACTGCTGAATAAAAGAATTAAATTAAACACCCTGCCCCCGGCTCCTAGAAATATGCGCGTTCAAATCCAAGTTACTCCTGAGCCCACATTAGCCTTTTCAGACACCTGGACCAGTTTTAAAAGGTCCGACGCTCAGCTAAGCGATACACTGCCCGATCGGCCGACCCGGGACCCGTGGGTCCACGATCTCTGATGGCCAATCTGGGCTTCCCTGAAGGTTCCTTGCAGAGAGGGAACCATGTTCTGCGAATTTGGTATGAAATGGACGGTCGGATTAGCTAAAATCGCGTTATCGCTTAAAATCCAAACCCTAGCCCCAGGGTTCGCGATTCCGGAGTATCCGGGACTCCGATTCGCAATCCGTCGAATCCTACACGATCCTGACATCGTGTAGACCGACATATCCAAAATTCAACGCGATCCGCCGATTTAACGACACTGCACCCACGGGTCGCGCGATATGGAAAATCCGTTCGGGGCTCAAACGGACTCCGAATCGAGATCCGCGAAATTCTACGCGCTCGTGACGACACGAGGATCTCAAAACTGTACATAATTACTTCACCACCCTCACCCACGCGTCCCAGCACGCGCGGTCAGATTTGGGTGTCTTAAGCGATTTCGGGTTGCCGAAAAATCTCGGAACCAAGACTCCAAACTCCTATCCTAGGTAAAAAACCCCATTTGGAGTCACTTTTGTTCTTGGACATACCCCAAAAAGTGACCGGAAACAGTAGTTTCGAGCGGCCGAAGTTTCGGCCAAACTTCAAGTCGAAAATTGATCGCTTTAGAGTTAAAATCGATCGAAACCCATACACCCATCAGCTAGAACGTGAAAAATAGCTGAGAAACCATACCTTACTCGATCGATTTGGTGGAGAATTGAAGGAGAAAATCGAGCTGGAAGTTCGAACTGGACCGTCGGCATCAATGGCAATTTCCGGCCGATTCCGGCCACGGCAGCGGCGGCCCAAGTTCGAGGATGGTTCGCCGTCGAGTGGTGGTTCATTTGGGACCGGTCTCGGAGATCGGCCCCAAGTGTGGTGGCCGGTACGACGGCTGGAAGGGAGGGAGGTCGCGGCGCAATCGAAACGGTATGAGAGAGAGAGCTGACGGGGGAGAGGAGAGAGAGGTATAAAAATCTGACTTTTTGACCAAATTACCATTTTGCCCTTCGCGGTTTTTAGACCATATCTTCTTCGTTATAGCTCCGATTCGGGTCTACTCCGTGTCTACGAACTCGTTTCGCCGCGCTCTACGCAATGACGCGAGTGATATTTCCAAATTCTTTCTCGATTAAAAAGTCAAATTGTCCCCCATTAAAATATGCGAGGGCAAACTTGTCTTTTTGCTAAAAGATATTTTCCTTACTTTTTAGATATTTTCTTTCTTTTTAGATATTTTGTTTTGAGTTCTTACATGGCAAGCTATTTTAAGCATTTTTTATTTTGATATTGAGTATCTAAGAGGAAGTGAGAATTGTATTCCTGCTTTTCTTATTAGAGAATTTCTACAGGACAAACAATGGCAGCCAGAAAAGATAAGCAAAAAGCACAAACTAGCCAGACCCAGTCACCCCAGTTGCCTAGAATCCTTCCCTCATCAATGAGTGTAGCTAAGCCCGAGTCTCCCTCTCAAATCATCCCCTGCCCAGGTTATTCTCCAATTCCAATTGCCAATAGATATTCAAGCCTAGGAACCACAGTAAGCCAGATTCGCCTTAATTACCAATCCGTTCTTGTTTCTAGTTATGACCCTTTTCAAATTTCTTCCCAAGTTGCTTCTCCCTCAGTTGCCTATAAGAAATCATCCCTTTATTTTGCAAGAAGCAATACCCATTTATTTGTTATTGAGCCCCATCTTAATGATATCATGTTCCCTGGCACAATGGCAAGAGAATATTTCCCCCCTAATTTCCATTATATGCCTCAAAGTCCTTATAAGTCCCTCAAGTTCTACTGAGACATTCTCCATGAGACTCAATCAGTTGACATTAAGCCCATTAAAGATAAAAATGATCCCCACAAAATCCTTTATCACTCCCTTTACATCCATCAAATTCTAAGCCAAAAAGATTGGGGTAAACACCCTTATGACCTAAGAACCCTTCAGTCGAATCTTCATTATAATTATTTTGATTATATTGAAGCCTGGTATTCAATTTTTCTACATCAAACAATTGACTTTTAGTCATTCCTGGTTTATCAATTTTGACAGTAAATTCAAAAGTTCTTTCCCTTATTGGTTCCTCCATTGGTGGGAAAAACATGGTCCAATTGCTAAGATTCTTCCTTCTCAAGTTCTTGAGTTAGTTGCTTATTTTGCAAAAAGATATAAATTTTCTGAGACAGAATTATTCTTTCCAAGTCAGCCTCATTTTATTGCTAAGTATAAAGTCCCTTGGATTTTAAAATGGAATTATTCTATTAACTAGTACACCAGAGTATTCTCCTGCCAATATTCTATTAACTGGTGGGATAAATTTCAAGTTGGTCGAATTGCTGAGTACGTCTACAAAGACTTTCCTCCAGTTACTCAGTTGCAGAAGTCTAAGCCCTCTTCCTCCAGCACTAATGAAGCCTCTCTCTCAGTTGAAGGAAAATCTAAAGCAGAGTTGCAAGAAATTGTCCGACAATTGATCATCCAAGCCTCTCAAATGGGTGTCGATGATGA

TGAGACATCCCCAAGATCACAGTCATCCACTAGCCAGCCTCATTTGCCCTCCAGTCAAAAGAGTCCTTCTCAAAAGCCACGATGGGTTGATTACCAAGACAGTCAAGACTCGTATGATCTTGATTCAGACTAAAGTTGAGATTCTCCGACAAGGCACTCAGTAAAAGCAATGCTTTCGCGTGCCTCTATAGGGAAAATTTGTTTTCCTCTAAGTTCAAGCAATAGTTGCCCTCCCGTGCCCCAACAGTTGAAATTTCAATTATTGTAATAGTTCTTGCCTTAGTCTATTTAAGCTGGCTCCGGCCTCAGTTGTAAGCATAATGTAATTTAGTAAACACTTTACTTTTCTCAGTTTCAAAGTTCTCTCAGAAACAATGCTCTGAAGAACCAAGTTGTAAGACGAGAGATTTTGTAAGTGGTTTTTGCATTCTCACAGTTATCAAAGTTATTTTCAATAAAATAATTATATTTCATATATATATATATATAAAGGGACAAGTTGTGTCCCCAATATATTATTTTGTCAGGCCGGTAGCATGAATTGCTATCACTTAAACACTTTCATATGATGCGGTCTTTTCGACACACACACACTACCAAACTCTAGTCCCTTTTCTCCCAAAAAAAACCCTTACACAGCTGTTTTCCCTTTGAGGGGGGAGGAAGCCATTGTTCTTCCTCCCATATCCCTTCTCCTCTCTTCTCTTCCTCCCTCTCTCTTTCCCTCATTTTCAGAGACAAAAAGTTTTCCCTATTTGTCTTCGTTTTATTATGATTTTCCTCCAATCATCATAGGCGGCTGCATCTCGATGTCGGATCTCCACCACCATCCTTTATGCGGTTTCTTTATGTTCAGAATAATTTACAGAGACTCTTTGGATTCTCTGTGTAGTTTTCACCTCTTCTTTGTGAGAGCTTTTCATCTCTCATTTGTGAGAGCTTTTCATCTCTCCTTTATGAGAGTTTTATTTGCTTTGTTATGGCTTATTTTTTTTCAACA

>Chr1-04; OZ022206.1; 8113065-8120855; rev; A;

AGTGGTGGATGTTCTGGCGTGTGAGACACTTTCCATATGCGGCGTTGGATGTTCCGGCGTATGGAAAGACTCGGTATAGTGTTTAGGGTCATCACCCATGGCGTTGGATGTTCCGGTGTGTGATGATCCAGTCTGCGCGCGTAGGACATAGTTTGGGAGCTACACATGGCGTTGGATGTTCCGGTGTGTGAGCTTTAGAGTTTCGTCCCCCGGTTGGACCGCTCATCCCTAGACGCAGGATAGTAATTGGAAATCCAGCGGACTAGTCAAACGATCCCCCAGTTGGATTGTTTCCTCGGTACCTAGGTAGTGTGATGAGTATATCGTATAGCTACGTATGGTTATATATATATATATATATATATACAGTTTATGCATGAAACAAATTAACGAATATATGAAAATAGATGCAATGAAAATAAGCATTAATCAAAATAAGTAATTCTCAAAATATCTCAAATATATTGATAACTTGGAGAATGCAAAACACTTACAAAGATCCTAGTTTCACCACTTAGTTCTTCAGAGTATTGGTTCCTGATAGAACTTTGAAACTAAGAAAATTATAGTGTACTATAATTACATTCTGCTCAAAACAGAGGCCGAAGCCTTCTGAAATACACAAATAAAACAGACTCAGATGACTTTTGGTGAGCTGGCAACTTTGGATGCTTTGTGAAAAGTAACTGTAGAAGCACGCGAAAGCTTTTGCTTTTACTGAGTGCAGTAACATTTACTTTTGTCGGGGAATCTAAAAAAGAACACGGCTTTCAAACTGATGAAGGCACGGGTTCAGATGCTTTTCTGCAAGGCAACTTTAACTTTAACTTTAATCATCTCCTAGATTGAAAGGACCAAAATCATAGGGGTCATGCTCATCAGGATAATCACTCCACCGCATTTTTGGGGCTGGAGTTTGACTGGCTGGCTTGGGCTGGCTAGTAGATGTCTGTGACGCAGGAGAGGCATCAGAGTCTGCATCATCATTCATCTGTGAGGC

TTGAAGCATTAGTTGGCGAGCAAGATCTTGTAATTCGGACTTAGACCTTCCCTCGATTGGGAGAGAAGAATGTGATGTGGCAAGGGAGATCTTGTAATCTCTGTGTTTACACTTGTCTTCAGTTGTGTTCTTCTCCCTCTCACTGGTTCTAGACCTCTTATGGTATCAGAGCCGTAAGTGTTGAAGAGTTAGTCTAAGTCTACAACTCATCCAACCCAAGTGGCTGGTTTAGGTTTTCTGAGTTGTAAATGTTCAGTTCCTTCTTTTCTTCTTCTGGTATCTCAGTTCACCTGTCCTTCTCTCTCTTGGTGTCCGAATCGTAAGACCCGTTCCAAACAGTAAGTTTCAGGGAGGCTTGAGCGGTAGTGGCATTAGTAGGAGGGCGAGAGAATTTTAGGTGTCCTGTTATCCATGGAGTCTGTATTAGAAGTTTTTGTTCACTTTCTCCTTTAGGAATCCAAACCCAGAACCTTGTGTAAAAAATAATGAGTCGTTTTCTTCGGACCAACTCTTACAGTTCTAGCAGCTCTAGAGCCAATAGTACTCTCCCAGAGATAGTAAATGAAGAACAAATTGAATATGAGAATAATAGTAATCTAGATTATAAAGATTGGAATATACCTCGAGTCCCTAGTCAAGATATTTATAATAAAAAAATGGTCATTATCTAGTTTTAAGTCAACCACCCATGTTAAAACAGTTGAACAAGTTTATGCCTTAAGTAAGAATCATGAGACATGCCAGTTACTTAATCCTGAGTCAATAAGAAAACACAAAACAGATGGAAACAATTTTCTTCATATTGGTTTGGTACAAGTTGCTGTTAAACCTTTAACCATATTAGGTTTAAAGGCTTCTATTCTTCTCTGTCTACGTGATGCTAGATTTAATAATTTTAACGACAGTACGTTAGGAATTATTGAGTCCAGTCTCGCTAATGGCCCATTTCATTTTGATTGTTATCCATATTTTACAGTCAGCCTGAGTGATCCACATATATTAAAAACTCTCACTTTAAATATTAAGACTGAAGGTTATGATGTTCTGCCAGGAAGCCAACCCTTAGCATTAGTTTATAGAATATATTATAAAGTCACAGGTACAAATATGAATTTTCAAGCCTTAAACAAGAGCCCAAAGAGTCAAACACTGTTAATTCAAAGTCATACGCCTGATGCTACTGTCAGAGTTCCTCACTCTATCAAATGGTTTGATGTCACTCTTCCCACTGATTGGACCCTAACCACTGAGACCCCTCCAGTACCTATCCAGCGTAGTCTAAGCGATTTAGACTACATACAACAATATTTAGGCGGTACAGTTAAAATCAATTTTGGTCACCAAGCCCCACAGAAATCAACAGTCCAGTTACAACAGTTCCCAACACCAGGTCAATCCCAGAGACATGTTCCTGCCAGACACTCTTTTGCAGCCTCTTCCTCTACCCTAGAACGAGACCTAGAGTTGGAGAAAGCTTTGATTGATCTAAAATTGGAATCACTAAGAAAAACTGCACAAGTCACCCATCCTGTCTATGGAAAACCAACAGAAGAAAATAAATCTGAGTCCCCTGAGTCACCCACTCAATCCGATTTTGTCGAAGCCGTTTCAAAGTAGAAAACCAACTCCGAACACTTAATAAGAAATTTGAAATTAATTGGAAAAAATTAGAACGTCACCTACATGCTCCTGAAAACCAAGCAAGAAAAGATAATTATCACCTAGTAATCCCAGATAGAGCCAGAAGACAACAGATATTTAATAAATGGAAAGAATACATGCGTATAAAGTCGAAATTTATTATCTTGATTTTGTAGAAAGCCGTCATCTATCTAATGACTTAAAAAGTTTAACTCAAGAAAATTGGGAAAAAATAGATCACACTAAAATCCTTGCCAGTCACCCTCCAGTAGAATCCATAGTTATACAACACCTGAATGCACAGATAATTGCCAGTCCTTTTAAGATCCCTGACCTAGAAAATAAGATGGTAATTGAACAAAATAATTATACAAATTAAAGCTTAGTTGTCATAGGAAAACAATTAGACAAAACAGAAACAAAAGTCGATAAATTACTTCCAAAAGAAATTTCAGTCAAGCCGCATAATCAGAAACCGTTAGTCACCTTCAAAGACTTACAATCAAGTCCAACTTTTAAAACAAAAACTACAATGACAAAAATTGAAAAAATGTTAGAAGAATTAACTCCAACCAAACGGAAAGATAAAGAGAAAGTTGAGTCAAGCGTTAAAGTATTAGGATCCCCTGAGTCACCTTTCATATTTCATCACTCCTCTGAGTCTGAATCCATCGATAGCACAGATACTGAGTCTAGTAATATCTCCAAAATTGAAAATGCGTTTAAAAATCTTGAGCTCGAACAAAGTCCACAAGTACAAAGATTAACAAACAAAGTTAACCCTACTAGCCTCACAAAAAATTGGTATCCCAAGCCTACACCACCAGACATTCAGTTTGAAGAACGAAATACTCAAAGTCAGTTTTCTGTTTCTTCTGATAAATTATATGAATGGAATATTGATGGCCTTTCTGAACAAGAAATCCTTAATAAGCTGCAACATATGTCTATGGTAGCCAACAGTTACATCACCAATCACAGTTTCAGACAGTCTGAAATTGTTCCCTTGTTAGAAACAGGTTTCACAGGAACTCTTCGTTCTTGGTGGGATAATCACTTAACTCATGAGTCTAAGCAACGAATAATTCATGCGCTAAAAGATGGTCTTCCTATTTATGATGAACAAATAGGTCAAGGTATTGAAGATGGCGTCAACACATTACTATACACAATAATCGATCATTTTATTGGAACACCTAGTAGTACTACCGCTAGAATACATGATCAATTAAGTAATCTAAGATGCCCTAAATTATCTGATTTTAGATGGTATAAAGATGTATTTATTTCCAGAGTCATGGGAAGAACAGATAGTAATCAGCCTTTTTGTAAAGAAAAATTTGTTAATGGATTACCAAATTTATTTGCTCATAAAATCCGATCAACCCTTAGTAATGAACAAGGTCATATTGATTGGAATAGTTTAACCTATGGAAATATAATTAGCACAATTAATCAAGTTGGTATGAGAATGTGCGTCGATATGAAAATTGGTAAACAAATCCAGTCTGAAAAAAATTCTGCTAAATATGAATTAGGAAATTTCTGCGAACAATATGGTTTGATGTCTATTCCTCCATCTAGAAAGAATAAGTCCAGCCGACAGTTTAGAAAAACCTGCCATTATTCGCGTAGAAAACAGTCATTCCAACATAATCATGCAGATAAAGAATTTTATAGTAAGAAAAGATTTTCCCCTCATAAAAATTGGAAAAAGAGTCACCATAAAGAAAAGAATAATAGCTTTAAGAAAAAACCCGACAAAAGAAAAATAAAATGTTATAAATGTCAAAAGTATGGTCACTATGCTAATAATTGTAAGGTTAAAGACACAATCAAACAATTAAAAATCACGGATGAAGATAAAATAAACTAATCCAAGTCCTTGAGTTAAGAAATTCTGAGTCCAGTGAAAATGAAACCATGGTTAGTCATACTGAGTCCTCTGAAATATATTCTTCTGAGTCACAACCCAGTTCCCCCGTAATCCAATCTGGTTGCCAAGATAATTGCTGTAAGTCTAAATCAATTAGTGTCCTCACTAAACAGGAAGAACAAGAAGAACTATTAATTGATTTAATAAGCAAGGTTGAGAATCCAAAATTAAAATCTGAGTATCTGAGAAAATTAAGAAAAGTCATCAGCCAAGAAGAACCGAAACAGTCTACCCAAAAGATAATCTGATGCAACCTTAGAAAAATTTAAGAAAAGGAAAGAAGTTACTTTACGGGACTTACAATTAGAAATAAAAACAGTTAAAAAAGAAATCATTGAATTAAGACAATTGAGTCCAAAATTACAATCTGAAAATTATGAGATAAAACAAGATCTTATTAATTTATTAGAAAAGAAAAATCCTGAGTCCACCCCAGAGAGTCCAACAAACTCAGATGAAGAAAGCCCTATTCACGAGCAAGCCGTTAATCTAATTAAACATGTAAATTTCAGAAAATGGTATGCTCGAGTCACTATATTTGTTCAAAATTTTGAGTTAAACATAGTCGCCCTATTTGATTCTGGAGCTGACCTTAACTGTATCCAAGAAGGCCTAATTCCAACTAAGTATTACAAAAAATCAAAAGAATCGTTAAGTACTGCTTCAGGAAAATCCCTCCAGTTAAATTATGAAATCCCTAAGGCCCATGTCTGTCAAAATAAAATCTACTTTAAAACTTCTTTTGTTTTAATCAAAAATATTACTGATGAAGTCATATTAGGTCTACCGTTCATTGCCCTATTGTACCCTTTCCAGGTTGAACATGATGGTGTCATTTCCACCTGTTTAGGAGACAATTAAGTTTGAATTTTTGAGTAAACTTGAGTTACATAGTTTAAAAGAATTACAGAAAAATGCAGTCACCAAAACTGTCCAGATTATCCAAAACAAAATAAACAATTAAGATTTCTCCAGGAAGAAATTAAAACCAAACGAATTGAGCAACAACTGAGTCATACGTCTTTTCAATTACAAATAACGATTTGAAGAAAAACTAAAACAAGAAGTCTGTTCTGAGTTGCCTACTACATTCTGGCATAGGAAACAACATGTTGTCAGCCTTCCCTATGTCAAATCCTTTAGTGAGAAAAATATTCCTACTAAAGCAAGACCCATTCAAATGAGCCAGGAATTAATGGAATTTTGCAAACTTGAGATTACTGATTTACTTAAAAAAGGAATAATTCGTAAGAGTAAGCCACCCTGGTCTTGTCCTGCTTTTTATGTCCAGAAAAATGCTGAATTAGAACGTGGTGTCCCAAGACTAGTCATCAATTACAAGCCACTTAATACTGTCTTAGAATGGATTAGATACCCAATTCCTAACAGAAGAGATTTAATCAACAGATTAGAAAAATCAGTCATCTTTTCTAAGTTTGATATGAAAAGTGGATTTTGGCAAATTCAAATTTCTGAGTCTGATAGGTATAAAACTGCTTTTGTTACTCCTTTTGGTCATTATGAATGGAATGTTATGCCTCTCGGCCTTAAGAATGCTCCTAGTGAATTCCAGAATATAATGAATGGATCTTTAATCCTTTCAGCCATTTTTCCATAGTTTATATTGATGATGTGCTTATATTCTCTGAGTCCATAGAACAACACTGGAAGCATCTGTATAAGTTTTTACAAATAGTTAAACAAAATGGTTTAGTTGTTTCTGCTAAGAAAATTAAGTTATTCCAAACTAATGTGAGATTTCTTGGGTTAAACATCTGCCAATCACAGATTAGTCCCATAGATCGAGTTATCCAATTTGCTGATAAATTTCCTGGCCAAATCCTAGACAAGAGTCAACTCCATAGATTTTTAGGATCTTTAAATTATGTTTATGCTTTTTATCAAAATCTGAGAAAACAATGTAAGCCTTTATTTGATCGTCTCCAAAATAATCCTCCTTCATGGTCATCCGCTCATACTGAAATTGTCAAACAAATCAAAGCCCATATCAAGACTCTTCCTTGTCTTGGTATCCCTTCTGCTAATTCATTTAAAATTGTTGAAACTGATGCTTCTAATGTAGGTTATGGAGGTATCCTCAAACAGAAAGTCCCTTCTGGTCAATTTGAGCAAATCATTCGATTTCACTTCGGCACTTGGTCCCAATCACAAAATAATTATAGCACTATTAAGAAAGAAATATTATCTATTATATTATGCATTAATAAATTCCAGTCTGATTTATTAAATCAAAAAATTTTAGTCCGAGTTGATTGTAAGTCTGCTAAGCATGTATTAGAAAAAGATGTTCAAAACATTGCATCTAAACAAATATTTGCACGTTGGCAAGCTATTTTAAGTATTTTTGATTTTGATATTGAATATTTGAGAGGAAGCGAGAATTCTATTCCTGATTTCCTTACTAGAGAATTTCTGCAGGAAAAACAATGGCAGAACGAAGAAAAGAAAGAGAAAAAGATGATAAACAAAAGAGTCAAATTAGTCAATCAAGTCAACCCCAGTCACTCCGAGTCCTCCCAACATCAATGAGTCAAGCCAAATATGAGCCTCCTTCTCAGATCGTCCCCTTCCCGGGATGCTCTCCTATCCAAATTAGTAACAGGTTTGCCAACCTAGGAGCCATAGTCGGCCAGGTTCGCCCTAGTTTCCAGTCTGCTTTAATTTCTAGTCATGATCCTTTTCAAGATATTGCTATCACTAACCCTCCAGTTGTTTCTTACCAGAAGACATCCCCATACATGGTCAAGAGTCATAGTCACCTATTTGTCATTGAATCCAAATACAATTCAATTGCCAATCCCATCACCATTGCTAAGTCTTATTTTCCACCCAATAGTCATTTCATTCCCCCAGCACCATTCAAGAATCTTAAATATTACAGAGATATCCTCCATGAGACCCATTCTATCATCATTAAGCCAATCAAAGATAGAGAAAATCCCCATATCATCCTCTATCATTCACTCTATATTCACCAAATTATCAATGAAAATGAATGGAGTAGTCACCCTTATGATCTCAAAGTTCTCCAATTAGAACTTCAATATAGTTATTATGATTATATCGAAGCATGGCATTCTATCTTACTTCACCAAACAGTTGATTTCAGTCACTCCTAGTTCTTAAACTCTAACAGCAAATTCAAGAGCTCTTTTCCTTACTAGTTCAATCACTGGTGGGAAAAACATGGTCCAGTCATTGATTTATTTCCTCCCCCCATGCAAGAATTAGTCAATTATTTTACTAATAAAAATAAATTCAAAGAATCTGAGTTGTTCTTCCCCAATCTGCTTCATTTTGTTGCCAAATATAAAGTCCCTTGGATCCTGAAATGGTCTTATCAGGTTAATTGGGAAACCAGAGTCCTATCTCGCCAGTTTTCCATCAAATGGTGGGATAAATTTAAACTTGAGCGTATTGCAGATTATGTTTATGCTGATTTTCCTCCAGTCAAGAATCCGCATTCTAAGAAAAAGAGTTCCCCTGCCACATCACATTCTTCTCTCCCAATCGAGGGAAGGTCTAAGTCCGAATTACAAGATCTTGCTCGCCAACTAATGCTTCAAGCCTCACAGATGAATGATGATGCAGACTCTGATGCCTCTCCTGCGTCACAAACATCTACTAGTCAGCCCAAGCTAGCCAGTCAAACTCCAGCCCCAAAAATGCGGTGGAGTGATTATCCTGATGAGCATGATCCCTATGATTTCGGTCTTTCAATCTAGGAGATGATTAAAGTTAAATTTGATGAGATGATTAAAGTTAAAGTTAAAGTTGCCTTGCAGAAAAGCATCTGAACCCGTGCCTTCATCAGTCTAAAAGCCGTGTTCTTTTTGAGATTCCCCAACAAAAGTAAATGCTACTGCACTTAGTAAAAGCAAAAGCTTTCGCGTGCTTCTATAGTTACTTTTCACAAAGCATCCAAAGTTGCCAGCTCACCAAAAGTCATCTGAATCTGTTTTATTTGTCTATTTAAGAAGGCTTCGGCCTCTGTTTTGAGCAGAATGTAATTATAGTACACTATAATTTTCTCAGTTTCAAAGTTCTATCGGGAACCAATACTTTGAAGAATTAAGTGGTGAAACTAGGATCTTTGTAAGTGTTTTGCATTCTCCAAGTTATCAATATATTTGAGATATTTTGAGAATTACTTATTTTGATTAATGCTTATTTTCATTGCATCTATTTTCATA

TATTCATTAATTTGTTTCATGCATAAACTGCGCCTGTTCTATAATCTCTGTGTTTACACTTGTCTTCAGTTGTGTTCTTCTCCCTCTCACTGGTTCTAGACCCCTTCAGTGTTTATATATATATATATATATATATGTATATGTATACTTGAGTGAGTGAAGAATTAGAGTTTTTCAGTGGTTTGGCAAGAGTTGCAAGATTGAGAGAATGGGGTTTATGAAAGAGTTGACTTGTGTTTTGAAGAATTTTTAAATGAATTATGTTTTGAGTAATTAAATAATGAATTGTGAACCTGCATGTCTTGAGCATTATTTTTACCCGGTGGGGTTACTATATTGTTTTTAACTGCAAATCTTGTTTTTGTCCACTCACATGTTTTCTGTTTTGCGTCCCCCCAGCCAGTAGCCGCTCCAGGAAGCGTGCAAGTGGTGTACAAGGCTCGAACTGTGAACCTTCCTAGTTAGGATTTGAGTAATTTCTTCTACTCAACTGTTCTTGTAATCTAAATTCAGTAGATGCTCTGTTATCGCCCCTGGTAGACTTTACTTGTGAGCCCGGAGGCCATTTAGTATGTTTTGTTGAATTATGAAAGCATGCTGCTGGTTGGGATTTTTATTTTATGTATGTTGCAGGTTGAAATTTTTGTTGGGTTTGCTTAAATTACAGGAGAGACTCTGCCAAAATTTTGATAGAGAGTCTCAGTTTTTAGTAAGTGGGCCCGGCATCGGGAAGATGTCGGAACAAAGACGGGGTCCGCTTCGGTTTTCGGGAAATTCCGGGGCGGGTCCTGTCATATTTGGGCAGTCTGAAACATTTTCTAAGAGTGGAAGTCACTCGATCTAAAGATGGCTTATTTCTTTCACAAAGGAAGTATGTCATGGACCTGCTAGCAGATACTGAAATGCTTGATTGTAAACCAGCTGATATACCTATTGTTGAGAATCATAAACTTGGTTTTATGTGGATCAGGTCCCAAATAACAAAGAAAGATACCAAAGG

>Chr1-05; OZ022206.1; 10426781-10432702; rev; D;

TCGATTCTTCTTATATGTTCCGTGACTTCCAAACAATTGGAGCTCGAGCAAATCCAGAATAAATTCAAAAACAAGAAAATTGCAGGTCCTTAATAATGGGGCCAGTTATAATGGTGGTTCACGACCAGCAAACAATTAAGACATTACGAAGCTTCCTTAAGATAAGAATCTTGTCCTTTTCAAAGTAAGCTGCTATGTTAATATAATAAGGGCGTGTTTGTTACACCGTGCAAGCTTGGATTGGCTTGGCTAAAGTTTTATCCCATGTTTGTTTCACCCTGTTCTAATTTTAAGTGGGATTAATTAATACGCAAGGAATAATGGACTTACATCACGATATCTTTTTCTTCCTCATTCTGATTTTGGTTTTCGTATCACGGATCTTGGTTTGCGCTTTACGGCATTTTCACTATAAAAAAAATCCAATCCCGCAAAGGATTGTTCATGGAGAGGAGGGACTAACAGTCCCGATGACCCAAGCAACTAGCAACCACACCCAATTGCCCCACAACCAACGACCCACCAAGCGGCAGCCACAAGCACCATCCCCAACCACCAGATACGACCACCCACCATGACCACCAACCATAGCCACCCAAGAAGGAGAAGACAGAATAGAAGAGGAAGAGGAGAGAAAAAAAAATTTCGAAAAACTAAACAAAGAAGAGGAGAGAAAATGGAGAAGAAGAGCTGAGAGAAATTTTTCGAAAAAAATAGAGAAGGAGAAAGAAAATGGAGACGAAGAGGAAAAAAAGTGGAGAAGAAGAGGAGAGAAAATTTTAGGAAAAAAAACAAAGAAGATGGGAGAAATTTTTTGAAAAAAAGAAAATAAAAGAAAAGTAGACGGAAGAAGATGAAAGATATGTATAGGGGAAGAAGAAGAAAGAGAAGTAGAGAGGTTCGTCCAAAAAAGAAAAAAAAAAGAAAAAAACGAGATGCAAAGAGGAAGAAAAAGATGGAGAGAGAAGAAAAGTCAAAAAAATATTAAAAAATCTTACTT

AAATTGTACCCCTGCTAGTCACAGGTTTTACAGGTACCCTCCGCAATTGGTGGGATAAACACCTCACACCTGAGTCTAAAAACTTAATTATTCATGCTGTAAAACTTAATGATGAAGGTCTCCCTATCTTTGATGAACAAATAGGTCAAGGAATAGAAGATGGAGTCAATACTCTGTTTTACACCATAATTGAGCATTTCATAGGAACCCCAAGCCATACTACCTCTAGAATTCATGACCAGCTTAGCAACCTTCGTTGCCCTAAACTGTCTGACTTTAGGTGGTATAAAGATGTTTTCATTTCGCGAGTAATGCTCCGAAATGATAGTAATCAGTCGTTTTGGAAAGAAAAATTCATTAATGGCCTTCCAAACCTATTTGCCCATAAGATTCGCACATCCCTTAGTAATGAACAAGGACATATTGATTGGAATAATCTGACCTATGGAAATCTAGTCAGTACCATCAACCAAGTTGGTATGAAGATGTGTGTTGACATGAAAATTGGTAAGCAGATACAGTCTGATAGAAAATCCGCTAAGTATGAGTTAGGAAATTTCTGCGAACAATATGGTCTTACTAGCCTACCTCCCTCCAGTCGAACTAAGTCACACCATAAGAGTCAATCCCGTTCTTACCATAATCGAAAAAATAGATACCCAAATAATGATAGAGAATTCTATAAGACTAAGAAACATAGCCCTAAAAAGAATTGGAAAAAATTTCCATCAAAAAATAAGAAAAACCGATTTCAGAACAATTCTGGAAAAGGAAAAGTTGTCTGCTGGAAGTGTCAAAAACCAGGACATTATGCTAATAAATGCAAGGTGAAAGACACAATCAAACAACTAAAAATTGAAGAAGTTGAAAAAGAAAATCTAATCCGACTTCTAGATCTGAAGGATTCCGAACCTAGTGATCTCGAGTCATCAGTTGACAGTGATTCCGAGAATTCTTATTCCTCGGACTCACAACCTAGTTCCCCCCATATACAACTCGGTTGTAAGGATAATTGTTGTAATTCTATGAAATCCATTTCTGTTCTTACTAAACAGGAAGAACAAGAAGAGCTTCTCATAGACTTAATCTGTAAAGTCGAAAACCCAGAGTTGAAGGCTGAGTATCTGAAGAAATTTCGGAAACTCCTTAGTCAGGAAGGTCCGAGTCAGTCTCCACCTCCTCAAAAGATAAGTTTAAATACAACCTTAGAAAGGTTCAGTCGCAAGAGAGACATCACCCTTCATGATTTGCATTCAGAAGTAAAATTAATCAAGAAGGAGATAGTTGACCTAAAACAATTAAGCCAGCAATTGCAATCTGAGAATCATGAAATCCGACAGGATTTAATGGCTCTCAAACCTGATTTACCTCAGAGTCCAACAAACTCAGATGATGAATCTTTGAATCCAGAACAAGCCTTATGTCTAGTCAAACGAATTAGCTTTAAGAAATGGTATGTTAAAATTACCATTTTTATTGATCAGTTTGAGTTTACCACAGTCGCTTTGCTTGATTCAGGTGCTGACCTTAACTGCATTCAGGAAGGTCTTATACCTACCAAGTATTATACTAAGTCCTGTGAATCTCTTAGAACAGCTTCAGGAAAATCCCTACAGCTGAACTATGAAATTCCCCGCGCCCATGTTTGTCAAAATAAAATTTGCTTTAAAACTTCATTCGTTCTAGTAAAAAACATTACTGATGAAGTTATTTTAGGATTACCTTTTCTTGCCTTGTTATATCCTTTCCAGGTGAACCATGATGGCATCATCTCTACTCACCTAGAAGAACAAGCCAAGTTTGAGTTCTTATCCAAGCCAGAGTTGCACCAGTTAAAAACCTTTCAGAAAAATTCAATTTCCAAAACTGTTGCTTTAATTTCCAGTAAGACTAAACATTTGACTTCTCTCCAGGAAGAAATTAGTCATACTAGAATCAATCAACAAATTAGGAATGAACCTTTCCAAAAACAAGTTCATGAATTTGAAGCTAAGTTAATTAATGATATTTGTTCTGATTTGCCTAATGCTTTTTGGTCCAGGAAACAACACATTGTCAGTCTTCCTTACGCCAAAACCTTTAGTGAAAAAATATCCCCACTAAAGCCAGACCAATCCAAATGAATCAGGAGACAATGGAATTCTGTAAAAAAGAAATTACAGAATTACTCTGAAGGGAATCATCCGTAAGTCTAAGTCCCCTGGTCTTGTCCTGCCTTCTATGTCCAGAAAAATGCTGAACTAGAAAGAGGAGTCCCTAGACTAGTTATCAACTACAAACCCCTTAATGCAGTCTTAGAATGGATTAGGTACCCCATACCTAATAAGCGAGACTTAATTAATCGGTTAGAAAAAGCAGTTGTCTTTTCCAAATTTGACCTTAAGAGTGGATTCTGGCAAATCCAAATAGAAGAGTCAGATCGGTACAAAACCGCCTTCGTCACTCCTTTTGGTCATTATGAATGGAATGTTATGCCTTTCGGTCTTAAGAATGCGCCTAGTGAATTCCAGAATATTATGAATGAAATTTTCAATGCTTATAGCCATTTTTCAATTGTCTATATTGATGATGTCTTAATCTATTCCCAATCGATAGAACAGCATTGGAAACATTTGTCTATATTCCTTCAAGTTGTTAAGCAAAATGGTCTAGTTGTTTCTGCTAAGAAAATCAAATTGTTCCAAACTAGTATTCGATTTCTTGGCTTCAATATCTGTCAGTCTCAGATTAGTCCCAATTGATCGAGTCATTCAGTTTGCTGATAAGTTCCCAGATGAAATTATTGATAAGAGTCAGCTCCAGAGGTTCTTAGGATCCCTCAATTATGTTTCTGATTTCTATCAAAATCTGAGAAAACAATGTAAGCCCTTGTTTGACAGGTTGCAGGAAAATCCTCCTCTTGGTCCTCTGTCCACACAGAAATTGTCAAACAAATCAAAATTCATGTTAAGACACTTCCCTGTCTTGGCATTCCTTCAGTTGGTTCCTTCAAAATAGTTGAAACTGATGCCTCTGACATTGGTTATGGAGGTATTCTTAAACAGAAATTGAGTCCCAATTCGCCAGAGCAAATTGTTCGTTTCCACTCTGGTATTTGGAATCAGTCGCAAAATAATTATATATACTATTAAGAAAGAATATTATCTATAGTTCTATGCATCTCTAAATTTCAAGATGATTTACTTAATCAAAAATTCTTAATCCGAGTTGATTGCAAATCTGCAAAACATGTTTTAGAAAAAGATGTTCAAAACATTGCATCCAAACAGATTTTTGCACGTTGGCAAAGTATTTTAAGTATCTTTGATTTTGACATTGAGTATTTGAGAGGCAGTGAGAATTTTGTTCCTGATTTTCTTACTCGAGAATTCTTGCAGGGAAAGCAATGGCACCCAAAAAAGACAAGCAGAAAGCAGGTGCCAGTCAGTCCAGCCAACCCAAAACCTCCCAAACACCAACCCAAGACCAGTCGCCCAAAAGAGTCCTCCCTGCATCCATGAGTCAATCCAGAATGGCCCATCCAGCCTCAATTGTCCCATATGCAGGTCATCCTCCAATCCCAATCGTCCCAATTGCAAACAGATTCACCAGCCTTGGAACCCCAATAGGATCCACCGTTGGCCCAATCCGCCCTAATTATTAGTCAGCCCTTGTTTCTGACTATGATCCATTTGCAATAGTTCCTCCAGTTATTCCAATTGCCCAAAATTCCCAGTCGTCCACTCAGTTCAGAAAGTCGTCCCCTTACCTCCCAAAAGATAAGGTTAATTTGTTCATAATCGAACCCAATTATGATGGGATTTCTGATCCGATAACAATTGTCAGGTCATATTTCCCTCCTAATTCCCATTTCCTTCCTCCTGCACCCTATAAAGACCTCAAATACTATAGGGATATCCTGCATGAGACCAAATCCGTTGAAATTAAGCCTATTAAGGATAGAGATAACCCCGCGGTCATCCTCTATCATTCCCTTTATATTCAGCAAATTCTGAGCCAAGCTGAATTCAGTCGCCACCCCTATGAGTTAAAACACCTTAAGTCCACCCATCCTTATACCTATGCAGATTACATTGATGCATGGTATAATAATCTTCCTCTCCTTCATCAACAGTTGATTTTTCCCACTCCTGGTTTCATTAATTTCGATGCCAAATTCAGGAGTCCTTTCCCCTACTGGTTTCTTCATTGGTGGGATCAACATGGCCCAACAGCTGATGTCCTTCCTACTGAAGTCCATAATCTGACCAATTATTATGCCAGTAAATCCCAATTTAATAAACAACAATTGTTCTTCCCCAAGTTGCTTCTTTTTATTGCCAGGTACAAAATTCCCTGGATTATGAAGTGGAATTATCATGTCAATTGGGAAACCAGAATCATGTCTCGCCAGTATTTTGTCAAGTGGTGGGATAAGTTCTCAATGGACAGAATTTCCATCTATGTCTTCAAGGATTTCCCCCAATCGGATCATCCTCCCAAGCCAAGCTCTCCCACAGGCTCAGTCAGTTCGGCCATCTCAATCGATGGAAAATCCAAAGCTGAACTCCGGGCCCTTGCTGCACAATTGCTTGACCAAGCTAGTCAGATGAATGATGATGAGGACAACGAATCTTCCCCATCTGCATCCAGCTGTCAGCCTGGCTCCAGCTCCTTTGATCCCAATATCCCTTTTGCGCAGGATCCTTATGACGCGTATGATTTGGGATCTGATTAGTCAAAAGTTGCCATCACTTCCTCTTTTCACCCCGGTGAAATGTTGGAATCAGAATATTTGTAACGTAAAATCAAAATTGCTTTTGCTTTTACCCTTACTTTACCCCAATAGGTAAAGAGAGTAAAACAAAAATACTTTTGCTTTTACCCCTGCTTTACCCCAATAGGTAAAGAGAAAGTTGTTTTCCGTGCTTGAAACCATGGTTACTCTCCAATCAGGGTTACTTTGGATTCAAACCTTAGTTGCTTTCTTTTCTTTTTAAGCTGGCTTCGGCCTCAGTTGTAAGCACTTGTAATTTTTATTTTCTTCTTCATTTTTCAAAGTTCTCTCTGAAACTCCTTCAGACATGAACTCTCCTGTAAATATCTGAGTGCTTTGCTTTCAATAAACAAGACTTTTTGTAAGTATTTTATCTTAAACTTATTTTTTTTTTGTATATGCATTATTTTCTTGCATTAGTCTGAAAAGTTGGTTTTAGGATTCCTTTTTTATTCAAATCCTCTTGTGCTTCTCTATCTATCTTCTGTTCTGCTATTGGCAACCATGGACATATGTTGAAGTTTATTGAAAACTTCTTGCTCAGAAAGACCATCAATGTTCCATTCATACAATTTATCAGAAGAGACAGAGAACTGGTTCTGAAAATGTCTTTCTTCAAATTGTATATCTGGAGGAGTTGGTCGAGTATACCAATTTTTGGTAAGACTAGTAGGGGTGACTTGATGAGTTAACCGGGTAACTCTGGGTTCAACTGCAAGTTCAGTGAAGGCTTTCTCAATTTTTGAGATATTACTGTCAACTGATTCTAGACTCTGAATAGATTCTTGGTCAGAATCCGGATTAATGGAGTCAAGAACTTTAACTCCAGATGGTTCACTCTTTACAGGGGTCAATTGTTCCAGCATTTCTTCAATTTTTTGAAGGGTAGGCTTGATCTTAAGAGTCGGATTTGTCTTAAGATCCTGGAATTGGACTAATGGTTTTTCAACTTTCATGTTGGATTTTTCAGAGGAAGGGATTAGCTTGTCTACTTTAGTTTCAATTTTGTCAAGCTGTTTTCCTATGACAATTAGACTCTGATTGGTGTAGTTGCTTTGTTCTATCAATTTCTTTTCTGAATCTAGTTTTTCAGGGAATTTGAAAGGACAAGCAGGGATCTGAGTTTTCTTAAAATTGATTAAGATGGACTCAGCAGGGGGATGACT

GGCAACTACTTGTGTGTCATTAGGTAAAGACCAGGTTTCTTTGGTAATTACCTTCAATTTTTTAGACATATATATATATATATATATATATATAGTCCTAGTCCAATCAAACATACACGAAACGCTGTACAAGTGTTATCCAGCTTATGCTAGGCCAAGCCAATCTAAGAAGTACCAATATTTAATCCAGTACATGACAATCCCCCGTACCAAACAACCCCTAATATGTAATGAATGAACAGCCTAGGGAGGAACAACATACAAAAGATGGGGTAGTGTAGGATACGGCTCTTATCTTAATTACCATAAGGTAAAAATGATCAATTCTCTTTCTGATTTGTTTGGTTTCTATAAATAAAGGGTGTCGTTAAACGAACCCTAAAATTTATTGAGTGCACTCTATAATTGTAATAACCCAAAACAAAATATCTAAAAAGAAATAAAATATCTAAAGAGTAGGATTAATATCTTCTGGAAAAAAGACAAGTTTGCCCTCGCATATTTTAATGAGGAAAATTTTGACTTTTTAATCGAGAAAGAATTTGGGAATTCCACTTACGCCATTGCGTAGAGCACGGCGAAATGAGTCCGTAGACACGGAGTAGACCCGAATCGGAGTTGTAACGAAGAAGTTACGATCAAAATACTGCGAAGGGCAAAACGGTAATTTGGAAAAAAGTCAGAATTTTTATCTCCTCTCTCCTCTCTCCCGTCAGCTCTCTCTCTCTCCCCCTCCCGCGCGATTCCTTGACCTTTCTCCCTTCCGATCGAGACAGCGGTCACCACAGGCCGTCTCGATCCCCGCCGTTGGTCCCAATAGCTCCGCCACTCACCCGCCGTTCTTTTCCGACCATTATCAGCCGCGGGGACGCCGGAACCTGGCCAGAATCTGCCATTGTTGCCGACTTTCGTGTTTGAACTTCCAGCTCGATTTTCTCCTTCAATTCTCCACCAAATCGATCGAGTAAGGTATGGTTTCTCAGCTATTTTTCGCGTTCTA

>Chr1-06; OZ022206.1; 13668674-13671197; rev; B;

CCAAGCTGTAAAGAAAAGAATGGATCTTACTTTTTCTTTGGGATAAATCATACACATTATTAGGATAGCCTCATCCCAGGGGGGGGAAGTCCATTCACTTCCAGGATAGCCTCATCTCAACTTGTACATGAGAAAATCATATTCCCTTACATGAAACAGAGAATACGTATTACGTACTGAGGAAACTTTGGCCTTTTGGTTTTTGTTTTTAAAACTAGGGTGACAGTTGGAAGATTTGGGAAAATCAGAATAACTGAGAGGGAGGTAACGAACAACCCAGGCTTATTGAGAGGCTTACTTTTAGAAATCAACAACCACAATCTTGCTTCCTCAGCACAGCACAATCGTTATGTTTTCCACACTACCCTCTAAAGTCCTATTTATAACTTAGGTTACAATTCCACTGAAGTAATAAGAAAATAAAATCCCAAAACACTACCATATGTAAATAGGAAATCCAATGACTTGGTGAGCAAGTTAGCAACCTAACTGCAACTTTCTCCGGAACTGTCTCTGTACATGTATCTCTCTCTTCCGTTGTTTCTGAATAGCAACCCCCAAATCTTACTTCTACTGAGATGAGGGAGATATCTGTCTCTCTATTTTTTTCACTCTTGTGTTTTAGACTGCTCTTTGTTTCAGCTGAGTTCACTTTGCAGTTCATGGGTTCACTCGAATTGAAACAACATACAGCTCATTGTGAGAGACAGTCTGTAGAAGTCAACGTTTTTTTTAATAAAAGCACGCTTATGCTTTTCTCATTCCAACTCATTTGCTTATATTTGCTTGAAATTAACTACTATATCCAAGAAATCAACTGATTAATAATGCAAAGCGCAAACCGATGAGGAACAAAGATCAACGAACAGAATAACTCTTCTCTCTCAAAGTACCTTTGTTATTACAAGAAAGGTTTTCAAATCAAACTCCAATGCAACCAAATGGAGACTAACAGTTATATAAAAATAGAATATGAAACTAGTAATGAAATTGACTACAA

AGATTGGAATATCCCCAAAGTCCCTAGTCAAGAAATTTACAAGAAAAAAATGGTCACTCTCAAGTTTCAAAAGTACAACCCATGTGAAAACAATCGAACAAGTCTATGCCTTAAGCAAAGAACATGAAACATGTCAATTGTTAAACCTAAAGTCAATAAAGAAACATAAGAGTGATGGAAGCAATTTCCTTCACATCGGCCTTGTCCAAGTTGCAGTCAAACCCCTTACCGGATTAGGACTTAATACCTTCATTTTACTTCGCCTACGTGATGCCAGATTTACCAACTTTAGTGATAGTACCCTAGGAATAATGGAGTCAAGTCTGTTTAATGGCCCAATTCACTTTGATTGTTACCCAGATTTTACAGTAAGCCTTAGTGATCCTCTATATTGAAAACTCTAACATTAAATATTAAAACTTCAGGGTGTAAAGTCTTACCAGGTACCCAATCTTTAGCTTTAGTCTATAAAATATATTATAAAGTCAGAGGAACCAATATGAATTTCCAAGCCCTTAACAAAAGTCCAAAAGACCAAACCTTGCTCCTTCAAAGTAGTAAAGCTGATGCTAATATAAGAGTTCCCCATACAATCAAATGGTCAAAAGTCACTCTACCTCTAGATTGGACACTAACCATAGAAAACCAACCTACTCCCATTCAACATAGTTTAAATAATTTAGATTACATCCAACAATACTTAGATGGCACAGTTAAAATCAATTTTGGAAATCAACCCCCTAAAAAGTCTTCAGTCCAACTATACCAACTCCCAACACCTAGTCAGTCCTTAAGACATACGCCTGCTTGACATTCCTTTGCAGCTTCCTCCTCTACTCTAGAATGAGACCTAGAGTTAGAAAAAAACTTTAATTGATTTAAAATTAGAATTTTTAAGAAAAACTTCCCAAGTCAACCATCTGTGTTATGGTAAGAAACGAGTTAAAGAAGATGATAAATCAGATTCTCCAGATTCCCCCACACAAACTAATTTTGAAGCTAAAACCATCCCTGTAGATAGCCAGTTACGAACCCTCAATAAAGAATTCAAAATTGATTGGAAAATCTTAGACAGTCATTTAAATGCCCCAGAAAATCTAGTCAGAAGAAATTATTATCACACAGTCTATGCAGATAAAGAACAAAGACAACAAATCTTTAATAAGTGGAAATTTTTTATGAAAAGCACTTAGTAGAAATTTCTACCTTGATTTTGTTGAAAGTCACTACATGCCCAGAGAGTTAAAAACTTTAACACAAGAAAAATGGAAAAGAGAAGATAACACTGAAGTCATAGCCAGTCACCCCTCGGTAGAAACAATAATTATTGGTCATAAAAATACTAAGATCACAGCCAGCCCCTTTAAAATTCCTAAACAAAATGATAAAAAAATAGTTGAGCAAAATAATTATACAAACCAGAGTCTAATAGTCATTGGGAAACAATTAGATAAGATCGATAGAAACGAAAATTGACAAAATAGTTCCCCAACAAATGTCCCTCAAGTCTCTGAAAGAGAACCTCTAGTCAAATTCCAAGACTTTAAAGTCAAGCCCCACCTTTAAGACAAAGCCCACAATGAAAAAAAAATAGAAGAAATGCTTGAACAACTAACACCAGTTAAAGAAAAACATGAGCCCTCTACTCTCAAAGTCCTTGAATCCTCTATTCTAGTCAATTCCTCTGACAATGAGTCCATAACTAATAATGAAACAGATTTCAGTACTATATCATAAATTGAAAATACCTTTAAAAATTTGGAAATAGATAGTGATTTGAAGCTTAATAGATTAAGAAACAAAGTTAATCCTACCAGTCTAACAAAAAATTAGTACCCCAAATCAACTCCTCCTGACGTCCAGTTTGAAGAAAGAAATTCCCAGAGTCAATTCTCATTTTCCTCAGATAAATTATACGAATGGAACATTGATGATGGCCTTTTTGAACAAGAAATCCTAAATAAACTCCAACATATGTCAATGGTAGCCAACAGTTCCATAACCAATCACAACTTCAGAGAGTCAAAAATCATTCCTCTTCTAGAAACAAAATTCACCAGAACCTTACGTTCTTGGTGGGAAAAACATTTAACTGAAGAGTCAAAACGACAAATCATACACGTAGTAAAACTAAATGAAGGTGGTCACCCTATTTTTGATGAACGTTTAGGTACAGGAATAGAAGACGGAGTCAATACCTTATTATACACTATTATTGAACATTTCATTGGAACACCTAGTAACACAACAGCTAGAATTCATGATCACCTTAAGTGATTTCAGATGGTATAAAGATGTCTTCATTTCTAGAGTCATCCTTAGAGACAATAGTAATAAGCCTTTTTGGAAAGAAAAATTTATTAATGGTCTTCCCAATCTGTTTGCCCATAAAATACGCACAGTCTTAAGTAATTATCAAGGCCATATATATTTTGATAGTTTAACTTATGGAAATATTATTAGTACAATTAATCTGTCCTTGTCTAGACATTTCATATTGTATTCATTGAGTCATTTCACAAAT

TGAAGCAGTGGAAGCAGTACTAAATTTCAAATGATAAGCTGGGGAGCTTTCCAAGTCAGTGCTAGTTTTTAACAGTAAGGGTTGTAAGATTCTTGCTGAGGTGGGTCGGAGCAGTGCTTCTTTTGTGTTGAAAGGGCTGTGGAGCAATACCTATTGATTGTATCAAGAACTCGTCTTCAGTGATATAGCCTTTATTGAGTAACTTGATAGCCTAAAATTGAAACTGTTTAAAGTCTAATAATTTCCTACTTGATATTATTGTTCTGCATATAATGTAAGTACAAAAGATTGGGGAGGGAGGGTTTACACAAATAGGGGAGGTGGAATGGGCTTAACCAACTGAGCTATACCCTATTCCTCACATATAAAACCTAACTGGAGAATTTTATTACCACCCCCAATCCATTGAAGGCACTTATCTTTCTAAGAACATCCCTTACAGACTTTTAACTGTATGTATTAAGTGAAACCACCCGCTCAACTTACCTTCATCAGAGTGACAAAGCTCATCTCTGTCCAATTAAAACATACTACATCTAATTATTTCAAATACATTCAATCTAATGTGGCTCTTTTTTTTTAAGCAGTGTAGCATTGTTGTCAAATTCAAGTTGTTTCTTTCGAAGTTCTCTATCCAAATGGAGCCTAATTCTTCCCATTAAAAAAAAAATTCCACTCCATCCACTTCGCCTTAAAATCTCTAGTTGTTTCCTTCCATCGAAAAATCTCCAACCACGTCATTAACCTTAACCATTCCAAGCACTTGGAAGCACCCTCACCAAGTTGCATTGTGGAAGGTCAAAGCAGAATCATTCTGCAACCATCTTGCTTTCTGTTTCGATGCATTGTTTAGGCTCTGCTTGGTTTCATTTTCAAAGTTACTTTCTGTTGTTTCCTTCCAAGTTATGATTCTGATTGAAGTTAACTTCCTTGTCACAAGGCATTATTCAAGGGTCCTTAACACTAATGTCGCACTGACAACGATCACATACAAAAAATT

>Chr1-07; OZ022206.1; 13768949-13770240; rev; B;

TCCCCGAGCCTGGCCAGTTCTCCTTTGAATCCCCAAGCAAGACTGACTTGTTCAGTGGCAAGTGCAGCTACCTTGGTCAGTATTCCCTCGGCAGCAAAAGTAAGCACAAACTCAGCGGCCATATTTCCCCTTTGATATGTACGCTCAAGACAAATAAAGAGAGGAATGCTTATTTAATTAAGGAAACTGAAAGACGCATATAGTTTGTTGTCTCTGCTATGAACGAATTTGTAGAGGCAGAAGGAATTTAGGTTTACCTAAAGAACACTCTACTTAACGTATTTAGAAGGTTAAGAATTTCAGCTTTCAACTTTAGCAAGACAGAGATGAGCACCAAGCTGTGAAGCAAAGGATAGCCTCAATCCCACAGATGCTTCGGCTTTTACACGAGAAAATCAGATTCCTCAATCAAATGAATAAGAGAATACATAGTGGCCTTTTCTTTTTTAAAAACTAGGGTGACAGTTGGAAGATTTCGGAAATGTAACGAACAAGCAAGGCTTATTGAGAATCTTAGTTTCAGAAATCAACGGCTACAATCTTGCCCCCTAAGCACATCACCATCGTTATGTTTTGCACATGACCCTCTAAGGTCCTGTTTATACCTTGGGTTACAATTAGACCAAAGTAAGAAACTGAACCCCAAAGAAATAGACAAAACACCACCCAATGTAATAGGAAATCCGATGACTCGGTTAGCAACCCGATACAACTTTCTCCTGAACTCTCTCTGTACATGTACTTTCTCTTCCGTAATAGCGACCCCAAAATTTTTCTTGTATTTTGTTCACTCTTGTGCTTTAGACTACTCTATGTTTCAGCTGAGTTCGCTTGTTGCAGTCCATGGGTTCAGTTTAAACTGTAGATGTTAATGCTTTTTAATAAATTCATGTTACATCTGATGTGTCAATGCAAAAGCACAGTAAGGAATTTATCACATTTAGCTGCTAGTCCGAGAGCACAAACCAAACGCAAGAAAAGTGATAGAAATGAAAACTCA

AGCAAACACAGAGAAATAAGTACCATATTTAAGTAGTTCACCCAAAATATGCATCTATGGGCAATGCTAATGATAATTCACTTAATCAAAAGAGTACACGAATATGACAACAAAGAACAAATAGGTTCTCCCGCAGTGGAACTAAATTAATCTCTCCCCTCATCCCATTTTAATTCCCAGTCACCCCTCGGTAGAAACAATAATTATTGGTCATAAAAATACTAAGATCACAGCCAGCCCCTTTAAAATTCCTGAACAAAATGATAAAAAAAAATAGTTGAGCAAAATAATTATACAAACCAGAGTCTCATAGCCATTGGGAAACAATTAGATAAGATAGAAACGAAAATTGACAAAATAGTTCCCCAACAAATGTCCCTCAAGTCTCTGAAAGAGAACCTCTAGTCAAATTCCAAGACTTTAAAGTCAAGCCCCACCTTTAAGACAAAGCCCACAATGAAAAAAAATAGAAGAAATGCTTGAACAACTAACACCAGTTAAAGAAAAACATGAGCCCTCTACTCTCAAAGTCCCTGACAATGAGTCCATCACTAATAATGAAACAGATTTCAGTACTACATCATAAATTGAAAATACCTTTAAAAACTTGGAAATACATAGTGATTTGAAGCTTAATAGATTGAGAAACAAAGTTAATCCTGCCAGTCTAACAAAAAATTGGTACCCCAAACCAACTCCTCCTAACATCCAGTTTGAAGAAAGAAATTCCCAGAGTCAATTCTCAGTTTCCTCAGATAAATTATACGAATGGAACATTGATGATGGCCTTTTTGAACAAGAAATCCTAAATAAACTCCACCATATGTCAATGGTAGCCAACAGTTACATAACCAATCACAACTTCAGAGAGTCAAAAATCATTCCTCTTCTAGAAATAGGATTCACCGGAACCTTACGTTCTTGGTGGGAAAAACATTTAACTGAAGAGTCAAAACGACAAATCATACACGCAGTAAAACTAAATGAAGAAGGTCACCCTATTTTTGATGAATGTTTAGGTACAGGAATAGAAGAGGAAGGTCACCCTAGTAACACAACAGCTAGAATTCATGATCACCTTAATAATCTTAGATGTCCCAGATGGTATAAAGATGTCTTCATTTCTAGAGTCATCCTTAGAGACAATAGTAATCAGCCTTTTTGGAAAGAAAAATTTATTAATGGTCTCCCCAATCTGTTTGCCCATAAAATACGCACAGTCTTAAGTAATTATCAAGGCCATATATATTTTGATAGTTTAACTTATGGAAATATTATTAGTACAATTAATC

CGTCCTTGTCTAGACATTTCATATTGTATTCATTGAGTCATTTCACAAATTGAAGCAGTGGAAGCAGTACTAAATTTCAAATGATAAGCTGCGGAGCTTTCCAAGTCAGTGCTAGTTTTTAACAGTATGGGTTGTAAGATTCTTGCTGAGGTGGGTCGGAGCAGTGCTTCTTTTGTGTTGAAAGGGCTGTGGAGCAACACCTATTGATTGTATCAAGAACTCGTCTTCAGTGATATACCCTTTATTGAGTAACTTGATAGCCTAAAATTGAAACTGTTTTAAGTCTAATAATTTCCTACTTGATATTATTGTTCTGCATATAATGTAATAAGTACAAAAGATTGGGGGAGGGAGGATTTACACAAATATTCACATGGTGCTTGGGGGTTTCAAACCTCTGTTCACATGAGTGGAGGTGGAATGGGCTTAACCAACTGAGCTATACCCAAAAGCACCCACTTTGTCATTCCTCACATATAAAAACCCAACTGGCGAATTTCATTACCACCCCAAATCCATTGAAGGCACTTATCTTTCTAAGAACATCCCTTACAGACTTTTAACTGTATGTATTAAGTGAAATCACCCGCTCAACTTACCTTCATCAGAGTGACAAAGCTCATCTCTGTCCAATTAAAACATACTACATCTAATTATTTCAAATACATTCAATCTAATGTGGCTTTTGTTTTTTAAGCAGTGTAGCATTGTTGTCAAATTCAAGTTGTTTCTTTCGAAGTTCTCTATCCAAATGGAGCCTAATTCTTCCCATTAAAAAAAAAATTCCACTCCATCCACTTCGCCTTAAAATCTCTAGTTGTTTCCTTCCATCGAAAAATCTCCAACCGCGTCATTAACCTTAACCATTCCAAGCACTTGGAAGCACCCTCACCAAGTTGCATTGTGGAAGGTCAGAGCAGAATCATTCTGCAACCATCTTGCTTTCTGTTTCGATGCATTGTTTAGGCTCTGCTTGGTTTCATTTTCAAAGTTACTTTCT

>Chr1-08; OZ022206.1; 13834329-13835617; rev; B;

ATCCCCGAGCCTGGCCAGTTCTCCTTTGAATCCCCAAGCAAGACTGACTTGTTCAGTGGCAAGTGCAGCTACCTTGGTCAGTATTCCCTCGGCAGCAAAAGTAAGCACAAACTCAGCGGCCATATTTCCCCTTTGATATGTACGCTCAAGACAAATAAAGAGAGGAATGCTTATTTAATTAAGGAAACTGAAAGACGCATATAGTTTGTTGTCTCTGCTATGAACGAATTTGTAGAGGCAGAAGGAATTTAGGTTTACCTAAAGAACACTCTACTTAACGTATTTAGAAGGTTAAGAATTTCAGCTTTCAACTTTAGCAAGACAGAGATGAGCACCAAGCTGTGAAGCAAAGGATAGCCTCAATCCCACAGATGCTTCGGCTTTTACACGAGAAAATCAGATTCCTCAATCAAATGAATAAGAGAATACATAGTGGCCTTTTCTTTTTTAAAAACTAGGGTGACAGTTGGAAGATTTCGGAAATGTAACGAACAAGCAAGGCTTATTGAGAATCTTAGTTTCAGAAATCAACGGCTACAATCTTGCCCATAAGCACATCACCATCGTTATGTTTTGCACATGACCCTCTAAGGTCCTGTTTATACCTTGGGTTACAATTAGACCAAAGTAAGAAACTGAACCCCAAAGAAATAGACAAAACACCACCCAATGTAATAGGAAATCCGATGACTCGGTTAGCAACCCGATACAACTTTCTCCTGAACTCTCTCTGTACATGTACTTTCTCTTCCGTAATAGCGACCCCAAAATTTTTCTTGTATTTTGTTCACTCTTGTGCTTTAGACTACTCTATGTTTCAGCTGAGTTCGCTTGTTGCAGTCCATGGGTTCAGTTTAAACTGTAGATGTTAATGCTTTTTAATAAATTCATGTTACATCTGATGTGTCAATGCAAAAGCACAGTAAGGAATTTATCACATTTAGCTGCTAGTCCGAGAGCACAAACCAAACGCAAGAAAAGTGATAGAAATGAAAACTCA

AGCAAACACAGAGAAATAAGTACCATATTTAAGTAGTTCACCCAAAATATGCATCTATGGGCAATGCTAATGATAATTCACTTAATCAAAAGAGTACACGAATATGACAACAAAGAACAAATAGGTTCTCCCGCAGTGGAACTAAATTAATCTCTCCCCTCATCCCATTTTAATTCCCAGTCACCCCTCGGTAGAAACAATAATTATTGGTCATAAAAATACTAAGATCACAGCCAGCCCCTTTAAAATTCCTGAACAAAATGATAAAAAAAAATAGTTGAGCAAAATAATTATACAAACCAGAGTCTCATAGCCATTGGGAAACAATTAGATAAGATAGAAACGAAAATTGACAAAATAGTTCCCCAACAAATGTCCCTCAAGTCTCTGAAAGAGAACCTCTAGTCAAATTCCAAGACTTTAAAGTCAAGCCCCACCTTTAAGACAAAGCCCACAATGAAAAAAAATAGAAGAAATGCTTGAACAACTAACACCAGTTAAAGAAAAACATGAGCCCTCTACTCTCAAAGTCCCTGACAATGAGTCCATCACTAATAATGAAACAGATTTCAGTACTACATCATAAATTGAAAATACCTTTAAAAACTTGGAAATACATAGTGATTTGAAGCTTAATAGATTGAGAAACAAAGTTAATCCTGCCAGTCTAACAAAAAATTGGTACCCCAAACCAACTCCTCCTAACATCCAGTTTGAAGAAAGAAATTCCCAGAGTCAATTCTCAGTTTCCTCAGATAAATTATACGAATGGAACATTGATGATGGCCTTTTTGAACAAGAAATCCTAAATAAACCACCATATGTCAATGGTAGCCAACAGTTACATAACCAATCACAACTTCAGAGAGTCAAAAATCATTCCTCTTCTAGAAATAGGATTCACCGGAACCTTACGTTCTTGGTGGGAAAAACATTTAACTGAAGAGTCAAAACGACAAATCATACACGCAGTAAAACTAAATGAAGAAGGTCACCCTATTTTTGATGAATGTTTAGGTACAGGAATAGAAGAGGAAGGTCACCCTAGTAACACAACAGCTAGAATTCATGATCACCTTAATAATCTTAGATGTCCAGATGGTATAAAGATGTCTTCATTTCTAGAGTCATCCTTAGAGACAATAGTAATCAGCCTTTTTGGAAAGAAAAATTTATTAATGGTCTCCCCAATCTGTTTGCCCATAAAATACGCACAGTCTTAAGTAATTATCAAGGCCATATATATTTTGATAGTTTAACTTATGGAAATATTATTAGTACAATTAATC

CGTCCTTGTCTAGACATTTCATATTGTATTCATTGAGTCATTTCACAAATTGAAGCAGTGGAAGCAGTACTAAATTTCAAATGATAAGCTGCGGAGCTTTCCAAGTCAGTGCTAGTTTTTAACAGTATGGGTTGTAAGATTCTTGCTGAGGTGGGTCGGAGCAGTGCTTCTTTTGTGTTGAAAGGGCTGTGGAGCAACACCTATTGATTGTATCAAGAACTCGTCTTCAGTGATATACCCTTTATTGAGTAACTTGATAGCCTAAAATTGAAACTGTTTTAAGTCTAATAATTTCCTACTTGATATTATTGTTCTGCATATAATGTAATAAGTACAAGAGATTGGGGGAGGGAGGATTTACACAAATATTCACATGGTGCTTGGGGGTTTCAAACCTCTGTTCACATGAGTGGAGGTGGAATGGGCTTAACCAACTGAGCTATACCCAAAAGCACCCACTTTGTCATTCCTCACATATAAAAACCCAACTGGCGAATTTCATTACCACCCCAAATCCATTGAAGGCACTTATCTTTCTAAGAACATCCCTTACAGACTTTTAACTGTATGTATTAAGTGAAATCACCCGCTCAACTTACCTTCATCAGAGTGACAAAGCTCATCTCTGTCCAATTAAAACATACTACATCTAATTATTTCAAATACATTCAATCTAATGTGGCTTTTGTTTTTTAAGCAGTGTAGCATTGTTGTCAAATTCAAGTTGTTTCTTTCGAAGTTCTCTATCCAAATGGAGCCTAATTCTTCCCATTAAAAAAAAAATTCCACTCCATCCACTTCGCCTTAAAATCTCTAGTTGTTTCCTTCCATCGAAAAATCTCCAACCGCGTCATTAACCTTAACCATTCCAAGCACTTGGAAGCACCCTCACCAAGTTGCATTGTGGAAGGTCAGAGCAGAATCATTCTGCAACCATCTTGCTTTCTGTTTCGATGCATTGTTTAGGCTCTGCTTGGTTTCATTTTCAAAGTTACTTTCT

>Chr1-09; OZ022206.1; 14241920-14242523; dir; B;

TTTAACGGCGTAGTGTTTTAGTTTCGTCGTTGTATGTCTATCTTGCGGCCTTGTTCTTTCAATATGTGTCATATTTTTCCTTTTTAACGACGGTGATTTGTTTGTTGGTCGTCTATTCTCATCCTCGACATTTTTAAAACTTAGCAGACTAAACACGTCTGTTTTTATTTGTTTTCGACGTTGTTTTATTTAACAACGTCTAATATTTATCGTCGTTGTTTGTTTCTGAGATAGGACGTATAAATTTTGTAATACGACTCCATATATTTGTAACCGACGTTGTTTCGTTTTTCAACGTCTACTCTATAAATACATACGTCGTAAATAATGACACTGACAACTATTTCCACGTCATCTTTTATAGAAAAATCGAAGTGGAAAATTTATTTTACGACGGCTGTTTTTATATAGGCGACGTAGTAGGAATAAATAACGTCGTCTTCTATGTATTTAAAGACGTCATATTTTTTATTGTTGAAAATGATATTTAACGGCGTCTTATTTTAATTTTCGTCGTTGTATGTCTATCTTGCGGCCTTGTTCTGTTAATATGTGTCATATTTTTCCTTTTTTACGACGGTTTGTTTAGAGAGTTGGTCGTCTATTCTCATCCCCGACTGCTTTTTTAGATCTTTTTGCAGTACAAAGACGTCGTTTTTTATTTGTTGATGACGTTGTATATTTTAACAACGACGGTGATTTAGCGTCGTGATTTATGAGGGAAAAGATGACGGTGGATTCCACGACCATTGAAAAACCGAATACACGACGGTGATTTACCGTCGTCTTTTTGCTTTTTTGTAGTAGTGGCAATATCCCAGCAAGAACAGGAACGGCTCAAGACTTCTGCTCTCTCTCAGAACCATACTATTATCAGTTCTTTCGAAATGACAGTAAAAATGAGTCAGAAATTCAGACTCTAGTTCTAAATTTCTGACTCATTTTTACTTCCTTTTTAGGAGAAAATGGTCCAATGCACCGAAGGATAAGAAAGTAGCCA

CTTTAAGAAAAGAGCCGACAAGAGTAAAGTAAAATGTTTTAAATGTCAAAAGTTTGGTCACTATGCTAAGGAGTGTAAAGTAAAAGATGCTATCCGACAATTACAAATTACAGAAGAGGAAAAAGCTAATCTAATTAAAGTCCTTGAATTACGTAACTCTGAGTCAAGTGAAAATGAAACCATGGTTAGCAATATTGAGTCAGATAACTATGAGTCCTCGAAATCCCAAAATAGTTCTCCCCGAGTCCAATTAGGATGTAAAGATAAATGCTGTAGCACTATAAAATCAATCTCTGTTGTAACTAAGCAAGAAGAACAAGAAGAGTTGTTAATTGATTTAATTAGTAAAATAGAAAACCTTGAGTTCAAATCTGAGTACCTAAAAAACTTAGGAAAATTATTACCCAGGAAACAAGTACGAGTCATCTGAGTCCCTCAATAAGTCTCAATACTACTTTAGAACGGTTTAATAAGAAAAAAGAATTGTCTTTACAAGATTTACAGTATGAGATAAAAATAATTAAGAAAGAAATAATTGAGTTAAAACAATTAAGTTACAAGTTACAAACTGAAAATTATGAAATTAAGCAGAGTTAACTGCCTT

GTTAGAAAAAGAGTTTAATTGCAAATTTTTTATAGTATTTTCGTATTAGATTATGCGAGGCCCCAATTTAAGTTTCGCACAGGGCCCTTAAAATCTCAGGTACGGCCCTGATCATCAATAAACGTCATAAACCACTTAAAACAGCTAATAGTGGGAACTCGTGATGGTCCCCAAACATCAGAATGGACAATCATGAAAGGCAAAGAACTTTTATTCAATCTTAATGGGTAAGAAATACGATGGCTTTTGGCCAAAATGCAAGTTTCACAATGAAAGTCTGATTCAGCAAACTGGGAAACAAATTTGGAAATAAAAGTTTCAGATAACCAAAAGAAGCATGCCCTAATCATCTATGCCATAGCCAAATTTTCTTCATCCTGATAGACTCATCTCCTCTCACATGATGTGTTTGACTCAATCTTGTACTGCTGTCTTCTGTCAAGTCCAGATAATACAACTTTCCCCTCCTAGTACCACATCCAATACGGTCTTGGTGAGAAGATCCTGAAATAGACAATACAATGGCCAAAAACACACGGTACAATTAAGGGAATCAATGATTTGAGGAACAGATAACAAGTCATAGTCGAGTGAAGGGACAACTAAGACATGATCAAGACTTAATGAAGATGTAAGAGATAAAGACCCTTCTCCCATAACTTGGGAGGTGGTTCCATTGGCACTGGTAATGTGTGTTTGGCTTGATGGTTTGGTAGAGTGCAACCCAATAAAACTAGATGTCATGTGATCAGTAGCGCTAGTGTCAATAATCCATGATCTATTACAAGTAAGTGAAGATGTGCAAGAGGTACCTGAGGTGGCCAAGGCCTTGGAGCGAGATGTACCTTATGGAGGTTTGATGCAGTCTCCTCGGATGACTGAGCGGATGCAAATGAGCTCTGGTAGAATCCTTTTTTCGCCCTTTGTGGACCCATCCTTCTGGATAACCAATGATCTCATAACAGCCCTTAATTGTGTGGTTATCTAAACCACATTTGGT

>Chr1-10; OZ022206.1; 18877447-18919859; rev; C;

TCATCTTCAAGCTCCAATTTTCAAATTTTTTCCTTCGGATTCTAGTTATGAAATCTTTCTTATACTTAAATCATTGGTTTTCTTTTTAGATGTTGGTTTGTTTTGTTCTTTCTTCATCCTTTCTTTGGTAGACATGCATTAATGACAATTATTAATCAACATGACAGTAAATAGCAGTGTTAATACCCTAGATTGGAACATTACTGCCACTAATCAAAATGACAGTAAATAGTAGTGTTAATACACTGGACTGGAACATTACAGTCATTAATCAACATGACAGTAAATAGCAATGTTAATACCATAGACTGGAACATTATTACAGTCATTAATCAACGTGACAGTAAACTAGCAGTAACATACAACCATTAATCAACGTGAAAGTAAATGTGTGGTTTTTAAATGTCTTACATTTGAACCATTTTCTGCTTGAAATTCTCCAGCTTTTTGTGTTGAATGGTTGCTTCTCTTTATTTTTTGTGATTTTGTAGGTGGTCGTTAGTTGTAGGTGTGGTGGTGTTCTTTTATTTTATTTTATAAAATTGGGTTGGGCTTGTTTTGGGTTTTTTTGTGCTGGGCTTCTTTCAATGGCAGTCACATCATTTATAGGAACTTAAACACTAATTTCTTATGTAATAAATGAGTTTTGGGTGTGTTTCTAAAGTGCATTCCATGTTTTTTTAATAATAATTTCACCACTATTTTGGGTATATTAATAAAGACCCCCCCTTTTTTTTTCTTCCAATTATTCCTTAAAATTTGTCTATAAAAGTAGTTAAGGGAGCTTATTGGCGGATGCTCCAAGTCCTTAGAAGTCATTTTATAAAAGGGTAAAATTTATTTTTAAAATTTTGAAAATAAAATAAGTGAGAAAGTACGATAGAGGTGGAAATAGCAACACTCATATATATATATATATATATATATATATATAAAGGTCACAAGAATGCCAAGGTTATAGCCAGCCCTTACAAAGTTGCTGAGTCAAAAGATTATTTAG

ATAAAAAGGTCATAGAACAAAATAATTGCACCAACATAAGCCTAATAGTCATCGGTAAACAATTAGATAAAATTGAAACCAAAATTGATAAGTTAATCCCTAGCGAGATTTCCTTAAAACCAAAGAAAGAAAAACCCTTAGTCAAATTTCATGAGTTAAAACCGGAGTTTGCTCTTAAGGTTAATTCAACCATGAAGAAAATTGAAGAAATGTTTCAGGAGTTAACCCCGGTTAAGCCAAAACCAAATAAGGATAAAGACAAAGTAAAAGACGAAGAAACTTCAGTTAAAGTTTTACAAGTTGAAGTTAATTCTGATAATGAGTCATCCATGTCTTTATAATCAACTTCTAGTCAAGAATCAGAAACTAGTAATATCTCTAAAATTGAACAAGCCTTTAGTAACCTACAAGTTAACCGGAATTCCCAGAATCTTAAAGTTAGTCGTTTAACAAGTAAAGTTAACCGCACAAGTCTTACAAAAAGTTGGTACCCTAAACCTACGCTGTAATAACCAAAAACAAAATATCTAAAAATTAGTATTAATTTATTCTAGCAAAAAGACGATTTTGCCATTGCATTATTTAATAGGGAAAAAGTTGACTTTTTGACCGAGAAAAAATTTGACAATTCCGCTTACGCCGTTGCGTAGAGCATGACGAAACGAGTCCGTAGATACGGAGTAAACTCGAATCGGAGTTGTAACGAAGAAAATACGGTCAAAATACCGCGAAGGACAAAACGATAATTTGGGAAAAAGTCAAATTTTTATCCCTTTTCTCCTCTCTCTCTCTCCTCCCTCCCGTCGTGCCGCAGCCCCTTCCCCTTCCATCCTTTCCAGCCGACGCCACACGCCGTCCCGATCTCCGGCACAGGTACCAACGAAACCACCTCGTCGCCGCCGTTCTTCCCCGACCCGTCGCCGCACTTGGGCCGCCCGGAACTGGCCGAAAAATCAAGTTTTCCGACCGGTTTTCACTTGAATTTCAGACCTCTCGTTCTCCTTCGTTTTCAAGGCACCAGAGGCCAAACTGAGCCACAGAAGGGACCTTCAAAAGGTCCAGCTAGCTCGGACCAGCAGTGAGTGGACTTTTCTTTTCTAAACGATATTTATAATTAAATTTCATTTTTGATCTTGAATAAGTATTATTGCATGTTTTTCCGAGCTTGAATTATATCGTAAACTATCTTTATTTCTATACTGCTTAGTTGAACATGTTAAAGTTATATGGACAGCAGGCTTTGAGATTTATATAACAGCAGACTTTGAGATTTATATAACAGAAATAGCAGCATAATTAAGATTATAGTTATTTATCAGGCTTTCCTACAGTAAATTAGTTTAAGACCACCATGTACCCGTTTTTTTGGTGATTACCCTTAGATGGACCGATGTCTACGGACATCCAGTCTATTTACCGTTCTATCCGTGCACTTGACATCGCCTCACAAGTTTCGGGGACGCTCGGACCGTGAGTTCCAGGATTTGCGGCTCGGCAGACTTGGTGTCCCGAGACCTGCCAAGATTTGCGGCTCGGCTGACTTCGTGTCCCCTAGACCTGGCAGGATTGCGGATCAGGCTGACTTCGGTCCCCTGCATCCTGCCAGAGCGGCTCGAGTCGACTCTGTGTCATCGAGACTTACCGGCGGATCAGGCTGACCATAGTCCCCTGAATCCTGCCAGTTTGCGGCTCGGATAGACTGTGTGTCGCCTAGACCTGCCGGGGGAGTTGACGGATTTTACAGGGGTGCACCTAGGTGGTAGTTTTAAAGGACTTCCAGTATTTTTATATCATTATTGCTTTCAGTCATTTTATATCAGTTTCTTGATATTCGTGCCCGTTTTATATCTGCATACATACCTATACATATACATGTTGATTTGACTGCCTTATAATTGAATACAGACGAACTTTAGATTTACATCCTTATTTATTTTTAAACGGGGGTTACTATGTTATAAATTGTTTTTAAGAACATTATTTTTGTCCACTCACACCTTCAAACTTGTTTTTCACCCCCAGGCCGTAGAAGTGCGAAGGAATCCACCACGGGCCACTCTTAGCTCCCGCGCCACCAAGAAAAGTAGGATTTTGTTGTAAAAGTTCTTGAAATCTTGTAAATTAGTAGAAAATGCTCTGATATCTAGTTTTAGTGGGAAATTTGAACTGGTGAATGCTTGGTTGTTTTATTCTGGCAGTTGGTGGAAAGTTTATTGCTTGTTTAACAGGTGGAAAAGTTTTGGGTTTGGACAATTTACAGGGGAGACTCTGCCGAATTTTCGGCAGAAGTCTAGGGAAAGTTTTAAACGTAATTGTAACGAGAAGGGTAAAACGGTCATTGTGCCCGACATTCGCCTGGTGTCGGACACGCACAGGACTTGGCTCGAATTCCAAAGTGGAATTGGGATCGGGTCCTGTCGTACGCCTCCAGATGTCCAGTTTGAAGAAAGAACCCAGAGTCATTTCTCAATATCTTCTGATAAGCTATACGAGTGGAATATCGATGGCTTGTCCGAGTAGGAGATACTCAACAAGTTACAACATATGTCTATGGTAGGCAATAGTTACGCCACCAATCATCAATTAAGACAATAAGAAATAGTTTCCCTTTTATAAACAGGCTTCACAGGAACACTTCGTTTCTGGTGGGATAAACATCTCACAGAAGATGCAAAATGAAGAATAATTAATGCTTTTAAAACAGATGAAGATAGTTACCCCATTTTTGATGAACGAATAGGAACAAGTATAGAAGACGGAGTCAATGTCCTTTTATACACAATAATTGAACATTTTATTGGTACACCCAGTAGCACAACAACTAGGATTCATGACCAGTTAAGTAATTTATGATGCCCTAAGCTAAGTGATTTTAGATGGTATAAAGATGTCTTTATTTCTAGAGTTATGTTACGAGATGACAGTAATCAACCATTTTAGAAAGAAAAGTTCATAAACGGTCTTCCTAATTTGTTTGCCCATAAGATTAGAACAGTCTTAAGTAATGAGTCGGGTCAAATTAATTATGATGAGTTAACTTATGGCAATATAATTAGCACAATTAATCAAGTTAGAATGAAAATGTGTGTAGATTTAGAATTTAGTAAATCAGTTCAAGCCGATAGAAAAAATGCTAAATATGAGCTAGGAAATTTCTGTGAACAATACGGTCTGGCCCCAGTTCCCACGTCTAGACAAGTCAAAAGAAGTAAAGGTAGATATTATTCCTGTAAAAAACATTCCTTTAAACCAAATGATGATTTTTATAGTAAAAAGAAGTTTACACCTCGGAAAAGTAAATGGTCAAAAGGGTACTCAAAAGGTTACCGACAAAGTAAAAATAATCAATTTAAGAAAAGTTCAGATAAAACAAAAGTTAAATGTTTTAAATGTAAAAGATTCGGTCATTTTGCTAATGAATGCCAAGTTAAAAACACAATTAAACAGTTAAAATTAAAATCTGATGATGAAGCCAAATTAATTAAAGTTCTTGTGTTAGCCGATACTGAGTCGAATCCTATGTCTTCAACCAGCTCAGTTAATTATGGTTCAGACAGCTCCCAGTCTAGTTCACCAAAAGTCCAGATATGTTGTAAAGATAAGTGGTGCAGAGTCTTAAGCAAATCAATTAGTGTTTTAACTAAGCAAGAAGAACAAGAAGAGTTACTAATCGATTTAATCAGTAAAGTAGAAAATTCAGAATTAAAAGCTGATTATCTAAAAAAACTCAGAAAAATCATAAGCCAAGAAGAACTCAGTCCTAGCCGAGCCCCTACGATTAGTCTTAATACTACTTTAGAAAGATTTAGTAAAAAGAAAGAAGTCACCTTTCAGGATCTCCAGTTAGAAGTTAAAACAATCAAAAAGGAAATCTTAAGTTTAAAACAAATTAGTCAAAAATTACAAACTAAGAATTACGAAATTAAACAGGATTTAGTTAAACTACTAGAGTCTAGAGCAGAGTCTAGTAATCCCTTTGGTAATGCCAGTTTTGGATCAAAACCTGAGTCTTCCGATAGTAACAAAAGTAACAAAGTCATCAAGTTAATTAAGCAAATTCATTTCAGAAAATGGTATTCCCGAGTTAGTATCTTTATTAATGATTTTGAGTTAAACACAGTTGCCTTATTTGATTCAGGAGCCGATTTAAATTGTATTCAAGAAGGTCTTGTTCAAACCCAGTTTTACAAAAAGTCAAAAGAAGTTCTAGGAACAGCATCCGGTAAAGCTTTAAAGTTAAACTATGAATTACCCAAAGCCCACGAATGCCAAAACAAAATATGTTTTAAAACGTCTTTTGTTTTAATTCAGAATATCACAAACGAAGTTATTCTAGGTTTACCCTTTATCTCATTGCTTTACCCTTTCCAAGTAGAATACAATGGAGTTACGTCTAATCATCTAAGTGAACGCGTTAAATTTGAAATCTTGACCAAACCAGAATTACACAATCTCAAGTCACTACAAAAGTCAGCAGTTTCTAAATCGGTCAAATTAATTCAGAATAAAACAAAACAAGTTAATTTTCTTAAAGAAGAAATTAAGTTCAAACGAGTTGAACAACAGTTAGCCGACAAATCACTTCAGTCTCAAATCATTAAGTTTAAAGAAAAATTAAAAAATGGAGTTTGTTCTGATTTACCTAATGCCTTTTGGCATCGAAAACAGCATGTAGTTACCTTACCTTATGTTAAGTCATTTTGTGAAAAGAACATTCCCACTAAGGCCCGTCCGATCCAGATGAGTCAAGAGTTAATGGAATTATGTAAACAGGAAGTCACTGAATTAGTTCAGAAAGGAATAGTTAGAAAAAGCAAGTCACTTTGGTCATGCCCAGCTTTTTATGTCTAGAAAAATGCAGAGTTAGAAAGAGGAGCCCCTCGTTTAGTTATCAATTATAAGCCCTTTAATGCAATCCTTGAATGGATTGGGTATCCAATTCCTAATAAAAGAGACTTAGTTAACAGATTAGAAAAATCAGTTATCTTTAGTAAATTTGATATGAAAAGTGGTTTTTGGCAAATCAAAATCAAAGAATCCGATAGATACAAAACTGCATTCGTAACCCTTTTCGGCCATTACGAAAGGAATGTCATGCTTTTTGGATTAAAAAATGCGCCTAGTGAATTCCAGAACATAATGAATGAAATTTTTAATCAATACAATCAATTTTCCGTAGTTTATATAGATGATGTCCTAATTTTCTCTGAGTCAATTGAGCAACATTGGAAACATTTGAATAAATTTCTTCAAATAGTTAAGCAAAATGGCCTAGTTATCTCAGCCAAGAAAATCAAATTATTTCAAGTACATGTCAGATTTTTAGGATTCAACACTGGTCAATCCAAAATAAGCCCAATCAATCGAGTTATCCAGTTTGCCGATAAATTTCCAGATCAGATTCTTGATAATAGCCAACTCCTGAGATTTTTAGGATCCTTGAATTATGTTTCTGATTTCTATCAAAATTTAAGAAAGTTATGTAAGCGCCTATTTGATTGTCTCCAAAGCAATCCTCCTGCTTGGACTCCGAGTCATACAGAAATAGTTAAACAAATCAAAGTTCATGTTAAGTCACTTCCATGCCTTGGTATTCTTTCAGTTAATTCTTTTAAAATTGTTGAAACTGATGCTTCTGAGTCTGATTATGGTGGTATCCTTAAACAAAGAATTACTTCTGAGCCACCTGAGCAAATTGACCGTTTTCATTCTGGAGTTTGGAGTCAGTCTCAAGCTAATTACAGTACTATTAAAAATGAAAATTTCTCTATAGTATTATGCATTAGTAAATTTCAAGATGATCTATTAAATCAAAATTTTTTAGTCAGAGTTGATTGCAAGTTAGCAAAACATGTTTTAGAACAAGATGTTAAAAATATAGCTAGTAAATAAATTTTTGCACGATGGCAAGCCATTCTGAGTGTTTTTTATTTTGAGTTAGAATATTTGAGAGGTAGTGAAAACAACATTCCTGATTTTTTTTACAATAGAGTTCCTGCAGGACAAGCAAGTAGAGTCATGTCCAGCAAAGAAAAAGACAAGCAGAAAATGCAGTATAACCAGCCGAGTCAACCCAGTCAACCAAGCCGACCCAGAATCCTCCCAGCTACCATGATGCCAGTCAAACAAGGGTCATCTTCCCAAATTGTGCCCTTCCTAGGTTATTCTCCAATTCCAGTCAATAATAGGTTTAATCCCTTGAGTCAACCCTCTTTACACCCTAATTATCAGTCAGCATTAACTTCCAATTATGATCCTTTCATAGTTAATCCTCCTGCTTCAGTTCCTTCGCCAAACCAAAATCCAGTAAAAAGGTCCAACAATGCCACTAGAAGCCATTGCCATCTTTTTATCATCGAATCAAATCTAGAAGACCTTTGTGACCCAATAAAAATTGCCATGAAATTCTTCCCTCCTAACCTTCATTATTTTCCAAGTCATCCATCAAAGTCAATTAAGTTTTACAGAGACATTCTCTATGAAACCCAGTCCATAGATATTAGGCCTATCAAAGACAAAGAAACAAATGCCACCATGTTTCATTCCCTTTACATACGCCAGATTCTAATGCCCCATATTTGGAATCGCAACCCTTATGATCAGTTACCCCTCCAATCAGGACTTACTTTCAATTACGGTGATTATATTGATGCCTGGTATACAATTTTTTTATCATCAACATCCTGATTTCAGTCACTCCTGGTTTATTAATTTCAATGCCAAGTTTGAAGTTCAGTTTTCTTTTTAGTTTCTCCAATGGTGGAATATGCATGGCCCCATTTCGGAAATCATTCCAGTCAATCTCCAGCAGTTAGTTAATTATTTTGGTCAAAAACACAAGTTCACAAAGTCATAAAAGTTTTTCCCTACGTTCCTCAATTTTGTAGCAAAATACAAAGTCCCATGGATTATGAAATGGAATTATCAAGTTAATTGGGATACCAGAGTTTTATCTCGTCATTTTGCAGTCAAATGGTGGGATAAGTTCGGGATTGAAAGAATTATCCAACAAGTCAACATAGAGTTCCCGCCCCTCCAAGCGCCAATCCATCTAGTCAAAGATACTTCCCATAGTGGTTCTAATGCCTCCCTCCAAGGAAAATCCAAAAGTATTTAAAAGAGTTAGCTGAGCAGATTCTTGCCCAAGTTACACAAATGAACGATGAGGATAGTGATGATGCCAGTCCAAAGTCTGAATCTTCTTCCAGTCAACAGCCAAGCCAGCTTCCTTTTAGTCAAAAGCCGAAGCACAGATGGTCTGATTATGCCCCCGACAGTTAGGATCCTTATGATTTAAATGAAGACTAAGCCTTTTTGCAAAGTTGTCGAAAGCAAAGATGCCCGAGAGTTAAAGCAAGAGTTAAAGCCAGATTTAAAGCAGATGCACAAGAGTTAAAGTGAGTGTTGAGACCCAAAAAAAATTCATGATTGGGCCCAAATAAATTACCAGCCCGAAACGCCGTTTTATTAAGAAAAGGCATAAAAAAAGAGGTACTCGAAAGCTTGCCACATGCGAATTGAAATTCATGCGCCATGCTAATTCACCATGCTAGCTCTACGAGCCCATGCCAAAATTAAATATCAAGGATGATTAAAGCACGCCAAGCGACATGCCATGCCAAGCATGAAATCGTTGTTCCACACCCAAACACACTACAAGCCTAAGTGGGCCCAAGCCACTCAAAATGCCCGGGGCTTGCTTGAGTGGGTTGTGGTATGGATGGTAACAGGTCGTCATGAGGCCCATTGATGGAACGAGAAATGGAGGTTTCGGGTTGCTTGGGAGCCTTGGGACCCAAGCCCATTTCTTAGGCCCAAATATATGGGTGAGCAAAAAGGGGAAAATAACTCAACCAAAATTAGCCTAGTCAAATTTTGGCCCAACCAAAAGCCCAATGTTTAAATAGCCCACTTATTCAATGAAGTACCTTAGCCCATACAAAATTTCACAATAAGCCATAAAAGCCTTAGCCCTTTGCTAGAAAATAGAAGCCACGTAGAAGAGGTCTGTAGGATCACTAAACAGAGCTGCTGGAAAATGCCAGCACATGGGAATGGATCAAGTTCCAAAACAAAACACCAAAGAAACCAGGGGATATTAGCATATAAGTTGTCAGGAAGGGGAGTACCCTTTCAAACCAGCATATATACCCAGTTGTTTTCCTTTATTAGTGCTGCCCCAGGAAAGAGAAGGAAAAAGGTAGCCAAGAATGGAGCTTCCTACTGAAGCAGCCGCGGGAAAGGGGGTCTTGAGCTCCAAAACTCCTGATTTTGTGCAAATAGATAAGGGGAAATCTCACCCAGATAGGAAAGTCAAGAGAGGATAGGAAAAAAGGATGGAAAGACTTGCCAAGATTGGGAAGAAACCATTAGGGTTTCTTGGGAAAGGATAATTTCCCGCAGCTATAAAAGGAGGTCCCCTCCACCTAGCAAAAACCAACCCAGGCTGAGAGAGCAAGCTTCCTCTCTTACTAGCAAAGTCCAGTAACCTTATTCACCCTTCTTCTTCATTCTACTCTGCTTGACAGATTATTTTCAGAATCTGTCAAGCTGCCGGAAGAAGTGATGTTCATCTTTTACCTCCTTTCAGCCAAGATCACCCTGCAAATCTGGTTTCCCTCATTTTAAACACTCACATTTCTCCTTAAGAAGCCTTATTTTCCTCTTTGGTGCTCAACTCATGCCGACCCTGCTCCCATGCCACAAGCTCTTCATGCTAAGCCAAAATCTTATCTGAAAGCAAGCACAAAACACCTGTAAGCAGCTCTCATTTTCATTGGTGAAGGTAACCAGAACACTTCCTAAGGTTTTGCTGCCCTTTCGAAGTGCTTTTGGGGCTTAATTTTTGAACTCCAGCACAGGGGGCAATTTCAGTGATTTCCCTCTTCTGGCAGCCCAGCCCTTTACAGCTGCCGGGAGAAAAAAAGACCCCCACAGTGAGTTTCCCCAGAGTTAAAGCAGATGCCCTCGAGTTTGAGATAAAGCAAGAGTTAAAGTCAGAGTTAAAGTATATGCCCAAGAGTTAAAGCGAGTTACCCCAGAGTAAAAGCAGATGCCCTCGAGTTAAAGCAAAGATGTCCAAGTTAAAGTTATTTTCAAAAGTTTTAAAAGTTATTTTGTCCTCTTTTGGATGCCAAGTCTTTAAGTAGAAGCCCCAAGTCATTTGTGATCCTCAAGCTTTTCTTTTTACTTTCTTCCTTCAGTTTTCAAAGTTCTTAAAGATAGGTGCGAGTGTTCATTAGAAGTATGAGTGTCCTGCGTGACATTGCTTTCTGTAAGTTTTTAATTTTCATGTTTTCAAAGTTTTATTAGTCAATAAAATTATCAAGTTAATCCTATTTTCGTTCCTAAGCATTAATTTAAATGATTAATTTTATTTTCATTCATGCAATTTGTGAATTGAGATTTTTCTGCATTTACCCTTTAATTAACTTTTATTTTGAGTTATTCCAATTCTATTCCTCAGTTCTCCCCTTTTTGAAGTTGTTCTCTATCTCTGTCTCTGGCTACAAAGTTGCCGAAAGCAAAGATGGCCAAGAGTTAAAGCAAGAGTTAAAGCCATAGTTAAAGCAGATGCCCTAGAGTTAAAGTGAGGTTCCCCAGAGTTAAAGCAGATGCCCTCGAGTTCGAGATAAAGCAAGAGTTAAAGTCAGAGTTAAAGCAGATGCCCAAGAGTTAAAGCGAGTTACCCCAGAGTTAAAGCAGATGCCCTCGAGTTAAAGCAAAGATGCCCAAGTTAAAGTTATTTTCTAAAGTTTTAAAAGTTATTTTGTCTTCTTCTGGATGCCAAGTCTTTAAGTAGAAGCCCCAAGTCATTTGTGATCCTCAAGCTTTTCTTTGTACTTTCTTCCTTCAGTTTTCAAAGTTCTTAAAGATAGGTGCGAGTGTTCAGTACAAGTGTGAGTGTCCTGCGTGGCATTGCTTTCTGTAAGTTTTAATTTTCATGTTTTCAAAGTTTTATTAGTCAATAAAATTATCAAGTTAATTTCAAGTTAATCCTATTTTCGTTCCTAAGCATTGATTTAAATGATTAATTATATTTTCATTCATGCAATTTGTGAATTGAGATTTTTCTGCATTTACGCTTTAATTAACTTTTATTTTGAGTTATTCTAATTCTATTCCTCAGTTCTCCCCTTTTTGTAGTTGTTCTCTATCTCTGTTTCTGGTTATCTCTAGTTCTCCCCTTTCTGGTATCAGAGCCGTAAGAGTTGAAGGAGTTGTGTCTAAGTCTGCTACTCATATCGCCCAAATGGCTGATTTAGGTTTTTTGAGTTAGCAAGATCCTCAGTTATTCGAAATAGTTTTCTTAGAGTTATCTGTTTCTTTAGGTTCCATCTCTCGAGAGTCTGAACCCTTAAGACCATGACCAAACAGTAAGTCCCAGGAGAGGCCTGAGCGGTTGCAGCAGGAGATCGGTGAGAGAGAGAATGCCTTAAGATACAGGTATTTTTGAGTTAACCCTAGGAATTTCTGTAGTTCTTCTATCTTTTGAAATCTTGAGTCAATTACTTGTAAAAATGAGTCGTTTATTTAGAACCAAATCAGTTAATTATTCCAGTTCTAGAACCAGTTTGAGTCAAGTTCCAAACATAGTTAATGAAGAACAATTAGAATTTGAGTCAACTAGTAGTGAATTACATTGTAATGATTGGAATATACCCAAAGTTCCTAGTAAAGAAATCTATAAGCAGAAATGGTTCATCAATGCATTTAAATCAACTACGCATATTAAGATAGTAGAACAAGTTTATGCCTTAGGTAAAGAACATGAGACATGCCAGTTACTTAATCTTGAGTCAATTAAGAAACATAGAAAAGAAGGTAATAATTTCTTGCATATAGGATTAGTCCAAGTTGCAGTTAAACCCTTAACTAAGTTAGGATTAAAAGCAGCAATTCTTTTAAGTTTAAGAGATGCTAGGTTTAACGATTTTCCAGGATGGAGTTTTAGGAATCATTGAGTCAAGCCTATGCCATGGTCCAGTTCATTTTGATTGTTACCCAGATTTCGCAGTTAGCCTAAATGATCCTCATATATTAAAAGTTTGAACTTTAAATATTAAAACACACTGTTATAATATTTTACCCGGTAGTCAACCCTTAGCTTTAATTTATAGAATTTATTACAAAGTCACAGGTACAAATATGAATTTCCAAGCCCTTAGTAAAAGTCCCAAGAACCAAACACTTTTAATTCAGAGTCATACTTCTAATGCCAATGTCATAGTACCCCAAATGATTAGATGGTCTAACATTAAGTTACCCCAAAATTGGTATTTAACAACCGAATGCCCTCAAAAGCCGATTCAACGTAGTCTGAATGATTTAGATTACATTCAACCCTATTTAGATGGTACAGTTAAAATTAGTTTTGATAGGCCAGTTAACTCCACAGTCCAACTAGAACAATTACCAACCCTTAGTCAATCTTTTAAAATTCCTGCTAGACATTTGTCTGCCGAGTCATCCTCAATTACAGAATGTGACCTAGAAGTAGATAGAGCCCTAATTGATTTCAAAACTAATGAATTAAGGAAACAAAAACAAGTAGTTCAACCAAGTTACGGAAAAGCCCTTGTTGAGCCAGTTCTGCCGGTTCAACCTGAATCACCTACTAGAACAAATTTCGAAGGTTCCACCTTTGAAGTTTGTGATGCCCAATTACGAACCCTTAGTAAGCCATTTAAAATTCGTTGATCAAAATTAGATGCCCATTTCCAAGCCCCAAAAAATGAATCACGAAATGCCAATTATCATGCTTCTTTCCCCAAAGTTCAAAGAAAAGAAATTTTCAATGAATGGAAAAAATATTTAAAAAAGACTAAGTCAGAATCACCTTATCTTGATTTTGTGGAAAGCAAATACATGAGTCAAAAGTTAGTTACTTTAACGCAAGAAAAATGGGAAAAAGCTGACAATACCAAAGTTATCAATACTCACCCCTCCAGAGATAGCCACAGACAGCGACAGAGAACAACTACAAAAAGGGGAGAACTGAGGAATAGAATTGGAATAACTCAAAATAAAAGTTAATTAAAGGGTAAATGCAGAAAAATCTCAATTCACAAATTGCATGAATGAAAATATAATTAATCATTTAAATCAATGCTTAGGAACGAAAATAGGATTAACTTGAAATTAACTTGATAATTTTATTGATTAATAAAACTTTGAAAACATGAAAATTAAAAGTTACAGAAAGCAATGCCACGCAGGACACTTACACTTCTACTGAACACTCGCACCTATCTTTAAAAACTTTGAAAATTGAAGGAAGAAAGTACAAAGAAAAGCTTGAGGATCACAAATGACTTGGGGCTTCTACTTAAAGACTTGGCATCCAGAAGAAGACAAAATAACTTTTAAAACTTTAGAAAATAACTTTAACTTGGGCATCTTTGCTTTAACTCGAGGGCATCTGCTTTAACTCTGGGGTAACTCGCTTTAACTCTTGGGCATCTGCTTTAACTATGACTTTAACTCTTGCTTTATCTCGAACTCGAGGGCATCTGCTTTAACTCTGGGGAAACTCACTTTAACTCATGGGCATCTGCTTTAACTCTGGCTTTAACTCTCGGGCATCTTTGCTTTCAGCAACTTTGCAGAAAGGCTTAGTCTTCATTTAAATCGTAAGGATCCTGAGTGTCGGGGGCATAATCGAACCATCTGTGCTTCGGCTTTTGACTAAAAGGAAGCTGGCTTGGCTGTTGACTGGAAGAAGATTCAGACTTTGGACTGGCATCATCACTATCCTCATCGTTCATTTGTGTAACTTGGGCAAGAATCTGCTCAGCTAGCCCTTTTAACTCCTTTTTGGATTTTCCTTAGAGGGAGGCATTGGAACCACTGGGGGAAGTATCTTTGACTGGATGGATTGGCGCTTGGAGGGGCGGGAACTCTATATTGACTTGTTGGATACTTCTTTCAATCCCGAACTTATCTTACCATTTGACTGCAAAATGATGAGATAAAACTCTGGTATCCCAATTAACTTGATAATTCCATTTCATAATCCATGGGACTTTGTATTTGACTACAAAATGAAGAAGCGTAGGGAAAAAACTTTTCTGACTTTGTGAACTTGTGTTTTTGACCAAAATAATTAACTGACTGCTAGAGATTGGCTGGAATGATTTCCCAAATGGGGCCATGGATATTCCACCATTGGAGAAACTAGAAAGGAAACTGGACTTCAAACTTGGCATCGAAATTAATAAACCAGGAGTGACTGAAATCAGGATGCTGATGATAAAAAATTGTATACCAGGCATCAATATAATCACAGTAATTGTAAGTAAGTCCTGACTGGAGGGGTAACTGATCATAAGGGTTGCGACTCAAAGCATGGGGCATTAGAATCTGGCATATGTAAAGGGAATGAAACATGGTGGCATTTGTTTCTTCGTCTTTGATAGGCCTAATATCTATGGACTGGGTTTCATAGAGAATGTCTCTATAAAACTTAATTGACTTTGATGGATGATTTGGAAAATAATGATGGTTAGGAGGGAAGAATTTCATGGCAATTTTTGCTAGGTCACAAAGGTCTTCTAGATTTGATTTGACGACAAAACGATGGCAATGACTTCTAGTGGCATAGTTGGACCTTTTTACTGGATTTTGGTTTGGCGAAGGAACTGAAGCAGGAGGATTAACTATGAAAGGATCATAATTGGAAGTTAATGCTGACTGATAATTAGGGCGTAAAGAGGGTTGACTCAAGGGATTGAACCTATTATTGACTGGAATTAGAGAATAACCTGGAAGGGCACAATTTGTGAAGATGACTCTTGTTTGACTGGCATCATGGTAGCTGGGAGGATTTTGGGTCGGCTTGGTTGACTGGGTTGACTCGGCTGGTTATACTGCATTTTCTGCTTGTCTTTTTCTTTGCTGGACATGACTCTGCTTGTTTGTCCTGCAGGAACTCTCTTGTAAGAAAATCAGGAATGTTGTTTTCACTGCCTCTCAAATATTCTAACTCAAAATAAAAAACACTCAGAATGGCTTGCCATCGTGCAAAAATCTATTTACTAGCTATATTTTTAACATCTTTTTCTAAAACATGTTTTGCTGACTTGCAATCAACTCTGACTAAAATTTTTTGATTTAATAGATAATCTTGAAATTTGCTAATGCATAATACTATAGATAAAATTTCCTTTTTAATAGTACTGTAATTAGCTTGATACTGACTCTAAACTCCGGAATGAAAACGGACAATTTGCTCAGGTGACTCAGAAGTAATGCTATGTTTAAGAATATGACCATAACCAGACTCAGAAGCATCAGTTTCAACAATTTTAAAAGAATTAACTGAAGGAATACCAAGGCATGGAAGTGACTTAACATGAACTTTAATTTGTTTAACTATTTATGTATGACTCGGAGTTCAAGCAGAAGGATTGCTTTGGAGACGATCAAATAGGGGATTACATAACTTTCTTAAATTTTGATAGAAATCAGAAACATAATTCAAGGATCCTAAAAATCTATGGAGTTGGCTTTTATCAAGAATCTGATTTGGAAATTTATAGGCAAACTGGATAACTCGATTGATTGGGCTTATTTGGGATTGACCAATGTTGAATCCTAAGAATCTGACATCGACTTGAAATAATTTGATTTTCTTGGCTGATATAACTAAGCCATTTTGCTTAACTATTTGAAGAAATTTATTCAAATGTTTCCAATGTTGCTCAATTGACTCAGAGAAAATTAGGACATCATCTATATGAACAATGGAAAATTGACTGTATTGATTAAAAAATTTCATTCATTATATTCTGGAATTCACTAGGCGCATTTTTTAATCCAAAAGACATGACATTCCACTCGTAATGGCCGAAAGGGGTTACGAATGCAGTTTTGTATCTATCAGATTCTTTGATTTGGATTTGTCAAAAATCACTTTTCATATCAAATTTACTAAAGATAACTGATTTTTCTAATCTGTTAACTAAGTCTCTTTTATTAGGGATTGGATACCGAATCCATTCAAGTACTGCATTAAGGGGCTTATAATTAATTACTGAACGAGGGGCTCCTCTTTCTAACTCTGAATTTTTCTGGACATAAAAAGCTGGGCATGACCAAGGTGACTTGCTTTTTCTAACTATTCCTTTATGAACTAACTCGGTGACTTCCTGTCTACCTAATTCCATTAGCTCTTGATTTATCTGGATTGGACGGGCCTTAGTGGGAATGTCTTTTCACTAAATGACTTAACATAAGGTAAAGTAACTACATGTTGTTTTCGGTGCCAAAAGGCATTAGATAAATAAGAACAAACTTCATTTTTTAAATTTTTCTTCAAAATTAATGATTTGAGACTGAAGTGACTTGTCGGCTAACTGTTGTTCAACTCGTTTGAACTTAATTTCTTCTTTAAGAAAATTAACTTGTTTTGTTTTATTCTAAATTAATTTGACCGATTTAGAAACTGCTGACTTTTGTAGTGACTTAAGACTGTGTAATTCTGGTTTGGTCAAGAATTCAAATTTAACTCGTTCACCTAGATGATTAGACATGACTCCATCATATTATACTTGGAAAGGGTAAAGCAATGAGATAAAGGGTGAACCTAGAATAACTTCGTCTGTGATATTCTGAAGTAAAACAGAAGACGTTTTAAAGCATATTTTGTTTTGGCATACGTGGGCTTTGGGTAATTCATATTTTAACTTTAAAGCTTTACCATATTTTGTTCTTAAAACTTCTTTTGACTTTTTGTAAAACTGGGTTGGAACAAGACCTTCTCGAATCCAATTTACATCGGCTCTTGAATCAAATAAGGCAACTGTGTTTAACTCAAATTCATCAATAAAAATACTGACTCGGGAATGCCATTTTTCTGAAATGAATTTGCTTAATTAACTTGATGACTTTGTTACTTTTGTTACTATCGGAAGACTGAGGTTCTGACTCAGAACTGGCTTTACCAAAGGAATTACTAGACTCTGCTCTAGACTCTAGTAGTTCAATTAAGTCTTGTTTAATTTTGTAATTCTCCGTTTGTAATTTTTGACTAACTGGTTTTAAACTAAAGATTTCCTTTTTTATTGTTTTAACTTCTAACTGGAGATCTTGAAGGGTGACTTCTTTCTTTTTACTAAACCGTTATAGGGTAGTATTAAGACTAATAGTAGGGACTTGGCTAAGGCGGGGTTCTTCCTGGCTTATGACTTTTCTAAGCTTTCTGAGATAATCGGCTTTTAAATCTGGATTTTCTACTTTACTGATTAAATCGATTAGTAATTCCTCTTGTTTTTCTTGCTTAGTTAGGACTTTACTGAGATAATCGGCTAAATCAAGAACTTTAATTAATTTGGCTTCATCATCGTATTTTAACTTTAACTGTTTAATTGTGTTTTTAACTTGGCATTCATTAGCAAAATGACCGAATCTTTTACATTTAAAACATTTCACATTTTTTTTATCTGAACTTTTCTTAAATTGATTCTTTTTACTTTGTCGGTAAACTTTTGAGTAGCCTTTTGACCATTTGCTTTTTCTAGGGGTAAACTTCTTTTTACTGTAGAACTCATCATTTGGTTTAATGGAATGTTTTTTACGAGAATAAAATCTACTTTTACTAAATTGTCGATCATTACTTTTTTTGACTTGTCTAGACGGGGGAACTGGGGTTAGACCATATTGTTCATAGAAATTTCCTAATTCATATTTAGCATTTTTCCTATCGACTTGAACTGATTTACTAATTTTTAAATCTACACACATTTTCTTCCTAACTTGATTGATTGTGCTGATTATATTGCCATAAGTTAACTCATTGTAATTAATTTGACCAGACTCATTACTTAAGACTGTCCTAATCTTATGGGCAAACAAATTAGGGAGACCGTTTATGAACTTTTCTTTCCAAAATGATTGATTACTGTAATCTCTTAACATAACTCTAGAAATGAAGACATCTTTATACAATCTAAAATCACTTAGCTGAGGGCATCGTAAATTACTTAAGTGGTCATGAATCCTGGCTGTTGTGCTACTGGGTGTGCCAATAAAATGCTCAATTATTGTGTATAAAAGGACATTGACTCCCTCTTCTATACTTGTTCCCATTCGTTCATCAAAAATGGGGTAACCATCTTCATCTGTGTTAAAGGCATTAATTATTCTTGATTTTGCATCTTTAGTGAGATGTTTATCCCATCAGAAATGAAGTGTTGCTGTGAAGCTTATTTGTAAAAGGGAAACTATTTCTTAGTCTTAATTGATGATTGGTGGCGTAACTATTGGTTACTGTAGACATATGTTGTAACTTGTTGAGTATCTTCTGTTAGGACAAGCCATCGATATTCCACTCGTATAGCTTATCAGAAGATACTGAGAATTGACTTTGGGTTCTTTCTTCAAACTGGACATCTGGAGGCGTAGGTTTAGGGTACCAATTTTTTGTAAGACTTGTGGGGTTAACTTTACATGTTAAACGACTAACTTTAAGATTCTGGGAGTTCCGGTTAACTTGTAGGTTACTAAGGGCTTGTTCAATTTAGAGATATTACTAGTTTCTGATTCTTGACTAGAAGTTGATTCTAAAGACATGGATGACTCATTATCAGAATTAACTTCAACTTGTAAAACTTTAATTGAAGTTTCTTCGTCTTTTACTTTGTCTTTGTCCTTATTTGGTTTTGGCTTAACCGGGGTTAACTCCTGAAACATTTCTTCAATTTTCTTCATGGTTGAATTAACCTTAAGAGCAAACTCTGGTTTTAACTCATGAAATTTGACTAAGGGTTTTTCTTTCTTTGGTTTTAAGGAAATCTCGCTAGGGATTAATTTATCAATTTTGGTTTCAATTTTATCTAATTGTTTACCTATGACTAGCTTGTGTTGGTGTAATTATTTTGTTCTATGACCTTTTTATCTAAATTATATTTTGACTCAGCAACTTTGTAAGGACTGGCTATAACATTGGCATTCTTGTGACTAATTACTATTGATTCAACTGGAGGGTGAGTACTGATAACTTTGGTATTGTCAGCTTTTTCCCATTTTTTCTGGGTTAAAGTAACTAACTTTTGACTCATATATTTGCTTTCTACATAATCAAGATAGGGTGACTCTGACTTAGTCTTTTTTCAATATTCTTTCCATTCATTGAAAATTTCTTTTATTTGAATTTTGGGGAAAGAGGCATGATAATTGGCTTTTCGTGATTCATTTTCTGGGGCTTGAAAATGGGCATCTAATTTTGATCAATGAATTTTAAATGGTTTACTAAGGGTTCGTAATTGGGCATCACAAACTTCAAAGGTGGAACCTTCGAAATTTATTATAGTAGGTGATTCAGGTTGAACCGGCAGAACTGGCTCAGCATGGGCTTTCTGTAACTTGGTTGAACTACTTTTTTTTGTTTCCTTAATTCATTAATTTTGAAATCAATTAGGGCTCTATCTGCTTCTAGATCACGTTCTATAATTGAGGATGACTCGGCAGGCGAATGTCTAGTAGGAATTTGAAAAGATTGACTAGGGGTTGTAATTGTTCTAGTTGGACTGTGGATTTAACTGGCCTATCAAAACTAATTTTAACTGTACCATCTAAATAGGGTTGAATGTAATCTAAATTGTTTAGACTACATTGAATCGGCTTTAGAGGGCATTCGGTTGTTAAATACCAATCTTGGGGTAACTTAATGTCAGACCATCTAATCATTTGGGGTACTGTGACATTGGCGTTAGAAGTATGATTCTGAATTAAAAGTGTTTGGTTCTTGGGGCTTTTACTAAGAGCTTGGAAATTCATATTTGTACCTGTGACTTTGCAATAAATTCTATAAATTAAAGCTAAGGATTGACTACCGGGTAAAATATTATAACCGTGCGTTTTAATATTTAAAGTTAAAACTTTTAATATATGAGGATCACTTAGACTAACTGTGAAATCTGGGTAACAATCAAAATGAACTGGACCATGGCATAGGCTTGACTCAATGATTCCTAAAACTCCATCCTGGAAATCGTTAAACTTAGTGAAAATACCTGTAGAATATCTATATATGCCTGGAGAATGTTAAGCAAGCTAATTCGAATTTTTTGCAATAAATAATGATCAACCTTTGAAGCAGGATTGATATGAGTTATCAATCCTGAGATGTGCAAAGCTTTAAAATAAACGAACACGCAGAGAATTATTTTACGTGGTAAAACCCCACCTCTGGGGAAAATCCACAGGACTCCTCAGTCCAACTCAAATCCACTATAGAACGATGATTACAAGAAGTACTCACACACTTATCCTAGACTCATTCTAGATAATGTTTACCGACTTCTTCGTAACTCAACTTCTGTCACGGGATGATCTTCGACACTCCTTATGTTGAACGCAATACTGGTCACGATTGCTTCAAGCTCGCCGGAGGAAAACTGCCACCACAGTCTTTGTGCCCTCAATCGTATGAGTCACCGATATGCATATGAATGCAATATGAATGATTAAAGTGTTTGATAAATCACCAAGCAAACATTAAACACATGATGATTTATGTCAAATAAATGCACAATAATTTTGTTAAGTATTTTAGATGCTCAAACAAAATATCAGAAAGCACACAAAAACAAAGAAGCACTTCGTCACCTTATACAAAGCACAATCTCCTTTTTGCAAGAGAAGAGCACAAAGCCACGTGCTCAATCTCTTGCCATTAATCATATTTGATTCACATAGAATCCTACCTCAATTCGTCAGCCCATGCAAACTTCCCATCATCAATATAAGATTTAGATGCATGCTGAATAATAGAGCCAAACTTCCTATATGGTTCTAAGTGATCACGCCAAACAGGAGACCACAAGTTCTATTCAATCATGAATATCATTTTTGATTCAGTATAGACTCTGAGTGATTGATGCCAATTAGCCACGCTTTTAGGGAGAAACAATATCTCTTAAAACAAATTAATTAGACCAAACAAAGATAAGATTTTAACTTGAATCAAAATAGAGTCCAACTAGGAAGCAATCTCAAGAGATAAAATCTAATCCTATTAAAAATAGGGCTAACATAAAAGTGCTTGAGTAAAAATAACTCCAACTAGGAATCCTATAATGAATGTATGATTATGTCATGATTTACCTGAAACCAGTTTCTCATATCGATCACGTCATACTTCAAAGATCCAGAATGAATGTGTTGCACCAAATCTTCCAGTAAAACAAGTTCACACACTAATTCCCAACCTATGCTAGAGTCTAGGAAATGACAATACAATTTTCATACTAAGAAATGTAATTCAAGGCAAAATTACAACACTACTCCCCCTTAATTATACAACACAATCTCTCCCCCTTTTTATTATAATTTCACAATAATCTCTCCCCCTTTTTGTCAGGAAATCCAAAGAGCAAGGAAAGAGTACAAGGGAGCATATATAAAGGCAAAAATAAGAGGAAAAAGAAATCAGTTGTGCCACAAGGCTGCAACTGGCAGGATTGATAATGATGCTGCAATCCTCAGGTTCAATTTATCATGTATCCGTCTTAGTAACATGAAGAAAAGCCTAACTGCACAATTAAACAAGAAGCATAAAACTACACTATGTTTGAAAACAAAAACAAATTCGTTTTTTTTTTTTTTTTTTTTTTTTTTTAAATAATGCTTAATAAGAACCCAAGACTCCTCCTTCATTCATGCCAAAAAAAAACTTGAGTACATGCCAACACTATGCAGAAGGATAGACAAGCAAAGCCAATACCAATATCTATCTAGAAGCATTTAGAAGCAAAGCCTTTCAAGATTGAAGGCACATCAAACCACAAAGGTTTAATCAAATACTAGGATTATAAAAAGAAAATCAAAAGTATTTGAAGAGATAAAGTCCATCAAAAGAGCAAATATTAAATCTCAATCTCGTGTTATGTGACCAAACTTATTTAATAACAAAGAAAAAATCCAATCAAGAACAAGCCAAGAACATAGAATAAACACATACAAAATGCATACAGAGGTCACCATTTTTTTTTTTATAATTATTTTATTTATTACTATTAATGCTAACTTTATTAAGCTTTCTACAAGACTCATGTAACGGGTGTTAAATCAATGTGCTCAAGTCAGATGACTTTAAGACAATGGGTGTTAAAGCACCCCTAAGATGTACTAACCCGAGTCATGTAGGCTACAAAGAAGAACAAAGTGTATAAGATCAAAGCTTTTTGACGTGAGCCTCGACGTTGGTTAGACAAGAGGCCCGGTTACTTGGCAAGGATCAAGCACTTATTTTACTTTTCTTTTAATTAAAGAGAAGTTACAAAATTGTGAGCTCAGTAGCAAAAAGTATAATGCAAATCTAGTATCAAGACAAGGGCAAGATGAGGATTCATTCAAAATTATTTAAACAAGATTAGAGTGTATGTGCTAAATGAGACACAAGAGATTTAATAAAAAGAGCATTTAATTGACTTTATAAAAACAAAGACAGTTGATAGCCATTCACAAAAACCCAATATTAATCTGCCCAAAGTGATCCAACGCCAAAAATCCAATCTAAGATCAGCAAAAACTTCCAGACGTCAGTAGGACTGATATTGCTTATCAATCCCTGCATATGCTGAACAAAAAACTTAGAATTTAATCCAAAAACAAAAACAGAGAAGATAATAGCTGGAGTCTTTTGGCATGTATACAAACAATTATTCAAAAGAACAAATACCAAAAGACTTTCTAAGAGTGTCAAATTGAAGATTATCCAAGGGTTTTGCAACAAGACATTTAAGATCCAAATTTTCTAAAGCAACGGGTTCATGCACAACATTAGTGGAGGCAAACATACAATTATGATTTTCCTTTTTCCTCCAAACTAACTTTGTCTTAGTTGATGGCAAACAATGAAAAGAAGATAGTTTGGATAAGCGGTTTACCTCATTCATAAGATTTGAAATCTTATGTTGGATACTTACTTTTACACCCATGCCATGAGAATTTGAACGAACAAATTCGTTTCGAAGCTTTTTGCATCTTGGTCTTATGTGCCCACGAGTTCCACAAAAGTGACAGATTGGAATAAATTTCTTGACTTCATGAGGGGCAGAAGGTTCAAAGAGTGAAGTGGATTTGACAAACTTTGTTTTAGACACAGCGGAAGACTTATTACTTGGTTGAAATCCCAAGCCTCGTTTGTCACCATCTCTTCGTCCCATGCTAATCATCTTGTCAATTTTTTCTGCACCAATGGTTAAAGAAACAATCCTTTCCTTTGAATTTTTAAGTTCAAGTTCAAGGGCTTTGTTGTCAGAAGTTAGAGAAGCAATTAATTTGTTCTGATCAGTCAATTTTTTCTGCAAAAACATCAATTTTTCGTTCATACCTGCACTTACCTTTTCAGAATCTGCATCAGGGGATTGAAACTCGTTTGCAATCCTGCAATTTTCCAGCTTCAGCACAGCAACTTCTTTACAATCATCATCCTCATGTGATTCATCCAAGCTAACTATGGTTATGAGTGCAACCGTCTTTTCATCATTCTCGCATTCCGATTCACTATCACTCCAAGTAATATGCATTGCCTATTCTTGCTATCCTTTTGTTTCTTTAAGGTGTTGGCACATTCCGAAGATATGTGTCCATAGCCTTCACATTCGAAACATTGGACCTTTTCTCTTGGATTCTTTCGTTCATTTGACCTGCGAAATTTAGACCCACCATCAGTATAGTTAACAAACCTATTTTTAGAATTTATACCTTTTGAATCTCGACTCCTTGGATCCTGATTCTTGAGAAAGTTCATGAATCGTCTTGTGAGAATCGTTAATTCCTCATCACTGCAATCTTCATTTGAATGCCCATCATCTTCTTCATTCACAACCTTGAAAGCAACAGTTTTGCTCTTTTTAAGGGCTAGATGTTTCATCTCATAGGTTTGTATGGACCCTATGAGTTCTTGAACTTTCAAGGTGTTCAAGTCACGGATCTCTTCAATAGCCGTGATCTTTGGTTGAAATCGTTGAGGCAAGGATCTCAAAATCTTTTTCACAACCCTGTCTTCTGGAATTTTTTCACCTAAACTTGAACAAGCATTCACAATTACACACAGTTTAGCATAAAATTCAGAAAAAGTTTCATTCTCATCCATCATGAGTGTCTCAAACTGTAACGTATGCATTTGAAGCTTGGCTCCCTTGACAGTATCTGTTCCTTCATGGGTAACCTGCAAAATATCCCAAGCTTCCTTAGAAGTATCACAACCAGATATATATTCAAATTGATCTGAAGAGACAGCAGTAAATAAAGCATTTAACCCCTTTTGATTATTAGTGCTGTGTGTGACTTCTGCAGTGGTCCACTCCTCTCTAGCTTTGAGGATTGTGGTTTCTTCATCTCCTTTTCCGATCTTCTTTGTGGGTTTAGGAAATCCATGAACCACTGTACTCCATACACGTTCATCTAAGGACCAAAGAAACGACTTCATTTTGGCCTTCCAAGCGCCATAGTTGTTGCCGTCAAAATATGGTGGATGTGCAATTGAGCCCGAGGCACGATCCATCCTAAGGTATATTAGATCTTACTGAGTATGTCCAGCAACCTGCTCTGATGCCAATTGAAAATACCTGTAGGATATCTATATATGCCTGGAGAATGTTAAACAAGCTAATTCGAATTTTTTGCAATAAATAATGATCAACCTTTGAAGCAGGATTGATATGAGTTATCAATCCTGAGATGTGCAAAGCTTAAAATAAACGAACACGCAGAGAATTATTTTACGTGGTAAAACCCCACCTCTGGGGAAAATCCACGGGACTCCTCAGTCCAACTCAAATCCACTATAGAACGATGATTACAAGAAGTACTCACACACTTATCCTAGACTCATTCTAGATAATGTTTACCGACTTCTTCGTAACTCAACTTCTGTCACGGGATGATCTTCGACACTCCTTATGTTGAACGCAATACTGGTCACGATTGCTTCAAGCTCGCCGGAGGAAAACTGTCACCACAGTCTTTGTGCCCTCAATCGTATGAGTCACCGATATGCATATGAATGCAATATGAATTATTAAAGTGTTTGATAAATCACCAAGCAAACATTAAACGCTTGATGATTTATGTCAAATAAATGCACAATAATTTTGTTAAGTATTTTAGATGCTCAAACAAAATATCAGAAAGCACACAAAAACAAAGAAGCACTTCGTCACCTTATACAAAGCACAATCTCCTTTTTGCAAGAGAAGAGCACAAAGCCACGTGCTCAATCTCTTGCCATTAATCATATTTGATTCACATAGAATCCTACCTCAATTCGTCAGCCCATGCAAACTTCCCATCATCAATATAAGATTTAGATGCATGCTGAATAATAGAGCCAAACTTCCTATATGGTTCTAAGTGATCACGCCAAACAGGAGACCACAAGTTCTATTCAATCATGAATATCATTTTTGATTCAGTATGGACTCTGAGTGATTGATGCCAATTAGCCACGCTTTTAGGGAGAAACAATATCTCTTAAAACAAATTAATTAGACCAAACAAAGATAAGATTTTAACTTGAATCAAAATAGAGTCCAACTAGGAAGCAATCTCAAGAGATAAAATCTAATCCTATTAAAAATAGGGCTAACATAAAAGTGCTTGAGTAAAAATAACTCCAACTAGGAATCCTATAATGAATGTATGATTATGTCATGATTTACCTGAAACCAGTTTCTCATATCGATCACGTCATACTTCAAAGATCCAGAATGAATGTGTTGCACCAAATCTTCCAGTAAAACAAGTTCACACACTAATTCCCAACCTATGCTAGAGTCTAGGAAATGACAATACAATTTTCATACTAACACTTAGCATCTCTTAAACTTAAAAGAACTGCTGCTTTTAATCCTAACTTGGTTAGGGTTTAACTACGACTTGGACTAATCCTATATGCAATAAATTATTACCTTCTTTTCCATGTTTCTTAATTGACTCAAGATTAAGTAACTGGTATGTCTCATGTTCTTTACCTAAGACATAAACTTGTTCTACTGTCTTAATATGCGTAGTTGATCTAAATGCATTGATTAACCATTTCTGCTTATAAATTTCTTTACTAGGGACTTTGGGAATATTCCAATCATTACAATGTAATTCACTAAGTTTGCAACTCATCCAACCCAAGTGGCTGGTTTAGGTTTTCTGAGCTGTAAGTTTTCAGTTTCTTCTCTTTCTACTTCTTACCTTTCAATTTACCTGTCCTTTTCTCTCTTGGTGTCCGAATTGTAAGACCCGTTCCAAACAGCAAGTCCCAAGGAGGCTTGAGTGGTTGTGGCATTAGTAGGAGGGCGAGAGAATCTTTAGGTATCCTGTTTCACTTAGAAGTCAGTCTTAGGAGTTTCTGTTCACTTTCTTCCTTAGAATTCCAACCCTAGAACCTTGAAAAATATGAGTCGCTTTTTTAGAACCAACCTTTACAATTCTAGCAGCTCTAGAGTCAGTATTGGTAAAGTTCCAGAGATAGTTAATGAAGAATAATTAGAATATGAGTCCAGTAATGAATTTGATTTTCGAGATTCGAATATACCTAAAGTCTCCAGTCAAAATATATATAAGAAAAAATGGTCACTCTCTAGTTTTAATACAACCACCCATGTAAAAACAGTTCAACAAGTTTATGCTTTGAGTCAAGAACATGAGACCTGTCAGTTACTTAATCTTGAGTCAATTAGGAAACACAAAACTGATGGAAATAATTTCCTTCATATTGGTCTAGTCCAAGTTGCTGTGAAGCCATTAACCAGATTAAGATTAAAAGCTTCTATCCTTCTCTATTTGCGTGATGCTAGATTCACTAATTTTAGCGACAGTACACTAGGAATTATTAAGTCAAGTCTTGCTAATGGTCCAGTTCATTTTGATTGTTATCCTGATTTTACTGTAAGTCTGAGTGATCCCCATATATTAAAAACTCTCACATTAAATATAAAAACTAATGGTTATGAAGTCTTGCCGGGTACCCAACCCTCAGCATTAGTATACAGAATCTATTACAAAGTCATAGGTACTAATATGAACTTCCAAGCCCCGAGTAAAAGTCCAAAAAATCAAACCCTGTTAATTCAGAGTCAAACACATGATGCCACTATAAGAGTCACATTCAATTAAATGGTTAGATGTGTCCCTTCCAGTTGATTGGACCTTAACAGCTGAGAGTCAGCCTGTTTCCATTCAGCGTAGTTTCAATAATTTAGGCTATATCCAACAATATCTTGATGGTACAGTCAAAATTAATTTTGGTAATCAAACCCCACAAACATCCACAGTCCAGTTACAACAGTTACCCACGCCTAGTCAATCCCAAAGACATATTCCTGCAAGGCATTCCTTTGAAGCCTCTTCCTCTACTCTAGAACGAGACCTAGAGTTAGAAAAAGCCTTAATTGATTTAAAATTAGAATCACTGAGAAAAACTTCACAGGTTACCTATCCTGTTTATGGAAAAACCCCAGTCCAAGAAGACACTAAGCCTGAGTCCCCTGATTCACTCACTCAATCTGATTTTGCCGAAGCTGGTTTCAAAATCGATAACCAGCTACGAACACTTAATAAAAAATTTATAATTAATTGGAAAAGATTAGAGAGTCATCTACATGCCCCTGAGAACCAAGCTAGAAAAGATAATTACCATTCAGTCATCTTTGATGAAGCCAAAAGACAACAAATTTTTAATGATTGGAAAGAATATATGCGCTCTGCTAAAGTCGAAATTTATTATCTTGATTTTGTAGAAAGCCGACATTTGCCCAATGAGTTACAAAACTTTAACCCAGGAAAAATGAGAAAAGATAGATCACACTGAGATTCTTGCTAGTCATCCACCTGTAGAAACAATAATTATTAAACATCAGAAATCAAAGATAGTCGCCAGTCCTTTCAAGATCCCTGATCCAGAGAATAAAATGATAGTTGAACAAAATAACTACACCAATCAAAGTCTAGTTGTCATAGGAAAAGAATTAGATAAAATCGAAAGAAGAGTCAATAAATTACTCCCTAGTAAAGTCAAAACAAAGCCTCAACATGAGAAACCACTAGTTACTTTCCAAGATTTAAAATCAAGCCCAACTTTTAAGACAAAAGAAACAATGTCAAAAATTGACAAAATGTTAGAAGAATTAACGCCAGTCAAAAGGAAAGACAAAGAGAAAATTGAAACAAGCGTTAAAGTCTTAGGATCCTCTGAGTCACAATTTCCTTTCCATAATTCCTCTAAGTCTGAATCAGCTGCCAGTATGGAAACAGAAACCAGTAATATCTCTAAATTGAAAATGCGTTTAAAAATCTTGAGTTAGAAAGTAATCCAAAGGTCCAGAGAATAATGAACAAAGTTAGCCCCACTAGTCTAACAAAAAATTGGTATCCTAAACCAACACCTCCGGATATACAATTTGAAGAACGAAATACCCAGAGTCAATTTCTAGTCTCTTCAAATAAGTTATATGAATGGAATATTGATGGTCTTTCTGAGCAAGAAATCCTTAATAAACTTCAACACATGTTCATAGTCGCCAATAGTTACATCACTAATCACAGTTTCAGACAGTCTGAAATTGTCCCCTTACTAGAAACAGGTTTCACCGGAACTCTTCGTTCTTAGTGGGATAAGCATTTAATCCATGAGTCTAAGCAACAAATAATTCATGCAGTCAAAGTTAGCGAAGACAGTCTTCCTATTTTTGATGAACAAATAGGTCAAGGCATTGAAGATGGAGTCAACACCTTGCTATACACAATTGTTGAGTATTTCATTAGAACACCCAGTAATACTACTGCTAGAATACATGATCAGTTAGTAACTTAAGATGCTCAAAATTATATGATTTTAGATGGTATAAAGATGTATTTATTTCCAGAATCATGCTTAGAGATGATAGTAACCAGTTATTTTGGAAAGAGAAATTTGTTAATGGTCTTCCTAATTTATTTGCCCATAAGATCCGAACAACCCTTAGCAATGAACAAGGTCATATTGATTGGGATAGTTTAACATACGGAAATATAATTAGCACTATTAATCAAGTCGGTATGAGAATGTGTATCAATATGAAAATAGGTAAACAAATTCAGTCAGAAAAGAATTCTGCTAAGTATAAGTTAGGTAATTTCTGTGAACAGTATGGTTTAATGTCTATTCCCCCATCTAGAAAGAATAAGTCAAGTCACCACAACGACAGTTTAGTAAACGTCGCCATTATTCACGAAGAAAACAGTCATTCCAACATAATAATGAAGATAAAGAATTTTATAGTAAAAATAGACTCTCCCCTAAGAAAAATTGGAAAAAGAGTCACGGCAAAAGTAAGAATAATCACTTTAAGAAAAGTACCGACAAGAGAAAAGTAAAATGTTATAAATGTCAAAAGTTTGGTCACTATGCTAATAATTGCAAAGTTAAAAATACAATAAAACAATTAAAAATCACGGATGAGGATAGAGACAAACTAATTAAAGTCTTTGAGTTAAGAAACTCCGAGTCTAGTGAAAATGAAACCATGGTTAGTAACACTGAGTCTTTAGAGTCTATCTCCTCATATTCCCAATTAAGTTCACCTAGAATTCAATTTGGTTGCCAAGACAATTGTTTGTAAGTCTAAGTCAATTAATGTCCTTACCAAGCAGGAAGAACAAGAAGAATTATTAATTGATTTAATAAGTAAAGTTGAAAATCCATAGTTGAAATATGAGTATTTAAAGAAATTAAGAAAAGTCATTAGTCAAGAAGAACCGAAACAATCTACCTCCCAAAAGATAAGGCTGAACACAACCTTAGAGAAATTTAATAAAAAGAAAGAAATCACTTTACAAGATTTACAACTACAGATAAAATTAGTCAAAAAAGAAATCATTGAGTTAAAACAAATAAGTCAAAAGTTACAAAATGAAAATTATGAGATAAAACAAGAGTTAGTTAACCTGATAGAAAAAGGAACTTCTGAGTCTACACCAGTAAGTCCCACAAATTCTGACGAAGAACCCTTCAATCAGGATCAAGTTGTCAACTTAATTAGACAAGTAAATTTCAAAAAATGGTATGCTAAAGTCACCATATTTGTCTAAAGCTTTGAGTTAAATATAATTGCCCTTTTTGATTTTGGAGCCGACCTTAACTGTATCCAAGAAGGTCTAATCCCCACTAAGTATTATAAGAAATCAAAAGAGTCTTTAAGCACAGCCTCAGGAAAGTCACTCCAGTTAAGTTATGAGATCCCTAAAGCCCATGTCTGTCAAGATAAAATCTGTTTTAAGACCTCTTTCATCTTAGTTAAAAATATTACTGATGAAGTCATCCTAGGTTTACCCTTTATTGCCATTTTATATTCTTTCCAGGTAGAACATGATGGTGTCATTTCCACCTATTTAGGAGAAAAGGTTAAGTTTGAATTCTTGAGTAAACCAGAGTTACATAATTTAAAAGAATTGCAAAAGAACGCAGTCACTAAAACAGTTAGAGTCATTCAAAATAAAGATAAGCAACTAAGATTTCTCCAGGAAGAAGTCAAGTCTAAAAGAATTGAGCAACAACTTAGTCATGAGTCTTTACAGTCACCAATTCGACAGTTTGAAGAAAAATTAAAACAAGAAGTTTGTTTTGAGTTACCCAATGCTTTTTGGCATAGGAAACAGCTTGTAGTCAGTCTTCCTTATGTCAAGTCCTTTAGTGAGAAAAATATCCCCACTAAAGCCAGACCAATCCAAATGAGCCAAGAATTAATGGAATTCTGTAAAACTGAGATTACAGAATTACTTGAAAAAGGAATAATCCGTAAGAGTAAGTCACCTTGGTCTTACCCTACTTTCTATGTCCAGAAAAATGCTGAATTAGAACGCGGAGTCTTGAGACTAGTCATTAATTACAAGCCGCTTAATACAGTCTTAGAATGGATTAGATATCTGATTCCTAATAAAAGAGATTTAATTAATAGATTAGAAAAGTCAATCATCTTTTCTAAATTTGATATGAAGAGTGGGTTTTGGTAAATTCAAATATGTGAGTCTGATAGATACAAAACTGCTTTTGTTACCCCATTTGGTCATTATGAGTGGAATGTTATGCCTTTCAGTCTTAAGAATGCGCCCGGTGATTTCAAAATATATGAATGAGATTTTTAATCCATTTAGTCATTTTTCCATAGTCTATATTGATGATGTGCTTATCTTTTCTCAGTCGATAGAGCAGCACTGGAAACATTTACATAAGTTTTTACAGATAGTCAAACACAATGGATTAGTTGTTTCTGCTAAGAAAATTAAGTTATTCCAAACTAATGTGAGATTTCTTGGGTTTAATATCTGCCAGTCACAGATTAGCCCCATAGATCGAGTCATCCAATTTACCAATAAGTTTCCTGATCAAATCCTAGACAAAAGTCAACTCCATAGATTTCTCGGATCTCTAAATTATGTTTCTGATTTTTATAAGAAGTTGAGAAAACAATGTAAGCCTTTATTTGATCATCTCTAGAGTAATCCTCCTCCTTGGTCATCTTCAAACAAATGAAAGCTCATATTAAGACACTTCCCTATCTTGGTATCCCCTCTGCTGGGTCATTTAAAATTGGTGAAACTGATGCCTCTAACATTGGTTACAGAGGTATCCTCAAACAGAGAATCAATTCTAGTCAATCTGAGCAAATCATTCGATTCCATTATGGTACTTGGTCTCAGTCTCAAAATAATTACAGTATTATTAAGAAAGAGATATTATCTATTATACTATGCATTAATAAATTTCAATCTGATTTATTAAATCAAAAATTTTTAGTCCGAGTTGATTGTAAGTATGCTAAGCATGTACCAGAAAAAGATGTTCAAAATGTTGCATCCAAACAAATATTTGCAAAATGGCAAGTTATTTTGAGTATATTTGATTTTGATTTTGAGTATTTGAGAGGAAGTGAGAATTATGTCCCTAATTTTCTTACCAGAGAATTTCTACAGGGAAAACAATGGCAGAAAGAAGAAAAGAAAAAGACAAGCAAAAGAGTCCAGTCGGTCAATCTAGTCAATCTATTCAACCCCAGTCACTCAGAGTCCTCCCTCCATCAATGAGTCAAGCTAAACACGAGTCACCTTCTCAGATCGTTCCCTTTTCGGGATGTTCTCCTATCCAAATTAGTAACAGATTTGCCAACCTAGGAGCCACAGTCGGCCAGGTTCGTCCCAGTTTTCAATCTGCCCTAATATCCAGTCATGATCCTTTCCAGGATATTGCTCCGTCTAATCCTCCAGTTGCCTCTTACCAAAAGACGTCCCCTTACATGGTCAAGAGTAATAGTCACCTATTCATAATTGAGTCAAAATACAATTCCTGTTTATGGAAAAACCCCAGTCCAAGAAGACACTAAGCCTGAGTCCCCTGATTCACTCACTCAATCTGATTTTGCCGAAGCTGGTTTCAAAATCGATAACCAGCTACGAACACTTAATAAAAAATTTATAATTAATTGGAAAAGATTAGAGAGTCATCTACATGCCCCTGAGAACCAAGCTAGAAAAGATAATTACCATTCAGTCATCTTTGATGAAGCCAAAAGACAACAAATTTTTAATGATTGGAAAGAATATATGCGCTCTGCTAAAGTCGAAATTTATTATCTTGATTTTGTAGAAAGCCGACATTTGCCCAATGAGTTACAAAACTTTAACCCAGGAAAAATGAGAAAAGATAGATCACACTGAGATTCTTGCTAGTCATCCACCTGTAGAAACAATAATTATTAAACATCAGAAATCAAAGATAGTCGCCAGTCCTTTCAAGATCCCTGATCCAGAGAATAAAATGATAGTTGAACAAAATAACTACACCAATCAAAGTCTAGTTGTCATAGGAAAAGAATTAGATAAAATCGAAAGAAGAGTCAATAAATTACTCCCTAGTAAAGTCAAAACAAAGCCTCAACATGAGAAACCACTAGTTACTTTCCAAGATTTAAAATCAAGCCCAACTTTTAAGACAAAAGAAACAATGTCAAAAATTGACAAAATGTTAGAAGAATTAACGCCAGTCAAAAGGAAAGACAAAGAGAAAATTGAAACAAGCGTTAAAGTCTTAGGATCCTCTGAGTCACAATTTCCTTTCCATAATTCCTCTAAGTCTGAATCAGCTGCCAGTATGGAAACAGAAACCAGTAATATCTCTAAATTGAAAATGCGTTTAAAAATCTTGAGTTAGAAAGTAATCCAAAGGTCCAGAGAATAATGAACAAAGTTAGCCCCACTAGTCTAACAAAAAATTGGTATCCTAAACCAACACCTCCGGATATACAATTTGAAGAACGAAATACCCAGAGTCAATTTCTAGTCTCTTCAAATAAGTTATATGAATGGAATATTGATGGTCTTTCTGAGCAAGAAATCCTTAATAAACTTCAACACATGTTCATAGTCGCCAATAGTTACATCACTAATCACAGTTTCAGACAGTCTGAAATTGTCCCCTTACTAGAAACAGGTTTCACCGGAACTCTTCGTTCTTAGTGGGATAAGCATTTAATCCATGAGTCTAAGCAACAAATAATTCATGCAGTCAAAGTTAGCGAAGACAGTCTTCCTATTTTTGATGAACAAATAGGTCAAGGCATTGAAGATGGAGTCAACACCTTGCTATACACAATTGTTGAGTATTTCATTAGAACACCCAGTAATACTACTGCTAGAATACATGATCAGTTAGTAACTTAAGATGCTCAAAATTATCTGATTTTAGATGGTATAAAGATGTATTTATTTCCAGAATCATGCTTAGAGATGATAGTAACCAGTTATTTTGGAAAGAGAAATTTGTTAATGGTCTTCCTAATTTATTTGCCCATAAGATCCGAACAACCCTTAGCAATGAACAAGGTCATATTGATTGGGATAGTTTAACATACGGAAATATAATTAGCACTATTAATCAAGTCGGTATGAGAATGTGTATCAATATGAAAATAGGTAAACAAATTCAGTCAGAAAAGAATTCTGCTAAGTATAAGTTAGGTAATTTCTGTGAACAGTATGGTTTAATGTCTATTCCCCCATCTAGAAAGAATAAGTCAAGTCACCACAACGACAGTTTAGTAAACGTCGCCATTATTCACGAAGAAAACAGTCATTCCAACATAATAATGAAGATAAAGAATTTTATAGTAAAAATAGACTCTCCCCTAAGAAAAATTGGAAAAAGAGTCACGGCAAAAGTAAGAATAATCACTTTAAGAAAAGTACCGACAAGAGAAAAGTAAAATGTTATAAATGTCAAAAGTTTGGTCACTATGCTAATAATTGCAAAGTTAAAAATACAATAAAACAATTAAAAATCACGGATGAGGATAGAGACAAACTAATTAAAGTCTTTGAGTTAAGAAACTCCGAGTCTAGTGAAAATGAAACCATGGTTAGTAACACTGAGTCTTTAGAGTCTATCTCCTCATATTCCCAATTAAGTTCACCTAGAATTCAATTTGGTTGCCAAGACAATTGTTGTAAGTCTAAGTCAATTAATGTCCTTACCAAGCAGGAAGAACAAGAAGAATTATTAATTGATTTAATAAGTAAAGTTGAAAATCCATAGTTGAAATATGAGTATTTAAAGAAATTAAGAAAAGTCATTAGTCAAGAAGAACCGAAACAATCTACCTCCCAAAAGATAAGGCTGAACACAACCTTAGAGAAATTTAATAAAAAGAAAGAAATCACTTTACAAGATTTACAACTACAGATAAAATTAGTCAAAAAAGAAATCATTGAGTTAAAACAAATAAGTCAAAAGTTACAAAATGAAAATTATGAGATAAAACAAGAGTTAGTTAACCTGATAGAAAAAGGAACTTCTGAGTCTACACCAGTAAGTCCCACAAATTCTGACGAAGAACCCTTCAATCAGGATCAAGTTGTCAACTTAATTAGACAAGTAAATTTCAAAAAATGGTATGCTAAAGTCACCATATTTGTCTAAAGCTTTGAGTTAAATATAATTGCCCTTTTTGATTTTGGAGCCGACCTTAACTGTATCCAAGAAGGTCTAATCCCACTAAGTATTATAAGAAATCAAAAGAGTCTTTAAGCACAGCCTCAGGAAAGTCACTCCAGTTAAGTTATGAGATCCCTAAAGCCCATGTCTGTCAAGATAAAATCTGTTTTAAGACCTCTTTCATCTTAGTTAAAAATATTACTGATGAAGTCATCCTAGGTTTACCCTTTATTGCCATTTTATATTCTTTCCAGGTAGAACATGATGGTGTCATTTCCACCTATTTAGGAGAAAAGGTTAAGTTTGAATTCTTGAGTAAACCAGAGTTACATAATTTAAAAGAATTGCAAAAGAACGCAGTCACTAAAACAGTTAGAGTCATTCAAAATAAAGATAAGCAACTAAGATTTCTCCAGGAAGAAGTCAAGTCTAAAAGAATTGAGCAACAACTTAGTCATGAGTCTTTACAGTCACCAATTCGACAGTTTGAAGAAAAATTAAAACAAGAAGTTTGTTTTGAGTTACCCAATGCTTTTTGGCATAGGAAACAGCTTGTAGTCAGTCTTCCTTATGTCAAGTCCTTTAGTGAGAAAAATATCCCCACTAAAGCCAGACCAATCCAAATGAGCCAAGAATTAATGGAATTCTGTAAAACTGAGATTACAGAATTACTTGAAAAAGGAATAATCCGTAAGAGTAAGTCACCTTGGTCTTACCCTACTTTCTATGTCCAGAAAAATGCTGAATTAGAACGCGGAGTCTTGAGACTAGTCATTAATTACAAGCCGCTTAATACAGTCTTAGAATGGATTAGATATCTGATTCCTAATAAAAGAGATTTAATTAATAGATTAGAAAAGTCAATCATCTTTTCTAAATTTGATATGAAGAGTGGGTTTTGGTAAATTCAAATATGTGAGTCTGATAGATACAAAACTGCTTTTGTTACCCCATTTGGTCATTATGAGTGGAATGTTATGCCTTTCAGTCTTAAGAATGCGCCCGGTGAGTTTCAAAATATAATGAATGAGATTTTTAATCCATTTAGTCATTTTTCCATAGTCTATATTGATGATGTGCTTATCTTTTCTCAGTCGATAGAGCAGCACTGGAAACATTTACATAAGTTTTTACAGATAGTCAAACACAATGGATTAGTTGTTTCTGCTAAGAAAATTAAGTTATTCCAAACTAATGTGAGATTTCTTGGGTTTAATATCTGCCAGTCACAGATTAGCCCCATAGATCGAGTCATCCAATTTACCAATAAGTTTCCTGATCAAATCCTAGACAAAAGTCAACTCCATAGATTTCTCGGATCTCTAAATTATGTTTCTGATTTTTATAAGAAGTTGAGAAAACAATGTAAGCCTTTATTTGATCATCTCTAGAGTAATCCTCCTCCTTGGTCATCTTCAAACAAATGAAAGCTCATATTAAGACACTTCCCTATCTTGGTATCCCCTCTGCTGGGTCATTTAAAATTGGTGAAACTGATGCCTCTAACATTGGTTACAGAGGTATCCTCAAACAGAGAATCAATTCTAGTCAATCTGAGCAAATCATTCGATTCCATTATGGTACTTGGTCTCAGTCTCAAAATAATTACAGTATTATTAAGAAAGAGATATTATCTATTATACTATGCATTAATAAATTTCAATCTGATTTATTAAATCAAAAATTTTTAGTCCGAGTTGATTGTAAGTATGCTAAGCATGTACCAGAAAAAGATGTTCAAAATGTTGCATCCAAACAAATATTTGCAAAATGGCAAGTTATTTTGAGTATATTTGATTTTGATTTTGAGTATTTGAGAGGAAGTGAGAATTATGTCCCTAATTTTCTTACCAGAGAATTTCTACAGGGAAAACAATGGCAGAAAGAAGAAAAGAAAAAGACAAGCAAAAGAGTCCAGTCGGTCAATCTAGTCAATCTATTCAACCCCAGTCACTCAGAGTCCTCCCTCCATCAATGAGTCAAGCTAAACACGAGTCACCTTCTCAGATCGTTCCCTTTTCGGGATGTTCTCCTATCCAAATTAGTAACAGATTTGCCAACCTAGGAGCCACAGTCGGCCAGGTTCGTCCCAGTTTTCAATCTGCCCTAATATCCAGTCATGATCCTTTCCAGGATATTGCTCCGTCTAATCCTCCAGTTGCCTCTTACCAAAAGACGTCCCCTTACATGGTCAAGAGTAATAGTCACCTATTCATAATTGAGTCAAAATACAATTCCATTGCCAACCCAATCACCATTGCCAAATCTTACTATCCACCAAACAGAAACATCCTCCATGAGACCTAATCCATCACAATTAAGCCAATTAAAGACAGAGATAATCCCCATATCATCCTCTACCATTCCCTCTACATCCATCGCATAATCAATGAGATTGAATGGAGTAGTTACCCCTATGATCTCAAAGTTCTTCAGTCAGAACTTCAATATAGTTACTATGATTATATCGAAGCATGGTATTCTATCTTGCTTCACCAAACAGTTGATTTTAGTCATTCATGGTTTCTTAATTTTAACAGCAAATTCAAGAGCCCTTTTCCCTATTGGTTCAATCACTGGTGGGAAAAACATGGTCCAGTCATTGATCTTTTTCCCCCTCCAATGCAAGATTTAGTCAATTTATTTCACTAATAAAAATAAGTTTAAAGAATCTGAGTTGTTCTTCCCAAATCTTCTCCATTTTGCCAAGTACAAAGTCCCTTGGATCCTCAAATGGTCATATCAAGTTAATTGGGAGAGTCCTATCTCGCCAATTTTCAATAAAATGGTGGGATAAATTTAAACTTGAGAGTATTGCAGATTATGTATATGCTGATTTTCCTCCAGTCTGGAATCTTCAACCAGAGAAAAATTCATCTCTTGCTACTTCGCATTCTTCTCTCCTAATCGAAGGAAAATCTAAGTCTGAATTGCAGGAAATTGCTAGCCAACTAATGTTTCAAGCCTCACAAATGAATGAAGACAATGATGATGATGCTTCCCCAAAATCACAGTCATCCATCAGTCACCAAAAATTGTCATCCAGTCAAAAAACTCCTTCCCCCAAAATGCGGTGGAGTGATTATCCCCCAGATGGTCAAGACTCTTATGATTTCAGTCCTTTCGATCTGGGTGAAGATTAAAGTTAACCAAAGTCATGAGTCCCGAAGTTAAAGCATTTGGAATCTTGGACCCATGCTTCAACAGTCTGATAGGCCGTGCTCCTTTAGTCAGATTCCCCGACAAAGTAAAAGTTACAGCACTCAGTAAAAGCAAAAGGTTTCGCGTGCTTCTACAGTCACTCTCCAAAGCATCCAAAGTTGCTAGAGTCTACTTTTATTTGCCTATTTAAGAAGCGAAGCCTCAGTTATCAACATAATGTAATTTTAGTCCACTTCTTTTCTTAGTTTCAAAGTTCTTTTAGTATCAAATCACTGACGAACTAAGTGGTGAAACAAAGATTTTGTCAGTGTTTTGCATTCTGCAAGTTATCAAGTTATCAATAATATTTTGAATATTTTGAGTAATGCTAATTTTAATTGCATCTATTTTCATCTATTCATTAATTTGTTTCATGCATAAACTGAGACTGTTCTATAATCTCTGTGCCTATAAATACTTCTGTCTTTATTCTATCTCTCACTGGTTCTAGACCCCTTATGGTATATATATATATATATATATATATATATATGTAGTCCCCTTCTAATGAGGGATGCCCTTTAGGGCACCCTATAATTTTTTTTTCCAATGATCCAAACATCTATTTTTTAGTTCTTCATTCATAGATCATCCTTACCAAAAATTAGACAAATCGGAAACCGTTTCGACATCCAATTGTGTCTTACAAAATCAATGAACACGTTCCTTCAAGAAAGTACTAAAATTTCAATAACTCAAATGAGTGGTCAAATGATATCGGATTCAAGTAATGTTTTGTAGAGATGATCTTTGAATGAGTATCTACAAAATAGACAGTTTGGATTAGTGAAATACAATCCGGAGTGGGGCCTACAAGGAGTGTCCCTCAAATAAGCTTATTTGAGGAATCCCTCAATGGAAGCTCTCTGTATATATATTATTCCCGTAGAAAATATTTCTTTAAACCAAATTATGAGTTTTACAGTAAAAAGAAGTTTACCCCTAGGAACAGTAAATGGTCAAAAGGCTACTCAAAGGGTTACCGACAAAGTAAAAAGAATCAATTTAAGAAAAGTGATGATAAAACAAAAGTTAAATGTTTTAAATGTAAAAGATTTGGTCATTTTGCTAATGAGTGCCAAGTTAAAGATACATTTAAACAGTTAAAGTTAAAATCTAATGATGAAGCCAAATTAATTAAAGTTCTTGAGTTAGCTGATACTGAGTCAGATCCCATGTCTTCAGCCAACTCAGTCAATTATAGTTTCTTTGTCTTTGATTGGCCTAATATCTATTGACTGGGTTTCATAGAGAATATCTCTGTAAAACTTAATTGACTTTGATGGATGACTTGGAAATAATGAAGATTAGTAGGGAAGAATTTCATGGCAATTTTTACGGGGTCATAAAGCTCTTCAAGATTTGACTCGACTACAAAAAGATGACAATGGCTTCTTGCGACATAGTTGGATCTTTTCATTGGACTTTGGTTTTGAGAAGGAACTGAAGCAGGAGGATTAACTATAAAAGGATCATAATTGGAAGTTAATGCTGACTGATAATTAGGGCGTAAAAAGGGTTGACTCAAGGGATTAAACCTATTATTGACTCGAATTGGAGAATAGCCTGAGAATGGCACAATTTGGGAAGACGGCTCTTGTTTAATTGGCATCATGGTAGCTGAGAAGATTCTGGGCTGACTTGGTTGACTGGGTTGACTCGGCTGACCATATTGCATTTTCTGCTTGTCTTTTTCTTTGCTAGACATGATTCGACTTGTTTGTCCTGCAGGAACTCTCTTGTAAGAAAATCAGGAATACTGTTTTCACTACCTCTCAAATATTCTAATTTAAAATAAAAAACACTCAGAATGGCTTGCCATCGTGCAAAAATATGTTTACTAGCTATATTTTTAACATCTTTTTCCAAAACATGTTTTGCTAACTTGCAATCAACTCTGACTAAAAATTTTTGATTTAATAAATCATCTTGAAATTTACTAATGCATAATACTGTAGATAAAATTTCTTTTTTAATAGTACTATAATTAATTTGAGATTGACTCCAAACTCCGGAATGAAAACGGACAATTTGCTCAGGTGAGTCAGGAGTAATTCTTTGCTTCAGAATACCTCCATAACCAGACACAGAAGCATCAGTTTCAACAATTTTAAAAGAATTAACTGAAGGAATACCAAGGCATGGAAGTGACTTGACATGAATTTTGATTTGTTTAACTATCTCTGTATGATATGGAGTCCAGACAGGAGGATTGCTCTGGAGACGATCAAACAGGGGTTTGCATAACTTTCTTAAATTTTTATAGAAATTAGAAATATAATTCAAAGATCCTAAAAATCTATGGAGTTGACTTTTATCAAGAATATGATTTGAAATTTTATCGACAAACTGGATAATTCGATTGATTGGGCTTATTTGGGATTGACCAATGTTGAATCCTAAAAATCTGACATTGACTTGAAATAATTTGATTTAAAGACTTAGAAAATACAACAGACGGAGCTTATGCATGAATGAAATTAATGAATAAATGAAGATACTCAAAATAAGCATGAATGAATGAAAATAAAATTAATTACTTCAAATTATTGCTTAGGAACGAAAATAGGATTAACATTTAAAATTAACTTGATAACTTTATTAACATAGAAACTTACAAATAATGTAAAAACTTATAGAAATCTTAGTTCTTAGGGAAAGCAATACCACGCAGGATACTCACACTTATCCGAGAAGTTTGAAAACTGAAAGAAATTACAAGACAAGCTTAGAAAGCTTGCCCCTTACAAATGATTTGGGGCTTCTACTTAAAAGACTTGGCATACAGAAGAAGACAAAATAACTTTTGAAATTAACTTCTATTTTAACTTGGGCATCTTTGCTTTAACTTTGGGGTAACTTGGACATCTTTGCTTTAACTCTGGGGTAACTTTGGGCATCTTTGCTTTAACTTTGGGGTAACTTGGACATCTTTGCTTTAACTCTGGGGTAACTTTGGGCATCTTTGCTTTAACTCTTGGGCATCTTTGACTCTGCTTTAACTCTGGGGAATTCTGCTTTAACTCTAGGGAACTTGCTTTAACTTTGGCATCTTACGCTTTAACTTTGGCATCTCTGCTTTAACTCTACTTTAACTCTGGGGAACTTGCTTTAACTTTGGCATCTTTGCTTTCGGCTACTTTGCAAAACACTTAGTCTTAATTTAAGCCATAAGGGTCTTGATTGTCAGGGGCATAATCTGACCATCTGGGCTTTAGCTTTTGACTAAAAGGCTGGCTTGGCTGTTGGCTTGAAGAAGACTTAGACTTTGGATTAACTTCAACTTGTAAAAGTTTAACTGAAGTTTCTTCTTGTTTTTCTTTGTTTTTATCTTTATTTGGTTTTGGTTTAACCGGGGTTAACTTCTGAAACATCTCTTCAAATTTTTTCATGGTTGAATTGACTTTAAGAGAAGACTCATGTTTTAACTCATGAAATTTGACTAAGGGTTTTTCTTTTTTAGTTTTTAAGGAAATTTCACTAGGGATTAACTTATCAATTTTGGTTTCAATTTTATCTAACTGTTTTCCTATGACTATTTAGGCTTGTGTTGGTGTAATTATTTTGTTCTATGACCTTTTTATCTAAATCATCTTTTGACTCAGCGACTTTGTAAGGACTAGCTATTACCTTGGCATTCTTGTGACTGATTACTATTGATTCAATTGGGGGGTGAGTACTGATAACTTTGGTATTGTCGGGTTTTTCCCATTTTTCCTGGGTTAAAGTAATTAACTTTTGACTCATGTATTTGCTTTCTATAAAATCAAGATAAGGTGATTCTGACTTAGTCTTCTTTAAATATTCTTTCCATTCATTGAAAATTTCTTTTCTTTGAACTTTGAGGAAAGAAGCATGATAATTCGCTTTTCTTGACTCATTTTCTGGGGCTAGGAAATGGGCATCTAATTTTGACCAATGAATTTTAAATGGTTTACTAAGGGTTCGTAATTGGGCATCACAAACTTCAAAGGTGGAACCTTCGAAATCTGTTTTAGTGGGTGATTCAGGCTGAACTGGCTCAGCATGGGCTTTTTCGTAACTTGGTTGGACTACTTGTTTTTGTTTTCTTAATTCATTAATTTTGAAATCAAGTGGGCTCTATCGACTTCTAGATCACGTTCTGTAATTGAGGATGACTCGGCGGATGAATGTCTAGCATGAATTTTAAAAGATTGACTAGGGGTTGGTAATTGTTCTAATTGAACTGTGGATTTAACTGGCCTATTGAAACTAATTTTAATTGTGCCATCTAAATAAGGTTGAATGTAATCTAAATCGTTCAGGCTACCTTAGAAAGGTTTAGCAAAAATAAAGAAGTCACCTTTCAAGATCTCCAGTTAGAAGTTAAAGCAGTCGAGAAGGAAATCTTAAATCTAAAACAAGTTAGTCAAAAATTACAAACTGAGAATTACGAAATCAAACAAGACTTAAATAAACTACTCGAGTCTAGAGCAGTGTCTAGTAATCCCTTTGGTAAAACCAGTTCCGAGTCAGAACCTGAGTCTTTCGATAGTAACAAAAGCGACAAAGTCATCAAATTAATTAAACAAATTAATATTATTAAATGGTATTCACGAGTCAGTATTTTTATTAATGAATTTGAGTTAAATATAATTGCCTTGTTTGATTCAGGAGCCGATTTAAATTATATTCAAGGTCTTGTTCCAACCCAGTTTTATAAAAAGTCAAAAGAAGTTTTAGGAATCGCATCAGGTAAAGCTTTAAAGTTAAAATATGAATTACCCAAAGCCCACGTATGCCAAAACAAAATATGCTTTAAACCGTCCTTTGTTTTAATTCAGAATATCACGGATGAAGTTATTTTAGGATTATCCTTTATCTCATTACTTTACCCTTTCAAAGTAGAATACGATGGAGTTACGTCTAGTCACTTAGGTGAATAGGTTAAATTTGAATTCTTGACCAAACCAGAGTTACACAGTCTTAAGTCACTACAAAAGTCAGCCGTTTCTAAATCAGTTAATTAATTCAGAGTAAAACCAAACAAATTAATTTTCTTAAGGAATAAATTAAATGCAAACGAGTTGAACAACAGTTAGCCGACAAGTCACTTCAGTCTCAAATAATTAAGTTTGAAGAAAAATTAAAAAATGAAGTTTGTTATGATTTACCTAATGCTTTTTGGCACCGAAAACAACGTGTAGTTACTTTACCTTACGTTAAGGCATTTAGTGAAAAGAACATTCCCACTAAAGCCCGTCTAATCCAGATGAGTCTAGGCTTA

GGATACCAATTTTTGGTAAGACTTGTGGGGTTGACTTTACCTGTTAGACTACTAACTTTAAGCTTGTGGGAATTCTGGTTAACTTGTAGATTACTAAAGGCTTGTTCAATTTTAGAGATATTACTAGTTTCTGATTCTTGACTAGAGGTTTAGATATATATATATATATATATATATATATATATATATAACACTACTAATATATTTAATGAATGCAATCCAACGGCAGAAATTAAAAGTATGCAACCAATCTTTAACAGAAAATACTTATTGAAAAATCTCCATATAAAAAGTAAAGGTAGAATTTGGTGAAACTAATTAAAATTCTAAAATTTAGCAACTTAATTAATAAGGGATAAAATAATAAATGCAAATCTTTTAAATTAAAAAAAATTAAAATAAACTTCCAAAGTGTTGGAAGAGTAGCGGGCAGATGTTATGTGGCCTGATTAAAAAAAAAAAAAAAAAATCAATGCTGTTGTAAAGGTTTTTGTGTGGAGCCCACTAACTATTCATTTGGAGACTTTGGGTAAAGTTTGTCTGCAACTTTCCCTTAGTGCACATTCACGTGGGTTGGCTACCTCTCCCAATCTGCCTATAAAAAAGGCATTATCCTGCCTTTGCACAAACACCTCCAGATGAGAGAAGAATGAGAGTCAGGCAAAGAAGAGGAAGAGAAGGAAGAAAGAGGGTGAGTGAGTGGAGAGGAAAAAGAGCAGAGAGAAATTCTCCAAAAGAGAAAATTCAGTGAGCCACACATATTGTTAACACTAAAGTTGTAACCCTATTATTTTATATAGTGGAAAAGTTACTGTTGATGCTCTCCGAGGACGTAGGCATAGCCGAACCTCGTTAAATGTTGTGTCTCATCTACTTTACGTGCAGCTCAATATTCGCACATATTCCAGTTCATTTTATAACACGTTATCAGCACGAGAAGGTCTCAGGTATAATTTTTTTGTGCTGCACTATTATTTATACTACTAACCATTATATTGAT

>Chr1-11; OZ022206.1; 19418907-19426489; dir; D;

ACCAGGAAGTTTTCCAGTGAAGCCATTTCGAGCAAAATCAAGAACCTGAAGTCCAGAAGCATTTGACAACGATGCAGGAATGTTCCCTGTGAAACTGTTGGCACCGCCAACAAAAACCTGGAGATTAGGAAGAGTAATGCCGGCATTTGGTGGCAGCTCTCCATGAAGCTGGTTCTGAGTGACACCAAAAATATATATGGAAGAAATATTATAGATTGAAGCAGGGACCATACCGGACAAATTATTCCTTCCAAGCTTGAATGATCCCCAGCTTTTCAGACGCCCGAGCTCATTCGGAATGCTTCCTTGGAGATTGTTGCTGCCAAGAGAGAGACTGTTCAAAGAAGAAAAGTTCCCTATCCAACTTGGGATTGTTCCACTGAGATTGTTTTCATCAAGCCATATGTGAGTTTAAGCTCAACATCAAACTGAGCTGCTTTCGGGATCGATCCAACAAGCTCATTTGAAACAAGACTGAGAACTTTCAGCTGCGTGCGGTGAGATATATTGGTTGGGATTTCCCCACCAAAGGAAATCATAGACAGGTTAAGATGCTGCAGATGTAGCAGACAACGGATTTCTTGAGGAATTTCGCCATGAAAGTTGTTGTCAATAAGGTTGATTCCTGTAAGATATGTTAGGTTTCCTATAGAAAGTGGTAAGGAGCCTGCCAGTTTTTGAGAATCCAGTTTCAAGATCACCACTCTTTTGCTCGAAACGCTGCACGTAACGCCTGTCCAGCTGCAGAGTTGAGTCGAATCATTCCATGAGCTCATGACATGGAGAGGATCTTCAGTTATTCTTTTCTTGAATTCGAGCAACGCCATGCAATCGGGTTCCTTTCTGAAATTGGTGGCTAGTTTTGCAGATGCCAGACCTACGCTCATGCATAAAAGAACAAACCATTCATTGAACAAAAAAATGTGCCTACGTCGAGCACCAGAATGCTGCATCTTGTAGTAGATGCACGTTTCAGATCATGACCATATATATATATATA

TACAGAAGAAGATAAGAACAAATTAATCCAAGTTTTAGAGTTGCGAGACTCTGAAATGAGTGAAGTTGAGACCTTTGCACCCAATTCTGAGTCTGAGTCTGCCCAGTCTTCAGATTCACAGCCCAGTTCTCCTAATATTCAAGTTGGTTGTACTGATAAATGTTGTAATAGGTTAAAATCTATTTTAGTTCTTACTAAACAAGAGGAACAAGAAGAGTTACTTGTAGATTTAATAGGAAAGGTTCAAAACCCTGAGTTAAAATCTGAATATTTGAAAAAATTAAAAAAGATCTTAAGTCAGGAAGGTCAGAGTTCACCATCTCCCATTCCTCAGCCAATCAGCATCAATACAACATTAGAAAAATTCAATCGTAGAAAAGATGTCACCCTTCATGACCTCCAAACGGAAGTCAAGCAGATTAAAAAAGAATTAGTTGAATTAAAACAGACAAGCCAACAATTGCAAGTCGAAAATAATATCATTAAGCAAGACTTATATGACCTAAAATAAGAAACCTGCAAGCCGAGTAACCTTTCTGAAAGTCCTCATCAATCTGATGATGAACCTTCTGAAGAACAACCAGTTGTCAATTTAATTAAACAAGTTCATCTTAGAAAATGGTATTCAAAAGTCACGATATTCGTTCATGATTTTGAATTTCCCACAGTGGCTTTATTCGATTCTGGTGCAGATCTTAACTGTATTCAGGAAGGTCTCATCCCTACTAAGTATTACCAGAAGTCAAGAGAATCCCTTAGCACTGCCTCCGGGAAGTCTCTCCAGTTAAATTTTGAAATACCAAAAGCCCATATTTGCCAAAACAAAATCTGTTTTAAAACTTCATTCGTTTTAGTTAAAAATATCATTGATGATGTCATTTTAGGACTACTCTTCCTAGCTTTGCTGTATCCTTTCCAGGTAGAATACGATAGCCTCACCTCTACCCATTTAGGAGAAACAGTCAAATTTAAATTCTTGTCAAAACCTGAGTTGCATAATTTGAAAGCTTTACAAAAACAGTCAGTAGCTAAGTCTATCCAAGTTATCCGAAAAACCAACCAATTGAATTTTCTTAAAGAAGAAATTAAGTTTAAAAGGGTTGAACAACAACTTACTGATAAGGCTCTACAGTTAAAAATCCAGTCGTTTGAAAACAAATTAAAAGAAGAAATATGTTCCGAATTACCTACTGCTTTTTGGTATAGAAAACAGCATGTTGTCTGTCTTCCTTATATCAAAACCTTTAGTGAGAAAAATATCCCCACAAAAGCCAGGCCAATACAAATGAATCAAGAGTTGATGGAATTTTGCAAGAAAGAAATTCATGAGTTGCTTCAGAAAAATATAATTAGCAAAAGTAAGTCCCCCTGGTCTTGTCCTGCTTTTTATGTCCAGAAAATTGCTGAACTAGAAAGAGGAGTCCCCAGGTTAGTCATCAATTATAAGCCCCTTAATTCGGTCCTAGAATGGATTAGGTATCCCATACCTAATAAAAGAGACCTTATTAATCGGTTAGGGAAAGCAGTTGTCTTCTCCAAATTTGATATGAAAAGTGGTTTCTGGCAAATCCAAATAGATGAGTCAGATAGGTATAAAACTGCATTTGTTACTCCTTTCGGTCATTATGAATGGAATGTCATGCCGTTTGGATTAAAAAATGCCCCCAGTGAGTTTCAGAATATTATGAATGATACCTTCAATCCTTACAGTCATTTCTCGATTGTCTATATTGATGATGTTCTGATTTTTTCTGAGTCAGTTGAACAGCATTGGAAACATTTGCATAAATTCCTTTACATAGTCAAACAAAATGGTCTAGTTGTCTCTGCTAAGAAAATTAAGTTGTTTCAAACAAATATAAGATTTCTTGGTTTCAATATTAGTCAATCTCATATCTCTCCAATTGATTGAGTCATTCAGTTTGCTGATAAATTCCCTGATCAGATTCTTGATAAAAGTCAACTCCAGAGGTTTTTAGGATCTCTAAATTATATCTCTGACTTCTATCCAAATCTGAGAAAACAATGTAAGCCCCTATTTGATAGGTTGAAAAATAATCCTCCTCCTTGGTCAAATATTCATACTGAAGTTGTCAAACAGATCAAAGCCCATGTCAAGACACTTCCCTGTCTTGGTATTCCTTCAGTCGATTCTTTCAAAATAATTGAAACTGATGCCTCTGACATTGGTTATGGTGGCATCCTCAAACAGAAAGTCAGTCCAGAATCTCCAGAACAAATTGTTCGTTTCCATTCTGGAACCTGGACTCAGTCGCAATATAATTATAGTACTATTAAAAAAGAAATATTAGCTATTGTATTATGCATTAGTAAATTTCAAGATGATTTACTTAATCAAAAGTTTTTAATACGAGTTGACTGCAAATCTGCAAAACATGTTTTAGAAAAAGATGTTCATAACATTGCATCTAAACAGATTTTTGCACGATGGCAAAGTATATTAAGTATATATAAATTTTGATATTGAATATTTGAGAGGCAGTGAGAATTCAGTTCCTGATTTTCTTACTCGAGAATTCCTGCAGGAACCACAATGGCTCCCAAAAAAGACAAAAACAAAGAAGTTGTCAGTCAGCCCAAGTCAAGCCAGTCGCAACCCAAATCATCCCAGTTGCAACCATCTCAGTCGCCATCCATGAGTCCAGCCAAGCCAGAAATGTCCAAACAGATAGTTCCCTATCCTGGTTGTACCCCCATCAAAGTTGCCACCCCTCTATCAGTTGCCAATAGATTTTCCTCTCTAGGATCCACAGTTGGCCAAATTCGCCCCAGTTACCAGTCTACTCTAGTTGCCAGTTATGATCCCTTTGCAGTTGATATCCCTGCTGAACCCTCGGTACCTGCAGTTATATATAAAAAATCATCCCCCTATTTGCCCAAAAGTAATTCCCATCTGTTTGTCATTGAGCCCATTTACGATATCCATACCAACCCAATTGAAATTGCCCGTCATTATTTCCCTCCTGGATTTCATTATATGCCTTCCAGTCCCTACAAATTTCTCAAGTATTATAGAGATATATTGCTTGAGACCAAATCAGTTGAAATCAAACCCATAAAGGACCGTGACAACCCTGGCACTATCCTTTATCATTCTTTGTACATCCATCAAATTATAAGCCAAGAAGCCTTCAGCAGTCGCCCTTATGAACTCAAGTTACTCCAGTCCCAATTGCAATACAATTATGCTGATTATATTGATGCCTGGTATACTATTTGTCTTCATCAATCTGAAGATTTTAGTCATTCCTGGTTCATTAATTTTGATAACAAGTTCAAATGTTCTTTCCCCTATTGGTTTCTCCATTGGTGGGAAAAACATGGTCCAGTTGAAGAAATTTTACCAGCATCAGTTCTTGCACTCATCCACCATTACACCAAGAAGGCCAAGTTCTCTGCAGTTGACTTATTTTTCCCCAAGCAATTGTTATTTATTGCCAGATACAAAGTGCCCTGGATATTGAAATGGTCCTACAGGGTCACTAAAGATGTTCGTTTTTTTTGCCCGCCAATTCTCTGTTAAATGGTGGGACAAATTTGAAGTTGACAGAATTCAACAGAGAAGAACACAGAGAAGATAAGGAAGACAACTAAATACAATACAGAGAGAAAGAAATTAGAAATTAGAAATGAATGCAACCTAAGCAACTTTCAGACTAATGCAGAAAATAATGATAATGCATCAAATTAGTGCTTTCAAGAAATAGATAATTTGAAAATAACTTTTATTTATACTCACAAAGATTGCAACTGCAATTCTTCAAAGCACAATTGCACGCTATTTACAAGAGTGTTTACAAAGATGAATTTCTCCGAGGGATGTATCAGAGAGATTCTTTAAAACTGAAGAAAGGAAAATTACATTCTCAACAGAGAAGAACACAGAGAAGATAAGGAAGACAACTGAATATAATACAGAGAGAAAGAAATTAGAAATTAGAAATGAATGCAACCTAAGCAACTTTCAGACTAATGCAGAAAATAATGATAATGCATCAAATTAGTGCTTTCAAGAAATAGATAATTTTAAAAGTTATTTTCTTAAAATTATCTATTTCTTGAAAGCACTAATTTGATGCATTATCATTATTTTCTGCATTAGTCTGAAAGTTGCTTAGGTTGCATTCATTTCTAATTTCTAATTTCTTTCTCTCTGTATTATATTCAGTTGTCTTCCTTATCTTCTCTGTGTTCTTCTCTGTTGGTATCAGAGCCGTAAGTGTTACGGTCTTTGTCTAAGTTTGCTACTCATCCCCCCCAAGTGGCTGGTTTAAGCATCTGAGTAGAAGGTTTTCATTCTTTCCTTTTCTTCCCTTTTAGCCCTCGAGTCCTCTGTTGGTTTTCTCTAAGGTCTGACCCGTGAGTCCTTTTTACTAGATCAGTAAGTCCTCAGGGAGGGCGTGTGTGCTAGGCAGGAGACAGAAAATTGAGAAAATTAATAGGTTACTTAGAGTCTAAACGGTCGGTTTAGGATATACTGTTCATCTTCTTCCTCAGTCTGCTGAATCCAGTTTCTTGAGTAGTCATGAGTCACTTATTTAGAACAAAGTCTTGTAATTCTGCCAGTTCTAGTGCCAGTATAAGTCGACTCCCAGAGATAGTTAATGAAGAACAATTTGAATATAATTCCAGCAGTAAAGAACAATTAGATTTCAGAGATTGGAACATACCTAAAGTCCCCAGCCAGAAAATCTATAAGAAACATTGGTTGCCTTCTAGTTTTAATAGCACAACACATGTCAAAACAGTTGAACAAGTATACGCCTTAAGTAAAGAACATGAAACTTGTCAGTTGCTTAATCTTGAGTCTATTTTAAAACATAAGAAAGATGGTAATAATTTCCTGCATATTGGCTTAGTTCAAGTTGCTGTTAAACCCCTGACACGATTAGGGTTGAAAGCCTCAATCCTACTTTGTCTACGTGATGCTAGATTCACCGAATTTAGCGATAGTACCCTCGGAGTCATTGAGTCCAGTCTGTGTAATGGCCCTGTCCATTTTGACTGCTATCCCGATTTCACTGTAAGCCTTAGTGATCCCCATATCCTGAAAACTTTGACTCTGAACATTAAGACTGAAGGTTATAATGTCCTTCCAGGAACCCAACCTTTAGCCTTAGTTTACAGAATCTATTATAAGGTCACAGGAACTAATATGAATTTCCAAGCCCTTAATAAAAGCCCAAAGGATCAAACCGTCTTGATCCAGAGTCACACCTCAGATGCCCATATACAAGTCCCCCATACTATCAAGTGGTCTGAAGTCGCCCTCCCAAAAGATTGGACCCTAGTTACTGAGAGCCACCCAGCTCCAATCCAGTGTAGTCTGAACAATTTAGACTACATCCAACAATACTTAGATGGTACAGTTAAAATCAAATTTGACAGTCTTCCCCTACGCAAGTCTAATGTCCAGTTACAACAGTTGCCAACTCCTGGTCAGTCCCAAAGGCATGTCCCTGCTCGACATTCCTTTGCTGCCTCTTCCTCCACTCTAGAACGAGACCTAGAGTTAGAAAAGGCCATGATCGATTTCAAACTTGAGTCCCTGAGAAAAACCTCTCAGGTTACCCAACCTTGCTACGGCATGATTCCAGTTCAAGAAGACAAGTCAGAATCACCAGAGTCACCTACCCAGTCTGACTTTGTAGTTGAAAATCAGCTTAGAACCCTTAATAAAGAATTTAAAATTGATTGGATTTACCTAAATCGTCATCTAAAAGCCCCCGAAAACAAGTCAAGGCGAGATCTTTATCACCAAGCCTATCCTAATTGCAAACACCGAGAAATGATCTTTAAACAATGGAAAGATTATATGCGGTCAGCCAAAGTTGAAATTTTCTACCTTGATTTTATTGAAAGCCGTTATATGTCTAGTGATGAATTAAAAACCCTGACTAAAGAAAAATGGAAATTAGTTGACAAATCTGTAGTTGAATCTAGTCACCCTCCAGTAGAAACTATAATTATAGAACATCAGAAAGCCCCAGTTCCTGCCACTCCTTTTCAAAACCTTTGAAAGCAATGATCCTCATAGGAAACTAATTGAACAAAATAATTATACGAACCAGAGTTTAATCACCATAGGTAAACAATTAGATACAATTGAAACCAAGGTTGACCTGCTGAGTCCGCCTGTTGCAGACTCCAGACCCAGACCCAAAGTTGAAAAACCAATAGTTCAGTTTCAAGAACTAAAGACAAGTCCAGTTTTAAAAGTTAATTCCACAATGAAAAAGATAGAAGACATGTTAGCACAATTGACCCCTGTCAAGCCCGAGAAAGGTGAACAATCTGGTATTAAAACGCTAGATGCTTTTGCCACTTCTCAATCTGAGTCAGAATCCGTCAGCTCCGAAACTAGTGACATTTCTAAAATTGAAAATGCCTTCAAAAATTTAGAAATCCAAACTGAGCCCAAAGTCAAAAGAATTGAAAAACATATAAGCCCTACTAGTCTTACAAAAAATTGGTACCCCAGGCCAACACCTCCAGATATCCAATTTGAAGAAAGAAACTTCCAAACTCAGTTTTCTGTTTCTTCTGATAAATTGTATGAATGGAATATTGATGGTCTCTCTGAGCAAGAAATTCTGAATAAATTGCAACATATGTCTATGGTCGCAAATAGTTACATCACCAATCACAGTTTCAGACAATCTGAAATTGTTCCTCTGTTAGTTACTGGATTTACTGGTACTCTCAGATATTGGTGGGATAAGCACCTCACCCCTGAGTCTAAAAATCGTGTCATCTATGCTGTAAAACTTAATGAAGACGGTCTCCCTATATTTGACGAACAAATAGGTCAAGGTATAGAAGACGGTGTCAACACTTTGTTTTACACAATAATTGAACATTTCATCGGAACCCCTAGTAACACCACCGCCAGGATCCATGATCAGTTAAGCAATCTTAGATGCCCAAAGTTGTCTGATTTCAGATGGTATAAAGACGTTTTCATTTCTAGAGTCATGCTTAGAGATGATAGTAATCAACCATTCTGGAAAGAAAAGTTCATTAATGGTCTACCTAATTTGTTTGCTCACAAAATACGCACGACACTAAGTAATGAACAAGGCCAAATTGATTGGGATAATTTAACGTATGGAAACATTATAAGCACAATTAATCAAGTCGGCATGAAAATGTGTATTGACTTTAAAATTAATCGTCAAATACAGTCTGATAGGAAGTCGGCCAAGTATGAGTTAGGAAACTTTTGTGAACAATATGGTCTCATTAGTACTCCTCCCTCCAGGAAACATAAGCCCAGTCACCTTAGAAAACGCCATCATCCGAAGAAGCAATTTCTCCGGAAAGATGAATTCTATAAGAAAAGAAAGTTCTCCCCTAAGAAGAATTGGTCAAAACACCCAAAGCAGAGTCGCCCTAAGAAAGATAAAAGCAAAGTCAAATGTTTTAAATGCCAAAAATTTGGCCATTATGCCTCTGAATGCAAAATAAAAGATGTTATTAAACAATTACAGATCACAGAAGAAGATAAGAACAAATTAATCCAAGTTTTAGAGTTGCGAGACTCTGAAATGAGTGATGTTGTA

TATATATATATATATATATATATATATATATATATACACACACACACGTATATAAAGAAAAACCAAGTTTCATTTTACAAAGTCTAAAGACAAGAAGTTTATGGCATATAGCAAAAATGTTGATGATATTGCAGCAGGACCACAAAAATTGTGATTGTAACTATGAGTTGTAGATGGTCCTGCAAGAGAAATGTGAAGAAATTTTGGTTCAATATTAATGCCTCTGGCTTCAATTTTGGTATGTGGATGTGAGATTGTAGTTGGGAAGCAATTAATATTCATAGGCCTGACCAATTTGGTCCAAATTTTTTATTTCATTATGGGGGATAAGATTTTTAGTTGTGAACTTGGAAGCATTTTCTTGTTCATTTTTTATTTATTTATTACAATGTGATTTATCAATCTGATATTGTTGGAACAGATCATGACTCCAATATTTACTTCCATGTTTCTATTTTACAACAATTTATATAATTAATATAGGGTTTTCTAACCAACAACAGCCCTTATAGCTCAGTGGTAGAGCGTCAGTCTTGTAAACTGAAGGTCCGTAGTTCGATCCTGCGTGAGGGCATCTTTTTTTTTTTTTTTTTCTTTATTAAACTATGTTGGAAAATGAATCAACAGCCACGTGATTTTTATTCAAGTTTGCTGAATTATTTTAAACTTATTTTTTAATAATGACGATATTCTAAGCTATTTTAATGTATGGGGAGAGAGTTCGAATTCAAGTGTAAGGGAGTCGAGGGGTGCCTGCATGTGCTTTTAAACCGGGAAGGTCCCTAGAAGGGACTAACCTGGGATATAGCCCATCTAAAATTTAATTGGGAGTTTCACTATTATGCATAATATAGGGACCCAAATTATAAAAAAAATCATATATGAAATGAACTTTAGAAACACATCCAAATTCCATTTACAATATAAAAATGAGGCTTCAAACTTTTTTTAAATTACAAAACTGTCATCGATTTTTTAAAACAAGCCTAACCCAAAAACC

>Chr1-12; OZ022206.1; 19659368-19662862; dir; B;

TATGAACGCGAAGTTACAGTTCTACCTCTTGCCTTTTTCACCATTGAACAAAGAGCAGTGAAGCATGACAGAGTAATTATGAACGCAATTGCACAAGTTAAAGGAACTACCACTTTTAAGGCAAGTAGTCCTTGAGATGAACGAGGCTTTTTGCTGGAGCACCTTGGTAGAAGTAATTTTGGGATGCCACCGCAGAGCCTATCATTTTGAGACACCACTAGCTTTTGAAAAGGATCCCTTCTTTCAGCAATTCACCCTTGAAATCATTGTGAGAAAGATTGAGATGCTTAAGAAATCTAAACTTGCTCAGAAACTTAGGAATCTGCCCAGATAAGTTGTTCCTTGAAATATATATTTCTTCCAATTCTCTTAAATCTTTAAGAGATTGAGGAATTGCTCCTGCAAATTCATTACCTTCCAAATGCAAGCGCGCCAAAGTAATACAACTGCCAAGGCTTCTTGGGATTTCACCAGATAACTTATTTCTTGATACATCTAGCTCCGTGAGATGTACCAAGTCACCCACGTCCGATGGTAGCGAACCAGTCAACGAATTTTTAGACATGCTCAAAGAAACCGAAAGAGATGAAAGCATAAGAAGCTGTTTATGTATGGTGCCAGATAGATTGTTACTAGAAAGGTTAAGCATTAGTAGATTTTGGCAGTTTCCAAGACTTTTAGGTATACTACCCTCAAACCTATTCTCTTGCATGTAAAGCCTTGTCAATGAAGTCAAATTTCCTATTGAGAATGGGATTAGACCAGAAAATTTGTTAGCATTTAAGAATAGTCCTTGTATCATATTGTTACCTTCCACTCCGAAAGCACTCAAATTTACAAGATTTCCAATGCCTCTGGGATGCTTCCATGTATCATATTGACGCCCAAAGTAAGAATTTTTAGCTGGGTAGAAAGGTTGGCTATGGATCCTGGAAATTCTCCTCCGAAACGATCACGGCTAAGACCTAACACCTGGAAATTCTTAAAATTATCTATTTCT

TGAAAGCACTAATTTGATGCATTATCATTATTTTCTGCATTAGTCTGAAAGTTGCTTAGGTTGCATTCATTTCTAATTTCTAATTTCTTTCTCTCTGTATTATATTCAGTTGTCTTCCTTATCTTCTCTGTGTTCTTCTCTGTTGGTATCAGAGCCGTAAGTGTTACGGTCTTTGTCTAAGTTTGCTACTCATCCCCGCCCAAGTGGCTGGTTTAAGCATATGAGTAGAAGGTTTTCATTCTTTCCTTTTCTTCCCTTTTAGCCCTCGAGTCCTCTGTTGGTTTTCTCTAAGGTCTGACCCGTGAGTCCTTTTTGCTAGATCAGTAAGTCCCCAGGGAGGGCGTGTGTGCTGGGCAGGAGACAAAAAATTGAGAAAATTAATAGGTTACTTAGAGTCTAAACGGTCGGTTTAGGATATACTGTTCATCTTCTTCCTCAGTCTGCTGAATCCAGTTTCTTGAGTAGTCATGAGTCACTTATTTAGAACAAAGTCTTGTAATTCTGCCAGTTCTAGTGCCAGTATAAGTCGACTCCCAGAGATAGTTAATGAAGAACAATTTGAATATAATTCCAGCAGTAAAGAACAATTAGATTTCAGAGATTGGAACATACCTAAAGTCCCCAGCCAGAAAATCTATAAGAAACATTGGTTGCCTTCTAGTTTTAATAGCACAACACATGTCAAAACAGTTGAACAAGTATACGCCTTAAGTAAAGAACATGAAACTTGTCAGTTGCTTAATCTTGAGTCTATTTTAAAACATAAGAAAGATGGTAATAATTTCCTGCATATTGGCTTAGTTCAAGTTGCTGTTAAACCCCTGACACGATTAGGGTTGAAAGCCTCAATCCTACTTTGTCTACGTGATGCTAGATTCACCGAATTTAGCGATAGTACCCTCGGAGTCATTGAGTCCAGTCTGTGTAATGGCCCTGTCCATTTTGATTGCTATCCCGATTTCACTGTAAGCCTTAGTGATCCCCATATCCTGAAAACTTTGACTCTGAACATTAAGACTGAAGGTTATAATGTCCTTCCAGGAACCCAACCTTTAGCCTTAGTTTACAGAATCTATTATAAGGTCACAGGAACTAATATGAATTTCCAAGCCCTTAATAAAAGCCCAAAGGATCAAACCGTCTTGATCCAGAGTCACACCTCAGATGCCCATATACAAGTCCCCCATACTATCAAGTGGTCTGAAGTCGCCCTCCCAAAAGATTGGACCCTAGTTACTGAGAGCCACCCTCCAATCCAGTGTAGTCTGAACAATTTAGACTACATCCAACAATACTTAGATGGTACAGTTAAAATCAAATTTGACAGTCTTCCCCTACGCAAGTCTAATGTCCAGTTACAACAGTTGCCAACTCCTGGTCAGTCCCAAAGGCATGTCCCTGCTCGACATTCCTTTGCTGCCTCTTCCTCCACTCTAAAACGAGACCTAGAGTTAGAAAAGGCCATGATCGATTTCAAACTTGAGTCCCTGAGAAAAACCTCTCAGGTTACCCAACCTTGCTACGGCATGATTCCAGTTCAAGAAGACAAGTCAGAATCACCAGAGTCACCTACCCAGTCTGACTTTGTAGTTGAAAATCAGCTTAGAACCCTTAATAAAGAATTTAAAATTGATTGGATTTACCTAAATCGTCATCTAAAAGCCCCCGAAAACAAGTCAAGGCGAGATCTTTATCACCAAGCCTATCCTAATTGCAAACACCGAGAAATGATCTTTAAACAATGGAAAGATTATATGCGGTCAGCCAAAGTTGAAATTTTCTACCTTGATTTTATTGAAAGCCGTTATATGTCTAGTGATGAATTAAAAACCCTGACTAAAGAAAAATGGAAATTAGTTGACAAATCTGTAGTTGAATCTAGTCACCCTCCAGTAGAAACTATAATTATAGAACATCAGAAAGCCCCAGTTCCTGCCACTCCTTTCAAAACCTTTGAAAGCAATGATCCTCATAGGAAACTAATTGAACAAAATAATTATACGAACCAGAGTTTAATCACCATAGGTAAACAATTAGATACAATTGAAACCAAGGTTGACCTGCTGAGTCCGCCTGTTGCAGACTCCAGACCCAGACCCAAAGTTGAAAAACCAATAGTTCAGTTTCAAGAACTAAAGACAAGTCCAGTTTTAAAAGTTAATTCCACAATGAAAAAGATAGAAGACATGTTAGCACAATTGACCCCTGTCAAGCCCGAGAAAGGTGAACAATCTGGTATTAAAACGCTAGATGCTTTTGCCACTTCTCAATCTGAGTCAGAATCCGTCAGCTCCGAAACTAGTTATATTTCTAAAATTGAAAATGCCTTCAAAAATTTAGAAATCCAAACTGAGCCCAAAGTCAAAAGAATTGAAAAACATATAAGCCCTACTAGTCTTACAAAAAATTGGTACCCCAGGCCAACACCTCCAGATATCCAATTTGAAGAAAGAAACTTCCAAACCCAGTTTTCTGTTTCTTCTGATAAATTGTATGAATGGAATATTGATGGTCTCTCTGAGCAAGAAATTCTGAATAAATTGCAACATATGTCTATGGTCGCAAATAGTTACATCACCAATCACAGTTTCAGACAATCTGAAATTGTTCCTCTGTTAGTTACTGGATTTACTGGTACTCTCAGATATTGGTGGGATAAGCACCTCACCCCTGAGTCTAAAAATCGTGTCATCTATGCTGTAAAACTTAATGAAGACGGTCTCCCTATATTTGACGAACAAATAGGTCAAGGTATAGAAGACGGTGTCAACACTTTGTTTTACACAATAATTGAACATTTCATCGGAACCCCTAGTAACACCACCGCCAGGATCCATGATCAGTTAAGCAATCTTAGATGCCCAAAGTTGTCTGATTTCAGATGGTATAAAGACGTTTTCATTTCTAGAGTCATGCTTAGAGATGATAGTAATCAACCATTCTGGAAAGAAAAGTTCATTAATGGTCTACCTAATTTGTTTGCTCACAAAATACGCACGACACTAAGTAATGAACAAGGCCAAATTGATTGGGATAATTTAACGTATGGAAACATTATAAGCACAATTAATCAAGTCGGCATGAAAATGTGTATTGACTTTAAAATTAATCGTCAAATACAGTCTGATAGGAAGTCGGCCAAGTATGAGTTAGGAAACTTTTGTGAACAATATGGTCTCATTTACTCCTCCCTCAGGAAACATAAGCCCAGTCACCTTAGAAAACGCCATCATCCGAAGAAGCAATTTCTCCGGAAAGATGAATTCTATAAGAAAAGAAAGTTCTCCCCTAAGAAGAATTGGTCAAAACACCCAAAGCAGAGTCGCCCTAAGAAAGATAAAAGCAAAGTCAAATGTTTTAAATGCCAAAAATTTGGCCATTATGCCTCTGAATGCAAAATAAAAGATGTTATTAAACAATTACAGATCACAGAAGAAGATAAGAACAAATTAATCCAAGTTTTAGAGTTGCGAGACTCTGAAATGAGTGATGTTG

TATATATATATATATATATATATATATATATATATATATATATATATATATATATATACGTATATAAAGAAAAACCAAGTTTCATTTTACAAAGTCTAAAGACAAGAAGTTTATGGCATATAGCAAAAATGTTGATGATATTGCAGCAGGACCACAAAAATTGTGATTGTAACTATGAGTTGTAGATGGTCCTGCAAGAGAAATGTGAAGAAATTTTGGTTCAATATTAATGCCTCTGGCTTCAATTTTGGTATGTGGATGTGAGATTGTAGTTGGGAAGCAATTAATATTCATAGGCCTGACCAATTTGGTCCAAATTTTTTATTTCATTATGGGGGATAAGATTTTTAGTTGTGAACTTGGAAGCATTTTCTTGTTCATTTTTTATTTATTTATTACAATGTGATTTATCAATCTGATATTGTTGGAACAGATCATGACTCCAATATTTACTTCCATGTTTCTATTTTACAACAATTTATATAATTAATATAGGGTTTTCTAGCCAACAACAGCCCTTATAGCTCAGTGGTAGAGCGTCAGTCTTGTAAACTGAAGGTCCGTAGTTCGATCCTGCGTGAGGGCATCTTTTTTTTTTTTTTTTTTCTTTATTAAACTATGTTGGAAAATGAATCAACAGCAACGTGATTTTTATTCATGTTTGCTGAATTATTTTAAACTTATTTTTTAATAATGACGATATTCTAAGCTATTTTAATGTATGGGGAGAGAGTTCGAATTCAGGTGTAAGGGAGTCGAGGGGTGCCTGCATGTGCTTTTAAACCGGGAAGGTCCCTAGAAGGGACTAACCTGGGCTATAGCCCATCTAAAATTTAATTGGGAGTTTCACTATTATGCATAATATAGGGACCCAAATTATAAAAAAAAATCATACATGAAATGAACTTTAGAAACACATCCAAATTCCATTTACAATATAAAAATGAGGCTTCAAACTTTTTTTAAATTACAAAACTGTCATCGATTTTTTAAAACAAGC

>Chr1-13; OZ022206.1; 20982235-20984681; rev; B;

TAAATTGTGAGAAAATCACTTATTATTAAAAATTGCTGTGAGAAAAAGCTATAAGGGAAAACAGTTAATGAGGTGCTTTCATTTTCTGATTATGTAGGAATCAGTTTTGAAGAGGCGCTGGGTTTAATGATTATGCGGCAATCATTTTCTAATTACACGTAAAATCAATACCAACAACACTTTTGACAAAAATTTTACCAAATCCCAAACTGCTATCTGTCACAGCAAATTTGACAGTGATTATTTTCACAATACAGCAATGCCAAACTGGCCCTAAGTGCCCATTTGGCATTGCTTATTTGGAGTGATAAGTGATTTTTAAAAAAATTTAAGAAGCCCAACCTGTTTGGTAAACTGTGAGAAAATCACTTATTGTTAAAAATTGTTGTGAGGTGCTTTCATGTTATGATTATGCAGGAATCAATTTCGAAGAGGCGCTGAGTTTAATGATTATGCGGCAATCATTTTCTGATTATGCGTAAAATCAATACCAACAACACTTTTAACAAAAATTTTACCAAACACCAAACTGCTTTCTCTCACAGCTGATTTCTCTCACAGCAAATTTGAAAGCGATTATTTTCATAGCACAGCAATGCTAGTATGTGGAGCTCACACATCCAATAATATAGAGCCACATATTTTTAAATAAAAGATAATCATTTAAAACAAAAAAATCCATTTTAAAAATTAAGATTAAAACATCAAATTTTAAATTTATTAAATATAAAATAAATAATAATAACAAAACCTACATTCATCCCTCCACCCACACCCCAGCCGCAACCAACCCTTCCCCACCCTTATTTCCGAGCCTTACCCTCGACCCAACGGCAGCCCCACCTCCCCAACCCACTCTCTGCCTCTCTCTCCCCTCCTTTTCCTCTCTCTTCCGCTCTCTCTCTCTCTTTCCATCATTGGCGGAGCCAATTAAGGCCCACGAGGGTCACTTGACCCTTCCCAACATTTTGAAAATAATATATATATATAGATATATATA

GGCGTGAGTGGTGGGCAGGAGACAGAAAATTGAGAAAGGTTAATAGGTTTCTTAGAGTTTAAACGGTCGGTCTAGGATTTACTGTTTATCTTCTTCCTCAGACTGCCATACCCAGTTATTTGAGTAGTCATGAGTCACTTATTTAGAACAAAGTCTTGTAGTTCAAACAGTTCTAATGCCAGTATAAGTCGACTCCCAGAAATAGTTAATGAAGAACAATTTGAATATAATTCCAGTAGCAAAGAACAACTAGATTTTAGAGATTGGAATATACCTAAAGTCCCCAGCCAGAAAATATATAAGAAACATTGGTTGCCTTTCAGTTTTAATAGCACAACACATGTTAAAACAGTTGAACAAGTTTACGCCTTAAGTAAAGAACATGAAACTTGTCAGTTGCTTAATCTTGAGTCTATATTAAAACATAAGAAAGATGGTAATAATTTCCTGCATATTGGTTTAGTTCAAGTTGCTGTTAAGCCCCTAACACGATTAGGACTAAAAGCCTCAATCCTGCTTTGTTTGCGTGATGCTAGATTCACTGAATTTAGCGACAGCACTCTAGGAGTCATTGAGTCCAGTCTGTGTAATGGCCCTGTCCATTTTGACTGCTATCCTGATTTCACTGTAAGCCTTAGTGATCCTCATATCCTGAGAACTTTGACTATGAATACTAAGACTGAAGGTTATAATGTTCTTCCGGGAACCCAACCTTTAGCCTTAGTTTACAGAATCTACTATAAGGTCACAAGAACAAATATGAACTTCCAAGCCCTCAATAAAAGCCCAAAGGATCAAACTGTCCTGATCCAGAGTCATACCTCAGACGCCCATATACAAGTTCCCCACACAATTAAGTGGTCAGAAGTTGCCCTTCCAAAAGATTGGACCTTAATCACCGAGAGCCAACCAGCTCCGACCCAACGTAGTCTAAACAATTTAGACTACATCCAACAATATCTAGACGGCACTGTAAAAATTAAATTTGATAGTCATCCTTTAAGAAAGTCCAATGTCCAGTTACAGCAGTTGCCAACCCCTGGACAATCACAAAGGCATGTCCCTGCTCGGCATTCCTTTGCTGCTTCGTCCTCCACTCTAGAACTAGACCTAGAGTTAGAAAAAGCCATGATAGATTTCAAACTTGAGTCCCTGAGAAAAACTTCTCAGGTCACACAACCTTGCTACGGCCAGGTTCCAGTTCCAGATGATAAGTCTGACTCACCTGAGTCTCCAACCCAGTCTGATTTCCTAGTGGATAACCAACTCAGAACTTTAAATAAGAAATTTGAAATTAATTGGATACGTCTAAATCGTCATTTAAAAGCCCCGGAAAACCAGTCCAGAAGAGATAAATATCACCAAGCCTATCCTGATAGTAAACGCCGTGAACAAATCTTTAGTGAATGGAAAGGTTATATGCGGTCAGCCAGAGTTGAAATTTTCTACCTCGATTTTGTTGAAAGCCGTTATATGTCTAATGATGAGTTAAAAACTCTAACTAAAGAAAAAAGGAATTTGGTTGATAAGTCTGTAGTTGAAGCCAGTCATCCCCCAGTCGACACTATAATAATAGATCATAAGAAAGCACCAATACCTGCCACTCCTTTCAAAACCTTTGAAAGCAGTGATCCAAATAGAAAATTAATCGAACAGAATAATTACACCAACCAGAGTCTAATAGTCATAGGAAAACAGTTAGACACAATTGAAACCAAAATTGACAAAATGAGTCCTCCGGAAACAGATCTCAAGCCTAAGCCCAGAATTGAAAAACCAATCGTTCAGTTTCAAGAGCTTAAGTCCAGTCCTGCACTTAAAGTCAATTCTACAATGAAAAAGATAGAAGAAATGTTAGAACAGTTGACCCCAACTAAAATTGAGAAAGCCGAAAAATCTGGTCTTAAAACTCTGGATTCCTTCGTTGCCTCACCTTCTGAGTCTGAGTCATTAATTTCCGAAACCAGCGACATTTCAAAAATTGAAAACGCTTTCAAAACCTTAGAAGTCCAAACTGAACCCAGAATCAAAAGATTAGACAAATATGTAAGTCCTACTAGTTTAACAAAAAATTGGTATCCCAGACCAACACCCCCAGACATCCAATTTGAAGAAAGAAGCTTCCAAACCCAGTTTTCAGTTTCTTCTGATAAATTGTATGAATGGAATATTGATGGTCTTTCTGAGCAAGAAATCCTAAATAAGTTACAACATATGTCCATGGTCACCAATAGTTACATCACCAATCACAGTTTCAGACAGTCTGAAAAATCGTATCACTTAAGCTGTAAAACTTAATGAAGACGGTCTCCCTATATTTGACGAACAAATAGGTCAAGGTATAGAGGATGGTGTCAACACTCTGTTTTACACAATAATTGAACATTTCATTGGAACCCCTAGTAATACCACTGCTAGGATACATGATCAGTTGAGTAATCTTAGAT

AGGGCAGAGGTGGAGAGAGATAGGGCAGAGGTGGAGAGAGACGGAGCGGAATGAGTTGGATGGGAAGTTGCAGAGGGACCTGCAACTTAAAAAAAAACTATTTTTTAAAAGCATTTTGTTTAGATTTAAAATATGAAACTATTTTTTAAATAAATTTTTTATTTAGATCCACGTGGTACCACATATGTGCCACGTCATTATTTTAACAGAATTTTTGATGAAACCTAACAGTATGACCAAATTGATGTGTGAGTTGTAACATAAAGGACCAATAATATCACAAAAAACATAAATGACCAAAGCTGATAATTTCACAAACCTCAAGGACCATTTGTGATTTAAAAAAAAACCTTTCATTTAATTAGTAACATTATGTGTTTTCACAATGCGAATAACCAAATAGTGTTAATACGATGCTAATCACTTGTTTTGGCATATTACAACTTTTAATTTCATATTTTGTATCACTCATTATTTTAGTTCTTTATTTAAAGATTGTAGACAAAGAAATTTTGGTGCAATAAATTCCATTGGACAAGAAAATAATTAGGGTTCGGTGGATTAAATCGTGGGCCCCGTAATTGCCATGTGCCGTGTGATTCATCAAAATCGGATTAGTTGTCAAAAATGACACATGGCGACATCCGACGGAGTGCATCAAATGGTTCAAGATGAAGACAAAATTAAAGGAAAGAATCTTCTGTGATTGACTTTGATTTAAATCAAATATTAATCAGGTTAATCAATTGAAGATAATCTGGTTAAATTGCCAGATTATGGGAAGTGATTTAAATCAAATCAAAATCCCATAAAACAAGGTTTTAAATAGGGAAAGTTATTTAGGTCAAGCATCCTACAAAAGCCTATAAATAGGAGGCCTCAAGATGAAAGAGGGTCAGAGAAAAAACCTAGCACTCAGAAGAAACAGAAAACTCTCTGCATTCTTCACCCTACTTTGGAAGAGCCACCAAGCACAACACATACGTGCCGGTTCCTTCAC

>Chr1-14; OZ022206.1; 22142519-22143981; rev; B;

AAGAATTATTTGAATCTGATACTGAAACTAGCTCAGATAGTGAAGACGAGTGCGCCTGTCAATTCAATGATCTTAGAATATGTGTCTTAACAAAAGAAGAAAGTCTGATAATAGACTTAATTGACAAGATAGAGGATCCAGAGAAAAAGAGAGAAGTGTTAGAAAGTTATATCTCTGTAGCTAAAGCAGGACCAAGTAACCCACAGCCAGTTATAACTAATGTGAGACAACCAGTTGAAAACTATAGCTTTAAAACTATAGTAAACAGAATGAATGATGAAAATCATAAGAAAGAACCTACTCTCAATGACCTTCAGTGCGAAGTTCATAATGTGAAAGAAGAACTTCGGAAATTGAAAATTAGGGTTAGAGTTCTAGAACTCTATCGCCCTTTTTTGGAAAAGGATACAGAAGAAAAATCTGAGCAATTAGAGTTTGAAAATCTGATGGGAGAATTTCAAGAGAAAATAGAACCTGTGAGAAGAGAAGCCCTACAAGAAGAGCAGGTTAGTACCATGAAATTCTTTAATAGCATAGATAGAGTAATAGCTCAGAAATGGTATGCGATGATAACTATTGTGATTAATAAAACCAATAAGTTTACAGTTGAAGGGCTGATAGACACAGGAGCCGGTCTTAACTGTATAAATGAAGGAGTAGTACCCACTAGGTATTTTCAAAAAACTACCCAGGTTCTACATACAACCGTTAATAGCCAGAAAATGGAAATTCAATTCAAATTATCAAACATTGTTGTACTTAATCAGGGTATTTATTTTGAAACTCCTTTTGTTCTGGTGAAAGGTTTAAACCAGTCTCTAATATTGGGCACACCATTCATTAATATGCTTTATCCTTTTTCTGTTTCTGAAGAAGTAATTAGAACTGAGGTAGAAGGTTAACCTATAATCTTTAGGTTTTCTCAACCACAAAAACATAAGTATATTAATGAAATTCAAGAAAAAATAAAAAGGGAAGAATACTATCTATATCATTTGAT

AGAAGAAATGCTGCACAAAAGAATAGAGGTGCAACTGCAAAATAATGAGATAAAAGAAAGAATTGATGAAATTAAAAAGATGTTCGAAAATCAAGTTTGTGCTGAAATACCCAACGCCTTTTGGAATAGAAAGAGGCACATAGTTGAATTGCCATATAAGCCTGATTTTGATGAAAAGAAAATCTCAACCAAAGTCAAGCCAATTCAAATGAATGCTAGGTTAATGGAAATTTGTAAAGCTGAAATACAAGATTTATTGAACAAAAGGTTAATTAGGAAAATCTCTTCTCCTTGGATATGTTCTGCCTTTTATGTTGAAAACACATCTGAATTAGAAAGAGGAGTACCCAAATTGGTGATAAACTATAAACCTCTAAATAAAGCCTTAAAATGGGTCAAATATCTGATTTCTAATAAAAAGGATCCTTTGGATAGACTAAATAAGGCAGTCATTTTTTCTAAATTTGATATGAAATTAGGATATTGGCAAATGCAGATCGCCAAAAAGGATAGGTACAAAACAACCTTTAATGTTCCCTTTGGACATTACGAATGGAATGTTCTACCTTTTGGATTGAAAAATGCCCCTTATGAATTTCAAAACATTATGAATGATATATTTAATCCTTACACTGCTTTTACTATTGTTTATATTGATGATGTTTTAGTTTATTCTAATTCTTTAGAACAACACTTTGAACATCTAAATATTTTCTTGAAAGTTGTAAAGCATGCAGGATTAGTAGTATCAGCAAAGAAAATGAAATTATTTTCTTCAAAAGTTCACTTTTTGGGACATAATATTAATAAGGGACTAATAGTTCCGATTAATCAAGCAATATCCTTTGTTGATAAGTTCCCTGATGAAATTAGGGACAAGCAACAATTACAAAGGTTCTTAGGAAGTCTCAATTACAGAAAATATTTTTATAAGGATTTAGCCCAAGACTCAAAATTATTATACCAAAGCTTGAAAAAGAACCCAGCTCCTTGGTCTGATGAACACACTAAAGCTGTTGGGAGACTAAAAGTTAAGGTCAAAGAACTTCCTTGTCTAGCTCTAACCAACCCAAAAGCCTTTAAAATCATAGAAACTGATGCTTCATAAATTGGGTACAGGGGAATCCTAAAACAAAAAGTTGATAACTTAGAACAACTAGTTAGGTTCACCTCGGGAATTTGGAAACCAGCTCAAAAAAATTATTCCACTACTAAGAAAGAAATGCTAGCCATTGTTCATGCTGTTTCTAAATTCGAAAGTGATCTTTTAAATAAAAGATTTTTTATAAGAGTGGATTGCAAGGCTGCAAAAGACATTTTGTTTAAATATGTAAAAAATTTAGTATCAAAACAGATTTTTGCAAGATGGCAAGGAATTCTTTTTGCTTTCAATTTTGATATTGAATTTATTAAAGTTGAAACTAACGCACTCTAGGACTTTTTAACTCGGGAATTTTTGCAGG

GTGCGAACCAGAAACTCTAGACAGATGACTCCCAATAAGAGTTCCTCTGCTCTTACTAAGTCATCCCCTCTTGATCCAAAGCCACTAATGTCTTCTTCTCCACATAATAAGGGTAAGGAGAAGCAGATTGCTAAAAATCCATTTCGTGAATATATGAATGTGTCTGTAGCAATCATTGAAATGAATGATCTAGATAAAACCCAAATTTTCGGGCCGATTAAGGGATAATCTTCCTCCCTTGAATAAGCCTCGTATTTTCTATGAAACTCTTCTAATTGAAACTAAGTCTGTTAATATTGTTCATCATCATAGAGGTGAAAATACCAACACAATAGAATTCTCTAAATCCAAATTCTAAATGTTATTTCCCTGGAAGAGTGGGGTCCTAGCCCTAAAATGGCCAAAAGATTTATCCATCCTGAAATTAGGCCATATTCCTATAATTTCTGGGATTATAAAAAAGCATGGGAATATGTTTTATGTTTTCAAAATCAAAACTATTCTCATACTTAGTTTATCTAGTTTAAGCTTCGAACTCTGAAAGAACTCCCAAATTGGTTGAAGAGCTTTTTTCTTATTTGGGGTCCTACTTTGGAATACCTTCCTCCTGAAATTAAGGAATTTTTTCAAATGAAATTTTGTAAATGGAATAAAAACCATCCTGATCAAAGGACCTTATTCATGTGGTTCATGATAATGTATGAACTACCATGGATATTAAAATGGGAATATGTTATTTTGGAGGATGTTGATGAGGAATGGACTTATATCCCGAATTTAGTCCGAAGTATCTTAATCAAATGGTGGGATAAATTCACTAGAGATCACTTGGCTCCTCTTAAGATCACCCCGACGCTTATCCCTTCCTTCGACCCTGCTGCTGCTCAACTTTTGGCCACTTCTTCTACTAATTCAAGTGTCTCCCAAAGGTTACAACTGTTGATAGACCAACTAGGCTCATCATCTTCCAAAGAAGACTTGAAAAAGAAAGTCCTTGAGA

>Chr1-15; OZ022206.1; 22173508-22178773; rev; B;

GCCTTTGGAGGTCCCTAGGCGTAGGTTTGCGGAGGTAATCCCTCGTGTAGAGAGTTTTGATTGCATCACAGAATCTAACCAAGCTTTTTAAGATAGTGGACTTCCCCATCCTAGCAATCTCATCCACCTAGTCTGCAGATGCACCAAACGCCAGCATATGTAAAGAAGCTGAAAGCTTTTGCTCTTGAAGAAGCTCCAAAACCCTAGTTGCATCACACTTCTGAACGAAGTATGTGTCGTAATTGCAAACATCATGCATGACTTTATTAAACAAATGGGGCTGCATTTTATATCGCCCTAAAAAATCAACAGTAGAGTACAACGAATTTGAGATATAGTAATCTTCCAAAAGATTCTTACTCCGAGAAAGCCTATGTTTGTTCACATTTGGGCCATGGCCGACTTGTGAACCACATTGGCAGCCTTGCCTTGATAGATCGAGCATACCCACTGCCATGGCAACTTATTCATCATCTTCTTCCTCCTCCCTTTCTTTTTCAGCTCTTCTGCGCATGTTTTCTTTAATTTCTCGCTCTTCCCTCTCCAAGCATCTCCTCATGTTCGACATTGAGAGTGGACTATGAAATCTTAAATCTGAGAAATAAAATGATAGAGAGGATATGATGTGAGAGGTCTGAGTTGAGCCCCTGGTTTTAAAAGGGATTTAGAAGATAGAGAGGACACGTTGCACAATTTTATATGGTGAAAATCTTATCTGAAATTTGAGAGGTGAATTCAATAATAAATATGGACACGTGGCAAGTAAAAATCTTATCATCCAAAAAATTCATTTGAATTATGAGATATGAATTTTATAATAAATAACACGTGGCAACAAGAAATATTATTCGAAAATCTTCTTCAAATTAATTGTTTAAGTATAAAATATATATATATAATAATAATAATAAATAAAAGTTAAAGTAAATAGTATTTGCCTTTTGCCATGACAATGAGTGGAAACACAACAAGTCTTTTGCAAGGGCCGTTACTATTCATG

AATAGCTCACTTTCAAGAACTTAAGCTTGGTCCTGCACTCAAGACCAATTCCACAATGGAAAAAATAGAAGAAATGCTAAAACAGTTGACTCCTGTCAAACCTGTCAAGACCGAGATAGGTGATCAATCTGGTATTAAAACACTAGACGCTTTTGCCTCTCATTCCGAAACCAAATCCGTTAGTTCAGAAACTAGTGATATTTCTGAAATTGAAAATGCTTTTCAAAATCTAGAAATACAACTTGAGCCCAGAGTCAAAAAGATCGAGAAACATATAAGCCCTACTAGCCTAACCAAGAATTGGTATTCCAGACCAACACCTCCAGATATCCAATTTGAAGAAAGAAACCTCCAAACCCAGTTTTCTGTTTCTTCCGACAAATTGTATGAATGGAATATTGATGGTCTTTCTGAGCAAGAAATCTTAAATAAATTACAACATATGTCGATGGTCGCCAATAGTTATATCACCAATCACAGTTTCAGACAGTCTGAAATTGTCCCCCTATTAGTTACTGGTTTCACCGGTACTCTCAGAAGTTGGTGGGATAAGCACCTTACCCCTGAAAATAAAAGTCGAATTATTTATGCTGTAAAACTTAATGAAGATGGTCTCCCTATATTTAACGAACAAATAGGTCAAGGTATAGAAGACGGTGTCAACACTCTGTTTTACACAATAATCGAACATTTCGTCGGAACCCCTAGCAACACCACATCTAGGATCCATGATCAATTAAGCAATCTTAGATGTCCCAAGTTGTCTGATTTCAGATGGTATAAAGACGTCTTCATTTCTAGAGTCATGCTTAGAGATGATAGTAATCAACCATTCTGGAAAGAAAAGTTTATTAATGGACTGCCAAACTTATTTGCTCATAAAATCCGCACAACCTTAAGTAATGAACAAGGTCAAATTGATTGGAATTCATTAACTTATGGTAATATTATAAGCACAATCAATCAAGTTGGTATGAAAATGTGTATTGATTTTAAAATTAATCGTCAAATCCAGTCGGATAGGAAGTCAGCCAAGTACGAGTTAGGAAACTTCTGTGAACAATATGGTCTCACTAGTATTCCTCCTTCTCGGAAACACAAACCTAGTCATCTTAGAAAACGCCATCATCCGAAGAAGCAATTTCCCCGGAACGATGAATTCTATAAGAAAAGAAAGTTCTCCCCTAAGAAAAGTTGGTCTAAACGCCCAAAACAAAGTTGCTCTAAGAAAGATAAAAGCAAAGTTAAATGTTTTAAATGTCAAAAATTTGGTCATTATGCTTCTGAATGCAAAGTTAAAGATACTATTAAACAGTTACAAATTTCAGAAGAAGATAAGAACAAATTGATCCAAGTTTTGGAGTTGCGAGACTCTGAAATAAGTAATGCTGAGTCTCCAATACCCAGTTCTGAATCTGAGCTGACCAGTCTTCAGATTCATCACTTAGTTCACCTAGGATTCAACTTGGTTGCACTGATAAATGCTGTAATAGGTTAAAGTCTATTTTTGTTCTTACTAAACAGGAAGAACAAGAAGAATTACTTGTAGATCTAATAGGAAGAATTCAAAACTCTGAATTAAAAGCTGATTATTTAAAAAAGTTGAAAAAGATCTTAAGTCAGGAAGGCCAGAGTACCCTCTCTCCTACTCCTCAGCCAATTAGCATCAACACTACGTTAGAAAAATTCAATCGTAGAAAAGATGTTACCCTACAAGATCTCCAGTCAGAAGTAAAACAGGTTAAAAAAGAAATCGTTGAGCTTAAGCAGATAAGTCAACAATTACAAGCTGAGAATTATACAATCAAACAAGATTTAGTTAATTTACAACAAGCCACTCGCCAACCTAGTACTCATTCGGAAAGCCCACATCATTCTGATGAGGAACCCTCTGAAGAACAACCAGTTGTCAATCTGATCAAACAAGTACACCTTAGAAAATGGTATTCCAAAGTCACGATTTTTGTTCATGATTTTGAATTTACCACAGTTGCCTTATTTGATTCTGGTGCAGATCTTAACTGTATTCAGGAAGGTCTCATCCCTACTAAGTACTACCAAAAGTCCAGAGAATCCCTTAGCACCGCTTCTGGGAAATCTCTCCAGTTAAATTTTGAAATACCAAAAGCCCATATTTGTCAAAACAAAGTTTGTTTTAAAACCTCATTCGTCTTAGTTAAAAATATTACTGATGAAGTCATATTAGGATTACCTTTCCTTGCTCTGTTGTATCCTTTTCAGGTAGAACATGATGGTCTCATTTCTACCCATTTAGGAGTAACGGTCAAGTTTAGTTTCCTATCTAAACCAGAGTTGCACAATTTAAAAACTTTACAAGAACAATCGATTTTTAAAGCTGTCCACATTATTCGAAAATCAGACCAGTTGAATTTTCTTAAAGAAGAAATTAAATATAAACGAGTTCAACAACTTAATGATAAAACTTTTCAGTTAAAAATCCAGTCTTTTGAGAATAAATTAAAAGAAGAAATATGTTCTGATTTGCCTACTGCTTTTTGGTATAGAAAACAGCATCTCGTCTGTCTTCCCTATATCAAAACCTTTAGTGAAAAAAATATCCCCACTAAAGCCAGGCCAATCCAGATGAGTCAAGAATTAATGGAAGTCTGTAAGAAAGAAATTCATGAGTTGTTACAGAAAAATATAATTCGCAAAAGTAAGTCCCCTTGGTCATGTCCTGCTTTTTACGTCCGGAAAAATGCTGAACTAGAAAGAGGAGTCCCTAGATTAGTAATCAACTATAAGCCTCTTAATTCGGTCCTAGAATGGATTAGGTATCCCATACCTAATAAAAGAGACCTTATTAATCGCTTAGGAAAAGCAGTTGTCTTCTCCAAATTTGATATGAAAAGTGGTTTCTGGCAAATCCAAATAGAGAGTCAGATAGGTATAAAACTGCATTTGTTACTCCTTTCGGTCATTACGAGTGGAATGTCATGCCGTTTGGTCTAAAGAATGCTCCTAGTGAATTCCAGAATATCATGAATGATATCTTCAATCCTTACAGTCATTTTTCGATTGTTTATATTGATGATGTCCTAGTATTCTCTGAGTCCGTTGAACAGCATTGGAAACACTTGCATCAATTCCTTTCCATTATCAAAAAGAATGGTTTAGCTATTTCTGCCAAGAAAATTAAATTATTCCAAGTCAACATCCGATTCCTTGGTTTTCATATATGTCAATCCCAAATTTCCCCAATAGACAGAGTCATCCAATTTGCTGACAAGTTCCCTGATCAAATTCTTGATAAGAATCAGCTCCAGAGGTTTTTAGGATCTCTAAATTATATTTCTGATTTTTATCCAAATTTGCGAAAACAATGTAAGCCTCTATTTGATAGATTAAAGGATAATCCTCTTCCTTGGTCTAATATTCATACTGAAGTTGTCAAACAGATTAAAGCTCATGTCAAGACACTTCCCTGTCTTGGTATCCCTTCAGTTGATTCCTTCAAAATAGTTGAAACAGATGCCTCTGACATTGGTTATGGTGGCATTCTCAAACAGAAAGTCAGTCCAGAATCCCCAGAGCAAATTGTTCGTTTCCACTCTGGAACCTGGACTCAGTCGCAATATAATTATAGTACTATTAAAAAAGAAATATTAGCTATTGTATTATGCATTAGTAAATTTCAAGATGATTTACTTAATCAAAAGTTTTTAATCCGAGTTGATTGTAAATCTGCAAAACATGTTTTAGAAAAAGATGTTCAAAACATTGCATCTAAACAGATTTTTGCACGATGGCAAAGTATATTAAGTATATTTGATTTTGATATTGAATATTTGAGAGGAAGTGAGAATTCAGTTCCTGATTTTCTTACTCGAGAATTCCTGCAGGAACCACAATGGCTCCCAAAAAAGACAAGAATAAAGAAGTTGCAAAAACAGTTGCTAGCCAACCCAAGTCATCCCAGTCGCAACCTTCTCAGTCGTCCAAACAAACCCAATCGACATCCATGAGTCCAGTTAAACCAGAAATACCCAAACATATAGTTCCCTATCCTGGTTGCAACCCCATCCCAGTTATTGCTACTCCGTTATCAGTTGCCAATAGGTTTTCCTCCTTAGGATCCACAATTGGCCAAATCCGCCCCAGTTACCAGTCCACCCTAGTTGCCAATTATGATCCCTTTTCAGTTGACATCCCTGCTGGACCTTCTGCTCCAGCTATCCGCAAAAAATCATCCCCTTATTTGCCCAAAAGCAATACCCATTTATTTGTTATTGAGCCCATCTACGACATAAATGCTAACCCAGTCGAAATTGCCCGTCATTATTTCCCTCCTGGATTTCATTATATGCCCTCTAGTCCATACAAGTCCCTCAAGTATTATAGAGATATACTACTTGAAACCAAATCAGTGGAGATTAAGCCCATAAAGGATCGTGACAACCCTGGAACCATCCTTTATCATTCTCTTTACATTCATCAGATCATAAGCCAAGCAGCTTTTGCCAGCCGCCCTTATGAACTGAAGTTGCTCCAATCCAAGTTGCAATACAATTATGCAGATTATATTGATGCCTGGTATACTATTTTCCTTCATCAATCTGAGGATTTTAGTCATTCCTGGTTCATCAATTTTGATAATAAATTCAAAAGTCCTTTTCCCTACTGGTTCCTCCATTGGTGGGAAAAGCATGGCCCAGTTGAAGAAATCCTGCCTGCATCAGTTATTCAACTCATCGTTCACTACACCAAAAAGACCAAGTTCTCTGAAGTTGATCTCTTTTTCCCCACACAGTTGCTTTTTATTGCCAGATACAAAGTTCCCTGGATCCTCAAATGGTCCTATCAAGTTGCTAAAGATGATCGTATCTTTGCCCGCCAATTCTCCGTTAAGTGGTGGGATAAATTTGAAGTTGACAGAATTGCTAAATATGTCTATAATGATCTACCAGTTGACCCAGTAGCCCCTGTTGTCTCTCCAACCGGATCTGTTCGATCTTCCCTCTCAGTCGAAGGAAAGTCCAAGGCAGAACTCCGCGAGATCGCCACTCAGTTGCTCATGCAAGCCTCCCAGATGGATGATGATGAAGACAACGGGTCCCCCTCGTCTTCCAGCTGTTAACCCATCCCAGTCAGGCCCAAGACTTGGTTTGAAGACAGTCAAGACCCATATGATGCCTATGACCTAGCCTCTGACTAGGCATCATTATGCTCCTGACCTCTTCACATGCCAAAAGTAGCCGAAAGCAGCAAAAGTCGCCAGTCGCCAGACACCATAATTGCCTCAGGA

TGTGAATAGTAACGGCCCTTATCTTGCCCTTACCTTTTATCATGAAAAATGAGTGGAAGTGCTCTTAACCTTAAAGTTTGAAAGCCGCTGTTGTTGTTTTGTTTTATCTTTAATTTTTATTTTATCGGGAGTTAACAGTGTTATTGTTGGAGTTAAGTGGGTGGACAAGTGGCAGACAGTTAGATGGGGAACACACACGGACAGTAAACTTGGAGACAGCAGTTCGGCTCCCGCACTGCTGAGACTCAGGGTCCTTTTCTCGTTTCGTGCTTCGGCTGTTGGGCGTCTATCTCGAACCGAAGAAGATAATGGAAATAGCCGTTAATTGTTCCCTCTTCTCGGGTTTAAATCCTAAGGTAACATAACCCTAACCCGTCCTCTCTTTTTTTCTTTCTTGTTCTACATATCAATTATATATGACATTGTCGATTCAATATTGCACAAGAAAAATTCAGATATCATCAGTACCCAACTGTTGGCTGGTGGGCTTTTTTGTTTTTTGTTTTTGTAACTCTAGTTCCCATGAACTATGAAATTGTCTGTGTACTTTTGTGAGTGGGTTGGTTTTTACTTTTTTTTCGTCATGGTTTTCTGGATGTGTTAATAGTATGTTGTTTGGATTTCCTTAGAACTCTGTTCTTGGGTGTAATCCTGTTCAGATCCTATTGAACCCAAAAGCCAATTATTTGTGTAATAATGTGTATTGTTGTTTCTGTCAGTGATGTTAAAAGGAAAAGAAAAAGGGTTTTTGCTCTGCTCTACAGCAAATTTTAGCTTTACTAAACCTTTTTCTTGGGTCTTTCTTCATGTCTTGTACCATTTCTCTTCTGATTTTTATTAGTGGTTTCATAGTTTACTACAATAACCCATTTGATTGCTGGAACTGAGAGTAAGTATATACATTTTGGAATGAACGCCCGGAGAATTATAAGTCACTTTTTACACTGAAAATGTTATCCAACAAAACAAATGTAGCCTTGAACTGACATAAGTTTTGTAC

>Chr1-16; OZ022206.1; 22487927-22495665; dir; A;

TTTGTGGGAAAAGATTAGTGTAGTCTGACAACTGATGTAGAACTGGCATTTTCAAAGTGACGAGTTGGTCATCAATTTGCTGTTCCATTGAACAAAAATAGACGTCTGAAACCATAAAATTTTCTTTTTTTGCACAATTTTTTGTGAGGATATCGATGTTAAGCAACGTTTTTAGTTATTACAGTGCTTATGACATAAAGTCTTTCCTTTGCCCCAAAAAAAGAGGGGAAAAAAAACACTAAAGCTTATTAGTTATGGAATCATTTTGTTGCGTTGGGATTTGATTATAGAAGGGCCCTGACCACCTCTACCACGATATTATGGTGAAGCCTCCACCACCAAATCTCAACCCCTCTCCACAATACACAAAAACTAGCTTTTCCTCATCACCAGGTCATTCTCGACCCAACCCAATCTGACCATACCTTAGAGCACTTATACTCATTTGTCATGGCAAAGTGCAAGGGCAAGGCAAGGGCTGTTACTATTCACGTGAATAGTAGTAGTCCTTGCAAAAGGCTTGTGGTATTTCCACCCATTGTCATGGCAACCTAAAGGCAACCACTATTCACATGAGATGTGAGGAGAGGAATTTGTATAGAGTTTTGGTGTGGGGAAGTGAAGAATATGACTAAGTATTTTGAAAAAAAAATTAGAAATTTTTAGTATTTTTTTTTATAGATTTTATAGATTTTTTGTTGTTGTTGCTGACGTCAACAAACCAGGCAACAGCTCAGGCTGTTTTAACCTTTGAGTTGGCTCGGTTTGCTGGGACCCATGCGCTTCCCGGGCTATAGATGCCCACTAGAACATCAAACTGGGGCTTGGGCCCCCTATTGCCCGGGTTGGGTGGCATCGCTGGAAGTGCTCTTACCACTTTAGATAATTTCCCATAAACGAACGTGGTCGGGTCCTACAAGGACCACTAGACCAAGACACTTGGTGAAAACTAACGACCATCCCATAAAAAATAAAAAATAAAACACCTTTGTTTTATTTA

GGTATCAGAGCCGTAAGTGTTGAAGAGTTAGTCTAAGTCTGCAACTCATCCAACCCAAGTGGCTGGTTTAGGTTTTCTGAGTTGTAAGTTTTCAGTTTCTTCTCTTGCTACTTCTGGTCTTTCAGTTTACCTGTCCTTCTCTCTCTTGGTGTCCGAATCGTAAGACCCATTCCAAACAATAAGTCACAGGGAGGCCTGAGCGGTAGTGGCATTAGTAGGAGGGCGAGAGAACTTTAGGTATCCTGTTATCCCATGAAGTCAGTCTTAGGAGTTTTTGTTCACTTTCTTCTTTAGAAGTCCAACCCCAGAACCTTGAGTAAAATGAGTCGCTTATTTAGAACAAATTCTTGCAATTCTAGCAGCCCTAGAGCCAATAGTTGTCTCCCAGAGATAGTTAATGAAGAACAAATTGAGTATAATAATAATAGTTTAGATTATAAAGATTGGAATATACCCAGAGTCCCCAGTCAGGACATCTATAAGAAAAAATGGTCATTATGCAGTTTTAAGTCAACTACCCATGTCAAAATAGTTGAACAAGTCTATGCCTTGAGTAAGAATCATGAGACCTGTCAATTGTTAAATCTTGAGTCCATAAGAAAACACAAAAATGATGGAAACAATTTCCTTCACATTGGTTTAGTTCAAGTTGCTGTTAAGCCCTTAACCAGATTAGGATTAAAAGCTTCAATCCTTCTTTGTTTGCGTGATGCTCGATTTACTAATTTCAACGACAGTACGTTAGGAATTATTGAGTCTAGTCTTGCTAATGGCCCTATTCACTTTGATTGCTATCCAGATTTTACAGTCAGTCTGAGTGATCCACACATATTGAAAACCCTCACTTTAAATATTAAAACTGAAGGTTATGAGGTCTTGTTGGGTTCCCAACCCTTAGCTTTAGTCTATCGAATCTATTATAAAGTCACAGGCACCAATATGAATTTTCAAGCCCTAAACAAGAGCCCAAAGAGTCAAACATTGTTAATCCAAAGTCATACGTCTGATGCTACTGTTAGAGTTCCTTACTTTATCAAATGGTCAGATGTTACCCTTCCTGCTGATTGGACCCTAACCACTGAGATTCCTTCAGTTCCCATCCAACGTAGTCTAAACTATTTAGACTACATCCAACAATACTTAGATGGCACAGTAAAAATTAATTTTGGTAATCAACCTCCACAAAAGTCAACCGTTCAATTACAACAGTTACCTACACCTGGTCAATCCCAGAGACATATGCCTGCAAGGCATTCCTTTGCAGCCTCTTCTTCCACTCTAGAACGAGACCTAGAGTTATAAAAAGCCTTAATTGATTTGAAATTAGAATCACTGAGAAAAACTTCCCAAGTCACCCATCCTGTTTATGGGAAAACTCCAGTCCAAAAAGACACTAAGTCTGAGTCCCCTGATTCACCCACTCAATTTGATTTCATAGGTAATCAGTTAAGAACACTTAATAAGAAATTTGTAATTAATTGGAAAAGATTAGATAGTCATTTGCATGCCCCTGAGAATCAAGCTAGAAGAGACTATTACCATTCAATCATTCCTGATAGAGTCAAAAGACAACAAATTTTTAATGATTGGAAAGAGTACATGCGCACTACTAAAGTTGAAATATATTATCTTGATTTTGTAGAAAGCCGATATTTGCCCAATGAGTTAAAAACTTTAACCTGGGAAAAATGGAAAAAAGTAGATCACACTGAGATTCTCGCTAGCCATCCACCTGTAGAAACAATAATCATTAAACATCAAGAAATCCAAAATAGTTGCTAGTCCCTTTAAGATCCCTGATCCAGAAAATAAAATGATAGTTGAGCAAAATAATTACACCAACCAGAGTCTAGTTGTCATAGGTAAACAATTAGATAAAATAGAAACAAAAGTTGACAAATTACTACCAAAAGAGCTCACACCTAAGCAACATGAGAAACCCTTAGTTACTTTCCAGGATTTAAAATCAAGTCCAACTTTTAAGACAAAAAAAACAATGACAAAAATTGAGAAAATGCTAGAAGAATTAAAGCCAGTCAAAAGAAAAGATAAAGATAAAGTTGAAACAAGCATTAAAGTCTTAGGATCATCTGAGTCACCATTTGCTTACCATAACTCCTCTGAGTCTGAATCAATTGCCAGTATGGAAACAGAAACTAGTAATATATCTAAAATTGAAAATGCGTTTAAAAATCTTGAGCTAGAAAGTGACCCACAAGTTCAAAGATTAACAAACAGAGTCAGCCCCACTAGTCTCACAAAAAATTGGTATCCTAAGCCAACACCTCCTGATATACAATTTGAAGAACGAAACACACAGAGTCAGTTAGTTTTCAATCTCTTCAGATAAGCTATATGAATGGAATATTGACGGTCTTTCTGAGCAAGAAATCCTTAATAAGCTTCAGCATATGTCCATGGTCGCTAATAGTTATATCACCAACCACAGTTTCAGACAGTCTAAGATTGTTACCTTGCTAGAAACTGGTTTCACTGGAACTCTTCGTTCTTGGTGGGATAAACATTTAACCCATGAGTCTAAGCAACAAATAATTCATGTAGTCAAACTTAGTGAAGATGGTCTTCCTATTTTCGATGAACAAATAGGTCAAGGCATTGAGGATGGAGTCAACACCCTACTGTACACAATAGTTGACCATTTCATTGGTACACCCAGTAACACTATTGCCAGGATACATGATCAGCTTAGTAACTTAAGATCCCAAAATTATCTGATTTTAGGTGATATAAAGATGTGTTTATTTCCAGAGTCATGCTTAGAGACGATAGTACTCAATCATTTTGGGAAGAGAAATTTGTTAATGGTCTTCCTAATTTATTTGCCCATAAAATCCGAACAACTCTTAGTAATGAACAAGGTCATATTGATTGGGATAGTTTAACATACGAAAATATAAATAGCACCATTAATCAAGTCGGTATGAGAATGTGCGTTGATATGAAAATTGGTAAACAAATTCAGTCTGAGAAAAATTCTGCTAAATATGAGTTAGGTAATTTCTGCGAACAATATGGTCTAATGTCTATTCCCTCATCTAGAAAGAATAAACCCAGTTACCACAACCGACAATTTAGTAAAAGACGTCATTATTCAAGCAGAAGACAGTCATTCCAACATAATAATGATGATAAAGAATTTTATAGTAAAAAGTGATTCTCCCATAAGAAAAATTGGAAAAAGAGTCACAGCAAAGATAAAGATAGTCACTCTAAGAAAAGAACAGACAAGAGAAAAGTAAAGTGCTATAAATGTCAAAAGTTTGGTCACTATGCTAATAATTGCAAGTTAAAGACACAATTAAACAATTGAAAATCACAGATGAGGATAAAGACAAACTGATTAAAGTCCTTGAGTTAAGAAATTTTGAGTCTAGTGAAAACGAAACCATGGTTAGTCACACTGAGTCTTCTGAAATCTATTCTTCTAAGTCACAACCTAGTTCACCTAGAATTCAATTTGGTTGCCAAGATAGTTGACAGGACCCGATCCAAATTTCGCTTTGGAATCCGAGCCGGACCCTGTGTGTGTCCGACACCTGGCAATGTTGGGCACAAATGACCTATTTGCCCTTCTATACAAAACACGATAAAATAACAAAGTTGACTTCTACCGAAAAACTAGCAGAGTTTCCCTTGTTACTGACTCAATACCAATCATTTACACCTGCCAAAACAAGTATATATATTTATACAACCAACTGCCAGCATACGTAAATATACATTCCAACAGTTCAATTCTCAATCTATGGCAAAATATCATAGCAACTGCTAAGAATTTACAAAGGAGTTAATCCTTTTACAAAACAAAAATCCTACATCAAAGGAAAGGCGCGGAAGATGGGAAACGGCCCGGTGCTGGATTCCTGTGCACGTCTACTGCCTGGGGGCGTAAAACAACAAAAAGGGTGAGTGGACCCAAAACAAAGTTTAGAAAAATAGCATATGAACATACTAACCCCACTGTAAAAACAGTTATGCAAAACTAAACGGTCCGGGCGTCCCCAAAACTCGTGAGGCAAATGTCAAGCGCGCTGACAAAACTGAAGGCAACCTGGATGTCCGTGGATATCATCATATCTGAAGGCAACATATACCGAAGGCAACTTGTTTCTATAACTGGGTACCTAAGGTGGCTTAAGAAAATATAAAATTTCTGAAAGGTCTGATGAATAACTGAATTTTTGTTAAATCTAGAACTCTGCTGCCATTTATCTGATATATATCTCAAGCTTTACTTTTCATATCTTTTTAGCATATTCAACTAGGCAGCATATAAATAAAGATATTTAACTTCAACAAATCATGCTAGGAAAACCATAATCAATAAAACTTATTCAAGATCAAATAATGAAATTCATTTGCATAAAATCATTTATAAAAGAAAAGTCCACTCACTCCTGGTCCGAGCTAGCTGGACCTCCTGAAGGTCCCTCATGCGGGTCTGCTTGGCCCCGGGTGGCTCGCTCGTCGAATCGGTGGCCGGAGAAGACACCGACCGCTTCTTCTCCATTTTCCGACGAAACCACGTCTGCTGGAGGCTGGAGTCGGCTGGGGCTGGAAGAGGACGACGCCTCGGTCATTTTGGTACTGGCCCCGTCCACAGCCATGGCCGGTGGCCGGAGTTTCGAAGAGAGAGAGAGAGAGAGAGAGAGAGAGAGAGAGAGAGAGAGAGAGAGAGAGAGAGAAATCTGATTTTTATAAAAATTCCATTTCGAAATAATTACGATTGTGCCACTGAGTTTCTTTTGACCATATCTCTCTCGTTACAACTCCGATTCGAGCCCACTACGTGTCTATGAACTCGTCTCAATACGACCTATCCAAAAATACTAGTCACTGCCCCAAACTCTCTCCGGGTAAGAAAATGACTAAAATACCCATATCTCAAGGGTAAATTTGTAATTTCCCTTAAATAATTAAATAAGGGATTAATTTGGGGTCGGGGTGTTACAATAATTGTTGTAAGTCTAAGTCTATTAATGTCCTTACCAAACAAGAAGAACAAGAAGAATTATTAATCGATTTAATAAGTAAAGTCGAAAACCCAGAATTAAAATCTGAGTATTAAAGAAATTAAGGAAAGTCATTAGTCAAGAAGAGTCGAAATAGTTTACCTCCCAAAAGATAAGTCTGAACACAACCTTAGAGAAATTCAATAAAAAGAAAGAAGTCACTTTACAGGATTTACAACTAGAGGTGAAGTTAGTCAAAAAAAAATCATTGAGTTAAGACAAATAAGTCAAAAGTTACAAAATGAAAATTATGAGATAAAACAAGATCTGATTAACCTATTAGAAAATAAAACCTCTAAGTCTACACCGGAAAGCCCTACAAATTCTGACGAAGAACCCTCCAATCAGGATCATGTCGTCAACTTAATTAAACATATAAATTTTAGAAAATGGTATGCCCGAGTCACTATATTTGTAAAAAGCTTTGAGTTAAACATAGTTGCCATCTTTGATTCAGGAGCCGACCTTAATTGCATCCAAGAAGGTCTAATCCCAACTAAATGTTACAAAAAGTCAAAAGAGTCGTTAAGCACAGCTTCAGGAAAATCACTTCAATTGAATTATGAGATCCCTAAAGCCCATGTCTGTCAAAATAAAATCTGTTTTAAGACCTCTTTTGTCTCAATTAAGAACATTACTGATGAAGTCATCCTAGGATTACCCTTTATTGCCCTTTTATATCCTTTCCAGGTAGAACACGATGGTGTCATTTCCACTTGTTTAGGAGAAAAGGTTAAGTTTGAATTCCTAAGCAAACCTGAGGTACATAATTTAAAAGAATTACAGAAGAATGCAGTCACTAAAACAGTCAGAGTCATGCAAAATAAAGATAAGCAACTAAAGTTCCTCCCGGAAGAAGTCAAATCTAAAAGAATTGAGCAACAAGTTAGTCATAAATCCTTACAGTTGCAAATCCAGCAATTTGAAGAAAAATTAAAACAAGAAGTTTGTTCTGAATTGCCCAATGCTTTTTGGCATAGGAAACAGCATGTTGTCAGCCTTCCTTATGTCAAGTCCTTTAGTGAGAAAAATATCCCACTAAAGCCAGACCAATTCAAATAAGCCAAGCATTAATGGAATTTTGTAAAACTAAGATTACAGAATTGCTTGAGAAAGGAATAATCCGCAAAAGAAAGTCACCCTGGTCCTACCCTGCTTTCTATATCCAGAAAAATACTGAATTAGAACGCGGAGTCCCGAGACTAGTCATTAATTACAAGCCACTTAATACTGTCTTAGATTGGATTAGATATCAAATCCCTAATAAAAGATATTTAATTAATATATTAGAAAAGTCAGTCATCTTTTCTAAATTTGACATGAAGAGTGGATTTTGGCAAATTCAAATTCATGATTCTGATAGATACAAAACTACTTCTGTTACCCCTTTCGGTCATCATGAGTGGAATGTCATGCCTTTTGGTCTTAAGAATGTGCCCAGTGAGTTTCAAAATATAATGAATGAGATTTTTAATCCTTTCAGTCATTTTTCCATAGTCTATATTGATGATGTGCTTATCTTTTCTGAGTTGATAGAACAGCACTGGAAACATCTGCATAAATTCTTACAGATAGTCAAACAAAATGGATTAGTTGTTTCTGCTAAGAAAATTAAGTTATTCCAAACCAATGTGAGATTTCTTGGGTTTAATATTTGCCAGTCACAAATTAGTCCCACAGATCGAGTTATCCAATTTGTTGATAAGTTTCCTGATCAAATCCTAGACAAAAGTCAACTCCAGAGATTTCTAGGATCTCTAAATTATGTTTCTGATTTCTACAAGAATTTGAGAAAACAACGTAAGCCTTTATTTGATCGTCTCCAGAACAATCCTCCTCCATGGTCATCTATTCATACTGATATAGTCAAACAGATCAAAGCCCATATCAAGAGCCTTCCCTGTCTTGGTATCCCTTCTGCTGATTCATACAAAATAGTTGAAACTGATGCCTCTAACATTGGTTATGGAGGTATCCTCAAACAAAGAGTCAATTCTAGTCAATTTGAGCAAATCATTCGCTTCCATTCTGGTACTTGGTCTCAGTCTCAAAATAATTATAGTACTATTAAGAAAGAGATATTATCTATTGTACTATGCATTAATAAATTTTAGTCTGATTTATTAAATCAAAAAATTTTAATCCGAGTTGATTGTAAGTCTGCTAAGCATGTATTAGAAAAAGATGTTCAAAATATTGCATCCAAACAAATATTTGCAAGATGGCAAGCTATTTTGAGTATCTTTGATTTTGATATTGATTATTTGAGAGGAAGGGAGAATTTTGTCCATGATTTTCTTACAAGAGAATTTCTGCAGGAAAAATAATGGCAGAAAGAAGAAAAGAGAAAGACAAGCAAAAGAGTCAAATTGGTCAATCTAGTCAACCCAGTCACCCCCAGTCGTCCCTCCATCAATGAGTCAAGCCAAACACGAGTCACCTTCTCAGATCGTCCCCTTCCCGGGATGTTCTACTATTCAAACCAATAACAGGTTTGCCAATCTAGGAGCCACAGTCGGCCAGGTTCGCCCCAGTTTTTAGTTTGCCCTAATATCCAGTCATGATCCCTTCCAGGATATTGCTTCGTCTAATCTTCTAGTTGCGTCTTATCATAAGACGTCTCCATACATGATCAAGAATAATAATCACCTATTTGTAATTAAGTCAAAATACAATTCCATGGCCAACCCAATCACCATTGCCAAA

TCCTACTCTCCACCAAATAGTCACTTCATTCCCCCAGAACCATTCAAAAATCTAAAATTTTACCGAGACATTGTTGTTAAAATGTTAACTCGCAATTGTACGGGCCAATTAGTAGTATAGCAAGACAAGTACGAGGTCGTACCCACGAGGACTGAGAAGTTAAAATTAGTTATGTACTTAGATTACCAAATTCAAGAAAGTAGGAGGTTAAGTCTAAAATAGAATTTAAAGCAAGAGTGGTTGTAATTGTTGAACAAATGAGGCAAAACGAAATTTCAAACAAAAGCAAGGTGAACAAATGTAACAAAGAGTACTTTTGTAGGCTGGAAAACAGATTGGGAGACTAGGGCATTCGATTCACCATAACAATTCAATTCTGGGTTATAAGGAGTCAAGTGACAAGTCTCATTCGGATTCAAAGATTGATTCTAGCTAAAGTATGATTAGGCCATGGTGTCTGGTCCGAACCTTGTGCTCCTAATTCCCTAACTTGATCATTTGTTGTCTAATCTAAATTAAGCATTAGGAACCCATTAAGCATAGAAGAACAATTTAAACAACCAAGCATGTATCTAATCCCTGTTGTCGAATGTCCATACATACTTTTCTTATTCATAGTTCAATTCAAATCAAAGCATGGTGTCTACTCTTGATTCAAACCAAGGTAGCTAATATACAAGTTCTAATGGTGATCAATCATCCAAACAAGCATATTAACTGATAAGCATAAAGAATAGGAATATGAATCATGAATCAAACTTGAAACTCAAAACTTCAACTTATAAACCCAGAATATTCATGTTAGGTTACATCATAGCCCTAGTTATGAATTTAGCTACTCATAATGGAAATGAAACTTAAGCAAAAATGAGAAAACATATTGAATGCAATAAGAGTAGAGAAACCCAATTCTGAAGTTGCTTGTGGTTTCCAAGGCTTCTCCAGGTGCTTCTCCACGTCTAATGCTACTCCAAGAACTTTCTCATGTCTAGAGTATGGC

>Chr1-17; OZ022206.1; 25739697-25742734; rev; B;

TCGTAGAGACTTGATCTTGATCAAACGGATAGCACAAGGCTTGTTTATTGATTAGATTTTTCTAGGGTGGAGGAGCCGGCACCTATTTGTTGTGCTTAGTGGCTGTTCCAAAGTAGGGTGAAAAATGCAGAAAGTTTTCTATTCTTCTGAGTGTTTCGTCTGTCTAAGCCTCATTTCCTTTGTCTTGAGGCCTCCTATTTAAAGGCTCTTGCAGGATTTTTGACCTAAATAGCTTTCCTTCTTTCGGACCTTGCTTTGTGGGATTTTGATTTGATTTAAAATCAATTCTGATAATTTGGAAATTTAACCAAATTATCTTTAATTGATTGACTTGATTAATATTTGATTTAAATCCAAGTCAATCATAGAAGATTCTTCCCTTTAATTTTTTCTTCACTTTGAACCGTTTGATGCACTCTGTCGGATATTGACATGTGTCACCTTGATGAGTCGTATGCCACGTGGGGCCCGCAATTTAATCCACAAAACCTTAATTATTTTCTTATTTTGAGGAATTTGATTTGCCAAAATTTTTTGCCTACAAATTGCCCCCACTTCACGATGCAGTGTAGATATGTGCTTGTCACTTGTAGGAGGTGCAACTTGAAGTGTTTATTTTTAAATTTTTGTTTTGGGTACAATATTATATATATATATATATATATAATGACCAAATTGGGTTACAAAAGCGGTTTTATATCTATCTGACTTTTGGATTTGAATTTTCCAAAATCCACTCTTTATATCAAATTTGGCGAACTCCATCCCAGATTTCCTTACTAGAGAATTTCTTCAGGAAAAACAATGGCAGAAAAACAAACAAAAAATTACTTAAAATAAATGCTTAATGAAATTACTTAAAATAAATGTGAAATTAAAAGTAATGCGTAAATAAAAATACTTACAGAAAAGCTTGAGATTTTATTTATGCAGATAACTATCTCTAGATCCCCTATTTATATTAGAGCCGTAAGCGTTGAAGAGTCACTCAATTCTGTAA

CCCTTTCAGTGTCTTCTTGTTTGCTGATCCTTTTCTCTCAACCCAAACAATAAGTCCTAGATTCCAAACAATAAGTTCCCGGGAGAGGCATGAGCGGAGAGAGGAAAACCATTCAGTCTTCAGGAATCCTTGAACCTGTTATAGGATTGTTAGCTTCCCTTTTTACCCTTTCTAAGAAAACCAATAATCTTTGTCCAAATGAGTCACCTTTTTAGAACAAACTCTTGTAACAGTAGCAGTTCTAGAGCCAGTATTAGTAAACTCCCAGAAATAGTCAATGAAGAACAAATAGAATACGAAACTAGTAATGAAATTGACTATAAAGATTGGAACATCCCTAAGATCCCTAGTACAAAAATTTACAAGAAAAAATGGTCACTTTCAAGTTTCAAAAGTACAATCCATGTGAAAACAGTCGAACAAGTCTATGCCTTGAGTAAACTTGTCAATTATTAAATCTTGAGTCAATAAAGAAACATAAGAGTGATGACAATTTCCTTCACATTGGCCTCGTTCAAGTAGCAGTCAAACCCCTTACCAGATTAGGACTAAATGCCTCAATTTTACTTTGCCTCCATGATGCCAGATTCACCAACTTTAGTGATAGCACTCTAGGAATAATTGAGTCAAGCTTGTTTAATGGTCCAATTTACTTTGATTGTTACCCAAATTTCACAGTAAGCCTAAGTGATCCCCATATATTAAAAACTCTGACATTAAATATAAAAACTTCAGGATATAAAGTCTTACAGCCTTGGTGTATAGAATTTATTATAAAGTCACAGGAACCAATATGAATTTTCAAGCCCTTAATAAGACTCTAAAAGACCACACTTTGCTTATTCAAAGTAGTAAAGCCGATGCTAATATAAGAGTCCCCCATACAATCAAATGGTCAGAAGTCACCCTGGCTCTAGATTGGACACTAACTACAGAAAATCAACCCACTCCTATCCTGCATAATTTGAATAATCCAGATTATATTCAACAATATTTATATGGTACAGTAAAGATAAATTTTGGAAATCAACCCCACAAGTCCTCGGTCCAAATACAACAGCTTCCAACACTTGGTTAATCGCTTAGACATACGCCTGCCAGACATTCCTTTGCAGCCTCCTCCTCTACTCTGGAAACAGACCTAGAGTTAGAAAAAAAAACGTTAATTGATTTAAACTTCGAATCTTTAAGAAAAACCTCCCAGGTCAACCATCCTAGTTATGGTAAGAAATTAGTCAAAGAAGATGAAAAATCAGATTCCCCAAATTCCCCTACACAAACTGATTTCGAAGCCGAAGCCATTCTAGTAGATAATCAATTACGAACCCTTAACAAAGAATTTAAAATTCATTGGAAAATTTTAGAGAAACATTTAAATGCCCCAGAAAATTTAGAAAGAATGAATAACTACCACATGGTCTACCCGAATAAAGAACAAAGATAGCAAATCTTTAATGAATAGAAAATTTTTATGAAAAATACTAAGTTAGAAATCTTTTATCTTGATTTTTTTTGAAAGCCACTACATGGCCAAAGAATTAAGAACTTTAACACAAGAAAAATGGAAAAGAGAAAATAACACCGAAGCCATAGCCAGTCATCCTCCAGCAAAAACGATAATCATTAATCATAAAATGTTAAAATCACTGCCAGTCCTTTTAAACTTCCTGAACAAAATGATAAAAAAAATTAGTTGAGCAAAATAATTATACAAACCAAAGACTAATAGTAATTGGAAAACAATTGGATAAAATAGAAACGAAGATTGACAAAATAGTCCCTCAACAGATATCCCTTAAGTCTCAAAAAGAAAACCTTTAGTAAAATTCCTAGATCTAAAGGAAAGCCCCACTCTCAAGTCAAGTCCCACAGTGGAGAAAATTGAAGAAATGCTTGAACAACTAACTCCAGTCAAAGAAAAACCTGAACCTTCTGGTCTCAAGGTCTTAGAATCCTCTATTCTAGCCAATTCATTTGACACCGAGTCTATCACCAGTATTGAAACAAGTTCTAGTAATATCTCAAAAATTGAGAATGCCTTTAAAAATGTAGAAATAGATAATAATCTGAAGCTTAATAGATTAAGGAACAAAGTCAATCCTGCTAGTCTAACAAAAAATTGGTATCCCAAACCAACCCCTCCAGACATTCAGTTTGAGGAAAGAAATTATCAAAGTCAATTCTCAGTCTCATCCGATAAATTATACGAATGGAATATTGATGGCCTTTCTGAACAAGAAATTCTAAATAAACTTCAACATATGTCTATGGTAGCTAATAGTTACATCGCCAATCACAATTTAAGGCTGTCAGAAATTGTTCCTTTGTTAGAAACTGGATTCACCGGAACCCTACGTTCTTGATGGGATAAACATTTAACTAAAGAGTCAAAACGACAGATCACACATGCAGTCAAATTAAATGAAGAAGGTCTCCCTATTTTTGATGAACGACTAGGAACAGGCATAGAAGACAGAGTCAATACCTTATTATATACGATTATTGAACATTTCATAGGAACACCTAGTAACACGACAGCTAGAATTCATGACCAACTTAGTAACATTAGATGTCCCAAACTAAGTGATTTTAGATGGTATAAAGATGTCTTTATTTCTAGAGTCATGTTAAGAGATGATAGTAATCAACCATTATGGAAAAAAAAAATTCATTAACGGTCTTCCCAATCTGTTTGCCCATAAAATACGAACAGTCTTAAGTAATGGCCAAGGCCATATAGATTTTGATAGTCTAACCTACAGTAATATTATTAGTACAATTAACCAAGTAGGAATGAAAATGTGTGTTGATATGAAAATAGGTAAACAAATTCAGTCAGAAAGAAATTCAGCCAAATAAGAACTAGGAGACTTTTGCAAACAATATGGTCTAGCTTCTATTCCCCTATCTACAAAAGTCAAGACCATGTACAATGACCGACAAACTAGTAAACGTAGACATTACTCACGCAGAAAACAGTCCTCTAGACATAATGATGAATTTTATGAAAAAGAAAATTTAACCCAAGAAAAACTAGG

GGTGGCAATTTCGGATACGACCCGATACACGACTCGAAACCCGCATGAGAAATTAGTGAGTTCAACCCGCACGATAAAAAAACAGGTCAATTTCGGGTCAACCCGTCCTGACCCGTTTAATAAACGGGTGGGTTTCGGGTCAACCCGCTAACCCGCTATAATACCTATCTAACTCGTTTATTTAACGGGTCGTGTCAACCCGCATCTACCCGTTTTATTTGCAAGTGATATGAACTTTTCTGTTCAATTTGGAGTTGAACTTGAACTTTTCTATTCAATTTGGAGTTGAACTTGAACTAATGAATATATATTTGTATTTAATTATTTACTTAGGTTGTATTTGGGGGAAGTTTTATCAAACTGTTAAAATTATTAATTTAGTTATAATAAAAAATGAACAGGTGGCGGGTTACATGGGTTGGGTCGTGTCAACCCGTTTATAAACGGGTCGTGTTCGGGTTGACAATTTCTGACCCATTTATTAAACGGGTCGTGTTCGGGTTGACCATTTCTGACCTGTTTAAAATCTCGACACGACACGAACCCGATACGACACGACCCATTGCCACGCCTAAGAAAAACTGGTCAAAAGCCCCCAGAAAAGACAAGAAAAGTAACTTTAAGAAAAATACAGATAAAAGAAAAGTTAAGTGCTGGAAATGTAAAAAACATGGTCATTATGCCAAAGAATGTAAAGTAAAAGACACAATAAAGCAACTTAAAGATGAAATCCAGAATGGGACTGCGCTATCAGTTTATTCAAATTGGAAGTCATGCTTAAATGTTGCCACTGCATCTACAATAACCCTACCTGCTAAGGACAATATCCAGGAATTTGAAGTCACCTCTGATTATGTTGTTTGGTGGTCAAAGGTGCATCACTTTGAATCAACAAAGTCAAAGACAATCTCAACTGTAGGCAAGTCGTCGTTGTTATCTCAGCCAAAATCTTTTAAGTCTCAAGACAACAGAACTTTAGTTCATAATAAATCCATACGTG

>Chr1-18; OZ022206.1; 26018229-26020263; dir; B;

TTCCGCGCTTTCATTTTTGATGCATGACTTTTTGTTTTGTAAAAGGATTGATTCCTTTGTAAATTCTTAGTTGTCGCTCTGATAATTTGCCCTATAATTAGAATGTAACTGTTGGAATGTATATGTACGTATGCTGGCAGTTGGTTGTGTATATATGCTTGTTTGGCAGGTGTAAATGTTTTGGTATTGATCCAATTACAAGGGAGACTCTGCCGATTTTTTTCGGTAGGAGTCAACCTTGTTATTTTATCGTGCTTTGTAGGGAAGGGCAATTAGGTCATTCATGCCCGACATCCGCTAGGTCTCGGACATGCACAGGGTTCAGCTCGGATTCCAAAGCAGAATTTGGGTCGGGTCTTGTCACATCCAACAATTTTTAGATGGCACATTTAAAATTAACTTTGGAAATCAACCAACTCAAAAATCTTCAATCCAACTACACCAACTTCCAACACCTAGTCAATCTCAAAGACATGTCCCGTCTAGACATTCCTTTACAACATCCTTCTCTACTCTAGAAAGAGATCTAGAGTTAGAAAAAACATTAATTGATTTAAAGTTAGAGTCCTTAAGAAAAACTTCTCAAGTCAACCATCCTTGTTATAAGAAAAGATGAAAAATCAGATTCCCCAAATTCTCCCACACAAACTGATTTTGAAGCAAAAGCCATCCCAGTAGACAACCAGTTAAGAGCCCTTAGGAAAGAATTCAAAATTAATTGGAAAATTTTAGATGATCATTTACATGCCCTTGAAAATCTAAAAAGAAGAAATGCTTATCATACAGCTTACCCAAGTAAAGAACAAAGACAACAAATTTTTAATGAATGGAAAAATTTTATGGAAAGCACTAAGTCAGAAGTCTTCTATCTTGATTTTGTTGAAAGTCATCACATGCCTAAAGAGTTAAAATAGTTCCCCAGCAAATGCCACTCAAGTCTCAGAAAGAAAAACCTTTAATCAAATTCCAAGATCTGAAGTCGACAAAACCCACCAAAAAA

AAAAAGAAAAAAAGAAGAAATGCTTGAACAACTTACACCAGTTAAAGACAAAACTGAGTCCTCTAGTCTTAAAGTTCTTGAGTCCTCCAATTTAGTCAATTCTTCTGATGACGAGTCTATAACCAGCAATGAGACAGATTTTAGTAATTTATCAAAAATTGAAAATGCCTTTAAAACCTTAGAAATTGATAATAATCTGAAACTTAATAAATTAAGAGACAAAGTCAATCCTACTAGTCTAACAAAAAATTGGTATCCTAGACCAACCCCTCTAGATATTCAGTTTTAAGAAAGAAATTCCCAGAGTCAATTCTCAGTCTCCTCTGATAAATTATATGAATGGAATATAGATGGCCTTTCTGAACAAGAAATCCTTAATAAACTTCAAAATATGTCCATGGTAGCCAACAGTTGCATAACAAATCAAAATTTCAGAGAGTCAGATATTGTTCCCCTCCTAGAAACAGGATTCACCGGAACCTTACGTTCTTGGTGGGATAAACAATTAACTGAAGAATCAAAACGACAAATTATTCATGTAGTCAAACTTAATGAAGAAGGTCTTCCTATTTTTGATAAACAATTAGGAAGAGGAATAGAAGATGGAGTCAACACTTTATCATACACAATAATTGAGCATTTCATAGGAACACCCAGTAACACTACAGCCCGAATTCATAATCAACTTAGTAATCTAAGATGTGCCAAATTAAGTGATTTCAGATGGTATAAAGATGTTTTCATTTCCAGAGTCATGCTTAGAGATGATAGTAATCAACCCTTTTGGAAAGAAAAATTTATTAATGGTCTCCCTAATTTATTTGCCTATAAAAAAAAATATAAAAAAAAAAGAACAGTCCTTAGTAATGATCAAGGTCATATAGATTCTGATAATTTAACTTATGGAAATATTATTAGCACAATTAACCAAGTAGGAATGAAAATGTGAGTTGATATGAAAATAGGAAAACAAATTCAGTCAGAAAGGAATTCAGCCAAATATGAATTAGGAAATTTTTGCGAACAATATAATTTAGCATCCATTCCTCCTTCCAGAAAAGCCAAAACTAGTCATCATGACCGACAAACTAGTAAACGAAGACATTATTCACGTAGGAAACAATTCTTCCGACATAATGATGAGTTCTATAGTAAAAGGACATACCACCTAAAGAAAAATTGGTCAAAAGTACGTGGAAAAGGAAAAAATGACAAACCTAAAAAGTCCACTGACAAAAGAAAATTTGAGTGCTGGAAGTGTAAAAAATATGGCCACTATGCTAATAAGTGTAAAGTAAAAGACACAATAAAGCAGTTGAAAATCACGGATGAATAAAAAGACAAATTAATTAAAGTCTTAGAATTAAGAAACTCTGAGTCTAGTGAAACCGACGTCACAGTAGCAAGCTCAAGCTCAGTAAATTACAACTCCTCAGATTCCCTACTAAGTTCCCCGAAAATCCAATTTGGTTGTAACGATAAGTGCTGTAAAACTCTAACTAAGTCAATTAATGTCTTTACATAACAAGAGGAACAAGAAGAATTATTAATTGATTTAATTAGTAAAGTTGAAAACCCAGAATTAAAATATGAGTATTTCAAAAAATTAAGAAAAGTCATTGGTCAGGAAGAACCAAGTCAACAAGCTCCCTCAAAGATAAGTCTGAACACTGTCTTAGAAAAATAGAATGAAAGAAAGAAGTAACTCTACAGGATTTGCAAATAGAAGTAAAATTAGTCAAAAAAAAAAGAAATAGTAGAATTAAAACAAATGAGCCACAAGTTACAAGCCGAGAATTTAGAAATTAAACAAGATTTGATTAATTTAATGGGAAAAGAAAACTCTGAGTCCAAGAGTCAACCAGAAAGTCCAGTAAATTCCGACGGTGAACCTTCAAATAATGATCAAACTATTAATTTAATAAAATAAGTAAATTTTAGAAAATGGTATTCCAAAGTCGCTATATTTGTAAAAAAAAATTTGAGTTAAATACAGTAGCTCTTTTTGATTCAGGAGCTGATCTGAATTATATAC

CTACCAAATATTATAAAAAGTCAAAAGAAAATCAAAAAATTTTGAGTTAAATACAGTGGCTCTTTTTTATTCATGAGCTGATCTTAATTGCAAAAAACAGATAATGGCAAAACATTCAAAATACTAGAAAATGCCATTGGTTAATGCAAATATTAAGAATTGAAATTATTAATTAAATGAGGATAATATTGTAAATTCACACTTTTTATATTAAAAAAATTAAAATTAAAAGAAAAATTAGATAATGGATCCTATTTTTATGGAACACAACTACCAATTTTCTTTTTGAATTCTAAAATAAATTTTAATTTTTTATATAAAAAAATGCTTCTCGCAGGCGCTGAAGCGCGTGCGGAGAGACTAGTATATTATAATATCATATATCATATATGGTGTATATATATAATTAGAAAAAATGAAGTGGGGGCCTGGGATCACACTAGTCCCTTAATGGCTCTGATACTGGGAGCACGTGTGGCCATTGGGCTTTCACACATGGGCAAATCTGCTCTGATACCATGAAGGAAGTTGAAGTTCCACTTAAAATCAACTGGCAATTGGGGGAGTAACCAAACTTCTTATGCATCCTTTTAGAGCAAGTCCACCAAGGAGCCCGACTCAAGGCAGAGGGGGGGCTGCACGAAAAACACAGTTCTAGTGGGCCACCCTTGCCCGAGCTGGCTGTGGAACCCACAAAGACGGGCTGGCCCGAAGACAAGTTTCGCCCCAGCTCGAGCCCGAGGGGAAGATGACGTCATCGCGCAGTTCAAATTTTACTGTAAAAAAAAAAATTTGCACCCGCATGCACTGATGTCAACCCTGCTCTTTTCCAACGGCTAGAAGACACGTGTCGCAACCTGGGCGCTCCGATCTGCTTCTCCTCCTCCAATCCAACGGTTGGCCTTTTTTTGGCAATAAAATTATGAAAAAAATTCAAAAGTTTTTTTAAAAAATACCAAAAAATTTTGATTTTTTTTCTTCTATAAATACCTATCAATAC

>Chr1-19; OZ022206.1; 26152627-26154067; dir; B;

AAGAAAGTCTGATAATAGACTTAATTGACAAGATAGAAGATCCAAAGAAAAAGAGAGAAACGTTAGAAAATTATATCTCGCTAGCTAAAGCAGGACCAAGTAACCCGCAACCATTTATAACTAACGTGAGACAACCAATTGAAAACTATAGCTTTAAAATTATAGTAAACAAGATGAATGATAAAATTCATAAAAAAGAACCTACTTCAACGACCTTCAGTGCAAAGTTCGTGATGTAAAAGAAGAACTTAGGGAGTTGAAAAATAGGGTTAGAGTTCTATAACTCTATCGTCCTTTTTCGAAAAAAGATACAGAAGAAAAATCTGAGCAATTAGAGTTTGAAAATCTAATGGGATAATTTCAAGAGAAAATAGAACCTGTGAGAAGAAAAGCCCTACAAGAAGAGCAGGTTAGTACCATTAAATTCTTTAATAGCATAAATAGAGTGATAGCACATAAATGGTATGCGATGATAACAATTGTGATGAATAAAACCTATAAGTTTACAGTTGAAGGGCTGATAGACACAGGAGCCGATCTTAACTGTATAAATGAAGGAGTGGTACCCACTAGGTATTTTCAGAAAACTACCCAGGTCCTACATACAACTATTAATAGCCAAAAAATGGGAATTCGGTTTAAATTATCTAACGCTGTTGTGCTTAATCATGGTATTTGTTTTGAAACCCCTTTTGTTATAGTGAAAGGTTTAAACCAGTCTCTAATATTAGGAACATCATTCATTAATATGCTTTATCATTTCTCTGTTTCTGAAGGAGGAATTAGAACTGAGGTAGAAGGTGAACCTATAATCTTCAGGTTTTCTCAACCATTGAAGCAAAAATATATTAATGAAATCCAAGAAAAAATAAAAAGGAAAGAAAATTATCTATATCATTTGAAAGAAAAAATGCATCACAAAAGAATAGAGGAGCAACTGCAAAATAATAAGATGAAAGAAAGAATTGATGAAGTCCAAAAGATGTTCGAAAATCAAGTT

TGTGCTGAAATACCTAACGCCTTTTGGAATAGAAAGAGGTACATAATTGAATTTCCATATGAGCCTGATTTTGATGAAAAGAAAATCACAACCAAAGCCAGGCCAATTCAAATGAATGCTAGGTTAATGGAAATTTGTAAATCTAAAATACAAGATTTACTGAACAAAAAGTTAATCAGGAAAAGCTCATCTCCTTAGAGTTGTTCTGCCTTTTATGTTGAAAACGCGGCTGAATTGGAAAAAGGATTACCCAGATTGGTTATAAACTATAAACCTCTCAATAAAGCATTGAAATGGGTTAGATACCCGATTCCTAATAAAAATAATCTCTTGGATAGATTAAATAAGGCAGTCATCTTTTCTAAATTTGATATGAAATCAAGATATTGGCAAAACCAGATCGCCAAAAAGGATAGGTACAAAACAACCTTTAATGTCCCCTTTGGACATTACAAATGGAATGTTCTACCTTTTGGATTGAAAAATGCCTCTTCTGAATTTCAAAATATTATGAATGATATATTTAATCGTTACACTGCTTTTATCATTGTTTATATTGATGATGTTTAAGTGTATTCTAATTCTTTAGAACAACAATTTAAACATCTAAATATTTTCTTGAAAGTTGTAAAGCATGAAAGTTATCGGCAAAGAAAATGAAATTATTTTCTTCAAAAATTCACTTTTTGGGACATAATGTTAATAAGGGACTGATAGTTTTGATTGATCGAGCAATATCCTTTGCTGATAAGTTCCCTGATGAAATTAGGGACAAGCAACAATTACAAAGGTTCTTAGGAAGTCTCAATTACATAAAAGATTTTTATAAGGATTTAGCCCAAGACTGAAAACCATTACACCAAAGGTTGAAAAAGAACCCGGTTCCCTGGTCTGATGAACACACTAAAGCTGTTAGGAGACTAAAAATCAAAGTCAAAGAACTTCCTTGTTTAGCTCTAGCCAACCCAGAAGCCTTTAAAATCGTAGAAACCGATGCTTCAGAAATTGGGTATGGGGGCATCCTAAAACAGAAAGTCTGTAACTCAGAACAATTGGTTAGATTTACCTCTGGAATTTGGAAACCAGCTCAAAAGAATTATTCCACTGTTAAGAAAGAAATGCTAGCCATTGTCCGTGCTGTTTCTAAATTCGAAAGTGATCTTTTAAACATTTTTTTTTTTTTTTATAAGAGTGGATTGCAAGGCTGCAAAAGACATTTTGTTTAAAGATGTAAAAAATTTAGCATCAAAATAGATTTTTGCAAGATGGCAAGGAATTCTTTCTACTTTTGATTTTGATATTGAATTTATTAAAGGTGAAACTAACGTACTCCCGGACTTTTTAACTCTGGAATTCTTGCAGGGTGCGAACCAGAAGCTCCGAATAGATGACTCCCAATAAAAGTCCCTCAACTCTTACTAAGTCATCCCCTCTTGATCCA

AAGCCACTGATGTCTTCTTCTCCATATAACAAGGGTAAGGAGAAGCAGATTTTTGAAAATCCATTCCGTGAATATATGAATATGTCTATAGCAATCATTGAAATGAATGATCTAGACAAAAACCCGAATTTTCTAATTGAAAAATATTTAAGGGATAACCTTCCTCCCTTGAATAAGCCTCGTATTTTCTATGAAACTCTTCTGATTGAGACCAAGTCTGTTACGAGGATTTTAGAGACCAAGAATATGGCCTAATAAGCCTCGTGTTGGTATTTTCTATGGATCTTTTCAAATGAAATTTTGTAAGTGGAATAAAAACCATCCGAATGAAAGGACCTTATTCATGTGGTTCATGATAATGTATGAACTACCCTGAATTCTGAAATGGGAATATGTTATTTCGGAATATGTTGATGATGAATGGACTTATATCCCTAATTTAGTCTGAAGTATCTTAATCAGATGGTGGGATAAATTCACTAAAGATCACTTGGCCCCCACCAAGATTACCCCGACGCTCATCCCATCTTTTGACCCTGTTGCTGCTCAACCTTTGGCCACTTCTTCTGCTAGTTTAAAGTCTCTCATAGGTTACAACTATTGATAGACCAACCAGGCTCAACTTCATCAAAAGAAGACTTGAAAAAGAAAGTTCTCGAGATCCTTGATCTAAATGACGAATCCATGGCATCAGCATCTGCCACTCAGCCTGAAGATGAAGTGGACCCGTTTGCTAACGAAGATATGCTAGACAATTATCATCCAGATCTATATGGTGGGCCTCAGTAATATTATGTCTGATAAGAAGACAATTTGTACTTATTGTTTACGTTAAAACATTTTTCTATTGCTTTAACTTTACTTTTCCTCTGGTTAAAGCAAGATGCTTCTACTTTAACCCTACTTTCACTTTACTTTTGTAAAAGTTTCTTTTTTTCTTTTGGCCATTTGCGTGAAAGTTCAGCAAGAATCATCAAAGTGAAGATGCCGCTCTACTGTA

>Chr1-20; OZ022206.1; 28117418-28120638; dir; B;

GAGGGTACCATAACGTTGCAAAGAGGATCCATAAACATGTACATTACAAGATTTGGGTCGCGAGTTACAAATTTATGGACTGGAATTACAAAGTTAGGGATCATCCAATCCATAATTTTGTAAATCATCATTTTACAAGGATATGGATCCACATTACAATGAATTGGTAAGTGTTGACCATGAAGATGTAAATTGGGGTCCTTATTCTAGTAAAATGAGGGGTTAAAAGATTAAGTGCCGATTCTACAATGTTTTGGATGCGACGGTACCATAAAATTGCAAAGAGGATCCATAAACATGTAAGTTACACGATTTGGGTAGGGAATTACATGATGATAGTGATGTCACTCCCAAGTCACAATCATCTACCAGTCAAAAAACTCTAGCCCCGAAAATGCGGTGGAGTGATTACCCTCATGATGATCAAATCCATGGTCAAGATCCCTATGATTTTAGTCCTCTTGACCTCGGAGATGATTAAAGTTGCCCAACATCATGAGCCCTGAGACAGGCAAAGCATTCGGAATCTTGGACCCGTACTTCAACGGTCTGATAGCCCGTGCTTCTTTAGTCAAATTCCCCGACAAAGTAAAAGTTATAGCACTCCGTAAAAGCAAAAGCTTTCACGTGCTTCTACAATCACTTTCCAGAGCATCCAAAGTTGCCAGCTCATCAATAGTTATCAGAGTTCACTTTATTTGCTTATTTAAGAAGGCCTCAGCTTCAGTTATCAACAGAATGTAATTTAGTACACTATAATTTTCTCAATTTCAAAGTTCTATCAGTACCAATGCTCTGAAGAACTCAGTAGTGAAACTAGGATCTTTGTAAGTGTTTTGCATTCTGCAAGTTAATAATAATATTTTGAGTATTTTGAGTAATGCTATTTTTAATTGCATCTATTTTCATCTTTTCTTAATTTGCTTCATGCATTCTACAATCTCTGTGCTTACACATACTTCTGTCTTTATTCTATCTCTTCCTGGTTCTAGAGCCCTTC

TGGTATCACAACCGTAAGTGTTGAAGAGTTAATCTAAGTTTGCAACTCATCCAACCCAAGTGGCTGGTTTAGGTTTTCTGAGTTGTAAGTTTTCAATTTCTTCTCTGTCTACTTCTGGTCTTTCAGTCTACCTGTTCTTCTTTCTCTTGGTGTCCGAATCGTAAGATCCGTAACAAACAGTAAGTCCCAGGGAGGCCTGAGAGGTAGTGGCATTAGTAGGAGAGCGAGAGAATTTTATGTATCCTGTTATCCCATGGAGTCAGTCTTAGAAGTTTCTGTTCACTTTCTTCATTAGAAGTCCAACCCCAGAACCTTGAGAAAAAATGAGTCGCTTATTTAGAACTAATTCTTGCAATTCTAGCAGCTCTAGAGCTAATAGTTGTCTCCCAAAGATAGTTAATGAAGAACAAGTTGAGTATGACAATAGTAATAATAATTTAGATAATAAAGATTGGAATATACCTAGAGTCCCCAGTCAGGACATCTAAAAGAAAAAATTGTCATTATCCAGTTTTAAGTCAACCACCCATGTCAAAATAGTCGAACAAGTCTATGCCTTGAGTAAGAATCATGAGACCTGTCAGTTGCTTAATCTTGAGTCAATAAGAAAACATAAAAATGATGGAAACAATTTCCTCCATATTGGTCTAGTCCAAGTTGTTGTCAAGCCATTAACCAGATTAGGGTTAAAGGCTTCAATCCTTCTCTGTTTGTGTGATGCTAGGTTTACTAATTTCAACGACGGTACGTTAGGAATTATTGAGTCTAGTCTTGCTAATGGCCCTATTCACTTTGATTGCTATCCAGATTTTACAGTCAGCCTGAGTGATCCACACATATTGAAAACCCTTACTTTAAATATTAAAACAGAAGGCTATGAGGTCTTGTCGGGTTCCCAACCCTTAGCTTTAGTCTATCGAATCTACTATAAAGTCACAGGCACCAATATGAATTTTCAAGCCCTAAACAAGAGCCCAAAGAGTCAAACTTTATTAATCCAAAGCCATACGCCTGATGCTATTGTTAGAGTTCCCCATTCTATCAAATGGTCAGACGTTACCCTTCCTGCTGGTTGGACCCTAACCACTGAGATCCCTCCAGTTCCCATCCAGTGTAGTCTAAACGATTTAGACTACATACAACAATATTTAGATGGCACGGTAAAAATTAATTTTGGTAATCAACCTCCACAAAAGTCAACAGTCCAATTACAACAGTTACCCACACATGGTCAATCCCAGAGACATGTTCCTGTAAGGCATTCCTTTGCAACTTCTTCCTCTACCCTAGAACGAGACCTAGAGTTAGAGAAAGCTTTAATTGATTTAAAATTAGAATCACTAAGAAAAACTTCCCAAGTTACCCATCCTGTTTATGGAAAAACCCCATTATAGGAAGACACTAAATTTGAGTCCCTTGATTCACCTACTCAATCTGATTTCATAGGTAATCAGTTAAGAACCCTAAATAAAAAATTTATAATTGATTGGACAGGATTAGAAAATCATTTGCATGCCCCTGAGAATCAAGCTAGAAGAGATTTTATGATTCAGTTATCCCTGATAGAGTCAAAATACAACAAATTTTTAATAATTGGAAAGAATACATGCGCACTGCTAAAGTTGAAATATATTATCTTGATTTTGTAGAAAGCCGACATTTGCCTAATGAGTTAAAAACTTTAACCTAAGAAAAATGGGAAAAAGTATATCACACTGAGATCCTTGCCAGTTTTCCACCTGTAGAAACAATAATCATAAAGCATCAGAAATCCAAAATAGTTGCCAGTCCCTTTAAGATCCCTGATCCAGAGAATAAAATGATAGTTGAGCAAAATAATTACACCAACCAAAGTTTAGTTGTCATAGGTAAGCAATTAGACAAGATAGAGACAAAAGTTGACAGATTATTGTCTAAAGAACTCACACCTAGGCAACATGAGAAACCCTTAGTCACTTTTCAGGATTTAAAATCAAGTCCAACTTTTAAGACAAAAGAAACAATGACAAAAATTGAGAAGATGCTAGAGGAATTAACGCCAGTCAAAAGAAAAGATAAAGAAAAGGTTGAAACAAGCATTAAAGTCTTAGGATCCTCTGAATCACCATTTGCTTTCCATAACTCCTCTGAGTCTGAATCAATTGCCAGTATTGAAACAGAAACTAGCAATATTTCTAAAATTGAAAATGCATTTAAAAATCTTGAGCTAGAAAATGATCCCCAAGTCCAACGATTAACAAACAAAGTCAGCCCTACTAGTCTCACAAAAAATTGGTATCCTAAGCCAACATCTCTGGATATACAATTTGAAGAACGAAACACACAGAGTTAGTTTTCAGTCAATTTTTAGTTTCTTCAAATAAGTTATATGAATGAAACATTGATGGTCTTTCTGAGCAAGAAATCCTTAATAAGCTTCAACATATGTCCATGGTCACCAATAGTTACATCACCAACCACAGTTTCAGACAGTCTGAAATTGTTCCCTTGTTAGAAACTGGTTTCACTTGAACTCTTCGTTCTTTGTGAGATGAACATTTAACCCATGAGTCTAAGCAACAAATAATTCATGCAGTCAAACTAAACGATGATGGTTTTCCTATTTTCGATGAACAAATAGGTCAATGCATTGAAGATTGAGTTAACACCTTACTATACACAATAGTTGATCACTTCATTGGAACACCTAGTAATACTACTGCCGGAATTCATGATCAATTAGTAATCTAAGATGCCCTAAGTTATCTAATTTTATGTGGTATAAAGATGTGTTTATTTCCAGAGTCATGATTAGAGATGATAGTAACCAGTCATTTTGGAAAGAAAAATTTGTTAATGGTCTTCCTAATTTATTCGCCCATAAGATCCGATCAACCCTTAGTAATGAACAAGGACATATTGATTGGGATCATCTCACATACGGTAATATAATTAGCACCATTAACCAAGTCGGTATGAGAATGTGTGTTGATATGAAAATTGGTAAACAAATCCAGTCTGAAAGAAATTATGCTAAATATGAGTTAGATAATTTCTGCGAACAGTATGGTCTAATGTCTATTTCCCCATCTAGAAAGAATAAGTCCAGTCACTCCAACCGATAGTTTAGTAAACATCGCCATTATTCATGCAGAAGACAGTTATTCCAACATAATAATGTGGATAAAGAATTTTATGGTAAAAAGAGATTCTCCCCTAAGAAAAATTGGAAAAATAGTCAC

GGCAAAGATAAGAAAAATTACTTTTCTATATATATATATATATATATCCTATAGCTGCACGGCAATACAAGATTTTTTTCAAATTTTTTAAGAAATATTATAGTCGTGCGGCTATACTCATTTTTCATTGTGTTTTTTAAAAATAGTATAGCCGCCCGGCTATATTGTTTAAAAAGGAAATTTAATCTAGAAATTTTGTTGGCATAGACGAGTCTGACGAACTTTTGGGAGAGGTTTTTTTTTTTTAAAGTTTTAAAAATATAAAATTAGTTTCTTTGCTCTTTTTTTTTTTTTTGGCATTCTTCTTTAATTTAATATATGTAACTAAGTAATATCTTAGCTTTTTTAATTACTTTAATTAGTAATTATATTTGAATTATTATTTAATATAAAAAATTAAAGAAAAAGTCCACAAAATAATTAATTTTTATTTATTAAGTTTTTTGTGAAATCATTTGGGTGTCTTAATTAAATTTCTCCATATCCCAAGAGCCCTATCCATTACTATTAGGCCATTACTAGAATATCTATTGTCCTTTTTTGATTTTTTACTTTTCCGGAGTTATTACGTATTTCTCATGTTTAATGCATGTATGTTTTACAAAGATTTCAATAAGACACACAAAATTTACGTATTATACACCTTATTCTCACATATTATTGAATAAAAATAATATATATTTAAAAAGGTAGCAGCATGGCTATACTCAGTTTTTTCCTAATTGTTTTTAAAAATAGTATAGCCGTCCGGCTAGACTCCTTTTTTCTAAATTTTTTTAAAAAAATAGTATAGTCGCACAGCTTTACTTGTTTGACACGTGGAGCATAGGCGCTGGGCGGGTCTTTTTATATATAAACTAATTTTGTATTTTTTTTTTTTAAAAAAAACCTCTACCGATGAAACTTCGTCGGCACAGAACTATGCCAGTAAAATTTTGCATATTAAAATTTTTTTTTTAAAGCCACGCGGCTATACTATTTATATAACTGTCTGTGGCCT

>Chr1-21; OZ022206.1; 29095792-29108862; dir; C;

GAAAGCAGTCGCAGTGGATGAAGACCTTGTCCATGGGGGGCCCCTAGAATCACTGGCGGACCTAGAAATTTTTTAGCAGAGGTGCAATATTGACTAATGATGAAAAATGATTTTTCGGAATAATCACCTTCTCAAGATAATTTCTGGCGGCTTTTATTCCTTACACATCAAATAAGAAAATTTGATTTTATGGGATTTTAGTATAAAATATATATTGACTTAATGAGCCATCATTTTTAGCCTAAATCGTATTTTGCACTAAAACCCATTTTTCATATTTTGATTTGGTGGGAATTTAATTTTCTAGTATTTTTATTTGAATCCAAATGGGGGTACTTCCTTATTTACATTATAAACTTAAGTAAATAGAGTACTATAAAAATAAATGAAAGTAAAAGGACAAATGACAGGACCCGATCCCAATTTCACTTTGGAATTCGAGCCAAGTCCTGTGCGTGTCCGACACCTGGCGAATGTCGGACACAAATGACCTTTTTACCCTTCTCGTTACAACTCCGATTCGGGTCTACTCCGTGTCTACGGACTCGTTTCGCCGTGCTCTACGCAACGGCGCAAGCGGAATTCCCAAATTCTTTCTCGATCAAAAAGTCAACTTTTTCTCTATTAAATTATGCGAGGGCAAAATCGCCTTTTTGGTAGAATAAATTAATCCTACTTTTTGGATATTTTGGTTTGGGTTATTACAACAAAGACATTTACTTAAATGTTGAAAATGAAAATAAGGTAATCTGTACACCAGTTGAGCAAATGGGCAATTTGAAACACTTACAATGATTTTTCCGGTGAGGGGAGGTTTGACTTTTTCTTCCTTTCTGTCTCTTTCTGTTCAATAAGCTTAGCCATGGAAAGTTTTACAGGTCATATTATTCAACCCTAGACCTCTTCTTTTTCTTTTTTTCTGTTGAGATAATTCAACCCTAGCCCTGGAAAGTTTTACAGGTCATATATATATATATATATTATATGAAAATAGATGCAA

TGAAAATAAGCATCAATCAAAATAATCAATTCTCAAAATATCTCAAATATATTGATAACTTGGAGAATGCAAAACACTTACAAAATCTTAGTTTCACCACTTGAGTTCTTCAGAGTATTAGTACTGATAGAACTTTTGAAACTGAGAAAATTATAGTGTACTAAAATTACATTCTGCCTAAGACAGAGGCCGAAGCCTTCTTATATAAGCAAATAAAAGGACTCAGATAACTTTAGGTGAGCTGGCAACTTTGGATGATTGTGAAAAGTAGCTGTAGAAGCACGCGAAAGCTTTTGCTTTTACTGAGTGCAGCCACTTTTACTTTGTCGGGGAATCTCAAAAAGAACACGGCTTTCAGACTGATGAAGGCACGGGTTCGGATACTTTTCTTCAGGGCTCATGATGCTAGGCAACTTTTGACTTTAACTTTTGCTTTAATCATCCCCTAGATTGAAAGGACCAAAATCATAGGGGTCTTGTTCATTAGGATAATCACTCCACCGCATTTTTGGGGCTGGAGCTTGACTGGTTGGCTTGGGTTGACTAGTAGATGTTTGTGACGCAGGAGAGGCATCGGAGTCTGCATCATCATTCATCTGTGAGGCTTGAAGCATTAGTTGGCGAGCAAGATCTTGTAATTCGGACTTAGACCTTCCCTCGATTGGGAGAGAGAAATGTGATGTGACAGGGGAACTCTTTTTCTCAGAATGCGGATTCCTGACTGGAGGAAAGTCAGCATAAACATAATATGCAATACGCTCAAGTTTAAATTTATCCCACCATTTGATTGAAAATTGGCGAGATAGGACTCTGGTTTCCCTATTAACCTGATAGGACCATTTAAAGATCCAAGGGACTTTATATTTGGCAACAAAATGAAGCAGATTGGGGAAGAACAACTCAGATTCTTTGAATTTATTTTTATTAGTAAAATAATTGACTAATTCTTGCATGGGGGGAGGAAATAAATCAATGACTGGACCATGTTTTTCCCACCAGTGATTGAACCAGTAAGGAAAAGAGCTTTTGAATTTGCTATCAAAGTTCAAGAACCAAGAGTGACTGAAATCAACTGTTTGGTGAAGCAAGATAGAATACCATGCTTCAATATAGTCATAATAATTGTATTGGAGTTCTGATTGGATAACTTTAAGATCATAGGGATGACTACTCCATTCATTTTCATTGATGATTTGGTGAATGTAAAGAGAATGATAAAGGATGATATGGGGGTTTTCTCTGTCTTTGATTGGCTTAATGGTGATAGAATGAGTCTCATGAAGGATGTCTCTGTAATATTTAAGGTTCTTGAATGGTGCTGGGGGAATGAAATGACTATTTGGTGGAAAATAAGACTTAGCAATGGTGATGGGATTAGCAATTGAATTATATTTGGACTCAATGACAAATAGGTGACTATGACTCTTGACCATGTATGGAGATGTCTTCAGGTAAGAAACAACTGGGGGGTTAGTAATAGCAATATCTTGATAAGGATCATGACTAGAAATTAAAGCAGATTGGAAACTGGGGCGAACCTGGCCGACTGTGGCTCCTAGGTTGGCAAACCTGTTACTAATTTGGATAGGAGAGCATCCCGGAAAGGGGACGATCTGGGAAGGAGGCTCAAATTTGGCTTGACTCATGGATGATGGGAGGACTCGGAGTGACTGAGGTTGACTGGGTTGACTTGATTGACCAATTTGACTCTTTGTTTATCATCTTTTTCTCTTTCTTTTCTTTGTTCTGCCATTGTTTTCCCTGCATAAATTCTCTGGTAAGGAAATCAAGAATAGAATTCTCACTTCCTCTCAAATATTCAATATCAAAATCAAAAATACTCAAAATAGCTTGCCATCGTGCAAATATTTGTTTGGATGCAGTGTTTTGAACATCTTTTTCTAATACATGCTTAGCAGACTTACAATCAACACAGACTAAAAATTTTTGATTTAATAAATCAGACTAAAATTTATTCATGCATAATACAATAGATAATATTTCTTTCTTAATAGTACTATAATTATTTTGTGATTGGGACCAAGTGCCAGAGTGAAATCGAATGATTTGCTCAGATTGACCAGAAGTGACTCTCTGTTTGAGGATACCTCCATAACCTACATTAGAAGCATCAGTTTCAACAATTTTAAATGAATTAGCAGAAGGAATACCAAGACAAGGAAGAGTCTTGATATGAGCTTTGATCTGTTTGACAATTTATGTATGAGCAGGTGACCATGGGGGAGGATTATTTTGGAGACGGTCAAATAGAGGCTTACATTGTTTTCTCATATTTTGATAGAAATCAGAAACATAATTTAGAGATCCTAGAAATCTCTAGAGTTGACTCTTGTCTAGGATTTGGTCAGGGAACTTATCGGCAAATTGGATAACTCGATCTATGGGGCTTATCTGTGACTGGCAAATATTAAACCCAAGAAATCTCACATTAGTTTGGAACAACTTAATTTTCTTAGAAGAAACAACTAAACCATTTTGTTTAATTATCTGTAAAAACTTATACAGGTGTTTCCAGTGTTGTTCTATGGACTCAGAGAATATAAGCACATCATCAATATAAACTATGGACAAATGACTAAAAGGATTAAAGATCTCATTCATAATATTCTGGAATTCACTAGGAGCATTCTTAAGACCAAAGGGCATAACATTCCACTCATAATGACCGAAAGGAGTAACAAAAGCAGTTTTATATCTATCAGACTCAGAAATTTGAATTTGCCAAAATCCACTTTTCATATCAAACTTAGATAAGATGACTGATTTTTCTAATCTGTTGATTAAATCTCTTTTATTAGGAATTGGGTATCTAATCCATTTTAAGACAGTATTAAGCGGTTTGTAATTAATGACTAGTCTTGGGACACCACGTTCTAACTCAGCATTTTTCTGGACATAGAATGCAGGACAAGACCAGGGTGACTTACTCTTACGGATTATTCCTTTTTAAAGTAAATTAGCAATCTCAAGTTTGCAAAATTCCATTAATTCCTGGCTCATTTGAATGGGTCTTGCTTTAGTAGGAATATTTTTCACACCAAAAGATTTGACATAGGGAAGGCTGACAACATGTTGTTTCCTATGCCAGAACGCATTAGGTAACTCAGAGCAGACTTCTTGTTTTAGTTTTTCTTCGAATTGTTGTATCTGTGATTGTAAAGACTGATGGCTTAGTTGTTGCTCAATTCTTCTAGCTTTAACTTCTTCCTGGAGAAATAGTAATTGGTTATCTTTATTTCGGATGATCTGGACAGTTTTGGTGGCTGGATTTTTTTGTAATTCTTTTAAACTATGTAACTCAGGTTTACTCAAAAATTCGAACTTAATTTTTTCTCCTAAACAGGTGGAAATGACACCATCATGTTCGACCTGGAAAGGGTACAGGAGGGCAATGAACGGCAGACCAAATATGACTTCATCAGTAATATTTTTAATCAAAACAAAAAAAGTTTTAAAACAGATTTTATTTTGACAGACATGGGCTTTAGGGATCTCATAATTCAATTGGAGGAATTTTCCTGAAGCAGTACTTAATGATTTTCTTGACTTTTTATAATATTTAGTTGGGGTTAAGCCTTCTCGGATACAATTAAGGTCGGCTCCTGAATCAAAAAGGGCAATTATATTTAGTTCAAAATTTTTAATAAATATGGTAACTCGTGCATACCATTTTCTAAAATTTACATGTTTAATTAAATTAACGGCTTGCTCTTGTATAGGGCTTTCTTCATCTGAGTTAGTTGGACTTTCTGGGTTAGACTCAGGAATTTTCTTTTCTAATAAATTAATCAGATCTTGTTTTATCTCATAATTTTCAGATTGCAATTTTTGACTCATTTGTCTTAACTCAATGATTTCTTTTTTTACTAATTTTATTTCTAATTGTAAATCCTGTAAAGTAACTTCCTTCTTTTTCTTAAATTTTTCTAGGGTTGTATTCAGATTTATTTTTTGGGCAGAATGTTTTGATTCTTCTTGGCTGATGACTTTTCTTTTAATTTATTTAAATATTCAGATTTTAATTCTGGATTCTCAACCTTACTTATTAGGTCGATTAATAATTCTTCTTGTTCTTCCTGTTTGGTGAAGACACTGATTGATTTAGACTTACAGCAATTATCTTGGCAACCGAATTGGATTCTGGGGGAACTGGGTTGTGACTCAGAAGAATATATTTCAGAAGACTCAGTACGACTAACCATGGTTTCATTCTCACTTGACTCAGTGTTATTTAACTTGAGGACTTGAATTAATTTATTTTTATCTTCATCCGTGATTTTTAATTGTTTGATTGTGTTTTTAACTTTGCAATTATTAGCATAGTGACCATACTTTTGACATTTATAGCATTTCACTTTTCTTTTGTCGGCCGTTTTCTTAAAGCTATTATTCTTTTCTTTATTGTGACTCTTTTTCCAATTTTTCCGAGAGGAAAATATTTTCTTACTATAAAATTCTTTATCTTCATTATTATGTTGGAATGACTGTTTTCTACGCGAATAATGGCGGGTTTTTCTAAACTGTCGACTAGAATTATTTTTTCTAGACGGAGGAATAGACATCAAATCGTATTGTTCGCAGAAATTTCCTAAATCATATTTAGTAGAATTTCTTTCAGATTGGATTTGTTTACCAATTTTCATATCAACACACATTCTCATACCAACTTGATTAATTGTACTTATTATATTTCCATAAGTTAGACTGTCCCAATCAATATGACCTTGTTCATTACTAAGGGTTGATCGGATTTTATGAGCGAATAAATTGGGTAATCCATTAACAAATTTTTCTTTCCAAAATGGCTGATTACTATCTGTTCTTCCCATGACTCTGGAAATAAATACATCTTTATACCATCTAAAATCAGATAATTTAGGGCATCTTAAATTACTCAATTGATCATGTATTCTGGCAGTAGTACTACTAGGTGTTCCAATAAAATGATCGACTATTGTGTAAAGCAATGTGTTGACTCCATCTTCAATACCCTTACCTATTTGTTCATCAAAAATAGGAAGACCATCTTCATTAAGTTTTAGCGCATGAATTATCCGTTGTTTAGACTCATGAGTTAAGTGATTATCCCACCAAGAACGAAGAGTTCCTGTGAAACCTATTTCTAACAAGAGAACAATTTCAGACTGTCTGAAACTGTGATTGGTGATGTAACTGTTAGCTACCATAGACATATGTTGAAGTTTATTTAGAATTTCTTGTTCAGAAAGACCATCAATATTCCATTCATATAATTTATCAGAAGAAACAGAAAACTAACTTTGGGTATTTCGTTCTTCAAACTGGATATCAGGCGGTGTAGGCTTAGGATACCAATTTTTTGTGAGGCTAGTAAGATTAATTTTGTTTGTTAATTTTTGTACCTGTGGACTTTGTTCAAGCTCAAGATTTTTAAATGCATTTTCAATTTTGGATATAGTACTAGACTCAGGATCGATGCTATCAATTGATTCAGGCTCAGAGGAGTGATGAAGTATGAAAGGTGACTTGGGGGATCCTAAAACTTTAACGCTTGATTCAACTTTCTCTTTATCTTTTCGTTTGGTTGGAGTTAATTCTTCTAACATTTTTTCAATTTTTGTCATTGTAGTTTTTGTTTTAAAAGTTGGGCTAGTCTTTAGATCTTGAAAAGTAACTAATGGTTTCTGGTCTCTCTGCTTAACAGAAGTTTCTTTTGGCAGTAATTTGTCAATTTTTGTTTCTATTTTGTCTAATTGTTTACCTATCATGACTAGAATTTGGTTTGTATAATTATTTTGTTCAATCACCATTTTATTTTCTAAGTCTGGGATCTTAAAAGGACTGGCAATAACTTGTGCATTTTGGTGTTGGATTACTATGGATTCAACTGGAGGATGACTAGCAAGGATCTTAGTGTGATCTATTTTTTCCAAATTTTCTTGAGTTAAAGTTTTTAATTTATTAGACAGATAATGGCTTTCTACAAAATCAAGATAATAAATTTCAACTTTGGCAGTGCGCATGTATTCTTTCCATTCATTAAATATCTGTTGTCTTGTTGTTCGGTCTGGGATTTTTGAGTGATAATTATCTCTTCTCGCTTGATTTTCAGGGGCATGTAAGTGATGTCCTAATTTTTCCCAATTAATTTCAAATTTCTTATTAAGTGTTCGAAGTTGGTTTTCTATTTTGGAATCGGCTTCGACAAAATCAGATTGAGTGGGTGACTCAGGGGACTCAGATTTATTTTCTTATGTTGGTTTTCCATAGACTGGATGGGTGACTTGTGCAGTTTTTCGTAGTGATTCCAATTTTAAATCAATTAAGGCTTTCTCTAACTTTAGGTCTCGTTCTAGGGTAGAGGAAGAGGCTGCAAAAGAGTGTCTGGCAGGGACATGTCTCTGGGACTGTCCTAGTGTTGACAACTGTTGTAACTAAACTGTTGATTTATGTGGAGTTTGATGACCAAAATTAATTTTAACTGTTCCGTCTAAGTATTGTTGTATGTAGTCTAAATCGCTTAGACTACATTGGATAGGAACTGGAGGGGTCTTAGTGGTTAAGGTCCAATCAGTGGGAAGAGTGACATCTGACCATTTGATAGAGTGAGGAACTCTAACAGTAGCATCAGGCGTATGACTTTGAATTAATAGTGTTTGACTCTTTGGGCTCTTGTTTAAGGCTTGGAAATTCATGTTTGTACTTGTGACTTTATAATATATTCTGTAAACTAATGCTAGGGGTTGGCTTCCTGGCAAAACATCATAACCTTCAGTCTTAATATTTAAAGTGAGTGTTTTTAATATATGTGGATCACTCAGGCTGACTGTAAAATCTGGATAGCAATCAAAATGAATTGGGCCATTAGCAAGACTGGACTCAATAATTCCTAACGTACTGTCGTTAAAATTATTAAATCTAGCATCACGCAAACAGAGAAGGATAAAAGCTTTTAATCCTAATCTGGTTAAAGGCTTAACAGCAACTTGTACCAAACCAATATGAAGAAAATTGTTTCCATCAGTTTTATGTTTTCTTATTGACTCAAGATTAAGTAACTGGCATGTCTCATGGTTTTTACTTAGGCATAGACTTGCTCGACTATCTTAACATGGGTGGTTGATTTAAAGCTGGATAATGACCATTTTTTCTTATAGATGTCTTGACTAGGGACTCTAGGTATATTCCAGTCTCTATAATCTAAATTACTATTATTCTCATATTCAATTTGTTCTTCATTAACTATCTCTGGGAGACTACTATTGGCTCTAGAGCTGCTAGAACTGTAAGAGTTGGTTCTAAGAAAACAACTCATTTTTTCTCAAGGTTCTGGGTTTGGATTCCTAAAGGAGAAAGTGAACAGAAACTTCTAATATAGACTCCATGGGTAACAGGACACCTAAAATTCTCTCGCCCTCCTACTAATGCCACTACCGCTTAAGCCTCCCTGGGACTTACTGTTTGGAACAGGTCTTACGATTCTGACACCAAGAGAGAGAAGGACAGGTGAACTAAGAGTCCAGAAGAAGAAAAGAAGAAACTGAATACTTACAACTCAGAAAACCTAAACCAGCCACTTGGGTTGGATGAGTTGCAGACGTAGACTAACTCTTCAACACTTACGGCTCTGATACCATAAGGGGTCTAGAACCAGCGAGAGGAAGAAGAACACAGAAGGCAAGTATGAACACAGGGATTACAGACACAATTTATGCATGGAGCAAAATAATGATTATATGAAAATAAGCATTAATCAAAATAATCAATTCTCAAAATATCTCAAATATATTGATAACTTGGAGAACGCAAAACACACACACACACACACATATATATATATATATATTCTTCTGATAAATTGGATGTCTGGAATATTGATGGTCTTTCTGAGCAAGAAATCCTGAATAAGTTACAACATATGTCCATGGTCGCCAATAGTTACATCACCAATCACAGTTTCAGACAATCTGAAATTGTTCCTCTGTTAGTTACCGGCTTTACTGGTACTCTCAGATATTGGTGGGATAAGCATCTTACCCCTGAGTCTAAAAATCGTATCACCTATGCTGTAAAACTTAATGAAGACGGTCTCCCTATATTTGACGAACAAATAGGTCAAGGCATAGAGGATGGTGTCAACACTTTGTTTTACACAATAATTGAACATTTCATTGGAACCCCTAGTAATACCACCGCTAGGATACATGATCAGTTGAGTAATCTTAGATGCCCAAAGTTATCTGATTTCAGATGGTACAAAGATGTTTTCATTTCTAGAGTCATGCTTAGAGATGATAGTAATCAACCATTCTGGAAAGAAAAATTTATTAATGGTTTGCCAAACTTGTTTGCCCACAAAATACGCACAACCCTGAGTAATGAACAAGGCCAAATAGATTGGAATAACCTGACATATGGAAATATTATAAGCACGATTAATCAAGTCGGTATGAAAATGTGTATTGATTTCAAAATCAATAGACAAATTCAGTCAGATAGAAAGTCAGCCAAATACGAATTAGGTAATTTCTGCAAACAGTACGGTCTTACCAGTATCCCTCCTTCGAGGAAAAATAAGCCCAGTCATCTTAGAAAAAATCGTTATTCTTTCCCTAAGAAACAGTTTTTTCAAAAGAATGAGTTTTATAAGAAAAGAAAATTCTCCCCCAAAAAGAATTGGTCGAAACACCCAGGCAGTAAGAAGAGTCGCCCTAAAAAAGATAAAAGCAAAGTTAAATGTTTTAAGTGTCAGAAGTTCGGTCATTATGCCTCTGAATGCAAAGTAAAAGATGTTATTAAACAATTGTAGATTACAGAAGAAGATAAAGAAAAGTTGATACAAGTTTTAGAGTTGCGAAACTCTGAAACAAGTTCTGTTGAAACCATAGTTTCCAGTTCTGAGTCTGAGTCAAACCAATCTTCAGAATCACAACCCAGTTCTCCTAGGGTCCAATTTGGTTGCACAGATAAATGTTGTAATAGATTGAATTCTATATTCGTTCTCACTAAACAGGAAGAACAAGAAGAACTATTAATAGATCTAATAGGAAAAGTTCAAAACCCTGAGTTAAAATCAGAATATTTGAAAAAATTAAAAAAGATCCTTAATCAAGAGCGTTTAACCCGTCCTACTACCCAGCCTATAAGTATCAACACCACCTTAGAAAAATTTAACTCCAAAAAAGAAGTAACCCTCCAAGACCTCCAGTTAGAAGTCAAGAAAGTTAAAAAAGAATTAGTTGAGTTAAAACAAATAAGTCAACAATTGCAAGTTGAGAATTATACGATCAAACAAGATTTAACTAATCTTTTAAGACAGGAATCTTTTGAGCCTAAAAGCCAATCAGAAAGCCCTCACCATTCTGATGATGAACCTTCTGAAGACCAACAAGTTTTCAATTTAATTAAACAAGTTCACCTTAGAAAATGGTATTCAAAAATAACAATTTTGTTCAAAACTTTGAACTCACCACAGTTGCTTTGTTTGATTCAGGAGCAGATCTTAACTGTATCCAGGAAGGTCTAATTCCCATTAGATACTATCAGAAGTCTAGAGAATCCCTTAGCACTGCCTCAGGGAAATCTCTTCAGTTAAATTTTGAATTACCAAAAGCCCATATTTGCCAAAACAAAGTCTGTTTTAAAACCTCATCTGTCCTACTCAAAAACATCACTGATGAAGTCATCTTAGGTTTGCCTTTTCTTGCCTTATTGTATCCTTTCCAGGTAGAATACGATGGTATCATTTCTACCTATCTAGGTGAAAAGGTTAAGTTCGAATTCTTGACGAAACCAGAGTTGCACAATTTAAAAGATTTACAAAAACAGTTAGTCTCTAAATCTATTCAAGTTGTTCAAAAAATTAACCAGTTGAAATTTCTTAAAGAAGAAATTAGATTTAAAAGAGTTGAACAACAGCTTGCTGATAAAGCCTTTCAGTTGCAGGTACAGTCCTTTGAAAATAATTTGAAAAATAAAATTTGTTATGAATTGCCTAATGCTTTTTGGTATAGAAAACAGCGTGTCGTCTGTCTTCCCTATATCAAAACCTTTAGTGAAAAGAATATCCCCACCAAAGCCCGGCCAATTCAGATGAATCAAGAGTTAATGGAATTTTGTAAACAAGAAATTCATGAGTTACTCCAAAAGAACATAATTCGCAAGAGTAAATCCCCTTGGTCGTGTCCTGCTTTTTACGTCCAGAAAAATGCTGAACTAGAAAGAGGAGTCCCAAGATTAGTTATCAATTATAAGCCCCTTAATTCAGTCCTAGAATGGATTAGGTATCCTATACCTAACAAGAGAGATCTCATTAACCGATTAGAAAAAGCAGTTGTTTATTCCAAATTTGATATGAAGAGTGGTTTCTGGCAAATCCAAATTGATGAGTCAGATAGATATAAAACTGCATTTGTTACTCCTTTCGGTCATTATGAATGGAATGTCATGCCGTTTGGTTTAAAGAATGCCCCTAGTGAATTTCAGAATATTATGAATGACATCTTCAATCCCTACAGTCATTTCTCGATTATTTATATTGATGATGTTCTTATTTTCTCTGAGTCAGTTGAACAGCATTGGAAGCATTTGCATAAATTCCTTCACATAATCAAAAAGAATGGTTTAGTTGTTTCTGCCAAGAAAATTAAGTTATTTCAAACCAATATCAGATTTCTTGGCTTCAATATCTGCCAATCCCAAATCTCCCCAATTGATCGAGTTATCTAATTTGCTGATAAATTCCTTGATCAAATCCTTGATAAAAAACCAACTCCAAAGGTTTTTAGGATCTCTAAATTATGTTTCTGACTTTTACAAAAATCTGCGAAAACAATGTAAGCCCATGTTTGATAGACTGCAGAATAATCCTCCCCTTTGGTCAAATCTCCATACTGAAGTTGTCAAACAAATCAAAGCCCATGTCAAGACACTCCCCTGTCTTGGTATTCCTTCAGTTGATTCTTTCAAAATAGTTGAAACCGATGCCTCTGACATAGGTTATGGTGGCATTCTCAAACAGAAAGTTAGCCCAGATTCTCCAGAACAAATTGTTCGTTTTCATTCTGGAACCTGGACTCAGTCGCAATATAATTATAGTACTATTAAAAAAGAAATATTAGCTATTGTATTATGCATTAGTAAATTTCAAGATGATTTACTTAATCAAAAGTTTTTAATACGAGTTGATTGCAAATCTGCAAAACATGTTTTAAAAAAAGATGTTCATAACATTGCATCTAAACAGATTTTTGCACGATGGCAAAGTATATTAAGTATATTTGATTTTGACATTGAATATTTGAGAGGTAGTGAGAATTCAGTTCCTAATTTTCTTACTCGAGAATTTTTGCAGGAACCACAATGGCCCCCAAAAAAGACAAACACAAAGAGTCAGCCAGTCAACCTAAGCCATCCCAGTTGCAGCCATCCCAAACTCCACCCATGAGTCAAAATAAGCCAGAAATTCCCAAATAGATAGTTCCCTATCCTGGTTGCACTCCCCTTACAGTTGCTACCCCATTATCAGTTGCTAACAGATTTTTCTCTTTAGGATCCATAGTTGGCCAGATTTGTCCCAGTTATCAATCCACACTAGATGCCAGTTATGACCCTTTTTTAGTTGAAACACCAGCCGGACCTTCAGTAGCATTTAAAAAGTCATCCCCTTATTTGCCCAAAAGTAATTCTCATTTATTTGTCATTGAGCCAATATACGATGTTCATGCCAACCCAGTTGAAATCGCCAAACACTATTTCCCTCCTGGATTCCATTATATGCCTTCCAGTCCTTACAAGTCTCTTAAGTATTATAGAGATATATTACTTGAAACCAAGTCAGTTGAAATTAAGCCAATAATGGATCGTGATCACCCTGAGGTCATTCTTTATCATTCCCTGTATGTTCACCAAATCATAAGCCAAGAAGCCTTCAGAAGTCGCCCTTATGAACTAAAGCTTCTTCAGTCGAAATTACAATATAATTATTCTGATTATATTTATGCCTGGTATACTATTTTTCTTCACCAATCTGAAGATTTTAGCCATTCCTGGTTTACCAATTTTGATAATAAGTTCAAATGTTCTTTCCCTTACTGGTTCCTCCATTGGTGGGAAAAACATGGTCCAGTCGATGAAATATTGCATGTTTCAGTTCATGAGCTAATCCGCCATTATACTAAGAAGGCCAAGTTCTCCAAAGTTGATTTATTCTTCCCAAAACAGTTGTTGTTTATTGCCATATATAAGGTTCCATGGATCCTCAAATGGTCCTACAGAATCACCAAAGATGCCCGGATCTTTGCCCGCCAATTCTCTATCAAATGGTGGGACAAATTTGAAGTTCATCGAATTGCCAAGTATGTCTATAATGATCTACCAGTCGAGCCAATGCCTCGTCCAGTTTCCCCAACTGGATCTGCCCGATCTTCCCTCTCAGTCGAAGGAAAATCCAAATCAGAACTCCAGGAAATTGCTCGCCAGTTGATTTTGCAAGCCTCCCAGATGGATGATGATGAGGACAACGATTCTCCTTCGTCGTCTAGTTGCCAGCCCATTCAGAATCCTGACAAGCCCAAATCATGGTATAAAGATAGTCAAGACCCCTATGACGCCTATGATTTGCACTCATACTAGACTCTGCAGACAATAATTACATGCCACAGTTGCCGACACCATAATTGCATGCCACAGTAGCCGACACCATAATTGCCACAGTACAAGCATCTGCCTTTTACTATTTGCTTTCCACCAAAAGATGCATCAATACAATCCGAATGAAGAAAAAATTGAAAGTTGAATTAGGTTGCACACCTGTGACTCTACAGTAGCAGTGAACCACAGTTAATTTTTCATAGTTAGTTGAACTCTAGTTGCTTTTCTCTATATAAGGAGGTTTCCGCCTCAGTTGTAATCAGAATACAATTTTAAGTTTTCTTCCTGTTTAAAGATTATCTCTGAAACATCTTTTGGAGAAATCACCCCTTTATTTAGAGAGTGAAGTTATTTGAAGTTGAAGTTGCTATCCTTGTGAGTATAAATAAAAGTTGTTATTTTGAAATTATCTATTTTCTG

TAAAACATTATTTTGATGCATTATCATTATTCTACTGCATTAGTCTGAAAGTTGTTCAGGTTTCCATACTCATAATCTGTTCTACTGTTGTTGATATTCAATCAGTTCTTCTTAATCTATCCTCTTGGTATATATATATATCACTCGAAAATGCCACTTGAGCGAGCTACCATGATATATAATGCATGGTTGAAAAATGAATGTAGGTTTTCATCATGTTCACGTGTTTAGTTTATTAAACTTGATCACATCTTAAATGTTCAACTCATCTGCTTTCACTTTTATATTAAATTTTCATATTTGTTTATGAAGTTATGTTCACCTAATCAGGATGGATTTGGAATTTTAAATTAGAGTGGGTTAAAAACTCGACATTATAAGTAAATTTAATTTATAAAAAAAAAACATAATCAGAATGACTTAATGAGTTTTACCCCTTAAAACACATCTAGTACACATCTCTTTTCTATTTTATTCAGATGTGTAAATTAAAACAGGGTTATTTTCTATATATATAAAAATTACAGGAGTTGTTTTCGACTTCGACCAAAAACTAAAAGAGGCAGGAGTTTGTGTTTTGCCTTTGGCCCCCTTCCTTTTTGTACCAAAAAAAGAAAACAGGGTGAAGGGGGGATTATGAGAATGCAGTGCATGGCTGTCCATTGGGTGGGTTGGGTATGTTAGTTTAGGCTTAAAAAGACAAAATCACCTGGGCTACAATACAACCCAGGTTAGTTAAAGGGTAATCCGTTCTGATTAGTATTGTTTCATGAAATGTTTACAAAATCAATTATATTAAACCGTACTTAACTGATAAATATAGCATTATTAGAAACAATTAAATAAATTACATTACTAACACAAATTCACAGCTCAGTCTTAGGAGAATTTATCAAAAATAGCCAAAACTTGATCTTTAATTTCAAAAATGTCAGTTTTTGTAAAATATTTCAAATATAGCAAAAAACTCACTTAAATAGACACAAATTCCCTCAAATTT

>Chr1-22; OZ022206.1; 29856375-29860830; dir; B;

GGGCCGGGGAGCTGTGGCTGCAATGTGCTGGCTGGTTCGGCTGAGGATTCCCAACGTTCTGGGTCAGGTTTTGTTGGGCATCCTGGGCGTGAGCCCTCTCTTGGTTTCGCTTTAGAGCACGAAAGTCATGCTCTAGCTCAACATTCCGGCTATGAAGATGCTCGTACTTCTCCGACATCTTGGTCATACTATCACGGAGGCGCTCCACTTCTGCCTGGAGAGAGGCTTCTCCCGGAGATTTCCTTCGGGAAGTACCCTCGCGGGGGGTCTGGTGCTGGGTATTGCGGCTATCCTCGGTCGGCATAGCAGAATGTGGGGTGAAGAAGAGGCGACGGGGCCTGAGGGGTGAAGGAGCCAGCTGGGTTGATAGAGAAAGAAGGGATTCAAGTGTCGAATTCCCACAGACGGCGCCAAACTGTTGATGCTCAAAATGGCTCGGCCACTATTTGAGTCCAACTTAGTGATTAGAGGTACTAGGTCTTCAGCAGGGAAGAGGGCTGAAGCCCTTGGGTGAAATGGATTGGTAAGACCTGCAGAAGGTAAAAAGAGGAGAAGCAACCGTTAAGCTTCGGACACCGGTGTGGTGCCGGCCGAAGGCTCTCCGATGCCTAAGTCAGTTACAGGTAGTAATTCTGGCAGAGTAACAGTGAAAGTAGGGGAATGGTCTCTCTGTTTTACCTGGGTACTGTTCCCTATTTATAGGGAAGTGAAAGTGGGAGCTTTGCACAAAGTTCCAATATGGGACTTTGTGCAAGCCGTTGTGGTGGCGACACGTGTCCTGAAGATTAGGTTTGGCATTTGAAAGCTTCGGCAGGTGTCTGGCACCTCGGAAGTGTGTCTTCCTTCGGCATGGGAGAAGGCATGGTTGCCTTGGTGCTAGTCGCTTTGTGGCTGGGTCTGCTCGACCTTGGAAGTGTGTCTTCCTTCGGCATGGGAGAAGGCGTGGTTGCCTTGGTGCTAGTTGCTTTGTGGCTGGGACTGCTCGGTTCATTATGGTGTA

AACATCCCTAAAATTCCTAGTCAAGAAATTTACGAAAAAAAAAAAAAATGGTCACTCTCAAGCTTCAAGTCAACTACCCATGTGAAAACAGTCGAACAAGTTTATGCCTTAAGTAAAGAACATGAAACATGCCAATTGTTGAATCTTGAGTCAATAAAGAAACACAAAAATGACGGAAACAATTTCCTTCACATTGGTCTAGTCCAAGTTGCAGTCAAACCTCTTATCAGATTAGGATTAAACGCCTCTATTCTACTCTGTCTAAGAGATGCTAGATTCACCAATTTTAGTGACAGTACCTTAGGAATAGTCGAGTCAAGTCTGTTTAATGGTCCAATTCATTTTGATTGTTACCCAAAATTTCACAGTAAGCCTTAGTGATCCTCACATATTGAAAACATTAACATTAAATATTAAAACTTCAAGATATAAAGTCCTACCAGGTACCCAACCTTTAGCTTTAGTTTATAGAATCTATTATAAAGTCACAAGAACCAACATGAATTTCCAAGCCCTTAATAAAAGTCCCAAAGATCAAACTTTGCTAATTCATAGTAGTAAAGCCGATGCCAATATAAGAGTCCCTTATACAATCAAATGGTCAGAAGTCACCCTACCTCTAGATTGGACATTAACCACAGAAAATCAACCTGCTCCCATTCAGCATAGTTTAAATAATTTAGATTACATTCAACAATACTTAGATGGCACAATCAAAATTAATTTTGGTAATCAACCATTCAAAAGTCTTCAGTTCAATTACACCAACACCCAACACCCAGTCAATCTCAGAGATATATTCCTGCCAGACATTCCTTTACATCGCCTCTACTCTAGAACAAGACCTAGAGTTAGAAAAAACATTAATTGATTTGAAGTTAGAATCCTTAAGAAAAATCTCTCAAGTCAACCATCCTTGTTATGGTAAAAAACCAGTTAAAGAAGATGAAAAATCAAATTCGCTAGATTCTCCCACACAAACCGATTTTGAAGTCGAAGCCATCCCTGTAGACAATCAGTTAAGAACTCTTAATAAAGAATTTAGAATTAATTGGAAAATTTTAAACAGTCTAGAGAGGAGAAATACCTACCATACAGTCTACCCATGTAAAGAACAAAGGCAACAAATCTTTAATGAGTGGAAATTTTTTATGAAAAGCACTAAGTCATAAGTCTTCTATCTTGATTTTGTTGAAAGTCACCACATGCCCGAAAGAGTTAAAAACTTTAACGCAAGAAAAATGGAAAAAAGAAGATAACACTGAAGTCATAGCCAGTCATCCCCCAGTAGAGACTATACTTATAAACCATAAAAGTGCCAAAATTACTGCCAGTCCTTTTAAAATTCCCGAGTCAAATGATAAAAAATTAGTTGAACAAAATAACTATGCAAACCATAGTCTAATCGTCATTGGAAAACAATTAGATAAGATAGAAACTAAAATTGACAAAATAGTTCCCCAACAAATGTCCCATAAGTCTCAGAAAGAAAAACCTTTAGTCAAATTCCAAGATTTAAATTCAAGCCCCACCTTTAAGACAAAACCCATGATGAAAAAAATAGACGAAATGCTTGAACAACTCACACCAGTCAAAGAAAAAATTGAATCCTCTACTTTCAAAGTCCTTGAATCTTTTGTTCTAGCCAATTCCTCTGATATTGAGTTTATAACTAGTGAAACAAATTCCAGAAATATATCAAAAATTGAAAATGCTTTTAAAAATCTAGAAATCGATAGTAATCTGAAGTTTAATAGATTAAGAAACAAGGTCAATCCTACTAGTCTAACCAAAAATTGGTATCCCAGACCAACTCCCCCAGACATTCAATTTGAAGAAATAAATTCCTAAAGTCAATTCTCAGTCTCCTCTGATAAATTATATGAATGGAATATTGATGGTCTTTCTGAAAAAGAAATCTTTAATAAACTTCAACACATGTCAATGTTGGCCAACAGTTACATAACCAATCACAATTTCAGAGAGTCGAAAATCATTCCCCTTCTAGAAACAGGATTCACCGGAAACCTACGTTCTTGGTGGGATAAACACTTAGTCAAGACGACAAATCATTCATGCAGTTAAATTAAATGAAGAAGGTCTCCCTATATTTGATGAACAATTAGGAAGAAAAATAGAAGAAGGAGTCAACACTTTACTTTATACAATTATTGAACATTTCATAGGAACACCTAGTAACACTGCAGCCAGAATCCATGATCAACTTAGTAACCTAAGATGCCCAAAGCTAAGCGATTTCCGATGGTATAAAGATTTTTTCATTTCCAAAATCATGCTTAGAGACGATAGTAATCAACCTTTTTGGAAAGAGAATTTCATTAACGGTCTCCCTAATCTGTTTGCCCATAAAATATGCACAGTCTTAAGTAATGATCAAGGTCATATAGATTTCGATAATTCAACTTATGAAAATATTATTAGCACAATCAACCAAGTAGGAATGAAAATGTGTGTTGACATGATAATAGGTAGACAAATTCAGTTAGAAAGGAATTCAGCCAAGTATGAATTAGGAAATTTCTATGAACAGTATGGTCTAACATCCATTCCCCCTTCCAGAAAAGCCAAAACTGGTTACCATGACCGACAGGTTAATAAACGTCGACATTACTCACGCAAAAAACAATTCGACATAATGATGAATTCTATTGTAAAAAGAAATTTAACCCCAAGAAAAACTGGTCAAAAGCCCCAAGAAAAGATAAGAAAAGTAACTTTAAGAAAAATACAGACAAAAGAAAAATTGAATGCTGGAAATGTAAGAAACACGATCACTATGCTAATGAGTGCAAAGTAAAAGACACAAAAAAGCAATTAAAAATCACGGATGAAGAAAAAGACAGACTAATTAAAGTTTTAGAATTAAGAAACTCTGAGTCTAGTGAAACCGACATCACAGTAGCAAATTCAAGCTCAGTAGATTATAGTTCCTCAAATACCCAGTCTAGCTCCCCAAAAGTCCAATTTGGATGTAAAGATAAATGCTGTAATGTCCTCACTAAGTCAATAAAAGTCCTTACAAAACAAGAGGAACAAGAAGAATTATTAATTGATTTGATTAGTAAAGTAGAAAACCCATAATTAAAATCCGAGTATTTAAAGAAATTAAGAAAAGTCATTAGCCAAGAAAACCCAAGTCAGCATATGCCCTCAAAGATAAGTCTAAACACTGTCTTAGAAAATTCAATAAAAGAAAAGAAGCCACTTTACAGGATTTATGAATGGAAGTAAAAATAGCCAAAAAAGAGATAATATAGTTAAAACAAATGAGTCACAAATTACAAACTGAAAATTTTGAAATTAAACAAGATTTGATTAATTTAATTGAAAGCAACTGTGAGTCCAAAATTCAGCCAGAAAGTCCAATAAATTCATACGTGAACCCTCAAATAATGATCAAACTATTAATTTAATAAAACAAGTAAATTTTAGAAAATGGTATTCTAAAGTCACAATATTTGTAAAAAAATTTGAGTTAAATACAGTAGCTCTTTTTTATTCAGGAACCGACCTTAATTGCATCCAAAAAGGATTAACTCCTATCAAATATTATAAAAAGTCAAAAGAAACATTAAGTACTGCCTTTGGCAAATCACTCCAATTAAATTATGAAATTCCAAAAAGCCCAAGTCTGTCAAAATAAGATATGTTTCAAAACCTCATTTGTCCTAGTCAAAAACATTACTGATGAAGTCATCTTAGGTCTACCTTTTATAGCCCTCTTATACCCTTTTCAGGTAGAATACGATGGAGTCATATCTACCCACTTAGGAGAAGAAGTCAAGTTTGAATTTTTAACTAAAGCTGAGTTGCATAATTTAAAAGCCTTACAGAAAAATGCAGTTTCAAAAATAGTTAAGGTAATCCAAAAGAAAAATAAACAATTAAATTTTCTCCAAGAAGAATTAGATTTAAAAGAATTGAACAGCAGCTTCGTGATAAGTCTTTGCAGTCACAAATTCATAAATTTGAAGAAAAATTAAAACAAGAAGTCTGTTTTGATCTCCCCAATGCCTTCTGGCATATGAAACAACACGTCGTCAGTCTGCCCTATGTCAAAACCTTTAGTGAGAAAAATATCCCTACTAAAGCCAGACTGATTCAAATGAGTCAAGAAATTATGGAATTTTGTAAACAAGAAATTACATAATTACTTCAAAAGGGGATCATCCGTAAAAATAAATCACCATGGTCATACCCAGCCTTTTATGTTCAGAAAAACGCTAAGTTAGAAATTAGTCATAAAGTATAAACCCCTTAATACGGTTTTGGAATGGATTAGGTATCTAATTCCCAATAAGAGAGACTTAATCAACAAATTAGACAAAGCAATCATCTTCTCTAAGTTTGATATGAAGAGTGGATTTTGGAAGATCAAAATCCGAGAGTCAGATAGGTATAAAACTGCTTTTGTAACCTCATTTGGTCATTATGA

GAAGAAATCTAAGACATTGAAGATCATTGTATCAAAGTTTCATAATTTTCTACCAAGCCAAATACTCCAGACTTGCAAGTCAAGACAACCTAAGTTGAAATCCAGTCAGAATTGCAGTGCTGTGGGAGATTGAAGATTTGAAGACTTTTATGATTTTTCCTAATGGCGTGTGATTTTTCCTTGTTTAATGTGGTGGGGATTACATGTACTTGGATGCCTATAAAAATTCAATCCTCATTCACATATGATATACTAATATAATACCATACCCCCACAATACCACATACTAGGTGTGGGCATCTAAAACCGAAATACCGAAACCGTACACTAATCATACCAATTTCATACCGTTTCATTTGGGATGGGGAGATAATCCGTACCGTACCGTACCGTACCAATATAGTATGGTATGGTAGCGGTATCAAGTATTTAGTACCAGTCAATACCGTACCGTACCAAAATATAAACATGTTTAAATAGAAACATATAGTTGTTGTGATGAACTGTGAAGGAAGGAATGAGATTTTCCAAAGTCCCATTTTGCAAATTTGTACAGATAAAAGAGAAGAATTTCTTGAGGCTGCTGGGGATTTGAGTAGTTGTTGTTTACTTTCGTTGATCAATGTCTATGAATTGTAATTTTCTTTTTGCATCTTCTGTCAAATTTTCTCGGATAATCATATCTGTAAGTTCATTTTCATGGCTTAATTAATGTAAATTTCATTTCCTCAATTTTTGTTTTCCCCATTTATGCAATTGAGTTGGGTTTTTTTTCCCCATAACTAATGTCTTGCACATATAAGAAAATGTTGTAATTTAATCTGGGTAGCCAGCATTATGATGAAAAAAGTTGTGCTTTGGATTCTCAGGGCATATCAGATGAATGAATTATGAAATTTAGTAGCTATTTTTATTGTCTTATTCATTCCGTCTTGCATTTTTTAGTTGTCATTTTGGTACCGAAATGGTACTGATACCATACCGATGTCGATTTGGCATG

>Chr1-23; OZ022206.1; 30332521-30343610; rev; C;

CATGTCAATTGTTTGGTGTAAACTTGTCTTCTTTAATTACCTGACTATGTTAAATGTGTTGGGCTTTTAATATTATCCTTACTTGACTAACTAATACAACCTACACAACCCGGTTTAGATAATACATGCTTAATTATAAAGCTAGGGAAAAAGAAAACACACTTTGTACACTCTTTTTATTGTCTTCTCTTTAGGGTTCTTCTTTGCTATCTTCTCTTTTCCTTGTATTCATCTCTCCCTCTCTCTCATACTCTCTCCAATATGCTTCTTCTTGCTTCTTCTCTTTCTTCTCTCTTCTTATGAGTTTTTCTTCAAAATCCATGTCGTTGCAATGGTTTGTTTGTGCATCCTTTGACTATACTTTCAAGTCTTATCAAACAAAGAGAACAATTAAAAAGACTAAAGAAAGAAAAAAAAAAGGCAATACGAAACTGCCAAAGCTTTGATAATTAAGGGTTTCTCATAATTTCAATTATTTAATAATTAATTTATCATTCTCGATCTCTTGGACCACAGGAGTTTAAGAGATTGTGGTCACCTACCGTTGGTTATTAATCCAATGGTTCAAAAAAGTTTCTTAAAAAGAGTGCAAGAATGAATGAATCGTTGAATTTACATCCAACGATGAGTGGCCACAAATCTCTTGGAGCCCTGTAGTCCAAGAGATCAGAACTGATTAATTTATATAGTGAAACAAATTTTGCAGTCCATATGCAATAACATCACATCGATCCTTCAATTTTTTATGTATTGGAAAAATTACTATAATTACAATGGTAAGCTAGGATGGCAAAAGCTAGAATGACCATGGGGAAATCGATTCATGCACATCAACCAAGTAAAATTAGGGATCTCTCATACACCTAACCCCTTTATTTTTGTCTGATATAAGTTTGTCAGACATGCTTCATGCTGCATGTCCATAAGAACACAAGAGAAAAGATTGATGATTGTATGTGATATATATATATATATACCAGGAAAAGAAAGAACAGTAGAG

TAGAGATGGATGCAACCTAAACAACTTTCAGACTAATGCAGAAAATAATGATAATGCATCAAAATAATGCTTTCAGAAAATAAATAATTTTGAAAATAGCAACTTTTATTTATACTTACAAAGGTTGCTACTGCAACTTCAAACAACTGCACTCCGTATTTACAAAGATGATTTCTCCGAGGGATGTTTCAGAGGAAATCTTTAAAACTGAAGAAAACTTAGAAAATGAAAAATTACATTCTGATTACAACTGAGGCCGAAGCCTCCTTATAAAGAGAGAAGCAACTATGATTTAGATTAACTATAGTAAAGTAACTGAGGTTTGCTGCAACTGTTGGGTCACGAAAAGCAACCTGATTCAACTTTGAATTTGGCTTTATTCGGAAGATTCTTTTACTGAGACAGCTTTCGGTGGAAAAGCGAACAGTAAAAGCAGATGCTTCTTCCTGTGGCAATTATGATGCTGGCAACATTGGCATGTGAAAGCGTCGGCAACCATAATGAAGCCTAGTCAGAGTGCATATCATAGGCATCATAAGGATCCTAACTGTCATCATACCAGGACTTAGGCTTAATAGGATTCGGAATGGGCTGACAACTGGACGACGAAGGAAAGCCGTTGTCTTCATCATCATTCATCTGGGAAGCTTGCATTATCAACTCGCGAGCAATTTCCTGGAGTTCTGCTTTGGATTTTCCTTCGACTGAGAGGGAAGATCGGATAGAACCAGTAGGGGAAATTGGGTGCGACATTGGCTCAACTGGGAGATCATTGTAGAGATATCTAGCAATTCGATCGACTTCAAATTTGTCCCACCACTTGACAGAGAATTAGCGGGCAAAGATTCGGGCGTCTTTGGTGATTCTGTAGGACCATTTGAGGATCCATGGAACCTTATATCTGGCAACAAACAGCAACTGTTTTGGGAAGAATAAATCAACTTTGGAGAACTTAGCCTTCTTAGTGTAATGACGGATTAACTCAAGGACTGAAACTGGTAATATTTCATCAACTGGACCATTCTTTTCCCACCAATGGAGAAACCAGTAGGGGAAAGAACTTTTGAACTTATTATCAAAATTGATAAACCAGGAATGACTAAAATCTTCAGATTGGTGAAGAAATATAGTATACCAAGCATCAATATAATCAGAATAATTATACTGCAACTTTAACTGGAGCAACTTGAGTTCGTAAGGGCGACTGCTGAATGCTTCTTGGCTTATAATCTGGTGAATATAGAGAGAATGATAAAGGATGATCTCTGGGTGGTCACGATCCTTTATCGGCTTGATTTCAACTGATTTGGTTTCAAGTAATATATCTCTATAATACTTGAGGGACTTGTAAGGACTAGAAGGCATATAGTGGAATCCAGGAGGGAAATAGTGCTTAGCGATTCCAACTGGGTTGGCATGAACATCATAAATTGGCTCAATGACAAATAAATGGGAATTACTCTTGGGCAAATAGGGGATGATTTTTTAACGGCTACTGAAGGTCCTGCTGGTACATCAACTGAAAAAGGGTCATAACTGGCAACTAATGTGGATTGATAACTGGGGCAAATTTGGCCAATTGTGGAACCTAAGGAAGAGAACATGTTTGCAACTGATAATGGGGTAGCAACTGTAAGAGGGGGATGATCGGGATAAGGAACTATCTGTTTGGGCAATTCTGGCTTATTTTGACTCATGGATGGAGATTGAGATGGTTGCGACTGGGTTGGCTTAGGTTGACTGGCTGACTCTTTGTGTTTGTCTTTTTTGGGAGCCATTGTGGTTCCTGCAAAAATTCTCGAGTAAGAAAATCAGGGACTGAATTCTCACTACCTCTCAAATATCATGTCAAAATCAAATATACTTAATACTTTGCCATCGTGCAAAAATCTGTTTAGATGCAATGTTATGAACATCTTTTTCTAAAACATGTTTTGCAGATTTGCAATCAACTCGTATTAAAAACTTTTGATTAAGTAAATCATCTTGAAATTTACTAATGCATAATACAATAGCTAATATTTCTTTTTAATAGTACTATAATTATATTGTGACTGAGTCCAGGTTCCAGAATGAAAACGAACAATTTGTTCTGGAAATCTGGACTGACTTTCTGTTTGAGAATGCCACCATAACCTATGTCAGAGGCATCAGTTTCAACTATTTTGAAAGAATCAACTGAAGGTATACAAAGACAGGGAAGGGTCTTGACATGGGCTTTGATTTGTTTGACAACTTCAGTATGAATATCTGACCAAGGAGGAGGATTATTCTGCAACCTTTCAAATAGGGGCTTACATTGTTTTCGCAGATTTTGATAAAAGTCAGAGACATAATTTAGGGATCCTAAAAACCTCTGGAGCTGACTTTTATCAAGGATCTGATCAGGAAATTTATCAGTAAACTGGATAACTCGATTAATTGGGGAAATTTGAGACTGGCAAATATTGAAACCAAGAAATCGAATATTGGTTTGGAACAACTTAATTTTCTTGGCAGAGACAACTAAACCATTTCGTTTGACTATGTGAAGGAATTTATGCAAATGCTTCCAATGCTGTTCAACTGACTCAGAAAAGATGAGGACATCATCAATATAGACAATAGAGAAATGACTGTATGGATTGAAGATATCATTCATAATATTCTGGAATTCACTCAGGGCATTTTTTAAACCAAACGGCATGACATTCCATTCATAATGGCCGAAAGGAGTAACAAATGCAGTCTTATATCTATCTGACTCATCTATTTGGATTTACCAGAAACCACTTTTCATATCAAATTTGGAATAAACAACTGCTTTTTCTAATTGATTAATAAGATCTCTTTTATTAGGTATAGGATACCTAATCCATTCTAATACTGAATTAAGAGGCTTATAGTTGATAACTAATCTCGGGACTCCTCTTTCTAGTTCAGCATTTTTCTGGACATAAAAAGCAGGACACGACCAAGGAGACTTACTTTTGCGTATTATGTTCTTTTGAAGCAACTCTTGAATTTCTTTCTTACAAAATTCCATTAGTTCTTGATTCATCTGAATTGGCCGGGCTTTAGTGGGGATATTCTTTTCACTAAAGGTTTTGATATAGGGAAGACAGACGACGTGCTGTTTTCTATACCAAAAAGCGGTAGGCAAATCAGAACAAATTTTATTTTTCAAATGGTTTTCAAAGGACTGTATTTGCAACTGAAAGGCTTTATTATTAAGCTGTTGTTCGACTCGTTTAAATTTAATTTCTTCTTTAAGGAATTTCAACTGATTAGATTTTTGGATAACTTGAACAGATTTAGAGACTTACTGTTTTTGTAAAGCTTTTAGATTGTGCATAAAAGTTGCTATTTTCAAAATTATTTATTTTCTGAAAGCATTATTTTGATGTATTATCATTATTTTCTGCATTAGTCTGAAAGTTGTTTAGGCTGCATCCATCTCTACTCTACTGTTCTTTCTTTTCCTAGTTCTTCTTTTGCCCTTCCTTTCTATTTCACTGGTTCTTCGTCTTGTTGGTATCAGAGCCGTAAGAGTTACGGTCTTTGTCTAAGTTTGCTACTCATTCCCGCCCAAGTGGCTGGTTTAAGCCTCTGAGTAGAAGGTTTTCATTTCTTCCTTTTCTTCCCTTTTATTCTTTGAGTCATCTGTTACTTTTCTCTAAGTTCTGACCCGTGAGTCCTTTTTTCTATATCAGTAAGTCCCCAGGGAGGGCATGTGTGGTGGGCAGGAGACAGAAAATTGAGAGAAGTTAATAGGTTCCTTAGAGTCTAGACGGTCGGTTTAGGATCTACTGTTTATCTTCTTCCTCAGACAGCCAAACCCAGTTATTTGAGTAGTTATGAGTCACTTATTTAGAACAAAGTCTTGTAGTTCAAACAGTTCTAATATCAGTCGACTCCCAAAAATAGTTAATGAAGAACAATTTGAATATAATTCCAGTAGCAAAGAACAATTAGATTTTAGAGATTGGAATATACCTAAAGTCCCCAGTCAGAAAATTTATAAGAAACATTGGTTGCCTTCCAGTTTTAATAGCACAACACATGTCAAAACAGTTGAACAAGTTTACGCCTTAAGTAAAGAACATGAAACTTGTCAGTTGCTTAATCTTGAGTCTATTTTAAAACATAAGAAAGATGGTAATAATTTCCTGCATATTGGTTTAGTTCAAGTGTCCGTTAAGCCCTTGACACGGTTAGGATTGAAAGCCTCAATCCTGCTCTGTCTGCGTGATGCTAGATTCACTGAATTTAGCGACAGTACACTTGGAGTCATTGAGTCCAGTCTGTGTAATGGCCCTGTCCATTTTGACTGCTATCCTGACTTCACTGTAAGCCTTAGTGATCCTCATATCCTGAGAACTTTGACTCTGAATATTAAGACTGAAGGTTATAATGTCCTTCCCGGGACACAACCGTTAGCCTTAGTTTACAGAATCTACTATAAGGTCATAGGAACAAATATGAACTTCCAAGCCCTCAATAAAAGCCCAAAGGATCAAACCGTCCTGATCCAGAGTCATACTTCAGACGCCCATATACAAGTTCCCCATACCATTAAGTGGTCTGAAGTTGCCCTTCCAAAAGATTGGACCCTAGTTACAGAGAGCCAACCAACTCCAATCCAGCATAGTCTAAACAATTTAGACTACATCCAGCAATACCTAGACAGCACAGTAAAAATTAAATTTGACAGTCATCCCTTACGAAAATCAAATGTCCAGTTACAACAGTTACCAACTCCTGGTCAGTCACAAAGGCATGTTCCTGCTCGGCATTCCTTTGCTGCTTCGTCCTCCACTCTAGAACGAGACCTAGAGTTAGAAAAAGCTATGATAGATTTCAAACTTGAGTCCCTGAGAAAAACCTCTCAGGTTACCCAACCTTGCTACGGCCAGGTTCCAGTTCCAGAAGATAAGTCTGACTTGCCTGAGTCTCCAACCCAGTCTGACTTCCTAGTAGAAAATCAACTTAGAACCCTAAATAAGAAATTTGAAATTAATTGGGTACGTCTAAATCGCCATTTAAAAGCCCCGGAAAACCAATCTAGAAGAGATAGTTACCACCAAGCCTATCCTGATAGTAAGCACCGTGAACAAATCTTTAGTAAATGGAAAGATTACATGCGGTCAGCCAGAGTTGAAATATTCTACCTAGATTTTGTTGAAAGCCGTTACATGTCTAATGATGAGTTAAAAACCCTAACTAAAGAAAAGTGGAATTTGATAGATAGGTCGGTAGTTAAAGCCAGTCATCCTCCAGTAGAAACTATAATAATAGATCATAAGAAAGCACCAATACCTGCCACCCCTTTCAAAACCTTTGAAAGTAGTGATCCAAATAGAAAACTAATCGAACAAAGTAATTACACCAACCAGAGTCTAATTGTCATAGGAAAACAGTTGGATACAATTGAAACTAAGATTGACAAGTTGCATCCCCAAGAGACAGATCTCAAGCCCAAATCTAGGGTTGAAAAACCAATAGTTCAGTTTCAAGAGCTTAAGTCCAGTCCTGCACTTAAAGTCAATTCTACAATGAAAAAGATAGAAGAAATGCTAGAACAGTTGACCCCAACTAAAACTGAGAAAGCCGAAAAATCTGGTCTTAAAACACTAGATTCTTTTGCTGTTGCTTCATCTTCTGAGTCTGAGTCAATAAGTTCTGAAACTAGTAACATCTCAAAAATTGAAAATGCTTTCAAAAATTTAGAAGTCCAAACTGAACCCAGAATCAAAAGATTAGACAAACATGTAAGTCCCACTAGCTTAACAAAAAATTGGTATCCCAGACCAACACCTCCAGATATGCAGTTTGAAGAAAGAAGCTTCCAAACCCAGTTTTCAGTTTCTTCTGATAAATTGTATGAATGGAATATTGATGGTCTTTCTGAGCAAGAAATCTTGAATAAGTTACAACATATGTCCATGGTTGCCAATAGTTACATCACCAATCACAGTTTCAGACAGTCTGAAATTGTTCCTCTGCTAGTTACTGGTTTTACCGGTACTCCCAGATATTGGTGGGATAAGCACCTCACCCCTGAGTCTAAGAACCAGATCACTTATGCCGTAAAACTTAATGAAGACGGTCTCCCTATATTTGACGAACAAATAGGTCAAGGTATAGAAGACGGTGTCAACACTCTGTTTTACACAATCATTGAGCATTTCATTGGAACCCCTAGTAATACCACTGCTAGGATCCATGATCAGTTGAGTAACCTTAGATGCCCAAAGTTGTCTGATTTCAGATGGTACAAAGATGTTTTCATTTCTAGAGTCATGCTTAGAGATGATAGTAATTAGCCATTCTGGAAAGAAAAATTTATTAATGGTTTGCCAAACTTATTTGCCCACAAAATACGTACAACCCTGAGTAATGAACAAGGTCAAATTGATTGGGATAATTTAACATATGGAAATATTATAAGCACAATTAATCAAGTCGGTATGAACATGTGTATTGACTTTAAAATTAATAGGCAAATCCAGTCTGATAGAAAGTCAGCCAAATATGAGTTAGGAAATTTCTGTGAACAATACGGTCTTACCAGCATCCCTCCTTCTAGGAAAAATAAGCCCAGTCATCTTAGAAAAAATCGTTATTCTTTTCCTAAGAAACAATTTTCCCGAAAAAATGAGTATTATAAGAAAAGAAAGTTCTCCCCCAAAAAGAATTGGTCGAAACACCCAGATAGTAAGAAAAGTCGCCCTAAGAAAGATAAAAGCAAAGTTAAATGTTTTAAATGTCAGAAGATCGGTCATTTTGCCTCTGAATGCAAAGTAAAAGATGTGATTAGACAGTTGCAAATTACTGAAGAGGAGAAGAACAAGCTGATACAAGTTTTAGAGTTGCGAGACTCAGAAGCAAGTTCTGCTGAAACCTTCGTTCCCAGTTCTGAGTCCGATTCTGACCAATCTTCATAATCACAACCCAGTTCTCCTAGAGTCCAAATTGGTTGCACAGATAAATGTTGTAATAGATTAAAATCTATATTTGTTCTTACTAAACAGGAAGAACAAGAAGAGCTGCTAATAGATTTAATAGGTAAAGTTGAAAATCCTGAGTTGAAATCTGAATATTTGAAGAAGTTAAAAAAGGTCCTTAGTCAAGACAGTTCAAACCACTCTAACCCCCAGCCTATAAGTATCAATACTACTTTAGAAAAATTCAATAGCAGAAAAGAAGTAACCCTCCATGACCTCCAGTTAGAAGTCAAGAAAGTTAAAAAAGAATTAGTTGAGTTAAAACAAATAAGTCAACAATTGCAGACTGAAAATTATACGATCAAACAAGATATAACTGCCCTTTTAAGACAGGAAGCCTTTAAGCTTAAAAGCCATTCAGAGAGCCCTCACCATTCTGATGATGAACCTCCTGAAGACCAACCAGTTATCAATTTAATTAAACAAGTTCACCTTAGGAAATGGTATTCGAAAGTAACGATATTTGTTCAAAATTTTGAACTCACCACAGTCGCCTTGTTTGACTCAGGAGCAGATCTTAACTGTATCCAAGAAGGTCTAATCCCCACTAAGTATTGTCAAAAGTCTAGAGAATCCCTTAGTACTGCCTTCGGAAAATCTCTTCAGTTGAATTTTGAGTTATCAAAAGCCCATATTTACCAAAACAAAGTCTGTTTTAAAACCTCGTTTGTCCTAGTCAGAAACATCACTGACGAAGTCATCTTAGGTTTGCCATTCCTTGCTTTATTGTATCCTTTTCAGGTAGAATACGATGGTATCATTTCTACCTATTTAGGTGAACAGGTTAAGTTTGAGTTCTTAACGAAACCCGAGTTGCACAATCTAAAAGCTTTACAAAAACAGTCAGTCTCTAAATCTGTTCAAGTTATCCAAAAATCTAATCAGTTGAAATTCCTTAAAGAAGAAATTAAATTTAAACGAGTCGAACAACAGCTTAATAATAAAGCCTTCCAATTGCAAATACAGTCCTTTGAAAACCATTTGAAAAATGAAATTTGTTCTGATTTGCCTACTGCTTTTTGGTATAGAAAACAGCACGTCGTCTGTCTTCCCTATATCAAAACCTTTAGTGAAAAGAATATCCCCACTAAAGCCCGGCCAATTCAGATGAATCAAGAACTAATGGAATTTTGTAAGAAAGAAATTCAAGAGTTGCTTCAAAAGAACATAATACGCAAAAGTAAGTCTCCCTGGTCGTGTCTTGCTTTTTATGTCCAGAAAAATGCTGAACTAGAAAGAGGAGTCCCAAGATTAGTTATCAATTATAAGCCTCTTAATTCGGTTTTGGAATGGATAAGGTATCCTATTCCTAACAAGAGAGATCTCATCAACCGATTAGGAAATGCAGTTGTCTATTCCAAATTTGATATGAAAAGTGGCTTCTGGCAAATCCAAATAGATGAGTCAAATAGGTATAAGACTGCATTTGTTACTCCTTTCGGTCATTATGAATGGAATGTCATGCCGTTTGGTTTGAAAAATGCCCCTAGTGAGTTTCAGAATATTATGAATGATATCTTCAATCCTCACAGTCATTTCTCGATTGTTTATATTGATGCTTTTCTCATCTTTTCTGAGTCAGTTGAACAGCATTTGAAGCATTTGCATAAATTCCTTCACATAGTCAAACGAAATGGTTTAGTTGTCTCTGCCAAGAAAATTAAGTTGTTCCAAACCAATATTCGATTTCTTGGTTTCAATATTTGCTAGTCTCAAATTTCCCCAATTGATCGAGTTATCCAGTTTGCTGATAAATTTCCTGATCATATCCTTGATATAAGCCCCTATTCGAAAGGTTGCAGAATAATCCTCCTCCTTGGTCAGATATTCATACTGAAGTTGTCAAACAAATCAAAGCCCATGTCAAGACCCTTCCCTGTCTTGGTATACCTTCAGTTGATTCTTTCAAAATAGTTGAACCAATGCCTCTGACATAGGTTATGGTGGCATTCTCAAACAGAAAGTCAGTCCAGATTCTCCAGAACAAATTGTTCGTTTTCATTCTGGAACCTGGACTCAGTCACAATATAATTATAGTACTATTAAAAAAGAAATATTAGCTATTGTATTATGCATTAGTAAATTTCAAGATGATTTACTTAATCAAAAGTTTTTAATACGAGTTGATTGCAAATCTGCAAAACATGTTTTAGAAAAAGATGTTCATAACATTGCATCTAAACAGATTTTTGCACGATGGCAAAGTATATTAAGTATATTTGATTTTGACATTGAATATTTGAGAGGTAGTGAGAATTCAGTCCCTGATTTTCTTACTCGAGAATTTTTGCAGGAACCACAATGGCTCCCAAAAAAGACAAACACAAAGAGTCAGCCAGTCAACCTAAGCCAACCCAGTCGCAACCATCTCAATCTCCATCCATGAGTCAAAATAAGCCAGAATTGCCCAAACAGATAGTTCCTTATCCCGGTCATCCCCCTCTTACAGTTGCTACCCCATTATCAGTTGCAAACATGTTCTCTTCCTTAGGTTCCACAATTGGCCAAATTTGCCCCAGTTATCAATCCACATTAGTTGCCAGTTATGACCCTTTTTCAGTTGATGTACCAGCAGGACCTTCAGTAGCCGTTAAAAAATCATCCCCTATTTGCCCAAAAGTAATTCCCATTTATTTGTCATTGAGCCAGTTTATGATGTTCATGCCAACCCAGTTGAAATCGCCAAACACTATTTCCCTCCTGGATTCCATTATATGCCTTCCAGTCCTTATAAGTCTCTCAAGTATTATAGAGATATATTACTTGAAACCAAGTCAGTTGAAATCAAACCAATAAAGTATCGTGATCACCCCGAGATCATCCTTTATCATTCTCTCTATATTCACCAAATTATAAGCCAAGAAGCATTCAGGTCGCCCTTATGAACTCAAGTTGCTCCAGTCAAAATTACAGTATAATTATTCTGATTATATTGATGCTTGGTATACTATATTTCTTCACCAATCTGAAGATTTTAGTCATTCCTGGTTCATCAATTTTGATAATAAGTTCAAAAGTTCTTTCCCCTACTGGTTTCTCCATTGGTGGGAAAAGCATGGTCCAGTTGATGAAATATTGCCTGTTTCAGTCCTTGAGTTAATCCGCCACTACACCAAGAAGGCTAAGTTCTCCAAAGTTGATTTATTTTTCCCAAAGCAGTTGTTATTTGTTGCCAGATATAAGGTTCCATGGATCCTCAAATGGTCCTACAGAATCACCAAAGACGCCCGAATCTTTGCCCGCCAATTCTCTGTCAAGTGGTGGGACAAATTTGAAGTTGATCGAATTGCTAGATATGTCTACAATGATCTCCCAGTTGAACCAATGCCGCGCCCAGTTTCCCCTTCTGGTTCTATCCGATCTTCCCTCTCAGTCGAAGGAAAATCCAAAGCAGAACTCCAGGAAATTGCTCGCCAATTGATAATGCAGGCCTCCCAGATGGATGATGATGAGGACAACGACTCTCCTTCGTCGTCCAGTTGTCAGCCCATTCAGAATCCTGCTAAGCCCAAGTCTTGGTACGAAGACAGTCAAGACCCCTATGATGCCTATGATCTGCACTCTGACTAGGCTTCATTATGGTTGCCGACACCTTCACATGCCAAAAGTTGCTGACACCTTCACATGCCAAAGTCGCCCAACATCATAATTGCCAAGAAGAAGCATCTGCTTTTACTGTTCGCTTTTCCACCGAAAAGCTGCCTCAGTAAAAGAATCTTCCGAATAAAGCCAAATTCAAAGTTGAATCAGGTTGCTTTTCCGTGACCCAACAGTTGCAGCAAACCTCAGTTACTTTACTATAGTTAATCTAAATCATAGTTGCTTCTCTCTTTATAAGGAGGCTTCGGCCTCAGTTGTAATCAGAATGTAATTTTTCATTTTCTAAGTTTTCTTCAGTTTTAAAGATTTCCTCTGAAACATCCCTCGGAGAAATCATCTTTGTAAATACGGAGTGCAGTTGTTTGAAGTTGCAGTAGCAATCTTTGTAAGTATAAATTAAAGTTGCTATTTTCAAAATTATTTATTTTCTGAAAGCATTATTTTGATGCATTATCATTATTTTCTGCATTAGTCTGAAAGTTGTTTAGGTTGCATCCATCTCTA

CTCTACTGTTTTTTCTTTTCCTGGTTCTTCTTTTGCCCTTCCTTTCTATTTCACTGGTTCTTCGTTATATATATATATATATATATATTCCCGATCTCTTGGACCACAGGAGTCCAAGAGATTGTGGTCACCCACCGTTGGATATTAATCCAATGGTTCAAAATGATATATATAAATGCAATATGACATAAATGATTATTACCCGATTCAAGTCAACCGTTGAATTTACATCCAACGGTGAGTGACTATAAATCTCTTGGACTACAGGGGTCCAAGAGATCAGGACTGTATATATATATATATATATATATATATATATATGGGGGTGGATCCGTGACATTATGTTTTTGACACAAGTTTTGACACAAATTTTCAACCATTAGATTGAAATGGATTGAATGGCTAAGATTAACTAAACTAATTATATTTTTTTGAAGAAAAATATATATATAAATAATTATTAATTGAATTTCTCTTTCTTATTTCTCTATCTCAATCAATGTTTTATAAATCTTCCTATTTCGTTGTTCTCTATCCTCTGGAATTCTTCAGTTAATGACTTTCCCATCCTCCTCCTTTCTTCTAAAGCTTTACACAAGTTTCTGATTCTTTTTCATCTCTATCCCTATCTTTGTTTTTTGCTTTTTTTTTTTATTGCTTAGTTTTTGGGTTATTACAGTTATACATAGATTATGACTGTCGAAGTAAAGGCTTGGGAATAACACCCCATCCACACAAACACACAATGAACTGCACAGCAAACTGTCTTTTGCCATATATTTTTTTAATGTTATTATTAATCATAATGAATAAAAGCTTGTAAGCCATTAAGATGTTAGAGGAAAAAAAAAAACAGAGAGAGAGAGAGAGAGAGAGAGAAACACAAAGTTAGATCAACCAAACGGTAGAGAAGAAATATTGCAACAAATCTGGGCTCTTTCTTTTTTGGTTAAATATTAATATCTTATTTTCTCTTCAATATTGGTAGCCTGAGACAGAT

>Chr1-24; OZ022206.1; 30532293-30537469; dir; D;

CGTGGTCATTTTGGGCTAGGGAAAAAAATAGGAAGAAGAAGAACAAGGGTTGTGGTTTTCTGTGGGGCCGTGGGTGGGCATGTTTTCCTTGTGGCCCACTGGGCCTGTTCAATTCGTCAACTTGGTCAATTTCATGTCAGTCTGCCACCGTATGCCATCGGCAGTCCCCACAAACCACAATTCATTAATTTGTTCCCTCTCTCTATCCATCTATATTTGTTAATTACTTGAAATTTAAAATTTCAATTGGCTCCCATATGAATTAACCGAGAGATGTTTGGAGTGGTGGAGTGCATCCATATGAACTCAAGAAGATTATGCAGAAATTGGACCCCATGGAGAATTGACATGGGAAATTGAATGTTAATTTTTCCAGTACATATATAATTGAATATTAATTTTGAATGAAGAAGAATATAAATTATGGTCATGACGAAGGAAAATCTTGTTTAGCTTTATTTGAGTTAACTATACTTTTTAAAACATGGCTACATATAATGGCCAAAATATTGCACCTCATCTAAAAAATTCCTGGGTCCGCCATTGACAGCGGCACCGGCTTCGTTCAGACATGACTTCTCCAACTTTGATGGCCAAGGTGGCCACATGGGTTAAGCTTTCTACGATAAGTTACAATGGTGAAGCCGAGGATTGAGGGGAGAAGGCACATTTGAACGAATAATTTTGTAGAGAGACTAGCATACAACTCGTGTATTAGTTTGAGGATTATTTGTGTAAATAACCCCAGATGTACTTGAATTGAAGGAGATGTGTGGTTTACAGAGCTAGGATTTGAAGGACTTTATTTGGGTCCCTTGTTGGAGAATACCACACAAGTTAAGCTGGAAAAAGTACATGCTGCAATTGTGAATAATTACTGTATGAGTTCCAACTTACAAGTTAAGTTGGAAAAAGTATATGATGCAAGACCTTGCCTGGATGTCCAATATCTTTTACATCGCTTCGCATTTAGATTCTTCTACTTCTCACAATCTCAACC

ACAAAAGCAGTTTTATATCTTTCAGACTCAGAGATTTGAATTTGCCAAAATCCACTTTTCATATCAAACTTAGAAAAGATGACTGATTTTTCTAATCTGTTGATTAAATCTCTTTTGTTAGGAATTGGGTATCTAATCCATTCTAAGACAGTATTAAGCGGTTTGTAATTAATGACTAGTCTTGGGACACCACGTTCTAGTTCAGCATTTTTTTGGACATAGAAAGCAGGACAAGACCATGGTGACTTACTCTTACGAATTATTCCTTTTTTAAGTAAATCAGTAATCTCAATTTTGCAAAATTTCATTAACTCTTGGCTCATTTGTATGGGTCTTGCTTTAGTAGGAATATTTTTCTCACTAAAGGTTTTGACATAGGGAAGGCTAACAATATGTTGTTTCCTATGTCAGAACGCGTTGGGTAACTCAGAATAGACTTCTTGTTTTAGTTTTTCTTTAAATTGTTGTATTTGTGATTGTAAAGATTTCTGACTTAGTTGTTGCTCAATTCTTTTAGCTTTAACTTACTTCCTGGAGAAATCTTAATTGTTTATCTTTGTTCTGGATGACCTGGACTATTTTGGTGACTGCATTTTTTTGTAATTCTTTTAAACTATGTAACTCAGGTTTACTCAAGAATTCGAACTTAATCTTTTCTCCTAAACAGGTAGAAATGACACCATCGTGTTCAACCTGGAAAGGGTACAGAAGGGCAATGAACGGTGGACCTAGTATAACTTCATCAGTAATGTTTTTGACTAAAACGAAAGAAGTTTTAAAGCAGATTTTATTTTGACAGACATGGGCTTTAGGGATTTCATAATTCAATTGGAGGGACTTTCCTGAAGCAGTGCTTAATGATTCTCTTGACCTTTTGTAATACTTAGTTGAGATTAGGCCTTCTTGGATACAATTAAGATCGACTCCGGAATCAAAAAGGGCAACTATGTTTAACTCAAAATTTTGAACAAATATAGGGACTCGAGCATACCATTTTCTAAAATTTACATGTTTAATTAAATTAACGACTTGTTCTTGAATACAGTTTTCTTCATCTGAATTAGTGGGACTTTCTGTTTTAGACTTAGAAATTTTCTTTTCTAATAGGGTAATCAGGTCTTGTTTTATCTCATAATTTTCAGTTTGTAATTTTTGACTTATTTGTCTTAACTCAATGATTTCTTTTTTGACTGATTTTATTTCTAATTGTAAATCCTGTAAGGTGACTTCTTTCCTTTTCTTAAATTTTTCTAAAGTAGTATTCAGATTTATCTTTTGGGTAGACTGTTTTGGTTCTTCCTGACTGATGACTTTTCTTAATTTCTTTAAATATTCGGATTTTAAATCTGGATTTTCAACCTTGCTTATTAAGTCAACTAATAGTTCTTCTTGTTCTTCTTGTTTGGTGAGGACACTGATTGACTTAGCTTTACAACAATTATCTTGGCAACCAAATTGGATTCAGGGGGAACTAGGTTGTGACTCAGAAGAATATATTTCAGAAGACTCAGTATGATTGACCACAGTTTCGTTTTCACTGGATTCAGAATTTCTTAACTCAAGGACTTGGATTAATTTGTTTCTATCTTCATCTGTGATTTTTAATTGTTTGATTGTTTCTTTAACTTGACAATTATTAGCATAATGACCATACTTTTGACATTTATAACATTTTACTTTCCTTTTGTCGGTCTTTTTCTTAAAGCTGCTATTCTTGTTTTTGTTGTAATTCTTTTTCCAATTTTTCTTATGGGAGAATCTCCTTTTGCTATAAAATTCTTTATCCTCATTATTATGTTGGAAGGACTGTTTTCTACGTGAATAATGACGCTTTTTGCTAAACTGCCGGTTGTGATGACTAGACCTATTTTTTCTGGATGGTGGAACAAACATTAAACCATATTGTTTGCAGAATTACCTAACTCGTATTTGGCAGAATTTCTTTCAGATTGGATTTGTTTACCAATTTTCATATCGACACACATTCTCATTCCGACTTGATTAATTGTGCTACCTATATTTCCATAAGTTAAACTATCCCAATCAATATGACCTTGTTCATTACTAAGGGTTGAGCCATACAAATGAGCCAAGAGTTAATGAAATTTTGCAAAATTGAGATTACTGATTTACTTAAAAAAGGAATAATTCGTAAGAGTAAGTCACCATGGTCTTGTCCTGCTTTCTATGTCCAAAAAAATGCTGAACTAGAACATGGTGTCCCAAGACTAGTCATTAATTACAAACCGCTTAATACTGTCTTAGAATGGATTAGATACCCAATTCCTAACAAAAGAGATTTAATCAACAGATTAGAAAAATCAGTCATCTTTTCTAAGTTTGATATGAAAAGTGGATTTTGGCAAATTCAAATCTCTGAGTCTGATAGATATAAAACTGCTTTTGTTACTCCTTTTGGTCATTATGAATGGAATGTTATGCCTTTCGGTCTTAAGAATGCTCCTAGTGAATTCTAGAATATAATGAATGAGATCTTTAATCCTTTCAGCCATTTTTCCATAGTTTATATTGATGATGTACTTATATTCTCTGAGTCCATAGAACAACACTAGAAACACCTGTATAAGTTTTTACAGATAGTTAAACAAAATGGTTTAGTTGTTTCTGCTAAGAAAATTAAGTTATTCCAAACTAATGTGAGATTTCTTGGGTTTAATATCTGCCAGTCACAGATTAGTCCCATAGATCGAGTCATCCAGTTTGCTGATAAGTTTCCTGACCAAATCCTAGACAAGAGTCAACTCCAGAGATTTCTAGGATCTCTAAATTATGTTTCTGATTTTTATCAAAATCTGAGAAAGCAATGTAAGCCTTTATTTGATCGTCTCCAAAATAATCATCCTCCATGGTCATCTGCTCATACTGAAATTGTCAAACAAATCAAAACTCATATCAAGACTCTTCCTTGTCTTGGTATTCCTTGTACTAATTCATTTAAAATTGTTGAAACTGATGCTTCTAATGTAGGTTATGGAGGTATCCTCAAACAGAAAGTCAATTCTGGTCAATCTAAGCAAATCATTCGATTCCATTCTGGCGCTTGGTCCCAGTCACAAAATAATTATAGTACTATTAAGAAAGAAATATTATCTATTGTATTATGCATTAATAAATTTCAATCTGATTTATTATAAATCAAAAATTTTAAGTCCGTGTTGATTGTAAGTCTGCCAAGCATATATTAGAAAAAGATGTTCAAAATATTGCATCCAAACAAATATTTGCACGTTGGCAAGCTATTTTGAGTATTTTTTATTTTGATATTGAATATATGAGAGGATGTGAGAATTCTATTCCTGATTTCCTTACCAGAGAATTTCTGCAGGGAAAACAATGGCAGAACGAAGAAAAGAAAGAGAAAAAGATGATAAGCAAAAGAGTCAAATCGGTCAATCAAGTCAACTCAGTCAACCCCAGTCACTCCGAGTCCTCCCACCATCAATGAGTCAAGCCAAACATGAGCCTCCTTCCCAGATCGTCCCCTTCCCGGGATGCTCTCCTATCCAAATAAGTAACAGATTTGCCAACCTATGAGCCACAGTCGACCAGGTTCGCCATAGTTTCCAGTCTGCTCTAATTCCAGTCATGATCCTTTCCAGGATATTGCCGTCACTAATCCCCCAGTTGCTTCTTACCATAAGACATCTCCATACATGATCAAGAGTCATAATCACCTGTTTGTAATTGAGTCCAAATATAATTCAATTGCTAATCCCATCACCATTGCTAAATCTTATTTTCCACCCAATAGTCATTTCATTCCCCCAGCACCATATAAGAATCTAAAATATTACATAGATATCCTCCATGAGACCCATTCTATCACCATTAAGCCAATTAAAGACAGAGAAAATCCTCATATCATCATCTATCATTCACTTTACATTCACCAAATTATCAATGAAAATGAATGGAGTAGTCATCCTTATGATCTCAAAGTTCTCCAAACAGAACTTCAATATAGTTATTATGATTATATGGAAGTATGGTATTCTATCTTGCTTCACCAAACAGTTGATTTCAGTCACTCCTGGTTCTTAAATTTTGATGGCAAATTCAAGAGTCCTTTTCCTTACTGGTTCAATCACTGGTGGGAAAAATATGGTCCAATTATTGATTTATTTCCTCCTCCTATGCAAGAAATAGTCAATTATTTTACTAATAAAAATAAGTTCAAAGAATCTGAGTTGTTCTTTCCCAACCTTCTCCATTTTGTTGCCAAATATAAAGTCCATTGGATCCTCAAATGGTCCTATCAAGTTGATTGGGAAACCAGAGTCCTATCTCGCCAGTTTTCAATCAAATGGTGGGATAAATTTAAACTTGAACGTATTGCAGATTATGTTTATGTTGATTTTCCTCCAGTCAGGAATCCGCACCCTGAGAAAAAGAGCTCTCTTGCCATATCACACTCTTCTCTCCCAATCGAGGGAAGATCTAAGTCTGAACTACAAGACCTTGCTCGACAATTAATGCTTCAAGCCTCCCAGATGAATGATGATGCAGACAGCGATGCCTCTCCTGCGTCCCAAACATCTACTAGTCAGCCAAACCAGTCAGTCAACAGGCTCCAGGCCCAAAGATGCAGTGGAGTAATTATCCAGATGAGCAAGACCCCTATGATTTTGGTCCTTTCAACCTAGGGGATGATTAAAGTTAAAAGTAAAAGTTGCCGCTCATCATGAGCCCTGAGACAAAGCATCCGAACCCGTGCATTCAACAGTCTGAAAGCCCGTGTTCCTTTTGAGATTCCCCCGACAAAGTAAAAGTTGCAGCACTCAGTAAAAGCAAAAGCTTTCGCGTGCTTCTACAGTTACTTTTCTTAAGCATCCAAAGTTGCCAGCTCATCAAAAGATATCTGAGTCCTTTATTTGCTTATTTAAGAAGGCTTCGGCCTCTGTTTTGTTCAGAATGTAATTTTGGTACACTATAATTTTCTCAGTTTCAAAAGTTCTATCAGTACTAATGCTCTGAAGAACTAAGGTGTGCTGAAACTAGGATCTTTGTAAGTGTTTTGCATTCTCCAAGTTATCAATATATTTGAGATATTTTGGGAATTGATTATTTTGACTAATCTATTTTCATTGCTAATCTATTTTCATTGCATCTATTTTCATAATCTCTGAGTTCTTCTTTTCTATATAAATATATGAGGA

TTCGTTTTTAGGTTGAGATCCGTGAGGTTGTGCTTTGTGATTTCCCTGCACTGGTATGAATGTCTGAGTCCCGGATTTGGGAAGTGTTGTTTGGCCACATACGTCTTGGATAGGGTATGGTTGCACGGAAATATTTGATAAAAGAAAGGAAATCATAAAGTGTTCAGGGGTTGCCTTGTTGAATTTGATATAATAAGGAGTCTAAAGTCATTCGTTGCTTATATTTAAAAGGAAAAAGATTGTATATTACCGGAGTTGTAATTGTTGTTGAAGAACTACGTGCAGCCGCGAACTAAAGTTATTTTGCTCTTCGGATAAATTTGGTTTCGACTTTGTCGACGGTCATTACACTCGTTGAAAAACGAGGCTTGATTTAATGATTATTCTTTTTCCCGTCGGACTAAGTGAGTTGTTAAAGTGTGGTTGTGGCCAAGACAGATTTGTAACCCGTCTTGGATAGTGTACGATTTGGGGTCGAGTGCTAAAACTACTTGTGTTTATGTTGGGTTGTTAAACATCGTATGACGGAAACCGAGAAGTTATTCTATATTAGTATGGTGGGCATGCATAACTTAATTCGAGTTCTTTATTTGCTTTATTAAATCTCATTATTTGGACTTAAGAGCTTCGTTGGTGGTTCGGTTTTAAATTGTTAAAGGCCAGAGGCCTAGTTTGTAAACTGTTTGAAGATTAACTTGTGCATGCTGCCAGTTGTGGATATTAATTGTATTTTTAACAAGTTGAATAATTTTGGGAAAAATCCAAAACTTCTTTTAACAAGTTGAATATATATCACATACAAGACATGAAGCATGTCGAACATAGTCAATATTTTCTCTTGTGTTCTTATGGACATGAAGCATGTCTGACAAACTTATATCAGACAAAAATAAAGGGGTAAAGTGTATGAGAGAACTCCCTAATTTTACTTGGTTGATGTGCATGAATCGATTTACTCATGATCATTCTAGCTTTTGCCATAGATGTGATGGTTATTGCA

>Chr1-25; OZ022206.1; 30542386-30542978; dir; B;

CCATTCGTACCAATCCCATCCGTCACAGAAGTTTGGCTCCACATTTTGGGGCACTTGATCATCATCTGCCAAAACTGCCCTCATGAAAATCCCCACGTACTGGTATGGCTTGGCCTCGTCTAGGAACACGTTGTTTCTCACCGTCATCATCTCTATATTCTTAATCTCCAAACCAGATTCCTCCTTCACTTCTCTTGCTGCACACTCCTCAAAGCTCTCACCTAAACCAACACAACAACACAAAAAATTCACTAGGAATTTGAACAAAAAGGAAAAAGAAAAGAGAAGCAAAATCCCATGCCATGGCCATGAAATTTTCTGTATATAATTAAGTACCGAATTCGAGGTGGCCACCGGGGAGGGAAAAGGTGGAGTCGCCAAGGGAGGAACGGCGCCGTCCCAACAGCACCGTTTTCCCTTTGATCAGGCACACCACTACCGCCACTCTCGGCACCGGTGTCGCCTCCGTTTCCGTTTGGTTTCCCATGCTTTAATTGGAGTTTCTTGTATTGTACGAGATATATGTACGTAGGCTTACTGCCCTCCGCCTTATATAGAAAATATTGGGTCTGCTTATACTCGCACTAGCCCTGATCCATAAATGGTGACACGAGAGAGAGCGCGCGCATCCTTTGACTTTTTTGTTTGGTCAAAAGCACTGTTCCCTGTTGATTCTATGTCTCTCTGACTCGCTGTTGCGGTCCATATGTTTGTCTTGTTTATCTGTCTCAACGACAACGTGTGTATTGAATAGTTCCCATGTTGTGACGCACAGCCTTGTTGTAATTGACGTAGGTTGGGTTGCACATTGATCTAACTAGATAATTAATTATTAATGTCCACAACGTAACTTGTAATAAGTTAATAACGTAATGCTTTCTCAGTTTCAAAAGTTTTATCAGTACTAATGCTCTGAAGAACAAAGGTGTGCTGAAACTAGGATTTTTGTAAGTGTTTTGCATTCTTCAAGTTATCAATATATTTGAGATATTTTGGGAAT

TGATTATTTTGACTAATCTATTTTCATTGCATCTATTTTCATAATCTCTGAGTTCTTCTTTTACTCACTGGTTCTAGACCCCTTCTGGTATCAGAGCCGTAAACGTTGAAGAGTTAGTCTAAGTCTGCAACTCATCCAACCCAAGTGGCTGGTTTAGGTTTTCTGAGTTGTAAGTTTTCAGTTTCTTCTTTTCTTCTTCTGGTCTTTCAGATCACCTGTCCCTCTCTCTCTTGGTGTCCGAATCGTAAGACCTGTTCCAAACAGTAAGTCCCAGGGATGCCTGAGCGGTAGTGGCATTAGTAGGAGGGCGAGAGAATTTTAGGTGTCCTATTACCCATGGAGTCTGTATTAGAAGTTTCTGTTCACTTTCTTCTTTAGGAATCCAAATCAAAACCTTGAGTAAAAATGAGTCGTTTTCTTAGAACCAACTCTTACAGTTCTAACAGCTCTAGAGCCAATAGTAGTCTCCCATAGATAGTAAATGAAGAACAAATTGAATATGAGAATAATAGTAATTTGGATTATAAAGATTGGAATATACCTAGAGTCCCTAGTTAGGACATCTATAAGAAAAAATGGTCATTATCTAGTTT

CAGCACACCTCCCACCATCATCATGTACCAATGAAAAACAATCTAATCCGGGGATCGTTTGACTAGACCACATGGAGGAGTCCTCAACAACTATGTGACTAGGGAACAAGCCGTCCAACTGAGGGACTAATTCTCAGCCAGCACGTACAACCGAGACCTTTAAGACTCGTACGCCTGTGACAAGTCCTACGCGAGCAGACTACCCAATCAAAGGTCCGGGTTCCAACATACCCACGTATCCATATCCATGTATCCAAGTATCAGTATCCAATGGAAGGTACATATGATTATGCACATCATGTACTAGAGGAAAACAATTCAACCGGGGGATTGTTTGACTAGTCCACATGAAAGAGTCCTCAATAGACCATGTGGCTAGGGAACGAACCGTCCAACTAGGGGACTAACTCTCAGCCAACACGTACAACCGAGACCCTCAAGACTCGTACGCCTGTGACCAGTCCTACGCGAGCAGACTACCTTGTTAAGGGTCAACGAACCCCTGTCTAGGAGTCCGAGTTCCAACATATATATTCAAGTATCTTCCATAATTCCAAATATCCATTTCCATTTCCATTCCAAATTCCATATCCAAGGAAGCCCACACGGCCGAAACACGGCACGTGTGTGTATAGTAACCAGGGAAGGCACGTATGATGGTGGAGTTCGTAACCCTTATAACAAAATTATAAAATAATATTTCCTTCCTTAATTTAATTGTTCCAAATATCTATTCATAGTGTAGACAATAAGCTCTCAAGCATATAATCCGTATCCGGTGATGTACATAAGGTAGTGCTAATAGCACTCAACCTAACATTCTAAACTCACATCTTTCCAACATCTTACCATAACACCCAAAGTACCCCACACAAAATCAACATAGATAGCTCTTTCTCAAAATATTATATATATAAACTCAAGATCCTCAAGTATGTCGAAACCCACATATATCACAAATACTTTACAAAAATGATAAATTTAACTATTAAATATCAAA

>Chr1-26; OZ022206.1; 34744869-34750619; dir; B;

GACAGGTGAAAACTTTTGGGAAACAAGCAAATTACATGAGAGACTCTGCCGAATTTTCGGCAGAAGTCTAGCTGATAGAAAAATTGGTTTCTAGTAAGAAGAACAATTTGGTTAATTATGCCCGGCATTCGCCAGGTGACGGACACGCATAGGGTTCGGCTTGGATTCCAAAGTGGAATTTGGGAAGGTCCTGTCATGTATGGTTTCATGTTCTTTACCAAGGACATATACTTGTTCAACCAAATTTAACATGGGTTGTTGACTTGAAAGAGGATAGTGACCAACGTTTTCTATAAATTTATTGACTAGGGGCTCTAGGTATATTCAAATCTTTAAAACTAATTTTCACTATCTGATTCAAATTCTACTTATTCTTCATTTACTATGTCTAGGATTTTACTAATACCAGCTCCAGAACTGCAGCTATTAACTGAGTTGATTCGAAGGAGGTGACTCATTGATACAAGTAAAACTGGAATGAAATTTTCTAGGAAAGAAGGACACGAGAACTTCTATTGAAAACTCATGAAAACCTGTAAAGCTGGGGATTCTCTCTCTCTCCGCTCATACCGCAACTGCTCATGTCTCTTCTTGGGACCTACTATTTGGATTCTAGGACTTATTGTTTGGGTTGAGAGAAAAGGACAGGTTAGCAGATAATCAAGAAAGAAGCAACATAAGAAATAATGACCATTAAACACAGAAAATCGGAACTAGTGACCTGTGAAAGAGAAGCATGCTAAGAACTGACTCAATACGGTTTAGTACCTATTCCTCCCTCTAGAAAAGTCAAAACTAGGAACAATGATCGACAAACTAGTAAAAATAGACATTATTCACATAAGAAACAGTCCTTTAGACATAATGATGAATTTTATAGTAAGAGAAAATTTAACCCTTGAAAAAAATTAGTCAAAAACACCCAGAAAGGACAAGAAAAGTAACTTTAAGAAAAATACAGACAAAAGAAAAGTTCGCTGCTGGAAATGTAAGAAATTTG

GTCACAAATTACAAAATGAAAATTTCGAAATTAAACAGGATTTAATTGATTTAATGGAAAGAAGAAATTCTGATTCCAAGAGTCAATATGAAAGTCAAGCAAATTCGAATGATGAACCTTCAAGCAGTGATAAAACAAATAAATTTTAGAAAATGGTACTCTAAAGTCACTATTTTTATTATATAAAAAAAATTGAGTTAAATACAATAGCCCTTTTTGATTCAGGAGCAGACTTTAATTATATACAAGAAGGTTTAATCCCCACTAAATATTACAAAAAGTAAAAAAAAAATGTTAGGCACTACCTTTGGTAAACCACTTCAGTTAGATTACGAAATTCCAAAAGCCCATGTCTGTCAAAATAAAATCTGTTTTAAAACTCTTTCGTACTAGTTAAAAATATTACAGATGAAGTCATCCTCGGTCTACCCTTCATAACTCTCTTGTGCCCTTTTCGAGTAGATTACGATAGAGTCACATCATATGGGAGAAGAAGTCAAGTTTGAATTTTTAACTAAACCCGAGTTACATAATTTAAAAGCTCTGCAGAAAAATGCAGTTTCAAAAACAGTCAAGATAATCCAGAATAAAAATAAATAACTAGATTTTCTTAAAGAAGAAGTCAAATTCAAAAGAATTGAACAACAACTTAATTATAAGTCTCTACAGTCATAGATTCAGAAATTTGAAGAAAAGTTAAAACAAGAAGTTCGTTCTGATCTCCCCAATGCCTTTTGGCATAGGAAACAACACGTAGTCAGTCTCCCCTATGTAAAAACCTTTAATGAGAAAAATATTCCTACTAAGGCCAGACCATCAAATGAGTCAATAAATTATGGAATTTTTTAAAAAAGAAAATGCAGAATTACTTCAAAAAGGGAAAATCCGAAAAGCAAGTCACATTGGTCCTGTCCAGCTTTTTATGTTTAGAAAAACTCTGAATTAGAAAGAAGAACCCCAAGATTAGTCATTAACTATAAACCCCTTAATACGATATTAGAATGGATTAGATATCTAATCCCCAATAAGAGAGATTTAATCAATAGATTAGATAAAGCAGTCATTTTCTCTAAATTTGATATGAATAGTGGATTTTAGCAAATACAAATCCAAGAATCAGATAAACATAAAACCGCATTTGTTACCCTCATTTGGTCATGGAATGTCATGTCTTTTGGTCTGAAAAATGCTCCTAGTGAATTCTAGAATATAATGAATGAACCCTTTCAGTGATTTCTCCATAGTTTATATTGATGATGTCCTTATTTTCTCTCAGTCAATAGAACAACATTGAAAACACCTGTATCAATTCCTTCAAATAGTCAAGAAAAATGGTTTAGTTATCTCAGACAAAAAAATCAAACTATTCCAAACCAATATTAGATTTCTTGGTTTTAATATATGTGACAGGACCCGCCCTAGATTTCCTCGGAAATCGGGACGAATCCCGTCTTGTTACTAACATCACCCCGATGCCGGCCCACTTACTAAGGATCAAGACTTCATACCAAAATTTTGGCAGGGTTTCCTATGTAAATTGAACAATCTCAACAAAATTCAACCTGCATAAAAACATAATATAGTCTCAACCAGCAGCATGCATTCAACATTTAACAGTACACTAAATGGCCTCCGACCTCACAAGTAAAACCTAACATGGGCAATAACAGAGCATCTATCGAATTTAGTTTACAAGAACATCCAGTTGAGTAAAAAGAAATTACTCGAAGCCTATTCTACTGATGTTCACGGTTCGGGGCCTCGAACACAGCTTGCAACTCTCCTTAAGCAACTACTGTCTGGGGGGGCACAAAACAGAAAACATGTGAGTGGACAAAAGCAAAGTTTGCATTCAAAACAACATAATAACTCCACCGTATAAAAATGATACTAAAGACATGCAGATTCATGCAGATTCATAACCTTTAAAACCAAACACTCTATCCAAAAACTTTATTTAATGATCACTTTTATTTATACTGCTCTTACCAAACCAATATACATATACTTTAACTGATTTACTTACTTACACACACACACACACACACACACACACACACACACACAAAAGTACATATATATCTATAAATATACATAATATTTATCACATTACTTATTTATCACATTATTTTGATTGGAATATTTGGCTAGCCCGCAGGATTTCCTATTGTTATCCTGCGTCTAGGGATGAGCGGTCTAACCGGGGGACGAAACTCAATAGCTCACACGCCGGAAATTCCAAAGCCACGTGTAGCTCCAATACTAAGTCCGACGCGCGAAGACTACATCATCACATGCCGGAAATTCCAACGCCACGTGTGATGATTCCTCATAATTCACATAATCTTCCCATACGCAGGAAATTCCTAACGCCAATATAGGAAGTGTCTCACATGCCGAAAATTCCAATGCCACGTGTGAGACTTAATCAACACATGTTCTTCCCACACGCCGAAAATTCTAATGCCACGTGTGGGAAGCCTAGTAACTAGGAATGGTACTTACATAGTGTAATCATAAATCTTCCCTCAATAACCCATCCTACTAAGTTCCCAGATCCCCCAAATTTCAACTTCAAATTTTCCAATCAAAATCACGTTCTCAATTTAATATTACTAATATCTTAACTTATTAATTTCTCAAACTTTAATTCAAAATATCTATATGTATGTAAATAAATACATTCCATGCATAACCTTTCAATCATAGAAAATAATGTATATTTAAAATAAATGTTCACTAACAAGTAGTCCCACGCTGCCTGACCCTGAGAGGGTCCCGCCTGCTGCGCAAGAGCGGCCGGATCACCTATTTTGAGATATGAACCCTTAATTAATATAAAATCACTTCGAAACGGAATTCACAAATTAAATACTTATTTTCCCAAGTTCCCGGTGCGTGTACATAGTACAAGGTATTCTAAGGTACGACTACGGGTTTAGAAAACACGTCCTGTAGGTTTAGTCAGAAATGAACGGTTATGTAAACCACAAACTTCAAGTTATTGAATCGGAACATCCGGGGCTCTGATTCTTGATCGGCGAATTCCTACACGATTCTAGGAGTGCCTAGATCAACATATTTTGATTTGGAAATGATCCAATGATTCAAACCTATCGAACTCGGATAACGCACAATAGGCATAAATTCATTCGATAGTCAAACAACATCCGAATTGAAATTCGAGCACGCCTACGTGCTCGTGACAACATGAGAATTACATCAGGACAAAATGTGGCTTCACTACCATTGCCCACGGGCCACCACACGCGCTGATAGCGCGTGGGTGTGTAGAGTAATTTTCGATGACGGAAAAACTCCACACCGCGATTCACCCTTCCTACCCTAGCTATTACTCGAGACGGGGATCATTTCATTCAACTACGGGGAGGTCCAATTTGGACGGCAACCGGCTAGAATTTCACTTTGAAATTCGGCCAAAACCTAGATTTCAAAATCGGAATTCTAGACGACAAATCGATCGTGCAACAGACAGCTAGAGCTCGGAAAAAGAGTAAGAAACCTCACTTGTTTCACCGGGATTGGACGGCGGTGGCAGCGGTGCAACGACGAAGGAAAACCAGTTCGAACCAGTCGAGGTTTTCGATGAAACTCAAGCTTTCCGGCCAGGAAAAATGGTGAGTTGAGTTCGTGGCTTCGAGAGGAATCGATCGGGATTGGTCTCGGCCCTTGTTGTGGCCGGAACTGAGAGAATCGAAGAGAGAGAGAGAGAGAGAGAGAGAGAGAGAGAGAGAGAGAGAGAGAGAGAAAGCCAGTTTCAAATTTTCTATACCCAAGTTTGAAATATTTACAAGTTTGCCACTGAACAAATTTTGGTCGGCATTTCTTTGTTTCAACTCCAATTCAAGTCTACCGCATGTCTACGAACTCGTCTCAGTACGACCTACGTAACCATACCACTTGTTGTCCCAAAATTCTTCCGGATAAGAAAATGACCAATTTACCCCTACCCCATGGGTAAATTCGTAATTTCAATTAAATAATTTAAATTTGGAATTAAATTTGGAGTCGAGGTGTTACAATCTGTCAGTCACAAATCAGTCCAATTGATCGAGTCATCCAGTTTGCCGATAAATTTTCAGATCAAATTCTTGACAAAAGTCAACTTCAAATGTTTCTAGGATCTCTTAACTATGTCTCTGATTTCTATCAGAATCTGCGAAAACAATGTAAACCCTTATTTGACAGACTCCAAAGTAATCCTCCTCCTTGGACCCCTGGTCATACAGAAATTGTTAAACAAATCAAAATCCATGTCAAAACACTACCCTATCTTGGTATTCCTTCAAAATAGTTGAAACTGATGCCTCAGAAAATGGTTATGGAGGAATCCTAAAAACGAAAGTCAGTTCAAACCAAACAAAGCAAATCGGTCAATTCTATTCTGGAATTTGGACACAGTCACATAGTAATTATAGTACTATAAAAAAAATAAAAATAAAAATATCTATAGTATTATGCATTTCAAAATTCCAAGATGACTTATTAAATCAAAAAAATTTAGTCAGAGTTGATTGTAAAAGTGCAAAACATGTTTTAGAAAAGGATGTTCAAAATTTTGCATCAAAACAGATTTTTGCACGTTGGCAAGCCATTTTAAGCATTTTTTATTTTGATATTGAATATTTGAGAAGTAGTGAAAATTCTATCCCAGATTTCCTTACCAAAGAATTTCTTCAAGAAAGACAATGGCAGAAAAAGAAACAAAAAAGTGAGGCAAGCAACCAGTCAGACAGCTCCCATCTTCAATGATCCCAGTCAAATCAGAACAACCTTCCCAAGTCATCCCTCATTCAGGATATTCACCCATCCAAATATCAAATAGGTTTACTACTTTAGGATCTACAGTTAGTAACATACGTGCCAATTATCAGTCAGCCCTTATTTCCAGTTATGATCCATTTCAAGTCTTGACCCCTTCACCATCCTATTCTCCAGTCAGTCAGAAAAAGTCTCCTTATTTCCCAAAATAGCCAAAGCCTATTTTCCTCCTAATTTCCATTACATACCCGCACACACTTCCAAGTCACTTAAATATTATCGAGATATTCTTCTCAAAACCCAGGCTTTAGAAATTAGACCAATCAAAGATAGAAATGACCCGAATATCAATCTCTATCACTCCATGTATATTCAAAAAATTCTCAGTCAAGCTGAATGGGGTGAACACCCTTACTCTCTTAAGACACTCAACTACAATCTCCAATACAATTACTATGACTACATCGAAGCTTGGCATACCATTTTCCTATACCAAACTGAAGATTTTAGCCATTCATGGTTCGTCAACTTTGATAGTAAATTTAGAGGTCCTTTTCCATACTGGTTTTCTTCCTGGGGGGAAAAACATGGTCCGACCCTTGACATTCTTCCTCCCAATCTTAAAGAGTCAGTCATGTACTTTTCCAGTAAATACAAATTCAAAGAAGAAAATCAATTTTTCCCAAATACAAAGTCCCCTGGATCTTAAAATGGTCATATCAAGTCGACTGGGAATCCCGTATTCTCTCTAGGCAAATCTCCATCAAATCGTGGGATTAAGTTCAAGTCAGAAAGAATTGTTGACCAGGTTAATACTGAATTCCCTCCCGTCAAGGTCCCTCAGTCAAAGCTTTCTTGCTCCGACACATCTCATTCTTCCTTCCCAATTGAAGGAACATCCAAGTTAGAAATAAAAGAATTAGCCAGACAATTAATGATTCAAGCCTCCCAGATGGATGATGATGAAGA

TGCTTCCCCAAGGTCTCAATCTACTCAAGCCTCCTCTTCTAGTCAAACACAAAATCCATCAATCAGTCAAAAAGATTCCATGCAGAAATTCAGATAGGCCGACTACCCCGACAGTCAAGACCCCTATGATTTGAATGAAGACTAAAGAAAAAGAAAAAGAACAAAGTAAAAATAAAAGTTAGAATAATGTCTTCAAGTCCCGTGCTTCTACTGTCAATATTTCCTACATAAAGACTAATAAAGTAACGGAAAAAGCAAAAGCAAAAGTAAATTTAGATGGAAGAAAAAGAAGTTAGCTCCGTGCCTCCACAGTCACCAAATGCTGCCGACATCTCAGTAGCCTGGCTATTTAAATAGAGGTTAATTTCATGTTGTAAAGTCAAGTTACATTTTTAATTTTCTAGTTTCAAAGTTCTTTCAGAAGTATCCCTTTGAAGAACATTTGTAAAATATGAGTGCTATCTATCAATAAAATCTTTCAATCAAGATTTTCTGTAAGTTTGCAATTTCATTATCATCATTATTTTCAGTTAAATATTATTGCAATTTCATTATCAGTTTTATTTAACTGCAGTTATATATAAAATATGTTTTCATCTCTCTACAGTTTGCTATTTTCTTCTTGTTCGTTTGTTTATTGTTTTGGTTCTACAAAGTTTCGTATATCAACAACAGCCACTTCGTGAACTAAATCAAAAGGCTATAACTCATTAGCAATTTAGAAATCAAAACGGAATTGAAGCTTTTAAAATTACTTTCATGGGTTAGGATCAGTTTGAGTTGTACATAACCCAAATCCATGATAAGCTTCTCTAACCTTAATTGAGAAGAATGAAATTGACAATAAAAATTAACCTTACATATCTCTTGATTCTCGTGGCTTCAAAAGAGTGCTTCAATAATCCAAGAAGAAAAATTGAAGAAAAAGAATGAGAAGAGTAGCTCTAGACTAAGGAACATGGAAGATGAGTTGTGAAGATGAACGATGGGAAGTAGAAGA

>Chr1-27; OZ022206.1; 36430521-36437771; rev; B;

TGTGAAAGCACAATAACCACGTAGGGTTATTCTAAATACAGAGAAATACACCACAAGGTATATCTCCATTAATAGAGGCATAAAACCAAACAAATTAGACATGGACCGCACCCAAAACTGCTAATGTACTACTACAACTTTTAAATTTTCACAAGTAAAAAATACAAGTAGAGACTCAAATACATACAGAATGCCCAACTCACAAAGAGTTGGTGTAATTATTACAACCCAAAAAATTGCAAAACAACCGAGATAACAAAAGGTAGAGTCGTCGTCGTCACCACAACGAAAAATAGGAATACCATCGTCGAAGCCCAAGTTGAGGGGAAAACCACAGCCATGCAAAAACCTACAACAAGAAAACACAAAACCCACAGATCTACAAAAAGAACCCAAACAACACAAAAATAAAGCCGCAAAACAAAGGGGCAATAGAAGGTGAGATATATTTGAAGAGACCCGAGGCAGCAAAAACCTATAGGAGATAGCACAACCAAACCCTAAAAGGGTCAGAATCACCACAAGTTCAACCAAAAAAACCAAAAGGAAGACAAGAGAAAAAAAGTGAGGAAGAGGAGGAGAGAAGATAGGGAAGGGGGAGGAAAGATGTCTTTCCTCCCTCCTCTCCTAGGATTTGAGAGGGAGAGAAGTGGAGAGTTTTCTTGCTATCTCTAAAACTCCTTTTTTCACACCTCTCATATATACAACATTTATTTATTTTTGGATGAACACATCCTTTCTTTGAAAATGCTAAAAACATGCATTTTTTCTTTTCATATAATAAATACAGACTTTCTTAATATGTTTTAAAAGAAACCTACATTTAAAATGCTAAAATAACAAACACTGCATTAATTGAATTGAAAAGTTTATTAGGGCACAAATTATTAAAATAGCAAATAAAAAATTTCAAATTGCACGTAGGTCAACAAACTACAATTTCAAGAGTTTGATTTACTATTATTATTGGAGATACTCTAACTTTGTAATTGATCAGGTA

TTAGAATATAATAAAACTGATGGAAACAACTTTCTTTATATTGGTTTAGTACAAGTTGCCGTAAAACCTTTAACCAGATTAGGATTAAAAGCTTCTATCCTTCTCTATCTGCGTGATGCTAGATTTAATAATTTTAATTACAGTACATTAGGAATTATTGAGTCCAGTCTTGCTAATGGCCCAATTCATTTTGATTGTTATCCAGATTTTACAGTTAGTCTGAGCGATCCACATTACTCTCACTTTAATATTAAAACTGAAGGTTATGATGTTTTGCCAGGAAGCCAACCCCTAGCATTAGTTTACAAATATACTATAAAGTCACAGGTACAAACATGAACTTCCAAGCCTTAAACAAGAGTCCAAAGAGTCAAACACTGTTAATTCAAAGTCATACGCCTGATGCTACTGTTAGAGTTCCTCACTCTATCAAATGGTCAGATGTCACTCTTCCCACTGATTAGACCCTAACCACTGAGACCCCTCCAGTGCCTATCCAGCGTAGTCTAAGCGATTTAGACTACATACAACAATACTTAGACGAAAAAGTTAAAATCAATTTTGGTCACCAAGCCCCACAAAAATCAACAGTCCAGTTACAACAGTTGCCAACACCTGGTCAATCCCAGAGACATGTTCCTGCCAGACTGCCTCTTCCTTTACCCTAGAACGAGACCTAGAGTTAGAGAAAGCTTTAATTGATTTGAAATTAGAATCACTAAGAAAAACTGCACAAGTCACCCATCCTGTCTATGGAAAACCAACAGAAGAAAATAAATCTGAGTCCCCTGAGTCACCCACTCAATCTGATTTTGTCGAAGCCGGTTTCAAAGTAGAAAACCAACTCCAAACACTTAATAAGAAGTTTGAAATTAATTGGAAAAAATTAGAACATCACTTACATGCTCCTGAAAACCAAGCAAGAAGAAATAATTATCACTCGATAATTCCAGATAGAGCCAAAAGATAATAGATATTTAATGAATGGAAAGAATACATGCGCACTGCTAAAGTCGAAATTTATTATCTTGATTTTGTAGAAAGCCGTCATCTCACTAATGAATTAAAAACCCTAACTCAAGAAAATTGGGAAAAAATAGATCACACCAAAATTCTTGCCAGTCACCCCCCTGTTGAATCCATAGTAATCCAGCACCAGAATACACAAGTTATTGCCAGTCCTTTTAAAATCCCAGACTTAGAAAATAAAATGATAATTGAACAAAATAATTATACAAACCAAAGCTTAGTCGTCATAGGTAAACAATTAGACAAGATAGAAACAAAAATTGACAAATTACTTCCACAAGAAATTTCTGTTAAACCGCATAACCAGAAACCTTTAGTCACCTTCAAAGACTTACAATCAAGTCCAACTTTTAAAACAAAAACTACAATGGCAAAAATTGAGAAAATGTTAGAAGAATTAACCTCAACCAAACGGAAAGATAAAGCGAAAGTTGAATCAAGTGTTAAAGTGTCACATCCCGGGATCAACTCCGTCGTAGCACGATATTGTCCGCTTTGGGCCTCCCTCTCTGCCCGCACGGTTTTGTTTCTGGGAACTCACGAGCAACTTCCTAGTGGGTCACCCATCCTGAGATTGCTCTGGCCCCCAACTCTCTTAACTTCAGAGTTCCTACGACTCTGAAGCCAGTGAGCTCCCAAAAGGCCTCGTGCTAGATGGATGCGGGTGTGCACATATAAGGCACATCACCCCCTCTCTGTTGGTTGATGTGGGATCTTACAATCTACCCTCCTTAAGGGCCCGACGTCCTCGTCGGCGCACTCGCACCACACGGCAGAGTGGCTCTGATACCAAATTGTCACATCCCGAGATCAACTCCGCAGTAGCACGATATTGTCCGCTTTGGGCCCCCCTCTATGCCCTCACGGCTTTGTTTCTGGAAACTCACGAGCAACTTCCCAGTGGGTCACCCATCCTGGGATTGCTCTAGCCCCCAACTCGCTTAACTTCGGAGTTCCTACGACTCCAAAGCCAGTGAGCTCCCAAAAGGCATCGTGCTAGATGGATGAGGGTGTGCACATATAAGGCACATCACCCTTTCTCCGTTGGTTGATGTAGGATCCTACATAAAGTCTTAGGATCCTTTGAGTCACCTTTCATACTTCATCACTCCTCTGAGTCTGATTCAATTGATAGCATAGATATTGAGTCTAGTAATATTTCAAAAATTGAAAATGCATTCAAAAATCTTGAGCTCAAACAAAGTTCACAAGTCCAAAGATTAACAAACAAAGTCAACCCTACTAGCCTCACAAAAAATTGGTATCCTAAGCCTACGCCACCTGATATCCAGTTCGAAGAACGAAACTCTCAAAGCCAGTTTTCTGTTTCTTCTGATAAATTGTATGAATGGAATATTGATGGTCTTTTTGAACAAGAAATCCTTAATAAACTTCAACATATGTCTATGGTCGCCAACAGTTACATTACCAATCACAGTTTCAGACAGTCTGAAATTGTTCCCTTGTTAGAAACAGGTTTCACAGGAACTCTTCGTTCTTGGTGGGATAATCACTTAACTCATGAGTCTAAGCAACGAATAATTCATGCGCTAAAACTTAATGAAGATGGTCTTCCTATTTTTGATGAACAAATAGGTCAAGGTATTGAGGATGGCGTCAACACATTGTTATACACCATAGTCGATCATTTTATTGGAACACCTAGTAGTACTACCGCCAGAATACATGATCAACTAAGTAATCTAAGATGCCCTAAATTATCTGATTTTAGATGGTAGAAAGATGTTTTCATTTCCAGAGTCATGGGAAGAACATATAGTAATAAACCTTTTTGGAAAGAAAAATTTGTTAATGGATTACCAAATTTATTTGCTCATAAAATCCGATCAACCCTTAGTAATGAACAAGGTCACATTGATTGGGATAGTCTAACTTATGGAAATATAATTAGTACAATTAATCAAGTTGGTATGAGGATGTGTGTCGATATGAAAATTGGTAAACAAATCCAGTCTGAAAGAAATTCTGCTAAATACGAATTAGGAAATTTCTGCGAACAATACGGTTTGATGTCTATTCCTCCGTCTAGAAAAAATAAGTTCAGCCAACAGTTAGAAAAATCCGCCATTATTCGCGTAGAAAACAGTCATTCCAACATAATAATGAAGATAGAGAATTTTATAGTAAGAAAAGATTTTCCCCTCGGAAAAATTGGAAAAAGAGTCACAATAGAGAGAAGAAAACAAACTTTAAGAAAAAGACCGACAAAAGAAAAGTAAAATGTTACAAATGTCAAAAGTATGGTCACTATGCTAATAATTATAAAGTTAAAGACACAATCAAACAATTGAAAATCACGGATGAAGATAAAAACAAACTAATCCAAGTCCTTGAGTTACATAACACTGAGTCAAGTGAGAATGAAACCATGGTTAGCCGTACTGAGTCTTCTGAAATATATTCTTCTGAGTCACAACCCAATTCCCCCAGAATCCAATTTGGTTGCCAAGATAACTGTTGTAAAAATAAGTCAATCAGTGTCCTCACCAAACAGGAAGAACAAGAAGAATTGTTAATCGATCTAATAAGTAAAGTTGAGAATCCAGAATTAAAATCTGAACATTTAAAGAAATTAAGAATAGTCATTAGCCAAGAAGGACCAAAACAGTCTACCCAAAAGATAAATAATACAACTTTAGAAAAATTTAAGAAAAAGAAAGAAGTTACCTTACAGGATTTACAACTAGAAATCAAATCAGTCAAAAAAGAAATCATTGAATTAAGACAAATGAGTCAAAAATTATAAACTGAAAATTATGAGATAAAACAAGACCTGATCAACCTATTAGAAAAGAAAATTCCTAAGTCTAATCCGGAAAGTCCCACTAATTCAAACGAAGAAAACTCTATTCAAGAACAAGCCGTTAATCTGATTAAACATGTAAATTTTAGAAAATGGTATGCTCGAGTCACTATATTTGTTCAAAATTTTGAGTTAAACATAGTCGCCCTTTTTGATTCCGGAGCTGATCTTAATTGTATCCAAGAAGGCCTAATCCCAACTAAGTATTACAAAAGTCAAGAGAATCTTTAAGCACTGCTTCAGAAAATCCCTCCAGCTGAATTATGAGATCCCTAAAACCCATGTCTGTCAAAATAAAATCTGCTTTAAAACTTCTTTGTTTTAATAAAAAAATATAACTGATGAAGTCATATTAGGTCTACCGTTCATTTCCCTCCTGTACCCTTTCCAGGTTAAACATGATGGTGTCATTTCCACCTGTTTAGGAGAAGAAATTAAGTTTGAATTTTTGAGTAAACCTAAGTTACATAGTTTAAAAGAATTACAAAAAAATGCAGTCACCAAAACTGTCCAAATCATCCAAAACAAAGATAAACAATTAAGATTTCTCCAGGAAGAGGTTAAAACCAAACGAATTGAGCAACAACTAAGTCATACGTCTTTTCAATCCCAAATACAACAATTTGAAGAAAAACTAAAACAAGAAGTCTGTTCTGAGTTACCCACTGCGTTCTGGCATAGGAAACAACATGTTGTCAGCCTCCCCTATGTCAAATCCTTTAGTGAGAAAAATATTCCTACTAAAGCAAGACCCATTCAAATGAGCCAGAATTAATGGAATTTTGCAAACTTGAGATTGCTGATTTACTTAAAAAAGGAATAATCCGTAAGAGTAAGTCACCCTAGTCTTGTCCTGCTTTCTATGTCCAGAAAAATGCTGAGTTAGAACGTGGTGTCCCAAGACTAGTCATTAATTACAAACCACTTAATACTGTTTTAGAATGGATTCGATATCCAATACCTAACAAAAGAGACTTAATCAACAGATTAGAAAAATCAGTCATCTTTTCTAAATTTGATATGAAAAGTGGATTTTGGCAAATTCAAATTTCTGAGTCTGATAGATATAAAACTTCTTTTGTTACTTCTTTCGGTCATTATGAGTGGAATGTTATGCCCTTTGGTCTTAAAAATGCTCCTAGTGAATTCCAGAATATAATGAATGAGATTTTTAATCCTTTTAGCCATTTTTCCATAGTTTATATTGATGATGTGCTTATATTCTCTGAGTCTACTGAACAACACTGGAAACATCTGTATAAGTTTTTACAAATTGTTAAACAAAATGGTTTAGTTGTCTCTACTAAGAAAATTAAGTTATTCCAAACTAAAGTGAGATTTCTTGGGTTTAATATCTGCCAGTCACAGATTAGTCCCATAGATCGAGTTATCCAATTTGCTGATAAGTATCCTGACCAAATCCTAGACAAAAGTCAACTCCAGAGATTTCTAGGATCTTTAAATTATGTTTCTGATTTTTATCAAAATATGAGAAAACAATGTAAGCCCATATTTGATCGTCTCCAAAATAATCCTCCTCCATGGTCATCTGCTCATACTGAAATTGTCAAACAAATCAAAGTTCATATCAAGACTATGCTATTCATTAAAATTGTTGAAACCGATGCTTCTAATGTAGGTTATGGAGGTATCCTCAAACAGAGAGTCCTATCTGGTCAATCTGAGCAAATCATTCGATTTCATTCCTGCACTTGGTCCCAGTCACAAAATAATTATAGTACTATTAAAAAAGAAATATTATCTATTGTATTATGCATTAATAAATTTCAATCTGATTTATTAAATAAATTTTTTTTAGTCCAAGTTGATTGTAAGTGTGCTAAGCATGTATTAGAAAAAGATGTTCAAAACATTGCATCAAAACAAATATTTGCACGTTGGCAAGCTATTTTGAGTAGTTTTGATTTTGATATTGAATATTTGAGAGGCAGTGAGAATTCTATTCCTGATTTCCTTACCAGATAATTTCTGCAAGGAAAAGAATGGCAGAAAGAAGAAAAGAAAGAGAAAAAGATGAGAAACAAAAGGGTCAATCAAGCCAACCCAGTCAACCCCAGTCACTCCAAGTCCTCCCGCCATCAATGAGTCAAACCAAATATGAGCCCTCTTCCCAGATCGTCCCTTTCCCGGGATGCTCTCCAATCCAAATTAGTAACAGGTTTGCCAACCTAGGAGCCACAGTCGGCCGGGTTCGCCATAGTTTCCATCTACTTTAATTTCTAGTCATGATCCGTTTCAAGATATTGCTGTTTCTAACCCCCCCCCCACCCCCCCCCCCCCCCCCCCCCCCCCCCCCCCCCCCCCCCCCCCCACCCCCCCCCCCCCCCCCCCCCCCCCCCCCCCCCCCCCCCCCCCCCCCCCCCCCCCCCCCCCCCCCCCCCCCCCCCCCCCCCCCCCCCCCCCCCCCCCCCCCCCCCCCCCCCCCCCCCCCCCCCCCCCCCCCCCCCCCCCCCCCCCCCCCCCCCCCCCCCCCCCCCCCCCCCCCCCCCCCCCCAAGTTGTTTCTTACCAGAAAACATCTCCATACATGATCAAGAGTCATAGTCACCTATTTGTCATTGAGTCCAAATACAATTCGATTGCTAATCCATCACCATTGCTAAGTCTTATTTTCCACCAAATAGTCATTTCATTCCCCCAGCACCTTTCAAGAACCTCAAATATTATAGAGATATCCTTCATGAGACCCATTCTATCACCATTAAGCCAATCAAAGACAGAGAAAACCCCATATCATCCTCTATCATTCTCTTTATATTCACCAAATTATCAATGAAAATGAATGGAGTAGTCACCCTTATGATCTCAAAGTTCTCCATCAGAACTTCAATACAGTTATTATGACTATATCAAAGCATGGTATTCTATCTTGATTCACCAAACAGTTGATTTCAGTCACTCTGGTTCTTAAACTTGGATAGCAAATTCAAGAGCTCTTTTCCTTACTGGTTCAATCACTGGTGGGAAAAACATGGTCCAGTCATTGATTTATTTCCTCCCCCCATGCAAGAATTAGTCAATTATTTTACTAATAAAAATAAATTCAAAGAATCTGAGTTGTTCTTCCCCAATCTGCTTCATTTTGTTGCCAAATATAAAGTCCCTTTGATCCTTAAATGGTCTTATCAGGTTAATTGGGAAACCAGAGTCCTATCTCGCCAATTTTCAATCAAATGGTGGGATAAATTTAAACTTGAGCGTATTGCAGATTATGTTTATGCTGATTTTCCTCCAGTCAGGAATCCGCACCCTGAGAAAAAGAGTTCCCCAGCCACATCACATTCTTCTCTCCCAATCGAGGGAAGGTCTAAGTCCAAATTACAAGATCTTGCTCGCCAACTAATGCTCCAAGCCTCTCAGATGAATGATGAGGCAGAC

TCTGATGCCTCTCCTGCGTCACAGACATCTACTAGTCAGCCCAAGCCAGCCAGTCAACAAGCTCCAGCCCCAAAAATGCGGAGTGATTATCTTGATGAACAAGACCCTTATGATTTTGGTCCTTTCAATCTAGGGGATGATTAAAGCAAAAGTAAAAGTCGCCTTGCATCAAGAGCCTTGAGACAAAGTATCCGGACCCATGCATTCATCAGTCTGAACGCCGTGGTCTTTTGAGATTCCCCGACAAAAGTAAAGGTTGCTGCACTCAGTAAAAGCTAAAGCTTTCGCGTGCTTCTACAGTTACTTTTCACAAAGCATCCAAAGTTGCCAGCTCACCAAAAGTTATTTGAGTCTGTTTTATTTGTCTATTTAAGAAGGCCCCGGCCTCTGTTTTGAGCACCGCCCTCTATTTTGAGCAGAATGTAATCTAGTACACTATAATTTTCTCAGTTTCAAAGTTCTATCAGGAATCAATACTCCGAAAGAACTAAGTGGTGAAACTAGAATTTTGTAAGTCTTTTGCATTCTCCAAGTTATCAATATATTTGAGATATTTTGAGAATTGATTATTTTAATTAATGTTTATTTTCATTGCATCTATTTTCATATATTCATTAATTTGCTTCATGCATAAACCTGCATGTGTTCTGTAATCTGTCCCTCTATCTCTCTGGTTCTAGACCCTTCTGGTATCATATATATATATATATATACTAAATTTCCTATTGTAATGGGCAATAATATACTAAATTCTCACACACTACACAGATACTAGAACTCGAAACGAGGACCTTGCCTGTGAAGCAATTACTCCAAACCACTATACTAGTAGATTCTTTGCGAAAATATATTATATTTGTGATAAGAAACAAATAAAAATATTGTATATACTATTAATTTGTCATTTTACACTTTTTTTATGTATCTATATCTCTATATATTTTTATAAAACAATAAGTTAATTTATTTACAATAATGGATTAGTCACAATTAGATATTA

>Chr1-28; OZ022206.1; 44710876-44718651; dir; A;

CGATTCTGCATGCTACGGCAACTAACAATTGCCCCCCGAATTGGAATCAGGCAAGCGACAATGGAGGATGGTGCAATCCACCACGCGAGCATTTTGACATAGCTAAGCCTGTATTCCTCAATATTGCCGAGTACAAGGCTGGAATCGTCCCAGTCGAATACCGCAGGTAAATTTCATGCATTAATTCATGGACAATTTAATAACAACATAACATGTTGATAAAAGAGAATATTATCGATCGACCTTTTCATTATTATATATCATCATGTTCAACATTGAATGAAATCTCAATATGCTTTGCAGGGTTCCATGCCAAAAGAAAGGAGGAATTCGATTTACGATAACCGGCAACCCTTACTTCAACGAAGTGTTGGTTTGGAATGTGGCAGGAGCTGGAGATGTGGTCAGCATGCAAGTGAAGGGCAACAAGTTGAAATGGACAATGATGAAGCGGATGTGGGGTCAGAGGTGGGTTACTGATGCCAAGTTGGTTGGTGAGTCACTCACCTTCCGAGTCCTAGCAAGTGATAAACGATACTCCACTTCATGGCATATAACCCCTAAGAATTGGCAGTTTGGCCAGACTTTCGAAGGAAAGAACTTTCGATAGGAATGAAGCTCCCCAATGTTTAACGTCATTCTTCTCAACCTTCTTGCACGAAAGAATAATTTTTTTGGCTTCTCGTCTTATATATATGTATGTTGACCAGTGGCCATGAAATCATAATTAATAGTACAAGAATGCTTAAAGGCTTTTGATTCTGCGGTGTATTGCATACTCTCAGATTAATATCTACTACATTTATCAAATTTTTCGAGTACTCGAAGGAAGGCATATATTATATTTTCCAAAAGTAAATAAAATAAAATCATAGCTACAAGTACAGTTGAAAATACTGAAATGCATTTTTGGTACCCTTTTGAAAATAAGTCACGGTGTGTATATATATATATATATATATATATTTATTTATTTATTTATTTTCAAAATTATCTATGT

TGAAAGCATTATTTTAAATGCATTATCATTATTTTGCTGCATTAGTCTGAAAGTTGCTTAGGTTGCATCCATTCCTAATTTCCTTCTGATCTTCGTCTCTGTATTATATTCAGTTGTCTTTCATATTCTCTTTCTGTTCTTTGTTCTTCCTCTGTTGGTATCAAAGCCGTAAGTGTTACGGTCTTTGTCTAAGTCTGCTACTCATCCCCGCCCAAGTGGCTGGTTTAAGCATCTGAGTAGAAGGTTTTCAATTCTTCCTTTTCTTCCCTTTTATTCTTTGAGTCCTCTGTTGGTTTTCTCTAAGGTCTGACTCGTGAGTCCTTTTTGCTAGATCAGTAAGTCCCCAGGGAGGGCGTGTGTGCTGGGCAGGAGACAGAAAATTGAGAAAATTAATAGGTTGCTTAGAGTCTAAACGGTCGGTTTAGGATCTACTGTTCATCTTCTTTCTCAGTCTGTTTAACCTAGTTTCTTGAGTAGTCATGAATAGTCTATTTAGAACAAAGTCTTGTAATTCTGCCAGTTCTAGTACCAGTATAGGTCGTCTTCCAGAGATAGTTAATGAAGAACAATTTGAATATAATTCCAGCAGTAGAGAACAATTAGATTTTAGAGATTGGAATATACCTAAAGTCCCCAGTCAAAAAATCTATAAGAAACATTGGTTGCCTTCCAGTTTTAATAGTACAACACATGTCAAAACAGTTGAACAAGTGTACGCTTTAAGTAAAGAACATGAAACCTGTCAGTTGCTTAATCTTGAGTCTATTTTAAAACATAAGAAAGATGGTAATAATTTCCTACATATTGGTTTAGTTCAAGTTGCTGTCAAACCCCTGACACGGTTAGGATTGAAAGCTTCGATCCTGCTATGTCTACGCGATGCTAGATTCACTGAATTTAGCGATAGTACCCTTGGAATCATTGAGTCCAGTCTGTGTAATGGCCCTGTCCATTTTGACTGCTATCCTGATTTCACTGTAAGTCTTAGTGATCCTCATATCCTGAGAACTTTGACTCTGAATATTAAGACTGAAGGTTATAATGTCCTCCCAGGAACCCAACCTTTAGCCTTAGTTTACAGAATCTATTATAAGGTCACAGGAACCAATATGAATTTCCAAGCCCTTAACAAGAGCCCAAATGATCAAACCGTCTTGATCCAGAGTCATACCTCAGACGCCCATATACAGGTCCCCCATACTATCAAGTGGTCCGAAGTTGCCCTTCCAAAAGATTGGACCTTAGTTACCGAGAGCCAACCAGCTCCCATCCAGCGTAGTCTAAACAATTTAAACTACATCCAACAATACTTAGATGGCACAGTTAAGATTCAGTTCGACAGTCATCCTTTACGAAAGTCTAATGTCCAGTTACAGCAGTTGCCAACCCCTGGTCAGTCCCAGAGGCATGCCCCTGCTCGGCATTCCTTTGCTGCCTCTTCCTCCACTCTAGAACGAGACCTAGAGTTAGAAAAGGCCATGATCGATTTCAAACTTGAGTCTCTGAGAAAAACCTCTCAGGTCACCCAACCTTGCTACAGCACGGTTCCAGTTCAAGAAGACAAGTCAGAGTCACCTGAGTCACCTACCCAGTCTGACTTTGTAGTTGAAAATCAGCTTAGAACCCTCAGTAAAGAATTTGAAATTAATTGGGTAAGTCTAAATCGTCATCTAAAAGCCCCTGAATACAAGCCAAGGCGAGATCTTTATCATCAAGCCTATCCTAATAGCAAACACCGAGATATGATCTTTAAACAATGGAAAGATTATATGCGGTCAGCCAAAGTTGAAATTTTCTACCTTGATTTTATTGAAAGCCGTTACATGTCTAATGATGAATTGAAAACTCTAAATAAAGAAAAATGGAAACTAGTTGACAAGTCTGTAGTAGAATCGAGTCACCCTCCAGAAGAAACAATTATAATAGAACACCAGAAAGCGCCAGTTGCCGCCACTCCTTTCAAAACCTTTGAAAGCAGTGATCCTCATAGAAAACTAATTGAACAAAATAATTACACGAACCAAAGTCTAATCACCATAGGAAAACAGTTAGACACAATCGAAACCAAGGTTGATCTGCTGAGTCCGCCTGTTGCAGACTCCAAACCCAAACCCAAAGTAGAAAAGCCCATAGTACAATTCCAGGAACTTAAGTCCAGTCCAGTCCTCAAAGTTAATTCCACAATGAAAAAGATAGAAGACATGTTAGCACAATTGACCCCCGTCAAATCCGAGAAAGGCGAACAATCTGGTATTAAAACACTAGATTCTTTTGCCGCTTCTCATTCTGAGTCCGAGTCAGAATCCGTAAGTTCAGAAACTAGTAACATTTCTAAAATTGAAAATGCTTTTCAGAATTTAGAAATCCAACCTGAGCCCAGAATTAAAAAGATAGAAAAACATATAAGCCCTGCTAGCCTAACAAAGAATTGGTACCCCAGACCAACACCTCCAGATATCCAATTTGAAGAAAGAAACTCTCAAACCCAGTTTTCGGTTTCTTCTGATAAATTGTATGAATGGAATATTGATGGTCTTTCTGAGTAAGAAATCTTAAATAAACTGCAACATATGTCTATGGTCGCAAATAGTTATATCACCAATCACAGTTTCAGACAGTCTGAAATTGTCCCTTTGTTAGTTACCGGTTTCACTGGTACTCTCAGATGTTGGTGGGATAAGCACCTCACTCCTAAGGCTAAAAATCGAATCATTTATGCTGTAAAACTTAATGAAGACGGTCTCCCTATATTTGACGAACAAATAGGTCAAGGTATAGAAGACGGTGTCAACACTCTGTTTTACACAATAATTGAACATTTCGTCGGAACCCCTAGTAACACCACTGCTAGGATCCATGACCAATTAAGCAATCTTAGATGCCCAAAGTTGTCTGACTTCAGATGGTATAAAGATGTTTTCATTTCTAGAGTCATGCTTAGAGATGATAGTAATCAACCATTTTGGAAAGAAAAGTTCATTAATGGTCTGCCTAATCTATTTGCTCACAAAATACGCACGACACTAAGTAATGAACAAGGTCAAATTGATTGGGATAGTTTAACTTATGGAAACATTATAAGCACAATTAATCAAGTTGGTATGAAAATGTGTATCGACTTTAAAATCAATCGCCAAATCCAGTCTGATAGAAAGTCAGCCAAATACGAGTTAGGGAATTTCTGTGAACAATATGGTCTCACCAGTATCCCTCCTTCTCGGAAACACAAGCCTAGTCATCTTAGAAAGCGTCATCTCCCGAAGAAACAATTTCTTCAGAAAGATGAATTCTACAAGAAAAGAAAGTTCTCTCCTAAGAAAAATTGGTCTAAACGTCCAAAACAAAGTCATTCAAAACAAAGTCGTTCTAAGAAAGATAAAAGCAAAGTTAAATGTTTTAAATGTCAAAAATTTGGTCATTATGCTTCTGAATGTAAAGTAAAAGATGTTATTAAACAATTACAGATTACAGAAGATGATAAGAACAAGTTAATTCAAGTTTTGGAGTTGCGAGACTCTGAAATGAGTGATGTTGAGTCTCTAGTATCCAGTTCTGAGTCTGAGTCTAGCCGGTCTTCAGATTCACAACCCGGTTCCCCTAAAGTTCAATTTGGTTGTACTGACAAATGCTGTAATAGATTAAAATCTGTCTTAGTCCTCACTAAGCAAGAAGAACTCTTAGTAGACATAATAGGTAGAGTCCAAAACCCTGAGTTAAAAGCTGAATATTTGAGAAAATGAAAAAAATCTTAAGCCAGGATAGCCAGAGTTCTCCCTCGCCTATTCCTCAGCCAATTAGCATCAACACTACGTTAGAAAAATTCAATCGTAGGAAAGATGTCACCATTCATGATCTCCAGTCAGAAGTCAAACAAGTTAAAAAAGAATTAGTTGAACTTAAACAGATAAGCCAACAATTGCAAACTGATAATTATAAGATCAAACAAGACCTGTTCGAATTAAAACAAGATTCCTGTAAACCTAGTAACCCTTCAGAAAGTCCGCATCATTCTGATGATGAACCTTATGAAGAACAGCCAGTTGTCAACTTAATCAAACAAGTTCACCTTAGGAAATGGTATTCCAAAGTCACGATTTATGTTCATGATTTTGAATTTACCACAGTTGCATTATTTGATTCCGGTGCAGATCTTAACTGTATTCAGGAAGGTCTCATCCCTACTAGGTACTTCCAGAAGTCTAGAGAATCCCTTAGCACCGCTTCAGGGAAATCTCTCCAGTTGAATTTTGAGTTACCAAAAGCCCATATTTGCCAAAACAAAGTTTGTTTTAAAACTTCATTCGTCCTAGTTAAAAATATTACTGATGAAGTCATTCTAGGATTACCCTTCCTTGCCCTACTGTATCCAATCCAAGTAGAATACGATGGTATCATTTCTACCCACCTTGGAGAGTCGGTCAAGTTTAGCTTTCTGTCTAAACTAGAGTTGCATAATCTAAAAGCTTTGCAGAAACAGTCAATTTCTAAATCTGTCCAAGTTTTATGAAAGACCAATCAGTTAAATTTTCTTAAAGAAGAAGTTAAGTTTAAACGAATTGAACAACAACTTAGTGATAAAACTTTACAGTTGAAAATCCAGACTTTTGAAAGCAAACTAAAAGAAGAAATATGCTCCGATTTACCTACTGCCTTTTGGTATAGAAAACAGCACGTTGTCTGTCTTCCTTATATCAAATCTTTTAGTGAGAAAAATATCCCCACTAAAGCAAGGCCGATACAGATGAGTCAAGAATTAATGGAAATTTGTAAAAAAGAAATTCATGAGTTGCTTCAGAAAAATATTATTCGCAAAAGTAAATCCCCCTGGTCGTGTCCTGCTTTTTATGTCCAGAAAAATGCTGAACTAGAAAGAGGAGTCCCCAGATTAGTTATCAATTATAATCCTCTTAATTCGGTCTTAGAATGGATTAGGTACCCCATACCTAATAAAAGAGACCTTATTAATCGCTTAGGAAAAGCAGTTGTCTTCTCCAAATTTGATATGAAAAGTGGTTTCTGGCAAATCCAAATTAATGAGTCAGATAGGTATAAGACTGCATTTGTTACCCCTTTCGGTCATTATGAATGGAATGTCATGCCGTTTGGTTTAAAAAATGCCCCTAGTGAATTCCAGAATATTATGAATGATATCTTCAATCCTCACAGTCATTTTTCGATTGTTTATATTGATGATGTTCTAATATTCTCTGAGTCAGTAGAGCAACATTGGAAGCATTTGCATAAGTTCCTTCATATAGTCAAACAAAATGGTCTAGTTGTCTCTGCTAAGAAAATTAAATTGTTTCAAACCAATATTAGATTTCTTGGTTTTAATATCAGTCAGTCTCAAATTACTCCAATTGATCGAGTCATCCAATTTGCTGATAAGTTCCCTGATCAGATTCTTGATAAAAATCAGCTCCAGAGGTTTTTAGGATCTCTAAATTATATTTCTGATTTTTATCCAAATTTACGAAAACAATGTAAGCCCCTATTTGACAGACTAAAGGATAATCCTCCTCCTTGGTCTAGTATCCATACTGAAGTTGTCAAACAAATCAAAACCCATGTCAAGACACTTCCCTGTCTTGGTATTCCTTCAGTCGACTCTTTCAAAATAGTTGAAACAGATGCCTCTGACATTGGTTATGGTGGCATTCTCAAACAGAAAGTCAGTCCAGAATCCCCAGAGCAAATTGTTCGTTTCCACTCTGGAACCTGGACCCAGTCGCAATATAATTATAGTACTATTAAAAAAGAAATATTAGCTATTGTATTATGCATTAGTAAATTTCAAGATGATTTATTAAATCAAAAGTTTTTAATACGAGTTGATTGCAAATCTGCAAAACATGTTTTAGAAAAGGATGTTCATAACATTGCATCTAAACAGATTTTTGCACGATGGCAAAGTATATTAAGTATATTTGATTTTGATATTGAGTATCTGAGAGGTAGTGAGAATTCAGTCCCTGATTTTCTTACTCGAGAATTTCTGCAGGAACCACAATGGCTCCCAAAAAAGACAAAAACAAAGAAGTTGCCAATCAATCCAAGTCATCCCAGTCGCAACCCAAGTCATCCCAGTTGCAACCATCTCAGTCGCCATCCATGAGTCCAGCCAAACCAGAAATGCCCAAACAGATAGTTCCCTATCCTAGTTGCACCCCCATCAAAGTTGCCACTCCTCTATCAGTTGCCAATAGATTTTCCTCCTTAGGATCTACAGTCGGCCAAATCCGCCCCAGTTATCAGTCTACTCTAGTTGCCAGTTATGATCCCTTTGCAGTAGACATCCCTGCCGGCACTGCTGGCCCCTCAGTTGCATTCAAAAAATCATCTCCTTATTTGCCTAAAAGTAATTCTCATTTATTTGTAATTGAGCCCTTGTATGACATCAATACCAGTCCAGTTGAAATTGCCAGACATTATTTCCCTCCTGGATTCCATTATATGCCTTCCAATCCTTACAAGTCCCTCAAGTATTATAGAGATATACTACTTGAAACCAAATCAGTTGAAATTAAGCCGATCAAGGATCGTGACAATCCTGCCACTATCCTTTATCATTCTTTATATATTCACCAAATTATAAGCCAAGAAGCCTTCAGCAGTCGCCCTTATGAACTCAAGTTACTTCAGTCCCAGTTGCAATACAATTATGCTGACTATATTGATGCATGGTATACTATTTTTCTTCATCAATCTGAAGATTTTAGTCATTCCTGGTTCATCAATTTTGATAACAAGTTCAAAAGCCATTTCCCCTACTGGTTCCTCCATTGGTGGGAAAAACATGGTCCAGTTGAAGAAATTTTACCGGTATCAGTCCTTGAGCTCATCCGCCATTACACCCAGAAGGCCAAGTTCTCTGCAGTTGATTTATTTTTTCCCAAACAGTTGTTATTTATTGCCAGATACAAGGTACCCTGGATACTAAAATGGTCTTACAGGGTCACTAAAGATGTTCGCATTCTTGCCCGCCAATTCTCTGTCAAGTGGTGGGACAAATTTGAAGTTGATAGAATTGCCAGATACGTCTACAACGACCTCCCAGTTGCACCAGTACCACTGCCGGTCTCTCCTTCTGCATCTGTTCGATCTTCCCTCTCAGTCGAAGGAAAATCAAAAGCAGAACTTCGAGAAATCGCCACCCAGTTGCTCAGACAAGCCTCCCAGATGGATGATGATGAAGACAACGGGTCCCCCTCGTCTTCCAGTTGTCAGCCCATCCCTGTCAAGCCAAAGACCTGGTATGAAGACAGTCAAGACCCCTATGACGCCTACGATTTGAACTCTGACTAGGCCTCATGATGTTCCTGATATCTTCACATGCCAAAGTAGCCGAAAGTAGCCGGTCCTACAAAAGTTGTCATTAGTCGCCGACATGATCATTGTCTTCTGTGAAGAATCTGCTTTTACTATCTACTTTCCACCGAAAGCTCCATCAGTAAAAGAATTCCCCACAAACAGACAAAATTGAAAGTTGAATTAGGTTGCTTTCTTGTGACCTAGCAGTAGCCTCCGTTCCAGTAACTCTACCATAGTTGAACCTTAGTTAATTTTCTCTTTATAAGCAGGCTTCGGCCTCAGTTGTAAAAGGAATGCAATTTTTCCTTTCTTCAGTTTCAAAGAAATCTCTCTGATACATCCTTCGGAGAATTTCACTTTTGTAAACACTCTTGTAAATAGCGTGCAGTTGTGCTTTGAAGAATTGCAGTTGCAATCTTTGTAAGTATAAATAAAAAGTTATTTTCAAAATTATCTATGTTGAAA

GCATTATTTTAAATGCATTATTTACACACACATATATATATATATATACACACAAAGAGCTTTTGTTGAGGAATTCTTTAAATTAGCTTATTTGAGGGACACCTTTGTAGGGCCGACTCCGTATTGTATTTCACTAATCCAAACCATCTATTTTGTAGATACTCATTCAAAGATCATCTCTACAAAAAATCACTTGAGTCCGATATCATTTGACCACTCAATTGAATTATTGAAATTTTAGTACTTTCTTGAAGCATCGTGTTCATTGATTTTGTAGGAAACAATTGGATATCGAAACGGTTTTCGATTTGTCTAATTTTTTTTGCAAGGATGATCTATGATCTATGAATGTATACTTAAAAAATAGATGGTTTGGATCGTTGAAAAAATATTTATAGGGGACCCTAAAGGGTGTGTGAATATAGAGGGGCTGAAACATGGGTTAAATAAACTTCTCTTATAAAAATAGCTCCAAAATTTTACAATATTGTACCAAAAATAGATAAAGTTCTACCATTCTCAACTAATTCAACCTCAACTCGCCTACCATCCGTCTTTTGGGTTTTAAACCTACAAGGTCTATAAGGGGGTGAGTGAAACCACAACTCAATAAGGACATAAACCATACTAAATTAAGTGACGTACAATCATACAATCAATGATAGTAATGCATAATGTATGCTCTTCTATTCCCGTTAATTCATATAAATGAACAAGCAGACCTCGCACGTCTCATACCATGCTTATACCAGTTGGTCTCGAATGCCCATTTGTAGGAACTAACACCCATCTAATAATCATCCCATAATTCCCATTTTGTTAGTCATCTTGTCTAAACCATTGATCTCCAAGCTCAAATCATCCAATTGACAATTATACCACTGATATCCTATTATACTGTGCACACGGGCTCACCTGAACATGGTCCCTCTTTCCCATAATTACTTTCTCAATCCAAGAGTTCCAACTATCACCGATAACATGTCAACATTAATCAAAT

>Chr1-29; OZ022206.1; 45812278-45833177; rev; C;

AGAGTCCCTCTTTGCAAATTCAAAAGGCCCTACGTAGTTTCGCAACTCCAAGCAATTCTCAACCAACACATGCGATTCATTCTGTTTCCGTCATAGGGTTGTTGAGTCAAGTCAAGAAGAAGAAAAGAAAGTCCCACCTTGTGGATTAAGGGGTCGTTTGGTACACATGCTTGGTTTTGACTAAATTTAGTATCATGTTTGTTTCATTTAATTCTAGTCTTAGATGAGATTGCTAATACCGGGTCCACCAAAAACACCCCCTTAGGAGGGGACTACTATGCCCATGTAGAAATGGAGGATTAACTAGTCCTATATTCACCAATGTATAAGATGCATATATTATATTGTAATACTAATAACATATATTACTATTATTATTATTATTATTACATACACATATACTATAATGTACATATATACACGTATATACACATATACATGTATATATATATAAACACATATACATATATAATATATATACATATAGATATATACACGTATATTATTATTATTATAATATACCCATATACACATACATATTAATATTATAATAATAGCATACATGTATATATGTAATATATATATATATATTATATTATAATATGTATATATATATTATACCCATGTATGTATTATAATATACATACATATATATAAAATATATGTATATGTATTATACCCATGTATATTATAATATACATATATACACCTATATACACTTATATATATATATGTTTATATTATTTATAATATATAATATTATTATAATATACATATATACATGTATATACACATATATGTTATATATATATTACCCTAAACCCTTAATCTGCAAAGTCGAAAATCCTGAGTTGAAGGCGGAGTATCTGAAGAAATTCAGAAAACTCCTTAGTCAGGAAGGTTCGAGTCAGTCTCCACCTCCTCAAAAGATAAGGTTAATTTATTCATAATCGAACCCAATTATGATGGGATTTCTGACCCGATAACAATTGTTAGGTCA

TATTTCCCTCCTAATTCCCATTTCCTTCCTCCTGCACCTTATAAAGACCTCAAATTTTACAGAGATATCCTGCATGAGACCAAATCAGTTGAAATTAAGCCCATTAAGGATAGAGATAACCTTGCGGTCATCCTCTATCATTCCCTTTATATTCAGCAATTTCTAAGCCAAGCTGAATTCAGTCGCCACCCCTATGAATTGAAACACCTTAAGTCCACTCATCCTTATACCTATGCAGATTACATTGATGCATGGTATAATATCTTCCTCCATCAAACAGTTGATTTTTCCCACTCCTGGTTCATTAATTTCGATGCCAAATTCAGGAGTCCTTTCCCCTACTGGTTTCTTCATTGGTGGGATCAACATGGCCCAACAGCTGATGTCCTTCCTACTGAAGTCCATAATCTGACCAATTATTATGCCAGTAAATCCCAATTTAATAAACAACAATTGTTCTTCCCCAAGTTGCTTCTTTTTATTGCCAGGTACAAAATTCCCTGGATTATGAAGTGGAATTATCATGTCAATTGGGAAACCAGAATCATGTCTCGCCAGTATTTTGTCAAGTGATGGGATAAGTTCTCAATGGACAGAATTTCCATCTATGTCTTCAACGATTTCCCCCAATCGGATCATCCTCCCAAGCCAAGCTCTCCCACAGGCTCAGTCAGTTCGGCCATCTCAATCGATGGAAAATCCAAAGCTGAACTCCGGGCCCTTGCTGCACAATTGCTTGACCAAGCCAGTCAGATGAATGATGATGAGGACAACGAATCTTCCCCATCTGCATCCAGCTGTCAGCCTGGCTCCAGCTCCTTTGATCCCAATATCCCTTTTGCGCAGGATCCTTATGACGCGTATGATTTGGGATCTGATTAGTCAAAAGTTGCCATCACTTCCTCTTTTCACCCCGGTGAAATGTTGGAATTAGAATAATTGTAACGTAAAAACAAAATTGCTTTTGCTTTTACCCTTACTTTACCCCAATAGGTAAAGAGAGTAAAAACAAAAATACTTTTGCTTTTACCCCTGCTTTACCCCAATAGGTAAAGAGAAAGTTGTTTTCCGTGCTTGAAACCATAGTTACTCTCCAATCAGGATTACTTTGGATTCAAACCTTAGTTACTTTCTTCTCTTTTTAAGCCGGCTTCGGCCTCAGTTGTAAGCACTTGTAATTTTTCTTTTCTTCTTCAGTTTTCAAAGTTCTCTCTGAAACTCCTTCAGACATGAACTCTCCTGTAAATATCTGAGTGTTTAAAGCTTTCAATAAAAGGCAGCTTTGTAAGTAGTTCCAAACCTATCTTATTTTCATTTATTCATTTATTTTCTTATGCATTAATTTCTTGCATCAGTCTGAAAGTTGCTTAGGTTTGTTATAATTCAAATTCTTTCTTGAATTCCCTAAACTATCCTCTGTTCTTCTATTAATGTTGGTCCTTCCCTTTTGGTATCAGAGCCGTAAGAGTTGTAAGAGTCTGATCTTAGGTTGCTACTCATCCAACCCAAGTGGCTGGTTTAGGATTTCAGAGTAGAAGTATTCAGTTGCTTTTCTTGTTGTTCTTCTTTCCGGTTTTGTTATCTGTATCTCTCTTAAATAGTGTCCGAAGAGTAAGGCCTAGAGTCCAAACAGTAAGTCCCGAGGTGAGGCATGAGCGGTTGCGGCATTGAGAGGGCTAGGAGAGAGAGGTCAGTTAATAGGAACGTATAGAAGTCAGTATTAGAAGTCCCTGTTTATTTTCTCCTTTGAAGTCAGAACCCAGTTGCCAAAGTAAAATGAGTCGTTTCCTTAGATCTAATTCTTGTAGTTCAAGCAGTTCAAGAACCAGTATTAGTCGTCTTCCAGAAATAGTAAATGAGGAACAAGTTGAATTTGAATCTAATAACAATGAATTAGATTACCGAGACTGGAATATACCTAGAGTCCCCAGTCAGGAAATATATAAGAAGAGGTGGTCTTTACCAAGCTTTAAGACCACTACCCATGTTAAAACAGTCGAACAAGTTTATGCCCTTGGCAAAGAACTTGAAGTCTGTCAGTTGCTCAATCTTGATTCCATCAAACGCCATAAGAATGAAGGAAACAATTTTCTTCATATTGGTTTAGTCCAAGTTGCCGTTAAGCCACTAACCAGAACAGGGCTTAAAGCCTCAGTGTTGCTCTGGTTAAGAGATGCTAGATTCACTAACAGTGAGGACAGTACTCTCGGAGTAATTGAGTCAAGTCTTTCCAATGGTCCGGTTCACTTTGATTGTTACCCTGACTTCACTGTCAGCCTTAGTGATCCCCACATTTTGAAACCTTGACGCTCAACATCCAGACTTCTGGATATGAGGTTCTTCCGGGAACCCAACCATTAGCCTTAATCTTTAGGATCTATTATAAGGTCACTGGAACCAATATGAACTTCCAAGCCCTTAATAAGAGTCCTAAGAATCATACCTTGTTATTCAAAGTAATAGACCTGAGGCCATTATCCGAGTACCTCAAACAATCAAATGGTCTGATCTCACTCCCAGTTGAATGGACACTCAAAAATGTCAATCCACCAACACCAATCCAACGTAGTCTAAATAATTTAGATTACATCCACCAATATTAGATGGATCAGTAAAAATCAATTTTGGAACCCAACCAATTCCAAAATCTACTGTCCAGCTACAACAGTTGCCAACCCCTGGTCAATCCCAGAGGCATGTCCCTGCCAGACATTCCTTTGCAGCCTCTTCTTCTACTCTAGAACGAGACCTAGAAGCAGAAAGAGCCCTGATTGATTTCCAATTAGAATCCCTCAGGAAAGCAGCACAGGTTAGCCAACCCTGTTATGGCAAACAACGTTGAAGAACAAAAGCCAAAATCTCCCGAGTCTCCAACTCAAACTGACTTTGAAGGTTTTGATTTTGTTGACCCTCAACTTAGAGTTCTAAATAAATTTCACATTAATTGGAAAAAATTACAAGCCCATCTCACAGCTCCTGAAAACCAGAAGCGCCGAGATGATTACCATAGTGCTTTTCCACTTAAGTACCGCCGTGACGCAATCTTTGTTGAATGGAAAGATTACCTACGGAAAGAACAGGTCGAAATGCCATTTAGATTTTGTAGAAACCCACTACATGTCTAAAAAATTGAAGGTAATCACCAAAGAAACATGGTCTTTACCCAATGACACACAAGTAGTTGCCAGTCATCCCCCGGTTGAGTCTATCTTAATTAATTTTAAGAAAACTCAGATCCCTGCCTGTCCTTTCAAATCCCTGAAAAATTAGATTCAGAGAAGAAATTGATAGAACAAAGCAACTACACCAATCAGAGTCTAATTGTCATAGGAAAACAGCTTGACAAAATTGAAACTAAAGTAGACAAGCTAATCCCTTCCTCTGAAAATCCAACATTAAAGTTGAAAAACCATTAGTCCAGTTCCAGATCTCAAGACAAATCCGACTCTTAAGATCAAACCTACCCTTCAAAAAATTGAAGAAATGTGAACAATTGACACCTGTAAAGAGTGAGCCATCTGGAGTTAAAGTTCTTGACTCCATTAATCCGGATTCTGACCAAGAATCTATTCAGAGTCTAGAATCAGTTGACAGTAATATCTCAAAAATTGAGAAAGCCTTTACTGAACTTGCAGTTGAACCCAGAGTTACCCGGTTAACTCACCAAGTCACCCCTACTAGTCTTACCAAAAATTGGTATACGACCAACTCCAGATATACAATTCGAAGAAAGACATTTTCAGAACCAGTTCTCTGTCTCTTCTGATAAATTGTATGAATGGAACATTGATGGTCTTTCTGAGCAAGAAGTTTTCAATAAACTTCAACATATGTCCATGGTTGCCAATAGTTATATTTCAAACCAGTTTTAGACAATCTGAAATTGTACCCTGTTAGTCACAGGTTTTACAGGTACCCTCCGCAATTGGTGGGATAAACACCTCACACCTGAGTCTAAAAACTTAATTATCATGCTGTAAAACTTAATGATGAAGGTCTCCCTATCTTTGATGAACAAATAGGTCAAGGAATAGAAGATGGAGTCAATACTTGTTTTACACCATAATTGAGCATTTCATAGGAACACCAAGCCATACTACCTCTAGAATTCATGACCAGCTTAGCAACCTTCGTTGCCTAAACTGTCTGACTTTAGTGGTATAAAGATGTCTTCATTTCGCGAGTAATGCTCCGAAATGATAGTACAGTCGTTTTGGAAAGAAAAATTCATTAATGGTCTTCCAAACCTATTTGCCCATAAGATTCGCACAACTCTTAGTAATGAACAAGGACATATTGATTGGAATAATCTGACCTATGGAAATCTTGTCAGTACTATCAACCAAGTTGGTATGAAGATGTGCGTTGACATGAAAATTGGTAAGCAATACAGTCTGACAGAAAATCCGCTAAGTATGAGTTAGGAAATTTCTGCGAACAGTATGGTCTTACTAGCCTACCTCCCTCCAGCCGAACTAAGTCACACCATAAGAGTCAATCCCGTTCTTACCATAATCGAAAAAATAGATACCAAAATAATGATAATAGAATTCTATAAACTAAGAAACATAGCCCTAAAAAGAATTGGAAAAAATTTCCATCAAAAAATAAAAAACCGATTCCAGAACAATCTGGAAAAGGAAAAGTTGTCTGCTGGAAGTGTCAAAAACCAGGTCATTATGCTAATAAATGCAAGGTAAAAGACACAATCAAACAACTAAAAATTGAAGAAGTTGAAAAAGAAAATCTAATCCGACTTCTAGATCTAAGGATTCTGAACCTAGTGATCTCGAGTCATCAGTGACAGTGATTCTGAGAATTCTTATTCATCGACTCACAACCTAGTTCCCCCCATATCAACTTGGATGTAATGATAAGTGCTAATTCTATGAAATCTATTTCTGTTCTTACTAAACAGGAAGAACAAGAAGAGCTTCTCATAGACTTAATCTGCAAAGTCGAAAATCCCAGTTGAAGGCGAGTATCTGAAGAAATTCGAAACTCCTTAGTCAGGAAGGTCGAGTCAGTCTCCACCTCCTCAAAAGATAAGTCTAAATACAACCTAGAAAGGTTCAGTCGCAAAAAAGATGTCACCCTTCATGATTTACATTCAGAAGTAAAATAATCAAAAAGATAATTGAGCTGAAACATTAAGCCAGAAATTGCAATCTGAGAATTATGAAATCCGACAGGATTTAATAGCTCTCAAACCTGATTTATCTCAGAGTCCAACAAACTCAGATGATGAATCTTTAATCCAGAACAAGCCTTATGTCTAGTCAAACGAATTAGCTTCAAAAATGGTATGTCAAAGTTACTATTTTTGTTGATCAGTTTGAATTTACCACAGTCGCTCTACTTGATTCAGGTGCTGACCTTAACTGCATTCAGGAAGGTCTTATACCTACCAAGTATTATACTAAGTCTTGTGAATCTCTTAGAACAGCTTCAGGAAAATCCCTACAGCTGAACTATGAAATTCCCCGAGCTCATGTTTGTCAAAATAAAATTTGCTTTAAAACTTCATTTGTTTTAGTGAAAAATATCACTGATGAAGTTATTTTAGGATTACCTTTTCTTGCCTTGTTATATCCTTTCAGGTAAACCATGACGGTGTCATCTCTACCCATTTAGGTGAACAAGCCAAGTTTGAGTTTTTATCTAAGCCCGAGTTGCATCAGTTAAAAACCTTTCAGAAAAGTTCAATTTCTAAAACAGTTGCTTTAATTTCCAGTAAGACTAAACATCTGTCTTCTCTCCAGGAAGAAATTAGTCATACTCGAATCAGTCAACAAATTAGGAATGAATCTTTCCAAAAACAAGTTCATGAATTTGAAACTAAATTGATTAATGTCATTTGTTCTGATTTGCCTAATGCCTTTTGGTCCAGGAAACAACACATTGTCAGTCTTCCTTATACCAAAACCTTTAGTGAAAGAAATATCCCCACTAAAGCCAGACCAATACAAATGAATCAGGAAACAATGGAATTCTGTAAAAAAGAAATTACAGAATTACTCCTGAAGGGAATCATTCGTAAGTCTAAGTCCCCCTGGTCTTGTCCTGCCTTCTATGTCCAGAAAAATGCTGAACTAGAAAGAGGAGTCCCTAGACTAGTTATCAATTACAAACCTCTTAATGCAGTCTTAGAATGGATTAGGTACCCCATACCTAATAAAAGAGACTTAATTAATCGGTTAGAAAAAGCAGTTGTCTTTTCCAAATTTGATCTTAAGAGTGGATTCTGGCAAATCCAAATAGAAGAGTCAGATCGGTACAAAACCGCCTTTGTCACTCCTTTTGGTCATTATGAATGGAATGTTATGCCTTTCGGTCTTAAGAATGCGCCTAGCGAATTCCAGAATATTATGAATGAAATTTTCAATGCTTATAGCCATTTTTCAATTGTCTATATTGATGATGTCTTGATCTATTCCCAATCGATAGAACAGCATTGGAAACATTTGTCTATATTCCTTCAAGTTGTTAAGCAAAATGGTCTAGTTGTCTCTGCTAAGAAAATCAAGTTGTTCCAAACTAGTGTTCGATTTCTTGGCTTCAATATCTGTCAGTCTCAGATTAGCCCAATTGATCGAGTCATCCAGTTTGCTGATAAGTTTCCAGATGAAATTATTGATAAAAGTCAGCTCCAGAGGTTCTTAGGATCCCTCAATTATGTTTCTGATTTCTATCAAAATTTGAGAAAACAATGTAAGCCCTTGTTTGATAGGTTGCAGGAAAATCCTCCTCCTTGGTCCTCTGTCCACACAGAAATTGTCAAACAAATAAAAATTCATGTTAAGACACTCCCCTGTCTTGGCATTCCTTCAGTTGATTCCTTCAAGATAGTTGAAACTGATGCCTCTGACATTGGTTATGGAGGTATTCTTAAACAAAAATTGAGTCCCAATTCGCCAGAGCAAATTGTTCGTTTCCACTCTGGTATTTGGAATCAGTCGCAAAGTAATTATAGTACTATTAAGAAAGAAATATTATCTATAGTACTATGCATCTCTAAATTTCAAGATGATTTACTTAATCAACCCAAAAAAGACAAACAGAAAATTGGTGCCAGTCAGTCCAGCCAACCCAAAACCTCCCAAACACCAACCCAAAACCAGTCGCCCAAAAGAGTCCTCCCTGCATCCATGAGTCAATCCAGAATGGCCCATCCAGCCTCAATTGTCCCATATGCAGGTCATCCTCCAATCCCAGTCGTCCCAATTGCAAACAGATTCACCAGCCTTGGAACCCCAATAGGATCCACCGTTGGCCCAATCCGCCCCAATTATCAGTCAGCCCTTGTTTCTGACTATGATCCATTTGCAATAGTCCCTCCAATTGTTCCAATTGCCCAAACTTCCCAGTCGTCTACCCAGTTCAGAAAGTCGTCCCCTTATTTCCCAAAAGATAAGGTTAATTTATTCATAATCGAACCCAATTATGATGGGATTCTGACCCGATAAACATTGTTAGGTCATATTTCCCTCCTAATTCCCATTTCCTTCCTCCTGCACCTTATAAAGACCTCAAATTTTACAGAGATATCCTGCATGAGACCAAATCAGTTGAAATTAAGCCCATTAAGGATAGAGATAACCCTGCGGTCATCCTCTATCATTCCCTTTATATTCAGCAATTTCTAAGCCAAGCTGAGTTCAGTCGCCACCCCTATGAATTGAAACACCTTAAGTCCACTCATCCTTATACCTATGCAGATTACATTGATGCATGGTATAATATCTTCCTCCATCAAACAGTTGATTTTTCCCACTCCTGGTTCATTAATTTCGATGCCAAATTCAGGAGTCCTTTCCCCTACTGGTTTCTTCATTGGTGGGATCAACATGGCCCAACAGCTGATGTCCTTCCTACTGAAGTCCATAATCTGACCAATTATTATGCCAGTAAATCCCAATTTAATAAACAACAATTGTTCTTCCCCAAGTTGCTTCTTTTTATTGCCAGGTACAAAATTCCCTGGATTATGAAGTGGAATTATCATGTCAATTGGGAAACCAGAATCATGTCTCGCCAGTATTTTGTCAAGTGGTGGGATAAGTTCTCAATGGACAGAATTTCCATCTATGTCTTCAAGGATTTCCCCCAATCGGATCATCCTCCCAAGCCAAGCTCTCCCACAGGCTCAGTCAGTTCGGCCATCTCAATCGATGGAAAATCCAAAGCTGAACTCCGGGCCCTTGCTGCACAATTGCTTGACCAAGCCAGTCAGATGAATGATGATGAGGACAACGAATCTTCCCCATCTGCATCCAGCTGTCAGCCTGGCTCCAGCTCCTTTGATCCCAATATCCCTTTTGCGCAGGATCCTTATGACGCGTATGATTTGGGATCTGATTAGTCAAAAGTTGCCATCACTTCCTCTTTTCACCCCGGTGAAATGTTGGAATTAGAATAATTGTGTTGGAATTAGAATAATTGTAACGTAAAAACAAAATTGCTTTTGCTTTTACCCTTACTTTACTCCAATAGGTAAAGAGAGTAAAAACAAAAATACTTTTGCTTTTACCCCTGCTTTACCCCAATAGGTAAAGAGAAAGTTGTTTTCCGTGCTTGAAACCATAGTTACTCTCCAATCAGGGTTACTTTGGATTCAAACCTTAGTTACTTTCTTCTCTTTTTAAGCCGGCTTCGGCCTCAGTTGTAAGCACTTGTAATTTTTCTTTTCTTCTTCAGTTTTAAAAGTTCTCTCTGAAACTCCTTCAGACATGAACTCTCCTGTAAATATCTGAGTGTTTAAAGCTTTCAATAAAAGGCAGCTTTGTAAGTAGTTCCAAACCTATCTTATTTTCATTTATTCATTTATTTTTGGTCCAGGAAACAACACATTGTTAGTCTTCCTTATACCAAAACCTTTAGTGAAAGAAATATCCCCACTAAAGCCAGACCAATACAAATGAATCAGGAAACAATGGAATTCTGTAAAAAAGAAATTACAGAATTACTCCTGAAGGGAATCATTCGTAAGTCTAAGTCCCCCTGGTCTTGTCCTGCCTTCTAATACTGACTTCTATACGTTCCTATTAACTGACCTCTCTCTCCTAGCCCTCTCAATGCCGCAACCGCTCATGCCTCACCTCGGGACTTACTGTTTGGACTCTAGGCCTTACTCTTCGGACACTATTTAAGAGAGATACAGATAACAAAACCGGAAAGAAGAACAACAAGAAAAGCAACTGAATACTTCTACTCTGAAATCCTAAACCAGCCACTTGGGTTGGATGAGTAGCAACCTAAGATCAGACTCTTACAACTCTTACGGCTCTGATACCAAAAGGGAAGGACCAACATTAATAGAAGAACAGAGGATAGTTTAGGGAATTCAAGAAAGAATTTGAATTATAACAAACCTAAGCAACTTTCAGACTGATGCAAGAAATTAATGCATAAGAAAATAAATGAATAAATGAAAATAAGATAGGTTTGGAACTACTTACAAAGCTGCCTTTTATTGAAAGCTTTAAACACTCAGATATTTACAGGAGAGTTCATGTCTGAAGGAGTTTCAGAGAGAACTTTGAAAACTGAAGAAGAAAAGAAAAATTACAAGTGCTTACAACTGAGGCCGAAGCCGGCTTAAAAAGAGAAGAAAGTAACTAAGGTTTGAATCCAAAGTAACCCTGATTGGAGAGTAACTATGGTTTCAAGCACGGAAAACAACTTTCTCTTTACCTATTGGGGTAAAGCAGGGGTAAAAGCAAAAGTATTTTTGTTTTTACTCTCTTTACCTATTGGGGTAAAGTAAGGGTAAAAGCAAAAGCAATTTTGTTTTTACGTTACAATTATTCTAATTCCAACACAATTATTCTAATTCCAACATTTCACCGGGGTGAAAAGAGGAAGTGATGGCAACTTTTGACTAATCAGATCCCAAATCATACGCGTCATAAGGATCCTGCGCAAAAGGGATATTGGGATCAAAGGAGCTGGAGCCAGGCTGACAGCTGGATGCAGATGGGGAAGATTCGTTGTCCTCATCATCATTCATCTGACTGGCTTGGTCAAGCAATTGTGCAGCAAGGGCCCGGAGTTCAGCTTTGGATTTTCCATCGATTGAGATGGCCGAACTGACTGAGCCGTAAGAGTTGTAAGAGTCTGATCTTAGGTTGCTACTCATCCAACCCAAGTGGCTGGTTTAGGATTTCAGAGTAGAAGTATTCAGTTGCTTTTCTTGTTGTTCTTCTTTCCGGTTTTGTTATCTGTATCTCTCTTAAATAGTGTCCGAAGAGTAAGGCCTAGAGTCCAAACAGTAAGTCCCGAGGTGAGGCATGAGCGGTTGCGGCATTGAGAGGGCTAGGAGAGAGAGGTCAGTTAATAGGAACGTATAGAAGTCAGTATTAGAAGTCCCTGTTTATTTTCTCCTTTGAAGTCAGAACCCAGTTGCCAAAGTAAAATGAGTCGTTTCCTTAGATCTAATTCTTGTAGTTCAAGCAGTTCAAGAACCAGTATTAGTCGTCTTCCAGAAATAGTAAATGAGGAACAAGTTGAATTTGAATCTAATAACAATGAATTAGATTACCGAGACTGGAATATACCTAGAGTCCCCAGTCAGGAAATATATAAGAAGAGGTGGTCTTTACCAAGCTTTAAGACCACTACCCATGTTAAAACAGTCGAACAAGTTTATGCCCTTGGCAAAGAACTTGAAGTCTGTCAGTTGCTCAATCTTGATTCCATCAAACGCCATAAGAATGAAGGAAACAATTTTCTTCATATTGGTTTAGTCCAAGTTGCCGTTAAGCCACTAACCAGAACAGGGCTTAAAGCCTCAGTGTTGCTCTGGTTAAGAGATGCTAGATTCACTAACAGTGAGGACAGTACTCTCGGAGTAATTGAGTCAAGTCTTTCCAATGGTCCGGTTCACTTTGATTGTTACCCTGACTTCACTGTCAGCCTTAGTGATCCCCACATTTTGAAAACCTTGACGCTTAACATCCAGACTTCTGGATATGAGGTTCTTCCGGGAACCCAACCATTAGCCTTAATCTTTAGGATCTATTATAAGGTCACTGGAACCAATATGAACTTCCAAGCCCTTAATAAGAGCCCTAAGAATCATACCTTATTAATTCAAAGTAATAGACCTGAGGCCATTATCCGAGTACCTTAAACAATCAAATGGTCTGACGTCTCACTCCCAGTTGAATGGACGCTCAAAAATGTTAATCCACCAACACCAATCCAACGTAGTCTAAACAATTTAGATTACATCCACCAGTACTTAGATGGATCAGTAAAAATCAATTTTGGGACCCAACCAGTTCCAAAATCTACTGTCCAGCTACAGCAGTTGCCAACCCCTGGTCAATCCCAGAGGCATGTCCCTGCCAGACATTCCTTTGCAGCCTCTTCTTCTACTCTAGAACGAGACCTAGAAGCAGAAAGAGCCCTGATTGATTTCCAATTAGAATCCCTCAGGAAAGCAGCACAGGTTAGCCAACCCTGTTATGGCAAACAACCGGTTGAAGAACAAAAGCCAAAATCTCCCGAGTCTCCAACTCAAACTGACTTTGAAGGTTTTGATTTTGTTGATCCGCAACTCAGAGTTCTAAATAAATTTCACATTAATTGGAAAAAATTACAAGCCCATCTCACAGCTCCTGAAAACCAGAAGCGCCGAGATGATTACCATAGTGCTTTTCCACTTAAGTACCGCCGTGACGCAATCTTTGTTGAATGGAAAGATTACCTACGGAAAGAACAGGTCGAAATGCCCTATTTAGATTTTGTAGAAACCCACTACATGTCTAAAAAATTGAAGGTAATCACCAAAGAAACATGGTCTTTACCCAATGACACACAAGTAGTTGCCAGTCACCCGGTTGAGTCTATCTTAATTAATTTTAAGAAAACTCAGATCCCTGCCTGTCCTTTCAAAATCCCTGAAAAATTAGATTCAGAGAAGAAATTGATAGAACAAAGCAACTACACCAATCAGAGTCTAATTGTCATAGGAAAACAGCTTGACAAAATTGAAACTAAAGTAGACAAGCTAATCCCTTCCTCTGAAACATCCAACATTAAAGTTGAAAAACCATTAGTCCAGTTCCAAGATCTCAAGACAAATCCGACTCTTAAGATCAAACCTACCCTTCAAAAAATTGAAGAAATGTTAGAACAATTGACACCTGTAAAGAGTGAGCCATCTGGAGTTAAAGTTCTTGACTCCATTAATTCGGATTCTGACCAAGAATCTATTCAGAGTCTAGAATCAGTTGACAGTAATATCTCAAAAATTGAGAAAGCCTTTACTGAACTTGCAGTTGAACCCAGAGTTACCCGGTTAACTCACCAAGTCACCCCTACTAGTCTCACCAAAAATTGGTATACTAGACCAACTCCTCCAGATATACAATTTGAAGAAAGACATTTTCAGAACCAGTTCTCTGTCTTTCTGATAAATTGTATGAATGGAACATTGATGGTCTTTCTGAGCAAGAAGTTTTCAATAAACTTCAACATATGTCCATGGTTGCCAATAGTTATATTTCAAACCACAGTTTCAGACAATCTGAAATTGTACCCCTGTTAGTCACAGGTTTTACAGGAACCCTCCGCAATTGGTGGGATAAACACCTCACACCTGAGTCTAAAAACTTAATTATCCATGCTGTAAAACTTAATGAAGAAGGTCTCCTATCTTTGATGAACAAATAGGTCAAGGAATAGAAGATGGAGTCAATACTTTGTTTTACACCATAATTGAGCATTTCATAGGAACACCAAGCCATACTACCTCTAGAATTCATGACCAGCTTAGCAACCTTCGTTGTCCTAAACTGTCTGACTTTAGATGGTATAAAGATGTCTTCATTTCGCGAGTAATGCTCCGAAATGATAGTACCCAGTCGTTTTGGAAAGAAAAATTTATTAATGGTCTTCCAAATCTATTTGCCCATAAGATTCGCACAACCCTTAGTAATGAACAAGGACATATTGATTGGAATAATCTGACCTATGGAAATCTAGTCAGTACTATCAACCAAGTCGGTATGAAGATGTGCGTTGACATGAAAATTGGTAAGCAAATACAGTCCGATAGAAAATCCGCTAAGTATGAGTTAGGAAATTTCTGCGAACAGTATGGTCTTACTAGCCTACCTCCCTCCAGTCGAACTAAGTCATACCATAAGAGTCAATCCCGTTCTTACCATAATCGAAAAAATAGATACCAAAATAATGATAATAGGGAATTCTATAAAACTAAGAAACATAGCCCTAAAAAGAATTGGAAAAAATTTCCATCAAAAAATAAAAAACCCCGATTCCAGAACAATCCTGGAAAAGGAAAAGTTGTCTGCTGGAAGTGTCAAAAACCAGGTCATTATGCTAATAAATGCAAGGTAAAGACACAATCAAACAACTAAAAATTGAAGAAGTTGAAAAAGAAAATCTAATCCGACTTCTAGATCTAAAGGATTCTGAACCCAGTGATCTCGAGTCATCAGTTGACAGTGATTCTGAGAATTCTTATTCATCAGACTCACAACCTAGTTCTCCCCATATCCAACTTGGATGTAATGATAAGTGCTGTAATTCTATGAAATCTATTTCTGTTCTTACTAAACAGGAAGAACAAGAAGAGCTTCTCATAGACTTAATCTGCAAAGTCGAAAATCCTGAGTTGAAGGCGGAGTATCTGAAGAAATTCAGAAAACTCCTTAGTCAGGAAGGTTCGAGTCAGTCTCCACCTCCTCAAAAGATAAGTCTAAATACAACCCTAGAAAGGTTCAGTCGCAAAAAAGATGTCACCCTTCATGATTTACATTCAGAAGTGAAACTAATCAAAAAGGAGATAATTGAGCTGAAACATTTAAGCCAGAAATTGCAATCTGAGAATATGAAATCCGACAGGATTTAATAGCTCTCAAACCTGATTTATCTCAGAGTCCAACAAACTCAGATGATGAATCTTTAAATCCAGAACAAGCCTTATGTCTAGTCAAACGAATTAGCTTCAAAAAATGGTATGTCAAAGTTACTATTTTTGTTGATCAGTTTGAATTTACCACAGTCGCTCTACTTGATTCAGGTGCTGACCTTAACTGCATTCAGGAAGGTCTTATACCTACCAAGTATTATACTAAGTCTTGTGAATCTCTTAGAACAGCTTCAGGAAAATCCCTACAGCTGAACTATGAAATTCCCCGAGCTCATGTTTGTCAAAATAAAATTTGCTTTAAAACTTCATTTGTTTTAGTGAAAAATATCACTGATGAAGTTATTTTAGGATTACCTTTTCTTGCCTTGTTATATCCTTTTCAGGTAAACCATGACGGTGTCATCTCTACCCATTTAGGTGAACAAGCCAAGTTTGAGTTTTTATCTAAGCCCGAGTTGCATCAGTTAAAAACCTTTCAGAAAAGTTCAATTTCTAAAACAGTTGCTTCAATTTCCAGTAAGACTAAACATCTGTCTTCTCTCCAGGAAGAAATTAGTCATACTCGAATCAGTCAACAAATTAGGAATGAATCTTTCCAAAACAAGTTCATGAATTTGAAACTAAATTGATTAATGTCATTTGTTCTGATTTGCCTAATGCCTTTTGGTCCAGGAAACAACACATTGTCAGTCTTCCTTATACCAAAACCTTTAGTGAAAGAAATATCCCCACTAAAGCCAGACCAATACAAATGAATCAGGAAACAATGGAATTCTGTAAAAAAGAAATTACAGAATTACTCCTGAAGGGAATCATTCGTAAGTCTAAGTCCCCCTGGTCTTGTCCTGCCTTCTATGTCCAGAAAAATGCTGAACTAGAAGAGGAGTCCCTAGACTAGTTATCAATTACAAACCTCTTAATGCAGTCTTAGAATGGATTAGGTACCCCATACCTAATAAAAGAGACTTAATTAATCGGTTAGAAAAAGCAGTTGTCTTTTCCAAATTTGATCTTAAGAGTGGATTCTGGCAAATCCAAATAGAAGAGTCAGATCGGTACAAAACCGCCTTTGTCACTCCTTTTGGTCATTATGAATGGAATGTTATGCCTTTCGGTCTTAAGAATGCGCCTAGCGAATTCCAGAATATTATGAATGAAATTTTCAATGCTTATAGCCATTTTTCAATTGTCTATATTGATGATGTCTTGATCTATTCCCAATCGATAGAACAGCATTGGAAACATTTGTCTATATTCCTTCAAGTTGTTAAGCAAAATGGTCTAGTTGTCTCTGCTAAGAAAATCAAGTTGTTCCAAACTAGTGTTCGATTTCTTGGCTTCAATATCTGTCAGTCTCAGATTAGCCCAATTGATCGAGTCATCCAGTTTGCTGATAAGTTTCCAGATGAAATTATTGATAAAAGTCAGCTCCAGAGGTTCTTAGGATCCCTCAATTATGTTTCTGATTTCTATCAAAATTTGAGAAAACAATGTAAGCCCTTGTTTGATAGGTTGCAGGAAAATCCTCCTCCTTGGTCCTCTGTCCACACAGAAATTGTCAAACAAATAAAAATTCATGTTAAGACACTCCCCTGTCTTGGCATTCCTTCAGTTGATTCCTTCAAGATAGTTGAAACTGATGCCTCTGACATTGGTTATGGAGGTATTCTTAAACAAAAATTGAGTCCCAATTCGCCAGAGCAAATTGTTCGTTTCCACTCTGGTATTTGGAATCAGTCGCAAAGTAATTATAGTACTATTAAGAAAGAAATATTATCTATAGTACTATGCATCTCTAAATTTCAAGATGATTTACTTAATCAAAAATTCTTAATACGAGTTGATTGCAAATCTGCAAAACATGTTTTAGAAAAAGATGTTCAAAACATTGCATCTAAACAGATTTTTGCACGATGGCAAAGTATTTTAAGTATTTTTGATTTTGACATTGAGTATTTGAGAGGCAGTGAGAATTCTGTTCCTGATTTTCTTACTCGAGAATTCTTGCAGGGAAAGCAATGGCACCCAAAAAAGACAAACAGAAAATTGGTGCCAGTCAGTCCAGCCAACCCAAAACCTCCCAAACACCAACCCAAAACCAGTCGCCCAAAAGAGTCCTCCCTGCATCCATGAGTCAATCCAGAATGGCCCATCCAGCCTCAATTGTCCCATATGCAGGTCATCCTCCAATCCCAGTCGTCCCAATTGCAAACAGATTCACCAGCCTTGGAACCCCAATAGGATCCACCGTTGGCCCAATCCGCCCCAATTATCAGTCAGCCCTTGTTTCTGACTATGATCCATTTGCAATAGTCCCTCCAATTGTTCCAATTGCCCAAACTTCCCAGTCGTCTACCCAGTTCAGAAAGTCGTCCCCTTATTTCCCAAAAGATAAGGTTAATTTATTCATAATCGAACCCAATTATGATGGGATTTCTGACCCGATAACAATTGTTAGGTCATATTTCCCTCCTAATTCCCATTTCCTTCCTCCTGCACCTTATAAAGACCTCAAATTTTACAGAGATATCCTGCATGAGACCAAATCAGTTGAAATTAAGCCCATTAAGGATAGAGATAACCCTGCGGTCATCCTCTATCATTCCCTTTATATTCAGCAATTTCTAAGCCAAGCTGAATTCAGTCGCCACCCCTATGAATTGAAACACCTTAAGTCCACTCATCCTTATACCTATGCAGATTACATTGATGCATGGTATAATATCTTCCTCCATCAAACAGTTGATTTTTCCCACTCCTGGTTCATTAATTTCGATGCCAAATTCAGGAGTCCTTTCCCCTACTGGTTTCTTCATTGGTGGGATCAACATGGCCCAACAGCTGATGTCCTTCCTACTGAAGTCCATAATCTGACCAATTATTATGCCAGTAAATCCCAATTTAATAAACAACAATTGTTCTTCCCCAAGTTGCTTCTTTTTATTGCCAGGTACAAAATTCCCTGGATTATGAAGTGGAATTATCATGTCAATTGGGAAACCAGAATCATGTCTCGCCAGTATTTTGTCAAGTGGTGGGATAAGTTCTCAATGGACAGAATTTCCATCTATGTCTTCAAGGATTTCCCCCAATCGGATCATCCTCCCAAGCCAAGCTCTCCCACAGGCTCAGTCAGTTCGGCCATCTCAATCGATGGAAAATCCAAAGCTGAACTCCGGGCCCTTGCTGCACAATTGCTTTTTCTAACCGATTAATTAAGTCTCTTTTATTAGGTATGGGGTACCTAATCCATTCTAAGACTGCATTAAGAGGTTTGTAATTGATAACTAGTCTAGGGACTCCTCTTTCTAGTTCAGCATTTTTCTGGACATAGAAGGCAGGACAAGACCAGGGGGACTTAGACTTACGAATGATTCCCTTCAGGAGTAATTCTGTAATTTCTTTTTTACAGAATTCCATTGTTTCCTGATTCATTTGTATTGGTCTGGCTTTAGTGGGGATATTTCTTTCACTAAAGGTTTTGGTATAAGGAAGACTGACAATGTGTTGTTTCCTGGACCAAAAGGCATTAGGCAAATCAGAACAAATGACATTAATCAATTTAGTTTCAAATTCATGAACTTGTTTTTGGAAAGATTCATTCCTAATTTGTTGACTGATTCGAGTATGACTAATTTCTTCCTGGAGAGAAGACAGATGTTTAGTCTTACTGGAAATTGAAGCAACTGTTTTAGAAATTGAACTTTTCTGAAAGGTTTTTAACTGATGCAACTCGGGCTTAGATAAAAACTCAAACTTGGCTTGTTCACCTAAATGGGTAGAGATGACACCGTCATGGTTTACCTGAAAAGGATATAACAAGGCAAGAAAAGGTAATCCTAAAATAACTTCATCAGTGATATTTTTCACTAAAACAAATGAAGTTTTAAAGCAAATTTTATTTTGACAAACATGAGCTCGGGGAATTTCATAGTTCAGCTGTAGGGATTTTCCTGAAGCTGTTCTAAGAGATTCACAAGACTTAGTATAATACTTGGTAGGTATAAGACCTTCCTGAATGCAGTTAAGGTCAGCACCTGAATCAAGTAGAGCGACTGTGGTAAATTCAAACTGATCAACAAAAATAGTAACTTTGACATACCATTTTTTGAAGCTAATTCGTTTGACTAGACATAAGGCTTGTTCTGGATTTAAAGATTCATCATCTGAGTTTGTTGGACTCTGAGATAAATCAGGTTTGAGAGCTATTAAATCCTGTCGGATTTCATAATTCTCAGATTGCAATTTCTGGCTTAAATGTTTCAGCTCAATTATCTCCTTTTTGATTAGTTTCACTTCTGAATGTAAATCATGAAGGGTGACATCTTTTTTGCGACTGAACCTTTCTAGGGTTGTATTTAGACTTATCTTTTGAGGAGGTGGAGACTGACTCGAACCTTCCTGACTAAGGAGTTTTCTGAATTTCTTCAGATACTCCGCCTTCAACTCAGGATTTTCGACTTTGCAGATTAAGTCTATGAGAAGCTCTTCTTGTTCTTCCTGTTTAGTAAGAACAGAAATAGATTTCATAGAATTACAGCACTTATCATTACATCCAAGTTGGATATGGGGAGAACTAGGTTGTGAGTCTGATGAATAAGAATTCTCAGAATCACTGTCAACTGATGACTCGAGATCACTGGGTTCAGAATCCTTTAGATCTAGAAGTCGGATTAGATTTTCTTTTTCAACTTCTTCAATTTTTAGTTGTTTGATTGTGTCTTTTACCTTGCATTTATTAGCATAATGACCTGGTTTTTGACACTTCCAGCAGACAACTTTTCCTTTTCCAGGATTGTTCTGGAATCGGGGTTTTTTATTTTTTGATGGAAATTTTTTCCAATTCTTTTTAGGGCTATGTTTCTTAGTTTTATAGAATTCCCTATTATCATTATTTTGGTATCTATTTTTTCGATTATGGTAAGAACGGGATTGACTCTTATGGTATGACTTAGTTCGACTGGAGGGAGGTAGGCTAGTAAGACCATACTGTTCGCAGAAATTTCCTAACTCATACTTAGCGGATTTTCTATCGGACTGTATTTGCTTACCAATTTTCATGTCAACGCACATCTTCATACCGACTTGGTTGATAGTACTGACTAGATTTCCATAGGTCAGATTATTCCAATCAATATGTCCTTGTTCATTACTAAGGGTTGTGCGAATCTTATGGGCAAATAGATTTGGAAGACCATTAATAAATTTTTCTTTCCAAAACGACTGGGTACTATCATTTCGGAGCATTACTCGCGAAATGAAGACATCTTTATACCATCTAAAGTCAGACAGTTTAGGACAACGAAGGTTGCTAAGCTGGTCATGAATTCTAGAGGTAGTATGGCTTGGTGTTCCTATGAAATGCTCAATTATGGTGTAAAACAAAGTATTGACTCCATCTTCTATTCCTTGACCTATTTGTTCATCAAAGATAGGGAGACCTTCTTCATTAAGTTTTACAGCATGGATAATTAAGTTTTTAGACTCAGGTGTGAGGTGTTTATCCCACCAATTGCGGAGGGTTCCTGTAAAACCTGTGACTAACAGGGGTACAATTTCAGATTGTCTGAAACTGTGGTTTGAAATATAACTATTGGCAACCATGGACATATGTTGAAGTTTATTGAAAACTTCTTGCTCAGAAAGACCATCAATGTTCCATTCATACAATTTATCAGAAGAGACAGAGAACTGGTTCTGAAAATGTCTTTCTTCAAATTGTATATCTGGAGGAGTTGGTCTAGTATACCAATTTTTGGTGAGACTAGTAGGGGTGACTTGGTGAGTTAACCGGGTAACTCTGGGTTCAACTGCAAGTTCAGTAAAGGCTTTCTCAATTTTTGAGATATTACTGTCAACTGATTCTAGACTCTGAATAGATTCTTGGTCAGAATCCGGATTAATGGAGTCAAGAACTTTAACTCCAGATGGCTCACTCTTTACAGGTGTCAATTGTTCTAACATTTCGTCAATTTTTTGAAGGGTAGGTTTGATCTTAAGAGTCGGATTTGTCTTGAGATCTTGGAACTGGACTAATGGTTTTTCAACTTTAATGTTGGATGTTTCAGAGGAAGGGATTAGCTTGTCTACTTTAGTTTCAATTTTGTCAAGCTGTTTTCCTATGACAATTAGACTCTGATTGGTGTAGTTGCTTTGTTCTATCAATTTCTTCTCTGAATCTAATTTTTCAGGGATTTTGAAAGGACAGGCAGGGATCTGAGTTTTCTTAAAATTAATTAAGATAGACTCAACCGGGGGATGACTGGCAACTACTTGTGTGTCATTGGGTAAAGACCATGTTTCTTTGGTGATTACCTTCAATTTTTTAGACATGTAGTGGGTTTCTACAAAATCTAAATAGGGCATTTCGACCTGTTCTTTCCGTAGGTAATCTTTCCATTCAACAAAGATTGCGTCACGGCGGTACTTAAGTGGAAAAGCACTATGGTAATCATCTCGGCGCTTCTGGTTTTCAGGAGCTGTGAGATGGGCTTGTAATTTTTTCCAATTAATGTGAAATTTATTTAGAACTCTGAGTTGCGGATCAACAAAATCAAAACCTTCAAAGTCAGTTTGAGTTGGAGACTCGGGAGATTTTGGCTTTTGTTCTTCAACCGGTTGTTTGCCATAACAGGGTTGGCTAACCTGTGCTGCTTTCCTGAGGGATTCTAATTGGAAATCAATCAGGGCTCTTTCTGCTTCTAGGTCTCGTTCTAGAGTAGAAGAAGAGGCTGCAAAGGAATGTCTGGCAGGGACATGCCTCTGGGATTGACCAGGGGTTGGCAACTGCTGTAGCTGGACAGTAGATTTTGGAACTGGTTGGGTCCCAAAATTGATTTTTACTGATCCATCTAAGTACTGGTGGATGTAATCTAAATTGTTTAGACTACGTTGGATTGGTGTTGGTGGATTAACATTTTTGAGCGTCCATTCAACTGGGAGTGAGACGTCAGACCATTTGATTGTTTGAGGTACTCGGATAATGGCCTCAGGTCTA

TATATTATATATATTATAAAATACATATATACATATATATAGATATTTTTGAAAAAATAAAAAAATCTAGTCCTAGTCCAAGCATACACCAAACGCAGGATAAATGTTATCCAACTTAGACCAAGCCAAGCCAAGCCAAGACAATCCCTAAACATAGTTCATATCAAGACAGTTCTGTATAACAAACGAGCCCTAAACTTTTGAGGACAAGCAAAAGTAGGGGCTCGCCATTAGAAAGAACGAAAAAAAAAAAAAAGGTTACTGTTTAGGTAGTTGCACTAGGACATCAAGCATTGGACACTCATGAGTAGCAAGTCGGCCATATGAGTACCTTATATGCAACAAACTCACACACAATAATTAATGTTGTGCAAGCAACAAATAATAAATGGCAACACATAAAATAGTTGTAAAGAGAACAATCTCACAAGGCAACACACGAGGTAACGAATGATTGAACAAAAGCTTCTTTATATTGATTGGCAAGAGAGTACAATGTATGATCTTAGGCTATCTCCAGTCATTGGCTATATCTCAAATATAGCCCTAAAATAATGCAAAATCCATTTCTAGTTATGGACTAAACAAAATTGTGGCCAAATTTGGAGGGCCGCATTTGGCCATTTAGCCCTCCAACCAGGCCACATTTAGCCCAGCTTTGGCTTAGGCCAAATTTTTTGTGGTCCCATACATGTGCTTTTGAAGAAGATAAATTATCAAGTGTGGGTAAAAGCACGAATCATCTCGTGCAAATGCATATCCAACAACCAACAATAAAAGTGTTGACGTCATCAATATATTTTTAAAAGCTTTTTTAAATGTTTTCAATTCGTGTTGTTTAAGAAAAATTTATAAAAATTATTGGTCATAACCATTGGAGGTTAGACTATTGAAACCCAACTATTTTTTATTATTATAAATGTGGTCACCTTTTTAAATATTTTTAATCAGTAAAAAAATTATCAAAAATTACTAGTCATGACCGTTGGAGGTCAAACCA

>Chr1-30; OZ022206.1; 47513054-47514324; rev; B;

ATAAAAACACAAGAAACTGCAATACTGCACTATGGGAATATTTTGTCAAGAAGCGCCTAAAAACAAAACAAAACAAAACAAAAAGAAAAACAAAAAAGGCAGGAGGGAATGAGGGAGGGAGGGAGGGAGGGAGAGAGAGAGTCACTGTTTTTCCAAGTAGCATGTCACCAATTGGGTCTCCAACCTTTCTCTGCTTTATAGCAATTTGCATCTCATTCTACAGTTGCAAAGAAAAAAAAAATTAGAGACAAATGAGCACACAAAAATATTCACAACTAAATTCAAGAGAATTAATGAATTATCATTTTGATAGGGAACATAAACTTTTATTAAATTAAGTAAAAGATAAATTACAGGAAACGCCAGAAATATTGCAACATATACATGAATAGCAGGCTAGCACCTTATAAATCACAACCAATTAAGATGAATATGAACAGAAGGGTATCTCTAGTATGACAGGGTGAATTTCAAGAAATTTTCTCTATTCAAGTTCTACCCAAATATTAAATTTCAAGAAATAGTATGAGAAGATGTAAGGAAGCAAGACAACACATCAATGCATTATGATTCAGAGATAACCAATATAAATAGGGGAACAAAGATTGGAAAGATAAATCACCATACCAAACATCTTTTCCAAAACTGAATTTTAGCACCATTTCCTAGCAGAAAATTAGCTGATACTAAGGAGAATAGCTAGGGCATTACCACTATTGCTTCCCAAATATTTAAACAAGGGTTACTCCAAAAAATCACAACCCAAAACCCTTCCTAAGCACAACAATTTCCTTTCTCAATGTTCATACCCACCATTAACTTTTGACGTTTAAATATGAGACTAACCCCAAATATGGAATATTTATAACCAAATTAAAATATATGTATATGTATCGTCCTTTGGTCTTCGGCCACCAGTATATCAACCATATATATATATATATATATATATATATATATATATATATATATATACCTACACCTGGTTCTAGATCCCTTC

TGGTATCAGAGCCGTAAGTGTTGAAGAGTTAGTCTAAGTCTGCAACTCATCCAACCCAAGTGGCTGGTTTAGGTTTTCCGAGTTGTAAGTTTTCAGTTTCTTCTCATTCTACTTCTGGTCTTTCAGTTTACCTGTCCTTCTCTCTCTTGGTGTCCGAATTATAAGACCCGTTCCAAACAGTAAGTCCCAGGGAGGCCTGAGCGGTAGTGGCATTATTAGGGGGGTGAGAGAATTTTAGGTATCCTGTTATCCCATGGAGTCAGTCTTAGGAGTTTCTGTTCACTTTCTTCTTTAGAAATCCAACCCCAGAACCTTGAGTAAAAATGAGTCGTTTATTTAGAACTAATTCTTGCAATTCTAGCAGCTCTAGAGCCAACAGTTGTCTCCCAGAGATAGTGAATGAAGAACAAGTTGAGTATGATAATAGTAATAATAATTTAGATTATAAAGATTGGAATATACCAAGAGTCCCCAGTCAGGACATCTATAAGAAAAAATGGTCTTTATCCAGTTTTAAGTCAACCACCCATGTTAAAACGGTCGAACAAGTTTATGCCCTGAGTAAGAATCATGAGACCTGCCAGTTACTTAACCTTGAGTCAATAAGAAAACATAAAAATGATGGAAACAATTTCCTCCATAATGGTCTAGTCCAAGTCGCTGTTAAGCCATTAACCAGATTAGGACTAAAAGCTTTAATCCTTCTCTGTTTGTGTGATGCTAGATTTACTAATTTCAACGACAGTACATTAGGAATTATTCAGTCTAGTCTTGCTAATGGTCCTATCCATTTTGATTGCTACCCAGATTTTACAGTCAGCCTGAGTGATCCAAACATATTAAAAGCCCTTACTTTAAATATTAAAACTGAAGGTTATAAGGTCTTGCCGGGTACCCAACCTGTAGCTTTAGTCTATCGAATTTATTACAAAGTCACAGGCACCAATAGGAATTTCCAAGCCCTAAACAAGAGTACAAAGATTCAAACATTGTTAATCCAAAGTCATACGCCTGAATGCTACTATTAGAGTTCCTCACTCTATCAAATGGTCAGATGTTACCCTTCCTGCTGATTGGACACTAACCACTGAGATTCCTGCTGTACCCATCCAGCATAGTCTAAACGATTTAGACTATAGACAACAATATTTAGATGGCACAATAAAAATCAATTTTGGTAATCAACCTTCACAAAAGTCATCAGTCCAGTTACAACAGTTACCTACAACTGGTCAATCCCAGAGACATGTTCCTGCAAGGCATTCCTTTGC

AAACTTCTTATTAAGTGTTCTTAATTGATTACCTATGAAATCGGATTGAGTAGGTGAATCAGGGGACTCAGACTTAGTATCTTCCTGGACTGGGTTTTTCCCATAAACAGGATAAGTAACTTGGGAAGTTTTTCTCAAAGATTCTAATTTTAAATCTATTAAAGCTTTCTCTAACTCTAGGTCTCGTTCTAGGGTATATATATATATATATATTGTTGTCCTTTCCCAGAGTAAAAAAGACTGTGTCATCTACAAACTGCGAATGAGATACGTGTGTTCACTCCCTCCCCACAACTAAACCCTTAATCAAGGTAAAATTTAACATCGTGAACAAACTACTTCAGAGATCACCCTGTATGAGACCCTTAGAAGTTTAAATCCATCCCTACCACTAATAGTAAATGAAACAAAAGTTGAACTCAAGCATCCACTCCTCCCCCTCCTCCATTACTATCAACATCTTTCCTTTCAAACAAACAATTTAGAGAACCGCAAACACCATGACCACTTTTGTAAACCAATTTTAATCACCACTCCCTCCTTATTTTTTTAAACTAGATATTATTGAACCACTTCACATAAAGAAAATGTCTAAATATGTCTACCCCTTCTTGAATGCCAAAGCCAAACACTGATTTGGGATGTAGCTAGTTACACTATACACACAGAAAAGTGAAGGAGTTAGGAAGCCTAGTGACATATCCCTCTAAGTACTAAATCATAAAATTTGGAGTTGGATTATTGTTTGTGATAGAGTGACTTGTGACATTATCCAAACGAGGAGGCCTAACTGAAGTCTAACCAGTGTTGGGCATAGTATTGTCTTAAGGCCATAGTGTCAAACACTAGCCTCAACAAAGTAAAGTTTTTCTACAATTTTTCTGCCTCTCTTACTTGGTATTTGTTGTATGTTGTAGAATTTTGATCTTTCAATTACATGTTTATTTGAGCTTTGTATTTTACAAATTAATGTAGCAATATTACATCTATTTTTCTAACA

>Chr1-31; OZ022206.1; 47952906-47960364; dir; A;

GGATCGATTTTTGCTTAAAATTAAAACCAATCCTGTACTATTTATCCGGTTTAGTTCGGTTTGATTTTAATAAAAGAATTTTAAAAATTGTCCGGTTCGAGTTGAACCAGGTTTCGGTCCGATTTGGTTTTGAACCGGATCATAAAAATTACTTTTTAATACAATTCTTAACTGAAATATTATTATTTTTACTTGAAATTTAACAATTTATTCAATTTCATATAAAAATAAATAGTAAAGTTAGAAAAGAGAGATATTTTGAAATTGAACTTGGATAAATATTTTTTTTTGTGTGAAATTAAAAATATAGCATTAAATATAAATATATATTGAAATTATATATTTTTTTAATTTAATCGGTTCGATTTGACCCAATCCGGTTTTTGAAGACATGGAATAGGATCCAAAACTAGTCCGGTTCGATTTTTGACCGGTTGTTGACTTTTTGGTCAACTCGATTTTTTTCCGGTTTGATTTGGTGCGGTTCGGCTCGGATTTTCAATTCGCCGGTTTGAGTGCCCACCCCTACTTCATATTTCCCTAATGTGTTGTATTTGTTGGTGGGCTATATAGGTTTTTTCTTTTGTTCTTCAAAGCCGTTTATTAAACAACTGCGTGACTTCATTTCCCTAATTAATGTGTTGTATTTGCTGGTGGTCCATATATATAGGTTTTTTCTTTCTTTAAAAAGCTACCACCATAACCGTGTGTGGTTCCGCCATTGGAGACGTGATTTGTTGGAGATAGTAAAACAATGCCTCCTTGTGATATGGAAGAATTTCTCCTTTGTTCATGATTTGTCCACTTTCATGTGTGTTAAACACTTAAGGGCTCGTTTGTTACACAGGATTGCCTTGGCATAGACTATACTTAGGAATTGTCTTGGCTTGGCTTGTCTTGGTATAAGTTGGATAACACTTATCTTGCATTTGGTGTATGCTTGGATTAGGACTAAATTTTTTTATTTTTTCAAAACAATCTATATATATATATATATATA

TATATCGAAGCATGGTATTCTATCTTGCTTCACCAAACAGTTGATTTCAGTCACTCCTGGTTCTTAAACTTTGACAGCAAATTCAAGAGTTCTTTTCCTTACTGGTTCAATCACCGGTGGGAAAAACATGGTCTAGTCATTGATTTATTTCCTCCCCCCATGCAAGAATTAGTCAATTATTTTACTAATAAAAATAAATTCAAAGAATCTGAGTTGTTCTTCCCCAATTTACTTCATTTTGTTGCCAAATATAAAGTCCCTTGGATCCTTAAATGGTCCTATTAGGTTAATTGGGAAACTAGAGTCCTATCTCGCCAATTTTCAATCAAATGGTGGGATAAATTTAAACTTGAGCGTATTGCATATTATGTTTATGCTGATTTTCCTCCAGTCAGGAATCCACACCATGAGAAAAAGAGTTCCCCAGCCACATTACATTCTTCTCTCTCAATCGAGGGAAGGTCTAAGTCCGAATTACAAGATCTTGCTCGCCAACTAATGCTCCAAGCCTCTCAGATGAATGATGATGCAGACTCTGATGCCTCTCCTGCGTCACAGACATCTACTAGTCAGCCCAAGCCAGCCAGTCAACAAGCTCCAGCCCCAAAAATGCGGTGGAGTGATTATCCTAATGAACAAGACCCCTATGATTTTGGTCCTTTCAATCTAGGGGATGATTAAAGCAAAAGTAAAAGTCGCCTTGCATCAAGAGCCCTGAGACAAAATATCCGGACCCATGTATTCATCAGTCTGAACGCCGTGGTCTTTTGAAGATTCCCCGACAAAAGTAAAAGTTGCTGCACTCAGTAAAAGCAAAAGCTTTCCCGTGCTTCTACAGTTACTTTTCACAAGCATCCAAAGTTCCAGCTCACCAAAAGTTATTTGAGTCTGTTTCATTTGTCTTTTTAAGAAGGCCCCGGCCTCTGTTTTGAGCAGAATGTAATCTAGTACACTAGAATTTTCTCAGTTTCATAGTTCTATCAGGAATCAATACTCCGAAAGAACTAATTGATTATTTTAATTAATGTTTACTTTCATTGCATCTATTTTCATATATTCATTAATTTGCTTCATGCATAAGCCTGCATCTGTTCTGTAGTTTGTCCTTGTTTTCTTCACTCTATCTCTCTCTGGTTCTAGACCCCTTCTGGTATCAGAGCCATAAGTGTTGAAGAGTTTGTCTAAGTCTGCAACTCATCCAACCCAAGTGGATGGTTTAGGTTTTCTGAGTTGTAAGGTTTTCAGTTTCTTCTTTTCTTTTTCTGGTTTTCAGTTCACCTGTCCTTCTCTCTCTTGGTGTCCGAATCGTAAGACCTGTTCCAAACAGTAAGTCCCAGGGAGGCTTGAGCGGTAGTGGCATTAATAGGAGGGCGAGAGAATTTTAGGTGTCCTGTTATCCATGGAGTCTGTTTTAGAAGATTCTGTTCATCTTCCTCTTTAGGAGTCCAAACCCAGAACCTTGAGTAAAAATGAGTCGTTTTCTTAGAACCAACTCTTACAGTTCTAGCAGCTCTAGAGCCAATAGTAGTCTCCCAGAGATAGTAAATGAAGAACAAATTGAGTATGAAAATAACAGCAATTTAGATTATAAAGATTGGAATATACCTAGAGTCCCCAGTCAGGACATCTATAAGAAAAAATGGTCATTATCCAGTTTTAAGTCAACCACCAATGTTAAGACAGTCGAACAAGTCTATGCCTTAAGTAAAAATCATGAGACATGTCAGTTACTTAATCTTGAGTCAATAAGAAAACATAAAACATATGGAAATAATTTTCTTCATATTGGTTTAGTACAAGTAGCTACTAAGCCTCTAACCAGATTAGGATTAAAAGCTTCAATCCTTCTCTGTCTACGTGATGCTAGATTTAATAATTTTAAAAATAGTACATTAGGAATTATTGAGTCCAGTCTTGCTAATAGCCCAATTCATTTTGATTGTTATCCAGATTTTACAGTCAGCTTGAGCGATCCACATATCTTGAAAACTCTCACTTTAAATATTAAAACTGAAGGTTATGATGTTCTGCCAGGTAGTCAACCCCTAGCATTAGTTTACAGAATATATTATAAAGTCACAGGTACAAACATGAATTTCCAAGCCTTAAACAAGAGTCCAAAAAGTCAAACATTATAAATCCAGAGTCATACGCCTGATGCTACAGTTAGGGTTCCTCACTCTATCAAATGGTCATATGTCACTCTTCCCTTTGGACCTTAACCACTGAGACCCCTCCAGTTCCTATCCAACGTAGTCTAAGCAATTTAGACTACATACAACAATACCTAGACGGAATAGTTAAAATCAATTTTGGTCACCAAGCCCCGCAAAAATCAACAGTCCAATTACAACAGTTGCCAACACCTCGTCAATCCCAGAGACATGTTCCTGCCAGACACTCTTTCGCAGCCTCTTCCTCTACCCTAGAACGAGACCTAGAGTTGATTTGAAATTAGAATCATTAAGAAAAACTGCACAAGTCACCCATTCTGTCTATGGAAAACTAACAGAAGATAATAAATCTGAGTCCCCTGAGTCACCCACTCAATTTGATTTTGTTGAGGCTGGTTTCAAAGTAGAAAACCAACTCAGAACACTTAATAAAAATTTTGAAATTAATTGAAAAAGATTAGAACGTCACTTACATGCTCCTGAAAATCAAGCAAGAAGAGATAATTATCACTCGATAATTCCAAATAGAGCCAAAAGACAACAGATATTTAATGAATGGAAAGAATACATGCGCACTGCTAGGCTAGAGTCGAAATTTATTATCTTGATTTTGTAGAAAGCCGTCATCTATCTAATGAATTAAAAACTCTAACTCAAGAAAATTGGGAAAAAATAGATCACACTAAGGTCCTTGCCAGTCATCCCCCTGTTGAATCCACAGTAATCCAGCACCAGAATGCACAAGTTATTGCCAGTCCTTTTAAAATCCCAGACTTAGAAAATAAAATGATAATTGAACAAAATAATTATACAAACCAAAGCTTAGTCGTCATAGGTAAACAATTAGACAAGATAGAAACAAAAGTTGACAAATTACTTCCACAAGAAATTTATGTTAAATCTCATAACCAGAAACCATTAGTCACCTTCAAAGACTTACAATCAAGTCCAACTTTTAAAACAAAAACTACAATGACAAAAATTGATAAAATGTTAGAAGAATTAACCCCAACCAAACGGAAAGATAAAGAGAAAGTGGAATCAAGTGTTAAAGTCTTAGGATTCTCTGAGTCACCTTCCATACTTCATCACTCCTCTGAGTCTGATTCAATTGATAGCATAGATACTGAGTCTAGTAATATTTCAAAAATTGAAAATGCATTCAAAGATCTTGAACTCGAACAAAGTCCACAAGTCCAAAGATTAACAAACAAAGCCAACCCTACTAGCCTCACAAAAAATTGGTATCCTAAGCCTACGTCACCTGATATCCAGTTCGAAGAACGAAACACTCAAAGTCAGTTTTCTGTTTCTTCTGATAAATTTTATGAATGGAATATTGATGGTCTTTCTGAACAACAAATTCTTAATAAACTTCAACATATGTCTATGGTCGCCAACAGTTATATTACCAATCACAGTTTCAGACAATCTGAAATTGTTCCCTTGTTAGAAACAGGTTTCACAGGAACTCTTCATTCTTGGTGGGATAATCACTTAACCCATGAGTCTAAACAACGGATAATTCATGCGCTAAAACTTAATGAAGATGGTCTTCCTATTTTTGATGAACAAATAGGTCAAGGTATTGAAGATGGAGTCAACACATTGCTTTACACAATAGTTGATCATTTTATTGGAACACCTAGTAGTACTACCGCTAGAATACATGATCAATTAAGTAATCTAAGATGCCCAAAATTATCTGATTTTAGATGGAACAAAGATGTTTTCATTTCCAGAGTCATGGAAAGAACAGATAGTAATCAACCTTTTTGGAAAGAAAAATTTGTTAATGGATTACCCAATTTATTTGCTCATAAAATCCGATCAACCCTTAGTAATGCACAAGGTCATATTAATTGGGATAGTTTAACTTATGGAAACATAATTAGCACAATTAATCAAGTCGGAAGGCGAATGTGTGTTGATATGAAAATTGGTAAAAAATCCAGTCTGAGAGAAATTCTGCTAAATATGAATTAGGTAATTTTTGTGAACAATATGGTTTGATGTCTATTCCTCCGTCTAGAAAGAATAAGAACAACCGACAGTTTAGAAAAACCCGCCATTATTCGAGTAGAAAACAGTCGTTCCAGCATAATAATGAAGATAAAGAATTCTATAGTAATAAAAGATTTTCCCCTCGGAAAAACTGGAAAAAGAGTCACCATAAAGACAAGAATAATAGCTTTAAGAAAAAGGCCGACAAAAGAAAAGTGAAATGTTATAAGTGTCAAAAGTATGGTCATTATGCTAATAATTGTAAAGTAAAAGACACAATCAAACAATTAAAAATCACAGATGAAGATAAAAACAAACTCATCCAAGTCCTTTACATGACACTGAGTCAAGTGAGAATGAAACCATGGTTAGCCGTACTGAGTCTTCCGAAATATATTCTTCTGAGTCACAACCCAGTTCCCCTATAATCCAATTTGGTTGCCAAGATAACTGTTGTAAAAATAAGTCAATCAGTGTCCTCACCAAACGGGAAGAACAAGAAGAATTGTTAATCGATCTAGTAAGTAAAGTTGAGAATCCATAATTAAAATCTAAATACTTAAAGAAATTAAGAAAAGTCATTAGCTAAGAAGAACCAAAACAGTCTACCCACAAGATAAATCTGAATACAACTTTAGAAAAATTTAAGAAAAAGAAAGAAGTTACCTTACAGGATTTACAACTAGAAATCAAATCAGTCAAAAAAGAAATCATTGAATTAAGACAAATGAGTCAAAAATTATAAACTGAAAATTATGAGATAAAACAAGACCTGATCAACCTATTAGAAAAGAAAATTCCTAAGTTTAATCCGGAAAGTCCCACTAATTCAGACGAAGAAAACTCTATTCAAGAACAAGCCGTTAATTTGATTAAACATGTAAATTTTAGAAAATGGTATGCTCGAGTCACTATATTTGTCTAAAATTTTGAGTTAAACATAGTCGCCCTTTTTGATTCCGGAGCTGATCTTAATTGTATCCAAGAAGGCCTAATCCCAACTAAGTATTACAGAAAGTCAAAAGAATCTTTAAGCACTGCTTCAGGAAAATCACTCCAGCTGAAATATGAGATCCCTAAAGCCCATGTCTGTCAAAATAAAATCTGCTTTAAAATTTCTTTTGTTTTAATAAAAAATATTACTGATGAAGTCATATTAGGTCTACCGTTCATTTCCCTCCTGTACCCCTTCCAGGTTGAACATGATGGTGTAATTTCCACCTGTTTAGGAGAAAACATTAAGTTTGAATTTTTGAGTAAACCTGAGTTACATAGTTTAAAATAATTACAAAAAAATGCAGTCACCAAAACTGTCCAGATCATCCAAAACAAAGATAAACAATTAAGATTTCTCCAGGAAGAGGTTAAAACCAAATGAATTGAGCAACAACTAAGTCATACGTCTTTCCAATCCTAAATACAAAAATTTGAAGAAAAACTAAAACAAGAAGTCTGTTCTGAGTTACCCACTGCGTTCTGGCATAGGAAACAACATGTTGTTAGCCTCTCCTATGTCAAATCCTTTAGTGAGAAAAATATTCCTACTAAAGCAAAACCCATTCAAATGAGCCAGGAATTAATGGAATTTTGCAAACTTGAGATTGCTAATTTACTTTAAAAAAGAATAATCCGTAAGAGTAAGTCACCCTAGTCTTGTCCTGCTTTCTATGTCCAGAAAAATGCTGAGTTAGAACGTGGTCTCCCAAGACTATTCATTAATTACAAACCACTTAATACTGTCTTAGAATGGATTCGATATCCAATACCTAACAAAAGAGACTTAATCAACATATTAGAAAAATCAGTCATCTTTTCGAAATTTGATATGAAAAGTGGATTTTGGCAAATTCAAATTTCTGAGCCTGATAGATATAAAACTGCTTTTGTTACTCCTTTCGGTCATTATGAGTGGAATGTTATGCCCTTTGGTCTTAAAAATGCTCCTAGTGAATTCCAGAATATAATGAATGAGATCTTTAATCCTTTTAGCCATTTTTCCATAGTTTATATTGATGATGTGCTTATATTCTCTGAGTCTATAGAACAACACTGGAAACATCTGTATAAGTTTTTACAAATTGTTAAACAAAATGGTTTAGTTGTCTCTGCTAAGAAAATTAAGTTATTCCAGACTAATGTGAGATTTCTTTGGTTTAGTATATGCTAGTCACAGATTAGTCCCATAGATCGAGTTATCCAATTTGCTGATAAGTTTCCTGACCAAATCCTAGACAAGAGTCAACTCCAGAGATTTCTAGGATCTTTAAATTATGTTTCTGATTTTTATCAAAATCTGAGAAAACAATGTAAGCCTCTATTTGATCGTCTCCAAAATAATCCTCCTTCATGGTCATCTGCTCATACTGAAATTGTCAAACAGATCAAAACTCATATCAAGACTCTTCCTTGCCTTGGTATCCCTTCTTCTAATTCATTTAAAATTGTTGAAACTGATGCTTCGAATGTAGGTTATGGAGGTATCCTCAAATAGAGAGTCACTTCTGGTCAATCTGAGCAAATCATTCGATTTCATTCTGGCACTTGGTCCCAGTCACAAAATAATTATAGTACTATTAAAAAAGAAATATTATCTATTGTATTATGCATTAATAAATTTCAATCTGATTTATTAAATCAAAATTTTGTAGTCCGAGTTGATTGTAAATATGCTAAGCATGTATTAGAAAAAGATGTTCAAAACATTGCATCCAAACAAATATTTGCACGATGGCAAGCTATTTTGAGTATTTTTTATTTTGAAATTGAATATTTGAGAGGCAGTGAGAATTCTATTCCTGATTTCCTTACCAGAGAATTTCTGCAGGGAAAAGAATGGTAGAAAGAAGAAAAGAAAGAGAAAAAGATGAGAAACAAAAGGGTCAATCAAGCCAACCTAGTCAACCCCAGTCACTTCGAGTCCTCCCGCCATCAATGAGTCAAACCAAATATGAGTCTCCTTCCCAGATCGTCCCTTTCCCGGGATGCTCTCCAATCCAAATTAGTAACAGGTTTGCCAACCTAGGGGTCATAGTCGGCCAGGTTCGCCCCAGTTTACAGTCTGCTTTAATTTCTAGTCATGATCCCTTTCAAGATATTGCTGTTTCTAACCCCCCAATTGTTCCTTACCAGAAAACATCTCCATACATGGTCAAGAGTCATAGTCACCTATTTGTCATTGAGTCCAAATACAATTCGATTGCTAATCCCATCACTATTGCTAAGTCTTATTTTCCACCAAATAGTCATTTCATTCCCCCAGCACCTTTCAAGAACCTCAAAAA

TATATATATATATATATATATATATGTATTATGGCATATGTGTATATACATGTATATATGTATATTATAAATAATAAATATATATAGCCGCTCGGCTATACCTTTAAAATATTCGAATATATAGTATAGCCGTGCGGCTATACTATTCTATACTATTTTAATATACAATTTTGTCATATTATTTTAGGGTGCAGGGCTAAATACCACTCAAGCTAATTGGGCTAGAGTTATTTCTTCAATTATTTTATTTATACAAGCGATATTGTGGAATGAGAATTTAAATTCTTTCCTATACCTCCCATTCTAATGATTTTCGGCTATTAGCCCATAGCTCAAGAATAAAAAAATCTGCTTTGAGATACATCAGTATATTCTCTGCATACGAAAAATTGGATGCAAAACATTCTAATGAGGCAACGAAGAGAAGAGTTCTGGAATAAATTGAAGTTATCAGGGGTCATATGGCATTGATATATATATGAACTATTCTAAATGAGATTAGATGAGAAGAAGCATCTCATTCTTACATATACCATACCCACATGCAGCTGAAATTCCCAATGTTAAGAGGGGAGAAAAGTGCAATACCTCTCTTGTATCTTGCAAGCGTAGGAGATCCAATATTTCATGAGGACACCGGTTTAATAAGGAAGCAAATTGCATCCAATCATACATTAATACCGGCAGTCACAGCCCACAATATACTCAAATTTAATAAAGAGAAATAGGGAGGAGAAGGAGGAAGGGAGGGGGGGGTGTTGGTGTCAAAATCAAAGGAGAAAGAGAAAGTTAGGAAAGACGCCAAAGTCAAGCCATGTACTACTATTGGCCATACAATGACGGCACTTACCCAGAAGCTGAAGGTTTGAGTCCTGCTGAAATGGCCCTATATTTTTTTAGGGTAATGTACTGTGGAAAGGAAAAACACAAAAGAAGAGCTTCTCTTGGAGGACAAAAGAAAAAAAAAAGAGCCAAAACAAGATAAACAATACAGGAAAGA

>Chr2-01; OZ022207.1; 800385; 807445; rev; D;

ATAATAACATCATATCAGACTATTTTTTGGTTTAAAAAAAATCTTTGATAATTTTAATGAAAATTGAGATTCAAATGACATGAAATTTTTTTAGTAAGATTCATATGACAAGATTTGTTTTTCAATTGTTTTTGCCAAGATTTATTTTTCCATATAACCCATTTTCAAAATTGTAGTAAGACTCTGTCACAATCTTAGTTATTACTTGATGGTTCAAATGCCTTTCGTCAAATATGGATAAGACTCAGTCAAAATTGATGTTAGAATTAGCTTCAGAAAAATCGTTTTCTTTTTTTGAGACGAAACTTATACAAAAGCCTAGCAACAATTTAACCCGAAGTTTTATTGAAGTTGATTTGTACGAACCTGAATGAGGGATGTATGTATTCAACTAAACTACCAACACTAGCACATTTGTTTGTTTTTGGACGAAAACTAGCACTACTCTAATACCAACAATCTAAATATTCCCACAACGTCGAATCACTATATCACATACAAAGAAAACATTCGATTAAAAAAATGTCAAAAAAGAAAAATTCATAACCTAATTTCCACAAAAATGTAATAAAAAATTGAAATATATAAAAAATAGAGAAGAAGCAAGAAGCAAATTGAAGAGAGTATGAAAAAGAGAGAAAGTGAGAGATGGATACCTTGAGAAGAGTGAAAAAATAACCTATGGAGAAGATAGCAGAGAAGAACCCTAGGGAGAAGAAGATGACAAGAGCGTCTAATTTTTTTGGTAGAACGCGTGTTCTTTTTCCTTAGCCGTATAATTAAACGTGTATTATCTAAACCGGGGTGTGAAGGTTGTATTAGGTCAGGTAAGGAGAGTATTAAAAGCCCAACCCGTATAAAATAGTCGGATAATTAAATAATACAAGTGGACACCAAACACCTGACATAAACTACTTACTCTTATCCAAAGTCTAATACGGTATATCAAACGAGCCCTACTTTATATATATATATACACATATATATATATATATATATA

TCTATATCCCCACTAAAGCCAGACCAATCCAAATGAACCAGGAGACAATGGAATTCTGTAAAAAAGAAATTACAGAATTACTCCTGAAGGGAATCATCCGTAAGTCTAAGTCCCCCTGGTCTTGTCCTGCCTTCTATGTCCAGAAAAATGCTGAACTAGAAAGAGGAGTCCCCAGGCTAGTTATCAACTACAAACCCCTCAATACAGTCTTAGAATGGATTAGGTATCCCATACCTAATAAACGAGACTTAATTAATCGGTTAGAAAAAGCAGTTGTCTTTTCCAAATTTGATCTTAAGAGTGGATTCTGGCAAATCCAAATAGAAGAGTCAGATCGGAACTGCCTTCGTCACTCCTTTTGGTCATTATGAATGGAATGTTATGCCTTTCGGTCTTAAGAATGCGCCTAGTGAATTCCAGAATATTATGAATGAAATTTTCAATGCTTATAGCCATTTTTCAATTGTCTATATTGATGATGTCTTAATCTATTCCCAATCGATAGAACAGCATTGGAAACATTTGTCTATATTCCTTCAAGTTGTTAAGCAAAATGGTCTAGTTGTTTCTGCTAAGAAAATCAAATTGTTCCAAACTAGTATTCGATTTCTTGGCTTCAATATCTGTCAGTCTCAGATTAGTCCAATTGATCGAGTCATTCAGTTTGCTGATAAGTTCCCAGATGAAATTATTGATAAGAGTCAGCTCCAGAGGTTCTTAGGATCCCTCAATTATGTTTCTGATTTCTATCAAAATCTGAGAAAACAATGTAAGCCCTTGTTTGACAGGTTGCAGGAAAATCCTCCTCCTTGGTCCTCTGTCCACACAGAAATTGTCAAACAAATCAAAATTCATGTTAAGACACTTCCCTGTCTTGGCATTCCTTCAGTTGATTCCTTCAAAATAATTGAAACTGATGCCTCTGACATTGGTTATGGAGGTATTCTTAAACAGAAATTGAGTCCCAATTCGCCAGAGCAAATTGTTCGTTTCCACTCTGGTATTTGGAATCAGTCGCAAAGTAATTATAGTACTATTAAGAAAGAAATATTATCTATAGTACTATGCATCTCTAAATTTCAAGATGATTTACTTAATCAAAAATTCTTAATACGAGTTGATTGCAAATCTGCAAAACATGTTTTAGAAAAAGATGTTCAAAACATTGCATCCAAACAGATTTTTGCACGCTGGCAAAGTATTTTAAGTATTTTTGATTTTGACATTGAGTATTTGAGAGGCAGTGAGAATTCTGTTCCTGATTTTCTTACTCGAGAATTCTTGCAGGGAAAGCAATGGCACCCAAAAAAGACGCAGAAAGTTGGTGCCAGTCAGTCCAGCCAACCCAAAACCTCCCAAACACCAACCCAAGACCAGTCGCCCAAAAGAGTCCTCCCTGCATCCATGAGTCAATCCAGAATGGCCCATCCAGCCTCAATTGTCCCTTATGCAGGTCATCCTCCAATTCCAATTGTCCCAATTGCAAATAGATTCACCAGCCTTGGAACCCCAATAGGATCCACTGTTGGCCCAATTCGCCCTAATTATCAGTCTGCCCTTGTCTCTGACTATGACCCATTTGCAATAGTTCCCCCAGTTGCTCCAATTGCTCAATCCTCTCAGTCGTCCACCCAATCCAGAAAGTCATCTCCCTATCTACCAAAAGATAAGGTCAATTTGTTCATAATCGAACCAATGTATGATGGGATTTCTGATCCGATCACAATTGTCAGATCATATTTCCCTCCTAATTCCCATTTCCTTCCTCCTGCACCCTATAAAGACCTCAAATTCTATAGGGATATCCTGCATGAGACCAAATCAGTTGAAATTAAGCCTATTAAGGATAGAGATAACCCCGCAGTCATCCTCTATCATTCCCTTTATATTCAGCAAATTCTGAGCCAAGCTGAATTCAGTCGCCACCCCTATGAGTTGAAACACCTTAAGTCCACTCATCCTTATACCTATGCAGATTACATTGATGCATGGTATAATATCTTCCTCCATCAAACAGTTGATTTTTCCCACTCCTGGTTTATTAATTTCGATGCCAAATTCAGGAGTCCTTTCCCCTACTGGTTTCTTCATTGGTGGGATCAACATGGCCCAACAGCAGATGTCCTCCCTACTGAAGTCCATAATCTGACCAATTATTATGCCAGTAAATCCCAATTTAATAAACAACAGTTGTTCTTCCCCAAGTTGCTTCTTTTTATTGCCAGGTACAAAATCCCCTGGATTATGAAGTGGAATTATCATGTCAATTGGGAAACCAGAATCATGTCTCGCCAGTATTTTGTCAAGTGGTGGGATAAGTTCTCAATGGACAGAATTTCCATCTATGTCTTCAAGGATTTCCCCCAATCGGATTATCCTCCCAAGCCAAGTTCTCCCACAGGCTCAGTCAGTTCGGCCATCTCAATCGATGGAAAATCCAAAGCTGAACTCCGGGCCCTTGCTGCACAATTGCTTGACCAAGCTAGTCAGATGAATGATGATGAGGACAACGAATCTTCCCCATCTGCATCCAGCTGTCAGCCTGGCTCCAGCTCCTTTGATCCCAATATCCCTTTTGCGCAGGATCCTTATGACGCGTATGATTTGGGATCTGATTAGTCAAAAGTTGCCATCAATTCCTCTTTTCCCCCCGGTGAAATGTTTGGAATCAGAATAATTGTAACGTAAAAACAAAATTGCTTTTGCTTTTACCCTTACTTTACCCCAATAGGTAAAGAGAGTAAAAACAAAAATACTTTTGCTTTTACCCCTGCTTTACCCCAATCGGTAAAGAGAAAGTTGTTTTCTGTGCTTTGAACCATGGTCACTCTCCAACCAGAGTTACCTTTGATTCAAACCTTAGTTGCTTTTCTTTCTTTATAAGCTGGCCTCGGCCTCAGTTGTAAGCACTTGTAATTTTTCCTCTGCTTTCTTAGTTTAAAAAAAAAAAAAAAAAAAAGTTCTCTCTGAAACTCCTTCAGACATGAACTCACTTGTAAATATATGAGTGTTTAGCTTTCAATAAACAAAGACAGCTTTGTAAGTAATTTCAAATCTATCTTATTTTCATTTATTTATTATCTTATGCATTATTTTCTTGCATTAGTCTGAAAGTTGGTTTTAGGATTCTTTTTTGTTATTCTAATTCTCTTGTGCTTATATATATATATATATATATATATATATATGGGTCAGGATCGAGGGGCACACAAAAAACCACACTTTTTGACACACTTTTTTTAACCATTAGATTTAAGTAGTTTCAATGGCTAAGATTGCATCTAAAATATATCATTAGTTTTTTGTTTTGTTTTTAGAAACAATTAATACACATTGGAAAAACAAAAAACTTTAAACTTTTTTTATTTATTATTTATTTTCTCTCTCCTCTCCTAGCTCTACATCTTCTTCATTACTCACACTGCATCTTATTCTTCATCTATGATGACATGAATCTCTGAAAAACAAGAAGACCAGAGAACTCATGCCATGAGTCTTTCTCATGAACAAAAAAAAAAAAAAAAAATCCAAAACAATTTTTATATCTCTCTCCTGCACAATTTCCTTCGTCCAAACTCTCATTTCTCATCAGTCTTAACAAAACAAACACTGAGTCGTAATTGAGCATCTGTTATGCCTTTCTCTTTTTTCTTTTTTTGGCATAATTGAGTGTCTATAATTTGAGGGTGGGTTTGTTTTCCACCATTTTCAGAAAATATAAGGCCATAAGTTTACCAATATAACAAAGTGGCCGAAATAACTCTTGTTGTTAAAATTAGACGACTTTAGGCAGAGATTCAAGCTAAGATCTGGGTTAAAATAAAGGCCTAGAAACAACAACGTAACTTTTCCAATGCCTATTTCCACCCATGACAATTGCTCCTGGGTTTGAACGAAAAAAGAAAAAACAGAGTCACCGACTTCAAACTAGGCGACAACGACTTCTACGTCATTGAAAATTATAATGATTAGACTAAACTAGTACATATATGGAAATTTATTGACAAACAATTTATTTAGATTAGATTGGTTGTTGTGTTTCAAAATTGGGTCAGGATTGAGGGATTGGACAACTGAGTTTGATCAAAAGAGAATTAAAGATTAGGAATATAATTAAAGATAAAAATGGTGGTGTCATTTCTTATGGGTCATATCCCTATGGGAAATTATCCTCTGAAATTTGTGCAGAAGGATAAGCTCTTTACACCGACATGAAATTAAAGAAACAGCTTGATAAGTAGAAGACCATAAGAAGAAAAGAATTATGAGACTTTTGTGAATAATTTGGTTTTGAACCAGTAAAATCATATTCACATAAACCACGTAAAATCATAATAAGAGTCTAAGTCTGAGCCTAAGAAACTGTATAGAACCTTTAGAAAAAATAAATAAGTAGGATAAACTAGTAAAAAAGCTAGTAAATGAAAGAAGGTCTGGTCCGAACATGCTATAAGTGTGGAAAAGTTGGACACTATAAATCTGAATGTAAAATGAAGATTAAATCAGTAACTTAAGCATTAGCGAAGAACTTAAGCAACAACTTTGCCAAATAATGCCTAATTCCTCTGGTTATGAGCAAGATAGTAATGACAAACTAGCTCAACTCGAAAATGAAGAATTATTTGAATCTGATACTGAAACTAGCTCAGATAGCGAAGACGAGTGCGCATGTCAATTCGATGATCTTAGAATATGTGTTTTAACAAAAGAAGAAGGTTTGATAATAGACTTAATTGACAAGATAGAAGATCCAGAGAAAAAGAGAGAAGGGTTAGAAAGTTATATCTCGCTAGCTAAAGTAGGACCAAGTAACCTGCAGCCATTTATAACTAATGTAAGACAACCAGTTGAAAACTATAGCTATAAAACTATTGTAAACATGATGAATGATCAAATTCATAAGAAAGAACCTACTCTCAATTACCTTCAGTGTGAAGTCATGATGTGAAAAAAGAACTTAGGGAATAGGGTTAGAGTTCTAGAACTCTATCATCCTTTTTCGAAAAAATATACAGAAGAAAAATCTGAGCAATTAGAGTTTGAAAATCTAATGGGAGAATTTCAAGAGAAAATAGAACCTACGAGAAGAGAAGCCCTGTAAGAAGAGCAGGTTAGTACAATGAAATTCGTTAATAGCATAGATAAAGTGATAGCATAGAAATGATATGCGATGATAACTATTGTGATTAATAAAACCTATAAGTTTACAGTTGAAGGGCTGATAGACACAAGAGCCGATCTTAACTGTATAAATGAAGGAGTGGTACCCACTAGGTGTTTTCAGAAAACTACCCATGTTCTACATACAACCGTTAATAGCTAGAAAATGGGAATTCAATTTAAATTATCTAACGTTGCTGCGCTTAATCAAGGAATTTGTTTTGAAACCCTTTTTGTTCTGGTGAAAGGTTTAAACCAGTCTCTAATATTACGAACACCCTTCATTAATATGTTTTATCATTTCTCTGTTTCTGAAGAAGGAATTAGAATTGAGGTAGAAGGTCAACCTATAATCTTTAGGTTTTCTCAACCACCGAAGCATAAGTATATTAATGAAATCCAAGAAAAAATAAAAAGGAAAGAAAATTATCTATATCATTTGAAAGAAGAAATGCAACACAAAAGAATAGAGGAGCAACTGCAAAATAATAAGATAAAAGAAAGAATTGATGAAATCCAAAAGATGTTCGAAAATCAAGTTTGTGCTGAAATACCTAATGCATTTTTAGAATAGAAAGAGGCACATAATTGAATTGCCATATGAGCCTAATTTTGACGAAAGGAAAATCCCAACCAAAGCCAGGCCAATTCAAATGAATACTAGGTTAATGCAAATTTGTAAATCTGAAATATCAGATTTATTGAACAAAAAGTTAATCAGGAAAGCTCATCTCCTTGGAGTTGTTCTGCCTTTTATGTGAAAAACGCAGCTGAATTGGAAAGAGGAGTACCCAGATTGGTGATAAACTATAAACCTCTCAATAAAGCCTTCAAATGGGTTAGATACTCGATTCCTAATAAAAAGGATCTCTTGGATAGATTAAATAAGGCAATCATCTTTTTTAGATTTGATATGAAATCAGGATTTTGGCAAATCCAAATCGCTGAAAAGGATAGGTACAAAACAGCCTTTAGTGTCCCATTTGGACATTATGAATGGAATATTCTACCTTTTGGATTGAAAATGCCCCTTCTGAATTTTAAAATATTATGAATGATATATTTAATCCTTACACTGCTTTTACCATTGTTAATATTGATGATGTTTTAGTTTATTCTAATTCTTTAGAACAACACTTTAAACATCTAAATATTTTCTTGAAAGTTGTAAAGCATGCTGGATTAGTGGTGTCGGCAAAGAAAATGAAATTATTTTCTTCAAAAGCTCGCTTTTTGGGACATAATATTAATAAGGGACTGATAGTTCCAATTGATCGAACAATATCCTTTGCTGATAAGTTTCCTAATGAAATTAGGGACAAGCAACAATTACTAAGGTTCTTAGGAAGTCTCAATTACATAAAGGATTTTTATAAGGATTTAACTCAAGACTCAAAATTATTATACCAAAGGTTGAAAAAGAACCCGATTCCCTGGTTTGATGAACACACTAAAACTGTTAGGAGACTGAAAGTCAAAGTCAAAGAACTTCCTTGTTTAGCTCGAGCCAACCTGGAAGCCTTTAAAATCATAGAAACTGATGCTTCAGAAATTGGGTATGGGGGAATCCTGAAACAAAAAGTCGATAACTCGGAACAATTGGTTAGGTTCACCTCTAGAATTTGGAAGCCAGCTCAAAAAATTTATTCCACTATTAAGAAAGAAATGCTAGCCATTGTCCATGCTGTTTCTAAATTCAAAAGTGATCTTTAAATAAAAGATTTTTCATAGAGTGGATTGCAAGGCCGCAAAAGACATTTTGTTTAAAGATGTAAAAAATTTAGCATCAAAACAAATTTTTGCAAGATGGCAAGGAATTCTTTTTGCTTTCAATTTTGATATTAAATTCATTAAAGGTGAAACCAACATACTCCCGGACTTCTTAACTCAGGATTTTTTGCAGG

GTGTGAACTAGAAGCTCCAGACAGATGACTCCTAGTAAGAGTCCTTCAGCTCTTACTAAGTCATCGCCTCTTGATCCAAAGCCACTGATGTCTTCTTTTCCATATAATAACGGTAAGGAGAAGCAGATTGCTGAAAATCCATTCCGTGAATATATGAATATGTCTGTAGCAATCATTGAAATGAATGATCTAGATAAACACCAGAATTTTCTCGCTGAAAAATATTTAAGGGATAACCTTCCTCCCTTGAATAAGCCTTATATTTTCTATGAAACTCTTCTGATTGAAACCAAGTCTGTTAGTATTGTTCATCGTCATAGGGGTGTGTAGGGGTCTTTTTGTTCCAGTAGCCTGACGAATGGGCTTGGACTGCTGTAAGAAGAAAATGAACAAAAATTCCCAAGTGCCTGAGTTCGAGAACCAAGCTCCCAAAAAAGTTTTTGAAAAGTTTGATGAAACTTTAGAAAAACACTCTTAGGTTCTTTTCACCAATAGCCAAATAAACATAATAAATCGATTAGTGACTTAGTTTGAGGAGCTTGTGGCATGGGAGCTAAGCTTTCATTAGTTTAGCAATTTGAGAGAGAGAGAGAGAGAGAGAGAGAGAGAGAGAGAGAGAGAGAGAAGCATGAAGTTCTTACAGAAAACGAAGGTTTGGAGAAGGGCAGAGGAAATTAGAACGATTTCTAACTTAAGGGAAGAAGAAAACAAACGAAGATGAAAATTAGGTGGCTTGAGTAAATTTTCAGTTGCTTGACAAAGCTTCTCAGGCCCTAGATCCTAATTTTGCTAGGTAGAAGAAGCTCCTTTTTATAGGCAGAAAGAATCCTTAATTTTGTCTAACCTCCTTCTTCCTTTTTCTTTGCTTCTTTCCATTTAACCCTATTCGGTGAAATTTTCCTATCGAAGGCACTTGGGTAAGGAGCCCTTTATGGTTCCAAGGTTTTGACACAGCTGGTCTTCTCTATAGGGGTAAGGCGCCACCAACTTTCTACTTGAT

>Chr2-02; OZ022207.1; 3211631-3214038; dir; B;

ATTTTAACGTACGAAATGACCAATTTGCCCTCATATTTAACAGCAATATTGACATAATGTAGCGGTTGGATCAAATGGGCGATTAAAATAGAATTTATGTACCATTTGGACTAATATTTTAATTTAGGGACCAAAATGTAAATCGCGTATAAGTTTGAGGACCATTTGAGTAATTTACCCTTAAAATAAGTATATGTAAAGTGAAAATAAAGGAAAATGTAAGGACCAAAGTGAAATTCTGGATAAGTCTGAAGACTGAAATGTATTGGAAAAAGTATAGGCGCCAAAGTGAAAATAAAGGAAAGTATAAGGACAAAAGTGAAATTATTGACAAGTATAAGGACTAAAGTGCAATTAAAGCTAGTTGTAATGACGGAATTGCAAATAAGGGTTTTTAGTAACATTTTTGAAATGTTGGCTTAGGAATCAGTTACCCAAGCAAGAACAATTTGAGTTCAAAGGTGTAGAATTCAAGTGAGGCATTAGCTTTGGATTCCAGGTAGGGGGGACTCACTCCCGGACTTGTATTTTTGTGTATGTATGACATTGTATATATTGTTGTGTGCGAAAACGAAAACTGTAATGTTTGTGATTGCCTAAATTAACACTTTAGTTTTCTTTATTTTCTTTACTTCTTTTTTTTTTGGTTGGGTGAGCAAAATACAAGTGGTTCCTTTACACTTTAGTAGGAAAACTCTTGTTCGTAACGTTGGTAATATTCTTTCTTTATTTGGGGATTAAGGTCCTATTTGTTTACTTTCTCCGGTTCCATTGCTTTACTTCCTTTGCTTGTTCTATTTCTCTCATCTTACAACACAATATATATATATATATATATATATATTTAAGTTTATGCCCGAAGCAAAGGAACCACCAGAAGATAGATCCTAGTCAGAGTGGTGAAAGGATCTTGGTACTAAACTCAGAGTGGTGAAACTAGGATCTTTGAGACAAACAAAATATGAAAGGTCACTAAATACTCTGAAGACCCAATACCCAA

AATTTTCAAGCCTTAAACAAGAGCCCAAAGAGTCAAACATTGTTAATCCAAAGTCATACGCCTGATGCTACTGTTAGAGTTCCCCACTCTATCAAATGGTCAGATGTTACTCTTCCCACTGATTGGACTCTAACCACTGAGAACCCTCCAGTACCTGTCCAACGTAGTCTAAGCGATTTAGATTACATACAACAATATTTAGACGGTACAGTTAAAATTAACTTTAATCACCAAGCCTCACAGAAATCAACAGTCCAGTTACAACAGTTACTAACACCTGGTTAATCCCAGAGACATGTTCTTGCCAGGCATTCTTTTGCAGCCTCTTCCTCTACTCTAGAACGAGCCTAGAATTAGAGAAAACTTTAATTGATTTGAAATTAGAATCACTAAGGAAAACTGCACAAGTCACTCAACCTGTCTACGACAAACCCTAAGATGATAATAAATCTGAGTCTCCTGAGTCACCTACTCAATCCGATTTTGTGGAAACAAGTTTCAAAGTAGAAAACCAACTCAGAACACTTAATAAAAAATTTGAAATCAATTGGAAAAAACTAGAAAGTCACCTACATGCTCCTGAAAATCAAGCAAGAAGAAATAATTATCATTCAACAATCCCTAATAGAGCCAAAAGACAACAGATATTTAATTAATGGAAAGAGTACATGCGCACTGCTAAAGTTGAAATTTATTACCTTGATTTTGTAGAAAGCCGTCATATATCTAACGAATTAAAAACTTTAACTCAAGAAAATTGGGAAAAAATAGATCACACCAAAATTCTTACTAGTCACCCCCCTGTTGAATCTATAGTTATCCATCACCAGAAAGCAGAAATCATTGCCAGTCCTTTTAAGATCCCTGACTTAGAAAACAAAATGGTAATTGAACAAAATAACTATACTAATCAAAGCCTAGTAGTCATAAGAAAACAGTTAGACAAAACAGAAATTTCAGTCAAACCACATAATCAGGAACCATTAGTTACCTTCAAAGACCTACAATCAAGTCCAACTTTTAAAACAAAAACCACAATGACAAAAATTGAGAAAATGTTAGAAGATTTAACTCCAACTAAACGTAAAGATAAAGAAAAAGTTTAGTCAAGCGTTAAAGTCTTAGGATACTCTGAGTCACCTTTCCTATTACATCAATCCTCTGAGTCTAAATCCATTGATAGCACAGATACTGAGCCTAGTAATATCTCCAAAATTGAAAATGCATTCAAAAATCTCGAGCTCGAACAAAATCCACAAGTCCAAAGATTAACAAACAAATTCAACCCTACCAGTCTCACAAAAAATTGGTATCCCAAACCTACACCACCAAACATTCAATTTGAAGAACGAAATACTCAAAGCCAGTTTTCTGTTTCTTCTGATAAATTGTATGAATGGAATATTGATGGTCTTTCTGAGCAAGAAATCCTTAATAAACTTCAACATATGTCTATGGTTGCCAATAGCTACATCACCAATCACAGTTTCAGCAAGTCTGAAATTGTTCCTCTATTAGAAAGGTTTCACAAGAACTCTTCGTTCTTGGTGGGACAATCATTTAACTCCTGAGTCTAAGCAACGAATAATTCATGCGTTAAAACTTAATGAAGATGGTCTTCCTATTTTTGATGAACAAATAGGTCAAGGTATTGAGGATGGAGTCAACACATTGCTTTACACAATAGTCGATCATTTTATTGGAACACCCAGTGGCACCATAGCCAGAATACATGATCAATTGAGTAATTTAAGATGCCCTAAATTATCTAATTTTAGATGGTATAAAGATGTATTTATTTCCAGAGTCATGGGAAAAACAGATAGTAATCAGCCTTTTTGAAAGAAAAATTTGTTAATGGATTACCGAATTTATTTGCTCATAAAATCCGATCAACCCTTAGTAATGAACAAGGTCACATTGATTGGGATAGTCTAACCTATGGAAATATAATTAGAATAATTAATCAAGTTGGTATGAGAATGTGCGTTGATATGAAAATTGGTAAACAAATCCAATCTGAAAGAAATTCTGCTAAATATGAATTAGGAAATTTCTGCGAACAATATGGTTTAATGTCTGTCCCCCGTCTAGAAAGAATAAGCCTAATTACCACAACCGACAGTTTAGTAAAAAGCGTTATTATTCACGTAGAAAACAGTCATTCCAGCACAATAATGATGATAGAGAATTTTATAAAAAAAAGGTTTTCCCCTAAGAAAAATTGGAAAAAGAGTCATCACAAAGAAAAAAGCCACAGCTTTAAGAAAAGAACCGACAAAAGAAAAGTGAAATGTTATAAATGTCAAAAGTATGGTCACTATGCCAATAATTGTGAGCTTAAAAACACGATTAAACAATTAAAAATTACAGATGAGGAAAAAGACAAATTAATTAAAGTCCTTGAG

ATAAGTCCAGTGAAAATGAAACCATGATTAGTCATACTGAGTTCTCTNNNNNNNNNNNNNNNNNNNNNNNNNNNNNNNNNNNNNNNNNNNNNNNNNNNNNNNNNNNNNNNNNNNNNNNNNNNNNNNN

>Chr2-03; OZ022207.1; 3369709-3388427; rev; D;

CATGACCCAATACCTTAGAAAAAAAGTGCAAAGCCTGTTAGAAAAGGCTTAAGACAATTAGAAAGCCTAAACGTGCTGTTAAAGTGCAATAGGCAAGCTGCTTCCGGCAGCTTGACGAAAAGCCCAGTAGATTGACCAGAATGACATATCACCAATTCAAAGCCTAGGAAAGAAAAGCCCAGCCCAAACTCTCTGTAAAATATTTGGCCAAGCATGTCTTGTCCCTATCCAAGAGTCAACAGCAACCATCTCAAAAACCAGAGGAAATCCACACGAGCAAGAGGTTTACTCGTTGGGTAAAACGTGGCTCCTTTCCCTCCCATTGTCCTGGCAGCTTGCATAGGAAAAGTTGAGAGAAAAGCAAGCGGCTCTTCATCTCTATGAGGAAGGGCCAGCAAAACCAAGACCTTGGAACCGCAAATGGGTTCCTTACCCATATGTCTTGGGCGGATAGGATTCATCAAAGAAGACGAAGTGGGGAAAAACAATGGAAAGAGGGAAGGAACTATGACGAAATTAGAACCCTGAGTGGCTATAAAAGGAGGACTTTTGCTACCCAGGAGAACCAACCCACTTCCAGAGCTCGACCAAGCATTGTCAAGCAGCTGGAAAATCTACTCAAGCCACCCCATCTCCCCCTCCATTCGTTTTCTTCCTCCATCAGGAAGGAAACCAGCTGAATCTTCCTTTGCTTGCTCTAAACCTCCATCCTCCATAGGAAAATTCATCTCTCTCTCTCAAAATTGCTAAACCTGTGAAAGCCTAGCTCCCATGCCACATGCTCTTCAAGCCAAGCTCTTCTGTAAAATTGGTTAAGTTTGTTTGGGCTATTAGTGAGGGAAGCCTGAGAGTGTTTTCGTAATAGTTTCATCAGGCTCTGCGAAAAACACTCTTCCAGGCGCTTGGTATCGAACCCAGGCACTTGGGGAATTTTGCTCACTTCTCTTCTTACGGCAGCCCAAGCCCATTCGTCAGGCTGCTGGAACAAAAGGCCCCAACA

GATCGCATATAATCTTTCCATTCACTAAAGATTTGTTCGCGGTGTTTACTATCAGGATAGGCTTGGTGGTAATTATCTCTTCTGGATTGGTTTTTCGGGGCTTTTAAATGACGATTGAGACGTTTCCTATCAATTTCAAACTTCTTATTTAAAGGTCTAAGTTGATTATCTACTGCAGCAAGGTTTTCAACTAAGAAATCAGATTGGGTTGGAGACTCGGGGGAGTCTGATTTATCTTCTGGAACCTGGCCGTAGCAAGGTTGGGTGACCTGAGAGGTTTTTCTCAGGGACTCAGGTTTGAAATCTATCATGGCTTTTTCTAACTCTAGGTCTCGTTCTAGAGTGGAGAACGAAGCAGCAAAGGAATGTTTGGCAGGGACATGCCTTTGGGACTGATCAGGAGTTGGCAATTGTTGTAACTGGACATTGGATTTTCGTAAGGGGTGGCTGTCAAATTTAATTTTTACTGTGCCGTCTAGATATTGCTAGATGTAGTCTAAATTGTTTAGACTGCATTGGATTGGAGCCGGTTGGCTCTCGATGACCAAAGTCCAATCTTTTGGAAGAGCAACTTTGGACCACTTAATGGTGTGGGGAACCTGTATATGAGCATCTTAAGTGTGACTCTGGATGAGGACAGTTTGATCTTTTAGGCTTTTATTGAGGGCTTGGGAATTCATATTTGTTCCTGTAATTTTATAGTAAATTCTGTAGACTGAGGCCAAAGGTTGGGTTCCTGGGAGGACATTATAACCTTAAGTCTTAATATTCAGAGTTAAAGTTTGCAGGATATAGGGGTCGCTTAGACTTACAGTAAAATCAGGATAACAATCGAAGTGAACTGGGCCATTACACAGACTGGACTCAATGACTCCTAGGGTGCTGTCACTAAATTCAGTGAACCTAGCATCATGCAGACAAAGCAACTGACATGTTTCATGTTCTTTACTCAAGGCATAAACTTGTTCAACTGTTTTGACATTTGTTGTGCTATTAAAGCTGGAAAGCAACCAATGTTTCTTATAATATATATATATATAGGATTTTGAGTATCTTCATTTATTCATTAATTTCATTCATCCATAAGCTCTACCTGTTTTATTATCTGAGTCTTTATACTTATCTATGCTTAAGCCGTAACTGTTGAAGAGTCAGTCTAAGTCTGCAATTCATCCAACCCAAGTGGTTGGTTTAAGATTTCTGAGTTGAAGTCCTCAGTTTCTTTTTCTATTAATTCATTGTTTTTCAGGTTTCCTGTCCTTTTCACTTTGGTCCAAACAGTAAGTCCTAGAGTCCAGACGATAAGTCCCAGGTAGAGGCACGAGTGGTTGCAACCTGAACGGAGAGAGAGAGAGAGAGAATCACTAGGTTTCCTGTTACGCTATGAGTTAGTATTAGAAGTTTCTGTTCATGTTCTTTCTTTAGAAGTCTGAACCCAGTACCTTGAGTAAAAATGGGTCGCTTATTTAGAACCAACTTTTATAACTCTAGTAGCTCTAGAACTAGTATCAGTCATCCCCCTGAGACAGTTAATGAAGAACAATTAGAATAGAATACTAGCAATGAGTTACATTTTAGAGATTGGAATATCCCTCAAATGGTAAAATGGTCAGAAGTCACCCTTCCCTTAGATTGGACCTTAACAAATGAAAGTCCACCGGTCCCAATTCAACATAGTCTAAACAATTTAGATTATATCCAACAATATCTAGATGGCACAGTCAAAATTAATTTCGAAAACCAACCACAACAAAAGTCCTCAGTCCAGTTATAACAACTTCCCACACCTGGTCAATCCTAGAGACACGTCCCTGCTAGACACCCCTTTGCAGCCTCCTCCTCTACTCAAGAACGAGACCTTGAGTTAGAGAAAGCTTTAATTAATTTAAAATTAGAATTGTTGAGAAAATCATCCAAAGTTAGTCAACCCTGTTATGGTAAAACACCAGTCAAAGAAGATACTAAATCGGAGTCCCTTGAGTCACCCACACAAACAAACTTCGAAGGCGGTTTTCAAATTGATAAACAATTAAGAACCTTTAATAGAAAATTTGAGATTAATTGAAAAGAAAAATACCATACAACCTATCCTGACAAAATCAAAAGACAACAAATTTTTAATGAATGGAAACAATATATGAGGACAGCCAAAGTTGAAATTTATTATCTTGATTTTGTAGGAAGCCGTTACATGACAAAAGAGTTAAAAACTTTAACTCAAGAAAAATGGGAAAAAGAAGACAAAACAGAAGTCTTAGCCAATCATCCCCCAGTTGAGACAATAGTTTTTCACCATAAAAAGTCAAAAATTGTGGCCAGTCCTTACAAAATTCTTGACGAAGAGAATAAGATGATTATAGAACAAAACAATTACACCAACCAAAGTCTAGTTGTCATAGGAAAACAGTGAGATAAAATAGAAACAAAAGTCGACAAATTACTCCCTAATGAAGTCAAACCCAAATTACAAAAACAAAAACCTTTAGTCAAATTCCAAGATTTAAAGTGAAGCCCAACCTTTAAGACAAAACTTACAATGAGAAAAATAGAAGAAATGCTTGAACAATTAACCCCTGAGAAAGCCGAGAAATCCGGTGTTAAAGTCTTAGGATCCTCTGAGTACTCCTCTGATTCTGAGTCAGTCACTAGTATGGAAACAGAAACTAGTAACATTTCAAAAATTGAAAATGCATTTAAGAATTTGGAATTAGAAAATGATTTAAAGGTTAAAAGATTAACTAATAGAATCAGTCCAACAAGTTTAACCAAAAATTGGTATCCCAGACCAACCCCTCTAGATATCCAATTTGAAGAACGAAACTCCTAGAGTCAGTTTTCCGTTTCTTCAAATAAGTTATATGAGTGGAATATAGATGGTCTGTCTGAGCAAGAAATTCTTAATGAACTTCAACATATGTCTATGGTTGCCAATAGTTACAATACGAATTACAGTTTTAGACAGTCTGAAATTGTGCCTTTGTTAGAAACTGGTTTCATCTGAACCTTACGTTCTTGGTGGGATAAACATTTAACGGATGAGTCTAAGCAACAAATAATCCATGCAGTCAAACTTAATGAAGAAGGTCTTCCTATTTTCAACGAACAAGTAGGTCGAAGTATAGAAGATGGAGTCAATACTTTGCTGTACACAATAATTGAGCATTTCATTGGAAAACCTAGTAACACCACTGCTAGAATTCATGATCAGCTTAGTAACCTTAGATGCCCTAAGTTATCTGATTTCCGTTGGTATAAAGATGTCTTCATCTCCAGAGTCATGATTAGAGATGATAGTAATCAACCATTTTGGAAAGATAATTCATTAATGGTCTCCCTAATTTGTTTGCACACAAAATACGCACAGTCCTTAGTAATGGTCAAGGTCATATAGATTGGGATAATTTAACCTATGGTAATATAATTAGCACAATTAATCAAGTCAGTATGAAAATATGTGTATATATGAAAATCGAAAAACAAATTCAGTTAGAAAAAAATTCAGCTAAGTATGAGTTAGGTCATTTCTGTGAACATTATGGTCTAATGTCCATTCCCCCATCTACAAAAGCCAAGTCTAGTCACCATGACCGGCAGTTTAGTAAGCGTCGTCATTATTCACGAAGAAATCAATCTTTCTGACAGAATAATGAGTTTTACAGTAAAAAGAGATTTCACCCCCTTAAAAATTGGAAAAAGAGTCACGGAAAATATAAGAAAAGTCACCTTAAGAAAACTACTGACAAAAGAAAAGTTAAATGTTATGAATGTCAAAAGTTTGGTCACTATGCTAATGAGTGTAAAGCAAATAACACAATCAAACAATTGAAAATCACGGATGAAGATAAAGAAAAGTTAATCAAAGTCTTTGAGTTAAGAAACTCATAGTCTAGTGAAAACGAGACCATGGTTAGAACACTGAGTCACCGAGTTTAATTCCTCAGTTTCCCAGATAAGTTCACCTAGAATTCAATTTGGTTGTCAAGATAATTGTTGTAAGTCTAAGTCAATTAATGTCCTTACTAAACAAGAAGAACAAGAAGAATTATTAATTGATTTAATAAGCAAAGTTGAAAACCCAGAGTTGAAATCTGAGTACTTAAACAAATTAAGGAAAGTCATCAGTCAGGAAGAACCGAGTCCGTCTACCCCTCAAAAGATAAGTCTGAACACTAGTTTAGAAAGATTCAACAAAAGAAAAGAAGTTACTTGACAAGATTTACACCTAGAAGTAAAATTACTCAAAAAAGAAATAGTGGAACTAAAACAGAGAAGTCAAAAGTTACAGGTCGAAAACTATGAGATTAAACAAGATTTAATTAATCTAATGGAAAAATTTGTGTCTACGCCAGTTAGCCCTACAAATTCAGATGGTGAACCCTCCAATCAGGATCAAGCCGTCAATTTAATTAAACAAGTAAATTTTCGAAAATGGTATTCTAAAGTCAACATATTTATCCAAATTTTTGAGTTAAACGCAGTTGTCCTTTTTGATTCAGGAGTCGACCTCAATTGTATCCAAGAAAGTCTAATTCCTACTAAGTATTACAAAATATTAAAAGAGTCATTAAGCACAGCCTCAAGAAAGTCACTCCAATTGAATTATGAGATTCTTAAAGCCCATATATGTCAAGATAAAATTTGCTTTAAAACCTCATTCGTCTTAGTCAAAAATATTACTGATGAAGCCATCTTAGGATTACCCTTTATTGCCCTACTGTATCCTTTCCAGGTAGAATACAATGGAGTCATTTCTACCCATTTAGGAGAAAAGGTTAAGTTTGAATTCCTAAGTAAACCAGAGTTACATAATTTAAAAGCCTTGCAAAAGAACGCAGTCACCAAAACAGTTAAAGTGATTGAAAATAAAGATAAGCAATTAAGATTCCTCCAAGAAGAAGTCAAATTCAAAAGAATTGACCAACAACTTAGTCTTGAGTCTTTGCAGACACAAATTCGCCAATTCAAAGAAAAATTAAAACAAGAAGTTTGCTCTGAGATACCCAATGCTTTCTGGCATAGGAAACAACATGTTGTTAGTCTTCCTTATATCAAATCCTTTAGTGAAAAGAATATCCTCACTAAAGCCAGACCATTCAAATAAGTCAAGAATTAATGGAATTTTGTAAAATTAAAATATTAGAATTACTTGAGAAAGGAATAATTCGTAAGAGTAAGTCACCCTAGTCCTGCCCTGCTTTCTATGTCTAGAAAAATGCTGAGTTAGAACGCGGAGCCCCAAGATTAGTTATTAATTATAAGCCTCTTAATACGGTATTAGAATGGATCAGATACCCAATACCCAATAAGAGAGATCAAATTAACAGGTTAGAAAAATCAGTCATCTTTTCTAAATTTGATATGAAGAGTGGTTTTTGCAAATCCAAATTCATGAGTCTGATAGATATAAAACTGCTTTTGTCACCCCATTTGGTCATTATGAGTGGAATGTCATGTCTTTCGGTCTTAAGAATGCCCCCAGTGAGTTTCAAAATATAATGAATGAGATATTTAATCCCTTCAGTCATTTCTCTATAGTTTATATTGATGATGTTCTTATCTTTTCCCAGTCGATAGAACAACATTGGAAACATTTGCATAATTTTATGCAGATAGTCAAACAAAATGGTCTACTTCTTTCTGCTAAGAAAATTAAGTTGTTTCAAACTAATATCTGATTCCTTGGTTTTAATATCTGCTAGTCACAGATTAGTCTAATTGATCGAGTTATCCAGTTTGCTGATAAATTCCCAGATTAAATCCTAGACAAAAGTAAACTCCAGAGATTCCTAGGATATCTAAATTACATTTATGATTTTTACAAAAATTTGAGAAAACAATGTAAGCCTTTATTTGATCGCCTCCAGAGTAATCCTCCTCCTTGGTCACCCATTCATACAGAAATAGTCAAACAAATCAAGGTCCATATCAAAACACTCCCTTGTCTTGGCATTCCTTCAGTTGATTCCTTCAAAATAGTAGAAACTGATGCCTCTAATATTGGTTATGGAGGTATTCTCAAGCAAAGAGTTGATTCTAGTCAAATTGAGCAAATCATCCGATTCTATTCCGGTATTTGGACACAGTCACAAAGTAATTATAGTACTATTAAGAAAGAGATATTATTTATAGTATTATGCATTACTAAATTTCAAGATGATTTATTAAATGAAATTTTTTTAGTTCGAGTCCATTGTAAGTCTGCTAAGCATGTATTAGAAAAAGATGTTCAAAATATTGCATCCCAACAAATATTTGCACGATGGCAAGCTATTATAAGTATATTTAATTTTAATATTGAATATTTGACAGGAAGTGAGAATTTTGTTCATGATTTCCTTACTAGAGAATTTCTACAGGAAAAACAATGGCAGACAGAAGAAAAGATAAATAGAAAGAACAAGCCAGTCAATCCAGTCAACCCCAGTCACTCAAAGTACTCCCCCCATCCAAGAATCCACCCAAAACAGAGTCACCATCCCAGATCGTCCCTTACACGGGATGTTCCCCAATCTAAATAAGCAACAAATTCGCCACTTTAGGAGCCACAATAGTCCAAATTCGTCCTAGTTTCCAGTCTGCCCTAGTCTCTAGTCATGATTCTTTCTAAGACACCACCCAGTTTACTCCTTTAGTTGCCTCATACCAAAAGTCACCCCCTACGTGCTCAAGAGTAATAGTCATCTCTTTATCATTGAGTCAAAATACAATGCTATTGCCAATCCAGTCACCATCGCCATGTCATACTTCCCTCCTAATAGCCATTTTATTCCCCAAGCGCCTTTTAAGAATTTGAAATTCTACAAAGACATCCTTCATGAGACCCAGTCCATCACAATCAAACCAATCAGAGACCGTGAGAATCCACACATAATCCTCTACTATTAATGTTACAAGCCTCCCAAATGAATGAAGAAGATGATAATGATTCTTCCTCAAAATCCTATTCATCCACTTGTCAACCAAGAATGCCATCCAATCAGAAGACTCCAATCCCCAAAATGCGGTGGAGTGATTATCCCTCTGACAGTCAAGACCCTTATGACTTCAGCCTTTTTGATGTGGGCAAAGATTAAGTTGCCAAAGTCATAATGAGCCCCCAAAAGAGAAAGAAAAGTTAAAGCAAGCAACCACTTTAGTAAAAGCATTTGGAATCTTAGACCCGTGCTTCAACAGTCTGATAGCCTGTGCTCCTTTAATAAGGTTCCCCGACAAAGTAAAAGTTACAACACTCAATAAAAGCAAAAGCTTTCGCGTGCTTCTACAGTCATCATCCAGAGCATCCAAAGTTGCCCATAGTCTACTTTATCTGTCTATTTAAGAAGGCTTCGGCCTCAGTTATCCTCAGAATAAATGGAGGATGACTGTAGAAGCACGCGAAAGCTTTTGTTTTTACTGAGTGCTGTAACTTTTACTTTGTCGAGGATCCTGACTAAAGGAGCAAGGGCTATCAGATTGTTGACGCACAGGTCCAAGATTCTAAATGCTTTTACTAAAGTGGTGGTTTGCTTTAACTTTTCTTTCTCTTTTGGGGGCTCATGATGACTTTGGCAACTTAATCTTTGCCCATATCAAAAGGGTTGAAGTCATAAGGGTCTTGACTGTCAGGGGGATAATCACTCCACCTCATTTTGGGGATTGGAGTCTTCTGACTGGATGGCATTCTTGGTTGACAAGTGGATGACTTGGATTTTGAGGAAGAATCAATATCATCTTCTTCATTCATTTGGGAGGCTTGTAACTTTAATTTGAGGCTTTTTTCCTGCTTAATTGGTGAGCAATTCCTGCATTCATACTTAGATTTTCCCTCGATTGGGAGAGAAGAATGGGAAGTAGTAGGAGATGACATTTTGTCTGACTGGGGATTCCTGACTGGAGGAAAGTGTGAATAAACATAATATGCAATTCCCTCAATTTTGAATTTATCCCACCATTGAATTGAAAATTGGCGAGACAGGACTCTCGATTCCCAATTGACTTGGTAGGACCATTTTAAGATCCAGGGGACTTTATATTTAGCAATGAAATGGAGTAGATTAGGGAAGAATAACTCAGACTCATTAAACTTGTGTTTATTGGTGAAATAATTGACTAGATCTTGCATTTGTGAAGGAAGGAGGTCAATAACTGAACCATGTTTTTCCCACCAATGAAGAAACCAAAAGGGAAAAGGACTCTTGAATTTACTGTCAAAATTAAGGAACCAGGAGTGACAAAATTGACTGTTTGATGAAGCAAGATGGAATACCAAGCTTCAATGTAATCATAGTAACTGTATTGGAGTTCTGACTGAAGAACTTTTAGATCATAAGGGTGACTGCTCCATTCATTTTCACTGAGGATTTGGTGGATGTATAAGGAATGGTAGAGGATTATGTGTGGATTCTCACAGTCTCTGATTGGTTTGATTGTGATGGACTGGGTCTCATGAAGGATGTCTTTGTACAATTTCAAATTCTTAAAAGGCGCTTGGGGAATAAAATGGCTATTAGGAGGGAAGTATGACGTGGCGATGGTGACTAGATTGGCAATAGCATTGTATTTTGACTCAATGATAAAGAGATGACTATTACTCTTGAGCATGTAGGGGATGACTTTTGGTATGAGGCAATTGGAGGAGTAAACTGGGTGGTGTCTTGGAAAGGATCATGACTAGAGACTAGGGCAGATTGGAAACTGGGACGGATTTGGCCTATTGTGGCTCCTAAAGTGGCGAATATGTTGCTTATTTGGATTGGAGAACATCCCGGATAAGGGATGATCTGGGATGGTGACTCTATTTTGGGTGGATTCATGGATGGGGGGAGTACTCTGAGTGACTAGGGTTGACTGAATCGACTGGATCTGGATTGACTGGTTTGTTCTTTCTGTTTATCTTTTCTTCTGTCTGCCATTGTTTTTCCTGTAGAAATTCTCTAGTAAGGAAATCAGGAACAAAATTCTCACTTCCTCTCAAATATTCAATATTAAAATCAAATATTCTGAAAATAACTTGCCATCGTGCAAATATTTGTTTGAATGCAATATTTTGAACATCTTTTTCTAATACATGCTTAGCAGACTTACAATCAACTCGGACTAAAAAATTTTGATTTAATAAATCATCTTGAAATTTAGTAATACATAATACTATAGATAATATCTCTTTCTTAATAGTACTATAATTACTTTGTGACTGTGTCCAAATACCGGAACGGAATAAGATGATTTGCTCAATTTGACTAAAATCAACTCTTTGCTTGAGAATACCTCCATAACCAATATTAGAGGCATCAGTTTCAACTATTTTGAAGGAATCAACTGAAGGAATGTCAAGACAATGGAGTGTTTTGACATGGACCTTGATTTGTTTGACTATTTCTGTATGAATGGGTGATAAAGGAGGAGGATTACTCTGGAGGCGATCAAATAAAGGCTTACATTGTTTTCTCAAATTTTTGTAAAAATCATAAACATAATTTAGATATCCTAGAAATCTCTAGAGTTTACTTTTGTCTAGGATTTGATCAGGGAATTTATTAGCAAACTGGATAACTCGATCAATTAGACTAATTTGTGATTGGTAGATATTAAAACCAAGGAATTGGATATTAGTTTGAAACAACTTAATTTTCTTAGCAGAAAGAACTAGACCATTTTGTTTGACTATCTGCATAAACTTATGCAAATGTTTCCAGTGTTGTTCTACCGACTGAGAAAAGATAAGAACGTCATCAATATAAACTATAGAGAAATGACTTAAGGGATTAAATATCTCATTCATTATATTTTGAAACTCACTGGGCGCATTTTTAAGACCGAAAGGCAGGAGATTCCACTCATAATGACCAAATGGGGTGACAAAAGCAATTTTATATCTATCAGACTCATGAATTTGGATTTGCCAAAAACACTCTTCATATCAAGTTTAGAAAAGATGACTGATTTTCCTGACCTGTTAATTAGATCTCTCTTATTGGGTATTGGGTACCTGATCCATTCTAATACCGTATTAAGAGGCTTATAATTAATAACTAATCTTGGGGCATCGCGTTCTAACTCAACATTTTTCTGGACATAGAAAGCAGGGCAGAACTAGGGTGACTTACTCTTACGAATTATTCCTTTCTCAAGTAATTCTGAAATTTCAATTTTACAAAATTCTATTAATTCTTGACTCATTTGAATTGGTCTGGCTTTAGTGGGGATATTCTGTTCACTAAAGGATTTGACATAAGGAAGACTAACAACGTGTTGTTTCCTATGCCAGAAAGCATTGGATATCTCAGAACAAACTTCTTGTTTAAATTTTTCTCCGAATTGGCGAATTTGTGTCTGCAAAGACTCAAGACTAAGTTGTTGCTCAATTCTTTTGAATTTGACTTCTTCCTGGAGGAATCTTAATTGCTTACCTTATTTTCAATTACTTTAACTGTTTTGGTGACTGTGTTCTTTTGCAAGGCTTTTAAATTATGTAACTCTGGTTTACTCAGGAATTCAAACTTAACATTTTCTCCTAAATGGGTAGAAATGACTCCATCGTATTCTACCTGAAAAAGATACAGTAGGGCAATAAAGGGTAATCCTAAGATGACTTCATCAATAATATTTTTGACTAAGACGAATGAGGTTTTAAAGCAAATTTTATCTTGACAGATATGGGCTTTAGGAATCTCATAATTCGACTTGATCCTGATTGGAGGGTTTACCATCTGAATTTGTAGGGCTTACTAGCGTAGACTCAGATTTTCCGATTAGATTAATTAAATCTTATTTAATCTCATAGTTTTCTACCTGTAACTTTTGACTTATCTGTTTTAGTTCCACTATTTCTTTTTTGACTAATTTTACTTCTGGGTGTAAATCTTGTAAAGTAACTTCTTTTCTTTTGTTGAATCTTTCTAAAGTAGTGTTCAGACTTTTCTTTTGAGGGGTAGACGGACTTGGTTCTTCCTGACTGATGACTTTCCTTAATTTCTTTAAGTACTTAGATTTCAACTCTGGGTTTTCAACTTTACTTATTAAATCAATTAATAATTCTTCTTGTTCTTCTTGTTTAGTAAGGACATTAATTGACTTAGACTTACAACAATTATCTTGACAACCAAATTGAATTACGTGAACTTATCTGGGAATCTGAGTAATTAAACTCTTTTGACTCAGTGTTACTCACCATGGTCTCGTTTTCACTAGACTTTGAATTTCTTAACTCTAAGATTTTGACTAACTTTTCTTTATCTTCATCCGTGATTTTCAATTGTTTGATTGTGTCTTTTACTTTACACTCATTAGCATAGTGACCAAACTTTTGACATTTATAACATTTAACTTTTCTTTTGTCGGTAGTTTTCTTAAAGTGACTTTTCATATCTTTTCCGTGACTCTTTTTCCAATTTTTACGCGGGTAAAATCTCTTTTTACTGTAAAACTCATTATTCTGTTTGAAAGATTGATTTCTTGGGAATAATGACGACGCTTACTAAACTGTCGTTCACGGTGACTAGACTTGGCTTTTCTAGATGGGGGAACGGACATTAGACCATACTGTTCGCAGAAATGACCTAACTCATACTTAGCTGAATTCTTTTCTGACTGAATTTGTTTTCCAATTTTCAAATCTACACACATTTTCATACCGACTTGATTAATTGTGCTAATTATATTACCATAGGTTAAGTTATCCCAATCTATATAACCTTGATCATTACTAAGGATTGTGCGTATTTTTTGTGCAAACAAATTAGGGAGACCATTAATAAATTTTTCTTTCCAAAATGGTTGACTACTATCATCTCTAATCATGACTCTGGAGATGAAGACATCTTTATACCAACGGAAATCAGATAACTTAGGGCATGTAAGGTTATGAAGCTGATCATGAATTCTAGTAGTGGTGTTACTAGGTGTTCCAATGAAATGCTCAATTATTGTGTATAATAAGGTATTGACTCCATCTTCTATACCTCGACCTACTTGTTCGTCGAAAATAGGAAGACCTTCTTCATTAAGTTTGACTGCGTGGATTATTTGTTGCTTAGACTCATCAGTTAAATGTTTATCCCACCAAGAACGTAAGGTTCCAGTGAAACTAGTTTCTAACAAAGGCACAATTTCTAACTATCTAAAACTGTGATTGGTATTGTAACTATCGGCGACCATAGACATATGTTTATGTTTATTAAGGATTTCTAAATTTTAAATTTTAAATCATTTTCTAATTCCAAATTTTTAAATGCGTTTTCAATTTTTGAAATGTTACTAGTTTCTGTTTCCATACTAATGACTGACTTAGAATCAGAGGAGTTAACTACAGCAAAAGGTAGCTCAGTGGATCCTAAGACTTTAACACCGGATTTCTTGGCTTTCTCAGGGGGTTAATTTTTCAAGCATTTCTTCTATTTTTCTCATTGTAGGTTTTGTCTTAAAGGTTGGGCTTGACTTTAAGTCTTGGAATTTAACTAAAAGTTTTTTTTTTTTTTTTTGGTAATTTGGGTTCGACTTTATTAGGTAGTAATTTGTCGACTTTTGTTTCTATTTTGTATAACTGTTTTCCTATGACAACTAGACTTTGGTTAGTGTAATTGTTTTGTTCTACAATCATTTTATTGTCTTCGTCAGGGATTTTGTAAGGGCTAGCCACTATTTTTGACTTTTTATGGTGAATAACTATTGTCTCAACTGGGGGATGACTGGCTAAGACTTCTGTTTTGTCTTCTTTTTCCCATTTTTCTTGGGTAAAAGTTTTTAACTCTTTTTTCATGTAACGGCTTTCTACAAAATCAAGATGATAAATTTCAACTTTGGCTGTTCTCATATATTGTTTCCATTCATTAAAAATTTGTTGTCTTTTGATTTTGTCAGGATAGGCTGTATGGTATTTTTCTTTTCTGGACTGGTTTTCTGGGGCACGTAGATGACTATCTAATCTTTTTCAATTAATCTTAAATTTTCTATTAAGGTTTCTTAATTGGTTATCAATTTGAAAACCGGCTTCAAAGTCTGTTTGTGTGGGTGACTCAGGGGACTCAGATTTAGTATTTTCTTTGACTGTTGTTTTACCATAACAGGGTTGACTAACTTGGGATGATTTCCTCAACTATTCTAACTTTAAATCAATTAAAGCTTTCTCTAACTCGAGGTCTCGTTCTTGAGTAGAGGAGGAGGCTGCAAAGGAGTGTCTAGCAGGGACGTGTCTCTGGGATTGACCAGGTGTGGGAAGTTGTTGTAACTGGACTGAGGACTTTCGTTGTGGTTGGTTTCCGAAATTAATTTTGACTGTGCCATCTAGATATTGTTGGATATAATCTAAATTGTTTAGACTATGTTGAATTGGGACTGGTGGACTTTCATTTGTTAAGGTCCAATCTAAGGGAAGGGTGACTTCTGACCATTTTACTATTTGAGGGACTCTTATTAGGGAATCGGTATTATTACTTTGAATTAATAGAGTTTGATCTTTTGGACTTTTATTAAGGGCTTGGAAATTCATATTGGTTCTTGTGACTCTGTAGTAAATTCTGTAGACTAAGGCTAATGGTTGGGTACCAGGTAAGACTTCGTATCCTGAGGTCTTAATATCCAATGTGAGGGTTTTTAAAATATGGGGATCACTAAGGCTCACCGTAAAGTCAGGGTAACAATCAAAGTAGACTGGGCCATTAGAAAAACTGGATTCAATAACTCCTAGCATACTGTCGCTAAAATTAGTGAATCTAGCATCACATAAGTAGAGAAGGATAGAAGCTTCTAATCCTAATCTGGTTAGTGGTTTAACTGCAACTTGGAATAGACCAATGTGAAGGAAATTGTTTCCATCATTTTTATGTTTCTAATAAATCTTTTTCTAATACATGCTTAGCAGACTTACAATAAACTCGGACTAAAAAATTTTGATTTAATAAGTCAATTGAAATTTATTAATCCATAATACAATAGATAATATTTCTTTCTTAATAGTACTATAATTATTTTGACTTTGTGACCAAGTACCAGAATGGAATCGAATGATTTGCTCAGACTGACCAGAAGGGACTTTCTGTTTGAGGATACCTCCATAACCTATATTAGAAGCATCAGTTTCAACAATTTTTAATGAAAGAGCATAAGGAATACCAAGACAAGGAAGAGTCTTGATATGGGTTTTGATTTGTTTGACAATTTCAGTATGAGTAGATGACCATGGAGGAGGATTATTCTGGAGACGATCAAATAAAGGCTTACATTGTTTTCTTAGATTTTGATAAAAATCATAAACATAATTTAAAGATCCTAAAAATCTCTGGAGTTGACTCTTGTCTAGGATTTGGTCAAGAAATTTATTAGCAAATTGGATAACTCGACTAATCTGTGACTGGCAGATGTTGAACCCAAGAAATCTCACATTGGTTTGTAATAAATTAATTTTCTTAGCAGAAACAACTAAACCATTTTGTTTAATTATTTGTAAAGACTTATACAAATGTTTCTAGTGTTGTTCTATAGACTCAGAGAATATAAGCACATCATCAATATAAACTATGGAAAAATGGCTGAAAGGATTAAAGATTTCATTCATTATATTCTGGAATTCACTACGCGCATTCTTAAGACCGAAGAGCATAACATTCCACTCATAATGACCAAATGGAGTAACAAAAGCAGTTTTATATTTATCAGACTCAGAAATTTGAATTTGCCAAAATCCACTTTTCATATCAAATTTAGAAAAGATGACTGATTTTTCTAACCTGTTGATTAAATCTCTTTTGTTAGGAATTGGGTATCTAATTCATTCTACGACAGCATTAAGTGGCTTATAGTTGATGACTAGTCTTGGGACACCGCGTTCTAATTCGGCATTTTTTTGGACATAAAAGGCAGGACAAGACCAGGGTGACTTACTCTTACGAATTATTCCTTTTTAAAGTAAATTAGTAATCTCAATTTTGCAAATTTCCATTAATTCTTGGCTCCTTTGAATGGGTCTTGCTTTAGTAGGGATATTTTTCTCACTAAAGTTTTTGACATAAGGAATGCTGACAACATGCTGTTTCCTATGCCAGAACGCATTGGGTAGCTCAGAACAGACTTCTTGTTTTAATCTTTCTTCAAATTGGTGTATTTGTAATTGAAAAGACGTATGACTCGGTTGTTGCTCAATTCGTTTGGTTTTAATTTCTTCCTGGAGAAATCTTAATTGTTTATCTTTGTTTTGGATAACCTGGATAGTTTTTGTGACTGCATTTTTTTGCAATTCTTTTAAACTATGTAACTCAGGTTTACTCAAAAATTCAAACTTGATCTTTTCTCCTAAACAGGTGGAAATGACACCATCATGTTCTACCTGGAAGGGGTACAGTAGGGCAATGAACGGTAGACCTAATATGACTTCATCAGTAATATTTTTTACTAGAACAAAAGAAGTTTTAAAGCATATTTTATTCTGACAGATATGGGCTTTAGGGATTTCATAGTTCAATTGAAGAGATTTTCCTGAAGCAGTACTTAATGATTCTCTTGATTTTTTGTAATACTTAGTTGGAATTAGGCCTTCTTGGATACAGTTAAGGTCAGCTCCGGAATCAAATAGGGCGACTATGTTTAATTCGAAATTTTGAACAAATATAGTGACTCGAGCGTACCATTTTCTGAAATTTACTTGTTTAATTAGGTTAACGGCTTGCTCGTGATTAGGGCTTTCTTCATCTGAGTTAGTTGGGCTCTCTGGGGTAGACTCAGGATTTTGCTTTTCTAATAAATTAGTCAGATCTTGTTTTATCTCATAATTTTCAGATTGTAATTTTTGACTTAATTGTCTTAACTCAATGATTTCTTCTTTGATTATTTTTATTTCTAATTGTAAGTCCTGTAAAGTAACTTCTTTCTTTTTCTTAAATTTTTCTAACGTTGTATTCAGATTTATCTTTTGGGTAGACTGTTTCGGTTCTTCTTGGCTGATGACTTTTCTTAATTTTTCAGATACTCAGATTTTAATTCTGGATTCTCAACCTTGGTTATTAAATCAATTAATAGTTCTTCTTGTTCTTCCTGTTTAGTGAGGACACTAATTGATTTAGACTTACAGCAATTATCTTGGCAACCAAATTGGATTCTGGGGGAACTGGGTTGTGACTCAGAAGAATATATTTCAGAGAACTCAGTATGACTAATCATGGTTTCATTTTCACTGGACTTAACTCAAGGACTTTAATTAATTTGTCTTTTTCCTCATCTGTAATTTTTAATTGTTTAATCGTGTTTTTAAGCTCACAATTATTGGCATAGTGACCATACTTTTGACATTTATAACATTTCACTTTTCTTTTGTCGGTTCTTTTCTTAAAGCTGTGGCTTTTTTCTTTGTGATGACTCTTTTTCCAATTTTTCTTAGGGGAAAACCTTTTATTTTTATAAAATTCTCTATCATCATTATTGTGCTGGAATGACTGTTTTCTACGTGAATAATAACGCTTTTTACTAAACTGTCGGTTGTGGTAATTAGGCTTATTCTTTCTAGACGGGGGGACAGACATTAAACCATATTGTTCGCAGAAATTTCCTAATTCATATTTAGCAGAATTTCTTTCAGATTGGATTTGTTTACCAATTTTCATATCAACGCACATTCTCATACCAACTTGATTAATTATTCTAATTATATTTCCATAGGTTAGACTATCCCAATCAATGTGACCTTGTTCATTACTAAGGGTTGATCGGATTTTATGAGCAAATAAATTCGGTAATCCATTAACAAATTTTTCTTTCCAAAAAGGCTGATTACTATCTGTTTTTCCCATGACTCTGGAAATAAATACATCTTTATACCATCTAAAATTAGATAATTTAGGGCATCTTAAATTACTCAATTGATCATGTATTCTGGCTATGGTGCCACTGGGTGTTCCAATAAAATGATCGACTATTGTGTAAAGCAATGTGTTGACTCCATCCTCAATACCTTGACCTATTTGTTCATCAAAAATAGGAAGACCATCTTCATTAAGTTTTAACGCATGAATTATTCGTTGCTTAGACTCAGGAGTTAAATGATTGTCCCACCAAGAACGAAGAGTTCTTGTGAAACCTATTTCTAATAGAGGAACAATTTCAGACTTGCTGAAACTGTGATTGGTGATGTAGCTATTGGCAACCATAGACATATGTTGAAGTTTATTAAGGATTTCTTGCTCAGAAAGACCATCAATATTCCATTCATACAATTTATCAGAAGAAACAGAAAACTGGCTTTGAGTATTTCGTTCTTCAAATTGAATGTTTGGTGGTGTAGGTTTGGGATACCAATTTTTTGTGAGACTGGTAGGGTTGAATTTGTTTGTTAATCTTTGGACTTGTGGATTTTGTTCGAGCTCGAGATTTTTGAATGCATTTTCAATTTTGGAGATATTACTAGGCTCAGTATCTGTGCTATCAATGGATTTAGACTCAGAGGATTGATGTAATAGGAAAGGTGACTCAGAGTATCCTAAGACTTTAACGCTTGACTAAACTTTTTCTTTATCTTTACGTTTAGTTGGAGTTAAATCTTCTAACATTTTCTCAATTTTTGTCATTGTGGTTTTTGTTTTAAAAGTTGGACTTGATTGTAGGTCTTTGAAGGTAACTAATGGTTCCTGATTATGTGGTTTGACTGAAATTTCTGTTTTGTCTAACTGTTTTCTTATGACTACTAGGCTTTGATTAGTATAGTTATTTTGTTCAATTACCATTTTGTTTTCTAAGTCAGGGATCTTAAAAGGACTGGCAATGATTTCTGCTTTCTGGTGATGGATAACTATAGATTCAACAGGGGGGTGACTAGTAAGAATTTTGGTGTGATCTATTTTTTCCCAATTTTCTTGAGTTAAAGTTTTTAATTCGTTAGATATATGACGGCTTTCTACAAAATCAAGGTAATAAATTTCAACTTTAGCAGTGCGCATGTACTCTTTCCATTAATTAAATATCTGTTGTCTTTTGGCTCTATTAGGGATTGTTGAATGATAATTATTTCTTCTTGCTTGATTTTCAGGAGCATGTAGGTGACTTTCTAGTTTTTTCCAATTGATTTCAAATTTTTTATTAAGTGTTCTGAGTTGGTTTTCTACTTTGAAACTTGTTTCCACAAAATCGGATTGAGTAGGTGACTCAGGAGACTCAGATTTATTATCATCTTAGGGTTTGTCGTAGACAGGTTGAGTGACTTGTGCAGTTTTCCTTAGTGATTCTAATTTCAAATCAATTAAAGTTTTCTCTAATTCTAGGCTCGTTCTAGAGTAGAGGAAGAGGCTGCAAAAGAATGCCTGGCAAGAACATGTCTCTGGGATTAACCAGGTGTTAGTAACTGTTGTAACTGGACTGTTGATTTCTGTGAGGCTTGGTGATTAAAGTTAATTTTAACTGTACCGTCTAAATATTGTTGTATGTAATCTAAATCGCTTAGACTACGTTGGACAGGTACTGGAGGGTTCTCAGTGGTTAGAGTCCAATCAGTGGGAAGAGTAACATCTGACCATTTGATAGAGTGGGGAACTCTAACAGTAGCATCAGGCGTATGACTTTGGATTAACAATGTTTGACTCTTTGGGCTCTTGTTTAAGGCTTGAAAATTTTGGGTATTGGGTCTTCAGAGTATTTAGTGACCTTTCATATTTTGTTTGTCTCAAAGATCCTAGTTTCACCACTCTGAGTTTAGTACCAAGATCCTTTCACCACTCTGACTAGGATCTATCTTCTGGTGGTTCCTTTGCTTCGGGCATAAACTTAAA

TATATATATATATATATATATTGTGTTGTAAGATGAGAGAAATAGAACAAGCAAAGGAAGTAAAGCAATGGAACCGGAGAAAGTAAACAAATAGGACCTTAATCCCCAAATAAAGAAAGAATATTACCAACGTTACGAACAAGAGTTTTCCTACTAAAGTGTAAAGGAACCACTTGTATTTTGCTCACCCAACCAAAAAAAAAAGAAGAAATAAAGAAAAAAAAAGAAAACTAAAGTGTTAATTTAGGCAATCACAAACATTACAGTTTTCGTTTTCGCACACAACAATATATACAATGTCATACATACACAAAAATACAAGTCCGGGAGTGAGTCCCCCCTACCTGGAATCCAAAGCTAATGCCTCACTTGAATTCTACACCTTTGAACTCAAATTGTTCTTGCTTGGGTAACTGATTCCTAAGCCAACATTTCAAAAATGTTACTAAAAACCCTTATTTGCAATTCCGTCATTACAACTAGCTTTAATTGCACTTTAGTCCTTATACTTGTCAATAATTTCACTTTTGTCCTTATACTTTCCTTTATTTTCACTTTGGCGCCTATACTTTTTCCAATACATTTCAGTCTTCAGACTTATCCAGAATTTCACTTTGGTCCTTACATTTTCCTTTATTTTCACTTTACATATACTTATTTTAAGGGTAAATTACTCAAATGGTCCTCAAACTTATACGCGATTTACATTTTGGTCCCTAAATTAAAATATTAGTCCAAATGGTACATAAATTCTATTTTAATCGCCCATTTGATCCAACCGCTACATTATGTCAATATTGCTGTTAAATATGAGGGCAAATTGGTCATTTCGTACGTTAAAATAAAAAAATAATAAAAATAAAATAAAAACTCCGTAATTGGAGTAGAGAGAAGAAATGGAAGGGAGGGGGTTTGGGTCTCATGATTTTTTCCTCTCTTTTTCTTCAATTATTTTCTAATTTTGTATTTATTTTAACCCTATTTTCTTTGTTAATTAAAA

>Chr2-04; OZ022207.1; 3400654-3409585; rev; D;

ATGCAAAAGTCATGGGCTAAATAAAATTTGGCCAAATTTGGAGGGCCTCATTTGGCCCTTTAGGCCATCTTCAGTCATAGAGCTAAATGTAGTCCAGGGCTAAAAATTTAGCCCTCCAAAATATTAATTTTTTAAATAATAGTGCAGGGCTAAATTTTTCCATCTCAAGCCATGCAGGGCTATATTATAGGATCCTTCAGCTTTCAATATATAGTTTTTCTCAAAATAATAAAATATGAAGGATACTAAAATATAACCCTGCATGGCTGGAGATGGAAAAATTTAGTCATGCACTATTCTTTAAAAAATTAATATTTTAGGGGGCTAAATTTTTAGCCCTGGACTACATTTAGCCCTATGACTGGAGATGACCTTAGGGGCGGTTTGGTATCCTACTTGGATCCAATTTTATAAACCCAAAAATATATTTTAAGTCCAAAACCCCAAAAACCCGTTTGGTAGGCCTATTTTTAAAAACCTAAACCCAAAAAAAAACTCAAAAACACCCAAATTATTGAAAAAAAAAAAGTGAGATTTTCCTTTTTATTTTTATTTATAGTACTGCTAAACGAAAGAGAGGTCCGGGCTTCTTTTGTGCTTTTCCTTTTATTTTTTGTATAAATTAAAAATAATAATTAATTGGAATAAGTTTTAAGATCTTAAAAATAGGTTTGAAGTTGAATACCAAACAAGTTTTCGATCTTAAGGCCCCGTTCGTTTATGTTTTTAGAGCCCATTTTCATTTTGAGAGAACAAAAACAAACAAAAAACCTGTTCGGTGGGCAAAAATAGAAAACACAGAGAATATTTTATGAAAACAAAACCACCGTCAATCGCTGCCAACAGAGAACCACACACAGAGCTTGAACCACCACCATCACCCAATCAAAATCACCACCGCCCCATTGAACCAGATTGACATCACCGCCACTCAATCGAACCCAACAAAATCTCTATGCATTTGAATTGAACCACCACCGTTGAACAACTAATTGCCGAC

GTGATTTTCAATTGCTTTATCGTGTCTTTTGCTTTACACTCATTAGCATAGTGACCATTCTTTTGACATTTCTAGCATTTAACTTTTCTTTTGTCGGTACTTTTCTTAAAGTTGCTTTTCTTATTTTTTCTGCGACCTTTAGACCAATTTTTACGCGGGTTAAATTTCTTTTTATTGTAAAATTCATTATTATCATTATTATGTATGAAGGACTATCTTCTTCGTGAATAATGACAACGTTTACTAGTTTGTTGTCATGATAACTAGACTTGGCTTTTCTGGATGGGGGAATGGGTGCTAAACTGTATTGTTCACAGAAATCTCCTAATTCATATTTGGCTGAGTTTCTTTCTGATTGAATTTGTCTACCAATTTTCTATCGACACACATTTTCATTCTGACTTGATTAATTGTACTGATAATATTTATGTAAGTTAAATTATCGAAATCTATGACCTTGATCATTACTAAGGACTGTTCTTATTTTATGAGCAAATGAATTGGGGAGATCTTTAATGAATTTTTCTTTCCAAAAGGGTTGATTACTATCATCTCTACGCATAACTCTGAAAATAAAAACATCTTTATACCAATGAAAATCAGATAATTTGGTGCATTTGAGATTACTATGTTGATCATGAATTCTGGGGAATATGCTAACTCGTTTCTTCCTGACTAATGACTTTTCTTAATTTCTTTTAATAACTCTTCTTGTTCTTCTTGTTTTGTAAGGACACTAATGGACTTGGATCATAGGGTGTACTAAGTTAATTTTAGGGTTCGTTTAGCAGCACTTTTTTTTTTCCTCTCTTCTTTCATGTCTCTATCCTCCCATTCTCATCTCTTTACTTACCAAAAATACCCCGGATACACCCTAATCCACCTTATCATACGGCAGCAAACCCATTCGAATCTGGTAACAACATTTATCAAGGTCAAGTGGCATCGAACTCATCCCCAGATTGGATCGACGAGATCGAGACCATTATGATGTAAGACGACGATGTCAATGACCCAGATCTTGTCGTCGCTGATAGCAGCTTCGGCCTAAATCTCGTCGGCGACGGCATCGTCTGTAACCTTTTTTTTGTTGTTGTTGTTACCTTTGCTTTCACTCTCTGCAAACCCACCCCAATTCCCCTCTCTCTCTCTCTCTCTCTCCAAAATTGAAGAATCAGACCAACCCCCCTCTTTTCCACTTTCTCTTCATGCTGTTGCACCCTCTCTCTCTCTCTCTCTCTCTCTCACTGCAACCTCACCTTAGCCTTCGGTAACAGATATCTAAATTCATGATTCAATATATGAGTTAATGGTAATCAATGGATGAAAGAAACAAGATTATAAGGTGGTATAATGTTTTGATTTGTCTATGAATAAAATTGAGTTTTTTGTTATTACTTTGTATTTTTTATGATTCAAAATTTGATTTTGGGAGTTGTAAGGTGAATGAAATATCACAAGAGGAGTTCCTTGGAATTTTTGGTCCTGGAGCTAGAAACAACCCTAATTGATCTAAAGTTAGAATCTCTGAGGAAAACTTCCCAAGTCAACCAACTTGTTGTGGTAAACAACCAGTCAAAGAAGAAAAAAATCAGGTTCCCCAAATTCTCCCACACAAACTGATTTCGAAGTCGGTTCTTTCAATATAGATAATCAATTACGAACCGTTAATAGAAAATTCAAAATCAATTGGAAAAATTTAGATAGTCATCTTCATGCCCCAGAAAATTTGGAAAGACGAAACAAGTATCATACAACGTAACCTAGTAAAGAACAGTGACTACAAATATTTAATGAATGGAAAGATTATATGCTAGCCGCTAAGTCAGAAATTTTTTATCTTGATTTTATTGAAAGTCGCTACATGTTCAAAGATTTGAAAACCCTAACACAAGAAAAATGGGAAAAATAAGATAATACTGAAGTCTTAGCTAGTCACCCCTTGTAGAAACAATAATTATTAATCACAAAAAGCCTAAAATAGCAGCCAGTCCCTTCAATGTCCCTGACCAAAATGATAGAAAATTAATTGAGCAAAATAAATATACAAACCACAGTTTAATAGTCATTAGAAAACAATTAGATAAGATTGAAAGATTTTATTGATCACTCAGACTATACAAGAGTTCATCAGGGTAGTAGTCCTGAGAGAACTTTGAAACTGAGAAAAGTAGAATTTACTTCTGATTACAAAGGAGGTCGAAGCCTCCTTAAAAAAACTAAGGCTACCAGACTGTTGGTAGAATTGGATGCTTGGAATCACGATATATATATATATATATATATATATCAGAGGCGTAAGGTGTTAATAGGTTTCTTATTGGTATAACACTTACGACCCTAAACCCAGGAACCTAACCTTTGGCCTTAGTCTACAGAATTTACTATAAAGTTACAGGAACAAATATGAATTTCCAAGCCCTCAATAAAAGCCCAAAAGATCAAACTGTCATAATCCAGAGTCACACTTCAGATGCCCATATACAGGTTCCCCACACCATTAAGTGGTCCGAAGTTGCTCTTCCAAAAGATTGGACTTTGGTCACCGAGAGCCAACTAGCACCAATCCAACGCAGTCTAAACAATTTAGACTACATCCAGCAATATCTAGACGGCACAGTAAAAATTAAATTTGACAGCCACCCCTTACAAAAATCCAATGTCCAGTTACAACAATTGCCAACTCCTGGTCAGTCCCAAAGGCATGTCCCTGCCAGACATTCCTTTGCTGCTTCGTCCTCCACTCTAGAACGAGACCTAGAGTTAGAAAAGCCATGATAGAATTCAAACTTGAGTCCCTGAGAAAAACCTCTCAGGTCACCCAACCTTGCTACGGCCAGGTTCCAGAAGATAAATCAGACTCCCCCGAGACTCCAACCCAATCTGATTTCTTAGTTGAAAACCTTGCTGCAGTAGATAATCAACTTAGACATTTAAATAAGAAGTTTGAAATTGATTGGAAATGTCTCAATCGTCATTTAAAAGCCCCGGAAAACCAATCCAGAAGAGATAATTACCACCAAGCCTATCCTGATAGTAAACACCGCGAACAAATCTTTAGTGGATGGAAAGATTATATGCGGTCAGCCAGAGTTGAAATCTTTTACCTAGATTTTGTTGAAAGCCGTTACATGTCAAATGATGAGTTAAAAACCTTAAGAAAAGTGGAATTTAATAGATAAGTCCGTAGTTGAAGCCAGTCATCCCCCAGTAGACACTATAATAATAGATCACAAGAAAGCACCAATACCTGCCACTCCTTTCAAAACCTTTGAAAGCAGTGACCCAAATAGAAAAATAATCGAGCAAAATAATTACACCAATCAAAGTCTAATCGTCATAGGAAAACAACTAGACACAATTGAAACCAAGATCGATAAGTTGAATCCTCCAGAAACAGATTTCAAGCCCAAACTTAGATTTGAAAAACAATAGTTCAGTTCCAAGAACTTAAGTCCAGTCCCGTACTTAAAGTCAATTCTACAATGAAAAAGATAGAAGAAATGCTAGAACAGTTGACCCCAACAAAAATTGAGAAAGTCGAAAAATCTAGTCTTAAAACACTAGATTTTTTTGTTGCCTCACATTCTGAGTCTGAGTCGATAAATTCCGAAACCAGTAATGTTTCAAAAATTGAAAACGTCTTTGAAAATTTAGAAGTCCAAACTAAACCAAAAATCAAAAGATTAGACAAACATGTAAGTCCTACCAGTTTAACAAAAAATTGGTATCCCAGACCAACACCTCCAGACATCCAATTTGAAGAAAGAAACTTCCAAACCCAGTTTTCAGTTTCTTTTGATAAATTGTATGAATGGAATATTGATGGTCTCTCTGAGTAAGAAATCTTGAATAAGTTACAACATATGTCCATAGTCGCCAATAGTTACATCACCAATCACAGTTTCACACAATCTGAAATTGTTCCTCTGTTAGTTACCGGCTTCACTGGTACTCTCAGATATTGGTGGGATAAAAATCTTACCCCTGAGTCTAAAAATCAGATCACTTATGCTGTAAAACTTAATGAAGACAGTCTCCCTATATTTGACGAACGAATAGGTCAAGGCATAGAGGATGAAGTCAACACTCTGTTTTACACAATAATCGAACATTTCATTGGAACCCCTAATAATACCACTGCTAGGATACATGACCAGTTAAGTAATCTTAAATGCCCAAAGTTATCCGATTTCAAATGGTACAAAGATGTTTTCATTTCTAGGGTCATACTTAGAGATGATAGTAATCAACCTTTCTGGAAAGAAAAATTTATTAATGGTTTGCCAAACTTATTTGCCCACAAAATACGCACAACCCTGAGTAATGAACAAGGTCAAATAGACTGGAATAATTTGACTTGTGGAAATATTATAAGCACAATTAATCAAGTTGGTATGAAAATGTGTATTGACTTTAAAATCAACCGACAAATTCAGTCTGATAGAAAGTCAGCCAAATACGAGTTAGGTAATTTCTGTGAACAATACGGTCTTACCAGTATTCCTCCATCCAGGAAAAATAAGCCCAGTCATCTTAGGAAAAATCATTATTCTTTTCCAAAGAAACAGTTTTTTTCAAAAAAATGAGTTTTATACGAAAAGAAAATTCTCCCCCAGAAAGAATTGGTCGAAACATCCAGTTAGTAAGACAAGTCACCCTAAGAAAGATAAAAGCAAAGTTAAGTGTTTTAAATGTCAAAAGTTCGGTCATTATGCCTTTGAATGCAAAGTAAAAGATGTTATTAGAAAATTACAAATTACAGAAGAAGATAAAGAAAAGTTGATACAAGTTTTAGAGTTGCGAGACTGTGAAACAAGTCCTGATGAACACCTAGTTCCTAGTTCGGAGTCCGATACTAACCAGTCTTCAGATTCACAACCTAGTTCTCCTGGTGTCCAAATTGATTGCACAGATAAATGTTGCAATTGATTAAAATCTATATGTGTTCTCACTAAACAGGAAGAGCAAGAAGAATTGTTAATAGATCTCATAGGAAAAGTCAAAAACCCTGAGCTAAAGTCAGAGTATTTGAAAAAATTAAAGAAGGTCCTTAGCCAGGAAAGCTCAAACCATTTTACTCCCCAGCCTATAAGTATCAATACCACCTTAGAAAAATTTAACTCCAAAAAAGAAGTAACCCTCTAAGACCTCCAGTTAGAAGTCAAGAAAGTCAAAAAAGAATTAGTTGAGTTAAAAAAATAAGTCAACAATTGCAAGCTGAGAATTATACGATCAAACAAGATATAACTAATCTTTTAAGACAAGAATCCTTTGAGCCTAAAAGCCATTCAGAAAGCCCCCACTATTCTGATGATGAACCTTCTGAAGATCAACAAGTCGTCAATCTAATCAAACAAATCCATCTTAGAAAATGGTATTCAAAAGTAACAATATTCGTCCAAAATTTTGAACTCACCACAGTTGCTTTATTTGATTCAGGAGCAGATCTTAACTGTATCCAAGAAGGATTAATCCCCACTAAATATTACCAAAAGTCTAGAGAGTCCCCTAGTACTGCCTCCCGGAAATCTCTCCAGTTGAACTTTGAATTACCAAAAGCCCATATTTGCCAAAACAAAGTCTGTTTTAAAACCTCATTTGTCCTAGTCAGAAATATCACTGACGAAGTCATTTTAGGTCTGTCTTTTCTTGCCTTATTGTATCCTTTCCAGGTAGAATATGATGGCATCATTTCTACCTATCTAGGTGAAAAGTTAAATTTGAATTCTTGTCGAAGCCAGAGTTGCACAATTTAAAAGCTTTACAAAAACAATCAGTCTCTAAATCTGTTCAAGTCATTCGAAAAATTAACCAGTTGAATTTTCTTAAAGAAGAAATTAGATTTAAAAGAATTGAACAACAGCTTGCTAATACAACCTTACAGTTGCAGATACAGTCTTTCGAAAACAATTTAAAAAATGAAATTTGTTCTGATTTACCTGCTGCATTTTGGTATAGAAAACAACACGTCGTCTGTCTTCCCTATATCAAAAACTTTAGTGAAAAGAATATCCTCACTAAAGCCCGACCGATTCAGATGAATCAAGAATTAATGAAATTTTGTAAGAAAGAAATTCATGAGCTCCTCCAAAAGAACATTATTCTCAAAAGTAAATCCCCTTAGTCGTGTCCTGCTTTTTATGTCCAGAAAAATGTTGAAATAGAAAGAGGAGTCCCGAGATTAGTTATCAATTATAAGCCTCTTAATTCAGTCCTCGAATGGATTAGAGATTGATTGTTTTTGTAAAGCTTTTAAATTGTGCAACTCTGGCTTCGACAAGAATTCAAATTTAACCTTTTCACCTAGATAGGTAGAAATGATACCATCATATTCTACCTGGAAAGGATACAATAAGGCAAGAAAAGGCAGACCTAAAATGACTTCATCAGTGATATTTCTGACTAGGACAAATGAAGTTTTAAAACAGACTTTGTTTTGGCAAATATGGGCTTTTGGTAATTCAAAGTTCAACTGGAGAGATTTCCCGGAGACAGTACTAAGGGACTCTCTAGACTTTTGGTAATATTTAGTGGGGATTAATCCTTCTTGGATACAGTTAAGATCTGCTCCTGAATCAAATAAAGCAACTGTGGTGAGTTCAAAATTTTGGACAATATTGTTACTTTTGAATACCATTTTCTAAGATGGATTTGTTTGATTAGATTGACGACTTGTTGATCTTCAGAAGGTTCATCATCAGAATGGTGAGGGCTTTTTGAATGGCTTTTAGGCTCAAAGGATTCTTGTCTTAAAAGATTAGTTATATCTTGTTTGATCGTATAATTCTCAGCTTGCAATTGTTGACTTATTTGTTTTAACTCAACTAATTCTTTTTTGACTTTCTTGACTTCTAACTGGAGGTCTTGGAATGTTACTTCTTTTTTGGAGTTAAATTTTTCTAAGGTGGTATTGATACTTATAGGCTGGGGAGTAAAATGTTTGAGCTTTCCTGGCTAAGGACCTTCTTTAATTTTTTCAAATACTCTGACTTTAGCTCAGGGTTTTCGACTTTTCCTATGAGATCTATTAACAATTCTTCTTGCTCTTCCTGTTTAGTGAGAACACATATAGATTTTAATCTATTGCAACATTTATCTGTGCAACCAATTTGGACACCAGGAGAACTAGGTTGTAAATCTGAAGACTGGTTAGTATCGGACTCAGAATTGGGAACCAGGTGTTCATCAGGACTTGTTTCAGAGTCTCGCAACTCTAAAACTTGTATCAACTTTTCTTTATCTTCTTCTGTAATTTGTAATTGTCTAATAACATCTTTTACTTTGCATTCAAAGGCATAATGACCGAACTTTTGATATTTAAAACATTTAACTTTGCTTTTATCTTTCTTAGGGTGACTTGTCTTACTAACTGGATGTTTCGACCAATTCTTTATGGGGGAGAATTTTCTTTTCGTATAAAACTCATTTTTTTGAAAAAACTGTTTCTTTGGGAAAGAATAATGATTTTTCCTAAGGTGACTGGGCTTATTTTTCCTGGATGGAGGAATACTGGTAAGACCTTATTGTTCACAGAAATTACCTAACTCGTATTTGGCTGACTTTCTATCAGACTGAATTTGTCGATTGATTTTAAAGTCAATACACATTTTCATACCAACTTGATTAATTGTGCTTATAATATTTCCATAAGTCAAATTATTCCAATCTATTTGACCTTGTTCATTACTCAGGGTTGTGCGTATTTTGTGGGCAAATAAGTTTGGCAAACCATTAATAAATTTTCTTTCCAGAAAGGTTGATTATTATCATCTCTAAGCATGACCCTAGAAATGAAAACATCTTTGTACCATCTGAAATCGGATAACTTTGGGCATTTAAGATTACTTAACTGGTCATGTATCCTAGCAGTGGTATTACTAGGGGTTCCAATGAAATGTTCGATTATTGTGTAAAACAGAGTGTTGACTCCATCCTCTATGCCTTGACCTATTCGTTCATCAAATATAGGGAGACCGTCTTCATTAAGTTTTACAGCATAAGTGATCTGATTTTTAGACTCAGGAGTAAGATGTTTATCCCACCAATATCTGAGAGTACCAGTGAAGCCGGTAACTAACAGAGGAACAATTTCAGAGTGTCTGAAACTGTGATTGGTGATGTAACTATTGGCGACCATGGACATATGTTGTAACTTATTCAGGATTTCTTGCTCAGAGAGACCATCAATATTCCATTCATACAATTTATCAAAAGAAACTGAAAACTGGGTTTGGAAGTTTCTTTCTTCAAATTGGATGTCTGGAGGTGTTGGTCTGGGATACCAATTTTTTGTTAAACTGGTAGGACTTACATGTTTGTCTAATCTTTTGATTTTTGGTTTAGTTTGGACTTCTAAATTTTCGCAGGCGTTTTCAATTTTTGAAATATTACTGGTTTCGGAATTTATCGATTCAGACTCAGAATGTGAGGCTGCAAAAAAATCTAGTGTTTTAATACCAGATTTTTCGGCTTTCTCAATTTTAGTTGGGGTCAACTGTTCTAGAATTGACTTTTTCATTGTAGAATTGACTTTAAGTACGGGACTGGACTTAAGTTCTTGGAGCTGAACTATTGGTTTTTCAAATCTAAGTTTGGGCTTGAAATCTGTTTCTGGAGGATTCAACTTATCGATCTTGGTTTCAATTGTGTCTAGTTGTTTTCCTATGACGATTAGACTCTGATTGGTGTAATTATTTTGCTCGATTATTTTTCTATTTGGGTTATTGCTTTCAAAGGTTTTGAAAGGAGTGCCAGGTATTGGTGCTTTCTTGTGATCTATTATTATAGTGTCTATTGGGGGATGACTGGCTTCAACTACGGACTTATCTATTAAATTCCACTTTTCTTTAGTTAAGGTTTTTAACTCATCATTTGACATGTAAGGGCTTTCAACAAAATCTAGGTAAAAGATTTCAACTCTGGCTGATCG

TGTTGAGGCCCAAAAATCCAAGGGGCTAGGCCCAAGTCGACTATTGGCCCAACAGGGACAAGCAAATACAAGAGAATTTAGGGAACGAATTAATACGAGCTACGGGCCACATGCCACGCCAAACAAGCACGTGCGGGAAGACCAAAAACATGCTAACTTTACAAGAAACATGCTCACTCCTATTGAACCTGGCTATAACCTATGATGATAGGATAAAAATCCAAGCACAAGCATAGGTGTCGGGACCGCCATGCCAACGCCAAGATATGTTACAAGCTTAGGTGGGCCCAAGCCACCAAAATTACCCGGGGCTTGCTTGAACGGGTTGTGGTATGGATGGCAACGGGCTTTCATAAAACGCATTGACGAGCCAGGGGAGTGGTAGTTTCGGGTCACTTGGGGGGCCCAAAACTCAAGCCTATTTCTTCGGCCCGAAATGCATGGGTTAGCATGAGAGAAGAGAGAGCATGACCCAATACCTTAGAAAAAAAGTGCAAAGCCTGTTAGAAAAGGCTTAAGACAATTAGAAAGCCTAAACGTGCTGTTAAAGTGCAATAGGCAAGCTGCTTCCGGCAGCTTGACGAAAAGCCCAGTAGATTGACCAGAATGACATATCACCAATTCAAAGCCTAGGAAAGAAAAGCCCAGCCCAAACTCTCTGTAAAATATCTGGCCAAGCATGTCTTGTCCCTATCCAAGAGTCAACAGCAACCATCTCAAAAACCAGAGGAAATCCACACGAGCAAGAGGTTTACTCGTTGGGTAAAACGTGGCTCCTTTCCCTCCCATTGTCCTGGCAGCTTGCATAGGAAAAGTTGAGAGAAAAGCAAGCGGCTCTTCATCTCTATGAGGAAGGGCCAGCAAAACCAAGACCTTGAACCGCAAATGGGTTCCTTACCCATATGTCTTGGGCGGATAGGATTCATCAAAGAAGACGAAGTGGGGAAAAACAATGGAAAGAGGGAAGGAACTATGACGAAATTAGAACCCTGAGTGGCTA

>Chr2-05; OZ022207.1; 3419598-3438144; dir; C;

CATGACCCAATACCTTAGAAAAAAAGTGCAAAGCCTGTTAGAAAAGGCTTAAGACAATTAGAAAGCCTAAACGTGCTGTTAAAGTGCAATAGGCAAGCTGCTTCCGGCAGCTTGACGAAAAGCCCAGTAGATTGACCAGAATGACATATCACCAATTCAAAGCCTAGGAAAGAAAAGCCCAGCCCAAACTCTCTGTAAAATATTTGGCCAAGCATGTCTTGTCCCTATCCAAGAGTCAACAGCAACCATCTCAAAAACCAGAGGAAATCCACACGAGCAAGAGGTTTACTCGTTGGGTAAAACGTGGCTCCTTTCCCTCCCATTGTCCTGGCAGCTTGCATAGGAAAAGTTGAGAGAAAAGCAAGCGGCTCTTCATCTCTATGAGGAAGGGCCAGCAAAACCAAGACCTTGGAACCGCAAATGGGTTCCTTACCCATATGTCTTGGGCGGATAGGATTCATCAAAGAAGACGAAGTGGGGAAAAACAATGGAAAGAGGGAAGGAACTATGACGAAATTAGAACCCTGAGTGGCTATAAAAGGAGGACTTTTGCTACCCAGGAGAACCAACCCACTTCCAGAGCTCGACCAAGCATTGTCAAGCAGCTGGAAAATCTACTCAAGCCACCCCATCTCCCCCTCCATTCGTTTTCTTCCTCCATCAGGAAGGAAACCAGCTGAATCTTCCTTTGCTTGCTCTAAACCTCCATCCTCCATAGGAAAATTCATCTCTCTCTCTCAAAATTGCTAAACCTGTGAAAGCCTAGCTCCCATGCCACATGCTCTTCAAGCCAAGCTCTTCTGTAAAATTGGTTAAGTTTGTTTGGGCTATTAGTGAGGGAAGCCTGAGAGTGTTTTCGTAATAGTTTCATCAGGCTCTGCGAAAAACACTCTTCCAGGCGCTTGGTATCGAACCCAGGCACTTGGGGAATTTTGCTCACTTCTCTTCTTACGGCAGCCCAAGCCCATTCGTCAGGCTGCTGGAACAAAAGGCCCCAACA

GATCGCATATAATCTTTCCATTCACTAAAGATTTGTTCGCGGTGTTTACTATCAGGATAGGCTTGGTGGTAATTATCTCTTCTGGATTGGTTTTTCGGGGCTTTTAAATGACGATTGAGACGTTTCCTATCAATTTCAAACTTCTTATTTAAAGGTCTAAGTTGATTATCTACTGCAGCAAGGTTTTCAACTAAGAAATCAGATTGGGTTGGAGACTCGGGGGAGTCTGATTTATCTTCTGGAACCTGGCCGTAGCAAGGTTGGGTGACCTGAGAGGTTTTTCTCAGGGACTCAGGTTTGAAATCTATCATGGCTTTTTCTAACTCTAGGTCTCGTTCTAGAGTGGAGAGAACGAAGCAGCAAAGGAATGTTTGGCAGGGACATGCCTTTGGGACTGATCAGGAGTTGGCAATTGTTGTAACTGGACATTGGATTTTCGTAAGGGGTGGCTGTCAAATTTAATTTTTACTGTGCCGTCTAGATATTGCTAGATGTAGTCTAAATTGTTTAGACTGCATTGGATTGGAGCCGGTTGGCTCTCGATGACCAAAGTCCAATCTTTTGGAAGAGCAACTTTGGACCACTTAATGGTGTGGGGAACCTGTATATGAGCATCTGAAGTGTGACTCTGGATGAGGACAGTTTGATCTTTTAGGCTTTTATTGAGGGCTTGGGAATTCATATTTGTTCCTGTAATTTTATAGTAAATTCTGTAGACTGAGGCCAAAGGTTGGGTTCCTGGGAGGACATTATAACCTTAAGTCTTAATATTCAGAGTTAAAGTTTGCAGGATATAGGGGTCGCTTAGACTTACAGTAAAATCAGGATAACAATCGAAGTGAACTGGGCCATTACACAGACTGGACTCAATGACTCCTAGGGTGCTGTCACTAAATTCAGTGAACCTAGCATCATGCAGACAAAGCAACTGACATGTTTCATGTTCTTTACTCAAGGCATAAACTTGTTCAACTGTTTTGACATTTGTTGTGCTATTAAAGCTGGAAAGCAACCAATGTTTCTTATAATATATATATATATAGGATTTTGAGTATCTTCATTTATTCATTAATTTCATTCATCCATAAGCTCTACCTGTTTTATTATCTGAGTCTTTATACTTATCTATGCTTAAGCCGTAACTGTTGAAGAGTCAGTCTAAGAATGCAATTCATCCAACCCAAGTGGTTGGTTTAAGATTTCTGAGTTGAAGTCCTCAGTTTCTTTTTCTATTAATTCATTGTTTTTCAGGTTTCCTGTCCTTTTCACTTTGGTCCAAACAGTAAGTCCTAGAGTCCAGACGATAAGTCCCAGGTAGAGGCACGAGTGGTTGCAACCTGAACGGAGAGAGAGAGAGAGAGAGAGAATCACTAGGTTTCCTGTTACGCTATGAGTTAGTATTAGAAGTTTCTGTTCATGTTCTTTCTTTAGAAGTCTGAACCCAGTACCTTGAGTAAAAATGGGTCGCTTATTTAGAACCAACTTTTATAACTCTAGTAGCTCTAGAACTAGTATCAGTCATCCCCCTGAGACAGTTAATGAAGAACAATTAGAATAGAATACTAGCAATGAGTTACATTTTAGAGATTGGAATATCCCTCAAATGGTAAAATGGTCAGAAGTCACCCTTCCCTTAGATTGGACCTTAACAAATGAAAGTCCACCGGTCCCAATTCAACATAGTCTAAACAATTTAGATTATATCCAACAATATCTAGATGGCACAGTCAAAATTAATTTCGAAAACCAACCACAACAAAAGTCCTCAGTCCAGTTATAACAACTTCCCACACCTGGTCAATCCTAGAGACACGTCCCTGCTAGACACCCCTTTGCAGCCTCCTCCTCTACTCAAGAACGAGACCTTGAGTTAGAGAAAGCTTTAATTAATTTAAAATTAGAATTGTTGAGAAAATCATCCAAAGTTAGTCAACCCTGTTATGGTAAAACACCAGTCAAAGAAGATACTAAATCGGAGTCCCTTGAGTCACCCACACAAACAAACTTCGAAGGCGGTTTTCAAATTGATAAACAATTAAGAACCTTTAATAGAAAATTTGAGATTAATTGAAAAGAAAAATACCATACAACCTATCCTGACAAAATCAAAAGACAACAAATTTTTAATGAATGGAAACAATATATGAGGACAGCCAAAGTTGAAATTTATTATCTTGATTTTGTAGGAAGCCGTTACATGACAAAAGAGTTAAAAACTTTAACTCAAGAAAAATGGGAAAAAGAAGACAAAACAGAAGTCTTAGCCAATCATCCCCCAGTTGAGACAATAGTTTTTCACCATAAAAAGTCAAAAATTGTGGCCAGTCCTTACAAAATTCTTGACGAAGAGAATAAGATGATTATAGAACAAAACAATTACACCAACCAAAGTCTAGTTGTCATAGGAAAACAGTGAGATAAAATAGAAACAAAAGTCGACAAATTACTCCCTAATGAAGTCAAACCCAAATTACAAAAACAAAAACCTTTAGTCAAATTCCAAGATTTAAAGTGAAGCCCAACCTTTAAGACAAAACTTACAATGAGAAAAATAGAAGAAATGCTTGAACAATTAACCCCTGAGAAAGCCGAGAAATCCGGTGTTAAAGTCTTAGGATCCTCTGAGTACTCCTCTGATTCTAGTCAGTCACTAGTATGGAAACAGAAACTAGTAACATTTCAAAAATTGAAAATGCATTTAAGAATTTGGAATTAGAAAATGATTTAAAGGTTAAAAGATTAACTAATAGAATCAGTCCAACAAGTTTAACCAAAAATTGGTATCCCAGACCAACCCCTCTAGATATCCAATTTGAAGAACGAAACTCCTAGAGTCAGTTTTCCGTTTCTTCAAATAAGTTATATGAGTGGAATATAGATGGTCTGTCTGAGCAAGAAATTCTTAATGAACTTCAACATATGTCTATGGTTGCCAATAGTTACAATACGAATTACAGTTTTAGACAGTCTGAAATTGTGCCTTTGTTAGAAACTGGTTTCATCTGAACCTTACGTTCTTGGTGGGATAAACATTTAACGGATGAGTCTAAGCAACAAATAATCCATGCAGTCAAACTTAATGAAGAAGGTCTTCCTATTTTCAACGAACAAGTAGGTCGAAGTATAGAAGATGGAGTCAATACTTTGCTGTACACAATAATTGAGCATTTCATTGGAAAACCTAGTAACACCACTGCTAGAATTCATGATCAGCTTAGTAACCTTAGATGCCCTAAGTTATCTGATTTCCGTTGGTATAAAGATGTCTTCATCTCCAGAGTCATGATTAGAGATGATAGTAATCAACCATTTTGGAAAGATAATTCATTAATGGTCTCCCTAATTTGTTTGCACACAAAATACGCACAGTCCTTAGTAATGGTCAAGGTCATATAGATTGGGATAATTTAACCTATGGTAATATAATTAGCACAATTAATCAAGTCAGTATGAAAATATGTGTATATATGAAAATCGAAAAACAAATTCAGTTAGAAAAAAATTCAGCTAAGTATGAGTTAGGTCATTTCTGTGAACATTATGGTCTAATGTCCATTCCCCCATCTACAAAAGCCAAGTCTAGTCACCATGACCGGCAGTTTAGTAAGCGTCGTCATTATTCACGAAGAAATCAATCTTTCTGACAGAATAATGAGTTTTACAGTAAAAAGAGATTTCACCCCCTTAAAAATTGGAAAAAGAGTCACGGAAAATATAAGAAAAGTCACCTTAAGAAAACTACTGACAAAAGAAAAGTTAAATGTTATGAATGTCAAAAGTTTGGTCACTATGCTAATGAGTGTAAAGCAAATAACACAATCAAACAATTGAAAATCACGGATGAAGATAAAGAAAAGTTAATCAAAGTCTTTGAGTTAAGAAACTCATAGTCTAGTGAAAACGAGACCATGGTTAGAACACTGAGTCACCGAGTTTAATTCCTCAGTTTCCCAGATAAGTTCACCTAGAATTCAATTTGGTTGTCAAGATAATTGTTGTAAGTCTAAGTCAATTAATGTCCTTACTAAACAAGAAGAACAAGAAGAATTATTAATTGATTTAATAAGCAAAGTTGAAAACCCAGAGTTGAAATCTGAGTACTTAAACAAATTAAGGAAAGTCATCAGTCAGGAAGAACCGAGTCCGTCTACCCCTCAAAAGATAAGTCTGAACACTAGTTTAGAAAGATTCAACAAAAGAAAAGAAGTTACTTGACAAGATTTACACCTAGAAGTAAAATTACTCAAAAAAGAAATAGTGGAACTAAAACAGAGAAGTCAAAAGTTACAGGTCGAAAACTATGAGATTAAACAAGATTTAATTAATCTAATGGAAAAATTTGTGTCTACGCCAGTTAGCCCTACAAATTCAGATGGTGAACCCTCCAATCAGGATCAAGCCGTCAATTTAATTAAACAAGTAAATTTTCGAAAATGGTATTCTAAAGTCAACATATTTATCCAAATTTTTGAGTTAAACGCAGTTGTCCTTTTTGATTCAGGAGTCGACCTCAATTGTATCCAAGAAAGTCTAATTCCTACTAAGTATTACAAAATATTAAAAGAGTCATTAAGCACAGCCTCAAGAAAGTCACTCCAATTGAATTATGAGATTCTTAAAGCCCATATATGTCAAGATAAAATTTGCTTTAAAACCTCATTCGTCTTAGTCAAAAATATTACTGATGAAGCCATCTTAGGATTACCCTTTATTGCCCTACTGTATCCTTTCCAGGTAGAATACAATGGAGTCATTTCTACCCATTTAGGAGAAAAGGTTAAGTTTGAATTCCTAAGTAAACCAGAGTTACATAATTTAAAAGCCTTGCAAAAGAACGCAGTCACCAAAACAGTTAAAGTGATTGAAAATAAAGATAAGCAATTAAGATTCCTCCAAGAAGAAGTCAAATTCAAAAGAATTGACCAACAACTTAGTCTTGAGTCTTTGCAGACACAAATTCGCCAATTCAAAGAAAAATTAAAACAAGAAGTTTGCTCTGAGATACCCAATGCTTTCTGGCATAGGAAACAACATGTTGTTAGTCTTCCTTATATCAAATCCTTTAGTGAAAAGAATATCCTCACTAAAGCCAGACCATTCAAATAAGTCAAGAATTAATGGAATTTTGTAAAATTAAAATATTAGAATTACTTGAGAAAGGAATAATTCGTAAGAGTAAGTCACCCTAGTCCTGCCCTGCTTTCTATGTCTAGAAAAATGCTGAGTTAGAACGCGGAGCCCCAAGATTAGTTATTAATTATAAGCCTCTTAATACGGTATTAGAATGGATCAGATACCCAATACCCAATAAGAGAGATCAAATTAACAGGTTAGAAAAATCAGTCATCTTTTCTAAATTTGATATGAAGAGTGGTTTTTGACAAATCCAAATTCATGAGTCTGATAGATATAAAACTGCTTTTGTCACCCCATTTGGTCATTATGAGTGGAATGTCATGTCTTTCGGTCTTAAGAATGCCCCCAGTGAGTTTCAAAATATAATGAATGAGATATTTAATCCCTTCAGTCATTTCTCTATAGTTTATATTGATGATGTTCTTATCTTTTCCCAGTCGATAGAACAACATTGGAAACATTTGCATAATTTTATGCAGATAGTCAAACAAAATGGTCTACTTCTTTCTGCTAAGAAAATTAAGTTGTTTCAAACTAATATCTGATTCCTTGGTTTTAATATCTGCTAGTCACAGATTAGTCTAATTGATCGAGTTATCCAGTTTGCTGATAAATTCCCAGATTAAATCCTAGACAAAAGTAAACTCCAGAGATTCCTAGGATATCTAAATTACATTTATGATTTTTACAAAAATTTGAGAAAACAATGTAAGCCTTTATTTGATCGCCTCCAGAGTAATCCTCCTCCTTGGTCACCCATTCATACAGAAATAGTCAAACAAATCAAGGTCCATATCAAAACACTCCCTTGTCTTGGCATTCCTTCAGTTGATTCCTTCAAAATAGTAGAAACTGATGCCTCTAATATTGGTTATGGAGGTATTCTCAAGCAAAGAGTTGATTCTAGTCAAATTGAGCAAATCATCCGATTCTATTCCGGTATTTGGACACAGTCACAAAGTAATTATAGTACTATTAAGAAAGAGATATTATTTATAGTATTATGCATTACTAAATTTCAAGATGATTTATTAAATGAAATTTTTTTAGTTCGAGTCCATTGTAAGTCTGCTAAGCATGTATTAGAAAAAGATGTTCAAAATATTGCATCCCAACAAATATTTGCACGATGGCAAGCTATTATAAGTATATTTAATTTTAATATTGAATATTTGACAGGAAGTGAGAATTTTGTTCATGATTTCCTTACTAGAGAATTTCTACAGGAAAAACAATGGCAGACAGAAGAAAAGATAAATAGAAAGAACAAGCCAGTCAATCCAGTCAACCCCAGTCACTCAAAGTACTCCCCCCATCCAAGAATCCACCCAAAACAGAGTCACCATCCCAGATCGTCCCTTACACGGGATGTTCCCCAATCTAAATAAGCAACAAATTCGCCACTTTAGGAGCCACAATAGTCCAAATTCGTCCTAGTTTCCAGTCTGCCCTAGTCTCTAGTCATGATTCTTTCTAAGACACCACCCAGTTTACTCCTTTAGTTGCCTCATACCAAAAGTCACCCCCTACGTGCTCAAGAGTAATAGTCATCTCTTTATCATTGAGTCAAAATACAATGCTATTGCCAATCCAGTCACCATCGCCATGTCATACTTCCCTCCTAATAGCCATTTTATTCCTTCAACCCCAAGCGCCTTTTAAGAATTTGAAATTCTACAAAGACATCCTTCATGAGACCCAGTCCATCACAATCAAACCAATCAGAGACCGTGAGAATCCACACATAATCCTCTACTATTAATGTTACAAGCCTCCCAAATGAATGAAGAAGATGATAATGATTCTTCCTCAAAATCCTATTCATCCACTTGTCAACCAAGAATGCCATCCAATCAGAAGACTCCAATCCCCAAAATGCGGTGGAGTGATTATCCCTCTGACAGTCAAGACCCTTATGACTTCAGCCTTTTTGATGTGGGCAAAGATTAAGTTGCCAAAGTCATAATGAGCCCCCAAAAGAGAAAGAAAAGTTAAAGCAAGCAACCACTTTAGTAAAAGCATTTGGAATCTTAGACCCGTGCTTCAACAGTCTGATAGCCTGTGCTCCTTTAATAAGGTTCCCCGACAAAGTAAAAGTTACAACACTCAATAAAAGCAAAAGCTTTCGCGTGCTTCTACAGTCATCATCCAGAGCATCCAAAGTTGCCCATAGTCTACTTTATCTGTCTATTTAAGAAGGCTTCGGCCTCAGTTATCCTCAGAATAAATGGAGGATGACTGTAGAAGCACGCGAAAGCTTTTGTTTTACTGAGTGCTGTAACTTTTACTTTGTCGAGGATCCTGACTAAAGGAGCAAGGGCTATCAGATTGTTGACGCACAGTCCAAGATTCTAAATGCTTTTACTTTGGGGGGGGGAGGAGGTTAGGGGAGGTCTCTTTTGGGGGCTCATGATGACTTTGGCAACTTAATCTTTGCCCATATCAAAAGGGTTGAAGTCATAAGGGTCTTGACTGTCAGGGGGATAATCACTCCACCTCATTTTGGGGATTGGAGTCTTCTGACTGGATGGCATTCTTGGTTGACAAGTGGATGACTTGGATTTTGAGGAAGAATCAATATCATCTTCTTCATTCATTTGGGAGGCTTGTAACATTAATTGGTGAGCAATTTCCTGCAATTCATACTTAGATTTTCCCTCGATTGGGAGAGAAGAATGGGAAGTAGTAGGAGATGACATTTTGTCTGACTGGGGATTCCTGACTGGAGGAAAGTGTGAATAAACATAATATGCAATTCCCTCAATTTTGAATTTATCCCACCATTGAATTGAAAATTGGCGAGACAGGACTCTCGATTCCCAATTGACTTGGTAGGACCATTTTAAGATCCAGGGGACTTTATATTTAGCAATGAAATGGAGTAGATTAGGGAAGAATAACTCAGACTCATTAAACTTGTGTTTATTGGTGAAATAATTGACTAGATCTTGCATTTGTGAAGGAAGGAGGTCAATAACTGAACCATGTTTTTCCCACCAATGAAGAAACCAAAAGGGAAAAGGACTCTTGAATTTACTGTCAAAATTAAGGAACCAGGAGTGACAAAATTGACTGTTTGATGAAGCAAGATGGAATACCAAGCTTCAATGTAATCATAGTAACTGTATTGGAGTTCTGACTGAAGAACTTTTAGATCATAAGGGTGACTGCTCCATTCATTTTCACTGAGGATTTGGTGGATGTATAAGGAATGGTAGAGGATTATGTGTGGATTCTCACAGTCTCTGATTGGTTTGATTGTGATGGACTGGGTCTCATGAAGGATGTCTTTGTACAATTTCAAATTCTTAAAAGGCGCTTGGGGAATAAAATGGCTATTAGGAGGGAAGTATGACGTGGCGATGGTGACTAGATTGGCAATAGCATTGTATTTTGACTCAATGATAAAGAGATGACTATTACTCTTGAGCATGTAGGGGATGACTTTTGGTATGAGGCAATTGGAGGAGTAAACTGGGTGGTGTCTTGGAAAGGATCATGACTAGAGACTAGGGCAGATTGGAAACTGGGACGGATTTGGCCTATTGTGGCTCCTAAAGTGGCGAATATGTTGCTTATTTGGATTGGAGAACATCCCGGATAAGGGATGATCTGGGATGGTGACTCTATTTTGGGTGGATTCATGGATGGGGGGAGTACTCTGAGTGACTAGGGTTGACTGAATCGACTGGATCTGGATTGACTGGTTTGTTCTTTCTGTTTATCTTTTCTTCTGTCTGCCATTGTTTTTCCTGTAGAAATTCTCTAGTAAGGAAATCAGGAACAAAATTCTCACTTCCTCTCAAATATTCAATATTAAAATCAAATATTCTGAAAATAACTTGCCATCGTGCAAATATTTGTTTGAATGCAATATTTTGAACATCTTTTTCTAATACATGCTTAGCAGACTTACAATCAACTCGGACTAAAAAATTTTGATTTAATAAATCATCTTGAAATTTAGTAATACATAATACTATAGATAATATCTCTTTCTTAATAGTACTATAATTACTTTGTGACTGTGTCCAAATACCGGAACGGAATAAGATGATTTGCTCAATTTGACTAAAATCAACTCTTTGCTTGAGAATACCTCCATAACCAATATTAGAGGCATCAGTTTCAACTATTTTGAAGGAATCAACTGAAGGAATGTCAAGACAATGGAGTGTTTTGACATGGACCTTGATTTGTTTGACTATTTCTGTATGAATGGGTGATAAAGGAGGAGGATTACTCTGGAGGCGATCAAATAAAGGCTTACATTGTTTTCTCAAATTTTTGTAAAAATCATAAACATAATTTAGATATCCTAGAAATCTCTAGAGTTTACTTTTGTCTAGGATTTGATCAGGGAATTTATTAGCAAACTGGATAACTCGATCAATTAGACTAATTTGTGATTGGTAGATATTAAAACCAAGGAATTGGATATTAGTTTGAAACAACTTAATTTTCTTAGCAGAAAGAACTAGACCATTTTGTTTGACTATCTGCATAAACTTATGCAAATGTTTCCAGTGTTGTTCTACCGACTGAGAAAAGATAAGAACGTCATCAATATAAACTATAGAGAAATGACTTAAGGGATTAAATATCTCATTCATTATATTTTGAAACTCACTGGGCGCATTTTTAAGACCGAAAGGCAGGAGATTCCACTCATAATGACCAAATGGGGTGACAAAAGCAATTTTATATCTATCAGACTCATGAATTTGGATTTGCCAAAAACCACTCTTCATATCAAGTTTAGAAAAGATGACTGATTTTCCTGACCTGTTAATTAGATCTCTCTTATTGGGTATTGGGTACCTGATCCATTCTAATACCGTATTAAGAGGCTTATAATTAATAACTAATCTTGGGGCATCGCGTTCTAACTCAACATTTTTCTGGACATAGAAAGCAGGGCAGAACTAGGGTGACTTACTCTTACGAATTATTCCTTTCTCAAGTAATTCTGAAATTTCAATTTTACAAAATTCTATTAATTCTTGACTCATTTGAATTGGTCTGGCTTTAGTGGGGATATTCTGTTCACTAAAGGATTTGACATAAGGAAGACTAACAACGTGTTGTTTCCTATGCCAGAAAGCATTGGATATCTCAGAACAAACTTCTTGTTTAAATTTTTCTCCGAATTGGCGAATTTGTGTCTGCAAAGACTCAAGACTAAGTTGTTGCTCAATTCTTTTGAATTTGACTTCTTCCTGGAGGAATCTTAATTGCTTACCTTATTTTCAATTACTTTAACTGTTTTGGTGACTGTGTTCTTTTGCAAGGCTTTTAAATTATGTAACTCTGGTTTACTCAGGAATTCAAACTTAACATTTTCTCCTAAATGGGTAGAAATGACTCCATCGTATTCTACCTGAAAAAGATACAGTAGGGCAATAAAGGGTAATCCTAAGATGACTTCATCAATAATATTTTTGACTAAGACGAATGAGGTTTTAAAGCAAATTTTATCTTGACAGATATGGGCTTTAGGAATCTCATAATTCGACTTGATCCTGATTGGAGGGTTTACCATCTGAATTTGTAGGGCTTACTAGCGTAGACTCAGATTTTCCGATTAGATTAATTAAATCTTATTTAATCTCATAGTTTTCTACCTGTAACTTTTGACTTATCTGTTTTAGTTCCACTATTTCTTTTTTGACTAATTTTACTTCTGGGTGTAAATCTTGTAAAGTAACTTCTTTTCTTTTGTTGAATCTTTCTAAAGTAGTGTTCAGACTTTTCTTTTGAGGGGTAGACGGACTTGGTTCTTCCTGACTGATGACTTTCCTTAATTTCTTTAAGTACTTAGATTTCAACTCTGGGTTTTCAACTTTACTTATTAAATCAATTAATAATTCTTCTTGTTCTTCTTGTTTAGTAAGGACATTAATTGACTTAGACTTACAACAATTATCTTGACAACCAAATTGAATTACGTGAACTTATCTGGGAATCTGAGTAATTAAACTCTTTTGACTCAGTGTTACTCACCATGGTCTCGTTTTCACTAGACTTTGAATTTCTTAACTCTAAGATTTTGACTAACTTTTCTTTATCTTCATCCGTGATTTTCAATTGTTTGATTGTGTCTTTTACTTTACACTCATTAGCATAGTGACCAAACTTTTGACATTTATAACATTTAACTTTTCTTTTGTCGGTAGTTTTCTTAAAGTGACTTTTCATATCTTTTCCGTGACTCTTTTTCCAATTTTTACGCGGGTAAAATCTCTTTTTTACTGTAAAACTCATTATTCATTTTGAAAGATTGATTTCTTGGGAATAATGACGACGCTTACTAAACTGTCGTTCACGGTGACTAGACTTGGCTTTTCTAGATGGGGGAACGGACATTAGACCATACTGTTCGCAGAAATGACCTAACTCATACTTAGCTGAATTCTTTTCTGACTGAATTTGTTTTCCAATTTTCAAATCTACACACATTTTCATACCGACTTGATTAATTGTGCTAATTATATTACCATAGGTTAAGTTATCCCAATCTATATAACCTTGATCATTACTAAGGATTGTGCGTATTTTTTGTGCAAACAAATTAGGGAGACCATTAATAAATTTTTCTTTCCAAAATGGTTGACTACTATCATCTCTAATCATGACTCTGGAGATGAAGACATCTTTATACCAACGGAAATCAGATAACTTAGGGCATGTAAGGTTATGAAGCTGATCATGAATTCTAGTAGTGGTGTTACTAGGTGTTCCAATGAAATGCTCAATTATTGTGTATAATAAGGTATTGACTCCATCTTCTATACCTCGACCTACTTGTTCGTCGAAAATAGGAAGACCTTCTTCATTAAGTTTGACTGCGTGGATTATTTGTTGCTTAGACTCATCAGTTAAATGTTTATCCCACCAAGAACGTAAGGTTCCAGTGAAACTAGTTTCTAACAAAGGCACAATTTCTAACTATCTAAAACTGTGATTGGTATTGTAACTATCGGCGACCATAGACATATGTTTATGTTTATTAAGGATTTCTAAATTTTAAATTTTAAATCATTTTCTAATTCCAAATTTTTAAATGCGTTTTCAATTTTTGAAATGTTACTAGTTTCTGTTTCCATACTAATGACTGACTTAGAATCAGAGGAGTTAACTACAGCAAAAGGTAGCTCAGTGGATCCTAAGACTTTAACACCGGATTTCTTGGCTTTCTCAGGGGGTTAATTTTTCAAGCATTTCTTCTATTTTTCTCATTGTAGGTTTTGTCTTAAAGGTTGGGCTTGACTTTAAGTCTTGGAATTTAACTAAAAGTTTTTTTTTTTTTTTTTGGTAATTTGGGTTCGACTTTATTAGGTAGTAATTTGTCGACTTTTGTTTCTATTTTGTATAACTGTTTTCCTATGACAACTAGACTTTGGTTAGTGTAATTGTTTTGTTCTACAATCATTTTATTGTCTTCGTCAGGGATTTTGTAAGGGCTAGCCACTATTTTTGACTTTTTATGGTGAATAACTATTGTCTCAACTGGGGGATGACTGGCTAAGACTTCTGTTTTGTCTTCTTTTTCCCATTTTTCTTGGGTAAAAGTTTTTAACTCTTTTTTCATGTAACGGCTTTCTACAAAATCAAGATGATAAATTTCAACTTTGGCTGTTCTCATATATTGTTTCCATTCATTAAAAATTTGTTGTCTTTTGATTTTGTCAGGATAGGCTGTATGGTATTTTTCTTTTCTGGACTGGTTTTCTGGGGCACGTAGATGACTATCTAATCTTTTTCAATTAATCTTAAATTTTCTATTAAGGTTTCTTAATTGGTTATCAATTTGAAAACCGGCTTCAAAGTCTGTTTGTGTGGGTGACTCAGGGGACTCAGATTTAGTATTTTCTTTGACTGTTGTTTTACCATAACAGGGTTGACTAACTTGGGATGATTTCCTCAACTATTCTAACTTTAAATCAATTAAAGCTTTCTCTAACTCGAGGTCTCGTTCTTGAGTAGAGGAGGAGGCTGCAAAGGAGTGTCTAGCAGGGACGTGTCTCTGGGATTGACCAGGTGTGGGAAGTTGTTGTAACTGGACTGAGGACTTTCGTTGTGGTTGGTTTCCGAAATTAATTTTGACTGTGCCATCTAGATATTGTTGGATATAATCTAAATTGTTTAGACTATGTTGAATTGGGACTGGTGGACTTTCATTTGTTAAGGTCCAATCTAAGGGAAGGGTGACTTCTGACCATTTTACTATTTGAGGGACTCTTATTAGGGAATCGGTATTATTACTTTGAATTAATAGAGTTTGATCTTTTGGACTTTTATTAAGGGCTTGGAAATTCATATTGGTTCTTGTGACTCTGTAGTAAATTCTGTAGACTAAGGCTAATGGTTGGGTACCAGGTAAGACTTCGTATCCTGAGGTCTTAATATCCAATGTGAGGGTTTTTAAAATATGGGGATCACTAAGGCTCACCGTAAAGTCAGGGTAACAATCAAAGTAGACTGGGCCATTAGAAAAACTGGATTCAATAACTCCTAGCATACTGTCGCTAAAATTAGTGAATCTAGCATCACATAAGTAGAGAAGGATAGAAGCTTCTAATCCTAATCTGGTTAGTGGTTTAACTGCAACTTGGAATAGACCAATGTGAAGGAAATTGTTTCCATCATTTTTATGTTTCTAATAAATCTTTTTCTAATACATGCTTAGCAGACTTACAATAAACTCGGACTAAAAAATTTTGATTTAATAAGTCAATTGAAATTTATTAATCCATAATACAATAGATAATATTTCTTTCTTAATAGTACTATAATTATTTTGACTTTGTGACCAAGTACCAGAATGGAATCGAATGATTTGCTCAGACTGACCAGAAGGGACTTTCTGTTTGAGGATACCTCCATAACCTATATTAGAAGCATCAGTTTCAACAATTTTTAATGAAAGCATAAGGAATACCAAGACAAGGAAGAGTCTTGATATGGGTTTTGATTTGTTTGACAATTTCAGTATGAGTAGATGACCATGGAGGAGGATTATTCTGGAGACGATCAAATAAAGGCTTACATTGTTTTCTTAGATTTTGATAAAAATCATAAACATAATTTAAAGATCCTAAAAATCTCTGGAGTTGACTCTTGTCTAGGATTTGGTCAAGAAATTTATTAGCAAATTGGATAACTCGACTAATCTGTGACTGGCAGATGTTGAACCCAAGAAATCTCACATTGGTTTGTAATAAATTAATTTTCTTAGCAGAAACAACTAAACCATTTTGTTTAATTATTTGTAAAGACTTATACAAATGTTTCTAGTGTTGTTCTATAGACTCAGAGAATATAAGCACATCATCAATATAAACTATGGAAAAATGGCTGAAAGGATTAAAGATTTCATTCATTATATTCTGGAATTCACTACGCGCATTCTTAAGACCGAAGAGCATAACATTCCACTCATAATGACCAAATGGAGTAACAAAAGCAGTTTTATATTTATCAGACTCAGAAATTTGAATTTGCCAAAATCCACTTTTCATATCAAATTTAGAAAAGATGACTGATTTTTCTAACCTGTTGATTAAATCTCTTTTGTTAGGAATTGGGTATCTAATTCATTCTACGACAGCATTAAGTGGCTTATAGTTGATGACTAGTCTTGGGACACCGCGTTCTAATTCGGCATTTTTTTGGACATAAAAGGCAGGACAAGACCAGGGTGACTTACTCTTACGAATTATTCCTTTTTAAAGTAAATTAGTAATCTCAATTTTGCAAATTTCCATTAATTCTTGGCTCCTTTGAATGGGTCTTGCTTTAGTAGGGATATTTTTCTCACTAAAGTTTTTGACATAAGGAATGCTGACAACATGCTGTTTCCTATGCCAGAACGCATTGGGTAGCTCAGAACAGACTTCTTGTTTTAATCTTTCTTCAAATTGGTGTATTTGTAATTGAAAAGACGTATGACTCGGTTGTTGCTCAATTCGTTTGGTTTTAATTTCTTCCTGGAGAAATCTTAATTGTTTATCTTTGTTTTGGATAACCTGGATAGTTTTTGTGACTGCATTTTTTTGCAATTCTTTTAAACTATGTAACTCAGGTTTACTCAAAAATTCAAACTTATCTTTTCTCCTAAACAGGTGGAAATGACACCATCATGTTCTACCTGGAAGGGGTACAGTAGGGCAATGAACGGTAGACCTAATATGACTTCATCAGTAATATTTTTTACTAGAACAAAAGAAGTTTTAAAGCATATTTTATTCTGACAGATATGGGCTTTAGGGATTTCATAGTTCAATTGAAGAGATTTTCCTGAAGCAGTACTTAATGATTCTCTTGATTTTTTGTAATACTTAGTTGGAATTAGGCCTTCTTGGATACAGTTAAGGTCAGCTCCGGAATCAAATAGGGCGACTATGTTTAATTCGAAATTTTGAACAAATATAGTGACTCGAGCGTACCATTTTCTGAAATTTACTTGTTTAATTAGGTTAACGGCTTGCTCGTGATTAGGGCTTTCTTCATCTGAGTTAGTTGGGCTCTCTGGGGTAGACTCAGGATTTTGCTTTTCTAATAAATTAGTCAGATCTTGTTTTATCTCATAATTTTCAGATTGTAATTTTTGACTTAATTGTCTTAACTCAATGATTTCTTCTTTGATTATTTTTATTTCTAATTGTAAGTCCTGTAAAGTAACTTCTTTCTTTTTCTTAAATTTTTCTAACGTTGTATTCAGATTTATCTTTTGGGTAGACTGTTTCGGTTCTTCTTGGCTGATGACTTTTCTTAATTTTTCAGATACTCAGATTTTAATTCTGGATTCTCAACCTTGGTTATTAAATCAATTAATAGTTCTTCTTGTTCTTCCTGTTTAGTGAGGACACTAATTGATTTAGACTTACAGCAATTATCTTGGCAACCAAATTGGATTCTGGGGGAACTGGGTTGTGACTCAGAAGAATATATTTCAGAGAACTCAGTATGACTAATCATGGTTTCATTTTCACTGGACTTAACTCAAGGACTTTAATTAATTTGTCTTTTTCCTCATCTGTAATTTTTAATTGTTTAATCGTGTTTTTAAGCTCACAATTATTGGCATAGTGACCATACTTTTGACATTTATAACATTTCACTTTTCTTTTGTCGGTTCTTTTCTTAAAGCTGTGGCTTTTTTCTTTGTGATGACTCTTTTTCCAATTTTTCTTAGGGGAAAACCTTTTTTTTATAAAATTCTCTATCATCATTATTGTGCTGGAATGACTGTTTTCTACGTGAATAATAACGCTTTTTACTAAACTGTCGGTTGTGGTAATTAGGCTTATTCTTTCTAGACGGGGGACAGACATTAAACCATATTGTTCGCAGAAATTTCCTAATTCATATTTAGCAGAATTTCTTTCAGATTGGATTTGTTTACCAATTTTCATATCAACGCACATTCTCATACCAACTTGATTAATTATTCTAATTATATTTCCATAGGTTAGACTATCCCAATCAATGTGACCTTGTTCATTACTAAGGGTTGATCGGATTTTATGAGCAAATAAATTCGGTAATCCATTAACAAATTTTTCTTTCAAAAAGGCTGATTACTATCTGTTTTTCCCATGACTCTGGAAATAAATACATCTTTATACCATCTAAAATTAGATAATTTAGGGCATCTTAAATTACTCAATTGATCATGTATTCTGGCTATGGTGCCACTGGGTGTTCCAATAAAATGATCGACTATTGTGTAAAGCAATGTGTTGACTCCATCCTCAATACCTTGACCTATTTGTTCATCAAAAATAGGAAGACCATCTTCATTAAGTTTTAACGCATGAATTATTCGTTGCTTAGACTCAGGAGTTAAATGATTGTCCCACCAAGAACGAAGAGTTCTTGTGAAACCTTTCTAATAGAGGAACAATTTCAGACTTGCTGAAACTGTGATTGGTGATGTAGCTATTGGCAACCATAGACATATGTTGAAGTTTATTAAGGATTTCTTGCTCAGAAAGACCATCAATATTCCATTCATACAATTTATCAGAAGAAACAGAAAACTGGCTTTGAGTATTTCGTTCTTCAAATTGAATGTTTGGTGGTGTAGGTTTGGGATACCAATTTTTTGTGAGACTGGTAGGGTTGAATTTGTTTGTTAATCTTTGGACTTGTGGATTTTGTTCGAGCTCGAGATTTTTGAATGCATTTTCAATTTTGGAGATATTACTAGGCTCAGTATCTGTGCTATCAATGGATTTAGACTCAGAGGATTGATGTAATAGGAAAGGTGACTCAGAGTATCCTAAGACTTTAACGCTTGACTAAACTTTTTCTTTATCTTTACGTTTAGTTGGAGTTAAATCTTCTAACATTTTCTCAATTTTTGTCATTGTGGTTTTTGTTTTAAAAGTTGGACTTGATTGTAGGTCTTTGAAGGTAACTAATGGTTCCTGATTATGTGGTTTGACTGAAATTTCTGTTTTGTCTAACTGTTTTCTTATGACTACTAGGCTTTGATTAGTATAGTTATTTTGTTCAATTACCATTTTGTTTTCTAAGTCAGGGATCTTAAAAGGACTGGCAATGATTTCTGCTTTCTGGTGATGGATAACTATAGATTCAACAGGGGGGTGACTAGTAAGAATTTTGGTGTGATCTATTTTTTCCCAATTTTCTTGAGTTAAAGTTTTTAATTCGTTAGATATATGACGGCTTTCTACAAAATCAAGGTAATAAATTTCAACTTTAGCAGTGCGCATGTACTCTTTCCATTAATTAAATATCTGTTGTCTTTTGGCTCTATTAGGGATTGTTGAATGATAATTATTTCTTCTTGCTTGATTTTCAGGAGCATGTAGGTGACTTTCTAGTTTTTTCCAATTGATTTCAAATTTTTTATTAAGTGTTCTGAGTTGGTTTTCTACTTTGAAACTTGTTTCCACAAAATCGGATTGAGTAGGTGACTCAGGAGACTCAGATTTATTATCATCTTAGGGTTTGTCGTAGACAGGTTGAGTGACTTGTGCAGTTTTCCTTAGTGATTCTAATTTCAAATCAATTAAAGTTTTCTCTAATTCTAGGCTCGTTCTAGAGTAGAGGAAGAGGCTGCAAAAGAATGCCTGGCAAGAACATGTCTCTGGGATTAACCAGGTGTTAGTAACTGTTGTAACTGGACTGTTGATTTCTGTGAGGCTTGGTGATTAAAGTTAATTTTAACTGTACCGTCTAAATATTGTTGTATGTAATCTAAATCGCTTAGACTACGTTGGACAGGTACTGGAGGGTTCTCAGTGGTTAGAGTCCAATCAGTGGGAAGAGTAACATCTGACCATTTGATAGAGTGGGGAACTCTAACAGTAGCATCAGGCGTATGACTTTGGATTAACAATGTTTGACTCTTTGGGCTCTTGTTTAAGGCTTGAAAATT

TTGGGTATTGGGTCTTCAGAGTATTTAGTGACCTTTCATATTTTGTTTGTCTCAAAGATCCTAGTTTCACCACTCTGAGTTTAGTACCAAGATCCTTTCACCACTCTGACTAGGATCTATCTTCTGGTGGTTCCTTTGCTTCGGGCATAAACTTAAATATATATATATATATATATATATATATTGTGTTGTAAGATGAGAGAAATAGAACAAGCAAAGGAAGTAAAGCAATGGAACCGGAGAAAGTAAACAAATAGGACCTTAATCCCCAAATAAAGAAAGAATATTACCAACGTTACGAACAAGAGTTTTCCTACTAAAGTGTAAAGGAACCACTTGTATTTTGCTCACCCAACCAAAAAAAAAAGAAGTAAAGAAAATAAAGAAAACTAAAGTGTTAATTTAGGCAATCACAAACATTACAGTTTTCGTTTTCGCACACAACAATATATACAATGTCATACATACACAAAAATACAAGTCCGGGAGTGAGTCCCCCCTACCTGGAATCCAAAGCTAATGCCTCACTTGAATTCTACACCTTTGAACTCAAATTGTTCTTGCTTGGGTAACTGATTCCTAAGCCAACATTTCAAAAATGTTACTAAAAACCCTTATTTGCAATTCCGTCATTACAACTAGCTTTAATTGCACTTTAGTCCTTATACTTGTCAATAATTTCACTTTTGTCCTTATACTTTCCTTTATTTTCACTTTGGCGCCTATACTTTTTCCAATACATTTCAGTCTTCAGACTTATCCAGAATTTCACTTTGGTCCTTACATTTTCCTTTATTTTCACTTTACATATACTTATTTTAAGGGTAAATTACTCAAATGGTCCTCAAACTTATACGCGATTTACATTTTGGTCCCTAAATTAAAATATTAGTCCAAATGGTACATAAATTCTATTTTAATCGCCCATTTGATCCAACCGCTACATTATGTCAATATTGCTGTTAAATATGAGGGCAAATTGGTCATTTCGTACGTTAAA

>Chr2-06; OZ022207.1; 3510523-3514316; rev; B;

CAGATGTGCTGGGTCTTCAGCAGGGAAGAGGGCTGAGGCCCTTGGTGAAATAGGTTGGTGAAAGACCTGCAGAAGATAACGTGGTAGAAGCAATCGTTAAGCTTCGGACACCGGTGTGGTGCCGGCCGAAGGCTCTCCGATGCCTAAGTCAGTTACAGGTAGTAATTCTGGCAGAATAACAGTAAAAGTGGGAGAATAGTTACCCTTTTTACTTGGGTACTGTTCCCTATTTATAGGGAGGTGAAAGTGAGCTTTGCACAAAGTTCCAATGTGGGACTTTGTGCAAGTCGTTGTGGTGGCGACACATGTCCTGGAGATTAGGTTTGACAGCTGGGAGCTTCGGCAGGTGTCTGGCACTTCGGAAGTGTGTCTTCCTTCGGCAGGGGAGAAGGCGTGGCTGCCTTGGTGCTAGTCGCTTTGATGCTGGGTTTGCTCGGTTCATTATGGTGTAAACAAAAACCACCCCAAAAGAATACAGATTTTGCACAAAGGGATGGATGCCTCTGTCTGTTGGGCCCAATGCTCATCTAGATTTAACACTTTTTTCAAGAAATGGAAAAACAGATAGAGGGAGAGAAGAGACTTTGAGGACGAGCGAGGTGGAGATGGTACTGCCGTCGGCGAAGTGGGATGGCTGATGGTGTTGGGTGCCGTCGGTTTGGATGGGGAAGGGGACGTTGGTGGTTTCTGGTGTTGGTTTGTTTTTGTTTTGTTTTCATTAAATTTGTTTTTTTTTTCCTCGGCAAAGTTATGAAGAATTAATGTGTCAAATTTCAAGTATCCCCTCACTTTTCTTTTCCCCGTGCATTAAGAAGGTATCTCGTAGAAATTCATTAATATAAAACTGGTAGCAAAAATATCCTTAAAATCCAAGTTAATTATTTGAAAAATTCCTTCGCAAGTTTCAGAGGAAATTGGGTTGGTGGAGAAGTGTGTTGTTACTGCCTGCCTTTTGTTTCCCAGCATATTTTGATATTCATAGTGAAATTTTATATATATA

CTCTAAGTCTGCAGCTCATCCAACCCAAGTGGCAGGTTTAGATTTTCTGAGTTGTAAGTTTTCATTCAGTTCTATTCCTACTTCTGGTTCTTTCAGTTTACCTGTCCTTCTCTCTCTTGGTGTCCGAACAATAAGACCCATTCCAAACAGTAAGTCCCAAAGAGGCTTGAGCGGTTGTGGCATTAGTAAGAGGGCGAGAGATTCACTAGGTATCATGTTTAATTAGAAGTCAGCCTTAAAAGTTTGTGTTTGCTTTCTTCCTTAGAACTCCAACCCCAGAACCTTGAGAAACATGAGTTGCTTATTCATAACCAACTCTTATAATTCTAGTAGCTCTAGAGTCAGTATTGGCAAAATGCTAGAGATAGTTAATGAAAAACAACTAGAATATGAGTCTAATAATGAACTAGATTTTCGAGATTGGAATATACCTAAGGTCCCTAGTCAAAATATTTATAAGGAAAAATGGTCGCTCTCTAGTTTCAATACAACCACCCATGTAAAAACAGTTGAACAAATCTATGCTTTAAGTCAAGAACATGAGACTTGCAATTACTTAATCTTGAGTCAATTAGAAAACATAAAAATGATGGAAACAATTTTCTTCACATTGGTCTAGTCCAAGTTGCAGTTAAGCCGTTAACTAGATTAGGATTAAAAGCTTCAATCCTTCTCTGCTTACGTGATGCTAGGTTCACTAATTTCAGCAATAGTATGCTAGGAATTATTGAATCCAGCCTTGCTAATGGTCCAATTCATTTTGATTGTTACCCTGATTTTATAGTAAGCCTGGGTAGTGATCCCCATATATTAAAAACCCTAAAATTAAATATAAAAACTGATGGCTATGATGTCCTGCCCGGCACACAACCTTTAGCTTTAGTTTACAAAATCTATTACAAAGTCACAGGTACTAATACGAATTTCCAAGCCCTGAGTAAAAGTCCAAAAAACCAAACTTTGTTAATCCAAAGTCATACACCTGATGCTACTGTTCAGTCCCTCACTCAATTAAATGGTCAGATGTATCCCTTCCAGTAGATTGGACCTTAACCGTTAAGAGTCAACCAGTTCCAATCCAGGTTAGTTTAAACAATTTGGACTATATCCAATAATATCTTGATGGCACTGTCAAAATTAATTTTAGTCATCAACCTCCACAAAAGTCAACAGTCCAGTAACAGCAGTTACCCATTCCAAGTCAATCCCAAAGACATGTTCTTGCTAGACATTCCTTTGATGCCTCTTCCTCTACTCTAGAACGAGACCTAGAGTTAGAAAAAGCATTGATTGACTTAAAACTAGAATCCTTAAGAAAAACTTCCCAAGTCACTCATCCTGTTTATGGAAAAAACCCCTGTCTCAGAAGACAATAAGCCTGAGTCCTCCGAGTCACCCACTCAATCTGATTTCATAGTCAAAAACTGCCCAGTTGACAACCAATTAAGAACTCTGAATAAATTTTTTTTAATTAACTAGAAAAGATTAGAAAATCATTTACATGCTCCTGAGAACCAAATTAGACGAAATAATTATCACACAGTCATCCTTGATAGAGTCAAAAGACAACAAATTTTTAATGATTGGAAAGATTATATGCGATCTGCTAAAATTGAAATTTTTTATCTTGATTTCGTAGAAAGCCGACATCTGTCCAATGAGTTAAAAACTTTAACCCAAGAAAAATGGGAAAAGATGGATCACGCTGAGATTCTCACTAGTCACCCCCTGTTGAAACAATCATTATTAACCATAACAAAACAAAACTAGTAGCAAGTCCTTTCAAAGTCCCTGATCCGGAAAATAAGATGTTAGTCGAGCAAAACAATTATACCAACCAAAGTCTAGTTGTCATAGGGAAACAATTAGATAAGATAGAAACAAAAATCAATAAAGTCAAAACTAAAAATGAGAAACCTTTAGTTACGTTCCAAGATTTAAAGTCAAGTCCAACTTTCCAGACAAAAGAAACAATGACAAAAATTGAAAAAATGTTTGAAGAATTAACACCAGTCAAAAGAAAAGATAAGGAAAAGATTGAAACTAGTATTAAAGTTCTAGGATCCTTCCAGTTACCCCTTACTTCAGATAAATCCTCTGAATCTGAGTCAACCTCCAGCATGGAAACAAAAATTAGTAATATTTCAAAAATTGAAAATGCATTTAGAAGTCTTGAGTTAGAACATAATCCAAAAGTAAAGCGATTAACAAACAGAGTCAGCCCCACTAGTCTCACAAAAAATTGGTATCCAAGGCCAACACCTCCTGATATTCAATATGAAGAAAGAAATTCACAAATCAATTCTCAGTTTCCTCTGATAAATTATACAAATGGAACATTGATGGTCTCTCTGAGCAAGAAATCTTAAATAAACTTCAACATATGTCCATGGTTGCCAATATTTATATCACCAATCACAGTTTCAGGCAATATGAAATTGTGCCTTTATTAGAAACTGGTTTCACTGGAACTCTTCGTTCTTGGTGGGATAAACATTTAACTAATGAGTCTAAACAACAAATAATTCATGCAGTCAAACTCAATGAAGATAGTCTCCAAATTTTTGATGAACAAATAGGTCAAGGCATAGAAGATGGAGTCAACACTTTATTATATACAATAGTCGAGCATTTCATTGGAACACCTCGTAATACTACTACTAGAATACACAATCAACTTCGTAACCTACGATGCCCTAAATTGTCTGATTTTAGATGGTATAAAGATGTGTTTATTTCCTGAGTTATGTTGAGAGATGATAGTAATCAGTCATTTTGGAAAGAAAAATTTGTTAATGGTCTTCCTAATTTATTTGCCCATAAGATTCGCACGACACTTAGTAATGAACAAGGTCATATCGATTGGGGTAGTTTAACACATGGTAATATCATTAGCACAATTAATCAAGTTGGTATGAGAATGTGTGTTGATATGAAAATCGGTAAACAAATCCGGTCTGAAAGAAATTCTGCTAAATATGAGTTAGGAAATTTTTGTGAACAATACGGGCTAATATCAGCTCCACCGTCTAGAAAGGATAAGTCTAGTCATCACAAACGACAGTTTAGTAAGAGCCGCCATTATTCTTGTCAAAAACAGTCATTCAAACATAATAATGACAATAATGAATTTTATAGTAAAAAGAAATTTTCCCCTCAGAAAAATTGGAAAAAGAGTCACGGAAAAGATAAGAATGGTCGCTTTAAGAAAAGTACCGACAAAAGAAAAGTAAAATGTTATAAATGTCAAAAGTTTGGTCACTACGCTAATAATTGCAAAATAAAAGACACAATCAAACAATTGAAAATCACAGACGAGGAAAAAGACACACTAATTAAAGTCCTTGAGCTTAGAAGTTCCGAGTCTAGTGAACATGATACCATAGACAGTAATACTGAGACTCCTGAATTTGATTCCTCAGATTCTCAATCCAGCTCTCCCAAAATACAATTTGGTTGCCAAGACAATTGTTGTAAGTCTAAGTCAGTTAATGTCCTCACTAAACAAGAAGAACAAGAAGAATTATTAATTGATTTAATTAGCAAAGTAGAAAATCCTGAGTTAAAATCCGAATATCTGAAAAAGTTAAGAAAGGTCATCAGTCAAGAAGAACCAAGACAGTTTACCTCTCAAAAGATAAGTTTGAACACAACTTTAGAAAAATTTAATAAAAAGAAGGAAGTTACGCTACAAGATTTGAAATCAGAAGTAAAGGTAGTCAAGAAGGAAATTTTAGAACTTAGGCAAATAAGTCAAAAGTTACAGGCTGAAAATTATAAGATAAAACAAAATTT

GAGTCACACCTCCTGGAAAAAGATGATGCATCCAAAGGTTTGCCTAAGAGTCCAACAAATTCAGAGGACAAGTCCCATGAACCTCCTAACCAAGAATCAACTGTCAATTTAATTAAACAAGTCATATATATATATATATATATATATTCTTTAAAGTTGATAAATCAAGAAAATGTGCTACAAAAACTTGGGCATGGCATGCATATGCTTGAGCTGGATGTTCAGGGAAATATTTAGGTTTAGCTTTTAAATATTCTAAAACCATCTTTTAGAAAAGTGTAGCAGGATCGCCTTTGCTTGAAGAAATATTTGACATACTTGAGTTGGGTTAGTATTATCAATAAGGAGAATACGTCAAGTATCCCCTCACTTTTATTTTCCTTGTTATAGAGGGCTAGTATTTATACCAGCCTTCCTCGTTAGTTAAGGTTAAGGAGGTATATACTCTCGCTTATCATTCTTTATTATTGAGTGGTTATTTATTAATAAATTTGGTAATTCATATCCTTAAAATCCATGTTAATTATTTGAAAATTCCTTCACATGTGTTAATGAGCATTAGGAAATATTGACCAATACGGTGTGGCAGCCCAAATTCAATCCATATTATCGTGTACATTAGAGTAGTTCCGAGTATTGGATCTTCAAAATGCAAAAAGAAATCCTAGAAATTTTTTATTTTTGGTAAAAAGATTCCTTGAAATTGGGTTGGTAGAGAGAGAGATGTGTGTTGTTACGTGCTACCTAGGTTTGCTTTTAAATATTCTAAAACCATCTTTGTAAACATGGCATCAACTTCTCTTGAAGTAATGCTCGTTATACATCTTCTCCTTGTCTCTTTTTATATATAGCTAACACAAACTCATCCTTTACCTCTTCTACATAGCGAATGTGACATACTCATTCACTTCCTCTGAGTTCTCAGTTCATATATTGTCCCTTTTGTGGTTATAATTAATTATATCATTCAAGCTTCTCGCAAGAGTTCACCAGTGTTTGC

>Chr2-07; OZ022207.1; 3839183-3846239; rev; A;

AATAGAAAATGTCGAAGAAGGCCTACACAATGCAGTTAATTGCTTGAAAATGCATCAACCAGCTCACACTTAAGAGTAAAGGTAACATCTGCTTTTATATTTACCACTTTTTCCCTTGCCCGAACTCCTTTAGAATGGCCTGAAAAATCTTTAAGACCACCAATACAAACACAGAACAGCGAACACGGATACCCTAGAGAGACTAAAATGTCTTCATTTCAGTATTTCACTCTCTTACCCTCTACTCTACCATTTTCTATTCCGTGGATGGAATTGAGGTTAGGAAAATCATCAAATCCCAGCAAACCGAAAAAACGAGCCACCTCCACTTTTTCTTTCATGACATCCTTAATGGCAAAAACCCTAGTGCTGTTAGAATTGCTGGGCCACTAAACAGTAACAATGTTTTGAAAGACGAGGCATAGAGGCAAAGCATTTTAATTCGCGCTATGTGAAGCGTAAGCCTTTCGAACATTAATTTATTTTAATAAAAAATTGATAAATAATACTACACAATTAAATTGTACTACTAATTATTATTGAACCCTAATAATGTATGGTCTCATTCATGTATAATAATCAATCCGTAAATCTAAAATATTTATTCAACATCAAAACAAAAACACAATTCAGTTCATATATTAGGGTTATACTATATATATATGAGCGTATTGCAGATTATGTTTATGCTGATTTTCCTCCAGTTAGGAATCCGCAGCCTGAGAAAAAGATATCCCCTACCACGTCACATTCTTCTCTCCCAATCGAGGGAAAATCTAAGTCTGAGTTGCAAGAAATTGCCCGCCAATTAATGTGTTCTGCATTCTGCAAGTTATCAATATATTTGAGATATTTTGAGAATTGATTATTTTGATTAATGCTTATTTTCATTGCATCTATTTTCATATATTCATTAATTTGCTTCATGCATAAATTGCGTCTGTTTTATAATCTCTGCGTTTTCGTTGTTCTTCTTTCTCTCACTGGTTCTAGACCCCTT

TTGGTATCAGAGCCGTAAGTGTTGAAGAGTTAGTCTAAGTCTGCAACTCATCATCCAACCCAAGTGGCTGGTTTAGGTTTTCTGAGTTGTAAGTTTTCAATTTCTTCTTTTCTTCTTCTGGTCTTTTAGTTTACCTGTCCTTCTCTCTCTTGGTGTCCGAATCGTAAGACCCGTTCCAAACAGTAAGTCCCAGGGAGGCCTGAGCGGTAGTAGCATTAGTAGGAGGGCGAGAGAACTTTAGGTATCCTGTTATTCCATGGAGTCTGTATTTGAAGTTTCTGTTCACTTTCTTCTTTAGAGGTCCAAACCCAGAACCTTGAGTAACAATGAGTCGCTTTCTTAGAACCAATTCTTACAGTTCTAGCAGTTCTAGAGCCAATAGTAGTCTCCCAGAGATTGTAAATGAAGAACAAATTGAATATGAGAATAATAATAACCTAGATTATAAAGATTGGAATATACCTCGAGTCCCTAGTCAAGACTATAAGAAAAAATGGTCATTATCCAGTTTTAAGTCAACCACCCATGTTAAGACAGTCGAACAAGTCTATGCCCTAAGTAAAAGTCATGAGACATGTCAATTACTTAATCTTGAGTCAATAAGAAAACATAAAACTGATGGAAACAATTTTCTTCATATTGGTTTATTCCAAGTTGCTGTTAAACCATTAACCAGATTAGGATTAAAAGCTTCTACCCTTCTCTGTCTGCGTGATGCTAGATTTAATAATTTTAACGACAGTATGTTAGGAATTATTGAGTCTAGTCTTGCTAATGGCCCAATTCATTTTGATTGCTATCCAGATTTTACAGTCAGCCTGAGTGATCCACATATTTTGAAAACTCTCACTTTAAATATTAAAACTGAAGGTTATGATATTTTGCCAGGTAGCCAACCTTTAGCATTAGTTACAGAATCTATTATAAAGTCACAGGCACGAATATGAATTTTCAAGCTTTAAACAAGAGCCCAAAGAGTCAAACATTGTTAATCCAAAGTCATACGCCTGATGCTACAGTTAGAGTCCCTCATTCCATAAAATGGTCAGATATTACACTCCCAACTGATTGGACTTTAATCACTGAGATTCCTCCAGTTCCTATTCAGTGTAGTCTAAACGATTTAAATTACATACATCAATATTTAGATGGCACAGTTAAAATCAATTTTGGTCACCAAGCCCCACAGAAATCAATAGTCCAGTTACAACAATTACCCACACCTAGTCAATCCCAGAGACATATTCCTGCAAAGCATTCTTTTGCAGTCTCTTCCTCTACCCTAGAACGAGACCGAGAATTAGAGAAAGCCTTAATTGATTTAAAATTGGAATCACTAGAAAAACTGCACAAGTCACTCATCCTATCTATGGAAAACCACCTGAAGATAATAAATCTGAGTCCGCCACTCAATCCGATTTTGTTGAAACAGGTTTCAAAGCAGAAAGCCAACTTCGAACACTTAATAAGAAATTTGAAATTAATTGGAAACGGTTAGAAAGTCACTTACATGCTCCTGAAAATCAAGCAAGAAGAGATAATTATCACTGAGTAATCCCTAATAGAGTCAAAAGACAACAAATATTTAATGAATGGAAAAACTATATGCGCACTGCTAAAGTTGAAATTTACTATCTTGATTACGTAGAAAGCAGTTATATATCTAATGAGTTAAAAACCCTAACACAGGAAAATTGGGAAAAAATGGATCACACTAAAATCCTTGCCAGTCATCCCCCTGTCGAATCAATAGTCATCCAGCACCAACAAGCACAAGTTATTGCTAGTCCTTTTAAGATCCCTGATTTAGAAAATAAAATGGTAATTGAACAAAATAATTATACTAACCAAAGCTTAATTGTCATAGGTAAACAACTAGACAAAAATAGAAACAAAAGTTGACAAATTAATTCCAAAAGAGATTGCACTTAAACCCCTTGATCAGAAACCTTTAGTCACTTTTCAAGATTTAAAGTCTAGTCCAACTTTCAAAACAAAAACTGCAATGTCAAAAATTGAGAAAATGTTGGAAGAATTAACACCAACCAAGAAGAAAGATAAAGAGAAAGTTGAATCAAGTGTTAAAGTCTTAGGGTCTTCAGAGTCACCATTCACCTTTCATCACTCCTCTGACTCTGAATCAATTATTAGTATAGATACTGAATCTAGTAATATTTCTAAAATAGAAAATGCGTTTAAAAATCTTGAGTTCGAAAATAATCCACAAGTACAAAAATTAACAAATAGAGTCGGCCCTTCTGGTCTCACAAAAAATTGGTATCCTAACCCTACGCCATTTGATATCCAATTCGAAGAACAAAATACTCAAAGTCAGTTTTTTGTTTCTTCTGATAAATTATATGAATGGAATATTGGTGGTCTTTCTGAACAAGAAATCCTTAATAAGCTTCAATATATGTCTATGGTCGCCAATAGTTATATCAATACAGTTTCAGGCAGTCTGAAATTGTCCCCTTGTTAGAAACAGGTTTCACAGGAACCCTTCGTTCCTGGTGGGATAATCACTTAACTCATGAGTCTAAACAACGAATAATTCATGCGCTTAAACTTAATGAAGAAGGTTTTCCTATTTTTGATGAACAAATAGGTCAAGGCATTGAGGATGGAGTCAACACCTTGCTTTACACAATAGTCGATCATTTTATAGGAACACCTAGTAATACTACTGCCAGAATACATGATCAATTAAGTAATTTAAGATGCCCTAAATTATCTGATTTCAGATGGTATAAAGATGTATTTATCTCCAGAGTCATGATTAGAGAAGACAGTAATCAGCCGTTTTGGAAAGAAAAATTTGTTAATAGATTGCCGAATTTATTTGCTCATAAAATCCGATAAACCCTTAGTAATGAACAAGGTCATATTGATTGGGATATTTTAACTTATGGAAATATAATCAGTACAATTAATCAAGTTGGTATGAGAATGTGTGTTGATATGAAAATTGTTAAACAGATCCAATCTGAAAGAAATTCTGCCAAATATGAGTTAGGTAATTTTTGCAAACAGTATGGTTTAATGTTTGTTCCCCCATCTAGAAAGAATAAGCTTAGTTATCACAACCGACGGTTTAGCAAAAAACGCCATTATTCACGTAGAAAACAGTCATTCCAACATAATAATGAAGATAAAGAATTTTAAGAAAAAAGATTCTCCCCTAAGAAAAATTAGAAAAAGAGTCATAATAAAGATAAGAATAGCAGTTTTAAGAAAAGAACCGACAAAAGAAAAGTGAAATGTTACAAATGTCAAAAGTATGGTCACTATGCTAATAATTGTAAAGTTAAAGACACAATCAAACAATTAAAAATTACGGATGAAGATAGAAACAAATTAATTAAAGTCCTTGAGTTAAGAAATTCTGAGTCAAGTGAGAATGAAACCATGGTTAGTCACATTGAGTCTTCTGAAATATATTCTTCTGAGTCACAACCCAGTTCCCCCAAAATCAAATTTGGTTGCCAAGATAATTGCTGTAAGTCTAAGTCCATCAACGTACTTACCAAACAGGAAGAACAAGAGGAACTATTAATCGATTTAATAAGTAAAGTTGAAAATCCAGAATTAAAATCTGAATACTTAAAGAAATTAAGAAAAGTTATTAATCGAGAAGAACCAAAACAGTCCACCCAAAAGATAAGTCTGAACACTACCTTAGAAAAATTTAACAAAAAGAAAGAGGTCACCTTACAGGATTTACAACTAGAAGTAAGACAGTAAAAAAAAAAATATTATTGAGTTAAGACAAATAAGTCAGAAACTACAAAATGAAAATTATGAGATGAAACAAGATCTGATTAACCTATTAGAAAAGAAAATCTCCGAGTCTAAACCAGAGAGTCCCACTAATTCATATGAAGAAAACTCTATTCAAGAACAAGCCGTTAATTTAATCAAACATGTAAATTTTAGAAAAATGGTATGCTCGAGTCACTATATTTGTTCAAAATTTTGAGTTAAACATAGTTGCCCTATTTGATTCAGGAGCCGACCTTAATTGCATTCAAGAAGGTCTAATCCCAACCAAGTATTATAAAAAGTCAAGAGAATTATTAAGCACTACCTCAGGAAAATCACTCCAATTGAATTATGAAATCTCAAAAGCCCATGTTTGTCAAAACAAGATTTGTTTTAAAACTTCTTTTGTTTTAATCAAAAACATTACTAGTGAAGTCATCCTAGGTCTACCATTCATCGCCCTTTTGTACCTTTTTCAGGTTGAACACGACGGTGTCATTTCCACCTGTTTAGGAGAAAAGATTAAGTTTGAATTTTTAAGCAAACTTGAGTTAAGTAATTTAAAAGAATTACAAAAAAATGCGGTCACTAAAACAATCAAGATTGTTCAAAATAAGGATAAGCAACTGAGATTTATCCAAGAAGAAGTTAAAGCTAAAAGAATTGAGCACCAACTAAGTCCAAAATCCTTACAATCACAAATTCAACAGTTTGAAGAAAAGTTAAAACAAGAAGTCTGTTCTGAGTTACCCAACGCGTTTTGGCATAGAAAGCAACATGTTGTCAATCTCCCTTGTGTCAAAACTTTTAGTGAGAAAAATATTCCTACTAAAGCAAGACCCATTCAAATGAGCCAAGAGTTAATGGAATTTTGCAAAATTGATATAACCGATTTGCTTAAAAAAGGAATCATTCGTAAGAGTAAGTCGCCCTGGTCTTGTCCTGCTTTTTATGTCCAGAAAAATACTAAATTAGAATGCGGTGTTCCAAGATTAGTCATTAATTACAAACCGCTTAATACTGTCTTAGAATGGATTAGATACCCAATTCCTAATAAAAGAGATTTAATTAATATATTAGAAAAATCAGTCATCTTTTCTAAATTTGATATGAAGAGTGGATTTTGGCAAATTCAAATTTATGAGTCTGATAGATATAAAACTGCTTTTGTAACCCCTTTTGGCCATTATGAGTGGAATGTTATGCCTTTTGGTCTTAAGAATGCTCCTAGAGAATTCCAGAATATAATGAATGAGATCTTTAATCCTTTCAGTCATTTTTCTATAGTTTATATTGATGAAGTGCTTATATTCTCTGAGTCAGTAGAACAACATTGGAAACACCTGTATAAGTTTTTACAGATAGTTAAACAAAATGGTTTAGTTGTTTCTGCTAAGAAAATTAAGTTATTCCAAACTAATGTGAGATTTCTTGGGTTTAACATCTGCCAGTCACAGATTAGTCCTATATATCAAGTTATCCAGTTTGCTGATAAGTTTCCTGACCAAATCCTAGACAAGAGTCAACTCCAGAGATTTCTGGGGCCTCTAAATTATGTTTCTGATTTTTATCAAAATCTGAGAAAACAATGTAAGCCTTTATTTGTCTCCAGAATAATCCTCCCCCCATGGTCGTCTACTCATACTGAAATTGTCAAACAAATCAAAGCTCATATCAAGACCCTTCCTTGTCTTGGTATTCCTCCTGCTAATTCATTCAAAATTGTTGAAACTGATGCTTCTAATGTAGGTTATGGAGGTATCCTAAAACAGAGAGTCACTTCTGGTCAATCTGAGCAAATCATTCGATTTCATTCCGGCACTTGGTCTTAGTCTCAAAAAAATAATAGTACTATTAAAAAAGATATATTATCTATTGTACTATGCATTAATAAATTTCAATCTGATTTATTAAACCAAACATTTTTAGTCAAAGTTGATTGTAAGTCTGCTAAGCATGTATTAGAAAAAGATGTTTAAAACATTGCATCCAAACAAATATTTGCACGATGGCAAGCTATTTTGATTTTGATATTGAATATTTGAGAGGAAGTGAGAATTCTATTCCTGATTTCCTTACCATAGAATTTCTGCAGGGTAAACAATGGCAGAACGAAGAAGAGAAAAGGAAAAAGAACAACAAAAGAGTCAAATTGGTTAATCTAGTCAGCCCAGTCAACCTCAGTCACTCTGAGTCCTCCCTCCATCAATGAGTCACACTACACATGAGCCTCCTTCCCAGATCGTCCCCTTCCTGGGATGTTCTCCTATCCAAATTAGTAACAGGTTTGCCAACCTAGGAGCCACAGTCGGCCAGGTTCGGCCTAGTTTTCAGTCTGCTTTAATTACCAGTTATGATCCTTTCCAGGATATTGCCTTAACCAATTCCCCAGTTGCCTCTTACCATAAGACATCTCCATATATGATCAAGAGTCATGGTCACCTGTTTGTCATTGAGTCTAAATACAATTCAATTGCTAATCCCATCACCATTGCTAAATCTTATTTTCCACCCAATAGTCATTTCATTCCCCCAGCACCATTTAAGAATCTGAAATATTACAGAGATATCCTCCATGAGACTCATTCCATCACCATTAAGCCAATCAAAGACAAAGAAAATCCTCACATCATCCTCTATCATTCTCTTTACATTCATCAAATAATCAATGAAAATGAGTGGAGTAGCCATCCTTATGACCTCAAAGTTCTCCAATCAGAAATACAATATTGTTATTATGATTATATCGAAGCATGGGATTCTATCTTACTTCATGAAACAGTTGATTTCAGTCACTCCTGATTCTTAAATTTTGACAGCAAATTCAAGAGTCCTTTTTCTTACTGGTTCAATCATTGGTGGGAAAAACATGGTCCAGTCATTGATATATTTCCTCCTCCCATTCAAGATTTAGTCAATTACTTTATAATAAAAATAAGTTCAAAGAATCTGAGCTATTTTTCCCCAACCTTCTCCATTTTGTTGCCAAGTATAAAGTCCCTTGGATCCTCAAATGGTCTTATCAGGTTAATAGGGAAACCAGAGTCCTATCTCGCCAATTTTCAATCAAATGGTGGGATAAATTTAAAGTTGAGCGTATTGCAGATTATGTTTATGCTGATTTTCCTCCAGTCAGGAATCCGCAGCCTGAGAAAAAGATATCCCCTGCCACATCACATTCTTCTCTCCCAATCGAAGGAAAATCTAAGTCTGAGTTGCAAGAAATTGCCCGCCAATTAATGCTTCAAGCTTCCCAAATGAATGATGATGCAGACAGTGATGCCTCTCCTGCGTC

ACAAACATCCACAAGTCAACCAAAGCCAACTAGTCAACAGGCTCCAGCCCCAAAAATGCGGTGAAGTGATTATCCAGATGAACAAGACCCCTATGATTTTGGTCCTTTCAACCTAGGCGATGATTAGCGTTGCCCTTCATCATGAGCCCTGAGACAAAGCATCCGGACCCGTGCATTCAACAGTCTGAAAGCTCGTGTTCCTTTTTGAGATTCCCCCGACAAAGTAAAAGTTGCAGCACTCAGTAAAAGCAAAAGCTTTCGCATGCTTCTACAGTTACTTTTCTTAAGCATCCAAAGTTGCCAGCTCATCAAAAGATATCTGAGTCCTTTTATTTGATTATTTAAGAAGGCTTCGGCCTCTGTTTTGTCCAGAATGTAATTTAGAACACTATAATTTTCTCAGTTTCAAAGTTCTATCAGTACCAATGCTCTGAAGAACTATAGTGGTGAAACTAGGATTTTGTAAGTGTTTTGCATTCTGCAAGTTATCAATATATTTGAGATATTTTGAGAATTGATTATTTTGATTAATGCTTATTTTCATTGCATCTATTTTCATATATTCATTAATTTGCTTCATGCATAAATTGCGTATGTTTTATAATCTCTGTGTTTACTCTGTTCTTCATTGTTCTTCTTTCTCTCACTGGTTCTAGACCCCTTCCGAATATATATATATATATATATATATATATATATATATCCATGTTAATATATTAATTTAGGGAAAGAAAAAAGGAAAAAAGGGCAAGTAGTTGTGTTTCAGTGAAAAAGGGTAAGTAGTGGTGTTTCAGTGGAGGCGTTAAGCACACTGTTTGTTAAAAACAAAAAATCATGATTTAAAAATAATAAAAAAGGCATGCTGAACGTCAGTGCTAAACGTGCGTTTCATTCCCAACGAACCTTAACAAAAGAACACGCGAGGTCATCTTCTTCCTCTCGCAGCCGCTTGCAGAACAGCCGCCCTTCGTCCGCTTCGCCACGCCTCTCCACGCCTCAC

>Chr2-08; OZ022207.1; 3856861-3858514; dir; B;

AAGAAAAAAGAACAGGTTGTATAATCTGCACAGACACCCATCCCGAGTTTTCAACTCCAAAACCCCCATTTTCTAAACCCTCAAACCTAAATCTCAAATCTCCACCCCCAACAAACCTTTAACCCTCTTCCCCACCAAGTCTTTAAGCCCCAACTCCTCCATTGCCGCCGCTGCCAGTAGCTCCGTTGCTTTCAATGTTATTTTAATTTTAAAAAAATTGAACTTTTACACTATGACCTTACGACATAAAATTCTGAGTCCGTCCCTGCACCCACCCAGCACCGTCAGCCAACCCACCTCGCCGGCAGTGGCACATTCTCCACCTTGCCGGCAGCGATACCTTCTTCACCTCGTTGGCGACAATACCTCCACCTCCTCAGCGATAACACCGTCTCCACCTAGACGGCGAGAACACCGCCTCCTCCTACCTCTCTTTCTCTTTCGTTCTTGAAGATAATCTCATATCTGGATCTAATTCAGCGAAGCTCATCATTCAATCCCTTACAAGTGAAGTGATGGATCTATAACAGGTATTTTGCATACAAAAATCTCAAACAAAACCACCCCAAAAGAATACAGATTTTGCACAAAGGGATGGATGCCTCTGTCTGTTGGGCCCAATGCTCATCCAGATTTAACACATTTTTCAAGAAATGGAAAAACAGATAGATGGAGAGAAGAGACTTTGAGGACGAGCGAGGTGGAGATGGTACTGCCGTCGGCGAAGTGGGATGGCTGATGGTGTTGGGTGCCGTCGGTTTGGATGGGGACGGGGACGTTGGTGGTTTCTGGTGTTGGTTTGTTTTTGTTTTGTTTTCATTAAATTTTTTTTTTTTTTTTCCTCGGCAAAGTTATGAAGAATGTGTCAAATTTCAAGTATCCCCTCAATTTTTTTTTCCCCGTGCATTAAGAAGGTATCTCGCAGAAATTCATTAATATAAACTAGATTAAGAAGTTTCTGTTTGCTTTCTTCCTTAGAACTCCAACCCCAGAACCTTGA

GATAGTTAATGAAGAACAACTAGAATATGAGTCTAATAATGAACTAGATTTTCGAGATTGGAATATACCTAAGGTCCCTAGTCAAAATATTTATAAGGAAAAATGGTCGCTCTCTAGTTTCAATACAACTACCCATGTAAAAACAGTTGAACAAATCTATGCTTTAAGTCAAGAACATGAGACTTGCCAATTACTTAATCTTGAGTCAATTAGAAAACATAAAAATGATGGAAACAATTTTCTTCACATTGGTCTAGTCCAAGTTGCAGTTAAGCCGTTAACTAGATTAGGATTAAAAGCTTCAATCCTTCTCTGCTTACATGATGCCAGGTTCACTAATTTCAACAATAGTATGCTAGGAATTATTGAATCCAGCCTTGCTAATGGTCCAATTCATTTTGATTGTTACCCTGATTTTATAGTAGGCCTGAGTGATTCCCATATATTAAAAACCCTAACATTAAATATAAAAACTGATGGCTATGATGTCCTGCCCGGCACACAACCTTTAGCTTTAGTTTACAGAATCTATTACAAAGTCACAGGTACTAATTTGAATTTCCAAGCCCTGAGTAAAAATCCAAAAAACCAAACTTTGTTAATCCAAAGTCATACACCTGATGCTACTGTTCAGTCCCTCACTCAATTAAATGGTCAGATGTATCCCTTCCAGTAGATTGGACCTTAACCGTTGAGAGTCAACCAGTTCCAATCCAGCTTAGTTTAAACAATTTGGACTATATCCAATAATATCTTGATGGCACTGTCAAAATTAATTTTAGTCATCAACCTCCACAAAAGTCAACAGTCCAGTTACAGCAGTTACCCATACCAAGTCAATCCTAAAGACATGTTCCTGCTAGACATTCCTTTGATGCCTCTTCCTCTACTCTAGAACGAGACCTAGAGTTAGAAAAAGCATTGATTGACTTAAAACTAGAATCCTTAAGAAAAACTTCCCAAGTCACTCATCCTGTTTATGGAAAAAACCCCTGTCTCAGAAGACAATAAGCCTGAGTCCTCCGAGTCACCCACTCAATCTGATTTCATAGTCAAAAACTGCCAAGTTGACAACCAATTAAGAACTCTGAATAAATTTTTTTTCATTAACTGGAAAAGATTAGAAAATCATTTACATGCTCCTGAGAACCAAATTAGACGAAATAATTATCACACAGTCATCCCTGATAGAGTCAAAAGATAACAAATTTTTAATGATTGGAAAGATTATATGCGATCTGCTAAAATTGAAATTTTTTATCTTGATTTTGTAGAAAGCCGACATATGCCCAATGAGTTAAAAACTTTAACCCAAGAAAAATGGGAAAAGATGGATCACACTGAGATTCTCGCTAGTCACCCCCCTGTTGAAACAATCATTATTAACCATAACAAAACAAAACTAGTAGCAAGTCCTTTCAAAGTCCCTGATCCGGAAAATAAGATGTTAGTCGAGCAAAACAATTATACCAACCAAAGTCTAGTTGTCATAAGGAAACAATTAGATAAGACAGAAACAAAAATCGATAAAGTCAAAACTAAAAATGAGAAACCTTTAGTTACGTTCCAAGATTTAAAGTCAAGTCCAACTTTCTAGACAAAAGAAACAATGACAAAAATTGAAAAAATGTTAGAAGAATTAACACTAGTCAAA

AGAAAAGATAAGGAAAAGATTGAAACACTACTACATTTTACACTTTGCGCGACGAAGAGAATTTCGTCGCGCAAAATACATTGTAGTCGCACAAATTTCAGCGCGACAAAAACTTCATCGCCCAGAATCCGTCGCGCAAAGATCGTGAAGAAAGACTTTGCGTGACGAAGACATTTCATTGGTCACACAAAGTGACTTTGCGCGATGAAAACATATTAGTCGCGCAAAGCGACTTGGATTGGTCGCGCAAAGTCTAGAAAAAATTTAAAAAAATAAAAAATTCCCGCGTCCAATTTTTGGAACCTGCCCAAATTTTTTGAGTTTTGCCCGACGAATACTATCGTCGCGCAAATATTTGCGCGACGAAATATTTTTTGTTTTAAATTTTTTAATTTAAAATAAACTAATTTAATAGTTTGCGCTACGAAATATTTTGTTGCGCAAGGTTTTGACTTTTTGTTTTATTATTTCTTTAACATGAATTTATTTATATGAATTTTTTCAAATTTTACTTTTTAAATTTAATTTAATTGTATGATTTCTTTTTAAAATTACTTTAATTTAAATTGATATTAATTTTTACTTTTTAAATTTAATTTAATTGTATGATTTTTTTAATTACTTTAATTTAAATTGACATATTCAAAATAAAATTACATATGAAAAATAGTGATTAAATTATCCAATAAATATATATATATATTCAAAATGTACACATAAATTAACAAAGTACAATAATAAAATCCTAATACAAGCGTATTGTGGTGGTAGTGGCGGAGGATATGTTTTGATAGCTTCCTAATCCTCAACTTTAGCCGTCAAAGTCTCGATGATTCCCTACAAAAAATATATATATATATGGTCAAATAACCACACACAAAAAAAAAAAAAAAGAAAAAAAAAAAGAAGGCATAATCTCCCCTAAAATTGTTATACCACAAATCAAACCCAAACTCCAATCCCTCCCTTTACTCAACCCCAAAACCCACACAAACTCAAA

>Chr2-09; OZ022207.1; 3889122-3890826; dir; B;

GTTGTCTTTGGAGACGAAAACTCTGGTCGCGCAAAATTTTTTTACCGCCATAAAAGGAGCCAATTTTCTGTCCATCTTTGCGCGACGAATAGCAAGAATTGTCGCGCAATGTTGTCATGCACGACGATCTCTGTCGTCGCACAAGATTTTGGCGCCAACAAAATAGACGGGGTGTTTTTTACCAACTTTGCACGACGAAATTTATTTACGTCGCGCATAGACTGTTTCCGCGACGAAATTATTTGTCGCGCTAGTTTTCTGTTGTATGCGAGACGGAATTTTTTTTTCGTCGCAAAAGTGCTTCTTGCGCGACGGCAACGACGAAAGTTTGTTCGTCGCGCAAACTTACGTCGTGGAAATAGTAAATTGTAGTAGTGAAAAATAAATTAGTTTCGGTGACGAAATGTTTGGATTACGTCATGCAAAGATTTTAAAATATATATATTTTTTATTTTTATAAGGACTTTGCGCGACGAAGTTATTGTTTCGTCGCGCAAAGTCACTTTGAGCGACGATGTATTGCTGTCGCGCAAAAGTGTGTCGCGCGAAGTTACTTTGCGCGATGAAACAATAACTTCGTCCCGCAAAATTTGGTCGCGCAAGACTTTGAGCGACGATGTATTGTCGTCGCGCAAAAGTTCGTCGCGCCAAGTCACTTTGAGCGACGATGTATTGTTGTCGCGCAAAGTTACTTTGCGCGACGAATGTTTTTTCGTCGAGCAAAATTTGCGCGCAAGACTTTAAGCGAAGATGTATTGTCGTCGCACAAAAGTTTGTCGCGCGAAGTGACTTTGCGCTACGAATTGTTTTTCGTCGCGCAAAATTTGGTCGCGCAATACTTTGAGCGACGAAGTTTTTTGTTTCGTCGCGCAAAGTGACTTTGTGCGACGAATAGTTTTTCGTCGCGCAAAATTTGGTCGCGCAAGGGGTTTTTTTTACTAGTGAAACTAGTATTAAAGTTCTAGGATCTTTCCAGTTACCCCTTACTTCAGATGAATCC

TCTGAATCTGAGTCAGCCTCCAGCATGGAAACAAAAATTAGTAATATTTCAAAAATTGAAAATGCATTTAGAAGTCTTGAGTTAGAAAATAATCCAAAAGTAAAGCGATTAACAAACACAGTCAGCCCCACTAGTCTCACAAAAAATTGGTATCCAAGGTCAACACCTCCAGATATTCAATACGAAGAAAGAAATTCACAAATCAATTTTCAGTGTCCTCTGATAAATTATACGAATGGAACATTGATGGTCTCTCTGAGCAAGAAATCTTAAATAAACTTCAACATATGTCCATGGTTGCCAATATTTATATCACCAATCACAGTTTCAGGCAGTCTGAAATTGTGCCTTTATTAGAAACTGGTTTCACTGGAACTCTTCGTTCTTGGTGGGATAAACATTTAACTAATGAGTCTAAACAACAAATAGTTCATGCAGTCAAACTCAATGAAGATGGTCTCCCTATTTTTGATGAACAAATAGGTCAAGGCATAGAAGATGGAGTCAACACTTTATTATATACAATAGTCGAGCATTTCATTGGAACACCTAGTAATACTACTACTAGAATACACGATCGACTTAGTAACCTAGGATGCCCTAAATTGTCTGATTTTAGATGGTGTAAAGATGTGTTTATTTCCCGAGTTATGTTGAGAGATGATAGTAATCAGTCATTTTAGAAAGAAAAATTTGTTAATGGTCTTCCTAATTTATTTGCCCATAAGATTCACACGACACTTAGTAATGAACAAGGTCATATCGATTGGGATAGTTTAACCCATGGTAATATCATTAGCACAATTAATCAAGTTGGTATGAGAATGTGTGTTGATATGAAAATCGGTAAACAAATCCAGTTCGAAAGAAATTCTGCTAAATATGAGTTAGGAAATTTTTGTGAATAATACGGGCTAATATCAGCTCCCCCGTCTAGAAAGGATAAGTCTAGTCATCACAAACGACAGTTTAGTAAGAGTCGCCATTATTCATGTCAAAAACAGTCATTCAAACATAATAATGACAATAATGAATTTTATAGTAAAAAAAATTTTCCCCTCAGAAAAATTGGAAAAAGAGTCACGGAAAAGATAATAATGGTCGCTTTAAGAAAAGTACCGACAAAAGAAAAGTAAAATGTTATAAATGTCAAAAGTTTGGTCACTACGCTAATAATTGCAAAATAAAAGACACAATCAAACAATTGAAAATCACAGACGAGGGAAAAGACACACTAATTAAAGTCCTTGAGCTTAGAAGTTCCGAGTCTAGTGAACATGATACCATAGACAGTAATACTAAGACTCCTGAATTTGATTCCTCGGATTCTCAATCCAGCTCTCCCAAAATACAATTTGGTTGCCAAGACAATTGTTGTAAGTCTAAGTCAGTTAATGTCCTCACTAAACAAGAAGAACAAGAAGAATTATTAATTGATTTAATTAGCAAAGTAGAAAATCCTGAGTTAAAATCCGAATATCTGAAAAAGTTAAGAAAGGTCATCAGTCTAGAAGAACCAAGACAGTCTACCTCTCAAAAGATAAGTTTGAACACAACTTTAGAAAAATTTAATAAAAAGAAGGAAGTTACGCTACAAGATTTGCAATCAGAAGTAAAGGTAGTCAAGAAGGAAATTTTAGAACTTAGGCAAATAAGTCAAAAGTTACAGGTTGAAAATTATGAGATAAAACAAAGGTTTGCCTGA

GAGTCCAACAAATTCAGAGGACAAGTCCCATGAACCTCCTAACCAAAAATCAACTGTCAATTTAATTAAACAAGTCATATATATATATATATATATATATATTCTTTAAAGTTGATAAATCAAGAAAATGTGCTACAAAAACTTGGGCATGGCATGCATATGCTTGAGCTGGATGTTCAGGGAAATATTTAGGTTTAGCTTTTAAATATTCTAAAACCATCTTTTAGAAAAGTGTAGCAGGATCGCCTTTGCTTGAAGAAATATTTGACATACTTGAGTTGGGTTAGTATTATCAATAAGGAGAATACGTCAAGTATCCCCTCACTTTTATTTTCCTTGTTATAGAGGGCTAGTATTTATACCAGCCTTCCTCGTTAGTTAAGGTTAAGGAGGTATACTCTCGCTTATCATTCTTTATTATTGAGTGGTAATTTATTAATAAATTTGGTAACTCATATCCTTAAAATCCATGTTAATTATTTGAAAATTCCTTCACATGTGTTAATGTGCATTAGGAAATATTGACCAATACGGTGTGGCAGCCCAAATTCAATCCATATTATCGTACATTAGAGTAGTTCCGAGTATTGGATCTTCAAAATGCAAAAAGAAATCCTAGAAATTTTTTTATTTTGGTAAAAAGATTCCTTGAAATTGGGTTGGTAGAGTTGCTTTTAAATATTCTAAAACCATCTTTGTAAACATGGCATCACCTTCTCTTGAAGTAATGCTCGTTATACATCTTCTCCTTGTCTCTTTTTATTTATAGCTAACACAAACTCATCCTTTACCTCTTCTACATAGCGAATGTGACATACTCATTCACTTCCTCTGAGTTCTCAGTTCATATATTGTCCCTTTTGTGGTTATAATTAATTATATCATTCAAGCTTCTCGCAACAGTTCACCAGTGTTTGCTAAAACATTGCATTGGAGAGCTAGGTAAGTCTTGAAATAGTTGTTTGCCTTCACACCTTAGCACAACACAAGTGGTTCGCTT

>Chr2-10; OZ022207.1; 5508146-5509420; rev; B;

ACCAAAGAAGAGAGTCTAATAATAGACTTAATGGACAAAATAAAGGATCCAGAGAAAAAGAGAGACACTTAGAAAGTTATATCTCACTAGCTAAAGCGGGACCAAGTAACCCACAGCCAGCTATAACTAATGTGAGACAACCAGTTGAAAACTATAGCTTTAAAACTATAGTAAACAGGATGAATGATAAAATTCACAAGAAAGAACCTACTCTCAATTACCTTCAGTATGAAGTTCATGATGTAAAAGAAGAACTTAGAGAGTTAAAAAATAAGGTTAGAATTCTAGAACTGTATCACCCTTTTTCGGAAAAAGATACAGAAGAAAAATATGAGCAATTAGAGTTTGAAAATTTAATGGAAGAATTTCAAGAGAAAATAGAACCTGTGAGAAGAGAATCCCTACAAGAAGAGCAGGTTAGTACCATGAAATTCGTTAATAGCATAGACAGAGTGATAGCTCAGAAATGGTATGCGATGATAACTGTTGTGATTAATAAAACCTATAAATTCACAGTTGAGGGGCTGATAGACACAGGAGCCGATCTTAACTGTATAAATGAGGGACTGATACCTACTAGATATTTTCAGAAGACTACCCAGGTTCTACATACAGCCGTTAATAGCCAAAAAATGGGAATTCAATTTAAATTATCTAAAGCTGCTGTGCTCAACAGGGTATTTGTTTTGAAACTCCTTTTATTCTGGTGAAAAGTTTAAACCAATCTCTTATATTAGGAACACCATTCATTAATATGTTTTATCCTTTTTCTGTTTCTGAAGAAGGAATTAAAACTGAGGTAGAAGGTCAACCTATAATCTTTAGGTTTTCTCAACCACCAAAGCATGAGTATATTAACGAAATTCAAGAAAAAAAAAAAGGAAAGAAAACTATCTATATCATTTGAAAGAAGAAATGCTGCATAAAAGAATACAGGAGCAACTGCAAAATAGCAAGATAAAAGAAAGAATTGATGAAATTCAAAAGGTGTTCGAAAGTC

AAGTTTGTGCTGAACTACCCAATGCCTTTTGGAATAGAAGGAGGCACATAATTGAATTACCTTATGAGCCTGACTTTGATGAAAAGAAAATCTCAACCAAAGCTAGGCCAATTCAAATGAATGCTAGGCTAATGGAAATTTGTAAATCTGAAATACAAGGTTTATTGAATAAGAAGTTAATCAGGAAAAGCTCATCTCCTTGGAGCTGTTCCGCTTTTTATGTTGAAAACACAGCTGAATTAGAAAGAAGAGTGCCCAGATTTGTGATAAACTATAAACCTCTAAATAAAGCCTTAAAATGGGTTAGATACCTGATACCTAATAAGAATGATCTTTTGGACAGAATAAATTAGGCAGTCATCTTTTCTAAATTTGACATGAAGTCAGGATATTGGCAAATCCAGATCACCAAAAAGGACAAGTACAAAACAGCTTTTAATGTCCCCTTTGGACATTACGAATGGAATGTTCTACCTTTTGGATCGAAAAATGCCCCTTCTGAATTTCAAAATATTATGAATGATATATTTAATCCTTACACTGCTTTCACCATTGTTTATATTGATGATGTTTTAGTCTATTCTAATTCTTTGGAACAGCACTTTAAACATCTAAATATTTTCTTGAAAGTTGTGAAACATGTAGGATTAGTAGTATGGCAAAGAAAATGAAACTATTTTCTTCAAAAGTTTGCTTTTTGGGACATAATTTTAATAAGGGACTAATAGTTCCGATTGATCGAGCAATATCCTTTGCTGATAAGTTCCCTAATGAAATTAGGGACAAGCAATAATTACAAAGGTTCTCAGGAAGTCTCAATTACATAAAATATTTTTACAAAGATTTAGCTCAAGACTCAAAATTATTATACTAAATGTTGAAAAAGAACCCGGTTCCTTAGTCTGATGAACACACTAAAGCTGTTAGGAGATTGAAAGTTAAAGTCAAAGAACTTCCTTGTTTAGCTTTAGCCAACCCAGAAGCCTTTAAAATCGTAGAAACGGATGCTTCAGAAATAGGGTATGGGGAAATCTTAAAATAGAAGGTTGATAACTCGGAACAGTTAGTTAGGTTCACCTCTGGAGTTTGGAAACCAGCTCAAAATAATTATTCCACTATTAAGAAAGAAATGCTATGGTCCATGCTATTTCTAAATTTGAAAGTGATCTTTTAAATAAGAGATTTTTTATAAGAGTTGATTGCAAGGCTGCCAAAGACATTTTGTTCAAGGATGTTAAAAATCTAGCATCAAAACAGATTTTTGCAAGACGGCAA

GGAATTCTTTCTGCTTTCGATTTTGATATTGAATTTATTAAAGGTGAAACTAATGTACTCCCGGACTTTTTAACTTGAGAATTTTTGCAGGGTGCGAATAAGAAATTCTGGACAGATGACTCCCAGTAAGAGCTCATCTGCTCTTACCAAGTCATCCCCTAATGATCCAAAGCCACTGATATCTTCTTCTCCATATAATAAGGGTAAGGAAAAGCAGATTGCTGAAAATCCATTCCATGAATATATGAAAGTGTCTGTAGCAATCATTGAAATGAATGATCTAGATAAACACCCAAATTTTCTAGCTGAAAAATATTTAAGGGATAATCTTCCTCCCTTGAATAAGCCTCGTATTTTCTATGAAACTCTTCTAATTGAAACTAAGTCTGTTAATATCGTTCATCGTCACAGAGATGAAAATGCCAACACCATAGCATTCTCCAAAATCCAAATTCTAAACGTCATTTCTTTGGAAGAGTGGGGTCCTAGCCCTGAAAGGGCCAAAAGATTTATCCATCCTGAAATCATGCCATATTCCTATAATTACTGGGATTATAAAAAGGCATGGGAATATGTTTTATGTTTTCAAAATCAAAACTATTCTCATACTTGGTCTATCCAGTTTAAGCATCGAACTCTGAAAGAACTCCCTAATTGGTTCAAGAGCTTTTTTCTAATTTGGGGTCCTACTTTGGAATACCTTTCTCCTGAAATTAAGGAATCTTTTCAAATTAAATTTTTTAAATGGAATAAAAACCATCCTAATCATAGGACCTTATTCATGTGGTTCATGATAATGTATGAACTAACATGGATATTAAAATGGGAATATGTTATTTCGGAGGATGTTGATGAAGAATGGACATATATTCCACATTTGGTCCTAAGTATCTTAATCAGATGGTGGGATAATTTCACTAAGGATCACCTGGCTCCCGCGAAGATCATCCCAACGCTTATCACATCCCCCGACTCTGCTGCTGCTCAGTCTTTGACCACT

>Chr2-11; OZ022207.1; 5893787-5903586; dir; B;

AGACGTGCTGGGCAGCAGAGGACAGTTTGAATGCCCACCCCTACTATATCCAACATGAGAGATAAGACCTTAAGTATAGTGCATACTATGAGTGAGAGTCCCCTAAATTAGGCCTCATCATTTGGCCCGCAGGCTCATGGGTCGATGGGGACCCGTTCGGTCCATCGAAAAAAAGTCTAGCCTGGTCCGTTATGGGCTTTGAGATGGCCCAGCCCGCCTCGGGCCGAGCTAGGTTTGGGTTTGGGTGTCTAAAGCTCGGCCCAGCCCATAGGCCCGGCCTGATTAAGCCCAAGAAAGCCCGACACGGCCTGCCCACAAAGCCCCACCCGTTAGGCCCGCATACATATATTTATGTATAAATAAGAGTTTAGGTTTTGGGATTATATCACTTAAATCACATATATAATTTGTTTCATTTTATTTACTTTTCTTTACTCATTCCTAGTTTATAATTGGATGATTAGCACATTAATTTGTGTCATTTATTAATTCAACAATTATATATATATATATATATATATATAACTAAGCTAGGATTCAAAGCAGCAATTCTTTTAAGTTTAAGAGATGCTAGATTTAACGATTTCCAGGATGGAGTTTTAGGAATCACTGAGTCAAGCTTATGCCATGGTACAGTTACAATTAGTTTCGATAGGCCAGTTAAATCGACAGTCCAACTAGAACAACTGCCAACCCCTAGTCAATCTTTTAAAATCCCTGCTAGACATTCAGTCATTCTAAACAGTTATCTTAGAGTTATCTGTCCCCCAAGGTTTACTCTCTCAAAAGAACACGGTAAGACTTATAGCTGAACAGTAAGTCTCGCGGTAGAGGCCTGAGAGGTGCGGCACGAGTGTGAGAGAGAGAATGCCTTAGGTTCCAGGTAATTATGAGTTAACTTTAGGAATTCCTATAGTTCTTCTACCTTTTGAAAACTTTGCGTCAAATACTTGTACGAATGAGTCGTTTTCTTAGAACCAATTCAGTAAGGTTTAATAAA

AAGAAAGAAGTCAACCTTCAGGATCTCCAGTTAGAAGTCAAAACAGTAAAAAAGGAAATATTAAGCCTAAAACAGGTTAGTCAAAAATTACAAACTGAGAATTATGAAATTAAACAAGATTTAATTAAACTCCTTGAGTCTAGAGCAGAATCTAGTAATCCCTTTAGTAAACCTAGTTTCGAGTCAGAACCCGAGTCTTCTGATAGTAACCAAAGCAACAAGGTTATCAAATTAATTAAGCAAATTAATTTCAGGAAATGGTATTCCCGAGTTAGTATTTTTATTAATGAATTTGAGTTAAATACAATTGCCTTATTTGATTCAGGAGCCAATTTAAATTGTATTCAAGAAGGTCTAGTTCCAACCCAGTTTTACAAAAAGTCAGAAGAAATTTTAGGCACAGCATCCAGTAAAGCTTTAAAATTAAATTATGAATTACCCAAAGCCCATGTATGCCAAAACAAAATATGCTTTAAAATGTCTTTTGTTTTAATTCAAAATATCACAGATGAAGTTATTTTAGGATTACCCTTTATCTCGTTACTTTACCCTTTCCAAGTAGAATATGATGGAGTTACATCTAGGCACCTAGGTGAAAGAGTTAAATTTGAGTTTTTAACCAAACTAGAGTTGCACAGTCTCAAGTCTTTACAGAAGTCAGCAGTTTCTAGATCAGTTAGATTAATTCAAAATAAGACAAAACAAGTTAATTTTCTTAAACACGAAATGAAGTTCAAATGCATCGACCAACAGTTAACAGATGAGTCACTACAGTCTCAAATAATTAAATTTGAAGAAAAGTTAAAAAATGAAGTTTGTTCTGATTTACCTAATGCCTTTTGGTACAGAAAGCAACATGTGGTAACTTTACCTTACGTTAAAACATTTAGTGAAAAGAATATTCACACTAAAGCCCGTCCAATCCAGATGAGGCAAGAGTTAATGGAATTATGTAAACAAGAAGTCACTGAATGAGTCCAAAAAAGAATAGTTAGGAAAAGCAAGTCACCTTGGTCATGCCCAGCTTTTTATGTTCAGAAAAATGCAGAGCTAGAAAGAGGAGCCCCTCATTTAGTTATCAACTATAAGCCCCTTAATGCAGTACTTGAATGGATTAGATATAATCCCTAATAAAAGAGATTTAGTTAATAGATTAGAAAAACCAGTTATCTTTAGTAAATTTGATATGAAAAGTGGTTTTTGGCAAATTCAAATCAAAGAATCAGATAAATATAAAACTGCATTCATTACCCATTTCGGTCATTACGAATGGAATGTCATGACAGGACCCGACCCAATTTCCACTTTGAAATTCGAGCCAAGTCCTGTGCGTGTCCGACACCTGGCGAATGTCGGGCACAAATGACCTTTTTGCCCTCTTACTTCAATTTTCTTTAAAATTCCTTAGACTTCTGCCGAAAATTCGGCAGAGTCTCCCCTGTATTTTGACCAGTCCCAAAATTTCACCTGTAAACAATCTCAAATATTTCCACCCAACTGCCAGAATAGGATAAACCAAAATTTGCCAGATTCAAATTTTCCAATAAACTCGATATCAGAGCAATTTTCTAAAGTTCACGGGTTTTCAGGAAAAATTTCTACAACTTTACCTGACTTTGAAGCGTGGAGGTTAAGAGTGGCCCGGTGGTGGATCCTATGCACTTCTACAACCTGGGGGCGAAAAACAAGTTTTAAACTGTGAGTGGACAAAAAGTGAGATTCTTAAAATCAGCTAAAATATAATTCCCCCATTTTGAAATAACTAGGGATATATATACATCGAAAATTTAACTATATCTCCATATAAAAGATTCTATAAGCATGAATTTGTATACTAACGAAGAAACTTACATAATCGATCTGATATAAAGATATTACTGCCTGGAAGAATCAAAGAACTGGTATACTGAATAAAATACCAAAAATACACATGTGCAATTACTGAGAGACCAATATAAAATGGCTGAAAATCATCCTTGAATAAAGAAAGCTCAAAATCCTTTAAAACTTCGTTCGACTGAATTTACTAAAACAAAAATGAGTAAACAATATCTGCAGTAAAAGCTTTAAAATCCTTTAAAACTGCCACCTAATTGTACCCCTGTTATATCCGTCAATTCCCCTGACAGGTCTCGGGCGCCACGCAGTCTACCCGAGCCGCAAACTGGCAAAATCAGGGGACTATGATCAGCCTGATCTGCCGGCAGATTCCTCGAGGACACCAAGTCAGCTCGAGTCGCTCTGGCAGGATGCAGGGGACCGTAGTCAGCCTGATCCGCAATCTTGGCAGGTCTCGGGGACACAGAGTCAGCCGAGCCGCAATCCTGGCAGGTCTCGGGACACCAAGTCTGCCGAGCCGCAAATCCTGGCACTCACGGTCCGAGCGTCCCCGAAACTCGTGAGGCAAAGTCAAGTGCACTGACTTAACTATAATCAGACTGGATGTCCGTAGACATCGGTCCGACTCTGGGTAATCACCATAAAGAAAATGGGTACAAGGTGGGTTTAAAATAGATTCCTTTAGAAAATCCGATTAAAACTGAAATCTCAAATTGTGCTGCTGTCTCATCTGATTTCAACCTCAAACTCTGTTTCTATAACTTTTAAATAAGCAGCACATATTTATAAAACTGTTACTGTACTGATCAAGTTTAGAACCGTAATAAAACTTACTCAAGATCAAATAAAGAAACTTATTCAAATAAATTCATTTCTAAAAACTTATTTATATAAAATCAATATAAACTCATTTATAAAACTTATTTATATAAAAGCACATCAATCATTCATAAAATCTGATTTATGAAAGAAGGTCCACTCACAGATGGTCCGAGCTACCTCGATCCCTCGAAGATCTCTTTTATAATTATGTCGGGCTCCTGGTGCCTGATTATCCCGAAAGATTAAGTTAATAAACTGCTGAATAAAAAGAATTTCAACTAAACACTCTGCCCCTGGTTCCTAGAAATACGCGCATCTCAATCCAACTACTCCTATGCCCACAATAGCCTTTTTCAATGCTTGGAACAGTTTTGGGAGGTTCGACGTTCAGCTTAAGCGATAAGCCTGTAAGGTCGGCCGACCCGGGACCCGTGGGGTCCACGGTGTCCGATGGCCAATTTGATCATCCCTGAAGGTTTCTCATAGGACAGGAACCATGTTCTGCGAATTTGGTTCGAAACGGACGGTCGGATTGGCCAAAATCGCATTATCGCTTAAAACCCTAACCCTAGCCTCAGGGTTCGCGATTCCGGAGTATCCGGGACTCCGATTCGCATTCCGTCGAATCCTACGCGATCCTGAAATCACGTAGACCGACATATCCAAAATTCAGTGCGATCCAACGATTACACGACACTGCACCCACGGATCGCGCAATATGGAAAATCCGTTCGGGGCTCAAACGGACTCCGAATCGAGATCCGCGAAATCCCACGCGCTCGTGGCGACACGAGGACCTCAAAACTGGCCAGAGCGGTGCCACCACCCTGGGCCACGCACCGCTACACGCGCGGGACAGATCGGGTGTCTAAAGCGATTTTGGGTGGCCGAAGAATCTCGGAACCAAGACTCCAAACTCCTACCCTAGGTAAAACACCCTATTTTAAGTCACTTTTGTTCTTGGACCACCCCCAAAAAGTGACCGGAAACAGTAGTTTCGAAGGGCCGAAGTTCGGCCGAATACTCAAGTTCAAAATCGAACTCTTTAGAGCTAAAATCGATCGAAGCCCACATACCCATCAGCTAGAGCACGAAAAATAGCTGAGAAATCATACCTTACTCGATCGATTTGGTAGAGAAACGAAGGAGAATTGCGAGCTGGAAGTTTGGGTAGCTTCGGACGAACCTCCGTCGGAAAACACGATTTTCCGGCCAGCTCAGGGCGGCACCGGGGTTAAGGTCGGTTGGAGAAGGTAGGAGACTCGATGGCGGTTCCAACGCCACCGGTCCCGTGGCCATTGGAGCTTGGAGGCGGCGCCAGCACCTTCTGGAAGTCGGCGGCCCGTTTCGCGCAGAGTCTGGGTTTATATAGATGCAACTTCGAAATATTTAGGGTTATGCCACTGAGACTCTTTTGACCATAACTTTTTCGTTACAACTCCGATTCGGGCCCACTCCGTGTCTACGGACTCCTTTCGCCGTGCTCTACGCAATGGCGCAAGCGGAATTCTCAAATTCTTTCTCGATTAAAAAGTCAAATTTTCCCCCATTAATAATGCAAGGGCAAAATGGTCTTTTCTAAAAGATATTTTCCTTACTTTTTAGATATTTTCTTTCTTTTTAGATATTTTGTTTTGGGTTCTTACAATCTACCCCCCTTATAAAAATTTCGTCCTCGAAATTTGCATCTCTGTCCTTTCGGAGGGGGAAAAACTGCTCCTGTACTTGCTACTCGGGTTACTATACTGCACATATCACTGAAAGCTAATTACTTTCCCTTCCAAAATCTGAATTGCTTGTGTTCTACACATTCTATAATCTCCTTGGGTCCGATATAGCTAGGATTGGCTCCCTTGCTTCCTTACCTTTCTCAGTCGATAAATATCGAGAATACTTATCTGAATCCTTCTTCTAGTATCTGCATAACTCTTGCCAATCCTGGTTACTTGAGGCTTTTTCTGCTTAATCCAATCAGGTTCTTTGTTTCCTACAATACTTCCAGATTCCAATGGACCTTGCTTAACATTCTATCTTCATACTTGAGTGATCCATACCATATCTCGAGTAAGGCATCTCTTGGAGACATCTAATCTAAGGAAATCCAACAACTGACACTTAAGGCATACTCTCAAACCAGCATCTTTTTCACCCAAATCATCCTGAAACTGTAGTAGATTATGTCTCCATAACCTTCCAACAACTGTCACTTAAGGTATTAAACTGAAATCAAGGCTCAAAAGCTTCTTTTACCTGAGTCCAACTGAAACCGAATCTGGATTTCTAATTCTGAATTATAGAGACTGGTATTTCACTAGCAGGGTCCAGAACGGTACCTACCACATTTGGCACACTAGGGCAACTTCTCCTGTTAAAGCCTATCAATACCTGTCTCTCAATACTTCTTCTACACCAACCTTAGAACGATTTGCAATATCACCATCCTGGCTGGTATCTCTACTGAAACTAACTCCAGTTCGATGGCTTTCAAGTCTGCGGCCATTTGATGATTGGAAACTGTGATCACCACTGTAGTATCTCTCTTAAACTCTTCCTAATTATTGTTACCTATCTCCAAATACTGTGCACAAAGTGCACCCCATAGTCTGCCCCTATTCTGACTGGGACAGACATCACCACAGCATCCACATTGACCAACTACTAACTAATCACAATACCTTGGATGGAGGCTTTTTAACCTTAACGCAACCAGGCACTATTTCTTACTTTCCGATCTCTATTAGTAAAACACAGTACGTGAAACCTTTTTCTCGTATTCGAGTTTAACCATTTCCTCTATTCTGCTGTAAAAACCCCTATATTCACACCTTCTGTAGACTTATAAATAAGATTGACTAACATATTCTGTTCCCAAAATGCTTACTCCGTACGAGTCAGAGGATTTTAACATCACTTCTTAACCAAATCCTGCCCAAAACTTTATTCTCCTGTGGAATAACCAAGATAACTCAATCCTCTTTTACCACTAGATACCATTATTATTCCAATTTATAGCTCTTACTCCTGTTTACTTGATCCACCCATCTTGCGGATGGATCTCTGGCTGACTATAGCCTGGGCTAACATTTCCAGGGTCTACTGAGTGTTAGGATCTCTCTGCCTACTAGATATAGTAGGTACGGTTCCTTACACACCCCTACTAAAGATTACTATGTCGTTCGAATACCGATGTAGTTCCTGTCTGACTTGGAAATTCATCCCCTAACGTCGGGATAACTATCTAATTTTCCTACGGATACCACCACCATTTGGGCCCAACTTGAGAAGAGAGCGAGGATGCACAGAGTGCGTAGTCTACAGGAAGTAAAACCTATGCTCTGATACCAAATTGACAGGACCCGACCCAATTTCCACTTTGAAATTCGAGCCAAGTCCTGTGCGTGTCCGACACCTGGCGAATGTCGGGCACAAATGACCTTTTTGCCCTCTTACTTCAATTTTCTTTAAAATTTCCTTAGACTTCTGCCGAAAATTCGGCAGAGTCTCCCCTGTATTTTGACCAGTCCCAAAATTTCACCTGTAAACAATCTCAAATATTTCCACCCAACTGCCAGAATAGGATAAACCAAAATTTGCCAGATTCAAATTTTCCAATAAACTCGATATCAGAGCAATTTTCTAAAGTTCACGGGTTTTCAGGAAAAATTTCTACAACTTTACCTGACTTTGAAGCGTGGAGGTTAAGAGTGGCCCGGTGGTGGATCCTATGCACTTCTACAACCTGGGGGCGAAAAACAAGTTTTAAACTGTGAGTGGACAAAAAGTGAGATTCTTAAAATCAGCTAAATATAATTCCCCCATTTTGAAATAACTAGGGATATATATACATCGAAAATTTAACTATATCTCCATATAAAAGATTCTATAAGCATGAATTAGTATACTAATGAAGAAACTTACATAATCGATCTGATATAAAGATATTACTGCCTGGAAGAATCAAAGAACTGGTATACTGAATAAAAATACCAAAAATACACATGTGCAATTACTGAGAGACCAATATAAAATGGCTGAAAATCATCCTTGAATAAAAGAAAGCTCAAAATCCTTTAAAACTTCGTTCGACTGAATTTACTAAAAAAAATGAGTAAACAATATCTGCAGTAAAAGCTTTAAAATCCTTTAAAACTGCCACCTAATTGTACCCCTGTTATATCCGTCAATTCCCTGGCAGGTCTCGGGCGCCACGCAGTCCACCCGAGCCGCAAACTGGCGAAATCAGGGGACTATGATCAGCCTGTCCCGCAGATTCCTCGGTGACACCAAGTCAGCTCGAGTCGCTCTGGCAGGATGCAGGGGACCGTAGTCAGCCTGATCCGCAATCCTGGCAGGTCTCGGGGACACAGAGTCAGCCGAGCCGCAATCCTGGCAGGTCTCGGGACACCAAGTCTGCCGAGCCGCAAATCCTGGCACTCACGGTCCGAGCGTCCCCGAAACTCGTGAGGCAAAGTCAAGTGCACTGACTGAACTATAATCAGACTGGATGTCCGTAGACATCGGTCCGACTCTGGGTAATCACCATAAAGAAAATGGGTACAAGGTGGGTTTAAAATAGATTCCTTTAGAAAATCCGATTCGATTAAAACTGAAATCTCAAATTGTGCTGCTGTCTCATCTGATTTCAACCTCAAACTCTGTTTCTATACTTTTAAATAAGCAGCACATATTTATAAAACTGTTACTGTACTGATCAAGTTCAGAACCGTAATAAAACTTACTCAAGATCAAATAAAGAAACTTATTCAAATAAATTCATTTCTAAAAACTTATTTATATAAAATCAATATAAACTCATTTATAAAACTTATTTATATAAAAGCACATCAATCATTCATGAAATCTGATTTATGAAAGAAGGTCCACTCACAGATGGTCCGAGCTACTTCGATCCCTCGAAGATCTCTTTTATAATTCTGTCGGGCTCCTGGTGCCTGATTATCCCGAAAGATTAAGTTAATAAACTGCTGAATAAAAAGAATTTCAACTAAACACCTGCCCCTGGTTCCTAGAAATACGCGCATCTCAATCCAACTACTCCTATGCCCACAATAGCCTTTTTCAATGCTTGGAACAGTTTTGGGAGGTTCGACGTTCAGCTTAAGCGATAAGCCTGTCAGGTCGGCCGACCCGGGACCCGTGGGGTCCACGGTGTCCGATGGCCAATATGATCATCCCTGAAGGTTTCTCATAGGACAGGAACCATGTTCTGCGAATTTGGTTCGAAACGGACGGTCGGATTGCCAAAATCGCATTATCGCTTAAAACCCTAACCCTAGCCTCAGGGTTCGCGATTCCGGAGTATCCGGGACTCCGATTCGCATTCCGTCGAATCCTACGCGATCCTGAAATCACGTAGACCGACATATCCAAAATTCAGAGCGATCCAACGATTACACGACACTGCACCCACGGATCGCGCAATATGGAAAATCCGTTCGGGGCTCAAACGGACTCCGAATCGAGATCCGCGAAATCCCACGCGCTCGTGGCGACACGAGGACCTCAAAACTGGCCAGAGCGGTGCCACCACCCTGGGCCACGCACCGCTACACGCGCGGGACAGATCGGGTGTCTAAAGCGATTTCGGGTGGCCGAAGAATCTCGGAACCAAGACTCCAAACTCCTACCCTAGTAAAACACCCCATTTGAAGTCACTTTTGTTCTTGGCCACCCCCAAAAAGTGACCGGAAACAGTAGTTTCGAAGGGCCGAAGTTCGGCCGAATACTCAAGTTCAAAATCGAACTCCATAGAGCTAAAATCGATCGAAGCCCATATACCCATCAGCTAGAGCACGAAAAATAGCTGAGAAGCCATACCTTACTCGATCGATTTGGTGGAGAAACGAAGGAGAATTTCGAGCTGGAAGTTTGGGTAGCTTCGGACGAACCTCCGTCGGAAAACACGATTTTCCGGCCAGCTCAGGGCGGCACCGGGGTTAAGGTCGGTTGGAGAAGGTAGGAGACTCGATGGCCGTTCCAACGCCACCGGTCCCGTGGCCATTGGAGCTTGGAGGCGGCGCCAGCACCTTCTGGAAGTCGGCGGCCCGTTTCGCGCAGAGTCTGGGTTTATATAGATGCAACTTCGAAATATTTACGGTTATGCCACTGAGACTCTTTTGACCATAACTTTTTCGTTACAACTCCGATTCGGGCCCACTCCGTGTCTACGGACTCCTTTCGCCGTGCTCTACGCAATGGCGCAAGTGGAATTCTCAAATTCTTTCTCGATTAAAAAGTCTGATTTTTCCCCATTAAAATATGCGAGGGCAATTTGGTCATTTCGCTAAAAGATATTTTCTTTACTTTTTAGATATTTTCTTTCTTTTTAGATATTTTGTTTTGGGCTCTTACATGTCATGCCCTTTGGATTAAAAAATGAGCCTAGTGAATTCCAGAATATAATGAATGAAATTTTTAATCAATAGTAATTTTTCCATAGTTTATATTGATGATGTCTTGATTTTTTTTGAGTCAATTGAGCAACATTGGAAACATCTGAATAAATTCCTTCAAATAGTTAAGCAAAATGGTCTAGTTATCTAGCCAAGAAAATCAAATTATTCCAAGCCAATGTCAAATTTTTAGGATTCAATATTCGTCAGTCTCATATAAGCCTAATTAACCCAGTTATCTAGTTTGCTGATAAATTTCTAGATCAAATTCTTGATAAAAGTCAATTACAGAGGTTTTTAGGATCATTAAACTATGTTTCTAATTTCTATCAAAATTTAAGAAAATTATGCAAGCCGCTGTTTGATCGTCTCCAGAGCAATCCTCCTGTTTGGACTCCGTGTCATACAAACCTAGTTAAACAAATCAAAGTTCATGTCAAGTCCCTTCCATGCCTTGGTATTCCTTCAGTTACTTCTTTTAAAATTGTCGAAACAGATGCTTCTGAGTCCGGTTATGGTGGTATCCTAAAACAAAGAGTTTCCCCTGAGTCACCTGAGCAAATTCTCCGATTTCATTCCGGAGTTTGGAGTCAATCTCAAGTTAATTATAGTACTATTAAAAAGGAAATTTTATCTATAGTATTATGCATTAGTAAATTCCAAGATGATTTATTAAATAAATTTTTTTTAGTCAGAGTTGATTGCAAGCCTGCAAAACATGTTTTA

TATATATATATATATATATATATATATAAGATGAACAACATATCACATCACCAAATATAATCATATCACGAAACGTAATTGTACCACCAAATATTTGCATTTTTATTCATACCATCCTTTAAAAATATATTTTTTTGATACTTATTTCAAAAATAATTCTTGGTTTTATTATTTTTGTGGAAAATCTAATAAGTTGTGTGACTTTATTGGGTGTTTTATGAATTTGGTTTGGTGTGAAAATATTGGAATGAAGTGAGTTTAATCTCAAATTTGGACTAAAATGCCTTTATGATTAAGTTTATGATTTTTTTTGAATAAAGTAGGGCCCGACCCGACCCGATGGGCTTTCTCAAGCCCAACTCATGGGTCGGGCTTGGGCTTCGAATGTTCTAAAAAACCCTGGCCCGGCCTGGCCTGATATTTTATCTCTCCTCTTTAAAGCCTGGCCCATTGACGAGCCCTACCCTGAATATTGATAGAGATATCTCCACATCTAATCCTAGCCGTTCATCACATAGTTGTTAATTTCTGTTTAGCTTTGCTTTAAAAAACTGTACAGCTGTCATTTGTACAGCTATATCAAGTGTATATAAGCAATTCAATATCATTATTGTTTTAATGAAATGTGAATACAATTCTAGTTTTCTCACATGGTATCACGAGCCCTAGTCTAACGACTTTTATCTCTGCAAATTTTCTTGCGATTTTCTTCCTTGCCAAACACCTTTCTTCGATCACTTTCTCTCTCTTTTTTCACTTTCTCATGGCTCCTTCGACCAATTCTGATGCGGCTCCTCTTCCACCGCTCTCTTCTCCCTCTTCCCCCAACTCTGATGCGACCAATTCTGTTAATTCGCCATTGTCAAACTCTGTGAGCTCGGTGACTGTCTAGAACATTGCTGATATGGTTCCTATTAAGCTGATATCTGGAAACAATCTGTTGTGGAAGACTCTTTTTCTTCCGGTGCTACGCAAATTCAAGCTTCTTGGTCTTATCAAT

>Chr2-12; OZ022207.1; 6728901-6735533; rev; A;

AATAAAAAGGGATTCACTGTCATAAACTTTTGATTAGAAAAGTACTAGACGCCAAAAAACAAAACAAAACAATATATATGTGTGTGTGTGTGTGTGTGTGTGTGTGTGTGTGTGTGTGTGCACGCACAAAAGCTATGCAGAGATGGTAATGCATGCAAAGAATGAGATGCAAGCCTGTTACCTCCAATCAAAGCAGAAGGAAAAAAATCTCAATGAAAGTCATGGTGGGGCCACTGTGTTGTCAGAAATCTGCACGAGAAGATTAAATTTTAGGGAGACTTTGAAAAAGGCCAAAGGAGAGAGAAATTTTTTAAAAGAAGCCAAAATGGACACAAATGTCCTTATATATTTTTGGATTTCTTAAGGAATCATTTACATTTGCATGTCTTTGGTTTGAAAGTTGAGAGGTTTTTATGTCTTTTTTTAATAAGCTTTGGCTTTTTCCAAAAATGAAAGTTTTTTTGTGGCTAAAGCCTAAATTTACTTAAATTTTAACCTGACTGGATTAAAAAAGACAAAAAGACAAAAAACTGACGCAAGCACAGAAAGAGAATGATGAAATCAAAAGTGGGTTCAATCTTGCTGCCAAGCGTATTTGCTATTGTTCCAAAGGAAATCAACAATCACACCAGCAAGTTTCTTGATAGGCCCAAAAAGGTGGGTTCTTGTGGATGTGCGGAGAAAGAAAAAAAAAAAAAAAAAAAAAAAACTCAATGTCAATTTTCTTTTTTTCTTGATAGGCTAAATCAGTTTCTCATTCCGCCAAAGTGGCTGGTATTTCTATTCTGCATTAGAATTCTGTCCTTTACTTTCTATTGTTCATTCATCATTCACCTGCTTACCTGTAACTCTCTTTTAATCCAAACAATAAGTCCTAGATTCCAAACACTAATCCCAAGTGAGGCATGAGCGGATGCGGCATGAGCGGAGAAAGAGAGAGTCCCTTACACTAGGAGTTTAGGATTACTTTCTACCCAGAAAAGAAAAGCCAGTGTCCTAA

TGAGTCGCTTTTTTAGATCAAACTCTTGCAGCAGTAGCAGTTCTGGAGCCAGTATTAGTAAGGTCCCAGACATAGTTAATGAAGAACAAATTGAGTATGAAACTAGTAATGAAGTCGACTGGAAAGATTGAAATATTCCCAAAGTCCCAACTACATAAATTTATAAGAAAAAATGGTATCTCTCCTCTTTCAAAAGCACAACTCATATTAAAATAGTTGAGCAAGTCTACGCCCTTAGTAAAGATCATGAAACTTGCCAATTACTTAATCTTGAGTCAATTAAGAAACACAGGAAAGATGGAAACAATTTCTTGCATATTGGTTTAGTTCAAGTAGCAGTCAAACCTCTAACAAGAACAGGTCTTAATGCTTCTATTTTGCTTTGCCTAAGAGATGCACGATTCACCAATTTTAATGACTGCACCTTAGGAATAATAGAATCTAGTTTGTTTAATGGTCCAGTTCACTTTGATTGCTACCCCAATTTCACAGTAAGCCTTAGTGATCCCCATATATTAAAAATCTTGACTTTAGACATTAAAACTGCAGGCTATGAGATCTTACCCGATCCTAACCTTTAGCCTAAGTCTATAGAATTTATTATAAAGTCACATAAACCAACATGAATTTCCAAGCCCATAATAAAAGTCCTAAAGATCAAACTTTACTAATCCAAAGTAGCCAAGCAAATGCTAACATAAGAATTCCCCATTCTATTAAATGGTCAGAAGTCACCCTTCCCTCATATTGGACCCTAGCAACTGAAAGCCAACCAACTTCGGTTCAACATAGCCTATCTAATTTGGATTATATCCAACAATATTTAGATGGCACAGTCAAAATCAACTATGGAAATCAACCTCTAAAGTCTTCAGTGCAGCTAAAACAATTACCAACCCCTAATCAGTCCCGTAGACATAATCCTGTCAGACATTCATTCACAGGATCTACTTCCACTACTGAAAGAGATCTAGAAATAGAAAAGGCCTTCATTGATTTAAAATTAGAATCCCTTAGGAAAAGCTCCCAAGTCGATCAACCCTGCTATTCTAATACAAAGAAGCCAACTGAAGAAAAAGAGCCAACCTCACCTACTCAAACAGAAGCCTTCCCAGTCAATGACCAATTAAGAACCCTTACTAAAGAATTCAAAATACATTGGGAAATTCTTGACCAACACCTAGACGCAGAAGAAAATATTAACCGAAGAAATCGTTATAGAACAGCCTATCCAAGTAAAGAACAAAGAATGCAAATCTTTAATGAATGGAAAGCCTTCATGTTAAAAACAAGATCAGAAATATTCTACCTTGATTTTGTTGAAAATCATTATCAAGGAAAAAAAGAAGTAAAAACTTTAACCCAAGAAAAATGGAAACGAGAAGATAACACGGAAATATTTTCCAGTCATCCCCGGTAGAAACAATAGTTGTGCGTCACAAAAACACCAAGATTGTAGCCAGCCCCTTTAAAATATCATATCGCAGTGACAGAAAGATAGTAGAACAGAATAACTACACAAATCAGAGTTTGATAATATTAGGAAAACAGTTAGACAAAATTGAAACAAAGATTGATACAGTCTTTAGCCAAGGTATACAAATCAAGCAAACAAATGAAAAACCTTTAGTAAAATTCCAAGAGTTAAAACCTGAATCAGCACTAAGAACAACTCCTACAATGAGAAAAATTGAACAAATGCTTGTACAATTAAGCAAAGGAAAAGCTGTCAAAACTGATTCTTCAATTAAGGTTTTACAAGCAGAGTCCTCAGATATAAATTACTCCTCTAACAACAATTCAGATCACGATTCTGATTCCAGCAATATATCAAAAATAGAAACAGCTTTTAACAATCTAAAACTCAATAGGTTGAAAAATAGAGTAAATCTTACTAGTCTGACAAAAAATTGGTACCCTAAGCCAACTCCTCCAGATATCCAGTTTGAAGAACGAACTTCCCAAAACCAATTCTTAGTTTCCTCAGACAAACTCTATGAATGGAACATAGATGGCCTGTCTGAACAAGAAATCGTGAACAAGTTACTACACATGTCTATGGTTGCCAATAGTTACATCACAAATCATAATCTAAGACAATCAGAAATAGTAACCCTCCTAGAAACAAGATTCACCGACACTTTACGTGCTTGGTGGGATAAGCATCTCACTGAAAAAGCTAAAGAAAGTGTCATACATGCAGTCAAAGTAGATGAAGAAGGTTTGCCTATATTTGATGAAAAATGTTAATATGGGAATTGAAGATGGAGTCAATACCTTGTTATATACGATAATAAAACACTTCATTGGAACACCTAGTAACACCACAACTAGGATTCATGACCAGCTTAGTAACCTTAGATGTCCAAAATTAAGTGATTTTAGATGGTACAAAGATGTTTTCATATCTAGAGTCATGCCAAGAGATGATAGTAACCAACCATTTTGGAAAGAAAAATTTATTAATGGTTTACCCAATTTATTTGCTCACAAAATAAGAACGGTCTTAAGTAATGACAAAGGTCATATAGACTTTGATAATCTTACTTATGGTAGTATAATCAGTACAATCAATCAAGTAGGAATGAAAATGTGTGTAGACAAGAAAATAGGTAAACAGATCCAATCAGAAAGAAACTCTGCTAAATATGAGTTAGGAAACTTCTATGAACAGTATGGTCTAACTTCAGTACCTCCTTCTAAGAAAATTAAGCATAAAAATTATGACGGTCAGACCAGTAAAACTAGACATTATCAAAGAAGAAGACATTCCTTTAAAGATAATAATGAATTTTATAAAAAACGCAAATATAATAAAAGAAGAAATTGGTCTAAAACGCCCAGGAAGAATGGGAAAAATAAAACTACATATAAAAACAAGATTGAATGTTGGAAATGCAAAAAATTTGGTCACTATGCTAATGAATGCAAGGTCAAGGATACAATAAAACAACTAAAAATCTCTGAGGAAGAAAAAGCTCAATTAATTAGAGCCCTTGAAATACAAAACTCCGAGTCAAGTGAAAGTGACATCTCAGTAACCAATTCAGATTACCATAGCTCTTCTGATTATCAGTCAAGCTCCCTCGATATCCAATTCGGATATAAGGATAATTGCTGCAGCACGAAATCAATAAATGTCCTTACAAAACAAGAAGAACAAGAAGAATTATTAATTGACCTAATTAGTAAAATGGAAAACCCAAAATTAAAATCGGAATACCTAAAAAAGCTAAAGAAAATAATTAGTCACAATGAAATCGGTCAAGTCATCCCAGCCCCAAAGATAAGTTTGACTACTACTGTAGAAAAGTTTGATCAAAAGAAAGAAATTTCCTTGCAAGACCTCCAACATGAAGTCAAAAACATAAAGAAGGAAATTATCACCTTACGACAAACAAGTCATGAACTACGAAATGAGAACCTTGAAATCAAAAAAATTCTTGTAGAAAAATTAGGATCCAGTACTCCAGAACAACCCTAAGAAGAAGATGAAAAGGAATCCTCCAGCAGTGACAAAGCAATTAATGTTACTAAACATTTGAATTTTAGAAAATGGTATTCAAAAGTCAGTATAATTATCAACAACTTCGAGCTTAAAACAGTAGCCCTTTTTCACTCAGGTGCAGACCTTAATATTGTATCGAAGAAGGTTTAATCCCCACTAAATATTATAAACAGTCTAAAGAATCCTTAGGCACTGCTTCTGGTAAACCTTTACACTTAAAATATGAAATCCCTAAAGCCCATTTATGCCAAAGTAATATTTGTTTTAAAAAATCTTTTGTTTTAGTAAAAGATATCACATATGAAGTCATCTTAGGACTACCTTTTATTGCCCTACTCTACCCCTTCAATGTAGAATACGTTGGAGTTACCTCTAAACACATGGGAGAAAAAGTTAAATTTGAATTCCTACAGCGAAACCAAAATTACAAGCTTTAAAAGCCCTCCAAAATGATGCAGTTTCAAAGACAACCAATCTACTTAAAAATAAAAGTAAACAAGTCAATTTCCTTAAAGAAGAAGTTAAATTCAAAAGAATTGAACGACAGCTTGAGGATAAAATATTACAAGAGAAAATCAAGAATCTGGAAGAAAAAATCAAACAAGAAATTTGTTCTGACCTTCCTAATGCCTTTTGGCATAGGAAACAGCACTCAGTCAGTCTTCCTTATATCAAAGACTTTAGTGAGAAAAATATCCCTACTAAGGCCAGACCGATACAAATGAGTCAAGAAGTTATGGAATTCTGCAAAAGTGAAATTACAAAATTATTACAAAAGAAAATAATTCGTAAAAGTAAATCCTCATGGTCTTGTCCGGCCTTCTATGTCCAGAAAAATACAGAATTAGAACATGAAGCACCCAGACTAGTTATTAACTATAAACCTCTTAATAGTGTGTTAGAATGGATAAGATACCCAATTCCTAATAAGAGAGATCTTATTCAAAGATTGAGCAACTCAGTTATCTTCTCCAAATTTGACATGAAGAGTGGATTCTGGCAAATACAAATTCAAGAATGCGATACGAACAAAACAGCCTTTGTTACCCCATTTGGACATTATGAGTGGAATGTCATGCCATTCGGTCTAAAAAATGCTCCTAGTGAATTTCAAAATATAATGAATGAGATCTTCAACCCATTTAGTGAATTTTCCATAGTCTATATTGATGAGTCCTCATTTCCTCCTGGTCAATAGAACAACATTGGAAACACCTACACAAATTCCTTCAGATAGTCAAAAACAACGGCTTAGTTGTTTCTGCCAAAAAGATTAAGCTATTTCAATCTAACGTTAGATTTTGAGGATATAATATTCATCAATCACAAGTCAACCCAATAGATAGAGCTATCCAATTCGCTGACAAATTTCCAGATGAAATTCTTAACAAAGGTCAACTTTAAATATTTGTAGGATCCCTTAATTATGTCTCTGATTTCTATCAAAATCTAAGAAAACAAAGCAAACCTTTGTTTGACTGACTTCAAATTAATCATCTTCCTTGGACTCCTCGTCATACAGAGATAGTCAAGAAAATCAAAATCCACGTCAAGACATTACCTTACCTTGGCATTCCAGCAATTGATTCTTTTAAAATTGTTGAAACTGATGCCTCTGAAAATGGTTATGGTGGTATCCTCAAACAAAGAATCTCCCCAAGTCAAACAAAACAAATTGTTCGATTCCATTTTGGAATTTGGACTCAGTCACAGAGTAATTACAGTACTATTAAAAAAGAAATTTTATCTATTGTATTATGCATTTCAAAATTCCAAGATGATTTACTTAATCAAAAATTTCTAGTAAGAGTTGATTGCAAAAGTGCAAAACATGTTTTAGAAAAAGATGTTCAAAACATTGCATCAAAACAGATTTTTGCACGTTGGCAAGCTATTTTAAGTATTTTTTATTTTGATATTGAATACTTGAAAGGAAGTGAGAATTGCATTCCCGATTTTCTTACACGAGAATTCCTGCAGGGAAAGAAATGGCAAGAAAAGAGTCTGAATCAGTCAAACAAAGTAGCCAATCAGTTACCAAAAAACTCCCAACTTCTATGAATCCAGTTAAAACAGAACCTTCTCAAATTGTCCCTTACCCGGGATATTCTCCAATACAAATTACCAATACGTTTACCAGTCTCGGCTCTACAACCCTTAATATTCGTCCTAATTACCAATCAGCCCTAATCCCCAGTTATGATCCTTTCCAAGTCACAATCCCTTCTTCTTCTCAGTTATCAATCAATTTCAAAAAGACCTCTCCCTATCTTCCCAGAAGTAACAACAACCTCTTCATAATTGAACCCTGTTATAATAACCTCTCAAACCCAGTCAAGATAGCCCAAGCTTATTTTCCTCCCAATTATCATTTTGTCCCTCACCATCCAGAAAAATCACTCAAATTTTATAGGGACATTCTCCTTGAAACCCAAACCCTTGAAACAAGGCCAATCAAAGATAGAAATGATCCAAATATAATCCTTTATCATTCCATGTACATTCGTCGAATCCTCAATCAATCTGAATGGGCTGATCGCCCTTACTCTCTCAAAACACTTAAGTCAAATCTTCAGCATATTTGTTATGATTATATTGAAGCCTGGAATATCATCTTTTTGCATCAAACCGAAGACTTTAGTCATTCATGGTTTATCAATTTTGATAGTAAATTTAAAAGTCCTTTTCCCTTTTGGTTTTCTTCTTGGTGGGAAAACCATGGCCCAGTCATTGAAATTCTTCCCTCTGATCTCAAAGATTCAATTACTTAGTTTACTAGTAAATATAAGTTTAAAGACGAGGACACTTTCTTCCCCAATCTCCTCATCTTTGTTGCCAAATACAAAGTCCCTTAGATTCTCAAATGGTCCTATCATGTCAACAGGGACTCCAGGACCCTGTCCAGAAGAATTGCTGCCAAATGGTGGGATAGATTTAATTCAGAAAGAATCACTGGTCAAGTTTACATAGAATTTCCCCCAGTAAAAGTTACTCCGCAAAAGCCATCAACTTCTAGAGCCTCTCAATCCTATCTCTCTCTCAATCGAAGGTAGGACCAAAGCAGAAATCAATGAATTAGCAAAGCAGCTCATGATTCAAGCCTCCCAAATGAGTGATTATGAAGA

TGCCTTTCCAAAGTCGCATTCCTCTCAGGCCTCATCCTCAAATTAGGAGCATAGTTCCAATCAAAAGAAAAGCTCTCCTAATCAAAAGATACGTTGTGCAGATTATCAAGACAGTAAAGACCCTTATGACCTGAATGATGACTAATTCCTCATAAAGTTCCAATCAGTCACTATTCACTTTTCTTTAAAAAGCAAAATGCATGATTCCACAGTAACCCCACTTAGAGCTCAACTATTCCAGTTCACGTGCAAAGGAAAGAATCCCTCTACCGACACTACAGTAAAGCACAAGCATCCAATGCTTCCGACACTGCAATACTTTAAGCCTTTTATATATCGGCCTTTGGACCTCGGTTGAGGCATGCTATCAATTTTCGCAAGTTAATATTCCAATTTCATATGGTTTCTCTGAACTCTCTTCTGAGAAACTATTGTAAATTATGAGTGCTACTATGAGTGCTACTCTGTATGCTACTACAAGCTATCAATAAAATCTTTCAATCAAGATTTTCAGTAAGTTTTATTGCATTAATTAACTTTATTGTATGCATTAATTTTAATTTAATTTCTTTATTTCCAATATTATCACTCTCTCTACCCCGAGACACCCTCCTAGTATATATATATATGAGAGATGAATCAAACCCTGAAGGAAAAGAAGCCAAGTCTCACCACTTTGGTTGCTGAGAATTCAGATGGTGCAAGATTCAAATTCGTAGACCTTCTTGCCAACAAGAGGAATTTTTGCTATTCTATTTGGTTAATATCAATATCCATGAGCGCAGTGCAATTTAAAGAAGCCAAAGGTCATCAAAGCTCCTGGTGGTGCTAGGGTTTCTTATTCCTAGTTTAGGAAAATATTAGATTTTGTTTCTTGCCACACAAGATCTTTTGGATATTATAAACCTTAATATTATCACCTTTTAAAAAAAAAAAAAAAAAACATATCTACAGTGCATCATGAAGTGGGGGCAATTTGTAGACAAAGAAATTTTGGCGG

>Chr2-13; OZ022207.1; 7155046-7161321; dir; D;

TTGGAGGTAGGAAAATTCACCCAAGCACTTTGGAATAGCGGCCGAGTGTGGGACTCCAAAGTTGGGCAAAGCTCAAGGTCAGATCTGCTGGGGTTCTTTGCGACCGGAATTGCTGGAAGTTTTTGGCGGGTCTCGGCCGGGTTTCAAGTGAAAGTTATGGTCGGGCTTGGAGTCTATTACGGCCGGGACTGCTGGGGGAGTTCTTGGAAAATCTCAGCTAAATTTTCGGATGGAGAGTTCGGCCGGACTTCTTAATCGCTGAGTTGGGCAGCTGTTGGAACAGGAGCTTGGGAGGTGCAGCCGGGCTAGGAGTTCAAAATCCGACCGGGGCCTTGCAGCTTGGAGACCGTAGCAGTCCGGGCTTCTTAAATTGCAGCCGGATTTTTGTTGCCTAAGGAGAGGCGCCCGGGTTTTCTCGAATGGTTGAATGCGGCTGGAGCTTTGTGTGGTCTCCAAAACTGGGCCAAAAGTGGTTTTGAGTTGGAGGATTGACTGACTTATTAATATATAAATATTACTATATATTAAATTTCTATATATATAATAGGGCAATGAGGTTTCACTTTAAAGAGCCCAACCACAGCATTTCGCCGCACTCATCTGATTCCATATGAATATATATATATATATATATATATATATTCAGAGAGCTTCTATTGAGGGATCCTTCAAATTAGCTTATTTGAGGGATCCCTCAAATTAGCTTATTTGAGGGATACCCTTGTAGGGCCCACCATGTATTGTATTTCACTAATCAAAACCGTCTATTTTGTAGATACTCATTCAAATATCATCTCTACAAAAAATCACTTGAATTCGATATCATTTAACCACTCAATTGAATTATTGAAATTTTAGTACTTTCTTGAAGCATCGTGTTCATTGATTTTGCAGGACATAATTGGATATCGAAACGGTTTCTGAATTGTCTAATTTTTTTTAAAGGATGATCTATGAATATAGACTTAATATATATATACATATATATATATATATTATA

TTATATTATCATAAGTTAAATTATTCCAATCAATATGACCTTGATCATTACTAAGGGTTGTTCTAATCTTGTGGGAAAATAGATTCGGAAGACCATTAATAAATTTCTCTTTCCATAATGGTTGATTACTATCTTCTCTAATCATGACTCTAGAAATAAAAAACATCTTTATACCATCTGAAATCAGATAACTTAGGACATCTAAGATTACTAAGTTGATCATGAATTCTAGCAGTAGTGCTACTAGGTGTTCCAATAAAATGCTCAATGATTGTATATAATAAAGTGTTAACTCCATCTTCTATTCCTCGACCTATTTGTTCGTCCAAAAAGGGAAGACCATCTTCATTATTGTTTTGACTCATCTGTTAGGTGCTTATCCCACCAAGAACGTAATGTTCCAGTCCTGTTTCTAATAGGGGAACTATCTCATATTGTCTAAAACTGTGGTTAGTAACGTAACTACTGGCTACCATAGACATATGTTCAAGTTTATTAAGGATTTCTTGCTCAAAAATACCATCTATATTTCATTCATATAACTTATCAGCAGAAACTGAAAATTGGCTTTGGTTATTTCTTTCTTCAAATTGAATATCTGGGGGCGTAGGCTTAGGATACCATTTTTTGTAAGACTCGTAGGGTTGACTTTGTTAGTTAATCTTTTAACTTTCAAATCATTTTCCAATTCTAGATTCTTAAATGCATTTTCAATTTTTGAAATATTGCTACTTTATGTGCTTAAACTAATGGTAGATTATGACTCAGAGGAATTATTGAAAGTATAGTTTAACTCAGAGGATCCTATAACTTTAATACTAGTTTCAGCTTTTTCTTTGTCTTTTCGTTTGGTTGGTGTTAATTCTTCTAACATTTTTTCTATCTTTCCTATTGTGGTGTTGGTCTTAAAGGTTAGTTTTGATTTTAAATCTTGAAATTTAACTAAAGGTTTTTCTTTTTGTAACTTGGGCGTAGTCTCTTAGGGAAGTAGATTGTCAACTAGACTTTGGTTGGTGTAATTATTCTGTTCAACTATCTTTATGTTGTCTTCATCAGGAATCATAAAGGGACTAGCAACTATTTTGGACTTCTTATGGCTAATAATAATTGACTCTATCAGAGGATGACTAGCTAGTACTTTTGTCTTATCTTCTTTTTCCCATTTTTCTTGGGTTAAAGTTTTTAACTCATTGGGTAAATGTTGGCTTTCTACAAAATCGAGATAAAATATTTCAACTATGGCAGAACGCATATAGTCTTTCCACTCAATAAAAATTTGTTGTCTTTTGGTTTTTTCAGGGATAACTTTGTGGTAATTATTTCTTCTGTCTTTATTTTCAGGGGCATTTAGGTGATCTTCTAATGTTTTCCAATTAATGTTAAACTAATCTTTTCCAATTAAGGGTTCTTAATTAATTATCAGCGCAAAAACTGGCTTCAAAATCTATTTGTGTGGAAGACTCAGGAGACTCAGACTTAGTCTCTTCTGTAACTGGTTTCTTACCATATCAAGGTTGGTTAACTTGGGACGATTTTCTTAATGATTCTAATTTTAAATCAATTAAATTTTTTTCTAACTCTAGGTCTCGTTCTAGAGTAGAGGAAGAAGCTATATAAGAATGTCGTGTAGGGGCATCTCTTTGGGAATGACTAGGTGTAGGTAACTGTTGTAGCTGAACTGAAGACCTATGAGGTTGTTGATTACCAAAATTAATCTTAACTGTACCATCTAAGTATTGTTGAATATAATCTAAATTATTTAAACTATGTTGAATAAGGGTTGGCTGACTCTTAGTAACTAAAGTCCAGTCTCAAGGCAGGCTTACTTCAGACCATTTAATAGTATGTGGTACTCGGATATTCGCATCAGCTTTACTACTATAAATTAAAAGGTTTGATCTTTGGGGCTCTTGTTTAGGGCTTGAAAATTCATATTGGTTCCTATGACTTTATAATAGATTCTAAAAACTAAGGCTAATCGCTGGGTACCTGGTATAACTTTGTATCCAACAGTCTTGATATTTAATGTCAATGCTTTTAAAATATGGGGATCACTAAGGCTTACTGTAAAATATGGGTAACAATCAACATGTATTGGGCCCTTAAATAAACTAGACTCGATCATTCCTAGAGTATTGTCTTTGAAATCAGTAAATCTAGCATCTCGCAAGCATAATAAAATTTAAGCTTTTAATCCTAATCTAGTAAGTGGCTTTACGGCAATTTGGACTAGACCAATATGCAGAAAATTATTACCATCGTTTTTATGTTTCTTAATTGACTCAAGATTAAGTAATTGACACGTCTCATGTTCTTTGCTAAGGGCATAAACTTGCTCAACTGTCTTAACATGTGTGGTTGACTTAAAACTAGATAGTGACCAATTTTTCTTATAAATTTTTTGACTCGGGATTTTAGGGATATTCCAATCTCTAAAATCTAATTGTTCATCATTACTAGAATCATATTCTAATTGTTCTTCATTAACTATCTCAGGGAGACGACTAATACTGGCTCTAGAGCTGCTAGAATTGTAAGAGTTAGTTCTAAATAAGCGACTCATTTTACTCAAGGTTTTGGGTTGGACTACTAAGAAGAAGGTGAACAGAAACTCCTAAAGTTACTTCACAAATTAACAAGAAACCTAGTGATTCTCTCTCTCTCCGCTCATGCCGCAACCGCTCGTGCCTCTCCTGGGACTTATCGTTTGGACTCTAGGACTTACTGTTTAGACCAGAGAGAAAAGGACCGGGAAACCTGAAAACTATGAGGCAAATAGAGGAGGAACTGAAAACTTTCAACTCAGAATCTTAAACCAGCCACTTGGGCGTGATGAGTTGCAACTTAGACTGACTCTTCAACACTTACGGCTCTGATACCATAAGGGGTCTAGAACCAGGATGATACCAGCACTATTCTAAAAGACAGTCCTTTTGACACAATAATGATAATAATGAGTTTTATAGTAAAAGAAAGTTTTACCCAAAGAAGAATTGGAAAAAGAATCACGGAAAAGATAAGAAAAGTAGCTTTAAGAAAAGAACTAACAAGAGAAAAGTAAAGTGCTACAAATGTCAAAAGTATGGCCACTATGCCAATGAGTGCAAAGTAAGAGACACAATAAAACAATTGAAAATCACGGATGAAGATAAAGAAAAATTGGTTAAAGTTTTAGAATTCAGAAATTTAGAGTCTAGTGAAAATGGGATACTGACTAGTAACACTGAGTCCTCAGAACCTTTGAGTTAAACACAATTGCCCTATTTGACTCCGGAGCCAATCTTAATTGCATACAAGAAGGATTAATTCATACTAAGTATTATAAGAAATCGAAAGAAACCTTAACTACAGCCTTAAGAAAATCACTTCAGTTGAATTATGAGATTCCCAAAGCCCATGTTTGTCAAAATAAGATTTAGTTTAAAACTTTATTTGTTTTAGTTAAAAATATTACTGATGAAGTTATTCGAGGTTTACCCTTCATAAGTCTATTATATCCCTTCCAAGTAGAATATGATGGAGTTATCTCTACCCATTTAGGAGAAAAGGTCAACTTTGAGTTCTTAACTAAGCCTGAATTACATAATTTACAAGCTTTACAAAAACATGCAGTCACCAAGACAATAAAAACAATTCAAAATAAAAATAAACAATTGAGTTTCCTCCAAGAAGAAGTCAAGTTCAAAAGGATAGAACAACAGCTTAGTAACAAGTCCTTACAGTCACAAATTCTAAGTTTTGAAGAAAAATTAAAATTGAAATTTGTTATGAGTTACCTAATGCCTTTTGGCACAGGAAACATCACGTGGTACGTCTACCCTACGTCAAAAGCTTTAGCGAAAAGAATATTCCCACTAAAGCCAAACCAATCCAAATGAGCCAAGAATTTATGGAATTTTGTAAGATATAAATCTCTGAGCTACTCCAGAAAGGAATAATCATGAAAAGTAAGTCTGCCTAGTCTTGCCCTGCCTTTTATGTCCAGAAAAACGCTGAGGTAGAACGAGGAACTCCTAAATTAGTTATTAATTATAAGCCACTTAATACAGTTCTTGAATGGATTAGGTATCCAATTCCCAATAAAAGAGATTTAATTAACATATTAGGAAAATCAGTTATCTTTTCCAAGTTTGATATGAAGAATGGATTTTGGCAAATACAAATTCATGAGTCTGATAGATACAAAACTGCGTTTGTTACACCATTTGGCCATTATGAATGGAATGTTATGCCTTTTGGCCTCAAAATTGCACCCAGTGAGTTTCAAAATATTATGAATGAGATATTTAACCCCTACAATCATTTTTCCATAGTTTATATTGATGATATTCTCATCTTTTCTGAGTCTTTAGAGCAACATTGGAAATATTTACACAAGTTCTTACAAGTAGTTAAACAAAATGGTCTAGTTGTTTCTGCTAAGAAAATCAAGTTATTTCAAACTAATATTCGTTGCCTTGGTTTCAATATACGCCAATCTCATATTAGCCCAATAGATCGAGTTATCCAATTTGCTGACAAATTTCCTGATCAAATCCTCGATAAAAGTCAGTTGCAAAGATTTTTAGGTTCTTTAAATTATATTTCTGATTTCTACCAAAACCTGAGAAAGTAATGTAAGCCTTTATTTGACAGATTGCAGTGTAATCCTCCCCCTTGGTCTTCCATCCATACTGAGATAGTCAAACAAATTAAAATCCATGTCAAGACACTTCCTTGTCTTGGTATTCCTTCAGTTGATTCATTCAAAATAGTCCAAACTGATGCTTCTAATATTGGTTATGGAGGCATACTCAAAGAAAGAGTCAATTCAAGTCATCCTGAGCAAATTGTCCGTTTTCATTCTAGTCTTTGGACCCAGTCGCAAAGCAATTATAGTACTATCAAGAAAGAAATATTATCTATTGTATTATGCATCAATAAATTTCAAGATGATTTATTAAATCAAAATTTTTTATTAAGAGTTGATTGTAAGTCTGCTAAGCTTGTATTAGAAAAAGATGTTCAAAATATTGCATCCAAACAAATATTTGCAAGATGGCAAGATATTTTAAGTATTTTTTTATTTTGATATTGAATATCTAAGAGGAAGTGAGAATTGCATTCCTGATTTTCTTACTAGAGAGTTCCTACAGGACAAACAATGGTAGCTCGAAAAGATAAACAAAAAACACAATCTCAGCCGCCCCAGTTGCCCAGAATCCTTCCTTCATCAATGAGTCCAACTAAGCCCGAGTCATCCTCTCAAATCATCCCTTGCCCAGGTTATGCTCCAATTCCAATTGCTAATAGATATTCAAGCTTAGGAACCACAGTAAGCCAAATTCGCCCTAATTATCAATCAGCTCTTGTTTCTAGTTACGACCCTTTTCAAATACCTTCCCAAGTTGCTTCTCCCTCAATTGTCTATAAGAAATCATCCCCTTATTTTCCAAGAAGCAGTACCCATTTATTTTTTATTGAGCCTCATCTTAATAATATCATGCTCCCAGGCACAATGGCCAAAGAATATTTTCCCCCTAATTTCCATTATATGCCTCAAAGTCCTTATAAGTCCCTCAAGTTCTACTGAGACATTCTCCACGAGACTCAATCAGTTGACATTAAGCCAATTAAAGATAAAAATGATCCCCAAAAAATCCTTTATCACTCTCTTTACATTCATCAAATTCTAAGCCAAAAAGATTGGGGTAAACACCCTTATGATCTAAGAACCCTCCAGTCAAACCTTCATTATAATTATTCTAATTATATTGAAGCTTGGTATTCAATTTTCCGGCACCAAACAGTTGATTTTAGTCATTCCTGGTTTATCAATTTTGATAGTAAATTCAAAAGCCCTTTCCCTCTCAAGTCCTTGAGTTAGTCGCCTATTTTGCAAAAAGATATAAATTTTCTGAGTCAAAGTTATTTTTTTCCAAGCCAACCTCATTTTATTGCTAAGTATAAAGTCCCTTGGATTTTAAAATGGAATTATTCTATTAACTGGGATACCAGAGATTTCTCTAGCCAATATTCTGTTAAGTGGTGGGATAAGTTTCAAGTTGAAAGAATTGCTAATTATGTCTATAAAGATTACCCTCCAATCGCTCAGTTACAAAAGCCCACGCCTTTGTCCTCCAGTTCTTCTCATGCCTCTCTCTCAGTTGAAGGAAAGTCTAAAACTGAGCTACAAGAATTAGCTCGTCAATTAATAATTCAAGCCTTCCAGATGAATGATGATGATGATGATGATGAGACCTCCCCAAGATCACAGTCTTCCACTAGTCAACCTCATT

TGCCCTCCAATCAAAAGAGTCCTTCTCAAAAGCACGATGGGCTGATTATCAAGACAGTCAATAACCATATGATCTAAGTTCATATTAAAGTTGAGATTCTCTGACAAGGCACTCAGTAAAAGCAATGCTTTCGCTTGCCTCTATAGGAAAATGTTGTTTTCTTCTGAGTTAAAGCAATAGTTTCTTATATATTTATAAATGAATATAAAGTTTGAATTAGGCAGACCAATTTTAGAGCTTCACGAATTCGGAAATTATGAAGGGCATCATCATGCCATTCCAAAGACTGAAGTTCTAAGCCACCAATTTCAAGCCCAAGATTCTCAGAGGATTATATGCGCGTGACAAAGGCCTCACAAGGAAGGAATTTGATAATAACTCCGAATGTTCCAAAGCAAAAAAGAATCAAGCATGTCAGAATATTTCAATCAATGCAATCCCACGGATTTAGGGGGGCTTTGAAAGGTAATATTTTCAAGAATAATTGACAAGAAGAGTTGACATTTGGCCATTCAATGGCCAGCCAATGGGCTGACAAGTGCCCTATATAAACCTTGCTCGATAAAGACATCATAGGACACAACTTCAGACAAACAAACCTACTTTTCGCCTTGCACTTTTCTTTTCCTAGACTTAGGATTTTATTTTTCAAGCACCTAGCGTAGTGAGTTCTTCCTTTTTTTTAGTGTAAGTTTATATTTTTCTTGTCATTTAAATTCTTGTTCGATTTATATTTCTTGTCATTTAAATTAAAGTTTATTTCGATTCCTTTACATTTCATTGTTATCACACTTCAATTAAGTTTAATTTCAAGTTTGTTTATTTTATCATTCTTATCACTTTTATTTTGTTCTTGCTTTATTTATCGCTTTGATTTGTTTTATCGCACTTCAATTCTTGTCTTTACGTTTATACGCATATTTAGTTTAATTGCTTTATTTTTACATAAACCCTAAAAATCACAAAAACATCCGTACGGAACCTAACCATCCTCGAGCCC

>Chr2-14; OZ022207.1; 7409676-7415117; dir; B;

TTGAGTCAATTAAGTAAGACTATAACTATAATTCAATAGTTAAATTATAATGAAAAGATTAATTATGGAATTAATATTGACATATAGTGACAGGATCTGACCCAAATTCCACTTTGGAATCTGAGCCGAACCCTGAGCGTGTCCGACACTTGGAGGGTGCCAGGCACAAATGACCGAATTGCCCTTCTAACTCGATTCCAATTTTATTACAAGTTTGACTTCTGCCAAAAATTCAGTAGAGTCTCCCCTGTAAATTGGTCATTTCCCAAAATTTTCACATGTACAAAAGCAATATATATGTTTCCCAACTTTTTAGCATGCATCAGCATAGTATTCTAACAACTCAACATCAAAATGAAATGGCCTCAGGCCTCTTTAAGACAAATAAATCATTCTGCCAACGGAAACTGACCCCAACTTTTGGGAACAACAAGAATATGTTAATAGATTTCAATATAACTAAACTAAACAGCTTTTCAATTACTTGGTCTTATGGCAGCACCTAGTAACTCTAGGTTGCCTACGTACCCTTAACAAGGAATCAAGCCACACGTAGTTCTTTCTCTTTGTATATTGCAGTTGTGTATCTCGTCAATTGTTATTGTCCATTGTGATTACAAATCAACCCAAGTTCTCATAGCATTGTGTATCTCGTCTTTATATAACAATTCACACGTTTAAAGCATTGACACGTCCTCGTAAGTCTCGGTAACACAAAGTCAACCCAAGCCGCAATCCTCTCAGGATTCATGGGACATTTAGTCAACTTGATCCGCATTCCTCGCTCCGCACGGTCCGGATATCCCTAAAACTCATGGAGCAACTGTCAACCGAACTGACATAACCGAAGGCAACCATTTATTCTGAAGGCAGCATCACCTATGGTGGTTTAAATAAATATTTCCTGGAAAATCATAAAAACTATTTCTCATCCATCCTTAAATTCTGTTGCCACTATCCTTCCTACTTTCCAAGCCATGCTCTCCTCTGAATCACCTTT

TGCTTTTCATAACTCCTCTGAGTCTGAATCAATTGCCAGTATTGAAATAGAAACTAGTAATATTTCTAAAATTGAAAATGCATTTAAGAATCTTGAGTTAGAAAATAATCCCTAAGTCCGAAGATTAACAAACAGAGTCAGCCCCACAAGTCTCACAAAAAATTGGTATCCTAAACCAACACCTCCTGATATGCAATTTGAAGAACGAAACACACAGAGTCAGTTTTCAGTCTCTTCAGATAAGTTATATGAATGGAATATTGATGGTGTTTCTGAGCAAGAAATCCTTAATAAGCTTCAACATATTTCTATGGTTGCCAATAGCTACATCACCAATCACAATTTCAGATAGTCTGAAATTGTTCCCTTATTAGAAACTGGTTTCACTGGAACTCTTCGTTCTTGGTGGGATAAACATTTAACCCATGAGTCTAAGCAACAAATAATTCATGCAGCCAAGCTTAATGATGATGGTCTTCCTATTTTTGATGAACAAATAGGTCAAGGCATTGAAGATGGAGTCAACACCTTGCTGTACACAATAGTTGATTATTTCATTGGAACACCTAGTAACACTACTGCTAGAATACATGATCAGCTTAATAACCTAAGATGCCCTAAATTATCTGATTTTAGATGGTATAACGATGTATTTATTTCCAGAGTCATGATTAGAGATGATAGTAATCAGTCATTTTGGAAAGAGAAATTTGTTAATGGTCTGCCTAGTTTATTCACTCATAAGATCCAATCAACCCTTAGTAATGAGCAAGGACACATTTTTGGAAACATAATAAGCACCATTAATCAGTCGGTATGAGAATGTGTGTTTATATGAAAATTGGTAAACATATCCAGTCTGAAAGAAATTATGCTAAATATGAGTTAGGTAATTTCTGCGAACAATACGGTCTCATGTCTATTCCCCCGTCTAGAAAGAATAAGCCCAGCCACCACAATCGATAGGTAAACGTCGCCATTATTCACGTAAAGGATAGTCATTCCAACATAATAATGATGAAAAAGAATTTTATAGTAAAAAGAGATTCTCTCCTAAGAAAATTGGAAAAAGAAAGGTAAAGATAGTTATTTTAAGAAAAAAATCCGACAAGAGAAAAGTAAAATGCTATAAATGTCAAAAATTCACTCACTATGCTAATAATTCCAAAGTTAAAGACACAATTAAACAATTGAAAATCACGGATGAGGAAAAATACAAGCTGATTAAAGTCATTGAGTTAAGAAATTCTGAGTCTAGTGAGAATGAAACCATGGTTAGTCACACTGAGTCTTTTGAAATCTATTCTTTTGGGTCACAACCTAGTTCACCCCGAATTCAATTTGGTCGCCAAGATAATTGCTGTAAGTCTAAGTCAACTAATGTCCTTACCAAACAGGAAGAACAAGAAGAACTATTAATTGATTTAATACGTAAAGTTGAAAACCCCGAATTAAAGTCTGAGTATTTAAGGAAATTAAGAAAAGTCATTAGTCAAGAAAAGCTGAAACAATCTACTTCCCAGAAAATAAGTCTACGCACAACCTTAGAGAAATTTAATAAAAAGAAAGAAGTCACTTTACAGGATTTACAGTTAGAGGTAAAGTTAGTCAAAAAAGAAATTATTGAGTTAAAACAAATGAGTCAAAACTTGCAAAGTGAAAATTATGAGATAAAACAAGATTTGATTAATTTATTAGAAAATAAGATCACTAAGTCTACACCTGCAAGCCCCTCAAATTGTGATGAAGAGCCCTCCAATCAGGATCAAGCCGTCGACTTAATTAAACATGTAAATTTCATAAAATGGTATGCCCGAGTCACTATATTTGTCAAAAATTTTGAGTTAAACATAGTTGCCCTCTTTGATTCAGGGGCCGACCTTAATTGCATCCTAGAAGGTCTAATCCCAACAAAGTATTATAAAAAGTCAAGGGAGTCATTAAATACTGCCTCAGGAAAGTCACTCCAGTTAAATTATGAGATTCCTAAAAGCCCATGTCTGTCAACATAAAATCTATTTTAAGACCTCTTTCGTCTTAGTCAAAAATATTACTGATAAAGTCATCTTAGGTTTACCCTTCATTGCCCTTTTATATCTTTTCCAGGTAGAACATGATGGAGTTATTTCCACATGTCTAGGAGAAAAGGTTAAGTTTGAGTTTATGAGTAAAGCTGAGTTACATAATTTAAAAGAATTACAGAAGAATGCTGTTATCAAAACAGTCAGAGTCATTTAGAATAAAAATAAGCACTAGAATTCCTTCAGGAAGATGTTAAGCCAAAAAGAATTGAGCAACAACTTAGTCAGGAGTCTTTACAATCACAAATCCGACAGTTTGAAGAAAAATTAAAACAAGAAGTATGTTCTGAGTTACCCAATGCTTTTTGTCATAGAAAACAACATGTAGTCAGCCTTCCTTATGTCAAGTCCTTTAGTGAGAAAAATATCCTTACTAAAGCCAGACCGATACAAATGAGCCAAGAATTAATGGAGTTTTGTAAAACTGAGATCACAGAATTGCTTGAGAAAGGAATAATCCGTAAGAGTAAGTCACCATGGTCTTGTCCTGCTTTCTATGTCCAGGAAAATGCTGAATTAGAACGCGGAGTCCCGAGATTAGTCATAAATTACAAGCCGCATAATACTATCTTAGAATGGATACGGTATCCAATCCCTAATAAAAGAGATTTAATTAACAAATTAGGAAAGTCAGTCATCTTTTCTAAGTTTGATATGAAGAGTGGATTTTGGCAAATTCAAATTCATGAGCCTGATCGATACAAAACTGCTTTTGTTACCCCTTTCGGTCATTATGAGTGGAATGTTATGCCCTTTGGTCTTAAGAATGCACCCACTGAATTCCAAAATATAATGAATGAGATTTTTAAATCTTATAGTCACTTCTCCATTGTTTATATTGATGATGTGCTTATCTTTTCTCAGTCGATAGAACAACACTGGAAACATCTGCATAAGTTCTTACAGATAGTCAAACAAATGTGAGATTTCTTGGGTTTAACATCTGCCAATCATAGATTAGCCCCATAGATCGAGTCATCCAGTTTGCTGATAAGTTTCCTGATCAAATCCTAGACAGAAGTCAACTCCAGAGATTTCTAGGATCTCTAAATTATGTTTCTGATTTCTATAAGAATTTGAGAAAACAATGTAAGCCTTTATTTGATCGCCTCCAAAATAATCCTCTACCTTGGTCATCTATTCATACTGATATTGTCAAACGGATCAAAGCTCATATTAAGACACTTCCCTGTCTTGATATCCCTTCTGCTGATGCATTCAAGATAGTTGAAATTGATGCCTCTAACATTGGTTATGGAGGTATTCTCAAACAAAGAGTCAATTCTAGTCAATCTGAGCAAATCATTCAATTCCATTCTGGTACTTGGTCGCAGTCTCAAAATAATTATAGTACTATTAAGAAAGAGATATTATCTATTGTATTATGCATTAATAAATTTCAATCTGACTTATTAAATCAAAATTTTTTAATCAGAGTTGATTGTAAGTATGCTAAGCATATATTAGAAAAAGATGTTCAAAATATTGCATCCAAACAAATATTTGCAAGATGGCAAGCTATTTTGAGTATATTTGATTTTGATATTGAGTATCTGAGAGGAAGTGAGAATTTTGTCCCTGATTTTCTTACCATAGAATTTCTGCAGGGAAAACAATGGCAGAAAGAAGAAAAGAAAAAGACAAGCAAAAGAGTCAAGTTGGTCAGTCTAGTCAACCTAAGTAACTCCGAGTCCTCCCTCCATCAATGAGTCAAGCCAAACATGAGTCACCTTCCCAAATCATCCTTTTCCCGGGATGTTCTCCTATCCAAATTAATAACAAATTTGCCAATCTAGGAGCCACAGTAGGCCAGGTTCGCCCCAATTTTTAGTCTGCCTTAATATCCAGTCATGATCCCTTCCAGGATAGTGCTCTGTCTAATCTTCCAGTTGCCTCTTACCATAAGACTTCCCCAAACATGATCAAGAGTAATAGTCACCTATTCATAATTGAGTCAAAATATAATTCCATTGCCAACCCAATCACCATTGCCAAATCCTACTTTCCACCAAATAGTCATTTCATTCCCCCAGCACCATTCAAAAATTTGAATTTTACAAAGACATCCTCCATGAGACTCAGTCAATCACAATTAAGCCAATCAAAGACAGAGATAATCTCGATATCATCCTCTATCATTCCCTTTACATCTACCAAATAATTAGTGAGAATGAGTAAAGCAGTCACCCCTATTATCTCAAAGTTCTTTAGTCAGAACTTCAATATAGTTACTATGATTATATCGAAGCCTGGTATTCTATCTTGCTTCACCAAACAGTTGATTTTTGTCATTCATGGTTCCTAAATTTTGACAGCAAATTCAAGAGTCCCTTCCCCTACTGGTTCAATCACTGGTAGGAAAAAACATGGTCCAGTCATTGATTTATTTCCTCCTCCAATGAAAGATTTAGTCAATTATTTCACTAATAAAAATAAGTTTAAAGAGTCTGAGTTGTTCTTCCCAAATCTTCTCCATTTTGTTGCCAAGTACAAAGTCCCTTGGATCTTCAAATGGTCATATCAAGTTAATTGGGAAACCAGAGTCTTATCCCGTCAATTTTCAATCAAATGCTGGGATAAATTTAAACTTGAGCGTATTGTAGATTATGTTTATGCTGATTTTCCTCCAATCAGGAATCCGCAGCCTATGAAACAGTCATCCCTGCTACTTCACATTATTCTCTCCCAATCGAGGGAAAATCTAAGTCTGAATTGCAGGAAATTGCTCGCCAATTAATGTTACAAGCCTCACAGATGAATGAAGATGATGATAGTGATCCCACTCCCAGGTCACAGTCATCTACCAGTCACCCAAAGTCATCCAGTCAAAAGACTCCAGCCCCCAAAATGTGGTGGAGTGATTATCGCCTAATGATCAAATCCATGGTCAGGACCCTTACGATTTCGGTCCCTTCAACCTAGGCGATGATTAAAGTTGTCAATCTTATGAGCCCTGAGACAAAAACATTTGGAATCTTGGACCCGTGCTTCAACAGTCTGATAGCCCGTGCTCCGTTAGTCAGATTCCCCGACAAAGTAAAAGTCACAGCACTCAGTAAAGCAAAAGCTTTTGCGTGCTTCTGCAGTCACTTTTCTAAGCATCCAATGTTGCGAGCTCATCAATAGTTACCAGAGTTCACTTTATTTGCTTATTTAAGAAGGCCTAGGCCTTATCCACAGAATGTAATTTAGTACACTATAAATTTCTCAGTTTCAAAGTTCTATCAGTATCAATGCTCTGAAGAACTAAGTGGTGAAACTAGGATCTTTGTAAGTGTTTTGCATTCTGCAAGTTATCAAGTTATCAATAATATTTTGAGTATTTTGAGTAATGCTTATTTTGATTGCATCTATTTTCATT

TATTCATTAATTTGCTTCATGCATAAACTGCGTCTATTCTATAATCTCTGTGCTTATTCATACTTCTGTCTTTATTCTATTTCTTTCTAGTTCTAGACCCCTTCTTATATATATATATATATATATATATAAGCAGAATGTGATAAAAATATTCAAAATCAAACAATCATGCTTGGAAAGTAAAATTCCTAACAACTCATTCAAGATCAATAGTAAAACTTAATTGCATAAAATCATTTATAAAACAATAGCCCACTCACTGGACTTCCTCTAATTCATAAATCATTTTATGTATTACTAGCTTAGATTAATATTTATTTATATATATAATAAAAATAAAAATAAACAAACAACCGCCGAAGACACTTTTAACCTTCCCTTTGAAACTTACATAAACCCAATTATAAATACATATATATATATATTATACAAATATATAAATAAATATATATATATGTGGATTTATAATTAACTTATCACAGATAAGTAAAAAAACGAGAAAATTATTAAAATAATATTGACCACGTGAAATCTTAATGTAAATAATAACAGTTTAACTGAAACTTTCATTTCCTCTTTTACTTACAAAACAAACTCATTGCTTAAGAAATTAATTGTGATCCGTCAATTCTAGTGATAATCCGTCAATTCCTTGTATCCCGTCAATTCCATCCTCGCAAGCCTCGGAAACACAAAGTTAACTGAGCCACAATCCTCGCAGGTCTCGGAGACACAAAGTCAATCCGAGCCTCAATCCTCGCACCACACGGTTTAGGTGTTCTTGAAACTCGTGGGGGATTATCAAAAATAGCAAAACTGTTTAAGGCAACTAGCGGGTACTCTTGTGGTGGTTTTGAAAACCATATCCAAACTTATCACAAACTTTCCTTTATCTCACAGATCCATTTTTCAAATAATTCCATTTCGATACTATAATAATGATCTTAATAGTAATAGATAACTAAATAGATAAATAATTAAATCAATCAATCAATACAAA

>Chr2-15; OZ022207.1; 7604580-7605893; dir; B;

AGCGCTAACCCTGATTGGGGATCTCCGTCTCTCTGCCTCCCGATCGTTAAGCCGGTGTCGGCAGTCGGAGTAGACCTTCTTCGGTATGTGCGGTTGGTCCCTAGGTGTCGGCACCACCAGTTGTTTGGAGGGCGCGGGCGTTGCCCTTGCTGCCCTTCGGTGCTTCCTCCTGTGAGGGGTTCCCAAGCTTTCGGAAGAGTGAGTGGTCTGGATGCCCTGCGGTTGCTGCTGGTGCAGCTGCCGTTGGGTTTCGTCCACTTTGGACGAAAGGTGGTGGTTGTGCTCCTGAAGCCTAGCCATGTCTTTTCGCAGGGATGCGATTTGGGCTACTAGCTCGGCCTCGGCGGTTGTTTGGGTTGAGGGGGCGTTTCCGTAGGGAGTGCATGGGGGTAGCGCTGGGCTAGCTCCCGCTTGCCTTCCGAACGGTCCCCCATCCTCCGATAGAAAAACGGGTACTGAAGTGGTCCCCATGTGTTTAGGGGATAGTGGGTTAATTGTCTTTCGATTCCCACAGACGGCGCCAAACTGTTGATGCGAAAAAGTGGTTTGACACGTGGTTCGCCCAACTTAGTGATATTGTGTTCTTCGGAAGGGAGTTAGTCTTCGGAAGTTCAAGTTCCTGCAAAAGGGAACGTTCGGTAAGACCTTGGGGTACCGGTGCGGTACCGGCCGAAGGCTCTCCGATGCTTAAATAAGTATGGGTTTGGTAAATATCAGATTACAGATCAAGCGATAGCGTACCGTTGATCGTTCTGTCTCCCTTTCTTTTGGGAATAGGGGTCTCCTTATATAGGCCTTGGGAGGTGTGCTTATTTTGGAATCTCCGATGTGGGACTGTAGGAGTGGATTGTGAGCATGACACGTGTCCAAGAGCAAAAGCACCATGATGCATATGATTCGGTAGGGTCCTTGCCGAAGGGCTTGCAGTGTTAAATGTTGACTCCGTCCACGTGTCAGGTGGTCATTGGTCGCTAATATGCGGTATAAACATATATATATA

TCCTTTGCAGCCTCTTCCTCGACCCTAGAACGAGACCTAGAGTTAGAGAAAGCTTTAATTGATTTAAAATTGGAATCACTAAGAAAAACTTCCCAAGTTACCCGTCTTGTTTATGGAAAAACTCCAGTACAAGAAGATACTAAGTCTGAGTCCCCTGAATCACCCACTCAATCCGATTTCATAGGTAATCAACTGAGAACACTTAATAAAACATTTGTAATTAATTGAAAACGACTAGATAGTCATTTGCATGCCCCTGAGAATCAAGCTAGAAGAGATTATTACCATTCAGTCATCCCTGATAGAATTCAAAGACAACAAATTTTTAATGATTGGAAAGAATACATGCGCACTGCTAAAGTTGAAATATATTAACTTGATTTTGTAGAAAGCCGACATTTTCCCAATGAGTTAAAAACTTTAACCCAAGAAAAATGGGAAAAAGTAGATCACACTGAGATTCTTGCTAGTCATCCACCCGTTGAAACAATAATCATTAAGCATCAGAAATCTAAAATAGTTGCAAGCCCCTTTAAGATCCCTGATCCAGAGAATAAAATCGTAGTTGAACAAAATAATTACACCAACCAAAGTCTAGTTGTCATAGGTAAACAATTAGATAAGATAGAGACAAAAGTCGACACGTTACTTCCAACAGAGCTCACACCTAAACATCATGAGAAACCCCTAGTTACATTCCAAGATTTAAAATCAAATCCAACTTTTAAGACAAAAGAAACAATGACAAAAATTGAGAAAATGTTAGAAGAATTAACGCCAGTCAAATGAAAAGACAAAGAAAAGGTTGAATCAAGCGTTAAAGTCCTAGGATCCTCTGAGTCACCATTTGTTTTCCATAACTCCTCTGAGTCTGAATCAATCGTTAGTATGGAAACATAAACTAGTAATATATCTAAAATTGAAAATGCGTTTAAAAATCTTGAGTTAGAAAATAATCCAAAAGTCCAAAGATTAACAAATAGTGTCAGTCCCAATAGTTTAACAAAAAACTGGTATCCTAAGCCAACACCTCCTGATATACAATTTGAAGAATGAAACACCTAGAGTCAGTTTTCAGTCTCTTCAGAAAAGTGATATGAATGGAATATTGATGGTCTTTCTGAGCAAGAAATTCTTAATAAACTTCAACATATGTCCATGGTTGCTAATAGTTACATCACCAATCACAGTTTTAGATAGTCTGAAATTGTTCCTTTGCTAGAAACTGGTTTCATCGGAACTCTTCATTCTTAGTGGGATAAGCATTTAACCCATGAGTCTAAGCAACAGATAATTCATGCAGTCAAACATAAT

TCATACAGATAATTCATGGCTTAGCATTAAAATAGTGATTCTTCTCATGGCTTTGCGTATTGATTTCATTGCATGATAATTTGGCCATGACCGGATCAAATAGCGTTGGATTAGGTGAGCTGAATTTACAAAGCTAGTAAATTTTGGTTCCTTTGTGCATGCTTGCTTGGCGTGACTCATGGCTCCAGGCCTATGTAAGCGTGTTCTTTGATTTATCTTATGCCCCGATCGAGCCTTTCTCGTGACTTATTTGAGTTGGGCTAAGAATGACCCTGGGCCTAGCCCTTGGACTTTTGAGCCTCAACAAGGTGCAATACTTTTTATTGAACTGGAAGGAAAAGGTAGCCCAGTCTATTTGGCCAAAGGATAAAAATCACAAACATGTGTAGGAAGTGAAATATCAGAAAAATGTTTAGACAAAGAGCGAAGTTTGGCATTGGAAGGGTGTCCTAATCGGAGATGCCAGAGGTTGTAGGAAGCAGACACATGATATGTGGAGGAAGAATGGAGCTGCGGAGCAAGGCGTTGTGGATTGAAATAATACAGGTTTCCATGCCTCTTACCCAACCCAATCATCTTCCTCGTGCGCAAGTCTTGTACATAACAAAAGGTGGGAAAAAAAACAAACACAACAATTTAGTGATGCAGTAAGGCCAGGGACAGACAATAAATTAACATTGAAAGAAGGGACGCATAAGACATCGGTAAGAAGAAAAATATCATCAAAGTTAACGGTGCCTATAGATGTGATGGGTGCCTTTTCCTCTGTGGGTCATTCTACCGGTGGAAGATGTGTTGGTGAATTTTCAGTTAGGAGTGCATGAGAATTGGTGATATGGTTTGTTGCGCCACTATCTATAACCCAGGCATGCGATAAATAAGAATTAGTTTGGGAACTAAGGCAAAAAGGATAACCCAGGCACACGTTGGTATATTGCCGGTTGCTTCCATGAGATTGGCGGTTTTGTGAAGGGCGACCACTGTCATCAAGTTTGGGTAC

>Chr2-16; OZ022207.1; 7650104-7655917; dir; B;

AATATGGTAAATTCACAGTTTTTCATGTTTAAAAAAATAAAATTAAAAGAAAAATTAGATAATGAATCATATTTTTATGGAACACAACTATCCATTATTTTTTTAATTCTAAAATAAATGTAATTTTTTTTTTAAAAAGCCTCTCATTGGCGCACAAGGGTGTGCGGAAAGGCTAGTATATATTAAGTTAAAGGCAGAGAATGCTAAAACATTCAAAATACTAGAAAACGCATTTGGTTAATCCAAACATTAAAAATTAAAATTATTAATTAAATGATGATAATATGGTAAATTCACATTTTTCATATTAAAAAATTAAAATTAAAAACAAAATCAAATAATAAGTCCTACTTTTATGAAACATAACTACCCATATTATCTTTTCTTAACTCTAAAAATAAAATAAAATAAAATAAAAACCAATTGAGCATGTGCTAAAGGGCTAGTATCAATAAAAAAATACATTTTAAGATCATCACTCAATTTTATTAAACCTCTAATGAATAACTTATATTAGGAAAACCACATACACAAAAAGATGGACGTTGGGGGTTGGAAAGAGGGGGGTACGAGCCTAAGGACAACCTGCGGCACTTGAGCGCGCATGAGGGGCAAGTGGGCCCTTGTCGGCGTGCCAATCTGGGAGCAAAGGAGGTAGGAAGAGGATTAGGTCGCCCTGAAGTGCAGGCCGTCAAGAATATTCTTCCGCCTTTTCTAATAGTTTTTGCGTTGTTCAACTAAATTATAAAACACCAAACAGATTGAACAAAATGGAGAAATCTATTGCTTGTTTTGTTTAGTTTCAAACTATACAAGATGGTTGCCAAGAGCAGTTTCGAGTGCCATTCCCAATGAAGATCCTGTTCACGTAGAAGAATCCAAAGAGTTGATTGATTTGCAGAGTCCAAATCTTATGCTTTTGCCTCGAGAATTCTGAGGTTTGGGATTTATACTTTGGCCTTCAGCTTCGACTAGATAAACAAGTTTATTGTTGACGCAT

CAACTCCCCCAGATATCCAATTTGAAGAAAGAAATTTTCAAAGTCAATTCTCAATCTCCTCTGATAGACTTTATGAATGGGATATTGATGGGATGTCTGAACAAGAAATTATGAATAAATTACAATATATGTCTATGGTCGCCAATAGTTACTGCTAAATATGAAAACTATGAGAGAATATTTGTTTTTGGGAATTTTTTTTTGTGTATTCTTCTCCTTGTAGGAGGTCATATTTATAATACAACGAAACCTGAATAAGCAAGTAAATAATAAATTCATAAATCTATTTACAGTAGGAAATCAGGAATTAAAGTAAATCAATTACAATTATAATAGGAATTCTTGGTGTGCAAGGAAAGTAAGTCAATATCCACAAGTCATGCCAACACTCCCCCTCACGTTGGTACATAGATGTCATTAATGCCCAACTGATCTAGTAAATTGTGGAATGCTTTACTGGAAATCATCTTTGTCAAAATATCCGCCAATTGATCCTCGGATTTCAGAAAAGAAAACTGAAACATTTTAGCCTCAAGCTTCTGTTTGATGAAGTGTCGATCCGCCTCAACATGTTTACGATCATGCTAAACCAGATTCTGTGCAATGGCTATAGCAGCCTTGCTGTCACAAAAGAGATTCATTGTGGATGTAGGTTTATACCCAAGTTCAGTAAGCAACTTTCTTAACCAAAGAAGTTCACAAATCCCTTTAGTCATGCCTCTGAACTCGGCTTTTGCACTGGATAAAGCTACCACATTCTGTTTCTTACTCCTCCATGTCACCAAATTACCTCCCACGAATGTGAAGTAACCTGATGTGGATTTTCTATTTGTTAAATTACCTACTCAATCTGCATATGAATAACCATCGATATTTAGATGACCATGCTTTGAGAACATAATTCCTTTTTCAGGTGCAAACTTCAAATATCTAAGTCTCCGAAGAGCTGCATTCATGTGGTCTTCACTTGGAGAGTGCAAAAATTTGACAACGCTCATCACATAAGCAATGTCTGGTCGAGTATGTGACAAATAGGTCAATCTTCCCACTAGCCTTTGGTATCTTTCTTTGTTAGTTGTAACTTGATCCTGATATTCTCCGAGACGATAATTTTGAACAATAGGAGTGTCCACAAGTTTGCAATCTAACATTCCTGTGTTTATCAATAAGTCCAAGACATATTTCCTTTGAGAGAGAAATATGTCTTGCTATGATCGCGCCACCTCAATTCCCAAGAAATACTTAGGTCTACCTAGATCCTTCATCTCTAACTCAGGAGCCAAATAGTCTTGTAGCTGTGATATTTCCTGTTTATCATTCCCTGTTAATCGTATCATCAACATAGATTATTAATGCTGTTACATTCCCTTTCCGATGTTTCAAGAATAGAGTATGATCTAAGTTGCATTGTTTGAAACCATTGTTCTTCATTGCCATGGTGAACCGTCCAAACCATGCATGTGGTGACTGTTTCAATCCATACAATGCTTTTCGTAACCTGCAAACTGCTTAAGTCTGAGTAGTATTATATCCAGGAGGAATGTCCATATACACCTCCTCTGTGAGTTCACCATGTAGAAAAGCATTCTTTACATCAAACTGGTGTAGTGGCCAATCCAAATTAGCAGCAAGAGACAGTAAGACTCTGACGGTGTTTAACTTTGCAACTGGTGCAAAGGTTTCTTCAGTTTATACCATAGGTCTGTGTGTACCCTTTTGCCACAAGTCTTGCCTTGTATCGCTCGATAGATCCATCTTCCTTGTGTTTCATAGTAAACACTCATCTACATCCGATAGTCTTTTTTCCTCGTGGTACAGTCTCCAATGTCCAAGTCTAATTTTTTTCAAGAGCTTTCATCTCCTCTTTTATAGCTTGGACCCACTTAGGATCTTTCAATGCTTCAGAGACCTTGGTTGGAATATGGATAGCAGACAACAGATGCACAAAATCCTTGAGTGGTTCAGATAGCTTCTCAGTGGATACATGATTTGCAATTGGATACTTGGATCTCTTGCCAATATCAGGTGAATAATGATTTGGTGGCTTCCCACGATTTTGCCTGAAAGGTAATTGATATCCAATAGTTTTATCCTCTAAATGCACAAGTCTAGTAGGAGTAGTTACCTCAAAGATGTTCTCAGGAGATTGGTCCGTTGGTACTGTTGAGTGAGAGGAGGATGGGGGGTCTTCATCTTGTTGTTCTGAGTTGTCTGTAGGAGTATAACTCGAATCAAAAACAGCTTCGCAAGGGTTAGGCGATCGATCGTTGTTTGGCACAGAGCATTCGCACGTGCCAGACTCGGGACAATCGATTGTTATTGTTTCTTGGCCGAGAGCAATCTTCATGACAGATGAATATCTTCATTTTCCAAGCTACTCCAATTGAGCTTTTCACTCCGCATCTCCCATTGAAGAGTAGAATTGGATGCTGGGTCATAGAAGAACATCTCTGATTCTAGAAAGGTGACATTCGAAGTGGCATAGGTTCGCCAGGTAGGTAGGTGATAATATTGGTAACATTTTTGATGAGTGGCATAACCCCAAACAAACGCAACGAAGTGCACATGGATCAAGTTTGCTTCCTTGATTTTTGTGGAGATGAACGAAGGCCACACATCCAAAAATGAGAGGGGTAAGCACCAAAACAGAGGGTAGAGGCCCGTGTTGCGCCAGCACTTCTAATGGAGTTTTAAAGTTCAAGACCCTAGAAGGCATGCGGTTGATAAGGTGGACAGTAGTGAGGACAGCATCATCCCAGTGATGACGAGGAACATGGCTACCAATGAGAAGAGCATGGACTGTTTCAAGGAGGTGATGATTCTTCAGTTTGGCAACACCATTTTGTTGTGGTATCTGGGGGCAAGTTGTCTCATGAATAATGCCATGTGTCTGGAAGTAAGTCTGAAAATCAAGATTAACAAATTCTCCACCGTTGTCAGAGCGAAGAATCTGTATTTGAGCATTAAACTTTGTTTCCATCTGTTTGTGAAAGAATTGGAATAGGGAAAACACTTCATTCTTATTTTTCATCAAATAAAGTCATGTCATCCGTGTACAATCATCTACGAATGTGACAAACCAACGAATACCGAAATGCGTAGAAATAGGTAAGGGTCCCCAAACATCATAATGAATTAAAACAAAAGGAATAGAACTTTTATTCATACTTAAAGGACATGATGCTCTGTGACTCTTGGCTAAAATGCAAGTGTCACATTTAAAATATGAGTCCTTAAATACATAAAACAACTTGGGTAGTAAGTGTCTTATATAACCAAAAGATGGATGCCCCAAATTTCAGTGCCAGAGCCAAATTTGTTGGTGCTTGTCATCAGATGGATGCTTCAAACTGTAGGCACGTCCAATACTGAAGTCATCCACATAGTACAGCCCCCCCTCTTTTAGTACCACACCCAACGATCTCCTTGGTGAGAATATCCTGTAGTAAACAAAAATTGGGATAAATTCGTACACAACAATTTAGTTGTTCAGTTGCCTGGCTTACAGACATTAATTTATTTGACAAAGATGGAACGAGTAGAGTATTGGAAAGGGACAGAGATGGTGAAAGGGTGACAGTACTGGCCCCTGTCATAGGATAGGTAACTCCATTCGCATGGCAATACTTATGTGGCCGGGTGTAACAGTGTGCAAAAAGTCATTCGGATAAAAGGTCATATGATCAGTGGCGCTAGAATCAATTATCCAACCAGTAGTATCTTGTAGATCAAAAGTATGAAATGCATAACCAAAGTTACTTGAGACATCCAGAAGTGCGGGACTATCAATAGAGTCTACTTGCGGAAACAAGGCTAATTGAGGTTCGGTAGAGACAAGAGCAGTACAACCGGAATTACTAGCAGTTTCTCTGAGCTTGCGAGCGCAGAGGTCCTATCACCAATCACGATAACCATTCAACTTAAAGCAGGTGTCGTAGGTATGTTTGGGGTTGCCACAGTACGTGCAACCACTTTCATCAATCGGAGCCTTGGGACGGGAATGTGGGATGGTTTTAGAAGGTGGCAGACTAGTAACAATAACATATGACTGGATCTGAAGGTGGGCCACAATAGAGGAATTCTGGGGCTGTTCGGCACGAGTAGAGGGACCTAATCGAGGTGAACGCTTGGCCGTTAAGCCAGTGCCAATAGGTGCAGAGGAACCTATCATCACAGCTTTTCAAACATCTTCACGGCGAACATAGGCATACGCTTGGTCCATAGTATGGAATGAAGTCATGTGAAGAACATCACTTCGAGGCTTATCAAGATGATCATCAAGGCCATCCAAAAATATGTAAACTCAATCTTCCTGCAGAATATCATTATAGCGCTTGATATCACTTTCATACTCCATCATATTTGGACGACATAAATCAATCTCTCCTCATAAGCTTTGGAGAGTATTGTAATAGGTTTCAATGGAGCCGCCAGCTTGTCGCATATGGGAGACTCATCGTTTCAGATCATAAACCTAAGAGGTGTCAGTGCCATCATAGTAAGTTATGGCAATTGCATCCCACACTGCTTTTGCCGTCGGAAAACGAATGGAATTGCCAATCAAGCTCTGCTCCAGAGATTTGATGAGTCATCCCTTCATAATAGAGTTCTCGGTGCGCTAGCAAGGAAATATTGGAGCGGTCGAAGGAGGTTGTGGAAGATCACCATTAACATATCCCAATTTGTCTTTGTCAGAGATATACAACTCGACAACTTGGGACCATAGGGCATAGTTGGTGCCAGCCAACTTTATGCCAATCTGTGTAGCGGAAGCTCCATAGGTAATCGTCATTGGAATCGTCGTTTGATTCGGGTTCAAGGTCGTCGTCTGATTCGAGTTCAGGGTCGTCGTCTGAGTCGAGTTCGGGGTCGTCTTGGTAGGATCCTGATTTAGGATCGAGTTTGAGGTCCGAGTCTGCATGTTCATTAGTTGAGCCACATGGGCACTCAACTCGGCTATGGTTGGTTGAGAATGAGAGGAGGAAGAAGTGGTGTTACCGCCATGAGGATTAGAAGTCATCCTCCCCCATGGTGGCGGCTAGGGTTTAGAAGCTAGAGTCAGTAATCCCAAGATCGCTGCTCTGATACCATGCTAAAATAAAACTATGAGAGAATATTTGTTTGTGGGAATTTTTTGCGTATTCTTCTTCTTGTAAGAGGTCATATTTATAATACAACGAAACCTGAATAGGCAAGTAAATAATAAATTCCTAAATCTATTTGCAATAAGAAATTAGGAATTTAAGTAAATCAATTACAATTATAATTGGAATTCTTGATGTGTAAGGAAAGTAAGTCAATATCCACAAGTCACGCCAACAGTTACATCACTAACCGTAACCTAAAACAGTCAGAAATAGTACCTCTCCTAGAAACAAGATTCATAGGAACCTTATGTTCTTGGTGGGATAAACACCTTACAGAAGAGTCAAAACGACAAATAATACATGCAGTAAAATTAAATGAAGATGGATTCCCTATATTCGATGAAAATATCAATATGGGAATTGAAGATGGAGTCAATACATTATTATACACAATAATTGAACATTTCGTACGAGCACCGAGTAACACTACAGCCAGAATTCATGATCACCTTAGCAATCTTAGATGCCCTAAGTTAAGTGACTTCAGATGGTACAAAGATGTCTTTATCTCTAGAGTCATGATAAGAGATGATAGTAATCAACCATTTTGGAAAGAAAACTTCATTAATGGTCTTCCCAATCTTTTTGCACACAAAATACTAACAGTCTTAAGTAATGATCAAAGCCATATCGATTTTGATAGTTTAACTTATGGAAATATATTTAA

TATATATATGTCAATCCCGATCTCTTGGACCACATGAGTCCAAGAGATTGTGGTCACCCACCTTTGGATATTAATCTAATGGTTGAAAAAAGTTTTTTAAAAAGAGTGCAAGACTGAGTGAACCGTTGAATTTACATCCAATAGTGAGTGACCACAAATCTCTTGGACCCTTATAGTCCAAGAGATCAGGACTGATATATATAATTAATTAAATTTCAAGTCCGAGAAAATTAATTAAATTTCAAGTCCGAGAAAATTAATTAAATTTCAAGTCAACTTCCAATCATAGCTGTAGCCAATTTCTAGAATGGTAAATGCCCAGTTTATTAAGCTCATGATTCACAACAATCTGTAGCTCAAAGAATGACTTTCCCCCCAAGGCTACCTGTATTTATTGTATAACCCCGATCAGGCCAGGTGCCATCTTGGACATTGTTCATATCTAATGTTTTGATATTCCTGTTCACATTGTATGAATACTATCTATAAGTTTTCAAGCATAAAAATCTTCATCAAAATTAAGCCATTTGTCTACTTTCAAAGATTTAAAGAATTTCAATAGTTCCAAAAGGATAGCTGCCAGTTGTAGAACATCATTTTTAATCATGAATGGTGATTCAAATTAAAGAAATTCGAAGCTCATCTAATTGGTAAAAGTGAAGGTTGATCAGTGGAACTTGCCTTCTTCATGAGATAATCAACAAGTGTTGTGGCATCAAATTTTTACTTTGTATTCTCTCTCTATTAAAACGATAAAATTTCTTAATGAAAAAAAGAAAAAAAAAACAGTAATAGTCAAAATTTTCAGATATGAGAAGATTAAAATCCCTAAATCAAACCCTCAAAAGAGATGGAGGTCTTTTCTAGTTTCATGAAAGAGAGGGAGGGAGGAGACCCTTGTTGCAGATAAAGAGAGAAAAAGAGATCGATGTTTTTTATGGTCTTATGAAAGAGAGTGAGAGAGATGTGTGCAAGAAAGAAGAGAGATTACCAAATTTAA

>Chr2-17; OZ022207.1; 8060306-8065790; dir; B;

ACTTGAGAAAAAAAAAAGAAGCAAAAGGGAGCAAGGGTATACTGGAACTAATACACACAGACATTTGTGGACCTTTTCCAACTCCAACTCATGAAGGGTTCAACTATTTTATAACTTTTATAGATGATTACTCTAGATATGGACATGTTTTTCTTATTAAAGAAAAAATTCAGTGCATTGGACATGTTCAAAATCTATAAAGTAGAGGGAGAAAAATATTTGGACTTAAAGATCAAAGTGGTTAGATCTGTCAAAGGAGGGGATTTCTATGAAAGGTTTGATGAAACTATGGTCATTTGCTAAGTTCCTGCAAGAAGAAGGGATTGTCGCACAGTATACCAACCTAGGCACTCCACAGCAGAATAGGATTGCAAAAAGAAGGAACAAAACCTTAAAAGATATGATAAGGAGCATAATGTGCTACACAAAATTGCCAATCTTTCTATGGGGAGAAGCATTGAAAACTGCAAACTATACATTGAACAGAGTACCTACTAAAAGTGGCTGAAAGAAAATGAAGTTTAAATCACCTCAGAGTATGGGCTATCTGGTTGGATTCAAGAACAATCCTATAGAAAAGAAGTTGGATTCGAGAACAGTCAGTGACTATTTTGTTGGTTATCTAGAAAGAACTAAGCATTGACACGTTTAAAGCATTTATATAACAATTCACACGTTTAAAGCATTGACACGTTCTCGCAGGTCTCGGTAACACAAAGTCGACCCGAGCCTCAATCCTCTCAGGATTCATGGGACATTTAGTCAACTTGATCCGCATTCCTCGCTCCACACGGTCCGGATATCCCTAAGACTCATGGAGCAACTATCAACTGCGCTGACATAACCGAAGGCAACCATTTATTCTGAAGGCAGCATCACCTATGGTGGTTTAAATAAATATTTCCTAGAAAATCATAAAAACTATTTCTCATCCATCCTTAAATTCTGTTGCCACTATCCTTCCTACTTTCCAAGCCATGCTCTCCTCTGAATCACCCTT

TGCTTTTCATAACTCCTCTGAGTCTGAATCAATTGCCAGTATTGAAATAGAAACTAGTAATATTTCTAAAATTGAAAATGCATTTAAGAATCTTGAGTTAGAAAATAATCCCCAAGTCCAAAGATTAACAAACAGAGTCAGCCCCACAAGTCTCACAAAAAATTGGTATCCTAAACCAACACCTCCTGATATACAATTTGAAGAACGAAACACACAGAGTCAGTTTTCAGTCTCTTCAGATAAGTTATACGAATGAAATATTGATGGTCTTTCTGAGCAAGAAATCCAACATATGTCTATGGTTGCCAATAGCTACATCACCAATCACAATTTCAGACAGTCTGAAATTGTTCCCTTATTAGAAACTGGTTTCACTGGAACTCTTCGTTCTTGGTGGGATAAACATTTAACCCATGAGTCTAAGCAACAAATAATTCATGCAGCCAAGCTTAATGATGATGGTCTTCCTATTTCTCATGAACAAATAGGTCAAGGCATTGAAGATGGAGTCAACACCTTGCTGTACACAATAGTTGATTATTTCATTGGAACACCTAGTAATACTACTGCTAGAATACATGATCAGCTTAATAACCTACGATGGTCTTCCTATTTATCCTATTTTAGATGGTATAACGATGTATTTATTTCTAGAGTCATGATTAGAGATGATAGTAATCAGTCATTTTGGAAAGAGAAATTTGTTAATCGTCTGCCTAGTTTATTCGCTCATAAGATCCAATCAACCCTTAGTAATGAGCAAGGACACATTTATGGAAACATAATAAGCACCATTAATCAAGTCGGTATGAGAATGTGTGTTGATATGAAAATTGGTAAACATATCCAGTCTGAAAGAAATTATGCTAAATATGAGTTAGGTAATTTCTGCGAACAATACGGTCTAATGTCTATTCCCCCGTCTAGAAAGAATAAGCTCAGTCACCACAACCGACAGTTTAGTAAACGTCGCCATTATTCACGTAAAGGACAGTCATTCCAACATAATAATGATGATAAAGAATTTTATAGTAAAAAGAGATTCTCTCCTAAGAAAAACTGGAAAAATAAAGGTAAAGATAGTCATTTTAAGAAAAAATCCGACAAGAGAAAAGTAAAATGCTATAAATGTCAAAAATTCGCTCACTATGCTAATAATTCCAAAGTTAAAGACACAATTAAACAATTGAAAATCATGGATGAGGAAAAATACAAGCTGATTAAAGTCATTGAGTTAAGAAATTCTGAGTCTAGTGAGAATGAAACCATGGTTAGTCACACTGAGTCTTCTGAAATCTATTCTTTTGAGTCATAACCTAGTTCACCCCGAATTCAATTTGGTAGCCAAGATAATTGCTGTAAGTCTAAGTCAATTAATGTCCTTACCAAATAGAAAGAACAAGAAGAACTATTAGTTGATTTAATACGTAAAGTTGAAAACCCCGAATTAAAGTCTGAGTATTTAAGGAAATTAAGAAAAGTCATTAGTCAAGAAAAGCCGAAACAATCTACTTCCCAGAAAATAAGTCTGCACAAACCTTAGAGAAATTTAATAAAAAGAAAGAAGTCACCTTACATGATTTACAGTTAGAGGTAAAGTTAGTCAAAAAAGAAATTATTGAGTTGAAACAAATAAGTCAAAAGTTGCAAAGTGAAAATTATGAGATAAAACAAGATTTGATTAATTTATTAGAAAATAAGATCACTAAGCCTACACCTGCAAGCCCCTCAAATTATGATGAAGAGCCTTCCAATCAGGATCAAGCCGTCAACTTAATTAAACATGTAAATTTCATAAAATGGTATGCCCGAGTCACTATATTTGTCAAAAATTTTGAGTTAAACATAGTTGCCCTCTTTGATTCAGGAGCTGACCTTAATTGCATCATAGAAGGTCTAATCCCAACAAAGTATTATAAAAAGTCAAGGGAGTCATTAAGTACTGCCTTAGGAAAGTCACTCCAGTTAAATTATGAGATTCCTAAAGCCCATGTCTGTCAACATAAAATCTATTTTAAGACCTCTTTCGTCTTAGTCAAAAATATTACTGATGAAGTCATCTTAGGTTTACCCTTCATTGCCCTTTTATATGTTTTCCAGGTAGAACATGATGGAGTTATTTCCACATGTCTAGGAGAAAAGGTTAAGTTTCAGTTTATGAGTAAAGCTAAGTTACATAATTTAAAAGAATTACAGAAGAATGCCGTTACCAAAACAGTCAGAGTCATTTAGAATAAAAATAAGCAACTAGAATTCCTTCAGGAAGATGTTAAGCCAAAAAGAATTGAGCAACAACTTAGTCAGGAGTCTTTACAATCACAAATCCGACAGTTTGAAGAAAAATTAAAACAAGAAGTATGTTCTGAGTTACCCAATACTTTTTGTCATAGAAAACAACATGTAGTCAGCCTTCCTTATGTCAAGTCCTTTAGTGAGAAAAATATCCTTACTAAAGCCAGACCGATACAAATGAGCCAAGAATTAATGGAGTTTTGTAAAACTGAGATCATAGAATTGCTTGAGAAAGGAATAATCCGTAAGAGTAAGTCACCATGGTCATGTCCTGCTTTCTATGTCCAGAAAAATGCTGAATTAGAACGCGGAGTCCCGAGATTAGTCATAAATTACAAGCTGCATAATACTATCTTAGAATGGATACGGTATCCAATCCCTAATAAAAGAGATTTAATCAACAAATTAGGAAAGTCAGTCATCTTTTCTAAGTTTGATATGAAGAGTGGATTTTGGCAAATTCAAATTCATGAGTCTGATCGATACAAAACTACTTTTGTTACCCCTTTCGGTCATTATGAGTGGAATGTTATGCCCTTTGGTCTTAAGAATGCGCCCACTGAATTCCAAAATATAATGAATGAGATTTTTAAATCTCATAGTCACTTCTCTATTGTTTATATTGATGATGTGCTTATCTTTTCTCAGTCGATAGAACAACACTGGAAACATATGCATAAGTTCTTACATATAGTCAAACAAAATGGATTAGTTGTTTCTGCTAAGAAAATTAAGTTATTCCAAACTAATGTGAGATTTCTTGGGTTTAACATCTGCCAGTCATAGATTAGCCCCATAGATCGAGTCATCCAGTTTGCTGATAAGTTTTCTGATCAAATCCTAGACATAATTAAACTCCAGAGATTTCTAGGATCTCTAAATTATGTTTCTGATTTCTATAAGAATTTGAGAAAACAATGTAAGCCTTTATTTGATCGCCTCCAAAATAATCCTCTACCTTGGTCATCTATTCATACTGATATCGTCAAACGGATCAAAGCTCATATTAAGACACTTCCATGTCTTGATATCCCTTCTGCTGATGCATTCAAGATAGTTGAAATTGATGCCTCTAACATTGGTTATGGAGGTATTCTCAAACAAAGAGTCAATTCTAGTCAATCTGAGCAAATCATTCGATTCCATTCTGGTACTTGGTCGCAGTCTCAAAATAATTATAGTACTATTAAGAAAGAGATATTATCTATTGTATTATGCAGTAATAAATTTCAGTCTGACTTATTAAATCAAAATTTTTTAATCAAAGTTGATTGTAAGTATGCTAAGCATATATTAGAAAAAGATGTTCAAAATATTGCATCCAAACAAATATTTGCAAGATGGCAAGCTATTTTGAGTATATTTGATTCTGATATTGAGTATCTGAGAGGAAGTGAGAATTCTATCCCTGATTTTCTTACCATAGAATTTCTGCAGGGAAAACAATGGCAGAAAGAAGAAAAGAAAAAGACAAGCAAAAGAGTCAAGTTGGTCAGTCTAGTCAACCCTAGTCACTCCGAGTCCTCCCTCCATCAATGAGTCAAGCCAAACATGAGTCACCTTCCCAAATCATCCCTTTCCCGGGATGTTCTCCTATCCAAATTAGTAACAAATTTGCCAATCTAGGAGCCACAGTAGGCCAGGTTCGCCCCAATTTTCAGTCTGCCTTAATATCCAGTCATGATCCCTTCCAGGATAGTGCTCTGTCTAATCTTCCAGTTGCCTCTTACCATAAGACTTCCCCATACATGATCAAGAGTAATAGTCACCTGTTCATAATTGAGTCAAAATATAATTCCATTGCCAACCCAATCACCATTGCCAAATCCTACTTTCCACCAAATAGTCATTTCATTCCCCCAGCACCATTCAAAAATTTGAAATTTTACAAAGACATCCTCCATGAGACTCAGTCAATCACAATTAAGCCAATCAAAGACAGAGATAATCTCGATATCATCCTCTATCATTCTCTTTACATCTACCAAATAATTAGTGAAAATGAGTAAAGCAGTCACCCCTATGATCTCAAAGTTCTTTAGTCAGAACTTCAATATAGTTACTATGATTATATCGAAGTCTGGTATTCTATCTTGCTTCACCAAACAGTTGATTTTTGTCATTCATGGTTCCTAAATTTTGACAGCAAATTCAAGAGTCCCTTCCCCTACTGGTTCAATCACTTGTAGGAAAAACATGGTCCAATCATTGATTTATTTCCTCCTCCAATAAAAGATTTAGTCAATTATTTCACTAATAAAAATAAGTTTAAAGAGTCTGAGTTGTTCTTCCCAAATCTTCTCCATTTTGTTGCCAAGTACAAAGTCCCTTGGATCTTCAAATGGTCATATCAAGTTAATTGGGAAACCAGAGTCTTATCCCATCATTTTTCAATCAAATGCTGGGATAAATTTAAGCTTGAGCATATTGCAGATTATGTTTATGCTGATTTTCCTCCAGTCAGGAATCCACAGCCTATGAAACAGTCATCCCCTGCTACTTCACATTATTCTCTCCCAATCGAGGGAAAATCTAAGTCTGAATTGCAGGAAATTGCTCGCCAATTAATGTTACAAGCCTCACAGATGAATGAAGATGGTGATAGTGATCCCACTCCCAAGTCACAGTCATCTACCAGTCACCTAAAGTCATCCAGTCAAAAGACTCCAGCCCCCAAAATGCAGTGGAGTGATTACCCCCTGATGATCAAATCCATGGTCAGGACCCTTATGATTTCGGTCCCTTCAACCTAAGCGATGATTAAAGTTGTCAATCTTATGAGCCCTGAGACAAAATCATTTGGAATCTTGGACCTATGCTTCAACAGTCTGATAGCTCGTGCTCTGTTAGTCAGAGTCCCCGACAAAGTAAAAGTCATAGCACTCAGTAAAGCAAAAGCTTTCGCGTGCTTCTGCAGTCACTTTTCTAAGCATCCAATGTCGCTAGCTCATCAATAGTTACCAGAGTTCACTTTATTTGCTTATTTAAGAAGGCCTAGGCCTCAGTTATCCACAGAATGTAATTTAGTACACTATAAATTTCTCAGTTTCAAAGTTCTATCAGTATCAATGCTTTGAAGAACTAAGTGGTGAAACTAGGATCTTTGTAAGTGTTTTGCATTCTGCAAGTTATCAAGTTATCAATAATATTTTGAGTATTTTGAGTAATGCTTATTTTGATTGCATCTATTTTC

ATTTATTCATTAATTTGCTTCATGCATAAACTGCGTCTAGTCTATAATCTCTGTGCTTATTCATACTTCTGTTTTTATTCTATTTCTTTCCAGTTCTAGACCCCTTCTGATATATATATATATATATATATATATATATATATATAAGCAGAATGTGATAAAAATATTCAAAATCAAACAATCATGCTTGGAAAGTAAAATTCCTAACAACTCATTCAAGATCAATTAGTAAAACTTAATTGCATAAAATCACTTATAAAACAAAAGCCCACTCACTGGACTTCCTCTAATTCATAAATCATTTTATGTATTACTAGCTTAGATTAATATTTATTTATATATATAATAAAAATAAAAATAAACAAACAACCGCCGAAGACTTTAACCTTCCCTTTGAAACTTACATAAACCCAATTATAAATATATATATATATATATATATATTATATACAAATATATAAATATATATATATGTGGATTTATAATTAACTTATCACAGATAAGTTAAAAAACGAAAAAATTATTAAAATAATATTGACCACGTGAAATCTTAATGTAAATAATAACAGCTTAACTGAAACATTCATTTCCTCTTTTACTTATAAAACAAACTCATTGCTTAAGAAATTAATTGTGATCCGTCAATTCTAGTGATAATCCGTCAATTCCTTGCATCCCGTCAATTCCATCCTCGCAAGCCTTGGAAACACAAAGTCGACTGAGCCACAATCCTCGCAGGTCTCGGAGACACAAAGTCAATCCGAGCCTCAATCTTCGCACCACATGGTTTAGGTGTTCCCGAAACTCGTGGGGGATTATCAAAAATAGCAAAACTGTTTAAGGCAACTAGCGGGTACTCTTGTGGTGGTTTTGAAAACCATATCCAAACTTATCACAAACTTTCCTTTATCTCACAGATCATTTTTCAAATAATTCCATTTCGATACTATAATAATGATCTTAATAGTAATAGATAACTAAATAGATAAATAATTAAATC

>Chr2-18; OZ022207.1; 8499951-8500717; rev; B;

CATACGTCGTAAATAATGACACAGACAGCTATATCTACGTCATCTTTTTTAGACCAATCGAAGTGGAAAACTTATTTCACGACGGTCTTTGTAAATAAGAGACGTAGTAGTTTTCAATAACGTCGTCTTCTCATGTATTTAAAGACGTAGTAGTTTTCAATAAGAGACAGAGTAGTTTTCATAACGTCGTGAAAATTATATTTAACGGCGTAATGTTTTAAATATCGTCGTTGTATGCCTATCTTGCGGCCTTTTTCTTCTAATATGTCATATTTTTCCTTTTTAACGACGGTGATTTGTTTGTGTTGGTCGTCTATTCTCATCCTCGACTATTTTTAAAAACTTATGCAGACTAAACACGTCTGTTTTTATTTTTTTTCGACGTTGTTTTATTTAACAACGTCTAATTTATCGTCGTTGTTGTTTCTGAAAATAGGACGTATAAATTTTGTAATACGACTCTCATATATTCGTAACCGACGTTGTTTGCTTTTTTCAACGTCTAATATATAAATACATACGTCGTAAACAATGACACTGACAACTATTTCCACGTCATGTTTTATAGAAAAATCGAAGTGGAAAATGTATTATACGACGGCTGTTTTTATATATTCGACGTAGTAGGAATAAATAACGTCGTCTTCTAATTTTCTTAAAGACGTCATATTTTTTGTTGTCGTGAAAATATATTTAACGGCGTATTATTTTAATTTGCGTCGTTGTATGTCTATTTTGCGGCCTTGTTCTGTTAATATGTGTCATATTTTTCCTTTCTAACGACGGTTTTTTTCGAGAGTTTGTCGTCTATTCTCATCCCCGACTGCTTTTTTCGTTCTTTTTGTAGAATAAAGACGTCGTTTTTTATTTGTTGATGACGTTTTATATTTTAACAACGACGGTGATTTATCGTCGTGGTTTATGAGGGAAAAGATGACGGTGGATTCCACGACCACTGGAAAACTCAATACACGACGGTGATTTACCGTCGTCCTTTTGC

TTATTTGTAGTAGTGAATTTTCATTATGTTCTCCAACATCCACCCAAGTCTCTTAAATACAATAGAGACATTCTCAATGAAACCCAGTCTGTGGAAATTAAGCCAATCAAAGACCGCAATAATCCTCACATCATCCTCTATCATTCCATGTATATTCATCAAATCTTGAGTGAAGAAGATTGGAGTAAACACCCTTATGATCTAAAAACCCTCCAATCAACCCTCCAATATTGCTATTATGATTACATCGAAGCCTGGTATACCATTTTCCTCCACCAAACAATTGATTTCAGTCACTCATGGTTTATTAATTTTGACAGCAAATTCAAAATTCCCTTTCTCTATTGGTTTCTTCATTGGTGGGAAAAACATGGCCCAATCATTGATATCCTTCCACCCCAAATGCAAGAGTCAGTTAATTATTTTTTCAATAAATATAAATTCATGGAGTCAGAATTATTTTTTTCCTAATCTTCTCATATTTGTAGCAAAATACAAAGTTCCCTGGATTTTAAAATGGTCTTACCATGTCAATTGGAATTCCTGAGTCTTATCCTGTCAATTTTCGGTCAAATGGTGGGATAAATTCAAAATCGAAAGAATCACATTGATCAAGTCAACATAGAGTTTCCCCCAGTCAATGCTTCCCAGAAGCCTTCCACCTCTGGCACATCTCACTCTTCCCTCCCAATCGAAGGAAGATCCAAGTTTGAGTTAAAAGAAATTGGCCGACAGTTAATGCTCCAAGCTTCCCAAATGGATGATGA

AGAAGATGATCCTGATGCTTCTCCAAAGTCCCAATCTTCTACCAGTTAGCCTCAAATTCCGCCAAGTCAAAAAGATTCTACCCCAAAACTTTGGTGGGCAGATTATCCTGATAGTCAAGATCCTTACAATCTCAGTGAAGACTAGTTAATAAAGGCAAAAGCCACACAGTTAATATTCTCCGACAGTCCTCATGAAAGTAGTCATCATGAAAGCAAGAAAAGGCAAAAGAAAAGTCAAAAGTAAGAATGATGCTCCAGTTAAAGCAATAGCGAAAGATCCATGCTTCAATAATAAAATTTCAAGCATCCAATGTTTGCCGACACTCTTGATTCGGCTCGTTAAATAGAGGCTTCAGTCTCAATTGTAGACAGATTGTAATCAAGTTTTATTTTTTCAGTCTTCAAAGTTTTTTCTGAGACTCCCTCAAGAAAACTACTATTGTAAAACAAAACAAAAGAGAGTGGGAGAGACACTCAACTCAACTGATTTTATGCTAGCTTTATAATTTTAGAGATAAAGTATTTTTGACTAAAACAAAAAATAAATAAATTGACTAAAAATAAAATAAATTGACTAAAACAAAAATAAAATAAAGTGATGTGGAAAATGTTTATTTAAAATTCACGTGAGTATTATTGTAAAAGACAAAAAATAAAGCAAAATAAATAAATTAATTAAAAACTGAAATCTACGCAGTCCGTGTACAGCATAGTGGGTTCTTAATTGTATGGTTTTTTCTTCAATTTCAATATAATCCGTGCCTGATTATTTTTCTTTTCCGATTCATTTACGAAGTTCAGTTCAGGCAGCTGACGATAGTAAAGAGAGTCTTTTTATATTATAACTGCTTTCCCTTCTAGGTTCATTTCCATTAATTTTCTTTCATTGTTGTTCTGAAATTTCATTTGTATTTTTCTTCACATTTCAGTTCTTGGCTCTACTGCTTTCTTCTGCTGGAATGGTTTTGGACAAAAGCATATCTGCAGCAGGCTTTGTTTC

>Chr2-19; OZ022207.1; 8512918-8524203; rev; D;

ACTAGCATTTTAATCATTTAACAGACAGAATTAGGTGTTTTGATCTTAAGGGAATTCCATCTCATATTGCCAATCATTTTGAGTTTCCGTTTAGTAATTTAAGACAGCATTCTGTACGTTTGACATTTGCAACGTTACACCCTATTGTACTGACTTGTTCTAACAGAAATACAATGTAACTATCATCTTATGTCGCTGGAAAACTGGTACTTTTAGGGTGAAAATGAAATATATTAAATAAACATACAAATAAAAATTTCCAAAGACTTCCAAAACTTCAAAAATAACCAAAACCAAGTACATAAACAGCTAGGGCACGAAAAATGGATGGAGAACCACACCTTTATTGACGACAATGGTTAGCGGAGTCGCCGGAAACGGCTACAGAAAAATCGCAAACCTCTCCGCCTTTCCGAGTCCGGCCGTCACAGGGAGGGGCTGTGCTGGTCGTAAAAGGTAAAGGATGAGGTCGCTGGTCAGTGGCCGTTATTTTCCGACTGGTCCCACGACCTGGCGTGGCCGGATGACGAGAATCGCTGCCAGTCTTGGTGCTGCGCGCAGAAGAAGAGAGAGGTATCGGGGAGAGGCAGAGGGAGAGAGAGAGAAGAGAGAATGACGCGTGCAGGGATGTAGGTATTTAGGGTTTCTTAAAACAAATTTTGAAATTCCCAACACGTCCTTTCCGTTTCTTGACTCGGATTCCACTGTTTTAACACGAAATTGAGCATGCCACCTGTCTACGGACTCGTATCGACGAGCTCTATGCAAATATGGGGCTCAATTTTCCAAATTCCTTCTCAAATAAAAAATGACTAAAATGCCCCTATACCCAAAGGAAAAAATGTAATTTCACAATTAAACAATTAAATAAATTGCTTAAATTGAGTTAGGGTATTACAACTATATATATATATTTGCCCACCGCAATTTCTGGGTCAAATCCTTTTTACTGGATGATTGTTGGGGTTGACTGGTAGATGTCTGAGACTTTGGGGAGGCA

TCATCTTCATCATCCATTTGAGAAGCTTGAAAAATTATCTGCCGGGCAATTTCTTGTAATTCTACCTTTGATCTTCCTTCGATTGGGAGGGAAGAATAAGACATTCCAGAAGTAGATGGCTTTTGGGAAGACTTTTGTGGAATTTTGACTGGAGGAAAATCTGCATTGACATAATCAGCAATTCTTTCGATTTTGAACTTGTCCCACCATTTGACTGAAAATTGATGGGAAAGGACTCTAGTATCCCAATTGATATTGTAAGACCATTTTGGTATACAAGGAACTTTATATTTAGAGATGAAAATAAGCAGATTGGGGAAAAATAATTCAGACTCTTTGAATTTGTTTTTATTGGAAAAGTAAGTGACTAATTCTTGCATAGGAGAGGGAGAAGATCAGTGACTGGGCCATGTTTTTCCCCCCAGTGGAGGAACCAGTAGGGAAAGGAACTTTTGAATTTACTATCAAAATTAATGAACCAAGGGTGACTGAAATCAATTGTTTGGTGAAGAAAAATTGTATATCAGGCTTCAATGTAATCATAATAACAATATTGTAGATCTGACTGGAGAATTTTTAGATCATAAGGGTGTTTACTCCATTCTTCTTCACTTAGGATGTGATGAATATACATGGAATGGTAGAGAATAATGTGGGGATTATTACGGTCTTTGATAGGCTTGATTTCAATTGACTGGGTCTCATGAAGGATGTACCTATAATATTTAAGGGATTTGTAAGGAGCTTGAGGACAAAAATGGCAATTTGGAGGAAAGTAGGCCTTTGCAATTGTGACTGGATTGGTAATATTGTTGTATTTGGATTCAATAATGAAAAGCTGAGTATTGCTCTTAGAAAGGTAAGGAGATGACTTGTGCAGAGGAATTGGAGATTGAGTCTCTTGGGTACTGATTTGAAAGGGATCATAACTGGAGACTAAAGCGGACTGATAATTTGGTCAAATTTGGCCGACTGTGGAACCAAGTGGATTGAACCTGTCACTAATTTGTATTGGCGAACATCTTGGGAAGGGGACGATTTGAGAAGGAGACTCTGATTTGACTGGGTTCATTGATACTGGAAGTATCCTGGGTGGCTAGGATGACTGGGGTTGGCTAGCTTGTTCTTTTTCTTTACTGGGTCTTCTTGCTGCCACGACTTTCCCTGTAAGAATTCTCTAGTAAGGAAATAAGGAATAGAATTCTCACTGCTTCTCAAATATTCAATATTAAAATTATAAAAAAAAAAAAAACTTAAAATAGCTTGCCATTATGCAAAAATCTATTTTGATGCAATGTTTTGAACATCTTTTCCTATGCTTCGCACTTTGCAATCGACTTTGACTAAAAAATTTTGATTTAATAAATCATCTTGAAATTTTGTAATGCATAATACTATAGATAAAATTTCCTTTTTAATAGTACTATAATTACGTTGTGCCTGAGTCTAAATTCCAGAATGAAATAGAACAATTTGCTCAGATAGACTGGGATTGATTCTTTCTTTAAGAATACCTCCATAACCTATTTCAGAAGCGTCAGTTTCAACTATCTTGAAGGAATTGACTGAAGGAATCCCAAGACAAGGTAGTGTCTTTACATAGATTTTAATCTGTTTTATAATTTCTGTAAGTACAGATGACCAAGGAGGGGGATTATTCTGGAGTCTATCAAATAAGGGCTTACATTGCTTTCGCAGATTCTGATAAAAATCAGACATAATTGAGAGAACCTAGAAACCTTTGGAGTTGACTCTTATCAAGAATTTGATCTGAAAATTTGTCAGAAATTGGATGACTCGATCAATCGGACTAATTTGTGACTGACAAATATTAAAGCCAAGGAATCGAATATTGGTTTGGAATAACTTGATCTTTTTGGCCGAGACAACTAAACCATTTTGTTTGATTATTTGGAGGAATTTATGCAGATGTTTCCAATCCTGTTCTATTGACTGCGAAAAAAATAAGAACATCATCAATATAAACTATGGAGAAATTATTGAAAGGATTGAATATCTCATTCATTATATTCTGGAAGTTACTATGCGCATTTTTTAGACCAAAATGCATAACATTCCACTCATAGTGACCAAATGGGGTTACAAAAGCGGTTTTATACCTATCTGACTCTTGAATTTGGATTTGCCAAAATCCACTCTTCATATCAAACTTAGATAAGATGACTGCTTTGTCTAATATGTTAATTAAGTTCTTTTATTGGGGATTGGGTACCTAATCCATGATCAACTTGGTAATCTCATATGTCCCAAATTATCTGATTTTCGTTGGTATAAAGATGTCTTCATTTCCAGAGTCATGCTTAGAGATGATAGTAACCAACCATTTTGGAAAGAAAAATTCATCAATGGTCTCCCCAATTTGTTTTGACAGGACCCGATCCAAATCCACTTTGGAATTCAAACCAAGCCCTGTGCGTGTCCGACACCTGGCAACTGTCGGGCACAAATGACCATTTTACCCTTCCTGCTACAGTTACTATTAAAACTTTCTTTGGACTCCTGCCGAAAATTCGGCAGAGTCTCCCCTGTATTTTGACCAAACCCAAAATCTTCCACCTGTTAAACAAGCAAATAACTTTTCACCAACTGCCAGAATAAACAACCAAACATTCACCAGTTCAAGTTTTCCACTAAAACTAGATATCAGAGCATTTTCTACTAATTTACAAGATTTCAAGAACTTTTACTATAAAATCCTACGTTTCTTGGCGGTGCGGAAGCTAAGAGTGGCCCGGTGATGGATCTCTGCGCACTTCTACGCCCTGGGGGAGGAAAACAAGTTGAAAGTGTGAGTGGACGAAAATAATATTCTTGAAACAAATTTATAATCATCATAACCCCCATGGTAAAAATAAAATGATAAGTAAAGCTACAGTTCGGCTGTACTCAAACACGAGGCCTTCATTTAAACATACCTAAATCTATATATATATATATATATATATATGTTCGTATAGCTGAACAAATATATGAAAATATAAAACATGCATGAATATCGAGAAAACTAGTTTAAAATGACTGAAAACAATAATTATATAAAAGTACTGAAATTCCTTTAAAATTACCACATAAGTATACCCCTGTAAATCCGTCAATTACCCTGGCAGGTCTCGGCGACACAAAGTCTTTTCGAGCCACAAACTGGTAGGATTCAGGGGACTAGGGTCAGCCTGATCCACCGGCAGGTCTCGATGACACCAAGTCAACTCGAGCCGCTCTGGCAAGATACAGTGACCGTAGTCAGCCTGATCCGCAATCCTGGCAGGTCTCGGGGACACAGAGTCAGCCGAGCCTCAAATCCTGGCAGGTCTCGGGACACCAAGTCTGCCGAGCCGCAAATCCTGGCACTCACGGTCTAAGCGTCCCGGAAACTCGTGAGGCAATGTCAAGTGCACTGACAGAACTGTAAAACAGACTGGATGTCCGTAGACATCGGTCCGTCTGAGGGTAATCACCAAATAAAAATGGGTACGTGGTGGTTTTAAAATAAATTACTTTAGAAAAATCTGATTAATAGCTGAAATCTCAAATTATGCTGCTACCTCATCTGATTCAAACCTCAAACTCTGTTTCTGTCACTTTTTAGCATGTGAAAGTAAGCAGCACAGAATTATAGAACGATTACTGTACTAATCATGCTCGGAAACATAATAAAACTTATTCAAGATTAATTAATAAAATTCACTCATATAAACTCATTTATAAAAACTTATTTATATAAAATCAAGATAAAATCATTTATGTAAACTCATTTATATAAATCATTTATATAACTCATTTATATAAAATCATTTATGAAAGAAAGTCCACTCACTGCTGGTCCGAGCTAGCTGGACCTTTTGAAGGTCCCTCTTGTGGTACAGTCTGGCCTCTGGTGCCTAGTTAACCATGAATATTATATTAATAAAACTACTCAATAACAGAATTAAATTAAACACCCTGCCCCCGGCTCCTAGAAATACGTGCACACATTTAAAAATCACTCATATGCCCACTTTAGTCTTTCGAAGAATTAGAACGGCCTTGAAAGACCCGAAGTTTTACTGAGCGATAAACTGCCAGACCAGGGGATCCGAAACCCAGTGGGGTCCATGATCTCCGATGGCCAATATGGGATTCTCTAAAGGTTCCTCACGGAGAAAGAACCATATTCTGTGAATTTGGTCCGAAACGGACGGTCAGATTAGCCAAAATCGCGTTATCGCTTAAAATCCAAACCTTAGCCCCAGAGTTCGCAATTTCAGAGTATCCGAGACTCCGATTCACAATCCGTCAAATCCTACACGATCCTGAAATCATGTAGAACGACATATCCAAAATTGAGTGCAATCTGACAACTCGATGACACTGCACCTCGGGATCGCGTAATATGGAAAATCCGTTCGGGGCTCAAACGGACTCCAAATCAAGGTCTGCGAAATCCTATGCGCTCGTAACAACACGAGGATCGCAAAACTACACAGAGTCACTTCACTACCCTCGCCCACGCGCAGCCACACGCGCGGGCAGTTCGGGTGTCCAAAGCGATAACGGGTCGTCGGAAAAACTCAAAACCAAGACTACTCCTATCCTAAGTAAAACACCCCATTTGGAGTCACTTTTGTTCTTGGACATGCCCCAAAAAGTGGCCGAAAATGGCCGACCACGGCGGTCGGAGTTCGGCCGATTTTCAAGTTGAAAATCGAGTGATCTACAGCAAAAATTGATCGAAACCCAGCCAACCATCAGTTAGAGCACGAAAAATAGCTAGAATCCATACCTTACTTGAATAATTTGGTTGAGAAACGAAGGAGATCGAAGAGCTTGAAGTTTCAAATGAAAACCCGCGGTTTTCTCCAGTTTCCGGCGAGCTCCGGCCGAGACAAGGGCGGCGACGGGTCGTGGCGTGACGGCGAGGGAGTGACTGTTCCGACGGTACCCTTGCGGGAGATCGAGCCGGCCTGTGGTAGTGACGGGAGCGGCTGGAAGGGACGGAGGTCGCGCGGGAGAGAGAGAGAGAGAGAGAGAGAGAGAGAGAGAGAGAGAGAGAGAGAGAGAGAGAGAGAGGAGAGAGAGAGCTGAGGGGAGGAGAGAGAGAAAGGGATAAAAATCTGACTTTTTGTCTAAATTACCGTTTTGTTCTTCGCGGTATTTTGACCGTATTTTCTTCGTTACAACTCCGATTCGGGTCTACTCCATGTCTACGGACTCGTTTAGCCACGCTCTACGCAACGACGTAAGCGGAATTACTAAATTCCTTCTCGATCAAAAAGTAAAATTTTTTCCTATTAAATAATGCGAGGGCAAAATCGTCTTTTTGCTAGAATAAATTAATCCTAATGTTTAGATATTTTGTTACTTTTTAGATATTTTGTTTTGGGTTATTACATGTTTGCCCATAAAATACACACAATCCTTAGTAATGATCAAGGTCACATAGATTTCGATAATCTAACTTACGGTAATATAATCAGTACAATTAATCTGTCGGAATGAAAATGTGTGTCGATATGAAAATAGGAAAACAAATACAGTCAAAAAGAATTTCAGCCAAATATGAATTAGGAGATTTCTGTGAGCAATATGGTCTAGTACCCATTCCTCAATCTAGAAAAGCCAAGCCTAGTTATCATGGCCGACAAACCAGTAATGATAATAATGAATTTTACAGTAAAAAGAAATTTCACCAGCGTAAAAATTGGTCAAAAGGTCACGGAAAAGATAAGAAAAGTACTGACAAAAGAAAAGTTAAATGCTGGAAATATCAAAAGAATGGTCATTAAGCTAATGAATGTAAAGTAAAAGACACGACAAAGCAGTTAAAAATCACGGACGAAGAAAAAGACAAACTAATCAAAGTTTTAGAATTAAAAAAAATTCTAAATCTAGTGAAGCCAACAACACAGTTACAAATTCTGAGTCAGTAGATTACAGTTCCTCAAATTCCCAAATAAGTTCACCCAGAATCCAATTTGGTTGTAAAGATAATTGTTGTAATATCCTAACTAAGTCAATTAATGTCCTCACAAAACAGAAAGAACAAGAAGAACTATTAATCGATTTGATCAGTAAAGTAGGAAACCCATAATTAAAATCTGAGTATTTAAAGAAATTACGGAAAGCCATTAGTCAGGAAGAATTGAGTCAGCACATTCCCCAGAAGATAAGTCTGAACACTACCTTAGAGAAATTTAATAAGAAACGAAAATAAGTTACCTTACAAGACTTACAATTGGAAGTAAAATTAGTCTAAAGGGAAATAGTAGAATTAAGACAAATAAGTCACAAATTACAAGCTGAGAATTCTGAAATTAAACAGGAATTGATTAACTTAATGGAAAAAGACGCCTCTGAAGCTAAAAGCCAAGCAGAAAGCCCAGCCAATCCAGATGGTGAACCCTCCAATAATGACCAGGCTATTAACTTGATAAAACATGTAAATTTAATGGTATTCCAAGGTCACCATAGTTGTAAAAGATTTTGAATTAAACGCAGTCGCCCTTTTTGATTCAGGAGCTGATCTTAATTGTATCCAAGAAGGTTTAATCCCAACTAAATATTACAAGAAGTCAAAAGAATCATTAAGCACAACCTCAAGAAAATCCCTCAGTTAAACTATGAAATACCAAAAGCCCATGTCTGTCAAAATAAAATATGTTTCAAAACTTCATTCATTTTAGTCAAAAACATTACGGATTCAGTCATACTTGGATTACCCTTTATAGCCTTATTGTATCCCTTTCATGTAGAATACGGTGGAGTCATATATACCCATTTAGGAGAAATAGTTAAATTCCAATTCTTAACTAAACCAGAGTTACACAATTTAAAAGCTCTACAAAAACATGCAATATCAAAAACAGTTCAAGTAATCCAGAATAAAAATAAGCAATTAGATTTCCTTAAAGAAGAAGTCAAATTCAAAAGAATTGAGAAACGACTTAATAATAAGTCCTTACAGTCACAAATTCAGAAATTTGAAGGATAGTTAAAACAAGAAGTCTGTTTTGATCTTCCCAATGCTTTCTAGCATAGGAAACAACACGCTATCAGTCTCCCTTACGTCAAGACCTTCAGTGAGAAAAATACCCCTACTAAAGCCAGACCAATCCAAATGAGTCAAAAAATTATGGAGTTTTGTAAACAAGAAATTACAGAATTACTTCAAAAGGGAATAATTCGAAAAAGTAAATCACCATGGTCGTGTCCAGCCTTTTATGTCCAGAAAAATGCTGAGTTAGAACGAGGAGCCCCAAGATTAGTCATAAATTATAAACCCCTTATACAGTTTTAGAATGGATTAGGTACCCAATCCCCAATAAAAGAGACTTAATTAACATATTAGACAAAGCAGTCATCTTCTCTAAGTTTGATATGAAGAGTGGATTTTGGCATATCCAAATTCAAGAGTCAGATAGGTATAAAACTGCTTTTGTAACCCTATTTGGTCACTATGAGTGGAATGTTATGCCTTTTGGTCTAAAAAATACGCCTAGTGAATTCTAGAATACAATGAATTAGATATTCAATCCTTTCAGTAATTTCTCCATAGTTTATATTGATCATGTTCTTATTTTTTCTCAGTCAATAGAATAGCATTGGAAACATTTGCATAAATTCCTCCAAATATTCAAACAAAATGGTTTAGTTGTCTCGGCCAAAAAGATCAACTTATTCCAAACCAATATTCGATTCCTTGGCTTTAATATTTGTCAGTCACAAATTAGTCCGATTGATCGAGTCATCCAATTTGCTGACAAATTTTCAGATCAAATTCTTGATAAGAGTCAACTCCAAAGGTTTCTAGGTTCTCTCAATTATGTCTCTGATTTTTATCAGAATCTGCGAAAACAATGTAAGCCCTTATTTGATAAACTCCAGAATAATCCTCATCCTTGGTCATCTGTACATACAGTGTAACACCCCGACTCCAAATTAAACCCCTTATTTAAATTATTTCAATTATTTAAGGGAAATTATAAATTTACCCTTGAGATATGAGTATTTTGGTCATTTTCTTACCCGGAGAGAGTTTGAGGCAGTGACTAGTATTTTTGGATAGGTCGTACTGAGATGAGTTCGTAGACACACAGTGGGCTCGAATTGGAGTTGTAACAGGAGAGATATGGTCAAAAGAAACCTAGTGGCACAATCGTAATTATTTTGAAATGAGATTTTTATAAAAAATCAGATTTCTCTCTCTCTCTCTCTCTCTCTCTCTCTCTCTCTCTCTCTCTCTTTCTCTCTCCCCTCTCTCTCGTGCGCGCAGTGGCAACCGGCCACGTTTTGGCCTGCCGTTCTCTCCTCCGACCACCGATGATGACAGGACCGGTACCAAAACGACTGGGGCGTCATCCTCTTCCAACCCCAGCCAGGTCCAGCCGCCACTCGTCGCGATTTCGTCGAAAACTGGAGAAAAAGTGGGTGGTGTCGTCCGAACTTCTCTGCCGTCGATCTCCTTCCTTCGGCCATCGATTCCGACGAGCAACGTATGGTTTCTCACCTACTTTTCATGCTCTAGCTACCTGTTGGTAGGATTGGATCGATTCTTAGCGTAGGTAATTCAATTTACAAATTGAAATTTGGCTGGTTTTGGGATCTCGATTTCGGCCACTTCCGGTCAGATTTTGGGCAGGTCCAAGAACAAAAGTGGCTCCAAATAGGTTGTTATACCTAGGGTAGGAGTTTGGAGCCGTGGTTTTGAGATTTTCCAGCGATGCATAATTGCTTTGGGCACCCAGCGCTGCGGGAGCATGGGCGAGGGTAGTGAAGTTGTACTGTGTCTCGATGAGATCCTTAGGTTGTCACGAGTGCGTACGATTTTGCGGATCTCAATTCGGACGTCGTTTGACTATCAAACGGATATCGCCTATTGCGCGTTATCTGGGTTCGATACGTTAGGACCGTTGGATGGTCTCAAATTTAATATGTTAATCTAGGTATCTCTAGGATCGTATAGGAATTCACGGATCGTGAATCGGAGCCCCGGATGTTCCGATTTAATAATTTAAAGTTTATGTTTTATATTAATCGTGCGATCGTGAGCGATCTGACCGTCCGATCTGGACCAAACTTCCAGGACAAGTGTCCTATACCTCGTAGAACCCAGAGGAACTTTCGGATCGGAATTTGGAGGTAGTGGGCCCCTTTGACCAGGGTTATGGTAGTCTGACCCTTGGTTGACCGTGAGTCTCCCGGAGTAATCTCGCCTTCCCAGGAGGATTATCGGGTGCTAGACTATTGTTGAGGTTTACACAATATTAAAATTAATATATATATATATTTATATATGGTTGTCAAGGTATAAAATGAGTCTAGAATGTTAGTTTGAAGTGCTATACGCACTACCTTAGGTACCTCCCCGGATTTTGGTTACACTACAAGTACCACCTTGTGAAAGTTAGGTCCCACATGGCGTAGGTTATCCAGCATGCGGACATATCCCATATATGGCATTGGTTGTACCGGCGTATGGGGAGATATTGTGATATGATGTTCAGGTCTTGGGTGGCGTAGGTTATCCGACGTTGAGACAGACCCCATACTTGGCGTTGGTTGTACCGGCGTATGAGGAGATTATGTGATATTATGTTGAGGTCGCCTGTGGCGTAGGTTATCCGGCGTGTCGACAAACCCCATACATGGCGTTGGTTGTACTGGCATATAGGGAGATGTTGTGATATGATGTTCAGGTCTCGCATGGCGTAGGTTATCCGGCGTTGAGACAGGCCCTATACGTGGCATTGGTTGTACTGGCTCATTGGGAGATGTTGTGATAGTATGGTATGGTCGCACGTGGCATAGGTTATCCGGTGTGTTGACAGGCCCCATACGTGGCGTTGGTTGTACCGGCATATTTGGAGATTATGTGAAATACAGAGAAATGAAATAAGGTATTGCCTATGTGTGGAATTGGGTTTTAAGAAGAACTATGTGTGGCTTGATCTCTCAGGGAGGGTACGTAGGCAACCTAAGGTTATTAAGTGCAGCCGCGAACATAGATGTGATTGTGTTAGGAGGTTAAAACCTCGTATGTTGAAATTGAAAAAGTTAGATGATGTATGTTGAAATTTAGTAGTCATAATTTATATTTGAATTTATCGTTTGCCTTGTTTAAGGCATGAATTGATTTGCTAGCATGCTTGGTAGTTGAGAATTATTGGAAGGCTGAAGACCGTTTGTGTGAATTGTATGAAATTATATTGTGCATGATGCCAGTTGTGGATTTTAAATGTGATTTTATACAGGTTGTAATTTTGGGAAATGTTCAATTTATAGGGGAGACTCTGCCGAAATTTAGGCAGAAAGTCTCGTACCCTTATTCCATTTTCGGAAAAAATGTATAACTTTAGAAGTGGACCCGACATCGGGGTGATGTCGGGAATTTCCACAAGATTTGCCTCAGGTTTTGGGAAATTCGGGGCGGATCCTTTCATACATAAATTATAAAATAGATTAAAATCCATGTCAAGACACTATCTTGTCTTGGGATTCCTTCAGTCAATTCCTTCAAGATAGTTGAAACTGACGCCTCTGAAATAGGTTATGGAGGTATTCTTACACAAAGAATCAATCCTAGTCTACCTGAGCAAATTGTTCGATTTCATTCTGGAATTTAGACTCTATCACAACGTAATTATAGTACTATTAAAAAGGAAATTTTATCTATAGTATTATGCATTAAAAATTTCAAGATGATTTATTAAATCAAATTTTTTTAGTCAGAGTCGATTGCAAGTGCGAAGCATGTTTTAGAAAAAGATGTTCAAAACATTGCATCAAAACATATTTTTGCACGATGGCAAGCTATTTTAAGTAGTTTTTATTTTAATATTGAATATCTGAGAGGAAGTGAGAACTCCATTCCATATTTTCTTACTAGAGAATTTCTCCAGGAAAAACAATGGCAGCAAGAAGACCCAATAAGCAAAAAGAGCAAATGTGTCAAACCAGTCAACCCCAGTCACTTAGAACACTCCCGGCATCAATGACTCCAGTCAAAACAGAGCCACCTTCTCAAATCGTCCCTTTCCCGGGATGCTCCCCCATCCAAATCAGAAATAGATTCACTACCCTTGGTTCCATAGTCAGTCAAGTCCGCCCAAATTATCAGTCAGCTTTAATTTCTAGTTATGATCCCTTTCAAGTCAATACCCAAATTTCCTAGTCTCTAGTGCCTTTACAAAAGTCATCCCCTTATTCCTCAAGAAGTAAAACTCATCTTTTTATCATTTAGTCCAAATATGACAACCTTTCCAATCCAATCACAATTGCAAAAGCTTATTT

TTCCCCCAATCACTACTACAAAAAGTGAAAAACACGACCAGAAAACAACGACGGTCTAAGTGAAAACCGCCGTGGTTTCTAAAAAGACGACGGTTCTAAAAACACTGTCGTGTAATACCACCTTAGACAACTAAAAAATGGAGATTCAAATGGCTGTTCAAAAACAACGACGTACTTAATTAAACAACCGACGTCTATTTGAAACAGATTTTCGCAGTGTAAAATCAAAGCGAACAAAGAACGGCAGAAGTTATGAATAGACCGACGTAGAAATTAAAGACGACGATTTCAATAAAAACCGCCGTTTTTACTCATAAAATAACACGACGCGGTTTTCGTATAAGACGCCGTATAATTACAAACAAATCCACGGCGTCAAAATACAAAATACCGCCGTATTAAAATAATTTACAACGACGGAAAATATAAATATATCGACGTCATTATTATATAGTTCTACGACGTTATCTTTAAATTGTTCTATAAAATTTAACGTCGTATTTATTCATTTTATACGTCGTACCTTTAATTTAAACCTAACGACGTTAATTTAAATATTAAACCTAACGACGTTAATTTAAATATTGAAAAAGCACCTTTTCGACTCACACATACACAGTTTCCAAACCCGATTAAAAATTTGAATCTACAAGAGAAAACTTTTCGTCTTTTTTCCTGTCAGAGCACTGTCAGAGTATCTCATCGTCTTTCTCTCGTCTTCTTCGTCGTCTCTGAACGCAAAAGATCGATCTTTTTCCTCTTGCTTTCATCGCACGCAAAAGGTAAGCTTTCATTTTCACTATTTTGGTTACTGTTTTGCATGCTCATACGTTTTTTGTTTGAAAAACCTTTTTACCTTTTTTCTGTCCAAAATCATTTTACTTCTTTCCAACTTTGAAGACAACTACCTCAACTAAATAAGACGACGGTAGTTCTTATTTACGTGGTTAAATATGTAGACCACGACGGTTCGTCAGTCGTTCTAGCGTCGTCTGAAAAT

>Chr2-20; OZ022207.1; 9627074-9640462; rev; C;

TCCCGCTGTCCTAGAAGGATATAGCGCTGCTGATGGAATACCCTGTCAAAGATTCAAAGGCTACTAGTGGATATATATTTAATATTGCTGGTGGTGCAGTTTCATGGAAGTCTAAGAAACAGACTATTCTGGCTCAGTCCACAATGGAGTCAGAGATGATAGCCCTAGCAATGGCTAGTGAGGAAGCAGGTTGGTTGAGAAACCTACTCTTAGATACCCCTTTGTGGGAGAGACTAATACCAGCTGTTCTTATCCACTGTGATAGTACTGCAGCTATTGCAAAAGTTCATAACCATTATTATAACGGCAAGAGGCGACAAATACGTCGCAAGCACAGTACTATTAGAGAGTTTCTTTCTAATGGGGCTATAAGAGTGGATTATATAGGTCTGGTGAGAATTTAGCTGATCCTTTGACGAAAGGACTAGCACGAGAAAAGGTTTGGAAAACCACAAAAGGAATGGGACTAAAACCCCTAGAAGGTTAAATCACTTGTGATGGTAACCCAACCTATAGATTGAAAATCCCAAGAAATAGGTTCAATGGGTAATAACAAGTCATTTAGTGATATGAGTGGAGTCATGCTAGTGTTCCATATCCATCCTTAGAAGCATGAAATCCTGAAGCGATTTAAGGTTGAGTTGTTTATAACTCTTAATGAGATCTATACTCTATATTGAGTGGGATATCTAGCTATAAGAATATCTCTGATTGTCCCACCTATGTGAATGTGAAAGTGAGGCCGCCTTCTATGAAAATTTTGGGGGGAAATTTTCTAGAGCATTCACTATACTGGGCTACAAGCACATGACCGTAAAGTGCTGGCTTTTGAACTTCACCTTAGTAGGGCTATGTGTGTGGTGCTATTAGAGATAGAGTTCAAAACTATATGTTACTCTTGTATATTCTGAAGCTCCGTCACTATGTAAAGGTTCAAGTTGAAAAACACCTCTGCTTATGCACAGTCTTACGATATGTTGCTTAATGAATTTATTTCTAA

GATTAACAAGCTAATTATATTTCCGTATGTTAAACTATCCCAATCAATATGACCTTGTTCATTACTAAGGGTTGATCGAATCTTATGGGCGAATAAATTTGGCAGACCATTAACAAATTTCTCTTTCCAAAATGGTTGATTACTATCATCTCTGATCATGACTCTAGAGATTAACACATCTTTATACCACCTAAAATCAGATAACTTAGGGCATCTTAAATTACTCAACTGATCATGTATCCTAGCAGTAGTGTTACTTGGTGTTCCAATGAAAAGGTATCCTGTTTTACTTAGAAGACAGTTTTAGAAGTTTCTGTTCACTTTCTTCTTTTGAAGATCAACCCAAGAACCTTGAGAAATATGAGTCGCTTATTTAGAACCAACTCTTGCAACTCTAGCAGCTCTACAGTCAGTATAGGTAAAGTCCCAGAGATAGTTAATGAAGAACAATTAGAATATGAGTCTAATAATGAACTAGACGTTCGAGACTGGAACATACCTAAAGTCCCTAGTCAAAATATTTATAAGAAAAAGTGGTCACTCTCTAGTTTTAATACAACCACCCATGTAAAAATGGTTGAACAAATCTATGCTTTAAGTCAAGAATATGAGACCTGTCAGTTGCTTAATCTTGAGTCAATTAGAAAACACAAAACTGATAGAAACAATTTCCTTCATATTGGTCTAGTCCAAGTTGCTGTTAAGCCATTAACCAGATTAGGATTAAAAGCTTCTATCCTTCTCTGTTTACGTGATGCTAGATTCACTAATTTTAGTGACAGTACGCTAGGAATTATTGAGTCAAGCATTGCAAATGGTCTAGTTCATTTTGATTGTTATCCTGATTTCACAGTAAGTCTGAGTAATCGCCACATATTAAAACTCTTAACTCTGAACATAAAAACTAATGGTTATGAAGTCTTGCCGGGTACCCAGCCCTTAGCATTAGTTTATAGAATCTATTATAAAGTCACAGATACTAATATGAATTTCCAAGCTTTGAGTAAAAGTCCAAAAAATCAAACCTTGTTAATCCAGAGTCATACACTTGATGCCACTATAAGAGTCCCTCATTCCATTAAATGGTCGGATGTGTCCCACCCAGCTGATTGGACCCTAACCTCCGAGAGTCAACCAGTTCCTATTCAGCGTAGTCTAAATAATCTAGATTATATCCAACAATATCTTGATGGCACAGTTAAAATTAATTTTGGTGATCCACCTCCACAAAAGTCGACAGTACAGTTACAACAGTTACCCACACCTAGTCAATCACAGAGACATATCCTTGCAAGGCATTCCTTCGCAGCCTCTTCTTCCACTCTAGAACGAGATCTAGAGTTAGAAAAAGCTTTGATTGACTTAAAATTAGAATCACTGAGAAAAACTTCCCAAGTTACTCATCTTGTTTATGGAAAACTTCCAGAAGACACAAATTTGAGTCTTCTGATTCACCCACTCAATCTGATTTTGTTGAAGCCGGTTTCAAAGTCAATAACCAGCTACGAACACTTAATAAAAATTTTATAATTAACTGGAAAAAATTAGATAGTCACCTACATGCCCCTGAGAACCAACCCAGAAGAGATAATTACCACTCAGCCATCACCGACAGAGTCAACAGGGAACGGATTTTTAATAAATGCAAAAAATATATGCGCACTGCTAAAACTGAAATTTATTATCTTGATTTTGTAGAGAACCGTTATTTGCCTAATGAGTTGAAAAATTTAACCCAGGAAAAATGGGAAAAAAAATAGATCACACTGAGGTCCTTACTAGTCATCCACCTGTTGAATCTATCGTTATTCAGCACCAAAAATAAAAAATAATTGCCAGTCCTTTTACGATCCCTGATTTAGAAAGTAAAATGATAAGTGAGCAAAATAACTACACCAACCAGAGTCTGATTATCATAGGTAAACAATTAGATAAGATAGAGACAAAAGTTGACAAATTATTACCAAGAGAAATTGCACCTAAGCCACATGATTAGAAACCTTTAGTTACTTTCCAGGATTTAAAGTCGAGTCCAACTTTTAAGACAAAAGAAACAATAACAAAAATTGAAAAAATGTTAGAAGAATTAACGCCAGTCAAAAGAAAAGATAAAGAAAAGATCGAAACAAGCATTAAAGTCATAGGATCCTTTGAGTCACCCTTTGCTTTCCATAATTCTTCTGAGTCTGAATCAATTACCAGTATGGACATAGAAACTAGCAATATTTTTAAAATTGAAAATGCGTTTAGAAATCTTGAGCTAGAAAATAATCCTCAAGTCCAAAGATTAACAAATAGAGTCAGCCTAACTAGCCTCACAAAAAACTGGTATGCTAAGCCAACACCTCCTGATATACAATTTGAAGAACGAAATACTCAAAGTCAGTTTTCTGTCTTTTCTGATAAGTTATATGTATGGAATATTGATGGCCTTGCTGAACAAGAAATCCTTAATAAGCTTCAACATAAGTCCATGGTAGCTAATAGTTACATCACCAACCACAGTTTCAGGCAGTCTGAAATTGTTCCCTTGTTAGAAACTGGTTTCACCAGAACTCTTTGTTCTTGGTGGGATAAACATTTAACCCATGAGTCTAAACAACAAATAATTCATGCAGTCAAACTTAATGAGGATGGTCTTCCTATTTTCGATGAACAAATAGGTCAAGGCATTGAGGATGGAGTTAATACCTTGCTTTACACAATAGTCGACCATTTCATTGGAACACCCAATAACACTACTACTAGGATACATGATCAGTTGAGTAATTTAAGATGCCCTAAGTTATCTAATTTTAGGTGGTATAAAGATGTGTTTATCTCTAGAGTCATGATCAGAGATGATAGTAATCAACCATTTTGGAAAGAGAAATTTGTTAATGGTCTGCCAAATTTATTCGCCCATAAGATCCGATCAACCCTTAGTAATGAACAAGGTCATATTGATTGGAATAGTTTAACATACGGAAATATAATTAGCACAATTAACCAAGTTGGTATGAGAATGTGTGTTGATATGAAAATTGGTAAACAAATCCAGTCTGAAAGAAATTATGCTAAATATGAGTTAGGAAATTTCTGCGAACAGTATGGAGTAATGTCTATTCCTCCGTCTAGAAAGAATAAGTCCAGTCACCACAACCGACAGTTTAGTAAAAATGCCATTATTCACGAAGAAGACAGTCATTCCAACATAATAATGAGGACAAAGACTTTTATAGTAAAAAGAGATTCTCTCCTAAGAAAAATGGGAAAAAAAGTCACACAAAGATAAGAATATTCACTTTAAAAAAAGAACCGACAAGAGAAAAGTGAAATGCTATAAATGTCAAAAGTTTGGTCACTATGCTAATAATTGCAAAGTTTAAGACACAATTAGACAATTAAAAATCACGGATGAGGAAAAAGACAAATTGATTAAAGTCCTTGAGTTAAGAAATTCTGAGTCGAGTGAAAATGAAACCATGGTTAGTCACACTGAATCTTCTGAGATATATTCTTCTGAGTCACAACCTAGTTCACCCAGAATTCAACTTGGTTGCCAAGATAATTGCTGTAAGCCCAAATCAATTAATGTCCTTACCAAACAGGAAGAACATGAAGAATTATTAATTGATTTAATAAGTAAAGTCGAAAATCCAGAGTTAAAATCTGAGTATTTAAAGAAATTAAGAAAAGTCATTAGTCAAGAAGAGCCAAAACAGTCTACGTCCCAGAAAATAAGTTTGAACACAACCTTAGAAAAATTTAATAAAAAGAAGGAAGTCACTTTACAGGATTTACAACTAGAGGTGAAGTTAGTCAAAAAAGAAATCATTGAGTGGAGACAAATAAGTCAAAAGTTAAAAAATAAAAATTATGAGATGAAACAAGATTTGATTAACTTATTAGAAAATAAAACCTCCAAATCTACACATGAGAGTCCCACTAATTCAGACGAAGAACCCTCCAATCAGGATCAAGTCGTCAATTTAATTAAACATGTAAATTTTAGAAAATGGTATGCCCGAGTCACTATATTTGTCAAAAGCTTTGAGTTAAACATAGTTTCCCTCTTTGATTCAGGAGCCGACCTTAACTGCATCCAAGAAGGTCTAATCCCAACTAAGTATTATAAAAAGTCAAAAGAATCATTAAGTACAGTTTCAGGAAAGTCACTCCAACTAAATTATGAGATCCCTAAAGCCCATGTTTGCCAAAACAAAATTTGTTTCAAGACCTCTTTCGTCTTAGTTAAGAACATTACTGATGAAGTCATACTAGGGTTACCCTTTATTTCCCTTTTATACCCTTTCCAGGTTGAACACGATGGTGTTATTTCCTCCTGTCTAGGAGAAGAAGTTAAATTTGAGTTTCTGAGTAAACCCAAGTTACATTATTTAAAAGAATTACACAAGAATGCAGTTACCAAAACAGTTAGAGTTATTCAAAATAAAGATAAACAACTAAGGTTTCCAGGAAGAAGTTAAATCTAAAAGAATTGAGCAACAACTTAGTCATCAGTCTTTACAGTCACAAATCCAACAATTTGAGGAAAAACTACAAAGAGAAGTCTGTTCTGAGTTGCCCAACGCCTTTTGGCATAAGAAACAACACATTGTTAGTCTTCCTTACATCAAGTCCTTTAATGAGAAAAATATCCCCACTAAGCTAGACCAATTCAAATGAGTCAAGAATTAATGGAATTCTGCAAAACTGAGATTACCGAAATGCTTGAAAAAAGAATAATACGTAAAAGTAAGTCACCTTGGTCTTGTCCTGCCTTCTATGTCCAGAAAAATGCTGAATTCGAACGCGGAGTCCCAAGATTAGTCATTAACTACAAGCCACTTAATACTGTCCTAGAATGGATTAGATATCCAATCCCTAATAAAAGAGATTTAATTAACAGGTTAGAAAAGTCAGTCATCTTTTCTAAGTTTGATATGAAGAGTGGATTTTAGCAAATTCAAATTCATGAGTCGGATCGATACAAAACTGCTTTTGTTACCCCTTTTGGTCATTATGAGTGGAATGTTATGCCATTTGGTCTTAAGAATGCGCTCAGTGAATTTCAAAATATAATGAATGCGATTTTTAATCCTTATAGTCACTTCTCCATTGTCTATATTGATGATGTGCTTATCTTTTCTCAATCGATAGAGCAGCACTGGAAACATCTGCATAAGTTCTTACAAATAGTCAAACAAAATAGATTAGTTGTTTCTGCTAAGAAAATTAAGCTATTCCAAACTAATGTGAGATTTCTTGGGTTTAACATCTGCCAGTCACAGATTAGCCCCATAGATCGAGTTATCTAGTTTGCTGACAAGTTTCCTGATCAAATCCTAGACAAAAGTCAACTCGAGAGATTTCTAGGATCTCTAAATTATGTTTCTGATTTCTATAAGAATTTGAGAAAAAAATGTAAGCCTTTATTTGATCGCCTTCAAAATAATCCTCCACCTTGGTCATCTATTCATACTGATATAGTCAAACAGATCAAAACTCATATTAAGACACTTCCTTGTCTTGGTATCCCTTCTGCTGGTGCGTTCCAGATAGTTGAAACTGATGCCTCTAACATTGGTTATGGAGTTATCCTCAAACAAAGGGTCAATTCTAGTCAATCTAAGCAAATTATTCGATTCCATTCTGGCACTTGGTCACAGTCCCAAAATAATTATAGTACTATTAAGAAAGAGATATTATCTATTGTATTATGCATTAATAAATTTCAGTTTGATTTATTAAATCAAAATTTTTTAATCAGAGTTGATTGCAAGTCTGCTAAGCATGTATTAGAAAAAGATGTTCAAAATATTCCATCCAAATAAATATTTGCAAGATGGCAAGCTATTTTGAGTATATTTGATTTTGATATTGAGTATTTGAGAGGAAGTGAGAATTCTATCCCTGATTTTCTTACCAGAGAATTTCTGCACAGAAAACAATGGCAGAACGAAGAAAAGAAAAAAGATAAGCAAAAGAGTCAAATTGGTCAATCTAGTCAACCCAGTCATCCCCAGTCACTCCGAGTCCTCCCTCCATCAATGAGTCAAACCAAACATGAGTTGCCTTCCCAGATCGTCCCTTTCCCGGGATGTCCCATTCAAATTAGTAACAAATTTGCCAACCTAGGAGCCACAGTCCGCCAGGTTCGGCCCAGTTTTCAGTCTGCCTTAATTTCTAGTCATGATCCCTTCCAGGATATTGCTCTGACTAATCCTCCAGTTGCCTCTTACCATAAGACGTCTCCATACATGATCAAGAGTAATAGTCACCTGTTCATTATTGAGTCAAAATATAACTCCATTGCCAACCCAATCACCATTGCCATGTGAGATCCCACATCGACCAAACGGAGAGGGGGTGATGTGCCTTATATGGCACACCTCGCATCAAAATAGTGGGAGGCCTCTTGGGAGATCACTGGCTTCGGGTTCGTAGGAACTCCGAAGTTAAGCGAGTTGGAGGCTAGAGCAATCCCAGGATGGGTGACCACCCTGGGAAGTTGCTTCGTGAGCTCCCAGGAACAAAACCGTGCGGGCTGGTGAGAATGTAGCCAGGCCCAAAGCGGACAATATCCTGCTACGGCGGAGCCGGTCCGGGGTGTGACAGTTTGGTATCAGAGCCACTCTGCCGTGTGGTGCGAGTGTGCCGACGAGGACGTCGGATCCCTTAAGGGGGGTGGATTGTGAGATCCCACATCGACCAAACGGAGAGGGGGTGATGTGCCTTATATGGCACAGCTCGCATCAAAATAGTGGGAGGCCTCTTGGGAGCTCACTGGCTTCGGGTTCGTAGGAACTCCGAAGTTAAGCGAGTTGGAGGCTAGAGCAATCCCAGGATGGGTGACCACCCTGGGAAGTTGCTTCGTGAGCTCCCAGGAACAAAACCGTGCGGGCTGGTGAGAATGTAGCCAGGCCCAAAGCGGACAATATCCTGCTACGGCGGAGCCGGTCCGGGGTGTGACATGCCAAATCTTACTTTCCACCAAATAGTCATTTCATTCCCCCAGCACCATTTAAAAATCTAAAGTATTACCGAGACATCCTCCATGAGACCCATCCCATCACCATTAAACCAATCAAAGACAGAGATAATCCCCACATCATCCTCTATCATTCCCTTTACATCCATCAAATAATCAGCGAGAATGAATGGAGTAGTCATCCCTATGATCTCATAGTTCTCCAATCAGAACTTCAATATAGTTACTATGATTATATCGAAGCATGGTATTCTATCTTGCTTCACCAATCAGTTTATTTTAGTCATTCATGGTTCCTAAATTTTGATAGCAAATTTAAGAGTCCTTTCCCTTACTGGTTCAATCACTGGTGGGAAAAACATGGTCCAGTCATTGAACCCTTCCCCCCTCCAATGCAAGATTTAGTCAATTATTTTACTAATAAAAATAAGTTCAAAGAATCTGAGCTGTTCTTCCCAAACCTTCTCCATTTTGTTGCCAAGTACAAAGTTCCTTGGATCCTCAAATGGTCATATCAGGTTAATTGGGAAACCAGAGTCCTATCTCGCCAGTTCTCAATCAAATGGTGGGATAAATTTAAACTTGAGCGTATTGCAGATTATATTTATGCTGATTTCCCTCCAATCAGGAATCCTCAGCCTGAGAAAAAGACATCCCCTGCTACTTCACATTCTTCTGTCCCAATCGAGGGAAAATCCAAGTCCGAATTGCAAAAAATTGCTCGTCAGTTAATGTTGCAAGCTTCACAAATGAATGAAGATAATGATATGATGCCACTCCTAAGTCACAGTCATCTACCAGTCAACTAAAGTCATCCAGTCAAAAGACTTCAGCCCCCAAAATGCGGTGGAGTGATTACCCTTCTGATGGTCAAGACCCCTATGATTTTAGTCCTCTTGACCTCGGAGATGATTAAAGCTGCCCAACATCATGAGCCCTAAGAAAAGGCATCCAAACCCATGCTTCAACAGTCTGATAGCCCGTGTTCCTTTTATGAGATTCCCCGACAAAGTAAAAGTTGCAGCACTCACTAAAAGCAAAAGCTTTCGCGTGCTTCTACAGTTACTTTCCATAAGCATCCAAAGTTACCAGCTCATCAAACAGTTCATTTTATTTGCTTATTTAAGAAGGCCTTGGCCTCAGTTCTCAGTGGAATGTAATTTAGTACACTATAATTTTCTCAGTTTCAAAGTTCTATCAATATTAATGCTCTGAAGAACTAAGTGGTGAAACTAGGATCTTTGTAAGTGTTTTGCATTCTGCAAGTTATCAAGTTATCAATAATATTTTACTTTAAGTAATGCTTATTTTAATTGCATCTATTTTCGTCTATTCATTAATTTGCTTCATGCATAAGTAGCATATGTTCTATAATCTTAGTGCCTATACATTCATATTGTCATCCTGGTATCATTCCCACCATTTGATTGAACCAGTAAGGGAAAGGACTCCTGAATTTGCTATCAAAATTTAGGAACCATGAATGACTAAAATCAACTAATTGGTGAAGCAAGATAGAATACCATGCTTTGATATAATCATAGTTTTCCTATACCCATAAAAGGAGTGGCCAAAGAAGACCTGAATAATCCTGATCTTTGATCAAATTCTCACGTGTTATTACGTTACGGATATGAGTTCGGAATCATAAGTAGGAATTTCTTGAAAGCCGAGAAGCGTTATAGGGCGAGGGGCCTAGCGTCTTTCTCGGAATAGCTATCTAAAGTACCCAAGCCAAGGGTAAGGTTGATTTTTCCTATAACTCTATCAATTCCGATCGAATGCCCATCTTTAGAAAGCATTTACCCCGCAAGAGAGGAGGATTGATGGACATAGGCTGCAGATGGACCAGAATATGTTGTGGGACTTCTGACGTTCGTTCCTCCTTATCTTGCCCGGTAGGTTGTTACAGAAAGAGCCAAAGCAAAGCAAAGCAGGGACTGATTCCACATGGGGAAAGAAAGGAGTCGGGCTAAATAGTGTAGATAAGAGTTTTCAAGTTAGGCTTTTTTGAAAGAAAATGCCTTTTGTTGTTGTCGCGTCTTAGGGTCTTATCGGCTTTGAAGCTGGTGACCTTCTCCGGGGTGTGATGGAAGCAATCTCTCTCCTGTCGGTTCGACTGTTAATGGTTTAATGAATATCTCCTTAGGAAGAAAAGGCTCTTTGGCTCTTTACAATAAGCGCTGTTCGAGGAATAACCACATCCGCTGCTGTGAAACAGTCTTACAGTAAGCGCTCTTAGGCTAATAACCGTTGTTCGTTAATCTAGCGTTTTCGATTTTCACTAATCCGAATCTAGGGGTTGTTCACGAATGTCCTCTTTCATAAGAGCATGGTGGTGAGTAGGCAACGTCGTTTTTAAGTCATTGATCTCCTACTATACAGAAAAAGAATCCAATTATGTTATGTGCATTTTGCCGGGCTTAGCCCTAGATACCCTCCCTACCCCTTTCGACGCAGCAATGAGAACAGCAGGGGTTGCTTTACTTCTTGGCAGAGGAGTGAGTGACTCTTCTTCTTTTAGGCTGGCATGGTCTTAGAATGCACGATAGAAGTGCAGACTAAGAGCTGTTTAGCGGGATAAACCCTGTGGGATAACGATTCTTTTAAGGCATACCTCTCTTTCTGATTCTGATTGTCTTGCGAACCTTGCTTTAAATCTTTCACGTGGGCAATTGATAATGTCAATATTCTAACTCCTTTTTCTTTTTCGGAGGAAGCTTAAACAGAAAGAAGACGATTTTCGGGCCTTTAGCAGACGATTTTTCCACTTTCATTAGACGCTCTCCTGGCTTTAGCGGAATAACAGCAGTTGGTAATATTATAGGTAAGAAAGAAGCCAATGTCCCTAGTATCAGGAAGAGGGTTGACGAAATCATAGGGTGCACATTCATATGATTCTAGAAATTCCTTTTTCATGTCCGTTCCCAGGCGAAAGACGGCTATTCTTCGCATCTCGAAATTGAATTCCGTCTTAGCCGTGGAAGGCAGAAATCCTATCCCTTCCAGCTCTCATCCTAGGATAGTCTCAAGCAGAGGTCTTATAGGGAAGAGAGTTAGCAAGCGAGTAAGGTTCATTGAAGTTATGATTGGAGACCTTTGAGATCATAGGGATGACTGCTCCACTCATTCTCACTGATTATTTGATGGATGTAAAGGGAATGATAGAGGATGATGTAGGGATTATCTCTGTCTTTGATTGGTTTAATGGTGATGGAATGGGTCTCATGGAGGATGTCTCGGTAATACTTTAGATTTTAAATGGCGCTGGGGGAATGAAATGACTATTTGGTGGAAAGTAAGATTTGGCAATGGTGATTGGGTTGGCAATGGAGTTATATTTTGACTCAATTATGAACAGGTGACTATTACTCTTGATCATGTATGGAGACGTCTTATGGTAAGAGGCAACTGGAGGATTAGTCATAGCAATATCCTGGAAGGGATCATGACTAGAAATTATCTGTAAGAACTTATGCAGATGTTTCCAGTGCTGCTCTATCGATTGAGAAAAGATAAGCACATCATCAATATAGACAATGGAGAAGTGACTATAAGGATTAAAAATCTCATTAATTATATTTTGAAATTCACTGGGCGCATTCTTAAGACCAAAGGGTATAACATTCCACTCATAATGACAAAAAGGGGTAACAAAAGCAGTTTTGTATCGATCCGACTCATGAATTTGAATTTGCCAAAATCCACTCTTCATATCAAACTTAGAAAAGATGACTAACTTTTCTAATGAAAGGACCCGCCCCGAATTTTCCCAAAATCCGAGACAAATCCTTTGGAATTCCAGACATCATCCCGATGCCAGGCCCACTTACTAAAGACCGAGACTTTCTGCCGAAAATTCGGCAGAGTCTCCCCTATAAATTAGACATTTCCCAAAATTTCACCTGCATAAAAACACATTTAATATCCCAACTGGCAGCATGCACAATATAAATTCATGCAATTCACACATATGGCCTTCGGCCTTCCATTAAATCTCAACCAACTACCAAACAATTCGAATACGAATTATGACTAATATTTGGTCTGCGGCTGCACCTAATGACCTTAGGCTGCCTACGTACCCTCCTTGAGGGATCAAGCCACACGTAGTTCTTCCATACAACCAATTCCACACTTAGGCGATACCTCATTTCTTTCTCTGTATTCCACATAATCTCCCCATACGCCGGTACAACCAACGCCACGTATGGGGTCTGTCAACACGCCGGATAACCTACGCCACGTGCGACCATGCCATACTATCACATATCTCCCCATACGCCGGTACAACCAACGCCACGTATGGGGTCTGTCTCAACGCCGGTTAACCTACGCCACGTGAGACCTACACGTCATATCACATAACTCCCCATACGCCGGTACAACCAACGCCACGTATGGGGCCTGTCCCAACGCCGGATAACCTACGCCACGCGGGACCTCAACATTATATCACAAATCTCCCCATACGCCGGTACAACCAACGCCACGTATGGGGCCTGTCGACACGCCGGATAACCTACGCCACGTGCGACCTCAACACAATATCACAATATCTCCCCATACGCCGGTACAACCAATGCCACGTATGGGGCCTGTCTCAACGCCGGATAACCTACGCCACGCGAGACCTGACTTTCACTTGGTTGTGCTTGTAGTGAATCCAAAATCCGGGGAGGTACTTAAGGTAGTGCATAAAGCACTCCAACTGACATTCTAGACACTTTTGTACCCTTGTACAAACATAATTCTAATTTATAAATATACAAATATTCTAATATTGCGTAAACCCAACATTAGTCTAGCACCAGATAATCCCCCTGGGAATGTGAGATTACTCCGGGAGACTCACGGTCAACCAAGGGTCAAACTACCCTAACCTTGGTCAAACGGGCCCACGGGGACCACGACCTCCAAACTCCGATCCGAAAGTTCCTATGGGTTCTATAAGGTATAGGACACTTGTCCTGCAAGTTTGGTTCAGATCGGACGGTCGGATCGCTCACGATCGCACGATCTAACGGTTAATATAAAATATAAACTTAAATTATTGAATCGGAACATCCGGGGCTCCGATTCACGATCCGTGAATTCCTACACGATCCTAGAATTACCCAGATTAACATATATTAAATTTGGGACCATCCGACGGTCCCAACCTATCGAACCCGGATAACGCCCGATAGGCGATATCCGTTCGATAGTCAAACGACGTCCGAATTGAGATCCGCGAAATCCTACGCGCTCGTGACGGCACGAGGACCTCAAAACTGCCCAGAGACACGCCACCACCCTCGCCCACGCGCCGCCACACGCGCGGGCAGACCGGGTGTCCAAAGCGATTACGGTCCGTCGAAAAATCTTGGAACCGAGACTCCAAACTCCTACCCTAGGTAAAACACCCCATTTGGAGTCACTTTTGTTCTTGGACAACCCCCAAAAAGTGGCCGGAAATGGCCGATCACGGCGGCCGAAGTTCGGCCGAATTTCAATTCGAAAATCGAATTGTCTACGCTACAAATCGATCAATTCCTACCCAACCATCAGCTAGAACATGTAGAAGGGATGAATTTCCATACCTTGATCGCCAGAAATGGCGGCCGGAGGAGGGAGATCGGAGCCGGCGAAGTTCCGGGCGGAAATCGTCATTTTCCGGCGGCTGGAGCGGCGGCCGATGTCGGGGGAGGGCTGGGTCGTGACGCCGAGGCCGAGCCGGTCCTTTTGGCACCGGTCCCGACCGCAGCCGCGGCCGGTGGCCGGAGATACGAAGAGAGAGAGAGAGAGGGCCGACGGGAGAGAGAGAGAAGTCGGAGAGAGAGAGAGAGAGAGAGAGAGAGAGAGAGAAAACCAGATTTTTATAAAAAAAATCCCATTTTGAAATATTTACGGTTGTGCCACTGGGTTTCTTTTGACCATACCTCTCTCGTTACAACTCCGATTCGAGCCCACTACGTGTCTATGAACTCGTCTCGGTACGACCTATCCAAAAATACAAGTCACGGTCCCAAATTCTCTCCGGTTAAAAATGTGACTAAAATACCCTTAAATAATTAAATAATTAAATAAGGGTTAAATTTGGGGAAATTTGGGGTCGGGGTGTTACATCTAACCTGTTAATTAAATCTCTTTTATTAAGGATTGGATATCTAATCCATTCTAGGACAGTATTAAGTGGCCTGTAGTTAATGACTAATCTTGGGACTTCACGTTCTAATTCAGCATTTTTCTGTTCATAGAAGGCAAGACAAGACCAAGGTGACTTACTTTTACATATTATTCTTTTTTCAAGTAATTCGATAATCTCGGTTTTGCAGTTCCATTAATTCTTGACTCATTTGAATTCGTCTGGCTTTAGTGGGGATATTTTTCTCAGTAAAGGACTTGATGTAAGGAAGACTAACAACGTGCTGTTT

CCACCATCGTCTATTTTGTGTCGTGGTTTTACAATTTTGTAGTAGGTTAAATTCTGCACAAGGTTTAGTTCTAGAAATTAAAATGTGATGTGAATTTTCTACAAAAGGTGGTAATAAAAAGGATAATCTGGTTACACCAGCTACTTTGATACAAAAATCAACAATACACATGCTAAAGTTTATATGAAAAGAAGTACTCAAATGACTACAATATACATATGGCACACGCAATTCAGATCATAATGAGTTGTTCAAGGGTAATATCATTTGATCTTCTATTGGAAGAAGCAGTTGGGAACGATAAAGCCTGAGAAACCATGTGCATTGTTGGCCTCGATTGTGGATGGGAATGTCTGCATGCAATTGATAGCCTTGCAATGGTTATGAGTTCATCTTGAAGTTGAGGTGTAGGAGGTGGAAGGCTTTGGTCCAACACATCCTTGAGAAATATGTTTGCATAGGTGGTGGATGGAAACGAAAAGGAGGAGACGAAATTGCCTAGCTGCTTTCCTATAATCACTTCCAGTGCCAGCACTCCAAAGCTATAAACATCACATTTTTCTGTTACCTTCATTGTGTAAGCCAGCTCTGCGTGCATATTAAAGAAGAGCAAACCATTAATATAGATATATCCAGAATTAATTTTTTTTTATAGAAAAAACTTCTATATTTTTATATTTTACCTGGTGCTACATATCCATATGTGCCTACAAGAGCAGTCCAATTCGACGAGTCTGGATTCAAAAGCTTAGCAGTGCCAAAGTCCGAAACGCAAGGCTCATAGTCACAATGCAGCAAAATGTTGCTGCTTGTTATGTCTCGATGCACGATAGGTGGCGCACAATCATGATGCATATAACACAAAGCATGAGCTACACCCTTTACGATTCTCACCCTTGTACTCCAGTCCAACTTTTTAGCTTCATGCTCGTTGCTCAAGATTGCTGCCAAGTTACCTTTTTCCAGATACTCATAGGCCAAAAACGAGTGATGGGAACTT

>Chr2-21; OZ022207.1; 12442894-12449293; dir; B;

CATTACCCTCTTTTTCATAATTTCTTTGTCAACCCAAGAATTCCAACCACCACCGACAACATATGAACATGTCAATATTAATCAAATTATGATGATAATTGATAACATGTAAACACCTAATATCAAGGATAATAATCAAATAATATTACCAAGCATAATATAGTATTCACAATATTTAATTAAATCAATTTATGTAAAGCCTCCCCAAGAAAACCCCCACCTCAAATCCAAAGCTATTGTATAACTCCCAGTCGCAAGTCCAAACACTTTGAGCTTCTCCAAAATCTCACCACACTTTAGTTCTCACTTTTTTTTTTCTTTGTCTATGATTTTTAGGAAATTAAAGCAATTTGAAGAACCTATGATACAAGAAGTATCAGGTAGTGCTATTTATAGGAAGTTAAACTCGACAAAGTCCAAATAATAAATATAGATGAAAGCTTAATATGATGCCACACAACTAGTCTATAGTTTCCTAGACTACTTGGGTGGCTTGAAAGTAATTTGCCACCCCAAATAGCCTTATGTGTTCGCTTTGTAGAGTGCTTAGTGAGCATTCTTCAATTAAGGTTATATATATAAGAATATCTACTGTGCTTACACGTCTTGCTTACATATCCCACCTCATGCTTTAACTGGATTTGAGGACTCTATATTTGCAACATGTCAATTTTTAACATAATGCTTAGTAAGCATAAAGGCTTTTCTAAATGCTTTTGTAAAGTATATATATACTTGAAGCCTTGTCTTCTTTCCGATGCCAAATCTTTAAATTGAAGCCCCAAGTCATTTGTAAGGAATAAGTTTTTCTTAAGACTTTTCTTCAGAAGTTTTCCTTTCAAGTTTTCTTTTGTAATTCTTAAACACGAGTGCGAGTGATCAATAGAAGTGTGAGTGTCCTACGTGGCATTGCTTTCTGTAAGTTTTATTTTCATGTTTCCAAAGTTTTATTAAGTCAATAAAATTATCAAGTTAATTTTATGATCCTATTTTCGTTCCT

AAGCATTAATTTAAATGATTAATTATATATTCTTCCATGCAATTTGTGAAATAAGGATTCTATGTATTTATGCTTTAACTAAATTCTATTTTGATCTATCTCATTTCTGTTTTTCTTCCCTTGGTCTTCTCTGATTTGACTGAGTCTTCGGTTCTCCCTTTTTCTGGTATCAGAGCCGTAAGAGTTACGGTTGTGTCTAAGTCTGCTACTCATATCGCCCAAGTGGCTGATTTAGGTTTTCTAAGTTAGCAAGCTCCTCAGTTATTCTAAACAGTTATCTAAGAGTTGTCTGCCTCCTTAGGATTACTCTCTCAAAAGAACACGGTAAGACCAATAGCTGAACAGTAAGTCCCGCAGTAGAGGCTTGAGAGGTGTGGCATGAGTGTGAGAGAGAGAGAATGCTTTAGGATCCAGATAATCTGCTGAGTCATCCTCCATTACAGAGCGTGATCTAGAAGCAGATAGAGCCCTAATTGATTTCAAAATGAATGAATTAAGGAAACAAAAGCAAGTAGTCCAACCAAGTTACGAAAAAGCCCATGATGAGCCAGTTTTACCTAATCAACTTGAATCACCTACCAGAACAGATTTTGAAGGTTCCACCTTTGAAGTTTGTGATGCCCAATTACGAACTCTTAGTAAACCATTTAAAATTCATTGGTCAAAATTAGATGTCCATTTCCGAGCCCCAGAAAATGAATCCAGAAAAGCCAATTATCATGCCACTTTCCCCAGAGTTCAAAGAAAAGAAATTTTCAATTAATGGAAAGAATATTTAAAAAAGACTAGGTTAGAGTCACCTTATCTTCATTATGTCGAAAGCAAATATATGAGTCAAAAGTTAGTTACTTTAACTTAGGAAAAGTGGGAAAAAGCCGACAGTACCAAAGTTATCAGTACTCACTCCCCAGTTGAATCAATAATCAATCACAAAAATGCCAAAGTCATAGCCAACCCTTACAAAGTTGCTGAGTCAAAAGATGATTTAGATAAAAGAGTCATAGAACAAAATAATTATACCAACGCAAGCCTAATAGTCATAGGTAAACAGTTAGATAAAATAGAAACTAAAATTGACAAGTTAACCCCTGGTGAAATTACATTAAAGCCTAAGAAGGAGAAACCTTTAGTCAAATTCAATGAGTTAAAACCCGAGTCTTCCCTCAAAGTAAATTCAACCATGAGAAAAATTGAAGAAATGTTTTAAGAGTTAACCCCCGTTAAACCAAAACCAAATAAAGATAAAGACAAAGGAAAGAAGAAGAAACTTCAGCTAAAGTTTTACATTTTGAAGTTAATTCTGGTAGTGAGTCAGCCATGTCTTTAGATTCAATCTCTAGTCAAGAGTATGAAGCAAGTAATATCTCTAAAATTGAACAAGCCTTTAATAATCTTCAAAGTAACCAGAATTCCCAGAGTCTTAAAGTTAGTCGTTTAACAGGAAAGGCCAATCCCACAAGTCTTACCAAAAATTGGTATCCTAAGCCTACGCCCCCAGATGTCCAGTTTAAAGAAAGAACCCAAAGTCAGTTCTTAGTATCTTCTGATAAGCTATATGAATGGAATATCGATGGCTTGTCCGAGCAAGAGATACTCAACAAGTTACAACATATGTCTATGGTAGCTAATAGTTATGCCACCAATCATCAGTTAAAACAATAAGAGATAGTTTCCCTTTTACAAACAGGCTTCATAGGAACACATTGTTTCTGGTGGGATAAACATCTCACAGAAGATGCAAAATTAAGAATAATCCATGCCTACAAAACAAATGATGATGGTTACCCTATTTTTTATGAACAAATAGGAACAAGTATAGAAGATGGAGTCAATGTCCTTTTATACACAATAATTGAGCATTTTATTGGTACACCCAGTAGCAAAACAGCCAAGATTCATGACCAGTTAAGCAATTTATGATGCCCTAAGCTAAGTGATTTTAGATGGTATAAAGATGTCTTCATTTCTAGAGTTATGTTAAGAGATGACAGTAATCAATCGTTTTGGAAAGAAAAGTTCATAAACGGTCTTCCTAATTTGTTTGCCCATAAGATTAGAACAATCTTAAGTAATGAGTCAAGTCAAATTAATTACGATGAATTAACTTGTGGCAATATAATCAGTACAATTAACGAAGTAGGAATGAAAATGTGTGCGGATTTAAAAATTAGTAAATCAGTTCAAGCCGATAGAAAAAAATGCTAAATATGAATTAGGAAATTTCTGTGCGCAATACAATCTAGCCCCAGTTACCCCGTCTAGACAAGTCAAAAGAAGTAATGATCGACGCTTCAGTACAAATAGATATTATTCCCGTAGAAAACATTCCTTTAAACCAAATGATGAGTTTTATAGTAAAAAGAAGTTCACCCCTAGGAAAAGCAAATGGTCAAAAGGTTATTCAAAAGGTTACCGACAAAGTAAAAAGAATCAATTTAAGAAAAGTTCAGATAAAACAAAAGTCAAATGTTTTAAATGTAAAAGATTCGGTCATTTTGCTAATGAGTGCAGAGTTAAAGATACCATTAAATAGTTAAAGTTAAAATGTGATGATGAAGCAAGTTAATTAAAGTTCTTGAGTTAGCCGATACTGAGTTAGATCCTATGTCTTCAGCCAGCTCAGTTAATTATAGTTCAGATAGTTCCTAGTCTAGTTCCCCAAAAGTCCAGATAGGTTGTAAAGATAAGTATTGCAAAGTCTTAAGCAAATCAATTAATGTCTTAACTAATCAAGAAGAACAAGAAGAGTTACTAATCAATTTAATAAGTAAGATAGAAAATCCTGAATTAAAAGCCCAATATTTAAGAACGTTTAGGAAAGTCATAAGTCAAGAATAACCCAGTCCTAGCCAAGTCCCTACGATTAGTCTTAATACTACTCTAGAAAGATTTAATAAAAAGAAAGAAGTCACCCTTCAGGATCTCCAGTCATAAGTCAAAACAGTCAAAAAAGAAATTTTAAGCCTAAAACAGGTTTGTCAAAAATTACAAACTGAGAATTACGAAATTAAACAAGACTTAATTAAAATGTTAGAATTTAGAACAGAGTCCAGCAATCCTTTTGGCAAAGCCAGTTCCGAATCAGAACCTGTGTCTTCTGATAGTAACAAAAACAAAAAAGTCATCAAATTAATTAAGCGAATTAATTTTAGGAAATGGTATGCTCGAGTCAGTATTTTTATTAATGAATTTGAGTTAAATACAATTGCCTTATTTGATTCAGGAGCCGATTTAAATTGTATTCATGAAGGTCTTGTTCCAACCCAATTTTACAAAAAGTCAAAAGAAGTTTTAGGAACAGCATCCGGAAAAGCCTTAAAGTTAAATTATGAATTACCCAAAGCCCACATTTGTCAAAACAAAATATGGTTTAAAACGTCTTTTGTTTTAATTCAGAATATCACAGATGAAGTTATTTTAGGTTTACCCTTTATTTCATTACTTTACCCTTTCCAAGTAGAATATGATGGAGTTACGTCTAGTCACCTAGGTGAAAGAGTCAAATTTGAGTTCTTAACCAAACCAGAGTTACATAGTCTCAAGTAGGGGTGGCAATTTCGGATATGACCCGATACATGACTCGAAACCCTCACGAGAAATTAGCGGGTTCAACCCGCACGATAAAAAAACAGGTCAATTTCGAGTCAACTCGTTCTGATCCATTTAATAAATGGGTGGGTTTCGGGTCAACCCGTGTACAACCCGCTAACCCACTAAAAAATAAAGACAAATGGAGACTTCAACACACACACAAGGGGTTTTGAACCCTTACCATCCTCATTTCTCTCAAACATCTTATTCACCATGCTACAAATAACTTTGTGATTTTATGGTATCCCTTTTGAAATTTATAATGTTTCTTTTTCTTTTGTGTTTTCCTTTCTTTTCCGTCTCTCTTCTCATCTTTTTGTTTCTTTTTTTTCCTTCTTTTTTCTTTCTCATCTCTCTTTTTTTTTCCCTATTTTCCTTTCCTCTTCTCTCTTTTTTTTTTCTTTCTTTTCCTTCTCTTTAGTTTTCTCTCATCTCTCTCTCTCTCTCTCTCTCTCTCTCTCTCTCTCTCTCTCTCTCTCTCTCTCTCTTTCTTTTCCTTCTCTCTTCTCTCCTCTCTCTATCTTTTTTTTCTTTTTTTTTCCTTATATTTTCTCTACTCTCTCTGTTTATTTTAATTTAAATTTTTTCGGTTGTGAGTTTTGTAACATGCATGTTATTATTTATGAACCCGTTACACGATAAAAGACGACCCGAACACGACACGACACATTTATTAAATGGGTTGGGTTCGGGTTGAGCATTCTTGACACGTTTATTATCTCGACACGACACGAACCCGACACGACAGGACCCATTGCCAGCCCTCTCAAGTCGCTACAAAAGTTAGCAGTTTCTAAATCAGTCAAATTAATTTAGAATAAAACAAAACAAGTTAATTTTCTCAAACAAGAAATCAAGTTCAAACGAGTCGACCAACAGTTAGCAGATATGTCATTACAGTCTCAAATAATTAAGTTTGAAGAAATGTTAAAAAATGAAGTTTGTTCTGATTTACCTAATGCCTTTTGGCACCGAAAGCAACATGTAGTTACTTTACCTTATGTTAAATCATTCAATGAAAAGAATATTCCCACTAAGGCCCGTCCAATACAGATGAGTCAAGAGTTAATGGAATTATGTAAGCAGGAAGTCACCGAATTAGTTCAAAAAGGAATAGTTAGAAAAAGCAAGTCACCTTGGTCATGCCCAGCTTTTTATGTCCAGAAAAATGCAGAGTTAGAAAGAGGAGCCCCTCGTTTAGTTATCAATTATAAGCCCCTTAATGCAGTACTTGAATGGATTAGGTATCCAATTCCTAATAAAAGAGACTTACTTAATAGATTAGAAAAAGCAGTTATCTTTAGTAAATTTGATATGAAAAGTGGGTTTTGGCAAATCCAAATCAAAGATTCAGATAGATACAAAACTGCATTCGTAACCCCTTTCGGTCATTATGAGTGGAATGTCATGCCCTTTGGATTAAAAAATGCGCTTAGTGAATTCCAGAATATAATGAATGAAATTTTTAATCAATACAGTCAATTTTCCTTAGTTTATATTGATGATGTCTTGATTTTTTCTGAGTCAATTGAGCAACATTGGAAACATTTGAATAAATTCCTTCAAATAGTTAAGCAAAATGGTCTAGTTATCTCAGCCAAGAAAATCAAATTATTCCAGGTCAATGTCAGATTTTTAGGATTTAATATTGGTCAGCCTCAAATAAGCCCAATCAATTGAGTTATCCAGTTTACAGATAAATTTCCAGATCAAATTCTTGATAAAAGTCAATTACAAAGGTTTTTTGGATCCTTGAATTATGTTTTTGATTTCTATCAAAATTTAAGAAAATTATGCAACCCGTTGTTTGATCGCCTCCAGAGCAATCCTCCTGCTTAGACCCCGAGTCATACAGAAATAGTTAAACAAATTAAAATTTATGTTAAGTCACTTCCATGCCTTGGTATTCCTTCACTTAATTCTTTTAAAATTGTTGAAACAGATGTTTCTGAGTTTGGTTATGGTGGTATCCTTAAACAAAAGCTTACTTCTGGGTCACCTGAGCAAATTGTTCGTTTTCATTCCGGAGTTTGGAGTTAGTCTCAAGCTAACTACAGTACTATTAAAAAGGAAATTTTATCTATAGTATTATGCATTAGTAAATTTCAAGATGATTTATTAAATCAAAAATTTTTAGTCCGAGTTGATTACCGGTCAGCAAACACATGTTTTGGAAAAAGATGTTAAAAATATAGCTAGTAAACATATTTTTGCACGTTGGCAAGCCATTTTGAGTGTTTTTTATTTTGAGTTAAAATATTTGAGAGGTAGTGAAAACAGTATTTCTAATTTTCTTGCAAGAGAGTTCTTGTAGGATAAACAAGCAGAGCCATGTCCAGCAAAGAAAAAGACAATATGGTCAACCGAGTCAACCCAGAATTCTCCCAGCTACCCTGATGCCAATCAAACAAGAGTCATCTTCTGAAAATATGCCTTCTCACATTGTGCCCTTCCCAGGTCATTTTTCAATTCCAGTAAATAATAGATTTAATCCCTTGAGTCAACCTTCTCTATGCTCCAATTATTAGTCAGCCTTAACTTCCAATTATGACCCTTTCATTATTAATCCTCCTGCTTCAGTTTCTTCGTAGAGCCAAAGCCCAGTTAAAAGGTCTAATTATGCCACCCGAAGTCATTGTCATCTTTTTATAGTTGAATCTTACCTAGAAGCGCATCGTGACCCAGTCAAAATTGCCCAGAAGTTCTTCCCTCCTAATCTTCATTATTTTCCGAGTCTTCCTTCCAAGTCCATCAAGTTCTACAGGGATATTCTTTATGAAACCCAGTCAATAGATATTAGGCCCATTAA

AAACAAAGAAACAAATGCCACAATGTTCCATTCTCTTTACAATGTTCCTACGTGGCATTGCTTTCTGTAAGTTTTATTTTCATGTTTCCAAAGTTTTATTAAGTCAATAAAATTATCAAGTTAATTTTATGATCCTATTTTTCCTAAACATTAATTTAAATGATTAATTATATATTCTTCCATGCAATTTGTGAAATAAGGATTCTATGTATTTATGCTTTAACTAAATTTTATTTTGATCTAAATCATTTCTGTTTTTCTTCCCTTGGTCTTCTCTGATTTGACTGAGTCTTCGGTTCTCCCTTTTTCTGGTATATATATATATATATATATATAATAAACATTAAATAAGGCTGAAACGAATTGATATAGTTAAAGCCATGCAATAAGATTTGATAGGCTATACTAGTTAATAGCTAGATTCACGTGACTTGACATACACCACACATATATATATATATATATATATATTATCAATTATATAGTTTGCAAACTAATAACTATAAATATTAATATACATATTTACAATATTACCACTTAAGTCCCTATGTGTAAGAAGAGAGGCTGGGGTATTTCTATGGTTTTTTAACAATTTAAGAAATTCCCTATGTGTCTTTTGCGTTTTCAGACCTTTTATCCTTCGTTACAACTCGGAAATTAGCGTGCTATCGACGAGCTCTATGCAATATTACCACTTAATTCCCCAAATTCCTTCCCAATAAAAAAAAAGTAACTAAAATGCCCCTACCTAAAGGACAGAAATGTAATATCACAAAATAAAAATAAGTAAAATTAAATAAAAAGGGTCAGGGCGTTACTATATATTAAAATTTTAATATATATATTTTTAATTGTCGTATCCATGACGTATCGTATCCTAGTTTTTACAAATTTCCAGTATCGCCGTATCATTTCGTGTCGTATCGCCATGTTCATATCCGTATCCATGCTACATAGGTTTTTAATCTTGTTAAGCTATATGTTGGGAAAGATTGCGAAA

>Chr2-22; OZ022207.1; 13763995-13768781; dir; B;

ACCTGCCATTATATTGACAATTTTTAAAAACTATTCATACTATGAACCTGTAGGCAAAGAATTGTAATTCCATTTGAACACGCGCTCCATAGCTGGAATATTGGTGTTATCTTATTTTATAGGAATTTCAATGTGGGCAGCCATCTTTTTTTTAAATTTTTTTCGTTTCAACAAATGCCCCCTCAAGCATTATGTGGGAAACAACTTTTTGCGTGCAAGGCTTGACAACTTTCCTCTCTATTTTTAAGATATTCATTCATAGATCATTCTTACAAAAAATTAGACAAATCGGAAACCGTTTCGACATCCAATTGTGTCTTACAAAATCAATGAACACAGTACTTCAAGAAAGTACTAAAATTTCAATAACTCAATTGAGTGGTTAAGTGATATCGGATTCAAGTGATTTTTTGTAAAGATGATCTTTGAATGAGTATCTACATAATACACAGTTTGAATTAGTGAAATATAATCCAGAGTGGGACCTACAAGGGGTGTCCCTCAAATAAGCTTATTTGAAGGATCCCTTAGTGGAAGCTCTCTGTATATATATATATATATATATATATATATATATATATATATATAAACGCATTTTCAATCTTAGAAATATTACTAGACTTTTCTGAATCATCTGTTTCTAGACTCATTTCAGAATCACTATTATTATACAAGGAAGAAGAACCTAATACTTTAACACCAGATTTTTCAGGTTTAACAGGAGTTAATTGTTCAAGCATTCCTTCAATTTTTTGCATAGTAGTGGAAGTTTTAAGTGCATGTGTAGGTTTTAAATCTTGAAAACAGACTAGGGGTTTTTCTTTAGACTTAAGTTTTGGCTCAGAAGGGACTAATTTATCAACTTTAGTTTCTATTTTATCTAATTGCTTTATATCTGTATGGATAGACGACCAAGGAGGAGGATTAGTCTGCAATCTATCAAATAAGGGTTTACATTGTTTTCTCAAATTTTTATAAAAATCAGAAACATAATAGTGGC

TCTGAACAAGAGATAATGAATAAACTTACACATATGTCAATAGTTGCTAATACATATATTGCAAATCATAGTTTACGACAATCTGAGGTAGTTCCTCTTTTAGAAACTGGATTTACAGGAACCCTACGTTCTTGGTGGGATAAACATTTAAGTAATGAGTCTAGAAATAATATTATACATGCTGTTAAATTAAATGAAGACGGTCTTCCTTTATTTGATACAAATATAGGCAGAGCCACTGAAGATGGAGTCAATACACTACTTTACACTATAATTGAGCATTTTGTAGGAACACCAAGCCATACGCAGTCTAGAATACATGATCAATTGAGTAATCTAAGATGTCCGCAATTAAGTGATTTTAGATGGTATAAAGATGTTTTTATTTCTCGTGTAATGTTAAGAGAAGATAGTAATCAAGCCTTTTGGAAAGAAAAATTTATAAATGGTCTACCTAATCTGTTTGCACATAAAATACGCACCGTTTTAAGTAATGAATTAGGACATATAGATTATGATAGTCTAACTTATGGTAATATTATAAGTACGATTAATCAAGAAGGTATGAAAATGTGTGTAGATATGAAACTAAATGGGCAAATACAATCTGAGCGAAAGTCTGCTAAATATGAGTTAGGAAATTTTTGTGAACAATATGGTCTAATCTCTACAGCTCCCTCTAGAAGAAACAGAAATAATTATCCGAGTCATATTCGTAAAAAACGCCATTATCGCAATTATAGAAAGTCTTTTAGATATAATGATGAGCCTGATGAATTTTACAAAAAGAAGAAATATTCCCCTAAGAAAAGATGGTCAAAAGCACCTCGATATAAGAAAGATGCTCGCTTTAAGTCTGGTAAAAATAAAGATAAAAGTAAAGTAAAATGTTTTAAATGTCAAAAGTATGGTCACTATGCAAATGATTGTAAAGTAAAAGATGCTATCCGACAACTGAAAATTACAGATGAAGAAAAAAACAATCTAATTAAAGTCCTTGAGTTACGGAACTCTGAGTCTAGTGAAAATGAAACTATGGTTATAAGTTCTGAGTCAGCTAGTCATGAGTATTCTGATTCCTTGATAAGTTCACCTAGAATACAAATAGGTTGCAGAGATAATTGTTGTAATACTCTTAAGCAAATTTCTGTTTTAACTAAGCAAGAAGAACAAGAAGAATTATTAATAGATTTGATTTCTAAAGTTGAAAACCCTGAGTTAAAATCTGAGTATTTAAGAAAATTAAGAAAAATTATTACACAAGATACAGGCACAAGTCACTCTAGCCCAGTTATTAATTTGAGTTCTACAATAGAAAGATTTAATAAGAAAAAAGATGTTTCTTTACAAGATCTACATGCTGAAGTAAAACTAATCAAAACAGAAATAGTTGAATTAAAACAATTAAGTCATAAATTACAAACTGAAAATTGTGATATTCAGCAAGCCTTAAACGCATTATTAGATAAAGAACCTACTGAGAAAAATAGTCCACCCAGTAGTCCTAAATATGAGTCTGGTAAAGAAGAACAAGCTGTTAATCTAATTAAACAAGTTAATTTTAGAAAATGGTATTCTAAAGTCACTATATCTGTAAAAGACTATGAGTTTAATGTTATTGCTTTGTTTGATTCTGGTGCAGATCTTAATTGTATTAAAGAGGGTTTAATTCCTACTAAGTATTATATAAAATCTACTGAGTCTTTACGTACTGCTTCTGGTAAATCATTGCAATTAAACTATGAAATTCCTAAAGCCCATGTTTGTCAAAATAAAATTTGTTTTAAAACTTCATTTGTTTTGATTAAAAATATAACTGATGATGTCATTTTAGGATTACCTTTTATAAGCATGCTTTACCCTTTCCAAGTTAAATATGATGGTGTTGTTTCTAATTATTTAGGTGAAAAAGTTAAATTTACTTTTTTAACTAAATCTGAGTTGCATGATCTAAAGCATTTGCATAATAATGATTTTACAAAAACTATTACTTTAATTAAAAATAAATCAAATCATTTGAGTCACCTACAGCAAGAAGTCAAAAATAAAAGGATTAAGTTTCAATTAAATGAACCTACTTTACAAAATGATATTCGTAATTTTGAACAAAAGTTGGTTTCTGAGATTTGTTCTGATCTTCCTACTGCTTTCTGGCATAGGAAAAATCATGTTGTTAATTTGCCTTACATAAAAGATTTTTCTGAGAAAAATATACCAACTAAGTCTAGACCTATACAAATGAATCATGAAATGATGGAATTTTGCAAAAATGAGATAAATGATCTATTACAGAAAAATATAATTAGGAAAAGTAAGTCTCCTTGGTCTTGCCCTGCCTTTTATGTTCAGAAAAATGCAGAATTAGAACGGGGATCTCCCCGACTGGTTATTAATTATAAGCCTTTAAATTCTGTTCTTCAATGGATTCGTTATCCTATTCCTAATAAAAGAGACTTGATTAAACGATTAGATAAAGCTGTGTTTTTTCTAAATTTGATGAAAAGTGGATTCTGGCAAATACAAATTCATGAGTCTGATCGTTACCAAACTGCTTTTGTTACTCCCTTTGGTCATTATGAATGGAATGTTATGCCTTTTGGTCTTAACAATGCTCCTAGTGAATTCCAAAATATTATGAATGAAATTTTCAATACTTATAGTCATTTCTCTATTGTTTACATCGATGATGTTTTAATTTTTTCTGAATCTATAGAACAACACTGGAAACATTTGCATACATTTCTGCATATCATCAAGACTAATGGATTAGTTGTTTCTGCTAAGAAAATTAAATTATTTCAGACTGCTATTAGATTTCTAGGATACAATATAAGTCAATCACAAATTCACCCTATAGATCGAGTTATCCATTTTGCAGATAAATTCCCTGATCAAATTACAGATAAAAATCAATTACAGAGATTTTTAGGTTCTCTTAATTATGTTTTTTATTTTTATAAAAATTTGAGAAAACAATGTAAACCCTTATTTGATAGATTGCAGACTAATCCTCCTCCTTGGTCGTCTATCCATACAGATATGTCTTACAGATTAAACGTCATGTTAAGACTCTTCCTTGTCTTGGCATTCCTACTTCTACTTCTTTTAAAATTGTTTGAGACTGATGCTTCTGATATAGGTTATGGTGGTATTCTTAAACAGAAATCTGAGTCTGATTCTTCTGAGCAAATAGTTCGTTTTCATTCTGGTATCTGGTCTTCCTCCCAGTTTAATTACAGTACTATTAAAAAAGAAATACTAGCTATTGTATTATGCATTACCAAATTTCAAGATGATTTATTAAATCAAAAGTTTTTATCCGTGTTGATTGCAAATCTGCTAAGCATGTATTAAAAAAGATGTTCAAAATATTGCATCAAAACAGATTTTTGCTCTGGCAAGCTTTTAAGTATTTTTGATTTCGATATAGAATATTTGAGAGGTAGTGAGAATTCTATTCCTGATTTTCTTTCTCGAGAATTTTTACAGGATAAGCAATGGCTGGATCACCCAAAGACAAGCAAAAAATGAGTCATAATCCCCCTAGAATTCTTCCTGCAACCTTGAGCCAAAAGCCTTCTCAATTCATAAAAAATGAGCCTGGATCTTTTTCTGTTAAATCTGAGCCTTTTACAATTACTCCCTATCCTGGTCATACACCCATACCAGTCATGAGCAATAGGTTTAGCCCCTTAGGATCTACAGTCACCCCTGCAAGACCCAACTACCAGTCTGCTTTAGTTTCTAGTTATGATCCGTTTCAGTTACCTCCCACTGCATCACCTATATCCTCTCCGTCTTTTGCCAAATCTTCTCCTTATGTCTCAAAAAATACATCTAATCTTGTCATTATTGATTCTCATATCCCTACATCTATGAGTCCTGCCCAAATTGCAGCTTATCATTTTCCTCCTGGCTTTCATTATCTTCCCCATAGCCCTTACAAGACCTTAAAATTTTACAGAGAAATTCTTCATGAAACCAAATCTGTTGAGATTAAGCCTATTCGTGACAAAACTGATCCTACAAAAATTATTTATCACTCCCTCTACATCCACCAAATTTTACATCCTAAGTCTTGGGGATCCCGTCCTCATGAGCTCCGTACCCTTCCTTCTAAATATCAATATAATTATGCAGACTATGTTGAGGCTTGGTACTACATTTTCTTACATCAAACCCCTGAGTTTTCCCACTCTTGGTTCATTAATTTTGATAACAAATTTAAAAATACTGAGCTTCCTTATTGGTTCCTTCATTGGTGGGACACCCATGGTCCCAAATCTGATATCATCCCTTCTGCAGTTCATGAGTTAATCACTTATTTTACCCAAAAACACAAAATTTCTGAGCGAGATCTATTTTTCCCTATTGATCTCATATTTTTCACAAAATACAGACTCCCTTGGATAATGAAATGGAATTATACTGTTAATTGGGAAACTCGTTTCTTCTCCCGACAGTTCTTTGTTAAATGGTGGGATCGTTTTGAAGTGCATCGTATTACAGATTATGTTTATAAAGACTTTCCCCAAGTCCCTCATCGTAACCCTGAATCCAAGCATGAGCCCTCTTCTTCTTCAGATACTATCCTTACAGTTGAAGGCAAATCTAAGACTGAGTTACAGGACTTAGCCCAGCAGCTCCTTCTTCGAGCCTCTCAAATGCATGATGATGATGCTGAGTCTGAAACTTCTTCTAGTTGTCAACCTGCACAGCCAACTCCTGAGCGTCCTGTGCGATGGGCTGATTATCTGCATGGTCAAGATCCTAATGATGCTTATGAT

GAAGCCTATAAAGCCTATGACTTGAATTCTGATTAGACTATTGCAGACAAGTTACCTGAGTTAAAGCAAGTTACCTGAGTAAAAGCCAGTTACCTGAGTTACTTTTGTAAACCTAAGTTACTTTTGTAAACCTGAGTTACTTTTAATGTTTGTCTTTAGTTACTTTTGTAAACCTGAGTTACTTTTAATGTTTGTCTTTAAAAGGAGGCTTCGGCCTCAGTTGAAGGCATCTCTTCATTTTTCTCTTCTTTTCTCTCCAGTTCCTTTCTCAGTTTCAAAAATGTTCTTTCAGAGATCATGTTCTGGAGAACAACCTTAGTTTGAGAATAAGCTTGTAAGTCTTATAAAAATTACTTTCAATGATAGATTATATTTGAAGTATCATCATGTTTTCTTATATCATGGTTTCTTATATACATGCAATTTATTTGTTTTATTTTATATATATATATATATATATAACTTACGGGCCCTGCACCTCTTATCCTTGAATTTTCCTAGACTATCCACAAAGAAATAACTTAATTTGAACCATCGCTCATCAGAAAAGAATGACCTATAAACAATGTAAAACGGGTATAACTCCCAGACGAAAATAAATAATAATTTCATCTTTCCTTGACAGAAGGGATGTTGATGAATGAATTTTTGAAAACAATGTGTACGGCTATGAGTTGGGGACAAAGGTGCCAACATAACCAACCCCCGCAATGAAGAATGCAACAGATTGGATGAATAGGTAGATAAAGTATCCGTTCTTTCCAAAGGCAAGGTCATAGCAGCCGCAGAAGAAGAGGTAGAATCCAACAATGAGCTCAAGCACATGCAGTCTGTTTATTAGTAATCACAAGAAAAGAAAATGTAAACTTCATATTCTTACATTTGACAAGATCTTGTGGCATCAAATGCTATCATAACAAATTGAAAAGTTATTCATTTTAAAGGAATTCTATCAACAGAACAGAAACCAAATGCATAGATTTATACCTTTCTCCAATTC

>Chr2-23; OZ022207.1; 14508109-14522271; rev; C;

CCTCATATGTGTTTGGTTTATTAATTTAGATATTAATATCTAAATAACATCAATTAATTCGAAACATAGATAATGAGAGGGAGACCTGTGCGGCGGAGCGTTCTTCCCAGAGAGCACAAGTGATGCATGCAAATGAGTGAGAGACAATATAGAGAATGCGGACGGCTAGCTACTGTTTGAAACAACTGGGCGACATATGGCAAATATTATAAACCAAAACAAACCAGCGACAAACCACCCAAACCCCAATTATATATATTTTATTTTATTTTATTGGTGGGCCTGTTAGCCAGACTTTGATGGGTGGGCTGGACTGAGTTTAAACTTTCATAATATATATATATATATATATATATATATTGGGAGACTCGTTTTTTCTCCCGCCAATTCTTTGTTAAATGGTGGGATCGTTTCGAAGTCCATCGTATTACAGATTATGTTTATAAAGATTTTCCCCAAGTCCCTGTTCGTTCCTCTGAGCCCAAGCATGAGCCCTCGTCTTCTTCGGATACCATCCTTACAGTTGAAGGTAAATCTAAGACTGAGTTACAGGATTTAGCCCAACAACTCCTTCTCCGTGCTTCCCAAATGCATGATGGTGATGATGATGCTGAGTCTGAGACTTCCTCCAGTTGCCAACCTGCACAGCCAACCCCTGAGCGCCCTGTTAGGTGGGCTGACTATCTGCATGGTCAAGATCCTAATGATGCCTATGACGAAGCCTATAAAGCCTATGATTTGAATTCAGATTAGACTACTACAGACAAGTTACCTGAGTTAAAGTAGAGTTAAGACAAGTTACCTGAGTTAAAGTAGAGTTAAAGCAAGTTACCTGAGTTACTTTTATTTACTTGAGTTACCTTTTGTAAACTTGAGTTACTTTCATTCTGTCTTTAAATAGAGGCCTGAGCCTCAGCTAGAATCAAGCTTTTCTTTTCAGAAGTTTTTTCAGTTTCAAAATGTTCTTTCAGAGATCATGTTCTGAAGAACAACCTTAGGA

TGAGAGTAAGCTTTGCACTTTGTAAGTTTTACTTTTAATGAACTTATTTTAAATTAAGTTATGAAGAATATTATTTTCATGTATTTCATTATTTTTATTATAATGTATACATTATCTTGCATAGCTTTGCTCCTTCTCTCCTTCTTCCCTTTCTGGTATCAGAGCCGTAAGCGTTGTAAGAATTAGTCTTAGGTCGCAACTCATCCAACCCAAGTGGCTGGTTTAGGATTTCTGAGTTGAAGTCTTTTGATTTCTTCTTTATCTTTCTTTTATCCTTTGGGCTTCTTGTTGGTTTTCCCCCTGGTCCAAACAGTGAGTCCTCTTTCCAAACGATAAGTCCCGGGGTAGGGCACGAGCGGTGGGCGTGAGCGGAGGGCGGGATTTCCAGCTAAGTTTCTTGTTGGGTAAAGGTTAGCTATCGAAGTTATTATCTTTCTTCTTCTTCAGTCTTCCTCTTCCATCCTCTTGAGTGAAAATGAGTCGCTTCCTTCGTACAAATTCTTATAGTTCTAGTAGCTCTAGAACCAATATAGGAAATCTTCCTGAGATTGTAAATGAAGAACAATTTGAGTATAATAATAATAATGAACAACTAGAATTTAAAGATTGGAATATACCTAAGGTCCCTAGTCATAATATATACAGAAAAAAATGGTCTATGACTGCATTTAATTCCACTACACATGTTAAAACCGTAGAACAAGTTTATGCCTTGAGTAAAGAACATGAGTCTTGTCAATTACTTAATTTAGATTCAATTAAGAAACATAAGCATGATGGTAATAATTTCTTGCATATCGGTTTAGTTCAAGTTGCTGTTAAACCCTTAACTAGATTAGGTTTACAAGCTTCTATTCTGCTATGTTTGCGTGATGCTAGATTTACAGATTTTAATGATAGTGTTCTTGGTATGATTGAGTCTAGTCTTTGCAATGGCCCTGTACATTTTGATTGCTATCCTGACTTTACTGTTAGCCTTAATGATCCTCATATCCTGAAAACATTAACTTTGAATATTAAGACATCTGGTTATAATGTTTTACCTGGTACTCAACCGTTAGCCTTAGTTTACAGAATTTATTATAAAGTTACAGGTACTAATATGAATTTTCAAGCTTTAAATAAAAGTCCTAAAGATCAAACGCTTTTAATACAAAGTTGTACGCCTGATGCTAATATTAGAATTCCTCATACTGTTAAATGGTCTGATATTACTTTGCCTACAGATTGGACATTAGTTACGGAATCTCAACCTATGCCTATACAACGTAGTTTAAATAATCTAGATTATATTCGTCAATATATGGATGGTAGTGTTAAAATTAATTTCCAGCACCAACCTAAGTCCACAGTTCATTTACAACAGTTGCCCACACCAAGTCCTTCTAAAAGACATGAACCTGCACGTCATTCTTCTGCCTCTTCTTCTACTACAGTTAGAGATTTAGAAATAGAAAAAGATTTAATCGATTTAAAAATTGCATCTTTAAAGAAAAAAGATCAAGTGAGTCATCCTTGTTATGGTCCTCCCCTAGTTGAAAATAATGAGCCTAAACAAGATTCGCCTACACAGTCTGATTTTCTTGCTAGTGAAATCGCTACTGCACATGTTTATGATATAAATAATTGCTTAAATGTTTTAAAAAGAGATAAGCCGTTTAGAATAGATTGGGATAAATTAGAAAAACACACTTTGGATCCTATTAACTCGTCCCGTAGAAGTAATTATCATAAAGCCATCCCTGATAGAAATAAAAGAAATGAAATACACCAAGCCTGGAAAGATTATATGAGAACCGCTAAAGTAGAAATTAATTACCTTGATTTTGTAGAAATGCGTTATATTAATAATGAATTAAAAACTATAACTAAAGAAAAATGGGTAAAATCGGATAAGTCTGAAGTAGTCTTGAGTCATCCTCCTGTAGAAACTATTTTGATAACTGCTGGCACTAAAACCATAACTGCTGCTCCCTTTAAGATACCTGATCCTAGTAATGAGCTTAATAAATGTTTAGTTGAACAAAACAATTATACTAATCAAAGTCTAGTTGTCATAGGAAAGCAATTAGATAAAATAGAAACCAAAGTTGATAAGTTACTCCCTTCTGAGCCGAAGCTTAAGTCTAAAGAAAAACCCTTAGTCTGTTTTCAAGATTTAAAACCTACACATGCACTAAAAACTTCCACTACTATGCAGAAAATTGAAGGCATGCTTGAGCAATTAACCCCTGTTAAAACTGAAAAATCTGGTGTTAAAGTATTAGGTTCTTCTTCCTTGTTTAATAATAGTGATTCTGAAATGAGTCTAGAAACAGATGATTCAGAAAAGTCTAGTAACATTTCTAAGATAGAAAATGCATTTAAAAATCTAGAATTAGAATCTGAATTAAAAGTTAAGAGATTAACTAATAAAATTAATCCTACTAGCCTAACTAAGAATTGGTATCCTAAACCTACACCGCCTGATATTCAGTTTGAAGAAAGAAATAATGTAAGCCAATTTTCGGTTTCCTCTGATAAATTGTATGAATGGAATATAGATGGTTTGTCTGAGCAAGAAATAATGAATAAACTTACACACATGTCAATGGTTGCTAATACTTACATTGCAAATCATAATTTAAGACAATCTGAGATAGTTCCTCTTTTAGAAACTGGATTTACAGGAACCCTACGCTCTTGGTGGGATAAACATCTAAGTAATGAGTCTAGAAATAATATTATACATGCTGTCAAATTAAATGAAGATGGTCTTCCTTTGTTTGATACAACTATAGGCAGAACCCCTGAAGATGGAGTCAATACACTGCTTTACACCATAATTGAGCATTTTGTAGGAACACCAAGCCATACGCAGTCTAGAATACATGATCAATTGAGTAATCTAAGATGTCCACAATTAAGTGATTTTAGATGGTATAAAGATGTTTTTATTTCTCGTGTAATGTTAAGAAAAGATAGTAATCAAGCCTTTTGAAAAGAAAAATTTATAAATGGTCTACCTAATCTGTTTGCACATAAAATACGCACCGTTTTAAGTAATGAATTAGGACATATAGATTATGATAGTTTAACTTATGGTAATATTATAAGTACAATTAATCAAGAAGGTATGAAAATGTGCGTAGATATGAAATTAAATAAGCAAATACAGTCTGAGCGAACGTCTGCCAAATATGAGTTAGGTAATTTTTGTGAACAATATGGTTTAATCTCTACAGCTCCCTCTAGAAGAAACAAGAATAGTTATCCGAGTCATATTCGTAAAAAACGCAATTATAGCAGGTATAGAAAATCTTTCCGATATAATAATGAGCCTGATGCATTTTACAAAAAGAAGAAATATTCCCCTAAGAAAAGATGGTCAAAAGCACCTCGGTTTAAGAAAGATGCTCGCTTTAAGTCTGGTAAAAATAAAGACAAAAGTAAAGTAAAATGTTTTAAATGTCAAAAGTATGGTCACTATGCAAATGATTGTAAAGTAAAAGATGCTATCCGACAACTGAAAATTACAGATGAGGAAAAAACCAATCTAATTAAAGTCCTTGAGTTACGGAACTCTGAGTCTAGTGAAAATGAAACTATGATTATAAGTTCTGAGTCTACCAATCATGAGTCTTCTAATTCCTCTTTGAGTTCACCTCGAATACAAATAGGATGCAGAGATAATTGTTGTAATACCCTTAAGCAAATTTCTGTTTTAACTAAGCAAGAAGAACAAGAAGAATTATTAATAGATTTGATTTCTAAAGTTGAAAACCCTGAATTAAAATCTGAGTATTTAAGGAAATTAAGAAAAATTATTACACAAGATACAGGAACAAGTCACCCTAGTCCAGTTATTAATCTGAGTTCTACAATAGAAAGATTTAATAAGAAAAAAGATGTTTCTTTACAAGATCTACATGCTGAAGTAAAACTAGTCAAAAAAGAAATAGTAGAATTAAAACAGTTGAGTCATAAATTGCAAACTGAGAATTATGATATTCGGCAAGTCTTAAACTCCTTATTAGATAAAGAACCTATTGAGAAAAATAGTCCACCTAGTAGTCCTAAGTATGAGTCTGGCAAAGAAGAATGCGCTGTTAATCTAATTAAACAGGTTAATTTTAGAAAATGGTATTCTAAAGTCACTATATCTGTAAAAGACTATGAATTTAATGTTATTGCTTTGTTTGATTCTGGTGCAGATCTTAATTGCATTAAAGAAGGCTTAATTCCTACTAAGTATTATACAAAATCTACTGAGTCTTTACGAACTGCTTCTGGTAAATCATTGCAGTTAAACTATGAAATTCCTAAAGCCCATGTATGTCAAAATAGAATTTGTTTTAAAACTTCATTTGTTTTGATTAAAAATATAACTGATGATGTTATTTTAGGATTACCTTTTATAAGCATGCTTTACCCTTTTCAAGTTGAGTATGATGGTGTCATTTCGAATTATTTAGGTGAAAAAGTTAAATTTACTTTTTTGACTAAATCTGAGCTGCATGATCAAAAGCATTTACATAATAATGATTTTACAAAAGCTATTACTTTAATTAAAAACAAAACAAATCATTTGAGTCACCTACAGCAAGAAGTCAAAAATAAAAGAATTGAATTTCAATTAAGAGAAACTAATTTACAAAATGATATTCGTAATTTTGAACAAAAGTTGGTTTCTGAAATATGTTCTGATCTTCCTACTGCTTTCTGGCATAGGAAAAATCATGTTGTTAAATTACCTTACATAAAAGAATTTTCTGAGAAAAATATATCAACTAAGTCTAGACCTATACAAATGAATCATGAAATGATGGAATTTTGCAAAAATGAGATAAATGATTTATTACAGAAAAATATAATTAGGAAAAGTAAGTCTCCTTGGTCTTGCCCTGCTTTCTATGTTCAGAAAAATGCAGAATTAGAACGAGGATCTCCTCGACTAGTTATTAATTATAAGCCTTTAAATTCTGTTCTTGAATGGATCCGTTATCCTATTCCTAATAAAAGAGATTTAATTAAACGATTAGATAAAGCTATTGTTTTTTCTAAATTTGATATGAAAAGTGGATTTTGGCAAATACAAATTCATGAGTCTGATCGCTATAAAACTGCTTTTGTTACTCCCTTTGGTCATTATGAATGGAATGTTATGCCTTTCGGTCTTAAAAATGCTCCTAGTGAATTTCAAAATATTATGAATGAAATTTTTAATCCTTACAGTCATTTCTCTATTGTTTACATTGATGATGTTTTAATTTTTTCTGAGTCAATAGAACAACACTGGAAACATTTGCATTCATTTCTGCATATCATTAAGACTAATGGATTAGTTGTTTCTGCTAAGAAAATTAAATTATTCCAAACTGCTATTAGATTTCTAGGATACAATATAAGTCAATCACAAATTCATCCTATAGACCGAGTTATTCAATTTGCAGATAAATTCCCTGATCAAATTACAGATAAAAATCAATTACAGAGATTTTTAGGTTCTCTTAATTATGTTTCTGATTTTTATAAAAACTTGAGAAAACAATGCAAACCCTTATTTGATAGACTGCAGACTAACCCTCCTCCTTGGTCGTCCATCCATACAGATATTGTCTTACAGATCAAACGTCATGTTAAGACTCTTCCTTGTCTTGGCATTCCTACTTCTACTTCTTTTAAAATTGTTGAGACTGATGCCTCTGATATAGGTTATGGTGGTATTCTTAAACAGAAATCTGAGTCAGATTCTTCTGAGCAAATAGTCCGTTTTTATTCTGGTATCTGGTCTTCCTCCCAGTATAATTACAGTACTATTAAAAAAGAAATACTAGCTATTGTATTATGCATTACTAAATTTCAAGATGATTTATTAAATCAAAAGTTTTTAGTCCGAGTTGACTGCAAATCTGCTAAGCATGTATTAGAAAAAGATGTTCAAAATATTGCATCAAAACAGATTTTTGCTCGTTGGCAAGCACTTTTGAGTATTTTTGATTTTATATTGAATATCTGAGAGGTAGTGAGAATTCTATTCCTGATTTTCTTACAAGAGAATTTTTACAGGATAAGCAATGGCTGGATTATCCAAGGACAAGCAAAAAATGAGTCACAATCCTACTCCCCGAATTCTGCCTGCTTCCCTAAGCCGGAAACCTCCTGTTTCTGTTAAAAATGAGTCTAGTACTTTTTCTGTTAAAACTGAGCCTTTTACAATTACTCCTTACCCTGGCCATACACCCATACCGGTCATGAGCAATAGATTTAGCCCCTTAGGATCTACAGTTGTCCCTGCAAGACCCAACTACCAGTCTGCTTTAGTTTCTAGCTATGATCCTTTCCAGTTACCACCCACTGCATCACCTATATCCTCCCCATCTTTTACTAAGTCTTCTCCTTATGTCTTAAAAAATACATCCAATCTTGTCATAGTTGATTCCCATATCCCTACATCCATGAGTCCTGCCCAAATTGCAGCCTTTCATTTTCCCCCTGGTTTTCATTATCTTCCCCATAGCCCTTATAAGTCCTTAAAATTTTACAGAGAAATCCTTCATGAGACCAAGTCTGTTGAAATTAAGCCTATTCGAGATAAAACTGATCCTACAAAGATAATTTATCACTCCCTTTACATTCATCAAATTTTGCATCTTAAGTCCTGGGGATCGTCCTCATGCTCGAACTCCAAATATCAATATAATTATGCAGACTATGTTGAAGCCTGGTATTACATTTTTCTTCATCAAACCCTGAGTTTTCCCATTCCTGGTTTATTAATTTTGATAATAAATTTAAAAATTCTGAGCTTCCTTACTGGTTCCTTCATTGGTGGGACACCCATGGTCCTAAATCTGATATTATCCATTCTGCAGTTCATGAGTTGATCACTTATTTTTCCCACAAATATAAAATTTCTGAGCGAGATCTATTTTTTCCTATTGATCTCTTATTCTTTACAAAATACAGACTCCCCTGGATAATGAAATGGAATTATACTGTTAATTGGGAGACTCGTTTTTTCTCCCGCCAATTCTTTGTTAAATGGTGGGATCGTTTCGAAGTCCATCGTATTACAGATTATGTTTATAAAGATTTTCCCCAAGTTCCTGTTCGTTCCTCTGAGCCCAAGCATGAGCCCTCGTCTTCTTCGGATACCATCCTTACAGTTGAAGGTAAATCTAAGACTGAGTTCACGGAGAAGGAGTTGTTGGGCTAAATCCTGTAACTCAGTCTTAGATTTACCTTCAACTGTAAGGATGGTATCCGAAGAAGACGAGGGCTCATGCTTGGGCTCAGAGGAACGAACAGGGACTTGGGGAAAATCTTTATAAACATAATATGTAATACGATGGACTTCGAAACGATCCCACCATTTAACAAAGAATTGGCGGGAGAAAAAACGAGTCTCCCAATTAACAGTATAATTCCATTTCATTATCCAGGGGAGTCTGTATTTTGTAAAGAATAAGAGATCAATAGGAAAAAATAGATCTCGCTCAGAAATTTTATATTTGTGGGAAAAATAAGTGATCAACTCATGAACTGCAGAATGGATAATATCAGATTTAGGACCATGGGTGTCCCACCAATGAAGGAACCAGTAAGGAAGCTCAGAATTTTTAAATTTATTATCAAAATTAATAAACCAGGAATGGGAAAACTCAGGGGTTTGATGAAGAAAAATGTAATACCAGGCTTCAACATAGTCTGCATAATTATATTGATATTTAGAAGGAAGAGTTCGGAGTTCATGAGGACGGGATCCCCAGGACTTAAGATGCAAAATTTGATGAATGTAAAGGGAGTGATAAATTATCTTTGTAGGATCAGTTTTATCTCGAATAGGCTTAATTTCAACAGACTTGGTCTCATGAAGGATTTCTCTGTAAAATTTTAAGGACTTATAAGGGCTATGGGGAAGATAATGAAAACCAGGGGGAAAATGAAAGGCTGCAATTTGGGCAGGACTCATGGATGTAGGGATATGGGAATCAACTATGACAAGATTGGATGTATTTTTTAAGACATAAGGAGAAGACTTAGTAAAAGATGGGGAGGATATAGGTGATGCAGTGGGTGGTAACTGGAAAGGATCATAGCTAGAAACTAAAGCAGACTGGTAGTTGGGTCTTGCAGGGACAACTGTAGATCCTAAGGGGCTAAATCTATTGCTCATGACCGGTATGGGTGTATGGCCAGGGTAAGGAGTAATTGTAAAAGGCTCAGTTTTAACAGAAAAAGTACTAGACTCATTTTTAACAGAAACAGGAGGTTTCCGGCTTAGGGAAGCAGGCAGAATTCGGGGAGTAGGATTGTGACTCATTTTTTGCTTGTCCTTGGATAATCCAGCCATTGCTTATCCTGTAAAAATTCTCTTGTAAGAAAATCAGGAATAGAATTCTCACTACCTCTCAGATATTCAATATAAAAATCAAAAATACTCAAAAGTGCTTGCCAACGAGCAAAAATCTGTTTTGATGCAATATTTTGAACATCTTTTTCTAATACATGCTTAGCAGATTTGCAGTCAACTCGGACTAAAAACTTTTGATTTAATAAATCATCTTGAAATTTAGTAATGCATAATACAATAGCTAGTATTTCTTTTTTAATAGTACTGTAATTATACTGGGAGGAAGACCAGATACCAGAATAAAAACGGACTATTTGCTCAGAAGAATCTGACTCAGATTTCTGTTTAAGAATACCACCATAACCTATATCAGAGGCATCAGTCTCAACAATTTTAAAAGAAGTAGAAGTAGGAATGCCAAGACAAGGAAGAGTCTTAACATGACGTTTGATCTGTAAGACAATATCTGTATGGATGGACGACCAAGGAGGAGGGTTAGTCTGCAGTCTATCAAATAAGGGTTTGCATTGTTTTCTCAAGTTTTTATAAAAATCAGAAACATAATTAAGAGAACCTAAAAATCTCTGTAATTGATTTTTATCTGTAATTTGATCAGGGAATTTATCTGCAAATTGAATAACTCGGTCTATAGGATGAATTTGTGATTGACTTATATTGTATCCTAGAAATCTAATAGCAGTTTGGAATAATTTAATTTTCTTAGCAGAAACAACTAATCCATTAGTCTTAATGATATGCAGAAATGAATGCAAATGTTTCCAGTGTTGTTCTATTGACTCAGAAAAAATTAAAACATCATCAATGTAAACAATAGAGAAATGACTGTAAGGATTAAAAATTTCATTCATAATATTTTGAAATTCACTAGGAGCATTTTTAAGACCGAAAGGCATAACATTCCATTCATAATGACCAAAGGGAGTAACAAAAGCAGTTTTATAGCGATCAGACTCATGAATTTGTATTTGCCAAAATCCACTTTTCATATCAAATTTAGAAAAAACAATAGCTTTATCTAATCGTTTAATTAAATCTCTTTTATTAGGAATAGGATAACGGATCCATTCAAGAACAGAATTTAAAGGCTTATAATTAATAACTAGTCGAGGAGATCCTCGTTCTAATTCTGCATTTTTCTGAACATAGAAAGCAGGGCAAGACCAAGGAGACTTACTTTTCCTAATTATATTTTTCTGTAATAAATCATTTATCTCATTTTTGCAAAATTCCATCATTTCATGATTCATTTGTATAGGTCTAGACTTAGTTGATATATTTTTCTCAGAAAATTCTTTTATGTAAGGTAATTTAACAACATGATTTTTCCTATGCCAGAAAGCAGTAGGAAGATCAGAACATATTTCAGAAACCAACTTTTGTTCAAAATTACGAATATCATTTTGTAAATTAGTTTCTCTTAATTGAAATTCAATTCTTTTATTTTTGACTTCTTGCTGTAGGTGACTCAAATGATTTGTTTTGTTTTTAATTAAAGTAATAGCTTTTGTAAAATCATTATTATGTAAATGCTTTTGATCATGCAGCTCAGATTTAGTCAAAAAAGTAAATTTAACTTTTTCACCTAAATAATTCGAAATGACACCATCATACTCAACTTGAAAAGGGTAAAGCATGCTTATAAAAGGTAATCCTAAAATAACATCATCAGTTATATTTTTAATCAAAACAAATGAAGTTTTAAAACAAATTCTATTTTGACATACATGGGCTTTAGGAATTTCATAGTTTAACTGCAATGATTTACCAGAAGCAGTTCGTAAAGACTCAGTAGATTTTATAATACTTAGTAGGAATTAAGCCTTCTTTAATGCAATTAAGATCTGCACCAGAATCAAACAAAGCAATAACATTAAATTCATAGTCTTTTACAGATATAGTGACTTTAGAATACCATTTTCTAAAATTATTAGGACTACTAGGTGGACTATTTTTCTCAATAGGTTCTTTATCTAATAAGGAGTTTAAGACTTGCCGAATATCATAATTCTCAGTTTGCAATTTATGACTCAACTGTTTTAATTCTACTATTTCTTTTTTGACTAGTTTTACTTCAGCATGTAGATCTTGTAAAGAAACATCTTTTTTCTTATTAAATCTTTCTATTGTAGAACTCAGATTAATAACTGGACTAGGTGACTTGTTCCTGTATCTTGTGTAATAATTTTTCTTAATTTCCTTAAATACTCAGATTTTAATTCAGGGTTTTCAACTTTAGAAATCAAATCTATTAATAATTCTTCTTGTTCTTCTTGCTTAGTTAAAACAGAAATTTGCTTAAGGTATTACAACAATTATCTCTGCATCCTATTTGTATTCGAGGTGAACTCAAAAGAATTAGAAGACTCATGATTGGTAGACTCAGAACTTATAATCATAGTTTCATTTTCACTAGACTCAGAGTTCCGTAACTCAAGGACTTTAATTAGATTGTTTTTTCTCATCTGTAATTTTCAGTTGTCGGATAGCATCTTTTACTTTACAATCATTTGCATAGTGACCATACTTTTGACATTTAAAACATTTTACTTTACTTTTGTCTTTATTTTTACCAGACTTAAAGCGAGCATCTTTCTTAAACCGAGGTGCTTTTGACCATCTTTTCTTAGGGGAATATTTCTTCTTTTTGTAAAATGCATCAGGCTCATTATTATATCGGAAAGATTTTCTATACCTGCTATAATTGCGTTTTTTACGAATATGACTCGGATAACTATTCTTGTTTCTTCTAGAGGGAGCTGTAGAGATTAAACCATATTGTTCACAAAAATTACCTAACTCATATTTGGCAGACTTCGCTCAGACTGTATTTGCTTATTTAATTTCATATCTACGCACATTTTCATACCTTCTTGATTAATTGTACTTATAATATTACCATAAGTTAAACTATCATAATCTATATGTCCTAATTCATTACTTAAAACGGTGCGTATTTTATGTGCAAACAGATTAGGTAGACCATTTATAAATTTTTCTTTTCAAAAGGCTTGATTACTATCTTTTCTTAACATTACACGAGAAATAAAAACATCTTTATACCATCTAAAATCACTTAATTGTGGACATCTTAGATTACTCAATTGATCATGTATTCTAGACTGCGTATGGCTTGGTGTTCCTACAAAATGCTCAATTATGTGTAAAGCAGTGTATTGACTCCATCTTCAGGGGTTCTGCCTATAGTTGTATCAAACAAAGGAAGACCATCTTCATTTAATTTGACAGCATGTATAATATTATTTCTAGACTCATTACTTAGATGTTTATCCCACCAAGAGCGTAGGGTTCCTGTAAATCCAGTTTCTAAAAGAGGAACTATCTCAGATTGTCTTAAATTATGATTTGCAATGTAAGTATTAGCAACCATTGACATGTGTGTAAGTTTATTCATTATTTCTTGCTCAGACAAACCATCTATATTCCATTCATACAATTTATCAGAGGAAACCGAAAATTGGCTTACATTATTTCTTTCTTCAAACTGAATATCAGGCGGTGTAGGTTTAGGATACCAATTCTTAGTTAGGCTAGTAGGATTAATTTTATTAGTTAATCTCTTAACTTTTAATTCAGATTCTAATTCTAGATTTTTAAATGCATTTTCTATCTTAGAAATGTTACTAGACTTTTCTGAATCATCTGTTTCTAGACTTATTTCAGAATCACTATTATTAAACAAGGAAGAAGAACCTGATACTTTAACACCAGATTTTTCAGTTTTAACAGGGGTTAATTGCTCAAGCATGCCTTCAATTTTCTGCATAGTAGTGGAAGTTTTTAGTGCATGTGTAGGTTTTAAATCTTGAAAACAGACTAAGGGTTTTTCTTTAGACTTAAGCTTCGGCTCAGAAGGGAGTAACTTATCAACTTTGGTTTCTATTTTATCTAATTGCTTTCCTATGACAACTAGACTTTGATTAGTATAATTGTTTTGTTCAACTAAACATTTATTAAGCTCATTACTAGGATCAGGTATCTTAAAGGGAGCAGCAGTTATGGTTTTAGTGCCAGCAGTTATCAAAATAGTTTCTACAGGAGGATGACTCAAGACTACTTCAGACTTATCCGATTTTACCCATTTTTCTTTAGTTATAGTTTTTAATTCATTATTAATATAACGCATTTCTACAAAATCAAGGTAATTATTTCTACTTTAGCGGTTCTCATATAATCTTTCCAGGCTGGTGTATTCATTTCTTTTATTTCTATCAGGGATGGCTTTATGATAATTACTTCTACGGACGAGTTAATAGGATCCAAAGTGGTTTTTCTAATTTCCCAATCTATTCTAAACGGCTTATCTCTTTTTAAAACATTTAAGCAATTATTTATATCATAAACATGTGCAGTAGCGATTCACTAGCAAAAATCAGACTGTGTAGGCGAATCTTGTTTAGGCTCATTATTTTCAACTAGGGAGGACCATAACAAGATGACTCACTTGATCTTTTTTTTAAAGATGCAATTTTTAAATCGATTAAATCTTTTTCTATTCTAAATCTCTAACTGTAGTAGAAGAAGAGGCAGAAGAATGACGTGCAGGTTCATGTCTTTTAGAAGGACTTGGTGTGGGCAACTGTTGTAAATGAACTGTGGACTTAGGTTGGTGCTGGAAATTAATTTTAACACTACCATCCATATATTGACGAATATAATCTAGATTATTTAAACTACGTTGTATAGGCATAGGTTGAGATTCCGTAACTAATGTCCAATCTGTAGGCAAAGTAATATCAGACCATTTATAACAGTATGAGGAATTCTAATATTAGCATCAGGCGTACAACTTTGTATTAAAAGCGTTTGATCTTTAGGACTTTTATTTAAAGCTTGAAAATTCATATTAGTACCTGTAACTTTATAATAAATTCTGTAAACTAAGGCTAACGGTTGAGTACCAGGTAAAACATTATAACCAGATGTCTTAATATTCAAAGTTAATGTTTTCAGGATATGAGGATCATTAAGGCTAACAGTAAAGTCAGGATAGCAATCAAAATGTACAGGGCCATTGCAAAGACTAGACTCAATCATACCAAGAACACTATCATTAAAATCTGTAAATCTAGCATCACGCAAACATAGCAGAATAGAAGCTTGTAAACCTAATCTAGTTAAGGGTTTAACAGCAACTTGAACTAAACCGATATGCAAGAAATTATTACCATCATGCTTATGTTTCTTAATTGAATCTAAATTAAGTTATTGACAAGACTCATGTTCTTTACTCAAGGCATAAACTTGTTCTACGGTTTTAACATGTGTAGTGGAATTAAATGCAGTCATAGACCATTTTTTTCTGTATATATTATGACTAGGGACCTTAGGTATATTCCAATCTTTAAATTCTAGTTGTTCATTATTATTATTATACTCAAATTGTTCTTCATTTACAATCTCAGGAAGATTTCCTATATTGGTTCTAGAGCTACTAGAACTATAAGAATTTGTACGAAGGAAGCGACTCATTTTCACTCAAGATGGAAGAGGAACTGAAGAAGAAGAAAGATAATAACTTCGATAGCTAACCTTTACCCAACAAGAAACTTAGCTGGAAATCCCGCCCTCCGCTCACGCCCACCGCTCGTGCCCTACCCCGGGACTTATCGTTTGGAAAGAGGACTCACTGTTTGGACCAGGGGGAAAACCAACAAGAAGCCCAAAGGATAAAAGAAAGATAAAGAAGAAATCAAAAGACTTCAACTCAGAAATCCTAAACCAGCCACTTGGGTTGGATGAGTTGCGACCTAAGACTAATTCTTACAACGCTTACGGCTCTGATACCA

GAAAGGGAAGAAGGAGAGAAGGAGCAAAGCTATGCAAGATAATGTATACATTATAATAAAAATAATGAAATACATGAAAATAATATTCTTCATAACTTAATTTAAAATAAGTTCATTAAAAGTAAAACTTACAAAGTGCAAAGCTTACTCTCATCCTAAGGTTGTTCTTCAGAACATGATCTCTGAAAGAACATTTTGAAACTGAAAAAACTTCTGAAAAGAAAAGCTTGATTCTAGCTGAGGCTCAGGCCTCTATTTAAAGACAGAATGAAAGTAACTCAAGTTTACAAAAGGTAACTCAAGTAAATAAAAGTAACTCAGGTAACTTGCTTTAACTCTACTTTAACTCAGGTAACTTGTCTTAACTCTACTTTAACTCAGGTAACTTGTCTGTAGTAGTCTAATCTGAATTCAAATCATAGGCTTTATAGGCTTCGTTGGTCAAGCCACATTAAAGTGACTAATTATCAAAACCATGCAAACCCAGAAAATATATAAGTCCTAATGTTCTATTTCATGCTTCTGTCACACCAAGTTTATTGTCTACTTTTAAAATTCAGCATCATATAGTAAGATCAACAACAGAGAAAGTATCTATTAATCCCAAGGAACAACTCATGCCTCCCTAAAAACTGTTGGTAGGTGGAACTGCTGCATATTGTTTGTCCAGAACCTCAATGAGCTTTTGGGCTATGGCACTTGCATACAAAGATGACCCTTCATACATCTGTAGATTTAAAACCAAATTAACCATAATTAATTAAAAACCGCACCACCTGTATGAAAGAAAAACGAACTGGGACAAATTGCAGACTACCAACCTTGGTGTTGAACAAGAAGCTGTAGGAATTGCCAATGTGTCCACCAGCAACATGATGCAGATCAAGCTGCAGCTCATCAAGAAGCTTGGACACATACAGCAACAGATTGATCTTCTTCCTCTGAACCAGTTTGATCGTAACTTCATCATCGATAATGCGGATATCGACCTCAGTGTCCT

>Chr2-24; OZ022207.1; 15239975-15245080; dir; B;

TCTGTTACTTATCTTTCTTCAATAAATACTATTGCTCTTTAAAAAAAAAAAAACCCTAAAAAAGCCAATAATTATATTATTTATAGGGGAGTAGTTAATCTCATACTCATTCACTCTCTTCCTTATTTTTGACTTTCCCGATTCATAGTTTCTCTCATTCCAAGTTTATATGCCATATACAAAAATTAAAGTTATAGAGTTTATAAAATATATATATATATATATATGTAAGTATTTAAAAGAAACAAGATCGACGAAGCGGTCAGACCAACGAAGCCAACCTAAATTCAGTTGGGATTAGGGTTTAGTCTCCACTAGGGGTGGGCATGATTTGGGTTAGTTCAGTTGTGGGCCCAATCGACCACCGAACCGTCCAAATCGATTGCGCAAAATTCCATATTGAAACCAAACCAACTAGGGTTCTAACTCACAAGGTCGATTCACCAACTATGATAAGTTGAATTAGGTTGGGTTGGTTCGTTTCAACGACATCCAACCAGGAGAAGAGAAGTGGCAAGGCAAGGCTCTGATGACGAGCGGAGCTTTGAATTGATCTACTAATTCATTGACGATGATGAAAGATTGAAAGAGAGAAATGAAGGTGAGAAAGAGAGATATATCAGGCAAGAGAGAAAAAGTGTAAGAAATGAGGAAAAAAGGAGATGAGAGTGCCATGGAGAGAGAGGTGAAGACTAAGTCACTGAAGAGGATGCGAGATTGACAGATTAATGATCAAGACGAGAGATAGAGAGAGAATGTGAGGATATATATATATATATATATATATATATATATATACAGTCCCGATCTCTTGGACCACAGGAGCCCAAAAGATTGTGGTCACCCACCATTGGATATTAATCCAATGGTTCAAAAAAGTTTTTTTAAAAGGAGTGCAAAAGTGAGTGAACCGTTGAATTTACATCCAACGGTGAGTGACCACAAATCTCTTGGACCCCTGTAGTCCAAGAGATCAGGACTGTATATATATATATATATA

TCTAATGAGTATAGAAACAGATGAGTCTAGTAATATTTCTAAAATTGAAAATGCATTTAAGAATTTAGAATTAGAATCTGAGTTAAAAGTCAAAAGATTAACTAATAAGATTAATCCTACTAGCCTAACTAAGAATTGGTATCCCAAACCTACACCTCCTGATATTCAATTCGAAGAAAGAAATAATTTGAGCCAATTTTCTGTTTCCTCTGATAAATTGTATGAATGGAATATTGATGGTTTGTCTGAGCAAGAAATAATGAATAAACTTACACACATGTCCATGGTTGCTAATAGTTATATTGCAAATCACAGCTTACGCCAATCTGAGATAGTTCCCTTGTTAGAAACTGGTTTTACAGGAGCCTTACGTTCCTGGTGGGATAGACATTTAACTGATGAGTCTAGAAATAATATTATACATGCTGTTAAATTAAATGAAGATGGTCTTCCTTTGTTTGATACAAATATAGGACGCGGTACAGAAGATGGAGTTAATACACTGCTTTACACTATAATTGAGCATTTTATAGGTACACCGAGTCATACACAATCTAGAATACATGATCAACTGAGCAATCTGAGATGCCCACAATTAAGTGATTTTAGATGGTATAAAGATATTTTTATTTCCCGTGTCATGTTAAGAGAAGATAGTAATCAAGCTTTTTGGAAAGAAAAATTTATTAATGGTTTGCCTAATTTGTTTGCACACAAAATTCGTACAGTCTTGAGTAATGAGTTAGGACATATAAATTATGATAGTTTAACTTATGGAAATATTATAAGTACAATTAATCAAGAAGGTATGAAAATGTGTATCGATATGAAATTGAATAAACAAATACAGTCTGATCGAAATTCTGCTAAGTATGAGTTAGGTAATTTCTGTGAACAATATGGTCTAATCTCTACAGCTCCTTCTAGGAAAAATAAGTACAGTCAATCTAGTTATATCCGTAAGAAACGTCATTATAGCAAACAAAGAAAGTCTTACAGACATTATAATGAGCCTAATGAGTTTTATAAGAAAATGAGATATTCCCCTAAGAAAAGATGGTCAAAAGCACCTAGGAATAAGAAAGATACTCGCTTTAGCACTAAGTCCAGTAAAAGTAAAAGCAAAGATAAAAGTAAAGTAAAATGTTTTAAATGTCAAAAGTATGGACACTATGCAAACGATTGTAAAGTTAAAGATGCTATCCGACAACTGAAAATTACAGATGAAGAAAAAGACAACCTGATTAAAGTCCTTGAGTTACGGAACTCTGAGTCTAGTGAAAATGAACCTATGGTTAGTAGTTCTGAGTCAGTTAACCATGAGTCCTCTGATTCCCAGTTAAGTTCACCTCGAATTCAATTAGGTTGCAGAGATAAATGTTGCAATACACTTAAGCAAATATCTGTTCTAACTAAGCAAGAAGAACAAGAAGAATTATTGATAGATTTAATTAGTAAAATTGAAAGTCCTGACTTAAAATCTGAGTACTTAAGAAAATTGAGAAAAATTATTACACAGGATACAAGCACGAGTCATCCTAGCCCCGTAATTAACCTGAGTTCTACTTTAGAAAGGTTTAATAAGAAAAAAGAAGTTTCTTTGCATGATTTACATTCTGAGGTAAAATTAGTCAAAAAGGAAATAATTGAGTTAAAACAATTGAGTCATAAATTGCAAACTGAGAATTATGATATTCGACAAGTTTTAAATACATTATTAGAAAAAGAACCCTCTGAGCCAAATAGTCCACCTGGTAGCCCTGTTCATAAATCTGAGTCTGATAAAGAAGAACAAGCTGTCAATTTAATTAAACAAGTTAATTTTAGAAAATGGTATTCTAAAGTCACTATAGTTGTTAAGAATTATGAGTTCAATACAGTTGCTTTGTTTGATTCTGGTGCAGATCTTAATTGTATAAAAGAAGGTTTAATTCCCACTAAGTACTATGAGAAATCTACAGAATCCTTACGAACTGCCTCTGGTAAATCACTTCAATTAAATTATGAAATTCCTAAAGCCCATGTTTGTCAAAATAAGATTTGTTTTAAAACTTCATTTGTTTTAATTAAAAATATAACTGATGAGGTTATTTTAGGATTACCTTTTACAAGCATGCTTTATCCTTTTCAAGTTGAATATGACGGAGTTGTTTCGAATTATTTAGGCGAGAAAGTTAAGTTTACTTTTTTAACTAAACCTGAGTTGCATAGTCTAAAGTATTTAAATAATAATGATTTTGCAAAAACAAATACTTTAATTAAAGATAAAACTAATCATCTGAGTCACCTACAGCTAGAAGTCAAAAGTAAAAGAACTGAGTCTCAATTAAATAATCCTGACTTGCAAAAAGACATTCGTAATTTTGAGCAAAAGTTAATTTCTGAAGTTTGTTCTGATCTTCCTACTGCTTTCTGGCATAGGAAAAATCATGTTGTTAATTTACCTTACATAAAGGATTTTTCTGAGAAAAATATACCAACTAAAGCTAGACCTATACAAATGAATCATGAAATGATGGAATTCTGTAAAAATGAGATTAATGAATTATTACAGAAAAAAATAATTAAGAAAAGTAAATCTCCCTGGTCTTGCCCTGCTTTTTATGTTCAAAAAAATGCAGAATTAGAAAGAGGATCTCCTCGATTAGTTATTAATTACAAGCCCCTGAATGCTGTTCTTGAATGGATTCGTTACCCTATCCCTAATAAAAGAGATTTGATTAAACGCTTAGATAAAGATGTTATTTTTTCTAAGTTTGATTTAAAGAGTGGATTTTGGCAAATACAGATTCATGAGTCTGATAGGTATAAAACTGCTTTTGTTACTCCTTTTGGGCACTATGAATGGAATGTTATGCCTTTCGGTCTTAAGAATGCTCCTAGTGAATTCCAAAATATAATGAATGAGATTTTCAATTCTTACAGTCATTTCTCTATTGTCTATATTGATGATGTTTTGATATTCTCTGAGTCTATAGAACAACACTGGAAACATTTGCATAAATTTTTGCATATCATTAAGACTAATGGACTAGTCGTCTCTGCTAAAAAAATTAAATTATTCCAGACTAGTATTCGATTCTTAGGATATAATATATGCCATGCACAGATTCACCCTATAGATCGAGTTATCAATTTTGCAGATAAGTTCCCTGATCAGATCATTGATAAAAACCAATTGCAGAGGTTTTTAGGATCCCTTAATTATGTTTATGATTTTTACAAAAATTTGAGAAAACAATGTAAACCCTTATTTGATAGATTACAGAATAATCCTCCCCCTTGGTCATCTATTCATACAGATATTGTTCAACAGATTAAGCGTCATGTTAAGACACTCCCTTGTCTAGGCATTCCTACTCCAAATTCCTTTAAAATAGTTGAGACTGATGCTTCTGATACAGGTTATGGTGGTATTCTTAAACAGAAATCTGAGTCTACTCCCTCTGAGCAAATTGTACGATTTCATTCTGGCATCTGGACTTCTTCCCAGTCCAATTATAGTACTATTAAAAAAGAAATATTATCTATTGTATTATGCATTACTAAATTTCAAGATGATTTATTAAATCAAAAATTTTTAGTCAGAGTTGATTGTAAATCTGCTAAGCATGTATTAGAAAAAGATGTTCAAAACATTGCATCTAAACAGATTTTTGCACGTTGGCAAGCTCTTTTAAGCATTTTTGATTTTGATATTGAATACTTGAAAGGCAGTGAGAATTCTATTCCTGATTTTCTTACTCGAGAATTTTTACAGGATAAGCAATGGCTGGATCATCAAAAGACAAACAAAAAATGAGTCAAAATCCTCCTCCCAGGATCTTACCTGCTACCCTGAGCCAAAAGCCTTCCAAATCAGTTAAAAATAAGTCTGCATCTTTTTCCGTTAAGACTGAGTCTCTTACTCCTTACCCTGGTCATATACCTATGCCCATCATGAGCAATAGATTTAGTCCTCTAGGATCTACAGTTGTACCTACAAGACCAAACTACCAATCTGCCTTAGTTTCTAGTTATGACCCATTTCAATTACCTCCAACTGTAACACCTGCATCCCCCCAGTTTTTTCAAAAATCTTCCCCTTATGTCTTAAAGCATACAGCTCACCTCTTCATTATTGATTCTCATATTTTTGCATCAATGAGCCCTGCCCACATTGCAGCTCATCATTTCCCTCCTGGATTTCATTTCTTACCCCACAGCCCTTACAAATCCTTAAAATTTTACAGAGAAATTCTGCATGAAACCAAGTCTGTAGAAATTAAGCCTATTCGGGATAAAACTGATCCCACTAAAATCATTTACCATTCTCTTTACATTCATCAAGTCTTACATCCTAAGTCTTGGGGATCTCGTCCTCATGAACTACGAACCCTACCTTCTAAATTCCAGTATAATTATGCAGATTACGTTGAAGCCTGGTATTACATATTCTTACATCAAACACCTGAGTTTTCTCATTCCTGGTTCATTAATTTTGATAATAAGTTTAAAAATACTGAGCTCCCTTATTGGTTTCTTCACTGGTGGGACACCCATGGTCCCAAATCTGATATTATTCCTCCTGCAGTCAATGAGCTGATCACTTATTTTACCCAAAAATCCAAGTTTGCTGAGCGTGATTTATTTTTCCCTCAAGATTTATTGTTCTTTACCAAATACAGAGTCCCTTGGATAATGAAATGGAATTATACTGCTGATTGGGCGACCCGTTTCTTCTCCCGTCAGTTTTTTGTCAAATGGTGGGATCGCTTTGAGGTCCATCGCATTACAGATTATGTCTACAGAGACTTTCCCCAAATTCAAGTTCATAAGCCTGAGCCCAAGCATGAGCCTGCCTCTTCTCACTCTTCGGATACTATTCTTACAGTTGAAGGTAAATCCAAAATTGAGTTGCAGGAATTGGCCCAGCAAATCCTTCTTCGAGCCTCACAAATGCATGATGATAATGTTGAAGATGATGCTGAGTCTGAAACCTCTTCCAGCTGTCAACCTGCACAGCCAACACCTGAAGGCCCTACAAGATGGTCTGACTATCTCCATGGTCAAGACCCAAATGAAGACTATGCAGAAGCCTACAAAGCCTATGATCTGAATTCTGATTAG

ATCACTTGAGTCAAAGCACGAGATTCCCTTCTGAGTTAAAGCATAAAGTAAAAGCAAGTTACCTGAGTAAAAGCACAAGAGCCTTACAGTTCTTACTTTTTCATGTAACCTAAGTTGCTTTAGTTTACAATAGTTTCTTTTGTATACCTGAGTTACTTTTGTTAAACCATAATCATTTTCTGTCTTTATAAGGAGGAAGTTGCCTCAGTTGTAAGCATCGCTTCTTATTACTTCTTTTCTTAGTTTTAAAATGTTCTCTCAGAGATGATGTTCTGAATAACACAAGTGAGCTATGAGCCTTTGTAAGCTACTTCTTTCATGAGTCTACTTTCAAATAAAATATTTTGAGTATTTTAAATATACCATTATGTTTCTCCATACAACCATTATGTTTATTTTCATCCATATAATGTTTCTATTTTTCCTATTTTTCCATCCTTGTATTATCCGCTGATTCCCTTTCTGGTGTATATATATATATATATATATATATATATATATATATATATATATATGAACTCAAGAGGCAATGAGCGGTGTGGTTTGGTTACAATTGAATCTATAAAATTAAACCAGCTCCAAACCAAATTAAACCGGTTTAATTTGGTTTTTTAATAAAATTATTAAAAAATCTAAAGGCCAAAATCGTTCGGTTCGGCCGGGTTTTCAGTGCATTTGCCCAGCCCTCGCTCAAACGGGATCAACCCGATCTGCACCGGCTCCATGGTTGATTCACATGAAAAAAAAAAAAAACTCCACCGTGTGTGTTTCTACGTGAACTGTTAGATATCAATCCTTAGATATTTCTTCATGTATTTTTCCTGAGAAGCATTAACAATTCTGCATCCATACTCCACATCCTTTTGAAAACTCATTCCTCAACGGCTATGACTGTACTTTCAAGTATGCGAACCCATCGGCTACTCTTTCAAAATCCAACTCCGAGAAACTTCAGAACCTTCACAAGGAAAAAACTAACACAACACTCACTCTTTCACTC

>Chr2-25; OZ022207.1; 16355023-16366105; rev; C;

ATGCGTTAAGGAAATTATTTGTGTAAAGCAAAACAGCGAAGAGCATGAGCCACATTGTTACACTATCTAAGTCGAAATCGAATTTCCTTTCAAGAAACTACATAAACAAGTCTCTCACATATGTCGAAATAGTCACATCATTGTCATTTTTACTCGTTTATATTTATTTTATGGTCATGTTTTGGTTTATAAGAGCATTTCCACAGGGAGGCCCCAACCCTCGTAGGAGGCCCCCAGGGCCCCAAATGTGTTTCCAGCGCCCAAGTCTTGCCAGGGCAAGAGCTGGGCTCCACCAACCTGGGCAAATTTAAAGGCAAGATTTGCATGGGCTGCTGCCAGGGCAAGTTGACCTAATGCTGACGTTAACAACCAATTTAAATATATAAAAATTCAAAAAAATAAGGAAAAAAACCAAAAATTTCAGAATTTTTTTAATAAATGCCTAGCCATATTCTTCACTTCCTACACCAAAACTCTATATAAATTCCTCTCCTCATATCTCATGTGAATAGTAATTGTCTTTAGATTTTATGGTAATGGGTGAAAAACCAATAAAAAAAATTGCTAGACATATACTATTCATGTGAATAGTAACAGTCTTTATTTTTTATCATAACAAATGAGTGAAAATGTTATAAATAGATGAAAGTAATAAAATCTACAATATGCTCAAGCAGCCCTTTGATGGACGAAGAGCAGCCCAGGATATATAAAACCGCTAATTAAGTTGAATTTTAGTCATGCTTATTTTAGTCCATTGTTTCTTCGGGTCAACTACTCATGTTTCTTTAAAAGCTGACATTCTGCAAAATATTATTTTAATCATAGGAAGCTGAAGATATCAAAGTTTTTTAGTTTCCTCTCATTAATGATAATAACCAATTAGTTACATAGCCAATTCGGAAAAGATAAACAATGTCACCAGTCAAAACAAAAGGATAAACACGGTCATGAGAAATGGCGAATCCTCTCTCTGTGTATATATATATATATATATATA

ATGCGTTTAAGACTTGCCGGATATCATAATTCTCAGTTTGCAATTTATGACTCAACTGTTTTAATTCTACTATTTCTTTTTTGACTAGTTTTACTTCAGCATGTAGATCTTGTAAAGAAACATCTGTTTTCTTATTAAATCTTTCTATTGTAGAACTTAGATTAATAACTGGACTAGAGTGACTTGTTCTTGTATCTTGTGTAATAATTTTTCTTAATTTTCTTAAATATTCAGATTTTAACTCAGGGTTTTCAACTTTAGAAATTAAATCTATTAATAATTCTTCTTGTTCTTCTTGTTTAGTTAAAACAAAAATTTGCTTAAGAGTGTTACAACAATTATCTCTGCAACCTATTTGTATTCTGGGTGAACTTAAATCAGAAGACTCATGACTAGCAGACTCAGAACTTATAACCATAGTTTCATTTTCACTAGACTCAGAGTTTCTTAACTCAAGGACTTTAATTAGATTGTTTTTTTCTTCATCTGTAATTTTCAATTGTCGGATAGCATCTTTTACTTTACAATCATTTGCATAGTGGCCATACTTTTGACATTTAAAACATTTTACTTTACTTTTATCTTTATTTTTACCAGACTTAAAGCGAGCATCTTTCTTATACCGAGGTGCTTTTGACCATCTTTTCTTAGGGGAATATTTCTTCTTTTTGTAAAATTCATCAGGCTCATCATTATGTCTAAAAGACTTTCTATATTTGCTATAATGGCGTTTTTTACGAATATGACTTGGATAACTATTCTTGTTTCTTCTAGAGGGAGCTGTAGAGATTAAACCATATTGTTCACAAAAATTACCTAACTCATATTTAGCAGACTTTCGCTCAGATTGTATTTGCTTATTTAGTTTCATATCTACACACATTTTCATACCTTCTTGATTAATTGTACTTATAATATTACCATAAGTTAGGTTATCATAATCTATATGACCTAATTCATTACTTAAAACGGTACGTATTTTATGAGCAAACAGATTGGGTAGACCATTTATAAATTTTTCTTTCCAAAAAGCTTGATTACTATCTTCTCTTAACATTACACGAGAAATGAAAACATCTTTATACCACCTAAAATCACTTAATTGCGGACATCTTAGATTACTCAATTGATCATGTATTCTAGACTGCGTATGGCTTGGTGTTCCTACAAAATGTTCAATTATAGTGTAAAGCAATGTATTAACTCCATCTTCAGTGGCTCTGCCTATATTCGTATCAAACAAAGGAAGACCGTCTTCATTTAATTTAACAGCATGTATAATATTATTTCTAGACTCATTACTTAAATGTTTATCCCACCAAGAACGTAAGGTTCCTGTAAATCCAGTTTCTAAAAGAGGAACTATCTCAGATTGTCTTAAACTATGATTTGCAATGTATGTATTAGCCACCATTGACATATGTATAAGTTTATTCATTATTTCTTGCTCAGACAAACCATCTATATTCCATTCATACAATTTATCTGAAGAGACAGAAAATTGACTTGTATTATTTCTTTCTTCAAACTGAATATCAGGCGGTGTAGGTTTAGGATACCAATTCTTAGTTAAGCTAGTAGGATTGATTTTATTAGTTAATCGCTTGACTTTTAATTCTAAATTTTTAAATGCATTTTCTATTTTAGAAATATTACTAGACTTATCTGATTCGTCATCTGTAGCTAAACTTATTTCCGAATCACTATTATATAGGGAAGAAGAACCTAATACTTTAACACCAGATTTTTCAGGTTTAACAGGGGTTAGTTGTTCAAGCATTCCTTCAATTTTTTGCATGGTAGTGGAAGTTTTAAGTGCATGTGTAGGTTTTAAATCTTGAAAACAGACTAGGGGTTTTTCTTTAGACTTAAGTTTTGGCTCAGAAGGGATTAATTTATCAACTTTAGTTTCTATTTTATCTAATTGCTTTCCTATGACAACTAGGCTTTGATTAGTATAATTATTTTGTTCAATTAAACATTTATTAAGCTCATTACTAGGATCAGGTATCTTAAAGGGAGCAGCAGTTATAGTTTTAGTGCCAGCAGTTATCAAAATAGTTTCTACAGGGGGATGACTTGTAACTACTTCAGACTTATCCAATTTTACCCATTTTTCTTTAGTTATAGTTTTTAACTCATTAGTAATATAACGCATTTCTACAAAATCAAGGTAATTAATTTCTACTTTAGCAGTTCTCATATAATCTTTCCAGGCTTGGTGTATTTCATTTCTTTTATTTTTATCAGGGATGACTTTGTGATAATTATTTCTACGGGATGAGTTAATAGGATCTAAGGTATGTTTTTCTAGTTTATCCCAGTCTATTCTAAACGGCTTATCTATTTTTAGTACATTTAGACAATTGTGTATATCATAAACATGTGCAGCAGCAATCTCACTTGCAAGGAAATCAGACTGTGTAGGCGAATCTGTTTTAGGCTCAGGTTCATTATTTTCAACTGGGGGAGGACCATAACAGGGATGACTTACTTGATCTTTTTTCTTTAAAGATGCAATTTTCAAATCAATTAAATCTTTCTCTATTTCTAAATCTCTAACTGTAGTAGAGGAAGAGGCAGAAGAATGACGTGCAGGTTCGTGTCTCTTAGAAGGGCTCGGTGTAGGTAATTGCTGTAAATGAACTGTAGACCTAGGTTGGTGCTGGAAATTAATTTTAACACTACCATCCATATATTGATGAATATAATCTAAATTATTTAAACTACGTTGTATAGGCATAGGTTGAGATTCCGTAACTAATGTCCAATCTGTAGGCAAAGTAATATCAGACCATTTAACAGTATGAGGAATTCGAATATTAGCATCAGGCGTACAACTTTGTATTAAAAGCGTTTGATCTTTAGGACTCTTATTTAAAGCTTGAAAATTCATATTAGTACCTGTAACTTTATAATAAATTCTATAAACTAAGGCTAACGGTTGGGTACCAGGTAATACATTATAACCAGATGTCTTAATATTCAAAGTTAATGTTTTCAGGATATGAGGATCATTCAGGCTAACAGTAAAGTCAGGATAGCAATCAAAATGTACAGGGCCATTGCAAAGACTGGACTCAATCATACTAAGAACACTATCATTAAAATCTGTAAATCTAGCATCACGCAAACATAACAGAATAGAAGCTTGTAAACCTAATCTAGTTAAGGGTTTAACAGCAACTTGTACCAATCCAATATGCAGAAAGTTATTACCATCATGCTTATGTTTCTTGATTGAATCTAAATTAAGCATTTGACAAGACTCATGTTCTTTACTTAAAGCATAAACTTGTTCTACGGTTTTAACATGTGTAGTAGAATTAAATGCAGACATAGACCATTTCTTTCTATATATATTATGACTAGGGACCTTAGGTATATTCCAGTCTTTAAATTCTAATTGTTCATTATTATTATTATACTCAAAATCATTTAAGTAATGAGTCTAGAAATAATATTATACATGCTGTTAAATTAAATGAAGACGGTCTTCCTTTGTTTGATACGAATATAGGCAGAGCCACTGAAGATGGAGTCAATACATTGCTTTACACTATAATTGAACATTTTGTAGGAACACCAAGCCATACGCAGTCTAGAATGCATGATCAATTGAGTAATCTAAGATGTCCGCAATTAAGTGATTTTAGGTGGTATAAAGATGTTTTCATTTCTCGTGTAATGTTAAGAGAAGATAGTAATCAAGCTTTTTGGAAAGAAAAATTTATAAATGGTCTACCCAATCTGTTTGCTCATAAAATACGTACCGTTTTAAGTAATGAATTAGGTCATATAGATTATGATAGTCTAACTTATGGTAATATTATAAGTACAATTAATCAAGAAGGTATGAAAATGTGTGTAGATATGAAACTAAATAAGCAAATAATCTGAGCGAAAGTCTGCTAAATATGAGTTAGGTAATTTTTGTGAACAATATGGTTTAATCTCTACAGCTCCCTCTAGAAGAAACAAGAATAGTTATCCAAGTCATATTCGTAAAAAACGCCATTATAGCAATATAGAAAGTCTTTTAGACATAATGATGAGCCTGATGAATTTTACAAAAAGAAGAAATATTCCCCTAAGAAAAGATGGTCAAAAGCACCTCGGTATAAGAAAGATGCTCGCTTTAAGTCTGGTAAAAATAAAGATAAAAGTAAAGTAAAATGTTTTAAATGTCAAAAGTATGGTCACTATGCAAATGATTGTAAAGTAAAAGATGCTATCCGACAACTGAAAATTACAGATGAAGAAAAAAACAATCTAATTAAAGTCCTTGAGTTACGGAACTCTGAGTCTAGTGAAAATGAAACTATGGTTATAAGTTCTGAGTCTGCTAGTCATGAGTCTTCTGATTTAAGTTCACCCAGAATACAAATAGGTTGCAGAGATAATTGTTGTAACACTCTTAAGCAAATTTCTGTTTTAACTAAGCAAGAAGAACAAGAAGAATTATTAATAGATTTGATTTCTAAAGTTGAAAACCCTGAGTTAAAATCTGAGTATTTAAGAAAATTAAGAAAAATTATTACACAAGATACAGGCACAAGTCACTCTAGCCCAGTTATTAATTTGAGTTCTACAATAGAAAGATTTAATAAGAAAAAAGATGTTTCTTTACAAGATCTACATGCTGAAGTAAAACTAGTCAAAACAGAAATAGTAGAATTAAAACAGTTGAGTCATAAATTGCAAACTGAGAATTATGATATCCGGCAAGTCTTAAACGCATTATTAGATAAAGAACCTACTGAGAAAAATAGTCCACCTAGTAGTCCTAAGTATGAGTCTGGAAAAGAAGAACACGCTGTTAATCTAATTAAACAAGTTAATTTTAGAAAATGGTATTCTAAAGTTACCATATCTGTAAAAAACTTTGAGTTTAATGTTATTGCTTTGTTTGATTATGGTGCAGATCTTAATTGTATTAAAGAAGGTTTAATTCCTACTAAGTATTATATAAAATCTACTGAGTCTTTACGTACTGCTTCTGGTAAATCATTGCAGTTAAACTATGAAATTCCTAAAGCCCATGTATGTCAAAATAGAATTTGTTTTAAAACTTCATTTGTTTTGATTAAAAATATAACTGATGATGTTATTTTAGGATTACCTTTTATAAGCATGCTTTACCCTTTTCAAGTTGAATATGATGGTGTCGTTTCGAATTATTTAGGTGAAAAAGTTAAATTTACTTTTTTGACTAAATCTGAGCTGCATAATCTAAAGCATTTGCATAATAATGATTTTACAAAAATTATTACTTTAATTAAAGATAAAACAAATCATTTGAGTCATCTACAGCAAGAAGTCAACAGTAAGAGAATTGAGTATCAATTAAATAATCCCACTCTACAAAATGATATTCGTAATTTTGAACAAAAGATGATTTCTGAGATTTGTTCTGATCTTCCTACTGCTTTCTGGCATAGGAAAAATCATGTTGTTAATTTGCCTTACATAAAAGATTTTTCTGAGAAAAATATACCAACTAAGTCTAGACCTATACAAATGAATCATGAAATGATGGAATTTTGCAAAAATGAGATAAATGATCTATTATAGAAAAATATAATTAGGAAAAGTAAGTCTCCTTGGTCTTGCCCTACTTTTTATGTTCAGAAAAATGCAGAATTAGAACGGGGATCTCCTCGACTAGTTATTAATTATAAGCCTTTAAATTCTGTCCTTGAATGGATTCGTTATCCTATTCCTAATAAAAGAGATTTGATTAAACGATTAGATAAAGCTGTTGTTTTTTCTAAATTTGATATGAAAAGTGGATTCTGGCAAATACAAATTCATGAGTCTGATCGTTACCAAACTGCTTTTGTTACTCCCTTTGGTCATTATGAATGGAATGTTATGCCTTTTGGTCTTAACAATGCTCCTAGTGAATTCCAAAATATTATGAATGAAATTTTCAATACTTATAGTCATTTCTCTATTGTTTACATCGATGATGTTTTAATTTTTTCTGAATCTATAGAACAACACTGGAAACATTTGCATACATTTCTGCATATCATCAAGACTAATGGATTAGTTGTTTCTGCTAAGAAAATTAAATTATTTCAGACTGCTATTAGATTTCTAGGATACAATATAAGTCAATCACAAATTCACCCTATAGATCGAGTTATCCATTTTGCAGATAAGTTCCCTGATCAAATTACAGATAAAAATCAATTACAGAGATTTTTAGGTTCTCTTAATTATGTTTCTGATTTTTATAAAAATTTGAGAAAACAATGTAAACCCTTATTTGATAGACTGCAGACTAATCCTCCTCCCTGGTCGTCCATCCATACGAATATTGTCTTACAGATTAAACGTCATGTTAAGACTCTTCCTTGTCTTGGCATTCCTACTTCTACTTCTTTTAAAATTGTTGAGACTGATGCTTCTGATATAGGTTATGGTGGTATTCTTAAACAGAAATCTGAGTCTGATTCTTCTGAGCAAATAGTTCGTTTTCATTCTGGTATCTGGTCTTCCTCCCAGTTTAATTACAGTACTATTAAAAAAAGAAATATTAGCTATTGTATTATGCATTACTAAATTTCAAGATGATTTATTAAATCAAAAGTTTTTAGTCCGAGTTGACTGCAAATCTGCTAAGCATGTATTAGAAAAAGATGTTCAAAATATTGCATCAAAACAGATTTTTGCTCGTTGGCAAGCGCTTTTAAGCATTTTTGATTTTGATATAGAATACCTGAGAGGCAGTGAGAATTTTATTCCTGATTTTCTTACAAGAGAATTTTTACAGGATAAGCAATGGCCGGAACATCAAGAGATAAGCAAAAAATGAGTCACAACCCTACTCCCTGAATTTTGCCTGCTTCACTGAGCCATAAGCCTCCTACTTCTGTTAAAAATGAGTCTGGTTCCTTTTCTGTTAAATCTGAGCCTTTTACAATTACTCCTTATCCTGGTCATACACCCATACCAGTCATGAACAATAGATTTAGCCCCTTAGGATCTACAGTCACCCCTGCAAGACCCAACTACCAGTCTGCTTTAGTTTCTAGCTATGATCCGTTTCAGTTACCTCCCACTGCATCACCTATATCCTCTCCGTCTTTTGCCAAATCTTCTCCTTATGTCTCAAAACATACATCTAATCTTGTCATTATTGATTCTCATATCCCTACATCCATGAGTCCTGCCCAAATTGCAGCTTATCATTTTCCTCCTGGTTTCCATTATCTTCCCCATAGCCCTTACAAGTCCTTAAAATTTTACAGAGAAATTCTTCATGAAACCAAGTCTGTTGAAATTAAGCCTATTCGAGACAAAACTGATCCTACAAAAATTATTTATCACTCCCTTTATATTCACCAAATTTTACATCCTAAGTCTTGGGGATCTCGTCCTCATGAGCTCCGTACCCTTCCTTCTAAATATCAATATAATTATGCAGACTATGTTGAAGCCTGGTACTACATCTTCTTACATCAAACCCCTGAGTTTTCCCACTCTTGGTTCATTAATTTTGATAACAAATTTAAAAATACTGAGCTTCCTTATTGGTTCCTTCATTGGTGGGACACCCATGGTCCCAAATCTGATATCATCCCTTCTGCAGTTCATGAGTTAATCACTTATTTTACCCAAAAATACAAAATTTCTGAGCGAGATCTATTTTTTCCTATTGATCTCTTATTTTTCACAAAATACAGACTCCCTTGGATAATGAAATGGAATTATACTGTTAATTGGGAGACTCATTTCTTCTCCCGTCAGTTCTTTGTTAAATGGTGGGATCGTTTTGAAGTCCATCGTATTACAGATTATGTTTATAAAGACTTTCCCCAAGTTCCTAATCGTAACCCTGAGCCCAAGCATGAGCCCTCTTCTTCTTCGGATACTATCCTTACAGTTGAAGGTAAATCTAAGACTGAGTTACAGGACTTAGCCCAGCAGCTCCTTCTTCGAGCCTCTCAAATGCATGATGATGATGCTGAGTCTGAAACTTCTTCTAGTTGTCAACCTGCACAGCCAACTCCTGAGCGTCCTGTGCGATGGGCTGATTATCTGCATGGTCAAGATCCTAATGATGCTTATGATGAAGCCTATAAAGCCTATGATTTGAATTCTGATTAGACTACTGCAGACAAGTTACCTGAGTAAAAGCAAGTTACCTGAGTTAAAGCAAGTTACCTGAGTTAAAGCAAGTTACCTGAGTTAAAGCCAGTCACCTGAGTTACTTTTGTAAACCTGAGTTACTTTCAATGTTTGTCTTTAAAAGGAGGCTTCGGCCTCAGTTGTATCCATCTTTCTTTCTCTTCTTTCTCTTTAGTTCTTTTCTTAGTTTTTAAAATGTTCTTTCAGAGACCATGTTCTGAAGAACAACCTTAGTATGAGAATGAGCTTGTAAGTTTTATGAAATTACTTTCAATGCTTTCCTATATTTGAAATATCATCATGATTTCTTATAACATGATTTCTTACATATATGCAATATATTTATTTTCTCTTATAAGATTTATGCATAAGTTGCATTTGTTCTACTTCCTATATTATATAACAAGCTTCCGCTCTTGATCTTCCTTTTGGTATCAGAGCCGTAAGAGTTGTGAGAGTTTGTCTAGGTCGCAACTCATCCAACCCAAGTGGCTGGTCTAGGATTTCTGAGTTGAAGTTATTTGATTTCTTCTTTATCTATCTTTTATCCTGTAAGAGTTTCTTGTTGGTTTTCTCCCTGGTCCAAACAGTGAGTCCTCTTTCCAAACGATAAGTCCCGGGGTAGGGCACGAGCGGTGGGCGTGAGCGGAGGGCGAGATTTCCAACCAGTCTCTTGTCGGGTAAAGGTTAGCCATAGAAGTTATCATCTTTCTTCTTCTTCAGTCTTCCTCTTCCATCCTCTTGAGAAAAGATGAGTCGTTTCCTTCGTACAAATTCTTATAGTTCTGGCAGCTCTAGAACCAATATAGGAAATCTTCCTGAGATTGTAAATGAAGAACAATTTGAGTATAATAATAATAATGAACAATTAGAATTTAAAGACTGGAATATACCTAAGGTCCCTAGTCATAATATATATAGAAAGAAATGGTCTATGTCTGCATTTAATTCTACTACACATGTTAAAACCGTAGAACAAGTTTATGCTTTAAGTAAAGAACATGAGTCTTGTCAAATGCTTAATTTAGATTCAATCAAGAAACATAAGCATGATGGTAACTTTCTGCATATTGGATTGGTACAAGTTGCTGTTAAACCCTTAACTAGATTAGGTTTACAAGCTTCTATTCTGTTATGTTTGCGTGATGCTAGATTTACAGATTTTAATGATAGTGTTCTTGGTATGATTGAGTCCAGTCTTTGCAATGGCCCTGTACATTTTGATTGCTATCCTGACTTTACTGTTAGCCTGAATGATCCTCATATCCTGAAAACATTAACTTTGAATATTAAGACATCTGGTTATAATGTATTACCTGGTACCCAACCGTTAGCCTTAGTTTATAGAATTTATTATAAAGTTACAGGTACTAATATGAATTTTCAAGCTTTAAATAAGAGTCCTAAAGATCAAACGCTTTTAATACAAAGTTGTACGCCTGATGCTAATATTCGAATTCCTCATACTGTTAAATGGTCTGATATTACTTTGCCTACAGATTGGACATTAGTTACGGAATCTCAACCTATGCCTATACAACGTAGTTTAAATAATTTAGATTATATTCATCAATATATGGATGGTAGTGTTAAAATTAATTTCCAGCACCAACCTAGGTCTACAGTTCATTTACAGCAATTACCTACACCGAGCCCTTCTAAGAGACACGAACCTGCACGTCATTCTTCTGCCTCTTCCTCTACTACAGTTAGAGATTTAGAAATAGAGAAAGATTTAATTGATTTGAAAATTGCATCTTTAAAGAAAAAAGATCAAGTAAGTCATCCCTGTTATGGTCCTCCCCCAGTTGAAAATAATGAACCTGAGCCTAAAACAGATTCGCCTACACAGTCTGATTTCCTTGCAAGTGAGATTGCTGCTGCACATGTTTATGATATACACAATTGTCTAAATGTACTAAAAATAGATAAGCCGTTTAGAATAGACTGGGATAAACTAGAAAAACATACCTTAGATCCTATTAACTCATCCCGTAGAAATAATTATCACAAAGTCATCCCTGATAAAAATAAAAGAAATGAAATACACCAAGCCTGGAAAGATTATATGAGAACTGCTAAAGTAGAAATTAATTACCTTGATTTTGTAGAAATGCGTTATATTACTAATGAGTTAAAAACTATAACTAAAGAAAAATGGGTAAAATTGGATAAGTCTGAAGTAGTTACAAGTCATCCCCCTGTAGAAACTATTTTGATAACTGCTGGCACTAAAACTATAACTGCTGCTCCCTTTAAGATACCTGATCCTAGTAATGAGCTTAATAAATGTTTAATTGAACAAAATAATTATACTAATCAAAGCCTAGTTGTCATAGGAAAGCAATTAGATAAAATAGAAACTAAAGTTGATAAATTAATCCCTTCTGAGCCAAAACTTAAGTCTAAAGAAAAACCCTAGTCTGTTTTCAAGATTTAAAACCTACACATGCACTTAAAACTTCCACTACCATGCAAAAAATTGAAGGAATGCTTGAACAACTAACCCCTGTTAAACCTGAAAAATCTGGTGTTAAAGTATTAGGTTCTTCTTCCCTATATAATAGTGATTCGGAAATAAGTTTAGCTACAGATGACGAATCAGATAAGTCTAGTAATATTTCTAAAATAGAAAATGCATTTAAAAATTTAGAATTAGACTCTGAATTAAAAGTCAAGCGATTAACTAATAAAATCAATCCTACTAGCTTAACTAAGAATTGGTATCCTAAACCTACACCGCCTGATATTCAGTTTGAAGAAAGAAATAATACAAGTCAATTTTCTGTCTCTTCAGATAAATTGTATGAATGGAATATAGATGGTTTGCCTGAGCAAGAAATAATGAATAAACTTACACATATGTCAATGGTGGCTAATACATACATTGCAAATCATAGTTTAAGACAATCTGAGATAGTTCCTCTTTTAGAAACTGGATTTACAGGAAC

CTTATGCAATATATTTATTTTCTCTTATAAGATTTATGCATAAGTTGCATTTGTTCTACTTCCTATGTTATATATATATATATATATATATATATATATATAGCTTCAGTTTAGGGATATTTTTGTAGGATTCATTATCTCTGTAAAAGAATCACTTGAATTTGATATCATTTGACCACTCAATTAAATTATTGAAATTTTAGTACTTTCTTGAAGCACCGTGTTCGTTGATTTTCAATAACACAATTAGATGTCTAAATTATTTCTGATTTATCTAATTTTTTGTAAAGATGATCTATGAATAAAGACTTAAAAAATAGATGGTTTCAATTGTTAGAAAAAAAAAATTAGTATGAAACCTTAAAGGACGTCCCTCAAATAAAATTATTTGAGAGATCCCTCAATAGAAGGGAAAAGTATAATGTATGCGGTATTTATCGATAAAGCCTATAAAGTCTCATTCTCAAGCCTTTTACAAAGACCATCGGACGAGTGATTATGCTTACTCTAAGTATTAGAACTCAAGATCCCACATTGGAAAAATTACATATCTTAGGATGGTTTAAGAGCTCTTGGTAGATGAGTGAAGTCACCTCAAATCACGAAAATAGCTACCACCAGAGCAACAATGGCGGACTTAGAGAGGCGCAAGATGCACCTCCCTTCGCCAGAAAACATGTTGGAGGTGGCAAAGTTTGCTTCTATGGTCTGCCTTGGAGCTGCCCACCAAGTGCTCGTGGAAATGCCGCAGTGCAGCTTCTATTTTGCGTTTCTATGCTGCGCACAGGCATATTGCCAAATTTTTTTATTTTTTATAATTTTAAAAAACTCGTGGTTAAAACGACGTCTTGGTCAAGGGAAAAATAAAAATAAAAATTTGCTGCAATCAATGTATACAACGGAAGGGAAGGAGTCCTCTTGCAAGATATCAACAACCATATAAATTAATTGAAGGCCCAATCCCAAATCCTTAAAACTTGAATAGCCTCTTCTCTTCCTT

>Chr2-26; OZ022207.1; 17843359-17846519; dir; B;

ATACATACGTCGTAAATAATGACACTGACACTATATCCACGTCATCATTTTTAGAAAAATCGAAGTGGAAAACTTATTTCACGACGGTCGTTTGTAAATAAGAGACGTAGTAGTTTTCAATAACGTCGTCTTCTCATGTATTTAAAGATGTCATATTTTTTCTTGTCGTGAAAATTATATTTAACGGCGTAGTGTTTTAGTTATCGTCGTTGTATGTCTATCTTGCGGCCTTGTTCTTCTAATATTGTCATATTTTTCCTTTTTAACGACGGTGATTTGTTTGTGTTGGTCGTCTATTCTCATCCTCGACTATTTTTTAAAACTTATGCAGACTAAACACGTCTGTTTTTATTTTTTTCGACGTTGTTTTATTTAACAACGTCTAATATTTATCGTCGTTGTTTGTTTCTGAAAATAGGACGTAAAATTTTGTAATACGACTCTCATATATTAGTAACCGACGTTGTTTGTTTTTTCAACGTCTATTATATAAATACATACGTCGTAAATAATGACACCGACAACTATTTCCACGTCATCTTTTATAGAAAAATCGAAGTGGAAAATGTATTATACGACGGCTGTTTTTATATAGGCGACGTAGTAGGAATAAATAACGTCGTCTTCTAATGTCTTAAAGACGTCATATTTTTTGTTGTCGTGAAAATGATATTTAACGGCGTATTATTTTAATTTTCGTCGTTGTATGTCTATTTTGCGGCCTTGTTCTGTTAATATGTGTCATATTTTTCCTTTTTAACGACGGTTTGTTTAGAGAGTTGGTCGTATATTCTCATCCCCGACTGCTTTTTTCGATCTTTTTGCAGTAAAAAGACGTCATTTTTTATTTGTTGATGACGTTGTATATTTTAACAACGACGGTGATTTAGCGTCGTGGTTTATTAGGGAAAAGATGACGGTGGATTCCACGACCGCTGAAAAACTGAATACACGACGGTGATTTACCGTCGTCTTTTTGCTTTTTTGTAGTAGTGTTCGT

TCTTGGTGGGACAATCACTTAACTCCTGAGTCTAAGCAACGAATAATTCATGCTCTAAAACTTAATGAAGATGGTCTCCCTATTTTTGATGAACAAATAGGTCAAGGAATTGAGGATGGAGTCAACACATTGCTTTACACAATAATCGATCGTTTTATTGGAACACCTAGTAGCACCACCACCAGAATACATGATCAATTGAGTAATTTAAGATGCCCTAAATTATCAGATTTTAGATGGTACAAAGATGTGTTTATTTCCAGAGTCATGAGTCGTGCAGACAGTAATCAACCTTTTTGGAAAGAAAAATTTGTTAATGGATTGCCGAATTTGTTTGCCCATAAAATCCGATCAACCCTTAGTAACGAACAAGGTCATATTGATTGGGATGGTTTAACTTATGGAAATATAATTAGCATAATTAATCAAGTTGGTATGAGAATGTGTGTTGATATGAAAATTAGTAAACAGATCCAATCTGAAAGAAATTCGCTAAATATGAGTTAGGTAATTTCTGCGAACAGTATGGTTTAATATCTGCCCCGCCGTCTAAGAAAAATAAGCCTAGTCACCACAACCGACTGTTTAGTAAAAAACGTTATTATTCACGCAGAAAACAGTCATTCCAACACAATAATGATGATAGAGAATTTTATAAAAATAAAAGGTTTTCACTTAAGAAAAATCAAAAAAAGAGTCATCACAAAGAAAAAAGTCACAGATTTAAGAAAAAAACCGACAAAAGAAAAGTGAAATGTTACAAATGTCAAAAGTATGGTCACTATGCCAATAATTGTGAAGTTAAAAATACGATTAAACAATTAAAAATTACAGATGAAGAAAAAGACAAATTAATCAAAGTACTTGAGTTAAGAAATTCTGAGTCCAGTGAAAATGAAACCATGATTAGTCATACTGAGTCCTTTGCAATATATTCTTCCGAGTCACAACCCATTTCCCCCAGAATCCAATTTGGTTGCCAAGATAATTGCTGTGCGTCTAAATCAATTAGTGTCCTCACAGAACAAGAAGAACAAGAAGAACTATTAATTGATTTAATAAGCAAGGTTGAAAGTCCATAATTAAAATCCGAATATCTGAAAAAATTAAGAAAAGTTATCAGCCAAGAAGAACCGAAACAGTCTACCCAAAAGATAAATCTAAATACAACCTTAGAAAAATTTAAGAAAAAGAAAGAAGTCACTTTATAGGACTTAAAAATGGAAATAAAAATAATCAAAAAAGAAATCATTCAGTTACGACCAAGGTCTAAAATATCGATGATATCGGAAATATCGGTAGTCTAAAAATATGGAAATTTCAATGGAAATATCGGGATATTATCGATATCGATAAAAATTGAATAAAAACCACGGAAATTGTAAGAAAAACTTGGAAATTTTCATTGAAACTTTGGAGAATGTTTATTTAGTCAATTATCTATGAGTTTATCACAAAAAATTAGAAGGAAATGCATTGCATGATGGATTTAACTAATTTTAAGTTGATTATACAGCAAGCGGGCAAACACTATGAGTGTAAAAAATATGTAATAATTAATGAAAAAGATTAAATACACCGTAATCATTTATATATAATGATTTACTATAATATTTTACACTTTATACATTGCATGGTAAGATACATGAGTGACTTAGTACCACATAGAGTTCCTACGAGGTTTAAAATTTTCACTATCTTCATCATCTCTATGTGTAGAATAAGTGTATTGTGAAGAGTAGTCATCAAATGATAAATCCCCAAAATAGTTTTGCATGTAATTGTTAACATACCACCCATATAAATATAAATATTTTAGCATATTATCACAAAAACATAAGTGATATGGTGTTTAAGGTGTTGGAGGAGTAAAATGGGAGGGGTTCATATCCCCTTTGTGCTTTTTTCTCCCATTTTTCCCTTTTTTTTAAAAAAAAACTTTTTTTTTCCTTCACATCGATATTTTCGATATTTTAGTGATATTACACCGATATTTTGACAAAATATTGCTGAAATGTGAGCGAAATTATCGACATCGATATTTTCCGATATTATCGTCGATATTGTCGATATTTATACGATATGTACATGGATATGTTCCGAAATATCCGTGATTCGAAAAAACTGAAATATCCTCGATATTATCGATATTTCAGACTATGGTTACGACAATCAAGTCAAAAATTACAATCTGAAAATTATGAGATAAAACAAGATCTGATCAATTTATTACAAAATCCAAATCCTGAGTCTACCCCAGAGAGCCCAACTAACTCAGATGAAGAAAGCCCTAATCACGAGCAAGCCGTTAATCTAATTAAACATTTAAACTTTAGAAAATGGTATGCCCGAGTCAATATATTTATTCAAAATTTTGAGCTAAACATTGTTGCCCTTTTTGATTCAGGAGCTGACCTTAATTGCATCAGAGAATGTTTAATCCCCACTAAGTATTATCAAAAATCAAAAGAATCTTTAAGTACTGCTTCAGGAAAATCCCTTCAATTAAATTATGAAATCCCCAAAGCCTATGTCTGTCAAAATAAAATTTGCTTTAAAACTTCTTTTGTCTTAATCAAGAATATTACTGATGAAGTCATATTAGGTCTACCGTTTATTGCCCTACTGTACCCATTCCAGGTTAAACATGATGATGTCATTACCACCTGTTTAGGAGAAAACGTTAAATTTGAATTTTTGAGTAAACCTGAATTGCATAATTTAAAAGAATTACAAAAGAATGCGGTCACTAAAACTGTCCAGGTCATCCAAAACAAAGATAAACAATTAAGATTTCTCCAAGAAGAAGTTAAAACCAAACGAATTGAGCATCAACTAAGTCATACGTCTTTTCAATTACAAATACAACAATTTGAAGAAAAACTAAAACAAGATGTTGTTCCAGTGACTTTATAGTAAATTCGATAGACTAAGGCTAGAGGTTGACTACCTGGTAGGATATCATAGCCATCAATCTTAATATTTAAAGTAAGAGTTTTCAGGATGTGTGGATCACTTAGGCTTACTGTGAAATCTGGATAGCAATCAAAATGAATTGGGTCATTGGTAAGACTAGACTCAATAATTCCTAATGTACTGTCACTAAAATTATTAAATCGAGCGTCACGTAAACAAAGCAGAATTGAAGCTTTTAATCCTAA

TCTTTAAGACTTAGCAATTATAACTGAATTATCATATTTCAATGACTTAAAGGGCTGGAGCCTGAATATATATGTAATTGGGGTTTAAGCTTAGCAATTTCGGATGATCTATGTAATTGCTAAGTCTTAAAGGGCTGGAGCCTGAATATATATATATATATCCTTATCCAATAAACTACCAACGTGGACTGGTCATAGCTGATTTTTGGCTTCAAAGTTATGTCATTGGAAAGAAAACAAGAACATGGCTATATGAGTAGAATAATGCACGCTTTAACAACTCCATCGGTTTCCTTTAGTTTTTACATAGTCAACAATTATTTTCCCAGTATAACAAACACCTTTTTCTATATTTGAAGAGGGGTGGTGGGTTCAAGGTTTACCTAATCTCTTGATGATGATGTTCCTTATCTCGTGTTAGGTGGCAATACAAAAGAAATTATAAAATAGAGTTATAAGTGGTGAAATGTTGTATGTATAAAGATAGGGGTGAGTATGGTCTGGATTGGACTGATTTTATCATAAAATTATAACTGAATCAATTACAATAAAATGGTTTAATTCAGTTTAGTATTGACAAAAATCACTAAGGAACCTAAACTAAACCAAACTAATTTTGGACAATTTGGTTCGGCAAGTTTTGGCCTATGTCCAATCCTTTTTTCTTTTATATATTTCAACTTTGTTAGCTCAATTACAACATAAAAAATTCATAACTTGCTATAAAATTTTCATTTTCTAACACATCAAACCACCCTAAAATTCAACATGCCATGAAACTTCAAAGTCCTGTAACTAATATTGTTACAATTCAAGGATCAATTTTCAAAGTTCCATTAAACTTCAAATTAATTAACATACTTATCTAAATATTATTCAATAAGAGAATTGCACATGTAAAATAAATACTAAAAAGGACAGTTCAATTCGATTTACGTCAGTTTACAAAAGCTTGCAACTGATCAAAACATATATGATTTTGTTCGATTTTAAACCGTTT

>Chr2-27; OZ022207.1; 19656500-19658045; dir; B;

ATTGCCTTCTGGCTTGCCCAGCCTTGATGGAGCCCATCATCTATCCATACTAGATTTGATTGGTGGAGTGTGTTTTTGCAATCCAGGGGGCCTTTTCCCTAACAATCCAAACCGGTGGAACCGCTCTTAGTAGCGTGTGTGAGAGAAATCTCCATTCCCTTGTAGTTTAGATTATCATTGTATTCAAAAAACAAAAAACAAACTTCATGCATGTTATGGTAAAAATTATAGAAACCGGCCAAATCTGATTGAATTAGATTAATCACTCTGTACTGATTAGATTCTGAATGCTTATAAAGATGGAAAATGTTATTTGGACACCCAGCTCAAAACTTTTTTTTGTCAAATATTTCCAATGTACCTATAAAAATACATTGAGCTGAAGTTGGAATTGGTTAGCAATTTTTGGATCAAGGCAAAACCCCACTTAAAATCTCAACTATAATTTGAATTTTGATGATCTTTCTTTGTTGGGCCAAAAACCGAAAGCAAAATTTGTTATAAGCAACTGGACTTTAAGCCCATATTCAAAAGAGAAAAATAAGAGAAGCTCACAGTGAAACAAAATAAAAACTCTCCATTACGTTTGGTATTCTATTCAAATAGGGAACTCAAAAACTTAATTTTTCTTAAAAACAAGGAGTTCTTAAATCTATTTTTAAGATTCAAAAACTTGTTTGGTATGCAACTTGAAAACTGTTTTCAAATCTTAAAAATTTGAGAACTTGTGGCTTGTTAAAAAAAAACTGAAAAAACAAATATTTTTTGTTTTTAATAATTGGGGTGTTTTGTAGTTGTTCTTGAGTTCAAGTTTTTAAAAATAGGGTTACTAAAAAGGTTTTCTTTGTTTTGAACTCAAATTTTATTTTTGAATTTATAAAGTTGGATTTAAATTGAATACCAAATATAGGCCCTAAATTTTCAGACCCATTTATCCATGGCACTCTCCTACCTACCCATTAGGTCCATTACAGAAAATGTATATATATATATATATATA

ACTAAATCTGAGTTGCATAGTCAAAAGCATTTGCATAATAATAATTTTACAAAAACTATTACTTTAATTAAAAATAAATCAAATCATTTGAGTCACCTACAGCAAGAAGTCAAAAATAAAAGGATTAAGTTTCAATTAAATGAACCTACTTTACAAAATGATATTCGTAATTTTGAACAAAAGTTGGTTTCTGAGGTTTGTTCTGATCTTCCTACTGCTTTCTGGCATAGGAAAAATCATGTTGTTAATTTGCCTTACATAAAAGATTTTTCTGAGAAAAATATACCAACTAAGTCTAGACCTATACAAATGAATCATGAAATGATGGAATTTTGCAAAAATGAGATAAATGATCTATTACAGAAAAATATAATTAGGAAAAGTAAGTCTCCTTGGTCTTGCCCTGCCTTTTATGTTCAGAAAAATGCAGAATTAGAACGGGGATCTCCCCGACTGGTTATTAATTATAAGCCTTTAAATTCTGTTCTTCAATGGATTCGTTATCCTATTCCTAATAAAAGAGACTTGATTAAACGATTAGATAAAGCTGTCGTTTTTTCTAAATTTGACATGAAAAGTGGATTCTGGCAAATACAAATTCATGAGTCTGATCGTTACAAAACTGCTTTTGTTACTCCCTTTGGTCATTATGAATGGAATGTTATGCCTTTTGGTCTTAAGAATGCTCCTAGTGAATTCCAAAATATTATGAATGAAATTTTCAATACTTATAGTCATTTCTCTATTGTTTACATCGATGATGTTTTAATTTTTTCTGAGTCTATAGATCAACACTGGAAACATTTGCATACATTTCTGCATATCATTAAGACTAATGGATTAGTTGTTTCTGCTAAGAAAATTAAATTATTTCAGACTGATATTAGATTTCTAGGATATAATATAAGTCAATCACAAATTCACCCTATAGATCGAGTTATCCATTTTGCAGATAAATTCCCTGATCAAATTACAGATAAAAATCAATTACAGAGATTTTTAGGTTCTCTTAATTATGTTTTTTATTTTTATAAAAATTTGAGAAAACAATGTAAACCCTTATTTGATAGATTGCAGACTAATCCTCCTCCTTGGTCGTCTATCCATACAGATATAGTCTTACAGATTAAACGTCATGTTAAAACTCTTCCTTGTCTTGGCATTCCTACTTCTACTTCTTTTAAAATTGTTAAAACTGATGCTTCTGATATAGGTTATGGTGGTATTCTTAAACAGAAATCTGAGTCTGATTCTTCTGAGCAAATAGTTCGTTTTCATTCTGGTATCTGGTCTTCCTCCCAGTTTAATTACAGTACTATTAAAAAAGAAATACTAGCTATTGTATTATGCATTACCAAATTTCAAGATGATTTATTAAATCAAAAGTTTTTAATCCGTGTTGATTGCAAATCTGCTAAGCATGTATTGGAAAAAGATGTTCAAAACATTGCATCAAAACAGATTTTTGCACGCTGGCAAGCTCTTTTAAGTATTTTTGATTTTGATATAGAATATTTGAGAGGTAGTGAGAATTCTATTCCTGATA

TATATATATATATATATATATAAGATACTCATTGCTCTCTCTCAAGAGGAAAAAAAACAAAAACCCAAGAATGAAAGAAAAAAAAAACAAAAACAAAAAAGCTAAACGTTGTTCCTTAACAAGAACAGCAAACACTTGGAACATTGACAGTAGTTATAAGCATCTTTCAGTGAGGGATGCGATAACGCCCTAAGCTGTGAGCCAAATTTATGTACAGGACCAAACTTTATTTTACCCTATACATCAAACTAATTCATTAGAATTTAGAAGTTGAGAAGGTTGGGCAAAGGGGTCAGCTTTTGAAGCCAAGGGCTATTCATTTGGGTCCAAAATGTGTCTTGGGTCCAGTCCATGCTCAATCGTGGCACTTGGAGCTCTGGTAATATGTACGTAGGAGAAGGGACTAGCGGACTCAAGAATTTTTTTTATAAATTTTTGTTTGTCATGAGTTGTGAGACTCTCTTTATATTGATGTTGCAAATGCATATGCAGGTCATTTCAAGGATTGAAATAGTAGTGAAAGTGGGTGGTGTTCGTAGAGTTGTTAATTATACCGCCATCACAAGGGTATCTGTGGTCTTCATGGGCACACTTGTGTCTAAAGTATAAACAGAGAGAGAGAGAGAGAGAGAGAGAGAGAGAGAGAGATGTTTTAATTTCTCAAAATTTTTTTAGAATTATTTTATTGTTGAGAAAGTTATACAAAATCTCCATTCATGTTAACACATGTAGCACCAGCACTTTTTGCCTTTGGGACCATCAGCACGGCAAAGTCCCATCTTTGCTTTCTCTCCTTTGCATTGTTTGTCACACTCTGGGTGGGCTGTGCAGGTGGTCATCGACTTGCTCGCCTGCTTGCATCCTCTAGTTCCTCCTTCAACCATTGGCAATTCTGTATATTATCACCCAAAAAAAAATATATAGTTTCGTAATTTCTTATATTCTTCTTAATAATACAGCTTTGTAAGACTAACAACAAAAACAAAGAAGGTATATTTGA

>Chr2-28; OZ022207.1; 23977183-23985538; rev; D;

GGCTTTGAAATTGCATTCAATCCCAACCCAATTTTGCTTGAATTTATTTACTATACTTGTTATATATATATATATGGGTGAATCCGTGACACTATATTTTTTACACAAGTTTTGACACAAATTTTCAATCATTAAATTGAAATGGATTGAATGGCTAGGATTAACTAAACTAATTATATATTTTTTAAAAATATATATATTAAAAGTAATTATTAATTGACTTTCTCTTTCTTATTTCTCTATCGTCAATCAATGTTTTATAAAATCTTCCTATTTCGTTGTTCTCTATCCTCTAGAATTCTTCAGTTAATAACTTTCCCATCCTCCTCCTCTTTCTTCTAAAGCTTTACACAAGTTTCTGATTCTTTTTTATCTCTATCCCTAACTTTGTTTTTTGCTTTTTTTTTACTGCTTAGTTTTTGAGTTATTACAGTTATACATAGATTATGACTCTCGAAGTAAAGGCTTGGGGATAACACCCCATCCACACAAACACACAATGAACTGCACAGCAAACTGTCTTTTGCCATATATTTTTTAATGTTATTAATCAACCAAACGGTAGAGATGAAATATTGCAACAAATCTGGGCTTTTTCTTTTTTGGTTAAATATTAATATCTTATTTTCTCTTCAATATTGGTAGCCTGAAACAGATTTTCTATGTCGTGGAAGAATGCTCGGTTGGTGTAGATGACAATGGCAAGGGGAAGGTAATGGTGAGGTTTCGCAGGTTATTAATTGAGAAGATCATGATGATAAGAAAACAAAAGGGGGGAACGATAATTGAGTGATTTTCGGGATTATCATAATTAATAAATAAAAATGAAATTTACTTGGCATTAGAGATTTAATTTAATTTAAGTAATTAATGAAACATAATCTCAACCATTCAATCCATTCTAATCTAATGGTTGAAAATTTGTGTCAAAAACATAGTGCGACGGATCCGCCTCCTATATATATATATATATATATATATATATATATATATATATATA

ATAAATTTCCTGATCAGATTCTTGATAAAAGTCAACTCCAGAGGTTTTTAGGATCTCTAAACTATGTTTTTGACTTCTACCAGAATCTGCGAAAGCAATGTAAGCCTCTGTTTGATAGATTACAGAATAACCCTCCCCCTTGGTCAACCATCCATACCGAAGTTGTCAAACAGATTAAGACTCATGTCAAGACACTCCCCTGTCTTGGTATTCCTTCAGTTGATTCCTTCAAAATAGTTGAAACTGATGTCTCTGACATAGGTTATGGTGGCATTCTCAAACAAAAAGTTTGTCCAGAATCTCCAGAACAAATTGTTCGTTTCCCTGGAACCTGGACCCAGTCGCAATATAATTATAGTACTATTAAAAAAGAAATATTAGCTATTGTATTATGCATTAGTAAATTCCAAGATGATTTACTTAATCAAAAGTTTTTAATACGAGTTGATTGCAAATCTGCAAAACATGTTTTAGAAAAAGATGTTCATAACATTGCATCTAAACAGATTTTTGCAAGATGGCAAAGTATATTAAGTATATTTGATTTTGATATTGAATATTTGAGAGGATGTGAGAATTCAATCCCTGATTTTCTTACTCGAGAATTTTTGCAGGAAACACAATGGCCCCCAAAAAAGACAAAAATAAAGAACCTACCAGCCAGCCTAAGCCATCTCAGTCGCAACCCAAGCCATCTCAGTTGCAGCAATCCCAGTCCTCATCCATGAGTCCTAATAAGCCAGAAATGCCCAAACAGATAGTTCCCTATCTTGGTTGCACCCCTATTTCAGTTGCTACCCCATTATCAGTTGCAAACAGATTTTCCTTTTTAGGATCCACTATTGGTCAAATTCGTCCTAGTTACCAATCTACATTAGTTGCCAGTTATGATCCTTTTTCAGTTGATGTCCCAGCCGGACCCTCAGCCGCACCCAAAAAGACATACCCTTATTTGCCTAAGAGTCAGTCTCACCTGTTTGTTATTGAGCCGTTGTATGATGTTCATCACAACCCAGTTGAAATTGCTAAACACTATTTCCCTCCTGGATTCCATTATATGCCTTCTAGTCCTTACAAGTCTCTCAAATATTATAGAGATATATTACTTGAAACCAAGTCAGTTGAAATTAAGCCGATACAGGATCGTGACCATCCTGAGATCATTCTTTATCATTCTTTGTATATTCACCAGATCATAAGCCAAGAATCCTTCAGTAGTCGCCCTTATGAGTTAAAAGTCCTTCAGTCAAAATTGCAGTATAATTATTCTGATTATATTGATGCCTGGTACACTATTTTCCTTCATCAATCTGAGGATTTTACTCATTCCTGGTTCATCAATTTTGATAATAAGTTCAAATGTTCTTTCCCATATTGGTTCCTCCATTTGTGGGAAAAACATGGTCCAGTTGATGAAATCCTACCGGTTTTAGTCCATGACCTAATCCGCCGCTACACTAAAACGGCCAAGTTCTCTAAAGTTGATTTATTTTTCCCAAAACAGTTGCTTTTTATCTCCACGTATAAGGTTCCACGGATCCTCAAATGGTCCTACAGAATCACCAAGGATGCAAGAATCTTTGCCCGCCAATTTTCAGTCAAATGGTGGGACAAATTTGAAGTTCAACGAATCGCCCGATATGTATACAATGATCTCCCTGCTGAGCCATAGGCAACTTCTCCCACTGGTTCTGTTCGATCTTCCCTCTCAGTCGAAGGAAAGTCCAAATCGGAGCTCCAGGAAATTGCCCGCCAGTTGATCATTCAAGCCTCCCGGATGGATGATGATGAGGACGACAATGTTTCTCCTGCATCGTCCAACTGTCAACCCCTGCCAGATCCAGTCAAGCCCAAATCATGGTATGAGGATAGTCAAGACCCCTATGATGCCTATGATTTGCATTCTGACTAGGCCCTGTAATCAAGAAGTCAAAAGTCGCCATAATTCCCGCTGGATATTGCATCTGCCCTTTGCTTTTCCACCGAAAGTAGCCATCAGTTCACCCTACCCGAATCAAGACATTTGGAAAGTCAGACCAGGTTGCCAGCTGTCGTCATAATTGCTTCAGCCAAAGCATCTGCCTCCTACTTTTCACCGAAAGTTGCCAACCGACAAATTTGAAAGTTGAATCAGGTTGCATGTAACTCCATTATAGCTGACAGGACCCGACCCAATTTCCGCTTTGGAATCCGAGCCGAATCCTGTGCGTGTCCGACACCTGGCGAATGTCAAGCACGAATGACCTTTTTACCCTTTCTTTTTTCCAATTTCTTTATAAAATTTCCCTCAGACTTCTGCCGAAAATTCGGCAGAGTCTCCCCTGTAATTTGACCAAACCCAAAATCTTCCACCTGTCAAACAATCTCAAAAAATCCTACCAACGGCCAGACTAATTCAACAAACAGATCTTAACTCCAGTTTCTCAAGTTCAACAAGATATCAGAGCATGTTTATACAAATTTACAGAACTAAGAAAAGTTAAAACGGGGTCCTACGCTTCGGTGGTGGGGCGGGAGCTAGGATGACGGCCCGGGTGGTTTCCTTCCTTCTTCTACGGCCTGGGGGCGAAAAACAAGTTAAAATTGCGAGTGACCAAAAATAATGTTCGTGAAAACATTTGAGAACATAGTAACCCCCGTTTGAAAAACATAGGGACATAAAAGCTACTATTTGATCATAATCAAACTCGAGATAATCATTTAAACATAGATAAACGTAACTATAGAAATGTTCACATACTAAACAAAGATATAACAGTATAACACATGTGGAGTATCGAAGATACTGATATAAATGAATGAAATAATAGCTGCATTAAAAAGTTTTAAAAAGAATCCTTAAAAAAACAGCCACCTACTTGTACCCAGTGATAACCGTCAATTTCCTGGCAGGTCTCGGGCGTCACACAGGCTACCCGAGCCATAAACTGGCAAAATCAGGGAACTATGATCAGCCTGATCCGCCGGCAGGTCCTCGATGACACCAAGTCAGCTCGAGTCGCTCAGGCAGGACACAGGGGACCGTAGTCAGCCTGACCCGCAATCCTGGCAGGTTTCGGGGACACAGAGTCAGCCGAGCCACAATCCTGGCAAGTCTTGGGACACCAAGTCTGCCGAGCCTCAAATCCTGGCACTCACGGTTCGAGCGTCCCCGAAACTCGTGAGGCAAGTCAAGTGCACTGACTGACTGAAATTGGACCGGACGTCCGTCGACATCAGTCCAACTCTGGGTAATCACCAAATAGAGAGTGGGTACATGGTGGTCTAAATAACTATCTCTGATAAGATCTGATACCCTCTGTACTCTGATAAATCTGAGCTAAACTACTATTTTGGCTCAAAAATCTCAAATCTGTTGCTGGACTAATTTTTATGAAAATAAAGATGTTTTCACAATAAAATTTATGCTATAACACTTATTCAAGATCAAATAGAAAATATAATTATAAAATCTTTTAAGAAAGAAAAGTCCACTCACTGCTGGTCCGAGCTAGCTGGGCCCTTTGAAGGTCCCTCCTGTGGGTTAGCTGGACTCCTGGTGTCTGATTATCCAAGAATAATGAATTAATAAACTACTGGATAAATAGAATTTAAATTAAACACCCTGCCCTCGACTCCTAGAATCACGTGCACTTCATCCCAAATTACTCCAATGCCCACATTAGCCTTTTCAAACACTTGGACCAGTTTTGGAAGGTCCGACACTCGGCTAAGCGGTAACCTGCCAGATCGGCCGACCAGGGACCCGTGGGGTCCACAATCTCCGATGGCCAATCTGGGCTTCCCTGAAGGTTTCTCACGGAGGAGGAACCATGTTCTGCAAATTTGGTCCGAATCGGACGGTCGGATTAGCCAAAATCGCGTTATCGCTTAAAATCCAAACCATAGCCCCAGGGTTCGCGATTTCGGAGTATCCGGGACTCCGATTCGCAATCCGTCGAATCCTACGTGGTCTTGAAATCACGTAGACCGACATATCCAAAATTCAGCCCGATCCAACGATTTAACGACACTGCACACACGGATCGCGCGATATGGAAAATCCGTTCGGGGCTCAAACGGACTCCAAATCGAGATCCGCGAAATCCTACGTGCTCGTGACGGCACGAGGACCTCAAAACTGCCCATAAACAAGCCACCAGCCTCGCCCACGCGCCGCCACACGCGCGGGCAGATCGGGTGTCCAAAGCGATTAGGGTGCGTCGAAAAATCTTGGAACCGAGACTCCAAACTCCTATCCTAGGTAAAACACCCCATTTGGAGTCACTTTTGTTCTTGGACAACCCCCAAAAAGTGGACGGAAATGGCCGATCACGGCGGCCGAAGTTCGGCCGATTTTCAATTAAAAAATTAAGCAACCTAGAGGCGAGATCGATGTAAACCCAGCCAACCATCAGTTAGAGCACGTAAAATAGGTGGAAAACCATACCTCACTCGAAAGATTTGGTTGAGAAACGAGAGAGAAAATCAAACTGGAAGTTTGATCACAACAATCGGCAACAATGGCCGATTCCGTCCAATATCCGGTGAGCTGAGGTCGGCGGCAGGTCCAGGGAGGTAGGCGGCGGCGGGGCGGACCTGACGGCACCGGTGGCGAGGGTCGCCGTGCCCTGTGGTGGCGGCGGCTGGAGCTGAAACGGGGGAGGGGGGCTGCGGGTCGCACGGGAGAGGAGAGAGAACTGAGGAGAGAGAAGGGAGAGGGAGAGATAAAAATCTGATTTTTCCAAATTACCGTTTTGCCCTTCGCGATGTTTTGATCGTATTTTGTTCGTTAGAACTCCGATTCAGGTCTACTTCGTGTCTACGGACTCATTTCGCCGTGCTCTACGCAACAGCGCAGGCGGAATTCTCAAATTCTTTTCCGATCAAAAAGTCAAATTTTCCCCTAATAAAATATGCGAGGGCAAAATCATCTTTTGACTAGAATAAAGAAATCTTAATTTTTAGATATTTTGGTTTGGGTTCTTACAATAGCAACCAACCACAGTTAGCCAACCATAGTTGAAAGAACTCTAGTTACTTTTTCCTTTATAAGCAGGCTTCGGCCTCAGTTGTAAGGGGAGGCAATTTAGAACATAAATTTCCTTTTCTTCAGTTTTAAAGATTCTCTCTGATACATCCTTCGGAGAAATCATTCTTGTAAATAGCTTGCAGTTGTGTTTGAAGTTGCAGTTGCAATCTTTGTGAGTATAAATAAAAGTTATTTTCAAATTATCTATTCGATGAAACGCATTATTTTGATGCATTATCATTATTTTGCTGCATTAGTCTGAAAGTTGTTTAGGTTGCATCCAATTCTATTCCTGTATTCTATTCTTCTGTGTTTATATTCAGCCTGTCCTTCAGACTGTCTCTGTCGTTCTCTTTTGTTGGTATCAGAGCAGTAAGTGTTACGGTCTTTATCTACGTTTGCTACTCATCCCCGCCCAAGTGGCTGGTTTAAGCATCTGAGTAGAAGGTTTTCATTTCTTCCTTTTCTTCCCTTTTATCCTTTAAGTCATCTGTTGCTCTTTCTCTAAGGTCTGACCCGTGAGTCCTTTTTCCTAGATCTGTAAGTCCCCAGAGAGGGCTTGAGTGGTGGGCAGGAGACAGAAAATTGAGAAAGATTAATAGGTTTCTTAGAGTCTAAACGGTCGGTTTAGGATCTACTGTTCATCTTCTTCCTCAGACAGCCAAACCCAGTTATTTGAGTAGTCATGAGTCGCCTATTTAGAACAAAGTCTTGTAATTCCAACAGTTCTACAGCCAGTATAAGTCGACTCCCAGAGATAGTTAATGAAGAACAATTTGAATATAATTCCAGTAGTAAAGAACAATTGGATTTTAGAGATTTGAATATACCTAAAGTCCCCAGTCAAAAAATATACAAGAAACACTGGTTGCCCTCTAGTTTTAATAGCACAACACATGTCAAAACAGTTGAACAAGTTTACGCCCTAAGTAAAGATCATGAAACTTGTCAGTTTCTTAATCTTGAGTCAATTGCAAAACATAAGAAAGATGGTAATAATTTCCTGCATATTGGCTTAGTTTAAGTTGCTATCAAACCCCTGACACGTCTAGGGTTAAAAGCCTCAATCCTTCTTTGTCTAAGAGATGCTAGATTCACTGAATTTAGCGATAGCACCTTAGGAGTCATTGAGTCCAGTCTGTGTAATGGCCCTGTCCATTTTGACAGTTATCCTGATTTCACTATAAGTCTAAGTGACCCCCATATCCTGAGAACTTTAACTCTGAATATTAAGACTGAAGGTTACAATGTTCTTCCAGGAACTCAACCTTTAGCCTTAGTTTACAGAATTTATTATAAAGTTACAGGAACAAATATGAACTTCCAAGCACTCAACAAAAGCCCAAAAGATCAAACTGTCTTAATCCAGAGTCACACCTCAAATGCTCACATACAGTTCCCCATACCATTAAGTGGTCCGAAGTTGCCCTTCCAAAAGATTGGACCCTAGTTACCGAAAGCCAACCGGCTCCAATCCAGCGTAGTCTAAACAATTTAGACTACATCCAGCAGTACCTAGATGGCACAGTTAAAATCAAATTTGACAGTCATCCCCTACGCAAGTCTAATGTCCAGTTACAACAGCTGCCAACTCCTGGTCAGTCTCAAAGGCATGTCCCTGCTAGGCATTCCTTTGCTGCCTCTTCCTCCACTCTGGAACGAGACCTAAAGTTAGAAAAAGCCATGATCGACTTCAAACTTGAGTCCCTGAGAAAAACCTCTCAGGTTACCCAATCGTGCTACGGTACAGTTCTAGTCCAAGACGATAAATCAGAATCACCTGAGTCACCTACCCAGTCTAATTTCCTAGTTGACAACCAACTCAAAACCCTAAATAAGAAATTTGAAATTAATTGGGTACGTCTAAATCGCCATTTAAAAGCCCCAGAAAATCAACCTAGAAGAGATAGTTATCACCAAGCCTACCCTGATAGTAAACGCCGTGAACAAATATTTAGTGAATGGAAAGATTATATGCGGTCAGCCAGAGTTGAAATTTTCTACCTAGATTTTGTTGAAAGCCGTTATATGTCTAAAGATGAGTTAAAGACCTTAACTAAGGAAAGGTGGAATTTGATAGATAAGTCTGTCATTGAAACCAGTCATCCTCCAGTAGACACCATAATCATATATCACAAGAAATCCTTAGTACCTGCCACCCCTTTCAAAACCTTTGAAAGCAGCGATCCAAATAGAAAACTAATTGAGCAGAATAATTACACCAATCAGAGTCTAATCACTATAGGAAAACAATTAGACACAATCGAAACCAAGATCGACAAGCTAGGTCTGCCAGAAACAGATTCCAGGCCTAGGTCTAAAATTGAAAAGCCAATAGTTCAGTTTCAAGAACTTAAGTCTAGTCCCGTACTTAAAGTCAATTCTACTATGAAAAAGATAGAAGAAATGCTAGAACAGTTGACCCCTACCAAACCTGAGAAAGGTGAAAAATCTGGTATTAAAACACTAGATTCTTTTGCTGCATTAGTCTGAAAGTTGTTTAGGTTGCATCCAATTCTATTCTTGTATTCTATTCTTCTATGTTTATATTCAGCATGTAAGATCCCACATCAACCAATGGAGAGGAGGTGATGTGCCTTATATGTGCACACCCGCATCCATCTAGCACGAGGCCTTTTGGGAGCTCACTGGCTTCGGGGTCGTAGGAACTCCGAAGTTAAGCGAGTTGGGGGCCAGAGCAATCCCAGGATGGGTGACCCACTGGGAAGTTGCTCGTGAGCTCCCAGAAACAAAACCGTGCGGGCAGAGAGGGGGGCCCAAAGCGGACAATATCGTGCTACGGCGGAGCCGATCCCGGGATGTGACAATTTGGTATCAGAGCCACTCTACCGTGTGGTGCGAGTGTGCCGACGTGGACGTCGGGCCCTTAAGGGGGGTGGATTGTAAGATCCCACATCAACCAATGGAGAGGAGGTGATGTGCCTTATATGTGCACACCCGCATCCATCTAGCACGAGGCCTTTTGGGAGCTCACTGGCTTCGGGGTCGTAGGAACTCCGAAGTTAAGCGAGTTGGGGGCCAGAGCAATCCCAGGATGGGTGACCCACTGGGAAGTTGCTCGTGAGCTCCCAGAAACAAAACCGTGCGGGCAGAGATGGGGGCCCAAAGCGGACAATATCGTGCTACGGCGGAGCCGATCCCGGGATGTGACACAGCATGTCCTTCAGACTGTCTCTGTCGTTCTCTTTTGTTGAGACCGAATTAAGGGGTTTATAATTGATTACTAATCTGGGGACTCCTCTTTCTAATTCAGCATTTTTCTGGACATAAAAAGCAGGACACGACCAAGGAAATTTACTTCTGCTTTATATATATATATATATTT

AGAGAGAGAGAGAGAGAGAGAGAGAGAGAGAGAGAGCTTTCATTGAGGGATTCCTCAAATAAGTTATTTGAGGGACACCTTTATAGAGCTCACTCCGTATTGTATTTTACTAATTCAAACTGTCTACAAAGAGAGAGAGCTTTCATTGAGGGATTCCTCAAATAAGTTATTTGAGGGACACCTTTATAGAGCTCACTCCGTATTGTATTTTACTAATTCAAACTGTCTATTTTGTAGATAGGCATTCAAAGATCATTTCTACAACAAAAAAAAAAAAAAAAAAAACCTCTTGAATCCGATATCATTTTACCACTCAATTAAAATATTGAAATTCTAGTACTTTCTTGAAGCACCATGTTCACTGATTTTGTATAACACAATTGGATGTCGAAACGGTTTATGAGTTGTCTAATTTTTTGCAAGGATGATCTATGAATGTAGACTTTAAAAAATAGATGGTTTGGATCGTTGAAAAAAAATTCGTAGGGGACCCTAAAAGGTTTCCCTTAAATAAGCTTATTTGAGGGACACGTCTCACTCCGCATTGTATTTTACTAATCCAAACTGTCTATGTGTCTATATTTTAGATAATCAATCAAAGATCATCTCTATAAAAATAAATCACTTAAATCGAATATCATCTGACCACTTAATTGTGTTATTGAAATTTTAGTACTTTCTTGAAGAACCGTGTTCATTGATTTTGTAAGATACAACTGGATGTTGAAACGGTTTCCGATTTGTCTAAATTTTTCCAAGGATAATCTATCAATGTAGACTTAAAAAATAGATAATTTGGATCGTTGGAAAAAAAATTTGTAGGGCACCCTAAAAAGTGTCCTTCAAATAAAATTATTTGAGGGATCCCTCAATAGAAGAGGATCAAAATTAGATTTAGAAATAAGTGGATCAAAATTTTCATTCCAAAAAGACATTAAAATAAGTGAAAACACTGTAGGAAAAACTTACATCCTACTTTTAAAAATCTAAATATTCATAT

>Chr2-29; OZ022207.1; 25198039-25205707; dir; A;

TTTTAATAAGTGTCATATTTTTCCTTTTTAACGACGGTTATTTGTTTGTGTTGGTCGTCTATTCTCATCCTCGACTATTTTTTAAAACTTATGCAGACTAAATACGTCTGTTTTTATTTGTTTTCGACGTTGTTTTATTTAACAACGTCTAATATTTACCGTCGTTGTTTGTTTCTGAAAATAGGACGTATAAATTTTGTAATACGACTTTCATATATTCGTAACCGACGTTGTTTAGTTTTTTCAACGTCTTCTCTGTAAATACATACGTCGTAAATAATGACACTGACAACTATTTCCACGTCATCTTTTATAGAAAAATCGAAGTGGAAAATGTATTTTACGACGTCTGTTGTTATATAGGCGACGTAGTAGGAATCAATAACGTCGTTTTCTAATGTATTTAAAGACGTCATATTTTTTGTTGTCGTGAAAATGATATTTAACGGCGTCTTATTTTAATGTTCGTCGTTGTATGTCTATCTTGCGGCCTTGTTCTGTTAATATGTGTCATATTTTTCCTTTTTAACGACGGTTTTTTTAGAGAGTTGGTCGTCTATTCTCATTCTCGACTGCTTTTTTCGTTCTTTTTGCAGTACAAAGACGTCGTTTTGTATTTGTTGATGACGTTGTATATTTTAACAACGACGGTGATTTAGCGTCGTGGTTTATGAGGGAAAAGATGACGGTGGATTCCACGACCATTGAAAAACTGAATACACGACGGTGATTTACCGTCGTCTTTTTGCTCTTTTGTAGTAGTGATATAATAAAGGCATGTGTGTAGTAATAAGATGTTTCTTCCCATCCGCGGCTACATGCATGCTTATTTACATACGTACTCTCGTTGTCTGATTGCTAGTATTTGATGATCTTGGTGCCAGGTTCTTGAAGAAATGGCAAGGAAGAGTGATGGCACGATTACTTGTCCAAGGACAGGTTTGGTTTGCAATTAAACAAATCTGGTGAAGGCATGTGTGTGTGTATATATATATATATA

AATTGCCAAGTATGTCTACAATGACCTTCTAGTTGAGCCAGCTCATCCAATTTCTCCTACTGGTTCTGTCAGATCTTCCCTCTCAGTCGAAGGAAAGTCCAAATCAGAACTACAGGAAATTGCACGCCAGTTGATCATGCAAGCTTCCCAGATGGATGATGATGAAGACAATGGGTCTCCTTCGTCGTCTAGCTGCCAGCCCATACAAGCCCAAGTCAAGCCCAAATCATGGTATGAGGATAGTCAAGATCCCTACGACGCCTATGATTTGCATTCTGACTAGAAGCCAAAATTTGCCGTAATTATCACCAGACAAAGCATTTGCTTTCCACCGAAAGAAGCCACCAGTCGTCCCAGATGCTAGCTGCTCCAGCCATTGCATCTGCCTTTTGTCAAAAGTTGCCAACCGACAGACTTAAAAGATGAATCAGCATCTGCTCCTTACTTTTCGCCGAAAGTTGCCAGAAGATGCCAACCGACAAAATTGAAAGTTGAATTAGGTTGCATGTGACTCCACTGTTGCAACCCTACCATAGTTAGCCAACTATAGTTGATTAAACCCAAGTTGATTTTCTCTTTATAAGTAGGCTTCGGCCTCAGTTGAAAACAGAATGTAATTTTCCCTTTCTTTAGTTTTAAAGATTTCCTCTGATACATCCTTCGGAGAAATCACCTTTGTAAACACCTTGTGAATAGCGTGCTGTTCGAAGAATTGCAGTTGCAATCTTTTGTGAGTATAAATAAAAGTTATTTTCAAATTATCTATTTTCTGAAAGCATTATTTTAAAAGCATTTTCATTATTTTGCTGCATTAGTCTGAAAGTTGTTTAGGTTGCATCCAATCTATTTATCAGTCACTCTCTCTCTTGTTTATATTCAGTTGTCTTGTTCTCTATTTCTGTTGGTCCTTCTTTTGTTGGTATCAGAGCCGTAAGTGTTGCGGTCTTTGTCTAAGTTTGCTACTCATCCCCGCCCAAGTGGCTGGTTTAAGCATCTGAGTAGAAGGTTTCATTTCTTCCTTTTCTTCCCTTTTATCCTTTAAGTCATCTGTTGGTTTTCTCTAAGGTTTGACCCGTGAGTCCTTTTTCCTAGATCAGTAAGTCCCCAGGGAGGGCGTGTGTGGTGGGCAGGAGACAGAAAACTGAGAAAATTAATAGGTTACTTAGAGTCTAAACGGTTGGTTTAGGATCCACTGTTTATCTTCTTCCTCAGATAGCCAAACCCAGTTATTTGAGTAGTCATGAGTCGTCTGTTTCGAGCAAAGTCTTGTAATTCTCCCAGTTCTAGTACCAGTATTAGTCGACTCCCAGAAATAGTTAATGAAGAACAATTTGAATATAATTCTAGTAGTAAAGAGCAATTAGATTTCAGAGATTGGAACATACCTAGAGTCCCAAGTCAGAAAATCTATAAGAAACATTGGTTGCCTTTTAGTTTTAATAGCACAACACATGTCAAAACAGTTGAACAAGTGTACGCCTTAAGCAAAGAACACGAAACATGCCAGTTGCTTAATCTTGAGTCAATTGCAAAACATAAGAAAGATGGTAATAATTTCCTTCACATTGGATTAGTTCAAGTTGCAGTCAAGCCACTGACACGGCTAGGATTGAAAGCTTCAATTCTACTTTGTCTTCGTGATGCTAGATTCACTGAATTTAGCGATAGCACCTTAGGAGTCATTGAGTCTAGCCTGTGTAATGGCCCTGTCCATTTGGATTGTTATCCTGACTTCACTGTAAGCCTTAGTGATCCTCATATCCTGAGAACCTTGACTCTGAATATTAAGACTGAAGGTTATAATGTTCTCCCGGGAACCCAACCTTTAGCCTTAGTCTACAGAATCTATTATAAGGTTACAGGAACAAATATGAACTTCCAAGCCCTTAACAAAAGCCCAAAGGATCAAACATTCCTGAGCCAGAGTCACACCTCAGATGCCCATATACAAGTTCCCCATACAATCAAGTGGTCCGAAGTTGCCCTTCCAAAAGATTGGACCTTAGTTACCGAGAGCCAACCAGCTCCAATCCAACGTAGTCTAAACAATCTAGACTACATCCAACAATATCTTGATGGCACGGTTAAAATAAAATTTGATAATCATCCTTTACGCAAATCTAATGTCCAGCTACAACAGTTGCCAACCCCTGATAGGCATTCCTTTGCTGCTTCCTCCTCCACTCTAGAACGAGACCTAGAGCTAGAAAAAGCCATGATTGATTTCAAACTTGAGTCCTTGAGAAAAACTTCTCAAGTGACACAACCGTGCTACGGTGCGGTTCCAACCCAAGATGACAAGTCTGACTCGCCCAAGTCTCCAACTCAGTCCGATTTCCTAGTTGAAAATCAATTAAGAACCCTTCATAAGAAATTTGAAATTAATTGGGTACGTTTAAATCACCACCTAAAGGCCCCAGAAAACCAAGCTAGAAGAGACAGTTATCACCAAGCTTATCCGAACAACAAACACCGAGAATTAATCTTTAAACAATGGAAAGATTATATGCGGTCAGCTAAAGTTGAAGTTTTCTACCTTGATTTTGTTGAAAGCCGTTATATGGCCACGGATGAATTAAAAACTATAAATAAAGAAAAATGGAAATTAGTAGACAAGTCGGTTGTTGAGTCCAGCCATCCTCCAGTAGAAACCATTATAATTGATCACCAGAAAGCCCCAGTTCCTGCTACTCCCTTCAAAACCTTTGAAAGCAGTGACCCCCATAGGAAACTAATTGAACAGAATAATTATACGAATCAGAGTCTAATCACAATAGGAACACAATTAGATGCAATTGAATCTAAGGTCGATTTGTTAAGTCCACCAGTTGCGGATTCCAGACCTAGATCTAAAATTGAAAAGCCGATAGTTCAGTTTCAAGAACTTAAGTCTAGTCCTGTCCTTAAAGTTAATTCCACAATGAAAAAGATAGAAGAAATGTTAGAGCAGTTAACCCCGGCCAAGTTAGAAAAAGGTGAAAAATCTGGTATTAAAACATTAGATTCTTTTGCTACTTCTCTATCTGAGTCTGAATTAGTAAATTCTGAAATCAGCGATATTTCAAAAATTGAAAGTGCCTTCAAAAATTTAGAAGTCCAAACTAAGCCCAGAATCCAAAAAGTTGACAGACATATAAGTCCTACTAGTTTAACAAAAAATTGGTATCCCAGACCAACACCACCAGACATCCAATTTGAAGAAAGAAACTTTCAAACCCAGTTTTCAGTTTCTTCTGATAAATTGTATAAATGGAACATTGATGGTCTTTCTGAACAAGAAATCTTAAATAAGCTGCAACATATGTCTATGGTCGCCAATAGTTATATCACCAATCACAGTTTCAGGCATTCTGAAATTCCTCTGTTAGTCACCGGTTTCACTGGTACTCTCAGATCTTGGTGGGATAAACATCGTACCCCTGAGTCTAAAAGTCACATCATTTATGCTGTAAAACTCAATGAAGACGGTCTTCCTATCTTTGATGAACAATTAGGTCAAGGCATAGAGGACGGTGTCAACACTCTGTTTTACACAATAATTGAACATTTCGTTGGAACCCCTAGTAACACTACTGCTAGGATACATGATCAGTTAAGTAATCTTAGATGCCCAAAGTTGTCTGATTTCAGATGGTACAAAGATGTCTTTATTTCCAGAGTCATGCTTAGGGAAGATAGTAATCAACCCTTCTGGAAAGAAAAGTTCATTAATGGTCTCCCAAACTTAGTCGCCCATAAAATACGTACAACCCTGAGTAATGAACAAGGTCAAATAGATTGGGATAATTTAACCTATGGAAACATTATAAGCACAATTAATCAAGTCGGAATGAAAATGTGTATTGACTTTAAAATCAATAAACAAATTCAGTCTGACAGAAAATCAGCCAAGTACGAACTAGGTAATTTCTGTGAACAATATGGCCTTACTAGTGTTCCTCCTTCTAGAAGAAGTAAGCCCAGTCATCTTAGAAAACGCCATCATCCGAAGAAACAATTTTTTCGGAAAAATGAATTCTATAAGAAAAGAAAGTTATCCCCTAAGAAGAATTGGTCGAAACACCCCAAACAGAGTCGCACTAAAAAAGATAAAAGCAAAGTTAAGTGTTTTAAATGTCAAAAATTTGGTCATTATGCTTCTGAGTGTAAAGTAAAAGATGTTATTAAGCAATTACAGATTACAGAAGAAGATAAGAACAAATTAATCCAAGTTTTAGAGTTGCAAGACTCTGAAATAAGTGATGCTGAATCCTTATTGTCCAGTTCTGAGTCTGAATTTATCCAGTCTTCAGATTCTCAACCCAGTTCACCTAGAATCTAATTTGGTTGTACAGATAAATATTGTAATAGACTGAAATCTGTCTCGGTTCTCACTAAACAAGAAGAACAAGAAGAACTTTTAATAGACCTAATAGGACGAGTCCAAAACCCCGAACTAAAAGCTGAATATTTGCAAAAGTTGAAAAAAGTCTTGAGTCATGAAAGCCCAAGTTTAGTTGCTCCTCCTCCCCAGGCCATTAGTATTAATACTACATTAGAAAAATTTTAAAAAAGAAATCACCCTTCATGATCTCCAATTGGAAGTCAAGAAAGTCAAACAAGAATTGGTTGAGTTAAAACAAGTCAGCCAACAACTGCAAAATGAGAATTACACGATCAAACAAGATTTATCTAGTCTTCCACATACAGGCCCTAGTTCGTTCAAAGCCCATTCTGAAAGTCCGCATCATTCTAATGACGAACCATTAGAGGAACAACCAGTCGTCAACTTAATTAAACAAGTTCATCTTAGAAAATGGTATTCAAAAGTAACGATATTTGTTAATGATTTTGAATTTTCCACAGTTGCCCTGTTTGATTCAGGTGCCGACCTTAATTGTATTCAAGAAAGCCTAACCCCACCAAATATTACCACAAGTCTAGAGAATCTTTGCGTACGGCCTCAGGGAAATCTCTCCAGTTGAATTATGAATTACCAAAGGTCCATGTTTGCCAAAACAAAGTCTGCTTCAAAACTTCATTTGTCTTAGTTAAAAATATAACTGACAGTGTCATCTTAGGATTACCCTTTCTACCTTTATTATATCCTTTCCAGGTGGAATACGATGGCCTCGTCTCTACCTATTTAGATGAAAAAATCAAGTTTCAATTTTTGTCTAAACCAGAGTTGCATAACGTGAAAGCTTTACAAGAACAGTCAGTCTCCAAGTCTATCCAAATAATTAGAAAAACTAATCAGTTGAATTTCCTTAAAGAAGAAATTAAATTTAAAAGAATTGAACGCCAACTTACTGATAAAGATTTACAGATGCAAATACAATCCTTTGAGGACAAAATAAAAGCAGAAATTTGTTCTGAGTTGCCTACTGCTTTTTGGTATAGAAAACAGCATGTTGTTTGTCTTCCTTATATCAAAACCTTTAGTGAAAAAAATATCCCTACAAAAGCCCGACCAATTCAGATGAGTCAAGAATTGATGGAAGTCTGTAAAGAAGAAATTCAAGAATTGCTACAAAAGAATATTATTCGCAAAAGTAAATCCCCGTGGTCATGTCCTGCTTTTTATGTTCAGAAAAATGCTGAATTAGAAAGAGGAGTCCCCAGATTAGTAATCAACTACAAACCCCTTAATTCGGTCTTAGAATGGATTAGGTATCCCATCCCTAATAAAAGAGACCTTATTAATCGATTAGAGAAAGCAGTTGTCTTCTCTAAATTTGATATGAAGAGTGGTTTCTGGCAAATCCAAATAGATGAGCCAAACAGGTATAAGACTGCATTTGTCACCCCTTTCGGACATTATGAATGGAATGTCATGCCGTTTGGTTTGAAAAATGCTCCTAGTGAATTCCAGAATATTATGAATGATATCTTCAATCCTTATAGTCATTTCTCCATTGTTTACATTGATGATGTCCTTATATTTTCTGAGTCAGTCGAACAGCATTGGAAACATTTGCACAAATTCCTTCACATAGTTAAACACAATGGTTTAGTTGTCTCTGTCAAGAAAATTAAGTTGTTTCAAACTACAGTTAGGTTTCTTGGTTTTAATATCAATCAATCCCAAATTTCCCCAATAGATAGAGTCATTCAATTTGCTAATAAATTTCCTGATCAGATTCTTGATAAAAGTCAACTCCAGAGGTTTTTAGGATCTCTAAATTTTGTTTCTGACTTCTACCAAAATCTGCGAAAGCAATGTAAGCCTCTGTTTGATAGATTGCAAAATAATCCGCCCCCTTGGACAAATATCCATACTGAGGTTGTCAAACAAATCAAAACCCATGTCAAGACACTTCCCTGTCTTGGTATTCCTTCAGTTAATTCCTTCAAAATAGTAGAAACAGATGCCTCTGACATTGGTTATGGTGGCATTCTCAAACAGAAAGTTAATCCAGATTCCCCAGAGCAAATTGTTCGCTTCCACTCTGGAACCTGGACTCAGTCGCAATACAATTATAGTACTATTAAGAAAGAAATATTAGCTATTGTATTATGCATTAGTAAATTTCAAGATGATTTACTCAATCAAAAGTTTTTAATACGAGTTGATTGCAAATATGCAAAACATGTTTTAGAAAAAGATGTTCATAACATTGCATCTAAACAGATTTTTGCACGATGGAAAAGTATATTAAGTATATTTGATTTTGAAATTGAATATTTGAGAGGTAGTGAGAACTCAGTTCCTGATTTTCTTACTCGAGAATTTTTTCAGGATACACAATGGCCCCTAAAAAAGACAAACACAAAGCAACCAGTCAACCTGAGCCATCCCAGTCGCAACCCAAGCCTTCTCAATTGCCACAACTCCAGTCCTCATCCATAAGTCCCATTAAGCCAGAATTGCCCAAACAGATAGTTCTGTATCCTGGTTGCACCCCCCTTTCAGTCGTCACCCCATTATCAGTTGCCAATCGATACTCCTCTCTAGGATCCACAGTTGGCCAAATTCGACCCAGTTATCAGTCTACCTTAGTGGCTAGTTATGATCCCTTTTCCATAGAAGTCCCTGCTAGACCATCAGTTGCATACAAAAAATCATCTCCCTATTTGCCCAAAAGTAACTCCTATTTATTTGTCATTGAGCCTGTATACGATGTTCATGCTAATCTAGTTGAAATTGCTCGACACTACTTCCCTCCTAGGTTTCATTATATGCCTTCCAGTCCCTACAAGTCTCTCAAGTATTATAGAGATATATTACTTGAAACAAAATCAGTTGAAATTAAGCCAATAAAGGATTGGGATCATCCCGAAATCATTCTTTATCATTCTTTGTATATCCACCAGATCATAAGCCAAGAATCTTTCAGCAGTCGCCCTTATGAATTAAAGTTGCTCCAGTCAAAACTGCAATATAACTATTCTGATTATATTGATGCCTGGTACACTATTTTCCTTCACCAATCTGAAGATTTTAGTCATTCCTGGTTCATCAATTTTGATAATAAGTTCAAGTGTTCTTTCCCTTACTGGTTCCTCCATTGGTGGGAAAAGCACGGCCCAGTTGATGAAATATTACCAGTATCAATTCATGAGTTAATTCGCCATTTTACCAAGAAAGCCAAGTTCTCTAAAGTTGATTCATTCTTCCCCAAACAGTTGCTGTTCGTCGCTAGGTACAAAGTGCCATGGATCCTGAAATGGTCCTATAAGATCACTA

TATATATATATATATATATAGGGGTCGATCCATGACACCATATTTTTGACACAAGTTTTAAACCCTTGGATTAGCCCACTATCAATGGATGTGATTAAATAAATAAATTATTTTATTCTTTAAATAAACAATTATAGAAAATAGATTTTTAATTGACTTTTGTATTTCCTCTCTCTTCATTTAATTAAGATTTGATGGCTTCTTGACACAAGGCACTTTTTCTTCTTCTCTTCTTCGTGCCTTCTCATTAACACACCTCCCTTCTTCTTCTTGCTTTTTCAGTAACCCTGAAATCACAAACTAGCCATCCAATTTCAGCAAACCATTCCATCTAAATCACAAACTAGCAATTAATCCTTAGAGTTAAGATTTATATTGATAAATAATTGTTTTAATAATTTACACAAGAAGTTGCAGAGTAGATCACATTAATCTAGCCAATTTTGTTAGAATATGCAGACCATTATCTTGCATATTAATTCAACCAAACATGTAATAGGTTTCAAAGAAAATGCATAGAGAATAGAGTAGTGAATTTCTGTAAGAGAGAGAGAGCTTGGAGAAGAAGCTGGCTATCTAAATTAGCTCAGCACCAAAATCATGCATATATTGTGATTTTGAAAAAACCGTTAGAGAAAGCATATTCCCTATTACATCACTCAATCTTAGCCATTTAATTATTACCTTATGAGATAATGACAAGTGGCATAAGCTATTACATTCTCTGCTGTACACTTGGACTATGATCCAACGCAGCCTTTCTAACTAGAATAGAACCTTCCTATTCTAATTACATTTCAGCAGCCACACTTATACACCAACACTCCCTTCTAAGTGTTGTACTGAAATAACTCCTAGAGCTTTCCTCAAATACTCAAATCTATCTCTTGCTAAAGACTTGGTGAAAATGTCAGCAAGCTGCCCTTTAATTTTGCAATATAGCAAATCGATCTCACCATTTTGAAGAGCATCTCTTATGAAGTGAAACCTGCAGTTGATG

>Chr2-30; OZ022207.1; 25567072-25574701; rev; A;

AGTATGCATTGCTACAGACAATGAGCCAAAAACTCATATATATATATATTTTGGTCATAAGTCAGGTGTTCATATTCAAAATAAATTCATTTAAAGTAAAAACTTACAATGTGCAAAGCTTACACTCATTCTAAGGTTGTTCTTCAGAACATGGTTTCTGAGAGAACATTTTGAAACTGAAAAAAGATGAAGAGAAAAAACGATCGATGTTTACAAATGAGGCAATAGCCTTCTTTTAAAGACAAAATAAAAGTAACTCAGGTTTACAAAAGTAACTCAAGTGAATAAAAGTAACTCAAGTAACTTTAGGTAACTTGCTTTAACTTCTAGTAACTTGCTTTAACTCAGGTAACTTGTCTGCAGTAGTCTAATCTGAATTCAAATCATAGGCTTTATAGGCTTCATCATAGGCATCATTAGGATCTTGACCATGCAGATAAGAAATTGAATTAACGGTTTATCTATAATTTTAAGCTTAGAAGGTAATATTTTATCAACTTTGGATTCTATATTGTCTAATTGTTTTCCTATAGTCACTAGACTTTGATTAGTATAATTATTTTGTTCAACTAAACATTTAGTAGACTCATTAGTAGGATCAGGAATCTTAAAGGGAGCAGCAGTGATAGTTTTTGTTCCAGCAGTTATCAGGATAGTTTCTACAGGGGGATGACTTGAAACCACTTCAGACTTATCTAGTTTTTTCCATTTTTCTTTAGTTATAGCTTTTAATTCATTAGGAATATAACGACTTTCTACAAAATCAAGATAATTGATTTCTACTTTTGCAGATCTCATATAATCTTTCCATGCATGGTGTATTTATCTCTTTCATTTTTATCAGGGACAACCTTATGATAAATATCTCGTCTGTAGGAATTAGCAGGGTCTAAAAGGTGTGATTCTAAGGCTTTCCAATTGATCTTAAAATTTTCAGGACGTCTTAAAACATTTAATTGATTATCTATATCATAAACATGTGCAGTAGCTATTTCACTAG

CAAGGTTGTAAACAAGAAATCAATTATCTTGATTTTGTAGAAAGTCGTTATATTCCTAATGAATTAAAAGCTATAACTAAAGAAAAATGGAAAAAACTAGATAAGTCTGAAGTGGTTTCAAGTCATCCCCCTGTAGAAACTATCCTGATAACTGCTGGAACAAAAACTATCACTGCTGCTCCCTTTAAGATTCCTGATCCTACTAATGAGTCTACTAAATGTTTAGTTGAACAAAATAATTATACTAATCAAAGTCTAGTGACTATAGGAAAACAATTAGACAATATAGAATCCAAAGTTGATAAAATATTACCTTCTAAGCTTAAAATTATAGATAAACCGTTAATTCAATTTCAAGATTTACAGCCTAAATTTGCTCTAAAAACCTCTTCTACTTCTAAAAAGATTGAGAAAATTGAAGAAATGCTTGAACAATTATCCCCGTTAAAAGGTGAGAAATCTGGTGTTAAAACCTTAGGAATTTCTCCTGTGAGTAATTCTGATTCCGAAATGAGTTTAGAAACTGATGAGTCTAGTAATATTTCTAAAATTGAAAATGCATTTAAAAACCTAGAATTAGAGTCTGAGTTAAAAGTTAAAAGATTAACTAACAACACCCGTAAGATAAACCCTACAAGCTTAACAAAAAATTGGTATCCCAAACCTACACCTCCTGACATTCAATTTGAAGAACGCAATAATCTGAGCCAATTTTCAGTTTCCTCTGATAAATTATATGAATGGAATATTGATGGCCTTTCTGAGCAAGAAATAATGAATAAACTTACACATATGTCCATGGTTGCTAACAGCTACATTACAAACCACAGTTTAAGACAATCTGAGATAGTCCCTTTGTTAGAAACTGGTTTTACAGGAACTCTCCGTTCTTGGTGGGATAAACACTTATCTGATGAGTCTCGAAATAAAATAATACATGCTGTTAAATTAAATGAGGATGGTCTTCCTTTGTTTGATACCAATATAGGACGAGGAATAGAAGATGGAGTCAATACACTTCTTTACACTATAATTGAGCATTTTATAGGTACACCAAGTCATACACAATCTAGAATACATGACCAGCTGAGTAATTTAAGATGTCCTCAATTAAGTGATTTTAGGTGGTATAAAGATGTTTTTATTTCTCGAGTAATGCTAAGAGAAGATAGTAATCAAGCTTTTTGGAAAGAAAAATTTATAAATGGTTTACCCAATTTGTTTGCACACAAAATACGAACTGTCCTAAGTAATGAATTAGGTCATATAGATTATGATAATTTGACTTATGGTAATATTATAAGCACAATAAATCAAGAAGGAATGAAAATGTGTATAGACATGAAGATAAATAAACAAATAGAGTCTGAACGCAGATCAGCTAAGTATGAATTAGGTAATTTCTGTGAACAATATGGTTTAATCTCCACTGCACCTTCCCGTAGGAATAAGCTTAGTTACCCTAGTCATCTCAAAAAGAAACGTCATTATAGCACCTTTAGGAAAACCTACAGAAACAATAATACACCTGATGAATTCTACAGGAAAAGAAAATATTCCCCTAAGAAAAGATGGTCAAAAGCACCTGCCTACAAGAAAGATGCTCGCTTTTACAAAAAGTCTGGTAAAGATAAAAGTAAAGTGAAATGCTTTAAATGTCAAAAGTATGGTCACTATGCAAATGATTGTAAAGTTAAAGAAACCATCCGACAATTACAAATCACAGATGCAGAAAAAGACAACTTAATTAAAGTCCTTGAGTTAAGAAACTCTGAGTCTAGTGAAAACGAATCCACAGTTAGTCATTCTGAGTCAGTTAATTATGAAACCTCTGATTCTCAGTTAAGTTCCCCTAAAATTCAATTAGGATGCAGAGATAAATGTTGCAGTACTCTTAAGCATATTTCTGTTTTAACTAAAAGTGAAGAACAAGAAGAGTTATTAATAGATTTAATTAGTAAAATTGAGAATCCTGATTTAAAATCTGAATATTTGAAAAAACTGAGACATATCCTTTCACAGGATGCTAGTACAAGTCAACCGAACCCTGTAATCAATTTGAGCACTACTCTAGAAAAATTTAATAAGAAAACAGAAATTACTTTACATGATTTACATTCTGAGGTAAAATTAATTAAAAAAGAAATAGTTGAATTAAAACAGCTAAGTTATAAACTACAAACTGAGAATTCTGACATCCGACAAGTTTTAAATACATTACTTGATAAAACACCTTCTGAGTCCCATAGTCCACCTAGTAGTCCTACTCATAAGTCAGAACCTGAACATGTTATTAACCTAATTAAACAAGTTAATTTCAGAAAATGGTATTCTAAAGTTACTATAATTGTAAAAAATTATGAATTTGATACAGTAGCTTTATTTGATTCTGGTGCAGATCTTAACTGTATAAAAGAAGGTTTAGTTCCTACTAAGTATTATCAGAAATCTACAGAATCCTTACATACTGCTTCTGGTAAATCGCTTCAATTAAATTATGAAATTCCTAAAGCCCATGTTTGTCAAAATAAAGTCTGTTTTAAAACATCATTTGTTTTAGTTAAAATATAACTGATGAAGTTATTCTAGGATTACCGTTTATAAGCCTGCTTTATATTTTCAAGTTGAATATGATGGTGTTGTCTCCACTTATCTAGGTGAGAGTGAAATTTACTTTTTATCAAAACCTGAGCTGTACAATTTAAAACATTCGACTAAAACTGATTTTGCAAAAACAATTACTTTGATTAAAGGAAAAACTAATCATCTGAGCCATTTACAACAAGAAATTAAAACTAAGAGAATTGAGTCTCAGTTAAATAGCCCTACTTTAAAAGAAGAAATTAGTTGCTTTGAGAAAAAACTGATTTCTGAAGTCTGTTCTGATTTCCCTACTGCTTTTTGGCATAGAAAAAATCATGTTGTACATTTGCCTATATCAAGGATTTTTCTGAGAAAAATATACCAACTAAAGCTAGGCCTACAGATGAATCATGAGATGATGGAATTCTGTAAAACTGAAATTAATGAGTTATTACAAAAAAATATAATCCGAAAAAGTAAATCTCCCTGGTCTTGTCCTGCTTTTTATGTACAGAAAAATGCAGAATTAGAAAGAGGATCTCCTCGATTAGTTATTAATTATAAACCTTTAAATTCTGTTCTTGCATGGATTCGTTATCCAATCCCTAATAAAAGAGATCTGATTAAACGGTTAGATAAAGCTGTTATTTTCTCTAAGTTTGATATGAAAAGTGGATTCTGGCAAATACAGATCCATGAGTCTAATCGATACAAAACTGCTTTTGTTACCCTTTTGGGCACTATGAGTGGAATGTTATGCCTTTTGGTCTTAAGAATGCTCCCAGTGAATTCCAAAATATAATGAATGATTTTTAACCCATACAGTCATTTTTCTATTGTTTACATTGATGATGTTTTGATTTTCTCTGAGTCCTTAGAGCAACACTAGAAACATCTGCATACATTCTTGCATATCATAAAAACCAATGGACTAGTTGTATCTGCAAAGAAAATTAAATTATTTCAAACTCATATTAGATTTTTAGGATATAATATACATCATTTACAGATTCATCCCATAGACCGAGCCATTCATTTTGCAGATAAATTCCCTGATCAAATTACAGATAAAAATCAATTACAAAGATTTTTAGGATCCCTTAATTATATATCTGAATTTTATAAGAATTTAAGACAACAATGTAAACCCTTATTTGATAGGCTACAGGTTAATCCTCCCCCTTGGTCGTCTATTCATACAAGCATTGTTAAACAGATTAAGTGTCATGTTAAGACTCTTCCTTGCCTTGGTATTCCTACTCCAAATTCTTTAAAATCGTTGAAACTGATGCCTCTGATTTAGGTTATGGTGGTATTCTTAAACAGAAAATTGAGTCTTCTTCTGAGCAAATTGTCCGTTTTCATTCTGGTATCTGGTCTCCTTCCCAATCTAATTACAGTACTATTAAAAAAGAAATATTATCTATTGTATTATGCATTACTAAATTTCAAGATGATTTATTAAATCAAAAATTTTTAGTTCGTATTGATTGCAAATCTGCTAAATATGTTTTAGAAAAGGATGTTCAAAATCTTGCATCAAAACAGATTTTTGCACGTTGGCAAGCTCTTTTAAGCATTTTTGATTTTGATATAGAATATCTGAGAGGCAGTGAGAATTCTATTCCTGATTTTCTTACAAGAGAATTTTTACAGGATAAGCAATGGCCGGCACATCAAGAGATAAGCAAAAAATGAGTCACGCCCCTACACCCCGAATCTTGCCTGCTTCTTTGAGTCAAAAGCCTACTCCTTCTATTAAAAATGAGTCTGATTCCTTTTCCGTAAAAGCTGAGCCCTATACAATCACCCCCTATCCTGGCCATCCACCTCTTCCAGTCTTGAGTAATAGGTTTAGCCCCTTAGGTACTACAGTCACCCCTGCAAGACCCAATTACCAGTCTGCATTAGTCTCTAGTTATGATCCGTTTCAGTTACCTCCCACTGCATCGCCTATATCTTCTCCCTCTTTCCAAAAGTCCTCCCCTTATGTTTTAAAGAATACAACCAACATAGTAATCATTGATTCTCATATTCCTACCTCCATGAGTCCTGCCCAAATTGCAGCCTTTCATTTCCCTCCTGGATTTCATTATCTTCCTCACAGTCCTTACAAATCTTTGAAATTTTACAGAGAAATTCTTCATGAAACTAAGTCTGTTGAAATTAAGCCCATTAGAGACAAAACTGACCCTACAAAAATTATATATCATTCCCTTTACATTCATCAAATTTTGCATCCTAAGTCCTGGGGTTCCCGTCCTCATGAGCTCCGAACTCTTCCTTCTAAATATCAATATAATTATGCAGACTATGTTGAAGCCTGGTATTACATTTTTCTTCATCAAACCCCTGAGTTTTCTCATTCCTGGTTTATTAATTTTGATAATAAGTTTAAAAATACTGAGCTTCCCTATTGGTTCCTGCATTGGTGGGGCACCCATGGTCCCAAATCTGATATTATTCCTCCTGCAGTCCATGAGCTAATTAATTATTTTACCAAGAAATACAAGATTCCTGAGCGAGATCTTTTCTTTCCTCCTGATCTCCTATTCTTTACAAAATACAGGCTCCCTTGGATTATGAAATGGAATTATATTGTTGATTGGGAATCTCGTTTTTTCTCCCGTCAGTTTCTTGTTAAGTGGTGGGACCGCTTTGAGGTCCATCGCATTACAGATTATGTTTACAAGGACTTTCCTCTGGTCCCTAGTCGAACTCCTAAGCCCAAGCATGAGCCCTCCTCTTCACATTCTTCTGATACTATGCTTACAGTTGAAGGCAAATCTAAAACTGAGTTACAAGATTTAGCTCAACAGCTCCTCCGTGCTTCCCAGATGCATGATGGTGATGATAATGATGCTGAGTCTGAAACCTCTTCTAGTTGTCAACCTATACAACCAACTCCTGAGCGCCCTGTCCATTGGGCTGACTATCTGCATGGTCAAGATCCTAATGATGCCTATGATGAAGCCTATAAAGCCTATGATTTGAATTCAGATTAGACTACTGCAGACAAGTTACCTGAGTTAAAGCAAGTTACTAGAAGTTAAAGCAAGTTACCTAAAGTTACTTGAGTTACTTTTATTCACTTGAGTTACTTTTGTAAACCTGAGTTACTTTTATTTTGTCTTTAAAAGAAGGCTATTGCCTCATTTGTAAACATCGATCGTTTTTTCTCTTCATTTTTTCAGTTTCAAAATGTTCTCTCAGAAACCATGTTCTGAAGAACAACCTTAGAATGAGTGTAAGCTTTGCACATTGTAAGTTTTTACTTTAAATGAATTTATTTTGAATATGAAGAACATTATATTCATGTATTACATTATTTTTATAATCATGTTTTCATTATCTTGTATAGTTCTGCTCCTCCTTTCTTTCTTTCCTTTTTGGTATCAGAGCCGTAAGCGTTGTAAGAATTAGTCTTAAGTCGCAACTCATCCAACCCAAGTGGCTGGTTTAGGATTTCTGAGTTGAAGTTCTTTGATTTTTCCTTTCCTTTCCTTTCCTCTGCAAAGTGTCTGTCGGTTTTCTCTCTGGTCCAAACAGTGAGTCCTTTTTCCAAACGATAAGTCCCGGGGTAGGGCACGAGGGTGGGCGTGAGGGAGAGAGAGTTTATCCTCAGATTCCTTGTCGGGTAAAGTTAAGTCCAGGATTTATCATTTTTCTTCTTCTCTGGTTATCCTATCCCATATCATTAGGAAAAGATGAGTCGCTTCTTACGCACAAATTCTTGTAACTCTAGCAGTTCTAGAGCCTCTATAGGAAGATTACCTGAGATAGTAAATGAAGAACAATTTGAGTACAATAGTACTAATAATGATGAACAATTAGACTTTAAAGATTGGAATATACCTAAGATTCCTAGTCATAATATATACAGAAAGAAATGGTCTTTATCTGCATTTAATTCTACTACGCATGTTAAAACTGTAGAACAAGTTTATGCTTTAAGTAAAGAACATGAGTCTTGTCAATTGCTTAATTTAGATTCAATTAAGAAACATAAGCATGATGGTAATAATTTCCTGCATATTGGTTTGGTTCAAGTCGCTGTTAAGCCTTTAACTAGATTAGGTTTACAAGCTTCAATTTTATTATGTTTACGTGATGCACGATTTACAGATTTTAATGATAGTGTGCTTGGTATGATTGAGTCGAGTCTTTGTAATGGCCTGTTCATTTTGATTGCTATCCTGATTTTACCGTAAGTCTGAATGATCCTCATATTCTTAAGACATTAACTTTGAATCTAAAAACATCTGGTTATAATGTTCTACCTGGTACCCAGCCTTTAGCCTTAGTTTATAGAATTTATTATAAAGTTACAGGTACTAATATGAATTTTCAAGCTTTGAATAAGAGTCCTAAAGATCAAACCCTGTTAATACAAAGTTGTACACCTGATGCTAATATTAGAATACCGCGTACTGTTAAATGGTCTGAAATTACTTTACCTACAGATTGGACTTTAATCACTGAGTCTCAGCCTATGCCTATTCAACGTAGCTTAGATAACTTAGATTATATTCATCAATATATGGATGGTAGTGTTAAGATTAATTTCCAACACCAACCTAAGTCTACAGTCCAATTACAACAATTACCTACGCCTAGCCCCTCTAAAAGACATGAGCCAGCACGTCATTCTTCTGCTTCTTCTTCCACTACAGTTAGAGATTTAGAAATAGAAAAAGATTTAATTGATCTAAAAATTGCATCCCTTAAGAAAAAAGACCAAGTTAGTCATCCTTGTTATGGTTCTCCACCCCTTGGAAACCCATATATTCCTGCCCCTGAGCCTAAAGTTAATTCCCCTACACAGTCTGATTTCCTTGCTAGTGAAATAGCTACTGCACATGTTTATGATATAGATAATCAATTAAATGTTTTAAGACGTCCTGAAAATTTTAAGATCAATTGGAAAGCCTTAGAATCACACCTTTTAGACCCTGCTAATTCCTACAGACGAGATATTTATCATAAGGTTGTCCCTGATAAAAATGAAAGAGATAAAATACACCATGCATGGAAATATATATATGGG

TCACGATCCGGGGACACACATTTTGACACAAAATATGACTCATTTTATTATACCTTTAGATTTCAGCACTTTTGGCGGGTAGATGAATGGAGTCTTAACCTTATAAAGAATAAAAATAAAATTGTTAGTTATCCTTTTGTCTTTTACGGGAAACTAAATTTTCTTTCCTAAAGCATTCTCTCTCCATCTCAGCATTCTCATTCTCTTCCTCTTCATTTGAAACTCAAACTTAAAACTATCTACCCAAAAAAAAAAAAAAAAAAAAAAAAAAATCAGACTGGAAACATAATGAAAGCAGACAGACTTTGATCAGGAGTGTGCGTAAAGCTTTTTATGGCTCGTATCATTTTTCTTAGAATCTGAAGATATGGAGCATAAATAAGTTTCAGCTTTTTTATGGCATGTTTAGGTTTTTACCTCTTCACGTTGCAGGGGGTTGAGTCGTGTATTTTATGAAAGAAAGAAAGTGGGAATCGGCTTGGGGTAGGTTTAGGCAAGGCAGTGAGAACTGCTTGTTTTCTTCCTTTTCTTTGTTCCCTCCAGCAATTATATAGTTGGAAAAATTGTTATATACGTGACAAAATATTCTGTGCAATGTTTATGAAAATGCCATTTGGAGGATTTGGTCTTTCTGGATACACATGTGGAATACCCTTTGATGATTTTAAAATCCTTTGTATAATATATTTATTTGATTGTCTGTTTTGCATCTTCAGTCACTGAAGGGATTGTCTGAAGAGATTGTTGAGACTGCAGAGACGGAGTCAGGATTCACTATGTTTCCAATGCCCATGGGCTTGAAAGGGAGATTCGTTTGTAACCCAGCACTGAATTTCCTGAGCTGCGGTTTTCCAAGGTAAAAATTAAAATGGATTTGGAATGTTATTCTTTGAGGCTGTTTGTCCTTGAAGATTGTAATTTATTTTGATGGTAAATTGATGTTTTTTTTATCAGCCTGGAAAAAGATGGTTGAGTTGAGACAGCAAATTTCCGGCAGAGA

>Chr2-31; OZ022207.1; 25588592-25596748; rev; C;

TCCGAAAGCATTCTCTCTCCATCTCTGCATTCTCATTCTCTTCCTCTTCATTTGAAACTCAAACTTAAAACTATCTACCCCAAAAAAAAAAAAAAAAATCAAACTGGAAACATAATGAAAGCAGACAGACTTTGATCAGGACTTTGCCTTAGTTGTTTCCCTGGATTTTTTATTGGCATATCTCGACCATTTTTCTTTCTTTCATGAGAATTTTCTGTGGTTTCTTTTTTCTATGTTTTTCGTTTTCTGCAAGTTAAGCTATATTTTAAAGGAGAAATCTGTTCATTTTATCTGGTTGTGGATAGTGGTGCCCTTTGGAACTTGAATCGTATGTGATGAATTATTGTCATGTGTTAGGAGTAGATCATACATACGAAAGTGTGCGTAGATCATATAGCGAACAAGTGAGTCTCACGAGCGAAACCCGATTAAGCGAGTGCGGAGAGGCTAGTACAAGATAAAATTCAGAAATCATATGCCCATTCCATTCCATCAGCTTGTCTACCACTAGTATGCATTGCTACAGACAATGAGCCAAAAACTCATATATATATATATTTTGGTCATAAGTCAGGTGTTCATATTCAAAATAAATTCATTTAAAGTAAAAACTTACAATGTGCAAAGCTTACACTCATTCTAAGGTTGTTCTTCAGAACATGGTTTCTGAGAGAACATTTTGAAACTGAAAAAAGATGAAGAGAAAAAACGATCGATGTTTACAAATGAGGCAATAGCCTTCTTTTAAAGACAAAATAAAAGTAACTCAGGTTTACAAAAGTAACTCAAGTGAATAAAAGTAACTCAAGTAACTTTAGGTAACTTGCTTTAACTTCTAGTAACTTGCTTTAACTCAGGTAACTTGTCTGCAGTAGTCTAATCTGAATTCAAATCATAGGCTTTATAGGCTTCATCATAGGCATCATTAGGATCTTGACCATGCAGATAAGAAATTGAATTAACGGTTTATCTATAATTTTAAGCTTAGAAGGTAATATTT

TATCAACTTTGGATTCTATATTGTCTAATTGTTTTCCTATAGTCACTAGACTTTGATTAGTATAATTATTTTGTTCAACTAAACATTTAGTAGACTCATTAGTAGGATCAGGAATCTTAAAGGGAGCAGCAGTGATAGTTTTTGTTCCAGCAGTTATCAGGATAGTTTCTACAGGGGGATGACTTGAAACCACTTCAGACTTATCTAGTTTTTTCCATTTTTCTTTAGTTATAGCTTTTAATTCATTAGGAATATAACGACTTTCTACAAAATCAAGATAATTGATTTCTACTTTTGCAGATCTCATATAATCTTTCCATGCATGGTGTATTTTATCTCTTTCATTTTTATCAGGGACAACCTTATGATAAATATCTCGTCTGTAGGAATTAGCAGGGTCTAAAAGGTGTGATTCTAAGGCTTTCCAATTGATCTTAAAATTTTCAGGACGTCTTAAAACATTTAATTGATTATCTATATCATAAACATGTGCAGTAGCTATTTCACTAGCAAGGTTGTAAACAAGAAATCAATTATCTTGATTTTGTAGAAAGTCGTTATATTCCTAATGAATTAAAAGCTATAACTAAAGAAAAATGGAAAAAACTAGATAAGTCTGAAGTGGTTTCAAGTCATCCCCCTGTAGAAACTATCCTGATAACTGCTGGAACAAAAACTATCACTGCTGCTCCCTTTAAGATTCCTGATCCTACTAATGAGTCTACTAAATGTTTAGTTGAACAAAATAATTATACTAATCAAAGTCTAGTGACTATAGGAAAACAATTAGACAATATAGAATCCAAAGTTGATAAAATATTACCTTCTAAGCTTAAAATTATAGATAAACCGTTAATTCAATTTCAAGATTTACAGCCTAAATTTGCTCTAAAAACCTCTTCTACTTCTAAAAAGATTGAGAAAATTGAAGAAATGCTTGAACAATTATCCCCGTTAAAAGGTGAGAAATCTGGTGTTAAAACCTTAGGAATTTCTCCTGTGAGTAATTCTGATTCCGAAATGAGTTTAGAAACTGATGAGTCTAGTAATATTTCTAAAATTGAAAATGCATTTAAAAACCTAGAATTAGAGTCTGAGTTAAAAGTTAAAAGATTAACTAACAACACCCGTAAGATAAACCCTACAAGCTTAACAAAAAATTGGTATCCCAAACCTACACCTCCTGACATTCAATTTGAAGAACGCAATAATCTGAGCCAATTTTCAGTTTCCTCTGATAAATTATATGAATGGAATATTGATGGCCTTTCTGAGCAAGAAATAATGAATAAACTTACACATATGTCCATGGTTGCTAACAGCTACATTACAAACCACAGTTTAAGACAATCTGAGATAGTCCCTTTGTTAGAAACTGGTTTTACAGGAACTCTCCGTTCTTGGTGGGATAAACACTTATCTGATGAGTCTCGAAATAAAATAATACATGCTGTTAAATTAAATGAGGATGGTCTTCCTTTGTTTGATACCAATATAGGACGAGGAATAGAAGATGGAGTCAATACACTTCTTTACACTATAATTGAGCATTTTATAGGTACACCAAGTCATACACAATCTAGAATACATGACCAGCTGAGTAATTTAAGATGTCCTCAATTAAGTGATTTTAGGTGGTATAAAGATGTTTTTATTTCTCGAGTAATGCTAAGAGAAGATAGTAATCAAGCTTTTTGGAAAGAAAAATTTATAAATGGTTTACCCAATTTGTTTGCACACAAAATACGAACTGTCCTAAGTAATGAATTAGGTCATATAGATTATGATAATTTGACTTATGGTAATATTATAAGCACAATAAATCAAGAAGGAATGAAAATGTGTATAGACATGAAGATAAATAAACAAATAGAGTCTGAACGCAGATCAGCTAAGTATGAATTAGGTAATTTCTGTGAACAATATGGTTTAATCTCCACTGCACCTTCCCGTAGGAATAAGCTTAGTTACCCTAGTCATCTCAAAAAGAAACGTCATTATAGCACCTTTAGGAAAACCTACAGAAACAATAATACACCTGATGAATTCTACAGGAAAAGAAAATATTCCCCTAAGAAAAGATGGTCAAAAGCACCTGCCTACAAGAAAGATGCTCGCTTTTACAAAAAGTCTGGTAAAGATAAAAGTAAAGTGAAATGCTTTAAATGTCAAAAGTATGGTCACTATGCAAATGATTGTAAAGTTAAAGAAACCATCCGACAATTACAAATCACAGATGCAGAAAAAGACAACTTAATTAAAGTCCTTGAGTTAAGAAACTCTGAGTCTAGTGAAAACGAATCCACAGTTAGTCATTCTGAGTCAGTTAATTATGAAACCTCTGATTCTCAGTTAAGTTCCCCTAAAATTCAATTAGGATGCAGAGATAAATGTTGCAGTACTCTTAAGCATATTTCTGTTTTAACTAAAAGTGAAGAACAAGAAGAGTTATTAATAGATTTAATTAGTAAAATTGAGAATCCTGATTTAAAATCTGAATATTTGAAAAAACTGAGACATATCCTTTCACAGGATGCTAGTACAAGTCAACCGAACCCTGTAATCAATTTGAGCACTACTCTAGAAAAATTTAATAAGAAAACAGAAATTACTTTACATGATTTACATTCTGAGGTAAAATTAATTAAAAAAGAAATAGTTGAATTAAAACAGCTAAGTTATAAACTACAAACTGAGAATTCTGACATCCGACAAGTTTTAAATACATTACTTGATAAAACACCTTCTGAGTCCCATAGTCCACCTAGTAGTCCTACTCATAAGTCAGAACCTGAACATGTTATTAACCTAATTAAACAAGTTAATTTCAGAAAATGGTATTCTAAAGTTACTATAATTGTAAAAAATTATGAATTTGATACAGTAGCTTTATTTGATTCTGGTGCAGATCTTAACTGTATAAAAGAAGGTTTAGTTCCTACTAAGTATTATCAGAAATCTACAGAATCCTTACATACTGCTTCTGGTAAATCGCTTCAATTAAATTATGAAATTCCTAAAGCCCATGTTTGTCAAAATAAAGTCTGTTTTAAAACATCATTTGTTTTAGTTAAAAATATAACTGATGAAGTTATTCTAGGATTACCGTTTATAAGCCTGCTTTATCCTTTTCAAGTTGAATATGATGGTGTTGTCTCCACTTATCTAGGTGAGAGTGAAATTTACTTTTTTATCAAAACCTGAGCTGTACAATTTAAAACATTCGACTAAAACTGATTTTGCAAAAACAATTACTTTGATTAAAGGAAAAACTAATCATCTGAGCCATTTACAACAAGAAATTAAAACTAAGAGAATTGAGTCTCAGTTAAATAGCCCTACTTTAAAAGAAGAAATTAGTTGCTTTGAGAAAAAACTGATTTCTGAAGTCTGTTCTGATTTCCCTACTGCTTTTTGGCATAGAAAAAATCATGTTGTACATTTGCCCTATATCAAGGATTTTTCTGAGAAAAATATACCAACTAAAGCTAGGCCTATACAGATGAATCATGAGATGATGGAATTCTGTAAAACTGAAATTAATGAGTTATTACAAAAAAATATAATCCGAAAAAGTAAATCTCCCTGGTCTTGTCCTGCTTTTTATGTACAGAAAAATGCAGAATTAGAAAGAGGATCTCCTCGATTAGTTATTAATTATAAACCTTTAAATTCTGTTCTTGCATGGATTCGTTATCCAATCCCTAATAAAAGAGATCTGATTAAACGGTTAGATAAAGCTGTTATTTTCTCTAAGTTTGATATGAAAAGTGGATTCTGGCAAATACAGATCCATGAGTCTAATCGATACAAAACTGCTTTTGTTACCCCTTTTGGGCACTATGAGTGGAATGTTATGCCTTTTGGTCTTAAGAATGCTCCCAGTGAATTCCAAAATATAATGAATGAGATTTTTAACCCATACAGTCATTTTTCTATTGTTTACATTGATGATGTTTTGATTTTCTCTGAGTCCTTAGAGCAACACTAGAAACATCTGCATACATTCTTGCATATCATAAAAACCAATGGACTAGTTGTATCTGCAAAGAAAATTAAATTATTTCAAACTCATATTAGATTTTTAGGATATAATATACATCATTTACAGATTCATCCCATAGACCGAGCCATTCATTTTGCAGATAAATTCCCTGATCAAATTACAGATAAAAATCAATTACAAAGATTTTTAGGATCCCTTAATTATATATCTGAATTTTATAAGAATTTAAGACAACAATGTAAACCCTTATTTGATAGGCTACAGGTTAATCCTCCCCCTTGGTCGTCTATTCATACAAGCATTGTTAAACAGATTAAGTGTCATGTTAAGACTCTTCCTTGCCTTGGTATTCCTACTCCAAATTCCTTTAAAATCGTTGAAACTGATGCCTCTGATTTAGGTTATGGTGGTATTCTTAAACAGAAAATTGAGTCTTCTTCTGAGCAAATTGTCCGTTTTCATTCTGGTATCTGGTCTCCTTCCCAATCTAATTACAGTACTATTAAAAAAGAAATATTATCTATTGTATTATGCATTACTAAATTTCAAGATGATTTATTAAATCAAAAATTTTTAGTTCGTATTGATTGCAAATCTGCTAAATATGTTTTAGAAAAGGATGTTCAAAATCTTGCATCAAAACAGATTTTTGCACGTTGGCAAGCTCTTTTAAGCATTTTTGATTTTGATATAGAATATCTGAGAGGCAGTGAGAATTCTATTCCTGATTTTCTTACAAGAGAATTTTTACAGGATAAGCAATGGCCGGCACATCAAGAGATAAGCAAAAAATGAGTCACGCCCCTACACCCCGAATCTTGCCTGCTTCTTTGAGTCAAAAGCCTACTCCTTCTATTAAAAATGAGTCTGATTCCTTTTTCGTAAAAACTGAGCCCTATACAATCACCCCCTATCCTGGCCATCCACCTCTTCCAGTCTTGAGTAATAGGTTTAGCCCCTTAGGTACTACAGTCACCCCTGCAAGACCCAATTACCAGTCTGCATTAGTCTCTAGTTATGATCCGTTTCAGTTACCTCCCACTGCATCGCCTATATCTTCTCCCTCTTTCCAAAAGTCCTCCCCTTATGTTTTAAAGAATACAACCAACATAGTAATCATTGATTCTCATATTCCTACCTCCATGAGTCCTGCCCAAATTGCAGCCTTTCATTTCCCTCCTGGATTTCATTATCTTCCTCACAGTCCTTACAAATCTTTGAAATTTTACAGAGAAATTCTTCATGAAACTAAGTCTGTTGAAATTAAGCCCATTAGAGACAAAACTGACCCTACAAAAATTATATATCATTCCCTTTACATTCATCAAATTTTGCATCCTAAGTCCTGGGGTTCCCGTCCTCATGAGCTCCGAACTCTTCCTTCTAAATATCAATATAATTATGCAGACTATGTTGAAGCCTGGTATTACATTTTTCTTCATCAAACCCCTGAGTTTTCTCATTCCTGGTTTATTAATTTTGATAATAAGTTTAAAAATACTGAGCTTCCCTATTGGTTCCTGCATTGGTGGGGCACCCATGGTCCCAAATCTGATATTATTCCTCCTGCAGTCCATGAGCTAATTAATTATTTTACCAAGAAATACAAGATTCCTGAGCGAGATCTTTTCTTTCCTCCTGATCTCCTATTCTTTACAAAATACAGGCTCCCTTGGATTATGAAATGGAATTATATTGTTGATTGGGAATCTCGTTTTTTCTCCCGTCAGTTTCTTGTTAAGTGGTGGGACCGCTTTGAGGTCCATCGCATTACAGATTATGTTTACAAGGACTTTCCTCTGGTCCCTAGTCGAACTCCTAAGCCCAAGCATGAGCCCTCCTCTTCACATTCTTCTGATACTATGCTTACAGTTGAAGGCAAATCTAAAACTGAGTTACAAGATTTAGCTCAACAGCTCCTTCTCCGTGCTTCCCAGATGCATGATGGTGATGATAATGATGCTGAGTCTGAAACCTCTTCTAGTTGTCAACCTATACAACCAACTCCTGAGCGCCCTGTCCATTGGGCTGACTATCTGCATGGTCAAGATCCTAATGATGCCTATGATGAAGCCTATAAAGCCTATGATTTGAATTCAGATTAGACTACTGCAGACAAGTTACCTGAGTTAAAGCAAGTTACTAGAAGTTAAAGCAAGTTACCTAAAGTTACTTGAGTTACTTTTATTCACTTGAGTTACTTTTGTAAACCTGAGTTACTTTTATTTTGTCTTTAAAAGAAGGCTATTGCCTCATTTGTAAACATCGATCGTTTTTTCTCTTCATCTTTTTCAGTTTCAAAATGTTCTCTCAGAAACCATGTTCTGAAGAACAACCTTAGAATGAGTGTAAGCTTTGCACATTGTAAGTTTTTACTTTAAATGAATTTATTTTGAATATGAAGAACATTATATTCATGTATTACATTATTTTTATAATCATGTTTTCATTATCTTGTATAGTTCTGCTCCTCCTTTCTTTCTTTCCTTTTTGGTATCAGAGCCGTAAGCGTTGTAAGAATTAGTCTTAAGTCGCAACTCATCCAACCCAAGTGGCTGGTTTAGGATTTCTGAGTTGAAGTTCTTTGATTTTTCCTTTCCTTTCCTTTCCTCTGCAAAGTGTCTGTCGGTTTTCTCTCTGGTCCAAACAGTGAGTCCTTTTTCCAAACGATAAGTCCCGGGGTAGGGCACGAGCGGTGGGCGTGAGCGGAGAGAGAGTTTATCCTCAGATTCCTTGTCGGGTAAAGTTAAGTCCAGGATTTCTCATTTTTCTTCTTCTCTGGTTATCCTATCCCATATCATTAGGAAAAGATGAGTCGCTTCTTACGCACAAATTCTTGTAACTCTAGCAGTTCTAGAGCCTCTATAGGAAGATTACCTGAGATAGTAAATGAAGAACAATTTGAGTACAATAGTACTAATAATGATGAACAATTAGACTTTAAAGATTGGAATATACCTAAGATTTCTAGTCATAATATATACAGAAAGAAATGGTCTTTATCTGCATTTAATTCTACTACGCATGTTAAAACTGTAGAACAAGTTTATGCTTTAAGTAAAGAACATGAGTCTTGTCAATTGCTTAATTTAGATTCAATTAAGAAACATAAGCATGATGGTAATAATTTCCTGCATATTGGTTTGGTTCAAGTCGCTGTTAAGCCTTTAACTAGATTAGGTTTACAAGCTTCAATTTTATTATGTTTACGTGATGCACGATTTACAGATTTTAATGATAGTGTGCTTGGTATGATTGAGTCGAGTCTTTGTAATGGCCCTGTTCATTTTGATTGCTATCCTGATTTTACCGTAAGTCTGAATGATCCTCATATTCTTAAGACATTAACTTTGAATCTAAAAACATCTGGTTATAATGTTCTACCTGGTACCCAGCCTTTAGCCTTAGTTTATAGAATTTATTATAAAGTTACAGGTACTAATATGAATTTTCAAGCTTTGAATAAGAGTCCTAAAGATCAAACCCTGTTAATACAAAGTTGTACACCTGATGCTAATATTAGAATACCGCGTACTGTTAAATGGTCTGAAATTACTTTACCTACAGATTGGACTTTAATCACTGAGTCTCAGCCTATGCCTATTCAACGTAGCTTAGATAACTTAGATTATATTCATCAATATATGGATGGTAGTGTTAAGATTAATTTCCAACACCAACCTAAGTCTACAGTCCAATTACAACAATTACCTACGCCTAGCCCCTCTAAAAGACATGAGCCAGCACGTCATTCTTCTGCTTCTTCTTCCACTACAGTTAGAGATTTAGAAATAGAAAAAGATTTAATTGATCTAAAAATTGCATCCCTTAAGAAAAAAGACCAAGTTAGTCATCCTTGTTATGGTTCTCCACCCCTTGGAAACCCATATATTCCTGCCCCTGAGCCTAAAGTTAATTCCCCTACACAGTCTGATTTCCTTGCTAGTGAAATAGCTACTGCACATGTTTATGATATAGATAATCAATTAAATGTTTTAAGACGTCCTGAAAATTTTAAGATCAATTGGAAAGCCTTAGAATCACACCTTTTAGACCCTGCTAATTCCTACAGACGAGATATTTATCATAAGGTTGTCCCTGATAAAAATGAAAGAGATAAAATACACCATGCATGGAAATATATATATGGG

TCACGATCCGGGGACACACATTTTGACACAAAATATGACTCATTTTATTATACCTTTAGATTTCAGCACTTTTGGCGGGTAGATGAATGGAGTCTTAACCTTATAAAGAATAAAAATAAAATTGTTAGTTATCCTTTTGTCTTTTACGGGAAACTAAATTTTCTTTCCTAAAGCATTCTCTCTCCATCTCAGCATTCTCATTCTCTTCCTCTTCATTTGAAACTCAAACTTAAAACTATCTACCCAAAAAAAAAAAAAAAAAAAAAAAAATCAGACTGGAAACATAATGAAAGCAGACAGACTTTGATCAGGAGTGTGCGTAAAGCTTTTTATGGCTCGTATCATTTTTCTTAGAATCTGAAGATATGGAGCATAAATAAGTTTCAGCTTTTTTATGGCATGTTTAGGTTTTTACCTCTTCACGTTGCAGGGGGTTGAGTCGTGTATTTTATGAAAGAAAGAAAGTGGGAATCGGGCTTGGGGTAGGTTTAGGCAAGGCAGTGAGAACTGCTTGTTTTCTTCCTTTTCTTTGTTCCCTCCAGCAATTATATAGTTGGAAAAATTGTTATATACGTGACAAAATATTCTGTGCAATGTTTATGAAAATGCCATTTGGAGGATTTGGTCTTTCTGGATACACATGTGGAATACCCTTTGATGATTTTAAAATCCTTTGTATAATATATTTATTTGATTGTCTGTTTTGCATCTTCAGTCACTGAAGGGATTGTCTGAAGAGATTGTTGAGACTGCAGAGACGGAGTCAGGATTCACTATGTTTCCAATGCCCATGGGCTTGAAAGGGAGATTCGTTTGTAACCCAGCACTGAATTTCCTGAGCTGCGGTTTTCCAAGGTAAAAATTAAAATGGATTTGGAATGTTATTCTTTGAGGCTGTTTGTCCTTGAAGATTGTAATTTATTTTGATGGTAAATTGATGTTTTTTTTATCAGCCTGGAAAAAGATGGTTGAGTTGAGACAGCAAATTTCCGGCAGAGAA

>Chr3-01; OZ022208.1; 2408959-2409696; dir; B;

GCTCTAAAAAGGAGCCACAAGCAGTCTCAAATGAGTATATCAAATCAATCACATGTGATATTTTCATGGAATAGATATTTATAGTTTTATCATATTCCTACATGTGTAATGCTACTTTTAATTAGCACAATATTACACCATGAGAAGTTGAGTTGGACCAAAAAGTACTGGAATCACTCCCACTCCTTTAATTGAGGGGCAAGGACTTGTGTCGAATATTGCATCTTAATTATTCCCTTAATTGGGTGGCATTGGGCATATTATACAGCTAATGCTTTCTTTCTTGCACGACAAGTATTAGTCTTAGGATGACATTACACACATTCATCAGCAAGCTTGCATATACAAGAATGACGATGAAATTCAAGGGGTATGATTTTCACTTTGATTAGTCAAACAGTATGATTTTTTTTTATTTTTTTTAAAGGGTTATAAATATGATTTATTTTCATAGCACTGGTGTGTATCATTCACATTGAGGTAAATGGTGTTGTGCCGGCATGTAGTTTAGTTAAGTTTAACTTCCGCAGATTCTTTTGAATGGTTTGAGATGCCCCATAACATTATTTGAGACGCTTTCTTAGTTGATGATCATAGTCTGAGTCAGGTTTTATGTTTCTCTGTTTTTTGAGTTTTAATTATGAATAAAATCTCCTCGTACAGTTGATGGATGGGACTCATAAATAAATAACAAGAGAACATTTCCGAAACATATAATATTATCTCGAGTTTTTTCATAACTGTATTGTGATTATCTTCTTTGATGGTAGAGATAATGATCTACAAACTCATTAATCACATCGCCGAAAGTTTTTGATTATTCATGACTAGTACACTAGAATATTGATCCCCAGATATGGCTTTGAAATTTGCCATAGAGTACCAATGATCTTCAAGCTCGTTAATCACACCCTCATAGTTGTGATTATTCAATATGGGATTACTATTTTCACACTAGAATATTGGTCCATATATATATATATATATATATATATATATA

TGCCCTTTCGTACCCTTTCCAGGTTGAACACGATGGTGTCATTTCTATATGTCTAGGAGAAAAAATTAAGTTTGAATTTCTGAGCAAATCCGAGTTACATAATTTAAAAGAATTACAAAAAAATGCAGTCACCAAAACAGTTCAGGTCATCCGAAACAAAGATAAACAATTAGGATTCCTCCAGGCAGAAGTTAAAGCTAAAAGAATTGAACAACAACTAAGTCATAAGTCTTTACAATCGCAAATACAACAATTTGAAGAAAAACTAAAACAAGAAGTCTGTTCTGAGTTACCCAACGCATTCTGGCATAGGAAACAACATGTTGTCAGCCTTCCCTATGTCAAATCCTTTAGTGAGAAAAATATCCCTACTAAAGCAAGACCTATTCAAATGAGCCAAGAATTGATGGAATTTTGTAAAATTGAGATTACCGATTTACTTAAGAAAGGAATAATCCGTAAGAGCAAGTCACCCTGGTATTGTCCTGCTTTCTATGTCCAGAAAAATGCTGAATTAGAACATGGAGTCCCAAGACTAGTCATTAATTACAAACCACTTAATTCTGTTTTGGAATGGATTAGATATCCAATCCCTAACAAGAGAGATTTAATTAACAAATTAAAAAAATCAGTCGTTTTTTCTAAGTTTGATATGAAGAGTGGATTTTGGCAAATTCAAATTTCTGAGTCTGATAGATATAAAACTGCTTTTGTTACCCCCTTCGGTCATTATGAGTG

AACAGTATGAACGACACGATCACGAACTGGTGCAGAAGGCAATGTCAAATAATATAACCCCCCTTATCCGTTTACCATGCCTAATCGTCTCGCAAGTCTTGAGATCCTGAAAAGAGTAATATGTAGGATAGAAAGTAGCAGAAGCATTAAGTGTATCAAGTAATCGGCCAACAGACAACAAGTTACAATGGATATTGGTAACGAGTAACGCACGAGACAAGGACAAAGTATGAGTGAGAGAGATTGTGGGCAAGCCATTAGCATTAGTTATGTATGGGTCACGTGTGTGACTAGACAACTCATAAAAAAAATGTAGCATCATAAGTCATATGATCAGAAGCACAAGTATCAATTATCCAAGTATTGGAGCCAGTACCTGAAATACAGTCTGAACCACATAAATTTCCAGAAGCAGCAGCAACAACACCAACAAAGGAGTTATCACCCATGATTGCAGCATAAATTCAACATTTCACGGTAGAGAGTACAATGGAACACAATAGATGCACAAATACAGCAGATGCACGAAGACAGGCACGAACACAGTAGAGGTATGAAACAGCAGAGTGCACAACGGAACACAGCAAAGGCGCAAATACGACAGATGCATGAAGACAGGAATGGCACAAACACGGCAACACAACTCACAAACCCAGAGGGGGGCATACACGGCACAAGTAGAGAAAAAAATATGGGATTAGAATACGGGTGGGCACAACACAATGGTCACTAGAAAAGTTTGCTCTGATACCATGTCAAACAAAGTAGGTAGAATATTTTCAAATTATATTTCATTCTCCAAAAGGAGGTATTTATAATACAAAAGTACAAATCCTAGTAGGGTTATACTAGGAAACTAAGATAAAGTCCTAATTACATAATATCTATACAAAAGGAAATAAATAGAATCATAATATATTAGGAAGTAAATCTCCTAATTACATTAGGATCTCCCTAACAAATCGAACCTAGGACCTTGAATGTAAAAATAAATATTCTT

>Chr3-02; OZ022208.1; 4426556-4432727; dir; B;

CGTTGGTCAGCCCAAAAATAAGCGTATACTTTTTAACTCATTGAACAAGAGGAGTCGTTCAGGTATCATTGGCTAATATGTAAATCCACATCTATAGTCTGTATATGGCACCCTCACTATAGAATTTCGCGAACAAAAAGAGGGAAACAAAATCAATGATTTTTGGACAAATAATAGAATCACAGCATATACACCTCTGTCGATCCAGATTGATTTTGAAATTGAAACTTAACATCATTGGCCAAAAACAATAGTAATTCTCGTACAAACAATAGAACCACAACACCTACATATTTGTCTTTTGAAGATTTTTGTTTTGTTATGTGAGATGAAGGTCCGTCACCAAAAGTCTGGCAAATGCTACAAAAGAGAAAACCAGGGAGGTGATGATTAACAATGGAGCTGTAAAGAAAGTGAGAACAATAGAAAAGAAAAACAAATCCCAAAAAGTGAAAGACCTTGTGTTGATCATTGATCATTCTTGGGAGGGATGTAGCTCAACCCCAAGACATGTCTTCATTGCTGCTCCTTGCTCCTTTTTTTCTTTTTCTTTAGCAACAAGAAAAGAAATATAAAGATGCATAGCCATAGAAGAACACAAGAAGAAGTAGAACAAGTGGGCTTACTTGCATCACTCCCCTCTAGTTTGTCCCAATGGCAGCCTCACCTCAACCCGTCTCTGAAAACCCGATTGATTTTCATGCCCCATCGCCTTCTCCCATTGCCTCAGGCCGCCGATCATTAGTCACAAACGATGAAGTCCTCACTGAATACCTCGAGCACTCCCTCCAAGTCCCTGACCTCGTTTTGCCAGACAAGTTATTCCCTAAACAAAATTCACTTGAAAGTATGAATTCATATTTTCGTGCCAATTATGATTTATAAGAAAAGAGAGAGGCATTCTTCTAAAGTATTTTTAGTGAAAACGTCATTACATACTAAATCAAAAATATCTAAACGTACCAAGTCTTAGGAATATATATATATATATATATATATA

AACCAGAAACTTGAGTTAAAATGAGTCGCCTATTTATAACAAATTCTTGTAATTCAAGTAGTTCTAGAGCCAGCATTAGTCACCTCCCAGAAATAGTTAATGAAGAACAAATAGAATATGAGTCTAATAGCGAATTAGATTATAGGGATTGGAATATACCTAGAGTCCCCAGTCAGGACATCTATAAGAAAAAATGGTCATTGTCTAGTTTCAAGTCGACCACCCATGTCAAAACAGTTGAACAAGTCTATGCTTTAAGTAAAGAACATGAAACATGTCAGTTGCTTAATCTTCAATCGATTAAAAAACATAAAAATGATGGAAACAATTTCCTTCACATTGGTTTAAACCAAGTTGTAGTTAAGCCCCTTACTAGACTAGGACTAAAAGCCTATGTCTGCCTATGTCTAAGAGATGCTAGATTCACTAATTTTAGCGACAGTACGCTAGGATTAATTGACTCAAGTTTATGTAATGGTCCAGTTCATTTTGATTGCTACCCAGACTTTACAGTCAGTCTCAGTGATCCTCAAATTTTGAAAACTTTAACTCTGAATATTAAAATTGATGGGTACCAAGTCCTCCCAGGAACACAACCATTAGCTTTAGTTTTTAGAATTTACTATAAAGTCACTGGAACAAATATGAATTTCCAAGCCCTTAATAAAAGTCCAAAGGATCAAACCCTATTAATTCAAAGTAGCAAACCCGAGGCCAATATACGAGTACCTCACACAATTAAATGGTCAGATATCACCCTTCCTACGAAATGGACACTCACCACTGAAAGTTATCCAACCCCAGTTCAGCATAATTTAAACAATTTAGATTATATTCAACAATATTTATATGGCACAGTTAAAATTAATTTTGGTAGCCAACCCGCACAAAAATTCACTGTCCAGTTACACCAATTGCCTACTCCTGGCTAGTCACAGAGACACATCCCTGCGAGACACTCATTTGCCGCTTCTTCCTCCACTCTAGAACGAGACCTAGAGTTAGAAAAAGCCATGATAGATTTCAAATTAGAATCCTTAAGAAAACCCTTCCAGGTTAATCAACCCTGCTACGGTAAACAACCAGTTGAAGATAACAAATCCAAGGCCCCTGAGTCTCCCACACAAACTGATTTTGAAGCCGAAGATTTTGCAGTTGTTAATAATCAATTATGGACACTTAACAAAAAATTCACAATCAATTGGAAAGCCTTAGATAGCCATTTAAGAGCCCCTGAAAACCAAGAAATAAAGAATTATTATCACATGGCCTACCCTAATAAAGAAAATCGCCAACAAATCTTTAATGAATGGAAAGAATATATGAGATCAGCCAAAGTAGAAATTTTCTACCTTGATTTGTAGAAAGTCATTACATGTCTAATAAGTTAACAACTTTAACTAAAGAAAAATGGAAATTACCTGATAATACAGAAGTTATAGCAAGTCACCCCCCAGGAGAAACAATAATAATTGAGCATTTAAAAACACAAATACTAGCTAGTCCTTTCAAAGCCCTTGAGAAAAATGATCCTGATTAAAAAAAATAATGGAACAAAGTAATTATACAAATCAAAGTCTAATTGTCTTAGGAAAACAGTTAGATAAAATCGAAACAAAAGTTGATAAACTGATTCCCCAGGATAGTAAAAATAAAGCCAAAGTGGAAAAACCATTCATTCAGCTCCAGGATCTTAAGCCAAATTCGACCCTTAAAATCAAACCAACTATGAAGAAAATTGAAGAAATGTTAGAACAGTTGACACAAGTTAAACCGGAAAAATCTGGTCTTAAAGTCCTAGATTCTTTTGTAAACCTTTCTGAGTCTGAGTATGAATCTATAATCAGTATGAAATCAGAAACTAGTTGTAATAACCCAAACCTAAATTAATATTTAATATTTAATTTATTAAGAGGAAAATACAATTTTGCCCTTGGAATAATTTATTAAAGAAAAATTGACTTTTTGACCGAGAAGGAATTTGACACTTCCACACACACCGTTGCGTAGAGTACGTCGAAACGAGTTTGTAGACACGAAGTGGGCTCGAATCGGAGCTTTAATTAAGAAAATACGATTAAAATACTATGAGGGGCAAAACGGTAATTTGAAAAAAAACAGATTTTAAAACTTCAATTCTCTCTCTCTCTCTCTCCCTCCCCAAGCGCTCTCTCCCTCTCTACGACAAAACACCCCCCGATCAAATTTTGCGGCGCTATCGCCACCACCGACCACCACTCATGGCGGGATCGGACTAGATAGAACCGTTGTCGCTCCCCCGTCCTTTCCCGACCAGCCCCGCCCCTGGAACCGCCGTATTCCGTCGGAAAAAGCCCAAAATCGCGCCGATTTTGACAGAAACTTCGCTGGCTGTATTCATCGTCGTCCGGCCACCATTTCCAACGATCCATGTATGGATTCTGAACCTCTCGAGCTGTTCTAGTTGCCTGTTGGGTAGGATTCAATCGATGCTTAGCTTAGGTAATTCGATTTGCGAATTGAAATTCGGCTGGTTTTGGGATCTCAATTCCGGCCACTTCCGGTCAGTTTTTGTGGTAGGTCCAAACAAAAGTGGTTCCAAATAGGGTGTTATACCTAGGGTAGGAGTTTGGAGCCGTGGTTTTGAGATTTTCTAGCGATACGTAATCGCTTTGGGCACCCAGCGCTGCTGGTGCGTGTGGTGGCGAGTGGGCGAGGGTAGTGAAGTTGCACTGTGTCTCGATGAGATCCTTAGGTTGTCAAGAGCGTGTAGGAATTCTCGGATCTCAATTTGGATACCGTTTGAGCCTCGAACGGATTGTTGCATATTGTGCGATTTTCGGGTTCAATATTGTTAAGCCGTTGGACCGTAATCTTTTTCAGATATATTACTCTAGGCAATTTCAAAATCGTTTAGTATTCGACGGATTGTGAATCGAAGTCCCGGATACTCCGAAATCGCGAACCCTAGGGCTAGGGTTTAAAAATCGTGCGATTGTACGATCGTGATCAATCCGACCGTCCGATCGAGACCAAACCCTCGGGACATGTGTTCTAGGTATATTGGAACTTCTGGAGGCTTCCGGATCATAATTGAGAGGTCATGGACTCGCGAGGCCCTTTGTTGACTTTGTGGGACTTTAGCGCTTTTTGAGTCCCAAGATACTGCTAAACTATTCCAAGTAGAAGAAAGCTTTTATGGCCATAGGAACAACTTTAATCTGACGCACGTACTTCTAGGAGCTGGGGGTCAGGGTTTTTAAATTAATACTACAGTTGACTCAGGCACGTACTTCTAGGAGCCGGGGGTCAGGGTTTTTAAATTAATACTACAGTTGACTCAAGCACGTACTTCTAGGAGCGGGGGGTCAGGGTATTGTATTAATATAATATTATGGTAATTTGGTAATTGAATCAGGCACCCGGGCACCAGTTGACTCGCAGGAGGGACCTGCAAGAGGTCCAGTGAGCACGAACCATCTGTGAGTGGACTCTTTGTTTTTATAAATGAATTTAAGCAGTTCAGTTACAGTATTTGATCTTGAATAAGTTTTATTCAAAGATTAGCGTTTTATAAGCTGTTATATGCTTTTAAATATTTTGTTTTGCATGCTGCCGAGTGAAATCTCCTTTAAAGGATATAGGAAAAGAAATTCTAGTTAATTATCAGATAAATGGCAGCAGAGTTTTAGAGTTTATAGAAATTCAGTTATTTAGCAGATTTTCTTCAGAAACTTTATATTTTCCTAAACCACCTTGTACCCAAATATACAAACGTTGCCTTCGGTAAATAAGTTGCCTTCAGATCAAATGTTGCCTTCGGATTTTATTGATAGCCTTCGGTTTAATCAGCATGCTTGACAGTTGCCCCACGAGTTTTGTGGTACCCAAACTCGTGTAGAGCGAGTAATGCGGATCAAACTGACTACGGTCCCCTGAATCCTGCTAGTTGCGGCTCGCACTGACTTTGTGTCACTGAGACCTGCGAGTACGTGTCACCCATGGTGATGCAAGGAATTGGCGGAAATAAAGGGTACACGTAGGTGGTAGTTTTAAACTGTTTTCAATGCCTTAAGAAATTAATGTTGACCATGAGTATGATTGGCTTATATACATGTATAATTATATGAGTGCATTGATTTCAGTAATAAAGAGAATAATTTAGTTTGTATAAATGTTTTTACAGTGGGGTTAGTATGTTCACATGCTATTTTCTAAACTTTGTTTTTGGGTCCATTCACCCTTTTTAATGTTTTGCGCCCCCAGGCAGTAGGCGTGCACAGGGATCCACCACCAGGCCGTTTTCCACCTTCCGCGCTTTCCTTGTTGATGTAGGACTTTTTGTTTTGTAAAAGGATTGACTCCTTTGTAAATTCTTAGTAGTCGCTCTGATAATTTGCCCTAGAATTAGAATGTAACTGTTGGAATGTATATATACATATGCTGGTAGTTGGTTGTGTATATATGCTTGTTTGGCAGATGTAAATGTTTTGGTATTGATCCAATTTACAAGGGAGACTCTGCCGATTTTTCGGTAGGAGTCAACCTTGTTATTTTATCGTGTTTAGTAGGAAAGGGCAATTAGGTTAATCGTGCCCAACATCCGCCAGGTGTCGGACACGCACAGGGTCCGGCTCGGATTCCAAAGCGAAATTTGGGTCGGGTCCTGTCACTAGTAATATTTCAAAAATTGAAAATGCTTATAAAAATTTGGAATTAGGAATTGAACCAGAGGTCAAACGATTAACTAACCATGTCAGCCCTACCAGCCTAACAAAAAATTGGTATTCTAGACCAACACCCCCAGACATTCAATTTGAAGAACGAAATTTCCAAACCCAGTTTTCAGTCTCTTTCGATAAATTGTATGAATGGAATATTGATGGTCTGTCTGAGCAAGAAGTCCTTAACAAACTTCAACATATGTCTATGGTTGCCAATAGTTCTGTCACTAATCACAGTTTTAGGCAGTCTGAAATAGTGCATTTACTAGTCACTGGTTTTACCAGAACTCTCCGGTACTGGTGGATAAACACTTAACCATAGAGTCAAAAAACCTAAAAATTCATGCGGTCAAAATCAATGAGGAAGGTCTCCCCATATTTGATGAACAAATAGGCCAAGGTGAACACCCAACAATTCATGATCAACTTAGCAAACTCTGATGCCCTAAGTTATCTGATTTTTGATGGTATAAGGATGTTTTCATCTCCCGAGTCATGCTTAGAGATGACAGTAATAGCCGTTTTGGAAAGAAAAATTTGTTAATGGTCTTCCAAGCTTATTTGCCCATAAAATTCGCACAGTCCGTAATAATGAACAAGGTCATATTGATTGGGATAGTTTAACTTATGGAAACATAATACGCATGATTAATCAAGTCGGAATGAAAATGTGTGTTGATATGAAAATTGGTAGATAGATACAGTCCGACAGAAAATTCACTAAGTATGAGTTAGGAAATTTCTGTGAACAATATGGTCTTACCAGTCTTCCTCCTTCCAGAAAAAATAAGCCTGGTCGACATATTAGCAAAAGTCGTCATTATTCACGCAGAAAACACGCCTTTAGAAATAATGATGAAAGTAGAGAATTTTACGGCAAAAAGAAATACAGTCCACGTAAAAATGGGAAAAAAACACCAAAGAAAAGTCATTTTAAGAAGAATACCGACAAGAAAAATGTTGAATGCTGGAAATGTCAAAAGAATGGTCACTATGGTAATGAGTGCAAAGTAAAAGATACTATTAAACAGTTGAAAATAACGGATGAAGAGAAAGACAAGTTGATTAAAATTTTAGAGTTAAGAAACTCCGAGTCTTGTGAAAACTGCCAGCACAAAGTCAGAAAGCTACCAGTCTTCAAATTCCCAGTCTAGTTCTCCCCAAGTTCAGTTTGGTTGTAAAGATAAATGTTGTAATAGATTAAAGTCTATTTATGTCCTCACTAAACAGGAAGAATTGTTAATTGATTTGATTAGTAGAGTCGAAAATTCATAGTTGAAGTCTGAATATCTAAAAAAATTAAGAAAAGTCATTAGTAAAGAAAGCACAAGCCAGTCTGTTCCCCAACCTATAAGTATGAATACTACTTTAGAAAAATTCAATCGAAAGAAGGATGTTACCCTACAAGATTTGCAACTAGAAGTCAAATTAGTTGTAACAACCCGGTCCTAAATAAATTAGTAAATACT

AATTTATTCAGAGAAAATACCATTTTGCCCATAGAATATTTTATTAGGTTTAAAGTTGACTTTTTGATCGGGAAGGAATTTGGGTATTGCGACTGTACTGTTGCGTAGAGCACGGCGAAATGAGTCCGTAGACACGTAGTAGGCTCGATTTTGAGTTGAAACGAAGAAGTTACAAGCAAAACAAGCTTAGTGGCAAAATCATAAATATTTTGAAATGAGTTTTTTTTTTTATAAAAATCAGATTTTCTCTCTCTATCTCTCTCCCGCGTGCTCTCTCTCTCTCTCTTCGAAACTCCGGCCACCGGCCGCGGCTGGGGACGGGACCGACGCCAAAAGGACCGACTCGACCTCGGCGTCACGACCCGGCCCTTCCCCGACATCGGCCGCCGCTCCAGCCGCCGAAAAAAGATGATTTTTGACCGGTTTTAGCTCGAACTTCACCGGCTCCGATCTCCCTCCTCCGGCTGCCATTGTTGGCGATCAAGGTATGATTTCGCACCTACTTTTCATGCTCTAGCTGCCTGTTGGGTTGGATTTGATCAATTCTTTGCGTAGGTAATTCGATTTAAGGATTGAAATTTTGCCGATCTTGGGATCGCGATTCCGGCAACCTCCGGTCAGTTTTTGGGGTAGCTCCAAGAACAAAAGTGGCTCCAAATATGGTGTTATACCTAGGGTAGGAGTTTAAAGCCGTGGTTTTGAGATTTTTCGTGATGCATAATTGCTTTGGGCTCCTACGCGCTGCCCGCGCGTGGGCGCTGTCGAAAAAGGGCACTATGTCCAAATGTAATCTCCTGGTTCTCACAAGTGTGTGGGTGTGCTCGGATTTCAATTTGGATACCGTTTGAGTCTCGAACGGATATCGTCTATTGCGCGTTATCCGGGTTCGATTGGTTGGGACCGAATTTATTATATGTTAATCTAGGCATTTCTAGGATCGTGTAGGAATTCACGGATCGTGAATCGGAGCCCCGGATGTTCAGATTTAATAATTTTTAAG

>Chr3-03; OZ022208.1; 4452551-4459473; rev; D;

ATCAAAGTTGCAACCTCTTTCTCTCTCTTTAGGATGGTAAGTTGATTTTGGCTATTCAAACATTTAATTTTACTTCGGAAAAACAAAACCTACCTTGAAAATGTTAAAAACATGTTTTTTTTTTTCTTTTCATATAATAAATACATAGACATTTTTCACATGTTTTCATATTTTTCATATTTTTCTATGGTATTTCATTAATTAAATTGAAGAGTATTTTGTCATCTCTTTCTCTCTCAAACCCTCAATTCTCCCTTCTCTTTAAGCAAGGAGTGGTAGGGGAGGGGGAGAAGGCCACTCCTCCTCCTCCTCCTTTTCCTCCTCTCTTCCTTTCTCAATCTCTTTTTCTCCTAACTTTCTACTCTGCTTGTTAGACAGTCAGACGTATCATGTTTTTAATGTCAATTTAATTACATATTCAAATAAGAATTCACACAATTAATAATTCAAGTTGAAAGCTTTGACAATGTAACAAACATTAAAAAAAAGTATTTTCTATTAAGTAGACGGTTTTCCTATACAACTGCGTACTTAGAGTCAGAGACTAGCTTTGTTATTTTAAGTCTTCTCAAGTAACCAACAGCAATCAACCAAGAAAACCAAAATTGGTCCAAACAAAAGACAGAGATACAAAAATTATCTACAACAAAGAAAAAGTATTTGGAATTTGGAGGGAGCTGAAATTGGACTTCATTTGAATTGTAGCCTACTCATTGCAACATCGAATCACATTTCAAACATCACCAATGCAACACAAAATATCACAAGAACCGAGAATCGTCACCATTACCATATATCTAGACAGCAATGTCATATCAAAGCCAAATAACTTTGTGTGAAAGAAAAAAGAGCATTTTCTTGATCAACTCTCAGAGGTCATACGTCGTTTATGGTTTTCAAGCTACAATGTCAAACAGTTGAAAGAGCACCTAAACCCTTATAATGAATGAAATACAACCATTAGCTTTAGTTTTACTATAAAGTCACCGGAATAAATATG

AATTTTCAAGCCCTTAATAAAAGTCCAAAAGATCAAATCCTATTAATTCAAAGTAGCAAACCCGAGGCCAATATACGAGTACCTCTCACACAATTAAATGGTCAGATATCACCCTTCCTACGGAATGGACACTTACCACTGAAAGTTATCCAACCCCAGTTTAGCATAATCTAAACAATCTAGATTATATTCAACAATATTTAGATGGCACAGTTAAAATTAATTTTGGTAGCCAACCCACACAAAAATCCACTGTCCAGTTACACCAGTTGCCTACTCCTGGCTAGCCCAAGAGACACATCCCTGCTAGAGACTCATTTGCCGCTTCTTCCTCCACTCTAGAACGAGATCTAGAGTTAGAAAAAGCCATGATAGATTTCAAATTAAAATCCCTAAGAAAAACCTCCCAGGTTAATCAACCTTGCTACGGTAAACAACCAGTTGAAGATAACAAATCCAAGTCCCCTGAGTCTCCCACACAAACTGATTTTGAAGCCAAAGATTTTGCAGTTGTTAATAATCAATTACGGACACTTAACAAAAAATTCATAATAAATTGGAAAGCCTTAGATAGCCATTTAAGAGCCCCTGAAAACCAAGAAAGAAAGAATTATTATCACATGGCCTACCCTAATAAAGAAAATCGCCAACAAATCTTTAATGAATGGAAAGAATATATAAGGTCAGCCAAAGTAGAAATTTTCTACCTTAATTTTGTAGTAAGTCGTTACATGTCTAATAAGTTAACAACTTTAACTAAAGAAAAATGGAAACTCTTGCTATTTTGGTGTGTAGAAGATCGTTTTTGGAGGCGGCATTGACAAAAAAGATACCTCTAGAAACTCTTGCTATTTTTTATTAACATATTACTACATATGCAACAGTGCAAGATTAGGGCCCAAACTCAGGTTGGGTGGGTTGGGTCAGCCCAATCCATTGATAGGCTTGAGCCAAGTTGTAACCCGTTGAATTGGACAAACTCGTTGGTCAGCCCAAAAATAATCATATATTTTTCGACTCATTGAACAAGAGGAGTCGTTCAGGTATCATTGACTAATATGTAGATTCACATCTATAGTATGTAAATGACACTCTCATTATAGAATTTCGCGAACAAAAAGAGGGAAACAAAATCAATGATTTTTGGACAAATAATAGAATCACATCATATACACCTCTGTCGATCCAGATTGATCTTGAAATTGAAACTTAACATCATTGGCCAAAAACAATAGTAATTCTAGTACAAACAATAGAACCACAACACCTACATATCTGTCTTTTGAAGATTTTTGTTTTGTTATGTGAGATGAAGGTCCCTCACCAAAAGTCTGGCAAATGCTACAAAAGAGAAAACCAGGGAGGTGATGATTAACAATGGAGCTATAAAAAAAGTGAGAACAATAGAAAAGAAAAGCAAATCCCAAAAAGTGAAAGACCTTGTGTTGATCATTGATCATTCTTGGGAGGGATATAGCTCAACCCCAAGACACGTCATCATTGCTGCTCCTTGCTCCTTTTTTTCTTTTTCTTTAGCAACAAGAAAAGAAATATAAAGATGCATAGCCATAGAAGAACACAAGAAGAAGTAGAACAAGTGGGCTTACTTGCATCACTCCCCTCTAGTTTTAGTCCCAATGGCAGGCTCACCTCAACCGTCTCTGAAACCCGATTGATTTTCGTGCCCCACCGCCTTCTCCAATCGTCTCAGGGCGCCGATCATTAGTCACAAACGATGAAGTCCTCACTGAATACCTCGAGCACTCCCTCCAAGTCCTTGACCTCATTTTGCCAGACAAGTTCTTCCCTAAACGAAATTCACTTGAAAGTATGAATTCATATTTTCGTGTCAATCATGATTTATAGGACAAGAGAGAGGCATTCTTCTAAAGTATTTTTAGTAAAAACGTCATTACATACTGAATCAAAAATATCTAAACGTACCAAGTCTTAGGAATATATATATAAACCAGAAACTTGAGTTAAAATGAGTCGCCTATTTAGAACAAATTCTTGTAATTGAAGCAGTTTTAGAGCCAGCATTAGTCGCCTCCCAGAAATAGTTAATGAAGAACAAATAGAATATAAGTTTAATAGAGAATTAGATTGTAGGGATTGGAATATACCTAGAGTCCCCAGTCAGGACATCTATAAGAAAAAGTGGTCATTGTCTAGTTTCAAGTCGACCACCTATGTCAAAACAGTTGAACAAGTCTATGCTTTAAGTAAAGAACATGAATCATGTCAGTTGCTTAATCTTGAATCGATTAAAAAACATAAAAATGATGGAAACAATTTCCTTCACATTGGTTTAGTCCAAGTTGTAGTTAAGCCCCTTACTAGACTAGGACTAAAAGCCTCTGTCTGCCTATGTCTAAGAGATGCTAGATTCACTAATTTTAGTGAGAGCTGACTCAAGTTTATGTAATGGTCCAGTTCATTTTGATTGCTACCCAGACTGTACAGTCAGTCTCAACGATCCTCATATTTTGAAAACTTTAACTCTGAATATTAAAACTGATGGGTACCAAGTCCTCCCAGGAACACAAGCATTAGCTTTAGTTTTTAGAATTTACTATAAAGTCACTAGAACAAATGTGAATTTCAAAGCCCTTAATAAAAGTCCAAAGGATCAAACCCTATTAATTCAAAGTAGCAAACCCGAGGCCAATATACGAGTACTTCACACAATTAAATGGTCAGATATCACCCTTCCTACGGAATGGACACTCACCACTGAAAGTTATCCAACCCCAGTTCAGCATAATCTAAACAATTTAGAATATATTCCACAATATTTAGATAGCACAGTTAAAATTAATTTTGGTAGCCAACCTGCACAAAAATCCACTATCCAGTTACACCAGTTGCCTACTCCTGGCTAGTCACAGAGACATATCCCTGCTAGAATCATTTGCCGCTTCTTCCTCCACTCTAGAACGAGACCTAGAGTTAGAAAAAGCCATGATAGATTTAAAATTAGAATCCTTAAGAAAAACCTCCCAGGTTAATCAACCCTGCTACGGTAAACAACCAGTTGAAGATAACAAATCCAAGGCCCCTGAGTCTCCCACACAAACTGATTTTGAAGCCGAAGATTTTGCAGTTGTTAATAATCAATTATGGACACTTAACAAAAAATTCACAATCAATTGGAAAGCCTTAGATAGCCATTTAAGAGCCCCTGAATACCAAGAAAGAAATAATTATTATCACATGGCCTACCCTAATAAAGAAAATCGCCAACAAATCTTTAATGAATGGAAATAATATATGAGGTCAGCCAAAGTAGAAATTTTCTACCTTTATTTTGTAGAAAGTCATTACATGTCTAATAAGTTAACAACTTTAACTAAAGAAAAATGGAAATTACCTGATAATACAGAAGTTATAGCAAGTCACCCCCAGCAGAAACAATAATAATTGAGCATTTAAAAACCCAAATACTAGCTAGTCCTTTCAAAGCCCTTGAGAAAAATGATCCTGATAAAAAAAATAATGGAACAAAATAATTATACAAATCAGAGTCTAATTGTCTTAGGAAAACAGTTAGATAAAATCGAAACAAAAGTTGATAAACTGATTCCCCAGGATAGTAAAAATAAAGCCAAAGTGGAAAAACCATTCGTTCAGTTCCAGGATCTTAAGCCAAATTCGACCCCTTAAAATCAAACCAACAATGAAGAAAATTGAAGAAATGTTAGAACAGTTGACACAAGTTAAACATGAGAAATCTGGTCTTAAAGTCCTAGATTCTTTTGTAAACCTTTCTGAGTCCGAGTCTGAATCGATAATCAGTATGAAATCAGAAACTAGTAATATTTCAAAAATTGAAAATGCTTTTAAAAATTTGGAATTAGGAATTGAACCAGAGGTCAAACGATTAACTAACCATGTCAGCCCTACAGCCTAACGAAAAATTGGTATTCTAGACCAACACCCCCAGACATTCAATTTGAATAACGAAACTTCCAAACCTAGTTTTCAGTCTCTTTCGATAAATTGTATGAATGGAATATTGATGGTTTGTCTAAGCAAGAAGTCCTTAACAAACTTCAACATATGTCTATGGTTGCTAATAGTTATGTCACCAACCATAGTTTTAGGCAGTCTGAAATAGTGCCTTTACTAGTCACTGGTTTTATCGGAACTCTCCGGTATTGGTGGATAAACATTAACCACAGAGTCAAAAAACCTAATAATTCATGCGGTCAAAATCAATGAAGAAGGTCTCCCCATATTTGATGAACAAATAGGCCAAGGTGAACACCCAGCAATACTACTGCTAGAATTCATGATCAACTTTGCAAACTCCGATGCCCTAAGTTATTTGATTTTAGATGGTATAAGGATGTTTTCATCTCCCGAGTCAAGCTTAGAGATGACAGTAATCAGCCGTTTTGGAAAGAAAAATTTGTTAATGGTCTTCCAAGCTTATTTGCCCACAAAATTCGCACAGTCCTTAGTAATGAACAAGGTCATATTGATTGGGATAGTTTAACTTATAGAAACATAATAAGCACGATTAATCAAGTTGGAATGAAAATGTGTGTTGATATGAAAATTGGTAGACAGATATAGTCCGACAGAAAATCCGCTAAGTATGAGTTAGGAAATTTCTGTGAACAATATGGTCTTACCAGTCTTCCTCCTTCTAGAAAAAATAAGCCTAGTCGACATATTAGCAAAAGTCGTCCTTATTCACGCAGAAAACACGCCTTTAGAAATAATGATGAAAGTAGAGAATTTTACAATAAAAAGAAATACAGTCCGCGTAAAAATGGGAAAAAAGCACCAAAGAAAAGTCACTTTAAGAAGAATACTGACAGAAAAATGTTAAATGCTGGAAATGTCAAAAGAATGGTCACTATGCCAATGAGTGCAAAGTTACTATTAAACATTTGAAAATAACGGATGAAGAGAAAGACAAGTTGATTAAAATTTTAGAGTTCAGAAACTCCGAGTCCAATGAAAACGAAACCATAGTTGCCAGCACAGAGTCAGAAAGCTACCAGTCTTCAAATTCCCAGTCTAGTTCTCCCCAAGTTCAGTTTGGTTGTCAATGTTGTAATAGATTAAAGTCTATTTTTGTCCTCACTAAACAGGAAGAACAAGAAGAATTGTTAATTGATTTGATTAGTAAAGTCGAAAATCCAGAGTTGATTAGTTAATTGAATTGATTTGTTAATCTGAATATTTAAAAAAAATTAAGAAAAGTCATTAGTAAAGAAAGCACAAGCCAGTCTGTTCCCCAACCTATAAGTCTGAATACTACTTTAGAAAAATTCAATCGAAAGAAGGATGTTACCCTACAAGATTTGCAACTAGAAGTCAAATTAGTCAAAAAAGAAATAGTTAATTTAAAACAAATAAGTCACAAGTTGCAAGTCGAGAATTATGAAATAAAGCAAGATTTAATCAATTTAATGGATAAAGCAAGTTCTAAGTCTAAAAGTCAGTCAGAGAGTCCAAATAACTCGGAAGATGAACCTTCAAACGATGAGCAAGCCATTAATTTAATAAAACAAGTTAATTTCAGAAAATGGTATTCCAAAGTTACAATATTTGTCAAAATTTTTGAATTAAATACAATTGCCTTATTTGATTCAGGAGCCGATCTTAATTGTATCCAGGAAGCTCTAATCCCCACTAGATATTATAAGAAGTGTAAAGAATCCTTAAGCACTGCTTCGGGAAAATCACTCCAGTTGAATATGAAATTCCCAGAGCCCATGTCTGTCAGAATAAAATTTGCTTTAAAACCTCATTCGTCTTAGTCAAAAATATCATTGATGAAGTCATTTTAGGATTACCTTTTATTGCCCTATTGTATCCTTTCCAGGTAGAATACGACGGTATCATATCTACCCATTTAGGAGAAAAAGTTAAGTTTGAATTTTTGTCAAAACCTGAACTGCATAATCTGAAAGCCCTGCAAAAGAGTTCAGTTTGAAAAACAATCAAATTAAATCATAATAAAAATAAGCAGTTAGTTTCTCTTAAAGAAGAAGTCAAATTCAAAAGAGTTGAACAGCAGCTAAATGATAAGTCTTTACAGCTACAAATACAACAATTCGAAAATAAGTTAAAAAATGAAGTCTGTTCTGACTTACCTAATGCCTTTTGGCACAGGAAACAACATGTTGTTAGTGTTCCTTATGTCAAAACCTTTAGTGAGAAAAATATCCCCACAAAAGCAAGGCCAATCCAAATGAGTCAGGAAATAATGGAATTCTGTAAAATTGAAATAACTGAGTTGCTCCAAAAAGGAATAATTCGTAAGAGTAAATCCCCTTGGTCTTGTTCCACTTTCTACGTCCAGAAAAATGCTGAGTTAGAAAGAGGAGCCCCAAGATTAGTTATTAATTACAAACCCCTTAATACGATCTTAGAATGGATTAGGTATCCCATACCTAATAAACGAGACTTAATTAACAGGTTAGAAAAAAGCAGTCGTCTTTTCCAACTTTGACATGAAGAGTGGTCTCTGGCAAATCCAAATAGATGAATCAGATAGGTATAAGACAGCATTTGTCACTCCTTTTCGTCATTATGAATGGAATGTCATGCCTTTTGGTTTGAAAAAGGCGCCGAGTGAGTTTCAAAATATAATGAATGAAATATTCAATCCCTTCAGTCATTTCTCTATTGTTTATATTCATGATGTCTTAATCTTTTCCCAGTCAGTAGAACAACATTGGAAGCATTTGCATAAATTTTTCCATATAGTCAAACACAATGGTCTAGTTGTATCCGCTAAAAAAATCAAGTTGTTTCAAACTAATGTTCTATTTCTTGGTTTCAATATCTGTTAGTCACAGATCAGCCTAATAGATCGAGTCATCCAGTTTACAGATAAGTTTCCTGATCAAAATTCTTGAAAAAATTCAGCTTTAGAGGTTCTTAGGATCTCTCAAATATGT

AATAACCCAAAACAAAATATCAAAAAAAAAAAAAAATATATCTAAAAAGAAGGATTAATATCTTCTAGCAAAAAGACAAGTTTGCCCTCATATATTTTAATAGGGAAAAAGTTGACTTTTCGATCGGAAAAGAATTTGGCAATTTCGTTTACGCCATTGTGTAGAGCACGGCGAAACGAGTCCGTAGACACGGAGTAGACCCGAATCGGGGTTGTAACGAAGAAAATGCGGTCAAAATACCACGAAGGGCAAAATGGTAATTTGGAAAAAGTCAGATTTTTATCTCTTCTCTCTCTCTCTCTCTCTCTCTCTCTCTCTCTCCCCCGCGCGACTTCGCCCCTCGACCTCCGCTTGTTCTCAGCGACGGCTCGGCCACCACAGGCCGGGTTGATCTCCGCCGTGGGTACCGTTGCAACCGCCACTCGCCCGCCGTTCTTCTTCGACCAACCTCAGCCACGAGGACGCCCGGAATCTGCCATTGTTGCCGACTTTCCAGTTTAAACTTCCAGCTCGTTTTTCTTCTTCAATTCTTCGCCAAATCGATCGAGTAAGGTATGGTTTCTCAGTTATTTTTCGTGTTCTAGCTGATGTATGTATGGGTTTCGATCGATTTTAGCTCTAAAGGGTTCGATTTTCGACTTGAAAATTGGCCGAAACTTTGGCCGCCGTGATCTACTGTTTCCGGTCACTTTTTGGGGTATGTCCAAGAACAAAAGTGACTCCAAATGGGGTGTTTTACCTAGGATAGGAGTTTGGAGTCTTGGTTCCGAGATTTTTCGGCAACCCAAAATCGCTTTGGACACACGAATCTGCCCGCTCATGAGGTAGCGCGTGGGCGAGGGTGGTGAGGTGACTCTGTGCAGTTTTGAGATCCTCGTGTCGTCACGAGAACGTAGGATTTTGCGGATCTCGATTCGGAGTCCGTTTGAGCCCCGAACGAATTTTCCATATCTTGCGATCCTTGGGTGCAGTGTCGTCAGATCGTTGGATCGCACTGAAT

>Chr3-04; OZ022208.1; 5054391-5061979; dir; D;

TGAGCCATTTACGTATCAAAATTCACTCTAACTTTTTTTCTGTTTCCCTGCCTGCTTAATTTAATTGTTTTCGGTGATAGGGGAATTGGAAATTAACTTCAACTCAAAAGGAAAGAAAGGTATTAACTAAAATACAATAGGATACGTGAAATATTTCTGACAAAGTAATACAAGTAATGTTCAAAGAGGGAAAATCGGACACAAGGCATCATGTACAGAGATAAACTTTCTAAATATTTATGTATTGTGACCGATCGAAACAATGTCTTATTATTTAAAGCAGTGAATGATCCAATCTGTAATGATATAAAAACTAAATTCTATATTATTATGTGAAAATGTAAGTGCGTTTTACAATTAAATCATTTTGGTAACAAGGCCTCTAAATTCTTGTTAGGTGCAATGAGGATTGGTAATTCAGGACAATATAAATATAATATAATGCGCTCATACCTTTAGGTTCATGTGGAGGCTCTCCGGGGAAAATCTCCACCACATCCTGTCGTTTGCCTCTCTTCCTCCTAAAGTAGATGATGAAAATGCGCAAAGCCAGCACAAGTAGTATGAAACCTATCACACACCCCGCAATCACTCCAGCCAAAATTACCTTCGATACTACGCACCTTTTCCCCAGGCGCAATCTCGGGTTGCATAATGGTGGTGGCGGTGGCGATGACAATGGTGATTGCGGTGGCGATGACAATGGTGATGGCGGTGGCGATGACAATGGTGATGGCAGTGGCGGTGGCGATGACAATGGTGATGACGCCGGTCCATATTGAGATTCAAGTGTTGTTGCCAGGGGCGATTCATATTGAAATTCAGGCGGTGTTGCCAGGGACGATTCATATTGAAATTCTGAGGGAGTTGGAGGGGAGGAATCTTGGTAGTACAAAAAAGGTGATGACATGTTTAGATTGCTAGCTTTTTTTCTTTTTTTTTTTAGGAATCCTTGAACCTATTTGTAGAATTTTTAGCTTTCCTTCCTACCCTTTGTAAG

AAAACCAGTAATCTTTGTCCCAATGAGTCGCCTTTTTAGAACAAACTCTTGTAACAGTAGCAGTTCTACAGTATTAGTAAACCCCCGGAAATAGTAAATGACCGGAAATAGTAAATGAACAAAAAATGGAAACAAAACTAGTAATGAAATTGACTATAAAGATTGGAATATCCCCAAAATCCCTAGTACATTAATTTACAAGAAAAAATTTTCACTTTCAAGTTTCAAAAGTACAACCCATGTGAAAACAGTCGAACAAGTCTATGCCTTGAGTAAAGAACACGAAACATGTCAATTATTAAACCTTGAGTCAATAAAGAAACATAAGAGTGATGGAAACAATTTCCTTCACATTGGCCTCATTCAAGTAGCAGTCAAACCCCTTAGCAGGTTTGGACCAAATGCCTCTATTTTACTACTTGCCTACGTGATGCCAGATTGACAAACTTTAGTGATAGTACCCTAGGAATAATATAGTCAAGCTTGTTTAATGGTCCTAACTTTAGTGGGATACATCAGAAGGTATCGATGATGAAGGTGGTGGTGGCGGAGATTCATATTGAAATTCAGGTGGTATTGCCAGGGCCGATTCATATTGGAATTCTTGAGGAGTTGGAGGGAGGGAATCTTGGTAGTACGAAAAAGGCAAATCCTCTGGTGATGACATATTTAGATTGCTAGTTATTTTTTTTCAGGAATCCTTGAACCTGTTTATAGAATTTTAGTTTTCCTTCCTACCCTTTGTAAGAAAATCAGTAATCTTTGTACCAATGAGTCGTCTTTTTAGAACAAACTCTTATAACAGTAGCAGTTCTAGAGCCAGTATTAGTAAACTCCCGGAAATAGTAAATGAAGCACAAATAGAATACGAAACTAGTAATGAAATTGACTATAAAGATTGGAATATCTTCAAAATCCCTAGTACATAAATTTACAAGAAAAAATGGTCACTTTCAAGTTTCAAAAGTACAACCCATGTGAAAACAGTCGAACAAGTCTATGCCTTGAGTAAAGAACACGAAACATGTCAATTATTAAACCTTCAGTCAATAAAGAAACATAAGAGTGATGGAAACAATTTCCTTCACATTGGCCTCGTTCAAGTAGCAGTCAAACCCCTTACCAGGTTCGGACTAAATGCCTCTATTTTACTTTGCCTACGTGATGCCAGATTGACCAACTTTAGTGATAGTACCCTAGGAATAATAGAGTCAAGCTTGTTTAATGGTCCTAACTTTAGTGGGATACATTAGAACGTATCGATGATTGCGGTGGCGGTGGAGGAGATTCATATTGAAATTCAGGTGGTGTTGCCAGGGCCGATTCATATTGGAATTCTGGAGGAGTTGGAGGGGGGAAATATTGGTAGTACGAAAAAGGCGAATCCGCTGGTGATGACATGTTTAGATTGCTAGCTATTTTTTTTCAAGATTCCTTAAACCTGTTTATAGAATTTTTAACTTTCCATCCTACCCTTTGGAAGAAAACCAGTAATCTTTGTCCCAATGAGTTGCCTTTTTAGAACAAACTCTTGTAACAGTAGCAATTCTAGAACCAGTATTAGTAAACTCTCGGAAATAGTAAATGAACAAACAAATAGAATACGAAACTAGTAATGAAATTGACTATAAAGATTAGAATATCCTCAAAATCCCTAGTACAGAAATTTACAAGAAAAAAAGGTCACTTTCAAATTTCAAAAGTACAACCCATGTGAAAACAGTTGAACAAGTCTATGCCTTGAGTAAAGAACACGAAACATGTCAATTATTAAACCTTGAGTCAATAAAGAAACATAAGAGTGATGGAAACAATTTCCTTCATATTGGCCTCTTTCAAATAGCAGTCAAACCCCTTACCAGGTTTGGACTAAATGCCTCTATTTTACTTTCCCTACGTGATGCCAGATTGACCAACTTTAGTGATAGTACCCTAGAAATAATAGAGTCAAGCTTGTTTAATGGTCCTAACTTTAGTGGGATACATTAGAAGGTATCGATGATGGCAGCGGCAGTGGCGGAGATTCATATTGAAATTCAGGTGGTGTTGCCAGGGCCGATTCATATTGGAATTCTGGAGGAGTTGAAAGGGGGAAATACTGGTAGTACGAAAAAGGCGAATTCGCTGGTGATGACATGTTTAGATTGCTAGCTATTTTTTTTCAAGAATCCTTGAACCTGTTTATAGAATTTTTAACTTTCCTTCCTACCCTTTGTAAGAAAACCAATAATCTTTGTCCCAATGAGTCGCCTTTTTAGAACTAACTCTTGTAACAGTAGCAATTCTAGAGCTAGTATTAGTAAACTCTCGGAAATAGTAAATGAAGCACAAATAGAATACGAAACTAGTAATGAAATTGACTATAAAGATTGGAATATCTTCAAAATCCCTAGTACATAAATTTACAAGGAAAAATAGTCACTTTCAAGTTTCAAAAGTACAACCCATGTGAAAACAGTCGATCAAGTCTATGCCTTGAGTAAAGAACACGAAACATGTCTATTATTAAACCTTGAGTCAATAAAGAAACATAAAAGTGATGGAAACAATTTCCTTCACATTGGCCTCGTTCAAGTTGCAGTCAAACACCTTACCAGGTTTGGACTAAATGCCTCTATTTTACTTTGCCTATGTGATGCCAGATTGACCAACTTTAGTGATAGTACCCTAGGAATAGTAGAGTCAAGCTTGTTTAATGGTCCTAACTTTAGTGGGATACATCAGAAGGTATCGTTGATGGCAGTGGCGGTGGCGGAGATTCATATTGAAATTCAGATGGTGTTGCTAGGGTCGATTCATATTGAAATTCTGGAGGAGTTGGAGGGGGGGAATATTGGTAGTACGAAAAAGGTGAATCCGCTGGTGATGACATGTTTAGATTGCTAGCTTTTGTTTCTTCAGGAATCCTTGAACCTATTTATAGAATTTTTAGCTTTACTTCATACCCTTTGTAAGAACACCAGTAATCTTTGTCCCAATGAGTCACCTTTTTAGAACAAACTCTTGTAACAATAGCAGTTCTAGAGCCAATATTAGTAAACTCTCGGAAATAGTAAATGAACAACAAATAGAATACGAAACTAGTAATGAAATTGACTATAAAGATTGGAATATCCCCAAAATCCCTAATACAGAAATTTACAAGAAAAAATGGTCACTTTCAAGTTTCAAAAGTACAACCCATGTGAAAACAGTTGAACAAGTCTATGTCTTGAGTAAATAACACGAAACATGTCAATTATTAAACCTTGAGTCAATAAAGAAACGTAAGAGTGACAGAAACAATTTCCTTCACATTGGCCTCATTCAAGTAGCAGTCAAACCCCTTACCAGGTTTGGACTAAATGCCTATATTTTACTTTGCCTACTTGATGCCAGATTTACCAACTTTAGTGATAGTACCCTAGGAATAATAGAGTCAAGTTTGTTTAATGGTCCAATTCGCTTTGATTGTTACCCAGACTTCACAGTAAGCCTTAGTGATCCTCACATATTAAAAACTCTAACATTAAACATTAAAACTTCAAAGTATAAAGTCTTGTCAGGTACCCAACCTCTAGCTTTAGTCTACATAATTTATTATGAAGTCACAGGAACTAATATGAATTTCCAAGCTCTTAATAAGAGTCCAAAAGACCAAACTTTGCGTATTCAAAGTAGTAAAGCCGATGCTAATATAAGAGTCCCCCATACAATTAAGTGGTCAAAAGTAACTCTACCCCTAGATTGGACACTAACAACATAAAATCAACCCACTCCCATTCAGCATAATTTGAATAATTTAGACTATATCCAACAATATTTAGACGGTATCGTAAAAATAAATTTTGGAAACCATCCCCACAAGTCCTCAGTCCAAATACAACAACTTCCAACACCCAGTCAATCCTTTAAAAATACCCCTGCTAGACATTCATTTGCAGCTTCCTCCTCTACATTGGAAAGAGACCTAGAGTTAGAGAAAATGTTAATTGATTTAAACTTAGAATCTTTAAGAAAAGCCTCCAAGGTCAACCACCCTTATTATGGTAAGAAACTAGTCAAAGAAGATGAGAAATCAGATTACCCAGACTCCCCCACACAAACTGATTTTGAAGCTGAAGCTATTCCAACAGACAATTTGTTGTGAACCCTCAATAAAGAATTCAAAATACGTTGGAAAATTCTAGACAAACACCTAGAGTCAAAAGAAAATGTTGAAAGACGGGAAAAATATAGGGCTACATACCCAAGTAAAGATCAAAGACAACAAATATTTAATGAATGGAAGGCTTACATGCTCCAAACCAAATCAGAAATTTTCTATCTTGATTTTGTTGAAAACCTTTACAAATAGAATGTTCACCCGTTTTAGATTCGTTCCTGTGATAGTAAGGTAAAGTTCCCCCATTGCCTCGAAAATCATTGACGGTCAACAAGTCAATTTTCTTTTGTGTGTGAGCGTGCCACTGCTAGCAACGAAAATCCTAGCAGACTTTTTAAAAATAGATAACTTAAGCCCCAAGTTACTGATTTCTAAACAAGCGATTGTGAATTTGGGTGAGGAATATCTCTCAAGTAAGTTCTTTTCTTGCCTCATACTGGTGAGGACTTCACAATTTTTCACAATACAAAATTGGTAGTTCTGCCCAGAATAAATGATAAGAAAGTGAACTGTTTCAGGCAGAATTGCCTTTTACAAAGCTCAAAAAGGAAGACCAACTTTAAAAAAAACAAAACAAAAAACGTTGGACTTCAATTTCTGCCTAGATGAATGCTTTTGATCTGGGCTGAACTGAGTATGTTTGTGCTGGATTAGACTATCAACATCTTCAACTGTCAAGTTTCAGCTATAAATTGATACCAACGTTCGAGTGTGGCAGAGCTTAAATCTATTTCAAGCCGTGGAAGGCTTTTTGAACTGGCAGAATTGGAACTTCTTCAGTCTGGCAGGGGCAGAAGTGGCTGAATTTGATGATTACTGAACTGGAACTGCACTGTAGTTCCTATTCTGCTTGATCAGGGCTCTCCATAAATTTGTTGAGGCTTTCATATTTGTAAAATCCGAACTCTGCTTGATTTGGCTTATCAAATTTGTAACGAGCGAAATCTCACTTTGAATGATTATTTCTCTGAAACTGAACTGAATTTAGAGATTATGGATTATAGCTGACTTGTTTCTTGTGGCTCAATGAGCTTTTGGTTGCTACAGTGTCTTGAAGGTTTTTGAGATCAAGTGATCATTTTGAGTTTTTGTTTTGATTGAATTTTTTTGCTGTCCTTGATCTGTTCATCTTGTCTCATATTTATAGAAAATGCTGGAGCGGGATTTCTAATCTTTTAATAATGGGCAGCAAATCTTCTTAAAAGATCTTTTTTCTTTTCTTAATGTCATAAGAGAAGAAAGCTACTATTCTGACAGAGAAGGGTTGTCAACAATCAGCTTCCATGGAGGTCTTTTCCTTGCTTTTTTCTGAAAGAAAACCAACTCTCTTTGTTCTCTGTTTGTTCCTTGTAAAGAGAAAAACTCAATCGACCATGATGGTCAGTTTGCTTTCCCTATTTTGAACAACTCCCTTGCTCCCTACTTATCTCTTGAAATAGTAGTCTGCTTATCTCTTGGAAGAGCCGTCTGCTTTGGTGCAGACTTTCTATATATATATACATATATATATAAGGGATTTTAATCTTCTTCCTCTGGCAGCAAAAATGGAGAAGTCCTCCTGTAAGACATGCCTTCATTAACTCTATATCAACTCTTCTGTGATAATTGAAAATTTTGACTTTTTCAACGTCAGGTAGTCACGTGGGGGCTTCATTGAGAAAAAGATATGATGTTTTTGCCTTTTTTGGGATAGAGACCAAGTGGTGAGATTTTCACAAAAGCTGGGCTTTTATCTAAAATTGTGTGTATCAGTTTTTAATTGCCAATAATGGTATTTGATTCTTTTCAGATCATGGCTACATTCGTAATTTGTTCAAAAGGGTCTGGGCTTTTCCAGTGTCTTGGTTTTCTTCTTCTGATTCCTGTAAATGAAAGCCCAAATTTTTTTGGTTCGTGGGCCTCTCGACGAAAATGGGCTGGCGTGCAGAATTTTGACCTAAACATAGAAAAAAGGAATAAAGACTATAACACAAGAAAAATGGAAAAGAGAAAACAAAACAGAAGTCATAGTTAGTCATCCCCAAGTAGAAACGATAATCATTTATCATAAAAAATACTAAAATTACTGTCAGTCCCTTTAAAATTCCTGAACAAAATGATAAAAAAAATTAGTTCAACAAAATAATTATACAAATCAAAGTCTAATAATTTTGGGAAAACAACTAGATAAAATAGAAACGAAATTTGATAAAATTCTTACCCGAGAAGTAAACCACAATAAAAGAAAGAAAGACCTTTAGTCAAATTCCAAGACCTAAAATCTGGTCAAACACTTAAAACAACCCCCAAGAAAATAGAAAAAATACTTGAACAATTGACACCCCTCAAAGAAAAACCCAAAACAGAGTCATCCTCTACCTTTAAAGTGGTCCAAGCAAATGAAAGTCGTCCCTTTGTCTTGAATCACACAGAAATAGAGTCACCAATTGACACTGACACTGATACTGACTCTAGTAATATATCCAAAATAGAAAACGCCTTTAACAATTTAAAACTCAATAGACTAAGGAGTAAAATAAATCCTACTAGTTTAACAAAGAATTGGTACCCTAAGCCAACTCCCCTAGATATCCAGTTTGACGAACGAAATTTCCAGAGTCAATTCTCAGTCTCTTCTGATAAACTTTATGAGTGGAATATAGATGGTCTGTTTGAACAAGAAATTATGAATAAACTACAGCACATGTCCATGGTAGCCAATAGTTACATCACTAATCATAACCTTAGACAGTCAGATATAGTACCCCTCATAGAAACAGGGTTTACAGGAACTTTACGTTTTTGGCGGGATAAACACCTCACAGAAGAGTCAAGACGTCAAATAATACATGCAGTAAAACTAAACGAAGATGGATTTCCTATATTTGAATAAAATGCTAATACAGGAATTGAAGACGGAGTCAATACCTTATTATACACGATAATTGAACACTTCATAGGTACACCTAGTAACACTACAGCCATAATTCATGATCAGCTTAGTAACCTTAGATGTTTCAAGTTAAATGATTTCAAATGGTACAAAGATGTCTTTATATCTAGAGTCATGCTAAGAGATGACAGTAATCAACCATTTTGGAAAGAAAAATTCATTAATGGTCTTCCCAATCTTTTCGCTCAGAAAATACGAACTGTTCTGAGTAATGATCAGGGTCACATAGATTTCGATAGTTTAACTTATGGAAATATTATCTGTACAATTAACCAAGTAGGAATGAAAATGTGTGTTAAAATGAAAATAGGTAAACAAATTCAGTCATAAAAAAATTCAGCTAAATACGAATTAGGAGATTTCTATGAGCAGTACAGTTTAGCTTCTATACCTCCATCTAGAAAAGTCAAGCATAAGAATTATGACCAAAACAATTGAACAAGACATTATGCTAGAAAACGACAACCTTTCAAAAATAATGATGAATTTTATAGTACAAATAAGTTTAACCCT

GTTGAGGCCCAAAAATCCAAGGGGTTAGGCCCAAGGTGATTATTGGCCCAATAGGGACCAAATGAACGCAAGAAAATTTAGGGAACAAATTAACACGAGCTACGGGCCACATGCTACGTCAAACAAGCACGTGCGGGGAGACCCAAAACATGCTAACTTTACAAAAACATGCTCACTCCTATTGAACTCGGCTATGGCTTATGATGATAGGATAAAAATCCAAGCACAAGCATAGGTGTCGGAACCGCCATGCCAACACCAAGATGTGTTACAAGCTTAGGTGGGCCCAAGCCACCAAAATCACCCGGGGCTTGCTTGAACGGGTTGTGGTATGGATGGCAACGGGTTTTCATAAAACGCATTGACGAGCCAAGGGAGTGGTAGTTTTGGGTCACTTGGGGGGCCCAAAACTCAAGCCTATTTCTTCGGCCCGAAATGCATGGGTTAGCATGAGAGAAGAGAGAGCATAACCCAATACCTTAGAAAAAAGATGCAAAGCCTGTTAGAAGAGGCTAAAACAATTAGAAGGCCTAAACATGCTGTTAAAGTGCAATTGGCAAGCTGTTTCCGGCAGCTTGACGAAAAGCCCAGTAGACTGACCAGAATCATATTACCAATTCAAAGCCTAGGAAAGAAAAGCCCAGCCCAAACTCTCTGCAAAATATCTGTCCAAGCACGTCTTGTCCCTATCCAAGAGTCAACAGCAACCATCTTAAAAACCAGAGGAAATCCACACGAGTAAGAGGTTTACTCGTTGGGTAAAACGTAGCTCCTTTCCCTCCCATTGTCCTGGCAGCTTGCATAGGAAAAGTTGAGAGAAAAGCAAGCGGCTCTTCACCTCTATGAGGAAGGGCCAGCAAAACCGAGACCTTGGAACCACAAATGGGTTCCTTACCCATATGTCTTGAGCGGATAAGATTCATCAAAGAAGACGAAGTAGGGGAAAACAATGGAAAGAGGGAGGGAACTATGACGAAATTAGAACCCTGAGTGGCTATAA

>Chr3-05; OZ022208.1; 5392816-5397033; dir; B;

AAAAAAGATGCAAAGCCTGTTAGAAGAGGCTAAAACAATTAGAAGGCCTAAACATGCTGTTAAAGTGCAATTGGCAAGCTGTTTCCGGCAGCTTGACGAAAAGCCCAGTAGACTGACCAGAATCACATATTACCAATTCAAAGCCTAGGAGAGAAAAGCCCAGCCCAAACTCTCTGCAAAATATCTGTCCAAGCACGTCTTGTCCCCATCCAAGAGTCAACAGCAACCATCTTAAAAACCAGAGGAAATCCACACGAGTAAGAGGTTTACTCGTTGGGTAAAACGTAGCTCTTTCCCCTCCCATTGTCCTGGCAGCTTGCATAGGAAAAGTTGAGAGAAAAGCAGGCGGCTCTTCACCTCTATGAGGAAGGGCCAGCAAAACCAAGACCTTGGAACCACAAATGGGTTCCTTACCCATATGTCTTGAGCGGATAAGATTCATCAAAGAAGACGAAGTAGGGGAAAACGATGGAAAGAGGGAGGGAAGTATGACGGAATTAGAACCCTTAGTGGCTATAAAAGGAGGACTTCTGCTACCCGGAAGAACCAACCCATTTTTCCAGAGCTCGACCAAGCATTGTCAAGCAGCTGGAAAATCTACTCAAGCCACCCCATCTCCTCTTCCGTTAGTTTTTTCTTCCTCCCTTAGGAAGGAAACCAGCCAAATCTTCCTTTGCCTGCTCTAAACCTCCCTCCTCCATAGGAAAAAACTCATCTCTCTATCTCAAAATTGCTAAACCCGTGAAAGCCTAGCTCCCATGCCACATGCTCTTCAAGCCAGGCTCTTTTGTAAAACTGGTTAAGTTTGTTTGGGCTATTAGCGAAGGAAGCCTGAGAGTGTTTGCGTAACAGTTTCATCGGATTCTGTGAAAAACACTCTTCCAGGCGCTTGGTATCGAACCCAGGCACTTGGGGAATTTTGCTCGCTTCTCTTCTTACGGCAGCCCAAGCCCATTCGTCAGGCTGCTGGAACAAAAAGGCCCCAACAAACCCAAAGAGA

AAAAATTGGTCAAAAACACCCAAAAGGGGAAGAAAAGATAAATTTAAAAAGAACACCGACAAAAGGAAAGCTGAAGGCTAGAAATGTAAACATTATGGTCATTATGCTAATGAATGGAAAGTTAAAGACACAATCAAACAATTGAAAATCACAGACGAAGAAAAGGCCCAATTAATTAAAGTTTAAGCATTAAGAAACTCTGAGTCTAGTGAAATTAGTGAACTAGAAACCTCGATAACAAGCTTAGCAGATTATAGTTCCTCAGGTTCCCAATCAAGTTCCCCAAAAATCCAATTTGGTTGTAGAGATAAATGTTGTAATGTTCTAACCAAGTCAATAAAAGTCCTTACAAAACAAGAAGAGCAAGAAGAATTATTAATTGATTTAATTAGTAAAGTAGAAGTACTTTACAAGATTTACAACTTGAAGTAAAAACTATCAAAAAGGAAATCATTGAGTTGAAACAATCCAACCGACAATTACAGAGTGATAATATTGACATTAAACAAGACTTAATCAATCTACTAGAAAAAAATGGAACCTAACAACAAAGGTAAAAAATAGTTTGAAGACCAGTTTGAAGATGAAGATGAACATTCAAGTACTGACAAAGCAATTATTTTAATCAAACAATTAAGTTTCCGAAAATGGATTCAAGAGTCAGCATTTATATCAACAACTTTGAACTAAAAACAATAGCCTTATTTGACTCAAGAGCCGACCTTAATTATATTCAAGAAGGACTAATTCCCACTAAATATTACAGAAAATCAAAAGAATCCTTAGGAATAGCCTCTGGTAAACCAGCACCTCAAATATGAAATCCCTAAAGCCCATTTATGCCAAAATAAAATAAGACATCCTTTGTCTTAGTCAAAGACATAACTGATGAAGTCATTCTAGGATTACCATTTATTGCCCTACTTTACCCTTTTAATGTAGAATACGACGGAGTCACCTCAATCCAAATGGGAGAAAAGGTTAAATGTCAAAGCTCTTCAAAAAAAATGTAGTCTCTAAATCAGTCAAACTAATTCAAATTAAAAGCAAGCAAGTCAACTTCCTTAAAGAATAAATCAACTTCAATAGAATTGAACAACAACTTGCAAATAAAAACTTGCAACTACAAGTCAAAATTTTTGAAGAAAAGTTAAAACAAGAAGTTTGTTCCGACCTACCTAATGCCTTTTGGTATAGAAAGCATGTAGTGAGTCTTCCTTACGTCAAAACCTTTAATGAGAAACATATTCCTACTAAAGCAAGACCAATCCAAATGAGTCAAGAAGTCATTGAATTTTGCAAAAATGAAATTACTGAACTACTTCAAAAAGGGATAATTCGTAAAAACAAGTCACCTTGGTCTTGTCCAGACTTTTATGTTCAGAAAAATGCTGAATTAGAAAGAGGGGCCCCAAGATTAGTCATCAACTATAAATCCCTTAATACGGCATTAGAATGGATTAGATATCTTATCCCCAGTAAAAGAGATCTAATTAATAGATTAGATAAAGCAATCATTTTCTCTAAATTTGATATGAAAAGTGGATTTTGGCAAATTCAAATCCAAGAGTCAGATAAATATAAAATCGTTTTTGTAACCCCATTTGGTCACTATGAATGGAATGTCATGCCTTTTGGTCTAAAAAATGCGCCTAGTGAATTCCAAAATATAATGAATGAAATATTCAACCCTTCAGTGATTTCTTCATAGTTTATATTGATGATGTCCTTATTTTCTCCCAGTCAATAGAACAACATTGGAAACATCTGTATCAATTCCTTCAAATAGTCAAGAGAAATGGTTTAGTTATCTCAGCAAAAACGATCAAGCTATTCCGAACCAATATTAGGTTTCTTGGTGTAAATATCTGTCAGTCACAAATCAGCCTAATTGATCGGGTCATCCAGTTTGCTGATAAGTTTCCAGATCAAATTCTTGATAAAAGTCAACTTCAAAGGTTCCTAGGATCTCTTAATTATGTCTCTGATTTTATCAGAATCTGCAAAAATAATGTAAACCCTTATTTGATAGACTCCAAAGTAATCTTCCTCCTTAGACTTCTGTTCATACATAAATTGTTAAGCAAATCAAAATTCATGTTAAAACACTACCCTGTCTTGGTATTCCTTTTGTTAATTCCTTCAAAATAGTTGAAACTGATGCCTTAGAAAATGGTTATGGAGGTATCCTAAAATAAAAAGTCAAACCACACCAAACAGAACAAATTGTTCGATGCCATTCTGGAATTTGGATACTGTCACAGCGTAATTATAGTACTATTAAAAAAGAAATTTTATCTGTAGTATTATGCATTTCAAAATTCCAAGATGATCTATTAAACCAATTTTTTTTTTTTTGTCAGAGTTGATTGTAAAAGTGCAAAACATGTTATAGAAAAAGATGTTCAAAATATTGCATCAAAACAGATTTTTGCACGTTGGCAAGCTATTTTAAGCATTTTTTATTTTGATATTGAATATTTGAGAGGTAGTGAGAATTTCATCCCAGATTTCCTTACCAGAGAATTTCTTCAAGAAAAACAATGGTAGAAAAACAAACAAAACAGTAAGGCAAGCAACTAGGCAGACAACTCCCAGCATCAATGATCCCAATTAAAACAGAGCAACCTTCCCAAGTCATCCCTTATCCGGGACATTCACCCATTCAAGTATCAAATGGGTTCACTACTTTAGGATCCACAGTCAATAACATTCGTCCCAACTATCAATCCGCTCTTATTTCCAGTTATGATCCATTTCAAGTCTTAACTCCTTCACCTGCCTGTTCTCCAGTTAGTCACAAAAAATCCTCTCCCTATCTCCCAAAAAGCAGTCACACCCCCTTCTTCATTGAACCATGTTTTAATCACTTGTCTAACCCAGTCAAAATAGCCAAAGCCTATTTTCCCCCCAATTTCCATTACATACCTCCACAACCTTTTAAGTCACTTAAATGTTATCGAGATATTCTTCTCGAAACCCAGACCTTAGAAATTAGACCAATCAAGGATAGAAATGACCCAAATATCATTCTCTATCACTCCATGTACATCCAAAAAATTCTCAGTCAAACTGAATGGGGTGAACACCCTTACTCTTTTAAGACGCTTAACTCCAGCCTCCAATACAATTACTATGTCTACTCGAAGCCTGACACCGTTTTCCTCCACCAAACTGAGGACTTTAGCCATTCATGGTTTGTCAACTTTGATAGTAAATTCAAAAGTCCTTTTCCTTACTGATTTTCTTCCTGGTGGGAAAAGCATAGCCCAACTCTTGAAATTCTTCCCCTCAATCTCAAAGAATTAGTCATGTACTTTTCCAATAAATACAAATTTAAGGAAGAACATCAATTTTTCCCAAAACTCTTAATCTTTATAGCCAAATACAAAGTTCCTTGGATTTTAAAATAGTCCTATCAAGTCACTGGGGATCCCATATTCTCTCCAGACAGATCTCCATCAAATGGTGGGATAAGTTCCAATCAGAAAGAATTATTGAACAAGTCAATACTGAATTCCCTGCAATCAAGGTTCCATAGCCAAAGCCCTCTTGCTCCTGCATGTCTCATTCTTCCCTCCCAATTGAAGGAAGATCCAAGTCAGAAATAAAAGAATTAGCCAGGCAATTAATGATTCAAGCCTCTTAAATGAATGACGATGATGAAGATGCTTCTCCAAAGTCTCAATCCACTCAAGCCTCCTCCTCCAGTTAGACACCAAATCAAGTCAAAAAGATTCCACGCAGAAATTCAGATGGGCCCATAGTCAAGACCCTTATGATTTGAATGAAGATTAAAGCAAAAGTGAAAGTAAAATCCAGAGGAAAGAAGTCCCATGACTCGTGCCTTTACAGTCAATATTCCCTGATAAAGGAAAAAAGCAGAAGTGAAAGAAATGAAAGAAAGCAAAAGTAAAGACAAGACAAAAGCAAGAATAATAGCTCTGGTTAAAGCAAAAGCACAAGACCCGTGCTCCAACAGTAAAGATTCAAAGCATTCAATGCTACTGACACTTCAGTAGTTTGGATCTTTAAATAGAGGCCTTTTGGCCAGATTAAAGCAGAATGTAATTTTTTAGTTTCTTACTTTTCAAAGTTTTCTCTGAGACTCACTCAAGAAAACCACCACTGTAAATCATGAGCGTTATCAATCAATAAAATCTTTCAATCAAGATTTTCTCTAAGTCTATTTTCTTCAAGTCTATTTTATCCATTTCC

AAGTTAATTGATGCATTAATTTTATTGTGATATATATATATATAGTGGCATGCATGCATGCAGTTGAGAGATTCAGTGTCATCATTTGAGGAATGAGGTGCTTAGTCGTTGGATCTATTTCAATGAAATCATGTGGTTGAGATTGATTTAAGTGTTTAAACTTGTTTGTGTCTAATGACTTGCCAAAAATCCACATGTATGTACGCTTCACTACGTCCACGTGTTATAATATGGATAAGCGATATGGTTAATATTTCTCACTTTTATGATATAATATCAATAAATATCCACTTATAGATTCTAACAAACGAGTTATACCATGTGACATACTCTCATACTACGTACACTATAATTACTCGCGCTTAAAGCATCCACAATGGCGTCCCTAAAGCACATCCTTAGACAAATTTTAGGGAGGAATTGGAAAAATACAACTCCAACCATGCTCCTTATTTACATCTTAAAATAAAGAGACCTCTAGGAACTCCTAAATCTGAGGAGAGAGAAATGACTCCTAGTGACTCCTTATAATTTAATGTTGTTTCGTTTTATGCATAGTTTAAACTTTTAATTAATACAAACTATTCTTTTTTATAGAGATGATAACCAATCATATTGAATTAAAAATAATTATAGTATTTATGACTTTCTAACTTAGAGAGTATGATTGGAGTTCAAATTTTATAGAGTTCCTAAAATAACTTGTGTATTTTTAGCTAAATTTTAAATAAAGAATAGGGAGCATGACCTTTGCCTACATAATTATTGACTAATTCCTTGTGATCGGGTATTTATTATTTCAGTATGTGGTTGTGAAAGTAGCTGGGATATTGAATGTTGGTGTTGACATCAGACAAGCTGCTTTAAACAGTGACTGCAACATTATGTGATTGACAGATAGCAGAGCAATTTTCATCTTTCAACGAAAACAATCAGTTTCAAAAAAAAAAAGAATTAAAATTTTAATTTGACCTTATTGTCATTCTGGTGGTTTTTCTGC

>Chr3-06; OZ022208.1; 5783606-5785441; dir; B;

CGCCTTTCCCTTTTTTTTCCTCTTAAAACAACATGAACCATGTAGAAGTACAGAATTAAAACCTGCTTGAAACAAAATGGCATTACTATCTGTCGTCCGTACAATATCATTCCTATTTTCTACGAGTTTGACTCTCAAATGCAACTTCATTTCCATGCTGCTTTAGATTTTTTTGTGCACCGGACCTTGAATAGAATCAAATGAATGATGTCATTTTATGGAGCAGGTGCTTGGAACAGAAGCTGGAGGCACAACAGAGATTGTTGAACACAATGTAACAGGTCTGCTTCATCCTGTTGGGCATCCCGGAACTCGTGTCCTAGCTGAAAATATCCGGTTTTTGCTTAAAAGCCCAAATGCAAGGAAGCAAATGGGATTGAAAGGAAGAGAGAAAGTAGAGAGGATGTACTTAAAGCGCCACATGTACAAGAGATTTGTAGACGTTCTTCTCAAGTGCATGAGACCCAAATAACGTCTCTCATTTGAGACATTCTCATTCTTTCATTTCTTCTCAAATTTGGAGGGTGCTGTGAGTATGGGAATTATGTTGCTCCGGCCAAATGATGTTGTAGATGCCAAGTTTGAGATTTTCTTGACGAAGTGTAGGAAACGTTTTTTATTGATTGAAAGGTTATTAGTTTAAAAATGAAGCTGAATGCTTTTTTCGTGTGATAATGTCAAATGGTTAAGCGCATTTATTCATCTACCGATGTATTGTTTTCGATTCCCTCCTCTCATTATCGATTTATAAATTTAAAAAAAAAAAAAGTTGTTTTACTAAACATTAAGTGCAGAATTTTTATCTCCAAATAACAGCCTGACTCAACATGACCTCTGTTCTCCTCAGGCACCTCCGCTCTTGTCCAACTGTAGCTATTCAAATATAGCCCGGTTTTATTAATGTTTCTTAAAAATAGTATAGCTATGTAGCTATATTCAATTTTTTTTTAAAAAAAGGGTATAGCCGAGCGATGTGTGTGTGTGTATATATATATATATA

TATTTAATTCAGGAGCCGATTTAAATTGTATTTAGGAAGGCCTAGTTCCAACCCAGTTTTATAAGAAATTAAGAGAAGTTTTAGGAACAGCGTCTGGTAAAGCATTAAAATTAAATTATGAATTACCCAAAGCCCATGTATGCCAAAATAAAATATGTTTTAAAACGTCTTTTGTTTTAATTCAAAATATCACGGATGAAGTCATTTTAGTTTACCCTTTATCTCTTTACTTTACCCTTTCCAAGTAGAATATGATGGCGTTACTTCCAGTCACCTAGGTGAAAGAGTTAAATTTGAGTTTTAAACCAAACCAGAATTACATAGTCTTAGGTCTTTACAGAAGTCAGCAGTTTCCAAATCAGTTAGATTAATTCAAAATAAGACAAAACAAGTTAATTTTCTCAAGCAAGAAATTAAGTTTAAACGTGTAGAACAACAGTTAGTAGATCAGTCTTTACAGTCTCAAATAATTGAATTTGAAGAAAAATTAAAAAATGAAGTTTGTTCTGATTCCCTAATGCTTTTTGGCACCGAAAGCAACATGTTGTTACTTTACCTTACGTTAAAACTTTTAGTGAAAAGAATATTCCCACTAAAGCCCCTCCAATCTAGATGAGTCAAGAGTTAATGGAATTATGTAAACAGGAAGTCACCGAGTTAGTCTAGAAAAGAATAGTTAGAAAAAGTAAATCACCCTGGTCATGTTCAGCTTTTTATGTTCAGAAAAATGCAGAGTTAGAAAGAGGAGCCCCTCGTTTAGTAATCAATTATAAGCCCCTTAATGCAGTACTTGAATGGATTATGTATCCAATCCCAAATAAAAGAGATTTAGTTAATCGGTTAGAGAAAGGGGTTATTTTTAGCAAATTCGATATGAAAACTGGTTTATGGCAAATTCAAATTAAAGAATCAGATAGATATAAGACTGCCTTCGTAACCCCTTTTGGTCATTATGAATGGAATGTCATGCCCTTTGGATTAAAAAATGTGCCTAGTGAATTCCAGAATATAATGAATGAAATTTTTAATCAATATGGTCAATTTTCCATAGTTTACATTGATGATGTCTTAATTTTTTCAGAGTCAATTGAGCAACATTGGAAACATTTAAATAAATTCCTTCAGATAATCAAGCAAAATGGTTTAGTTCTTTCAGCAAAGAAAATTAAGTTATTTCAAACCAATGTCAGATTTTTAGGATTTAATATTAATCAATCTCAGATAAGCCCAATTGATCGAGTTATCCACTTTGCTGATAAGTTCCCAGATCAAATTTTAGATAAGAATCAATTACATAGATTTTAAAGATCATTGAATTATATTTCTGATTTTTATAAAAATTTAAGAAAATTATGCAAGCCCCTGTTTGATCGTCTCCAGAGCAATCCTCCTGCTTGGACTCCTGGTCATATAGAAATAGTTAAAGAAATTAAAGTTCATGTCAAGTCTCTTCCATGCCTTGGTATTCCTTCAGCCAATTCTTTTAAAATTGTCAAGACAGATGCTTCTGAGTCTGGTTATGGTGGTATCCTAAAGCAAAGAGTTTCCCTTGGGTCACCAGAGCAAATTGCCCGATTTCATTCTGGAGTTTGGAGTCAATCTCAAATTAATTATAGTACTATTAAAAAAGAAATTTTATCTATAGTATTATGCATCAGTAAATTTCAAGATGATCTTTTAAATCAAAATTTTTTAGTTAGAGTTGATTGCAAATCTGCAAAGCATGTTTTAGAAAAAGATGTTAAAAATATAGCTAGTAAACAGATTTTTGCACGCTGGCAAGCCATTCTAAGTGTTTTTGATTTTGAGATTGAATATTTGAGAGGTAGTGAAAAT

AGTATACCTGTTTATATATATATTGTAGAATCCTTTTTTTCTTTTAAATCTTTTCCTTGATTTTCGTTTATCAATAAGAGTAGTCTAGAGCATTATCCATTACTATTTTGTTAGTGCCCCCCAGTCTCTATTATCCCCTTTTTATTTTTCATTTTTCTAGAGTCATTACTTGTATAAAGAATTTAACACTTTCTCGTTCAATAATCTATTATACATGTATGTACTTATTTTTTAATAATACATCCGTGTTCTGTACACGTCTTAAGGGCTCGGTAAATTTAAGCTTTTTCAAAAGCCGCACGGCTATACTTATTTTGACACGTGGCTACCTATTCGCTAGGCGGATTTTCTTTAAAAAATATATATAGAATATTCGCGCGGCTATACTTAGTTTTTTTCTGATTTTTTTTATAAAAATAGTACAACGCCATGGCTATATTGGGTTCTATTAATGTTTCTGAACTGCTTAAATTAATACACATATTTTAATAAGATACCCAGGTTCATGTTAAATTAATCCGGCAGAGTATGATACTTTGGGTTTCTAGTATCCAGGTTCATGTTAACCCGTGCTTGTGCCATAACAATGATGGGTTGGCTATAACCGGCGGCTTTGCGCAGCAGGAGAAACTCCGGTGGCTCAACCTCCGCCTTCCAATCCTTTCATACAGATGCATATTGTGACAAGTAGTTGTACGGTACACGAAATAAGCATTCTTGGGCAATCTCATCGGGTATACCCGGGAGTAGCCATCATGAAGGAAGAAAATCAATTCAATCAAATAATTGTGCTTAATGAAAATTAGATATCATTAAAAAATGAGAATCTAATTTTCTAATAATTATTATAACATGAAGGAAGTTGAGGTTTCATCCCAAAATCAATTGGCAATGGGGGTAGTAGTCTAAGCACGTTCTTCATAAATATATGCTCATTAAACTTGTGTCACAGATGCAAATCCTATCATTGCACCAGTGGAGGCAACAAAATGACAGTTTA

>Chr3-07; OZ022208.1; 6450994-6457272; rev; A;

GTTTTCGTTTTTCATCTCAACTTTCCTGTTCCAAGCTTCCTAGCATTTCTCACATGGCAATATATAATCAAACAAAGAAATAAAAACATCTATATCAGGTAAGCATGCTTGGAAAGCCATAACCATCAATTCCTCGTGACACCAATGATGACACGTCCTGACCTGTAATTCTCGCTGGATTCAGGAGACATCTGGTCAATTTGGTCTGCAATTCTCGCTCCCACAGTCCAGATAACTCTAAGATTCGTGGGGCAAAATATGCTAGAAAAACTTAAGATATTTAATACTATATAAAATAATAAAAGATAAAGATAAATAAACATACTAAAATAAAATAAAATGAATAAAATAAGTTAAAGATTTATAAGCTAAGAACACCCTTGTATCGAACTCAAGTAAAGTTCATATATATATATATATATACATATATCTCCATCTATCGCTTGTAAAACAAATGTGACGCTTAAGAAAATAATGGTAACTGTATAAAATCCTCAATTTAGTTTAAATGCATCTCAGAACTTAAATTCTCTCCCTAGGCGTACCCCCATTTCTGATTCGTCAATTCCCTTAATCCGTCAATTCCATATATGCCATCAATTCCATATATCGTCAATTCCAATAATAATCCGTCAATTCCAATGTTAGTCCATCAATTCCATCTCGCAAGTCTCGGAACCATAAAGTTAACCGAGCCGCATTCCTCGCAGGTCTCAGAGATTACACTTCAATTTGAGCCGTAATCCTCGCAGGTCTCAGAGATAACACTTCAATCTGGCCACAATCCTCGCACCACACGGTTCAGGCGTCCCCGAATGTGGGCATCATCAAAATAAACAAACTATCTCAAGGCAACTTGAGGTACCCTGCGGTGGGTTTGAAAACCATAACTCGTCATTTATATAAACTTGAAATAGATTGGTATATATAAACTTGAAATAGATTGGTTCTCAACTAAACTTATATATATATAGTTCCAATAAAGTGATCGACTATTGTG

TAAAGTATGTGTTGATTTCCAGAGTCATGATCAGAGAAGACAGTAACCAACCATTCTGGAAAGAAAAATTTGTTAATCGATTGCCAAACTTATTTGCCCATAAAATCCGATCAACCCTTAGTAATGAACAATCATATTGATTGGGATAGCTTAACTTATGGACATAATCAGTACCATTAATCAAGTCGGAATGCGAATGTGTGTGGATATGAAAATTGGTAAACAAATCCAATCTGAAAGAAATTCTGCCAAATATGAGTTAGGTAATTTGTGCGAACAATATGGTGTTTAATGTCTGTTCCCCCATCTAGAAAAAATAAATCTAGTCATCCGAGCCGGCAGCTTAGAAAAAACCGCCATTATTCGCGTAGAAAGCGGTCATTCCAACATAATAATGAAGATAAAGAATTTTATAGTAAAAAAAGGTTCTCCCCTCGGAAAAATTGGAAAAAGAGTCATAATAGAGATAAGAATAATAGCTTTAAGAAAAAGACCGACAAAAGAAAAGTGAAATGTTACAAATGTCAAAAGTATGGTCATTATGCTAATAATTGTAAAGTTAAAGACACAATCAAACAATTAAAAATCACGGATGAAGATAAAAACAAATTAATCCAAGTCCTTGAGTTAAGAAATTCTGAATCCAGTGAGAACGAAACTATGATTAGTCATACTGAGTCTTCTGAAATATATTCTTCTGAGTCACAACCCAGTTATCCTAGAATCCAATTTGGTTGCCAAGATAATTATTGTAAATTTAAGTCAATCAGTGTCCTCACCAAACAGGAAGAACAAGAAGAACTATTAATTGATTTAATAAGCAAAGTTGAAAATCCAGAATTAAAATCTGAGTACCTGAAGAAATTAAGAAAAGTTATCATTCAAGAAGAACCAAAACAGTCTACCCAAAAGATAAATCTGAATACAACTTTAGATAAATTTAAGAAAAAGAAACAAGTCACTTTACAGGATTTACAATTAGAAATAAAAACGGTCAAAAAAGAAATCATTGAGTTAAGACAAATAAGTCAAAAATTACAAACTGAAAATTATGAGATAAAACAAGATTTGGTTAACCTCTTAGAAAAGAGAATTTCTGAGTCTAAACCAGAAAGTTCCACTAACTCAGACGAAGAAAACTCTATACAATAACAAGCCGTTAATTTAATTAAACATGTAAATTTTAGAAAATGGTATGCTCGAGTCACTATATTTGTTAAAAATTTTGAGTTAATACAGTTGCCCTTTTTGATTCCGGAGCCGACTTTGATTGTATTCAAGAAGGCCTAATCCCAACTAAGTATTACAAAAAGTCAAGAGAATCATTAAGTATTGCTTCAAGAAAATCCCTCCAATTGAACTATGAGATCACTAAAGCCATGTCTGTCAAAATAAAATCTGCTTTAAAACTTTTTTTATTTTAATCAAAAACATTACTGATGAAGTTATACTAGGTCTGCCGTTGATTGCCCTTCTATACCTTTTCCAGGTTGAACACGATGGCGTCATTTCCACCTGTTTAGGAGAAAAGATCAAGTTCGAATTTTTGAGTAAACCTGAGTTACATAATTTAAAAGAATTACAAAAAAATGCAGTCACCAAATCTGTCCAGGTCATCCGAAACAAAGATAAACAATTAAGATTTCTCCAGGAAGAAGTTAAAACTAAAAGAATTGAGCAACAACTAAGTCATAAGTTTTTACAATCACAAATACAGCAATTTGAAGAAAAACTAAAACAAGAAGTCTGTTCTGAGTTTCCCAACGCATTCTGGCATAGGAAACAACATGTTATCTGCCTTCCCTATGTCAAAACCTTTAGTGAGAAAAATATTCCTACTAAAGCAAGACCCATTCAAATGAGCCAAGAGTTAATGGAATTTTGTAAAATTGAGATTACCGATCTACTAAAAAAGGGAATAATCCGTAAGAGTAAATCGCCTTGGTCTTGTCCTGCCTTCTATGTCCAGAAAAATGCTGAATTAGAACGTGGTGTTCCAAGACTAGTCATTAATTACAAACCGCTTAATACTGTCTTAGAATGGATTAGATACCCAATTCCTAACAAAAGAGATTTAATCAACAGATTAGAAAAATCAGTCATTTTTTTAAGTTTGATATGAAAAGTGGATTTTGGCAAATTCAAATTTCTGAGGCTGATAGATATAAACTGCTTTTGTTACTCCTTTTGGTCATTATGAGTGGAATGTTATGCATTTTGGTCTTAAAAATGCGCCTAGTGAATTCCAGAATATAATGAATGAGATCTTTAATCCTTTCAGCCATTTTTCCATAGTTTATATTGATGATGTGCTTATATTCTCTGAGTCCATAGAACAACACTGGAAACACCTGTATAAGTTTTTACAGATAGTTAAACAAAATGGTTTAGTCATTTCTGCTAAGAAAATTAAGTTATTCCAAACAAATGTGAGATTCCTTGGGTTTAATATCTGCCAGTCACAGATTAGTCCATAGATCGAGTCATCCAGTTTGCTGATAAGTTTCCTGACCAAATCGTAGACAAGAGTCAGCTCCAGAGATTTCTAGGATCTCTAAATTATGTCTCTAACTTTTATCAAAATCTGAGAAAACAATGTAAGCCTTTATTTGATCGTCTCCAAAATAATCCTCCTCCATGGTCGTCTGCTCATACTGAAATTGTCAAACAGATCAAAACTCATATCAAGACTCTTCCATGTCCTGGTATTCCTTTTGCTAATTCATTTAAAATTGTTGAAACTGATGCTTCTAATGTAGGTTATGGCGGCATCCTCAAACAAAGAGTCAACTCCAATCACTCTGAGCAAATTATTCGATTCCATTCTGGCACTTGGTCTCAGTCTCAAATTAATTATAGTACTATTAAGAAAGAAATACTATCTATTGTATTATGCATTAACAAATTTCAGTCTGATTTATTAAATCAAAATTTTTTAGTCCGTGTTGATTGTAAGTCTGCTAAGCATGTATTAGAAAAAGATGTTCAAAATATTGCATCTAAACAAATATTTGCGAGATGGCAAGCTATTTTGAGTATTTTTTATTTTGATATTGAATATTTGAGAGGAAGTGAGAATTCTATTCCTGATTTCCTTATCAGAGAATTTCTGCAGGGAAAACAATGGCAGAAAGAAGAAAAGAAAGAGAAAAAGATGATAAACAAAAGAGTCAAATTGGTCAATCAAGCCAACCAAGTCAACCCCAGTCACTCCGAGTCCTCCCACCATCAATGAGTCAAACTAAATATGAGCCTCCTTCCCAGATCGTCCCCTTCCCGGGATGCTCTCCTATCCAAATCAGTAACAGATTTGCCAACTTAGGAGCCACAGTCGGCCAGGTTCACCCTAGTTTCCAGTCTCATTTCCAGTCATGATCCTTTCCAGGATATTGCCGTCACTAATCCCCCAGTTGCTTCTTACCATAAGACATCTCCATACATGATCAAGAGTCATAGTCACCTGTTTGTCATTGAGTCCAGATATAATTCAGTTGCTAATCCCATCACTATTGCTAAATCTTATTTTCCACCAAATAGTCATTTCATTCCCCTTGCACCATTTAAGAATTTAAAATATTATAGAGATATCCTTCATGGACCCATTTCCATCACCATTAAGCCAATTAAAGATAGAGAAAATCCTCATATCATCCTCTATCATTCACTTTACATTCACCAAATTCTCAATGAAAATGAATGGAGTAGTCACCCCTATGATCTCAAAGTTCTCCAATCAGAACTTCAATATAGCTATTATGATTATATCGAAGCATGGTATTCTATCTTGCTTCACCAAACAGTTGATTTTAGTCACTCCTGGTTCTTAAATTTTGATAGCAAATTCAAAAGTCATTTTCCTTACTGGTTCAATCACTGGTGGGAAAAACATGGTCCAGTCATTGATCTATTTCCTCCTCCTATGCAAGACTTAGTCAATTATTTTACTAATAAAAATAAGTTCAAAGAATCTGAACTGTTCTTCCCCAACCTTCTCCATTTTGTTGCCAAATATAAAGTCCCTTGGATCCTCAAATGGTCCTATCAGGTCAATTGGGAAACCAGAGTCTTAGCTCGCCAGTTTTCAATCAAATGGTGGGATAAATTTAAACTTGAGCGTATTGCAGATTATGTTTATGCTGATTTTCCTCCAGTCAGGAATCCGCACCCTGAGAAAAAGAGCTCTCCTGCCACATCACACTCTTCTCTCCCAATCGAGGGAAGATCTAAGTCTGAACTAAAAGATCTTGCTCGCCAGTTAATGCTTCAAGCCTCTCAGATGAATGATGATGCAGACAGTGATGCCTCTCCTGCGTCACAAACATCTACTAGTCAACCAAAGCCAGCCAGTCAATAAGCCAGCCCCAAAAATGCGGTGGAATGATTATCCAGATGAGCAAGACCCCTATGATTTTGGTCCTTTCAATCTAGGGGATGATTAAAGCTAAAGTAAAAGTCGCCTCGCATCATGAGCCCTGAAGAAAAGCATCCGAACCCATGCATTCATCAGTCTGAAAGCTGTGTTCTTTTCGAGATTCCCCGACAAAGTAAAAGTTGCAGCACTCAGTAAAAGCAAAAGCTTACGTGTGCCTCTACAGTTACATTTCACAAGCATCCAAAGCTGCCAGCTCACCAAAAGTTATCAGAGCCTGTTTTATTTGTCTATTTAAGAAGGCCCCGACCTCTATCTTCTCTCAGAATGTAATTTTAGTACACTATAATTTTCTGAGTTTCAAAAGTTCTATCAGTACCAATCTTTTGAAGAACTAAAGTGGTGAAACTAGGATCTTTGTAAGTGTTTTGCATTCTCCAAGTTATCAATTTATTTGAGATGTTTTGAGAATTGATTATTTTGATTAATGCTTATTTTCATTGCATCTATTTTCATATATTCATTAATTTGCTTCATGCATAAGTTGCGTCTATTCTATAATCTATGTGTTTACACCAGCCTTCATTGTTCTTCTTCTTGTAACTGGTTCTAGACCCCTTCTGGTATCAAAGCCGTAAGTGTTGAAGAGTTAGTCTAAGTTTGCAACTCATCCAACCCAAGTGGCTGGTTTAGGTTTTCTGAGTTGTAAGTGTTCAGTTTCTTCTTTTCTTCTTCTGGTCTTTCAGTTCACCTGTCCTTCTCTCTCTTGGTGTCCGGATCGTAAGACCCATTCCAAACAGTAAGTCCCAGGGAGGCCTGAGCGGTAGTGGCATTAGGAGGAGGGCGAGAGAATTTTAGGTGTCCTGTTACCTATGGAGTCTGTATCAGAAGTTTCTGTTCATCTTCTTCTTTAGGAATCTAAACCCAGAACCTTGAGTAAAAATGAGTCGTTTTCTTAGAACCAACTCTTATAGTTCTAGCAGCTCTAGAGCCAATAGTAGTCTCCCAGAGATAGTAAATGAAGAACAAATTGAATATGAGAATAATAGTAATCTAGATTATAAAGATTGGAATATACCTCGAGTCCCTAATCAAGACATCTATAAGAAAAAATGGTCATTATCCAGTTTTAAGTCAACCACCCATGTTAAAACAGTTGAACAAGTTTATGCCCTAAGTAATAATCATGAGACATGTCGGTTACTTAATCTTGAGTCAATAAGAAAACATAAAACTGATGGAAACAATTTTCTTCATATGGTCTAGTACAAGTTACCTTTAACCAGATTAGGATTAAAAGCTTCAATCCTTCTCTGTCTGCGTGATGCTAGATGTAATAATTTTAACGACAGTACATTAGGAATTATTGAGTCCAGTCTTGCTAATGGCCCAATTCATTTTGATTGTTATCCAGATTTTGCAGTCAGTCTGAGCGATCCACATATATTAAAAACACTCACTTTAAATATTAAAACTGAAGGTTATGATGTTTTGCCAGGAAGCCAACCCCTAGCATTAGTTTACAGAATATATTATAAAGTCACAGGTACAAACATGAATTTCCAAGCCTTAAACAAGAGCCCAAAGAGTCAAACATTGTTAATCCAAAGTCATACGCCTGACTCTACTGTTAGAGTTCCTCACTCTATCAAATGGTCAGAAGTTACTCTTCCCACTGATTGGACCCTAACCACTGAGATTCCTCCAGTTCCTATCCAACGTAGTCTAAGCGATTTAGACTACATACAACAATATTTAGATGGTACAGTTAAAATCAATTTTGGTCACCAAACCCCACAGAAATCAACAGTCCAGTTACAACAGTTACCAACACCTGGTCAATCCCAGCGTCATATTCCCACGAGACATTCCTTTGCAGCCTCTTCTTCCACCCTAGAATGAGACCTAGA

CTGGGCCACTTGCATGACTAAACCAAACTATTCTATTTCCAAGAGTTTGTCCTGTAAATTTCAACTCTAGTGTTATAAAGACCTCCAAGGAACTCTAGTGTTCCAATGTGGGACTAATTTCAACTCAAACATGTGGGACTGTTGATGAGAGTTCACCATACGAACTTGGTTTCTCTAATTGGGTATTGCGATGAAGGTGACACTGTGGCACTTGTTTATGAATAAGTGGCCAATGGAAATTTGCAACACCATATATTTTCTGCAGGTACAATACTCATATATTAATATTAATATATTTTGGACTTAATTATTGCAATGGTCTCTAAACTTGTACACCTGAGTTGTATTTTCGTCCATGATATCCAAAAATAGCTACAACCACCATCCACTAATTAGTCTAGTATTGCATGACTGGTATAGTTGCTCCTTTTTCTCTTTAATTTTAAAGAAATACAGAATAATTTTTAATAGTAAGTTTTGGTATAATCGTAAACGTGGTTGGATGTAGAAAGTAAATTCCTGGATCATGAAAAATTGAGTGTCTAAAAAGTACTATTTTTTGGGTTTTCATAAAATTTCCAAGTTTGTTAACAATAAAAGTTTGGGTTTCCTTAACTAGTCCTTACAATTAAATTTTCTATTCATCATTATCATTTGTCTCACATATACTGGACTTCAGGTGCCAAATTTTAACAATAGATATGAACAGTCATGTGGTGGAAAGTGTATGTTTGGTTTATTTTCTCTCGTTTGTTTCCTTTGTAAATTGCATTATTACTGATAATACACTCTTGGATTGGATGTGTGCAGATATAACTAAAAAGGTTTTGACTTGGAAAGAGAGACTTTTAACTAAAAAGGTTATACACCCGCAAGCGGTAAAGTACTAGTAGTTGTATGAATAGTAAGGATACAATGTGTGAGGAAGAAAGTAAAGCTTGGCTCCTTAACTCCTCCTTTCCACCAATAATTAAAAAAAAACAGTTTACATTCAGAATCA

>Chr3-08; OZ022208.1; 8818898-8819639; dir; B;

CTCCCCAAAACAAAATCGTGAGGGCAAATAGGAGGGCCCAAAGTAGACAATATTGTGCTACGGCGGAGCTGATCCCGGGATGTGATAATTTGGTATTAGAATCAATCTGCCGTGTTGAGCAAGTGTGTCGACGAGGACGTCGGGCCCTTAAGGAGGGTGGATTGTAACATCCTACATCAACCAACGAAGAGGGGGTGATGTGCCTTATATGTATATGCCTGACTCCATCTAACACGAGGCCTTTTGGGAGCTCACTGGCTTCGGAGTCATGAGAACTCCGAAGTTAAGCCAGTTGGAGGCTAGAGCAATCCTAGGATGGGTGACCCACTGGGAAGTTGCTCGTGAACTCCCAAAAATAAAACCGTGAGGGCAGAGAGGAGGGCCCAAAGCGAACAATATTGTGCTACAGTGGAGCCGATCCCAGGATGTGACAATTTGGTTCCCAGTGGGTCACCCATCCTGGGATTGCTCTAGCCACAACTCGCTTAACTTCGGAGTTTCCATGACTCCGAAGCCAGTAAGCTCTCAAAAACAAAACCATGAGGGCAGAGAGGGGGCCCAAAGCGGAAAATATTGTGCTATGGCAGAGCCGATCTCGGGATGTGACAATTTGGTATCAAAGCCACTCTGTCGTGTGGTGCGAGTGTGCCGACGAGGACGTCGGGCCCCTAAGGGGGGTGGATTGTAACATCCCACATTGACCAACGGAGAGGGGGTGATGTGCCTTATACATGTCTGCCTCCATCTAGCACGAGACCTTTTGGGAGCTCACTGGCTTCGGAGTCATGGGAACTCCGAAGTTAAGCGAGTTGGGGGTTGGAGCAATCCCAAGAGTGGTGACCCACTGGGAAGTTGCTCGTAAGCTCCCAAAAATAAAACCGTGAAGGCAGAGAATGAGGCCCAAAGCTGACAATATTGTGCTACGGTGGAGTCGATCCCGGAATGTGACAACAATACTCACCTTTTCATTGTTGAATCCAAATATAACAATATTGCCAAT

CCAGTCACAATTGCAAAAGCTTATTTTCCCCCAAATTGCCATTTTATTCCTCAAGCTCCTTACAAATCCCTTAAATACTATAGAGATATCCTTCATGAGACCCAGTCAATTGAAATTAAGCCTATCAAAGACCGTAATCACATTATTCTCTATCATTCTTTGTATATTCATCACATCCTAAGTGAAGACAAATGGAGTAATATGATCTAAAAATTCTCCAATCCGATTTACAATATTGCTATTATGATTACATCGAAGCCTGGTATACAATCTTCCTTCACCAAACAATTGATTTCAGTCACTCATGGTTCATTAATTTTGACAGTAAATTCAAAAGTCATTTTCTCATCTGGTTCCTCCACTGGTGGGAAAAACATGGCCCAGTCATTGACCTTCTTCCTCCTCAAATGCAAGAATTAGTAAATTATTTTTCCAATAAAAACAAATTCAAAGAATCAGAATTATTCTTTCCCAATCTACTTATTTTCATTGCTAAATACAAAGTTCCTTGGATCCTGAAATGGTCTTACAATGTCAATTGGGATTCTAGAGTCCTATCCCGTCAATTTTCAGTCAAGTGGTGGGATAAGTGCAAAATCGAAAGAATTGTTTATTATGTCAATTCAGATTTTCCTCCAGTCAAAATTCCACAAAAGTCTTCCCAGAAGCCATCTTCCTCTGGAACGTCTCATTCTTCCCTCCCAATAGAAGGAAGATCAAAGGCCGAGTTACAGGAAATTGC

CCGGCAATTAATTACACTCATAGTCAACAGTAAAAGTTGTTTCGTTGTTTCCCCAACAAGGTTAAATCACAAAGTAAAAGCAAAAGTCTTTTCGTGCTTCAACAGTCACCCTCCAAAGTATCCAATGTTGCCGACACTCTTTAATCCGACTCTTTAATTAGAGGTTCAGGCCTCAGTAATTGCAAGTTAAATTTTAGTTTTTAGTTTTCAAAATTTTCTCTTAGACTCCCTCAAAAAAACTACTATTGTAAAATATGAGTGTAATCCTGTACGTTACTGCAAGTTATTGATAAAATCCTTCAATCAGGATTTTTCCTGTAAGTTCAATGTTTTCAATTAAACATATTTTCAATTATTCATTACTTTCATTTCTGCATTATTTTCACATATTCTATTATCTTTGCTTCATTACTATCACAGTTAACTTGTCCTTCTGGTTCTAAATCACTATATATATAAACGTGCCTTTTGTCTTTTTTATATAGCCCATGTTGGCTGTCAGCTTTTTTGATTTTTTTTTTCTCTTTCTTCCTTCACTGGTTGTACAGGGTGGTGGGATTCAATTTCAGTGTTTGTTAAGAGCCATAGATTCAAAGTGATCTAATGGCTGAAATTTGGGCGCATCCATGCTCCTGGCGCATGGTAGAATTTTCGCTGGAGATATTTCAAACAAAGATGCGGAACTGACATGACTCAGACCAAGTCACCACAAGATTCTTTTCACTAACCTAAAAAAATAAAATGGAAATAAAAGAGTGGAGGAGGGAAATGCCCTCAGCAGGCAACACTTTTGGGTTCACAAATTATGTTCAAAGCGATCATGTTCGATTATGTCACTACTTTTAATAATTATAGATAAATATCAATCCTGCAGACGACTTTGACTTCAAGTCTTGGAGTGATTGATTTGGCTTCAATTATTTTGGTTGAGTTAGTTGGATGCATAAAAATTCTAGGAACATCATTAATCCAATGTAATTATTTTGGTTGAGTTAGTTGG

>Chr3-09; OZ022208.1; 10249006-10253102; dir; B;

ATTATAAAAAAAAAAATTTCAAAAAAAAAATTTCAAAAAAAACTTTAATTTCCTATCCCTTTGTTAGGTTAACATTGTCATTTGGAGATTTTTGTGTGATGACATCAACAATTTTTAGGTGGAAAACCAACGGGAGGGGTTTTGAATCTTAAGCAACGTGAGTTGGAAAACCAGCATGGGGAACTGCGTGCCCTCCTTCTTGCCCAAGGTGTTTCTGCTGACAGTGCCGGCTGTGTGGCAGAAGCAGAAACTAGATCCTCTCATAAGGCCTCCTCAGTTCTCTTTTAGGTCCGTGACTCTTCATCCCTTCTCACTTTACTCCTATTTTTTTCTTCTTTAGTAGCCTTGTCCTGCACCAGCCTGTTTTCTTCTGACATTTACCGTTACTCTGCCTTGTAGGTTACGTTTGCTTGGTGTGAGACTGACTGGCTTGACACATTTTAGAATTCATTTTGAAACCGAAGAACACATTAATCTTTGGCTTGTATAAGTTAATTTTACTTAGGCTGTATTTCCTTTGGATGTCCCAGAACTTATACTGTGGCAAGTTTGTACTGTAATGTTGTTGTGTTTAAGCTGCTGGAACCTATGTCCCCTTTTTTTTTTGGATGCTAGAGACACATTGTAATTCCTGTTTGGTATATATATATATATATGCTGCCTGGTTGGTGGAATTCTTTGCTTGAGATCTTCTTACAGGTTGGGTACCCTTTTAAAACAAGGTAGATGCTGCCAAAATTTTGATAGGATCCTTGTTCAAACTTCTCGATACCTCCTTTTTTTTAAAAAAAAAAATTACCCCCAAAGGCTCTTGGACGTTGTGGGCATGCTAGTTTCTTTGTAAATGCTCAACTTTGCGCGCTCCATTTTATTTTGCTTAAGCATGAAGCTTGTTAATGATCTGGGATTCATACTAGTGACTTTGCTTGGCATGCTTTCTGCTAAGTAGCGTTGTTTTTTTTGTATATATATATATATAATAGGTAAGCAAATACAGTCA

GAAAGAAATTCCCAGAGTCAGTTTTTAGTTTCCTTAGATAAATTATATGAATGGAATATAGATGGTCTGTCTAAGCAAGAAATATTAAACAAGTTAACTGATATGTCCATGGTTGCCAATAGTTATGTCACCAATCACAATTTTAGGCAGTCAGAAATAGTGCCTTTATTAGAAGTTGGTTTCACCGGAACCTTACATTCTTGGTGGGATAAACATTTGACAGAAGAATCTAAACGTCAAATAATTCATGCAGTTAAACTTAATGAAGAAGGTCTCCCAATATTTGATGAAAATTTAGGTCAAGGTATAGAAGACGGAGTCAATACTTTGTTATATACCATAATTGAGCATTTTATTGGAACACCGAGCCATACCACTACTAGAATCCATGATCAACTTAGCAATCTAAGATACCCAAAATTGTCTGATTTTAGATGGTATAAAGATGTCTTTATTTCCTGAGTCATGTTAAGAGAAGATAGTAACCAAGCTTTTTGGAAAGAAAAATTTATTAATGGCCTTCCAAATTTATTTGCCCATAAAATACGCACAGTCCTTAGTAATGATCAAGTTCGCATAGATTATGATAATTTGACTTATGGTAATATTATTAGCACAATTAACCAAGTTGGTATGAAAATCTGTGTTGATATGAAAATAGGTAAGCAAATATAGTCAGAAAGAAATTCCGCTAAGTATGAATTAGGAAATTTCTGTGAACAATATGGTTTAGCATCGATTCCCCCATCTAGAAAAGATAAGTCTAGCCGACAGTTTAGTAAAAATTGCCACTACTCACGTAAAAGAAAGTCATTCCGACATAATAATAACAATAATGAGTTTTATAGTCAAAAGAAGTTTCACCCGAAGAAAAACTGGAAAAAGAGTCACGAGAAAAATAAGCAAAGTAGCTTTAAGAAGAATACCGACAAAAGAAAAGTAAAATGTTACAAATGTCAAAAGTTTGGTCATTATGCTAATGAGTGTAAAATTAAAGATACAATAAAGCAGTTGAAAATCACGAATGCAGATAAAGAAAAGTTAATTAAAGTTTTAGAATTAAGAAATTCTGAGTCCAGTGAAAATGAAACCATAGTTACCGACACTGAGTCAGCAGCTTATATGTCTACGGATTCCCAACCTAGTTCACCCAATATCCAGTTAGGTTGTAAAGATAAATGTTGTAATGTCTTAAGTAAGTCAATTAGTTAATTTAATAGAAAAAAGAAGTTATGGTATTCTAAAGTCACTTTATTTATTAATAATTTTGAATTAAACACAGTTGCCTTATTTGATTTAGGAGCTGACCTTAATTGTATTAGAGAAGGTTTAGTCCCCACTAAATATTACAAAAAGTCAAAAGAGTCATTAAGCACTGCCTCAGGAAAATCACTCCAGTTAAATTATGAAATCCCTAAAGCCCATGTTTGTCAAAACAAAATTTGCTTCAAAACCTCATTCGTCTTAGTAAAAAATATTACTGATGAAGTCATTTTAGGATTACCCTTTATTGCACTTTTATATCATTTCCAAGTAGAATACGAAGGAGTCATTTCTACCTATTTAGGTGAAAAGGTTAAGTTTGAGTTTTTAACTAAGCCGGAATTGCACAATTTGAAAGCCCTACAGAAAAGTGCAATTTCAAAGACAGTTAAAATAATTGAAAGTAAGAATAAACAACTAAATTTTCTTAAAGAAGAAGTTAACTTTAGGAGAATTGAACAATAGCTTAATGATGTGTCTGTCTAGTCACAGATTCAAAAGTTCGAAGACAAATTAAAAATAGAAGTCTGTTCTGAGTTACCTAACGCTTTTTGGCATAGGAAGCAACATGTAGTCAGTTTACCCTATGTTAAAAACTTAGTGAACAGAATATTCCCACTAAGGCTAGACCGATTCAAATGAATCAAGAGATAATGGAATTTTGCAAAACTGAAATTTCTGAATTACTCCAAAAAGGAATAATTAGCAAAAGCAAGTCACCATGGTCTTGTCCTGCTTTTTATGTCCAAAAAAATGCTGAGTTAGAACGTGGAGCCCCAAGATTAGTTATTAATTACAAACCTCTTATTATAGTCTTAGAATGGATTAGGTACCCAATACCTAATAAACGAGACTTAATTAATAGGTTAGGAAATGCAGTTGTCTTCTCTAAGTTTGATTTGAAAAAAGGATTTTGGCAAATTCAAATTAGTAAGTCAGATAGGTATAAAATCGCTTTTGTTCCCCCATTCGGTCACTATGAGTGGAACGTTATGCCTTTCGGTCTTAAGAATGCACCCAGTGAATTTTAAAATATAATGAATGATATATTTAGTCAATATAGTCATTTTTCCATAGTTTACATTGATGATGTTTTAATCTTTTCCCAGTCAATAGAACAGCATTGGAAGCATTTGCATAAGTTTCTTCATATAGTTTAACAAAATGGTTTAGTTGTCTCTGCTAAGAAGATTAAGTTGTTCCAAACGAATATCCGATTTCTTGGTTTTAATATTTGTCAATCTCAAATAAGTCCAATTGATCGAGTCATCCAGTTTGCAAAACAATTCCCTGATCAAATTTTAGACAAGAGTCAACTTCAAAGATTCTTAGGATCTCTTAATTATGTGTCTGATTTCGATTAGAATCTGAGATAACAATGTAAGCCATTATTTGATCGTCTCCAGAGTAATCCTCCTCCCTGGTCATCTACTCATACAGAAATAGTCAAACAAATCAAGGGACCGATGCCTCTGAAATTGGTTACGGAGGTATTCTCAAACAAAAAATCAATTCAAGTCAATCTGAGCACCTTCCCAAATTGTCCCAGTCATACAGAATACTTCCCCCATCAATGAATACACCCAAATCAGAGTCACCTTCCCAGATCGTCCCTTACCCGGGATGTTCTCCTATTCAGATCAGTAACAGATTTGCCACCTTAGGAGCCACAGTAGGCCAAATCCGTCCTAGTTTTCAGTCAGCCTTAGTCTTCAGTCATGATCCATTCCAGGCCAATACTCAGTTTACTCCTCTAGTTGTCTCTTTCCAAAAGTCATCCCCTTACATGGTTAAAAGTGATAGTCATCTCTTTATTGTTGAGTCTAAATATGATAATATTTCTAATCCAATTACAATCGCAAGGTCATATTTCCCCCCTAATTGCCACTTCATTCCTCAAGCCCCCTATAAGAATTTAAAATATTACAGAGATATTTTTCTTGAGACCCAATCAATTGAAATCAAGCCAATTAAAGATCGAAATAATCCAGCTATTATCTTTTATCACTCCCTATACATCCATCGAATTCTTAGTGAAAACGAATGGAGCAAGCACCTTTATGACCTCAAACTTCTTTAGTTAGAAGTCCAATTTAGTTACTATGATTATATTGAAGCCTGGTATTCAATTTTGCTTCATCAAACAGTTGATTTCAGTCACTCTTGGTTCATCAATTTTGATAGTAAATTCAAGAGTCCTTTTCCCTTCTGGTTCCTCCACTGGTGGGAAAAACATGGTCCAGTTATTGAGCTTCTTCCCCCTTATATGCAAGAACTAGTCAGTTATTTCACCAATAAGCATAAGTTCAATGAGTCAGAGTTGTTCTTCCCTAATTTACTCCATTTCATTGCTAGGTACAAAGTCCTATGGATTCTTAAATGGTCATACCAAGTCAATTGGGATTCTAGATATCTCTCCCGTCAATTTTCAATCAAATGGTGGGACAAGTTTAAAATAGAAAGAATTGCTAATTATCTCTATTCAGATTACCCCCTTGTGAAGCCTCTCCAGTCAGAGAAAAAGGTGTCCCTTGCAGTGTCCCATTCTTCTCTCCTAGTCGAGGGAAAATGAGAGTCAGAATTACAAGAAATTGCTAGACAGTGGATGGCTCAAGCCTCCCAAATGAATGATGATGAAGATAATGATGCTAACAGTGATGCTACCCCTAAATCTCAGTCTTCTACTATCCAGTCTCGGATCCCTCCAAGTCAAAAGAAGCGGTGGAGTGATTATCCTCCTAATAGTCAAGCCCCTATAATTTCAGTCCATTTGATCTCGGCAAAGATTAGCAGTTTTTAAGACAGTCATCATGAGCCCTAACAAAAGCAAAAGT

ATTTCAGCACTCAGTAAAAGCAGATGCTTTCGCGTGGTTCTACAGTCAAAAGTTTATTTCAGTCAAAAGTTGTATGATTTCCAGAGTAGTTCCCCCGTGGTTGAATAGTCAAGTTATCTAGTCTATTTAAGAAGGCTTCGGCCTTAGTTATCCTCGGAATGTATAATTTACTGTTCTCAGTTTTATAAGTTCTCTCAGGAGTACTACCCTGATGAATTCTTGTACTTTCTGAGGGATCGAAATCTCTTCAGTCAAGATTTCTTGTAAGTTTATACCTATTTTTGCTTATATTCTATCTCCTCCTGGATCTCTCTCTCTCTCTCTCTCTCTCTCTCTCTCTCTCTCTATATATATATATATATATATACACATATATGTATACATACACAGGAGTTTGGGATGCAAAAAGACAAATTCCCAGAGAGTTTTTTTTTTTCTTCTCAAAACACTCATTCATTTTCATTTCAAATCCCATGGTACCATGACGGTACATCTTACAAAAGTTTCCCAGAGCAAAAGGCGGAATATAGAAGGAGACCATATTTAAAAAGGAAATGAGATAACACAAAAGGTAAGATACGATGTCTACTTAGTAGTAGTAAGGCTTGAGACACTTTCCATTGATGGGATCCATTAGCATGCCTTCCTTGACGGGGATCAAGCGATAATAGCTACTGCTATGTACTTCCTTGACTGCAAACATGCCTTCCCATTGTGGAGCAAACTTGGAAGGTCCCGACACTAGTCTTCTCACATGCTCCACAACTTTTAGCACAGGATCTCCTCGTTTGAAGGTTTGAGGCTTGACGGTCCTGTTATATGCTTGGGAACAGTGTGGTAAAGTGCCATTTTTCTTTCGGCTTCTACTCTTTTTTCTGTCTAATAAGCGCCAGTCCGAGCATGACTTCACATCCCATTCAAGATCATTGACAGCCGATACTCTGGCCGTGGGAATGGTGATTTCCACAGGTGAGATGGCATCAAACCCATACACAAGCGA

>Chr3-10; OZ022208.1; 11717711-11726102; dir; A;

ATATGTTGAATAAAAAGGTATTTTATTGAATGCTTTGTTTATTAAATAAAGAAAGCCAATACAAGTCATTAAGAATTACTACTACTAAAATAAAATACATGAATTACAACAACTTTAATGAAAAACATGAAAAATACAAATACATAAAAGGTATACTAACTAATTTAATGGTTTTGATCATTTAACCAATCCGTGTTGCTAGGCCCATCATCCTGAAAAAGTCTTCTTCTCATACATCCCTTCGTTCTAGCTTCCAAAATTATTTTGTTTCAGGGGATATATGGCTTGTATCCATCGTCATGGTTTCCCGATCTGTCTTGTCCATCTCTTTTTTGCTCAAATACTCCCTTTCTTCTCCATATTATGCTCGAATTGCCATCTCGTTCTCTTGGCATTTCAATTCTTATGGCGCTCTGCCTTACAATTTCCTCCAAATTTTTTGATGTATTACTGCTCGAATGACCCCCTTTCTTTGCCTTCCTTCACCGCCTTTCGGCCAATGGGCCTCGGCTCCTTTTCGATAGTGAGTCTTGACTCATAGGGGAATCGAGAGGTGAATCCGATGTCGTTAAATCACGGAGTGGCGTCTCGTTTAACATAACGGCGGGACCCGTGGGAATAATTCTGAAGTGTTTGCAATTTTTCACCACCTCCCAATATTCATGATGGTTGAAAGTTTTTTTGCCTTGCCCAATTGCACCAAACCACATCTGTACTTGAATCATCTATTAAAAAAAAAATGAAAATGCAATTAACCTTACAAATATATTGGAAGTGCAAGAAATATATACAAAATATTAAAAATGCAATAAACATTAATATATATATATATTAATGTTTATTGCATTTTTAATATTTTTAATATATATATATTTAGTACACTATAATTTTCTCAGTTTCAAAATTCTGTTAGTATCCGTGCTCTGAAGAATTAAATGGTGAAACTAGGATCATTGTAAGTGTTTTGCATTCTGCAAGTTATCAATAAATCTTGAGATAT

TTTGAGTATTTTGATTAATGCTTATTTTCATTGCATCTATTTTCATATACTCATTAATTTGCTTCATACATAAGTTGCGTCTATTCTATAATCTCTGTGCTTATACCTACTTCTGTACTTCTTCCATCTCTCACTGGTGCTAGACCCTTTCTGGTATCAGAGCCATAAGTGTTGAAGAATTAGTCTCAGTCTGCAACTCATCCAACCCAAGTGGCTGGTTTAGGTTTTCTGAGTTGAAAGTTTTTAGTTTCTTCTTTTCTTCTTCTGGTCTTTTCAATTTACCTGTCCTTCTCTTTCTTGGTGTTCGAATTGTAAGACTCGTTCCAAACAGTAAGTCCCAGGGAGGCCTAGCGGTAGTGGCATTAGTAGGAGGGCGAGAGAACTTTAGGTATACTGTTATTCCTAGGAGCCTGTATTAGAAGTTTCTGTTCACCTTCTTCTTTAGAGATCCCAAACCCAGAACCATGAGTCATAATGAGTCGCTTTCTTAGAACCAATTCCTACAATTCTAGCAACTCTAGAGTCAGTATAGGCAAAGTCTCAGAGATAGTTAATGAAGAACAACTAGAATATGAGTCTAATAATGAATTAGATTTTCGAGACTGGAATATACCTAAAGTCCCTAGTCAAAATATATATAAGAAAAATTGGTCAGTCTCTAGTGTTAATACAACCACCCATGTAAAAACAGTTGAACAAGTTTATGCTTTGAGTCAAGAACATGAGAACTGTCAGTTGCTTAATCTTGAGTCAATTAGAAAACATAAATCTGATGGAAACAATTTTCTTCATATTGGTCTAGTCCAAGTTGCTGTTAAACCCCTGACCAGATTAAGATTAAAAGCTTTTATCCTTCTCTAGATTCACTAATTTTAGTGACAGTACACTAGGAATTATTGAGTCAAGTCTTGCAAATGGTCCAGTTCATTTTGATTGTTATCCTGATTTCACAGTTAGTCTGAGTGATCCCCAAATATTAAAAACTCTAACTTTGAACATAAAAACTAATGGTTATGAAGTCCTGCCAGGTACCCAACCCTTAGCATTAGTTTATAGAATTTATTATAAAGTCACAGGTACTAATATGAATTTCCAAGCTTTGAGTAAAAGTCCAAAAAATCAAACCCTGTTAATCCAGAGTCATACACCTGATGCTACTATAAGAGTCCCTCATTCCATTAAGTGGTCAGATGTGTCTCTTCCAGCTGATTGGACCCTAACCTCTGAGAGTCAGCCAGTTCCCATCCAGCGTAGTGTAAACGATTTAGATTACATACAACAATATTTGGATGGTACTGTAAAAATTAATTTTGGTCATCCTCAAAAATCAACAGTCCAGTTACAGTAGTTACCCACACCTAGTCAATCTCAGAGACATGCCCCTGCAAGGCATTCCTTTGCAGCCTCTTCCTCTACCCTAGAACGAGACCTAGAGTTAGAGAAAGCTTTAATTGATTTAAAATTAGAATCACTAAGAAAAACCTCCCAAGTTACTCATCTTGTCTATGCAAAACCTCCAGAAGACAACAAATCTGAGTCCCCTGAATCACCTACCCAATTTGATTTTGTTGAAGCCGGTTTCAAAATAGATAACCAGCTACGAACACTTAATAAGAAATTTGAAATTAATTGAAAAAAATTAGAAAGTCACCTACATGCCCCCCAAAAACCAAGCTAGAAGAAATAATTACCACTCAGTAATCCCCGACAGAGTCAAAAGACAACAAATATTTAATGAATGGTAAGAATATATGTGCACTACTAAAGTTGAAATTTATTATCTTGACTTTGTGGAAAGTCGCCATTTGCCGAATGAGTTAAAAACTTTAACCCAGGAAAATTGGGAAAAAATAGATCATACTAAAGTCCTTGCTAGTCATCCCCCTGTCGAATCTATAGTTATCCAGCACCAGAAATCACAAGTCATTGCCAGTCCTTTTAAGATCCCTGACTTAGAAAATAAAATGGTAATTGAACAGAATAATTACACCAACTAGAGCTTAATTGTTATAGGAAAACAATTAGATAAAATAGAAACAAAAGTTGACAAATTACTACCAAAAGACATTGCCTCTAAGTCACATGATCAGAAACCTTTAGTTACTTTCCAGGATTTAAAGTCTAGTCTGACCTTTAAAACAAAAGATACAATGACAAAGATGTTAGAAGAATTAAAGCCAACCAAAAGAAAAGATAAGGAAAAAGTCGAATCAAGCGTTAAAGTTTTAAGATCCTTTGAGTCACCCTTTGTTTTCCATAAGTCCTCTGAGTCAGAATCGATCACCAGCATAGATACAGAAACTAGTAATATTTCAAAAATTGAAAATGCGTTCAGAAATCTTGAGTTAGAAAATAATCCACAAGCACAAAGATTAACAAATAGAGTTAACCCTACTAGCCTCATAAAAAATTGGTATCCTAAGCCTACACCACCTGACATCCAATTTGAAGAACGAAATACTCAAAGTCAGTTTTCTGTTTCTTCCGATAAGTTATATGAATGAAATATTGATGGTCTTTCTGAATAAGAAATCCTTAATAAACTCCAACATATGTCTATGATCGCCAATAGTTACATTACCAACCACAGTTTCAGACAGTCTGAAATTGTTCCCTTATTAGAAACTGGTTTCACCGGAACTCTTCGTTCTTGGTGGCATAAACATTTAACCCATGAGTCTAAACAAAGAATAATTCATGCTCTTAAACTTAATGAAGATGGTCTTCCTATTTACGATGAACAAATAGGTCAAGGCATTGAGGATGGAGTTAATACCTTGCTTTACACAATAGTCGATCATTTCATTGGAACACCTAGTAATACTACTGCCAGGATACATGATCAATTAAGTAATTTAAGATGCCCAAAATTATTTAATTTTCATTGGTATAAAGATGTGTTTATTTCCAGAGTCATGATTAGAGAAGATAGTAATCAGCCATTTTGGAAAGAGAAATTTGTTAATGGTCAGCCGAATCTATTTGCACACAAGATCCGATCAACCCTTAGTAATGAACAAGGACATATTGATTGGGATAATTTAACATATGGAAATATAATTAGCACAATTAACCAAGTCGGTATGAGAATGTGCGTTGATATGAAAATTGGTAAACAAATCCAGTTTGAAAGAAATTCTGCTAAATATGAGTTAGGTAATTTATGTGAACAATACGGTCTAACATCTATTCCCCCATATAGAAAGAATAAGTCTAGTTATCACAACCGATAGTTTAGCAAAAAACGCCATTATTCAAGTAGAAGACAATCATTCCAACATAATAATGAAGATAAAGAATTTTATAGTAAAAAGAGATTTCCCCTAAGAAAAATTGGAAAAAGAGTCATAACCAAGATAAAGATAATCGCTTTAAGAAAAAACCTGACAAGAGAAAAGTGAAATGTTATAAATGTCAAAAATATGGTCACTATGCTAATAATTGCAAAGTTAAAGACACAATTAAACAATTAAAAATCACAGATGAAGATAAAGATAAATTGATTAAAGTACTTGAGTTAAGAAATTCTGAGTCAAGTGAAAATGAAACCATGATTAGTCACACTGAGTCCTTTGAAATGTATTCTTCTGAGTCACAACCTAGTTCCCTCAGAATCCAATTTGGCTGCCAAGATAATTGCTGTAAGTCTAAGTCAATGAATGTCCTCACCAAACAGGAGGAACAAGAAGAATTATTAATTGATTTAATAAGTAAAGTAGAAAACCCAGAATTAAAATCCGAGTATTTAAAGAAATTAAGAAAAGTCATAAGTCAGGAAGAGCCGAGACAGTCTACCTCCCAGAAGATAAGTCTAAACACAACCTTAGAAAATTTTAATAAAAGGAAAGAAATTACTTTACTGGATTTACAACTAGAGGTCAAGTTAGTCAAAAAAGAAATTATTGAATTAAGACAAATAAGTCAAAAATTACAAAATGAAAATTATGAGATAAAACAAGATTTGATTAATCCATTAGAAAATAAAACCCCCAAGTCTACACCTGAAAGTCCCATTAATTCTAACGATGAACCCTCCAATCAGGATCAAGTCGTCAATCTGATTAAACATGTAAATTTTAGAAAATCGTATGCCCGAATCACTATATTTGTCAAAAGCTTTGAGTTAAACATAGTTGCCCTTTTTGATTCAGGAGCTGACCTTAATTGCATCCAAGAAGGTCTAATCCTAACCAAATCTTACAAAAAATCAAGAGTCTTTAAGCACTGCCTTAGGAAAATCACTCCAGTTAAATTATGAGATCCTTAAAGCCCATGTCTGCCAAAATAAAATTTGTTTCAAAACCTATTTCGTTTTAGTTAAAAACATTACTGATGAAGTCATCCTAGGATTACCTTTTATTGCCCTTTTATACCCTTTTCATGTTGAACACGATAATGTCATTTCCACCTGTTTAGGAGAAAAAGTTAAGTTTGAATTCCTAAGTAAACCTGAACTACATAGTCTAAAAGAATTACAGAAAAATGCAGTCACCAAAACAGTTAGAGTGTCACGCCCCGGACCGGCTCTACCGTAGCACGATATTGTCCGCTTTGGGCCTAGGCCCGCACGGTTTTATTTTTGGGAGCTCACGGAACAATTTCCCAGGGTGGTCACCCATCCTGGGATTGCTCCAGCCTCCCACTCGCTTAACTTCGGAGTTCTTACAACTCCGAAGCCAGTGAGCTCCCAAAAGGCCTCGCGCTAGGTAGATGCGAGTGTGCACATATAAGGCACATCGCCCCCTCTCCGTTTGGTCGATGTGGGATCTCACAATCCACCCCCCTTAAGGGATCCGACGTCCTCGTCGGCACACTCGCACCACACGGCAGAGTGGCTCTGATACCAAATTGTCACGCCCCGGACCGGCTCTACCGTAGCACGATATTGTCCGCTTTGGGCCTAGGCCCGCACGGTTTTGTTTTTGGGAGCTCACGGAACAACTTCCCAGGGTGGTCACCCATCCTGGGATTGCTCCAGCCTCCCACTCGCTTAACTTCGGAGTTCTTACAACTCCGAAGCCAGTGAGCTCCCAAAAGGCCTCGCGCTAGGTAGATGCGAGTGTGCACATATAAGGCACATCGCCCCCTCTCCGTTTGGTCGATGTGGGATCTCACATAGAGTCATTTAGAACAAAGGTAAACAACTGAGTTTTCTCCAGGATGAAGTTAAAACCAAGAGAATTGAGCAACAATTAAGTCATAAGTCCTTACAATTACAAATTCAGCAGTTTGAAGAAAAGCTAAAACAAGAAGTTTGTTCTGAGTTACCCAACGCCTTCTGGCATAGGAAACAACATTTTGTCAGCCTTCCCTATGTCAAATCTTTTAGTGAGAAAAATATCCCCACTAAAGTAAGACCCATTCAAATGAGCCAAGAACTAATGGAATTTTGTAAAACTGAGATTACCGATTTGCTTAAAAAAGGAATAATTCGTAAGAGCAAGTCACCCTGGTCTTGTCCTGCTTTCCATGTCCAAAAAAATGCTGAATTAGAATGCGGAGTCCCGAGACTGGTCATTAATTGCAAGCCACTTAATATTGTCTTAGAATAGATTAGATATCAAATCCCTAACAAAAGAGATTTAATTAATCGATTAGGAAAATCTGTCATCTTTTCTAAGTTTGATATGAAGAGTGGATTTTGGCAAATACAAATTTCTGAGTCTGATAAATACAAAACTGCTTTCGTTACCCCTTTCGGTCATTATGAGTGGAATGTTATGCCTTTTGGCCTTAAGAATGCGCCCAGTGAATTCCAAAATATAATGAATGAGATTTTCAATCCTTTCAGCCATTTTTCCATAGTATAAATTGATGATGTGCTTATCTTTTCTGAGTCTATAGAACAACACTGGAAACATCTACATAAATTTTTAGAAATGGTTAAACAAAGTGGATTAGTTGTCTCTGCTAAGAAAATTAAGTTATTTCAAACTAATGTGAGATTTCTTGGGTTTAACATTTGCTAGTCACAGATTAGCCCCATAGATCGAGTTGTTCAGTTTGCTGATAAGTTTGCTGATCAAATCCTAGACAAAAGCCAACTCCAACGATTTTTAGGATCTCTAAATTACGTTTCTGACTTCTACCAAAATCTGAGAAAACAATGTAAGCCTTTATTTGATCGTCTCCAGAACAACCACCCTCCTCCCCCCATGGTCACCTATTCATACTGATATAGTCAAACAGATCAAAACTCTTCCCTGTCTTGGTATCCTTTCTGCTGATTCATTCAAAATTGTTGAAACTGATGCTTCTAATATTGGTTATGGAGGTATCCTTAAACAAAGAGTCAATACTAGTCAATCTGAGCAAATTATTCAATTCCATTCTGGTACTTGGTCACAGTCTCAAAATAATTTAGTACTATTAAGAAGGAGATATTGTCTATTGTACTATGCATTAATAAATTTCAGTCCGATTTATTAAATCAAAATTTTTTGGTCAGAGTTGATTGTAAATCTGCTAAGCATGTATTAGAAAAAAATGTTCAAAATATTGCATCCAAACAAATATTTGCATGTTGACAAGCTATTTTGAGTATTTTTGATTTTGATATTGAATATTTGAGAGGAAGTGAGAATTCTATTCCTGATTTTCTTACTAGAGAATTCCTGCAGGGAAAACAATGGCAGAAAGAAGAAAAGAGAAAGACAAACAAAAGAGTCAAGTTTGTCAATCTAGTCAACCCCAGTCACTCCGAGTCTTCCCGCCATCAATGAGTCAAGCTAAACATGAGTCTCCTTCCCAGATCGTCCCCTTCCTGGGATGCTCTCCTATACGAATTAGTAACAGATTTTCCAACTTAGGAGCCACAGTCGGCCAGGGTCGCCCTAGTTTTCAGTCTGCTTTAATTTCTAGTCATGATCCTTTCCAGAATATTGCCCTAACTGTCCCTCCAGTTGCCTCTTACCATAAGACGTCTTCATACATGATCAAGAGTAATAGTCACCTGTTCATTATTGAGTCCAAATACAATTCCATTGCCAACCCAATCACCATTGCCAAATCTTATTGTCCAAAAAATAACCATTTCATTCCTCCAACACCATTTAAAAATCTGAAATACTATAGAGACATCTCCATGAGACCCATTCCATCACCATTAAGCCAATCAAAGACAGAGATAATCCCCACATCATCCTCTATCATTCCCTATACATCCATCAAATAATCAATGAGAATGAGTGGAGCGGCCATCCCTATGACCTCAAAGTTCTCCAATCAGAACTTCAATATAGTTACTATGATTATATCGAAGCATTGTATTCTATCTTGCTTCACCAAACAGTTGATTTCAGTCATTCCTGGTTCTTAAATTTTGACAGGAAATTCAAGAGTCCTTTTCCTTACTGGATTAACCACTGGTGGGAAAAACATGGTCCAGTCATTGATTTATTTCCTCCTCCAATGCAAGACTTAGTTAATTATTTTACCAACAAAAATAAGTTCAAAGAATCTGAGTTGTTTTTCCCAAACCTTCTCCATTTTGTTGCCAAGTACAAAGTCCCTTGGATCCTCAAATGGTCTTACGAGGTTAATTGGGAAACCAGAGTCCTATCTCGCCAATTTTCAATTAAATGGTGGGATAAATTTAAACTTGAGCGTATTGCAGATTATATTTATGTTGACTTTCCTCCGGTCAGGAATCCACAACTTGAGAAAAAGACATCTCTTGCTACTTCACATTCTTCTCTCCCAATCGAGGGAAAATCCAAGTCTGAGTTACAAGAAATTGCTCGCCAGCTGATGCTTCAAGCCTCATAAATGAATGATGACGCAGACAGTGATGCCTCCCCAAAGTCGCAGACATCTACTAGTCAGCCAAGTCAGCCAAAATCATCCAATCATAAGGCTCCAGTCCCAAAAATGCGGTGGAGTGATTATCCTGATGATCCTATTCCTGAGCAATTCCCAATTCATGGTCAAGACCCCTATGATTTTGGTCCTTTCAACCTAGATGATGATTAAAGCTGTACAACATCATGAGACCTGAGACAAAGAATCAAGACCCGTGCTTTCAACAGTCTGAAAACTCGTGATCCTTTCCCCTGACAAAGTAAAAGTTGCAGCACCCAGTAAAAGCAAAAACCTTTCGCGTGCTCCTACAGTTACTTTTCACAAGCATCCAAAGTTGCCAACTCATCATTAGTTATCTGAGTCCTCTTTATTTGCTTATTTAGGAAGGCCCCGGCCTCAGTTATCATCAGAATGTAATTTAGTACACTATAATTTTCTCTGTTTCAAAATTCTATCAGTACCCATGCTCTGAAGAATTAAGTAGTGAAACTAGGATCGTTGTAAGTGTTTTGCATTCTGCAAGTTATCAATAAATCTTGAGATATTTTGAGTATTTTGATTAATGCTTATTTTCATTGCATCTATTTTCATA

TACTCATTAATTTGCTTCATACATAAGTTGCGTCTATTCTATAATCTCTGTGCTTATACCTACTTCTGTACTTCTCCCATCTCTCACTGGTGCTAGACCCTTTATATATATATATATATATATATAGGACATTAAAACTTAATAAAACGAAAATTACAAAATACTTACCTCTCTACTAAGATTTTCCCCGCTTCTATAGTTGTCCCTCACTTTTGCCAAGGCATCTCTCCATTTTCCCAATTCTTTGTTGAGAATTTTCCATCTACTAGATAATGCCATTTCCTTAAGAGTCGAGATTGGAGCCAAGCCATTTCGGCATGAATTTTTTTCAACATATGACAAAATTTAATCTCATTGCCTGACATGGAACAATGACTAACTTGGAGCCAAGCCTCAGACAAGAAAACATCTTCCATGGTGCTCCAATCCCCTCCAGTTTCGATAGAAGAAGCCAGAAAGATAAGAAATAAAAATACAATTTGAAAGAGAGAAAAACGTTGAGTAAAGAGTGAATAATAGTAGAAGTAGAAGAAAATGTATGAGGATTGGTGTTGAAAGTGAAGGGTATTGATAGGTATTTATAGAAAAAAAAAAAAAGGAATTTTTTTAAAAAAATTTCGATTTTTTTTTCTTCATAATTTTATTGCCAAAATAATGCCAGCCGTTGGATTGGAGGAGGAGAAGTAGATCGGAGCGCCTGGGCCTTGACACCTGTCTTCTACCCGTTAGAGATTGCGCTGGGCTGACGTTAGCGCACGCGAGTTCAATTTTTTTTTACCGTTGGCTCGTGCATTGCACGTGCCAATGGTAAAAAAAATTCAAACTGCGAGATGACGTCGCGCCTGACGTCATCCCCCCTTGGGCTTCAGCTCGGGCTAAACTAGCCTTCGGGCCAGTCCGTCTTCGTGGGTCCCACTCCCAGTCCAGGCAAGACTGGACCACTAGAAGCTCGTTTTTCGTGCAGCCCACCCCTAGCCTCGTGCTTGGGCTGAACGGTGGA

>Chr3-11; OZ022208.1; 11992835-11994046; rev; B;

AAACCTTTACAATAAGATGACACTTTTTTAATTTGGTGCAGCCAGCTACAACTAATGTGAGACAACCAGTTGAAAACTATAGCTTTAAAACTATAGTGAACAGGATGAATAATAAAATTCACAAGAAAGAACCTACTCTCAATGATCTTCAGTGCGAAGTTCATGATGTGAAAGAAGAACTTAGAGAGTTAAAAAAAATAAGATTAGAATTCTAGAACTCAATCGCCCATTTTCGGAAAAAGATACAAAGGAAAAATCGGAGCAATTAGAATTTGAAAATCTAATGGAAGAATTTCAAGAGAAAATAGAATCTGTGAAAGAATCCCTACAAGAAGAACAGATTAGTACCATGAAATTCGTCAATAGCATAGATAGAGTGATAGCTCAGAAATGGTATACGATGATAATTGTTGTAGTTGATAGAACCTATAAATTTACAGTTGAAGGGCTAATAGATATAGGGGCCGATCTTAACTGTATAAATGAGGGAGTGGTACCCACTAGGTATTTCAAGAAGACTACCCAGGTTCTACATACAGTCATTAATAGCCAAAAAATGGGAATTCAATTTAAATTATCTAACGCTACTATGCTCAATCAGGGTATTTGTTTTGAAACTCCTTTTGTTCTGGTGAAAGGTTTAAACCAGTCTCTTATATTAGAAACACCATTCATTAATATGCTTTACCCTTTTTCTGTCTCTGAAGAAGGAATTAGAACTGAGGTAGAAGGTCAACCTATAATCTTTAAGTTTTCTCAACCACCAAAGCATAAGTATTTTAATGAAATCCAAGAAAAAATAATAAGGAAGGAAAACTATCTATACCATTTGAAAGAATAAATGCAGCATAAGAGAATAGAAGAGCAGCTGCAATATAGCAGGATAAAAGAAAAAATTGATGAAATCCAAAAGATGTTCGAAAGCCAAGTTTGTGCTGAAATACCCAATCCCTTTTGGAACAAAAAGAGGCACATAATTGAATTACCCTATGAGCCTGAT

TTTGATGAAAAGAAAATCCCAACCAAAGCCAGGCCAATTCAAATGAACGCTAGGCTAATGGAAATTTGTAAATCTGAAATACAAGATTTATTAAATAAGAAGTTAATCAGGAAAAGCTCATCTCCTTGGAGCTGTTATGCTTTTTATGTTGAAAATGCAGCTGAATTAGAAAGAGGAGTACCCATATTGGTGATAAATTACAAACCTCTAAAGAAAGCCTTAAAATGGGTTAGATATCCAATACCTAATAAGAAAGATCTTTTGGACCGACTAAATAAGGCAGTTATCTCTTCTAAATTTGACATGAAATCGGGATATTGGCAAATCCAGATCACCGAAAACGACAGGTACAAAACAGCTTTTAATGTCTCTTTTGGACATTACGAATGGAACGTTCTACCTTTTGGATTGAAAAATGCCCCTTCTGAATTTCAAAAGATTATGAATGATATATTTAATCCTTACACTGCTTTCACCATTGTTTATATTGATGATGTTTTAGTCTATTCTAATTCTTTGGAACATCACTTTAAACATTTGAATATTTTCTTGAAAGTTGTGAAACATGCAGGATTAGTGGTATTGGCAAAGAAAATGGAACTATTTTCCTCAAAAGTTCACTTTTTGAGACATAATATTAATAAGGGACTAATAGTTCCGATTGATAGAGCAATATCCTTTGCTGATAAGTTCCCTGATAAAATTAGGGACAAGCAACAGTTACAAAGATTCTTAGGAAGTCTTAGTTACATAAAGGATTTTTATAAAGATTTAGCTTAGGACTCAAAATTATTATATCAAAGGTTGAAAAAGAACTTGGTTTCTTGGTCTGATGAACACACTAAGGCTGTTAGGAGATTGACAGTTAAAGTTAAAGAACTTCCTTGGTTAGCTTTAGCCAACCTTGAAGCCTTTAAAATCGTAGAAACCGATGATTCAGAAATAGGGCATGGGGGAATCTTAAAACAGAGGGTTGATAACTCGGAACAATTAGTTAGGTTCACCTCTGGAGTTTGGAAACCAGCTCAAAAGAATTATTCCACTGTTAAGAAAGAAATGTTAGCCATTGTCCATGCTGTCTCTAAATTTGAAAGTGATCTTTTAAACACGAAATTTTTGATAAGAGTAGATTGCAAGGCGGCCAAAGACATTTTGTTCAAGGATGTTAAAAACCTAGCATCTAAAGAATTTTTTGCAAGATGGCAAGGAATT

CTTTCTGCTTTCGATTTTGATATTGAATTTATTAAAGGTGAAACTAACACACTCCCGGACTTTTTAACTCGAGAATTTTTGCAAGGTGCGAACCAGAAGTTCCAAACAGATGACTCCAAGTAAGGGCTCTTCTGCTCTTACCAAGTCATCCCCTAATTATCCAAAGCCACTGATGTCTTCTTCTCCATTTAATAAGGGTAAGGAGAAGCAGATTGCTGAAAATCCATTCCGTGAATATATGAATATGTCTGTAGCAATCATTGAAATGAATGCTCTAGATAAACACCCAAATTTTCAGGCTGAAAAATATTTAAGGGATAATCTTCCTCCCTTGAATAAGCCTCATATTTTCTATGAAACTCTTTTAATTGAAACTAAGTATGTTAATATCGTTCATCGTCATAGAGGTGAAAATGCCAACACCATAGCATTCTCCAAAATCCAAATCCTAAATGTCATTTTCTTGGAAGAATGGGCCTCTAGCCTTGAAAGGGCCAAAAGATTTATCCATCCTGAAATTAGGCCATATTCCTATAATTTCTGGGATTATAAAAAGACATGGGAATATGTTTTATGTTTCCAAAATCAAAATTATTCTCATACTTGGTTTATCCAATTTAAGCTTAGAGCTCTGAAAGAACTCCCTAATTAATTGGTTCAAGAGCTTCTTTCTGATTTGGGGTCCTACTTTGGAATACCTTCCTCCTGAAATTAAGGAATCTTTTCAAATGAAATTTTGCAAATGGAATAAAAACCATCATGATCAAAGGACCTTATTTATGTGGTTCATGATAATGTATGAACTACCATGGATATTAAAATGCGAATATGTTATTTCGGAGGATGTTGATGAGGAATGGACTTATATTGCAAATTTAGTCCGAAGTATCTTAATCAGATGGTGGGATAAATTCACTAGGGATCACCTGGCTCCCACCAAGATCGTCCTAGCTCTTATCCCATCCCCCGACTCTGCTGCTGCTCAGCCTTTGGTCACTTC

>Chr3-12; OZ022208.1; 12589046-12593097; dir; D;

CCAATTGCTATGAAATCAACAAGAATTCTATAGTGAGGGCATTCTGTATGGCCTTTAGGTGAGGCTCTATTTCTTAATCATTGGATTAAATTGGTTAGCCTAATCCAACGGTTAGGAAATATGACCTCACCTAAAGGCCACATATAAGGAGAAAAACTTCTCGGGCATGATGGTGCCAGGGTAAGGATTCCAACTAAGTATCCCCAACCAATCAGTTATCAATAGTTGTCACCACACTTTGAAAGAATTATTTTTTGGCCACATCTGCTCATCCTCTGCTAGTCCATCATTGCAACTCAAAACCCCTCTTCATTAATTTTGTAGGACACAATTAGATATTGAAACGATTTCCGATTTGTCTAATTTTTCAATAGTTCAATTGAGTGGTCAAATGACATCAGATTCAAGTGATTTTTTTGTAAAGATGATTTTTGAATGAGTATCTACAAAATAGACAGTTTGGATTAGTAATACGGAGTGGGCCCTACAAGGGTGTCCCTCAAATAAGCTTATTTGAGGGATCACTCAATTGAAGCTCCCTGTATATATAGTACCTTCTTGAAGCATGGTGTTCATTGATTTTGTAGGACACAATTCTTTATCAAAATAGTTTTTGATTTGTCTAATTTTTTGCAAGGAGGATCTATGAATGTAGACTTAATAAATAGATGGTTTGAATCGTTGAAAAAAAAATTTGTAGGGAACCCCAAAGGGTGTCCTTCAATAACATTATTTGAGAGATCCCTCAACAAAAGGGGAATACATATATATATTTATTTTATTCTTAACAAACAAAACGGGTCATTTCTTGTGATTCGCGAATTGTAATAGGTCGTGTTCATATCTGGTACTTTTCATCCATTACAAACATGACCTGTTTGCTAGGTCTACTTAAACCCTATAATCATTTCATAGGTCATGTTCAATTATTGGAATACTCTGGGGATATTCCAATCTTTATAATCATTTCATAGGTACACCTAGTCAATACCCTATTATA

CCATCTAAATATTGTTGAATATAATCCAGATTGCTTAAATTATGCTGAATAGGAGCAGGTTAATTTTCTGTTGCTAGTGTCCAATCTCGGGGTACAGTTACTTCTAACCACTTAATTGCATGGGGGACTCTTATGTTGGCATCGGCTTTATTGCTTTGGATAAGAAAGGTTTGGTCTTTTGGACTTTTATTTAGAGCTTGGAAATTCATATTAGTTCCTATGACTTTATAATAAATTCTATAGACTAAAGCTAGAGGTTGGGTACCTGGTAAGACTTTATACCCTGAAGTTTTAATGTTTAATGTTAGAGTTTTTAATATGTGGGGATCACTAAGGCTTACTGTGAAGTCTGTTTAACAGTCAAAGTGAATTGGACCATTGAATAAGCTTGACTCTATTATTCCTATGGTACTATCACTAAAGTTGGTAAATCTGGTGTCACGTATGCAAAGTAAAATAGAGGCATTTAGTCCTAGCCTGGTAAGGGATTTTACTGCAACTTGAACAAGGCCAATGTGGAGGAAATTGTTTCCATCATTCTTATGTTTCTTTATTGACTCAAGGTTTAGTAATTGACATGTTTCGTGTTCTTTACTCAAGGCATAGACTTGTTCGACTGTTTTCACATGGGTTGTACTTTTGAAACTTGAAAGTGACCATTTTTTCTTGTAAATCTTTGTACTAGGAACTCTGGGGATGTTCCAATCGATTTCATTATTAGTTTCGTATTCGATTTGTTCTTCATTTACTATTTCTGGGAGTTTATTAATACTGGCTCTAGAACTGCTACTGTTACAAGAGTTTGTTCTAAAAAGGCCACTCATGTAAGCAAGCTTACGGGTTCTTCACTTCTAGGTGAGAAGAAAACCTGAAATTCTACTGTAGACTCAAACTTGTAGACTCTCTTTCTCTCCGCTCATGCCGCATCCGCGCATGCCTCACATGGATCTTATTGTTTGAAATCTAGGACTTATTGTTTGGGTTGGAAGAGAGTGACAGACAAACAAGTAAACGTTGAAATAACATTATGAACTTACGGATGAACTTCTAACTCAGAGGGAAAGCTACCAGCCACTTGGGTGGAATGAGAAGCAAGTTTAGAATGACTCTTCAAAGCTTACGGCTCTGATACCAGGAGGTGTCTAGGGCCAGAGAGAATGACAATATTGGAAATCAAGAAATTAAATTAAAATAAATGCATGCAATAAATTTAATCAATGCAATAAAACAAACTTACTGAAAATCTTGATTGAAAGATTTTATTGATAGCTTGTAGTAGCACACAGAGTAGCACTCATATTTTACAAAGAGAGAATTTTCTTTGAGGAAGTCTCAGAGAAAATTTTGAAAACTAAGGAAACTAGTCAGCCCCACAAGCCCTCAGTACAGATACAACAACTCCCAACACCTAGTCAGTCCTTTAAAGATACTCCTGGTAGACATTCCTTTGCAGCTTCCTCCTCTACTCTAGAAAGAGATTTAGAATTAGAAAAAACCCTATTCAACTTGAAGCTAGAATCACTTAGGAAAAGCTCCCAGGTTAGTCACCCATGTTACAATAAAAAACTAATCGAAGAAAATGAAGGACCAAACTCTCCTACGCAAACAGATTTCGAAGTCGAAGCCATCCCAGTAGATAACCAATTAAGAACCCTAAATAAGAAATTTAAAATACATTGGAAAACTCTTGACAAACATCTCGAGTTAGAAGAAAATATTGAAAGACGCAATAGATATAGAACAACCGATCCCAGTAAAGAACAAAGACAACAAATTTTTAATGAATGGAAAACTTGCATGTTGCAAACTAAGTCAGAAATATACTTTCCTGATTTTGTTGAAAATATTTATCAGCAAAGGAAAGAAATAAAGACTTTAACGCAAGAAAAGTGAAAAAAAGAAGACAACAAGTCATAGCTAGTCATCCACTAGTAGAAACAATTATTATTAATCATAAAAATGCTAAAATCACTGCCAGTCCCTTTAAAATCCCTGAACAAAATGATAAAAAATTGATTGAGCAAAATAATTATACAAATCAAAGTCTAATAATTTTAGGAAACAACTAGATAAGATAGAAACAAAATTTGATAAAATTCTTACCCAAGAAGTGAACCTCAAACCTAAGAAAGAAAAACCTTTAGTCAAATTCCAAGACCTAAAACCTGGTCAAACACTTAAGACAATTCCCACAATGAAGAAAATATAAGAAATGCTTGAACAATTGATATCAGTCAAAGAAAAACCCACTAAACCAGAGTCATCCCCAGATATACAGTTTGAAGAAAGAAATTTTCAAAGCCAATTCTCAATCTCCTCTGATAAACTTTATGAGTGGACTATAGATGGCATGTCTGAACAAGAAATTATGAATAAATTGCAGCACATGTATGTGGTCGCCAATAGTTACATCACTAATCACAACCTTAGACAGTCAGAAATAATGCCCCTCTTAGAAACAGGATTTACAGGAACTTTATGTTCTTGGTGGGATAAACATCTCACAGAAGAGTCAAAACGACAAATAATACATGCAGTAAAACTAAATGAAGATGGATTCCCTATATTTGATGAAAATGTCATTACGGGAATAGAAGAAGGAGTCAATACCTTATTATACACAATAATTGAACATTTCATAGGAACACATAGTAACACTACAGCCAGAATTCATGATCAACTTAGCAACCTTTGCTGTCCCAAGTTAAGTGATTTCAGATGGTACAAAGATGTCTTTATATCTAGAGTCATGCTAAGAGATGATAGTAATCAACCATTTTGGAAAGAAAAATTTATTAACGGTCTTCCCAATGTTTTCGCACACAAAATATGAACAGTTCTAAGTAATGATCAAGGTCACATAGATTTCGATAGTTTAACTTATGGAAATATTATTAGTACAATTAACCAAGTAGGAATGAAAATGTGTGTTGACATGAAAATAGGTATAAAAAAAATTAGTCATAAAGAAATTAAGCTAAATATGAATTAGGAGATTTCTGCGGACAATATGGTTTAGCATCCATTCCTCTATCTAGAAAAGTCAAGCATA

AGAATTATGACCAAAACAGTCGAACTAGATGACAGGACCAGTCCCAAATTCCTCAGAACCCGGGACGAATTCTGTGGAATTCCCGACATCCCACAGATGCTGGGCCCACTTTAGAAATTATATCATATTTTCCAAAATTAAGGAAAAAGGGTACGAGACTTTCTGCCGAAATTTTAGTAGAGTCTCCCCTGTAAATCGAATTTTCCCCAAAAAAATTCAACCTATTCAAAATTCACAATTAATATCAAGAACTGGTAGCATGCACAATATAATAATACGCAATTCAAATAAACGGCCTCCGGCCTTCAAATAATCCGAAACCAAACCACCAACAAAACTCTTAAGTCAAATACTACTCCAAACAATGCAAATAACTAATTCAAACAATTTTATGAATGCCCACTGTAATAAACATAAAATGACTTCCCAATTTCCATCATGTGATGTTTAATATTCTAACACAAACACAAGTAGTTTAAGGCCTCAGTCCAAACCATACAACAATCCACATGGGTTTTACTCCAGTTTTGACCATAACACATTTACACAACCCTCTCTTAGCCAAACGGGAGTATGAACAATTATCTAATCCCACCTCAGTTTCAACCGAGTTTCAGGACCAACGACAAGGTCAGAACCAAACTCAATAAAACAACACAAGTAAATTTAGTCCGCGACTGCACCTAGGTAAGACCTTAGGCTACCTACGTACCCTTACCGAGGGATCAAGCCACACGTAGTTCTACATCAACCGTCACAGCTCCAATGATATATAATTTTTCTTTTAAGTAACACCAACGAATAACTTTAGATTCCTTAATATATCAATTCTTCCCGCGCAACTATACCCTATCCAAGCCTTATGTTGCCAAACAACACTTCCCAATGTAGGACTCAGACATCCATATGAGTGCAGGGTAATCACAAAGCACAACCTCACGGATCTCAATCCGGAAACAAATCCACATATACCTATGTAATATGCGTGTCCATATATTTA

>Chr3-13; OZ022208.1; 12596217-12604800; dir; D;

TACCGGTCCCGTCCATTACCATGGCAGGTGGCCGGAGTTTTGAAGAGAGAGAGGGAGAGGGCTGTCGTCGGGTAGAGAGAGAGAGAGAGAGAGAGAGAGATTTTATCAAACCCTCTTCGAAATAATTACGATTTTGCCACTGGTGATGTTTTGCTCGTAACTTCTTCGTTACAACTCCGATTCGAGCCCACTACATGTCTACGGACTCATCTCAGTATGATCTGCTTAAAAATACCACTCATTACTCCAAAAACTCTTCGGATAAAAAAATAACCAAAATACCCCTACCCCAAGGGTAAATTTGTAATTTCATATTTAATTAAATTAAATAATTCAAATAGGGAAGTTAATTGGAGTTGGGGTGTTACACCAGACGTTACACTAGAAAACGACAATCCTTCAAAAATGATAATGAATTGTATAGTAAAAACAAGTTTTCCCCAAAGACAAAAAATTGGTCAAAAGTACCCAAAAAAGATAGAAAAGACAAATTTAAAAAGAACACCGACAAAAGAAAAGTTGAGTGCTGGAAATGTAAAAAACATGGTCATTATGCTAATGAATGTAAAGTAAAAGACACAATCAAACAATTAAAAATCACAAACGAAAAAAAAGCCCAATTGATTAAAGTTTTAGAATTAAGAAACTTTGAGTCTAGTGCAATTGAGACCCCAGTAACAAGCTCAGTAGACTATAGTTCCACTGATTCTCAATCAAGTTCCCCAAAAATCCAATTCGGTTGTATTGTTACTAGGTGTACCTATGAAATGTTCAATTATTGTCTATAATAAGGTATTGACTTCATCTTCTATAATCATTTCATAGGTATTTACATGGCACATTACTACATGGCACCTTCTATAATAAGTTAATTACATGACCTGTTTGCTAGGTAAACGTCGAAACTAGTAATGAAATTGATTATAAAGATTGGAATATCCCCAGAGTTCCTCGTACAGAGATTTACAAGAAAAAATGGTCACTTTCAAGTTTCAAAAGT

ACAACCCATGTGAAATCAGTCGAACAAGTCTATGCCTTGAGTAAAGAACACGAAACATGTCAATTACTAAACCTTGAGTCAACAAAGAAACATAAGAATGATGGAAACAATTTCCTCCACATTGGCCTCGTTCAAGTAGCAGTAAAACCCCTTACCATGCTAGGACTAAATGCCTCTATTTTACTTTGCCTATGTGACGCCAGATTTACCAACTTAGTGATAGTACCCTAGGAATAATAGAGTCAAGCTTATTTAATGGTCCAATTCACTTTGACTGTTACACAGACTTCACAGTAAGCCTTAGTGATCCCCACATATTAAAAACTCTAACATTAAACATTAAAACTTCAGGGTATAAAGTCTTACCAGGTACCCAACCTCTAGCTTTAGCCTATAGAATTTATTATAAAGTCACAGGAACTAATATGAATTTCCAAGCTCTAAATAAAAGTCCAAAAGACCAAACCTTGCTTATCCAAAGTAGTAAAGCCAATGCTAACATAAGAGTCCCCCATGCAATTAAGTGGTTAGAAGTAACTCTACCCCTAGATTGGACACTAGCAATAGAAAGTCAACCCACTCATATTCAGCATAATTTGAACAATCTGGATTATATTTAACAAAATTTAGACGGTACAGTAAAAATAAATTTTGGAAATGAGCCCCACAAGTCCTCAGTGCAGATACAACAGCTCTCAACACCTAGTCAATCCTTTAAACATACTCTTGCTAGACATTCCTTCACAGCTTCCTCCTCTACTCTAGAAAGAGATTTAGAATTAGAAAAAACTTTAATTGACTTAAAACTAGAATCACTTAGGAAAAGCTCTCAAGTTAGTCAACCTTGTTGCAACAAAAAATCGATAGAAGAAAATGAAAGACCCAATTCTCCACAATCCCCCACTCAAACAGACTTTGAAGCAGAAGCCATTCCAGTCGACAATTAGCTAAGAACACTTAATAAAAAGTTCAAAATACATTGGAAAATCCTAGACAAACATCTAGAGTCAGAAGAAAATATTGAAAGACGCAATAACTATAGGACAACATACCCAAGTAAAGACCAGAGACAACAAATCGTCAATGAATGGAAGGCTTACATGCTACAAACTAAATTAGAAATATTCTATCTTAATTTTGTTGAAAATGATTATCAACAGAAAAATGAAATAAAAACATTAACACAAGAAAAATGGAAAAGAGAAAACAACACAGAAGTCATAGCTAGCCATCCCCCGATAGAAACAATAATCATTGATCACAAAAATGCTAAAATCATTGCCAGTCCCTTTAAAATCCCTGAATAAAATGATAAAAAAATTGGTTGAGCAAAATAATTATACAAATCAAAGTCTAATAATTTTAGGAAAACAACTAGATAAAATAGAAACAAAATTTGATAAAATTCTTACCCAAGAAGTGAACCCCAAACATAAGAAAGAAAAACCTTTAGTCAAATTCCAAGACCTAAAACCTGGTCAAACACTTAAGACAACCCCCACAATGAAGAAAATAAAAGAAATGCTTGAACAATTGACACCAGTCAAAGAAAAACCCACTAAACCAGAGTCATCCTCAACCCTTAAGGTCATCCAAGTAAACCCAATAGATACAGAGTCAAGCCCTGACACTGATACTGATTTTAGTAATATATCAAAAATAGAAAACGCCTTTAACAATTTAAAACTCATTAGACTAAGGAGTAAAATAAATCCCACTAGTTTAACAAAGAACTGGTATCCTAAGCCAACTCTCCCAGATATCCAGTTTGAAGAAAGAAATTTTCAAAGCCAATTCTCAGTCTCTTCTGATAAACTTTATGAGTGGAATGTAGATGGCCTGTCTAAACAAAAAATTATGAATAAATTGCAGCACATGTCTGTGGTCACCAATAGTTACATCACTAATTACAACCTTAGACAGTCAAAAATAGTGCCCCTCTTAGAAACAGGATTTACAAGAACTTTACATTCTTGGTGGGTTAACATCTCACAAAAGAGTCAAAACGACAAATAATACATGCAATAAAATTAAATGAAGATGGATTCCCTATATTTGATGAAAATGTCCATATGGGAATTGAAGATGGAGTCAATACCTTATTATACACAATAATTGAACATTTCATAGGTACACCTAGTAACACTACAGCCAGAATTCATGATCAGCTTAGCAACCTTAGATGTCCCACTTTAAGTGATTTCAGATGGTACGAAGATGGTTTTATATCTAGAGTCATGCTAAGAGATGACAGTAATCAACCATTTTGAAAGAAAAATTCATTAACGGTCTTCCCAATCTTTTCACACAGAAAATACGAACAGTTCTAAGTAATGATCAGGGTCACATAGATTTCAATAGTTTAACTTATGGAAATATTATTAGTATAATTAACCAAGTAGGAATGAAAATGTGTGTTGACATGAAAATAGGTAAACAAATTCAGTCAGAAAGAAATTCAGCTAAATATGAATTAGGAGACTTTTGTGAACAGTATGGTTTGGCATCTATTCTTCCATCTAGAAAAGTCAAGCATAAGAATTATGACCAAAACAGTCAAACCAAACGTTACACTAGAAAACGACAATCCTTCAAAAATGATAATGAATTTTATAGTAAAAACAAGTTTAACCCGAAGAGAAAAAATTGGTCAAAAGTACCCAAAAAAGATGAAAAAGACAAATTTAAAAAGAACATCGACAAAAGAAAAGTTGATTGCTGGAAATGTAAAAAACATGGTCTTTATGCTAATAAATATAAAGTAAAAGACACAATCAAACAATTAAAAGTCACAGACGAAGAAAGAGCCCAATTGATTAAAGTTTTAGAATTAAGAAACCCTGAGTCTAGTGAAATTGACACCCTAGTAACAAGCTCAGTGGACTATAGTTCTACAGATTCCCAATCAAATTCCCCAAAAATCCAATTCGGTTGTAGAGATAATTGTTGTAATGTCTTAACTAAGTCAATAAAAGTCCTTACAAAAGAAGAAGAGCAAGAAGAATTGTTAATTGATTTAATTAGTAAAGTAGAAAATCCAGAATTAAAATCAGAATATTTAAAGAAATTAAGAAAAGTCGTTGGTCAGGAAGAAACGAGTCAACACATCCCCCCGAAAATAAGTTTAAATACTACCTTAGAAAAATTCAACAAAAGAAAAGAAGTCACTTTACAAGATTTACAACTTGACGTAAAAACCATCAAAAAGGAAATCGCTGAGTTAAAACAGTTCAACCGACAATTACAAAGTGATAATGTTGAAATCAAACAGGACCTCATTAATCTACTAGAAAAAATAGAACCTAACAACAACGGTAAAAGACAGTTTGAAGACCAATTTGGAGATGAAGATGAACATTCAAGTACCAACAAAGCTATTAATTTAATCAAACAATTAAATTTCCATAAATGGTATTCAAAAGTCAGTATTTATATCAACAATTTTGAACTAAAAACAATAGCTCTCTTTGACTCAGGAGCCGACCTTAATCATTCAAGAGGGATTAATTCCCACTAAATATTACAGGAAATCAAAAGAATCTTTAGGAACAGCCTCTGGTAAACCACTACACCTTAAATATGAAATCCCTAAAGCCTATTTATGCCAAAATAAAATATGCTTTAAGACATCCTTTGTCCTAGTCAAAGATATAACTGATGAAGTCATTTTAGGATTACCATTTATTGCCCTACTCTACCCTTTTAAAGTAGAATATGACGGAGTCACCTCTATCTAAATGGGAGAAAAGGTTAAATTTGAATTCTTAGCCAAGCCAGAATTACAAGCGTTAAAAGCCCTAAAAAAAAAAATGCAATTTCCAAATCAGTCAAATTAATTCAAATTAAAAGCAAGCAAGTCAACTTCCTTAAAGAAGAAATCAACTTTAATAGAATTGAACAACAGCTTGCAAATGAAAACTTGCAACTACAAGTCAAAAGTTTTGAAGAAAAGTTAAAACAAGAAATTTGTTCTGACCTACCTACTGCCTTTTGGCATAGAAAACAACATGTCGTGAGTCTTCCTTACGTCAAAACCTTTAATGAGAAACACATTCCTACTAAATCAAGACCAAACCAAATGAGTCAAGAAGTCATGGAATTTTGCAAAAAGGAAATGGTAGAGCTACTTTACAAAGGAATAGTACGTAAAAGCAAGTCACCATGGTCCTGCCCAGCCTTTTATATTCAAAAAAATGCTGAGCTAGAAGAGGAGCACCCAGATTAGTCATTAATTTTAAACCCCTTAATACCGTTTTAGAATGGATTAGATATCCAATTCATAATAAAAGAGATTTAATTAATAGACTAAGTAGTGCGGTCATTTTTTCCAAATTTCACATGAAAAGTGGATTTTGGCAGATACAAATCCAAGAAAGCGATAGGTACAAAACTGCTTTTGTCACCCCCTTTGGTCATTATGAATGGAATGTCATGCCTTTTGGCGTAAAAAATTCCCCTATTGAATTTCAGAATATAATGAACGAGATTTTTAATCCTTTTAGTGAATTTTCCATAGTCTATATTGATGATATTCTTATCTTCTCTTCCTCAGTAGAACAACATTGGAATCACTTACACAAATTCCTACAAATAGTCAAGAAAAATGGTCTAGTACTTTCGGCCAAGAAAATTAAGTTATTTCAAACCAACATCAGATTTCTTGGATTCAATATTCATCAGTCACAGATCAGTCCAATTAATAGAGTCATCCAATTTGCTGAGAAATTCCCTGATGAAATTCTTGATAAGAGTCAACTCCAAAGATTCTTAGGTTCTCTCAATTACGTTGCTTATTTCTATCATAATCTAAGAAAACAATGTAAACCATGTCAAGACTTTGCCTTGTCTTAGTATTCCTTCAGTCGATTCTTTCAAAATTGTCGAAACGGATGCCTCTAAAATTGGTTATGAAGGTATCCTCAAACAAAGAGTCTCTTCCAATCAAGCAAAGCAAATAGTTAGATTCCATTCTGGAGGCTAGACCCAGTCACAAAGTAATTGTAGTACTATTAAAAAAGAAATTCTAGCTATTGTATTATGCATTGTGAAATTTCAAGATGATTTACTTAATCAAATTTTTTAATAAGGGTTGACTGTAAAAGTGCCAAACATGTTTTAGAAAAAGATGTTCAAAATATTGCATATAAGCAAATTTTTGCAAGGTGGCAAACTATTCTAAGTATTTTTTATTTTGAAATTGAGTATTTGAGAGGGAGTGAGAATAGTATTCCCGATTTTCTTACCCGGGAATTTCTACAGGGAAACAGATTGCAGGAAAATAACCAAGTAAACAAGAAAGCAAATCAGTCAGAAGCCTCCCAGCTTCTATGAACCCAATCAAATCAGAGCTTTCCCAGATTGTCCCCTCCTGGAACACTCACCAATTCAAATTACCAACAGATTCACTAGTCTAGGATCCACTATTAGTAGTATTCGTCCTAATTACCAGTAAGCTCTAATTTCTAGCTACTATCCTTTTCAAGTCCCTACTCCACCTTCAACCCAGTCAACCATTAGTCACAAAAAATCCTCTCCTTATCACCCCAAAAGTAACACCAACCTCTTTATTATAGAATCATGCTACAATAATCTTTCTAATCCTGCAAAAATTGTCAAAGTCTATTTTCCCCCCAATTTCCATTTCATCCCTCAACACCCATCAAAGTCACTTAAATTTTATAGAGATATTTTACTTGAAACACCAACTCTTGAAATACGGCAAATCAAATATAGAAATAACCCAGAAATCATCCTTTATCACTCCATGTATATTCACAAAATCCTTAGTCAATCTGATTAGGGTGATCGCCCCTACTCTCTCAGAACCCTTCAATCAAAAATCCAGTATAGTTATTACGATTATGTTGAAGCCTGGAATGTTATTTTCCTACATCAAATAGAAGACTTCAGTCACTCTTGGTTTGTCAACTTTGATAGTAAATTTCGAAGCCCTTTCCCATTCTGGTTTTCTTCCTTGTGGGAAAAGCATGGTCCAGTTACTGAAATTCTTCCTCCCGATCTCAAAGACTCAGTCACCTATTTCTCCATTGAATACAAATTCAAAGAAGAAGACACTTTCTTCCCAGTCCTATTAATTTTTGTTTCCATAGTCTATATTGATGATATTCTTATCTTCTCTTCCTCAGTAGAACAACATTGGAATCACTTACACAAATTCCTACAAATAGTCAAGAAAAATGGTCTAGTACTTTCGGCCAAGAAAATTAAGTTATTTCAAACCAACATCAGATTTCTTGGATTCAATATTCATCAGTCACAGATCAGTCCAATTAATAGAGTCATCCAATTTGCTGAGAAATTCCCTGATGAAATTCTTGATAAGAGTCAACTCCAAAGATTCTTAGGTTCTCTCAATTACGTTGCTTATTTCTATCATAATCTAAGAAAACAATGTAAACCATGTCAAGACTTTGCCTTGTCTTAGTATTCCTTCAGTCGATTCTTTCAAAATTGTCGAAACGGATGCCTCTAAAATTGGTTATGAAGGTATCCTCAAACAAAGAGTCTCTTCCAATCAAGCAAAGCAAATAGTTAGATTCCATTCTGGAGGCTAGACCCAGTCACAAAGTAATTGTAGTACTATTAAAAAAGAAATTCTAGCTATTGTATTATGCATTGTGAAATTTCAAGATGATTTACTTAATCAAATTTTTTAATAAGGGTTGACTGTAAAAGTGCCAAACATGTTTTAGAAAAAGATGTTCAAAATATTGCATATAAGCAAATTTTTGCTAGGTGGCAAGCTATTCTAAGTATTTTTTATTTTGAAATTGAGTATTTGAGAGGGAGTGAGAATAGTATTCCCGATTTTCTTACCCGGGAATTTCTACAGGGAAACAAATGGCAGGAAAATAACCAAGTAAACAAGAAAGCAAATCAGTCAGAAGCCTCCCAGCTTCTATGAACCCAGTCAAATCAGAGCTTTCCCAGATTGTCCCCTACCTGGAACACTCACCAATTCAAATTACCAACAGATTCACTAGTCTAGGATCCACTATTAGTAGTATTCGTCCCAATTACCAGTAAGCTCTAATTTCTAGCTACTATCCTTTTCAAGTCCCTACTCCACCTTCAACCCAGTCAACCATTAGTCACAAAAAATCCTCTCCTTATCACCCCAAAAGTAACACCAACCTCTTTATTATAGAATCATGCTACAATAATCTTTCTAATCCTGCAAAAATTGTCAAAGTCTATTTTCCCCCCAATTTCCATTTCATCCCTCAACACCCATCAAAGTCACTTAAATTTTATAGAGATATTTTACTTGAAACACAAACTCTTGAAATACGGCAAATCAAATATAGAAATAACCCAGAAATCATCCTTTATCACTCCATGTATATTCACAAAATCCTTAGTCAATCTAATTAGGGTGATCGCCCTTACTCTCTCAGAACCCTTCAATCAAAAATCCAGTATAGTTATTACGATTATGTTGAAGCCTGGAATGTTATTTTCCTACATCAAATAGAAGACTTCAGTCACTCTTGGTTTGTCAACTTTGATAGTAAATTTCGAAGCCCTTTCCCATTCTGGTTTTCTTCCTTGTGGGAAAAGCATGGTCCAGTTACTGAAATTCTTCCTCCCGATCTCAAAGACTCAGTCACCTATTTCTCCATTGAATACAAATTCAAAGAAGAAGACACTTTCTTCCCAGTCCTATTAATTTTTGTTGCCAAATACAAAGTCCCTTGGATCCTCAAATGGTCCTATCAAGTCAATCGCGAATCCAGAGTTCTTTTCAGACAAATCTCAGTAAAATGGTGGGATAAATTCAAGTCAAAGAGAATTATTAACCAAGTCAACCAAGAATTTCCCTCAATCAAAGTTCCTCAGAAGCCTTCTACTTCAGGAACATCCCATTCATCATTACCAATCGAGGGAATATCAAAATCCGAAATTAAAGAATTGGCAAGACAACTAATGATTCAAGCCTCTCAGATGGATGATGAGGATGTCTCTCCGCGGTCTCCTTCATCTTTGACCTCATCTTCCAGTCAAAAAGAATCAACTTAGAAGTTCAGCAGATTACCCGAATAGTCAAGATCCATATGATCTCAATGAAGACTAGTAAAAGCAAAAGCAAGAAAGCAGAAGTAAAAGACAAGGTAAAAGAAAAGCAGAAGTGAAAGAAAGGAAAAAGCAAAAGTAAAGACAAAATGAAAGCAAGAATAATATTCTGAAGTCCCGTGCTCCAACAATTAAAGTAAATAGCTAAAGCAGAAGTCAGAAGACCGGTGCTCCAACAGTCAAAATTCTCAGCATCTAATGCTACCGACACTTCAGTAGTTTGGATCTTTAAATGGAGGCCTCTTGGCCAGAGTCAAGCAGAATGTAATTTTAGTTTCTTAGTTTCAAAGTTTTCTCTGAATCACCTTCAAAGAAAGCTCTACTTGTAAAATATGAGTGCTACTTTCTATCAATAAAATCTTTTAGTGAATATTTTCTGTAAGTTCTCTCATTTAAGCATTATTTTCATTTACGCATTTTATTTTAATATCATTATTATTACATGGCACCCTTTTGGTTCCTATATATATATATATCAATTTATCTATTT

ATTTTATTCTTAACATATAAAACAGGTCGTTTCGTGTTATTCGTGAATTGAATAGGTCGTGTTCGTATTAGGTATTTTTCATCCATTAACTTAACGGCTCGTATTGTGTCAACCCGTTAATGACACGAATACTACATTACAAACATGACTTGTTTGCTAGGTCTACTTAAACCCTATCGGTCAGAAACAGCAACGTAAAACCCAAACAAGTGTACATAACTCGGCCATTTCTTTCAGTCTATGTTGCACAGATACAGATACAGGTACACCAATACAACAAATCTCCAAAAACTAGGATACAATTACAGATACAACACCAATACGGCAATTAAAATAATATATATTTTAAAAAATAATATAAATAAGTCAATAATGGGCCAAAGAATTATCAAAAAGTCTGGGCATCTGGGTTGGGCTTACTTATAATAACCCAAATTGGTTCTCATGCTTCTTATAGTGGTCTTTTTAGTGAAACAGACCTAGGCTCTAGCCCATTGTGAAACTTACTGTCTCTTTCACTCAAAGTATGCAAGTAAAACGATGCGTATTGTAATTAGGGCACCTAACATCATCTTCTTCACGAGTTAGACTCCTCTTCTTCGCAGAGCAACTGCAACTGGAGACTCGCAGCAGCTGCAAGCGCAACTAAGTTCTTTTCTTCTTCCTTCTTCTTCTTTCTTCTCTACTCCGAAACCAGTAAAAACTAGCCTCTGTCGAATCCAGAAGAAGAAGCTAAGTATATTGCAAAATAAACCCCGCCATATCCTTGACCGTATCGCTCGTGTGTCCATGGCGTACCGAAGGCGTATCGGATGGTCAAGATAACAGAAAAGTTTGCCGTATCCTCAGTATCCGATACGAGCATACCCCTTCTTCCGTCTCAGTTTTTAGAAATGGTGGGGGTGCTTCGCTCTCTCTGGTCCTCCACCCGAAACTCTTTCTCTTCCTCAGCTTCATCCATCTCTCCCAAACCGTGCCTACTGCCCCCACGCGCCTCAGC

>Chr3-14; OZ022208.1; 14490535-14495226; rev; D;

GGGACCCTCTCAGGTCCAACTATGATTTTATTTTAAATATGCATTATTTTCTATGATTGAAAGGTTATACATGAAATGCATTTATTTATATACATATAGATATTTTGGATTAAAGTTTGAGAAATTAATAAGTTGAGATATTAGTAATATTAAATTGAGAATGTGATTTTGATTGGAAAATTTGAAGTTGAAATTTGGGGGATCTGGGAATTTAGTATGATGGGTTATTGAGGGAAGATATATGATTATACTATGTAAGTACCATCCCCTAGTTACTAGGCTTCCCATACGTGGTGTTGGAATTTCCGGTGTGTGAGACACTTCCCATACATGGCGTTGGAATTTCTGGCGTATGGGAAGATTATGTGAATTGCTAGAAATCATCACACGTGGCGTTGGAATTAACGGCTTGTTAGCTATGGAGTTTCGTCCCCGGATTGGACCGCTCATCCCTAGACGCAGGATAGCAACAGAAAATCTTGCGGGCTAGCCAAACAATCCCCCTGTTGGATTGTTTCCCCGATACCTAAGTAGTGTGATAAATACTATGTGTGTGTGTGTATATATATATATATATATAGTCCCCTTCTATTGAGGGACACCCTTTAGGGTTTCCTACGAAATTTTTTTCAACGATCGAAATCATCTATTTTTTAAGTATACATTCATAGATCATCCTTGCAAAAAATTAGACAAATCGGAAACCGTTTCGACATCCAATTATGTCCTACAAAATCAATGAACACAGTGCTTCAAGAAAGTACTAAAATTTCAATAACACAATTGAGTGGTCAAACAATATCGGATTCAAGTAATTTTTTGTAGAGATGATCTATGAATGATTATCTAAAACATAAATGGTTTGGATTAATGAAATACAATTCGAAGTGGACCCTACAAGAGTGTCCCTCAAATAAGCTAATTTGAGGGATACCTCAATAGAAGCTCTCTGTATATATATATATATATATATATATATATATATATATATATATATATA

GTTTGGTTAAAAACTCAAATTTAACTCTTTCACCTAAATGACTAGACGTAACTCCATCATATTCTACTTGAAAAGGGTAAAGCAAAGAGATAAAGAGTAAACCGAGAATAACTTCGTCTGTGATATTTTGAATTAAAACAAAAGACGTTTTAAAGCATATTTTGTTTTGGCATACATGGGCTTTGGGTAATTCATAATTTAATTTTAAGGCTTTACCAGACACTGTTCCTAAAACTTCTCTTGATTTTTTATAAAACTGAGTTGGAACTAGACCTTCTTTAATACAATTTAAATCTGCTCCTGAATCAAATAAAGCAATTGTATTTACAAATTCATTAATGAAAATACTAACTCGGGAGTACCATTTTCTAAAATTGATTTGCTTAATTAATTTAATGACTTTATCGCTCTTGTTATCATTTGAAGACTCAGGTTCTGACTCTGAACTGTGTTTACCAAAGGGGTTACTGGACTCTACTCTAGATTCTAATAATTTAATTAGATCTTGTTTAATTTCATATTCTCAATTTGTAATTTCTGACTAACTTGTTTTAAACTTAAAATTTCTTTTCTGACTGTTTTGACTTCTAACTGAAGATCCTGAAAGGTGACTTCTTTCTTTTTATTGAATCTTTCTAAAATAGTATTAAGACTAATCGTAGGGACTTGGCTAGGAACAAGTATAGAAGACGGAGTTAATGTCCTTTTGTATACGATAATTGAGCACTTCATAGGTACACCCAGTAGCACAACAGGTAGGATTCATGATCAATTAAGTAATTTATGATGCCCTAAATTAAGTGATTTTAGATGGTATAAAGATGTTTTTATTTCCAGAGTCATGTTAAGAGACGATAGTAATCAATCGTTTTGGAAAGAAAAATTCATAAATGGTCTTCCAAATTTATTTGCTCACAAAATTAGAATAGTTTTAAGTAATGAGTCAGGACAAATTAATTATGATGAATTAATGTATGGCAATATAATTAGCACAATTAATCAAGTCGGAATGAAAATGTGTGTTGATTTAAAAATTAGTAAATCAATTCAAGCCGATAGCAAAAATGCTAAATATGAATTAGGTAATTTCTAAAATAGTATTAAGACTAATCGTAGGGACTTGGCTAGGACTGGGTTCTTCTTGACTTATGACCTTTCTAAGCTTTCTTAAGTAATCGGCTTTTAACTATGGATTTTCTATTTTACTGATTAAATCTATTAGCAACTCTTCTTGTTCTTCTTGCTTAATTAAGACACTAATTGATTTATTTAAGACTCTGCAACACTTATCTTTACATCCTATCTGTACCTTTGGTGAACTAGACTGGGAGCTGTGAGGGTCTTGCGTGGCCTTGCTTGCTGTAAGTTTTATTTTCATGTTTTCAAAGTTTTATGAAGTCAATAAAATTATCCAGTTAATTTTCAGATCCTATTTTCGTTCCTAAGCATTATTTTAAATGATTAATTGTATTTTCATTCATGCAATCTGTGAGTTGAGAATCTTATGCATCTACGCTTTACTTAAATTCTATTTTGATTTATTTCATTTCTGTTTTTCTTGCCTTGGTCTTCCCTGAGTCTTGTTCTTCCCTCTGAGTCTTGGATCTCTCTCAATCTTGGTATCAGAGCCGTAAAAGTTACAGTTGTGTCTAGGTCTGCTACTCATATCGCCAAAGTGGCTTATTTAGGTTTTCTGAGTTAGCAAGTTCTTCAGTTCTTCTAAACAGTTATCTTAGAGTTGTCTGTCATCTTCGGTTCACTCTCTCAAAAGAACACGGTAAGACCTATAGTTGAACAGTAAGTCCCGCAGTAGAGGCCTGAGAGGTGCGGCACGAGTGTGAGAGAGAGAATGCCTTAGGTTCCAGGTAACAATGAGTTAACTCTTAGGAGTCCCTGTAGATCTTCTACCTTTTAAAACTTTGAGTAAAACACTTGTAAGAATGAGTCATTTTCTTAGAACCAATTCAGTTAATTCTTCCAGTTCTAGAACTAGTTTGAGTCAAGTTCCAGACATAGTTAATGAAGAACAATTAGAATATGAGTCCACAAGTAGTGAGTTACATTGTAATGGCTGGAATATTCCCAATGTCCCTAGTAAAGAAATTTATAAGCAAAAATGGTCAATGAATGCATTCAAGACAACTACGCATGTTAAAACAGTAGAACAAGTCTATGCCTTAGGAAAAGAACATGAAACATGCCAATTACTTAATCTTGAATCAATTAAGAAGCATAGAAAAGAAGGTAATAATTTTTTACATATAGGATTAGTTCAATTCGCAGTTAAACCCTTAACTAAGTTAGGATTAAAAGCAGCAATTCTTTTAAGTTTAAGAGATGCTTGATTTAACGATTTCCCGGATGGAGTCTTAGGAATCATAGAGTCAAGCTTATTCCATGGTCCAGTTCATTTTGATTGTTACCCAGATTTCACAGTCAGTTTAAGTGATCCCCACATACTAAAGGTTTTAACTTTAAATATTAAAACTCATGGTTATAATGTTTTACTCGGTAGTCAACCTTTAGCTTTTACTTACACAATTTATTACAAAGTCACAGGTACAAATATGAATTTTCAAGCCCTTAAGAAAAGCCCAAAGAATCAAACACTTTTAATTCAAAGTCATACTTCAAATGCGAATGTCACAGTACCTCAAATGATTAGATGGTCTGATGTTAAGTTACCCTAGGATTGGTATTTAACAACTGAATGCCCTCCAAAGCCGATTCAACGTAGTCTGAACGATTTAGATTACATTCAACCTTATTTAGATGGAACGGTTAAAATTAGTTTTGATAGGCCAGTTAAATCCACAGTCCAGTTAGAACAATTGCCAACCCCTAGTCAGTCTTTTAAAATACCTGCTAGACATTCATCCGCAGAATCATCCTCAATTACAGAACGCAATTTAGAAGCCGATAGAGCCCTAATTGATTTCAAAATTAATGAATTAAGAAAGCAAAAACAGGTAGTTCAACCAAGTTACGGAAAAGTCCCAACTGAACCAATTTTGCCGGTCCAACCAGAGTCACCTACACGAACAGATTTTGAAGGTTCCACCTTTGAAGTTTGTGATGCCCAATTAAGAACTCTTAATAAACCATTTAAAATTCATTGGTCCAAATTAGTTGCCCATTTCCAAGCCCCAGAAAATGAATCCAGAAAAGCCAATTATCATGCTTCTTTCCCCAAAGTTCAAAGAAAAGAAATTTTCAATGAATGGAAAGAATACTTAAAAAAAACTAGGTCAGAATCACCTTATCTAGATTACATAGAAAGTAAATACATGAGTCAAAAGTTAGTTACTTTAACTCAGGAAAAATGGGAAAAAGCCGACAACACCAAAGTCATTAGTACACACCCCCCAGTCGAGTCAATAATAATCAATCATAAAAATGCCAAAATCATAGCTAGCCCTTATAAGGTTAATGAGTCAAAAGATGATTTAGATAAAAGAGTCATGGAACAAAATAATTACACCGACACAAGTCTGATAGTCATAGGCAAACAATTAGATAAAATAGAAACTAAAATTGACAAGTTAAACCCCAGTGAAATTACTTTAAAACCAAAGAAAGAAAAACCTTTAGTTAAGTTCAATGGGTTAAAACCCGAGTCTTCCTTCAAAGTAAATTCAACCATGAAAAAGATTGAAGAAACGTTTCAGGAATTAACCCTAGTTAAACCTAAGAAAGACAAAGGAAAAGAAGAAGAGGAAACTTCAGTTAAAGTCTTACAGTGTGAAGTTAATTCTGATAGTGAGTCAGCCATGTCATTAGAATCAATCTCTAGTCAAGAGTCAGAAACTAGTAATATTTCTAAAATTGAACAAGCCTTTAATAATCTACAAATTAATCAGGATTCCCGGAGTCTTAAAGTTAGTCGTCTAACAAGTAAAATCAATCCCACAAGTCTTACCAAAAATTGGTACCCTAAGCCTACGCCTCCAGATGTCCAATATGAAGAAAGAACCCAGGGTCAGTTTTCGGTATCTTCTGATAAGCTATACGAATGGAATATCGATGGCTTGTCCAAACAGGAGATATTCAACAAATTACAACACATGTCCATGGTAGCCAATAGTTATGCCACTAATCATCAATTGAAGCAATCAGAAATAGTTTCCCTTTTACAGACAGGCTTCACAGGAACACTTCGTTTCTGGTGGGATAAACATCTCACAGAAGACGCAAAATTAAGAATAATCCATGCCTATAAAACAAATGATGAAGGTTATCCTATCTTTGATGAACGCATAGGAAGAAGTATAGAAGACGGAGTTAATGTCCTTTTGTATACGATAATTGAGCACTTCATAGGTACACCCAGTAGCACAACGGCTAGGATTCATGATCAACTAAGTAATTTACGATGCCCTAAATTAAGTGATTTTAGATGGTATAAAGATGTTTTTATTTCCAGAGTCATGTTAAGAGATGATAGTAATCAATCGTTTTGGAAAGAAAAATTCATAAACTGTCTTCCGAATTTATTTGCTCACAAAATTAGAACAGTTTTAAGTAATGAGTCAGGACAAAGTAATTTTGATGAATTAACGTATGGCAATATAATTAGCACAATTAATCAAGTCGGAATGAAAATGCGTGTAGATTTAAAAATTAGTAAATCAATTGAAGCATAGAAAAAATGCTAAATATGAATTAGGTAATTTCTGCAAACAGTATGGTCTA

AGCTCCTATACCCCCGTCTAGGCAAGTCAAAAGAAGTAATGATCGACGCTTTAATAAAAGTAGATATTATTCCCGTAGAAAACATTCTTTTAAATCAAATGATGAGTTTTACAGTAAAAAGAAGTTATTCCTACAAATGACTTGAGGCATTAATTTAAAGACTTGGCATCTGGAAGAGGACAAAATAACTTTTGCAAATAAAATTACTTTAACTTGGGCATCTGTTGCTTTAACTCTGGGCATCTGCTTTAACTCTAAGTAACTGCTTTAACTTTGGGTAAACTACTTTAACTCGGCATCTTTTGACTGTAGGCATCTTTGACTTGGGCATCTGTTGCTTTAACTCTGGGCATCTGCTTTTAACTCTGAGTAAACTGCTTTAACTTTGGGTAAACTACTTTAACTCGGCATCTTTTGATTGTAGGCATCTTTGCATCTTTGCTTTCGGCTACTTTGAAGAAAAGACTAATCTGCATTCAAATCAAAGGGATCCTGACTGTCGGGCATAGGATCCTGACTGTCAGGGGCATAGTCGGACCATCTCTGCTTGGGCTTTTGACTAAATGGAAGTTGACTGGGCTGCTGACTGGATGAAGATTCGAATTTTGGGCTGGCATCATCGCTATCATCATCATCATGCATTTGAGTGACTTGGGCTAGGATTTGTTCTGCTAATTCTTTTAACTCTCTTTTTGATTTGCCTGGAGGGAGGTACTGGAACTACTTGGAGAAGTAACTTTGACTGGTGGCATTTGAAGTGGTGGAAAATCTGACTAAACTTGGTGAATAATTATGTCGACTCCGAACTTATCCCACCATTTGACTGCAAAATGAGTATATATATACGTATGAGTATAAATTAGTTAAAGTATATGTAGTGGTTTGGTGGGAGCAATATAAATTTTGTGAAGAAGGTTTTTGGATAGAGTGTTTGGTTTTAAAGGGTTTCGGAGAATGATGTTTTTTATCATTAATTAAAAGTATGAATCTGCATGTCTTG

>Chr3-15; OZ022208.1; 15552944-15558922; dir; B;

ATCCTTTCAACGTGATCTAGTTGATGGTTGGGTTGGCTATGATCGATTTTGAAGTTTTCAAACTCAAATTTGGCCCTTTTTTGCTCGAGTTTCTGGCAAGCTCTGGCCACTTTTTGGCTGTGGCCCATGAACAAAAGTTGCTCCAGTAGTTGTCCTGATCACAAAGATAAAGGTATTGTTTGGTGTTTTGAAAATTTTCCGGTAGCCGATGTCATCCCATTTGGGTGGCCCATTTGGTTCTAAAATGTCCCCAATGTTGCCTCGAGCGAGACGATATGCTCGGATTTGAATTTGGTTATCATTTGATGGTCGATCGGATTTACGCATATTAGGCGTTATCTGGGTTCGATATGTTCGGGCTCTTTGATCGACTCCATTTCCAAATATGTGATCTAGGTACCTCTAGGATCATGTTGGAATTTGCGGATTGAGAATCGGAGTCCCGGATGCTCGGATTCAATGATTCAAAGATTATGCAAAACAGTGACCTTCCGATCATGCAATCGAGAGCAATCCAACCGCCTGATCTGGACCACACTTGCAGGATGTGTTAAGTATATTTTGTCGAACCTGTAAGAACTTTCGGATCATAATCGAATGTCATGGGACTCACAGGCCCGGTTAACCAAGTTTGGGAAATTTGACCACTCGGTTGACTGAGCGTCTTCCGAGGTTAGGAACCTCCCATTCGTCGTACACATACTTTGAATCCTTGGAAAATTAGGAGTTGTTTTTAATGTTCTCGTTTGAAGTATTTTAAGTAATCTACTCCTCGTATTCTTCAATAGGTACTTCGGCTCTTCCTACTGAGAAGGCGGGACCTTCTCAGGGTCAAGGAGATTTAGATTATCTGTGAGTGTACTTTATTTTAAAATAGCATTATTTTCAATGATTAAATTACTATGCATTTAAAATGGGTTACCCTGACTATGGAGTTATTATATATATATATATATAATTAGCACAATTAATCAAGTCGGTATGAGAATGTGTGTTGATA

TGGAAATTGGTCTAGTCCAAGTTGCGGTTAAGCCATTAACCAGATTAGGATTAAAAGCTTCAATCCTTCTCTGTTTGCGTGATGCTAGATTCACTAATTTTAGCGACAGTACGCTAGGAATTATTGAGTCAAGCCTTGCAAATGGTCCAGTTCATTTTGATTGTTATCCTGATTTCACAGTAAGTCTGAGTGATCCCCACATATTAAAAACTCTAACTCTGAACATGAAAACTAATGGCTATGAAGTCTTGCCAGGTACCCAACCCTTAGCATTAGTTTATAGAATCTATTATAAAGTCACAGGAACTAATATGAATTTCCAAGCTCTGAGTAAAAATCCAAAAAATCAAACCATGTTAATCCAGAGTCATACACCTAATGCCACTAATAAGAGTCCCTCATTCCATCAAATCGTCAGATGTGTCCCTTCCAGCTGATTGGACCCTAACCTCTGAGAGTCAGCCAGTTCCTATTCAGCGTAGTCTAAACAATTTAGATTACATCCAACAATATCTTGATGGCACAGTTAAAATTAATTTTGGTAATCAACCTCCACAAAAGTCAACAGTCCAGTTACAACAGTTACCCCACACCTAGTCAATCACAAAGACATATCCCTGCAAGGCATTCCTTGGCAGCTTCTTCTTCCACTCTAGAACTAGACCTAGAGTTAGAATCACTGAGAAAAACTTCCCAAGTTACTCATCCTGTTTATAGAAAACTTCCAGAAGACAACAAATCTGAGTCTCCTAATTCACCCACTCAATTTGATTTTGTTGAAGCTGGTTTCAAAGTCGATAAGCAGCTACGAACACTTAATAAAATTTCTATAATTAACTGGAAACAAATTAGATAGTCACCTACATGCCCCTGAGAACCAAGCCAGAAGAGATAATTACCATTTAGCCATCCCTGACAGAGTCAACAGACAACAAATTTTTAATGAATGGAAAGAATATATGCGCACTGCTAAAGCTGAAATTTATTATCTTGATTTTGTAGAAAGCCTTTATTTTCCTAATGAGTTAAAAATTTAACCCAGGAAAAATGGGAAAAAATAGATCACACTGAGGTCCTTGCTAGTCATCCACCTGTCGAATCTATAGTTATTCAGCACCAGAAGTCAAAAATAATTGCCAGTCCTTTTAAAATCCTTGATTTGGAAAATAAAATGATAATTGAGCAAAATAACTACACCAACTAGAGTTTGATTGTCATAGGTAAACAATTAGATAAGATAGAGACAAAAGTTGACAAATTATTACCAAGAGAAATTGCACCTAAGCCACATGATCAGAAACCTTTAGTTACTTTCCAGGATTTAAAGTCAAGTCCAACTTTTAAGACAAAAGAAACAATAACAAAAATTGAAAAAATGTTAGAAGAATTAACGCCAGTCAAAGGAAAAGATAAAGAAAAAATCGAAACAAGCGTTAAAGTCTTAGGATCCTCTGAGTCACCCTTTGTTTTCCATAACTCCTCTAGGTCTGAATCAATTGCCAGTATGGACATAGAATCAAGTAATATTTCTAAAATTGAAAATGCGTTTAGAAATCTTGAACTAGAAAATAATCCACAAGTACTAAGATTAACAAACAGAGTCAGCCCCACTAGCCTCACAAAAAATTGGTATCCTAAGCCAACATCTCCTGATATCCAATTTGAAGAATGAAATACTCAAAGTCAGTTTTCCGTCTCTTCAGATAAATTATACGAATGGAATATTGATGGCCTTTCTGAGCAAGAAATCCTTAATAAGCTTCAACATATGCCTATGGTCGCCAATAGTTACATTACCAACCACAGTTTTAGACAGTTTGAAATTGTTCTCTTGTTAGAAACTGGTTTCACCAGAACTCTTCGTTCTTGGTGGGATAAACATTTAACCTATGAGTCTAAACAAAAAATAATTCATGTAGTCAAACTTAATGAGGATGGTCTTCCTATTTTCGATGAACAAATAGGTCAAGGCATTGAGGATGGAGTTAACACCTTGCTTTACACAATAGTCGATCATTTCATTGGAACACCCAGTAACACTACTGCTAGGATACATGATCAGTTGAGTAATTTAAGATGCCCTAAGTTATCTGATTTTAGGTGGCATAAAGATGTGTTTATCTCTAGAGTCATGATCAGAGATGATAGTAATCAACCCTTTGGGAAAGAGAAATTTGTTAATGGTCTGCCAATTTATTCGCCCATAAGATCCGATCAACCCTTAGTAATGAACAAGATCATATTGATTGGGATAGTTTAACATATGTAAATATAATTAGCACAATTAATCAAGTCAGTATGAGAATGTGTGTTGATATGAAAATTGGTAAACAAATCCAGTCTGAAAGAAATTCTGCTAAATATTAGTTAGGTAATTTCTGCGAACAGTATGGTCTGATGTCTATTCCCCCATCACAACCGACAGTTTAGTAAACGACGCCATTATTCACGTAGAAGACAATCATTCCAACATAATAATGAAGATAAAGAATTTTATAGCAAAAAGAGATTCTCTCCTAAAAAGAATTGGAAAAAGAGTCACAACAAAGATAAGGATAGTCACTTTAAGAAAACAACTGACAAAAGAAAAGTAAAATGTTATAAATGTCAAAAGTTTGGTCACTATGCTAATAATTGCAAAGTTAAAGACACAATTAAACAATTAAAAATCACGGATGAAGAAAAAGACAAACTCATCAAAGTCCTTGAGTTAAGAAATTCTGAGTCAAGTGAAAATGAAACCAAAGTAAATCAGACTGAGTCTTCTGAGATGTATTCTTCTGAGGCACAACCCAGTTCACCCAGAATCCAATTTGGTTGCCAAGATAATTGTTGTAAGTCTAAAACAATTAATGTCCTCACCAAGCAAGAAGAACAAGAAGAATTATTAATTGATTTAATAAGTAAAGTTGAAAATCCAGAATTAAAATCTGAGTATTTAAAAAAAACTGAGAAAAGTCATTAGTCAAGAAGAGCCAAAACAATTTACCTCCAAGAAAATAAGTCTAAACACAACCCTAGAAAAATTTAATACAAAGAAGGAAGTCACTTTACAGGATTTACGACTAGAGGTAAAGTTAGTCAAAAAAGAAATCATTGAGTTAAGACAAATAAGTCAAAAGTTACAAAATGAAAATTATGAGATAAAACAAGATTTGATTAACTTATTAGAAAATAAGATCACTAAGTCTACACCAGTAAGTCCCTCAAATTCTGATGATGAACCCTCCAATCTGGAGCAAGCCGTCAATTTAATTAAATATGTAAATTTTAGGAAATGGTATGCCCGAGTCACTATATTTGTCAAAAGCTTTGAGTTAAACATAGTTGCCCTCTTTGATTCAGGAGCCGACCTTAATTGTATCCAAGAAGGTCTAATCCTAACTAAGTATTACAAAAAGTCAAGAGAATCGTTAAGCACTGCCTCAGGAAAATCACTCCAATTGAATTATGAGATCTCTAAAGCCCATGTTTGCCAAAATAAAATTTGTTTCAAGATCTCTTTCGTCTTAGTTAAGAACATTACTGATGAAGTCATCCTAGGGTTACCCTTTATTGCCCTTTTATACCCTTTCCAAATTGAACACGATGGTGTCATTTCCACCTGTTTAGGAGAAAAGGTTAAGTTTGAATTCCTGAGTAAACTTGAGTTACATAATTTAAAAGAATTACAGAAAAATGTAGTCACTAAAACAGTCAGAGTCATCCAAAATAAAGATAAGGAACTAAAATTTCTCTGGGAAGAAGTTAAATCTAAAAGAATTTAGCAACAAATTAGTCATGAGTCTTTACAGTCACAAATCCGACAGTTTGAAGAGAAGTTAAAACAAGAAGTCTGTTCTGAGTTACCCAATGCCTTCTGGCATAGGAAACAACATGTAGTCAGCCTTCCTTATGTCAAATCCTTTAGTGAGAAAAATATCCCCACTAAAGCCAGATCAATTCAAATGAGCCAAGAATTAATGGAATTTTGTAAAACTGAGATCACAGAATTGATTGAGAAAAGAATAATCCGTAAGAGTAACTCACCCTGGTCTTGTCCTGCTTTTTATGTCCAAAAAAATGCTGAATTAGAACGTGGAGTCCCGAGACTAGTTATTAATTACAAACCACTTAATATTGTCCTAGAATGGATTAGATATCCAATTCCTAACAAAAGAGATTTAATTAATAGATTAGAAAAATCAGTCATCTTTTCTAAGTTTGATATGAACAGTGGATTTTGGCAAATTCAAATTCATGAGTCTGATCGATACAAAACTGCTTTTGTTACCCCTTTTGGTCATTATGAGTGGAATGTTATGTCCTTTGGTCTTAAGAATGCGCCCAATGAATTTCAAAATATAATGAATGAGATTTTTAATCCTTATAGTCACTTCTCCATTGTCTATATTGATGATATGCTTATCTTTTCTCAATTGATAGAACAGCACTGGAAACATCTGCATAAGTTCTTACAGATAGTCAAACAAAATGGATTAGTTGTTTCTGCTAAGAAAATTAAGCTATTCCAAACTAATGTGAGATTTCTTGGGTTTAACATCTGCCACACTCAGATTAGCCCCATAGATCGAGTTATCTAGTTTGTTGTTAAGTTTCCTAATCAAATCCTAGACAAAAATCAACTCCAGAGATATCTAGGATCTTTAAATTATGTTTCTGATTTCTACAAAAATTTGAGAAAACAATGTAAGCCTTTATTTGATCGCCTCCAGAACAATCCTCCTCCATGGTCATCTATTCATATTGATATAGTCAAACAGATCAAAGCCCACATCAAGACTCTTCCCTACCTTGGTATCCCTTCTACTGATTCATTCAAAATAGTTGAAACTGATGCCTCTAGCATTGGTTATGGAGGTATCCTCAAACAAAGAGTCAATTCTAGTCAATCTGAGCAAATTATAGGATTCTATTCTGGTACTTGGTCACAATCTCAAAATAATTATAGTACTATTGAGAAAGAGATATAATCTATTGTACTATGCATCAATAAATTTCAGTCTGATTTATTAAATCAAATTTTTTTAATCCGAGTTGATTATAAGTTTGCTAAGCATGTATTAGAAAAAGATGTTCAAAATATTGCATCCAAACAAATATTTGCAGTTGGCAAGCTATTTTGAGTATTTTTCATTTTGTTATTGAGTATTTGAGAGGAAGTGAGAATTCTGTCCCTGATTTTCTTACCAGAGAATTTCTGCAGGGAAAACAATGGCAGAACGAAGAAAAGAAAAAGACAAACAAAAGAGTCAAATTGGTCAATCTAGTCAACCCAGTCATCCCCAGTCAGTCCGAGTCCTCCCTTCATCAATGAGTCAAGCTAAACATGAGTCTCCTTCCCAGATCGTCCCCTTTCCGGGATGTTCTCCTATCCAAATTAGTAACAGATTTGCCAACCTAGGAGCCACAGTCAGCAGATTCGCCCCATTTTTCAATTTGCCTTAATTTCTAGTCATGATCCCTTCCAGGATGTTGCTCTGACTAATCCTCCAGTGCCTCTTACCATAAGACGTCTCCATACATGATCAAGAGTAATAGTCTCCTGTTCATAATTGAGTCAAAATATAATTCCATTGCCAACCCAATCACCATTGCCAAATCTTACTTTCCACCAAATAGTCATTTCGTTCCCCAGCACCATTTAAAAATCTAAAGTATTACTGAACATCCTCCATGAGACCCATTCCATCACCCTTAAGCCAATCAAAGACAGAGATAATCCCCACATCATCCTCTATCATTCCCTTTACATCCATCAAATAATCAGTGAGAATGAGTGGAGCAGTCATCCCTATGATCTCAAAGTTCTCCAATCAGAACTTCAATATAGTTACTATGATTATATCGAAGCATGGTATTCTACTTGCTTCACTAAACAGTTGATTTTAGTCATTCATGGTTCCTAAATTTTGATAGCAAATTCAAGAGTTCTTTCCCTTATTGGTTCAATCACTGATGGGAAAAACATGGTCCATT

CATTAACCTCTTCCGCCCTCCAATGCAAGATTTAGTCAATTATTTCACTAATAAAAATAAGTTCAAAGTCCCTTGGATCCTCAAATGGTCATATCAAGTTAATTGGGAATCAAATAGTGGGATAAATTTAAACTTGAACGTATTGCAGATTATATTTATGCTGATTTCCCTCCAGTCAGGAATCCTCAGCCTGAGAAAAAGACATCCCCTGCTACTTCACATTCTTCTCTCCCAATCGAGGGAAAATCCAAGTCCGAATTGCAGGAAATTGCTCATCAGTTAATGTTGTAAGCCTCACAAATGAATGAAGATGATGATAGTGATGCCACTCCCAAATCACAGTCATCTGCCAGTCAACCAAAGTCATCCAGAGCCTCCAAAATGCGGTGGAGTGATTACCCTCCTGGTGGTCAAGATCCCTATGATTTTATTCCTCTTGACCTCGGAGATGATTAAAGTTGCCCAACATCATAAGCCCTGAGAAAAGCATCTGGGCCCGTGCTTCAACAGTCTAATAGCCAGTGTTCCTTTTGTAAGATTCCTCGACAAAGTAAAAGTTGCAGCACTCAGTAAAAGCAAAAGCTTTCGCGTGCTTATACAGTTACTTTCGCATGTTTAAATGTAAAAATATCCACGTCACATTTTTACTGTTTCAAGACACTACTACAAAAAATGAAAAAGACGATTAGAAAACAACGATGGTAGATCAAAAAACCATCATTGTGTTTGAAAAGACGATAGTAATAGAAAATTGTGTTTGAAAAGCAAAAGCTTTTGCATGCTTAATATAGCGAAGTAAAAATTCTAAAAGCAGAACTCAAAGCAGTTCCTTAGCTTCTTCCTTTTGTTTCTCTAATTGTCTTAAAATTCCTCTTGTTCCCTTATTCCTATAAATTTGGAATTCATAGTTCTATCAACTAGAAGTTTTGGATCAGTGGTGTCAAGGTACATGGTGAATCCGGGCAAACAACATTGGACATCAGTAAAGTGGATTCTCAGG

>Chr3-16; OZ022208.1; 16233387-16238909; rev; B;

TTTGCAAATGGTATTAATTATGCTTTGCATTAATTGTTATGTTTCTATTTGCATATGAAACTGTTTGTTTTCAATTTGGGGTTCTTTCACTTTTAGTAGGATTGATTAAGTGCAATAGTTCTTTTCTTTTTTGATTAGTAGATATGGATCAAAGACGAATGTTGTTGATATTATTGTTGGAGATGTGGCATTTAGAGACCACGTGTGCTTGCACGATTCTTGTGGACAATGCTTGGCCAAGCTAAATTTGATGCATCTATCTTTTTGGAAACACCACAAATGATGCATTGATAAGATTGGCTTCATTTTGGAAACACCAAAAATTGTATAACATATTGCTAGCATTAAAGCCAGATAGTTCATAGTTATGGGGCTTATTAATTCATTTTGAAATTGTGAATGTTTATTTGTATGTATTAATGGTAGAATTGTGTGTAGTAAACCTCTAATTTTTGAGTCATGTAGATGACTTTGGCATATATGAAGAATTTCTAACTTTTGCATATATGAAATGTACATGCCAATTTGGTTTAGATGAAAAGAAATGTATATGTCAATTTGGTAATTCTAATAACAAAAAAACTTACATTTAATGATTTAAAAATTATTATTTTTAAATAATTTTTTTTTTTTTTAGTCCCACACAGCACCAAACATAGGTCTTCACTATTTTTACAATCCAGCTTCTTAGTCCGACACAGCACCAAACGTAGGTCAGTACTTAATCCAGCTTAGTCCAATCCGAGCTAATCCAAGACAGTCCCAGAATTTAGTCTAATCCATAACAGTCAGCCGTACCAAACGACACAGGACTGTCTTGACATGGACTATACTTAGGGATTGTCTTGGCTTGGTTTGGTTTGGTCTAAGTTAGATAACACTTATCCTGTGTTTGGTGTATGCTTGGACTAGGACTAAATTTTTTTTATTTTTTCAAAAATATATATGTATATGTATATATGTATTTATATATGTATATATGTATATATATATATATATA

ATTCGCCCATAAGATCCGAACAACCCTTAGTAATGAACAAGGTCATATTGATTGGGATAGTCTCACATACGGAAATATAATTAGCACAATTAATGCAGTTGGTATGAGAATGTGTGTTGATATGAAAATTGGTAAACAAATACAGTCTGAGAGAAATTCTGCTAAATATGAATTAGGTCATTTTTGCGAACAGTATGGTCTAATGTCTATTCCTCTGTCTAGAAAGAGTAAGCCCAGTCACCACAGCCGACATTTTAGCAAACGTCGCCATTATTCACGAAGAAAACAGTCATTCCAACATAATAATGAAGATAAAGAATTTTATAGTTAAAAGAGATTCTCCCCTAAGAAAAATTGGAAAAAGAGTCACGGCAAAAACAATCGCTTTAAGAAAAGTACCGACAAGAGAAAAATAAAATGCTATAAATGTCAAAAGTTTGGTCACTATGCTAATAATTGCAAAGTTAAAAACACAATTAAGCAATTAAAAATCACGGATGAAGATAAAGACAAATTAATTAAAGTCCTTGAGTTAAGAAATTCTGAGTCTAGTGAAAATGAAACCATGATTAGTCATATTGAGTCTTTTGAAATCTATTCTTCTGAGTCACAACCCAGTTCGCCTAGGATTCAATTTGGTTGCCGAGATAGTTGTTGTAAGTCTAAATCAATTAATGTCCTTACCAAACAAGAAGAACAAGAAGAATTATTAATTGATTTAATAAGTAAAGTTAGTCTGAGTATTTAAAGAAATTAAGAAAAGTCATTAGTCAAGAAGAGCCGAGACAGTCAACCTCCTAAAAGATAAGACTGAACACAACCTTAGAAAAATTTAATAAAAAGAAAGAAGTCACTTTACAGGATTTACAACTAGAGGTAAAGCTAGTCTAAAAAGAAATAATTGAGTTAAAACAAATAAGTCAAAAGTTACAAAATGAAAATTATGAGATAAAACAAGATCTGATTAACCTATTAGAAAATAAAATCCCTAAGTCTACACCGGAAAGCCCTACAAATTCGGACGAAGAACCCTCTGATCAGGATCATGTCGTCAACTTAATTAAACACGTAAATTTTAGAAAATGGTATGCCCGAGTCACTATATTTGTCAAAAGCTTTGAGTTAAACATAGTTGCCCTCTTTGATTCAGGAGCCGACCTTAATTGCATCCAAGAAGGTCTAATCTCAACTAAGTATCACAAAAAGTCAAAAGAATTATTAAGCACTGCCTCAGGAAAGTCACTTCAATTGAATTATGAGATCCCTGATGCCTATGTCAGCCAAAATAAAATTTGTTTTAAGACCTCTTTCGTCTTAGTTAAAAACATTACTGATGAAGTCATTCTAGGTCTACCATTTATTGCCCTGTATATCCTTTCCAGGTGGAACATGATGGTGTCCTTTCTACTTGTTTAGGAGAGAAAGTTAAATTTGAATTCTTGAGCAAACCTGAGTTACACAATTTAAAAGAATTACAAAAGAGTGCAGTCACCAAAACGGTCAGAGTTATTCAGAATAAAGATAAACAGTTAAGATTTCTCCAGGAAGAAGTCAAATCCAAAAGAATTGAGCAACAGCTTGCTCATGAGTCTTTACAATCACAAATTCGACAGTTTGAAGAAAAATTAAAACAAGAAGTCTGTTCTGAGTTACCCAACGCTTTTTGGCATAGGAAACAGCATGTTGTCAGTCTTCCTTATGTCAAGTCTTTTAGTGAGAAAAATTTCCCCACCAAAGCCAGACCAATTCAAATAAGCCAAGAATTAATGGAATTTTGTAAAACTGAGATCACAGAATTACTTGAGAAATGAATAATTCGTAAGAGTAAGTCACCATGGTCTTGTCCTGCTTTCTATGTCCAGAAAAATGCTGAATTAGAATGTGGAGTCCCGAGATTAGTCATTAATTACAAGCCACTTAATACTATCTTAGAATGGATTAGATATCCAATTCTTAATAAAAGAGATTTAATTAATCGATTAGAAAAGTCAGTCATCTTTTCTAAATTTGATATGAAGAGTGGATTTTGGCAAATTCAAATTCATGAGTCTGATAGATACAAAACTGTTTTTGTTACCCCTTTCGGTCATTATAAGTGGAATGTTATGCCATTTGGTCTTAAGAATGTGCCCAGTGAGTTTCAAAATATAATGAATGAGATTTTTAATCCTTTTAGTCATTTTTTCATAGTCTATATTTATGATGTGCTTATCTTTTCTCAGTCGATAGAGTAGCACTGTAAACATCTGCATTAGTTCTTACAGATAGTCAAACAAAATGGATTAGTCGTTTCTGCTAAGAAAATTAAGTTGTTCCAAACTAATGTGAGATTTCTTGGGGTTTAATATCTGCCAATCACAGATTAGTCCCATAGATCAAGTTATTCAATTTGCTTATAAGTTTCCTGATCAAATCCTAGACAAAAGTCAACTCTAGAGATTTCTAGGATCTCTAAATTATGTTTCTGATTTCTATAAGAATTTGAGAAAACAATGTAAGCCTTTATTTGATAGTTTGTAGAGTAATCCTCCCCCATGGTCATCTATTCATACTAATATAGTCAAACAGATCAAAGCTCATATTAAGACACTTCCTTGTCTTGGTATCCCTTCTACTGATGCATTCAAAATAGTTGAAACTGATGCCTCTAACATTGGTTATGGAGGTATCCTCAAACAAAGAGTCAATTCTATTCAATTTGAGCAAATCATTCGATTCCATTCTGGTACTTGGTCACAGTCTCAAAATAATTATACGACTATTAAAAAAGAAATACTATCTATTGTACTATGCATTAATAAATTTCAGTCTGATTTATTAAATCAAAATTTTTTAATCCAAGTTGATTGTAAGTCTACTAAGCATGTATTAGAAAAAGATGTTCAAAATATTGCATCCAAACAAATATTTGCACGATGGCAAGCTATTTTAAGTATATTTGATTTTGATATTGAGTATTTGAGAGGAAGTGAGAATTCTATCCCTGATTTTCTTACTAGAGAATTCCTGCAGAGAAAACAATGGCAGAAAGAAGAAAAGAAAAAGACAAGCAAAAGAGTCAAATTGGTCAAACTAGTCAACCCAGTCATCCCCAGTCACTCTGAGTCCTCCCTCCATCAATGAGTCAAGCCAAATGCGAGTCACCTTCTCAGATCGTCCCCTTCTCGGGATGTTCTCCTATCCAAATTAGTAACAGATTTGCCAACCTAGAAGCCACAATCGCCCAGGTTCGCCCCAGTTTTCAATCTGCCTTAATATCTAGTCATGATCATTTCCAGGATATTGCTCCGTCTAATCTTCCAATTGCCTCCTACCATAAGACGTCCTCATACATGATCAAGAGTAATAGTCACCTGTTCATAACTGAGTCAAAATAAAATTCTATTGCCAACCCAATCACCATTGCCAAATCCTACTTTCCACCAAATAGTCACTGCATTCCCCTAGCACCATTTAAAAATTTAAAGTTTTACAAAGACATCCTCCATGAGACCCAATCCATCACCATTAAGCCAATTAAAGACAGAGATAATCCCCATATCATCCTCCATCATTCCCTCTACATCCATCAAATAATCAATGAGAATGAATGGAGTAGTCACCCCTATGATCTCAAAGTTCTTCAGTCAGAACTTCAATATAGTTACTATGATTATATCAAAGCATGGTATTCTATCTTGCTTCACCAAACAGTTGATTTTAGTCATTCATGGTTCCTTAATTTTGACAGCAAATTCAAGAGTCTCTTCCCCTACTGGTTCAATCACTGGTGGGAAAAACATGGTCCAGTCATTGATCTATTTCCTCCTCCAATGCAAGATTTAGTCAATTATTTCACTAATACATATAAGTTCAAAGAGTTTGAGCTATTCTTCCCAAATCTTCTCCATTTTGTAGCCAAGTACAAAGTCCCTTGGATCCTCAAATGGTCATATCAAGTTAATTGCGAAACCAGAGTCTTATCCCGTCAATTTTCAATCAAGTGGTGGGATAAATTTAAACTTGAGCGTATTGCAGATTATGTTTATGCTGATTTTCCTCCAATCAGGAATCCGCAGCCTGATAAAAAGTCATCCCCTGCTTCTTCACATTCTTCTCTCCCAATCGAGGGAAAAACTAAGTCTGTGTTGCAGGAAATTGCTCGCCAACTAATGTTACAAGCCTCCCAAATGAATGAAGACGATGATAGTGATGCCACTCCTAAGTCGCAGTCATCTACCAGTCAACAAAAGTCATCCAGTCAGAAGACTCCAGCCCCCAAAATGCTGTGGAGTGATTATCCCCCAAATAGTCAAGACCGTTATGATTTTGGTCCTTTCAATTTGGGTGAAGAGTAAAAATTGCCATCATCATGAGCCCCAAGTCAAAAGCATTTGGAATCTTGGACCCGTGCTTCAACAGTCTGATAGCCCATGCTCTTTTAGTCAGATTCCCCGACAAAGTAAAAGTCATAGCACTCAGTAAAAGCAAAAGCTTTCGCGTGCTTCTACAGTCACTCTCCAAAGCATCCAAAGTTGCCAGCTCATCAATAATTGCTAGAGTTTACTTTATTTGCTTATTTAAGAAGGCCTTGGCCTCAGTTTTCGACATAATGTAATTTAGTCCACTTTCTTTTCTTAGTTTCAAAGTTCTTTCAGTACCAATGCTCTGAAAAACTAAGTGCTGAAACAAAGATTTTGTAAGTATTTTGCATTCTGCAAGTTATCAATAATATTTTGAGTACTTTGAGTAATGCTATTTTTTGCATCTATTTTCATCTATTCATTAATTTGCTTCATGCATAAACTGCGTCTATTCTATAATCTCTTTGCTTATACATACTTCTGTTTTACTTCTATCTCTTTCTGGTTCTAGACTCCTTTTGGTATCAGAGCCGTAAGTGTTGAAGAATTAGTCTAAGTCTGTAACTCATCCAACCCAATTGGCTGGTTTAGGTTTTCTGAGTTGTAAGTTTTCAGTTTCTTTTCTCTCTACTTCTGGTCTTTCAGTTTAGCTGTCCTTCTCTCTCTTGTGTCCGAATCGTAAGACCCGTTCCAAACAGTAAGTCCCAGGAAGGCCTGAACGGTAGTGGCATTAGTAGGAGGGCGAGAGAATTTTAGGTATTCTGCTATCCCATGGAGTCAGCCTTAGGAGTTTCTGTTTACTTTCTTCTTTAGAAATCCAACCCCAGAACCTTGAGTAAAAATGAGTCGCTTATTTAAAACTAATTCTTGCAATTCTAGCAGCTCTAGAGCCAATAGTTGTCTCCCAGAGATAGTTAATGAAGAACAAGTTGAGTATGATAATAGTAATAATCTAGATTATAAAGATTGGAATATACCTAGAGTCCCCAGTCAGGACATTTGTAAGAAAAAATGGTCATTATCCAGTTTTAAGTCAACCACCCATGTCAAAACAGTCAAATAAGTCTATGCCTTGAGTAAAAATCATGAAACCTGTTAGTTGCTTAATCTTGAGTCAATACGAAAACATAGAAATGATGGAAACAATTTCCTCCATGTTGATCTAGTCCAAGTTGCTGTA

TATATGTTATATAAATATATATATATATATATGGGCATGTTATAATAATAATAATATACGTGTATATATGTATATTATATATGTATGTATATATACATATATATATTATAAAATACATATGTATATATACAGTTCTGATCTCTTGGACTATAGGGGTCCAAGAGATTTGTGGTCACTTACCGTTAGATGTAAATTCAACGGTTCACTCACTCTTGCACTCCTTTTAAGAAACTTTTTTGAACCATTGGATTAATATCCAACGGTGGGTGACCACAATCTCTTGAACTCCTGTGATCCAAGAGATCGGGACTGTGTATATATAATATATATAATATATGTATATTATATATGTATAATATATATATATATTATATATTATAATACATATATATATATACATATATACATGTATATAATGTATATGTGTATAAATGTGTATATGTATATTATAATACGTACATGGGTATAATTTATATACACATACATGTATATATATGTGTATATATATATATATATATATAGAGCTTCCATTGAGGGATCCCTCAAATTAGCTTATTTGAGGGATACTATTGTAGGGCCCACTCTGTATTGTATTTCACTAATCCAAACCGTCTCTTTTGTAGATACTCATTCAAAGATCATCTCTACAAAAAATCACTTGAATCCAATATCATTTGACCACTCAATTGAATTATTGAAATTTTAGTACTTTCTTGAAGCACCGTGTTCATTGATTTTGTAGGACACAATTGGATATCGAAACAGTTTCCGATTTGTCTAATTTTTTTCAAGGATGATCTATGAATATAGACTTAAAAATGAGATGGTTTCGATCGTTGAAAAAAATTTGTAGGGAACCCTAAAGGGTGTCCCTCAAATAAAATTATTTGAGGGATCCTTCAATAGAAGGGGACTGTATATGTATATATATTACATATATACATGTATGCTATTATTATAATATTAATATGTATGTGTATATGAGTACATATATTATACA

>Chr3-17; OZ022208.1; 16591915-16599977; dir; D;

ATTACATACTTCCTGTCTACCTTGGATTTTTGTAAATCCAATTGCATTTCAAGTTTGCGTAATGTACATTATTTTGTTTTAATGTACCATTATTCTAGCTGTGTGCTAAGTAAATCCTTGGCCATTTGATTGATTGTTGGGAGTATGTTTAGTTTTTTCAACTTGTCTAGTGCATTCCACTCCATTGATGCAATTAGATTTCACATATTCAATTACCTGCTTTCTGTCTGCCAGTTTGTAAATTCAATTCTATTTCAATTACCTCATGATCATTAATGTTTGTTATTTTTACTTGTGACTAGGATATTAACACTTGCAATGAAAGTAAAAGTTAATTGATATGTTAATATTTATATATGTTTTAAATAATATTTCCCTTAGGTTTTGTGTGATTAGAAAGGTTAAAAGCACTAAATTTTATTGTTTTAACATATTTGTTTCTTTTCAAAAAAAAAAAAAGAGAAAAAAAAAAGATTAACAATTACATAGTGCATGTTTGCCAATTGGCAACATAACGTGGCTATGTTGTAGCAAAATAAAGGAATTTTGTATGAGCTTGTTGATGTAAGGCATGGGGCTGAATTGAAGATGAAAGTGATGAGAGAAAGTCCTAGTCTTTGTTTTTTTGTTTTTTTGTGAATTGCAGTTCCGTTTTCTTTTCTTTCTATTTCTATTGTTGTTATTCTTCAGCCACGTCAAGTTGTTAATGAACAAACAGGAACCACATAATTGCATTTTCAATTTGAGAAAACAGACTACCTGAGTGGCAACCGTGCATGGCCTTGGAGATAATGCAATTATGTGGTTCCTCAAATGTGCGTGTCCGACACCTGGCGGGTGCCGACCACAATGACTAAATTGCCCTTTTAGTTAAAAATTCTAGTTTATTATCAAATCGCCTTCTGCCTAAAATTCGGCAAAAACCTCCCTTGTAATTTGATCATTTTCAACATTTCAACCTGTCAAAAGCCTATATATATACTTCAAGTTGGACTGTGGT

TTGGCAACTGTTCTAGTTGGACTGTGGATTTAACTGGCCTATCAAATCTAATTTTAACTGTGTCATCTAAATAAGGTTGAATGTAATCTAAATCGTTTAGACTACGTTGAATCGGCTTTGGAGGGCATTCGGTTGTTAAATACCAATCTTGGGGTAACTTAATATCAGACCATTTAATCATTTGGGGCACTGTAACATTTGCATTAGAAGTATGGCTTTCAATTAAAAGTGTTTGATTCTTTGGGCTTTTACTAAGAGCTTGGAAATTCATATTTGTACCCGTGACTTTGTAATAAATTCTATAAATTAAAGCTAATGGCTGACTACCTGGTAAAACATTATAACCATGTGTTTTAAGATTTAAAGTTAAAACTTTTAATATATGAGGATCACTTAGGCTTATTGTGAAATTTGGGTAGCAATCAAAATGAATTGGACCATGACATAGGCTTGACTCAATGATTCCTAAAACTCCATCTTGGAAATCGTTAAATCTAGCATCTCTTAAACTTAAAAGAGTCGTAAGAGTTATGGTTGAGTCTAAGTCTGCTACTCATATCGCCCAAGTGGCTGATTTAGGTTTTCTGAGTTAACAAGTTCTTCAGTTATTCTAAACAGTTATCTTAGAGTTGTCTGTCACCTTAGGATTACTCTCTCAAAAGAACACGGTAAGACCTATAGCTAAACAGTAAGTTCGGCGGTAGAGGCCTGAGAGATGCGGCACGAGTGTGAGAGAGAGAATGCCTTAGGTTCCAGGTAATTTTACTGTCGATCTTCTACCTTTTGAACCATTGAGTCAAACACTTGTAAGTATGAGTCATTTATTTAGAACCAATTCAGTTAATTCTTCTAGTTCTAGAACCAGTTTGAGTCAAGTTCCAGACATAGTTAATGAAGAGCAGTTAGAATATGAGTCTACTAGTAGTGAGTTGCATTGTAATGATTGGAATATACCCAAAGTCCCTAGTAAAGAAATCTATAAGCAAAAATGGTCAATTAATGCATTTAAGACAACACGCATATTAAGACAGTAGAACAAGTTTATGCCTTAGGAAAAGAACATGAAACATGCCAGTTACTTAATCTTGAGTCACTAAAGAAACACAGAAAAGAAGGTAACAATTTCTTGCACATAGGATTAGTTCAAGTTGCAGTTAAACCCTTAACTAAGTTAGGATTGAAAGCAGCAATTCTTTTAAGTTTAAGAGATGCTAGATTTAACGATTTCCAAGATGGAGTTTTAGGAATCATTGAGTCAAGCCTATGTCATGGTCCAGTTCATTTTGATTGTTACCCAGATTTCACAGTAAGCCTAAGTGATCCTCATATATTAAAAGTTTTAACTCTAAATCTTAAAACACATGGTTATAATGTTTTACCAGGTAGTCAGCCATTAGCTTTAATTTATAGAATTTATTACAAAGTCAACAGGTACTAATATGAATTTCCAAGCTCTTAGTAAAAGCCCAAAGAATCAAACACTTTTAATTCAAAGCCATACTTCTAATGCAAATGTTACAGTGCCCCCAAATTATTAAATGGTCTGATGTTAAGTTACCCCAAGATTGGTATTTAACAACCGAATGCCCTCCAAAGCTGATTCAACGTAGTCTAAACGATTTAGATTACATTCAACCTTATTTAGATGGCACAGTTAAAATTAGATTCGATAGGCCAGTTAAATCCACAGTCCAACTAGAACAGTTGCTAACCCCTAGTCAATCTTTTAAAATTCCTGCTAGACATTTGTCTACTGAGTCATCCTCAATTACAGAACGTGATCTAGAAGCAGATAGAGCCCTAATTGATTTCAAAATTAATGAATTAAGAAAACAAAAACAAGTAGTCCAACCAAGTTACGGAAAAGCTGCTGCTGAGCCAGTTTTACCAGCTCAACCTGAATCACCTACCAGTACAGATTTTGAAGGTTCCACCTTTGAAGTTTGTGATGCCCAATTACGAACTCTTAGTAAACCATTTAAAATTCATTGGTCAAAATTAGATGCTCATTTCCAAGCCCCAGAAAATGAATCACGAAAAGCCAATTATCATGCATCTTTCTCCAAAGTTCAAAGAAAAGAAATTTTCAATGAATGGAAAGAATATTTAAAAAAGACTAGGTCAGAGTCACCTTATCTTGATTATGTCGAAAGTAAATATATGATTCAAAAGTTAGTTACTTTAACTTAGGAAAAATGGAAAAAAAGCCGACAATACCAAAGTTATTAGTACTCACCCCCTGGTTGAATCAATAATAATAAATCACAAGAATGCCAAAGTCATAGCCAGCCCTTACAAAGTTGCTGAGTCAAAAGATGATTTAGACAAAAGAGTCATAGAACAAAATAATTTTACCAACACAAGCCTAATAGTCATAGGTAAACAGTTAGATAAAATTGAAACTAAAATTGACAAGTTAACCTCTGGTGAAATTATCTTAAAGCCTAAGAAAGAGAAACCTTTACTCAAATTCAATGAGTTAAAACCCGAGTCTTCCCTCAAAGTAAATTCAACCATGAGAGAAATTGAAGAAATGTTTCAAGAGTTAACCCCCGTCAAACCAAAATCAAATAAAGACAAAGGAAATGAAGAAGAAACTTCAGTTAAAGTTTTACAGTTTGAAGTTAATTCCGATAGTGAGTCAGCCATGTCTTTAGATTCAATCTCCAGTCAAGAGTCCAAAACAAGTAATATCTCTAAGGTTGAACAAACAAGTCTCTAATAACCTTCAAGTTAACCAGAATTCCAGAGTCTTAAAGTTAGTCGTTTAACAGGAAAGGTCAATCCCACAAGTCTTACCAAAAATTGGTTTCATAAGCCTACGCCCCCAGATGTCCAGTTTGAAGAAAGAACCCAGAGTCAATTCTCAGTATCTTCTGATAAGTTATATGAGTGGAATATCGATGGCTTGTCCGAACAGGAGATACTCAACAAGTTACAACATATGTCTATGGTAGCTAATAGTTATGCCACCAATCATCAGTTAAAACAATCAGAGATAGTTTCCCTTTTACAAACAGGCTTCACAGGAACACTTCGTTTCTGGTGGGATAAACATCTCACAGAAGATGCAAAATTAAGAATAATCCATGCCTATAAAACAAATAATGATGGTTACCTTATTTTTGATGAACGAATAGGAACAAGTATAGAAGATGGAGTCAATGTCCTTTTATACACAATAACTGAGCATTTTATTGGTACACCCAGTAGCACAACAGCCAGGATTCATGACCAGTTAAGTAATTTGCAATGCCCTAAACTAAGTGATTTTAGATGATATAAAGATGTCTTTATTTCTAGAGTTATGTTAAGAGATGACAATAATCAATCATTTTGGAAAGAAAAGTTCATAAACGGTCTTCCTAATTTGTTTGCCCACGAATGGTCTTCATAAACGGGCAATATAATCAGTACAATTAACCAGGTAGGAATGAAAATGTGTGTAGATTTAAAAATTAGTAAATCAGTTCAAGCCAATAGAAAAAATGCTAAATATGAATTAGGAAATTTCTGTGAGCAATACGATCTAGCCCCAGTTCCCCTGTCTAGACAAGTCAAAAGAAGTATAATGATCGACACTTTAGTAAAAGTAGATATTATTCCCGTAGAAAACATTCCTATAAACCAAATGATGAGTTTTATAGTAAAAAGAAGTTAAACCCTAGGAAAAGCAAATGGTCAAAAGGTTATTCAAAAGGTTACTGAAAAAATAAAAAGAATAAAAAGAATAAATTTAAGAAAAGTCCAGATAAAACAAAAATTAAATGTTTTAAATGCAAGAGATTTGGTCATTTTGCTAAGGAGTGCAAAGTTAAAGATACAATTAAACAGTTAAAGTTAAAATTTGATGATGAAGCCAAATTAATTAAAGTTCTTGAGTTAGCCGATACTGAGTCAGATCCTATGTCTTTAGCCAGCTCAGTTAATTACAGTTCAGACAGTTCCCAGACTAGTTCACCAAAAGTCCAGATAGGTTGTAAAGATAAGTGTTGCAAAGTCTTAAGCAAATCAATTAATGTCTTAACTAAGCAAGAAGAACAAGAAGAGTTACTAATCGATTTAATCAGTAAGATAGAAAATCCAGAATTAAAAGCCAAATATTTAAGAAAACTTAGAAAAGTCATAAGCCAAGAAGAACCTAGTCCTAGCCAAGTCCCTACAATTAGTCTTAATACTACCCTAGAAAGGTTTAATAAAAAGAAAGAAGTCACCCTTCAGGATCTCCAGTTAGAAGTTAAAACAGTTAAAAAGAAATCTTAAGCTTAAAAAGAGTCAGTCAAAAATTACAAACTGAGAATTACGAAATTAAAGAAGACTTAATTAAATTGCTAGAATCCAGAGCAGAGTCTAGCAATCCTTTTGGTAAACTCAGTTCCAAGTCAGAACCTGAGTCTTCCGATAGTAACAAAAGCAACAAAGTCATCAAATTAATTAAGCAAATCAATTTTAGGAAATGGTACTCCCGAGTCAGTATTTTTATTAATGAATTTGAATCAAACACAGTTGCCTTATTTGATTCAGGAGCCGATCTCAATTGTATTCAGGAAGGTCTTGTTCCAACCCAGTTTTACAAAAAGTCAAAAGAAGTTCTAGGAACATCATCTGGTAAAGCTTTAAAGTTAAATTATGAATTACCCAAAGCCCATGTTTGCCAAAACAAAATATGCTTTAAAACATCTTTTGTCTTAATTCAAAATATCATAGATGAAGTTATTTTAGGTTTATCCTTTATCTCTTTACTTTACCATTTCCAGGTAGAATACGATGGAGTTATATCTAGTCACTTAGGTGAAAGAGTTAAATTTGAGTTCTTAACCAAACCAGAGTTACACAGTCTCAAGTTACTACAAAAGTCAGCAGTTTCTAAATCAGTTAAATTAATTCAGAATAAAACAAGACAAGTTAATTTTCTTTAAAACGAAATTAAATTCAAACGCGTCGAACAACAGTTAGCCGACAAGTCGCTTCAGTCCCAAATAATTAAGTTTGAAGAAAAGTTAAAAAATGAAGTTTGTTCTGATTTACCTAATGCCTTTTAGCACCGAAAGCAACAGTTACTTTACCTTATGTTAAAACATTCTGTGAAAAGAACATTCCCACTAAAGCCCATCCAATCCAGATGAGTCAAGAGTTAATGGAATTATGTAAGCAGGAAGTCACCGAATTAGTCCAAAAAGGAATAGTTAGAAAAAGCAAGTCACCCTGGTCATGCCCAGCATTTTATGTTCAGAAAAATGCAGAGTTAGAAAGAGGAGCCCCTCGTTTAGTTATCAATTATAAGCCCCTTAATGCTGTCCTTGAATGGATTAGGTACCAATCCCCTAATAAAAGAGACTTAGCTAATAGATTAGAAAAAGTAGTTATCTTTAGTAAATTTGATATCAAAAGTGGATTTTGGCAAATCCAAATCAAAGAATCAATTAGATACAAAACTGCATTCGTAACCCCTTTCGGTCATTACGAGTGGAATGTCATGCCTTTTGGATTAAAAAATGGGCCTAGTGAATTCCATAATATAATGAATGAAATTTTTAATCAATACAGTCAATTTTTCATAGTTTATATTGATAATGTCTTGATTTTTTCTTAGTCAATTGAGCAACATTGGAAACATTTGAATAAATTCCTTCAAATAGTCAAGCAAAATGGTCTAGTTATCTAAGCCAAGAAAATCAAATTATTTCAAGTCAATGTCAGATTCCTAGGATTTAATATTGGTCAGTCTCAAATAAGCCCAATAAATCGAGTTATCTAGTTTGCCGATAAATTTCCAGATCAAATTCTTGATAAAAGTCAATTACAGAGGTTTTTAGGATCATTGAACTATGTTTCTAATTTTTATCAAAATTTAAGAACATTATGCAAGCCCTTGTTTTATCGCCTCCAGAGCAATCCCCCTGCTTGGACCCCGAGTCATACAGAAATAGTTAAACAAATCAAAGTTCATGTCAAGTCCCTTCCATGTCTTGGTATTCCTTCCGTTAATTCTTTTAAAATTGTTGAAATAGATGCTTCTGAGTCTGGTTATGGCGGTATCCTTAAACAAAAGCTTACTTCCGAGTCACCTGAGCAAATTGTCCGTTTCCATTCTAGAGTTTGGAATCAGTCTCAAGCTAATTACAGTACTATTAAAAAGGAAATTTTATCTATAGTATTATGCATTAGTAAATTTCAAGATGATTTATTAAATCAACAATTTTTAGTAAGAGTTGATTGCAAGTCAGCAAAATATGTTTTGGAAAAAGATGTTAAAAAATAGCTAGTAAACAGATTTTTGCACGTTGGCAAGCCATTTTGAGTGTTTTTGATTTTGAGTTAGAATATTTGAGAGGTAGTGAAAACAATATTCTTGATTTTCTTACAAGAAAGTTCCTGCAGGACAAGCATGCTGAATCATGTCTAGCAAAGAAAAAGACAAGCAAAAAATGCAATATGGTCAACCGAGTCAACCCAGAATTCTCTCAGCTACCCTGATGCTAGTTAAACAAGAGTCATCTTCTCAAATCATGCCTTCTCACATTGTGCTCTTCCCAGATGATTTTCCAATTCCAGTCAATCATAGGTTTAATCCCTTGAGTCAACCGTCTTTGCACCCCAATTATCAGTCAGCATTAACTTCCAATTATGATCCTTTCATAGTTAATCCTCCTACTTCAGTTTCTTCGCAGAGCCAAAGCCTAGTTAAAAGATCCAATTATGCCACCCGAAGTCATTGTCATCTTTTTATAGTTGAGTCATACCTAGAAGAGCTTTGTGACCCAGTCAAGATTGCCTAGGAATTCTTCCCTCCTAATCTTCACTATTTTCCAAGCCATCCTTCCAAGTCAATTAAGTTTTACAGGGATATTCTCTACGAAACTCAGTAAATAGATATTAGGCCTATCAAAGACAAGGACACAAATGCCACCATGTTCCATTCCTTTACATACGCCAAATTCTTATGCCTCAAGTTTGGAGTCGCAATCCCTATGACCAGAAGCCCCTCCAGTCAGGACTTACTTATAATTATGGTGATTATATTGATGCCTGGTATACCATTTTTTATCATCAACACCCTAATTTCAATCACTCATGGTTTATTAATTTTGATGCCAAGTTCGAAGTCCAGTTTCCTTTCTGGTTTCTCCAATGGTGGAATATCCATGGCCCTATTTGGGAAATCATCCCAGTCAATCTCCAGCAGTCTGTAAATTATTTTGGTTAGAAACACAAGTTAACAAAGTCAGAAATTTTTTTCCCTACACTCCTCCATTATGTCACCAAATACAAAGTCCCATGGATTATGAAGTGGAATTATCAAGTTAATTGGGATTCTAGAGTTTATCTCGTCATTTTGCTGTCAAATAGTGGGATAAGTTTGGAACATACTGAATTATCCAACAAGTCAATGCAGAGTTTCTACCTCTCCAAGTGCCAATCCATCCAGTCAAAGATGCTTCCCCAAGTGGCTCCAATGTCTCCCTCCAGGGAAATCCAAAAAAGAGTTACAAGAGTTAGCTGAGCAAATCCTTGCCCAAGTTACCCAAATGAATGATGAAGATAGTGATGATGCCAATCCAAAGTCTGAATCTTCTTCCAGTCAACAGCCAAGCCAACTTCCTTTTAGTCAAAAGCCGAAGCCCAAATGGTCAGACTATGCCCTCGATAATCAGGATCCCTATGCTTTGGATGAAGACTAGGTTAGGCCTCCTTTCTGCAAAGTAGCCGAAAGCAAAACATGCCCCGAGTTAAAGCAAAAGATGCCCAAGTTAAAGAAAATTACTTAGAGTTAAAGCGATGCCTCAGTTAAAGTAAAGCTAAAGCAGATGCCCCGAGTTAAAGTAAGTTTACTTAGATGCCCAAGTTAAAGTAGAGCTAAAGCAGATGCCTAGAGTAAAAGCAAGTTTCCCCAGAGTTAAAGCAAAGATGCCAAGTTAAAGTAGAAGTTAATTTCAAAAGTTATTTTGTCTTCTTCTCGATGCCAAGTCTTTAAGTAGAAGCCCCAAGTCATTTGTAATACAGATCATTAAAAAAAAAGCTTTTCTTTAAGCCTTCCTTGGTAAGTGTTCAGAGAGTTCTCT

AAGCTAAGAAGTGTCCAATAGAAGTGAGAGTATCCTTCGTGGCATTGCTTTCTGTAAGTTTTACTTTCATGTTTTCAAAGTTTTAAGGCTGGCTGGTTTTATAAATCCAACTTCAAAATATTTACGGTTGTGCCACTGAGACTCTTTTGACCATAACTCTCTCGTTACAACTCCGATTCGGCCCCACTACGTGTCTATGAACTCATTTCACCGTGCTCTACGCAACGGTGTAAGTGGAATTGTCAAATTCCTTCTCGATCAAAAAGTCACCTTTTTCTTAATAAAATATTCCGAGGGCAAAATTGTCTTTTCTCCAAATAAATTACTAATTAAATAAGAATTTTTATTTTGGGTTATTACAATCTACTCCCCTTACAAAAATTTCATCCTCGAAATTTACATGCCTGTCACTTGAAGAGGGGAAGGGACTACTCCCGCATCTACTACTCGGGTTACCACAAAGCCTTTTTCACTGAAAGCTAACAGTTCCCATTTCAAAATCTAAAGTGATTGCTTTCCATAAGTGAAATCAGCCTCCAATTTTGCTGATTATTTGTCTAACACATGAGATGGATCAGATTTTTCTTTTCGGATCATGGGAATATGAAAAACATCAAGTCACTGTGATAGATCTGTAGGCAAAGTAAGCCTGTAAGTTCCTTGGCCCACACGTTCTATAATCTCATTGGATCCGATATAGCGAGGACTTAGTTCCCTCGCTTCCTCAAATCTCACTACACTTTCCTAAGGTGATAAATTTTGAAAAATACCCAACTGAAGATCTTTCTTCTATTATCTGCATAACTCCTTGTCAATCCTGGGTCACTTGAGTCTTTTCCTAATTAATCCAATATATTTCTTTCTTTTCTACCAAACTTTCAGATATCAATAACCCTGCTTACCATTCCATCTTCATACTGAGATGGTCTATACTATGTCTGGTATAAGGCATCAATTGGAGACATCTCAATTATAGGCACCAATGACAAACTTGAGATTT

>Chr3-18; OZ022208.1; 18749086-18755025; rev; D;

TGCCGTATAGTACAACACAAGAACCCTATTACATGGCATGCATCCAACCATTTCAACTACTTTAGTATCAGAGGTAATAAGCTCCAATATGTTACCATCCTTTTCTCCAGCCTTTCTATACCTAATCTCCATAGAAAGTCATCAACAACATTATCTAAATAACAGACCTTACCACCTTTATAAACCCCATTTTCAAACATACCCCTATGGTGTAGTTCAAGAGTGACTCTGTCAGAGATAGCTGCAAACAGTACATATCAAATTTCAGAGTTAGCTGCACACACATCTCCTTTACTAATGCTACAGGTTTAGGGTTTTAATTTTTTTTACCGTTTCACTCAATGCTAATAGTGAATTAAGTGTGAAGTAGACAACAAAGGGACCACACCAAACATAAAAACCTATACTTTGCAGTCAATCCCAGCATAAAGGAAATAAAATGCCAGAGTATCTTTTCAACATAAAGAAACTAACCTTGGACCACACACTTGCAAACAAAAAACATCGATTTGCAAAACAAGATGAATATAAAATACAAACAGAGAATCCCTTACCGTATTTGGGATTCTCTCCACCCCAACGGAACTCAAACTCCATCAGTGATGAGCATTCACAATGATCGAAATCCAGAAATTCCGGCAAAATCCCCAAATAGGAACCCTAACAACTCCAAAATCTCTGTTATTGCCTTCGCACCTCCTTTTTGGATGCTCACGAACTGAATATGACGTTTTGGCTAATCGGAGAAGACATTGAGGTTTAAAAAGACGCATATACCCTTTCAGATTTAAAAAGACGCATATACCCATCCTAGACCCCATCACTGGCGTTGACTTGTTAAAAACTCACAGAAAATGTAACGGTTGGATCAAATGGGCGATTAACATAGAGTTTATGTACCATTTGGACTAATAATTTAATTTAAGAACCAAAATGTAAATCTCATACAAGTTTGAGGACCATTTGAATAATTTACCCTATATATATATATATATATATA

TGCCTTGAAGAATTGCAGTTGCAATCTTTGTGAGTATAAATAAAAGTTATCTTCAAATTATCTATTTTCTGAAAGCATTATTTTGATGCATTATCATTATTTTCTGCATTAGTCTGAAAGTTGTTTAGGATTGCATCCATTTCTAATTTCCTTCTGGTCTCTCTTCTGTATTATATTCAGTTGTCCTTCTTTTATTTTTATCTCTCTTTGTTCTTCTTGAGTTGGTATCAGAGCCGTAAGTGTTACGGTCTTTGTCTAAGTCTGCTACTCATCCCCGCCCAAGTGGCTGGTTTAAGCATCTGAGTAGAAGGTTTTCATTTCTTCCTTTTCTTCCCTTTTATTCTTTGAGTCCTCTGTTGGTTTTCTCTAAGGTCTGACCCGTGAGTCCTTTTTGCTAGATCAGTAAATCCCCAGGGAGGGTGCGTGTGCTGGGCAGGAGACAGAAAATTGAGAAAATTAATAGGTTGCTTAGAGTTTAAACGGTCGGCTTAGGATCTACTGTTCATCTTCTTCCTTAGTCTGCTAAACCCAGTTTCTTGAGTAGCCATGAATAGTTTATTTAGAACAAGGTCTTGTAATTCTGCCAGTTCTAGTGCCAGTATGAGTCGACTTCCAGAGATAGTAAATGAAGAACAATTTGAATATAATTCCAGTAGTAAAGAACAATTAGATTTTAGAGATTGGAACATACCTAAAGTCCCAAGTCAGAAAATTTATAAGAAACATTGGTTGCCTTCCAGTTTTAATAGTACAACACATGTCAAAACAGTTGAACAAGTATACGCTTTAAGTAAGGAACATGAAACTTGCCAGTTGCTTAATCTTGAGTCTATTTTAAAACATAAGAAAGATGGTAATAATTTCTTGCATATTGGTTTAGTTCAAGTTGCTGTCAAGCCCCTGACACGGTTAGGGTTGAAAGCCTCAATCCTACTATGTCTGCGAGATGCTAGATTCACTGAATTTAGCGATAGTACCCTCGGAATTATTGAGTCCAGTCTGTGTAATGGCCCTGTCCATTTTGACTGCTATCCCGATTTCACTGTAAGCCTTAGTGATCCTCATATCCTGAGAACTTTGACTCTGAATATTAAGACTGAAGGTTATAATGTCCTCCCAGGAACCCAACCTTTAGCCCTAGTTTACAGAATCTACTATAAGGTCACAGGAACTAATATGAATTTCCAAGCCCTTAATAAAAGCCCGAAGGATCAAACCGTCCTGATCCAGAGTCATACCTCAGACGCCCATATACAAGTCCCCCATACTATCAAGTGGTCTGAAGTTGCCCTTCCAAAAGATTGGACCTTAGTTACCGAGAGCCAACCAGCTCCGATCCAGCGTAGTCTAAACAATTTAGACTACATCCAACAATACTTAGATGGCACAGTTAAGATTCAGTTCGACAGTCATCCTTTACGAAAGTCTAATGTTCAGTTACAACAGTTGCCAACTCCCGGTCAATCCCAGAGGCATGCCCCTGCTCGGCATTCCTTTGCCGCCTCTTCCTCCACTCTAGAACGAGACCTAGAGTTAGAAAAGGCTATGATTGATTTCAAACTTGAGTCTCTGAGAAAAACCGCTCAGGTTACCCAACCTTGCTACGGCACGGTTCCAGTTCAAGATGACAAGTCAGAATCACCTGAGTCACCTACCCAGTCTGACTTTGTAGTTGAAAATCGGCTTAGAACCCTTAATAAAGAATTTAAAATTGATTGGGTCTACCTAAATCGTCATCTAAAAGCCCCTGAGAATAAGTCAAGGCGAGATCTTTATCATCAAGCCTATCCTAATAGCAAACACCGAGAAATGATCTTTAAACAATGGAAAGATTATATGTGGTCAGCCAAAATTGAAATTTTCTACCTTGATTTTATTGAAAGCCGTTATATGACTAATGATGAATTAAAAACTTTGACTAAAGAAAAATGGAAACTAGTTGACAAATCCGTAGTTGAGTCTAGTCATCCTCCAGTAGAGACCATAATTATAGAACATCAGAAAGCTCCAGTTCCTGCCACTCCTTTCAAAACCTTTGAAAGTAATGATCCTAATAGGAAACTAATTGAACAAAATAACTATACGAACCAGAGTCTAATCACCATAGGTAAACAACTAGATACAATCGAAACCAAAGTTGATCTTCTGAGTCCGCCTGCTGCAGACTCCAGACCCAGACATAAAGTTGAAAAACCAATAGTTCAATTTCAAGAATTAAAAACGAGTCCAGTTTTAAAAGTTAATTCTACAATGAAAAAGATAGAAGACATGTTAGCACAATTGACCCCTGTCAAGCCCGAGAAAGGTGACCCGTCTGGTATTAGAACACTAGATGCGTTTGCTGCTTCTCAATCTGAGTCAGAATCAGTAAGTTCAGAAACTAGTGATATTTCTAAAATTGAAAATGCCTTCAGAAATCTAGAAGTCCAAACTGAACCCAGAGTTAAAAAGATAGAAAAACATATAAGCCCTACTAGCCTAACAAAGAATTGGTATCCCAGACCAACACCTCCAGATATCCAATTTGAAGAAAGAAACTTTCAAACCCAGTTTTCTGTTTCTTCTGATAAATTGTATGAGTGGAATATTGATGGTCTTTCTGAACAAGAAATCCTGAATAAATTGCAACATATGTCTATGGTCGCCAATAGTTATATCACCAATCACAGTTTCAGACAATCTGAAATTGTCCCTCTGTTAGTTACTGGTTTCACCGGTACTCTCAGATATTGGTGGGATAAACACCTCACTCCTGAGTCAAAAAATCGAATCATTTATGCTGTAAAACTTAATGAAGATGGTCTTCCTATGTTTGGTGAACAAATAGGTCAAGGTTTTGAAGACGGTGTCAATACTCTGTTTTACACAATAATTGAACATTTCATCGGAACCCCTAGTAACACCACCGCTAGGATTCATGATCAATTAAGCAATCTTAGATGCCCAAAGTTGTCTGATTTCAGATGGTATAAAGATGTTTTCATTTCTAGAGTCATGCTTAGAGATGATAGTAATCAACCATTCTGGAAAGAAAAGTTCATTAATGGTTTACCTAATTTGTTTGCTCATAAAATACGCACGACACTAAATAATGAACAAGGACAAATTGATTGGGATAATTTAACGTATGGAAACATTATAAGCACAATTAATCAAGTTGGTATGAAAATGTGTATTGACTTTAAAATCAATCGCCAAATCCAGTCTGATAGAAAGTCAGCCAAGTATGAGTTAGGAAACTTTTGTGAACAATATGGTCTCACTAGTATTCCTCCCTCCAGGAAACATAAGCCCAGTCATCTTAGAAAGCGCCATTATCCGAAGAAACAATTTCTCCGGAAAGATGAATTCTATAAGAAAAGAAAGTTCTCCCCTAAGAAAAATTGGTCTAAACGTCCAAAACAAAGTCGTTCTAAGAAAGATAAAAGCAAAGTTAAATGTTTTAAATGTCAAAAATTTGGTCATTATGCTTCTGAATGTAAAGTAAAAGATGTTATCAAACAATTGCAGATTACAGAAGATGATAAGAACAAATTAATCCAAGTTTTGGAGTTGCGAGACTCTGAAATGAGTGACGTTGAGTCTCTGGCATCTAGTTCTGAGTCTGAGTCTAGCCAGTCTTCAGATTCACAACCCAGTTCCCCTAAAGTTCAATTTGGTTGCACTGATAAATGCTGTAATAGGTTAAAATCTGTCTTCGTCCTCACTAAGCAAGAAGAACAAGAAGAACTCCTAGTAGATCTAATAGGCAGAGTTCAAAACCCTGAGTTAAAATCTGAATATTTGAGGAAGTTGAAAAAGATCTTAAGCCAGGATAGCCAAAGTTCTCTTTTGCCTACTCCTCAGCCGATTAGTATCAACACTACGTTAGAAAAATTCAATCGTAGGAAAGATGTCACCATTCATGATCTCCAGTCAGAAATCAAACAAGTTAAAAAAGAATTAGTTGAACTTAAACAGATAAGCCAACAAATGCAAACTGATAATTATAAAATCAAACAAGACTTATTCGAATTAAAACAAGAGTCCTATAAACCCAATAACCATTCAGAAAGTCCGCATCATTCTGATAATGAACCTTCTGAAGAACAGCCAGTCATCAATATGATTAAGCAAGTTCACCTCAGGAAATGGTATTCCAAAGTCACGAATTTTGTCCATGATTTTGAATATACCACAGTTGCATTATTTGATTCCGGTGCAGATCTTAACTGTATCCAGGAAGGTCTCATCCCTACTAAGTACTACCAGAAGTCTAGAGAATCCCTTAGCACCGCTTCAGGGAAATCTCTCCAGTTGAATTTCGAATTACCAAAAGCCCATATTTGTCAAAACAAAGTTTGTTTTAAAACTTCATTCGTCTTAGTTAAAAATATTACTGATGAAGTTATTTTAGGATTACATTTCCTTGCCCTGCTCTATCCTTTCCAGGTAGAACACGATGGTATCATTTCTACCCATCTTGGAGAGTCGGTCAAGTTTAGCTTCCTGTCTAAGCCAGAGTTGCATAATCTAAAAGCCTTGCAAAAACAGTCAGTCTCTAAATCTGTCCAAATTGTCCAAAAAATTAACCAGCTGAATTTTCTTAAAGAAGAAATTAAGTTTAAAAGAGTTGAACAACAACTTAGTGATAAAACTTTACAGTCGAAAATCCAGTCTTTTGAAGACAAATTAAAAGAAGAAATATGTTCTGATTTGCCTACTGCCTTTTGGTATAGAAAACAGCATGTTGTCTGTCTTCCTTATATCAAAACCTTTAGTGAAAAAAATATCCCCACTAAAGCCAGGCCAATACAAATGAATCAAGAGTTAATGGAATTTTGCAAAAAAGAAATTCATGAGTTGCTTCAGAAAAATATAATTCGCAAAAGTAAGTCTCCCTGGTCTTGTCCTGCTTTTTATGTCCAGAAAAATGCTGAACTAGAAAGAGGAGTCCCCAGATTAGTTATCAATTATAAGCCCCTTAATTCGGTCTTAGAATGGATTAGGTATCCCATACCTAATAAAAGAGACCTTATTAATCGATTAGGGAAAGCAGTTGTCTTCTCTAAATTTGATATGAAAAGTGGTTTCTGGCAAATCCAAATAGATGAGTCAGATAGGTACAAAACTGCATTTGTTACCCCTTTCGGTCATTATGAATGGAATGTCATGCCGTTTGGTTTAAAAAATGCCCCTAGTGAATTCCAGAATATTATGAATGATATCTTCAATCCTTACAGTCATTTCTCGATTGTCTATATTGATGATGTTCTGATATTCTCTGAGTCAGTTGAACAGCATTGGAAACATTTGCATAAATTCCTTCACATAGTCAAACAAAATGGTCTAGTTGTCTCTGCTAAGAAAATTAAGTTGTTTCAAATCAATATTAGATTTCTTGGTTTTAATATCAGTCAGTCTCAAATTACTCCAATTGATCGAGTCATTCAATTTGCTGATAAGTTCCCTGACCAGATTCTTGATAAAAATCAGCTCCAGAGGTTTTTAGGATCTCTAAATTATATCTTTGATTTTTATCCAAATTTGCGAAAAGAATGTAATTTTCCCTTTCTTCAGTTTTAAAGAATCTCTCTGATACATCCTTCGGAGATATTCATCTTTGTAAACACTCTTGTAAATAGCGTGCAGTTGTGTGCCTTGAAGAATTGCAGTTGCAATCTTTGTGAGTATAAATAAAAGTTATCTTCAAATTATCTATTTTCTGAAAGCATTATTTTGATGCATTATCATTATTTTCTGCATTAGTCTGAAAGTTGTTTAGGATTGCATCCATTGCAATCTTTGTGAGTATAAATAAAAGTTATCTTCAAATTATCTATTTTCTGAAAGCATTATTTTGATGCATTATCATTATTTTCTGCATTAGTCTGAAAGTTGTTTAGGATTGCATCCATTTCTAATTTCCTTCTGGTCTCTCTTAGGTATTATATTCAGTTG

TCTTTCTTTATATATATATATATATATATATATATATATATATATATATATAAGAAATAGGGAACTAAATTAGCCTTTTAACTTTTAAAGGCCCAAGATGAAATGTAACCCGATGATAAATTCAAGACTCATTACTCCAAATTAACCAAAATTAAGCCATCTTAATTTGTTTTCAGGTCTAAAAATTTACCCATCTTACACAAAAAAGTTATAATATAAAAATTAAATTAAAGAACAGCACAACTTTTACCCAAGACTATTACAACATTCCTCGCATAAAAATAAAAATAAAAAATTAACAACATTCCTTTGAATCTTTGTTGATGGTTGATACTTGTCGAGTTGTCCTACTTGCAATCTCAAAGCTTAATGACGTGGCTCTCCACATGCACGTTCCGGACACCTGTAATGCCAACAAAGCAAAGACACAAGCTTTCCTTTTCTTTTCTCTCTCTCTCTCTCTCTCTCTCTCCCTCTCTTTTTGCTAAAACTCTTGCTTAAAGCCTCAAATCTCAGCTTAGCACACACTCCGAAAGTCGCCTTCGCTCTACTAGAAACCCACAGATCTCTCTGCAACACAAATATGGAGACTTCAGCATCGCCTCCGCCACCAACCACCAAACCAAAGCTTCGCATTATGTGCAGCTATGGCGGCCACATAGTCCCCAATCCACGCTCCAAGTCCCTCTGCTACATTGGCGGCGACACTCGCATACTCTCAATCGACCGAACCACCGCCTCAACTCTCTCTTCTCTCACATCCCACCTCGCAGCCACTCTCTCTCTCCCCGCACCTCTGTTTCTCAAGTACCAACTTCCCCGCCACGTCCTCCTCTCCTTAGTCTCTCTCTCCACCGACGACGACCTTCTCATCTTCCTCGACGAGCTTCGCCGCCTCTCCAATTCCGCCCCTCGGATCAGATTGTTTGTGTTTTTCGACAAGACCCATCATCAGTGTAAGGCCATTCAGCACCCAAAGACTGAGTCTTGGTTCATTGAT

>Chr3-19; OZ022208.1; 21614159-21615332; rev; B;

AAATACATATATTTGAGGATTTGTTTTTGGGTTGAGATCCGTGAGGTTGTGCTTTGTGATTACCCTACACTGGTATGAACATCTGAGTCTCGGATTTGGGAAGTGTTGTTTAGCAACATATGCCTTGGATAGGGTATAGTTGCACGGAAAGAATTGATAAAAGAAAGGAAATCATAAAGTGTTTAGGGGTTTCCTTGTTGAATTTGATATAATAAGGAGTCTAAAGTCATTCGTCGATTTTATTTTAAAGGAAAAAGATTGTATATTACTGGAGTTGTGTCTGTGGATGAAGAACTACGGGTGGCTTGATCCCTTAGTAAGGGTACATAGGTAGCCTGGGTCCCACCCAAGTGCAATTACGAACTAAAGTTATTTGGGTCCTGGGATGATTTCGGTTTCGACCTTGTCGTTGGTCATTAAACTCGTTTGAAAACGAGGCTTGATTGATTGATTTTTCGTTTTCCCATTTTACTAGTTGGGTGTTGTTGAAATGTGGTTGTGATCAAGACAGTTTTGGAACCCTTTGTGGATCGTTGTACGTTTTGGGCTGAGGCCTTAAATCTACTTGCAGTTGTGTTAGGAGGTTAAACCTCGCTTGATTGAAATTTGGAAAGTTAATTTTATATTGATTACAACAGTATTCAAAAAATGATTTGAGTTCCTTAATTGCATTTCTAGTTGTCATGCCTTGATTTATTTGAAGGCTGGAAGCCTTGTCCGTAAATTGTGTGTTATTACATTGTGCGTGCTGGCAATTAGCCTCCTAAATTGTAATTTTTGAAAGGTTGAAAATTTGGGGAAAGTCCGATTTACAGGGAAGACTCTGTCAAAATTTCGGCACAAAGCCTCGTGCCCCTATTCCTATTTGGGAAAGGAGGTAATAGTTTTAGTAAGTGGGCCCGGCATAGGTGCAATGTCGGAAATTCCACAGGATTCGTTTTGGGTTTCCGAGGAATTCAGGGTGGGTCTTGTCATTACTTGCAACTCTCTTTCACCCCAA

ACAATAAGTCCTAGACTCCAAACACTAAGTCCCATGTGAGGCATGAGCAGAGAAAGAGAGAGCTCCTGGGTCTTCAGATAACCTTGAGTCTACACTAGGATTTTAGGTTTACTTTCTACCCAGAAGAGAAAAGCCAGTAAACTTGTCCTAATGAGTCTCATTTTTAGATCAAACTCTTGCAGCAGTAGCAGTTCTAGAGCCAGTATTAGTAAGGTCCCATACATAGTTAATGAAGAACAAACTGAGTATGAAACTAGTAATGAAGTCGACTGGAAAGATTGGAATATTCCTAAAGTCCCCACTATATAAATTTATAAGAAAAGATGGTCTCTCTCTTCTTTCAAAAGCACAACCCATGTTAAAACAGTTGAGCAAGTCTATGCCCTTAATAAAGAACATGAAACCTGACAATTACTTAATCTTGAGTCAATTAAGAAACATAGGAAAGATGGAAACAATTTCTTGCATATTGGTCTAGTTCAAGTAGCAGTCAAACCTCTAACAAGAACAGGTCTTAATGCTTATATTTTGCTTTGCCTAAGAGATGCACGATTCACCAATTTTAGTGACAGTACCTTAGGAATATTATAATCCAGTTTGTTTATTGGTCCAGTTCACTATGATTGCTACCCTGATTTCACAGTAAGCCTTAGTGATCCCCATATATTAAAAATCTTGACTTTATTAAAACTGCAAGTTATGAGATCTTACCCGGATCCCAACTTTTAGCCTTAGTCTATAGAATTTATTATAAAGTCACAAGAACCAATATGAATTTCCAAGCCCTTAATAAAAGTCCTAAAAATCAAACTTTACTAATCCAGAGTAGCCAAGCAAATGCTAACATAAGAATTCCCCATTCTATTAAATGGTCAGAAGTCACCCTTCCCTCAGATTGGACTCTAGCAACTTAAAGCCAACCCACTTCGGTTCAACATAGGCTATCTAATTTGGATTATATCCAACAATACTGAGATGACACAGTCAAAATCAACTTTGGAAATCAATCTCTAAAGTCTTCAGTACAGCTAAAACGGTTACCAACCCCTAGTCAATCCCATAAGCATAATTCTGCGAAACATTCATTCACAGGATCTACTTCCACTATAGAAAGAGATCTAGAAATAGAAAAGGCCTCAATTGATTTAGAATTAGAATCCCTTAGAAAAAACTC

ATCTCATGATGAAACATCTTTACATACATCATGTGCACGTTCACGGTGGTGTTTCTAACATTGCTTTGATTACTTCTTTTTCATTTTTGCCAGGTGACGTCATGCATTTGCTGGCATTCGTTTAGTCAAGATTTGATAAACTTATTTAATTGGAAACCAAGCCAAAACAAAAGTTATCATAACTTGTTTTGGTCTGACAAGTCGTCGTGGACTTGGCAGTAGAGAGTCGGCATGCCCTCAAATCATGGCTCTTGGTCTCTGATACTTGACCACACAACTTCACAAGACTCATCAACAAGGATGAGGTCAGAATTTAATGAGTAAAAACATGTTAGGAATTGTAGACCAAACACACAACTTCACAGGACTTTTCATAAGCGTTTACCACATTTTACGTTGATTCTCCTGTAGATTCTATGGATTGCCACTTCGTTCGAGGAGTTTTTCTTTTTCAAATTCAAAACAGTATTTAACAAAGTGGAAAAGGTTACTGTTGCTCTATCTTTGGGGATGTTCACACCACTTGCTGAATCTCGATGAATATTATATCTTATTTATTTTAGTACAACTCGGTATTCCCGCATCATATACCATTCATTTAACAAGATTTTTCTTTTTTTGGTATGCACCACAAAATTGAAGTAGAAAGTTGAAGAATAGAAATTGAATAAATATATATCAACCTCAGTTCATGTTGTGAAGAAGGAGCATATATATATAGAGAAAATTATTAGAATTAAGAACTTTATTAAATTCATCTACTCAAGACAGAAACAAAAACAGTAGGAATTTCTCATTTTTCTAGGACTTTCTAATATATCAAAAGAAAATCTGATCAAGAAAATGTCCCAATGAAGAGTGCGAGGAGCCACCTTCCATCAAGGCTTTTTTGCTCTTCTCACTCATCTCTTTCACCCTCTTCCTCACATCACTATCATGCTCCATCACCTCCCTTATCCCTCTCTCTATCTCCTCCGCACTCACCACCACTTGGGTGTCC

>Chr3-20; OZ022208.1; 24166294-24167726; dir; B;

TTATAATTATTACTTATATTTAATTATATTTGATTGATTATTAAGCTTCAATTAATGGTTCAGTTTAATTATATTGTATTATTTATCAATGTTCTTATAAAAATAATTCATGTCTCAAAAGGCTTACGCCTCAACGCTTCAAGGCTTGCGCCTCACCTTACGAGACCAAAAACGCCTTGCATCTACACCTCCCCTTTCAAAACATAATGCATAACATATAGGTTAATTAGAGCTTGAACATCTAGGTAAGCATTTGTATAAAGTCTATGATTTCAAATGTGCTGGATACTAGTCCCTTCTGCATCTGCATTGATATGAAACTTTCCACAGCGTCCTCCTCTGTCGTATCTGGGCCGACAGGTTCATAGTGATGCACTTTCGAAATAGTTGTGTTGTGCCGCTGATGTTTATGCAGGATGTTTGTATTTAGAATTAGGCAGTTTTTTTTGTGCCAGTTTTGCCTCACTTCTACAAAGAAGCTGTTTAGCCTCCTGATTGTTTTCCTTCCGCCTGTCCTGCACTCTTGGACGCGGCAGTAATAATTACTGGCAATTCTGCCACGGTTTACTTATGGAGCCGGCCAAGCTCTCCCCCAAGCAGAATTTAGCTTGCCTTTCTGGTTCTTGGGCGGGAGTCTATATTCTTGAAAAGTCGGCAACCGACAATTATATGTCCGTACTTTCAGTATTGTTTGAAAGGAGGTGTTATTGAAACTTAAAAATACTCCTCCTTCGTAGAGAAAAAACATGTGGGAGAAATTACGTATTGAGCCAACAAGAGTTCCCTTGTTTGTTTGTTTGAATCGTTGAGTTGACCCTAATGAAGAAATTGTAGAGATTCCTAAATTCATAGATACCAATGAACCAGTGCTTAGGTTTTTTATCGTCTTTTGGCACATGCTTCCTTCCTAGACTGAGCTTCTGCAAAACGTGCGTAATCAGATATATATATATATATATATATATTACATTTATGTCTATTGAAAACCCCTTGTTGATGT

CTATTCCTCCGTCTAGAAAGAATAATTTTAGCCGACAATTTAGAAAAACTCGTCATTATTCTCGTAAAAAACAGTCATTTCAACACAATAATGAAAATAAAGAATTTTATAGTAAAAGGAGATTTTCCCCTAAGAAAAATTGGAAAAAGAATTACAGTAAAGATAAGAATAGCAGCTTTAAGAAAAAGACCGACAAAAGAAAAGTAAAATGTTATAAATGTCAAAAGTATGGCCATTACGCTAATAATTGTAAAGTTAAAGATACAATCAAACAATTGAAAATCACGAATGAAGATAAAAACAAATTAATCCAAGTCCTTGAGTTAAGAAACTCTGAGTCTAGTGAAGACAAAACCATGGTTAGCCATACTCAGTCCTCTGAAATATATTCTTCTGAGTCACAACTCAGTTCCCCCAGAATCCAATTTGGTTGCCAAGATAATTGCTGTAAATCTAAGTCAATCAATGTCCTTACCAAACAGGAAGAACAAGAAGAATTACTAATCGATCTAATAAGTAAAGTTGAAAATCCAGAATTAAAATCTGAATACCTAAAGAAATTAAGAAAAGTCATTAGCCAAGAAGAACCGAAACAGTCTACTCAAAAGATAAATCTGAACACAACCTTAGAAAAATTTAAGAAAAAGAAAGAAGTTACTTTACAGGACTTACAATTAGAAGTAAAATCAGTTAAAAAAGAAATCATGAGTTAAGACAAATAAGTCAAAAATTACAAACTGAAAATTATGAGATAAAATAATATCTAATTAACTGCTTAGAAAAAGAAAATTTGAGTCTAAACCAGAAAGTCCCACTAACTCAGATGAAGAAAACTCAAAGCCTAGTCGTCATAGGTAAACAATTAGACAAGATAGAAACAAAAGTTGACAAATTGCTTCCAAAAGAGATTTCTATTAAGCCACATGACCAGAAACCACTAGTCACCTTTCAGGACCTAAAATCCAGTCCAACTTTTAAAAAAAAACTACAATGACAAAAATCGAAAAAATGTTAGAAGAATCAACTCCAACCAAACGGAAGGATAAAGAGAAAGTCGATTCAAGCGTCAAAGTTTTAGGAACTTTTGAGCCATCATTCACCTTTCATCACTCCTCTGAGTCTGAATCAATTAGCATAGATACTGAGTCTAGTAATATTTCCAAAATTGAAAATGCGTTTAAAAATCTTGAGCTCAAACAAAGTCCACAAGTACAAAGATTAACGAACAGAGTCAGCCCTACTAGCCTCACAAAAAATTGGTATCCTAAGCCCACGCCACCTGATATCCAGTTCGAAGAACGAAATACTCAAAGCCAATTTTCTATATCTTTCGATAAATTATATGAATGGAATATTGATGGTCTTTCTGAACAAGAAATTCTTGATAAACTTCAATATATGTCTATGGTAGCCAACAGTTACATCTCCAATCA

TATATATATATATATATATATATGTATTTCTGCGCATGAAGAAATATAAAATAAAAAAAAGTTATAGGCCTCCCTGAAAATCAAATGAAAAATCTAAATTGTATGGAATTGTTATAAACTAAGACTGCCAGCAGGAGATGAAACAGAAACAACCAAGTAGATTGCCCTGCTGGGAGATCCAACCAATAATTAAGAGCCTTGTAGGGACGGATCAGGACAATGATAACAAAAAAATTACACAGGGTACATAATGAGCCTTATTTCTCAAAAATCTCGTATCTGATGCCGCTATTAAAATAACATGTATTTAAAATAATATACATTCAAAATAGTCATTTTCTAAATTAGCATAATTGTTATTAGAATTCCCCAAAAAATAATAGCCCAATACAAAGGTAGAGGAAAAGGACCTCTATGGAGTTGAGAAGAAGCAACCTTGGAACTGGTGCAGTCGATCGATAAAAGTGTTGGTTTTTTTTTTTTTTTTGTTTGGTTAGCCAGCCCTGTGTAGATTAGTTAATAACATTGGCCTAGGCTAGTGTATTATTTTAGTTTTTTTTTTTAAAAAAAAAAAGTCTCCTAGGCTATAGCCCTGGTTAGCTTGTTAGTAGTACAGTACCTGAAACCTTGTCTTTCATAGAGACATGAATGGTCTTTTCCGTCCTATCTCAAATATATATATATTTTTTTTTGGTGAAAGTTAAGATCGAAGTTAGGATAAAATAGAGTTTTTCTCACATGTACTACGATGTATTATTGAAGTTCGATCCTAACATCATTCATTTACAAATGAAAATCTTTTTTTACGCTTAGGTTAGACTCCATTAGCCTATTTTTTTTATTTTTTATTTTTATCAACGAGTACATTCAACCCAAACTGTTCATTACTTGAATGATTATCCCGTCGAACACCTACAATCTTCTTCCTCTCCCCCAATTAATACGATTTGCAGAAGATGTGTCTCCTAGTGGAGCTAGTCCAAGTAAGAGGACTTTCAAA

>Chr3-21; OZ022208.1; 26223513-26226158; rev; B;

ATTTATGGGGTTAAAAACGACTTGCCAAAGTCGCTTTCAAGTCGCTTTGGTCTTCGTCAACTTTATTTTAAAGGAGACTATTGCAAAATGAATTTGGAGTTTAGCTACTTAATTAAGTAGCAGTTGAGGTGTACTGATTTGTTAGTTTGAAATGTTGGTATGATTATGCATTAACATATTATAATTCCTTAACAACCAAACTATAATATTCTGTTTTGAAGATCCTTGAGACTGTAAGTAATGGTGGCAAAAGATGGTCTCACAGAAGCATCTCCATCCCATGAAAGGCAAATTAGGTCAATTAGCTTCCGAAATTGCCCATCATCATCAGGAAGCCTTGGCCTTAGGTTAGCCTCCACAACTTCCATCGCAATCTACAAAAAATAATTAAGCACAATTAACAATCATGTTTAATTTGATTATTGTTTGCTTATAGTTTTAAAAAATAAATAAATACAAACGATAGTCTAAACTACTCTAAATTAAGCAAATGATCCACTAGTGTAGTGGCTTGGAGTAATTACTCCCTCTAGGAAAGGTCCTGAGTTTAAATCTTAACATCTGTGTGGTGTGTATGTAAGTTTAGTACAGGTATGGACCATAAAAGGTACTAACCTGAGCTATAACCCAGACAATTTTTAATTTTTTAAAGCCCAAAATACATATAGCATAGCACATCACCAATTCAAAATATATTATACCCTTTTCTAACCTATTCTCTCTCTTCTACCGAAGAATCCACTAACCTATTCTCTTCTACTGAATTTGCAAGTCGTTACACTGTGGCATACAACAATTTTATTTTTTAAAGTGAAAAATAATAATCAATTATTTGCATGCTAATTAGCAATCACAAATCAAATTTATAAATAAATTTGGCGTTTCAATTTGAGAAACTTAAATACCAGTATGTTCAGCATAAAGACTAGGTTTTCCTTTCAATGCTTGTTAACTATATGAAGACTTGTATATAATATAATCATATATATATATATATATA

TTACGTACTGCTTCTGGTAAATCTTTACAGTTAAATTATGAAATTCCTAAAGCTCATGTATGCCAAGATAGAATTTGTTTTAAAACTTCATTTGTTTTGATTAAAAATATAACTGATGATGTTATTTTAGGATTACCTTTTATAAGCATGCTTTATCCTTTTCAAGTTGAATATGATGGTGTCGTCTCTAATTATTTAGATGAGAAAGTCAAGTTTACCTTTTTAACTAAATCTGAGCTGTATAATCTAAAACATCTTGATAAAAATAATTTTGCAAAAACTAATATTTTAATTAAAGACAAAACCAATCATCTGAGTCATCTGCAGCATGAAATTAAAAGTAAAAGAATTGAGTCTCAATTAAATGACCCTACTTTACAAACAGATATACATCATTTTGAGCAAACGTTAATTTCTGAAGTATGTTCTGATTTTCCTACTGCATTTTGGCATCGTAAGAATCATGTTGTTAATCTGCCTTACATAAAGGGGTTTTCTGAAAAGAATATACCAACTAAGTCTAGACCTATACAAATGAATCATGAAATGATGGAGTTCTGCAAAAATGAGATAAATGATTTATTAAAGAAAAATATAATTAGGAAAAGTAAGTCTCCTTGGTCTTGCCCTGCTTTTTATGTTCAGAAAAATGCAGAACTAGAAAGAGGATCTCCTCGCCTAGTTATTAATTACAAGCCTTTAAATACTGTTCTTGAATGGATCCGTTACCCTATCCCTAATAAAAGAGACCTGATTAAACGATTAGATAAAGCAGTTGTTTTCTCTAAGTTTGATATGAAAAGTGGATTCTGGCAAATACAAATTCATGAGTCTGATCGCTATAAAACTGCTTTTGTTACTCCTTTTGGTCATTATGAATGGAATGTTATGCCTTTTGGTCTTAAGAATGCCCCTAGTGAATTTCAAAATATTATGAATGACATTTTTAATCCTTATAGTCATTTCTCCATTGTTTACATCGATGATGTTTTAATCTTTTCTGAATCCATAGAACAGCACTGGAAACATTTGCATACATTTCTGCATATCATTAAGGCTAATGGATTAGTTGTATCTGCTAAGAAAATTAAATTATTTCAGACTGTTATTAGATTTCTAGGATACAATATAAGTCAATCACAAATTCACCCTATAGATCGAGTTATTTATTTTGCAGATAAGTTCCCTGATCAAATTACAGACAAAAATCAATTGCAGAGATTTTTAGGGTCGCTTAACTATGTATCTGATTTTTATAAAAACCTAAGGAAACAATGTAAACCCTTATTTGATAGATTACAGGCTAATCCTCCTCCCTGGTCGTCCATCCATACAGATATTGTAATACAGATTAAACGCCATGTTAAGACTCTTCCTTGTCTTGGCATTCCTACTTCTACTTCTTTCAAAATTGTTGAGACTGATGCTTCTGATATAGGTTACGGTGGTATTCTTAAACAGAAATCTGGGTCTGATTCCTCTGAGCAAATAGTCCGTTTTCACTCCGGTATCTGGACTTCTTCCCAGTTTAATTACAGTACTATTAAAAAAGAAATATTAGCTATAGTTTTATGCATTACTAAATTTCAAGATGATTTATTAAACCAAAAATTTTTAGTTCGTGTTGATTGCAAATCTGCTAAGCATGTATTAGAAAAAGATGTTCAAAATATTGCATCCAAGCAAATTTTTGCACGCTGGCAAGCACTTTTAAGTATTTTTGATTTTAATATAGAATATCTGAGAGGAAGTGAGAATTCTATTCCTGACTTTCTTACAAGAGAATTTTTACAGGATAAGCAATGGCTGGATCATCCAAAGACAAGCAAAAAATGAGTCACAATCCTCCTCACTGATCCTACAAAAATTATTTATCACTCTCTTTACATTCACCAAATTTTACATCCTAAGTCTTGGGGTTCTCGTCCTCATGAGCTCCGAACCCTTCCTTCTAAGTATCAATATAATTATGCAGACTATGTTGAAGCCTGGTATTACATCTTCTTACATCAAACCCCTGAATTTTCCCATTCCTGGTTTATTAATTTTGATAACAAGTTTAAAAACACTGAGCTCCCTTATTGGTTCCTTCATTGGTGGGACACCCATGGTCCCAAATCTGATATCATCCCCTCTGCAGTCCATGAGTTGGTCTCCTATTTTACCCAGAAATACAAGCTTCCTGAACGTGATTTATTTTTCCCTATTGATCTCTTGTTTTTTACAAAGTACAAGCTCCCTTGGATTATGAAGTGGAATTACAAAGTTGATTGGGAGACACGTTTCTTCTCCCGACAGTTCTTTGTTAAATGGTGGGATCGTTTTGAAGTCCACCGCATTACAGACTATGTTTATAAAGATTTTCCCCAAATGACTGACCGTACTCCTAAGCCTAAGCATGAGCCTACATCCTCTTCAGATACTATTCTTACTGTTGAAGGCAAGTCTAAGACCGAGCTGCAAGATTTAGCCCAGCAGCTCCTTATTCGCGCTTCTCAAATGCATGATGATGATGCTGAGTCTGAGACTTCCTCCAGTTGTCAACCTGCACAGCCCACTCCTGAGCGCCCTGCCAGGTGGAGTGATTATTTACATGGTCAAGATCCTAATGATGCCTATGAAGCCTATGATCTGAACTCTGATTAG

ACTACCCGACAAGTTACCTAAGTAAAAGCAGATTCCCTGAGTTAAATTTTACCTGAGTTACTTTTGTAAACCTAAGTTACTTTCCTTGATTATTTAAAGAGGCTTTGGCCTCAGTTGTAAGCATCGAATTCTTTCCTTTTCTTAGTTTCAAAATGTTCTTTCAGAGATCATGTTCTGAAGAACAACCTTAGGAATGAGAGTAAGCTTTGTACATTGTAAGTTTTAAGTTTTTAAGAAAATGAAGAATATTATTTTCATGTATTTCATTATTTTTATAATTATGTATGCATTATCCTGCATAGTCCTTCTTTTACTTGCTTACTTCCCTTTTTGGTATCAGAGCCGTAAGCGTTGTAAGAATTAGTTCTTAGGTTACAACTCATCCAACCCAAGTGGCTATATATATATATATATATATAACACACATATGAGCGTGATGAGATAGGGGGGCCTCGAACCCACGCCATAAGCAGAAGTTAAAGTGCAGCACTTCGCTGCCAAACCCGTCCTCATGTAAATGTCCTATGCGGGCTTTATATCGATAATATTTAAAATATTAACATATATAACATATAAGTTTTTTATAAAAAAAAATTTGGGGTTCTGTGCAGTGGCACTTGCACACATTCAGGACCGCCTCTACCTGCACGTAAGGAGGCTGCAAAGGCTTTAGAATAGCGTCTCTGACGTACTTCACCATACCAGACTTGCTTTCTTAATTTTAATTTTTTTTTATGTGTTTCTTTAGTTTTACTTTACCTTAAAAACCTTTCTATATATTATTTATAATATAAATATTTTTACATATGACTTTACAGGAGAAAAAAAAAAGAAAAAAAAGGGGGAAAACTATAATTTTTATAACATCTAGCTCCTCCATGTTCTGTTTCCATGCAGTGTAATCGAAATTGGTACATAGTTTTGTTGTGCTAGTTTCCTACTCTCTTTTGCAGTTGTTACGTTTACTTTTGGGATATTTCTTTCCCTCTTTCTAGGAAAG

>Chr3-22; OZ022208.1; 28270802-28272340; rev; B;

AGACCTCGTTCATCGGCGCAGTCAAACAGTCAGGTCACTGCTTTGACAGCACAGGTGGCCACTCTTCAGAGCCAGATGTCGGTCATTCTGCAGTCCCTCGCACAGTCCGGCATTCCAGTCCCGCATTTTGATGCGCCGACCTCCGAGCCTGTCCATCCCGAGCATCCCCACCAGACCACTGCCCCGGTGAACGCCCAGACCTCCGAGCCGCATACTGGCGACGACTCGGTTGATTTTGATTCATTATTTGATTAGTGTATTTATTTTCATCATAAATATTTATTTTATTTGTAATACATTTATTTTATTTTATAACAAAATTTTATTCTAATTTATTTTTATTGCAATTTCATTGTTTAATCAATAAAAAAAAACCAATTTTATTTAAAATAAAAAATAAAATAAAATTTTTGTAATATTATTTTTGCGCGACGGCAAATATTTCGTCGCGCAAACCCACATGTAACAAAAAAATAAAAAATCAACTTTTTTATTACTTTTAATTCTTGCGCGACGAATACATACGTCTCGCAAACACGTTTTGCGCAACGAATCTTTTCGTCGCGCATCTTCTTCGGCGCGACGACAGAGATTTCGTCGCGCAAATATATGAGCGACGAAAACTTCTCATCGCGTGAATGGTTCTGCGGCGAACATCTACGTCTTCCGAACAAGTTTTGCGCGACGAATTATTTTTCGTCGCGCTATTCTAGTCGTCGCGCAAATATACATGCGCCGAATACTTGTTCGTCGAGCAAATTTTATGCGACGAACAGTTGTTTCGTCTCCATACCGTTGGTGCAACGATGGTTTACCCGTCGCACATTTTTGCGCGACGAATGGTTGTACCTCGTCGCGCAAAGGCTTTCTTCACGATCTTCGCGCGACGGAATATGCGCCACGAATTGTTTGTCGCGCTGAAGTTAGTGCGACTAAATGAGACTTTGCGCGACGTTTTGTTATTCGTCGCGTAAAGGGTAAAATGTAGTAGTGTTAGTTG

TCTCTGCAAAGAAAATTAAGTTATTCCAAACCAATATTAGGTTTCTAGGGTTCCAAATCTGTCAATCCCAAATTTCCCCAATTTATGTATTCATCCAATTTGCTGATAAGTTCCCTGATCAAATTCTTGATAAAAGTCAGCTCCAGAGGTTTTTAGGATCCCTAAATTATGTCTCTAACTTTTACCAAAATCTGCGAAAACAATGTAACCCCTATTTGACAGATTGCATAATAATCCTCCTCCTTGGTCGAATCTCCATACTGAAGTGGTCAAACAAATCAAAGCCCATGTGAAGACACTTCCCTGTCTTGGTATTCCTTCAATTGATTCTTTCAAGATAGTTGAAACTGATGCCTCTGACATAGGTTATGGTGGCATTCTCAAACAGAAAGTCAGTCCAGATTCTCCAGAACAAATTGTTCGTTTCCATTCTGGAACCTGGACTCAGTTGCAATATAATTATAGTACTATTGAAAAAGAAATATTAGCTATTGTATTATGCATTAGTAAATTTCAAGATGATTTACTTAATCTAAAGTTTTTAATATGAGTTGATTGTCAATCTGCAAAACATGTTTTAGAAAAAGATGTTCATAACATTGCATCTAAACAGATTTTTGCACGATGGCAAAGTATATTAAGTATATTTGATTTTGACATTGAATATTTGAGAGGTAGTGAGAATTCAGTCCCTGATTTTCTTACTCGAGAATTTTTGCAGGAACCACAATGGCTCCCCAAAAAAGACAAAAACAAAGAATTAGCTAGTCAGCCTAAGCCTTCCCAGTCGCAACAGTCGCAACAAGCCCAATCCATGAGTCCATCTAAGCCAGAAGTCCCCAAACAAATAGTTCCCTATCTTGGTTGCACTCCTCTTACAGTTGCTACCCCGTTATCAGTTGCCAATAGATTCTCCTCTCTAGGATCTATAGTTGGTCAGATTCGACCCAGTTATCAGTTTACTCTAGTTGCCAGTTATGATCCCTTTTCAGTTGATGTCCCTGCCGGGCCATCAGTTACTTATAAAAAATCATCCCCCTATTTGCCCAAAAGTAATTCTCACTTATTTGTTATTGAACCAGTTTATGATGTTCATGCCAACCCAGTTGAAATTGCCAGACATTATTTCCCTTCTGGATTTCATTGCATGCCTTCTAGTCCTTACAAATCTCTCAAGTGCTATAGAGATATATTACTTGAAACCAAGTCAGTTGAGATCAAGCCCATAAAGGATCGTGATCATCCTGAAACCATCCTTTATCATTCTTTATATATTCACCAGATTGTAAGCCAAGAAGCCTTCAGTAGCCGCCCTTATGAATTAAAGTTGTTACAATCAAAATTACAATATAATTATTCTGATTATATTGATGCCTGGTATACTATTTTCCTTCACCAATCTGAAGATTTTAGTCATTCCTGGTTTATCAATTTTGATAATAAGTTCAAATGTTCTTTCCCATATTGGTTCCTCCATTGGTGGGAGAAACACGGTCCAGTTGATGAAATATTACCAGTATCAGTTCTTGAGTTGATCCT

CTTTGGGTTTTATTTCTTGTCGTTCTTCTTATCGTTCTTGTTGGTATCATTCTTGTTGTGGTATCGTTCTTGTGGCATCAGCTTATATATATGTAACTTACTTAGGGTAACCCAGATTGCTCCTCGTCGGGTTTCATTGTGAAGCAGCGGGCAATGATCATGTTCTTCAGATACTCTATCAAATTTTCACAGGTGTGAACAATTCCTCCAACTGCACTTGTAAACCATGTTTTGATGTATGGGTACAAGTAAACAGCTACAAAGCTCACAAACCAAATGAGGAGGGCAGCTGACCCTAAATATGGGACAAGGATCATCAATTCCAAAATGAAGGTAAGAGCTCCAAAACTGAGGCTAAGCGAGCTCACGAATTTTCCAAAGTTTGAGTTAGAGTGAGCTTTCAGTATATATGTAGCACCAATTAGTGCCCCGCAGTAAACGGTTAAAGCAACAACAAGCATCACAAAAATCAGATGGTTTGTGTCAAATAGAGATGTCATTCCAGCTCTTTGCTTTAGCGAGAGGAGTACGCCTAGCATAGCGATGAAAGAAAGTATATATGTGTGCAATGCCTTTGTAGAAGGTTCTTCGCTTGTGCTGTTATAATTAATGACAAAGCTTAAATGGTCTTGCGACAAAGGATTATATATATAAATATATTTATAACTAATAAGAAAAAGTCTGAGTAATATTTTTGAGAATAAATAACAGACCATAGCTAGAAATAAGCCGAATAATTTTCGGGAAGCAATTGTGCCCAAAAGAATAAAAAAGAAAGGAGATGTCTGAAAATGAAAACAAAGAGAGACACAACATTTCCCCCACAAAGAAAGACCAAGTACCTCAACTTTTTTTCTCCAACAACCTTTCTCATCAAACCATGCAGGATTTCATACCTCAACTTTATGTTTTTGGAGAGGAATTCTTCTCCAAGAACAATTTTTGAGTCAGGTTGGTTTTTCTGTTTTCCAGAGACCCATATGGCATAACAGAAGGAGAA

>Chr3-23; OZ022208.1; 28282660-28286588; rev; B;

GGGAGAATTAGGGATTTTGGGTTTGGGGAATGTCGAGCTGGGCTGCTGGAGGGAAAATGGAGAAGACAGTGCTCTTTTGACATTTTTTGAAATAAATAAATAAATTCGTGGCTCAAAACGACGTTGTTTGAGCCAGAAATTTTAAAAACGAAATAAAAAAAAAGAAGCTGCGCGCCAAAGCGCTGGCGGGACAGCCCAGCATTGGCATTAGACAATCTCTTTTCTAGAACCGGACCATCTGCTTTCTGTGGAAGATTTGCCATTGCAAAGTGTAGTGGCGGAGCCACTCCTAGACTTGGAGTGGCCCTGGCCCCTCCATATTTTTATCACTTTTTTATTATATTAAATATGATATATAGAATTTGTAATAAACAATTTAGAATTTAAGTTAAAATTTCTTCTAAAACAAATTAGACATGACTCAATTAGAAATATGTAGCTATAAACATGTAAGTATTGAATACTTAGGTGTTTTATGTACTTGTATTTTCTACTTCGGTCCCCCCACTTTTACAATTCTAGCTCCGTCTTGGCAAAGTGGTACCCCCTCAAGCCGATGTAATCTTCCTTGTGGTATCCTAGCTTCAGTTTCTGATGAAGGAAACATTTATAGTAATAGTCTTGTGCTTTATTAGCAGTAAAGTAAACAATGAAGGATTGTCTTGGTGAGCGTGGCAACCAAATAGAAAACAACTCACAAGGGGTATTAACTGCCTCATACTAATTCTTCATAAAGCAAGATTAGCATAAACATTGTCGATTATGAACAAGTAAATTACAAAAAGACTAATACTACCACTTGTACCTCCTCAAAGTAGTATATATGCATTGTCAGCACAATTTGGGCAAAAGTAGCTACAACAGTATGAAATCAAAGAAGTAATATGAGAAATCCACATGCACATTAATAAGGCCCATTCTCAGCACAATTTGGGCCAAAGCACAAAAACGAGGAACGCAAAAAGCCTTCGCGGCTACAAACTATATATATATATATATA

TTTGAATATAATTCCAGTAGTAAAGAACAATTAGATTTTAAAGATTGGAACATACCAAAAGTTCCCAGTCAAAAAATTTATAAGAAACATTGGTTGCCTTCTAGTTTTAATAGCACAACGCATGTCAAAACAGTTGAACAAGTATACGCCTTAAGTAAAGAACATGAAACATGTCAGTTGCTTAATCTTGAGTCTATTTTAAAACATAAGAAAGATGATAATAATTTCTTGCATATTGGTTTAGTTCAGGTTGCAATTAAGCCTCTGACACGATTAGGATTGAAAGCCTCAATTCTATTATGTCTTCGAGATGCTAGATTTACTGAATTTAGCGATAGTACTCTTGGAGTCATTGAGTCCAGTCTGTGTAATGGCCCTGTCCATTTTGACTGCTATCCAGACTTTACTGTAAGCCTTAGTGATCCCCATATCCTGAAAACTCTGACCCTAAATATTAAGACTGAAGGTTATAATGTCCTTCCTGGTACCCAACCATTAGCCTTAGTCTACAGAATTTATTATAAGGTCACAGGAACAAATATGAACTTCCAAGCCCTTAATAAAAGCCCAAAGGATCAAACCGTCCTGATCCAAAGTCATACCTCAGACGCCTATATACAAGTTCCTCATACTATCAAGTGGTCAGAAGTTGCCCTTCCAAAAGATTGGACATTAGTCACTGAGAGCCAACCAGCTCCAATCCAACGCAGTCTAAATAATTTAGACTACATCCAGCAATACCTAGATGGCACAGTTAAAATTAAGTTTGATAGTCATCCCTTAAGAAAATCTAATGTCCAGTTACAACAGTTGCCAACTCCCGGTCAATCACAAAGGCATGTCCCTGCCAGGCATTCCTTTGCTGCTTCATCTTCCACTCTAGAACGAGACCTAGAGTTAGAAAAAGCAATGATTGATTTTAAACTTGAGTCCCTGAGAAAATCCTCTCAGGTTACCCAACCTTGCTATGGCCAGGTTTCAGAAGACAAGTCAGAATCCCCTGAGTCACCAACTCAGTCAGATTTTGTAGTTGAAAATCAACTCCGAACCCTTAGTAAGAAATATGACATTAACTGGATATACGTCTAAATCGTCATCTAAAAGCTCCAGAAAATCAGGCAAGACGAGATAGTTATCACCAAGCCTATCCAAATAGCAAACACCGCGAACTAATCTTTAAAAAATGGAAAGAATATATGCGGTCAGCAAAAATTGAAATTTTTTATCTTGATTGAAAGCCGTTATATGTCCACAGATGAGTTAAAAACTATAAATAAAGAAAAATGGATATTAGTAGATAAGTCGGTTGTTGAGTCTAGTCATCGTCCAGTAGAAACAATCATAATAGATCACCAGAAAGCCCCAATTCCTGCCACTCCTTTCAAAACCTTTGAAAGCAGTGATCCACATAGGAAATTAATTGAGCAGAACAATTACACGAACCAGAGTTTAATCACTATAGGGAAACAGTTAGACACAATAGAAACAAAAGTCGATAAATTAAATCTCGCAGAAACAGGATTCAAACCTAAACCCAGAATTGAGAAACCAATAGTCCAATTTCAAGAACTTAAGTCCAGTCCTGCACTCAAAGTAAACTCTACAATGAAAAAAATAGAAGAAATGTTAGAACAGTTGACACCAACTAAAACTTAGAAAACCGAGAAATCTGGTCTTAAAACACTAGATTCTATTGCTGCCTCACATTCTGAGTCCAAGTCAGTTAGTTTTGAAACTAGTAATATTTCCAAAATTGAAACTGCTTTTAAATGTCTAGAAGTCCAAACTGCGCCTAGAATTAAAAGATTAGACAAACATGTAAGTCCTACTAGTCTCACAAAAAATTGGTACCCCAGACCAACACCTCCAAATATCCAATTTGAAGAACGAAACTTCCAAACCAGTTTTCAGTTTCTTCTGATAAATTGTATGAATGGAATATTGATGGTCTTTCTGAACAAGAAATCTTAAATAAACTCCAACATATGTCTATGGTCGCCAACAGTTATATCACCAATCACAGTTTCTGACAATCTGAAATTGTCCCTCTGTTAGTAACTGGTTTTACCGGTACTCTCAGATACTGGTGGGATAAGCACCTCACCCCTGAATCCAAAAGTCGAATCACCTATGCTGTAAAACTTAATGAAGATGGTCTCCCTATCTTTGATGAACAAATAGGTCAAGGTATAGAAGATGTCATCAACACTTTGTTTTACACAATAATTGAACATTTTATCGGAACCCCTAGTAACACTACTGCTAGGATCCATGACCAATTGAGTAATCTTAGATGCCCAAAGTTGTCTGATTTCAGGTGGTATAAAGATGTTTTCATTTCTAGGGTCATGCTTAGAGATGATAGTAATCAGCCATTCTGGAAAAGAAAAATTTATCAATGGCCTGCCTAATTTGTTTGCTCACAAAATACGCACAACACTAAGTAATGAACAAGGCCAAATAGATTGGGATAATCTGACCTATGGAAACATTATAAGCACAATTAATCAAGTTGGTATGAAGATGTGTATTGACTTTAAAATCAATAGACAAATTCAGTCTGATAGAAAGTCAGCCAAGTATGAGTTAGGAAACTTCTGTGAACAATATGGTCTAACTAGCATCCCTCCTTCTAGGAAGAACAAGCCCAGTCATCTTAGGAAAAAACGTTATTCATCCCCGAAGAAACCGTTTTTCCGTCAAAATGAATTTTAGAAGGGAAAAAAAAAAGTTCTCCCCTAAAAAGAATTGGTCGAAACATCCGGAAAATAGGAAGAAAAGCTCTAAGAAAGATAAGAGCAAAGTTAAACGTTTTAAATGTCAAAAGTTCGGTCATTATGCTTCTGAATGCAAAGTAAAAGATGTTATTAGACAATTGCAGATAACAAAAGAGGAACAAGAAAAGTTGATCAAAGTTTTAGAGTTGCGAGACTCTAAAACAAGTTCTGTTGAAACTATAGTTCCCAGTTCTGATTCTGATTCTGACCAGTCTTCAGAGTCACCACCCAGTTCCCCTAATGTTCAATTTGGTTGTACAGACAAATGTTGTAATAGGATCAAGTCTATTCTTGTTCTTACCAAACAAGAAGAGCAAGAAGAGTTGTTGGTCGATCTAATTGGCAAGGTTGAAGATCACAAGTTAAAATCTGAATACTTGAAAAAATTAAAAAAGATCCTCCGCAAGAAAACCCAAGTCATAGTACTCCCCAACCTATCAGCATCAATACCACTTTAGAGAAATTCAACCGCAAGAAGGAAGTGACCCTCCAAGATCTCCAATTGGAAGTTAAGACAATTAAAAAAGAATTAGCCGAACTAAAACAAATAAGTCACCAATTGCAAACTGAGAATTATGAAATCAAACAAGAGATTTCTAGTTGTCAAAAACAAAAGTCTTTTAAGCCCCGTAGCCCTTCAGAGAGTCCTCATCATTCTGATGAAGAACTTGCTGAAGAGCAGCCAGTTGTCAATTTAATCAAACAAGTCAATCTGAGAAAATGGTATTCCAAAGTCACTATATTTATCAAACATTTTGAGTTTACCACAATTGCTTTATTTGATTCTGGGGCAGATCTTAACTGTATCCAGGAAGGTCTAATCCCCACTAAATATTATCAGAAATCTAGAGAATCCCTTAGCACTGCTTCCGGAAAATCTCTCCAGTTGAATTATGAGTTGCCAAAAACCCATATTTGCCAAAACAAAGTCTGTTTTAAAACTGCATTTGTCACTCCCTTCGGTCATTATGAGTGGAATGTCATGCCGTTTGGTTTAAAAAATGCCCCTAGTGAGTTTTAGAATATTATGAATGATATCTTCAATCCTTACAGTCATTTCTCGATTGTTTATATTGATGATGTCCTCATCTTCTCTGAATCAGTTGAACAACATTGGAAACACTTGCATAAATTCCTTCATATAGTCAAACA

AAACGGTTTACACTACTACGATCTACTATTTGCGCGAAGGAAGTTTGTGCGACGAACACACTTTCGTCGCGCAAGAAACACTGTTGCAACGAAAAAATAATTCTGTCGCGCATACAACAGAAAACTAGCACGGCAAATATTTTCGTCGTGGAAACAGTCTATGCGCGACGGAAATAAATTTCGTCACGCAAAGGTGGGTGTTGGCGCCAAAATCTTGCACGACGACAGAAGTCGTCGCGCATGAAAATATTGCGCGACAATTCCAACTATTCGTCGCGTAAAGATGGACAGAAAATTGGCTCCGTTTATGGCGGTAAAAAAATTTTGCGCGACGAGAGTTTTCGTCACAAAAGACAACTTAGCGCGACGAAAAAATCTACCGTCGCGTAAGAAAACGAGGGAAACTTATTTTTGGCGCCAAAATGCAGGGTGGATTAAGTTGTTTTTGCGCGACGGAAGTTCCTTCATCGCGTAAAGTTTAATAGCGGTGCGTTCTTGCGCGACGAAATAGTGTTATTTGCGCGACGGAAACTGCGCTTTGCGCGACGTCATATAGTATTTGCGCGACGGAAGTATGTGCGACGAATAATTAATTTCGTCGCGCAAAAAATCAGATGTCGACACGTGATTGCGCGACGGAAATGTTGTTTTGTGCGACGGAAAAGTGGATTTTCGCGACGGCATTTTGCGCGACGAATTTTGTTTTTCGTCGCCCAAAGTCCTTCTTCTTCATATTAGTATCTGTCATTGCAGAGGCAGAATGCAAAAATTGCATTATGCCTCTCTCTCACTCTCCCTGCCGCTGCTCTGCTGTGAATTCAGTACGTTTATTGGGTATGTATTTTTTGTCGTTAGTTTAAATGTATTAATGTAAATTCGTACTAATACTATTAATTTTGTTCTCGTTAGGGATATTTGTGATTAGTGATTAGTGGAGAACGATTGAGAAGGTATGTTATTGTAAAAGTTTGTTTGTTATGTTTGTAATTTTTTTGTGAAG

>Chr4-01; OZ022209.1; 1379780-1386818; rev; A;

ACAAATATATAACAAAGAAAATAATACGCGTTCATGTAAGGATATATACGTTGACAAATAATTGATATTGTAGGTGATATCCATATGACTGTGATGCATCATTAATTCTAGCAGGAAATTAATTAATTACCTTGTAAACAGAGCCAAATCCTCCTTGTCCGAGTTTATTAACTTCAGAAAAGTCATCTGTGGCTAATCTAATGGTGTCAAAGTCCATTTGCAAGTATTCCGCATTTCCAATCTCATCTACATCTTCGCCTGAGGAACAATTTAAGAAAAATTAAATAGGTTCTTTGTTTAAGGAGCATCCAAAAATGATTATATTATAACAACAATAAGCTTCAGCAAATCAAATGGAATTTTGATGTTCACTAGAGTTCACTTTGTTCCAGTACCTGGAAGTATTTCAAGCTTTCCCTTTGTCTTTCTCACTCTTAAACAAATGCAGATGGATATAATTAGTAGCACAGAAACAACGATTGACACAACAATAATCACGACATTCCGAGATCTGTTGGATCCTGCAAAAATCACCAAATCTTCTATAATCAAAACATTCTGCATATATATATATATATATACAGTCCCCTTCTATTGAGGGATCCCTCAAATAAAAGGGACACCCTTTAGGGTCCCCTACGAATTTTTTTTCAACAATCGAAACCATCTATTTTTTTAAGTCTATATTCATAGATCATCCTTGAAAAAAATTAGACAAATCGGAAACTGTTTCGATATCCAATTGTGTCCTACAAAATAAATGAACATGGTGCTTCAAGAAAGTACTAAAATTTCAATAATTCAATTGAGTGGTCAAATGATATCGGATTCAAGTGATTTTTTGTAGAGATGATCTTTGAATGAGTATATACAAAAGAGACGGTTTGGATTAGTGAAATACAATACAGAGTGGGCCCTACAATGGTATCCCTCAAATAAGCTAATTTGAGAGATCCCTCAATGGAAGCTCTATATATATATATATATATATATATATATA

AGTGTTGCGGTCTTTGTCTAAGTTTGCTACTCATTCCCGCCCAATTGGCTGGTTTAAGCATCTGAGTAGGAGGTTTTCATTCTTTCCTTTTCTTATCTTTTATTCTTTGAGTCCTCTGTTGGTTTTCTCTAAGGTCTGACCCGTGAGTCCTTTTTGCTAGATCAGTAAGTCCCCAGGGAGGGCGTGTGTGCTGGGTAGGAGACAGAAAATTGAGAAAATTAATAGGTTGCTTAGAGTCTAAACGGTTGGTTTAGGATCTACTGTTTATCTTCTTCCTCAGACAGCCAAACCCAGTTATTTGAGTAGTCATGAGTCGTCTATTTAGAACAAAGTCATGTAATTCTGCCAGTTCTAGTACCAATATAAGCCGACTCCCAGAGATAGTTAATGAAGAACAATTTGAATATAACTCCAGTAGTAAAGAACAATTAGATTTTAGATATTGGAATATACCTAAAGTCCCCAGTCAGAAAATTTATAAGAAACATTGGTTGCCTTCTAGTTTTAATAGCACAACACATGTCAAAACAGTTGAACAAGTTTATGCATTAAGCAAAGAACATGAAACTTGCCAGTTGCTTAATCTTGAGTCTATTTTAAGACATAAGAAAGATGGTAATAATTTTCTGCATATTGGATTAGTTCAAGTTGCCGTAAAACCTCTGACACGGTTAGGATTGAAAGCTTCAATTCTGCTATGTCTCCGCGATGCTAGATTCACTGAATTTAGCGATAGTACCTTAGGAGTCATTGAGTCCAGTCTGTGTAATGGTCCTGTCCATTTTGACTGCTATCCTGATTTCACTGTAAGCCTAAGTGATCCTCATATCCTGAGAACTTTGACTCTGAACATTAAGACTGAAGGTTATAATGTTCTTCCAGGACTCAACCTCTAGCCTTAGTATACAGAATCTATTATAAGGTCACAGGAACAAATATGAATTTCCAAGCCCTCAACAAAAGCCCAAAGGATCAAACCGTCCTGATTCAGAGTCATACCTCAGACGCCCATATACAAGTTCCTCATACGATTAAGTGGTCTGAAGTTGCCCTTCCAAAAGATTGGACCCTAGTCACCGAGAGCTAACCAGCTCCAATCCAGCGTAGTCTAAACAATTTAGACTATATCCAGCAATACTTAGATGGCACAGTTAAGATTCAGTTTGATAGTCATCCTTTACGAAAGTCTAATGTCCAGTTACAACAATTGCCAACCCTGGTCAATCATAAAGGCATGTCCCTGCCAGACAATCCTTTGCGGCCTCTTCCTCCACTCTAGAACGAGACCTAGAGTTAGAAAAGGCTATGATCGACTTCAAACTTGAGTCCCTGAGAAAAATCTCTCAGGTCACCCAACCTTGCTACGGCCAGGTTCCAGTTCCGGACGATAAGTCAGAATCACCTGAGTCACCTACCCAGTCTGACTTCGTAGTTGAAAATCAACTTAGAACCTTGAATAAGAAATTTGAAATTAATTGGGTCAGCCTAAATCGTCATCTAAAAGCCCCCGAAAACAAGCCAAGGCGAGATCTTTATCATCAAGCCTATCCTAATAGCAAGCACCGTGAAATGATCTTTAAACAATGGAAAGATATGCGGTCAGCCAAAGTTGAAATTTACTACCTTGATTTTATTGAAAGCCGTTATATGACTAATGATGAGTTAAAAACATTGACTAAAGAAAAATGGAAATTAGCCGATAAGTCTGTTGTAGAGTCTAGTCACCCTCCAGTAGAAACCATCATAATAGAACACCAGAAAGCCCCAGTTCCTACCACTCCTTTCAAAACCTTTGAAAGCAGTGACCCTCATAGAAAACTAATTGAACAAAATAATTATACGAACTAAAGCCTAGTCACCCTAGGAAAACAATTATACACAATTGAAACCAAAGTTGATCTGCTGAGTCCGCCAGTTGCAGACTCCAGTCCCAAACCTAAAATTGAAAGACCAATAGTTCAGTTCCAAGAACTTAAGTCTAGTCCAGCTTTAAAAGTCAATTCTACAATGAAAAAGATAGAAGATATGTTAGCACAGTTGACCCCTGTTAAACCCGAGAAAGATGATCAGTCTGGTATTAAAACACTGGATGCTTTTGCCGTTTCTCAATCTGAGTCGGAATCTTTAAGTTCAGAAACTAGTGATATCACTAAAATCGAAAATGCTTTCAAATATCTAGAATTCCAACCTGAGCCCAGAGTCAAAAAGATAGAAAAACATATAAGTCCTACTAGTCTAACAAAAAATTGGTATCCCAGACCAACACCTCCAGATATCCAATTTGAAGAAAGAAACTTCCAAACCCAGTTTTCTGTTTCTTCTGATAAATTGTATGAATGGAATATTGATGGTCTTTCTGAGCAAGAAGTCTTGAACAAATTACAACATATGTCTATGGTTGCCAATAGTTACATCACCAATCACAGTTTCAGGCAGTCTGAAATTGTCCCTCTGTTAGTTACCGGGTTTACCGGTACTCTCAGATATTGGTGGGATAAGCACCTTACCTCTGAGTCTAAAAGTCAAATTATTTATGCCGTAAAACTTAATGAAGATGGTCTCCCTATATTTGACGAACAAATAGGTCAAGGTATAGAAGACGGTGTCAACACTCTGTTTTACACAATAATTGAACATTTTATTGGAACCCCTAGCAACACTACGGCTAGAATCCATGACCAGTTGAGTAATCTTAGATGTCCAAAGTTGTCTGATTTCAGATGGTATAAAGATGTTTTCATTTCTAGAGTCATGCTTTGAGATGATAGTAATCAACCATTCTGGAAAGAAAAGTTTATTAATGGTCTGCCTAATCTGTTTTCCCACAAAATACGCACAACGTTAAGCAATGAACAAGGTCAAATTGAATGGGATAATTTAACTTATGGTAATATTATAAGCACAATTAATCAAGTTGGTATGAAAATGTGTATTGACTTTAAAATTAATCGACAAATTCAATCTGATAGAAAGTCAGCCAAGTATGAGTTAGGAAACTTTTGTGAACAATATGGTCTCACTAGTATTCCTCCCTCCAGGAAACATAAGCCTAGTCATCTTAGAAAACGTCATTACCCGAAGAAGCAGTTTCTCCGGCAAGACGAATTCTATAAGAAAAGAAAGTTCTCCCCTAAGAAAAATTGGTCGAAACGCCCCAAGCAGAGTCGCCATAAGAAAGATAAAAGCAAAGTTAAATGCTTTAAATGTCAAAAATTTGGTCATTACGCTTCTGATTGTAAGGTAAAAGATGTAATTAAACAGTTGCAGATTACAGAAGAAGATAAGAACAAATTGATCCAAGTTTTGGAGTTGCGAAACTCTGAAATGAGTGATGTTGAGTCCCTAGTATCCAGTTCTGCGTCTGATTCTGACCAGTCTTCAGAATCACAATCCAGTTCCCCTAATGTCCAATTTGGTTGTACTGATAAATGTTGTAATAGGTTAAATTTTGTTTCAGTTCTTACTAAGCAGGAAGAACAAGAAGAGTTGCTTGTAGACATAATTGGTAAAGTCCAAAACCCTGAGTTAAAATCTGAATATTTGAAAAAGCTAAAAAAGATCCTAAGTCGAGATAGCCAGAGTTCACCCTCTCCTACTCCCCAGCCAATTAGCATCAACACTACGTTAGAAAAATTCAACCGTAGAAAAGATGTCACCCTTCATGACCTCCATTCGGAAGTCAAGCAGGTTAAAAAAGAAATAGTTGAGCTTAAACAATTAAGTCAACAATTGCAAGCCGAGAATTATGTTATCAAACAAGATTTATTTGACTTAAGACAAGAAACCTATAAGCCTAGCAACCACTCGGAAAGTCCGCATCACTCTGATGAGGAACCTTCTGAAGAACAGCCAGTTGTCAATTTAATTAAACAAGTTCATCTTAGAAAATGGTATTCACAAGTCACGATATTTGTTCATGATTTTGAATTTACCACAGTTGCTTTATTTGATTCTGGTGCCGACCTTAACTGTATTCAGGAAGGTCTCATCCCTACTAAGTACTACCAGAAGTCTAGAGAATCCCTTAGCACTGCCTCTGGGAAATCTCTCCAGTTGAATTTTGAAATACCAAAAGCCCATATTTGCCAAAATAAAGTCTGCTTTAAAACTTCATTCGTCTTAGTTAAAAATATTACTGATGAAGTTATTCTAGGATTACCTTTCCTAGCCTTATTATATCCTTTCCAGGTAGAATACGATGGCCTCATTTTTACCCATTTAGGGGAACAAGTTAAATTCAAATTCCTGTCAAAACCTGAGTTGCATAATTTAAAAATTTTACAAAAACAATCAGTTTCTAAATCTGTTTAAATTATTCGAAAAACCAACCAATTGAATTTCCTTAAAGAAGAAATTAGATTTAAACGAATTGAACAACAACTTTCTGATAAAGCCTTGCAATTGAAAATTCAGTCTTTTGAGAATAGATTACAAGTTGAAATATGTTCTAATTTGCCTACTGCTTTTTGGTATCGAAAACAGCATGTTGTTTGTCTTCCTTATATCAAGACCTTTAGTGAGAAAAATATCCCCACTAAAGCCAGGCCAATCCAGATGAATCAAGAATTGATGGAAATCTGTAAGAAAGAAATCCAAGAGTTGCTTCACAAAAATATCATTCGCAAAAGTAAATCCCCCTGGTCATGTCCTGCTTTTTATGTCCAGAAAAATGCTGAACTAGAAAGAGGAGTTCCCAGATTAGTTATCAACTATAAGCCTCTTAATTCGGTCTTAGAATGGATTAGGTACCCTATACCTAATAAAAGAGATCTTATTAATCGGTTAGAAAAAGCAGTTGTCTTCTCTAAGTTTGATATGAAAAGTGGTTTCTGGCAAATCCAGATAGATGAGTCAGATAGGTATAAGACTGCATTTGTCACTCCTTTTGGTCATTATGAATGGAATGTTATGCCATTCGGTTTAAAAAATGCCCCTAGTGAATTCTAGAATATTATGAATGATATCTTCAATCCTTACAGTCATTTCTCGATTGTTTATATTGATGATGTTCTAATATTCTCTGAGTCAGTTAAACAACATTGGAAGCATTTGCATAAATTCCTTCACATAGTCAAACAAAATGGTTTAGTTGTCTCTGCTAAGAAAATCAAGTTATTACAAACCAATATTAGATTTCTTGGTTTTAATATTTCCCAGTCCCAAATTGCTCCAATTGATCGAGTCATTCAGTTTGCTGACAAATTCCCTGACCAGATTCTTGATAAAAGTCAACTCCAGAGGTTTTTAGGATCTCTAAATTATGTTTTTGACTTCTACCAAAATCTGCGAAAACAATGTAAGCCGCTATTCGATAGATTACAGAATAATCCTCCTCCTTGGTCGAATATCCATACTGATGTGGTTAAACAAATTAAGTCCCATGTCAAGACACTTCCCTGTCTTGGTATTCCTTCAGTTGATTCTTTCAAAATAGTTGAAACTGATGCCTCTGACATTGGTTATGGTGGCATTCTTAAACAGAGAGTCAGCCCAGAATCTCCAGAACAAATTGTTCGTTTCCATTCTGGAACCTGGACCCAGTCGCAATATAATTATAGTATGATTAAAAAAGAAATATTAGCTATTGTATTATGCATTAGTAAATTTCAGGATGATTTATTAAATCAAAAGTTTTTAATCAGAGTTGACTGCAAATCTGCAAAGCATGTTTTAGAAAAAGATGTTCATAACATTGCATCTAAACAGATTTTTGCACGATGGCAAAGTATATTAAGTATATTTGATTTTGATATTGAATACTTGAGAGGTAGTGAGAATTCAATCCCTGATTTTCTTACTCGTGAGTTTTTACAGGAACCACAATGGCTCCCAAAAAAGACAAGAATAAACAAGTCGCCAGTCAGCCAATTGCCAGCCAGCCAAAACATTCCCAGTCGCCACAGTCACAACAGGCCCAATCCATGAGTCCATCTAAGCCAGAAATTCCTAAGCAAATAGTTCCCCATCCTAGTTGCACCCCTCTTTCCATTGGCTCCCCCTTATCAGTCGCAAACAGATTTTCCTCCTTACGATCCACAGTTGGCCAAATTCGTCCTAGTTATCAGTCAACCCTAGTTGCCAGTTACGACCCCTTTTCAGTTGATGTCCCTGCCGGACCCTCAGTCACGTATAGAAAGTCATCTCCTTACTTGCCCAAAAGTGAGTCGCATCTATTTGTCATCGAACCCGTATATGACATTATTGCCAATCCAGTTGAAATTGCCAGACATTATTTCCCTCCTGGATTTCACTATATGCCTCCAAGTCCTTACAAATCCCTCAAGTATTATAGAGATATATTACTTGAAACCAAATTAGTTGAAATTAAGCCCATAAAGGATCGTGACAACCCTGGAACTATCCTTTATCATTCTCTATATATCCATCAGATTATAAGCTAAGCAGCCTTTGCAAGTCGTCCTTTTGAGTTAAAGTTGCTCCAATCAAAATTGCAATATAATTATTCTGATTATATTGAAGCCTGGTACACTATCTTTCTTCACCAATCTGAAGATTTTAGTCATTCCTGGTTCATCAATTTTGATAATAAATTTAAATGCCCTTTCCCCTACTGGTTCTTCCATTGGTGGGAAAAGCATGGTCCAGTTGATGATATCCTGCTAGTATCAGTTCATGAGTTGATTATCCATTATACTAAGAAGGCCAAGTTCTCCGCAGTTGACTTATTTTTTTCCAAAGCAAATGTTATTTATTGCCAGGTACAAAGTTCCCTGGATTCTGAAGTGGTCTTATAGAGTCACCAAAGATGCTCGCATTTTTGCCCGCCAATTTTCAGTCAAATGGTGGGATAAGTTTGAAGTTGACAGAATTGCCAAATATGTCTACAATGATCTCCCAAATGAACCAATACCGCATCCTATTTCTCGTACTGGATCTGTTCGATCTTCCCTCTCAGTCGAAGGAAAATCCAAATCAGAGCTTCAAGAAATTGCCTGCCAGTTGATCCTACAAGCCTCTCAAATGGATGATGATGAAGACAATGGGTCTCCTTCGTCCTCTAGTTGCCAGCCCATATCTGA

CAGGACCCGACCCAATTTCCACTTTGGAATTCGAGTCAAGTCCTGTGCGTGTCCGACACCAGGCGAATGTCGGGCACAAATGACCTTTTTACCCTTCTTACTTCAATTTCTCTTTAAATTTCCTGAGACTTCTGCCGAAAATTCGGCAGAGTCTCCTCTGTATTTTGATCAATCCCAAAAATTTTCATCTGTCAACAATCTCAAATATTTTCACCCAACTGCCAGAATGTTTCTAGTAATCAATTCGATTCTAAACACCAAATTTTCAACTGGATCTCAGAGCATATTCTCAAGTTCTCAGGGTTTAAAGGATTTTCTACAACGCTACCTTACTTGATGGTGCGGAAGCTATGATTGGCCCGGTGGTGGATCCCGCGTACCTCTATGGCCTGGGGGCGAAAAACAGTTTGAAAAATGTGAGTGGACCAAAAATAATGTTCTTCAAAGCAATTCTCATAAACATAATATCCCCCATTGTAAAAAAAGAAATTTAACTAAATAAAACTGAATACTTACTTTCTACATCACGCATAGTCAATTCAAGGCATTCTATATCAGTCATATTCAAGCTCGAAAGTTTCATACAAAAATCACTGAATTCAAAGCTTTCATAAAAGTACTGAAATCAAACGCGTATAAAAACTCTGTACTCAGACCTTTCATAACTGAATAAATATAAAGGGATCTATGTCCGAAAAATCAGAAAAAACTGATTTATAATAATTGAAAATCAACATTTAATAAAGAAACTCAGAATCCTTTGAAAATACCACCGGTATGTACCCCTGTCATTTCCGTCAATTCCCTGGCAGGTCTCGGGCGTCACACAGACTTACCCGAGCCGCAAACTGGAGAAATCAGGGGACTATGATCAGCCTGTCCCGCCGGCAGATTCCACGATGACACCAAGTCAACTCGAGTCGCTCTGGCAGGATGCAGGGGACCGTAGTCAGCCTGATCCGGAATCCTGGCAGGTCTCGGGGACATAAAGTCAGCCGAG

>Chr4-02; OZ022209.1; 2906100-2914784; dir; C;

AGCATGTCGAATCAATGGGGAAGTCAACTACACCCCGTATGTTCTGCAAAAATCCTTACTGCTTCAGATACTAGCAGTCACGGAGGCTTCTCTGTTCTTCGTCGCGCTGCTGAAGATTGCTTTCCTCCCCTGGTATTCGTAGTTCATTTTGCCCCCCCCACATGTAGTTCAGCATAACCTGTTTGAGATTTCCATTACCTGAAATGATTTTTATAAATGCTTCATAAGTCTAATCAAGTATAAGTTCGTATTTTATTTTGAGACAACTAAATTATGCTTGTAGGATTACAATCAACAAAGGCCTTCACAAGAACTCATAGCGAAGGATCTGCATGGCCTGGAGTGGAGGTTCAGACATATATACAGGGATATGCAAAAAGGTATCTCATGTTTTGTTTTCATTATTTGGTGGTTTATCTCTTCATGAAACAATAGCAATCTGATTAGGGTTTTATTATGCACTTTGTAAATGGAATATTTTAGATTCAGTGAGTTTCTGATTTTTATTCTTCTTTCCATTGGCTTGAGTGGGGCAGCCACAAAGGCATTTTCTTACCACTGGGTGGAGTGCGTTTGTGAACAAGAAGAAGCTCGTATCCGGAGATGCAGTGCTGTTTCTTAGGGATATGAATCCTTCGGTTCAAGAAACTTTGGATGCCCTTATTCATGATTCTCATACAATGTTCATCCAACTCTTCGTAGCCAGCAATTAAACTATAGTACTATCACAGATGTGATGAATGCGGTTCAAAATGCATTTCGAAACTGATGCAGCAGAGCGAAGGTTTGACATTCTTAAATTTGACATAGTTGAAGTATTTAAAGTTCAATTTTAATTAAAATTTATTTTTGGCAGATACACTGGGCTGATAACTGGGATTAGTGAATTGGATCCTGTAAGATGGCCTGGTTCAAAATGGAGGTGTCTAGTTGTACGTTACTTTTGATAGAGCTTTTCTATATATATATATATATATATATATATATATATATATATATA

CTCATAGACTTAATTTGTAAAGTCGAAAACCCAGAGTTAAAGGCTGAGTATCTGAAGAAATTTCGGAAACTTCTTAGTCAGGAAGGTCCGAGTCAGTCTCCACCTCCTCAAAAGATAAGTTTAAACACAACCTTAGAAAGGTTCAGTCGCAAAAGAGATATCACCCTTCATGATCTACATTCAGAAGTAAAATTAATCAAGAAGGAGATAATTGAGATGAAACAATTAAGCCAGAAATTGCAATCTGAGAATTATGAAATCCGACAGGATTTAATAGCTCTCAAACCTAATGATTTATCTCCGAGTCCAACAAACTCAGATAATGAATCTTTGAATCCAGAACAAGCCTTATGTCTAGTCAAACGAATTAGCTTCAAGAAATGGTATGTCAAAGTTACTATTTTTGTTGATCAATTTGAGTTTACCACAGTTGCTCTTCTTGATTCAGGTGCTGACCTTAACTGCATTCAGGAAGGTCTTATACCTACCAAGTATTATACTAAGTCTTGTGAATCTCTTAGAACAGCTTCAGGAAAATCCCTACAGCTGAACTATGAAATTCCCCGAGCCCATGTTTGTCAAAATAAAATTTGTTTTAAGACTTCCTTTGTTCTAGTGAAAAATATCACTGACGAAGTCATTTTAGGATTACCCTTTCTTGCCTTGTTATATCCTTTCCAGGTGAACCATGATGGTATCATCTCCACTCATTTAGGAGAACAAGCCAAGTTTGAATTCTTATCCAAACCAGAGTTGCACCAGTTGAAAACTTTCCAGAAAAGTTCCATTTCCAAAACCATTGCCTTAATTTCCAGTAAGACTAAACACTTGTCTTCCCTCAAAGAAGAAATTCGTCATACTAGAATAAGTCAACAAATTGGTAATGAATCCTTTCAAAACCAAGTTCGTGAGTTTGAAAACAAATTGATATTTGATATTTGTTCTGATTTGCCTAATGCTTTTTGGTCCAGGAAACAACATATTGTCAGTCTTCCTTACGCCAAAACCTTTAGTGAGAAAAATATCCCCACTAAAGCCAGGCCAATTCAGATGAATCAAGAGACAATGGAATTCTGTAAACAAGAAATTACAGAACTACTCTTGAAAGGAATAATCCGTAAGTCTAAGTCCCCCTGGTCTTGTCCTGCTTTCTATGTCCAGAAAAATGCTGAACTAGAACGAGGAGTCCCCAGATTAGTCATCAATTATAAACCCCTTAATGCAGTCTTAGAATGGATTAGGTACCCCATACCTAATAAACGAGACCTAATTAATCGGTTAGAAAAAGCAGTTGTCTTTTCCAAATTTGATCTTAAGAGTGGATTCTGGCAAATCCAAATAGAAGAGTCAGATCGGTACAAAACCGCCTTTGTCACTCCTTTTGGTCATTATGAATGGAATGTTATGCCTTTCGGTCTTAAGAATGCACCCAGTGAATTCCAGAATATTATGAATGAAATTTTCAATGCTTATAGCCATTTTTCAATTGTCTATATTGATGATGTCTTAATCTATTCCCAATCGATAGAACAGCATTGGAAACACTTGTCTATATTCCTTCAAGTTATTAAGCAAAATGGCCTAGTTGTCTCTGCCAGGAAAATCAAGTTGTTCCAAACTAGTGTTCGATTTCTTGGCTTCAATATCTGTCAGTCTCAGATTAGCCCAATTGATCGAGTCATCCAGTTTGCTGATAAATTCCCAGATGAAATTGTTGATAAAAGTCAGCTCCAGAGGTTCTTAGGATCCCTCAATTATGTTTCTGATTTCTATCATAATCTGAGAAAACAATGTAAGCCCTTGTTTGACAGGTTGCAGGAAAATCCTCCTCCCTGGTCTTCTGTCCATACAGAAATTGTCAAACAGATCAAAATTCATGTTAAGACACTCCCCTGTCTTGGCATTCCTTCAGTTGATTCCTTCAAAATAGTTGAAACTGATGCCTCTGACATTGGTTATGGAGGTATTCTTAAACAGAAATTGAGTCCCAATTCGCCAGAGCAAATTGTTCGTTTCCACTCTGGTATTTGGAATCAGTCTCAAAATAATTATAGTACTATTAAAAAAGAAATTTTATCTATAGTATTATGCATCTCCAAATTTCAAGATGATTTACTTAATCAAAAATTCTTAATACGAGTTGATTGCAAATCTGCAAAACATGTTTTAGAAAAAGATGTTCAAAACATTGCATCTAAACAGATTTTTGCACGATGGCAAAGTATTTTAAGCATTTTTGATTTTGACATTGAGTATTTGAGAGGCAGTGAGAATTCTGTTCCTGATTTTCTTACTCGAGAATTCTTGCAGGGAAAGAAATGGCACCCAAAAAAGACAAACAGAAAGCTGGTGCCAGTCAGTCTAGCCAACCCAAAAACTCCCAAACACCAACCCAAGACCAGTTGCCCAAAAGAGTCCTCCCTGCATCCATGAGTCAATCCAGAATGGCTCATCCAGCCTCAATTGTCCCTTATGCAGGTCATCCTCCAATTCCAATTGTCCCAATTGCCAATAGATTCACCAGCCTTGGAACCCCAATAGGATCCACTGTTGGCCCAATTCGCCCTAATTATCAGTCAGCCCTTGTCTCTGACTATGATCCATTTGCAATAGTTCCTCCAGTTGCTCCAATTGCCCAATCCTCTCAGTCGTCCACCCAATACAGAAAGTCATCCCCCTATCTCCCAAAAGATAAGGTTAATTTGTTCATAATCGAACCAATGTATGATGGGATTTCTGATCCGATAACAATTGTCAGATCATATTTCCCTCCTAATTCCCATTTCCTTCCTCCTGCACCCTATAAAGACCTCAAATTCTATAGGGATATCCTGCATGAGACCAAATCAGTTGAAATTAAGCCTATTAAGGATAGAGATAACCCCGCGGTCATCCTCTATCATTCCCTCTATATTCAGCAAATTCTAAGCCAAACTGAGTTCAGTCGCCACCCCTATGAGTTGAAACACCTTAAGTCCACTCACCCTTATACCTATGCAGATTACATTGATGCATGGTATAATATCTTCTTACATCAAACAGTTGATTTTTCCCACTCCTGGTTCATTAATTTTGATGCCAAGTTCAGGAGTCCTTTCCCCTACTGGTTTCTTCATTGGTGGGATCAACATGGCCCAACAGCAGATGTCCTCCCTACTGAAGTCCATAATCTGACCAATTATTATGCCAGTAAAAGCCAATTTAATAAGCAGCAGTTGTTCTTCCCAAAGTTGCTTCTTTTTATTGCCAGGTACAAAATCCCCTGGATTATGAAGTGGAGTTATCATGTCAATTGGGAAACCAGAATCATGTCTCGCCAGTATTTTGTCAAATGGGGGGATAAATTCTCAATGGACAGAATTTCCATCTATGTCTTCAAGGATTTTCCCCAATCGGATCATCCTCCCAAGCCAAGTTCCCCTACAGGCTCAGTCAGCTCGGCCATCTCAATCGATGGAAAATCCAAAGCTGAACTTCGGGCCCTTGCTGCTCAATTACTCGACCAAGCTAGTCAGATGAATGATGATGAGGACAACGAATCTTCCCCATCTGCATCCAGCTGTCAGCCTGGCTCCAGCTCCTTTGATCCTAATATTCCTTTTGCGCAGGATCCTTATGACGCGTATGATTTGGGATCTGATTAGTCAAAAGTTGCCTTCAATTTCTCTTTTCACCCCGGTGAAATGTTGGAATCAGAATAATTGTAATGTAAAACAAAAATACTTTTGCTTTTACCCTTACTTTACCCCAATAGGTAAAGAGAGTAAAACAAAAATACTTTTGCTTTTACCCCTGCTTTACCCCAATAGGTAAAGAGAAAGTTATTTTCCGTGCTTTGAACCATGGTCACTCTCCAATCAGGGTTACTTTTGATTCAAACCTTAGTTGCTTTTCTTCCTTTTTAAGCTGGCCTCGGCCTCAGTTGAAAACACTTGTAATTTTTCTTCTGCTTTCTTAGTTTTCAAAGTTCTCTCTGAAACTCCTTCAGACATGAACTCTCTTGTAAATATCTGAGTGTTTAATCTTCAATAAACAAGACAGCTTTGTAAGTAGTTTTAAATCTATCTTATTTTCATTTATTCATTTATTTTCTTATATGCATTAATTTCTTGCATTAGTCTGAAAATTGTTTAGGAACACTGTTATTATTCATTCAATCCTCTTCTTGTATTCTTTAAGGAATCTACTTGTTCTGGAAGACGACTAATACTGGTTCTTGAACTGCTCGAACTACAAGAATTAGATCTAAGGAAACGACTCATTTTACTTTGGCAACTGGGTTTTGACTTCAAAGAAGAAAATAAACAGGGACTTCTACTGCTGATTTCTATACGTTTCTATCAACTGACCTCTCTCTCCTAGCCCTCTCAATGCCGCAACCGCTCATGCCTCACCTCGGGACTTACTGTTTGGACTCTAGGCCTTACTCTTCGGACACTATTTAAGAGAGATACAGACAACAGTAACGGAAAGAAGAACAACAGTAAAAGCAACTGAATAATTTCTACTTAGAGATCCTAAACCAGCCACTTGGGTTGGATGAGTAGCAACCTAAGATCAGATTCTTACAACTCTTACGGCTCTGATACCAAAAGGGAAGAACCAACATTAATGAAAACCAGGAGATAGATTAAAGAATACAAGAAGAGGATTGAATGAATAATAACAGTGTTCCTAAACAATTTTCAGACTAATGCAAGAAATTAATGCATATAAGAAAATAAATGAATAAATGAAAATAAGATAGATTTAAAACTACTTACAAAGCTGTCTTGTTTATTGAAGATTAAACACTCAGATATTTACAAGAGAGTTCATGTCTGAAGGAGTTTCAGAGAGAACTTTGAAAACTAAGAAAGCAGAAGAAAAATTACAAGTGTTTTCAACTGAGGCCGAGGCCAGCTTAAAAAGGAAGAAAAGCAACTAAGGTTTGAATCAAAAGTAACCCTGATTGGAGAGTGACCATGGTTCAAAGCACGGAAAATAACTTTCTCTTTACCTATTGGGGTAAAGCAGGGGTAAAAGCAAAAGTATTTTTGTTTTACTCTCTTTACCTATTGGGGTAAAGTAAGGGTAAAAGCAAAAGTTTTTTGTTTTACATTACAATTATTCTGATTCCAACATTTCACCGGGGTGAAAAGAGAAATTGAAGGCAACTTTTGACTAATCAGATCCCAAATCATACGCGTCATAAGGATCCTGCGCAAAAGGAATATTGGGATCAAAGGAGCTGGAGCCAGGCTGACAGCTGGATGCAGATGGGGAAGATTCGTTGTCCTCATCATCATTCATCTGACTAGCTTGGTCGAGTAATTGAGCAGCAAGGGCCCGAAGTTCAGCTTTGGATTTTCCATCGATTGAGATGGCCGAGCTGACTGAGCCTGTAGGGGAACTTGGCTTGGGAGGATGATCCGATTGGGGAAAATCCTTGAAGACATAGATGGAAATTCTGTCCATTGAGAATTTATCCCACCATTTGACAAAATACTGGCGAGACATGATTCTGGTTTCCCAATTGACATGATAACTCCACTTCATAATCCAGGGGATTTTGTACCTGGCAATAAAAAGAAGCAACTTTGGGAAACCAGACCTTTTGGTATCAGAGCCGTAAGAGTTGTAAGAATCTGATCTTAGGTTGCTACTCATCCAACCCAAGTGGCTGGTTTAGGATCTCTAAGTAGAAATTATTCAGTTGCTTTTACTGTTGTTCTTCTTTCCGTTACTGTTGTCTGTATCTCTCTTAAATAGTGTCCGAAGAGTAAGGCCTAGAGTCCAAACAGTAAGTCCCGAGGTGAGGCATGAGCGGTTGCGGCATTGAGAGGGCTAGGAGAGAGAGGTCAGTTGATAGAAACGTATAGAAATCAGCAGTAGAAGTCCCTGTTTATTTTCTTCTTTGAAGTCAAAACCCAGTTGCCAAAGTAAAATGAGTCGTTTCCTTAGATCTAATTCTTGTAGTTCGAGCAGTTCAAGAACCAGTATTAGTCGTCTTCCAGAAATAGTAAATGAGGAACAAGTAGAGTTTGAATCTAACAACAGTGAGCTAGATTACCGTGACTGGAATATCCCTAGAGTCCCCAGTCAGGAAATCTACAAGAAGAGGTGGTCTTTACCTAGTTTCAAGACCACTACTCATGTTAAAACAGTTGAACAAGTTTATGCCCTTGGCAAAGAACTTGAAGTCTGTCAGTTGCTTAATCTTGAATCCATTAAACGCCATAAGAATGAAGGAAACAATTTTCTTCATATTGGTTTAGTCCAAGTTGCCGTTAAGCCACTAACCAGAACAGGGCTTAAAGCCTCAGTTTTGCTCTGGTTAAGAGATGCTAGATTCACTAACAGTGAGGACAGTACTCTCGGAGTAATTGAGTCAAGCCTTTCCAATGGTCCGGTTCACTTTGATTGTTATCCTGACTTTACCGTCAGTCTTAGTGATCCCCACATTCTTAAGACCTTGACGCTCAACATCCAGACTTCTGGATATGAGGTTCTTCCGGGAACCCAACCATTAGCCTTAATCTTTAGGATCTATTATAAGGTCACTGGAACCAACATGAACTTCCAAGCCCTTAATAAGAGTCCTAAGAATCATACCCTGTTGATTCAAAGTAATAGACCCGAGGCCGTCATCCGAGTACCTCAAACAATTAAATGGTCTGATGTCTCGCTCCCAGTTGAGTGGACACTCAAAAATGTCAATCCACCAACACCAATCCAACGTAGTCTAAATAATTTAGATTACATCCACCAATACTTAGATGGATCAGTAAAAATCAATTTTGGGACCCAACAAGTTCCAAAATCTACTGTCCACCTACAACAGTTGCCAACCCCTGGTCAATCCCAGAGGCATATCCCTGCCAGACATTCCTTTGCAGCCTCTTCCTCTACTCTAGAACGAGACCTAGAAGCAGAAAGAGCCTTGATTGATTTCCAATTAGAATCCCTCAGGAAGGCAGCACAGGTCAGTCAACCCTGTTATGGCAAACAACCAGTTGAAGAACAAAAGCCAAAATCTCCCGAGTCTCCAACTCAAACTGACTTTGAAGGTTTTGATTTTGTTGATCCTCAACTTAGAGTTTTAAATAAGTTTCACATCAATTGGAAAAAATTACAAGCCCATCTCACAGCTCCTGAAAATCAGAAGCGTCGAGATAATTACCATAGTTCTTTTCCTCTTAAGTATCGCCGTGATGCAATCTTTAATGAATGGAAAGATTACTTACGGAAAGAACAAGTTGAAATGCCTTATTTAGATTATGTAGAGACCAACTACATGTCTAAAAAATTGAAGGTAATCACCAAAGAAACGTGGTCTTTACCTAATGACACACAGGTAGTTGCCAGTCACCCCCCTGCAGAGTCTATCTTAATTAATTTTAAGAAAACTCAGATCCCTGCTTGTCCTTTCAAATTCCCTGAAAAATTAGATTCAGAAAAGAAATTGATAGAACAAAGCAACTACACCAATCAGAGTCTAATTGTCATAGGAAAACAGCTTGACAAAATTGAAACTAAAATAGACAAGCTAGTCCCTTCCTCTGAAAAATCCAATACAAAAATTGAAAAACCACTGGTCCAGTTCCAAGATCTTAAGACAAATCCGACCCTTAAGATCAAACCAACTCTTCAAAAAATCGAAGAAATGTTAGAACAATTGACACCTGTTAAAGGAGAACAATCTGGAGTCAAAGTTCTTGACTCTGCTAATCTTGATTCTGACCAAGAATCTGTCCAGAGTCTAGATTCAGTTGACAGTAACATCTCAAAAATTGAGAAAGCCTTTACTGAACTTACAGTCGAACCCAGGGTTACCCGGTTAACCCACCAAGTCACCCCTACTAGTCTTACCAAAAATTGGTACACTCGACCAACACCTCCAGATATACAATTCGAAGAAAGACATTTTCAGAACCAGTTCTCTGTCTCTTCTGATAAATTGTATGAATGGAATATTGATGGTCTTTCTGAGCAGGAAGTTTTCAACAAACTTCAACATATGTCCATGGTTGCCAATAGTTATATTTCAAACCACAGTTTTAGACAGTCTGAAATTGTACCCCTACTAGTTACAGGTTTTACAGGTACCCTCCGCAATTGGTGGGATAAACACCTCACGCCTGAGTCTAAAAACTTAATCATTCATGCTGTAAAACTCAATGATGAAGGTCTCCCTATCTTTGACGAACAAATAGGTCAAGGTATAGAAGATGGAGTCAATACTCTGTTTTACACCATAATTGAGCATTTCATAGGAACACCAAGCCATACTACCTCTAGAATTCATGACCAGCTTAGCAACCTTCGTTGCCCTAAACTGTCTGACTTTAGGTGGTATAAAGATGTCTTCATCTCTCGCGTAATGCTCAGAAATGATAGTAATCAGTCGTTTTGGAAAGAAAAATTTATTAATGGTCTTCCAAATCTATTTGCCCATAAGATTCGCACTACCTTTAGTAATGAACAAGGACATATTGATTGGAATAATCTGACCTATGGAAATCTAGTCAGTACTATCAACCAAGTCGGTATGAAGATGTGCGTTGACATGAAAATTGGTAAGCAGATACAGTCTGACAGAAAATCCGCTAAGTATGAGTTAGGAAATTTCTGTGAACAGTATGGTCTTACTAGCCTACCTCCCTCCAGTCGAACTAAGTCACACCATAAGAGTCAATCCCGTTC

TATATATATATATATATATATATATATATATATATATCCTATTTTTGTCTTCAAAAGCATTTGGAACTTATTTATCTTAAACTTTTACATGAGCATGCATTACTGCTTTGCTGCCGCACCTTCGGTTCTCTAAACTAATATATATGGTAGGGGTAGAATTAAATCCCCCTCCTTGTTAGTTGTTTGTGTATTTACTTCGTTGATTTTACATTTAAGATTCGCAAAACCATTTCCTGAACAAATCTGGTGTAAGCCTTTTTAACAAGAGTTGAAGCATTTGGGGCACTGGACAAGAGGGCAAAAAATAACGAAAATTGTATGACATCTTCACCATGTTAAAACTAAAATGAATTGCTAACACTTCTTTGTATTTGTCATGAAATGTCATGAGGGTTTAGCACATTTTTTTGGCTAAATCACGCTATGAAGGGATAAATCTGCTCTTGTATTTGTCACAAAATTTGTCCTGTGAAAGAATAACAGAGAGGAAAGCAATAATTCTTTCATTGATATAGGGACCATATATATACAAGAGAGAGAGCAGCCAATATGGCTTGCTGCACAAGTACTGAATTACAGGAAGGAATAAAGGCCTTGGAGTTGCAAAAAAACGTGTACACAATAATCAAGGTTTGATTTCCTGATTTGTAGGCAATAGTGTAGATTCACCTCTATTATGTTGATCAAAACACCGTACTCATAGTCCTGTGGATAGAAGCTCAGGGCCTTAATTATTTGAACCCTATCATGTTCAGAAATATTTTTAATGTTTCATAGACATCGGTGTTGCATATTGTAGCTGTCAAGTCTTTAGAACTGCAAAAGGATTTGTTGAGTTATTTTTTACTGCCTTGAAAGCTGTATTAACCTAAATATCACAAATGATGTTTTCTCTGCTCTGTAGTATGCCAATGCTAACTTACAAGGTGATAAGTTCATGCCATTAGCACTAAATACATTGGTTTCAAACTCTTGCAGGTAAGGTGCGATGATATAGAC

>Chr4-03; OZ022209.1; 9495272-9501490; rev; B;

TTATTTTTTTTTTGAGCTCTTAGCTTTTAAAAATTGCACCTTCGTTGAAAAATTCCTGGGTCCGCCGCTGATAATATGATGTAAGTTTTTCCATATATTTTGTGAGTTTTTCTTTTCATAATTGTGAGGTGTCATATAAATAATAAAAAGAGTTATATTTAAATTAAGATATTATGAGACTTTCATTCATATAATGACTAAAGGGAATAACTATTGCAGGAGGTGGATATCATCCATTATCTCCACGTTTTGGCTGGCAGTTGTCTCTTAATAGGAGAATCAGTTGTAAAGAAACTTATATAAAAGGCTTGGTCTTTTTCCTCATATCCATATGTTCATATTATGTATGCTTGTCCAATTGTAAGCCTTGACATTCTGGAATACTGAGAGGAGAAGATCATCTTGTATATTTGATTATGGCTAATTCAAGTAAGTCTTATCTCTAATAATTGTTTTGTTCAATTGTCTTTCTGAGTTAAGATGTGGTTTAAGTCCTCAATTGGTTTAGAAATATTTTGCATATAGCAAACTCTTACAATAGAAACTAAAGTCTTGTAACTCGATGGGAATAAGGGTGTACAAGGAAAATGAAAGGAAAGAAGTTGGAGATTTCCGATGGATCATGTGAAAAGTACAACTATTTTCGAGCCCTATAGCTAGTTTCTTCAATACGCACGGCACTTGTTTATAGGGTGAGAGAATCTGTTTAAAGAAAAAAATATGCTTTTTGATTCACCACTCTATAGCCTGTAATGCCTCTATCCTTGTTATAGAGGGTGAGAGAAACAAACATTTATGTGATGTATGTATTTGGACTAGCTCTAGCGATGGCAATTTTTCCCGCAGGGAATGGTTTTCGTGGGGATGCGACTCTATAAAGGGTTAGGTATGAAGGATAAAATATGGGTTAGGACGGGTACAGAGAATTTTTGGGTATGGTGTTCGGAGAGGGGTGAGTATGGTATTTGAATCCTCGACCCAATATATATATATATATATA

TTAGATGAATTGAAAACTCTGACTAAAGAAAAATGGAAACTAATTGATAAATCTGTAGTTGAGTTGAGTCCCTCCAGTAGAAACTATCATAATAGAACACAGAAAGCACCAGTAGCTGCCACCCCTTTCAAAACCTTTGAAAGCAGTGACCCGAATAGAAAAATGATTGAGCAAAACAATTACACCAACAGAGTTTAATCACCATAGGAAAACAATTAGATACAATCGAAACCAAGGTCGACATGCAGAGTCCGCCTGTTGCAGACTCCAAACCCAGACCTAAAATTGAAAAGCCGATAGTTCAATTCCAAGAGCTTAAGTCCAGTCCAGTCTTAAAAGTCAATTCCACAATGAAAAAGATAGAAGAAATGCTAGAACAATTGATCCCTCCAAGCCCGAGAAAGGTGACAAATCTGGTATTAAAACACTAGATTCTTTTGCTGCTTCTCAATCTGAGTCCGAATCAGTAAGTTCAGAAACCAGTGACATTTCCAAAATTGAAAACGCCTACAAAAATTTAGAAGTCCAAACTAAGCCCATAGTCAAAAGAATAGAAAAACATATAAGTCCCACTAGCCTAACAAAGAATTGGTATCCCAGACCAACACCTCCAGATATCCAATTTGAAGAACGAAACTTTCAAACCCAGTTTTCAGTTTCTTCTGATAAATTGTATGAATGGAATATTGATGGTCTTTCTGAACAAGAAATCCTGAATAAATTGCAACATATGTCTATGGTCGCTAATAGTTATATCACCAATCATAGTTTCAGACAGTCTGAAATTGTTCCTCTATTAGTTACCGGTTTCACTGGTACTCTTAGATATTGGTGGGATAAACATCTTACCCCTGAGTCTAAAAATCGAATAATCTATGCTGTAAAACTTAATGCAGACGGTCTCCCTATATTTGACGAACAAATAGGTCAAGGTATAGAAGACGGTGTCAACACTTTGTTTTACACTATAATTGAACATTTCATAGGAACCCCTAGTAATACCACTGCAAGGATACATGATCAGTTGAGTAATCTTAGATGTCCAAAGTTGTCTGATTTCAGATGGTACAAAGATGTCTTTATTTCCAGAGTCATGCTTAGGGACGATAGTAATCAACCCTTCTGGAAAGAAAAGTTCATTAATGGTTTACCAAACTTATTCGCTCACACAAAATCCCACAACCCTAAGTAAAAACAAGGTCAATTGATTGGGATAATTTATCTTATGGTAACATTATAAGCACAATTAATCAAGTTGGTATGAAAATGTGTATCGACTTTAAAATCAATCGCAAAATCCAGTCTGACAGAAAGTCAGCTAAGTATGAATTAGGGAATTTCTGTGAACAATACGGTCTCACTACTATCCCTCCTTCTAGGAAACATAAGCCTAGTCACCTTAGAAAGCGTCATTACCCGAAGAAGCAATTTCTCTGGAAAGATGAATTTTATAAGAAAAGAAAGTTCTCCCCTAAAAAGAATTGGTCGAAACGCCCTAAGAAAGATAAAAGCAAAGTCAAATGTTTTAAATGCCAAAAATTTGGTCATTATGCTTCTAAATGCAAAGTAAAAGATGTTATTAAACAATTGCAGATTACAGAAGAGGATAAGAACAGATTAATCCAAGTTTTGGAGTTGCGAGACTCTGAAATGAGTGATGTTGAGTCCCTGATATCTAGTTTCGAGTCTGAGTTCAGTCAGTCTTCAGATTCACAACCCAGTTCTCCTAAAGTCCAATTTGGTTGTACTGATAAATGTTGTAATAGGTTAAAATTTGTCTTAGTTCTCACTAAGCAAGAAGAACAAGAAGAACTTTTAATAGACTTAATAGGAAGAGTTCAAAACCCTGAGTTAAAAACTGAATATTTGAAAAAATTGAAAAAGGTCCAAAGTCAGGATAGCCAGAGTTCACTCTCTTCTACTCCCCAGCCAATTAGCATCAACACTACTTTAGAAAAATTCAATCGAAAAAAAGATGTTACCCTCCATGATCTCCAGTCAGAAGTTAAACAGGTTAAAAAAGAAATAGTTGAGCTAAAACAGATAAGTCAACAATTGCAAACCGAGAATTATACTATCAAGCAAGACTTATTTGATCTAAAGCAAGAAACCTGTAAGCCTAACAACCACTCAGAAAGTCCGCATCACTCTGATGATGAACCTTCTGAAGAAAAGCCAGTTGTCAATCTAATTAAACAAGTTCACCTTAGAAAATGGTATTCAAAAGTAACAATATTTGTCCAAGATTTTGAGTTTACCACAGTCGCCTTATTTGATTCTGGTGCAGACCTTAACTGTATTCAGGAAGGTCTCATCCCCACTAAGTATTACCAGAAGTCCAGAGAATCCCTTAGCACTGCCTCAGGGAAGTCCCTCCAGTTAAATTTTGAATTACCAAAAGCCCATATTTGTCAAAACAAAGTCTGTTTTAAAACCTCATTCGTCTTAGTTAAAAACATTACTGACGAAGTCATCTTAGGTCTGCCCTTCCTTGCCTTGTTGTATCCCTTCCAGGTAGAATACGATGGTATCATTTCTACCTATCTAGGTGAGAAGGTCAAGTTTGAATTTTTGTCGAAACCCGAGTTGCATAACCTGAAAGCTTTACAAAAACAGTCAGTCTCTAAATCTGTCCAAGTTATCCGAAAAACTAATCAGTTGAATTTTCTTAAAGAAGAAATTAAGTTTAAAAGAATTGAACAACAGCTTACTGATAAAGCTTTACAGTTAAAAATCCAGTCCTTTGAAACCAAACTAAAAGATGAAATATGTTCTGATTTGCCTACTGCCTTTTGGTATAGAAAACAGCACGTCGTCTGTCTTCCTTATATCAAAACCTTTAGTGAAAAGAATATCCCCACTAAAGCCCGGCCAATTCAGATGAATCAAGAACTAATGGAATTTTGTAAAAAAGAAATTCAAGAGTTACTTCAAAAGAACATAATACGCAAAAGTAAGTCTCCTTGGTCGTGTCCTGCTTTTTATGTCCAGAAAAATGCTGAACTAGAAAGAGGAGTCCCCAGGTTAGTCATCAATTATAAGCCCCTTAATTCGGTCCTAGAATGGATTAGGTATCCCATACCTAATAAAAGAGACCTCATTAACCGACTAGAAAAAGCAGTTGTCTTCTCCAAATTTGATATGAAAAGTGGTTTCTGGCAAATCCAGATAGATGAGTCAGATAGGTATAAAACTGCATTTGTTACTCCTTTCGGTCATTATGAATGGAATGTCATGCCGTTTGGTTTAAAAAATGCCCCCAGTGAGTTTCAGAACATTATGAATGATATCTTCAATCCTTACAGTCATTTCTCGAATGTTTATATTGATGATGTTCTCATTTTTTCTGAGTCAGTTGAACAGCATTGGAAGCATTTGCATAAATTCCTTTACATAGTCAAACAAAATGGTCTAGTTGTCTCTGCTAAGAAAATTAAGTTGTTTCAAACCAATATTAGATTTCTTGGTTTCAATATCTGCCAGTCTCAGATTTCCCCAATTGATAGAGTTATCCAATTCGCTGATAAATTCCCTGATCAGATTCTTGATAAAAGTCAACTCCAGAGGTTTTTAGGATCTCTAAATTATATCTCTGACTTCTATCCAAATCTGCGAAAACAATGTAAGCCCCTATTTGATAGGTTGCAGAATAATCCTCCTCCTTGGTCAAATATTCATACTGAAGTTGTCAAACAGATCAAAGCCCATGTCAAGACACTTCCCTGTCTTGGTATTCCTTCAGTTGATTCTTTCAAAATAGTTGAAACCGATGCCTCTGACATAGGTTATGGTGGCATTCTCAAACAAAAAGTCAGTCCAAATTCTCCAGAACAAATTGTTCGTTTCCATTCTGGAACCTGGACTCAGTCGCAATATAATTATAGTACTATTAAAAAAGAAATATTAGCTATTGTATTATGCATTAGTAAATTTCAAGATGATTTATTAAATCAAAAGTTTTAATACGATTGATTGCAAATCTGCAAAACATGTTTTAGAAAAAGATGTTCATAACATTGCATCTAAACAAATTTTTGCACGATGGCAAAGTATATTAAGTATATTTGATTTTGACGTTGAATATTTGCGTGGTAGTGAGAATTCAGTTCCTGATTTTCTTACTCGAGAATTCCTGCAGGAACCACAATGGCCCCCAAAAAAGACAAGCACAAAGAAACAGCCAGTCTGCCTAAGCCAACCCAGTCGCAACAACCAACCCAGTCGCAACAGTCGCAACCGTCCCAATCCCCATCCATGAGTCAAAATAAGCCTGAACAGATAACTCCCTATCCTGGTCATCCCCCGCTTGCAGTTGCCACCCCATTATCAGTTGCAAACAGATTTTCCTCTTTAGGATCCACAGTTGGCCAAATTCGTCCTAGTTACCAGTCAACCCTAGTTGCCAGTTATGACCCTTTTTCAGTTGACATCCCAGCAGGTCCTTCAGTAGCCATTAAAAAATCATCCCCCATTTGCCTAAAAGTAACTCCCATTTATTCGTCATTGAGCCAGTTTATATATTCATGCCAATCCAGTTGAAATCGCCAAACACTATTTCCCTCCTGGATTCCATTATATGCCTCTAGTCCTTACAAGTCCCTCAAGTTATAGAGATATAATTACTTGAAACCAAGTCAGTTGAAATCAAGCCTATATCATTCTCTCTATATTCACCAGATTATAAGCCAAGAAGCCTTCAGCAGTCGCCCTTATGAACTCAAGTTGCTCCAGTCAAAATTGCAGTATAATTATTCTGATTATATTGATGCCTGGTATACTATATTTCTTCACCAATCTGAAGATTTTAGTCATTCCTGGTTTATCAATTTTGATAATAAATTCAAATGTTCTTTCCCCTATTGGTTTCTCCATTGGTGGGAAAAACATGGTCCAGTTGATGAAATATTGCCAGTTTCAGTCCTCGAATTAATCCGCCATTACACCAAGAAGGCCAAGTTCTCTAAAGTTGAATTATTTTTCTCCAAACAGTTGTTGTTTCTTGCCAGGTACAAGGTGCCGTGGATCCTTAAATGGTCCCTACAAAAATCACCAAAGATGCCAGGATCTTTGCCCGCCAATTCTCTGTCAAGTGGTGGGAGAAATTTGAAGTGATCGAATTGCTAGATATGTCTACAATGATCTCCCAGTTGAACCAATGCCGCGCCCAATTTCCCCTACTGGTTCTACCCGATCTTCCCTCTCAGTCAAAGGAAAATCCAAAGCTGAACTCCAGGAAATTGCTCGCCAGTTAATCTTACAGGCTTCCCAGATGGATGATGATGAGGACAACGACTCTCATTCGTCGTCTAGTTGTCAGCCCATTCCGAATCCTGTTAAGCCCAAGTCCTGGTATGATGACAGCAGGATCCTTATGACGCCTATGATCTACATTCTGACTAGGCCTCATAATTTCTGCCGACATCTTCACATGCCAAGTTGCTGACACCTCCACATGCAAAGTTGCCCCGACACCATAATTACCACAGGAAGAAGCATCTGCTTTTACTGTTCGCTTTTCCACCGAAAGCTGCCTCAGTAAAAGAATCTTCCGAATAAAGCTAAATTCAAAGTTGAATCAGGTTGCTTTTCCGTGACCCTACAGTTGCAGCAAACCTCAGTTACTTTACTATAGTTAATCTAAACCATAGTTGCTTCTCTCCATATAAGCAGGCTTAGGCCGCAGTTGTATTCAGAATGTAATTTTCCCTTTCTTCAGTTTTAAAGATTCTCTCTGATACATCCTTCGGAGAAATCATCCTTGTAAATAGAGTGTGAGTTTGAAGTGTTGCAGTTGCAATCTTTGTGAGTATAATAAAAGTTATTTTCAATTATCTATTTTCAGAAAGCATTATTTGATGCATTATCATTATTTTGCTGCATTAGTCTGAAAGTTGTTTAGGTTGCATCAATCTGCGTTCTTTCTTCTATTGTTCTATGCTCTTGATAATTTTATTCACTTGTTTTTCTGCTACTCTGTTATTCTTCTTATTTGGTATCAGAGCCGTAAGTGTTACGGTCTTTGTCTAAGTTTGCTACTCATCCCCGCCCAAGTTGGGTTTAAGCATCTGAGTAGAAGGTTTTCATTCCTTCCTTTTCTTCCCTTTTATTCTTTGAGTCCTCTGTTGGTTTTCTCTAAGGTCTGACCCCTTGAGTCATTTTTTCCTAGATCAGTAAGTCCCCAGGGAGGGCATGTGTGGTGGGCAGGGGACAGAAAAATTG

TATATATAATATATAGTCCCTTTCTATTGAGGGACTTTAGGGTCCCCTACAAATATTTTTTGAACGATACAAACTAGCTTTTTTAAGTCTACATTCATAGATCATCCTTGCAAAAAATTAGACAAATTGGAAACCGTTTCGATATCCAATTGTGTCCTACAAAATCAATGAACACGGTGCTTGAAGAAAGTACTAAAATTTCAATAATTCAATGGAGTGGTCAAATGATATCGGATTCAAGTGATTTTTTGTAGAGATGATCTTTGAATTAGTATCTACAAAATCGACATTTGGATTAGTGAAATACAATACGGAGTGGGCCCTATAAGGGTGTCCCTCAAATAAGCATATTTGAGGGATACCTCAACTAAAGCTTCCTATATATATATATAGGATACCGAGCCTTTACATTTAGCATTCACGCATTTATGCGCATTTAAAAGAATCTTAACCATTTTATTAATCTAATGGAGATTAAGTATTACACCTCATAGTAGTTATATGTTATTAAAGTGATTGTGCTAACTAAGTTTTACATTTCGTATTATTATATTATATATTTATGTCATTATACGAATCTTAAGCCATACGAAATATATATTTAGTTAACTTCCAGTCAATATGTAAATATTCTTATATTGTCTTATACGTCTATATTATATATATTTATTATCTTACCCTAGTCTTATTTTTTTCTTTCAACAAAGTAGTTTTATATTGGTTTTTTCTTTCGTAATGGGGCAGGTCGGGAACTCATACCCCAACGAAGGTGGGGATGGGGAATATCCAATATGGATATTGAGGCTATGAAGGCGGGGATGGGGGAAAAAACTTAACGAGAACGGGGATGGGAATGGCATACTCGCCCCCGATTTGACCCATTGTCAGCTCTCCCTCTTATATGCTTAATTGTATGCTTAAATTCCCTTTTTCTTTTTCATTTTGTGGAATATTTTGGGTACCCCATTTTTCGCTTATTTATACCTACCCTGTATGCCTT

>Chr4-04; OZ022209.1; 12437942-12445139; dir; A;

AAGTAAGGAGGGCATATGAAGGAAATTACTTAAATATAGCATAAATTTTAAGAAAATACAATTTTTTTTAATTTGATATTTATTTGGAGGTGATCGTTTTACTTTAAATAAAGAAAACAAAAATGTAGTTATCTTGGTGATTTTCAAAGCAATTGCTTCTCCAATTAGCCAACTTATTTGCTTTCGAACATTTTTCAAAAGAGTATAGCCTCACATTCCATTCACATTTTGTTCATCTTTTATCCACATGTAGGAAAAGATATCAAAGCAGCTGTCCTTTTTTTTTTTTTCGGTGTGGTAGACAAAAGTTTAGGAGGAGGGGAGACTACCCCATTCCAGGACTAGGGCATACTAAGGCCTCTTTGAGGACTTACAAAGGGGATCTTAGCTCTATTTTGGTTTTTACAAATTACCCACCAAATTGTCAGCAACGGGACTTGAATATAAACCTTGATAGCGAAGAGAACGATCCTTACCAACTCAACCAACTTCATTGGCAGTTGTCCTCCTCATTTCTACTCAACCTTCTCATGTCAAAGTCAAAAATTGTGAAATTTAAAGCGAAAATATTTCAAATTTCTTAATTTTGTAATTTCATTAATTCTTGGTTAATAATATAGAAGGAGCTAATAAACTTGACCACTAAAGCTATATTGAAATCATGGCTTGGATTCTTCAATGCGATTGGTCCTAGTCAATGGAAAATAGAAAATTGACATGAGCAACTGAACCCACTCATCCTCATATGAGCTTGAAGCCATGCCTTGACTTCAATGGTTGATAAACTAATGCCTTTCAATTTCGAGGGTCCTCCCCAAAATAGACAAACTTCTTTTTGTTTTGTGAAAGTAATCGACCAATTCTTTTTATTTTTATTTTTTTCTAGATATATGTATCGACAATTTTGCCTTTGAACAAGTTTAATTTCATTGACATTTAGAAGCTTTTGTTACTTCTATATATATATATATATATATATATATATATATATATATATATA

TGAAACTAAATAAGCAAATACAATCTGAGCGAAAGTCTGCTAAATATGAGTTAGGTAATTTTTGTGAACAATATGGTTTAATCTCTACAGCTCCCTCTAGAAGAAACAAAAATAGTTATCCGAGTCATATTCGTAAAAAACGCCATTATAGCAAGTATAGAAAGTCTTTTAGACATAATAATGAGCCTGCTGAATTTTATAAAAAGAAGAAATATTCCCCTAAGAAAAGATGGTCAAAAGCACCTCGGTATAAAAAAGATGCTCGCTTTAAGTCTGGTAAAAATAGAGATAAAAGTAAAGTAAAATGTTTTAAATGTCAAAAGTATGGTCACTATGCAAATGATTGTAAAGTAAAAGATGCTATCCGACAATTGAAAATTACAGATGCAGAAAGAGACAATCTAATTAAAGTACTTGAGTTAAGAAACTCTGAGTCTAGTGAAAATGAAACCATGATTATAAGTTCTGAGTCTGCCCGTCATGAGTCTTCTGATTCCTTGTTAAGTTCACCAGAAATACAAATCGGTTGCAGAGATAATTGTTGTAATACTCTTAAGCAAATTTCTGTTTTAACTAAACAAGAAGAACAAGAAGAATTATTAATAGATTTAATTTCTAAAGTTGAAAACCCTGAATTAAAATCTAAGTATTTAAGAAAATTAAGAAAAATTATTACACAAGATACAAAAACATGTCACTCTAGTCCAGTTATTAATCTGAGTTCTACCATAGAAAGATTTAATAAGAAAAAAGATGTTTCTTTACAAGATCTACATGCTGAAGTAAAACTAGTCAAAAAAGAAATAGTAGAATTAAAACAATTGAGCCATAAATCGCAAACTGAGAATTATGATATTCGGCAAGTCTTAAATGCATTATTAGATAAAGAACCTACTGAGAAAAATAGTCCACCTAGTAGTCCTGAGCATGAGTTTAGAAAAGAAGAACACGCTGTTAATCTAATTAAACAAGTTAATTTTAGAAAATGGTATTCTAAAGTTACCATATCTGTAAAAGACTATGAGTTTAATGTTATTGCTTTATTTGATTCTGGTGCAGATCTTAATTAAAGAAGGTTTAATTCCTACTAAGTATTATGTGAAATCTACTGAGTCTTTACGTACTGCTTCTGGTAAATCATTGCAATTAAATTATGAAATTCCTAAAGCTCATGTATGTCAAAATAGAATTTGTTTTAAAACTTCATTTGTTTTGATTAAAAATATAACTGATGATGTTATTTTAGGATTACCTTTTATAAGCATGCTTTACCATTTCCAAGTTGAATATGATGGTGTCGTTTCGAATTATTTAGGTGAGAAAGTTAAATTTACTTTTTTAACTAAATCTGAGTTGCATAATTTAAAGCATTTGCATAATGATGATTTTACAAAAACTATTACTTTAATTAAAAATAAAACAAATCATCTGAGTCATCTACAGCAAGAAGTCAACAATAAAAGAATTGAGTTTCAATTAAATAATCCCACTTTACAAAATGATATTCATAATTTTGAACAAAAGTTGATTTCTGAGATTTGTTCTGATCTTCCTACTGCTTTTGTTACTCCCTTTGGTCATTATGAATGGAATGTCATGCCTTTTGGTCTTAAGAATGCTCCTAGTGAATTTCAAAATATTATGAATGAAATTTTCAATACTTATAGTCATTTTTCTATTGTTTACATCGATGATGTTTTAATTTTTTCTGAGTCTATAGAACAACACTGGAAACATTTGCATACATTTCTGCATATCATTAAGACTAATGGATTAGTTGTTTCTGCTAAGAAAATTAAATTATTTCAGACTGCTATTAGATTTCTAGGATACAATATAAGTCAATCACAAATTCACCCTATAGACCGAGTTATCCATTTTGCAGATAAGTTCCCTGATCAAATTACAGATAAAAATCAATTACAGAGATTTTTAGGTTCTCTTAATTATGTTTCTGATTTTTATAAAAATTTGAGAAAACAATGTAAACCCTTATTTGATAGATTGCAGACTAATCCCCCTCCTTGGTCGTCCATCCATACAGATATTGTTTTACAGATTAAACGTCATGTTAAGACTCTTCCTTGTCTTGGCATTCCTACTCCTACTTCTTTTAAAATTGTTGAGACTGATGCTTCTGATATAGGTTACGGTGGTATTCTTAAACAGAAATCTGAGTCTGGTTCTTCTGAGCAAATAGTTCGTTTTTATTCTGGTATTTGGTCTTCCTCCCAATTTAATTATAGCACTATTAAAAAAGAAATACTAGCTATTGTATTATGCATTACTAAATTTCAAGATGATTTATTAAATCAAAAATTTTTAATCCGTGTTGATTGCAAATCTGCTAAGCATGTATTAGAAAAAGATGTTCAAAATATTGCATCAAAACAGATTTTTGCACGTTGGCAAGCTCTTTTAAGTATTTTTGATTTCGATATAGAATACTTGAGAGGTAGTGAGAATTCTATCCCTGATTTTCTTACTCGAGAATTTTTACAGGATAAGCGATGGCTGGATCATCCAAAGACAAGCAAAAAATGAGTCATTATCCTCCTCCTCGAATTCTTCCTGCAACCCTGAGCCAAAAGCCTTCTCAATCCATAAAAAATGAGCCTGGATCCTTTTCTGTTAAATCTGAGCCTATTACGATTACTCCTTATCCTGGTCATACACCCATACCAGTCATGAGCAATAGATTTAGCCCCTTAGGATCTACAGTCACCCCTGCACGACCCAACTACCAGTCTGCTTTAGTTTCTAGTTATGATCCGTTTCAGTTACCTCCCACTGCATCACCTATATCCTCTCCGTCTTTTGCCAAATCTTCTCCTTATGTCTCAAAAAATATATCTAATCTTGTCATTATTGATTCTCATATCCCTACATCCATGAGTCCTGCTCAAATTGCAGCTTATCATTTTCCGCCTGGTTTCCATTATCTTCCCCATAGCCCTTACAAGACTTTAAAATTTTACAGAGAAATTCTTCATGAAACCAAGTCTGTTGAAATTAAGCCTATTCGAGACAAAACTGATCCTACAAAAATTATTTATCACTCCCTTTACATTCACCAAATTTTACATCCTAAGTCTTGGGGATCTCGTCCTCATGAGCTCCGTACCCTTCCTGCTAAATATCAATATAATTATGCAGACTATGTTGAAGCCTGGTACTACATCTTCCTACACCAAACCCCTGAGTTTTCCCACTCCTGGTTTATTAATTTTGATAACAAATTTAAAAATACTGAGCTTCCTTATTGGTTCCTTCATTGGTGGGACACCCATGGTCCCAAATCTGATATCATCCCTTCTGCAGTTCATGAGTTAATCACTTATTTTACCCAAAAATACAAAATTTCTGAGCGAGACCTATTTTTTCCTATTGATCTCTTATTCTTCACAAAATACAGACTCCCTTGGATAATGAAATGGAATTATACTGTCAATTGGGAGACTCGTTTCTTTTCCCGTCAATTCTTTGTTAAATGGTGGGATCGTTTTGAAGTCCATCGTATTACAGATTATGTTTATAAAGACTTTCCCCAAGTTCCTAATCGCAACCCTGAGCCCAAGCATGAGCCCTCTTCTTCTTCAGATACTATCCTTACAGTTGAAGGCAAATCTAAGACTGAGTTACAGGACTTAGCCCAGCAGCTCCTTCTTCGAGCCTCTCAAATGCATGATGATGATGCTGAGTCTGAAACTTCTTCTAGTTGTCAACCTGCACAGCCAACTCCTGAGCGTCCTGTACGATGGGCTGATTATCTGCATGGCCAAGACCCTAATGATGCTTATGATGAAGCCTATAAAGCCTATGATTTGAATTCTGATTAGACTACTGCAGACAAGTCACCTGAGTTAAAGCAAGTTACCTGAGTTAAAGCAAGTTACCTGAGTTACCTGAGTTAAAGCAAGTTACTTGAAGTTACTTTTGTAAACCTAAGTTACTTTTGTAAACCTAAGTTACTTTTGTAAACCTGAGTTACTTTCAATGTTTGTCTTTAAAAGGAGGCTTCGACCTCAGTTAGAGGAATCCTTTTCTTTCTCTTCTTTTCTTAGTTCTAAAAATGTTCTTTCAGAGTTCATGTTCTGAAGAACAACCTTAGCATGAGAGTAAAATTGTAAGTTGTAAGTTTTATGAAATTATTTTCAAATACATTGTTATATATTTGAAGTATCATCATGTTTTCTTACACTATGATTTCTTACATACATTCAATATTTTTATTTTCTCTTATAAGATTTATGCATAAGTTACATTTGTTCTATTTCCTATATTATATAACAAGCTTCCGCCTTTGATCTCCTTTTGGTATCAGAGCCGTAAGAGTTGTGAGAGTTTGTCTAGGTTGCAACTCATCCAACCCTAGTGGCTGGTTTAGGATTTCTGAGTTGAAGTCATTTGATTTCTTCTTTGTTTTCTTTTATCCTGTAAGTTTCTTGTTGGTTTTCTCCCTGGTCCAAACAGTGAGTCCTCTTTCCAAACGATAAGTCCCGGGGTAGGGCACGAGCGGTGGGCGTGAGCGGAGGGCGAGATTTCCAACCAGTTTCTTGTCGGGTAAAGGTTAGCCAAAGAAGTCATCATCCTTCTTCTTCTTCAGTCTTCCTCTTCCATCCTCTTGAGAAAAGATGAGTCGCTTCCTTCGTACAAATTCTTATAGTTCTAGCAGCTCTAGAACCAATATAGGAAATCTACCTGAAATTGTAAATGAAGAACAATTTGAGTATAATAATAATGAACAATTAGAATTTAAAGACTGGAATATACCTAAGGTCCCTAGTCATAATATATATAGAAAGAAATGGTCTATGTCTGCATTTAATTCCACTGCACATGTTAAAACTGTAGAACAAGTTTATGCTTTGAGTAAAGAACATGAGTCTTGTCAATTACTTAATTTAGATTCAATTAAGAAACATAAGCATGATGGTAATAACTTTCTGCATATTGGATTGGTACAAGTTGCTGTTAAACCCTTAACTAGATTAGGTTTACAAGCTTCTATTTTGCTATGTTTGCGTGATGCTAGATTTACAGATTTTAATGATAGTGTTCTTGGAATGATTGAGTCTAGTCTTTGCAATGGCCCTGTACATTTTGATTGCTATCCTGATTTTACTGTTAGCCTGAATGATCCTCATATCCTGAAAACATTAACTTTGAATATTAAGACATCTGGTTATAATGTTTTGCCTGGTACCCAACCTTTAGCCTTAGTCTACAGAATTTATTATAAAGTTACAGGTACTAATATGAATTTTCAAGCTTTAAATAAGAGTCCTAAAGATCAAACGCTTTTAATACAAAGTTGTACGCCTGATGCTAATATTCGAATTCCTCATACTGTTAAATGGTCTGATATTACTTTGCCTACAGATTGGACATTAGTTACAGAATCTCAACCTATGCCTATACAACGTAGTTTAAATAATTTAGATTACATTCGTCAATATATGGATGGTAGTGTTAAAATTAATTTCCAGCACCAACCTAGGTCTACAGTTCATTTACAGCAATTACCTACACCAAGCCCTTCTAAGAGACACGAACCTGCACGGCATTCTTCTGCCTCTTCCTCTACTACAGTCAGAGATTTAGAAATAGAGAAAGATTTAATTGATTTGAAAATTGCATCTTTAAAGAAAAAAGATCAAGTGAGTCATCCTTGTTATGGTCCTCCCCCAGTTGAAAATAATGAACCTGAGCCTAAAACAGATTCGCCTACACAGTCTGATTTTCTTGCAAGTGAGATTGCTACTGCACATGTTTATGATATACACAATTGTATAAATGTACTAAAAAGAGATAAGCCGTTTAGAATAGATTGGGATAAACTAGAAAAACATACATTAGATCCTATTAACTCATCCCGTAGAAATAATTATCATAAAGTCATCCCTGATAGAAATAAAAGAAATGAAATACACCAAACCTGGAAAGATTATATGAGAACTGCTAAAGTAGAAATTAATTACCTTGATTTTGTAGAAATGCGTTATATTACTAATGAATTAAAAACTATAACTAAAGAAAAATGGGTAAAGATGGATAAGTCTGAAGTAGTTGCGAGTCATCCCCCTGTAGAAACTATTTTGATAACTGCTGGCACTAAAACTATAACTGCCGCTCCCTTTAAGATACCTGATCCTAGTAATGAGCTTAATAAATGTTTAGTTGAACAAAATAATTATACAAATCAAAGCTTAGTTGTCATAGGAAAACAATTAGATAAAATAGAAACTAAAGTTGATAAATTAATTCCTTCTGAGCCAAAACTTAGGTCTAAAGAAAAACCCTTAGTCTGTTTTCAAGATTTAAAACCTACACATGCACTTAAAACTTCCACTACTATGCAGAAAATTGAAAGAATGCTTGAACAATTAACCCCCATTAAACCTGAAAAATCTGGTGTTAAAGTATTAGGTTCTTCTTCCTTGTATAATAATAGTGATTCTGAAATGAGTCTAGATACAGATAATGAATCAGATAAGTCTAGCAATATCTCTAAGATAGAAAATGCATTTAAAAATTTAGAATTAGAATCTGAGTTAAAAGTCAAGAGATTAACTAATAAAATTAATCCTACCAGCTTAACTAAGAATTGGTATCCTAAACCTACACCACCTGATATTCAGTTTAAAGAAAGAAATAATATGAGTCAATTTTCTGTCTCCTCAGATAAATTGTATGAATGGAATATAGATGGTTTGTCTGAACAAGAAATAATGAATAAACTTACACATATGTCAATGGTGGCTAATACATACATTGCAAATCATAGTTTAAGACAATCTGAGATAGTTCCTCTTTTAGAAACTGGATTCACAGGAACCTTACGTTCTTGGTGGGATAAACATTTAAGTAATGAGTCTAGAAATAATATTATATATGCTGTTAAATTAAATGAAGACGGTCTTCCTTTGTTTGATACAAATATAGGCAGAGCCACTGAAGATGGAGTCAATACATTGCTTTATACTATAATTGAACATTTTGTAGGAACACCAAGCCATACACAGTGTAGAATACATGATCAATTGAGTAATTTAAGATGTCCGCAATTAAGTGATTTTAGATGGTATAAAGATGTTTTCATTTCTCGTGTAATGTTAAGAGAAGATAGTAATCAAGCTTTTTGGAAAGAAAAATTTATAAATGGTCTACCCAATCTGTTTACACATAAAATACGCAC

CGTGTACGTATAAGCTCAATAACTAAAACTAATATGAAAATCTTAATATTGTTTAATCAATTCAAAAGGTTTATCTTAAGAAAACAAATTTAAAACGATGTACGTTTTTTGATGTGTTAGGTTTACAAATCATCCAAAATATAGACTTGTGTTTTTGCACCGGTCATCAACCAGTGAGAAATGTTTTCGAATTTGACAAGAAACTTAAGTAGAGTCAATTTATTTATTTGATAAGAGATATTGGAGACGGAGAATTTGAACATAAAACCTCATATGCAAAGATAACTGCTCTTAACTACTTAAGCTAAAGTCTATACGAGTCCATTTCTTTATTAAGTACACTCTTTTGGTACCAAATGTGAGACATGCCATTTTTCCGTTGGAAAGATAATGGGTGACAAAATAAGCTTGTTTGGATAAGCTATAGTTGGATTTATTTCCTAGATCAATTGGAACTTATTCTACTAATAAATGTTGATCATAGGTTACCTAACTTAGAAACTCCATAACCAAATCCTTTTAGATGCAGAATGCTTATAACCTCTCTAAATCTCTGCTAACTTTCTCAGCAATATTACACAATTTTATAAGCTAATGCATAACTTGTTCTTGTTTTCTTGGTTGGTTTTTTTTTTTTTTTTTGGGTGGTCCTCAACAAGAGCATGAAACATAGATTGACAACCAAGTAGTTTCTCTCAAAGTAACATAGGCCAGATCTCATGAACATATTGGGCCCATACGTTGGAGGTCCATTGAAGTTGAGGCCATTTACACCCAAAGCTCTTTTCTAGACACCCGGCTCCAAATTTCTTTTTCCAATTTTGTCTCTACACCATGAAAAACCAAAGGAAAACTATTGGAAAAAATAATAATTTCACAAACCTCCCCGCACAATCTCGACCTCGACCGCCTTGTTGCACCGTCGGCCGCCAACCTGCCGAGGTGACTTCAAATTTTATGGATCAAGAGTATACTTGTCCTAACATGTTAAGCATAGCAA

>Chr4-05; OZ022209.1; 14569426-14577629; dir; C;

GATTAAATAAAAGAAAATAATAAATGTAATTTGCTTTAAAAAAATAAATATACTTCAATTTAACGTAATTTGTTATCGAAAAGTGGGTTCGTCATTGGGCCGACCTGGTCGAAACTTTTTGAGCTCAGGCCGGACCTCTTCGTGTCTTTTCTTTTTTTAGGTTGGATCAGGCCGGGTCGAGATTTCAAAAGATTCGACTTAGCCCACTCATTGAGTCTGGCTTATAGGGCTTTTCGAGTTGGGTCAGACAGGGCTGTCGATGGGCCTAAACGGGCTGAGCCTTTTATTTTATTTTATAATACTTTTTAGAGGAATGCTAGCAACCTTCTCTTTTGGACCTTCTTTCTTTTCCTCCTGACATGTGTCGCTTTTTTTACACTTCAAAGAGTGTCATTAATATATCACATACAAATTTGTAGTAATGGTCCCTCGATTAAGACCAAACTTTACTTTTCGCCCCTCATATTGTTAAATTGTTACGATGGTACTTTAATTATGCCTTTACTTTCAGAAATCGTCTTTTCCGTCATCTCAGTCAACTTTTCCGTTAAAAATGATGACAAGGCAATGACAAGTCCTACTTAGCACGGGCAAAAATGTCTTTTCACACAAAAAATTTAAAAAATAAATCTCTCTCTCTCTCTCTCTCTCTCCAGCCCCGATTCCCCTCATGAATTTCTTCCCCCCCCCCCCCCCCCCCCCCCCCCCCCCCCCCCCCCCCCAAATAGCTACAATTTTGACAATAATTGTCAGTTGTAAACAATTCACCCAGACTGACCAAGGAAGCAAACCAAATAGGAATAGCAATGACATCGGACTCCTCAACAACAATAGCAAAGGTAGAATATTAGATCCTTCTCAGCTCATCACACTGCCAGTTTTGTGTGCTTTGGTTTATAGTTTGTCGTGCTTTGATTGCATATGGCGGGTTGTGGGTGGCAAAATTCACACACACACACACACACATATATATATATATGTGTATATATATATATATATA

ATCCAATTGTTCTTTACTACTGGAATTATATTCAAATTGTTCTTCATTAACTATCTCTGGGAGTCGACTTATATTGGTACTAGAATTGGCAGAATTACAAGACTTTGTTCTAAACAAACGACTCATAACTACTCAAGAAACTGGGTTTGGCAGACTGAGGAAGAAGATGAACAGTAGATCCTAAACTGACCGTTTAAACTCTAAGCAACCTATTAATTTTCTCAATTTTCTGTCTCCTGCCCACCACACACACCCTCCCTAGGGATTTACAGTTCTAGGAAAAAGGACTCACGGGTCAGACCTTAGAGAAAACCAACAGAGGACTCAAAGAATAAAAGGGAAGAAAAGGAAGAAATGAAAACCTTCTACTCAGATGCTTAAACAAGCCACTTGGGTGGGGATGAGTAGCAAACTTAGACAAAGACCGTAACACTTACGGCTCTGATACCAATTAAGAAACATTGGTTGCCTTCCAGTTTTAATAGTACAACACATGTCAAAACAGTTGAACAAGTGTACGCCCTAAGCAAAGAACACGAAACTTGCCAGTTGCTTAATCTTGAGTATATATTAAAACATAAGAAAGACGGTAATAATTTCCTGCATATTGGTTTAGTTCAAGTAGCTGTCAAACCCTTGACACGGTTAGGATTGAAAGCCTCAATCCTACTCTGTCTACGTGATGCTAGATTCACCGAATTTAGCGATAGTACCCTTGGAGTTATTGAGTCCAGTCTGTGCAATGGCCCTGTCCATTTTGACTGCTATCCTGATTTCACTGTAAGTCTTAGTGATCCTCATATCCTGAGAACTTTGACTCTGAATATTAAGATTGAAGGTTATAATGTCCTTCCAGGAACCCAACCTTTAGCCTTAGTTTACAGAATCTACTATAAGGTCACAGGAACGAATATGAATTTCCAAGCCCTTAATAAAAGCCCAAAGGATCAAACCGTCCTGATCCAGAGTCACACCTCAGACGCCCATATACAAGTTCCCCATACTATCAAATGGTTCGAAGTTGCCCTTCCAAAAGATTGGACCTTAGTCACTGAGAGCCAACCGACTCCGATCCAGCGTAGTCTAAACAATTTAGACTACATTCAACAATACTTAGATGGCACAGTTAAAATTAAATTCGACAGTCATCCCTTACGAAAGTCTAATGTCCAGTTACAACAATTGCCAACTCCTAGTCAATCCCAGAGGCATGTCCCTGCTCGACATTCCTTTGCTGCCTCTTCCTCCACTCTAGAACGAGACCTAGAGTTAGAAAAGGCCATGATCGATTTCAAACTTGAGTCCCTGAGAAAAACCTCTCAGGTTACCCAACCTTGCTACGGCACGGTTCCAGTCAGAGACGACAAATCAGAATCCCCTAAGTCCCCGACCCAGTCTGATTTCCTAGTTGACAATCAACTCAGAACCCTAAATAAGAAATTTGAAATTAATTGGGTAAGTCTAAATCGCCATTTAAAAGCCCCTGAAAATCAGTACAGAAGAGATAATTATCACCAAGCCTACCCTAATAGCAAACACCGCGAAATGATCTTTAGTAAATGGAAAGATTACATGCGGTCAGCCAAAGTTGAAATTTTCTACCTTGATTTCGTTGAAAGCCATTACATGTCTAGTGATGAATTAAAAACTCTGACTAAAGAAAAGTGGAAACTAATGGATAAATCTGTAATCGAGTCGAGTCATCCTCCAGTAGAAACTATCATAATAGAACATCAGAAAGCACCAGTAGCTACCACCCCTTTCAAAACCTTTGAAAGCAGTGACCCGAATAGAAAAATAATTGAACAAAATAATTACACCAACCAGAGTCTAATCACTATAGGACAGCAATTAGACACAATAGAAACCAAGGTCGACATGCTGAGTCCGCCTGTTGCAGACTCCAGACCCAGACCTAAAATTAAAAAGCCGATAGTTCAATTCCAAGAGCTTAAGTCTAGTCCCGTCTTAAAAGTCAACTCTACAATGAAAAAGATAGAAGAAATGCTAGAACAATTGACCCCTGCCAAGCCCGAGAAAGGTGACAAATCTGGTATTAAAACATTAGATTCTTTTGCTGCTTCTCAATCTGAGTCCGAGTCAGTTAGTTCAGAAAGTGACATTTCCAAAATTGAAAACGCCTTCAAAAATTTAGAAGTCCAAACTAAGCCCAGAGTCAAAAGAATAGAAAAACATATAAGTCCCGCTAGCCTAACAAAGAATTGGTATCCCAGACCAATACCTCCAGATATCCAATTTGAAGAACGAAACTTTCAAACCCAGTTTTTAGTTTCTTCTGATAAATTGTATGAATGGAATATTGATGGTCTTTCTGAACAAGAAATCCTGAATAAGTTGCAACATATGTCTATGGTCGCCAACAGTTATATCACCAATCACAGTTTCAGACAATCTGAAATTGTCCCCCTGTTAGTTACCGGTTTCACTGGTACTCTCAGAAATTGGTGGGATAAACATCTTACCCTTGAGTCAAAAAATCGAATAATCTATGCTGTAAAACTTAATGAAGACGGTCTCCCTATATTTGACGAACAAATAGGTCAAGGAATAGAAGACGATGTCAACACTCTGTTTTACACGATAATTGAACATTTCATAGGAACCCCTAGTAATACCACTGCTAGGATACATGATCAGTTGAGTAATCTTAGATGTCCGAAGTTGTCTGATTTCAGATGGTATAAAGATGTCTTTATTTCCAAGGTCATGCTTAGGGACGATAGTAATCAACCCTTCTGGAAAGAAAAGTTCATTAATGGTTTGCCAAACTTATTCGCTCACAAGATTCGCACGACCCTAAGTAATGAACAAGGCCAAATTGATTGGGATAATTTAACTTATGGTAATATTATAAGTACGATTAATCAAGTTGGTATGAAAATGTGTATCGACTTTAAAATCAATCGCCAAGTCCAGTCTGATAGAAAGTCAGCTAAGTATGAATTAGGGAATTTCTGTGAACAATACGGTCTCACTAGTATTCCTCCTTCTAGGAAACATAAGCCTAGTCACCTTAGAAAGCGTCATTACCCGAAGAAGCAATTTCTCCGGAAAGATGAATTCTATAAGAAAAGAAAGTTCTCCCCTAAAAAGAATTGGTCGAAACGCCCTGAGCAAAAACAAAGTCGCCCTAAGAAAGATAAGAGCAAAGTCAAATGTTTTAAATGCCAAAAATTTGGTCATTATGCTTCTGAATGCAAAGTAAAAGATGTTATTAAACAATTGCATATTACAGAAGAAGACAAGAACAAATTAGTCCAAGTTTTGGAGTTGCGAGACTCTGAAATGAGTGATGTTGAGTCTCTGATATCTAGTTCTGAGTCTGAGTTCAACCAATCTTCAGATTCACAACCCAGTTCCCCTAGAGTGCAATTTGGTTGTACTGATAAATGTTGTAATAGGTTAAAATCTGTCTTAGTCCTCACTAAACAAGAAGAACAAGAAGAACTCCCAATAGACTTAATAGGGAAAGTTCAAAATCCAGAATTGAAGTTAGAAAATTTGAAAAAAATTAAAGAAGGTCCTGAGTCAGGAAAGCTCAAGCCTGAGTCATTCTACTCCCCAGCCAATTAGCATCAACACTACTTTAGAAAAATTCAATTGTAGAAAAGATGTCACCCTCCAAGATCTCCAGTCAGAAGTTAAACAGATCAAAAAAGAAATAGTTGAACTGAAACAAATCAGTCAACAATTGCAAACCGAAAATTATACAATTAAACAAGAATTATTTAATTTATCCAAGCAGGAACCTAGTACCCCTAAAAGCCAGTCAGAAAGCCCTCACCAATCTGATGATGAGCCTTATGTTGAACAACCCGTTGTCAATTTAATTAAGCAAGTTCACCTTAGAAAATGGTATTCAAAAGTAACAATATTTGTCCAAGATTTTGAGTTTACCACAGTCGCCTTATTTGATTCTGGTGCAGACCTCAACTGTATTCAGGAAGGTCTCATCCCACTAAGTATTACCAGAAGTCCAGGGAATCCCTTAGCACTGCCTCAAGGAAGTCTCTCCAGTTAAATTTTGAAATACCAAAAGCCCATATTTGCCAAAACAAAGTCTGTTTCAAAACTTCATTCGTCTTAGTCAAAAACATTACTGATGAAGTCATTTTAGGATTACCATTCCTAGCCTTATTATATCCTTTCCAGGTAGAATACGATGGTATAATTTCTACCCGCTTAGGAGAAAAAATTAAGTTCCAATTTTTGTCAAAACCTGAGTTGCATAACCTGAAAGCTTTACAAAACAGTCAGTCTCTAAATCTGTCCAAGTTATCCGAAAAACTAATCAGTTGAATTTTCTTAAAGAAGAAATTAAGCTTAAAAGAATTGAACAACAGCTTACTGATAAAGCCTTACAGTTGAAAATCCAATCCTTTGAAAACAAATTAAAAGATGAAATTTGCTCTGAATTGCCTACTGCTTTTGGTATAGAAAACAACATGTTGTCTGTCTTCCCTATATCAAAACCTTTAGTGAAAAAAATATCCCCACTAAAGCTCGGCCAATCCAGATGAATCAAGAGTTGATGGAAGTCTGTAAAAAAGAAATTCATGAGTTACTCCAAAAGAATATTATTCGCAAAAGTAAATCCCCTTGGTCGTGTCCTGCTTTTTATGTCCAGAAAAATGCTGAAGTAGAAAGAGGAGTCCCCAGGCTAGTCATCAATTATAAGCCCCTTAATTCGGTCCTAGAATGGATTAGGTATCCCATACCTAATAAAAGAGACCTCATTAATCGACTAGAAAAGCAGTTGTCTTCTCCAAATTTGATATGAAAAGTGGTTTCTGGCAAATCCAGATTGATCAGTCAGACAGGTATAAAACTGCATTTGTTACTCCTTTCGGTCATTATGAATGGAATGTCATGCCGTTTGGATTAAAAAATGCCCCCAGTGAGTTTCAGAATATTATGAATGATATCTTCAATCCTTACAGTCATTTTCGATTGTTTATATTGATGATGTTCTCATTTTTTCTGAGTCAGTTGAACAGCATTGGAAGCATTTGCATAAATTCCTTCACATAGTCAAACAAAATGGTCTAGTTGTCTCTGCTAAGAAAATTAAGTTGTTTCAAACCAATATTAGATTTCTTGGTTTCAATATATGCCAGTCTCAGATTTCCCCAATTGATAGAGTTATCCAATTCGCTGATAAATTCCCTGATCAGATTCTTGATAAAAGTCAACTCCAAAGGTTTTTAGGATCTCTAAATTATATCTCTGACTTCTATCCAAATATGCGAAAGCAATGTAAGCCTTTATTTGACAGATTACAAAATAATCCTCCTCCATGGTCCAATATTCATACCGAAGTCGTCAAACAGATTAAAACTCATGTCAAGACACTTCCCTGTCTTGGTATCCCTTCAGTCGATTCCTTCAAAATAGTTGAAACTGATGCCTCTGACATTGGTTATGGTGGCATTCTCAAACAGAAAGTCAGTCCAGAATCTCCAGAACAAATTGTCCGTTTCCATTCTGGAACCTGGACTCAGTCGCAATATAATTATAGTACTATTAAAAAAGAAATATTAGCTATTGTATTATGCATTAGTAAATTTCAAGATGATCTACTCAATCAAAAGTTTTTAATACGAGTTGACTGCAAATCTGCAAAACATGTTTTAGAAAAAGATGTTCATAACATTGCATCTAAACAGATTTTTGCACGATGGCAAAGTATATTGAGTATATTTGATTTTGATATTGAATATTTGAGAGGTAGTGAGAATTCAGTTCCTGATTTTCTTACTCGAGAATTCCTGCAGGAACCACAATGGCTCCCAAAAAAGACAAACACAAAGAATCAGCCAGTCTGCCTAAGTCAATCCAGTCGCAACAGTCGCAAGCATCCCAATCCCCATCCATGAGTCAAAATAAGCCTGAACAGATAGTTCCCTATCCTGGTCATCCCCCGCTTACAGTTAGGGGTGGGCGCGGTCCGGTTCGGTCCGGTTCACACCTCAAACCGGAACCGAAACCGGTACTATTTAACCGGTCCGGTTCGGTTCGGTTTAGGCAAAAAAAATTTCAAAAACCGGCCGGTTCGGTTCTGACCGGTTCCGGTCCGGTTCTGACCGGTTCCGGTTTTGAACCGGATTATTAATTTTTTATTAATTTAATTTTTTTTGAAAAAAAAAATTATTTTTTTAATTTTTTTTTAAAAAAATTATAATAAATGACTTATTTTTTTAGCTTAAAAATGAACAAATAATCCAATTCATACATTTAGAAATTTTTTGAAGTTGAACTTGGAAAATTAGTGATTATAAAAGTTAAAATATGGCATTAGGTTTTAAAAATTTGTAAAAATATAAAATATAAAATATATGACCGGTCCGGTTCGGTCCGGTCCGGTGCCGAAAGATGTAAAACCGGAACCGGAACCGGTTGGAACCGGTTCGGTCCGGTTCCGGTCCGGTTTGTTGACTTTTTTGGTCAACCGGTTTTTTTCCAGTTTTTTTTTCACCGGTTCGGTTCGGTGTTCCGATTTTCCGGTCCCGATGCCCACCCCTACTTACAGTTGCCACCCCATTATCAGTTGCCAATAGATTTTCCTCTCTAGGATCCACAGTTGGCCAAATCCGTCCTAGTTATCAATCCACACTAGTAGCCAGTTATGATCCCTTTTCAGTCGATGTCCCAGCCGGACCTTCAGTTGCATTCAAAAAATCATCTCCTTATTTGCCAAAGAGTAATTCCCATCTATTCGTCATTGAGCCACTATACGATGTTAATACAAACCCAGTTGAAATTGCCAAACACTACTTTCCTCCTGGATTTCATTATATGCCCTCTAGTCCCTACAAATCCCTCAAGTATTATAGAGATATATTACTTGAAACCAAATCAGTTGAAATCAAGCCGATAAAGGATCGTGATCATCCCGAGTTCATTCTTTATCATTCTTTGTATATTCATCAAATAATAAGCCAAGAAGCCTTCAGTAGCCGCCCTTATGAGTTGAAAGTCCTTCAGTCCAAATTACAGTACAATTATGCTGATTATATTGATGCCTGGTATACTATTTTTCTTCATCAGTCTGAAGATTTTAGTCATTCCTGGTTCATTAATTTTGATAACAAGTTCAAATGTTCTTTCCCCTATTGGTTCTTCCATTGGTGGGAAAAGCATGGTCCAGTTGACGAAATATTGCCAGTTTCAGTCATTGAGTTAATCCGCCATTACACCAAGAAGACCAAGTTCTCCAAAGTTGAATTATTCTTCCCCAAGCAGTTGCTGTTTCTTGCCAGGTACAAGGTGCCGTGGATCCTTAAATGGTCCTACAGAGTCACCAAAGATGCCAGGATCTTTGCCCGCCAATTCTCTGTCAAGTGGTGGGACAAATTTGAAGTTGATCGAATTGCTAGATATGTCTACAATGATCTCCCAGTTGAACCAATGCCGCGCCCGATTTCCCCTACTGGTTCTACCCGATCTTCCTTCTCAGTCGATGGAAAATCCAAGGCTGAACTCCAGGAAATTGCTCGCCAGTTGATCTTACAGGCTTCCCAGATGGATGATGATGAGGACAACGACTCTCCTTCGTCGTCTAGTTGTCAGCCCATTCCAAATCCTGTTGAGCCTAAGTCCTGGTATGACGACAGCCAGGATCCTTATGACGCCTATGATCTGCATTCTGACTAGGCTTCATAATTGCTGCCGACACTTTCACATGCCAAAGTTGCCGCCCCATAATGTCAGTTGACGACACCATAATTGCTACAGGAAGAAGCATCTGCTTTTACTGTTCGCTTTTCCACTGAAAGCTGTCTCAGTAAAAGAATCTTCCGAATAAAACCAAATTCAAAGTTGAATCAGGTTGCTTTTCGTGACCCAACAGTTGCAGCGAACCCCAGTTACTTTACTATAGTTAATCCAAACCAGAGTTGCTTCTCTCTATATAAGCAGGCTTCGGCCTCAGTTGTAAGCAGAATGTAATTTTCCCTTTCTTCAGTTTTAAAGATTCTCTCTGATACATCCTTCGGAGAAATCATCTTTGTAAATAAGTTTGTGAGCTTTGAAGTGTTGCAGTTGCAATCTTTGTGAGTATAATAAAAGTTATTTTCAATTATCTATTTTCTGAAAGCATTATTTTGATGAATTAACATTATTTTCTGCATTAGTCTGAAAGTTGTTTAGGTTGCATCCATTCCTAATTTCCTTCTGATCTTCTC

TATATATATATAAGCAAAAGAAAAAATGCCTCTGCTTCTCTGTTTTGATATGATATGGGGTGGAGGATGGGGGCTGAGGTTCTGGGTTTGTGGGTGCTGGTGTTGCAGGCTAGGGTAGAGGGTTCTGGGTTTGTGGGTGCTGGGGTTAGGTTCCCAAATTGCCCTTAATTTTGACAGAAAATTTAACTAAGTTGACAAAAAGACCAATGATACAACAAGATATCTAATTGAAACACCACCGTAACAATTTAACAATATGAGGGACGAAAAGTAAAGTTTGGTCTTAGTCGAGGGACCATTGATACAATTTTGCCTATATCACACAAACTAATTTTTGTTTTTCAAATTTACCCTTATTAAATGTATTGACATTTTTTTTTTCTTTTACAACAAACTAGCCTTTTTACATCTTTGTTTCCATCTTGTTAAAAAATTAAATAAGAAAAAAAACATAATTTTTTTAAAATATAAAATGTCTGCCTTTTTTCTTTTTTTTTTCCAAGAAAACAAACATTGCTAACATGCTAATTTTTTTTAACAGACGCTTTATGATTACCATGAATCTATTGTTGCTAATAAACTATTCTATCATTTCCTATTTTTAAAATCAATTTTTATTTGTCAATGGATATTGGTAATGTTTATCTTCCACTGTTTGGTATTATTGATGTTTATCTTCCACTGTTTGGTATTATTGATATTTATCTTCTACTGTTATGTTTTCTTTTCCTTATCTTAACATTTAACTGCCTATTGTGTTTAAAAACAAGACGTTCCCATTGGATTACAAAAAATTTTAAAAAAAAACTAACTGATGTTTTTTGTTGAATTTTTTAAACAAAGCGTTAAGAAAGTTGTAAAAAAAAAAAAAACCAATAGATTTAATAAGGATAAACTTGGAAAGTAGAAACTAGTTTGTATGACACATTAATGGGTATTGTTTCAAGTGCAAGGAAAGTGACACATGTCATGAGGAAAATAAAGAATGTCCAAAAGAGAA

>Chr4-06; OZ022209.1; 15113124-15129491; dir; C;

CCCTACATGAAACGAACTTTAGAAATACAAGTCAAACTCATTTACAACTTAACAAAGAAACTTTAAACTTTTTATAAATTACAAAACTGGCACCGTGGATGATTAGGTTAAAGCACAAATAAGGGATTGGTCTTCGACCGTACTTTTTATAGAAGAAGCCCCAAAACCCTAACCCTCCTGTTGGAACTTGAGCAGTCCTGGGAAATCGAAGACAAGGATGTCAGTCCACTTCCCTGACGAAATTATAGACAAAATCCTTGTCAGGTTGGACCCCAAATCTTTGATCAAATTCAGTGCAGTGTGCAAGTCATGGAGGTCTCTAATCAAAAGCCCTACCTTCAATCCACACCCACCTCAGCCGCACAATCCAACCCAACAATCAAAACGACTCTCACCCGGCTTATCGACCGTTTTGCTGACAATGAAGAACGTTCAGGCCGGGATCTCAACGTGGTTGGGACTTGTATGCCTTTCCTTAGGTTACAATAAACTAAGAATTTGGAATCCCAGCGTTAGAAAGTTTGTGATTTTGCCATGCCCTATTTCTGAGTATATAGCTAGGTATGCCTTTGGCTATGATTCGCGTAACAAGCAGAGAGTGGGTTGGTCCTTAGCTCGGGGCTCGTGGAAGAGTCTTGGAGTTGGAAGTACTGGTCGAGGCTTTTTATCTGAACTCATCCAGCAGCCTGTTTTTGTCAATGGTGCTCTGCATTGGGTTCAACTCAATACGATGAAAGGGGAAAATGGGATTATGTCGTTTGATATGGTGAGTGAATCGTTTGGCGAGACTAGGGTTCCCCAAAAATGCTAATGTGTACCTTAAGACATGAGGAGAACCTTGCCTCGCTTACATGGGATTCTGCAAGCGTATACCTTCCTTTTTCTTATGACATATGGGTGCTGAAAGAGTATGGTGTCGAGGAGTCGTGGACTAAACTATTCAGTCCATAATTTGACCAACTATTATGTCAGTAAAAGCCAATTTAACAAACACCAGTTG

TTTTTCCCCAAGCTGCTTCTTTTTATTGCCAGGTACAAAATCCCCTGGATTATGAAATGGAGCTATCATGTTAATTGGGAATCCAGAATTATGTCTCGCCAATATTTTGTCAAATGGTGGGATAAATTCTCAATGGACAGGATCTCAATTTATGTCTTCAGGGATTTTCCCCAATCGGATCATCCCCAGCCTAAGCCAAGTTCTCCTACCGGCTCAGTCAGTTCGGCTATCTCAATCGATGGAAAATCCAAAGCTGAGCTCAGAGCCCTCGCAGCACAGTTGCTTGACCAAGCTTCTCAGATGAATGATGATGAGGACAAAGATTCTTCCCCATCAGCATCCAGCTGTCAGCCTGGTTCCAGTTCCTTTGATCCTAATCTGCCTTACGCTCAAGATCCTTATGACGCGTATGACTTGGGATCAGATTAGTCAAAAGTTGCCACCGAAAGTTGTCCAAACAGTTGCCATCTTTTCTCTTTTCACCCTGGTGAAATGTTGGAGTAAAACAAAACCACTTTTGCTTTTACTTCTACTTTACCCCAATAGGTAAAGAGAAATGTCGAAGGAAAAAGTTGACATCATAATTGTATCATACCATGGTCACTCTCCAATCAGAGTTACTTTTGGTCTAAACCATAGTTGCTTTCTTTTCTTTATAAGCTGGCTTCGGCCTCAGTTGAAGACACTTGTAAATTTTACTAAGCTTTCTCAGTTATCAAAGTTCTCTCTGAAACTTCTTCAGCTAGAACTCTCATGTAAATATCTGAGTGCTTAGCTATCAATAAACAAGGCTTTTGTGAGTATCAAAATGTCTTTAATTTCAATATCCGTAATTTTATTTATTGCTATTTTAATTATTTTCATCTATGCATTAATTTGTTCATTATTAATTTGTTCCTGTTTAAATTATTTCTGTTCTTGTTTCCTATCCATGGTTCTTCCTTTCTTTGGTATCAGAGCCGTAAGAGTTGTAAGAGTCTGTTCTTAAGTCGCTACTCATCCAACCCAAGTGGCTGGTTTAGGATTTCTGAGTAGAAGTTTTACAGTTGCCTTTCTTATTGATCTTTTATTCAGTACTGTTTTCTGTATCTCTCTTAAATAGTGTCCGAAGAGTAAGGCCTAGAGTCCAAACAGTAAGTCCCGAGGTGAGGCATGAGCGGTTGCGGCATTGAGAGGGCTAGGAGAGAGAGGTCAGTTGACTGTAATAAATAGAAGTCAGCCTTAGAGGTCCCTGTTTATCTTCTTCTTTGAAGTCAGAACCCAGTTGCCAAAGTAAAAATGAGTCGTTTCCTTAGAACAAATTCTTGTAGTTCGAGCAGTTCAAGAACCAGGGTCGATCGACTACCAGAAATAGTTAATGAAGAACAAGTTGAATTTGAGTCTAATAACAATGAGTTAGATTACAAAGACTGGAATATCCCTAAGATCCCCAGTCAAGAAATTTATAAGAAACGATGGTCTTTACCAAGTTTCAAGACCACTACCCATGTAAAAACAGTTGAACAAGTCTATGCCCTCGGCAAAGAACTTGAAGTCTGTCAGTTGCTCAATCTTGAATCAATCAAAAGACATAAGAATGAAGGAAACAATTTCCTGCATATTGGTCTTGTTCAAGTCGCTGTCAAACCACTAACCAGAACAGGGCTTAAAGCCTCAGTTTTGCTCTGGTTAAGAGATGCTAGATTCACTAACAGTGAGGACAGTACTCTCGGAGTAATTGAGTCAAGTCTTGCTAATGGTCCAGTTCACTTTGATTGCTACCCAGACTTCACGGTCAGCCTTAGTGACCCCCACATTTTAAAGACTTTGACATTGAACATCCAGACTTCTGGATATGAGGTTCTCCCAGGAACCCAACCATTAGCTTTGATTTTTAGAATCTATTATAAGGTCACAGGAACCAATATGAATTTCCAAGCCCTTAATAAAAGTCCTAAGAATCATACTTTGTTGATTCAAAGTAATAGACCCGAGGCCATCATACGAGTACCTCAAACAATCAAATGGTCTGATGTCTCACTCCCAGTTGAGTGGACACTCAAAAATGTTAATCCACCAACACCAGTTCAACATAATCTAAACAATTTAGATTACATCCACCAATACTTAGATGGATCAGTAAAAATCAATTTCGGTACCCAACCAGTTCCAAAATCTACTGTCCAACTACAACAATTGCCAACCCCTGGTCAATCCCAGAGACACATTCCTGCCAGACATTCCTTTGCAGCCTCTTCCTCTACCCTAGAAAGAGACCTAGAAGCGGAAAGAGCCCTGATTGATTTCCAATTAGAATCTCTCAGAAAAACTGCACAGGTTAGTCAACCCTGTTATGGCAAACAACCAGTTGAAGAACACAAGCCAAAATCTCCCGAGTCTCCTACCCAAACTGACTTTGAAGGTTTTGATTTTGTTGATCCTCAACTCAGAGTTTTGTCCAAATTTACAATTAATTGGAAAAAATTACAAGCCCATTTCACAGCTCCTGAAAATCAGAAGCGCCGAGACAATTATCATAGTTCTTTTCCTCTTAAGTATCGCCGTGATGCAATCTTTAATGAATGGAAAGATTACTTGCGAACAGAACAAGTAGAAATCCCTTACTTAGATTTTGTAGAAACCAACTACATGTCTAAAAAATTGAAGGTAATCACCAAAGAAAAATGGTCGCTACCAAATGATACTGAAATAGTAGCTAGTCACCCTCCTGCTGAGTCTATCTTAATCAATTTTAAGAAAACTCAGATTCCCGCCTGTCCTTTCAAATTCCCTGGAAAATTAGATTCAGAGAAGAAATTGATCGAACAGAATAATTATACCAACCAGAGTCTAATAGTCATAGGAAAACAGCTTGACAAAATCGAAACTAAAGTCGACAAGCTAGTCCCTTCCTCTGAGAAATTAAAAACAAAAGTTGAAAAACCATTAGTTCAGTTCCAGGATCTTAAGACAAATCCGACCCTTAAGATCAAACCTACATTACAAAAAATTGAAGAAATGTTAGAACAGTTGACACCCATCAAAGGTGAACAATCTGGAGTCAAAGTTCTTGACTCTGTTCAAACCAGTTCTGACTCTGAACACGAATCCGTCCAGAGTATGGTTTCAGTTGATAGTAATATTTCAAAAATTGAAAAAGCCTTCATTGAACTCACAGTTGAACCAAGGGTCACTCGGTTAATTAACCATGTCACCCCTACAAGCCTTACCAAAAATTGGTATACCAGACCAACACCCCTGACATACAATTTGAAGAAAGACATTTTCAGAACCAGTTCTTTGTCTCTTCTGACAAATTGTACGAATGGAATATTGATGGTCTTTCTGAGCAAGAAATCTTTAATAAACTTCAACATATGTCCATGGTTGCCAATAGCTATATCTCAAACCACAGTTTTAGACAATCTGAAATTGTGCCGTTACTAGTAACAGGTTTTACAGGTACCCTCCGCAATTGGTGGGATAAACACCTCACACCTGAATCTAAAAACTTAATTGTTCATGCTGTAAAACTCAATGATGAAGGTTTCCCTATATTTGACGAACAAATAGGTCAAGGTATAGAAGATGGAGTCAATACTCTGTTTTACACCATAACTGAACATTTTATTGGAACACCAAGCCATACCACTGCTAGAATCCATGACCAGCTAAGCAACCTTCGTTGTCCTAAACTGTCTGATTTCAGGTGGTATAAAGATGTCTTCATCTCTCGCGTCATGCTCAGAGATGATAGTCACCAATCCTTTTGGAAAGAAAAATTCATTAATGGTCTTCCAAATCTATTTGCTCATAAAATTCGCACAACCCTTAGTAATGAATAAGGTCATATTGATTGGGATAATCTGACCTATGGAAACATAGTCAGTACTATCAACCAAGTCGGTATGAAAATGTGTGTTGATATGAAAATTGGTAAGCAAATACAGTCTGACAGAAAGTCTGCTAAGTATGAATTAGAAAACTTCTGCGAACAGTATGGTCTCACTAGTCTTCCTCCGTCTAGTAGAAATAAGTCTTCTCATAAGCATAAGTCTCGTTCTCATCCCAGTAGAAAAAACACTTACAAACAGACTAATGATAAAGAATTCTATACACACAGAAAGCTTAATCCGAAAAAGAATTGGAAGAAATTTCCACACAAAAATAAGAAAACCCGATTCCAGAACAATTCTGGAAAAGGAAAAATCATTTGCTGGAAGTGTCAAAAACCAGGTCATTATGCTAATAAATGCAAAGTTAAAGACACAATCAAACAATTAAAAATTGAAGAAATAGAAAAAGAAAACCTCATTAGACTTTTAGATTTACAGGATTCTGGATCCAGTGATCTTGAGTCTTTAGTTGACAGTGATTCTGAGATTTCTTATTCTTCTGATTCACAACCCAATTCCCCTCATATTCAATTTAGTTGTCAAGATAACTGCTGTAGTTCTATGAAATCTGTTTCTGTTCTTACAAAACAGGAAGAACAAGAAGAGCTTCTCATAGATCTAATCTGCAAAGTAGAAAACCCAGAGTTGAAAACTGAGTATCTGAAAAAATTTCGGAAACTCCTTCGCCATGAAGGACCAAGTCAGTCTCCACCTCCTCAAAAAATCTGCCTAAATACAACCTTAGAAAAGTTTAGTCGCAAACGAGATATCACCCTTCATGATTTACATTCAGAAGTAAAGATTATCAAGAAGGAAATAGTTGAATTAAAACAATTAAGTCAGAAACTGCAATCTGAGAACAATGATATTCGCCAAAACTTCATTGCTTTCAAAATTGATTCGAATTCTCAACATTCATCTCAGAGTCCAACCAACTCAGATGATGAATCTTTTCCAGACCAAACCTTATGCCTAGTTAAACGAATTAGCTTTAAAAAATGGTATGTCAAAATCACCATTTTTGTCGAACAATTTAAGTTCACTACAGTTGCTTTACTTGATTCGGGTGCTGACCTTAACTGCATTCAGGAAGGCCTTATACCTACCAAGTACTATACTAAGATATGCGAATCTCTTAGAACAGCTTCAGGAAAATCCTTACAGCTGAACTATGAAATTCCCCGAGCCCATGTATGCCAAAATAAAATCTGTTTTAAAACTTCATTTGTCCTCGTAAAAAACATTACTGATCAAGTTATTCTTGGATTACCTTTCCTTGCCTTGTTATACCCTTTCCAGGTAAATCATGATGGTGTCATCACTACCTATTTAGGAGAACAAGCCAAGTTTGAGTTTTTATCCAAGCCTGAGTTACACCAGTTGAAAAATTTCCAGAAAAGTTCAATTTCAAAAACTGTTGCCCTAATTTCTAGTAAGACTAGACATTTGTCTTCCCTCAAGGAAGAAATTAGTCATACCCGGACATGTCAACAAATCGAAAATGAATCTTTTCAAAAACAGGTACATGATTTTGAAAATAAGTTGATACAGGATATTTGTTCTGATTTGCCTAATGCTTTTTGGTCTAGGAAACAACATATTGTCAGTCTTCCTTACACCAAAACCTTTAGTGAGAAAAATATCCCCACTAAAGCCAGGCCAATCCAGATGAATCAGGAGACAATGGAATTCTGTAAAAAAGAAATTACAGAATTACTCCTGAAAGGAATAATCCGTAAGTCTAAGTCACCTTGGTCTTGCCCTGCTTTCTATGTCCAGAAAAATGCTGAACTAGAACGAGGAGTCCCTAGGCTAGTCATCAACTACAAACCCCTCAATGCAGTCTTAGAATGGATTAGGTACCCCATACCTAATAAAAGAGACCTAATTAACCGATTAGAAAAAGCAGTCGTCTTTTCCAAATTTGATCTTAAGAGTGGATTCTGGCAAATCCAAATAGAAGAGTCAGATCGGTACAAAACCGCCTTTGTCACTCCTTTTGGTCATTATGAATGGAATGTTATGCCCTTTGGTCTTAAAAATGCACCTAGTGAATTCCAGAATATTATGAATGAAATTTTCAATGCTTACAGCCATTTTTCAATTGTTTATATTGATGATGTCTTGATCTATTCTCAATCGATAGAACAGCATTGGAAACATTTGTCCATATTTCTCCAAATCATTAAACAAAATGGCCTAGTTGTTTCTGCTAAGAAAATCAAACTGTTTCAAACTAGCATTCGATTCCTTGGCTTCAATATCTGTCAATCACAGGTTAGTCCAATTGATCGAGTCATCCAGTTTGCTGATAAGTTCCCTGATGAAATTATTGATAAGAGTCAGCTTCAGAGGTTCTTAGGATCTCTTAATTATGTTTCTGATTTCTATCAAAATCTGAGAAAACAATGTAAGCCTTTGTTTGATAGATTGCAGGACAGTCCTCCTCCCTGGTCCTCTGTCCATACTGAAATTGTCAAACAAATCAAAATCCATGTTAAGACACTTCCCTGTCTTGGCATTCCTTCAGTTGATTCTTTCAAAATAGTTGAAACTGATGCCTCTGACATTGGTTATGGAGGTATTCTTAAACAAAAATTGAGTCCCCATTCGCCAGAACAAATCATTCGTTTCCACTCTGGCATCTGGAATCCATCACAAAGTAATTATAGTACTATTAAAAAAGAAATTTTATCTATAGTACTATGCATTTCTAAATTTCAAGATGATTTACTTAACCAAAAATTCTTAATACGCGTTGATTGCAAATCTGCAAAACATGTTTTAGAAAAAGATGTTCAAAACATTTGCATCCAAACAGATTTTTGCACGATGGCAAAGCATTTTAAGTATTTTTGATTTTACTATTGAATATTTGAGAGGCAGCGAGAATTCTATTCCTGATTTTCTTACTCGAGAATTCTTGCAGGGAAAGCAATGGCACCCAAAAAAGACAAGCAAAAATTGGATGCCAGTCAGTCCAGCCAACCTAAACCCTCCCAAACCCCTGCACCACCCCAGTCGCCCAAAAGAATCCTTCCTGCATCCATGAGTCAATCTAGAATGGCCCATCCATCCTCAATTGTCCCATTTGCAGGCTACCCCCAATCCCAGTTGCAAACAGATTTACCAGCCTTGGAACCCCAATAGGATCCACTGTTGGCCCAATTCGCCCCAATTGCCAGTCAGCCCTAGTTTCTGACTATGATCCTTTTGCAATTGTTCCCCCAGTTGCCCCAGTTGCTCAGTCCTCCCAGTCGTCCACCCAGTTCAAAAAATCGTCCCCTTATCTCCCAAAAGATAAGACCAATTTATTCATAATTGAACCCTTGTGTGATGGGATTTCTGATCCCATAACAATTATTCGATCATATTTCCCTCCTAATTCTCATTTTCTTCCTCCTGCACCTTATAAAGACCTTAAATTCTATAGGGATATCCTGCATGAGACTAAATCAGTTGAAATTAAGCCTATTAAGGATAGAGATGATCCCACTGTCATCCTCTATCATTCTCTTTACATTCAGCAAATTCTGAGCCAATCCGAATTTAGTCGCCACCCCTATGAGTTGAAACACCTTAAGTCCACTCACCCTTACAACTATGCAGATTACATTGATGCATGGTATAACATCTTCCTTCACCAAACAGTTGACTTTTCCCACTCCTGGTTTCTTAATTTTGAATTTGGCCTCAAAATTAAGAAACCAGGAGTGGGAAAAGTCAACTGTTTGGTGAAGGAAGATGTTATACCATGCATCAATGTAATCTGCATAGTTGTAAGGGTGAGTGGACTTAAGGTGTTTCAACTCATAGGGGTGGCGACTAAATTCGGATTGGCTCAGAATTTGCTGAATGTAAAGAGAATGATAGAGGATGACAGTGGGATCATCTCTATCCTTAATAGGCTTAATTTCAACTGATTTAGTCTCATGCAGGATATCCCTATAGAATTTAAGGTCTTTATAAGGTGCAGGAGGAAGAAAATGAGAATTAGGAGGGAAATATGATCGAATAATTGTTATGGGATCAGAAATCCCATCACACAAGGGTTCAATTATGAATAAATTGGTCTTATCTTTTGGGAGATAAGGGGACGATTTTTTGAACTGGGTGGACGACTGGGAGGACTGAGCAACTGGGGCAACTGGGGGAACAATTGCAAAAGGATCATAGTCAGAAACTAGGGCTGACTGGCAATTGGGGCGAATTGGGCCAACAGTGGATCCTATTGGGGTTCCAAGGCTGGTAAATCTGTTTGCAACTGGGATTGGGGGGTAGCCTGCAAATGGGACAATTGAGGATGGATGGGCCATTCTAGATTGACTCATGGATGCAGGAAGGATTCTTTTGGGCGACTGGGGTGGTGCAGGGGTTTGGGAGGGTTTAGGTTGGCTGGACTGACTGGCATCCAATTTTTGCTTGTCTTTTTTGGGTGCCATTGCTTTCCCTGCAAGAATTCTCGAGTAAGAAAATCAGGAATAGAATTCTCGCTGCCTCTCAAATATTCAATAGTAAAATCAAAAATACTTAAAATGCTTTGCCATCGTGCAAAAATCTGTTTGGATGCAATGTTTTGAACATCTTTTTCTAAAACATGTTTTGCAGATTTGCAATCAACGCGTATTAAGAATTTTTGGTTAAGTAAATCATCTTGAAATTTAGAAATGCATAGTACTATAGATAAAATTTCTTTTTTAATAGTACTATAATTACTTTGTGATGGATTCCAGATGCCAGAGTGGAAACGAATGATTTGTTCTGGCGAATGGGGACTCAATTTTTGTTTAAGAATACCTCCATAACCAATGTCAGAGGCATCAGTTTCAACTATTTTGAAAGAATCAACTGAAGGAATGCCAAGACAGGGAAGTGTCTTAACATGGATTTTGATTTGTTTGACAATTTCAGTATGGACAGAGGACCAGGGAGGAGGACTGTCCTGCAATCTATCAAACAAAGGCTTACATTGTTTTCTCAGATTTTGATAGAAATCAGAAACATAATTAAGAGATCCTAAGAACCTCTGAAGCTGACTCTTATCAATAATTTCATCAGGGAACTTATCAGCAAACTGGATGACTCGATCAATTGGACTAACCTGTGATTGACAGATATTGAAGCCAAGGAATCGAATGCTAGTTTGAAACAGTTTGATTTTCTTAGCAGAAACAACTAGGCCATTTTGTTTAATGATTTGGAGAAATATGGACAAATGTTTCCAATGCTGTTCTATCGATTGAGAATAGATCAAGACATCATCAATATAAACAATTGAAAAATGGCTGTAAGCATTGAAAATTTCATTCATAATATTCTGGAATTCACTAGGTGCATTTTTAAGACCAAAGGGCATAACATTCCATTCATAATGACCAAAAGGAGTGACAAAGGCGGTTTTGTACCGATCTGACTCTTCTATTTGGATTTGCCAGAATCCACTCTTAAGATCAAATTTGGAAAAGACGACTGCTTTTTCTAATCGGTTAATTAGGTCTCTTTTATTAGGTATGGGGTACCTAATCCATTCTAAGACTGCATTGAGGGGTTTGTAGTTGATGACTAGCCTAGGGACTCCTCGTTCTAGTTCAGCATTTTTCTGGACATAGAAAGCAGGGCAAGACCAAGGTGACTTAGACTTACGGATTATTCCTTTCAGGAGTAATTCTGTAATTTCTTTTTTACAGAATTCCATTGTCTCCTGATTCATCTGGATTGGCCTGGCTTTAGTGGGGATATTTTTCTCACTAAAGGTTTTGGTGTAAGGAAGACTGACAATATGTTGTTTCCTAGACCAAAAAGCATTAGGCAAATCAGAACAAATATCCTGTATCAACTTATTTTCAAAATCATGTACCTGTTTTTGAAAAGATTCATTTTCGATTTGTTGACATGTCCGGGTATGACTAATTTCTTCCTTGAGGGAAGACAAATGTCTAGTCTTACTAGAAATTAGGGCAACAGTTTTTGAAATTGAACTTTTCTGGAAATTTTTCAACTGGTGTAACTCAGGCTTGGATAAAAACTCAAACTTGGCTTGTTCTCCTAAATAGGTAGTGATGACACCATCATGATTTACCTGGAAAGGGTATAACAAGGCAAGGAAAGGTAATCCAAGAATAACTTGATCAGTAATGTTTTTTACGAGGACAAATGAAGTTTTAAAACAGATTTTATTTTGGCATACATGGGCTCGGGGAATTTCATAGTTCAGCTGTAAGGATTTTCCTGAAGCTGTTCTAAGAGATTCGCATATCTTAGTATAGTACTTGGTAGGTATAAGGCCATCCTGAATGCAGTTAAGGTCAGCACCCGAATCAAGTAAAGCAACTGTAGTGAACTTAAATTGTTCGACAAAAATGGTGATTTTGACATACCATTTTTTAAAGCTAATTCGTTTAACTAGGCATAAGGTTTGGTCTGGAAAAGATTCATCATCTGAGTTGGTTGGACTCTGAGATGAATGTTGAGAATTCGAATCAATTTTGAAAGCAATGAAGTTTTGGCGAATATCATTGTTCTCAGATTGCAGTTTCTGACTTAATTGTTTTAATTCAACTATTTCCTTCTTGATAATCTTTACTTCTGAATGTAAATCATGAAGGGTGATATCTCGTTTGCGACTAAACTTTTCTAAGGTTGTATTTAGGCAGATTTTTTGAGGAGGTGGAGACTGACTTGGTCCTTCATGGCGAAGGAGTTTCCGAAATTTTTTCAGATACTCAGTTTTCAACTCTGGGTTTTCTACTTTGCAGATTAGATCTATGAGAAGCTCTTCTTGTTCTTCCTGTTTTGTAAGAACAGAAACAGATTTCATAGAACTACAGCAGTTATCTTGACAACTAAATTGAATATGAGGGGAATTGGGTTGTGAATCAGAAGAATAAGAAATCTCAGAATCACTGTCAACTAAAGACTCAAGATCACTGGATCCAGAATCCTGTAAATCTAAAAGTCTAATGAGGTTTTCTTTTTCTATTTCTTCAATTTTTAATTGTTTGATTGTGTCTTTAACTTTGCATTTATTAGCATAATGACCTGGTTTTTGACACTTCCAGCAAATGATTTTTCCTTTTCCAGAATTGTTCTGGAATCGGGTTTTCTTATTTTTGTGTGGAAATTTCTTCCAATTCTTTTTCGGATTAAGCTTTCTGTGTGTATAGAATTCTTTATCATTAGTCTGTTTGTAAGTGTTTTTTCTACTGGGATGAGAACGAGACTTATGCTTATGAGAAGACTTATTTCTACTAGACGGAGGAAGACTAGTGAGACCATACTGTTCGCAGAAGTTTTCTAATTCATACTTAGCAGACTTTCTGTCAGACTGTATTTGCTTACCAATTTTCATATCAACACACATTTTCATACCGACTTGGTTGATAGTACTGACTATGTTTCCATAGGTCAGATTATCCCAATCAATATGACCTTATTCATTACTAAGGGTTGTGCGAATTTTATGAGCAAATAGATTTGGAAGACCATTAATGAATTTTTCTTTCCAAAAGGATTGGTGACTATCATCTCTGAGCATGACGCGAGAGATGAAGACATCTTTATACCACCTGAAATCAGACAGTTTAGGACAACGAAGGTTGCTTAGCTGGTCATGGATTCTAGCAGTGGTATGGCTTGGTGTTCCAATAAAATGTTCAGTTATGGTGTAAAACAGAGTATTGACTCCATCTTCTATACCTTGACCTATTTGTTCGTCAAATATAGGGAAACCTTCATCATTGAGTTTTACAGCATGAACAATTAAGTTTTTAGATTCAGGTGTGAGGTGTTTATCCCACCAATTGCGGAGGGTACCTGTAAAACCTGTTACTAGTAACGGCACAATTTCAGATTGTCTAAAACTGTGGTTTGAGATATAGCTATTGGCAACCATGGACATATGTTGAAGTTTATTAAAGATTTCTTGCTCAGAAAGACCATCAATATTCCATTCGTACAATTTGTCAGAAGAGACAAAGAACTGGTTCTGAAAATGTCTTTCTTCAAATTGTATGTCAGGGGTGTTGGTCTGGTATACCAATTTTTGGTAAGGCTTGTAGGGGTGACATGGTTAATTAACCGAGTGACCCTTGGTTCAACTGTGAGTTCAATGAAGGCTTTTTCAATTTTTGAAATATTACTATCAACTGAAACCATACTCTGGACGGATTCGTGTTCAGAGTCAGAACTGGTTTGAACAGAGTCAAGAACTTTGACTCCAGATTGTTCACCTTTGATGGGTGTCAACTGTTCTAACATTTCTTCAATTTTTTGTAATGTAGGTTTGATCTTAAGGGTCGGATTTGTCTTAAGATCCTGGAACTGAACTAATGGTTTTTCAACTTTTGTTTTTAATTTCTCAGAGGAAGGGACTAGCTTGTCGACTTTAGTTTCGATTTTGTCAAGCTGTTTTCCTATGACTATTAGACTCTGGTTGGTATAATTATTCTGTTCGATCAATTTCTTCTCTGAATCTAATTTTCCAGGGAATTTGAAAGGACAGGCGGGAATCTGAGTTTTCTTAAAATTGATTAAGATAGACTCAGCAGGAGGGTGACTAGCTACTATTTCAGTATCATTTGGTAGCGACCATTTTTCTTTGGTGATTACCTTCAATTTTTTAGACATGTAGTTGGTTTCTACAAAATCTAAGTAAGGGATTTCTACTTGTTCTGTTCGCAAGTAATCTTTCCATTCATTAAAGATTGCATCACGGCGATACTTAAGAGGAAAAGAACTATGATAATTGTCTCGGCGCTTCTGATTTTCAGGAGCTGTGAAATGGGCTTGTAATTTTTTCCAATTAATTGTAAATTTGGACAAAACTCTGAGTTGAGGATCAACAAAATCAAAACCTTCAAAGTCAGTTTGGGTAGGAGACTCGGGAGATTTTGGCTTGTGTTCTTCAACTGGTTGTTTGCCATAACAGGGTTGACTAACCTGTGCAGTTTTTCTGAGAGATTCTAATTGGAAATCAATCAGGGCTCTTTCCGCTTCTAGGTCTCTTTCTAGGGTAGAGGAAGAGGCTGCAAAGGAATGTCTGGCAGGAATGTGTCTCTGGGATTGACCAGGGGTTGGCAATTGTTGTAGTTGGACAGTAGATTTTGGAACTGGTTGGGTACCGAAATTGATTTTTACTGATCCATCTAAGTATTGGTGGATGTAATCTAAATTGTTTAGATTATGTTGAACTGGTGTTGGTGGATTAACATTTTTGAGTGTCCACTCAACTGGGAGTGAGACATCAGACCATTTGATTGTTTGAGGTACTCGTATGATGGCCTCGGGTCTATTACTTTGAATCAACAAAGTATGATTCTTAGGACTTTTATTAAGGGCTTGGAAATTCATATTGGTTCCTGTGACCTTATAATAGATTCTAAAAATCAAAGCTAATGGTTGGGTTCCTGGGAGAACCTCATATCCAGAAGTCTGGATGTTCAATGTCAAAGTCTTTAAAATGTGGGGGTCACTAAGGCTGACCGTGAAGTCTGGGTAGCAATCAAAGTGAACTGGACCATTAGCAAGACTTGACTCAATTACTCCGAGAGTACTGTCCTCACTGTTAGTGAATCTAGCATCTCTTAACCAGAGCAAAACTGAGGCTTTAAGCCCTGTTCTGGTTAGTGGTTTGACAGCGACTTGAACAAGACCAATATGCAGGAAATTGTTTCCTTCATTCTTATGTCTTTTGATTGATTCAAGATTGAGCAACTGACAGACTTCAAGTTCTTTGCCGAGGGCATAGACTTGTTCAACTGTTTTTACATGGGTAGTGGTCTTGAAACTTGGTAAAGACCATCGTTTCTTATAAATTTCTTGACTGGGGATCTTAGGGATATTCCAGTCTTTGTAATCTAACTCATTGTTATTAGACTCAAATTCAACTTGTTCTTCATTAACTATTTCTGGTAGTCGATCGACCCTGGTTCTTGAACTGCTCGAACTACAAGAATTTGTTCTAAGGAAACGACTCATTTTTACTTTGGCAACTGGGTTCTGACTTCAAAGAAGAAGATAAACAGGGACCTCTAAGGCTGACTTCTATTTATTACAGTCAACTGACCTCTCTCTCCTAGCCCTCTCAATGCCGCAACCGCTCATGCCTCACCTCGGGACTTACTGTTTGGACTCTAGGCCTTACTCTTCGGACACTATTTAAGAGAGATACAGAAAACAGTACTGAATAAAAGATCAACAAGAAAGGCAACTGTAAAACTTCTACTCAGAAATCCTAAACCAGCCACTTGGGTTGGATGAGTAGCGACTTAAGAACAGACTCTTACAACTCTTACGGCTCTGATACCAAAGAAAGGAAGAACCATGGATAGGAAACAAGAACAGAAATAATTTAAACAGGAACAAATTAATAATGAACAAATTAATGCATAGATGAAAATAATTAAAATAGCAATAAATAAAATTACGGATATTGAAATTAAAGACATTTTGATACTCACAAAAGCCTTGTTTATTGATAGCTAAGCACTCAGATATTTACATGAGAGTTCTAGCTGAAGAAGTTTCAGAGAGAACTTTGATAACTGAGAAAGCTTAGTAAAATTTACAAGTGTCTTCAACTGAGGCCGAAGCCAGCTTATAAAGAAAAGAAAGCAACTATGGTTTAGACCAAAAGTAACTCTGATTGGAGAGTGACCATGGTATGATACAATTATGATGTCAACTTTTTCCTTCGACATTTCTCTTTACCTATTGGGGTAAAGTAGAAGTAAAAGCAAAAGTGGTTTTGTTTTACTCCAACATTTCACCAGGGTGAAAAGAGAAAAGATGGCAACTGTTTGGACAACTTTCGGTGGCAACTTTTGACTAATCTGATCCCAAGTCATACGCGTCATAAGGATCTTGAGCGTAAGGCAGATTAGGATCAAAGGAACTGGAACCAGGCTGACAGCTGGATGCTGATGGGGAAGAATCTTTGTCCTCATCATCATTCATCTGAGAAGCTTGGTCAAGCAACTGTGCTGCGAGGGCTCTGAGCTCAGCTTTGGATTTTCCATCGATTGAGATAGCCGAACTGACTGAGCCGGTAGGAGAACTTGGCTTAGGCTGGGGATGATCCGATTGGGGAAAATCCCTGAAGACATAAATTGAGATCCTGTCCATTGAGAATTTATCCCACCATTTGACAAAATATTGGCGAGACATAATTCTGGATTCCCAATTAACATGATAGCTCCATTTCATAATCCAGGGGATTTTGTACCTGGCAATAAAAAGAAGCAGCTTGGGGAAAAACAACTGGTGTTTGTTAAATTGGCTTTTACTGACATAATAGTTGGTCAAATTATGGACTTCAGTGGGGAGGATATCGGCTGTTGGGCCGTGTTGATCCTACCAATGAAGGAACCAGTAGGGGAAAGGACTCCTGAATTTGGCATCAAAATTAAGAAACCAGGAGTGGGAAAAGTCAACTGTTTGGTGAAGGAAGATGTTATACCATGCATCAATGTAATCTGCATAGTTGTAAGGGTGAGTGGACTTAAGGTGTTTCAACTCATAGGGGTGGCGACTAAATTCGGATTGGCTCAGAATTTGCTGAATGTAAAGAGAATGATAGAGGATGACAGTGGGATCATCTCTATCCTTAATAGGCTTAATTTCAAATGATTTAGTCTCATGCAGGATATCCCTATAGAATTTAAGGTCTTTATAAGGTGCAAGAGGAAGAAAATAAGAATTAGGAGGGAAATATGATCGAATAATTGTTATGGGATCAGAAATCCCATCATACAAGGGTTCAATTATGAATAAATGGGTCTTATCTTTTGGGAGATAAGGGGACGATTTTTTGAACTGGGTGGACGACTGGGAGGACTGAGCAACTGGGGCAACTGGGGGAACAATTGCAAAAGGATCATAGTCAGAAACTAGGGCTGACTGGTAATTGGGGCGAATTGGGCCAACAGTGGATCCTATTGGGGTTCCAAGGCTGGTAAATCTGTTTGCAACTGGGATTGGGGGGTAGCCTGCAAATGGGACAATTGAGGATGGATGGGCCATTCTAGATTGACTCATGGATGCATGAAGGATTCTTTTGGGCGACTGGGGTGGTGCAGGGGTTTGGGAGGGTTTAGGTTGGCTGGACTGACTGGCATCCAATTTTTGCTTGTCTTTTTTGGGTGCCATTGCTTTCCCTGCAAGAATTCTCGAGTAAGAAAATCAGGAATAGAATTCTCGCTGCCTCTCAAATATTCAATAGTAAAATCAAAAATACTTAAAATGCTTTGCCATCGTGCAAAAATCCGTTTGGATGCAATGTTTTGAACATCTTTTTCTAAAACATGTTTTGCAGATTTGCAATCAACGCGTATTAAGAATTTTTGGTTAAGTAAATCATCTTGAAATTTAGAAATGCATAGTACTATAGATAAAAATTTCTTTTTTAATAGTACTATAATTACTTTGTGATGGATTCCAGATGCCAGAGTGGAAACGAATGATTTGTTCTGGCGAATGGGGACTCAATTTTTGTTTT

TATATATATATATATATATATATATATATATATATATATATATATATTACTCGTTTGGTATCCTACTCAAAAAAATAACTAAAAATCATGATTCTTTTGGTCAAATATGAGTTTTCAATTTGGTCAGAAATCCTAATTATTCCGTAACATGAATGCATATGCCAGAAGTGACTGACGGGTTCAGTTTTGAGTTTTCTTCTTCATCCTTTGCACCAAGCCCATCTTACCATTAATAATCATAGACCATTCAATGTATTCAACACACGAGGTTGGAGGCAAAGAAGAAAATAAAAAAATTATCAGTTTGAAGTGACAGTTTTGGTAGCACATTAATTAATTGAGATATTTAGTCATATATCTATTTTTAGCATTAAAACTATAAATAAATTCTACACTATTTAATTTCATACCCAAAAAAACTCCAAAAATATACAGCTGGTCCTATTGAATTTAATTTTGATTATTAAATTACTTTAATACCCTATTGAGTGTTTTGGGCTTTTTTATGAGTTTTTGGGTTGAACTTGTTTTAAGAAATTAATAGCAGTTTTGTAATTTAAAAGAAGTTAAAAGCCTTTTTGTTATGTTGTAAATGAATTTTGGGTGTGTTTCTAAAGTCCATTTTATATATGATATTTTTATAATTTGAGCTCCTATATTTGGTATAATAGTGAATCTCCGTAATTAATTTCATTGGTTATATCTCTGACATAGGTTTGAGTATCTAATAAGGATGACAAATCAAGAAGAAAAAAAAAAACAAAAAACAAAGGAGGCTTTAGTGACATGCAAAAGCATATTCCACATAAAAGCTTTGGGATGAGGACAATCAAATTAAAAGCACTTTAGGACTTAGGGTAAATTACTCAAATGATATTTAAATTTGTACCCGATTTACATTTTGGTCTATAAACTAAATTATTCATTCATATGGTCCACCAACTCTTTATTAATCGGCACTTTGGTCCTACCGTTACATTATCTGTTAAATTTAGTCATG

>Chr4-07; OZ022209.1; 18435586-18442274; rev; A;

TTTCTCATTGTGTTAGTGGTATGAAAAATATTGGTCATTTCCTAAGGACAGACAGTTTAAAACCATATAGAGCTCATCCAGATTTACCGGGCATAGATGGTTTCAATTTCTTAGGAATACCTTTGCCCAAAAACCAATCAGATGATGCATCTTTTAAAAAATTTTGTCAAGATGCTGTAGCTTCATATTGGCATTACCATGGTGGATGCCTTGTTGGGAAGGTTGTTGATGACGGTCTGCGTGTTATGGGGATCGACGCATTACGTGTTGTTGATGCCACTACATTCCCTTCCATGCCAGCAAGCCACCCTCAAGGCTTCTATATGATGTTAGGCAGGTATGTGTTGCAAACCACTAGAAATTCTGAAGAATTATTTTGTTGAAATCGCTTGCCTGAAATGAATTCTATGCCATATTTCCCTTTTTCACCCCTTTCAATTTTTGTGCTTTGGGCAGGTATATGGGCAGTAAAATTATGCAAGATAGATGAGCTTCAAGAGAGGCTATTTATAACATCTTGGACTTCCATATGGCAGCATTATCCTTTTGCTTATATATATATATATATATATATATATATATATATATATATAAAGTCTTAGGTTGCAACTCATCCAACCCAAGTGGCTGGTTTAGGATTTCTGAGTTAAAGTAAAGTTAAAGCAAGTTACCTGAGTTACTTTCCTGAATTACTTTTGTAAATCTGAGTTACTTTCACTGTCTGTTTGTTTATTTAAGGAGGCTTTGGCCCCAGTTGTAAGAAATCGGGTTTTTTTTTGAAATCGATCAATTTTTTCTTAGTTTCAAAATGTTCTTTCAGAGATCATGTTCTGAAGAACAACCTTAGGAATGAGAGTAAGCTTTACAGATTGTAAGTTTTATATGAATTTAATTTAAAATAGTTATAATGAAGAATATTATTTTCATGTACTTCATTATTTTTATTATAATGTATATACTATCATGCATAGTTCTGCTCCTTCTTTCTTTTTTCCCTTTC

TGGTATCAGAGCCGTAAGCGTTGTAAGAATTAAAGTCTTAGGTTGCAACTCATCCAACCCAAGTGGCTGGTTTAGGATTTCTGAGTTGAAGTCTTTTGATTTCTTCTTTGTCTTCCTTTTATCCTGTAAGTTTCTTGTTGGTCTTCTCCCTGGTCCAAACAGTGAGTCCTCTTTCCAAACGATAAGTCCCGAGGGTAGGGCACGAGCGGTGGGCGTGAGCGGAGGGCGAGATTTCCAACTAGTTTCTTATCGGGTAAGGGTTAGTCATTTGAAGTTATCATTTTTCTCCTTCTCCAGTCTTCCTCTTCCATCCTCTTGAGTGAAAATGAGTCGCTTCCTTCGCACAAATTCTTATAGTTCTAGCAGCTCTAGAACCAATATAGGAAACCTTCCTGAGATTGTAAATGAAGAACAATTTGAGTTTAATAATAATAGTGAACAATTAGAATTTAAAGATTGGAACATACCTAAGGTCCCTAGTCATAATATTTATAGAAAGAAATGGTCTATGTCTGCATTTAATTCCACTACACATGTTAAGACTGTAGAACAAGTTTATGCTTTGAGTAAAGAACATGAGTCTTGTCAATTGCTTAATTTAGATTCAATTAAGAAACATAAGCATGATGGTAATAACTTTCTGCATATTGGATTGGTACAAGTTGCTGTTAAACCTTTAACTAGATTAGGTTTACAAGCTTCTATTCTGCTATGTTTGCGTGATGCTAGATTTACAGATTTTAATGATAGTGTTCTTGGAATGATTGAGTCAAGTCTTTGTAATGGCCCTGTACATTTTGATTGTTATCCTGATTTTACTGTTAGTTTGAATGATCCTCATATTCTGAAAACATTAACTTTGAATATTAAAACATCTGGTTATAATGTTTTGCCTGGCACACAACCGTTAGCCTTAGTTTACAGAATTTATTATAAAGTTACAGGTACTAATATGAATTTTCAAGCTTTAAATAAGAGCCCTAAAGATCAAACACTTTTAATACAGAGTTGCACGCCTGATGCTAATATTAGAATTCCCCATACAGTTAAATGGTCTGATATTACTTTACCTACAGATTGGACGTTAGTCACTGAGTCTCAACCTATGCCTATACAACGTAGTTTAAATAATCTAGATTATATTCGTCAATATATGGATGGTAGTGTTAAAATTAATTTCCAGCACCAACCTAAGTCTACAGTTCATTTACAGCAATTGCCTACACCAAGTCCTTCTAAAAGACATGAACCTGCACGACATTCTTCTGCCTCTTCTTCTACTACAGTTAGAGATTTAGAAATTGAGAAAGATTTAATTGATTTAAAAATTGCATCTTTAAAGAAAAAAGATCAAGTAAGTCATCCTTGTTATGGTCCTCCCCCAGTTGAAAATAATGAACCTGAGCCTAAACCAAATTCGCCTACACAGTCTGATTTTCTTGCTAGTGAAATTGCTACTGCACATGTTTATGATATACATAATTGTTTAAATGTTTTGCACAGAGACAAGCCATTTAGGATAGACTGGGATAAATTAGAAAAACATACATTAGATCCCATTAACTTACCCCGTAGAAGTAATTATCATAAAGTCATCCCTGGTAGAAATAAAAGAACTGAGATACACCAAGCCTGGAAAGATTATATGAGAACTGCTAAAGTAGAAATTAATTACCTTGATTTTGTAGAGATGCGTTATATTAATAATGAGTTAAAAACCATAACTAAAGAAAAATGGGTAAAATTAGATAAGTCCGAAGTAGTTTCGAGTCATCCTCCTGTAGAAACCATTTTGATAACTGCTGGCACTAAAACTATAACTGCTGCTCCCTTTAAGATACCTGATCCTAGTAATGAGCTTAATAAATGTTTAGTTGAACAACATAATTATACTAATCAAAGTCTAGTTATCATAGGAAAACAATTAGATAAAATAGAAACTAAAGTTGATAAATTAATCCCTTCTGAGCCAAAACTTAAGTCTAAAGAAAAACCCCTAGTCTGTTTTCAAGACTTAAAACCTACACATGCACTTAAAACTTCTACTACTATGCAAAAAATTGAAGGAATGCTTGAACAATTAACCCCTATTAAACCTGAAAAATCTGGTGTTAAAGTATTAGGTTCTTCTTCCTTGAATTATAATAGTGATTCTGAAATGAGTCTAGATACAGATGATTCAGAAAAGTCTAGTAATATTTCTAAGATAGAAAATGCATTTAAAAATTTAGAATTAGAATCTGAATTAAAAGTCAAGAGACTAACTAATAAAATTAATCCTACCAGCTTAACTAAGAATTGGTATCCTAAACCTACACCGCCCGATATTCAGTTTGAAGAAAGAAATAATATGAGTCAATTTTCTGTCTCCTCAGATAAATTATATGAATGGAATATAGATGGTTTGTCTGAGCAAGAAATAATGAATAAACTTACACATATGTCAATGGTGGCTAATACATATATTGCAAATCATAGTTTAAGACAATCTGAGATAGTTCCTCTTTTAGAAACTGGATTTACAGGAACCTTACGATCTTGGTGGGATAAACATTTAAGTAATGAGTCTAGAAATAATATTATACATGCTGTTAAATTAAATGAAGACGGTCTTCCTTTGTTTGATACAAATATAGGCAGAGCCACTGAAGATGGAGTTAATACATTACTTTACACTATAATTGAACATTTTGTAGGAACACCAAGCCATACACAGTCTAGGATACATGATCAATTGAGTAATTTAAGATGTTCACAATTAAGTGATTTTCGATGGTATAAAGATGTTTTTATTTCTCGTGTAATGTTAAGAGAAGATAGTAATCAAGCTTTTTGGAAAGAAAAATTTATAAATGGTTTACCCAACTTGTTTGCACATAAAATACGTACCGTTTTAAGTAATGAATTAGGTCATATAGATTATGATAGCCTAACTTATGGAAATATTATAAGTACAATTAATCAAGAAGGTATGAAAATGTGCGTAGATATGAAACTAAATAAGCAAATACAATCTGAGCGAAAGTCTGCTAAATATGAGTTAGGTAATTTTTGTGAACAATACGGTTTAATCTCTACAGCTCCCTCTAGAAGAAACAAAAATAATTATCCGAGTCATATTCGCCGAAAGCGCAATTATAGCAGGTATAGAAAGTCTTTTAGACATAATAATGAGCCGGAAGAATTTTACAAAAAGAAGAAATATTCCCCTAAGAAAAGATGGTCAAAAGCGCCTCGTTATACAAAAGATACTCGCTTTAAGTCTGGTAAAAATAAAGATAAAAGTAAAGTAAAATGTTTTAAATGTCAAAAGTATGGTCACTATGCAAATGATTGTAAAGTAAAAGATGCTATCCGACAACTGAAAATTACAGATGAAGAAAAAGACAATTTAATTAAAGTCCTTGAGTTACGGAACTCTGAGTCTAGTGAAAATGAAACTATGGTTATAAGTTCTGCGTCTACTATTCATGAAACTTCTGATTCCTTAAGCTCACCTAGAATACAAATAGGTTGCAGAGATAATTGTTGTAATACTCTTAAGCAAATTTCTGTTTTAACTAAGCAAGAAGAACAAGAAGGGTTATTAATAGATTTAATTTCTAAAGTGGAAAACCCTGAATTGAAATCTGAGTATTTAAGAAAATTAAGAAAAATTATTACACAAGATACAGGCACAAGTCACTCTAACCCAGTCATTAATCTAAGCTCTACAATAGAGAGATTTAATAAGAAAAAGGATGTTTCTTTACAAGATCTACATGCTGAAGTAAATCTAGTCAAAAAAGAAATAGTAGAATTAAAACAGTTAAGTCATAAACTACAAACTGAGAATTATGATATTCGGCAGATTCTAAACGCTTTATTAGATAAAGAACCCACTGAGAAAAATAGTCCACCTAGTAGTCCTAAGTATGTGTCTGATAAGGAAGAACCTGCTGTTAATCTAATTAAACAAGTTAATTTTAGAAAATGGTATTCTAAAGTTACCATATCTATAAAAGACTATGAGTTTAATGTTATTGCTTTGTTTGATTCTGGTGCAGATCTTAATTGTATTAAAGAAGGATTGATTCCTACTAAGTATTATATGAAATCTATTGAGTCTTTACGTACTGCTTCTGGTAAATCTTTGCAGCTAAATTATGAAATTCCTAAAGCCCATGTTTGTCAAAATAGAATTTGTTTTAAAACCTCATTTGTGTTAATTAAAAATATAACTGATGATGTTATTCTTGGATTACCCTTTATAAGCATGCTTTACCCCTTTCAAGTTGAGTATGATGGCGTCGTTTCTAATTATTTAGGTGAAAAAGTTAAATTTACCTTTTTGACTAAATCTGAGTTGCATGATCTAAAGCATTTGAATAATAATGATTTTACAAAAACAATTACTTTAATAAAAAATAAATCAAATCATTTGAGTCACCTTCAGAATGAAGTTAAAATTAAAAGAATTGAATCTCAATTAAGTAATCCTGCTTTACAAAAAGATATTTATAGCTTTGAGCAAAAGTTAATTTCAGAAGTTTGTTCTAATCTCCCTACTGCTTTCTGGCATAGGAAAAATCATGTTGTTAATTTACCTTACATAAAGGATTTCTCTGAGAAAAATATACCAACTAAGTCTAGACCCATACAGATGAATCATGAAGTGATGGAATTTTGCAAAAATGAGATTAATGATTTACTACAGAAAAATATAATTAGGAAAAGTAAGTCTCCTTGGTCTTGCCCTGCTTTTTATGTACAAAAAAATGCAGAACTGGAACGAGGTTCTCCTCGATTAGTTATTAATTATAAGCCTTTAAATTCTGTTCTTGAGTGGATTCGTTATCCTATTCCTAATAAAAGAGATTTGATTAAGCGTCTTGATAAAGCTATTATTTTTTCTAAGTTTGATATGAAAAGTGGCTTCTGGCAAATACAGATTCACGAGCATGATAGATATAAAACTGCTTTTGTTACTCCCTTTGGTCATTATGAATGGAATGTTATGCCTTTTGGTCTTAAGAATGCTCCTAGTGAATTCCAGAATATTATGAATGAAATTTTCAATACTTATAGTCATTTCTCTATTGTTTACATTGATGATGTTTTAATTTTTTCTGAGTCTATAGAACAACACTGGAAACACTTGCATACATTTTTGCATGTCATTAAGTCTAATGGATTAGTTGTTTCTGCTAAGAAAATTAAACTATTCCAAACCAGTATTAGATTTTTAGGATACAATATAAGTCAATCACAAATTCATCCTATAGATCGAGTTATACATTTTGCAGATAAATTCCCTGATCAAATCACAGATAAAAATCAATTACAGAGATTTTTAGGTTCTCTTAATTATGTGTCTGATTTTTATAAAAACTTGAGAAAACAATGTAAGCCCTTATTTGATAGATTGCAGACTAATCCTCCTCCTTGGTCATCCATCCATACAGATATTGTCTTACAGATTAAACGTCATGTTAAGACTCTTCCTTGTCTAGGCATTCCTACTTCTACTTCTTTTAAAATTGTTGAAACTGATGCTTCTGATATAGGTTATGGTGGTATTCTTAAGCAGAAATCTGAGCCTGATTCTCCTGAGCAAATAGTCCTTTTTCATTCTGGTGTCTGGTCTTCCTCCCAGTTTAATTACAGTACTATTAAAAAAGAAATTCTAGCTATTGTATTATGCATTACTAAATTTCAAGATGATTTATTAAATCAAAAGTTTTTAATTCGTGTTGATTGTAAGTCTGCTAAGCATGTATTAGAAAAAGATGTTCAAAATATTGCATCAAAACAGATTTTTGCACGTTGGCAAGCGCTTTTAAGTATTTTTGATTTTGACATAGAATATCTGAGGGGTAGTGAGAATTCTATTCCTGATTTTCTTACAAGAGAATTTTTACAGGACAAGCAATGGCTGGATCATCCAAAGACAAGCAAAAAATGAGTCATAATCCTCCTCCTAGAATTCTTCCTGCAACCTTGAGTCAAAAGCCTTCCCAATCCGTGAAAAATGAGCATGGATCCTTTTCTGTCAAATCTGAGCCTTTTACAATTACTCCCTATCCTGGTCATACACCCATACCAGTCATGAGCAATAGGTTTAGTCCCTTAGGATCTACAGTCACCCCTACAAGACCCAACTACCAGTCTGCTTTAGTTTCTAGTTATGATCCTTTCCAGTTACCTCCCACTGCATCACCTATTTCCTCTCCCTCTTTTCCCAAGTCTTCCCCTTATGTTTTAAGAAATACAGCTAATTTGCTTATAATTGATTCCCATATCCCTACATCCATGAGTCCTGCCCAAATTGCAGTTTATCATTTTCCTCCTGGTTTTCATTATCTACCCCATAGCTCTTACAAGTCATTAAAGTTTTACAGAGAAATTCTACACGAAACCAAGTCTGTTGATATTAAGCCTATTCGCGATAAAACTGATCCTACAAAAATTATTTATCACTCCCTTTACATTCATCAAATTTTACACCCTAAGTCCTGGGGATCTCGTCCTCATGAGCTCCGTACCCTTCCTTCAAAATATCAATACAATTATGCAGACTATGTTGAAGCCTGGTATTATATCTTCCTGCATCAAACCCCTGAGTTTTCCCATTCCTGGTTCATTAATTTTGATAATAAATTTAAAAATACTGAGCTTCCTTATTGGTTCCTTCATTGGTGGGACACCCA

TATATATATATATATATATATATATATATATATATATATAATCAATAAACCCATGAGTTGCGACTCAAATGGGATAAGGGTTTGGTTGGGAAAAAGGAGGCACTTATCTACACCCCCACCCCACCAACCCAACTCCTTGTGTACCTCTTTATTTCATATTAAATAAATTTCCCTTGTAATTCTGAGTTATGTAAAAAATAACAAAATAAAAACACTCTTCCTCCGTACAGCTTGTCTTTGGAAAACAAGGACATCGAAAGTCTCAATTCCCAAAGAGCAACAAATTAATATGTTAAAATAAAACTAAAGTCGCACTAAAACCATTCGCTCACAACACCATATTTAATCATAAGCTCAAACAAAACACACATACTCAACATAACCGAAACCCTAAACCAATTCATGAGCTTAAACGAACTCCACCGTCACTACCTGAAGAAATGCTATTGTGACCACACCCACCACCACATCCGTAACAAATCTAGGGAAAACATGGTTTCCAGCTATCTACCTGAGTATAAGAGCAAACAAATAAACAAAAACAACTACCTTGACAAAGAACCACTGGGTGATTTGCAGCAACCTCCATGAAAGACGATGTAGACCTAAATTTAGAGAAAATGAGCACCTCCATCAAAGCGGGTTTTGAGTGACGGATTTGTTTGAGAAAGCCTAAAAGGGGTGATGTGGAACTTCAATCTGAGCATGGCTCAGAAGAAATCACTACAAGGGGCTTCTTATTCGAAACACCCCAGTTGACTTATTCACTAACCAAAACAAAAAAACAAACAAAAAAGGCTAGGAGAGAGGATGGGAGGAGGGGCAATGACGCTTCTTCCTCCTCCCCCTATGTAGAGTTTTTCTTTGAGACTCATAGAAGGAGAGAATGGCTAGGATTTTTCTACTTTCTCTTAATCATAAATTATGCCTGAAGTTTATATATGACAATTTTTTTTTTTCAATGTGGATTTTTAGGATCAAAATAGTGTTGGGTTGCCAA

>Chr4-08; OZ022209.1; 20336104-20343713; rev; A;

GACCCTACCTACCAATGTAATGGGAACCAAAGCCTATTATAAGTCACACCCTTTGGATATTAAACGTCGCCTTTATATATCTACCGCATAAGTTATGTCCTAAACACCGATCCTAAAGTGCCTTCCATGTGCCAAAAAATCATTGACCTCCGATGCCTAAACATGCAACGACAGCGGTAGTTTTAATAATTTCGATGTTAAATACTGTTTACTACATTAGAATTCTGTACTACATTGATACTTTTTCTGACATACAACATCACATAATACAATAGTGATGATGCCACTTCCAATTATAGGCAACAATTGTCTTAGAGTGCTGTTGTTATTTTAAATTTTTAACTTTTGTTGTTTGTGCAAAATTGAGGTTGTTATGAACAATATCATGTAGTGTTGTCCATTGCACATCAAAAAATATAGATAAATTTAAATATATCCATACATCAAATATATATCCAACACTTCCTAGAAAATATCTCGCCCTCCTACTAATGCCACAACTGCTCAAGCCTCCCTGGGACTTACTGTTTGGAACGGGTCTTACAATTCAGACACCAAGAGAGAGAAGGACAGGTAAACTGAAAGACAAGAAGTAGAAGAAGAAGGAACTGAAACTTACAACTTAGAAAACCTAAACCAGCCACTTGGGTTGTATGAGTTGCAGACTTAGGCTAACTCTTCAACATTACGGCTCTGATACCAGAAGGGGTCTATTTGGTAGAAAGTAAGATTTGGCAATGATGATTAGGTTGGAAATGGAATTGTATTTTGACTCAATTATGAATAGGTGACTATTACTCTTGATCATGTAAGGGGATGTCTTGTGGTAGGAGGCAATTGGAGGATTACTAGCAGCAATATCCTGGAAAGGATCATGACTATAAATTAGGGCAGACTAAAAACTGGGACGAACCTGGCCGACTCTGGCTCCTAGTCTAAGTCTGCAACTCATCCAACCCTAGTCATGTTATTCTATCTCTCACTGGTTCTAGACCCCTTC

TGGTATCAGAGCCGTAAGTGTTGAAGAGTTAGCCTAAGTATGCAACTCATCCAACCCAAGTGGCTGGTTTAGATTTTCTGAGTTGTAAGTTTCAGTTCCTTCTTCTTCTACTTCTTGTCTTTCAGTTTACATGTCCTTCTCTCTCTTGGTGTCCGAATTGTCAGACCCGTTCCAAACAGTAAGTCCCAGGGAGGCTTGAGCGGTTGTGGCATTAGTAGGAGGGCGAGAGATTTTCTAGGTATCCTGTTCCACGTAGAAGTCAGTCTTAAAAGTTTCTGTTCACTTTCTTCCTTAGATTTCCAACCCCATAACCTTGAGAAATATGAGTCGCTTATTTAGAACCAACTCTTACAATTCTAGCAGCTCTAGAGTCAGTATCGGTAAAGTCCCTGAGATAGTTAATGAAGAACAATTAGAATATGAGTCTAATAATGAATTAGATTTTCGAGATTAGAATATACCTAAAGTCCCCAGTCAAAATATATATAAGAAAAAATGGTCACTCCCTAGTTTTAATACAATTACCCATGTAAAAACAGTTGAGCAAGTTTATGCTTTGAGTCAAGAACATGAGACCTGTCAGTTGCTTAATCTTGAGTCAATTAAAAAACATAAAACTGATGGAAACAACTTCCTTCTTATTGGTCTAGTCCAAGTTGCTGTTAAGCCATTAACCAGACTAGGATTAAAAGCTTCTATCCTTCTCTGTTTGCGTGATGCCACTAATTTTAGCGACAGTACACTAGGAATTATTGAGTCAAGTCTTGCTAATGGACCAGTCCATTTTGATTATTATGCTGATTTCACAGTAAGCCTGAGTGATCCTCACATATTGAAAACTCTCACATTTAATATAAAAACTAATGGTTATGACGTCTTACCGGGTACCCAATCCTTAGCATTAGTCTATAGAATCTATTTCAAAGTCACAGGTACTAATATGAATTTCCAAGCTCTGAGTAAAAGTCCAAAAAATCAAACCCTGTTAATTCAGAGTCATACACCAGATGCCACTGTATGAGTCCCACATTCAATTAAATGGTCTGATGTGTCCTTGCCAGTTGATTGGACCCTAATTGCCGAGAGTCAGCCAATTCCCATCCAGCGTAGTCTAAACAATTTAGACTATCCAACAGTATCTTGATGGTACAGTCAAAATTAATTTTGGTAATCAAGCTCCACAAAAATCGACAGTCCAGTTACAACAGTTACCTACGCCTAGTCAATCCCAAAGACATATTCCTGCCAGGCATTCCTTTGCAGCCTCTTCTTCCACTCTAGAATGAGACCTAAAGTTAGAAAAAGCCTTAATTGATTTAAAATTAGAATCACTGAGAAAAACTTCGCAAGTTACCCATCCTGTTTATGGAAAAGCCCCAGTGCAGGAAGACATTAAGTCTGAGTCCGCTGATTCACCCACTCAGTCTGATTTTGTCGAAGCTGGTTTCAAAATCGATAACCAGCTACGAACACTTAATAAAAAGTTTACCATTAATTGGAAAAAATTAGGTAGTCATCTACATGCCCCTGAGAATCAAGCTAGGAGAGATAATTACCATTCAGTCATCCCTGATAAAGTCAAAAGACAACAAATTTTTAATGATTGGAAAGAATACATGCGTACTGCCAAAGTTGAAATATATTATCTTGATTTTGTAGAGAGCCAACATTTACCTAATGAGTTAAAAACTTTAACCCAAGAAAAAATGGGAAAAGATAGACCACACTGAGATTCTCGCTAGTCATCTACCTGTAAAAACAATAATTATTAAACATTAGAAATCAAAACTAGTAGCCAGTCCTTTCAAGATCCCAGATCCAGAGAATAAAATGATAGTTGACCAAAATAATTACACCAACCAGAGTCTAGTTGTCATAGGTAAACAATTAGATAAAATAGAAACAAAAGTTGACAAATTACTTCCAACATAGTTCACATCTAAACATCATGAGAAACCTTTAGTTACATTCCAAGATTTAAAATCAAGTCCAACTTTTAAGACAAAAGAAACGATGACAAAAATTGAGAAAATGTTAGAAGAATTAACACCAATCAAAAGAAAAGACAAAGAAAAGGTTGAAACAAGCTTTAAAGTCATAGGATCCCCTGAGTCACCTTTTGCTTTCCATAGCTCCTCTGAGTCTGAATCAATCGCTAGTATGGAAACAGAAACTAGTAATATCTCTAAAATTGAAAATGCGTTTAGAAATCTTGAGTTAGAAATTCAAAAGTCCAAAGATTAACGAATAAAGTCAGTCCTAATAGTCTAACAAAAAACTAGTATCCTAAGCTAACACCTCCCGATATACAATTTGAAGAACAAAACACCTAGAGTTAATTTCCAGTCTCTTTGGATAAGTTATATGAATGGAATATTAATGGTCCTTCTGAGCAAGAAATCCTTAATAAGATTCAACATATGTCCATGGTCGCCAATAGTTACATCACCAATCACAGTTTTAGATAGTCTGAAATTGTTCCTTTGCTAGAAACTGGTTTCACCGGAACCCTTCATTCTTGGTGGGATAAACATTTAACCCATTTGTCTAAGCAACAAATAATTCATGCAGTCAAACTTAGTGAAGACGGTCTTCCTATTTTTGATGAACAAATAGGTCAAGGCATTGAAGATGGAGTCAACACCCTGCTATATACAATAGTTGAACATTTCATTGGAACACCCAGTAACACCACCGCTAGAATACATGATCAAGTTAGTAATTTAAGATGCCCTAAGTTATCAGATTTTAGATGGTATAAAGATGTGTTTATTTCCAGAATCATGCTTAGAGATGATAGTAATCAGTCATTTTGGAAAGAAAAATTTGTTAATGGTCTTCCTAATTTATTCGCCCATAAGATCCGAACAACGCTTAGTAATGAACAAGGTCATATTGATTGGGATAATTTAACATATGGAAATATAATTAGCACAATAAATCAAGTCAGCATGAGAATGTGTGTTGATATGAAAATTGGTAAACAAATTCAGTCTGAGAGAAATTCTGCTAAGTATGAGCTAGGTAATTTCTGCGAACAGTATGGTCTAAAGTCTATTCCCTCGTCTAAAAAGAATAAGTCCAGTCATCACAATCGACAGTTTAGTAAAAAACGCCATTATTCACGTAGAAGACAGTCATTCCAACATAATAATGAAGATAAAGAATTTTATAGTGAAAAGAGATTCTCTCCTAAGAAAAATTGGAAAAAGAGTCACGGCAAAGATAAGAATAATCGCTTTAAGAAAAGTACCGACAAGAGAAAAGTAAAATGTTATAAATGTCAAAAGTTTGGTCACTATGCTAATAATTGTAAGGTGAAAGACACAATCAAACAATTAAAAATCACAGAAGAAGATAAAGATAAATTAATTAAAGTCCTTGAGTTAAGAAACTCTAAGTCTAGTGAAAATGAAACCATTGTTAGCAATACTGAGTCTCCTGAAATCTATTCTTCTGAGTCACAACCCAACTCGCCTAGAATTCAATTCAATTGTCAAAACAATTGCTGTAAGTCTAAGTCAATTAGTGTCCTTATTAAACAAGAAGAACAAGAAGAATTATTAATTGATTTAATAAGTAAAGTCGAAAATCCAAAGTTGAAATATGAGTACTTAAAGAAATTAAGGAAAGTCATTAGTCAAGAAGAGCCAAAACAGTCTACCTCCCAAAAGATAAGTCTGAACACAACCTTAGAAAAATTTAATAAAAAGAAAGAAGTCACTTTACAGGATTTACAACTAAAGGTAAAATTAGTCAAAAAAGAAATCATTGAGCTAAAACAAATAAGCCAAAAGTTTACAGAGTGAAAATTATGAGATAAAACAAGAGTTAGTTAAATTATTTGAAAAAAGAACTTCTGAATCTACACATGTAAGTCCCACAAATTCTGACGAAGAGCCCTCCAATCAGAACCATGTCGTCAATTTAATCAGACAAGCCAATTTTAGGAAATGGTATGCTAAGGTCACCATATTTGTAAAACATTTTGAATTAAACATAGTTGCCCTTTTTGATTCAGGAGCCGACATTAACTGCATCCAAGAAGGCCTAATTCCTACTAAGTATTACAAAAAGTCAAAAGAGTCTTTAAGCACAGCCTCAGGAAAGTCACTCCAATTGAATTATGAGATTCCCAAAGTCCATGTCTGTCAAAATAAAATTTGCTTTAAAACTTCATATGTCTTAGTTAAAAATATTACTGACGAAGTCATCCTAGGTTTACCTTTTATTGCTCTTTTATATCCTTTCCAGGTAGAACATGATGGAGTCATTTCCACCTGTTTAGGAGAAAAGGTTAAGTTTGAATTCCTAAGTAAACCAGAGTTACATAATTTAAAAGAATTACAAAAGAATGCAGTCACTAAAACAATCAGAGTCATTCAAAATAAAGATAAGCAACTACGATTCCTCCAGGAAGAAGTCAAAACTAAAAGAATTGAGCAACAACTTAGTCATGAGTCTTTACAGTCACAAATTCGACCGTTTGAAGAAAAATTAAAACAAGAAATTTGTTCTGAGTTACCCAATGCTTTCTGGCATAGGAAACAGCATGTAGTCAGTCTTCCTTATGTCAAATCCTTTAGTGAAAAGAATATTCCCACTAAAGCCAGACCAATTCAAATGAGCCAAGAATTAATGGAATTTTGTAAAACTGAAATTACAAAATTACTTGAGAAGGGAATAATCCGTAAAAGTAAGTCACCCTGGTTTTGTCCTGCTTTCTATGTTCAGAAGAATGCTGAACTAGAACGTGGAGTCCCAAAATTAGTCATTAATTATAAGCCTCTTAATATAGTCTTAGAATGGATCAGATACCCAATTCCTAATAAAAGAGATTTAATTTAATAGATTAGAAAAGTTAGTCATCTTTTCTAAATTTGATATCAAGACTGGTTTGTGGCAAATTCAAATCCGTGAGTCTGATAGATACAAAACTGCTTTTGTCACCCCATTTGGTCATTATGAGTGGAATGTTATGCCTTTCGTTCTTAAGAATGCGCCCAGTGAGTTTCAAAATATAATGAATGAGATTTTTAATCCATTTAGTCATTTTTCCATAGTCTATATTGATGATGTGCTTATCTTTTCCCAGTCGATAGAACAACACTAGAAACATTTGCATAAGTTCTTACAGATAGTCAAACAAAATGGATTAGTTGTTTCTGCTAAGAAAATTAAGTTATTCCAAACTAATGTAAGATTTCTTGGGTTTAATATCTGCCAGTCACAGATTAGCCCCATAGATCGAGTCATCCAGTTTGCAGATAAGTTTCCTGATCAAATCTTAGACAAAAGTCAACTCCAGAGGTTTCTAGGATCTCTAAATTATGTTTATGATTTTTATAAGAATTTGAGAAAACAATGTAAGCCTTTATTTGATCGTCTCCAGAGTAATCCTCCCCCTTGGTCATCTATTCATACTGATATAGTCAAACAAATCAAAACTCATATTAAGACACTTCCCTGTCTTGATATCCCCTCTGCTGGTTCATTCAAAATTGTTGAAACTGATGCCTCTAACATTGGTTACGGAGGTATCCTCAAACAGAGAATCAATTCCAGCCAATCTGAGCAAATCATTCGATTCCATTCTGTACTTGGTCTCAGTCTCAAAATAATTACAGTAATATTAAGAAAGAGATATTATCTATTGTACTATGCATTAATAAATTTCAATCTGATTTATAAATCAAAAATTTTTAGTCCGAGTTGATTGTGAGTCTGCCAAGCATGTACTAGAAAAAAAAATGTTCAAAATATTGCATCCAAACAAATATTTGCAAGATGGCAAGCTATTTTGAGTATATTTGATTTTGATATTGTGTATTTGAGAGGAAGTGAAATTATGTCCCTGATTTTCTTACCAGAGAATTCCTGTAGGGAAAACAATGGCAGAAAGAAGAAAATAAAAAGACAAGCAAAAAAGTCAAGTTGGTCAATCTAGTCAACACAGTCAACCCCAGTCACTCCGAGTCCTCCCTCCATCAATGAGTCAAGCCAAACATGAGTCACCTTCTCTAATCGTCCCCTTTTCGGGATGTTCTATCCAAATTAGTAACAGATTTGCCAACCTAGGAGCCACAGTCGGCCAGGTTCGCCCCAGTTTTCAGTCTGCCCTAATTTCTAGTCATGATCCTTTTCAGGATATTGCTGTTAGTAATCCTCAATTGCCTCCTATCACAAGACATCCCCTTACATGATTAAGAGTAATAGTCAACTATTCATAATTGAGTCAAAATACAATTCCATTGCCAACCTAATCACCATTGCCAAATCTTACTTTCCACCAAATAGTCACTTCATTCCCCCAGCTCCATACAAGAATCTAAAATTTTACAGAGACATTCTCCATGAGACCCAATCCATCACAATTAAGACAATTAAAGACAGAGATAATCCTCATATCATCCTCTACCATTCTCTCTACATCCATCAAATAATCAATGAGAATGAATGGAGTAGTCACCCTTATGATCTCAAAGTTTTTCAGTCAGAACTTCAATATGGTTACTATGATTATATCGAAGCATGGTATTCTATCTTGCTTCAACAAACAATTGATTTTAGTCATCCATGGTTCCTTAATTTTGACAGCAAATTCAAGAGTCCTTTTCCCTATTGGTTCAATCACTGGTGGGAAAAACATGGTCTAGTCATTGATCTATTTCCTCCTCCAATGCAAGATTTAGTCAATTATTTTACTAATAAAAATAAGTTTAAAGAGTCTGAGTTGTTCTTCCCAAATCTTCTCCATTTTGTCGCTAAGTACAAAGTCCCTTGGATCCTCAAATGGTCATATCAAGTTAATTGGGAAACCAGAGTCTTATCCCGACAATTTTCAATCAAATGGTGGGATAAATTTACTTGAGCGTATTGCAGATTATGTATATGTTGATTTTCCTCCAGTCAGGAATCCACAACCAGAGAAAAAGTCATCCCCTGCTACTTCACATTCTTCTCTCCTAATCGTGGGAAAATCTAAGTCTGAATTGCAGGAAATCACTCACCAACTGATGTTACCAGCCTCACAAATGAATGATGATGATGATAGTGATGCTACTCCAAAATCGCAGTCATCAACCAGTCAACCAAAATTGTCATCCAGTCAAAAGACTCCAACCCCCAAAATGTGGTGGAGTGATTATCCCCCAGATTGTCAAGACCCTTATGATTTCAGCCCTCTTGATCTGGGTGAAGATTAAAATTACCAAAGTCATGAGCCCCAAAGGTAAACGCATTTGGAATCTTGGACCCGTGCTTTAACAGTCTGATAGCCCGTGCTCCTTTAGTCAGATTCCCGACAAAGTAAAAGCAAAAGCTTTTGTGTGCTCCTACAGTCACCCTTCAAGCCACCCTCCAGATTATCCAAAGCTGCCAGCTCATCCAAAGTTGCTATAGTCTACTTTTATTTGCTTATTTAAGAAGGCTTCGGCCTCAGTTATTAGTGGAATGTAATTTAGTCCACTTTCTTTTCTTAGTTTCAAAGTTCTTTCAGTATCAATGCTCTGAAGAACTAAGTGGTGGAACAAAGATTTTGTAAGTGTTTTGCATTCTGCAAGTTATCAAGTTATCAATAATATTCTGAGTATTTTGAGTAATGCTATTTT

TAACTGCATCTATTTTTATCTATTCATTAATTTGCTTCATGCATAAGTTACGTCTATTCTACAATCTCTGTGCTTATACTTACTTTTGTGTATATATATATATATAATAGGACAAAAGCTTTTCAACCCAAATTTTGGAGACCAACAGCCGCATCCTCCAAAACTGTGTTTGCTCAAAACCAAATATCTTGGCCACAATTTCTAACAAAACCCAACCGCATATTCATCTGCCCGAAAAATCTATATTGAGCATCTCTTGGGCATCTCCGGCCGCTCTAGATCTCGATTGCAATACTCTTTTGAAACCCGGCCAAGGTAGTCTCAGCTGTGCCCAGCCACTTTCGAGCACAGCCCAACCAGCCGGCTTCAAATGACGCCCGACCCAGATCTCCAACACCCCTGCCCGCGAACTTCTCCAATCCCGGCCGAGATTTCCAGCTCCATTCAAACTGCCCAGCCGCTATCTCTTCGCAAGCTTCAAATCCGGCCGTGATTTTCAACAGCCCCCAAACACAAATATCCCCACGGGGGCGGGTCCCCATCGGGTCCCCGACCCTAACGGGGGAACATTTCGAGGCCAAATTGGTATTGGGTATGGGGATCCCCGAACATGAAAAATCCTCGACGGGTATGTGGAGGGGATGGTATTGGGGTTCCCATCCTCGAACCCGACTAGAAAAAATATATGTTTATATTTAAATAAATAAATATATAATTATAATATTTATGTTTAACCCTAAGTTAACCTCCCTCTCCTAATTCTTCATAATCTTCTTTGGAATTTCATATTGAAGAGATTTTGAATAGTTGAGTTGAACTTTATAGTTAATTTGGATATGTTGAATGATAAATTAATATGAAACTTAATATTAAAATGTATGATAAAAAAAATTTATGCAAAAAAAAAAATCGGGATTCGGGAGGGTATGGGGATAGTCCCCCGACGAACGGAGACGGGGACGGGGATTCCCAAAACCTATTAAACAGAGATGGGTTCGGG

>Chr4-09; OZ022209.1; 21206317-21213389; rev; D;

AAATCACCAAGTAAATATGGAACACTTGATGATTTATCTCAAATAAATGCACAATAGCTTTCTTAGCAATTTCAAAATATGGGAAAGCTCCCTCTTTGAAAAGAAAGGCACGCCTTTTATTCAAAATTAGTTCTCTCTCATAAGAGAGAAACACAGGAGTTCACCATTCCTATTCAATCATGACTTTCCAATATGATCTGATATGGACTTTTGTTAAAGACGTCAAGCAGTAGACCACCAATGCTATTCAAACATGAATAATAGTTTTGATTCAATTATAATATTATGTGATGACGCCAACTAATGCAAGACGTGAGGAAACCTAATAGCTTCCACTACAGCCAATCTTTCAAAACAATATCTACCTAAACAAATTCATTAGACCGAGAAGATAATATTTTAACTTAAATCAAAATAGAGCCCAACTAGGAAAGAAATCTCAAGAGATAAAATATAATCCTATTAAAAATAAGATTAACATAAAAATACTTGAGTGAAAAAACTCCAACTAGGAATCCTATAATGAATGCATGATTATGTCATGATTTACCTGAAGTCAAGTTTCTCATATCGGTCAAGTTATGCCTCGGATGTATGAGGTTGAATGAGTTGTGCCAAAACGTCCAGTAACAATTCTTTACACACTAATTTTCAACCTATACTAGAGTCTAGGAAATGAAACATTCTCTCTTCCTCATTAGAACCACACCGAAAACATAGATTTTGAGATTAAAATTGATTGGCTTCTGGACATGAATTGTATGAGTTTCAGAGTGACATGAATGGACTTGCACACTTTCTTCCGTCAGAGATGTTTTTATATCTTTCATCTTGTTTGCTGTCATCTCGGAGTGTACAAGAAGCCAAGGATTGAATGTTTCCAGTGTCATGTTATTGGGTGTACATAAAAAGCAGGACAAGACCAGGGAGATTTACTTTTTCGGATTATATTTTTTTGTAATAAATCATTGATTTCAGTTTTACCAGAAGCAGTATGTAA

GGATTCTGTAGATTTCTGATAATACTTAGTAGGGACTAAACCTTCTTTTATACAATTGAGATCTGCACCAGAATCAAATAAAGCTACTGTATTGAACTCATGATTTTTTATAATTATAGTGACTTTAGAATACCATTTCCTAAAATTAACTTGTTTAATGAGGTTAATAGCTTGTTCAGGTTCTGGTTCTGACTTATGAGTAGGACTACTAGGTGGACTGTGGGACTCAGAGGGTTCCTTATCAAGTAATGTATTTAAAACTTGTCGGATGTCAGAATTATCAGTTTGTAATTTATGACTTAGCTGTTTTAATTCAACTATTTCTGTTTTGATTAATTTTACTTCAGAATGTAAATCATGTAAAGTAATTTCTTTTCTTTTATTAAATTTTTCTAGAGTTGTGCTCAAATTGATTACAGGGTTCGATTGGCTCGTACTAGTATCCTGGGAAAGGATATGCCTGAGTTTTTTCAAGTAGTCAGATTTTAATTCAGGATTCTCGATTTTACTAATTAATTCTATCAGAAGTTCTTCTTGTTCTTCAATTTTAGTTAAAACAGAAATATGCTTAAGAGTATTACAACATTTATCTCTACATCCTAATTGAATTCTAGGTGAACTTAACTGGGAACCAGAGGTTTCATAATTAATTGACTCAGAATGACTATCAATGGATTCGTGTTCACTAGACTCAGAGTTTCTTAACTCAAGGACTTTAATTAAGTTGTCTTTTTCTGCATCTGTGATTTGTAATTGTCGGATAGTTTCTTTAACTTTGCAATTATTTGCATAATGTCCATACTTTTGACATTTAAAACATTTTATTTTACTAGAGATCAAACCCTGCTAATACAAAGTTGTACACCTGATGCTAATATTAGAATACCACGTACAGTTAAATGGTCTGAAATTACTCTGCCTACAGATTGGACTTTAATCACTGAGTCTCAGCCTATGCCTATTCAACGCAGCCTAGATAACTTAGATTATATACACCAATATATGGATGGTAGTGTTAAAATTAATTTCCAACACAAGTCCACAGTTCAATTACAACAATTACCTACGCCTAGTCCTTCTAAAAGACATGAGCCAGCACGTCATTCCTCTGCTTCTTCTTCCACTACAGTTAGAGATTTAGAGATAGAAAAAGATTTAATTGATCTAAAAATCGCATCCCTTAAGAAAAAAGACCAAGTTAGTCATCCTTGTTATGGTTCTCCACCTCTTGGATACCCATATATTCCTGCCCCTGAGCCTAAAATAAATTCCCCTACACAGTCCGATTTCATTGCTAGTGAAGTAGCTGCTGCACATATTTATGATATAGATAATCAATTAAATGTTTTAAGACGTCCTGAAAATTTTAAGATCAATTGGAAAGCCTTAGAATCACACCTTTTAGACCCTGCTAATTCCTACAGACGAGATATTTATCATAAGGTTGTTCCTAACAAAAATGAAAGAGATAAAATACACCATGCTTGGAAAGATTATATGAAGACTGCAAAAGTAGAAATTAATTATCTTGATTTTGTAGAAAGTCGTTATATTCCTAATGAATTAAAAGCTATAACTAAAGAAAAATGGAAAAAACTAGATAAGTCTGAAGTAGTTTCAAGTCACCCCCCTGTAGAAACTATCCTGATAACTGCTGGAACAAAAACTATAACTGCTGCTCCCTTTAAGATTCCTGATCCTACTAATGAGTCTACCAAATGTTTAGTTGAACAAAATAATTATACTAATCAAAGTTTAGTGATCATAGGAAAACAATTAGACAATATAGAATCTAAAGTTGATAAAATATTACCTTCTACGCCTAAGCTTAAATTTGTAGATAAACCGTTAATTCAATTTCAAGATTTACAGCCTAAATTTGCTCTAAAAACCTCCTCTACTTCTAAGAAAATTGAGAAAATTGAAGAAATGCTTGAACAATTATCTCCGTTAAAAGGTGAGAAATTCGGTATCAAAACCTTAGGAACTTCTTCTGTGAGTAATTCTGATTCAGAAATGAGTTTAGAAAATGATGAGTTTAGTAATATTTCTAAAATTGAGAATGCGTTTAAAAACCTAGAAATAGAGTCTGAGTTAAAAGTTAAAAGATTAACTAACAACACCCGTAAGATAAACCCTACAAGTTTAACAAAAAATTGGTATCCCAAACCTACACCCCCTGATATTCAATTTGAAGAGCGCAATAATTTAAGCCAGTTCTCTGTTTCCTCTGATAAATTATATGAGTGGAACATTGATGGACTTTCTGAGCAAGAAATAATGAATAAACTTACACATATGTCTATGGTTGCCAATAGTTACATTACAAATCACAGCTTAAGACAGTCTGAGATAGTCCCTTTATTAGAAACTGGTTTTACAGGAACTCTCCGTTCTTGGTGGGATAAACATTTATCTGATGAGTCCCGAAACAATATAATACATGCTGTTAAATTAAATGAGGATGGTCTTCCTTTGTTTGATACAAATATAGGACGAGGAATAGAAGATGGAGTCAATACACTTCTTTACACCATAATTGAGCATTTTATAGGTACACCAAGTCATACACAATCTAGAATACATGATCAACTGAGTAATTTAAGATGTCCTCAATTAAGTGATTTTAGGTGGTATAAAGATATTTTTATTTCTCGAGTAATGCTAAGAGAAGATAGTAATCAAGCCTTTTGGAAAGAAAAATTTATAAATGGTTTACCTAATCTATTTGCACATAAAATACGAACTGTCTTAAGTAATGAATTAGGTCATATAGACTATGATAAATTGACTTATGGTAATATTATAAGTACAATAAATCAAGAAGGAATGAAAATGTGTATAGACATGAAAATAAATAAACAAATACAGTCTGAACGCAGATCGGCTAAGTATGAATTAGGTAATTTCTGTGAACAATATGGTTTAATCTCCACTGCACCTTCTCGTAAGAATAAGCCTAGTCATCTTAAAAAGAAACGCCATTATAGCACTTTTAGGAAAACCTACAGACACAATACTACACCTGATGAATTCTACAGAAAAAGAAAATATTCCCCTAAGGAAAAATGGTCAAAAGCACCTCGATTCCATAAAGATGCTCGCTTTTACAAAAGGTCTGAAAAAAGTAAAGATAAAAGTAAAATAAAATGTTTTAAATGTCAAAAGTATGGACATTATGCAAATAATTGCAAAGTTAAAGAAACTATCCGACAATTACAAATCACAGATGCAGAAAAAGACAACTTAATTAAAGTCCTTGAGTTAAGAAACTCTGAGTCTAGTGAACACGAATCCATTGATAGTCATTCTGAGTCAATTAATTATGAAACCTCTGGTTCCAGTTAAGTTCACCTAAATTCAATTAGGATGCAGAGATAAATGTTGTACTCTTAAGCATATTTCTGTTTTAACTAAAATGAAGAACAAGAAGAATTCTGATAGATTAATTAGTAAAATTGAGAATCCTGATTTAAAATCTGAATATTGAAAAAACTGAGACATATCCTTTCCAGGATACTAGTACAGCAACGAACCCTGTAATCAATTTGAGCACACTCTAGAAAAATTTAATAAGAAAAGAAATTACTTTACATGATTTACATTCTGAGTAAAATTAATCAAAAAGAAATAGTTGAATTAAAACAGCTAAGTCATAAATTACAAACTGAGAATTCTGACATCCGACAAGTTTTAAATACATTACTTGATAAGGAACCCTCTGAGTCCCACAGTCCACCTAGTAGTCCTACTCATAAGTCAGAACCAGAACCTGAACAAGCTATTAACCTCATTAAACAAGTTAATTTTAGGAAATGGTATTCTAAAGTCACTATAATTATAAAAAATCATGAGTTCAATACAGTAGCTTTATTTGATTCTAGTGCAGATCTCAATTGTATAAAAGAAGGTTTAGTCCCTACTAAGTATTATCAGAAATCTACAGAATCCTTACATACTGCTTCTGGTAAATCGCTTCAATTAAATTATGAAATTCCTAAAGCCCATGTCTGTCAAAATAAGGTCTGTTTTAAAACATCATTTGTTTTAGTTAAGAATATAACTGATGAAGTTATTCTAGGATTGCCCTTTATAAGCCTGCTTTATCCTTTCCAAGTCGAATATGATGGTGTTGTCTCCACTTATTTAGGTGAGAAAGTAAAATTTACCTTTTTATCAAAGCCTGAGCTGTACAAATTAAAACATTCGACTAAAACTGATTCTGCAAAAACGATCACTTTGATTAAAGGAAAAACTGATCATCTAAGTCATTTACAGCAAGAAATTAAAACTAAGAGAATTGAGTCTCAGTTAAATAGCCCTATTTTAAAAGAAGAAATTAGTCGCTTTGAGAAAAAACTGATTTCTGAAGTCTGTTCTGATTTCCCTACTGCTTTTTGGCATAGAAAAAATCATGTTGTGCATTTGCCTTATATCAAGGATTTTTCTGAAAAAAAATATACCAACTAAAGCTAGGCCTATCCAAATGAATCATGAGATGATGGAATTCTGTAAAACTGAAATCAATGATTTATTACAAAAAAATATAATCCGAAAAAGTAAATCTCCCTGGTCTTGTCCTGCTTTTTATGTACAGAAAAATGCAGAATTAGAAAGAGGATCTCCTCGGTTAGTTATTAATTATAAACCTTTAAATGCTGTTCTTGAATGGATTCGTTATCCAATCCCTAATAAAAGAGATCTGATTAAACGATTAGATAGAGCTGTTATTTTCTCTAAGTTTGATATGAAAAGTGGATTTTGGCAAATACAGATTCATGAGTCTGATAGATATAAAACTGCTTTTGTTACTCCTTTTGGGCATTATGAGTGGAATGTTATGCCTTTTGGTCTTAAGAATGCTCCTAGTGAATTTCAAAATATAATGAATGAAATTTTTAATCCATACAGCCATTTTTCTATTGTATATATTGATGATGTTTTGATTTTCTCTGAGTCTTTAGAACAACATTGGAAACACTTGCATACCTTCTTGCATATCATGGATTAGTTGTGTCTGCAAAGAAAATTAAACTATTCCAAACTCATATCAGATTTTTAGGATATAATATACATCATTCACAGATTCATCCTATAGATCGAGTTATTCATTTTGCAGATAAATTCCCTGATCAAATTACAGATAAAAATCAATTACAAAGATTCTTAGGATCCCTTAATTATATATCTGATTTCTATAAGAATTTAAGACAACAATGTAAACCTTCATTTGATAGGCTAAAGGTTAATCCTCCCCCTTGGTCGTCTATTCATACAAGTATTGTTAAACAGATTAAGTGTCATGTTAAGACTCTTCCTTGTCTTGGTATTCCTACTCCAAATTCTTTTAAAATCGTTGAGACTGATGCTTCTGATTTAGGTTATGGTGGTATCCTTAAGCAAAAAACTGAGTCTTCTTCTGAGAAAATTGTCCGTTTTCACTCTGGTATCTGGTCTCCTTCCCAGTCTAATTATAGTACTATTAAAAAAGAAATATTATCTATTGTATTATGCATTACTAAATTCCAAGATGATTTATTAAACCAAAAATTTTTAGTTCGGATTGATTGCAAATCTGCTAAATATGTATTAGAAAAGGATGTTCAAAACCTTGCATCAAAACAAATTTTTGCACGTTGGCAAGCCATTTTAAGTATTTTTGACTTTAATATAGAATATCTGAGAGGCAGTGAGAATTCTATTCCTGATTTTCTTACAAGAGAATTTTTACAGGATAAGCAATGGCCGGAACATCAAGAGACAAGCAAAAAATGAGTCACAACCCTACTCCCCGAATCTTGCCTGCTTCTTTGAGTCACAAGCCAACTACTTCTGTTAAAAATGAGTCTGATTCCTTTTCCGTTAAAACTGAGCCCTTTACCATTACCCCCTATCCTGGCCATCCACCTCTACCAGTCTTGAGCAATAGATTTAGCCCCTTAGGTACTACAGTCACCCCTGCAAGGCCCAATTACCAGTCTGCATTAGTTTCTAGTTATGATCCGTTTCAGTTACCCCCCACTGCATCGCCCATATCCTCTCCGTCTTTTGCTAAATCTTCTCCTTATGTCTTAAAAAATACATCAAATCTTGTTATTATTGATTCTCATATTCCTACATCCATGAGTCCAGCCCAGATTGCAGCTTATCATTTTCCTCCTGGATTACATTATCTTCCCCATAGCCCTTACAAGTCCTTAAAATTTTACAGAGAAATTCTTCATGAAACCAAGTCTGTTGAAATTAAGCCCATTAGGGACAAAACTGATCCTACAAAAATTATTTATCACTCCCTTTACATTCACCAAATTTTACATCCTAAGTCTTGGGGATCTCGTCCTCATGAGCTTCGTACCCTTCCCTCTAAATATCAATATAATTATGCAGATTATGTTGAAGCCTGGTATTACATCTTCTTACATCAAACCCCTGAGTTTTCCCACTCCTGGTTCATTAACTTTGATAACAAGTTTAAAAATACTGAGCTTCCTTATTGGTTTCTTCATTGGTGGGACACCCATGGTCCCAAATATGATATCATCCCTTCTGCAGTTCATGAGCTCATCGGTTATTTTTCCCAAAAATACAAAGTTCCTGAGCGAGATTTATTTTTTCCTCTTGATCTCTTATTTTTTACAAAATACAGACTCCCTTGGATAATGAAATGGAATTATAAAGTTGATTGGGAGACTCGTTTCTTTTCCCGTCAGTTCTTTGTTAAATGGTGGGATCGTTTTGAGGTCCATCGCATTACAGATTATGTATATAAAGACTTTCCACAGGTTCCACGTTGCACTCCTGAGCCCAAGCATGAACCTTCTTCCTCTTTGGATACTATCCTTACAGTAGAAGGAAAATCTAAGACTGAGTTACAGGACTTAGCCCAGCAGCTCCTTCTCCGAGCTTCGCAAATGCATGATAATGATGACGCTGAGTCTGAAACCTCTTCTAGCTGTCAACCTGCACAGCCAACTCCTGAGCGCCCTGTTCGTTGGGCTGATTATTTGCATGGTCAAGATCCAAATGATGCCTATGATGAAGCCTATAAAGCCTATGATTTGAATTCTGATTAG

ACTACTGCTAGCAAATTACCTGAGTTAAAGCAAGTTACCTGAGTTAAAGCTACTTGAGTTACTTTTATTTACATTGAGTTACTTTTGTAAACCTGAGTTACTTTCAATATTTGTCTTTAAAAGGAGGCTTCGGCCTCAGTTGTAAGGGGACCTTCCTTTTTCTTCTCTTCTCTTCAGTTCTTTTCTTAGTTTTAAAATGTTCTTTCAGAGATCATGTTCTGAAGGACAATCTTAGAATGAGAGTAAGCTTAATGAGAGTAAGCTTTGCACATTGTAAGTTTTTATAATGAATTTATTTTTAAATAAATGTTTTCTTTTATATTTGAAGTATCATTATGTTCTATACATTTATATATATATATATATATATGTGTGTGTTCGATTCAACCGGTCCAATCAAATTATAATGGATTGATTGGATTTGATCTGTATATTTACATGGAACTTGATTGAATTTTATTACGGAGATTAGAATAGATTGAATTTGTCAACTTCTAGTGTTCTAATCAGACTTTGGAAGTGTTCTAATCAATTCATGGCCCACATATATTTGTTAATTAAATATCCAGGCCAATAGATGGTCAATATAAGAATTATTTCATGAATAATTCAACATGTATTTACTTGACGTGAAAAGCAAGTAAGATCGGCTTAATTGATCCTGATCCATTTTCATCCTAACCTAGTAGGAGAAGGTTCTCCTGCGGAGATTAGGGTTATGAGCTATTATATAAAGAAGATTTCTACAAGCTCAACGACACACAACTCTTCAACAACAAACTTTACACTAAAGTCAACATTTTTAGTCAAAGACACTTGTCTCTCTCTGCTATTTGTTATGCCAAGCCTCCTTGGTATTGATTGCCGATTGACTCCTTCAGTTGAAGACCATTATGGTGGCACTCTTGCCTTGTTCTAATCCATTAGAACCGCACAGTTCCAATCCTCACTCGATTGTCACCCGTCATTTAGTTCTGTTTTGAATTAGAATTGATACAAG

>Chr4-10; OZ022209.1; 21479519-21481293; dir; D;

AGCGACTCGAGCTGACTTGGTGTCCTCGAGGAATCTGCCGGCAGATCAGGCTGATCATAGTCCCCTGATTCTGCCAGTTTGCGGCTCGGGTAGACTGCGTGGCGCCCGAGACCTGCCAGGGGAATTGACGGATATGACAGGGGTACAAATAGGTGGTATTTTCAAAGGATTTTGAATTTCTTTTATTCAAGTCCAACTTTCAGTTATTTTATATCGGTTTCTTTGATATTCGTGCATTATATTCTATATATTTGTTCAGTTATACGAGTATATTCTTCAGTAGCTTTTACCTGCTTATGGTTTTCAGCTATTTCAAACCAGTTTCTTTGATATTTACGCATTTTATATCTTCATATAATTGTTCAGTTTTACGAAGCTACATACATACGATTATATATATATTCGGATGAATACCTTACATTAAACACAGGTGAACTTTAGCCTTACTTATCAAGTTATTTTTACTATGGGGGTTATTATGATTTTTAACTGTTTTCATAAATCTTATGTTTGGTCCACTCACATTTTCAACCTGTTTTTTTCGCCCCCAGGCCATAGAAATACGCGGGATCCACCACCGGGCCATTCAAAGCTTCCGCCTCACCACGTAAGGTAAAGTTTTGTAGTTGGAACCCTGAAACTTAGAAAATGCTCTGATATATATAGTTGAAGTTGGGAAACTGGAGTGGAGATCTGTTATTTGGAATATTCTGGCAGTTGGTGTGAATTTATGGGTTGTTTAACATGTGAAAATTTTTGGGATTAGTCAAAATACAGGGGAGACTCTGTCGAAATTTCGGCAGAAGTCTAAGGGAAGTTAAAAAGAATTTGAGACAAGAAGGGTAAAAAGGTCATTTGTGCCCCGACATTGCCAGGTGTCGGACACGCACAGGACTTGGCTCGAATTCCAAAGTGGAAATTGGGTCGGGTCCTGTCAATTACTATATATACATACATATACATATATACACACACATATATATATGTATATATATATATA

ATTCTTCTGAGTCACAACCTAGTTGCCTAGGATTCAATTTGGTTGCCAAGATAATTGTTGTGAGTCTAAGTCAATAAATGTCCTTACCAAACAAGAAGAGCAAGAACATTTATTAATTGACTTAATAAGTAAAGTTGAAAACCCATAGTTAAAATCTAAGTATTGAAAGAAATTAAGAAAAGTCATTACTCAAGAAGAGCCGAAACAACTTACCTCTAAAAAGATAAGTCTGAACACAACCTTAGAAGAATGTAATTTTAGTACACTACAATTTTCTCAGTTTCAGAGTTCTATCCATATCAATGCTCTGAAGAACTAAGTGGTGAAACGCTTTGTAAGTGTTTTGCATTCTATAAGTTATGAAGTTATCAATAGTATTTTGATTATTTTGAGTAATGCTATTTTGATTGCATCTATTTTCATATATTCATTAATTTGCTTTATTGCATCTATTCTATAATCTTTGTGCCTATATATGCCTATACATACTTCTGTCTTTATTCTATCTCTCACTGGTTCTAGACTCCTTATGGTATCAGAGCCGTAAGTGTTGAAAAGTTAGTCTAAGTTTACAACTCATCCATCCCAAGTGGCTGGTTTAGGTTTCCTGAGTTGTAAGTTTTCAGTTTCTTCAGATAAGTTATATGAATGGAATATTGATGGTCTTTCTGAGCAAGAAATCCTTAATAAGCTTCAACATATGTCCATGGTCACCAATAGTTACATCACCAACCACAGTTTCAAACAGTCTGAGATTGTTCCCCTGCTAGAAACTCGTTTCACTGGAACTCTTCGTTCTTGGTGGGATAAGCATTTAACCCATGAGTCTAAGCAACAAATAATTCATGCAGTCAAACTTAATGAAGACGGTCTTCCTATTTTTGATGAACAAATAGGTCAAGATGGAGTCAACACTTTGCTATACACAATAGTTGACCATTTCATTGGAACACCCAGTAACACTACTGCTAGGATACATGATCAGCTTAGTAACTTAAGATGCCCAAAATTATCTGATTTTAGGTGGTATAAAGATGTGTTTATTTCCAGAGTCATGATTAGAGATGATAGTAACCAATCATTTTGGAAGAAAAATTTGTTAATGGTCTTCCTAATTTATTCGCCCATAAGATCCGAACAACCCTTAGTAATGAACAAGGTCATATTGATTGGGATAGTCTCACATACGGTAATATAATTAGCACCATTAATCAAGTCGGTATGAGAATGTGTGTTGATATGAAAATTGGTAAACAAATCCAGTCTGAAAGAAATTCTGCTAAATATGAGTTAGGAAATTTCTGCGAACAATATGGTCTAATGTCTATTCCCCCATCTAGAAAGAATAAGTCTAGTCACCACAGCCGACAGTTTAGTAAACATTGCCATTATTCACGCAGGAGATAGTCATTCCAACATAATAATGATAATAAAGAATTTTATTGGTAAAAAGAGATTCTTCCCTAAGAAAAATTGGAAAAAGAGTCACGGCAAAGGTAAGGATAGTCACTTTAAGAAAATAACCGACAAGAGAAAAGTAAAGTGCTATAAATGTCAAAAGTTTGGTCACTATGCTAATAATTGCAAAGTTAAAGATACAATTAAACAATTAAAAATCACGGATGAAGATAAAGACAAACTGATTAAAGTCCTTGAGTTAAGAAATTTTGAGTCTAGTGAAAACGAAACCATGATTAGTCATATTGAGTCTTCTGAAATCTATTCTTCTGATTCACAACCTAGTTTGCCTAGGATTCAATTTGGTCGCCAAGATA

CACACACACACACACACACACATATAAGTAATTGTTAATGTATATAATGTATCAATATACTAATATGTGAGCTATATAGCATATTATATAAAATATATAATCAGGCAGGCAAAGAAAGTGATCAATATCACGCTTGCCACTGCCTTTCAATTTGACCTGCCACCAGCACATGCACTAATATTAATATTAAATAATATATAATAATAATAGAAAAGGTGATGGGAAGCTCATTATTTCGGTGGGTGATGTTTTTGTAGGATATTAATAATATTAATAAAATTAAAGGAGAAGATAAAAAGGCAGAGATCATCTTATCATGTTTTGTAGGTATGATCTTTTCTCTCCTTGTGGGTATGATCTTATCATGTTTTGTGGGAAGCTCAAGAACTTGTTTTGTTGGGTTTGTGTTTTGAAGCTATATATATATATATATATATAGAGAGAGAGAGAGAGAGAGAGAGAGAGAGAGAGAGAGAGTAAAGAGCTGAGACAGTGATAACAAAGTTTCGCAGAAGCTATATCGATACACAGAAGAGCATGAGCAACTCAATCAAACCCGATAAAGAAAAGCAATGAAGCAGAGTGTTTTGATCGAATATCCCAACTGGTATTTTTGAGATATTCGGGCTGATAACGTGTTATAAAACTAAAGAGATTATGGAGAGAATTGAGAGAATTCAAATAGCAATAGGAATGATGTTCTGATGTTTTCATTCAATCTTTTGTTATGTATATTACATATAAGGAGGATACCTTATTTTATAGGCAAAGGCCTTGGCTAGGGAAAGGAAAGCATCTGAAGGTGCAGGAAGTTTCCTTCCGACATATGAGCTCCACTTTCATCATTTTACAACAAAGGACATCTTTTGTAATGACTTATTAAGTATTTATGGGCAAGAACCATTGTTTTCAAGTCATGATTCCTATTCTATTATCATAAATGTAAAACAACAATATTCCTAGGGATATGAAAGAGGACATAAGAACTATGTTATGAGAT

>Chr4-11; OZ022209.1; 22171255-22172245; dir; B;

AGTTTATTTCTGGAGGCGATAATTTTGCAATGATCTGCAACTGAGGAGAGGTCCTCCTTTTAAAGCCTCGGGGCTGTTGTCGTGTCAAATAAAAACAAGAAGTCAAAAGCAGCCTACAGTAGAGCGGCATCTTCACTTTGAGGATTCTCGCTGAACTTTCCGCAAACAGCCAAAAAGAAAAAGCAAACTTTTTACAAAAGCAAAAGCAAAAGAATCTTGCTTTAACCAGAGGAAAAATACAGTAAAACGACAAGAAAAATTATCTAGCATGTCTTCATTAGCAAAAAGGTCAACCTCATCTTCAGGCTGGGTGGCAGATGCTGAAGCCATAGATTCGTCATCCAAATCGAGAATCTCGAGGACTTTCTTTTTCAAGTCTTTTTTAGAAGATGATGATCCTGGCTGGTCTATCAACAGTTACAACCTTTGGGAAACACTCAGATTAGAATGAGTGACCAAAGGCTGAGCAGCAACAGAGTCGGGAGATGGGATAAGAGCTGGGATGATCTTGGTGGGAGCCAGGTGATCCCTAGTGAATTTATCCCACCATCTGATGAAGATACTTCGGACTAAATTTGGAATATAAGTCCATTCCTCATCGACATAAGCTGAATTAACATATTCCCATTTTAATATCCATGGTAGTTCATACATTATCATGAACCACATGAATAAGGTCCTTTGATCAGGATGGTTTTTATTCCATTTTCAAAATTTCATTTGAAAAGATTCCTTAATTTCAGGAGGAAGGTATTCCAAAGTAGGACCCCAAATCAGAAAGAAGCTCTTGGACCAATTAGGGAGTTCTTTCAGAACTCTAAGCTTAAACTGGATAAACCAAGTATGAGAATAGTTTTGATTTTGGAAACATAAAACATATTCCCATGCCTTTTTATAATCCATAAATTATAGGAATATGGCTAATTTCAGGATGGATAAATCTTTTGGCCCCTTCAGGGCTAGGGCCCCATTCTTCCAAGGAAATGACATTTAGGATTTG

TGAAATCGGGATATTGGCAAATCCAGATCGTCGAAAAGGACAGGTACAAAACAGCTCTTAATGTCCTTTTTGGACATTACGAATGGAACGTTCTACCTTTTGGATTGAAAAATGCCCCTTCTGAATTTCAAAATATTATGAGCGATATATTTAATCTTTACACTGCTTTCACCATTGTTTATATTGATGATGTTTTAGTCTATTCTAATTTTTTGTAATGGCACTTCAAACATCTGAATATTTTCTTGAACGTTGTGAAACATGCAAGATTAGTGGTATCGGCAAAGAAAATGAAACTATTTTCTTCAAAAGTTTACTTTTTGGGACATAATATTAATAAGGGACTAATAATTCCTATTGATAGAACAATATACTTTGCTGATAAGCTCCCTAACGAAATTAGGGACAAGCAACAGTTTCAAAGATTTTTAGGAAGTCTTAATTACATAAAGGATTCTTATAAAGATTTAGCTCATGACTCAAAATTATTATATCAAAGGTTGAAAAAGAACCCGATTTCTTGGTCTGATGAACACACTAAAACTGTTAGGAGATTGAAATTTAAAGTTAAAGAATTTCCTTGTTTAGCTTTAGCCAACCCAGAAGCCTTTAAAATCGTAGACCGACGCTTCAAAAATAGGGTATGGGGGAATCTTAAAACAGAGGGTTGATAACTCGGCACAATTAGTTAGGTTCACCTCTGGAGTTTGGAAACCAGCTTAAAAGAATTATTCCACTGTTAAGAAAGAAATGCTAGCCATCGTCCATGCTGTCTCTAGATTTGAAAGTGATCTTTTAAACAAGAAATTTTTGATAAGAGTAGATTGCAAGGGGGCCAAAGACATTTTGTTCAAGGATGTTAAAAATCTTGCATCTAAACAAATTTTTGCAAGATGGCAAGGAATTCTTTCTGCTTTCGATTTTGATATTGAATTTATTAAAGGTGAAACTAACGCACTCCCGGACTTTTTAACTCGAGAAGTTTTGCAGG

GTGCGAACTAGAAGTTCCGGATAGATGACTCGAAGTAAGAGCTCTTCTGCTCTTACCAAGTCATCCCCTAATGATCCAAAGCCACTGATGTCTTCTTCTCCATATAATAAGGGTAAGGAGAAGCAGATTGCTGAAAATCCATTTCGTGAATATATGAATATGTCTATAGCAATCATTGAAATGAATGATCTAGATAAACACCCAAATTTTCTGGCTGAAAAATGTTTAAGGGATAATATTCCTTTCTTGAATAAGCCTCGTATTTTCTATGAAACTCTTCTAATTGAAACTAAGTCTGTTAATATCGTTCATTGTCATAGAGGTGAAAATACCAACACCATAGTATTCTCCAAAATCCAAATCCTAAATGTCATTTCCTTGGAAGAATGGGGCCCTAGCCCTCAATGGGCCAAAAGATTTATCCATCCTGAAATTAGGCCATATTCCTATAATTTATGGAATTATAAAAAGGTATGGGAATATGTTTTATGTTTCCAAAATCTAAACTATTCTCATACTTGGTTTATCCAGTTTAAGCTTAGAGCTCTAAAAGAACTCCCTAATTGGTTCAAGAGCTTCTTTCTGATTTGGGGTCCTACTTTGGAATACCTTCCTCCTGAAATTAAGGAATCTTTTCAAAAGAAATGTTGCAAATGAAATAAAAATCATCCAGATCAAAGGACCTTATTCAAGTGGTTCATGATACTGTATGAACTACCATGGATATTAAAATAGGAATATGTTATTTCGGAGGATGTTGACGAGGAATAGACTTATATTCCAAATTTAGTCCGAAGTATCTTAATCAGATGGTGGGATAAATTAATTGGGGATCACCTGGCTCCCACCAAGATCGTCCCAGCTCTTATCCCATCTCCCGACTCGGCTGCTGCTCAGCCTTTAGTCACTCCTTCTAATCTGAGTGTTTCCCAAATGTTGCAACTGTTGATAGACCAGCTAGGATCATCATCTTCTAAAGAAGACTTGAAAAAGAAAGTCC

>Chr4-12; OZ022209.1; 22344315-22347815; rev; B;

AACCAAGGTAGCTAATATACAAGTTCTAATGGTGATCAATCATCCAAACAAGCATATTAACTGATAAGCATAAAGAATAGGAATATGAATCATGAATCAAACTTGAAACTCAAAACTTCAACTTATAAACCCAGAATATTCATGTTAGGTTACATCATAGCCCTAGTTATGAATTTAGCTACTCATAATGGAAATGAAACTTAAGCAAAAATGAGAAAACATATTGAATGCAATAAGAGTAGAGAAAACCAATTCTGAAGTTGCTTGTGGTTTCCAAGGCTTCTCCAGGTGCTTATCCACTTCTAATGCTACTCCAAGAACTTTCTCATGTCTAGAGTATGGCAACTAAAGAGGTGATTTGGGATGAGTTTGGGTGGCTGGTGATGGCAGAGAAAGAACAAATTCGTAGGAATATGGAAGGAAGGCTTGCTGGAGTTGTCCTTCTGATTTTGGGCTGAATGAAGTGATATAGGATGGCTGGAGTGATTTTTGAATGGAGTGACTCCTCTGCACTTGAGTAGCTGACACACCTCCCTTAATTGCTCCCTTAATTGCTCAACATGTCAGGTCTAAAGTCAAGGAAAGACCTCCCTTAATTGCTCCCTCAATTGCTCCCTTAATTGCTCCCTCAATTGCTCCCTTAATTGCTCAACATGTCAGGTCTGGTGTGTACATGGGCTCACGAATTGGGCTCAACTTCATTATCTGATGCATTTGAGTAGTCCATCTTGAAGTTCAGACTCTCATCTTTCCAACCACAATTTATTTGGGCAAATCCAAGTTCGGAGGAAGATGTTAAGGCCGAAATGAGTTGCTAACCCAGTCTGCTCGTTTTTGCACCATTTTGCATCTTTTCTTATCCAAAGACACCATTGTTACCTAAGGCCCAATGATCTGAATGATAAACAGAAAAAGGGATTAAAAGCACATGTTTGACTCAATAACATAACCAAATCATAAGCAAGAAGGGTACAAAATGATATACAATTGTGAACTAATC

AAGCCCTAGCAGTCTTACAAAAAATTGGTACCCTAGACCAACACCACCGGATATCCAATTTGAAGAAAGAAACTCCCAAACCCAGTTTTCTGTTTCTTCTGATAAATTGTATGAATGGAATATTGATGGTCTTTCTGAGCAAGAAATCCTAAATAAGTTGCAACATATGTCTATGGTCGCCAATAGTTACATCACCAATCACAGTTTCAGACAATCTGAAATTGTTCCTCTGTTAGTTACCGGTTTCACTGGTACTCTCAGATGTTGGTGGGATAAACACCTTACTCCTGAGAATAAAAGTCGAATCATTTATGCTGTAAAACTTAATGAAGACGGCCTCCCAATATTTGACGAACAAATAGGTCAAGGTATAGAAGACGGTGTCAATACTCTGTTTTACACAATAATCGAACACTTCGTTGGAACCCCTAGTAATACCACTGCTAGGATCCATGATCAGTTGAGTAACCTTAGGTGCCCAAAGTTCTCTGATTTCAGATGGTATAAAGATATTTTCATTTCTAGAGTCATGCTTAGAGATGATAGTAATCAACCATTTTGGAAAGAAAAGTTCATTAATGGTCTGCCTAATCTGTTTGCTCACAAAATACGAACAACCCTAAGTAATGAACAAGGTCAAATTGATTGGAATTCATTAACCTATGGAAACATTATAAGCACAATTAATCAAGTTGGTATGAAAATGTGTATTGACTTTAAAATTAATCGCCAAATCCAATCTGATAGAAAGTCTACCAAGTACGAGTTAGGAAATTTCTGTGAACAATATGGTCTCACTAGTATCCCTCCTTCTCGGAAACACAAGCCTAGTCCTCTTCGAAAGCGTCATTCCCCGAAGAAGAAATTTCTTCAAAAGGATGAATTCTATAAAAAGAAAAAGTTCTCTCCTAAGAAAAATTGGTCTAAACGCCCGAAACAGAGTCGCTCTAAGAAAGATAAAAGCAAAGTTAAATGTTTTAAATGTCAAAAATTTGGTCATTATGCTTCTGAATGTAAAGTAAAAGATATTATTAAACAAATACAGATTACAGAAGATGATAAGAACAAGTTAATTCAGGTTTTGGAGTTGCGAGACTCTGAAATGAGTGATGTCGAGTCTCTAGTATCCAGTTTTGAGTCTGAGTCTAGCCAGTCTTCAGATTCACAACCCAGTTCCCCTAAAGTTCAATTTGGTTGCACTGATAGATGCTGTAATAGATTAAAATCAGTCTCAGTTCTCACTAAGCAAGAAGAACAAGAAGAACTCTTAGTAGATTTAATAGGCAGAGTCCAAAACCCTGAGCTAAAATCTGAATATTTGAGAAAATTAAAAAAGATCTTAAGCCAGGATAGCCAGAGTTCTCTTTTGCCTACTCCTCAGCCAATTAGCATCAATACTACGTTAGAAAAATTCAATCGTAGGAAAGATGTTACCATTCATGATCTTCAGTTAGAAGTCAAACAAGTTAAAAAAGAATTAGTTGAACTTAAACAGATAAGTCAACAATTGCAAACTGATAATTATAAGATCAAACAAGACTTATTTGAGTTAAAACAAGAATCTTGTAAACCTAGTAACCCTTCAGACAGTCCGCATCATTCTGATGATGAACATTATGAAGAACAGCCAGTTGTCAACCTAATCAAACAAGTTCAACTTAGAAAATGGTATTCCAAAGTCACGATTTATGTTCATGATTTTGAATTCACCACAGTTGCATTATTTGATTCTGGTGCAGATCTTAACTGTATTCAGGAAGGTCTCATCCCTACTAAGTACTATCAGAACTCTAGAGAATCCCTTAGCACCGCTTCAGGGAAATCTCTCCAATTGAATTTCGAGTTACCCAAAGCCCATATTTTCCAAAACAAAGTTTGTTTTAAAACTTCATTCGTCCTAGTTAAAAATATTACTGATGAAGTTATTTTAGGATTGCCTTTCCTTGCCTTGATCTATCCTTTTCAGGTAGAATACGATGGTATCATTTCTACCCACCTTGGAGAATCGGTCAAGTTTAGTTTTCTGTCTAAACCAGAGTTGCATAATCTACAAGCCTTGCAAAAACAGTCAGTTTCTAAATTCGTCCAAGTTATCCGAAAAACTAACCAGTTGAATTTTCTTAAAGAAGAAGTTAAGTTTAAACGAATTGAACAACAACTTAGTGATAGAACTTTACAGTTGAAAATCCAGTCTTTTGAGAACAAATTAAAAGAAGAAATATGTTCTGATTTACCTACTGCTTTTTGGTATAGAAAACAGCATGTTGTCTGTCTTCCTTATATCAAATCCTTTAGTGAGAAAAATATCCCCACTAAAGCAAGGCCGATACAGATGAGTCAAGAATTAATGGAAATTTGTAAAAAAGAAATTCATGAGTTGCTTCAGAAAAATATTATTCGAAAAAGTAAATCCCCCTGGTCATGTCCTGCTTTTTATGTCCAGAAAAATGCTGAACTAGAAAGAGGAGTCCCCAGATTAGTTATCAATTATAAGCCCCTTAATTCGGTCTTAGAATGGATTAGGTATCCCATACCTAATAAAAGAGACCTTATTAATCGATTAGGAAAAGCAATTATCTTCTCCAAATTTGATATGAAAAGTGGTTTCTGGCAAATCCAAATTGATGAGTCAGATAGGTACAAGACTGCATTTGTTACCCCTTTCGGTCATTATGAATGGAATGTCATGCCGTTTGGTTTTAAAAATGCCCCTAGTGAATTCCAGAATATTATGAATGATATCTTCAATCCTTACAGTCATTTTTCGATTGTTTATATTGATGATGTTCTAATATTCTCTGAGTCAGTTGAACAGCATTGGAAACATTTGCATAAGTTCCTTCATATAGTCAAACAGAATGGTCTAGTTGTTTCTGCCAAAAAGATCAAGTTGTTTCAAACCAATATTAGATTTCTTGGTTTTAATATCTGTCAGTCTCAAATTACTCCAATAGACAGAGTCATACAATTTGCTGATAAGTTCCCTGATCAGATTCTTGATAAAAATCAACTCCAGAGGTTTTTAGGATCTCTAAATTATATCTCTGATTTTTATCCAAATTTGCGAAAACAATGTAAGCCCCTATTTGACAGACTAAAGGATAATCCTCCTCCTTGGTCTAATATTCATACTGAAATCGTCAAACAAATCAAAACCCATGTCAAGACACTTCCCTGTCTTGGTATTCCTTCAGTCGATTCTTTCAAAATAGTTGAAACAGATGCCTCTGACATTGGTTATGGTGGCATTCTCAAACAGAAAGTTAGTCCAGAATCCCCAGAGCAAATTGTTCGTTTCCACTCTGGAACCTGGACCCAGTCGCAATATAATTATAGTACTATTAAAAAAGAAATATTAGCTATTGTATTATGCATTAGTAAATTTCAAGATGATTTACTTAATCAAAAGTTTTTAATCCGAGTTGATTGCAAGTCTGCAAAACATGTTTTAGAAAAAGATGTTCATAACATTGCATCTAAACAGATTTTTGCACGATGCCA

GCATACATATATATACATTCCAATAGTTCAAGTCTCAGTCTAGGGCAAAACATCAGAGCAACTACTAAGAATTAACAAAGGAATCAATCCTTTTACAAAACAAAAATCCTACATCAAAAGAAAGGAGTGGAAGATGGGAAACGGCCCGGTGGTGGATTCCTGTGCACGTCTACTGCCTGGAGGCGCAAAACAACAAAAAGGGTGAGTGGACCCAAAACAAAGTTTAGAAAATAGCATACGAACATACTAACCCCACTGTAAAAACAGTTATACAAACTAGCTTATTCTCTTTATTTCTGAAATCAATGCATGTATATAATTATACATTTTGAAAACAGTTTAAAACTACCACCTAGGTGTACCCTTATTTCCGTCAATTCCTTATATCCGTCAATTCCTTGCATCACCATGGGTGACACATACTCGCAGGTCTCAGTGACACGAGGTCAGTCCGAGCGCAACTCGCAGGATTCAGGAGACCGTAGTCCACCTTTATCCGCATTACTCGCTCCACACGAGTTCGAGTACCAAGGAACTCGTGGGGCAACTGTCAAGCATGCTGACTAAACCGAAGGCAACCAATATAATCCGAAGGCAATATTTAATCTCTGGGTACAAGGTGATTGAGGAAAATATAACATTTTTGAAGAAAATCTGCTGGATAACTGAATTTCTGTAACCTTTAAAATTCTACTGCCATTTATCTGATAATTAACGAGAATATCTTTTCCTATATTCTTTAAAGGAGATTTCACTCGGCAGCATGCAACATAAAATATTTAACATAATAAAACAGCTTATAACTAAAACTGAACTGTTTAAATTCATATATTAAAACAAAGAGTCCACTCACTAATAGTCCGAGCTCACTGGACCTCTTGAAGGTCCCTCCTGCGGGTCAACAGGTGCCCGGGTGCCTGATTCAATGACCATAATATTTTATTAATACAATATAGTATTAAATTAAAAACCCTACCCCCAGCTCCTAAAAGTACGTGCG

>Chr4-13; OZ022209.1; 22367159-22369525; rev; B;

ACACAAAAAACTTTAAAAGGATAGTAGTTCTTTTCTTATTCGGATATTTGGATTTTCTTATTGTACGAACACCATTGTTGCAGCTATATTTCTTACATCTAAAGCTTGTTTTGTATACCCACTCTAGCTCGTTGAAGCATATGAATACAAAAGACAAGGAGGAAAACTAAGAAAGGGAGAAAAGTATGGAGGAAATTGAAAAAACAATGATGTCCGTACGATAAGAAATGCTAGACATCCTGATAAGAGAAAATAAACCTATCCACCCATGGCACAAAGTAAATCTCACGTCTTTTACTCAATAAGGCTATTTGCTCTCATTTATTGAACTGCAATCTGAAATCCTAAATGGAGAACAGATGACATTGTTGAGGAAAAATATGAGTTTTAGGACTAAAATGTGTATTTTGATGAAAATGCACATATAAACTCGTTCAAGAGATGACAAGTGAACAATTGTAATGAAATTTTTTTGGAATGCGACATCATGGATCAAATAGGTGTGTAGACCAAAAGATGAAAGACCACATTAATTGATTTTAGAATTGAAGACTAAAATGAAGACTTTTAAAAAGTTCAAGAACCACTTTTAAATATTTAGTCTATATTATGCATTATTTTTAGTCTGAATTGTAATATATTTAACTTAGAATACATTAGATTTAGTCTATAGTATGTAAAATAGTTTATTATTACAAATTAAGATAAAAAAAATTGAATATATTTTTGTTTTAAAAAAGTTCATTTTCATTGCCCAACCCTCGTGGGTGGACTTGGGTTGGTGACAAGACCCGATCCAAATTCCGCTTTAGAATCCAAGCCGGACTCTGTGCGTGTTCGACACCTGGCGGATGTCGGGCACAAATGACCTATTTGCCCTTCTATGCAAAGCACGATAAAATAACAAGGTTGACTTCTACCAAAAATTCGGTAGAGTTTCCCTTGTAACTGGCTGAATACCAAACCATTTTCACCTGCCAAACAAGTATATATATATATA

TCATTATTTTGCTGCATTAGTCTGAAAGTTGTTTAGGTTGCATCCATTTCTAATTTCTTTCTGATCTTCACTCTATTTTATATTCAGTCGTCTTCTATTTATCTCTCTTTGTTCTGTTGGTATCAGAGCCGTAAGTGTTGCGGTCTTTGTCTAAGTCTGCTACTCATCCCCGCCCAAGTGGATGGTTTAAGCATCTGAGTAGAAGGTTTTCATTTTTTCCTTTTCTTCCCTTTTATTCTTTGGGTCCTCTGTTGGTTTTCTCTAAGGTCTGACCCGTGAGTCCTTTTTGCTAGATCACGTAAATCCCAGGGAGGTGGTGTGTGCTGGGCAGGAGACAGAAAATTGAGAAAATTAATAGGTTGCTTAGAGTCTAAACGGTCGGTTTAGGATCTACTGTTTATCTTCTTCCTCGGTCTGTTTAACCTAGTTTCTTGAGTAGTCATGAATAGTTTATTTAGAACGAAGTCTTGTAATTCTGCCAGTTCTAGTGCCAGTATAGGACGTCTTCCAGAGATAGTTAATGAAGAACAATTTGAATATAATTCCAGTAGTAAAGAACAATTAGATTTTAGAGATTGGAATATACCTAAAGTCCCCAGTCAGAAAATCTATAAGAAACATTGGTTGCCTTCTAGTTTCAATAGCACCACACATGTTAAAACAGTTGAACAAGTATACGCCTTAAGCAAAGAACATGAAACTTGCCAGTTGCTTAATCTTGAGTCTATTTTAAAACATAAGAAAGATGGTAATAATTTTCTGCATATTGGTTTAGTTCAAGTTGCTGTCAAACCTCTGACACGGTTAGGATTGAAAGCCTCAATCCTGCTATGTCTTCGCGATGCTAGGTTCACTGAATTTAGCGATAGTACCCTTGGAATCATTGAGTCCAGTCTGTGTAATGGCCCTGTCCATTTTGACTGCTATCCTGATTTCACTGTAAGCCTTAGTGATCCTCATATCCTGAGAACTTTGACTCTGAATATTAAGACTGAAGGTTATAACGTTCTCCCGGGAACTCAACCTCTAGCCTTAGTATACAGAATCTATTATAAGGTCACAGGAACCAATATGAACTTCCAAGCCCTTAATAAAAGCCCGAGGGATCAAACCGTCCTGATCCAGAGTCACACCTCAGACGCCCATATACAAGTACCTCATACCATTAAGTGGTCCGAAGTTGCCCTACCAAAAGATTGGACCTTAGTTACCGAGAGCCAACCAGCTCCCATCCAACGTAGTCTAAACATTTTAGACTACATTCAACAATACTTAGATGGCACAGTTAAGATTCAGTTCGACAGTCATCCTCTACGTAAGTCTAATGTCCAGTTACAACAGTTGCCCACACCTGGTCAGTCCCAGAGGCATGCCCCTGCTCGGCATTCCTTTGCTGCCTCTTCCTCCACTCTAGAACGAGACCTAGAGTTAGAAAAGGCTATGATCGATTTCAAAATTGAGTCCCTGAGAAAAACCTCTCAGGTTACCCAACCTTGCTACGGCACGGTTCCAGTTCAAGAAGACAAGTCAGAATCTCCTGAGTCCCCTACCCAGTCTGACTTTGTAGTTGAAAATCAGCTTAGAACCCTCAATAAAGAATTTGAAATTAATTGGGTAAGTCTAAATCGTCATCTAAAAGCCCCTGAAAACAAGCCAAGGCGAGATCTTTATCATCAAGCCTATCCTAATAGCAAACACCGAGATATGATCTTTAAACAATGGAAAGATTATATGCGGTCAGCCAAAGTTGAAATTTTCTACCTTGACTTTATTGAAAGCCGTTACATGTCTAATGATGAATTGAAAACCCTAAATAAAGAAAAATGGAAATTGGTTGATAAATCTGTAGTTGAGTCGAGTCACCCTCCAGTAGAAACAATTATAATAGAACACCAGAAAACACCAGTTGCCGCCACTCCTTTCAAAACCTTTGAAAGCAGTGATCCTAATAGGAAATTAATTGAACAAAATAATTACACGAACCAAAGTCTAATCACCATAGGAAAACAGTTAGACACAATTGAAACCAAGGTTGATCTGCTGAGTCCGCCTGTTGCAGACTCCAGACCCAAACCCAAAGTAGAAAAGCCCATAGTACAGTTCCAAGAACTTAAGTCCAGTCCAGTTCTCAAGGTCAATTCCACAATGAAGAAAATAGAAGACATGTTAGCTCAGTTGACCCCGGTCAAACCCGAGAAAGGTGATACATCTGGTATTAAAACGCTAGATGCTTTTACCGCTTCTCAATCTGAGTCAGATTCAGTAAGTTCAGAAACTAGTGACATTTCTAAAATTGAAAATGCTTTCAAAGATTTAGAAATTCAAACTAAGCCCAGAGTTAAAAAGATAGAAAAACATGTAAGCCCT

GTTGTTAAAATGTTAACTCGCAATTGTACGGGCCAATTAGTAGTATAGCAAGACAAGTACGAGGTCGTACCCACGAGGACTGAGAAGTTAAAACTAGTTATGTACTTAGATTACCAAATTCAAGAAAGTAGGAGGTTAAGTCTAAAACAGAATTTAAAGCAAGAGTGGTCGTAATTGTTGAACAAATGAGGCAAAACGAAATTTCAAACAAAAGCAAGGTGAACAAATGTAACAAAGAGTACTTTTGTAGGCTGGAAAACAGGTTGGGATACTAGGGCATTCGATTCACCATAACAATTCAATTCTGGGTTATAAGGAGTCAAGTGACAAGTCTCATTCGGATTCAAAGGTTGATTCTAGCTAAAGTATGATTAGGCCATGGTGTCTGGTCCTAACCTTGTGCTCCTAATTCCCTAACTTGATTATTTGTTGTCTAATCTAAATTAAGCATTAGGAACCCATTAAGCATAGAAGAACAAGTTAAACAACCAAGCATGTATCTAATCCCTGTTGTCGAATGTCCATACATACTTTTCTTATTCATAGTTCAATTCAAATCAAAGCATGGTGTCTACTCTTGATTCAAACCAAGGTAGCTAATATACAAGTTCTAATGGTGATCAATCATCCAAACAAGCATATTAACTGATAAGCATAAAGAATAGGAATATGAATCATGAATCAAACTTGAAACTCAAAACTTCAACTTATAAACCCAGAATATTCATGTTAGGTTACATCATAGCCCTAGTTATGAATTTAGCTACTCATAATGGAAATGAAGCTCAAGCAAAAATAAGAAAACATATTGAATGCAATAAGAGTAGAGAAAACCAATTCTGAAGTTGCTTGTGGTTTCCAAGGCTTCTCCAGGTGCTTATCCACTTCTAATGCTACTCCAAGAACTTTCTCATGTTTAGAGTATGGCAACTAAAGAGGTGATTTGGGATGAGTTTGGGTGGCTGGTGATGGCAGAGAAAGAACAAATTCGTAGGAATAT

>Chr4-14; OZ022209.1; 24107108-24114200; rev; A;

GTAGGATGAGAAGATAGGAGATGGTTTGAATGAAAGTTAGTGTGAGTAGAAAAGCAGCTGCAATGACAAAAATGCCTGCTCAAGATGTCCTGAAATACTTTTGTATCTAAATTGCATTCCATCTGTTCCACAGGTTCCTCTAGTATTTCTTTAGTTCCTCACATATATAAGCATAATAAAAGTTATGAGTATTTACAATAAGCCCATTCACGCCGTTGATCATTGATGCAGTATATTTCTTTAATATTTCGCTTAACACTTTATCTTCAAGTAGGACTTTACTCTAAACCAGCAACTCCAGATCTCTTGGAGTTTTCACCAAATTACCTGTACAGTAAGCATAATCACTCATTTCAAATGCAATGATATTTTTGATAACTTCAAGTTCAGTACCTTATTGAAATGGTGGGATCTTTAGAACCCCTTTGCTCTGCCTGAATTCACAGGACCCGATCCAAATTCCACTTTGGAATCTGAGCCAAACCCTGTGTGTTTCCGACACCTGGCAGTGCCGGGAACAAATGACCTAATTGCCCTTCTTCTCAAAACTCAAATTCATTTTAAGTTTGACTCCGGCCGAAAATTCGGTAGAGTTTCCCCTGTAATTCGAACATTCCCGAAATTTACACTTGTCAACAAGCATTTAAATATATACACCAACTACCAGAATACATATATAAATACGTCCTAGGGCAATATTAAAGCAACTATACAGAATAAGTTACAAGTGTAATTTAACTATACAAGACCCTACATCAGCAAGAGATATGGAGGGAGGCGAACTCTGCCCGGTGGTGGCTCACTGCTCACAAATTACTGCCTATGGGCACAAAACATTGAAAATATGAGTGGACCAAGAACAAAGTTTAGAAAATAGCATATGAACATACTAACCCCACTGTAAAAACAGTTATAAAAGCTTAAACAAGTTTTTCAGCTATACTCGTACTGAAATCAATACATTCTTATATATATATATATATATATATATATATATATA

AGCTTCTGAGTAGCAGGTTTTCATTTCTTCCTTTTCTCCCCTTTTATTCTTTGAGTTATCTGTCACTTTTCTCTAAGGTATGACCCGTGAGTCCTTTTTCCTAGATCAGTAAGTCCCCAGGGAGGGCGTGTGTGGTGAGCATGAGACAGAAAATTGAGAGAAGTTAATAGGTTCCTTAGAGTCTAGACGGTTGGTTTAGGATCTACTGTTTATCTTCTTCCTCAGACAGCCAAACCCAGTTATTTGAGTAGTCATGAGTCACTTATTTAGAACAAAGTCTTGTAGTTCAAACAGTTCTAATATCAGTCGACTCCCAGAAATAGTTAATGAGGAACAATTTGAATATAATTCCAGTAATAAAGAACAATTAGATTTTAGAGATTGGAACATACCTAAAATCCCCAGTCAGAAAATCTATAAGAAACATAGGTTGCCTTCCAGTTTTAATAGCACAACACATGTCAAAACTGTTGAACAAGTATATGCCTTAAGTAAAGAACATGAAACATGCCAGTTGCTTAATCTTGAGTCTATTCTGAAACACAAGAAAGATGGTAATAATTTCCTGCATATTGGTTTAGTTCAAGTTGCCGTTAAGCTCTTGACACGGTTAGGATTGAAAGCCTCAATCCTGCTCTGTCTGCGTGATGCTAGTTTCACTGAATTTAGCGACAGTACACTTGGAGTCATTGAGTCCAGTCTGTGTAATGGCCTTGTCCATTTTGACTGCTATCCTGACTTCACTGTAAGCCTTAGTGATCCTCATATCCTGAGAACTTTGACTCTGAATATTAAGACTGAAGGTTATAATGTCCTTCCCGGGACACAACCGTTAGCCTTAGTTTACAGAATCTACTATAAGGTCACAGGAACAAATATGAACTTCCAAGCCCTCAATAAAAGCCCAAAGGATCAAACCGTCCTGATCCAGAGTCATACTTCAGACGCCCATATACAAGTTCCCCACACCATTAAGTGGTCTGAAGTTGCCCTTCCCAAAGATTGGACCCTAGTTACAGAGAGCCAACCAGCTCCAATCCAGCATAGTCTAATCAATTTGGACTACATCCAACAATACTTAGACGTCACAGTCAAAATTAAATTCGATAGTTATCCTTTACGAAAATCTAATGTTCAGTTACAACAGTTGCCAACTCCAGGTCAATCACAAAGGCATGTCCCTGCTCGGCATTCCTTTGCTGCTTCGTCTTCCACTCTAGAACGAGACCTAGAGTTAGAAAAAGCCATGATAGATTTTAAACTTGAGTCCCTGAGAAAAACCTCTCAGGTTACCCAACCTTTCTACGGCCAGGTTCCAGTCCAAGAAGATAAGTCAGATTCTCCTGAGTCCCCGACTCAGTCCGATTTTTTTGTCGAAAACCCTGCAGCAGTTGATAACCAACTTAGAACCTTAAATAAGAAATTTGAAATTGATTGGATACGTCTAAATCGCCATTTAAAAGCCCCGGAAAACCAATCTAGACGAGATAGTTACCACCAAGCCTATCCTGATCGTAAAAGTCGTGAACAAATCTTTAGTAAATGGAAAGATTACATGCGGTCTGCCAGAGTTGAAATTTTCTACCTTGATTTTGTTGAAAGCCGTTATATGTCTAATGATGAGTTAAAAACATTAACCAAAGAAAAGTGGAGTCTGTTAGATAAGTCTGTAGTTGAAGCCAGTCATCCCCCGGTAGAAACCATAATAATAGATCACAAGAAAGCACCAATACCCGCCACTCCTTTCAAAACCTTTGAAAGTAGTGATCCCAATAGAAAGCTAATCGAACAAAGTAATTACACCAACCAGAGTCTAATCGTCATAGGGAAACAATTAGACACAATTGAGACTAAAATTGACAAAATAAATGCCCCAGAAGTAGAACTCAAGCCTAAACCCAGAGTTGAAAAACCCATAGTCCAGTTTCAAGAGCTTAAGTCCAGTCCTGCACTTAAAGTCAATTCTACAATGAAAAAGATAGAAGAAATGCTAGAACAGTTGACCCCGACTAAGCCTGAGAAAGCCGAAAAATCTGGTCTTAAAACACTAGGTTCCTTTGCTGCCTCACAATCTGAGTCTGAGTCAATAAGTTCCGAAATTAGTGATATTTCAAAAATTGAAAATGCCTTCAAAAATCTAGAAGTCCAAACTGAACCCAGAATCAAAAGATTAGACAAACACATAAGCCCCACTAGTTTAACAAAAAATTGGTACCCCAGACCAACACCTCCAGATATGCAGTTTGAAGAAAGAAGCTTCCAAACCTAGTTTTCAGTTTCTTCTGATAAATTGTATGAATGGAATATTGATTGTCTTTCTGAGCAAGAAATTCTCAATAAATTGCAACATATGTCTATGGTCGCCAATAGTTATATCACCAATCACAGTTTCAGACAGTCTGAAATTATTCCTCTGTTAGTTACCGGTTTCACTGGTACTCTCAGATATTGGTGCGATAAGCATCTTACCCCTGAGTCTAAAAATCGTATCACTTATGCTGTAAAACTTAATGAAGATGGTCTCCCTATATTTGACGAACAAATAGGTCATGGTATAAAAGACGGTGTCAACACTCTGTTTTACACAATAATTGAGCATTTCATTGGAACCCCTAGTAATACCACTGCTAGGATCCATGATCAGTTGAGTAACCTTAGATGCCCAAAGTTGTCTGATTTCAGATGGTACAAAGATGTTTTCATTTCTAGATTCATGCTTAGAGATGATAGTAATCAGCCATTCTGGAAACAAAAATTTATTAATGGTTTGCCAAACTTATTTGCCCACAAAATACGTACAACCCTGAGTAATGAACAAGGTCAAATTGATTGGGATAATTTAACATATGGAAATATCATAAGCACAATTAATCAAGTCGGTATGAAAATGTGTATTGACTTTAAAATCAATAAGCAAATCCAGTCTGATAGAAAGTCAGCCAAGTATGAGTTAGGTAATTTTTGTGAACAATACGGTCTTACCAGTATCCCTCCTTCTAGGAAAAATAAGCCCAGTCATCTTAGAAAAGATCGTCATTCTAAGAAACAATTTTCTCAAAAAAAATGAGTATTATAAGAAAAGAAAGTTCTCCCCCAAAAAGAATTGGTCGAAACACCCAGATAGTAAGAAGAGTCGCCCTAAGAAAGATAAAAGCAAAGTTAAATGTTTTAAATGCCAAAAGTTCGGTCATTATGCCGCTGAATGCAAAGTAAAAGATGTTATTAGACAATTGCAGATTACAGAAGAAGAAAGAAACAAGTTAATACAAGTTTTAGAGTTGCGAGACTCTGAGACAAGCTCTATTGAAACCTTAGTTCCCAGTTCTCAGTCCGATTCTGACCAGTCTTCAGGATCACAACCCAGTTCCCCCAGAGTCCAAATTGGTTGCACAGATAAATGTTGTAATAGATTAAAATCTATATCTGTTCTCACTAAACAGGAAGAACAAGAAGAACTATTAATAGACCTAATAGGAAAAGTTGAAAATCCTGAATTAAAATCAGAATATTTGAAGAAGTTGAAAAAGGTCCTTAGTCAAGAGAATTCAAGTCATCCTACACCTCAGCCTGTAAGTCTCAATACTACTTCAGAAAAATTTAACTCCAGAAAAGAAGTAACCCTCCAAGACCTCCAGTTAGAAGTCAAGAAGGTTAAAAAAGAATTAGCCGAATTAAAACAAATAAGTCAACAATTGCAAACTGATAATTATACGATCAAACAAAATATAGCTAGCTTTTTATCCCCTGAGCATAAAAATCATTTAGAAAGCCCTCACCATTCTGATGAGGAACCTTCTGAAGATCAACCAGTCGTTAACCTGATCAAACAAGTTCATCTTAGAAAATGGTATTCGAAAGTCACCATATTTGTTCAAAATTTTGAGCTTACCACAGTTGCTTTATTCGATTCAGGAGCAGATCTTAATTTTATCCAGGAAGGTCTAATCCCCACTAGATATTACCAGAAGTCTAGAGAGTCCCTTAGCACTACCTCAGGGAAGTCACTCCAGTTAAATTTTGAATTACCAAAAGCCCATATTTGCCAAAACAAAGTCTGTTTTAAAACCTCATTTGTCCTGGTCAAAAACATCACTGACGAGGTCATCTTAGGTCTGCCTTTCCTTGCCTTGTTGTATCCTTTCCAGGTAGAATACGATGGTATCATTTCTACCTATCTAGGTGAGAAGGTTAAGTTTGAATTCCTGTCAAAACCTGAGTTGCACAATTTAAAAGCTTTACAAAAATAGTCGGTCTCTAAATCTATTCAAGTTGTCCGAAAGTCTAACCAGTTGAAATTTCTTAAAGAAGAAATCAGATTTAAAAGAATTGAACAACAACTTGCTGATAAAGCCTTTCAGTTGCAGGTTCAGTCCTTTGAAAACAAATTGAAAAATGAAATTTGTTCTGAATTACCTACTGCTTTTTGGTATAGAAAACAGCACGTCGTCTGTCTTCCCTATATCAAAACTTTTAGTGAAAAGAATATCCCCACTAAAGCTCGGCCAATTCAGATGAATCAAGAATTAATGGAATTCTGTACAAAAAGAAATCCAAGAGTTGCTCCAAAAGAACATAATACGCAAAAGTAAGTCTCCTTGGTCGTGTCTTGCTTTTTATGTCCAGAAAAATGCTAAACTAGAAAGAGGAGTCTCGAGATTAGTTATCAACTATAAGCCCCTTAATTCAGTATTAGAATGGATTAGGTATCCTATACCTAATAAAAGAGATCTTATTAATCGATTAGAAAAAGCAGTTGTTTATTCCAAATTTGATATGAAAAGTGGTTTCTGGCAAATCCAAATAGATGAGTCAGATAGATATAAGACTGCATTTGTTACTCCTTTCGGCCATTATGAATGGAATGTCATGCCGTTTGGTTTAAAAAATGCCCCGAGTGAGTTCCAGAATATTATGAATGATATCTTCAATCCATACAGTCATTTCTCTATTGTCTATATTGATGATGTCCTCATTTTCTCTGAGTCAGTTGAACAGCATTGGAAACACTTGCATAAATTCCTTCACATAGTCAAACGAAATGGTTTAGTTGTCTCTGCCAAAAAAATTAAGTTGTTCCAAACCAATATTTGATTTCTTGGTTTCAATATTTGCCAGTCTCAAATTTCTCCAATTGATCGAGTTATCCAGTTTGCTGATAAATTTCCTGATCAGATCCTTGATAAAAGTCAGCTCCAGGGGTTTTTAGGATCTTTAAATTATGTCTCTGACTTCTATCAAAATCTGCGAAAACAATGTAAGCCCTTATTTGAAAGGTTGCAGAATAATCCTCCTCCTTGGTCAGATATTCATACTGAAGTTGTCAAACAAATCAAAGCCCATGTCAAGACCCTTCCTTGTCTTGGTATACCTTCAGTTAATTCTTTCAAAATAGTTGAAACCGATGCCTCTGACATAGGTTATGGTGGCATTCTCAAACAGAAAGTCAGTCCAGATTCTCCAAAACAAATTGTTCGTTTCCATTCTGGAACCTCGACCCAGTCGCAATATAATTATAGTACTATTAAAAAAGAAATATTAGCTATTGTATTATGCATTAGTAAATTTCAAGATGATTTACTTAATCAAAAGTTTTTAATACGAGTTGATTGCAAATCTGCAAAACCCAGTTGAAATCGCCAAACACTATTTCCCTCCTAGATTCCATTATATGCCTTCCAGTCCTTCTAAGTCTCTCAAGTATTATAGAAATATATTACTTGAAACCAAGTCAGTTGAAATCAAACCGATAAAGGATCGTGATCACCCCGAAATCATCCTTTATCATTCACTCTATATCCACCAGATTATAAGCCAAGAAGCCTTCAGCAGTCGCCCCTATGAACTCAAGTTGCTCCAGTCAAAATTGCAGTATAATTATTCTGATTATATTGATGCCTGGTATACTATATTTCTTCACCAATCTGAAGATTTTAGTCATTCCTGGTTTATCAATTTTGATAATAAGTTCAAAAGTTCTTTCCCTTATTGGTTCCTCCATTGGTGGGAAAAACATGGTCCAGTTGATGAAATTTTGCCTGTTTCAGTCCTTGAGTTAATCCGCCACTACACCAAGAAGGCTAAGTTCTCCAAAGTTGATTTATTTTTCCCAAAGCAGTTGTTATTTATTGCCAAATACAAGGTTCCATGGATCCTCAAATGGTCCTACAGAATCACCAAAGACGCCCGAATATTTGCCCGCCAATTCTCCGTCAAATGGTGGGACAAATTTGAAGTTGATCGAATTGCTAAGTATGTCTACAATGATCTCCCAGTTGAGCCAATGCCTCGCCCAATTTCCCCTACTGGTTCTATCCGATCTTCCCTCTCAGTAGAAGGAAAATCCAAGGCAGAACTCCAAGAAATTGCTCGCCAGTTGATATTGCAGGCCTCCCAGATGGATGATGATGAGGACAACGACTCTCCTTCATCGTCCAGTTGTTAGCCCATTCAGAATCCTGCTAAGCCAAAGTCTTGGTACGAAGACAGTCAAGACCCCTATGATGCCTATGATCGCACTCTGACTAGGCTTCATTATGGTTGCCGACACCTTCACATGCCAAAAGTTGCCGACGCTTTCACATGCCAAAGTTGCCAGCATCATAATTGCCACAGGAAGAAGCATCTGCTTTTACTGTTCGCTTTTCCACCGAAAGCTGTCTCAGTAAAAGAATCTTCCGAATAAAGCCAAATTCAAAGTTGAATCAGGTTGCTTTTCCGTGACCCAACAGTTGCAGCAAACCTCAGTTACTTTACTATAGTTAATCTAAACCATAGTTGCTTCTCTCTTTATAAGGAGGCTTCGGCCTCAGTTGTAGACAGAATGTAATTTTCCTTCTTAAGTTTTCTTCAGTTAAAAGATTCTCTCTGAAACATCCTTCGGAGAAATCATCCTTGTAAATACGGAGTGAAGTTGTTTGAAGTTGCAGTAGCAATCTTTGTGAGTATAAATAAAGTTGCTATTTTCAATTATCTATTTTAAAGCATTATTTTGATGCATTATCATTATTTTCTACATTAGTCTGAAAGTTGTTTAGGTTGCATCCATCTCTATTCTGTTGCTCTTTGTCTAAAGTTTGTTCATCTAGTTCTTCCC

TATATATATATATATATATATATATATATATATGTCAATCATACTCATGGTCAATATTAATCTCTTAAAAGCATTGAAATCAATTTAAAACTACCACCTAGGTGTACGCCTTTATTTCTGTCAATTCCTAATATCCGTCAATTCCTTATATGCCGTCAATTCCTGATATCAGTCAATTCCTTATATGCCTTCAATTCCATCCTCGCAGGTCTCGGAAACATAAAGTTAACCAAGCCGCATTTCTCGCAGGTCTCAGAGATAACACTTCAATCTAAGCCACAACACTCGCAGGATTCAGGGGACATTAGTCAGCCTGATCCGCATTCCTTGCTCCGCACGAGTCCGGGTACCAACAGAACTTGTGGGGAAACTATAAAGCATGCTGACTAAATCGAAGGCAACCAATAAATTCAAAGGCAACATAATCACCGAAGGCAACAAATTTATCTGAAGGCAACTTAATTACCGAAGGCAACATCTTTATATCTGGGTACCTAAGGTGGCTTAAGAAAATATAAATTTCTGAAAGATCATATGAATAACTGAATTTTTGTTAAATCTAGAACTACTCTGCCATTTATATGATATTTAGCTAGAATTTCCTTTTACTATATCCTTAAAGGATATCCCACTTGGCAACATATAAATTAAAATATTCAAAAGCACATATCAAATATTGGAAACCCTAATCTTTGAATAAACCTTATTCAAGATCAAATAATGAAATTGAATTGCATAAATTATTTATAAAACAAAAAGTCCACTAACTCCTGGTCCGAGCTAGCTCGACCTCTTGAAGGTCCATCCTGCGGGTCTGCCTGGCCCCGGGTGCTTAATTAATCCATCATGAATATTTGATTAATAAAACTACTCTTTATAAGTATTTAGTTAAAACCCTAACCCTCAGCTCCTAGAAGTGCGTGCACTAACTTTAAAGTCATTCCTATGCCCATAGAAGCTTTCCTTATGGTTAGAACGGTCTGAAAAGACCCGAGACTCG

>Chr4-15; OZ022209.1; 24418725-24422747; rev; B;

TTTGTGAAAACTCAAACTCCGCTTGTCTTGGCTTTTGCTGAACCAAATTTGTAACGAGAGAAGTTTCGTTTTGAATGACTTATTTCTCTAAGCCTGAACTTTGGAAGTTTTGATCTATAGCTAGCTTCTTTTGTGGATGAAATGAGCTTTTGGTTGCTCCAGTTTATTTGAAGGTTCTCAGTGATCAAGTGATCATTTTGAGTTTTTCGCCTTTGGTTGATTTATGATTGTTTGATTGATTGTTTTATTGATCCTTTCTTTGTCTTCACCCTCTCCTATATTTATGAGAAATGTTTTGGAGTAGAGTTTTCTGATTTAAAATGGGCGGCAGATTTTCTAAAAAGATCTTTCATTTTTTAAATGCAAGGAAAGAGGGGTTTTATCAATTCTGGTAGATAAGCTCTCAATAGTCAATTCTCATGGCTATCACTTCCCTGTTTTTCTTGAAGGAAAACCTAATTCCTTTTTCCAATTTTAACTGCCCCTCATGAGAGCTCAAGCTGTGATGGTTAGTTTGATCTTCCAGTTGAACAACTCTCTGTTTTCCTGCTTATCTCTTGAAAATAGTGTCTTTCTTAGGAGCAGAAAATATCTTTGAATATATATATAAAAATGGATTTTTTGATCTTCTGGTGGTAGAGTTTCAGAGAAATCTCTTCATAGTTGCCTACTTTTATCAACTCTTTATCAATTTAGTTGAAATTGTTGACTTTTCAAAGTTTATTTCAGATTAGGGCCTCTTCAGAAAAGAGCTGTCGATTTGTCCCTGGGCCTTTTGAGAGAAATGGTAGATTTTGTAGACTGGGCCCAATCTGATTTATAATCGCCATTTGACTTGGCAATTTTTACTTTTCTGCCATTTTCTAAAAAAGACGTGGGTTGGGCTTGCTCCTGTTCTGATATTTGAAGCCCATTTTTCTATTCCTTTTGTTTCGTGGGCTTTTGAGAAGTGGGTCGACATGTATTTGCTTTTGGCCCAAACATTAAGTCAATAAACATG

CTCAGAAAACAGGAAGAACAAGAAGAGTTATTAATAGATCTAATAAGTAAGGTTGACAACCCAGAGTTAAAATCTAAGTACTTAAAAAAATTACAAAAAGTTATAGGACAAGAAGACGCAAGTCAAAGTAATTTTTCCAAAATAAGCTTTAACACAACTTTAGAAAGAAAGATGTAACTTTACAAGATTTACAAATGGAAGTAAAGACAGTGAAAAAGGAGATAATAGAATTACGACAAATGAGTCACAAATTACAAAATGAAACTTTGAAATTAAACAGGATTTAATTAATTTAATGGAAAGAAGAAATTCTGATTCTAAAAGTCAACCTGAAAGCCCAGCAAATTCAGATGATGAAACCCCAAAAAACGATAAAGCCATTAATTTAATCAAACAAATAAATTTTAGAAAACGGTACTCTAAAGTCACTATTTTTATAAAAAAAAAAAAAAATTTGAATTAAATACAGTAGCCCTTTTTGATTCAGGAGTAGACCTTAATTGTATACAAGAAGGTTTAATCCCCACCAAATATTACAAAAAGTCAAAAGAAACGTTAGGCACTGCCTCTGGTAAACCACTCCAGTTAGATTACGAAATCCCAAAATCCTATATCTATCAAAATAAGATATGTTTTAAAACCCCTTTCGTTTTAGTTAAAAATATTACAGATGAAGTCATCCTTGGTCTACCCTTCATAGCCCTCCTATATCCTTTTCGAGTAGAATACGATAGAGTCACATCCACCCATATGGGAGGAGAAGTCAAGTTTGAATTGCTAACTAAACCTGAGTTACATAATTTAAAAGCTTCGCAGAAAAATGCAGTTTAAAAAACAATGAAGATTATCCAGAATAAAAATAAACAACTAGATTTTCTTAAAGAAGAAGTCAAATTCAAGAGAATTGAACAACAACATACTGATAAGTCTTTGTAGTCACAAATTCAGAAATTTGAAGAAAAGTTAAAGCAAGAAGTTTGTTCTGATCTCCCCAATGCCTTTTGGCATCGAAAACAACACGTAGTCAGTCTCCCCTATGTAAAAACCTTTAATGAAAAAAATATTCCTACTAAGGCCAGACCCATTCAAATGAGCCAAGAAATTATGGAATTTTGTAAGAAAGAAATTGCATAATTATTTCAAAAAGGGATAATCCGTAAAAGCAAGTAACCTTGGTCTTGTCCAGCCTTTTATGTTCAGAAAAATGTTGAATTAGAAAGAGGAGCCCCAAGACTAGTCATAAACTATAAACCCCTTAATACAGTTCTAGAATGGATTAGATATCCAATCCCCAATAAGAGAGATTTAATCAATAGATTAGACAAAGCATTCATTTTCTCTAAATTTGATATGAAAAGTGAATTTGGCAAATCCAAATCCAAGAGTCAGATAAATATAAAACCGCTTTTGTTACCCCATTTGGTCACTATGAATGGAATGTCATGCCTTTTGGTCTAAAAAATGCACCTAGTGAATTCCATAATATAATGAATGAAATATTCAATCCTTTCAGTAACTTCTCCATAGTTTACATTGATGATGTTCTTATCTTCTCTCAGTCAATAGAACAACATTGGAAACACCTGTATAAATTCCTTCAAATAGTCAAGAAAAATGGTTTAGTTATCTCAGCAAAGAAAATCAAGTTATTCCAAACCATTATCTGATTTCTTGGTTTTAATATATGTCATTCACAAATCAGCCTAATTAATCGAGTCATCCAGTTTGCCGATAAATTCCCGAATCAAATTTTTGATAAAAGTCAACTTCAAAGGTTTATAGGATCCCTTAACTATGTCTCTGATTTCTATCAGAATCTACGAAAGCAATGTAAACCCCTATTTGACAGACTCCAAAGTAATCCTCCTCCTTGGACCTCTATTTATACAGAAATTGTTAAAAAAATCAAAGTCCATGTCAAAACACTACCTTGTCTTGGTATTCCTTCAGTCAATTCCTTCAAAATAGTTGAAACTGACACTTCAGAAAATGGTTATGAAGGCATCCTCAAACAAAAAATTAGTCCAAACCAAATTGAGCAAATCGTTCGATTCCATCCTGGAATTTGGACACAGTCACATCGTAATTATAGTACTATTAAAAGGAAATTTTATCTATAGTATTATGCATTTCAAAATTCGAATATGACTTACTAAATCAAAAAAATTTAGTCAGAGTTGATTGTAAAAGTGCAAAACATGTTTTAGAAAAGGATGTTCAAAATATTGCATCAAAAAAAAATTTTGCACGTTGGCAAACTATTTTAAGAATTTTTTATTTTGATATTGAATATTTGAGAGGTAGTGAGAACTCCATCCCAGATTTCCTTACTAGAGAATTTCTTCAGGAAAAACGATGGCAGAAAAAATAAAATAGCAAGGTAAGCAACTAGTCAGACAACTCCTAGCATCAATGACCCCAATCAAAACAGAGCAACCTTCCCAAGTCATCCCTTATCCGGGATATTCACCCATTCAAGTATCAAATAGGTATACTACTTTAGGATCTACATTTAATAACATTCGTCCCAATTAGCAGTTAGCTCTTATTTCCAGTTATGATCCATTTCAAGTCTTAACCCCCTCACCTGCTTGTTCTCCGCTTAATCACAAAAAATCCTCTCCCTATTTCCCAAAAAGCAGTCACACCCTCTTCTTCATTGAACCACGATTTAATCACTTGTCTAACCCAGTCAAAATAGCCAAAGCCTATTTTCCCCAAATTTCCATTACATACCTCCACACCTTTCTAAGTCACTTAAATATTATCGGGATATTCTTCTCGAAACCCAGGCCTTAGAAATCAGACCAATCAAGGATAGAAATGACCCAAATATCATTCTCTATCACTCCATGTATATCCAAAAAATTCTCAGTCAAACTGAATGGGGTGAACACCCTTACTCTTTTAAGACACTTAACTCTAGCCTCCAATACAATTACTATGATTACATCGAAGCTTGGCACATCATTTTCCTCCACCAAACTGAGGATTTTAGTCATTCATGGTTTGTCAACTTTGATAGTAAATTCAAAAGTCCTTTTCCTTACTGGTTTTCTTCCTGGTGGGAAAAGCATGGGCCAACCCTTGAAATTCTTCCCCCCAATCTCAAAGAGTCAGTCATGTACTTTTCCAATAAATACAAATTTAAGGAGGAACATCAATTTTTCCCAAAACTTTTAATCTTCATAGTCAAATACAAAGTCCTATGGATTTTAAAATGGTCCTATCAAGTCAACTGGGAATCCCGTGTTCTCTCTAGGCAGTTCTCCATCAAATGGTGGGATAAGTTCAAGTCATAAAGAATTATTGAACAAGTCAATACTGAATTCCCTCTAGTCAAGGGCCCCCAGTCAAAGCCCTCTTGTTCCGGCACGTCTCATTCTTCCCTCCCAATTAAAGGAAGATCCAAGTCAAAAATAAAAGAATTAGCCATACAGTTAATGATTCAGGCCTCTCAAATGAATGATGATGACGATGCCTCCTCCAGGTCTCAATTCACTCAAGCCTCCTCCTCCAGTCAGACACAAAATCCATCGAACAATCAAAAGGATTCCACTCAGAAATTCAGATGGGCCGACTACCCCGACAATCAAGACCCTTATGATTTGAATGAAGACTAAAGAAAAGTTAAAGTAAGACGTTGCATGATGCCTCCATGACCCGTGCCTCTACAATCAATATTCCCTGATAAAGGAAAAAGCAAAAGAACATGTGAAAGCAAGAAAGCAAAAGTAAAAGACAAAGAAAAAGCAAAAGCAGAAGTCTGAAGTCCCGTGCTCCAACAGTCAAGATTCTCTACCAACACTTCTATAGTTTGAGTCTTTATATAGAGGCCTTTTGGCCAGAGTCAAGCAGAATGTAATTTTTTGTTTCTCAGTTTCAAAGTTTTCTCTGAATCACCGTCGAAGAAAACTCTACTTGTAAAATATGAGTGCTACTTTCTATCAATAAAACTCTTTCAGTCAAGGTTTTCTGTAAGTACTCTCATTCACGCATTACTTTCAGTTAGCATTATTTTCA

TTATCATTATTTTCATTATATATATATATATTTATTTATTTATTTATGCACATGTGGGATGAGAAGAAGATTGTTCTAACCTCAATAGCCCTTGATTAAGTTTCAATGGATTAATCTGAATTATTTATGTAGGCAACCTAGAGTTACTTGGTGAAGCCACAAGATCAAGTGTTGAAAATTTGTTCAGTTAAGTTATTTTTGAACTTGATAACGTATTCATGTTGTTGTAAGAGTTGAGACCGGTTTCAGATGGTAGAATGATTTATTTTGGTATAAAGTGGCCTGAGGCCATTCAAAGTTATTTGTCTGATGCATGCTGAACGATTTGGATTTGATTTTCAATATTAATTGTTTATTTACAGGTGGAAGTTTTTGGGAAAATGTCTGTTTTATAAGGGAGATTCTGCCGATTTTTCGGCAGAAGTCAAACCTTAAAAGAATTGTTGAGTCTTAGTTAGCAGGGCAATTTGGTCAGGTGTGCCCGACACCTGGCAGGTGTCGAGCGCGCACAAGGTTCAGCGCAAATTCCAAAGTGGAATTTGGGTCTGGTCCTGTCAGTTTATATGGTTTTTTTTTAAAAATCATAAACATATATTTGATGACATGACCCGACCCAAATTCCACTTTGGAATCTGAGCCGAACCCTGTGTGTGTCTGACACTTGGCGGGTGCCGAGCACAATGACTAAATTGCCCTTCTAGTTAAAATTCAAGTTTATTACAAGTTCAACTTCTACCGAAAATTCGACAAAGTCTCTCCTGTAATTTGAACATTTCCCAAAATTTCCACCTGTCAAAAGCGTAAATATACATATCCCAACTGCCAGTATGCAATCAATCTACATCCAAACTATTCAACTTAACTAAACGGCCTCAAGCTATACAGAAAGTCCTAGGGCAATTCATCAGAGCAACTAATTCAAGAATTTACAACAGCACAGTTTGATTTCAAAAGAAAATCCTACGAAGAGAGAGAGAGTGGAGTGAAGTATGGAATCTGC

>Chr4-16; OZ022209.1; 24556415-24558514; rev; B;

AAATTTTATAAGGAGCTCCTAAAATAGCTTTCTAGTGTTTTTAGCTACATTTTAACTAAAACATAAGGAGCATGATTGTGGATGCTCTAAGTAAGGTGTATGTATTAAAATTACATTTATTTCACAATTCAATATTATTTTTTTGTTCATTTTCTATTAGGTTAAATTAGTAATTCAATAAATAGTTTGGTAGTAAGGTGTGCTGAGTTAATTTTAGGGTTCGCTTAATATAGCACCCTTTCCAATATTGGAAGAGATCCCAATATTGAATTCGCTATTGATAAAAAGATTGATATCCATAAAATAGGCAAGTGGTATCCCCATATAACACTCATTAAATGAAAATTCACAAATCCTTTTCTAACTTGACTCTAATTTCATGAAAGAAATTGAGGTTACACTCCAAAACCAATTGGCAATGAAGGAAATAGCTCAACTCCTAATAAACCCATGCTAGGTCTTAACTTTTCAACATGGGACAATACTCTCACCACACTCAACACTTGATCATTCTTATATTGAAATTAAATATTCTTTGACAAGTATAAAAAATATAAAATAAAAAGAAATAATCAGTTGGTTGAGTCCTTTCCACCTATAACCAAGTGGGCAGATGTTTGAAACCTCCAAGCACTCAACCATCACTTGTACTAAAAAAATATACATATAAGAAATAATCCATATGTATATGTGTATATAACTTTGAATACATTCTTTAGTCTAACAACTATTTCAAAAGTATAGTTTTATTTCCGATATATAACTATATATATATATATATATATATATACTCTAGTATCAGCGTACTTCTTTTCCTTCTATAGTTGGTTCTTAGTTTGTATGTTAACTTGTTTTCCTCTCTGGTCCAAACAATAAGTCCTAGGTAAGATATGAGTGGAGAGAGAGAGAGGATTCCTTAGGTTCCCAGATAATCTTCCACCCACTATAGCGGCCATAGTTTTATGCTCCCCTACTTAGATAACCTTCCTGCTTGCCAAAA

TGAGTCTCCTTCTTAGAACCAACTCAGCTAATAGCTGCAGTTCTAGAGCAAGTGTTAGCAAAATCCCAAACATAGTTAATGAAGAATAAGTAGAATATGAAATAGATAGTGAAGTCAGCTTTAAAGATTCGAATATACCCAGAGTCCCTAGTCAAGAAATCTATAGAAAACGTTGGTCACTTACTTCTTTCAAGTCAACTACCCATATTAAAACAGTTGAACAAGTCTATGCCCTTAGTAAAGAACACGAAACTTGTCAGTTACTTAATCTTGAATAAATTAAAAAACACCGTAAAGATGGGCATAATTTTTTGCATATTGGTGTAGTACAAGTAGCAGTCAAACCCCTAACCAAAATAGGACTTAATGCCTCCATTCTTCTCTGCCTTATAGATGGTAGATTCACCAACTTTAGTGATAGTACCCTAGGAATAATAGAGTCAACTCTATTTAACGGTCCAATCCATTTTGATTGGTACCTAGATTTCACAATAAGCCTCAGTGATCCTCACATACTAAAAACTCTAACATTAAATATTAAAACCTCTGGTATGAAGTCCTACCAGGCACCCAACCTTTAGCCTTAGTTTATAGAATTTATTATAAGGTCACAGGCACTAATATGAACTTCCAAGCCCTTAATAAAGCAAATGCCAATATTCAAGTTCCCCATGCAATAAAATGGTCTGATGTTAAACCACCTTCAGATTGGACACTAGCAACCGAAAGTCATCCTGCTCCTATTCAACATAATCTGAATAATTTAGATTACATTCAACAATATCTAGATGGCAAAGTCAAAATTAATTTTGGAAATCAACCTCTTCCAAAGTCTTCAGTTCAGTTACAACAACTTTTCACACCTAGTCAATCTCTTAGACATACCCCTGTTAGGCATTTTTTTGTAGCTTCTTCTTCCACTCTAGAGCGAGACCTAGAGCTAGAAAAAACTCTTATTGATCTTAAATAAGAATCTGTATGAAAAAACTCTCAAGTTAACCATCTTTGTTATGGTAAAAAACTGGTCAAAGAAGACGAAAAGTCAAACTCTCCAGATTCTCCTACACAAACTGATTTTGAAGCCGATGCCATTCCAGTAGACAACCAATTAAGAACCTTACATAAGAAATTTAAAATACATTGGAAAATTCTTGATGAACATCTTGAGTCAGAAGAGAATATTGAAAGACGCAATAAGTATAGAACAACTTACCCCAATACAGACCAAAGACAGCAAGTTTTTAATGCATGGAAAAATTATATGTTGGAAACTGAGTCAGAAATATTTTATCTTGATTTTATTGAAAATATTTATCAAAAGAAAGAAATAAAAACTTTGAGTCACTAATCACAATTTAAGGCAGTTGGAAATTGTACCCCTTTTAGAAACAGGATTCACCGGAACCTTACGTTCTTGGTGGGATAAACACTTAACTGAAGAGTCAGAACGACAAACGACCCATGCAATCAAACTAAATGAAGAAGGTCTCCCTATTTTTGATGAACGATTAGGTACATGAATAGAAGACGGAGTGAACACTTCATTATATACGATAATTGAGCATTTCATAGGAACACCTAGTAACACTACAGCCAGAATTCATGATCAACTCAGTAACCTTAGATGTCCCAAGTTAAGTGATTTCAGATGGTACAAAGATGTCTTTATATCCAGAGTCATGCTTAGAGACGACAGTCCAATAGATCGAGTTGTTCAATTTGCTAGTAAATTCCCAAATCAAATTCTTGATAAGAGTCAACTTCAAAGATTTCTAGGTTCTCTCAACTATGTTTCTGATTTCTATCATAATCTAAGAACTCTATGTAAACCCTTGTTCGAAAGACTACAAAGTAACCCTCCTCCTTGGACTTCTAATCATACAGAAATAGTCAAACAAATCAAAGTTCATGTTCAGACTCTTTCTTGTCTTGGTATTCCCTTAGTCAACTCCTTGAAAATCATAGAAACAGATGCCTTTGAAATTGGTTATAGAAGCATTCTCAAACAAAAAACTTCTCCAGATCAACTAGAACAACTTGTTCGTTTCCATTCCGGCATCTGGACTCAGTCACAAAGGAATTATACACAACTACAAAAAG

TGAAAAAGACGACTAGAAAAAAATGACGGTTTAAAGAAAAAACGTCGTTGTGTTTGAAAAGACAACGGTGTTATAAAACTGCTATGTATGATTAACTTAAACACATAAAACATGGAGATTCCAATTGGTGTTTTTAAAAAACGACGCACCAAATAAAAAAAGTGTCGTCTTATTGAAAAGGTTTTCCTCAAAGGTAAACTCTGAGCCCAAAAACAACGGCATAAGGTATGAATCCACTGACGTAGAAAGTAAAGACGACGATGTTCAACAACAGCCGCCGTTTTTACTCCATAATAAACACGACCAAGTTTTCGAATTCGGCGCCGTATAATTTAAAAAAAAATCCACATCGGTATATTACAAAATACTGCTGTATTAAATAAATGTACAATTACAGTAAAGATAAATTTATCGACTTATTTTTTGTAGCGTACTACGACGGTATCTATAACTCTAATGACGTTGGTTTGTTCAATTAATGCCATATAATAAAATTTCCCCCGTCGTGAAGTTAAAATTACCATCGTATTTACCTTATATCTAAATCGTCGTCTTAATATTAATTTTTTTTTACTATTTATTTATTGGATTTTGGTATCCAACGACGGTAGATCAACATAATGACAAGAATTGAGAATAACCACGACGGTTTATATAGAAAACCGCCGATAGAATCAGATTATTCTGAGATCCCAAGAGATACGAAATCTTACTTGAGGGAACTCTGTTCCTCATCATAATCGGACCACCAATGTGAGAGAAAGACAATATTTTAGAAGTTGATGATGTGTGTATCTTTGTGTCTTTGAGATACTCAAAGGCACACACACATCAACATCCAAAAAATTTGCTTCTAAAAATTAACGACGGCAGCCACAGATTTGCATTCAGAGAGACAATTTTTTGGAAGTTAATGATGTATGTGTGTTTGTGTCTTTCAGGTACACAAACGCACACACACCAACATCCAAACAATTGCCTCCTAAGAGGTGAGACAGAG

>Chr4-17; OZ022209.1; 25383714-25385178; rev; B;

AAGGAAAGAAAGGTGCGGTCCCAACATGCTATAAATGTGGAAAAGTTGGACACTATAAATCCGAATGTAAAATGAAAGATAAAATTAGTAACTTAAGCATTAGCGAAGAACTTAAGCAACAGCTTTGCCAAATAATGCTTAATTCCTCTAATTATGAGCAAGATCATGATAACGAACTTGCTCAGCTCGAAAATGAAGAATTATTTGAATCTGATACTGAAATTAGTTCAGATAGTGAAAATGAGTGTGCCTATCAATTCAATGATCTCAAAATATGTTTTTTTAACTAAAGAAGAGAGTCTGATAATAGACTTAATTGACAAAATAGAGGATCTAGAGAAAAAGAAAGAGGCGTTAGAAAGTTATATCTCACTAGCTAAAGTAGGACCAAGTGACCCACAGCCAGCTACAATACAGAAGAAAAATCGGAGCAATTAGAATTTGAAAATCTAATGGAAGAATTTCAAGAGAAAATAGAACTTGTGAAAGATTCCTTACAAGAAGAACATGTTAGTATTATGAAATTCGTCAATAGCATAGATAGAGTGATAGCTCATAAATGGTACCGATGATAACTGTTGTGGTTGATAGAACCTACAAATTTATAGTTGAAGGGATAATAGATACAAGGGCTGATCTTAACTATATAAATGAGGGAGTAGTACCCACTAGGTATTTCAAGAAGACTACCCAGGTTCTACATACAACCGTTAATAGCCAAAAAATGGGAATTCAGTTTAAATTATCTAACGTTGCTGTGCTCAATCAGGGTATTTATTTTGAAACTCCTTTTGTTCTGGTGAAAGGTTTAAACCAGTCTCTTATATTAGGAACACCATTCATTAATATGCTTTACCCTTTTTCTCTCTCTGAAGAAGGAATTAGACCTGAGGTAGAAGGTCAACCTATAATCTTTAGGTTTTCTCAACCACCCAAGCATAAGTATATTAATGAAATCCAAGAATAAATAAAAAGGAATGAAAACTATCTATACCATTTG

AAAGAAGAAATGTAGCATAAGAGAATAGAAGAGCAGCTGCAAAATAGCAAGATAAAAGTAAAAATTGATGAAATCCAAAAGATGTTGGAAAGCCAAGTTTGTGCTGAAATACCCAATGCCTTTTGGAACAGAAAGAGGCACATAATTGAATTACACTATGAGCCTGATTTTGATGAAAAGAAAATCCCAACCAAAGCCAGGCCAATTCAAATGAATGCTAGGCTAATGCAAATTTATAAATATGAAATACAAGATTCATTAAATAAGAATTGAATCAGGAAAAGCTCATCTCCTTGGATTTGTTCCGCTTTTTATGTTGAAAATGCAGCTGAATTAGAAAGAGGAGTACCCAGATTGGTGATAAATTACAAACCTCTAAATAAAGCCATAAAGTGGGTTAGATATCCGATACCTAATAAGAAAGATCTTTTGGACAGACTAAATAAGGCAGTTATCTTTTCTAAATTTGACATGAAATCGGGATATTGGCAAATCCAGATCGCCGAAAAGGACAGGTACAAAACAGCTTTTAATGTCCTTTTTGGACATTACGAATGGAACGTTTTACCTTTTGGATTGAAAAATGCCCCTTCTGAATTTCAAAATATTATGAATGATATATTTAATCCTTACACTGCTTTTTCCATTGTTTATATTGATGATGTTTTAGTCTATTCTAATTCTTTGGAACAGCACTTTAAACATCTGAATATTTTCTTGAAAGTTGTGAAACATGCAGGATTAGTGGTATCGGCAAAGAAAATGAAACTATTTTCTTCAAAAGTTCACTTTTTGGGACATAATATTAATAAGGGACTAATAGTTCCGATTGATAGAGCAATATCCTTTGCTGATAAGTTCCCTGATGAAATTAGGGACAAGCAACAGTTACAAAGATTCTTAGGAAGTCTTAATTACATAAAGGATTTTTATAAAGATTTAGCTCATGATTCAAAATTATTATATCAAAGGTTGAAAAAGAACCCGGTTTCTTGGTCTGATGAACACACTAAAGCTGTTAGGAGATTGAAAGTTAAAGTTAAAGAACTTCCTTGTTTAGCTTTAGCCAACCCAGAAGCCTTTAAAATCGTAGAAACCGATGCTTCAGAAATAGGGTATGGGGGAATATTAAAACAGAGGGTTGATAACTCAGAACAATTAGTTAGGTTCACCTCTGGAGTTTAGAAACCAGCTCAAAAGAATTATTCCACTGTAAAGAAAGAAATGTTAGCCATCGTCCATGCTGTCTCTAAATTTGAAAGTGATCTTTCAAACAAGAAATTTTTGATAAGAGTAGATTGCAAGGCGGCCAAAGACATTTTGTTCAAGGATGTTAAAAATCTAGCATCTAAACAAATTTTTGCAAGATGGCAAGGAATTCTTTATGCTTTCGATTTTGATATTGAATTTATTAAAGGTGAAACTAACGCACTCCCGGACTTTTTAAGTCGAGAATTTTTGCAGG

GTGTGAACCAGAAGTTCCGGACAGATGACTCCAAGTAAGAGCTCTTTTGCTCTTACCAAGTCATCCCTTAATGATCCAAAGCCACTGATGTCTTCTTCTCCATATAATAAGGGTAAGGAGAAGCAGATTGCTGACAATCCATTCCGTGAATATATGAATATGTCTGTAGCAATCATTGAAATGAATGATCTAGATAAGCACCCAAATTTTCTGGCTGAAAAATATTTAAGGGATAATCTTCCTCCCTTGAATAAGCCTTGTATTTTGTATGAAACTTTTCTAATTGAAACGAAGTCTGTTAATATCGTTTATTGTTATAGAGGTGAAAATGCCAACACCATAGCATTCTCCAAAATCCAAATCCTAAATGTCATTTCCTTGGAAGAATGGGGCCCTAGCCCTGAAAGGGCCAAAAGATTTATCCATCCTGAAATTAGGCCGTATTCCTATAATTTTTGGGATTATGAAAAGGCATGGGAATATGTTTTATGTTGCAAAAATAAAAAATATTCTGTTTATTCTCTACATTTCTACACTACCTTCCGCGCATAAACTAAAGCAAAAAATTGATTAAAAATTTGCTCTTAAGAGGCAGGGCCAGTACCTTGTATTAGAGCCTAAACCCTAAACCCTATATAAGTTTGAATGGTTTTCGAAACAGTTTTTAAACGGTGGGGTTAGTATGTTCGTATTATGTTTAATAATATTTATTTTTTATCCACTCACATCTTTATTGTTTTGCGCCCCCCTAGACAGTCGACGAGTATTGGACCACCACTTGAGGGCATCTCTTCTTCTGCTAGTCCTCCAATTGTCCTCGTGTAAGATTTCTTCTTGTTGTTGTAGTTTCTCTAGTTGTAAACTTAACTTGACTAGATACTGATATAGCCTGGAATTTTTGTGAGGCCTGTTGTCACAACCTGATTCAAGAATAAGGAATATTCCTGAATCAAGGTTGGAGTCATACTATAGTCATAAGAATAAAACCTTACAACCATGA

>Chr4-18; OZ022209.1; 25454147-25456491; rev; B;

TTCGTTAATGTAAGGCTAATTTCTTGTATTTTCCATTATGTTGTTTTAAGATTCAATTTTATAGCTTTATTGTTAATTGTTATCGAATTTGCTTTAAGCTTAATTTAATTTCAAATTGCTTTATTTTATTGCCCTTATAATTTATATCGTTCTTATTTCGTTCTTCACTTAATTTAAGTTTATATATCGCTTTGATTTACTTTATCGTACTTCAATTTTCATCTTTAAGTTTAATTTGCATCGTTATCATTAAAAATCTAAAAAATACTAAAATACCTATACGAAACCTAACCATCCTCGAACCCGAGGAAGTGAAAGAACCTAGGCCAAGAGGAGGATCCTTGCCTCGCCGATCAATCGGTTGGTTAGAAGTCAGAAGCATGAACACAACAATCCAACCTTTTCCATTCAAGCCAGTCAGTGGCTTGGTGGTCGCATGCCTACGCAATCTAAAAGAAGAAGGACCAAAAACTCCTTCAACTGGCCTACCCGGCCGAAAAGAGGGAAACAGAGAGCAAAAGCAAGAATAATATTCTAAAAGCTCTAGTCAACGCAAAAGCAGAAGACCCGTGCTCCAACAGTAAAGATTCCAAGCATCCAATGTTGCCGACACCTTCATAGTTAAGTTCTTTAAATAGAGGCTTTGGGCCTCGGTCGTAGTCAGAATGTAATTTTTAGTTTTCAGTTTCAAAGTTCTTTCAGAAATATCTCTCTGAAGTACACTTGTAAGATATGAGTGCTATGCATGAATAAAATCTCACGCTTTTCTGTAAGTCTGCAGTTCAATACCTTCATTTAAAGTTATTTTCATTTGCGTATTGCATTAATTTTATAATTTCTCATCATTTAAAGTTATTTTCATTTCATATGCATTACTCTAGCTCCAGATCCCCTCCTGGTATGCGTATTGCATTAATTTTATAATTTCTCATCATTTAAAGTTATTTTCATTCCATATGCATTACTCTAGCTCTAGATCCCTTCCTGGTATCAGATCCCC

TCTTGGTATCAAAGCCATAAGTGTTGAAGAGTCATTCTAATTATGCTTCTCATTTTGTCGAAGCAACTGGTCATTTCACTATGTGTTTGTAGTTTTGCTATTTAAATATGTAACTCTTTCTGTGTCTTCCTGAATACTAATACTTTTCTCTCAACCCAAACAATAAGTCCTAGATTCCAAACAATAAGTCCTAGGGAGAGGCATGAGCGGAGAAAGAGAAAACCCTTTAGTCTTCCAGAATCCTTGATTTCATTATAGGTTTATTAGCTTTCCTTCTCATCCTTTGTGAGAAAACTAGTAATCTTTGTCCAGATGAGTCGCCTTTTTAGAACAAACTCTTGTAACAGTAGTAGTTTTAGAGCCAGTATTGGTAAACTCCCAGAAATAGTAAATGAAGAACAAATAGAATATGAAACTAGTAATGAAATTGACTATAAAGATTGGAATATCCCCAAAATCCCTAGTACAGAAATTTACAAGAAGAAATGGTCACTTTCAAGTTTCAAAAGTACAACCCATGTGAAAATAGTTGAACAAGTCTATGCCTTGAGTAAAGAACATGAAAAGAAACATAAGAGTGATGGAAACAATTTCCTTCACATTGGCCTCATTCAAGTAGCAGTCAAACCCCTTACCAGTTTAGGACTAAATGCCTCTATTTTACTTTGCCTACGTGATGCCAGATTTACCAACTTTAGTGACAGTGCCTTAGGAATAATTGAGTCAAGCTTGTTTAATGGTCCAACTTTAGTGACAGTACTTTTATTGCTATCCAGACTTCACAGTAAGCCTTAGGGATCCCCACATATTGAAAAATTTAACATTAAATATTAAAACTTCAGGGTATAAAGTCTTACCAAGTACCCAACCTCTAGCTTTAGTCTATATAATTTATTATAAAGTCACAGGGACTAATATGAATTTCCAAGCCCTCAATAAAAGTCCAAAAGACCAAACCTTGCTTATTCAAAGTAGTAAAGCCGATGCTAATATAAGAGTTCCTCATACAATTAAGTGGTCAGAAGTAACTCTACCTCTAGATTGGACACTAACAACAGAAAATCAACCTACTCCTATTCAGCATAATTTGAATAATCTAGATTACATTCAACAATATTTAGACGGAACAGTAAAAATAAATTTTGGAAATCAGCCCCACAAGTCCTCAGTCTAGTTACAACAACTCCCAACACCCAGTCAATCCCTTAGGCATACTCCTGCCAGACATTTCTTCGCAGCTTCCTCCTCTACTTTGGAAAGAGACTTAGAATTAGAAAAAACATTAATTAATTTAAAATTAGAATCTTTAAAAAAAACCTCCTAGGTCAACCATCCTTGCTACAGTAAAAACCAGTCAAAGAAGATGAGAAATCCAATTCGCCAAACTCTCCCACTCAAATTGATTTTGAAGCCGAAGCCATTCCATTAGACAATTAGTATCGAACCCTTAACAAAGAATTTAAAATTCATTGAAAATTTTTAGACAGTCATCTAAATGCCCTAGAAAACCTGGAAAGACGAAATAACTACCACACAGTCTACCCAAATAAAGAACAAAGAGATCAAATATTTAATGAATGGAAATTTTTTATGAAAAATACTAAATCAGAAGTCTTTTACCTTGATTTCGTTGAAAGTCATTACATGTCCAAGGAGTTAAGAACTTTAACACAAGAAAAATGGAAAAGATAAAATAACGCACAAGTCATCGCTAGTCATCCCCCAGTAGAAACAATAATCATTGATCATAAAAATAATACAATCACTGCTAGTCCTTTTAAGATCCCTGAACAAAATGATAAAAAATTAGATGAACAAAATAATTACGCGAACCAGAGTCTAATAGTAATTGGAAAACAATTAGATAAAATAGAAACAAAAATAGACAAAATAATTCCGCAACAAATATCTCTTAAGTCTTAGAAAGAGAAACGTCTAATAAAATTCCAAGATTTAAAGTCAAGTTCCACCCTCAAGAAAAGTCCCACAACGGAAAAGATTGAAGAAATGTTTAAACAGTTAACCCTGGTTAAAGAAAAACCAGAACCTTCTAGTCTCAAAGTCCTAGAATCCTCTGCTTTAGTCAATTCTACTGAAACCAAGTCTATAACGAGCAATGAAACCAATTCCAGTGATATATCAAAAATTGAAAATGCCTTTAAAAATTGAGAAATAGATAGTGATCTGAAACTTAATAGATTAAGGAACAAAGTCAACCCTACTACTCTAACAAAAAATTGGTACCCCAAACCAACTCCTCCTAACATCTAGTTTGAGGAAAGAAATTCCCAAAGTCAATTCTCAGTCTCATATGATAAACTTTACAAATGGAATATCGATGGTCT

AGGAATAAGTAAAGTCATGAAAACATAAGGCTCCTTCATGCGCATCCCCGGAGGAAGATTATATGGGTATATGAGAACCGGCCAAATACTATAGAGCAAATTCATGTTCCCAAAAGGGTTAAATCCATATGTTGCCAAACCAAGTCTTACATTTCGAGGTTCCATACCAAATGACTCATGAATTTGATTAAACGATTTCCAAGCCTTAGCATCTGCTGGATGCCTCAACACTCCATCTTTTACACGATCCTTAAAATGCCACCTCATATCTACAGCTGTCTTGGATGACATGAATAATCTTTGAAGTCTTGGTGTGAGTGAAAAATATCTAAGCACCTTCCATGGAACCCTTTTACCTTTCTTTTTGCTACCCTTTCCATGTCTCTTCCATCTCGAAAACTTACAAGTAGGACACTGCGTTGCATTTGCATGCTCCTTATAATAAATGATGCAATCATTATTGCATGCATCTATTTTATCATAACGAAGACCTAAATCTGAAATAATTTTCTTTGCACTGTTGGTGGTTATAGGTAACTTCTCACAAAAAGGAAATGCATTTTTCAAAACATACCAAATGCTTTATTGCTCATAGTACCGATACCCTTGATATGCATCAAGTTCACAACAAAAGACAAATTAGAATATGACTCACAACCAACGTACAATGGTTGTTCTGCCTTTTCTAACAATTTATAAAACTTGTGCGCATCTCCATTTGGATCTTGTGGACAACTTTCTTCTATTAAACCATGCATATCATCGTCTTGCTCCATATTGGCCACATCATGCCCATCATTATCACTATCACTTTCATCACTATCCTCATCCTCACCCTCACTGTGCAAATACCATGTAGTATAAGTTAGTTTAATTCCGTTATAAAGAATATGCTTGTGTACCTTATACCTCAGATGTCTACATCTATTACTGCATTTAGAGCAAGGACAAAAGATCTTAGAATCATCCGGCTTAACATGGTTGTATGCATAATTGAGAA

>Chr4-19; OZ022209.1; 25474272-25475348; rev; B;

ACAATTAAGGAAACAAATTAGGAAAGAATCCTTCTTTGACTTGGAAACACGTCAGCCAACTTGCACGCACATTTTAAGGTCGTTCCACTTCCTAGAAAATTCTGGAAAAATATGAGAAACGTAGCTTGATCTCTAAGATTTCCAAGCATATATCACACGCCACTAGGAAAAATCGGAAAGGTCTTCATATCTTCAAAGTCTGGAGCATTTGCCTTAGTAGAAAATTATGAAACTTTGACACATCAAAGTCGATTTTCAATCAAAACCCTTCCAAGAGCGCATAATAGCTAAAAATAAGAAAATTAAGGTAATAATGCTATTTTTAATTAACTTTCCATACAAAAACCCAATTAATAGGTAGACAAATATAGGAAATAATGCACTTATCACCCTGCATCTTATCTATTATGCAAAACTCAACCCTGCATCTAGTTCGTTCATCTTTTTGCAGGTTGGTTGCTAGTTTGTCAAGCGAGCCAGCTTTGCTTCATAATTTTATTTTACCCCCTTGCTCATTTTGGAGCCATCGTATGATAAACTTGTAAACAAATAACTTGCGACAATTATATACCCACAATGCAATTCATTTGGTTTCAATCTTTTTTTTCCTTTTTGTTTTCATCTATGGTATCAACTAATATAAAATAGACTGAATTTTCTTTTAGGGAGTACATAGTGCACACACAGTGTGCATGCTCCCACTTTCCTTTTAGGGTGAACACATGTAAAATATGAAAAACATAATCAACTAAAAATGCTAATATCGTGTTACTGAATTGTCATCGAACTTAAAGTCTTCTTCATTCTTATGTTGTTTACAAGGCTACAATGGGATGGCAATACTTTATCACCTTGTTTGTTATATAGAACTGTCTTCGCATGGACTATGCTTAGGGGCTTATCTTGGCTTGGTAAGCTTAGTTTAAGTTGAATGACACTTATCTTGTGTTTGGTTTATGCTTGTTCTAGGACTAGATTTTTTTATTTTTCAAAGCATATA

TATACACACATATATTGTTAAACAAATCAAAGTCCATGTCAAAACACTACCCTGTCTTGGTATTCCTTCAGTCAATTCCTTCAAAATAGTTGAAACTGACGCCTCAGAAAATGGTTATGGAGGCATCCTCAAACAGAATGTTAGGCCAAACCAAACCGAGCAAATCGTTTGATTCCATTCTGGAATTTGGACACAGTCACAGCATAATTATAGTACTATTAAAAAAGAAATTTTGTCTATAGTATTATGCATCTCAAAATTTCAGGATGATCTATTAAACCAGAAGCTTTTAGTCAGAGTAGATTATAAAAGTGTAAAACATGTTTTAGAAAAGGATGTTCAAAATATTGCATCAAAACAAATTTTTGCACGATGACAAGCTATTTTAAGCATTTTTTATTTTGATATTGAGTATTTGAGAGCTAGTGAGAACTCCATCCCAGATTTCCTTACTAGAGAACTCCTTTAGGAAAAAAGATGGCAAAAAAAGCAAACAAAACAGCAAGACAAGCAACCAGTCAGACAACTCCTAGCATCAATGATCTCAGTCAAAATAGAGCAACCTTCCCAAGTCATCCCTTATCTAGGACATTCACCCATTTAGGTATCAAACAAGTATACCACTTTAGGATTCACAGTCAGTAACATACGTCCCAATTACCAGTCAGCTCTCATCTCCTGTTATGACCAATTCCAAGTCCTAACCCCCTTTCCTGCTTGCTCCCCAATTAGTCATACCAAATCTTCTCCCTACTTCCCCAAAAGCAGTCATACCCTCTTCCTTATTGAACCATGCTTTAATCATTTGACCAACCCAGCTAAAATAGCCAAAGCCTATTTGCCTCCCAATTTCCATTACATACCTCCACATCCTTCTAAGACACTCAAATATTATCGTGACATCCTTCTTCAAACCCAGACTTTAGAAATCAGGCCAAACAAAGATAGAAATGATTCAAATATCATTTTCTATCATTCCATGTATATCAAGAACATTCTCAGTCAAACTGAATGGGGCGATCACCCATACTCTTTCAAGACACTTAATTCCAGTCTCCAATATAATTACTGTTTACA

CCATAATCAACTGAGCATATCCAGCATCAAAACGACTAGCACCAAGGCATACATGCCTTCTCCCTTGCCGAAGGAGGACACTCTTCTGAGGTGCCAGACACCTGCTGAAGCTCCCGACTGCCGAAGCTCTCGACTGCCGAACCTAATCTCCAAGACATGTGTCACAAACACAGCAACTTGCACAAAGTCCCACATCGAAACTTTGTGCAAACCTCCCACTTTCACCTTCCTATAAATAGGGAATAGTACCCTAAGTAAAAAGGGTAACTACTTTCTCACATTTACTGTTACTCTGCCAAATTTACCACTCTTAGCTGACTTAAGCATCGGAGAGCCTTCGGCCGGCTCCACACCGGTGTCTGAAGCTTAACGACTGCTTCTCCTACTTTGTTTCTACAAGCTCTTCCCTTACCGAAGGTCCTCACCTTCTCCCCTGCTGAAGACTACCTACTTCTCTCTAAGTTGACCCTCCCAAAAAGAGCATCAACAGTTTGGCGCCGTCTATGGGAATCGAACTTGAATCCCATTTCTCTTTACCAGGCCTCGTCGCCTCTTCTTTACCCAACATTCTGCTATGTCGACCGAAGATAGCCACGATACTCCGCACCATACCCCCTGCGAGGGTACCTCCCGAAGGAAACCTTCGGCATATGTCAATTTCCAGGCAGAAGTGGAGTTCCTCCGCACCAGCGTCGCCAAGATGTTTCGAGAAATACGAACACCTTTATACCAAGAACATCGAGCTGGAACATGACTACCGCACCCTGAAGTGTAACGGGGAGAAGGCTCAGGCCCAGGGCTCCCAACATGAGTTTACCCAGGATGTTGAGCGTACCTAACCGAACCAGCCAGTTCCCTCTCACCACAGTGCCCCAGTCCAGTCTAGGCTCGGCAAGGGTAAATGCCCTCTTTACCCAGAGCCAACTAAGTTGGACTTCTTCACATCTCCCCACCGAAGGATAGCCCCCATGAACCGACGAAGATCTATAGGGATTGCAGG

>Chr4-20; OZ022209.1; 27484346-27489762; dir; D;

TCAAGAACTTTCTTCTTCAAGTCTTCTTTTGATGAAGTTGAGCTTGGCTGATCTATCAACAGTTGGGAGACACTTAAACTAGCCGAAGAACTTACCAAAGGCTGAGCAGCAACAAGGTCAAAAGATGGGATGAGTGTCGATGTGATCTTGGCGGGGGCCAAGAGATCTTTAGTGAATTTATCCCACCATCTGATCAAGATACTACGGACTAAATTAGGGATATAAGTCCATTCATCATCAACGTCTTCCAAAATAACATACTCCCATTTTAGTATCCAAGGTAGTTCATACTTTATCATGAGCCACATAAATAAGGTCCTTTCATCAGGATGATTTTTATTCCATTTACAAAATTTCATTTGAAAATATTCCTTAATTTCAGGAGGAAGGTATTCCATAGTAGGACCTCAAGTAAGGAGCAAGCTCTTGAACCAATTAGGGAGTTCTTTCATAGCTTAAAGCTTAAACTGGATAAACCAAGAATGAGAATAATTTTGATTTTGAAAACATAAGACATATTCCCACAATTTTTTATAATCCTTGAAATTATATGAATATGGCCTAATTTCAGGCTGGATAAATCTTCTGGCCCTTTCAGGGCTAGGACCCCATTTTTCCAAAGAGATGATATTTAGAATTTGGATTTTTGAGAATGCTATCGTATTGGTATTTTCACCCCTATGACGATGAACAATATTAACAGACTTGATCTCAATCAGAAGAGTTTCATAGAAAAGATAGGGCTTATTTATGGGAGGAAGGGTATCCCTTAAATACTTTTCAACTACAAAATTCTGGTGTTTGTCCAGATCACTCATTTTAATGATCGCTATAGACATATTCATATATTCACATAATGGATTATCAGCAATTTGCTTCTCCTTACCCTTATTATATGGAGAAGAAGACATCAATGGCTTTGGATTAGGTAGGGGGGACTTAGTAAGAGTTGATGGGCTCTTACTGGGAGTCATCTGTCCGGCGCTCCTGATTCGCACCT

TGCAAGAATTCCCGAGTTAGAAGTCCAGGAGTGTGTTAGTTTCACCTTTAATAAATTCAATATCAAAAATTGAAAGAAGAAAGAATTCCTTGCCATCTTGCAAAAATCTGTTTTGAAGCTAAATTTTTGACGTCTTTAAACAAAATGTCTTTTGTAGCCTGGCAATCTACTCTTATAAAAAAAATCTTTCGTTTAAGAGATTGCTTTCGAATTTAAAAACAGCATGGACAATGGCTAGCATTTCTTTCTTAAGAATGGAATAGTTCTTTTGAGCTGGTTTTTGAGTCCCAGAGGTAAACCTAACCAATTGTTCTAAGTTATTGACTTTCTATTTTAGGATTCCCCATACCCAATCTCTTGAAGCATCGGTTTCTAAGATTTTAAAGGCTTCTGCGTTGGCTAAAGCTAAACAAAGAAGTTCTTTGACCTTGACATTCAGTCTCCTAACAGCTTTAGTGTGTTCATCAGCCTAGGGAACTGGGTTCTTTTTCAGCCTTTGGTATAACAATTTTGAGTCTTGGGCTAAATCCTTATAAAAACCCTTTATGTAATTGAGACTCCTTAAGAACCTCTGTAACTATTGCTTGTCCCTAATTTCATCAAGGAACTTATCAGCAAAGGATATTGCTCGATCAATCGAAACAATCAATCCCTTACTAATATTGTGTCTCAAAAAGCGAATTTCAAAGAAAATAATTTCATTTTCTTTGCAGACACCACTAATCCTGCACACTTCACAACTTTCAAGAAAATATTTAGATGTTTAAAATGTTGCTGTAAAGAATTAGAATAAACCAAAACATCATCAATATAAACAATGGGAAAGCAGTGTGAGGATTAAATATGTCATTCATAATACTCTGAATTTCAATTTTTGATATTGAATTTATTAAAGGTGAAACAAACGCACTGCCAGACTTCTTAACTTGGGAATTCTTGCAGGGTACGAACCAGGAGCTCTGGACAAATGACTCATAGTAAGAGCCCCACAGCTCTTACTAAGTCACCCCTGCCTAATCCATAGCCATTGATGTCTTCTTCTCCATAAAATAAGGGTAAGGAGAAGCAGATTGCTGATAATCCATTCCATGAATATATGAAAATGTCCGTAGCGATCATTAAAATGAGTGATCTGGACAAACACCCGAATTTTCTAGCTAAAAGTATTTAAGGGATAAACCTTACACCCTTAAATATGCCTCATCTTTTCTATGAAACTCTTCTGATGACCAAATTTGTTAATATTGTTCATCATCATAGAGGTGAAAATACCAATACGATAGCATTCTCAAAAATCCAAATTCTAAATGACAAAGTTCATATAGATTTTGATAATCTCACTTATGGTAGTATAGTTAGTACAATTAATCAAGTATGAATGAAAATGTGTGTAGATCTGAAAATAGGTAAACAAATCCAGTCAGAAAGAAACTCTGCCAAATATGAGTTAGGAAATTTTTGCAAGCAATATGGTTTAATTTTTTTTCAGTACCTCCCTCCAAAACAATTAAGCATAGAAAATATGAAGGCCAAACTAGTAAAACTAGACATTACCAAAGAAGAAAACATTCCTTTACAAATAATACCGAATTTTACAAAAAAACCCAGATATAACAAAAAGAAAAATTGGTCAAAAAACCTTGGAAAAATAAGAAAAGTAGACACACAGGGAAAAGTAAGGTTGAATGTTGGAAATGCAAAATATTTGTCACTATGCTAATGAATGCAAAGTTAAGGATACAATAAAACAGCTAAAAACCTCTTATCACAATTAATTAAAGTACTTGAACTACGAAACTCTGAGTCTAGTGAAAGTGAAATCTCAATAGCCAGTTCAGATGATAACTCTCCAGCCTACCAGTCAAATTCCCCCAATATTCAATTTGGATGTAAAGATAATTGCTGCAACACAAAATCAATAAATGTCCTCACAAAACAAGAAGAACAAGAAGAATTACAAATTGACTTAATTAGCAAAATAGAAAACTCAGAATTAAAATCAGAATATCTAAAAAAGCTCAAGAAACTAATTAGCCATTGTGAAGCCAATCAAGTTATTCTAACCCCAAAGATAAGTCTAACCACTACTTTAGAAAAATTTAATCAAAAGAAGTAAATAACCCTAGAAGACCCAACTAGAAGTCAAAAATATAAAAAAGGAAATTATTACTCTACGACAAATAAGTTATAATTTACAAAATGAAAATTATGAAAAATAGACCCAATTAACTTCATGGAAAGACTAGGGTCTAGTTGTGGGCCATATCTAGAGCATCATAAAGAAGAAGAAGATGAGCCTTCCAGTAATGATAAAGCTATTTATCTAATCAAACAAATAAATTTTACAAAATGGTATTCAAAAGTAAGTATTTTCATTAATAATTTTTGAATTAAAAACTGTGGCTTCCTCAGGTAGACTTTCATCAACTATTCAAGAAGGATTAATCCCATTAAATATTATTAAAAAAATCCAAAGAAATCTTGGGGACTGCCTCAGGTAAACTTTTACAGCTAAGATATGAAATCCCTAAAGTCCATTTATGCCAGAATAACATCTGTTTTAAAACCTCTTTTGTTCTAGTGAAAGATATCATAGACAAAGTCATCCTAGATTTACCTTTCATTGCTTTACTCTACCCCTTCAATGCTGAATGTGATGGAGTGACTTCTAAACACATGGGAGAAGAAGTCAAGTTTAAATTCCTAGCAAAACCAGAATTGAGAGCTCTAAAAGCCCTTCAGAGTGATGCAGTCTCAAAAACAGTCAATTTAATTAGAAATAAAAGTAAACAAATCAACTTCCTCAAAGAAGAAGTCAAGTTCAAAAGAATTCAACATCAACTTGAAGATAAAACATTACAAGAGAAAATCAAGAATTTTGAAGAAAAAATCAAGCAAGAAATTTGTTCTGACCTCTCTAATGCCTTTTGGCACAGGAAAAATCACTCAGTTACCCTTCCTTATATCAAAGAATTTAGTGAGAAAAATATTCCTACTAAAGTTAGACCTATCCAAATGAGTCAAGAAGTTATGGAATTTTGCAAAAGTGAAATTACAGAATTACTACAAAAGAAAATAATCCGTAAAAGTAAATCCCCTTGGTCTTGCCCAGCCTTTAATGTTAAGAAAAATGCAAAATTGGAACAAGGGGGCACCCAGACTAGTCATCAATTACAAACCTCTGAATAGCATTTTAGAATGGATAAGGTACCCAATTCCTAATAAGAGAGACCTTATTAAAAGATTAAGTAACTCAGTCATCTTCTCCAAATTTTACACGAAGAGTGGTTTCTGGCAAATACAAATCCAGGAATGTGATAGATATAAGACTGCCTTTGTTACACCTTTTGGTCATTCTAAATGGAATGTTATGCCATTTGGTTTGAAAAATGCGCCTAGTGAATTTCAAAATATAATGAATGAGATTTTCGACCCATTTAGTGAATTCTCCATAGTCTACATTGACGACTTCCTCATTTTCTCTCAGTCAATAGAACAACATTGGAAACACTTACATAAATTCCTACAAATAGTCAAAAGTAATGGATTAGTTGTTTTTGCCAAGAAAATTAAGCTATTCCAATCCAATGTCAGATTTCTTGGATACAACATTCATCAATCACAAATCAGTCCAGTAGATAGAGTTATCCAATTTGGTAATAATTTCCCATATGAAATCCTTGAAAAAAGTCAACTTCAAGGTTCTTAGGATCTCTTAGTTATGTCTCTGATTTCTACCAAACCTGAGAAAACAATGCAAACTTTTATTCGATAGACTCTAGAGTAACCTCTTTCTTGAACTCCTAGTCATACAGAGACAGTCAAGAAAATCAAAATCCATGTCAAGACATTACCTTGCTTTGGCATTCCATCAATTGATTCTTTCAAAATTGTTGAAACTAATGCCTCAGAAAATGGTTATGGTGGTATCCTCAAACAAAAAATTTCCCCAACTCATACAGAACAAATTGTTCGATTTCATTCTAGAATTTGGCCTCAATCACAAAGTAATTATAGTACTATTAAAAAAGTAATTTTATCTATTGTATTATGCATCTCAAAATTCCAAGGTGATTTACTCAATCAACAGTTTTTAGTGAGAGTTGATTGCAAAAGCGCAAAGCATGTTTTAGAAAAAGATGTTCAAAACATTGCCTCAAAATAGATTTTTGCACGATGGCAAGCTATTTTAAGTATTTTCGATTTTGATATTGAATATTTGAAAGGCCGTGAGAATTGTATTCTCGATTTTCTTACACGGCAATTTCTGCAGGGAAAGAAATGGCAGGAAAAGAATCCGAATCAGTCAAACAAGGTAGTCAATCAATTACCAAAAAACTTCCCATTTCTATGAATCCAGTAAAAACATAGCCTTCCCAAATCGTCCCTTATCCGGGATATTCTCCAATTCAAATTACCAATAGATTCACCAATCTTGGCTCTACAGTCCCCAGCATTCGTCCCAATTATAAGTCAGCCCTAATCTCCAGTTATGATCCCTTCCAAGTCACAACCCCTTCTTCTTCTCAGCCATCAATTTCTTACAAGTAAACTTCTCCTTGTCTCCCCTGCAGTAACAACAACCTCTTCATGATTGAACCCTACTATAATAATCTCTCCAATCCAATCAAAATAGCTAAAGCCTACTTTCCCCACAACTATCACTTTGTTCCTCATCACCCAGAAAAGTCACTCAAATTCTATAGAGATATTCTTCTCGAAACCCAAACCCTAGAAATCAAACCAATCAAAGAAAGAAATGATCCAAACATAATCCTTTATCATTCTATGTATATCCACAAAATTCTCAGCCAATCCGATTGGGGAGACCGCCCCTACTCTCTTAAAATTCACAAATCAAACATCCAATGCAGTTATTATGATTATATTGAAGCCTGGAATAACATATTCCTTCACCAAACTGAAGATTTTAGTCATTCATGGTTTGTCAACTTTGATAGTAAATTCAGAAGTCTTTTTCCCTCCTGGTTTTCTTCTTGGTGGGAAAAGCATGGCCCAGTCATTGAAATTCTTCCTCATGATCTCCAAGATTATGTTACTTATTTTACTAGAAAATACAAATTCAGAGAAGAAGATGCTTTCTTCCCCAGTCTCCTATTTTTGTCGCCAAATACAAAGTTCCTTGGATCCTCAAATGGTCTTATCAAGTTAATTAGGAACCAAGAACTCTTTCCCGACAGATTTCAATCAAATGGTGGGATAGATTCAATTATGAAAGAATCATTGGTCAAGTTCATACCGAATTTCCTCTAGTCAAAGTCACACAACGCAAGTCATCAATATCTGAAGCCTCTTAGTCTTCCATCCCAATTGAAGGAAGAACCAAATTAGAAATTAAAGAATTAGCAAGGCAACTCATGATTCAAGCCTCCCAAATGAGTGATGATGAAGACACC

TCTCCAAAGTCACACTCCTCCCAAGCCGCCTCCTTAAATCAGGAGCGTAATGCCAGTCAAAAGAAAAGCTCTTCCAGTCAAACAAAGCAGTGGGCAGACTACGTACCAAGACAGTCAAGATCCCTATGATCTCAATGATGACTAAGCCCTCAAAAGTCTCGATTAGTCAATATTTGCTTTTCCTAAAAGCATACGCATGAATCTATAATAACCCCACTCAGAGACAATTATTCAGTTCACATGATAAAATATAATTGTGCCGACACTACAGTAAGGCATCCAATGCTACCAACATCTAAGTTTTAGGCCCTTTAAATATTGGCCTCTTGGCCTCCTTGGAACTAGGCTTTTTTTTTTTTCAGCAAGTGAATTTTCAATTTTCATAAATTTTCCCTGAGACCTCCTCTGAGAAACTGTTGTAAATTATGAGTGTTACTATGTATGCTACTACAAGCTATTAATAAGATCCTTCAATCAAGATTTTCTGTAAGTTTTATTGCATTAATTTTACAACAGTTTTATTTAATTTTACATTAGTTTTATTGCAACCATTTTCGCTTAGTTTTACTTATATATATTAATTAAAGCATAAATATAAATATATGGCATGTGTTGTAAAATGGGTGAAGCCCACATAGCAAACTAACATTCAAGTTCAACACATGCTCTAACATTCAAGTTGAACACAAAATCAAACCACACATCCACCAAATCAAACCACACACCCATCAAACCAAACCACATACCTACCAAACCAAATCACACACCCACCAAACCAAGCTATTTGCTTTGCCTATAAATAGGCATTATATTTGCTTGTAATAAAATGATGAAATGAGAGAAGATGAAGAGAAGAAAGAAAGAGGGTGAGTGTGTGAAGAGAAAGAGAGCAAAGAGAAATTCTCCTAGAGAGAAAATTAGGTGAGCATACATATTGTAAACACTAGAGTTGTAGCCATATTATGTTATGAATGAAAGGTTACTGTTGCTGCTCTCTGAG

>Chr4-21; OZ022209.1; 27499777-27501659; rev; B;

ATTTTACAGGCTATAGTACTCGAGCAAATGAACAACTTTCATCGAGGAAGTTTTCTGATCTAAGCTTCCGAAATATGACTTATGTACTGATGCACAATACCCACGGGTTTGGGTCTTGGAGAAGAAGAAGAAGAAGAAAGAAGAGTGAGAGAAAATCAGAAAATAAAGCATGCCATGCCAAATGAAAGGAAAAGAAAGCAGTGACTTGCAAAGAACTCAAGCTGTTGTAAGCCAAAATATGCTTGCAGTAACCAAGATTCGAGTCAAGCAAAACATGGAAGATAATTATGCGTTTTACGAGATGCTCTTGGAAAATAACACAAGCTCTATATTCATGTCGTTGGAAGAGTGAACCCTCATGCCAGAGCAAGCTATATCTTGATTCAACTCAACCATTAGATTATCCACATCCTCATAGCAAATCCAAGCTAATCATGTCAAGCCAAGTCACGTTTTTCCAGCACCTATTCCATGCCAAACAAAACATTTTCCAGCACCTGCCCTATTTCAAACATCCTTCCAGCATACATTCTGTTTCAAACATCCTCCCAGCATACACTCTGTTTCAAACATTCTTGTAGTATCTAGCCATCATGTCGCACGTTATCACCTTCACTGAGGTTTTGCCCGCCAAATTCAAAGTGTTTGGAGGCTGGTGTTGTAAACTCAAACACAATGGTGAATCAAAGCTCATTGCATCCGCAACTCCAAGATTGGATCCCCACCGTCCACTAGCACGTCTGGCTACTGTCAAATGACATCCACCATGCTAGATGTCCCAAAAGCCATATTAAATCATGTTACCTTAGCCAAAATCAATTATCCAAGCATACAAGCCGAAGCCAAGTGTACATGCCAAAACCAAGCATCCAAGCATACAAGCCAAAGCCAAGCGTACATGCCAATACCAAGCATCCAAGCATACAGGCCAAAGCCAAGCGTACAAATCAATACCAAGCATACAAGCCAAAATCAAGCGTACACGCCAAGCATATAGACC

ACACTTGAATAAATTCCACCATATAGTCAAATAAAATGGTCTAGTTGTCTTTGCCACACAAATTAAATTATTCCAAACCAATTTTAGATTCCTTGGATTCAGTATTCAATAATCTCGAATTAGCCCTATAGATCAAATCATCTAATTTTCTGATAACTTCCCAGATGAACTCCTTTATAAAAATCAGCTCTAGCGATTCTTAGGATCCCTCAACTATGTCTCTGATTTTTATCAAAACGTAAGACAATATTGTAAACCTTTATTTGAAAGACTTTAAAATAACCCTCCACCTTGGACTTCCATTCATACTGAAATAGTCAAAAAAATCAAGATCCATGTTAAGACTCTTCCGTGTCTTGGCATTCCTTTAGTTAATGCTTTTAAAATTGTTGAAACTGATGCCTTTGAAAATGGTTATGGAGGTATTTTCAAACAAAAAATCTCTCCAAATCAACTAGAAAAACTTGTTCTCTTTCATTATGGAATATGGACTCAATCACAGAGTAACTACAGTATTATCAAAAAAGAAATTTTATCTGTTGTTTAATTTATCTCGAAATTTCAAGATGATTTACTCAATCAAAAGTTTTTAGTAAGAGTTGACGGCAAACTGCTAAGCATGTTTTAGAAAAAGATGTTCAAAATATTGCATCCAAATAGATTTTTGCCCAATGGCAAGCTATTTTAAGCAATTTTTATTTTGATATTGAGTATTTGAGAGGAAGTGAGAATAGTATTTCTGACTTTCTTACCAGGGAATTTCTAGAAAGAAACAAATGGCAGAAAAAGAGTCAAGCAAGAAAGAAAGCCAACCACTCAGAAAACTCCCAGCTTCCATGAACTTAGTCAAAATAGAACCCTCCCAAATTGTCCCCTACCCGGGATACTCACCTATTCAAATTACCAACATATTCACCAGTCTAGGTTCCACTGCTAGTAGTATTCATCCCAATTACCAGTCAACTCTAATTTCTAGCTACGATCCTTTTCAAGTCTCTACTTCACCCTCAACCCAGTCAACCATTAGTCACAAAAAATCCTCCCAAAAGTAACTCCAATCTCTTCATTATAGAACCATGCTGCAATAATCTTTCTAATCCTACCAAAATCGCCAAAGCCTATTTCCCCCCCAATTTCACTACATCCCTCAACACTCATCAAAGTCACTAAAATTCTACATAGATATTCTACTTGAAACCCAAACCCTAGAATTTCGTCCAATCAGAGACAAATAATCCATACATAATTCTTTACCACTCCATGTATATCCACAAAATTATGAGCCAGCCCGAATTGGGAGATCTCCCCTACTCACTCAAAACCCTTTAGTCCAAAATTCTGTATAGTTTTTATGATTACATTGAAGCCTGGAATGTCATCTTTTTACACAAAACCAAAGATTTTAGTCATTCCTGGTTCGTCAATTTTGATAGTAAATTTCGTAGCACTTTCCCCTCCTGGTTCTCTTCTTGGTGGGAAAAACATGGTCCAATTATTGATATTCTTCCTTTAGACCTCAAAGAATCGTCACTTATTTCTCCAGTAAATACAAAATTAAAGATGAAGACACCTTTTTTCCTAGTCTGCTACATTTTGTTGCCAAATACAAAGTCCCTTGGATTCTTAAATGGTCCTTTCAAGTCAACCATGACTCCATAATCCTATTCAGACAAATTCCAGTAAAATGGTTGGATAAATTCAAGTCAGAAAAGGTCATTGATCAAGTCAATCAGGAATTTCCCTTAGTCAAAGCTCCTTAGAAGCCTTCAACCTCAGGCACTTCTCACTCTTCTCTCCTAATTGAAGGAAGATCAAAGTCAAAAATTAAAGAATTAGCTAGACAACTCATGATTCAAGCCTCCCAAATGGACGATGACGATGAC

GCCTCCCCAAGATCTCATTCCCCTCAGGCCTCATCTTCTAGTCAAAAGGAATCTACTCACAAGTTTCGGTGGGCAGATTACCCTGACAGTCAAGACCCTTACTATCTTAATGAAGACTGACTCAGAAAGGTCAGAAAAGCAAAAGTAAAAGTAAAGTAGAAATGATTGAAGTGAAAGAAAGGAAAAAGAAAAAGTGAAAAAGTAAAGATAAAAGTAGAAAAGAAAATAGTCAAAATCCCGTGCCTCCACAGTCACCATAAGCTGCCAAATAGTCAAGATTCCCATACCTTCAGAATTTGCCGACATCACAGTAGTCTGGCTCTTTAAATAGATGCCTCTCGGCCTTAGCAAACTAGTATGTAATTTTTCAGTTTTCTCAGTTTCAAAATTTGCTCTGAGACTCCCTCAAAGAAAATTCTCTCTTTGTAAAATATGAGTGTTATCAACTATCAATAAAATCTTTCAGTCAAGATTTTCTGTTAGACCATAATTCTTCTAAGCCTATTTGTTGAAATTTTCATTTTAGTACTTTTTTAAATATATTCATGCTGTTATAAAACGAACTGGAATATGTGCAAATATTGAACTGCACGTAAAGTAGATGAGACACAGTATTTAACGAGGTTCGGCTATGCCTACGTCCTCAGAGAGCAGCAACAGTAACCTTTCATTCATAACATAATATGGCTACAACTCTATTATTTACAATATATATGCTCACCTAATTTTCTCTCTAGGAGAATTTCTCTTTGCTCTCTTTCTCTTCACACACTCACCCTCTTTCTTTCTTCTCTTCATCTTCTCTCATTTCATCATTTTATTACAAGCAAATATAATGCCTATTTATAGGCAAAGCAAATGGCTTGGTTTGGTGGGTGTGTTATTTGGTTTGGTAGGTATGTGGTTTGGTTTGATGGGTGTGTGGTTTGATTTGGTGGGTGTGTGGTTTGATTTTGTGTTCAACTTGAATGTTAGAGCATGTGTTGAACTTGAATGTTAG

>Chr4-22; OZ022209.1; 27506936-27518560; dir; B;

CTGGTAGGAGAAATGAGTGGGTCATGGCGTTGGTCTAAAGGGTGTTTTGCTCAAGTATCCTTTGGTTTTTGGATGACTTGTGTTGGGATTTGTCTTTGGCCATGTGCTGGACATTCCCAGCAGCCTGCAGACGTTAGTGACCCTTGGATTTTGTCATGGTTTTGTCTCCAGCCATGTGCTGGAGACTTTCAGATATTTTATAAGTCACTTGGGCCTTTCTTTAAGGTAATGGGCTAGTTCACTAAGAGGATGGGCCAATGGACTATTTTTACTAAGGTAATGGGCCAATTTTCTCTCTTTTATGCTTACCTACATATTTGAGCTCAAGACATGGGCTCGAGTTTTGGGGCTCCCAAGCAGCCCAAAACTTCCACCTCCTGACCCATTAATGGGCCTCGTCAATGCACGTAACCATTCATACCACAACCTGCTCAACAAGCCCCAGGCATTCGCGTGGCTCGAGCCCACTTAAGCTTGTAGCGTGGCGTGTTGCTTATTTGCTTGGCGTGGCACGTGTGCAAACGTATATGATGAACTCTTGCATACCCAAGTTGGGTTTCTTCCCAAGTTAGGTGTCGTACTGGAATAGAAATATATGTGGGCCCACCCTTGGATTTTTTGGGCCTCAACAGATCCTTTTCAAGTCTCTACCAGTCAACCATTCATCACCAAAAAAATCCTCTCCTTATCACCCCAAAAGTAACTCCAATCTCTTCATTATAGAACCAAGCTACAATAATGTTTCTAATCCTGCCAAACTCGCCAAAGCCTATTTTCCCCCCAATTTCCACTACATCCCTCAACACCCATCAAAGTCACTAAAATTCTACACAGATATTCTACTTGAAACCCAAACCCAAGAATTTCGTCCAAACAAAGACAAATAATCCAGACATAATTCTTTACAACTCCATGTACATCCACAAAATTATGAGCCAGCCCGAATGGGGAGATCGCCCCTACACAGTCAAAACCCTTCAGTCAAAAATTCAGTACAGTT

ATTAGATAAGATAGAGACAAAAATTGACAAAATAATCCCTCAACAAATATCCCTTAAGTCTCAGAAAGAGAAACCTCTAATAAGATTCCAAGATTTAAAGTCAAGCACCACTCTCAAGACAAGTCCCACAATGGAAAAGATTGAAGAAATGCTTAAACAATTAACTCCAGTTAAAGAAAAACTTCAACCTTCTGGTCTCAAAGTCCTAGAATCCTCTGTTTTAGTCAATTTCTCTGAAACCGAGTCTATAACCAGTAATGAAACAAATTCCAGTAATATATCAAAAATTGAAAACGCCATTAATAACCTAGAAATAAACAATGCTAAGAAACAAAGTCAATCCTACTAGTCTAACAAAAAATTAGTACCCCAAACCAACTCCTCCTAACATCCAGTTTGAAGAAAGAAATTCTCAAAGTCAATTCTCAGTCTCATCTGATAAACTATACGAATGGAATATCGATGGTCTATCAAAACAAGAGATTCTGAATAAACTTCAACACATGTGTATGGTAGCCAACAGTTATATAACCACTCACAATTTAAGACAGTAAGAAATTGTTCCCCTTTTAGAAACTGGATTTACCAGAACCTTACGTTCTTTGTGGGATAAACACTTAACCGAAGAGTCAACACGACAAATCACACATGCAGTAAAAATAAATGAAGGTCTCCATATTTTTGATGAGCAATTAGGAACGGGAATAAAAGATGGAGTCAATACTTAATTATATACCATAGTTCAACACTTTATAGGAACACCTAGTAATACTACTGCCAAAATCCATTACCAGCTCAGTAATCTTAGATGCCCCAAATTAAGTAATTTTTTAGATGGTATAAAGATGTCTTTATTTCTAGAGTTATGTTAAGAGATGATAGTAATCAACCATTCTGGAAAGAAAAATTCATTAACGGTCTTCCCAATTTGTTTGCTCATACAATTCGAACAGTTTTAAGCAACAACCAAGGCCATATAGATTTCGTTAATCTAACTTATGGTAATATAATTAGCACGATTAACCAAGTAGGTATGAAAACGTGCATGGATTTAAAAATAGGAAAACAAATACAGTCAAATACAAAGTCAGCCAAATATGAATTAGGAGATTTTTCTGAACAATACGATTTAGTACCTATTCCTCCCTCTAGAGAGGTCAAGACTAGGAATAATGACCGACAAACTAGTAAAAATAGACATTATTCACATAGGAAACAATATTTTAGACATAATGATGAATTTTATAGTAAGAGAAATTTGACCCTAAAAAATTTTGGTCAAAAACACCCAAAAAAGACAAGAAAAGTAACTTTTAAAAAAAATACAGACAAACGAAAAGTTGAGTGCTGAAAATGTAAGAAATTTGGTCACTATGCTAAAGAATGCAAAGTAAAAGATACAATAAAGCAGTTAAAGATTTCAGAAGAAGACAAAGCCCAGTTGATCAAAGTTTTAGAATTAAAAAATTCTGAGTCTAGTGAAAATGAAATCTCAGTAACAAGCTCAGAAGACTATAGCTCCTCAAACTCTCAGTCAAGCTCTCCACAAATTCAGTTTGGATGTAATGATAAATGTTGTAATACCTTAACCAAGTCAATAAGTGTGCTCACGAACAGGAAGAACGCAAGTCAAAGTAATTTCCCCAAAATAAGCTTAAACACAACTTTAGAAAGATTTAATAGAAAGAAAGAAGTTACTTTACAAGATTTACAAGTGGAAGTAAAAACAGTCAAAAAGGAGATAATAGAATTACGATAGAAAACACACAAATTTCAAAATGAAATTTTCAAAATTAACCAAGATTTGATTAATTTAATGGAAAGAAGAAATTCTAATTCTAAGAGTTAGTCTGAAAGTCAAGCAAATTTAGATGATGAACCCTCAAGTAGTGATAAAGCCATTAATTTAATAAAACAAATAAGTTTTAGAAAATGGTACTCTAAAGTCACTATTTTTATAATAAAAAATTTTGAGTTAAATACAACAAGGTTTAATCCCCACCAAATATTACAAAAAGTCAAAAGAAACGTTAGGCACTGCTTCTGGGAAACCACTTCAGTTAGACTACAAAATTCCAAAAGCCCATGTTTGTCAAAATAAGATCTGTTTTAAAACTTCTTTCATATTAGTTAAAAATATTACAGTTGAAGTCATCCTCGGTCTATCCTTTATAGCTCTCCTGTCCCCTTTTCGAGTAGATTACGATAGAGTCACATCCACTCATATGGGAGAAGAAGTCAAGTTTGAATTTTTAACTAAACCTGAGTCGCATAATTTAAAAGCTCTGCAGAAAAATGCAGTTTCAAAAATAGTGCTTGTGAAATATGGAAGGATATCAAATTTGAATTCCAAGCAATGGATACTTTGTAATCCAGTAATTACTTACTGTTAGTTCTCTTAATTGAATTGAAATTAAACTCGGCCCAATCTTTTACTAAAAGGATTGAGCCGAATACAATAAAAAGATCCTATTGTATAGATGTATAGATTTTTGATAGATACATACTTATATAGATATACAAGATCTTAAATACAAAAATATAAGACGAAACAACTAATTCTTTTATTGTTGGGTTGGATCCACAATTAATCCTATGGATCCTTAGGATTGGCGTATTCTTATAATATTTTTTTTTATATTAGTTTAGTATATTCTTTTTTTATATATTATTATATCCTGAATATCCTGATCTTGCCAAGTATCACAACTTCTCCCATCCTGTATATTGTCCTTTTCATTCTGTGTTGGAATATAAATTTATTAGTAGGTGATATTTTTATGAAAAAGGTTCTTTATATTCATAGCATAGGAGAAACAACTCTTTTTTTTTTTTTCAATATACCCTAGTGATTGGATTTATATGTTTATTCTGATCGGAATATTCAAATGATTTTCATCGAATGACTATTCATTTATTGTATTTTCATGTAAATAGGGCAAGAAAGTTCTATGGAAAAATGGCGATTCCATTCGATGTTGTTTAATGGGGAGTTAGAATACAGGTGTAGGCTAAGTAAATCAATGGACAGCCTCGGTCCTTTTGAAAATACCAGTGTAAGTGAATATCCAATTATAAATGATATGGATAAAGACATTTTAAATTGTAATGACAAGTCTAATTACAGTAATGTTGATCATTTTGTCAGCGACAGATACATTCGGAATTTCATATCTGCTGACACTTTTTTCGTTAGGGATAGTAATAGGGACAGCTATTCCATATATTTTGATATTGAAAATAAAAATTTTGATATTGACAACGACCGTTCTTTTCTAAGTGAACTAAAAAGCTCTTTTTATAGTTATCAGACTTCTAGTTATATAAATAATGCACCTAAATTGTTACGTGTATGATACTAATTCTCATTATATTTGGAATAATCACATTAATAGTTGCATTGACAGTTATCTTCGTTCTCAAATTTGTATTGATAGTTATATTTTATGCAATAGTGACAATTACAGTGACAGCTACATTTATAGTTACATTTGTAGCGAAAGCGTAAATAGTAGTAAAAGCGAGAGTTCCAGTATAAAAACTAGCACAGATGGTAGTGATTTTACTATAAGTTCTAATAATTTAAATGTAACTCAAAAATACAGGCATTTGTGGATTCAATGCGAAAATTGTTATGGATTAAATTATAAAAAATTTTTAAAATTAAAAATGAATATTTGTGAACAGTGTGGATGTCATTTGAAAATGAGTAGTTTCGATAGAATCGAACTTTCGATTGATCCCGGTACTTGGGATCCTATGAACGAAGACATGGTCTCTCTGTATTTTATTGAATTTCATTCGGAGGAGGAACCTTATAAAAATCGTATTGATTCTTATCAAAAAAATACAGGATTAATTGAGGCCGTTCAAACAGGCACAGGCCAACTAAATGGTATTCCCGTAGCAATTGGAGTTATGGATTTTCAGTTTATGGGGGGTAGTATGGGATCTGTAGTGGGCGAAAAATAGATCGGGATCGGGGGGAATTTCTTAAATGTAAAGTCAATATATCTATATGTATTCGGAAATCATGTGAAAGGGATGTTACTATTTTAGTTTGGATCTAAGAAATTCGATGAATTACCCCTAAACGTTCACATCATAATAGTGCTGGTTGATGCGAGTTACTTCGGAAACAAAAAAAGTAAAATAAAATTTATTTTTGTTATTCTCCCAATTCCAATAAAATGCAACTAGGTCTAGTTATAGTATGAGTCGGTGATCAGAACGTATATGGATAGAACTTATAGCAGGGTCTCGAAAAACAAGTAATTTCTGCTGGGCCTTTATTCTTTTTTTAGGTTCATTAGGGTTTTTATTGGTTGGAACGTCCAGTTATTTTGGTAGGAATTTTATATCTTTATTTCCTTCTCAGCAAATAATTTTTTTTCCACAAGTTATCGTGATGTCTTTCTATGTTATCGCAGGTCTTTTTATTAGCTCTTATTTGTGGTGCACAATTTCGTGGAATGTAGGTAGTGGTTATTATCGATTCGATATAAAAGAGGGCGTAGTGTGTATTTTTCGTTGGGGATTTCCTGGAAAAAATCGTCGCATATTCCTCCGATTCCTTATGAAAGACATTCAGTCCATCAGAATAGAAATTAAAGAAGGTATTTATGCTCGTCGTGTCCTTTATATGGAAATCAAAGGCCAGGGGGCCATTCCCTTGTCTTGTACTGATGAGAATTTTTCTCCTCGAGAAATTGAGCAAAAAGCTGCTGAATTGTCCTATTTCTTGCGTGTACCAATTGAAGTATTTTGAAATTCTTGCGTGTACCAATTATTGTATTTTTAAAAAAGGAACTGAAGAATGAATACTTTGCAAGAGAGAAAAAACTCAAGAATCCTCGTTTTTTTATATAGCATAACTTAACTGAAGTTTCTTCAGAACGCTTATTCGAGCCAAAGCAAACGTTACGAAATAATTCTAAAAAAAAATAGTTTGTGTCCACAATTTTTTTTATGCGTATGTAAACCCACTTAACTCAATTCAGTAACAAATCTAACTACTAGATTCATCATATATTAAAACAAAACATTTCATAATAAATCATAATATAATAAAGTAAAGAATTTTTTTTTAATAGAAATGTTTGATATATTTTGATAGAACGGGAGTTCTTTCGTCTCGCAATCCGACTACTATTAATTTGAAGTGGGCTTTTTCTTATTCTATTTCTGTATTCTTTCTAAATTCAAGGACTAACCACTGTACTGAATCACAAAAAAAAAAATAGGAAATAGATTCATGGGCTCGATAACTTGTAATAGAACTTATGCTTGATAGAAATATTAGTATCGTATAGAGTCGACGAACGAGGTGCGTTCAGTAAAAATTAAAAAATTCACAGACGAAAAAATGGCAAAAAAGAAAGTATTAATTTCCCTTCTATATCTTGCATCTATAGTATTTTTGCCTTGGTGGATCTCTCTCTCATTTAATAAAAGTTTGGAATCTTGGATCTCTTATTCTAAAAAAATTCATAGACTTAGAGGAACTCCTACGTTTGGACGAAATGTTAAAGGAATACCCGGAAGCACTTTTACAAAAGCCTCGCATCGAAATCTACAAAGAAACGATCCAATTGATCAAGATGCACAATGAGGATCGCACGCATACAATTTTACACTTCTCGACAAATATAATCTGTTTCGTTATTTTAAGTGGTTATTCTATTATAGGTAATGAAGAACTTGTTATTCTTAACTCTTGGGTTCAAGAATTCCTATATAACTTAAGCGACACAATAAAAGCTTTTTCTATTCTTTTATTAACTGATTTATGTATAGTATTCCATTCGCCCCACGGTTGGGAACTAATGATTGGCTCTGTCTACAAAGATTTTGGATTTGCTCATAATGATCAAATTATATCCGGTCTTGTTTCTACTTTTCCAGTCATTTTAGATACAATTTTTAAATATTGGATCTTTCGTTATTTAAATCGTGTATCTCCTTCACTTGTAGTGATTTATCATTCAATGAATGACTGAAAAATGATCGAACTTTTCAATTATAATATTTGTTACTTTGTGGATAAGCAAAACATTCAAAATTGTACTTATTCTTTCTACCCATCCAAGGGATTCCTCCAATATTCCAGTAAAATTATTTATATTTAAGTAAATAGCAAAATTATAGATAGGGAACTATACTAGCGACCTGCCTAATTTTATTGTATAAATTTTCGTTTTTTTTTTTTTGACTTTGCAATCTAAAAATATCTTTTCTTGGATAATGAAAGAGATTACTCGATACATTTCCGTATCTCTCATGATATATTTAATATCCCAAGCACCTCTTTCAAATGCATATCCCATTTTTGCACAGCAGGGTTATGAAAATCCACGAGAAGCGACTGGCCGTATTGTATGTGCCAATTGCCATTTAGCTAATAAACCCGTGGATATCGAGGTTCCACAAGCGGTACTTCCTGATACTGTATTTGAAGCAGTTGTTCGAATTCCTTATGATCTGCAACTGAAACAAGTTCTTGCTAATGGTAAAAAAGGAGCTTTGAATGTAGGGGCTGTTCTTATTTTACCCGAGGGGTTTGAATTAGCCCCTCCCGATCGTATTTTGCCCGAGATTAATGAAAAGATAGGAAATCTGTCTTTTCAGAGCTATCGCCCCACTAAAAAAAATATTCTTGTGATAGGTCCTGTTCCTGGTCAGAAATATAGTGAAATCACCTTTCCTATTCTTTCCCCGGACCCCGCTATTATGTAAGATGTTCACTTCTTTTAATATTTCATATACGTAGGCGTGAACAGGGGAAGGTGTCAGATTTATCCCGACGGGAGCAAGAGTAACAATAATGTTTATAATGCTACAGCAGCAGGTATAGTAAGCAAAATCATACGAAAAGAAAAAGGGGGGTACGAAATAACCATAGCGGGTGCATCGGATGGACGTCAAGTGGTTGATATTATCCCTCCAGGACCGGAACTTCTTGTTTCAGAGGGCGAATCCATCCAACTTGATCAACCATTAACGAGTAATCCTAATGTGGGTGGATTTGGTCAGGGGGATGCGGAAATAGTACTTCAAGATCCATTACGTGTCCAAGGTCTTTTGCTATTCTTGGCATCTGTTATTTTGGCACAAATCTTTTTGGTTCTTAAAAAGAAACAGTTTGAGAAGGTTCAATTGTCCGAAATGAATTTCTAGATTCGCGGATTTATCAACATCAAGCTCTAAAAATAAACAAATTTTTGTTGGCAATTATGTTATGTAATTATGTATAATCAAAAAAATTTTGCTTGTTTATATTCTTTCTTCTACCGGATTTCGGGAATTACTTATACCGCATTACTGGTCATAGTATATTGTGAAGAAGACTATTTGATTTTACTTATCTCTTCTTTATTTATTTGTTTTTTCAATCCAAATTCAAACGGTATGATATAGAACGAATCCATAGTTTTATATTTGAATATAATCAAAACAAAAGATAAATAAGCGGAGAATATAAACCAAAGTATAAATGAGAGGAACAAAGGATTTTAGGGGGGATTGTTCGTCTACTAGTCCTTGACACAAGAAACGGAATTTTCCACAACCCTTTCTTGTGTCGAATTAATAATAATTCTTGATCCCGTTCGTAAAAAATTTCTATTTTTAGTTTTTATTTTTTTATAGGTTTATCATAATCTTTTTTAGATTTATTACGTAAATAGTGGTAGTGGACAAACAAAAATAAATATAGGTAAGTTTATTGAGCAAATAGAACTTCTTCAATTTCAAAAAAAAAAAATTTTTAACTTTTATTAGATTAAATATTTGTTAGATTAAATAGAGTAAGGTTCTATGCTATTAGTATAGAAAGGGTTTGCATGATATCTGATCGATAGAAATATATAGTATATATAATTGTGAGTCATCCAAAAAGAGTAAATAGAGTGATTCCTTCTTCGTTTTAAGTATTGACAGATACTATTGAGTAACAGATGTTCTGAATCCCTTAACGAAGTTAATTTTTCAAAAACATTATCAGAAAAAGTGGAGGTTTTTGAAACATCTATAAAAAAATATTATGATTATTAAGAACTCAACGGGACCTACCCCCTCTTTCCTTGTCTGATTCGAGGGGGATCCCGTTGAGTTCTTATGCTTTCATGTCTACAACTCAGTTCATCCGATTATTACAGGGATGAACCTAATCCAGAATAGGAACCATAAAAGAAAATACCGATTAAACCGATCACAAGAATACCCGCTACAGTACCTATTATCCAAAGAGTAATCCTTCCAGTAGTATCGGCCATTTGTCCTACTTTCCTCCACATTTTATCAAGTGGTCATGCTCGAGACATAAACAGCCATAGATAATTATGAGATGATATCTTTCCGAATGGGATAAGAGAATTCTTATATTCTTTTTTTTAATTAAAGAAATAATTGGAAAATAAAACAGCAAGTACAAAAATGAGTAATAACCCCCAGTAGAGACTGGTACGATTCAATTCAACACTTTGTTCGTTCGGGTTTGATTGTGTCGTAGCTCTATTATTCGGATTAGGTTTATCGTTGGATGAACTGCATTGCTGATATTGACCCTAAAAAAGAAACGGTAGGTACAGCTAGTCCGTGAACAGCCAACCATCGCACTGTAAAAATTGGATAGGTTCTATCTATAGTCATTGGGGCCTCCTAAAAAGATCTACTAAATTCATCGAGTTGTTCCAAAGGATCAAAACGGCCAGTTATTAATGAAATTCCTTGTCGGCTTTCTGTAAAATACTCGTTTGGACGAGGGCTTCCAAATACATCGTAAGCTAATCCCGTGCTGTCGAATAACCAACCCGCAATAAATAAGGAAGGTATAGTAATGCTATGAATGACCCAGTATCGAATACTGGTAATAATATCAGCAAAAGAACGTTCTCCTGTGCTTCCAGACATGTTGAGCTCCGCATATTCTTGTACAGTCAGGGGGGGGGGGGGTCGATTCCGTAAAAGATGAAATCAGTAAATGGAAATTCACTGAAAAAAAATCTTTGTGAGATCGTCAATATTGTACCAAGGGTGTCTTTAGAGTATACCTAATCAGTATAGCTATCCTTCTTCTGACACAGCAATGCAATTTCACAATCAGTATCGAAATGAAGTGTTATATAATTTCTTTCTTCTTTTCTTGTTGCTTGTCGATGTAGAATTTTGTACCATTTAATAGAAAATTCCTCAAATTCCTGTAGTATAAGGGTTTTCTTCATTATTGACTTCGGACTAGAAACTGAAGATCAGGTAAAAAACAAAAATTGTAGAATTACTTCAAAAAGGGATAATTCATAAAAGCAAGTCACCTTGGTCCTGTCTAGCTTTTTATGTTCAGAAAAATGTTGAATTAGAAAGAGGGGCCCTAAGATTAGTCATTAATTATAAACCCCTTAATACGGTATTCGAATGGATTAGATATCCGATCCCCAATATGAGAGATTTAATCAATAGATTAGATAAAGCAGTCATTTTCTCTAAATTTGATATGAAGAGTGGATTTTGGCAACTCCAAATCCAAGAGTCAAATATAAAACTGCTTTTCTTACCCCATTTGGTCACTGTGAATGGAATGTCATGCCTTTTGGTCTAAACAACGCACCTAGTGAATTCCAGAATATAATGAATGAAATATTCAACACATTCAGTGATTTCTCCATAGTTTATATTGATGATGTCCTTATTTACTTCCAGTCAATAGAACAACATTGGAAACACCTGTATAAATTCCTTCACATAGTCAAGAAAAATAGTTTAGTTATCTCAGCCAAAAACATCAAGCTTTTCCAAACCAATATTAAATTTCTTGGATTTAATATATGTCAGTCACAAATCAGCCCAATGGATCAAGTCATTCAATTTGCCAATAAATTCCCGGATCAAATTCTTGATAAAAGTCAACTTCAAAGGTTTCTAGGATCTCTTAACTATCTCTCTGATTTTTATCAGAATCTGCAGAAGCAATGTAAACCCTTATTTGATAGACTCCAAAGTAACCATCCTCCATGGACCTCTACTCATACAGAAATTGTTAAACAAATCAAAATCCATGTCAAAACACTACCCTATCTTGGTTTTCCTTCAGCCAATTCCTTCAAAATAGTTGAAACTGACGGCTCAGAAAATGGTTATGGAGGTATCCTAAAACAGAAAGTCAATCCAAATTAAACAGAGCAAATCGTTCGATTCCATTCTGGAATTTGGACACAGTCACAACATAATTATATACTATTAAAAAAGAAATTTTATCTATAGTATTATACATTTCAAAATTCCAAGATGACCTACTAAATCAAAAAAATTTAGTCAGAGTTGACTGTAAAAGTGCAAAACATGTTTTAGAAAAGGATGTTCAAAATATTGCATCAAAACAGATTTTTGCACGTTGGCATGCTATTTTAAGCATTTTTTATTCTGATATTGAATATTTGAGAGGTAGTGAAAATTCTATCCCAGATTTCCTTACCAGAGAATTTCTTCAGGAAAAAAACAATGGTAGAAAAAGAATCAAAAAAGCAAGGCAAGCAATCAGCCAGACAACTCCCAGCTTCAATGATCCCAGTTAAAACAGAACAACCTTTCCAAGTCATCCCTCATCCAGGATATTCATTTATTCAATAGGTTTACTACTTTAGGATCTATAGTCAGTAACATTCGTCCCAATTATCAGTCAGCTCTTATTTCCAGTTATGATCCATTTCAAGTCTTGACCCCCTCACCATCCTGTTCCCCAAATAGTCAGAAAAATCCTCTCCTTATTTCCCAAAAAGTAGTCACACCCTCTTTCTCATTGAACCATGCTTTAATCACTTATCTAGCCCAGTCAAAATAGCCAAAACCTATTTTCCCCATAATTTCCATTACATACCCCCATACCCTTCTATGTCACTTAAATATTATTGAGATATTCTTCTCGAAACCCAAACCTTAGAAATTAGACCAATCAAAGATAGAAATGACTCGAATATCATTCTCTACCACTCCATGTATAATCAAAAATTCTTAGTGAAACTGAATTGGGTGAACACCCTTACTCTCTTAAGACACTTAACTCCAGTCTTCAATATAATTACTATGATTACATTGAAGCATGGCACATTATTCTCCTCCACCAAACTGAGGATTTTAGTCATTCATGGTTTATCAACTTTGATTTGCTAGTGGGAAAAGCATGGTCCAACCCTTGAAATTCTTCCCCCCAACCTTAAAGAGTCAGTCATGTACTTTTCCAATAAATACAAATTTAAAGAAGAAAAATCAGTTTTTCCCAGAACTTTTAATTTTCATAGCCAAATACAAAGTCTCATGGATTTTAAAATGGTCCTATCAAGTCAACTAGTAATCCCGTATCCTCTTTAGGCAGATCTCCATCAAATGGTGGGACAAGTTCAAGTCAGAAAGAATTGTTGATCAGGTCAATATTGAATTCCCTCCAGTCAAGATCCTCAGTCAAAGCCTTCTTGCTGCGGCACATCTCATTCTTCCCTCCAAATTGAAGGAAGATCCAAGTCAGAAATAAAAAAATTGGCCAGACAATTAATGATTCAAGCCTCCCACCCAGATGGATGATGATGAAGATGCTTCCCCAAGGTCTCAATCCACTCAAGCCTCCTCTTCCAGTTA

GACGCAAAATCCATCAATCAATCAAAAAGATTCCACGCAAAAATTAAGATGGGCCGACTACACTGACAGTCAAGAACCCAGAGATTTGAATGAAGACTAAAATAAAGTAAATATAAAAGCTAGAATAATGTCCTCAAGCCTCATGCCTCTACAGTCAATATTCCCCGACAGTCAAGCCCTGTGGCCAAACAATTAAATATTCTAAACCCTAATATTCCATATTTTTTATCACGGGGCTTGACTGTCGAGGAATATTGACTGTAGAGGCACGGGGCTTGAGGACATTATTCTAGCTTTTATATTTACTTTATTTTAGTCTTCATTCAAATCATAGGGGTCTTGACTGTCAGGGTAGTCGGCCCATCTTAATTTCTGCGTGGAATCTTTTTGACTAATTGATCGATTTTGTGTCTAACTGGAAGAGGAGGGTTGAGTGGATTGAGACCTTGGGGAAGCATCTTCATCATCATCCATCTGGGTGGGAGGCTTGAATCATTAATTGTCTGGCCAATTTTTTTATTTTTGACTTGGATCTTCCTTCAATTAGGAGGAAGAATGAGATGTGCCGGAGCAAGAAGGCTTTGACTGAGGGATCTTGACTAGAGGGAATTCAGTATTGACCTGGTCAACAATTCTTTCTGACTTGAACTTGTCCCACCATTTGATGGAGATCTGCCTACAGATGATACGGGATTCCCAATTGACTTCATAAGACCATTTTAAAATCCAAGGGACTTTGTATTTGGCTATGAAAATTAAAAGTTTTGGGAAAAACTGATTTTTCTTCTTTAAATTTGTATTTACTGGAAAAGTACATGACTGACTCTTTAAGGTTGGGGGGGGGGGGGGAAGAATTTCAAGGGTTGGACCATGCTTTTCCCACCAGGAAATCAAAGTTGATAAACCATGAATGACTAAAATCCTCAGTTTGGTGGAGGAAAATAATGTGTCATGCTTCAATGTAATCGTAGTAATTATGTTGAAGCTAGAGTTAAGTGTC

>Chr4-23; OZ022209.1; 27520751-27529956; dir; D;

AGGGGGAAAATAGGCTTTGGCTATTTTGACTGCGCTAGACAAGTGATTAAAGCATGGTTCAATGAGAAAGAGGGTGTGACCGCTTTTTGGGAAATAAGGAGAGGATTTTTTATGACTAATTGGGGAACAGGATGGTGAGAGGGTCAAGACTTGAAATGGATCATAACTAGAAATAAGAGCTAACTGATAATTGGGACGAATGTTACTGACTGTAGATCCTAAAGTAGTAAACCTATTTGATATTTGAATAGATGAATATCCAGGATGAGGGATTACTTGGGAAGGTTGCTCTATTTTGACTGGGATCATTGAAGCTGTGAGTTGTCTGACTGATTGCTTGCGTTGCTTTTTTTAATTCTTTTTCTACCATTGTTTTTCCTGAAGAAATTCTCTAGTAAGGAAATCTGGGATAGAATTTTCACTACCTCTCAAATATTCAATATCAAAATAAAAAAATGCTTAAAATAGCTTGCCAAAGTACAAAAATCTGTTTTGATGTAATATTTTGAACATAATTTTCTAAAGAGTGCTGCTAAACGAACCCATTAAATTAATTAAGTACACCCTACTATAAAAGTATATATTAAATTACTGATTTACCCTAATATAAAATGACCAAAAAAGATATATGGAATTATGAAACAAATATAATTTTAATAAGTATACCATACTAACCATATTCAACCCTAATCAAGAGATTGCTTGTCCTATTATGTTTTTAACAAAAGAGGTGGTGATTGCAAGAACTTAGAATTGTAAAAAAAGTGTTAACAATTTTAGTAGAAGCATAAATTCATATGAAACGTCAAAGATTTATAAACACTATTAAAAAAATAAAGCTGAAATTCAACTATAAAATAATGCTACAAGTATTAGGGTGTATTATGGGTATTTTTGGTAGTTTTATAAGGTGTACTTAGTTAATTTTATATTTGAATTAAAATATTATGGTGTTCTTAAATCATTGGGTGTACTCAGTTAATTTTAGGGTTCATTTAGC

AACACTCTTTTCTAAAACATGTTTTGCACATTTACAGTCAACTATGACTAAAAAATTTTGATTTATTAGGTCATCTTGGAATTTTGAAATGCAGAATACTACATATAAAATTTCTTTTCTCATAGTACTATAATTACTTTGTGACTGTGTCCAAATTCCATAATGGAATCGAAGATTTGCTCTGTTTGATTTGGATTGACTTTCTATTTTAGGATACCTCTATATCCATTTTCTGGGGTCTCAGTTTCAACTATTTTGAAGGAATTGGCTAAAGGAATGCCAAGACAGGGTAGTGTTTTGACATGGATTTTGATTTGTTTAACAATTTCTGTATGAGTAGAGGTCCATGGAGGAGGATTACTTTGGAGTCTATCAAATAAGGGTATACATTGCTTTCGCAGATTCTGATAAAAATCAGAGACATAGTTAAGAGATCCTAGGAACCTTTGAAATTGACTTTTATCAAGAATTTGATCCGGGAATTTATCGGCAAACTGAATGACTCGATCAATTGGGCTGATTTGTGACTGACATATATTAAATCCAAGAAATCTAATATTGGTTTGGAATAGCTTGATTTTTTTGGCTGAAATAACTAAACCATTTTTCTTGACTATTTGAAGGAATTTATACAGGTGTTTCCAATATTGTTCTATTGACTGGAAGAAAATAAGGACATCATCAATATAAACTATGGAGAAATCATTGAATGGGTTGAATATTTCATTCATTATATTCCGGAATTCGCTAGGCGCATTGTTTAGACCAAAATGCATGACATTCCATTCATAGTCAACAAATGGGGTAACAAAAGTAATTTTATATTTGTCTGACTCTTGGATTTGAATTTTCCAAAATCCACTCTTCATATCAAATTTAGAGAAAATGACTGCTTTATCTAATCTATTGATTAAATCTCCCTTATTGGGGATCTAATATCTAATCCATTCTAATACCGTATTAAGGAGTTTATAATTAATGACTAATCTTGGGGCTTCTCCTTCTAATTCAGCATTTTTCTAAAAATAAAAAGCTAGACAGGACCAAGGTGACTTGCTTTTACAGATTATCCCATTTTGAAGTAATTATACAATTTCTTTTTTACAAAATTCCATAATTTCCTGACTCATTTGAATTGGCCTGGCCTTAGTAGGAATAATTTTTTCATGAAAGGCTTTTACATAGGGGAGACTGACTACGTGTTGTTTCCTGTGCCAAAAGGGATTGAGGAGATCATAACAAACTTCTTGTTTTAACTTTTCTTCAAATCTCTGAATCTGTGACTGTAAAGACAGATTATTAAGTTGTTCAATTCTTTTGAATTTGGCTTCTTCTTTAAGAAAATCTGGTTGTTTATTTTTATTCTGGATTATCTTCAATGTTTTCGAAATTGCATTTTTCTGCAGATCTTTTAAATTATGCAACTCAAGTTTAGTTAAAAATTCAAACTTGACTTCCTCTCCCATATGAGTGGATGTGACACTATCGTAATCTACTCGAAAGGGGTACAGGAGAGCTATGAAGGGTAGACCGAGGATGACTTTATCTGTAATATTTTTAATTAATACGAAAGAAGTTTTAAAATAGATCTTATTTTGACAAACATGGGCTTTTGAAATTTCGTAGTCTAAATGAAGTGGTTTCCCAGAGGCCGTGCCTAACGTTTCTTTTGACTTTTTGTAATATTTGGTGGGGATTAAACCTTCTTGTATACACTTGAGGGTTCATCATCTGAATTTGCTTGACTTTTAGATTGACTCCTAGAATTGGAATTTCTTCTTTCCATTAAATTAATTAAATCCTGTTTAATTTCAAAATTTTCATTTCGTAATTTATGACTTATTTGTCATAATTCTATTATCTCCTTTTTGACTGTTTTTACTTCCAATTGTAAATCTTGTAAAGTAACTTCTCTCTTTCTATTAAATCTTTATGAAGTTGTGTTTACGCTTATTTTGGGAAATTACTTTGATTTGCGTCTTCTTCTCCTATAACTTTTCACAATTTTCTTAGATATTCATATTTCAATTCTGGATTGTCAACCTTACTAATTAGATCTATTAATAACTCGTCTTGTTCTTCATGTTTCTTGAGCACACTTATTGACTTGGTTAAGGTATTACAACATTTATCATTACACCTAAACTGAATTTTTGGAGAGCTTGACTGAGAGTTTGAGGATCTATAGTCTTCTGTAATTTCACTAGACTCAGAATTTTTTAATTCTAAAACTTTGATCAACTGGGCTTTGTCTTCTTCTGAAATCTTTAACTGCTTTATTTGTACTTTACTTTGCATTCATTAGCATAGTGACCAAATTTCTTACATTTCCAGCACTCAACTTTTCTTTTGTCTGTATTTTTCTTAAAGTTACTTTTCTTGTCTTTTTTGGGTGTTTTTGACCAAAATTTTCGAGGGTTAAATTTCTCTTACCATAAAATTCATCATTATGTCTAAAGGATTGTTTCCTACATGAATAATGCCTATTTTTACTAGTTTGTCGGTCATTATTCCCAGTCTTGACCTTTCTAGAGGGAGGAATAGATACAAAACCGTATTGTTCACAAAAATCTCTTATTTCATATTTGGCTGACTTTCTATGTGACTGTATTTGTCTTCCTCTTTTTAAATCAATGCACATTTTCATGCCTGCTTGGTTAATCGTGCTAATTATATTACCATAAGTTAGATTATCGAAATTTATATGGCCTTGGTCGTTGCTTAAAACTGTTCGAATCTTATAAGCAAACAAATTGGGAAGACCATTAATGAATTTTTCTTTCCATAATGGTTGATTACTATCATTTCTTAACATAACTCTAGAAATAAAAGACATCTTTATACCATCTAAAATCACTTAATTTGGGGCATCTGAGATTACTGAGCTGGTCATGGATTGGAACTCTCAGATATTGGTGGGATAAACATCTTACCCCTGAGTCTAAAAATCGATTCACATATGCTGTAAAACTTAATGAAGACGGTCTCCCTATATTTGACGAACAAATAGGTCAAGGTATAGAAGACGGTGTCAACACTCTGTTTTACACTATAATTGAACATTTCATTGGAACTCCTAGTAATACCACTGCTAGGATACATGATTAGTTGAGTAATCTTAGATGTCCGAAGTTGTCTGATTTCAGATGGTACAAAGATGTCTTTTTATCCAGAGTCATGCTTAGAGACGATAGTAACCAACCCTTCTGGAAAGAAAAGTTCATTAATGGTTTGCCAAATTTGTTTGCTCACAAAATACGCACAACTCTGAGTAATGAACAAGGTCAAATAGATTGGAATAATTTGACGTATGGAAATATTATAAGCACGATTAATCAAGTCGGTATGAAAATGTGTATAGACTTTAAAATCAATAGACAAATCCAGTCTGATAGGAAGTCTGCCAAATACGAATTAGGAAATTTCTGTGAACAATATGTTCTTACCAGTGTCCCTCCTTCCAGGAAAAATAAGCCTAGTCATCTTAGAAAAGGTCATTATCTGAAGAAACAATTTTTTCGAAAAAAATGAATTTTATAAGAAAAGAAAGTTCTCCCCCAAAAAGAATTGGTCGAAACACCCAAATCGCCAGAAGAGTCGCCCTAAGAAAGATAAAAGCAAAGTCAAATGTTTTAAATGTCAAAAGTTCGGTCATTATGCTTCTGAATGCAAAGTAAAAGATGTTATTAGACAATTACAGATCACAGAAGAAGATAAAGAAAAGTTGATACAGGTTTTAGAGTTGCGAGACTCTGAAACAAGCTCTATTGAAACAATAGTACCCAGTTCAGAATCTGAGTCTATCCAGTCTTCAGATTCACAACCCAGTTCTCCTGAAGTCCAATTCGGTTGTACTGACAAATGTTGTAATAGATTAAAATCTATATTAGTCCTCACTAAACAGGAAGAACAAGAAGAGCTTTTAATAGACTTAATCGGAAGAGTTCAAAACCCTGAGTTGAAATCAGAATATTTGAAAGAATTGAAAAAGATCCTTAACCAAGAAAGTCCGAGTTCAACCTCTTTTCCCCAGCCGATTAGTATCAATACCACATTAGAAAAATTTAACCACAGAAAAGAAGTCACCCTCCAAGACCTCCAGTTAGAAGTCAAGCAGGTTAAAAAAGAATTAGTTGAGTTAAAACAAGTCAGTCAACAATTGCAAGCTGAGAATTATACGATCAAACAAGATTTATTTAATCTTTTAAAACAAGAACCTAGTACGCCTAAAAGCCATTCAGAAAGCCCACATCATTCCGATGATGAACCTGTTGAAGACCAACCAGTTGTCAATTTAATTAAACAAGTCTATCTCAGAAAATAGTATTCAAAAGTCACAATTTTCGTTCAAAATTTTGAGCTTACCACAGTTGCTTTATTTGATTCTGGAGCAGATCTTAACTGCATTCAGGAAGGTCTCATTCCCACTAAGTATTACCAGAAGTCCAGAGAATCCCTTAACACTGCCTCAGGGAAGTCCCTTCAGTTGAATTATGAATTACCAAAAGCCCATATTTGTCAAAACAAAGTCTGCTTTAAAACCTCATTTCTCTTAGTCAAGAACATTACTGACGAAGTCATCTTCGGTTTACCTTTTCTTGCCTTACTATATCCCTTTCAGGTAGAATACGATGGTATCATTTCTACCTATCTAGGTGAAAACGTTAAGTTTGAATTTTTGTCAAAACCAGAGTTGCACAACTTAAAAGCCATGCAAAAACAGTCAGTTTCTAAATCTGTTCAAGTTATCCGAAAAACTAACTAGTTGAATTTCCTTAAAGAAGAAATTAGATTTAAAAGAGTTGAACAACAGCTTGCTAATAAAGCCTTACAGTTGCAAATTCAATCCTTTGAAAACAAATTAAAAAACGAAATTTGTTCTGAATTGCCTACTGCTTTTTGGTATAGAAAACAGCACGTCGTCTGTCTTCCCTATATCAAAACCTTTAGTGAGAAAAATATCCCCATTAAAGCACGACCAATCCAGATGAATCAAGAGTTGATGGAATTCTGTAAAAAAGAAATTCATGAGTTACTCCAAAAGAACATTATTCGCAAGAGTAAATCCCCTTGGTCATGTCCTGATTTTTATGTCCAGAAAAATGCTGAACTAGAAAGAGGAGTCCCCAGACTAGTTATCAATTATAAGCCTCTTAATTCAGTTTTGGAATGGATTAGGTATCCTATACCTAATAAGAGAGATCTCATTAATCGATTAGAAAAAGCAGTTGTCTATTCCAAATTTGACATGAAGAGTGGATTCTGGCAAATCCAAATAGATGAGTCAGATAGATATAAGACTGCATTTGTTACTCCTTTCGGTCATTATGAATGGAATGTCATGCCGTTTGGTTTAAAAAATGCCCCTAGTGAATTCCAGAATATTATGAATGATATCTTCAATCCCTACAGTCATTTTTCGATTGTTTATATTGATGATGTTCTTATATTCTCTGAGTCAATTGAACAGCATTGGAAGCACTTACAGAAATTCCTTCATATAGTCAAACAGAATGGTCTAGTTGTTTCTGCCAAAAAAATTAAACTATTCCAAACCAATATAAGATTTCTTGGTTTCAATATCAGTCAATCCCAGATTGCCCCAATTGATCGAGTCATTCAATTTGCTGATAAATTTCCTGATTAGATTCTTGATAAAGTCAACTCCAGAGATTCTTAGGATCTCTAAATTATGTTTCTGACTTCTACAAAAATCTGCGAAAGCAATGTAAGCCCTTGTTTGAAAGATTACAGAATAATCCTCCCCCATGGTCAAATCTCCACACTGAAGTTGTCAAACAGATTAAAGCCCATGTCAAGGCACTCCCCTGTCTTGGTATTCCTTCAGCCGACTCCTTCAAGATGGTTGAAACTGATGCCTCTGACATAGGTTATGGTGGCATTCTTAAACAGAAAGTCAGTTCACGACCTCCCGAACAAATTGTTCGTTTCCATTCGAGAACCTGGACTCAGTCGCAATACAATTATAGTACTATTAAAAAAGAAATATTAGCTATTGTATTATGCATTAGTAAATTTCAAGAAGATTTACTTAATCAAAAGTTTTTAATACGAGTTGATTGCAAATCTGCAAAATATGTTTTAGAAAAAGATGTTCATAACATTGCATCTAAACATATTTTTGCACGATGGCAAAGTATATTAAGTATATTTGATTTTGACATTGAATATTTGAGAGGTAATGAGAATTCAGTCCCTGATTTTCTTACTCGAGAATTTTTGCAGGAATCACAATGGCCCCCAAAAAAGACAAACACAAAGAATCTATCAGCCAGCCTAAGCCATCCCAATCGCAACCCAAGCCATCCCAGTTGCAGCCATCTCAGTCTCCATCCATGAGTCAAAATAAGCCAGAATTGCCCAAACAGATAGTTCCCTATCCTGGTTGCACCCCCATTTCAGTTGCTACCCCATTATCAGTTGCAAACAGATTTTCCTCTTTAGGATCCACTGTTGGTCAAATTCGCCCAAGTTATCAATCAACATTAGTAGCCAGTTATGATCCGTTTTCAGTTGATGTCCCAGCCGGACCTTCAGTAGCATTTAAAAAATCATCTCCTTATCTACCCAAGAGTCAGTCTCATTTATTCGTCATTGAGCCGTTGTAGGATGTTCATACCAACCCAGTTGAAATTGCCAAAAACTATTTCCCTCCTGGATTCCATTATATGCCCTCCAGCCCTTACAAGTCCCTCAAATATTATAGAGATATATTACTTGAAACCAAGTCAGTTGAAATTAAGCCGATAAAGGATCGTGATCATCCGGAGATCATTCTTTATCATTCCTTGTATATTCATCAAATCATAAGCCAAGAATCCTTCAGTAGTCGCCCTTATGAGTTAAAGGTCCTTCAGTCAAAATTGCAATATACTTATTCTGATTATATTGATGCTTGGTATACTATTTTTCTTCACCAATCTGAAGATTTTAGTCATTCCTGGTTTATCAATTTTGACAATAAGTTCATATGCTCTTTCCCATATTGGTTCCTCCACTGGTGGGAAAAACATGGCCCAGTTGACGAAATCCTGCCCATTTCAGTCCATGAGTTAATCCGCCATTACACCGAAATCGCCAAGTTCTCCAAAGAACGAATTGCCAAATATGTCTACAATGATCTCCCTGCTGAGCCCCAGGCAACTTCTCCCACTGGTTCTGTCCGATCTTCCCTCTCAGTCGAAGGAAAGTCCAAATCAGAACTCCAAGAAATTGCCCGCCAGTTGATCATTCAAGCCTCCCAGATGGATGATGATGATGAGGACAATGACTCTCCTGCATCGTCCAGCTGCCAGCCCATGCAAAATCCTGTCAAGCCCAAATCATGGTATGAAGATAGTCAAGACCCCTATGATGCCTACGATTTGCACTCTGACTAGGCCCTGCAGACAAAAGTCAAAAGTTGCTATAATTTATGCTGATCATTGCATCTGCCCTTTGCTTTTCGCCGAAAGTTGCCATCAGTTCAACCTACCTGAATGAAGACAAAATTGAAAGTTTAATCAATCCCCAAATTGCTTCAGCCGCCAACATCATAATTGCTTCAGAGCATCTGCTTTTTACTTTCCGCCGAAAGTTGAAAGTTGAATCAGGTTGCATGTGACTTCACTGCAGCAACCAACCACACCAGGAAAAGGGGAAATGAAACCTTCTACTCAGATGCTTAAACTAGCCACTTGGGCGGGGATGAGTAGCAGACTTAGACAAAGACCGTAACACTTACGGCTCTGATACCAGGAAAAGGGGAAACAACAGAGAAGAAGTGAAGAACTGGCTGAATATAAACAGAGAAGAACAAATAGTATTCAGATTGAATGCAACCTAAACAACTTTCAGACTAATGCAACAAAATAATGATAATGCATCAAAATAATGCTTTTCAGAAAATAGATAATTTGAAAATAACAACTTTAATTTATACTCACAAAGATTGCTACTGCAACTTCAAACACAACTGCACGCTATTTACAAGGATGATTTCTCCGAAGGATGTATCAGAGAGAATCTTTAAAACTGAAGAAAAGGAAAATTACATTCTGATTACAACTGAGGCCGAAGCCTGCTTATAAAGAGAAAATTAACTAAGGTTCATTCAACTATGGTAAACTAACTGTGGTTGGTTGCTGCAGTGAAGTCACATGCAACTTGATTCAACTTTCAACTTTCAACTTTCGTCGGAAAGTAAAAATCAGATGCTCTGAAGCAATTATGATGTTGGCGGCTGAAGCAATTTGGCGATTGATTAAACTTTCAATTTTGTCTTCATTCAGGTAGGTTGAACTGATGGCAACTTTCGTCGAAAAGCAAAGGTCAGATGCAATGATCAGCGGAAATTATAGCAACTTTTAACTTTTGTCTGCAGGACCTAGTCAGAGTGCAAATTGTAGGCATCATAGGGGTCTTGACTATCTTCATACCATGATTTGGGCTTGACAAGATTCTGCATGGGCGCTGCTGGACGATGCAGGAGAGTCATTGTCCTCATCATCATCATCCATCTGGGAGGCTTGAATGATCAACTGGCGGGCAATTTCTTGGAGTTCTGATTTGGACTTTCCTTCGACTGAGAGGGAAGATCGGACAGAACCAGTGGGAGAAGTTGCCTGGGGCTCAGCAGGGAGATCATTGTAGACATATTTGGCAATTCGTTGAACTTCAAATTTGTCCCACCATTTGACTGAAAATTGGCGGGCAAAGATTCTGGCATCCTTAGTGATTCTGTAGGACCATTTGAGGATCCATGGAACCTTATATCTAGCAATAAAAAGCAACTGTTTTGGGAAAAATAAATTAACTTTGGAGAACTTGGCGATTTTGGTGTAATGGCGGATTAACTCATGGACTGAAATGGGCAGGATTTCGTCAACTGGGCCATGTTTTTCCCACCAGTGGAGGAACCAATATGGGAAAGAGCATATGAACTTATTGTCAAAATTGATAAACCAGGAATGACTAAAATCTTCAGATTGGTGAAGAAAAATAGTATACCAAGCATCAATATAATGAGAATAATTATATTGCAATTTTGACTGAAGGACCTTTAACTCATAAGGGCGACTACTGAAGGATTCTCGGCTTATGATTTGATGAATATACAAGGAATGATAAAGACTGATCTCAGGATGATCACGATCCTTTATCAGCTTAATTTCAACTGACTTGGTTTCAAGTAATATATATATATA

TATTCAGTCCTGATCTCTTGGACCCTTGTAGTCCAAGAGATTTATGATCACTCACCGTTGGATGTAAATTCAACGGTTCACTCATTTATGACTTGAATCGGGTGATAATCATTTATGTCATATTGCATTTATATATATCATTTTGAACCATTGGATTAATATCCAACGGTGGGTGACCACAATCTCTTGGACTCCTGTGGTCCAAGAGATCGGGACTGTATATATATTTATATATATATATATATATATCACATGCACAAATGAATACTTCAATTCCTTTTTACTCCCATATAGTGCCAAAAAAACCCTTATAATTCCCGCATGCAAACATGCATAAATTTCCAACATTTCCCATATCCAAGTAAAAACAAATATAAGTTTAAAAATACCTTTTTATGCATGAAATTTAAACTTTAAAACAATTCTTATTTAAAACAAAGCCCACTCACAGAAAGTCCAGGCTGGTACTGCCAGATGGGGTCTCGCCTGCTCGCTAGCCAGAGCCTAAGCACCTATTGAAGAAATATTTAGATATGTTAAAAATTGTATTTCGAACACGAACTTTAAGTTAGAAACAAAACCCTAATTCCCGGAATTTAATGCGTAATTCCTGTTGGAGAGAATATGAAAAATAGGAGTGAGAGATGGATACCTGGAGAAGATTGATGAGAATAACCTATTGAGAAGATAGCAGAGAACCCTAGGGAGAAGAATGAAAAGAGCGTTTAATTTTTCATGGTAGAACACGTGTTATTTTCCCTAGTCTTATAATTAAGCGTGTATTATCTAAACTGGAGTGTGTGAGTTGAATTAATTAGTTAGATAAGGAGAGTATTAAAAATCCAACCCGTTTAAAATAGTCAGGTAATTAAATAATATAAGTTGACACCAACATAACATGGACTATTTACTCCTATCCAGAGTCTAATACGGTGTATCAAATGAGCCCTTGGTACTCTCCTCACCTGACTAACTAATACAATCCACATACTCCGGTTTA

>Chr5-01; OZ022210.1; 1621012-1623517; dir; B;

AAGCGAGTATTGAAGGAAAACAGGTGGTTGGCCCTTAGCTGGGGATATTGAGAGGTGGAAACTATATCACCCCAACTTGGAATCAAGAGCAAGAGATGTGTCTTTGTTAAGAAAGAAGCATAGATTTCCTTATATTGTGATCAAGTCTTCTTCAGAAGAGATGTTGTCTGCCGAGAAGACTAACATTGAAACTTCTTCTTCGTCGTCTGCTAGGGTTAAGCACCTTTTAGGTGCAAACAGTAAGAAGATTGATGGCATGAAGAACATTCAGGATATTCTGCTCAAGTCTTCAGCTTAAAAGCTCAAAATTTATGATCTTCCCTGCGAAGATGTCTACTCTAGACCTGGTTCGTCTGCCGAGAAACATAGGGCAGTTGACTTTATTCACAAGAGCTCGAATCATCTAGTCATCTGCATGAGTCAAGGGATGAGGATTGAACTGAAAAGCCTGTTCCTCTATCGAGCGAGTGTGCGCCATCTGCCGAGTTACGAGAAATCAAGCATGGAGCTGATTTAACGTCGTTGACTCCTTTCGAGGCGAGGATGGTTACGGCTAAGAAGGCCAAAGAGTTGTCTACTTAGGCAAAAAGTTCATCAAGTGCACGAGCTGATGGACCAAAAGTTGACAAGCCTAGCTCTGTTGGGCATGTAAGCATGTCTGATCTTCTCAAAATGAACTTCTCGTCGACCTCATCTGTTTGCGCCAAACTGGTTGATCATTTACATCAAGCTAACGATCTTGGTGCATTTCCGAGCCTTTCTCTGAAGAAGTAGAAGGATGCAGTTATCGCTTTACTTTAAAAAGGGGTGATTTTTGCTACTGAGGCTATCTGGAGCTCGTATGTGTTAGTAATGTTAAGGATATCTTATGTAATGACTTATTAAGTTATACATGAGCAAGACTAGTTGTTTTCAACCATGATATTTGGTTTATTATTATAACAGTAAAACAACTACAATTCTAGGAATATATATATATATATATATATATGTATATATA

ATTTCTGTATATTCTATTATCTGAGTCTAATTACCATTGTTGCTAATCCCTCCTGGTTCTAGATCCTTAGATCTCTTCTTGTATCAGAACCTGAAGTTGAAGAGTCAGTCTAAATTTTCTTCTCCAAGTTCAAAGTCACTTATCTTCTCTGTATTTGAAGTTTGTTTTCTATTCTTTGACTTTTTCAGAGTCTTCCTTTTGTCTGAGTCTTCTCTCCCAGTCCAAACAATAAGTCCTAGATTCCAAACAGTAAGTCCCAAGGTGAGGCCTAAGCGGAGAAAGAGAGAACCCTTTGGTCCTTAGGAAGCCTTTGAATTTGTTCTAGGATAATTAACCTTCTTCTTACACCTTGTAAGAAAATCCAGTATCTTTGTCCAGATGAGTCGCTTGTTTAGAACCAACTCTTGTAACTCTAGCAGCTTTAGAGCCAGTATTAGTAAACTCCCAGAGATAATTAATAAAGAACAAATAGAATATGATTCCAGTAATGAAATAGATTGTAAAGATTGGAATATCCCTAAAATCTCTAGTCAAGAAATTTATAAGAAAAAATGGTCACTCTCTAGTTTCAAGTCAATCACCCATGTAAAAACATTCGAACAAGTTTATGCTTTAAGTAAAGAACATGAAACATGCTAGTTATTGAATCTTGAGTCAATTAAGAAACCTAAAAATGATGGAAACAATTTCCTTCACATTGGTTTAGTTCAAGTAGCAGTCAAGCCACTTACCAAATTAGGATTAAATGCTTTTAGGATTAAATGCTTCAATCATTCTCTGTCTCCGTGATGCTAGATTCAATGAATTCAGTGGCAGCACTTTAGGTATAATTGAGTCAAGTCTGTTTAATGATCCAATTCATTTTGATTGTTACCCAGATTTCACAGTAAACCTTAGTGATATCCTGAAAACTTTAACTTTAAATATTAAAACTTTAGGGTATAAAGTCTTACCTGGGACCCAGCCTTTAGCCTTAGTTTATCAAATCTATTATAAAGTCACAGGAACTAATATGAATTTCCAAGCCCTTAATAAAAGTCCAAAAGATCAAACCTTGTTAATTCAGAGTAACAAGATCAAACCTTGTTAATTCAGAGTAACAGAGCCGATGCCAACATAAGAGTCCCCAACACAATTAAATGGTCAGAAGTCACCTTACCTCTAGATTGGACACTAACCACTGAGAATCAACCTACCCCTATCCAACATAGTTTAAATAATTTAGATTATATTCAACAATACTTAGATGGCACAGTTAAGATCAACTTTGGAAGTCAGCCACCCCAGAAATCTTCAATCCAAATACATCAATTACCTACACCTAGTCAATCCCAGAGACATGCCCCTGCAAGACATTCCTTTAAAGCATCATCTTCCACTCTAGAACGAGACCTAGAATTAGAAAAAACTTTAATTTGTTTGAAATTAGAATCCTTGAGAAAAACTTCCCAAGTCAACCAACCTTGTTATGGTAAAAAACCAATCAAATAAGAAGATAAATCTGATTCCCCGAATTCTCCCACACAAACTGATTTTGAAGTCGGATCCTTTGATATAGATAATCAATTACGAACCCTTAATAGAAAATTCAAAATCAATTGGAAATATTTAGATAGTCACCTACATGCCCCAGAAAATGAAGAAAGATGAAATAATTACCACACAGCCTACCCAGATAAGACTAAAAGGCAACAAATCTTTAATGAATGGAAAGATTATCTACGATCTGCAAAGACAGAAATTTTCTATCTTGATTTTATTGAAAGCCGCTATTTGTCCAAGAAAAATAGCAAAAATTAAAAACCTTAACACAGGAAAAATGCGAAAAAGAAGATAATACTGAAGTCATAGCTAGTCACCCCCCAGTAGAAACCATAGTTATAAGTCACAAAAAAGCAAAAATTATTGCCAGTCCCTTCAAAGTCCCTGACCAAAATGATAGGAAGATAATTGAACAGAATAATTATGCAAACCAGAGTCTGATAGTTATAGGAAAACAATTAGATAAAATTGAAACTAAAATTGATAAAATAATTCCCCAGGAAGTAAAAAACAAATCACAGAAAGAAAAACCCTTAATTAAATTCCAAGACCCAGAGTCAAGTCCAACCTTTAAGACTAAACCCACAATGAGAAAAATAGAGAAAATGCTAGAACAATTAACACCTGAGAAACCTGAAACTTCTACCATTAAAGGTTTAGGATCTTTTGTCAATCTCTCCAGTACTGAGTCTAACGCTAGTATGGAAACAGAGTCCAGTAATATTTCAAAAATTGAAAATGCTTTCAAAAACTTGGAAATAGAAAATGATTTAAAGCTTAAATGGTTAACAAATAGAGTTAACCCTACTAGTTTAACAAAAAATTGGTACCCTAAACCAACTCCTCCAGATATCTAGTTTGAGGAAAGAAATGCCCAAAGTCAATTCTCATTCTCCTGTGATAAACTTTACTAATGGAACATTAATGGTTTGTCAGAACAAGAAATTTTAAATAAATTATGAGT

GAGAGAGGAGAGAGAGAGAGAGAGATTTTTCTAGGATTTTAGAATTACCTCAAGTGTGATGAGATGCCATCTATTTATAGCCTCCTTGCTTGGCTAGGGTTTGAAGAATAATCCTTATTTAATTAGGATTATTCTTATCCAAAAATAGGTGATAGGATAAATCAATGAGATTTGTTGAAATCAGATCCTTGATTTAGGGAGATATTTCTATCTTAAATCATGGATAATTGCCTAATTAAATGAGATTTAATTAGCATAATTAGTTGAGCTAAGAAATATTTCTTTTTCCATGTGTCACCTTTTTAATGGTTGTCAAAAATGTGTTCTCACAGTGGTGACCGCCATACTTTTTGGCCCGTCTTCCTTTTTTTCCCCTCTTTCCTCTCCACCTTCTCCCCATCCCGTCCTCTTTGCCTCCCCCTTCTACATCATCTCTCCCTTGTTTTGTTGGCTCGTTTATGGCGTAATTCTATAACAATCATAGTGAATCTATCATTTTTCGATGTATTTGCTCTATTTTCCTAAGAGGATAGTTGATTGGTGCGAATATGTTATCTTTTGGTGTTTGTGGACAAGTTGGCTTTGTGACAACGAAGTTGCAATCTGACATGTGTAGTTTTTATATTTTTGGAATACGTCACAGAAACTCTCTCTGAGTTTTCTGCATAGTTTCCACCTATCTTTTGTGAGAGTTTTTATACTCTCTTTTGTTGTGAGAGTTTTTCACTTTTCAGGGGAAAATAATTAGTTTAGCCTTAAAATAATGAAGTTGCCATTGTGTCTTGATTAGTTATTGTTTCATTTTAGGGAAAAAGAAATTAAAAAAATCATATTGAGTTGATTAACCATATAATCTCAATATAGTTTCAATATGGTGTCGGATTTAGGGGCAGGCACTGAGACACCAGATTGGAAAAAAGGGCCAAAAATTTAGTTGACTAAAAAGTCAATAAAAGTATTATAAAAAAAAAAAGAAGGGATTCAAACCCGGTTCCAATTC

>Chr5-02; OZ022210.1; 3959885-3960426; dir; B;

AAATGGTGAAACTTGCAAAAGAAGGCTACATGGTTTATGCCGGCCAGGTTAAAAAATAAATAAATTTGTACGCTGTATAAGGGTTGGACTATGTTGTATAAGTATATTTACTGTGTCTGGCGTGGAAAATTTCTTTTCGGTTTGCAATGGCAAACCTTATACAAAATTGGTCAAAGGACATTGTTTTCTTTTTGTGCTCTGTTAGTGTTCTTTGACTTTTAAAGCACAAATTAATAACCATGTTTAACAATTAAACATTTGTGGTAACATGTTTTACTCTTGCACACCACTTTTTTTTTTTTCTTTTTTGGGTGCTCTTATTATTTTTGATGCGGTTTTGGGTGCTCTTATGCCCGTGTTTTTTCCCAGTTTTTAAGTAGCGTAATATATATAAATGAAAATAATGCTTAAATGAGAGTACATAAATCAAAATAATGCTTAAATGAGAGTACTTACAGAAAATCTTGACTGAAAGATTTTATTGATAGAAAGTAGCACTAATTTAAGCATTATTTTATTGATAGACAATCTTGATTGAAAGGCAGTGAGAATCGTATTCCCGATTTTCTTACAAAGGAATTTCTGCAGAGAAAGAAATGACAGGAAAAGAATCCGAATCAGTCAAACAAGCTAGTCAATCAGTTACCAGAAAACTTCCCCCTTCTATGAATCCAGTAAAAACAGAACTTTCCCAAATCGTCCCCCATTCAGGATATTCTCCAATTCAAATTACCAATAGATTCACTAGTCTTTGTTCTACAGTCCCCAACATTCGTCCCAATTATCAGTCAACCCTAATCTCCAGTTATGATCCCTTCCAAGTCACAACCCTACTTCTTCTCAGCCATCAATTTCTTACATGAAAACTTCTCCCTATCACCCCTGCAGTAACAATGACCTCTTCATAATTGAACCTTGCTATAATAATCTCTCCAATCCAGTCAAAATAGCTAAAGCCTACTTTCCCCCCAACTATCACTTTGTTCCTCATCACCCAGAA

AAGTCACTCAAATTCTACAGAGATATTCTTCTTGAAACCCAAACCCTAAAAATTAGACCAATCAAAGACAGAAATGATCCAAACATAATCCTTTATCATTCTATGTATATCCACAAAATTCTCAGCCAATCCGATTGGGGAAACCGCCCCCACTCTTTCAAAATTCTCAAATCAAACATCCAATACAGTTATTATGATTATATTGAAGCCTGGAATAACATATTCCTTCACCAAACTAAAGATTTCAGTCATTCATGGTTTGTCAACTTTGATAGTAAATTTAGAAGTCCTTTTCCCTCCTGGTTTTCTTCTTAGTGGGAAAAGCATGGCCCAGTCATTGAAATTCTTCCTCATGATCTCCAAGATTTTGTTACTTATTTTACTAGTAAATACAAATTCAGAGAAGAAGATGCTTTCTTCCCCAGTCTCCTAATTTTTGTCGCCAAATATAAAGTTTCTTGGATCCTCAAATGGTCTTATCAGGTTAACTGGGAAACAAGAACTCTTTCCCGCCAGATTTCAGTCAAATGGTTGGATAGATT

CAATTATGAAAGAGTCATTGGTCAAGTTTATACAGAATTTCCTCTAGTCAAAGTCGCACAACACAAGTCATCAATTTCTGAAGCCTCTCAGTCTTCCTCCGAATTGAAGGAAGAACCAAATTATAAATTAAAGAATTAACAAGGCAACTCATGATTCAAGCCTCCCAAATGAGTGATGATGAAGACGCCTCTCCAAAGTCACATGCCTCCCAAACCTCCTTCTCAAATCAGGAGCGTAATGCCAGCCAAAAGAAAAGCTCTTCCAGTCAAACAAAGCGGTGGGTAGACTACCAAGACAGTCAAGATCCCTATGATCTCAATGAGGACTAAGCCCTCAAAAGTCTCAATCAATCACTATTTGCTTTTCCTAAAAGCAGATGCATGACTCTATAGTAACCCCACTCAGAGACAATTATTCAATTCATATGATGAAATATAATTGTGCTGACACTACAGTAAGGCATCCAAGGCTACGGACACCTAAGTTTTAGGCCCTTTAAATATTGGCCTCTTGGCCTCTTTGGAAGAAGGCTGTTCTTTTCAGCAAGTGAATTTTCCAATTTTCATAAGTCTTCTCTGAGCCCTCCCCTAAGAAACTATTGTAAAGTATTGAGTGTTACTAGTAATAACCATATATTCATATATTATATTGAATAGCACGTGTTCTGTTTGTTAAGAGAGTCTGTTAGAGTGTTAAGAGAATCTATTAGTTTGTTAAGAGAGTCTGTTAGTTTACTATATAAGCACTATTTCATTGTAATCAGTTTTACAGATAATCAATACAAAGCCTTCTATTCTCAGACTTCTTCTTCTTCCTTCTTCTTCTTTCCTCAATTTTCTCTTACTGTTATCATGGTATCACGAGCTTAGGTTCGATTCTGAGGTCTATTCACTTCCGCATTCTCTTCTTTCCCATTTCTCTGCTTCTTTCTTCTGTCAATCTCAAATCCTGATCTCTCAATTTCTCTACAATTTTCTCCAAACCAAATTTTCTTCTGTG

>Chr5-03; OZ022210.1; 4236977-4241596; dir; D;

TGAGAAATGTCCGGCATAGACAACTGGACGGAGGTGATGCACAGTCAGTCCTTACTTACTTTAGGAAGAAGCAAGCTGAGAATCCTCAATTTTTTTATGCCATTCAATGTGATAAAAACGGAAGGGCAACTAATTTCTTTTGGGTGGATGCTCGATCACGCATGGCGTACCACTATTTGGAGATGTAGTCACATTTGAAACAACCTATAGGACAAACAAATATGATATGCCATTTGCACCATTCACAGGAGTAAACCACCATTTGCAATCAATACAATTTGGATGTGCACTTTTACAAGATGAGACAGAGGTGACATATATGAGAAGATTAGTATTCTGTCCCTATTAATTGGGGAAAAAAAAGTAATAGTCAAAATTTTCATATATGAGAAGATTAAAATCCCTAAATCAAACCCTCAAAATAAGAAATCCTACCAACATTTTGAAACAAATTGAAATGAATAGCTTAATCATAGATACCTAAGAAATAAACTTAATCCCCTTGGAAACTATTAATTCTTTAAATTCCTAATATAGAGATCCTAATTTAATTCCAACAAAAAATAAAATAAAAACAAACCAAATATGATCAAGAAAATCGAAAATTGAAATATGTACCTGGAGCAAAGCTTTTCTCCCAACAAAGCAGGCAAAAATTGAGAGAGAGAGAGAGAGAGAGAGAGAGAGGTGCAAATTTGGGGTTTGGGTATTTAACTTTTGGTGTGATTCTTTATTTATTAGACCATAAAAAATATTTGAGATTAAAGAGGCATGAGAGATTACATCTTCCAGCAAAAAGGTCGGAGAGAGAAAAATTTCCTTTTATTTTTATGAGAGAGAAAATAAAATTAATGATTGTCTATTTTAAAAAAATTAAAACTACTTACCATATAATATTTAATTTCAACCATTGAAAGTGGAGTAATCTAATGTCAAAACTTGGGTCAAAAATGTAGTGCCACGGATCTGCCCCCATAATATATATATATATATATATATA

AATCTCTTTTATTGGGAATTGGATATCTAATCCATTCAAGTACTGCATTAAAGGGCTTATAGTTGATAACTAAACGAGGGGCTCCTCTTTCTAACTCTGCATTTTTATAAAAAAAGTTGGGCACGACCAAGGTGACTTGCTTTTTCTGACTATTCCCTTCTGTACTAACTCAATGACTTCCTGCTTACATAATTCCATTAATTCCTTACTCATCTGAATAGGACGGGCCTTAGTGGGAATGTTCTTTTCACTGAATGTTTTAACTTAAGGTAAAGTAACTACATATTGCTTTCGGTGCCAAAAGGCATTAGGTAAATCAGAACAAACTTCATTTTTTAACTTTTCTTCAAACGTAATTATTTGAGACTGAAGTGACTTGTTGGCTAATTGTTGTTCGACTCGTTTGAACTTAATTTAATTTTAAAGAAAATTAACTTGTCTTGTTCTATTCTGAATTAATTTAACTGATTTAGAAACTGCTGACTTTTGTAGTGACTTGAGACTGTGTAACTTTGGTTTGGTTAGGAACTCAAATTTAACTTCTTCTCCTAGGTGACTAGATGTAATTCCATCAATAGATATTAGGCCCATTAAAGACAAAAAAAACAAATGCCACAATGTTCCATTCCCTTTATATACGCCAAATTCTTATGGCTCAAGTTTGGAGTCGTAATCCTTATGATCAGAAACCCTTCAAGTCAGGCCTTACTTACAATTATGGTGACTATATTGATGCCTGGTAAACAATTTTTTATCACCAGCACCCTGATTTCAGTCACTCATGGTTTATTAATTTCGATGCCAAGTTTGAAGCCTAGTTTCCTTTCTGGTTTCTCCAATGGTGGAATATCCATGGCCCCATTTGGGAAATCATTCCAGTCAATCTCCAGCAGTCAATAAATTATTTTGGTCAAAAACACAAGTTAACAAAGTCAGAAAAGTTTTTTCCTACACTCCTCCATTATGTCGCTAAATACAAAGTCCCATGGATTATGAAATGGAATTATCAAGTTAATTGGGATACCAGAGTTTTATCTCATCATTTTGCTGTCAAATGGTGGGATAAGTTTGGAACAGACATAATTATTCATCAAGTCAATGCAGAGTTTCCACCCCTCCAGGTGCCAATCCATCCAGTCAAAGATGCTTCTCCAAGTGGTTGCAATGCCTCCCTCCAGGCAAAATCTAAAAAAGAGTTAAAAGAGTTCGTTAAACAAATCCTTGCCCAAGTTACACAAATGAATGATGAAGATAGTGATGATGCCAGTCCAAAGTCTAAATCTTCTTCCAGTCAACAGCCCAGCCAACTTCCTTTTAGTCAAAAGCCGAAGCCCAGATGGTCAGATTATGCCCCCGACAGTCAACATCCTTATGATTTGAATGAAGACTAGGTTTTTCTGCAAAGTGGCCGAAAGATGCCCAAAGTTAATATGATGCCCAGAGTTAAAGTTAAAGTCAGTAGATGCCCATAGTTAAAGCAAGTTACTCAGAGTTAAAGCAAGAGTTAAAGTAGATGCCCAGAGTTAAAGCAAAGATGCCCAAGTTAAAGCAGATGTCAATTTCAAAAGCAAAGATGCCCAAGTTAAAGCAAATGTTAATTTCAAAAGTTATTTTGTCTTCTTTCCGATGCCAAGTCTTTAAATAGAAGCCCCAAGTCATTTGTAATCCTTAAGTTTTTTCACAAGTTTTTCTTTCAAGCTCTTCTTTGTAATTTTCTTCAGTTTTCAAAGTTCTTAAGGAAGAGTGCGAGTGATCAGTGGAAGTGTGAGTGTCCTACGTGGCATTACTTTCTGTAAGTTTTATTTTCATGTTTTCAAAGTTTTATTAAGTCAATAAAATTATCAAGTTAATTTTATGTTAATCCTATTTTCGTTCCTAAGCATTATTTTAAATGATTAATTGCATTTTCATTCATGCAATTTGTGAGATAAGAATTTTATGTATCTAAGCTTTAGTTAAATTCTACTTTGATTTATTTCAATTCTGTTTTTCTTGTCTTGGTCCCCTCCTCTAATTTCTTCCTTGGTTCTTCTCCTCTTATGGTATCAGAGCCGTAAGAGTTACGGTTGAGTCTAAGTCTGCTACTCATATCGCCCAAGTGGCTGATTTGGGTTTTCTGAGTTAGCAAGTTCTTCAGCTATTCCGAACAGTTATCTTATAGTTGTCTGTTCGTTTAGGTCTCATCTCTCGAGAGTCTGAACCTTTAAGTCTAGGACCAAACAGTAAGTCCTAGGAGAGGCCTGATCAGTTACAGCAGGAGATCGGTGAGAGAGAGGATGCCTTAGGTTACAGGTACCTTTGAGTTAACCTTAGGAGTTTCTGTAGTTCTTCTACCTTTTGAAACTTTGAGTCAAACACTTGTAAGAATGAGTCGTTTTCTTAGAACCAATTCAGTTAATTCTTTTAGTTCTAAAACCAGTTTGAGTCAAGTTCCAGACATAGTTAATGAAGAGCCATTAGAGTATGAGTCCACTAGTAGTGAATTACATTGTAATGATTGGAATATACCCAAAGTCCCTAGTAAAGAAATCTATAAGCAAAAATGGTTAATTAATGCATTTAAGACAACCACGCATATTAAGACAGTAGAACAAGTTTATGCTTTAGGAAAAGAACATGAGACATGCCAATTACTTAATCTTGAGTCAATTAAGAAACACAAAAAAGAAGGTAATAATTTCTTGCATATAGGATTAGCTCAAGTTACAGTTAAACCCTTAACTAAGTTAGGATTAAAAGCAGCAATTCTTTTAAGTTTAAGAGATGCTAGATTTAACGATTTCCAGGATGGAGTCTTAGGAACCATTGAGTCAAGTTTATGCCATGGTCCAGTTCATTTTGATTGTTACCCAGATTTCATAGTTAGCTTAAGTGATCCTCATATATTAAAAGTTTTAACTTTAAATATTAAAACGCATGGTTATAATGTTTTACCTGGTAGTCAGCCATTAGCCTTAATTTATAGAATTTATTACAAAGTCACAGGTACAAATATTACTTTCCAAGCTCTTAGTAAAAGCCCCAAAAATTAAACACTTTTAATTCAAAGTCATACTTCTATTAACAATGTTACCGTACCCCAAATGATTAAATGGTCTGATGTTAAGTTACCCCAAGATTGGTATTTAACAACCGAATGCCCTCCAAAGCCGATTCAACGTAGTCTAAACGATTTAGATTACATTCAACCATACTTAGATGGCACAGTTAAAATTAGTTTTGATAGGCCAGTTAAATCTACAGTCCAATTAGAACAATTACCAACCCCTAGTCAATCTTTTAAAATCCCTACTCGACATTCATCCGCAGAGTTATCCTCAATTACAAAACGTGATCTAGAAGCCGATAGAGCCCTAATTGATTTCAAAATTAATGAACTAAGGAAGCAAAAGAAAGTAGTCCAACCAAGTTACGGAAAAGCCCCTGTTGAGCCAGTCCTGCCAGTTCAACCTGAATTACCTACTAGGACAGATTTTGAAGGTTCCACCTTGAAGTTTGTGATGCCAATTATGAACTTTCAATAAACCATTTAAAATTTATTCGTCAAAATTAGATGCCCATTTCCAAGCCCCAGAAAATGAATCATGAAAAGCCAATTATCATGCGTCTTTCCCTAAAGTTCAAAGAAAAGAAATTTCAATGAATGGAAAGAATATTTAAAAAAGACTAGGTGAGAGTCACCTTATCTTGATTATGTAGAAAGCAAATACATGAGTCAAAAGTTAGTTACTTTAACCCAGGAAAAATGGGAAAAACCCGACAATACTAAAGTTATCAGTACTCACCCCCAGTTGAATCAGTAATAATTAGTCACAAGAACGCCAAAATCATAGCTAGCCCCTACAAAGTTGCTGAGTCAAAAGATGATTTAGATAAAAGAGTCATAGAACAAAATAATTACACCAACACAAGCCTCATAGTCATAGGTAAATAGTTAGATAAAATTGAAACTAAAATTGACAAGTTAAACCCTGGTGAAATTATCTTAAAGCCTAAAAAAGAGAAACCTTTAGTCAAATTCAATGAGTTAAAACCCGAGTCTTCCCTTAAGGTAAATTCAACCATGAAGAAAATTGAAGAAATGTTTCAGGAGTTAATCCCGGTTAAACCAAAACCAAATAAAGATAAAGACAAAGGAAAAGAAGAAGAAACTTCAGTTAAAGTTTTACAGTTTGAAGTTAATAACGGTAGTGAGTCAGCCATGTCTTTAGATTCAATCTCCAGTCAAGAGTCAGAAACCAGTAATATCTCTAAAATTGAACAAGCCTTTAGTAACCTACAAGTTAACTAGGATTCCCAAAGTCTTAAAGTCAGTCGTCTAACAGGTAAAGTCAGTCCCACAAGTCTTACCAAAAATTGGTATCCTAAGCCTACACCTCCAGATGTCCAGTTTGAAGAACGAAGCCAGAGTCAATTCTCAGTATCTTCTGATAAGCTATATGAGTGGAATATAGATGGCTTGTTCGAACAGGAGATACTCAACAAATTACAACACATGTCAATGGTAGCCAATAGTTATGCCACCAATCACCAGTTAAAGCAATCAGAAATAGTCTCCCTTTTACAAACAGGCTTTACAGGAATTCGAACACTTCATTTCTGGTGGGATAAACATCTCAC

AGAAGACGCAAAATTAAGAATAATCCATGAAGGGGAGAAGAACCAAGGAAGAAATCAGAGGAGGAGACCAAGACAAGAAAAACAGAATTGAAATAAATCAAAGTAGAATTTAACTAAAGCGTAGATACATAAAATTCTTATCTCACAAATTGCATGAATGAAAATGTAATTAATCATTTAAAATCATGCTTAGGAACGAAAATAGGATTAACATAAAATGAACTTGATAATTTTATTGACTTAATAAAACTTTGAAAACATAAAAATAAAACTTACAGAAAGCAATGCCACGCAGGACACTCACACTTCTACTGATCACTCGCACTCTTCCTTAAGAACTTTGAAAACTGAAGAAAATTACAAAGAAGAGCTTGAAAGGAAGCGGTATCTCTGAGGCCTGCTAGGATTCCGACCCGGCCTGCGAGGAGGGTATTAACAGTATTAATGGAGTTGACGGTTCAGGATTAGAGGTACGCCTAGGTGGAGAGAGTTTCAGTTATAAGATATTCTTAAAGTAAGATAAGGGAGTTCAGTGTATGGCATACTTATATTCAGTGTGTGACTGGTATGAGTAGAGAGGAGAGCTACGTGTGGCTTGATCCCTCAGTAAGGGTACGTAGGTAGCCTAAGGTTACAAGGTGGAGCCACGAGTTGGAGTCTTTGGGTTTTGCAGAATGTTTTATTTGATTGGCTAAGGCCTGAGGCCAGAATGTTATAATGTTGTTTTCTGGGATGTTATTTAATGCATGCTAGCAATTGGGTTATATGTGTGTATATTTACGCTTGTTTTACAGGTGGAAAACTTTTGGGAAACTTTATTTTACAGGAGGGACTCTGCCGAATTTTCCGCAGAAGTCGGACTTGAATTTCTTTTGGATTGGGTTTTGAAGGGTTTTTTGGTCATTTGTGCTCGGCACCTGCCAGGTGTCGGACACGCACAGGGTTCGGCTCAGATTCCAAAGTGAAATTTGGGTCGGGTCCTCTCATTGGTGGTTATAAC

>Chr5-04; OZ022210.1; 5455555-5469818; rev; D;

AAGTCACCTAGGGAGTCCGCAACACAATTTGCCGGGAGCAACCTGCCTGACTTGGACAGGACTCAGTTGGAGCCGTCGAGGTTGCTTGACATCCTCACCAAGAAATCCTCCTTCCCCGGAAGCTTCATCTCGATCCCTGAGATACAAGCTCAAAACAAGGTCTTGAAGCACTGTGGATTGACTGATGATGAGTACCTTGTTCTCTTCACCCCAACCTACAGGGATGCCATGGTGTTAGTAGCAGAGAGCTACCCTTTCTTCAAGGGAAACTATTACTTGACCATTCTTGGGGAACAAGACTGCATAAAGGAGTTTGCCGGTTACAAGGAATCCAAAGTGATTTTGGCACCAGAGACTTGGTTGGATTTGAGGATCAAGGGTTCACAATTAAGCCAGTATTTCAGAAAGAAGAGTAAGCTCAAGGTCAAAGGGTTGTTCTCTTATCCAGTTTTTGCAAATGGGGTTCATCACTCACTACATTGGGTTTCCGAAGCTCACCGGAGCTCGTGGCATGTTCTTCTTGATGCAACCGAACTGGTTTTTGGGAAGGATCACTTGAATCTCAGGCTTCACAGGCCTGATCTTGTTCTGTGTAGCATTGACAATTCTCATGGCCAGACTTCGAAGATCACTTGCCTCTTGGTCAGGAAGAAATCCTTTGACACTTCAATGCCCTAGCCTCAAGTCAATGAATGAGGGCTCTTTGTGTGTTTATGTACAATATGTTAAACGGTTCTAAGAAACACCAGTAAGCAGTAACATTTTTTAACTGTGGAAAAGGGAAGATGAACACGAACATGGGCGGGTATAATCACTAAATTGAAATTTTAATTGATCGAAGTACAATAAAACTTATGAATGTTGTGAGTTTCTGAATATATACAAAATTCATTCTGAATATACATATATATATATATATAGTCAATGGAAGCTCTCTCTCTCTCTCTCTCTCTCTCTCTCTCTCTCTATATATATATATATATATATATATCTATATATA

TTCACTCAAAAATGTTAATCCACCAACACCAATCCAACGTAGTCTAAATAATTTAGATTACATCCACCAATACTTAGATGGATCAGTAAAAATCAATTTTGGGACCCAACCAGTTCCAAAATCTACTGTCCAACTACAGCAGTTGCCAACCCCTGGTCAATCCCAGAGGCATGTCCCTGCCAGACATTCCTTTGCAGCCTCTTCCTCTACTCTAGAACGAGACCTAGAAGCAGAAAGAGCCCTGATTGATTTCCAATTAGAATCTCTCAGGAAGGCAGCACAGGTTAGTCAACCCTGTTATGGCAAACAACCAGTTGACGAACAAAAGCCAAAATCTCCCGAGTCTCCAACCCAAACTGACTTTGAAGGTTTTGATTTTGTTGACCCTCAACTTAGAGTTTTGAATAAGTTTCATATTAATTGGAAAAAGTTGCAAGCCCATCTCACAGCTCCAGAGAACCAGAAGCGCCGAGATGAATACCATAGTTCTTTTCCTATTAAGTATCACCGTGATGCAATCTTTAATGAATGGAAAGATTACTTACGGAGAGAACAAGTTGAAATACCCTACTTAGATTATGTAGAAACCAATTACATGTCTAAGAAATTGAAAGTAATCACCAAAGAAAAATGGTCTTTACCTAATGACACACAAGTAGTTGCCAGTCATCCCCCTGCTGAGTCTATCTTAATTAATTTTAAGAAAACTCAGATCCCTGCCTGTCCTTTCAAAATCCCTGAAAAATTAGATTCAGAGAAGAAGTTGATCGAACAGAGCAATTTTACAAACCAAAGCCTAATCGTCATAGGAAAACAGTTAGACAAAATTGAAAGACACTTTGGAAGACCATTTTGGTATCAGAGCCGTAAGAGTTGTAAGAGTCTGCTCTTAGGTTGCTACTCATCCAACCCAAGTGGCTGGTTTAGGATTTCTGAGTAGAAGTATTCAGTTGCTTTTATTGTTGTTCTTCTTTCCGGTACTGTTGTCTGTATCTCTCTTAAATAGTGTCCGAAGAGTAAGGCCTAGAGTCCAAACAGTAAGTCCTGAGGTGAGGCATGAGCGGTTGCGGCATTGAGAGGGCTAGGAGAGAGAGGTCAGTTGATAGGAACGTACAGGAGTCAGCAGTAGAAGTCTCTGTTTATTTTCTTCTTTGAGGTCAGAACCCAGTTGCCAAAGTAAAATGAGTCGTTTCCTTAGAACAAATTCTTGTAGTTCGAGCAGTTCAAGAGCCAGTATTAGTCGTCTTCCAGAAATAGTAAATGAGGAACAAGTTGAATTTGAGTCTAATAACAGTGAGTTAGATTACCGAGACTGGAATATCCCTAGAATCCCCAGTCAGGAAATCTATAAGAAGAGGTGGTCTTTACCCAGTTTTAAGACCACTACCCATGTTAAAACAGTTGAACAAGTCTATGCCCTTGGCAAAGAACTTGAAGTCTGTCAGTTGCTCAACCTTGATTCCATCAAACGCCATAAGAATGAAGGAAACAATTTTCTTCATATTGGTTTAGTTCAAGTTGCAGTTAAGCCACTAACCAGAACAGGGCTTAAAGCCTCAGTGTTGCTCTGGTTAAGAGATGCTAGATTCACTAACAGTGAGGACAGTACACTCGGAGTAATTGAGTCAAGTCTTTCAAATGGTCCGGTTCACTTTGATTGTTATCCTGACTTTACTGTCAGTCTTAGTGATCCCCACATTCTTAAGACTTTGACGCTGAATATCCAGACTTCTGGATATGAGGTTCTTCCGGGAACCCAACCATTAGCCTTAATCTTTAGGATCTATTATAAGGTCACTGGAACTAATATGAATTTCCAAGCCCTTAATAAGAGTCCTAAGAATCATACCTTGTTGATTCAAAGTAATAGACCCGAGGCCGTCATCCGAGTACCTCAAACAATTAAATGGTCTGATGTCTCGCTCCCAGTTGAGTGGACACTCAAAAATGTTAATCCACCAACACCAATCCAACGTAGTCTAAATAATTTAGATTACATCCACCAATACTTAGATGGATCAGTAAAAATCAATTTTGGGACCCAACCAGTTCCAAAATCTACTGTCCAACTACAGCAGTTGCCAACCCCTGGTCAATCCCAGAGGCATGTCCCTGCCAGACATTCCTTTGCAGCCTCTTCCTCTACTCTAGAACGAGACCTAGAAGCAGAAAGAGCCCTGATTGATTTCCAATTAGAATCTCTCAGGAAGGCAGCACAGGTTAGTCAACCCTGTTATGGCAAACAACCAGTTGACGAACAAAAGCCAAAATCTCCCGAGTCTCCAACCCAAACTGACTTTGAAGGTTTTGATTTTGTTGACCCTCAACTTAGAGTTTTGAATAAGTTTCATATTAATTGGAAAAAGTTGCAAGCCCATCTCACAGCTCTGGAGAACCAGAAGCGCCGAGATGAATACCATAGTTCTTTTCCTATTAACTATCGCCGTGATGCAATCTTTAATGAATGGAAAGATTACTTACGGAGAGAACAAGTTGAAATACCCTACTTAGATTATGTAGAAACCAAATACATGTCTAAGAAATTGAAAGTAATCACCAAAGAAAAATGGTCTTTACCTAATTTTTCAGGGATTTTGAAAGGACAGGCAGGGATCCGAGTTTTCTTAAAATTAATTAAGATAGACTCAGCAGGGGGATGACTGGCAACTACTTGTGTGTCATTAGGTAAAGACCATTTTTCTTTGGTGATTACTTTCAATTTCTTAGACATGTAATTGGTTTCTACATAATCTAAGTAGGGTATTTAAACTTGTTCTCTCCGTAAGTAATCTTTCCATTCATTAAAGATTGCATCACGGCGATACTTAATAGGAAAAGAACTATGGTATTCATCTCGGCGCTTCTGGTTCTCCGGAGCTGTGAGATGGGCTTGCAACTTTTTCCAATTAATATGAAACTTATTCAAAACTCTAAGTTGAGGGTCAACAAAATCAAAACCTTCAAAGTCAGTTTGGGTTGGAGACTCGGGAGATTTTGGCTTTTGTTCGTCAACTGGTTGTTTGCCATAACAGGGTTGACTAACCTGTGCTGCCTTCCTGAGAGATTCTAATTGGAAATCAATCAGGGCTCTTTCTGCTTCTAGGTCTCGTTCTAGAGTAGAGGAAGAGGCTGCAAAGGAATGTCTGGCAGGGACATGCCTCTGGGATTGACCAGGGGTTGGCAACTGCTGTAGTTGGACAGTAGATTTTGGAACTAGTTGGGTCCCAAAATTGATTTTTACTGATCCATCTAAGTATTGGTGGATGTAATCTAAATTATTTAGACTACGTTGGATTGGTGTTGGTGGATTAACATTTTTGAGTGTCCACTCAACTGGGAGCGAGAGCCGTAATGCTCAAAATTTTACCATGCTAATAAATGCAAGGTGAAAGACACAATCAAACAACTACAAATTGAAGAAGTTGAAAAGGAAAATCTAATCCGACTTCTAGATCTGAAGGATTCTGAACCTAGTGATCTTGAGTCGTCAGTTGACAGTGATTCTGAGAATTCTTATTCCACGGATTCACAACCTAGTTCCCCCCATGTTCAACTTGGATGTAAAGATAATTGCTGCAATTCTATGAAATCTATTTCTGTTCTTACTAAGCAGGAAGAACAAGAAGAGCTTCTCATAGATTTAATCTGCAAAGTCGAAAATCCTGAGTTGAAGGCTGAGTATCTGAAGAAATTTCGGAAACTCCTTACTCAGGAAGGTCCAAGTCAGTCTCCACCTCCTCAAAAGATAAGTCTCAATACAACCTTAGAAAGGTTCAGTCGCAAACGAGATATCACCCTTCATGATTTACATTCAGAAGTAAAATTAATCAAGAAGGAGATAGTTGATCTAAAACAATTAAGCCAGAAATTGCAAACTGAGAATTATGAAATCCGACAGGATTTAATAGCTCTCAAACCTGGTTCTATTTCATCTCAGAGTCCAACAAACTCAGATGATGAATCTTTGAATCCAGAACAAGCCTTATGTCTAGTTAAACGAATTAACTTTAAGAAATGGTATGTCAGAGTGACTATCTTTGTTGATCAATTTGAGTTTACCACAGTTGCTTTACTTGATTCAGGTGCTGACCTTAACTGCATTCAGGAAGGCCTTATACCTACCAAGTATTATACTAAATCCTGTGAATCTCTTAGAACAGCTTCAGGAAAATCCCTACAGCTGAACTATGAAATTCCCCGTGCCCATGTTCGTCAAAATAAAATTTGCTTTAAGACTTCATTTGTTCTAATAAAAAACATTACTGATGAGGTTATCTTAGGATTACCTTTTCTTGCCTTGTTATATCCTTTCCAGGTAAACCATGACGGTGTCATCTCTACCCATTTAGGAGAACAAGCCAAATTTGAGTTTTTGTCTAAGCCGGAGTTGCACCAGTTAAAAACCTTTCAGAAAAGTTCAATTTCCAAAACTATTGCTTTAATTTCCAGTAAGACTAAACATTTGTCCTCTCTCCAGGAAGAAATTAGTCATACTAGAATCAGTCAGCAAATTAGGAATGAACCTTTCTAGAAACAAGTTCATGAATTTGAAACCAAGTTGATTAATGATATTTGTTCTGATTTGCCTAATGCTTTTTGGTCCAGGAAACAACACATTGTCAGTCTTCCTTACGCCAAAACCTTTAGTGAGAAAAATATCCCCACTAAAGCCAGACCAATCCAGATGAATCAGGAGACAATGGAATTCTGTAAAAAAGAAATTACAGAATTACTCCTGAAGGGAATCATTCGCAAGTCCAAGTCCCCCTGGTCTTGTCCTGCTTTCTATGTCCAGAAAAATGCTGAACTAGAAAGAGGAGTCCCTAGACTAGTTATCAATTATAAACCTCTTAATGCAGTCTTAGAATGGATTAGGTACCCCATACCTAATAAGCGAGACTTAATTAATCGGTTAGAAAAAGCAGTTGTCTTTTCCAAATTTGATCTTAAGAGTGGATTCTGGCAAATCCAAATAGAAGAGTCAGATCGGTACAAAACCGCCTTCGTCACTCCTTTTGGTCATTATGAATGGAATGTTATGCCTTTCGGTCTTAAGAATGCGCCTAGTGAATTCCAGAACATTATGAATGAAATTTTCAATGCTTATAGCCATTTTTCAATTGTCTATATTGATGATGTCTTAATCTATTCCCAATCGATAGAACAGCATTGGAAACATTTGTCTATATTCCTTCAAGTTGTTAAGCAAAATGGTCTAGTTGTTTCTGCTAAGAAAATCAAGTTGTTCCAAACTAATGTTCGATTTCTTGGCTTCAATATCTGTCAGTCTCAGATTAGCCCAATTGATCGTGTCATCCAGTTTGCTGATAAATTCCCAGATGAAATTGTTGATAAGAGTCAGCTCCAGAGGTTCTTAGGATCCCTCAATTATGTTTCTGATTTCTATCAAAATCTGAGAAAACAATGTAAGCCCTTGTTTGACAGGTTGCAGGAAAACCCTCCTCCTTGGTCCTCTGTCCATACAGAAATTGTCAAACAGATCAAAATTCATGTTAAGACACTTCCCTGTCTTGGCATTCCTTCAGTTGATTCCTTCAAAATAGTTGAAACTGATGCCTCTGACATTGGTTATGGAGGTATCCTTAAACAGAAATTGAGTCCCAATTCGCCAGAGCAAATTGTTCGTTTCCACTCTGGAATTTGGAATCAGTCGCAAAGCAATTATAGTACTATTAAGAAAGAAATATTATCTATAGTACTATGCATCTCTAAATTTCAAGATGATTTGCTTAATCAAAAATTCTTAATCCGAGTTGATTGCAAATCTGCAAAATATGTTTTAGAAAAAGATGTTCATAACATTGCATCTAAACAGATTTTTGCACGTTGGCAAAGTATTTTAAGTATTTTTGATTTTGATATTGAGTATTTGAGAGGCAGTGAGAATTCTGTTCCTGATTTTCTTACTCGAGAATTCTTGCAGGGAAAACAATGGCACCCAAAAAAGACAAGCAGAAAGTTGGTGCCAGTCAGTCCAGTCAACCCAAAACCTCCCAAACACCAACTCAAGCCCAGTCGCCCAAAAGAGTTCTCCCTGCATCCATGAGTCAATCCAGAATGGCCCATCCAGCATCCATTGTCCCATATGCAGGTCATCCTCCAGTCCCAGTTATCCCAATTGCAAACAGATTCACCAGCCTTGGAACCCCAATAGGATCCACCGTTGGCCCAATTCGCCCTAATTATCAGTCAGCCCTTGTTTCTGACTATGATCCATTTGCAATAGTTCCTCCAGTTGCCCCAATTGCCCAAACATCCCAGTCGTCCACCCAGTTCCGAAAGTCGTCCCCTTATTTCCCAAAAGATAAGGTTAATTTGTTCATAATTGAACCTCTGTATGACGGGATTTCTGATCCGGTAACAATTGTCAGATCATATTTCCCTCCTAACTCCCATTTTCTTCCTCCTGCACCCTATAAAGATCTCAAATTCTATAGGGATATCCTGCATGAGACCAAATCAGTTGAAATTAAGCCTATTAAGGATAGAGATAACCCTGCGGTCATTCTCTATCATTCTCTTTATGTTCAGCAAATTCTGAGCCAACCCGATTCCAGAACAATTCTGGAAAAGGAAAAGTCGTCTGTTGGAAGTGTCAAAAACCAGGTCATTATGCTAATAAATGCAAGGTGAAAGACACAATCAAACAACTACAAATTGAAGAAGTTGAAAAGGAAAATCTAATCCGACTTCTAGATCTGAAGGATTCTGAACCTAGTGATCTTGAGTCGTCAGTTGACAGTGATTCTGAGAATTCACTAGGCGCATTCTTAAGACCGAAAGGCATAACATTCCATTCATAATGACCAAAAGGAGTGACGAAGGCGGTTTTGTACCGATCTGACTCTTCTATTTGGATTTGCCAGAATCCACTCTTAAGATCAAATTTGGAAAAGACAACTGCTTTTTCTAACCGATTAATTAAGTCTCGCTTATTAGGTATGGGGTACCTAATCCATTCTAAGACTGCATTAAGAGGTTTATAATTGATAACTAGTCTAGGGACTCCTCTTTCTAGTTCAGCATTTTTCTGGACATAGAAAGCAGGACAAGACCAGGGGGACTTGGACTTGCGAATGATTCCCTTCAGGAGTAATTCTGTAATTTCTTTTTTACAGAATTCCATTGTCTCCTGATTCATCTGGATTGGTCTGGCTTTAATGGGGATATTTTTCTCACTAAAGGTTTTGGCGTAAGGAAGACTGACAATGTGTTGTTTCCTGGACCAAAAAGCATTAGGCAAATCAGAACAAATCATTAATCAACTTGTTTCAAATTCATGAACTTGTTTCTGGAAAGGTTCATTCCTAATTTGTTGACTGATTTAGTATGACTAATTTCTTCCTGGAGAGAGACAAATGTTTAGTCTTACTGGAAATTAAAGCAATAGTTTTGGAAATTGAACTTTTCTGAAAGGTTTTTAACTGGTGCAACTCGGCTTAGATAAAAATCAAACTTGGCTTGTTCTCCTAAATGGGTAGAGATGACACCATCATGGTTTACCTGGAAAGGATATAACAAGGCAAGAAAAGGTAATCCTAAAATAACTTCATCAGTAATGTTTTTTACTAGAACAAATGAAGTCTTAAAGCAAATTTTATTTTGACAAACATGGGCTCGGGGAATTTCATAGTTCAGCTGTAGGGATTTTCCTGAAGCTGTTCTAAGAGATTCACAGGACTTAGTATAATACTTGGTAGGTATAAGACCTTCCTGAATGCAGTTAAGGTCAGCACCTGAATCAAGTAAAGCACTGTGGTAAACTCAAACTGATCAACAAAAATAGTAACTTTGACATACCATTTCTTAAGTTAATTCGTTTACTAGACATAAGGCTTGTTCTGGATTCAAAGATTCATCATCTGAGTTTGTTGGACTCTGAGATGAAATAGAACAGGTTTGAGAGCTATTAAATCCTGTCGGATTTCATAATTCTCAGTTGCAATTTCTGGCTTAATTGTTTTAGATCAATTATCTCCTTCTTGATTAATTTTACTTCTGTATGTAAATCATGAAGGGTGATATCTCTTTTGCAACTGAACTTTTCTAGTGTTGTATTTAAGCTTATCTTTTGAGGAGGTGGAGACTGACTCGGACCTTCCTGACTAAGGAGTTTCCGAAATTTCTTCAGATACTCAGCCTTTAACTCAGGATTTTCAACTTTGCAGATTAAGTCTATGAGAAGCTCTTCCTGTTCTTCCTGTTTAGTAAGAACAGAAATAGATTTCATAGAATTACAGCAATTATCTTACATCCAAGTTGATATGGGGGGAACTAGGTTGTGAGTCCGTGGAATAAGAATTCTCAGAATCACTGTCAACTGACGACTCGAGATCACTAGGTTCAGATCCTTAATCTAGAAGTCGGATTAGATTTTCTTTTCAACTTCTTCAATTTGTAGTTGTTTGATTGTGTCTTTCACCTTGCATTTATTAGCATAATGACCTGGTTTTTGACACTTCCAACAGACGACTTTTCCTTTTCCAGAATTGTTCTGGAATCGGGTTTTCTTTCTTGATTGAAATTTTTTCCAATTCTTTTTAGGACTATGTTTCGTAGTCTTATAGAATTCTCTACTATTGTTATTTTTGTACGTATTTTTTCGACTATGGTAAGGACGGGACTGGCTCTTATGGTGTGACTTAGTTCGACTGGAGGGAGGTAAGCTAGTAAGACCATACTGCTCGCAGAAATTTCCTAATTCATACTTAGCGGATTTTCTGTCGGACTGTACCTGCTTGCCTATTTTCATGTCGACGCACATTTTCATACCGACTTGGTTAATAGTACTGACTATGTTTCCATAGGTCAGATTATTCCAATCAATATGTCCTTGCTAATTACTAAGGGTTGTGCGAATCTTATGGGCAAATAGATTTGGAAGACCATTAATGAATTTTTCTTTCCAAAACGACTGATTACTATCATCTCGGAGCATTACGCGCGAGATAAAGACATCTTTATACCACCTGAAGTCAGACATTTTAGGGCAACGAAGGTTGCTAAGCTGATCATGAATTCTAGAGGTAGTATGGCTTGGTGTTCCTATGAAATGCTCAATTATGGTGTAAAACAGAGTATTGACGCCGTCTTCTATTCCTTGACCTATTTGTTCGTCAAAGATAGGGAGACCTTCGTCATTAAGTTTTACAGCATGAACAATTAAGTTTTTAGACTCAGGTGTAAGGTGTTTGTCCCACCAATTGCGGAGGGTTCCTGTGAAACCTGTGACTAACAGAGGTACAATTTCAGATTGTCTAAAACTGTGGTTTGAGACATAACTATTGGCGACCATGGACATATGTTGAAGTTTATTGAAAACTTCTTGCTCAGAAAGACCATCGATATTCCATTCATACAATTTGTCAGAAGAGACAGAGAACTGGTTCTGAAAATGTCTTTCTTCGAATTGTATATCTGGAGGTGTTGGTCGAGTATACCAATTTTTGGTAAGGCTAGTAGGGGTGACTGGGTTGGTTAACCTGGTAACTCTGGGTTCAACTGTAAGTTCAGTAAAGGCTTTCTCAATTTTTGAGATGTTACTATCAACTGAATCTAGACTCTGGACGGATTCTTGGTCAGAATCAAGATTGATAGAGTCAAGAACTTTGACTCCAGATTGTTCACCTTTTATGGGTGCCAACTGTTCTAACATTTCTTCAATTTTTTGAAGGGTAGGCTTGATCTTAAGGGTCGGATTTGTCTTAAGATCCTGGAACTGGACTAATGGTTTTTCAACTTTTGTATTAGTTCTTTCAGGGGAAGGAACTAATTTGTCAACTTTAGTTTCAATTTTGTCTAACTGTTTTCCTATGACGATTAGGCTTTGGTTTGTAAAATTGCTCTGTTCGATCAACTTCTTCTCTGAATCTAATTTTTCAGGGATTTTGAAAGGACAGGGAGGGATCTGAGTTTTCTTAAAATTAATTAAGATAGACTCAGCAGGGGGATGACTGGCAACTACTTGTGTGTCATTAGGTAAAGACCATTTTTCTTTGGTGATTACTTTCAATTTCTTAGACATGTAATTGGTTTCTACATAATCTAAGTAGGGTATTTCAACTTGTTCTCTCCGTAAGTAATCTTTCCATTCATTAAAGATTGCATCACGACGATACTTAATAGGAAAAGAACTATGGTATTCATCTCGGCGCTTCTGGTTCTCCGGAGCTGTGAGATGGGCTTGCAACTTTTTCCAATTAATATGAAACTTATTCAAAACTCTAAGTTGAGGGTCAACAAAATCAAAACCTTCAAAGTCAGTTTGGGTTGGAGACTCGGGAGATTTTGGCTTTTGTTCGTCAACTGGTTGTTTGCCATAACAGGGTTGACTAACCTGTGCTGCCTTCCGGAGAGATTCTAATTGGAAATCAATCAGGGCTCTTTCTGCTTCTAGGTCTCGTTCTAGAGTAGAGGAAGAGGCTGCAAAGGAATTTCTGGCAGGGACATGCCTCTGGGATTGACCAGGGGTTGGCAACTGCTGTAGTTGGACAGTAGATTTTGGAACTGGTTGGGTCCCAAAATTGATTTTTACTGATCCATCTAAGTATTGGTGGATGTAATCTAAATTATTTAGACTACGTTGGATTGGTGTTGGTGGATTAACATTTTTGAGTGTCCACTCAACTGGGAGCGAGACATCAGACCATTTAATTGTTTGAGGTACTCGGATGACGGCCTCGGGTCTATTACTTTGAATCAACAAGGTATGATTCTTAGGACTCTTATTAAGGGCTTGGAAATTCATATTAGTTCCAGTGACCTTATAATAGATCCTAAAGATTAAGGCTAATGGTTGGGTTCCCGGAAGAACCTCATATCCAGAAGTCTGGATATTCAGCGTCAAAGTCTTAAGAATGTGGGGATCACTAAGACTGACAGTAAAGTCAGGATAACAATCAAAGTGAACCGGACCATTTGAAAGACTTGACTCAATTACTCCGAGTGTACTGTCCTCACTGTTAGTGAATCTAGCATCTCTTAACCAGAGCAACACTGAGGCTTTAAGCCCTGTTCTGGTTAGTGGCTTAACTGCAACTTGAACTAAACCAATATGAAGAAAATTGTTTCCTTCATTCTTATGGCGTTTGATGGAATCAAGGTTGAGCAACTGACAGACTTCAAGTTCTTTGCCAAGAGCATAGACTTGTTCAACTGTTTTAACATGGGTAGTGGTCTTAAAACTGGGTAAAGACCACCTCTTCTTATAGATTTCCTGACTGGGGATTCTAGGGATATTCCAGTCTCGGTAATCTAACTCACTGTTATTAGACTCAAATTCAACTTGTTCCTCATTTACTATTTCTGGAAGACGACTAATACTGGCTCTTGAACTGCTCGAACTACAAGAATTTGTTCTAAGGAAACGACTCATTTTACTTTGGCAACTGGGTTCTGACCTCAAAGAAGAAAATAAACAGAGACTTCTACTGCTGACTCCTGTACGTTCCTATCAACTGACCTCTCTCTCCTAGCCCTCTCAATGCCGCAACCGCTCATGCCTCACCTCGGGACTTACTGTTTGGACTCTAGGCCTTACTCTTCGGACACTATTTAAGAGAGATACAAACAACAGTACCGGAAAGAAGAACAACAATAAAAGCAACTGAATACTTCTACTCAGAAATCCTAAACCAGCCACTTGGGTTGGATGAGTAGCAACCTAAGAACAGACTCTTACAACTCTTACGGCTCTGATACCAAAAAGGAAGAACCAACATTAATGGAAGAACCAAGAAATAGAACAAAGAGTATTTTTTCTATCAAGATATATGCAGATTACATTGATGCATGGTACAACATCTTCCTCCACCAAACAGTTGATTTTTCCCACTCCTGGTTTATCAATTTCGATGCCAAATTCAGGAGTCCTTTCCCTTGCTGGTTTCTTCATTGGTGGGATCGACATGGCCCAACAGCCGATGTCCTTCCTACTGAAGTCCATAATCTGACCAATTATTATGCCAGTAAAAGCCAATTTAATAAACAGCAGTTGTTCTTCCCCAAGTTGCTTCTTTTTATTGCCAGGTACAAAATCCCCTGGATTATGAAGTGGAATTATCATGTCAATTGGGAATCCAGAATCATGTCTCGCCAGTATTTTGTCAAATGGTGGGATAAGTTCTCAATGGACAGAATTTCCATCTATGTCTTCAAGGATTTTCCCCAATCGGATCATCCTCCCAAGCCAAGTTCTCCCACAGGCTCAGTCAGTTCGGCCATCTCAATCAATGGAAAATCCAAAGCTGAACTCCGGGCCCTTGCTGCACAATTGCTTGACCAAGCTAGTCAGATGAATGATGATGAGGATAACGAATCTTCCCCATCCGCATCCAGCTGTCAGCCTGGTTCCAGTTCCTTCGACCCCACTATCCCCTTCGCGCAGGATCCTTATGACGCGTATGATTTGGGATCTGATTAGTCAAAAATTGCCATCAAGTCTTCTTTTCACCCCGGTGAAATGTTGGAATAATTGTAATGTAAAACAAAAATACTTTTGCTTTTACCCTTACTTTACCCCAATAGGTAAAGAGAGTAAAACAAAAATACTTTTGCTTTTACCCCTGCTTTACCCCAATAGGTAAAGAGAAAGTTGTTTCCCGTGCTTTGAACCATGGTCACTCTCCAACCAGAGTTACTTTGGAATCAAACCTTAGTTGCTTTTCTTTCTTTATAAGCTAGTTTCAGTCTCAGTTACAAGGGACTCGAAATCTAGTATTTTTTTTTTCTCTTCTTTCAAAGTTCTCTCTGAAACTCCTTCAGACATGAACTCTCTTGTAAATATCTGAGTGTTTATCTTTCAATAAACAAGACAAGCTTTGTAAGTTATTCTTGTCAGTTATTTTGAATTTATCTTATTTTGATTATTTTCTTTTATGCATTAATTTCTCACATTAGTCCGAAAGTTGCTTAAGAACTTTATTATTATTATTCAAATTTCTTCTTGTACTCTTTGTTCTATTTCTTGGTTCTTCCATTAATGTTGGTTCTTCCTTTTTGAAGTTGCTTAAGAACTTTATTATTATTATTCAAATTTCTTCTTGTACTCTTTGTTCTATTTCTTGGTTCTTCCATTAATGTTGGTTTTTCCTTTTTGGTATCAGAGCCGTAAGAGTTGTAAGAGTCTGTTCTTAGGTTGCTACTCATCCAACCCAAGTGGCTGGTTTAGGATTTCTGAGTAGAAGTAATTCAGTTGCTTTTATTTGTGTTTTCTTCTTTCCGGTACTGTTGTCTGTATCTCTTAAATAGTGTCCGAAGAGTAAGGCCTAGAGTCCAAACAGTAAGTCCCGAGGTGAGGCATGAGCGGTTGCGGCATTGAGAGGGCTAGGAGAGAGAGGTCAGTTGATAGAGAACGTACAGGAGTCAGCAGTAGAAGTCTCTTTATTTTCTTCTTTGAGGTCAGAACCCAGTTGCCAAAAGTAAAATGAGTCGTTTCCCTTAGAACAAATTCTTGTAGTTCGAGCAGTTCAAGAGCCAGTATTAGTCGTCTTCCAGAAATAGTAAATGAGGAACAAGTTGAATTTGAGTCTAATAACAGTGAGTTAGATTACCGAGACTGGAATATCCCTAGAATCCCCAGTCAGGAAATCTATAAGAAGAGGTGTCTTTACCCAGTTATTAAGACCACTACCCATGTTAAAACAGTTGAACAAGTCTATGCTCTTGGCAAAGAACTTGAAGTCTGTCAGTTGCTCAACCTGATTCCATCAAACGCCATAAAGAATGAAGGAAACAATTTTCTTCATATTGGTTTAGTTCAAGTTGCAGTTAAGCCACTAACCAGAACAGGGCTTAAAGCCTCAGTGTTGCTCTGGTTAAGAGATGCAGATCACTAACAGTGTGGACAGTACACTCGGAGTAATTGAGTCAAGTCTGTCAAATGGTCCGGTTCACTTTGATTGTTATCCTGACTTTACTGTCAGTCTTAGTGATCCCCACATTCTTAAGACTTTGACGCTGAATATCCAACTTCTGATATGAGGTTCTTCCGGGAACCCAACCATTAGCCTTAATCTTAGGATCTATTATAAGGTCACTGGAACTAATATGAATTTCCAAGCCCTTAAATAAGAGTCCTAAGAATCATACCTTGTTGATTCAAAGTAAAGACCCGAGCCGTCATCCGAGTACCTCAAACAATTAAATGGTCTGATGTCTCGCTCCCAGTTGAGTGGACACTCAAAAATGTTAATCCACCAACACCAATCCAACGTAGTCTAAATAATTTAGATTACATCCACCAA

TATATATATAGATATATATATATATATACACACTTGAGTTCTCATCTCTGCAACTATTTTTTGTTCAGATTGGTATTTCATTCTTAGTAAACATTTTTGGCTAATGTATGTAATTTATGCAACTTTCCTTTCCTGCATCTTTGTTGTACAAGGTTTTTAAGCGAGTAATTGTCAGTTACATTTGCATTGGATGCTTCTTCAATCATTTCAAAAAAGCGAAATTGGAACCGTGTTGAGGTTGCCGGTAAAATGAGCCAAACTAATCCTGGCCATTTATGTTTAAAAGAAACGAAAAGACACATGGAATGAGGTTCTAACTTGGTATATTAAGGCAGAGGAATGCTTATGCAGGTTACATAACATAATAATGCACATAATGTACCTCAAATAGGAAAATATGTTATGGGAAATGAAACTTGCCAAGGATAAGAACATGACCCCGCTTGTCTGCTTCTCAAATAACTCACAAAAGAGTGCCAGAAGCACCAAATGCAGGACAATAGAATAGGAACAAGCCAGGGCCCCTGAAAGTATAAATTAGTTCCTCCAAGGTGCACGCAAATTTGGCATCTTGTCAGATGCGGTCATCAAAATGAAATTCAAGAGATTTAGGAGATCACATTAAGTTGGTCAAGCAGCATGGTTTCTTTCCACTTTATGTTAAGAAAAAGAAAGAGAACAACTTCATACTGAGTCTACTGGTACTATCCCTCAGTGTGTTTATGTACATTGAGATCACATATAATTTACTCTGACCAGTCTCAACAAGAACTCCAAAGGTTCATTTTGACAACCATTATGTTACATCCGCCGATATAGAACAAAATGGGTACATAGTTTGGATAGTTAGCCCACCTTTGGAGGTTGATTTTCCACCTCATGTTCCTGAGAGGGGAAAAATAAGGAATATAAAATAAGGAAATGCCAATGAAAGCTAAGGACTTCTAGGGAACAAATTGCGGCAGAAAGATTTTTGCTAACCTAGAAATAATATCCCATG

>Chr5-05; OZ022210.1; 5785574-5791901; dir; D;

GTCGGAATCGGTGGCCGAAGGAGGGAGATCGACGGCGGCGAAGTTCGGACGACACCGGCCGCTTCTTCTCCATTTTTCGGAGAAACTGCGGCGGCTAGAGTAGGGAGCTGGCTGGGGCTAGAAGAGGACGACGGCCCGGTCATTTTGGTACTAGCCCCGTCGTCATCGGTGGCTGGAGGAGAGAACGGCCGGCCAAAACGTGGCGCGCGAGAGAGAGGAGATAGAGAGAGAGAAAAAAAAATCTGATTTTGAAAAAATTATGATTGTGCCACTGGGTTTCTTTTGACCATAACTCTCTCGTTACAACTCCGATTCGAGCCCACCACGTGTCTACGAAGTACGACCTATCTAAAAATACTAGTCACTGCCCCAAACTCTCTTTGGGTAAGAAAATGACCAAAATACCCTTATCTCATGGGTAAATTTGTAATTTTCCTTAAATAATTTAAATAATTTAAATAAGGGGTTTAATTTGAAGTTGGGGTGTTACAGCTCAGTCAATTACTGGCTGGGGGGCGCAAAACATAAAAGTGTGGGTGGACAAAAATAAGATTCAATTCAATGTAAAACAACATAATATAAACCCACCGTTTAGAAAACCATGCAAGAAATCCAAATGCAACCCAAAACCATCATAATCAAGAGTATATATATATATATATATAATATATATATATATATATCTCAAGTTAAATCAATACCGGATGCCAAGTCTGATATCCATAAAGAACACTTTACAAACCCACTATTCAAATATTATAAATCTCGAAAGCGTACCCATTGGCACGACCGGCAAGGAAGTATCCTCACTGAGAAACAAGAACGAATGAATGGTTATATATACATATAATATATATATATATATAATGCATCAAAATAATGCTTTTCAGAAAATAGATAATTGAAAATAACTTTTATTAGACTCACAAGGATTGCAACTGCAACACTTCAAGCTCACACTCTATTTACAAAGATGATTTCTCCGAAGAATGTATCAGAG

AGAATAAAAGGGAAGAAAAGGAAGGAATGAAAACCTTCTACTCAGATGCTTAAACCAGCCACTTGGGCGGGGATGAGTAGCAAACTTAGACAAAGACCGTAACACTTAGGGCTCTAATACCAATTAAGAGACAAACACAATGGCTCTCAATTAAGAGACAAACATAAAAAGAAGTGTTGCAGTTGCAATCCTTGTGAGTATAATAAAAGTTATTTTCAATTATCTATTTTCTGAAAAGCATTATTTTGATGCATTATCATTATTTTACTGCATTAGTCTGAAAGTTGTTTAGGTTGCATCCATTCTTTCTTCTATTGTTCTATGCTCTTGAGTTTTTATTCAGTCTGTTCTTCTCCCTACTCTGTTATTCTCTTAATTGGTATCAGAGCTCTAAGTGTTACGGTCTTTGTCTAAGTTTGCTACTCATCCCCGCCCAAGTGGCTGGTTTAAGCATCTGAGTAGAAGGTTTTCATTCCTTCCTTTTCTTCCCTTTTATTCTTTGAGTCCTCTGTTGGTTTCTCTAAGGTCTGACCCGTGAGTCCTTTTTCCTAGATCAGTAAGTCCCCAAGGAGGGCGTGTGTGGTGGGCAGGAGACAGAAAATTGAGAAAATTAATAGGTTGCTTAGAGTTTAAACGGTCAGTTTAGGATATACTGTTTATCTTCTTCCTCAGACAGCCGAACCCAGTTATTTGAGTAGTTATGAGTCGTCTGTTTAGAACAAAGTCTTGTAATTCTGCCAGTTCTAGTACCAGTATAAGTCGACTTCCAGAGATAGTAAATGAAGAACAATTTTAATATAATTCCAGTAGTAAAGAACAATTGGATTTTAGAGATTGGAACATACCTAAAGTCCCCAGTCAAAAAATCTATAAGAAACATTGGTTGCCTTCCAGCTTTAATAGTACAACACATGTCAAAACAGTTGAACTATACGCCTTAAGTAAAGAACACGAAACTTGTCAGTTGCTTAATCTTGAGTCTATTTTAAAGCATAAGAAAGATGGTAATAATTTCCTGCATATTGGTTTAGTTCAAGTAGCTGTCAAAACCTTGACACGGTTAGGGTTGAAAGCCTCAATCCTACTCTGTCTGCGTGATGCTAGATTCACCGAATTTAGCGATAGTACCCTTGGAGCCATTGTGGTTCCTGCAGGAATTCTCGAGTAAGAAAATCAGGGACTGTATTCTCACTACCTCTCAAATATTCAATATCAAAATCAAATATACTCAATATACTTTGCCATCGTGCCAAAATTTGTTTAGATGCAATGTTATGAACATCTTTTTCTAAAACATGTTTTGCAGATTTGCAATCAACTCGTATTAAAAATTTTTGATTTAATAAATCATCTTGAAATTTACTAATGCATAATACAATAGCTAATATTTCTTTTTTAATAGTACTATAATTATATTGCGACTGAATCCAGGTTCCAGAATGGAAACGAACAATTTGTTCTGGAGAATTTGGACTGACTCTCTGTTTGAGAATGCCACCATAACCTATGTCAGAGGCATCAGTTTCAACTATTTTGAAAGAGTCAACTGAAGGAATACCAAGACAGGGAAGTGTCTTGACATGGACTTTGATCTGTTTGACAACTTCAGTATGAATATTTGACCAAGGAGGAGGATTATTCTGCAGCCTATCAAATAGGGGCTTACATTGTTTTCGCAGATTTGGATAGAAGTCAGAGATATAATTTAGAGATCCTAAAAACCTCTGGAGTTGACTTTTATCAAGAATCTGATCAGGAAATTTATCAGCGAATTGGATAACTCTATCAATTGGGGAAATATGAGACTGGCAGATATTGAAACCAAGAAATCTAATATTGGTTTGAAACAACTTAATTTTCTTAGTAGAGACAACTAGACCATTTTGTTTGACTATGTGAAGGAATTTATGCAAATGCTTCCAATGCTGTTCAACTGACTCAGAAAAAATGAGAACATCATCAATATAAACAATCGAGAAATGACTGTAAGGATTGAAGATATCATTCATAATATTCTGAAACTCACTGGGGGCATTTTTTAAACCAAACGGCATGACATTCCATTCATAATGACCGAAAGGAGTAACAAATGCAGTTTTATACCTATCTGACTCATCTATCTGGATTTGCCAGAAACCACTTTTCATATCAAATTTGGAGAAGACAACTGCTTTTTCTAGTCGATTAATGAGGTCTCTTTTATTAGGTATGGGATACCTAATCCATTCTAGGATCGAATTAAGGGGCTTATAATTGATGACTAGCCTGGGGACTCCTCTTTCTAGTTCAGCATTTTTCTGGACATAAAAAGCAAGACACGACCAATTGGTTTAGTTCAAGTAGCTGTCAAACCCTTGACACGGTTAGGGTTGAAAGCCTCAATCCTACTATGTCTGCGTGATGCTAGATTCACCGAATTTAGCGATAGTACCCTTGGAGTCATTGAGTCCAGTCTGTGTAATGGCCCTGTCCATTTTGACTGCTATCCTGATTTCACTGTAAGCCTTAGTGATCCTCATATCCTGAGAACTTTGACTCTGAATATTAAGACTGAGGTTATAATGTCCTTCCGGGAACCCAACCTTTAGCCTTAGTTTACCGAATCTACTATAAGGTCACAGGAACAAATGAATTTCCAAGCCCTTAATAAAAGCCCGAAGGATCAAACCGTCCTGATCCAGAGTCACACCTCAGACGCCCATATACAAGTTCCCCATACCATCAAGTGGTCTGAAGTTGCCCTTCCAAAAGATTGACCTTAGTTACTGAGAGCCAACCAGCTCCGATCCAGCGTAGTCTAAACAATTTAGACTGCATCCAACAATACTTAGATGGCACAGTTAAAATTAAGTTCACAGTCATCCCCTACGCAAGTCTAATGTCTAGTTACAACAATTGCCAACTCCTAGTCAATCCCAGAGGCATGACCCTGCTCGGCATTCCTTTGCTGCCTCTTCCTCCACTCTAGAACGAGACCTAGAGTTAGAAAAGGCCATGATCGATTTCAAACTTGAGTCCCTGAGAAAAACCTCTCAGGTTACCCAACCTTGTTACGGCACGGTTCCAGTCCGAGACGATAAATCAGAATCCCCTGAGTCTCCGACCCAGTCTGATTTCCTAGTTGACAACCAATTAAGAACCCTAAATAAGAATTTGAAATTAATTGGGTAAGTCTAAATCGCCATTTAAAAGCCCCTGAAAACCAGCACAGAAGAGATAGTTATCACCAAGCCTACCCTAACAGTAAACATCACGAAAATGGAAAGATTACATGCGTCTGCCAAAGTTGAAATTTTCTACCTTGATTTCGTGAAAGCCATTACATGCTAGTGATGAATTGAAAACTCTGACTAAAAAAAAAAGGAAACTAATTGATAAATCTGTAGTTGAGTCGAGTCATCCTCCAGTAGAAACTATCATAATAGAACATCAGAAAGCACCAGTAGCTGCCACCCCTTTCAAAACCTTTGAAAGCAGTGACCCGAATAGAAAAATGATTGAACAAAACAATTACACCAACCAGAGTCTAATCACTATAGGAAAGCAATTAGATACAATCGAAACCAAGGTCGACATGAGTCCGCCTGTTGCAGACTCCAGACCCAGACCTAAAATTGAAAAGCCGATAGTTCAATTCCAAGAGCTTAAGTCCAGTCCAATCTTAAAAGTCAATTCTACAATGAAAAAGATAGAAGAAATGCTAGAACAATTGACCCCTGCCAAGCCCGAGAAAGGTGACAAATCTGGTATTAAAACACTAGATTCTTTTGCTGCTTCTCAATCTGAGTCCGAATCAGTAAGTTCAGAAACCAGTGACATTTCCAAAATTGAAAACGCCTTCAAAAATTTAGAAGTCCAAACTAAGCCCAGAGTCAAAAGAATAGAAAAACATATAAGTCCCACTAGCCTAACAAAGAATTGGTATCCCAGACCAACACCTCCAGATATCCAATTTGAAGAACGAAACTTTCAAACCCAGTTTTCAGTTTCTTCTGATAAATTGTATGAATGGAATATTTGGTCTTTCTGAGCAAGAAATCCTAATAAGTTGCAACATATGTCTATGGTCGCTAATAGTTATATCACCAATCACAGTTTCAGACAATCTGAAATTGTCCCTCTGTTGGTTACCAGTTTCACTGGTACTCTCAGATATTGGTGGGATAAACATCTTACCCTCGAGTCTAAAAATCAGATCATTTATGCTGTAAAACTTAATGAAGACGGTCTCCCTATATTTGACGAACAAATAGGTCAAGGTATAGAAGATGGTGTCAACACTCTGTTTTACACGATAATTGAACATTTCATAGGAACCCCTAGTAATACCACTGCTAGGATACATGATCAGTTGAGTAATCTTAGATGCCGGAAGTTGTCTGACTTCAGATGGTACAAAGATGTCTTTATTTCCAGAATCATGCTTAGGGACGATAGTAATCAACCCTTCTGGAAAGAAAAATTTATTAATGGTTTACCAAACTTATTCGCTCACAAAATCCGCACAACCCTAAGTAATGAACAAGGTCAAATTGATTGGGATAATTTAACTTATGGTAACATTATAAGCACAATTAATCAAGTTGGTATGAAAATGTGTATCGACTTTAAAATCAATCGTCAAATCCAGTCTGATAGAAAGTCAGCTAAGTATGAATTAGGAAATTTCTGTGAATAATACGGCCTCACTACTATCCCTCCTTCTTGGAAACATAAGCCTAGTCACCTTAGAAAGCGTCATGACCCGAAGAAGCAATTTCTCCGGAAAGATGAATTCTATAAGAAAAGAAAGTTCTCCCCTAAAAAGAATTGGTCGAAACGTCCTGAGCAAAAACAAAGTCTCGCTAAGAAAGATAAGAGCAAAGTCAAATGTTTTAAATGCCAAAAATTTGGTCATTATGCTTCTGAATGCAAAGTAAAAGATGTTATTAAACAATTACAGATTACAGAAGAGGATAAGAACAAATTAATCCAAGTTTTGGAGTTGCGAGACTCTGAAATGAGTGATGTTGAGTCTCTAATATCCAGTTCCGAGTCTGAGTTCAGCCAGTCTTCAGATTCACAACCCAGTTCTCCTAGAGTCCAATTTGGTTGTACTGATAAATGTTGTAATAGGTTAAATTCTGTCTTAGTCCTCACTAAACAAGAAGAGCAAGAAGAACTTTTAATAGACTTAATAGGAAGAGTTCAAAACCCTGAGTTAAAATCTGAATATTTGAAAAAATTGAAAAAGGTCCTAAGTCAGGATAGCCAGAGTTCCCTCTCTTCTACTCCCCAGCCAATTAGCATCAACACTACTTTAGAAAAATTCAATCGTAGAAAAGACGTCACCCTCCATGATCTCCAGTCAGAAGTTAAACAGGTTAAAAAAGAAATAGTTGAGCTAAAACAGATAAGTCAACAATTGCAAACCGAGAATTATACTATCAAACAAGACTTATTTGATCTAAAACAAGAAACCTGTAAGCCTAACAACCACTCAGAAAGTCCGCATCAGTCTGGTGATGAACCTTCTGAAGAACAAGTAGCTGTCAAACCCTTGACACGGTTAGGGTTGAAAGCCTCAATCCTACTCTGTCTGCGTGATGCTAGATTCACCGAATTTAGCCATAGTACCCTTGGAGTCATTGAGTCCAGTCTGTGTAATGGCCCTGTCCATTTTGACTGCTATCCTGATTTCACTGTAAGCCTTAGTGATCCTCATATCCTGAGAACTTTGACTCTGAATATTAAGACTGAGGGTTATAATGTCCTTCCGGGAACCCAACCTTTAGCCTTAGTTTACAGAATCTACTATAAGGTCACAGGAACAAATATGAATTTCCAAGCCCTTAATAAAAGCCCGAAGGATCAAACCGTCCTGATCCAGAGTCACACCTTAGACGCCCATATACAAGTTCCCCATACCATCAAGTGGTCTGAAGTTGCCCTTCCAAAAGATTGGACCTTAGTTACTGAGAGCCAACCAGCTCTGATCCAGCGTAGTCTAAACAATTTAGACTACATCCAACAATACTTAGATGGCACAGTTAAAATTAAGTTCAACAGTCATCCCCTACGCAAGTCTAATGTCCAGTTACAACAATTGCCAACTCCTAGTCAATCCCAGAGGCATGACCCTGCTCAGCATTCCTTTGCTGCCTCTTCCTCCACTCTAGAACGAGACCTAGAGTTAGAAAAGGCCATGATCGATTTCAAACTTGAGTCCCTGAGAAAAACCTCTCAGGTTACCCAACCTTGCTACGGCACGGTTCCAGTCCGAGACGATAAATCAGAATCCCCTGAGTCTCCGACCCAGTCTGATTTCGTAGTTGACAACCAATTAAGG

TATATATATATATATATGACAATCACCTAGTTGTACCAGGGAAACAATCCAACTGGGGGATTGTTTGACTAGTCCACATGGAAGAGCCCGCATAAAAGAAATTCCTCAATTGACCATGTGGCTAGGGAACCAGCTGTCCAACTGGGGGACTAACTCTCAGCCAGTACGTATAGTTGACACTCTCAAAACTTGTACGCCTGAAAACAGTCCTACGCGCGCAGACTACCGTCAAGGATGCCCCGGGTTCCAACATTTTCAAGTATCTCAAGTTTCCATATCCAAGTTCCAAATCCGATGAGGTACATAGGGTAGTGCTTATAGCACTCCAAACTGACATTCTGTACTCAAATCTTTCCACAATCTCATCTCAACAACCCAAGTACCCAACACATAGACAATTATAGTTAGAATTTCTCAAAATCCATAAATACATACTCAAGATACTCAAATATGTCAATTGCCAATGCATCATAAAAATATCAATTTCAACAATTGAATAAATAATATATCCAAAACACCATTAAAATATTATGCATAATTTTTCCCATCATAAAACCATGCATAAGATTTGAAAACAAAGTCCACTTACAGGAAGTCCCAAGCTGCTTGACCCTGAGAGGGTCCCGCCTATTGCGTATGCGTGGTCGGAATACCTATTTCAGAATACGAACACGAGATTAACACAAAGTGTTTCGAATGAGAACTTTGAATTAACATCTTAGTTTTCTGGGTTTCTAGTGAGTGTACGAGGAACGAGACAATTTGAAAATTCCTAAGGGTTCCACAAAGTTTAGGATACACGTCTTGTAAGTTTGGTCCGAAACGGACGGTCAAATCGCTCACGCTCGCACAATCTGAAGGTTATCGTAAAAATCCAAACTTTAAGTTATTGAATTGGAACATCAGGGGTTCCGATTCATTTTTGCGAAATTCCTATACAATCCTAAGAATGTCTAAAGTAACGTATATTGGAATGGGGTCAATCCAACGGTCCGAAAAT

>Chr5-06; OZ022210.1; 6222876-6224195; dir; B;

TAAATTCTTTTTTTTCTCTTCTTCTTTTTGACATCTCAAAAAGAAGACATACAAATAACATAATTGAAAAAAACTAGAAAATAAAAAACAAATGTTGTCCTTGCTTTGCTTGCGACCATATAATTATCATCCCACCAAACATACGTGGTAAATGAGGAGGAGCCCATGGGACATGGTTGAAAACAGAAATAATTGCATAAATAAATTGAATATATCATATCACTAACGGACACATATATTTGAGTAATTCTTAGAATCCTAGAGAAACTTGCCAAAACCAAGAGACACATATACAGACCAAGAGAAACTTGCCAAAACCAAAGGGCACTTGGTTTCTTGTTTATTTTTTTTGTTTGGAATTGGTCAGTTCTGCCATGAGTGATGGTTTGGGAAAACCAAAAAATCCAACATAACTGGCAATGCCATCTCACTAAATCAAAAGCAAGCAGGCATCACATGCCGAAGTGAAAGAAATAAATGAGAGATGTACTATGATTCACATGTGACACCCAATCACAATCATGCTACAGTAACAAGACGCAAGGCACTTCAATCTTATATATTCAACCACCCCCTCACCCCTACTTCCCCACTGCCTCCACATCAAAACTCTCTCTCTCTCTCTCTCTCTCTCTCTCTTCAGCAAGCAACCATCTTGAGGTACATAATTTATACATTTCAATACATGAATTCTTTTTCTTTCATTTTTGACATTTCTTGCTACTAAAAGGAATATTGATGTTGTATTTTGCAGATGAAGGCCATGAAAAAGAAAAAGAAAAAGAAGAGGCATGATATCGTGGGTTAGCAGAAAGTTGGAATGAGAGGAATCGGTAAAAAGTGGTATAAATTTTTTACATCTCTTGTTTGTTGCTTTGTGATATTACACTTTTGGAATGTGTTTTTATCCATTCAAGCCCAAGTAAACGATAATAACAAGTAACCTACTTGTATTCAGAACAATCTACAAAATCCAGATAGAAAACAAGTACTTGTCGAT

AGAAAAACGACCAAGTACAAATTAGGAAACTTTTGGGAACAATATGGTCTAACATCGATTCCCCAGTTCATAAAAAAATAAAATCTATGATGGTCAAGCTAGTAAAACTAGACATTACAGAAGAAAAAGACATTACACTAAAAACAATATATTATTTTATAATAAAACAAAAGGAAAAATTGGTCTAAAAAACTCAAAATTTTTAAGAAAAATAAATTCAGCAAAAACATTGATAAACGAAAAATTGAGTGCTGGAAATGTAAAAGATTTGGTCATTATGCTCATCATGAATATAATGTTAAAAATACAATAAAACAGTTAAAAATATATGAGGAAGAAAAGACTCAGTTAATTAAAGCTTTTGATTTAAGAAGCTCAGAAGGTTATAGTTCTTTTGATTACCAGTCAAATTCCCCAAATATTCAATTTGGTTGTAAAGATGATTGTTGTAACACTAAGACAATAAATGTTCTTACAAAACAAGAAGAACAAGAAGAATATTAATTGACTTAATTAGTAAAGTTGAAAACCCATAATTAAAGTTTGAGTATTTCAAGAAACTTAAAAAGGTAATAAGGGAGAGAGAAACAAAGCATGAAAAAACCCCTCCTCCAAAAATAAGTCTAAGCTCAATCTTAGAAAGAAAGAAATCACCTTACAAAATCTACAGCTTGAAGTAAAAAATGTCGAAAGAGAAATTATAACTTTAAAACAAATAAGTCATGAACTACAAAATGAAAAAATAAAACAAGATTTAATCAACCTAATGGAAAGATTAGGACAACCCTCTAACCAAGAACTGTCATAACAGTCTGATGAAGAATCAGTTAAGACGAGAAGAAGAAGAGCAACCTAAAACACACTCTGACAAAGAAGAACAGTCAAGTAATCAAAAAACTATCAGCCTAATTAAACAGTTAAAACATAAAAAATGGTATTCGAAAGTCAACATTTTTATTGGCGACTTTAAATTCAACACCATAGCCCTATTTGATTCGGGTGCAGATCTTAATTGTATACAAAATGGTTTGTTTCCTACCAAATATTATAAGAAGTCAAAAGAATCCTTAGGAACTGCTTCTAGTAAACCACTTCAACTACAGTATGAAATTCGTAAAGCTCATCTGTGTTAAAATAAGGTCTATTTCAAAACATCTTTTGTCCTAGTAAAAGATATAACAGACGAAGTCATTTTAGGTTTACCATTCATCACCTTACTCTACCCCTTTATGGTAGAATATGATGATATTACCTCTAAGTACATGGGAGAAAAGATAAAGTTTGAATTTCTAGCAAAACATGAATTACAAGTCTTAAAGG

GAAAATGATTGCACAGCAAACAACACGATGCCAAAAGAAGAAACCTACATGCAGCAACCAAACGGAGAATGATTGCACAGCAAACAACACGTGAGCAGAAGATGTGGCTGCAGCTAAAGTTATTAAAGAAGAACAACTGGCAATAAACAAAATAAAACAAAGAGCTTGTAGGCATGTGAAGATTGGCAGCAGTACAGAAGAAAGAGAAAAGCAAGAGCATTGGCTCCGTTCGCTCTCTATAAATAGGGAACTCAATAAGAGGATGGGGAAAAGACGAGTGTGCATATTTTGGTGAAGACTTGGGCACCAAATAGAGTTAGAGTTAGAGACCCTGGGGATCCGAGAGGCAAGGATCCAGGGGCATCTACTATTCTAGTTTTCCTTTTAATTTCTCAATTGTTGTAATAAAATTATTTTGCTAAATTTTCCTTTATAAATTTTTTTTTCTTAGTTGTATTTTATTTTTCATTGTCATGTAATTTCTTATTCTTAAGTTTTCAAGTTTGAATAGTTTACAAGTTTCAATTACGTTTCTTGTTCTTTTTCATTATGCTTATAAGTTAATATGCTTGTTTGAATGATTGATCTGTAATAACCCAAAACAAAATATCTAAAAAGAAATAAAATATCTGAAAAGTAGGATTAATATCTTCTAGCAAAAAGACAAGTTTGCCCTCGCATATTTTAATAGGGGAAAGATTGATTTTTTGATCGAGAAAGAATTTGGAAATTTCTCTTGCGCCGTTACGTAGAGCACGGCGAAACGAGTCTGTAAACACGGAGTAGACCCGAATTGGAGTTGTAACAAAGAAAATACGATCAAAATACCACGAAGGGAAAAATGGTAATTTGGTCAAAAAGTCAGATTTTTATCTCTTTCTCCCTTCTCTCCCCTCATTTCTCTCTCCTCTCTCTCTCTCTCCTCCCGATCCCGCGCGCAGGCCCGACCTCCGCTGGCTTTCGACGTCGCCCCGGCCGCCACAGGTGGAGCCGATCTTCG

>Chr5-07; OZ022210.1; 6237051-6238190; dir; B;

CTCACATTTTCAACCTGTTTTTCGCCCCCAGGCCGTAGAAGTGCGCAGGATCCACCACCGAGCCATTCCTAGCTTCCACGCCACCACATCAGGTAGAGTTTTGTGGAAAAATCCTTGAAACCCTGTAAACTTTAGAAAATGCTCTGATATCTAGTTTTAGTGGAAAACTGGAGCTGGTGAATACTTGGTTATTCTATTATGGTAGTTGGTGTAGATTTATTTGATTGTTTGACAGGTGGAAAAATTTGGGTTTGGTCAAAATACAGGGGAGACTCTGCCGAATTTTCTGCAGAAGTCCAAAGAAAGTTTTCCTTGTAACTGTAGCAGGAGGGGTAAAATGGTCATTTGTGCCCGACAATTGCTAGATGTCGGACACGCACAGGGCTTGGTTCGAATTCCAAAGTGAAATTGGGATCGGGTCCTGTCAGATCACCATTAAGACTTGTATGTTAGCTACCTTGGTTGGAATTAAGAATAGACACCATATGCCTTAATTCGAATTGAACTATGAATAGGAAAAGTATGTATGGGCATTCGACAATGGGGATTAGATACATGCTTGGTTGTTTAAAACATTCTTCTATACTTAATGGATTTCTAATGCTTAATTTAGGTTTGACGACAAATGATCTAATTAGGGAATTAGGCATACAAGGTTAGGACCAAATACCATGGCTTAATCATACTTTAGCTAGAATCAATCTGTGAATCTGAATTGAACTTGTTAACTTGATTGTTTTAGATTTTTTTCTTTCATTTACGTGTTTACTTTTGTCGTTTAAGTTTAGTTCATGTTTTCCTTACCTAATCAAAACACCTCATTTCTATTTTGTACAAGGAATCGAATTGAATCAATCTATCATCAATTCACGTGAGATCAACCTTGCACTTACATATGCTATACTAGGATGCAGTTGAATCAATTTGGAGCAGCTGAATCGACACAACATTACATATAAAAAAAATAAAGAATTTCAAAGAAAAGATCGAACAAGACGTC

TGTTCTTATCTTCCTAATGCCTTCTGGCATAGAAAACAACATGTAGTCAATCTTCCATATATCAAGGAGTTTAATGAGAAAAATATTCCTGGATCCATTCAAATGAGTCAAGAGATTATGAAATTCTGTAAAAAAGGAAATTATAGAATTACTTCATAAGAAAATAATTTGTAAAAGTAAATCTCCTTGGTCTTGTCTAGCCTTCTATATTCAGAACAGAGCAAAGTTAGAACGAGGAGCACCCAGACTAGTCATAAATTACAAACCTCTCAATAATGTGTTGGAATGGATTAGATACCCAATCCCTAACAAAAGAAATCTAATCAAAAGATTAAGCAACTTAGTCATTTTTTCAAAATTTGACATGAAGTTGGTTTTGGGAAATACAAATCCAAGAAAGTGATAGGTACAAAAAACTGCTTTCACTACCCCTTTTGGTCATTACGAATGGAATGTCATGCCATTCGGCTTAAAGAACGCTCCTAGTTATTTTCAGAATATAATGAATGAAATCTTCAATCAATTCAGTGAATTTTCTATAGTATATATTGATGATGTTCTCATCTTCTCCCAGTCAATTGAACAACATTGGAAAATTCCTTCAAATAGTCAAGAGTAATGGCTTAGTAGTTTCAGCCACTAAGATTAAGCTGTTCCAAATGAATGTTAGGCTTCTCGGATTTCATATCCATCAGTCACAAATCAGCCTAATAAATCGAGTTATTCAATTTGCTGATAAATTTCCAAAAGAACTTTTTGACAAGAGTAAGCTCTAAAGATTCTTAGGATCTCTTAACTATGTAGCTGATTTCTATCAAAACTTAAGAAAGCAATGCAAACCCTTATTTGATAGACTTCAAAGCAATCCTCCTCCTTGGTCTCCTAGTCATATAGAGATAGTTCAGAAAATCAAACTACATGTCAAAACATTACCTTGTCTTGGCATTCCCGTTGACAACTCATTCAAAATTATTGAAACTGATGCCTCTGAAATTGGTTAAGAAGGTATTCTTAAACAGAAAAAATCTCATAGTCAATTAGAACAAATTGTTCAATTTCATTCCAAAGTTTGGACTCAGTCACAGTAAAACTATAGTACTATAAAAAAGGAAATTGTATCTATATTGTACTATTCTCTTA

TCATACGTATGCATGAACTCTTCACAATTAATACATTTTTTTTCCCCGAATATTTTGTAGTGTTTTCCTATTTTTATGCTTTCAGTTAGAGTCCTTATTTGTATAATAAATTTCGATAGCTTTTCATTAATGGATTATTATATATATTATCCCATATTGTTAGCTTGCCTAAATTGGAGGTTATTGGGTTCCACTTCTCTTTTGACTCTAAGTTATTAGTTTCAAGATAGGAAGAGGGAAGAAGAAGACTCTAGCACCAGATCAATCTCCCTTGGTTATTCGTCCAGTTACTCGAGCTATGGCACGTAACAGAAATATAAAAGGTTTTAGATTGTTTCTTCTGCAGTTTTGAAACTCCATAAAGATAAAAAAAAATCCAATGCTTATCGAGCTTCCAAAGACATAATATAGGGCTGGGGGGATATTTGAAGATATCAGAAGTCTCCTGCAACATCACTCTGGTTCTGTTTTTCAATTCATTGGGACAAGCCAATCGCTAGACATGGACTACTTAATGCTTTTTCATGTGTTTGGTGAGGTGCAAATTGTTTATTCACGACGCCCTTAATTTTGATTCGATGATTTGAGGTTAGGCTCCATGCCCCCTAGTGTATTTCTTAAATATTCAATCAAAATAATTCCATGTTTAATTAAAAAAGGCTTAACTGTTAAAATAGTCTCTGTGTTTTATCATGTTGGCCAATTTAATTTCTATGTTTTTAATTTGGCTAATTTGATCTCTGTATTTAGGACCGTTAGCCAATATGATCCAATCCGTTAAATTTATGTTTAATTTTTTTTAAACGGTTTAAATATTAATAAAAAAAATAGATATATTTGAATTCAATTTAAAAAATAGATATATTTGAATTCAATTTAAAAAATAGATAATTAAATAAGATCTAAAATTTTGGTCTAGCATCCACAATGGGCTCCCTAAACCACCTTCTTAGACAAATTTTAGGGAGGATTTGGAAAAATACAACTCCAACCATGCTCC

>Chr5-08; OZ022210.1; 6458184-6464619; dir; B;

GGAATAATTTGTAGTTAAGGTAAACTTCCAAATCTTGCACCCAACTACAAGATCCTGTGAACTAATTGTTTTGTACTCCGTTGTAATACATTTTATCAATTACTCTTCCTTTTCTAAAGCTGGCCATAATACTGGATAATTCAAATGTAATCTCAAACATAGCAGTGTTGATATGTTCTATATCAGACTTCACAAATCTATGGGTTGCATTCAAAACTCAAAATAAACAAATAAAATTGAGTTATTCAAATTACTTGCATATTTTAATTTAGGAGTCAAATATGTAAGTACCAAAGAGTTTGATTTTGATTTGTGCATTCCTACAAATATATATTTTTTTTTGTTGCCAACATCACAATGTACATACACATCAAAATAATCCCAAATTAGGAACTAATCATGCCCTTATGTTATTTTACATTCTTCTCATATTTTTTTTTTCTCATAAAACTATTTAATAGATGCTAAAGCTTGCATCTGAAAATACCAACATTTGACCTAATTTGACTTGGATTATTTCCCATGCTACTTTATTTATTTATCTATTGCTATCACAAATAATCATACATTCCAACCAGCTTTGCTAGGTCCACATTTGTTATAAGGTCATCATCTGATGCTAGTATAACTTTATCATGGTCATTATCTTCATACTACACAATTTTCCACCATTTAAAAGAAAATTAGGATGAGAGAACTCACAAGTCAAATTCAAAAACCTTTAAACATGTAATTTTTTATCTAAAAAATACCAAAATTGAAGGAAGGTTGTTGTGGTCAATGTCATCACTCATTCACTGAATAATTGCGGTTATAAGGTTTGTCAAACTCTTAATATCTGAAAAGAAAGCCATAATAGAGAAAACTCATTGAAGCGATAATGTTAGCTAGTCAAACTATTCTTGTTGAAGAAAAAAAAAATGGTGTTATTTCAAAGCTACCATACACACCCAAAAAATAAATAAAAAAAATTAAATAAAAAAAAATATATATATATATA

TAATCTTGAGTCGATTAAAAAACATAAAAATGATGGAAACGATTTCCTTCACATTGGTTTAGTTCAAGTTGCAGTCAAGCCTCTTACCAGATTAGGATTAAAAGCTTCTACCCTTCTCTGTCTTAGAGATGCTAGATTCACTAATTTCAGCGACAGTATGCTAGGATTAATTAAGTCAAGCTTGTTTAATGGTCCAGTTCATTTTGATTGCTACCCAGACTTTACAGTCAGTCTCAGTGATCCCCATATTTTGAAAACATTAACTTTAAATATTAAGACTTCCGGGTACCAAGTCCTCCCAGGAACACAGCCATTAGCCTTAGTCTACAAAATTTATTATAAAATCACTGGAAGTAATATGAATTTTCAAGCCCTTAATAAAAGTCCAAAAGATCAAACTCTGTTAATTCAAAGTAGCAAACCCGAGGCCAATATAAGAGTACCTCAAACAATCAAATGGTCAAATGTCACCCTTCCATTAGATTGGACACTCACCAATGAGAATCAGCCAACCCCAATTCAGCATAATCTAAACAATTTAGATTATATTCAACAATATTTAGATGGCACAGTTAAAATTAATTTCGGTAACCAGCCTGTACCTAAATCTACAGTCCAGTTACACTAGTTGCCTAACCCTGGTAAATCCCAGAGACATATTCCTGCAAGGCATTCCTTCACAGCTTCTTCCTCAACCCTAGAATGAGACCTAGAGGTAGAAAAAGCAATGATAGATTTCAAATTAGAAACTTTAAGAAAAACTTCCCAAGTAAATCAACCCTGTTATGGCAAAATACCAGTCCAGGAAGAAAAATCTGAGTCTACTGAGTCACCCACACAAACTGATTTTGAAGAAGCCGGTTTCCAAATCGATAACCAACTAAGAACTCTTAATAGAAAGTTCACAATTAATTGGAAAATTTTGGATATTCATCTTAGAGCTCCTGAAAACCAAGAACGAAAAGATTAATATCATGAAGCCTATCCCAGTGAAGAACAGCGTCAGCAAATCTTTAATGAATGGAAAGATTATTTGCGCACAGCTAAGTTAGAAATTTTTTATCTTGATTTTGTAGAAAACCGCTACATGTCTAATAAGATAAAAACTTTAAACAAAGAAAAATGGAAATTATCTGATGATACAGAAGTCGTAGCAAGTCACCCTCCAGTAGAAACAATAATAATTGAACACAGGAAAACCCAGATACCTGCTAGTCCTTTCAAAGTCCCTGAGAAAAATGATCCAGACAAAAAACTAATTGAGCAGAATAATTATGCCAACCAGAGTCTAATAGTCATAGGAAAACAGTTGGATAAAATTGAAACAAAAGTTGATAAGTTGATACCCCAGGATAACAAAACCAAAGCTAAAGATGAAAACCCTTAGTTCAATTCCAGGATCTTAAAACAAGTCCTACCCTTAAGATAAAGCCGACAATGAAAAAAATAGAAGAAATGCTTGAACAATTGATACCAGTTAAACATGAGAAATCTGGTCTTAAAGTCCTAGATTCTTTTGCAAACCCTTCTGGTTCCGAATCTAAATCAATAGTCAGTATGGAAACAGAAGCTAGTAATATTTCAAAAATTGAAAATGCTTTTAAAAATTTGGAATTAGATAACTAGTTGAAAGTCAAACGGTTAACTGACCAAGTCAATCCTACTAGTCTAACCAAAAATTGGTATCCTAGGCCAACACCCCCAGACATCCAGTTTGAAGAACGAAATTTCCAGAGTCAATTTTCAGTTTCTTCAGATAAATTGTATGAATGGAACATTGATGGTCTGTCTGAACAAGAAATATTTAATAAACTTCAACACATGTTCATGGTTGCTAATAGTTATATCACCAACCACAGCTTTAGGCAGTCAGAAATAGTGCCTTTACTTGTGACAGGATTTATTGGAACTCTCCGGTATTGGTGGGATCAACACTTAACCACAGAGTTGAAAAATCTCATAATTCATGCAGTCAAACTTAATGAAGATGGTCTCCCTATATTTGAAGAACAAATAGGTCAATGTATAGAAGATGGAGTCAATACTTTGTTTTACACAATAATTGAACATTTTATTGGAACCCCCCCCAGTAATACTACTGCTAGAATCCATGATCAACTGAGTAATCTCCATTGTCCTAAGTTGTCTTATTTTAGATGGTAGAAAGATGTTTTCATTTCCAGTGTCATGCTAAGAGATGATAGTAATCAGCCGTTTTGGAAAGAAAAATTCATTAAATACGCACAAACCTCAGTAATGATCACGGTTTCATAGATTGGGATAGTTTAACCTATGGAAACATAATTAGCACAATTAATCAAGTTGGTATGAAAATGTGTGTTGATATGAAAATTGGTAGACAAATACAGTCTGACAGAAAATCAGCTAAGTATGAGTTATGAAATTTCTGTGAACAATACGGTCTTACCAGTATTCCTCCCTCTAGAAAAGATAAGTCTCGATACCCTAGTCACATTAGAAAAAGTCGCCATTATTCATGTAGGAAACAATCTTTCCAACATAATTTATAATAATGAGTATAATTATGAGTTTTACAGGAAAAACAAATTTCACCCTAAGAAAAGTTGGTCAAAAGCACCGTAAAATAAAAAGAATAGTCGCTTTAAGGAAAGCACCGACAAAAGAAGAGTTAAATGTTATAAATGTCAAAAGTTCAGTCACTATGCTAATGAATGCAAAGTAAAATACACAATTAAACAGTTGAAAATCACGGACGAAGACAAAGATATGTTAATAAAAGTATTAGAACTAAGGAATTCTGAGTCTAGTGAAGTTGAAACTATAGCTGTTAGCACTGTGTCAGATAGTTATCATTCTTCAGATTCCCAGCCTAGTTCGCCTAAAGTCCAGTTCGGTTGTACAGATAAATGTTGTAGTAGACTAAAATCTGTTTATGTCCCCACTAAACAGGAAGAACAAGAAGAGTTGAATTGATTTAATTAGTAAAGTTGAAAACCCCGAGTTAAAGTCTGAATATTTGAAGAAGTTAAGAAAAGTCATAAGCCAAGAAAGTCCGAGTCCAAGCCAGTTTGTTCCCCAAAAAATCAGTCTAAATACAACTTTAGAAAGGTTCAATAAGAAAAAGGAAGTGACCTTACAGGATTTACAATCAGAAGTTAAAATAGTCAAAAGAGAAATAGTTGAATTAAAACAGATGAGTCATAAGTAGCAAGTCGAGAATTATGAAATTAAACAAAATTTGATAAGGTTAATCGAAAAAGAAAGTTCTGAGTCTAGAAGCCAAGCCGAGAGTCCAAACAACTCAGATGAAGAACCTTCAAATAATGATCAAGCAATTAACCTAATCAAACACGTCACTTTCAGAAAATGGTATGCTAAAGTCACAATATTTATCAAAAAATTTGAGTTCAACACAGTTGCCCTATTTGACATAGGAGCCGACCTTAATTGTATTCAGGAAGGTTTAATTCCCACTAAATATTATCAGAAGTCTAAAGAATCGCTTAGAACTGCTTTAGGAAAATCGCTGCAGTTGAATTATGAAATTCCCAAAGCCCATGTTTGTCAAAACAAAATTTGCTTCAAAACTTCATTTGTCTTAGTAAAAAATATCACTGATGAAATCATTTTAGGATTACCTTTTATTGCCATGTTATATCCTTTCCAGGTTGAATATGATGGTGTTATCTCTACCCATTTAGGAGAAAAGGTCAAATTTGAATTCTTAACTAAGCCCGAGTTGCATAATTTGAGAGCTCTCCAAAAGAGTTCAGTTTAAAAAACAATTCAATAAATTCATAGAAAAAATAATCAGTTGAATTTTCTTAAGGAAAAAGTCAAATTCAAAAGAGTCAAACAACAACTTATTAATGAATCCTTACAGTTGCAAATCCAACAATTTGAGAATAAATTAAAGAATGAAGTATGTTCTGATTTGCCTAACGCCTTTTGGCATTGGAAACAACATGTTGTTAGTCTTCTTTACGTCAAAACCTTTAGTGAGAAAAATAGCCCCACTAAAGCCAGGGCAATCCAAATGAGTAAAGAAATAATGGACTTCTGTAAAGTTGTAATAACTGAGTTGCTCCAAAAAGGAATAATCCAAAAGAGTAAATCGCCTTGGTCTTGTCCCGCTTTCTATGTCCAGAAAAATGCAGAGTTAGAAAGAGGAGCCGCAAGATTAGTTATCAATTATAAACTCCTTAATTCAGTCTTAGAATGCATTAGGTATCCCATACCTAATAAGAGAGACTTAATCAACAAATTAGAAAAAGCAGTCGTCTTTTCCAAATTTGATATGAAGAGTGGTTTCTGGCAAATTCAAATAGATGAGTCAGATAGGTATAAAACTGCATTTGTCACTCCTTTTGGTAATTATGAATGGAATGTAATGCCTTTTGGTTTGAAAAATGCGCCGAGTGAGTTTCAAAATATAATGAATGAAATATTCAATCCCTTCTGTCATTTTTCCATTGTTTATATTGATGATGTTCTAATCTTTTCCCAGTCGATAGAACGACATTGGAAAGATTTGCATAAGTTTGTCCAAATAGTCAAGCACAATGGTCTAGTTGTCTCTGCTAAGAAAATCAAGTTGTTTCAAACTAATGTTCGATTTCTTCGATTTAATATTTGCCAGTCACAGATTAGTCCAATCAACCGAGTCATTCAATTTGCTGATAAATTCCATGATCAAATTCTTGATAAGAGTCAGCTTCAGAGGTTTTTAGGATCTCTCCATTATGTGTCTGATTTTTATCAAAATCTGAGAAAACAATGTAAGCCTTTGTTTGATAGGTTGCAGGATAACCCTCCTCCTTGGTCTTCTGTCCATACAGAAATCATCAAATAAATCAAAATCAATATCAGGACACTTCCCTGTCTTGGTATTCCTTCAGTTGATTCCTTCAAAATAGTTTAAACTGATGCCTCTGATATTGGTTAAGGAGGTATTCTCAAACAAAGAGTCAATCCAAATCTCCCAGAACAAATCATTCGCTTTCATTCTGACATTTGGACACAGTCGCAATTTAATTATAGTACTATTAAAAAAGAAATATTATCTATAGTACTATGCATTTCCAAATTTCAAGATGATTTGCTTAATCAAAAAATTTTAGTTAGAGTTGATTGTAAGTCTGCCAAGCATGTTTTGGAAAAATATGTTCATAATATTGCATCCAAACAAATCTTTGCACGTTGGCAAGCTATTTTAAGTATTTTTGATTTTGATATTGAATATTTGAGAGGTTGTGAGAATTCAATTCCTGATTTTCTTACTCGAGAATTTCTGCAGGAAAAACAATGGCACCCAAAAAAGACAAGCAAAAAGAGTCAGCCAGTCAGCCTAAGCCACCTCAGTCACTCCGAGCCCTTCCTTCTTCCATGAATCCAGTTAAACCAGAGTCACCCTCTCAGATTGTCCCTTTCCCAGGATGCTCTCCTATTCAAGTCAGTAATCGGTTCTCCAGTCTTGGAACCACAGTTGGCCAAATTCGCCCCAATTACCATTCTGCCTTAGTCTCCAGTTATGATCCTTTCCAGATCATTCCTTCAGTTGCTCAGTCTTCCCAGTCGCCTACTTCTTCTAGAAAGTCATCTCCTTACCTCCCTAAAGATAAGTCTCACCTCTTCATAATTGAACAAGTTTATGATGGTATTCCTGACCCAATTACAATTGTCAGATCTTATTTTCCGTCCAATTGTCATTTTCTTCCTCCCTGCCCTTATAAGAATCTGCAGTACTATAGGGACATTCTTTATGAGACAGGATCAGTTGAAATTAAGCCCATTAAAGATCGTGAAAAGCCCCACATCATTCTCTACCATTCGCTCTACATTCACAAAATTATAAGTCAAGAAGAGTTCAGTTGCCATCCTTATGAGTTGAAACACCTCCAATCCACTCATCCCTACAACTATGCTGATTACATTGAGGCATGGTACACCATATTTCTCCATCCAATTGTTGACTTCAGCCACTCTTGGTTCATCAATTTTGATTCTAAATTCAAGAGTCCATTTCCTTATTGGTTCCTTCATTGGTGGGAAAAACATGGCCCAATAGCCGAAACCCTCCCCACTGAAATCCATAATTTGACCAATTATTTTTCCAGCAAATACAAATTTAATAAGCAAGAGTTGTTTTTCCCCAAATTACTCATTTTCATCGCCAGGTATAAAGTTCCTTGGATTTTGAAATGGTCTTATCATGTAAATTGGGAATTCAAAATTATGTCTCGCCAATTTTCAGTCAAATGGTGGGATAAATTCAAAGTTGATAAAATAGCAGAATATGTTTATAGGGATTTTCCACAAATCACAAATCCCCCAGTTTCTCCAGTTGGTTCCACTCACTCTTCTCTCTCATTCGAGGGAAAATCAAAATCTGAGTTGCAGGAACTAGCCAGACAATTGATTTTCCAAGCTTCTCAAATGGATGATGATGAGGACA

GTGATGCTACTCCCAAATCCCCGTCATCTGCCAATCAACCCATTTTGCCTAGCAGCCTTCAGCACCCTTCTTAGTTTTAGAGGTGGGCTGATTATCAAGATAGTCAGGATCCGTATGACCTTGGATCTGATTAGCCACAAGCGCCATTATTTGTTTCCCCGACAAGCATGAGTTAAAGCATTCAGTAAAAGCAATAAAAGCTTTCGCATGCTTCTACAGTCATTATTCCTCGACAAGTAAAAGTTAAAGTTGAAGTTGCCAGAGTTAAAGTATCCCGCTGAAATCCCGTGCTTCTACAGAAAGATTCTCAGCATCCTATGTTCCAAATAGTTCAGTTGTTTTCTTATTTAAGAAGGCTTAGGCCTCAGTTGTAAGCAGACTGCTTTTGAAGTTATTTTTTCCAAAGTTTTTCAGTTGCAAAGTTTTCTTTGAGACTCTCTCAAAGAAAACTATCAGTGTAAATAATGAGTGATGTCTATCAATAAAAGCAAGACTTTTGTAAGTATCAAAATGTCTTCTATTATGTCTTCTATTATATTTATTTTTCAGTATTTTCATTTATATTTATTTTAAGTATTTTCATTTATGCATTAATTTCATTCATGCATAAGCTCCACATATTTTATTATCTAAGTGCTTATACTTATTTCTTTTTATTTTCTATCTCTTCCTGGTTCTAGTCCCCTTTTGGTAGAAACATTTGACGACGTAAAAAAAAGCAACCTCATCGTCTTTGCTCCCTAGAAACAACACTAAGATGACAATGAAGTTGCGTAAATACATATATATGTAAAAACGAAGAAGTCAAACATCATGTTAAAATAGAGAACATTATGTTAAAAATGGAGTTGGTATGATCCACCTTTGGTATCCAACTATTAAAAAATGACAAATACAGCTTTAACTAGCAAAATGTTATGTCCTAGTAAAAATCCGATTCACAAAATAGTACATCCTCATCTAATCAAAAAGACAATACTTAAACCTATCATTGAAGGAT

>Chr5-09; OZ022210.1; 7000696-7004424; dir; D;

TCTAATAACACTTCAAAATCTTTGTCTGATCTAAAAACCTGATTAGTTGCATTTCAAAGTCTTCGTCCAATTGTGGAATAGTACAATGTCAAACACTTTCTACGAAACTTGAAGTTCCTGAAAAGATTAAAAAAAACAAAGTTACTGTGATTTCAAAGAAATTTCACATATACATTAAGAAACATCTTCTAATAAGTATGAAAACATCTTCTATTAAGTATGAAAGTAAAGAGAATACTCTCAAAACCTTATTCACTTTCAAATTATATATGCTTAGCTTGTGAGATTACAGTTACTATTTAATTGGTTATTAGGAAAAAACAAGTAAACCAATCCTCTTGTCACCTAACATCCAAAAGAATCACAAAAGAAAAAGCAAGTTAGCTGAGATTTATAACTATGCAATACGTTATTTAATGCCATACTAGAATAAAAGAACCATCAAACCATATGGCAGCTGACTTGTCACAAAAAACCTACAAATAATAATAAACCATGGCAGCCAGCAAGAGTCCAGGAAAAGACCCCTCTGAAAAGAACACGAAAAAAGAAACAGGAAGTTAATGGAGCCATAGATATATATAAGCATCAATTCAACGTGGAATGCACATTTTCTAATATTGATTGACCACCTATAGTTTTATCCAGGGTAATGTTCTATCAGATCTATCCAGATTTCTTCTCCCCAGAGAAAATAAAGTTCAATATATTGGTTAGACTTGAACTACATTCATTGCTTTTACAATATATATATATATATATATATATATATATATATATATATATATGAAATGCACACCTCTCATGTACAACTCTAGTAAGAAACTTTCACACATTTGGACTACTAGCATCAAATATTGAATTTTTACAATCCCAAATATTTTATCCATTAAAGCATAGCAATGGAAAATAAAATAGGAAATAAAGAGCCAAAATCTTAACAATCAATCCCTGCTCATTAACGACCTCAAGTATACTTATATATATATATATATATATA

GATCAGCCTTCTCTATTTCAAAACCTTTAGTGAGAAAAATATCCCTACTAAAGCAAGACCCATTCAAATGAGTCAAGAATTAATGGAATTTTGCAAAATTGAGATTACCAATTTACTAAAAAAGGGAATAATACGTAAGAGTAAGTCACCATGGTCTTGTCCTGCTTTCTATGTCCAGAAAAATGCTGAATTAGAACATGGTGTCTCAAGACTAGTCATCAATTACAAACCACTTAATACTGTCTTAGAATGGATTAGATACCCAATTCCTAACAAAAGAGATTTAATCAATAGGTTAGAAAAATCAGTCATCTTTTCTAAGTTTGATATGAAAAGCGGATTTTGGCAAATTCAAATTTGAGTCTGATAGATATAAAACTGCTTTTGTTACTCCCTTTGGTAATTATGAGTGGAATGTTATGCCTTTCGGTCTTAAGAATGCTCCTAGCGAATTCCAGAATATAATGAATGAGATCTTTAATCCTTTCAGCCATTTTTCCATAGTTTACATTGATGATGTGCTTATATTTTCTGAGTCCATAGAACAACAATGGAAACATCTATATAAGTTCTTACAAATAGTTAAACAAAATGGTTTAGTTGTTTTTGCCAAGAAAATTAAATTATTCCAAACTAACGTGAGATTCCTTGGATTTAATATCTGCCAGTCACAGATTAGTCCCATAGATCGAGTTATCCAATTTGTTGATAAGTTTCCTGACCAAATCCTAGACAAGAGTCAACTCCAGAGATTTTTAGGATCCCTAAATTATGTTTCTGATTTTTACCAAAATTTGAGAAAACAATGTAAGCCGCTATTTGATCGTCTCCAAAATAATCCTCCTTCATGGTCATCTATTCATACTGAGATTGTCAAACAAATCAAAGCTCATATCAAGACTCTTCCCTGTCTTGGTATTCCTTCTGCTAATTCATTTAAAATTGTTGAAACTGATGCTTCTAATATAGGTTATGGAGGTATCCTCAAACAGAGAGTCTCTTTTGGTCAATCTGAGCAAATTATTCGATTCCATTCTGGCACTTGGTCCCAGTCCCAAGCTAATTATAGTACTATTAAGAAAGAAATATTATCTATTGTATTATGCATTAATAAATTTCAGTATGATCTATTAAATCAAAAAATTTTAGTACGAGTTGATTGTAAATCTGCTAAGCATGTATTAGAAAAAGATGTTCAAAACATTGCATCCAAACAAATATTTGCACGTTGGCAAGCTATTTTGAGTATTTTTTATTTTGATATTGAATATTTGAGAGGAAGTGAGAATTCTATTCCTGATTTCCTTACTAGAGAATTTCTGCAGGGAAAACAATGGCAGAAAGAAGAAAAGAAAGATATAAAGATGAGAAACAAAAAAGTCAAATTGGTCAATCCAGCCAACCTAGTCAACCCCAATCACTCCGAGTCCTCCCACCATCAATGAGTCAAGCCAAATATGAGCCTCCTTCTCAAATCGTCCCCTTTCCGGGATGCTCTCCTATCCAGATTAGTAACAGGTTTGCCAACCTATGAGCCACAGTCAGCCAGGTTCGCCCTAGTTTCCAGTCTGCTTTAATTTCCAATCATGATCCTTTTCAAGATATTGCTATTACTAACCACCCCCCCCCCCCCCCCCCCCAATTGTTTCTTACCAGAAAACATCTCCATACATGGTCAAGAGTCATAGTCACCTATTTGTCATTGAGTCCAAATATAATTCAATTGCTAATCCCATCACCATTGCTAAGTCTTATTTTCCACCAAATAGTCATTTCATTCCCCCAGCACCATTCAAGAACCTCAAATATTACAGAGATATCCTTCATGAGACCAATTCCATCACCATTAAGCCAATCAAAGACAGAGAAAACCTCCATATCATCCTCTATCATTCTCTTTACATTCACCAAATCATCAATGAAAATGAATAGAGTAGTCATCCCTATGATCTCAAAGTTTTCCAATCAGAACTTCAATACAGTTATTATGATTATATCGAAGCATGGTATTCTATCATGCTTCACCAAACAGTTGATTTCAGTCACTCTTGGTTCTTAAACTTTGATAGCAAATTCAAGAGCTCTTTTCCTTACTGGTTCAATCACTGGTGGGAAAAACATGGTCCAGTCATTGATTTATTTCCTCCCCCCATGCAAGAATTAGTCAATTATTTTACTAATGAAAATAAATTTAAAGAATCTGAGTTGTTCTTCCCCAATCTGCTTCATTTTGTTGCCAAATATAAAGTCCCTTGGATCCTTAAATGGTCCTATCAAGTTAATTGGGAAACCAGAGTCCCTTCTCGCCAATTTTCAATCAAATGGTGGGATAAATTTAAACTTGAGCGTATTGCAGATTATGTTTATGCTGATTTTCCTCCAGTCAGGAATCCGCACACTGAGAAAAAGAGTTCTCCTGCCACATCACATTCTTCTCTCCCAATCGAGGGTAGGTCCAAGTCAGAATTACAAGATCTTGCTTGCCAGCTTATGCTCCAAGCCTCTCAGATGATTGATGATGCAGACTCTGATGCCTCTTCTGCGTCACAAACATCTACTAGTAAGCCCAAGCCAACCAGTCAACAAGCTCCAGCCCCAAAAATGCGGTGGAGTGATTATCCAGATGAGCAAGACCCCTATGATTTTGGTCCTTTCAATCTAGGGGATGATTAAAGTAAAGTTAAAGTTGCCTCGCATCATGAGCCCTGAAGAAAAGCATCCGAACCCGTGCATTCATCAGTCTGATATATGCATTAATTTGAGAATTGCTTATTTTGATTAATGCTTATTTTCATTGCATCTATTTTCATATATGCATTAATTTTCTACATGCATAAGTTGCGTCTATTTTCTTAGTCTACTTGTTTATACTCAGCTTTGTTCTTCCTTCACATTTCCTTGGTTCTAGACCCCTTCTGGTATCAGTGTTTTGCATTCTGTAAGTAATCAATATATTTGAGATTTTGAGAATTGCTTATTTTGATTAATGCTTATTTTCATTGCATCTATTTTCATATATGCATTAATTTGCTACATGCATAAGTTGCGTCTATTTTCTTAGTCTACTTGTTTATACTCAGCTTTGTTCTTCCTTCATATTCCTTGGTTCTAGACCCCTTCTGGTAGGAAACTTCTGCGAACTATACGGTTTAATATCTATTCCTCCGTCTAGAAAGAATAAGTCCAGTCATCACAGCCGACAGTTTAGAAAAACCCGCCATTATTCGCGTAGAAAACAGTCATTCCCATATAATCATGAAGATAAAGAATTTTATAATAAGAAAAGATTTTCCCCTTGGAAAAACTGGAAAAAGAGTCACAATGAAGAAAAGAAAAATAGCTTTAAGAAAAAGACCGACAAAAGAAAAGTGAAATGTTATAAATGTCAAAAGTATGGTCACTATGCTAATAATTGTAAAGTAAAAAACACAATCAAACAATTAAAAATCACGGATGAAGAAAGAAACAAATTAATCCAAGTCCTCGAGTTAAAAAATATTGAGTCAAGTGAGAATGAAACCATGGTTAGCTGTACTGAGTCTTCTGAAAATACTCTTCTGAGTCACAACCCAGTTCCCCCAGGATCCAATTCGATTGCCAAGATAATTGCTGTAAGTCTAAATCAATCAGTGTCCTCACCAAACAAGAAGAACAAGAAGAATTATTAATTGATTTAATAAGTAAAGTTGAGAATCCAGAATTAAAATCTGAATACTTAAAGAAATTAAG

TATATATATATAATTATGCCAAAAAAAAAAAAAAAGAGAGGCAAAGAATGCATAATTGTCAAGGCATAACAGATCCGGGATATCAAAGAGGCTCAGTGAATTATACCTCATCATAGTTGATGCTTAATTACATAACCAAGCAAATGCATCTTAAACCAATAGCTAAACAGCAACTTGAGTATAAAAGCAATGAAAGCACCAGTGCATAAGAGAATCAAACATATACATAAATAATAATAAAAATTCTAAAATCAAAGTAAACCAACCTGTCAAATTGCTGCATTCCCATTCTTCATCCATAGCAAGAAACCAAGTAAATCAATCCTCTTGTCACCCAATATCTAAAAGAAACTTTGAGGCAATACCAGAAAAGAAACATGATTAAATTTGAGGCAATCTTCAGTTAATCCATAAAAAGAAATATGATTAAAAATATTTGGATTACCAACAGCAATAGCAAAGAGAAGCCAAAAGGGTCATATGGTGTGTCAAGTGTGTTCACCGACTGCAATGGCAGACAAGATTTCTTAACAGCAAACTCTTACAGCTGCTCAAGCCTAGTCCCCCTTCAAAAGTCATGATCGAAAATCCACTAACTGACTAGTCTCTCAAATCTACCAAAATATTTCATTCATTGATAGAATCTAAGATATTTAATGAGTATACTTCCATACAAAGTCATCAATTATTTTCATCAAATGCATAATGACATGTCAACCATTGTGTTTTCACCCCACAAATTTGCTAATTTGAAGCACAATGACATCTTCTAAATATACACTCTAGCTATATTCAACTATATGTCTGTTATGTATAAGCTTTTCATAATTTCTTGTCATTTAAAAACTTTTGTTGGTATTGAACTCAAGACACGAATAAAAACATTTTGAGGCAGTTTGAATACGTTACAACAGAGCAAACTATCACAATTGAAGAATACCTCTTACCCAAGATGTGTGACATAGATACACTGCTGGCATACAAGACATTTCAAATTTTA

>Chr5-10; OZ022210.1; 9240839-9246203; dir; B;

GGCCGACATATCCAAAATTCAGCGCGATCCAACGATTACACGACACTGCACCCACGGATCGCGCGATATGGAAAATCCGTTCGGGGCTCAAACGGACTCCGAATCCAGATCCGCGAAATCCCACGTGCTCGTGACGACACGAAGACCTCAAAACTGGCCAGAGCCATGCCACAACCATGGGCCACGCGCCGCCACACGCGCGGGACAGATCGGGTGTCCAAAGCGATTTCGGGTGGCCGAAAAATCTCGGAACCAAGACTCCAAACTTCTACCCTAGGTAAAACACCCTATTTGGAGTCAGTTTTGTTCTTGGACCACCCCCAAAAAGTGACCGGAAACAGTAGTTTCGAAGGGCCGAAGTTCGGCCGAATTTTCAAGTTCAAAATCGAACCCCTTAGAGCTAAAATCGATCGAAGCCCATATACCCATCAGCTAAATCACGAAAAATAGCTGAGAAACCATACCTTACTCGATCGATTTGGTGGAGAAACGAAGGAGAATTTCGAGCTGGAAGTTTGGGTAGCTTCGGACGAACCTCCGTAGGAACACACGATTTTCCGGCCAGCTCAGGGCGGCCCCCGGGGTTGAGGTCGGTTGGGGAAGGTAGGGGACTCGATGGCGGTTCCAACGCCACCGGTCCCTTGGCCATTGGAGCTCGGAGGCTGCGCCAGCACCCTCTGGAAGTCGGCGGCCCGTTTCGCGCAGAGTCTGGGTTTATATAGATGCAGCTTCGAAATATTTACGGTTATGCCACTAAGACTCTTTTGACCATAACTTTTTCGTTACAACTCCGATTCGGGCCCACTCCGTGTCTACGGACTCCTTTGGCCGTGATCTACGCAATGGCGCAAGCGGAATTCCCAAGTTCTTTCTTGATCAAAAAGTCAAATTTTCCCCCATTATTAATGCAAGGGCAAAATGGTCTTTTCACTAAAAGATATTTTCCTTACTTTTTAGATATTTTCTTTCTTTTTAGATATTTTGTTTTGGGTTCTTACAG

TTTAACTTATGGAAACATAATTAGCATAATTAATCAAGTCAGAATGCGAATGTGTGTCGATATGAAAATTGGTAAACAAATTCAATCTGAAAGAAATTCAGCTAAATACGAATTAGGAAATTTCTGCGAATAATACGGTTTGATGTCAATTCCTCCGTCTAGAAAGAATAAGTCTAGCAGACAGTTTAGAAAAACCCGTCATTATTCACGAAAAAAACAGTCATTCCAACATAATAATGAAGATAAAGAATTTTATAGTAAGAAAAGATTTTCCCCTCGGAAAAACTGGAAAAAGAGTCATAATAAAGAAAAGAATAATAGCTTTAAGAAAAAGGCCGACAAAAGAAAAGTGAAATGCTATAAATGTCAAAAGTATGGTCACTATGCTAATAATTGTAAAGTTAAAGACACAATCAAACAATTAAGAATCACGGATGAAGATAGAAATAAATTAATCCAAGTCCTTCAGTTAAGAAATACTGAGTCTAGTGAAAACGAAACCATGGTTAGCCACACTAAGTCTTCTGAAATGTATTCTTCTGAGTCACAACCCAGTTCCCCCAGAATTCAATTTGGTTGCCAGGATAATTGCTGTAAATCTAAGTCAATCAATGTCCTTACCAAATAGGAAGGACAAGAAGAACTATTAATTGATCTAATAAGTAAAGTTGAGAATCCAGAATTAAAATCTGAATACCTAAAGAAATTAAGAAAAGTCATCAGCCAAGAAGAACCAAAACAGTCTACCCAAAAGATAAATCTGAATACAACTTTAGAAAAATTTAAGAAAAAGAAAGAAGTTACTTTACAGGATTTACAATCAGAAATAAAAACGGTCAAAAAAGAAATCATTGAGTTAAGACAAATAAGTCAAAAATTACAAACAGAAAATTATGAGATAAAACAAGATCTAATCTATTAGAAAAGAAAATCTCTGAGTCCAAACCAGAAAGTCCCACTAACTCAGATGAAGAGAATTCTATCCAAGAACAAGCAGTTAATTTAATTCTATCCAAGAGAATTCTATCCAAGAACAACCACTAACTCAAATGGTATGCTCGGGTCACTATATTTGTTCAAAATTTTGAGTTAAACATAGTTGCCCTTTTTGATTCAGGAGCCGACCTTAATTGTATTCGAGAAGGATTAATCCCAACTAAGTATTATAAAAAGTCAAGAGAATCATTAAGTACTGCTTCAGGAAAGTCCCTCCAATTGAATTATGAGATCCCTGAGGCCCATGTCTGTCAAAATAAAATTTGCTTTAAAACTTCTTTTGTTTTAATCAAAAATATTACTGATGAAGTCATATTAGGTCTGCCGTTCATTGCCTTACTGTACCCTTTCCAGGTTGAACATGATAGCGTTATTTCCACCTGTTTAGGAGAAAAGATTAAGTTCGAATTCTTGAGTAAACCTGAGTTACATAATTTAAAAGAATTGCAAAAAAAATGCAGTCACCAAAGCAGTCCAAGTAATCCAAAACAAAGATAAACAATTAAAATTTCTCCAGGAAGAAGTTAAAGCTAAACGAATTGAGCAACAACTAAGTCATCAGTCTTTACAATCACAAATACAACAATTCGAAGAAAAACTAAAACAAGAGGTCTGTTCTGAGTTACCCAATGCGTTCTGGCATAGAAAACAACATGTTGTCAGCCTTCCCTATGTCAAATCCTTTAGTGAGAAAAACATTCCTACCAAAGCACGACCCATTCAAATGAGCCAAAAGTTAATGGAATTTTGCAAAATTGAGATTACTGATTTACTAAAGAAGGGAATAATCCGTAAGAGTAAGTCACCCTGGTCTTGTCCTGCCTTCTATGTCCAGAAAAATGCTGAATTAGAACGTGGTGTTCCAAGACTAGTCATTAATTACAAACTGCTTAATACTGTCTTAGAATGGATTAGATATCTAATACCTAAAAAAAGAGATTTAATCAACAGATTAGAAAAATCAGTCATCTTTTCTAAATTTAATATGAAAAGTGGATTTTGGGAAATTCAAATTTCTGAGTCTGATAGATATAAAACTGCTTTTGTTACTCCCTTTGGTCATTATGAGTGGAATGTTTGCCCTTTGGTCTTAAGAATGCTCCTAGTGAATTCCAGAATTTAATAAATGAGATCTTTAATCCTTTCAGCCATTTTTCCATAGTTTATATTGATGATGTGCTTATATTCTCTGAGTCCATAGAACAACACTGGAAACATCTGTATAAGTTTTTACAGATAATTAAACAAAATGGTTTAGTTGTTTCTGCTAAGAAAATTAAGTTATTCCAAACTAATGTGAGATTTCTTGGGTTTAATATTTGCCAGTCACAGATAAGTCCCATAGATCGAGTCATCCAATTTGCTGATAAGTTTCCTGACCAAATCCTAGACAAGAGTCAGCTCCAGAGATTTTTAGGATCCTTAAATTATGTTTCTGATTTTTATCAAAATCTGAGAAAACAATGTAAGCCTTTATTCGATCGTCTCCAAAATAATCCTCCTCCATGGTCATCTGCTCATACTGAAATTGTCAAACAGATCAAAGCCCATATCAAGACTCTTCCTTGTCTTGGTATTCCTTCTGCTAATTCCTTTAAAATTGTTGAAACTGATGCTTCTAATGTAGGTTATGGCGGTATCCTTAAACAAAGAGTCAATTCCAATCACTCTGAGCAAATTATCTGATTTCATTCTGGCACTTGGTCCCAGTCTCAAATTAATTACAGTACTATTAAGAAGGAAATATTATCTATTGTATTATGCATTAATGAATTTCAATCTGATTTGTTAAATCAAAAATTTTTAGTCCGAGTTGATTGTAAGTCTGCTAAGCATGTATTAGAAAAAGATGTTCAAAACATTGCATCCAAACAAATATTTGCACATTGGCAAGCTATTTTAAGTATTTTTGATTTTGATATTGAGTATTTGAGAGGAAGTGAGAATTCTATTCCTGATTTCCTTACCAGAGAATTTCTGCAGGGAAAACAATGGTAGAACGAAGAAAAGAAAGAGAAAAAGATGATAAACAAAATAGTCAAATTGGTCAATCAAGTCAACCCAGTCAACCCCAGTCACTCCGAGTCCTCCCATCATCAATGAGCCAAGCCAAACATGAGCCTTCTTCCCAGATCGTCCCCTTCCCGGGATGCTATCCTATCCAAATCAGTAACAGATTTGCTAACCTAGGAGCTACAGTCGGCTAGGATCGCCCTAGTTTCCAGTCTGCTTTAATTTCCAGTCATGATCTTTTCTAGGATATTGCCGTCACTAACCCCCCAGTTGTTTCTTACCGTAAGATGTCCCCGTACATGATCAAGAGTCACAGTCACTTGTTTGTCATTGAGTCCAAATACAATTCAATTGCTAATCCTATCACCATTGCTAAGTCCTATTTTCCACCCAATATTCATTTCATTCCCCCGGCACCATTCAAGAATCTCAAATATTACAGAGATATCCTTCATGAGACCCATTCTATCACCATTAAGCCAATTAAAGACAGAGAAAACCCCCATATCATCCTCTATCATTCACTCTATATTCACCAAATTATCAATGAAAATGAATGGAGTAGTCACCCTTATGATCTCAAAGTTCTCCAATCAGAACTTCAATATAGTTATTATGATTATATCGAAGCATGGTATTCTATCTTGCTTCACCAAACAGTTGATTTCAGTCACTCTTGGTTCTTAAACTTTGATAGCAAATTCAAGAGCCCTTTTCCTTACTGGTTCAATCACTGGTGGGAAAAACATGGTCCAATCATTGATTTATTTCCTCCCCCCATGCAAGAATTAGTTAATTATTTTACTAATAAAAATAAATTCAAAGAACCTGAGTTGTTCTTCCCCAACTTGCTTCATTTTGTTGCCAAATATAAAGTCCCTTGGATCCTTAAATGGTCCTGTCAGGTTAATTGGGAAACCAGAGTCCTATCTCGCCAGTTTTCAATCAAATGTGGGATAAATTTAAACTTGAGCGTATTGCAGATTATGTTTATGCTGATTTTCCTCCAGTCAGGAATTCGCATTCTGAGAAAAAGAGTTCCCCTGCACATCACAATCTTCTCTCCCAATCGAGGGAAGGTCTAAGTCCGAATTACAAGATCTTGCTCGCCAACTAATGCTTCAAGCCTCACAGATGAATGATGATGCAGACTCTGATGCCTCTCCTGCGTCACAGACATCTACTGGTCAGCCCAAGCCAGCCAGTCAAGCTCCAGCCCCAAAGATGTGGTGGAGTGATTATCCTGACGAACAAGACCCCTATGATTTCGGTCCTTTCAATCTAGGAGATGATTAAAGCAAAAGTTAAAGTTGCCTTGCATTATGAGCCCTGAAGAAAAGCATCTGAACCCGTGCCTTCATCAGTCTTAAAGCCGCGTTCTTTTTGAGATTCCCCGACAAAGTAAAAGTTGCTGCACTCAGTAAAAGCAAAAGCTTTCGCGTGCTCCTACAGTTACTTTTATAAGCATCCAAAGTTGCCAGCTCATCAAAAGTTATCTGAGTCTGTTTTATTTGCTTATTTAAGAAGGCTTCAGCCACTTCTTTTGACAGAATGTAATTTAATACACTATAATTTTCTCAGTTTCAAAACTTTATCAGTTTTCAACTCTCTCAAGAACTAAAGAGGTGAAACTAGGATCTTTGTAAGTGTTTTGCATTCTCCCAGTTATCAATATATTTGAGATATTTTGAGAATTGATTATTTTGATTAATGCTTATTTTAATTGCATCTACTTTCATATATTCATTAATTTGCTTCATGCATAAGTTGCGTCTATTCTACAATCTCTGTGTTTACACTTATCTTCATAGTTCCTATATCTCTTTCTGGTTCTAGACCCTTTCTGGTATCAGAGCCGTAAGTGTTGAAGAGTTAGTCTAAGTCTACAACTCATCCAACCCAAGTGGATGGTTTAGGTTTTCTGAGTTATAAGTTTTCAGTATCTTCTTTTCTTCTTCTGGTCTTTCAGATCACCTGTCCTTCTCTCTCTTGGTGTCTGAATCGTAAGTCCCGTTCCAAACAGTAAGTCCCAGGGAGGCCTGAGCGGTAGTGGCATTAGTAGGAGGGCGAGAGAATCTTAGGTGTCCTATTACCCATGGAGTATGTATTAGAAGTTTCTGTTCACTTTCTTCTTTAGGAATCCAAACCCAGAACCTTAAGTAAAAATGAGTCGTTTTCTTAGAACCAACTCTTACAGTTCTAGCAGCTCTAGGGCCAATAGTAGTCTCCCAGAGATAGTAAATGAAGAACAAATCGAATATGAGAATAGTAGTAATTTGGATTATAAAGATTGGAATATACCTCGAGTCCCCAGTCAAGACATCTATAAGAAAAAATGGTAATTATCCAGTTTTAA

TATATATATATATATACAGTCCCCTTCTATTTATTTGAGGGATGCCCTTTAGGGTACACTACATTTTTTTTTCCAATGATCCAAACTATCTATTTTTTAGGTCTTTATTTATAGATCATCCTTACAAAAAATTAGACAAATTGGAAACCGTTTCAACATCCAATTGTGTCCTACAAAATCAATGAACACGGTGCTTCAAGAAAGTACTAAAATTTCAATAACTCAATAGAATGGTCAAATGATATCGGATTCAAGTGATTTTTTGTAGAGATGATCTTTGAATGTGCATCTGCAAAATAGACGGTTTAGATTAGTGGAATACAATCGGGAGAGGGCCCTAAAAGGGGTGTCCCTCAAATAAACTTATTTGAGAAATCCCTCAATGGAAGCTCTTTGTATATATATATATATATACACTATTTTTTGTATGTATTGTGAGTTCGAACTTTTGATTCTTTATAATTTGTAAGAGGCCCCCACACACATGAAATTCTGGTTTCGCCACGGTTAGGGAGTGTATGTATTTGCCACATTATTTAGAGACCAAAATTTGAGTATGCTCGAAACTTCATTCATACTTATTATTTGAGTTAATTCGTTCGATAATCGCTTTGGTGAACATCACTAATTAAAAACCACCAACACGATAGTCAATAAAAACTTGAGTTAGTTAGGCCAATTGATATACATGGAGATTTACAAATATATATATATATATATATACACAGAGAGCTTCCATTGAGGGATCCTTCAAATAATTATTTGAGGAATACCCCTTGTAGGCCCCACTCCGGATTGTATTTCACTAATCCAAACCGTCTATTTTGTAGATACTCATTCAAAGATCATCTCTACAAAAAATCACTTGAATCAGATATCATTTGACCACTCAATTGAGTTATTGAAATTTTAGTATTTTCTTGAAGCACCGTAGTCATTGATTTTGTAAGATACAATTGGATGTCGAAATGGGTTCCAATTTGTCTAATTTTTTGTAAGG

>Chr5-11; OZ022210.1; 11122245-11124286; rev; B;

TGATTCAAAAAGAGAGGTTGTAATCGAGTTGGTTGACGAATATGGTCGTGATGTGTCTTCTTCTGGTTCGAATAGGTATTTTAATATGATATTTCACTATGTTGTCTTTGGTTTCTTTTATGGGTTTTTTTTATAGTTGCTGTTATATGAACATTTTTCTTTTGTGTTCGAATTCAATATGATAGGTAAGCTATGTAATGTGAAAGACAACCAATTCTCGATAGAACTTAAAGAAAACTTTTCCTCTCATACTAAATAGTCTACATATATACCTACTATCTCCCTATCGCACTAAACTGTCATCTCTTCTCTCCTCACTCACCATTCCCTCTTCCCTCCTCCCTCCCAAAACATGCATAAGCCTCCGTTGAAATCCATGGCTATTAAGCTTTGGCTTTCGGACATACTAAAATTCAGTAAGTTAGGTAAGCCCTGTGAGGTGAAAGCCAACAACGTATCTGTGAAACATGAGGAAATATTTTTCTCTCATACGAAACAATATGTATACACACCCACTCTCTCACATACACACACTAAACTGTCATCTCTTCTCATCTCTCTCTCCTCCTCCAAAAAAAAATATATGCATAGGTCTCAGTCGAGGTCCATGACTATCAATCTTTGGCTTCTGGACAAGCGATGATTCGGTAAGCCTTGTCAAGTGAAAGCCAACCACTTTTTGGTGAAACTTGGAAAAAAAAATTCACTTTCTTTACGATGTAGATCAGTTATGTTAAATACAACAAAAAAAGGAATAGTTGCTCCTATCATTAAGCTCTTATGGGAGTGAACACACCCAATGGGATCCTGACATCTGTAATTGATACAAAGAAGAATGTAGTAGATTCTCTAGCTATCAGTAAAGAAAAGATATGAACGAGACTACAACTGAAATGGTTGTGGCCCGAATGGATATTGGGCCTAACACAAAGATATATATATAAACTGATTTGAACTTATTGTCACTAAAGTTGCTATTTTCAAATTATCTATTTTATGA

AAAGCATTATTTTGATGCATTATCATTATTTTGCTGCATTAGTCTGAAAGTTGTTTAAGTTGCATCCATATCTATTCTACTGTTCTTCGTTCTTGTTCTGGTTTCCCCTCTCTTGGTATCAGAGCCATAAGAGTTATGGTCTTTGTCTAGGTCTGCTACTCATTCCCGCCCAAGTGGCTGGTTTAAGCATCTGAGTAGAAGGTTTCATTTCTTCCTCTTCTTCCCTTTTATTCTTTGAGTTATCAGTTGATCTTTCTCTAAGGTCTGACCCGTGAGTCCTTTTTCCTAGATCTGTAAGTCCCCAGTGTAGAGGGCGTGAGTGGTGGGCAGGAGACAGAAAACTGAGAAAGGTTAATAGGTTCTTAGAGTCTAAACGGTCGGTTTAGGATTTACTGTTTATCTTCTTCCTCAGACAGCCAAACCCAGTTATTTGAGTAGTCATGAGTCACTTATTTAGAACAAAGTCTTGTAATTCAAACAGTTCTAAAGCCAGTATGAGTCGACTCCCATAAATAGTTAATGAAGAACAATTTGAATATAATTCCAGTAGTAAAGAACAATTAGATTTTAGAGATTGGAACATACCTAAAGTCCCCAGTCAAAAAATATATAAGAAACATTGGTTGCCTTCCAGTTTTAATAGCACAACACATGTCAAAACAGTTGAATAAGTATACGCCTTAAGTAAAGAACATGAAACATGTCAGTTGCTTAATCTTGAATCAATTAAGAAACATAAGAACGATGGTAATAATTTCCTGCATATTGGTTTAGTTCAAGTTGCCGTTAAGCCCCTAACACGTTTAGGACTAAAAGCCTCAATCCTTCTTTGCCTGCGAGATGCTAGGTTCACTGAACTTAGTGACAGCACCTTAGGAGTCATTGAGTCCATTTTGTGTAATGGCCCAGTTCATTTTGATTGTTATCCTGATTTCACTATAAGTCTAAGTGATCCTCATATCCTGAGAACTTTAACTCTGAATATTAAGACTGAATGTTACAATGTCCTCCGAGGAACCCAACCTTTAGCCTTAGTTTACAGAATTTATTATAAAGTTACAGGAACAAATATGAATTTCCAAGCCCTAAATAAGAGCCCAAAAGATCAAACTGTCCTAATCCAGAGTCACACCTCAGACGCCCATATACAAGTTCCCCACACCATTAAGTGGTCAGAAGTTGCCCTTCCAAAAGAATGGACCCTAATTACCGAGAGCCAACCGGCTCCAATCCAACGTAGTCTAAACAATTTAGACTACATCCAGCAATACCTAGACGGCACAGTAAAAATTAAATTTGACAGTCACCCCTTACGAAAATCCAATGTCCAGTTATAGCAGTTGCCAACTCCTGGTCATTCGCAAAGACATGTCCCTGCTAGGCATTCCTTTGCTGCTTCGTCCTCCACTCTAGAACGAGACCTAGAGTTAGAAAAAGCCATGATAGACTTCAAACTTGAATCCCTGAGAAAAACCTCTCAGGTCACCCAACCTTGCTACGGCCAGGTTCCAGAAGCCAAGTCAGACTCACCTGAGTCCCCAACCCAATCCGATTTCCTAGTTGAAAATCTTGCAGAAGTTGATAACCAACTAAGATCCCTAAATAAGAAATTTGAAATTAATTGGCTACGTTTAAATCGTCATTTAAAAGCCCCGGAAAACCAGTTCATAAGAGATAATTATCACCAAGCCTACCCTGATAGTAAACGCCGCGAACAAATCTTTAGTGAATGGAAAAATTATATGTGGTCAGCCAGAGTTGAAATTTTCTACCTAGATTTTGTTGAAAGCCGTTACATGTCTAATGATGAGTTGAAAACTTTAACTAAAGAAAAATGGAATTTGATAGATAAGTCTGTCGTTGAAGCCAGTCATCCCCCAGTAGAAACTATAATAATAGATCATAAGAAAGCACCAATACCTGCCACTCCTTTCAAAACCTTTGAAAGTAGTGATCCAAATAGAAAACTAATCGAGCAGAATAATTACACCAATCAGAGTCTAATCGTCATAGGAAAACAGTTAGGTATTTGGAATGCAAGC

GATAGAAGAAAAAAAGAAAAGTATTTTCATCCGTTTTACCCCCTGAAGTACGACCCTTGTAGAAATTAACCCCGTGAACTAAAAAATAAGCCAATTTAGCACCCGAATTAATTAAAATCACCAATTTAGCCATTGACGTTAATTTTTTCGAATTTCATCCATTTTTCCGTCCAAAAAGTCACGTGTTATCAACGTGACTTACTTTTTTTTTTTTTTTCAATTATTATAAAACCTAAAATAGTTAGAAAATAGAAAATGAAAAAAAAAAACTGACTTACTCTCTCTCTCTCTCTCTCTCATATGTTCCTTCCCTAAACCCTAAACCCTTCTAACCCAAAAAAAAAAAAAAAGCATTAAAAAACCCTCTAAACCCAGCCCCTCCGAGACCCAGATTATCGAGCGACATACAGATTACCTAGTCCCACCCTCATCCTCCTACCTCCAAAACCCAGCCCCTTGAAACCCATATTACCTACCCTCTCCAAAACCCAAAATTTTCAGTTTCTCTCTCTCTCTCTCTCTCTCTCTCTCTCCTCCTCCAAAGTTTCCAAATTTACAAACAAAGCTTCAATCTATCTCCTTCTTAAAGACACACCTCAATCTAGATTGACACAAAGCTTCAAGAAGAGAAAGGATGTCGATCAACGCTAGAGTCATTGTTGGTGATGTGAACGTGTCGGTGGTGTTGCGGTTGGTTTCGCCAAAGAATGAGGGTCCTGAGGTTTGTGATGATCTATCTCATATCTGGGTTGACTTCATACTAGCACAACTCAGGGGAATTGAATTGATTGCTGGGTCTTGAATATTGCTAGTTGATTATATTGATGGCTTGATTGGGAATTGATTGCTTGGTTGAAGAAGAGTTGTGCCTCTAGCCCTTTTTTTAATTTAGGGTTTTTATCACAAATGGTCTTAGAGGTTTGCCAAATTATCACCTTCGGTCCTTTATGTTTTTTTATTATATATTATTGGTCCCTTATGTTACACTTCACACATCAAT

>Chr5-12; OZ022210.1; 11212311-11217792; rev; D;

ATAAATTACCTATAAAATCCACCCGGTCCTTTTTTTTCTTTTTCATATCCCCATAATTACCCTATGGCCAGCATTTAATCCAAAGATCCAAAAACTTTGTAAAGTAAATACATAACCATCAAATGGTCCAAAAAATTCAAAAGTAAATCTTATCGGAATTTTAAAATATTTAAGTGGATTTGTTTAGAAAAAAAAATATATTATAGCACAAGAAAAAATTAACTAAAAAATAAATTTATACAAGAAAATCATATTTACATCTTTTCCTCATCTTTTCCATGAGAGGAAAAACATGATTTACATCTGCCATAAATGTAAAAGGGAATGTAAAAGTGGCTTTACACCCTCAAATTTAACTACAGTGTTATTATTAAACAGTTACCATTAATTATGATTGCCAAATATAAGAAAAATTCTAGAAAAGAAAGTAGTAAAAATTAAAGTTGAAGGGGGCTCTCTCTCCTATTTGCATCAATGTCATTCAGTTGGTAACTTGAACCTGCAAACGGGGCAGGTATGACTCAGCTTTAGCCATTTGACAACGCAGTTTCCATGAAACACATGAGAGCATGTCAAGCATGTGACTTGATCCCCACGCACCATCTCCTTCATGCAATGCAAACCACACACTGATAATCTACTTCTCCATCAAACACCAACTTCTCCAAGACCTCAACCGATGCTTTGCTTGCAGGTTTTGGTCTCAACAACACTGCAGCTTGATCTAACGGTTCTCTCACAGCAGCCTCTTCTGCTTGTGTTTGTATACCCAACGCAATACTCAATAATTCTTCAGTTTCTGTGAGTAGCCACTCTATTTCTTGAAGATATCCTCTAGCTGTCGAATAGCATCAGCATATAATGAGGCCCAACTTTGCCTCATTCCTTGATTTCAAGAATTGTGCAAGTTCTAATGATGTAACGGACAAACATCATGAATCAATTGAAGTAAAAAAGAATACAAGATATATATATATATATATATATATATATATATATA

TTACCCGGGGAAAATTAGAAAGTCATTTACATGCTCCCGAGAACCACGCTAGAAGAAATAATTACCGCTCTGTAATCCTTAACAGAGTTAAAAGGCAACAAATATTTAATGAATGGAAAGAATATATGCGCACTGCTAAAGTTGAAATTTATTATCTTGATTTTGTAGAAAGTCGATACTTGCCTAATGAGTTAAAAACTTTAACCCAGGAAAATTGGGAAAAAATAGATCATACTAAAATCCTTGCTAGTCATCCCCTTGTCGAATCTATAGTTATCCAGCACCAAAAATCACAAGTCATTGCCAGTCCTTTTAAGATTCTTGATTTAGAAAATAAAATGATAATTGAACAAAATAACTACACCAACCAGAGCTTAATTGTTATAGGAAAACAATTAGATAAAATAGAAATTAAAGTTGACAAGTTACTACCAAAAGAAATTGCCTCTAAATCGCATGATCAAAAACCTCTAGTCACTTTTTAGGATTTAAAGTCTAGTCCAACTTTTAAAACAAGAGATACAATGACAAAGATTGAAAAAATGTTAGTAGAATTAACGCCAACTAAAAGAAAAGATAAGGAAAAAATTGAATCAGAGTTGATTGTAAGTCTGCTAAGCATGTATTAGAAAAAGATGTTCAAAATATTGCATCCAAACAAATATTTGCACGTTGGCAAGCTATTTTGAGTATATTTGATTTTGATATTGAATATTTGAGAGGAAGTGAGAATTCTATTCATGATTTTCTTATCAGAGAATTCCTGCAGGGAGAACAATGGCAGAAAGAAGAAAAGAAAAAGATAAACAAAAGAGTTAAGAAAGCGACTCATTAATACTCAAGGTTCTGGATCTGGACTTCTAAGGAAGAAAGTGAACAGAAACTCCTAATACAGACTTCATGGGATAACAGTATACCTAAAGTTCTCTCGCCCTCCTATTAATGCCACTACCGCTCAGGCCTCCACGGGACTTACTGTTTGGAACGGATCTTACAATTTGGACACCAAGAGAGAGAAGGACAGGTAGATTGAAAGACCAGAAGAAGAGAAGAAGAAATTGAAAACTTACAACTCAGAAAACCTAAACCAGCCACTTGGGTTGGATGAGTTGCAGACATAGACAAATTCTTCAACACTTACGGCTCTGATACCAGAATGGATCTAGAGCCAATAGTTGTCTCCCAGAGATAGTTAATGAAGAACAAGTTGAGTACGATAATAATAGTAATTTAGATTATAAAGACTAGAATATACCTAGAGTCCCCAGTCAAGACATTTATAAGAAAAAATGGTCATTATCCAGCTTCAAGTCGACCACTCATGTCAAAACAGTAGAACAAGTCTATGCCCTAAGTAAGAATCATGAGACATGTCAATTGCTTAATCTTGAGTCAATTAGAAAACATAAAAATGATGGAAACAACTTCCTCCATATTGGTCTAGTCCAAGTTGCTGTCAAACCATTAACTAGATTAGGATTAAAAGCGTCAATCCTTCTTTGTTTGCGTGATACTAGATTTACTAATTTCAACGACAGCACGTTAGGAATTATTGAGTCCAGTCTTGCTAATGGCCCGATTCATTTTGATTGCTACCCAGATTTCACAATCAGCCTGAGTGATCCACATATTTTAAAAACTCTCACTTTAAATATTAAAACCGAAGGTTATGAGGTCTTGTGGGATAATCACTTAACCCATGAGTCTAAGCAACGAATAATTCATGCACTTAAACTTAATGAAGAAGGTCTTCCTATTTTTGATGAACAAATAGGTCAAGGCATTGAGGATGGAGTCAACACTTTGCTTTATACAATCGTCGATCATTTCATAGGAACACCCAGTAATACTACTGCCAGAATACATGATCAATTGAGTAATTTGAGATGCCCTAAATTATCTGATTTCAGGTGGTATAAAGATGTGTTTATCTCCAGAGTCATGCTTAGAGAAGATAGTAATCAACCGTTTTGGAAAGAGAAATTTGTTAATGGTCTATCGAATCTATTTGCTCATAAGATCCAATCAACCCTTAGTAATGAACAAGGTCATATTAATTGGGATAGTTTAACATATGGAAATATAATTAGCACAATTAATCAAGTCGGTATGAGAATGTGTGTTGACATGCAAATTGGTAAACAAATCCAGTCTGAAAGAAATTCTGCTAAATATGAGTTAGGTAATTTCTGTGAATAGTATGGCCTACTGTCCATTCCTCCGTCTAGAAAGAATAAGTCTAGTCATCACAACCGACAGTTTAGTAAACGACGCCATTATTCACGCAGAAAACAATCATTCCAACATAATGATGAAGATAAAGAATTCTATAGTCAAAAAGGATTCTCCCCTAAGAAAAATTGGAAAAAAAGTCATAAAGATAAGAATAGTAGCTTTAAGAAAAGAACCGACAAGAAAAAGGTGAAATGTTACAAATGTCAAAAGTATGGTCACTATGCTAATAATTGCAAAGTTAAAGACACAATCAAACAATTAAAAATCACGGATGAAGACAGAGACAAACTGATTAAAGTTCTTGAGTTAAAAAATTCTCAGTCAAGTGAAAATGAAACGATGGTTAGTCACACTGAGTCTTCCGAGATATATTCTTCTGATTCACAACCCAGTTCCCCCATAATTCAATTTGGTTGCCAAGATAATTGCTATAAGACTAAATCAATTAATGTCCTCACCAAACAAGAAGAACAAGAAGAATTGTTAATTGATTTAATAAGTAGAGTCGAGAATCCAGAGTTAAAATCTGAGTATTTAAAGAAATTAAGAAAAGTCATTAGTCAGGAAGAATCAAAACAATCCACCTCCCAAAAGATAAGTCTGAACACAACCTTAGAAAAATTTAACAAAAAGAAAGAAATCACTTTACAGGATTTACAATTAGAAGCAAAGACAGTCAAAAAAGAAATCATTGAGCTAAGACAAATAAGTCAAAAGTTACAAAGTCAAAATCATGAAATAAAACAAGATCTAATTAATCTGTTAGAAAACAAAACCTCCAAGTTCACACCTGAAAGTCCCACTAATTCTGACGAAGATACCTTTAATCATGACCAAACAGTCAATTTAATTAAACATGTAAATTTCAGAAAATGGTATGCCCGAGTTACTATATTTGTCAAAAGCTTTGAGTTAAACATAGTTGCCCTCTTTGATTCAGGAGCCGACCTTAATTGTATCCGAGAAGGTCTAATCCCAACTAAGTATTACAAAAAGTCAAGAGAATCATTAAGTATTGCCTCAGGAAAGTCACTCCAATTGAATTATGAGATCCCTAAAGCCCATGTCTGCCAAAATAAGATTTGCTTTAAAACTTCTTTTGTCTTAATCAAAAACATACTGATGAAGTTATCTTAGGTCTTCCATTTATTGCCCTTTTATATCCCTTCCAGGTTGAACACGATGGTGTCATCTCCACCTGTTTAGGAGAAAAGGTTAAGTTTGAATTTTTGAGTAAACCTGAGCTACATAATTTAAAAGAATTACAAAAAAATGCAGTCACCAAAACAGTCAATGTTATTCAAAAGAAAGATAAACAACAGAAATTTCTCCAAGAAGAAGTTAAATTTAAAAGAATTGAGCAACAACTAAGTATAAATCATTACAAACACAAATTCAACAGTTTGAAGAAAAGCTAAAACAAGAAGTCTGTTTTGAGTTACCCAACGCCTTCTGGCATAGGAAACAACATGTTGTCAGCCTTCCTTATGTCAAATCCTTTAGTGAAAAAAATATCCCCACTAAGGCAAGACCCATTCAAATGAGCCAAGAACTAATGGAATTTTGTAAAACTGAAATTACCGATATACTTAGAAAAGGAATAATTCGTAAGAGTAAGTCACCATGGTCTTGTCCTGCTTTTTATGTCCAGAAAAATGCTGAATTAGAACGAGGAGTCCCAAGACTAGTCATTAATTACAAGCCGCTTAACACTGTCTTAGAATGGATTAGATATCCAATCTCTAACAAAAGAGATTTAATTAACATACTAGAAAAATCAGTCATTTTTTCTAAGTTTGATATGGAGAGTGGATTTTGGCAAATTCAAATTTCTGAGTCTGATAGATACAAAACTGCTTTTGTTACCCCTTTTGGTCATTATGAGTGGAATGTAATGCCTTTCGGTCTTAAGAATGCACCTAGCGAATTCCAGAATATAATGAACGAGATTTTTAACCCTTTTAGTCAATTTTCCATAGTTTATATAATGGTCTACCGAATCTATTTGCTCATAAGATCCGATCAACCCTTAGTAATGAACAAGGTCATTTTAATTGGGATAGTTTAACATATGGAAATATAATTAGCACAATTAATCAAGTCGGTATGAGAATGTATGTTGACATGGAAATTGGTAAACAAATCCAGTCTGAAAGAAATTCGGCTAAATATGAGTTATGTAATTTCTGTGAACAGTATGCATACTGTCTATTCCTCTGTCTAGAAAGAATAAGTCTAGTCATCACAACCGACAGTTTAGTAAACGACGCCATTATTCACACAGAAAACAATCATTCCAACATAATGATGAAGATAAAGAATTCTTACAATTCTTGCAGCTCTAGAGCCAATAGTTGTCTCCCAGAGATAGTTAATGAAGAACAAGTTGAGTATGATAATAATAGTAATTTTCTGGTATCAGAGCCAGAAATGTTGAAGAATTTGTCTAAGTCTGCAACTCATCCAACCCAAGTGGCTGGTTTAGGTTTTCTGAGTTGTAAGTTTTCAGTTTCTTCTTCTCTTCTTCTGGTCTTTCAGTCTACCTGTCCTTCTCTCTCTTGGTGTTCGAATTGTAAGACCCGTTCCAAACAGTAAGTCCCAGGGAGGCCTGAGCGGTAGTGGCATTAGTAGGAGGGCGAGAGAAATTTAGGTATACCGTTATCCCATGAAGTTTGTATTAGGAGTTTCTGTTCACTTTCTTTTTAGAAGTCCAGATCCAGAACCTTGAGTATTAATGAGTCGCTTTCTTAGAATCAATTCTTACAGTTCTTGCAACTCTAGAGCCAATAGTTGTCTCCAAGAGATAGTTAATGAAGAACAAGTTGAGTATGATAATAATAGTAATTTAGATTATAAAGACTGGAATATACCTAGAGTCCCCAGTCAAGACATTTATAAGAAAAAACGGTCATTATCCAGCTTCAAGTCGACCACTCATGTCAAAACAGTAGAACAAGTCTATGCCCTAAGTAAGAATCATGAGACATGTCAATTGCTTAATCTTGAGTCAATTAGAAAACATAAAAATGATGGAAACAACTTCCTCCATATTGGTCTAGTCCAAGTTGCTGTCAAACCATTAACTAGATTAGGATTAAAAGCTTCAATCCTTCTCTGTTTGCGTGATGCTAGATTTACTAATTTCAACGACAGTACGTTAGGAATTATTGAGTCCAGCCTTGCTAATGGCCGGATTCATTTTGATTGCTACCCAGATTTCACAGTCAGCCTGAATGATCCACATATC

TTAATAGATCAAACACCAACTTCTCCAAGGCCCTCGACCAATTGCACAATTGAGGCGTCATCCATCGGCCCAAAAACACAATTCAAGCTTTCAGGGGACAGAGTGGTAATTATCCCATCCCGAATGCCGAAGAGTTTTTGCAAAGCATACCGGAGTTGGACAAGACCAGCAGAAACGCAACGCAAACGTTGTAATCCTTTATCAGTAAGTCCCACTCTTCCTTGAGTGCCCTGGCCTTGATCTCCTCTTTCATAGCTTGGTACTCTTTCATGATCCTCTCGTGGTCATAGAGGTTGCCGGGTATCGAGGAAAGCCAGCACCTCTTACCCATCATGTTGCCGACGAAGACCGGAGAGAGAAGGGAAGCTATCTGCCCAAGAATTAGGGTTGTTTGCCGCGTGTTTTTTTGTCTTTTTGAAAACAGTATACTCTGTAAAAAAAACAACTGGAAAAACAAATTACAATATCTATGGCTAGAGTATAGCCTTGCGGCTATACTATTTATAAAAATAAAACGATCATCGCCGCTCGGCTATACAATTTATAAATTTATAAAAAGACCCGCCTAGCGCCTGGCGCGTGTTTCGGTGAAATAGTACAGCCCTATTTACAAAAGTATAGCCGTAAGTCACCGTCTGTCAGCTGGTACCTGAAAAAGCCACCGGCAGCAGCCGACTCAAACACGTGGTCGCTTCTGAGCTGTCTGTGAGGCACTTGTATGAGATCGCCAAAGTGAAGCAATCTGACCCGTATTGCCAGTACTTGCCCTTGGAGTCCATTTGTAAGTCCATCAATGTACGGCTAATTCTATGGGGATTAAGATTGTTAAGGACTTAGATTGATTTTGGGGAAAAGGTCACATTTTGAAACCATCATCGTTATTTTGAGAAAAAGATTGGATTTTTGTTTGGTTTGAGAGGTTTTGCTTGTGACTTGTTAGTTCTATAGTGATTTTGTCAGTGTTGTTCATGAGCTTTGTGTAATTGGGTAGCTTCTCTTG

>Chr5-13; OZ022210.1; 11706744-11717361; rev; D;

GTCGGAAAACACGATTTTCCGGCCAGCTCAGGGCGGCCCCAGGGTTGAGGTCGGTTGGAGAAGGTAGGGGACTCGATGGCGGTTCCAACGCCACCGGTCCCGTGGCCATTGGAGCTCGGAGGCGGCGCCAGCACCTTCTGGAAGTCGGTGGCCCGTTTCGCGCAGAGTCTGGGTTTATATAGATGCAACTTCGAAATATTTACGGTTATGCCACTGAGACTCTTTTGACCATAACCTTTTCGTTACAACTCGGATTCGGGCCCACTCCGTGTCTACGGACTCCTTTCGCCGTGCTATACGTAATGGTGCAAGCGGAATTCCCAAATTCTTTCTCGATCAAAAAGTCAAATTTTCCCCCATTAATAATGCAAGGGCAAAATGGTCTTTTTACTAAAAGATATTTTCCTTACTTTTTAGATATTTTCTTTCTTTTTAGATATTTTGTTTTGGGTTCTTACATCAGGTATGTATGTATTCAACTAAATTACCAACACTAGCACAGTTATTTGTTTTTGGACAAAAACTGGCAATACTCTGTTTAATACCAATAATCTAAATATTCCCACAACGTCGAATCACTATATCACATACAAAGAAAACATTCGATTAAAAAAAATGCCCAAAAAAAAAAATTCATAACCTAATTTCCACTAAAAATTTGAAATATATAAGGCATATTGGAGAAAGTATGAAAAAGAGAGAGAGAGTGAGAGATGGATACCTGGAGAAGAGTGAAGAAAATAACCTGTGAAGAAGATAGTAGAGAACAACCCTAGGGAGAAGAAGATGAAAAGAGCGTATGATTTCTTTCGTAGAACGCGTGTTTTTTTTCCCAAGCCGTATAATTAAGCATGTATTATCTAAACCGGGGTGTGTGGGTTGTATTAGTTAGTTAGGTAAGGAGAGTATTAAAAGCCCAACCCGTATAAAATAGTCGGGTAATTAAATAATACAAGTTGACACCAAACACCTAACATAGACTATTTACTCATATCCAGAG

TCTAATACGGTGTACCAAACGAGCCCATAGTTACTTGTTTGTTATTGAGTCCAAATATTATTCAATTGCTAACCCCATCACCATTGCTAAATCACATTTTCCACCTAATAGTCATTTCATTCCCCCAGCACCATTCAAGAATCTCAAATACTACAGAGATATCCTCCATGAGACCCATTCTATCACCATTAAACCAATTAAAGATAGAGAAAACCCTCATATCATCCTCTATCATTCACTTTACATTCACCAAATCATCAATGAAAATGAATGGAGTAGTCATCCTTATGATCTTAAAGTTCTCCAATCAGAACTGCAATACAGTTATTATGATTATATCGAAGCATGGTATTCTATCTTGCTTCACCAAACAGTTGATTTCAGTCACTCCTAGTTCTTAAACTTTGACAGCAAATTCAAGAGTCCTTTTCCTTACTAGTTCAATCATTGGTGGGAAAAACATGGTCCAGTCATTGATTTATTTCCACCTCCTATGCAAGAATTAGTCAATTATTTTACCAATAATAATAAGTTCAAAGAATCTGAGCTATTCTTCCCCAACCTTCTCCATTTCGTTGTCAAATATAAAGTCCCTTGGATTCTCAAATGGTCCTATCAGGTTAATTGGGAAACCAGAGTCTTATCTCGCCAGTTTTCAATCAAATGGTGGGATAAATTTAAACTTGAACGAATTGCAGATTATGTTTATGCTGATTTTCCTCCAGTCAGGAATCCGCATCCTGAGAACAAGAGTTCCCCTGCCACATCACATTCTTCTCTCCCAATCGAGGAAAGGTCTAAGTCCGAATTACAAGATCTTGCTCACCAACTAAAGCTTCAAGCCTCACAGATGAATGATGATGCAGACTCTGATGCCTCTCCTGTGTCACAAACATCTACTAGTCAGCCCAAGCCAGCCAGTCAACGAGCTCCAGTCCCAAAAATGCAGTGGAGTGATTATCCTGATGAGCAAGACCCCTATGATTTTGGTCCTTTCAATCTAGGGGATGATTAAAGCAATTAAAGATAGAATTAAAGCTGCCTTGCATCATGAGCCCTGAAGAAAAGTATCCGAACCCATGCCTTCATCAGTCTGAAAGTTGTGTTCTTTTTGAAATTCCCCATATATATATATATATAATAAAATAAATGTAACTTATGCATGAATCTTATTAATAAAAATAGAAATATTGCATGGATGAAAGTAAACATAATGCTATATTTTTTTTAAAATACTCAAAATATTTTATTTGAGAGTAGGCTCATGAAAGAAGTAGCTTACAAATGCTCATAGCTCACTTGTGTTCTTCAGAATATCATCTCTGAGAGAACATTTTTGAACTAAGAAAAGAAGCTATAAGAAGCGATGCTTACAACTGAGGCGAATGCCTCCTTTTAAAGACAAAAAATGACTATGGTTTAACAAAAGTAACTCAAGTATACAAAAGAAACTAGGGTAAGGCTAAAGTAGGAAAGAACTGTAGCGCTTTTGTGCTTTTACTCAGGTAACTTGCTTTTACTGTATGCTTTAACTCAGGAGGGAATCTCATGCTTTGACTCAAGTGATCTAATCAGAATTCAGATCATAGGCTTTGTAGGCTTCTGCATAGTCTTCATTTGGGTCTTGACCATGGGGATAGTCAAACCATCTCATTGGACGCTCAGGTGTCGGTTGTGTAGGTTGACAGCTGGAAGAAGTCTCAGACTCAGTGTCGTCTACAACATTGTCATCATGCATTTGGGAGGCTCTAAGAAGGATTTGTTGAGCCAATTCCTGCAACTCAGATTTAGATTTACCTTCAACTGTAAGAGTAGTATCCGAGGAGTGAGAAGAGGTAGGCTCATGCTTGGGCTCAGGCTTATGGACTGGCGCTGGGGGGAAATCTCTGTAAACATAATCTGTAATGCGGTGGACCTCAAAGCAATCCCACCATTTGACAAAAAATTGACGGGAGAAGAAACGGGTCTCCCAATCAGCTGTATAATTCCATTTCATTATCCATGGGACTCTGTATTTGGTAAAAAACAATAGATCTTGAGGAAAAAATAAATCTCGCTCAGCAAACTTGAATTTTTGAGTGAAATAAATGATCAGCTCATTGACTGCAGGAGGAACAATATCAGATTTGGGACCATGAGTGCCCCACCAATGAAGAAACCAATAAGGGAGCTCAGTATTCTTAAACTTATTATCAAAATTAATGAACCAGGAATGAGAAAACTCAGGTGTTTGATGCAGGAAAATGTAATACCAGGCTTCAACATAATCTGCATAATTATACTGGTATTTAGAAGGTAGGGTTCGCAGTTCATGAGGGCGTGGCCCCCAAGACTTTGGATGTAGGATTTGATGAATGTAAAGGGAGTGGTATATGATTTTAGTGGGATCAGTTTTATCCCGAATAGGCTTAATGTCTACGGACTTAGTTTCATGCAAAATTTCTCTGTAAAATTTCAAGGACTTATAAGGGCTATGAGGTAAGAAATGAAATCCAGGAGGGTAATGATGGGCTGCAATTTGGGCAGGGCTCATGGATGTAGAAATATGAGAGTCAATAATTACAATGTTTGTTGTATTTTTTAAAACATAAGGGGAAGATTTTTGAAAAAACTGGGGGGACACAGGCGTTGCAGTTGGAGGTAATTGAAATGGATCATAACTAGAAACTAAAGCAGAATGATAATTTGGTCTTGTAGGTGTAACTGTAGATCCTAGAGGACTAAATCTATTGCTCATGATAGGTATGGGTGTATGACCAGGGTATGGAGTAATTGTAAAAGGCTCAGTTTTAACGGAAGAAGATCCAGACTCATTTTTGACAGATTTGGAAGGCTTTTGGCTCAGGGTGGCAGGTAAGATCCTGGGAGGAGGGTTGTGACTCATTTTTTGTTTGTCTTTTGATCCAGCCATTGCTTATCCTGTAAAAAACTCGTGTAAAGAAAATCAGGAACAGAATTCTCACTTCCTTTCAAGTATTCAATATCAAAATCAAGATACTTAAAAGAGCTTGCCATCGTGCAAAAATCTGTTTTGATGCTATGTTTTTAACATCTTTTTCTAGTACAAGCTTAGCAGATTTACAGTCAACTCGAACCAAAAATTTCTGGTTTAACAAATCATCTTGAAATTTAGTAATACATAATACAATAGATAATATTTCTTTTTTAATGGTACTATAATTGGACTGGGAAGGAGTCCAAATTCCAGAATGAAAACGTACAATTTGCTCAGAGGGACTAGACTTAGATTTCTGTTTAAGAATACCACCATAACCTACATCAGAAGCATCAGTCTCAACAATTTTAACAGAATTTGGAGCAGGAATGCCAAGACAAGGAGTCTTAACATGACGCTTAATCTGTTGAACAATACAGTATGAATAATGACCAAGGGGAGGATTATTCTGTAATCTATCAAACAAGGGTTTACATTGTTTTCTCATATTTTTGTAAAAATCAGAAACATAATTAAGGGAACCTAAGAACCTCTGCAATTGGTTTTTATCAATAATCTGATCAGGGAACTTATCTGCAAAATATGATAACTCGATCTATAGGATGAATTTGTGATTGGCGTATATTGTATCCTAAGAATCGAATATTAGTCTGAAATAATTTAATTTTCTTAGCAGAGACAAATAATCCATTAGTCTTAATGATATGCAGAAATTTATGCAAATGTTTCCAGTGTTGTTCTAGGGACTCAGAAAATATCAAAACATCATCAATATACACAATAGAGAAATGACTATAAGAATTGAAAATTTCGTTCATTATATTTTGAAATTCACTAGGGGCATTCTTAAGACCAAAAGGCATAACATTCCACTCATAGTGACCAAAAGGAGTAACAAAAGCAGTTTTGTATTTATCAAACTCATGAATCTGTATTTGCCAAAATCCACTCTTCATATCAAACTTAGAAAAAATAACAGCTTTATCTAAGCGTTTAATCAGATCTCTTTGTTAGGGATAGGATAACGAATCCATTCTAAAACAGTATTCAATGGCTTATAATTAATAACTAATCGAGGAGATCCTCTTTCTAATTCTGCATTTTTTTGAACATAAAAAGCAGGGCAAGACCAAGGAGACTTACTTTTTCTAATTATGTTTTTCTGTAATAAATCATTAATCTCATTTTTACAAAATTCCATCATTTTATGATTCATTTGTATAGGTCTAGCTTTAGTTGGTATATTTTTCTCAGAAAAATCCTTTATGTAAGGTAAATTAACAACATGATTTTTCCTATGCCAGAAAGCAGTAGGAAGATCAGAACAGACTTCAGAAATCAACTTTTGCTCAAAGTTACGAATATCTTTTTGTAAAGTAGGATTGCTTAATTGAGACTCAATTCTTTTACTTTTGACTTCTTGCTGTAAATGACTCAGATGATTAGCTTTATCTTTAATTAAAGTAATTGTTTTGGCAAAATCATTATTATTCAAATGCTTTAAATTATGCAACTCAGGTTTAGTTAAAAAAGTAAACTTAACTTTCTCACCTAAATAATTTGAAACAACACCATCAAATTCAACTTGAAAAGGATAAAGCATGCTTATAAAAAGGTAATTCTAAAATAACCTCATCAATTATATTCTTAATTAAGACAAATGAAGTTTTAAAACAAATTTTATTCTGGCAAACATGGGCTTTAGGAATCTCATAGTTTAAATGAAGTGATCTACCAGAGGCAGTTCGTAAAGACTCTGTAGATTTCTTATAATACTTAGTGGGAATTAATCCTTCTTTTATACAATTAAGATCTGCACCAGAATCAAACAAAGCAACTGTATTGAACTCATAATTTTTTACAACTATAGTGACTTTAGAATACCATTTTCTAAAATTAACTTGTTTAATTAAATTGACAGTTTGTTCTTCTTTATCAGACTCAGACTTATGAACAGGACTACCAGGTGGACTATTTGACTCAGAGGGTTCTTTTTCTAATAATGCATTTAAAACTTGCCGAATATCATAATTCTCAGTTTGCAATTTATGACTTAATTGTTTTAACTCAACTATTTCTTTTTTGACTAATTTTACTTCAGAATGTAAATCATGCAAAGAAACTTCCTTTTCTTATTAAATCTTTCTAAAGTAGAACTCAGGTTAATTATAGGATGACAAGTGCTTGTATCCTGTGTAATAATTTTTCTTAATTTTCTTAAGTACTCAGATTTTAACTCAGGACTTTCAATTTTACTAATTAAATCTATCAATAATTTTTCCTGCTCTTCCTGTTTAGTTAGAACAGAAATTTGCTTAAGTGTATTACAACATTTATCTCTGCAACCTAATTGGATTCTAGGTGAACTTAACTGGGAATCAGAGGACTCATAGTTAACTGACTCAGAACTACTAACCATAGGTTCATTTTCACTAGGCTCAGAGTTCCGTAATTCCAGGATTTTAATCAAGTTGTCTTTTTCTTCATCTGTAATTTTTAGTTGTCGGATAGCATCTTTAACTTTACAATCATTTGCATAGTGTCCATACTTTTGGCATTTAAAACATTTTACTTTACTTTTATCTTTACTTTTACCTTTACCAGACTTAGTGCTAAAGCGAGCATCTTTCTTATATCTAGGTGCTTTTGACCATCTTTTCTGAGGGGAATATTTTCTTTTCTTGTAAAATTCATCAGGCTCATCATAATGTCTGTAAGGCTTTCTTCGTTTGCTATAATGACGTTTCTTACGGATATGACTAGGATAACTGTATTTATTTTTCCTAGAAGGAGCTGTAGAGATTAGACCATATTGTTCACAAAAATTACCTAACTCATATTTAGCAGATTTTCGTTCAGACTGTATTTGTTTATTAAGTTTCATATCTATACACATTTTCATACCTTCTTGATTAATTGTACTTATAATATTGCCATAAGTTAAACTATCATAATCTATATGACCTAACTCATTACTTAAGACTGTACGAATTTTGTGTGCAAATAAATTGGGCAAACCATTAATAAATTTTTCTTTCCAAAAGGCTTGATTACTATCTTCTCTTAACATAACACGGGAAATAAAAACATCTTTATACCATCTTTATACCATCTGAAATAAAAACATCTTTATACCATCTGAAATAAAAACATCAAGTGATTTCAGATGGTATAAAGATGTTTTTATTTCCCGTGTTATGTTAAGAGAAGATAGTAATCAAACCTTTTGGAAAGAAAAATTTATTAATGGTTTGCCCAATTTATTTGCACACAAAATTCGTACAGTCTTAAGTAATGAGTTAGGTTATATAGATTATGATAGTTTAACTTATGGCAATATTATAAGTACAATTAATCAAGAAGGTATGAAAATGTGTATAGATATGAAACTTAATAAACAAATACAGTCTGAACGAAAATCTGCTAAATATGAGTTAGGTAATTTTGTGAACAATATGGTCTAATCTCTACAGCTCCTTCTAGGAAAAATAAATACAGTTATCCTAGTCATATCCGTAAGAAACGTCATTATAGCAAACGAAGAAAGCCTTACAGACATTATGATGAGTCTGATGAATTTTACAAGAAAAGAAAATATTCCCCTCAGAAAAGATGGTCAAAAGCACCTAGATATAAGAAAGATGCTCGCTTTAGCACTAAGTTTGGTAAAGGTAAAAGTAAAGATAAAAGTAAAGTAAAATGTTTTAAATGCCAAAAGTATGGACACTATGCAAATGATTGTAAAGTTAAAGATGCTATCCGACAACTAAAAATTACAGATGAAGAAAAAGACAACTTGATTAAAGTCCTGGAATTACGGAACTCTGAGCCTAGTGAAAATGAACCTATGGTTAGTAGTTCTGAGTCAGTTAACTATGAGTCCTCTGATTCCCAGTTAAGTTCACCTAGAATCCAATTAGGTTGCAGAGATAAATGTTGTAATACACTTAAGCAAATTTCTATTCTAACTAAACAGGAAGAGCAGGAAGAATTATTGATAGATCTAATTAGTAAAATTGAAAGTCCTGAGTTAAAATCTGAGTACTTAAGAAAATTAAGAAAAATTATTACACAGGATACAAGCACTTGTCATCCTATAATTAACCTGAGTTCTACTTTAGAAAGATTTAATAAGAAAAAGGAAGTTTCTTTGCATGATTTACATTCTGAAGTAAAATTAGTCAAAAAAGAAATAGTTGAGTTAAAACAATTAAGTCATAAATTGCAAACTGAGAATTATGATATTCGGCAAGTTTTAAATGCATTATTAGAAAAAGAACCCTCTGAGTCAAATAGTCCACCTGGTAGTCCTGTTCATAAGTCTGAGTCTGATAAAGAAGAACAAACTGTCAATTTAATTAAACAAGTTAATTTTAGAAAATGGTATTCTAAAGTCACTATAGTTGTAAAAAATTATGAGTTCAATACAGTTGCTTTGTTTGATTCTGGTGCAGATCTTAATTGTATAAAAGAAGGATTAATTCCCACTAAGTATTATAAGAAATCTACAGAGTCTTTACGAACTGCCTCTGGTAGATCACTTCGTTTAAACTATGAGATTCCTAAAGCCCATGTTTGCCAGAATAAGATTTGTTTTAAAACTTCATTTGTCTTAATTAAGAATATAACTGATGAGGTTATTTTAGGATTACCTTTTATAAGCATGCTTTATCCTTTTCAAGTTGAATTTGATGGTGTTGTTTCAAATTATTTAGGTGAGAAAGTTAAGTTTACTTTTTTAACTAAACCTGAGTTGCATAATTTAAAGCATTTGAATAATAATGATTTTGCCAAAACAATTACTTTAATTAAAGATAAAGCTAATCATCTGAGTCATTTACAGCAAGAAGTCAAAAGTAAAAGAATTGAGTCTCAATTAAGCAATCCTGCTTTACAAAAAGATATTCGTAACTTTGAGCAAAAGTTGATTTCTGAAGTCTGTTCTGATCTTCCTACTGCTTTCTGGCATAGGAAAAATCATGTTGTTAATTTACCTTACATAAAGGATTTTTCTGAGAAAAATATACCAACTAAAGCTAGACCTATACAAATGAATCATGAAATGATGGAATTTTGTAAAAATGAGATTAATGATTTATTACAGAAAAACATAATTAGAAAAAGTAAGTCTCATTGGTCTTGCCCTGCTTTTTATGTTCAAAAAAATGCAGAATTAGAAAGAGGATCTCCTCGATTAGTTATTAATTATAAGCCATTGAATACTGTTTTAGAATGGATTCGTTATCCTATCCCTAACAAAAGAGATCTGATTAAACGCTTAGATAAAGCTGTTATTTTTTCTAAGTTTGATATGAAGAGTGGATTTTGGCAAATACAGATTCATGAGTTTGATAAATACAAAATTGCTTTTGTTACTCCTTTTGGTCACTATGAGTGGAATGTTATGCCTTTTGGTCTTAAGAATGCCCCTAGTGAATTTCAAAATATAATGAACGAAATTTTCAATTCTTATAGTCATTTCTCTATTGTGTATATTGATGATGTTTTGATATTTTCTGAGTCTTTAGAACAACACTGGAAACATTTGCATAAATTTCTGCATATCATTAAGACTAATGGATTAGTTGTCTCTGCTAAGAAAATTAAATTATTTCAGACTGATATTCGATTCTTAGGATACAATATACGCCAATCACAGATTCATCCTGTAGATCGAGTTATCAATTTTGCAGATAAGTTCCCTGATCAGATTATTGATAAAAACCAATTGCAGAGGTTCTTAGGTTCCCTTAATTATGTTTCTGATTTTTACAAAAATCTGAGAAAACAATGTAAACCCTTGTTTGATAGATTACAGAATAATCCTCCCCCTTGGTCATCTATTCATACTGATATTGTTCAACAGATTAAGCGTCATGTTAAGACACTCCATTGTCTTGGCATTCCTGCTCCAAATTCTGTTAAAATTGTTGAGACTGATGCTTCTGATGTAGGTTATGGTGGTATTCTTAAACAGAAATCTGAGTCTAGTCCCTCTGAGCAAATTGTACGTTTTCATTCTGGAATTTGGACTCCTTCCCAGTCCAATTATAGTACCATTAAAAAAGAAATATTATCTATTGTATTATGCATTACTAAATTTCAAGATGATTTGTTAAACCAGAAATTTTTGGTTCGAGTTGACTGTAAATCTGCTAAGCATGTACTAGAAAAAGATGTTAAAAACATAGCATCAAAACAGATTTTTGCACGATGGCAAGCTCTTTTAAGTATCTTTGATTTTGATATTGAATACTTGAAAGGAAGTGAGAATTCTGTTCCTGATTTTCTTACACGAGAATTTTTACAGGATAAGCAATGGCTGGATCAAAAGACAAACAAAAAATGAGTCACAATCCTCCCCCCAGGATCTTACCTACCACCCTGAGCCAAAAGCCTTCCAAATCTGTCAAAAATGAGTCTGGATCTTCTTCCGTTAAAACTGAGCCTTTTACAATTACTCCATACCCTGGTCATACACCCATACCTATCATGAGCAATAGATTTAGTCCTCTAGGATCTACAGTTACACCTACAAGACCAAATTATCATTCTGCTTTAGTTTCTAGCTATGATCCATTTCAATTACCTCCAACTGCAACGCCTGTGTCTCCCCAGTTTTTTCAAAAATCTTCCCCTTATGTTTTAAAAAATACAACAAACATTGTAATTATTGACTCTCATATTTCTACATCCATGAGCCCTGCCCAAATTGCAGCCCATCATTTCCCTCCTGGATTTCATTTCTTACCTCATAGCCCTTATAAGTCCCTGAAATTTTACAGAGAAATTTTGCATGAAACTAAGTCCGTAGACATTAAGCCTATTCGGGATAAAACTGATCCCACTAAAATCATATACCACTCCCTTTACATTCATCAAATCCTACATCCAAAGTCTTGGGGGCCACGCCCTCATGAACTGCGAACCCTACCTTCTAAATACCAGTATAATTATGCAGATTATGTTGAAGCCTGGTATTACATTTTCCTGCATCAAACACCTGAGTTTTCTCATTCCTGGTTCATTAATTTTGATAATAAGTTTAAGAATACTGAGCTCCCTTATTGGTTTCTTCATTGGTGGGGCACCCATGGTCCCAAATCTGATATTGTTCCTCCTGCAGTCAATGAGCTGATCATTTATTTCACTCAAAAATCCAAGTTTGCTGAGCGAGATTTATTTTTTCCTCAAGATCTATTGTTTTTTACCAAATACAGAGTCCCATGGATAATGAAATGGAATTATACAGCTGATTGGGAGACCCGTTTCTTCTCCCGTCAATTTTTTGTCAAATGGTGGGATCGCTTTGAGGTCCAACGCATTACAGATTATGTTTACAGAGATTTCCCCCCAGCGCCAGTCCATAAGCCTGAGCCCAAGCATGAGCCTACCTCTTCTCACTCCTCGGATACTACTCTTACAGTTGAAGGTAAATCTAAATCTGAGTTGCAGGAATTGGCTCAACAAATCCTTCTTAGAGCCTCCCAAATGCATGATGACAATGTTGTAGACGACACTGAGTCTGAGACTTCTTCCAGCTGTCAACCTACACAGCTGACACCTGAGCGTCCAATGAGATGGTCTGACTATCCCCATGGTCAAGACCCAAATGAAGACTATGCAGAAGCCTACAAAGCCTATGATCTGAATTCTGATTAG

ATCACTTGAGTCAAAGCATGAGATTCCCTCCTGAGTTAAAGCATACAGTAAAAGCAAGTTACCTGAATAAAAGCACAAAAGCGCTACAGTTCTTTCCTACTTTAGCCTTACCCTAGTTTCTTTTGTATACTTGAGTTACTTTTGTTAAACCATAGTCATTTTCTGTCTTTATAAGGAGGCATTCGCCTCAGTTGTAAGCATCGCTTCTTATAGCTTCTTTTCTTAGTTCAAAAATGTTCTCTCAGAGATGATATTCTGAAGAACACAAGTGAGCTATGAGCCTTTGTAAGCTACTTCTTTCATGAGCCTACTCTCAAATAAAATATTTTGAGTATTTTAAAAAAATATAGCATTATGTTTACTTTCATCCATGTAATATTTCTATTTTTATTAATAAGATTCATGCATAAGTTACATTTATTTTATTATTGTTGGTTCTAGCCCCTTTTGGTATCAGAGCAATAGATTTAGTCCTCTAGGATCTACAGTTACACCTACAAGACCAAATTATCATTCTGCTTTAGTTTCTAGCTATGATCCATTTCAATTACCTCCAACTGCAACGCCTGTGTCTCCCCAGTTTTTTCAAAAATCTTCCCCTTATATATAGAGGATAGGCTAAGCAAACTTATTTAATTTTTTGTTAGGCAACATGGGAAAAAGATATAACTACCTTTTATTTATTATTATTTTTTTATAACACACAATAGGACATATACAAAGATATTTTTATAACTAGTTTATACACCCATTACTGTTAGGTTAACACAATGGGGCTGATTAGAGATCAAACTTATCATATATAAATATGTCCCTCAAATAAAATTATTTGAAAGATTCCTCGATAGAAGGGGACAACTCCATGTGAATCAAAGCATGCATGAGTCTTCCTTCTACTAAGTGGAGATACATCTACCACTCAATCGAAAGAAAGTTGATAGAAAAACTAATATCGTGTAAGTTTATTTTCTTTGTTCAAATGCAGCTTTATGGAACAGAA

>Chr5-14; OZ022210.1; 11727401-11734313; dir; D;

AATCCACCTCGGGCCATCCTCCCAGCTTCCGCACCACCACAAAATGTAGGACTCCGTTGTAAATTTCTTAAAGTTATGTAAATTTGTAGAAAATTAAAGTTCTGTAAATTTGTAGAAAATGCTCTGATATCTTGTTGAAACTGAGAACCAGAGTTGAGACTTGTTTCTTTGAAATATTCTGGCAGTTGGTGTGGATTTATGTGCTTGTTTGACATGTGGAAGATTTTGGGTTTGGACAAAATACAGGGGAGACTCTGCCGAATTTTCGGCAGAAGTCTAAGGGAAATTTTTAAAAGAATTTGAAACGAGAAGGGTAAAAAGGTCATTTGTGCCCGACATTCGCCAGGTGTCGGACACGCACAGGACTTGGCTCGAATTCCAAAGTGGAATTGGGATCGGGTCCTGTCAAGAAAAGCATTTGTTCTTCTCTTACTTGGGGCGAGGGAGTTGTGATTCACTCTTTGATGTAGTTTGGTTTATGAACAACTTTCTCTTTTGCTCTTATCACCTTAATATGTGATCACTTTGAATTATACATTATTTGGTAATATTTTTATTAACGTATGCGAGTATGTTGGGATTTTTATGAGATACTACAATTATGATAATGGGTTATATGAAAAAAAATCTTGGCAAAAACAATTGAAAAACAAATCTTGTCATATGAATCTTACCGAAAAAAAATTTGTGTCATTTGAATCTCAATTTCCATTAAAATTATCAAAGATTTTATATATATATATATAAATAGTCTGATCTGATGTTATTCGAAACACCACCAAACACAGTATAACTTAGTCAGATTATTTATCCGGAACAGCACCAAACGCCTTATAATATTATGGATAAATAGTCAGTACTCTCCAGAACAGATTAATACTATCCGACGGAAATTAATCAGTACAGTCCGACGTACCAAACGAGCCCTATGGCTCTTGATGATGTATGGGGATGTCTTATGATAAGAAACAACTGGGGATTAGTGACGGCAATATCTTGG

AAAGGATCATGACTGGAAATTAAAGCAGACTGGAAACTAGGGCACCTAGCAGACTGTGGCTCCTAGGTTGGCAAATCTGTTACTGATTTGGATAGGAGAACATCCCGGGAAGGGGACGATCTGGGAAGGAGGCTCATATTTGGCTTGACTCATTGATGGTGGGAGGACTCGGAGTGACTGGAGTTGACTGGGTTGACCAATTTGACTTTTTTGTTTTTCATCTTTTTCTCTTTCTTTTCTTCTTTCTGCCATTGTTTTCCCTGCAAAAATTCTCTGGTAAGGAAATCAGGAATAGAATTCTCACTCCCTCTCAAATATTCAATATCAAAATCAAAAATACTCAAAATAGCTTGCCATCGTGCAAATATTTGTTTGGATGAAATATTTTGAACATCTTTTTCTAATACATGCTTAGCAGACTTACAATCAACATGGACTAAAAAATTTTGATTTAATAACTCAGATTGAAATTTATTAATGCATAATACAATAGATAGAATTTCTTTCTTAATAGCACTATAATTATTTTGGGACTGAGACCAAGTGCCGGAATCAAATCGAATGATTTGCTCAGATTGACCAGAAGTGACTCTCCATCAGTTTTATGTTTTCCTATTGACTCAAGATTAAGTAACTGACATATCTCATGATTTTTACTAAGGTATAGACTTGTTTAACTCTCTTAACATGTGTGGTTGACTTAAAACTAGATAATGACCATTTTTTCTTATAGACGTCCTGACTGGGGACTTGAGATATGTTCCAATCTTTATAATCTAAATTACTATTATTTTCATATTCAATTTGTTCTTCATTTACTATCTCTGGGAGACTACTATTGGCTCTAGAGCTGCTAGAACTGTAAGAGTAGGTTCTAAGCAAACGACTCATTTTTACTCAAGGTTCTGGGTCTGGACTCCTAAAGCAGAAAGTGAACAGAAACTTCTAATACAAACTCCATGGGTACAAATCAAAGCTTAGTCGTCATAGGTAAACAATTAGACAAGATAGATACAAAAGTTGACAAATTGCTTCCAAAAGAAATTTCTATTAAGCCGCATGACCAGAAACCATTAGTCACCTTTCAAGACCTAAAATCCAGTCCAACTTTTAAAACAAAAACTACAATGACAAAAATCAAGAAAATGTTAGAAGAATTAACTCCAACCAAACGGAAGGACAAAGAGAAAGTTGAATCAAGCGTCAAAGTTTTAGGAATTTCTGAGCCATCATTCACTTTTCATCATTCCTCTGGGTCTAGTAACATTTCAGATACTGAGTCTAGTAACATTTCCAAAATTGCAAATGCGTTTAAAAATCTTGAGCTCGAACAAAGTCCACAAGTACAAAGATTAATGAACAGAGTCAGCCCTACTAGCCTCACAAAAAATTGGTATCCTAAGCCCACACCACCTGATATCCAATTCGCAGAACGAAATACTCAAAGCCAGTTTTCTGTTTCTTTTGATAAATTATATGAATGGAATATTGATGGTCTTTCTGAACAAGAAATCCTTGATAAACTCCAACATATGTCTATGGTCACTAATAGTTACATTACCAATCACAGTTTCAGACAATATGAAATTCTTCCCTTGTTAGAAACAGGTTTCACAGGAACTCTTCGTTCTTGGTGGGATAATCACTTAACTCATGAGTCTAAGCAACGAATAATTCATGCCCTAAAACTTAATGAAGATGGTCTTAGCATTTTTTATGAACAAATAGGTCAAGGCATTGAGGATGGAGTCAACACATTGTTGCACACCACAGTCGATCATTTTATTGAATCACCTAGTAATACTACTGCCAGAATACCTGATCAATTGAGTAATTTAATATGCCCAAATTATCCGATTTTTTTTTTGAGGACTTTTCCTTTTGAAAGGGGCGATAGCATATTAAACTCACACACACAACACGGATGCTAGGACCCGAACCCAAGACCTTGCCTGATGGAGTAAATACTCCAAACCACTACACTGGTGAGTCCTTTGCCAAATTATTTGATTTTAGATGGTATAAAGAATAAATAAAGATGTGTTTATTTCCAGAGTCATGATTAGAGAAGATAGTAATCAACCATTTTGGAAAGAAAAATTTGTTAATGGACTACCAAATTTATTTGCTCATAAAATCCGATCAACCCTTAGTAATGAACAAGGTCACATTGATTGAGATAGTATAACTTATGGAAATATAATCAGTACAATTAATTAAGTTGGTATGAGAATGTGTGTTGATATGAAAATTGGTAGACAAATCCAATCTGAAAGAAATTCTGCTAAATACGAATTAGGTAATTTCTGTGAACAATACGGCTTGATGTCTATTCCTCCGTCTAAAAAAATAAGTCTAGTCATCCTAGCCGACAACTTAGAAAAAACAGCCATTATTCGCGAAGAAAGCAGTCATTCCAACATAATAATGAAGATAAAGAATTTTACAGTAAAAAAAAGATTCTCCCCTCGTAAAAATTGGAAAAAGAGTCATAATAAAGATAAGAATAATAGCTTTAAGAAAAGGACCGACAAAAGAAAAGTAAAATGTTATAAATTTCAAAAGTATGGTCATTACGCTAATAATTGTAAAGTTAAAGACACAATCAAACAATTAAAAATCACGGATGAAGATAAAAAGAAATTAATCCAAGTCCTTGAGTTAAGAAATTCTGAGTCCACTGAAAACGAAACCATGATTAGTCATACTGAGTCTTCCAAAATATATTCTTCTGAGTCACAGCCCAGTTCTCCCAGAATCCAGTTTGGTTGCCAAGATAATTGCTGTAAATCTAAGTCAATCAATGTCCTTACCACACAAGAAGAACAAGAAGAATTATTAATCGATCTAATAAGTAAAGTTGAGAATCCAGAATTAAAATCTGAATACCTAAAGAAATTAAGAAAAGTCATCAACCAAGAAGAACCAAAACAGTCTATCCAAAAAATAAATCTGAATACAACTTTAGAAAATTTTAAGAAAAGGAAAGAAGTCACCTTACAAGATTTACAATTAGAAATAAAATCAGTCAAAAAAGAAATCATTGAGTTAAGACAAATAAGTCAAAAATTACAAACTAAAAATTATGAGATAAAACAGGACCTGATTAACCTATTCGAAAAGAAAATTTCTGAGTCTAAACCAGAAAGTCCCACTAATTCAGATGAAGAAAACTCTATTCAAGAACAAGCCGTTAATTTAATTAAACATATAAATTTTAGAAAATGGTATGCTCGAGTCACTATATTTGTTCAAAATTTTGAGTTAAACATAGTTGCCCTTTTTGATTCCGGAGCTGATCTTAATTGTATCCAAGAAGGCCTAATCCCAACTAAGTATTACAGAAAGTCAAAAGAATCGTTAAGTACTGCTTCAGGAAAGTCCCTCCAATTGAATTATGAGATCCCTAAAGCCCGTGTATGTCAAAATAAAATATGCTTTAAAACTTCTTTTGTTTTAATCAAAACTATTACTGATGAAGTCATATTAGGTATGCCGTTCATTGCCCTTCTGTACCCTTTTCAGGTTGAACATGATGGTGTCATTTCTACCTGCTTAGGAGAAAAAATTAAGTTCGAATTTTTGAGTAAACCTGAGTTACATAGTTTAAAAGAATTACAAAAAAATGCAGTCACCAAAACAGTCCAGGTCATCCGAAACAAAGATAAACAATTAAGATTTCTCCAGGAAGAAGTTAAAGCTAAAAGAATTGAGCAACAACTAAGTCATACGTCTTTTCAATTACAAATACAACAATTTGAAGAAAAACAAAAACAAGAAGTCTGTTCTGAGTTACCCAATGCGTTCTAGCATAGGAAACAACATGTTGTCAGCCTTCCCTATGTCAAATCCTTTAGTGAGAAAAATATCCCTACTAAAGCACGATCCATTCAAATGAGCCAAGAATTAATGGAATTTTGCAAAATTGAGATTACCGATTTACTTAAAAAAGGAATAATACGTAAGAGTAAGTCACCTTGTTCTTGCCCTGCTTTCTATGTTCAGAAAAATGCTGAATTAGAACGTGGTGTCCCACGACTAGTCATTAATTACAAGCCACTTAATACTGTCTTAGAATGGATTAGATACCCAATTCCTAACAAAAGAGATTTAATTAACAGATTAGAAAAATCAGTCATCTTTTCTAATTTTGATATGAAAAGTGGATTTTGGCAAATTCAAATTTCTGGGTCTGATATATATAAAAATGCTTTTGTTACTCCTTTTGGTCATTATGAATGGAATGTTATGCCTTTCGGTCTTAAGAATGCTCCTAGTGAATTCCAGAATATAATGAATGAGATCTTTAACCCTTTCAGTCATTTTTCCATAGTTTATATTGATGATCTGCTTATATTCTCTGCGTCCATAGAACAACACTGGAAACATCTGTATAAGTTTTTACAAATAGTGAAACAAAATGGTTTAGTTATTTCTGCTAGGAAAATTAAATTATTCCAAACTAATGTGAGATTTCTTGGGTTCAATATCTGCCACAGATTAGCCCCATAGATCGAGTCATCTAGTTTGCTGATAAGTTTCCTGACCAAATCCTAGACAAGAGTCAACTCCAAAGATTTCTAGGATCTCTAAATTATGTTTCTGATTTTTATCAAAATATGAGAAAACAATGTAAGCCTTTATTTGATCGTCTCCAAAATAATCCTCCTCCATGGTCATCTGCTCATAATGAAACTGTCAAACAGATCAAAGCCCATATCAAGACCCCCTCTGTCTTGGTATTCCTTCTGCTAATTCATTTAAAATTGTTGAAACTGATGCTTCTAATGTAGGTTATGAAGGTATCCTCAAACAGAGTCACTTCTGGTCAATCTGAGCAAATCATTCGATTTCATTCCGGCACTTGGTCTCAGTCCCAAAATAATTATAGCACTATTAAGAAAGAAATACTATCCATTGTATTATGCATTAATAAATTTCAGTCTGATTTATTAAATAAAAATTTTTAGTCCGTGTTGATTGTAAGTCTGCTAAGTATGTATTAGAAAAAGATGTTCAAAATATTGCATCCAAACAAATATTTGCACGATGGCAAGCTATTTTGAGTATTTTTTTATTTTGATATTGAATATTTGAGAGGGAGTGAGAATTCTATTCATGATTTCCTTACCAGAGAATTTCTGCAGGGAAAACAATGGCAGAAAGAAGAAAAGAAAGAGAAAAGGATGAAAAATACAAGAGTCAAATTGGTCAACCCAGTCAACCCCAGTCACTCCGAGTCCTCCCACCATCAATGAGTCAAGCCAAATATGAGCCTCCTTCCCAGATCGTCCCCTTCCCTGGATGTTCTCCTATCCAAATTAGTAACAAATTTGCCAACCTATGAGCCACAGTCGGCCAGGTTCGCCCTAGTTTCCAGTCTGCTTTAATTTCCAGTCATGATCCTTTCCAAGATATTGCCGTCACTAATCCCCCAGTTGTTTCTTATCATAAGACATCCCCATACATGATCAAGAGCCATAGTCATTTGTTTGTTATTGAGTCCAAATATAATTCAATTGCTAACCCCATCACCATTGCTAAATCATATTTTCCACCCAATAGTCATTTCATTCCCCCAGCACCATTCAAGAATCTCAAATACTACAGAGATATCCTCCATGAGACCCATTCTATCACCATTAAACCAATTAAAGATAGAGAAAACCCTCATATCATCCTCTATCATTCACTTTACATTCACCAAATCATCAATGAAAATGAATGGAGTAGTCATCCCTATGATCTCAAACTTCTCCAATGAGAACTCTAATACAGTTATTATGATTATATCGAAGCATGGTATTCTATCTTGCGTCACCAAATAGTTGATTTCAGTCACTCCTGGTTCTGAAACTTTGACAGCAAATTCAAGAGTCCTTTTCCTTACTAGTTCAATCACTGATGGGAAAAACATGGTCCAGTCATTGATTTATTTCCACCTCCTATGCAAGAATTAGTCAATTATTTTACCAATAAAAATAAGTCAAAGAATCTGAGCTATTCTTTTCCCTCCAACCTTCTCCATTTCGTTGCCAAATATAAAGTCCCTTGGATTCTCAAATGGTCTTATCAGGTTAATTGGAAAACTAGAGTCCTATCTCGCCAGTTTTCAATCAAATGGTGGGATAAATTTAAACTTGAACGAATTGCAGATTATGATTATGCTGATTTTCCTCCAGTCAGGAATCCGCATCCTGAGAACAAAGTTCCCCTGCCACATCACATTCTTCTCTCCCAATTGAGGGAATCTAAAGTCCGAATTACAAATCTTGCTAGCCAACTAATGCTTCAAGCCTTATAGATGAATGATCGTGCAGACTCTGATGCCTCTCCTGCGTCCTAAACATCTACTAGTCAGCCCAAGCCAGCCAGTCAACACTCCCAGTCCCAAAAGCGGTGAGGATGATCCTGATGAGCTAGACCCTATGATTTTGGTCCTTTCAATCAGGGATGATTAAAGCAATTAAAGATAGAGTTAAAGCTGCCTTGCATCATGAGCCCTGAAGAAAATTACCGAACCCATGCCTTCATCAGTCTGAAAGCTTGTTCTTTTTGAGATTCCCCAACAAAGTAAAAGTTGCTGCACTCAGTAAAAGCAAAAGCTTTCGCGTGCTCCTACAGTTACTTTTCACAAGCATCCAAAGTTGCCAGCTCACCGAAAGCTATCTAAATTTGTATTATTTGCCTATTTAAGAAGGCCTCGGCCTCTGTCTTCTTCATAATGTAATTTAGTACACTATAATTTTCTCAGTTTCAAAGTTCTATCAGTTTCAATGCTCTGAAGAACGAAAGTGGTGAAACTATGATCCTTGTAAGTGTTTTGCATTCTCTAAGTTATCAATATATTTGAGATATTTTGTGAAGTGATTATTTATTTTGATTAATGCTTATTTTCATTGCATCTATTTTC

ATATATTCATTAATTTGCTTCATGCATAAGTTGCGTCTATTTTACAATCTCTGTATTCATACTTGTTTCATTATTCTTCTTTCTCTCATTGGTTCTAGACCCCTTCTGGTATATATATATATATATATATAATCGTACGGGAGGGCCTTTTTTTCTTCTGGCAGCTACAGATGGGCTGGGGTGCCGTAAGGAGCAAACCGAAGAAAATATCTCATGTGTCTGAGTTCAAATATCAGACCCCAAAATGTTTTTGAAAGATAGTAAAGTCTTAGGAAACACCTTGGTACCTTCACCAACAAACAATAACGGCTGCTTACGATAAATTCTTAGTGCTTACAGGTAGAATCTTAGTTTGGCATGAGAGGCTTGTGCAATGAGAGCAAAACAATCATGAGTTTAGCATAAACAGAGAAAGAGTTGTGAGTTCTTATGGAAGACAGGGGTTCTAAGGAAATGGGGAATGGATTCTAAGAGGGTTTATTAAAGAGGAGAGAAATGGGAGATAATAGGTGTGTTGAGTAGTTTCCGGCAGTTTGACGAAGACTCTGCAAGCTGTGGAAGCGATTCACCATAAGAACAAAATGGGAAAATATGGAGGAATGGGCTCGAATTTAACATAGGTCTCAGGGGTGAGAGATGAAGCTTGCTCTCTGAAAGTGGTTGAGTGAATTTTCAGTTGCTTGACAAAGCTTGGCTGCACCCTGGGATTATGATTTTGTCTGGGAGGAAAGATGCCTCCTTTTACAGGCCTTGAAGCTCCTTCTTCCAAAGAAACCCTAATAGTTCTATCCAATTTGGCCAAGTCTTTCCCTCATTTCTCTTCTCTTCCTTTGACTCATCCTATTTGGTGAAATTCTCTCTGCCCAATTGCACGAAAACAGGGTTTTGGGAGCTCTAGGCTTTAGCTGTTTCTGTAATAACCCAAAACAAAAATTCTAAATTAATTATTAATTTATTCTGAAAGAAAGACGATTTGCCCTCACCATATTTAATTGGAAAA

>Chr5-15; OZ022210.1; 12237503-12238109; dir; B;

AGCCGAGGGACTTGGAAGAGTGCGACCGTATATATCTTCTTGACCCAATTGTACATGTCAACTACTTGGAAAACCCTCTACGGATGTCTTGAAAGCCAAGTCACCACCCCCTCTAGTCGTGAAACGCATGCGCACTTCTCTTGGACGTTGCAGAATTTATTAATTTTTATCAAAAGCTTCTCCAAGATGAGCCGACTCTTGTGGCATCTCCTTGCCAAGGATCACACCAACACATTACTTCACAGGGCTATTGTTCACAAGGAGTTCGGACAACAACCCAAGTAGTACATAGGAGAAGTAGCATTCTTTCCTACTTCCGCACCACCATAGGCATTCGAAAAGTCTAGCTCATGACTATAAAGAAAAGCTTCTTGGGAGGTAACCCAAGCATTCTCAACTTTTGTTTTCGCTTTTGCTTTTGGTTTATGTGTGATTTCTAGTACATTTTTCATGTTTTTGTTTTTGTTAAGTTTTGCATCATCAGACAAAGGATTTTAGTAAACACATTCTTTGTCCAGCCTCTTCGCCCTCCTAATCACAAGAAGCAAGCCTCAAAGAAGGCTTGAAATTTGAGCAGCAACCGCAGTGTTGGTGTCGACTTGCACTCTCCTATGGCCGGCCATGGGTAGAAGTGTAGGTTTGAGTTGGGTCCTTGGATTTTTGTTGCCATACAACACTTATGTCAAAGTTAATTCCATAATTTACTCTTAATATGAATTATGATTTAACCCTTCAATCACTATTATAGTCTTACTAATTTGACTTGGTCATTTGCTCAGGTCATATATGTTGACTCTGACTTGATCGTTGACTTTGACCTTTGATTTTTGACTCCTAGTTTCTTTTCTTTTCATTTGTTTCTCTAATCTCATTTCTCGGTGTGCGATCCATGGGTTCCTTTTAGCAAGATGTGGGAGGGCTCTACTTCTATGAGTTGACTTGTGAATTAAATCACACCATTTAATTCTTCATTTTGGGGAATTGAGTGTCACCGTTGGGT

ATACTGCCTTTGTAACACCTCTCGGTCATTATGAATCGAATGTCATGCCCTTTGGTTTTAAAAATGCGCCTAGTGAATTTTAGAATATAATGAATGAAATTTTTAATCAATATAGCCAATTTTCCATAGTTTATATTGATGATGTCTTCAGTTTTTCAGAGCCGATTGAACAACATTGGAAGCATTTAAATAAATTCTTCCAGATAATCAAGCAAAATGGTTTAGTTCTTTCAGCTAAGAAAATTAAGTTATTTTAAACCAATGTCAGATTCTTAGGATTTAATATTAGTCAAGCCCAGATAAGCCCAATTGATAGAGTTATTCAATTTGCTGATAAATTCCCAGATCAAATTCTAAATAAGAATCAATTACAGAGATTTCTAGGATCATTGAATTATGTTTCTGATTTCTACAAAAATCTAAGAAAATTATGCAAGCCCTTGTTTGATCATCTCCAGAGCAATCCTCCTGCCTGGACTCCGAGTCATACAGAAACAGTTAAACATATTAAAATTCATGTCAAGTCTCTTCCATGTCTTGGTATTCCTTCAGTAAATTATTTTAAAATTGTCGAAACAGATGCTTCTGAGTCTGGTTATGGTGGTAT

CTAGTTATCGTTCAATAGTCGAAAGGATACTGAACTTATTATGCGATACCCGATTTTGATAGGTTTGGACCATTCGATCAGTCCCAATTTCAAATGTTTTGTTCCTCGTACCTCTAAGATTGTGTAGGAACTAGTTGATCGAGAATTGGAGTCCCGAATACACCGAATCAATGATTCTAGGGTTAAGTAAATCGATCATTGTACGATCGTGCGATCTAAAGTGATCCAACCGCTCGATTGAAACCAAGCTCTCAGAACTTGTTAATTAGACGTTGGACTTATAGGAATGTTTGATTGGAAATCGGAGGTTGTGGGCCTCGTAGACTAAATTGGTCAAATTTCACGATCAGAGTCAAACCGATCGTTGGATCGTGATGAGACTTCTTAGATGAGTTCAACTTATGGTTATGCACCAATAGGAATTATCATATAAAAAATTGACGATGTGAGCCCTACGAGCCCAGTTGACTTAGGTAGTACTCTATACATACTCGGTTGAGGCTATTTAGTAGTGGTAATTGTAATTGGAGGTTTGTTTTAAAATGGTATTATGTTATGAGAGTTTCATAGAATGCTTATGAATTATTATACAATCGCTTTTACCTTCCAAGCATGTTATATTATGTTTAATTGTGTGGAGTTGGGAATGGCTTGGATAACTGGGTTTTGCAATGAATAAGAAATTAATTAAATGACAGATTATATGTCTATATTTAAATATATGCTCAGTGGCTTGAAATCGATGAATTATGAACACTAGTTTTCTCAATAGTATGTTGTACGTAGTTGAATGTAGTTTGAAAATAGTTTTGAGAGATTTTGCATGGTCAACTTCAAATTAAGGAATGTTAAGATTGATCTCGTTTTAAAAAAGGGATTTTTGGTTATTTATTTAAGAAATGATTTTTGAAAAGTTGAGAAAGGATTTGGAAATGGATTTTTGAAAGTGGATTTGTAGAAAAAGATGAATTTTATGATAAGTTTCGGAAA

>Chr5-16; OZ022210.1; 12355521-12362693; rev; A;

ATTTGCTCTTTACGGCAGCCCAGGCCCATCAGTAGCTGCCAGAAAGAAAAAGCCCCCTCATAAATTGGCGACTTCACTGGGGATCAGGTCATTATCTTCCCCAACGCCTCAAAAGAAGAAGACTAGAAGCACTAAACCAGAGAAAATAAAGAGATAAGAACATTTCCTCTTTTCTTTTTTTTTTTTTCTTCTGTTGGCTATGTACTTTGCATAACAAATATAATATGGACTGCTGACATGCGCCCTTTGTTTGTGTCTTAGATCTCCATTCCCAAGCAAAACAAGACCTTGTAATTGAGCTGGGGACTTTGGCAAATCCATTATTCATGCCATTGAGCAAACTATACATGCAAACTGCAAAATACTTGGGAACTTCATGCCAAGCAAATTTTTCCAAAAAGAAAAAGAAAGAGCCAAACACAGGCCAAGCATCTGATAAAGAGAATTGAACTTCTTTTTCAGTTTTCTACTTGAGATATTCCTGGAAGATAACATAAGCTCTATATTCATACTGCCGAAAGAATGAACCCTCATGCTAGACCGAGCTATATCTTGGTTCAACTCAACCATTAGATTATCCGCATAGCAAGTCCAAGCTAATCATGCCAAGCCAAGTCACGTTCTTCCAGCACCTATTCCATGTCAAAACAAATTTTTTCCAGCACATTCCATATTTCAAACAACCTTCCAGCACATGCCCTATTTCAAACATCCTTCCAGCACATGCCTTATTTCAAGCATTCTTCCAATATCTAGCAATCATTCCGCACGTCATCACCTCCACATAGGTTTTGTCCACTAATTTCAAGTGTTCTGGCGCTAGTGTTGTAAACTCAAACACAGTAGAAGTGTGAGGGTCTTTCGTGGCCTTGCTTGCTGGAAGTTTTAATTTTCATGTTTTCAAAGTTTTTTATTAAGTCAATGAAATTATCGAGTTAATTTTACGATCCTATTTTTGTTCCTAAGCATTATCTTAAATGATTAATTGTATTTTCATTCA

TGCAATCTGTGTGTTAAGAATTTTATGCATTTACGCTTTACTTAAATTCTATTTTGATTTATTCCATTTCTGTTTCTCTTGCCTTGGTCTTCTCTGAGTATTAATTGAACTTCTTTTTCTTCTGGTTCTTCCCTCTTTCTGGTATCAGAGTCGTAAGAGCTGCGGTTGTGTCTAGGTCTGCTACTCATATCGCCCAAGTGGCTGATTTAGGTTTTCTGAGTTAGCAAGTTCTTCAGTTATTTCAAAACAGTTATCTTAGAGTTGTCTGTCATCTTCGATTTACTATCTCAAAAGAACACGATAAGACCTATAGCTGAAAGTAAATCCCGTGGTAGGGGCCTGAGAGGTGCGGCATGAGTGTGTGAGAGAGAATGCCTTAGGTTCCAGGTAATAATGAGTTAGCTATTAGGAATCCCTGTAGATCTTCTACCTTTTAAAACTCTGAGTCAAACACCTGTAAGTATGAGTCGTTTTCTTAGAACCAATTCAGTTAATTCTTCCAGTTCTAGAACTAGTTTGAGTCAAGTCCCAAACATAGTTAATGAAGTACAATTAGAATATGAATCAACAAGTAGTGAATTACACTATAATGACTGGAATATTCCCAAAGTCCCTAGCAAAGAAATTTATAAGCAAAAATGGTCAATGAATGCTTTCAAGACAACTACGCATGTTAAAACAGTAGAACAAGTCTATGCCTTAGGAAAAGAACATGAAACATGCCAATTACTTAATCTTGACTCAATTAAGAAGCATAGAAAAGAAGGTAATAATTTTTTACATATAGGACTAGTTCAGGTTGCAGTTAAACCCTTAACTAAGCTAGGATTAAAAATAGCAATTCTTTTAAGTTTAAGAGATGCTAGATTTAACGATTTCCAGGATGGAGTCTTAGGAATCATAGAATCAAGCTTATGCCATGGTCCAGTTCATTTTGATTGCTACCCAGATTTTACAGTCAGTTTAAGTGATCCCCATATATTAAAAGTTTAAACATTAAATATTAAAACGCATGGTTATAATATTTTACCAGGTAGTCAACCTTTAGCTTTAATTTATAGAATATATTACAAAGTCACAGGTACAAATATGAATTTCCAAGCCCTTAGGAAGAGCCCAAAGAATCAAACACTTTTAATTCAAAGTCATACATCAAATGCGAATGTTACAGTACTTCAAATGATTAGATGGTCTGATGTTAAATTACCCCAGGATTGGTATTTAACAACTGAATGTCCGCCAAAGCCGATTCAACGTAGTCTAAACGATCTAGATTATATCCAGCCCTATTTAGATGGAACGGTTAAAATTAGTTTTGATAGGCCAGTTAAATCAACAGTCCAATCAGAACAATTGCCAACCCCTGGTCACTCTTTTAAAATTCCTGCTAGACATTCATTCGCAAAGTCATCCTCAATTACAAAACGCGATTTAGAAGCCGATAGAGCCTTAATTGATTTTAAAATTAATGAATTAAGAAATAAAAAACAGGTAGTTCAACGAAGTTACGGAAAAGTCCCAACTGAGCCCGTTTTACCAGTACAGCCTGAGTCACCTACACGAATAGATTTTGAAGGTTCCACCTTTGAAGCCTATGATGCTCAGTTAAGAACTCTTAATAAACTTTTTAAAATTCATTGGTCAAAATTAGATGCCCATTTCCAAGCCCTGGAAAATGAATCCAGAAAAGCCAATTATCATGCTTCTTTCCCCAAAGTTCAAAGAAAAGAAATTTTCAATGAATGGAAAGAATACTTAAAAAGACTAAGTCAGAATCACCTTATCTCGATTACGTAGAAAGTAAATACATGAGTCAAAAGTTAGTTACTTTAACTCAGGAAAAATGGGAAAAAGCCGACAATACCAAAGTCATCAGTACACACAACTCCAGTCGAGTCAATAATAATCAATCATAAAAATGTCAAAATCATAGCTTGCCCTTATAAAGTGCTGAGTCAAAAGATGATTTAGATAAAAGAGTCATGGAACAAAATAATTACACCAACACGAGTCTAATAGTCATAAGTAAACAATTAGATAAAATTGAAACTAAAATTGACAAGTTAACCCCCAGTGAAACTACTTTAAAGCCAAAAAAAGAAAAACCTTTAGTTAAGTTCAATGAGTTAAAACCCGAGTCTTCCCTTAAGGTAAATTCAATGATGAAAAAGATTGAAGAAATGTTTCAGGAATTAACCCCAGTTAAACCTGAGCCAAACAAAGACAAAGGGAAAGAAGAAGAAGAAACTTCAGTTAAAGTTTTACAGTGTGAAGTCAATTCTGATAGTGAGTCAGCCATGTCATTAGATTCAAGGCTTATTCAAGAGTCAGAAACTAGTAATATTTCTAAAATTGAACGAGCCTTTAACAATTTACAAGTTAACCAGGATTCCCAGTGTCTTAAAGTTAGTTTTTTGACAGGTAAAATTAATCCCACAAGTCTCACAAAAAATTGGTACTCTAAGCCTATAGCTCCAGATGTCCAATATGAAGAAAGAACCCAGTGTCAGTTTTCTGTGTCTTCTAATAAGCTATATGAGTGGAATATCGATGGCGTGTCCGAACAGGAGATATTTAACAAATTACAACACATGTCCATGGTAGCCAATAGTTTTGCCACTAATCATCAATTAAAGCTATCCGAAATAGTTTCCCTTTTACAAATAGGCTTCACAGGAACACTTCATTTCTGGTGGGATAAACATCTCACAGACGATGCAAAATTAAGAATAATCCATGCCTATAAAATAAATGATGAAGGTTATCCTATCTTTGATGAACACAAAGGAACGAGTATAGAAGACGGAGTTAATGTCTTTCTGTATACGATAATTGAGCATTTCATAGGTATACCCAGTAGCACAACAGCTAGGATTCATGATCAATTAAGTAATTTACGATGCCCTAAATTAAGTGATTTTAGATGGTATAAAGATGTTTTTATTTCTAGAGTCATGTTAAGAGATGATAGTAATCAATTGTTTTGGAAAGAAAAATTCATAAACAGTTTTTCGAATTTATTTGCTCACAAAATTTGAACAGTTTTAAGTAATTACTCAGGACAAATTAATTATGATGAATTAACATGTGGCAACATAATTAGCACAATTAATCAAGTTGGAAAGAAAATGTGTGTAGATTTAAAAATTAGTAAATCAATTCAAGACGATAGGAAGAATGCTAAATATGAATTAGGAAATTTCTGTGAATAGTATGGTCTAGCTCCTATACCCCCATCTAGACAAGTCAAAAGAAGTAATGATCGTCGATTTAGTAAAAGTAGATATTATTCCCGTAGAAAACATTCATTTAAACCAAATGATGTGTTTTACAGTAAAAAGAAGTTTACTCCTAGGAAAAGCAAATGGTCAAAAGGTTATTCGAAAGGTTACCGACAAAGTAAAAAGAGTCAATTTAAGAAAAGCTCAGATAAAACAAAAGTTAAATGTTTTAAATGTAAATGATTCGGTCATTTTGCTAATGAGTGCAGAGTTAAGGATATGATTAAACAGTTAAAGTTAAAATCTGATGATGAAGCCAAATTAATTCAAGTTCTTGAGTTAGCTAACACCGAGTCAGATCCTATGTCTTCTGCTAGTTCAATTCATTATGGTTCCGATAGTTCCAAATCTAGTTCACCAAAAGTTCAGATAGGTTGTAAAGACAAGTGTTGCAGGGTCTTAAATAAATCAATTAGTGTCCTAACTAAGCAAGAAGAACAAGAAGAATTATTGATAGATTTAATTAGTAAAATAGAAAATCCAGAATTAAAAGCCGATTATTTAAGAAAACTGAGAAAAGTCATAAGTCAAGAAGAACCCAGTCCGAACCAAGTCCCTATGATTAGTCTTAGTACCATTTTAGAAAGGTTTAATAAAAAGAAAGAAGTCACCCTTCAGGATCTCCAGTTAGAAGTCAAAACAGTCAAAAAAGAAATTTTAGACTTAAAACAAGTTAGTTAGAAATTACAAACTGAGAATTATGAAATTAGACAAGACTTAGTTAAATTGTTAGAATCTAGAGCAGAATCCAGCAATCCTTTCGGTAAACACAATTCAGAGTCAGAACCTGAATCATCCGATAATGACAAGAACGACAAAGTTATTAAACTAATTAAGCAAATTAATTTTAGAAAATGGTACTCCCGAGTTAGTATTTTCATTAATGAATTTGAATTAAATACAATTGCTTTATTTGATTCAGGAGAAGATTTAAATTGCATTCAAGAAGGTCTAGTTCCAACCCAGTTTTACAGGAAGTCAAGAGAAGTCTTAGGAACAGCGTCTGGTAAAGCATTAAAATTAAATTATGAATTACCCAAAGCCCATGTATGCCAAAATAAAATATGTTTTAAAATCTCTTTTGTTTTAATTCAAAATGTTACAGATGAAGTTATTTTAGGTTTACCCTTTATCTCTTTACTTTATTCTTTCCAGGTAGAATATGATGGAGTTATGTCTAGTCATTTAGGTGAAAAAGTTAAATTTGGATTTTTAACCAAACCAGAATTACACAATCTTAAGTCCTTACAGAACTCAGCAGTTTCTAAGTCAGTGCGGTTAATTCAAAATAAGACAAAACAAGTTGATTTTCTCAAGCAAGAAATTAAATTCAAACATGCAGAACAACAGTTAACCAATAAGTCTTTACAGTCTCAAATAATTGAATTTGAAGAAAAGTTAAAAAATGAAGTTTGCTCTGATTTACCTAATGCTTTTTGGCACCGAAAGCAACATGTAGTTACTCTACCTTACGTTAAAACATTCAGATGAGTCAAGAGTTAATGGAATTGTGTAAACAGGAAGTCACCGAATTAGTCCAAAAAGGAATAGTTTGGAAAAGTAAGTCACCATGGTCATGCCCAGCTTTCTATGTTCAAAAAAATGCAGAGTTAGAAAGAGGAGCCCTCCTCTAGTAATCAATTATAAGCCCCTTAATACAGTTCTTGAATGGATTAGGTATCCAATCCCAAATAAAAGAGATTTAGTTAACCAATTAGAGAAACAAGTTATTTTTAGCAAGTTTGATATGAAAAGTGGTTTCTGGCAAATTCAAATTAAAGAATCTGATAGATATAAGACTGCCTTTGTAACCCCTTTCGATCATTATGAATGGAATGTCATGCCCTTTCGTTTAAAAAATGCGCCTAGTGAATTTCAGAATATAATGAATGAAATTTTTAATCAATATAGCCAATTTTCCATAGTTTATGTCGATGATGTCTTGATTTTTTGAGAGTCGATTGAACAACATTGGAAGCATTTAAATAAATTCCTCCAGATAATCAAGCAAAATGGTTTAGTTCTTTCAACTAAGAAAATTAAGTTATTTCAAACCAATGTCAGATTCTTAGGATTTAATATTAGTCAAGCCCAGATAAGCCCAATTGATAGAGTTATTCACTTTACTGATAAATTCCCAGATCAAATTCTAGATAAGAATCAATTACAGAGATTTCTAGGATCATTGAATTATGTTTCTGATTTCTACAAAAATTTAAGAAAATTATGCAAGCCCTTGTTTGATCATCTCCAGAGCAACCCTCCTGCCTGGACTCCGAGTCATGTCAAGTCTCTTCCATGTCTTGGTATTCCTTCAGCAAATTCTTTTAAAATTGTCGAAACAGATGCTTCTGGGTTTGGTTATGGTGGTATCTTAAAGCAAAGAGTTTACCCTGGGTCACCTGAGCAAATTGTCCGATTTCGTTCCGGAGTTTGGAGTCAGTCTCAAGTTAATTATAGTACTATTAAAAAAGAAATTTTATCTATAGTATTATGCATTAGTAAATTTGAAGATGATCTTTTAAATCAAAAATTTTTAGTTAGAGTTAATTGCAAATCTGCAAAACATGTTTTAGAAAAAGATGTTAAAAATATAGCTAGTAAACAGATTTTTGCACGTTGGCAAGCCATCCGGAGTGTTTTTGATTTTGAGATTGAATATTTGAGAGGTAGTGAAAATAGTATTCTTGATTTTCTTACAAGAGAATTCCTACAGAACAAACAAGCAGAATCATGTCAAGTAGAGAAAAAGAAAAACAAAAAATGCAGTATGGTCAAAGCCAATCAAGCCAAATGAGTCAACCCAAGATACTTCCAGCCACCCTGATGCCTATCAAACAAGAGTCTTCTTCTCAAGCCATGCCTTCTCATATTGTGCCCTACCCAGGTCTTCTCCAATTCCAGTAGGTAATAGATTTAATCCTTTGAGTCAACCTTCTTTACGCCCCAATTATCAGTCTGCATTAACTTCTCAGTATGATCCATTTATTATTCATCCGCCTGCTCCAGTTTTTTTTCGCAGAGCCAAAGCCCAGTCAAAAGGTCTAATTATGTCACACGAAGCCATTGTCATCTTTTCGTAGTTGAGTCCTACCTTGAAGAACTTGGAGATCCAATTAAAATTGCCCATAAGTTTTTCCCTCCTACTCTTCATTATTTTCCGAGTCATCCTTCCAAGTCTATTCAGTTTTATAGGTATACTCTTTATGAAACCCAGTCTATAGATATTAAGCTCATTAAAGACAAAGAAACCAATGCCACAATGTTTCATTCCCTCTACATATGCCAAATTCTCATGCCCCAAGTCTGGAGTCGTAATCCTTATGACCAAAAGCCCCTTCAATCAGGCCTTACTTATAATTATGGTGATTATATTGATGCATGGTACACAATTTTTTATCATCAGCATTCAGATTTCAGTCATTCATGGTTCCTTAATTTTGATGCCAAGTTTGAAGCTCAGTTTCCTTTCTGGTTTCTCCAATGGTGGAATATTCATGGCCCCGGCTGGGAACTCATCCCCTCCAATCTCCAACTGTCAATCAATTATTTTAGCCAGAAGCTTAATTTATCCAAATCTGAAATTTTTTTTTCCTACAATCCTTCATTTTGTAGCTAAATACGAAGTTCCTTGTATTATGAAATGGAATTATCAAGTTAATTGGGATTCTCGAGTTTTATCCCGACATTTTGCAGTCAAATGGTGGGATATGTTCGGAATTGACAGAATTATCCAACAAGTTCATGTAGATTTTCCATCACTCCAAATGCCTCCAGTCAAAGTTACTTCTCCAAGCAGTTCCAGTACTTCCCTCAAAGGCAAATCAAAAAGAGAGATAAAAGAATTAGCTGAGCAAATTCTCGCCCAAGTCACTCATATGCATGATGATGA

TAGCGATGACGCTAGTCTGAAATCCGAATCTTCATCTAGTCAGTAGCCCAGTCAACTTCCATTCAGCCAAAAGCCAAAGCAGCGATGGTCCGACTATGCCCCTGACTGTCAGAATCCTAACCCCGACAGTCAGGATCCCTTTCATTTGAATGCAGACTAGTTTTCTACAAAGTAGCCGAAAGCAGAAGTTAATTTTCCCAAAGATGCCCAAAGTTAAAGCAGTTTACCCAGAGTTAAAGCAGATGCCAGATGCCTTTAGTCAAAATATGCCAAGTTAAAGAAGTTTACCCAAGGTTAAAGCAACAGATGCCCAAGTTATTTTCAAAAGTTATTTTGTCTTCTTCCAGATGTCAATTCTTTAAATTAATGCCCCAAGTCATTTGTAAGAATAACTTCTTTTTCAGAACTTCTTTCAAGCTTTCCTTACTTGTAATCTTCCAGTTTTCTAAACAAGAGTGTAAGTGATCGGTAGAAGTGTGAGGGTCTTTCGTGGCCTTGCTTGCTGTAAGTTTTAATTTTCATGTTTTCAAAGTTTTTTATTAAGTCAATAAAATTACCAAGTTAATTTTATGATCCTATTTTCTTTCCTAAGCATATCCTAAATGATTAATTTTATTTTCATTCATACAATCTGTGAGTTAAGAATTTTATGCATTTACGCTTTAATTAAATTCTATTTTGATTTATTCCATTTCTGTTTCTCTTGCCTTGGTCTTCTCTGAGTCTTAATTCTTGTTCTTCAAGATTCAATAGAACCATACTACAGCTCATCAAAACCACTTGTTAGGACATTACATGATTATTTGCATCCTGCTCGTACAAGTGTGCCATCTTGCATCATTTTAGAATTAAATGGTCAAACTTTTGATCTTAAACCGGGCATGATACAACTTTTACCAACTTTTCATGGAATGGAATCTGAAAATCCATATTCACATGTGCGTGAGTTTGAGGAAGTTTGTGGAACATTTCCAACACAAGGGTGTAGGCTTGATACAGT

>Chr5-17; OZ022210.1; 12438521-12440396; rev; B;

TGAAATTGTTACATCTCGGGCTGAAAAAGTGTTAAAATATTTAAAGTAAGAGAGAATAAATTTAAAAGAGGAAATAAACTGAAATAAACATTAAAATAAAATGATTTGAATTAATTAAGGTTTTTGAGAAATTAAAATATTATTTAATATTACAAGGGGCAAAAAGGTCATTTTAAGTTTGGGAAGGAATTTTGAAAATTCCTATTGTACTACTGTGTAGGGCACATCAAAATGAGTTCGTAGACACGTACTGGGCTCAAATCGGAGCTGTAGCGAAGAAGTTACGATTAAAAGAAGTTCAGGGGCATAACTGTGAATATTTCAAAGTTCAGTTTTAAAACCCAGATTTTCTCCTTCCCCTTAGAAACTTCACGATCAGCCAACTTCTTTCCTCCACCATTGCCACCACACACCATCGTTCTTGGCGGCACCGGTCCTGCTAGAACCGCCTTACTTCCCCCTTTGATTCCCAGCCGGTCCTAGTCTTGATCCGCCAGCGTGGTCACTGGAATCAGCCACGGAACAAGCGGTGTTGGATAGTTTTTTAGTAAATTTTCAGGAGCTCGTTCAGAAGCCTCCGGCTACCAAATCTTTCGAATCTGGTATATTTTCCATCCTTTCAACGTGTTCTAGCTGTTGGTTGTGTTATTTTTGAGAAATTTCTGTGTTTAATTGGTTCATTAAGCGTAGCGTACTTGTCCTTGGGGGTTGCAAGATATAAATTTCTATAATTCATACAAACCATTAGAGCTCCAGTATTTTTCAACACTGGCACAATGTTGGCCAGCCACTCAACATATCGGGCGGTATACATATAGCCGGCTTTGAGAAGATGTTTAATTTCTTCTTTGACTTTTAATTGTACCTTGGCAGAAAATCGCAGGTGGTGGTTGTTCGAAAGGTTTGAACCTGTCTTTGATTTTCAATTTGTGTTCTACTAAACCTCTTGGTAATCCAAGAAATTTATAAGCAAAAATGGTCAATGAATGCTTTCAAGACA

ACTACGCATGTTAAAACAGTAGAACAGGTCTATTCCTTAGGAAAAGAACATGAAACATGCCAATTACTTAATCTTGAGTCAATTAAGAAGCATAGAAAAGAAGGTAATAAGTTTTGCATATAGGATTAGTTCAGGTTGCAGTTAAACCCTTAAATAAGCTAGGATCAGCAATTCTTTTAAGTTTAAGAGACGCTAGATTTAACGATTTCTAGGGTGGAGTCTTAGGAATCATAAAGTCAAGCTTGTGCCATGGTCCAGTTCATTTTGATTGCTACCCAGATTTTACAGTCAATTTAAGTGATCCCCATAAATTAAAAGTTTTAACTTTAAATATTAAAATGCATGGTTATAATATTCTACCAGGTAGTCAACCTTTAGCTTTAGTTTATAGAATTTATTACAAAGTCACAGGTACAAATATGAATTTCCAAGCCCTTAGCAAAAGCCCAAAGAATCAAACACTTTTAATTCAAAGTCATACGTCAAATGTGAATGTTACAGTACCTCAAATGATTAGATGGTCTAATGTTAAATTACCCCAGGATTGGTATTTAACAACTGAATGTCCGCCAAAGCCGATTCAACGTAGTCTAAACGATCTAAATTATATCCAGCCCTATTTAGATGGAACGGTTAAAATTAGTTTTGATAGGCCAGTTCAATCAACAGTCCAATTAGAACAATTGCCAACCCCAGGTTAGTCTTTTAAAATTCCTGCTAGACATTCATCCGCAGAGTCATCCTCAATTACAGAACGCGATTTAGAAGCCAATATAGTCCAAATTGATTTTAAAATTAATGAATTAAGAAAGCAAAAGCAGGTAGTTCAACAAAGTTACGGAAAATTCCCAACTGAGCCCGTTTTATCAGTACAGCCTTAAGTCACCTACAAACAGATTTTGAAAGTTCCACCTTTGAAGCCTGTGATGCCCAGTTAAGAACTCTTAATAAAATTTTTAAAATTCTTGGTTCAAAATTAGATGCCCATTTCCAAGCCCTGGAAAATGAATCTAGAAAAGCCAATTATCATGTTTCTTTCCCCAAAGTTCAAAGAAAAGAAATTTTCAATGAATGGAAAGAATGCTTAAAAAAGACTAAGTCAGAATCACCTTATCTCGATTACGTAGAAAGTAAATACATTAGTCAAAAGTTAGTTACTTTAACTCAGAAAAAATGGGAAAAAGCCGACAATACCAAAGTCATCAGTACACACCCTCCAGTCGAGTCAATAATAATCAATAATAAAAATGCCAAAATCATAGCTAGTCCTTATAAAGTTGCTGAGTCAAAAGATGATTTAGATAAAAGAGTCATGAAACAAAATAATTACACCAACACGAGTCTAATAGTCATAGGTAAACAATTAGATAAAATTGAAACTAAAATTGACAAGTTAACCCCCAGTGAAATTACTTTAAAGCCAAAGGAAGAAAAACCTTTAGTTAAGTTCAATGAGTTAAAACCCGAGTCTTCCCTCAAGGCAAATTCAACAATGAAAAAGATTGAAGAAATGTTTCAGGAATTAACCCCAGTTAAACCTAAGCCAAACAAAGACAAAGGGAAAGAAGAAGAAGAAGAAACTTCAGTTTAAGTTTTACAGTGTGAAGTCAATTCTGATAGTGAGTAAGCCATGTCATTAGATTCAAGCCATAGTTAAGAGTCAGAAACTAGTAATATTTCTAAAATTGATAACAATTTACAAGTTAAGCAGGATTCCCAGTGTCTTAAAGTTAGTCGTTTGACAGGTAAAATTAATCCCACAAGTCTCACAAAAAATTGGTACCCTAAGCCTACAGCTCCAGATGTCCAATATGAAGAAAGAACCCAAAGTTAGTTTTCTGTATCTTTTGATAAGCTATATGAGTTGAATATCGAT

TGCTTGTCCAAAAGAATCGTCTATCTCCTTTACTTGACTGACCCATTTTTTCCGATCGAAAACCAGAGTAGCGCTGAAGATATTGACTTGAAAACTGGGCAGATTTTTGAAGCCATTTTCGGAGATTCCGACGGTTGTTTTCTTTGATCTAGGTACACATCTTCATCCCCACCACTTGCTCTAGCTATCTATGCTGATACCTTGAATCAGTTTTGAGGTTTTACCGATCATTCAATATACCCACTTTGGCCGACGGTTTCGATTTCGATTCGTCTACCTCTGTCTTGTTTTTGGCCGTGGTCCAAGAACAAAAGTCGATCCCCTAGTTAGCATGAATCTATTGGCAAAGTGGTTTGGATATTTTCTGTGGCAGAAAAATCTCTCAAGACACCCACCTTTGCCAGTGATGGGTGGGGGCGTGTGGGCCATTGTGTGGCAGCCTGTTGAGCTAGTTATCGTTTGACAGTCGAAATGATATTACATTATTATGCGATACTTGGTTTCGATAGGTTTGGACCATTCGATCGGTCCCAATTTCAAAAATTTTGTTCCTCGTACATCTAGGATTGTGTAGGAACTGGTACATCAAGAATCAGAGTCCCAGATACACCGAATCAATGATTCTAGTGTTAAGTAAATCGATCATTGTACGATCGTGCAATCTACAATGATCCAACCGCTCGATCGAGACCTAGCTCTCTGAACTTGTTAATTAGACCTTGTTGGACTTATAGGAATGTTTGAATCAAAAATCGGAGGTTGTGGGGCCCGTAGACCAAATAGGTCAAATTCCAGGAAAGGGGCCAAACCGATGTTGGATCATGATGAGACTTCTTCGATGAATTCAACTTATGGTTATAGAACAATAGGAATTATCATATAACAAATTGAGGATGTGGGCCCTACGAGCCCAGTTGACTTAGATAGTGGTCGATGTCGACTCGGTTGAGGCTATTTAGTAGTTGTGATTGTAATTGGAGGTTTGTTTTAAAATGTATTA

>Chr5-18; OZ022210.1; 12695027-12697470; rev; B;

AACGCTCCAGAAAGAAAAGACTAGAAGCACTAAACCAGAGAAAATAAAGAGATAAGAACAATTCCTCTTTTTTTTTTTTTTTTTTGGTTGGCTATCTACTTTGCAGAACAAACATAATATGGATTGCTGACATGCACCCTTTGTTTGTGTCTTAGATCTCCATTCCCAAGCAAAACAAGACCTTGTAAAAAGGGTCGGGGGCTTTGGCAAATCCATTATTCATGCCATTGAGCAAATATACATGCAAACCCCAAAATACTTGGGGGCTTCGTACCAAGCAAATTTTTCCAAAAAGAAAAAAAATAGCCAAACACAAGTCAAGCATCTGATAAAGAGAATTGAACTTCTTTTTCAGTTTTTTGCCTGAGATATTCTTGGAAGATAACACAAGCTCTATATTCATGTTGCCGAAAGAGTGAACCCTCACGCTAGACCAAGCTATATCTTGGTTCAACTCAACCATTAGGTTATCCGCATAGCAAGTCCAAGCTAATCATGCCAAGCCAAGTCACGTTCTTCTAGCACCTATTCCATGTCAAACAAAATATTTTCCAGCACATGCCCTATTTCAAACATCCTTCCAGCACATGCCCTATTTCAAACGTCCTTTCATCACATGCCCTATTTCAAGCATTCTTCCAGTATCTAGCAATCATGCCGCACGGCATCACCTCCACAGAGGTTTTGTCCGCTAATTTCAAGTGTTCTGGGGCTAGTGCTGTAAACTCAAACACAGTGGAAGTGTGTGGGTCTTTCGTGGCCTTGCTTGCTGTAAGTTTGAATTTTCATGTTTTCAAAGTTTTTTATTAAGTCAATAAAATTATCAAGTTAATTTTATGATCCTATTTTCGTTCCTAAGCATCATCTTAAATGATTAATTGTATTTTCATTCATGCAATCTGTGAGTTAATCATTTTATGCATTTACGCTTTACTTAAATTCTATTTTGATTTATTCCATTTCTGTTTCTCTTGCCTTGGTCTTCTCTGAGTCTTAATTC

TTGTTCTTCTTCTTCTGGTTCTTCCCCCTTTCTGGTATCAGAGCCATAAGAGTTGCGGTTGTGTCTAGGTCTGCTACTCATATTGCCCAAGTGGCTGATTTAGCTTTTCTGAGTTAGCAAGTTCTTCAGTTATTTCTAAATAGTTATCATAGAGTTGTCTGTCATCTTCGGTTTACTATCTCAAAAGAACACGGTAAGACCTATAGCTGAATATAAGTCCTGCAGTAGAGGCCTGAGAGGTGCGGCACGAGTGTGAGAGAGAGAATGCCTTAGGTTCCAGGTAATAATGAGTTAACTATTAGGAATCCCTATAGATCTTCTTCCTTTTAAAACTCTGAGTCAAACATCTGTAAGTATGAGTCGTTTTCTTAGAACCAATTCAGTTAATTCTTCCAGTTCTCGAACTAGTTTGAGTCAAGTCCTAGACATAGTTAATGAAGAACAATTAGAATATGAATCAACAAGTAGTGAATTACACTGTAGTGATTGGAATACTCCCAAAGTCCCTAGTAAAGAAATTTATAAGCAAAAATGGTCAATGAATGCTTTTAAGACAACTACGCATGTTAAAACAGTAGAACAGGTCTATGCCTTAGGAAAAGAACATGAAACATGCCAATTACTTAATCTTGAGTCAATTAAGAAGCATAGAAAAGAAGGTAATAATTTTTTGCATATAGGATTAGTTCAGGTTGCAGTTAAACCCTTAACTAAGCTAGGATTAAAAGCAGCAATTCTTTTAAGTTTAAGAGATGCTAGATTTAACGATTTCCAGGATGGAGTCTTAGGAATCATAGAGTCAAGCTTATGCCATGGTCCAGTTCATTTTGATTGCTACCCAGATTTTACAGTCAGTTTAAGTGATCCCCATATATTAAAAGTTTTAACTTTAAATATTAAATGCATGGTTATAATATTTTACGCGGTAGTCAACCTTTAGCTTTAATTTATAGAATTTATTACAAAGTCACAGGTACAAATATGAATTTCCAAGCCCTTAGCAAAGCCCAAAGAATCAAACACTTTTAATTCAAAGTCATACGTCAAATGCGAATGTTACAGTACCTCAAATGATTAGATGGTCTAATGTTAAATTACCCCAGGATTGGTATTTAACAACTGAATGTCCGCCAAAGCCGATTCAACGTAGTCTAAATGATCTAAATTATATCGAGCCCTATTTAGATGGAACAGTTAAAATTAGTTTTGATAGGCCAGTTCAATCAACAGTCCAATTAGAACAATTGCCAACCCCTGGTCAGTCTTTTAAAATTCCTGCTAGGCATTCATCTGCAGAGTCATCCTCAATTACAGAACGCGATTTAGACACCGATAGAGCCGTAATTGATTTTAAAATTAATGAATTAAGAAAGCAAAAACAGGTAGTTCAACCAAGTTACGGAAAAGTCCCAACTGAGCCCGTTTTACAATACAGCCTAAGTCTCCTACACGAACAGATTTTGAAGGTTCCACCTTTGAAGCCTATGATGCCCAGTTAAGAACTCTTAATAAAATTTTTAAAATTCATTGGTTAAAATTAGATGCCCATTTCCAAGCCCTGGAAAATGAATCTAGAAAAGCCAATTATCATGCTTCTTTCCCAAAAGTTCAAAGAAAAGAATTTTCAATAAATGGAAAGAATACTTAAAAAAGACTAAGTCAGAATCACCTTACCTCGATTACGTAGAAAATAAATACATGAGTCAAAAGTTAGTTACTTTAACTAAGGAAAAATGGGAAAAAGCCGACAATACCAAAGTCATCAGTACACACCTTCCAGTTGAGTCAATAATAATCAATCATAAAAATGCCAAAATCATAGCTAGCCCTTATAAAGTTGCTGAGTCAAAAGATGATTTAGATAAAAGAGTCATGGAACAAAATAATTACACCAACACGAGTCTAATAGTCATAGGTAAACAGTTAGATAAAATTGAAACTAAAATTGACAAGTTAACCCCTAGTGAAATTACTTTAAAGCCAAAGAAAGGAAGACCTTTAGTTAAGTTCAATGAGTTAAAACCCGAGTCTTCCCTTAAGGTAAATTCAAAAATGAAAAAGATTGAAGAAATGTTTCAGGAATTAACCACAGTTAAACCTAAGCCAAACAAAGACAAAGGGAAAGAAGAAGAAGAAACTTCAGTTAAAGTTTTACAGTGTGAAGTCAATTCTAATAGTGAGTCAGCCATGTTATTAGATTCAAGCCTTAGTCAAGAGTCAGAAACTAGTAATATTTCTAAAATTGAACGAGTCTTTAACAATTTACAAGTTAACCAGGATTCCCAGTGTCTTAAAGTTAGTTATTTGACAGGTAAAATTAATCCCACAAGTCTCACCAAAAATTGGTACCCTAAGCCCACAGCTCTAGATGTCCAATATGAAGAAAGAACCCAGGGTCAGTTTTCTGTATCTTCTGATAAGCTATATGAGTGGAATATTGATGG

CTTGTCCAAAAGAATCGTCTATTTCCTTTACTTGACTGACCCATTTTCTCCAATCGAAAACCAGCGTAGCACTGAAGATATTGATTTAAAAACTAGGCAGATTTTTGAAGCCGTTTTCGAAAATTTCGACAGTTGTTTTCTTCGATCTAGGTACACATCTTCATCCCCTCCACTTGCTCTAGTTGTCTATGCTGATACCTTGAATCAATTTTGAGGTTTTACCGATCGTTTAATATACGCACTTTCGCCAACGGTTTCGATTTCGATTCGGTTGCCTCAGTCTAGTTTTTGGCCCTGGTCCAAGAACAAAAGTCGATCCCCTAGTTAGCATGAATCTATTGGCATAGGTTTTGGCTAGTGGTTTGGATATTTTCTGTGGCAGAAAAATCTCTCAAGACACCCACCTTTGCCAGAGATGGGTGGCGGCGCGTGGGCCATTGTGTGGCAGCCCATTGAGCTAGTTATCGTTTCACAGTCGAAAGGATATTACACTTATTATGCGATACCCTGTTTCGATAGGTTTGGACCATTCGATCTGTCCCAAATTCAAAAATTTTGTTCCTCGTACATCTAGGATTGTGTAGGAACTGGTTGATCAAGAATCAGAGTCCCGGATACACCAAATCAATGATTCTAGGGTTAAGTGAATCAATCACTGTACGATCGTGCGATCTATAGTGATTCGACCGCTCGATTGAGATCAAGCTCTCAGAACTTGTTAATTAGACCTTGTTGGACTTATAAGAATGTTTGAATGGAAATCGGAGGTTGTGGGCCCTGTAGACCAAATAGGTCAAATTCCACGATAGGAGCCAAATCGATCGTTGGATCGTGATGAGACTTCTTCGATGAATTCAACTTATGGTTATAGAACAAGAGGAATTATCATATAACAAATTGAGGATGTGGGCCCTACTAGCCCAGTTGACTTAGATAGTGGTCTATGTCGACTCGGTTGAGGCCATTTAGTAGTTGTGATTGTAATTGGAGGTTTGTTTTA

>Chr5-19; OZ022210.1; 12660093-12662652; rev; B;

AGATTGTAACGACCCGGTCCTAAACTAATAATTAAATACTAAATTATTAAAGAAAAAGACAATCTTACCCCAGAATATTTTAATAGGTTTACATGTTGACTTGTCGACCGGGGAGACTTTGGTGAAATTGTTACATCTCGGGCTTAAAAAGTGTTAAAATATTTAAAGTAAGAGAGAATAAATTTAAAAAAGGAAATAAACTGAAATAAACATTAAAATAAAATGATTTGAATTAATTAAGGTTTTTGAGAAATTAAAATATTATTTAATATTACAAGGGGCAAAAAGGTCATTTTAAGTTTGGGAAGGAATTTTGAAAATTCCTATTGTACTACTGTGTAGGGCACATCAAAATGAGTCCGTAGACACGTACTGGGCTCAAATCGGAGCTGTAGCGAAGAAGTTACGATTAGAAGAAGTTCAGTGGCATAACTGTAAATATTTCAAAGTTCAGTTTTAAAACCCAGATTTTCTCCTTCCCCTTAGAAACTTCACGATCAGCCAACTTCTTTCCTCCACCACTGCCACCACACACCATCGTTCTTGGCGGCACCGGTCCTGCTAGAACCGCCTTTGATTCCCAGCCAAATCTTTCGAATATGGTATATTTTCCATCCTTTCAACGTGTTCTAGCTGTTGGTTGTGTTATTTTTGAGAAATTTCTGTGTTTAATTGGTTCATTAAGCGTAGGGTACTTGTCCTTGGGGGTTGCAAGATATAAATTTCTATAATTCATGCAAACCATTAGAGCTCCAGTATTTTTCAACACTGGCACAATGTTGGCCAGCCACTCAACATATCGGGCGGTATACATATAGCCGGCTTTGAGAAGATGTTTAATTTCTTCTTTGACTTTTAATTGTACCTTGGCAGAAAATCGCAGGTGGTGGTTGTTCGAAAGGTTTGAACCTGTCTTTGATTTTCAATTTGTGTTCTACTAAACCTCTTGGTAATCCAAGAAATTTATAAGCAAAAATGGTCAATGAATGCTTTCAAGACA

ACTACGCATGTTAAAACAGTAGAACAGGTCTATTCCTTAGGAAAAGAACATGAAACATGCCAATTACTTAATCTTGAGTCAATTAAGAAGCATAGAAAAGAAGGTAATAAGTTTTGCATATAGGATTAGTTCAGGTTGCAGTTAAACCCTTAAATAAGCTAGGATTAAAAGCAGCAATTCTTTTAAGTTTAAGAGACGCTAGATTTAACGATTTCTAGGGTGGAGTCTTAGGAATCATAAAGTCAAGCTTGTGCCATGGTCCAGTTCATTTTGATTGCTACCCAGATTTTACAGTCAATTTAAGTGATCCCCATATATTAAAAGTTTTAACTTTAAATATTAAAATGCATGGTTATAATATTCTACCAGGTAGTCAACCTTTAGCTTTAGTTTATAGAATTTATTACAAAGTCACAGGTACAAATATGAATTTCCAAGCCCTTAGCAAAAGCCCAAAGAATCAAACACTTTTAATTCAAAGTCATACGTCAAATGTGAATGTTACAGTACCTCAAATGATTAGATGGTCTAATGTTAAATTACCCCAGGATTGGTATTTAACAACTGAATGTCCGCCAAAGCCGATTCAACGTAGCCTAAACGATCTAAATTATATCCAGCCCTATTTAGGGCCAGTTTGGCATTGCTGTGCTGTGAAAATAATCGCTGTCAGATTTGCTGTGAGAGAAAGCAGTTTGGTGTTTGGTAAAATTTTTGTTAAAAGTGTTGTTGGTATTGATTTTATGCATAATCAGAAAATGATTGCTACATAATCATTAAACCCAGCGCCTCTTCGAAACTGATTCCTGCATAATCAGAAAATGAAAGCACCTCATTAGCTGCTTTCTTTTATAGCTTTCTCTCGCAGCAATTTTTAACAATAAGTGATTTTCTCACAGTTTACCAAACAGGTTGAGCTTCTCAAAATTTTTAAAAAATCACTTATTACTCCAAATAAGCAATGCCAAACGGGCACTTAGATGGAACGGTTAAAATTAGTTTTGATAGGCCAGTTCAATCAACAGTCCAATTAGAACAATTGCCAACCCCAGGTTAGTCTTTTAAAATTCCTGCTAGACATTCATCCGCAGAGTCATCCTCAATTACAGAACGCGATTTAGAAGCCAATAGAGTCCTAATTGATTTTAAAATTAATGAATTAAGAAAGCAAAAACAGGTAGTTCAACAAAGTTACGGAAAATTCGCAACTGAGCCCGTTTTATCAGTACAGCCTTAAGTCACCTACAGAACAGATTTTGAAAGTTCCACCTTTGAAGCCTGTGATGCCCAGTTAAGAACTCTTAATAAAATTTTTAAAATTCATGATTCAAAATTAGATGCCCATTTCCAAGCCCTGGAAAATGAATCTAGAAAAGCCAATTATCATGTTTCTTTCCCCAAAGTTCAAAGAAAAGAAATTTTCAATGAATGGAAAGAATGCTTAAAAAAGACTAAGTCAGAATCACCTTATCTCGATTACGTAGAAAGTAAATACATTAGTCAAAAGTTAGTTACTTTAACTCAGAAAAAATTTGAAAAAGCCGACAATACCAAAGTCATCAGTACACACCCTCCAGTCGAGTCAATAATAATCAATCATAAAAATGCCAAAATCATAGCTAGCCCTTATAAAGTTGCTGAGTCAAAAGATGATTTAGATAAAAGAGTCATGAAACAAAATAATTACACCAACACGAGTCTAATAGTCATAGGTAAACAATTAGATAAAATTGAAACTAAAATTGACAAGTTAACCCCCAGTGAAATTACTTTAAAGCCAAAGGAAGAAAAACCTTTAGTTAAGTTCAATGAGTTAAAACCCGAGTCTTCCCTTAAGGCAAATTCAACAATGAAAAAGATTGAAGAAATGTTTCAGGAATTAACCCCAGTTAAACCTAAGCCAAACAAAGACAAAGGGAAAGAAGAAGAAGAAGAAACTTCAGTTAAAGTTTTACAGTGTGAAGTCAATTCTGATAGTGAGTCAGCCATGTCATTAGATTCAAGCCTTAGTCAAGAGTCAGAAACTAGTAATATTTCTAAAATTGATAACAATTTACAAGTTAAGCAGGATTCCCAGTGTCTTAAAGTTAGTCGTTTGACAGGTAAAATTAATCCCACAAGTCTCACAAAAAATTGGTACCCTAAGCCTACAGCTCCAGATGTCCAATATGAAGAAGGATCCCAAAGTTAGTTTTCTGTATCTTTTGATACGCTATATGAGTGGAATATCGATGGCTTGTCCCAAAGAATCGTCTATCTCCTTTACTTGACTGACCCATTTTTTCCGATCGAAAACCAGAGTAGCGCTGAAGATATTGACTTGAAAACTGGGCAGATTTTTGAAGCCGTTTTCGGAGATTCCGACGGTTGTTTTCTTTGATCTAGGTACACATCTTCATCCCCACCACTTGCTCTAGCTATCTATGCTGATACCTTGAATCAGTTTTGAGGTTTTACCGATCATTAAATATACCCACTTTGGCCGACGGTTTCGATTTCGATTCGTCTACCTCTGTCTTGTTTTTGGCCGTGGTCCAAGAACAAAAGTCGATCCCCTAGT

TAGCATGAATCTATTGGCAAAGTGGTTTGGATATTTTCTGTGGCAGAAAAATCTCTCAAGACACCCACCTTTGCCAGTGATGGGTGGGGGCGTGTGGGCCATTGTGTGGTAGCCTGTTGAGCTAGTTATCGTTTGACAGTCGAAATGATATTACATTATTATGCGATACTTGGTTTCGATAGGTTTGGACCATTCGATCGGTCCCAATTTCAAAAATTTTGTTCCTCGTACATCTAGGATTGTGTAGGAACTAGTACATCAAGAATTAGAGTCCCAGATACACCGAATCAATGATTCTAGTGTTAAGTAAATCGATCATTGTACGATCGTGCAATCTACAGTGATCCAACCGCTCGATCGAGACCTAGCTCTCTGAACTTGTTAATTAGACCTTGTTGGACTTATAGGAATGTTTGAATCGAAAATCGGAGGTTGTGGGGCCCGTAGACCAAATAGGTCAAATTCCAGGAAAGGGGCCAAACCGATCGTTGGATCATGATGAGACTTCTTCGATGAATTCAACTTATGGTTATAGAACAATAGGAATTATCATATAACAAATTGAGGATGTGGGCCCTACGAGCCCAGTTGACTTAGATAGTGGTCGATGTCAACTCGGTTGAGGCTATTTAGTAGTTGTGATTGTAATTGGAGGTTTGTTTTAAAACGGTATTATGTTATGAGAGTTTCATTGAATGCTTATGAATTACTATACAATCGCTTTTACGTTCCAAGCATGTTATATTATGTTTAATTGTGTGGAGTTGGTAATGGCTTGGATAACTGGGTTTTAGAATGAATAATAAATTAATTAAATGCCTGATTATATGTCTATATTTAAAGATATACTCAGTGGCTTGAAATCGATGAATTATGAATACTAGGTTTCTCAATAGTATGTTGCACCTATTTGAATGTAGTTTGAGAATAGTTTTGAGAGATTTTGCATGGTCAAATTCAAATTAAGGAATGTTGGGATTGATCTCGTTTAAAAGAGGGA

>Chr5-20; OZ022210.1; 13358685-13360006; dir; B;

ACGAGAACAAAATTAATAGCATTAGTACGAATTTACATTAATGCATTTAAACTAACGACACAAAATACATACTTAATACACGTACAGAATTCACAGCAGAGCAGCAGCAGGGAGGGAGAGTGAGAGAGAGGCAGAATGCAAGAATTAACCCACTTTTCCGCCGCACAAAACAAAATTTCCGTCGCGCAATCACGTGTCGACATCTGCTTTTTTGCGCGACGAAAATAATTATTCGTCGCATAAAATTCCGTCGCGCGTATACAATATAGCATCGCGCAAAGTATACATTTCGTCGCGCAAATGTAACTACTTCGTCGCGCAAGACTGCACCGCTATTACACTTTACGCGACGACGGGATTTCCGTCGCGCAAAAACCCACCTGTATATTGGCGCCAAAAATAAGTTTCCCTCGTTTTCTTACGCGACGGTTGATTTTGTCGTCGCGCTAAGTTGTCTTTTGAGACGAAAACTCTCGTCGCGCAAAATTTTTTTACCGCCATAATAGGGGCCAATTTTCTGTCCATCTTTGCGCGACGAAGACGTGTCATTGTCGCGCAATGTGGTCATGCGCGACGACATGTGTCGTCGCGCAAGGATGTGGCGACGAAATATATTTACGTCGCGCATAGCCTCTTTCCGCGACGAAATTTTTTGTCGCGCTAGTTTTCTGTAGTATGCGCGACGGAATATTTTTTTCGTCGCATTTGTGCTCCTTGCGCGACGAAATGTCCTTCGTCGCGCAAACGTCCGTCGCGCAAATAGTAAATCGTAGTAGTGTTCTTAGGAGGGGACTACTAAACCCATGTGAAAATGAAGGATTAATTAGTCTCATGCTCACCAACCAACCCAGTCAAATTGGTCAATCAAGCCAACCCAATCAACCCCAGTCACTCCGAGTCCTCCCATCATCAATGAGTCAAGCTAAATATGAGCCTCCTTCCCAGATCGTCCCCTTTCCGGGATGCTCTCCTATCCAAATCAGTAACAAATTTGCCAACC

TAGGAGCCACAGTCGGCCAGGTTCGCCCTAGTTTCCAGTCTGCTTTAATTTCCAGTCATGATCCTTTATAGGATATTGCCGTCACTAACCCCCCAGTTGCTTCTTACCATAAGACATCTCCATACATGGTCAAAAGTCATAGTCACTTGTTTGTCATTGAGTCCAAATATAATTCAATCGCTAATCCCATCACCATTGCTAAATCCTATTTTCCACCCAATGGTCATTTCATTCCCCCAGCACCATTTAAGAATCTAAAATATTACATAGATATTCTTCATGAGACCCATTCTATCACCATTAAGCCAATTAAAGACAGAGAAAATCCCCATATCATCCTCTATCATTCACTTTATATTCACCAAATTGTCAATGAAAATGAATGGAGTAGTCACCTCTATGATCTCAAAGTTCTCCAATCATAACTTCAATATAGCTATTATGATTATATCGAAGCATGGTATTCTATCTTGCTTCACCAAACAGTTGATTTCAGTCACTCCTGGTTCTTAAATTTTGATAGCAAATTCAAGAGTCCTTTTCCTTACTGGTTCAATCACTGGTGGGAAAAACATGGTCTAGTCATTGATTTATTTCCTCATCCTATGCAAGAATTAGTCAATTATTTTACTAATAAAAATAAGTTCAAAGAATCTGAGCTGTTCTTCCCCAACCTTCTCCATTTTGTTGCCAAGTATAAAGTCCCTTGGATCCTCAAATGGTCATATCAAGTTAATTGGGAAACCAGAGTCCTATCTCGCCAGTTTTCAATCAAATGGTGGGATAAATTTAAACTTGAGCGTATTGCAAATTATGTTTATTCTGATTTTCCTCCAGTCAGGAATCCGCATTCTGAGAAAAAGAGTTCCCCTGCTACATCACATTCTTCTCTCCCAATCGAGGGAAGATCTAAGTCTGAGTTACAAGAAATTGCTCGCCAATTAATGCTCCAAGCATCCCAGATGAATGATGATGCAGATAGTGATGCCTCCCCTGCGTCACAAACATCTACTAGTCAGCCAAAGCCAATCAATCAACAGACTTCTGCCCAAAGTTGCGGTGGAGTGATTACCTAGATGAGCAAGACCCCTATGATTTCGGTCCTTTCAATCTAGGGGATGATTAAAGTAAAAGTAAAAGTTGCCGAGCATCATGAGCCCTGAAGAAAATCATCCGAACCCGTGCATTCATCAGTCTGAAAGTCGTGTTCTTTTTGAGATTCCCCGACAAAATAAAAGTTGTTGCACTCAATAAAAGCAAAAGCTTTCGCGTGCTCCTACAGTTACTTTTTCACAAGCATCCAAAGTTGCCAGCTCACCAAA

AGCTATATGAATTTGTATTATTTGTCTATTTAAGAAGGCCCCGGCCTTTGTCTTCTTCAGAATGTAATTTAGTACACTATAATTTTCTCAGTTTCAAAAGTTCTATCAGTACCAATACTCTGAAGAACTAAGGTGGTGAAACTAGGATTTTTGTAAGTGTTTTGCATTCTCCAAGTTATCAATATATTTGAGATATTTTGAGAATTAATTATTTTGATTAATGCTTATTTTCATTACAGCTATTGTCATATATATATATATATATATATATATATATTTGGGATGAGTGGCTCTCAACAAGGCCTAGGAACTGATATTATCAAATTCCAATATTTAATATTATACTGCGTCAACAGCAAAAATAAACCTACAAAATCATAAATGAAAAATCTGCAAAATAGAGAGAAGGAATTCTGATGTACACCATATGCAATTCAAGAAGCAACAGTGTCTATAATTTATAAAATTAATACATTAAAAGAGATAATTTATTTGTATGAGACATGTCGAATTGGATATCAGTTTTTAATCAAATTCAAGGTTTTGATAATTATGAAAATGATTTAATTGTGGGAGAGAAGGGGGAAATGCCGCATGGGAGAGGAAGATGTGGGTAAGTGATACAGTGCAAAAAAAGGAGTTTTAATCTAATACAATTATGATGAGGTGGAATAATTAAATTCCATTGGATTAATTAAAGAGTAATGCTACATTTATCATATTTTTATTCTACATTTCTATACCATATAATGTGGCATGTCCATATCATATGCCACATCAATTAAATCGATGAAGTGTATTTTAATAAATAAAGTTTAATTAACAGTTTTTTTAAAGTATTAATAAAAAATCATAAAAGAAAAGAAAATATTAAATAATTTTAATTGATGTGGCTATCCACCTCATCTGCCACATGAGGTGGTATGCGAATGTTGTATACAAATGTGGTAAGTGTAGCATTATTCTAATTAAGATTTTTTTAAATGCATGTAAATACACC

>Chr5-21; OZ022210.1; 14881166-14887787; rev; B;

AATCAAATAGCTAAATTTATTGTGAAGTAGTTTTTGCATGGTTGGTCTTTGAAGGAGAATTTAAAAAGATAACGGTTCGGATCAACAAATTCCAGATAAGATATTCATATCCATAGATACATATAAACTCCTCTATGATATATAAATGTCAATCATCATATTCTAAATATTGTCTTACTTATCCTGAACTAATGGAAATTATTGTGATTTCAGGCTTTTCAAGTCTGCCAAGAGCTGCAAGGTATGGCATAGTGATAGGCGTGGGAATTCCAGGGCTACTGTGCATCATAGGGCTTGCAGTTTATGTTTGTAACAAGACAAGGGTTCCTGGTCAGGTTCACCAGCCCACCATCACACAAGTGTTATCCATACCCGATCGACAACCTTCGACAGTCAGAATTGGCCTTGATGATCCGACCATCGAATCATATCCCGAGATTCAACTTGGTGAGAGCTGGGAATTGCCAAAACTCAATGACAGCACTTGCCCAATATGTTTGTCTGAGTACCAGCCCAAAGAAACACTAAGGACCATACCAGAATGCAGCCATTATTTCCATGCTAATTGCATAGATGAGTGGCTTAGAATGAATGCAACTTGCCCAGTTTGTAGAAACCCACAAAAGAGATAACAATATGTTTATGTAGTACTGGTTATTCATATATAATTGTTCCCTATGTTGAGTCCTTACTTAGTTAGGATTTTGGTTACTTACCCATTTTTCTAGTCCTATTATAATTGAGATTTATTTCCTATTGTAATTCATATTTATTTTCTATTGTAATTTGGATTTTTTTCCTATTTTATTTAGGGTTGTATTTTTTTATATGAATTCCTCCATATTGGAGAAGAATAAAGATATTTAGGGTTGTACCTTTTTTTATAAATACCTTCATATTGGAAAAGAATAAAGATATGAGCATTTATGAGCATAACTTAATATTTTTCCTAATTTGTTTGACTTATATATATATATATATATATATATATATATATATA

ACAAGTCTATGCCTTGAGTAAAGAACACGAAACATGTCAATTGCTAAACCTTGAGTCAATAAAGAAACGTAAGAGTGATGGAAACAATTTCCTTCATATTGGCCTAGTTCAAGTAGCAGTAAAACCCCTAACCAGATTAGGACTAAATGTCTCTATCTTACTTTGCCTACGTGATGCCAGATTTACCAACTTTAGTTATAGTACCCTAGGAATAATAGTCAAGCTTGTTTAATGGTCCAATTCATTTTGACTGCTACTCAGACTTCACAATAAGCCTTAGTGATCCCCACATATTAAAAACTCTAACATTAAACATTAAAACTTCAGGGTATAAAGTCTTACCAGGAACCCAACCTCTAGCCCTGGTCTATAGAATATATTATAAAGTCACAGGAACTAATATGAATTTTCAAGCCCTTAATAAAAGCCCAAAAGACCAAACCTTACTTATACAAAGTAGTAAAGCTGATGCTAACATAAGAGTCCCCCATACAATTAAATGGTCAAAAGTAACTCTACTCCTAGATTGGACACTAACAACAGAAAATCAACCCACTCCTATTCAGCATAACTTGCATAATCTGGATTATATTCAACAACAATCCTGATCTCTTGGACTACCGGGGTCCAAGAGATTTGTGGTCACTCACCGTTGGATGTAAATTCAACGGTTCACTCACTCTTGCACTCTTTTTAAGAAACCTTTTTAAACCATTAGATTAATATTCAACGGTGGGTGACCACAATCTCTTGGACTCTTGTGGTCCAAGAGATCGGGACTGTATTCAACAATATTTAGATGATACAATAAAAATAAACTTTGGAAACCAGCCCCAAAAGTCCTCCGTCCAAATACAACAACTCCCAACGCCTAGTCAATCCTTTAAACATACTCCTGCTAGACATTCCTTTGCAGCTTCCTCCTCTACTTTAGAAAGAGATCTAGAGTTAGGAAAAAAAAAAAACATTAATTGATTTAAAATTAGAATCTTTGAGGAAAACCTCTCAAGTCAACCATCCTTGTTATGGTAAAAAATCAGTAAAAGAAGATGGAAACTAATTTTGAAGCCGAAGTCATCCCAGTAGATAATCAGTTACGAACCCTCAACAAAAAGTTCAAAATACATTGAAAAATCCTAGACAAACATCTTGAGTCAGAAGAAAATATTGAAAGACGCAATAGATATAGAACAACCTATCCAAGTAAAGAACAAAGACAACAAATTTTTAATGAATGGAAAACTTACATGTTGCAAACTAAGTCTGAAGTATACTTTCTTGATTTTGTTGAAAATCTTTATCAGCAAAAGAAAGAAATAAAGACTTTAACGCAAGAAAAGTGGAAAAAAAAGACAACACAAAAGTCATAGCTAGTCATCCACCAGTAGAAACAATTATTATTAATCATAAAAATGCTAAAATCACTGCCAGTCCTTTTAAAATCCTTGAACAAAATGATAAAAAATTGGTTGGGCAAAATAATTATACAAATCAAAGTCTAATAATTTTAGGAAAACAACTAGATAAAATAGAAACAAAATTTGATAAAATCTTTACCCAAGAAGTGAACCCCAAACCCAAGAAAGAAAAACCTTTAGTCAAATTCCAAGACCTAAAACCTGGTCAAACACTTAAGACAACCCCCACAATGAATAAAATAGAAGAAATGCTTGAACAATTGACACTGGTCAAAGAAAAACCCACTAAACCAGAGTCACCCTCAACCCTTAAGGTCATCCAAGTAAACCCAATAGATACATAGTCAAGCCCTGACATTGATACTACACTGATACTGATTCTAGTAATATATCAAAAATAAAAAATACCTTTAACAATTTAAAACTCAGTAGACTAAGGAGTAAAATAAATCTCACTAGTTTAACAAAGAACTGGTACCCTAAGCCAACTCCCCCATATATCCAGTTTGAAGAAAGAAATTTTCAAAGCCAATTCTCAGTCCCTTCTGATAAACTTTATGAGTGGAATTTAGATGGCTTGTCTGAACAAGAAATTATGAATAAATTGCAGCACATGTCTATGGTCGCAAATAGTTACATCACTAATCACAACCTCAGACAATCAGAAATAGTGCCCCTCTTAGAAATAGGATTTACAGGAACTTTACGTTCTTGTGGGATAAACATCTCATAGATGAAAAAGACAAATAATACATGCAGTAAAACTAAATAAAGATGGATTCCCTATATTTGATGAACATGTCAATACGGGAATTGAAGATGGAGTCAATACCTTATTATACACAATAATTGAACATTTCATAGGTACACCTAGTAACACTACAACCAGAATTCAAGATCAGCTTAGCAACCTTAGATGTCCCAAGTTAAGTGATTTCAGATGGTAAAAAGATGTCTTTATATCCAGAGTCATGCTAAGAGATGACAATAATAAACCATTTTGGAAAGAAAAATTCATTAACGGTCTTCCCAATCTTTTCGCACACAAAATACGAACAGTTCTAAGTAATGATCAAGGTCACATAGATTTCGATAGTTTAACTTATGGAAATATTATTATTAGTACAATTAATCAAGTAGGAATGAAAATGTGTGTTGACATGAAAATAGGTAAACAAATTCAATCAGAAAGAAATTTAGCTAAATGTGAATTAGGATATTTCTGTGAACAGTATGGTTTAGCATCTATTCGTCCATCTAGAAAAGTCAAGCATAAGAATTATGACCAAAACAGTCGAACCAGACGTTACACTAGAAAACAACAATCCTTCAAAAATGATAATGAATTTTATAGTAAAAACAAGTTTAACCCAAAGAGAAAAAATTGGTCAAAAGTACCCAAAAAAGATAGAAAAGACAAATTTAAAAAGAACACCGACAAAAGAAAAGTTGAGTGCTGGAAATGTAAAAAACATGGTCATTATGCTAATGAATGTAAAGTAAAAGACACAATCAAACAATTAAAAATCACAGACGAAGAAAAAGCCCAATTGATTAAAGTTTTAGAATTAAGAAACTCTAAGTCTAGTGAAATTAAGACCCTAGTAACAAGCTCAGTAGACTATAGTTCCACAGATTTCCAATCAAGTTCCCCCAAAATCCAATTCGGTTGTAAAGATAATTGTTGTAATGTCTTAACTAAGTCAATAAAAGTCCTTACAAAACAAGAAGAGCAAGAAGAATTATTAATTGAATTAATTAGTAAAGTAGAAAATTCATAGTTAAAATCAGAATATTTAAAGAAATTAAGAAAAGTCATTGGTCAAGAAGAAACGAGTCAACACATCCCCCCGAAAATAAGTCTAAATACTACTTTAGAAAAATTCAACAAAAGAAAAGAAGTCACTTTACAAGATTTACAACTTGAAGTAAAAACCATAAAAAAAAGGAAATCGCTGAGTTAAAACAGTTCAACCAACAATTACAAAGTGATAACATTGAAATCAAACAAGACCTGATCAGTCTACTAGAAAAAATAGAACCTAACAACAACGGTAAAAGACAGCTTGAAGTCCATTCTGAAGATGAAGATGAACATTCAAGTACTGACAAAGCTATTAATTTAATCAAACAATTAAGTTTCCATAAATGGTATTCAAAAGTCAGTATTTATATCAACAATTTTGAACTAAAAACAATAGCTCTCTTTGACTCAGGAGCCGACCTTAATTGCATTCAAGAAGGATTAATTCCCATTAAATATTACAGGAAGTCAAAAGAATCTTTAGGAACAGCCTCTGGTAAACCACTGCACCTTAAATATGAAATCCCTAAAGCCCACTTATGCCAAAATAAAATATGCTTTAAGACTTCCTTTGTCCTAGTCAAAGACATAACTGATGAAGTCATTTTAGGATTACCATTTATTGCCTTACTCTACCCTTTTAATGTAGAATATGACGGAGTCACCGCTATCCAAATGTGAGAAAAGGTCAAATTCGAATTCTTAGCCAAGCCAGAATTACAAGCGTTAAAAGCCCTCCATAAAAATGCAGTCTCCAAATCAGTCAAATTAATTCAAATTAAAAGCAGACAAGTTAACTTCCTCAAAGAAGAAATCAACTTTAATAGAATTGAACAACAGCTTGCAAATGAAAACTTGCAACTACAAGTCAAAAGTTTTGAAGAAAAGTTAAAACAATAAATTTGTTCTGACCTTTTGGCATAGAAAACAACATGTCGTGAGTCTTCCTTACGTCAAAACCTTTAATGAGAAACACATCCCTACTAAAGCAAGACCAATCCAAATGAGTCAAGAAGTCATGGAATTTTGCAAAAAGGAAATTGCATAGCTACTTCACAAAGGAATAATATGTAAAAGCAAGTCACCATGGTCCTGCCCAGCCTTTTATGTTCAAAAAAATGCTGAGCTTGAAAGCGGAGCACCTAGATTAGTCATTAATTATAAACCCCTTAATATTGTTTTATAATGGATTTTATATCCAATTCCTAATAAAAGAGATTTAATTAATAGGCTAAGTAGTGCAGTCATCTTTTCTAAATTTGACATGAAAATCGGATTTTGGCAGATACAAATCTAAGAAAACAATAGGTACAAAGCTGTTTTTGTCACCCAATTTGGTCATTATGAATGGAATGTCATGCCTTTTGGCTTAAAAAATGCCCCTAGTGAGTTTCAAAATATAATGAACGAGATTTTAATCCTTTTAGTGAATTTTCCATAGTCTATATTGATGATATTCTTATATTCTCTTCCTCAATATAACAACATTGGAAACACTTACACAAATTCCTACATATAGTCACGAAAAATGGTCTAGTACTTTCAGCCAAGAAAATTATGTTATTTCAAACCAACATCAGATTTCTTGGATTCAATATTCATTAGTCACGGATCAGTCCAATTGATAGAGTCATCCAATTTGCTGACAAATTCCTTGATAAAATTCTTGATAAAAGTCAACTCCAAAGATTCTTAGGTTCTCTCAATTGCGTTGCTGATTTCTATCATAATCTAAGAAAACAATGTAAATCCTTATTTGATAGACTTCAAAGTAATCATCCTCCCTGGACCCCCATTCATACAGAAATTGTCAAACAAATCAAAATCCATGTCAAGACTTTGCCTTGTCTTGGTATCCCTTCGGTCAATTCTTTCAAAATTGTTGAAACTGATGCCTCTGAAATTGGTTATGGAGGTATCCTCAAACAAAGAGTCTCTCCCAATCAAGCAGAGCAAATAGTTAGATTCCATTATGGAATCTGGACCCAGTCACAAAGTAATTATAGTACTATAAAAAAGGAAATTTTATCTATTGTATTATGCATCTCAAATTTTTAAGATGATTTACTTAACCAATAGTTTAGTCAGAGTTGATTGTAAAAGTGCAAAACATGTTTTAGAAAAAGATGTCCAAAATATTGCATCAAAACAAATTTTTGCACGGTGGCAAGCAATTTTGACTGTTTTTTATTTTGATATTGAATATTTGAGAGGAAGTGAAAATAACATTCCCCTCTTTCTCACCCGGGAATTCTTACAGGGAAAACAATGGCTGAAAAAAGATCAGACAAACAAAAAGAACAAGCCAGTCAACCAGTCAGAACACTCCCAACATCCATGAATCCAGTCAAATCCAAGATTGTCCCTTTTCCCGGATGTTCTCCCATCCACATTAGTAACAGATTTACCAGTCTTGGTTCCACGGTCAGCCATGTCCGCCCCAATTATCAGTCAGCCCTGGTATCTAATTTGATCACTTTCAGGTCTCAACCCCTCCTCCATTAACTCAGTCAACCATAAGCCACAGAAAATCCTCTTCCTACGTCCCAAAGTTTACTAACAAACTCTTCATCATCGAGTCATGCTACAATAATCTTTCCAGTCCTACAAAAATTGCCAAAGCTTGTTTTCCTCCTAATTTCCACTTCATCCTTCAGCACCAGTCAAAATCACTTAAATACTATCGAGACATTCTTCTCGAAACCCAAACCCAAACCCTTGAAATCTGCCCTATCAAAGACAGAAATAACTCGAAAATCATCCTCTATCATTCTATGTACATCCACAAGATCATTAGTCAATTTGAATGAGGCGACCACCCTTATTCACTAAAAACCCTTAAATCAACCCTTCAATACAGTTACTATGATCATATTAAAGCCTGGGATGCCATCTTTCTTCATCAAACTGAAGATTTCAATCATTCCTGGTTTGTAAATTTTGACAGTAAATTCTGAAGCCCCTTTCCACTTTGGTTTCTTTCCTGGTGGGAACAACATGGTCTAGTCATGGAAATTCTTCCCACAGACCTCCAAAATTCAGTCGCTTATTTTTCCACAAAATACAAATTCAGAAATGAAGACACTTTCTTCCCAAGTATTTTAATTTTTGTTGCAAAATACAAAGTTCCTTGGATTCTTATGTGGTCTTATCAAGTTAATTGGGAATCAAGAATCCTTTCCAGAAAAATCTCAGTAAAATGGTAGGATAAGTTCAAGTCAGAAAGAATTATTGAACAAGTCAATACTGAATTCTGCCCAGTTAAGATCTCCCAGTCAAAGCCCTCTTGTTCTGGCACGTCTCATTCTTCCCTCCCAATTGAAGGAAGATCCAAGTCAGAAATAAAAGAATTAGCCAAACAATTAATGATTCAAGCCTCTCAGATGGATGATGATGATGA

TGTTTCCCCAACGTCACATTCCACTCAAGCCTCCTCCTCCAGTCAAACAAAAAATCTTTCAAGCAGTCAAAAGGATTCCACGCAGAAATTCAGATGGGCCAACTATCCGGATAGTCAAGACCCTTATGATTTGAATGAAGACTAAAATAAAAAATTGCATAATGCCTCCAAGACATGTTCCCCTACCGTCAATATTCCTTGATAAAGGAAAAAGCAAAAGCACATGTGAAAGCAAGAAAGCAAAAGTAAAAGCATAAGCAAAAGGGCCAGAAAACCCGTGCTTCAACAGTCAAGATTCTCCACCGACACTTTTGAGTCTTTAAATAGAGGCCCATTTGGCCATAGTTAGGAAGAATGTAATTTTGAGTTTTCTCAGTTTTAAAGTTTTCTCTGAATCACCTTCAAAGAAAACTCTACTTGTAAGTTCATTTTTAGTTAAGATTTTCTATAAGTTCATTTTCTGTAAGTTCTCTCATTTAAGTATTACTTTCATTTACGCATTATTTTCCTGGCATTGGCACCCTCCTGGTGTGTATATATATATATATATACACACATATACAGTCCCCTTCTATAGAGGGACACCCTTTAGGGAACCCTATGAACTTTTTTCCAACTATCCAAACCATTTATTTTTTTAAGTCTACAATCATAGATCATCTTTGCAAAAAATTAGACAAATCGGAAACCCTTTCGACATCCAATTGTATCTTACAAAATTAATGAACACGATGCTTCAAGAAAGTACTAAAATTTCAATAACATAATTGAGTGATCAAACGATATCGGATTCAAGTATTTTTTGTAGAGATGATCTTTGAATGATTATATAAAAAATAGATGGTTTAGATTAGTAGAATACAATGTGGAGTAAGGCCCTACAAGAGTGTCCCTCAAATAAGCTTATTTAAAGAATCCCTATATATATCCCTTATATTTTTGAGGCCTGGGGCGAGACCAAAGAAAAGGCCTTGTATATACAATAATATTTGTTTGTGTG

>Chr5-22; OZ022210.1; 19140260-19147945; rev; A;

TGGCACTTCATGGAGTCTCAAATAATGGGAGCAACAATCCTGCTGCTACTTGGGTTCTGCTGACCAATGTTATCTAATCTGTTGTAAAAGAATAAAAAGATTATTACCGTGACATATCTTCTGTAGAATAAATTCATTTTTTTTTCTTCTTCTTCTTCTTTTTGACATCTCAAAAAGAAGACTTACAAGTAAACATAATTGGAAAAAAAACTAAAAAATCAAAAGCAAATGTTGTCCTTACAAGAATAAGCAAATGGTTTAAACTATTATTATCATCCCACCAAACATACGTGGTAAATGAGGAGGAGCCCATGCTACATGGTTGAAAACAGAAATAATTGCATAAATACCTTTCTTTATCTTTTTCCTTGGCTAACCACTTAAAAAATCCAACATAAAGAAGGCTGTCATTTCACTAAATCAAAAGCAAGTAGGCATCACATGCGGTGTTTTTAATTTATATGAATGATACATGCCAAAGTGAAAGAAATATATTATTCACATGTGACACCCAATCACAATCATGCTATAGTAACACTGCCTCCAGATCAAAACTCTCTCTCTCTCTCTCTCTCTCTCTCTCTCTCTCTCTTCAGCAAGCACAAAACTGAAACCAAGTCTCCTCCTCCATACTCCTTAGCAAATTCCACACAGAACCTCAAAACTCTCAACCATCATGCCTAACGATCAACTTGAGGTACGTAATTTATACATTTCAATACATGAATTCTTTTTCTTTCATTTTTGAAATTTCTTGCTTACTAAAAGGAATATTGATGTTGTATTTTGCAAATGAAGGCCATGAGAAAGAAAAAGAAAAAGAAGAGGCATGGTATCGTGGGTTAGTAGAAAGTTGGAATGGGAGGAATCGGTAAAAAGAGGTATAAATTTTTACATCTGTTGTTTGTTGCTTTGTGGTATTACACTTTTGGAATGTGTTTTTTTCCATTTTAACCTAAATTATGTTTGAAACTCATATATATATATATATATATATATA

TCCTTTGTGAGTATAAATAAAAGTTATTTTCAAATTATCTATTTTTATTTTGATGCATTATCATTATTTTGCTGCATTAGTCTGAAAGATGTTTAGGTTGCATCCAAATCTCCTCCTGTATTCTATTCTGCTGTGTTTTATTCAGTCTGTTCTTTAGATTGTCTCTGTTGTTCCTTATTTGGTATCAGAGCCGTAAGAGTTACGGTCTTTGTCTAAGTTTGCTACTCATCCCCGCCCAAGTGGCTGGTTTAAGCTTCTAAGTAGAAGGTTTTCATTTCTTCCTTTTCTTCCCTTTTCTCCTTTGAGTCATCTGTTGCTCTTTCTTTAAGGTCTGACCCGTGAGTCATTTTTCCTAGATCTGTAAGTCCCCAGGGAGGGCGTGAGTGGTGGGCAGGAGACAGAAAATTGAGAAAGATTAATAGGTTTCTTAGAGTTTAAACGGTCGGTTTAGGATCTACTGTTTATCTTCTTCCTCAGACAGCCAAACCCGGTTATTTGAGTAGTCATGAGTCGTCTGTTTAGAACAAAGTCTTGTAATTCCAACAGCTCTACTACCAGAATAAGTCGACTCCTAGAGATAGTTAATGAAGAACAATTTGAATATAATTCCAGTAGTAAAGAACAATTGGATTTTAGAGATTGGAATATCCCTAAAGTCCCCAGTCAAAAAATATATAAGAAACACTGGTTGCCTTCTAGTTTTAATAGCACAACACATGTCAAAACAGTTGAACAAGTATACGCTTTAAGTAAAGAACATGAAACTTGTCAGTTACTTAATCTTGAGTCAATCGCAAAACATAAGAAAGATGGTAATAATTTCCTGCATATTGGTTTAGTTCAAGTTGCTGTTAAACCCTTAATACGTTTAGGGTTAAAAGGCTCAATCCTTCTGTGTCTGCGAGATGCTAGATTCACTGAATTTAGCGACAGCACCTTAGGAGTCATTGAGTCCAGTCTGTGCAATGGCCCTGTCCATTTTGACTATTATCCTGATTTCACTGTAAGTCTAAGTGACCCCCATATCCTGAGAACTTTAACTCTGAATATTAAGACTGAAGGTTATAATGTTCTTCCGGGAACCCAACCTTTAGCTTTAGTATACAGAATTTATTATAAAGTTACAGGAACAAACATGAACTTCCAAGCCCTCAACAAAAGCCCAAAGGATCAAACTGTCCTGATCCAGAGTCACACCTCAGACGCCCATATACAGGTTCCTCACACCATCAAGTGGTCCGAAGTTGCCCTTCCGAAAGATTGGACCTTAGTTACCGAGAGCCAACCGGCTCCAATCCAACGTAGTCTAAACAACTTAGACTACATCCAGCAATACCTAGATGGCACAGTTAAAATCAAATTTGACAGTCATCCCCTACGCAAGTCCAATGTCCAGTTACAACAGCTGCCAACTCCTGGTCAATCTTAAAGGCATGTCCCTGCTAGGCATTCCTTTGCTGCCTCTTCTTCCACTCTGGAACGAGACCTAGAGTTAGAAAAAGCTATGATCGATTTCAAACTTGAGTCCCTGAGAAAAACCTCTCAGGTTACCCAACCGTGCTACGGTACGGTTCCAGTCCAAGACGATAAATCAGAATCTCCTGAGTCACCTACCCAGTCTGATTTCCTAGTTGATAATCAACTCAGAACCCTAAATAAGAAATTTGACATTAATTGGGTATATCTAAATCGCCATTTAAAAGCCCCAGAAAATCAACCTAGAAGAGATAGGTATCACCTTGCCTACCCTGATAGTAAACGCCGTGAACAAATCTTTAGTGAATGGAAAGATTACATGCGGTCAGCCAGAGTTGAAATTTTCTATCTTGATTTTATTGAAAGCCGTTATAAGTCTAATGACGAGTTGAAAACTTTAACCAAAGAAAAGTGGAATTTAATCAACAAAACTGTAATTGAAACCAGTCATCCTCCAGTAGACACCATAATCATAGATCACAAGAATTCCCTAGTACCTGCCACCCCTTTCAAAACCTTTGAAAGCAGTGACCCAAATAGAAAATTAATTGAGCAAAATAATTACACCAATCAGAGTCTAATCACTATAGGAAAACAACTAGATGCAATTGAAACCAAAGTTGACAAGATAAGTCCACCAAAAACAGATTTCAAGCCCAGACCCAAAGTTGAAAAACCAATAGTTCAGTTTCAAGAGCTTAAGTCCAGTCCCGTACTTAAAGTCAATTCTACAATGAAAAAAATAGAAGAAATGCTAGAACAGTTGACCCCGGCTAAGTCAGAGAAAGCCGAAAAATCTGGTATTAAAACACTAGATTCTTTTGCTGCTCCACAATCTGAGTCCGAGTCAGTAAGTTCTGAAACTAGTGATATTTCAAAAATTGAAAATGCCTTCAAAAATTTGGAAGTCCACAATGAACCTAGAGTCAAAAGATTAGACAAACATGTAAGCCCTACCAGCTTAACAAAAAATTGGTATCCCAGACCAACACCACCAGACATCCAATTTGAAGAAAGAAACTTTCAAACCCAGTTTTCAGTTTCTTCTGATAAATTGTATGAATGGAATATTGATGGTCTTTCTGAGCAAGAAATCTTGAATAAGTTGCAACATATGTCCATGGTAGCCAATAGTTATATCACCAATCACAGTTTCAGACAATTTGAAATTGTTCCTCTGTTAGTCACCGGTTTCACTGGTACTCTCAGATATTGGTGGGATAAACATCTCACCCCTGAGTCTAAAAATAAGATCACTTATGCTGTAAAACTGAATGAAGACGGTCTCCCTATATTTGACGAACAAATAGGTCAAGGTATAGAAGACGGTGTCAACACTCTGTTTTATACAATAATTGAACACTTCATTGGAACCCCTAGTAATACCACTACTAGGATATATGATCAGTTAAGTAACCTTAGATGCCCAAAGTTATCGGATTTCAGATGGTACAAAGATGTCTTCCTTTCCAGAGTCATGCTTAGGGACGATAGCAACCAACCCTTCTGGAAAGAAAAGTTCATTAATGGTCTGCCAAACTTATTTGCCCATAAAATCCGCACAACCCTGAGTAATGAACAAGGTCAAATATATTGGAATAATTTGACTTATGGAAACATTATAAGTACAATTAATCAAGTCGGTATGAAAATGTGTATTGACTTTAAAATCAATCGACAAATCCAGTCTGATAGAAAGTCAGTCAAATATGAATCAGGAAATTTCTGTGAACAATACGGTCTTACCAGTATTCCTCCTTCTGGGAAAAACGAGCCCAGTCATCTTAGGAAACGTCATTTTCCGAAAAAACAGTTCTTTCGGAAAAATGAATTCTATAAGAAGAGCAAGTTCTCCCCTAAAAAGAATTGGTCTAAACACCCTGAACGAAGACAGAGTCGCCCTAAGAAAGATAAAAGCAAAGTTAAATGTTTTAAATGTCAAAAGTTCGGTCATTATGCTTCTGATTGCAAAGTAAAAGACGTTATCAGACAATTGCAAATTACAGAAGAAGAAAAGAACAAGTTAATCCAAGTTTTAGAGTTGCGAGACTCTGATATGAGTAATGCTGAATCCTTAATATCTAGTTCCGAGTCTGAATCTATCCAGTCTTCAGATTCACAACCTAGTTTTCCCAGAGTCCAATTTGGTTGTACTGACAAATGTTGTAATAGATTAAAATCTGTATTAGTTCTCACTAAACAGGAAGAACAAGAAGAACTTTTAATAGACTTAATAGGAAGAGTTCAAAACCTTGAGTTGAAATATGAATATTTGAAAAAGTTGAAAAAAGTTCTTAACCAAGAAAGTTCAAGTTCAAACTCTCCTACTCCCCAGCCGATTAGCATCCACACCACATTAGAAAAATTTAACCTTAGAAAAGAAGTCACCCTCCAAGACCTCCAGTTAGAAGTCAAGAAGGTTAAACAAGAATTAATTGAGTTAAAAGAAGTAAGTCAACAATTGCAAGCCGAGAATTATACAATCAAACAAGATCTATTTAATCTTTCCAAACAGGAACGTAGTACACCTAAAAGCCAGTCAGAAAGCCCTCACCATTCTGATGAAGAGCCTTCTGCAGATCAACTAGTTGTCAATTTAATTAAACAAGTCCATCTCAGAAAATGGTATTCAGAAGTAACAATATTTGTTCAAGATTTTGAATTTACCACAATTGCTTTATTTGATTTTGGAGCCGACCTTAATTGCATTCAGGAAGGTCTTATCCCCACTAAGTACTATCAGAAGTCCAGAGAATCCCTTAGCACTGCCTCAGGGAAATCCCTTCAGTTAAATTTTGAATTACCAAAAGCCCATATTTGCTAGAACAAAGTCTGTTTTAAAACCTCATTTGTCCTAGTCAAAAACATTACTGACGAAGTCCTCTTAGGTCTGCCTTTCCTCGCCTTATTATATCCTTTCCAGGTAGCATATAATGGTATCATTTCTACCTATCTTGGCGAAAAGGTTAAGTTTGAATTCTTGTCAAAACCAGAATTGCATAACTTAAAAGCTTTACAGAAACAATCAGTCTCTAAGTCTATTCAAATTATCTGCAAAACTAACCAGTTGAATTTCCTTAAAGAAGAAATCAGGTTTAAAAGAATTGAACAACAACTTGCTGATAAAAACTTACAGTTGCAAATGCAATCTTTTGAAAACAAGTTGAAAAATGAAATTTGTTCTGAATTGCCTACTACTTTTTGGTATAGAAAACAGCATGTCGTCTGTCTTCCTTATATCAAAACCTTTAGTGAGAAAAATACCCCACTAAATCACGGCCAATCCAGATGAATCAAGAATTAATGGAAGTCTGTAAAAAAGAAATCCTTGAGTTGCTCCAAAAGAATATTATTCGCAGAAGTAAATCTCCTTGGTCATGTCCTGCTTTTTATGTCCAGAAAAATGCTGAATTAGAAAGAGGAGTCCCCAGATTAGTAATCAATTATAAACCCCTTAATTCGGTCTTAGAATGGATTAGGTATCCTATACCTAATAAAGAGACCTCATTAATCGGTTAGAGAAGGCAGTTGTCTATTCCAAATTTGATATGAAAAGTGGTTTCTGGCAAATCCAAATTGATGAGTCGGATAGGTATAAGACTGCATTTGTCACCCCTTTTGGTCATTATGAATGGAATGTCATGTCATTTGGTTTGAAAAATGCTCCTAGTGAGTTCCAGAATATTATGAATGATATCTTCAATCCTTACAGTCATTTTTCGATTGTTTATATTGATGACGTTCTAATATTCTCTGAGTCAGTTGAACAGCATTGGAAGCATTTATACAAATTCCTTCATATAGCTAAACAGAATGGTCTAGTTGTTTATGCCAAGAAAATTAAGTTATTTCTGCCAAGAAAATTAAGTTATTTCAAACCAACATAAGGTTTCTTGGTTTCAACATCAGTCAGTCTCAAATTTCCCCAATTGATCGAGTCATTCAATTTGCTGATAAATTCCCTGATCAGATTCTTGATAAAAGTCAACTCCAGAGGTTTTTAGGATCTCTAAACTATGTCTTTGACTTCTACCAGAATCTGCGAAAGCAATGTAAGCCTTTGTTTGATAGATTACAGAATAATCCTTCCCCATGGTCAAACGTCCATACCGAAGTTGTCAAGCAGATTAAGACCCATGTCAAGACACTCCCTTGTCTTGGTATTCCTTCAGTTGATTCCTTCAAAATAGTTGAAACAGATGCCTCTGACATTGGTTATGGTGGCATTCTCAAACAGAAAGTTAGCCCAGAATCTCCAGAACAAATTGTTCGCTTCCATTCTGGAACCTGGACTCAGTCGCAATATAATTATAGTACTATTAAAAAAGAAATATTAGCTATTGTATTATGCATTAGTAAATTTCAAGATGATCTGCTTAATCAAAAGTTTTTAATACGAGTTGATTGCAAATCTGCAAAACATGTTTTAGAAAAAGATGTTCATAACATTGCATCTAAACAGATTTTTGCACGATGGCAAAGTATATTAAGTATATTTGATTTTGACATTGAATATTTGAGAGGTAGTGAGAATTCAGTCCCTGATTTTCTTACTTGAGAATTTTTGCAGGAAACACAATGGCCCCCAAAAAAGACAAAAACAAAGAACCTGCCAGCCAGCCTAAGCCATCTCAGTCGCAACCTAAGCCATCCCAGTTGCAGCATTCCCAGTCCTCATCCATGAGTCCCAATAAGCCAGAAATGCCCAAACAGATAGTTCCCTATCCTGGTTGCACCCCTATTTCAGTTGCTACCCCATTATCAGTTGCAAACAGATTTTCCTCTTTAGGATCCACTATTGGTCAAATTCGTCCAAGTTACCAATCTACATTAGTCGCCAGCTATGATCCTTTTTCAGTTGATGTCCCAGCCGGACCCTCAGTTGCACCCAAAAAGACATCCCCTTATTTGCCTAAGAGTCAGTCTCACCTGTTTGTCATTGAGCCGTTGTATGATGTTCATCACAACCCAGTTGAAATTGTCAAACACTATTTCCCCCCTGGATTCCATTATATTCCTTCCAGTCCTTACAAGTCTCCCAAGTATTATAGAGATATATTACTTGAAACTAAGTCAGTTGAAATCAAGCCGATAAAGGATCGTGATCACCCTGAGATCATTCTTTATCATTCTTTGTATATCCACCAGATCATGAGCCAAGAAGCCTTCAGTAGTCGCCCTTATGAGTTAAAAGTCCTTCAGTCAAAATTGCAGTATAATTATTCTGATTATATTGATGCCTAGTACACTATTTTCCTTCACCAATCTGAGGATTTTAGTCATTCCTGGTTCATCAATTTTGATAATAAGTTCAAATGTTCTTTCCCATATTGGTTCCTCCATTGGTGGGAAAAACATGGTCCAGTTGATGAAATTTTGCCCATTTCAGTCCATGAGCTAATCCGCCACTATACTAAAACGGCCAAGTTCTCCAAAGTTGATTTATTTTTCCCAAAACAGTTGCTTTTTATTGCCATGTATAAGGTTCCATGGATCCTCAAATGGTCCTACAGAATTACCAAAGACGCCCGAATCTTTGCTCGCCAATTTTCAGTCAAATGGTGGGACAAATTCGAAGTCCAACGAATTGCCAAGTATGTCTACAATGATCTCCCGATTGAGCCCCCGACAACTTCTCCTACTGGTTCTTTCGATCTTCCCTCTCAGTCGAAGGAAAGTCCAAATCAGAGCTCCAAGAAATTTCCCGCCAGTTGATCATTCAAGCCTCCCAGATGGATGATGATGATGAAGACAATGTTTCTCCCGCATCGTCCAGCTGTCAGCCCTTGCAAGATCCAGTCAAAACCAAATCATGGTATGAGGATAGTCAAGACCCCTATGATGCCTATGATTTGCATTCTGACTAGGCCTTGCAATCAACCAGTTGTCAAGAAGTCACAAGTCGCCTTAATTCCCGCTGGAAATTGCATCTGCCCTTTGCTTTTCCACCGAAAGTTGCCATCAGATCAACCTGCCTGCCAGTTGTCCTCATAATTGCCTCCGCCAAAGCATCTGCCTTTACTTTTCACCGAAAGACACTAACAGACAAAATTGAAAGTTGAATCAGGTTGCATGTGACTCCATTATTGTAACCAACCACAGTTAGCTGACCACAGTTGAAAGAACTCTAGTTACGTTTCCCTTTATAAGCAGGCCTCGGCCTCAGTTGTAGATAGAATGTAATTTTCCTTTCTTCAGTTTTAAAAGATT

ATGTAAGAGCCGTAAATAAAAGTTATTTACAATTGTGTATATATATATATATATATATTCTCAGCTCTTGGAAAAACAATTGTAGATGAAAGTGATGCATCACTTGAAGAAAATGCAAAAACTATGATTTTTCTTCACCGTCACATTCATGAAGCGCTGAAAACAAGTTAGTGTCTGAGTACAACTCAGCAATGTACAGAATCACCTCCCTGATGAAATTATGTGGTGAAAATATCCCTGAGGAGGATATGCTTGAAAAAACTCTGAGTACCTTTCATGCCTCAAATGTGCTCCTTCAGGTGCAGCAGCAATATAGACAGTGGTTTTACGAAATACGTAGAACTGGTATCTTACATTTTGTTGGCTGAGTAGAACAATGAGCTTTTATTGAAAAATCACCAATCTTGCCCAATAGACTCAGCACCATTTCCTGAAGTAAATGATGCATCTTAGGAAGTGAATGCCACCTCATCTCGTGGCAGTTCCAACAGACGTGGGCGAGGAGGCAAGCGTGGCCACTGACAAGGGAATAGAGAAGATCGCGGTACCCATTCATATGGCTTGGGTCCAAGGAGTAATCCATCCACGAGGGGCAAAAATGCATCCCGGAATAAGGGAAAAACGTAGACAAGCCATGTGCCTAGGTCATATGTTCAAACCCCACTTTCGTCAGTATTATTTGTATAAAAAAATCATACAAATACTCATAAAATACATACTAAAAAAGTCCTATCGTATGTTGACAATTGACCGTAACCCAGTGTGTTGTTGGTCAATAAACTTATCAATGCTTTTATCTGGTCAATAAACTCGACTGCTTTCATTATTGAATGCTCTAAGAAACATATCATTGCTTTTATCTGGTCAATAAACTTGACCCTGTATGTAGTTGGTAGAGATTTGGAAGATAGAATTGATTTCATTATAATATGATAATATGATATTCTAATTGTTAGGAACAAGAAAATTGTTTAACCATTGGAGTCTGTAAACTACAATC

>Chr5-23; OZ022210.1; 19335522-19338224; rev; B;

TGGTTGAAAACAGAAATAATTGCATAAATAAATTGAATACACGTACCTGAACTAAATAGATGCAAAGAGAGCTAGCAGCAGATATAGAGAGAGAAACTTGCCAAAAGCAAAGGGCACTTGGTTTGTTTTTTTGTTTGGAATTGGTCACTACTGCCATGAGTGATGGATGGGAACAACCAATAAATCCAACATAAAGAAGGCTGCCATTTCACTAAATCAAAAGCAAGCAGGCATCACATGCGGTGTTTTTAATTTCTATGAATGCTACATGCCAAAGTGAAAGAAATATATTATTCACATGTGACACCCAATCACAGTCATGCTATACTAACAAGACCCAAGGCACCTCAATAATTATATATTCAACCCTCACCCACCCCTACTTCCCCACTGCCTCCAGATCAAAACTCTCTCTCTCTCTCTCTCTCTCTCTCTCTCTCTCTAGCAAGCACAAAACTGAAACCAAGTCTCCTCCTCCCTACCCCTTAGCAAATTCCACACAACACCTCAAAACTCTCAACCATCATGCCTAACGATCAACTTGAGGTACATAATTTATACATTAATTTCAATACATGAATTTTCTTCTTTCATTTTTGACATTTCTTGCTACGAAAAGGAATATTGATGTTGTATTTTGCAGATGAAGATCATGAAAAAGAAAAAGAAAAAAAAGAGGCGTGGTATCGTGGGTTAGCATAAAGTTGGAATGGGAGGAATCGGTAAAAAGAGGTATAAATTTTTTACATCTCTCGTTTATTGCTTTGTGATATTACACTTTGAAACTCAGATATATATATATATATATATATATATATATATATATATATATATATATACAGTCCCGATCTCTTGGACCACAGGAGTCCAAGAGATTGTGGTCACCCACCGTTGGATATTAATCCAATGGTTCAAAATGATATATATAAATGCAATATGACATAAATGATTATTACCCGATTCAAGTCAACCGTTGAATTTGGTTCATTAATTTTGATAA

CAAATTCATGAGTCTGATCGTTATAAAACTGCTTTTGTTACTCCCTTTGGTCATTATGAATGGAATGTTATGCCTTTTGGTCTTAAGAATGCTCCTAGTGAATTCCAAAATATTATGAATGAAATTTTCAATACTTATAGTCATTTCTCTATTGTCTACATCGATGATGTTTTAATTTTTTCTGAGTCTATAGAACAACACTGGAAACATTTGCATAAATTTCTGCATATCATTAAGACTAATGGATTAGTTGTCTCTGCTAAGAAACTTAAATTATTTCAGACTAATATTAGGTTTCTAGGATACAATATAAGTCAGTCATAAATTCACCCTATAGATCGAGTTATCCATTTTGCAGATAAGTTCCCTGATCAAATTACAGATAAAAATCAATTACAGAGATTTTTAGGTTCTCTTAATTATGTTTCTGATTTTTATAAAAATTTGAGAAAACAATGTAAACCCTTATTTGATAGATTGCAGACTAATCCTCCTCCTTGGTCGTCCATCCATACAGATATTGTCTTACAGATTAAACGTCATGTTAAGACTCTTCCTTGTCTTGGCATTCCTACTTCTACTTCTTTTAAAATTGTTGAGACTGATGCTTCTGATATAGGTTATGGTGGTATTCTTAAACAGAAATCTGAGTCTGATTCTTCTGAGCAAATAGTTCGTTTTCATTCTGGTATCTGGTCTTCCTCCCAGTTTAATTACAGTACTATTAAAAAAGAAATATTAGCTATTGTATTATGCATTACTAAATTCCAAGATGATTTATTAAATCAAAAGTTTTTAGTCCGAGTTGATTGCAAATCTGCCAAGCATGTTTTAGAAAAAGATGTTCAAAATATTGCATCAAAACAGATTTTTGCACGTTGGCAAGCTCTTTTAAGTATTTTTGATTTCGATATTGAATATTTGAGAGGTAGTGAGAATTCTATTCCTGATTTTCTTACTCGAGAATTTTTACAGGATAAGCAATGGCTGGATCATCCAAAGACAAGCAAAAAATGAGTCATAATCCTCCTCCTAGAATTCTTCCTGCAACCCTGAGCCAAAAGCCTTCTCAATTCATAAAAAATGAGCCTGGATCTTTTTCTGTTAAATCTGAGCCCTTTACAATTACTCCCTATCCTGGTCATACACCCATACCAGTCATGAGCAATAGATTTAGCCCCTTAGGATCTACAGTCACCCCTGCAAGACCCAACTACCAGTCTGCACTAGTTTCTAGTTATGATCCGTTTCAATTACCTCCTACTGCATCGCCTATATCCTCTCCGTCTTTTGCCAAATCTTCTCCTTACGTCTCAAAAAATACATCTAATCTTGTCATTATTGATTCTCATATCCCTACATCCATGAGTCCTGCCCAAATTGCAGCTTATCATTTTCCTCCTGGTTTTCATTATCTTCCCCATAGCCCTTACAAGTCCTTAAAATTCTACAGAGAAATTCTTCATGAAACCAAGTCTGTTGATATTAAGCCTATTCGAGACAAAACTGATCCTACAAAAATTATTTATCACTCCCTTTATATTCACCAAATTTTACATCCTAAGTCTTGGGGATCTCGTCCTCATGAGCTCCGTACCCTTCCTTCTAAATATCAATATAATTATGCAGACTATGTTGAAGCCTGGTACTACATCTTCTTACATCAAACCCCTGAGTTTTCCCACTCTTGGTTCATTAATTTTGATAACAAATTTAAAAATACTGAGCTTCCTTATTGGTTCCTTCATTGGTGGGACACCCATGGTCCCAAATCTGATATCATCCCTTCTGCAGTTCATGAGTTAATCACTTATTTTACCCAAAAATACAAAATTTCTGAGCGAGATCTATTTTTTCCTATTGATCTCTTATTTTTCACAAAATACAGACTCCCTTGGATAATGAAATGGAATTATACTGTTAATTGGGAGACTCGTTTCTTCTCCCGTCAGTTCTTTGTTAAATGGTGGGATCGTTTCGAAGTCCATCGTATTACAGATTATGTTTATAAAGACTTTCCCCAAGTTCCTAATCGTACCCCTGAGCCCAAGCATGAGCCCTCTTCCTCTTCGGATACTATCCTTACAGTTGAAGGTAAATCTAAGACTGAGTTACAGGACTTAGCCCAGCAGCTCCTTCTTCGAGCCTCTCAAATGCATGATGATGATGCTGAGTCTGAAACTTCTTCTAGTTGTCAACCTGCACAGCCAACTCCTGAGCGTCCTGCGCGATGGGCTGATTTTCTGCATGGTCAAGATCCTAATGATGCTTATGATGAAGCCTATAAAGCTTATGATTTGAATTCTGATTAGACTACTGCAGACAAGTTACCTGAGTTAAAGCAAGTCACCTGAGTTAAAGCAAGTTACCTAAGTTAAAGCAAGCTACCTGAGTTAAATTTATTTACTTGAGTTACTGTTGTAAACCTGAGTTACTTTCAATGTTTGTCTTTAAAAGGAGGCTTCGACCTCAGTTGTAATCAATCCTTTTCTTTCTCTTCTTTTCTCTTTAGTTCTTTTCTTAGTTTTAAAAATGTTTTTTCAGAGATCATGTTCTGAAGAACAACCTTAGTATGAGAGTAAGCTTATAAGTTTTATAAATTTATGAATTTACTTTTATATATTTGAAGTATCATCATGTTTTCATACATCATGATTTCTTACATACATGCAATATTTTTATTTTCTCTTATAGAAACTCAGA

TATATATATATATATATATAACATATAGAAATAAATCTAAATACTCTCAAATGTTGTAAAGGAAATAATGAAGCCCACATGAAGAAAAGAATGAGATATTAATCAAAAAGTTAATAATGATGTTGGATAGAAACATCATGGTGTCTCCCTTACCACTCACGAGTGCCATTTACTTTGTCTATAAATAGGCATCCTTTCTACTTGCAACGACATTGTTGACTATTTGACTCAAGATTGCACCCACATGCATTGTTGTTGTCGGCTATTTAAATTTGTAGTTGGAAATGTTGTAAAGAAGGATGGGTATTTGTAGTTGGAAATGTTGTAAAGAAGGATGGGTGAAGCCCACATAGCCAACCAACATTCAAGTTCAACACAAAATCAAACCACACACCCACCAAACCAAGCCTATCTATATTGAAAAGCCATTTGCTTTGCCTATAAATAGGCATTATATTTGCTTGTAATGAGAGAAGAAAGAGAGAAGAGGAAGAGAAGGAAGAAAGAGGGTGAGTGAGTGAAGAGAAAGAAAGCAGGAGAAATTCTATCAGTGTAAACACCACAAAGAGAAATATAATTCCACTCCCAATATTACAGTGGTACTTCTCATCCTCTTATTATTCTAGTTTTATAACACGTTATCAGCACGATAGTCTCTCCTTTTATTTCTGAAAATTTTCAACTCAACACTATGTGGTTATTTCTCTGTCTCCTCTCTGTCATATGTCTACTCTCACTTTACTACTACAACGACATGCAAGGTTTCTTTTTTGTGTTCTCACAATTTCTATGTTGTTTAATCCATGCTTGTTTAATTTACTTCTTTGTAACGTAATTTGTAATTTCTATGTTGTTTAATCCGTCCTGTTATTTTATGGTGGTGTAATTTTTTTTACCCATCGCGTTGTAACACAAATTATTGTAATAAAATGATGGTGTGATCTTAAAACCCATCTATTATATCTTGAATAGTGGCGCAGTTATATTGCCCACAATATAT

>Chr6-01; OZ022211.1; 3250087-3257419; rev; A;

ACCAAATATCCTTGCTTAAGTAATTTGTATATAATTAACTCAACAATTAATTGAGGGGTTTTATTCATTTCAGCAACTCAAAGTTGAGCCTCGAGAGGGAGGCTTTCTTATCAAGCTGTTCGCTGAAAGGAGCTGCAGTGGGTTGCTTGTTTTCATATTGGAAGCTTTTGAGGAGCTAGGCCTTGATGTGCATCAAGCTAGGGTTTCTTGTTCCAACAATTTTCTTCTAGAAGCAGTTGGAACAATTAATGTAAGAATATATACATGCAGCACGTATAGCACTGCACGTACTCAATTGAAACAGACAATTAAAGTTACGCAAACGTGGCATCTATATATACATATATTTATATATATGTGAATTTATTTTTGACATGTTTGATGATCAAATATTGTGTTATTTTCTGTTGAATTTGGTGTTGCAGGATAATCAAAGTGCTGACCAGAAAGATGCTCAGGCAGTGAAAGAAGCGGTGTTGCAAGCCATTCAAAACTGGAGTGAAGTTACCCAACAAGAATAATAATGCAAGCAGACTCTCAATTCTTGAACTCATCGTTTTCTACTTTCCTTTTTCAGAATTAATGTCCTCTCTCTCTCTCTCTCTCTCTCTCTCTCTCTCTCTCTCTCCCGTTTTTCTTTTCTTCTTTCCACTCTCCCCTAATGTTTTTGTACGTCCAAATCAGTGGTTGTCTCAAGCATATATATATATATATATATATATATATATATATATATATATAGGTTACAGATTTATTAATTGTTTTGGTTTATGAATGGATGAGACATATAGCCTATATCTAAAGAGCCCTAAAATTAATAATTAAAACCAACTTACATATCTGATGAGAATTTGCTTTGCTTTTAAATAAATAAATAAATATATATAAATATAAATATATATATATATATATATATATATATATATATATATATATATATATAAAGAACAGAGAGAGAGAAATGAAAGACAACTGAATATAATACAGAGATGAAGATCAG

AAGCAAAGTTAAATGTTTTAAATGTCAAAAATTTGGTCATTATGCTTCTGAATGTAAAGTAAAAGACGTTATTAAACAGTTGCAAATTACAGAAGATGATAAGAATAAGTTAATCCAAGTTCTGGAGTTGCGAGACTCTGAAATGAGTGATGTTGAGTCCCTCGTATCCAGTTCTGAATCTGAGTCTAGCCAGTCTTCAGATTCACAACCCAGTTCCCCTAAAATTCAATTTGGTTGCACTGATAAATGCTGTAACAGACTAAAATCTGTCCTAGTTCTCACTAAGCAAGAAGAACTCCTAGTAGACCTAATAGGCAGAGTCCAAAACCCTGAGTTAAAATTCGAATATTTGAGAAAATTGAAAAAGATCTTAAGCCAGGACGGCCAGAGCCCTCTTTCGCCTACTCAGCCGATTAGCATCAATACTACGTTAGAAAAATTCAACCGTAGGAAAGATGTCACCATCCATGACCTTCAGTCAGAAATCAAGCAAGTCAAAAAAGAATTAGTTGAACTCAAACAGATAAGCCAACAATTGCAAACTGATAATTATAAGATCAAACAAGACTTATTCGAATTAAAACAAGAATCTTGTAAACCTAGTAACCCTTCAGAAAGTCCGCATCATTCTGATGATGAACCTTATGAAGAACAGCCAGTTGTCAATCTGATCAAACAAGTTCACCTTAGGAAATGGTATTCCAAAGTCACGATCTTTGTTCATGATTTTGAATATACCACAGTTGCATTATTTGATTCTGGTGCAGATCTTAACTGTATTCAGGAAGGTCTCATCCCTACTAGGTACTACCAAAAGTCTAGAGAATCCCTTAGCACCGCTTCAGGGAAATCTCTCCAGTTGAATTTTGAATTACCAAAAGCCCATATTTGTCAAAACAAAGTTTGTTTTAAAACTTCATTCATCTTAGTTAAAAATATTACTGATGAAGTAATATTAGGATTGCCTTTCCTTGCCCTGCTCTATCCGTTCCAGGTAGAATATGATGGTATCGTTTCTACTCATCTTGGAGAATCGGTCAAGTTTAGCTTCCTGTCTAAGCCAGAGTTGCATAATCTAAAAGCCTTGCAAAAACAGTCAGTCTCTAAATCTGTCCAAGTTATCCGAAAAACTAATCAGTTGAATTTTCTTAAAGAAGAAGTTAAAATTAAACGAGTTGAACAACAACTTAATGATAAAACTTTACAGTTGAAAATCCAGTCTTTTGAAAACAAATTAAAAGAAGAAATATGTTCTGATTTGCCTACTGCCTTTTGGTATAGAAAACAGCATGTTGTCTGTCTTCCCTATATCAAAACCTTTAGTGAGAAAAATATCCCTACTAAAGCTAGGCCAATTCAGATGAATCAAGAATTAATGGAAATTTGTAAAAATGAAATCCATGAGTTGCTTCAGAAAAACATCATACGCAAAAGTAAATCCCCCTGGTCATGCCCTGCTTTTTATGTCCAGAAAAATGCTGAACTAGAAAGAGGAGTCCCCAGATTAGTTATCAATTATAAACCCCTTAATTCGGTCCTAGAATGGATTAGGTATCCCATACCTAATAAGAGAGACCTTATTAATCGCTTAGGAAAAGCAGTTGTCTTCTCCAAATTCGATATGAAAAGTGGTTTCTGGCAAATCCAAATAGATGAGTCAGATAGGTATAAAACTGCATTTGTTACTCCTTTCGGTCATTATGAATGGAATGTCATGCCCTTTGGTTTAAAAAATGCCCCTAGTGAATTCCAGAATATTATGAATGATATCTTCAATCCTTACAGTCATTTTTCGATTGTTTATATTGATGATGTTCTAATATTCTCTGAGTCAGTCGAACATCATTGGAAACATTTGCATCAATTCCTTTCCATTATCAAAAAGAATGGTCTAGTTGTCTCTGCCAAAAAGATTAAGTTATTCCAAACTAACATCCGATTCCTTGGTTTTCACATAAGTCAATCCCAAATCTCTCCAATAGACAGAGTCATTCAATTTGCTGATAAGTTCCCTGATCAGATTCTTGATAAAAATAGCTCCAGAGGTTTTTAGGATCTCTAAATTATATTTCTGACTTCTATCCCAATCTGAGAAAACAATGTAAGCCCCTATTTGATAGACTGAAGGATAATCCTCCTCCTTGGTCTAGTATTCATACTGAAGTTGTCAAACAAATCAAAGCCCATGTCAAGACACTTCCCTGTCTTGGTATCCCTTCAGTTGATTCTTTCAAAATAGTTGAAACAGATGCCTCTGACATTGGTTATGGTGGCATTCTCAAACAAAAGGCCAGTCCAGAATCCCCAGAGCAAATTGTTCGTTTCCACTCTGGAACCTGGACCCAGTCGCAATATAATTATAGTACTATTAAAAAAGAAATATTAGCTATTGTATTATGCATTAGTAAATTTCAAGATGATTTACTAAATCAAAAGTTTTTAATCCGAGTTGATTGCAAGTCTGCAAAACATGTTTTAGAAAAAGATGTTCATAACATTGCATCTAAACAGATTTTTGCACGATGGCAGAGTATATTAAGTATATTTGATTTTGATATTGAATATTTGAGAGGCAGTGAGAATTCAGTTCCTGATTTTCTTACTCGAGAATTCTTGCAGGAACCACAATGGCTCCCAAAAAAGACAAAAACAAAGAAGTTGCCAGTCAACCCAAGTCAAGCCAGTCGCAAACCAAACCATCTCAGTCGCCATCCATAAGTCCAGCCAAACCAAAAATGCCCAAACAGATAGTTCCCTATCCTGGTTGCACCCCCATCAAAGTTGCCACACCTCTATCAGTTGCCAATAGATTTTCCTCTTTAGGATCCACAGTTGGCCAAATCCGCCCCAGTTACCAGTCTACTCTAGTTGCCAGTTATGATCACTTTGCAGTAGACATCCCTGCAGGCCCCTCAGTTACATTTAAAAAATCATCTCCTTATTTGCCCAAAAGCAATTCCCATTTATTTGTCATTGAGCCCTTGTATGATATTAATACCAATCCAGTTGAAATCGCCAGACACTATTTTCCCCCTGGATTCCATTATATGCCTTCCAACCCTTACAAGTCCCTCAAGTATTATAGAGATATATTACTTGAAACCAAATCAGTTGAAATCAAGCCGATTAAGGATCGTGACAATCCTGCCACTATCCTTTATCATTCTTTATATATTCACCAAATTATAAGCCAAGAAGCCTTCAGCAGTCGTCCTTATGGCTCAAGTTACTTCAGTCCCAATTGCAATACAATTATGCTGATTATATTGATGCATGGTATACTATTTTTCTTCATCAATCTGAAGATTTTAGTCATTCCTGGTTCATCAATTTTGATAACAAGTTCAAAAGTTCTTTCCCCTACTGGTTCCTCCATTGGTGGGAAAAACATGGCCCAGTCGAAGAAATTTTACCAGCATCAGTTCTTGCACTCATCCACCATTACACCCAAAGGCCAAGTTCTCTGCAGTTGATTTATTTTTTCCCAAACAGTTGCTTTTTATTGCCAGATATAAAGTACCCTGGATACTGAAATGGTCTTACAGGGTCACCAAAGATGTTCGCATTCTTGCCCGCCAATTCTCTGTCAAGTGGTGGGACAAATTTGAAGTTGATAGAATTGCCAGATACGTCTACAATGACCTCCCAGTTGCACACCAGCACCCCTGCCGGTTTCTCCCTCTGCTTCTGTTCGATCTTCCCTCTCAGTCGAAGGAAAATCAAAAGCAGAACTCCGAGAAATTGCCACCCAGTTGCTCAGACAAGCCTCTCAGATGGATGATGAAGACAACGGGTCCCCCTCGTCTTCTAGCTATCAGCCCATCTCTGTCAAGCCAAAGACCTGGTTTGAAGACAGTCAAGACCCCTATGACGGCTATGATCTAAACTCTGACTAGGCCTCATGATGCTCCTGACATCTTCACATGCCAAAGTAGCCGAAAGTAGCCAGTCTTGCAAAAGTTGTCGTTAGTCGCCGACATGATCATTGTCTTCTGTGAAGAATCTGCTTTTACTATCCACTTTCACCGAAAGCTGCATCAGTAAAAGAATTCCCCACAAACAGACAAAATTGAAAGTTGAATTAAGTTGCTTTCCTGTGACCCAACAGTTGACTCAGATTCCGTAACTCTACCATAGTTGAACTTTAGTTACTTTTCTCTTTATAAGCAGGCCTCGGCCTCAGTTGAAACCAGAATGTAATTTTCCTTTTCTTCAGTTTTCAAAGAATCTCTCTGATACATCCTTCGGAGAAATTCATTTTTGTAAACACTCTTGTAAATAGCGTGCAGTTGTGCTTTGAAGAATTGCAGTTGCAATCTTTGTGAGTATAAATAAAAAGTTATTTTCAAATTATCTATGTTGAAAGCATTATTTTAAATGTATTATCATTATTTTACTGCATTAGTCTGAAAGTTGCTTAGGTTGCATCCATTTCTAATTTCCTTCTGATCTTCATCTCTGTATTATATTCAGTTGTCTTTCATTTCTCTCTCTCTGTTCTTTGTTCTTCCTCTGTTGGTATCAAGCCGTAAGTGTTACGGTCTTTGTCTAAGTCTTCTACTCATCCCCGCCCAAGTGGCTGGTTTAAGCATCTGAGTAGAAGGTTTTCATTTCTTCCTTTTCTTCCCTTTTATTCTTTGAGTCTTCTGTTAGTTTTCTCTAAGGTCTGACCCGTGAGTCCTTTTTGCTAGATCAGTAAGTCCCCAGGGAGGGCTTGTGTGCTGGGCAGGAGACAGAAAATTGAGAGAATTAATAGGTTACTTAGAGTCTAAACGGTCGGTTTAGGATCTACTGTTCATCTTCTTCCTCAGTCTGTTTAACCTAGTTTCTTGAGTAGTCATGAATAGCTTGTTTAGAACAAAGTCTTGTAATTCTGCCAGTTCTAGTTCCAGTATTGGTCGTCTTCCAGAGATAGTTAATGAAGAACAATTTGAATATAATTCCAGTAGTAAAGAACAATTAGATTTTAGAGATTGGAACATACCTAAAGTCCCAAGTCAGAAAATCTATAAGAAACATTGGTTGCCTTCTAGTTTTAGTAGTACAACACATGTCAAAACAGTTGAACAAGTTTACGCTTTAAGTAAAGAACACGAAACTTGCCAGTTGCTTAATCTTGAGTCAATTTTGAAACATAAGAAAGATGGTAATAATTTCCTGCATATTGGTCTAGTTCAAGTTGCTGTCAAACCTCTGACACGGTTAGGGTTGAAAGCTTCAATCCTGCTATGTCTTCGTGATGCTAGATTCACTGAATTTAGCGATAGTACCCTTGGAATCATTGAGTCCAGTCTGTGTAATGGCCCTGTCCATTTTGACTGCTATCCTGATTTCACTGTAAGCCTTAGTGATCCTCATATCCTGAGAACTTTGACTCTGAATATTAAGACTGAAGGTTATAATGTTCTCCCAGGAACCCAACCTCTAGCCTTAGTATACAGAATCTACTATAAGGTCACAGGAACTAATATGAATTTCCAAGCCCTTAATAAAAGCCCGAAGGATCAAACCGTCCTGATCCAGAGTCATACCTCAGACGCCCATATACAAGTTCCTCATACCATTAAGTGGTCCGAAGTTGCACTTCCAAAAGATTGGACCTTAGTCACCGAAAGCCAACCAGCTCCGATCCAGCGTAGTCTAAACAATTTAGATTACATCCAACAATACTTAGATGGCACAGTTAAAATTCAGTTCGATAGTCATCCTTTACGTAAGTCCAATGTTCAGTTACAACAGTTGCCAACTCCTGGTCAATCCCAGAGGCATGCCCCTGCTCGGCATTCCTTTGCTGCCTCTTCCTCCACTCTAGAACGAGACCTAGAGTTAGAAAAGGCTATGATTGATTTCAAACTTGAGTCCCTGAGAAAAACCGCTCAGGTTACCCAACCTTGCTACGGCACAGTTCCAGTTCAAGAAGATAAGTCAGAATCACCTGAATCACCTACCCAGTCTGACTTTGTAGTTGAAAATCAGCTTAGAACCCTTAATAAAGAATTTGAAATTAATTGGGTAAGTCTAAATCGTCATCTAAAAGCCCCTGAAAACAAGCCAAGGCGAGATCTTTATCATCAAGCCTATCCTAATAGCAAACACCGAGATATGATCTTTGAACAATGGAAAGATTATATGCGGTCAGCCAAAGTTGAAATTTTCTACCTTGATTTTATTGAAAGCCGTTATATGACTAGTGATGAATTAAAAACCCTGACTAAAGAAAAATGGAAACTAGTTGATAAATCTGTAGTAGAGTCGAGTCACCCTCCAGTAGAAACAATTATAATAGAACACCAGAAAGCGCCAGTTGCCGCCACTCCTTTCAAAACCTTTGAAAGCAGTGATCCTAATAGAAAACTAATTGAACAAAACAATTACACGAACCAGAGTCTAATCACCATAGGTAAACAATTAGATACAATTGAAACCAAAGTTGATCTACTGAGTCTGCCTGTTGCAGACTCCAGACCCAGGCACAAAGTTGAAAAACCCAGTTCAATTTCAGAACAAAAACAAGTCCAGTTTTAAAAGTTAACTCCACAATGAAAAAGATAGAAGACATGTTAGCACAATTGACCCCGGTCAAGCCCGAGAAAGGTGATACATCTGGTATTAAAACACTAGATGCTTTTGCCACTTCTCATTCTGAGTCCGATACAATAAGTTTCAGAAACCAGTAATATTTTCTAAAATTGAAAACGCCTTTAGAAATTAGAAGTCCAAACTGAGCCTAAAGTCAAAAAAATCGAGAAACATATAAGTCCTACCAGTCTACAAAAAATTGGTACCCCAGACCAACACCTCCAATATCCGATTTGAAGAGAGAAACTTTCAAACCCAGTTTTCTGTTTCTCTGATAAATTGTATGAATGGAATATTGATGGTCCTTTCGAGCAAGAAATCCTAAATAAATTGCAACATATGTCCATGGGTCCGCCAATAGTTACATCACCAATCACAGTTTCAGACAGTCTGAAATTGATCCCTTTGATAGTTACCCGTTTCACTGGTACTCTCAGATATTGGTGGGATAAGCACCTACCCTGATCTAAAAATCGAATCATTTGCTGTAAAACTTAATGAAGACGGTCTCCCTATATTTGAACAAATAGGTCAAGGTATAGAAGACGGTGTCAACACTCTGTTTTACCACGATGAATTGAACATTTCATAGGAACCCCTAGTAATACCACTGCTAGGATCCATGATCATTGAGTAACCTAGATGCCCAAAAATTATCTGATTTCAGATGGTATAAAAGATGTTTCATTTCTAGAGTCATGCTTAGAGATGATAGTAATCAACCATTCTGGAAAGAAAAGTTCATTAAAATATT

TATATATATAAATATATATTTATATATACAATTATTCTTTTATTCGGATGTCCGGCACATTGTTACCATGTGAACGAGAGAGGAAAGCAAAGAGAGATAGTATAAAATACATGGTGTCCATAAGAATAATAACGTCCAGACGTTCGCGTAGGAGAAAATACGCAAACATTTGATGTGAATCCGTTTGTCACTCACCTTTGTTTCTCTTCACAAATTTGTAGAAAAGGGAAAATGTAGTTGGGAATGAAAGGCCTTTCGTACCTTAAAGTTCAGTATTATTCATTTCGTATGTATATTTTCATGAATGCCAACCAAAAAGAAAGGTTAGTCCACCAATAAAGGTGATCTGAATAACATGTGCCTTATTATTTTGAGGACCTTTTCTATTGAGAGGAAGCGAAACATATTAGACTCACACACATTACATGAATGCTAGAACTTGAACTTGAGCTTTCTGGACGGAACATGTGACTATAATGGCATGAGAGTATGAGAATCGTTAAATTAACCATGGTGGTGGTGGGGCATATACATGCATGTCATAAATATAACACCTATTGGAACTTTGGGTGCCTCTCTTGAGAACCTGAATATCCAACTTGAAACGTATTGAGTGTGAAAAAGAGAAAAACTTAACTTGTTTCTCAATCTTCTCCTGTCAGAAACATCAAAGATGGATTTGGCCATAAAACTTTGCACATAACTCAATGTTAATCGCTGCCTTTACAGAAGATTAAGGGGAAATAAAAGAAATTTGTGAAGGGAAAAAAGTGGGCATTTTAAGGGAAAGATTATAAGGTATAATCATCAAATTGGGAGGGTTCTATAGAATTTTCCCATTGAAAATTTATTTTTAAGATGGGTGAGCAAAGAGATGGTCGCAACATAGCACCACTCCAATTTTGGGCAAAATTGGACGATACTGTTCCCACGTCCTTTTGGGCTTTAAGAATCTGAAATAAGGCAAAAATACTCCTGATGCATGACACTCCCT

>Chr6-02; OZ022211.1; 6173442-6195128; dir; C;

CCACACCTAAAATCAAAGACCTGCGTTGCATTTCCATAACTGGGTGTTTGTGTTGATCCAGATGATTGATTGCCTTTACCTGTCATAAACCATGGCATTATATAGAGGGAGTACGTGTTGAGATAGCGTCTCGAATTAGTGATCAAAAGCAAATTAAGTTCTTTTTATTTTTTATTTTTTATAGTTTGAGTCATATTTGCGTATGTGTTTGGGATTACTTGTTGTTTTGGTCTTGGGTATTCACATGGATTGCGAGTAGTGAAATTACTTAAGTGTGTGATTGAAGATGCTATTTCCTCTTCCTTGTAATTTTGCTACTTTCTTGGTGGAACGTAGTTGCCGTTAAAGCTTTTTTAAGAAGTGATTAATTTCAAAAAAGGTAGGGAGAGTGCCAGGGATTGGGGGGTGTGGGTGGTTTTGGGGGAATAATGTACACGGTCCTTTCATGATCATTGAAGCTTTTTTTAGAAGTGATTAATTTTATATTTAAAAAAAGAGTAGGGGTAGTTAGTTATGTACACGGTCCCTGCATTGAAGCTTGTTTTAGTGGTGATTAGTTTGGGAAAAGGGAGAGGGATTACTTCTTAATTAATAACACAATGATATAAGGGATAATTTTTTCACGTATCATTATTTTATTGATCGGGAATAACAAAAAATGTTATCCCGTTACAAGAAAGTTTTTTTTCTACCTTTGACTTAGAGGCCAAGTGTAGCCCCAGCCATGCACCTAAACAAATGGGTTGCATGAGGGAGAAAACGTTGTTATTGTAAGTAGATGCATTTAAAATTTAGCTACCTAAAAAGTTTAGGGAAGTAGAATTCTACTTAGGTATCTAAAATGCGCCTACTTTCAATATAGGTTTAAATTTATTAGAATTCTACTTAGGTACCTAATTAGAGGTTAATTATGTACTTTGTGCTTGTATAGTTTCAATTGTCTAGATTAGTTTATTAATTGTTACAATTTAAGGACAATATATATATATATATATATATA

GAATCTTAGACTTACGGATGATTCCCTTTAGAAGTAATTCTGTAATTTCTTTTTTACAGAATTCCATTGTCTCCTGATTCATTTGGATTGGTCTGGCTTTAGTGGGGATATTTTTCTCACTAAAGGTTTTGGCGTAAGGAAGACTGACAATGTGTTGTTTCCTGGACCAAAAAGCATTAGGCAGATCAGAACAGATATCATTGATTAATTTAGTTTCAAACTCATGAACTTGTTTCAGGAAAGGTTCATTCCTAATTTGTTGGCTGATTCTAGTATGACTAATTTCTTCCTGGAGAGAAGACAGATGTTTAGTCTTACTGGAAATCAAAGCAATAGTTTTGGAGATTGAACTTTTCTGAAAGGTTTTCAACTGGTGCAACTCTGGCTTAGATAAAAATTCAAACTTGGCTTGTTCTCCTAGGTGAGTAGAGATGATGCCATCATGGTTCACCTGGAAAGGATATAACAAGGCAAGAAAAGGTAATCCTAGAATAACTTCATCAGTAATGTTTTTTACTAGAACAAATGAAGTTTTAAAGCAAATTTTATTTTGACAAACATGGGCGCGGGGAATTTCATAGTTCAGCTGTAGGGATTTTCCTGAAGCTGTTCTAAGAGATTCACAGGACTTAGTATAATACTTGGTAGGTATAAGACCTTCCTGGATGCAGTTAAGGTCAGCACCTGAATCAAGCAAAGCGACTGTGGTAAAATCAAACTGATCAACAAAAATAGTAACTTTGACATACCATTTCTTGAAGTTAATTCTTTTGACTAGACATAAGGCCTGTTCTGGATTCAAAGATTCATCATCTGAGTTTGTTGGACTCTGAGATAAATCATCAGGTTTGAGAGCTATTAAATCCTGTCGGATTTCATAATTCTCAGCTTGCAATTTCTGGCTTAATTGTTTTAGATCAACTATCTCCTTCTTGATTAATTTTACTTCTGAATGTAAATCATGAAGGGTGATGTCTTTTTTGCGACTGAACCTTTCTAAGGTTGTATTTAAACTTATCTTTTGAGGAGGTGGAGACTGACTTGGACCTTCCTGACTAAGGAGTTTCCGAAATTTCTTCAGATACTCAGCCTTCAACTCTGGGTTTTCGACTTTACAGATTAAGTCTATGAGAAGTTCTTCTTGTTCTTCCTGTTTAGTAAGAACAGAAATGGATTTCATAGAATTACAACAATTATCTTTACAACCGAGTTGTATATGGGGGGAACTAGGTTGTGAGTCCGAGGAATAAGAATTCTCAGAATCACTATCAGTCGATGACTCGAGATCACTAGGTTCGGAATCCTTAAGATCTAGAAGTCGGATTAGATTTTCTTTTTCAACTTCTTCAATTTTTAGTTGTTTGATTGTGTCTTTCACCTTGCATTTATTAGCATAATGACCTGGTTTTTGACACTTCCAGCAGACAACTTTTCCTTTTCCAGAATTGTTCTGGAATCGGTTTTTTTTATTTTTTGATGGAAATTTTTTCCAATTCTTTTTAGGGCTATGTTTCTTAGTCTTATAGAATTCTCTATCATTATTTTGGTATCTATTTTTTCGATTATGGTAAGAACGGGATTGACTCTTATGGTGTGACTTAGTTCGACTGGAGGGAGGTAGGCTAGTAAGACCATATTGTTCGCAGAAATTTCCTAACTCATACTTAGCGGATTTTCTGTCAGACTGTATCTGCTTACCAATTTTCATGTCAACACACATCTTCATACCGACTTGGTTGATAGTACTGACTAGATTTCCATAGGTCAGATTATTCCAATCAATATGTCCTTGTTCATTACTAAGGGTTGTGCGAATCTTATGGGCAAATAGGTTTGGAAGGCCATTAATGAATTTTTCTTTCCAAAACGACTGATTACTATCATTTCGGAGCATTACTCGCGAAATGAAACATCTTTATACCACCTAAAGTCAGACAGTTTAGGGCAACGAAGGTTGCTAAGCTGGTCATGAATTCTAGAGGTAGTATGGCTTGGGGTTCCTATGAAATGCTCAATTATGGTGTAAAACAGAGTATTGACTCCATCTTCTATTCCTTGACCTATTTGTTCATCAAAGATAGGGAGACCTTCATCATTAAGTTTTACAGCATGAATAATTAAGTTTTTAGACTCAGGTGTGAGGTGTTTATCCCACCAATTGCGGAGGGTACCTGTAAAACCTGTGACTAGCAGAGGTACAATTTCAGATTGTCTAAAACTGTGTTTGAAATATAACTATTGGCAACCATGGACATATGTTGAAGTTTATTGAAAACTCTTGCTCAGAAAGACCATCAATGTTCCACTCATACAATTTATCAGAAGAGACAGAGAACTGGTTCTGAAAATGTCTTTCTTCGAATTGTATATCTGGAGGTGTTGGTCGAGTGTACCAATTTTTGGTAAGACTAGTAGGGGTGACTTGGTGAGTTAACCGGGTAACTCTGGGTTCAACTGCAAGTTCAGTAAAGGCTTTCTCAATTTTTGAGATATTACTGTCAACTGATTCTAGACTCTGAATAGATTCTTGGTCAGAATCCGGATTAATGGAGTCAAGAACTTTAACTCCAGATGGCTCACCTTTTACAGGTGTCAATTGTTCCAGCATTTCTTCAATTTTTGAAGGGTAGGTTTGATCTTAAGAGTTGGATTTGTCTTGAGATCCTGGAACTGGACTAATGGTTTTTCAACTTTAATGTTGGATTTTTCAGAGGAAGGGTTAGCTTGTCTACTTTAGTTTCAATTTTGTCAAGCTGTTTTCCTATGACAATTAGACTCTGATTGGTGTAGTTGCTTTGTTCTATCAATTTCTTCTCTGAATCTAATTTTTCAGGGATTTGAAAGGACAGGCAGGGATCTGAGTTTTCTTAAAATTAATTAAGATAGACTCAACCGGGGGATGACTGGCAACTACTTGTGTGTCATTGGGTAAAGACCATGTTTCTTTGGTGATTACCTTCAATTTTTTAGACATGTAGTGGGTTTCTACAAAATCTAAATATGGCATTTCGACCTGTTCTTTCCGTAGGTAATCTTTCCATTCAACAAAGATTGCGTCACGGCGGTACTTAAGTGGAAAAGCACTATGGTAATCATCTCGGCGCTTCTGGTTTTCAGGAGCTGTGAGATGGGCTTGTAATTTTTTCCAATTAATGTGAAATTTATTTAGAACTCTAAGTTGAGGGTCAACAAAATCAAAACCTTCAAAGTCAGTTTGAGTTGGAGACTCGGGAGATTTTGGCTTTTGTTCTTCAACTGGTTGTTTGCCATAACAGGGTTGGCTAACCTGTGCTGCTTTCCTGAGGGATTCTAATTGGAAATCAATCAGGGCTCTTTCTGCTTCTAGGTCTCGTTCTAGAGTAGAAGAAGAGGCTGCAAAGGAATGTCTGGCAGGGATATGCCTCTGGGATTGACCAGGGGTTGGCAACTGTTGTAGCTGGACAGTAGATTTTGGAATTGGTTGGGTTCCAAAATTGATTTTTACTGATCCATCTAAATATTGGTGGATGTAATCTAAATTATTTAGACTACGTTGGATTGGTGTTGGTGGATTGACATTTTTGAGTGTCCATTCAACTGGAAGTGAGACATCAGACCATTTGATTGTTTGAGGTACTCGGATGACGGCCTCGGGTCTATTACTTTGAATCAACAAGGTATGATTCTTAGGACTCTTATTAAGGGCTTGGAAGTTCATATTGGTTCCGTGACCTTATAATAGATCCTAAAATTAAGGCTAATGGTTGGGTTCCCGGAAGAACCTCATATCCAGAAGTCTGGATATTGAGTGTCAAGGTCTTAAGAATGTGGGGATCACTAAGGCTGACAGTGAAGTCAGGATAACAATCAAAGTGAACCGGACCATTGGAAAGACTTGACTCAATTACTCCGAGAGTACTGTCTTCACTGTTAGTGAATCTAGCATCTCTTAACCAGAGCAAACTGAGGCTTTAAGCCCTGTTCTGGTTAGTGGTTTGACGGCAACTTGGACAAGACCAATATGAAGAAAATTGTTTCCTTCATTCTTATGGCGTTTGTAGAATCAAGATTGAGCAACTGACAGACTTCAAGTTCTTTGCCAAGGGCATAAACTTGTTCAACTGTTTTAACATGGGTAGTGGTCTTAAACTTGGTAAAGACCACCTCTTCTTATAAATTTCCTGACTGGGGACTCTAGGTATATTCCAGTCTCGGTAATCTAAATCATTGTTATTAGATTCAAATTCTACTTGTTCCTCATTTACTATTTCTGGAAGACGACTAATACTGGTTCTTGAACTGCTCGAACTACAAGAATTAGATCTAAGGAAACGACTCATTTTACTTTGGCAACTGGGTTCTGACTTCAAAGAAAAATAAACAGGGACTTCTACTACTGACTCCTGTACGTTCCTATCAACTGACCTCTCTCTCCTAGCCCTCTCAATGCCGCAACCGCTCATGCCTCACCTCGGGACTTACTGTTTGGACTCTAGGCCTTACTCTTCGGACACTATTTAAGAGAGATACAGATAACAGTACCGGAAAGAAGAACAATAATAAAAGCAACTGAAAACTTTCTACTCAGAAATCCTAAACCAGCCACTTGGGTTGGATGAGTAGCAACCTAAGATCAGATTCTTACAACTCTTACGGCTCTGATACCAAAAGGGAAGAACCAACATTAATAGTAGAACAGGAGATAGATAAAGAAGCACAAGAGAAAACAGAGAATCTTACAACCAACTTTCAGACTAATGCGAGAATATAATGCATAAATGAAAATAGTAAAATAAGATTAATTTCAAAATAAGAGAAATTCAAGATAACTTACAAAGCTGTCTTGTTTATTGAAAGCTAAACACTCAGATATTTACAAGTGAGTTCATGTCTGAAGGAGTTTCAGAGAGAACTTTTGAAAACTAAGAAAGGAGAAAAAAAAAACCAAGTGCTTACAACTGAGGCCGAAGCCAGCTTAAAAAGAAAGAAAGCAACTAAGGTTTGAATCCAAAGTAACCCTAATTGGAGAGTAACCATGGTTTCAAGTACGGAAAACAACTTTCTCTTTACCTATTGGGGTAAAGCAGGGGTAAAAGCAAAAGTATTTTTGTTTTACTCTCTTTACCTATTGGGGTAAAGTAAGGGTAAAAGCAAAAGCAATTTTGTTTTTACGTTACAATTATTCTGATTCCAACATTTCACCGGGGTGAAAAGAGGAAGTGATGGCAACTTTTGACTAATCAGATCCCAAATCATACGCGTCATAAGGATCCTGCGCAAAAGGGATATTGGGATCAAAGGAGCTGGAGCCAGGCTGACAGCTGGATGCAGATGGGGAAGATTCGTTGTCCTCATCATCATTCATCTGACTAGCTTGGTCAAGCAATTGTGCAGCAAGGGCCCGGAGTTCAGCTTTGGATTTTCCATCGATTGAGATGGCCGAGCTGACTGAGCCTGTAGGAGAACTTGGCTTGGGATGATGATCCGATTGGGGAAAATCCTTGAAAACATAGATGGAAATTCTGTCCATTGAGAACTTATCCCACCACTTGACAAAATACTGGCGAGACATGATTCTGGTTTCCCAATTGACATGATAATTCCACTTCATTATCCAGGGGATTTTGTACCTGGCAATAAAAAGAAGCAACTTGGGGAAAAACAACTGTTGTTTAGTAAATTGGCTTTTACTGGCATAATAATTGGTCAGATTATGAACTTCAGTAGGGAGGATATCAGCTGTTGGACCATGTTGATCCCACCAATGAAGAAACCAGTAGGGGAAAGGACTCCTGAATTTGGCATCGAAATTAATAAACCAGGAGTGGGAAAAATCAACTGTTTGATGGAGGAAGATATTATACCATGCATCAATGTAATCTGCATAGGTATAAGGATGAGTGGACTTAAGGTGTTTCAATTCATAGGGGTGGCGACTAAATTCAGCTTGGCTTAGAATTTGTTGAATATAAAGAGAATGATAGAGAATGACTGCGGGGTTATCTCTATCCTTAATGGGCTTGATTTCAACTGATTTGGTCTCATGCAGGATATCCCTATAAAATTTGAGGTCTTTATAGGGTGCAGGAGGAAGGAAATGGGAGTTAGGAGGGAAGTATGATCTGACAATTGTTATCGGATCAGTAATCCCATCATATATTGGTTCGATTATGAACAAATTAACCTTATCTTTTGGGAGGTAAGGGGACGACTTTCTGAACTGGGTGGACGACTGGGATGTTTGGGCAATTGGGACAACTGGAGGAATTATTGCAAATGGATCATAGTCAGAAACAAGGGCTGACTGATAATTAGGGCGGATTGGGCCGACGGTGGATCCTATTGGGGTTCCAAGGCTGGTGAATCTGTATGCAATTGGGACGTAAGAGTTGTAAGAATCTGATCTTAGGTTGCTACTCATCCAACCCAAGTGGCTGGTTTAGGATTTCTGAGTAGAAAGTTTTCAGTTGCTTTTATTATTGTTCTTCTTTCCGGTACTGTTATCTGTATCTCTCTTAAATAGTGTCCGAAGAGTAAGGCCTAGAGTCCAAACAGTAAGTCCCGAGGTGAGGCATGAGCGGTTGCGGCATTGAGAGGGCTAGGAGAGAGAGGTCAGTTGATAGGAACGTACAGGAGTCAGTAGTAGAAGTCCCTGTTTATTTTCTCCTTTGAAGTCAGAACCCAGTTGCCAAAGTAAAATGAGTCGTTTCCTTAGATCTAATTCTTGTAGTTCGAGCAGTTCAAGAACCAGTATTAGTCGTCTTCCAGAAATAGTAAATGAGGAACAAGTAGAATTTGAATCTAATAACAATGATTTAGATTACCGAGACTGGAATATACCTAGAATCCCCAGTCAGGAAATCTATAAGAAGAGGTGGTCTTTACCAAGCTTTAAGACCACTACCCATGTTAAAACAGTTGAACAAGTTTATGCCCTTGGCAAAGAACTTGAAGTCTGCCAGTTGCTCAATCTTGATTCTATCAAACGCCATAAGATGAAGGAAACAATTTTCTTCATATTGGTCTTGTCCAAGTTGCCGTCAAACCACTAACCAGAACAGGGCTTAAAGCCTCAGTTTTGCTCTAGTTAAGAGATGCTAGATTCACTAACAGTGAAGACAGTACTCTCGGAGTAATTGAGTCAAGTCTTTCCAATGGTCCGGTTCACTTTGATTGTTATCCTGACTTCACTGTCAGCCTTAGTGATCCCCACATTCTTAAGACCTTGACACTCAATATCCAGACTTCTGGATATGAGGTTCTTCCGGGAACCCAACCATTAGCCTTAATTTTTAGGATCTATTATAAGGTCACGGGAACCAATATGAACTTCCAAGCCCTTAATAAGAGTCCTAAGAATCATACCTTGTTGATTCAAAGTAATAGACCCGAGGCCGTCATCCGAGTACCTCAAACAATCAAATGGTCTGATGTCTCACTTCCAGTTGAATGGACACTCAAAAATGTCAATCCACCAACACCAATCCAACGTAGTCTAAATAATTTAGATTACATCCACCAATATTTAGATGGATCAGTAAAAATCAATTTTGGAACCCAACCAATTCCAAAATCTACTGTCCAGCTACAACAGTTGCCAACCCCTGGTCAATCCCAGAGGCATATCCCTGCCAGACATTCCTTTGCAGCCTCTTCTTCTACTCTAGAACGAGACCTAGAAGCAGAAAGAGCCCTGATTGATTTCCAATTAGAATCCCTCAGGAAAGCAGCACAGGTTAGCCAACCCTGTTATGGCAAACAACCAGTTGAAGAACAAAAGCCAAAATCTCCCGAGTCTCCAACTCAAACTGACTTTGAAGGTTTTGATTTTGTTGACCCTCAACTTAGAGTTCTAAATAAATTTCACATTAATTGGAAAAAATTACAGCCCATCTCACAGCTCCTGAAAACCAGAAGCGCCGAGATGATTACCATAGTGCTTTTCCACTTAAGTACCGCCGTGACGCAATCTTTGTTGAATGGAAAGATTACCTACGGAAAGAACAGGTCGAAATGCCATATTTAGATTTTGTAGAAACCCACTACATGTCTAAAAAATTGAAGGTAATCACCAAAGAAACATGGTCTTTACCAATGACACACAAGTAGTTGCCAGTCATCCCCCGGTTGAGTCTATCTTAATTAATTTTAAGAAAACTCAGATCCCTGCCTGTCCTTTCAAATCCCTGAAAAATTAGATTCAGAGAAGAAATTGATAGAACAAAGCAACTACACCAATCAGAGTCTAATTGTCATAGGAAAACAGCTTGAAAATTGAAACTAAAGTAGACAAGCTAACCCTTTCCTCTGAAAAATCCAACATTAAAGTTGAAAAACCATTAGTCCAGTTCCAGGATCTCAAGACAAATCCAACTCTTAAGATCAAACCTACCCTTCAAAAATTGAAGAAATGCTGGAACAATTGACACCTGTAAAGGTGAGCCATCTGGAGTTAAAGTTCTTGACTCCATTAATCCGGATTCTGACCAAGAATCTATTCAGAGTCTAGAATCAGTTGACAGTAATATCTCAAAAATTGAGAAAGCCTTTACTGAACTTGCAGTTGAACCCAGAGTTACCCGGTTAACTCACCAAGTCACCCCTACTAGTCTTACCAAAAATTGGTACACTCGACCAACACCTCCAGATATACAATTCGAAGAAAGACATTTTCAGAACCAGTTCTCTGTCTCTTCTGATAAATTGTATGAGTGGAACATTGATGGTCTTTCTGAGCAAGAGGTTTTCAATAAACTTCAACATATGTCCATGGTTGCCAATAGTTATATTTCAAATCACAGTTTTAGACAATCTGAAATTGTACCTCTGCTAGTCACAGGTTTTACAGGTACCCTCCGCAATTGGTGGGATAAACACCTCACACCTGAGTCTAAAAACTTAATTATTCATGCTGTAAAACTTAATGATGAAGGTCTCCCTATCTTTGATGAACAAATAGGTCAAGGAATAGAAGATGGAGTCAATACTCTGTTTTACACCATAATTGAGCATTTCATAGGAACCCAAGCCATACTACCTCTAGAATTCATGACCAGCTTAGCAACCTTCGTTGCCCTAAACTGTCTGACTTTAGGCGGTATAAAGATGTTTTCATTTCGCGAGTAATGCTCCGAAATGATAGTAATCAGTCGTTTTGGAAAGAAAAATTCATTAATGGCCTTCCAAACCTATTTGCCCATAAGATTCGCACATCCCTTAGTAATGAACAAGGACATATTGATTGACATAATCTGACCTATGGAAATCTAGTCAGTACTATCAACCAAGTCGGTATGAAGATGTGTGTTGACATGAAAATTGGTAAGCAGATACAGTCTGACAGAAAATCCGCTAAGTATGAGTTAGGAAATTTCTGCGAACAATATGGTCTTACTAGCCTACCTCCCTCCAGTCGAACTAAGTCACACCATAAGAGTCAATCCCGTTCTTACCATAATCGAAAAAATAGATACCAAAATAATGATAGAGAATTCTATAAGACTAAGAAACATAGCCCTAAAAAGAATTGGAAAAAATTTCCATCAAAAATAAAAAAAACCGATTCCAGAACAATTCTGGAAAAGGAAAAGTTGTCTGCTGGAAGTGTCAAAAACCAGGTCATTATGCTAATAAATGCAAGGTGAAAGACACAATCAAACAACTAAAAATTGAAGAAGTTGAAAAAGAAAATCTAATCCGACTTCTAGATCTTAAGGATTCCGAACCTAGTGATCTCGAGTCATCACTGATAGTGATTCTGAGAATTCTTATTCCTCGGACTCACAACCTAGTTCCCCCCATATACAACTCGGTTGTAAGATAATTGTTGTAATTCTATGAAATCCATTTCTGTTCTTACTAAACAGGAAGAACAAGAAGAGCTTCTCATAGACTTAATCTGTAAAGTCGAAAACCCAGAGTTGAAGGCTGAGTATCTGAAGAAATTTCGGAAACTCCTTAGTCAGGAAGGTCCGAGTCAGTCTCCACCTCCTCAAAAGATAAGTTTAAATACAACCTTAGAAAGGTTCAGTCGCAAAAAGACATCACCCTTCATGATTTACATTCAGAAGTAAAATTAATCAAGAAGGAGATAGTTGATCTAAAACAATTAAGCCAGAAATTGCAATCTGAGAATTATGAAATCCGACAGGATTTAATAGCTCTCAAACCTGATTTATCTCAGAGTCCAACAAACTCAGATGATGAATCTTTGAATCCAGAACAGCCTTATGTCTAGTCAAAAGAATTAACTTCAAGAAATGGTATGTCAAAGTTACTATTTTTGTTGATCAGTTTGATTTACCACAGTCGCTTTGCTTGATTCAGGTGCTGACCTTAACTGCATCAGGAAGGTCTTATACCTACCAAGTATTATACTAAGTCCTGTGAATCTCTTAGAACAGCTTCAGGAAAATCCCTACAGCTGAACTATGAAATTCCCCGCGCCATGTTTGTCAAAATAAAATTTGCTTTAAACTTCATTTGTTCTAGTAAAAAACATTACTGATGAAGTTATTCTAGGATTACCTTTTCTTGCCTTGTTATATCCTTTCCAGGTGAACCATGATGGCATCATCTCTACTCACCTAGGAGAACAAGCCAAGTTTGAATTTTTATCTAAGCCAGAGTTGCACCAGTTACAAACCTTTCAGAAAAGTTCAATCTCCAAAACTATTGCTTTGATTTCCAGTAAGACTAAACATTTGTCTTCTCTCCAGGAAGAAATTAGTCATACTAGAATCAGTCAACAAATTAGGAATGAACCTTTCCAGAAACAAGTTCATGAATTTGAAACTAAATTAATAATGATATCTGTTCTGATTGCCTAATGCTTTTTGGTCCAGGAAACAACACATTGTCAGTCTTCCTTACGCCAAAACCTTTAGTGAGAAAAATATCCCCACTAAAGCCAGACCAATCCAAATGAATCAGGAGACAATGGAATTCTGTAAAAAAGAAATTACAGAATTACTCTGAAGGGAATCATCCGTAAGTCTAAGTCCCCCTGGTCTTGTCCTGCCTTCTATGTCAAGAAAAATGCTGAACTAGAAAGAGGAGTCCCTAGATTAGTTATCAATTACAAACCCCTCAATGCAGTCTTAGAATGGATTAGGTACCCCATACCTAATAAACGAGACTTAATTAATCGGTTAGAAAGCATTGTCTTTTCCAAATTTGATCTTAAGAGTGGATTCTGGCAAATCCAAATAGAAGAGTCGGATCGGTACAAAACTGCCTTCGTCACTCCTTTTGGTCATTATGAATGGAATGTTATGCCTTTCGGTCTTAAGAATGCGCCTAGTGAATTCCAGAATATTATGAATGAAATTTTCAATGCTTATAGCCATTTTTCAATTGTCTATATTGATGATGTCTTAATCTATTCCCAATCGATAGAACAGCATTGGAAACATTTGTCTATATTCCTTCAAGTTGTTAAGCAAAATGGTCTAGTTGTTTCTGCTAAGAAAATCAAATTGTTCCAAACTAGTATTCGATTTCTTGGCTTCAATATCTGTCAGTCTCAGATTAGTCCAATTGATCGAGTCATTCAGTTTGCTGATAATTCCCAGATGAAATTATTGATAAGAGTCAGCTCCAGAGGTTCTTAGGATCCCTCAATTATGTTTCTGATTTCTATCAAAATCTGAGAAAACAATGTAAGCCCTTGTTTGACAGGTTGCAGGAAAATCCTCCTCTTGGTCCTCTGTCCACACAGAAATTGTCAAACAAATCAAAATTCATGTTAAGACACTTCCTGTCTTGGCATTCCTTCAGTTGGTTCCTTCAAAATAGTTGAAACTGATGCCTCTGACATTGGTTATGGAGGTATTCTTAAACAAAAATTGAGTCCCAATTCGCCAGAGCAAATTGTTCGTTTCCACTCTGGTATTTGGAATCAGTCGCAAAGTAATTATAGTACTATTAAAAAGAAATATTATCTATAGTACTATGCATCTCTAAATTTCAAGATGATTTACTTAATCAAAAATTCTTAATCCGAGTTGATTGCAAATCTGCAAAACATGTTTTAGAAAAAGATGTTCAAAACATTGCATCCAAACAGATTTTTGCACGTTGGCAAAGTATTTGAAGTATTTTTGATTTTGACATTGAGTATTTGAGAGGCAGTGAGAATTCTGTTCCTGATTTTCTTACTCGAGAATTCTTGCAGGGAAAGCAATGGCACCCAAAAAAGAAACAGCAAAGCAGGTGCCAGTCAGTCCAGCCAACCCAAAACTTCCCAAACACCAACCCAAGACCAGTCGCCCAAAAGAGTCCTCCCTGCATCCATGAGTCAATCCAGAATGGCCCATCCAGCCTCAATTGTCCCATATGCAGGTCATCCTCCAATCCCAATCGTCCCAATTGCAAACAGATTCACCAGCCTTGGAACCCCAATAGGATCCACCGTGGCCCAATCCGCCCTAATTATCAGTCAGCCCTTGTTTCTGACTATGATCCATTTGCAATAATTCCTCCAGTTGTCCAATTGACCAAACATCCCAGTCGTCCACCCAGTTCAGAAAGTCGTCCCCTTACCTCCCAAAAGATAAGGTTAATTTGTTCATAATCGAACCAATATATGATGGGATTTCTGATCCGATAACAATTGTCAGATCATACTTCCCTCCTAACTCCCATTTTCCTCCTGCACCCTATAAAGACCTCAAATTTTATAGGGATATCCTGCATGAGACCAAATCAGTTGAAATCAAGCCCATTAAGGATAGAGATAACCCCGCAGTCATTCTCTATCATTCTCTTTATATTCAACAAATTCTAAGCCAACTGAATTTAGTCGCCACCCCTATGAATTGAAACACCTTAAGTCCACTCATCCTTATACCTATGCAGATTACATTGATGCATGGTATAATATCTTCCTCCATCAAACAGTTGATTTTTCCCACTCCTGGTTTATTAATTTCGATGCCAAATTCAGGAGTCCTTTCCCCTACTGGTTTCTTCATTGGTGGGATCAACATGGTCCAACAGCTGATATCCTCCCTACTGAAGTTCATAATCTGACCAATTATTATGCCAGTAAAAGCCAATTTACTAAACAACAGTTGTTTTTCCCCAAGTTGCTTCTTTTTATTGCCAGGTACAAAATCCCCTGGATAATGAAGTGGAATTATCATGTCAATTGGGAAACCAGAATCATGTCTCGCCAGTATTTTGTCAAGTGGTGGGATAAGTTCTCAATGGACAGAATTTCCATCTATGTTTTCAAGGATTTTCCCCAATCGGATCATCATCCCAAGCCAAGTTCTCCTACAGGCTCAGTCAGCTCGGCCATCTCAATCGATGGAAAATCCAAAGCTGAACTCCGGGCCCTTGCTGCACAATTGCTTGACCAAGCTAGTCAGATGAATGATGATGAGGACAACGAATCTTCCCCATCTGCATCCAGCTGTCAGCCTGGCTCCAGCTCCTTTGATCCCAATATCCCTTTTGCGCAGGATCCTTATGACGCGTATGATTTGGGATCTGATTAGTCAAAAGTTGCCATCACTTCCTCTTTTCACCCCGGTGAAATGTTGGAATCAGAATAATTGTAACGTAAAAACAAAATTGCTTTTGCTTTTACCCTTACTTTACCCCAATAGGTAAAGAGAGTAAAACAAAAATACTTTTGCTTTTACCCCTGCTTTACCCCAATAGGTAAAGAGAAAGTTGTTTTCCGTACTTGAAACCATGGTTACTCTCCAATTAGGGTTACTTTGGATTCAAACCTTAGTTGCTTTCTTTCTTTTTAAGCTGGCTTCGGCCTCAGTTGTAAGCACTTGGTTTTTTTTTTCTCCTTTCTTAGTTTTCAAAAGTTCTCTCTGAAACTCCTTCAGACATGAACTCACTTGTAAATATCTGAGTGTTTAGCTTTCAATAAACAAGACAGCTTTGTAAGTTATCTTGAATTTCTCTTATTTTGAAATTAATCTTATTTTACTATTAATGTTGGTTCTTCCCTTTTGGTATCAGAGCCGTAAGAGTTGTAAGAATCTGATCTTAGGTTGCTACTCATCCAACCCAAGTGGCTGGTTTAGGATTTCTGAGTAGAAAGTTTTCAGTTGCTTTTATTATTGTTCTTCTTTCCGGTACTGTTATCTGTATCTCTCTTAAATAGTGTCCGAAGAGTAAGGCCTAGAGTCCAAACAGTAAGTCCCGAGGTGAGGCATGAGCGGTTGCGGCATTGAGAGGGCTAGGAGAGAGAGGTCAGTTGATAGGAACGTACAGGAGTCAGTAGTAGAAGTCCCTGTTTATTTTCTCCTTTGAAGTCAGAACCCAGTTGCCAAAGTAAAATGAGTCGTTTCCTTAGATCTAATTCTTGTAGTTCGAGCAGTTCAAGAACCAGTATTAGTCGTCTTCCAGAAATAGTAAATGAGGAACAAGTAGAATTTGAATCTAATAACAATGATTTAGATTACCGAGACTGGAATATACCTAGAATCCCCAGTCAGGAAATCTATAAGAAGAGGTGGTCTTTACCAAGCTTTAAGACCACTACCCATGTTAAAACAGTTGAACAAGTTTATGCCCTTGGCAAAGAACTTGAAGTCTGCCAGTTGCTCAATCTTGATTCTATCAAACGCCATAAGAGTGAAGGAAACAATTTTCTTCATATTGGTCTTGTCCAAGTTGCCGTCAAACCACTAACCAGAACAGGGCTTAAAGCCTCAGTTTTGCTCTAGTTAAGAGATGCTAGATTCACTAACAGTGAAGACAGTACTCTCGGAGTAATTGAGTCAAGTCTTTCCAATGGTCCGGTTCACTTTGATTGTTATCCTGACTTCACTGTCAGCCTTAGTGATCCCCACATTCTTAAGACCTTGACACTCAATATCCAGACTTCTGGATATGAGGTTCTTCCGGGAACCCAACCATTAGCCTTAATTTTTAGGATCTATTATAAGGTCACGGGAACCAATATGAACTTCCAAGCCCTTAATAAGAGTCCTAAGAATCATACCTTGTTGATTCAAAGTAATAGACCCGAGGCCGTCATCCGAGTACCTCAAACAATCAAATGGTCTGATGTCTCACTTCCAGTTGAATGGACACTCAAAAATGTCAATCCACCAACACCAATCCAACGTAGTCTAAATAATTTAGATTACATCCACCAATATTTAGATGGATCAGTAAAAATCAATTTTGGAACCCAACCAATTCCAAAATCTACTGTCCAGCTACAACAGTTGCCAACCCCTGGTCAATCCCAGAGGCATATCCCTGCCAGACATTCCTTTGCAGCCTCTTCTTCTACTCTAGAACGAGACCTAGAAGCAGAAAGAGCCCTGATTGATTTCCAATTAGAATCCCTCAGGAAAGCAGCACAGGTTAGCCAACCCTGTTATGGCAAACAACCAGTTGAAGAACAAAAGCCAAAATCTCCCGAGTCTCCAACTCAAACTGACTTTGAAGGTTTTGATTTCGTTGACCCTCAACTTAGAGTTCTAAATAAATTTCACATTAATTGGAAAAAATTACACGCCCATCTCACAGCTCCTGAAAACCAGAAGCGCCGAGATGATTACCATAGTGCTTTTCCACTTAAGTACCGCCGTGACGCAATCTTTGTTGAATGGAAAGATTACCTACGGAAAGAACAGGTCGAAATGCCATATTTAGATTTTGTAGAAACCCACTACATGTCTAAAAAATTGAAGGTAATCACCAAAGAAACATGGTCTTTACTCAATGACACACAAGTAGTTACCAGTCATCCCCCGGTTGAGTCTATCTTAATTAATTTTAAGAAAACTCAGATCCCTGCCTGTCCTTTCAAACTCCCTGAAAAATTAGATTCAGAGAAGAAATTGATAGAACAAAGCAACTACACCAATCAGAGTCTAATTGTCATAGGAAAACAGCTTGACAAGATTGAAACTAAAGTAGACAAGCTAACCCTTTCCTCTGAAAAATCCAACATTAAAACTGAAAAACCATTAGTCCAGTTCCAGGATCTCAAGACAAATCCAACTCTTAAGATCAAACCTACCCTTCAGAAAATTGAAGAAATGCTGGAACAATTGACACCTGTAAAGGGTGAGCCATCTGGAGTTAAAGTTCTTGACTCCATTAATCCGGATTCTGACCAAGAATCTATTCAGAGTCTAGAATCAGTTGACAGTAATATCTCAGAAATTGAGAAAGCCTTTACTGAACTTACAGTTGAACCCAGAGTTACCCGGTTAACTCACCAAGTCACCCCTACTAGTCTTACCAAAAATTGGTACACTCGACCAACACCTCCAGATATACAATTCGAAGAAAGACATTTTCAGAACCAGTTCTCTGTCTCTTCTGATAAATTGTATGAGTGGAACATTGATGGTCTTTCTGAGCAAGAGGTTTTCAATAAACTTCAACATATGTCCATGGTTGCCAATAGTTATATTTCAAATCACAGTTTTAGACAATCTGAAATTGTACCTCTGCTAGTCACAGGTTTTACAGGTACCCTCCGCAATTGGTGGGATAAACACCTCACACCTGAGTCTAAAAACTTAATTATTCATGCTGTAAAACTTAATGATGAAGGTCTCCCTATCTTTGATGAACAAATAGGTCAAGGAATAGAAGATGGAGTCAATACTCTGTTTTACACCATAATTGAGCATTTCATAGGAACCCCAAGCCATACTACCTCTAGAATTCATGACCAGCTTAGCAACCTTCGTTGCCCTAAACTGTCTGACTTTAGGTGGTATAAAGATGTTTTCATTTCGCGAGTAATGCTCCGAAATGATAGTAATCAGTCGTTTTGGAAAGAAAAATTCATTAATGGCCTTCCAAACCTATTTGCCCATAAGATTCGCAACCCTTAGTAATGAACAAGGACATATTGATTGGAATAATCTGACCTATGGAAATCTAGTCAGTACTATCAACCAAGTCGGTATGAAGATGTGTGTTGACATGAAAATTGGTAAGCAGATACAGTCTGACAGAAAATCCGCTAAGTATGAGTTAGGAAATTTCTGCGAACAATATGGTCTTACTAGCCTACCTCCCTCCAGTCGAACTAAGTCACACCATAAGAGTCAATCCCGTTCTTACCACAATCGAAAAAATAGATACCAAAATAATGATAGAGAATTCTATAAGACTAAGAAACATAGCCCTAAAAAGAATTGGAAAAAATTTCCATCAAAAAATAAAAAAAACCGATTCCAGAACAATTCTGGAAAAGGAAAAGTTGTCTGCTGGAAGTGTCAAAAACCAGGTCATTATGCTAATAAATGCAAGGTGAAAGACACAATCAAACAACTAAAAATTGAAGAAGTTGAAAAAGAAAATCTAATCCGACTTCTAGATCTTAAGGATTCCGAACCTAGTGATCTCGAGTCATCGACTGATAGTGATTCTGAGAATTCTTATTCCTCGGACTCACAACCTAGTTCCCCCCATATACAACTCGGTTGTAAAGATAATTGTTGTAATTCTATGAAATCCATTTCTGTTCTTACTAAACAGGAAGAACAAGAAGAACTTCTCATAGACTTAATCTGTAAAGTCGAAAACCCAGAGTTGAAGGCTGAGTATCTGAAGAAATTTCGGAAACTCCTTAGTCAGGAAGGTCCAAGTCAGTCTCCACCTCCTCAAAAGATAAGTTTAAATACAACCTTAGAAAGGTTCAGTCGCAAAAAAGACATCACCCTTCATGATTTACATTCAGAAGTAAAATTAATCAAGAAGGAGATAGTTGATCTAAAACAATTAAGCCAGAAATTGCAAGCTGAGAATTATGAAATCCGACAGGATTTAATAGCTCTCAAACCTGATGATTTATCTCAGAGTCCAACAAACTCAGATGATGAATCTTTGAATCCAGAACAGGCCTTATGTCTAGTCAAAAGAATTAACTTCAAGAAATGGTATGTCAAAGTTACTATTTTTGTTGATCAGTTTGATTTTACCACAGTCGCTTTGCTTGATTCAGGTGCTGACCTTAACTGCATCCAGGAAGGTCTTATACCTACCAAGTATTATACTAAGTCCTGTGAATCTCTTAGAACAGCTTCAGGAAAATCCCTACAGCTGAACTATGAAATTCCCCGCGCCCATGTTTGTCAAAATAAAATTTGCTTTAAACTTCATTTGTTCTAGTAAAAAACATTACTGATGAAGTTATTCTAGGATTACCTTTTCTTGCCTTGTTATATCCTTTCCAGGTGAACCATGATGGCATCATCTCTACTCACCTAGGAGAACAAGCCAAGTTTGAATTTTTATCTAAGCCAGAGTTGCACCAGTTGAAAACCTTTCAGAAAAGTTCAATCTCCAAAACTATTGCTTTGATTTCCAGTAAGACTAAACATCTGTCTTCTCTCCAGGAAGAAATTAGTCATACTAGAATCAGCCAACAAATTAGGAATGAACCTTTCCTGAAACAAGTTCATGAGTTTGAAACTAAATTAATCAATGATATCTGTTCTGATCTGCCTAATGCTTTTTGGTCCAGGAAACAACACATTGTCAGTCTTCCTTACGCCAAAACCTTTAGTGAGAAAAATATCCCCACTAAAGCCAGACCAATCCAAATGAATCAGGAGACAATGGAATTCTGTAAAAAAGAAATTACAGAATTACTTCTAAAGGGAATCATCCGTAAGTCTAAGTCCCCCTGGTCTTGTCCTGCCTTTTATGTCCAGAAAAATGCTGAACTAGAAAGAGGAGTCCCTAGACTAGTTATCAATTACAAACCCCTCAATGCAGTCTTAGAATGGATTAGGTACCCCATACCTAATAAACGAGATTTAATTAATCGGTTAGAGAAAGCAATTGTCTTTTCCAAATTTGATCTTAAGAGTGGATTCTGGCAAATCCAAATAGAAGAGTCAGATCGGTACAAAACCGCCTTCGTCACTCCTTTTGGTCATTATGAATGGAATGTTATGCCCTTCGGTCTTAAGAATGCGCCTAGTGAATTCCAGAATATTATGAATGAAATTTTCAATGCTTATAGCCATTTTTCAATTGTCTATATTGATGATGTCTTAATCTATTCCCAATCGATAGAACAGCATTGGAAACATTTGTCTATATTCCTTCAAGTTGTTAAGCAAAATGGTCTAGTTGTTTCTGCTAAGAAAATCAAATTGTTCCAAACTAGTATTCGATTTCTTGGCTTCAATATCTGTCAGTCTCAGATTAGTCCAATTGACCGAGTCATTCAGTTTGCTGATAAATTCCCAGATGAAATTATTGATAAGAGTCAGCTCCAGAGGTTCTTAGGATCCCTCAATTATGTTTCTGATTTCTATCAAAATCTGAGAAAACAATGTAAGCCCTTGTTTGACAGGTTGCAGGAAAATCCTCCTCCTTGGTCCTCTGTCCACACAGAAATTGTCAAACAAATCAAAATTCATGTTAAGACACTTCCTTGTCTTGGCATTCCTTCAGTTGGTTCCTTCAAAATAGTCGAAACTGATGCCTCTGACATTGGTTATGGAGGTATTCTTAAACAAAAATTGAGTCCCAATTCGCCAGAGCAAATTGTTCGTTTCCACTCTGGTATTTGGAATCAGTCGCAAAGTAATTATAGTACTATTAAAAAAGAAATATTATCTATAGTACTATGCATCTCTAAATTTCAAGATGATTTACTTAATCAAAAATTCTTAATCCGAGTTGATTGCAAATCTGCAAAACATGTTTTAGAAAAAGATGTTCAAAACATTGCATCCAAACAGATTTTTGCACGTTGGCAAAGTATTTTAAGTATTTTTGATTTTAACATTGAGTATTTGAGAGGCAGTGAGAATTCTGTTCCTGATTTTCTTACTCGAGAATTCTTGCAGGGAAAGCAATGGCACCCAAAAAAGACAAGCAGAAAGCAGGTGCCAGTCAGTCCAGCCAACCCAAAACTTCCCAAACACCAACCCAAGACCAGTCGCCCAAAAGAGTCCTCCCTGCATCCATGAGTCAATCCAGAATGGCCCATCCAGCCTCAATTGTCCCATATGCAGGTCATCCTCCAATCCCAATCGTCCCAATTGCAAACAGATTCACCAGCCTTGGAACCCCAATAGGATCCACCGTCGGCCCAATCCGCCCTAATTATCAGTCAGCCCTTGTTTCTGACTATGATCCATTTGCAATAATTCCTCCAGTTGTCCCAATTGCCCAAACATCCCAGTCGTCCACCCAGTTCAGAAAGTCGTCCCCTTACCTCCCAAAAGATAAGGTTAATTTGTTCATAATCGAACCAATATATGATGGGATTTCTGATCCGATAACAATTGTCAGATCATACTTCCCTCCTAACTCCCATTTCCTTCCTCCTGCACCCTATAAAGACCTCAAATTTTATAGGGATATCCTGCATGAGACCAAATCAGTTGAAATCAAGCCCATTAAGGATAGAGATAACCCCGCAGTCATTCTCTATCATTCTCTTTATATTCAACAAATTCTAAGCCAAACTGAATTTAGTCGCCACCCCTATGAATTGAAACACCTTAAGTCCACTCATCCTTATACCTATGCAGATTACATTGATGCATGGTATAATATCTTCCTCCATCAAACAGTTGATTTTTCCCACTCCTGGTTTATTAATTTTCGATGCCAAATTCAGGAGTCCTTTCCCCTACTGGTTTCTTCATTGGTGGGATCAACATGGTCCAACAGCTGATATCCTCCCTACTGAAGTTCATAATCTGACCAATTATTATGCCAGTAAAAGCCAATTTACTAAACAACAGTTGTTTTTCCCCAAGTTGCTTCTTTTTATTGCCAGGTACAAAATCCCCTGGATAATGAAGTGGAATTATCATGTCAATTGGGAAACCAGAATCATGTCTCGCCAGTATTTTGTCAAGTGGTGGGATAAGTTCTCAATGGACAGAATTTCCATCTATGTTTTCAAGGATTTTCCCCAATCGGATCATCATCCCAAGCCAAGTTCTCCTACAGGCTCAGTCAGCTCGGCCATCTCAATCGATGGAAAATCCAAAGCTGAACTCCGGGCCCTTGCTGCACAATTGCTTGACCAAGCTAGTCAGATGAATGATGATGAGGACAACGAATCTTCCCCATCTGCATCCAGCTGTCAGCCTGGCTCTAGCTCCTTTGATCCCAATATCCCTTTTGCGCAGGATCCTTATGACGCGTATGATTTGGGATCTGATTAGTCAAAAGTTGCCATCACTTCCTCTTTTCACCCCGGTGAAATGTTGGAATCAGAATAATTGTAACGTAAAAACAAAATTGCTTTTGCTTTTACCCTTACTTTACCCCAATAGGTAAAGAGAGTAAAACAAAAATACTTTTGCTTTTACCCCTGCTTTACCCCAATAGGTAAAGAGAAAGTTGTTTTCCGTACTTGAAACCATGGTTACTCTCCAATTAGGGTTACTTTGGATTCAAACCTTAGTTGCTTTCTTTCTTTTTAAGCTGGCTTCGGCCTCAGTTGTAAGCACTTGGTTTTTTTTTCTCCTTTCTTAGTTTTCAAAAGTTCTCTCTGAAACTCCTTCAGACATGAACTCACTTGTAAATATCTGAGTGTTTAGCTTTCAATAAACAAGACAGCTTTGTAAGTTATCTTGAATTTCTCTTATTTTGAAATTAATCTTATTTTACTATTTTCATTTATGCATTATATTCTCGCATTAGTCTGAAAGTTGGTTGTAAGATTCTCTGTTTTCTCTTGTGCTTCTTTATCTATCTCCTGTTCTACTATTAATGTTGGTTCTTCCC

TATATATATATATATATATATATATATATATATATATATTTGGACAGAATCACCATGCCTCACAATATATGCGAGGGACATATTTGAGGGTAAAGTAGTATTTTTCCCATGGTGTCAAAACACCAACACACACATGCCTATGTGAGGGACATATTTGAGGGTAAAGTAGTCTCTTTTCCATGGTGTCAAAACACCAACACACACAAGCCTCTCAATTGCTAGTCATCGTTTGGTGGGCATGAGTGCTTGTCATGATTTTTCGGGTGAAATTCAATGTTCTAATAGATTAGGTTGTGGGAATCTTTGCATCTTCTACGTTATGGAAGCTAGGAAATCGACAGATTCTGGATTAAGGACAACGAATATCTTTGGTATTTTTTCGAAGTTTCTAATTTTTTTTTTAAAAATTTAATGTTCGGTCCAATCTTGCACCAAACAATGGACATAATTCAAGTCTGGTCCCAACCAATCCAATCCAAAAATTGAAGTGTCTTGTCTAGTCTTGGTCACCAAACGGGGCCTATATCTTATATCGGATAGGAATGATTGAAACTCTATGGACCAAACTAGAACTTTGATCAAACTATAAGGACCAAAAAATTAACTTCTATTAAGCTACCTCGAAGTTTTTATTTTTGGTAATTAAATAAATATAGAACCAAAATCAACTTCTCTGGCATATACGACTGTACACAATCTGTTTTCAACATTTCATTCTCGAACTGCTAATGGAATTGCTAATGCTACTGCCAAGCCAATACTAAAATTGTATCCAGTGCTCCGCACTCATGCCACTTGACTTGAGTGCTTGTCCTGCTAGAGTTGCATTTTCAAAACTCAATCACTCCAACATTTCCATCATCAAGCCCTAAATCCTTGTATCACGTTAGAGTGCCTTGAGCTAGGCTTGATTATAGTCTAAAATATTGATAATATCGACAAAATATCAAGGATATTATCTTTTTTTCGAATCACGGATATTTCGAAACGTATCCATGCA

>Chr6-03; OZ022211.1; 10551352-10551696; dir; B;

AAAGGCTTTGGTAGTGTGTGTGATAATCCCTTCTCTCGGATCCCTCAAATAAACACCTTCTAGGGTCCCCTACGAATTTTTTTTCTCAACGATCCAAACCATCTATTTTTTAAGTCAACATTCATAGATCATCCTTGTAAAAAATTAGACAAATCGGAAACCATTTCGACATCCAATTGTATCCTAAAACATCAATGAACACGGTGTTTCAAGAAAGTACTAAAATTTCAATAACTCAATTGAGTGGTCAAATGATAATAGGTTCAAGTGATTTTTTTTGTAGAGATTATCTTTGAATGAGTATATACAAAATAGACGGTTTGGATTAGTGAAATACAATCCAGAGTGGGGCAGAAGGGGTATCCCTTATTTGAGGGACACCCCTATAGGGCCCACTCTGCATTGTATTTCATTCATCCAAATAATCTATTTTGTATATATTCATTCAAAGATCATCTCAACAAAAAAAATTACTTGAATCCGATATCATTTGACCACTCAATTGAGTTACTGAAATTTTAGTACTTTCTTGAAGCACCGTGTTCATTGATTTTGTAAGACACAATTGGATGTCGAAATGATTTCCGATTTGTCTAATTTTTTGTAAGGATGATCTATGAATGAATACCTAAAAAATAGATAGTTTGGATCATTAGGAAAAGAATTGTAGGATACCATAAAGGGCGTCCCTCAAATAAATATTTGAGGGATCCTCAATAGAAGGGGACTGTATATATATATATATATATATATATATTTATTTATTTATTTGAGGGATCCCTCAATAGAAGGGGACTGTATATATATATATATATATATATATATATATTTATTTGAGGGATCCCTCAATAGAAGGGGACTGTATATATATATATATATATATATATATTTATTTATTTGAGGGATCCCTCAATAGAAGGGGACTGTATATATATATATATATATATATTTATTTGAGGGATCCCTCAATAGAAGGGGACTGTATATATATATATATATA

GTTATTCAATTTTCTGATAAATTTCCAGAAGAAATTTTTGACAAAAGTCAACTCCAAAGATTCTTAGGATCTTTCAATTATATTGCTGACTTCTATCAAAACTTAAGAAAGCAGTGCAAACCCTTGTTTGATAGACTTCAAAGCAATCCTCCTCCTTGGTCTCCTAGTCACACAGAGATAGTTCAGAAAATCAAGCATTATGTCAAAACATTACCTTGTCTTGGCATTCCGCAGACAACTCATTCAAAATAGTTGAAACTGATGCCTTTGGAATTGGTTATAGATGTATTCTTAAACCAAAAAATCCCTCCTAGTCAATCAGAACAAATTGTTCGATTAGATAGA

TATATATATATATATATATATATATTTTGACCATCTATGAATTTGTGCTCGATTCGCATTTTGGTCTTCGAATTAGAAAAATGGTTGTTGTATTCCCTCAACTGCAATTTCTCTCGTAATGTTGGTCATAGTGTCAATTCTATTACATTTTATGTCAATTTTGAGCACGTGCCATGCATGTGGTTTACCGTTGAAGGTCAAAATTGACTTTTAACACAATTTCAAATCTCCAATTGCTCATCAGTAGGGAAATTGAAGATGTTTCGTAAATTGGGGCCGATTAAAGTTCTAAGGTTTTAGAAATTCACCAATTTGATTGCAATTTGAGTGGATTTGTTGGGGGTAATTGTTCTAGTAGTGCCAATTTAAGGTTCAAGGGAGGGCTTGATTAGGGTTTGATTATGGTATTAGGGCAATTTTTTAAGACATTATGACGCTAGGACCAACGGTGCAAGAGACACTGAGGTTGAGAGACTACAACAATCATTTATCAAATTCAAGGACCAAAATGTGAATTTGGTACAAATTGATGGACTATTGCTCTAAAAAGCTTTAAAATTAAGTTATAGTTAGTTTGCGAGCAACTGGCTCTTAGTTTACCCTGTTGCTTTATCAAAATGAAATGTTAGATTTATTTTTTATTTTTGATACAAACGATATTTGGAGGAATGGGAAATCGAACCTAGATTAGGTGCATGAGTAAATGTTCTTAACCACTTGAGCTACAAGTCTCTTGCAAAGGTTAGGAAATTCGGAATGAAAGATCAGCTACAAAATTGTCAACTCACAAAATTTTGTCAGCTACAAAACGAATTCTATGATAATGAGTTCACTTCATTTTATAGCACACAATAAATCCAAAACACAACATTCCGAACAAAAACAGCGCAAAGACGCAAATGGGTTTTTGTCAAATCATGGGTTACTTTTCCTTGTAACAAAAAACAGGCATAAAATAAAAGCAAAAATGGCAAATGAGTTTTCAATCTTGAATATGAAG

>Chr6-04; OZ022211.1; 10831841-10837805; dir; D;

TAGAAATAGAAATTTATTATTTACTTGGCCACTAAGGCTCCGTTATATTATGAATATGACCTCCTACAAGGAGGTGAATATACAGAAAATTCCCACAAACAAATATTCTCATATTAGGTTTCATATTTTAGTAGGCTTTAAATACGATCTACAAGAAGAGCAATGGTGACTATGGCCTCGAAATAGTCAGATCTTCTATTTCTCCTTCTTCATACAAAGTATTACTCAAAGACCCTAAATCCCAGAGAAAGGTAATCAAGTTTTAGCCAATGAAGAGAGAAATATGAGGGGCTAAGATAGAGAAATCTAAGGCTGAGAGAGAATAACATGATGGAGTCTGAACAGACGAGGGTATTTTGGCGTTTCAGAAAAAAGAGAGAGGGTATTTTTGCAATATCAAAACCTTCATTAAAGGAAACCAAATGCTTCCTCTATATTTGCGTTGTATTTGCCTGGCGCAGCACTTCTATGAAGAAGCACCTTATATTATTGCTAGTTTTGGCTACAGATCAATTTGTGACTAAAATTAATCGAGGTTTTCTAGTTGCCATATCTAAATTAGACTACTTAATTAGCTTGTATATATATATATATATATATATATATAGTCCATTTCTATTGAGGGATCCCTCAAATAATTTTATTTGAGGGACGCCCTTTAGGGTACCTTACAAATTTTTTTTTCCAATGATCCAAACAATCTATTTTTTAGGTCTTCATTCATAGATCATCCTTACAAAAAATTAAATAAATCGAAAACCGTTTCAACATCCAATTGTGTCCAACAAAATCAATAAACACGGTGCTTCAAGAAAGTACTAAAATTTCAGTAACTCAATAGAATGGTCAAATGATATCGGATTCAAGTGATTTTTTGTAGAGATGATCTTTGAATGTGTATCTACAAAATAGACGGTTTGAATTAGTAGAATACAATTCAGAGTGGGCCCTACAAGGGGTGTTCCTCAAATAAGCTTATTTGAGGGCTCAATGGAAGCTC

AAATCTGTAGTCGAGTCTAGTCACCCTCCAGTAGAAACTATAATTATAGAACATCAGAAAGCCCCAATTCCTGCCACTCCTTTCAAAACCTTTGAAAGCTGTGATCCTCATAGGAAATTAATTGAACAAAATAACTACACGAACCAGAGTCTAATCACCATAGGTAAACAATTAGATACAATTGAAACCAAGGTTGATCTGTTGAGTCCGCCTGTTGCAGACTCCAGACCCAAACCTAAAGTTGAAAAACCAATAGTTCAATTCCAAGAACTTAAGTCCAGTCCAGTTTTAAAAGTTAATTCCACAATGAAAAAGATAGAAGATATGTTAGCACAATTGACCCCTGTCAAGCCCGAGAAAGGTGATCAGTCTGGTATCAAAACACTAGATGCTTTTGCTGCTTCTCAATCTGAGTCAGAATCCGTAAGTTCAGAAACTAGTGACATTTCTCAAATTGAAAATGCTTTCAAAAATTTAGACGTCCAAACTGAGCCCAAAGTCAAAAGAATTGAAAAACATATAAGCCCTACTAGTCTTACGAAAAATTGGTACCCCAGGCCAACACCTCCAGATATCCAATTTGAAGAAAGAAACTTCCAAACCCAGATTTCTGTTTCTTCCGACAAATTGTATGAATGGAATATTGATGGTCTTTCTGAACAAGAAATTCTAAATAAATTGCAACATATGTCTATGGTCGCAAATAGTTACATCACCAATCACAGTTTTAGACAGTCTGAGATTGTTCCTCTGTTAGTTACTGGTTTTACTGGTACTCTCAGATATTGGTGGGATAAACACCTCACTCTTGAGTCAAAAAATCGAATCATCTATGCTGTAAAACTTAATGAAGACGGTCTCCCTATTTTTGACGAACAAAGTGAGTATAAATAAAAGTTATTTTAAATTATCTATTTTCTGAAAGCATTAGTTTATGCATTATTCATTAATTTGCTGCATTAGTCTGAAAGTTGTTTAGGTTTTCATCCAATCTGCATTTTCTGTTCTTCCTCTGACTCTATTCATTGTCTTCTTCAATTACTCTGTCTGTTGTTCTTCCCTTGTTGGTATCAAAGCCGTAAGTGTTACGGTCTTTGTCTAGGTCTGCTACTCATCCCCGCCCAAGTGGCTGGTTTAAGCCTCTGAGTAGAAGGTTTTCATTTCTTCCTTCTCTCCCCTTTTAGTCTTCGAGTCCTCTGTTGGTTTTCTCTAAGGTCTGACCCGTGAGTCCTTTTTGCTAGATTAGTAAGTCCCCAGGGAGGGCTGTGTGCTGGGCAGGAGACAGAAAATTGAGAAAATTAATAGGTTGCTTAGAGTCTAAACGGTCGGTTTAGGATCTACTGTTCATCTTCTTCCTCAGATAGCCAAACCCAGTTATTTGAGTAGTCATGAGTCGTCTATTTAGAACAAAGTCTTGTAATTCTGCCAGTTCTAGTACTAGCATAAGTCGACTCCCAGAGATAGTTAATGAAGAACAATTTGAATATAATTCCAGTAGTAAAGAACAATTAGATTTCAGAGATTGGAACATACCTAAAATCCCAAGTCAGAAAATCTATAAGAAACATTGGTTGCCTTCTAGTTTTAATAGTACAACACATGTCAAAACAGTTGAACAAGTATACGCTTTAAGTAAAGAACATGAAACTTGCCAGTTGCTTAATCTTGAGTCTATTCTGAAACATAAGAAAGATGATAATAATTTCTTGCATATTGGTCTAGTTCAAGTTGCTGTCAAGCCTTTGACACGGTTAGGGTTGAAAGCCTCAATCCTGCTATGTCTTCGTGATGCTAGATTCACCGAATTTAGCGATAGTACCCTTGGAATCATTGAGTCCAGTCTGTGTAATGGCCCTGTCCATTTTGACTGCTATCCTGATTTCACTGTAACCCTTAGTGATCCTCATATCCTGAGAACTTTGACTCTGAATATTAAGACTGAAGGTTATAATGTTCTTCCGGGAACCCAACCTCTAGCCTTAGTTTACAGAATCTACTATAAGGTAACAGGAACAAATATGAACTTCCAAGCCCTTAATAAAAGCCCAAAGGATCAAACAGTCCTGATCCAGAGTCACACCTCAGACGCCCATATACAAGTTCCCCATACTATTAAGTGGTCAGAAGTTGCCCTTCCAAAAGACTGGACCTTAGTTACTGAGAGCCAACCGGCTCCCATCCAGCGTAGTCTAAATAATTTAGACTACATCCAACAATACTTAGATGGCACGGTCAAAATCCAGTTCGACAGTCACCCCTTACGCAAGTCTTGTTTTACACAATAATTGAACATTTCATTGGAACCCCTAGTAATACCACTGCTAGGATCCAATGTCCAGTTACAACAATTGCCTACTCCTGGCCAATCCCAGAGGCATGCCCCTGCTCGGCATTCCTTTGCTGCTTCTTCCTCCACTCTAGAACGAGACCTAGAGTTAGAAAAGGCCATGATTGATTTCAAACTTGAGTCCCTGAGAAAAACCTCTCAGGTCACCCAACCTTGCTACGGCCAGGTTCCAGCCCAAGAAGACAAGTCAGAATCACCTGAGTCACCTACCCAGTCTGACTTTATAGTTGAAAATCAGCTTAGAACCCTTAATAAAGAATTTAAAATTGATTGGGTTCACCTAAATCGTCATCTAAAAGCCCCTGAAAACAAGCCAAGGCGAGATTTTTATCATCAAGCCTATCCTAATAGCAAACACCGTGAAATGATCTTTAAACAATGGAAAGATTATATGCGGTCAGCCGAAGTTGAAATTTTCTACCTTGATTTTATTGAAAGCCGTTATATGTCCAGTGATGAATTAAAAACCCTCACTAAAGAAAAATGGAAACTAGTTGACAAATCTGTAGTCGAGTCTAGTCACCCTCCAGTAGAAACTATAATTATAGAACATCAGAAAGCCCCAATTCCTGCCACTCCTTTCAAAACCTTTGAAAGCTGTGATCCTCATAGGAAATTAATTGAACAAAATAACTACACGAACCAGAGTCTAATCACCATAGGTAAACAATTAGATACAATTGAAACCAAGGTTGATCTGCTGAGTCCGCCTGTTGCAGACTCCAGACCCAAACCTAAAGTTGAAAAACCAATAGTTCAATTCCAAGAACTTAAGTCCAGTCCAGTTTTAAAAGTTAATTCCACAATGAAAAAGATAGAAGATATGTTAGCACAATTGACCCCTGTCAAGCCCGAGAAAGGTGATCAGTCTGGTATCAAAACACTAGATGCTTTTGCCGCTTCTCAATCTGAGTCAGAATCCGTAAGTTCAGAAACTAGTGACATTTCTAAAATTGAAAATGCTTTCAAAAATTTAGACGTCCAAACTGAGCCCAAAGTCAAAAGAATTGAAAAACATATAAGCCCTACTAGTCTTACGAAAAATTGGTACCCCAGGCCAACACCTCCAGATATCCAATTTGAAGAAAGAAACTTCCAAACCCAGTTTTCTGTTTCTTCCGACAAATTGTATGAATGGAATATTGATGGTCTTTCTGAACAAGAAATTCTAAATAAATTGCAACATATGTCTATGGTCGCAAATAGTTACATCACCAATCACAGTTTCAGACAGTCTGAGATTGTTCTGTTAGTTACTGGTTTTACTGGTACTCTCAGATATTGGTGGGATAAACACCTCACTCTTGAGTCAAAAAATCGAATCATCTATGCTGTAAAACTTAATGAAGACGGTCTCCCTATTTTTGACGAACAAATAGGTCAAGGAATAGAAGACGGTGTCAACACTCTGTTTTACACAATAATTGAACATTTCATTGGAACCCCTAGTAATACCACTGCTAGGATCCATGATCAGTTGAGTAATCTTAGGTGTCCGTTGTCTGATTTCAGGTGGTATAAAGATTTTTTCATTTCTAGAGTCATGCTTTGAGATGATAGTAATCAACCATTCTGGAAAGAAAAGTTCATTAATGGTCTGCCTAATTTATTTGCTCACAAAATACGCACCACACTAAGTAATGAACAAGGTCAAATTGATTGGGATAATTTAACATATGGAAACATTATAGGCACAATTAATCAGGTTGGTATGAAAATGTGTATTGACTTTAAAATCAATCGCCAAATCCAGTCTGATAGAAAGTCAGCCAAGTATGAGTTAGGAAACTTTTGTGAACAATATGGTCTCACTAGTGTTCCTCCCTCCAGAAAACATAAGCCTAGTCACCTTAGAAAACGCCATTATCCGAAGAAGCAGTTTCTCCGGAAAGATGAATTCTATAAGAAAAGAAAGTTCTCCCCTAAAAAGAGTTGGTCGAAACATCCCAAACAGAGTCGCTCTAAGAAAGATAAAAGCAAAGTTAAATGTTTTAAATGTCAAAAATTTGGTCATTATGCTTCTGAATGCAAAGTAAAAGATGTTATTAAACAGTTGCAGATTACAAAAGAAGATAAGGATAAATTGATCCAAGTTTTAGAGTTGCGAGACTCTGAAATGAGTGATGTTGAGTCTCTCATACCTAGTTCCGAATCTGAGTCCAGCCAGTCTTCAGATTCAAAACATAGTTCACCTCAAGTCCAATTTGGTTGCACTGATGAATGTTGTAATAGGTTAAAGTCTGTTTTAGTTCTTACTAAACAAGAAGAACAAGAAGAGTTACTTGTAGATTTAATAGGAAAAGTTCAGAACCCTGAGTTGAAATCTGAATATTTGAAAAAGTTAAAAAAGATCTTAAGTCAGGAAGGTCAGAGTTCATCCTCTCCTGCTCCCCAGCCAATCAGCATCAATACTACGTTAGAAAAATTCAACCGTAGAAAAGATGTTACCCTTCATGACCTCCAATCGGAGGTCAAGCAGATTAAAAAAGAATTAGCTGAATTGAAACAAATAAGCCAACAATTGCAAGCCGAGAATTATACCATTAAGCAAGACTTATATGACCTAAAGCAAGAAACCTGCAAGCCTAATAATCTTTCAGGAAGTCCTCATCAGTCTGATGATGAACCTTCTGAAGAACAGCCAGTTGTCAATTTAATTAAACAAGTTCATCTCAGAAAATGGTATTCAAAGGTCACGATATTTGTTCATGATTTTGAATTTACCACAGTTGCTTTATTCGATTCTGGTGCAGATCTTAACTGTATTCAGGAAGGTCTCATCCCCACTAAGTATTACCAGAAGTCTAGAGAGTCCCTTAGCACTGCCTCCGGGAAGTCTCTCCAATTAAATTTTGAAATACCAAAAGCCCATATTTGCCAAAACAAAATCTGTTTCAAAACTTCATTCGTCTTAGTTAAAAACATCACTGATGAAGTCATCCTAGGATTGCCTTTCCTAGCTTTACTTTATCCTTTCCAGGTAGAATATGATGGCCTCATTTCTACCCATTTAGGAGAAATAGTCAAATTTAAATTTCTGTCAAAACGTGAGTTGCATAATCTGAAAGCTTTATAAAAACAGTCAGTTGCTAAATCTATTCAAGTTATCCGAAAAACTAACCAATTGAAATTTCTTAAAGAAGAAATTAAGTTTAAGAGGGTTGAACAACAACTTGCCGATAAAGCTTTACAGTTAAAAATCCAGTCGTTTGAAAATAAATTAAAAGAAGAAATATGTTCTGATTTGCCTACTGCCTTTTGGTATAGAAAACAGCACGTTGTCTGTCTTCCTTATATCAAAACCTTTAGTGAGAAAAATATCCCCACTAAAGCCAGGCCCATACAGATGAACCAAGAGTTAATGGAATTTTGTAAAAAAGAAATTCATGAGTTGCTTCAGAAAAATATAATTCGCAAAAGTAAATCCCCTTGGTCTTGTCCTGCCTTTTATGTCCAGAAAAATGCTGAACTAGAAAGAGGAGTCCCCAGGTTAGTCATCAATTATAAGCCCCTTAATTCGGTCCTAGAATGGATTAGGTATCCCATACCTAATAAAAGAGACCTCATTAATCGACTAGGAAAAGCAGTTGTCTTCTCCAAATTTGATATGAAAAGTGGTTTCTGGCAAATCCAGATAGATGAGTCAGATAGGTATAAAACTGCATTTGTTACTCCTTTTGGCCATTATGAATGGAATGTCATACCGTTTGGTTTAAAAAATGCCCCCAGTGAGTTTCAGAATATTATGAATGATATCTTCAATCCTTATAGTCATTTCTCGATTGTTTATATTGATGATGTCCTCATTTTTTCTGAGTCAGTTGAACAACATTGGAAACATTTGCATAAATTCCTTCACATAGTCAAACAAAATGGTCTAGTTGTCTCTGCTAAGAAAATTAAATTGTTTCAAACCAATATTAGATTTCTTGGTTTCAATATCTGCCAGTCCCAGATTTCCCCAATTGATCGAGTCATTCAGTTTGCCGATAAATTCCCTGATCAGATTCTTGATAAAAGTCAACTTCAGAGGTTTTTAGGATCTCTAAATCATATCTCTGACTTCTATCCAAATCTGCGAAAACAATGTAAGCAATGAAAACCTTCTACTCAGAGGCTTAAACCAGCCACTTGGGCGGGGATGAGTAGCAGACCTAGACAAAGACCGTAACACTTACGGCTCTGATACCAACAAGGGAAGAACAACAAACAGAGTAATTGAAGAAGACAATGAATAGAGTCAGAGGAAGAACAGAAAATGCAGATTGGATGAAAACCTAAACAACTTTCAGACTAATGCAGCAAATTAATGAATAATGCATAAACTAATGCTTTCAGAAAATAGATAATTTAAAATAACTTTTATTTATACTCACAAAGATTGCAACTGCAATGCTTCAAAGCACAACTGCACGCTATTTACAAGAGTGGTTACAAAGATGAATTCTCCGAAGGATGCATCAGAGGAATTCTTTGAAAACTGAAGAGAGAAAAAATTACATTCTACTTACAACTGAGGCCGAGGCCATCTTCTATAGAGGAAAGTAACTAAGGTTCCACTAACTATGGTAAAGTAACTGAGTCTGAGGCAACTGTTAGATCACGGGAAAGCAACCTAATTCAACTTTCAATTTTGTCTTTATTCGAAAGATTCTTTTACTGAGGCAGCATTCGGTGGGAAAGCAAACAGTAAAAGCAGATGTTTCTTCCTGTGGCAATTATGGTGTTGGGCAACTTTGGCATGTGAAGGTGTCGGCTACTTTCGGCTACTTTTGGCATGTGAAGATGTCATAAAACAATAATGAGGCCTAGTCAGAATGCAGATCATAGGCATCATAGGGATCTTGACTGTCTTCATACCAGGACTTTGGTTTGACGGAGATGGGATGACAGCTAGAAGACGAGGGGGACCCGTTGTCTTCATCATCATCCATCTGCGAGGCTTGTCTGAGCAACTGGGTAGCGATTTCTCTGAGTTCTGCCTTGGATTTTCCTTCGACTGAGAGGGAAGATCGAACAGATGCAGAGGGAGAGACCGACTGGGGTACTGGTTCAACTGGAAGGTCATTGTAGACATATCTGGCAATTCTTTCAACTTCAAATTTGTCCCACCATTTAATAGAGAACTGGCGGGCAAAAATACGGACATCTTTAGTGACCCTGTAAGACCATTTTAGTATCCAGGGCACCTTGTATCTGGCAATGAATAGCAACTGCTTAGGGAAAAATAAATCAACTGCAGAGAACTTGGCCTTCTTGGTGTAATGGCGGATGAGCTCAAGGATTGCTACTGGAAGTATTTCATCAACTGGACCATGCTTTTCCCACCAATGGAGAAACCAGTATGGGAAAGGACTTTTGAACTTGTTATCAAAATTGATGAACCAGGAATGACTAAAATCTTCAGATTGGTGAAGAAAAATAGTATACCAGGCATCAATATAATCAGAATAATTATATTGTAGTTGGGATTGGAGCAACTTGAGTTCATAAGGGCGACTGCTGAAGGCTTTTTGGCTTATAATCTGGTGAATGTACAAGGAATGATAAAGGATAGTGCCAGGGTTGTCACGATCCTTTATGGGCTTGATTTCAACTGATTTGGTCTCAAGCAGTATATCTCTATAATACTTGAGAGATTTGTAAGGACTGGAAGGCATATAATGGAATCCAGGAGGGAAATAATGACGGGCAATTTCAATTGGGTTGGTATGGATGTCATAAATGGGCTCAATGACAAACAGATGGGAATTACTTTTGGGCAAATAGGGAGATGATTTTTTGTGTATGACTGTGGGTACCGAGGGTCCGGCAGGGATATCGACTGCAAATGGATCATAACTGGCAACTAGGGTAGACTGGTAACTGGGACGAATTTGGCCAACTGTGGATCCTAGAGAGGAGTACCGGTTTGCAACTGACAATGGGGTGGTAAACCCTCTCTC

TATATATATATATATATATATATATATATATATATATATATATCTCGAATGTATACTCACAAATCTAACTTATTATCTTATACGTACTTCATGCTCAAAGAAAGACTGAAAATATTTCCTTCTGTGTCTAGCGTTTCCATACGACCCAAAAAAAGACGAGTGTTTCCCGAAAATTAGGTAGATTGCAGAGGTTCAAAAATTCAAGATTCAAAATTAGTAGGAAAAAAATGCATACATAGGATTATGAAATTCTAATAACTCATCTTTCGAACTATAGTAATCTAAAACCCCCTTTCAGATAATGTTGTTGATTCAAGCACTCAAAGTACGAAGACTTATTGAATCAATATCACCATTACTAAAGTACTTTATTACAAATTTTTCCTAAATTATCATGCATAGATACACATTATTCTGGGGGTAAGGTTGAACATTGAGGACTTGGCTTTCTATTGGATCATATGTTTTCCTGGCTGAAATATAAGACTTGAATAACATGTTGACAAGAACATATCGCACAAGAGTGGACATTGCAAGAAAACGAACAAGACAACTGGAACAAATGCAAAAATAAATATAAATTCCGAGATGAAACGTAATCCGTAGTGACAAGATAAATAGAAGGCTGTGCTGAAGGCTATCATCATGGATGTCATGGAGATGAAGAGTGATGCGAGTCCTACCATTAACTTCAACGGTAAGGATTTGAGAAAATCGTTTTCTGCATACCGCGAGGTGAGGATGTACAAGAACATCAGCATGGAAGTTGAAGAGGAAAAGAGTGCTACTCCATCTGATATGGCAAAGATGAGAAATGCTGTATCTTTTAGGAATTTTGGTGCTCCTGTATTATCAGCTATACCACCTGGTATGCTAAATGCAGCTGAAAACACGACAGTGGTGATAATGGTAGCAACAAGCATGCACAAATTTGCAGTGTTCTTCATCCATGATTCTCCCTCACACAATAACCCCTCATGCTCACTAGTG

>Chr6-05; OZ022211.1; 10858877-10918228; rev; C;

GGATACTTCATCATATCAGACTAAGATACTTTATTCAATGGGAAACTAGAATACTTTATTGAATAGGAAATAGACCACACAAGAAATAGCATAAAATAATAATAAAATAATAAAAAGACAACAACCAGCCACCAATGGTCCAATGCATTGGCCACGTAACCCACGCTGGCCCTCATAAATTAAACACAACCACATCTAATTTTTTTTTTTTAAATTTAAAAAAAGGTCCTTGGGCTAGATAGTTCCGCCCCTAGATCCGAGGTTGTGTTGGATTACCCATCCTTGATATTGCTTGTATAAAAAATAAAATTATTTACATCAAAAGAAGATATTTTAAGCACCAAACTTAGTCTACTAAAAAAATTAGGCTTAATATCACAAAACGTCTTTAAGGTTTACAAAACTATCAGATTGTATTCTTAATGTTTTTTTTTTTTTTTTTTGTCATTCATGGTCCTCAAGGTTAGTATGTGTGATCACAAATGATCTTTGCCATATATATACACTTACGGCTGTGATACCAGGAGGGATCTAGAACTAGGAGGGATTAGCAACAATAGTAATTAATCTCAAATAATAAAATATGCTGAAATAATGTATGAATGAAATTAATGAATGAATGAAAATAATTACTAACATAAATTTGATTGATAACTTTGAGAATGTAAACTTACAGGAAATCTTGATTGAAAGATTTTATTGATCACTTAGATTGTACAAGAGTTCATTAGGATAGTAGTCCTGAGAGAACTTTGAAACTGAGAAAAGTAAAATTTACATTCTAAAAACAAATGAGGCTGAAGCCTCTTTAAATAGACTCAGGCTACCAGACTGTTGGCAGCATTGGATACTTGGAATTATGACTGTTGAAGCACGGGACTTTAACTTTTAGTTTAGCTTTTAACTTTGCCGGACTTTTACTTTAGCTGGCTCATGATAACTGTAGGGAAATGTGCTTTTAACTAATCTGAATTGAGATCATACGGATCTTGACTGTCTC

CAATAGAGTTACTCTGATTATATTGAAGCCTGGTACACAATCTTCCTCCACCAGACAATTGATTTTAGTCATTCCTGGTTTATTAATTTTGATAGCAAATTCAAAAGCCCTTTTTCCTTCTGGTTTCTCCACTGGTGGGAAAAACATGTCCAGTCATTGATTTGCTTCCCCCTCAAATGCAAGAATTAGTCAATTATTTTTCCATTAAAAACAAGTTCAAAGAATCAGAACTGTTGTTCACCAATCTGCTTATTTTCTGTGCTAAATATAAAGTTCCTTGGATCCTCAAATGGTTCTATCACATTAATTTGGATTCCAGAGCCCTTTCTCGCCAATTTTCAGTCAAATGGTGGGATAAGTTTAAAATTGACAAAACTGTAGATTACGTCCATTCATATTTCCCTCTAGTTATTGTACCTCCTCAATCCTCCCAGAAGCCATCTTCTTCTGGATCATCTCTTGTTTCCTTCTCAGCCGAAGGAAAGTCAAAAGCAGAATTACAAGAAATTGCCCGGCAATTAATCATTCAAGCCTCCCAAATGGAAGATAATGATGACACAGCCTCTCCAAGATCCCAGTCATCTACCAGTCAGCCTCATCAGCCATCCAGTCAGAAGACTAGTCAAAAGGATTCCACCCCAAAATTGCAGTGGGCAGATGATGAAGACAGTCAAGATCCGTATGATCTCAATTCAGATTAGTCAAAAGCAGATTCCCCGACAGTCATCATGAGCCAGCTAAAGCAAAAGTCCGGCAAAGTTAAAAGCTAAAGTAAAAGTTAAAGTCCCGTGCTTCAACAGTCATGATTCCAAGTATCCAATGCTACCAACAATCTGGTAGCCTGAGTCTATCTAAAGAGGCTTCGGCCTCATTTTTTTTCAGAATGTAAATTTTACTTTTCTCAGTTTCAAAGTTCTCTCAGGACTACCACCCTGATGAACTCTTGTACAATCTGAGTGATCAATAAAATCTTTCAATCAAGATTTCCTGTAAGTTTGCATTCTCAAAGTTATCAATCAAATTTATATTAGTAATTATTTTCATCAATAAAATATATATATATATGCAATTAAAATTAGCATTACTCAAAATATTCAAAATATTATTGATAACTTGATAACTTGCAGAATGCAAACCACTTACAAAATCTATGTTTCACCACTTAGTTCTTCAGAGCATTGATACTGAAAGAAGTTTGAAACTAAGAAAAGAAAGTGGACTAAAATTACATTTTGCCGATAACTGAGTCCGAAGCCTTCTTAAATAAGCAACTAAAAGTAAACTTTGGCAACTTTGGATGCTTTGGAGAGTGACTGTAGAAGCATGCGAAAGCTTTTCCTTTTACTGAGTGCTGTAACTTTTACATTGCCAGGGAATCTGACTAAAGGAGTACGGGCTATCAGACTGTTGAAGCACGGGTCCAAGATTCCAAATGCTTTATCTTCGAGGCTCATGATGTTGGGGCTTATGATGTTAGCAACTTTAATCTTCACCCAGATTGAAAGGACTGAAATCATAAGGGTCTTGACCATCTGGGGGATAATCAATCCACCGTATTTTGGGGGTAGGAGTCTTTTGACTGGATGACAATTTTTGGTGACTGGTGGATAACTGTGACTTTAGGGAAGCATCATCATCATTGTCTTCATTCATTTGTGAGGCTTGAAACATTAGTTGGCGAGCAATTTCCTGCAATTCAAATTTAGACTTTCCCTTGATTGGGAGAGAAGAGTGAGAAGTAGCAGGAGATGACTGTTTGACTAGTTGGGGATTCCTGACTGGAGGAAAATCCGAATAGACATATTCTGCAATTCGCTCAATTTTAAACTTATCCCACCATTTGATTGAAAATTGGCACGAAAGGACTCTGGTTTCCCAATTAACTTGATAAGACCATTTTAAGATTTAAGGGACTTTGTACTTGGCATTAAAATGGAGTAAATTAGGGAAAAATAACTCAGCCTCCTTGAACTTATTTTTATTAGTGAAATAATTGACTAAATCTTGCATTGGGGGAGGAAAAAGATCGATGACAAGACCATGTTTTTCCCACCAATGATTGAACCAATAGGGGAAAGGACTCTTGAATTTGCTGTCAAAATTAAGGAACCATGAATGACTAAAATCAACTGTTTGGTGAAGCAAGATAGAATACCATGCTTCGATATAATCATAGTAACTATATTGAAGTTCTGACTGAAAAACTTTGAGATCATAGGGGTGACTGCTCTATTCATTCTCACTGATTATTTGATGGATGTAGAGGGAATGGTAAAGGATGATATGGGGATTATCTCTGTCCTTGATTGGCTTAATTGTGATAGATTGGGTCTCATGGAGGATGTCTCTGTAAAATTTTAAATTTTTAAATGTTGCTGGGGAAATGAAATGACTATTTGGTGGATAGTAAGATTTGGCAATGGTGATTGGGTTGGCAATGAAACTGTATTTTGACTCAATTATGAATAGGTGGCTATTACTCTTGATCATATAGGGAGACGTTTTTTGGTAAGAGGCAACTGGAGGATTACTTATAGCAATATCCTGGAAAGGATCATGACTAGATATTAGGGCAGACTGAAAACTGGGGCGAACCTGGCTGACTGTGGCTCCTAGGTTGGCAAATTTGTTACTAATTTGGATAGGAGAACATCCAGGAAATGGGACGATCTGAGAAGGTGACTCATGTTTGACTAGACTTATTGATGAAGGGAGGACTCGGAGTGATTGGGGTTGACTGTGTTGACTACATTGACCGACTTCACTCTTTTGCTTGTCTTTTTCTTTTCTTCTTTCTGCCATTATTTTCCCTGCAGGAATTCTCTGGTAAGGAAATCAGGGACATAATTCTCACTTTCTCTCAAATATTCAATATTAAAATCAATTATACTCAAAATAGCTTGCCATCGTGCAAATATTTGTTTTGATGCAATATTTTGAACATCTTTTTCTAATACATGCTTAGTAGATTTACAATCAACTCGGATTAAAAAATTTTGATTTAATAAATCAGACTAAAATTTATTAATGAATAGTACAATAGATAATATCTCTTACTTAATAGTACTATAATTATTTTGGGACTGAGACCAAGTACCAGAATGGAATCTAATGATTTTCTCATATTGACTGCAATTGATTCTCTGTTTGAGGATACCTATGTAACCAATGTGAGAGGCATCAGTTTCAACTATTTTGAATGAATCAGCAGAGGGGATACCAAGATAGGGAAGTGTCTTAATATGAGCTTTGATTTGTTTGACTATATCAGTATGAATAGATGACCATAGGAGACGATTACTCTGGAGGCGATCAAATAAAGGCTTACATTGTTTTCTCAAATTTTTATAGAAATCAAAAACATAATTTAGAGATCCTAGAAATCTCTGGAGTTGACTTTTGTCTAGGATTTGATCTGGAAATTTGTCAGCAAACTGGATGACTCGATCTATGAGGCTAATCTGTGACTGGCAGATATTAAACCCAAGAAATCTCACATTAGTTTGGAATAATTTAATTTTCTTAGCAGAAATAACTAATCCATTTTGTTTGACTATCTGTAAGAACTTATACAAGTGTTTCCAGTGCTGCTCTATCGACTAAGAAAAGATAAGCACATCATCAATATATACTATGGAAAAATGACTAAATGGGTTAAAAATCTCATTCATAATATTTTGAAACTCACTTGGCGCATTCTTAAGACCGAAATGCATAATATTCCCCATAATGTCCAAATGGGGTAACAAAAGAAGTTTTGTATCTATCAGACTCACGAATTTGGATCTGCCAAAATCCACTCTTCATATCAAATTTAGAAAAGATGATTGACTTTTCTAATCTATTAATTAAATCTCTTTTATTAGGAATCAGATATCTAATCAATTCTAAGACTGTATTAAGCGGCTTGTAATTAATGACTAGTCTCGGGACTCCGCGTTCTAATTCAGCATTTTTTTAGACATAGAAGGCAAAGCAGGACGAGGGTGACTTACTCTTGCGGATTATTCCTTTCTCAAGTAATTCTGTAATCTCAGTTTTACAAAATTCCATTAATTCTTGGCTCATTTGAATTGGTCCGGCTTTAGTGGGGATATTTTTCTCACTAAAGGACTTGACTTAAGGAAGGCTAACTTCATGCTGTTTCCTATGCCAAAAAGCATTGGGTAACTCGGAACAAACTTCTTGTTTTAATTTTTCTTCAAACTGTCGAATTTGTGACTGTAAAGACTCATGACTAAGTTGTTGCTCAATTCTTTTAGTTTTGCCTTCTTCCTGGAGGAATCTTAGTTGCTTATTTTAAAACTGAATTATTTTGACGGTTTTGGTAACTGGGTGTTTTTGCAAGGCTTTTATATTGTGTAGCTCTGGTTTACTTAAAAATTCAAACTTAACCTTTTCTCCTAAACAGGTGGAAATAACACCATCGTGTTCTACCTGGAAAGGATATAAAAGGGCAATAAAAGGTAACCCTAGGATGACTTCATCAGTAATGTTCTTAACTAAGATGAATAAGGTTTTAAAGCAAATTTGATTTTGACAGACATGGGCTTTAGGGATCTCAAAATTTAACTGGAGTGACTTTCCTGAGACTGTGCTTAAAGACTCTTTTAACTTTTTGTAATACTTAGTAGGGATTGGACCTTCTTGGATGCAATTAAGGTCGGCTCGTTAATCAAAGAGGGCAACTTTATTTAACTCAAAATTTTGGACAAATATAGTGACTTTAGCATACCATTTTTTAAAGTTTACTTGTCTAATTAAGTTGACGACGTGGTCCTGATTGGAGGGTTCTTCGTCAGAATTTGTGGGACTTATAGGTGTAGACTTGGAAGTTTCTTTTTCTAATAAGTTAATTAACTCTTGTTTTATCTCATAATTTTCACTCTGTAATTTTTGACTTCTTTGTTTTAGCTTAATGATCTCTTTTTTGACTAATTTTACCTCTAGTTGTAAATCCTGTAAAGTGACTTCTTTCTTTTTATTAAATTTTTCTAAGGTTGTGTTCAGACTTATCTTTTGGGAGGTAGATTGTTTCGGTTCTTCTTGACTAATGATTTTTCTTAATTTTTTTTAAATACTCAGATTTCAACTATGGATTTTCAACTTTACTTATTAAATCAATTAATAATTCTTCTTGTTCTTCCTGTTTAGTAAGGACATTAATTGACTTCGACTTACAGCAATTATCTTGGCAACCAAATTGAATTATGGTGAGCTTAATTGGGAATCTAAGGAAATAGATTCTAAAGACTCAGTGTTACTAACCATAGTTTCATTTTCACTAGACAGAATTTCTTAACTCAAGAACTTTAATCAGTTTGTCTCTATCCTCATCCATAATTTTTAATTGTTTGATTGTATCTTTAACTTTGCAATTATTAGCATAGTGACCAAACTTTTGACATTTATAACATTTTACTTTTCTTAAAGCGATTATTCTTATCTTTGCCGTGACTCTTTTTCCAATTTTTCTTAGGAGAGAATTTCTTTTTACTATAAAATTCTTTATCTTCATTATTATGTTGGAATGACTGTTTTCTTTGTGAATAATGGCGACGTTTACTAAACTGTCGGTTGTGGCTACTGGACTATTCTTTCTAGACGGGGAAATAGACATTAGACCCTATTGTTCGCAGAAATTACCTAACTCATACTTAGCAGAATTTCTTTCAGACTGAATTTGTTTACCAATTTTCATATCCACACACATTCTCATACCGACTTGATTAATAGTGCTAATTATATTTCCATATGTTAAACTATCCCAATCAATATGACCTTGTTCATTACTAAGGGTTGTTCGGATCTTATAGGCAAATAAGTTTGGAAGAGCATTAACAAATTTTTCTTTCCAAAATGACTAGTTACTATCATCTCTAAGCATGACTCTGGAAATGAACACATCTTTATACCATCTAAAATCTGATAATTTAGGGCATTTTAGGTTACTAAGCTGATCATATATTCTGGCAGTAGTGTTACTGGGTGTTCCAATGAAATGCTCAACAGTTGTATGTAGCAAAGTGTTGACTCCATCTTCAATGCCTTGACCTATTTGTTCATAAAAAATAGGAAGACCATCTTCATTAAGTTTGACTACATGAATTATTTGTTGCTTAGACTCATGGGTTCAATGCTTATCCCATCAAGAACGAAGAGTTCCGGTGAAACCTGTTTCTAGTAAAGGGACAATTTCAGACTGTCTGAAACTGTGGTTGGTTATGTAACTATTGGCGACCATGGACATGTGTTGAAGTTTATTAAGAATTTCTTGCTCAGAAAGACCATAAATGTTCCATTCATATAATTTATCTGAAGAGACTGAAAACTGATTCTAAGTGTTTCGTTCTTCAAATTGTATATTAGGAGGTGTTGGCTTAGGATACCAATTCTTTGTAAGACTAGTGGGGCTGACTTTGTTTATTATTCTCTGGAATTTTGGATTACTTTCTAACTCAAGATTTTTAAATGTACTTTCAATTTTAGAGATATTACTGGTTTCTGTTTCCATACTAGCGATTGATTCAGACTCAGAAGAGTTATGGAAAGCAAAGGGTGGCTCAGAGGATTCTAAGATTTTAACCCTTGTTTCAACCTTTTCCTCATCTTTTCTTTTGACTGGCGTTAATTCTTCTAACATTTTCTCAATTTTTGTCATTGTTTCTTTCGTCTTAAAAGTTGGACTTGATTTTAAATCTTGGAAAGTAACTAGTGGTTTCTCATTTTAAGACTTTTTTACCTCACTAGGGAGTAACCTATCTACTTTTGTTTCTATTTTATCTAATTGTTTACCTATGACAACTAGACTTTGGTTGGTGTAATTATTCTGTTCAACTATCATTTTATTCTCTGGATCAGGGATTTTGAAAGGATTGGCGACTATCTTTGATTTTCTGATGTTTAATAAGTATTGTTTCTACAAGTGGATGACTAGCAAGAATCTCAGTGTGATCTATCTTTTCTCATTTTTCCTGGGTTAAAGTTTTCAACTCATTGGGCAAATGTCGACTTTCTACAAAATCAAGATAATATATTTCAACTTTAGCAGTGCGCATATATTCTTTCCAATCATTAAAAATTTATTGTCTTTTGACTCTGTCAGAGATGACCGAATGGTAATTATCTCGTCTGGTTTGATTCTCAGGGGAATGTAGATGATTATCTAATCTTTTCCAATTAATCACAAATTTTTTATTAAGTGTTCTTAATTGATTACTGACTTCTACAAAATCGGATTGTTTGTGTGAATCAGGAGACTCAGATTTGTTGTCTTTTAGAGGTTTTCCGCAGACATAATGGGTAACTTGAGACATTTTTCTTTAGGATTCCAATTTTAAATCAATTAAGGCTTTTTCTAACTCTAGGTCTCATTCTAGAGTGGAAGAAGAAGCTGTAAAGGAATGCCTTGCAGGAAAATGTCTCTGGGATTGACTAGGTGTAGGTAACTGCTGTAACTGGACTGTCGAATTTTGTGGGGCTTGATTACTAAAGTTAATTTTGACTGTGCCATCTAAATATTTTTGAATATAGTCTAAATTGTTTAGACTACGCTGAATAGGAACTGGGTGATTTTCGGCGATTAGGGTCCAATCTATTGGCAAGAACACATCAGACCAGTTAATTGAATGTGGGACTCTTACAATGGCATCTGGTGTATGACTCTGAATTAACAGAGTTTGATTTTTTGGACTTTTACTCGGGGCTTGGAAATTCATATTAGTACCTGTGGCTTTGTAATAGATTCTATAGACTAAAGCTAAGCGTTGGGTACTCGGCAAGACTTCATAACCATTAGTTTTTATATTTAATGTGAGAGTTTTTAATATATAGGGATCACTCAGGCTTACTGTGATATCAGGATAACAATCAAAATGGACTGGACCATTAACAAGACTTGACTCAATAATTCCTAGCATATGTTTTCTAATTGACTCAAGATTAAGCAACTGACAGGTCTCATGTTCTTGACTCAAAGCATAAACTTGTTCAACTGTTTTTACACGGGTGGTTTAATTAAAACTATAGAGTGACCATTTTTTCTTATATATTTTGACTGGGGACTTTAGGTATATTCCAATCTCGAAAATCTAATTCATTATTAGACTCATATTCTAATTGTTCTTCATTAACTATCTCTTGGACTTTACCTATACTGACTCTAGAGATGCTGGTTCTAAATAAGCAACTCATATTTCTCAAGGTCCTGGGGTTGGATTTCTAAGAAAGAAAGTGAACAGAAACTCCTAAAACTGACTTCTAAGTGGAACAGGATACATAGTAGATCTTTCGCCTTCCTACTAATGCCACAACCGCTCACCCCTCCGTGGGACTTATTTTTTGGAACAGGTCTTACAATTTGGACACCAAGAGAGAGAAGGATAGGTACATTGAAAGACTAGAAGGAGTAAAAGAAGAAACTGAAAACTCACAACTCAGAAAACCTAAACCAGCCACTTGGGTTAGATGAGTTGCAGACTTTTACTCCTTCTAGTCTTTCAATGTACTTGTCCTTCTCTCTCTTGGTATCCAAATTGTAAGACCTGTTCCAAACAGTAAGTCCCAAGGAGGCGTGAGTGGTTGTGGCATTAGTAGGAGGGCGAGAGAAGTACTAGGTTCCTGTTCCACTTAGAAGTCAATTTTAGGAGTTTCTGTTCACTTTCTTCCTTAGAATCCAACCCCAGGACCTTGAGAAATATGAGTTGCTTATTTAGAACCAACTCTTGCAATTCTAGCAGCTCTAGAGTTAGTATAGGTAAAGTCCGAGAGATAGTTAATGAAGAACAATTAGAATATGAGTCTAATAATGAATTAGATTTTCGAGATTGGAATATACCTAAAGTCCCCAGTCAAAATATATATAAGAAAAAATGGTCACTCTCTAGTTTTAATAAAACCACCCATGTAAAAACAGTTGAACAAATTTATGCTTTGAGTCAAGAACATGAGACATGTCAGTTGCTTAATCTTGAGTCAATTAAAAAACATAAATCTGATGGAAACAACTTCCTTCATATTGGTCTAGTCCAAGTTGACGTTAAACCATTAACCAGATTAGGATTAAAAGCTTCTATCCTTCTCTGTTTGCGTGATGCTAGATTCACTAGGTTTAGCGACAGTACGCTAGGAATTATTGAGTCAAGTCTTGCTAATTGTCCAGTCCATTTTGATTGTTATCCTGATTTCACAGTAAGCCTGAGTGATCCCTATATATTAAAAACTCTCACATTAAATATAAAAACTAATGGTTATGAAGTCTTGTCGAGTACCCAACCCTTAGCTTTAGTCTATAGAATCTATTACAAAGTCACAGGTACTAATATGAATTTCCATGCCCTGAGTAAAAGTCCAAAAAATCAAACCCTGTTAATTCAGAGTCATACACCAGATGCCACTGTAAGAGTCCCACATTCAATTAAATGGTCTGATGTGTCCTTGCCAGGAGATTGGACCCTAATCGCCGAAAATCACCTAGTTCCCATTCAGCGTAGTGTAAACAATTTAGACTATATTCAAAAATATTTAGATGGCACAGTCAAAATTAACTTTAGTAATCAAGCCCCACAAAATTTGACAGTCCAGTTACAGCAGTTACCTACACCTAGTCAATCCCAGAGACATTCCTGCAAGGCATTCCTTTGCAGCTTCTTGTTCCACTCTAGAACGAGACCTAGAGTTAGAAAAAGCCTTAATTGATTTAAAATTGGAATCCTAAAGAAAAACGTCTCAAGTTACCAATTCTTTCTACGGAAAACCTCTAAAAGACAACAAATCTGAGTCTCCTGATTCACACAAACAATCCGATTTTGTAGAAGTCAGCAATCAATTTAAAACACTTAATAAAAAATTTGTAATTAATTGGAAAAGATTAGATAATCATCTACATTCCCCTGAGAATCAAGCTAGAAGAGATAATTACCATTCGGTCATCTCTGACAGAGTCAAAAGACAATAAATTTTTAATGATTGGAAAGAATACATGCGCACTGCTAAAGTTGAAATATATTATCTTTATTTTGTAGAAAGTCGACATTTGCCCAATGAGTTAAAAACTTTAACCCAGGAAAAATGGGAAAAGATAGATCACACAGATTCTTGCTAGTCATCCACTTGTTGAAACAATAATTATTAAACATCAGAAAATCAAAGATAGTCGCCAGTCCTTTCAAAATCCCTGATCCAGAGAATAAAATGATAGTTGAACATAATAATTACACCAACCAAAGTCTAGTTGTCATAGGTAAACAATTAGATAAAATAGAAACAAAAGTCGATAGGTTACTCCCTAGTGAGGTAAAAAAGTCTTAAAATGAGAAACCACTAGTTACTTTCCAAGATTTCAAATCAAGTCCAACTTTTAAGACGAAAGAAACAATGACAAAAATTGAGAAAATGTTAGAAGAATTACCGCCAGTCAAAAGAAAAGATAAGGAAAAGGTTGAAACAAGGGTTAAAGGCTTAGGATCCTTTGAGTCACCATTTGCTTTCCATAACTCTTCTGAGTCGGAATCAATCGTTAGAATGGAAACAGAAACCAGCAATATCTCTAAAATTGAAAATAAGTTTAAAAATCTTGAGTTAGAAAGTAATCCAAAAGTCTAGAGAATAATAAACAAAGTCAGCCCCAGTAATCTTACAAAGAATTAGTATCCTAAGCCAACACCTCCTGATATACAATTTGAAGAACGAAACACTCAGTGTCAGTTTTCAGTCTCTTCAGATAAGTTATATGAATGGAACATTGATGGTCTTTCTGAGCAAGAAATTCTTAATAAACTTCAACACATGTCCTTGGTCGCCAATAGTTACATCACCGATCACAGTTTCAGACAGTCTGAAATTGTCCCTTTACTAGAAACAGGTTTCACCGAAACTCTTCGTTCTTGGTGGGATAAGCATTTAACTCATGAGTCTAAGTAATAAATAATTCATGCAGTCAAACTTAATGAAGATGGTCTTCCTATTTTTGATGAACAAATAGGTCAAGGCATTGAAAATGGAGTCAACACTTTGCTATACACAATTGTTGAGCATTTCATTGGAACACCCAGTAACACTACTGCCAGAATACATGATCAGCTTAGTAACCTAAGATGCCCTAAATTATTAGATTTTATATGGTATAAAGATGTGTTCATTTCCAGAGTCATGCTTAGAGATGATAGTAACCAGTCATTTTGGAAAAAAATAAAATTTGTTAATGGTCTTCCAAACTTATTTGCTCATAAGATCCGAACAACCCTTAGTAATGAACAAGGTCATGTTAATTGGGATTGTTTAACATACGGAAATATAATTAGCACTATTAATCAAGTCGGTATGAGAATGTGTGTGGATATGAAATTGGTAAACAAATTCAGTCTGAAAGAAATTCTGCTAAGTATGAGTTAGGTAATTTCTGTGAACAATATGGTCTAATGTCTATTTCCCCGTCTAGAAAGAATAAGTCCAGTCACCACAACCGACAGTTTAGTAAACGTCGCCATTATTCATGAAGAAATCAGTCATTCCAACATAATAATGAAGATAAAGAATTTTATAGTAAAAAGAAATTCTCTCCTAAGAAAAATTGGAAAAAGAGTCACGGCAAAGATAAGAATAATCGCATTAATAAAAGTACCGACAAAAGAAAAGTAAAATGTTATAAATGTCAAAAGTTTGGTCACTATGCTAATAATTGCAAAGTTAAAGATACAATCAAACAATTAAAAATCACGATGAGTATAGAGACAAACTGATTAAAGTCCTTGAGTTAAGAAATTCTGAGTCTAGTGAAAATGAAACTATGGTTAATAACACTGAGTCTTTAGAATCTACATCTCCTCAGATTCCCAATTAAGCTCACCCAGAATTCAATTCGGTTGCCAAGATAATTGCTGTAAGTCTAAGTCAATTAATGTCCTTACTAAACAGGAAGAACAAGAAGAATTATTAATTGATTTAATAAGTAAAGTTGAAAATCCATAGTTGAAATTTGAGTATTTAAAAAAAAATTAAGAAAAGTCATTAGTCAAGAAGAACCGAAACAATCTACCTCCCAAAAGATAAGTCTGAACACAACCTTAGAAAAATTTAATACAAAGAAAGAAGTCACTTTACAGGATTTACAACTAGAGGTAAAATTAGTCAAAAAAGAGATCATTGAGCTAAAACAAAGAAGTCAAAAATTACAGAGTGAAAATTATGAGATAAAACAAGAGTTAATTAACTTATTAGAAAAAGAAACTTCTAAGTCTGCACCTATAAGTCCCCCAAATTTTGACGAAGAACCCTCCAATCAGGATCACGTCGTCAACTTAATTAGACAATTACATTTTATTAAATGGTATGCTAAAGTCACTATATTTGTCCAAAATTTTGAGTTAAATACAGTTGCTCTCTTTGATTCAGGACCGACCTTAATTGCATCCAAGAAGGTCTAATCCCTACTAAGTATTACAAAAAGTTCAAAGAGTCTTTAAGCACAGCCTCAGGAAAGTCACTCCAATTAAATTTTGAGATCCCCAAAGCCCATGTCTGTCAAAATCAAATTTGCTTTAAAACCTCATTCGTCTTAGTTAAGAACATTACTGATGAAGTCATCCCAGGGTTACCTTTTATTGCCCTTTTATATCATTTCCAGGTAGAACACGATGGTGTTATTTCCACCTGTTTAGGAGAAAAGGTTAAGTTTGAATTCTTAAGTAAACCAGAGTTACACAATATAAAAGCCTTGCAGAAACACGCAGTTACCAAAATCGTCAAAATAATTCAGTTTAAAAATAAGCAACTAAGATTCATCCAGGAAGAAGTCAAAACTAAAAGAATTGAGCGATAGCTTAGTCATGAGTCTTTACAGTCACAAATTCGACAGTTTGAAGAAAAATTAAAACAAGAAGTTTGTTCTGAGTTACGCAAAGCTTTTTGGCATAGGAAACAACATGAAGTCAGCCTTCCTTATGTCAAGTCCTTTAGTGAGAAAAATATCCCCACTAAAGCTAAACCAATTCAAATGAGCCAAGAATTAATGAAATTTTGTAAAATTGAGATTACAGAATTACTTGAGAAAGGAATAATCCGCAAGCGTAAGTCACCCTGGTCCTACCCTGCCTTCTATGTCCAGAAAATGCTGAATTAGAACGCGGAGTCCCAAGACTAGTCATTAATTACAAGCCGCTTAATACAGTCTTAGAATGGATTAGATATCTGATTCCTAATAAAAGAGATTTAATTAATAGATTAGAAAAGTCAATCATCTTTTCTAAATTTGATATGAAGAGTGGATTTTGGCAGATCCAAATTCGTGAGTATGATAGATATAAAACTGCTTTTGTTACCCCATTTGGACATCATGAGTGGAATGTTATGCCTTTCGGTCTTAAGAATGCCCCAGTGAGTTTCAAAATATTTTGAATGAGATTTTTAACCCATTTAGTCATTTTTTCATAGTATATATTGATGATGTGCTTATCTTTTCTCAGTCGATAGAGCAGCACTGGAAACACTTGTATAAGTTCTTACAGATAGTCAAATAAAATGGATTAGTTATTTTTGCTAAGAAAATTAAATTATTCCAAACTACTGTGAGATTTCTTGGGTTTAATATCTGCCAGTCACAGATTAGCCCCATAGATCGAGTCATCCAGTTTGCTAACAAATTTTTAGATCAAATACTAGGCAAAAGTCAACTCCAAAGATTTCTAGGATCCCTATATGATCAAGAGTAACAGTCACCTATTCATAATTGAGTCAAAATACAGTTCCATTGTCAACCCAATCACCATTGTCAAATCTTACTATCCACCAAATAGTCATTTCATTCCCCCAGCACCATTTAAAAATTTAAAATTTTACAGAGACTTCCTCCATGAGACCCACTCTATCACAATTAAGCCAATCAAGGACAGAGATAATCCCCATATCTTCCTTTACCATTCCCTCCACATCCATCAAATAATCAGTGAGAATGAATGGAGCAGTCACCCCTATGATCTCAAAGTTCTTCAGTCAGAACATCAATATAGTTACTATGATTATATCGAAGCATGGTATTCTATCTTGCTTCACCAAACAGTTGATTTTAGTCATTCATGGTTCCTTAATTTTGAAAGCAAATTCAAGAGTCCTTTCCCCTATTGGTTCAATCACTGGTGGGAAAAACATGGTCCTGTCATCGATCTTTTTCCTCCCCCAATGCAAGATTTAGTCAATTATTTCACTAATAAAAATAAGTTCAAAGAGTCTGAGTTATTTTTCCCTTATTTACTCCATTTCAATGCCAAGTACAAAGTCCCTTGGATCTTAAAATGGTCTTTTCAAGTTAATTGGGAAACCAGAGTCCTTTAGCACTAATTTTCAATCAAATGGTGGGGTAAGTTTAAATTGAGCGAATTGCAGAATGTCTATTAAGATTTTCCTCCAGTCAGGAATCCCCATTCAGTCAACCAATCATCTCCTGCTACTTCTCACTCTTCTTTCCTAATTGAGGGAAAGTTTAAATCTGAATTGCAGGAAATTGCTCGCCAACTAATGTTTCAAGCCTCACAAATGAATGAAGAGAAAGATGATGATGCTTCCCCAAAGTCAGTCATCCACCAGTCACCAAAAATTGTCATCCAGTCAAGACTCGTACCCCCAAAATGCGGTAGAGTGATTATCCCCCAGATGGTCAAGACCCTTATGATTTCAGTCCTTTCGATCTGGGTGAAGATTAAAGTTGCTAACATCGTGAGCACCAACATCATGAACCCCGAAGATAAAGCATTTTGAATCTGGGACCCGTGCTTCAACAATTTGATAGCCCGTGCTCCTTTAGTCAGATTCCCCAACAAAGTAAAAGTTACAGCACTCAGTAAAATCAAAAGCTTTCGCGTGCTTCTACAGTCACAATCCAAAGCATCCAAAGTTGCCAGCTCATCCAAAGTTGCCAGAGTCTACTTTTTGTTGCTTATTTAAGAAGGCTTCGGCCTCCGTTATCAGCAGAATGTAATTTTAGTCCATTTTCTTTTCTTAGTTTCAAACTTCTTTCAGTATCAGTGCTCTGAAGAACTAAGTGGTGAAACATAGATTTTGTAAGTGTTTTGCATTCTGCAAGTTATCAATAATATTTTGAATATTTTGAGTAATGCTAATTTTAATTGCATATATATACACATATACTCAAATAATAAAATATGCGAAAATAATGTATGAATGAAATTAATGAATAGATGAAAATAATTACTAATATAAATTTGATTGATAACTTTGAGAATGCAAACTTGCAGGAAATCTTGATTGAAAGATTTTATTGATCACTCAGATTGTACAAGAGTTCATCAGGGTGGTAGTCTTGAGAGAACTTTGAAACTGAGAAAAGTAAAATTTACATTATGAAAACAAATAAGGCAGAAGTCTCCTTAAATAGGCTCAGGCTACCAGATTGTTGGTAGCATTGGATACTTGGGATCATGACTGTTGAAGCACAGGACTTTAACTTTTACTTTAGCTTTTAACTTAACCTATGGAAATATAATTTGGACAATTAATCAAGTTGGAATGAAAATGTGTGTTGATATGAAAATTGGTAAACAAATACAGTCTGACAGAAAATCCGCTAAGTATGAATTAGGAAATTTCTGTAAACAATACGGTCTTACTAGTATTCCTTCTTCTAAGAAAGATAAGCCTAGTCACATTAGAAAAAGTCGCCATTATTCACGTAAAAAACAGTCATTTAGACACAATAATGATAATAATGAGTTTTACAAGAAAAAGAAGTTTCACCCTAAGAAAAGTTGTTCAAAAGCACCGAAAAATGAAAAGAATGGTCGCTTTATGAAAACCTCAGACAAACGAAAAGTTAAATGTTATAAATATCAAAAGTTCAGTTACTATGCCAATGAATGCAAGGTAAAAGATGCAATCAAACAATTAAAAATCATAGTTGAAGAAAAAAATAAGTTAATAAAAGTTTTAGAATTAAAAAACTATGAGTCAAGTGAAGTTGAAAACAAAGTTACCGATACCGAGTCAGCCGGCTACAATACCTCTGATTCCTCAACCAGTTCACCCAGAATCCAGTTCGGTTGTCAAGATAAATGCTGTAATAGATTAAAATCTGTTTTTGTCGTCACTAAACAGGAGGAACAAGAAGAGTTACTAATTGATCTAATCAGTAAAAGCTAAGAAAAGTCATAAGTCAGGAAAGTCCAAGTCCAAACGAGTCTACCCCTCAAAAAATCAGCTTGAACACAACCATAGAAAGGTTTAATAAGAAAAAAGAAATTATTTTACAAGATTTACAATCAGAAGTTAAAATAGTCAAAAAAGAAATAGTTGAATTAAAACAGATAAGTCAAAAACTACAAGTTGAGAATTATGAAATTAGACAAAATCTGATAAGTTTAATGGAAAAAGAAAGTTCTGAGTCCAGAAGCCAAGCTGAGAGTCCAAATAACTCAGATGATGAGCCTTCAAATAATGATCAAGCAGTTAACCTAATGAAGCACATTACTTTCAGAAAATGGTATTCTAAAGTAACCGTATTTGTCAAAAAATTTGAGTTCAACACAGTCGCCTTATCTGATACAGGAGCCGATTTTAATTGTATTCAGGAAGGTTTAATACCCACTAAATATTATCAGAAATCTAAAGAATCGCTTAGAACTGCTTCAGGAAAATCACAGCAGTTAAATTATGAAATCCCCAAAGCCCATGTCTGCGAAAACAAAATTTGCTTCAAAACTTCATTGTTCTTAGTCAAAGATATCACTGATGAAGTCATTTTAGGACTACCTTTTATTGCCCTATTATATCCTTTTCAGGTAGAATATGATGGTGTCATCTCTACCCATTCAAGAGAAAAGATAAAATTTGAATTCTTAACTATGCATGAGTTGCACAACCTGAAAGCCCTCCAAAAGAATTCAGTATCCAAAACAATCCAGTCAATTAATCAAAAAAATCATCAATTGAACTTCCTTAAGGAAGAAGTCAAATTCAAAAGAGTCGAACAACAACATACTGATAAGTCTTTATAGTTGCAAATTCAACAATTTGAAAATCAATTGAAATATTAGTCTGTTCTGATTTGCCTAATTCCTTTTGACACAAGAAACAACATGTTGTTAATCTTCTTTATGTCAAAACCTTTAGTAAGAAAAATATCCCCACTAAAGCCAGGCCAATCCAGATGAGTCCAGAAATAATGGAGTTCTGTGAAATTGAAATAACTGAGTTGCTTCAAAAAGGAATAATCCGAAAAAGTAAATCACCCTGGTCTTGTCCTGCTTTCTATGTCCAAAAAAATGCTGAGTTAGAAAGAGGAGCCCCAAGATTAGTTATCAATTATAAACCCCTTAATACAGTCTTAGAATGGATCAGGTACCCTATACCTAATAAAAGAGACTTAATCAATAGATTAGAAAAAGCAGTCGCCTTTTCCAAATTTGATATGAAGAGTGGTTTCTGGCAAATCCAAATAGATGAGTTAGATAGGTATAAAACTGCATTTGTCACTCCTTTTGGCCATTATGAATGGAGTGTTATGCCTTTCGGTTTGAAAAATGTACCGAGTGAATTCCAAAATATAATGAATGAAATATTCAATCCTGTCAGTCATTTCTCTATTGTTTATATTGACGATGTTCTAATCTTTTCCCAGTCGATAGAGCAACATTGGAAGCATTTGCATAAATTTCTCCAGATAGTAAAAAACAATTGTCTAGTTGTCTCTGCTAAGAAAATCAATTTGTTTCAAACAAATGTCCAATCCTTGGTTTTAGTATTTGTTAGTCACAGATCAGTCCAATTGACAGAGTAATCCAATTTCCTGATCAAATCCTCGATAAGAGTCAGCTCCATAGGTTTTTAGGATCTCTCAATTATGTGTCTGATTTTTATCAAAATATGAGAAAACAATGCAAGCCCTTGTTTGACAGGTTACAAGATAACCCTCCTCCTTGGTCTTCTGTCCATACAGAAATTGTCAAACAAATCCAAATTCATGTCAAGACACTTCCCTGACTTGGTATTCCTTCAGTCGATTCCTTCAAAATAGTTGAAACTGATGCCTCCGACATTGGATATGGAGGTATTCTTAAACAGAAAGTCTATCCAAATCACCCAGAACAAATCATTCGCTTTCATTCTGGCATTTGGACTCAGTCGCAATTTAATTAGTCCTATTAAAAAGGAAATATTATCTATAGTACTATTCATTTCCAAATTTCAAGATGATTTGCTTAATCAAAAATTTTTAGTCAGAGTTGATTGTAAGTCTGCTATGCATGTTTTAGAAAAAGATGTTCAAAACATTGCATCCAAACAAATTTTTGCAAGATGGCAAGCTATTTTTAAGTATTTTTGATTTTGACATTGAATATTTAAGAGGAAGTGAGAATTCCATTCCTGATTTTCTTAAACGAGAATTCCTGCAGGAAAAACAATGGCAACCAAATAAGATAAGCAAAAAGAGCCAGCCAGTCAACCTAAGCCACCCCAGTCGCTCCGAGCCCTTCCTTCTTCCATGAATCCAATCAAACCAGAGTCACCCTCTCAGATCGTCCCTTTCTCAGGATGCTCTCCTATTCAAGTCAGTAACAGGTTCTCCAATCTTGGAACCATAGTTGGCCAAATTCGTCCCAATAACTAGTCTGCCCTAGTCTCCAGTTATGATCCCTTCCAGATCATTCCCCCAGTTGCCCAGTCTTCCCATTCATCTACTCCTCTCCGAAAGTCATCTCCGTACCTCCCGAAAGATCTCATATTTTCATAATTGAACCAGTTTATAATGGTATTTCTAACCCAATTACAATTGTCAAGTCTTATTTTCCTCCAAATAGTCATTTTCTTCCTCCCTGCCCTTATAAAAATCTCTAGTACTATAGGGACATTCTCCATGAAACAGGATTAGTCGAAATTAAGCCCATTAAAGACTGAGAAAGACCTCACGTCATTCTATAAGACTCGCTTTACATTCACAAAATCATGAGTCAAGAAGAGTTCAATCGCCATCCTTATGAGTTGAAACACCTCCAATCCACCCATCCTTACAACTATGCTGACTACGTTGAAGAATGGTACACCATATTTCTCCATCAAATAGTTGACTTCAGTCACTCTTGGTTCATCAATTTTGATAGCAAATTCAAGAGTCCATTCCCTATTGGTTCCTTCATTGGTGGGAAAAACATGGCCCGATCGCCGAAACTCTCCCCATTGAAATCCATAATTTGTCCAATTATTTTTCCAGCAAAAAAAAATTTAATAAGAAAGAGTTGTTTTTTCCCAAATTACTCCTTTTCATCGCCAGGTATAAAGTTCCCTGGATCCTGAAATGGTCATATCATGTAAATTGGAAATCCAGAATTATGTCTCGCCAATTGTCAGTCAAATGGTGGGATAAATTCAAAGTTGATAGAATAGAAGATTATGTTTATAGGGATTTTTCAGAAGTAACTAATCCCCTTAAACCTGAACCAGTTTCTCCTGTTGGTTCCACTCACTCTTCTCTCTCAATCGAGGGAAAATCAAAATCTGAGTTGCAGGAACTAGCCAGACAGTTGATTCTCCAAGCCTCCCAAATGGATGATGATGAAGACAGAGATGCTACTCCCAAGTCCCTGTCATCTACCAACCAGTTGCCCAACAGCCAACAACGCTCTTCTCAGTCGAAGCGGTGGGCAGATTATCAAGATAGTCAAGACCCGTATGATCTCGAATCTGATTAGTCTTAAAAACCATTATTTGATTCCCCAACAAGTCATCATTAAAGCCTGAGTAAAAGCATCTAGAAAAAGCAGTCAATCAGCTTTCGCGTGCTTCTACAGTCATTATTCCCCGACAAGTAAAAGTTAAAGTTAAAGTTGCATAAAAGTTAAAGTTGCCTGCGTTAAAGCATCTCTCTGAAAATCCCGTTCTTCAAATTGGATGTCTGGGGGTGTTAGCCTAGGGTACCAATTTTTGGTTTGGGTTGTCAGACTGATTTGATTGGTTAACCGTTTGACCTTCAATTGGTTATGTAATTCCAAATTTTTAAAGGCATTTTCAATTTTTGGAATATTACTAGTTTCTGATTTGATTCAGATTCAGACTCAGAAGGATTTGTAAAGACAGAATCTAGGACATTGAGACCAGATTTCTCAGGTTTAACTGGTGTCAATTGTTCTAGTATGTCTTCTATTTTTTTCATTGTTGGTGTTATCTTAAGGGTAGGACTTGATTTAAGATCCTGGAATTGGACTAAGGGCTTTTCAACTTTGACTTTGGATTTGTTATCCTGGGGTGCCAACGTGTCAACCTTTGTTTCAATTTTGTCTAACTATTTTCCTATGATTATCAGACTTTGGTTTGCATAATTATTGTGCTCAATTAGTTTTTTATCTGAATCATTTTTCTCAGGGATTTTGAAAACCGGCATCTTCAAAATCAGTTTGCGTGGGTGACTCAGGAGACTCAAGCTTTTCTTCCTGGGTTGGTATTTTACCATAACAGGGAGCAGTTACTTGGGAAGTTTTTCTCAAGGTTTCTAATCTAAAGTCTATCATTGCCTTTTCTAACTCTAGGTCTCGTTCTAGGGTTGAGGAGGAAGCTGCGAAGGAATGCCTTTCTGGAATATGTCTCTGGGATTGACCAGGGGTAGGCAACTGGTGTAACTGGACTGTGTTTTTTGGTACAGACTGATTACCAAAATTGATTTTTAACTGTGCCATCTAAATATTTTTGAATATAATCTAAATTATTTAGATTATGTCGAATTGGGGTTGGCTGATTTTCATTGGTGAGTGTCCAATCTAATGGAAGGGTGACATCTGACCATTTGATTGTTTGAGGTACTCTTATATTGGCCTCAGGTTTGATACTTTGAATTAACAGAGTTTGATCTTTTGGGCTTTTATTAAGGGCTTGGAAATGCATATTAGTTCCAGTAACTTTATAATAAATTCTGTAAACTAGGGCTAATGGCTGTGTTCATGGGAGGACTTGATACCCGGAAGTCTTAATATTTAAAGTTAATGTTTTCAAAATATGGGGATCACTAAGGCTGACTGTGAAGTCTGGGTAGCAATAAAAATGAACTGGACCATTAAACAAACTTGACTCAATTACTCCTAGCGTACTGTCACTAAAATTAGTGAATCTAGCATCTCTAAGGTAGAGAAGGACAGAGGCTTTTAATCCTAATCTGGTAAGAGGTTTGATTGCAACTTGAACTAAACCAATAAGAAGGAAATTGTTTCCATCATTTTTATGTTTCTTAATCCACTCAAGATTAAGCAACTGGCATGTTTCATGTTCTTTACTTAAGGTGTAGACTTGTTCAACTGTTTTGACATGGGTGGTTGACTTGAAACTGGACAAAGACCATTTTTTCTTATAGATGTCCTGACTGAGGACTCTAGGTATGTTCCAATCTTTATAATCTAATTCATTATTGGATTCATATTCTAGTTGTTCTTCATTAACTATCTCTAGGAGACGACTAATACTGGCTCTAGAGCTACTCGAATTACAAGAGTTTGTTCTAAATAGGTGACTCATTCTTATAAGTTCTGGGTTTGGCTCCTAAAGAAGAAGATAAACAGGGACCTCTAAAACTGACCCTTTACTCTACAAGAAACCTAATGCTTTTCTCACCCTCCGCTCATGCCGCAACCGCTCATGCCTCACCTGGGACTTACTGTTTGGACTCTAGGACTTACTGTTTGGACACCAATAGAGAAAAACGCCGGAACCTTGAAAAATAAAAAGATAGTAAAAGAAGAGACTGAAAACTTCAACTGTAGAAACTTTCAACCAGCCAATTGGGCGGAATGAATCGAAGATGTAGAACTGACTCTTACAACTCTTACGACTCTGATACCAGAAGGGTCTAGAACCAGGAAGAGATAGAGTAAAGAGTGAAATAAGTATATGCAACTAAATGGTAAAATATGCGGAGCTTATATATGAATGAAATTAGTGTGTATATATATATATATATAGTCCCTTTGAAAAGCAAAAGTTATTCAACACTCAGTAAAAGCAGATGCTTTCGCGTGCTTCTACAGTCAAGATTCCAAAGCAAAAGCAAAAGTTTATTTTAGTCAAAATTTGTATGATTTACACAATAGTTCTCCCGTGGTTGAATAGTCAAGTTATCTATTCTATTTAAGAAGGCTTCGGCCTTAGTTATCGACATAATGTAAAATTTACTTTTTCTTAGTTTCAAAGTTCTCTCAGAAACAATGCTCTGAAGAACCAAGTTGGGAGACAAGAGATTTTGTAAGTGTTTTTGCATTCCCATAGTTATCAAAGTTATTTTCAATAAAAATACTTATGTTTCAAAGACATCTCTATGTTCATTCTATTTTCATTTATTCATTAATTTCATTCATGCATAAGCTCCGCATATTTTATTATCTAAGTGCTTATACATATTTTTGCTTATGTTCTATCTCCTCCTGGTTCTAGATCCCTTCTAGTATCAGAGCCGTAAGTGTTGAAGAGTCAGTCTAAGTTTGCAACTCATCCAACCCAAGTGGCTGGTTTAGGATTTCTAAGTTGAGGTTTTCAGTTTCTTCTTCTACTCCTCCTTGTCTTTCGAGTTACATGTCCTTTTCCCTCTGGTCCAAACAGTAAGTCCTAGAGTCCAAACAGTAAGTCCCAGATAGAGGCCTGAGCGGTTGCGACCTGGACGGAGAGAGAGAGAGAATCACTAGGTGTTAGTCTAATTAGGGTCAGTTTTAGGAGTTCCTGTTTACTTTCTTCTTAGTAATCCAAACCCAGAACCTTGAGTGTAATGAGTCGCTTATTTAGAACAAACTCTTGTAACTCTGGCAGCTCTAGACCTAGTATTAGTCGCCTCCCTGAGATAGTTAATGAAGAACAATTAGAATATGATCCTAATAATGATGAACAATTAGATTTTAGAGACTAGAATATCCCCAAAATACCTAGTCAAAAAATTTATAAGAAACATTGGTCATTATCTAGTTCAAGTTGCTAATATATGAATATGGTTTGAATATCTTAGGTTGTTCTTTATTCTTCTCCATAAGGAGGTATATATAGGAGTTTCAAAATGAAGGTTTTACAAAGAATTAGATATAGTAAAGAATTAGGAAAGTATCTCCTAATTGTACAATGCTTTATCACTTATATAGAAAATAGGAAAGTAAATCCCTTAATTAATCAAGGAAGTAAATCCCCTTGATTAATTAGGAGACTCACGTCAACACTCCTCCTCAAGTTGGTGCATATATGTCACGAATGCCCAACTTGGTAAGTGAGTCATGAAATACTCTTCCACACTCACAATATATGCAATATCTGATCTTGGAGGACGTATGAAGAGTAGAACCTTTGGTGGTGTTGATAGAAGAAGGAGTCTGGGTTGACCGCATGCTTCTGTGAGTCTGCAATAAGATGGAGAACCGTATGGGTTCTTGACATCACAACGGAAGTAAGATGTGATATGAGAAGGTGAGGAAGGTGATGTGAGAAATTTAATGTAGGAATTTGATGTAGGAATTGGATTTAAGAGGTATGTATATAAGATTTGATGGTATGTGTTTAGGGATTTGGATAAGAGGATTTGAGGACTTTAGATTTGAGGGATTTGGATTTGGGGAAAAAAAAAGAGGAATTTGATTTAGGGTTTTAGATTTGAATATGAGAATTTGAAGAAATTTGAGATAAGGGTTTGGATTTGAATATGAGAAATTTGAAGAAATTTGAGAAATTTGAGATAAGGGTTTAGAGACAAAACAACTTCTCTTCACTCCCCTTCTCCCCTTGAAGAGAAGGGGTCGTCAGAGTCAAGAAATAAGCAACATCTTCTTGAGAAAGAGAAACATATCTTTTTCACTCCCCACTCCCCCCCTTGAAGAGGAGGGGAGGAGAGCACTTAAATTGCAAGAAGAACGCAAGCACAGGTCACAAGAGAACTAGGCTCTGATACCATGCTAAAATATGAATATGGTTTGAATATCTTAGGTTGTTCTTTATTCTTCTCCATAAGGAGGTATATATAGGAGTTTACATATGAAAGTTTTACAAGGAATTAGATATAGTAAAGAATTAGGAAAGTATCTCCTAATATATAAAAAATAGGAAAGTAAATCCATTAATTAATCAAGGAAGTAAATCTCCTTGATTAATTAGGAGACTCACGTCAACACAAGTCAACCACACATGTTAAGACAGCTGAGCAAGTTTACGCCCTTAGCAAAGAACATGAGACTTGTCAGTTATTTAATCTTGAGTCAATTAAGAAACATAAAAATGATGGCAATAATTTTCTGCATATTGGTTTAGTTCAAGTTGCAGCTAAGCCCCTCACCAGATTAGGATTAAAAGCTTATATCCTTCTCTATCTTCGTGATGCCAAATTCACTGAGTTTAGTGACAGTACTTTAGGAATGATTGAGGCTAGTCTGTTTAATGGCCCAATACATTTTGACTGTTATCCAGATTTTACTGTGAGTCTTAGTGATCCTCATATTTTAAAGTCATTAACTTTGAATATCAAGACCTCTGGTTACAAGGTTTTACCAGGTACCCAGCCATTAGCCTCAGTTTAAAGAATTTATTATAACGTCACAAGAACTAATATGAATTTTCAAGCCCTTAATAAAAGCTCAAAAGATCAAACTCTATTAATCCAAAGTCATACAGCTGATGCTAATATAAGAGTGCACAAACAGTTAAGTGGTCTGAGATTAGTCTGCCATTAGATTGGACTTTAGCCACTGAAGTTCCACCAGTCCCAATCCAACATAGTTTAAGTAATTTAGACTATATTCAACAGTATTTAGATGGCACAGTTAAGATTAATTTTGGAAATCAACAGAAGTCTTCAGTTCAGTTACAACAATTACCAACACCTAGCCAATCCAAGAGACATGCCCCTGCAAGACATAGTTACACTGACAGGACCCGACCCAATTTCCACTTTGAAATTCGAGCCAAGTCCTGTGCGTGTCCGACACCTGGCGAATGTCGGGCACAAATGACCTTTTTGCCCTCTTACTTCAATTTCTCTTTAAAATTTCCTTAGACTTCTGCCGAAAATTCGGCAGAGTCTCCCCTGTATTTTGACCAGTCCCAAAATTTCACCTGTAAACAATCTCAAATATTTCCACCCAACTGCCAGAATAGGATAAACCAAAATTTGCCAGATTCAAATTTTCCAATAAACTCGATATCAGAGCAATTTTCTAAAGTTCACGGGTTTTCAGGAAAAATTTCTACAACTTTACCTGACTTTGAAGCGTGGAGGTTAAGAGTGGCCCGGTGGTGGATCCTATGCACTTCTACAACCTGGGGGCGAAAAACAAGTTTTAAACTGTGAGTGGACAAAAAGTGAGATTCTTAAAATCCAGCTAAAAATAATTCCCCCATTTTGAAATAACTAGGGATATATATACATCGAAAATTTAACTATATCTCCATATAAAAGATTCTATAAGCATGAATTTGTATACTAATGAAGAAACTTACATAATCGATCTGATATAAAGATATTACTGCCTGGAAGAATCAAAGAACTGGTATACTGAATAAAAATACCAAAAATACACATGTGCAATTACTGAGAGACCAATATAAAATGGCTGAAAATCATCCTTGAATAAAAGAAAGCTCAAAATCCTTTAAAACTTCGTTCGACTGAATTTACTAAAAAAAATGAGTAAACAATATCTGCAGTAAAAGCTTTAAAATCCTTTAAAACTGCCACCTAATTGTACCCCTGTTATATCCGTCAATTCCCTGGCAGGTCTCGGGCGCCACGCAGTCCACCCGAGCCGCAAACTGGCGAAATCAGGGGACTATGATCAGCCTGTCCCGCCGGCAGATTCCTCGGTGACACCAAGTCAGCTCGAGTCGCTCTGGCAGGATGCAGGGGACCGTAGTCAGCCTGATCCGCAATCCTGGCAGGTCTCGGGGACACAGAGTCAGCCGAGCCGCAATCCTGGCAGGTCTCGGGACACCAAGTCTGCCGAGCCGCAAATCCTGGCACTCACGGTCCGAGCGTCCCCGAAACTCGTGAGGCAAAGTCAAGTGCACTGACTGAACTATAATCAGACTGGATGTCCGTAGACATCGGTCCGACTCTGGGTAATCACCATAAAGAAAATGGGTACAAGGTGGGTTTAAAATAGATTCCTTTAGAAAATCCGATTAAAACTGAAATCTCAAATTGTGCTGCTGTCTCATCTGATTTCAACCTCAAACTCTGTTTCTATGACTTTTAAATAAGCAGCACATATTTATAAAACTGTTACTGTACTGATCAAGTTCAGAACCGTAATAAAACTTACTCAAGATCAAATAAAGAAACTTATTCAAATAAATTCATTTCTAAAAACTTATTTATATAAAATCAATATAAACTCATTTATAAAATTTATTTATATAAAAGCACATCAATCATTCATGAAATCTGTTTTATGAAAGAAGGTCCACTCACAGATGGTCCGAGCTACTTCGATCCCTCGAAGATCTCTTTTATAATTCTGTCGGGCTCCTGGTGCCTTACTCGATCGATTTGGTGGAGAAACGAAGGAGAACTTCAAGCTGGAAGTTTGGGTAGCTTCGGACGAACCTCCGTCGGAAAACACGATTTTCCGGCCAGCTCAGGGCGGCCCCAGGGTTGAGGTCGGTTGGAGAAGGTAGGGGACTCGATGGCGGTTCCAACGCCACCGGTCCCGTGGCCATTGGAGCTCGGAGGCGGCGCCAGCACCTTCTGGAAGTCGGTGGCCCGTTTCGCGCAGAGTCTGGGTTTATATAGATGCAACTTCGAAATATTTACGGTTATGCCACTGAGACTCTTTTGACCATAACTTTTTCGTTACAACTCCGATTCGGGCCCACTCCGTGTCTACGGACTCCTTTCGCCGTGCTCTACGCAATGGCGCAAGCGGAATTCCCAAATTCTTTCTCGATCAAAAAGTCAAATTTTCCCCCATTAATAATGCAAGGGCAAAATGGTCTTTTTACTAAAAGATATTTTCCTTACTTTTTAGATATTTTCTTTCTTTTTAGATATTTTGTTTTGGGTTCTTACATACACAACCTCCTCCTCTACTCTAGAACGAGACCTAGAAGTAGAAGAAGCATTAATAGATTTAAAATTAGAGTCATTAAGGAAATCGTCCCAAGTAAACCAACCCTGTTATGGTAAACAACCAGTTACAGAAAAAGCCAAGTCTGAATCTCCTGAGTCACCTACACAAATAGATTTCGAAACTAGTTTTCAAATCGATAACCAGTTACGAACTCTTAATAAGAAATTTAAGATTAATTGGAAACACCTAAATAATCATTTACATGCCCCCCTGAAAATCAAAAAAGAAGAGACAATTATCACAAAACCTACCCCATTAGAGAAAGACGCCAGACGATCTTTAATAAATGGAAAAGATTACATGCGTACAGCTAAGTTAGAAATTTTCTATCTTGATTTTGTTGAAAGCCGTTATCTACCTAATGAGTTGAAAACTTTAACCCAAGAGAAATGGGAAAAAGAAGATAGCTCCAAAGTTATAGGCAGCCACCCCCCCAGTAGAAACAATAGTCATTACCCATAAGAAGTCAAAAATACTTGCCAGCCCTTTCAAAGTCTCCGACAACAATGATAAAAAGATAATTGAACAAAACAATTACACCAATCAAAGTCTAATAGTTATAGGAAATCAATTGGATAAAATAGAAACAAAAATTGACAAAATAGTCCCTTTTGAAATTACTTCCAAATCACAAAAAGAGAAACCTTTAGTTAAATTCCAAGACCTAGAGTCAAACCCAGCCTTTAAAACAAAACCAACCATGAAAAAAATAGAACAAATGTTAGAAGAATTAACCCCAAAGAAAGAGAAAAAAGATAAACAAATTGAGGAAACATCTGGTCTTAAAGTCTTAGGATCCAATACCTTAGTTAATGCCTCTGAGTCTGAGTCAGAAACCAGTAATATTTCAAGAAATGAGAATGCTTTTAAAAATTTAGAATTAGAAAATGATCCAAAAATTAAAAGATTAATCAACAGAGTTAACCCCACAAGCCTAACTAAAAATTGGTACCCTAAGCCAACTCCCCCTGACATTCAATTTGAAGAAAGGAACAATCAGAGTTAGTTTTCAGTTTCCTCTGATAAATTATATGAATGGAATATAGATGGTCTGTCTGAGCAAGAAATATTAAACAAGTTATCCCGCATGTCTATGGTTGCCAACAGCTATGTCGCCAATCACGGTTTTAGGCAGTCTGAAATAGTGCCTTTATTAGAAGTCGGTTTCACCGGAACGTTACGTTCCTGGTGGGATAAGCATTTGGTAGTAGAATCCAATTGTCAAATAATTCATGCGATTAAGCTTAATGAAGAGGGTCTCCCAATATTTGACGAAAATTTAGGCCAAGGTATAGAAGACGGAGTCAATACTTTGTTATATACCATAATTGAACACTTCATTGGAACACCTAGTCACACTACTACTAGAATACATGATCAGCTTAGTAGCCTAAGATTCCCAAAGTTGTCTGACTTTAGATGGTATAAAGATGTCTTTCTTTCCAGAGTCATGCTAAGAGAAGATAGTAACCAAGCTTTTTGGAAAGAAAAATTTATTAATGGCCTTTTGAATTTATTTGCCCATAAAATACACACAGTTCTTAGTAATGATCAAGGTCACATAGATTATGATAATTTGACTTATGGTAATATTATTAGCACAATTAATCAAGTTGGTATAAGAATGTGTGTGGATATGAAAATAAGTAAGCAAATACAATCAGAAAGAAATTCTGATAAGTATGAATTAGGAAATTTCTGTGAGCAATATGGTTTAGCACCAATTCCCCCATCTAGAAAAGATAAGTCTAGTTACCACTACCGACAGTTTAGCAAAAGTCGTCCTTATTCACGTAAAAGAAAGTCCTTCCAACAAAATAATAACAATGATGAGTTCTATAGTAAAAAGAAAATTCACCCTAAGAAAAACTAGTCAAAAGGTCACAACAATAATAAAAGTAGCTTTACGAAAAATACCGATAAAAGAAAAGTAAAATGTTACAAATGTCAAAAGTATGGTCACTATGCCAATGAGTGAAAGTTAAAAACACAATAAAGCAGTTGAAAATCACGGATGAATATAAAGAAAAATTGATTAAAGTTTTAGAGTTAAGAAACTCTGAGTCCAGCGAAAATGAAACCACAGTTACCGACACTGAGTCAGCCGCTTATATGTCTACAGATTCCCAACCTAGTTCCCCAAATATCCAATTAGGTTGTAAGGATAAGTGTTGTAATGTTTTAAGAAAGTCAATTAATGTTCTCACTAAGCAAGAAGAACAAGAAGAATTATTAATTGATTTAATTAGCAAAGTTAAGAACCCTGAATTAAAATATGAATATTTAAAGAAATTAAGAAAAGTCATTAGTCAAGAAGAATCCAGTCAGTCAATACCCCAAAAGATAAGTTTACATACTACTTTAGAAAATTTTAACAAGAAAAGAAAAGAAGTTACTTTACAGGATTTGCAATTAGAAGTTAAATTGGTCAAAAAAGAAATAGTAGAATTAAGACAAATTAGTTATAAGCTGCAAGCTGAGAATTTTGAAATTAAACAGGATTTAATTAATTTAATGAGAAAAGGAAGTTCTGAGCCTAATGGTATTACAGAGACCCCTACAAATTCCCCTACACATTCAGACGAAGAGCCTTCTTCTCATTCCAATAATGAGCAAGCCATCAACTTAATCAGGCAAGTAAGTTTTAGAAAATGGTACTCTAGAGTCTCTATATTTGTCAACAAGTTTGAGCTCAACACAGTTGCCTTTTTTGATTAAGGAGCCGACCTTAATTGCATTAGAGAAGGTTTAGTTCCACTAAGTATTATAAAAAGTCAAAAGAATCATTAAGTACTGCCTTAGGAAAATCACTTCAGTTGAATTATGAATTACCAAAAGCCCATGTCTATCAAAATAAAATTTGTTTTAAAACCTCATTCTTCTTAGTTAAAAATATTACTGATGAAGTCATTTTAGGATTACACTTTATTGCTCTTTTGTATCCTTTCCAGGTAGAATATGATAGTGTCATCTCTACCCATGTAGGAGAAAAGGTTAAGTTTGAGTTCCTAACCAAACTTGAGTTGCACAATTTAAAATCTTTACAGAAAAGTGCAGTTTCAAAGACAGTTAAGATAATTAAAAGTAAGAGTAAGCAAGTAGATTTTCTTAAAGAAGAAATCAAATTTAGGAGAATCGAACAGCAGCTTAATAATGTGTCTGTCCAGTCACAGATCCAACAATTTGAAGGTAAGTTAAAAATTGAAATATGTTATGAGTTACCTAACGCCTTTTGGCATAGGAAACAAGATGTATTCAGTTTACCCTATATTAAAAACTTTAGTGAAAAGAATATACCCACTAAAGCCAGACCAATTCAAATGAAACAAGAGATAATGGACTTTTGCAACACTGAAATTTCCGAGTTACTCCAAAAAGGAATAATTAGCAAAAGCAAGTCACCATGGTCTTGTCCTGCTTTTTATGTTCAGAAAAATGCTGAGTTAGAACGTAGAGTCCCAAGACTAGTTATCAATTACAAACCCCTTAATACAGTCTTAGAATGGATTAGGTGCCTGATACATAACAAACGAGAGTTAATTAATAGGTTAGGAAATGCAATTATTTTCTCTAAGTTTGATTTGAAAAGTGGATTTTGGCAAATTCAAATTAATAAGTCAGATAGGTATAAGACCGCTTTTGTTACCCCATTTGGTCACTATGAGTGGAATGTTATGCCTTTCGGTCTTAAGAAAGCGCCCAATGAGTTTCAAAATATAATGAATGAGATATTCAATCCTTATAATCATTTCTCCATAGTTTATATTGATGATGTTCTAATCTTTTCCCAGTCATTAGAACAACATTGGAAACACTTGCATAAGTTTCTCCAAATAATTAAACAAAATGGACTAGTTGTCTCTGCTAAGAAAATAAAGTTATTCCAAACTAATGTCTGATTCCTTGATTTCAATATTAGTCAGTCTCAAATCAATCTAATCGATCGAGTCATTCAGTTTTCAGAAAAATTCTCAGATCAAATTCTAGACAAGAGTCAACTCCTAAGATTCTTATGATCTCTGAATTATGTATCTATTTTTTATCAAAATCTGAGAAAACAATGTAAGCCATTATTTGATCGTCTTCAAAGCAATCCTCCTCCCTGGTCATCTATTCATACAGAAATAGTTAAACAGATTAAAGCCCATGTCAAGACACTTCCCTGTCTTGGTATTCCTTCAATTATAGCACTATTAAGAAAGAGATAATATCTATAGTATTATGCATTAGTAAATTTCTAGATGATTTATTAAATCAAATTTTTTTAGTCAGAGTTTATTGTAAGTCTGCAAAGCATGTATTAGAAAAAGATGTTCAAAATATTGCATCCAAACAAATATTTGCACGATGGCAAGCAATTTTAAGCAATTTTAAGCATTTTTTTATTTTGATATTGAATATTTGAGAGGAAGTGAGAATTCTGTTCCTGATTTTCTTACTCGAGAATTTCTTCAGGATAAACAATGGCAGACAGAAGAAAAAAAAAGAAAAAAAAAAGAGCAAGCTAGTCATTCCCAATCATCCCAGACATACAGAATACTCCCCCCATCAATGAATGCACCCAAATCATAGTCACCCTCCCAGATCGTCCCTTACCCGGGATGTTCCCCTATTCAAATCAGTAACAGGTTTGCCACCTTAGGAGCCACAGTAGGCCAAGTTCGTCCCTGTTTCCAATCAACCTTAGTCTCGAGTCATGATCCATTCCAAGTCAATACCCAGTTTACTCCTCCAGTTGTCTATTTCCAAAAGTCATCTCCTTACATGGTTAAAAGTGATAGTCATCCTTTTATTGTTGAGTCTAAATATGATAGTATTGCTAATCCAATCACAATTGCAAAATCATATTTCCCTTCTAATTGCCACTTTATACCTCAAGCCCCTTATAAGAACTTAAAATATTATAGAGATATTCTTCTTGAGACCCAATCAATTGAAATCAAGCCAATTAAAGATTGAAATAATCCAGCCATTATCCTTTATCATTCCTTCTATATCCATCGAATTCTCAGTGAAAACAAATGGAGTAAGCACCCTTATGACCTCAAACTTCTTCAGTCAGAAGTCCAATGTAGTTATTATGATTATATTGAGGCCTGGTATTCAATTCTGCTTCATCAAACAGTCGATTTCAGTCACTCTTGGTTCATCAATTTTGATAGTAAATTCAAGAGTCTTTTTCCCTTCTGGTTTCTTCACTAGTGGGAAAAACATGGTCCAATTATTGATCTCCCCCCACCCCCCCCCCCCCCCCCCAAATGCAAGAATTAGTTAATTACTTCACCAATAAACGCAAATTCAATGAGTCAGAATCTTTCTTCCCCAATTTGCTCCACTTCATTGCCAGATACAAAGTCCCCTGGATCCTAAAATGGTGTTATTAAGTCAATTGGGATTCTAGATATCTCTCCTGTCAATTTTCAATCAAATGGTAGGATAAGTTTAAAATTGAAAGAATTGCTGATTATGTCTATTTGGATTTCCCCCCTGTGAAACCTCTCCAATCAGAGAAAAAGGTGTCCCCTACAGTCTCCCATTCTTCTCTCCCAGTTGAGGGAAAAACAAAATCAGAATTACAAGACCTTGCTAGACAGTTGATGGCTCAAGCCTCCCAAATGAATGATGATGATGATGCAAATGATGGCTCAAGCCTCTCACAGTTGATCAGAATTACAAGACCTTATTAGCATCACTATAAGCATCACTATTAGCGTCACTTTCAGATATCCAATTTGAAGAAAGGAACTCCCAAAGTCAATTCTCAATCTCCTCTGATAAATTATATGAATGGAATATTGATGGCCTTTCTGAACAAGAAATCCTTAATAAACTCCAACATATGTCCATGGAAGCCAACAGTTACATAACCAATCATAATTTCAGAGAGTCAGAAATCGTTCCCCTTCTAGAAACAAGATTCACCGAAACCTTACGTTCTTGGTGGGATAAACATTTAACTGAAGAATCAAAAAGACAAATTATTCATGCAGTCAAACTTAATGAAGAAGGTCTCCCTATTTTTTTATGAACAATTAGGAAGAGGAATAGAAGACGGAGTCAGCACTTTATTATACACGATAATTGAGCATTTCATAAGAACACCCAGTAACACTACAGCCCGAATCCATGATCAACTTAGTAATCTCCGATGTCCAAAGTTAAGCAAATTCAGATGGTATAAAGATGTTTTCATTTCCAGAGTCATGCTTAGATATGATAGTAATTAACCCTTTTGGAAAGAAAAATTTATTAATGGTCTCCCTAATTTATTTGCCCATAAAATAAGAACAATCTTTAGTAATGATCAAGGTCACATAGATTTCGATAACTTAACTTACGGAAATATTATTAGCACAATTAACCAAGTAGGAATGAAAATGTGTGTTGATATGAAAATAGGAAAACAAATTCAGTCAGAAAGAAATTCAGCCAAATATGAATTAGGAAATTTTTATGAACAGTATGGTCTAGCTTCGATTCCACCATCTAGAAAAGCCAAGACTAGGTATAATGGTCGACAAACTAGTAAACAAAGACATTATTCACATAGAAAACAATTCTTCCAACATAATGATAAGTTCTATAGTAAAAGGAAATACCACCCAAAGAAAAATTGGTCAAAAGTACGTGGAAAAGGAAAACATGACAAACCTAAAAAGTCCCCCGGCAAGAGAAAACTTAAGTGCTGGATGTGTAAAAAAAACATGGTCACTATGCTAATGAGTGTAAAGTAAAAGACACGATAAACCAGTTGAAAATCACGGATGAAGAAAAAGACAAATTAATTAAAGTCTTAGAATCAAGAAACTCTGAGTCTAGTGAAACCGACGTCATAGTAGCAAACTCAAGCTCAGTAAATTACAACTCCTCTGATTCCCAACTAAGTTCCCCGAAAATCAAATTTGGTTGTAATGATAAGTGCTGTAATACTCTAAGTAAGTAAATTAATGTCCTTATAAAACAAGAGGAACAAGAAGAATTATTAATTGAATTAATTAGTAAAGTTGAATACCTAGAACTAAAATCTGAGTACGTATTTAAAGAAATGAAGAAAAGTCATTGGTCAGGAAGAACCGCAACCGGTCAACAAGTTCCCTCAAAGATAAGTCTGAACCTGTCTTTAGAAAAATTCAATAAGAGAAAAGAAGTAACTTTACAGATCTGCAAATAGAAGTAAAATTGGTCAAAAAAGAAACAGTAGCACTAAAACAAATGAGCCACAAGTTACAAGACGAGAATTTTGAAGTTAAACAAGATTTGATTAATTTAATGAAAAAAGAAAACAATAAGTCCAAGAGTCAACCAGAAAGTCCAGTAAATTCAGATGGTGAACATTCAAATAATGATCAAACTATTAATTTAATAAAAACAAGTAAATTTTAGAAAATGGTATTCTAAAGTCACTATATTTATAAAAAAAATTTGAGTTAAATACAGTAGCTCTTAATTGTATACAAGAAGGATTAATCCCTACCATTTTATACCATTTCCAAGTAGAATACGATGGAGTCATATCCACTCATTTAGGTCAAAAAGTCAAGTTTGAATTTTTAACTAAACTTTAGTTACACAATTTAAAAGCTTTGCAGAAAAGTGCAGTTTCAAAAACAGTCAAAATAATTGAAGATAAAAGTAAACAACTTCATTTTCTTAAAGAAGAAGTGAAGTTTAAAAGAACTGAACAGCAGCTTAGTGATAAGTCCTTACAGTCACAAATTCAGAAATTTGAAGAAAAGTTAAAACAAGAAGTCTGTTCTGATCTTCCCAATGCTTTCTGGCATAGGAAACAACACGTTGTCAGTCTCCCTTATGTCAAGACCTTTAGTGAGAAAAATATCCCTACTAAAGCCAGACCAATCCAAATGAGTCAAGAAATTATGGAATTTTGTAAACAAGAAATTACAGAATTACTTCAAAATGGAATAATTCGGAAAAGTAAATCACCATGGTCGTGTCTAGCCTTTTATGTCCAGAAAAATGCTGAGTTAGAACAAGGAGCCCCAAGATTAGTCATAAATTATAAACCCCTTAATACAGTTTTAGAATGGATTAGGTACCCAGTCCCTAATAAAAGAGACTTAATCAACAGATTAGACAAAGCAGTCATCTTCTCTACGTTTGATATGAAGAGTGAATTTTGGCAGATCCAAATTCAAGAGTCAGATAGGTATAAAACCGCTTTTGTAACCCCATTTGGTCACTATGATTGGAATGTTATGCATTTTGGTCTAAAAAATGCGCCTAGTGAATTCCAGAATATAATGAATGAGATATTCAATCCTTTTAGTAATTTCTCCATAGTTTATGTTGATGATGTTCTTATTTTTTCTCTGTCAATAGAACAGCATTGGAAACATATGCATAAATTCCTCCAAACAATAAAACAAAATGGTTTAATTGTCTCAGCCAAAAAGATCAAGTTATTCCAAACCAATAATCGATTCCTTGGCTTTAATATTTGTCAGTCACAAATTAGTCCGATTGATCGAGTCATCCAATTTGCTGATAAATTTCCAGATCAAATTTTGATAAGAGTCAACTCCAAAGGTTTCTAGGTACTCTCACTTATGTCTCTGATTTTTATCAGAATCTGCGAAAACAATGTAAGCCCTTATTTTATAGACTCCAGAATAATCCCCCTCCTTGGTCATCTGTACATATAGAAATTATAAAACATATCAAAATCCATGGCAAGACATTACCTTGTCTTGGTATTCCTTCAGTCAATTCCTTAAAGATAGTTAAAACTGATGCCTCTGAAATAGGTTATGGAGGTATTCTTAAACAGAGAGTCAATCCTAGTCAACCTAAGCAAATTGTTCGATTCCATTATGGAATTTGGACTCAGTCACAACGTATTTATTGTACTATTAAAAAGGAAATTTTATCTGTAGTATTATGCATTACAAAATTTCAAGATGATTTAGAACCAACTCTTGTAACTCTAGCAGCTCTAGAGCCAGTATTAGTAAACTCCCAGAAATAATAAATGAAGAACAAATAGAATATGATTCTAGTAATGAAATTGATTACAAAGATTGGAATATCCCAAAAATTCCTAGTCAATAAATTTACAAGAAAAAATGGTCACTCTCTAGTTCAAATCAACTACCCATGTGAAAACAGTCGAGCAACTCTATGCCTTAAGTAAAGAACATGAAACATGCCAATTGTTAAATCTTGAGTCAATAAAGAAACACAAAAATGATGGAAACAATTTCCTTCACATTGGTCTAGTCCAAGTTGCAATCAAACCTCTTACCAAATTAGGATTAAACGCATCTATTCTACTCTATCTAAGAGATGCCAAATTCACCAATTTTAGTGACAGTACCTTAGGAATAATTGAATCAAGCCTGTTTAATGGTCCAATTTATTTTGATTGCTACCCAGATTTCACAGTAAGCCTTAGTGATCCTCATATTTTAAAAACTTTAACATTAAATATTAAAACCTCAGGGTATAAAGTTTTAGCAGGTACACAACCCTTAGCCTTAGTCTATAGAATTTATTATAAAGTACTAGAACCAACGTACCAAAAGGAATCTAGAACCAGAAGGACAAGTTAACTGTGATAGTAATGAAGCAAAGATAAATAGAATATGCGAAAATAATGCATAAATGAAAGTAATGAATAAATGAAAATTTGTTTAATTGAAAACATTGAACTTACAGAAAATCTTGATTGAAAGATTTTATTGATAACTTCAGTAGCATACATGATAGCACTCATATTTTACAATAGTAGTTTTCTTGAAGGAGTCTCAGAGAAAACTTTGAAAACTAAAAACTAAAACTTAGTTACAATCTGCTTACAATTGAGGCAAAGGCCTCTATTTAAAGAGCCGAATCCAAGAGTGTCGGCAACATTGGATGCTTGAAACTTTACTATTGAAGCACGGGTATTTCACTATTGCTTTAACTGGAGCATCATTCTTACTTTTGACTTTTCTTTTGCTCTTTCTTGCTTTCATGATGGCTACTTTCATGAGGACTGTCGGAGAATATTAACTATGTACTTTTGTCTTTGTTGACTAGTCTTCATTGAGATCTTAAGGATCTTGACTATCAGGATAATCTGCCCACTGAAATTTTGGGGTAGAATCTTTTTGACTTGGCAAAATTTGAGGCTGATTGGTAGAAGATTGGGACTTTGGAGAAGCATCAGGATCATCTTCTTCATCATCCATTTGGGAAGCTTGGAGCATTAACTGTCGGGCAATTTTTTTTAACTCAGACTTGGATCTTCCTTCGATTGGGAGGGAAGAGTGAGATGTGCCAGAGGTGGAAGGCTTCTGGGAAGCATTGACTGGGGGAAAGTCTATGTTGACTTGATCAATGATTCTTTCGATTTTGAACTTATCCCACCATTTGACCGAAAATTGATGGGATAAGACTCGGGAATTCCAATTGACTTGGTAAGACCATTTTAAAATCCAGGGAAATTTGTATTTTGCTACAAATATGAGAAGATTAGGAAAAAATAATTCTGACTCCTTGAATTTATATTTATTGGAAAAATAATTAACTGACTCTTGCATTTGGGGAGAAAGGATATCAATGATTGGGCCATGTTTTTCCCACCAATGAAGAAACCATTAGGGAAAGGGACTTTTGAATTTGCTGTCAAAATTAATAAACCATGAGTGACTGAAATCAATTGTTTGGTGGAGGAAAATGGTATACTAGGCTTTGATGCAATCATAATAGCAATATTGGAGGGTTGACTGGTGGGTTTTTAGATCATAAGGGTGTTTACTCCAATCTTCTTCACTCAAGATTTGATGAATATACATGGAATGACAGAGGATGATGTGAGGATTATTGCGGTCTTTGATTGGCTTAATTTCCACAGATTGGGTTTCATTGAGAATGTCTCTATAGTATTTAAGAGACTTGGATGGATATTGTGGAACATAATGAAAATTTGGGGGACAATAAGCTTTTGCAATTGTGACTGGATTGGAAAGGTTGTCATATTTGGACTCAATGATAAAAAGATGAGTATAACTTCTTGAGGAATAAGGGGATGACTTTCGTAAAGGCACTGGAGACTGGGAAATTTGGGTATTGATTTGAAAGGGATCATAATTAGAAATTAAAGCTGACTGATAATTTGGGTGGACTTGGCTGACTGTGGAACGAAGGGTAGTTACTGATTTGGATCGGGAATCATCCCGGGAAAGGGACGATTTAAAAAAGTGACTCTGTTTTGACTGGAGTCATTGATGTCGGGAGTGTTCTGAGTGACTGGGGTTGACTGGTTTGACTAATTTGCTCTTTTTGCTTGTCGGGTCTTCTTGCTACCATTGTTTTTCCTGGAGAAATTCTCTAGTAAGAAAATATGGAATGGAGTTATCTCTCAGATATTCAATGTCAAAATAAAAATACTTAAAATAGCTTGCCATTGTGCAACAATCTGTTTTGATGCAATGTTTTGAGCATCTTTTTCTAAAACATGTTTTGCACTTATGCAATCAACTATAACTAAATTTTTTTCATTTAATAGATCATCTTGAAATTTCGTAATGCATAATACTATAGATAATATTTCTTTTTTAATAGTACTATAATTTCGTTGTTACTGAGTCCAAATTCCAGAATGGAATTGAACAATTTGCTCAGGTTGACTTTGATTGACTCTCTGTTTAAGGATGCCTCCATAACCAGTGTCTGAGGCGTCAGTTTCAACAATTTTGAAGGAATTGGCTGAAGGAATACCTAGAAAAGGTAGAGTCTTGACATGAGTTTTGATTTGTTTTGACAATTTAGAGTCTTTCTGAAAAATCGTATCTCTATACATCGAGAATTGCCCATTATATGGACAGAATACAAGTTAATTAGGAAAGAACACAGGCTGATATTCAGCCCCTGAAAAACAGCTCACCTTCCACATATACATGTATCTAATTATAGCTAACCTACCACATATACATGTATATAATTAATGAAAGAATCTGCTGGCATTTAAATCTGATTTCCTTTGACATCCCCCCTCAAATTGATGCTGGTAAATCGATAAGCATTAATTTGCTAACGAGGAAATGACGGCGCTGACGTGGCATGGATTTGGTGAAGATATCGGCAATCTGCAGAGTAGTGGAGACATGTGGAAGGGTGATAACCTGATGATCATATGCTTCTCGGATGGAGTGACAGTCAACCTCGATGTGCTTGGTGCGTTCGTGATAGACGGGGTTGGCTGCAATTTGGATGGCACTTGTGTTGTCAGCATGCAGTGGAGTAGGATGAAGTGGACTGAACCGAAGCTCTGTGAGGAGACCACGCAGCCAAATGATCTCGGAGCACGTAGCAGACATGGCGCGATACTCTGCCTCAGTGGATGATTTGGAGACGTGGTCTTGCTTTTTGCATTTCCAAGAGATAAGGGCATCACCAAGAAACATACACCAGCCAGTAGTAGATCTCCTGGTGTCTGGGCAACCAGCCCAATCAGCGTCACTATAGGACTGAAGCTGAAGAGAAGAACCTGTAGGGAAGAACAAGCCACGACTAGGTGTGCCAAGAATATAGCGAATGATGCGGCGAACGGCAGAGAGATGGTGATGCCTCGGGGTTTGCATGAACTTGTTGACAGTGTGAACAGCATAAGAGATGTCTGGTCGGGTGATGGTTAGATAGATAAGACTACCAACCAGCTTCCGATAGGTAGTAGGATCCTCAAGAAGCTCCCCGTTGTCACGTCGATATTTCACATTAAGTTCCATGGGGGTGTCAACGGAAGTAGTGTTGGTGAGGCCGGCTAACTGAACCAGAGCTTGACTGTACTTGTGCTGAAGCAAGAAGAGACCATGAGGTTGATGATGGACCTCTAAACCCAAGAAATAAGTGAGCTGACCAAGATCTTTCATGTGGAAAGTAGAAACTTTTCAAACCATACTCGCAGGGCCTGCTTCAAACCATACAAAGAATGTTTCAGTTTGCAAACATCATTAGGTGAGGAAGTAGGCATACCAGAGGGAAGTTTCAAGTAAACTTCTTCCTTGAGATCACCGTGGAGAAATGCGTTCTTGACATCCATTTGATGTAAGGGCCAGGATTGGGATGCAGCAAGGGCGAGTATGGTGCGGACAGTGGTCATTTTGGCAACTGGTGCGAAGGTCTCATCATAGTCGAGACCATATTCTTGTTTGTTGCCAAGTGCCACTAACGGAGCCTTATAACGGTCTATGGACCCATTAGAACGAAGCTTGACAGAAAACACGAACTTACTGCCAAGGGGTTTCACGGAGGAGGGACATGGAACAACGTCCCAAGTTTGGTTTTCCTCAAGTGCGAGAAGCTCAGTTTCAACCGCTGTCCGCCAACACTCATGCTTCATGGCCTGTGTATAAGACGAAGGAAGAGAAATAGAGGACAAGGTGGCAGTGAAGAGGGAAAGAGGAGAGGAACCAAACCTATCTGGTGGTTTACGAACCCGAGTACTCTGTCTGAGGAGGGGAGGTGCAGTTGCTGGAACAGGAGCAGCAGTCGGGGAAGGAGCTGGAGGGGGCTGGGGAGGTGCTGGAGGTTGATGGGAAGTTTCGGAGGAGCGTCGTTGATACACGAAGAGAGGCTGAGAAGGGACTGGAGCTGGAGAAGAGATATTTAAAACAGGAGCTGCAGAAGAGTTAGTAAACATAGGCAAAACAGAAAATGAAGGAGAAACGGGGTCGTGATGCGTGGGAAAAAAATACTGATTTTCTAGAAAGATTACATTTCTAGAAACTCAAATGCGTCGCAAATTTGGATCGTAGCAAAGAAAACCTTTGGTGGACTGAATATCCTAGGAAAGCACACTGGATCGATTGAGCCATGAGTTTTGTGCGTTCATGTGCAGGAAGATGAACATAACAGACACAACCAAATGTACGAAGGTTGGAATAAGATGGAGGGCGACCAAATAACTTGGTGAAAGGAGAGTTGTGGTGCAATGTGGGAGAAGGCAACCTGTTGATGAGATGGACAGCAGTGGAGAGAGCTTCACACCAAAAACGAGAGGGCGTAGATGACTCTAACAGGAGAGTGCGAACAACATCAAGAAGGTGACGGTTCTTCCTTTCAGCCACCCCATTTTGTTGAGGTGTTGAAGGGCAAGACCGTTGAGAGATGATGCCATGAGTCTGTAGGAAGTCCTGAAACAAATGAGACATATACTCACCCCCATTGTCAGAACGAAGGATTTTGATATGGGCCGAAAATTGGGTCTGAATATATGCATGAAAAATCTTAAAAACAGAAAAGACTTCATCTTTAGAATGTAGAAAATATATCCATGTGAAACGGCTATAGTCGTCAATAAAAGTGACGAAGTATTTGTAATTAGCATGAGAAGTGACTGGTGCCATACCCCACACATCACTATGTATCAAATCAAAAGGTTGGACCACATGGGGTGTGTGAATAGGAAATGGCAGAGTCTTACTTTTGCCAAGTCTGCAAGAATTGCAATCAAATTGAACAGCACTAAGAGATGGGATTCTTTATTCCCAAGAAAACCAGATTTCAGTAAATCATGAAGTACAGTAGAGTTTGGATGTCCGAGACGTTTATGCCACGCTCGGTAATCAACATGAGCAGAATTACAAGAGACAAAGGGTGAAAAAGAAAACAAAGGAAATTGAAGAGGAAAAAGACGTCCCACTTTAGGCCCCCTCGCGATCATCCTCCCTGACTGTTGATCCTGCACAACACAACCAGATTTAGAGAATTCCACTTTGCAATCATTATCAACCAACTGACCGACAGACACAAGATTGGTGGTAAGACCAGGAGATACAAAGACATCAGTGAGAGAGGAAGAAACATCACCAGTGGCCGTGATAGGTAAATGATTGCCATCAGCAGTATGAATTTTGAGATTTCCAAAATATTTGTTTACATTGGTAAGAGAATCAGCATTGTTTGTCATATGATGGGAAGCCCCGGAATCAAAGTACCAAGGAGATGATGTAGAGAAGGGTTTACCTGAGAGTCCTAAAGCTGAAAATGCAGAAATGATCATTTGTTGAATCATGTCAGGGGTCACAGGTTGAACAGGAGCACTTTGTGTCAGCGGTACTGCAGTCACAGAACTTCCAGCACTAGAAGAGCCAACGGAGACAGTATATGCGGTCTCTGATTTCTTGGGGGGTCGAGTAGGACATTCCTTGATGATATGGCCATCCTTTTTGCAGTAGTGGCAGAATTTTCGGGGACAATTTGCAGCATAATGACCAAATCCCTTGCAACAAAAACACTGAACAGTGCTCATATCCCTGCCTCGTGGCTTACCTTGTGCTGCATAGGCCACAGGAACAGATGCAGACCGCCGTTGTTCCATGGTGGTTTGAGTGAGAAGGCGCTGTTCCTCGCGAAGGAGATCATTGAGGCATGTATCCAAGGAGGGGACAGATTCTCGGTTCATAAGATTGGATCTGGTGCCTTCAAATTCAGGTCTCAGTTTCATTAGAAATTGATCCCTCTGGGTAGTTTCATGGACAGATTGAACAGAACTAAGACCTTCGGATGGTAAGTCAGCATAAACTATATCAGTGTATTCACCCCAAAGATTAGTGAATCTAGAGTAAAAATCAGAGATAGAAAGGCTATCCTGTTGGAGAGCGGCCAATTCATGTTCAAGCTGGAACCGACGAGCAGTGTTTCGTTGACTATAGATCTTCTTCAGGTAAGTCCACATCTGAGCTGCAGTCTGAGATGATCGAAGGTTCAAGATGATATTAGGTTCGACAGATCCTAAAATCCATGTCATAACTTGAGCGTCCTTGACTTCCCACTTGGCGTGCTGGTCTTTGTCTTTGTTTTTGTCAGGAGCTGGGTTACTTCCATCAACATGTCCCCACGCCTCCTTACCTTTGACGAAAATCCGAAGTGAAACGCCCAAGCAGAGTAGTTCTTGCCATTGAAACGAATCAAAGAAACCTCAGACTTATCCGAGACATGATGTCAAGAGAACTTGATCAAGAATAACCGAGACTAAGAGACTTGAGAGCAAGAGATAACTAAGAGTAAGAGACTTGGGAGCGAGAGATAATTCAGAGCTATAGCTCTGATACCATGACAATTTAGAGTCTTTCTGAAAAATCGTATCTCTATACATCGAGAATTGCCCATTATATGGACAGAATACAAGTTAATTAGGAAAGAACACAGGCTAATATTCAGCCCCTGAAAAACAGCTCACCTTCCACATATACATGTATCTAATTATAGCTAACCTACCACATATACATGTATCTAATTAATGAAAGAATCTGCTGGCATTTAAATCTGATTTCCTTTGGAGCCTGTCAAATAGAGGTTTAACATTGCTTACGCAGATTCTGATAAAAATCAGAGACATAATTAAGAGAACCTAGAAACCTTTGGAGTTGACTCTTATCAAGAATTTGACTTGGAAATTTATCAACGAATTGGATGACTCGATCAGTCAGACTAATTTGTGACTGACAAATATTAAAGCCAAGGAATTGAATATTGGTTTAGAATAACTTGATCTTTTTGGCTGAGACTACTAAACCATTTTGTTTGACTATTTGGAGGAATTTATGCAGATGTTTCCAATGCTGTTCTATTGACTGCGAAAAAAAAAGAACATCATCAATATAAACTATGGAGAAATTACTAAAATGATTCAATATCTCATTCATTGTATTCTGGAATTCACTAGGCGCATTTTTTAGACCAAAATGCATAACATTCCACTCATAGTGACTAAATGGGGTTACAAACGCGGTTTTAACCTATCTGACTCTTGAATTTGAATATGCCAAAATCCACTCTTCATATCAAACTTAGAGAAGATGACTGCTTTGTCTAATTTGTTGATTAAGTCTCTTTTATTGGGGATTGGGTACCTAATCCATTCTAAAACTGTATTAAGGGGTTTATAATTTATGACTAATCTTAGGGGCTCCTCGTTCTAACTCAGCATTTTTCTGGACATAAAAGGCTGGACACGACCATGGTGATTTACTTTTATGAATTATTCCCTTTTGAAGTAATTTTGTAATTTCTTGTTTCCAAAATTCCAAATTTCTTAACTCATTTGGATTGGTCTGGCTTTAGTAGAGATATTTTTCTCAATAAAGGTCTTGACATAAGGGAGACTGACAACTTGTTGTTTCCTATGCCATAAAACATTAGGAAGATCAGAACAGACTTCTTGTTTTAACTTTTCTTCAAATTTCTGAATTTGTGACTGTAAGGACTTATCACTAAGCTATTGTTCAATTCTTTTAAACTTGAGTTCTTCTTTAAGAAAATCAAGTTGTTTAGTTTTACTTGAATTATTTTGACTGTTTCTGAAACTGTACTTTTCTGCAAAGCTTTTAAATTGTGTAACTCAGGTTTAGTTAAAAATTCAAACTTGACTTTTTCACCTAAATGAGTGGATATGACTCCATTGTATTCTACTTAGAAAGGGTATAAAAGGGCTATGAAGGGTACCCCGAGGATGACTTCATCTGTAATATTTTTGACTAAGACGAATGAGGTCTTGAAACAGATCTTGTTTTGACAGACATGGGCTTTTGGAATTTCATAGTTTAATTGGAGTGACTTTCTTGAGGCAGTGCTCAATGTTTCTTTTGACTTCTTATAATATTTTGTAGGGATTAATCCTTCTTGTATACAATTAGGAGCAGCTCCTGAATCCAAAAGAACTATTGCATTTAACTCAAATTTTTTTACAAATATAGTGACTTTAGAATACCATTTTCTAAAATTTACTTGTTTTATTAAACTAATAGTTTGATCATTATTTGAAGGTTCACCGTCTGAATTTACTAGACTTTCTGGTTGACTCTTGGACTCAGAGTTTTCTTTTTCCATTAAATTAATCAAATCTTGTTTAATTTCAAAATTCTCGGCTTGTAACTTGTGGCTCAGTTGTTATAGTGCTACTATTTCTTTTTTGACCAATATTACTTCTATTTGTGGATCTTGTAAAGTTACTTCTTTTCTTTTATTGAATTTTTCTAAGACTGTTTTCAGACTTATCTTGTGGGAACTTGTTGACTCGATTCTTCCTGACTAATGAATTTTCTTAATTTCTTTAAATACTCAGATTTTACTTCTGGGTTTTCAACTTTACTAATTAAATGAATTAATAATTCTTCTTGTTCCTCTTGTTTTGTAAGGACATTAATTGACTTACTTAGAGTATTACAGCACTTATCGTTACAACCAAATTTGATTTTTGGGGAACTTAGTTGGGAATGAGGGAGTTGGAATTAACTGAGCTTGAGTTTGCTACTGTGACGTTGGTTTCACTAGACTCAGAGTTTCTTAATTCTAAGACTTTAATTAATTTGTCTTTTTATTCATCCGTGATTTTCAACTGCTTTATCGTGTCTTTTACTTTACACTCATTAGCATAGTGACCATGTTTTTCACACTTCCAGCACTCAATTTTTCTCTTGTCGGTGGACTTTTTAGGTTTGTCATGTTTTCCTTTTCCACATACTTTTGACCAATTTTTCTTTGGGTGGTATTTCCTTTTACTATAGAACTCATCATTATGTCGGAAGAATTGTTTTCTACGTCAATATTGTCTTTGTTTACTAGTTTGTTGATCATTATACCTTGTCTTGGCTTTTCTAGATGGGGGAATCGAAGCTAGACCATACTGTTCACAAAAATTTCATAATTCATATTTGGCTTAATTTCTTTATGACTGAATTTCTTTTCCTATTTTCATATCAACACACATTTTCATTCCTATTTGGTTAATTATGCTAATAATATTTCCGTAAGTTAAGTTATCGAAATCTATGACCTTGATCATTACTAAGGACTGTTCTTATTTTATGGGCAAATAAATTAGGGAGACCATTAATAAATTTTTCTTTCTGAAAGGTTGATTACTATCATCTCTAAGTATGACTCTGTAAATGAAAACATCTTTATACCATCTGAAATTGCTTAACTTTGGACATCGGAGATTACTAAGTTGATCATGGATTCCAGCAGTAGTGTTACTGGGTGTTCTCTTGAAATGCTCAATTATCATGTATAATAAAGTGTTGACTCCGTCTTCTATTCCTCTTCCTAATTGTTCATCAAAAATAGGGAGAACTTCTTCATTAAGTTTGACTACATGAATAATTTGTCATTTTGATTCTTCAGTTAAATGTTTATCTCACCAAGAATGTAAGGTTTTGGTGAATCCTGTTCCTAGAAGGGGAACGATTTCTGACTCTCTGAAATTATGATTGGTTATGTAACTGTTGGCTACCATGGACATATGTTGGAGTTTACTAAGGATTTCTTGTTCAGAAAGGCCATCAATATTTCATTCATATAATTTATCAGAGGAGACTGAGAATTGACTTTGGGAGTTCTTTTCTTCAAATTGGATATCTGGAGGAGTTGGTTTGGGAGACCAATTTTTGGTTAGACTAGTAGGGTTGACTTTATTTTTTAATCTATTAAGTTTCAAATTATGATCAATTTCTAAGGTTATAAAAGCATTTTCAATTTTCGATATATTACTAAAATCTGTCTCATTGCTGGTTATAGACTCAAAATCGAGGAATTGACTAAATTGGAGGACTCAAGAACTTTAAGACTAGAGGACTCAGTTTTTTCTTTAACTGGTGTGAGTTGTTCAAGCATTTCTTCTATTTTTTTCCATTGTGGGTTTTGTCTTAAAGGTGGGGCTTGACTTCAGATCTTGGAATTTAACTAAAGGTTTTTCTTTCCGAGACTTAAGGGACATTTGCTGGGGGACTATTTTGTCAATTTTAGTTTCTATCTTATATAATTGTTTTCCAATGACGATTAGACTTTGGTTTGCATAGTTATTTTGTTCAACTAATTTTTTATCATTTGACTCGGGGATCTTAAAAGGACTGGCAGTAATTTTGGCATTTTTATGGCTTATAAGTATAGTTTCTACCGGTGGATGACTGGCTATGACTTCAGTGTTATCTTCTTTTTTCCATTTTTCGTGCGTTAAAGTTTTTAACTCTTTGGGCACGTAGTGGTTTTCAACAAAATCAAGATAAAATATTTCTGACTCAGTATTTCTCATGGAGTTTTTCCATTTATAAAAAATTTGTTGTCTCTGTTTTTTATCTAGGTAGGCTGTGTGATAATTATTTCTTCTGACAAGATTTTCTGGTGCATGTAAATGATGATCTAAAATTTTCCATTTAATTTTGAATTCTTTATTAGGGGTTCTTAATTGGTTGTCTACTAGGATGGCTTCTGCTTCAAAATCAGTTTGTGTAGGAGAATCTGGGGAGTATGATTTTTCATCTTCTTTGACTGGTTTTTTTATAACAAGGATGGTTGACTTGAGAATTTTTTCTTAAAGATTCTAACTTTAAATCAATTAATGTTTTTTTTTCTAACTCTAGGTCTCTTTCTAGAGTAGAGGAGGATGCAGTAAAAAAATGTCTAGCAAGGACATGTCTCTGAGATTGACTAGGTGTTGGAAGTTGGTGTAGTTGGACTGAGGATTTTTGAGGTGGTTGAATTCCAAAGTTAATTTTAACTGTGCCATCTAAATATTGTTGGATGTAATTAAATTATTGAAACTATGTTGAATAGGAGCAGGTTGATTTTTTGTGGTTAATGTCCAATCTAAAGGTAGGGTGACTTCTGACCACTTAATTATATGAGAGACTCTTATATTGGCATCGGCTTTACTACTCTGAATTAGTAAGGTTTGATCTTTTGGACTTTTGTTGAGGGCTTGGAAATTCATGTTGGTTCCAGTGACTTTATAATAAATTCTATAGACTAAGGGTAAGGGTTTGGTACTCGGTAAAACTTTATACCCTGAGGTTTTAATATTTAATGTTAAAGTTTTTAAACTATGAGGATCACTAAGGCTTACTGTGAAATCTGGGTAGCAATCAAAATGAATTGGACCATTAAACAGGCTTGACTTAATTATTCCTAAGGTACTGGTAAGAGGTTTGACTGCAACTTGGACTAGACCAATGTGAAGGAAATTGTTTCCATCATTTTTGTGTTTCTTTATTGACTCAAGATTTAACAATTGGCATGTTTCATGTTCTTTACTTAAGGCATAGACTTGCTCAACTGTTTTCACATGGGTAGTTGATTTGAAACTAGAGAGTGACCATTTTTTCTTGCAAATTTATTGACTAGGAATTTTTGGGATATTCCAATCTTTGTAATCAATTTCATTACTAGAATCATATTCTATTTGTTCTTCATTTACTATTTATTGGAGTTTACTAATACTGGCTCTAGAGCTGCTAGAGTTACAAGAGTCGGTTCTAAATAGGCGACATCTGGACAAAGTTACCGGTTTTTCTTACAAAGGGTAAGAAGAAGGCTAACTATCCTATAACAAATTCAAAGGTTCCTGGGAACTAAAGGATTCTCTCTCTCTCTCTCTCTCCGCTCATGCCTCACCTGGGACTTATTGTTTGGAATCTAAGACTTATTGTTTGGACCAAAGAGAGAAGACTTAGTTGACAGGAAGACGCTGAAAAAGTTACAGAATAAGAAAGCAAACTTCCAAACACAGAGAAGGTGACCGGTGACTATGAACACAGAGAAGCTTAATTAGAATGACTCTTCAACACTTAAGGCTCTAATACCAAACGGGATCTAGAACCAAAAGGACAAGTTAACTGTGATAGTAATGAAGCGAAGATAATAGAATATGTGAAAATCATGCGTAAATAAAAATAATGAATAAATGAAAATATGTTTAATCGTCATTGTATATGTATATACACACATTACCAGTCTAAAAAAAATTGGTATCCCAAACCAACTCCTTCTGACATCTAGCTTGAAGAAAGAAATTTCCAGATCAAATTCTTGATAAAAGTCACCTCCAAATATTTTTAGGTTCTCTTAATTATGTCTCTGATTTCTATCAGAATCTATGAAAACAATGTAGGCCCTTATTTGATAGGCTCCAGAGTAATCCCCCTCCTTGGACCTCTATTCATACAGAAATTGTAAAACAAATCAAAGTCCCTGTCAAAACACTACCCTGTCTTGGTATTCCTTCGGTCAATTCTTTCAAAATAGTTGAAACTGATCCCTCAGAAAATGGTTATGCAGGTATCCTTAAACAGAGAGTTAATCCAAACCAACCAGAGCAAATCGTTCGATTCCATTCTAGAATTTGGACACAGTCACAGCATAATTATAGTACTGTTAAGAAATAAATTTTATCTATAGTATTATGCATCTCAAAATTTCAGGGTGATCTATTAAATCAAAAAAATTTAGTTAGAGTCGATTGTAAAAGTGCAAAACATGTTTTAGAAAAAGATGTTCAAAATATTGCATCAAAACATATTTTTGCACGTTGGCAAGCTATTTTAAGCATATTTGATTTTGATATTGAATATTTGAGAGGAAGTGAGAACTTCATCCCAGATTTCCTTACTAGAGAATTTCTCCAGGACAAAAGATGGCAGAAAAGCAATCAAAACAGCAAGGCAAGCCACCAATCAGACAACTACCAGCTACAATGATCCCAGTCAAGATAGAGCAACCTTCCCAAGTCATCCCTTATCCAGGTTATTCACCCATTCAAATATCAAATAGGTATGCCACTCTAGGATCCACAGTCAGCAACATGCGCCCTAATTACCAGTCAGCTCTCATATCTAGCTATGATGCATTCCAAGTTGTAACCCATTCTCCTACTTCTTCCCCAATTGGTCACACCAAATCTTCTCCTTACTTCCCCAAAAGTAGTCAAATCCATTTTCTTATTGAGCCATGTTTTAATCATCTAGCCAACCCAGTCAAAATTGCCAAATCTATTTTCCTCCTAATTTCCACTATATGCCACCACACTCTTCTAAGTCACTCAAATATTATCGTGATATTCTCCTCGAAACTCAATCACTAGAAATGAGGCCAATTAAGGATAGAAATTATCCAAACATCATTCTCTATCATTCCATGTATATCAAAAGCATTCTCAGTCAATCTGAATGGGGTGAACACCCCTACTATTTCAAAGCATTTAATTCCAGTCTTCAATACAGTTACTATGATTACATTGAAGCTTGGCATGCCATTTTCCTCCATCAAACCAAGGATTTTAGTCATTCATGGTTTGTCAATTTCGATAGAAAATTCAAAAGCCCTTTTCCCTACTGGTTTTCTTCATGGTAGGAAAAACATGGTCCAACTATTGATATTCTTCCCCCCAGTCTCCAAGAATTAGTCAAATACTTTTCCAGTAAATATAATTTTATGCAAGAAAGTCAATTTTTCCAAAACTCTTAGTTTTCATTGCTAAATACAAAGTCCCCTGAATTCTAAAATGGTCATATGAAGTCAATTGGGAATCACGTATTCTCTCCAGGCAATTCTCCATCAAATGGTGGGATAAGTTCAAGACAAAGAATTATTGAACAAGTCGACGTTGAGTTCCCCTCAGTCAAAGCCCCTAAGAAGACGTCTACCTCTGGCACGTCTCACTTTTCCCTCCCAATCGAAGGAAGATCCAAGTCAGAATTAAAAGAATTGACATCCAAGCCTCTCAAATGAATGATGAAGATGATGATGACGATGCTTCTCCAAAGTCTCAATCCTCTTCCAGTCAGCCTCAAAATCCATCAAGTCAAAAAGATTTCACCCAAAAATTTCGCTGGGTAGATTATCCAGACAGTCAAGAGTCAAGACCCATATGATCTCAATGAAGACTAAGTAAAAACAGGAGTGAAAGCAGAAAGCAAAAGTAAAAGCATGCCTCTACAGTCAATATTCCCTGATAAAGAAGAAAGTAAAAGTAAAGACAAGTCAAAAGAAAGAATAGTAGCTCCGTTAAAGCAAATGCAGACAACCCGTGCTTCAATAGTAAAACATCCAATCTTGACTGAAAGATTTTATTAATTGAAAGTAGCACTCATATTTTACAAGTGTTCTTCAGAGTGATATTTCTGAGAGAACTTTGAAACTGAGAAAACTAAAAATTACATTCTACTTACAGCTGAGGCCTAAGCCTCTATTTAAAGGGCTTGACTATGAAAGTGTCGGTAATATTGGAAGCTTTACTGTTAGAGAAAATGATTGCTTTATCTAATCTATTAATTAAATCTCTTTTATTGGGGATCTGATATCTATTCCATTCTAAAACCGTATTAAGGGGTTTATAATTGATGACCAATCTTGGGGCTCCTTTTTGTAATTCAGCATTTTTCTGGACATAAAAAGCTGGACAAGACCAAGGTGACTTGGTCTTACGGATTATACCTTTTTGAAGTAATTCTGTAATTTCTTGTTTACAAAATTCTATAATTTCTTGACTCATTTGAATTGGTCTGGCCTTGACAGGGATATTTTTCTCAATAAATGTTTTTACATAGGGTAGACTAACTATGTGTTTCCTATGCCAAAAGGCATTGGGGGGATTAGAACAAACTTCTTGTTTTAACTTTTCTTTAAATTTCTAAATCTGGGACTGTAAAGGCTTATCATTAAAGCTGTTCAGTTCTTTTGAATTTGACTTCTTCTTTAAGGAAATCTAATTGTTTATTTTTTATTTTGAATTATTTTGACTGTTTTTGAGATTGCCTTTTTCTGTAAGGCTTTTAAATTACGTAATTCAGGCTTGGTTAGGAATTCAAACTTGACTTCTTCTCCCAAGTGGGTAGATGTGACTCCATCATATTCAACTTGGAAAGGATATAGAAGGGCTGAATGGTAGACCTAGGATGATTTCGTCAGTAATGTTCTTACTAAAACGAATGAGGTTTTGAAGCAGATCTTATTTTGACAGACATGGGCTTTTGGTATTTCGTAATCTAACTAGAGTGGCTTACCAGAGGTCGTACCTAATGTCTCTTTTGACTTTTTGTAATATTTAGTAGGGATTAATCCTTCTTGTATACAATTAAGGTAGGCTCCTGAATCAAAAAGAGCTACTGTGTTTAACTCGAAATTTTTTATAAAAATTGTGACTTTAGAATACCATTTTCTAAAATTTATTTGTTTGATTATATTAATGGCTTTATCATCGTTTGAAGGTTCATCATCTGAATTTGTTGGACTCTCAGATTGACTCTTGGAATCAGGATGTCTTCTTTCCATTAAATTAATTAAATCTTGTTTAATTTCAAAATTTTCATTTTGTAACTTGTGACTCATTTGTTTTAACTCTATTATCTCCTTTTTGACTATTTTTACTTCCATTTGTAAATCCTGTAATGTTACTTCTTTTCTTTTGTTGAATTTTTCTAAGGTAGTATTTAGACTTATTTTTGGGGGAATTTGTTGACTCGTTTCTTCTTGACCAATGATTTCTTAATTTCTTTAAATATTCAGATTTTAATTCTGGATTTTCTACTTTGCCGATTAAATCAATTAATAATTCTTCTTGTTCTTCTTGTTTTGTAAGGACCTATATTGACTTAGTTAGGACATTACAACACTTATCTTTGCATCCAAATTGGACTTTTGGGGAACTTAGTTGAGACTCTAAGGAACTATAATCTGCTGAGCTTGTTTCTGCTTCAATTTCGCTAGAGTCAGAGCTTCTTAATTCTAATCCCTTAATTAATTGTGCTTTTTCTTTCATCCGTGATTTTTTATTGCTTTATCGTGTCTTTTACTTTACATTCATTAGCATAATGACATGTTTTTTACACTTCCAGCACTCAACTTCTCTTTTATCTGTATTTTTCTTAAAGTTACTTTTCTTGTCTTTTCTGGGGGCTTTTCACCAGTTTTTTCTCGGGTTAAATTTTCTTTTACCATAAAATTCACCATTATGTCTAAAGGACTATTTCCTGTGTGAGTAATGTCTATGTTTACTAGTTTGTCGGTTATTGTACCTAGTCTTGACTTTTCTAGATGGGGGAATGGATGCTAGACCATATTGTTCACAAAATTCTCCTAGTTCATATTTGGTTGAATTTCTTTCTGACTGAATTTGTTTACCTATTTTCATGTCAACACACATTTTCATTCCTACTTGGTTAATTGTACTAATGATATTCCCATAAGTTAAATTATCGAAATCTATGTGGCCCTTGTCATTACTTAGAACTGTTCTTATTTTGTGAGCAAACAAATTAGGGAGACTATTAATGAACTTCTCTTTCCAGAATGGTTGGTTACTATCATCTCTTAACATAACTCTAGAAATGAAGACATCTTTATACCATCTAAAATCACTTAATTTGGGACATCTGAGATTACTGAGCTGGTCATGAATCATAGCAGTAGTAGTACTAGGTGTACCTATGAAATGTTCAATTTATTGTGTATAATAAAGTATTGACTCCATCTTCTATTCCCATTTCTAATCGTTCATCAAAAATAGGGAGACCTTCTTCATTTAACTTTACTGCATGTGTGATTTGTCGTTTTGACTCTTCAGTTAAGTGTTTATCCCACCAAGAACATAAGGTTTCGGTGAATCTTGTTTCTAAAAGGGGGACAATTTCTGACTGTCTTAGATTGTGATTGGTGATGTAACTATTGGCGACCATAGACATGTGTTGAAGCTTATTCAGGATTTCTTGTTTTGAAAGACCATCGATATTCCATTCATATAACTTATTAGATGAGACTGAGAATTGACTTTGATAATTTCTCTCTTCAAACTGGATGTCAGGAGGAGGTGGTTTGGGGTACCAATTTTTTGTTAGGCTAGTAGGATTGACTTTGTTCTTTAATCTATTAAGCTTTAGATCATTATCTATTTCTGAATTTTTAAAGGCATTCTCAAATTTTGATATATGACTGGAATTTGTTTCATTACTGGTTATAGACTCAGTGTCAGAGAAATTGACTAAAACAGAGGATTCGAGGACTTTGAGACCAAAAGGTTCAGGTTTTTCTTTGACTGGAGTTAGTTGTTTAAGCATTTCTTCAATTTTTTCCATTGTAGGACTTGTCTTAAGAGTGGGGCTTGACTTTAAATCATGGAATTTTACTAGAGGTTTCTCCTTCTGAGACTTAAGAGTATTTGTTGGGGACTATTTTGCTTATTTTTGTTTCAATTTTATCTAATTGTTTTCGTAGGATTATTAGACTTTGATTTGTATAATTATTTTGCTCAATTAATTTTTTATCATTTTGCTCAGGAATTTTAAAAGGACTAGCAGTGATTTTGACATTTTTATGGTTTATGACTATAGTTTCTACCGGGGGGTGACTAGCAGTAACTTCGGTATTATCTTTCTTTTTCCATTTTTCTTGTGTTAATGTTTTTAACGCTTTGGGTATGTAATGACTTTCATCAAAATCAAGATAAAAAATTTCTAACTTAGTATTTTTCATAACATTTTTCCAATCATTAAAGATTTGTTGTCTTTGTTCTTTACTTGGGTAGGCTGTGTGATAGTTATTTCTTCGTTCCAGGTTTTCTAGAGCATTTAAATGATTATTTAGAATTTTCCAATGAATTTTAAAGTCTTTGTTGAGGGTTCGTAATTGATTGTCTACTGGAATGGCTTCGGCTTCAAAGTCAGTTTGTATGGAAGAATCTGGGGAATCTGACTCTTCATCTTCTTTGATTGGTTTCTTTCCATAACAAGGATAGTTGACCTAGGAGGTTTTTCTTAAAGTTTCTAGCTTTAAATCAATTAACGTTTTTCTAACTCTAGGTCTCTTTCCAAAGTAGAGGAGAGGAAGCTGCGAAGGAATGTCTAGCAGGAGTATGTCTAAGGGATTGACTAGGTGTTGGGAGGTGTTGTATCTAGACTAAAGACTTGTGGGGTTGATTTCCAAAATTTATTTTTACTGTACCGTCTAAATATGTTGAATATAATCTAAATTATTCAAATTATGCTGGATAGGAGTGGGTTGATTTTCTGTTGTTGGTGTCCAATCTAGAGGTAAAGTGACTTCTGACCATTTGATTGTATGGGGGACTCTTATATTGGCATCAGCTTTACTACTTTGAATAAGCAAGGTTTGGTCTTTTGGACTCTTATTAAGGGCTTGGAAATTCATATTGGTTCCTGTGACTCTATAGTAAATTCTATAGACTAAGGGTAGAGGTTGGGTACCTGGTAAGACTTTATACCCTGGAGTTTTTATATTTAATGTTAGAGTTTTTAGTATGTGGGGATCACTAAGGCTTACTGTGAAGTCCGGGTAACAATCAAAGTGAATTGGTCCATTAAACAAGCTTGACTCTATTATTCCTAAGGTACTATCACTAAAGTTGGTAAATCTGGCATCACGTAGATAGAGTAAAATAGAGGCATTTAGTCCTAACCTAGTAAGGGGTTTGACCGCCACTTGTACGAGGCCAATGTGAAGGAAATTGTTTCCATCATTCTTGTGTTTCTTTATTGACTCAAGGTTTAATAATTGACATGTTTCGTGTTCTTTACTCAAGGCATAGACTTGTTCGACTGTTTGCACATAGGTTGTACTTTTGAAACTCAGTTAAACATTATTAAATTATTAAACTCAGTTAAGTTACCTAATTCCATTTTTCACATGGTCAGATGTCAAGCTACCTTTAGATTGGACACTAGCAACCGAAAGTCACCCTGCTCCCATCCAGCATAATTTGAATAATTTAGACTATATTCAACAATATCTAAATGGCACAGTTACAATTAATTTTGGAAATCAACCCCTTCCTAAGACCTCAGTTCAGTTACAGCAACTCCCTACACCTAGTTAGTCTTTTAAACACAATCCTACTAGACATTCCTTTACTGCATCATCTTTCACTCTAGAATGAGATTTAGAGTTAGAAAAAACACTAATTGATTTAAAACTAGAATCTCTAAGAAAGACCTCTCAAGTCAACCATCCTTGTTATGGAAAGCAACCGGTAAAAAGAGATGAAAAGTCAAATTATCCAGATTCCCCCACTTAAATTGATTTTGAAGCCGATGTCATCCCAATAGACAATCAGCTACGAACCCTTAGTAAAAAAATTAAGATTCATTGGTCAAACCTGGACAAACATCTTGAATACCCAACCAATCTAGAAAGACGTAGCCACTATAGAACAACCTACCCTAGTAAAGAATAAAGACAAGAAGTTTTTAATGAGTGGAAAAATTATATGGTAAAAACTGAGTCAGAAATATTTTATCTAATTGAAAGCATTTATCAAAAGAAAGCAATAAAAACCTTAACTCAAGAAAAATTGAAAAAAGAAGATAATACAGAAGTCACCGCTAGTTATCCCCCGGTAGAAACTGTAGTTATAAACCATAAAAATGCCAAAATTACTGCCAGTTCTTTCAAAATTCCTGAGCAAAATGATAAAAAGTTAATTGAGCAAAATAACTATACAAATCAAAGTCTGATAATTTTAGGAAAACAATTAGATAAAATTGAAACAAAGGTCGACAAGTTACTCCCTCATGAAATAAAAGCCAAGCTCCTAGCAAAATTCCAGGAACTAAAGCCAAGCTCTACTCTCAGAAAAACCCCTACAATAGAAAAAATTGAAGAAATGCTTGAACAGTTAACCCCAGTAAAGGAAAAACCTAAACCCTCTGGTCTCAAAGTTTTAAATCCTCTGCTCTAATAAGTTGCTTTGACACTGAGTCACATCCAGCAAGTAACATAGATTCTAGTGATATAACAAAGATTGAAAAGCATTTAATAATCTAGAAATAAACAATTATCAAAATCTAAAACTTAATAGGCTAAGAAACAAAGTCAATCCTACTAGTCTAAGAAAAAATTGATACCTTAAACCAACTCCCCCTGACATCCAGTTTGAAGAAAGAAATTCCCAAAGTCAATTCTCAGTCTCATCTGATAAACTTTACGAGTGGAATATGGATGGTCTATCAAAACAAGATTCTGAATAAAACTTTAACACATGTCTATGGTAGCCAATATTTATATAATCAATCACAATTTAAGACAGTCAGAAATCGTTCCCCTTTTAGAAACTGAATTTATCGAAACCCTACTTTCTTGGTGGGATAAACACTTAACCGAAGAGACAAAAAGACAAATCACACATCCAGTAAAACTAAATGAAGGTGGTCTCCCTATTTTTGATGAACGACTAGGTACTGAATAGAAGATGGAGTCGATACTTTATTATATACCATAACTGAACATTTTATAGGAACACCTAGTAACACTACAGCCAGAATCCATGATCAATTCAGTAACCTTAGATGTAACAAGTTAAGTATTTCAGATGGTACAAAGATGTCTTTATATCTAGAGTTATGCTTAGAGACGATAGTAATCAATCCTTTTGGAAAGAAAATTTTATTAATGGTCTTTCCAATTTGTTTGCCCATAAAATACGTACAGTCTTAAGTAATGATCAGGGTCATATAGACTTTGATAGTCTAACCTACGGTAATATAATTAGTACAATTAATCAAGTAGGAATGAAAATATGTGTTGATATGAAAATTGGTAAACAAATTCAGTCAGATAGAAAGTCAGCCAAATATGAGTTAGGAGATTTTTGTAAACAATATGGCTTAGCATCTATTCCACCCTCCAGGAAAATCCAACATAGAAATCATGAGCGTCAAACAAGAAAATATAGACATTTTCAAAGAAAACGACATTCCTTTAAACATAATGATGAGCTTTATGGTAAAAGGAAATTTCACCCAAAGAAAAATTGGTCAAAAGAACGTGGAAAAGGTAAAGATAACAAACCTAAAAAGACCACCGACAAAAGAAAAGTTGAGTACTGGAAGTGTAAGAAATATGGTCACTATGCTAATGAGTGTAAAGTAAAAGACACGATTAAACAATTAAAAATCTCAGAAGAAGACAAGGCTCAATTAATTAAAGTCCTCGAACTACAAGATTCTGAGTCTAGTGAAAACATGACCTCTATCTCAAGTTCTCCCAATTATAGTTCGTCCAATACCCAGTCAAGCTCCCCAAAAATTCAGTTTGGATGTAAAGATAAATGTTGTAATGTCTTAATTAAGTCAATCTAAGTCCTCACAAAACGAGAAGAACAAGAAGAATTGCTCATAGATTTTATTAGTAAAGTCGAAAATCCAGAGTTAAAATCTGAATATCTGACAAGATTAAGAAAAGTCATTAGTCAAGAAGAACCAAGTCAAACCAGCCTCCCCAAGATTAGCCTAAACACTACTTTAGAAAAGTTCAACAAAAGCAAGGAAATTACTCTTACAAGATCTTCAACTTTAGATCAAAACCATCAAAACAGAAATAATAGATTTGAAACAATTTAATTGTAAATTACAAAATGAAAATTTTGAAATAAAACAAGATCTAATCACTTTAATAGATAGAATAGATTCGATACCCAAAGGTCAACCAAAAAGTCGTTCCGATGATGAACCTGAACCTTCAAGTAATGATAAAGCCCTTAACTTAATTAAACAGATAAATTTTAGAAAATGGTATTCAAAAGTCAGTATTTTTATTAATGATTTTGAACTACAAATAGTAGCCCTTTTTTATTCAGGAGCCTATCTTAACTGTATTCAAGAAGGATTAATTCCAACCAAATACTACAGAAAGTCAAAAGAAACCTTAGGAAAAGCTTCAGGTAAACCACTTCAATTAAACTACGAAATTCCTAAAGTCCATGTTTGTCAAAATAAAATTTGTTTTAAAACGTCTTTTGTCCTAGTCAGGAATATTACCAACGAAGTCATTTTAGGCCTTTCCTTTTTAGCCCTACTTTACCCATTCCAAGTAGAAAATGATGGAGTCACCTCTATCCATATGAAAGAAAAGGTTAAGTTTGAATTCCTAACAAAGCCAGAATTGCATGCCCTAAAAGCCCTTCAAAAATGCTCCATTTCTAAAACAGTTAAAATCATTCAAAATAAAAACAAATAGGTAAACTTCCTTAAAGAAGAAATTAAATTCAAAAGAATTGAACAGCAACTCAGTAATGAATCCTTACAGTTACAAATTAAAAAATTCGAAGAAAAATTAAAACAAGAAATTTGTTCAGATGTTCCTAACGCTTTTTTGCATAGGAAACAGCACGTTGTCAGTCTACCTTATATCAAAACTTTTGGTGAAATGAACATTCCCACTAAAGCTAGACCCATTCAAATGAGTTAAGAAATTATGGAATTTTGCAAAACAGAAATTTCAGAATTACTCCAAAAAGGAATAATCAGGAAAAGTAAGTCATTTGGTCATGCCCTGCCTTCTATGTTCAAAATAGCGCCGAATTAGAAAGAGTTGCTCCCAGATTAGTCATCAACTACAAACCCCTCAATGCTTTTCTTGAATGGATCAGATACCCCATTCCCAAAAAAAGAGACCTCATTAAAAGAATAGGAAATTCAGTCATCTTCTCTAAATTCGATATGAAAAGTGGATTTTGGCAAGTTCAAATCAAAGAGAGTGAGAGATACAAAACTGCCTT

CCTCAACCCCTTTGGATTTTTTACTCTACGTGATTCATATTCTCAAATAGAGAATCCAATGGTTGGTTAATAAATGCATCACAATGGTATAGTTTTCAAATAATGGCTCACTCTCTAACATGATATAAAACTTAAGCCTTACTCTGTCTATTCTAAAACATGGTGACGATCAAAGATTTTGAGCCCATTATTCTGTTTAAGGTTCAGCCACCAATATTAAAAGCCGAAATTCAAGCTCTAAAATAAATAATTTAGTACAAATCATGCTTTTTCTGGGCCAAATTTATTATTGTTAGGCTAATGCAGATGATACAGAAATGATTATAAATGTTGGCAGCAAAACATCACAACTAATCACAACAAAATTTTACTAATTAGGGATTTCGGTTGTGCAGTCATCCAAACATGGTCAAGAAATAATCTTAGCAATGGTGGCTTGCTTTCTTGCCCTGCCCCCACCACCATTTTTTTTCTTTTTGGGTTCTATGTTTGACTCTGTGCTTGTGTCTTTCCAGCATTGTATGCTCTTTGGATGTGGCTCATTTTGAGAAGGGGGGTCACTACTACTAGTCACAGTAGTAGCTAATCTTTTTAATTGTGTTAATCGTCATAATTAATTAATTAAGTACCCTTTCGATCTAAAGCATGACCTAATTTTAATATACTTGACCTCAATTATTGTTGGAGTGTAAAGTTCTGTGAATGTATTACTACTAGGTTCTGTAATGTTCATTGTGATTTTGAAATAACAAGGCTTAAGTTAAATATGCATGTGGTATGTAATTCATGTAACATCATTGCAATTGAGCTGAATTGCATGCAATTGGCTGCTGCCCTTTTTGTTTTGAATATAAACTAGTGTTTCCTCTAACTTTATTGTGTATTGGTAAAAATGATGGGCTAAGCAATTATGGAAAAATCTTGGATGCCTGCCTATAGGAGAAGCAAAGACTATGAAGAGGGAGTTGAAGAATTTCTAAGGATATCATTAGCGAATGCT

>Chr6-06; OZ022211.1; 10937439-10944581; dir; A;

ATCGTGTAGGACTTCACGGATCATGAATTGGAGCCCCGGATGTTTCGATTCAATAATTCAAAGTTTGTGGGTTAACGATAACCGTACAATCGTGAGCGATTCCTAAACCTGTAACTGGACCTTAGGATACCTCGTTCAACGTACACGTACTGGGAACATGGGGATTTAAGTATTTAAATTATGATTTCACTTCGAAGTAATTTTATATTAATTAAGGATTCGTATCTCGAAATAGGTGCTCCGACTGCACGTACTCAGCAGGCAGGACCCTCTCGTGGTCAGGCAGCGTGGGAATACTTGTGAGTAGACTTTGTTTTCAATCATATGCATGGTTTTATTGATCAAAGATTTATACATAATGTTTTTAATGGTTTATTGAATATATTATATTCATGGGTTGAATTTGATGTTTGTATAGCGCTTTGGCAATATATTGTGGGTTTGGCATATTTGGGTATCGAGAGTGTTATATATATTGTCTATAGGTGGGATACTTGGGTTGTTGAGGTGAGTTTGTGGAAGAACTTGAAGAATATGAGGGGTTGTTGAGAGAAGTTGTATGATTACACTATGTAAATACCTTCCCCAATTATTAGGCTTCTCACACGTGGCGTTGGATATTCCGGCGTGTGGGAAGAATAGGTTATCAGTCTCACACGTGGCGTTGGATGTTCCGGCGTACGGGAAGACTCTGTATATTGCTTGGGATCATCACACATGGCGTTGGATGTTCTGATGTGATGATCCAGTTTACGCGCGTAGGACTTAGTATTGGAGCTACACATGGCCTTGCATGTTCTGGTGTGTAAGCTATGGAGTTTCGTTCCCTGGTTGGACTGCTCATCTCTAGACGCAAGATAGCACCTGAAAATCCTGCATGCTAGTCAAACGATCCCCCGGTTGGATTGTTTCCTCGGTACCTAGGTAGTGTGATGAGTATATTGTATAGTTATATATACATATATATATATATATATATATATATATATATATATATATA

CCAAGTGGCTGGTTTAGGTTTTCTGAGTTGTAAAGTGTTCAGTTTCTTCTTTTCTTCTTTTGGTTTTCAGTTCACCTGTCCTCTCTCTCTTAGTGTCCAAATCGTAAGACCCGTTCAGTAAGTCCCAGGGAGGCCTGAACGGTAGTGGCATTAGTAGGAGGGCGAGAGAATTTTAGGTGTCCTGTTACCCATGGAGTCTGTATTAGAAGTTTCTGTTCATTTTTTTCTTTAGGAGTCCAAACCCAGAACCTTGAGTAAAAATGAGTCGATTTCTTAGAACCAACTCTTACAGTTCTAGCAGCTCTAGAGCCAATAGTAGTCTTCCAGAGATAGTAAATGAAGAACAAATTGAATATGAGAATAATAGTAATCTAGATTATAAAGAGTGGAATATACCTAGAGTCCCCAGTCAAGACATCTATAAGAAAAAATGGTCTTTATCCAATTTTAAGTCAACCACCCATGTTAAGACAGTCGAACAAGTTTATGCCCTAAGTAAAAATCACGAGACATGTCAGTTACTTAATCTTGAGTCAATAAGAAAGCATAAAACTGATGGAAACAATTTCCTCCACATTGGTTTAGTTCAAGTAGCCGTCATTAAAAGCTTTTATCCTTCTCTATTTGCGTGATGCTAGATTTAATAATTTTAACGACAGTACGTTAGGAATTATTGAATCCAGTCTTGCTAATGGCCCAATTCATTTTGATTGTTATCCAGATTTTACAGTCAGCCTGAGCGATCCCCATATATTAAAAACACTCACTTTGAATATTAAAACTGAAGGTTATGATGTTTTGCTCGGTAGTCAACCCTTATCATTAGTCTACATAATTTATTATAAAGTCACAGGTACGAATATGAACTTCCAAGCCTTAAACAAGAGCCCAAATAGTCAAACATTGTTAATCCAAAGTCATACACCTGATGCTACTGTCCGAGTCCCTCACTATCAAATGGTCAGATGTCACCTTACCTACTGATTGGACCTTAACCACCGAGATTCCTCCAGTCCCTATCCAACGTAGTCTAAACGATTTAGATTACATACAAAAATATTTAGACGGTACATTTAAAATCAATTTTGGTCACCAAGCCCCACAAAAATCAACAGTCCAGTTACAACAGTTACCAACACATGGTCAATCCCAGCGTCATGTCCTCGCAAGGCATTCCTTTGCAGCTTCTTCTTCTACCCTAGAACGAGACCTAGAGTTAGAAAAAGATTTAATTGATCTATAATTAGAATCACTAAGAAAAACTGCACAAGTCACTCAGCCCGTCTATGGTAAACCAACAGAAGATAATAAATCTGAATCCCCTGAATCACCTACTCAATCTGATTTTGTTGAAGCCAGTTCCAAAATGGAAAACCAACTACGAGCACTTAATAAGAAATTTGAAATTAATTGGAAAAAATTAGAACGTCACTTACATGCTCCTGAAAATCAAGCAAGAAGAGATAACTATCACTCAATAATCCCAGACAGAACCAAAAGACAACAGATATTTAATGAATGGAAAGAATACATGCGCACTGCCAAAGTTGAAATTTATTATCTTGATTTTGTAGAAAGCCGTCATCTATCTAATGAATTAAAGACCTTAACTCAAGAAAATTGGGAAAAAATAGATCACACTAAGATCCTTGCTAGTCATCCTCCAGTTGAATTCATAGTAATCCAGCACCAGAATGCACAAGTTATTGCCAGTCCTTTTAAAATCCCAGACTTAGAAAATAAAATGGTAATAGAACAAAAAAATTATACGAATCAAAGCCTAGTCGTCATAGGTAAACAATTAGACAAAATAGAAACAAAAGTCGACAAATTGCTTCCAAAAGAAATTTCTGCTAAGACACATGACCAGAAACCATTAGTCACCTTTCAAGACCTAAAATCTAGTCCAACCTTTAAAACAAAAACCAAAATGACAAAAATTGAAAAAATATTAGAAGAATTAACTCCAACCAAACAGAAAGATAAGGAAAAAGTAGAAACAAGCGTCAAAGTCTTAGGAGTTTCAGAGTCCTCTTTCATCTTTAATCACTCCTCTGAGTCTGAATCAATTGCTAACATAGATACTGAGTCTAGTACTATTTCCAGAATTGAAAATGCGTTTAAAAACCTTGATCTTGAACAAAGTCCACAAGTATAAAGATTAACAAACCGAGTCAGCCCTACTAGCCTCACAAAAAATTGGTATCCTAAGCTTACGCCACCTGATATCCAGTTCGAAGAACGAAATACTCAAAGTCAATTTTCTGTCTCTTCTGATAAATTATATGAATGGAATATTGATGGCCTTTCTGAACAAGAAATTCTTAATAAGCTTCAACACATGTCTATGGTAGCCAACAGTTACATCACCAATCACAGTTTCAGACAGTCTGAAATTGTACCATTGTTAGAAACAGGTTTCACCGGAACTCTTCGTTCCTGGTAGGATAATCACTTAACCCATGAGTCTAAGCAAAGAATAATTCATGCCCTTAAACTTAATGAAGATGGTCTTCGAATTTTTTATGAACAAATAGGTCAAGGCATTGAAGATGGAGTCAACACATTGTTATACACAATAGTCGATCATTTTATTGGAACACCTAGAAATACTACTACCAGAATACATGATCAATTGAGTAATTTAAGATGCCCTAAATTATCTGATTTTAGATGGTATAAAGATGTGTTTATTTCCAAAGTCATGGTAAGGGAAGATAGTAACCAACCATTTTGGAAAGAAAAATTTGTTAATGGATTACCAAATTTATTTGCTCATAAAATCCGATCAACCCTTAGTAATGAACAAGGTCATATTGATTGGGATAGTTTAACTTATGGAAACATAATTAGCACAATTAATCAAGTCGGAATGCGAATGTGTGTCGATATGAAAATTGGTAAACAAATTCAGTCTGAAAGAAATTCAGCCAAATATGAATTAGGAAATTTCTGCGAACAATATGGTTTACAGTCTGTTCCCCCATCCAGAAAAAATAAGTCTAGTCATCACAACTGACAGTTTAGCAAAAAGCGTCATTATTCACGTAGAAAACAGTCCTTTCAACACAATAATGAAGATAAAGAATTTTATAGTAAAAGGAGATTCTCCCCTAAGAAAAATTGGAAAAAGACTCACAACAAAGATAAGAATAGTAGCTTTAAGAAAAAGACCGACAAAAGAAAAGTAAAATGTTATAAATGCAAAAGTATGGTCATTATGCTAATAATTGTAAAGTTAAAGATACAATCAAACAATTAAAAATCACGGATGAAGAAAGAAACAAACTAATCCAAGTCCTTGAGTTAGGAAATTCTGAGTCCAATGAAAACGAAACCATGATTAGCCATACTGAGTCTTCTGAAATATATTCTTCTGAGTCACAACCCAGTTCCCCCAGAATTCAATTTGGTTGCCAGGATAATTGCTGTAAGTCTAAATCAATCAGTGTTCTCACCAAACAGGAAGAACAAGAAGAACTATTAATTGACTTAATAAGCAAAGTTGAAAATCCAGAATTAAAATCTGAATACTTAAAAAAACTAAGAAAAGTTATCAGCCAAGAAGAACCAGAACAGTCCATCTCCCAGAAGATAAGTCTGAACACAACCTTAGAAAAATTTAACAAAAGGAAAGAAGTCACCTTACAGGATTTACACCTAGAAGTAAAGTCTGTCAAAAAAGAAATTATTGAGTTAAGGCAAATAAGTCAAAAATTACAAAGTGAGAATCATGAAATAAAACAAGATTTGATTAACCTGTTAGAAAAAAGGCCTCCGAGTCTAAACCAGAAAGTCCCACTAATTCAGATGACGAAAACTCTATCCAAGATCAAGCTGTTAACTTAATCAAACACGTAAATTTTAGAAAATGGTATGCCAGAGTTACTATATTTATCAAAAGCTTTGAGTTAAACATAATTGCTTTATTCGATTCAGGAGCCGACCTTAATTGCATTCAAAAAGGTTTAATCCCAACTAAATATTACCAAAAGTCAAGAGAATCGTTAAGTACTGCCTCAGGAAAGTCCCTCCAATTGAACTATGAAATTCCTAAAGCTCATGTCTGCCAAAACAAAATCTGCTTTAAAACTTCTTTTGTCTTAATCAAAAACATTACTGATGAAGTTATACTAGGACTGCCATTCATCTCTCTGCTATACCCCTTCCAGGTCGAACATGATTGTGTCATTTCCACTTGTCTAGGAGAAAAGATTAAGTTTGAATTTTTAAGCAAACCTAAATTACATAATTTAAAGGAATTACAGAAAAATGCAGTCACCAAATCAGTCAAAATTATTCAAAATAAAGATAAACAACTAAGATTTCTTCAGGAAGAAGTTAAAGCCAAAAGAATTGAGCAACAACTAAGTCATGAGTCTTTACAATCACAAATACAGCAATTTGAAGAAAAACTAAAACAAGAAGTCTGTTCTGAACTAGCTAACGCATTCTGGCATAGGAAACAACATGTTGTTAACCTTCCCTATGTCAAAACCTTTAGTGAAAAAAAATATTCCTACTAAGGCAAGACCCATACAAATGAGCCAAGAACTAATGGAATTTTGCAAAATTGAGATTACTGATTTACTTAAAAAAGGAATAATCCGTAAAAGTAAGTCACTCTGGTCTTGTCATGCCTTCTATGTCCAGAAAAATGCTGAATTAGAACGTGGTGTTCCAAGATTAGTCATAAATTATAAACCGCTTAATACTGTCTTAGAATGGATTAGATACCCAATTCCTAACAAAAGAGATTTGATTAACAGACTAGAAAAATCAGTCATCTTTTCTAAGTGCCCGTTTGGTATTGCTTATTTGGGGTAATAAGTGATTTTTTAAAAATTTTGAAAAGCCCAATCCGTTTGGTAAACTATGAGAAAATCACTTATTGTTAAAAATTGCTGTGAGAGAAAGCTATAAAGGAAAGCAGCTAATGAGGTGCTTTCATTTTCTGATTATGCAGGAATCAGTTTCGAAAAGGCGCTGGGTTTAATGATTATGCGGCAATCATTTTTTGATTATGCATAAAATCAATACGAACAACACTTTTAACAAAAATTTTACCAAACGCCAAACTGCTTTCTCTCACAGCTGATTTCTCTCACTGCAAATCTGACAGCGATTATTTTCACAGCACAGCAATGCCAAACTGGCCCTAAGTTTGATATGAAAAGTGGATTTTGGCAAATTCAAATTTCTGAATCCGATAGATATAAAACTGCTTTTGTTACTCCCTTTGGTCATTATGAATGGAATGTTATGCCTTTCGGTCTTAAGAATGCTCCTAGTGAATTCCAGAATATAATGAATGAGATTTTTAATCCTTTCAGCCATTTTTTCATAGTTTATATTGATGATGTGCTTATATTCTCTGAGTCCATAGAACAACACTGGAAACACCTGTATAAGTTTTTACAAATAGTTAAACAAAATGGTTTAGTTGTTTCTGCTAAGAAAATTAAGTTATTCCAAACTAATGTGAGATTTCTTGGGTTTAATATCTGCCAATCACATATTAGTCCCATAGATCGAGTCATCCAGTTTGCTGATAAGTTTCCTGACCAAATCCTAAACAAGAGTCAGCTCCAGAGATTTTTAGGATCTCTAAATTATGTTTCTGATTTTTACCAAAATCCGATAAAGCAATGTAAGCCTTTATTTGATCGTCTCCAAAATAATCCTCCCCCATGGTCATCTGCTCATATTGAAATTGTCAAACAGATCAAAGCTCATATCAAGACTCTTCCCTGCCTTGGTATTCCTTCTGCTAATTCATTTAAAATTGTTGAAACTGATGCTTCTAATGTAGGTTATGGAGGTATCCTCAAACAAAAAGTCCCTTCTGGTCAATCTGAGCAAATCATTCGATTCCATTCTGGCACTTGGTCTCAATCACAAAATAATTATAGTACTATTAAGAAAGAAATACTATCTATTGTATTATGCATTAATAAATTTCAGTCTGATTTACTAAATCAAGAATTTTTAGTCCGTGTTGATTGTAAGTCTACTAAGCATGTATTAGAAAAAGATGTTCAAAACGTTGCATCCAAACAAATATTTGCACGTTGGCAAGCTATTTTGAGTATTTTTTTATTTTGATATTGAATATTTGAGAGGAAGTGAGAATTCTATTCCTGATTTCCATACCAGAGAATTTCTACAGGGAAAACAATGGCAGAACAAAGAAAAGAAAGAGAAAAAGATGATAAACAAAAGAGTCAAATTAGTCAATCAAGTCAACCTAGTCAACCCCAATCACTCCGAGTCCTCCCACCATCAATGAGTCAAGCCAAATATGAGCCTCCTTCCCAGATTGTCCCCTTCTCGGGATGCTTTCATATCCAAATCAGCAACAGATTTGCCAACCTAGGAGCCACAGTCGGCCAGGTTCGCCCTAGTTTCCAGTCTGCTTTAATTTCCAGTCATGATCCTTTTCAGGATATTGCAGTCACTAACCCCCCAGTTGTTTCTTACCATAAGACGTCCCAATACATGATCAAGAGTCACAGTCACCTGTTTGTCATTGAGTCCAAATATAATTCAATTGCTAATCCCATCACCATTGCTAAGTCTTATTTTCCACCCAATAGTCATTTCATTCCCCCAGCACCATTCAAGAACCTCAAATATTATAGAGATATCCTTCATGAGACCCATTCTATCACCATTAAGCCAATTAAAGACAGAGAAAACCCCTATATCATCCTCTATCATTCACTCTGCATTCACCAAATTATAAATGAAAATGAATGGAGTAGTCACCCTTATGATCTCAAAGTTCTCCAATTAGAACTTCAATATAGCTATTATGATTATATCGAAGCATGGTATTCTATCTTGCTTCACCAAACAGTTGATTTCAGTCACTCCTGGTTCTTAAACTTTGATAGCAAATTCAAGAGTTCTTTTCCTTACTGGTTCAATCACTGGTGGGAAAAACATGGTCCAGTCATTGATTTATTTCCTCCCCCCATGCAAGAATTAGTCAATTATTTCACTAATAAAAATAAATTCAAAGAATCTGAGTTATTCTTCCCCAACCTGCTTCATTTTGTTGCCAAATATAAAGTTCCTTGGATCCTCAAATGGTCCTACCAGGTTAATTGGGAAACCAGAGTCCTATCTCGCCAGTTTTCAA

TATATATATATATATATAGTTGTGAGAGTGAAGAAATTAGAGTTTATCAGTGGGTTGGCAGGAGCAGCAAAATTGTGGGAATGAGGTTTATGGATGGAGTGATTTGTGTTTTTAAAGAATTTTAAATGAAATATGTTTTTGAGCAATTAAATTATGAATTGTAAGCCTGCATGTCTTGAACATTATTTTTACTCGGTGGAGTTACTATGTTGTTTTAACTGCAAATCTTGTTTTTGTCCACTCACATGTTTTCTGTTTTTTGCCCCCCCAGCTAGTAGTCGCCCCAAGAAGGTTGCTAGCAGTGTTTGAAGGCTGAATAATTTGCATCAGTAGATTAGGCTTCGAGTAATTTCCTTTTACTCAACTGTTCTTGTAAACAAAATTCAGTAGATGCTCTGTTATCGCCCATGTTAGATTTTACTTTTGAGGCCGGAGGCCATTTAGTATGTTTTGTTGTATGATGAAATCATGCTGCTGGTTGGGATCATTTTTATGTATGTTGCAGGTTGAAATTTTTTTGTTGGGTTTGCTCAATTTACAGAGGAGACTTTGCCAAAATTTTGGTAGAGAGTCTCGGTTTTTAGTAAGTGGGCCCGGCATCGAGGTGATGTTGGAACAAAGACGGGGTCCGCTCTGGTTTCCGAGAAATTCCGGGGCGGGTCTTGTCAAGCCAAATTTGTTTTCAAATTTATTTTGAATTTTGTCCAATAATACACATGTATATTTTATGCCTAATTTTTCTATGTCCTATCTTACTACAAACTATGTGTTAGGTTTAACTACTTTTTAGTTTAGTGTATTTGTTTCGAATATAGTATCAGAGATGCCTTCTTGCCTTCATGCCTTCTTGCCTTCTTGCCTTCTTGCCTTTTCCAAAATAAATCACCACAAATACAAGCCATTTCACATATATTAAAACTTTTTATATAATTACTAATGTTTCTTTTATAAAATTATAA

>Chr6-07; OZ022211.1; 11870214-11879148; rev; D;

AAAAGTCATGGGCTAAATAAAATTTGGCCAAATTTGGAGGGCCTCATTTGGCCCTTTAGGCCATCTTCAGTCATAGAGCTAAATGTAGTCCAGGGCTAAAAATTTAGCCCTCCAAAATATTAATTTTTTAAATAATAGTGCAGGGCTAAATTTTTCCATCTCAAGCCATGCAGGGCTATATTATAGGATCCTTCAGCTTTCAATATATAGTTTTTCTCAAAATAATAAAATATGAAGGATACTAAAATATAACCCTGCATGGCTGGAGATGGAAAAATTTAGTCATGCACTATTCTTTAAAAAATTAATATTTTAGGGGGCTAAATTTTTAGCCCTGGACTACATTTAGCCCTATGACTGGAGATGACCTTAGGGGCGGTTTGGTATCCTACTTGGATCCAATTTTATAAACCCAAAAATATATTTTAAGTCCAAAACCCCAAAAACCCGTTTGGTAGGCCTATTTTTAAAAACCTAAACCCAAAAAAAAACTCAAAAACACCCAAATTATTGAAAACAAAAAAAGTGAGATTTTCCTTTTTATTTTTATTTATAGTACTGCTAAACGAAAGAGAGGTCCGGGCTTCTTTTGTGCTTTTCCTTTTATTTTTTGTATAAATTAAAAATAATAATTAATTGGAATAAGTTTTAAGATCTTAAAAATAGGTTTGAAGTTGAATACCAAACAAGTTTTCGATCTTAAGGCCCCGTTCGTTTATGTTTTTAGAGCCCATTTTCATTTTGAGAGAACAAAAACAAACAAAAAACCTGTTCGGTGGGCAAAAAAAGAAAACACAGAGAATATTTTATGAAAACAAAACCACCGTCAATCGCTGCCAACAGAGAACCACACACAGAGCTTGAACCACCACCATCACCCAATCAAAATCACCACCGCCCCATTGAACCAGATTGACATCACCGCCACTCAATCGAACCCAACAAAATCTCTATGCATTTGAATTGAACCACCACCGTTGAACAACTAATTGCCGACGTG

ATTTTCAATTGCTTTATCGTGTCTTTTGCTTTACACTCATTAGCATAGTGACCATTCTTTTGACATTTCTAGCATTTAACTTTTCTTTTGTCGGTACTTTTCTTAAAGTTGCTTTTCTTATCTTTTCTGCGACCTTTAGACCAATTTTTACGCGGTTAAATTTCTTTTTATTGTAAATTCATTATTATCATTATTATGTATGAAGGACTATCTTCTTCGTGAATAATGACAACGTTTACTAGTTTGTTGGTCATGATAACTAGACTTGGCTTTTCTGGATGGGGGAATGGGTGCTAAACTGTATTGTTCACAGAAATCTCCTAATTCATATTTGGCTGAGTTTCTTTCTGATTGAATTTGTCTACCAATTTTCTATCGACACACATTTTCATTCTGACTTGATTAATTGTACTGATAATATTTATGTAAGTTAAATTATCGAAATCTATGACCTTGATCATTACTAAGGACTGTTCTTATTTTATGAGCAAATGAATTGGGGAGATCTTTAATGAATTTTTCTTTCCAAAAGGGTTGATTACTATCATCTCTACGCATAACTCTGAAAATAAAAACATCTTTATACCAATGAAAATCAGATAATTTGGTGCATTTGAGATTACTATGTTGATCATGAATTCTGGGGAATATGCTAACTCGTTTCTTCCTGACTAATGACTTTTCTTAATTTCTTTTAATAACTCTTCTTGTTCTTCTTGTTTTGTAAGGACACTAATGGACTTGGATCATAGGGTGTACTAAGTTAATTTTAGGGTTCGTTTAGCAGCACTTTTTTTTTTTCCTCTCTTCTTTCATGTCTCTATCCTCCCATTCTCATCTCTTTACTTACCAAAAATACCCCGGATACACCCTAATCCACCTTAATCATACGGCAGCAAACCCATTCGAATCTGGTAACAACATTTATCAAGGTCAAGTGGCATCGAACTCATCCCCAGATTGGATCGACGAGATCGAGACCATTATGATGTAAGACGACGATGTCAATGACCCAGATCTTGTCGTCGCTGATAGCAGCTTCGGCCTAAATCTCGTCGGCGACGGCATCGTCTGTAACCTTTTTTTGTTGTTGTTGTTACCTTTGCTTTCACTCTCTGCAAACCCACCCCAATTCCCCTCACTCTCTCTCTCTCTCTCCAAAATTGAAGAATCAGACCAACCCCCCTCTTTTCCACTTTCTCTTCATGCTGTTGCACCCTCTCTCTCTCTCTCTCTCTCTCTCTCTCACTGCAACCTCACCTTAGCCTTCGGTAACAGATATCTAAATTCATGATTCAATATATGAGTTAATGGTAATCAATGGATGAAAGAAACAAGATTATAAGGTGGTATAATGTTTTGATTTGTCTATGAATAAAATTGAGTTTTTTGTTATTACTTTGTATTTTTTATGATTCAAAATTTGATTTTGGGAGTTGTAAGGTGAATGAAATATCACAAGAGGAGTTCCTTGGAATTTTTGGTCCTGGAGCTAGAAACAACCCTAATTGATCTAAAGTTAGAATCTCTGAGGAAAACTTCCCAAGTCAACCAACTTGTTGTGGTAAACAACCAGTCAAAGAAGAAAAAAAATCAGGTTCCCCAAATTCTCCCACACAAACTGATTTCGAAGTCGGTTCTTTCAATATAGATAATCAATTACGAACCGTTAATAGAAAATTCAAAATCAATTGGAAAAATTTAGATAGTCATCTTCATGCCCCAGAAAAATTTGGAAAGACGAAACAAGTATCATACAACGTAACCTAGTAAAGAACAGTGACTACAAATATTTAATGAATGGAAAGATTATATGCTAGCCGCTAAGTCAGAAATTTTTTATCTTGATTTTATTGAAAGTCGCTACATGTTCAAAGATTTGAAAACCCTAACACAAGAAAAATGGGAAAAAAATAAATACTGAAGTCTTAGCTAGTCACCCCTTGTAGAAACAATAATTATTAATCACAAAAAGCCTAAAATAGCAGCCAGTCCCTTCAATGTCCCTGACCAAAATGATAGAAAATTAATTGAGCAAAATAAATATACAAACCACAGTTTAATAGTCATTAGAAAACAATTAGATAAGATTGAAAGATTTTATTGATCACTCAGACTATACAAGAGTTCATCAGGGTAGTAGTCCTGAGAGAACTTTGAAACTGAGAAAAGTAGAATTTACTTCTGATTACAAAGGAGGTCGAAGCCTCCTTAAAAAAACTAAGGCTACCAGACTGTTGGTAGAATTGGATGCTTGGAATCACGATATATATATATAATATATATCAGAGGCGTAAGGTGTTAATAGGTTTCTTATTGGTATAACACTTACGACCCTAAACCCAGGAACCTAACCTTTGGCCTTAGTCTACAGAATTTACTATAAAGTTACAGGAACAAATATGAATTTCCAAGCCCTCAATAAAAGCCCAAAAGATCAAACTGTCATAATCCAGAGTCACACTTCAGATGCCCATATACAGGTTCCCCACACCATTAAGTGGTCCGAAGTTGCTCTTCCAAAAGATTGGACTTTGGTCACCGAGAGCCAACTAGCACCAATCCAACGCAGTCTAAACAATTTAGACTACATCCAGCAATATCTAGACGGCACAGTAAAAATTAAATTTGACAGCCACCCCTTACAAAAATCCAATGTCCAGTTACAACAATTGCCAACTCCTGGTCAGTCCCAAAGGCATGTCCCTGCCAGACATTCCTTGCTGCTTCGTCCTCCACTCTAGAACGAGACCTAGAGTTAGAAAAGCCATGATAGAATTCAAACTTGAGTCCCTGAGAAAAACCTCTCAGGTCACCCAACCTTGCTACGGCCAGGTTCCAGAAGATAAATCAGACTCCCCCGAGACTCCAACCCAATCTGATTTCTTAGTTGAAAACCTTGCTGCAGTAGATAATCAACTTAGACATTTAAATAAGAAGTTTGAAATTGATTGGAAATGTCTCAATCGTCATTTAAAAGCCCCGGAAAACCAATCCAGAAGAGATAATTACCACCAAGCCTATCCTGATAGTAAACACCGCGAACAAATCTTTAGTGGATGGAAAGATTATATGCGGCAGCCAGAGTTGAAATCTTTTACCTAGATTTTGTTGAAAGCCGTTACATGTCAAATGATGAGTTAAAAACCTTAAGAAAAGTGGAATTTAATAGATAAGTCCGTAGTTGAAGCCAGTCATCCCCCAGTAGACACTATAATAATAGATCACAAGAAAGCACCAATACCTGCCACTCCTTTCAAAACCTTTGAAAGCAGTGACCCAAATAGAAAAATAATCGAGCAAAATAATTACCCAATCAAAGTCTAATCGTCATAGGAAAACAACTAGACACAATTGAAACCAAGATCGATAAGTTGAATCCTCCAGAAACAGATTTCAAGCCCAAACTTAGATTTGAAAAACCAATAGTTCAGTTCCAAGAACTTAAGTCCAGTCCCGTACTTAAAGTCAATTCTACAATGAAAAAGATAGAAGAAATGCTAGAACAGTTGACCCCAACAAAAATTGAGAAAGTCGAAAAATCTAGTCTTAAAAACACTAGATTTTTTTGTTGCCTTGCTCACATTCTGAGTCTGAGTCGATAAATTCCGAAACCAGTAATGTTTCAAAAATTGAAAACGTCTTTGAAAATTTAGAAGTCCAAACTAAACCAAAAATCAAAAGATTAGACAAACATGTAAGTCCTACCAGTTTAACAAAAAATTGGTATCCCAGACCAACACCTCCAGACATCCAATTTGAAGAAAGAAACTTCCAAACCCAGTTTTCAGTTTCTTTTGATAAATTGTATGAATGGAATATTGATGGTCTCTCTGAGTAAGAAATCTTGAATAAGTTACAACATATGTCCATAGTCGCCAATAGTTACATCACCAATCACAGTTTCACACAATCTGAAATTGTTCTCCTCTGTAGTTACCGCTTCACTGGTACTCTCAGATATTGGTGGGATAAAAATCTTACCCCTGAGTCTAAAAATCAGATCACTTATGCTGTAAAACTTAATGAAGACAGTCTCCCTATATTTGACGAACGAATAGGTCAAGGCATAGAGGATGAAGTCAACACTCTGTTTTACACAATAATCGAACATTTCATTGGAACCCCTAATAATACCACTGCTAGGATACATGACCAGTTAAGTAATCTTAAATGCCCAAAGTTATCCGATTTCAGATGGTACAAAGATGTTTTCATTTCTAGGGTCATACTTAGAGATGATAGTAATCAACCTTTCTGGAAAGAAAAATTTATTAATGGTTTGCCAAACTTATTTGCCCACAAAATACGCACAACCCTGAGTAATGAACAAGGTCAAATAGACTGGAATAATTTGACTTGTGGAAATATTATAAGCACAATTAATCAAGTTGGTATGAAAATGTGTATTGACTTTAAAATCACACCGACAAATTCAGTCTGATAGAAAGTCAGCCAAATACGAGTTAGGTAATTCTGTGAACTACGGTCTTACCAGTATTCCTCCATCCAGGAAAAATAAGCCCAGTCATCTTAGGAAAAATCATTATTCTTTTCCAAAGAAACAGTTTTTTTTTTCAAAAAAATGAGTTTTTATACGAAAAGAAAGTCTCCCCCACAAAGAAAGAATTGGTCGAAACATCCAGTTAGTAAGACAAGTCACCCTAAGAAAGATAAAAGCAAAGTTAAGTGTTTTAAATGTCACAAGTTCGGTCATTATGCCTTTGAATGCAAAGTAAAAGATGTTATTAAAAATTACAAATTACAGAAGAAGATAAAGAAAAGTTGATACAAGTTTTAGAGTTGCGAGACTGTGAAACAAGTCCTGATGAACACCTAGTTCCTAGTTCGGAGTCCGATACTAACCAGTCTTCAGATTCACAACCTAGTTCTCCTGGTGTCCAAATTGATTGCACAGATAAATGTTGCAATTGATTAAAATCTATATGTGTTCTCACTAAACAGGAAGAGCAAGAAGATTGTTAATAGATCTCATAGGAAAAGTCAAAACCCTGAGCTAAAGTCAGAGTATTTGAAAAAATTAAAGAAGGTCCTTAGCCAGGAAAGCTCAAACCATTTTTACTCCCCAGCCTATAAGTATCAATACCACCTTAGAAAAATTTAACTCCAAAAAAGAAGTAACCCTCTAAGACCTCCAGTTAGAAGTCAAGAAAGTCAAAAAAGAATTAGTTGAGTTAAAAAAAATAAGTCAACAATTGCAAGCTGAGAATTATACGATCAAACAAGATATAACTAATCTTTTAAGACAAGAATCCTTTGAGCCTAAAAGCCATTCAGAAAGCCCCCACTATTCGATGATGAACCTTCTGAAGATCAACAAGTCCGTCAATCTAATCAAACAAATCCATCTTAGAAAATGGTATTCAAAAGTAACAATATTCGTCCAAAATTTGAACTCACCACAGTTGCTTTATTTGATTCAGGAGCAGATCTAACTGTATCCAAGAAGGATTAATCCCCACTAAATATTACCAAAAGTCTAGAGAGTCCCCTAGTACTGCCTCTGCCGGAAATCTCTCCAGTTGAACTTTGAATTACCAAAAGCCCATATTTGCCAAAACAAAGTCTGTTTTAAAACCATTTGTCCTAGTCAGAAATATCACTGACGAAGTCATTTTAGGTCTGTCTTTTCTTGCCTTATTGTATCCTTTCCAGGTAGAATATGATGGCATCATTTCTACCTATCTAGGTGAAAAGTTAAATTTGAATTCTTGTCGAAGCCAGAGTTGCACAATTTAAAAGCTTTACAAAAACAATCAGTCTCTAAATCTGTTCAAGTCATTCGAAAAATTAACCAGTTGAATTTTCTTAAAGAAGAAATTAGATTTAAAAGAATTGAACAACAGCTTGCTAATACAACCTTACAGTTGCAGATACAGTCTTTCGAAAACAATTTAAAAAATGAAATTTGTTCTGATTTACCTGCTGCATTTTGGTATAGAAAACAACACGTCGTCTGTCTTCCCTATATCAAAAACTTTAGTGAAAAGAATATCCTCACTAAAGCCCGACCGATTCAGATGAATCAAGAATTAATGAAATTTTGTAAGAAAGAAATTCATGAGCTCCTCCAAAAGAACATTATTCTCAAAAGTAAATCCCCTTAGTCGTGTCCTGCTTTTTATGTCCAGAAAAATGTTGAAATAGAAAGAGGAGTCCCGAGATTAGTTATCAATTATAAGCCTCTTAATTCAGTCCTCGAATGGATTAGAGATTGATTGTTTTTGTAAAGCTTTTAAATTGTGCAACTCTGGCTTCGACAAGAATTCAAATTTAACCTTTTCACCTAGATAGGTAGAAATGATACCATCATATTCTACCTGGAAAGGATACAATAAGGCAAGAAAAGGCAGACCTAAAATGACTTCATCAGTGATATTTCTGACTAGGACAAATGAAGTTTTAAAACAGACTTTGTTTTGGCAAATATGGGCTTTTGGTAATTCAAAGTTCAACTGGAGAGATTTCCCGGAGACAGTACTAAGGGACTCTCTAGACTTTTGGTAATATTTAGTGGGGATTAATCCTTCTTGGATACAGTTAAGATCTGCTCCTGAATCAAATAAAGCAACTGTGGTGAGTTCAAAATTTTGGACAATATTGTTACTTTTGAATACCATTTTCTAAGATGGATTTGTTTGATTAGATTGACGACTTGTTGATCTTCAGAAGGTTCATCATCAGAATGGTGAGGGCTTTTTTGAATGGCTTTTAGGCTCAAAGGATTCTTGTCTTAAAAGATTAGTTATATCTTGTTTGATCGTATAATTCTCAGCTTGCAATTGTTGACTTATTTGTTTTAACTCAACTAATTCTTTTTTGACTTTCTTGACTTCTAACTGGAGGTCTTGGAATGTTACTTCTTTTTTGGAGTTAAATTTTTCTAAGGTGGTATTGATACTTATAGGCTGGGGAGTAAAATGTTTGAGCTTTCCTGGCTAAGGACCTTCTTTAATTTTTTCAAATACTCTGACTTTAGCTCAGGGTTTTCGACTTTTCCTATGAGATCTATTAACAATTCTTCTTGCTCTTCCTGTTTAGTGAGAACACATATAGATTTTAATCTATTGCAACATTTATCTGTGCAACCAATTTGGACACCAGGAGAACTAGGTTGTAAATCTGAAGACTGGTTAGTATCGGACTCAGAATTGGGAACCAGGTGTTCATCAGGACTTGTTTCAGAGTCTCGCAACTCTAAAACTTGTATCAACTTTTCTTTATCTTCTTCTGTAATTTGTAATTGTCTAATAACATCTTTTACTTTGCATTCAAAGGCATAATGACCGAACTTTTGATATTTAAAACATTTAACTTTGCTTTTATCTTTCTTAGGGTGACTTGTCTTACTAACTGGATGTTTCGACCAATTCTTTATGGGGGAGAATTTTCTTTTCGTATAAAACTCATTTTTTTGAAAAAACTGTTTCTTTGGGAAAGAATAATGATTTTTCCTAAGGTGACTGGGCTTATTTTTCCTGGATGGAGGAATACTGGTAAGACCTTATTGTTCACAGAAATTACCTAACTCGTATTTGGCTGACTTTCTATCAGACTGAATTTGTCGATTGATTTTAAAGTCAATACACATTTTCATACCAACTTGATTAATTGTGCTTATAATATTTCCATAAGTCAAATTATTCCAATCTATTTGACCTTGTTCATTACTCAGGGTTGTGCGTATTTTGTGGGCAAATAAGTTTGGCAAACCATTAATAAATTTTCTTTCCAGAAAGGTTGATTATTATCATCTCTAAGCATGACCCTAGAAATGAAAACATCTTTGTACCATCTGAAATCGGATAACTTTGGGCATTTAAGATTACTTAACTGGTCATGTATCCTAGCAGGTATTACTAGGGGTTCCAATGAAATGTTCGATTATTGTGTAAAACAGAGTGTTGACTCCATCCTCTATGCCTTGACCTATTCGTTCATCAAATATAGGGAGACCGTCTTCATTAAGTTTTACAGCATAAGTGATCTGATTTTCAGACTCAGGAGTAAGATGTTTATCCCACCAATATCTGAGAGTACCAGTGAAGCCGGTAACTAACAGAGGAACAATTTCAGACTGTCTGAAACTGTGATTGGTGATGTAACTATTGGCGACCATGGACATATGTTGTAACTTATTCAGGATTTCTTGCTCAGAGAGACCATCAATATTCCATTCATACAATTTATCAAAAGAAACTGAAAACTGGGTTTGGAAGTTTCTTTCTTCAAATTGGATGTCTGGAGGTGTTGGTCTGGGATACCAATTTTTTGTTAAACTGGTAGGACTTACATGTTTGTCTAATCTTTTGATTTTTGGTTTAGTTTGGACTTCTAAATTTTCGCAGGCGTTTTCAATTTTTGAAATATTACTGGTTTCGGAATTTATCGATTCAGACTCAGAATGTGAGGCTGCAAAAAAATCTAGTGTTTTAATACCAGATTTTTCGGCTTTCTCAATTTTAGTTGGGGTCAACTGTTCTAGAATTGACTTTTTCATTGTAGAATTGACTTTAAGTACGGGACTGGACTTAAGTTCTTGGAGCTGAACTATTGGTTTTTCAAATCTAAGTTTGGGCTTGAAATCTGTTTCTGGAGGATTCAACTTATCGATCTTGGTTTCAATTGTGTCTAGTTGTTTTCCTATGACGATTAGACTCTGATTGGTGTAATTATTTTGCTCGATTATTTTTCTATTTGGGTTATTGCTTTCAAAGGTTTTGAAAGGAGTGCCAGGTATTGGTGCTTTCTTGTGATCTATTATTATAGTGTCTATTGGGGGATGACTGGCTTCAACTACGGACTTATCTATTAAATTCCATTTTCTTTCTTTAGTTAAGGTTTTTAACTCATCATTTGACATGTAAGGGCTTTCAACAAAATCTAGGTAAAAGATTTCAACTCTGGCTGATCG

TGTTGAGGCCCAAAAATCCAAGGGGCTAGGCCCAAGTCGACTATTGGCCCAACAGGGACAAGCAAATACAAGAGAATTTAGGGAACGAATTAATACGAGCTACGGGCCACATGCCACGCCAAACAAGCACGTGCGGGAAGACCAAAAACATGCTAACTTTACAAGAAACATGCTCACTCCTATTGAACCTGGCTATAACCTATGATGATAGGATAAAAATCCAAGCACAAGCATAGGTGTCGGGACCGCCATGCCAACGCCAAGATATGTTACAAGCTTAGGTGGGCCCAAGCCACCAAAATTACCCGGGGCTTGCTTGAACGGGTTGTGGTATGGATGGCAACGGGCTTTCATAAAACGCATTGACGAGCCAGGGGAGTGGTAGTTTCGGGTCACTTGGGGGGCCCAAAACTCAAGCCTATTTCTTCGGCCCGAAATGCATGGGTTAGCATGAGAGAAGAGAGAGCATGACCCAATACCTTAGAAAAAAAGTGCAAAGCCTGTTAGAAAAGGCTTAAGACAATTAGAAAGCCTAAACGTGCTGTTAAAGTGCAATAGGCAAGCTGCTTCCGGCAGCTTGACGAAAAGCCCAGTAGATTGACCAGAATGACATATCACCAATTCAAAGCCTAGGAAAGAAAAGCCCAGCCCAAACTCTCTGTAAAATATCTGGCCAAGCATGTCTTGTCCCTATCCAAGAGTCAACAGCAACCATCTCAAAAACCAGAGGAAATCCACACGAGCAAGAGGTTTACTCGTTGGTAAAACGTGGCTCCTTTCCCTCCCATTGTCCTGGCAGCTTGCATAGGAAAAGTTGAGAGAAAAGCAAGCGGCTCTTCACTCTATGAGGAAGGGCCAGCAAAACCAAGACCTTGGAACCGCAAATGGGTTCCTTACCCATATGTCTTGGGCGGATAGGATTCATCAAAGAAGACGAAGTGGGGAAAAACAATGGAAAGAGGGAAGGAACTATGACGAAATTAGAACCCTGAGTGGCTAT

>Chr6-08; OZ022211.1; 15142215-15149144; dir; B;

CTAACTTCCGATTACACAAAACGCAAGCAAAATTTAATAATAGTTACGTCATCTCTTGTGAAATAATCTGGAAAAAAAATAACATTAGAACCAACATGTTCAACTAAGAACACAAATGTAATTCAAACCACCAATTATAGAACTTCACCCAATATCCTCTCCAAATGCTTACTCCTAATGTCATTAAGCCTAAGTTTAGGATCTTTTACCTACATTTCATAATTTTCCACCCCCACCAAAAACAATCAACTAAGTATCTTGAATATTCATAAGGAAATGACACATTGTTGATTCCAAAAATGACGCATTATTGATGCCACAAATGACGAATTTCCCACCCTCACAAAAAATAAACCAAACCCAATAAAATCAATCCAGCAAGTGAACTTACCTCTCAAAATCAATCGGGAGCTCTGGAGTCAGTCAGCTTGAGAGAATGTGGTGGACATTAGAGAAGACTCTTTGGTATTTGAGACTCCATCTCTACCAAAATCGAGAAACAACTGCTGCTGCGGCGTCTAGCTACAAAAAGAGATATGCTACTTTGCTTTCATCTAGAATCTTGTCAGCTAAATGAAAAAGAGAAGGGATTTTCACTTGAGCCATAGAATCAAAACAGGGCAACAGCTAGGTAAGAGCCATTCATCGTAATACCGAGGATACCATAGATCGCAGGCCTCCCAAGGAGCTAATGACAATCGAGTGACTATAAGGTTTGGGAGATTTGGATCCTTCTAATTGTGGGTTTGCTTTACATTCAAAAAGAGAGGATTGTGTTCTTGAGTTTTTCACAGAGAAGGAAAGAGGGGAAGATCTTCAACTGAAATTGGGTTTCTAACGGGTTAGATTTCAACCCGATTCTACATGCACCCTCTTTTAAAAACTCACCGTTGGATGAAATCCAACGGCTCTTACATACCCCTCAAAAACACCCCTTATAACTTTCCACTCACTATAGAAGCTCCCGATAGTTTAATGGTAAAAGTATTTATCTGGTTAG

TGATCTCAAGCCTATGTCATGGTCCAGTTCATTTTGATTGTTACTCAGATTTTACAGTTAGTCTAAGTGACCCTCATATATTAAAAGTTTTGACTTTAAATATTAAAATGCATGGATACAACATTTTACCTGGTAGTCAACCATTAGCTTTAATTTATAGAATTTATTACAAGGTCACAGGTACAAATATGAATTTTCAAGCTCTTAGTAAAAGCCCCAAGAATCAAACACTTTTAATTCAAAGTCATACTTCTAACGCCAATGTTACCGTACCCCAAATGGTTAGATGGTCTGATGTGAAGTTACCCCAAGATTGGTATTTAACAACCGAATGCCCTCCGAAGCCGATCCAACGTAGTCCAAACGATTTAGATTACATTCAACCTTATTTAGATGGTACAGTTAAAATTAGTTTTGATAGGCCAGTCAAATCCACAATCCAACTAGAACAATTACCAACCCCCAGTCAATCTTTTAAAATCCCTGCTAGACATTCTTCAGCAGAGTCATCCTCCATAACAGAACGTGATCTAGAAGCCAATAGAGCCCTAATTGACTTCAAAATTAATGAATTAAGGAAACAAAAACAAGTAGTCCAACCAAGTTACGGGAAAGCCCCTGCTGAGCCAGTTTTGCCAGTTCAACCTGAATCACCTACTAGAACAGATTTTGAAGGTTCCACCTTTAAAGTTTGTGATGCCCAATTACGAACCCTTAGTAAACTATTTAAAATTCATTGGTCAAAATTAGAGGCCCATTTCCAAGCCCCAGAAAATGAATCACGAAAAGCCAATTATCATGCCTCTTTCCCCAAAGTTCAAAGAAAAGAAATTTTCAATGAATGGAAAGAATATTTAAAAAAGACTTGGTCAGAGTCACCTTATCTTGATTATGTAGAAAGCAAATATATGAGTCAAAAGTTAGTTACTTTAACCCAGGAAAAATGGAAAAAAGCCGACAATACCAAAGTTATCAGTACTCACCCCCTAGTTGAATCAATAATAATTAGTCACAAGAACGCCAAAATCATAGCTAGTCCCTACAAAGTTGCTGGGTCAAAAGATGATTAAGATAAAAGAGTCATAGAACAAAATAATTACACCAACACAAGTCTAATAGTCATAGGTAAACAGTTAGACAAAATTGAAACCAAATTTGATAAGTTAACCCCTAGTGAGATTACTTTAAAACCTAGGAAAGAGAAACCTTTAGTAAAATTCAATGAGTTAAAATCCGAGTCTTCCCTTAAAGTAAATTCAACCATGAAAAAAATTGAAGAAATGTTTCAGGAATTAACCCCAGTTAAACCAAAACCAAATAAAGATAAAGACAAAGGAAAAGAAGAAGAAACTTCGGTTAAAGTTTTACAATTTGAAGTTAATTCCGATAGTGAGTCAGCCACGTCTTTAGATTCAATCTCCAGCCAAGAGTCCGAAACTAGTAATATCTCTAAGATTGAACAAGCCTTTAGTAAACTACAAGTTAACCAGGATTCCCAAAGTCTTAAAGTTAGTCGCCTAACAAGTAAAGTCAATCCCACAAGTCTTACCAAAAATTGGTATCCTAAGCCTACGCCTCCAGATGTCCAGTTTGAAGAAAGAACCCAGAGTCAATTCTCAGTATCTTCTGATAAGTTATACGAGTGGAATATCAATGGCTTGTCCGAACAAGAGATACTCAACAAATTACAACACATGTCAATGGTAGCCAATAGTTATGCCACCAATCACTAGTTAAAACAATCAGAAATAGTTTCCCTTTTACGAACAGGATTTACAGGAACACTTCGTTTATGGTGGGATAAACATCTCACAGAAGACGCAAAATTAAGAATAATCCATGCCTATAAAACAAATGATGATGGTTACCCTATTTTTGATGAGCGAATAGGAACAAGTATAGAAGATGGAGTCAATGTCCTTTTATACACAATAATTGAACATTTTATTAGCACACCCAGTAGCACAACAGCCAGAATTCATGATCAATTAAGTAATTTATGATGCCCTAAGCTAAGTGATTTTCGATGGTATAAAGATGTATTTATTTCTAGAGTTATGTTAAGAGATGACAGCAATCAATCATTCTAGAAAGAAAAGTTCATTAACGGTCTTCCTAATTTGTTTGCTCACAAAATTAGAACAGTCTTAAGTAATGAGTTAGGTCAAATTAATTATGATGAATTAACATATGGCAATATAATTAGCACAATTAATCAAGTTGGAATGAAAATGTGTGTAGATTTAAAAATTAACAAATCAGTTCAAGCCGATAAAAAAAATGCTAAATATGAATTAGGAAATTTCTGTGAGCAATACGGTCTAGCCCCAGTTACCCCGTCTAGACAAGTCAAAAAAAGTGATGATCGACGCTTTAGTAAAACTAGATATTATTCCCATAGAAAACATTCCTTTAAACCAAATGATGAGTTTTATAGTAAAAAAGAAGTTTACCCCTAGGAAAAGTAAATGGTCAAAAGGCTACTCAAAAGGTTACCGACAAAGTAAAAAGAATCAATTTAAGAAAAGTTCGGATAAAACAAAAGTTAAATGTTTTAAATGTAAAAGATTCGGTCATTTTGCTAATGAGTGTCGAGTTAAAGATACAATTAAACAGTTAAAGTTAAAATCCGAAGATGAAGCCAATTTAATTAAAGTTCTTGAGTTAGCTAATACTGAGTCAGATTCTATGTCTTCAGTCAGTTCAGTTAATTATAATTCAGACAGTTCCCAGTCTAACTCACCAAAGGTCCAGATAGGTTGTAAAGATAAGTGTTGCAGAATCTTAAGCAAATCAATTAGTGTCTTAACTAAGCAAGAAGAACAAGAAGAGTGGCTAATCGATTTAATCAGTAAAATAAAAAATCCAGAATTAAAAACTGAATATTTAAGAAAGCTTAGGGAAGTCATAAGTCAAGAAGAACCCAGTCCTAGCCAAGTCCCTACTATTAGTCTTAATACTACTCTAGAAAGGTTTAACAAAAAGAAAGAAGTCACCTTTCAAGATCTCCAGCTAGAAGTTAAAACAATCAAAAAAGAAATCTTAAATTTAAAACAAATTAGTCAACAATTACAAACTGAGAATTACGAAATTAAAAAAGACTTAATTCAATTATTAGAGTCTAGAGCAGAGTCTAGTAATCCCTTTGGTAAAGCCAGTTCCGAGTCAGAACCTGAATCTTCCGATAAAGATGACAAAAATCCTCAAGATAATTAAGCAAATTAATTTTAGAAAATGGTATTCCCGAGTCAATATTTTTATTAATGAATTTGAATTAAACACAGTTGCCTTATTTGATTCAGGAGCCGATCTAAATTGTATTCAGGAAGGTCTTGTTCCAACCCAGTTTTACAAAAAATCAAAAGAAGTTTTAGGAACAGCATCCGGTAATGCCTTAAAGTTAATATATGAATTACCCAAAGCCCACATATGCCAAAACAAAATATGCTTTAAAACGTCTATTGTTTTAATTCAAAATATCACAGATGAAGTTATTTTAGGTTTACCCTTTATCTCATTACTTTACCCTTTTCAGGTAGAATACGATGGAGTTACATCTAGTCACTTAGGTGAAAGAGTTAAATTTGAGTTCTTAACCAAACCAGAGTTACACAGTCTCAAGTCACTACAAAAGTTAGCAGTTTCTAAATCAATTAAATTAATTCAGAATAAAACAAAACAAGTTAATTTTCTTTAAAACGAAATTAAGTTCAAACGAGTCGAACAGCAGTTAGTCGACAAGTCACTTCAGTCTCAAATAATTACGTTTGAAGAAAAGTTGAAAAATGAAGTTTGTTCTGATTTACCTAATGCCTTTTGGCATAAAAAAACATGATAAGTCATTTAATGAAAAGAGCATTCCCACTAAGACCCATCCTATCTAGATGAGTCAAGAATTAATGGAATTATGTAAACAAGAAGTTACCGAGTTAGTACAGAAAGGAATAGTGAGAAAAAGCAAGTCACCTTGGTCATGCCTAGCTTTTTATGTTAGAAAAATGCAGAGTTAGAAAGAGGAGCCCCTTGTTTAGTAATCAATTATAAGCCCCTCAATGCAGTACTTGAATAGATTAGGTATCTCATTCCTAATAAAAGAGATTTAGTTAACATATTAGAAAAAGGCAGTTCTCTTTAGTAAATTTGATATGAAAAGTGGTTTCTGGTAAATCCAAATCAAAGATTCAGATAGATACAAAACTGCATTCGTAACCCCTTTCGGTCATTACGAGTGGAATGTCATGCCTTTTGGACTGAAAAATGCGCCTAGTGAATTACAGAATATAATGAATGAAATTTTTAATCAATACAGTCAATTTTCCATAATTTATATTGATGATGTCTTAATTTTTTCTGAGTCAATTGAGCAACATCGGAAACATCTGAATAAATTCCATCAAATAGTTAAGCAAAATGGTCTAGTTATCTCAGCCAGGAAAATCAAATTATTTCAAGTCAATATCAAATTTTTAGAATTTAATATTGGTCAATCTCAAATAAGCCCAATCAATCGAGTTATCCAATTTGCCGATAAATTTTCAGATCAAATTCTTGATAAAAGTCAATTACATAGGTTTTTAGGATCCTTGAACTATGTTTCTTATTTCTACCAAAATTTAAGAAAATTATGCAAGCCCCTGTTTGATCGTCTCAAGAGCAATCCTCCTGCTTGGACTCCGAGTCATACAGAAATAGTTAAACAAATCAAAGTTCATGTCAAGTCCCTTCCATGCCTTGGTATTCCTTCAGTTGATTCTTTCAAAATTGTTGAAACAAATGCTTCTGAGTCTAGTTACGGTGGTATTCTAAAGCAAAGAATTTCCCCTAAGTCACCTGAGCAAATTGTCCGTTTTCATTCCGAAGTTTGGAGTCAGTCTCAAGCTAATTATAGTACTATTAAAAAGGAAGTTTTATCTATAGTATTATGCATTAGTAAATTTCAAGATGATTTATTAAATCAAAAAATTTTAGTCAGAATTGATTGTAAGTCTGCAAAACATGTTTTAGAAAAAGATGTTAAAAATATAGCTAGTAAACAGATTTTTGCATTTTGGCAAGCCATTCTGAGTGTCTTTGATTTTGAGTTAGAATATTTGAGAGGTAGTGAAAACAGTATTCCTGATTTTCTTACAAGAGAGTTCCTGCAGGACAAACAAGCAGAATCATGTCCAGCAAAGAAAAAGACAAGCAAAAAATGCAATATGGTCAACCGAGTCAACCCAGAATCCTCCCAGCTACCCTAATGCCAGTTAAACAAGAATCATCTTCTCAAATCATGCCTTCACATATTGTGCCCTTCCCAGGTCATTCTCCAATTCCAGTCAATAATAGGTTTAATCCCTTGAGTCAACCCTCTTTACGCCCCAATTATCAGTCAGCATTAACCTCCAATTATGATCCTTTCATAGTTAATCCTCCTGCTTCAGTTCCTTCGCAGAGCCAAAGCCCAGTTAAAAGGTCTAACTATGCCACCAGAAGCCATTGCCATCTTTTTATAGTTGAGTGATACCTAGAAGAGCTTTGTGACCTTGTCAAAATTGCCCAGAAATTCTTCCCTCCTAATCTTCACTATTTTCCAAGCCATCCTTCAAAGTCAATTAAGTTTTACAGGGATATTCTCTACGAAACTCAGTCAATAGATATTAGGCCCATCAAAGACAAAGAAACAAATGCCACCATGTTCCATTCCCTTTACATATGCCAAATTCTTATGCCTCAAGTTTGGAGTTGCAATCCTTATGATCAGAAACCCCTCTAGTCATGACTTACTTAAAATTATGGTGATTATATTGATGCCTGGTATACAATTTTTTATCACCAGCACCCTGATTTCAGTCACTCATGGTTTATTAATTTCGACGCCAAGTTCGAAGTCCAGTTTCCTTTCTGGTTTCCCCAATGGTGGAATATCCATGGCCCCATTTGGGAAATCATTCCAATCAATCTCCAGCAGTCAGTAAATTATTTTGGTCAAAAACACAAGTTAACAAAGTCAGAAAATTTTTTCCCTACACTTTTCCATTATGTCGCCAAATACAAAGTCCCATTGATTATGAAATGGAATTATCAAGTTAATTGGGATACCAAAGTTTTATCTCGTCACTTTGCTGTCAAATGGTGGGATAAGTTTAGGACAGAGAAAATTATCCATCAAGTCAATGCAGAGTTTCCACCCCTCCAAGTGCCAATCCATCCAGTCAAAGATGCTTCCCCAAGTGGCTCCAATGCCTCCCTCCAGGGAAAATCCAAAAAAGAGTTAAAAGAGTTAGCTGAGCAAATCCTTGCCCAAGTTACACAAATGAATGACGAAGATAGTGATGATGCCAGTCCAAAGTCTGAATCTTCTTCCAGTCAACAGCCAAACCAACTTCCTTTTAGTCAAAAGTCGAAGCCCAGATGGTCAGATTATGCCCCCGACAGTCAGGATCCTTATGATTTGAATGAAGACTAGGACTTTTTGCAAAGTGGCCGAAAGCAAAAGATGCCCAAAGTTAAAGCAAATTACCCATAAGTTAAAGCAGGTGCCCAGAGTTGAAGCAAGGTCCCCAAGAGTTAAAGTAAAGATATGCCCAGAGTTCAAGCAAATACCCAGAGTTAAAGCAGATGCCCAGAGTTAAAGCAAGTTCCCCAGAGTTAAAGAAAAGATGCCCAAGTTAAAGTAAAGATGCCAAAGTTAATTTCAAAAGTTATTTTGTCTTCTTCCCAATGCCAAGTCTTTAAATAGAAGCCCCAAGTCATTTGTAATCTCCAAGCTTTTCCTTCCAACTTTCCTTTGTAATTTCCTTTCAAAGTTCTTCAAGCTAGGATTCGAGTGTTTAGTAGAAGTGTGAATGTCCTACGTGACATTGCTTTCTGTAAGTTTTATTTTCATGTTTTCAAAGTTTTATTAAGTCAATAAAATTATCAAGTTAATTTTATGTTAATCCTATTTTC

GTTCCTAAGCATTCATTTAAATGATTAATTATATTTTCTTTCATGCAATTTGTGAGATAAGAATTTTATGTATTTACGCTTTAGTTAAATTTTACTTTGACTTATTTCAATTTTGTTTTTCTTATCTTGGTCATCTCTAAGCCCCTATTCTGTTTCTCCTTTAGCCCCTCTTTTTTGCTATTCCTTAACTATTCCTTGGTTCTTCCCCCTTTCTATATATATATATATATATATATATATATATATATATACAAGCAAGTTGTTAAATGGGTAATTTATTCAATGGGCCAACTCTAGATGGGCCGGGCCGGCACCACCCGTTTGACACTCTAGATGTGACACTTCTAAGTTACAACTTTTGAGTTCCATTTTTTTTTTCATTCATATTCAATGATGACATCAAGGATCATCATTAGGCTTTTCTTAAGATTTTGTTTTGCAAAATACATAATATATTAAATTCATCTAATTAGAGTGTAACGATTTTGAAGTAATGAAATAAAATGTTAAAAAATTAATATATCTCTACAAGCAAGTTGTTAAATGGGCAATTTATTAAATGGACCGACTCTAGACGGGCCGGGCCAGGACGACTCTTTTGACACTCTAGTTGTAACACTTTTGAGTTGCAACTTTTGAGTTTTTTTTATTTTATTTTTATTTTATTCATATTAAATGAGTACATCAAGGATCATCATTAGGCTTTTCTTAAGATTTTGTTTTGCAAAATACATAATATATTAAATTCATCTTATTAGAGTGTAAAGATTTTTAAGTAATGAAACAAAATGTTAAAAAATTAATATATCTCTACAAGGAAGTTGTTGCTCCACAAATCTTTTGGCCCTTATTTAATTATGTAACCGAGTAAATAATTAAAGGTGGCCGAACGGGAATGGCAATATAGTCATCCTTCTTGGAGTGTATTCGTGCGGCATGCCAATTCTCACTATTGTTGGTGACCGTGAAAAGAAAGAGAGTGATGTTGTGGGCTTTGGTA

>Chr6-09; OZ022211.1; 15530317-15537321; rev; B;

ATGTTTGAAGTTTGGGGAAGGACCCAAACCACGATGGAATGGATCCCATAAAATTGTTGAATCTGAATCTAATCAACTTCAGCCGACGCAAATAAGACAATTCCTTGGGCAGGGTGCCATGGAAGCTGTTGTTTCTTAAGTCCAGCTCAACAAGAAATGAGAGGTTGCCGAGGGGTGGAGGAGTGCCGAAGAGTCCCATGTAAGAGAGGTTCAAGACTGAAACTCTGAGGCGGCGAGCGCCACAAGTAACTCCAACCCAATTGCAAACGGAGGTGGTGGTTGACCAGTTGACCAAGATGTTGTGAGGGTCACTAGTGATATGGGATTTGAGAGCAAGAAGAGCAGACTGGTCCGTGGTGATGTTGGTTTGTGCTGCACCTACTGTGCCTAGAGAACTGCACTGCAGCTGCAACACCAGCAAGAAGGTGATTGACAAGAGGAAACAACTTCTCTCCATGACCATGAACACCGAAAACAAAGAAGTTCCTTCCAACTTCAAATTGTTTTTCGTTTGTTTTTTCCTTTCCGATTTAGAACTTGAAATCATTGAATAATTTGTTGCAGTGCCAATGTATGTAGGCAGAACAAGTTGCCAAAGAGTGGGGCACATGATTGCACCCTCGGGGAAGAAATTCGCAAGTAGCCAAATGCAACGACTTGTAATGAAGGAAGAATGGACAGTTGGGCATCTTCACAAATGTAGAAGAACTTGCCGTACATCTATATATTCCGTCAATCAGATATGGATGGTCAATCCAATCTCCTCCGCATAATAAGGACCGTTTTGCTTTGATTTTTTTTTTTTTCAGAAACAAAGCATATACACATACATATTCCAGGATAGCAAGCTGGTAGACTAAATTTGCAAGCAGTTAATTGCTCAGACCACGGCCAATTTCCGTTAGTTTTTCGCTTTGTTGGGCTTAGGCCTGTACATACTTGATAATGCTGCCAAAAGGTTGATAGTTTAATGGTAAAAGTATTTATCTGGTTAGTGATC

TCAAGCCTATGTCATGTTCCAGTTCATTTTGATTGTTACTCAGATTTTACAGTTAGTCTAAGTGATCCTCATATATTAAAAGTTTTGACTTTAAATATTAAAACGCATATACAACATTTTACCTGGTAGTCAACCATTAGCTTTAATTTATAGAATTTATTACAAGGTCACAGGTACAAATATGAATTTTCAAGCTCTTAGTAAAAGCCCCAAGAATCAAACACTTTTAATTCAAAGTCATACTTCTAACGCCAATGTTACCGTACCCCAAATGATTAGATGGTCTGATGTGAAGTTACCCCAAGATTGGTATTTAACAACCGAATGCCCTCCGAAGCCGATCCAACGTAGTCCAAACGATTTAGATTACATTCAACCTTATTTAGATGGTACAGTTAAAATTAGTTTTGATAGGACAGTCAAATCCACAGTCCAACTAGAACAATTACCAACCCCCAGTCAATCTTTTAAAATCCCTGCTAGACATTCTTCAGCAAAGTCATCCTCCATAACAGAACGTGATCTAGAAGCCGATAGAGCCCTAATTGACTTCAAAATTAATGAATTAAGGAAACAAAAACAAGTAGTCCAACCAAGTTATGGGAAAGCCCCTGCTGAGCCAGTTTTGCCAGTTCAACCTGAATCACCTACTAGAACAGATTTTGAAGGTTCCACCTTTAAAGTTTGTGATGCCCAATTACGAACCCTTAGTAAACTATTTAAAATTCATTGGTCAAAATTAGAGGCCCATTTCCAAGCCCCAAAAAATGAATCACGAAAAGCCAATTATCATGCCTCTTTCCCCAAAGTTCAAAGAAAAGAAATTTTCAACAAATGGAAAGAATATTTAAAAAAGACTTGGTCAGAGTCACCTTATCTTGATTATGTAGAAAGCAAATATATGAGTCAAAAGTTAGTTACTTTAACCCAGGAAAAATGGAAAAAAGCCGACAATACCAAAGTTATCAGTACTCACCCCCTAGTTGAATCAATAATAATTAGTCACAAGAACGCCAAAATCATAGCTAGTCCCTACAAAGTTGCTGAGTCAAAAGATGATTTAGATAAAAGAGTCATAGAACAAAATAATTACACCAACACAAGTCTAATAGTCATAGGTAAACAGTTAGACAAAATTGAAACCAAATTTGATAAGTTAACCCCTAGTGAGATTACTTTAAAACCTAGGAAAGAGAAACCTTTAGTAAAATTCAATGAGTTAAAACCCGAGTCTTCCCTTAAAGTAAATTCAACCATGAAAAAAATTGAAGAAATGTTTCAGGAACTAACCCCAGTTAAACCAAAACCAAACAAAGATAAAGACAAAGACAAAGGAAAAGAAGAAGAAACTTCGGTTAAAGTTTTACAGTTTGAAGTTAATTCCGATAGTGAGTCAGCCACGTCTTTAGATTCAATCTCCAGCCAAGAGTCTGAAACTAGTAATATCTCTAAGATTGAACAAGCCTTTAGTAAACTACAAGTTAACCAGGATTCCCAAAGTCTTAAAGTTAGTCGCCTAACAGGTAAAGTCAATCCCACAAGTCTTACCAAAAATTGGTATCCTAAGCCTACGCCTCCAGATGTCCAGTTTGAAGAAAGAACCCAGAGTCAATTCTCAGTATCTTCTGATAAGTTATACGAGTGGAATATCGATGGCTTGTCCGAACAAGAGATACTCAACAAATTACAACACATGTCAATGGTAGCCAATAGTTATGCCACCAATCACTTGTTAAAACAATCAGAAATAGTTTCCCTTTTACGAACAGGATTTACAGGAACACTTCGTTTCTGGTGGGATAAACATCTCACAGAAGACGCAAAATTAAGAATAATCCATGCCTATAAAACAAATGATGATGGTTACCCTATTTTTGATGAGCGAATAGGAACAAGTATAGAAGATGGAGTCAATGTCCTTTTATACACAATAATTGAAAATTTTATTGGCACACCCAGTAGCACAACAGCCAGAATTCATGATCAATTAAGTAATTTACGATGCCCTAAGCTAAGTGATTTTAGATGGTATAAAGATGTATTTATTTCTAGAGTTATGTTAAGAGATGACAGCAATCAATCATTCTGGAAAGAAAAGTTCATTAACGGTCTTCCTAATTTGTTTGCTCACAAAATTAGAACAGTCTTAAGTAATGAGTCAGGTCAAATTAATTATGATGAATTAACATATGGCAATATAATTAGCACAATTAATCAAGTTGGAATGAAAATGTGTGTAGATTTAAAAATTAACAAATCAGTTCAAGCCGATAAAAAAAATGCTAAATATGAATTAGGAAATTTCTGTGAGCAATACGGTCTAGCCCCAGTTCCCCCGTCTAGATAAGTCAAAAAAAGTGATGATCGACGCTTTAGTAAAAGTAGATATTATTCCCATAGAAAACATTCATTTAAACCAAATGATGAGTTTTATAGTAAAAAGAAGTTTACCCCTAGGAAAAGTAAATGGTCAAAAGGCTACTCAAAAGGTTACCGACAAAGTAAAAAGAATCAATTTAAGAAAAGTTCGGATAAAACAAAAGTTAAATGTTTTAAATGTAAAAGATTCGGTCATTTTGCTAATGAGTGCCGAGTTAAAGATACAATTAAACAGTTAAAGTTAAAATCCGAAGATGAAGCCAATTTAATTAAAGTTCTTGAGTTAGCTAATACTGAGTCAGATTCTATGTCTTCAGTCAGTTTAGTTAATTATAATTCAGACAGTTCCCAGTCTAGCTCACCAAAGGTCCAGATAGGTTGTAAAGATAAGTGTTGCAAAATCCTAAGCAAATCAATTAGTGTCTTAACTAAGCAAGAAGAACAAGAAGAGTTGCTAATCGATTAAATCAGTAAAATAAAAAATCCAGAATTAAAAACTGAATATTTAAGAAAGCTTAGGAAAGTCATAAGTCAAGAAGAACCCAGTCCTAGCCAAGTCCCTACTATTAATCTTAATACTACTCTAGAAAGGTTTAACAAAAAGAAAGAAGTCACATTTCAAGATCTCCAGCTAGAAGTTAAAACAATCAAAAAAGAAATCTTAAATTTAAAACAAGTTAGTCAACAATTACAAACTGAGAATTACAAAATTAAAAAAGACTTAATTCAATTATTAGAGTCTAGAGCAGAGTCAAGTAATCCCTTTGGTAAAGCCAGTTCCGAGTCAGACCTGAATCTTCCAATAAAGATGACAAAATCCTCAAGATAATTAAGCAAATTAATTTTAGAAAATGGTATTCCCGAGTCAATATTTTTATTAATGAATTTGAATTAAACACAGTTGCCTTATTTGATTCAGGAGCCGATCTAAATTGTATTCAGGAAGGTCTTGTTCCAACCCAGTTTTACAAAAAATCAAAAGAAGTTTTAGGAACAGCATCTGGTAAAGCCTTAAAGTTAAGATATGAATTACCCAAAGCCCACGTATGCCAAAACAAAATATGCTTTAAAACGTCTTTTGTTTTAATTCAAAATATCACAGATAAAGTTATTTTAGTTTTACCCTTTATCTCATTACTTTACCCTTTTCAGGTAGAATACGATGGAGTTACATCTAGTCACTTAGGTGAAAGAGTTAAATTTGAGTTCTTAACCAAACCGGAGTTACACAGTCTCAAGTCACTACAAAAGTTAGCAGTTTCTAAATCAATTAAATTAATTCAGAATAAAACAAAACAAGTTAATTTTCTTTAAAACGAAACTAAGTTCAAACGAGTCGAACAACAGTTAGTCGACAAGTCACTTCAGTCTCAAATAATTACGTTTGAAGAAAAGTTGAAAAATGAAGTTTGTTCTGATTTACCTAATGCCTTTTGGCATAAAAAACAGCATGTAGTTACCTTACCCTATGATAAGTTATTTAATGAAAAGAACATTCCCACTAAGACCCATCCTATCCAGATGAGTCAAGAATTAATGGAATTATGTAAACAAGAAGTCACCAAGTTAGTACAGAAAGGAATAGTGAGAAAAAGCAAGTTACCTTGGTCATGCCTAGCTTTTTATGTTCAGAAAAATGCAGAGTTAGAAAGAGGAGCCCCTTGTTTAGTAATCAATTATAAGCCCCTCAATGCAGTACTTGAATAAATTAGGTATCTCATTCCTAATAAAAGAGATTTAGTTAACAGATTAGAAAAAGGCAGTTCTCTTTAGTAAATTTGATATGAAAAGTGGTTTCTGGTAAATCCAAATCAAAGATTCAGATAGATACAAAACTGCATTCGTAACCCCTTTCGGTCATTACGAGTGGAATGTCATGCTTTTGGACTGAAAAATGCGCCTAGTGAATTCCAGAATATAATGAATGAAATTTTTAATCAATACAGTCAATTTTCCATAGTTTTGATGATGTCTTGATTTTTTCTGAGTCAATTGAGCAACATTGGAAACATCTGACTAAATTCCTTCAAATAGTTAAGCGAAATGGTCTAGTTATCTCAGCCAGGAAAATCAAATTATTTCAAGTCAATATCAGATTTTTAGGATTTAATATTGGTCAGTCTCAAATAAGCCCAATCAATCGAGTTATCCAGTTTGCCGATAAATTTTCAGATCAAATTCTTGATAAAAGTCAATTACATAGGTTTTTAGGATCCTTGAACTATGTTTCTTATTTCTACCAAAATTTAAGAAAATTATGCAAGCCCCTGTTTGATCGTCTCCAGAGCAATCCTCCTGCTTGGACTCCAAGTCATACAGAAATAGTTAAACAAATCAAAGTTCATGTCAAGTCCCTTCCATGCCTTGGTATTCCTTCAGTTAATTCTTTCAAAATTGTTGAAACAAATGCTTCTGAGTCTAGTTACGGTGGTATTCTAAAGCAAAGAATTTCCCCTAAGTCACCTGAGCAAATTGTCCGTTTTCATTCCGAAGTTTGGAGTCAGTCTCAAGCTAATTATAGTACTATTAAAAAGGAAGTTTTATCTATAGTATTATGCATTAGTAAATTTCAAGATGATTTATTAAATCAAAAATTTTTAGTCAGAATTGATTGTAAGTCTGCAAAACATGTTTTAGAAAAAGATGTTAAAAATATAGCTAGTAAACAGATTTTTGCACGTTGGCAAGCCATTCTGAGTGTCTTTGATTTTGAGTTAGAATATTTGAGAGGTAATGAAAACAGTATTCTTGATTTTCTTACAAGAGAGTTCCTGCAGGACAAACAAGCAGAATCATGTCTAGCAAAGAAAAAGACAAGCAAAAAATGCAATATGGTCAATCGAGTCAACCGAGAATCCTCCCAGCTACCCTAATGCCAATTAAACAAGAATCATCTTCTCAAATCATGCCTTCACATATTGTGCCATTCCCAGGTCATTCTCCAATTCCAGTCAATAATAGGTTTAATCCCTTGAGTCAACCCTCTTTATGCCCCAATTATCAGTCAGCATTAACTTCCAATCATGATCCTTTCATAGTTAATCCTCCTGCTTCAGTTCCTTCGCAGAGCCAAAGCCCAGTTAAAAGGTCTAACTATGCCACCAGAAGCCATTGCCATCTTTTTATAGTTGAGTCATACCTAGAAGAGCTTTGTGACCTTGTCAAAATTGCCCAGAAATTCTTCCCTCCTAATCTTCACTATTTTCCAAGCCATCCTTCAAACTCAATTAAGTTTTACAGGGATATTCTCTACGAAACTCAGTCAATAGATATTAGGCCCATCAAAGACAAAGAAACAAATGCCACCATGTTCCATTCCCTTTACATATGCCAAATTCTTATGCCTCAAGTTTGAAGTTGCAATCCTTATGATTAGAAACCCCTCTAGTCAGGACTTACTTAAAATTATGGTGATTATATTGATGCCTGGTATACAATTTTTTATCACCAGCACCCTGATTTCAGTCACTCATGGTTTATTAATTTCGACGCCAAGTTCGAAGTCCTGTTTCCTTTCCATGGCCCCATTTGGGAAATCATTCCAATCAATCTCCAGCAGTCAGTAAATTATTTTGGTCAAAAACACAAGTTAACAAAGTCCCATTGATTATGAAATGGAATTATCAAGTTAATTGGGATACCAGAGTTTTATCTCGTCACTTTGCTGTCAAATGGTAGGATAAGTTTAGGACAGAGAAAATTATCCATCAAGTCAATGCAGAGTTTCCACCCCTCCAGGTGCCAATCCATCCAGTCAAAGATGCTTCCCCAAGTGGCTCCAATGCCTCCCTCCAGGGAAAATCCAAAAAAGAGTTAAAAGAGTTAGCTGAGCAAATCCTTGCCCAAGTTACACAAATGAATGACGAAGATAGTGATGATGCCAGTCCAAAGTCTGAATCTTCTTCCAGTCAAAGCTAAACCAACTTCCTTTTAGTCAAAAGTCGAAGCCCAGATGGTCAGATTATGCCCCCGACAGTCAGGATCCTTATGATTTGAATGAAGACTAGGCCTTTTTGCAAAGTGGCAAAAAGCAAAAGATGCCCAAAGTTAAAGCAAATTACCCAGAAGTTAAAGCAGGTGCCCAGAGTTAAAGCAGGTGCCCAGTGTTGAAGCAAGTTCCCCAAGAGTTAAAGTAAAGACATGCCCAGAGTTCAAGCAAATTACCCAGAGTTAAAGCAGATACCCAGAGTTAAAGTAAAGATGCCCAAGTTAAAGCAAAAGTTAAAGCAAAGATGCCAAAGTTAATTTCAAAAGTTATTTTGTCTTCTTCCCAATGCCAAGTCTTTAAATAGAAGCCCCAAGTCATTTGTAATCTCCAAGCTTTTCCTTCCAACTTTCCTTTGTAATTTCCTTTCAAAGTTCTTCAAGCTAGGATTCGAGTGTTTAGTAGAAGTGTGAATGTCCTGCATGACATTGCTTTCTGTAAGTTTTATTTTCATGTTTTCAAAGTTTTATTAAGTCAATAAAATTATCAAGTTAATTTTATGTTAATCCTATTTTCGTTCCTAAGCATTCATTTAAATGATTAATTATATTTTCTTTCATGCAATTTGTGAGATAAGAATTTTATGTATTTACGCTTTAGTTAAATTTTACTTTGACTTATTTCAATTTTGTTTTTCTTGT

CTTGGTCATCTCTGAGCCCCTATTCTGTTTCTCCTTTAGCCCCCTTTTTTTGCTATTCCTTAACTATTCCTTGGTTCTTCCCCCTTTCTATATATATATATCTACAAGCAAGTTGTTAAATGGGTAATTTATTCAATGGGCCAACTCTGGATGGGCCGGGCCGGCACCGCCCGTTTGACACTCTAGATGTGACACTTCTGAGTTACAACTTTTGAGTTCCATTTTTTTATCATTCATATTCAATGATGACATCAAGGATCATCATTAGGCTTTTCTTAAGATTTTGTTTTGCAAAATACATAATATATTAAATTCATCTCATTAGAGTGTAACGATTTTGAAGTAATGAAATAAAATGTTAAAAAATTAATATATCTCTACAAGCAAGTTGTTAAATGGGCAATTTATTAAATGGGCCGACTCTAGACGGGCCAGGCCAGGACAACTCTTTTGACACTCTAGTTGTAACACTTTCGAGTTGCAACTTTTGAGTTTTTTTATTATTTTTATTTTATTCATATTAAATGATCACATCAAGGATCATCATTAGGCTTTTCTTAAGATTTTGTTTTGCAAAATACATAATATATTAAATTCATCTAATTAGAGTGTAAAGATTTTAAAGTAATGAAACAAAATGTTAAAAAATTAATATATCTCTACAAGGAAGTTGTTGCTCCACAAATCTTTTGGCCCTTATTTAATTATGTAACCGAGTAAATAATTAAACGTGGCCGAACGGGAATGGCAATATAGTCATCCTTCTTGGAGTGTATTCGTGCGGCATGCCAATTCTCACTATTGTTGATGACCGTGAAAAGAAAGAGACTGATGTTGTGGGCTTTGGTCTGGACTCCGTCAAACAATTCAAGTTGTGTTTACCTTAAAAATATCCAAGCATTAGAAATAGGCTCTAGATGATGTAAGTGGCAAACGTGCCACTATTAGCAATGTAAAAAACATAATAGACTTGAAAACTAAATAAATAGCTAAATAACTT

>Chr6-10; OZ022211.1; 19715393-19722607; dir; A;

TAATTTTCGGCCACTAGACATACTCCAAAACACCTGCCTAAACTCCTATCCTAGGTGTAACACCCTATTTAGAACCACTTTTGTTCTTGGACCTACCCCAAAAACTGGCCGAAAATGGCCGGAATCAGAGGTCGAAAATCGACAGATTTTCAATCGCTTATTTACCCTCCAAAACTCAGAATTTTCTCCTAACCAACACAACTAGCAGCAAGAACAGCTCGAGAGGTTGAAAAATCATACCTGGATCGAAGCAATTGGTAGTCGGAATTGCCCAAACGAGCGATGGAGATTCTAGGTAAAAATCGGACAATTTCTTGCCTTTTCCGGCAGTTCAAACGGCGGTTGGCGGTGGCTGAGATGTGAGGCTGGTAGAGGACAACGTGCTGGTTCTTTTGGTACACATGTCGTCACTTGGGGTGGCCATATGAGGAGATGGCGGCCCAAAAACTGTTCGAATGAACAGTAACAAAGGGGAGGGGCTGCTCTTCTCCAATGGGAGGGGGAAGGAGAAGAAAAAAGGAAATTTTTAAAACAGCCCGATTACTGTAGCTAACAAATTTAAAAATCTCCATTACGTCCTTTTCGTTTCTAGACATTTTCTCCTTCGTTACAACTCGGAATTGGGCTTGCCACATGTCTATGAACTCGTATCGTCGAGCTCTACGCAATGGTACCATCTAAATCCCCAAATTCCTTCCCCATTAAAAAGTCAACTTAAAATCTAATAAAACATTCTAGGGGTATAATGGTCTTTTACTAGAATAAATTAATAATTAACTATTAATTTTAGGATCGAGTCGTTACACATATAGTACATTTAATCCTAGTAGTTGTACAATATTCATGTTCTAACATAATAAGATTCTTTTGTTTGATTTTCCTATGATAATAACATTCTAGTTATATGTTAATTCAGATTTCACAACTCATTTAAAACGGGTAATTATTGTTGCTTGGGATATATATATATATATATATATATTGTAAGACTCGTTCCAAA

CAGTAAGTCCCAGGGAGGCCTGAGCGGTAGTGGCATTAGCAGGAGGGTGAGAGAACTTTAGGTAACATGTTATCACATGGAGTCAGTCTTAGGAGTTTCTGTTCACTTTCTTCTTTAGACGTCCAACCCCAGAACCTTGAGTAAAATGAGTCGCTTATTTAGAACTAATTCTTGCAATTCTAGCAGCTCTAGAGCCAATAGTTGTCTCCCAGATATAGTTAATGAAGAACAAGTCAAGTATGATAATAATAATAATAATTTAGATTATAAAGATTGGAATATACCAAGAGTCCCCAGTCAGGACATCTACAAGAAAAAATGGTCATTATTTAGTTTTAAGTCAACCACCCATGTTAAAACAGTCGAACAAGTTTATGCCCTGAGTAAGAATCATGAGACATGTCAGTTGCTTAATCTTGAGTCCATAAGAAAACATAAAAATGATGGAAAAAATTTTCTCCATACTGGTTTAGTCCAAGTTGCTGTTAAGCCATTAACCAGATTATGATTAAAAGCTTCAATCCTTCTCTGTTTGCGTGATGCTAGATTTACTAATTTCAACGACAGTACGTTAGGAATTATTGAGTCCAGTCTCGCTAATGGCCCTATTCACCTATTCACTTTGATTGTTACCCAGATTTTACAGTCAGCCTGAGTGATCCACACATATTAAAAGCCCTCACTTCGAATATTAAAACTGAAGGTTATGAGGTCTTGTCGGGTACTCTACCCTTAGCTTTAGTATATATAATTTATTATAAAGTCACAGGCACTAATATGAATTTTCAAGCCCTAAACAAGAGTCCAAAGAGTCAAACATTGTTAATCCAGAGTCATACGCCTGACGCCACTGTTAGAGTTCCTCACTCTATCAAATGGTTAGATGTTACCCTTCCTGCTGATTGGACCTTAACCACTGAGATTCCTCCAGTGCCTATCCAACGTAATCTAAACGATTTAGACTACATACAACAATACTTAGATGGCACAGTAAAAATTAATTTTGGTAATCAACCTCCACAAAAATCAACAGTCCAGTTACAACAGTTATCTACACCTGGTCAGTCCCAGATACATGTTCCTGCAAGGCATTCCTTTGCAGCTTCTTCCTCTACCCTAGAACGAGACCTAGAGTTAGAGAAAGCTTTAATTGATTTAAAATTAGAATCATTGAGAAAAACTTCCCAAGTTACCCATCCTGTTTACGGAAAAACTCCAGTACGGGAAGACACTAAGTCTGAGTAGCCTGATTCACCTACTCAATTTGATTTCATAGTAATCAACTGAGAACACTTAATAAAAAATTTGTAATTGATTGGAGAAAATTAGATAGTCATTAACATGCCCCTGAGAACCAAGCTAGAAGAAATTACTACCATTCAGTCATTCCTGATAGAGTCAAAAGACAACAGATTTTTAATGATTGGAAAGAATATATGTGCACTGCTAAAGTTGAAATATATTATCTTGATTTTGTAGAAAGACGACATTTGCCTAATGAGTTAAAAACTTTAACCCAGGAAAAATGGGAAAAAGTAGATCACACTGAGATCCTTGCTAGTCATCCACCTGTAGAAACGATAATTATTAAGCACCAAAAATCCAAAATAGTCGCCAGTCCTTTTAAGATCCCTGATCTAGAGAATAAAATAATATTTGAGCAAAATAATTACACCAACCAGAGTTTAGTTGTCATAGGTAAACAATTAGATACCTCTTCTGAGTCTGAATCAATTGCCAGTATGGAAACAGAAACTAGTAATATTTCAAAAATTGAAAATGCGTTTAAAAATCTTGAGCTAGATAGTAATCCACAAGTCCAAAGATTAACAAACAGAGTTAGCCCCTTAGTCTCAAAAAATTGGTTCCTAAGCCAACACCTCCTGATATACAATTTGAAGAACGAAACACGCAGAGCCAATTTTCAGTCTCTTCAGATAAGCTTTATGAATGGAATATTGATGGTCTTTCAAAGCAAGAAATTCTTAATAAGCTTCAACATATGTCTATGGTTGCCAATAGTTACATCACCAATCACAGTTTCAGACAGTCTGAAATTGTTCCCTTGTTAGAAACTGGTTTCACCGGAACTTTTCGTTCTTGGTGGGATAAACATTTAACCCATGAGTCTAAGCAACAAATAATTCATGCAGTCAAGCTTAGTGAAGATGGTCTTCCTATTTTCAATGAACAAATAGATCAAGGCATTGAGGATGGAGTCAACACCCTACTGTACACAATAGTTGACCATTTCATTGGAACACCCAGTAACACTACTGCTAGAATTCATGATCAGCTTAGTAATTTAAGACGCCCTAAATTATTTGATTTTAGATGGTATAAATATGTGTTTATTTCCAGAGTCATGCTTAGAGACGATAGTACTCAATCATTTTGAAAAGAGAAATTTGTTAATGGTCTTCCTAATTTATTCGCCTATAAAATTCGAACAACCCTTAGCAATGAGCAAGGTCATATTGATTGGGATAGCCTAACATATGGAAATATAATTAGCACAATTAATCAAGTCGGTATGAGAATGTGTGTTGGTATGAAAATTGGTAAACAGATCCAGTCTGAAAGAAATTATGCTAAATATGAGTTAGGTAATTCTGCGAACAGTATGGTCTAATGTATATTCCCCGTCTAGAAAGAATAAGTCAAGTCACAACAACCGACAATTTAGTAAACGTCGCCATTATTCACGCAGAAGCCAGTCATTCCAACATAATAATGATGATAAAGAATTTTATAGTAAAAAGAGATTCTCCCCTAGAGAAAATTGGAAAAAGAGTCACGGCAAAGATAAGAATAATCGCTTTAAGAAAAGTACCAACAAGAGAGAAGTAAAATGTTATAAATGTCAAAAGTTTGGTCACTATGCTCATAATTGCAAAGTTAAAGACACAATCAAACAATTAAAAATCACGGATGAAGATAAAGACAAACTGATTATAAAGTCCTTGAGTTAAGAAATTCTAAGTTTAGTGAAAACGAAATCATGCTTAGTCACATTGAGTCTTCTAAAATCTATTCTTCTGAGTCACAACCTAGTTCACCTAGAATTCAATTTGGTTGCCAAGATAATTGCTGTAAGTCTAAGTTAATTAATGTCCTTACTAAACAAGAAGAGCAAGATAAATTATTAATTGATTTAATAAGTAAAGTTGAAAACCCAGAGTTAAAATCTGAGTATTTAAAGAAATTAAGAAAAGTCATTAGCCAAGAAGAGCCGAAACAATTTACCTCCCAAAAATAAGTCTGAATACAACCTTAAAGAAATTTAATAAAAAGAAAGAAGCTACTTTACAAGATTTACAACTAGAGGTGAAGTTGGTCAAAAAAGAAATCATTGAGTTGAGACAAATAAGTCAAAAGCTACAAAGTGAAAATTATGAGATAAACAAGATTTGATTAACCTATTAGAAAATAAAACCTCTAAGTCTACACCTGAAAGCCCTACAAATTCTGACGAAGAACCCTCCAATCAAGATCATGTCGTCAACTTAATTAAACATTTAAATTTTAGAAAATGGTATGCCAGAGTCACTATATTTGTCAAAAGCTTTGAGTTAAACATAGCCGCCCTCTTTGATTCAAGACCCGACCTTAATTGCATCCAAGAAGGCCTAATCCCAACTAAGTATTACAAAAAGTCAAGAGAGTCGTTAAGCACAGCTTCAGGAAAGTCACTCCAATTGAATTATGAGATTCCCAAAGCCCATGTCTGTCAAAACCAAATTTACTTTAAAACTTTATTTGTCTTAGTTAAAAATATTACTGACGAAGTCATCCTAGGTTTACCCTTTATTACTCTTTTATATCCTTTCCAGATAGAACACGATGGAGTCATTTCCACCTGTTTAGGAGAAAAGATTAAGTTTGAATTCCTGAGTAAACCAGAATTACATAATTTAAAAGAATTACAAAAGAATGCAGTCACTAAAACAGTCAGAGTCATTCAAAATAAAGATAAGCAACTAAAATTCCTCTAGGAAGAAGTTAAATAAAAAAAAATTGAGCAACAACTTAGTCATGAGCCTTTACAGTCACAAATTCGACAGTTTGAAGAAAAATTAAAACAAGAAGTTTGTTCTGAGTTACCCAATGCTTTTTGGCATAGGAAACAGCATGTTGTTAGCCTTCCTTATGTCAAGTCCTTTAGTGAGAAAAATATCCCCACTAACACCAGACCAATTCAAATGAGCCAAGAATTAATAGAATTTTGTAAAACTGAAATTACTGAATTACTTGAGAAAGGAATAATCTGTAAGAGTAAGTCACCCTGGTCTTGCCCTGCTTTCTATGTCCAAAAAAATGCTGAATTAAAATGCGGAGTCCCGAGACTAGTCATTAATTACAAGCCGCTTAATACAGTTTTAGAATAGATCAGATACCCAATTCCTAATAAAAGAGACTTAATTAATCGATTAGAAAAGTCAGTCATCTTTTCTAAATTTGATATGAAGAGTGAGTTTTGGCAAATTCAAATTCGTGAGTCTGATAGATACAAAACTGCTTTTGTCACTCCTTTCGGTCATTATGAGTGGAATGTTATGCCTTTTGGTCTTAAAAAAGAGCCCAGTGAGTTTCAAAATATAATGAATGAGATTTTTAATCCTTTTAGTCATTTTTCCATAGTCTATATTGATGACGTGCTTATCTTTTCTGAGTCGATAGAACAGCACTGGAAACATCTGCATAAGTTCTTACAGATAGTCAAACAAAATGGATTAGTTGTTTCTGCTAAGAAAATTAAGTTGTTCCAAACTAATGTGAGATTTCTTGGGTTTAATATCTGCTAGTCACATATTAGTCCCATAGATCGAGTTATCCAGTTTACTGATAAGTTTCCTGATCAAATCCTAGACAAAAGTCAACTCCAGAGATTTCTAGAATCTCTAAATTATGTTTCTGATTTCTATAAAAATTTGAGAAAACAATGTAAGCCTCTATTTGATCGTCTCCAAAATAACCCTCCTCCTTGGTCATCTATCCATACTGATATAGTCAAATAGATCAAAGCTCATATTAAGACACTTCCCTGTCTTGGTATCCCTTCTGCCGATGCATTCAAAATAGTTGAAACTGATGCCTCTAACATTGGTTATGGAGGTATCCTCAAACAAAGAGTCAATTCTAATCAACCTGAGCAAATTATTCGATTTCATTCTGGTACTTGGTCTCAATCTCAAAATAATTATGGTACTATTAAGAAAGAGATATTATCTATTGTACTATGCATTAATAAATTTCAGTATGATTTATTAAATCAAAAATTTTTAATCCGAGTTTATTGTAAGTCTGCTAAGCATGTATTAGAAAAAGATGTTCAAAATATTGCATCAAAACAAATATTTGTAAGATGGCAAGCTATTTAAGTATATTTGATTTTGATATTGAGTATTTGAGAGGAAGTGAGAATTATGTCCCTGATTTTCTTACCAGAGAATTTATGCAGGGAAAACAATGGCAGATAGAAGAAAAGAAAAAGACAAGCAAAATAGTCAAATTGGTCAAACTAGTCAACCCAGTCATCCCCAATCACTCCGAGTCCTCCCTCCATCAATGAGTCAAGCCAAACATAAGTCACCTTCTCAGATCGTCCCCTTCCTGGGATGTTCTCCTATCCAGATTAGTAACAGATTTGCCAACCTAGGAGCCACAGTAGACTAGGTTCGTCCCAGTTTTCAGTCTGCCCTAATATCTAGTCATGATCCTTTCCAGGATATTGCTCCGCCTAATCTTCCACTTGCCTCTTACCATAAGACGTCTCCATACATGATTAAGAGTAATAGTCACCTGTTCATAATTGAGCCAAAATACAATTCCATTGCCAACCCAATCACCATTGCAAAATCCTACTTTCCACCAAATAGTCACTTCATTCCCCCACCATTCAAAAATTTAAAATTTTATATAGACATCCTCCATGAGACCCAGTCAATCACAATTAAGCCAATCAAAGACAGAAATAACCCCCATATCATCCTCTATCATTCCCTTTACATCCATCAAATAATTAATGAGAATGAATGGAGTAGTTACCTCTATGATCTCAAAGTTCTTCAGTCAGAACTTCAATATAGTTATTATTATTATATCGAAGCATGGTATTCTATCTTGCTTCACCAAACAGTTGATTTCAGTCATTCATGGTTCCTCAATTTTGATAGCAAATTCAAGAGCCCTTTTCCCTATTGGTTCAATCACTGGTGGGAAAAACATGGTCCAGTCATTGATTTATTTCCTCCTCCAATGCAAGATTTAGTCAATTATTTCACTAATAAAAATAAGTTTAAAGAATCTGAGTTGTTCTTCCCAAATCTTCTCCATTTTGTAGCCAAGTATAAAGTCCCTTGGATACTTAAATGGTCTTATCAAGTTAATTGGGAAACCCGAGTCCTATTCCGTCAATTTTCAATCAAATGGTGGGATAAATTTAAACTTGAGCGTATTGCAGATTATGTTTATGCTGATTTTCCTCCAGTCAGGAATCCACAGCCTGAGAAAAAGTCATCCCCTGCTACATTACATTCTTCTCTCCCAATCGAGGGAAAATCTAAGTCTAAGTTGCAAGAAATTGCTCGCCAGCTAATGTTACAAGCCTCACAAATGAATGAAGATGAGGATAGTGATGCTGCTCCAAAATCGCAGTCATCTACCAGTCAACCAAAGTTGCCACCCAGTTAGAAGACTCCAACACCCAAGATGCGGTGGAGTGATTATCCCCCTGATGATCAAATCCATGGTCAAGACCCCTATGATTTTAGTCCCCTTGACCTAGGTGATGATTAAAATTGCCAAGATCATGAGCCCTGAGACAAAGATATTTGGAATCTTGGACCCGTGCTTCAACAGTCTGATAGCCTGTACTCCTTTAGTCAGATTCCTCAACAAAGTAAAAGTCACAAGCACTTAGTAAAAGCAAAAGCTTTCGCGTGTTTCTACAGTCACTTTCCATAGCATCCAAAGTTGCCAACTCATCAATAGTTACCAGAGTTCACTTTATTTGCTTGAAGGCCTCGACCTCAGTTATCCTCAGAATGTAATTTAGTCCACTTTCTTTTCTTAGTTTCAAAGTTCTTTCAATATCGATGCTCTGAAGAACTAAGTGCTGAAACAAAGATTTTGTAAGTGTTTTGCATTATGCAAAGTTATCAATAATATTTTGAGTATTTTGAGTAATGCTATTTTTAATTGCATCTATTTTCA

TATATATATATATATATATGAATTCTCTTATGCGGACGTCTTAATGTTATTATTGTACCTACGCCATGTAGTTTTTACCACCCATCTTGCTTTCTTTCCTGTGTACAATGGCATCCGCACTATAATAACGTACGGATATCCGAATAAGAGAATAACTGTATATATATACAGTCCATTTCTATTGAGGGACGCCCTTTAAGTTACCCTACGGAATTTTTTTCCAACGATCCAAACTATCTATTTTTTAGGTCTTGATTCATAGATCATCCTTATAAAAACTGTTTAGATATCTAATTGTGTCCTACAAAATCAATGACACAGTGTTTCAAGAAAATACTAAAATTTCAATAAAATAATTGAGTAGTCAAATGATATCGGATGATTTTTTACAGAGATGATATTCGAATGAATATCTACCAACTAGACATTTGGATTAATGAAATAAAATGTATGGTGGGCCCTACAAAAGGATCCCTCAACAGAAGCTCACTGTATATATATAGTTTATTTGACGGAAATTCTGGTGGGCCCCACCACGCATTGTATTTTAATGATCTGAACTATCTAGTTTTTAGATATTCACTCAAAAATTATCTTTGCAAAAGACGACTTGAATACAAAACCATTTGGCCACTCAATGTTCATCTATTTTGTTGGTCCTTACAAGATGCCTTAATGATTTTCAATTTTCTTGAATTTTTGCAAGGATGATTTATGAATGAAGACTTGAAAATAGACGATTTGTATTGTTGGAAAAAAAATCCGTAGAGACCCGAAATTGGTGTCCCTCAAATAAAATTATTTGAGAAACCTCTCAAAATAGACGAACACTATGCTTCAAGGAACTAGGCAATACAAATAGTAATTGAGAGAGAGGGGGGATGGGTGACATAAGCATAATTTTGAAGGGTGTTTCTATTGAAAATCTATCGACTGCATGCACAACATTTGTTAAAGGGTTGGAGAAAATTTAGCTCTACCACTACACCTCACAATCTGA

>Chr6-11; OZ022211.1; 20360737-20362580; dir; B;

AGGGAACGTAGAAGAGGGAGGCACGCGCGGGATCCCGCTTGCGGCAGGGATGGTTCTCCACACGTGCGTGGAAGATCATCTCCGCGGTGAACTGGTTGGTGGCGAACCAGCCGCCCTGGAAATTGGCTGGCCGAGACCGGAGTTGGCGACGTGGGGACACATGTCTGTGTGCACGTTGAGGTTGTGGCAGTGGCGAAGGAGGCCGAGGTTGAACTTGGCCGGCAGAGGGTATACATAGACTGACATGTTTTTGTCGCACGTGGTGGCTGCGGATGGTGATTTGTTGACTTGGTTGTCGTGTTGGGTTTTGTTAAGAAGAGGATGTTCATGTTGTTGTTGGATAACGTTAACGTCAGTGAACGGGTACCAAAAAACGAGCCAAGCAGCCGATACAAAGAGAAAAAGGAAGCCACCGCGCCTCGTTAGAGAGGGAGGTCTTTGCTTTTCGGAAAACATGAAGAACTGAAACAGCAAGAGAGAAGAGAGACGAGAAGACAATTTTGTTTTTAGAAACAGAAATTCTCAGTCGTTTGATGCAGGTTCAAGGCAAATAGATGTTTGATCTGCTATTGGTTTGATCTACGTGTGAGATTCTAATTGGTCGAGGGTTTACCGCATGAATATCAATAAATGTATGAAGGTTGTTTTATGTTGGTCTATTTTGTCAGCTGAGGCCGAACGCCAGCTTTATTATTATGTTATATATATATATATATATATATATATATATATATATATATATATATATTATGTTTCTCATTTTGTCCAAATAACTGGTTATTCAACCTTGTGTTTGAAGCTTTGCTATTCAATTCTACAACCTTTTCAGTGTCTTCCTGGATATTGTTCCTTTTTCTCTCAACCCAAATAATAAGTCATAGATTCCAAACAATAAGTCCCAGGAGAGGTATGAACGGATACGGCATGAGCAGAGAGAGAGAAAACTCTTCAGTCTTCAGGATTCTTTGAACCTGTTATAGGATTATTAGCTTTTCTTTTT

ACAACCCATGTGAAAACAGTCGAACAAGTCTATGTCTTGAGTAAAGAATACAAAACATGTCAATTGCTAAACCTTGAGTCAATAAAGAAATACAAGAGTGATGGAAACAATTTCCTTCACATTGGCCTAGTCCAAGTAACAATAAAACCTCTTACCATATTAGGACTAAATGCCTCTATTTTACTTTGCTTACGAGATGCCAAATTTACCAAATTTAGTGATAGTCTCTAGGAATAATAGAGTCAAGCTTGTTTAATGGTCCAATTCACTTTGACTGTTAGTCAGACTATACAGTAAGCCTTAGTGATCCTCACATATTAAAAACTCTAATGTTAAACATCAAAACTTCAGGGTATAAAGTCTTGCCAGGTACCCAACCTTTAGCCTTAGTCTATAGAATTTATTATAAAGTCACAGGAACTAATATGAATTTCCAAGCTCTTAATAAAAGTCCAAAAGACCAAACCTTGCTTATTCAAAGTAGTAAAGCCGATGCTAATATAAGAGTTCCCCATACAATTAAGTGGTCAGAAGTAACTCTACTCCTAGATTGGACAATAACAACAGAAAAATCAACCCATTCAACATAATTTGAATAATCTAGACTACATTCAACAATATTTAGACGGTATGGTAAAAATAAACTTTGGTAACCAGCCCCCCAAGTCCTCAATACAGATACAACAACTCCCAACACACCTAGTCAATCCTTTAAACATACTCCTGCAAGACATTCCTTCGCAGCCTCCTCTTCTACCCTGGAAAGAGATTTGGAGTTAGAAAAAACATTAATTGATTTAAAGTTAGAATCTTTAAGAAAAACCTCCCAAGTCAACCATCCTTGTTATGGTAAGAAACCAGTCAAAGAAGACGAGAAATCAAATTCCCCAGGTTCTCCCACACAAACTGATTTTGAAGCCGAAGCCATTCCAGTAGACAATCAGTTACGAACTCTCAATAAAGAATTCAAAATACATTGGAAAACCTTAGACAAGCACCTAGAGTCAAAAGAAAATGTTGAAAGACGCAATAAATATAGGACAACATATCCAAGTAAAGATCAGAGACAACAAATCTTTAATGAATGGAAAACTTTACATACTCCAAACCAAATCAGAAATTTTCTATCTCGATTTTGTTGAAAACCTTTACCAACAGAAAAAAGAAATAAAAACTTTAACACAAGGAAAATGGAAAAGAGAAAACAACACAGAAGACATAGCTAGTCATCCCCCAGTAGAAACAATAATTATTGATCATAAAAATACTAAAATCATTGTCAGTCCTTTTAAAATTCCTGAACAAAATGATAAAAAATTGGTTGAGCAAAATAATTATACAAATCAAAGCCTAATAATTTTAGGAAAACAACTAGACAAAATAGAAACAAAATTTGATAAAATACTTACCCAAGAAGTGAACCCCAAACCTAAGAAAGAAAAACCTTTAGTCAAATTCCAAGACCTAAAACCTAGTCAAACACTTAAGACAACCCCTACGATGAAGAAAATTGAGAAAATGCTTGAACAATTGACGCCGGTTAAAGAAAAACCCAAAACAGAGTCATCCTCTACCCTTAAAGTCATCCAAGCACATGAAAGTCATCCCTTTGTCTTGAATCAAACAGATACAGAGTCACCCATTGACACTGATACTGATTCCAGTAACATATCAAAAATAGAAAATGCCTTTAACAATCTCAAACTCAGTAGACTAAGGAGTAAGATAAATCCTACTAGTTTAACAAAGAACTGGTATCCTAAGCCAACTCCCCCAGATATCCAGTTTGAAAAAAGAAATCTTCAAAGGCAATTCTCAATCTCTTCTGATAAACTTTATAAATA

TATATATATATATATAAGCATATCCATAGTCCAAAATGGCAGACGAGTCTTTTTAGAACCATGAAATGAATTAAATTAATGGAGAAAATTATAGCATGATGGATGAAAAATGGTGAAATTGAGTAGAGGTAATTGAGGCTTGATGGAGTTGACCAAACTAATAGAGTGAGATAAAGCATGATTACTACATAATTCACATTCCTGCGTATCCTAATTTGTCAGGCCTGTTGTAACTTGTTGAAACGAAACGATTAACAAAATAATATAAATAAAAAAGCTTTGGAAAAGGAATATAATTATATAAAAAAGGAGTTGGTTCGCTGAACATGTTGTACAAGCTAGGTATCTTTGATTGGGGGAAATAATTAAATAATAAACACACAAGGTGAGCCAGAGATACATAAATAAATAAATGAATACATAACTCCTAGTAGCTAGAAAGGTGAGCCACGCATATGTACTAGGGCTCTATCCAGGTCCATGGATACAGAAACAGCTTCGGCTTGGCTTTATAATTTAGAGCCCAAAATGCATGTCATTGGCATTGGGGATGCCTTTTCTCTCTTAACCTTATGTTTAATCGTTTGCATTAATTACTTTCTAAGATCTTGGGTTTCTTATAAGATCTTAGATTGACTTATAACAAACTCAAGATCTTAGAGCAAGTTCATCCTAAAAGGCTAAGGTAAGGCAAAGGTAAGATAAGGGCTGCTACTATTCACGTGAATAGTGACACCCCTGCCTTTTTTGGTTATACCTCAAAAGGCTAGGGTAAGAGCAAATATTATTCATTGTACTTTATTTCCTATTTTTTTAAAATACTTTAAACCATTAATTTTGATAAGCTTTTCAGATAATATTTTTGGTTGCCACGTGTCACTATTTATTATAAAATTATCACAAAAAATTTCTAGATAACATTTTCAGATAAAATTTTTGAATAAAATTTTCGGATTAGATTTTTGATTGCCACGTGTCCATCATTTTTCAGAAAAGATTA

>Chr6-12; OZ022211.1; 22443999-22451536; dir; A;

TTGGCACAACTATCACAAGATTGGCAAGATCCCACCATGCATCCAACACCTACCTTGTCTCCAACTTTGAATTTTTCTACTTTGCTTCCTAGCTCTGTCACTACACCAACAACCTCATACCTGAATTCAATCAATCAGTTACACATTTGGTTAGTTAAATGGACATCACAATGAATTCCACATACTAAGTTCAATAACATAAAAATAATGGTAAAAAATGTTGTGCTGTTGACATTCAAACCATTAATTAGATGGATATTAACATGAGATGAAACTAATTAACTCTTAGTCTAATGATATTTCTTCTTTCAGTGTCAGAAGAGGTTGGACCTCATTCCCACGTCGTTTATCGAGATTCCCAAAAAATAAAATTTGTATATTTGTGAGACTTTGTATACGTACCCAGGAACAATAGGATAGCTAGTGGTCCCCCATTCATTTTTGACCTTGTGAAGGTCCGAATGGCATATCCCACAGTACAAAACTCTTAAAGCCACGTCTTTCTCTCCCGTTTTCCTGCAACCAAATCCCTTCAAAAACTGAGTTCATTGGAAGGAAAGATTGTACCAAACTCAAAATAAAGCATGGAGCTTATACGTGTTCAATATAATATTTTGTTTTAATTTTTGAAGAAACCAAAGAAAGACTGGTATTCCTAGAGTGAAAAGAAAGCGGCAAATAAATAACAGGCGAGAAGATATAGAATATATAACAACCTTCTTGAGATTTTGAAGGGAGAGAGAACGCCAGAAGAATCTGTGGCAGCCCATGCAAAGGCCTTATTTGGGTGTTCTTGCTCTGGAGATACCATTTCCATTATCTCCCTCTTACAATATAATATAGATGAGAGTCTGAGGTGGGTAGAGAAGAGATTGTGAAAGTAGCAATTGCGATGTTGTTCATATATATATATATATATATATATATATAAATCAGGGTAAAACTCTGATTGTTCTTTTCTCCGCCGAAAGTTGCCTCAGTTAAAGAAAATTCATATGAA

GACAAAATTGAAAGTTGAATTAGGTTGCTTTCAGGTGACTCCACTGTGACAAGCAAACTGCAGTTGCTTCACCATAGTTAGCAAACCCTAGTTGCTTATCTATTTAAGCTGGCTTCGGCCTCAGTTGCAGGCAGAATGTAATTTTCCTTTCTTCAGTTTTTTTACAGAATCTCTCTGATATATCCTTCGGAGAAATTCACTTTTGTAAACACTCTTGTAAATAGCGTGCAGTTGTGTTTGAAGAATTGCAGTTGCAATCTTTGTGAGTATAAATAAAAGTTATTTTCAATTATCTATTTTCTGAAAGCATTATTTTAATGCATTATCATTATTTTCTGCATTAGTCTGAAAGTTGCTTAGGTTGCATCCTTTTCTAATTTCTTTCTGATCTTCATTCTGTATTATATTCAGTTGCTTGTTTGTTCTATCTCTTTGTCCCTCTTTCTTGGTATCAGAGCCGTAAGTGTTACGGTCTTTGTCTAAGTCTGCTACTCATTCCCGCCCAAGTGGCTGGTTTAAGCTTCTGAGTAGAAGGTTTTCATTTCTTCCTTTTCTTCCCCTTTATTCTTTGAGTCCTCTGTTAGTCTTCTCTAAGGTCTGACCCGTGAGTCCTTTTTGCTAGATCACGTAAATCCCAGAGAGGTAGTGTGTGTTGGGCAGGAGACAGAAAATTGAGAAAATTAATAGGTTGCTTAGAGTCTAAACGGTCAGTTTAGGATCTACTGTTCATCTTCTTCCTCAGACAGTTCAACCCAGTTTCTTGAGTAGTCATGAGTCGTTTCCTTAGGTCAAGGTTTAGTAATTCTGCCAGTTCTAGTGCCAGTATAAGTCGACTCCCAGAGATAGTAAATGAAGAACAATTTGAATATAATTCCAGTAGCAAAGAACAGTTAGATTTTAGAGATTGGAACATACCTAAAATCCCTAGTCAGAAAATCTATAAGAAACATTGGTTGCCTTCTAGTTTCAATAGCACCACACATGTCAAAACAGTTGAACAAGTATACGCCTTAAGCAAAGAACACGAAACTTGCCAGTTGCTTAATCTTGAGTCTATTTTAAAACATAAGAAAGATGGTAATAATTTCCTGCATATTGGTCTAGTTCAAGTTGCAGTAAAGCCTCTGACACGGTTAGGGTTGAAAGCCTCAATCCTGCTATGTCTTCGTGATGCTAGATTCACTGAATTTTGCGATAATACCCTTGGAATCATTGAGTCCAGTCTGTGTAATGGCCCTGTCCATTTTGACTGCTATCCTGATTTCACTGTAAGCCTTAGTGATCCCCATATTCTGAGAACTTTGACTCTAAATATTAAGACTGAAGGTTATAATGTTCTCCCGGGAACCCAACCTCTAGCCTTGGTATATAGAATCTACTATAAGGTCACAGGAACTAATATGAACTTCCAAGCCCTTAATAAAAGCCCAAAGGATCAGACAGTCCTGATCCAGAGTCATACCTCAGATGCCCATATCCAAGTTCCCCATACTATTAAGTGGTCAGAAGTCACCCTTCCAAAAGATTGGACCTTAGTCACTGAGAGCCAACCAGCTCCCATTCAGCGTAGTCTAAACAATTTAGACTATATCCAACAATATTTAGACGGCACAGTTAAGATTCAGTTTGATAGTCATCCCTTACGCAAGTCCAATGTCCAATTACAACAGTTGCCAACCCCTGGTCAGTCCCAAAGGCATGCCCCTGCTCGACATTCCTTTGCTGCCTCTTCCTCCACTCTAGAACGAGACCTAGAGTTAGAAAAGGCTATGATTGATTTTAAACTTGAGTCCCTGAGAAAAACCTCTCAGGTTACCCAACCTTGCTACGGCCAGGTTCCAGTTCCTGATGATAAGTCAGAATCACCTGAGTCACCTACCCAGTCTGACTTCGTAGTTGAAAATCAGCTTAGAACCCTCAATAAAGAATTTAAAATTGATTGGGTTTACTTAAATCGTCACCTAAAAGACCCTGAGAACAAGCCCAGGCGAGATCTTTATCATCAAGCCTACCCTAATAGCAAACACCGAGATATGATCTTTCAACAATGGAAAGATTATATGCGGTCAGCCAAAGTTGAAATATTTTACCTTGATTTCATAGAAAGCCGTTATTTGACTAATGATGAGTTAAAAACTTTGACTAAAGAAAAGTGGAAATTAGCTGATAAATCTGTTGTAGAGTCTAGTCACCCTCCAGTAGAAACTATAGTCATAGAACACCAGAACACCAGAAAACCTTTGAAAGCAGTGACCCTCATAGGAAATTAATCGAACAAAATAATTACACCAACCAGAGTCTAATCACCATAGGAAAACAGTTAGACACAATTGAAACCAAAGTTGATCTGCTGAGTCCGCCTGTTGCAGACTCCAGACCCAAACCTAAAGTTGAAAAGCCGATAGCTCACTTCCAAGAACTTAAGTCTGGTCCTGCACTCAAGACCAATTCCACGATAGAAAAAATAGAAGAAATGCTAAAACAATTGACTCCTGTCAAGTCTGAGAAAGGTAATCAGTCTGGTATTAAAACACTAGACGCCTTTGCCGCTTCTCAATCTGAGTCTGAATCTGTAAGTACTGAAATTAGTGATATTTCTAAAATTGAAAAAGCTTTTCAAAATATAGAAATCCAACCTGAGCCTAGAGTCAAGAAAATTGAGAAACATGTAAGTCCTACTAGCCTTACCAAGAATTGGTACCCCAGACCAACACCTCCAGATATCCAATTTGAAGAAAGAAACTTTCAAACCCAGTTTTCTGTTTCTTCCGACAAATTGTATGAATGGAATATTGATGGTCTTTCTGAGCAAGAAATATTAAATAAATTGCAACACATGTCAATGGTCGCCAATAGTTATATCACCAATCACAGTTTTAGACAGTCTGAAATTGTCCCTCTGTTAGTTACTGGTTTCACCGGTACTCTCAGATATTGGTGGGATAAACACCTTACCCTTGAAAATAAAAATCAAATTATTTATGCTGTAAAACTTAATGAAGACGGTCTCCCTATTTTTGACGAACAAATAGGACAAGGTATAGAAGACGGTGTTAACACTTTGTTTTACACGATAATTGAACATTTCATTGGAACCCCTAGTAACACCACTGCTAGGATCCATGATCAGCTGAGTAACCTTAGATGTCCCAAGTTGTCTGATTTCAGATGGTATAAAGACGTTTTCATCTCTAGAGTCATGCTTAGAGATGATAGTAATCAACCATTCTGGAAAGAAAAGTTTATTAATGGACTGCCAAACTTATTTGCTCATAAAATCCGCACAACTCTAAGTAATGAACAAGGTCAAATTGATTGGGATTCATTAACTTATGGTAATATTATAAGCACAATTAATCAAGTTGGTATGAAAATGTGTATCGATTTTAAAATTAATCGTCAAATCCAGTCTGATAGAAAGTCAGCCAAATATGAGTTAGGAAACTTCTGTGAACAATATGGTCTCACTAGTGTTCCTCCCTCTAGAAGACAAAAGCCTAGCCATCTGAGAAAGCGTCATTTCTCGAAGAAGCAATTTCTTTGAAAAGACGAATTCTACAAGAAGAGAAAGTTCTCTCCTAAGAAGAGCTGGTCTAAACGCCCTAAGCAGAGTCGCTCCAAGAAAGATAAAAGCAAAGTTAAATGTTTTAAATGTCAAAAATTTGGTCATTATGCTTCTGAATGTAAAGTTAAAGATACTATTAAACAGTTGCAGATTTCAGAAGAAGATAAGAACAAATTGATCCAAGTTTTGGAGTTGCGAGACTCTGAAATGAGCGATGTTGAGTCTCTAGTACCCAGTTCTGAATCTGAGTCAAGCCAGTCTTCAGATTCACAACCCAGTTCACCTAGAATTCAATTTGGTTGCACTGATAAATGCTGTAATAGATTAAAGTCTGTTCTTGTTCTTACTAAACAGGAAGAACAAGAAGAGTTGCTTGTAGATCTAATAGGAAGAGTTCAGAACCCTGAATTAAAAGCTGAATATTTAAAGAAGTTGAAAAAGATCCTAAGTCAGGATAGTCAGAGTTCATCCTCTCCTAATCCTCAGCCAATTAACATCTCCACTACGTTAGAAAAATTCAATCGTAGGAAAGATGTTACCCTGCATGATCTCCAGCTGGAAGTCAAGCAGGTTAAGAAAGAAATAGTTGAACTCAAACAGATAAATCAACAATTGCAAGCCGAGAATTATACAATCAAACAAGACTTAGTTGATTTACAACAAGCCACTCGCCAGCCTAGTACCCATTCGGAAAGCCCACATCATTCTGATGAGGAACCTTCTAAAGAACAACCAGTTGTCAATCTGATCAAACAAGTACACCTTAGAAAATGGTATTCCAAAGTCACGATTTTTGTTCATGATTTTGAATTTACCACAGTCGCTTTATTTGATTCTGGTGCAGATCTTAATTGTATTCAGGAAGGTCTCATCCCTACTAAGTACTACCAAAAGTCCAGAGAAGCCCTTAGCACCGCTTCAGGGAAATCTCTCCAATTGAACTTTGAGCTACCAAAAGCCCATATTTGTCAAAACAAAGTTTGTTTTAAAACCTCATTCGTCTTAGTTAAAAATATTACTGATGAAGTCATATTAGGATTGCCTTTCCTTGCTCTGTTATATCCTTTCCAGGTAGATTACGATGGCCTCATTTCTACCCATTTAGGAGAAACGGTCAAGTTTAGTTTCCTGTCTAAACCAGAGTTGCATAATTTAAAAACCTTACAAAAACAATCAGTCTTTAAATCTGTCCAAGTTGTTTGGAAAACCAACCAGTTGAATTTTCTTAAAGAAGAAGTTAAATTTAAACGAGTTGAACAACAACTTAGTGATAAAACTTTACAGTTGAAAATTCAGTCTTTTGAGAACAAATTAAAAGAAGAAATATGTTCTGATTTGCCTACTGCCTTTTGGTATAGAAAACAGCATGTCGTCTGTCTTCCCTATATCAAAACCTTTAGTGAGAAAAATATCCCTACTAAAGCCAGGCCAATCCAGATGAGTCAAGAGTTAATGGAAGTCTGTAAGAAAGAAATTCATGAGTTGTTACAGAAAAATATAATTCGCAAAAGTAAGTCCCCCTGGTCATGTCCTGCTTTTTACGTCCAGAAAAATGCTGAACTAGAAAGAGGAGTCCCTAGATTAGTAATCAACTATAAGCCTCTTAATTCGGTCCTAGAATGGATTAGGTATCCCATACCTAATAAAAGAGACCTTATTAACCGATTAGGTAAAGCAGTTGTCTTCTCAAAATTTGATTTGAAAAGTGGTTTCTGGCAAATCCAAATAAATGAGTCAGATAGGTATAAAACTGCATTTGTTACTCCTTTCGGTCATTATGAATGGAATGTCATGCCGTTTGGTTTAAAAAATGCCCCTAGTGAATTCCAGAATATTATGAATGATATCTTCAATCCTTACAGCCATTTTTCGATTGTTTATATTGATGATGTTCTAATATTCTCTGAGTCAGTTGAACAACATTGGAAACATTTGCATAAGTTCCTTCATATAGTCAAACAGAACGGTCTAGTTGTTTCTGCCAAAAAGATCAAGTTGTTTCAAACCAATATTCGATTTCTTGGTTTTAACATTTGCCAGTCTCAAGTTGCTCCAATCAATCGAGTCATTCAGTTTGCTGATAAGTTCCCTAATCAGATTCTTGATAAAAGTCAACTCTAGAGGTTTTTAGGATCTCTAAATTATGTTTCTGACTTCTACCAAAATCTGCGAAAGCAATGTAAGCCTTTATTTGACAGATTGCAAAGTAATCCTCCTCCGTGGACAGAGCTCCACACTGAAATTGTCAAACAGATTAAGTCCCATGTCAAGACACTTCCCTGTCTTGGTATTCCCTCAGTTGATTCCTTCAAGATAGTTGAAACAGATGCCTCTGACATTGGTTATGGTGGCATTCTCAAACAGAGAGTCAGTCCAGATTCTCCAGAACAAATCGTTCGTTTCCATTCTGGAACCTGGACCCAGTCGCAATATAATTATAGTACTATTAAAAAAGAGATATTAGCTATTATATTATGCATCAGTAAATTTCAAGATGATTTACTAAATCAAAAGTTTTTAATCAGAGTTGATTGTTTATCTGCAAAACATGTTTTAGAAAAAGATGTTCAAAACATTGCATCTAAACAGATTTTTGCACGATGGCAAAGTATATTAAGCATATTTGATTTTGATATTGAATATTTGAGAGGTAGTGAGAATTCAGTTCCTGATTTTCTTACTCGAGAATTCCTGCAGGAACCACAATGGCTCCCAAAAAAGACAAAAACAAAGAAGTTGCTAAGTCAAGCCAGTTGCCCAAACCATCCTAGTCGCAACAATCTCAGTCGCCATCCATGAGTCCAGCCAAGCCTGAATTGCCCAAACAGATAGTTCCCTATCCTGGTTGCCCCCCTATCAGAGTTGCTACCCCACTATCAGTTGCCAACAGATTTTCCTCTTTAGGATCCACAGTTGGCCAAATCCGTCCTAGTTATCAGTCCACTCTAGTTGCCAGTTATGACCCTTTTGCAGTTGACATCCCTACTGGATCCTCTGCACCTACAGTTGTCCATAAAAAATCGTCTCCCTATTTGCCTAAGAGTAACTCCCATTTATTTGTCATTGAGCCCATTTATGATATTAATACCAACCCAGTCGAAATCGCCAGGCATTATTTTCCTCCTGGTTTTCATTATATGCCCTCTAATCCCTATAAGTCCCTCAAGTATTATAGAGATATTTTGCTTGAAACCAAATCAGTTGAAATCAAGCCCATAAAAGATCGTGATAATCCCAGCACTATCCTTTATCATTCATTGTACATTCACCAGATTGTAAGCCAAGAAGCCTTCAGCAGTCGCCCTTATGAACTTAAGTTGCTTCAATCTCAGTTGCAATACAATTATGCAGATTATATTGATGCATGGTATACTATTTTCCTTCATCAGTCTGAGGATTTTAGTCATTCCTGGTTCATCAATTTTGATAATAAGTTCAAAAGTCCTTTCCCATATTGGTTTCTCCATTGGTGGGAAAAACATGGTCCAGTTGAAGAAATTCTACCAGCTTCGGTTCTCCAGCTCATTCACCATTACACTCAGAAGGCCAAGTTCTCCGCAGTTGATTTATTTTTCCCCAAACAGTTATTATTTATTGCCCGATACAAGGTGCCCTGGATACTAAAATGGTCTTACAGGGTTACTAAAGATGCTCGCATTTTTGCCCGCCAATTTTCAGTCAAATGGTGGGATAAATTTGAAGTTGACAGAATCGCCAAATATGTTTACAATGATCTTCCAGTTGCACAAGTCCCTCTGCCGGTCTCTCCCTCTGCATCTGTCCGATCTTCCCTCTCAGTCGAAGGAAAATCCAAGGCAGAACTCCGAGAAATCGCCACTCAGTTGCTCCGACAAGCCTCCCAGATGGATGATGATGAAGACAACGGGTCCCCCTCGTCTTCCAGCTGTCAGCCCATCTCTGTCAAGCCCAAAACCTGGTTTGAAGACAGTCAAGATCCTTATGATGCCTATGA

TATATATATATATATATATATATATATATAAGGAGAGAGGATGTGGGTCCTATCTACCTCAATTTGATAGGGACTCGTCCAAAGTAAACTGCTTCATGACGCATTGGGTGAGCTAAGGATGATAAGTCAAGAGATTAATTAGACCAGAAGAAAAACTCCTCACCAAATTTAAGAAGACATATTTTAATGGTTTTTGCAACTTCTTTTTAACAAGACAAATTTTACTCGTCAAAACCATTGTGGCGAGGCAAATGTGAGTTTCCTTCCAATTTTCTTCTCCCTTGTGTTAGAAGCTGCGTAAACCATAAAATACATATGCTCTTGTTGTCAATCGTCGTCGATCATGTTGTTGCCATGATAAATAGTTTTGAATGATAAAATGAATGTTCATTATTGAAAAGTCCTAATAAGATCTGTTCATCTAATAAATCACCTTTTAAAAATCATGAATCATCATTTAACTATCATCTCTTCAAAAAATCAATCGTACCTCACCCGAAAATTTGATTCCAAGATACACACACCATTCCATTTTATTTAAGATCCATTCTATCACATGTGCTGACAGACAGTCTGATATGTTTACACAACAGCCGCAAACAACAGTTAAGACCACAAAATCGAACTTTTAATTTCGATTGCATGATGCACAACTTAAAAGGCAGGAGGCTTCAATGTATTTCCAAAGTCGATAACAAATCGGTATCTGACGTCCGCTTTCGCTAGGCGCTCCATAGCAGTGCTCACATAGTCAATAGGTATAACCTCAACCTCGGCTGTTATGTTGTGCTTGGCTGCAAAATCAATCATCTCTTGTGTCTCCTTGACACCTCCAATGTTGCTACCACCTACCATCTTTCTTCCTATATAATTGAAAAATAATGTTAATACACACTCATCAAATTCTCTCAATCAAAATTATAGCTTTCTTTTGTCCAATCCAATGTTTGAACTTTGAAGTGAGTTACGCCATTTATATGCGCCTTACCCATTAGCAAAG

>Chr6-13; OZ022211.1; 22622323-22622488; dir; B;

AATAGAATTCATGCAACCAATTATCACATCTTTATGGCTTCAAGCCTCTTTAATCCAAATAAATCGTTCTACCATCTTTATCTGACCTCAATTTTCAGAACAATATGAATATGTTATCAACTTTCAAAATAACTTAACTGAACAACTTGTCAATCGCTTTATCTTGTGGCTGCACCTAGTGACTCTAGGTTGCCTACGTACCCTTACCAAGGGATCAAGCCACACGTAGTTCTCTCAATGTGATTGCAAAACAATTCGGCTCATGTTAGTGATACGCTCACCATTGTTTACAAGCTCTAAAGTATATAGTCTTCAACTTTCCCAAATCTCAGAACAGTTCATATTCAGAACATAAGTAGGCCACTCACTGAATTTCCTCTAATTCATAAATCAATTTGTATATTATTGGCTTAAATTATATATATAATAATAATAATAAAATACAAATACAAATACAAATAAAACCAACAAGCTCCGAAGACACTTTTATCCTTTCATTCGAAACTTACATAAGGCTCATTATATAAATATATTTATATGTCAACAACTTAACTAGAATATATATATTTTCCTCTTTTACTTGTAAAACAAACTCATCGCTTAAGAAAATAATGGTGAATTGTATAAAATCCATAAAATTTAGGAAATTATCAATCCTTGTCTCTTAATCATAAAATAGGTGTTAAACTTTTACTCCAAAAATACCAAAATAATATCATCAATATTAGCTAAACATCTTATACCCAAATACTCTTCCACCTAGGCGTACCCCCATTTATATTATGTCAATTCCTTTAATATGCCGTCAATTCCGATAATATTCCGTCAATTCCAAGTATCCTGTCAGTTTCATCCTCGCAGGCCTCGGAAACACCAAGTTAACATAGCCGCATTCCTCGCAGGCCTCGGAGACACCAAGTCAACCTCAGCCGCAATCCTCGCATCACACGGTTTAGGCGTGCCCTAAACTCGTGGGGCATTATAAAAAATAACCAAACTA

TTTCTAGGATCTTTAAATTATGTTTCCGATTTCTACAAAAATTTGAGAAAACAATGTAAGCCTTTATTTGATCGCCTCTAGAACAATCCTCCTCCATGGTCATCTATTCATATTGATATAGTCAAACAGATCAAAGTCCATATCAAGACTCTTCCCTGCCTCGGTG

TATATATATATATATATATATATATATATACTCTCATGTAATTAGATATTTAGTTAATTTTATTAATCATGTCGAAATCCAATTAACAAATCATTCCATTTCGATACTATAAAAATAATCTTAATAGTAATAGATAACTAAATAGATAAATAATTAAATAACTATTATCCCCAGAAATTTCATCACCTCCAATAATGCATATAAATCCATAGTAGATTGTATGGTAATTTAATCAATGATTAGTATTAACCTTTAAAGAAAATCCCTTATCGAATCACTTTCCAATCTAAACTTATTAAACAAGAAATTCCATACTAAAAGAACCATGTACTCTTAATAACAAATGTCCACTGATCTACGAATAATCATAACCGAGCCCAAGGCTCACTTTCACAACCCACCAATCCGATATTCCAAGCTCGCCCATTCCATAATTTCAATTATCAATAATATCCAGGAATTTCCATAAAACACTATAAAACAATTTCCTAATCTCAACATAATCAGAATAACTCGACTACCGGTCAAGACTCGTTCACAGGGTCATTTTAGTACTTCTCGAAAAGTAAAACTACATTGGAAGACTCACGGTCATCTGAGGAGTCAGACTGCCCAAACTTGGTCAACCGGGTCCGTGGGGCCCACAACCTCCCATTTCCCAACCGAGAATTCCTACGGGTTTGACAAGGTTTACTAAACATGTCTTGCAAATTTGGTCCTAATCGAACAGTCGGATTGCACTATTGGTTTATCTAAACTATAAATTATTGAACCAAAGTATCTGGGACTCCAATTCTCAATCCGCGAATTCCTATATGATCCTAGAAATGTCTAGATCAACATATTTGAAAACCAAAACGATCAACGCTCCGAACATATCAAACCCGGATATCGCCTAATATGCGATATCTGATCAGCAGTCAAACAATACCCAAAATAAAATCCAAGCATACCGTCGTGCTTGGGACAACAAGAGGATCAATTTAGGACCAAGAGAGTC

>Chr6-14; OZ022211.1; 23627930-23628206; rev; B;

TTTTCTTTTCTTTTTCCAAATATTTACCGTCGTCCTACTAATTTCTAGTGACTAGTGTTAATACTCCATAGTCTATAATAAATAAATAAATAAACCTAACAACAACAACTCGACTGAGTCCTTTACACTGGTTGTATCAGAAAAATTAGGGGTTTCTTGTTATAAGATAAAAGATTCTTGTTTGGTGTTAATCTATACGAAAATGGGAAATTTTTTTAATGAAGAAAATAATGGAAGATAAGAACTAGCAATGCGTTTGGATGAATGAATTGAAAATTGTATAGAATTCTTAAATTTCGGAATTTGGATCTTTATAATTTAAATTTCTTACAATTAATAATTTAAAGTGTTTGGATTTATTATGTATGTCAAATTTTTTAAATTACAGAAAAAAAGAAAAAGAAATTTTTGCTCAATATAAATTGCAAAATGACGTCTTTTTTAGCATAAATTAAACTCGGAACTCTCGGTCCCCATATTCCACGGTTTTTTTTTTGCGAGTCATTTAATAAGTTTTATAAATAATTCAGTATAAGTCCAATTCTTCTATATATATATATATATATAAATAGAGTTTCCATTGAGGGATCCCTCAAATAAGCTTATTTGAGGGACACCCCTTGTAGGCCCCTCTCTAGATTGTATTTCACTAATCCAAAACCGTCTATCTTGTAGATACTCATTCAAAGATCATTTCTACAAAAAAAAATTACTTGAATCCGATATCATTTGATCACTTATTTGAGTTTTTGAAATTTTAGTACTTTCTTGAAGCATTGTGTTCATTGATTTTGTAAAACACAATTAGATGTCAAAACGGTTTCCGATTTTTCTAATTTTTTGTAAGGATGATCTATAAATGAAGACCTAAAAAATAGATGGTTTGGATCATTTGAAAAAAAAAATTGTAGAGTACCCTAAAGGGCATTCCTCAAATAAAATTATTTGAGGGATCCATCAATAGAAGGGGACTACGTATATATATACCAATAGTAGTCTT

CCAGAGATAGTAAATGAAGAATAGATTGAGTATGATAATAATAGTAATTTAGATTATAAAGATTGAAATATACCTAGAGCCCCAGCCAGGACATTTATAAGAAAAAATGGTCATTATCCAGTTTTAAGTCAACCACCCATGTTAAGACAGTCGAACAAGTCTATGCCCTAAGTAAAAGTCATGAGACATGGCAATTACTTAATCTTGAGTCAATAAGAAAACATAAAACTGATGGAAACAATTTCCTTCATATTGGTCTAGTTCAAGTAGCCGTTAA

ATTTATTTTCATGTTCCAATTATGCCTTTATGATTTAAATCCGAAAAGACTAATTTATATAATAATAATTAGCTAATTAATACAATTATTGATTCTTATTGCAATTCACAAACAGGTAGGAAATTAATTTTAGGCCAATAGTTACTATATTTGAACTTTTAGTTAATTATGATTTGAATGTATAAAATATTTTCTGTCTATCCATTTTAATTTTGAAAATCTCATAAATTTCCTATAATCTTTCTTCTTCATATTGTTCATCTTCTTGTTTCTTCTTCCTTCTTTCTCTTCTTACAATCTTATGGAGGAGGTATAATTATCTATGAATAGGAATATACATGAATGTTAATTAGGAATGGATAGGTAAATTACCTATGGAGGAGGTATACATATGAATGTTATTTATCTATGGAGGAGGTATACATTTGAATGTTAATTACCTATGGAGGAGATATATGATATGAAATTACCTATGAAGGAGGTATACACAAACTTGCAAAGCAGGTAAATCAAACCACCCAAAGACATACATACCTCACCATAAGGTATTCACTGGTTGAAAGCGTTTGATAAATTGAGATTAATAGTATAAATGTGATATTAATTTATTGATTACAGTAATTAATAAAATTTAGAAGATTGACTGTAGAATGGCAACATGTGTAAATGTTTTAAAAACAGTGGCCAAGGCTTTGCATAGTTGGCTTGCCTATTGAGTCAATGGATCCAATTCTACGTCAAAAGATAGATGAACTGCATTCATAGTGCAAACTCAAATTTGGACCTATATCGAAAGACCCCTAAGTTTTATGTTAAGTTGCCAAACAACGGAAATATTAACTTCTCCTTTTCAATTTTTGACTTTTAGCTAAATTCCAAATTATTTTCCTCCATCTAAACAGAGAGTAGGAGAATATTAATGAAAACAAATAGAATAGCTTCGGTCATAAAGGAGAAAAACTTCTATTTTGTGGGAATAGACCTATTTTGTTATTTCCAA

>Chr6-15; OZ022211.1; 23904270-23910590; dir; B;

GAGGGGACTGTGAAAGAGTATTTGGAATTCAGTCTTGGCTATGCCCTTGGGTTGCTTGGAGTTTTAGCTTATGTGCTAGTGAAAAATGGCCAGAAAGTCGGCTACGGCTAGCGAAAAAAATAATTAAACATATCAAGTATAAATCAAGAGTATTTCGGTCTAGACTAGAACTGGCTCAAAGACTTTTAAATTGTACAAGTTATACATACTATCATGGGTCAAAATAGTTGAAGAGTAGAAATCAAAATGAGTTATTTGTACCCTTTTCGGCCCCGTTTGATTCATGGGAAAGGAGACTTGAGGATGAGAAATCAATTGATTTCTCATGTTTGGTAAGTCCATGTTGAAGAATACACAATCCCATATCAAAAACTTAACAAAATAAAAAATCCAATATAATGAATGGCTCCAATTCTAATAACATTGAGGCATTTTGTATAAAAACAGGTGGTAAAGTAGGGAACAATATTGATATTGCTAGTAGTGGGTCAACAATCTAAGCATGAAAAATCGTTGCCTTGCATATGGGAAAGTAGGCCTCATCATTTGGCCCGCGGGCTCATGGGGCAATAGGAGCCCGCCCAGCCCATCGAAAAAAAGCCCGACCCAGCCGGTTATGGGCTTTGAGATGGCCCGGCACACCTTGGGTTGGGCTAGGCTTGGGCTTGGGTGTCTGAAGCCTAGGTAGCCCGACCCATAGGCCCGACCCGATTAAGCCCAAGAAAGCCCGGCTCGGCCCGCCCACAAAAGCCCGCTCCGTTAGGCCTGCATAAATATATAATTATGTATAAATAAAAGTTTAGGTTTAGAATTATATCACTTAAATCACATATATAATTTGTTTCATTTCATTTAATTTTCTTTACTCATTCCTAGTTTATAATTGGATGATTAGCATATTAATTTATGTTAATTATTAATTCAACAATTATATATATACATTCATGCATAAGCTCCGCCTATTTTATTATCTAAGTGCCTATAACTATTTCTGCTTGTA

TTCTATCTCTTCCTGGTTCTAAATCCCTTCTAGTATCAGAGCCGTAAGTGTTGAAGAGTCAGTCTAAATCTGCAACTCATCCAACCCAAGTGACTGGTTTAAGATTTTTGAGTTGAAGATTTCAGTTTCTTCTTCTACTCCTCCTTGTCTTTCGGGTTACCTGTCCTTTTCTCTTTGGTCCAAATAGTAAGTCCCAAATAGAGGCCTGAATGGTTGCTGCCTGAGAGGAGAGAGAGAAAATCCCTAGGTGTCCTGTCTTACTAGGAGTCAGTTTTAGGAGTCCCTATTTACTTTCTTCTTAGTAATCCAAACCCAGAACCTTGAGTTTAATGAGTCGCTTATTTAGAACCAACTCTTGTAACTCTGGCATCTCTAGAGCCAGTATTAGTCGCCTCCCTGAGATAATTGTTGTGAAATTATTGCAAGCGCACAATCCGTACAAGTAATATAGAAATAAGTAGAGTATCGTTCCCACGAAGATTGTAATTTCCTATTAATTACCAATTATGATTAATGCATAAGTTTATTTGAACAGTTGATTAAGAAGATTGATTAATTTTAAGAAAACTAAAACAATTATGAAAAATAACAATTTAATTAACGATATAAAATTAAGATGATGAGACACTAGAGCATCCGACTTCACCATAACCAATTCTACTTAATTCTTATAGCCAATTAACCTTAATTACTATCCATGTTGACAGTTGGTTTCTCCTAATTCATTTGATATCCCCTCTCGGGCTCAACCAAAAGTATTCTCAATTAACAACCCATTCTCTCTCGAGCAACGGATTTCGAAAACCAAGAACCCATTAAGTTCTATGAGAACCTACAAGAAAACTGCATGAGTCATGTTAGTCCCTCTCGGGCACTCATTCAAGTCATGGTATTTGTTATAAGAAGCAAACACCAAACATCTCCTCTCGGTCTCCGCTTGGATCATCAATTAAATATTAGCTAGATATTTAAATCATTAAGAACAGTAACAAATACTCAACATGAGATAATAATCAGAAATTAATATAACAACAAAAATTAAGTATTCAAGGTTAGGCTACATCGTAGCCCTAGCAAGGGAAATTAGTTCATGACAGCAGAAAATATAAACAAGATAATTGTTGTCATAAAACCAATTTGGCCGATGGACTTGATCTTCAAAATCTTCACAGGAACACCTTCAAAAGCTCCTCAAATAAAGCAGCAGCAAATTCTTGGCTCTCTCTGATATTTCTCTAGTTGAATAAAAGTGGGTGGAAAAATATGGTAGAGAAGACGATGGTGGAAAAAGATGATGGGTAATGGATCCTTTCAACAATGACCTAAACTCACTATTTATAAAACTCCTAGAAATCCTACCTAAGGAATAATTACAATTGTTATAAGAATAGGATTCCTTCTAAAATAAGGTCTTCTTTATTTCTCCTTATTTGACTTGATAAGACTTGCACCATCTTGAACTAGGAAATTTGACAACATTCCTTGCAACAAAAGGAATGACTTGGCTTCTTTTTAATTGATCTTCATCTTCTTGCCTTTTTTAGCACACTTTCTCCTATTTCACAATTCCTTCACAAATCTCACAAAACCAATTAAAAACAACCAATTTAATCTGAGAAATTAATAAATAAATAAGTAAATGAGGCATAAAATATATATAAAATATAGACTTATCAAATACCCCCAAACCTATCTTTTGCTAGTCCTCGAGCAAAACTGAAAATCAAAAATAAATTAAAACAAACTAACTAAATTAAACTACTTTCGCAGGTAATCTCGATGGCACTTAGCATGTGCCACAAGCCTTTAAACCTCTAGGTGTCCCTAGTAGGAGGAGTTGTGTCTCCTGAGGGTTTACCAGTCACGACACCCACAAACACTTCTTGCAAAACAAATTCGTTAAAGCAATACTTCCAACAAACTAATAATAACCAATGAATGCCAATCACAATTCAAAAACTAAAGTTATGCCACTAAAAAAAACAGCACCATAGTGTAAAGTGTAAAGCCAAAACTCCAAGCATTCAAACATAAAGATAACCACATTCGGCGTGTGTACAAATTCGTCTCGACTCTAGGCCTCACTGGATAACTCTCAATTTTCTCACAGAACCTAGGGTTGCACTCTTACCCCGCATATGTGAATGAAGTTAGGTGAAGAATTCAAAATATAAGACACATATGCATCCACAAAATGAATAAAAGGCAATTTCTAAAAATAGATCTCATGGAAAGGAAATCAAATACTAAAAACCATAGATGAAGTACATACCTCTACTTCCGAGTAAGAAGTCCCCAAAGATCCGTTAGGTCTTTACTATGGTTGTAATGAAGGGCTAGGTTATGGGTTATGAAAGAAAGGGAAGGAAATACTAGAACTTGAGACATACCAAGGCATTTATCACGTGATAAGGCTCGGCTCTTCAAGTTTACTTCACAGAGGCGTCCAGGATTTTTGATATGCTTCACTTTTTTCTTTTTTTTTTTCTTTTTTTTTTAAATTATTTATTTTTCTTTATTTTTTTTATTTTTTTTATAAAGCACATATTTCCTCGGTACACGCCACCTCTTTTGTTGATGTAGAGGTCAACCTTCATCCTTGTCTTCATGCCATGGCATATCCCAAGTTATAGGGTATGGCTAAAAATAGGGTGAAAAAGGTAAGGAAATATTGTGGGTGATGAAAGAAAAGGCTGAGTGTTTGGCTTCAAAGGGTTGATGGCACACACAATGGGTAGGACATGTGGTTTTATTTAGTGGCTAGAACATGCATAGCCTAACATCATCTCTCAAGGTCCATCCAACTCAAAAGTAAAAGCATGAGATGATTCCAAAATAAAGATTAGTTCTTAGTATTCAATTCAAAAGCCTAATTGAGATCAATTAAAATTTTAAAAAAAAAAAATAGTGAATAAGACTAATCCCAAGTACTCTCTCAATGAATGGCAATTTGGCTCAAGTATCTCACAATGGTAGTCTCCACTATTTCTCCACAATCATCCAAGTATAGCTAGCTTGTCCATTAAAATACTTGAAGCATGGCTATGTGAAGTGCTATACTTATGATGCATAAACTTTCATGACAACACTTTAGTTAAAGGAATATGATCAAACATCACATATACATCACTATTAGTAAGAGAAAGTAAAAGCTTTAACAAATATTTTATTTTTATTTTTATTTTTTAAAAGTTAAAGGCATTTTGTGATTTTTTTCAAACAAATGTGAGTGAATTTATGTGCCATAATTGCTTAGGGATAAAGAAATTTCATCAAAAGGAAGAGGACACACCTGGAATAGCTCCTAGAAACCTCTGATCACAAATGGAATGGATCATAATTGATTTGGAATGAAATTAGAAAATTCTGGAAAATTGACCTTTAGCGACGAAAAATCTGATGAACACCGTCGCTGAAAATCCTTTAGCAACGAAAAATCTGATGAACACCGTCGCTAAAAACCATTTTGTGACGAAAAATCTTCATATTTCTGGGTAGATAGCAGCTAGTTTTTTTGGGTTTTAGGCCACTTTTTTAACTTCAAATCGTCCTAAAACGTCATGTAAAGCACTTGAGACCCCCAAAACTGTTTCAAATCATCATAATAACCATTCTAAACATGTTGGTAGCTTCAAATTTGCCTGAAAAACATCAAAAAGGAAAATAGGCAGAATTTTTGATTTTGACCAAAATAAGAACATAATAACCTCTTTTGCTATTTTCTGGGTTTTAAAGTCATCTAATGGCCAAAACAACATCTGGAAAACATCTGGAACATGTTTGCAATCATCACAGGGCTAGAAAAAAATCGAGAAATGTTCTTGGAAACACAAAATCCTTTCTTTGGAACACAAAATCTGCCATTTTTTTTTTCTATGACTCAAAACTCAAACAAAAACAACCAAACTCCACACCCCCAAACCTAAACTAAGCATTGTCCTCAATGTTTAAAGTAAATGCATGTAACAATTAGTAAAGGCATAGAAGGATGGAAAATAAAGGGAAAACAAACAAAATAAAATAAAAAGGAACGAAGGTACCTAGTTGGGTTGCCTCCCAACAAGCGCTTGGTTTAGAGTCACTAGCCCGACTTTAAGGTGCGTTTACTTGGGGTCATCAAGGAGGATAGTGGTTTCCTCCTCAAGAAATGCAGCTGCCACATAAGGTTTGAGTCGATGGCCATTAACTTTGAAAGTTGTACCGTTCACGATCATTCTTGATTTCACTGTCCCATAAGGATACACTGTTAACACCGTGAATGGCCCAGACCAACGAGAGCGAAGTTTCCCAGGAAAAAGTTTCAACCGGGAGTTGTAGAGGAGAACACTTTGACCCACATAAAATTCCTTCTTCAAGATGTGCTTGTCATGCCACTTCTTTGTACAGTCCTTGTAAATTTTTGCATTCTCATACGATTCATTTCGAAGTTCCTCTAGCTCATTCAATTGTAACTTCCTCTTCTCCCCTGCCGAACTCATGTCAAAATTGAGCGTTTTAATAGCCCAAAACGCTTTATGCTCCAATTCCACTGGAAGGTGGCATGCTTTCCCAAAAACCAGTCTATATGGTGACATCCCAATTGGCGCTTTGAACGCCGTTCTATAAGCCCATAGTGCATCATCCAATTTTAATGACCAATCTTTCCTAGAGGCACTCACCGTCTTTTCCAAGATTTTCTTCAATTCCCTATTCGAAACTTCAACTTGCCCACTAGTTTGCGGATGGTAGGGAGTAGACACTTTATGGGTGATGCCATATTTTGCAAGAAGGGAGTTAAATTGACGATTGCAAAAATGCGTACCTCCATCACTAATAATTGCACGAGGGACCCCAAATCGTGTAAAAATATTCTTTCGTAGAAATCTGACCACAACCTTGGCGTCATTCGTGGGTAGGGCAGCTGCTTCGACCCATTTGGATACATAATCCACTGCAACCAAAATATACAAATTCCCATATGATGCCGGAAACGGACCCATGAAGTCAATACCCCAAACATCAAACATCTCCTCTCGGTCTCCGCTTGGATCATCAATTAAATATTAGCTAGATATTTAAATCATTAAGAACAGTAACAAATACTCAACATGAGATAATAATCAGAAATTATATACAATAGAAATTAAGTATTCAAGGTTAGGCTACATCGTAGCCTTAGCAAGGGAAATTAGTTCATGACAGCAGAAAATATAAACAAGAGAATTATTGTCATAAAACCAATTTGGCCGATGGACTTGATCTTCAAAATCTTCACAGGAACACCTTCAAAAGCTCCTCAAATAAAGCAGCAGCAAATTCTTGGCTCTCTCTGATATTTCTCTAGTTGAATAAAAGTGGGTGGAAAAATATGGTAGAGAAGACGATGGTGGAAAAAGATGATGGGTAATGGATCCTTTCAACAATGACCTAAACTCCCTATTTATAAAACTCCTAGAAATCCTACCTAAGGAATAATTACAATTGTTATAAAAATAGGATTCCTTCTAAAATAAGGTCTTCTTTATTTCTCCTTATTTGACTTGATAAGACTTGCACCATCTTGAACTAGGAAATTTGACAACATTCCTTGCAACAAAAGGAATGACTTGGCTTCTTTTTAATTGATCTTCATCTTCTTGCCTTTTTTAGCACACTTTCTCCTATTTCACAATTCCTTCACAAATCTCACAAAACCAATTAAAACCAACCAATTTAATCTGAGAAATTAATAAATAAATAAGCAAAGGAGGCATAAAATATATATAAAATATAGACTTATCAATAATTAATGAAGAACAATTAGAATATGATTCTAGTAATGATGAACAATTAGATTTTAGAGACTAGAATATCCCTAAAATCCCGAGTCAAAAAATTTATAAGAAAAATTGGTCATTATCTAGTTTCAAGTCAACCACACATGTTAAGACAGTTGAACAAGTTTATGCCCTTAGCAAAGAACATGAGACTTGTCAGTTACTTAATCTTGAGTCAATTAAGAAACATAAAAATGATGGTAATAATTTCCTACATATTGGTTTAGTTCAAGTCGCAGTCAAGCCCCTCACCAGATTAGGATTAAAAGCTTCTATCCTTCTCTATCTTCATGATGCTAGATTTACTGAGTTCAGTGATAGTACTTTAGGAATGATTGAGTCTAGTCTGTTTAATGGCCCAATACATTTTGATTGTTACCCAAATTTTACTGTGAGTCTTAGTGATCCTCATATCTTAAAGTCATTAACTCTACATATTAAGACCTCTGGTTATAAGGTTTTACCAGGTACCCAACCATTAGCTTTAGTCTATAGAATCTATTACAAAGTCACAGGAACTAATATG

AATTTTCAACACTACTACAAAAAGTGAAAAAGACGACCAGAAAAAAACGACGGTCTAATTGAAAACCGTCGTGGTTTTTAAAAAGACGACGGTTCTAAAAACACCGTCGTGTTATACCACCTTAGACAACCAAAAATGAAGATTCAAATGGCTGTTCGAAAATAACGACGTACCTAATTAAACAACCGACGTCTATTTGAAACAGATTTCCTCAGTGTAAAATCAAAGCCAACAAAGAACGGCAGATGTTATGAATCGACCGACGTAGAAAGTAAAGACGACGTTTTCAATAAAAGCCGCCGTTTTTACTCATAAAATAACACGACGAGGTTTTCGTATAAGACGCCGTATAATTACAAACAAATCCACGGCTTTAAAATATTATACCTAACGTCGTTTAAAATTTGCAATCAGAGAGACAATTTTCAACGACGGTTATATTATACCTAACGACGTTTAAAAAACAAATCAACGTCGCTCAACAAATATATTACGTCGTCTTAGTCTTACTTAAGACGACGAAAAACGTCAACAGTAATTCAAAAACAGTCGTTGTTTTTATACTCTTTTTTTTTTAAATCTGTCATCCAAAAAAGAAAATTTACATATTCCAGAACATTAATTTCCTTCATTTTAAATTTCTTTGATTTTCAGATTTCTTCATTCCAGCACATCTGATCTTCAGATTTCCCTGAGAAGCTAACTTTCCTTCGACAGCGAGTGGAGGCCAGGCTTGCATCTCTTTTGATGGAAAGCAAGGAGTATTCAGAAGCACTAAGTGTCCTATATCAGGCTTGATCAAGGAAGTGAGAAGGCTAGTTGCAAGCTTCTTCTTGTGGACATAGATTTGATGCGAGTAAGCTCAATTTCTCTTTGAGAAAGACATCCAAATTATTGTCTTTGAGATGTGAGAGACAGTGTCTCTGAGAGAGTAAGAACCTGGAACCCTAAATGTAAACCCAAAATGAATGAAAGCGACAAATTTATAGAATAAATTTTT

>Chr6-16; OZ022211.1; 23928972-23933904; dir; B;

NNNNNNNNNNNNNNNNNNNNNNNNNNNNNNNNNNNNNNNNNNNNNNNNNNNNNNNNNNNNNNNNNNNNNNNNNNNNNNNNNNNNNNNNNNNNNNNNNNNNAATCCATTTAATGTTATTGATTATTTTATTATTCATTATAAGTACTTATATTACTAAATTCCCCTTCAAATTGTTGCTTAATTACTTAAAACTCTATAATTATTTACTTTGTTTACTACTTAATTCTTCATTTGATAAATTTATTTATGTTCTTAGTTAATTTTCAATCTTTACTATTATTTTTTAAGTATGGTAAAGTGCATAATTTTCATGAAAAGTCTAGTTGGTTTAATTATTTTTCAATTTTTGTTAAATAATAAATATAAGAACGTGTACCATGTCCAATGAATAATGTGAAATAAGGTTACACATGTATCTTACCATGCAATTTATAACGTGTAAAATATTGTACTAAAAAATGATAAATAAATTACTATGGGGTGTTTAATTCTCTTCATTAATTACTCCATATTTTCTAAGTCACGGAGTTTGTCAGCTCGCAATATAATCAATTTAAATCACTCAAACCCACTATGCAATGCATTTTCTTCCAATTTTTTGTGTCAAAATAATAGATGATTGACTAAATAAACTTCTTCTAAAGTTTCATAAAAAATTTCCATGTATTTATCACAATTTATGTGGTTTTTATCCAATTTTTATCGATATCGATATTTTCCCGATATTTTCATCGATATTTCCGTATTTTTGAACCACCGATATTTCCAAAACTATC

AATATTTAAGACCTTGGTAATGATTGAGGTCACATAGATTATGATAATTTGACTTATGGTAATATTATTATCACAATTAATCAAGTTGGTATGAAAATGTGTGTCGACATGAAAATAGGTAAGCAAATACAGTTAGAAAGAAATTCCGCTAAATATGAATTAGGAAATTTCTGTGAACAATATGGTTTAGCGCTGATTCCCCCGTCTAGAAAAGATAAGTCTAGTCACCACAGCCAACAGTTCAGTAAGAATCGCCACTACTCACGTAAAAGAAAGTCATTCCGACACAATAATGATCATAATGAGTTTTATAGTAAAAAGAAATTTCACCCTAAGCAGAATTGGTCCAAAAGTCACGGGAACAATAAGAAAAGTAGCTTTAAGAAAAATACCGACAAAAGAAAAGTAAAATGTTACAAATGTCAAAAGTTTGGTCATGCTAATGAGTGTAAAGTAAAAGATACAATAAAGCAGTTGAAAATCACAGATGAAGATAAAGAAAAGTTGATTAAAGTATTAAAGCTAAGAAATTCTAAGTCTAGTGAAAATGAGACTATAGTCACCGACACTGAGTCAGCATTTTATATGTCAACAGATTCCCAACCTAGTTCTCCCAATATTCAGTTAGGATGTAAAGATAAGTGTTGTAATATTTTAAGTAAGACAATTAATTAATGTTCTCACTAAGCAAGAGGAACAGGAAGAACTATTAATTGATTTAATCAGTAAAGTGGAAAACCCTGAGTTAAAATATGAGTATTTGAAAAAATTAAGAAAAGTCATTAGTCAGGAATAACCTAGTCAGTCAATTCCCCAAAAGATAAGTCTACATGCTACTTTAGAAAAATTCAACAAGAAGAGAAAAGAAGTTACTTTACAAGATCTACAATTAGAAGTTAAACTAGTCAAAAAAGAAATTATAGAGTTAAGACAAATGAGTCATAAGTTGCAAGCCAAGAATTTTGAAATTAAACAAGACTTAGTTAATCTAATGGAAAAAGGAAGCTCTGAGTTTAGAAATAATACAGAAACCCCCACAAATTCCCCTATACATTCAGACGATGAGCCTTCCCATCACTCCGATAATGAGCAACCATTAACTTAATCAAATAAGTAAGTTTTAGAAAATGGTACGCTAAAGTCTCAATATTTGTCAATAATTTTGAGTTAAACACAGTTGCCCTTTTTGATTCAGGACCCGACCTTAACTGCATTAGAGAAGGTTTAGTTCCCACTAAATACTATAAAAAATCAAAAGAATCATTAAGTACCGCATCAGGAAAGTCACTTCAGTTAAACTACGAAATTCCAAAAGCCCATGCATGTCAAAATAAAATCTATTTTAAGGCCTCATTCGTCCTAGTTAAAAATATCACTGATGAAGTTATTTTAGGATTACCCTTTATTGCCCTTTTGTATCATTTCCAAGCAGAATATGATGGAGTCATTTCTATCCATTTAGGTGAAAAGGTCAAATTTGAGTTTTTAACCAAACCTGAGTTGCACAATTTAAAAGCTTACAGAAAAGCGCAGTTTCAAAGACAGTTAAAATAATTGAAAGTAAGAATAAGCAATTAGATTTTCTCAAAGAAGAAGTTAAATTTAGGAGAGTTGAACAACAGCTTAATGATGCCTCTGTCCAATCATAGATTCAAAAATTTGAAGACAAGTTAAAAAATGAAGTCTGTTCTGAGTTACCTAACGCTTTTTGGCATAGGAAATAACATGTTTTCAGTTTACCCTATGTTGAAAACTTTAGTGAAAAGAATATTCCCACTAAAGCCAGGCCAATCCAAATGAGTCGAGAGTTAATGGAATTATGTAAAATTGAAATTTCTGAATTACTCCAAAAAGGAATAATTAGCAAAAGCAAGTCACCATGGTCTTGTCCTGCTTTTTATGTCCAGAAAAATGCTGAGTTAGAATGCGGAGCCCCAAGACTAGTTATCAATTACAAACCTCTTAATACAGTCTTAGAATGGATTAGGTACCCAATACCTAATAAATGAGACTTAATTAACAGATTAGGAAATGCAGTCATTTTCTCAAAGTTTGATTTGAAAATTGGATTTTGGCAAATTCAGATTAATAAGTCAGATAGGTATAAGACCGCTTTTGTTACCCCATTTGGTCACTATGAATGGAATGTTATGCCTTTCGGTCTTAAGAATGCGCATAGTGAGTTCCAAAATATAATGAATGAGATATTCAATCCTTATGTAATTTTCTCCATAGTTTATATTGATGATGTCCTAATTTTTTCCCAATCATTAGAACAACATTGGAAACATTTGCATAAGTTCCTCCAAATAATTAAACAAAATGGACTAGTTGTCTCGGCTAAGAAAATTAAGTTATTTCAAACTAATATCCGATTCCTTGGTTTCAATATTTGTCAGTCGCAAATTAGTCCAATCGATCGAGTCATTCAGTTTGCAGAAAAATTCCCAGATCAAATTCTAGACAAGAGTCAACTCCAAAGATTTTTAGGATCTCTGAATTATGTATCTGATTTTTATCAAAATCTGTGAAAACAATGTAAGCCACTATTTGATCGTCTTCAAAGCAATCCTCCTTCCTAGTCATCGATTCATACAGAAATAGTCAAACAGATTAAAATCCATGTCAAGACACTTCCCTGTCTTGGTATTCCTTCAGTAGATTCCTTCAAAATAGTCAAAACTGATGCCTCTGAAATTTGTTACGGAGGTATTCTCAAGCAAAGAGTCAATCCGAGTCATTCTGAGCAAATCGTTTGGTTCCATTCTGGAATTTGGACACAGTCACAAAGTAGTTATAGCACTATTAAGAAAGAAATATTATTTATAGTATTATGCATTAGTAAATTTCAAGATGATTTATTAAATCAAAATTTTTTAGTCAGAGTTCATTGTAAGTCTGCAAAGCATGTATTAGAAAAAGATGTTCAAAACATTGCATCCAAACAAATATTTGCACGATGGCAAGCAATTTTAAGTATTTTTTTTTATTTTGATATTGAATATTTGAGAGGAAGTGAGAATTATGTTCCTGATTTTCTTACTCGAGAATTTCTCCAGGAAAAACAATGGCAGACAGAAAGAAATACAAGCAAAAAGAGCAAGTTAGTCACTCCCAGTCATCCCAGTCATACAGAACACTCCCCCCTTCCATGAATACACCCAAATCAGAGTCACCCTATCAGATCGTCCCTTACCCGGAATGTTCTCCCATTCAAATCAGTAATAGGTTTGCCACCTTAGAAGCCACAGTAGGCCAAGTCCGTCCCAATTTCCAGTCAGCCTTAGTCTCCAGTCATGACCCATTTTAAGTCACTACCCAGTCTACCCCTCCGGTTGTCTCTTTCCAAAAGTCATCCCCTTACATGGTCAAAAGTGACAGTCATCTCTTTATTGTTGAATCCAAATATGATAATATTGCTAATCCAATCACTATCGCCAAGTCATATTTCCCTCCTAATTGCCACTTCATTCCTCAAGCCCCATATAAGAATTTAAAATATTACAGAGATATTCTCCTTGAGACCCAATCAATTGAAATAAAACCAATCAAAGATCGAAATAATCCAGCTATTATCCTTTATCACTCCATGTACATCCATCGAATTCTCAGTGAAAACGAATTTAGCAAGCACCCCTATGATCTCAAACTTCTCCAGTCAAAAGTCCAATTCAGTTACTATGATTATATTGAAGCCTGGTATTCGATTATGCTTCACCAAATAGTCGATTTCATTCACTCTTGGTTCATCAATTTTGATAGTAAATTTAGGAGTCCTTTTCCCTTCTGGTTCCTTCACTGGTGGGAAAAACATGGTCCAGTTATTGAGCTCATTCCCCCTCAAATGCAAGAACTAGTCAGTTATTTTACCAATAAGCATAAATTCAATGAGTCAGAATTATTCTTCCCCAATTTACTCCATTTCATTGCTAGATACAAAGTTCCATGGATCCTAAAATGGTCATATCAAATCAATTGGAATTCTCGATATCTTTCTCATCAATTTTCAATCAAATGGTGAGATAAATTTAAAATTGAGAGAATTGCTTATTATGTTTTTTCGGATTTCCCCCTGTAAAACCTCTCCAGTCAGAGAAAAAAGTATCCCCTGTAGTATCCCAGTCTTTTCTCCCAATCAAGGGAAAATCAAAATCATAGTTACAGGACATTGCCAGACAATTGATGGCCCAAGCGTCTCAAATGAATGATGATGAAGATAGTGATGCTAATAGTGATGCTACCCCTAAGTCCCAGTCTTCTACTAGCCAGTCTCGGATCCCTCCAAGTCAAAGAAGCGGTGGATAGTCAAGACCCATATGATTTCAATCCTTTTGATCTTGGCAAAGATTAATTGCCATCATGAGCCCCGACATAGTTAAAGCAAAAGTTGTTCCGCACTCAGTAAAAGCAGATGCTTTCGCGTGCTTCTACAGTCAAGATTCCAAAGCAAAAGAAAAAGTTATTTTCAGTCAAAAGTTGCAGCACAATAGCTCGCCCGTGATTGCATAGTCAAGTTATCTAGTCCATTTAAGTTGGCTTCAGCCTCAGTTGTAAGCAGAATGTAAATTTTACTTTTCTCAGTTTCAAAGTTCTATCAGCTCTACAATTCTGATGAACTCTTGTACTTTATGAGAGATCAAAATCTTCCAGTCCAGATTTTGTAAGTTTACATTTTCTCAAAGTTATCAAAGTTTTTTTCAATAAAAATATTTATGTTTCAAAGTTATCTATATGTTCATTTATTCACTAATTTCATTCATGCATAAGCTCAGCCTATTTTATTATCTAAGTGCCTATACCTATTTCTGCTTGTATTCTATCTCTTCCTGGTTCTAAATCCCTTATGGTATCAGAGCCGTAAGTGTTGAATAGTCATTCTAAGTCTGAAACTCATCCAACCCAAGTGGCTGGTTTAAGATCCCTTCTAGTAACA

TATATATAAATAAGATGAACAACATATCACATCACAAAATATAATCATATCACGAAATATAATTGTACCACCAAATATAATCATGCTTTTCTTGAACACATCACCAAATATTTGCATATTTATTCATACCATCCTTTAAATTTTTTTTTTTTTGATACTTATTATTTTTTAAAACGTATTACGATCTAATTATGTAATAACCTAAAAAATAATACTTGGTTTTATTATTTTTGTGGAAAGTCTTATTAAGTTGTGTGACTTTATTGGGTGTTCTATGAATTTGGTTTGATGTGAAAATATTGGAATTAAGCGAGTTTAATCTCAAATTTGGACTAAAATGCCCTTATGATTAAGTTGATGATTTTTTTTTTTAATGAAGTAGGCCCAATTAGGCCCAACCCGTTGGGCTTAGCCCGCCTTGGCCCGATGGGCTTTGAATTTTCTAAAAAAATCCGGCTCGATATTTTCTATCTTCTCTTTAAAGCCCGGCCTGGCCCAAGCCCATTAAATTTGGGCTGGGCTAGCCCGGCCCGGCTTATTGACAAGCCCTATGGGAAAGTAGGGGGAAAGTGAAGCCCTCTCATCTTGAGTGTTGTTTTCCTCCTTATGGGAAAGTTTGGGAAAATGAGCCAACATTAAATACATATTTTCATGACCATATTGTCCCCATTAACAATTTAGAATTACAATATAGCATTAAATGCATTCTTTTTTGGGAGCTTCACTAATATACCCAAAATAAGGGTTGAAATTATATTTAAAAAACTCCACATGGAATGCACTTTAGAAACACACCCAAAACTCATTTACTACATAAGAAATTGGTGTTTAAGTTTCTATAAATTACGTGACTGTCACTGATCGAGGTTCGTTCGAGGTTTCAAACAATCCAAAAAAAAAATGAATAGAAGCACGATTTTAGAACAAGAAATCGAAGGTGAAATATTGAAAATTGTAGATCAGAGATGAAGAATATAGTTGGACCTGAGATTAAGCATCA

>Chr6-17; OZ022211.1; 28271778-28273865; rev; B;

AAGGGTAGAATTGTCTTATCAGTTGTTATTTTTTACCTGGCCCAACGAATCTGGACCTCTCTTTGGGCTAATCCAAAATAGTAGTTTTTTCCTAGGTATTAAAACTAAAACCAAAATATCCAATATCTTACAAACTAATTAATAAAGTTAATTATGTCTAAAACCTAATTTTTCTTAAAATAAAATAGTTTGTTTAGCTATCCTGGTCATTTAAGGCCATGTTTGGTGTGTTGGAATTTTTGTGAGTATTTTAAAATACAAAATTATTTTAGAGTGCTGCTATTTTCACAAACATGTCCTATTTTTCCCTAATCAACTTCATTTTATTGCTAAATATAAAGTCCCCTGGATTCTTAAATGGTCCTACAAAGTTACCTGGAAAACCAAAGTCTTTTCCCATCAATTTTCTGTAAAATGGTGGGATAAATTTCAAATTGATAGAATTGCTGAATATGTCTTCCGAGATTTTCCTATGGTTAAGCCTATTCATAAACCTTCTTCTTCTGGAGGATCTCATGCTTCTCTCTCAGTCGAGCAAAAATCTAAGTTTGAGTTGCAAGAAATTGCCAGACAATTAATTATTCAAGCCTCCCAAATGGATGATGAGACTTCTCCAAGATCACAGTCATCTACTAGTCAACCCAATCTGCCAGGTAGTCAACAAAGCGCTTCTCAATAGAAGCGGTGTGCAGATTACCAAGATAGTCAAGATCCATATGATCTCGGTTTTGATTAAGTCACATGTGCCACGGTCATCATTCCCCAACAGTCATCATAAAAGCCTGAGTTAAAGTTGAAGTAAAAGAGAAAGTTAAAGCAGCACTCAGTAAAAGCAAAAGCTTTTGCGTGCCTCTACAGTTACCTTTGGTAGAATCCAATGTTGAATAGGCTAGTTGTTTTCTTTATTTAAGAAGGCTAAGGCCTCAGTTGTAAGCAGAATGTAAATTTTATTTTCTCAGTTTCAAAGTTCTATTAGGACCACTACCTTGACTCAAGGGAC

TCAGATTTTGTATCTTCTATCTCGCACTAGTTCTAGCCCCCTTTTGGTATCAGAGCTGTAAGTGTTGAAAAGTCAGTCTAAATTTGATATTCATCCCAGTGGCTGGTTTACGATTTCTGAGTCGAAGTTTTTCAATTCTTTCTTCCACTCCTTCACTGTTTTTCAGGTTACCTGTCCTTTTCTCTCTGGTCCAAACAGTAAGTCCTAGAGTCCAAACAGTAAGTTCCAGATAGAGGTCTGAGCGGTTGCGGCCTGAGTAGAGAGAGAGAGAATCACTAGGTGTCATGTTTAGCTTTGGTTGAGTTTTAGGAGTTTCTGTTCATCTTCTTCCTTAGAAGTCTAGCCCAGAACCTTGAGCAATGAGTCGCTTATTTAGAACCAACTCTTATAACTCTAGCAGCTCTAGAGCCAGTATTAGTAAGTTACCTGAGATAGTTAATGAAGAACAAGTAGAATATGATTCTAGTAATGAATTATATTTTCGAGATTGGAATATCCCTAAAATCCCTAGTCAAGAAATTTATAAGAAAAAATGGTCAATTTCTAGTTTTAAGTCTACTACCCATGTTAAGACAGTTGAACAAGTTTATGCTTTAAGTAAAGAACACGAAACATGTCAATTGCTTAATCTTGAGTCAATTAAGAAACATAAAAATGATGGAAACAATTTCCTTCACATTGGTTTAGTTCAAGTTGCAGTTAAGCCACTCACTAGATTAGGATTAAAAGCTTTTATCCTTCTCTGTCTTCATGATGCTAGATTCACTAATTTCAGTGACAGTACGCTAGGAATAATTGAATCAAGTCTGTTCAATGGTCCAGTTCATTTTGATTGTTACCCAGATTTTACAGTAAGTCTTAGTGATCCCCATATTCTGAAAACCCTAACATTGAATATTAAAACTTCAGGACACGAAGTATTACCAGGCACCCAGCCATTAGCCTTATTTTATAGAATCTATTATAAAGTCACATACACTAATATGAATTTCCAAGCCCTTAATAAAAGTCCGAAAGATCAAACTCTGTTAATTCAAAGTAACAAAGCCGATGCCAATATTAGAGTCCCTCACACTATTAAGTGGTCAGAAGTAACCCTACCTTTAGATTGGACATTAGCCACTGAGATTCCACCAGCTCCAATTCAACATAGTTTCAATAATTTAGATTACATTCAACAATACCTAGATGGTACATTTAAAATTAATTTTGGAAATCAACCTCCTCAAAAGTCTTCAGTCCAGTTACACCAGTTGCCTACACCTAGTCAGTCCTAGAGACATGCCCCTGCACGACATAGTTACACAACTTCTTCCTTTACCCTAGAACGAGACCTAGAGTTAGAGAAAACATTAATTGATTTAAAATTAGAATATTTAAGAAAGTCTTCCCAAGTTAACCAACCGTTTTATGGTAACAAACCCATAAAAGAAGATACAAAATCTGAGTCCCTTGAGTCACCCACACAAACTGATTTTGAAGTCGGTTTCCAAGTTGATAACTAGCTGAGAACCCTTAATAAAAAGTTCAAGATTAATTGGAAGAGTCTAGATAATCATTTACATGCCCCTGAGGATAAAAAAAGAAGGGACAATTATCACACAGCCTACCCTAGTAGAACAAGACGTCAACAAATCTTTAATGAATGGAAAGATTATATGCGCACAACTAAGTTAGAAATTTTCTACCTTGATTTCATTGGAAGTCGTTATTTACCAAAAGAGTTAAAAACTTTAACGCAAGAAAAATGGGAAAAAGAAGATAATACCGAAGTTTTAGCAAGTCACCCCCCAGTAGAAACAATAGTTATTACTCATAAGAAGACAAAAATAGTTGCCAGTCCCTTTAACGTTCCCGACCAAAATGACAGAAAGTTAGTAAAACAAAATAATTACACCAACCAAAGTCTGATAGTCATAGGGAATCAATTAGATAAAATAGAGACTAAAATCGACAAAATAGTCCCTTCAGAAAGAGCCTTTAAGTCACCAAAAGAAAAACCTTTAGTAAAATTCCAGGATTTGCAATCAAGTCCTACCCTTAAGTCAAAACCAACAATGAGAAAAATAGAGAAAATGCTAGAAGAAT

GTAAATTTTATTTTCTATCAGTTTCAAAGTTCTATCAGGACCACCACCCTGACGGACGTTTGTAATGAGAGCCTAAAATCTTTCAATCAAGATTTTCTGTAAGTTTGCATTCTCAAAGTTAATAATATAATATTTTCAAGTTATCTCTATGTTTTCAATCATTCATATTTTCATATTAGCTTATTTGAGGGACATCCTTGTAGGGCCCACTCCACATTGTATTTCACTAATCCAAACCTTCTATTTTATAGATATTCATTCAAAGATCATCTCTACAAAAAATCACTTGAATCCGATATCATTTGACCACTCAATTAAATTATTAAAATTCAAATACTTTCTTGTAGAACTGTGTTTATTGATTTTGTGAGTCTCAAATACTTTCAAGGCACAAACATACAAAGGACTCATCTTGAGCTCCAATTATTTCTTTATATTTAGCATGTCACTGCTTTGAGACACATAAATGAAGTCTATAAGAAAATAATTAGTCAGCCTCAACTTGAAGTTTCATTCCATGCTTGCAATAAGATATATCAAAAGGTACGAAGGCATTTGGGCCTTTCTCTAATACCACGTGATCGTTTCCGGAAGTATATATGTCCTTTGCAACAGGATAAGGGTCACATGCCTCGTAGTATCCTGGATAATTGATTAGGGCAACTACTTCGAATGAGGGATTGGTGTACTTGAAATCTGTTTAAATAAATAAATAAATAAAATAAGTAACCAATCCAATTATTTAAAAAATGAAACGCTAAAAGAATAAATTATAAGAGGGATAAAGAAAAAGAAACGAAACCAGTATATCTTACTCAGAACATCTCCAGCCTTGAACTTCTTGCCCTCAGGCCAACTAGATACGTTATGATCTGGACTCCATCCTTTTTCATCCCCCACTACATATTCTTTTCCATAGATTTCTTCACATTGGAACAGCAAGCATACAAAAAGACTAATTCCAATCACCAAAAGTTTGCTCATTCCAATAGACATCTTT

>Chr7-01; OZ022212.1; 479237-479901; dir; B;

GTTCTCATAATCCTTAATGTTCTTACTTTACAAGGATATGGATCCACATTACAAAAAATTGGTAAGTGTGGACCACGAGATGTAAATTAGGTCCTTATTTTAGTTATAATGAGGGTTTACAAGATTAATGACTGATTTTACAATTCTGGGATACGAGGGTATCATAACATTGTAAAGAGGATCCATAAACATGTAGGTTATACGATTTGGGTAGTGATTTACAGGTTTTTGCACCACATTACAACATTAGGGTTCTCATGATCCTTAATGTTGTAAATCCTTATTTTACAAAGATATGGATTCACATTACAAAGAATTGGTAATAAGAACTATGAGGATGTAAATTGGGTCATTATTATAGTAAAATGAGGGTTTACAAAATTAAGGGCAGATTTTACAATTTTGGGATACGAGGGTACCATAACATTGTAAAGAGAATCCATAAACATGTAAGTTACATGATTTGGGTAGTGATTTACAGGTTTTTGCACGACCATTACAACATTAGGGGTGTCATAATCCTTAATGTTGTAAATCCTAATTTTATATGGATCCACATTACATAGAATTGATATTAGGTCCTGATTCCAGTAAAATGAGGGTTTACAAGATTAAGAGCTGATTTTATAATTTTGGGATACGAGGGTATCATAACATTGTAAAAAGGATCTATAAACATGCAGGTTACATAATTTGAGTAGTAATTTACAAGTTTTTGCACAACGATTACAACATTAGGGTTCCCATGATCTTTAATGTTGCAAATCCTTATTTTACAAGGATATAGATCCACATTACAAAGAATTATAAGTCTGAACCCCGAGGATGTAAAATTGGGTCTTTATTCTAGTAAAATGAGGGTTTACAAGATTAAGAGCCCTTTTTGCAATTTTGAGACTCAAGGGTACCAAAACATTGTAAAAAAGATCCATATCCCCCAATATTCAATTTGGATGTAAGGACAATTGCTGCAACACAAAATCAATAAATGTCCTTACAA

AACAAGAAGAATTATTAATTGACCTAATTAGTAAAGTGGAAAACCCATAATTAAATTCAGAATACCTACAAAAGCTAAAGAAAATAATTTGCCACAATGAAATCGGTCAAGTCATCTCAGCCCTAAAGATAAGTCTGACTACTACTTTAGAAAAGTTTGATCAAAAGAAAGAAATTATCTTGCAAGACCTCCAACTTGAAGTTAAAAACATAAAGAAGGAAATTATCACCTTACCGCAAACAAGTCATGAACTACGAAATGAGAACTTTGAAATAAAACAAATTCTTGTGGAAAAATTAAGATCCAGTGGTCCAGAACAACCCCAAGAAGAAGATGAAAAGGAATCCTCTAGTAGTGACAAAGCAATTAATCTTATTAAACATTTGAATTTTAGAAAATGGTATTCAAAAGTCAGTATAATTATTAACAACTTCAAGCTAAAAACAGTAGCCCTTTTCGACTCAGGTGCAGACCTTAATTATATTCAAGAAGGACTAATCCCCACTAAATATTATAAAAAGTCTAAAGAATCCTTAGGCACTACTTCTGGTAAACCTTTACAACTGAAATATGAAATCCCTAAAGCCCATTTATGCCGAAGTAATATTTGTTTTAAAACATCTTTCGTTTTAGTAAAAGATATCACAGATGAAGTCATTCTAGGT

CTGCTTTTTATTGCTCTACTCTAGATGGAGGTGGGCATGTACATATAAGGTACATCACCCCCTCTCCGTTGCTCGATGTGGGATGTTACATGTGGGCATAGTGGGATTACCATATAAGCCCAAGGAGCCCAATAGAGAGCTCATAACCAGTTTTACCTAGAGGGAGACTTAGGTCTCTTTCTTGTGGCTCTATACATATAAACTTTGTTTTTTTTAGAAAAGTTAGGGTTTTTGAGCTAGTTTCAAGAGACGAGTTTGAACACTATCAAGCTCCTCCCACCTAGCCAAAAATCGGCCACCCTAAGAAGTGAGCACTAGCATATTTTGTTTCTAAGTTTGAATCATATCCTCCATCTTCCTTGGTGTAGAAAAGTTAGAGATCACCTCCATTGGTGATTTGATGAATCTAGAAGGCCTTCCATAAGGTGATCAACTCAAAGGATGTCAACCATGAATAGTGCTTCAAGGGTAATTTTTCTACTATCTCTTTCTCTTAGTCTAAAATGTCTGGGAGTGGAACAATCAAAACTAAGTCTCAAGATACTTGGGGTTAGTTATGTTAAAGCTAAAAGATTAAATTTTAAAACTTCCATTGCATAAGGTTAAACTTCTGTTTGTGGCTAAATATAACTAGGTTATCATTTTAGCCATACTGAGAGTTAACGATTGGTAGGTGGTTTAAGTCAATCGGTTCGGTCACTGATTTAAACTCAACCCTGGTCCCTTGAGTTTGGACCTTTTGTCCACCTGACCTCGAAAAATTTAAAGGTTGGCCCTTTCGACCACCTGAACTAAACAAATTTACAAGGTTAGACTTTTCGTACACATGAGCTCAACAAATTTGCAAGGTTGGACCTTTCGTCTACCTGGCCAACTATTAGAGAAACATATAAGTCCCTACTCGGGGGTTGTTAAAGAATCAGATAAAAATTAACCTCGAATAGTCACATAATCGACTCAATTGTATAATTTTTTTGGTAGAAACATTTGTAGAATCAAG

>Chr7-02; OZ022212.1; 875154-875593; dir; B;

CTAGTTTGTGTAACTTGCCAAATGATTGGCTCCTTGAGGTGATTTCCAGACCCCTTACATAACGCTTTAAAATCTTGGGCACTAGCGCAGAGGGGCAAACCTAGCACTTCCATGGCTTTTTTCAGACGAGGGGAGGTGTAGGTGCACCTCCTTGTGCCTCTATAAGTATGCATTGAATATATATATATATATAGCCGCCTGACTATACTATTTTTAAGAAACATTAATAGAACCCGATATAGCCGTGGGGCTGTACTATTTTTTAAAAAAAATCAGAAAAAACTAAGTATAGCCGCGCGGCTATTCTATATATATATTTTTTAAAAGACAACCTGCCTAGCGAATAGGCAGCCACGTGTCAAAATAAGTATAGCCGCGCGGCTTTTTAAAAAGCTTAAATTTTACCTAGCCTTTAAGACGTGTATAGAACACGGATGTATTATTAAAAAATAAGTACATACATGTATAATAGATTATTGAATGTGAAAGTGTTAAATTCTTTATACAAGTAATGACTCTAGAAAAATGAAAAATAAAAAGGGGATAATAGAGACTGGGGCACTAACAAAATAGTAATGGATAATGCTCTTAACTACTCTTATCAATAAACGAAAATCAGGGAAAAGATTTAAAAGAAAAAAAGGATCCTAAATATATATATATATATATACATATATATATACACATCGCTCGGCTATACCATTTAAATTAAAAAAAAATTGAATCTAGCCGCATAGCTATACTATTTTTAAGAAACATTAATAAAACCGGACTATATTTGAATAGCTACAGTTGGACAAGAGCGGAGGTGCCTGAGGAGTACATAGGTCATGTTGAGTCAGGCTGTTATTTGGAGATATAAATTCTGCACTTAGTGTTTAGTAAGACAACTTTTTTTAAAAATTTATAAATCGATAATGAGAGGAAGGAATCTAAAACAATAGATCGGTAGATGAATAAATGCGCTTAACCATTTGACATTATCACACGAAAAAAGCAT

TAGGATTTAATATTGGTCAGTCTCAGATAAGCCCAATTGATCGAGTTATCCAATTTGCTGATAAATTCCCAGATCAAATCCTTAATAAAAATCAATTACAAAGGTTTTTAGGATCATTGAATTATATTTCTGATTTCTATCAAAATTTAAGAAAATTGTGCAAGCCCCTGTTTGATCGTCTCCAGAGCAATCCTCTTGCTTGGACTCCGAGTCATACAGAAATAGTTAGACAAATCAAAGTTCATGTCAAGTCCCTTCCATGCCTTGGTATTCCTTCAGTTAATTCTTTCAAGTTAATTACAGTACTATTAAAAAGGAAATTTTATCTATAGTGTTATGCATTAGTAAATTTCAAGATGATTTATTAAATCAAAATTTTTTAATCCGAGTTGATTGCAAGTCAGCAAAACATATTTTGGAAAAAGATGTTAAAAACATAG

GTAGTAGGGGTCAAAGAAGCCACCCTTGAGGTTTGCCTAGGTTTAACGTTCGAATTATAATATATCAAGCATAAATTTCTTTTTCATGATAACAAAAATAAAAATTGAGAGAAGTATTTTACCATCATATAAAACAAGAAGGAGCACAATGAGAAAAGCTTACTTGAACGAACCTTTCTCCATAGAGATATTTGTTGAAATGAACTTATATCTTAGTTTTAATTTGTCACATGAAAAGAAATTTAAGAGAAATGTTACCTGAACTTTTCAAAATCTATAATTTGTATCCAATTTTCCATCCATAATTAACGATATTTTAGTCTTAAAAAATCATTATACAAGCCTTTCCTATCAAACATTAAAAAAATTTAAAAATGCATTTTTTTTCCATCCCCCTGTCGTCTCTCTCTATTCTCCCTCCCTCCCCAACCACACCTCCCCTCTCCTCTGTTTTTTTTTTCTCCCCCCTAGACCTGAACATGGAAGGGATGGGCTGCCATATCTGTTTGTGCAAATAATCTCACGGGAGAATATTCGCTTCTGGATCCTTCAACCTTCGATCTTCCTTATCTCTCTTCCTCGATTCCTGTAAAAAAGATTGGAGTAAATAGACCACACCCGGGGGGTGTTGGCCAAAGGCCCTCCGATGCCTAAGTTAGTTCGAGTGTTTGTAGGAAAACAATAGCTAAGCAAAGGGTACGGAGTTGTATGTAGGGTGTAAACCGGGTGGCCGGAGCCGTGTGTGGTGGCCGGAGCCGTGTGGAGAAAATATGGAGAGTGGAGAGGGAGAGAGAGCTTCGGGTATTTTTCTCGGGTATAAGGAGTAGGTTTAGATGTACCTTGAATGATGAATGAGGTTGTCTATTTATAGGAGCCTCGGGGCTAGGGTTTCGTAGGAATCGAGTTGGGCCTGATAATATCTGAATTAAATATATATTATCTCTTAAGAAGATAATATCTGAATTAAATGATATTATCTTCTCTTTATTAGGATAATATC

>Chr7-03; OZ022212.1; 1044965-1055684; rev; D;

GTCTCCTCTATAATTGGAACAATTCCCAAATTACAATACCTGCAAACAAGTACATAAATAACCCCAATTTTCAGCATGCACAAGATAATGGCACACACAACAACTAAAATTAAGGAAATGGGCCTCATGCCCTCTACCCAAACTTATATGAGCAACCAGAACAACTAAAGATTTGATTTATAACCCGGGAAGAAAGAAAGTAGGAAAGATAAAGAAGAAGTTCCTACAGAGCAAGCAGCCACGATGCCTCGACGTAGTGGTCTCAACCTTGGATATTTAGAACCTGGGGGCCGCAAAACATAAAATGTGAGTAGATGAAAACAAAGTGTTTAACATAATTTATATGCAACATAAATAACCCCTCTGTTTTAGAAAATAATGCCAACCAACAAAATCCCTTGTATGTGTACATATATACATACTTGAAAATAGTCCAATTTAGTATACTTATAACAAAATATATGATAAAGTAATCCATTTAATTAATATTTCTCACTTTTTTTGGAAAACACGAACATAATCATTTAGTCTTTATTGTAATACCATATAATTGAAACTTCCAAAATAATCCCGTTAATGTAAAATATAAACATCAAAACCCAAAATATCCTTTTTCTTCTGCAACCATACCCTTTCTTAGGCATATGCTTGCAAACATAAAATGTCAAATAATTGTTTGCAAATAGCCAATCTGAAATCCAAACTTCATTTCAAAAATCCATACCTGTGTAGAGCAGTCTCAATGCACATGCTCAGAAAACTCAACCTGGAAACAAGTCCACAAATGTACTCACACAAATACCCATATGCATATTTATATAACAAAATCGACTTTAAAACCAAGATATCGCATAATCCCAGTATAGCATATAACACAAACTCAAACAGGACCTTTTTCCCTTCCCAGACCACTCTCTTCCAAAATGCATGCATGGCGATGAAGGATCTTCACCAGTAAATAAGAATAAAAGTGCATATATATATATATATAAATGATCAA

GGTTGATTTTCTGTTGTTAATGTCCAATCTAGGGGTAAGGTTACTTCTGACCACTTAATTTTATGGGGAACTCTTATATTAGCATAAGCTTTACTACTTTGAATAAGCAAGGTTTGGTCTTTTGGGCATTTATTAAGAGCTTGGAAACTCATATTAGTTCCTGTGACTTTATAATAAATTATATGGACTAAAGCTAGAGGTTGGGTACCTAGCAAGACTTTATACCCTAAAATTTTTAATGTTTAATGTCAGATTTTTTAATATGTGGGGATCGCTTAGGCTTATCGTGAAGTCTGGGTAACAATCAAAGTGAATTGGACCATTAAACAAGCTTGACTCAATTATTCCTAAGGTACTATCACTAAAGTTGGTAAATCTAGCATCACGTAGGCAAAGTAAAATAGAGGCATTTAGTCCTAACCTTGTAACGGGTTTGACTGCTACTTGAACGAGGCCAATGTGAAGGAAATTGTTTCCATCACTCTTATGTTTCTTTATTGACTCAAGGTTTAAAAGTTGACATGTTCCGTGTTCTTTACTCAAGGCATAGACTTGTTCGACTGTCATGGGTTGTACTTTTGAAACTTGAAAGTGACCATCTCTTCTTGTAAATTTCTGTACTAGGGATCTTAGGGATGTTCCAATCTTTATAATCAATTTCATTACTAGTTTCGTATTCTATTTGTTCTTCATTGACTATTTCTGGGAGTTTACTAATACTGGCTCTAGAATTGCTACTATTACAAGAGTTTGTTCTAAAAAAGTGACTCATCTGGACAAAGATTACTGGTTTTCTACAAAGGGTAAAAAGGGAAGCTAATAATCCTATAACAGATTCAAGGATTCCTGAAGATTGAAGGGTTATCTCTCTCTCCGTTCATGTCGCATCCGCTCATGCCTCTCCCTGGGACTTATTGTTTGGAATCTAGGACTTATTGTTTGGGTTGAGATAAAATGAACAGTATACAGGAAGACACTAAAAGGGTTACAAAATTGAATAGCAAAGCTTTAAACACAGAGTAAGATGACCAATGACTTTGTACACAGAGAAGCAGAATTAGAATGACTCTTCAACACTTACGGCTCTGATACCAGAAGAGATCTAGAGGCAGAGAGAATAATAATGCATAATGAAATAATTTAAAATAAATACGAAATTAAAAGTAATGTGTAAATGAAAATACTTATAGAAAATCTTGATTGAAAGATTTTATTGATTGATAGTAGCACTCATATTTTACAAATAGAGTTTTCTTTGACAGTGATTCAGAGAAAACTTTGAAACTGAGAAAACTAAAAATTACATTCTGACTACAACTGAGGACAGAGCCTCTGTATAAAGAGCCAAAAAAGTGTTGGCAACATTGGATGCTTGGAATTTTTACTGTTGGAGCACAGGTCTTCTGCTTTTGCTTTAACTGGAGCTTTAGGACTTGACTTCACCTTTTCCTTTCTTCTTTTTCAGGGAATATTAACTGTTGAGGCATGGGGCTTGGAATCATGCTAAGTTTTGCTTTCGCTTTCTTTCACATATGCTTTCCTACTTTTACTAGTCTTCATTGAGATCATAAGTATCTTGACTGTCTGGGTAATCTGCCCATCTGAACTTTTGTGTGGAATCTTTGTGACTTGATGGATTTTGAGTCTAACTAGAGGATGAGGCTTGAGTGGAGTGAGACTTTGGGGAAGCAACATCATTATCATCATGCATTTGTGAGGCTTGGATCATTAATTGCCTGGCTAATTCCTTTAATTCTGATTTGGATCTTCCTTCGATTGGGAGGGAAGAGTGAGATGTACCAAAGGAACTAATTCCTTAATTGTATGGGGAACTCTATAGAATTTATTATAAAGTCACAGGAACTAATATGAATTTCCAAACTCTTAATAAAATCCCAAAAGACTACACCTTGCTTATTCAAAGTAGTAAAGCTGATGCTAATATTAGAGTTCCTCATACAATTAAGTGGTTAGAAGTAATCTTACCCCTAGATTGGACATTAATAATAGAAAATCAACCCGCTCCTATTCAACATAATTTGAATAATCTAGATTATATCCAACAATATTTAGATGGTATAGTAAAAATAAATTTTGGAAACCAGCCTCACAAGTCCTTAGTCCAAATACAACCACTCCCAACACCTAGTCAATCCTTGAGACATATGCTTGCTAGACATTCCTTTGCAGATTCCTCCTCTACTCTAGAAAGAGACCTAGAGTTTGAAAAAAACGTTAATTGATTTAAAGTTAGAATCTTTAAGAAAAACTTTCCAAGTCAACCATCCTTATTATGGTAAGAAACCAGTGAAAGAAGATGAAAAATCAGATTCCCCCACACAAACTGATTTTGAAGCCGAAGCCATTCCCACAGACAATCAGTTACGAACCCTCAACAAAGAATTCAAAATTCATTGGAAAATTTTAGATAGTCATTTAAATGCCTCAGAGAATCTGGAACGAAGAAATAACTACCACACAGCCTACCCAAATAAAGTCCAATGCTAGCAAATCTTTAATGAATGGAAAAATTTTATGAAAAATACCAATTCATAAGTTTTTTTTTATCTTGATTATGTTGAAAGTCACTACATGACAAAAGAGTTAAAAACTTTAACCCAAGAAAAATGGAAAAGAGAAGATAACACCGAAGTTCTAGCCAGTCATCCTCTTACTGAAACTATAGTTATCACCCATAAAAATACTAAACTCCAGGCAAGTCCTTTCAAAATTCCATAACAAAATGACAAGAAAATAATTTAGCAAAATAATTGTACAAACCAAAATCTAATATTCATTGGAAAACAATTAGATAAAATAGAAACAAAAATTGACAAAATAGTCCCTCAACAAATATCCCTTTAGTCTCAAAAAGAAAAACCTTTAGTAAAATTCCAGGACCTAAAGTCAAGCCTCACTCTCAAGTCGAGTCCCAGAATGGAAAAGATTGAAGAAATGCTTAAACAATTAACCCCAATTAAAGAAAAACCTGAAGTTTTTGGTCTCAAAGTCCTTGAGTCCTCCATTTTAGTCAATACCTCTGAAACCGAGTCTATAACCAGTAATGAAACATATTCTAGTGATATATCAAAAATTGAGAAAGCCTTTAATAGTTTGGAAATATATAATGATTTCAAGCTTAATAGATTAAGGAACAAAGTCAATCCTACTAGTCTAACAAAAAATTGGCATCCCAAACCAACCCCTCCAGACATTCAGTTTGAAGAAAGAAATTATCAAAGTCAATTCTCAGTCTCATCCGATAAACTATACGATGGAATATTGATGGCCTTTCCGAACAAGAAATCCTAAATAAACTTCACATATGTCTATGGTAGCTAATAGTTATATAACCAATCACAATTTAAGACAGTCAGAAATTGTTCCGTTTTTAGAAACAGGATTCACCGGAACCTTACAGTCTTGGTGGGATAAACATTTAACTGAAGAGTGAAAACGACAAATCACATATGCAGTCAAATTGAGTGAAGAAGATCTCCCTATTTTTTATGCACGCTTAGGAACATGCATAGAAGACAGAGTCAATACTTTATTATATACCATAATTGAACATTTCATAGGAACACCACACTACAGTCAAAATTCACGATCAACTTAGCAATCTCATATGTCCCAAATTAAGTGATTTTAGATGGTATAAAGATGTCTTTATTTCTAGAGTCGACATCATGTTAAGAGATGATAGTAACCAACTGTTTTGGAAAAAAAAAAAATTTAGTAACGGCCTTCCTAATTTGTTTGCTCACTAAATACGAACAGTCCTAAGTTATGATTAGAGTCACATTGATTTCGATAATTTAACTTATGGAAATATTATTAGTACAATTAACCAAGTAGGAATGAAAAAGTGTGTTGACATGAAAATAAGTAAACAAATTCAGTCATAAAGAAATTCAGCCAAATATGAACTAGGAGACTTTTGTGAACAATATGGTCTAGCATCTATTCCCCCATCTAGAAAGGTCAAGACTAGGTACAATGACCAACAAACTAGTAAACATAGTCATTCCTCACGTAGGAAACAATCCTTTAGACATAATGATGAATTTTATGGTAAAAGAAAATTTAACCCACGAAAAAACTGGTCAAAAACCCCCAGAAAAGACAAGAAAAGTAACTTTAAGAAAAATACAGATAAAAAGAAAGTGGAATGCTGGAAGTGTAAAAAACATGGTCATTATGCTAATGAATGTAAAGTAAAAGACACAATAAAGCAATTAAAAATCACCGATGAAGAAAAAGCCCAATTAATTAAGGTATTAGAATTAAGAAACTCTGAGTCTAGTGAAGTTGAAACCACAGTAACGAGCTCAGTAGATTATAGTTCCTCAAATTCCCAATTAAGTTCCCCAAAAGTCCAATTTGGTTGTAAAGATAAGTGTTGTAATGTCCTCACTAAGTCAATACAAGTCCTCACAAAACAAGGAGAGCAAGAAGAATTATTGATTGATTTAATCAGTAAAGTAGAAAATTTAGAATTAAAATCTGAATATTTAAAGAAATTAAGAAAAGTCATTGGTCAAGAAGAGACGAGTCAACACATTTCCCCAAAAATAAGTCTAAATACTACCTTGGAAAAATTCAACAAAAGAAAAGAAGTAACTTTATAGGATTTACAGCTGGAAGTAAAAATGGTAAAAAAAGAGATAATAGAGTTAAAACAAAGGAGTCATGAGTTACAAAATGAAAATTTCGAAATTAAACAAGATTTGATTAATTTAATGGAAAAAAAGGAATTCTGATACTAAGAGTCAATCTGAAAGTCCAACAAATTCATATGATGAACCTTCAAACAATGATAAAGCCATTAATTTAATATAACAAATAAATTTTAGAAAATGGTACTCTAAAGTAACAATTTTTATATAAAAAAATTCGAGTTAAACACAGTAACTCTTTTTTATTCAGGAGCAGACCTTAATTGTATACGAGAAGGTTTAATCCCCACCAAATATTACAAAAAGTCAAGAGAAACATTAGGCACTACCTCAAGTAAATCATTTCAGTTAGATTATGAAATCCCAAAAGCCTATGTCTGTCAAAATAAGATATGTTTTAAAACTTCTTTCGTTTTAGTAAAAAATATCATAGATGAAGTCATCCTCTATCTACCTTTCATAGCCCTCTTATACCCTTTTCAAGTTGAATACAATGGAGTCACATCTACCCACATGGGAGAAAAAGTCAATTTTGAATTTTTAACCAAACCAGAATTACATAATTTAAAAGCTTTATAGAAAAATGCAATTTCAAAAACAGTCAAGATAATCCAGAATAAAAGTAAACAACTAGATTTTCTTAAAGAAGAAGTTAAATTCAGAAGAATTGAACAGCAGCTTACTGATAAGTCCTTGCAGTCACAAATTCAAAATTTTGAAGATAAACTAAAACAAGAAATTTGTTCCGACCTACCTAATGCGTTTTGGCATAGAAAACAACATGTGGAAAAAAAAAACATTCCTACTAAAGCAAGACCAATCCAAATGAGTCAAGAAGTCATGGAATTTTGCAAAAAGGAAATTGCAGAGCTACTTCACAAAGGAATAATCCATAAAAGCAAGTCACCTTGGTCCTGCCCTGACCGTGTCCGACACCCGGCGGATGCCGGGCTCAAATGACCGAAATGCCATTCTAACTAAAAACCAAATTCTTTTCAAGTTTGACTTCTATCGAAAATTCGGCAAAGTCTCCCTTGTAACTTGCTCGTTTCCCAAAAGTTTCCACCTATCCACAAACATTTATATATCCCAATCGTTCAACATGCATTAAATTATATCCAGGCAATCAAGCATCATATCATTCTGGCCTTAGGCCTCAACAAATCAAATAAATCATTATGCTAATAAAATTCATCTCAACTTTCAGAACATCAATAGTACACTAGCAATCTCAAACACTCCAACTCGTGGCTGCACCTTGTAACATTAGGCTGCCTACGTACCCTTATTGAGGGATCAAGCCACACGTAGTTCTTTTCCTTTAATAATGTACTTGAATATCTTATTCCTTCATCATATTGCCATGTGTTTTGTAAATCAAACTGAATTCTCATGTGCGTGAAGTACTCAATCATATTCACAACTCTAAAACAATACTTTGCAACTTCCCAATACTCGTAACAATCATATACTGATTATAGATAAGCCATACACTGAACTCCCTCTATTTTAGTTTAAGAGCATCTCAGAACTGAAATTCTTTCCATCCAGGCGTACCCCAATTCCTGATCCGTCAATTCCATTAATACCGCCAATTCCATCCTCATAGGCCTCAGAGACACCAAGTCTACTCGAGCCGTAATCCTCACAGGCCTCAAAGATACCACTTCTATCAGAGTCGCAATCCTCGCAGGCCTTGAAGATACCACTTCTATCCGAGCCGCAATCCTCGCAGGCCTCAGGGGACCTTTGGTCAGCCTAAGCCGCATTCCTTGCTCGGCATGATTCAGGAGTTCCCAAAACACGTGGGGCATTATTTATAAATGACATTGTTTCAAAGAAATTAGGGGGTACCCTTGTGGTGGGTTTGAAAAACCATATCCCAAACTTATATCAATACTTTCCTTTTATTTTACAATCCATTTCCCGACCTTTATATCATCTTTCTAATAAATACCAAGGGCCATGATTCATCATTTCCAACTGGGTAAATTAGCAATCTATATATAACTATCTATACTCAATTCGTAACATGACCCAGATAACACAGTCTCCGTCAGGACTCGTTTACATGGTCATTTTAACACTTCTCAAAGGATATAACTGCGTTGGAAGACTCACGGTCAACCAAGAGGGCAAACCGCCTAAACTTGGCCAGCGAGGCCCACAACCTCCGATCACAATTCCAGGAGTCCCTATAGGTTCAACGCATTCTTCTGAACCTACCCTGCAAGTTTGGTCCAAATCGAACTGTCGGATTGATCACAATCGCACGATCGGACAACTACAATTTATCTTGCCCTAGGTTTCTCGATTTTGAGGTATCCGGGACTTTGATTCACGATCCGTCGAATCCTATACGGAGGTACAACATATATGAATTTGAGTTCAATCCAAACCTATCGAACCCAAAAATCACACAATATACGAAATTCGTTTGAAGCTCAAACGGTATCCAAATTGGAATCCGAATATACCTACACACTCGTGACAACTAGAGGTCGCATCGGGACAATGCCCCTTTTCTACAGTGCCCAAAGCGATAACGTTCACTGGAAAATTCTCAAAATTAAAAGCCAAGCTTCCTACCCTAGGTTCAGGACATCAATTGGAGCCACTTTTTGCTCTTGGACCTACCCCGAAAAATGGCTGGAAGTGGCCAGAATTTCAACCTGAAATCCGGCCAAACTCTGAGTTACAAATCGAACCACCTAGGCTAGAAATCGATCAAATCTTTCCCAACCATTAGCTAGAGCACGAAAAATAGGTGTGAAACCATCCTTACACGTCAAGTTTGGTGGCTGGGGGAGAGAGAACAAGGCTGGCGAAGTTCTATCAAAAATCGACTGTTCTCAAGTGGTTTCCGGCGGCTGCCGAGGTCTGGGCTACGGCGGAATCGGTTGCTGAGGTGATGGCGACTGTGCGGAACAAAAGGGGAGGGGAACGACGACTTGGAGAAAGAGAGAGAGAGAGAGAGAGAGAGAGAGAGAGAGAGAGAGAGAGAGAGAGAGAAATCTGGATTTTAAAAATCCAATTTCATAAAATTACCATTTTACCCCTGCATAGGTTTTAATCATGTATTTCTCAATACAACTCGGATTTGAGCCCACTACGTGTCTACGGACTCATCTCAACATGCTCTAGGCAACGGTATAACTGAAATTTCCGAATTCCTTCCCGGTCAAAAACTCAATTTTTAACCTAGTAAAATATTCTTGGGGCAGAATGGTCTTTTAAAAGAATAAATTAATAATTACGACTTGATTTAGGATAGGGTCGTTACATGCCCAGCTTTTTATGTTCAGAAAAACGCTGAGTTGGAAAGAGGAGCCCCAAGACTAGTCATTAATTATAAACCCCTTAATACAATATTAGAATGGATTAAATATCCAATCCCCAATACGAGAGATTTAATCAATAGATTAGACAAAGTAGTCATTTTCTTTAAGTTTTATATGAAGAGTGGATTTTGGCAAATTCAAATCCAAGAGTCAGATAAATATAAAACCGCTTTTGTAACCTCATTCGATCATTATGAGTGGAATGTCATGCCTTTTGGTCTAAAAAAATGCGCCCAATGAATTCCATAATATAATGAATGACATATTCAACAATTTCAGTGATTTCTCCATAGTTTATATTGATGATGTCCTTATTTTTTCCCAGTCATTAGAGCAACATTGGAAAAATCTGTATAAATTCCTTCACATAGTCAAGAAAAATGGTTTAGTTGTCTCAGCTAAAAAGATCAAGCTATTCCAAACCAACATTAGATTTCTTGGTTTTAATATTTGTCAGTCACAAATCAATCCAATTAATCGAGTCATTCAGTTTGCTGATAAATTTTCAAATCAAATTCTTGACAAAAGTCAACTCCAATGGTTTATGGGATCTCTTAATTATATCTATGATTTCTATCAAAATTTGCGAAAGCAATGTAAACCCCTATTTGACAGACTCCAAAATAATCCTCCTCCTTGGACCTCTATTCATACAGAAATTGTCAAACATATCAAAGTCCATGTCAAAACACTACCTTGTCTTGGTATTCCTTTAGTCAATTCCTTCAAAAAATTTGAAACTGATGCCTTAGACAACGGTTATGGAGGAATCCTAAAACAAAGAGTCAATCCCCACCAAACAGAGCAAATCATTCGATTCCATTCTAGAATTTGGACAGAGTCACAGCATAATTGCAGTACTATTAAAAAAGAAATTTTATCTATAGTATTATGCATCTCAAAATTTCAAGATGATTTATTAAATCAAAAAAATTTAATCAGAGTCAATTGTAAAAGTGCAAAACATGTTTTAGAAAAAGACGTTCAAAATATTGCATCAAAATAGATTTTTGCACGTTGGCAAGCTCTTTTAAGTATTTTTGATTTTGATATTGAATATTTGAGAGGTAGTGAGAATTCTATCACAGATTTCCTTGCTAGAGAATTTCTTCAAGATAAGTGATGACAGAAAAACAAACAAAACAGCAAGGCAAGCAACTAGTCAGACAATTCCCAACGTAAATGACTCCAGTAAAAATAGAGCAACCTTCCCAAGTCATCCCTTACCCAAGATTTTCTCCCATTCAAGTATCAAATAGGTATGCCACTTTAGGATCCACAGTCAGTAACATATGACCTAATTACCAGTTAGCTCTCATCTCTAGTTATGATCCATTCTAAGTCTTAACCCTCTTTCCTGCTTCCTCCCCAATTAGTCATACCAAATCTTCTCCTTATTTCCCCAAAAGCAGTCATACCCTCTTCTTTATTGAACCATGCTTTAACCATTTGACCAATCCAGTCAAAATAGCCAAAGCCTATTTCCCTCCCAATTTCCATTACATACCTCCACATCCTTCTAAGTCACTCAAATACTACCGTGATATTCTTCTTGAAACCTAGACTTTAGAAATCAGGCCAATCAAAGATAGAAATTATCCAAATATCATTCTCTATCATTCCATGCATATCAAAAACATTCTCAGTCAATCTGAATAGGGCGATCACCCCTACTCTTTCAAGACACTTAATTCCAGTGTCCAATATAATTACTATGATACATTGAAGCTTGGCACATCATTTTCCTCCATCAAACCAAAGATTTTAGTCATTCATGGTTTGTCAATTTCAATAGTAAATTCAAAAGCGACATGGTCCAACTATTGATATTCTTTCCCTAAATCCCAAAGAATCAGTCAAGTACTTTTCTAGTAAGTATAAATTTATGCAAGAAAGTCAATTTTTTCCGAAGTCCTTAATTTTCATTGCCAAATACAAAGTTCCCTGGATTATAAAATGGTCCTATCAAGTCACTGGGAATCACGTATTCTCTCCAGGCAATTCTCCATCAAATGGTGGGATAAATTCAAGACGGAAAGAATTATTGAACAAGTCAAAAATGAGTTTCCCCCAGTCAAAGCTCCCCAGAAGCCTTCCACGTCTGGTACATCTCACTCTTCCTTTCCAATCGAAAGAAGATCCAAATCAAAATTAAAGGAACTAGCTAGACAATTAATGATCCAAGCCTCACAAAGGAATGATGATGATGATGATGATGATGCTTCCCCAAAGTCTCACTCCACTCAAGCCTCATCCTGCAGTCATACTCAAAATCCATCAAGTCAAAAAGATTCCACACAAAAGTTCATATGGCAAATTACCCAGACAGTCAAGTCCTAAAGCTCTAGTTAAAGCAAAAGCAGAAGACCCATGCTCCAACAGTAAAAATTTCAAGCATCCAATGTTGCTGACACTTTTTTGGCTCTTTATATAGAGGCTCTGGCCTCAGTTGTACTGAAAATGTAATTTTTAGTTTTTAAGTTTCTAAGTTTTCTCTGAATCACCTTCAAGGAAAACTTTATCTGTAAAATGTGAGCGCTACTTTCTATTAATAAAATCTTTCAGTCAAGATTTTCTGTAAGTTTGCAATTTTCTTATTTTCATTTAAGTTTCATTAATTTTATGCACTGTTATTTCCTTATTATTTATTTCATTCCCATTTATTATGATTATGTCATCACTGGTTCTACACCCTTCTGG

TATATATATAAGTATACATATAGGTATGACCACTATCAAACTTGTATCGGGGAAACAATCCAACCGGAGGATTGTTTGACTAAACCACATGGAAGAATCCTCAATAACCATGTGGCTAGGGACGAGCTGTCAAATTGGGGGACTGGGCTCCCAAACACACATAGCAGAATCCTGAATACTCGTGCAGTTGAACTAAGTCCTACGAGAGTAGACTACCTGGCCAAGGGCCTATGAAACACCTCATAAATGGTGTCTGGGTTCCGACATATCCAAGTATCTATATCCAAGTAGTTAAGTGCCAAGGAGGTACATAGGGTGGTGTTATCAACACTCCAATATAACATATCCTACATCCCAACTCATGTACCATAACTCTAACCATAACCAATTCCCAAATAATCCCAATGTACCCAAGCCCATATACATAGACTTCCGGATAATAATAGCGTCAATAACTCAATACCCAATATTTATAGGTAATATAGATATCAAACTCTCAAAAGCATAAATTTCATAACCCAAAATATTCATAAATTCCAAATGTCAAAATAAACTCCAATAATTCCATAAAGCACTATAATCCACAAAATAAACAATATTATAACTTAATAAACTCAATAATCCAAATAATCTAAATTATATAAATATGCAAGACAATCCATATACTTTAATAATGACCATAATAACAATAATAATTCTTGAAAAACCCATGTCACATTCAGATAAAATCATAGTCAAAACATATAAAATCCCAATAAACCAAATAAGCCAAGTTCTAAATCAAATCTCATTTATGCATGCAATTCCATATTGGAAAAATAATGCTCATTTAAAACAATTTTCACTCACAACAAGTCCACGCTGCTCAATCCTGAGAGGGCTTTTCCTTTGGAATACGCGAAGTAAAAGTACCTTGGTTGACGGAAACAGCCGCCGAAATAAATCTGCAAAAAATTCAAATTGTTATAGTCGCTAAACATCGGGTTGTTACAGTCGCTGA

>Chr7-04; OZ022212.1; 1128195-1128582; rev; B;

TCTAGTTAACAAGGCAACAAATAGTACGAGATGTCCCTGCTCTTGTACTATGTCTCTTCTAAATAATTCTTTGATCTCCCATTCCTTCTTATTGCAACATTAGGTACTATATACTAAATCCCCAATGTTTATTTCAAAGTTATTCGGAAGCTCCTAATGAAATGATGCATTTGGTTACACATAGAGTTTTGAAATATGTACATGTATCTGCATCTCATACTCACAACTAGAAATTTGAAAAGGCTCGGTTCTAATTCATTCCATATGTGCACTTTTACTAATTTTTGGATTAGATGGTACCTTTCAGATAATTACGGTATTCTTTATGTGATGGACATGTAATATAGTCAGTGCTGGATTACCTAAATGGGTAGGAAATTTCATATCTTTCTCTCATTATATGTATATTTGTTTATATGGTTAAACTCAGATAACTGTTGGAATTAATGCACTTCCTTAATTCTCTAGAATATCTCTATTTATTACAGTAGCTGTAGCTGTCCTTAATTAATTTGGTAGAGTTCCTATGCAATGTCTTTTAGGTTCTCTAGGAAGAATTCTCTAGAATATTTGTTCTCTTTAAAAAGCCTTTGAGCTTATCATTGTGACAACAACACAAGCTAAGAGAATACAAAAGAACTCTGCTGAAGCAACTCATATTTCCAGCCAGCACCCCATATTCCATCACAAAACCAACAATAACATGGATTTTTGTGTAAAGATGGGTGTTTTCTTTTTTGCTTTGTTATACCAAATTCAATATTATTTGTTAGCATATTTATAGTTTTTTTTTTTTTTGGATGACAGCATATTTATAGTTTTGGAAGCAGGAAAAGGCATTTTCTCATTACTTTGGGTTTCCCGTTTTCAGTTTCCCATGGTTGCTTCTTGAGATATTGTAGTGTTCAACGCTCTCTGAGGTGTAGCTTTGGGTACTCTTAATGATCTTGTGATTTATGAGAGCTACAGTAAGTATGTTTCAAAGTATTTGGGACTTCCT

AGTCACTGAGAGCCAACCAGCTCCAATCCAACGTAGTCTAAGCAATTTAGACTATATTCAACAATATTTAGACGGCACAGTAAAAATTAAATTTGAAAATCAACCTTTGAGAAAATCTAATGTCCAGTTGCAACAGTTGCCGACCCCTGGTCAATCTCAAAGGCATACCCCTGCCAGGCACTCCTTTACTGCTATGATCCTTTTTCAGTAGACACCTCAGCTGGTCCCTCAGTTGCTTTCAAAAAGTCGTCCCCTTATTTGCCCAAAAGTAATTCTCACCTGTTTGTTGTTGAGCCTGTTTATGATATCCATGCAGATCCCATTGAAATAGCCAAACACTATTTCCCTCCTGGATTTCATTACATGCCACCCAGTCCATACAAGTCTC

AAGTATGTTTCAAAGTATTTGGGACTTTTAGCTTTTATATCTTAAACAATATGAAAGATCTATTTGTTTGCTCGAATAGGAACTTGAGATGGTTGGATTGGGTAGTTGTAATGAACAATCTGTGTTTTTGTTATGTAATTCGTCTTCTTAATGTTGTGAAGTTTGTGTGCGTGTGTGCTATTCTGGCTTAAAATCCAAGTTCTACACTAAACATGTTAATGATGCTCTGATAATGGAGAGAACATAGAGAAAGTATGAGACATAGAAATGGGATGGACAAATTAAGGGAGGTAAAAGGATTTTTTTTTTTTTTTTTTTTGGTGTGTCATGAGGCTCATTTGTGAATAGTAATCCTTACACTACTTATGTATTGCATATCAGGTGTCTATTGTACTTATATTGCTGATATATCCCTTGGATATTGCTTTGATGTTGTTCATTTGGAATTTCCCTATTTTCGTAGGTTTAATGTTTTAAATTTTCAATTATTTCAAAAAATTACCTAAATTCCACTTTTGCAAATTTCCCAGCAAGGTATTTTTCCAATTTTCCAATTTTACTGTCTATGAATTGGATCTGATTTGGTGACATGATGAGCTTAAGAAGAAGCAAACGCTAGATTGTGCTTGATGTCCACCACTTACTTTAATTAGACTCATGTACAATGTAGAACACTTTTTGCAAAGTTAGATTGTGCTTGTAGAGGAATTGGATAATGGATTCACCAATTACAAGACTGCTTTGTTCATTCCACTCTGGAATTTCTCTCCAATTCTTATAACCTTCCTTGTTTCTCTTTCTAAAATTATGTTTCATTGCTGCAGGTGGATGTAGGCAGATGCCAATAAACCATATCAGTTATCTCTTTTGCAGGGTTCACTTGCAGGCATCTAAAACTTTTTTGTTTTGCTATCTCTTTTGCAGTGTTCACTACCAGGGTTCATTTTTTTTCATGATAGTTGGACCTAGATTTTTGGCTTCTGGAAGATTGTTGTGATTG

>Chr7-05; OZ022212.1; 4630796-4637911; rev; A;

CATGTATGGTGATACGGACATGGGTATAGCAACCTTTAAAGATGTATGCTAATACCCATGCTTTGAGGGTTTGATTGGTTTGATTGTAGAGTTGACACAGACCTTTAGATCTCCTCATACACAAGAAGTCTTAAACGGCTATTTCTCTTCAAGGACTTTGGTTTTATGGCTGCTATTTCATCTAAATTTTTTTTATTCTTTTTCCTTTTTGGATGGATTTTTTTTATAAGTATTATTTCTTATGTATGTTTGTTTTTAATTATGACATGGGTTCTTTTGTGATTATAAATGGCAGCCATGGCAAAAAAGAAAAAGAGCAATAATAGAGCATCACATGATCATGAAATGCAAGAGAAAAATCAAACATGTATGAGTTATTTGCGTGTTATTATCGATTAAATTCTTGTTTCTATTATTTTTTTAAGTTGTATTTCTAATATATGAGTTTGTAATTTTTCAATTATGTTTTGTGTTTGTATTTGTACAAATGATAGCCGTGGCAAGAAAGGGAAAGAGTCAAGATAGAGCATCAAGCACTGCACATCCCAATCATCTAGACTCTCCTTAGATTGCCATATTTCCAATGAGTCATCACATGATAACGAAATGCAAGAAACATGTATGATTTATTTTGAATTTCCTTTGGTATATATATATATTTCAATTATATGATTAGTTCTCAATTAATTTTCAATGTTTTGCTCTATTATAAATTGTAGTCGGGGCAAGAAAGAGAAAGAATCAGAATAGAGCATCTGACATTACACATCCTCAGTCATCTAGACCTTCCTTAGACCAACATGATGACCCCATTGAGTCACCACATGATAATGCTATGGAAGACATACCTCTTGAGACTGAACCATCACATGATTATTTTATTAATGCTTATTTTCATTGCATCTATTTTTCATATATTCATTAATTTGCTTCTTGCATAAATTACTTATGTTCTCTGTCTTTAATGTTCTTCTTCCTCTCACTGGCCCTAGACCCCTTC

TGGTATCAGAGCCATAAGTGTTGAAGAGTTAGTCTAAGTTTGCAACTCATCCAACCCAAGTGGCTGGTTTAGGTTTTCTGAGTTGTAAGTGTCCAGTTTCTTCTTTTCCTCTTCTGGACTTCCAGTTCACCTGTCCTTCTCTCACTTGGTGTCCGAATTGTAAGACCCGTTCCAAAGAGTAAGTCCCAAGGAGGCCTGAGCGGTAGTGGCATTAGCAGGAGGGCGAGAGAATTTTAGGTGTCCTGTTATCCATGGAGTCTGTATTAGAAGTCTCTGTTCATTTTCTTCTTTAGGAATCCAGACCCAGAACCTTGAGAAATAATGAGTCGTTTTCTTAGAACCAACTCTTACAGTTCTAGCAGCTCTAGAGCCAATAGTAGTCTCCCAGAGATAGTAAATGAAGAACAAATTGAATATGAGAATAATAGTAATTTAGATTATAAAGATTGGAATATACCTCGAGTCCCCAGTCAAGAGATCTATAAGAAAAAATGGCCATTATCCAGTTTTAAGTCAACTACCCATGTTAAGACAGTTGAACAAGTCTATGCCCAAAGTAAAAATCATGAGACATGTCAGTTACTTAATCTTGAGTCAATAAGAAAACATAAAACTGATGGAAATAATTTTCTTCATATTGGTCTAGTACAAGTTGCTGTCAAACCATTAATCAGATTAGGATTGAAAGCATCTATCCTTCTCTGTCTGCGTGATGCTAGATTTAACAATTTTAACGACAGTACGTTAGGAATTATTGAGTCTAATCTTGCTAATGGCCCAATTCGTTTTGATTGTTATCCGGATTTTACAGTAAGCCTGAGCGATCCACATATCTTAAAAACTCTCACTTTGAATATAAAAACTGAAGGTTATGATGTTCTGCCAGGAAGTCAACCCCTAACATTAGTCTATAGAATTTATTCTAAAGTCACAGGTACAAATATGAATTTCCAAGCCTTAAACAAGAGTCCGAAGAGTCAAACATTATTAATCCAAAGTCATACGCTTGACGTTACGGTTAGAGTTCCTCACTCTATCAAATGGTCAAATGTCACTCTTCCTAATGATTGGACCCTAACCACTGAGATTCCTCCAGTTCCTATCCAACGTAGTCTAAGCGATTTAGACTACATACAACAATACTTGACAATACAGTTAAAATCAATTTTGGCCACCAAGCCCCACAAAAATCAACAGTCCAGTTACAATAGTTGCCAACACCTGGTCAATCCCAGAGACATGTTCCTGCCAGACACTCTTTTGCAGCCTCTTCCTCTACCCTAGAACGAGACCTAGAGTTAGAGAAAGCTTTAATTGATCTAAAATTAGAATCACTAAGAAAAACTGCACAAGTCACTTAGCCTGTCTATGGTAAACCAACAGAAGATAATAAATCTGAGTCTTCTGAGTCACCCACTCAATCTGATTTTGTTGAAGCCGGTTTCAAAATAGAAAACCAACTCCAAAGACTTAATAAGAAATTTGAAATTAATTGGAAAAAAATTAGAACGCCACTTACATGCTCACGAAAATCAAGCAAGAAGAGATAATTATCACTCGATAATCCCAGACAGAACCAAAAGACAACAGATATTTATTAAATGGAAAGAGTATATGCGCACTGCCAAAGTTGAAATTTATTATCTTGATTTTGTAGAAAGTCGTCATCTATCTAATGAATTAAAAACTTTAACTCAAGAACATTAGAAAAAAATAGATCACACTAAGATCCTTGCTAGTCACCCTCCAGTTGAATCCATAGTTATTGAGCACCAAAATGCACAAGTTATTGCCAGTCCCTTTAAAATCCCAGACTTTGAAAATAAAATGGTGATTGAACAAAATAATTATACAAATCAAAGCTTGGTTGTCATAGGTAAACAGTTAGACAAGATAGAAACAAGAATTGACAAATTACTTCCAAAAGAAATTTCTATTAAACCGCATGACCAGAAACCATTAGTTACCTTTCAAGACCTGAAATCCAGTCCGACTTTTAAAACAAAAACTACAATGACAAAAATCGAGAAAATGTTAGAAGAATTAACTCCAACCAAACGAAAAGATAAAGAGAAAGTTGAATCAAGCGTTAAAGTTTTAGGAATTTCTGAGCCATCATTCACCTTTCATCACTCCTCTGAGTCTGAATCAATTATTGGCATAGATACTGAGTCTAGTAATATTTCCAAAATTGAAAATGCATTTAAAAATCTTGAGTCAGCCCTACTAGCCTCACAAAAAATTGGTATCCTCAGCCCACGCCACCTAATATCCAGTTCGAAGAACGAAAAACTCAAAGCCAGTTTTCTGTTTTTTCTGATAAACTATATGAATGGAATATTGATGGTCTTTCTGAACAAGAAATCCTTAATAAGCTTCAACATATGTCTATGGTCGCCAATAGTTACATTACCGATCACAGTTTCAGACAGTCTGAAATTGTTCCCTTTGCTAGAAACAGGTTTCACAAGAACTCTTCGTTCTTGGTGGGATAATCACTTAACTCATGAGTCTAAGCAACGAATAATTCATGCGCTAAAACTTAATGAAGAAGGTCTTCCTATTTTTGATGAACAAATAGGTGAAGGCATTGAAGATGGAGTCAACACATTGTTATACACAATAGTCGATCATTTTATTGGAACACCTAGTAATACTACTGCCAGAATACATGATCCATTGAGTAATTTAAGATGCCCCAAATTATCTGATTTTAGATGGTATAAAGATGTGTTTATTTCCAGAGTCATGGTCAGAGAAGATAGTAATCAACCATTCTGGAAAGAAAAATTTGCCCAATTCGTTTGCTCACAAAATCCGATCAACCCTTGGTAATGAACAAGGTCATATTGATTGGGATAGTTTAACTTATGAAAACATAATTAGCACAATTAATCAAGTCGGAATGTGAATGTGTGTTGATATGAAAATTGGTAAACAAATCCAGTCTGAAAGAAATTCTGCTAAATATGAATTAGGTAATTTCTGCGAACAATATGGTTTGATGTCTATTCCCCCGTCTAGAACAAATAAGTTTAGTTATCCTAGCCGACAGCTTAGAAAAAACCGCCATTATTCGCGTAAAAAGCAATCATTCCAACACAATAATGAAGATAAAGAATTTTATAGTAAAAGGAGATTCTCCCCTAAGAAAAATTGGAAAAAAAATTACAATAAAGACAAGAATAAAAGCTTTAAGAAAAAGACCGACAAAAGAAAAGTAAAATGTTGTAAATGTCAAAAGTATGGTCATTATGCTAAAAATTGTAAAGTTAATGACACAATCAAACAATTAAAAATCACTGAAGAAGATAGAAACAAATTAATCCAAGTCCTTCAGTTAAGAAATTCTGAGTCTAGTGAAAACGAAACCATGGTTAGCCATACTGAGTCTTCCGAAATATATTCTTCTAAGTCACAACCCAGCTCCCCCAGAATTCAATTTGGTTGCCAAGATAATTGTTGTAAGTCTAAATCAATCAGTGTCCTCACCAAACAAGAAGAACAAGAAGAATTATTAATCGATCTAATAAGTAAAGTTGAGAATCCAGAACTAAAATCTGAATACAAATTAAGGAAAGTCATCACCCAAGAAGAACTGAAACAGTCTACCCAAAAGATAAATCTGAATGCAACTTTAGAAAAATTTAAGAAAAAGAAAGAAACCACCTTACAGGATTTACAATTAGAAATAAAATCAGTCAAAAAAGAAATCATTGAGTTAAGACAAATAAGTCAAAAATTACAAACTGAAAATTATGAGATAAAACAGGATCTGATTAATTTATTCGAAAAGAAAATTTCTGAATCTAAACCAGAAAGCCCTACCAACTCAGATGAAGAAAATTCTGTTCAAGAACAAACCGTTAATTTAATTCAACATATAAACTTTAGAAAATGGTATGCTCGAGTCACTATATTTGTCCAAAATTTTGAGTTAAACGTGGTTGCCCTTTTTGATCCTGGAGCTGATCTTAATTGTATTCAAGAAGGCCTAATCCCAACTACGTATTACAAAAAGTCAAGAGAATCATTACGCACTGCTTCAGGAAAATCCCTCCAATTAAACTATGAGATCCCTAAAGCCCATGTCTGTCAAAATAAAATCTGCTTCAAAACTTTTTTTGATTTAATCAAAAACATTACTGATGAAGTTATACTAGGTTTACCGTTCATTGCCCTCCTGTACCCTTTCCAGGTTGAACATGATGGTGTCATTTCCACCTGCTTAGGAGAAAAGATTAACTTTGAATTTCTGAGCAAACCCGAGTTACATTATTTAAAAGAATTTCAAAAAAATGCAGTCACCAAAGTAGTCCAGTCATCCAAAACAAAGATAAAGAATTAAGATTTCTCCAGGAAGAAGTTACCACTAAAAGAATTGCGCAACAACTAAGTCGTCAGTTCTTACAATCACAAATACAACAATTTGAAGAAAAACTAAAACAAGAAATCTGTTCTGAGTTACCCGATGCGTTCTGGCATAGGAAACAACATGTTGTCAGCCTTCCCTATGTCAAATCCTTTAGTGAGAAAAATATTCATATTAAAGCAAGACCCGTTCAAATGAGCCGAGAATTAATGGAATTTTGCAAACTTGAAATTGCTGATTTACTTAAAAAAGGATTAATTCGTAAAAGTAAGTCACCCTGGTCTTGTCCTGCTTTCTATGTACAGAAAAATGCTGAATTAGAATGTAGTGTCCCAAGACTAGTCATAAATTACAAACCACTTAATACTGTCTTAGAATGGATTAGATACCCAATCCCTAACAAAAGAGATCTAATAAACATATTAGAAAAATCAGTCATCTTTTCTAAGTTTGATATAAAAAGTGGATTTTGGCAAATTCAAATTTCTGAGTCTGATAAATATAAAACTACTTTTGTCACTCCCTTTGGTCATTATGAGTGGAATGTTATTCCTTTCGGTCTCAAGAATGCTCCTAGTGAATTCCAGAATATAATGAATGAGATCTTTAACCCTTTCAGTCATTTTTCCATAGTTTGATGATGTGCTTATATTCTGAGTCTATAGAACAACACTGGAAACATCTGTATAAGTTTTTACAAGTAGTTAAACGAAATGGTTTAGTTGTCTCTGTTAAGAAAATTAAGTTATTCCAGACTAATGTGAGATTTCTTGGGTTTAATATCTGCCAGTCACAGATTAGTCCCATAGATCGAGTCATCCAGTTTGCAGATAAGTTTCCTGATCAAATCCTAGACAAGAATCAACTCCAGAGATTTCTAGGATCTCTAAATTATGTTTCTGACTTTTATCAAAATCTAAGAAAACAATGTAAGCCTTTATTTGATCGTCTCCAAAATAATCCTTCTCCATGCTCATCTGCTCATACTGAAATTGTCAAACAGATCAAAGCCCATATCAAGACTCTTCCTTGTCTTGGTATTCCTTCTGCTAATTCATTTAAAATTGTTGAAACTGATGCTTCTAATATAGGTTATGGAGGTATCCTCAAACTGAGTCACTTCTGGCCAATCTGAGCAAATCATTCGATTTCATTCCGGCATTTGGTCTCAGTCCCAAAATAATTATAGTACTATTAAGAAAGAAATACTATCTATTGTATTATGCATTAATAAATTTCAGTTGGATTTATTAAATCAAAAATTTTTAGTCCGCGTTGATTGTAAGTCTGCTAAGCATGTATTAGAAAAATATGTTCAAAACATTGCATCCAAACAAATATTTGCACGTTGGCAAGCTATTTTGAGTATTTTTTATTTTGATATTGAATATTTGAGAGGAAGTGAGAATTCTATTCCTGTTTTCCTTACCAGAGAATTTCTGCAGGGAAAACAATGGCCAAAAGAAGAAAAGAAAGAGAAAAAATGACAAACAAAAGAGTCAAATTGGTCAATCAAGCCAACCCACTCAACCCCAGTCACTCCGAGTCCTTCCACCATCAATGAGCCAAGCTAAATATGAGCCTCCTTCCCAGATCATCCCCTTTCCGGGATGCTCTTCTATCCAAATCAGTAACCGATTTGCCAACCTAGGAGCCACAATCGGCCAGGTTCGCCCTAGTTTCCAGTATGCTCTAATTTCCAGTCATGATCCTTTCCAGGATATCGCCGTCACTAACCCCCCAGTTGCTTCTTACCATAAGACATCTCCATACATGGTCAAGAGTCATAGTCACTTGTTTGTCATTGAGTCCAAATATAATTCAATTGCTAATCCCATCACCATTGCTAAATCTTATTTTCCACCCAATAGTCATTTCATTCCACTAGCACCATTCAAGAACCTCAAATATTACAGACATATCCTTCATGAGACCCATTCTATCACCATTAAGCCAATTAAAGACAGAGAAAACCCTCATATCATCCTCTACCATTCACTCTACATTCACCAAATTATCAATGAAAATGAATGGAGTAGTCACCCTTATGATCTCAAAGTTCTCCAATCAGAACTTCAATATAGCTATTGTGATTATATCGAAGCATGGTATTCTATCTTGCGTCACCAAACAGTTGATTTCAGTCACTCCTGGTTCTTAAACTTTGACAGCAAATTCAAGAGTTCTTTTCCTTACTAGTTCAATCACTGGTGGGAAAAACATCGTCCAGTCATTGATTTATTTCCTCCTCCTATGCAAGAATTAGTCAATTATTTTACTAATAAAAATAAATTCAAAGAGTCTGAATTATTCTTCCGCAACTTGCTTCATTTTTTTGCCAAATATAAAGTCCCTTGGATCCTCAAATGGTCCTATCAGGTTAATTGGGAACCAGAGTCCTATCTCACCAGTTTTCAATCAAATGGTGGGATAAATTTAAACTTGAGCGTATTGCAGATTATGTTTATGCTGATTTTCCTCCAGTCAGGAAGCCGCATTCTGAGAAAAAGAGTTCCCCTGCCATATCACATTCTTCTCTCCCAATCGAGGGAAGGTCTAAGTCCGAATTACAAGATCTTGCTCGCCAACTAATGCTTCAAGCCTCACAGATGAATGATGATGCAGACTCTGATGCCTCTCTTGCGTCACAAACATCTACTAGTCAGCCCAAGCCAGCCAGTCAACAAGCTCCAACCCCAAAAATGCGGTGGAGTGATTATCCTGATGAGCAAGACCCCTATGAT

TTTGGCCCTTTCAATCTAGAGGATGATTAAAGCAAAAGTTAAAGTTAAAGTTGCCCTTCATCATGAGCCCTGAGACAAAGCATCCGGACCCATGCATTCAACAGTCTGAAAGCCCATGTTCCTTTTAAGATTTCCCCGACATAGTAAAAGTTGCCGCACTCAGTAAAAGCAAAAGCTTTCGCATGCTTCTACAGTGACTTTTCTTAAGCATCCAAAGTTGCCAGCTCATCAAAAGTTATCTGAGTCTTTTTATTTGCTTTTTTAAGAAGGCTTCGGCCTCTATTGGTATCAGAATGTAATTTTAGTACACTATAATTTTCTCAGTTTCAAAGTTCTATCAGTATCAATGCTCTGAAGAACTAAAGTGGTGAAACTAGATCTTTGTAAGTGTTTTGCATTCTCCAAATTATCAATATATCTGAGGTATCCTTGGGATATATACAAGGAGATTATTGGTGGGCCAAGTCACGGTCGTATCCTTGGCTTAGGTTATGGCTATAGTACAAAAGATGTGTATCCCTCAGATGACCAAGGTTATAATCAACGTATATGCTTAGAATGTGAAGAAGAGCATGAGAAATTAAAGGAGAAAGTGAAGGACTTGAAAAGTGTGTTGTATGAATTGAAAGATCTGGTTCAAAGTATGAGGAAAAACAACTCAAATACTTATGCACGCTCAAGACAATTTGCTTCAATGGTGAACTCTTTTATTCTCATTTCAATAATTTGCTCACGGTTAAATATTGTATTTGTTATTTTTATTCTCAAATGGTTTATGTCTTATTAATTATTATCTTTTATTCGATAGGACAATGTAGAAAGTTGTTCAGATGCTCATCTCGAAGACAAGTAGTGCAACATCAATTTTATTTTCCTACAATATGTATTTTGAATTTCAAGGTTTTGATATATTTGCGGATTGCTTATATATTTTAAGGATGATGGGGATTGTTTATGTGTTAACTATTTAGAATAATTTTAATTGAACGCTTAAAATTTT

>Chr7-06; OZ022212.1; 4840745-4845205; dir; B;

GGCTCCAATTTGTGGAGGAATTGACTCAAAAAGTATATATATTCATTGTTCTTTTTCTAAATGGGTGTCGTTAGATATTTGTTGGGTGTTTAATTTGTATATATAGGTAATTATGCAATAAATACATATAGTTGTGTACTTTGATAATAATTATAGTATTATTATAATAATAATTTAGATGTTTTTATATTTATAATAATATTTAATTACTACAACAAATAAGTTTTGTGTTATTCACCTCCTGTATGTTAAATAGCTAAGTCCAAAAGTTGTATAATCGTTTTTAATCTTGGTAGGGACAAATCAATGTTGGGGGCTATATGGTCTATAGCCCGTACTGATTTTTCAAATTTTTGAAAAATGTGATATAGTATTTACACACAATGAAAAAAAAAAATAGTTGATAATATCGATAATGAATCAATAATGAATTATTTTAATTCTTTAAAGACTCGAAGGATGCAACTTAAATAGTTTTATTTTGTATTTGCATTGCATGTTTATTTACTATTTTCTTTTTTTACTAGCTTATATCTTATGTATTAGATGCATTTTAAGAAGTATATGCCTCATTATGTTCCTAGGTGTATCATTTTTTGTTGTCAATAAAGTTTGCTTGTGCCGTAGAGTTTCTTTGGTAAATCCAGTCCAGTCCATTTTAAAATTATGGATCCGTTAAACTTGGCCATTAGGAACATAATCACCTCATCTAAATCCCTTGCATGCAAAACCTCTAGCTATTAATTAGTGTAGTTGGCAGCCTTACATTTGTTGCATGCTAATTAATCAACATGGAAACGTATTTGCAATTATTTGATGCACACTAAGACTCACATGTGATATAAGAATGCAATTACTTGAAGTACACTAAGACTTACATGTGTCAAGCTGGTTCATTAATTTTGGAGGCCTCGTGCAAAGAGAGATGTTGAGTGTTGAGATATATATATATCATTTAACAGAAGAGTCAAAACGAGTTATCATCCACGCAGTCAAGTTA

AATGAAGAAGGACTCCCTATTTTCGACGAACAATTAGGTCGAGGTATAGAAGACGGAGTCAATACTTTGTTATACACCATAATTGAGCAGTTCATAGGAACACCCAGTAATACAACAGCCCGAATTCATGATCAACTTAGTAATCTCAGATGTCTGAAATTATCTGATTTTCATTGGTATAAAGATGTTTTCATTTCTAGAGTCATGCTTAGAGATGATAGTAACCAACCTATTTGGAAAGAAAAATTTATTAATGGTCTTTCCAATTTATTTGCTCATAAAATACGCACAGTCCTTAGTAATAATCAAGGTCATATAGATTTCGATAATTTGACTTATGGTAATATAATCAGTACATTTAACCAAGTAGGAATGAAAATGTGTGTTGATCTGAAAATCATTAAACAAATTTAGTCATAAAGGAATTCAGCCAAATATGAGTTAGGAAATTTTTGTGAATAGTATGGTCTAGTGTCAGTTCCCCCATCCAGAAAAGCCAAAACTAGTTATCATGACCAACAAACCAGTAAACATCGTCATTATTCCTGAAAAAGACAGTCTTCTAGACATAATAATGATAATAATGAGTTTTATAAAAATTTCACCCGCGAAAAAATTGGTCAAAAGGTCACGGAAAAAATAAGAAAAGTAACTTTAAGAAAAGATTCGACAAAAGACAAGTTAGGTGCTGGAAATGTCAAAAGAACGGTCACTATGCTAATGAATGTAAGGTAAAAGATGCAATAAAGCAATTGATAATCACGGATGAAGAAAAAGAAAATTTGATTAAAGTTTTAGAATTAAAAGACTTTGAGTCTAGTGAAGCCGACAACACAGTTATGAGTTGTTCTGAGTCAGCAGATTACAGTTCCTCAGATTCCCAACTAAGTTCCCCAAAAATCCAATTAGGTTGTAAAGACAAGTGTTGTAATGTCATCACTAAGTCAATACGACTCCTTACCAAACAGGAAGAGCAAGAAGAAATGTTAATAGGTTTAATCAGTAAAGTAGAAAACCCATAGTTAAAATCTGAGTATCTAAGAAAACTGACAAATATCATTAGTCAGGAAGAGCCAAGTCAGTCCGTTCCCCAGAAGATAAGTCTAAATACAACATTAGAAAAATTCAACAAGAAAAGAAAAGAAGTAACTTTGCAAGATTTAAAAATAGAAGTAAAATTAGTCAAAAAAGAGATAGTTGAATTAAGACAAATGAGTCAAAAGTTACAAGCCGAGAATTATGAGATTAAGCAAGATCTGGTTAATTTAAAGGAAAAAGAAGCCTCTGAATCTAAAAGTCAACCAGACAGTCCAGCCAATTCAGACAAAGAACCCTCTCATAATGATTAGACCATTAATTTAATTAAACAAGTAAATTTTAGGAAACGGTATTCAAAAGTCACTATATAAAAAATTTTGAGTTAAATACAGTTGCCCTTTTTGATTCATGTGCTGACCTTAATTGTATTCAAGAAGGATTGATCCCTACTAAATATTACAAGAAGTCGAAAGAATCATTAAGCATAGCCTCAAGAAAGTCACTTAAGTTAAATTATGAAATACCAAAAGCCCATGTTTGTCAAAATAAAATTTGTTTCAAAACCTCATTCGTCTTAGTCAAAAATATTACTGATGAGGTTATCCTAGGATTACCTTTCATTGCCCATTTGTATCCTTTCCAGGTAGAATACAATGGAGTCTTATCTACCCATTTAGGAGAAAAGGTCAAATTTGAATTCTTAACCAAACATGAGTTGCACAATTTAAAAGCCTTACAGAAGAGCGCAGTTTCCAATACAGTCAAAGTAATTCAAAATAAGGATAAACAGTTAGAATTCCTTAAGGAAGAAGTCAGATTTAAAAGAATTGAACAAAAGCTTAATTTTACAGTCACAGATTCAAAATTTTGAAGAAAAGTTAAAACAAGAAGTCTGTTCTGATTTACCCAATGCCTTTTGGCACAGGAAACAACATGTAGTCAAGTTACCTTATGTCAAAAACTTTAGTGAAAAGAATATTCCCACTAAAGCCAGACCAATCCAAATGAGTCAAGAAATAATGGAATTTCTCAAAACAGAAATTACAGAGTTACTCCAAAAAGGAATAATCCGAAAAAGCAAGTCACCCTGGTCATGTCCTGCTTTCTATGTTTAGAAAAATGCTGAGTTAGGACGAGGAGCCCCTAGATTAGTCACCAACTATAAACCTCTTAATACAGTCTTAGAATGGATTAGATATCCAATTCCTAATAAAAGAGATTTAATTAATCGATTAGACAAAGCAGTCATCTTTTCTAAGTTTGATATGAATAGTGGTTTCTGGCAAATCCAAATCCAGGAATCTGATAGGTATAAAATCGCTTTTCTAACCCCATTCGGTCACTATGAGTGGAATGTTATGCCTTTCGGACTTAAGAATGCCCCTAGTGAATTTCAAAATATAATGAATGAGATATTCAATCCTTTCAGTCATTTCTCCATAGTCTATATTGATGATGTTCTTATATTCTCTCAGTCCATAGAACAACATTGGAAGCATTTGCATAAGTTCCTCCAGATAGTTAAACAAAATGGCTTAGTTGTCTCTGCTAAGAAAATGAAGTTATTTCAGACTAATATCCGCTTTCTTGGTTTTAATATTTATTAGTCTCAAATCAGTCCCATTGATCGAGTCATTCAATTTGCTAATAAATTCCCAGATCAAATTCTTGACAAGGGTCAACTCCAAAGATTCTTGGGATCTCTAAATTATGTTTATGATTTTTATCAAAGTATGAGAAAACAGTGTAAGCCCTTATTTGACCGACTTCAGAGTAACCCTCCGCCCTGGTCATTCATCTATACAGACATAGTCAAACAAATCAAAACCTATGTCAAAACACTTCCTTCTCTTGGCATTCCTGCATTCAGTTCTTTCAAAATAGTTGAAACTGATGCCTCTGAAACAGGTTATGGAGGTATTCTCAAACAGAAAGTGAATCCAAGTCAATTTGAGCAAATTGTTCGATTTCATTCTGGAATTTGGACTTAGTCATAGAGTCATTATAATACTATTAAAAAAGAAATATTATCTATAGTCCTATGCATCAGTAAATTTCAAGATGATTTATTAAATCAAAAATTTTTAGTCAGAGTCGATTGCAAAAGTGCGAAGCATGTTTTAGAAAAAGATGTTCAAAACATTGCATCAAAACAGATTTTTGCACGTTGGCAAGTAATTTTAAGTAATTTTTATTTTGAAATTGAGTATTTAAGAGGTAGTGAGAATTCTATTCCTGATTTTCTTACAAGAGAATTTCTTCAGGATAAACAATGGCAGCCAGAAGGCCTGGCAAAGAAAATGAACAAATCAATCAACCCCAGTCACTAAGAGCTCTCCCAACATCAATGATCCCAGTTAAAATAGGGTCACCTTCACAGATCGTCCCCTTCCCGAGATGCTCTCCAATCCAGATTAGTAATCGATTCAATCCACTTGGTTCCTCAGTCTAACAAGTTTGTCCAAATTATCAGTCAGCCCTAGTTTCAAGTTATGATCCCTTCCAAGTCAGTACCCAAGTCACCCAATCTTTTACTCCATCTCAAAAGTCTTCTCCTTACCTTCAAAAGAGCAGTTGCCATTTGTTCATAATTGAATCCAAATATAATAACATTACCAACCCAGTCACCATTGTAAAGTCATATTTTCCTCCAAATCATCATTTTATCCCCCAAGCTCCCTACAAGTCACTTAAATATTACAGGGATATCCTTCATGAGACACAATCAATTGAAATAAAGCCAATAAAAGATCGCAATCAACCTCATATTATTCTCTACCATTCCCTTTACATTCATCGTTTGATTAGTGAAGATGAATGGAGTCGCCACCCTTATGATCTCAAAGTCCTTCAGTCAGATCTCCAATATAGTTACTATGATTATATTGAAGCATGGTATACAATCTTCCTCCATCAAACAATTGATTTCAGTCATTCTTGGTTTATTAATTTCGATAGTAAATACAAAAGCCCTTTTCCTTTCTGGTTCCTCCACTGGTGGGAAAAGCATGGTCCAGTCATTGATTTGCTTCCCTCTCAAATGCAAGAACTAGTCAATTATTATTCCATTAAAAATAAGTTCAAAGAGTCAGAGTTGTTCTTTCCTAATCTACTTATTTTCTGTGCTAAATATAAAGTTCCCTGGATCCTTAAATGGTCCTATCATATTAATTGGGATTCCATAGTCTTTTCTCCCCAATTTTCAGTCAAATGGTGGGATAAGTTCAAATTGACAGAATTGCAGATTATGTCCATTTAGACTTCCCTCCAGTTACTGTTCCTCCACAGTCCGCCCACAAGCCTTCTTCATCCGGATCATCTCTTGCCTCCTTCTCAGCCAAAGGAAAGTCTAAAGCCGAATTGCAAGAAATTGCCCGACAGTTAATCATTCAAGCCTCCCAAATGGACGATGACGATGAGACC

TCTCCCATATCTCAATCTTCTTCCAGTCAACCCCATCAGCCATCTAGTCAAAAGACTAGTCAAAAGGATTCCACCCCGAAATTGCGGTGGGCAGATTATGAAGACAATCAAGATCCCTATGATCTCAATTCAGATTAATCAAAAGCAGATTCCCCGACAGTCATCATAAGCCAGTAAAAGATAAAGTTCCCTAACAGTCATCAATAAAAGCAAAAGTTAAAGTCCTCTAATTCTACAGTTCTCCCATGCCTATCTACAGTCATGATTTCAAGCATCCAATGCTACCAACAGTCTGGTAGCCTTAGTCTATTTAAGGAGCTCCTGCCTCATTTAGAATTCAGAATGTAAATTTTACTTTTCTCAGTTTCAAAGTTCTCTCAGGACTATTACCATGATGAACTATTGTATGAGTGATCAATAAAATCTTTCAATCAAGATTTTCTGTAATTTTGCATTCTCAAAGTTATCAATTCTATGTTTTAATAGTTATCAAATAATTTCATGCTTTTAATTATCCATATATAATCCATAAATAGAACCAAACACACCCTTTATTGACATCAATTACATCATAATCTATAAATAGAACCAAGTCAATCCTTAAATAAAGTCAATTACATTTACTTTCTTATATTCATAACCCACTCAATTGTCTTTTACATACAAAAAATGTGAAGGTAGTTAAAAAAACTAAATAAAAAAAAAGCGACCAGCGACCAACACTTCATCCAATAACCAAAACAACCACAGAACCAACAACTAGCAACCAACAGTTCAAAAATCATCTAAAAACTATATTCTTGCATTTTTAGTTTCAAAATCATTAATTAAACGAGTACAATCAATTTCGTCCAAAAAATCACTTTCAATAGATATCAAAGTTACACTATTTAATCTTTCTTGTGACATGGTTGATCGCAAGTAAGACTTCAATACTTTCAATTTGGAAACACTTCTTTTAGCTGAAGCAACTGTAACAGGTAAAGTTCATAATACTCTA

>Chr7-07; OZ022212.1; 6205886-6219857; dir; C;

AAAGATATCAAATTATCAAATAATATAAAAAAGTTTGATTTCCCTCATCATCATATCGAAAGAATTAAGCGAAATTACTAATGTAAACGAATTTAATTTGGATTACATTGGCCTAATTTGGATTAAGTGAAATTACTAATGTAAACGAATTTGGATTGTATTCGAATTTTCAAATTAAACTCTTTCGTACTATAAGACCATCAATTATAGCTCGATCCTATTGTTTTATAATTGAATATGTGTTTCTTCTAACAATAACCAAAAGGATTTTAATTGTAATTTCAATTGTTATGAGTTCATCTTTCAAATCTTATTGGACGTCTAGGAGCAATTCTTAGTGAAATGTAAAACTTATCCAACTCCATTGACCTTTCTTGTTAATCATTCTTTCTTGGTTGATTGGTTCTCAATAACATAGCGACAAATCTTAAATTCAAAAATATATAGGTATTATATATATATATATATATTTATATATACCCAATCAAAATTGCCCAGAAATTCTTCCCTCCTCATCTTCACTATTTTCCAAGCCATCATTCAAAGTCAATCAGATTTTACAGAGATATCATCTACGAAACACAGTCAATAGATATTAGGCCCATTAAGGACAAAGAAACAAATGCTACAATGTTCCATTCCCTTTAGTATTTTTATTAATGAATTTGAGTTACAATTACTTTATTTGATTCAGGAAGGTCTTGTTCCAACCCAGTTTTACAAAAAGTCAAAAGAAGTTTTTGGAACAGCATCCGGTAAAGCTTTAAAGTTAAATTATGAATTACCCAAAGCCCATGTATGCCAAAACAAAATATGCTTTAAATCAGCCACTTGGGTGATATGAGTAGCAGACTTCTTTTCACTAAATGTTTTAACGTAAGGTAAAGTAACTACATGTTGCTTCCGGTGCCAAAAGGCAGTAGGTAAATCAGAACAAACTTCATTTTTTAATTTTTCTTCAAACATAATTATTTGGGACTGAAGTGACTTATCGGCTAAC

TGTTGTTCGACTCGTTTGAACTTAATTTCATTTTTAAGAAAATTAACTTTTCTTGTTTTATTCTGAATTAGTTTAACTGATTTAGAAACTGCTGACTTTTATAATGACTTGAGGCTGTGTAACTCTGGTTTGGTTAGAAATTCAAATTTAACTTTTTCACCTAAATGACTAGAAGTAACTCCATCATATTCTACCTAGAAAGTGTAAAGCAATGAAATAAAGGGTAAACCTAAAATGACTTCATCTGTGATGTTCTGAACTTCTTTGTGATATAAAACTCATCATTTGGTTTAAAGGAATGTTTTCTACGGGAATAATATCTACTTTTACTAAAGCGTTGATCATTACTTCTTTTGACCTGTTTAGACGGGGGAACAGGGGCTAGACCGTATTGCTCACAAAAATTTCCTAATTCATATTTAGCATTTTTCCTATCGGCTTGAACTGATTTGCTAATTTTTAAATCTACACACATTTTCATTCCAACTTGGTTGATGGTGCTAATTATATTGCCATATGTTAATTCATCATAATTAATTTGCCAGATTCATTACTCAAGACTGTTCTAATTTTGTGGGCAAATAAATTGGGAAGAACCAAGGACCAGAAAGGGGAAGAACCCAGGAGTCAGAAGTCAGAAGAGATCAGAGCAGACCAAGACAAGAAAAACAGAATTTAAATAACTCAGAGTAGAATTTAGCTAACGTGTAAATACATAAAATTCTTAACGCACAGGTTGCATGAATGAAAATGTAATTAATCATTAAAAATAATGCTTAGGAACGAAAATAGGATCATAACATTAACTTGATAATTTTATTGACTTAATAAAACTTTGAAAACATGAAAATAAAACTTACATTAAGCAATGCCACAGACACTCACACTTCTACTGATCACTCACACTCTTGCTTAAGAACTTTGATAACTGAAAATTACAAGGCGGAACTTGAAAGAAAAGCTTATTTTTAAAAACCCTTTTTTCTCACAAATGACTTGGGGCATCTATTTAAAGTCTTGGTCTGAAAGAAGACAAAATAACTTTTGAAATTAACTTGTGCATCTTTTGCTTTAACTCTAGGCATCTGCATTATATCTACTTTAACTTTGGCATCTTTGCTTTAACTCTGGGGTAACTTGCTTTAACTCTAGGCATCTTTGCTTTCGACCACTTTGCAAAAAGGCTTAGTCTTCACTCAAATCATAAGGATCTTGATTGTCGGGGGAATAGTTCGAGGCATGATCGGACCATCGGGGCTTTGGCTTTTGGCTAAAAGGAAGGTGGCAAGGTTGTTGACTGGAAGAAGACTCGGATTTGGGGCTGGCATCATCACTATCTTCTTCATTCATCTGTTTTACTTGGGCAAGGATTTGTTCAGCTAACTCTTTTAACTCTTTTTTGGATTTTCCTTTGAGGGAGGCATTGGTTAATGGTGCTGATTATATATATATATATAAGTGTAAAAAATTTCAATTTCAGCAGTTCGCATATAATCTTTCCATCCATTAAAGATTTGTTGTCTTTTGACTTTGTCAGGGATGACTTTGTGATAATTATCTCTTCTAGTTTGATTCTCGAGACCATGCAGATGGCTATCTAGGTTTTTCCAATTGATCCTAAATTTTTTATTAAGTGTTCTTAATTGATTATCAACTTGAAAATTGTTAACAACGAAATCGGATTGAGTTGCTGATTCAGAGGACTCAGATTTATTGTCTTCTTGGACTGGGGCTTTTCCATAGACTGGATGGGTAACTTGGGATGTTTTTCTTATGTATTTTAATTTTAAATCAGTTATGGCTTTTTCTAACTCTAGGTCTCGTTCTAGAGTAGAAGAAGAGACTGCAAAGGAATGTCTTGCAGGAACATGCCGTTGGGACTGACCAAGTGTGGGTAATTGTTGTAACTGGACTGTTGACTTTTGTGGAGGTTGGTTACCAAAATTAATTTTGACTGTACCGGCTGACTTTCAGCTGTTAGGGTCCAATCAACAGGAAGGGAAACATCTGACCATTTAATTGAGTGGGGAACTCTTACAGTAGCATCGGGTGTATGACTCTGAATTAACAATGTTTGGTTTTTTGGGCTTTTACTCAAGGCTTGGAAATTCATATTAGTGCCTGTGACTTTGTAATAGATTATGTAGACTAAGGCTAAGGGTTGGGTACTTGGCAGAACTTCATAACCATTAGTTTTTATATTTAATGTAAGGATTTTTAATATATGGGATCACTCAGACTTACAGTGAAATCAGGATAACAATCAAAATGAATCGGACCATTAGCAAGGCTTGACTCAATGATTCCTAGCGTACTGTCGCTAAAATTAGTGAATCTGGCATGACGTAAACAAAGAAGGATATAAGTTTTTAATCCTAATGTGGTTAATAGCTTAACTGCGACTTGAACTAAACCAATGTGAAGGAAATTGTTTCCATGATTTTTATGTTTTCTAATTGACTCAAGATTAAGTAACTAACATGTCTCATGTTCTTGACTCAAGCATAAACTTGTTCAACTGTTTTTACATGGGTGGTTGTATTAAAACTAGAAAGTGACCATTTTTTCTTATAAATATTTTGACTAGGGATTTTAGGTATATTCTAATCTCGAAAATCTAGTTCATTATTAGACTCATACTCTAGTTGTTCTTCATTAACTATCCCTGGGACTTTACCAATACTGACTCTAGAGCTGCTAGAATTAAAAGAGTTGGTTCTAAATAAGCGACTCATTTTTTCTCAAGGTTCTGGGGTTGGAATTCTGAGGAAGAAAGTAAATAGGAACTCCTAAGACTGACTTCTAGATGAAACAACAACATACCATTTTCTAAAATTTACTTGTCTAATTAAGTTGACGACATTGTCCTGATTGGAGGGTTCATCATCTGCATTTGTAAACTTACTGGTGTGGACTCAGAAATTCCTTTTTCTAATAAGTTAACTAAATCTTGTTTTATCTCATAATTTTCACTTTGTAATTTTTGACTTATTTGTTTTAACTCTATTATTTCCCTTTTTACTATTTTTACTTCTAGTTGTAAATCTTGTAAAATAACTTCTTTCTTTCTATTAAATTTTTCCAAGGTTGTGTTCAGACTTATCTTTTGGAAGGTAGATTGTTTCGGTTCTTCTTGACAGGTGATTTTCCTTAATTTCTTAAAGTACTCGGATCTCAACTATGGGTTTTCAACTTTACTTATTAAATCGATCACTACTTCTTCCTGTTCTTCCTGTTTGGTAAGGACATTAATTGATTTAGACTTACAACAATTATCTTGACAACTAAATTGAATTCGAGGGGAACTAGTTTGGGAATTTGAGGAACTAGACTCTGGTGACTCAGTGTTGCTAACCATAGTTTCATTTTCATTGGATTCAGAATTTCTTAATTCTAAGACTTTAATTAGTTTATCTCTATCTTCGTCCGTGATTTTTAATTGTTTGATTGTGTCTTTAACTTTACACTCATTAGCATAGTGACCAAACTTTTGACATTTATTACATTTCACTTTTCTCTTGTCGGTACTTTTCTTAAAGCGACTATTCTTATCTTTTCCGTGATTCTTTTTCCATTTTTTCTTAGAGGAAAATCTCTTTTTACTATAAAACTCATTATCATTCTTATTATGTTGGACTGACTGTCTTTTGCGTGAATAATGGCGACGTTTGCTAGACTGTCGATTGTGGTGACTAGACATATTCTTTCTAGAAGGGGGAATAGAGCTAAGGCCATACTGTACACAGAAATTACCTAACTCATACTTAACAGAATTCCTTTCTGACTGAATTTGTTTACCAATTTTTATATCAACACATATTCTCATACCGACTTGATTAATTGTGCAAATGATATTACCATATGTTAAATTATCCCAATCTATATGACTTTATCATTACTCAGGGTTATTCGAATTTTATGGGCGAATAAATTAGGGAGACCATTAACAAATTTTTCTTTCCAAAAAGACTGGTTACTATCATCTCTGATCATGACTTGGGAAATAAATACATCTTTATACCACCTAAAATTAGATAATTTAGGGCATCTTAAGTTACTAAGCTGATCATGAATTCTAACAGTAGTGTTACTTGGTGTTCCAATGAAATGTTCGACTATTGTATATAATAAGGTGTTGACTCCATCTTCAATGCCTTGTCCTATTTGTTCATCAAAAATAGGAAGACCATCTTCGTTAAGTTTGACTGCATAGATTATTTGTTGCTTAGACTCACTAGTTAAGTGTTTATCCCACCAAGAACGAAGAGTTCCAGTGAAACCAGTTTCTAACAAAAGAACAATTTCAGACTATCTAAAATTGTGGTTTGTGATATAACTATTGGCTACCATGGACATATGTTGAAGCTTATTAAGGATTTCTTGCTCAGAAAGACCATCTATATTCCATTCGTACAATATATCAGAGGAAACTGAAAATTGACTCTCTGAATTTCTTTCTTCTTGTTGGATATCCGGAGGTGTTGGCCTAGGATACCAGTTTTTTGTTAGACTAGTTGGGCTGACTCTGTTGGTTAATCACAAGAAACAATAATTATTAGCCTCAAGAAAACAAAGTTAGTAGCAAGTCCTTTCAAAATCCTTGATCCAGATAATAAAATGGTAGTTGAGCAAAATAATTACACCAACCAAAGTCTAGTTGTCATAGGAAAACAATTAGATAAGACAGAAACAAAAATTGACAAAATTCTCCCGAAAGAGACCACACTTAAGCATCATAATGAGAAGCCTCTAGTTAAATTCCAAGATTTAAAGTCAAGTCCAACTTTTAAAACAAAAGAAACAATGACAAAAATTGAGAAAATGTTCGAAGAATTAACACCAGTCAAAAGAAAAGACAAAGAAAAGATTGAAACAAGTGTTAAAGTTTTAGGATCCTCTCAGTTACCCCTAGATAAATCCTCTGATTCTGAGTCAGTCACTAGCATGGAAACAGAGACCAGTAATATTTCTAAAATTGAAAATGCGTTTAGAAATCTTGAGTTAAAAAGTAATCCAAAGGTAAAAAGATTAACCAACAAAGTTGTTGGTAATTTTTAACAACAATATTAAAATCTTGCAAGTGCACAAATCAAATGATAGTATAGTAAGCAAGGAAGGGTCGATTCCACAGAGATTGTGATCTAAAGAGAGTTTATCAATGTCACACAATGAAAACTAAAACACAATTAAACACGAAAACAAAATAAAGATTGAAATCACAAGGACTTCCACACACACGCCAACACCAAGGGGGGTTTTAATTTGATTTTGTGAATAAAACAAAATAAGGAAAACAAAACAAAATAGAATTTAAATTAGAGTTTAAGAGAAATTGGTTTAAAATCAATCAAGAACAATAGCTAGGACTTACTATGCACCTCCAAACAATCAATCAATCCATAAACAACAATAGATAATCAATTTCTAGCAAGCATATGATGACGACACTGACAACAAGATCATGGCATTTGACTCGAGTCGTATGATTCTCTCTTAAGTGTATGTAAAGTGTATGGCATTTACATCAATACGTGTATTCTTAATTGAAATCTAGTATGGCATTTACTCAAATTAACAATCAAGAATCCATTAAGATCAAAGAGAATATGAGTGTCACAAAAACCCTAAAACATGGCATTTGTTATTAGGAATTCAAGAATCGATTTCTCATAACTAGTTCTTAACCCAATTATTCGTTGCAAACAAGAACACATTCAATATGGAAATTGACCTATGTTCTACAACAATTTAATTCCAATACATGCTTCCAAGTTAGCTACTCAAAGAAACAAAATATCAGAATTCACCTAACATAGAGAAACCGAAATCATCACGCTTGTAGAAACTAAAAATCCATTAAACTTGTTTATAAAATTGAAATCATGTTTAGGACTTCAAGATTAAGCCCTAACTAACACGAAATTAGTTACTCATGGTCTTGGATGAACTCAAAAATAAATACATAAGAACTGGAAATTAAAAGAAGGTAGGAAAAGAAAGAACTCTTTGGCAACTTTCTCCTCTTGCTCGGTTGATCTCTTTAGCACGTGAATCCTTTGTATGGGGGCCTTGGCTGGCCTTTTATATCACTTAAATCAGAGTTATAAACCCTAAAATTCACTGAAAATTCATCCAAGAAGTCTTGGTAAACAAGGAATCCAAATAGGAAAGTAAAAATCACAATTCCTAACTCCATAGGGAAATACAGCTCAGCCCAATTGTCACATGATTTGGGGATCTTCGGCCTCCTAGAAAAATCAGTAAAAATATATTTTAGAAATATAAATGTCTTATCTTTCCAACCATATATAGCACGTAACTTGAAAAATTTGGGAAGGCTTCCAGTTCTTCATATTCACGTAACATGTACGAAACTGTCATGTCTGCACGTGGGCTAACTTTACTTGTAATACTGTACCAATTGGCTAGGGGGAAAATTCTAGAAAAATCACAGATTGAACTTCAAGATATTAGCTTTCATCTTCAATTAGCCTTGCACCAAAATTCCTCCAAAAAGTTAATTTTTCCTTAAAAACCTTCTAAGAGCGCGGAAAAGCTAAAAATCAGAAAAGTAAAGTAATAAGCCCTTTTAAAATCTAATTCTCTTACAAAACCTAAGTAAAAAGAGTACATAAATGAGTATATTTATGGACTTATTAAGAGTTAGCCCCACTAGTCTAATAAAAAACTGGTATCCTAACCTCCGGATATCCAATATGAAGAAAGAAATTCACAGAGTCAATTTTCAGTTTCCTCTGATAAATTATACGAATGGAATATAGATGGTCTTTCTGAGCAAGAAATCCTTAATAAGCTTCAACATATGTCCATGGTCGCCAATAGTTATATCACCAACCACAGTTTCAGATAGTCTGAAATTGTTCCTTTGTTAGAAACTGGTTTCACCTGAACTCTTCGTTCTTGGTGGGATAAATACTTAACTAGTGAGTCTAAGCAACAAATAATCTATGCAGTCAAACTTAATGAATATGGTCTTCCTATTTTTGATGAACAAATAGGACAATGCATTGAAGATGGAGTCAACATCTTATTATATACAATAGTCGAACATTTCATTAGAACACCAAGTAACACTACTACTAGAATTCATGATCAGCTTAGTAACTTAAGATGCCTTAAATTATCTGATTTTAGGTGGTATAAAGATGTATTTATTTCCCGAGTCATGATCAGAGATGATAGTAACCAGTCTTTTTGGAAAGAAAAATTTGTTAATGGTCTCCCTAATTTATTCGCCCATAAAATCCGTACAACCCTTAGTAATAAACAAGGTCATACCGATTGGGATAATTTAACATATGGTAGTATCATTAGCACAATTAATCAAGTCGGTATGAGAATATATGTTGATATGAAAATTGGTAAACAAATTCAGTCAGAAAGGAATTCTGCTAAGTATGAATTAGGTAATTTCTGTGAACAGTATGGCCTCAGCTCTATTCCCCCGTCTAGAAAGAATATGTCTAGTCACCACAACCGACAGTCTAGTAAACGTCAGCATTATTCACGCAAAAGACAGCCATTCCAACATAATAATATTGATAATGAGTTTACAGTAAAAAGAGATTTTCCCCTAAGAAAAAATAGAAAAAGAATCACGGAAAAGATAAGAATAGTCGCTTTAAGAAAAGTACCGACAAGGGAAAAGTGAAATGTTATAAATATCAAAAGTTTGGTCACTATACTAATGAGTGTAAAGTTAAAGACACAATCAAACAATTAAAAATCACGGACGAAGATAGAGATAAACTAATTAAAGTCGTAGAATTAAGAAATTCTGAATCCAGTGAAAATGAAACCATGGTTAGTAACACTGAGTCACCAGAGTCTAGTTCCTCAGATTCCCAAACTAGTTCCCCTCGAATTCAATTTGGCTGTCAAGATAATTGTTGAAAGTCTAAATCAATTAATGTCCTTACCAAACAAGAAGAACAAGAAGAATTATTGATCGATTTAATAAGTAAAGTTGAAAACCCAGAGTTGAAATCCGAGTACTTAAAGAAATTAAGGAAAATCGTCAGTCAAGAAGAATCGAAACAATCTACCTTCCAAAAGATAAGTTTGAACACAACCTTGGAAAATTTAATAGAAAGAAAGAAGTTACTTTACAAGATTTACAACTAGGAGTAAAAATAGTAAAAAATGAAATAATAGAGTTAAAAAAATAAGTCAAAAATTACAAAGTGAAAATTATGAGATAAAACAAGATTTAGTTAACTTATTAGAAAAAGGAATTTCTGAGTCCACAAATTCAGATGATGAACCCTCCAATCAAGACAATGTCGTCAACTTAATTAGACAAGTAAATTTTAGAAAATGGTATGCTAAAGTCACCATATTTGTTAAAAACTTTGAGTTAAACACAGTTGTCCTTTTTGATTCAGGAGCCGATCTTAATTGCATCCAAGAGGATCTAATTCCTACTAAATATTACAAAAAGTCAAAAGAGTCTTTAAGCACAGCCTCAGGAAAGTCACTCCAATTGAATTATGAGATTCCTAAAGCCCATATCTGTCAAAATAACATTTGCTTTAAAACTTCATTTTTCTTAGTAAAGAATATTATTGATGAAGTCATCCTAGGGTTACCCTTTATTGCCCTTTTATATCATTTTCAAGTAGAATACGATGGAGTCATTTCTACCCATTTAGGAGAAAAGGTCAAATTTGAGTTCATGAGCAAACCAGAGTTATACAATTTAAAGGCTTTACAGAAAAATGCAATTACTAACACAGTCAAAATAATTCAAAATAAAGATAAACAATTGAATTTCCTTCAAAATGAAGTCAAATCCAAAATAATTGAACAATGCCTTAGTCATAAGTCTCTACGGTCACAAATTCGCCAATTTGAAGAAAAGTTAAAACAAGAAGTTTCTTCTGAGTTACCTAAAGCTTTCTGGCATAGGAAACCACATGTTGTCAGTCTGCCTTATGTCAAATCCTTTAGTGAAAAGAATATCCCCACTAAAGCTAGACCAATTCAAATGAGCCAAGAATTAATGAAATTTTGTAAAATTGAAATTTTAGAATTACTTGAGAAAGGAATAATTCGTAAGAGTAAGTCACCCTGCTCCTGCCTTGCTTTCTATGTCCAGAAAAATGCTGAATTAGAACGCGGAGCCCCAAGATTAGTTATTAATTACAAACCCCTTAATACAGATTTAGAATGGATCAAATACCCTATTCCTAATATAAGAGACTTAATTAGTAGTTAGAAAAGTCAGTCATCCTTTCTAAGTTTATGAAGAGTGGTTTTTGGCAAATCCAAATTCCTGAGTCTGATAGATACAAAACTGCTTTTGTTACCCCATTTGGTCATTATAAGTGGAATGTTATGCTTTTTGGTCTTAAGAATGCACCCAATGAGTTTCAAAATATAATAAATGAGATTTTTAATTCATTTAGTCATTTTTCCATAGTCTATATTGATGATGTTCTCATCTTTTCTCAATCGATAGAACAACATTAGAAGCATTTGCATAAATTCTTACAGATAGTCAAACAAAATGGATTAGTTGTTTCTGCTAAGAAAATTAAGTTATTCCAAACTAATGTGCGATTCCTTGGGTTTAATATCTGCCAGTCACAGATTAGTCCAATAGATCGTGTCATCCAGTTTGCTGATAAGTTTCCTGATCAAATCCTAGACAAAAGTCAACTCCAGAGATTTCTAGGGTCTCTATATTATGTTTCTGATTTCTACAGAAACTTGAGAAAACAATGTAAGCCTTTATTTGATCGTCTTCAGAGTAATCCCCCTCCTTGGTCATCTATGCATACTGAAACTGACGCCTCTAACATTGGTTACGGAGGTATTCTCAAACAAAGAGTCAATTCTAGTCAATCTGAGCAAATCATTCGATTCCATTCTGGTACTTGGTCACAGTCCCAAAATAATTATAGTACCATTAAAAAGGAGATATTATGTATTAGAAAAAAATGTTCAAAATATTGCATCCAAACAAATAGTTGCACGATGGCAAGCTATTTTGAGTATATTTGATTTTGATATTGAATATTTGAGAGGAAGTGAGAATTATGTTCCTGATTTCCTTACTAGGGAATTTCTCCAGGGCAAACAATGGCAGACAGAAGAAAAGAAAAAGATAAGCAAAAAGAACAAGCCAGTCAATCCAGTCAATACAGTCAACCCCAGTCACATAGAGTACTCCCCTCATCCATGAATCCATCCAAAACAGAGTCACCATCACAGATTATCCCCTACCGGGGATGTTCTCCAATTCAAATGAGCAACAGATTCGCCAGTCTGGGCCAAATTCGTCCCAGCTTCCAGTCTGCATTAATATTCAGCCATGATCCTTTCTAGGAAATCACCCAGTCTACCCCCAAGTTGTCTCCTATCAAAAGTCATCCCCTTACATGATCACGAGTAATAGTCTCCTCTTCATGATTGAGTCCAAATACAATGCTATCTCCAACCCAGTCACTATCGCCAAGTCATACTTCCCACCTAATAGCCATTTCATTCCCCCAGCACCTTTTAAGAACCTAAAGTTTTACAGAGATATCCTCCATGAGACCCAATCCATCACAATTAAACCAATCAAAGACCGAGATAATCCACTTGTAATCCTTTACCATTCCCTTTACATCCATCAAATCATCAGTGAAGATCAATGGAGCAATCATCCTTATGATCTAAAAGTTCTTCAGTCAGAACTTTAATACAATTACTATGATTACATCGAAGCCTGGTATTCCATCTTGCTTCACCAAACAGTTGATTTTAGTCACTCATGGTTCCTTAATTTTGACAGCAAATTCAAGAGTCCTTTTCCCTACTGGTTCCTCCACTGGTGGGAAAAACATGGTCCAGTCATTGATCTCCTTCCCCCCCCCCCCCCCCCCCCCCCCAAATGAAAGATTTAATCAATTATTTTACTAATAAAAATAAGTTTAAAGAATCTGAGTTATTCTTCCCTGATTTACTCCATTTTATTGCTAAGTACAAAGTCCTCTAGATCCTAAAATGGTCCTACCAAGTCAATTGGGAAACCAGAGTCCTATCTCGCTAATTTTCAATTAAATGGTGGGATAAATTCAAAATAGAAAGAATTGCATATTATGTCCATACAGACTTTCCTCCAATCAGGAATCCTCAGTCAGACAAAAACTCAACTCCTGCTACTTCCCATTCTTCTCTCTCAATCGAGGGAAAATCTAAATATGAATTGCAGGAAATTGCTCGCCAACTAATGTTACAAGCCTCCCAAATGAATGAAGAAGATGATAGTGATTCTTCCTCAAAATCTCAGTCATCTACTAGTCAACCAAGAATGCCATCCAGTCAGAAGACTCCAATCCCCAAAATGCGGTGGAGTGATTATCCCCTAGACAGTCAAGACCCTTATGACTTCAGTCCGTTTGATTTGGGCAAAGATTGAAGTTGCCAAAGTCATCATGAGCTCCGAAGGTAAAAGCATTTGGAATCTTGGACCTGTGTTTCAACAGTTTGATAGCCCGTGCTCCTTTAGTCAGATTCCCTGAGAAAGTAATAGTTATAGCACTTAGTAAAAGCAAAAAGCTTTCGTGTGCTTCTACAGTCACCATCTAGAGCATCCGAAGTTGCCACAGTCTACTTTTATTTGTTTATTTAAGAAGGCTTCGGCCTCAGTTATCTTCAGAATGTAATTTACACTTATAGTTTCTCAATTTCAAATGTTCTTTAGCAAAACAATATTATGAAGAACACTAGTTTGGAGCTATTTTTCTGTAAGTTTACATATCTCATAGTTATCAAAGTTATCAATAATATTTTCAAGTTATCAATGCTTTTTTTGAGTATTTTCATTTATTCATTAATTTCATTCATGCATAAGCTCCGCCTGTTTTATTATCTGAGCCTCTATACATATCTGTGCCTTCGTTCTATCTCTTCCGAGTTCTAGACCCCTTCTAGTATCAGAGCCGTAAGTGTCAAAGAGTCAGTCTAAGTCTGTAGCTCATCCAACCCAAGTGGCCGGTTTAGGTTTTCTAAGTTATAAGTTTTCAATTTCTTCTTCCACTACTTCTGGTCTTTCAGTTTACCGGTCCTTCTCTCTCTTGGTGTCCAAATAGTAAGTCCTAGGGAGGCCTGAGCGGTTGTGGCATGAGCGGAGGGCGAGAGATTTATTAGGTATCCGGTTTCATCTAGAAGTCAGTCTTAGAAGTTCCTGTTTACTTTCTTCCTTAGAATTCCAACCCCAGTACCTTGAGAAAAAATGAGTCGCTTATTTAAAACCAACTCTTTTAATTCTAGCAGCTCTAGAGTCAGTATTGGTAAAGTCCCAGAGATAGTTAATGAAGAACAACTAGAGTATGAGTCTAATAATGAACTAGATTTTCGAGATTGGAATATACCTAAAATCCCTAGTCAAAATATTTATAAGAAAAAATGGTCACTTTCTAGTTTTAATACAACCACCCATGTAAAAACAGTTGAACAAATTTATGCTTTGAGTCAAGAACATGAGACATGCTAGTTGCTTAATCTTGAGTCAATTAGAAAACATAAAAATGATGGAAACAATTTCCTTCACATTGTTTTAGTCCAGGTTGCAGTTAAGCCATTAACCAGATTAGGATTAAAAGCTTCTATCCTTCTCTGTTTTCATGATGCTAGATTCACTCATTTTAGCGACAGTACGCTAGGAATTGTTGAGTCAAGCCTTGCTAATGGTCCGGTACATTTTGATTGTTATCCTGATTTCACTGTAAGTTTGAGTGACCCCCATATATTAAAAATCCTTAGATTAAATATGAAAACTAATGGTTATGAAGTTCTGCCAGGTACCCAACCCTTAGCCTTAGTCTACCGAATCTATTACAAAGTCACAGGCACTAATATGAATTTCCAAGCCTTGAGTGAAAGCCCAAAAAATCAAACCTTGTTAATTCAGAGTCATACACCCGATGCTACTGTAAGAGTTCCCCACTCAATTAAATGGTCAGATGTTTCCCTTCTTGTTGATTGGACGCTAACAGCTGAGAGTCAGCCGGTTCCCATCCAATGCAGTCTAAACAATTTAGACTATATACAGCAATACTTAGATGGTACAGTCAAAACTAATTTTGGTAACCAACCTCCACAAAAGTCAACAGTCCAGTTAGAACAATTACCCACACTTGGTCAATCCCAACGGCATGTTCCTGCAAACATTCCTTTGCAGCCTCTTCTTCTACTCTAGAACGAGACCTAGAGTTAGAAAAAGCCTTAATTGATTTAAAATTAGAATCCTTAAGAAAAACATCCCAAGTTACCCATCCAGTCTATGGAAAAGCCCCAGTCCAAGAAGACAATAAATCTGAGTCCTCTGAATCACCCACTCAATCCGATTTCGTTGTTAAAAAATTTCAAGTTGATAATCAATTAAGAACACTTAATAAAATATTTAGGATCAATTGGAAAAACCTAGATAGCCATCTGCATGCTCCTGAGAATCAAACTAGAAGAGATAATTATCACAAAGTCATCCTTGACAAAGTCAAAAGACAACAAATCTTTAATGAATGGAAAGATTATATGCAAACTGCTGAAGTCGAAATTTTTTATCTCGATTTCGTAGAAAGCCGACATATGCCCAATGAGTTAAAAACTTTAACCCAAGAAAAATGGGAAAAGACAGATCAAACTGAGATTTTTGCCAGTCGCCCCCTTGTAGAAACAATAATTATTAACCACAAGAAAACAAAGTTAGTAGCAAGTCCCTTCAAAATCCCTGATCTAGATAATAAAATGGTAGTTGAGCAAAATAATTACACCAATCAAAGTCTAGTTGTCATAGGAAAACAATTAGATAAGATAGAAACAAAAATTGACAAAATGCTCCCAAAAGAGCCCACACATCATAATGAGAAACCTCTAGTTACATTCCAAGATGTCAAGTCCAACTTTTAAGGCAAAAGAAACATGACAAAAATTGAGAAAATGCGGTAGAGTGATTATCCCC

CAGACAGTCAAGACCCTTATGACTTCAGTCCATTTGATTTGGGCAAAGATTAAAGTTTCCAAAGTCATCATGAGCCCCGAAGGTAAAAGCATTTGGAATCTTAGACTCGTGCTTCAACAGTTTGATAGCCCGTGCTTCTTTAGTCAGATTTTCCCGTGCTTCTACAGTCACCTTCCAGAGCATCCGAAGTTGCCATAGTCTACTTTTATTTGTTTATTTAAGAAGGCTTCGGCCTCAGTTATCTTTAGAATGTAATTTACACTTATAGTTTCTCAGTTTCAAATGTTCTTTAGGAAAACAATATTATGAAGAACGCTTGTTTGGAGCTATTTGTCTGTAAGTTTACATATCTTATAGTTATCAAAGTTATCAATAATATTTTCAAGTTATCAATGCTTATTTTGAGTATTTTCATTTATTCATTAATTTCATTCATGCATAAGCTCTGCCTGTTTTATTATCTGAGCCTCTATACATATCTATGCCTTTGTTCTATCTCTTCCTAGTTCTAGACCCCTTCTGGTATACATATATATATATATATATATAGCAGATTATATGTCCTTTGTGGCTTAAGCTTGACTACAGCCCTTTGTAACAGATTATATATATATATATATATGGCAAATCTCCATTGACTATTGTATATACAAGAAATGAACCTGCTGTGGCTTAAGCTTTTTCGTTTGGAAAACAAAATCCCAAAAAAAAGCCCTTTGTTTTATTTTCTTAGATTAATTAATTAATTTAACCACAATACATTAAACAAATCAGCAATTAAACTCCCGCTTGCCCAAAAAAAGTGGGGGAAAGCTAAAATTAATTATTTGTTTTGCCGCTCAAGCCTCTTTTTTAATTTAGGTTTTTTGCTAACCCTAACAGTTATCAGTTTTCAACAGCAGCCACAAAATTGAAAAAATCCTCCATACGTCTTCTCTGCAGCAATATCAAAGAAGCCTTACAGGTACCTACTACATTATTGTTATAGTGATATAGAAAT

>Chr7-08; OZ022212.1; 6885891-6887183; rev; B;

TTACGAACTTACCCTTGGGGTAGGGGTATTTTGTTTATTTTCTTATTCGGAGAGATATTGGGGCAACGAGTGGTATTTTGAAGTAGATCGTACTGAGATGAGTCCGTAGACACGTAGTAGACTCGATTCGGAGTTGTGACGAGGAAGTTACGATCAAAAGAAGCTCAGTGGCACGATCATAATTATTTCGAAAAGAGTTTGATAAAATCTGATTTCTCTCTCTCCCCGCGCGCAACCCCCTCCCCCTTTGTTTTTCTCACAGCCGCCGCCCTCCAAGACCACCTTCGCCGCCACAGCTCAAAGCCGGCCATGACTCCGAATAGGGTGTTACACCTAGGATAGGAGCCTTGAGCCGTAATTTTGAGAATTTTCGGTGAAGTGTTATCGCTTTGGACACCCAAGCGCTGCTGGCGCTTGGGCACTGTAGAAATGGGGCATTGTGTCCCAATGAGATCTTCTAGTTGTCACTAGTGCGTAGGTGTGCTTGGATTCCATTTTGAATATCGTTTGAGCGTCGAAAGGATTTCACCTAGTGTGCGATTTTCGGGTTTGATAGGTTCGAACCGTTGGATCATTTCGAATGTAATATATGTTACTCTAGACATTCCTAGGAATTCGCGGATAGTGAATCGGAGCCCTGGATGTTCCGATTCAATAACTTAAAGTTTGCGTTTTGTGATAACCATCCGATCGTGCGATCGTGAGCGATCCGACCGTCCGTTTCGGACCAAACTTGCATGACGTGTATCCTAGACCTTGTGAAACCTTTAGGAACTTTCGGATCGTCGTGGGCCCACTTGACTCGAATTGGGAAGTTTGACAACTGGTTCACATAGGTTAGTTGGACCTTAGAATGTCTCTTTCCTCGTGCACTTACTTGAAACTCAGGAAATTAGAATTTTAGTTTAAAGTTCTCGTTCGAAGCGATTTGTATTAATCTCATATTTGTATTCTGAAATAAGTACTCCGACCATACAAATGTAGCAGGCGGGACCCTCTC

AGGAAACAATATATTGTGAGTCTTCCTAAAGTCAAAACCTTTAATGAAAAACACATTCCTACTAAAGCAAGACCAATCCAAATGAATCAAGAAGTCATGGAATTTTGCAAAAAGGAAATTGCAGAACTACTTCACAAAGGAATAATCCGTTAAAGCAAGTCACCTTGGTCCTGCCCAGCTTTTTATGTTTAGAAAAACGCTGAATTAGAAGACGAGCCCCAAGACTAGTCATTAATTATAAACCCACAATGTTAGAATGGATTAGATATCCAATCCCCAATAAGAGAGATTTCATCAATAGATTAGATAAAGCAGTCTTTTTCTCTAAGTTTGATATGAAGAGTGGATTTTGGCAAATTCAAATCCGAGAGTCAGATAGGTATAAAACCGCCTTTGTAACCCCATTCGGCCATTATGAGTGGAATGTCATGCCTTTTGGACTAAAAAATGCGCCTAGTGAATTCCAGAATATAATGAATGAAATATTCATTCCTTTCATGAACTTCTCCATAGTTTATATTGATGATGTTCTTATCTTCTCTCAGTCGATAGAACAACATTGGAAACACTTGTATAAGTTCCTTCGCATAGTCAAGAAAAATTGTCTAGTTGTTTCGGCCAAAAAGATCAAGCTATTCCAAACCAACATCAGATTTCTTGGTTTTAATATTTGTCAGTCACAAATCAGTCCAATTGATTGAGTAATCCAGTTTGCTGATAAATTTTCTAATCAAATTCTTGATAAAAGTCAACTCCGAAGGTTTCTAGGATCTCTTAATTATGTCTCTGATTTCTATCAGAATTTGCGCAAACAATGTAAACTCTTATTGGACAGGCTCCAGAGTAATCCTCCTCCTTGGACCTCTATTCATACAGAAATTGTAAAACAAATCAAAGTCCATGTCCAAACACTACCCTGTCTTGGTATTCCTTCAGTCAATTCCTTCAAAATAGTTGAAACTGATGCCTCAGAAAATGCTTATGGAGGAATCCTAAAACAAAAGAGCCAATCCACACCAAACAGAGTAATTATAGTATTATTAAAAAAGAAATTTTATCTATAGTATTATGCATCTCAAAATTTCAGGATGATCTATTAAACCAAAAGTTTTTAGTTACAGTAGATTGTAAAAGTGCAAAACATGTTTTAGAAAAGGGTGTTCAAAATATTGAATCAAAACAGATTTTTGCACGATGGCAAGCTATTTTAGGCATTTTTTATTTTGATATTGAATATTTGAGAGGTAGTGAGAATTCCATCCCAGATTTCCTTACTAGAGTATTTCTTCAGGA

CAAACGATGGTAGAAAAACAAACAAAACAACAAGGGAAGCAACCAGTCAGACAATTCCTAGCACCAATGATCCCAATCAAAACAGAGCAACCTTCCCAAGTCATCCCTTATCCAAGATATTCACCCATTCAAGTATCAAATAGGTATGCCACTCTAGGGTCCACAGTCAGTAACATATGTCCTAATTACCAGTCAGCTCTCATCCAGTTATGATCCATTCCAAGTCTTAACCCCCTCCCCTGCTTCCTCCCCAATTAGTCATCCCAAATCTTCTCCCTATTTCCCCAAAAGCAGTCATACCCTCTTCCTTATTGAACCATGCTTTAATCATTTGACCAACCTAGTCAAAATAGCCAAAGCCTATTTGTAACAACCCGATCCTAAATAAAATTTTAATTATTAATTTATTATGAGAAAAGACTATTTTATCCCGGGAATATTTTATTGGGTCAAAAGTTGACTTTTCGCTCCAGAAGGAATTTAGGAATTTCAATTACACCGTTGTGTAGAGCACGTCGAGATGAGTCCATAGACATGTAGTGGACTCAAATCGGAGTTGTAACGAAGAAGTCACGATCAAAAGAAGTTCAGTGGCAAAATAGTAAATTTTTCAGAGTTCAGAATTTTTAAACCCAGATTTCTTCTTTCTCTCACGAATTTGACTAGCAGCAGTTTTCAAACTCCGATTTCTCCTTCATCTAGCCACCAATTCCAACGCCATAACTTGAAAACGCTTCGTATCTCTCTCCTCTTTCCATGCATATCCATATCCAGCCTTGATTCCAAGTTGAGGTCGAAAACGGGCAGTTTCGAAACCGGAATTTTCAGTGGACATAGACGCCATTTCCGGCCAATTCCGGCCGCCACAACTCATGTAATTACACCCAAACTACTCTCCTACGTTCCCTTTGTGTTATTCCTTGAAAACCTGAGCTGAATCCATTCCAATTTCTTTTATTTCGAAACTAAAATTTTCTAGTTTTTTTTTTTTTTAGACAGA

>Chr7-09; OZ022212.1; 8340359-8347596; dir; A;

GTAAGGTTCATAAATAAATAAATATATATATATATATCAAAGAGCTTCTATTGAGGGATCCCTCAAATTATTTGAGGGACACCTTTTGTAGGCCCCACTTCGGATTGTATTTCACTAATCCAAACCGTCTATTTTGTAGATACTCATTCAAAGATCATCTTTACAAAAAAATCACTTGAATCCGATATCATTTGACCACTCAATTAAGTTATTGAAATTTTAGCATTTTCTTGAAGCATCGTGTTCATTGATTTTGTAAGACACAATTAGATGTCGAAACGATTTCCGATTTGTCTAATTTTTTGTAAGGATGATCTATGAATGAAGACCTAAAAAATAGATGGTTTGGATCATTGGGAAAAAAATTGTAGGGTACCCTAAAGGGCGTCCCTCAAATAAAATTATTTGAGGGATCCCTCAATAGAAGGGAACTGTATATATATATATATATATATATATATATCAACAACTTAATTAGAATCTATTTGTATATTTCAATCTAACACTTGTAAAACAAATGTGACCTTAAGAAAATAATGGTAACCATATAAAATCCTCAATTTAATTTAAAAGCATCTCAGGACTTAAATTCTCTCCACTTAGGCGTACCCCCATTTCTGATCCGTCAATTCCTTTAATACTGTCAATTCCATATATGCCATCAATTCCATATATCCGTCAATTTCAATAATAATCTGTCAATTCCAATGTTAGTTCGTCAATTCCATCCTCGTAGGTCTCAGAATCATAAAGTTAACCGAGCCGCATTCCTCGCAGGTCTCAAAGATAACACTTCAATCTGAGCCGCAATCCTCGCACCTCACGGTTCAAGCATCCCCGAAACTCGTGGGACATCATCAAAATAACAAACTGTCTCAAGACAACTGAGGGTTACCCTCACGGTGGGTTTGAAAACCATAACTCGTCATTTATATAAACTTTAAATAGATTGGTTCCCAACTAAACTTAAAAACAAAATATATACACACATATATATATA

GAGAGAACAAAGTCTTGTAGTTCAAACAGTTCTAATATCAGTATGAGCCGAGTCCCAGAAATAGTAAATGAAGAACAATTTGAATATAATTCTGGTAGTAAAGAACAATTAGACTTTAGAGATTGGAACATACCTAAAGTCCCCAGTCAAAAAATTTATAAGAAACATTGGTTGCCTTCCAGTTTTAATAGCACAACACATGTCAAAACAGTTGAACAAGTTTACGCCTTGAGTAAAGAACATAAAACATGTCAGTTGCTTAATCTTGAATCAATTAAGAAACATAAGAAAGACAGTAATAATTTCCTACATATTGGTCTAGTCAAGTTGCTGTTAAGCCCCTAACACGATTAGGACTAAAAGCTTCAATTCTGCTTTGTTTGCGTGATGCTAGGTTCACTGAATTTAGTGACAGCACCCTAGGAGTCATTGAGTCCAGTCTGTGTAATGGCCCAGTTCATTTTGATTGTTATCCTGATTCCACTGTAAGTCTAAGTGACCCCCATATCCTGCGAACTTTAACTCTGAATATTAAGACTGAAGGTTATAATGTCCTCCCAGTAACCCAACATTTAGCCTTAGTTTACAGAATCTACTATAAAGTTATAGGGACAAATATGAATTTCCAAGCTCTCAATAAAAGCCCAAAAGATCAAACTGTCCTAATCCAGAGTCACACTTCAGATGCCCATATACAGGTTCCCCACACCATTAAGTGGTCCGAAGTTGCCCTACCAAAAGATTGGACCTTAGTTACCGAGAGCCAACCGGCTCCAATCCAACGCAATCTAAACAATTTAGACTACATCCAACAATACTTAGATGGCACAGTAAAAATTAAATTTGATAGTCATCCCTTACGAAAATCTAATGTCCAGTTACAACAATTGACAACTCCTGGTCAGTCCCAAAGGCATGTCCCTGCTAGGCATTCCTTTGCGGCTTCGTCCTCTACTTTAGAACGAGACCTAGAGTTAGAAAAAGCCATGATAGACTTCAAACTTGAATCCCTGAGAAAAACCTCTCAGGTCACCCAACCTTGCTACGGCCAGGTTCCAGTTTAAGAAGATAAGTCGGAATCGCCAACCCAATCTGACTTCTTAGTTGAAAACCTTGCAACAGTTGATAACCAACTTAGAACTTTAAATAAGAAATTTGAAATTGATTGGAAACGTCTAAATCGTCATTTAAAAGCCCCAGACAACCAGTCTAGAAGAGATAAATATCACCAAGCCTATCCTGATAGTAAACACCGCGAGCAAATCTTTAGTGAATGGAAAGATTATATGCGGTCAGCCAGAATCGAAATTTTCTACCTAGATTTTGTTGAAAGCCGTTACATGCCTAATGATGAGTTAAAAACCTTAACTAAAGAAAAGTGGTGTTTGATAGATAAGTCTGTCGTTGAAGCCAGTCATCCCCCAGTAGACACTATAATAATAGATCATAAGAAAGCACCAATACCTGCCACTCCTTTCAAAACCTTCGAAAGCAGTGACCCAAATAGAAAATTAATCGAGCAAAATAATTACACCAATCAGAGTCTAATCGTCATAGGAAAACAACTAGACACAATTGAAACCAAGATCGACAAGTTAAATCCTCCAGAAACAGATTCCAAGCCCAAACCTAGATTTGAAAAACCAATAGTTCAATTTCAAGAACTTAAGTCCAGTCCCGTACTTAAAGTCAATTCTACAATGAAAAAGATAGAAGAAATGCTAGAACAGTTGACCCCAACCAAAATTGAGAAAGCCGAAAAATCTGGTCTCTAAACACTAGATTCTTTTGTTGCCTCACATTTTGAGTCTGAGTCGATAAATTCCGAAACTAGTAATATTTCAAAAATTGAAAACGCCTTCAAAAATTTAGAAGTCCAAACTGAACCCAGAATTAAGAAATTAGACAAACATATAAGCGCTACCAGTTTAACAAAGAATTGGTATCCCAGACCAATACCCCCAGACATCCAATTTGAAGAAAGAAGCTTCCAAACCCAATTTTCAGTTTCTTCTGATAAATTGTATGATTGGAACATTGATGGTCTTTCTGAGCAAGAAATTCTGAATAAGTTACAACACATGTCCATGGTCGCTAATAGTTACATCACCAATCACAGTTTCAGACAATCTGAAATTGTTCCTCTGTTAGTTACCGGCTTTACTGGTACTCTCAGATATTGGTGGGATAAGCATCTTACCCCTGAGTCTAAAATTCAAATCTCTTATGCTGTAAAACTTAATGAAGATGGTCTCTCTATATTTGACGAACTAATAGGTCAAGGCATAGAAGATGGAGTCAACACTTTGTTTTACACAATAATTGAACATTTCATTGGAACCCCTAGTAATACCACCTCCAGGATACATGATCAGTTGAGGAATCTTAGATGCCCAAAGTTATCCGATTTCAGATGGTACAAAGATGTCTTCATCTCTAGAGTCATGCTGAGAGATGATAATAATCAACCATTCTGGAAAGAAAAATTTATAAATGGTTTGCCAAACTTGTTTGCCCACAAAATACGCACAACCCTGAGTAATGAACAAGGTCAAATAGATTGGAACAATTTAACTTATGGAAATATTATCAGCACAATTAATCAAGTTGGTATGAAAATGTGTATTGACTTTAAGATCAATAGGCAAATTCAGACTGATAGAAAGTCAGCCAAATATGAGTTAGGTAATTTCTGCGAACAATACGGTCTCACCAGTATTCCCCCATCCAGGAAAAATAAGCCTAGTCATCTTAGAAAAAATCATTATTCGAAGAAACAGTTTTTTGGAAAGAATGAATTTTATAAGAAAAGAAAGTTCTCCCCCAAAAAGAATCAATCAAAACACCAGGACAATAGAAAGGGTCGCCCTACAAAAGATAAAAGCAAAGTTAAATGTTTCAAATGTCAAAAATTCGGTCATTATGCCTCTGAATGCAAAGTAAAAGATGTTATTAAACAGTTATAGATTACAGAAGAAGATAAAGAAAAGTTGATACAAGTTTTAGAGTTGCGAGACTCTGAAACAAGTTCTGTTGAAACCATAATTCCCAGTTCAGAGTCTGAGTCTGACTAGTCTTCAGAATCACAACCCAATTCTCCTGGAGTCCAAATTGGTTGTACAGATAAATGTTGTAGTAGACTAAAATCTATATTCGTTCTCACTAAACAGGAAGAACAAGAAGAGCTTTTAATAGAACTAATAGGAAAAGTCCAAAACCCTGAGTTAAAATCAGAGTATCTAAAAAAATTAAAAAAGGTCTTTAACCAAGAAAGTTCAAGTTCAAACCATCTTGCTCCCCAGCCTATAAGTATCAATACCACCTTAGAAAAATTTAACCACAAAAAAGAAGTAACCCTCCAAGATCTCCAGTTAGAAGTCAAGAAAGTTAAAAAAGAATTAGTTGAATTAAAACAAATGAGTCAACAATTGCAAGCTGAAAATTATACGATCAAACAAGATATAACTAATCTTTTAAGGGCCTAAAAGCCAATCAGAAAGCCCTCACCATTCTGATGATGAACCTTCTGAAAATCAACAAGTTGTCAATTTAATTAAACAAATACATCTTAGAAAATGGTATTCAAAAGTTACAATATTTGTTCAAAATTTTGAATTCACCACAGTTGCTTTATTTGATTCCGGAGCTGATCTTAATAGTATCCAGGAAGGTCTAATCCCCACTAAATATTACCAGAAGTCCAGGGAATCCCTTAGCACTGCCTCAGGGAAGTCTCTCCAGTTAAATTTTGAATTACCAAAAGCCCATATTTGCCAAAACAAAGTCTGTTTTAAAACCTCATTTGTCCTAGTCAGAAACATCACTGACGAAGTCATCTTAGGTCTCCCTTTTCTTGCCCTATTGTATCATTTCCAGGTAGAATACGATGCATCATTTCTACCTATTTAGGTGAAAAGGTTAAGTTTGAATTCTTGACAAAACCAGAGTTGCACAATTTAAAAGCCTTACAAAAACAGTCAGTCTCTAAATCTGTTCAAGTTATTCAGAAAATTAACCAGCTGCATTTTCTTAAAGAAGAAATTAAATTTAAAAGAGTTGAACAACAACTTGCTGATAAAACCTTGCAGTTGCAAATACAGTCCTTTGAAAACAACTTGAAAAATGAAATTTGTTCTGAATTGCCTAACGCTTTTTGGTATAGAAATCAGCATGTCGTCTGTCTTCCCTATATCAAAACCTTTAGTGAAAAGGACATCCCCACTAAAGCCCGGCCAATTCAGATGAATCAAGAGTTAATGGAATTTTGTAAAAAAGAAATTCATGAGTTACTCCAAAAGAACATTATTCGCAAAAGTAAGTCCCCTTGGTCATGTCCTACTTTTTATGTCTAGAAGAACGCTGAACTAGAAAGAGGAGTCCCGAGATTGGTTATCAATTATAAGCCCCTTAATTCAGTCCTAGAATGGATTAGGTATCCCATACCTAACAAGAGAGATCTCATTAACCTGTTAGAAAAAGCAATTGTTTATTCCAAATTTGATATGAAGAGTGGTTTCTGGCAAATCCAAATTGATGAGTCAGATAGATATAAGACTGCATTTGTTACTCCTTTCGGTCACTATGAATGGAATGTCATGCCGTTTGGTTTAAAGAATGCCCCTAGTGAATTCCAGAATATTATGAATGATATCTTCAATCCCTACAGTCATTTCCCAATTGTTTATATTGATGATGTTCTTATTTTCTCCGAGTCAGTTGAACAGCATTGGAAGCATTTGCATAAATTCATTCATATAGTCAAGAAGAATGGTTTAGTTGTTTCTGCCAAGAAAATTAAGTTGTTTCAAACCAATATCAGATTTCTTGGCTTCAATATCTGCCAGTCTCAAATCTCCCCAATTGATCGAGTTATTCAATTCGCTGATAAATTCGCTGATCAAATCCTTGATAAAAGTCAACTCCAGAGGTTTTTAGGATCTCTAAATTATGTTTATGACTTTTACCAAAATTTGAGAAAACAATGTAAGCCCCTGTTTGATAGACTGTAGAATAATCCTCCCCCTTGGTCAAATGTCCATACTGAAGTTGTCAAGCAGATCAAAGCCCATGTCAAGACACTCCCCTGTCTTGGTAGTCCTTCAGTTGATTCTTTCAAAATAGTTGAAACCGATGCCTCTGACATAGGTTATGGTGGCATTCACAAACAGAAAGTTAGCCCAGATTCTCCAGAACAAATTATTCATTTTCATTCTAGAACCTGGACTCAGTCGCAATATAATTATAGTACTATTAAAAAAGAAATATTAGCTATTTGTATTATGCATTAGTAAATTTCAAGATGAATTACTCTTGGGCTAATACGAGTTGATTGCAAATCTGCAAAACATGTTTTAGAAAAAGATGTTCAAAACATTGCATCTAAACAGATTTTTGCACGATGACAAAGTATATTAAGTATATTTGATTTTGACATTGAATATTTGAGAGGTAGTGAGAATTCAGTCCCTGATTTTCTTACTCGAGAATTTTTGCAGGAACCACAATGGCCCCCAAAAAAGACAAACACAAAGAGTCAGCCAGTCAACCTAAGCCATCCCAGTCGCAACCATCCCAAACTCCGTCCATGAGTCAAAACAAGCCCGAATTGCCCAAACAGATAGTTCCCTATCCTGGTTGCACCCCTCTTATAGTTGCTACCCCATTATCAGTTGCAAACAGATTCTCCTCTTTAGGATCCACAGTTGGTCAGATTCGTCCCAGTTATCAATCAATACTAGTTGCCAGTTATGATCCTTTTTCAGTTGATATACCAGCCGGACCTTCAGTAGCATTTAAAAAATCATCCCCTTATTTGCCCAAGAGTAATTCTCATTTATTTGTCATTGAGCCGCTGTATGATGTTCATACCAATCCAGTTGAAATCGCCAAACACTATTTCCCTCCAGATTCCATTACATGCCCTCCAGTCCTTACAAGTCTCTCAAGTATTATAGAGAGATATTACTTGAAACTAAGTCCGTTGAAATTAAGCCAATAAAGGATCGTGATCACCCTGAGATCATTCTTTACCATTCCTTGTATATTCACGACATCATAAGCCAAGAAGCCTTCAGCAGCCACCCTTATGATCTAAAGCTCCTTCAGTCGAAATTGCAATATAATTATTCTGATTATATTGATGCCTGGTATACTATTTTTCTTCATCAATCTGAAGATTTTAGCCATTCCTGGTTTATCAATTTTGACAATAAATTCAAATGTTCTTTCCCTTATTGGTTTCTCCATTGGTGGGAAAAACATGGTCCAGTTGATGAAATCCTACCTGTTTCAGTCCATGAGCTAATCCGCCATTACATTAAGACGGCCAAGTTCTCCAAAGTTGATTTATTCTTCCCAAAACAGTTGCTATTTATTGCCAGATACAAGGTTCCATGGATCCTCAAATGGTCCTACAGAATCACCAAAGACGCCAGAATCTTTGCCCGCCAATTTTCTGTTAAATGCTGGGACAAATTTGAAGTTCACCGAATTGCCAAGTATGTCTACAATGATCTTCTTGCTGAGCCACAACCTCAGGCAACCTCTCCTACTGGTTCTGTCTGATCCTTCCTCTCAGTCGAAGGAAAATCCAAGTCAGAACTCCAGGAAATTGCTCGCCAGTTGATTCTTCAAGCCTCCCAGATGGATGATGATGAGGACAATGATTCTCCTGCGTCATCCAGCTGCCAGCCCTTACAGAATCCTGTCAAGCCCAAGTAATGGTATGAAGATAGTTAAGACCCTTATGATGCCTATGACTTCCACTCGGACTACACTCTGCAGACAATAATTACGTGCCACAGTTGCCGACACCATAATTGCCACAGTACAAGCATCTGCCTTTTACTATTCGCCACAGTTGCCGACACCATAATTGCCACAGTACAAGCATCTGCCTTTTAATATTCGCTTTCCACCGAAAGTTGCATCAGTACAATCCAAATGAAGAAAAAATTGAGAGTTGAATCAGGTTGCATACCCGTGACCCTACAGTAGCAGCAAACCACAGTTAATTTATCATAGTTAGTTGAACCCTAGTTGCTTTTCTCTATATAAGGAGGCTTCTACCTCAGTTGTAATCAGAATACAATTTTAGTTTTCTTTCAATTTAAAGATTCTCTCTGAAACATCTTTTGGAGAAATCAACCCTGTACAAAGAGTGAAGTTATTTGAAGTTGCAGTTGCTATCATTGTGAGTATAAATAAAAGTTGCTATTTTCAAATTATCTATTTTATGA

AAAGTATTATCATTATTTTGCTACATTAGTTTGAAAGTTGTTTGGGTATGCTTCTCTTCTCTTTGTTTCTTTGTTTCTGTTTCCGTTGTCCCCTCTTATATATATATATATATATATATATATATATATAGACACACACACACTAAACACTTGGCTAGTTTGTTAACTATCTCGATCTCTGCTTAGCATACCATTCCAATATTATATTTATACTCCTACCACACAGTTAACCATATGAACATATATAAACAGGTGATAAATAAACAGATAATTAAATACTCATCTCGAAATCACATAAAATCATGAAATTAGAACTACAACTAAACCAAGTGAACGCTCACACGCGCATGAGCTTTCTAAAGGCTCAAACATATTACATGGAGCTCATCCTCGTTATTTAATCCAAAGTCCTGGCCCTACGCTCCTACAAGTGCGTGCATTGTATTTGAAGTCAACACAATTTGTTACTGTAAATTTAGCCCTAGGATTGGCCTTTTAAGAGTATCCGGGACTCCGATTCCCAATCCGTTGAATCCAACACAATTCAGGAATTTTCTAGATTGACATGTACAAGAATAACTACGATCCAACGGTTCAAATGTATCAAACCTGGAAATAGCATAATATGCAAGATCCGATCGAGGCTCAAACGTTATTCAAATTGAGATCCGTAAATTCCTACACGCTCGTGACAACACGAGGATCATTCTAGGACTAAGAGTGACCTCACGATGCAGCCCACGCTCCCCCACATGCGCTGGCAATGAGTGGGTGGCTTGATCAATTTTCGGCAGCCGGAAATACTCCAAACCACCAGCCCAAACTCCTATCCTAGGTATAACACCCCATTTGGAACCACTTTTGTTCTTGGACCTACCTCGAAAAATGACTGAAAGTGGCCGGAATCTTATTCCAAAATTCGGCCAAATTTTGGGTTGCAAATCAAGTTGCCTACACTAGAAATTGATCAAATCCTACCCAACCATCAGCTAGAAAACAT

>Chr7-10; OZ022212.1; 8574482-8582769; dir; A;

ACCTGCCAGGGGAATTGACGAAATTACAGGGATACATCCGAGTGGTAGTTTTGATTAATTGCAGTGCTTTAATTCAAGTTTTGAGTACATTGCTTTTATGCATGTTTGATTTCAGTATTTTTATGCGAGTTTTGATTTCAGTGCTTTTAGGCAAGAATTAATTACTGTGATTTCATGCAAGTCTTCTCGATATTGATAGCATAGTTCAAGCGCATTTTACATATTCAGCTTTTGTTACTACTATTTTACAGTGGGGGTTATTATATTCACAAAATTGTTTTCTGAAACCTTATTTTGTCCACTCACACTTTCAATTGTTTTGCGCCCCCAGGCTATAGCGTGCACAAGACATCCACCACCGGGCCATCTTAACTTCCGTGCCTTTTGGTTCAAAAGTGTTGGTAAGTAAAATAATTAAGTGATCCGTAAACTATCAAAATTGCTCTGATATGTTGCCCGAAAGCGGGGATGGAACTTTTGACCTGTTTATCGAAGTGCTGGCTGCTGGTTGTTGGATAATTATGCTCGTTTTGACAGGTGTAAGTTTTGGGATTGAACAAAATATAGGGGAGACTCTGCCGAATTTTCGGCAGGAGTCAAGGTTGATATTTCAATTAAAAGGGCAATTAGGTCAATTGTGCCCGACACTCGCCATGTGTCGGACACGCACAGGGTTCGACTCGAATTCCAAAACGAAATTCAGTTCGAGTCCTGTCAGTTTGTCCAAGTTAACTTGCTCAGAAAGATAAAAATAGATTTTTAGCTATTTAATACACGGGAGGTAAATAACACAATTCTAACAATTGAATGTTATTATAAATATATATATATAGTTATATAAGTTAAATACGCAAGAAATATCTAAGGGTACGTGCCCATTTAGAAAAACAAATAATAAATATATTATTTATAGTAATTTCAAATGAACATATATATATATATAATTCATCTCTATTCTACTGTTCTTTAAATTCTTGGAATCTCAGTTCTTGTTCTTCCT

TTCTGTTTCTCTGGTTCTTCCTTTGTTGGTATCAGAGCCGTAAGCATTACGGTCTTTGTCTAAGTTTGCTACTCATTCCCGCCCAAGTGGCTGGTTTAAGCATCTGAGTAGAAGGTTTTCATTCCTTCCTTTTCTTCCCTTTTATTCTTTGAGTCCTCTGTTGGTCTTCTCTAAGGTCTGACCCGTGAGTCCTTTTTCCTAGATCAGTAAGTCCCCAGGGAGGGCGTGTGTGGTGGGCAGGAGACAGAAAATTGAGAAAATTAATAGGTTGCTTAGAGTTTAAGCGGTCGGTTTGGGATCTACTGTTTATCTTCTTCCTCAGATAGCCAAACCGAGTTATTTGAGTAGTTATGAGTCGTCTGTTTAGAACAAAGTCTTGTAATTCTGCCAGTTCTAGTACCAGTATAAGCCGACTCCCAGAGATAGTTAATGAAGAACAATTTGAATATAATTCCAGTAGTAAAGAACAATTGGATTTTAGAGATTGGAATATACCTAAAATCCCGAGTCAAAAAATATATAAGAAACATTGGTTGCTGTGGGAGCAAATTTCCCCACGATAATATTCGCCCAAGATTCCTTCGTCTTCTCCGCCTCTCTTTCTCCCTGCAAAACAGATCGGAGTAAAAGGACCACACCCGGGGGGTGTTGGCCAAAGGCCCTCCGATGCCTAAGTTAGATAGGGTAATTCAAGGAAAACCGGGTGGCCGGAGCCGTGTGTGGTGGCCGGAGCCTTGTGTAAGAAAGAGAAAGAGAGGAGGTGGCTAGGGTTTTTGAGAAATAACTTATGCAGAGTTTGAGTAGGAATTCTGACGTACCTCATCCTTGTGCGTAACCAAGTATTTATAGTGGCCTTGAAGAGGTTGGTGTAGTTGGTGAGGTTGGTGTGGTTGGTGTAGTTGGTGAGGTTGGTGTGGTTGGTGAGGTTGGTGTATTTAGGGATTATGGATTTAACCGAATCCCTAATTAAGGCGACGATCAAGTAACCCCCAATTTGATTAGGTAATTCCCAATTAGGTTAGGTAACTCCCAATTAGGTTAGGTAACTCATAATTAGGTCAAATAATTCCCAAATTGATTGGCCTAATTATTTAACCTAGGGTTTTAATTAGGTTCCCACAGTTGCCTTCCAGTTTTAATAGCACAACACATGTCAAAACAGTTGAACAAGTATACGCCCTAAGTAAAGAACATGAAACTTGTCAGTTGCTTAATCTTGAGTCTATTTTAAAATATAAGAAAGATGGTAATAATTTCCTGCATATTGGTTTAGTTCAAGTAGCTGTCAAACCCTTGATACGGTTAGGGTTGAAAGCCTCAATCCTACTCTGTCTGCGTGATGCTAGATTCACCGAATTTAGCGATAGTACCCTTGGAGTCATTGAGTCCAGTATGTGTAATGGCCCTGTCCATTTTGACTGCTATCCTGATTTCACTGTAAGCCTTAGTGATCCTCATATCCTGAGAACTTTGACTCTGAATATTAAGACTGAAGGTTATAATGTCCTTCCGGGAACCCAACCTTTAGCCTTAGTTTACAGAATCTACTATAAGGTCACAGGAACTAATATGAATTTCCAAGCCCTTAATAAAAGCCCGAAGGATCAAACCGTCCTGATCCAGAGTCACACCTCAGACGCCCATATACAAGTTCCCCATACCATCAAATGGTCTGAAGTTGCCCTTCCAAAAGATTGGACCTTAGTTACTGAGAGCCAACCGGCTCCGATCCAGCGTAGTCTAAAAAATTTAGACTACATCCAACAATACTTAGATGGCACAGTTAAAATTAAGTTCGACAGTCATCCCCTCCGCAAGTCTAATGTCTAGTTACAACAATTGCCAACTCCTGGTCAATCTCAGAGGCATGCCCCTGCTCGGCATTCCTTTGCTGCCTCTTCCTCCACTCTAGAACGAGACCTAGAGTTAGAAAAGGCCATGATCGACTTCAAACTTGAGTCCCTGAGAAAAACCTCTCAAGTTACCCAACCTTGCTGCGGCACGATTCCAGTCCAAGACGATAAATCAGAATCCCCTGAGTCTCCGACCCAGTCTGATTTCCTAGTTGACAACCAACTCAGAACCCTAAATAAGAAATTTGAAATTAATTGGGTAAGTCTAAATCGCCATTTAAAAGCCCCTGAAAACCAGCACAGAAGAGATAATTATCACCAAGCCTACCCTAATAGCAAACACCGCGAAATGATCTTTAGTAAATGGAAAGATTACATGCGGTCTGCCAAAGTTGAAATTTTCTACCCTGATTTCGTTGAAAGCCATTACATGTCTAGTGATGAATTGAAAACTCTGACTAAAGAAAAATTGAAACTAATTGATAAATCTGTAGTTGAGTCGAGTCATCCTCTAGTAGAAACTATCATAATAGAACACCAGAAAGCACCAGTAGCTGCCACCCCTTTCAAAACCTTTGAAAGCAGTGACCCGAATAGAAAAATGATTGAACAAAACAATTACACCAATCAGAGTTTAATCACCATAGGAAAACAATTAGATACAATCGAAACCAAGGTCGACATGCTGAGTCCGCCTGTTGCAGACTCCAGACCCATACCTAAAATTGAAAAGCCGATAGTTCAATTCCAAGAGCTTAAGTCCAGTCCGGTCTTAAAAGTCAATTCTACAATGAAAAAGATAGAAGAAATGCTAGAACAGTTGACCTCTGCCAAGCCCGAGAAAAGTGAAAAATCTGGTATTAAAACACTAGATTCTTTTGCTGCTTCTCAATCTGAGTCCGAATCAGTAAGTTCAAAAACCAGTGACATTTCCAAAATTGAAAACGCCTTCAAAAATTTAGAAGTCCAAACTAAGCCCAGAGTCAAAAGAATAGAAAAACATATAAGTCCCACTAGCCTAACAAAGAATTGGTATCCCGGACCAACACCTCCAGATATCCAATTTGAAGAACGAAACTTTCAAACCCAGTTTTCAGTTTCTTCTAATAAATTGTATGAATGGAATATTGATGGTCTTTCTGAGCAAGAAATTCTAAATAAGTTGCAACATATGTCTATGGTCGCTAATAGTTATATCACCAATCATAGTTTCAGACAGTCTGAAATTGTTCCTCTGTTAGTTACCGGTTTCACTGGTACTCTCAGATATTGGTGGGATAAGCATCTTACCCCTGAGTCTAAAAATTGAATAATCTATGCTGTAAAACTTAATGAAGACAGTCTCCCTATATTTGACGAACAAATAGGTCAAGGTATAGAAGACGGTGTCAACACTTTGTTTTACACTATTATTGAACATTTCATAGGAACCCCTAGTAATACCACTGCTAGGATACATGATCAGTTGAGTAATCTTAGATGCCCAAAGTTGTCTGATTTCAGATGGTACAAAGATGTCTTTATTTCCAGAGTCATGCTTAGGGACGATAGTAATCAACCCTTCTGGAAAGAAAAGTTCATTAATGGTTTACCAAATCTATTCGCTCATAAAATACGCACAACCCTGAGTAATGAACAAGGTCAAATTGATTGGGATAATTTAACTTATGGTAACATTATAAGCACGATTAATCAAGTTGGTATGAAAATATGTATCGACTTTAAAATCAATCACCAAATCCAGTTTGATAGAAAGTCAGCTAAGTATGAATTAGGAAATTTCTGTGAACAATATGGTCTTACTACTACCCCTCCTTCTAGGAAACATAAGCCTAGTCAACTTAGAAAGCGCCATTTCCCGAAGAAGCAATTTCTCCGGAAAGATGAATTCTATAAGAAAAGAAAGTTCTCCCCTAAAAAGAATTGGTCAAAACGTCCCGAGCAAAAACAAAGTCGCCCTACGAAAGATAAAAGCAAAGTCAAATGTTTTAAATGCCAAAAATTTGGTCATTATGCTTCTGAATGCAAGGTAAAAGATGTTATTAAACAATTGCAGATTACAGAAGAGGATAAGAACAAATTAATCCAAGTTTTAGAGTTGCGAGACTCTGAAATAAGTAATGTTGAGTCTCTGCTTTCCAGTTCTGAGTCTGATTCTGACCAGTCTTCAGATTCACAACCCAGTTCTCCTAGAGTCCAATTTGGTTGTACTGATAAATGTTGTAATAGGTTAAAATCTGTCTTAGTCCTCACTAAACAAGAAGAACAAGAAGAACTTTTAATAGATTTAATAAGAAGAGTTCAAAACCCTGAGTTAAAATCTGAATACTTGAAAAAATTGAAAAAGGACCTAAGTCAGGATAGCCAGAGTTCACTCTCTCCTACTCCCCAGCCAATTAGCATCAACACTACTTTAGAAAAATTCAATCGTAGAAAAGATGTCACCCTCCATGATCTCCAGATAGAAGTTAAATAGGTCAAAAAAGAAATAGTTGAGCTAAAACAGATAAGTCAACAATTGCAAACCGAGAATTATACTATCAAACAAGAATTATTTGATTTAAAACAAGAAACCTGTACGCCTAACAACCACTCAGAAAGTCTGCATCACTCTGATGACGAACCTTCTGAAGAACAGCCAGTTGTCAATTTAATTAAATAAGTTCACCTTAGAAAATGGTATTCAAAAGTCACAATATTTGTCCATGATTTTGAATTCACCACAGTCACCTTATTTGATTCAGGAGCAGACCTTAACTGTATCCAGGAAGGTCTCATCCCCACTAAGTATTACCAGAAGTCCAGAGAATCCCTTAGCACTGCCTCAGGGAAGTCCCTCCAGTTAAATTTTGAAATACCAAAAGCCCATATTTGCCAAAACAAAGTCTGTTTTAAAACTTCATTCGTCTTAGTTAAAAACACTACTGATGAAGTCATCTTAGGTTTGCCTTTCCTCGCCCTGCTTTATCCTTTCCAGGTAGAATACGATGGTATCATTTCTACCCATCTTGGAGAAAAGGTCAAATTTGGCTTCCTGTCGAAACCTGAGTTGCATAACCTGAAAACTTTACAAAAACAGTCAATCTCTAAATTTGTCCAAGTTATCAGAAAAACTAATCAGTTGAATTTTCTTAAAGAAGAAATTAAGTTTAAAAGAATTGAACAACAGCTTACTGATAAAGCCTTACAGTTGAAAATCCAGTCCTTTGAAACCAAACTACAAGATGAAATATGTTCTGATTTACCTACTGCTTTTTGGTATAGAAAACAACATGTTGTCTGTCTTCCCTATATCAAAACCTTTAGTGAGAAAAATATCCCCACTAAAGCTAGGCCAATCCAGATGAATCAAGAGTTTATGGAAGTCTGTAAAAACGAAATTCATGAGTTGCTCCAAAAGAATATTATTCGAAAAAGTAAATCCCCTTGGTCGTGCCCTGCTTTTTATGTTCAGAAAAATGCTGAATTAGAAAGAGGAGTCCCCAGATTAGTTATCAATTATAAACCCCTTAATTCGGCCTTAGAATGGCTTAGGTACCCTATACCTAATAAAAGAGATCTCATTAATCGGTTAGAAAAAGCAATTGTCTTCTCCAAATTTGATATGAAAAGTGGTTTCTGGCAAATCCAAATAGATGAGTCAGATAGGTATAAGACTGCATTTGTTACTCCTTTCGGTCATTATGAATGGAATGTCATGCCGTTTGGTTTAAAAAATGCTCCTAGTGAATTCCAGAATATTATGAATGACATCTTCAATCCTTACAGTCATTTTTCGATTGTTTATATTGATGATGTTCTTATTTATTCTGAGTCAGTTGAATAGCACTGGAAGCATCTGCATAAATTCCTTCATATTGTCAAACAGAATGGTCTAGTTGTTTCTGCCAAGAAAATTAAGTTGTTTCAAACTAATATAAGGTTTCTAGGTTTCAATATTAGTCAATCTCAAATTTCCCCAATTGACAGAGTCATTCAATTTGCCGATAAATTCCCTGATCAGATTCTTGATAAAAGTCAACTCCAGAGGTTTCTTGGATCTCTAAATTATGTTTCTGACTTCTACCAAAATCTGCGAAAGCAATGTAAGCCTCTGTTTGATAGATTACAAAATAATCCCCCTCCATGGTCGAATGTCCATACTGAAGTTGTCAAACAGATTAAGACCCATGTCAAGACACTTCCCTGTCTTGGTATTCCTTCAGTTAACTCTTTCAAAATAGTTGAAACTGATGCCTCTGACATAGGTTATGGTGGCATTCTCAAACAGAAATTCAGTCCAGAATCTCCAGAACAAATTGTTCGTTTCCATTCTGGAACCTGGACTCAGTCGCAATATAATTATAGTACTATTAAAAAAGAAATATTAGCTATTGTATTATGCATTAGTAAATTTCAAGATGATTTATTAAATCAAAAGTTTTTAATACGAGTTGATTGCAAATCTGCAAAACATGTTTTAGAAAAAGATGTTCATAACATTGCATCTAAACAAATTTTGCACGATGGCAAAGTATATTAAGTATTTTTGATTTTGATATTGAATATTTGAAAGGTAGTGAGAATTCGGTCCCTGATTTTCTTACTCGAGAATTTTTGCAGGAACCTCAATGGCTCCCAAAAAAGACAAACACAAAGAAGTTGCCAGTCAACTCAAGTCACCCCAGTTGCAACCATCCCAGTCACCCCAGTTGCAACCATCCCAGTCACCATCCATGAGTCCAGCCAAACCAGAAATGCCCAAACAGATAGTTCCTTATCCTGGTCATCCCCCTCTTCCAATTGCCACCCATTATCAGTTGCAAACAGATTCTCTTCCTTAGGATCTACAGTTGGCCAAATCTGCCCCAGTTACCAATCCACATTAGTTGCCAGTTATGACCCTTTTTCAGTAGATGTCCCAGCAGGACCGTCAATTGCTTTGAAAAAATCTTCTCCTTATTTGCCAAAGAGTAATTCCCATCTATTTGTCCTTGAGCCTTTGTATGATATTCATACTAACCCAGTTGAAATTGCCAAACATTATTTCCCTCCTGGATTCCATTATATGCCTTCCAGTCCTTACAAGTCCCTCAAGTATTATAGAGATATATTGCTTGAAACTAAGTCAGTTGAAATTAAGCTGATAAAGGATCGAGATCACCCTGAGATCATTCTTTATCATTCAATGTACATCCACCATATCATAAGCCAAGAAGCCTTCAGTAGTCGCCCTTATGAGTTAAAAACCCTTCAATCCAAATTACAGTATAATTATGCTGATTATATTGATGCCTGGTACACTATTTTCCTTCACCAATCTGAGGATTTTAGTCATTCCTGGTTCATCAATTTCGATAATAAGTTCAAATGTTCTTTCCCCTATTGGTTCCTCCATTGGTGGGAAAAACATGGCCCAGTTGATGAAATTCTGCCTGTATCAGTTCTTGAATTAATCCGCCATTATACGGAGACTGCCAAGTTCTCCACCGTTGATTTATTCTTCCCCAAACAATTGCTTTTCCTTGCCAGATAAAAAGTTCCTTGGATCCTCAAATGGTCCTACAGAGTCACTAAGGATGTTCGTATCTTTGCCCGCCAATTTTCAGTCAAATGGTGGGACAAATTTGAAGTTCAAAGAATAGCCAAGTATGTTTACAATGATCTCCCTGCTGAGCCTCAGTCAACTTCTCCAGTTGGTTCTGTTCGATCCTCCCTCTCAGTCGAAGGAAAGTCGAAAGCAGAACTCCAAGAAATTGCTCGCCAGTTGATGATGCAGGCCTCTCAGATGGATGATGATGAGGACAACGACTCTCCTTCGTCGTCCAGTTGTCAACCCATTCCTGATCCTGTTAAGCCTAAGTCCTGGTATGATGACAGCCAGGATCCCTATGATGCCTATGATCTGCATTCTGACTAGGCCTCATAATTTCTGCTGACATCTCCACATGCAAAAGTTGCCCGACACCATAATTGCCACAGGAAGAAGCATCTGCTTTTTCTGTTTACTTTTCCACCGAAAGAAGCCATCAGTTCCAGACAAAGTCGCTCAGACCGCCAGCTGTCATCTGCCTTTCCACCGAAAGGAGCCAGCCGACAAAATTGAAAGTTGAATCAGGTTGCATGTGACTCCACTGTTGCAACCAACCATAGTTTAGCCAACCATAGTTGAAAGAACTCTAGTTGATTTTTCTCTTTATAAGCCGGCTTCGGCCTCAGTTGAAAACAGAATGTAAATTTTCCCTTCCTTCAGTTTCAAAGATTCTCTCTGATACATCCTTCGGAGAAAATCATCTTTGTAAATAGCGTGTGAGTTTGAAGTGTTGCAGTTGCAATCCTTTGTGAGTATAATAAAAGTTATTTTCAATTATCTATTTTCTGAAAAGCATTATTTTGATGCATTATCATTATTTTGCTGCATTAGTCTGAAAGTTGTTTAGGTTGCATTCATCTCTA

TTCTACTGTTCTTTAAATTCTTGGAATCTCAGTTCTTGTTCTTCCTTTCTGTTTCTCTGGTTCTTCCTTTATATATATATATATATATATATATATATATATATAAACGTTTAATCCTCTTTTTTAAAAAAACTTTAAAGAATACAGATTCTCACAGTTAGCGGCAGAGAGGATCTAATCATCTAATAATACGAAGATTCTACGTAGTGAACTTAAATTGATTCTTGTGTTGAATTATTATCTTGCCTTGAAATTTGAAGAGGCTATTTCTATGTTCTTGCTGCGGACCACAAGGTGAAGATATTTTACCATGTTTGCTCAATTATTAAGTACGAGATGATTGTGTTGCTTTTTTTTTTTTTTTTTTCCTCATCACCATTATTTCTCTGTTGCTTCTTTATTAGCTCCTCATAACTCAATGTGTCATGTTTGTCACTGATTCACTCCACTGCATTTTTTTTTCTCTTTTTCCTTCTAGTTTTTGGTCGGTTGATAGTTTTTGAAGGTTTTTTTTTTTTTTCCCCAAGCTTAGTCATTCATATAGTAGAGCATTCAAATTAGAAGTTGAAAGGACATAATGACCATGTTGTTTGTGATGTTGAGAGTTGCCTGCTTCACTAAATTAATTAGAAAGTTCAAAATCTGAATAGAGATCATGTAAAAATAGAAATATGTGATAACGTGTATTAGGGTATTTTTCGTAGTTAAATAAGGTGTACTTAGTTAATTTTACATTTTGATTTAAATATTAGGGTGTACTTTGATTATAGGGTTTGTTTAGCAACACTCTATATATTTTGGTAATTATTTTGCTAATTAATGCATGTATATATAGTTGGTAAATATATATTGTCAAAAAAAGGGTTTTGTATATGGATCTCTTTTTACGAGCGTAGAGATCATTAACATGACCAAACAACTTCTGAATTAAATGAACTACTTTTTTTTTTTCCACACATTTTAGATGACCGAACTAATAAATTTTTTTTGCATGGATAAT

>Chr7-11; OZ022212.1; 8848122-8854967; rev; A;

AGCTCTCCTAAGACCACGCGTTTCTTGGTGGCCGGCTGCTCTTGGCCCATGGCAGCTTCGGCCCTCCTCTTCATTGACCTTGTGACCCGGGCGCAGTTCTCCTTCTCCGACATGGCCCTAGCGAGTCTGTGAGTGTCGAGGTTTTGGTTGGGTTAGAGAGCGAGAAAGGTTTTGAGGGGTGAAAGAGAAAGAAGAGAGTTTTGTAGTGAGTAAAATGGGAGCGCCACTTTGAAAACTAAAGTTGGTGGTTTGAAGCAGGAGGGGGTTTTGTAGCATATATGGTGGTTTGAAGAGAGTTTTGTAGCATATATGGTGGTTTGAAGCAGGAGAGGGGCTTGAGGCTTCAAACCTGAGGGAGGCTTCAAACGAGATCCAAAACTAATTTAATGCTAAAAAGGGTAATAAAGATATTTCATCTTATTAATTAAATTAAAAAATATACAATTGTTGATTTTTGGGTTTATTTTGGGTGTGTTTCTATGTTTTAAATAGTGTAGGGTATATTTATAGTTTCATGTCTAGGAATGGGTGTTTCACTAATTTTGTATTTTTTATATATAAAAATCCTGGTATAGGCGCCTGGCTATACTATTTAATTAAAAAAATTAATAATAAAAAAAGACCTTCCTAGTTGCATTAGTTTGTACTTTATAGATATTAATTTGCAACATATCCAAAGTATATATTAATCTTCCACTATGTGATATTTTAATATAATTTTTATAACACATGTTATTCTTATTCAATAATATGTGAGAATTTTTTGTATAATACGTGAATTTTGTGTGTCTTACTGAAAATTTTGTACAAAATATGTCTATAAAATAGTAAAAATACGTACGTACACGTACAATACGACAATTGTATGTTTGCTTTTTAAAGAAAACGTGAGATGTGTATAGAACTCGAATGTATTGTTGAAAAATACATATGCATTACTTTCGTATTTTCTATTATTTGTGCTATCTGTGTTAATCCCTCCTGGTTCTAAATCCCCTTC

TGGTATCAGAGCCCTAAGTGTTGAAGAGTCAGTCTAAATCGGCTTCTCAAAGTCTAAACTCACTGGTCATCTTCTCAGTGTTTGAAGTTTACTTTATTCTTTAGCTTTTTCAGATTCTTCCTGTCAACCAAGTCTTCTCCCCCGGTCCAAACAATAAGTCCTAGGTTCCAAACAGTAAGTCCCAGGTGAGGCATGAGTGGATGCGGCATGAGCGGGAGAAACAGAGAACCCTTTAGTTCCCAGGAACCTTTGAGTCTGTTATAGGATAGCTACCTGCTTCTTACCCTTTGTAAGAAAAACCAGTAGCCTTGATGAGTCGTTTATTTAGAACTAACTCTTGTAACTCTAGCAGTTCTAGAGCCATTATTAGTAAACTCCCAGAGATAGTTAATGAAGAACAAATAGAATACGATTCTAGTAATGAAATGGATTATAAAGATTGGAATGTTCCTAAAATTCCTAGTCAAGAAATTTATAAGAAAAAACGGTCACTTTCTAGTTTCAAGTCAACCCACCCATGTGAAAACAGTCGAACAAGTTTATGCTTTAAGTAAAGAACATGAAACATGCCAGTTGCTTAATCTTGAGTTAATTAAAAAACATAAAAATGATGGAAACAATTTCCTTCACATTGGTCTAGTTCAAGTCGCAGTTAAGCCTCTCACCAGATTAGGATTAAAAGCTTTTATCCTTCTCTGCCTTCGAGGTGCCAGATTCACTAATTTTAGTGACAGTACTTTAGGAATAATTGAGTCAAGTCTATTCAACAGTCCAATTCATTTTGATTGTTGACCAAATTTCACACTAAGTCTTAGTGATCGTCATATCTTAAAAACCCTCACATTAAACATTAAAACTTCTGGGTATAAAGTCTTACCGGGTACCCAACCATTAGCCTTAGTTTACAGAATTTACTACAAAGTCACAGGTACTAATATGAATTTCCAAGCCCTCAATAAAAGTCCAAAGGATCAAACCTTGTTAACTCAAAGCAATAAAGCCAATGCCAATATAAGAGTTTCTCATACTATTAAGTGGTCAGAAGTCACCCTACCTTTAGATTGGACACTAACCACTAAGAATCAACTAGTTCCCATTCAACATAGTTTAAATAATCTAGATTATATTCAGTAGTATTTAGATGGCATAGTCAAAATTAATTTTGGAAATCAACCTCCCCAAAAGTCTTCAGTTCAATTACATCAACTTCCTACACCTAGTCAATCTTAGAGACATGCCCCTGCAAGACATTCCTATACAACTTCTTCCTCTACTCTAGAACGAGACCTAGAGTTAGAAAAAACCTTAATAGATTTAAAATTAGAATCTTTAAAGAAAACTTCCCAAGTAAACCAGCCCTGTTATGGAAAGAAACCAGTAAAAGAAGACACCAAATCCGAGTCTCCAGAGTCACCCACACAAACTGATTTCGAAGCTAGTTTTCAGATTGACAACCAGCTACGAACCCTTAGTAAAAAATTCAAAATTAATTGGAAAAGTTTACATAGTCATCTACATGCCCCTGAGAATCAAAAAAGAAGAGATCTTTACCACATAGCTTACCCCAGTAAAGAAAAACGCCAACAAATCTTTAATGAATGGAAAGATTACATGCGTACCGCCAAGTTAGAAATTTTTTATCTTGATTTTATTGAAAGTCGTTATTTGCCTAAAGAGTTAAAAACTTTAACCCAAGAAAAATGGGAAAAGGAAGATAACACCGAAGTCTTAGCCAGTCATCCCCCTGTAGAAACGATTATTATTAACCATAAAAAGTCTAAAATACCGGCTAGTCCCTTCAAGATCCCTGACCAAGATAATAAAAAATTAATGAGAAAAATAATTACACCAACCAAAGTCTAATAGTCATAGGAAAGCAATTAGACAATATAGAAACAAAAGTTGACAAATTACTCCCTCATGAAGTTAAACCCAAGTCCCAGATAAAGTCCTAGGATCCTCCCCTTTAGTCAATATCTCTGATTCTGAGTCTATTTCCAGTATGGATACAGAAACTAGTAATATATCAAAAATTGAAAATGCCTTTAAAAATTTAGAAATAGACAATGATCTAAAACTTAAAAGATTAAGAAGTAAAGTCAATCCCACAAGTCTAACCAAAAATTGGTATCCTAGACCAACTCCTCCAGACATTCAATTCGAAGAAAGAAATTCCCTGAGTCAATTCTCAGTCTCCTCCGATAAATTATACGAATGGAATATAGATGGTTTGTCTGAGCAAGAAATCTTAAACAAGTTAACTCATATGTCTATGATAGCCAATAGCTACATAACCAATCACAGTCTTAGACAGTCAGAGATTATTCCTCTATTAGAAACATGATTCACTGGAACCCTGCGTTCTTGGTGGGACAAACATTTAACTGAAGAGTCAAAACAACAAATAATTCATGCAGTCAAATTAAATGAAGAAGGTCTCCCTATCTTTGAAGAACAAATAGGTCGAGGTATAGAAGATGGAGTCAAGACTTTATTATACACAATAATTGAGCACTTCATAGGCACACCTAGTAACACAACATCCAGAATTCATGACCAACTAAGTAATCTCAGATGTCCCAAATTATTTGATTTTCGTTGGTATAAAGATGTTTTTATTTCCAGAGTCATGCTTAGAGATGATAGTAACCAACCATTTTAGAAAGAAAAATTCATTAATGGTCTTCCCAATCTGTTTGCCCATAAAATATGCACAGTTCTTAGTAATGATCAAGGTCATATAGATTTCGATAATTTAACCTATGGTAGTATAATCAGTATAATTAATCAAGTAGGTATGAAAATGTGTGTTGATATGAAAATTGGTAAACAAATTCAGTTAGAAAGAAATTCAGCCAAATATGAGCTAGGAGATTTCTGTGAACAATACGGTCTAGCTTCCATTCCTCCATCTAGAAAAGCCAAAAATAGTCACCATGACCGACAGGTTAGTAAACAACGACATTATTCAATGATAATAATGATAATAATGAGTTTTATGGTAAAAGGAAGTTTTACCCGCGTAAAAAATGGTCAAAAGGTCATGGAAAAGAGAAGAAAAGTCACTTTAAGAAAAGCAACGACCAAAAAAGAAGAAGTTAAATGCTGGAAATGTCAAAAGAACGGTCACTATGCTAATAAATGTAAAGTAAAAGACACAATAAAATAATTGAAAATCTCGGATGAAGAAAAAGACAAATTGATTAAAGTCTTAGAGTTAAGAAACTTCGAGTCTAGTGAAAATGAAACCATGGTAACCAACACTGAGTAGATAGATTATAACTCCATGGATTCACAACTCAGTTCCCCTAGAGTACAGCTCAGTTGCAAAGATAAATGCTGTAATAGCTTAAGCAAGTCAATTAGTGTCCTCACCAAACAAGAAGAACAAGAAGAATTGTTAATTGATTTAATCAGTAAAATAGAAAACCCAGAGTTAAAATCAGAGTATTTAAAGAAATTAAGAAAAGTCGTTAGTCAAGAAGAACCAAGTCAGTCTATTCCTCAGAAAATAAGTCTAAACACAACTTTAGAAAAATTCAATAAAAGAAAAGAAGTCACCCTGCAGGATTTACAAATGGAAGTAAAATTAGTCAAAAAGGAAATAGTATAGTTAAGGCAAATAAGTCACAAATTACAAGTCGAGAATTATGAAATAAAGCAAGACTTAATTAATTTAATAGATAAAGAAACCTCCGAGTCAAAAAATAATTCTGAGAGCCCTACAAACTCAGATGGTGAACCTTCAAACAATACTCAAGCCATTAATTTAATTAAACAAGTAAATTTTCGGAAATGGTATTCTAAAGTCACCATTTTTGTCAAAAATTTCGAGTTAAATACAGTCGCCCTTTTTGATTCAGGAGCTGATCTTAACTGTATTATAAGAAGTCAAAAGAATCGTTAAGCACTGCATCAGGAAAGTCACTCCAATTAAACTACAAAATTCCCAAGGCCTATGTTTGTTAAAATAAAATTTGTTTCAAAACCCTCATTCGTCTTAATAAAAAAATATCACTGATGAAGTCATTCTAGGGTTACCCTTCATTGCCCTTTTGTATCCATTTAGGAGAAAATGTCAAATTCGAATTCCTAGCCAAACCCGAGTTACATGATCTAAAAGCCTTACAAAAGAGTGCAGTTTCAAAAACAGTCAAAATAATTCAAAATAAAGATAAATAATTAAATTTTCTTAAAGAAGAAGTTCGATTCAAAAGAATGGAACAACAACTTGCTGATGAGTCCTTACAATCACAAATTCAAGAGTTTGAAGAAAAGTTAAAACAAGAAGTCTGTTCTGATTTACCTAATGCTTTTTGGCACAGGAAACAACATGTAGTCAGTTTACCTTATGTGAAAAACTTTAGTGAAAAGAATATTCTCACTAAAGCCAGACCAATCCAAATGAGTCAAGAAATAATGGAATTCTATAAAACAGAAATTACAGAGTTACTCCAGAAAGGAATAATCCATAAAAGCAAGTCTCCCTGGTCCTGCCCAGCCTTCTATGTTCAGAAAAATGCTGAATTAGAAAGAGGTGCCCCAAGAATAGTCATCAATTATAAGCCTCTCAATACAGCTTTAGAATGGATTAGGTGTCCAATTCCTAACAAAAGAGACTTAATTAACAGGTTAGATAAATCAGTCTTTTCTAAATTTGATATGAAGATTGGTTTTTGGCAAATCCAGATTCAAGAGTCAGATAGGTATAAAACCGCTTTTGTAACCGCATTCGGTCATTATGAGTGGAATGTTATGCCTTTCGGTCTTAAAAATGCGCCTAGTGAATTTCAAGATATAATGAATGAAATATTCAATCCTTTCAGTCATTTTTCCATAGTCTATATTGATGATGTTCTTATTTTTTCTCAGTCAATAGAACAACATTGGAAACATTTGCATAAGTTCCTTCAGATAGTCAAACAAAATGGTCTAGTTGTCTCAGCTAAGAAGATCAAGTTATTTCAAACCAATATTCGTTTCCTTGGTTCCAATATCTATCAGTCACAAATCAGTCTAATTGATCGAGTCATTCAGTTTGCTGATAAATTTTCATACCAGATACTTGATAAAAGTCAACTTCATAGATTTTTAGGTTCTCTTAACTATATGTCTCTGATTTCTATCAAAATTTGAGAAAACAATGTAAGCCTTTATTTGATAGACTCCAGAGTAACCCTACTCCTTGGCCACCTATTCACACAAATATAGTAAAGCAAATCAAAATCCATGTTAAAACACTTCAATGTCTTGGCATTCCTTCAGTAAACTCCTTTAAAATTGTTGAAATTGACGCCTTTGATGTTGGTTACGAAGGTATCCTTAAACAAAAAGTTAATCCAAGTCAAACAGAGCAAATCATTCCATTCCATTCTGGAATTTGGACCCAGTCACAACGTAATTATAGTACTATTAAAAAGGAAATATTATCTATAGTATTATGCATTACTAAATTTCAAGACGATTTATTAAATCAAAAATTTCTAGTCAGAGTTGATTGTAAAAGTGCAAAGCATGTTTTAGAAAATGATATTCGAAACATTCCATCAAAGCAGATTTTTACACGTTGGCAAGCAATTTTAAGTATTTTTTATTTTGATATTGAATATTTGAGAGGTAGTGAGAATTCTATTCCTGATTTCCTTACTAGAGAGTTTCTTCAGGAAAAACAATGGCAGATAAAAAGAAAAGAAAAGCAGAAAGAGCAACCCAGTCAACCCCAGTCACTCAGATCACTCCCTCCATGAATGAATCCAGTTAAAACAGAGTCCCCTTCCCAGATCGTCCCTTTCCCGAGATGTTCTCCAATTCAGATCAGTAACAGATTCACTACTCTTGGCTCCACAATTAGTTAAGTCCGCCCAAATTACCAGTCAGCCTTAGTCTCTAGTTATGATCCCTTTCAAATTAGTACCCAATCACAATTGCAAAGTCATATTTCCCCCCAAATTGCCATTTCGTTCCTCAAGCTCCTTATAAATCCCTCAAATATTATAGGGATATCCTTCATGGAACCCAATCAATCGAAATTAAGCCCATTAAAGATCGTAATAATCCCCACATTATTCTATATCATTCCATGTACATTCATCGCATTCTAAGTGAAGAAGAATGGAGTAAAAACCCCTATGACCTAAAAGTTCTCCAGTCAGATCTCCAATACAGTTACTGTGATTATATTGAAGTTTGGTATACAATCTTCCTCCATTAGACAATTGATTTTAGTCACTCTTGGTTCATTAATTTTGATAGTAAATTTAAAAGTCATTTTCCCTTCTGGTTCCCCACTGGTGGGATAAAAATGGTCCAGTCATTGATCTTCTTCCTTCCCAAATGCAAGGGTTAGTTAATTATTTCTCCAATAAAAACAAATTTAAGAGTCAGAGTTATTCTTTCCCAATCGGCTTATTCTCATTGCTAAATACAAAGTCCCTTGGATCCTGAAATGGTCTTACCAAATCAACTGGGATTCTAGAGTCCTTTCCCATCAATTTTCAGTCAAATGATGGGATAAGTTCAAAATTGAAAGAATTGCTGATTATTTAATTCAAATTTTCCTCCAGTCAAAATTTCACAAAAGCTTTCCCATAAGCCATCTTCTTCTGGAACGTCTCATTCTTCCCTCTCAATCGAAGGACGAACAAAGGCAGAGTTACAAGAAATTGCCCGGCAGCTAATCATTCAAGCTTCCCAAATGGAAGATGAGGATAATGATGCTTCTCCCAAGTCACAAACATCTACCAGTCAACCTCATCAATCACCCACTCAAAAGGATCCATCCCAGAAATTGAGGTGGGCAGATTATCAAGACAGTCAAGACCCTTATGATCTTGATTCAGATTAGTC

AAAAGCAGTCACCATGAGCCAGTTAAAAGCAAGGTAAAAGTGGATTCCCTAACAAAGTTAAAAGCAGCACTCAATAAAAGCAAAAACTTTTGTGTGCTTCCACAGTCACTCTCTTGAGCATCCAATGTTGCCGACACTTCTACTTCGGCTCTTTAAATAGAGGCTTCAACCTCACTTGTAGGCAGAATGTAACTTAGTTGATGTAAAATATGTTATCACTGTAAAATATGTGTGCTGTCAATGAATAAAATCTTTTAGTCAAGATTTTCTGTAAGTATTGCATTCTCAACAATTTTATGTTTTCAGTTATCCATATTTTCATCTATTCATTAATTTCATTTATGCATTACTTTCATATTTTCTACTGTTTGTGCTATCTATGTTAATCCCGCCTGGTTCTAGATCCCCTTCTGTATTGCATTCTCAACAATTTTATGTTTTCAATTATCCATCTTTTCATCTATTCATTAATTTCATTTATGCATTACTTTCGTATTTTCAATTATTTTTGTGTATACATATATATGTAAAAGACTATAGCCACTCGTCTATACTATTTATTAAGGATAATTTATAAATAAATTATATATTAAAAAAAAACCAGTAATGCTTCATTGCTATACTAATTTTTTTAAACAAATAGAAAAAAAATCAAGTATAGCCGCACGGCTATACTATTTTCTAAACAAAAAACATAGAAGCCCACTCTGTGAAAATAGTATAGTCGGGTGGCTATAAATTTTTTTAAAGTTTTAAAAACTTAGTATAGCCCGAGCGGCTATACAATTTTTTAATATAAAAAAGAGGATCCCCCAACCATTGAGTGCCATGTGTCACCCCATGTATTTTTAAAAATAAAAAATAAAAAACAAGGAGCATAGGCGGATAGCTGTACTCTTTTTTTTTAAATAAAGAAGAACCTCTATCGGTTAAAGCCACGTGTCAAAAACTAGTATAGCCGCCTGGCTATACTGTTTTTTATATAATAAAAATCGCACCC

>Chr7-12; OZ022212.1; 9380045-9382478; rev; B;

TTTCCCTCTTTTCGGCAAGGGAGGCCGACGGAAGGAACTTTTGGTCATTCTTCTTTTGGATGTGGGGCGTGCCACCACCAAGCCCTGAATTTGGGCTTGAATGGAATGTGAATAGATGGATGTGTTGCGCTTCTAACTTCTAACCAAACGATTGATCGGCCAAGCAAGGAACCTCCTATTGGCCTAGATTCTTTCACTTCCTCGGGCTCGAGGATGGTTAGGTTCCGTACGGATGTTTTTGTGATTTTTAGGGTTTATGTAAAAATAAAGCAATTAAACTAAATATGCGTATAAACGTAAAGACAAGAATTGAAGTGCGATAAAACAAATCAAAGCGATAAATAAAGCAAGAACAAAATAAAAGCGATAAGAACGATAAAATAAACAAACTTGAAATTAAACTTAATTGAAGTGTGATAACAATGAAATGTAAGGAATCGAAATAAACTTTAATTTAAATGACAAGAAATATAAATCGAACAAGAATTTAAATGACAAGAAAAATATAAACTTACACTAAAAAAAGGAAGAACTCACTACGCTAGGTGCTTGAAAAATAAAATCCTAAGTCTAGGAAAAGAAAAGTGCAAGACGAAAAGTAGGTTTGTTTGTCTGAACTTGTGTCCTATGATGTCTTTATCGAGCAAGGTTTATATAGGGCACTTGTCAGCCCATTGGCTGGCCATTGAATGGCCAAATGTCAACTCTTCTTGAAAATATTACCTTTCAAAGCCCCCCTAAATCCGTGGGATTGCATTGATTGAAATATTCTAACATGCTTGATTCTTTTTTGCTTTGGAACATTCGGAGTTATTATCAAATTCCTTCCTTGTGAGGCCTTTGTCACGTGCATATAATCCTCTGAGAATCTTGGGCTTGAAATTGGTGGCTTAGAACTTCAGTCTTTGGAATGGCATGATGATGCCCTTCATAATTTCCGAATTCGTGAAGCTCTAAAATTTGTCTGCCTAATTCAAACTTTATATTCATTTATAAGTCA

ACCACACATGTTAAGACAGTTGAGCAAGTTTATGCCCTTAGCAAAGAACATGAGACGTGTCAATTACTTAATCTTGAGTCAATTAAGAAACATAAAAACGATGGTAATAATTTTCTGCATATTGGTCTAGTCCAAGTTGCCGTAAAGCCACTTACTAGATTAGGATTAAAAGCTTCAATTTTATTATGCTTGCGAGATGCTAGATTTACTGATTTAAAAGACAATACTCTAGGAATGATCGAGTCTAGTTTATTTAAGGGCCCAATACATGTTGATTGTTACCCATATTTTACGGTAAGCCTTAGTGATCCCCATATTTTAAAAGCATTGACATTAAATATCAAGACTGTTGGATACAAAGTTATACCAGGTACCCAGCCATTAGCCTTAGTTTTTAGAATCTATTATAAAGTCATAGGAACCAATATGAATTTTCAAGCCCTAAACAAGAGCCCCAAAGATCAAACCTTTTAATTTATAGTAGTAAAGCTGATGCGAATAACCGAGTACCACATACTATTAAATGGTCTGAAGTAAGCCTACCTTGAGACTGGACTTTAGTTACTAAGAGTCAGCCAACCCTTATTCAACATAGTTTAAATAATTTAGATTATATTCAACAATACTTAGATGGTACAGTTAAGATTAATTTTGGTAATCAACAACCTCATAAGTCTTCAGTTCAGCTACAACAGTTACCTACACCTAGTCATTCCCAAAGACATGCCCCTGCACGACATTCTTATACAGCTTCTTCCTCTACTCTAGAACGAGACCTAGAGTTAGAAAAAAATTTAATTGATTTAAAATTAGAATCATTAAGAAAATCGTCCGAAGTTAACCAACCTTGATATGGTAAGAAACCAGTTACAGAAGAGACTAAGTCTGAGTCTCCTGAGTCTTCCACACAAATAGATTTTGAAGCCAATTTTTGCACTGATAATCAATTAAGAACCCTTAATTGGAAAAGATTAGTTTAACATTAATTGGAAAACATTAGAAGATCACCTAAATGCCCCTGAAAATAAAGACAGAAGAAATAATTACCACAAAGTTATCCCTGAAAAAACCAAAAGACAACAAATTTTTATTGAGTGGAAAGACTATATGCGTTCTGCCATAGTTGAAATATTTTGTCTCGATTTTGTAGAAAGCCAATATTTACCCAATGAGTTAAAAACTTTAACCCAAGAAAAATGGGAAAAAGAAGATAAGACAAAAGTACTAGCTAGTCATCCTCTGATAAAGTCAATTATTATTAGCCATAAGAAGTCCAAAATAGCTGCTAGTCCCTTTATGATTCCTGATGAAGACAACAGAAAGATAGTTGAACAGAATAATTACACCAACCAAAGTCTAGTTGACAATCTACTTCCCTAAGAGACTACGCCCAAGTTACAAAAAGAAAAACCTATAGTTAAATTTCAAGATTTAAAATCAAAACTAACCTTTAAGACCAACACCACAATAGGAAAGATAGAAAAAATGTTAGAAGAATTAACACCAACCAAACGAAAAGACAAAGAAAAGGCTGAAACTAGTATTAAAGTTATAGGATCCTCTGAGTTAAACTATACTTTCAATAATTCCTCTGAGTCAGAATCTACCATTAGTTTAAGCACAGAAAGTAGCAATATTTCAAAAATTGAAAATGCATTTAAGAATCTAGAATTGGAAAATGATTTGAAAGTTAAAAGATTAACTAACAAAGTCAACTCTACAAGTCTTACAAAAAATTGGTATCCTAAGCCTATGCCCCCAGATATTCAATTTGAAGAAAGAAATAACCAAAGCCAATTTTCAGTTTCTGCTGATAAGTTATATGAATGAAATATAGATGGTATTTTTGAGCAAGAAATCCTTAATAAACTTGAACATATGTCTATGGTAGCCAGTAGTTACGTTACTAACCACAGTTTTAGACAATCTGAGATAGTTCCCCTATTAGAAACAGGACTGGAACATTACGTTCTTGGTGGGATAAGCACCTAACAGATGAGTCAAAACAATAATGAAGATGGTCTTCCCTTTTTGGACGAACAAATAGGTCGAGGAATAGAAGATGGAGTTAACACTTTATTATATACAATCATTGAGCATTTTATTGGAACACCTAGTAACACTACTGCTAGAATTCATGATCAACTTCGTAATCTTAGATGTCCTAAGTTATCTGATTTCAGATGGTATAAAGATGTTTTTATTTCTAGAGTCATGATTAGAGAAGATAGTAATCAACCATTATGGAAAGAGAAATTTATTAATGGTCTTCCGAATCTATTTTCCCACAAGATTAGAACAACCCTTAGTAATGATCAAGGTCATATTGATTGGAATAATTTAACTTATGGTAATATAATATAATATATATATATATATTAAGGTTATATTCATAGATCATCCTTTAAAAAAATTAGACAATTCAGAAACTGTT

TCGATATCCAATTATGTCCTGCAAAATCAATGAAAACGATGCTTCAAGAAAGTACTAAAATTTCAATAATTCAATTGAGTGGTCAAATGATATCGAATTCAAGTGATTTTTTTGTAGAGATGATATTTGAATGAGTATCTATAAAAGAGACGGTTTGGATTAGTGAAATACAATACATGGTGGGCCCTACAAGGGTATCCCTCAAATAAGCTAATTTGAGGGATCCCTCAATAGAAGCTCTCTGTATATATATATATATATATATATTCATATGGAATCAGATGAGTGCGGCGAAATGCTGTGGTTGGGCTCTTTAAAGTGAAACCTCATTGCCCTATTATATATATAGAAATTTAATATATAGTAATATTTATATATTAATAAGTCAGTCAAACTCAAAACCACTTTTAGCCCAGTTTTGGAGACCACACAAAGCTCCAGCCGCATTCAACCATTCGAGAAAACCCGGGCGCAACTCCTTGGGCAATAAAAATCCGGCTACAATTTAAGAAGCCCGGACTGCTACGGTCTCCAAGCTGCAAGGCCCCGGTCGGATTTTGAACTCCTAGCCCGGCTGCACCTCGCAAGCTCCTGTTCCAACAGCTGCCCAACTCAGCGATTAAGAAGTTTGGTTGAACTCTCCATCCGAAAATTCAGCTGAGATTTTCCAAGAACTCCCCCAGCAGTCCCGGCCGTAATGGACTCCAAGCCCGACCATAACTTTCACTTGAAACCCGACCGAGACCCGCCAAAAAATTCTAGCAATTCCGGTCGCAAAGAACCCCAGCAGATCTGACCTTGAGCTTTGCCCAACTTTGGAGTCCCACACCCGGCCGCTATTCCAAAGTGCTTGGGTGAATTTTCCTACCTCCAAACATTGCCCCCTCGGCTTGCTTGGCTCGGGTTCATTGTAGAGGTAAGCAAGTCGAAACCCAAGGCCATTTAATTTTTTGAATTTTGAAACTCTTTGCTTTCCTTGCACGTTATTCAATTTTGAATT

>Chr7-13; OZ022212.1; 12435045-12439457; rev; B;

GAACAGCAGGAAATGTTTTTGGACACGGCATGATGCATGATTTACTACAGCAACTGGGCAGAGAAATTGTTCGCGGAGAACCCAAAAAGGAAGGAGAATGCAGCAGATTGTGGCTTCATACAGACGCCCTTCCTGTACTGAAGAAAAATAAGGTAAGAATTTAGTATAACCATATAGTACTCCCTAAAAAATGCATTTTGATTTTACCAATGCTAATATTTAATGGATTTTGAGAAACAAATTTTCTTTTGAATGCCTATGTTTCATATTCACTTAGATTAAAAAGCTGTTTAGTTATTATTTTAAAGGTAGATGTCTACTTCATTTGAATTTCAAATTTTTGGTTTGCTGAAACGAGCTTTCAAAGTTGCTAATACTCTATGGCATGAATACTTTTCTATTATGTGTTGGGGACTCCTTATTTAGTTAGGATTTCGATTACTTATCCGTTTGTTTAGTCCTATTGTAATTGAGATTTATTTCCTATTTGATCTCTTTTAGGGTTGGCACATCTTTCTACTTCTTTATACATAACTTTATATTGGGAAAGAAAACATATCTGAAAATTATCATATACTTTGTTTGGCATGGTTGCATTTAACTCTCTTACCTTGATATATTCCCATGAGGATATGGCCTATATCTTGGCTCCGACGAGTGCTACTCTTCAGGGGGAGGGAGATCAAAGAGAAGTTGTGTTTGCGTGAATTAGGTCAAGTTGATATAGAAGAATTCTCCTTTAACGTTGCTGTTTATAACTTTGCCTTTGTCCTTGCCTTTGCTCTTACTTTGCTTTGCCTTGCCAGTGCTTTGTGTTGGGTTACGTACTATGATATTATATGGGGTAATGCTTATCATTTTAGGATTGGTTTATTTATGACATGTGGTTAATGTTAAAAATAGTCGTAGGGTATTTTCTATTGGCGTGAGCCATAATTCAGGGATCTCGTGATCCTTGATTTCCTGATGTAATTCACGTTACCTAATTAGAATAATTACC

TAGCTAGGATTCTTTGTAATTTGTTAATTACTTTTTCTTGTAGGAAAAGTCAACTTGTACTTGTATATATATATATATATATATATTTTGAACTCACCACAGTTGCTTTGTTTGATTCAGGAGCAGATCTTAACTGTATCCAGAAAGGTCTAATCCCCACTAAGTATTACCAGAAGCCTAGAGAGTCCCTTAGTACTGCCTCCGGGAAATCTCTTCAGTTAAATTTCGAATTACCAAAAGCCCATATTTGCCAAAACAAAGTCTGTTTCAAAACCTCGTTTGTACTCGTTAGAAACATCACTGACGAAGTCATCTTAGGTTTACCTTTTCTTGCTTTATTGTATCCTTTCCAGGTAGAATACGATGGTATCATTTCTACCTATTTAGGCGAAAAGGTCAAGTTTGAATTCTTAACGAAACCAGAGTTGCATAATTTAAAAGCCTTACAAAAACAATCAGTCTCTAAATCTGTTCAAGTTGTCCGAAAAACTAATCAATTGAAATTTCTTAAAGAAGAAATTAGGTTTAAAAGAGTCGAACAACAACTTGCTGATAAGGCCTTACAATTGCAGATACAGTCTTTTGAACATAAATTGAAAAATGAAATTTGTTCTGAATTGCCTACTGCTTTTTGGTATAGAAAACAGCACGTCGTCTGTCTTCCCTATATCAAAACCTTTAGTGAAAAGAATATCCCCACTAAAGCACGGCCAATTCAGATGAATCAAGAATTAATGGAATTTTGTAAAAAAGAAATCCAAGAGTTGCTCCAAAAGAACATAATTCGCAAGAGTAAGTCCCCTTGGTCGTGTCCTGCTTTTTATGTCCAGAAAAATGCTGAACTAGAAAGAGGAGTCCCGAGATTATTTATCAATTATAAGCCTCTTAATTCGGTTTTGGAATGGATTAGGTATCCTATTCCTAACAAGAGAGATCTCATAAACCGATTAGGAAACGCAGTTGTCTATTCCAAATTTGACATGAAAAGCGGTTTCTGGCAAATCCAGATAGATGAGTCAGATAGATATAAGACTGCATTTGTTACTCCTTTCGGTCATTATGAATGGAATGTTATGCCGTTTGGTCTAAAAAATGCCCATGGTGAATTCCAGAATATTATGAATGATATCTTCAATCCATACAGTCATTTCTCGATTGTTTATATTGATGATGTTCTCATTTTCTCTGAGTCAGTTGAACAACATTGGAAACACTTGCATAAATTCCTTCATATAGTCAAACAGAATGGTTTAGTTGTCTCTGCCAAGAAAATTAAGTTGTTCCAAACCAATATTAGATTTCTCGGTTTCCAAATCTGCCAGTCTCAAATTTCCCCAATTGATCGAGTCATCCAATTTGCTGATAAATCACCTGATCAGATCCTTGATAAAAGTCAACTCCAGAAGTTTCTAGGATCTCTAAATTATGTCTCTGACTTCTATCACAATCTGCGAAAACAATGCAAGCCCCTATTTGATCGATTGCAGAATAATCCTCCTCCTTGGTCAAGTCTCCATACTGAAGTTGTCAAACAAATCAAAGCCCATGTCAAGACACTCCCCTGTCTTGGTATTCCTTCAGTTGATTCTTTCAAAATAGTTGAAACGGATGCCTCTGACATAGGTTATGGTGGCATTCTCAAATAGAAAGTCAGTCCAGAGTCTCCAGAACAAATTGTTCGTTTCCATTCTGGAACCTGGACCCAGTCGCAATATAATTATAGTACTATTAAAAAAGAAATATTAGCTATTGTATTATGCATTAGTAAATTTCAAGATGATTTACTTAATCAAAAGTTTTTAATAAGAGTTGATTGCAAATCTGCAAAACATGTTTTAGAAAAAGATGTTCATAACATTGCATCTAAACAGATTTTTGCACGATGGCAAAGTATATTAAGTATATTTGATTTTGACATTGAATATTTGAGAGGTAGTGAGAATTCAGTCCCTGATTTTCTTATTCGAGAATTTTTGCAGGAACCACAATGGCTCCCAAAAAAGACAAACACAAAGAGTCAGCCAGCCAACCCAAGTCAACCCAGTCGCAACCATCCCAATCTCCATCCATGAGCCAAAATAAGCCAGAATTGCCCAAACAGATAGTTCCCTATCCTGGTTGCCCCCCTCTTACAGTTGCTACCCCATTATCAGTTGCAAACAGATTCTCCTCTTTAGGATCCACAGTTGGCCAAATTCGCCCCAGTTATCAATCAACATTAGTTGCCAGTTATGATCCTTTTTCAGTTGATATACCTGCCGGACCCTCAGTAGCACCCAAAAAGTCGTCTCCTTATTTGCCTAAGAGTAATTCTCACTTATTTGTCATTGAGCCAGTATATGATGTTCATGCCAACCCAGTTGAAATCGCCAAACACTATTTCCCTCCTGGATTCCATTATATGCCTTCCAGTCCTTATAAGTCTCTCAAGTATTATAGAGATATATTACTTGAAACCAAGTCAGTTGAAATCAAGCCAATAAAGGATTGTGATCATCCCGAGATCATCCTTTATCATTCTCTCTATATCCACCAGATTATAAGCTAAGAAGCCTTCAGCAGTTGCCCTTATGAACTCAAGTTGCTCCAGTCAAAATTGCAGTATAATTATTCTGATTATATTGACGCTTGGTATACTATATTTCTTCACCAATCTGAAGATTTTAGTCATTCCTGGTTTATCAATTTTGATAATAAGTTCAAGTGTTCTTTCCCCTATTGGTTTCTCCATTGGTGGGAAAAGCGTGGTCCAGTTGATGAAATATTGCCTGTTTTAGTCCATGAGTTAATCCGCCATTACACCAAGAAGGCCAAATTCTCCAAAGTTGAATTATTCTTCCCCAAACAGTTGCTGTTTCTTGCCAGGTATAAGGTGCCTTGGATCCTCAAATGGTCCTACAGAATCACCAAAGATGCCCGGATCTTTGCCCGCCAATTCTATGTTAAATGGTGGGATAAATTTGAAGTTGATCGAATTGCCAAGTATGTCTATAATGATCTACCAGTGGAGCCAATGCCTCGCCCAATTTTCCCTACTGGTTCTATCCGATCTTCCCTCTCAGTCGAAGGAAAATGCAAAGCAGAACTCCAGGAAATTGCACGCCAGTTGATAATGCAAGCTTCCCAGATGGATGATGATGAGGACAACGACTCTCCTTCGTCGTCCAGATCAGCCCATTCCGAATCTTGTTAAGCCAAAGTCGTGGTATGATGACAGTCAGGACCCTTATGATGCCTATGATCTGCATTCCGACTAGGCCTCATAATTGCTGCCGACATCTTCACATGCCAAAGTTGCCGACACCTTCACATGCCAAAGTTGCCCGACACCATAACTGCCACAGGAAGAAGCATCTGCTTTTACTGTTTGCTTTTCCACCGAAAGCTGCCTCAGTAAAAGAATCTTCCAAATAAAGACAAAATTGAAAGTTGAATCAGGTTGCTTTCCCGTGCCACTACAGTTGCAGCAAATCTCAGTTACTTTACTATAGTTAGTCTAAACCATAGTTGCTTTTCTCTATATAAGCAGGCTTCAGCCTCAATTGCAGGCAGAATGTAATTTTCCATTTCAAGTTTTCTTCAGTTTAAAGATTCACTCTAAAACCTTTTTTAGAGAAATCATCCTTGTAAATACAGAGTGAAGTTGTTTGAAGTTGCAGTAGCAATCTTTGTGAGTATAAATAAAAGTTGCTATTTTCAATCTATCTATTTTATGAAAAGCATTATTTTGATGCATTATCATTATTTTGTTGCATTAGTCTGAAAGTTGTTTAGGATTGCATCCACTCTATTCTTCTGTTCTTCCCTTCTCTTATATTTAGTCTGTTCTTCATTCTGTTTCTCTGGTTCTCCCTCTGTTGGTATCAGAGCCGTAAGTGTTACGGTCTTTGTCTAAGTCTGCTACTCATCCCCGCCCAAGTGGCTGGTTTAAGCTTCTGAGTAGAAGGTTTCATTCCTTCCTTTTCTTCCCTTTTATTCTTTGAGTTATCTGTTACTTTTCTCTAAGGTCTGACCCGTGAGTCATTTTTCCTAGATCAGTAAGTCCCAAGGGAGGGCGTGTGTGGTCGGCAGGAGACAGAAAATTGAGAGAAGTTAATAGATTTCTTAGAGTCTAACCGGTCAGGTTAGGATTTACTGTTTATCTTCTTCCTCAGACAGCCAAACCCAGTTATTTGAGCAGTCATGAGTCACTTATTTAGAACAAAGTCTTGTAATTTGAACAGTTCTAATATCAGTATGAGTCGACTCCCAGAAATAGTTAATGAAGAACAATTTGAATATAATTCCAGTGGTAAAGAACAATTAGATTTTAGAGATTGGAACATACCCAAAGTCCCCAGTCAGAAAATCTATAAGAAACATTGGTTGCCTTCCAGTTTTAATAGCACAACACATGTCAAAACAGTTGAACAA

ATATTGTTACTTTTGAATACCATTTTCTAAGATGGACTTGTTTGATTAGATTGACAACTTGTTGATCTTCAGAAGGCTCATCATTAGAATGATGGGGGCTTCCTGAATGGCTTTTAGGCTCAAAAGCTTTCTGTTTGAGAAGATCTTTAAACTTGTCAGTTGCTCCAGTATACGCTCCAGTACTGGATGTACTGCCACTCCCTTCTCTTATATATATATATATATATATATATATATATATCATCCTCTTGGAGAAATATAATATCAACCACAATTATTCAATCCTGTGTTTAGTTTGAGTTGGTATCAGAGTCAGTTAACTCTCAAACAGAAATACAAACCCTACCCATCGACGTCCTCTAGCGTCGAATATAGAAAAATAGAAAAACATAGCTGCTGCAAAACAGCAGCTAAGCCCTTATCGCCACAAACCTAGTGCGGCAAAAGACTCCTACATCACCAACCTATCCAAACTTGTTCTCTCTCCTCCATGGCTTTCAAAGATACTCCCGACAACCACACCACAGCTGCAAAATCTATGCTTCCTGTGCTTCAGAAACCCATAAAAATCTTGATATGGTGCAACCAATTGTTACAGTTCGCAATGATACTTTTGCTGCCCCATTAACCATACGGTTGAATGGAAAAAACTATAACACTTGGTTGAAGATGATGTTGCTCAATGTGTCAGGCCAGGGAAAAAGAGGTTATCTAGCAGGGAAGGTGGCTTAAGTGGAGGAAGATGCTTCGGGCTTCGATTCGTGGTGTATTGAGGACTTCGTTGTTAAGGGATGGTTGATAAAAACCGTGGAACCTGATTTGGTAGAATTGTTCTTAGATCTACCAATAGCTAAAGATGTTTGGGTGAGTACAACCTAGATGTATTATGACGTGTCTGATGAGTTGCAGATTTATGAATTACGGTGTGAAGCAACTCGTATTACTCAAGGAGGTCACTTTAATGCATCATACTCTGCTGAGCACAAGAGCGTTTGGTTGG

>Chr7-14; OZ022212.1; 12611081-12614177; dir; B;

GCAATACAAAAGCAATACTACTACAATACAATCGCAATAAAAACACAATACGATTGCAATAGCAAATCAACAATGCTACCTCACTATGATATAATTATACGTTCATTGAAAATCCAAATACCAAATGCAAGAATCAATCAACAAAGAACAAACACATTAATTTGTTGTTTCAGACAAACAAAATCAATCCCAATACTTCACTTTCTACACAAAATACAGACGACATCAATCACAATTCCAATTTCAGTAATTAAATTCAATTTAACATCAAAACTTACATATTCCCAACAATTCATTTTCTGGAGAAGGTGTTCTTGAAGAGGGCATGTAAACCCAGACTTCAAAACCCCCCAAAATTGGACCAAAATGGTCAAACAATGGCCACCTAATGCCAAGACATGCTGTCATATTCAGAAAAGAGGGTTTTGCTTGGGTTTGTTCAGGTTGCAAAAGGGATTCGAAGAGAGACGAAGTTGGAGTTTGGATTTGGGTAGAGAAGAAAACATAGAGGAAGAGGAAGAATTGGGTTTTGAGGGCAAAGCTGTGGGTTTTATAGGTTGTTCTTGATCTTCTTCTTCGTGAGAAGAGGCGTAGTTTGCTAGAGAGAGAGAGAGAGAGAGAGAGAGAGAGCCGCGCTAGAAAGAGAGCAAAGAGAGAGACAGAGAGAGAACTAAATTTCAGAAAAAGTGATATGTGTGCAATAAGTGGACAATAAATATATATTTATAGGTGATAGTTGCAAAAAAATTGGGGAGGGGTGGTATTTTCAGTTAAAATTATAAAAAGGGTGGCATTTTGACCTATTTCCCAAAAAAAAAAAGGCCAGTTAGGTGGCCTTGACGCCAAAGTCATCGAAATGGCCTTGCAGCCACGAAGAAATTCAAATGTTAAGATGAAAGTATGCAGCCTAACAAAATTAGAAGTAATGGCAACATAGTTTAATTGGAGATGTTAACAGGGCAATATTTGTGTGAGAAATATATATATATATATATATATA

GTCAAACTGAGCAAATTGTTCGATTCCATTCTGGTATTTGGTCACAATCCCAAAATAATTATAGTACTATTAAAAAAGAAATATTATGTAATAACCCAAAACAAAATATCCTAAAATTAGTATTAATCTATTCTAGAAGAAAGACGATTTTGCCCTCACATTATTTAATAGGGAAAAAGTTGACTTTTTGACAGAGAATGAATTTGGCAATTTCGCTTATGCCGTTGCGTAGAACACGACGAAACGAGTCCGTAAACATGGAGTGGACTCGAATCGGAGTTGTAACGAAGAAAATACGGTCAAAATATTGTGAAGGGCAAAACGGTAATTTGGACAAAAATTCAGATTTTTATCCCTTTCCTCTCTCTCTCTCTCTCTCTCTCTCCTCAGTTTCTCTCTCCTCTCTCTCCTTCCGTCGCGCGACCGCCCCTCCCCTCCTCCCTTCCAGCCGTTCCCGCCGCCACCACAGGCCGTCTCGATCTCCGGCACGGGTACCATCGGAACCGCCTAGGCCCCGCCGTCCTTTCCCGACCCGTCTCCTCCCTTGGGTCGCCCGGAGTTGGCCGGAAACGGGAGAAAACCGACCGGTTTTCATCGAATTTTCACGCCTTTCGTTCTCCTTCGTTTCTCAACCAAATCGTTCGAGTAAGGTATGGATTCTCACCTATTTTTCATGCTCTAGCTGATGGTTGGATAGGTTTTCATCGATTTTAGCTGTAGATCACTCGATTTTCGAATTGAAAATTGGTCGAACTTTGACCTCCTTGATCGGCCTTTTTCGGCCACTTTTTGGGGTATGTCCAAGAACAAAAGTGGCTCCAAATGGGGTGTTTTACCTAGGATAGGAGTTTGGAGCCTTGGTTTTGAGTTTTTCCGGCGAGCCGTTATCACTTTGGACACCCAAACTGCCGGCGCGTGTGGCGGCGCATGGGGTGAGGGTAGTGTAGTAACTCTGTGTATTTTTGCGATCCTTGTGTCGTCACGAGTGCATAGGATTTTTCATATTGTTCGATTCCCGGGTGCAGTGTCGTCGAGCCGTTGGATCGCACTCAATTTCAGATATGTCATTCTACATGATTTTAGGATCGTGTAGGATTCAACGGATCCCGGATCGGAGTCCCGGATACTCCGAAATCGCGAACCCTGGGGTTCGGGTTTGGAATTTAAGCGATAACGCGATTGTGGCCCATCTGACCGTCCAATTCGGGCCAAACTCGCAGGATATGGGTATTCCTCTGTAAGGAACCTCTAAAGAATCCCAGATCAGCCATCGGAGATTGTGGACCCCACGGGGTTCCGGATCGACCGGTCTAACAGTTTATCGCTTAGTAAAACTTCAGGTCTTTTAAGACCGTTTTAATTGTCTGAAATGCTAAAGTGGGCATAGGATTAACTTTAAAATAAGTGCACGCAATTCTAGGAGTCGGGGTCAGGATTTTAAATTTAAATCTTTTATTGAGCAAGTTTATTAATTTAATATTCATGGTTAATCAGGCACCAGAGGCCCGACCGACCCCCAGGAGGGACCTTCAAGAGGTCCAGCTAGCTCGGACCAGCAGTGAGTGGACTTTTCTTTTCTAAATGGGTTTTATAAATAAAATTGCATTATTTGATCTTGAATAAGTATTATTCATTGTGATTTTCTAGCATGTCTCTGAAACTAAATATCGTATTTATACATTGCTTAGCTGAGTATACCAAAGTGATATAAAAAGCAGAGTTTGAGAATTTTACCAGAGGAAATAGCAGTTTAATTTCAGATTTACTGAAATGCAGTTATTTAGCAGACTTTTCTAAGATATTATATTTTTAAACCACCATGTACCCAGTTATGGTGATTACCCTCAGTTGGACCGATGTCTACGGACATCCAGTCTATTTTTAGTTTTGTCAGTGCACTTGACATTGCCTCACGAATTTCGGGGACGCTCGGACCGCGAGTGCCAGGATTTGCAGCTCGGCTGACTTGGTGTCCCGAGACCTGCTAGGATTTGCTGCTCGAGTTGACTCTGTGTCACCGAGACTTGCTAGGATTGCGGATCAGGCTAACTACGGTCCCCTGAATCCTGCCAGAGCGGCTCGAGTCGACTTTGTGTCATCGAGACCTGCCAGCGGATCAGGCTGACTATAGTCCCCTGAATCCTGCCAGTCTGCTGCTCGGATAGACTGTGTGTTGCCGAGACCTGCCAGGGGAATTGACGGATTTACAGGGGTACACCTAGGTGGTAGTTTTAAAGGAATTTGAGTACTTTTATGCAATTATTGCTTTCAGTTATTTTATACAAGTTTTCTCGATATTCATGCATGTTTTACATTTCCATAGATACGAATATATATATATATATATATATGTTTAGATGAATACCTTGTATTTGAATACAGTCGAACTTTAGCTTTAACTACTTATTTATTTTTACAATGGGGGTTACTATGATTATAACTTGTTTTAAGAACATTATTTTTGTCCACTCACACTTTCAAATTGTTTTGCGCCCCTAGACAGTAGAAGTGCACAGGAATCCACCACCAGGCCACTCCTAGCTCCCGCGCCACCCACGACACGTAGGATTTTATCGTAAAGCACTTAAAGTCTTGTAAATTATTAGTAACTGCTCTGATATCTTGTTGGACTGGAGAACCAGAACTGGTGAATGTTTTATTTACTTATTCTGGCAGTTGGTGTGAAGATTATTGCTTGTTTAACAGGTGAAAAAATTTTGGGTTGGACAAATTACAAGGGAGACTCTGCCGAATTTTCGGCAGGAGTCTAATGAAAGTTTTAATCGTAGTTGCAACAGGAAGGGTAAAAATGTCATTTGTGCCCGACATTCGCCAGGTGTCATATTATCATGAGCATTAATAAATTTCAGTCAGATTTACTAAATCAAAAAATTTTAGTCCGAGTTGATTGTAAGTCTGCTAAGCATGTTTTGGAAAAAGATGTTCAAAATATTGCATCCAAACAAATATTTGCACGATGACAAGCTATTTTAAGCATATTTGATTTTGATATTAAATATTTGAGAGGAAGTGAGAACTCTATCCCTGATTTCCTTACTAGAGAATTTCTACAGGGAAAACAATGGCAGAAAGAAGA

AGAGAAAAAGATAAACATAAAGAACATAAAGCCCAGTCCCAGAGATAGTTAATGAAAAACAACTAGAATATGAGTCTAATAATGAATTAGATTTTCGAGATTGGAATATACCTAAGGTCCCTAGTCAAAATATTTATAAGAAAAAATGGTCACTTTCTAGTTTCTTGGTGTCCGAACAGTAAGACTTGTTCCAAACAGTAAGTCCCAGGGATTCCTGACTGGAGGAAAGTCCGAATAAACATAATATGCAATTCTCTCAATTTTGAACTTATCCCACCATTTGATTGAAAATTGGTGAGATAGGACTCTAGTTTCCCAATTGACTTGATAAGACCATTTGAGGATCCATGGGACTTTGTATTTGGCAATAAAGTGGAGTAAATTAGGGAAGAATAACTCATACTCTTTGAATTTATTTTTATTAGTGAAATAATTGACTAAATCTTGCATTTGGGGAGGAAGGAGGTCAATGACTGGACCATGTTTTTCCCACCAGTGGAGAAACCAGTAGGGAAAAGGGCTTTTGTATTTGCTGTCAAAATTAAGGAACCATGAGTGACTAAAATCAACTGTTTGGTGAAGCAAGATGGAATACCAGGCTTTGATGTAATCATAGTAATTGTATTGGAGTTCTGACTGAAGAACTTTCAGATCATAAGGATGATTGCTCCATTCATTTTCACTAAGAATTTGGTGGATGTAAAGGGAGTGGTAAAGGATGATATGAGGGTTGTCACGATATTTGATTGGCTTGATCGTGATGGATTGGGTCTCATGGAGGATATCTCTGTAAAATTTCAGATTCTTAAAAGGTGCTGAAGGGATGAAATGACTATTAGGCGGGAAATATGACTTGGTGATGGTAACTGGGTTGGAAATAGCATTATATTTGGACTTAATTATGAAAAGGTGACCATTACTCTTGATCATGTAGGGGGATGACTTTTGATATGAGACAACTGGAGGGGTAGACTGGGTAGTATCTTGGAAAGGATCATGA

>Chr7-15; OZ022212.1; 13109607-13115851; rev; A;

TCTGAGTGTGAATGAAAATAAAGAGCAGAACCTTCTTTAATTAATCCATCTAATCAATCTTCAAATTCAAATTACAAGTAGTAGGAAAAGAAGAAATTGGAGATTTCTCAATCTAGTTAGATGATTAATAAAGTGAAATAGATCACTTTGAAAGAAAATCATACCATGAAGATGACAGAGCGACGAGAGAGATAAACACAGGAGTTGTCAACTGGTTATTTAGAAAGGAAGGCAGATTGTTATCTTGAGCTTTTTCAAGTTTTTGAGACGTTGCGTCTGGTTTTGGAGAGAGAGCCCTCTTCTTTTGAGGTGGAACCTCTTTATTTATAGACTAAGACATAACCCGTAATGATGGAAACTGCGAAAAGACACTTGGCTTGTGGTTGAAAGTCAGCTGCTGGTTCGTTGGTCTTAACTGGTTCATAGAGGGAAGGATGGAAGGCTTCTGGCAGGAACCGTCGGTTCTTTGCGAGATCCTTTTGATTTGATCCCCTCGTAAATCTGGCTGGCAACTTGATCTTTCTCTAGTTTTGGTGGTTTATGTACGACTTGCTTTCTTGCTGGGCAAGAAGGAGGTCAAGTCGTCATTAATGGTGATAATCCCCTTCAGTCCCTCACGAGGGCTTAGAAGACACGTTTATCTATTTTTATCTTTTAGTGCGAAATAAATCTATGGGCCTCCTAATTTTGGGCCTTTTCATTAGTTTCTTCCAAAAAGCCCATTAGAATGATGACCCTCTGTTTTTAAAGAAAAAGACAAGTGCTACTGGGCCCGGGCTCTCTTTAAAAATATTCTTTCAGACCATTAGACCATTTACTAAAATTGGGTACAAACAACAATCAATTGAATGACAGCCCTCATTTTTGACTTGTTGAAGTTCTCAGCACAACATAAGCTGCAGCCAGCAAACCTAAAGTTTTATAGATTTGGCTCACTGTCTTGAGTGGATATATATATATATATATATATATATATATATATAAGAGTCTGATCTTTGGT

TGCTACTCATCCAACCTAAGTGGCTGGGTTAGGATTTCTGAGTAAAAATATTCAGTTGCTTTTACTATTTTTCTTCTTTCCGGTACTGTTGTCTGTATCTCTCTTAAATAGTGTCCGAAGAGTAAGGCCTAGAGTCCAAACAGTAAGTCCCGAGGTGAGGCATGAGCGGTTGCGGCATTGAGAGGGCTAGGAGAGAGAGGTCAGTTGATAGGAACGTACAGGAGTCAGTAGTAGAAGTCCCTGTTTATTTTCTTCTTTGATTTCAGAACCCAGTTGCCAAAGTAAAATGAGTCGTTTCCTTAGATCAAGTTCTTGTAGTTCAAGCAGTTCAAGAACCAGTATTAGTCGTCTTCCAGAAATAGTAAATGAGGAACAAGTAGAGTTTGAATCTAATAATAGTGAGTTAGATTACCGAGACTGGAACATCCCTAGAGTCCCCAGTCAAGAAATCTATAAAAAGAGGTGGTCTTTACCTAGTTTTAAGACCACTACCCATGTTAAAACAGTTGAACAAGTTTATGCCCTTGGCAAAGAACTTGAAGTCTGTCAGTTGCTTAATCTTGAATCCATTAAACGCCATAAGAATGAAGGAAACAATTTTCTTCATATTGGTCTAGTTCAAGTTGCCGTTAAGCCACTAACCAGAACAGGGCTTAAAGCCTCAGTTTTGCTCTGGTTAAGAGATGCTAGATTCACTAATAGTGAAGACAGTACTCTCGGAGTAATTGAGTCAAGTCTTTCCAATGGACCGGTTCACTTTGACTGTTATCCTGACTTTACCGTCAGCCTTAGTGATCCTCACATCCTTAAGACCTTGACGCTCAACATCCAGACTTCTGGATATGAGGTTCTCCCGGGAACCCAACCATTAGCCTTAATCTTTAGGATCTATTATAAGGTCACTGGAACCAACATGAACTTCCAAGCCCTTAATAAGAGTCCTAAGAATCATACATTGTTGATTCAAAGCAATAGACCTGAGGCCGTCATCCGAGTACCACAAACAATCAAATGGTCTGATGTCTCACTCCCAGTTGAGTGGACACTCAAAAATATCAATCCACCAACACCAATTCAACGTAGTCTAAATAATTTAGATTACATCCACCAATACTTAGATGGATCAGTAAAAATCAATTTTGGGACCCAACCAGTTCCAAAATCTACTGTCTAATTACAACAGTTGCCAACCCCTGGTCAATCCCAGAGGCATATTCCTGCCAGGCATTCCTCTGCAGCCTCTTCTTCTACTCTAGAACGAGACCTAGAAGCAGAAAGAGCCCTGATTGATTTCCAATTAGAATCCCTCAGGAAAGCAGCACAGGTCAGTCAACCTTGTTATGGCAAACAACCAGTTGAAGAACAAAAGCCAAAATCTCCCGAGTCTCCAACCCAAACTGACTTTGAAGGTTTTGATTTTGTCGATCCTCAACTTAGAGTTCTAAATAAATTTCATATCAATTGGAAAAATTTGCAAGCCCATCTCACAGCTCCTAAAAACCAGAAGCGCCGAGATGATTACCATAGTGCTTTTCCACTTAAGTATCGCCGTGACGCAATCTTTGTTGAATGGAAAGATTACCTACGGAAAAAACAAGTCGAAATGCCTTATTTAGATTTTGTAGAAGCCCACTACATGTCTAAAAATCTGAAGGTAATCACCAAAGAAACATGGTCTTTACCTAATGACACACAAGTAGTTGCCAGTCATCCCCCTGCTGAGTCTATCTTAATTAATTTTAAGAAAACTCAGATCCCTGCTTGTCCTTTCAAATTCCCTGAAAAATTAGATTCAGAAAAGAAATTGATAGAACAAAGCAACTACACCAATCAGAGTCTAATTGTCATAGGAAAACAGCTTGACAAAATTGAAACTAAAGTAGACAAGTTAGTCCCTTCCTCTGAAAAATCCAATACAAAAATTGAAAAACCATTAGTCCAGTTCCAGGATCTTAAGACAAATCCGACCCTTAAGATCAAGCCTACCCTTCAAAAAATCGAAGAAATGCTGGAACAATTGACACCTGTAAAAGGTGAACCATCTGGAGTTAAAGTTCTTGACTCCATTAATCCGGATTCCGACCAAGAATCCATTCAGAGTCTAGACTCAGTTGATAGCAGTATCTCAAAAATTGAGAAAGCCTTTACTGAACTCACAGTTGAACCTAGGATCACCCGGTTAGCCAACCAAGTTACCCCTACAAGCCTTACCAAAAATTGGTATACTCGACCAACTCCTCCAGATATACAATTTGAAGAAAGACATTTTCAGAACCAGTTCTCTGTCTCTTCTGATAAATTGTATGAATGGAACATTGATGGTCTTTCGGAGCAAGAAGTTTTCAATAAACTTCAACATATGTCCATGGTTGCCAATAGTTATATTTCAAACCACAGTTTTAGACAATCTGAAATTGTACCCCTGCTAGTCACAGGTTTTACAGGCACCCTCCGCAATTGGTGGGATAAACACCTCACGCCTGAGTCTAAAAACTTAATCATTCATGCTGTAAAACTTAATGATGAAGGTCTCCCTATCTTGGAACAAATAGGTCAAGGTATAGAAGATGGAGTCAATACTCTGTTTTACACCATAATTGAGCATTTCATAGGAACACCAAGCCATACTACCTCTAGAATTCATGATCAGCTTAGCAACCTACGTTGCCCTAAACTGTCCGACTTTAGGTGGTATAAAGATGTCTTCATTTCGCGAGTAATGCTCCGAGATGATAGTAATCAGTCGTTTTGGAAAGAAAAATTCATTAATGGTCTTCCAAACCTATTTGCCCATAAGATTCGCACAACCCTTAGTAATGAACAAGGACATATTGATTGGAATAATCTGACCTATGGAAATCTAGTCAGTACTATCAACCAAGTCGGTATGAAGATGTGCGTTGACATGAAAATAGGTAAGCAGATACAGTCCGACAGAAAATCCGCTAAGTACGAGTTAGGAAATTTCTGCGAGCAGTATGGTCTTATTAGCCTACCTCCCTCCAGCCGAACTAAGTCACACCATAAGAGTCAATCCCGTTCTTACCATAATCGAAAAAATAGATACCAAAATAATGATAGTAGAGAATTCTATAAGACTAAGAAACATAGCCCTAAAAAGAATTGGAAAAAATTTCCATCAAAAAATAAGAAAACCCGATTCCAGAACAATTCTGGAAAAGGAAAAGTTGTTTGTTGGAAGTGTCAAAAGCCAGGCCATTATGCTAATAAATGCAAGGTAAAAGACACGATCAAACAATTAAAAATTGAAGAAGTTGAAAAAGAAAATCTAATCCGACTTCTAGATCTTAAGGATTCCGAACCTAGTGATCTCGAGTCATCAGTGATAGTGATTCTGAGAATTCTTATTCGGACTCACAACCTAGTTCCCCCCATATACAACTCGGTTGTAAGGATAATTGTTGTAATTCTATGAAATCCATTTCTGTTCTTACTAAACAGGAAGAACAAGAAGAGCTTCTCATAGACTTAATCTGTAAAGTCGAAAACCCAGAGTTGAAGGCTGAGTATCTGAAGAAATTTCGGAAACTCCTTAGTCAGGAAGGTCCGAGTCAGTCTCCACCTCCTCAAAAGATAAGTTTAAATACAACCTTAGAAAGGTTCAGTCGCAAGAAAGACATCACCCTTCATGATTTACATTCAGAAGTAAAATTAATCAAGAAGGAGATAATTGATCTAAAACAATTAAGCCAGAAATTACAATCTGAGAATCATGAAATCCGACAGGATTTAATGGCACTCAAACCTGATTCTATTTCTCATCAGAGTCCAACAAACTCAGATGATGAATCTTTGAATCAGAACAAGCCTTATGTCTAGTCAAAAGAATTAACTTCAAGAAATGGTATGTCAAAGTTACTATTTTTTTGATCAGTTTGAGTTTACCACAGTCGCTTTGCTTGATTCAGGTGCTGACCTTAACTGCATTCAGGAAGGTCTTATACCTACCAAGTATTATACTAAGTCCTGTGAATCTCTTAGAACAGCTTCAGGAAAATCCCTACAGCTGAACTATGAAATTCCCCGAGCCCATGTTTGTCAAAATAAAATTTGCTTTAAGACTTCATTTGTTCTAATAAAAAACATTACTGATGAAGTTATTCTAGGATTACCTTTTCTTGCCTTGTTATATCCTTTCCAGGTGAACCATGATGGTATCATCTCTACTCACCTAGGAGAACAAGCCAAGTTTGAATTCTTATCCAAGCCAGAGTTGCACCAGTTAAAAACCTTTCAGAAAAGTTCAATTTCCAAAACTGTTGCTTTAATTTCCAGTAAGACTAAACATTTGTCTTCTCTCCAGGAAGAAATTAGTCATACTAGAATCAGCCAACAAATTAGGAATGAACCTTTCCAGAAACAAGTTCATAAATTTGAAACTAAGTTAATTAATGACATTTGTTCTGATTTGCCTAATGCTTTTTGGTCCAGGAAACAACACATTGTCAGTCTTCCTTACGCCAAAACCTTTAGTGAGAAAAATATCCCCACTAAAGCCAGACCAATCCAAATGAATCAGGAGACAATGGAATTCTCTAAAAAAGAAATTACTCTTGCAGGGAATCATCCGTAAGTCCAAGTCCCCCTGGTCTTGTCCTGCCTTCTATGTCCAGAAAAATGCTGAACTAGAAAGAGGAGTCCCTAGACTAGTTATCAACTACAAACCCCTCAATGCAGTCTTAGAATGGATTAGGTACCCCATACCTAATAAGCGAGACTTAATTAATCGGTTAGAAAAAGCAGTTGTCTTTTCCAAATTTGATCTTAAGAGTGGATTCTGGCAAATCCAAATAGAAGAGTCAGATCGGTACAAAACCGCCTTCGTCACTCCTTTTGGTCATTATGAATGGAATGTTATGCCTTTCGGTCTTAAGAATGCGCCTAGTGAATTCCAGAATATTATGAATGAAATTTTCAATGCTTATAGCCATTTTTCAATTGTCTATATTGATGATGTCTTAATCTATTCCCAATCGATAGAACAGCATTGGAAACATTTGTCTATATTCCTTCAAGTTGTTAAGCAAAATGGTCTAGTTGTTCTGCTAAGAAAATCAAATTGTTCCAAACTAGTGTTCGATTTCTTGGCTTCAATATCTGTCAGTCTCAGATTAGCCAATTGATCGAGTCATCAGTTTGCTGATAAGTTCCCAGATGAAATTATTGATAAGAGTCAGCTCCAGAGGTTCTTAGGATCCCTCAATTATGTTTCTGATTTCTATCAAAATCTGAGAAAACAATGTAAGCCCTTGTTTGACAGGTTGCAGGAAAATCCTCCTCCTTGGTCCTCTGTCCACACAGAAATTGTCAAACAAATCAAAATTTATGTTAAGACACTTCCCTGTCTTGGCATTCCTTCAGTTGATTCCTTCAAAATAGTTGAAACTGATGCCTCTGACATTGGTTATGGAGGTATTCTTAAACAGAAATTGAGTCCCAATTCGCCAGAGCAAATTGTTCGTTTCCACTCTGGTATTTGGAATCAGTCGCAAAGTAATTATAGTACTATTAAGAAAGAAATATTATCTATAGTACTATGCATCTCTAAATTTCAAGATGATTTACTTAATCAAAAATTCTTAATCCGAGTTGATTGCAAATCTGCAAAACATGTTTTAGAAAAAGATGTTCAAAACATTGCATCCAAACAGATTTTTGCACGTGGCAAAGTATTTTAAGTATTTTTGATTTTGAATTGAGTATTTGAGAGGTGAGAATTCTGTTCCTGATTTTCTTACTCGAGAATTCTTGCAGGGAAAGCAATGGCACCCAAAAAAGACAAACAGAAAAGGTGCCAGTCCAGTCCAGCCAACCCAAAACCTTCCCAAACACCACCAAGCCAGTCGCCCAAAAGTCCTCCCTGCATCCATGAGTCAATCCAGAATGGCCCATCCAGCCTCAATTGTCCCATATGCGGGTCATCCCCCAATCCCAATCGTCCCAATTGCAAACAGATTCACCAGCCTTGGAATTGTCCCATATGCGGGTCATCCCCCAATCCCAATCGTCCCAATTGCAAACAGATTCACCAGCCTTGGAACCCCAATAGGATCCACCGTTGGCCCAATCCGCCCTAATTATCAGTCAGCCCTTGTTTCTGACTATGATCCATTTGCAATAGTTCCTCCAGTTGCTCCAATTGCCCAAACATCCCAGTCGTCCACCCAGTTCAGAAAGTCGTCCCCTTACTTCCCAAAAGATAAGGTTAATTTGTTCATAATCGA

TATATATATATATATATATATATATTAGGCCCTCAGTTTTGGCACTCCACCTTTGGCCACTCAAATGATGTTCCAATGGATTCCACATTTTTATTTTTCCAATTGTAGTTATATATAAGTGAATTAGTAACATCATATGAGGATATTACGCTGAATCCAGGCCTTTAGTAAATGAGAGAGACTCTATTAAATTGGCCATTCAAATCATGTTCTATTGGATTCCACATGTTTGAATGTACTTACAGAACAAAATATCGATAATTTCGCCGATCGTTTTGTTGGGGGAGCGATATTTTAGCATGTTTCCATTCATTTATCGTCAATTTATCGATAATTTTGCGATAATTTTGGTTGCAATATTTTCATTCAAATGGCAGCTGTAAATAGTCAACATATCGCGATATTATCGATATTTCCCAATTTTTGAACCACCGATATTTCCGAAACCCTCGATATTTAAGAACTTGAATGTACTACATGGGGCAAGGCATGGTACTTCTGTGATATCCATTAATGCCTCCTATAAGGCCTAGGCCACGTGACCAAACTCCAAAACCTTTCGTCAATATTATAACTTATCAGCACAATGCAATACCTATAAATATGCAACACTTCTTTGAATCACAATCACATTTCTCATCCAAAACCCAAAAAATGGACCTCAAGCTCTCAACCCTCCTCTGCTTAACCCTACTCTTCTCCACCATCCTAAACCCAGCTCTCTCAGAGCTCTGCAACCCGAAAGACAAGAAAGTCCTCCTACAAATCAAGAAAGCCTTCAACAACCCCTACATCTTGTCCTCATGGAAACCAAAGAGAGACTGCTGTGACTGGTACTCTGTCACCTGTGACTCCACCACAAACCGCGTCAACTCCTTCACCCTCTTCTCCGGCGGACTCTCCGGTCAAATCCCGCCTCAGGTCGGAGACTTGCCATATCTTGAACTCCTTCAGTTCCACAAGCAACCCAATCTCACTGGCCCAATCCAACCCTCCATT

>Chr7-16; OZ022212.1; 14806244-14808879; rev; D;

AGAAAGAAAGAGCATGCAATCTATGCTGTCAGATTAATTTTTGCAATAATTTGTACTTCTTATGAGAATCTTGGCATTAACTTATAGTATTGTTGTTTAAAGGCAAATTACCTTGACCCATCTTCTCTTTTTTCGAACTCTTTTTGAGTAATAACATATGGCATCCAAAAATTTATTTCAAGATCTCTTTGAAATGTAAGGGACTTATAGCTTAAGTAGTTAAGAACATTTATTCGTGCATTCTGTGAGAATGTGAAGGTAAAGAGTCCCACATTGAAAAGTTGAGAAACCTAGTAAGGGCTTATGAGGAATTAGGTTACTCCCCCCATTGCCAATTGGTTTTTGGGTATAATCTCAACTTCCTTCATGATATCAGAGCAGGTTAGCTCACATGTGAAACCTAGCAAGGGCTTGAAAATTCGTCACACATGCGGGGCGTGTGCGAATGTTAAGGTAAAGAGTTCTACATTGGAAAGTTGAGAAACCTAATAAGGACTTATAAAGAGTTGGATTGCTCCCCCCATTATCAATTGGTTTTTGAGGTGGAACCTCAACTGCTTTCACATTCCTAGTCATGTATTCATTTTCCTCCCAACATTGTTTGTATGAAGAAGACTTCTCTAAAAAATTTAATGTATTAAGGAGATTTAGCATTTGTAATTTCTTAAACCTCTCAAAGGTGCTTTTTGCAATTTACTAAAGAACCATCGTACATTCATACAATTGAAGGGGCTAATTATTAGTGGTCATATTAGAGGTCGTAAATTCAAATATACAAAGATCTAAATGGTGTAGTTCATTAAAAAAAATTCGTATATATATTTGTGAGAATGTTGTTAAGGTTTCCAAATCATGGATACAACATTTGAACCGACGGTTGACTCAATTAAAGAAATTGCTTCATCTAGAAGCTAAGCTAGTACTACTTTTCTTCTCAGTATTGAAAGATGTCTCCAGTTCGAATCGTGTCTTGATGAAAATATATATATATATATATATA

AGTAAACCTGAGTTGCATAGTTTAAAAGAATTACATAAAAATGCGGTCACCAAAACTGTCCAGGTCATCCAAAACAAAGATAAACAATTAAGATTTCTCCAAGAAGAAGTTAAAGCCGAATTGAGCAACAACTGAGTCATACGTCTTTTCAATTACAAATACAACAATTTGAAGAAAAGCTAAAACAAGATGTCTGTTCTGAGTTACCCAATGTGTTCTGGCATAGGAAACAACACGTTGTCAGCCTTCCTTATGTAAAAACCTTTAATGAGAAAAATATTGCTGGATTGGTACACATTGGTCTGGTTCAAGTAGCCGTTAAGCCATTAACCAGATTAGGATTAAAAGCTTCAATTCTTCTCTGTTTACGCGACGCTCTATTTAATAATTTTAGTGACAGTACACTAGGAATTATTGAGTCTAGTCTTACAAATGGCCCAATTCATTTTGATTGCTATCCAGATTTCACAGTAAGCCTGAGTGATCCACACATCCTGAAAACTCTTACTTTAAACATTAAGACTGATGGTTATGATATCCTACCAGGTAGTCAACCTCTAGCCTTAGTCTACCGAATTTACAATAAAGTCACTGGAACAAACATGAATTTTCAAGCCTTAAACAAGAGTCCAAAGAGTCAAACATTGTTAATCTTAAGTCATACGCCTGATGCCATTGTTAGAGTTCCCCACTCTATCAAATGGTCAGATGTTACTCTACCCACTGATTGGACCCTAACCACTGAGACTCCTCCAGTACCAATCACTGATTTACTTAAAAAAGAAACAATTCGTAAGAGTAAGTCACCCTGGTCTTGCCCTGCCTTTTATGTCCAGAAAAACGCTGAATTAGAACGAGGTGTCCCGAGACTAGTCATCAATTACAAGCCTCTTAATACGGTCTTAGAATGGATTAGATACCCAATTCCTAACAAAAGAGATTTAATCAACAAGTTAGAAAAAGTAGTCATCTTTTCTAAGTTTGATATGAAAAGTGGATTTTGGCAAATTCAAATTAAAGAGTCTGACAGATACAAAACTGCTTTTGTTACTCCTTTTGGCCATTATGAATGGAATGTTATGCCTTTCGGTCTTAAGAATGCACCTAGTGAATTCCAGAATATAATGAATGAAATTTTCAACCCTTACAGTCATTTTTCCATCGTTTACATTGATGATGTGTTAATCTTCTCTGAATCCATAGAACAACATTGGAAACATCTGCACAAGTTTTTACAGATAATTAAGCAAAATGGTTTAGTTGTTTCTGCTAAGAAAATTAAGTTATTCCAAACCAATGTGAGATTTCTTGGGTTCAACATCTACCAGTCACAGATTAGTCCCATAGATCGAGTTATTCAATTTGCTGATAAATTTCCTGACCAAATCTTAGACAAAAGTCAACTCCAGAGGATTTTAGGATCTTTAAATTATGTTTCTGATTTCTATCAAAATATAAGAAAACAATGTAAGTCTTTTTTTGATCGTTTCCAAAATAATCATCCTCCATGGTCATCTGCTCATACTGAGATTGTTAAACAAATCAAAGTTCATATCAAGACTCTTCCCTGTCTTGGTATTCCTTCTGTTAATTCATTTAAAATCGTTGAAACTGATGCTTCTAATATAGGTTATGGTGGAATCCTCAAACAGAGAGTCTCTTCTAATCAATCTGAGCAAATTATCCGATTTCATTCTGGTACTTGGTCACAAAGTCAAAATAATTATAGTACTATTAAGAAAGAAATACTATCTATTGTATTATGCATTAATAAATTTCAATATGATTTATTAAATCAAAATTTTTTAGTCCGAGTTGATTGTAAGTCTGCTAAGCATGTATTAGAAAAAGATGTTCAAAATATTGCATCCAAACAAATATTTTCACGTTGGCAAGCTATTTTAAGTATTTTTGATTTTGATATTGAATATTTGAGAGGAAGTGAGAATTCTATTCCTGATTTTCTTACCAAAGAATTCCTGCAGGGTAATCAATGGCAGAAAGAAGAAAAGAAAAAGAAAAAGATAAACAAAAAATTGATCAGTCAAGCCAACCAAGTCAACCACAGTCACTCCGAGTCCTCCCATCATCCATGAGTCAAACCAAATATGAATCTCCTACCCAGATTGTCCCCTTCTCGGGATGCTCTCCTATTCAAATTAGTAACAGGTTTGCCAACCTAGGAGCCACAGTCGGCCACGTTCGCCCTAGTCTGCTTTAATTTCTAGTCATGATCCTTTTCAAGATATTGCTGTCACTAACCGCCTAGTTGCTTCTTATCAAAAGACGTCCCCATACATGATCAAGAGCCATAGTCACCTATTTGTCATTGAGTCTAAATACAATTCAATTGCTAACCCAATCACCATTGCTAAGTCTTATTTTCCACCAATGAGTCATTTTATTCCCCCAGCACCTTACAAGAATCTCAAATACTACCGAGATATCCTTCATGAGACCCATTCTATCACCATTAAGCCAATAAAAGACAGAGAAAACCCACATATAATCCTCTATCATTCGCTCTATATCCACCAAATCATCAATGAAAATGAATGGAGTAGTCATCCTTATGATCTCAAAGTTCTCCAATCAGAACTTCAATATATTTATTATGATTATATTGAAGCATGCTA

GAACTGTAAGAGTTGGCTCTAAGAAAACGACTCATTTTTACACAAGGTTCTGGGTTTGGATTCCTAAAGGAGAAAGTGAACAGAAACTTCTAAAACAGACTTCATGGATAACAGGATACCTAAAGTTCTCTCGCCCTCCTACTAATGCCACTACCGCTTAGGCCTCCTTGGGACTTACTGTTTGGAATGGGTCTTACGATTCGGACACCAAGAGAGAGAAGGACAGGTAAACTGAAAGACCAGAAGAAGTAAAGAAGAAACTAAAAACTTACGGAAAACCTAAATCAGCTACTTGGGTTGGATGAGTTGTAGACTTAGATAGACTCTTCAACACTTACGGATCTGATACCAAAAGGGCTAGAACCACCAGAAGATAGATGAAGGAAGATTAAAAGAGGAGAGAATAATGGAGGAACACAGATATGAAAAAATAAAATATTCTCAATTCAAGCATGAAGCAAATTAATGAGTATATGAAAATAGATGCAATGAAAATAAGCATTAATCAAAATAGGCAATAGGCAATTCTCAAAATATCTCAAAATATATTGATAACTGGGGAATGCAAAACACTTACAAAGATCCTAGTTTCACCACTTAGTTCTTCGGAGTATTGGTTCCTGATAGAACTTTGAAACTGAGAAAATTATAATGTATTAAATTACATTCCTGTAAACATAACGGCCGAAGCCTTCTTAAATAGAAAAGACTCAAGGTTTCTGATGATTTGTGGTGAGCTGGCAACATTGGATGCCTTGAGAAAAGAAACTGTAGAGGCATGCGAAAGCTTTTGCTTTTTCTGAGTGCAGCAATAGTAACATCAGCAACAGTGACATGTCGGGGAATCTCAAAAGAACACGGCTTTCAGATTGATGAAGGCACGGGCATGGGTACTTTTCTTCAGTGCTTACTTTTGACTTTATCTTTTGGTTTAATCATCGCCTAGGTTGAAAGGACCGAAATCGTAGGGATCTTGCTCATCAGGATAATCACTCCACCG

>Chr8-01; OZ022213.1; 4378761-4378919; dir; B;

TACACATGTTAGTAGGACGAATTGGCTGTCTTTGACGTAGGCAAAGGCCATTACCTCTGATAAAAAGAATATGTAACCCCGTATTGATAGACGGGAGTTCTCCCAACAGTGTGAAAAGAAAGGTCAACTACGTCGAACCTTCCGATCAATAAAATAATAAAGTATGAAAAACTGGATTAAGAGACCAAAATTCTTCAAAGTCTTGTGGCGCCCGCTAATCCAATAACGCGATCCGGGGGGTGGCTTTGTTGTCTCTATTTTGATCTATTGTTGAAGCAAAAAGAAAGGGAAACTGTTCTCGAATCAGCAATGCCACATCTGACTCAATCAAAAAATCGTGGCTTCTTGTGCTATACTCTAATTGTGAGGAGAAATTCCATCCGATGGAAGGAAGAGAAATTCTTTCTGATCAAGGGCTTGGCCAGGTGAAGAAGAAGAGTGAGAAAAGGAAGAATGGGGCTTATAAGGCCCAAAAGTGCTGAGTGTTATACATGCGAGTCTTAAGCGTCAAAAAGAAGAGCCAATCTCTTTAAAAGCCCTAAGAAATAGGCTTTTAGTCCCCTTATATTGATGGATTTCTCAAATATTTTATTTGAGGGACACCTTTTAGGGTCTCTTATGAATTCTTTTTGAACGATTCAAACCATCTATTTTTCACATTTTCATTCAAAGATCATCCTTGCAAAAAATCATGCAAATTGAAAATTATTAAGGCATCTAATTGAGACCAACAAAATCAAAGAACACGGTGCTTCAAAATAGTACTTGAATTTCAATAATTTAATTGAGTGGTCAAATGATATCGGATTCAAATAATTTTTTGTAGAGGTGATCTTAAAATGAATATCTATAAAATAAATAGCTTTGATTAGTGAAATACAATGCGGAGTGGACCCTACAAAGGTGTCCCTACATGGTGTTCTCACGATAATAATGTGCGCATGTCTGCATAGGAGAATTTATGTATATATATATATATATATATATATATATATATATA

GCTCTAGTTTATAGAATTTACTACAAAGTCACAGGTACTAATATGAACTTCCAAGCCCTCAATAAAAGTCCAAAAGATCAGACCATAATTAATTCAAAGTAGCAAAGCTGATGCCAATATAAGAGTACCTCATACAATCAAATGGTCAGAAGTCATCCT

ACCTTTTTTTTTTTCCCACCAGTGGAGGAACCAAAAGGAAAAAGGGCTTTTGAATTTGGTATCAAAATTAATGAACCAAGAATGACTAAAATCAATTGTCTGGTGGAGGAAGATTATATATATATATTTATTTATTTATATCAAGAGCACTCAAGCATTTATCTGTTAGTAAGCTAATACAGCGATGACCACTTGTTTGATCTCACTCCACAATATCATGTCATAATCCAAACCGTTCATTTGTTAGGTCATCATTCAGAAATCATCCTTTTTAAAAAACCAAAAAAAAAAAATAAATTAAAATCATTAACTTAATCATTTCCATATTATCATGAATATTTTCATTGTTTATAGAAAGTATTATATTTGTCTATTTATTTAATAATAGTGAGCGCCGTATTAACTTACTAACCAGTTAGGGGTGCAAATAGATATATGTTCTATTGGGCTCTTTCTAGAACAAATTAGCAAATACATAAATAATATAGAACTATCATATTGTAATATCACTTAAAGCTCTTATTTTTAGAATATGTATTTTTTTGTTGCCAGTCATTTGCGCATGATTTATAAGTTGCTGAGATATATAATATGTAAGATATAAATTAGTTTTGGTCCATTCGTCCCCAAAAGAGTCATGTTTATATATAATACTTAATAAGCTGATGGGGTGAGAGAATTCATTTTTCCGTTTGCTAAAAGTATATATATCCCGTTGAGTATAATGATGGGATATTGCTTGCATCTCTTAGAAAAAAAAAAAAAAGTAAAAAAAGAAAAACAAAGGTCAATCACTACAAAACGAAAGAAAAAAGAAAGAGAGGTTATTTGAGGATATTGCTGTATTTTAATAACATATATGTATGGGTTAATTGCCGTAGACATCCCTGAATTTGGCCCCCATTTGCAATTGGGCTTGTAAACTTTTTTTTTTCGGCAATTACATCCGTTAACTAGATAAATAGTTCCAATTGGATCCTGCCGTCCAATTCTGTCAATT

>Chr8-02; OZ022213.1; 4542076-4545101; dir; B;

CAAACTGACAGCGAACCGAGTCTAAGAAAGATTCCTCTTGTGTCGAACCTATGGTTCAACCTTCCAATCTTGTTTGCTCCTTTGGGAAGGTGCAAAGATTCATCATTTCAGCACTTACCCTTTTTCATTTTTGTTTCAAAAGTCTACAAATTTTAGCACCCATGAGTCAGATTTAGTGGCTCTTGCAAAAGTGTTGGGTTTTAAATTGGTGTATTTGAATTGGGTTCGAAGATTATGGAAGACCGATGTGATTTAGGAGAAGGGTTGGTCTCGATACCCAGATTGGATTCAGAGGTTGGTTTTTCTTCCTACGCGTATTTTTTGGGTTGTTTTAGATTGTTGGGAAAAAGCATAAGAGTACTTAATTGGGACAAGAACGTGAGTTGGGTTCTCGTAAGCGGCTTAAAATGCGAGATCTTGAATCTGTTTGCTGGTTTGAAGGTATATATTATAAATATTTGAAAATATTCTTGGTGTTGTGCGGCATCTTTTTAATTGAATTTTTGGGTGTCGGCAAGGATTAGGCGGCAATGGAGGTTTGTGGAGGAAAGGGAGGGTTGTTGATGAATTTCCAAATTTATCCTCACTTTTGACAGAAAATTTAACAGAATTTGAAGGAATGACTAATAGTGCAACACAACACCTAATTCAGTGACGTCTACAACTAATAATTGAGTTCAGTGATCCAAATGTAACTACAGGTATATTTGAGGGACCATTGCTGCGAATAACCCTATTAACTTTTATTGTTAAACTCTTTTTTGTTAAGTCAGATCTCACTTTTAAATGACGATGAGAAATTTGTCTCTTGTTTGGCAATTTCCATTGATATATATATATATATATATGTATAGTCTTATGTGTCCCTTCCAATAGATTGGACCTTAACCGCTGAGAGTCAACCAATTCCAATCCAACGTAGATTAAACAATTTAGATTATATCTAACAATATCTTGATGGCACTGTCAAAATTAATTTTGGTCATCAACCTCCACAAAA

GGCAATCGTCTAGCGAGTTTCAGAATATAATGAATGAGATATTCAACCCTTACAGTCATTTCTCCAAAGTCTATATTGATGATGTCCTTATTTTTTCTCATTCAATAGAACAACATTGGAAACACTTGTATAAGTTTTTGCAGATAGTCAAACAAAATGGTTTAGTCGTCTCTGCCAAGAAGATCAAGTTATTCCAAACCAATATCAGATTCGTTGGTTACAATATCTGTCAGCCATAAATTAGTCCAATTGATCAAGTCATTCAATTTGCTGATAAATTCCCTGATCAAATCATTGATAAAAGTCAATTACAGAGGTTTTTAGGATCTCTTAATTATGTTTCTGAATTTTATCAAAATCTGAGAAAACAATGTAAGCCTTTGTTTGATCGCCTCCAGAGTAATCCCCCACTCCCCCCACTGACAGGACCCGACCCAATTTTCACTTTGGAATTCGAGCCAAGTCCTGTGTGTCCGACACCTGGCGAATGTCGGGCACAAATGACCTTTTTACCCTTCTTATTTCAATATCGCTTTAAAATTTCCTTAGACTTCTGCCGAAATTTCGGCAGAGTCTCCCCTGTATTTTGACTAATCCCAAAGTTTTCCACCTGTTAAACATGCTAATAATTCTTCACCAACTGCCAGAATAGTCAATCAACCAATTTCAACTCTAAGCACTACATTTCCATCTGGATATCAGAGCGTTCTCTGAGTCCCTCAGGGTTGTGAGGATTTCTACAATACTATACCTGGTTTGAGACATGGAAGCTAAGAATGGCCCGGTGGTGGATCCCGCGTACTTCTATGGCCTGGGGGCGAAAAACAGGTTGGAAATGTGAGTGGACAAAAAATAATGTTCTTCAAAACAATTTTCATAACATAATATCCCCCATTGTAAAAAGAAAATTTAGCTAAATAAAATTGAATACGTACTTTCTGCATCAGGCATATTCAACTCAAGGCATTCTATATCAGTCATATTCAAGCTCGAAAGTTTCATACAAAACCACTGAAATCAAAGCTTTCATAAAAGTGCTGAATTCAAACGTGTATAAAACCTCTGTACTCAAACCTTGCATAACTGAACAAATATAAAGGTATCTATGCCTGAAAAATCAGAAAAACTGATTTAAAATAACTGAAAATCATAATTTAATAAAAGAAACTCAAAATCCTTTGAAAATACCACCAGTTTGTACCCCTGTCTTTTCCGTCAATTCCCTGGCAGGTCTCGGGCGTCACACAGGCTACCCGAGCCGCAAACTGGCGAAATCAGGGGACTATGATCAGCCTGTCCCGCCGGCAGATTCCTCGATGACACCAAGTCAGCTCGAGTCGCTCTGGCAGGATGCAGGGGACCGTAGTCAGCCTGATCCGCAATCTTGGCAGGTCTCGGGGACACAGAGTCAGCCGAGCCGCAATCCTGGCAGGTCTCGGGGACACAGAGTCAGCCGAGCCGCAAATCCTGGCACTCACGGTCCGAGCGTCCCCGAAACTCGTGAGGCAAAGTCAAGTGCACTGACTGAACTATAATCAGACTGGATGTCCGTAGACATCGGTCCGACTCTGGGTAATCACCATAAAGAAAATGGGTACAAGGTGGGTTTAAAATAAAAGCCTTTAGAAAAATCTGAATAACAACTGAAATCACAAATTGTGCTGCTGTCTCATCTGATTTCAACCTCACTGTTTCTATAACTGGTAAATAAGCAGCACAGATTTATAGAACTGTTACTGTACTGATCATGTTCCGAACCGTAATAAAACTTACTTAATATCAAATAAAGAAACTTATTCAAATAAATTCATCTCTAAAAACTTATTTATATAAAATCAATATAAACTCATTTATAAAACTTATTTACATAAAAGCACATCAAATCATTCATGAAATCTGATTTATGAAAGAAAGTCCACTCACAGATGGTCCAAGCTACTGCGACCCTTCGAAATTCTCCTTTGCAATTCTGTCGGGTTCCTGGTGCCTTACTCGATCGATTTGGTGGAGAAACGAAGGAGAATTTCAAGCTGGAAGTTTGGGTAGCTTCGGACGAACCTCCGTCGGAAAACACGATTTTCCGGCCAGCTCAGGGCGGCCCCGGGGTTGAAGTCGGTTGGAGAAGGTAGGGGACTCGATGGTGGTTCCAACGCCACCGGTCCCGTGGCCATTGGAGCTTGGAGGCGGCGCCAGCACCCTCTGGAAGTCGGCGGCCCGTTTCGCGCAGAGTCTGGGTTTATATAGATGCAACTTCGAAATATTTACGGTTATGCCACTGAGACTCTTTTGACCATAACTTTTTCGTTACAACTCCGATTCAGCCCCACTCCGTGTCTACGGACTCCTTTCGCCTTGCTCTATACAATGGCGCAAGAGAAATTCCCAAATTCTTTCTCAATCAAAAAGTCAAAATTTCCCCTATTAAATAATACAAGGGCAAAATTGTCTTTTCGCTAAAAGATATTTTCCTTACTTTTTAGATATTTTCTTTCTTTTTAGATATTTTGTTTTGGGTTCTTACACCCACCCTCCCCGCGGGTCATCTATTCATACTGACATAGTCAAACAAATCAAGATCCATGTCAAAACACTTCCATGTTTAGGCATCCCTTCTGCCAATTCATTCAAAATTGTTGAGACGGATGCTTCTAATGTTGGTTATGGTGGTATCCTCAAACAAAGAGTCAATTCTAGTCAAACTGAGCAAATCATTCGATTCCATTCTGGTACTTGGTCACAATCCCAAAATAATTACAGTACTATTAAAAAAGAAATATTATCCATAGTATTATGCATTAATAAATTTTAGTCATATTTATTAAATGAAAACTTTTTAGTCCAAGTCGATTGTAAGTCTGCTAAGCATGTTTTGAAATAAGATGTTCAAAATATTGCATCCAAACAAATATTTGCACGATGGCAAGCTATTTTGAGCATATTTGATTTTGATATTGAATATTTGAGAGGAAGTGAAAATTCTATCCCTGATTTCCTTACTAGAGAATTTCAACAGGGTAATCAATGGC

AGAAAGAAGAAGAGAAAAAGATAAACAAAAAGAGCATGTTTTACCGTCAATTACCCTATTTGCTTCACTTTTGACTTCGTTTTCAACAAGATTGCACTTCCCATTCCTAATGCTTTTGTAACCCCAAAGTGAATAAGTTTTTTTTTTGGGTAAAAAACATGTGATTGTTGGCCTTTGTAAATATGTTGTAAACATGTATTTAAAATATACTAAAATTAATAAAATAAATTTATAAAGGGAAGGTGAGATATTAGAGAAGTAAATTAGGTCTTTATCCTTGTAAACCCAAATTAATAAAATAAATTTATAAAGGGAAGGTGAGATATTAGAGAAGTAAATTAGGTCTTTATCCTTGTAAACCAAAGGGTTTACAATAGTAAGGACTCATGTGCAATTGACAAACAGTTAGTGGGAAATTGAAACTGAATAATGCAATTGACAAATAGTGAGCTCAAAATTGCAATTGACAAACAGTAAAATGGAACATTCAGAAGGTAAATTGGGTCCTTATACTTATAAATACAAACGTTTACAATATTAAGGTCTCGTATTACTCTATGTTAAACAGGTGTGTTCCATAAAAGTATAAAACCCATCCTTAATATTGTAAAATCGTAATTTACAAAGATAAAGACATATAGTACAATATTAAGGTAACTTGGGGCATTAGGAAAGTAAATTGGACTTGTATAAGAATAGGGTAAGAGTGGACCTTGGTGGAATAAATTTGGTCTTTGTCATTATAAACAAAGGAGTTTACAACATATAGGACTCCCGTTATATTGTTATGGACAGAGGGGTTCCATAAAGTTATAAAAGAGATCCTTAATCTTGTAATATCGTAATTTACAAAACTGATAATGACCCCCAATACAATGTTAAGGTAAGAATGAACCTTGGAGTAGTAAGTTGAGTCATTATCCTTGTAAACAAGGTGGTGGTTACAACATTAAGGATTCGTATTATACTAAAATGGACATAGTCATTCCATTACATTGTA

>Chr8-03; OZ022213.1; 4622969-4637732; dir; C;

NNNNNNNNNNNNNNNNNNNNNNNNNNNNNNNNNNNNNNNNNNNNNNNNNNNNNNNNNNNNNNNNNNNNNNNNNNNNNNNNNNNNNNNNNNGGATGCAATCCAATATCGATTGTTCCATGAAATTTGAAAATTCAATTTCGTACGAAGTTCGTTATGAGATGGAGTGGTTGGTGTATTGCATAGTTTGAGATATTGGATTTCACTCAAAGGGCACCAAATGACTCCTCCCACCATACTATGATTTTCCCCAAGTTTTGGACTCAATCTGATATCAATTGGTCCATGAAATTGGACAATTCAGGTTCGTACGAAGTTCGTTCTGGAATGGAGTGGTTGGTGTTTTGCATTGTTTTAGACTTTGAATTTCACTCAGAACTTACCAAATGACTCCTCCCACCAAATTATGATGTTCCCCAAGTTTTGGGCTCAATCCGGTATCGATTGGTCCTTGAAATCGGACAATTCAAGTTTGTACGAAGTTCGTTCTGAAATAGAGTGGTTGGTGTATCGCATTGTTTTACATATTGGATTTCGCTCGAAACTCACCAAATGACTCCTCCCACCAAATTATGATGTTCCCCAAGTTTTGGACTCAATCCGATATCGATTGGTCCATGAAATTGGAGAATTCAGGTTCGTACAAAATTCGTTCTGAAATATAGGGGTTTGTGTTTTACATTGTTTAAGATATTGGTAGGGGAGGATCTCTAAATTATATTTCTGATTTT

TATCCAAATTTGCGAAAACAATGTAAGCCTCTATTTGATAGATTAAAGGATAATCCTCTTCCTTGGTCTAATATTCATACTGAAGTTGTCAAACAGATTAAAGCTCATGTCAAGACACTTCCCTGTCTTGGTATCCCTTCAGTTGATTCCTTCAAAATAGTTGGCATCTAAACAGATTTTTGCACGATGGCAAAGTATATTAAGTATAAAACAGATGCCTCTGACATTGGTTATGGTGGTATTCTCAAACAGAAAGTCAGTCCAGAATCCCCAGAGCAAATTGTTCGTTTCCACTCTGGAACCTGGACTCAGTCGCAATATAATTATAGTACTATTAAAAAAGAAATATTAGCTATTGTATTATGCATTAGTAAATTTCAAGATGATTTACTTAATCAAAAGTTTTTAATCCGAGTTGATTGTAAATCTGCAAAACATGTTTTAGAAAAAGATGTTCAAAACATTGCATCTAAACAGATTTTTGCACGATGGCAAAGTATATTAAGTATATTTGATTTTGATATTGAATATTTGAGAGGAAGTGAGAATTCAGTTCCTGATTTTCTTACTCGAGAATTCCTGCAGGAACCACAATGGCTCCCAAAAAAGACAAGAATAAAGAAGTTGCAATTTAGACTACGCTCAGCAATATCTAGACGGCACAGTTAAGATTCAGTTTGAAAGCCATCCCTTACGCAAATCTAATGTCCAGTTGCAACAGTTGCCAACCCCTGGTCAGTCCCAAAGGCATGCCCCTGCTCGGCATTCCTTTGCTGCCTCTTCCTCCACTCTAGAACGAGACCTAGAGTTAGAAAAGGCTATGATTGATTTCAAACTTGAGTCCCTGAGGAAAACCTCTCAGGTAACCCAGCCTTGCTACGGTCAGGCTTCAGTTCCAAAAGATAAGTCAGATTCACCTGAGTCACCTACCCAGTCTGACTTTGTAGTTGAAAATCAGCTCAGAACCCTTAATAGTAAATTTAAAATTAATTGGGTCAATTTAAATCGTCATCTAAAAGCTCCAGAGAACAAGCCCAGGCGAGACCTTTATCATCAAGCCTACCCTAATAGCAAACATCGAGATATGATTTTTCAACAATGGAAAAATTATATGCGATCAGCCAAAGTTGAAATTTTTTACCTAGATTTCATAGAAAGCCGTTATTTGACTAATGATGAGTTAAAAACTTTAACTAAAGAAAGATGGAAATTAGCTGATAAGTCTGTAGTAGAGTCTAGTCACCCTCCAGTAGAAACCATAGTAATAGAACACCATCAAGCCCCAGTTCCCGCCACTCCTTTCAAAACTTTTGAAAGCAGTGACCCGCATAGGAAACTAATCGAACAAAATAACTATACCAATCAGAGTCTAATTACCATAGGTAAACAGTTAGACACAATTGAAACCAAAGTCGATCTGCTGAGTCCGACTATTGCAGACTCTAGACCCAAATCAAAAATTGAAAAACCAATAGCTCACTTTCAAGAACTTAAGCTTGGTCCTGCACTCAAGACCAATTCCACAATGGAAAAAATAGAAGAAATGCTAAAACAGTTGACTCCTGTCAAACCTGTCAAGACCGAGATAGGTGATCAATCTGGTATTAAAACACTAGACGCTTTTGCCTCTCATTCCGAAACCAAATCCGTTAGTTCAGAAACTAGTGATATTTCTGAAATTGAAAATGCTTTTCAAAATCTAGAAATACAACTTGAGCCCAGAGTCAAAAAGATCGAGAAACATATAAGCCCTACTAGCCTAACCAAGAATTGGTATTCCAGACCAACACCTCCAGATATCCAATTTGAAGAAAGAAACCTCCAAACCCAGTTTTCTGTTTCTTCCGACAAATTGTATGAATGGAATATTGATGGTCTTTCTGAGCAAGAAATCTTAAATAAATTACAACATATGCTCATTCTATTTTCCCATAGTGGCATTCTCCTTCCTGATTCCAGAGCAGAGAGAGGAAATCTGTCTCATTCGGGGAGATAGTAAAGCATTTTGATACCGGGCTCAGAGAAGAAAGATGCAAAGGCAGGGATTGAGTGAAGCTCTTCCCCTCTCTCCAACTAGTCGCTTTTTCTACTCTAGTTCTTTATATACTCGCTTCAAGTATTCCGCTCGCTCTAGCTATCGCTTTTTCTTTGAAAAAGCTCTTACGCTACGTAAACTACGCTCTTACGCTTAATCGCTCTTACGTTACTTCGTAACTCGATAGTATTCTCATAAAAAAGCTAAGATAGATCTAATAGTTCGCGGAGAGGAACTGGCCAGGAGAATAAGAAGATTTGAGAAAAAGAATTCTTATCCCAAGTCGAGTGACAGGGCTTGACCTTTACGGATGGCTTAACTGATCTAATTAGAAGTACATCGCTAACAGGTTTGCTTGAAATACGCCTTTCAATCTCTTACCTGTTTTCCTAAGCAAACTCTCATTGGCTGGCAGGAAACTTGCCTATCGATGGTCGCCAATAGTTATATCACCAATCACAGTTTCAGACAGTCTGAAATTGTCCCCCTATTAGTTACTGGTTTCACCGGTACTCTCAGAAGTTGGTGGGATAAGCACCTTACCCCTGAAAATAAAAGTCGAATTATTTATGCTGTAAAACTTAATGAAGATGGTCTCCCTATATTTAACGAACAAATAGGTCAAGGTATAGAAGACGGTGTCAACACTCTGTTTTACACAATAATCGAACATTTCGTCGGAACCCCTAGCAACACCACATCTAGGATCCATGATCAATTAAGCAATCTTAGATGTCCCAAGTTGTCTGATTTCAGATGGTATAAAGACGTCTTCATTTCTAGAGTCATGCTTAGAGATGATAGTAATCAACCATTCTGGAAAGAAAAGTTTATTAATGGACTGCCAAACTTATTTGCTCATAAAATCCGCACAACCTTAAGTAATGAACAAGGTCAAATTGATTGGAATTCATTAACTTATGGTAATATTATAAGCACAATCAATCAAGTTGGTATGAAAATGTGTATTGATTTTAAAATTAATCGTCAAATCCAGTCGGATAGGAAGTCAGCCAAGTACGAGTTAGGAAACTTCTGTGAACAATATGGTCTCACTAGTATTCCTCCTTCTCGGAAACACAAACCTAGTCATCTTAGAAAACGCCATCATCCGAAGAAGCAATTTCCCAAGAACGATGAATTCTATAAGAAAAGAAAGTTCTCCCCTAAGAAAAGTTGGTCTAAACGCCCAAAACAAAGTCGCTCTAAGAAAGATAAAAGCAAAGTTAAATGTTTTAAATGTCAAAAATTTGGTCATTATGCTTCTGAATGCAAAGTTAAAGATACTATTAAACAGTTACAGATTTCAGAAGAAGATAAGAACAAATTGATCCAAGTTTTGGAGTTGCGAGACTCTGAAATAAGTAATGCTGAGTCTCCAATACCCAGTTCTGAATCTGAGCTGAGCCAGTCTTCAGATTCATCACTTAGTTCACCTAGGATTCAACTTGGTTGCACTGATAAATGCTGTAATAGGTTAAAGTCTATTTTTGTTCTTACTAAACAGGAAGAACAAGAAGAATTACTTGTAGATCTAATAGGAAGAATTCAAAACTCTGAATTAAAAGCTGATTATTTAAAAAAGTTGAAAAAGATCTTAAGTCAGGAAGGCCAGAGTACCCTCTCTCCTACTCCTCAGCCAATTAGCATCAACACTACGTTAGAAAAATTCAATCGTAGAAAAGATGTTACCCTACAAGATCTCCAGTCAGAAGTAAAACAGGTTAAAAAAGAAATCGTTGAGCTTAAGCAGATAAGTCAACAATTACAAGCTGAGAATTATACAATCAAACAAGATTTAGTTAATTTACAACAAGCCACTCGCCAACCTAGTACTCATTCGGAAAGCCCACATCATTCTGATGAGGAACCCTCTGAAGAACAACCAGTTGTCAATCTGATCAAACAAGTACACCTTAGAAAATGGTATTCCAAAGTCACGATTTTTGTTCATGATTTTGAATTTACCACAGTTGCCTTATTTGATTCTGGTGCAGATCTTAACTGTATTCAGGAAGGTCTCATCCCTACTAAGTACTACCAAAAGTCCAGAGAATCCCTTAGCACCGCTTCTGGGAAATCTCTCCAGTTAAATTTTGAAATACCAAAAGCCCATATTTGTCAAAACAAAGTTTGTTTTAAAACCTCATTCGTCTTAGTTAAAAATATTACTGATGAAGTCATATTAGGATTACCTTTCCTTGCTCTGTTGTATCCTTTTCAGGTAGAACATGATGGTCTCATTTCTACCCATTTAGGAGTAACGGTCAAGTTTAGTTTCCTATCTAAACCAGAGTTGCACAATTTAAAAACTTTACAAAAACAATCGATTTTTAAAGCTGTCCAGATTATTCGAAAATCAGACCAGTTGAATTTTCTTAAAGAAGAAATTAAATATAAACGAGTTGAACAAAACTTAATGATAAAACTTTTCAGTTAAAAATCCAGTCTTTTGAGAATAAATTAAAAGAAGAAATATGTTCTGATTTGCCTACTGCTTTTTGGTATAGAAAACAGCATCTCGTCTGTCTTCCCTATATCAAAACCTTTAGTGAAAAAAATATCCCCACTAAAGCCAGGCCAATCCAGATGAGTCAAGAATTAATGGAAGTCTGTAAGAAAGAAATTCATGAGTTGTTACAGAAAAATATAATTCGCAAAAGTAAGTCCCCTTGGTCATGTCCTGCTTTTTACGTCCAGAAAAATGCTGAACTAGAAAGAGGAGTCCCTAGATTAGTAATCAACTATAAGCCTCTTAATTCGGTCCTAGAATGGATTAGGTATCCCATACCTAATAAAAGAGACCTTATTAATCGCTTAGGAAAAGCAGTTGTCTTCTCCAAATTTGATATGAAAAGTGGTTTCTGGCAAATCCAAATAGAAGAGTCAGATAGGTATAAAACTGCATTTGTTACTCCTTTCGGTCATTACGAGTGGAATGTCATGCCGTTTGGTCTAAAGAATGCTCCTAGTGAATTCCAGAATATCATGAATGATATCTTCAATCCTTACAGTCATTTTTCGATTGTTTATATTGATGATGTCCTAGTATTCTCTGAGTCCGTTGAACAGCATTGGAAACACTTGCATCAATTCCTTTCCATTATCAAAAAGAATGGTTTAGCTATTTCTGCCAAGAAAATTAAATTATTCCAAGTCAACATCCGATTCCTTGGTTTTCATATATGTCAATCCCAAATTTCCCCAATAGACAGAGTCATCCAATTTGCTGACAAGTTCCCTGATCAAATTCTTGATAAGAATCAGCTCCAGAGGTTTTTAGGATCTCTAAATTATATTTCTGATTTTTATCCAAATTTGCGAAAACAATGTAAGCCTCTATTTGATAGATTAAAGGATAATCCTCTTCCTTGGTCTAATATTCATACTGAAGTTGTCAAACAGATTAAAGCTCATGTCAAGACACTTCCTTGTCTTGGTATCCCTTCAGTTGATTCCTTCAAAATAGTTGAAACAGATGCCTCTGACATTGGTTATGGTGGCATTCTCAAACAGAAAGTCAGTCCAGAATCCCCAGAGCAAATTGTTCGTTTCCACTCTGGAACCTGGACTCAGTCGCAATATAATTATAGTACTATTAAGAAAGAAATATTAGCCATTGTATTATGCATTAGTAAATTTCAAGATGATTTACTTAATCAAAAGTTTTTAATCCGAGTTGATTGTAAATCTGCAAACATGTTTTAGAAAAAGATGTTCAAAACATTGCATCTAAACAGATTTTTGCACGATGGCAAAGTATATTAAGTATATTTGATTTTGATATTGAATATTTGAGAGGAAGTGAGAATTCAGTTCCTGATTTTCTTACTCGAGAATTCCTGCAGGAACCACAATGGCTCGCAAAAAAGACAAGAATAAAGAAGTTGCAAAAACAGTTGCTAGCCAACCCAAGTCATCCCAGTCGCAACCTTCTCAGTCGTCCAAACAAACCCAATCGACATCCATGAGTCCAGTTAAACCAGAAATACCCAAACATATAGTTCCCTATCCTGGTTGCAACCCCATCCCAGTTATTGCTACTCCCGTTATCAGTTGCCAATAGGTTTTCTCCTTAGGATCCACAATTGGCCAAATCCGCCCCAGTTACCAGTCCACCCTAGTTGCCAATTATGATCCCTTTTCAGTTGACATCCCTGCTGGACCTTCTGCTCCAGCTATCCGCAAAAAATCATCCCCTTATTTGCCCAAAAGCAATACCCATTTATTTGTTATTGAGCCCATCTACGACATAAATGCTAACCCAGTCGAAATTGCCCGTCATTATTTCCCTCCTGGATTTCATTATATGCCCTCTAGTCCATACAAGTCCCTCAAGTATTATAGAGATATACTACTTGAAACCAAATCAGTGGAGATTAAGCCCATAAAGGATCGTGACAACCCTGGAACCATCCTTTATCATTCTCTTTACATTCATCAGATCATAAGCCCAGCAGCTTTTGCCAGCCGCCCTTATGAACTGAAGTTGCTCCAATCCAAGTTGCAATACAATTATGTAGATTATATTGATGCCTGGTATACTATTTTCCTTCATCAATCTGAGGATTTTAGTCATTCCTGGTTCATCAATTTTGATAATAAATTCAAAAGTCCTTTTCCCTACTGGTTCCTCCATTGGTGGGAAAAGCATGGCCCAGTTGAAGAAATCCTGCCTGCATCAGTTATTCAACTCATCGTTCACTACACCAAAAAGACCAAGTTCTCTGAAGTTGATCTCTTTTTCCCCACACAGTTGCTCTTTATTGCCAGATACAAAGTTCCCTGGATCCTCAAATGGTCCTATCAAGTTGCTAAAGATGATCGTATCTTTGCCCGCCAATTGTCCGTTAAGTGGTGGGATAAATTTGAAGTTGACAGAATTGCTAAATATGTCTATAATGATCTACCAGTTGACCCAGTAGCCCCTGTTGTCTCTCCAACCGGATCTGTTCGATCTTCCCTCTCAGTCGAAGGAAAGTCCAAGGCAGAACTCCGCGAGATCGCCACTCAGTTGCTCATGCAAGCCTCCCAGATGGATGATGATGAAGACAACGGGTCCCCCTCGTCTTCCAGCTGTCACCCATCCCAGTCAGGCCCAAGACTTGGTTTGAAGACAGTCAAGACCCATATGATGCCTATGACCTAGCCTCTGACTAGGCATCATTATGCTCCTGACCTCTTCACATGCCAAAAGTAGCCGAAAGCAGCAAAAGTCGCCAGTCGCCAGACACCATAATTGCCTCAGGAGACAGCATCTGCTTTCCCTGTTCTCTTTTCCACCGAAAGTTGCCTCAGTTAAAGAATCTTCCGAGTAAAGACAAAATTGAAAGTTGAATTAGGTTGCTTTCTCGTGATTCCACAGTGACAAGTGAACCTCAGTTGATTTACCATAGTTAGTTGAACTCTAGTTGCTTTTCTATATAAGCTGGCCTCGGCCTCAGTTGTAAGCAGACTCCTTTGGGAGAATGCAATTTTCTTTCTTCAGTTTTAAAGAATCTCTCTGATACATCCTTCGGAGAATTCATCTTTGTAAACACTCTTGTAAATAGCCGTGTGAGTTGAAGTTTGCAGTTGCTCTCTTTGTGAGTATAAATAAAAGTTATTTTCAATCATCTATTTTCCGCAAACATTATTTTAATGCATTATCATTAATTTCTGCAATAGTCTGAAAGTTGCTTAGGCTCCATCCTTTTCTAATTTCCTTCTGATCTTCACTCTGATTTATATTCAGTTGCCTTTCCTTGCTATCTTTGTTGTTTTTCCTTAGTTGGTATCAGAGCCGTAAGTGTTACGGTCTTTGTCAAAATTGATGAACCAGTAGGGAAAAGGACTTTTGAATTTATTATCAAAATTAATGAACCAGGAATGACTAAAATCCTCAGATTGATGAAGGAAAATAGTATACTAGGCATCAATATAATCTGCATAATTGTATTGCAACTTGGATTGGAGCAACTTCAGTTCATAAGGGCGGCTGGCAAAAGCTGCTTGGCTTATGATCTGATGAATGTAAAGAGAATGATAAAGGATGGTTCCAGGGTTGTCACGATCCTTTATGGGCTTAATCTCCACTGATTTGGTTTCAAGTAGTATATCTCTATAATACTTGAGGGACTTGTATGGACTAGAGGGCATATAATGAAATCCAGGAGGGAAATAATGACGGGCAATTTCGACTGGGTTAGCATTTATGTCGTAGATGGGCTCAATAACAAATAAATGGGTATTGCTTTTGGGCAAATAATGGGATGATTTTTTGCGGATAGCTGGAGCAGAAGGTCCAGCAGGGATGTCAACTGAAAAGGGATCATAATTGGCAACTAGGGTGGACTGGTAACTGGGGCGGATTTGGCCAATTGTGGATCCTAAGGAAGAAAACCTATTGGCAACTGATAACGGAGTAGCAATAACTGGGATGGGGTTGCAACCAGGATAGGGAACTATATGTTTGGGTATTTCTGGTTTAACTGGACTCATGGATGTCGATTGGGTTTGTTTGGACGACTGAGAAGGTTGCGACTGGGATGACTTGGGTTGGCTAGCAACTGTTTTTGCAACTTCTTTATTCTTGTCTTTTTTGGGAGCCATTGTGGTTCCTGCAGGAATTCTCGAGGAAGAAAATCAGGAACTGAATTCTCACTTCCTCTCAAATATTCAATATCAAAATCAAATATACTTAATATACTTTGCCATCGTGCAAAAATCTGTTTAGATGCAATGTTTTGAACATCTTTTTCTAAAACATGTTTTGCAGATTTACAATCAACTCGGATTAAAAACTTTTGATTAAGTAAATCATCTTGAAATTTACTAATGCATAATACAATAGCTAATATTTCTTTTTTAAAGTACTATAATTATATTTGCGACTCCAGGTTCTAGAGTGGAACGAACAATATTGCTCTGGGGAATCTGACTACTTTCTGTTTAGAAATGCCACCATAACCAATGTCAGAGCATCTGTTTCACTATTATGAAGGAATCAACTGAAGGGATACACAAGACAGGGAAGTGTCTTGACATGAGCTTTAATCTGTTTGACAACTTCATATGAATATTAGACCAAGAAGAGGATTATCCTTTAATCTAATCAAATAGAGGCTTACTTTGTTTTCCCGCAAATTTGGATAAAAACAGAAATATAATTTAGAGATCCTAAAAACCTCTGGAGCTGATTCTTATCAAGAATTTGATCAGGTAACTTGTCAGCAAATTGGATGACTCTGTCTATTGGGAAATTTGGGATTGACATATATGAAAACAAGGAATCGGATGTGACTTGAATAATTTAATTTTCTTGGCAGAATAGCTAAACCATTCTTTATTGATAATGGAAAGGAATGATGCAAGTGTTTCCAATGCTGTTCAACGACTCAGAGAATACTAGACATCATCAATATAAACCAATCGAAAAATGACTGTAAGGATTGAAGATATCATTCATGATATTCTGGAATTCACTAGAGCATTCTTTAGACCAAACGGCATGACATTCCACTCGTAATGACTGAAAGGAGTAACAAATGCAGTTTTATACCTATCTGACTCTTCTATTTGGATTTGCCAGAAACCACTTTTCATATCAAATTTGGAGAAGACAACTGCTTTTCCTAAGCGATTAATAAGTCTCATTTATTAGGTATGGGATACTAATCCATTCTAGGACCGAATTAAGAGGCTTATAGTTGATTACTAATCTAGGGACTCCTCTTCTAGTTCAGCATTTTTCCGGACTAAAAAGCAGGACATGACCAAGGGGACTTACATTTGCGAATTATATTTTTCTGTAACAACTCATGAATTTCTTTCTACAGACTTCCATTAATTCTTACTCATCTGGATTGGCCTGGCTTTAGTGGGGATATTTTTTTCACTAAAGGTTTGATATAGGGAAGACAGACGAGATGCTGTTTTTCTATACCAAAAAAGCAGTAGCAAATCAGAACAAATTCTTCTTTTAATTTATTCTCAAAGACTGGATTTTAACTGAAAAGTTTTATCAAGAAGTTTGTGAACTCGTTATATTAATTTCTTCTTTAAGAAAATTCAACTGGTCTGATTTTTCGAATAATTGGACAGCTTTAAAAAATCGATTGTTCTTGTAAAGTTTTTAAATTGTGCAACACTGGTTTAGATAGAAACTAAACTTGACCGTTACTCCAAATGGGTAAAATGAGACCATCATGTTTCTACCTGAAAAGGATACAACAGAGCAAGGAAAGGTAATCCTAATATGACATTCATCAGTAATATTTTTAACTAAGACGAATGAGGTTTTTAAAAACAAACTTTGTTTTGACAAATGGGCTTTTGGTATTTCAAAATTTAACTGGAGAGATTTCCCATAAGCGGTGCTAAGGATTCTCTGGACTTTTGGTAGTACTTAGTAGGATGAGACCTTCCTGAATACAGTTAAGATCTGCACCAGAATCAAATAAGGCAACTGTGGTAAATTCAAAATCATAACAAAATCGTGACTTTGGAATACCATTTTCTAAGGTGTACTTGTTTGATCAGATTGACAACTGGTTTGTTCTTCAGAGGGTTCCTCATCAGAATGATGTGGGCTTTCCGAATGAGTACTAGGTTGGCGAGTGGCTTGTTTAAATTAACTAAATCTTGTTTGATTGTAATAATTCACAGCTTGTAATTGTTTGACTTATCTGCTTAAGCTCAACGATTTCTTTTTTAACCTGTTTTACTTCTGACTGGAGATCTAGTAGGTAACATCTTTTCTACGATTGAATTCTAACGTAGTTTGATGCTAATTGGCTGAGGAGTAGGAGAGAGGGTACTCTGGCCTCCTGACTTAAGATCTTTTTCAACTTTTTTAAATAATCAGCTTTTAAATAGAGTTTTGAATTCTCCTATTAGATCTACAAGTAAATCTTCTTGTTCTTCCTGTTTAGTCAAAACAAAATAGTCTTTAACCTATTACAGCATTTATCAGTGCAACCAAGTTGAATCCTAGGTGAACTAAGTGATGAATCTGAAGACTGGCTCAGCTCAGATTCAGAACTGGGTATTGGAGACTCAGCATTACTTATTTCAGAGTCTCGCAACTCCAAAACTTGGATCAATTTGTTCTTATCTTCTTCTGAAATCTGTAACTGTTTAATAGTATCTTTAACTTTGCATTCAGAAGCATAATGACCAAATTTTTGACATTTAAAACATTTAACTTTGCTTTTATCTTTCTTAGAGCGACTTTGTTTTGGCGTTTAGACCAACTTTTCTTAGGGAGAACTTTCTTTTCTTATAGAATTCATCGTTCTTGGGAAATTGCTTCTTCGGATGATGGCGTTTTCTAAGATGACTAGGTTTGTGTTTCCGAGAAGGAGGAATACTAGTGAGACCATATTGTTCACAGAAGTTTCCTAACTCGTACTTGGCTGACTTCCTATCCGACTGGATTTGACGATTAATTTTAAAATCAATACACATTTTCATACCAACTTGATTGATTGTGCTTATAATATTACCATAAGTTAATGAATTCCAATCAATTTGACCTTGTTACTTAAGGTTGTGCGGATTTTATGAGCAATAAGTTTGGCAGTCCATTAATAAACTTTTCTTTCCAGAATGGTTGATTACTATCATCTCTAAGCATGACTCTAGAAATGAAGACGTCTTTATACCATCTGAAATCAGACAACTTGGGACATCTAAGATTGCTTAATTGATCATGGATCCTAGATGTGGTGTTGCTAGGGGTTCCGACGAAATGTTCGATTATTGTGTAAAACAGAGTGTTGACACCGTCTTCTATACCTTGACCTATTTGTTCGTTAAATATAGGGAGACCATCTTCATTAAGTTTTACAGCATAAATAATTCGACTTTTATTTTCAGGGGAAGGTGCTTATCCCACCAACTTCTGAGAGTACCGGTGAAACCAGTAACTAATAGGGGGACAATTTCAGACTGTCTGAAACTGTGATTGGTGATATAACTATTGGCGACCATCGATAGGCAAGTTTCCTGCCAGCCAATGAGAGTTTGCTTAGGTAAAACAGGTAAGAGATTGAAAGGCGTATTTCAAGCAAACCTGTTAGCGATGTACTTCTAATTAGATCAGTTAAGCCATCCGTAAAGGTCAAGCCCTGTCACTCGACTTGGGATAAGAATTCTTTTTCTCAAATCTTCTTATTCTCCTGGCCAGTTCCTCTCCGCGAACTATTAGATCTATCTTAGCTTTTTTATGAGAATACTATCGAGTTACGAAGTAACGTAAGAGCGATTAAGCGTAAGAGCGTAGTTTACGTAGCGTAAGAGCTTTTTCAAAGAAAAAAGCGATAGCTAGAGCGAGCGGAATACTTGAAGCGAGTATAAAAGAACTAGAGTAGAAAAAGCGACTAGTTGGAGAGAGGGGAAGAGCTTCACACAATCCCTGCCTTTGCATCTTCTTCTCTGATCCGGTATCAAAATGCTTTACTATCTCCCCGAATGAGACAGATTTCCTCTCTCTGCTCTGGAATCAGGAAGGAGAATGCCACTATGGGAAAATAGAATGAGCATATGTTGTAATTTATTTAAGATTTCTTGCTCAGAAAGACCATCAATATTCCATTCATACAATTTGTCGGAAGAAACAGAAAACTGGGTTTGGAGGTTTCTTTCTTCAAATTGGATATCTGGAGGTGTTGGTCTGGAATACCAATTCTTGGTTAGGCTAGTAGGGCTTATATGTTTCTCGATCTTTTTGACTCTGGCTCAAGTTGTATTTCTAGATTTTGAAAAGCATTTTCAATTTCAGAAATATCACTAGTTTCTGAACTAACGGATTTGGTTTCGGAATGAGAGGCAAAAGCGTCTAGTGTTTTAATACCAGATTGATCACCTATCTCGGTCTTGACAGGTTTGACAGGAGTCAACTGTTTTAGCATTTTCTTCTATTTTTTCCATTGTGGAATTGGTCTTGAGTGCAGGACCAAGCTTAAGTTCTTGAAAGTGAGCTATTGGTTTTTCAATTTTTGATTTGGGTCTAGAGTCTGCAATAGTCGGACTCAGCAGATCGACTTTGGTTTCAATTGTGTCTAACTGTTTACCTATGGTAATTAGACTCTGATTGGTATAGTTATTTTGTTCGATTAGTTTCCTATGCGGGTCACTGCTTTCAAAAGTTTTGAAAGGAGTGGCGGGAACTGGGGCTTGATGGTGTTCTATTACTATGGTTTCTACTGGAGGGTGACTAGACTCTACTACAGACTTATCAGCTAATTTCCATCTTTCTTTAGTTAAAGTTTTTAACTCATCATTAGTCAAATAACGGCTTTCTATGAAATCTAGGTAAAAATTTCAACTTTGGCTGATCGCATATAATTTTTCCATTGTTGAAAAATCATATCTCGATGTTTGCTATTAGGGTAGGCTTGATGATAAAGGTCTCGCCTGGGCTTGTTCTCTGGAGCTTTTAGATGACGATTTAAATTGACCCAATTAATTTTAAATTTACTATTAAGGGTTCTGAGCTGATTTTCAACTACAAAGTCAGACTGGGTAGGTGACTCAGGTGAATCTGACTTATCTTCTGGAACTGAAGCCTGACCGTAGCAAGGCTGGGTTACCTGAGAGGTTTTCCTCAGGGACTCAAGTTTGAAATCAATCATAGCCTTTTCTAACTCTAGGTCTCGTTCTAGAGTGGAGGAAGAGGCAGCAAAGGAATGCCGAGCAGGGGCATGCCTTTGGGACTAACCAGGGGTTGGCAACTGTTGCAACTGGACATTAGATTTGCGTAAGGGATGGCTTTCAAACTGAATCTTAACTGTGCCATCTAGATATTGTTGAATGTAATCTAAATTGTTTAGACTACGCTGGATTGGAGCTGGTTGGCTTTCAGTGATTAAGGTCCAATCTTTTGGAAGGGCAACTTCTGACCACTTAATTGTATGGGGTACTTGGATATGGGCATCTGAGGTATGACTCTGGATCAGGACTGTTTGATCCTTTGGGCTTTTATTAAGGGCTTGGAAGTTCATATTAGTTCCTGTGACCTTATAGTAGATTCTGTATACTAAGGCTAGAGGTTGGGTTCCCGGGAGAACATTATAACCTTCAGTCTTAATATTCAGAGTCAAAGTTCTCAGGATGTGAGGATCACTAAGACTTACAGTGAAATCAGGATAACAGTCAAAATGGACAGGGCCATTACACAGACTGGACTCAATGATTCCTAGGGTACTATCGCTAAATTCAGTGAATCTAGCATCACGAAGACATAGCAGGATTGAGGCTTTCAACCCTAACCGTGTTAGAGGCTTTACGGCAACTTGAACTAGACCAATATGCAGGAAATTATTACCGTCTTTCTTATGTTTTAAAATGGACTCGAGATTAAGCAACTGGCAGGTTTCGTGTTCTTTGCTTAAGGCGTAAACTTGTTCAACTGTTTTGACATGTGTGGTACTGTTGAAACTAGAAGGCAACCAATGTTTCTTATAAATTTTCTGACTAGGGATTTTAGGTATGTTCCAATCTCTAAAATCTAATTGTTCTTTACTGCTGGAATTATATTCAAATTGTTCTTCATTAACTATCTCTGGGAGTCGACTTAGACTGGCACTAGAACTGGCAGAATTACTAGACCTTGACCTAAGGAAACGACTCATGACTACTCAAGAAACTGGGTTGAACTATCTGAGGAAGAAGATGAACAGTAAATCCTAGACCGACCGTTTAGACTCTAAGTAACCTATTAATTCTCTCAATTTTCTGGCTCCTGCCCAGCACACACTACCTCCCTGGGATTTACGTGATCTAGCAAAAAGGACTCACAGGTCAGACCTTAGAGAAAACTAACAGAGGACTCAAAGCATAAAAGGGAAGAAAAGGAAGAAATGAGAACCTTCTACTCAGAAGCTTAAACCAGCCACTTGGGCGGGAATGAGTAGCAGACTTAGACAAAGACCGTAACACTTACGGCTCTGATACC

TTGGCTGACGTTGGTGTATTGCATCGTTCAAGAGATTGGATTTCACTCAAAACCTACCAAATGACTCCTCCCACCATACTATGTTGTTCCCCAAGTTTTGGACTCAATACGATATCGATTGGTCCATGAAATTGGACAATTCAGGTTCGCACGAAGTTCATTCTGAAATGAAGTGGTTGGTGTACTGCATAGTTCTAGATATTGGATTTCACTCAAAACCTACCAAATCACTCCTCCCACCATACTATGATGTTCCCCAAGTTTTGGACTCTATCCGATAACGATTGGTCTATGAAATTTGACAATTCAGGTTCGTACGAAGTTCATTCGGAAATGGAGTGGTTGGTGTATTGCATAGTTCTAGATATTGGATTTCACTAAAAACTCACCACATGACTCCTCCCACCATATTATGATGTTCCCCAAGTTTTGGACTCAATCCGATATCGATTGGTCTGTGAAATTGGATAATTCAGGTTCGTACAAAGTTCGTTCTGAAATGGAGTGGTTGGTGTAATGCACAGTTCTAGAGATTGTGTTTCACTCAAAACCTACCAAATAACTCCTCACATCATACTATGATGTTCCCCAGGCTTTGGACTCAAACCGATATCGATTGGTCCACGAAATTGGACAATTCAAGTTCGCACGATGTTCGTTATGAACTGGAGTGGTTGGTGCGTTGCATAGTTTAAGATATTGGATTTCACTCAAAGAGCACCTAATGACTCCTCACACGATATTATAATGTTCCATTAGTTTTTGGCTCAATCTGATATCGATTTGTCCATGAAATTGAACAATTTAGGATCGTACAAAGTTCGTTCTGAAATGGAGTGGCTTGTGTTTTGCATAGTTTTAGAGATTGGATTTCACTCAAAACTCATCAAATGACTCCTCCCACAATATTATGATGTTCCCCAAGTTTTGGACTCAATCCGATATCGATTGGTCCTTGAAATTGGACAATTCAAGTTCGCACAAAGTTCATTCTGAAATG

>Chr8-04; OZ022213.1; 5048608-5055675; rev; A;

CCTGAAAAAAGAAAACAAACAATAGTAAGTAATACAACAGTAAAATGATAAAAGAAGCAACATCACATCACGAAATTTGATGACAATGGTTGGCTAGCTGGCAAAAGATGACTAGCCCTTGAAACCAAAGCATGGCTCACACTCCCCAATTTCTGAACATACCCAGCAACCTCTTTACCCACCTTCCAAACTTTCTGGTCCGCTGATAGAAACTTGTTCATCCTCAGGTTTTCATATTTTTAATTTGTTTAAGGAAAAAAAATCAGAGTCCAAGAAATATTCATCTCTCAATGCTTTAAGTAATCTTAAAGATCTTAGAAATTCTGTTCAAATAGGATTTCCTTACCTTTTCGTTAAAATTATTCTCTTCAACTTCCATTCTCTTGTTTTCTTCTTCATAAACAATTTGATTGAATGAATAGAAAAGAGAAGAAAATTATTTCAGAATTCAGTTATGAGCACTGAAAAGTCATAAGAGGAATCATGAGTAAGGAGTCATAGCTTCCTTGTGTTGTTTTTTTTTCCTGTCAAACGACAAGGTTACGTGAGAACAGAAGTCTGCGTCTTTTTTTTTTTTTTTTTTTCCTCTTTCTTTTTCCTTAAGTCCTTTTAAGTCATTTTAAAATAAGGTTAAATTTGTCTTTAAAAGTTTAAAGATAAGGTGGGTGGGAAAATAGGACATATGTGTGGAAATAGCAGCACTCTTAGCAAAAGGTATATAATAACAAAAAGGCTTTTTATCACAAAATGTCCCTAACGTTTGCGAAACTATCAGATTGCATCCTTAAAGTTTTTTTGTATTACTCATGGTCCCTAATGTTAATATAGGTGCTCTTAAATAGTCCCTCTGTCAAAAAAACTGTTAAATCCAAAGATATAATCGTCAAATCAATCCAAAATTATAATATATATTCATACATAAGTTCCGCATATTCTATCAATTAGTTGCTTCCACTTACTTCTGTCTTTATACTATCTCCCACTGGTTCTAGTTCCCTTC

TGGTATCAGAGCCGTAAGAGTTGTAAAAGTCAGTTCTAAGTCTGTAATTCATTCCGCCCAAGTGACTGGTTTAAGTTTCTTGAGTTGAAGTTTCCAGTTCCTTCTTTTACTTTCTTTTTATTCTTCCAGTTGTCTGTTGGTTCTCTTTCTTGGTGTCCAAACAGTGAGTCCTTTTTCCAAACAGTAAGTCCTGAGAGAGGCATGAGCGGTGGGCGTGAGTGGAGGCCAAGAGAATTAGCAGGTTTCTGGTAAGAGTAAAGGGTCAGTCTTAGGAGTCCCTGTTTATCTTCTTCATTTAAAGCCAAGCCCAGTTCTTGTAAGAATGAGTCGCCTATTTAGAACAAACTCTTGTAATTCGAGCAGCTCTAGATCCAGTATGAGTCGTCTCCCTGAGATAGTTAATAAAGAACAAGTTGAACATGATCCTAATAGTAATTTAGATCATAATGATTGGAATATACCCAGAGTCCCCAGTCAAGACATCTATAAGAAAAAATGGTCTTTGTCTAGTTTCAAGTCGACCACCCATGTCAAAACAGTTGAACAAGTCTATGCCCTTAGTAAAGATCATGAAACATGTCAATTGCTTAATCTTGAGTCAATTAAAAAACATAAAAATGATGGAAATAATTTCCTTCACATTGGTTTAGTCCAAGTTGCAGTTAAGCCATTAACCAGATTAGGATTAAAAGCTTCTATCCTTCTCTATCTACGCGATGCTAGATTCCTTAATTTCACCGACAATACGCTTGGAATTATTGAGCCCAGTCTAGCAAATGGTCCAGTTTACTTTGATTGCTATCCCGATTTCATGGTCAGTCTTAGTGATCCACATATTTTAAAAATTCTTACGCTAAATGTCAAAACTGAAGGTTATGAAGTCCTCCCAAGAGCCCAACCTTTAGCATTAGTCTATAGAATTTATTACAAGGTCACAGGCACGAATATGAATTTTCAAGCCTTAAGCAAAAGTCTAGAAAATCAGACATTGTTAATCCAGAGTCACACGTCTGATGCCATAATAAGAGTCCCTCATACAATCAAATGGTCTGATGTGTCCCTTCCTACGGATTGGACCTTAACCACTGAGATTAAACCGACTCCCATCCAGCATGGTCTAAACAATTTAGACTATATCCAACAGTACCTTGATGACACAGTTAAAATTAGTTTCGGTAACCAGCCTGTACAAAAGTCTACAGTCCAGTTACATCAGTTGCCTACCCCTAGTCAGTCCCAGAGCCACATTCCTGCAAGGCATTCCTTTGCAGCCTCTTCCTTCACTCTGGAACGAGACCTAGAGTTAGAGAAAGCCATGATTGATTTTAAATTAGAAACATTAAGGAAAAATTCCTAAGTCAATCAACCCTGTTATGGTGCGATACCAGCACATGAGGAAAAACCTGAGTCTCCAGAGTCACCCACTCAATCTGACTTTGTCAAAGCCGGTTTCCCAATTGATAACCAGCTAAGAACACTTAATAAAAAATTTGTAATTAATTGGAAATATTTGGATAATCATCTTAGAGCCCCTGAGAACCAGGAACGAAAAGATAATTATCATAAAGCCTATCCTAGTAGAGAAAAGTGTCAATTAATCTTTAATGAATGGAAAGATTATATACGCACAGCCAAGTTAGAAATTTTTTATCTAGATTTTGTCGAAAGTCGCTATATGTCTAATAAGTTAAAAACTTTAACTAAAGAAAAATGGAAATTATCTTATGACACAGAAGTCGTAGCAAGTCATCCACCAGTTGAAACAATAATAATTGAACATAAGAAATCCCAAGTTCCTACTAGTCCGTTCAAAGTCCCTGAGAAAAATGATCGAGATAAGAAACTAATTGAACAAAATAATTACTCCAATCAGAGTCTAATTGTCATAGGAAAACAGTTGGATAAAATTGAAACAAAAGTTGATAAGTTGCTACCACAGGATGTCAAAACCAAAACTAAAGTTGAAAAACCCCTAGTCCAATTTCAGGATCTTAAGTCAAGTCTGACCCTGAAGATAAGTCCAACCATAAAGAAAATTGAAGAAGTCTGGTCTTAAAGTTCAATTTACACCAGTTAAACCTGAGAAGTCTTCTGAGTCCGAATCTGAATCAATAGTCAGTATGGAAACAGAAACTAGTAATATTTCAACATTTGAAAATGCTTTTAAAATTTTAGAATTAAATAATCAATTGAAAGTCAAACGGTTAATTCACCAGGTCAGCCCTACGAGTCTAACCAAAAATTGGTATCCTAGGCCAACACCCCCAAAAATTCAATTTGAAGAACGAAACTTCGAGAGTCAGTTTTCAGTTTCTTCAGATAAATTGTATGAATGGAACATTGATGGTCTGTCTGAACAAGAAATCCTTAATAAACTCCAGCACATGTCCATGGTTGCTAATAGTTATATCACCAAAGACAGCTTTAGGCAGTCAGAAATAGTGCCGTTGCTTGTCACTGGTTTCACCGGTACACTTCGGTACTGGTGGGATAAACATTTAAGCTTGATGAAGATGGTCTTCCTATATTTGACGAACAAATAGGTTCAGGTATAGAAGATGGAGTCAACACTTTGTTTTATACAATAATAGAACACTTTGTTGGAACCCCCAGTAATACAACTGCTAGAATCCATGATCAACTTAGCAATCTTCGCTATCCTAAGTTGTCTAATTTTAGATGGTATAAAGATGTTTTTATTTCCTAAGTCATGTTAAGAGATGATAGTAATCGGCCGTTTTGGAAAGAAAAATTTATTAATGGTCTTCCAAACCTGTTTGCTCATAAGATTTGAACTGTCCTTAGCAATGATCACAGTTTCATTGATTGGAATAGTTAAACATATGGAAATATAATTAGCACAATTAATCAAGTTGGTTTGAAAATGTGTGTTGACATGAAAATTGGTAAACAAATACAGCCCGACAGAAAATCTGCTAAGTATGAGTTAGGAAATTTCTGTGAACAGTATGGTCTTGCCAGTATTCCCCCTTCCAGGAAAAATAAGTCCAGTTATCCTAGTCACATTAGGAAAAGTCGTCATTATTAGCGTAGGAAACAATCATTCCGACACAATAATGATAATAATGAGTTTTATAAGAAAAGAAAATTTCACCCTAAGAAGAGTTGGTCAAAAGCACCAAAAGGTGAAAAGAATAGTCGCTTTAAGAAAACCTCCGACATGAGAAAAGTAAAATGCTATAAATGTCAAAAGTCTGGTCACTATGCTAATGAATGCAAAGTAAAAGACACAATTAAACAATTAAAAATCATGAATGAAGAAAAAGAAAAATTAATTAAAGTTTTAGATTTAAAAAATTCTGAGTCTAGTGATAATGACAACCTAGTTGCCAGCACTGAGCCAGATAGCTATCAGTCTTCCGATTCCCAGTCTAGCTGGCCTAAAGTCCAATTTGGTTGCACAGATAAATGTTGTAATAGATTAAAATCCGTTTCTGTCCTCACTAAACAGGAAGAATAAGAAGAGTTGCTAATTGATTTAATCAGTAAAGTAGAAAACCCAGAGTTGAAATCTGAGTATCTAAGAAAGTTACGAAAAGTCATTAGCCAAGAAGGCCCAAGCTCTAGTAGTCAGTCTACTCCCCAAAAAATTAGTGTAAACACAACCTTAGAAAGGTTTAATAGAAAGAAAGAGGTCACCCTCCAAGACCTACAGTCCGAAATCAAAGTAATCAAAAAAGAAATAGTTGAATTAAAACAAATAAGTCATAAGTTGCAAGCCAAGAATTATGAGATAAACAGAATTTAATCAGTTTAATGAAAAATGATGGCTCTGAGTCTAGAAGTCAAGCAGAGAGTCCTAATAACTCAGATGGTGAGCCTTTAAACAATGATCAAGCAATTAATTTAATTAAACATGTTACTTTCAGGAAATGGTATGCTAAAGTAACAATATTTGTCAAAAATTTTGAATTCAACACAGTTGCCTTGTTTGATTCAGGAGCCGACCTAAACTGTATTTAGGAAGGTCTAATCCCTACTAAATATTATCAGAAGTCTAAAGAGTCGCTTAGAATTGCCTCAGGAAAATCGTTGCAGTTGAATTATGAAATCCCTAAAGCCCATATTTGCCAAAATAAAATTTGCTTCAAAGCATCATTTGTCTTAGTCAAAGATATTACTGATGAGGTTATCTTAGGATTACCTTTCATCGCCTTACTGTATCCTTTCCAGGTACATTGCGATAGTGTAATCTCTACCCACTTAGGAGAAAATGTTAAATTTGAATTTTTAAGTAAGCCTGAGTTGCACAATTTGAAAGCCCTTCAAAAGAGTTCAGTCTCAAAAACAATTCGAACAATCCACAGGAAGAATAGTCAGTTAAACTTCCTTAAGGAAGAAGTCAAGTTTAAAAGAGTCGAACAACAGCTTAGTAATGAGTCTTTACAGTTGCAAATTCAACAGTTTGAAAATAAATTAAAGAATGAAGTCTGTTCTAATTTGCCTAACGTGTTTTGGCACAGGAAACAACACGTTGTTAATCTTCCCTACATCAAAACCTTTAGTGAGAAAAATATCCCCACTAAAGCTAGGCCAATCCAAATGAGTCAAGAAATAATGGAATTCTGTAAAAATGAAATTGCTGAGTTGCTTCATAAAGGAATAATTAGAAAAAGTAAGTCACCCTGGTCTTGCCCTGCATTCTATGTTTAGAAAAATGCTGAATTAGAAAGAGGAGCCCCCAGACTAGTCATAATTATAAACCCCTTAATTCGGTCTTAGAATGGATTAGGTATCCCATACCTAATAAAAGAGACTTAATTAACATATTAGAAAAAGCAGTTGTCTTCTCTAAATTTGATATGAAGAGTGGTTTCTGGAAAATCTAAATAGATGAGTCAGATAGATATAAGACTGCATTTGTCACTACTTTTGGTCATTATGAATGGAATGTAATGCCTTTTGGTTTGAAAAATGCACCGAGTGAGTTTCAGAATATTATGAATGAGATTTTTAATAGCTTTAGTCATTTTTCTATTGTCTATATTGATGATGTTCTCATCTTCTCCCAGTCAGCAGAACAACATTGGAAACATTTGAACAAATTTCTCCAAATTGTCAAACACAATGGTCTAGTTGTTTCTGCTAAGAAAATTAAGTTGTTCCAAACTAATATCAGATTTCTTGGATTTAATATCTGCCAATCACAGATCAGTCTAATAGATCGAGTCATCCAGTTTGCTGATAAATTTCCAGATCAAATTCTTAATAAAAGTCAACTCCATAAGTTTTTAGGATCTCTTAATTATGTGTCTGACTTCTACCAAAATCTGCGAAAACAATGTAAGCCACTGTTTGACAGGTTGCAAGATAACCCTCCTCCTTAGTATTCTGTCTATACAAAAATTGTCAAACAGATCAAAGTCCATGTCAGGACACTTCCCTATCTTGGTATTCCCTCAGTTGATTCTTTCAAAATAGTTGAAACTGATGCCTCTGATATTGATTATGGAGGTATTCTCAAACAGAAAATCGATCCAAATCAACCAGAACAAATCATTCGTTTTCATTCTGGTATTTGGACTCAGTCACAATATCTATAGTACTATGCATATCTAAATTTCAAGATGATTTGCTTAATCAAAAAATTTTAGTCAGAGTTATTGTAAGTCTGCAAAGCATGTTCTGGAAAAAGATGTTCAAAACATTGCATCAAAACATATTTTTGCACGTTGGCAAGCTATTTTGAGTATTTTTTATTTTAATATTGAATATTTGAGAGGTAGTGAGAATTTAATCCTTGATTTTCTTACTCAAGAATTCCTGCAGGAAAAACAGGATCAATGGCACCTAAAAAAGACAAATAGAAAGAGTCGGCTAGTCAGCTCAAGCCAATCCAGTCAACCCAGTCGCTCAGAGCCCTTCCATCGTCCATGAATCCAGCCAAATCAGAGTCACCTTCTCACATCGTCCCTTTCCCGGGATGTTCCCCAATCCAAGTCAGTGACTGGTTCTCTACCTTGGGAACCACAATCGGTCAAATATGCCCCAACTGCCAGTCTGCCTTAGGCTCCAGTTATGATCCATTCCAGATCATTCCTCCAGTTGCTCAGTCCTCCTAGTCATCCCCTTAACTTCCAAAATAAAAGTCGCACCCCTTCGTAATTGAACCAGTTTAAGATGGTATTTCTGACCCCATCACAATTGCTAAGTCTTATTTTCCACCTAATTGTCATTTCTTTCCTCCATGTCCTTACAAAAATTTGCAGTTTTATAGGGATATCTTAGTTGAAATAGATTCAGTCAAAATTAAGCCAATCAAAGATAAAGATAAGCCCCATATTATTCTTTATCATTCTCTTTATATCCATAGAATTATAAGTAAAGCAGATTTCAGTCGCCATCCTTATGAGTTGAAACATCTTAAGTCCATCCATCCGTATACTTATGCTGATTATATTGAGGCATGGTATACTATTTTCCTCCACCAAACAGTTGATTTCTCTCACTCTTGGTTTATTAATTTTGACAATAAATTCAAGAGTCAGTTTCCTTATTTGTCCCTCCATTGGTGGGAAAAACATGGCCCAATCGCCGAGTCTCACCCCGCTGAAATCCATAATTAACCAATTATTTTGCCAATAAATATAAATTTAATAAGCAAGAATTGTTATTTCCCAAGTTGCTTTTTTTCATTGCCAAGTACAAAGTCCCCTGGATATTGAAATGGTCCTATCATGTTAATTGGGAATCCAGAATTTTGTCTCGCCAATTTTTAGTCAAATGGTGAGATAAGTTCAAAGTTGACAGAATTGTAGAATATGTTTATCAGGATTTTCCTCAAATTGTTAACTCCCCCAAACCTCAACCAGTTTCTCCAGTTGGTTCCACGCATTCTTCTCTCTCAATCGAGGGAAAATCTAAATCTGAATTGCAGGAACTTGCAAATCAGTTGCTTCTCCAAGCTTCCCCGTATGGATGATGATGAGGACAGTGATGCTGATCCAATTTCCACATCATCCTCCAGTCAGTTGCCTCTTGGTCATCCTTGTCCTGCTCCAAAAAAGCCCTGGGCAGATTACCAAGACCGTCAAGATCCTTATGATC

TCGGATCTGACTAAAGCCATAAAGTTGCCAAGGTGAAAGTGAATGTAAAAGCATCCAGTAAAAGCAGCCAAAAGTTTTCGCGTGCTTCTGCAGTCGTTATTCCACAACAAAATCAAAGTTAAAGTTGCCTCGACAAAAGTTAAAGTTAAAGTTGCCAGAGTTAAAGCATTTTGCTGATTTCTCGTGCCTCTATAGGAAGATTCTCAGCATCCTATGCTCAATAGTTGCTCAGTTGATTTCTTATTTAAGCAGGCTTCGGCCTCAGGTTTTTAAAAAAATTTCTCAGTTCCAAAGTTTTCTCTGAGACTTCCTCATTGAGAACTCTTAATGTAAATTATGAGTGCTACTATCTATCAATAAAACAAGACTTTTGTAAGTATCAAAATGACAACAACTATGTCTTCAATTATTGATATTTTAATTATTTTCATATATGCATTAATTTCATTCATACATAAGTTCCGCATATATATATATATATATCTAGAAACCTGTAACCATAACAAGGATCGGGGAAATACAAAAAATCAGGAAATTTGGAACATCCGTCGCAATTGGCCAGAGCCCAAGATATAAGTCTGAAGTTGATCTGGAAATTTGACTTTTAGTGACGAAAAATCTTAAGAATACTGTCGCAGAAAATGTTTAGCCACGAAAAATCTGATGAACATCTGTCACTAAAAACTTGTGGTGACGAAAAATCTTCAAATTTCTGAGCAAAACAGCAGCTGGTTTTTTATAGTTCAATTCTTTTATGTTATTCCCTTCCATTTTCAGTGCTTACCTTTAGTTTTCCAACAAGAAATGGAGGGAAATGGTTCCATAAGTGTGCATCTTGTTCAGTTGATTTTCTTTTTCTTCTCTTTGTCTCACATTTTACAAGAACATGTGAATTTTCTCCCAATGTTTCAGTTTCATAAGAGGCAGGTCCTTCCTCTTTACCATTACTAGGCTTCCTTACCTGGCCAGCAGAAGATATGCCCAGCATCCGCTGTAGCAG

>Chr8-05; OZ022213.1; 7211648-7213254; dir; D;

GTGTACTCTTCCCTCGCTTTGGAGGGTTCCATGAATGAGCATGATTTCGACCAAAAAGGGGAGGAGGAGATTCTGGGAGTTTCCCCCTCTGCTTTTATGTTATATTATTATATTTGCATCGCCAGAAAAATGGCACAAACCACTGTTGCTGCCCATCGCCATTGATCGACATCAAACGGACGCGCAATGGCTAAACAACAAACAACTTTTACCAATGTTGTACTTGATCTGTGGCTTACATGGAGCATTGGCACATTTGTTTTTGAAGATATTTAGTCATATATTTATTTTTAGTATTAAAATTATAAATAAATTCTATACTATTTAATTTTTATAAACAAATCTAAAAACTAATAGCTGGTCCTATTGAATGTAATTTTAATTTTTAAATTACTTTGATGCCCCATTGAGTGTTTTGAGATTTTTTATGAGGTTTTTTGGTTGTGTTTATTTTAAGAAATCAATGACAATTTTGTAATTTAAAAGAAGTTAAAACCCTTTTTGTTATGTTGTAAATAGACTTTGGGTGTGTTTGTTTGTACTCAGTACATTCATTCATGTACTGAGTACAAACAAACACTTTAAAATATATTTTCGTTGACGGACTATATATATATATAGTTCTTCCATTGAGGGATCTCTCAAATTAGCTTATTTGAGGGATACCATTGTAGGGCCCACTCTGTATTGAATTTCACTAATCCAAACTGTCTCTTTTGCAGATACTCATTCAAAGATTATCTCTACAAAAAATCACTTGAATCCGATATCATTTGACAACTCAATTGAATTATTGAAATTTTAGTACTTTCTTAAAGCACAATGTTCATTGATTTTGTAGGACACAATTGGATATCGAAACAGTTTCTAATTTGTCTAATTTTTTTCAAGGATGATCTATGAATATAGACTTAAAAAATAGATGGTTTCGATCGTTGAAAGAAAATTCGTAGGAGACCTTAAAGGGTGTCCCTCAAATAAAATTATTTGAAAGAATAAG

TCTAGTTAAAAGAGCTTGCCAACGTGCAAAAATCTGTTTTGATGCAAGGTTTTGAACATCTTTTTCTAATACATATTTAGCAGATTTACAATCAATGCGGACTAAAATTTTTTGATTTAATAAATCATCTTGACATTTAGTAATGCATAATACAATAGATAGGATTTCTTTTTTAATAGTACTGTAATTAGACTGGGAAGGAGTCCAAACACCAGAATAAAAACGGACAGACTACGTTGAAGCCTGGTATTACATCTTCTTACATCAAACCCCTGAGTTTTCTCACTCCTGGTTCATTAATTTTGATAATAAATTTAAAAACACTGAGCTTCCCTATTGGTTTCTTCATTGGTGGGGCACCCATGGTCCCAAATCCGAAATTATTCCTTCTGCAGTCCATGAATTAATCAGTTATTTTACCCAGAAATACAAAACAATTTTAAAGGAATTTGGAGTAGGAATACCAAGGCAAGGGAGAGACTGAACATGACGCTTAATCATTTGAACAATATCAGTATGAATAGACGACCAAGGAGGAGGATTATTCTGTAATCTATCAAATAAAGGTTTACATTGCTGTCTCAGGTTTTTATAAAAATCAGAAACATAATTAAGGGATCCTATAGATTATGATAATTTAACCTATGGCAATATTATAAGTACAATAAACCAAGAAGGAATGAAAATGTGTATCGACATGAAAATTAATAAACAAATACAGTCTGAGCGTAGATCGGCTAAGTATGAATTAGGTAATTTAGTCACCATAATTGTAAAAAATTATGAGTTCAATACGGTTGCTTTATTTGATTCCGGTGCAGATCTTAATTGTATAAAAGAAGGTTTAGTTCCTACTAAGTATTATCAGAAATCTACAGAATCTTTACATACTGCTTCTGGTAAATCACTCCAATTAAATTATGAGATTCCTAAAGCCCATGTTTGTCAAAATAAAGTTTGTTTTAAAACATCATTCGTTTTTGTTAAAAATATAACTGATGAAGTTATTTTAGGATTACCCTTTATAAGCATGCTTTATCCTTTTCAAGTAGAATTTGATGGTGTAATTTCAACTTATTTAGGTGAGAAAGTCAAATTTACTTTTTTAACAAAGCCTGAGTTGTACAATTTAAAACATTCAATTAAAACTGATTTTGCAAAAACAATTGCTTTAATTAAGGGAAAAACTGATCATTTGAATCATTTACAGCAAGAAATTAAAAGTAAAAGAATTGAGTCCCAGATAAGTAATCCTACTTTACAAAAAGAAATTATTCACTTTGAGAAAAAACTAATTTCTGAGGTCTGTTCTGATTTTCCTACTGCTTTCTGGCATAGAAAAAATCATGTTGTTCATTTACCTTACATCAAAGACTTTTCTGAGAAAAATATACCAACTAGAGCTAGGCCTATACAAATGAATCATGAAATGATGGAATTTTGTAAAACTGAGATTAATGATTTATTGCAGAAAAATATAATTCGAAAAAGTAAGTCTCCTTGGTCTTGTCCTGCCTTTTATGTTCAGAAAAATGCAGAATTAGAAAGAGGATCTCCTCGCCTAGTTATTAATTATAAACCTTTGAATGCTGTTCAAGAACAGCATT

CAAGAAACAAAAAAAATACATACATACATGCCAGTAAAACACGAGTGGAGAAAGGAAAAAAAACATATAAACCTTCTCGTAAAAGAAGTGGAAGAGACCTGATAAGAGAGTAAATGTTATTAGAGCATCCACAATTATGCTCTCTATTTTTTAGTTAAAATTTAGCTAAAAACACTAGAAAGCTATTTTAGGAGCTCTCTATAAAATCCCAACTCCAAGCATACTCCCTAGTTTAAAGAGTCATAAATGTTTAAATTTTAATTCAAATTTTGAATAAAGTGTAGACACAAAAGCGATAATAAAAAATAGTTGGCATTAAATAAATACTTAAAATTGTTAGCGAGATCCGAACTTAATTAGGATATTTATTTCGTACAAGTTGCTCTTCTTAGATTCGTTTACTTTGTCATGGTATTTCTTTATTTCGATCAAATATTCAAAGACTTTCAATGGAATATATATATATATATATATATATATATATATATATATATTGTTGCTGCTCTTCTCAAGTCTTTCAGTGATTGGGGCTCATTCTACGTACAAATATAAAACAAATATCGAAACACTTGCGAGTCGCATCGCATGCACCTCTCAGTCTTCTGGTTTTGATTTTATTTATTTATAAAATATGTAAGAAATTAGTCTTCGTCTTTTTATTTTTATAATTAAGAAAAAGTCTTCGTCTTTTTTTATTTAAAAATTTAAGAAAAAGAAGAGAAAGTCTTCTCTTCTCTTTTCATCAAAAACAACAATTGAAAAAAAAGAGGAAGTCCTCTTTTTGGTTGCTATTACAATGGTGGCTTACTATATATATATCTCTCTCTCACTTGCCTCTTCCACACTGCCTAAACAAACTTCCTTTGCATTAGTTTTTCTCACAGAGTGCACCTTCTCCTCACCTCTCTCTCTCTCTCTCTCTCTCGCGCGCGCGCACATTCACACTCACAGAGTGCATCTTCCTTTGCATTATATGTTTTCATTGCTATTATTTTTGGCT

>Chr8-06; OZ022213.1; 7423244-7425923; dir; B;

GAACCAATTAAAAAATGACAAGTGACTAATTTTGGGGTTTCTCTCCAAATAGGCTTTATATGAGTTTTTGAATAAATATTTCTTCATTTTAATAGAATTTCAATTTTTTTAAGGTTAAAATGTTTGAAAAAATAAATAAATAAGTTATAAACAATAATAAACTAAAGAAAAATTGAACATTAAAAAAATAAAAATAAAAGTTAAATTTTTAAACAATTTAATAAAACTTCATACTCAAATTTTGAGTCGTTAGTATTGGGAGAAAAACTATTTGAGTCCGGACAATACATATGTATTTTGGAGGGGGAAATTTAGGTTGGTGATGGTCGTAAGGTCCTCATTGTAAATGCTCTTTAGAGAGTTGTTAACATGACTTGTTCAAAAGAATCTTTGCATGAACTTACTCTCACCTCATAATTGTGGCTCAAAGATTTAAGTTAAGCTTGACCCATTTTATATTAGGGCTCTTTAGTTTTAACTCATAAAAAACTTAACTAATTTGGAAGAAAGATAATTAATTTTGCTTAATTAATATAAAACCAAAGCCACCTAATTTTACCAAAAGGACATCCAACAACCCTAAAATTACTACATGTGCCATTGTGCTTAACTGTCAAATACAGTTTATTTAATTAAGAAGAAGGGTAGAATTGTCTTATCAGTTGTTATTTTTTACCTGACCCAACGAATCTGGGCCTCTCTTTGGGCTAATCCAAAATAGTAGTTTTTTCCTAGGTATTAAAACTAAAACCAAAATATTCAATATCTTACAAACTAATTAATAAAGTTAATTATGTCTAAAACTTAATTTTTCTTTTATATTATAAGCATACTAGCCCCTTGGCACACACCAATTGGTTTTTATTTTTATTTTAGAATTAAAAAAATAATGGGTAGTTGTGTTCTATAAAAATAGGATCCATTACCTGATTTTATTTTTAATTTTAAAATTTTTTAATAAAAAAGTGTGAATTTACCATAATACCCTCATTTAATTAAT

AATTTCAATTCAAAATGTTTAAAAGTCCAAAAGACCAAACCTTGCTTATTCAAAGTAGTAAAGCCGATATAAGAGTTCCCCATACAATCAAATGGTCAAAAGTCACTCTACCGCTAGATTGGACACTAACAACAGAAAATCAGCACGTTCCTATTGAACATAATTTGAATAATCTGGATTATATTCAACAATATTTAGATGGTACAATAAAAATAAATTTTGGAAACCAGCCCTATAAGTCCTTAGTCCAAATACAACAACTCCCAACATCTAGTCAATCCTTAAGACATACGCCTGCTAGACATTCCTTTGCAGCTTCCTTCTCTACTCTAGAAAGAGACCTAGAGTTAGAAAAAAAAAACGTTAATTGATTTAAAATTAGAATCTTTAAGAAAAACCTCCCAGGTCAACCATCCTTGTTATGGTAAGAAACCAGTCAAAGAAGACGAAAAATCAGATTCCCCAAATTCACCCACACAAACTGATTTTGAAGCAGAAGCCATTCCATTAGATAATCAATTACGAACCCTTAACAAAGAATTTAAAATTCATTGAAAAATTTTAGACAGACATTTAAATGCAGCAGAAAACCTGGAAAGAAGAAATAACTACCACACAATCTACCCAAATGAAGAACAAAGGCAACAAATCTTTAATCAATTGAAAAATTTTATGAAAAATACTAAGTCAGAAGTCTTTTATCTTGATTTTGTTGAAAGTCACAACATGTCCAAAGAATTAGGGTTAATGGTTTTATTAGTTCCCAAAATTTGACCCGATTTGCATTTGAGTACCTCAATTTTCAAAATGGTTCCCGTGGTCCTTTAACTTTAGTTTCGTTTGGACAAATGGTCCTACCGTCAATTTTGTTAACTTTTTCTATTAAATGCAGGGGCAAAATGGTATTTTTATATTAAAACTAAAAAAATGAATAAAAAATCATATCTTTAAAAATAACAATAAAAGAAAATCAAATAAAAACCCAACAACAAAAAAAAAAAATAATCAAGTTTTGGGGGAGTAGAAATCGGAATAGAGAGGGAGGGAGGGAGAGAGTATAATTTTAATTTTTTTTTAAAAAAAATTTTATTCATATTTTGATACAAAAATACTATTTTGCCCCTACACTTAACAGAAAAGTTAACAAAATTGACGGTAGGACCATTTGTCTTAACGAAACTAAAGTTAAAGGACCACGGGAACCATTTTGAAAATTGAGGTACTCAAATGCAAATCGGGTCTAAATTCAGGGACTAATAAAACAGTTAACCCAAAGAATTAAGAACTTTAACACCAGAAAAATGGAAAAGAGAAAACAACACAGAAGTCATAGCTAGTCATCCCCTAGTAGAAACAATAATCATTGATCACAAAAATACTAAAATCACTGCCTGTTCTAACCAGGGAGCACGCAGATCCCAGAAACGCATAGTTTCTCCTCCAAAAATAACTTCCCCGGTCGGAGAGCGCATTAGATATTTACCTAAACCGGTATCCCTTTAAAATCTCTGAACAAAATGATAAAAAATTGGTTGAACAAAATAATTACATGAACCAGAGTCTAATAGTCATTGGAAAACAATTAGATAAGATAAAAATAAAAATTGACAAAATAGTCCCTCAACAAATATCCCTTAAGTCTCAAAAAGAAAAACTTTTAGTAAAATTCCAAGACCTAAAATCAAGCCCCACTCTCAAGTCGAGTCCCACAATGGAAAAGATTGAAGAAATACTTAAACAATTAACCCCAATTAAAGAAAAACCTAAAGTTTTTGGTCTCAAAGTCCTTGAGTCCTCCATTTTCGTCAATACCTCTGAAACCGAGTCTATAACCAGTAATGAAACGGATTCCTGTAATATATCAAAAATTGAGAATACCTTTAAAAATTTAGAAATAGATAATAATTTGAAGCTTAATAGATTAGGGAACAAAGTCAATCCTACTAGTCTAACAAAAAATTGGTATCCCAAACCAACCCCTCTAGACATTCAGTTTGAAGAAAGAAATTATCAAAGTCAATTCTTAGTCTCATTCGATAAATTATACGAAAGGAATATTGATGGTCTTTCCGAACAAGAAATCCTAAATAAACTTCACATATGTCTATGGTAGCTAATAGTTATACAACCAATCACAATTTCAGACAGTTAGAAATTGTTCCCCTTTTAGAAACAGGATTCACCGGAACCTTACGATCATGGTGGGATAAACATTTCACTGAAGAGTCAAAATGACAAATCACACATGCTGTCAAACTAAATGAAGAAGGTCTCCCTATTTTTGATGAATGCTTAGGTACAGGCATAGAAGACGGAGTCAACCTTTTATTATATACCATAATTGAACATTTCATAGAAATACCTAGTAACACTACAGCCAGAATTCACGATCAACTTAGTAATCTAAGATGTCCCAAATTAAGTTATTTTAGATGGTATAAGGATGTCTTTATTTCAAAGAGTCATGTTAAGAGATGATAGTAACCAACCATTTTGGAAAGAGAAATTTATTAACAGCCTTCCTAATTTGTTTGCTCACAAAATACGAACAGTCCTAAGTAATGATCAGGGTCACATAGATTTCGATAATTTAACTTATGGAAATATTATTAGTACAATTAACCAAGTAGGAATGAAAATGTGTGTTGACATGAAAATAGGTAAACAAATTCAGTTAGAA

AGAAACTCAACCAAATATGAACTAGAAGACTTTTGTGAACAATATGAACTAGGAGACTTTTGTGAACAATATGAGTCTTTAATGTTGTAAACCCTTGTTTTTATAAGAATAAGGAACAATTTTACTTGTTCAAGGTCCAACATTACATTAATATTGTAATTGAGATCCTTCTCATTGTAAATTAGGCCATAACAATATTATGGATTAAAGTGAACCTAAATGTTGTAATCACCATACGAAATCTCATAAGTTGGAACCCAAATCTTGTAACCTACAAGATTAGGGATATACTTTACAATATTATGGAATGCTTGTATCCCAAACACTGTAAAATGAGTCCTTAATGTTATAAACCCCTGTTTTTACAACATTAGAGATAAGCTTTATAATGTTATCGAACTCTCATGTCCCAAATACTGTAACATGATCCCTTAATGTTGTAAACCATTATTTTTTACAAGATTAGGATAAACTTTAAATGTTATGGACTCCTCAGGTCCCAACTATTGTAAAATGAGTCCTTAATGTTGTAAATCCTTGTTTTTACAACAATAAGGAATACTCTTTTACAACGTTATGGTACCCTCGGTCCTTAAACTTTTAACAAAATTATTTACTAGAATAAGAACCCATTTTACATCGCTAAGGTCCACCATTACCAACTCCATGTAATGTGGATCCATATCCTTGTAAACATATGATTTACAACATTATGGATTGGGTAAACCTTAACGTTGTAATCCAAGAGCAAAACCGTGTAAATTGTTAACAAAAACTTGTAAGGTACGAGTTTAGTGATTCTCTTTACAACGTTATGGTACCCTCAATCCTTAATCTTGTAAAGCATTGTTTACTAGAATAATAACCCATTTTACATCACTAAGGTCCACCATTACCAATTCCTTTTAATGTGGATCCGTATCCTTGTAAAATGGTGATTTACAACATTATGAATTAGGTAAACCCTAATGTTGTAATTGAGGTGCAAAACCCAGCAAAT

>Chr8-07; OZ022213.1; 7981285-7987584; rev; B;

GGTTTTTGCAATGGCTTACCATTTGACTTGGTTCTGAAGCTTTTGACAACACTAGATACGATGCTAGATATTTTATAAGGTGCAGGGCTGGGCTTTTTTCTAAGGTATTGGGTCATTGGCATTTTATTTCCAGCAGCCTGTGAGTTTGGTTTCGTCAAACTACTGGAAGTAGCTTATTTGCTTGATTTTTTGACAAGGTGATGGATGTCTTAGCTGATGTAGGATTTTTCTAAATGTTGCAGACCTTTGGATATTCTCCCAAGGCATTGGACCACTTTTCTATCTCTCTTGCTTACCCATATATTTGGGCTCAAAAAATGGGCTTGAGTTTTGATGCTTCCAAGTAACCCAAAACTTCCATCCATACCACAACTTGCTTAGCAAGCCCCAGCCATTCGCGTGGCTTGGGCCCACTTAAGCTTGTAGCGTGACTTGGTGTCAAAATAAAGGTCTTTCGCTTGACGTTGCGTGCTATTTGGCGTGCTTATGCTCGAGTTCTACCGCATTGTAATGGGCTATGGCCGGGCCAAATGGCACTTAGCATGTCAGTAAGCTTGGGTTCACAGAGTCAATATGCTTTATGGGCTTGTTTTGGCCTTTCTGCGTGGCGGGTTATGTGTTTGCTTGGCATGGCATGTGGCCCATAACTTCTGCAAGTGCGTACAACAAATTCTTGTGTATGCCAGTTAGGCTCCAGTTAAGTGTTGTACTGGGTTAGAAATATGTTTGGGCCCAACCTTAGATTTTTTGGGCCTCGACACCAGGCTTACTGTAGACTGCACAGATTTTTCTCAAGCAAAATAATGTGGGCCATAAGGTATTGATTACTTTTAATAAGTGGTCCAATTCGGTTTCGTTAGATTTTAGACTTAAATTGTAAATCGGACTAAATATGAGTCCTCCTAGAAAAACACTTCGACTCATAAGCATTTTTTGTTAGAAGCCCTTCTACTAGAACTTCAAAAAAATTATTTTTGGAAAAACACATGAAAGTGTTTTT

ATGACTCATGACCTCGTTTGTTACTCAGGATTGTTTTGGTATGGATTGTGCTTAGGGATTGTCTTGGCTTTGCTTAACTTGGTCTAAGTTGGATAACACTTATCCTGCATTCGGTGTATGCTTGTACTAGGACTAGATTTTTTTATTTTTCCAAAAATATCCTTACCAGGTTAGGATTAAATGCTTCTATTTTTCTTTGTCTACGTGATGCCAGATTTACCAACTTTAGTGATAGTACCCTAGGAATAATAGAGTCAAGCGTGTTTAATGGTCCAATTCACTTTGATTGTTACCCAGATTTCACAGTAAGCCTTAGTGATCCCCACATGCTAAAAACTCTGAATTAAATATTAAAACTTCAGGGTATAAAGTCTTACCAGGTACCCAACCTCTTGCCTTAGTCTATAGAATTTATTATAAAGTTACCGGAACCAATATGAATTTCCAAGCTCTTAATAAAAATCCAAAAGACCAAACCCTGCTTATTCAAAGTAGTACAGCCGATGCTAATATAAGAGTCCCCAATACAATTAAGTCGTCGGAAGTAACTCTACCCCTAGATTGATCACTAACAACAAAAAATCAACCCACTCCTATTCAGCATAATTTGAGTAATTTGGATTATATTCAACAATATTTAGACGGTACAGTAAAAATAAATTTTGGAAACCAGCCCCACAAGTCCTCATTCTAGATACAACAGCTCCCAACACCTAGTCAATCCTTTAAACATACTCCTGCTAGACATTCCTTCACAACTTCCTCCTCTACTCTGGAAAGAGACGTAGAGTTAGAAAAAACTTTAATTGATTTAAAATTAGAATCTTTAAGGAAAACCTCCCAGGTCAACCACCCTTGTTACGGTAAAAAAAAACCAGTAAAAGAATATGAGAAATCAGATTCCCCAGATTATCCCGCACAAACTGATTTTGAAGCCGAAGCTATTCCAGTAGACAACCAGTTATGAACCCTCAACAAAGAATTCAAACTTCATTGGAAAATTTTAGACAGTCATCTAAATGCCGCAGAAAATCTTTAATGAATGGACAAATTTCATGGAAAACACCAAGTCAGAAATATTTTACCTTGATTTTGTTGAAAGTCACTACTTGTCCAAAGAATTAAAAACTTTAACCCAAGAAAAATGAAAAAAAGAAGACAACACCGAAGTTTTACCAGTCGCCTCTCGGTCGAAACTATAGTTATCACCCATAAAAATGTTAAACTCCAGGCCAGTACTTTCAAAATTCCAGAACTGAATGACAAAAAACTAATTGAGCAAAATAATTATACAAACCAAAGTCTGATAGTAATTGGAAAACAATTAGACAAAATAGAAACAATAATTGACAAAATAATTCCTCAACAAATATCCCTTAAGTCTCAAAGAGAAAAACCTCTAGTAAAATTCCAAGATTTAAAGTCAAGCTTCACCCTCAAGACCACTCCCACAATGGTAAAAATTGAAGAAATGCTTAAACAGCTAACTCCAGTTAAAGAAAAGCCTGAGCCTTTTAGTCTTAAAATCCTAGAATTCTCTGTTTTAGTCAATTCATCTGAAGCCGAGTCCATAACCAGTAATGAAACAAACTCCAGTAATATATCAATAATTGAAAACGCCTTTAAAAATTTAGAAATGACCAATGATCTGAAACTTAATAGGCTAAGAAACAAAGTCAATCTCACTAGTCTCACAAAAAATTGGTACCCCAAACCAACTCCTCCTAACATCCAGTTTGAATAAAGAAATTCTCAGAGTCAATTCTCAGTCTCATCTGATAAACTATACGAATGGAATATTGATGGTCTTTTAGGGCAAGAAATCTTGAATAAACTTCAAAACATGTCTACGGTAGCCAATAGTTATATAACCAATCACAATTTCTGACAGTCAAATTATTCCTCTTTTAGAAACAGGATTCATCGGAACCTTACGTTCTTGGTGGGACAAGCACTTAACTGAAGAGTCAAGACTACAAATCACACATGCAGTGAAACTAAATGAAGAAGGTCTCCCTATTTTTGATGAACGATTAGGTACAGGAATAGAAGACAGAGTCAATACTTTACTGTATACCATAATTGAACACTTTCTAGGAACACCTAGTAATATTACTGCTAGAATCCATGACCAGCTCAGTAATCTCAGATGTCCCAAATTAAGTGATTTCATATGGTATAAAGATGTCTTTATTTCTAGAGTTATGTTAAGAGATGATAGTAACCAACCATTATTAATGGTCTTCTTAATTTGTTTGCTCACAAGATACGAAAAGTTCTAAGTAATGATCAGGGTCACATAGATTTCGATAATTTAACTTATGGAAATATTATCAGTATAATTAACCAAGTAGGAATGAAAATGTGTGTTGACATGAAAATAGGTAAACAAATTCAATCAAAAAGAAATTTAGCTAAATATGAATTAGGAGACTTTTGTGAACAATATAGTTTAATATCTATTCCTCCCTCTAGAAAAGCCAAGACTAGTTACAATGACCCACAAACTAGTAAAAATAGGCATTATTCACGTAAAAAACAGTCCTTAGGACATAATGATGAATTTTACAGTAAAAAGAAATTTAACCCTCGCAAAAATTGGTCAAAAGCACTGAAAAGAGACAAGAAAAAGAAAAATTGAGTGCTGGAATTGTAAAAAACATGGTCATTATGCTATGAATGTAAAGTTAAAGACACAATCAAACAATTGAAAATCACATACGAAGAAAAAGCCCAATTAATTAAGGTGTTAGAATTAAGAAACTATGAGTCTAGTGAAATCGAGACCCAAGTAACAAGTTCAGCAGACTATAGTTCCTCATATTCTCAATCAAGTTCCCCAAAAGTCCAATTTGGTTGTAAAGATAATTGTTGTAATGTCTTAACTAAGTCAATAAAAGTCCTTACAAAACAAGAAGAGCAAGAAGAATTATTAATTGATTTAATTAGTAAAGTAGAAAATCCAGAATTAAAATCAGAATATTTAGTCAGGAAGAAATGACATTCCCCCAAAAATAATTCTAAATAGTACCTTAGAAAAATTCAACAAAAGAAAAGAAGTAACTTTACAAGATTTACAATTGGAAGTAAAAGCAGTCAAAAAAGAGAGACTAGAGTTAAGACAGATGAGTCACAAGTTACAAAATGAAAACTACGAAATTAAACAAGATTTAATTAGTTTAATGGAAAAAAGAAAGTCTGATTCCCATAGTCAATCTGAAAGCCCAGTAAATTGAGATGAGGAACCCTCACGCAGAGATAAAGCAGCCATTAATTTAATAAAACAAATAAATTTTAGAAAATGGTACTCTAAAGTCACTATTTTTATAAAAAATTTTGAGTTAAACAAAATAGCTCTTTTGATTCAGGAGCCGACCTTAATTGTATACGAGAAGAAGTGATCCCCACCAAATATTACAAAAAGTGAAAAGAAACGTTAGGCACTACCTCTGGCAAACCACTTCAGTTAGATTATGAAATTCCAAAAGCCCATATTTTTCAAAATAAAATATGTTTCAAAATTTCTTACGTTTTAGTTAAAAATATTACAGACGAAGTCATCCTTGGTCTACCATTCATAGCTCTCCTATATCCTTTCCAAGTAGAATATGACAAAGCCACGTCTACCCATATGGGAGAAGAAGTCAAATTTGAATTTCTAACAAAACCTGAGTTAAATAATTTAAAAGCCTTGCAGAAAAATGCAGTTTCAAAAACAGTCAAAATAATCCAGAATAAAAATAAGCAACTAGATTTTCTTAAAGAAGAAGTCAAATTCAGGAGAATTGAACAACAGCTTACTGATAAGTCTTTACAGTCACAAATTCAAAAATTTGAAGAAAAATTAGAGCAAGAAATTTGTTCTAATCTCCCCAATGCCTTCTGGCATAGGAAACAACATGTAGTGAGCCTTCCTTACGTCAAAACCTTTAATAAAAAAATATTCCCACTAAAGAAAGACCAATCCAAATGAGTCAAGAAGTCATGGAATTTTGCAAAAAAGAAATTGCAGAATTACTTAAAAAAAGGGATAATACGTAAAAGCAAGTCACCTTGGTCCTGTCCAGCCTTTTATGTTAAAAAAAAAGCCAAATTAGAAAGAGGAGCCCCAAGATTAGTTATTAATTATGAACCCCTTTATACGGTATTATAATGGATTCGATATCCAATTCCCAACAAGGGACATTTAATCAATAGATTAGATAAAGCAGTCATTTTTTATAAATTTGATATGAAAAGTGGATTTTGGCAAATTCAAATCCAAGAGTCAGATTGATATAGAACCGCCTTTGTCACCCGATTCAGTCACTATGAATGGAATGTCATGCCTTTTGGCCTAAAAAAATGCGCCTATTGAATTCCAGAATATAATGAATGAGGTATTCAACCCTTTCAGTGATTTCTCCATAGTTTACATTGATGATGTCCTCATTTTTTCTCAGTCAATAGAACAACATTGGAAACACCTGTACAAATTCCTTCAAATAGTCAAGAAGAATGGTTTAGTTGTCTTCGCTAAAAAGATCAAGTTATTCCAAACCAACATTAGATTTCTTAGTTTCAATATCTGTCAGTCACAAATCAGTCCAATTGATCGAGTCATCCAATTTGCTGATAAATTTCCAGATCAAATTCTTGACAAAATTCAACTTCAAAGGTTTCTAGGATCTCTTAACTATGTCTTTGATTTTTATCAGAATTTGCGAAAACAATGTAAACCTTTATTTGACAGACTCCAAAGTAACCCTCCTCCTTGGACTTGTACTCATACAGAAATTGTCAAACAAATCAAAATTCATGTCAACACTTTGCCTTTATCTTGTTATTCCTTCAGTCAATTCCTTCAAAATTATCGAAACCAATGCTTTTGAAATTGGTTATGGAGGTATCCTCAAACAATGAGTCTCTCCTAGTCAAACAGAACAAATAGTTAGATTCCATTCTGGAGTATGGACTCAGTCACAAAGTAATTATAGTACTATTAAAAAACAAATTCTATCTATTGTATTATGTATTTCAAAATTTCAAGATGATTTACTTAATCAATTTTTTTTAATAAGAGTTGACTGTGAAAGTGCTAAACATGTTTTAGAAAAAGATGTTCAAAATATTGTATCCAAACAAATTTTTGTACGGTGGCAAGCTATTCTAAGTATTTTTTTATTTTGAAATTGAAAATTTGCGAGGAAGTGAGAATAGTATTCCCGATTTTCTTACCCGAGAATTTCTACAGGGAAACAAATGGCAAGCAAAGAACCAAGTAAACAAGACGACAAATCAGTCAGAAGCCACCCAGCTTCCATGAACCCAGTCAAAACAGAGCCCTTCCAAATAGTCCCCTACCCCGGACACTCACCTATTCAAATCACCAACAGATTCACCAGCCTAGGATCCACTGCTAGTAGTATTTGTCCTAATTACCAATCAGCCTTAATTTCCAATTATGATCCTTTTCAAGTCTCTACTCCACATTCAACCCAGTCACCCATTAGTCATAAAAAAAGACCTTTTCCTTATCCCCCCAAAAGTAACACCAACCTTTTTACCATGGAACCCTGCTACAATTATCTTTCCAATCCTACAAACCGCCGCCCCCCCACCCTTGGCCCCCCATTTCCATTTCATCCCTCAACAAATTAAAAACATAAATAACCCAGAAATTATTCTTTACCACTCCATGTATATTCATAGAATCCTTAGTCAATCTGATTGAGGTGATCGCCCCTGCTCTCTCAAAACCCTTCAATCAAAAATCCAGTATAGTTATTATGATTAAATTGAAGCATGGAATGTTCTTTTCTTACATCAAACTGAAGACTTCAGTCACTCTTTGTTTGTCAACTTTGATAGTAAATTTCGATGCTCTTTCCCATTTTGTTTTCTTTCTTGGTGGGAAAAGCATGGTCCAGTCATTGAAATTCTACCTCCTAATCTCAACGACTCAGTCAACTATTTCTCCACTAAATACAAATTCAAAGAAGAAGACAATTTTTTCCCAGTCCTATTAATTTTTGTTGCCAAATATAAAGTCCATTGGATCCTCAAATGGTCCTATCAAGTCAATCTTCAATCGAGAATCTTATTAAGAAAAATCTCAGTAAAATGGTGGGATAAATTCAAGACTGAAAGAATTATTGACCAAGTCTACCATGAATTTCTCCCAGTCAAGGTTCCTTAGAAGCCTTCTATTTCAGGAGCATCTCATTCATCATTATCAATTGAGGGCAAGTCAAAGGCTGAACTTAAAGAATTAGCAAGACAACTAATGATTCAAGCCTCCCAAATGGATGATAATAAGGA

TGCCTCTCCTCAGTCTCATTCATCTCAAGCCTCATCTTCCAGTCAAAAGGAGTCAACTCAGAAGTTCAGATGGGCAGATTACCCAGACAGTCAAGATCCATACGATCTCAATGAAGACTAGTAAAAGGAGAAAAGCAGAAGTAAAAGACAAGTTAAAAGAAAAGCAGGCGTGAAAGAAAAGAAAAAAGCCAAAGTAAAAGAATAATATTCTGAAGTCGTGTGTCTCTACAGTTAAAGCAAGACCTAAAGCAATAGTCAAAAGACCTGTACCCCAATAGTCAAGATTCTCAAGCATCCAATGCTACTGACGCTTCAATAGATTGGATCTTTAAATAGAGGCCTCTTGGATAGAGTAAAGGCAGAATGTACTTTTTAGTCTCTCTGTTTAAAGTTTTCTCTGAATCACCTTCGAAGAAAACTCTACTTGTAAAATATGAGTGCTACTTTCTATCAACAAAATCTTTTAATCAAGATTTTTTGTAAGTACTCTTATTTATGCATTACTTTCATTATTTTCATCATTGCATTATTTTCATTTAATTATCTTCATGATTGCTACTCTAATGCTACTGGTACTATATATATATATATATATATACAAAGACCTTTGTTGATGTGGAAAAGTGATTCTGCACTTTTGGGATCAACTTAGTGATGTCTGGGCCTTCGGATTGCTCTGGGTCTCTTGGAGGTGGATCTTGCAAAAAGAGTATCTGCTCGACAAGACTTAGGGCACTGGTTTGGTACTAGCCGAAGGCTCTCCGATGCTTAAGTAAGTACGGATTTAGTAAGCGAAATGAACAAATCTATGGACCGTATGCCGAGAGTTCAGATTATCTCCATTTTGGGGAATTGTGCTTCTTTTATAAACCTTGGGAGGTGTGCAATTGTGTAGAATTTCAATGTGGGACTGTGGATATTCACAGTGGATGCGCCACGTGTCCAGTGGGCAATTTGTGCTTTTATACTGCGAACGGTAGGTGTAGGTGTCGAGGGCTTC

>Chr8-08; OZ022213.1; 11153026-11153751; rev; B;

TTGGGGCGGGACAGGTTGTTGATTGGAGGGAGCCTGGAAACTAATAGCTTAATTGGTTGACAATTATATGCAAATCATTTAAATTTTAAGTGCAAGAATAACATATATTAGTTCTTTAACCCAAATAAAGAAGTTGAATTTGCCATTTAAATATAGTGCTGCTATTTTCACCCTCTATCCTATTTCCCTACCCATCTCATCTTCAAAAATTTTAAGTCAAATTTACTCCTTTGTAAAATGACTTCTAAGAACTTAGGCAAAAATCTCAACTCACATAACCCTACACAGGATAATTACTATGGGAATCAACAGACCATTCAGGGGTTGTTTGGCACGGCGGATTATCATGGGCTAGATTAAATCCTGGGACTGTCTCAAATTAAATCAGATTGGTCTAAGCTGGATTAAGTACTGACCTACGTTTGGTGCTGCGTCGGACTAAGAAGCTGGATTGTAAAAATAGTGAAGACCTATGTTTGGTGATGTGTAGGACTAAAAAATAATTCTTATAATATTTAAATTTAATTGGGGTTTTTGACCTAAAAGATAAACAAATGCTACAAAGAAACCCAATTCAAAGATTTATAACAATGACAGGACCCGACCCAAATTCTACTTTGGAATCTTTGTCGAACCCTGTACATGTCTAACACCTGGCAGGTGCCGGGCACAATTGACCAAATTGCCCTTCTAAGACTCAAAATTTCTTTAGGGTTTGACTTCTATCGAAAATTCGGCAGAGTCTCCCCTGTAATTTGCTCGTTTTCCAAAATTTTCACCTATACAAAATGCAACTCACATATCCACAAACGTTTAGAAAGCATCAGACAACTAAATCTGAATGGCCTCAGCCCTCATTATACCACATAAATCATTTAGCCATCTGAATCTGATTTCAACTCTCAGAACAGCATGGATACGTTATCAATTGCCAATATGACCTAACTGAGCAAATTTTCAACACTAGATTCTGGGGTTGCACTTGGTAACTCTAGTAATG

AAATTGATTATAAAGATTGGAATATCCCCAAAATTCCTAGTCAAGAAATTTATAAGAAAAAATGGTCACTTTCTAGTTTCAAGTCAACCACCCATGTGAAAACAGTCGAACAAGTCTATGCCTTAAGCAAAGAACATGAAATATGCCAATTGTTAAACCTTGAGCCAATAAAGAAACACAAAAATGTTGGAAACAATTTCCTTCACATTGGTCTAGTTCAAGTAGCAATCAAACCTCATACCAGATTAGGATTAAACGCTTCTTTTCTACTCTGTCTAAGAGATGCTAGATTTACCAACTTTAGTGACAGTACCCTAGGAATAATTGAGTCAAGTTTGTTTAACGGTCCAATCCATTTTGACTGTTACCTAGACGTCACAGTAAGCCTTAGTGGTCCTCATATTTTGAAAACCCTAACATTGAATATTAAAACTTCCGGGTATAAAGTCTTACCAAGTACCCAGCCTTTAGCTTTAGTTTACAGAATTTATATAAAGTTACAGGCACGAACATGAATTTCCAAGCCCTAAATTAAAGTCCAAAAGTTCAGACCTTGCTTATACAAAGTAGTAAAGCCAATGCCAACATAAGGGTTCCCCATACAATCAAGTGGTTAGATGTCACTCTTCCCTTAGATTGGACACTAACCACAGAAAATCAGCCAACTCCCATTCAACATAGTCTAAATAATTTAGATTATATTCAACAGTATTTAGATGGTATAGT

TATATATATATATAGTTCCCTTCAGTTGAGGGATGTGGCGCTCAGCCGTTGATCTATTTTAATGACCTTGTGTGGTTGAGATTGGATTACATCTTTTAACTCTTGACCCACCACAAACTCCACTTACGCTTGGGAACGACGTACGTTGGGAAACCAAACGTCTTTCTTCTTCCTCCAACGCTTGGAGACCTTTCACACAGACCAAACGAAGTGAAGCAGAAGACTCACCATCGTCAGTTGAAGAAAAAGACCCACCATCGTCAGTTGAAGAAGAAGACCCACCATCTATGCGACAGAGGTATGCTATCATGTGTACGAAGTACAAAATTTTTGGATGAGTTCTGATATGGTTTTAATGGGTTTGAAGTTCACTCCTCCAGATTTTTAATGATTTTTTGAAATTTTGGAATATCATGTGTATGAAGTACAAAAAAACATAATTTGATTAATCGAATTTCGATTTTGGGTTTCTGTGTGATGAGTTTCAATTTGGGTTCTATTGAGTAATTCTTAATTTGGGGGTTCTTGCTAATTATTGTTAGTTTTTTCAGTGTTAACCCCAATTTATTTATTCAGGGACAAAGTGCTGCTAGAACAAGATGTCAACCCAATTCAGGATGAGGGTCCAATAATTCACAACAGTACTTCTCAGTTACAAAGTGGTAAAAGATGATTGTTGATTTTGCTATACTAGTTCAGTATCTTAGTTCAATCTCATGGGGCTCTGTTTTTTGTACATTATATAAAGTAGAAGGTAAAAAGAAAATGAAATGTAGATGTTTCATACACTTTTTTCCTTGACAGATAGTTTTGATATGTATAGAACTATATCAGACATTTTGCTGCTTTCAGGAGAAAAAAAAAAGAAAAAAGAAAAAAGAAAAAAAGAACAGCATTTTGCTAATGTGCTACCCAATAATAATGTTACTGGCTTTCTATCAATGTGAAACATTCAAAGTGATAATGATTGTACTACTTTTTGTTTAGATGAATACTTGCT

>Chr8-09; OZ022213.1; 13596448-13610805; rev; C;

TTGATTGAAAATCTGATTCCTTGATTCTCTCCTTCTTAGGAATGTTCCAAGTTTACATTGATTTCTTTGATTGCTTTCCTAAAGTGGATCTCGTTACGTGCATGAAAACATCTGTTTTAGGCCTACTTTCGTGGGTTCGGCTTGAAACTCTGAGGACTTGGCTTGGAATCTAGAGTTCTAATCTTGAAAAAATTATCAATGATGTTGGGTGACTTGGAAACCCAATGAATCCACATACATCCTTAAGCTGTCTTAATACATAATATGCATGAATTATCACCTAAAATATAATATATTTATCAAATAGTCCGCTTAATGGCCATTGAGAATGCACAAGCCATAAGGCCAAGACCTGCATGTATGTATAAGAAAAATTCAAGCCATAAAGGCCTAAGGTAAATGCTACACATGCTAAAACTCAAGTCAAGAGTTTGTCTTCACTTTGCCAAAGTCAAACAGTATATATATACTGTCACATCCCGAAATCGGCTCCGCTGCAACATGATATTGTCCACTTTGAGCCTTCCTCTCTGCCCTCACAGTTTTGTTTCTGGTAAACTATCCTGGGATTGCTCTAGCCCTAACTCCCTTAACTTCGGAGTTCCCATGACTATGAAGCCAATGAGCTCCCAAAAGGCCTCGTACTTGGAGGCAGACATGTACATATAAGGCGCATCACCCCTCTCCGTTGGTCGATGTGGGCATGTACATATATATATAAGGCTTCGGCCTCAGTTGTAAGCATAATGTAAAATTTTACTTTTCTCAGTTTCAAAGTTCTCAGGACCACTACCCTGAAGAACTCTTGTAAGATATGAGTGCCTATCAATAAAAATCTTTCAATCAAGATTTTCTGTAAGTTTGCATTCTCACAGTTATTTATGTTTTCAGTTACTCATACTTTCATCTATTCATTAATTTCATTCATGCATAAGTTATGCCTATTGTATATCTTTTTTCACTACTATCTCTGCTAATCGTTTCTGGTTCTAGACCCCTT

TTGGTATCAGAGCCGTAAGTGTTGAAGAGTCGGTCTAAGTTTGCAATTCATTCGGCCAAGTGGCTGGTTTAAGATTTCTGAGTCGAAGTCTTCAGTTTCTTCTTCTACTCCTTCTTGTCTTTCAGTTTACCTGTCTTTTTCTCTCTGGTCCAAACAATAAGTCCTAGAGTCCAAACAGTAAGTCCTAGGTAGAGGCATGAGCGGTTGTGGCTTGAGCAGAGAGAGAGAGAGAGAATCACTAGGTGTCCTGTAAAGCCATAAATGAGTGTTAGGAGTTTCTGTTTATCTTCTCCTTTAGAAGTCTAGTCCAGAACCTTGAGTAATGAGTCGCTTATTTAGAACCAACTCTTGTAACTCTAGCAGTTCTAGAGCCAGTATAAGTAAACTTCCAGAGATAGTTAATGAAGAACAAGTTGAATTTGAGTCTAGTAATGAATTAAATTACAAAGATTGGAATATATCGAAAATCCCTAGTCAAGATATTTATAAGAAAAAAGGTCGCTTTCCAGTTTCAAGTCATCCACCCATGTAAAAACAGTCGAACAAGTTTATGCTTTAAGTAAAGAACATGAAACATGCCAGTTGCTTAATCTTGAGTCAATTAAGAAACATAAAAATGATGGAAACAATTTCCTTCACATTGGTTTAGTTCAAGTTGCAGTTAAACCTCTTACCAAATTAGGATTAAAAGCTTCTATCCTTCTCTGTCTTAGAGATGCTAGATTAACCAGTTTTAGTGACAGTACCCTAGGAATAATTTAATCAAGTCTGTTTAATGGTCCAATTCATTTTGATTGTTATCCAGATTTTACAGTCAGTCTCAATGATACTCACATATTAAAAACCTTAACTTTAAATATCAAAACTTCTGGGTATGAAGTCTTACTAGGTACCCAGCCATTAGCTCTAGTCTACCGAATTTACTACAAAGTCACAGGTACTAATATGAATTTCCAAGCTTTAAATAAAAGTCCAAAATATCAAACCTTGTTAATTCAAAGTAACAAAGCCGATGCCAAAATTAGAGTTCCCCATACAATTAAATGGTCAGAAGTCACCCTTCCATTAGATTGGACGTTAACCACTGAGAATCAACCAGTACCCATTCAACACAGTTTAAACAATTTAGATTACATTCAACAATATTTAGATGGCACAGTCAAAATTAATTTTGGAAGTCAACCTCTCCACAAAAGTCCTCAGTTCAGTTACACCAATTGCCTACACCTAGTCAATCCCAGAGACATGTCCCTGCAAGACATTCTTACACTATTTCTTCCTCTACTCTAGAACGAGATCTAGAATTAGAAAAAACCTTAATTGATTTAAAATTAGAATCCTTAAGAAAAACTTCCCAAGTTAACCAGCCATGTTATGGTAAAAAACTAGCAAAAGAAGATACTAAGTCAGAATCTTCAGAGTCACCCACACAAACCGATTTCGAAGCCGGTTTTCAAGTTGATAACCAATTAAGAACCCTCAGTAAAAAATTTAAGATTAATTGGAAAAGTCTAGATAATCATTTACATGCCCCAGAGAATAAAAAAAAGAAAAGACAATTATCACAAAGCCTACCCTAGTCGAGAAAAACGACAACAAATTTTTAATGAGTGGAAAGATTATATGTGCACAGCTAAGTTAGAAATTTTCTGCCTTGATTTTATTGAAAGTCATTATTTACCTAATGAGTTAAAAACTTTAACCCAAGAAAAATGGGAAAAAGAAGATAACACCGAAGTCATAGCCAGTCATCCTCCAGTAGAAACAATAGTCATTACCCATAAAAAGTCAAAAATAGTTGCCAGTCCCTTCAAAGCTCCTGACCAAAATGATAGAAAATTATTTGAACAGAATAATTACACCAACCAAAGTCTAATAGTAATAAGGAATCAATTAGATAAAATAGAAACTAAAATTGACAAAATACTCCTTCAGAAACCACCTCTAAGTCACAAAAAGAAAAACCTTTAGTCAAATTCCAAAATTTAGAGTCAATCCCAACTTTTAAGAAAAACACCAACAATGAAAAAAATAGAAGAAATGCTACAACAGTTAACCCCAAAGAAAGAAAAAGAGAAAATTGAAAAATCTGGTCTTAAGGTCTTAGGATCCTCTGCTTTAGTCAATACCTCTGATTCCGAGTCAGTAACCAGTATGGAAACAGAAACCAGTAATATCTCTAAAATTGAAAATGCATTCAAAAATTTAGAATTAGAAAATGATCTAAAAATTAATAGATTAACAAACAGAGTCAGCCCCACGAGTCTAACCAAAAATTGGTACCCTAAGCCAACTCCTTCATACATCCAATTTGAAGAGAGAAATTTCCAAAGTCCATTCTCAGTCTCCTCCGATAAGTTATACGAATGGAATATTGATGGTCTTTCTGAACAAGAAATTCTTAATAAACTCCAACACATGTCAATGGTAGCCAATAGTTATGTAACCAATCACAGTTTTAGGCAGTCAGAAATCGGTCCTTTGTTAGAAACATGTTTCACCGGAAATTTACGTTGTTGGTGGGATAAACACTTAACAGAAGAGTCAAAAAGACAAATAATTCATGCAGTCAAACTTAATGAAGAAGGTCTTCCTATCTTTGACGAACAATTAGGTCAAGGTATAGAAGATGGAGTCAATACTTTGTTGTATACAATAATTGAACATTTCATTGGAACACCCAGCAATACCACTACTAGAATTCATGATCAGCTTAGTAACCTTAGATGCCCCAAATTGTTTGACTTTAGATGGTATAAAGATGTTTTTATTTCCAAAGTCATGCTTAGAGACGATAGTAACCAACCATTTGGAAAGAAAAATTTATTAATGGTCTTCCAAATTTATTTGCCCATAAAATATGCACAGTCCTTAGTAATGATCAAGGTCATATAGGTTGGGATAATTTAACCTATGGTAATATCATTAGTACAATTAATCAAGTTGGAATGAAAATGTGTGTCGACATGAAAATTGGTAAACAAATACAGTCAGAAAGAAATTCAGCTAAGTATAAGTTAGGAAATTTCTGTGAACAATATGGTCTAGCATCAATACCACCGTCTAAAACCAAGTCTAGTCATCATAACCGACAATTTAGTAAAAGTCGCCATTATTCACGTAGGAAACAATCCTTTTGATGTAATAATGATAATAATGAATTTTATAGTAAAAAGAAGTTTCACCCGCGAAAAAATTGGTCAAAAGGTCACGGAAAAGCTAAGCAAAGTAACTTAAGAAAAGAACCGACAAAAGGCAAGTTAAATGCTGGAATGTCAAAGGAATGGACACTATGCTGATGAGTGCAAAGTAAAAGACACAATAAAGCAGTTAAAAATGACGGATGAAGATAAAGAAAAGTTGATTAAAGTTTTAGAGTTAAGAAACTCCGAGTCAAGTGAAAATGAAACCATGGTTACCAATCATATATAGTTCCACATATTCCCAACCTAGTTCACCCAGAATCCAATTAGGGTGTAAAGATAAATGCTGTAATGTCTTAAGTAAGTCTATCAATGTCCTTACTAAACAAGAAGAATAGGAATAGTTGTTAATTAACTTAATCAGTAAAGTAGAAAACCTAGAGTTAAAATCCAAGTATTTGAAGAAGTTGAGAAAAGTCATAAGCCAAGAAGAGCTATGTCAGTCCATTCCCCAGAAGATAAGTCTGAACATTACCCTAGAAAAATTCAACAAAAGAAAAGAAGTCACTTTACAGGATTTACAATTGGAAGTTAAATTAGTCAAGAAAGAAATAGTAAAGTTAAGACAAATAAGTCACAAGTTACAAACCGAGAATTTTGAAATAAAACAAGATTTAATTGGAAAAAGAAAGTTCTGAGTCTAAAGGCCATTCAGAGAGCCGTACAAATTCAGATTATGAACCTTCCAATCACTCTATTAATGACCAAGCCATTAACTTAATTAAACAAGTAAATTTTAGAAAATGGTATTCTAAAGTCACTATTTTTATAAACAATTTTGAGTTAAACACAGTAGCCCTTTTTGATTCAGGAGCCGACGTTAATTGTATAAGAGAATGTCTAGTCCCTACCAAATACTACAAAAAATCAAAAGAGTCATTAAGCACTGCCTCAGGAAAGTCACTCCAATTAAATTATGAGATTCCAAAAGCCCATGTTTGTCAGAATAAAATTTATTTCAAAACCTCATTTGTCTTAGTTAAAAATATTATTGATGAAGTCATCCTAGGATTACCCTTTATTGTCCTTCTGTACCCTTTTCAGGTAGAATACGATGGAGTCATATCTACCCATTGAGGAGAAAATGTTAAGTTTGAATTTTTAACCAAACCTGAGTTACATAATTTAAAAGCTTTACAAAAGAGTGCAGTTTCAAAAGCAGTCAAAGTAATTCAAAGTAAAAATAAACAGTTAGATTTCCTTAAATAAGAAGTTAAATTTAAAAGAATAGAACAACAGCTTAGTGATACGTCCGTACAGTCACAGATTCAAAAGTTTGAAGAAAAGTTAAAACAAGAAGTCTGTGCTTATTTACCTAATGCTTTTTGGCACAGGAAACAACACGTAGTCAGTTTACCTTATGTCAAAAACTTTAGTGAAAAGAATATTCCCACTAAAGCCAGACCAATCCAAATGAGTCAAGAGTTAATGGAATTTTGTAAAAAAGAAATCACAGAGTTACTTCAAAAAGGAATAATTAGTAAAAGCAAGTCACCCTGGTCTTGTCCTGCTTTCTAGGTCCAAAAAAATGCTGAGTTAGAACGAGGAGCCCCAAGACTAGTAATAACTACAAACCTCTTAATACAGTCTTAGAATGGATTAGGTACCCAATACCTAATAAACGAGACTTAATTAACATATTAGGAAATGCAATTGTTTTTTTTCTAAGTTTGATATGAAAAGCGGATTTTGGCAAATTCAGATAGGTATAAAACCGCTTTTGTAACTCCATTTGGTCACTATGAGTGGACCGTTATGTCTTTTGGTCTTAAGAATGCGCCCAATGAGTTTCAAAATATAATAAATGAAATATTTAATCCTTTCAGTCATTTTTTCATAGTCTATATTGATGATGTTCTAATCTTTTCCCAGTCGTTAGAACAACATTGGAAATATTTGCATAAGTTTCTTCAAATAGTCAAACAAAATGGTTTAGTTGTTTCTCCTAAAAAGATTAAGTTATTCCGAACCAATATTCGATTTCTTGGTTTTAATATCTGTCAGTCGCAAATTAGTCCTATTGACCAAGTCATTTAGTTTGCAGAAAAATTCCCAGATGAAATCTTAGACAAGAGTCAACTTCAAAGATTATTAGGATCTCTTAATTATGTTTCTGATTTTTATCAAAATCTGTGAAAACAATGTAAGCCATTATTTGACCGTTTATAGAGTAATCCTCCTCCTTGGTCATCCACTCATACAGAGTTAGTCAAACAAATCAAAGTCCATGTTAAGACACTTCCTAGTCTTGGTATTCCTTCAGTTGATTCCTTCAAAATAGTTTAAACTGATGCCTCTAAAATAGGTTATGGAGGTATTCTTAAGCAAAAAGTCATTTCAAGTCAATTCGAGCAAATCGTTCGATTCCATTCTGGAATTTGGACATTGTCACAAAGTAACTATAGTACTATTAAGAAAATAATGTTATCTATAGTACTATGCATTAGTAAATTTCAAGATGATTTATTAAATAAAAAAATTTTAGTTAGAGTTGATGGTAAAAGTTCAAAACATTTTTGGAAAAGGATGTTCAAAACATTGCATCAAAATAGATTTTTGCATGATGGCAAGCAATTTTAAGTATTTTTTATTTTGATATTGAGTACTTGAGAGGAAGTGAGAATTCAATTCCTGATTTCCTTACCAGAGAATTTCTATAGGAAAAAGAATTGCAGAAAAAAGAACAGATAAGCAAAAAGAAAAGCAAAAAGAGTCTCAGTCATTTAGAGCCCTCCCGGCATCAATGAACCCAGTAAAAACAGAGTCATCTTCAAAGATCGTCCTCTTCCTGGGATGTTCTCGAATCCAAATTAGTAACAGATTCACCCCACTTGGTTCCACAGTCGGCCAAGTTCGTCCTAGTTTCCAGTCTGCCTTAGTCTCTAGTCATGATCCTTTCCAAATCAATACCCAAGTTGCCCAAACTCCAGTTTCTTACCAAAAGTCATCTCCTTATTTGCCAAGTAGTTGCCACCTATTCATTGTTGAGCCCAAATACAATAACATCACTAATCCAGTCATAATTGCAAAGTCATACTTTCCCCCGAATTTCCATTTTGTTCATCCTGCTCCTTACAAGTCACTTAAAAATTACAGAGATATCCTCCATGAGACACAATCAATTGAAATTAAGCCTATTAAATATCGCAATAATCCCCAAGTTATCCTTTTCTATTCCTTGTACATTCATCAAATCCTGAGTGAAGAAGAATGGAGTAGTCACCCATATGACCTCAAAATCCTTCAGTCAAAACTTCAATATTGTTATTATGACTATATTGAAGCTTGGTATTCAATATTCCTCCACCAAATGGTCGATTTCAGTCATTCTTGGTTCATTAATTTTGACAATAAATTTAAAAGTCCTTTCCCTTGTTGGTTCCTTCACTGGTGGGAAAAACATGATCAAATCACCAATCTTTTTCCCCAGCAAATGCAAGAGTTGCTTTTCTTTTTTTTTTAATGTTAAAAATAAATTCAAAAATCAGAGTTATTTTTCCCTAATTTACTCATATTCATTGCCAAGTATAAAGTTCCTTGGATCCTAAAATCGTCGTATCATGTCAATTGGGATTCCAGAGTTCTCTCTCGCCAATTTTCAGTCAAATGGTGGGATAAGTTTAAAATTGACAGAATTGCAGATTATGTCCACAATGATTTTCCTCCAGTCATCATCCCTGCAAAGCCTTCCCTCTCAACCAAAGGAAAATCCAAAGCAGAGTTGCAGGAAATTGCCAGACAATTGATTCTTCAGGCTTCTCAAATGGAAGATGAAGAAGACAGTGCCTCCCCGAGGTCCTAGTCATCCACTAGTCAACCTCATCAGTCGCCTGGTCAAAAGGATTCCACTCAGAAGATGCGGTGGGCAGACTACCAAGACAGTCAAGATCCTTATGATCTCAATTCGGATTAATCCCCAAAAGGCAAAAGTTACACAGTCATTATTCCCCGACAAAAGCAAGGGAGTAAAAGCAATTTTCAAAAGTGAAAGCATAAGTAAAAGTCTGTCAAAAGCAGGCATGATAGCCACAGTAGGCTGCCCGTGCTTCAATAGTCAGATTTTCAAGTATCCAAAGTGGCCTAGTTGTTTTACCTATTTAAGAAGGCTTCGGCCTCAGTTGTAAGCATATTGTAAATTTTACTTTTCTCAGTTTCAAAGTTCTCCCAGGACCACTACCCCGAAGAACTCTTGTAAGATATGAGTGCCTTTCAATAAAAATCTTTCAATCAAGATTTTTTGTAAGTTTGCATTCTCATAGTTATTTATGTTTTCAGTAACTCATATTTTCATCTATTCATTAATTTCATCCATGCATAAGTTACATCTATTTTATTATCTTTGTTTCCAATAATACATATTTTCATCTATTCACTAATTTCATTGATGCATAAATTCCACATATTTCATTATCTTTGCTACATTACTAATTCTGGGAATCCCTCCTGGTTCTAGATCCTTTATGGTATCAGAGCCGTAAGTGTTGAAGATTCAGTCTAAATTTGCTATTATGAGTCCAAAGTCCCTGGTCATCTTCTTTGTGCTTGAAGTCTGCTTGTTATTCTGTAACTTTTTCGGAGTCTTCTTGTTGTCCGAGTCTTCTCTTCTGGTCCAAACAATAGGTCCTAGATTCCAAACAGTAAGTCCCAGGGTGAGGTCTGAGCGGATGCAGCATGAGCGAAGAAAGAGAGAATCCTTTGGTCTTTAAGAAGCCTTTGAGCCTGTTATAGGATAGTCAGCCTACTTCTTACCCTTTGTAAGAAAACCCAGTAGCTTTGTCCAGATGAGTCGCTTATTTAGAACCAACTCTTGTAACTCTAGCATTTCTAGAGCCAGCGTTAGTAAACTCCCAGAGATAGTTAATGAAGAACAAATAGAATATGATTCTAGTAATGAAATAGATTTTAAGGATTGGAATATCCCTAAAATTCCTAGTCAAGAAATTTATAAGAAAAAATGGTCACTCTCTAGCTTCAAGTCAACCACCCATGTGAAAACAGTAGAACATGTTTATGCTTTAAGTAAAGAACATAAAAAACATGCCAGTTGCTAAATCTTGAGTCAATTAAGAAACATAAAAATGATGGAAACAATTTCCTTCACATTGGTCTAGTTCAAGTTTAGTCAAACCACTAACCAGATTAGGATTAAATGCCTCTATCCTTCTCTGTTTACATGATGCTAGATTTACCGATTTCAGTGACAGTACCTTAGGAATGATTTAGTCAAGTCTGTTTCACGGTCCAATTCATTTTGATTGTTATCCAGATTTCACAATAAGTCTTAGTGATCCTCATATTTTGAAAACTCTCACATTAAATATTAAAACTTCTGGGTATAAAGTCTTACTAGGAACCCAGCCATTAGCCTTAGTCTACATAATATATTACAAAGTCACAGGTATGAACATGAATTTCCGAGCTCTTAATAAAAGTCCAAAAGATCAGACCTTGTTAATTCAAAGTCACAAAGCCGATGCCAATATAAGAGTTCCGCACACTATTAAGTGGTCAAAAGTCACCCTTCCATTAGATTGGACATTAACCACTGAAAATCAACCAGTTCCTATTCAACATAGTTTAAATAATTTAGATTACATTCAACAATATTTAGATGGCACAGTCAAAATTAATTTTGGAAATCAACCTCCTCAAAAATCATCTGTTCAGTTACAACAACTTCCTACTCTGAGTCAATCCCAGAGACAAGCCCCTGCAAGACATTCTTTTACAGCCTCTTCTTCTACCCTAGAACGAGACCTAGAGTTAGAGAAGACATTAATTGATCTAAAATTAGAATCCTTAAGAAAAACTTCCTAAGTAAATCAGCCTTGTTACTGTAAGAAATCAATAAAAGAAGACACCAAGTCAGAATCTCCAGAGTCACCCACACAAACTGATTTCGAAGGAGGTTTTCAAGTTGATAGTTATGAACCCTTAGTAAAAAATTTAAAATTAATTGGAAAAGTCTAGATAGTCATTTACATGCCCTTGAGAATTAAAAAAGAAGACTTAATTATCACACAGCCTACCCCAATAAAGAAAAACGCCAACAAATCTTTAATGAGTGGAAAAACTTTATACGATCCACTAAGTCAGAAATATTCTACCTCGATTTTGTCAAAAGTCATCACATGTCAAAAGAGTTAAAAACTTTAACCCATGAAAAATGGGAAAAAAAAGATAACACCGAAGTCCTAGCCAGTCATCCCCCTGTAGAAATAATAATTATTAACCATAAAAAGTCCAAAATAGTGGCTAGTCCCTTCAAAATCCCCGGCCAGGATAATAGAAAGTTAGTTGAGCAAAATAATTACACCAACCAAAGTCTGACAGTAATAGGAAACCAGTTAGATGAAATAGAAACTAAAATTGACAAAATAGCCCCTTCAGAAATATTCTCTAAGCCACCAAAGGAAAAGCCTTTAGTTAAATTCCAAGACATACAGTCAAGCCCAACCTTTAAGACAAAACCAACAATGAAGAAAATAGAAGAAATGCTAGAACAGTTAACTCCGAAGAAAGAGAAAGAGAAAATTGAAATATCTTGACTTAAGGTCTTAGGATCCTTTGCTCCAGTTAGCCCCTCCGATTCTGAGTCTATTACCAGTATGGATACAAAAACCAGCAATATCTCCAAAATTGAAAATCCCCAGTAATATCTTCTAGGTCGAGGAATAGAATACGGAGTCAACACTTTATTATACACAATAATTGAGCACTTCATAGGTACACCCAGTAACACAGCAGCCAGAATTCATGATCAACTTAGTAACCTCAGATGCCCCAAGTTATCTGTTTTTCGTTGGTATAAAGATGTCTTCATTTCCAGAGTCATGCTTAGAGATGATAGTAACCAGCCCTTTTGGAAAGAAAAATTCATTAATGGTCTCCCCAATTTATTTGCCCATAAAATACGCACAGTCCTTAGTAATGATCAAGGTCACATAGATTTCAATAATCTAACCTACGGGAATATAATCAGTACAATTAACCAAGAAGGAATGAAAATGTGTGTTGATATGAAAAGTGGTAAACAAATTCAATCAAAAAGAAATTCAGCTAAATATGAATTAGGAGATTTCTGTGAACATTATGGTTTAGCTTCTATTCCCCCATCCAGAAAAGCCAAGTCTAGTTATCATGATCGTCAACGTAGTAAACATCGTCATTATTCATGAAGAAAATAGTCCTTCCGACATAATAATGATAATAATGAGTTTTATAGTAAAAAGAAGTTTCACCCGCGTAAAAAATGGTCAAAAGCACAGAAAAAAGATAAGAAAAGTTACTTCAAGAAAAGCACTGACAAAAGAAAAATTAAATGCTGGAAATGTCAAAAGAATGGTCACTATGCTAATGTTGTAATGTCCTATGTAAATCAATTAGTGTCCATACAAAACAAGAAGAACAAGAAGAGTTATTAATGGATTTAATTAGTAAAGTAGAAAATCCATAGTTAAAATCTGAGTATTTAAAGAAATTAAGAAAAATCATTAGTCAAGAAGAACCAAGTTAGTCTGTTCCCTAGAAAATAAGTATGAATTCTACCTTAGAAAAATTCAACAAAAGAAAAGAAGTCACATTTCAGGATTTACAATTGGAAGTAAAATTAGTCAAAAAAGAAATAGTAGAGTTAAGAGAGATAAGTCACAAATTAAAAGCAAAAAATTTTGAAATTAAACAAGATTTGGTTAATTTAATGGAAAAAGAAAGTTCTGAGTCAAAGAGTCAATTAGAAAGCCCCACAAACTCAGACGATGAACCCTCCAACAATGACCAAGCTATTAATTTAAGAACAATGCTACTCTTACCACATTTGTATACCACATTCTTATACAATCTTAAGTGACAGTTGAGGTGGACAGCCACATCAATTAAAATTATTTAATATTTTCTTTTCTTTTATGATTTATTCCAATATTTTTTTTTCTAATTGTCTTAATTAAAATACACTTCATTGATTTAATTGACGTGGCATATAATATGGACATGCCACATCATGTGGTATAGAAATGTGGGACAAAAATGTGGTAAGTGTAGCATCACTCTTAATTTAATAAAACAAGTAAATTTTAGAAAATAGTATTCTGAAGTCACCATCTTTGTCAAGAATTTTCAGTTAAATACAGTTGCCCTTTTTGATTCAAGAGCCGACCTTAATTGTATCCAAGAAGGCTTAATTCCTAAATATTATAAGAAGTCAAAAGAATCGTTAAGCACTGCCTCAGGAAAGTCACTCCAATTAAATTATGAAATTCCTAAAGCCCATGTCTGTTAGAATAAAATTTGTTTCAAAACCTCATTCGTCTTAGTAAAAAAATATTACAGATGAAGTCATCTTAGGGTTACCATTTATCGCCCTTTTGTATCCTTTCCAGGTAGAATACGATGGAGTCATATCTATCCATTTAGGAGAAAAGGTCAAATTTGAGTTCTTAACTAAACCTGAGTTACATAATCTAAAAGCTTTACAGAAGAGTGCAGTTTAAAAAACAGTCAAAATAAAGATAAACAATTAGATTTTCTTAAAGAAGAAGACAAATTCAAAAGAATTGAACAACAGCTTAATGATAAGTTTTTACAGTCACAGATTCAAAAATTTGAAGAAAAGTTAAAACAAAAAGTCTGTTCTGATTTACCTAATGCTTTTTGGCACAGGAAACAACATGTAGTCAGTTTACCTTATGTCAAAAACTTGAGTGAAAAGAATATTCCCACAAAAGCCAGACCAATTCAAATGAGTCAAGAAATAATGGAATTTTGTAAAACAGATATTACAGAGTTACTTCAGAAAGGAATAATTCGTAAAAGCAAGTCACCATGGCCTTGTCCTGCTTTCTATGTTCAGAAAAATGCTGAGTTAGAATGCGGAGCCCCAAGATTAGTCATCAATTATAAGCCTTTTAATACGGTTTTAGAATGGATTAGGTATCCAATTCCTAATAAACGAGACTTGTTTACAGGTTAGGAAATGTAGTCATCTTCTCTAAGTTTGATATGAAGAGTGGTTTCTGGCAAATCCAAATTCAAGAGTCGGATAGGTATAAAACCGCTTTTGTAACCTCATTCGGTCACTATGAGTGGAACGTTATGCCTTTCGGTCTTAAGAATGCGCCTAGTGACTTTCAAAATATAATGAATGAAATATTCAATCCTTTCAATCATTTTTCCATAGTCTATATTGATGATGTTCTTATTTTTTTCTTAGTCAATAGAGCAACATTGGAAACATTTGCACACGTTTCTTCAGATAGTCAAACAAAATGGTTTAGTTGTCGCTGCTAAGAAAATTAAGTTATTCCAAACTAATGTTCGATTCCTTGGTTTTAATACTTGTTAGTCCAATTGATTGAGTTATTCCGTTTGCAGAAAAATTCACAGATCAAATATTAGACAAGAATCAACTCCAAAGATTCTTAGGATCTCTTAATTACGTTTCTGATTTCTATCAAAATCTGAGAAAACAATGTAAACCCTTATTTGATCGCCTTCAGAGTAATCCTCCTCCCTGGTCATCCATCCATACAGACATAGTCAAACAAATCAAAATTCATGCCAAAACAATCCCCTGTCTTGGCATTCCTTTAGTTAATTCCTTCAAAATTGTTGAAACTGATGCCTCAGATTTTGGTTATGGTGGTATCCTTAAACAAAGATTCAATTTGAGTCAATTTGAGCAAATCGTTCGATTTTATTCTGGAATTTGGTCACAGTCACAAAGTAATTATAGTACTATTAAGAAAGAAATATTATCTATAGTACTCTGCATTAGTAAATTTCAAGATGATTTGTAAAATCAAAATTTTTTAGTCATAGTTGATTGTAAAAGTGCAAAGCATGTTTTAGGAAAGGATGTCCAAAACATTGCATCAAAATAGATTTTTGCACGATGGCAAGCAATTTTAAGTATTTTTTATTTTGATATTGAGTATTTGAGAGGTAGTGAGAATTCAATTCCTGATTTCCTTACTAGAGAATTCCTAGAGGAAAAACAATGGCAGAAAGAAGATCAGAAAAGCAAAAAGAAAAGCAAAAAGACAAGCAAAAAGAGCCTCAGTCATTTAGAGCCCTCCTGTCATCAATAAATCCGGTTAAATCAGAGTCACCTTCTCAGATCGTCCCCTTCCCAGGGATGTTCTCCAATTCATATCAGTAACAGATTCACCTTACTTGGTTCCACAGTCGGCCAAGGTCGCCATACCTTCCAGTCAGCCTTAGTCTTTAGTCATGATCCCTTCCAAATCAGTACCCAAGTCGCCTAATCTCTAGTTTCCTTCCAAAAGTCATCTACTTATTTGTCAAGAAGCAGTTGCCACCTTTTTATTGTTGAACCCAAATACAACAGCATCACTAATCCAGTCACAATTGCAAAGTTATATTTTCCCCCGAATTTCCATTTTGTTCCTCCTGCGCCTTACAAATCTCTTAACTATTATAGAGACATCCTACATGAGACCCAATCAATTGAAATTAAGCCCATTAAAGATCGAAATAATCCCTAAGTTATTCTTTTTCATTCTTTGTATATTCATCAAATCTTAAGCGAAGAAGAATGGAGTAAAAACCCCTATGATCTAAAAGTTCTTCAGTCAGAACTTCAATATTGTTATTATGATTATATCGAAGCTTGATATACAATTTCCTCCATCAAACAGTCGACTTCAGTCACTCTTGGTTCATTAATTTTGATAGTAAATTCAAAAGTCCATTTTCCTGCTGGTTCCTCCACTTGTGGGAAAAACAAGGTCCAGTCACTAATCTCCTTCCCCAACAAATGCAAGAGTTAGTCAATTAATTCTCCATTAAAAATAAATTCAAAGAGTCAGAGTTATTCTTTCCCAATCTGTTACTCTTCATTGCTAAATATAAAGTCTCTTGGATCCTGAAATGGTCTTGTCATGTCAATTGGGATTCCAAAGTTCTTTCCCGTCAATTTTCAGTCAAATGGTGGGATAAGTTCAAAATTGAAAGAATTGCAGATTATGTAAATAATAATTTCCCTCCAGTCAAAATCCCTCAAAAGTCTTCCTATAAGAAATCTTCTTCTAGTTCATCTCATGCCTCCCACTTAATCGAAGGAAAATCCAAAGTAGAGCTACAAGAAATTGCCCCGCCAATTGATTCTTCAGGCTTCTCAAATGGAAGATGAAGATGA

TAGTGCCTCCCCAAGGTCCCAGTCATCCACCAGTCAGCCTCATCAATCACCAAGTCAAAAGGATTCCACTCAGAAAATGCGGTGGGCAGATTACCAAGACAGTTAGGACCTTTATGATCTCGAATCAGACTAGTATTCAAAAGTTAAAAATCCAACAGTCATTATTCCTCGACAAAAGCAAGGAAGTAAAAGCAAAAGTAAAAGTAAAAGCTGAAGTAAAAGCAAATCTCAATCAAAAGTCTAAATGATAGCCCCAATAGGCTACCTGTGCTTCAACAGTCAGGTTTCCAAGCATCCAATGCTGCCATCAGTCTGGTAGTGTAGGTCTATTTAAGGAGGCTTCAACCTTAGTTGTATCCAGAATGTGACTTAGTTTTAATTTTCAGTTTTTAAAGTTTTCTCTGAGACTTCCTCAAGGAAACTACTATTGTAAAATATGGGTGCTATCTATCAATAAAATCTTCCAGTCAAGATTTTCTGTAAGTTTGTTGCATTCTCAAAGTTATTATATGTTTTCAATAATACATATTTTCATCTATTCACTAATTCCATTCATGCATAAATTCCTCATATTTTATTATCTTTATATATATCTATATATATATAAGCTAAAGAGTGAACAAAGTTCAGCCACAAAATCACGCCAGCACACCTAATATTCCTTTGCAACTCGAAGACCTAACTAGCCATCCTCACAGCAGCAAACCTATGCACACATCCATCTCAAAAACTAGCCAAGTTTGCTTGCCCAGCTAGCACTCACACACCTCCAGCAAAAAGGCATCGGGGTAGTGAGACCATTTCGAATTTTTCTTGTTTTGGCCTCTTAGAATTGGGTTCCGCCTCTACTTGGAGTTGGGTTCCAACTTGACCTCTGATTGGATTTTAGACCACAGTCTGCATATAAGGCAAAGTTCACAATTTCTTCATCCGAGGCATCATTGCCTGCACCCATTTGATAATCTTAGAAGCGAGATGACAATCGGTGCTCACATGGTGG

>Chr8-10; OZ022213.1; 16032085-16039164; rev; D;

TATTGTCTAGCCCTTTATAGATTTCAATCATTTGATTTTCAAAAAATAGAACAAAAATCATGATTATTCTGCCCCATTTCTCAATAAAACTGTTGATCATGATGAATTTAGGGAACGGATGTCCTGCAAGTTGCAACTCAAAGCTCTTTTCAAGGTCCCTTCTCTTACTCACCCACTGCAATCCACCCCCTCTTTCCTACAACCCAACGAACAGCCAACACTACCACCTATTCAAATCTCACGATCTCTCTCTCTCTCTCTCTCTCTCTCTCTTAAGCTTCAACCCCAAATCCAATTGATCCACACAACCCAACATTAGGCATCTCCTCCCTCTGTCTCTAAGGCCAAACGAAAATTCAATCCCAAGAGTGCAACATTGTGCATCCCTGTAAATTGAATTTGTTTTCCTCGATTCTGTGTTGCTTTTTCACTTCCGAAAAAAAAAACAAAAAAAACAGAGTTTCAAGATGTTTTCTTTTAACATAATTTTTTTTATTTTCCTATCAATTTTCTATGTTTCTGTCCACTGTTTATGGTTCCAAGATTTCTTCTGTTCTTTCAATTTTTCTACCCTGAACTTATTATGCTGTAATGTACCTGCAGTCTTGGTTAGTAGAAAGGTCTGTCTCCATCCTTTGCCATCAACGAGGCCGACCACACATTCTTCTCTTCTGCAATCGCTCCACAAGGTATTTTCCTTTTTTTCTTTTTCTTTTTTCTTTAGCCCAGCGAAAAAAAATTGAAAAGAAAGAAAAGGAAACTATCTCTAGCTGCTATCAAAAACAAAGAGAATAGAGAAGGGAAAGAAAGAGGACGAAGAAGAAGAGAGAAAACAAACAAGAGGGAAATAGAGAAAGGTCTTGATCTTAGGAGGGATGTGGAGTTTCCAAAACTGAGGGAGGGAGTAAAGTCTGACAAACAAACCCCGATACAAACATAGAACTTCCTCAGAACAACAAGATTACATATAATTTTTCGTTCTTTTTCTAGTTTTAATTCT

ACTGCACACGTTAAGACAGTAGAACAAGTTTATGCCCTAAGTAAAGAACATGAGTCGTGTCAATTACTTAATCTTGAGTCAATTAAAAAACATAAACAAGATGGTAATAATTTCCTGCACATAGGCCTTGTTCAAGTTGCTGTTAAGCCTCTAACCAGATTAGGATTGCAAGCTTCTATTCTATTATGTTTACGTGATGCTAGATTTACTGAGTTTAATGACAGTGTACTTGGAATGATTGAGTCAAGTCTGTGAATGGCCCTGTCCATTTTGATTGCTACCCTGATTTCACAGTAAGTCTAAATGATCCACATATTCTTAAGATTTTAACTTTGAATATTAAGACTTCTGGTTATAATGTTCTTCCAGGTACATAGCCTTTAGCCTTAGTTTATCGAATTTATTATAAAGTCACAGGCACTAATATGAATTTCCAAGCTTTAAATAAGAGTCCTAAAGATCAAACACTTTTAATACAAAGTTGCACACCTGATGCTAACATTAGGATACCACATACAGTTAAGTGGCCTGAGATTAGTTTACCTGTTGATTGGACTTTAGTTACTGAGAGTCAACCTGCACCAATTCAACGGAGTATAAATAATTTAGATTTTATTCAACAATATCCAGATGGTACAGTTAAAATTAATTTTGGACATCAACGCGAACCTTTAGCTCAACCTAAATCTAACATCCAGTTGCCTACACCAAGCCAATCTAAAAGACATGTTCCTGCTCGACATTCTTTTACTGCTTCCTCTTCTACCGATATTAGGGATCTAGAAATAGAAAAGACTTTAATTGATTTAAAATTGGGATCCCTAAGAAAAGCAGCCCAAGTAAGTCATCCCTGTTACGGAACTCCTCCAGCTCTTCCAATTCAAAATGAGCCTAAAACTGAGTCCCCTCCTACTTCACGTACTCAGTCAGATTTCCTTGCTAGTGAAATTACTGCCCACGTGTTAGATCAACAATTAAGAGTTCTTAAGAAATCCAAGCCTTTCAAGATTAATTGGAAAAAATTAGATGATCACTTTCATCATCCTGAGCATTAGTCTAGAAAAGATAACTACCATAGGATAATTTCTGATAAAAATGAAAGACAAAAAATCTTTAATGCTTGGAAAGATTATGTGCGATCTGCTAGAGTTGAGATTTGTTACCTTGATTTCGTAGAAAGCCGTTATATTTCAAATAAGTTAAAAACTTTAACTAAAGAAAAATGGGTAAAAGAAGACCAATCTGAAATTATTTCTAGTCACCCCCTAGTTGAAAAAATAGTCATTCCTTATGGCACGTCCAAAGTCATTGCAGCCCCCTTCAAAATTGCTGGCCAAGACAATGATAAAAAATTAATTGAGCAAAATAATTACACCAACCAAAGCTTAGTTGTCATAGGAAGACAACTAGACAAGATAGAAACCAAAGTTGATAAGTTTCTCCCTTCTGAGCCAAAACTTAAGATTAAAGAAAAACCTTTAGTTCAATTCCAAGATTTGAAACCTGCACATTCGCTTAGGACCTCTACCACTATGAAAAGAATTGAAGGAATGCTTGAACAATTAACTCCTGTTAAACCTGAGAAGTCTGGTCTTAAAATCCTAGAATCCTCTACAGCCTTAGTCATTCATTCGGATAGTGAGTCTAATGACAGCCAGTCTAATGACAGCATGGAAACCATTGAGTCTAGTAATATTTCCAAAATTGAAAATGCATTCAAAAATTTAGAATTAGAGTCTGAGTTAAAAGTCAAAAGATTAACTAATAGAGTTAATCCTACTAGTTTAACAAAAAATTGGTATCCTAAGCCTACACCTCCTGATATCCAATTAGAAGAAAGAAATAATTTGAGTCAATTCTCTGTTTCTTCTGATAAATTATATGAATGGAATATAGATGGTTTATCTGAGCAAGAGATAATAAATAAATTAACTCATATGTCCATGGTCGCTAATAGTTATATAACTAATCACAGTTTGCGCCATAGTCTGAAATATCGATAATATCGAGGAAATATTGATAATATCGAGGAAATATCGAGGATATTTCGGTTTTTTCGAATCACGGATATTTCGGAACATATCCATGTACATATCGTATAAATATCGACAATATCGATGATAATATCAGAAAATATCGATGTCGATAGAAAAGCACAAAGGGGATATGAACCCCTCCCATTTTACTCCTCCAACACCTTAAACACCATATCACTTATGTTTTTGTGATAATATGCTAAAATATTTATATTTATATGGGTGGTATGTTAACAATTACATGCAAAACTATTTTGGGGATTTATCATTTGATGACTACTCTTCACAATACACTTACTCTACACATAGAGATGATGAAGATAGTGAAAAATTTGAACCTCGTAAGAACTCTATGTGGTACTAAGTCACTCATGTATCTTACCATGCAATGTATACAGTATAAAATATTATAGTAAATCATTATATATAAATGATTACGGTGTATTTAATCTTTTTTCATTAATTATTACATATTTTTTACACTCATATTGTTTGCCCGCTTGCTGTATAATCAACTTAAATTAGTTAAATCCATCATGCAATGCATTTCCTTCTATTTTTTTGTGATAAACTCATAGATAATTGACTAAATAAACATTCTCCAAAGTTTCAATAAAAATTTCCAAGTTTTTCTTACAATTTTCGTGGTTTTTATTCCATTTTTATCGATATCGATAATATCCCGATATTTCCATCGAAATTTCTATATTTTTGGACTATCGATATTTCTGTTATTATCGATATTTTAGACCTTGGTTTGCGCCAATCTGAGATAATTCTGTTATTAGAAACTGGTTTCACCGGAATCTTACGTTCTTGGTGGGATAAGCATTTAACCAATGAATCACGAAATAATATAATTCATGCAGTTAAATCTGATGAAGAAGGTCTCCCTTTATTTGACCAAAATATAGGTCGCGGTACAGAAGACGGAGTCAACTGTCTTTTGTGCACTATAGTTGAAAATATACCTACTAAAGCCAGACCAATTCAAATGAGCCACGAAATGATAGAATTTTGCAAAACTGAGATTAATGATTTATTATAGAAAAATATAATTAGGAAGAGTAAGTCTCCTTGGTCCTGTCCTGCTTTTTATGTCCAGAAAAATGCTGAGTTAGAAAGAGGATCACCACGATTAGTTATTAATTATAAGCCACTCAATACTGTGTTAGAATGGATTCGTTACCCTATTCCTAATAAAAGCTGTTGTTTTCTCCAAGTTTGATATGAAGAGTGGATTCTGGCAAATACAGATTGATGAGTCTGATAAATATAAAATTGCATTTGTTACTCCTTTCGGTCATTATGAATGGAACGTTATGCCTTTTGGTCTTAAGAATGCACCGAGTGAATTTCAAAATATAATGAATGAGATTTTTAATTCTTACAGTCACTTTTTCATAGTCTGTATCGATGATGTTCTTATTTTTTCAGAGTCTATAGAACAACACTGGAAACATCTGCATAAATTCTTGCACATAATTAAGTCAAATGGTTTAGTAGTCTCTGCTACAAAGATTAAGTTATTTCAGAATAATATTAGATTTCTTGGTTTTAACATTTGCAAAGCCCAGATTCACCCAATAGACCGAGTCATCCAATTTGCCGATAAATTTTCCTATCAAATAATTGATAAAAATCAATTGCAAAGATTCTTAGGATCCCTTAACTATGTTTCTGATTTTTATCAAAATCTAAGAAAACAGTGTAAGCCTTTGTTTGACAGATTGCAGAGTAATCCTCCTCCTTGGTCAGCTGTCCATACTAAGATTGTTAAACAAATCAAGAGCCATGTCAAGACACTTCCCTGCCTTGACATTCCTACTCCAAAAGGCATAACGTTCCATTCATATCATAATCAATGTGTCCAGACTCATTACTCAAGACTGTTCTGATTTTATGGGCAAATAAATTTGGTAGACCATTAATAAATTTTTCTTTCCAAAAGGCTTGATTACTATCTTCCCTTAACATTACTCTGGAAATAAAAACATCTTTATACCATCTAAAATCGCTTAATTGGGGACATCTTCAATTACTTAATTGATCATGTATTCTGGCTTGTGTATGACTAGGTGTTCCTATAAAATGTTCGACTATAGTGTACAAAAGACAGTTGACTTCGTCTTCTGTACCGCGACCTATATTTTGGTCAAATAAAGGGAGACCTTCTTCATCAGACTTAACTGCATGAATTATATTATTTCGTGATTCATTGGTTAAATGCTTATCCCACCAAGAACGTAAGGTTCCGGTGAAACCAGTTTCTAATAACAGAAATATCTCAGATTGGCGCAAACTGTGATTAGTTATATAACTATTAGCGACCATGGACATATGAGTTAATTTATTTATTATCTCTTGCTCAGATAAACCATCTATATTCCATTCATATAATTTATCAGAAGAAACAGAGAATTGACTCAAATTATTTCTTTCTTCTAATTGGATATCAGGAGGTGTAGGCTTAGGATACCAGTTTTTTGTTAAACTAGTAGGATTAACTCTATTAGTTAATCTTTTGACTTTTAACTCAGACTCTAATTCTAAATTTTTGAATGCATTTTCAATTTTGGAAATATTACTAGACTCAATGGTTTCCATGCTGTCATTAGACTGGCTGTCATTAGACTCACTATCCGAATGAATGACTAAGGCTGTAGAGGATTCTAGGATTTTAAGACCAGACTTCTCAGGTTTAACAGGAGTTAATTGTTCAAGCATTCCTTCAATTCTTTTCATAGTGGTAGAGGTCCTAAGCGAATGTGCAGGTTTCAAATCTTGGAATTGAACTAAAGGTTTTTCTTTAATCTTAAGTTTTGGCTCAGAAGGGAGAAACTTATCAACTTTGGTTTCTATCTTGTCTAGTTGTCTTCCTATGACAACTAAGCTTTGGTTGGTGTAATTATTTTGCTCAATTAATTTTTTATCATTGTCTTGGCCAGCAATTTTGAAGGGGGCTGCAATGACTTTGGACGTGCCATAAGGAATGACTATTTTTTCAACTAGGGGGTGACTAGAAATAATTTCAGATTGGTCTTTTTTACCCATTTTTCTTTAGTTAAAGTTTTTAACTCATTTGAAATATAACGGCTTTCTACGAAATCAAGGTAACAAATCTCAACTCTAGCAGATCGCATATAATCTTTCCAAGCATTAAAGATTTTTTGTCTTTCATTTTTATCAGAAATTATCATATGGTAGTTATCTTTTCTAGACTTATGCTCAAGATGATGAAAGTGATCATCTAATTTTTTTCCAATTAATCTTGAAAGGCTTGGATTTCTTAAGAACTCTTAATTGTTGATCTAACACGTGGGCAGTAATTTCACTAGCAAGGAAATCTGACTGAGTAGGTGAAGTAGGAGGGGACTCAGTTTTAGGCTCATTTTGAACTGGAAGAGCTGGAGGAGTTCCGTAATAGGGATGACTTACTTGGGCTGCTTTTCTTAGGGATTCCAATTTTAAATCAATTAAAGCCTTTTCTATTTCTAGATCCCTAATATCGGTAGAAGAGGAAGCAGTGAAAGAATGTCGAGCAGGAACATGTCTTTTAGATTGGCTTGGTGTAGGCAACTGTTGCACCTGGATATTAGATTTAGGTTGAGCTAAAGGTTCGCGTTGATTTCCAAAATTAATTTTAACTGTACCATCTGGATATTGTTGAATAAAATCTAAATTATTTATACTTCGTTGAATTGGTGGAGGTTGACTCTCAGTAACTAAAGTCCAATCAACAGGTAAACTAATCTCGGGCCACTTAACTGTATGTGGGATCCTAATGTTAGCATCAGGTGTGCAACTTTGTATTAAAAGTGTTTGATCTTTAGGACTCTTATTTAAAGCTTGGAAATTCATATTAGTGCCTGTGACTTTATAATAAATTCGATAAACTAAGGCTAAAGGCTATGTACCTGGAAGAACATTATAACCAGAAGTCTTAATATTCAAAGTTAAAATCTTGAGAATATGTGGATCATTTAGACTTACTGTGAAATCAGGGTAGCAATCAAAATGGACAGGGCCATTACACAGACTTGACTCAATCATTCCAAGAACACTGTCATTAAACTCAGTAAATCTAGCATCACGTAAACATAACAGAATAGAAGCTTGCAATCCTAATCTGGTTAGAGGCTTAACAGCAACTTGAACAAGGCCTATGTGCAGGAAATTATTACCATCTTGTTTATGTTTTTTAATGGACTCAAGATTAAGTAATTGACACGAATCATGTTCTTTACTTAGGGCATAAACTTGTTCTACTATCTTAACGTGTGTAGTAGAATTAAAACTAGAAAAAGAACGAAAAATTATATGTAATCTTGTTGTTCTGAGGAAGTTCTATGTTTGTATCGGGGTTTGTTTGTCAGACTTTACTCCCTCCCTCAGTTTTGGAAACTCCACATCCCTCCTAAGATCAAGACCTTTCTCTATTTCCCTCTTGTTTGTTTTCTCTCTTCTTCTTCGTCCTCTTTCTTTCCCTTCTCTATTCTCTTTGTTTTTGATAGCGGCTAGAGATAAAGCGACCATTTTTTCTTATATATATTTTGCCTTGGAACTCTAGGTATATTCCAGTCTCGAAAATCTAATTGTTCATCATTACTAGAATTATATTCAAATTGTTCCTCATTTACAATCTCAGGCAAATGACCTATAGAGGTTCTAGAGCTGCTCGAACTAAAAGAATTTGTTCTAAAGAAGCGACTCATTTTTACACAAGTGTTTGGGAAAGGATGACTGAAGAAGAAGATAAATAGTGACTTCTAAAACTTACTAATTATTCAACAAGAAATTTACTGGATTCCTCTCTAGTTTTCTGTCTCCTACCCACCGCTCGGGCCCTAACCTGAGGACTTATCGTTTGGAAAAAGGATTCACGGGTCAGACCTTAAGAGAAGAAACCAAGAAATAACTTGAAGAATAAAAGAAGATGAAGAAGAAACTAAAAGAACTTCAACTTAGAAACCTAAACCAGCCACTTGGGTTGGACGAGTTGCGACGTAGACAAATTCTTACAACTCTTACTGCTCTGATACCA

GAAGGGGTCTAGAACCAGAGAAGCATATTAATGTGAGTATAATAACAAAGATAATAAAAAAGTTGCAAATTATGCATGAATCTTATTAATGAAAATATAAACATTTAGATTATAAACATAATGATATTTGCTCAAATTACTCAAAATATATTGATATATATATATATATGTACATATTATAAACATACAGCTAGTCAAATGAGATGGCACTTTGATAAGTATCACATTCATTACCTCCAAAAGTATCACATTCTCCATGCAAATCTTGATTCATGTCTCGAGGAAATGCATCATTTAAGATTTCATAAGTAATATCATTGGGTCCAAGTGCCTCTACACCATTTGGAAGAGTTGCATCTTGTCTGGGTTTCTCAAATTTTTCCCTATAGTATATCCATGTGTGATAAATAGCAAACATCCCATACTCCCATAAATGTGCATGGACAGTTTGAAGAGCATGCCATCGCGTATTCAGACATTTCTTGCAAGGAAACCTTGTCTCATTTTTTTCATTCACATGCTGAATTGCTACTTCCATAAATGATGCAATATCATCCATATATTCCTTACTCAACCTATTTTTCACTTTAATCTATTGTCTATCTGTGTGAACGTCCTGTAACATAATATAGGTGATGTCACATTCTTTCCGAACATGAAATGTCTCCTTATAACATATAAAGTTTTGTATATCTATGTTATTTTAAATATTATGCATAGTTAGTTAGTTATCCTATTATTCATGTATTACGTCTGTATTCCGTACACTTATGAAATATATGGAAAATTTTGTGTTTGACATATTTTATCAAAGATATGCAAACATGTCATTCACAAATTATGCACGTATTGACATATTTTCTAAACAGGGGCCCAAGGTATTGTATATGCATAAATTCCAAGTTGAAACAACTATCGAAGCCTACAAATTTTAGGGCCAAGTAAATCTAGAAAGTTCCTAGATACGTACCTCAGTGATTATTGAACCCTTGACCACATA

>Chr8-11; OZ022213.1; 16953610-16953997; rev; B;

TAATGTAAAACAACTAGTACAACAAGTTTGAGTTCATATCAAGGACAAAGGACAGTTCTTTTATTACGAATTACAGTCCTCATACAATACATCATAAATAAATTGAGGGGGAGTTGAGGAGGAGTTGGATTGAGAAGAATTAGATTCACGATTCTTATTGAAGTTGTTTACTAAACCATATGGATTGAGAAGAGTTAGATTCTGGATTCCCGTTGAAGTTGTTTACTACACCATATGGAATTCGGGTATGAATGGTACTCATTACTAAAATGTCCTTGTTGTTGGGTTAAAGTAGATGGCAAATTTAGAATTTTAAATTTGTGTGAGGATATAATGGATAAAAAAAATGAATTCATAATAGGTTAGTTCTCCGTGAATTAAAATATCACCTCCCACCCAAGATTTGAATTACTCCAAAATCAGGAATTCAATTCCTAATTTTGTGTGAATCCCACTTACTTTTTAATTACTTGATGTGAAGTGAACTAATTAATAAACACACCATTTAACTAGCCCCTATTTTTAATAACAAATTACACCATTTGCCGTTGAATTTAATGAACCAATACTCTTTTCACCTAACAACGGATTCCCTAAACTATAGCACACGCTTAATAACCTATATATATACATATATTACGATCTTTGATGGGCTTAATTTCGATTGACTGGGTCTCATGAAGGATATCCCTATAATATTTGAGGGATTTGTAAGGAGCTTGAGGCACAAAATGACAATTTGGAGGAAAATATGACTTTGCAATTGTGACTGGATTGGTGATGCTATTATATTTGGATTCAACAATAAAAAGGTGACAACTACTTCTGGAAAAATAAGAGGATGACTTTTGGAAGGGAACTGGAGATTGAGTGGTTTGCTTCTCTGCATTTCAAAGTCACTGGTCATCTTCTTTGTGTTTGAAGTTTGCTTTTTATTCTGTAGCTTTTTCAAAGTCTTCCTGTCAATCGAGTCTTCTCTGCCGGCCCAAACAATAAGTCT

GAATCACTAAGTACTGCTTCAGGAAAGTCACTCCAATTAAACTTTGAAATTCCCAATACCCATGTCTGTCAAAATAAGATTTGTTTTAAACCCTCATTCGTCTTAGTCAAGAATATCACTGATGAAGTTATCTTAGGATTACTCTTCATAGCCCTTTTGTATCCTTTCCAAGTAGAATACGATGGAGTCATATATCTACCCATTTAAGAGAAAAAGTCAAATTTGAATTTTTAACCAAACACGAGTTACATAATCTAAAAGCTCTACTAAAAAGTGCAGTTTCAAAAATAGTCATTGAACCAAACATTGAAAATAACATGGCTTCTTTTTTTTTTCCCCAATCTTGATACGCTCAGATATTTGGTACTCCAGAATTTCACTATCACTT

TTAAGTGAGACCCTATCACAATACATTATACATGTCAACTCAGTTCGGAACTCCCTAAATTTGAAAGTGGTGTAAACATCTTGAGCTTGCTTTTCCATGTCATACTCGGTTGCACAGTTAAAACTTTCAGTGAAAGCCTTTGAATCTTCGTGTTTCTCTTTTTTTCACCTTAGTCCTCAATGCTTTCTCATATTGCTCCACAAATTGTTTTAAAGTAGTCTTAGAGTTTACATATAGCCATCAAAAAATGAATTCATACTCTCACTACGTTGTGTGGTAGACATTCCCGCCAAAAAATGATTTTTTACAAATATTGGCACCCAATGATGTCTCTCTTTATATATGATGTTTAACCAATCATTATTATGCTACCCATATTTGGCAATCATCTCAACCCAACGCTCTTCAAATTCATAACGAGTCGATGAGTCATACACGATGTTATCCAAAGAAAGTCTAATGGACTCATATTTCTTGTAACTTTTCAACTTTTCAGGAACTTTCTTCATTATATGCCACAAGCACAATCGATGCCTAGTCTTTTACTAGGAACTTGTCAGGAACTTTTCAGGAAAGACTTCTGCAATTGCATTTTTCATGGCATACGGATAAATCCCCTTACAATAACTAATACTACTTTAAGAAAGAGTTTGCTTATATATTGAATGAATTATATTCTATTCCTCTCTCACTCTCTCCCAGTCGCTGCTCGCTCTCTGATACTTGTCTCTCTCTCTCTCTCTCTCTCTCTCTCTCTCTCTCTCTCTCTCTCTAGATCGAGATGGCTGACCCGATCGCTGAAGACTTTGATCCGCCACAGAAACCCAAGAGAAACAAATTCGCCATTGCCTGCTCCCTTATGGCTTGCACGACATCAATTTTACTGGGCTACGGTAATTATCCTATTAGCACTCGAAATCGTTTTCTCTTACTTTTTTTAATCTTAATTTTGCAAGAAAAAATTGTTGGTTTTGGAGTGTATGAACTGAGCGTGGTCACT

>Chr8-12; OZ022213.1; 25487597-25488783; dir; B;

TTTATATATAAACCACAAACCCCTCTTGCTTATACCAAAAAAAAAAACCCATTACCAAGAACTTGTTCATCATTTCACATTTCACAGAAATATAGTGTCCCTTGCAAATCATAGCCACTCTCACTCAAGAATAAAAAAATGGCAGTTAGGGGGATTGAGATTGTGCTAGCTTTGGTCCTTGCAGTGGTTCTGCGGGAAGGGACTGTTGATGCTCAATCAAGCTGCACCAATGTGATCATCAGCATGTCCCCATGCCTTGATTACGTAACAGGAAACTCATCAACCCCATCTTCCTCATGTTGCTCACAGTTGGCTAATGTGGTCAGCTCACAGCCAAAGTGCCTCTGTGAGGTTCTTAATGGAGGCCCCTCTTCCGTTGTTGTGAAAGTCAATCAGACTCAGGCATTGGCCCTCCCCTCTGCTTGCAAAGTCCAAACACCCCCGCTTAGCCGATGCAGCGGTATATAAAAAAAATTTCAAATTTCAAAAAACATAATGTATGTGATTCTGCAGGTTCTATAAACCTATGAACTAACCTCTGGTCATGATTTTGCAGGGTCATCTCCAACAGGTTCTCCCTCAGGGGCACCAACTTCTCCTTCAGGTAACCAATTTGATTCCCAAACTCACATTTCTTTACAATAGATTCTAATCGATTTACAATTTGACATATTAATCACTGTAGATGTGTGTGTGTGTGTGCAGGGAGTGGATCTAAAACTGTACCATCACCATCACATGGAAATTCTAACAAGATGGCATTTCCTCTGCTGTTCTTCTTGCTCTTCATTGCATCATATGCTTCAACTTCCACAGCCAACTAAGCTCCTCTTCCACGAATGTTTATGTAGCTAGATTCATGGGACTATATAGTATAGAGTGCCATAAACTTTTTCTTATTTTTGCATATTGTTGGGTGGCTGATTGGTTTCTGGTTTTTGTAAATCCTTTGCTTGGGGATTCAGCCCCTCATGGAATTCAAATAAACTAGTCACAAATT

TTTTACTTTGATATATATAATTAGTACAATCAATCAAGTATGAATGAAAATGTGTGTAGACATGAAAATAGGTAAACATATCCAATTAGAAAGAAACTCCGCTAAATATGAGTAAGGAAACTTCTGTGAACAGTATGGTCTAACTTCAATACCTTCTTCTAAGAAAATTAAGCATTAAAATTGTGACGATCAGACCAGTAAAACTAGACATTATCAAAGGAGAAGACATTCCTTTAAAGATAATAATGAATTTTATATATAAAAAAATGCAAATATAATAAAAGAAGAAATTGGTCTAAAATGCCCACGAAGAATGGGAAAAATAAAACTACAGATAAAAGTAAGATTGAATGTTGGAAATGCAAAAAATTTGGTCACTATGCTAATGAATGCAAGGTCAAAGGATACAATAAAACAGCTAAAAATCTATGAGGAAGAAAAAGCTCAATTAATTAAAGTCCTTGAAATACGAAACTCTGAGTCAAGTGAAAGTGACATCTCAGTAACCAATTCAGATTACTATAGCTCTTCTGATTATCAGTCAAGCTCCCCCGATATTCAATTCGGATGTAAGAATAATTGCTGCAACACGAAATCAATAAATGTCCTTACAAAACAAGAAGAACAAGAAGAATTATTAATTGACCTAATTAGTAAAGTGGAAAACCCAGAATTAAAATCAGAATACCTTAAAAAGCTAAAGAAAATAATTAGCCACAATAAAATCGATCAAGTCATCCCAGCCCCAAAGATAAGTTTGACTACTACTCTAGAAAAATTTGATCAAAAGAAAAAAATTACCTTGCAAGACCTCCAACTTGAAGTCAAAAACATAAAGAAGGAAATTATCACCTTACGACAAGCAAGTCATGAACTACGAAATGAGAACCTTGAAATAAAACAAATTCTTGTAGAAAAATTAGGATCCAGTACTCCAGAACAACTCCAAGAAGAAGATGAAAAGGAATCCGCCAACAGTGACAAAGCAATTAATGTTTAATAACATTTGAATTTTAGAAAATGGTATTCAAAAGTCAGTATAATTATTAATAACTTTAAGCTTAAAACAGTAGCCTTTTTTGACTCAGGTGCAGACTTTAACTCTAAAGGCACGTCCACTCACAACCCTCATATGGACCAAGAACCAAGTGTGCAAAAGAAGGCAATTTGACCCGCCTTAGCACTAC

AAACCCATCTTCATCATTTTCCGATGTACTATTTCTTTGACACTTCAATAGGGTCTTCCTAGAACACCTATCTAATCAATTTTCTCCCATTCATTGGCCTCAGTACATTGGTTGAAATCCACCTATACAATTGCAATTCAAAACCGAATCATCCAATGCACTACACACCACAAACTTGTCACAACTATTGACAATTGTAAAGTTCACAATCACTAAACTTTTCTCCGTCTAAACAAAAAAGGAGAAAATGAAGACGGAAAAGAAAATATTGAACTTGGAAACGAGGTTTTTAAACTAGGTTTTTGCAAGAGATGAGTAAAGTTCCCATCATCTAATCTGACAAACTCTTTTCAGACATACTTTATTTGGTCTAGCTAATTTATAACCACATTGAGAATTGATGATAATATAGTTCATATTCACATCATCTAGGTAGTTGAGAGTGGTTGTGTCTAGCTCATATTAACATTGAGATAATTGATGATAATATAGCTCATATTCACATTGATCCGTACATTGAATAATAATGTAAAATATTAGTTTCATCTTACATAATAAGGAAAATCCCATTTAATTTACCAAAATTAACAACTTGTTATCTCATTAGTCATTTTCTTGTTATTGGCCCTTAAGGGTTGAATCGCTCGGGCCAGACCAATTCTATCTCAAAAATGCTAAGCCTAGGCCCTACCCCAAGGGCCAAGAATCTTTGAGTTTATGGGTGGGCCGATGGGCCGGGCCTATATGACCATATCAAATAGGCCACATATATTATATTTAAGAGTGTTGCTAAATGAACTCTAAAATTAACTGAGTACACCCTATGATCTAAGTTCACCATAATATTTAAATCAAAATATAAAATTAACTAAGTATATCCTATTTAACTACCAAAAATACCCATAATACACCCTCTTTAAATAAATTTAAAAACTAATAATACATATTTACAAATGAAATAATTAAAAAAGATTTATTTATAAAAATAAAATACTTAAAC
